# Delayed and Accelerated Aging Share Common Longevity Assurance Mechanisms

**DOI:** 10.1371/journal.pgen.1000161

**Published:** 2008-08-15

**Authors:** Björn Schumacher, Ingrid van der Pluijm, Michael J. Moorhouse, Theodore Kosteas, Andria Rasile Robinson, Yousin Suh, Timo M. Breit, Harry van Steeg, Laura J. Niedernhofer, Wilfred van IJcken, Andrzej Bartke, Stephen R. Spindler, Jan H. J. Hoeijmakers, Gijsbertus T. J. van der Horst, George A. Garinis

**Affiliations:** 1 Department of Genetics, Erasmus University Medical Center, Rotterdam, The Netherlands; 2 Department of Internal Medicine, Erasmus University Medical Center, Rotterdam, The Netherlands; 3 Institute of Molecular Biology and Biotechnology, FORTH, Heraklion, Crete, Greece; 4 University of Pittsburgh Cancer Institute, Department of Molecular Genetics and Biochemistry, University of Pittsburgh School of Medicine, Pittsburgh, Pennsylvania, United States of America; 5 Department of Molecular Medicine, University of Texas Health Science Center at San Antonio, San Antonio, Texas, United States of America; 6 Barshop Institute for Longevity and Aging Studies, University of Texas Health Science Center at San Antonio, San Antonio, Texas, United States of America; 7 Integrative Bioinformatics Unit, Institute for Informatics, Faculty of Science, University of Amsterdam, Amsterdam, The Netherlands; 8 National Institute of Public Health and the Environment (RIVM), Laboratory of Toxicology, Pathology, and Genetics (TOX), Bilthoven, The Netherlands; 9 Erasmus Center for Biomics, Erasmus University Medical Center, Rotterdam, The Netherlands; 10 Department of Internal Medicine, Geriatrics Research, School of Medicine, Southern Illinois University, Springfield, Illinois, United States of America; 11 Department of Biochemistry, University of California Riverside, Riverside, California, United States of America; Stanford University Medical Center, United States of America

## Abstract

Mutant dwarf and calorie-restricted mice benefit from healthy aging and unusually long lifespan. In contrast, mouse models for DNA repair-deficient progeroid syndromes age and die prematurely. To identify mechanisms that regulate mammalian longevity, we quantified the parallels between the genome-wide liver expression profiles of mice with those two extremes of lifespan. Contrary to expectation, we find significant, genome-wide expression associations between the progeroid and long-lived mice. Subsequent analysis of significantly over-represented biological processes revealed suppression of the endocrine and energy pathways with increased stress responses in both delayed and premature aging. To test the relevance of these processes in natural aging, we compared the transcriptomes of liver, lung, kidney, and spleen over the entire murine adult lifespan and subsequently confirmed these findings on an independent aging cohort. The majority of genes showed similar expression changes in all four organs, indicating a systemic transcriptional response with aging. This systemic response included the same biological processes that are triggered in progeroid and long-lived mice. However, on a genome-wide scale, transcriptomes of naturally aged mice showed a strong association to progeroid but not to long-lived mice. Thus, endocrine and metabolic changes are indicative of “survival” responses to genotoxic stress or starvation, whereas genome-wide associations in gene expression with natural aging are indicative of biological age, which may thus delineate pro- and anti-aging effects of treatments aimed at health-span extension.

## Author Summary

To identify mechanisms that regulate mammalian longevity, we have quantified the expression parallels of a number of long-lived mice that show delayed aging and DNA repair mutants that age and die prematurely. Unexpectedly, we found significant, genome-wide similarities and a widespread overlap of over-represented biological processes in the transcriptomes of these disparate mouse strains. Subsequent analysis revealed that similar responses are triggered constitutively in a number of organs in aged mice. Thus, both intrinsic and environmental stressors (e.g., aging, genome instability, or food shortage) induce survival responses aimed at overcoming crisis and extending lifespan. Such survival responses are likely to allow assessment of biological age as well as provide valuable targets for therapies aimed at health-span extension.

## Introduction

The complexity of the aging process, as well as the conspicuous lack of tools to study it, has hindered hypothesis-driven reductionist approaches to identifying the molecular mechanism of aging in mammals. In mice, recent progress has revealed that several aspects of aging could be accelerated or delayed by single gene mutations. Mouse models for progeroid syndromes are invaluable for studying accelerated aging [1]. In humans, defects in the genome maintenance mechanisms can lead to a variety of progeroid disorders [2] suggesting a causative role of DNA damage in aging [3],[4],[5]. Prime examples are Cockayne syndrome (CS; affected proteins: *CSB*, *CSA*), XPF-ERCC1 syndrome (XFE; affected proteins: *XPF*, *ERCC1*) or trichothiodystrophy (TTD; affected proteins: *XPB*, *XPD*, *TTDA*) that are caused by defects in the transcription-coupled subpathway of nucleotide excision repair (TC-NER) [6],[7]. NER removes a wide range of helix-distorting DNA damage such as UV lesions, and is divided into global genome (GG-NER) that recognizes helical distortions throughout the genome and TC-NER that removes transcription-blocking lesions on the transcribed strand of active genes [2],[8]. Defects in TC-NER lead to progeria but are not associated with increased cancer predisposition, whereas defects in GG-NER lead to high susceptibility to skin cancer but not to significant accelerated aging. Recently, we applied genome-wide expression profiling to characterize the severe progeroid *Csb^m/m^*;*Xpa^−/−^* and *Ercc1^−/−^* [7],[9] and the phenotypically milder progeroid *Ercc1^−/Δ−7^* mouse models (GAG/LJN unpublished data), and uncovered a systemic attenuation of the growth hormone/insulin-like growth factor 1 (GH/IGF1) somatotropic axis along with the thyrotropic (*e.g.* deiodinases I and II; thyroid hormone receptor) and lactotropic (*e.g.* prolactin receptor) axes, suppression of oxidative metabolism (glycolysis and the Krebs cycle) and upregulation of anti-oxidant and detoxification defenses early in life [10].

On the other side, long-lived mouse models provide valuable insights into the biology of delayed aging [11],[12],[13] and point to an important role of the GH/IGF1 axis in determining lifespan. Ames and Snell dwarf mice [14],[15], GH releasing hormone (GHRH) defective little mice (*Ghrhr^lit/lit^*) [16], GH receptor/binding protein null mice (*Ghr/bp^−/−^*) [17] and IGF1 receptor heterozygous mice (*Igf1r^+/−^*) [18] all show suppressed GH/IGF1 signaling, dwarfism and a significantly increased lifespan compared to wild type (wt) control mice. Likewise, calorie restriction (CR) results in decreased insulin/IGF1 signaling, prolonged lifespan and delays several age-associated diseases in mammals [19]. Intriguingly, attenuation of the somatotropic axis and oxidative metabolism also occur in naturally aged mice [7],[9].

Defective DNA damage repair mechanisms can lead to lifespan shortening, whereas suppression of the somatotropic axis can lead to lifespan extension. The relation between these two distinct aspects of longevity, however, is unclear. The suppression of the GH/IGF1 axis in both progeroid and long-lived mice lies in contrast to the opposing nature of accelerated and delayed aging. We, therefore, sought to determine whether, and to what extent, these two extremes of murine lifespan are inter-related and further, how both relate to natural aging. Importantly, comparing genome-wide expression profiles across accelerated, delayed and normal aging could allow delineating markers indicative of biological age.

## Results

### Genome-Wide Expression Similarities between NER Progeroid and Long-Lived Mice

**Figure 1:**
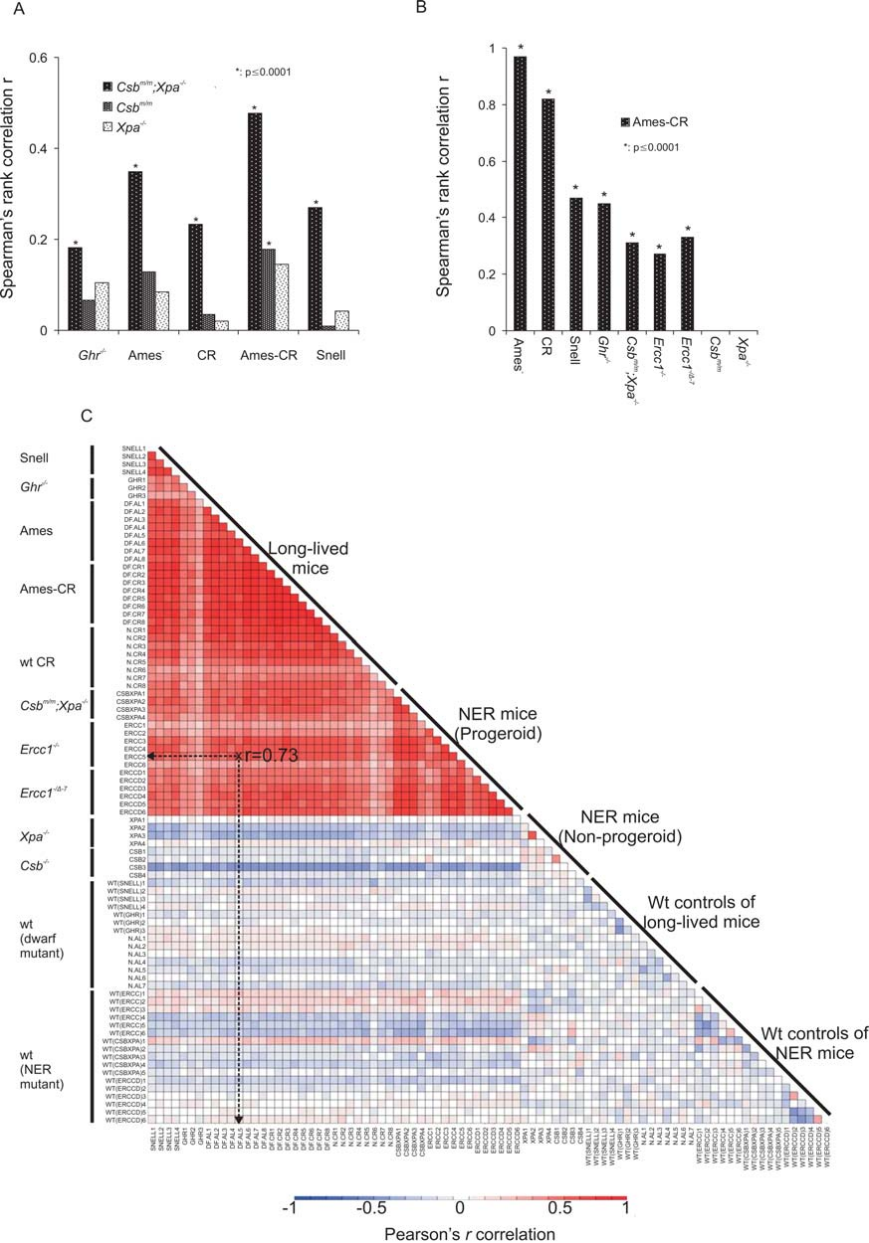
Genome-wide expression correlations between mouse models of accelerated and delayed aging. (A) Spearman's rank correlation r between the expression profiles of the significantly differentially expressed genes of the progeroid *Csb^m/m^*;*Xpa^−/−^* or non-progeroid *Csb^m/m^* and *Xpa^−/−^* mouse groups (as compared to their respective wt control mouse group) and those of the long-lived mouse groups (relative to corresponding wt controls). The comparison examines whether the genes that have significantly changed expression in the livers of short-lived *Csb^m/m^*;*Xpa^−/−^* progeroid mouse groups relative to their respective wt control mice show the same or opposite direction of expression change in the liver of long-lived mouse groups relative to their corresponding wt controls. (B) Spearman's rank correlation between the significant expression profiles of the Ames-CR mouse group relative to the group of respective wt controls and each of the long-lived (Ghr^−/−^, Ames, CR and Snell), progeroid NER (Csb^m/m^;Xpa^−/−^, Ercc1^−/−^, Ercc1^−/Δ−7^) and non-progeroid NER (Csb^m/m^ and Xpa^−/−^) mouse groups relative to their respective wt controls. (C) The correlation heat-map depicts the Pearson's rank correlation between the transcriptomes of individual mice to one another (mice are plotted on the x and y axis; e.g., indicated in a dotted line) on the basis of a predefined data set. This data set includes the genes whose expression changed significantly in all progeroid mice (*Csb^m/m^*;*Xpa^−/−^*, *Ercc1^−/−^* and *Ercc1^−/Δ−7^*) as compared to their respective controls and the genes whose expression changed significantly in all long-lived mice (*Ghr* ^−/−^, Ames, CR, Ames-CR and Snell) as compared to their corresponding wt control mice (Figure S1A, two-sided t-test, p<0.01). The deep red color indicates a strong positive correlation between the transcriptomes of NER progeroid and long-lived mice. Blue and white indicate a negative or no correlation, respectively, as seen for all wt mice and non-progeroid mutants.

To analyze genome-wide expression profiles across the extremes of murine lifespan, we compared independent liver microarray datasets from a series of short-lived DNA repair-deficient mouse mutants with severe (*Csb^m/m^*;*Xpa^−/−^*, *Ercc1^−/−^*), intermediate (*Ercc1^−/Δ−7^*), mild (*Csb^m/m^*) or no significant progeria (*Xpa^−/−^*) [7],[9] with mice that show lifespan extension either due to genetic alteration (Ames and Snell dwarf, growth hormone receptor knockout *Ghr^−/−^* mice)[20],[21],[22], calorie restriction (CR) [21] or a combination of both (Ames-CR) [21] (Figure 1A). In addition, we generated extensive microarray datasets of lung, liver, kidney and spleen of 13- and 130-week old naturally aged wt mice. Information on the groups of mice, the wt control mice, genetic background, number, gender, age and tissue for which the expression profiles were generated is summarized in Figure S1A.

Using these datasets, we asked whether the genes that have significantly altered expression in the livers of short-lived *Csb^m/m^*;*Xpa^−/−^*, *Ercc1^−/−^* and *Ercc1^−/Δ−7^* progeroid mouse groups as compared to their corresponding wt control mouse groups (Figure S1A) have the same or opposite direction of expression change in the liver of long-lived mouse groups relative to their respective wt control mouse groups (expression changes relative to corresponding wt controls; ≥1.2 fold change, two-tailed t-test p≤0.01, Tables S1–S2).

To do this, we first classified all significantly differentially expressed genes derived from the short-lived *Csb^m/m^*;*Xpa^−/−^* mice relative to their respective wt controls in terms of having increased or decreased expression. We then asked how many of those genes show the same or opposite direction in expression in the long-lived mouse groups relative to their own wt control mouse groups using the non-parametric Spearman's rank correlation coefficient. This correlation method tests the direction and strength of the relationship between two variables resulting in values ranging from perfect similarity (*r* = +1.0) to no correlation (*r* = 0.0) or dissimilarity (*r* = −1.0) (Figure S1B and Methods). As depicted in Figure 1A, unlike the non-progeroid wt and *Xpa^−/−^* mice, the significant expression changes identified in the progeroid *Csb^m/m^*;*Xpa^−/−^* mice relative to their respective wt control mice showed a positive correlation with the expression changes in all long-lived mouse groups (*Ghr* ^−/−^, Ames, *CR*, Ames-CR and Snell; *r* = 0.20 to 0.50, p≤10^−4^) as compared to their corresponding wt control groups (Figure S1A), despite the difference in age, genetic background and gender. This was also confirmed by comparing the significant expression changes of 2- and 16-week old *Ercc1^−/−^* and *Ercc1^−/Δ−7^* progeroid mouse groups relative to their corresponding wt control mice to those of long-lived mouse groups (Figure S2A–B; Table S3). As the expression profiles of NER progeroid mice are more comparable to the group of calorie restricted Ames dwarf mice (Ames-CR) -previously known to result in synergistic lifespan extension- than to the groups of Ames dwarf or CR mice alone (Figure 1A and Figure S2), our findings suggest that NER progeroid mice (*Csb^m/m^*;*Xpa^−/−^*, *Ercc1^−/−^* and *Ercc1^−/Δ−7^*) share similar gene expression changes with both the somatotropic mutants (*Ghr* ^−/−^, Ames, Ames-CR and Snell) and dietary restricted mice (CR) [21].

We next asked whether the correlations between the expression profiles were reciprocal. Following the same approach, the expression profiles from the significantly differentially transcribed genes of Ames-CR mice (as compared to their wt controls) also correlated significantly with those of progeroid *Csb^m/m^*;*Xpa^−/−^*, *Ercc1^−/−^* and *Ercc1^−/Δ−7^* mice (*r*: 0.15 to 0.34, p≤10^−4^, Figure 1B) but not with those of mild progeroid (*Csb^m/m^*), or non-progeroid (*Xpa^−/−^*) mice relative to their respective wt controls (Figure S1A).

To further challenge the strength of correlation between NER progeroid and long-lived mice, we then compared the transcriptomes of individual mice to each other on the basis of a predefined gene set. To do this, we first pooled all progeroid mice together (*Csb^m/m^*;*Xpa^−/−^*, *Ercc1^−/−^* and *Ercc1^−/Δ−7^*) and derived the genes whose expression changed significantly as compared to the group of all their respective controls (i.e. all the wt mice of *Csb^m/m^*;*Xpa^−/−^*, *Ercc1^−/−^* and *Ercc1^−/Δ−7^*; Figure S1A, two-sided t-test, p<0.01). Next, we pooled all the long-lived mice together (*Ghr* ^−/−^, Ames, CR, Ames-CR and Snell) and derived the genes whose expression changed significantly relative to the group of all their corresponding wt control mice (i.e. all the wt mice of *Ghr* ^−/−^, Ames, CR, Ames-CR and Snell; Figure S1A, two-sided t-test, p<0.01). This approach generated two sets of genes representing the progeroid and long-lived gene sets. We then used all the significantly differentially expressed genes of both progeroid and long-lived gene sets to ask whether the significantly differentially expressed “progeroid” or “long-lived” genes have the same or opposite direction of expression change between the livers of any two mice employed in this study when each mouse was compared to the group of its respective wt controls (Figure S1A). In Figure 1C, the deeper color of each cell indicates the positive (red), negative (blue) or lack of correlation (white) between the transcriptomes of two mice. Unlike wt controls and non-NER progeroid mutants, long-lived and NER progeroid expression profiles correlated strongly to one another (*r*: 0.40–0.95) forming a red triangle at the top of the plot (Figure 1C). Thus, failure to repair DNA damage causes gene expression changes that are associated with hyposomatotropism and CR, either of which in itself promotes longevity.

### Overlapping Biological Features in NER Progeria, Endocrine Dwarfism, and CR

**Figure 2:**
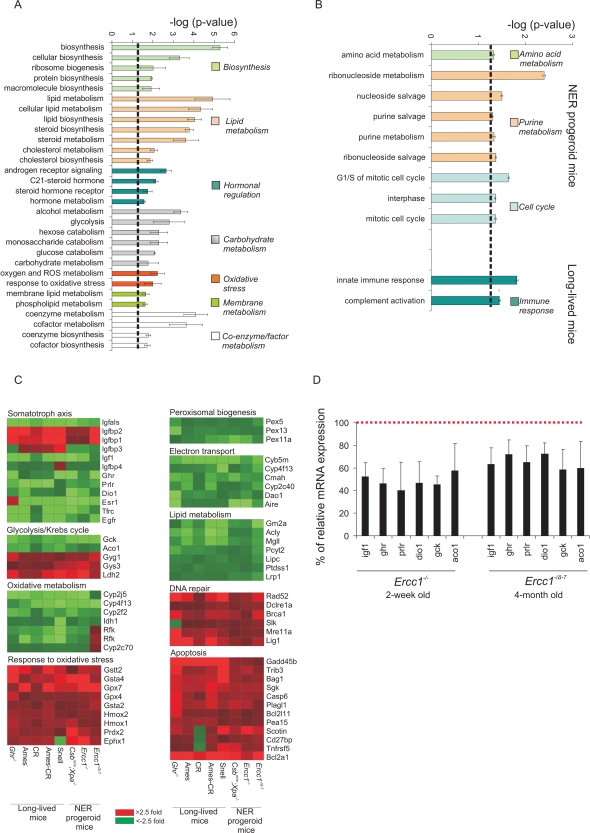
Significantly over-represented biological processes in NER progeria, dwarfism and CR. (A) Shared and (B) distinct biological processes associated with NER progeria, dwarfism and CR. All significant differentially transcribed genes derived from each mouse group (*Csb^m/m^*;*Xpa^−/−^*, *Ercc1^−/−^* and *Ercc1^−/Δ−7^*, *Ghr* ^−/−^, Ames, Ames-CR and Snell) against their corresponding controls (Figure S1A) were grouped into functional GO categories and tested for overrepresentation. Among others, lipid and carbohydrate metabolism, hormonal regulation and a response to oxidative stress were significantly over-represented in both progeroid and long-lived mouse groups. Amino acid metabolism, purine metabolism and cell cycle were only identified in NER progeria, whereas immune responses were limited only to long-lived mice. (C) Expression of genes associated with over-represented biological processes. The average gene expression (upregulation in red; downregulation in green) in each of the mouse groups (X-axis) is compared to that of the corresponding wt controls. (D) Quantitative real time PCR evaluation of % of relative mRNA expression levels of genes associated with the GH/IGF1 somatotropic axis (Igf1, Ghr, Prlr, dio1) and carbohydrate metabolism (gck, aco1) in 15-day old *Ercc1^−/−^* and 16-week old *Ercc1^−/Δ−7^* mice as compared to corresponding age matched wt controls. For each biological process, p-values represent the average of p-values obtained for each over-represented process that exceeded the significance threshold (−log (p-value)>1.3) when each progeroid and long-lived mouse group was compared relative to its respective wt control group (error bar = SD).

**Figure 3:**
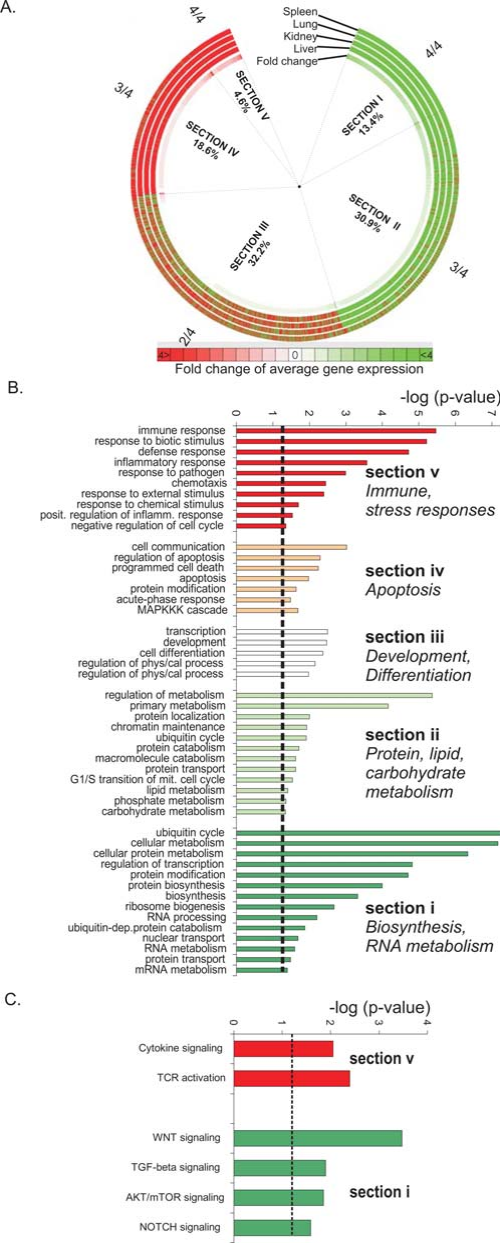
Age-related expression changes and significantly over-represented biological processes. (A) Circular map with age-related expression changes in the spleen, lung, kidney and liver of 2.5 year-old mice relative to 13 week-old young adult mice. Each ring of the map depicts the expression of the complete mouse transcriptome in each tissue. The innermost ring depicts the average fold change in expression across all four organs. A large fraction of genes have similar (i.e., 3/4 organs; section II and IV) or identical expression (i.e. 4/4 organs; section I and V) in the four organs with aging. Green and red arrows indicate down- and up-regulated genes respectively. Percentages indicate the fraction of probe sets associated with each section over the entire mouse transcriptome. (B) Over-represented biological processes within each section of the circular map. Sections IV and V contain the genes that are significantly upregulated with aging and include immune, stress and apoptotic responses whereas sections I and II, which are genes significantly downregulated with aging, contain processes associated with metabolism. (C) Over-represented signaling pathways within sections I and V of the map.

The seemingly paradoxical genome-wide associations prompted us to explore the biological processes that underlie the gene expression parallels between accelerated and delayed aging. We first grouped all genes according to their known or predicted biological function into gene ontology (GO) categories. Next, we asked which GO terms are significantly over-represented among the significantly differentially expressed genes in either the NER progeroid or long-lived mouse groups as compared to the group of their respective control mice (Figure S1A; Methods). This approach revealed seven common biological processes that were significantly over-represented in each of the short-lived and the long-lived mice relative to their respective control mice (Figure 2A). These processes were ranked by their relative enrichment score (see Methods) and included: (i) macromolecular biosynthesis, (ii) lipid metabolism (iii) hormonal regulation, (iv) carbohydrate metabolism, (v) response to oxidative stress, (vi) membrane metabolism and (vii) co-enzyme and co-factor metabolism. The identification of shared biological processes does not necessarily imply that expression of the same genes is altered (*e.g.*, distinct sets of genes may elicit the same biological outcome) or that the direction of the change in expression is the same. However, when we analyzed the expression changes of genes associated with the previously identified shared biological processes, we found a substantial suppression of the GH/IGF1 axis, oxidative metabolism (suppression of glycolysis and Krebs cycle with upregulation of glycogen synthesis) and peroxisomal biogenesis, coupled with a widespread up-regulation of a response to (oxidative) stress in long-lived and short-lived mice (Figure 2C). Whereas the majority of genes associated with the somatotropic, thyrotropic and lactotropic axes, electron transport and oxidative metabolism were downregulated, genes associated with the response to oxidative stress, DNA repair and apoptosis were upregulated. Therefore, processes related to growth, stress and metabolism might be most responsible for the genome-wide expression parallels between NER progeroid and long-lived animals. Interestingly, reduced protein biosynthesis as well as enhanced detoxification mechanisms have been recently identified as evolutionary conserved mechanisms controlling lifespan in multiple animal species [23]. Few processes were only enriched in either the progeroid (amino acid, purine metabolism and cell cycle; Figure 3B) or long-lived animals (innate immune response). There was no significantly over-represented process enriched in both mouse groups with opposite direction in gene expression. Importantly, the suppression of the GH/IGF1 axis seen already in the extremely short-lived NER progeroid mice (*Csb^m/m^*;*Xpa^−/−^*, *Ercc1^−/−^*) is not likely the result of a developmental defect as 16-week old adult *Ercc1^−/Δ−7^* mice showed a similar response at a later stage in life (Figure 2C and D).

### Identification of Systemic and Tissue-Specific Expression Changes with Age

We next sought to examine whether biological processes altered in NER progeria, pituitary dwarfism or CR are also similarly altered in naturally aged mice. As the age-related functional decline is ubiquitously manifested in all tissues and stochastic DNA damage likely affects the function of most organs, we also asked whether there might be similar, systemic gene expression changes with aging.

We analyzed the kidney, lung, and spleen transcriptomes in addition to liver, in a cohort of adult 13-week old and naturally aged 2.5 year-old wt mice (C57BL/6J; n = 3 per age group per organ). We included all ∼45,000 probe sets to avoid any potential introduction of bias (Table S4). The transcriptome of 2.5 year-old mouse liver, kidney, lung and spleen tissues was compared to that of 13 week-old mice and the results compiled in a circular heat map (red and green color indicate up- and downregulated genes, respectively; Figure 3A). The average fold change for each gene across all four organs was used to sort the expression profiles in a clockwise direction from the most negative (deep green) to the most positive (deep red) fold change. Subsequently, all genes were sorted by their consistency of expression changes across all four organs and plotted in a clockwise direction in five sections beginning with those downregulated in all organs (Section I) to those consistently upregulated (Section V). Sections II and IV include genes for which expression was different in one of the four tissues, whereas section III includes genes for which the direction of expression changes was different in two of the four organs in aged mice. Approximately 70% of the total transcriptome represented genes with identical (∼20%; section I and V) or similar (∼50%; section II and IV) direction of expression in all four organs. The remaining ∼30% of the genome showed greater variance, likely reflecting previously described tissue-specific expression changes with age [24] or expression changes with no apparent relevance to aging. These data reveal a remarkably homogeneous expression with aging across mouse tissues with distinct physiology and marked differences in age-related pathology.

### Biological Features of Natural Aging

To delineate which biological processes are significantly over-represented in each of the five sections of the circular map, we identified in each section, those genes whose expression was significantly altered in all organs examined (spleen, kidney, lung and liver) of 2.5 year-old relative to 13 week-old mice (two-sided t-test; p≤0.01). Then, using the set of significant genes in each section of the circular map, we identified all GO terms with an unequal distribution between this gene set and the remainder of the genome. Processes related to energy utilization and oxidative metabolism, growth, ubiquitin cycle and ATP synthesis were significantly over-represented in sections I and II, which include genes that are systemically downregulated (Figure 3B). Immune and stress responses as well as programmed cell death (apoptosis) were significantly enriched in sections V and IV, containing genes that are upregulated with age. Biological processes such as cellular differentiation and tissue development were significantly over-represented in section III of the circular map, which contains genes with variable expression changes in the different organs. Interestingly, a large fraction of the common responses previously identified in accelerated and delayed aging also occur with normal aging. Genes that are associated with immune, stress and defense responses were overrepresented in the upregulated genes, whereas genes associated with growth, energy utilization, lipid and carbohydrate metabolism were overrepresented in the downregulated genes in aged liver, kidney, spleen and lung.

A subsequent analysis to identify over-represented gene networks, representing pathways rather than biological processes, revealed genes related to the WNT, NOTCH, TGF-β and AKT/mTOR signaling pathways to be significantly enriched in section I, indicating significant down-regulation with aging (Figure 3C). Interestingly, both WNT and AKT (a downstream target of IGF-1) signaling have been previously implicated in longevity regulation [18],[25],[26]. In contrast, genes related to signaling in response to cytokines and activation of T-cell receptors were significantly enriched in section V, indicating significant up-regulation with aging. These data reveal a similar down-regulation of genes associated with growth, energy utilization and metabolism in aged mice as observed in progeroid NER mutant and long-lived mice and a similar up-regulation of genes associated with stress and defense responses.

**Figure 4:**
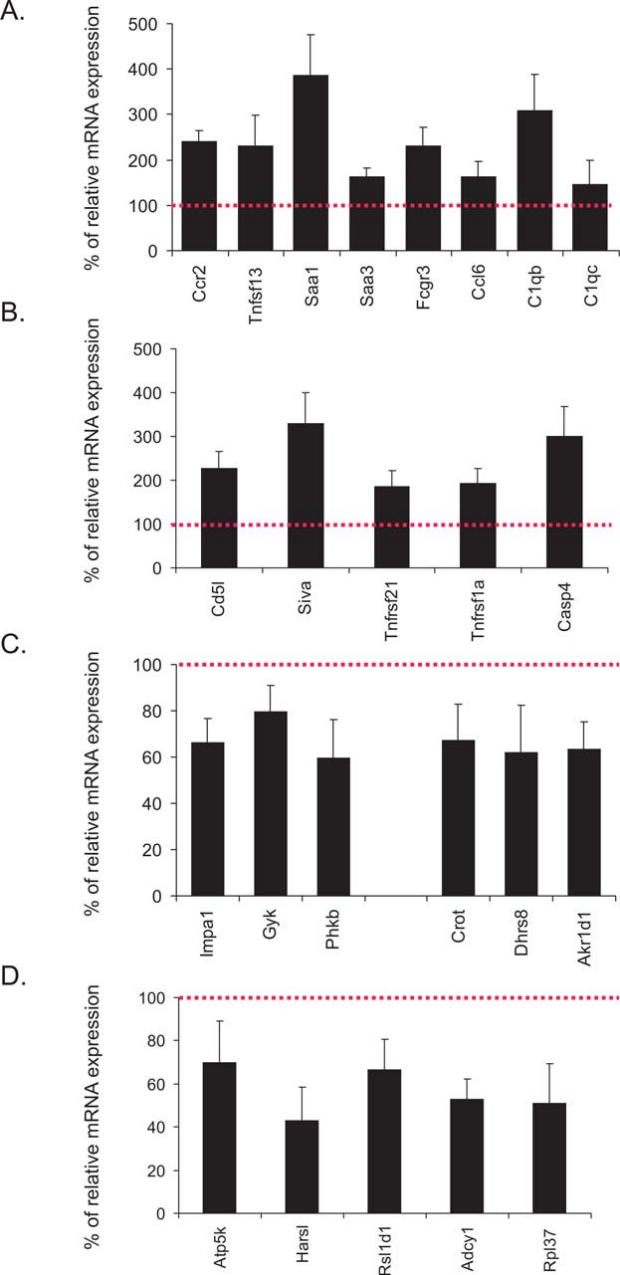
Relative mRNA expression levels of genes associated with significantly over-represented biological processes. (A–D) % of relative mRNA expression levels of genes associated with immune responses (A), apoptosis (B), carbohydrate (Impa12, Gyk, Phkb) and lipid metabolism (Crot, Dhrs8, Akr1d1) (C), and energy derivation and biosynthesis (D) in the liver of an independent aging cohort of 130-week old compared to 13-week old wt male mice. For each gene, expression levels in the 130-week old livers are plotted relative to those of 13-week old tissues (red colored dotted line). Error bars indicate S.E.M between replicates (n≥6).

To independently assess the relevance of these processes to natural aging, we examined the expression levels of several genes relevant to immune responses (Ccr2, Tnfsf13, Saa1, Saa3, Fcgr3, Ccl6, C1qb, C1qc), apoptosis (Cd5l, Siva, Tnfrsf21, Tnfrsf1a, Casp4), carbohydrate and lipid metabolism (Impa1, Gyk, Phkb, Crot, Dhrs8, Akr1d1) and ATP and protein biosynthesis (Atp5k, Harsl, Rsl1d1, Atp5k, Rpl37) in livers derived from an independent aging cohort of male mice (n = 6) by means of quantitative real time PCR (Figure 4A–D). Confirming the gene expression changes of the first aging cohort of female mice (Figure S3), we found that all examined genes associated with immune and apoptotic responses were significantly upregulated in the 130-week mouse livers (compared to 13-week old mouse livers) whereas the expression of those genes associated with energy utilization and metabolism was substantially downregulated.

### Genome-Wide Expression Similarities between NER Progeria and Natural Aging

**Figure 5:**
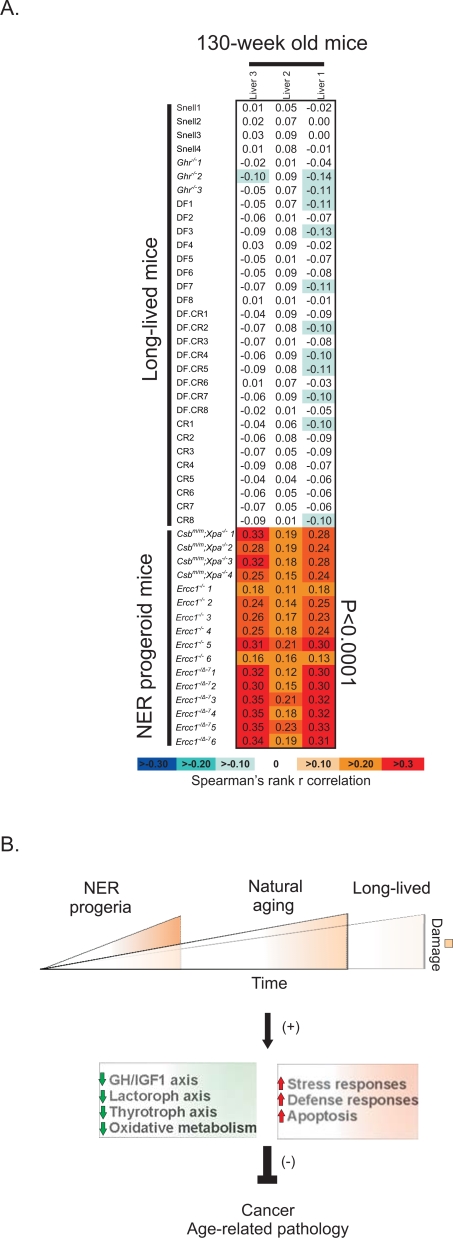
Genome-wide expression correlations between progeroid, long-lived and naturally aged mice. (A) Correlation between the transcriptomes of individual NER progeroid or long-lived mouse livers as compared to their respective wt controls and naturally aged mouse livers. The analysis was performed by pooling all progeroid (*Csb^m/m^*;*Xpa^−/−^*, *Ercc1^−/−^* and *Ercc1^−/Δ−7^*) and long lived mice (*Ghr* ^−/−^, Ames, CR, Ames-CR and Snell) into two separate groups and derive the genes whose expression changed significantly as compared to the group of all their respective controls (i.e. all the wt mice of either the progeroid or long-lived mice; Figure S1A, two-sided t-test, p<0.01). Using the non-parametric Spearman's rank correlation coefficient, we then asked whether the “progeroid” or “long-lived” genes have the same or opposite direction of expression change with those seen in the livers of 130-week old as compared to 13-week old mice. Note the significant positive correlation (red) between NER progeroid but not long-lived and naturally aged mouse liver expression profiles. (B) A model for the pro-survival/life extension response that occurs with aging: DNA damage accumulates with age or rapidly in organisms with defects in DNA repair. This triggers a systemic response that includes the suppression of the GH/IGF1 axis and oxidative metabolism and up-regulation of stress responses. Similar transcriptional changes are seen in long-lived organisms (dwarf mutants or as a consequence of CR) and with aging. This response shifts resources from growth to somatic maintenance, thereby protecting against cancer and delaying the onset of age-related pathology.

The identification of overlapping biological processes between NER progeroid, long-lived and naturally aged mice, prompted us to measure the extent to which each of the progeroid and long-lived transcriptomes relate to those of natural aging. To facilitate this, we pooled all progeroid mice together (*Csb^m/m^*;*Xpa^−/−^*, *Ercc1^−/−^* and *Ercc1^−/Δ−7^*) and derived the genes whose expression changed significantly as compared to the group of all their respective controls (i.e. all the wt mice of *Csb^m/m^*;*Xpa^−/−^*, *Ercc1^−/−^* and *Ercc1^−/Δ−7^*; Figure S1A, two-sided t-test, p<0.01). Next, we also pooled all the long-lived mice together (*Ghr* ^−/−^, Ames, CR, Ames-CR and Snell) and derived the genes whose expression changed significantly relative to the group of all their corresponding wt control mice (i.e. all the wt mice of *Ghr* ^−/−^, Ames, CR, Ames-CR and Snell; Figure S1A, two-sided t-test, p<0.01). This approach generated two sets of genes representing the progeroid and long-lived gene sets respectively. Then, using the non-parametric Spearman's rank correlation coefficient, we asked whether the “progeroid” or “long-lived” genes have the same or opposite direction of expression change with those seen in the livers of 130-week old as compared to 13-week old mice. This approach revealed a significant positive correlation between the significant expression profiles of short-lived NER-deficient mutants but not those of long-lived mice with aged mice (Figure 5A). Importantly, implementing the reverse approach to compare the significant expression profiles of naturally aged 130-week old mice relative to 13-week old mice to those of the short- or long-lived mouse groups showed that this correlation was bi-directional (Figure S4). Thus, despite a number of shared biological processes, the gene expression profiles of naturally aging mice correlate, on a genome-wide scale, with the progeroid but not with the long-lived mutant dwarfs or CR mice.

## Discussion

Genome instability promotes aging and shortens lifespan and, therefore, *a priori* is not anticipated to be associated with lifespan extension. Contrary to these expectations, we found significant genome-wide, reciprocal associations between mice with accelerated aging due to DNA repair defects and those with delayed aging. The genome-wide expression responses in the NER progeroid mouse models (*Csb^m/m^*;*Xpa^−/−^*, *Ercc1^−/−^* and *Ercc1^−/Δ−7^* mice) included the suppression of the GH/IGF1 somatotropic axis and oxidative metabolism along with upregulated stress responses. Paradoxically however, the systemic suppression of the GH/IGF1 axis and energy metabolism (i.e. glycolysis, tricarboxylic acid cycle and oxidative respiration), together with the upregulation of antioxidant and detoxification defense genes are all associated with increased longevity rather than with the short lifespan of progeroid mice. For instance, *Ghr^−/−^* mice, as well as Ames and Snell dwarf mice that have a primary, congenital defect in the pituitary gland responsible for producing GH, thyroid stimulating and prolactin hormones, benefit from delayed age-related pathology and substantially prolonged lifespan [27],[28]. Conversely, transgenic mice over-expressing GH show pathology early in life, increased tumor incidence and die prematurely [29]. However, CR mice show a similar longevity response, including suppression of the GH/IGF1 axis, but carry neither a pituitary defect (as in the dwarf mutants) nor are they exposed to rapid accumulation of irreparable DNA damage (as in the NER progeroid mutants). Thus, whereas pituitary dwarf mutants reveal the biological significance of these processes in lifespan extension, the similar expression patterns in NER progeroid mice and CR mice suggest that intrinsic and environmental stressors, such as genome instability or food shortage can trigger similar transcriptional changes. Suppression of genes associated with the somatotropic, lactotropic and thyrotropic axes together with the upregulation of genes associated with stress and defense responses are indicative of a shift from growth to somatic maintenance [30]. This reallocation of the organism's resources from growth to somatic preservation might have evolved as a mechanism to overcome crises such as food shortage, infection or other disease states (Figure 5B). For instance, the *C. elegans* long-lived dauer larvae are formed during starvation periods by suppressing Insulin/IGF1-like signaling [31]. In mammals, a shift from growth to somatic preservation may also function as a tumor suppressor mechanism [32],[33] and accordingly may explain the diverse outcomes of distinct DNA repair deficiencies in human diseases. Whereas irreparable DNA damage accumulates in both TC-NER and GG-NER deficiencies, only TC-NER leads to progeroid syndromes though without associated cancer predisposition. Indeed, not only CR or mutant dwarf mice but also progeroid *Xpd^TTD^* mice, which have a milder TC-NER defect than *Csb^m/m^*;*Xpa^−/−^*, *Ercc1^−/−^* and *Ercc1^−/Δ−7^* mice, are protected from tumorigenesis and despite showing segmental progeria in a variety of tissues they show a number of paradoxically improved histopathologic changes related to a CR-like phenotype in other tissues such as lower incidence and/or severity of de-myelination of the peripheral nerve, cataract, thyroid follicular distension, pituitary adenomas and ulcerative dermatitis [34].

Interestingly, a similar shift in gene expression towards somatic preservation as seen in NER progeria, mutant dwarfism and CR also occurs with natural aging. For example, the majority of upregulated genes was associated with immune, stress and defense responses as well as programmed cell death, whereas the majority of downregulated genes was associated with growth, energy utilization, lipid and carbohydrate metabolism in aged liver, kidney, spleen and lung. A number of recent consonant findings suggest evolutionary conservation of our findings. For instance, Tower et al., have found that immune genes are induced with age and in fact are predictive of remaining lifespan in Drosophila [35] whereas others have recently showed that NFkappaB activity, a key driver of immune and stress response genes, is induced with age in multiple murine and human tissues [36]. Importantly, however, a genome-wide correlation analysis, which extends to far broader expression changes than those linked to longevity-associated biological processes, revealed a strong association between naturally aged and progeroid but not long-lived mice. The correlations we found between certain groups of mice are most likely due to distinct groups of differentially expressed genes, i.e. there might be one large set of genes similarly affected in short-lived and long-lived mice and a separate large group of genes similarly affected in progeroid and naturally aged mice. This appears indeed to be the case. Nonetheless, there are also groups of genes, such as genes of the somatotropic axis that are similarly affected in accelerated, delayed and natural aging. However, these are smaller sets of genes that cannot influence genome-wide correlations in gene expression. In terms of the expression similarities, the resemblance of progeroid mice to both naturally aged and long-lived dwarfs suggests that progeroid mice harbor “expression signatures” associated with either age-related pathology or lifespan assurance mechanisms that likely attempt to counteract it. However, as age-related pathology is most likely absent in long-lived mice at this age (6 months), it might also explain the lack of any substantial positive correlation between the expression profiles of long-lived and naturally aged mice. It also suggests that both accelerated and natural aging trigger a common gene expression response, likely in response to accumulating (DNA) damage [37],[38]. However, the NER progeroid and long-lived animals employed in this study had a biological age of ∼50% and 10–15% of their lifespan respectively. Thus, these findings also indicate that a genome-wide correlation analysis may serve as a powerful tool to determine the biological age of animals and might hence allow prognosis of longevity. In contrast, the expression of particular genes (i.e. specific biomarkers) or groups of genes associated with e.g. somatic growth or oxidative metabolism are not indicative of the biological age as they are similarly affected in natural, accelerated and delayed aging. Determination of biological age is indispensable for the assessment of anti-aging treatments. Although reliable biomarkers of aging are long sought after, they have yet remained elusive [39]. To this end, single genes or limited sets of genes used as biomarkers of aging may poorly reflect a true biological age; instead these markers likely indicate survival responses that can be beneficial upon intrinsic or extrinsic challenges (e.g. macromolecular damage or limited food availability) but futile when the detrimental effects of DNA damage accumulation become too severe, as in the case of progeroid syndromes. In diagnostic terms, a CR treatment might, therefore, equally induce a similar age-related biomarker, as treatment with a DNA damaging agent does. We, therefore, propose the facilitation of comprehensive genome-wide correlation analyses to evaluate pro- and anti-aging effects of treatments aimed at health-span extension. It will also be of utmost importance to identify biological parameters based on e.g. the previously obtained genome-wide expression profiles, which may also be applicable to easily accessible samples such as sera.

## Methods

### Animals

The generation and characterization of NER-deficient *Csb^m/m^*, *Xpa^−/−^*, *Csb^m/m^*;*Xpa^−/−^*, *Ercc1^−/−^* and *Ercc1^−/Δ−7^* mice has been previously described [7],[9],[40],[41],[42]. With the exception of *Ercc1^−/−^* and *Ercc1^−/Δ−7^* mice which were generated in an FVB∶C57BL/6J (50∶50) genetic background, all mice were in a C57BL/6J genetic background. Animals were kept on a regular diet and housed at the Animal Resource Center (Erasmus University Medical Center) and the National Institute of Public Health and the Environment (RIVM), which operate in compliance with the “Animal Welfare Act” of the Dutch government, using the “Guide for the Care and Use of Laboratory Animals” as its standard. As required by Dutch law, formal permission to generate and use genetically modified animals was obtained from the responsible local and national authorities. All animal studies were approved by an independent Animal Ethical Committee (Dutch equivalent of the IACUC).

### Microarray Hybridizations

Standard procedures were used to obtain total RNA (Qiagen) from the liver, kidney, spleen and lung of naturally aged wt mice (3 animals per age group per organ) at 13 and 130 weeks of age as well as from the liver of 16-week old *Ercc1^−/Δ−7^* mice and wt control mice (6 mice per group). Synthesis of double-strand cDNA and biotin-labeled cRNA was performed using the GeneChip Expression 3′-Amplification IVT Labeling kit according to the manufacturer's instructions (Affymetrix, USA). Fragmented cRNA preparations were hybridized to full mouse genome oligonucleotide arrays (Affymetrix, mouse 430 V2.0 arrays), using Affymetrix hybridization Oven 640, washed, and subsequently scanned on a GeneChip Scanner 3000 (Affymetrix, USA). Initial data extraction and normalization within each array was performed by means of the GCOS software (Affymetrix). Expression intensities were log transformed and normalized within and between arrays with the quantile normalization method using the R open statistical package (http://www.r-project.org/).

### Data Collection

Figure S1A provides detailed information on the different mouse groups, the number of animals, their gender, genetic background, age and tissue of each mouse group. Data were collected from cited sources or generated in this study:

1. 2 week-old DNA repair-deficient *Csb^m/m^*;*Xpa^−/−^* (n = 4), *Csb^m/m^* (n = 4), *Xpa^−/−^* (n = 4) and age-matched wt control mice (n = 5; Figure S1A)[9].
2. 2 week-old DNA repair-deficient *Ercc1^−/−^* (n = 6) and age-matched wt control mice (n = 6)[7].
3. 16 week-old adult DNA repair-deficient *Ercc1^−/Δ−7^* (n = 6) and age-matched wt control mice (n = 6).
4. Ames dwarf (n = 8), calorie restricted (CR; n = 8) or calorie restricted Ames dwarf (n = 8) and aged-matched wt control mice (n = 7; derived from mice fed ad libitum) [21].
5. Growth hormone receptor knockout (*Ghr^−/−^*; n = 3) and aged-matched wt mice (n = 3)[20].
6. Snell dwarfs (n = 4) and aged-matched wt mice (n = 4) [22].
7. 13- and 130-week old wt mouse liver, spleen, kidney and lung (n = 3 per age group per organ).

To facilitate the comparison, analysis was restricted to the 8,524 probe sets (Table S3) that were present in both Affymetrix microarray platforms used in this and previous studies with long-lived mice [20],[21],[22] (Affymetrix Mouse Genome 430 Av2 and Affymetrix Murine genome UV74 sets). When the comparison did not include the long-lived mice, the analysis was extended to include the full mouse transcriptome covering all known and predicted genes in the Affymetrix Mouse Genome 430 Av2 platform (Table S4). All microarray experiments complied with the standards set by the “minimum information about microarray experiments; (MIAME)” and are available through ArrayExpress, a public repository for microarray experiments. The accession codes are: E-MEXP-835 for *Csb^m/m^*;*Xpa^−/−^*, *Csb^m/m^*, *Xpa^−/−^* and wt, E-MEXP-834 for *Ercc1^−/−^* and wt, E-MEXP-1503 for *Ercc1^−/Δ−7^* and wt and E-MEXP-1504 for 13- and 130-week old wt mouse liver, spleen, kidney and lung.

### Data Analysis

Two-tail, pair-wise analysis or a two-way analysis of variance was used to extract the statistically significant data from each group of mice by means of the Spotfire Decision Site software package 7.2 v10.0 (Spotfire Inc., MA, USA). The criteria for significance were set at p≤0.010 and a ≥±1.2-fold change in gene expression. We used the bivariate correlations procedure to compute Spearman's rho, and Pearson's correlation coefficient with their two-tailed significance levels by means of the statistical package SPSS 12.0.1. (SPSS Inc. IL, USA). All correlations reported were calculated by Spearman's rank correlation (except the heatmap visualization where correlations were calculated by Pearson's r correlation; Figure 1C). The Spearman's rank correlation coefficient (rho) is a non-parametric measure of correlation that assesses how well an arbitrary monotonic function describes the relationship between two variables without making any assumptions about the frequency distribution of the variables. The Spearman rank correlation coefficient is defined as: 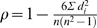 where *d_i_* = the difference between each rank of corresponding values of *x and y*. The correlation coefficients were derived from comparisons of expression profiles between two mouse genotypes (as in Figure 1). These coefficients were calculated on coordinates assigned to genes in each of the following categories: upregulated in both mouse groups (1,1), upregulated in one mouse group and down in another ([1, −1] or [−1,1]), or downregulated in both mouse groups (−1, −1). Genes having no direction of change (+1.2>fold change>−1.2) when all mice of the same genotype were compared against their own wt controls were discarded, because these genes lack any information about changes associated with progeria, long-lived dwarfism, calorie restriction or aging. In addition, by scoring for qualitative rather than quantitative similarities, this approach disregards variations in the magnitude of gene expression that might originate from, for example, differences in genetic background, sex or animal housing conditions. In addition, when examining the p-values derived from the Spearman rank correlation analysis, one should note that such an analysis is based on the assumption that all probe sets represent statistically independent pairs of variables: each pair has a value derived from either the long- or short-lived mice and there is one pair per probe. However, for a number of genes, their expression changes can be highly correlated with each other because genes are functionally interrelated such as being part of the same pathway. As a result, the data used in the correlation application may not be as statistically independent as it was originally assumed. In fact, these values could be mutually correlated because “correlated” genes in a small group of mice might drive them all. The Pearson correlation is defined as: 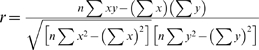


### Gene Ontology Classification and Overrepresentation of Biological Themes

All significant gene entries were subjected to GO classification (http://www.geneontology.org). Significant overrepresentation of GO-classified biological processes was determined by comparing the number of genes in a given biological process that were significantly differentially expressed in a particular mouse strain to the total number of the genes relevant to that biological process printed on the array (Fisher exact test, p≤0.01 False discovery rate (FDR) ≤0.1) using the publicly accessible software Ease and/or DAVID (http://david.abcc.ncifcrf.gov/summary.jsp). Due to the redundant nature of GO annotations, we employed kappa statistics to measure the degree of the common genes between two annotations and heuristic clustering to classify the groups of similar annotations according to kappa values (http://david.abcc.ncifcrf.gov/summary.jsp). Significant overrepresentation of pathways and gene networks was determined by DAVID (http://david.abcc.ncifcrf.gov/summary.jsp; through BBID, BIOCARTA and KEGG annotations) as well as by means of the ingenuity pathway analysis software (www.ingenuity.com).

### Circular Heat Map Visualization

The expression data from naturally aged mice is summarized using a visualization that sorts all probe sets present on the Affymetrix GeneChip™ by their pattern of expression across all four tissues (Figure 3A). It was created as PNG file using a combination of Perl, the GD.pm graphics module and the gdlib graphics library (http://www.boutell.com/gd/; http://search.cpan.org/dist/GD/). The circular map maximizes the display area by plotting data around a series of concentric circles. Probe sets were sorted by their consistency of expression across all four tissues and plotted in a clockwise direction: those downregulated in all tissues towards the top right; those upregulated in all tissues towards the top left and those with mixed expression states towards the middle. This results in five sectors. Probe sets within each sector are ordered by the most extreme average fold change observed for that probe set in each of the four tissues. Due to the high density of the Affymetrix GeneChip (∼45,000 Probe sets), it was necessary to combine data from individual probe sets prior to plotting. The direction of the probe sets were counted in the minimum length of the arc that was practical to plot, then colored red if the majority of probe sets were upregulated and green if the majority were downregulated. The innermost circle denotes the average fold change across all tissues. The color ramp ranges from green to white to red representing -4-fold change to no change to +4-fold change. A detailed representation of all expression changes depicted in the circular map is shown in Table S4.

### Quantitative Real Time PCR Evaluation

Total RNA was isolated from liver, heart, kidney, spleen and lung of 13- and 130-week old mice as well as the livers of 2-week old Ercc1^−/−^ and 16-week old day *Ercc1^−/Δ−7^* mice using a Total RNA isolation kit (Qiagen) as described by the manufacturer. Quantitative PCR (Q-PCR) was performed with a DNA Engine Opticon device according to the instructions of the manufacturer (MJ Research). Primer pair designed to generate intron-spanning products of 180–210 bp were as follows: *Ghr*: 5′-ATTCACCAAGTGTCGTTCCC-3′ and 5′-TCCATTCCTGGGTCCATTCA-3′; *Igf1*: 5′-TGCTTGCTCACCTTCACCA-3′ and 5′-CAACACTCATCCACAATGCC-3′; *Prlr*: 5′-GCATCTTTCCACCAGTTCCG-3′ and 5′-GCTCGTCCTCATTGTCATCC-3′; *Dio1*: 5′-CCCTGGTGTTGAACTTTGGC-3′ and 5′-TGAGGAAATCGGCTGTGGA-3′; Saa1: 5′-CATTTGTTCACGAGGCTTTCC-3′ and 5′-TGTCTGTTGGCTTCCTGGT-3′; Saa3: 5′-AGCCTTCCATTGCCATCATT-3′ and 5′-CTTCTGAACAGCCTCTCTGG-3′; Fcgr3: 5′-TGATGTGCCTCCTGTTTGC-3′ and 5′-GAGCCTGGTGCTTTCTGATT-3′; C1qb: 5′-TGTCCAACAGCAAGCAGGTC-3′ and 5′-TCAGGAAAGAGCAGGAAGCC-3′; C1qc: 5′-AGCACACAGTCAGGACCAA-3′ and 5′-AGTCAGGGAAGAGCAGGAAG-3′; Tnfrsf21: 5′-TGTGAACAAGACCCTCCCGA-3′ and 5′-ACACCACGATGACCACCAA-3′ Tnfrsf1a: 5′-AAAGTGTGGAGATGGGCAAA-3′ and 5′-CTGGCTGACATTTATCGCAC-3′; Impa1: 5′-CCAGAGCACCAGAGACTGTA-3′ and 5′-CCCACCTGTCACATCCATT-3′; Ccr2: 5′-ATTCTCCACACCCTGTTTCG-3′ and 5′-CCTTCGGAACTTCTCTCCAAC-3′; Ccl6: 5′-ATGAGAAACTCCAAGACTGCC-3′ and 5′-TGCTGATAAAGATGATGCCCG-3′; Tnfsf13: 5′-ATCTAAGGAGAGAGGTGGCTC-3′ and 5′-ACCGAGTGCTTCTTCTTCTGT-3′; Cd5l: 5′- CGACACAACAGCAGCAGAA-3′ and 5′-CTGGAAACCCACATACGACTC-3′; Casp6: 5′-ACATCAGACAGCACATTCCTG-3′ and 5′-GTAGACCTGGACAGTGGCAA-3′; Siva: 5′-CGCTCCAACTCAAAGTCCA-3′ and 5′-GCCATCAGGTCCAATCAACA-3′; Gyk: 5′-GAGGGAGGAATAGGTTGGAGA-3′ and 5′-GACAAGGGATAGCAATGACCA-3′; Phkb: 5′- ACATTCTCCAGCCTCAACAGA-3′ and 5′-ACCATTAGGTGTGCGTTCCA-3′; Crot: 5′-ATGTATCCCAAGCCAAAGCC-3′ and 5′-AAGGTATCAGGGTGAAGGGC-3′; Dhrs8: 5′-CTTCTTGCTGGCTTACTGCT-3′ and 5′-TGGTGCTTGGGTTCTTGATG-3′; Akr1d1: 5′-TTTCAACATCCAGCGAGGG-3′ and 5′-AGCAACTCCACATAGCGGA-3′; Atp5k: 5′-TTCAGGTCTCTCCACTCATCA-3′ and 5′-TATTCTCCTCTCCTCCTCTGC-3′; Harsl: 5′- CTATCCCAGAACAAGCAGGC-3′ and 5′-CAGGCTGAGGTCAAAGGAGA-3′; Rsl1d1: 5′-AATGCGGGCTCAAGACATC-3′ and 5′-CTGACTTCCCAGTTTCCACAA-3′; Rpl37: 5′-GGTCGGATGAGGCACCTAAA-3′ and 5′-AAGAAGAACTGGATGCTGCG-3′; Adcy1: 5′-TTACTGGTCACAGCCGCCTT-3′ and 5′-ATCCGCACGAAGACGCCATA-3′. The generation of specific PCR products was confirmed by melting curve analysis (which measures product specificity by the decrease in fluorescence signal when the PCR product is denatured) and gel electrophoresis (using Roche Agarose MS for analyzing small PCR products). Each primer pair was tested with a logarithmic dilution of a cDNA mix to generate a linear standard curve (crossing point (CP) plotted versus log of template concentration), which was used to calculate the primer pair efficiency (E = 10^(−1/slope)^). Hypoxanthine guanine phosphoribosyltransferase1 (*Hprt-1*) mRNA was used as an external standard. For data analysis, the second derivative maximum method was applied: (E_1gene of interest_^ΔCP (cDNA of wt mice - cDNA of *genetically modified or treated mice*) gene of interest^)/(E_hprt-1_^ΔCP (cDNA wt mice- cDNA of *genetically modified or treated mice*) hprt-1^).

## Supporting Information

Figure S1 (A) Groups of mice in the study. The respective wt mice were controls of the mutant or CR mice within each color-coded group. (B) Schematic representation of approach used to calculate bi-directional Spearman's rank correlation *r*. Red and green colored arrows indicate direction of expression for up- and down-regulated genes respectively. (C) Spearman's rank correlation *r* between the significantly expressed genes of NER progeroid *Csb^m/m^*;*Xpa^−/−^* and *Ercc1^−/−^* mice. On the y-axis, a value of 1 indicates perfect correlation whereas 0 indicates no correlation. Fc: fold change. To test for the validity of the approach, we examined the similarity of the expression profiles of *Csb^m/m^*;*Xpa^−/−^* and *Ercc1^−/−^* mice that are both DNA repair-deficient and progeroid but, like the long-lived mice, have a different genetic background (C57B L/6J vs. hybrid C57BL/6∶FVB, respectively; Figure S1A) and gender (only males vs. males and females, respectively). We selected all significantly differentially expressed genes from the *Csb^m/m^*;*Xpa^−/−^* dataset (522 genes, Table S1) and measured the Spearman's rank correlation to the *Ercc1^−/−^* mouse dataset. Next, the reciprocal analysis was performed using the *Ercc1^−/−^* dataset (833 genes; Table S2 and figure 1B). This approach revealed both progeroid NER mutants to possess a significant degree of similarity to each other in terms of their expression profiles (r = 0.76 and r = 0.83 respectively, p = 10−4). This confirms the overruling nature of the transcriptional response to NER progeria, as well as our ability to measure the response at the fundamental level of gene expression.
(0.03 MB PDF)

Figure S2 Spearman's rank correlation r between the significantly expressed genes in (A) 2 week-old *Ercc1^−/−^* and (B) 16 week-old *Ercc1^−/Δ−7^* mutants and those of long-lived mice (*Ghr^−/−^*, Ames, CR, Ames-CR and Snell). The strongest Spearman's rank r correlation was between the transcriptome of 2 week-old *Ercc1^−/−^* mice or 16 week-old *Ercc1^−/Δ−7^* mice and Ames mice that were calorie restricted (Ames-CR). Although highly significant, the correlations between the expression profiles of *Ercc1^−/−^* and *Ercc1^−/Δ−7^* mice and long-lived mice is weaker than those for the *Csb^m/m^*;*Xpa^−/−^* mice. This is likely due to the fact that although all progeroid NER mutants are hypersensitive to UV-induced lesions (that is reflected by the substantial genome-wide similarity in gene expression between the *Csb^m/m^*;*Xpa^−/−^* and *Ercc1^−/−^* mice; Figure 1C), *Ercc1^−/−^* and *Ercc1^−/Δ−7^* mice are also hypersensitive to DNA interstrand crosslinks. As a result, these mice show prominent pathology in the liver and kidney not seen in *Csb^m/m^*;*Xpa^−/−^* mice or any of the long-lived mutants.
(0.03 MB PDF)

Figure S3 Significant mRNA expression levels of selected gene targets as detected by microarrays. The expression levels of this set of genes were also verified in an independent aging cohort of male mice by means of quantitative real time PCR (Figure 4).
(0.01 MB PDF)

Figure S4 Correlation between the significantly differentially expressed genes of 2.5 year-old mouse livers to the expression profiles for the same set of genes in each of the progeroid DNA repair-deficient or long-lived mouse livers. There is a significant positive correlation between all NER progeroid and naturally aged mouse livers (indicated with deeper red) but not between long-lived and naturally aged mouse livers (indicated with white to deeper blue).
(0.02 MB PDF)

Table S1 A list of all probe sets with significant transcriptional changes in the liver of *Csb^m/m^*;*Xpa^−/−^* as compared to littermate controls. FC: fold change, P: p-value.
(0.05 MB PDF)

Table S2 A list of probe sets with significant transcriptional changes in the liver of *Ercc1^−/−^* as compared to littermate controls. FC: fold change, P: p-value.
(0.07 MB PDF)

Table S3 A list of all expression profiles of long-lived mice. FC: fold change, P: p-value. Expression profiles from these tables as well as all previously unpublished microarray data are available in public repository Array Express (www.ebi.ac.uk/arrayexpress/) and comply with the MIAME regulations.
(0.78 MB PDF)

Table S4 Expression changes in the kidney, liver, lung and spleen of 130-week old as compared to 13-week old naturally aged mice. Expression profiles from these tables as well as all previously unpublished microarray data are available in public repository Array Express (www.ebi.ac.uk/arrayexpress/) and comply with the MIAME regulations.
(3.7 MB PDF)
