# Supplementary material for: Delayed and Accelerated Aging Share Common Longevity Assurance Mechanisms
Source: PLoS Genet. 2008 Aug 15;4(8):e1000161. doi: 10.1371/journal.pgen.1000161 (PMC2493043; doi:10.1371/journal.pgen.1000161)
Supplement: Figure S1 — (A) Groups of mice in the study. The respective wt mice were controls of the mutant or CR mice within each color-coded group. (B) Schematic representation of approach used to calculate bi-directional Spearman's rank correlation r. Red and green colored arrows indicate direction of expression for up- and down-regulated genes respectively. (C) Spearman's rank correlation r between the significantly expressed genes of NER progeroid Csbm/m;Xpa−/− and Ercc1−/− mice. On the y-axis, a value of 1 indicates perfect correlation whereas 0 indicates no correlation. Fc: fold change. To test for the validity of the approach, we examined the similarity of the expression profiles of Csbm/m;Xpa−/− and Ercc1−/− mice that are both DNA repair-deficient and progeroid but, like the long-lived mice, have a different genetic background (C57B L/6J vs. hybrid C57BL/6∶FVB, respectively; Figure S1A) and gender (only males vs. males and females, respectively). We selected all significantly differentially expressed genes from the Csbm/m;Xpa−/− dataset (522 genes, Table S1) and measured the Spearman's rank correlation to the Ercc1−/− mouse dataset. Next, the reciprocal analysis was performed using the Ercc1−/− dataset (833 genes; Table S2 and figure 1B). This approach revealed both progeroid NER mutants to possess a significant degree of similarity to each other in terms of their expression profiles (r = 0.76 and r = 0.83 respectively, p = 10−4). This confirms the overruling nature of the transcriptional response to NER progeria, as well as our ability to measure the response at the fundamental level of gene expression. (0.03 MB PDF) [file pgen.1000161.s001.pdf]

A.

LONG-LIVED OR NER-DEFICIENT MICE

Genotype	Age	Sex	No of mice	Tissue	Background
Long-lived mice and respective controls					
Ames dwarfs $^{(df/df)}$	6 months	F	8	Liver	Mixed
CR	6 months	F	8	Liver	Mixed
Ames dwarfs $^{(df/df)}$ + CR	6 months	F	8	Liver	Mixed
Wt control (N.AL.)	6 months	F	7	Liver	Mixed
Snell $^{(Pit1\ dw/dw)}$	3 months	F/M	4	Liver	Mixed
Wt control	3 months	F/M	4	Liver	Mixed
Ghr $^{-/-}$	ns	ns	3	Liver	Mixed
Wt control	ns	ns	3	Liver	Mixed
NER-deficient mice and respective controls					
Csb $^{m/m}$;*Xpa* $^{-/-}$	15 days	M	4	Liver	C57Bl/6J
Csb $^{m/m}$	15 days	M	4	Liver	C57Bl/6J
Xpa $^{-/-}$	15 days	M	4	Liver	C57Bl/6J
Wt control	15 days	M	5	Liver	C57Bl/6J
Ercc1 $^{-/-}$	15 days	F/M	6	Liver	C57Bl/6J:FVB (50:50)
Wt control	15 days	F/M	6	Liver	C57Bl/6J:FVB (50:50)
Ercc1 $^{-/\Delta-7}$	4 months	F/M	6	Liver	C57Bl/6J:FVB (50:50)
Wt control	4 months	F/M	6	Liver	C57Bl/6J:FVB (50:50)

NATURALLY AGED MICE

Young adult	13 weeks	F	3 mice/organ	liver, kidney, lung, spleen	C57Bl/6J
Old	130 weeks	F	3 mice/organ	liver, kidney, lung, spleen	C57Bl/6J

B.

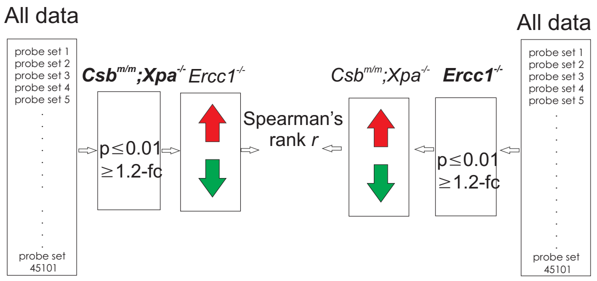

C.

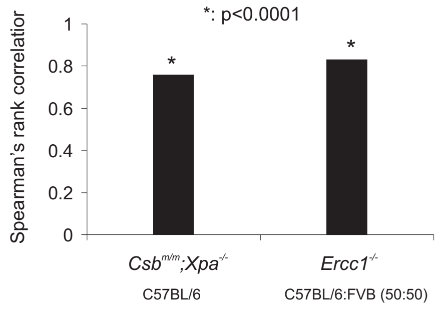

Schumacher et al. Supplementary figure 1
