# Supplementary figures and images for: Delayed and Accelerated Aging Share Common Longevity Assurance Mechanisms
Source: PLoS Genet. 2008 Aug 15;4(8):e1000161. doi: 10.1371/journal.pgen.1000161 (PMC2493043; doi:10.1371/journal.pgen.1000161)

A.

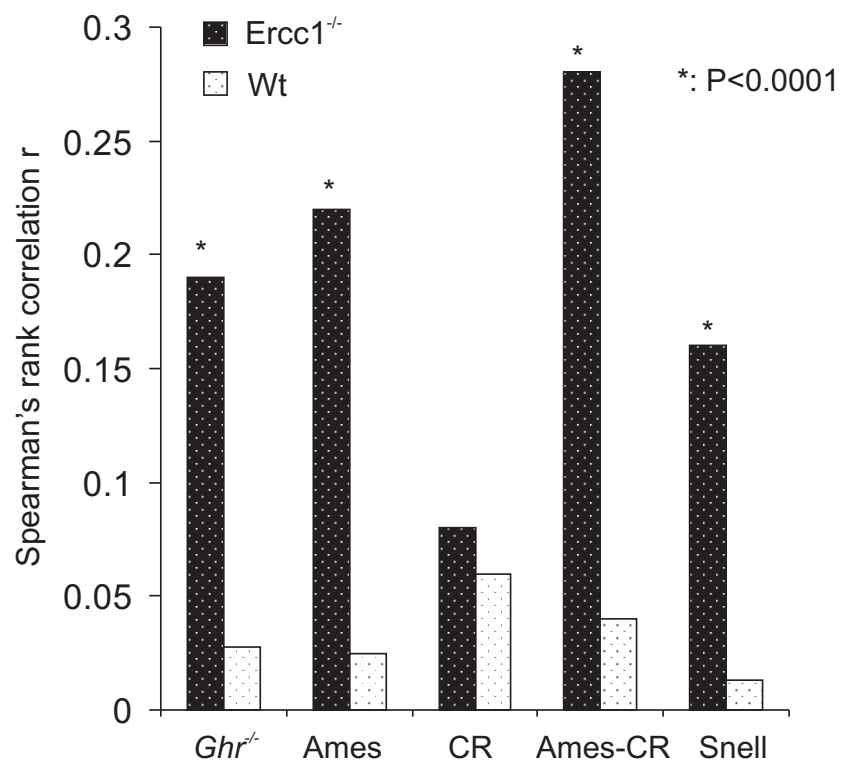

B.

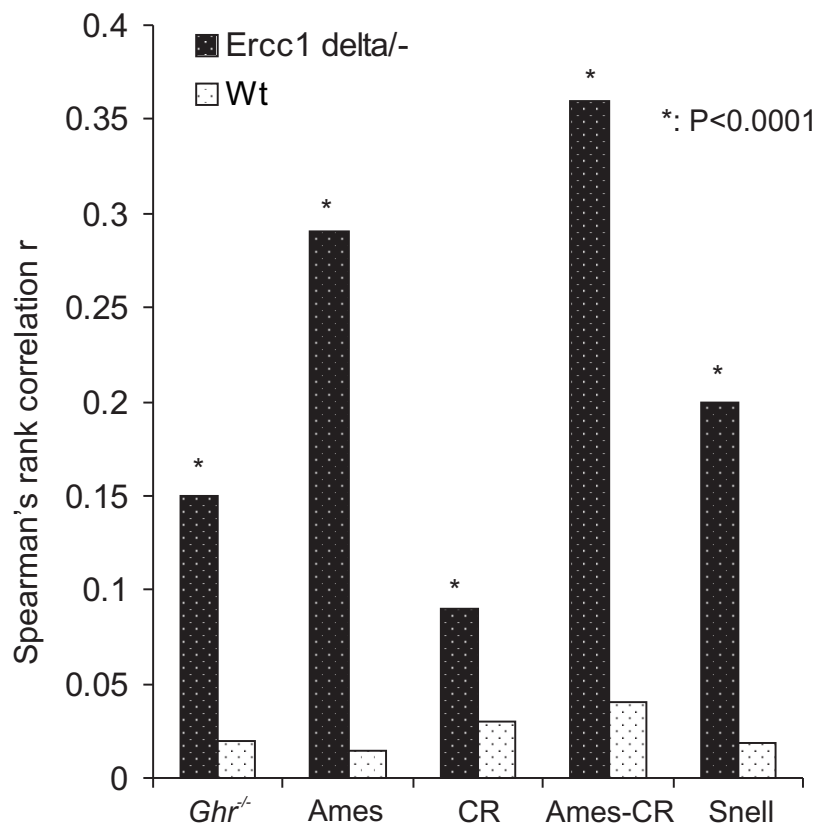

Supplement: Figure S2 — Spearman's rank correlation r between the significantly expressed genes in (A) 2 week-old Ercc1−/− and (B) 16 week-old Ercc1−/Δ−7 mutants and those of long-lived mice (Ghr−/−, Ames, CR, Ames-CR and Snell). The strongest Spearman's rank r correlation was between the transcriptome of 2 week-old Ercc1−/− mice or 16 week-old Ercc1−/Δ−7 mice and Ames mice that were calorie restricted (Ames-CR). Although highly significant, the correlations between the expression profiles of Ercc1−/− and Ercc1−/Δ−7 mice and long-lived mice is weaker than those for the Csbm/m;Xpa−/− mice. This is likely due to the fact that although all progeroid NER mutants are hypersensitive to UV-induced lesions (that is reflected by the substantial genome-wide similarity in gene expression between the Csbm/m;Xpa−/− and Ercc1−/− mice; Figure 1C), Ercc1−/− and Ercc1−/Δ−7 mice are also hypersensitive to DNA interstrand crosslinks. As a result, these mice show prominent pathology in the liver and kidney not seen in Csbm/m;Xpa−/− mice or any of the long-lived mutants. (0.03 MB PDF) [file pgen.1000161.s002.pdf]

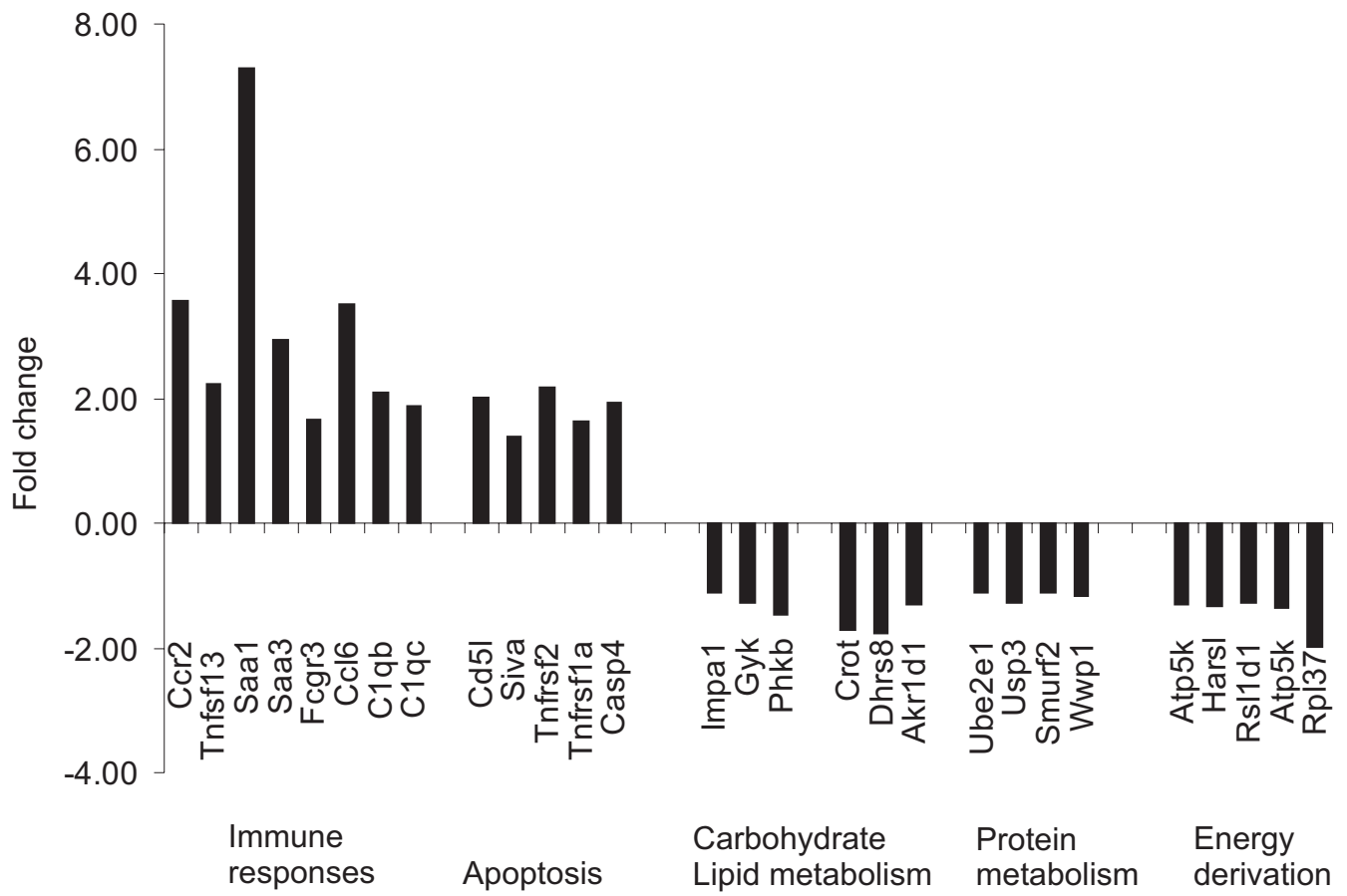

Supplement: Figure S3 — Significant mRNA expression levels of selected gene targets as detected by microarrays. The expression levels of this set of genes were also verified in an independent aging cohort of male mice by means of quantitative real time PCR (Figure 4). (0.01 MB PDF) [file pgen.1000161.s003.pdf]
