# Supplementary material for: Delayed and Accelerated Aging Share Common Longevity Assurance Mechanisms
Source: PLoS Genet. 2008 Aug 15;4(8):e1000161. doi: 10.1371/journal.pgen.1000161 (PMC2493043; doi:10.1371/journal.pgen.1000161)
Supplement: Figure S4 — Correlation between the significantly differentially expressed genes of 2.5 year-old mouse livers to the expression profiles for the same set of genes in each of the progeroid DNA repair-deficient or long-lived mouse livers. There is a significant positive correlation between all NER progeroid and naturally aged mouse livers (indicated with deeper red) but not between long-lived and naturally aged mouse livers (indicated with white to deeper blue). (0.02 MB PDF) [file pgen.1000161.s004.pdf]

130-week old mice

		Liver 1	Liver 2	Liver 3
Long-lived mice	Snell1	0.01	0.05	-0.02
	Snell2	0.02	0.07	0.00
	Snell3	0.03	0.09	0.01
	Snell4	0.01	0.08	-0.01
	Ghr1	0.17	0.17	0.17
	Ghr2	-0.03	-0.03	-0.03
	Ghr3	0.02	0.01	0.02
	DF.AL1	0.07	0.07	0.07
	DF.AL2	-0.09	-0.09	-0.09
	DF.AL3	-0.12	-0.12	-0.12
	DF.AL4	0.24	0.24	0.24
	DF.AL5	-0.08	-0.08	-0.08
	DF.AL6	0.08	0.08	0.08
	DF.AL7	-0.18	-0.18	-0.18
	DF.AL8	0.26	0.26	0.26
	DF.CR1	-0.05	-0.04	-0.04
	DF.CR2	0.02	0.02	0.02
	DF.CR3	0.06	0.06	0.06
	DF.CR4	-0.22	-0.22	-0.22
	DF.CR5	-0.20	-0.20	-0.20
	DF.CR6	0.26	0.26	0.26
	DF.CR7	-0.19	-0.19	-0.19
	DF.CR8	0.25	0.25	0.25
	N.CR1	-0.18	-0.18	-0.18
	N.CR2	-0.06	-0.06	-0.06
	N.CR3	-0.16	-0.16	-0.16
	N.CR4	-0.01	-0.01	-0.01
	N.CR5	-0.21	-0.21	-0.21
	N.CR6	-0.10	-0.11	-0.11
	N.CR7	-0.14	-0.14	-0.15
	N.CR8	-0.20	-0.19	-0.19
NER progeroid mice	CSBXPA 1	0.36	0.36	0.36
	CSBXPA 2	0.41	0.41	0.41
	CSBXPA 3	0.42	0.42	0.42
	CSBXPA 4	0.28	0.28	0.28
	ERCC 1	0.25	0.26	0.26
	ERCC 2	0.33	0.33	0.33
	ERCC 3	0.33	0.33	0.34
	ERCC 4	0.37	0.37	0.36
	ERCC 5	0.41	0.41	0.41
	ERCC 6	0.21	0.21	0.22
	ErccD 1	0.28	0.28	0.28
	ErccD 2	0.30	0.30	0.30
	ErccD 3	0.39	0.39	0.39
	ErccD 4	0.37	0.37	0.37
	ErccD 5	0.45	0.45	0.45
	ErccD 6	0.40	0.40	0.40

$P<0.0001$

Schumacher et al. Supplementary figure 4
