# Supplementary material for: Delayed and Accelerated Aging Share Common Longevity Assurance Mechanisms
Source: PLoS Genet. 2008 Aug 15;4(8):e1000161. doi: 10.1371/journal.pgen.1000161 (PMC2493043; doi:10.1371/journal.pgen.1000161)
Supplement: Table S1 — A list of all probe sets with significant transcriptional changes in the liver of Csbm/m;Xpa−/− as compared to littermate controls. FC: fold change, P: p-value. (0.05 MB PDF) [file pgen.1000161.s005.pdf]

Supplementary table S1. A list of all probe sets with significant transcriptional changes in the liver of Csbm/m;Xpa-/- as compared to littermate controls FC: fold change, P: p-value

**Independent Affymetrix platforms**				**CSBm/m-XPA-/-**		**CSBm/m**		**XPA-/-**	
74code	430code	Gene Symbol	Gene Title	FC	P	FC	P	FC	P
100078_at	1436504_x_at	---	---	3.78	0.001	-1.29	0.949	1.11	0.123
100491_at	1418445_at	---	---	-1.19	0.006	1.09	0.489	1.15	0.088
101363_at	1460710_at	---	---	-1.31	0.002	-1.25	0.055	-1.07	0.350
101471_at	1438711_at	---	---	-1.49	0.003	-1.08	0.544	-1.16	0.689
101647_at	1421767_at	---	---	-1.59	0.008	-1.1	0.505	-1.2	0.260
101930_at	1436364_x_at	---	---	-1.31	0.007	-1.18	0.170	-1.18	0.110
102195_at	1448050_s_at	---	---	2.88	0.004	1.47	0.323	1.62	0.205
102353_at	1450678_at	---	---	1.72	0.010	1.31	0.302	1.3	0.387
104069_at	1460677_at	---	---	1.46	0.007	1.12	0.481	-1	0.656
104213_at	1451548_at	---	---	-2.26	0.003	-1.04	0.623	-1.14	0.196
104533_at	1435458_at	---	---	1.76	0.005	1.17	0.269	1.09	0.538
160173_at	1436713_s_at	---	---	4.15	0.001	1.29	0.142	-1.06	0.582
160375_at	1449434_at	---	---	-1.63	0.005	1.17	0.252	1.22	0.195
160489_at	1438855_x_at	---	---	2.32	0.005	1.47	0.048	1.33	0.016
93283_at	1451703_s_at	---	---	1.57	0.002	-1.08	0.252	-1.05	0.610
93351_at	1419905_s_at	---	---	-1.26	0.001	1.07	0.214	1.28	0.276
94535_at	1450054_at	---	---	1.21	0.003	1.09	0.245	1.07	0.218
94769_at	1449366_at	---	---	1.28	0.001	-1.07	0.503	-1.01	0.173
95291_r_at	1419846_at	---	---	-2.14	0.004	-1.44	0.009	-2.19	0.005
95505_at	1448848_at	---	---	1.18	0.005	-1.09	0.460	-1.03	0.087
97867_at	1449038_at	---	---	-1.23	0.001	1.01	0.695	1.08	0.301
98540_g_at	1437039_at	---	---	1.12	0.007	-1.07	0.811	-1.05	0.713
98951_at	1437205_at	---	---	-1.17	0.007	-1.8	0.199	-1.43	0.577
160807_at	1450504_a_at	Agpat3	1-acylglycerol-3-phosphate O-acyltransferase 3	1.08	0.002	-1.13	0.037	-1.18	0.003
160711_at	1419367_at	Decr1	2,4-dienoyl CoA reductase 1, mitochondrial	1.19	0.009	1.01	0.875	1.06	0.450
97279_at	1435967_s_at	Hibadh	3-hydroxyisobutyrate dehydrogenase	-1.29	0.007	-1.06	0.430	-1.09	0.895
103939_at	1451512_s_at	Hibch	3-hydroxyisobutyryl-Coenzyme A hydrolase	-1.23	0.004	-1.09	0.047	1.04	0.577
102259_at	1420816_at	Ywhag	3-monooxygenase/tryptophan 5-monooxygenase activation protein, gamma polypeptide	-1.42	0.004	-1.22	0.138	-1.32	0.017
96713_at	1434510_at	Papss2	3'-phosphoadenosine 5'-phosphosulfate synthase 2	-1.2	0.007	1.03	0.876	-1.06	0.143
93464_at	1455151_at	Akap9	A kinase (PRKA) anchor protein (yotiao) 9	-1.89	0.007	-3.11	0.008	-2.24	0.098
96744_at	1448445_at	Acp6	acid phosphatase 6, lysophosphatidic	-1.25	0.005	-1.07	0.123	1.13	0.170
101213_at	1419441_at	Arbp	acidic ribosomal phosphoprotein P0	1.11	0.001	-1.06	0.276	1.02	0.028
100147_at	1423644_at	Aco1	aconitase 1	-1.29	0.001	-1.02	0.749	1.04	0.567
93288_at	1437148_at	Arpc2	actin related protein 2/3 complex, subunit 2	1.37	0.004	-1.01	0.846	-1.08	0.302
95434_at	1448279_at	Arpc3	actin related protein 2/3 complex, subunit 3	1.16	0.009	-1.31	0.125	-1.71	0.031
160762_at	1433477_at	Abr	active BCR-related gene	1.84	0.000	1.37	0.072	1.62	0.031
98976_at	1419476_at	Adamdec1	ADAM-like, decysin 1	1.43	0.005	1.43	0.050	1.47	0.001
97526_at	1448308_at	Ap3m1	adaptor-related protein complex 3, mu 1 subunit	-1.51	0.005	1.07	0.037	-1.22	0.001
104261_at	1435077_at	Asxl1	additional sex combs like 1 (Drosophila)	1.21	0.006	1.02	0.337	-1.03	0.979
93512_f_at	1438292_x_at	Adk	adenosine kinase	-1.22	0.005	-1.07	0.731	-1.16	0.042
103392_at	1456307_s_at	Adcy7	Adenylate cyclase 7	1.72	0.001	1.41	0.088	1.36	0.025
104605_at	1434329_s_at	Adipor2	adiponectin receptor 2	-1.37	0.007	1.04	0.161	1.06	0.122
98589_at	1448318_at	Adfp	adipose differentiation related protein	1.41	0.001	1.2	0.115	1.28	0.010
98084_at	1417112_at	Arl2bp	ADP-ribosylation factor-like 2 binding protein	1.41	0.000	-1.24	0.006	-1.17	0.191
99993_at	1421424_a_at	Anpep	alanyl (membrane) aminopeptidase	-1.64	0.002	-1.02	0.676	-1.17	0.364
97450_s_at	1460167_at	Aldh7a1	aldehyde dehydrogenase family 7, member A1	-1.14	0.006	1.04	0.198	1.12	0.028
93781_at	1416460_at	Aldrl6	aldehyde reductase (aldose reductase)-like 6	1.97	0.002	1.23	0.983	1.31	0.686

102826_at	1423556_at	Akr1b7	aldo-keto reductase family 1, member B7	6.45	0.002	-1.18	0.192	1.24	0.812
160215_at	1420619_a_at	Aes	amino-terminal enhancer of split	-1.63	0.009	-1.03	0.961	-1.02	0.782
94954_at	1423931_s_at	Anapc4	anaphase promoting complex subunit 4	1.57	0.000	1.27	0.145	1.23	0.190
100569_at	1419091_a_at	Anxa2	annexin A2	1.8	0.004	1.27	0.131	1.34	0.002
96529_at	1457695_at	Ap1gbp1	AP1 gamma subunit binding protein 1	-2.22	0.009	-1.72	0.056	-1.46	0.205
97887_at	1418069_at	Apoc2	apolipoprotein C-II	1.24	0.008	1.03	0.350	1.05	0.220
93840_at	1419096_at	Apom	apolipoprotein M	1.24	0.001	1.08	0.608	-1.02	0.085
93559_at	1416135_at	Apex1	apurinic/apyrimidinic endonuclease 1	1.12	0.008	1.06	0.211	1.01	0.953
104328_at	1424011_at	Aqp9	aquaporin 9	1.22	0.003	1	0.408	1.05	0.515
97130_at	1420651_at	Ate1	arginine-tRNA-protein transferase 1	-1.3	0.007	-1.07	0.116	-1.07	0.029
95563_at	1427188_at	Arih1	ariadne ubiquitin-conjugating enzyme E2 binding protein homolog 1 (Drosophila)	-1.21	0.008	-1.36	0.002	-1.18	0.022
160666_at	1428502_at	Actr6	ARP6 actin-related protein 6 homolog (yeast)	-1.38	0.000	-1.07	0.565	-1.07	0.106
95439_at	1448813_at	Aadac	arylacetamide deacetylase (esterase)	-1.05	0.007	1.13	0.237	1.16	0.241
95133_at	1451095_at	Asns	asparagine synthetase	2.73	0.001	1.3	0.398	1.28	0.103
95725_at	1448539_a_at	Acy3	aspartoacylase (aminoacylase) 3	-1.3	0.005	-1.02	0.995	1.04	0.442
103899_at	1456388_at	Atp11a	ATPase, class VI, type 11A	1.48	0.006	1.28	0.108	1.03	0.719
96281_at	1423256_a_at	Atp6v1g1	ATPase, H+ transporting, V1 subunit G isoform 1	1.14	0.000	-1.04	0.029	-1.03	0.020
94733_at	1449818_at	Abcb4	ATP-binding cassette, sub-family B (MDR/TAP), member 4	1.15	0.002	-1.03	0.393	-1.04	0.475
103689_at	1428988_at	Abcc3	ATP-binding cassette, sub-family C (CFTR/MRP), member 3	1.55	0.001	-1.13	0.477	-1.12	0.549
93045_at	1416679_at	Abcd3	ATP-binding cassette, sub-family D (ALD), member 3	-1.24	0.000	1.01	0.762	1.03	0.623
160612_at	1423570_at	Abcg1	ATP-binding cassette, sub-family G (WHITE), member 1	1.33	0.004	1.19	0.015	-1.09	0.686
93626_at	1422906_at	Abcg2	ATP-binding cassette, sub-family G (WHITE), member 2	1.32	0.008	1.04	0.087	1.05	0.191
102943_at	1428612_at	Apg7l	autophagy 7-like (S. cerevisiae)	-1.48	0.007	-1.11	0.450	-1.01	0.886
103091_at	1417856_at	Relb	avian reticuloendotheliosis viral (v-rel) oncogene related B	1.86	0.005	-1.16	0.454	1.2	0.126
160876_at	1417077_at	Bcap29	B-cell receptor-associated protein 29	1.34	0.003	1.03	0.295	1.18	0.378
96146_at	1449007_at	Btg3	B-cell translocation gene 3	1.73	0.000	1.12	0.425	1.01	0.441
161129_r_at	1423948_at	Bag2	Bcl2-associated athanogene 2	-1.48	0.003	-1.08	0.528	-1.07	0.649
100988_at	1435448_at	Bcl2l11	BCL2-like 11 (apoptosis facilitator)	1.72	0.001	1.03	0.443	-1.11	0.778
94363_at	1433636_at	Bms1l	BMS1-like, ribosome assembly protein (yeast)	1.42	0.001	1.14	0.037	-1.02	0.728
97489_at	1433504_at	Pygb	brain glycogen phosphorylase	1.72	0.008	1.04	0.415	1.07	0.618
103636_at	1418746_at	MGI:1930773	brain protein 17	-2.61	0.000	1.01	0.470	1.05	0.368
92381_at	1420975_at	Baz1b	bromodomain adjacent to zinc finger domain, 1B	-1.32	0.001	-1.08	0.166	1.09	0.364
102852_at	1418815_at	Cdh2	cadherin 2	-1.37	0.004	-1.22	0.436	-1.35	0.089
96203_at	1424713_at	Calml4	calmodulin-like 4	1.88	0.000	-1.24	0.977	-1.14	0.452
97943_at	1450429_at	Capn6	calpain 6	1.47	0.002	1.15	0.393	-1.19	0.049
93523_at	1456170_x_at	Calr	calreticulin	-1.26	0.009	-1.02	0.326	-1.14	0.075
92835_at	1418013_at	Cml1	camello-like 1	-3.24	0.002	-1.02	0.641	1.43	0.030
102672_g_at	1452529_a_at	Creb1	cAMP responsive element binding protein 1	-1.38	0.005	1.05	0.607	-1.16	0.304
97259_at	1422608_at	Arpp19	cAMP-regulated phosphoprotein 19	1.31	0.010	1	0.517	-1.01	0.487
98127_at	1423057_at	Capza2	capping protein (actin filament) muscle Z-line, alpha 2	1.19	0.001	1.07	0.098	1.04	0.208
160106_at	1450355_a_at	Capg	capping protein (actin filament), gelsolin-like	1.62	0.001	1.32	0.009	1.13	0.157
93824_at	1455540_at	Cps1	carbamoyl-phosphate synthetase 1	-1.08	0.007	1	0.039	1.02	0.138
98079_at	1450725_s_at	Car14	carbonic anhydrase 14	-1.76	0.000	1.06	0.549	1.39	0.225
98137_at	1419525_at	Car5a	carbonic anhydrase 5a, mitochondrial	-3.11	0.001	-1.34	0.876	-1.38	0.588
161042_at	1427912_at	Cbr3	carbonyl reductase 3	1.98	0.003	1.33	0.286	1.03	0.654
103882_at	1417745_at	Cpn1	carboxypeptidase N, polypeptide 1	1.22	0.001	1.04	0.517	1.04	0.482
93076_at	1428537_at	Csnk1a1	casein kinase 1, alpha 1	1.08	0.007	-1.06	0.716	-1.14	0.388
100291_at	1450457_at	Cbl	Casitas B-lineage lymphoma	-1.57	0.008	-1.42	0.018	-1.4	0.156
94458_at	1415995_at	Casp6	caspase 6	1.37	0.000	-1.01	0.084	1.3	0.015
95608_at	1417492_at	Ctsb	cathepsin B	1.46	0.001	1.32	0.100	1.41	0.020
98254_f_at	1455316_x_at	Ccrn4l	CCR4 carbon catabolite repression 4-like (S. cerevisiae)	1.52	0.005	1.37	0.104	1.35	0.049
95373_at	1418770_at	Cd2	CD2 antigen	2.28	0.001	1.59	0.017	1.88	0.003
104023_at	1428018_a_at	Cd300c	Cd300C antigen	1.57	0.005	1.25	0.387	1.48	0.032

103611_at	1419554_at	Cd47	CD47 antigen (Rh-related antigen, integrin-associated signal transducer)	1.47	0.006	1.36	0.080	1.15	0.507
93445_at	1449193_at	Cd5l	CD5 antigen-like	2.01	0.009	1.68	0.085	1.33	0.436
95661_at	1416066_at	Cd9	CD9 antigen	1.95	0.000	-1.16	0.180	-1.11	0.510
95920_at	1448048_at	BC016495	cDNA sequence BC016495	-1.71	0.004	-1.1	0.596	-1.24	0.105
160815_at	1456014_s_at	BC032204	cDNA sequence BC032204	1.78	0.004	1.33	0.172	1.27	0.299
97179_at	1460713_at	BC048355	CDNA sequence BC048355	-1.65	0.002	1.02	0.978	-1.07	0.450
98516_at	1424117_at	BC056474	cDNA sequence BC056474	-1.4	0.009	-1.05	0.636	1.02	0.658
99237_at	1450362_at	U55872	cDNA sequence U55872	-1.87	0.007	-1.05	0.124	1.51	0.995
102806_g_at	1425675_s_at	Ceacam1	CEA-related cell adhesion molecule 1	-1.63	0.003	-1.01	0.071	1.18	0.880
102804_at	1460681_at	Ceacam2	CEA-related cell adhesion molecule 2	-1.31	0.000	1.07	0.841	1.17	0.306
94105_at	1460708_s_at	Cdc42	cell division cycle 42 homolog (S. cerevisiae)	1.25	0.002	1.08	0.984	1.03	0.303
160699_at	1448466_at	Cdca5	cell division cycle associated 5	-1.41	0.004	1.04	0.678	-1.1	0.228
102659_at	1417551_at	Cln3	ceroid lipofuscinosis, neuronal 3, juvenile (Batten, Spielmeyer-Vogt disease)	1.19	0.000	1.26	0.004	1.15	0.005
103012_at	1419426_s_at	Ccl21b /// Ccl21	chemokine (C-C motif) ligand 21b (serine) /// chemokine (C-C motif) ligand 21a (leucine) /// chemok	-1.8	0.006	1.31	0.607	1.27	0.557
162198_f_at	1420249_s_at	Ccl6	chemokine (C-C motif) ligand 6	1.74	0.003	1.17	0.384	1.12	0.549
104443_at	1423466_at	Ccr7	chemokine (C-C motif) receptor 7	1.82	0.007	1.27	0.323	1.75	0.215
92481_at	1422747_at	Chek2	CHK2 checkpoint homolog (S. pombe)	-1.29	0.003	1.09	0.558	-1.08	0.148
104059_at	1437297_at	Chd8	chromodomain helicase DNA binding protein 8	1.06	0.004	-1.3	0.178	-1.15	0.373
100580_at	1421062_s_at	Clta	clathrin, light polypeptide (Lca)	1.05	0.004	-1	0.110	-1.11	0.493
102915_at	1448931_at	F2rl1	coagulation factor II (thrombin) receptor-like 1	2.88	0.003	-1.48	0.399	-1.19	0.590
96501_at	1418993_s_at	F10	coagulation factor X	1.2	0.001	-1.02	0.686	1.03	0.107
94410_f_at	1419803_s_at	Ccdc12	coiled-coil domain containing 12	1.17	0.007	1.17	0.010	1.17	0.014
93284_at	1416332_at	Cirbp	cold inducible RNA binding protein	2.74	0.002	-1.11	0.763	1.12	0.204
93547_at	1460716_a_at	Cbfb	core binding factor beta	-1.17	0.009	-1.07	0.166	-1.04	0.276
96648_at	1455269_a_at	Coro1a	coronin, actin binding protein 1A	2.49	0.001	1.06	0.950	1.39	0.071
98107_at	1449660_s_at	Coro1c	coronin, actin binding protein 1C	1.48	0.008	1.1	0.834	1.09	0.960
94054_at	1433908_a_at	Cttn	cortactin	1.39	0.006	1.14	0.117	1.04	0.924
95161_at	1451075_s_at	Ctdsp2	CTD (carboxy-terminal domain, RNA polymerase II, polypeptide A) small phosphatase 2	1.37	0.008	-1.03	0.667	-1.07	0.953
95951_at	1420804_s_at	Clec4d	C-type lectin domain family 4, member d	1.68	0.006	1.12	0.548	-1.24	0.650
99179_at	1431145_a_at	Cuedc2	CUE domain containing 2	1.48	0.002	1.19	0.160	1.18	0.120
93630_at	1425932_a_at	Cugbp1	CUG triplet repeat, RNA binding protein 1	-1.5	0.008	-1.29	0.042	-1.32	0.009
100444_at	1450674_at	Cdk5	cyclin-dependent kinase 5	-1.12	0.006	1.08	0.384	1.21	0.229
100278_at	1419497_at	Cdkn1b	cyclin-dependent kinase inhibitor 1B (P27)	-1.55	0.002	-1.08	0.128	-1.28	0.079
160330_at	1460645_at	Chordc1	cysteine and histidine-rich domain (CHORD)-containing, zinc-binding protein 1	-1.29	0.003	-1.03	0.491	-1.02	0.272
103391_at	1455991_at	Ccbl2	cysteine conjugate-beta lyase 2	1.52	0.005	-1	0.747	1.04	0.869
96346_at	1448842_at	Cdo1	cysteine dioxygenase 1, cytosolic	-1.28	0.005	-1.07	0.553	-1.07	0.259
99184_at	1427981_a_at	Csad	cysteine sulfinic acid decarboxylase	2.36	0.002	-1.28	0.279	-1.25	0.349
160652_at	1448111_at	Ctps2	cytidine 5'-triphosphate synthase 2	1.82	0.001	1.05	0.958	1.08	0.523
100059_at	1454268_a_at	Cyba	cytochrome b-245, alpha polypeptide	1.93	0.004	1.5	0.122	1.49	0.104
100965_at	1455167_at	Cox8c	cytochrome c oxidase, subunit VIIIc	-1.36	0.001	-1.18	0.173	-1.24	0.011
96228_at	1448804_at	Cyp11a1	cytochrome P450, family 11, subfamily a, polypeptide 1	1.52	0.003	-1.07	0.756	9.11	0.083
103670_at	1418821_at	Cyp2a12	cytochrome P450, family 2, subfamily a, polypeptide 12	-1.38	0.000	-1.04	0.353	1.06	0.124
102701_at	1425645_s_at	Cyp2b10	cytochrome P450, family 2, subfamily b, polypeptide 10	-3.09	0.001	-1.46	0.308	-1.34	0.808
102820_at	1449479_at	Cyp2b13	cytochrome P450, family 2, subfamily b, polypeptide 13	-2.33	0.005	-1.29	0.930	-1.22	0.834
101862_at	1419590_at	Cyp2b9	cytochrome P450, family 2, subfamily b, polypeptide 9	-1.56	0.000	-1.04	0.583	-1.06	0.879
93585_at	1417651_at	Cyp2c29	cytochrome P450, family 2, subfamily c, polypeptide 29	-1.45	0.007	-1.33	0.086	-1.29	0.132
95043_at	1424273_at	Cyp2c70	cytochrome P450, family 2, subfamily c, polypeptide 70	-1.48	0.003	-1.12	0.993	-1.03	0.021
162174_at	1419349_a_at	Cyp2d9	cytochrome P450, family 2, subfamily d, polypeptide 9	1.76	0.006	1.02	0.880	1.18	0.419
100069_at	1448792_a_at	Cyp2f2	cytochrome P450, family 2, subfamily f, polypeptide 2	-2.41	0.003	1.28	0.478	1.68	0.056
92814_at	1417532_at	Cyp2j5	cytochrome P450, family 2, subfamily j, polypeptide 5	-3.29	0.000	-1.34	0.357	-1.52	0.090
103704_at	1435376_at	Ddhd2	DDHD domain containing 2	1.25	0.007	-1.06	0.763	1.02	0.318
92226_at	1417875_at	Ddx50	DEAD (Asp-Glu-Ala-Asp) box polypeptide 50	1.23	0.004	1.06	0.103	1	0.767
93965_r_at	1424598_at	Ddx6	DEAD (Asp-Glu-Ala-Asp) box polypeptide 6	-2.68	0.010	-2.42	0.038	-1.93	0.195

96008_at	1418528_a_at	Dad1	defender against cell death 1	1.13	0.000	-1.05	0.612	-1.09	0.893
100882_at	1419492_s_at	Defb1	defensin beta 1	1.52	0.003	1.12	0.315	1.17	0.539
102370_at	1434642_at	Dhrs8	dehydrogenase/reductase (SDR family) member 8	1.22	0.001	-1.27	0.120	-1.33	0.048
95552_at	1417991_at	Dio1	deiodinase, iodothyronine, type I	-2.15	0.002	-1.03	0.330	-1.01	0.333
96115_at	1438001_x_at	Dp1	deleted in polyposis 1	1.65	0.001	1.23	0.147	1.18	0.461
160769_at	1448438_at	Derl2	Der1-like domain family, member 2	-1.14	0.010	1.11	0.921	1.13	0.904
96742_at	1418511_at	Dpt	dermatopontin	-1.24	0.009	1.05	0.643	-1.02	0.180
97768_at	1421156_a_at	Dsc2	desmocollin 2	-1.33	0.003	1.06	0.528	1.2	0.312
97248_at	1422432_at	Dbi	diazepam binding inhibitor	1.65	0.005	1.33	0.235	1.29	0.373
160490_at	1415853_at	Def8	differentially expressed in FDCP 8	-1.57	0.008	-1.02	0.469	-1.22	0.085
94956_at	1452434_s_at	Dgcr6	DiGeorge syndrome critical region gene 6	-1.55	0.003	-1.26	0.092	1.08	0.073
98966_at	1449118_at	Dbt	dihydrolipoamide branched chain transacylase E2	-1.27	0.008	-1.4	0.058	-1.39	0.015
92887_at	1416457_at	Ddah2	dimethylarginine dimethylaminohydrolase 2	1.26	0.008	1.21	0.063	1	0.499
96757_at	1449000_at	D10Jhu81e	DNA segment, Chr 10, Johns Hopkins University 81 expressed	-1.73	0.004	-1.53	0.105	-1.51	0.034
93232_at	1435259_s_at	D2Ertd217e	DNA segment, Chr 2, ERATO Doi 217, expressed	1.18	0.005	-1.18	0.325	-1.19	0.272
95934_at	1445485_at	D7Ertd187e	DNA segment, Chr 7, ERATO Doi 187, expressed	-2.76	0.004	-1.72	0.052	1.09	0.613
93529_at	1419041_at	D8Wsu49e	DNA segment, Chr 8, Wayne State University 49, expressed	-1.22	0.001	-1.07	0.309	1.05	0.083
95613_at	1448339_at	D9Wsu20e	DNA segment, Chr 9, Wayne State University 20, expressed	-1.41	0.009	-1.09	0.081	-1.08	0.038
160522_at	1450839_at	D0H4S114	DNA segment, human D4S114	-1.71	0.001	1.2	0.067	1.27	0.087
97261_at	1460179_at	Dnaja1	DnaJ (Hsp40) homolog, subfamily A, member 1	-1.26	0.000	-1.06	0.544	-1.03	0.753
94343_at	1433887_at	Dnajc3	DnaJ (Hsp40) homolog, subfamily C, member 3	-1.29	0.005	-1.09	0.586	-1.4	0.012
95700_r_at	1452683_at	Dnajc8	DnaJ (Hsp40) homolog, subfamily C, member 8	1.19	0.009	1.15	0.053	1.18	0.016
97428_at	1423116_at	Dom3z	DOM-3 homolog Z (C. elegans)	1.17	0.006	-1.05	0.308	1.08	0.154
97755_at	1422830_s_at	Drd4	dopamine receptor 4	1.38	0.005	-1.03	0.642	-1.04	0.626
102374_at	1434027_at	Dscr1l2	Down syndrome critical region gene 1-like 2	1.47	0.001	1.26	0.036	1.15	0.118
160327_at	1448368_at	Dctn6	dynactin 6	1.17	0.009	1.06	0.183	1.09	0.150
101109_at	1426778_at	Dag1	dystroglycan 1	-1.15	0.010	1.03	0.606	1.01	0.825
104174_at	1459546_s_at	Enpp1	ectonucleotide pyrophosphatase/phosphodiesterase 1	-1.33	0.002	-1.13	0.191	-1.08	0.328
95468_at	1423785_at	Egln1	EGL nine homolog 1 (C. elegans)	-1.14	0.008	1.03	0.239	1.02	0.315
160667_at	1434920_a_at	Evl	Ena-vasodilator stimulated phosphoprotein	2	0.005	1.54	0.199	1.46	0.206
102698_at	1449888_at	Epas1	endothelial PAS domain protein 1	-1.52	0.009	-1.08	0.473	1.01	0.915
93754_at	1448491_at	Ech1	enoyl coenzyme A hydratase 1, peroxisomal	1.27	0.003	1.01	0.288	1.03	0.872
103240_f_at	1422411_s_at	Ear1 /// Ear2 ///	eosinophil-associated, ribonuclease A family, member 1 /// eosinophil-associated, ribonuclease A f	2.19	0.008	1.87	0.113	1.71	0.026
103980_at	1421151_a_at	Epha2	Eph receptor A2	-1.57	0.000	1.15	0.089	1.1	0.281
94125_at	1416237_at	Eva1	epithelial V-like antigen 1	-1.51	0.002	-1.01	0.123	1.05	0.411
101587_at	1422438_at	Ephx1	epoxide hydrolase 1, microsomal	2.12	0.004	-1.14	0.011	1.13	0.355
93608_at	1449222_at	Ebi3	Epstein-Barr virus induced gene 3	2.27	0.003	1.73	0.139	1.65	0.081
104065_at	1424065_at	Edem1	ER degradation enhancer, mannosidase alpha-like 1	-1.37	0.000	-1.06	0.467	-1.03	0.341
101500_at	1433490_s_at	Epb4.1l2	erythrocyte protein band 4.1-like 2	1.34	0.001	1.28	0.117	1.19	0.097
99941_at	1451600_s_at	LOC13909 /// E	esterase 31-like /// esterase 31	-3.57	0.005	1.26	0.314	1.44	0.061
93945_at	1421244_at	Esr1	estrogen receptor 1 (alpha)	-1.47	0.006	-1.45	0.041	-1.06	0.057
160129_at	1449506_a_at	Eef1d	eukaryotic translation elongation factor 1 delta (guanine nucleotide exchange protein)	1.08	0.005	-1.22	0.293	-1.2	0.268
96883_at	1417718_at	Eif3s4	eukaryotic translation initiation factor 3, subunit 4 (delta)	1.23	0.000	-1.04	0.765	-1.08	0.361
99101_at	1416100_at	Eif3s7	eukaryotic translation initiation factor 3, subunit 7 (zeta)	1.1	0.010	-1.06	0.611	-1.07	0.582
101072_at	1437733_at	Eif4ebp2	Eukaryotic translation initiation factor 4E binding protein 2	-2.43	0.008	-1.14	0.551	-1.39	0.436
160265_at	1415723_at	Eif5	eukaryotic translation initiation factor 5	1.23	0.008	1.08	0.323	-1	0.881
96755_at	1424013_at	Etf1	eukaryotic translation termination factor 1	1.19	0.003	-1.02	0.825	1.08	0.176
92625_at	1448808_a_at	Nme2	expressed in non-metastatic cells 2, protein	1.14	0.007	-1.01	0.411	1.06	0.028
97918_at	1427243_at	AA536743	expressed sequence AA536743	1.71	0.003	1.18	0.636	1.16	0.875
95577_at	1452122_at	AI314180	expressed sequence AI314180	-1.24	0.001	-1.12	0.077	-1.02	0.527
161857_r_at	1427176_s_at	AI428936	expressed sequence AI428936	1.89	0.007	1.36	0.134	1.36	0.220
104034_at	1435417_at	AI464131	expressed sequence AI464131	-1.72	0.010	-1.09	0.879	1.16	0.257
93963_at	1436212_at	AI661017	expressed sequence AI661017	2.96	0.006	1.44	0.244	1.93	0.018

104260_at	1435326_at	AW112037	expressed sequence AW112037	1.4	0.007	1.12	0.274	1.01	0.430
93747_at	1454698_at	AW742319	expressed sequence AW742319	1.28	0.000	1.06	0.277	1.02	0.357
102135_at	1442211_at	C78893	expressed sequence C78893	1.89	0.010	-1.32	0.232	1.1	0.867
96367_at	1442494_at	C79242	expressed sequence C79242	-1.15	0.003	-1.02	0.066	-1.04	0.093
94689_at	1446148_x_at	C79248	expressed sequence C79248	1.24	0.003	-1.91	0.101	-2	0.085
103816_at	1424595_at	F11r	F11 receptor	1.21	0.008	-1.01	0.862	1.12	0.055
160098_s_at	1434370_s_at	Faf1	Fas-associated factor 1	-1.35	0.008	-1.11	0.100	-1.06	0.209
160544_at	1416022_at	Fabp5	fatty acid binding protein 5, epidermal	2.39	0.000	1.38	0.633	1	0.158
104109_at	1452828_at	Fbxo21	F-box only protein 21	1.36	0.003	-1.01	0.332	-1.1	0.506
92188_s_at	1452410_a_at	Fes	feline sarcoma oncogene	1.64	0.005	1.27	0.151	1.71	0.002
97949_at	1421855_at	Fgl2	fibrinogen-like protein 2	1.87	0.004	1.38	0.054	1.21	0.171
97964_at	1417267_s_at	Fkbp11	FK506 binding protein 11	1.81	0.006	1.13	0.397	1.32	0.408
101991_at	1417429_at	Fmo1	flavin containing monooxygenase 1	-1.4	0.000	-1	0.771	1.04	0.281
104421_at	1449525_at	Fmo3	flavin containing monooxygenase 3	-14.37	0.000	-2.31	0.570	-2	0.787
95095_at	1448559_at	Flot1	flotillin 1	1.46	0.010	1.08	0.512	1.01	0.924
160850_at	1460673_at	Fpgs	folylpolyglutamyl synthetase	-1.53	0.002	1.01	0.598	1.01	0.650
92958_at	1434832_at	Foxo3a	forkhead box O3a	-1.69	0.006	-1.19	0.485	-1.36	0.055
99835_at	1417487_at	Fosl1	fos-like antigen 1	-1.66	0.006	-1.89	0.000	-1.45	0.029
160226_at	1416570_s_at	Gfm1	G elongation factor 1	-1.12	0.008	1.07	0.591	1.15	0.439
103332_at	1426782_at	Gpr125	G protein-coupled receptor 125	-1.24	0.001	1.05	0.543	1.16	0.157
94184_at	1451708_at	Gpr33	G protein-coupled receptor 33	1.1	0.003	1.01	0.324	-1.26	0.182
102787_at	1433485_x_at	Gpr56	G protein-coupled receptor 56	3.24	0.008	1.38	0.785	1.22	0.934
103270_at	1416969_at	Gtse1	G two S phase expressed protein 1	2.42	0.006	1.99	0.175	-1	0.907
97820_at	1417177_at	Galk1	galactokinase 1	1.41	0.008	-1.12	0.114	-1.08	0.331
99342_at	1449980_a_at	Gabrd	gamma-aminobutyric acid (GABA-A) receptor, subunit delta	-1.52	0.009	1.04	0.213	-1.26	0.103
102552_at	1422339_at	Gja3	gap junction membrane channel protein alpha 3	-2.31	0.002	-1.66	0.042	-2.87	0.000
94697_at	1419526_at	Fgr	Gardner-Rasheed feline sarcoma viral (Fgr) oncogene homolog	1.23	0.003	1.09	0.099	-1.09	0.891
103667_at	1434513_at	Gm542	gene model 542, (NCBI)	-1.28	0.001	-1.11	0.769	-1.19	0.005
160225_at	1451135_at	Gtf2b	general transcription factor IIB	1.28	0.005	1	0.080	1.07	0.840
99441_at	1426462_at	Gphn	gephyrin	-1.41	0.009	-1.1	0.907	-1.18	0.200
102651_at	1425303_at	Gck	glucokinase	-5.13	0.010	-1.05	0.495	-1.19	0.539
98931_at	1433546_at	Gns	glucosamine (N-acetyl)-6-sulfatase	1.11	0.002	1.15	0.013	1.12	0.002
160194_at	1448717_at	Gcdh	glutaryl-Coenzyme A dehydrogenase	-1.32	0.010	-1.1	0.115	1.05	0.592
160646_at	1421817_at	Gsr	glutathione reductase 1	1.15	0.005	-1.1	0.615	-1.11	0.548
96670_at	1452823_at	Gstk1	glutathione S-transferase kappa 1	-1.67	0.005	-1.09	0.294	1.07	0.022
101872_at	1421041_s_at	Gsta2	glutathione S-transferase, alpha 2 (Yc2)	1.85	0.002	-1.72	0.409	-1.26	0.194
93009_at	1416411_at	Gstm2	glutathione S-transferase, mu 2	-1.36	0.007	1.05	0.909	1.3	0.223
100629_at	1416842_at	Gstm5	glutathione S-transferase, mu 5	1.25	0.002	-1.05	0.139	1.01	0.759
99583_at	1449575_a_at	Gstp1	glutathione S-transferase, pi 1	1.35	0.002	1.06	0.674	1.06	0.855
104603_at	1417883_at	Gstt2	glutathione S-transferase, theta 2	2.78	0.002	-1.34	0.038	1.16	0.178
95603_at	1416049_at	Gldc	glycine decarboxylase	-1.32	0.005	-1.04	0.539	-1.07	0.348
96828_at	1417422_at	Gnmt	glycine N-methyltransferase	-1.21	0.004	1.05	0.953	1.12	0.269
92217_s_at	1420394_s_at	Gp49a /// Lilrb4	glycoprotein 49 A /// leukocyte immunoglobulin-like receptor, subfamily B, member 4	3.78	0.000	1.69	0.089	1.84	0.032
96925_at	1433495_at	Glt25d1	glycosyltransferase 25 domain containing 1	1.25	0.009	1.07	0.288	1.16	0.072
160158_at	1450990_at	Gpc3	glypican 3	2.52	0.007	-1.03	0.200	1.04	0.331
92672_at	1453413_at	Gnas	GNAS (guanine nucleotide binding protein, alpha stimulating) complex locus	-1.47	0.007	-1.27	0.063	-1.33	0.080
102003_at	1415709_s_at	Gbf1	golgi-specific brefeldin A-resistance factor 1	-1.17	0.004	1.01	0.625	-1.08	0.052
99107_at	1451871_a_at	Ghr	Growth hormone receptor	-2.32	0.006	-1.06	0.311	1.09	0.650
160172_at	1426758_s_at	Gtl2	GTL2, imprinted maternally expressed untranslated mRNA	1.63	0.002	1.64	0.049	1.13	0.165
99340_at	1455168_a_at	Gnb2-rs1	guanine nucleotide binding protein, beta 2, related sequence 1	1.15	0.003	1.04	0.354	1.05	0.071
101582_at	1426579_at	Gnl2	guanine nucleotide binding protein-like 2 (nucleolar)	1.13	0.005	-1.09	0.849	-1	0.260
100708_at	1420376_a_at	H3f3b	H3 histone, family 3B	1.4	0.000	1.1	0.211	-1.05	0.249
98111_at	1425993_a_at	Hsp105	heat shock protein 105	-2.07	0.009	-1.02	0.779	1.01	0.696

160101_at	1448239_at	Hmox1	heme oxygenase (decycling) 1	2.43	0.001	-1.18	0.127	-1.53	0.036
103533_at	1450736_a_at	Hbb-bh1	hemoglobin Z, beta-like embryonic chain	1.21	0.007	1.44	0.000	1.2	0.007
93118_at	1433829_a_at	Hnrpa2b1	heterogeneous nuclear ribonucleoprotein A2/B1	1.28	0.010	1.06	0.316	-1.17	0.145
94303_at	1425142_a_at	Hnrpd	heterogeneous nuclear ribonucleoprotein D	1.39	0.002	1.04	0.390	-1.08	0.996
96084_at	1428224_at	Hnrpdl	heterogeneous nuclear ribonucleoprotein D-like	1.32	0.001	1.11	0.013	-1.14	0.687
94041_at	1423684_at	Hnrpk	heterogeneous nuclear ribonucleoprotein K	1.22	0.003	-1.02	0.689	-1.06	0.483
93095_at	1416176_at	Hmgb1	high mobility group box 1	-1.1	0.004	-1.01	0.559	-1.03	0.171
160447_at	1423195_at	Hiat1	hippocampus abundant gene transcript 1	-1.1	0.000	-1.03	0.016	-1.07	0.056
101681_f_at	1450531_at	H2-Bl	histocompatibility 2, blastocyst	1.13	0.007	1.1	0.013	-1.04	0.250
92866_at	1452431_s_at	H2-Aa	histocompatibility 2, class II antigen A, alpha	1.25	0.008	-1.22	0.445	-1.01	0.072
103671_at	1451814_a_at	Htatip2	HIV-1 tat interactive protein 2, homolog (human)	1.79	0.008	-1.02	0.520	1.09	0.810
96672_at	1428662_a_at	MGI:1916782	homeobox only domain	-3.61	0.001	-1.43	0.918	-1.32	0.645
95057_at	1448185_at	Herpud1	homocysteine-inducible, endoplasmic reticulum stress-inducible, ubiquitin-like domain member 1	-1.48	0.002	-1.23	0.252	-1.19	0.057
97551_at	1425553_s_at	Hip1r	huntingtin interacting protein 1 related	1.37	0.000	1.12	0.028	1.01	0.511
100042_at	1424172_at	Hagh	hydroxyacyl glutathione hydrolase	-1.28	0.000	1.07	0.138	1.09	0.247
94276_at	1450011_at	Hsd17b12	hydroxysteroid (17-beta) dehydrogenase 12	-1.04	0.010	1.12	0.625	1.03	0.038
160768_at	1423879_at	D030056L22	hypothetical protein D030056L22	-1.37	0.001	1.04	0.600	1.11	0.833
101094_at	1416480_a_at	MGI:1930666	hypoxia induced gene 1	-1.14	0.003	1.01	0.706	1.03	0.922
95336_at	1421572_at	Hif3a	hypoxia inducible factor 3, alpha subunit	1.29	0.000	1.04	0.166	1.02	0.145
101054_at	1425519_a_at	Ii	Ia-associated invariant chain	1.58	0.009	-1.24	0.449	-1.06	0.427
104500_at	1418793_at	Idua	iduronidase, alpha-L-	1.24	0.002	-1.05	0.294	-1.05	0.338
160820_at	1460675_at	Igsf8	immunoglobulin superfamily, member 8	1.38	0.010	1.1	0.364	-1.14	0.270
95034_f_at	1436420_a_at	Ipo4	importin 4	1.47	0.010	1.18	0.155	1.1	0.330
104163_at	1426760_at	Ipo8	importin 8	-1.15	0.006	1.07	0.465	1.08	0.310
101501_r_at	1415911_at	Impact	imprinted and ancient	1.55	0.002	1.07	0.427	1.03	0.719
100050_at	1425895_a_at	Id1	inhibitor of DNA binding 1	2.13	0.001	1.12	0.786	-1.19	0.219
93013_at	1435176_a_at	Id2	inhibitor of DNA binding 2	2.23	0.001	1.25	0.704	1.24	0.766
92614_at	1416630_at	Id3	inhibitor of DNA binding 3	1.43	0.004	1.08	0.834	1.07	0.731
101498_at	1436848_x_at	Impa1	inositol (myo)-1(or 4)-monophosphatase 1	-1.39	0.006	-1.04	0.498	-1.05	0.397
160290_at	1423120_at	Ide	insulin degrading enzyme	-1.11	0.005	-1.18	0.105	-1.12	0.171
95546_g_at	1452014_a_at	Igf1	insulin-like growth factor 1	-1.96	0.001	1.02	0.894	1.21	0.133
95082_at	1423062_at	Igfbp3	insulin-like growth factor binding protein 3	-1.39	0.002	1.03	0.611	-1.07	0.385
97987_at	1422826_at	Igfals	insulin-like growth factor binding protein, acid labile subunit	-2.43	0.000	1.05	0.245	1.12	0.198
96283_at	1415961_at	Itm2c	integral membrane protein 2C	1.5	0.002	1.13	0.841	1.06	0.569
104386_f_at	1452784_at	Itgav	integrin alpha V	1.18	0.008	1.01	0.304	-1.09	0.669
99904_at	1421511_at	Itgb3	integrin beta 3	-1.31	0.006	1.07	0.794	-1.02	0.797
100906_at	1418741_at	Itgb7	integrin beta 7	1.8	0.001	-1.19	0.512	-1.04	0.958
98014_at	1417973_at	Itih1	inter-alpha trypsin inhibitor, heavy chain 1	-1.1	0.007	1.05	0.953	1.03	0.570
96752_at	1424067_at	Icam1	intercellular adhesion molecule	1.61	0.001	1.08	0.483	1.19	0.054
102149_f_at	1422404_x_at	Ifna1 /// Ifna2 ///	interferon alpha family, gene 1 /// interferon alpha family, gene 2 /// interferon alpha family, gene 4 /	-2.17	0.010	-1.35	0.305	-1.38	0.162
100007_at	1451252_at	Irf2bp1	interferon regulatory factor 2 binding protein 1	-1.28	0.009	-1.2	0.194	-1.44	0.004
104760_at	1451016_at	Ifrd2	interferon-related developmental regulator 2	-1.45	0.000	-1.08	0.034	1.08	0.889
99491_at	1419455_at	Il10rb	interleukin 10 receptor, beta	1.46	0.009	1.45	0.017	1.67	0.002
101905_at	1415769_at	Itch	itchy	-1.3	0.003	-1.12	0.841	-1.16	0.286
98911_at	1433803_at	Jak1	Janus kinase 1	1.13	0.000	1.11	0.096	1.13	0.170
93601_at	1450746_at	Keap1	kelch-like ECH-associated protein 1	-1.22	0.000	-1.15	0.017	-1.08	0.194
94270_at	1448169_at	Krt1-18	keratin complex 1, acidic, gene 18	2.24	0.001	-1.01	0.662	1.05	0.948
101009_at	1435989_x_at	Krt2-8	keratin complex 2, basic, gene 8	1.86	0.004	-1.05	0.105	1.04	0.328
96938_at	1416833_at	Keg1	kidney expressed gene 1	-2.68	0.001	1.05	0.292	1.14	0.088
94772_at	1450296_at	Klrb1a	killer cell lectin-like receptor subfamily B member 1A	2.65	0.004	1.8	0.107	1.62	0.185
93677_at	1460245_at	Klrd1	killer cell lectin-like receptor, subfamily D, member 1	1.75	0.001	1.14	0.078	1.19	0.026
97976_at	1455434_a_at	Ktn1	kinectin 1	1.3	0.009	-1.08	0.214	-1.01	0.657
93837_at	1416676_at	Kng1	kininogen 1	1.1	0.008	-1.05	0.717	-1.08	0.562

96072_at	1419737_a_at	Ldh1	lactate dehydrogenase 1, A chain	1.23	0.003	1.16	0.066	1.12	0.183
93103_at	1415847_at	Ldh3	lactate dehydrogenase 3, C chain, sperm specific	-1.89	0.003	1.12	0.595	1.35	0.175
98059_s_at	1421654_a_at	Lmna	lamin A	1.31	0.007	-1.12	0.339	1.04	0.534
97750_at	1448245_at	Lamr1	laminin receptor 1 (ribosomal protein SA)	1.25	0.002	-1.1	0.220	-1.05	0.757
95706_at	1426808_at	Lgals3	lectin, galactose binding, soluble 3	2.39	0.002	1.69	0.079	1.28	0.626
100431_at	1425644_at	Lepr	leptin receptor	2.96	0.004	-1.15	0.278	-1.72	0.095
93600_at	1424438_a_at	Leprot	leptin receptor overlapping transcript	1.16	0.007	-1.06	0.485	-1.14	0.896
104659_g_at	1425107_a_at	Lifr	leukemia inhibitory factor receptor	-2.41	0.001	-1.06	0.874	1.09	0.140
98962_at	1419560_at	Lipc	lipase, hepatic	-1.31	0.000	1.04	0.706	1.09	0.313
99452_at	1451255_at	MGI:1927471	liver-specific bHLH-Zip transcription factor	-1.8	0.002	-1.14	0.386	-1.03	0.589
100086_at	1452148_at	Lrpap1	low density lipoprotein receptor-related protein associated protein 1	1.35	0.007	1.06	0.556	1.26	0.015
96186_at	1416836_at	Lrp10	low-density lipoprotein receptor-related protein 10	-1.38	0.003	-1.27	0.022	-1.29	0.003
104093_at	1417756_a_at	Lsp1	lymphocyte specific 1	1.46	0.010	1.14	0.314	1.03	0.862
100611_at	1423547_at	Lyzs	lysozyme	1.86	0.000	1.19	0.242	1.43	0.028
99071_at	1427076_at	Mpeg1	macrophage expressed gene 1	2.35	0.007	1.9	0.051	1.44	0.245
102974_at	1449498_at	Marco	macrophage receptor with collagenous structure	5.02	0.003	1.82	0.211	1.44	0.821
94792_at	1448061_at	Msr1	Macrophage scavenger receptor 1	1.53	0.006	1.1	0.054	1.09	0.080
102984_g_at	1448208_at	Smad1	MAD homolog 1 (Drosophila)	1.32	0.001	1.02	0.484	-1.02	0.180
160550_i_at	1416212_at	Magoh	mago-nashi homolog, proliferation-associated (Drosophila)	1.28	0.004	1.15	0.238	1.27	0.030
94549_at	1424129_at	Mfsd1	major facilitator superfamily domain containing 1	-1.43	0.005	-1.08	0.687	1.06	0.231
93195_at	1429005_at	Mfhas1	malignant fibrous histiocytoma amplified sequence 1	-1.51	0.000	-1.02	0.380	-1.04	0.496
101474_at	1419578_at	Mbl1	mannose binding lectin, liver (A)	-1.66	0.004	1.01	0.376	1.05	0.284
92847_s_at	1416385_a_at	M6pr	mannose-6-phosphate receptor, cation dependent	-1.11	0.009	1.02	0.843	-1	0.598
160580_at	1417110_at	Man1a	mannosidase 1, alpha	-1.42	0.001	-1.15	0.090	-1.12	0.220
99562_at	1416340_a_at	Man2b1	mannosidase 2, alpha B1	1.2	0.003	1.12	0.544	1.13	0.114
97436_at	1423687_a_at	Man2c1	mannosidase, alpha, class 2C, member 1	1.24	0.004	-1.01	0.861	-1	0.739
96012_f_at	1438368_a_at	Matr3	matrin 3	1.12	0.007	-1.08	0.842	-1.15	0.299
94289_r_at	1426306_a_at	Maged2	melanoma antigen, family D, 2	1.87	0.002	1.44	0.175	1.37	0.174
99604_at	1428843_at	5-Mar	membrane-associated ring finger (C3HC4) 5	1.2	0.000	-1.02	0.577	-1.02	0.591
160103_at	1420609_at	7-Mar	membrane-associated ring finger (C3HC4) 7	1.28	0.008	-1.05	0.071	-1.03	0.322
102022_at	1438477_a_at	Mcee	methylmalonyl CoA epimerase	-1.15	0.001	1.03	0.137	1.28	0.133
99613_at	1448486_at	Mut	methylmalonyl-Coenzyme A mutase	-1.11	0.001	1.06	0.650	1.11	0.176
160929_at	1423099_a_at	Mettl3	methyltransferase-like 3	-1.04	0.004	1.07	0.817	1.14	0.065
104742_at	1452592_at	Mgst2	microsomal glutathione S-transferase 2	4.63	0.005	1.33	0.200	1.11	0.390
160258_at	1416765_s_at	MGI:1913699	mitochondria-associated protein involved in granulocyte-macrophage colony-stimulating factor sign	-1.15	0.006	-1.07	0.335	-1.07	0.185
101834_at	1427060_at	Mapk3	mitogen activated protein kinase 3	1.18	0.005	1.12	0.182	1.09	0.186
100391_at	1420931_at	Mapk8	mitogen activated protein kinase 8	-1.55	0.008	-1.21	0.106	-1.46	0.048
92585_at	1416351_at	Map2k1	mitogen activated protein kinase kinase 1	-1.16	0.005	1.16	0.420	1.18	0.344
99960_at	1426233_at	Map2k4	mitogen activated protein kinase kinase 4	-1.34	0.008	-1	0.448	-1.07	0.103
103020_s_at	1424850_at	Map3k1	mitogen activated protein kinase kinase kinase 1	1.2	0.003	1.03	0.599	-1.1	0.741
104272_s_at	1421450_a_at	Map3k4	mitogen activated protein kinase kinase kinase 4	-1.2	0.007	-1.04	0.282	1.11	0.059
94007_at	1456142_x_at	Morf4l1	mortality factor 4 like 1	1.05	0.007	1.08	0.142	1.05	0.011
96783_at	1426014_a_at	Mucdhl	mucin and cadherin like	-2.47	0.007	-1.12	0.212	-1.34	0.804
97451_at	1424024_at	Mcfd2	multiple coagulation factor deficiency 2	-1.12	0.007	-1.01	0.937	-1	0.383
92837_f_at	1417835_at	Mug1	murinoglobulin 1	-2.38	0.001	-1.12	0.671	1.12	0.422
97680_at	1448854_s_at	Mug-ps1 /// Mu(	murinoglobulin, pseudogene 1 /// murinoglobulin 1 /// murinoglobulin 2 /// similar to Murinoglobulin 1	-2.33	0.001	1.05	0.490	1.33	0.048
99089_at	1417275_at	Mal	myelin and lymphocyte protein, T-cell differentiation protein	1.63	0.001	1.32	0.023	-1.23	0.759
160337_at	1415977_at	MGI:1919030	myo-inositol 1-phosphate synthase A1	2.74	0.001	1.18	0.730	1.22	0.737
94537_at	1428608_at	Mylc2b	myosin light chain, regulatory B	1.18	0.004	1.11	0.111	1.11	0.002
94713_at	1421385_a_at	Myo7a	myosin VIIa	1.42	0.009	1.24	0.360	1.19	0.349
103264_at	1421879_at	Mtmr1	myotubularin related protein 1	-1.45	0.002	-1.04	0.255	-1.03	0.319
96865_at	1415973_at	Marcks	Myristoylated alanine rich protein kinase C substrate	1.52	0.001	1.24	0.113	1.21	0.369
100575_at	1417118_a_at	Ard1	N-acetyltransferase ARD1 homolog (S. cerevisiae)	-1.04	0.004	-1.11	0.015	1.48	0.112

96280_at	1417368_s_at	Ndufa2	NADH dehydrogenase (ubiquinone) 1 alpha subcomplex, 2	1.13	0.003	1	0.990	-1.01	0.837
96273_at	1423231_at	Nrgn	neurogranin	1.41	0.003	-1.05	0.082	-1.48	0.994
161032_i_at	1419325_at	Nmu	neuromedin U	-1.42	0.001	-1.08	0.094	1.24	0.445
102326_at	1448561_at	Ncf2	neutrophil cytosolic factor 2	1.77	0.004	1.35	0.116	1.22	0.369
100120_at	1416808_at	Nid1	nidogen 1	1.55	0.004	1.17	0.496	-1.05	0.085
94236_at	1452156_a_at	Nisch	nischarin	1.16	0.003	1.06	0.150	1	0.833
92190_at	1418605_at	Nr2c1	nuclear receptor subfamily 2, group C, member 1	-1.18	0.009	-1.02	0.127	1.3	0.183
102796_at	1423522_at	Npm3	nucleoplasmin 3	-4.58	0.004	-2.17	0.155	-2.23	0.140
94828_at	1416750_at	Oprs1	opioid receptor, sigma 1	-1.25	0.009	1.14	0.291	1.21	0.089
160193_at	1424235_at	Ormdl2	ORM1-like 2 (S. cerevisiae)	-1.22	0.008	1.03	0.475	1.07	0.121
98003_at	1424302_at	Pirb	paired-Ig-like receptor B	1.77	0.003	1.14	0.624	1.34	0.088
101451_at	1417355_at	Peg3	paternally expressed 3	2.37	0.002	-1.48	0.076	-1.4	0.208
104080_at	1434019_at	Pdap1	PDGFA associated protein 1	-1.16	0.006	-1.13	0.384	-1.29	0.018
104094_at	1417928_at	Pdlim4	PDZ and LIM domain 4	4.43	0.007	2.51	0.224	4.34	0.006
94505_at	1417371_at	Peli1	pellino 1	-1.15	0.003	1.17	0.019	1.06	0.921
94491_at	1422471_at	Pex13	peroxisomal biogenesis factor 13	-1.17	0.007	-1.13	0.068	1.06	0.174
97926_s_at	1420715_a_at	Pparg	peroxisome proliferator activated receptor gamma	2.49	0.003	1.11	0.690	1.87	0.018
97825_at	1416271_at	Perp	PERP, TP53 apoptosis effector	-1.18	0.002	1.05	0.825	1.08	0.693
95407_at	1454638_a_at	Pah	phenylalanine hydroxylase	-1.12	0.004	-1.04	0.436	-1.04	0.362
96627_at	1416667_at	Ebp	phenylalkylamine Ca2+ antagonist (emopamil) binding protein	-1.44	0.001	1.01	0.771	1.12	0.003
161074_at	1438011_at	Pcyt1a	phosphate cytidylyltransferase 1, choline, alpha isoform	-1.35	0.004	-1.08	0.151	-1.04	0.509
96662_at	1429514_at	Ppap2b	phosphatidic acid phosphatase type 2B	-1.42	0.008	-1.18	0.266	-1.21	0.100
104208_at	1435003_at	Pik4ca	phosphatidylinositol 4-kinase, catalytic, alpha polypeptide	1.29	0.002	-1.09	0.398	-1.03	0.862
95358_at	1449404_at	Pip5k2a	phosphatidylinositol-4-phosphate 5-kinase, type II, alpha	1.35	0.009	-1.02	0.857	1.03	0.619
97965_at	1422147_a_at	Pla2g6	phospholipase A2, group VI	1.36	0.000	-1.25	0.720	-1.07	0.172
100607_at	1416013_at	Pld3	phospholipase D3	1.32	0.004	1.1	0.541	1.06	0.530
102839_at	1453181_x_at	Plscr1	phospholipid scramblase 1	1.65	0.000	1.13	0.992	1.17	0.570
96295_at	1451064_a_at	Psat1	phosphoserine aminotransferase 1	1.97	0.005	1.35	0.499	1.33	0.634
93039_at	1416441_at	MGI:1889205	plasma glutamate carboxypeptidase	-1.09	0.010	1.01	0.233	1.02	0.347
94445_at	1423725_at	Pls3	plastin 3 (T-isoform)	-1.55	0.001	-1.05	0.614	-1.09	0.352
104138_at	1448489_at	Pafah2	platelet-activating factor acetylhydrolase 2	-1.49	0.004	-1.11	0.529	-1.04	0.870
101064_at	1448282_at	Plrg1	pleiotropic regulator 1, PRL1 homolog (Arabidopsis)	1.17	0.001	1.13	0.279	1.18	0.005
92867_at	1437239_x_at	Phc2	polyhomeotic-like 2 (Drosophila)	1.2	0.007	1	0.732	-1.09	0.363
100408_at	1421015_s_at	Pole3	polymerase (DNA directed), epsilon 3 (p17 subunit)	-1.35	0.007	-1.22	0.012	-1.26	0.026
97651_at	1419501_at	Polk	polymerase (DNA directed), kappa	-1.14	0.007	-1.11	0.053	-1.17	0.265
97332_at	1416378_at	Pnkp	polynucleotide kinase 3'- phosphatase	-1.2	0.009	1.04	0.460	1.15	0.508
104735_at	1434881_s_at	Kctd12	potassium channel tetramerisation domain containing 12	1.77	0.003	1.31	0.331	1.34	0.206
97100_at	1449536_at	Kcnn1	potassium intermediate/small conductance calcium-activated channel, subfamily N, member 1	-2.15	0.004	-1.45	0.032	-1.01	0.956
102892_at	1416956_at	Kcnab2	potassium voltage-gated channel, shaker-related subfamily, beta member 2	2.33	0.001	1.47	0.283	1.63	0.109
93968_at	1450638_at	Pdcd5	programmed cell death 5	1.54	0.006	1.14	0.082	-1.03	0.458
93926_at	1421382_at	Prlr	prolactin receptor	-2.02	0.001	1.08	0.023	1.18	0.034
95030_at	1448556_at	Prlr	prolactin receptor	-2.16	0.002	-1.11	0.650	-1.29	0.086
100125_at	1450854_at	Pa2g4	proliferation-associated 2G4	-1.57	0.008	1.05	0.290	-1.39	0.020
95597_at	1436448_a_at	Ptgs1	prostaglandin-endoperoxide synthase 1	1.78	0.008	1.4	0.186	1.34	0.334
93803_at	1418078_at	Psme3	proteaseome (prosome, macropain) 28 subunit, 3	-1.84	0.001	-1.03	0.382	-1.18	0.748
93735_f_at	1416282_at	Psmc3	proteasome (prosome, macropain) 26S subunit, ATPase 3	1.18	0.010	1.03	0.967	1.13	0.016
96952_at	1416506_at	Psma6	proteasome (prosome, macropain) subunit, alpha type 6	1.15	0.008	-1.03	0.334	1.07	0.088
101055_at	1448128_at	Ppgb	protective protein for beta-galactosidase	1.25	0.001	1.06	0.386	1.11	0.195
99510_at	1460419_a_at	Prkcb1	protein kinase C, beta 1	1.37	0.004	1.1	0.421	1.18	0.073
94161_at	1449956_at	Prkce	protein kinase C, epsilon	-1.54	0.008	-1.32	0.119	-1.43	0.018
103559_at	1450519_a_at	Prkaca	protein kinase, cAMP dependent, catalytic, alpha	-1.48	0.009	1.02	0.397	1.02	0.357
101482_at	1450149_a_at	Ppp1cc	protein phosphatase 1, catalytic subunit, gamma isoform	1.07	0.007	-1.03	0.940	-1.06	0.439
104525_at	1428351_at	Ppm1m	protein phosphatase 1M	-1.17	0.010	1.05	0.837	1.18	0.052

92638_at	1456390_at	Ppp2ca	protein phosphatase 2a, catalytic subunit, alpha isoform	1.35	0.004	1.06	0.263	1.15	0.098
160821_r_at	1424346_at	Ppp6c	protein phosphatase 6, catalytic subunit	-1.2	0.009	-1.16	0.011	-1.09	0.023
94929_at	1417068_a_at	Ptpn1	protein tyrosine phosphatase, non-receptor type 1	1.32	0.000	-1.05	0.571	-1.24	0.063
92273_at	1419125_at	Ptpn18	protein tyrosine phosphatase, non-receptor type 18	1.77	0.009	1.21	0.466	1.41	0.039
101048_at	1422124_a_at	Ptprc	protein tyrosine phosphatase, receptor type, C	1.76	0.004	1.4	0.114	1.36	0.141
93485_at	1435537_at	Ptprd	Protein tyrosine phosphatase, receptor type, D	-1.48	0.002	-1.07	0.984	-1.15	0.217
93896_at	1424886_at	Ptprd	protein tyrosine phosphatase, receptor type, D	-1.22	0.000	-1.17	0.219	-1.27	0.006
160480_at	1426794_at	Ptprs	protein tyrosine phosphatase, receptor type, S	1.22	0.008	1.15	0.205	1.08	0.393
92802_s_at	1425467_a_at	Plp1	proteolipid protein (myelin) 1	-1.57	0.002	-1.38	0.068	-1.59	0.002
102807_at	1434138_at	Prune	prune homolog (Drosophila)	1.07	0.003	1.01	0.205	1.04	0.023
101472_s_at	1421259_at	Pklr	pyruvate kinase liver and red blood cell	-1.92	0.005	1.16	0.784	1.16	0.725
97415_at	1418890_a_at	Rab3d	RAB3D, member RAS oncogene family	1.76	0.001	1.03	0.326	1.24	0.266
96262_at	1424684_at	Rab5c	RAB5C, member RAS oncogene family	-2.36	0.001	-1.38	0.234	-1.54	0.127
104108_at	1424015_at	Rab6ip1	Rab6 interacting protein 1	1.17	0.010	1.05	0.307	1.03	0.537
92410_at	1422964_at	Rad23a	RAD23a homolog (S. cerevisiae)	-1.38	0.000	1.08	0.110	1.26	0.707
101858_at	1460704_at	Rfng	radical fringe gene homolog (Drosophila)	-1.22	0.010	-1.03	0.772	-1.08	0.416
93070_at	1426946_at	Ranbp5	RAN binding protein 5	1.39	0.006	1.02	0.921	1.03	0.857
92659_at	1421622_a_at	Rapgef4	Rap guanine nucleotide exchange factor (GEF) 4	-2.13	0.003	-1.35	0.077	-1.55	0.014
96056_at	1448605_at	Rhoc	ras homolog gene family, member C	1.54	0.001	1.18	0.562	1.07	0.683
103231_at	1429319_at	Rhoh	ras homolog gene family, member H	1.99	0.004	1.97	0.058	2.18	0.004
92368_at	1420401_a_at	Ramp3	receptor (calcitonin) activity modifying protein 3	2.21	0.003	1.08	0.291	1.55	0.459
103606_r_at	1417786_a_at	Rgs19	regulator of G-protein signaling 19	1.48	0.009	1.33	0.102	1.32	0.055
93733_r_at	1422763_at	Rgs19ip1	regulator of G-protein signaling 19 interacting protein 1	1.3	0.001	1.14	0.141	1.33	0.001
98336_s_at	1451920_a_at	Recc1	replication factor C 1	-1.66	0.008	-1.3	0.330	-1.18	0.210
93839_at	1418101_a_at	Rtn3	reticulon 3	1.3	0.003	1.1	0.964	1.02	0.169
93081_at	1415775_at	Rbbp7	retinoblastoma binding protein 7	2.16	0.005	1.14	0.866	1.04	0.129
104716_at	1448754_at	Rbp1	retinol binding protein 1, cellular	1.23	0.006	1.06	0.725	1.02	0.814
101702_at	1421085_at	Rs1h	retinoschisis 1 homolog (human)	-1.82	0.004	-1.31	0.112	-1.58	0.014
104450_at	1425971_at	LOC277923	reverse transcriptase	-1.29	0.005	-1.03	0.674	-1.34	0.042
94258_at	1426454_at	Arhgdib	Rho, GDP dissociation inhibitor (GDI) beta	1.6	0.001	1.2	0.226	1.12	0.503
98342_at	1448157_s_at	Rpl10	ribosomal protein 10	1.18	0.004	1.05	0.619	1.07	0.782
100711_at	1431177_a_at	Rpl10a	ribosomal protein L10A	1.17	0.005	-1.03	0.118	1.03	0.940
102109_at	1460581_a_at	Rpl13 /// LOC2{	ribosomal protein L13 /// similar to 60S ribosomal protein /// similar to 60S ribosomal protein L13	1.23	0.002	-1.07	0.820	-1.06	0.957
99653_at	1438626_x_at	Rpl14	ribosomal protein L14	1.18	0.001	-1.11	0.321	-1.24	0.131
99335_at	1423855_x_at	Rpl17	ribosomal protein L17	1.29	0.001	-1.04	0.073	1.17	0.000
160476_f_at	1436699_x_at	Rpl18	ribosomal protein L18	1.25	0.008	-1.08	0.258	-1.02	0.563
97483_at	1416219_at	Rpl19	ribosomal protein L19	1.15	0.003	-1.04	0.043	-1.02	0.305
94823_at	1426660_x_at	Rpl23a	ribosomal protein L23a	1.19	0.002	-1.01	0.036	1.01	0.345
100727_at	1416074_a_at	Rpl28	ribosomal protein L28	1.07	0.002	-1.11	0.278	-1.16	0.194
100734_at	1449323_a_at	Rpl3	ribosomal protein L3	1.13	0.001	-1.05	0.860	-1.07	0.541
92628_at	1416520_x_at	Rpl36	ribosomal protein L36	1.33	0.004	-1.06	0.079	1.05	0.876
160081_at	1416807_at	Rpl36a	ribosomal protein L36a	1.5	0.006	1.07	0.881	1.06	0.847
100213_f_at	1455578_x_at	Rpl41	ribosomal protein L41	1.16	0.000	1.07	0.117	1.08	0.280
101129_at	1423665_a_at	Rpl5	ribosomal protein L5	1.13	0.001	1.01	0.113	1.02	0.040
99093_at	1456497_x_at	Rps10	ribosomal protein S10	1.25	0.001	-1.03	0.111	1.01	0.841
94767_at	1424000_a_at	Rps11	ribosomal protein S11	1.18	0.009	-1.07	0.193	-1.05	0.311
93730_at	1453467_s_at	Rps15a	ribosomal protein S15a	1.25	0.006	-1.05	0.063	-1.01	0.191
99590_at	1459986_a_at	Rps17	ribosomal protein S17	1.2	0.002	1.1	0.259	1.07	0.137
98333_at	1448739_x_at	Rps18	ribosomal protein S18	1.15	0.001	1	0.550	1.02	0.249
100686_at	1431766_x_at	Rps2	ribosomal protein S2	1.07	0.003	1	0.659	1.01	0.897
96358_at	1460175_at	Rps23	ribosomal protein S23	1.28	0.001	1.05	0.823	1.12	0.033
98564_f_at	1415876_a_at	Rps26	ribosomal protein S26	1.19	0.001	1.01	0.049	1.03	0.785
93030_at	1449196_a_at	Rps27a	ribosomal protein S27a	1.13	0.005	-1.07	0.641	-1.06	0.888

98085_f_at	1454778_x_at	Rps28	Ribosomal protein S28	1.21	0.003	1.01	0.415	1.02	0.723
101137_at	1435151_a_at	Rps3	ribosomal protein S3	1.24	0.007	1.03	0.831	1.17	0.003
101664_at	1422475_a_at	Rps3a	ribosomal protein S3a	1.13	0.010	-1	0.119	1.07	0.321
99336_at	1416054_at	Rps5	ribosomal protein S5	1.22	0.002	-1.06	0.186	1.02	0.533
100732_at	1436760_a_at	Rps8	ribosomal protein S8	1.21	0.009	-1.01	0.126	1.09	0.520
100694_at	1416277_a_at	Rplp1	ribosomal protein, large, P1	1.15	0.000	1.01	0.778	1.07	0.298
92850_at	1452767_at	Rrbp1	ribosome binding protein 1	1.13	0.006	1.16	0.052	-1.08	0.511
94518_at	1416245_at	0610033H09Ril	RIKEN cDNA 0610033H09 gene	-1.05	0.005	1	0.079	1.11	0.794
160156_at	1423829_at	0910001A06Ril	RIKEN cDNA 0910001A06 gene	1.43	0.009	1.1	0.994	1.05	0.666
98033_at	1448786_at	1100001H23Ril	RIKEN cDNA 1100001H23 gene	1.52	0.000	1.07	0.205	1.02	0.344
95041_at	1449046_a_at	1110007C05Ril	RIKEN cDNA 1110007C05 gene	-1.46	0.001	1.2	0.087	1.25	0.018
95409_at	1415733_a_at	1110019J04Rik	RIKEN cDNA 1110019J04 gene	-1.28	0.005	1.04	0.844	1.24	0.118
160184_at	1423334_at	1200007D18Ril	RIKEN cDNA 1200007D18 gene	-1.51	0.003	-1.09	0.627	-1.31	0.111
103531_f_at	1434714_at	1300013B24Ril	RIKEN cDNA 1300013B24 gene	-1.3	0.000	-1.06	0.159	-1.03	0.879
160260_at	1448461_a_at	1500006O09Ril	RIKEN cDNA 1500006O09 gene	1.08	0.002	1.03	0.191	1.01	0.642
93533_at	1425674_a_at	1500011L16Rik	RIKEN cDNA 1500011L16 gene	1.24	0.007	1.03	0.441	1.15	0.092
104726_at	1451625_a_at	1700013L23Rik	RIKEN cDNA 1700013L23 gene	-1.39	0.002	-1.04	0.853	1.05	0.154
97514_at	1435864_a_at	1810063B05Ril	RIKEN cDNA 1810063B05 gene	-1.13	0.009	-1.08	0.087	1.05	0.974
104000_at	1424968_at	2210023G05Ril	RIKEN cDNA 2210023G05 gene	-2.28	0.000	1.06	0.435	1.1	0.457
100321_f_at	1449910_at	2210418O10Ril	RIKEN cDNA 2210418O10 gene	-1.29	0.000	1.18	0.674	1.15	0.235
102783_at	1452799_at	2310009E04Ril	RIKEN cDNA 2310009E04 gene	-1.37	0.001	1.17	0.034	1.18	0.120
94366_at	1417779_at	2310079N02Ril	RIKEN cDNA 2310079N02 gene	1.27	0.007	1.04	0.936	1.09	0.446
100562_at	1448740_at	2400006H24Ril	RIKEN cDNA 2400006H24 gene	-1.42	0.006	-1.18	0.176	-1.07	0.615
160727_at	1460672_at	2410002F23Rik	RIKEN cDNA 2410002F23 gene	1.34	0.010	1.07	0.573	-1.11	0.448
160425_at	1454116_a_at	2410017I18Rik	RIKEN cDNA 2410017I18 gene	-1.18	0.004	-1	0.389	-1.08	0.029
96779_f_at	1420113_s_at	2410022L05Rik	RIKEN cDNA 2410022L05 gene	1.25	0.004	1.18	0.007	1.17	0.041
96329_at	1416438_at	2410104I19Rik	RIKEN cDNA 2410104I19 gene	1.06	0.003	-1.04	0.692	-1.03	0.894
93591_at	1448644_at	2600002E23Ril	RIKEN cDNA 2600002E23 gene	-1.15	0.009	-1.05	0.322	-1.03	0.651
95723_r_at	1428708_x_at	2610009E16Ril	RIKEN cDNA 2610009E16 gene	-1.61	0.005	1.11	0.101	1	0.171
100902_at	1428357_at	2610019F03Rik	RIKEN cDNA 2610019F03 gene	1.34	0.001	-1.07	0.595	-1.04	0.461
104119_at	1434620_s_at	2610024E20Ril	RIKEN cDNA 2610024E20 gene	1.31	0.009	1.11	0.083	1.07	0.092
100115_at	1426289_at	2610028H07Ril	RIKEN cDNA 2610028H07 gene	1.09	0.002	1.03	0.172	1.04	0.154
160293_at	1459985_at	2700038L12Rik	RIKEN cDNA 2700038L12 gene	-1.09	0.009	1.11	0.382	1.25	0.064
104089_at	1428529_at	2810026P18Ril	RIKEN cDNA 2810026P18 gene	1.2	0.003	-1.15	0.308	-1.16	0.336
98756_at	1430195_at	2810043O03Ril	RIKEN cDNA 2810043O03 gene	1.66	0.002	1.44	0.088	1.03	0.524
97292_at	1452167_at	2810407C02Ril	RIKEN cDNA 2810407C02 gene	-1.09	0.003	-1.01	0.081	1.07	0.645
103852_at	1423958_a_at	2900001O04Ril	RIKEN cDNA 2900001O04 gene	-1.39	0.003	-1.16	0.022	-1.08	0.212
103412_at	1435321_at	3732412D22Ril	RIKEN cDNA 3732412D22 gene	1.38	0.003	-1.51	0.050	-1.2	0.375
93801_at	1418173_at	4631426H08Ril	RIKEN cDNA 4631426H08 gene	2.42	0.007	1.17	0.751	1.29	0.465
102030_at	1420948_s_at	4833408C14Ril	RIKEN cDNA 4833408C14 gene	-1.3	0.006	-1.05	0.051	-1.01	0.035
160322_at	1423759_a_at	4930403O06Ril	RIKEN cDNA 4930403O06 gene	-1.32	0.003	-1.09	0.824	-1.02	0.516
94243_at	1437349_at	4930432B04Ril	RIKEN cDNA 4930432B04 gene	-1.26	0.008	1.06	0.502	-1.03	0.057
103716_at	1455072_at	4933409L06Rik	RIKEN cDNA 4933409L06 gene	-1.11	0.010	1.01	0.026	-1.01	0.134
96614_at	1454606_at	4933426M11Ril	RIKEN cDNA 4933426M11 gene	-1.35	0.008	-1.13	0.139	-1.36	0.035
103557_at	1426227_s_at	5730409F24Rik	RIKEN cDNA 5730409F24 gene	1.36	0.001	1.18	0.247	1.02	0.540
104206_at	1453287_at	5730557B15Rik	RIKEN cDNA 5730557B15 gene	-2.02	0.000	-1.43	0.091	-1.25	0.169
103584_at	1453015_at	5830471E12Rik	RIKEN cDNA 5830471E12 gene	1.4	0.008	1.08	0.655	-1	0.465
96207_at	1434005_at	Rbms1	RIKEN cDNA 6030432P03 gene	1.65	0.001	1.17	0.174	1	0.473
100511_at	1428242_at	6330406L22Rik	RIKEN cDNA 6330406L22 gene	1.36	0.008	1.19	0.039	1.08	0.368
103200_at	1460555_at	6330500D04Ril	RIKEN cDNA 6330500D04 gene	1.39	0.000	1.05	0.712	-1.06	0.297
103198_at	1455239_at	6330512M04Ril	RIKEN cDNA 6330512M04 gene	1.49	0.003	1.04	0.231	-1.06	0.552
95523_at	1428333_at	6530401D17Ril	RIKEN cDNA 6530401D17 gene	2.2	0.008	1.13	0.845	1.07	0.595
95887_at	1448068_at	8430426K15Ril	RIKEN cDNA 8430426K15 gene	1.49	0.001	1.34	0.102	1.2	0.142

103435_at	1434184_s_at	9430080K19Rik	RIKEN cDNA 9430080K19 gene	2.34	0.000	1.26	0.203	1.21	0.284
94995_at	1435695_a_at	A030007L17Rik	RIKEN cDNA A030007L17 gene	-1.32	0.009	1.17	0.250	1.3	0.056
104362_at	1433765_at	B230113M03Ri	RIKEN cDNA B230113M03 gene	-1.7	0.002	-1.4	0.081	-1.45	0.040
103331_at	1451085_at	C030006K11Ril	RIKEN cDNA C030006K11 gene	-1.7	0.009	-1.11	0.613	1.04	0.136
96859_at	1423102_a_at	Rnf10	ring finger protein 10	-1.46	0.001	-1.37	0.132	-1.11	0.324
160757_at	1428101_at	Rnf38	ring finger protein 38	1.43	0.003	1.25	0.087	1.11	0.750
101421_at	1422888_at	Rnf5	ring finger protein 5	-1.22	0.001	1.05	0.771	1.18	0.065
102782_at	1449346_s_at	Riok1	RIO kinase 1 (yeast)	1.34	0.005	1.07	0.582	-1.1	0.298
96041_at	1422660_at	Rbm3	RNA binding motif protein 3	1.84	0.001	1.08	0.099	1.08	0.299
97765_g_at	1425970_a_at	Ros1	Ros1 proto-oncogene	-1.63	0.005	-1.4	0.010	1.14	0.588
160422_at	1422482_at	Ruvbl2	RuvB-like protein 2	-1.27	0.001	-1.06	0.253	-1.01	0.620
98600_at	1460351_at	S100a11	S100 calcium binding protein A11 (calizzarin)	2.02	0.003	1.29	0.527	1.22	0.652
101861_at	1420688_a_at	Sgce	sarcoglycan, epsilon	1.47	0.004	1.08	0.762	1.06	0.440
96763_at	1448426_at	Sardh	Sarcosine dehydrogenase	-1.47	0.006	1	0.663	1.03	0.959
96759_r_at	1424530_at	Sec14l2	SEC14-like 2 (S. cerevisiae)	-1.25	0.002	1.08	0.191	1.18	0.048
99617_at	1452147_at	Sec24c	SEC24 related gene family, member C (S. cerevisiae)	1.19	0.003	1.01	0.463	1	0.206
104365_at	1416611_at	Scamp2	secretory carrier membrane protein 2	1.14	0.007	1.07	0.125	1.03	0.129
100596_at	1450699_at	Selenbp1	selenium binding protein 1	-1.32	0.005	-1.08	0.877	-1.14	0.247
160836_at	1420824_at	Sema4d	sema domain, immunoglobulin domain (Ig), transmembrane domain (TM) and short cytoplasmic dc	1.12	0.002	1.1	0.059	-1.1	0.949
96227_at	1448506_at	Serpina6	serine (or cysteine) proteinase inhibitor, clade A, member 6	-1.86	0.000	1.11	0.022	1.13	0.073
96060_at	1450138_a_at	Serpinb6a	serine (or cysteine) proteinase inhibitor, clade B, member 6a	2.2	0.000	1.16	0.741	1.4	0.074
104700_at	1422198_a_at	Shmt1	serine hydroxymethyl transferase 1 (soluble)	-1.46	0.007	1.02	0.398	1.36	0.205
92242_at	1419319_at	Saa4	serum amyloid A 4	-2.7	0.010	1.17	0.763	1.26	0.544
103755_at	1449084_s_at	Sh3d19	SH3 domain protein D19	-1.12	0.008	-1.22	0.135	-1.21	0.117
92360_at	1416861_at	Stam	signal transducing adaptor molecule (SH3 domain and ITAM motif) 1	-1.32	0.004	-1.05	0.486	-1.11	0.047
160869_at	1417892_a_at	Sirt3	sirtuin 3 (silent mating type information regulation 2, homolog) 3 (S. cerevisiae)	1.2	0.010	1.01	0.885	1.21	0.046
92578_at	1416486_at	Scye1	small inducible cytokine subfamily E, member 1	1.35	0.004	1.09	0.976	1.23	0.085
97200_f_at	1451294_s_at	Snrpe	small nuclear ribonucleoprotein E	1.22	0.002	1.02	0.605	1	0.485
95424_at	1422457_s_at	Sumo3	SMT3 suppressor of mif two 3 homolog 3 (yeast)	-1.21	0.005	1.05	0.474	-1.01	0.709
100339_at	1424898_at	Slc10a1	solute carrier family 10 (sodium/bile acid cotransporter family), member 1	-1.37	0.000	-1.04	0.137	1	0.285
100341_g_at	1450261_a_at	Slc10a1	solute carrier family 10 (sodium/bile acid cotransporter family), member 1	-1.42	0.001	1.06	0.336	1.07	0.558
103218_at	1451227_a_at	Slc10a3	solute carrier family 10 (sodium/bile acid cotransporter family), member 3	1.37	0.003	-1.15	0.399	-1.02	0.805
94419_at	1448132_at	Slc19a1	solute carrier family 19 (sodium/hydrogen exchanger), member 1	-1.74	0.003	-1.39	0.082	-1.45	0.034
101397_at	1450628_at	Slc2a8	solute carrier family 2, (facilitated glucose transporter), member 8	-1.45	0.006	-1.34	0.034	-1.01	0.342
100916_at	1418118_at	Slc22a1	solute carrier family 22 (organic cation transporter), member 1	-1.39	0.010	-1.12	0.609	-1.06	0.832
94797_at	1451239_a_at	Slc26a1	solute carrier family 26 (sulfate transporter), member 1	-3.51	0.000	-1.24	0.479	-1.18	0.456
95733_at	1451782_a_at	Slc29a1	solute carrier family 29 (nucleoside transporters), member 1	-1.91	0.002	-1.18	0.332	-1.1	0.840
93304_at	1448741_at	Slc3a1	solute carrier family 3, member 1	-1.45	0.001	1.03	0.331	1.16	0.840
103510_at	1449382_at	Slc6a12	solute carrier family 6 (neurotransmitter transporter, betaine/GABA), member 12	-1.24	0.008	1.03	0.899	1.07	0.670
95625_at	1417116_at	Slc6a8	solute carrier family 6 (neurotransmitter transporter, creatine), member 8	1.42	0.006	-1.12	0.147	-1.1	0.224
103934_at	1424338_at	Slc6a13	solute carrier family 6 (neurotransmitter transporter, GABA), member 13	-1.23	0.002	1.02	0.689	1.18	0.045
94663_at	1440874_at	Slco5a1	solute carrier organic anion transporter family, member 5A1	2.16	0.005	-1.53	0.762	1.46	0.201
98351_g_at	1422256_at	Sstr2	somatostatin receptor 2	1.64	0.009	1.13	0.415	1.19	0.118
100401_at	1420952_at	Son	Son cell proliferation protein	1.22	0.010	-1.2	0.106	-1.3	0.014
97839_at	1429497_s_at	Snx6	sorting nexin 6	1.2	0.010	1.1	0.334	1.11	0.254
93609_at	1424876_s_at	Spg20	spastic paraplegia 20, spartin (Troyer syndrome) homolog (human)	-1.31	0.002	-1.09	0.235	-1.17	0.034
98428_at	1460400_at	Spg4	spastic paraplegia 4 homolog (human)	-1.21	0.007	1.06	0.867	1.08	0.684
96657_at	1420502_at	Sat1	spermidine/spermine N1-acetyl transferase 1	1.24	0.000	1.11	0.018	1.22	0.016
100099_at	1448621_a_at	Smpd1	sphingomyelin phosphodiesterase 1, acid lysosomal	-1.24	0.005	-1.01	0.661	-1.04	0.471
94872_at	1416635_at	Smpdl3a	sphingomyelin phosphodiesterase, acid-like 3A	1.38	0.006	1.26	0.091	1.27	0.025
160843_at	1460164_at	Spin	spindlin	-1.38	0.003	-1.07	0.171	1.07	0.835
96027_at	1449333_at	Sf3a1	splicing factor 3a, subunit 1	-1.25	0.007	1.06	0.478	-1.01	0.775
102012_at	1418895_at	Scap2	src family associated phosphoprotein 2	1.4	0.006	1.12	0.882	1.06	0.584

100279_at	1421890_at	St3gal2	ST3 beta-galactoside alpha-2,3-sialyltransferase 2	1.48	0.006	1.21	0.693	1.06	0.892
95758_at	1415822_at	Scd2	stearoyl-Coenzyme A desaturase 2	2.16	0.001	1.15	0.715	1.17	0.423
95787_s_at	1426219_at	Scp2	sterol carrier protein 2, liver	-1.09	0.005	1.04	0.074	1.08	0.008
103421_at	1423465_at	Sdfr2	stromal cell derived factor receptor 2	1.12	0.003	1	0.372	1.16	0.008
104484_at	1419528_at	Sult2a2	sulfotransferase family 2A, dehydroepiandrosterone (DHEA)-preferring, member 2	-1.52	0.005	-1.18	0.359	-1.08	0.795
100538_at	1451124_at	Sod1	superoxide dismutase 1, soluble	1.16	0.001	1.12	0.112	1.21	0.021
99111_at	1416541_at	Skd3	suppressor of K+ transport defect 3	-1.28	0.004	-1.22	0.016	1.13	0.698
93586_at	1448577_x_at	Syngr2	synaptogyrin 2	-1.27	0.008	-1.03	0.874	1.02	0.201
96734_at	1417834_at	Synj2bp	synaptojanin 2 binding protein	-1.37	0.001	1.05	0.134	1.06	0.248
160190_at	1415844_at	Syt4	synaptotagmin 4	-1.89	0.002	-1.49	0.021	-1.6	0.025
103781_at	1451573_a_at	Stx4a	syntaxin 4A (placental)	1.29	0.003	1.14	0.272	1.06	0.506
102890_at	1416825_at	Snta1	syntrophin, acidic 1	-1.33	0.000	-1.04	0.970	-1.09	0.104
160331_at	1420663_at	Thpok	T helper-inducing POZ/Krueppel factor	-1.44	0.005	-1.23	0.103	-1.17	0.092
103542_at	1427051_at	Tnks1bp1	tankyrase 1 binding protein 1	-1.58	0.001	1.02	0.007	-1.06	0.043
160377_at	1423723_s_at	Tardbp	TAR DNA binding protein	-1.21	0.003	1.04	0.763	-1.08	0.266
94403_at	1420196_s_at	Tbc1d14	TBC1 domain family, member 14	1.24	0.001	1.09	0.441	1.06	0.939
93105_s_at	1452205_x_at	Tcrb-V13	T-cell receptor beta, variable 13	1.82	0.009	1.08	0.376	1.11	0.189
94259_at	1417998_at	MGI:1929282	telomerase binding protein, p23	1.06	0.010	-1.08	0.851	-1.12	0.362
102916_s_at	1450798_at	Tnxb	tenascin XB	-2.53	0.001	-2.13	0.013	-1.38	0.136
101551_s_at	1460378_a_at	Tes	testis derived transcript	1.55	0.001	-1.03	0.346	-1.12	0.844
102418_at	1417482_at	Tex19	testis expressed gene 19	-1.27	0.004	-1.09	0.612	-1.35	0.020
95326_at	1460717_at	Tspyl1	testis-specific protein, Y-encoded-like 1	-1.33	0.000	-1.12	0.103	-1.24	0.002
95120_at	1418643_at	Tspan13	tetraspanin 13	1.2	0.005	-1.22	0.664	-1.15	0.928
93621_at	1434400_at	Tgif2	TGFB-induced factor 2	1.84	0.007	1.13	0.290	1.04	0.686
104025_at	1448907_at	Thop1	thimet oligopeptidase 1	-1.08	0.006	-1.12	0.120	1.08	0.922
96773_at	1423247_at	Txndc4	thioredoxin domain containing 4 (endoplasmic reticulum)	-1.15	0.006	-1.01	0.178	1.07	0.675
101356_at	1426100_a_at	Tk2	thymidine kinase 2, mitochondrial	1.19	0.009	-1.05	0.744	1.12	0.041
96426_at	1415906_at	Tmsb4x	thymosin, beta 4, X chromosome	1.53	0.006	1.31	0.061	1.2	0.305
94261_at	1460545_at	Thrap3	thyroid hormone receptor associated protein 3	1.18	0.001	1.08	0.122	-1.03	0.686
94948_at	1449041_a_at	Trip6	thyroid hormone receptor interactor 6	1.37	0.001	-1.06	0.392	-1.03	0.414
101464_at	1460227_at	Timp1	tissue inhibitor of metalloproteinase 1	1.57	0.004	-1.09	0.591	-1.03	0.331
103255_at	1448861_at	Traf5	Tnf receptor-associated factor 5	1.21	0.000	1.23	0.008	1.2	0.026
104755_at	1427689_a_at	Tnip1	TNFAIP3 interacting protein 1	1.26	0.005	1	0.403	-1.02	0.840
93145_at	1455505_at	---	Transcribed locus	1.42	0.002	1.04	0.610	1.14	0.301
103958_g_at	1422966_a_at	Tfrc	transferrin receptor	-2.26	0.002	-1.14	0.115	-1.17	0.028
98937_at	1452648_at	Tbrg1	transforming growth factor beta regulated gene 1	1.25	0.010	1.03	0.530	-1.04	0.760
93352_at	1455900_x_at	Tgm2	transglutaminase 2, C polypeptide	1.49	0.009	1.26	0.012	1.11	0.442
101036_at	1455357_x_at	Tomm20	translocase of outer mitochondrial membrane 20 homolog (yeast)	1.16	0.009	-1.09	0.594	-1.07	0.556
160136_r_at	1433704_s_at	Tloc1	translocation protein 1	-1.2	0.003	1.03	0.425	-1	0.727
92531_at	1455698_at	Tloc1	translocation protein 1	-1.82	0.003	-1.02	0.666	1.01	0.514
103017_at	1449670_x_at	Tm7sf1	transmembrane 7 superfamily member 1	1.5	0.003	1.12	0.214	-1.15	0.542
92437_at	1460684_at	Tm7sf2	transmembrane 7 superfamily member 2	-1.19	0.001	1.07	0.973	1.06	0.771
96894_at	1448422_at	Tmed4	transmembrane emp24 protein transport domain containing 4	1.2	0.010	1.05	0.709	1.13	0.101
103315_at	1434898_at	Tnrc6a	trinucleotide repeat containing 6a	1.18	0.008	1.02	0.301	-1.01	0.784
101449_at	1424275_s_at	Trim41	tripartite motif-containing 41	1.22	0.002	-1.04	0.611	-1.01	0.450
93827_at	1449337_at	Tdo2	tryptophan 2,3-dioxygenase	-1.7	0.000	-1.24	0.188	-1.22	0.045
104682_at	1419518_at	Tuba8	tubulin, alpha 8	2.44	0.001	-1.18	0.310	1.05	0.632
160246_at	1416950_at	Tnfaip8	tumor necrosis factor, alpha-induced protein 8	1.88	0.005	1.27	0.598	1.22	0.851
160376_at	1452646_at	Trp53inp2	tumor protein p53 inducible nuclear protein 2	-1.63	0.003	-1.05	0.764	-1.21	0.157
100397_at	1450792_at	Tyrobp	TYRO protein tyrosine kinase binding protein	1.85	0.009	1.61	0.116	1.6	0.070
98053_at	1455815_a_at	Ywhab	tyrosine 3-monooxygenase/tryptophan 5-monooxygenase activation protein, beta polypeptide	1.27	0.002	1.31	0.073	1.27	0.015
95601_at	1419385_a_at	Ubqln1	ubiquilin 1	-1.11	0.006	-1.05	0.063	1.04	0.113
92715_at	1419762_at	Ubd	ubiquitin D	4.34	0.003	1.22	0.556	1.4	0.112

92820_at	1417169_at	Usp2	ubiquitin specific protease 2	-2.3	0.002	-1.88	0.138	-2.24	0.001
92821_at	1417168_a_at	Usp2	ubiquitin specific protease 2	-2.36	0.009	-1.43	0.258	-1.7	0.027
96563_at	1448014_s_at	Usp24	ubiquitin specific protease 24	-1.37	0.008	-1.05	0.394	-1.02	0.705
95858_at	1445116_at	Usp25	Ubiquitin specific protease 25	-1.31	0.009	-1.03	0.756	-1.56	0.040
96286_at	1423185_a_at	Ubap1	ubiquitin-associated protein 1	-1.33	0.008	-1.1	0.713	-1.11	0.584
93509_at	1423106_at	Ube2b	ubiquitin-conjugating enzyme E2B, RAD6 homology (S. cerevisiae)	-1.34	0.002	-1.06	0.154	1.05	0.983
93312_at	1415688_at	Ube2g1	ubiquitin-conjugating enzyme E2G 1	-1.25	0.001	-1.01	0.745	-1	0.577
97479_at	1417908_s_at	Ube2l3	ubiquitin-conjugating enzyme E2L 3	-1.29	0.000	-1.15	0.166	-1.08	0.228
102936_at	1460329_at	B4galt6	UDP-Gal:betaGlcNAc beta 1,4-galactosyltransferase, polypeptide 6	1.98	0.002	1.66	0.042	1.41	0.129
93978_at	1454842_a_at	B3galnt2	UDP-GalNAc:betaGlcNAc beta 1,3-galactosaminyltransferase, polypeptide 2	-1.7	0.007	-1.03	0.935	-1.17	0.440
103367_at	1418655_at	Galgt1	UDP-N-acetyl-alpha-D-galactosamine:(N-acetylneuraminyl)-galactosylglucosylceramide-beta-1, 4-l	-1.11	0.002	1.1	0.183	1.04	0.034
161016_at	1455915_at	Galnt4	UDP-N-acetyl-alpha-D-galactosamine:polypeptide N-acetylgalactosaminyltransferase 4	-1.13	0.002	1.19	0.045	1.1	0.863
95555_at	1418123_at	Unc119	unc-119 homolog (C. elegans)	2.05	0.001	1.17	0.605	1.28	0.005
96847_at	1448447_at	Vps28	vacuolar protein sorting 28 (yeast)	1.17	0.000	-1.03	0.770	1.04	0.329
104165_at	1418486_at	Vnn1	vanin 1	1.85	0.003	1.17	0.307	1.02	0.678
99799_at	1422932_a_at	Vav1	vav 1 oncogene	1.68	0.006	1.36	0.155	1.4	0.098
93305_f_at	1420624_a_at	Vamp8	vesicle-associated membrane protein 8	1.21	0.004	1	0.597	1.15	0.023
94264_at	1425419_a_at	Raf1	v-raf-1 leukemia viral oncogene 1	1.25	0.010	1.08	0.598	1.08	0.530
102356_at	1448418_s_at	Wdr23	WD repeat domain 23	-1.15	0.009	1	0.393	1.03	0.079
100523_r_at	1451230_a_at	Wbp5	WW domain binding protein 5	1.75	0.003	1.08	0.645	-1.05	0.170
98767_at	1435824_at	Yy1	YY1 transcription factor	-1.39	0.003	-1.09	0.408	-1.1	0.279
99020_at	1457834_at	Yy1	YY1 transcription factor	-1.59	0.006	1.06	0.989	-1.01	0.634
95792_at	1450190_at	Zfp106	zinc finger protein 106	1.08	0.005	1.01	0.171	1.03	0.150
92974_at	1419207_at	Zfp37	zinc finger protein 37	1.62	0.003	1.48	0.001	1.18	0.044
161084_at	1427104_at	Zfp612	zinc finger protein 612	1.76	0.002	1.16	0.472	1.06	0.750
104572_at	1433910_at	Zcchc6	zinc finger, CCHC domain containing 6	1.16	0.010	1.12	0.030	1.05	0.418
