# Supplementary material for: Delayed and Accelerated Aging Share Common Longevity Assurance Mechanisms
Source: PLoS Genet. 2008 Aug 15;4(8):e1000161. doi: 10.1371/journal.pgen.1000161 (PMC2493043; doi:10.1371/journal.pgen.1000161)
Supplement: Table S2 — A list of probe sets with significant transcriptional changes in the liver of Ercc1−/− as compared to littermate controls. FC: fold change, P: p-value. (0.07 MB PDF) [file pgen.1000161.s006.pdf]

Supplementary table S2. A list of probe sets with significant transcriptional changes in the liver of Ercc1-/- as compared to wt littermate controls. FC: fold change, P: p-value

**Annotation data (from two independent Affymetrix platforms)**				**ERCC1-/-**	
74code	430code	Gene Symbol	Gene Title	FC	P
95509_at	1451228_a_at	Sycn	syncollin	18.4	0.006
160744_r_at	1431763_a_at	Ctrl	chymotrypsin-like	9.01	0.007
92873_f_at	1435507_x_at	Prss2	protease, serine, 2	8.5	0.009
99939_at	1417257_at	Cel	carboxyl ester lipase	7.21	0.008
92811_at	1422846_at	Rbp2	retinol binding protein 2, cellular	6.56	0.002
104742_at	1452592_at	Mgst2	microsomal glutathione S-transferase 2	6.09	0.001
102787_at	1433485_x_at	Gpr56	G protein-coupled receptor 56	4.84	0.000
102826_at	1423556_at	Akr1b7	aldo-keto reductase family 1, member B7	4.8	0.001
160173_at	1436713_s_at	---	---	4.72	0.001
92601_at	1415777_at	Pnliprp1	pancreatic lipase related protein 1	4.27	0.004
104603_at	1417883_at	Gstt2	glutathione S-transferase, theta 2	4.23	0.000
103279_at	1449393_at	Sh2d1a	SH2 domain protein 1A	3.56	0.002
93020_at	1448595_a_at	Rex3	reduced expression 3	3.5	0.004
101872_at	1421041_s_at	Gsta2	glutathione S-transferase, alpha 2 (Yc2)	3.35	0.001
160172_at	1426758_s_at	Gtl2	GTL2, imprinted maternally expressed untranslated mRNA	3.32	0.002
101990_at	1455235_x_at	Ldh2	lactate dehydrogenase 2, B chain	3.31	0.006
101451_at	1417355_at	Peg3	paternally expressed 3	3.21	0.005
92252_at	1421195_at	Cckar	cholecystokinin A receptor	3.02	0.005
104094_at	1417928_at	Pdlim4	PDZ and LIM domain 4	3.01	0.000
98035_g_at	1449580_s_at	H2-DMb1 /// H2-DMb2	histocompatibility 2, class II, locus Mb1 /// histocompatibility 2, class II,	3.01	0.000
92357_at	1423378_at	Adam23	a disintegrin and metalloprotease domain 23	2.92	0.002
101587_at	1422438_at	Ephx1	epoxide hydrolase 1, microsomal	2.91	0.000
100078_at	1436504_x_at	---	---	2.9	0.001
99370_at	1426182_a_at	Klrc1	killer cell lectin-like receptor subfamily C, member 1	2.9	0.004
94186_at	1423602_at	Traf1	Tnf receptor-associated factor 1	2.78	0.002
100016_at	1417234_at	Mmp11	matrix metalloproteinase 11	2.75	0.005
97926_s_at	1420715_a_at	Pparg	peroxisome proliferator activated receptor gamma	2.74	0.000
93284_at	1416332_at	Cirbp	cold inducible RNA binding protein	2.66	0.003
98967_at	1450779_at	Fabp7	fatty acid binding protein 7, brain	2.64	0.010
160828_at	1426858_at	Inhbb	inhibin beta-B	2.61	0.000
97793_at	1420563_at	Gria3	glutamate receptor, ionotropic, AMPA3 (alpha 3)	2.59	0.000
102751_at	1417455_at	Tgfb3	transforming growth factor, beta 3	2.58	0.009
95523_at	1428333_at	6530401D17Rik	RIKEN cDNA 6530401D17 gene	2.56	0.005
92537_g_at	1455918_at	---	---	2.55	0.002
103434_at	1431707_a_at	Pscd3	pleckstrin homology, Sec7 and coiled-coil domains 3	2.54	0.002
96765_at	1433924_at	Peg3	Paternally expressed 3	2.51	0.000
160594_at	1418799_a_at	Col17a1	procollagen, type XVII, alpha 1	2.5	0.005
102925_at	1454737_at	Dusp9	dual specificity phosphatase 9	2.42	0.005
96203_at	1424713_at	Calml4	calmodulin-like 4	2.39	0.001
102195_at	1448050_s_at	---	---	2.37	0.004
97828_at	1418377_a_at	MGI:1353606	Cd27 binding protein (Hindu God of destruction)	2.33	0.001
104267_at	1417329_at	Slc23a2	solute carrier family 23 (nucleobase transporters), member 2	2.3	0.001
92715_at	1419762_at	Ubd	ubiquitin D	2.27	0.006
160158_at	1450990_at	Gpc3	glypican 3	2.19	0.000
103833_at	1448631_a_at	Hipk2	homeodomain interacting protein kinase 2	2.14	0.005
97316_at	1448382_at	Ehhadh	enoyl-Coenzyme A, hydratase/3-hydroxyacyl Coenzyme A dehydrogen	2.14	0.001
93384_at	1421455_at	Sntb1	syntrophin, basic 1	2.12	0.008

93898_at	1419667_at	Sgcb	sarcoglycan, beta (dystrophin-associated glycoprotein)	2.05	0.007
161029_at	1426316_at	6330416G13Rik	RIKEN cDNA 6330416G13 gene	2.02	0.001
162174_at	1419349_a_at	Cyp2d9	cytochrome P450, family 2, subfamily d, polypeptide 9	2.02	0.000
95555_at	1418123_at	Unc119	unc-119 homolog (C. elegans)	2.02	0.000
102974_at	1449498_at	Marco	macrophage receptor with collagenous structure	2	0.000
97352_f_at	1435275_at	Cox6b2	cytochrome c oxidase subunit VIb polypeptide 2	2	0.000
99937_at	1424234_s_at	Meox2	mesenchyme homeobox 2	1.99	0.003
97474_r_at	1448254_at	Ptn	pleiotrophin	1.98	0.000
104165_at	1418486_at	Vnn1	vanin 1	1.94	0.003
160127_at	1420827_a_at	---	---	1.94	0.000
160547_s_at	1415996_at	Txnip	thioredoxin interacting protein	1.92	0.005
96630_at	1415938_at	Spink3	serine protease inhibitor, Kazal type 3	1.92	0.005
100348_at	1456319_at	X83313	EST X83313	1.91	0.000
160544_at	1416022_at	Fabp5	fatty acid binding protein 5, epidermal	1.89	0.000
160670_at	1448147_at	Tnfrsf19	tumor necrosis factor receptor superfamily, member 19	1.88	0.000
94758_s_at	1427767_a_at	Cftr	cystic fibrosis transmembrane conductance regulator homolog	1.88	0.000
100434_s_at	1420713_a_at	Mdfi	MyoD family inhibitor	1.87	0.006
99842_at	1421698_a_at	Col19a1	procollagen, type XIX, alpha 1	1.87	0.001
96818_at	1421720_a_at	Dtx2	deltex 2 homolog (Drosophila)	1.85	0.001
103435_at	1434184_s_at	9430080K19Rik	RIKEN cDNA 9430080K19 gene	1.83	0.001
104011_at	1419435_at	Aox1	aldehyde oxidase 1	1.81	0.004
160489_at	1438855_x_at	---	---	1.81	0.001
104692_at	1420558_at	Selp	selectin, platelet	1.79	0.002
95621_at	1426284_at	Krt20	keratin 20	1.79	0.001
98033_at	1448786_at	1100001H23Rik	RIKEN cDNA 1100001H23 gene	1.79	0.000
104310_at	1428245_at	G6pc3	glucose 6 phosphatase, catalytic, 3	1.78	0.002
99157_at	1427895_at	2310004N24Rik	RIKEN cDNA 2310004N24 gene	1.78	0.000
101975_at	1449939_s_at	Dlk1	delta-like 1 homolog (Drosophila)	1.76	0.005
103005_s_at	1452483_a_at	Cd44	CD44 antigen	1.75	0.004
104688_at	1439323_a_at	Map4k1	mitogen activated protein kinase kinase kinase kinase 1	1.75	0.005
92730_at	1418350_at	Hbegf	heparin-binding EGF-like growth factor	1.75	0.004
99949_at	1424428_at	AI225782	expressed sequence AI225782	1.75	0.005
100050_at	1425895_a_at	Id1	inhibitor of DNA binding 1	1.74	0.003
104156_r_at	1449363_at	Atf3	activating transcription factor 3	1.74	0.002
101190_at	1450352_at	Mtnr1a	melatonin receptor 1A	1.73	0.001
103391_at	1455991_at	Ccbl2	cysteine conjugate-beta lyase 2	1.73	0.000
93287_at	1449836_x_at	Biklk	Bcl2-interacting killer-like	1.72	0.000
93691_s_at	1425457_a_at	Grb10	growth factor receptor bound protein 10	1.72	0.010
102875_at	1434563_at	Rps6kc1	ribosomal protein S6 kinase polypeptide 1	1.71	0.005
103040_at	1416111_at	Cd83	CD83 antigen	1.71	0.005
101116_at	1420109_at	Pip5k2a	Phosphatidylinositol-4-phosphate 5-kinase, type II, alpha	1.7	0.008
98056_at	1449002_at	Phlda3	pleckstrin homology-like domain, family A, member 3	1.7	0.004
160897_at	1451359_at	BC005662	cDNA sequence BC005662	1.69	0.002
94270_at	1448169_at	Krt1-18	keratin complex 1, acidic, gene 18	1.69	0.000
95758_at	1415822_at	Scd2	stearoyl-Coenzyme A desaturase 2	1.69	0.000
160337_at	1415977_at	MGI:1919030	myo-inositol 1-phosphate synthase A1	1.67	0.000
93869_s_at	1419004_s_at	Bcl2a1a /// Bcl2a1b /// Bcl2	B-cell leukemia/lymphoma 2 related protein A1a /// B-cell leukemia/lym	1.67	0.007
95373_at	1418770_at	Cd2	CD2 antigen	1.67	0.008
96085_at	1416368_at	Gsta4	glutathione S-transferase, alpha 4	1.67	0.000
96207_at	1434005_at	Rbms1	RIKEN cDNA 6030432P03 gene	1.67	0.001
98435_at	1449383_at	Adssl1	adenylosuccinate synthetase like 1	1.67	0.007
98495_at	1460361_at	5033414D02Rik	RIKEN cDNA 5033414D02 gene	1.67	0.001
102035_at	1430889_a_at	Tpmt	thiopurine methyltransferase	1.66	0.009

95133_at	1451095_at	Asns	asparagine synthetase	1.66	0.002
102630_s_at	1420353_at	Lta	lymphotoxin A	1.65	0.005
160667_at	1434920_a_at	Evl	Ena-vasodilator stimulated phosphoprotein	1.65	0.000
92262_at	1449353_at	Wig1	wild-type p53-induced gene 1	1.65	0.001
94020_at	1431292_a_at	Ptk9l	protein tyrosine kinase 9-like (A6-related protein)	1.65	0.004
95927_f_at	1439516_at	2610201A13Rik	RIKEN cDNA 2610201A13 gene	1.65	0.000
101173_at	1420983_at	Pctp	phosphatidylcholine transfer protein	1.64	0.007
94231_at	1417419_at	Ccnd1	cyclin D1	1.64	0.008
95738_at	1415836_at	Aldh18a1	aldehyde dehydrogenase 18 family, member A1	1.64	0.007
94057_g_at	1415964_at	Scd1	stearoyl-Coenzyme A desaturase 1	1.63	0.000
96600_at	1441618_at	B130017I01Rik	RIKEN cDNA B130017I01 gene	1.63	0.000
102910_at	1419759_at	Abcb1a	ATP-binding cassette, sub-family B (MDR/TAP), member 1A	1.62	0.000
92934_at	1449126_at	Zfp90	zinc finger protein 90	1.6	0.000
96060_at	1450138_a_at	Serpinb6a	serine (or cysteine) proteinase inhibitor, clade B, member 6a	1.6	0.001
101206_at	1420289_at	T25656	expressed sequence T25656	1.59	0.010
94176_at	1425094_a_at	Lhx6	LIM homeobox protein 6	1.59	0.000
102013_at	1420541_at	Rdh6	retinol dehydrogenase 6	1.58	0.001
103200_at	1460555_at	6330500D04Rik	RIKEN cDNA 6330500D04 gene	1.58	0.001
92310_at	1427005_at	Plk2	polo-like kinase 2 (Drosophila)	1.58	0.000
94289_r_at	1426306_a_at	Maged2	melanoma antigen, family D, 2	1.58	0.000
102831_s_at	1449858_at	---	---	1.57	0.001
160626_at	1425350_a_at	Myef2	myelin basic protein expression factor 2, repressor	1.57	0.000
92348_at	1454675_at	Thra	thyroid hormone receptor alpha	1.57	0.002
95887_at	1448068_at	8430426K15Rik	RIKEN cDNA 8430426K15 gene	1.57	0.003
103671_at	1451814_a_at	Htatip2	HIV-1 tat interactive protein 2, homolog (human)	1.56	0.010
100279_at	1421890_at	St3gal2	ST3 beta-galactoside alpha-2,3-sialyltransferase 2	1.55	0.004
103250_at	1417903_at	Dfna5h	deafness, autosomal dominant 5 homolog (human)	1.55	0.007
103025_at	1416380_at	Mov10	Moloney leukemia virus 10	1.54	0.000
160808_at	1424119_at	Prkab1	protein kinase, AMP-activated, beta 1 non-catalytic subunit	1.54	0.000
93702_at	1427128_at	Ptpn23	protein tyrosine phosphatase, non-receptor type 23	1.54	0.001
99057_at	1423135_at	Thy1	thymus cell antigen 1, theta	1.54	0.001
99669_at	1419573_a_at	Lgals1	lectin, galactose binding, soluble 1	1.54	0.000
101048_at	1422124_a_at	Ptprc	protein tyrosine phosphatase, receptor type, C	1.53	0.008
160101_at	1448239_at	Hmox1	heme oxygenase (decycling) 1	1.53	0.003
98018_at	1420664_s_at	Procr	protein C receptor, endothelial	1.53	0.000
100915_at	1417472_at	Myh9	myosin, heavy polypeptide 9, non-muscle	1.52	0.003
160767_at	1417696_at	Soat1	sterol O-acyltransferase 1	1.52	0.007
93188_at	1417312_at	Dkk3	dickkopf homolog 3 (Xenopus laevis)	1.52	0.003
96041_at	1422660_at	Rbm3	RNA binding motif protein 3	1.51	0.000
100626_at	1426040_a_at	Odf2	outer dense fiber of sperm tails 2	1.5	0.008
100988_at	1435448_at	Bcl2l11	BCL2-like 11 (apoptosis facilitator)	1.5	0.006
104445_at	1429159_at	4631408O11Rik	RIKEN cDNA 4631408O11 gene	1.5	0.002
92941_at	1436187_at	1110054M08Rik	RIKEN cDNA 1110054M08 gene	1.5	0.009
97820_at	1417177_at	Galk1	galactokinase 1	1.5	0.000
97228_at	1451421_a_at	MGI:1913299	leucine zipper domain protein	1.49	0.002
97943_at	1450429_at	Capn6	calpain 6	1.49	0.000
98790_s_at	1450992_a_at	Meis1	myeloid ecotropic viral integration site 1	1.49	0.001
103293_at	1448256_at	Gosr1	golgi SNAP receptor complex member 1	1.48	0.001
93968_at	1450638_at	Pdcd5	programmed cell death 5	1.48	0.000
100611_at	1423547_at	Lyzs	lysozyme	1.47	0.004
101009_at	1435989_x_at	Krt2-8	keratin complex 2, basic, gene 8	1.47	0.000
160334_at	1421993_a_at	2410001H17Rik	RIKEN cDNA 2410001H17 gene	1.47	0.005
160652_at	1448111_at	Ctps2	cytidine 5'-triphosphate synthase 2	1.47	0.003

160762_at	1433477_at	Abr	active BCR-related gene	1.47	0.009
160826_at	1416412_at	Nsmaf	neutral sphingomyelinase (N-SMase) activation associated factor	1.47	0.004
93021_at	1428209_at	LOC406217	RIKEN cDNA 2410004M13 gene	1.47	0.001
94088_at	1423470_at	Ptbp2	polypyrimidine tract binding protein 2	1.47	0.002
96109_at	1448890_at	Klf2	Kruppel-like factor 2 (lung)	1.47	0.006
97489_at	1433504_at	Pygb	brain glycogen phosphorylase	1.47	0.000
97889_at	1418438_at	Fabp2	fatty acid binding protein 2, intestinal	1.47	0.000
101588_at	1415802_at	Slc16a1	solute carrier family 16 (monocarboxylic acid transporters), member 1	1.46	0.004
102333_at	1427226_at	Epn2	epsin 2	1.46	0.001
102370_at	1434642_at	Dhrs8	dehydrogenase/reductase (SDR family) member 8	1.46	0.002
94351_r_at	1423627_at	Nqo1	NAD(P)H dehydrogenase, quinone 1	1.46	0.003
95608_at	1417492_at	Ctsb	cathepsin B	1.46	0.003
100928_at	1423407_a_at	Fbln2	fibulin 2	1.45	0.000
103574_at	1454708_at	Ablim1	actin-binding LIM protein 1	1.45	0.002
97518_at	1448130_at	Fdft1	farnesyl diphosphate farnesyl transferase 1	1.45	0.000
104003_at	1434518_at	Phka2	phosphorylase kinase alpha 2	1.44	0.001
160415_at	1437932_a_at	Cldn1	claudin 1	1.44	0.000
100507_at	1426852_x_at	Nov	nephroblastoma overexpressed gene	1.43	0.002
102957_at	1418641_at	Lcp2	lymphocyte cytosolic protein 2	1.43	0.007
104322_at	1434748_at	Ckap2	cytoskeleton associated protein 2	1.43	0.001
92441_at	1417552_at	Fap	fibroblast activation protein	1.43	0.006
94055_at	1421313_s_at	Cttn	cortactin	1.43	0.010
95643_at	1415770_at	Wdr6	WD repeat domain 6	1.43	0.000
100949_at	1417688_at	BC004044	cDNA sequence BC004044	1.42	0.002
103818_at	1417392_a_at	Slc7a7	solute carrier family 7 (cationic amino acid transporter, y+ system), mer	1.42	0.001
160232_at	1418074_at	St6galnac4	ST6 (alpha-N-acetyl-neuraminyl-2,3-beta-galactosyl-1,3)-N-acetylgalac	1.42	0.009
160789_at	1423695_at	9530090G24Rik	RIKEN cDNA 9530090G24 gene	1.42	0.001
93081_at	1415775_at	Rbbp7	retinoblastoma binding protein 7	1.42	0.001
103016_s_at	1449164_at	Cd68	CD68 antigen	1.41	0.004
92256_at	1417491_at	Ctsb	cathepsin B	1.41	0.001
93454_at	1419589_at	C1qr1	complement component 1, q subcomponent, receptor 1	1.41	0.000
94024_at	1424144_at	Ris2	retroviral integration site 2	1.41	0.002
94278_at	1415983_at	Lcp1	lymphocyte cytosolic protein 1	1.41	0.002
98007_at	1417542_at	Rps6ka2	ribosomal protein S6 kinase, polypeptide 2	1.41	0.002
98555_at	1448361_at	Ttc3	tetratricopeptide repeat domain 3	1.41	0.001
98990_at	1450622_at	Bcar1	breast cancer anti-estrogen resistance 1	1.41	0.004
100523_r_at	1451230_a_at	Wbp5	WW domain binding protein 5	1.4	0.000
104735_at	1434881_s_at	Kctd12	potassium channel tetramerisation domain containing 12	1.4	0.003
160317_at	1416591_at	Rab34	RAB34, member of RAS oncogene family	1.4	0.007
93220_at	1425475_at	Col4a5	procollagen, type IV, alpha 5	1.4	0.006
94493_at	1460569_x_at	Cldn3	claudin 3	1.4	0.000
96056_at	1448605_at	Rhoc	ras homolog gene family, member C	1.4	0.000
96069_at	1417294_at	Akr7a5	aldo-keto reductase family 7, member A5 (aflatoxin aldehyde reductase	1.4	0.002
97887_at	1418069_at	Apoc2	apolipoprotein C-II	1.4	0.000
98589_at	1448318_at	Adfp	adipose differentiation related protein	1.4	0.000
100607_at	1416013_at	Pld3	phospholipase D3	1.39	0.007
93536_at	1416837_at	Bax	Bcl2-associated X protein	1.39	0.000
99416_at	1439283_at	Osbpl9	Oxysterol binding protein-like 9	1.39	0.007
100629_at	1416842_at	Gstm5	glutathione S-transferase, mu 5	1.38	0.000
101437_at	1449336_a_at	Slk	STE20-like kinase (yeast)	1.38	0.010
101850_at	1417635_at	Spa17	sperm autoantigenic protein 17	1.38	0.001
160081_at	1416807_at	Rpl36a	ribosomal protein L36a	1.38	0.004
160612_at	1423570_at	Abcg1	ATP-binding cassette, sub-family G (WHITE), member 1	1.38	0.005

94850_at	1449968_s_at	MGI:1928939 /// MGI:1928	acyl-Coenzyme A thioesterase 2, mitochondrial /// acyl-Coenzyme A th	1.38	0.001
94954_at	1423931_s_at	Anapc4	anaphase promoting complex subunit 4	1.38	0.001
95471_at	1417649_at	Cdkn1c	cyclin-dependent kinase inhibitor 1C (P57)	1.38	0.001
95674_r_at	1428572_at	Basp1	brain abundant, membrane attached signal protein 1	1.38	0.000
98351_g_at	1422256_at	Sstr2	somatostatin receptor 2	1.38	0.001
103557_at	1426227_s_at	5730409F24Rik	RIKEN cDNA 5730409F24 gene	1.37	0.004
104280_at	1417788_at	Sncg	synuclein, gamma	1.37	0.000
92368_at	1420401_a_at	Ramp3	receptor (calcitonin) activity modifying protein 3	1.37	0.001
94792_at	1448061_at	Msr1	Macrophage scavenger receptor 1	1.37	0.001
97915_at	1460341_at	Plekhb2	pleckstrin homology domain containing, family B (evectins) member 2	1.37	0.002
103896_f_at	1418918_at	Igfbp1	insulin-like growth factor binding protein 1	1.36	0.006
161083_at	1455733_at	A130052D22	RIKEN cDNA A430105I05 gene	1.36	0.007
92280_at	1427385_s_at	Actn1	actinin, alpha 1	1.36	0.000
92555_at	1448501_at	Tspan6	tetraspanin 6	1.36	0.002
93330_at	1416203_at	Aqp1	aquaporin 1	1.36	0.006
97918_at	1427243_at	AA536743	expressed sequence AA536743	1.36	0.001
100059_at	1454268_a_at	Cyba	cytochrome b-245, alpha polypeptide	1.35	0.002
103817_at	1448592_at	Crtap	cartilage associated protein	1.35	0.000
104471_at	1448928_at	Hdac6	histone deacetylase 6	1.35	0.003
160506_at	1423959_at	Ropn1l	ropporin 1-like	1.35	0.001
92230_at	1424981_at	Nln	neurolysin (metallopeptidase M3 family)	1.35	0.007
95531_at	1454890_at	Amot	angiomotin	1.35	0.004
97203_at	1415922_s_at	Mlp	MARCKS-like protein	1.35	0.000
92608_at	1425811_a_at	Csrp1	cysteine and glycine-rich protein 1	1.34	0.000
92632_at	1426710_at	Calm3	calmodulin 3	1.34	0.007
94504_at	1450410_a_at	4930570C03Rik	RIKEN cDNA 4930570C03 gene	1.34	0.010
94733_at	1449818_at	Abcb4	ATP-binding cassette, sub-family B (MDR/TAP), member 4	1.34	0.003
98594_at	1433581_at	1190002N15Rik	RIKEN cDNA 1190002N15 gene	1.34	0.003
98600_at	1460351_at	S100a11	S100 calcium binding protein A11 (calizzarin)	1.34	0.001
93780_at	1417316_at	Them2	thioesterase superfamily member 2	1.33	0.001
95693_at	1450048_a_at	Idh2	isocitrate dehydrogenase 2 (NADP+), mitochondrial	1.33	0.002
96936_at	1415670_at	Copg	coatomer protein complex, subunit gamma	1.33	0.010
97123_at	1449854_at	Nr0b2	nuclear receptor subfamily 0, group B, member 2	1.33	0.001
100023_at	1417656_at	Mybl2	myeloblastosis oncogene-like 2	1.32	0.010
102374_at	1434027_at	Dscr1l2	Down syndrome critical region gene 1-like 2	1.32	0.001
103095_at	1448227_at	Grb7	growth factor receptor bound protein 7	1.32	0.008
93674_at	1416865_at	Fgd1	FYVE, RhoGEF and PH domain containing 1	1.32	0.002
95506_at	1419649_s_at	Myo1c	myosin IC	1.32	0.004
100511_at	1428242_at	6330406L22Rik	RIKEN cDNA 6330406L22 gene	1.31	0.004
92567_at	1422437_at	Col5a2	procollagen, type V, alpha 2	1.31	0.002
93733_r_at	1422763_at	Rgs19ip1	regulator of G-protein signaling 19 interacting protein 1	1.31	0.001
94872_at	1416635_at	Smpdl3a	sphingomyelin phosphodiesterase, acid-like 3A	1.31	0.002
95703_at	1416443_a_at	Uble1a	ubiquitin-like 1 (sentrin) activating enzyme E1A	1.31	0.003
96747_at	1449027_at	Rhou	ras homolog gene family, member U	1.31	0.005
96774_at	1434180_at	Plekhc1	pleckstrin homology domain containing, family C (with FERM domain) r	1.31	0.002
99559_at	1415776_at	---	---	1.31	0.009
100958_at	1454656_at	Spata13	spermatogenesis associated 13	1.3	0.006
100974_at	1449511_a_at	Ssbp4	single stranded DNA binding protein 4	1.3	0.002
104222_f_at	1419805_s_at	Ggps1	geranylgeranyl diphosphate synthase 1	1.3	0.001
94440_at	1435007_s_at	AI132487	expressed sequence AI132487	1.3	0.005
95597_at	1436448_a_at	Ptgs1	prostaglandin-endoperoxide synthase 1	1.3	0.001
99009_at	1416105_at	Nnt	nicotinamide nucleotide transhydrogenase	1.3	0.003
99962_at	1450053_at	Kif2a	RIKEN cDNA D930043N17 gene	1.3	0.003

160107_at	1448736_a_at	Hprt1	hypoxanthine guanine phosphoribosyl transferase 1	1.29	0.003
160657_at	1460669_at	Ilf3	interleukin enhancer binding factor 3	1.29	0.000
160807_at	1450504_a_at	Agpat3	1-acylglycerol-3-phosphate O-acyltransferase 3	1.29	0.001
93288_at	1437148_at	Arpc2	actin related protein 2/3 complex, subunit 2	1.29	0.001
93306_at	1450740_a_at	Mapre1	microtubule-associated protein, RP/EB family, member 1	1.29	0.001
93465_at	1453076_at	9130211I03Rik	RIKEN cDNA 9130211I03 gene	1.29	0.001
94712_at	1419417_at	Vegfc	vascular endothelial growth factor C	1.29	0.002
95090_at	1455752_a_at	2410001H17Rik	RIKEN cDNA 2410001H17 gene	1.29	0.002
95514_at	1426897_at	2610510H01Rik	RIKEN cDNA 2610510H01 gene	1.29	0.002
96075_at	1450851_at	Wdr1	WD repeat domain 1	1.29	0.002
97444_at	1422476_at	Ifi30	interferon gamma inducible protein 30	1.29	0.002
98434_at	1449066_a_at	Arhgef7	Rho guanine nucleotide exchange factor (GEF7)	1.29	0.002
98580_at	1415678_at	Ppm1a	protein phosphatase 1A, magnesium dependent, alpha isoform	1.29	0.009
103816_at	1424595_at	F11r	F11 receptor	1.28	0.005
160757_at	1428101_at	Rnf38	ring finger protein 38	1.28	0.005
160869_at	1417892_a_at	Sirt3	sirtuin 3 (silent mating type information regulation 2, homolog) 3 (S. cer	1.28	0.000
92216_at	1423389_at	Smad7	MAD homolog 7 (Drosophila)	1.28	0.002
92365_at	1449528_at	Figf	c-fos induced growth factor	1.28	0.007
93013_at	1435176_a_at	Id2	inhibitor of DNA binding 2	1.28	0.000
94359_at	1417965_at	Plekha1	pleckstrin homology domain containing, family A (phosphoinositide binc	1.28	0.000
96020_at	1417063_at	C1qb	complement component 1, q subcomponent, beta polypeptide	1.28	0.010
97818_at	1460182_at	Snx4	sorting nexin 4	1.28	0.001
99335_at	1423855_x_at	Rpl17	ribosomal protein L17	1.28	0.000
103216_f_at	1454690_at	Ikbkg	inhibitor of kappaB kinase gamma	1.27	0.006
160245_at	1453013_at	1110034O07Rik	RIKEN cDNA 1110034O07 gene	1.27	0.009
94054_at	1433908_a_at	Cttn	cortactin	1.27	0.003
94061_at	1416326_at	Crip1	cysteine-rich protein 1 (intestinal)	1.27	0.004
96284_at	1423370_a_at	Csnk1g2	casein kinase 1, gamma 2	1.27	0.000
96358_at	1460175_at	Rps23	ribosomal protein S23	1.27	0.001
96360_at	1451168_a_at	Arhgdia	Rho GDP dissociation inhibitor (GDI) alpha	1.27	0.006
97274_at	1421751_a_at	Psmd14	proteasome (prosome, macropain) 26S subunit, non-ATPase, 14	1.27	0.009
97527_at	1417458_s_at	Cks2	CDC28 protein kinase regulatory subunit 2	1.27	0.002
99168_at	1433429_at	Pigs	phosphatidylinositol glycan, class S	1.27	0.008
101778_at	1422192_at	Gja5	gap junction membrane channel protein alpha 5	1.26	0.001
102065_at	1418243_at	Fcna	ficolin A	1.26	0.002
102217_at	1449514_at	Gprk5	G protein-coupled receptor kinase 5	1.26	0.006
160171_f_at	1418073_at	MGI:1928939	acyl-Coenzyme A thioesterase 2, mitochondrial	1.26	0.000
92607_at	1423294_at	Mest	mesoderm specific transcript	1.26	0.005
93271_s_at	1450186_s_at	Gnas	GNAS (guanine nucleotide binding protein, alpha stimulating) complex	1.26	0.006
93753_at	1416303_at	Litaf	LPS-induced TN factor	1.26	0.007
94535_at	1450054_at	---	---	1.26	0.001
96122_at	1451322_at	2310016A09Rik	RIKEN cDNA 2310016A09 gene	1.26	0.006
97106_at	1419208_at	Map3k8	mitogen activated protein kinase kinase kinase 8	1.26	0.001
97456_at	1428082_at	Acsl5	acyl-CoA synthetase long-chain family member 5	1.26	0.000
99340_at	1455168_a_at	Gnb2-rs1	guanine nucleotide binding protein, beta 2, related sequence 1	1.26	0.000
99491_at	1419455_at	Il10rb	interleukin 10 receptor, beta	1.26	0.008
100425_at	1418261_at	Syk	spleen tyrosine kinase	1.25	0.005
102331_at	1428372_at	St5	suppression of tumorigenicity 5	1.25	0.001
104014_at	1422645_at	Hfe	hemochromatosis	1.25	0.001
160917_r_at	1422498_at	Mageh1	melanoma antigen, family H, 1	1.25	0.002
161009_at	1434436_at	Zcwcc2	zinc finger, CW-type with coiled-coil domain 2	1.25	0.004
93071_at	1415869_a_at	Trim28	tripartite motif protein 28	1.25	0.001
95034_f_at	1436420_a_at	Ipo4	importin 4	1.25	0.009

96172_at	1424375_s_at	Gimap4	GTPase, IMAP family member 4	1.25	0.005
96426_at	1415906_at	Tmsb4x	thymosin, beta 4, X chromosome	1.25	0.001
101137_at	1435151_a_at	Rps3	ribosomal protein S3	1.24	0.000
102344_s_at	1424531_a_at	Tcea3	transcription elongation factor A (SII), 3	1.24	0.002
103027_at	1433867_at	1810030O07Rik	RIKEN cDNA 1810030O07 gene	1.24	0.000
104750_at	1417292_at	Ifi47	interferon gamma inducible protein 47	1.24	0.002
93051_at	1448499_a_at	Ephx2	epoxide hydrolase 2, cytoplasmic	1.24	0.004
95675_at	1426759_at	Map4k3	mitogen-activated protein kinase kinase kinase kinase 3	1.24	0.004
99649_at	1455959_s_at	D9Wsu168e	DNA segment, Chr 9, Wayne State University 168, expressed	1.24	0.005
100893_at	1460243_at	Sptlc2	serine palmitoyltransferase, long chain base subunit 2	1.23	0.006
102321_at	1418128_at	Adcy6	adenylate cyclase 6	1.23	0.008
93999_at	1448357_at	Snrpg	small nuclear ribonucleoprotein polypeptide G	1.23	0.007
94442_s_at	1418396_at	Gpsm3	G-protein signalling modulator 3 (AGS3-like, C. elegans)	1.23	0.008
97412_at	1428004_at	3300001G02Rik	RIKEN cDNA 3300001G02 gene	1.23	0.007
99119_at	1448346_at	Cfl1	cofilin 1, non-muscle	1.23	0.002
101393_at	1460330_at	Anxa3	annexin A3	1.22	0.001
103584_at	1453015_at	5830471E12Rik	RIKEN cDNA 5830471E12 gene	1.22	0.008
160078_at	1450914_at	Ppp1r14b	protein phosphatase 1, regulatory (inhibitor) subunit 14B	1.22	0.000
160389_r_at	1450925_a_at	Rps27l	ribosomal protein S27-like	1.22	0.000
160682_at	1433685_a_at	6430706D22Rik	RIKEN cDNA 6430706D22 gene	1.22	0.003
160814_at	1424052_at	Thap4	THAP domain containing 4	1.22	0.009
94079_at	1448729_a_at	4-Sep	septin 4	1.22	0.008
96894_at	1448422_at	Tmed4	transmembrane emp24 protein transport domain containing 4	1.22	0.005
96913_at	1426522_at	Hadhb	hydroxyacyl-Coenzyme A dehydrogenase/3-ketoacyl-Coenzyme A thio	1.22	0.001
98333_at	1448739_x_at	Rps18	ribosomal protein S18	1.22	0.001
100953_at	1417586_at	Timeless	timeless homolog (Drosophila)	1.21	0.004
160538_at	1422441_x_at	Cdk4	cyclin-dependent kinase 4	1.21	0.003
160876_at	1417077_at	Bcap29	B-cell receptor-associated protein 29	1.21	0.000
92192_s_at	1451406_a_at	Pcsk5	proprotein convertase subtilisin/kexin type 5	1.21	0.003
94853_at	1417432_a_at	Gnb1	guanine nucleotide binding protein, beta 1	1.21	0.005
96865_at	1415973_at	Marcks	Myristoylated alanine rich protein kinase C substrate	1.21	0.001
96911_at	1450623_at	Gnb2	guanine nucleotide binding protein, beta 2	1.21	0.000
97385_at	1419509_a_at	Nagk	N-acetylglucosamine kinase	1.21	0.001
98550_at	1426853_at	Set	SET translocation	1.21	0.003
160318_at	1416580_a_at	Stub1	STIP1 homology and U-Box containing protein 1	1.2	0.006
160602_at	1416843_at	Pde6d	phosphodiesterase 6D, cGMP-specific, rod, delta	1.2	0.002
160711_at	1419367_at	Decr1	2,4-dienoyl CoA reductase 1, mitochondrial	1.2	0.006
92578_at	1416486_at	Scye1	small inducible cytokine subfamily E, member 1	1.2	0.007
93626_at	1422906_at	Abcg2	ATP-binding cassette, sub-family G (WHITE), member 2	1.2	0.000
95593_at	1415698_at	Golph2	golgi phosphoprotein 2	1.2	0.006
99583_at	1449575_a_at	Gstp1	glutathione S-transferase, pi 1	1.2	0.007
95646_at	1416772_at	Cpt2	carnitine palmitoyltransferase 2	1.19	0.008
98038_at	1416155_at	Hmgb3	high mobility group box 3	1.19	0.010
98571_s_at	1416669_s_at	Naca	nascent polypeptide-associated complex alpha polypeptide	1.19	0.001
100561_at	1417380_at	Iqgap1	IQ motif containing GTPase activating protein 1	1.18	0.009
101213_at	1419441_at	Arbp	acidic ribosomal phosphoprotein P0	1.18	0.001
103070_at	1416985_at	---	---	1.18	0.003
104328_at	1424011_at	Aqp9	aquaporin 9	1.18	0.002
93588_at	1419462_s_at	Gtl3	gene trap locus 3	1.18	0.006
93789_s_at	1424355_a_at	Sin3b	transcriptional regulator, SIN3B (yeast)	1.18	0.001
96666_at	1423538_at	Ntan1	N-terminal Asn amidase	1.18	0.005
97360_at	1448316_at	Cklfsf3	chemokine-like factor super family 3	1.18	0.000
97803_at	1450919_at	Mpp1	membrane protein, palmitoylated	1.18	0.002

98168_at	1422613_a_at	Rpl7a	ribosomal protein L7a	1.18	0.000
98335_at	1418342_at	Recc1	replication factor C 1	1.18	0.010
99179_at	1431145_a_at	Cuedc2	CUE domain containing 2	1.18	0.005
100732_at	1436760_a_at	Rps8	ribosomal protein S8	1.17	0.002
102644_at	1427040_at	Mdfic	MyoD family inhibitor domain containing	1.17	0.003
103007_at	1416895_at	Efna1	ephrin A1	1.17	0.001
103452_at	1432099_a_at	Prodh2	proline dehydrogenase (oxidase) 2	1.17	0.002
103507_at	1451161_a_at	Emr1	EGF-like module containing, mucin-like, hormone receptor-like sequen	1.17	0.002
94789_r_at	1416256_a_at	Tubb5	tubulin, beta 5	1.17	0.001
96696_at	1452787_a_at	Hrmt1l2	heterogeneous nuclear ribonucleoproteins methyltransferase-like 2 (S.	1.17	0.005
96962_at	1416546_a_at	Rpl6	ribosomal protein L6	1.17	0.000
103717_at	1448146_at	Wwp2	WW domain containing E3 ubiquitin protein ligase 2	1.16	0.009
104579_r_at	1428585_at	Actn1	actinin, alpha 1	1.16	0.007
160461_f_at	1416431_at	Tubb6	tubulin, beta 6	1.16	0.007
160477_at	1424085_at	Ndufa4	NADH dehydrogenase (ubiquinone) 1 alpha subcomplex, 4	1.16	0.004
93830_at	1448103_s_at	Nono	non-POU-domain-containing, octamer binding protein	1.16	0.000
96526_at	1437885_at	D030029J20Rik	RIKEN cDNA D030029J20 gene	1.16	0.003
96940_at	1448519_at	Tead2	TEA domain family member 2	1.16	0.004
97450_s_at	1460167_at	Aldh7a1	aldehyde dehydrogenase family 7, member A1	1.16	0.001
98993_at	1425542_a_at	Ppp2r5c	protein phosphatase 2, regulatory subunit B (B56), gamma isoform	1.16	0.001
99093_at	1456497_x_at	Rps10	ribosomal protein S10	1.16	0.005
99176_at	1422516_a_at	Fibp	fibroblast growth factor (acidic) intracellular binding protein	1.16	0.008
99188_at	1451130_at	2010315L10Rik	RIKEN cDNA 2010315L10 gene	1.16	0.003
99651_at	1460697_s_at	2610209M04Rik	RIKEN cDNA 2610209M04 gene	1.16	0.005
100711_at	1431177_a_at	Rpl10a	ribosomal protein L10A	1.15	0.010
100734_at	1449323_a_at	Rpl3	ribosomal protein L3	1.15	0.008
101104_at	1415745_a_at	Dscr3	Down syndrome critical region gene 3	1.15	0.002
101984_at	1460639_a_at	Atox1	ATX1 (antioxidant protein 1) homolog 1 (yeast)	1.15	0.000
104716_at	1448754_at	Rbp1	retinol binding protein 1, cellular	1.15	0.002
160225_at	1451135_at	Gtf2b	general transcription factor IIB	1.15	0.002
160371_at	1423818_a_at	Arl6ip1	ADP-ribosylation factor-like 6 interacting protein 1	1.15	0.000
93362_at	1450894_a_at	Ap2m1	adaptor protein complex AP-2, mu1	1.15	0.000
93754_at	1448491_at	Ech1	enoyl coenzyme A hydratase 1, peroxisomal	1.15	0.008
94377_at	1448942_at	Gng11	guanine nucleotide binding protein (G protein), gamma 11	1.15	0.007
94397_at	1426700_a_at	Usp52	ubiquitin specific protease 52	1.15	0.009
97318_at	1451040_at	Hars2	histidyl tRNA synthetase 2	1.15	0.004
97538_at	1448124_at	Gusb	glucuronidase, beta	1.15	0.009
100535_at	1428362_at	E130105L11Rik	RIKEN cDNA E130105L11 gene	1.14	0.000
101992_at	1448822_at	Psmb6	proteasome (prosome, macropain) subunit, beta type 6	1.14	0.008
103299_at	1433678_at	AI132321	expressed sequence AI132321	1.14	0.009
103402_at	1452664_a_at	Tm7sf3	transmembrane 7 superfamily member 3	1.14	0.007
104708_at	1455042_at	Tbl1x	Transducin (beta)-like 1 X-linked	1.14	0.006
160082_s_at	1423052_at	Arf4	ADP-ribosylation factor 4	1.14	0.000
93840_at	1419096_at	Apom	apolipoprotein M	1.14	0.001
95308_at	1418098_at	Adcy4	adenylate cyclase 4	1.14	0.002
96082_at	1423857_at	Mrpl30	mitochondrial ribosomal protein L30	1.14	0.002
96831_at	1424650_at	Pdia5	protein disulfide isomerase-associated 5	1.14	0.006
100213_f_at	1455578_x_at	Rpl41	ribosomal protein L41	1.13	0.007
100332_s_at	1423223_a_at	Prdx6	peroxiredoxin 6	1.13	0.002
103205_at	1420635_a_at	Tcirg1	T-cell, immune regulator 1	1.13	0.001
104300_at	1434998_at	Iqgap1	IQ motif containing GTPase activating protein 1	1.13	0.004
104756_at	1435431_at	2310047M15Rik	RIKEN cDNA 2310047M15 gene	1.13	0.002
93333_at	1417652_a_at	Tbca	tubulin cofactor a	1.13	0.007

93787_f_at	1448373_at	Mrpl18	mitochondrial ribosomal protein L18	1.13	0.006
94240_i_at	1455348_x_at	Rpl29 /// LOC240367 /// LC	ribosomal protein L29 /// similar to ribosomal protein /// similar to riboso	1.13	0.004
94537_at	1428608_at	Mylc2b	myosin light chain, regulatory B	1.13	0.002
94843_at	1427885_at	Pold4	polymerase (DNA-directed), delta 4	1.13	0.009
95715_at	1452674_a_at	Eif3s12	eukaryotic translation initiation factor 3, subunit 12	1.13	0.001
96899_at	1423737_at	Ndufs3	NADH dehydrogenase (ubiquinone) Fe-S protein 3	1.13	0.000
100120_at	1416808_at	Nid1	nidogen 1	1.12	0.002
103882_at	1417745_at	Cpn1	carboxypeptidase N, polypeptide 1	1.12	0.001
160162_at	1426529_a_at	Tagln2	transgelin 2	1.12	0.004
160308_at	1421814_at	---	---	1.12	0.004
160696_at	1416812_at	Tia1	cytotoxic granule-associated RNA binding protein 1	1.12	0.010
93293_at	1423807_a_at	Calm2	calmodulin 2	1.12	0.001
96052_at	1450721_at	Acp1	acid phosphatase 1, soluble	1.12	0.008
98521_at	1433916_at	Vamp3	vesicle-associated membrane protein 3	1.12	0.003
100729_at	1448109_a_at	Rpl26	ribosomal protein L26	1.11	0.008
102961_at	1449242_s_at	Hrg	histidine-rich glycoprotein	1.11	0.010
93270_at	1423784_at	Gars	glycyl-tRNA synthetase	1.11	0.002
93605_r_at	1417376_a_at	Igsf4a	immunoglobulin superfamily, member 4A	1.11	0.010
93839_at	1418101_a_at	Rtn3	reticulon 3	1.11	0.001
160140_at	1428282_at	Tbce	tubulin-specific chaperone e	1.1	0.000
92872_at	1448106_at	1200016B17Rik	RIKEN cDNA 1200016B17 gene	1.1	0.004
94834_at	1418365_at	Ctsh	cathepsin H	1.1	0.002
95420_at	1423706_a_at	---	---	1.1	0.005
96072_at	1419737_a_at	Ldh1	lactate dehydrogenase 1, A chain	1.1	0.006
98075_at	1416763_at	G431001I09Rik	RIKEN cDNA G431001I09 gene	1.1	0.005
99128_at	1416278_a_at	Atp5o	ATP synthase, H+ transporting, mitochondrial F1 complex, O subunit	1.1	0.001
160844_at	1450660_at	Pts	6-pyruvoyl-tetrahydropterin synthase	1.09	0.007
92544_f_at	1448442_a_at	Psma3	proteasome (prosome, macropain) subunit, alpha type 3	1.09	0.007
94823_at	1426660_x_at	Rpl23a	ribosomal protein L23a	1.09	0.010
96641_at	1433478_at	Psarl	presenilin associated, rhomboid-like	1.09	0.007
101834_at	1427060_at	Mapk3	mitogen activated protein kinase 3	1.08	0.001
93496_at	1415840_at	Elovl5	ELOVL family member 5, elongation of long chain fatty acids (yeast)	1.08	0.002
93540_at	1448333_at	Adprh	ADP-ribosylarginine hydrolase	1.08	0.010
104313_at	1451149_at	Pgm2	phosphoglucomutase 2	1.07	0.003
92854_at	1449256_a_at	Rab11a	RAB11a, member RAS oncogene family	1.07	0.010
98113_at	1448166_a_at	Psmb1	proteasome (prosome, macropain) subunit, beta type 1	1.07	0.003
98342_at	1448157_s_at	Rpl10	ribosomal protein 10	1.07	0.001
93028_at	1448194_a_at	---	---	1.06	0.004
100589_at	1433470_a_at	Immt	inner membrane protein, mitochondrial	1.05	0.003
93603_at	1448849_at	Mrpl40	mitochondrial ribosomal protein L40	1.05	0.005
95097_at	1417157_at	Actr10	ARP10 actin-related protein 10 homolog (S. cerevisiae)	1.05	0.005
96047_at	1426225_at	Rbp4	retinol binding protein 4, plasma	1.05	0.003
99512_at	1434251_at	Cnot1	CCR4-NOT transcription complex, subunit 1	1.05	0.001
92863_at	1448121_at	Wbp2	WW domain binding protein 2	1.04	0.006
93281_at	1422449_s_at	Rcn2	reticulocalbin 2	1.04	0.007
103707_at	1419483_at	C3ar1	complement component 3a receptor 1	1.03	0.002
103807_at	1418202_a_at	Wiz	widely-interspaced zinc finger motifs	-1.01	0.003
160402_at	1428263_a_at	Tceb2	transcription elongation factor B (SIII), polypeptide 2	-1.01	0.002
93585_at	1417651_at	Cyp2c29	cytochrome P450, family 2, subfamily c, polypeptide 29	-1.02	0.002
101389_at	1460235_at	Scarb2	scavenger receptor class B, member 2	-1.04	0.008
98480_s_at	1448975_s_at	Ren1 /// Ren2	renin 1 structural /// renin 2 tandem duplication of Ren1	-1.04	0.005
94794_at	1448771_a_at	Fth1	ferritin heavy chain 1	-1.05	0.002
93373_at	1417706_at	Naglu	alpha-N-acetylglucosaminidase (Sanfilippo disease IIIB)	-1.06	0.007

94400_at	1428501_at	1110051M20Rik	RIKEN cDNA 1110051M20 gene	-1.06	0.002
101058_at	1417765_a_at	Amy1	amylase 1, salivary	-1.07	0.005
101123_at	1417999_at	Itm2b	integral membrane protein 2B	-1.07	0.007
104072_at	1419059_at	Apcs	serum amyloid P-component	-1.07	0.003
160088_at	1450332_s_at	Fmo5	flavin containing monooxygenase 5	-1.07	0.004
94318_at	1416677_at	Apoh	apolipoprotein H	-1.07	0.005
97446_at	1416140_a_at	Dhx30	DEAH (Asp-Glu-Ala-His) box polypeptide 30	-1.07	0.004
99610_at	1419360_a_at	Ss18	synovial sarcoma translocation, Chromosome 18	-1.07	0.006
104181_at	1420723_at	Vnn3	vanin 3	-1.08	0.002
104318_at	1426770_at	Pex5	peroxisome biogenesis factor 5	-1.08	0.006
160355_at	1427099_at	Maz	MYC-associated zinc finger protein (purine-binding transcription factor)	-1.08	0.005
97998_at	1421149_a_at	Drpla	dentatorubral pallidoluysian atrophy	-1.08	0.002
100634_at	1448723_at	Rdh7	retinol dehydrogenase 7	-1.09	0.001
102720_at	1418788_at	Tek	endothelial-specific receptor tyrosine kinase	-1.09	0.008
92879_at	1416792_at	Ppm1g	protein phosphatase 1G (formerly 2C), magnesium-dependent, gamma	-1.09	0.001
93278_at	1449686_s_at	Scp2	sterol carrier protein 2, liver	-1.09	0.002
96864_at	1433878_at	Mrps26	mitochondrial ribosomal protein S26	-1.09	0.006
99587_at	1415734_at	Rab7	RAB7, member RAS oncogene family	-1.09	0.007
104110_at	1451197_s_at	Gatad2a	GATA zinc finger domain containing 2A	-1.1	0.005
160661_at	1454621_s_at	5730472N09Rik	RIKEN cDNA 5730472N09 gene	-1.1	0.009
94049_at	1450624_at	Bhmt	betaine-homocysteine methyltransferase	-1.1	0.002
95978_at	1442145_at	Gm542	gene model 542, (NCBI)	-1.1	0.005
96158_at	1451125_at	BC017133	cDNA sequence BC017133	-1.1	0.003
97367_at	1449019_at	Akap1	A kinase (PRKA) anchor protein 1	-1.1	0.001
98908_at	1451073_at	Sppl3	Signal peptide peptidase 3	-1.1	0.005
98921_at	1451134_a_at	2410018G23Rik	RIKEN cDNA 2410018G23 gene	-1.1	0.002
100579_s_at	1434540_a_at	Clta	clathrin, light polypeptide (Lca)	-1.11	0.005
102002_at	1450021_at	Ubqln2	ubiquilin 2	-1.11	0.009
103667_at	1434513_at	Gm542	gene model 542, (NCBI)	-1.11	0.009
104121_at	1426873_s_at	Jup	junction plakoglobin	-1.11	0.006
94014_at	1416979_at	2510048O06Rik	RIKEN cDNA 2510048O06 gene	-1.11	0.006
96628_at	1452157_at	Eprs	glutamyl-prolyl-tRNA synthetase	-1.11	0.008
96898_at	1433562_s_at	Atp5f1	ATP synthase, H+ transporting, mitochondrial F0 complex, subunit b, i	-1.11	0.000
97279_at	1435967_s_at	Hibadh	3-hydroxyisobutyrate dehydrogenase	-1.11	0.000
99990_at	1426487_a_at	Rbbp6	retinoblastoma binding protein 6	-1.11	0.003
101013_at	1436292_a_at	Oaz1	ornithine decarboxylase antizyme	-1.12	0.002
101025_f_at	1439016_x_at	Sprr2a	small proline-rich protein 2A	-1.12	0.010
102271_at	1460739_at	D11Bwg0280e	DNA segment, Chr 11, Brigham & Women's Genetics 0280e expressed	-1.12	0.004
102317_at	1422895_at	Vamp4	vesicle-associated membrane protein 4	-1.12	0.001
104271_at	1418554_at	Admr	adrenomedullin receptor	-1.12	0.000
160742_at	1415901_at	Plod3	procollagen-lysine, 2-oxoglutarate 5-dioxygenase 3	-1.12	0.005
161864_f_at	1441866_s_at	Ptdss1	phosphatidylserine synthase 1	-1.12	0.002
96956_at	1416381_a_at	Prdx5	peroxiredoxin 5	-1.12	0.005
97451_at	1424024_at	Mcfd2	multiple coagulation factor deficiency 2	-1.12	0.004
98118_at	1422241_a_at	Ndufa1	NADH dehydrogenase (ubiquinone) 1 alpha subcomplex, 1	-1.12	0.005
98538_at	1426820_at	2610507B11Rik	RIKEN cDNA 2610507B11 gene	-1.12	0.008
104364_at	1417016_at	Mapkapk5	MAP kinase-activated protein kinase 5	-1.13	0.004
104629_at	1417361_at	Asb3	ankyrin repeat and SOCS box-containing protein 3	-1.13	0.007
104741_at	1454787_at	Zdhhc9	zinc finger, DHHC domain containing 9	-1.13	0.009
95445_at	1448100_at	4833439L19Rik	RIKEN cDNA 4833439L19 gene	-1.13	0.002
95902_at	1440964_s_at	AI465270	expressed sequence AI465270	-1.13	0.003
96157_at	1429615_at	Zfp91	zinc finger protein 91	-1.13	0.002
98912_at	1434597_at	D13Wsu64e	DNA segment, Chr 13, Wayne State University 64, expressed	-1.13	0.003

99469_at	1451226_at	Pex6	peroxisomal biogenesis factor 6	-1.13	0.002
100482_at	1460691_at	Zfp598	zinc finger protein 598	-1.14	0.003
101572_f_at	1420553_x_at	Serpina1a	serine (or cysteine) proteinase inhibitor, clade A, member 1a	-1.14	0.005
103378_at	1448572_at	Prlpa	prolactin-like protein A	-1.14	0.001
160885_at	1433519_at	8430423A01Rik	RIKEN cDNA 8430423A01 gene	-1.14	0.005
93601_at	1450746_at	Keap1	kelch-like ECH-associated protein 1	-1.14	0.007
96048_at	1428326_s_at	Hrsp12	heat-responsive protein 12	-1.14	0.000
96757_at	1449000_at	D10Jhu81e	DNA segment, Chr 10, Johns Hopkins University 81 expressed	-1.14	0.003
100151_at	1455550_x_at	---	---	-1.15	0.008
101887_at	1423396_at	Agt	angiotensinogen	-1.15	0.007
103490_at	1450772_at	Wnt11	wingless-related MMTV integration site 11	-1.15	0.000
103739_at	1428374_at	MGI:2136405	glucuronyl C5-epimerase	-1.15	0.006
103893_at	1452255_at	Fbxo38	F-box protein 38	-1.15	0.001
104126_at	1415920_at	Cstf2t	cleavage stimulation factor, 3' pre-RNA subunit 2, tau	-1.15	0.000
104151_at	1451522_s_at	Lrch4	leucine-rich repeats and calponin homology (CH) domain containing 4	-1.15	0.004
104296_at	1434551_at	1110031M08Rik /// LOC38	RIKEN cDNA 1110031M08 gene /// similar to hypothetical protein	-1.15	0.010
160198_at	1433442_at	Klhl9	Kelch-like 9 (Drosophila)	-1.15	0.007
160423_at	1420846_at	Mrps2	mitochondrial ribosomal protein S2	-1.15	0.000
160693_at	1416387_at	---	---	-1.15	0.002
94886_at	1415692_s_at	Canx	calnexin	-1.15	0.009
96919_at	1435732_x_at	Atp6v0c	ATPase, H+ transporting, V0 subunit C	-1.15	0.008
98022_at	1417180_at	Pcsk7	proprotein convertase subtilisin/kexin type 7	-1.15	0.007
101367_at	1435414_s_at	---	---	-1.16	0.006
102683_at	1460654_at	---	---	-1.16	0.005
103222_at	1422824_s_at	Eps8	epidermal growth factor receptor pathway substrate 8	-1.16	0.004
103578_at	1431188_a_at	Tom1	target of myb1 homolog (chicken)	-1.16	0.001
103900_at	1423910_at	Centg3	centaurin, gamma 3	-1.16	0.003
103947_at	1424347_at	Ppp6c	protein phosphatase 6, catalytic subunit	-1.16	0.001
94003_at	1433676_at	Prkwnk1	protein kinase, lysine deficient 1	-1.16	0.006
94852_at	1426236_a_at	Glul	glutamate-ammonia ligase (glutamine synthase)	-1.16	0.006
95359_at	1416364_at	Hspcb	heat shock protein 1, beta	-1.16	0.007
95437_at	1426912_at	Rfwd2	ring finger and WD repeat domain 2	-1.16	0.001
95723_r_at	1428708_x_at	2610009E16Rik	RIKEN cDNA 2610009E16 gene	-1.16	0.005
96019_at	1422881_s_at	Sypl	synaptophysin-like protein	-1.16	0.002
96073_at	1416534_at	Dpf2	D4, zinc and double PHD fingers family 2	-1.16	0.001
96098_at	1422819_at	Mrpl36	mitochondrial ribosomal protein L36	-1.16	0.000
98147_at	1448311_at	Usp5	ubiquitin specific protease 5 (isopeptidase T)	-1.16	0.002
100088_at	1433540_x_at	Ppp1cb	protein phosphatase 1, catalytic subunit, beta isoform	-1.17	0.001
101866_at	1425507_at	Arfrp1	ADP-ribosylation factor related protein 1	-1.17	0.004
102262_r_at	1422866_at	Col13a1	procollagen, type XIII, alpha 1	-1.17	0.010
102789_at	1450333_a_at	Gata2	GATA binding protein 2	-1.17	0.009
103670_at	1418821_at	Cyp2a12	cytochrome P450, family 2, subfamily a, polypeptide 12	-1.17	0.000
104375_at	1435026_at	Spock2	sparc/osteonectin, cwcv and kazal-like domains proteoglycan 2	-1.17	0.004
160672_at	1423612_at	AI462438	expressed sequence AI462438	-1.17	0.002
160678_at	1454604_s_at	Tspan12	tetraspanin 12	-1.17	0.002
93054_at	1428063_at	1110054N06Rik	RIKEN cDNA 1110054N06 gene	-1.17	0.000
95320_at	1420788_at	Klrg1	killer cell lectin-like receptor subfamily G, member 1	-1.17	0.004
99365_at	1436351_at	Coq3	coenzyme Q3 homolog, methyltransferase (yeast)	-1.17	0.005
100474_at	1420377_at	St8sia2	ST8 alpha-N-acetyl-neuraminide alpha-2,8-sialyltransferase 2	-1.18	0.001
102340_at	1423595_at	Mina	myc induced nuclear antigen	-1.18	0.001
102943_at	1428612_at	Apg7l	autophagy 7-like (S. cerevisiae)	-1.18	0.008
103585_at	1454794_at	---	---	-1.18	0.007
104528_at	1416651_at	Znhit2	zinc finger, HIT domain containing 2	-1.18	0.005

92816_r_at	1434985_a_at	Eif4a1	eukaryotic translation initiation factor 4A1	-1.18	0.010
93434_at	1449816_at	Sult5a1	sulfotransferase family 5A, member 1	-1.18	0.000
93599_at	1423408_a_at	2500003M10Rik	RIKEN cDNA 2500003M10 gene	-1.18	0.005
94552_at	1448642_at	Pcbp1	poly(rC) binding protein 1	-1.18	0.009
95603_at	1416049_at	Gldc	glycine decarboxylase	-1.18	0.008
95875_at	1459220_at	C78651	expressed sequence C78651	-1.18	0.001
96341_at	1424104_at	MGI:1915842	GCIP-interacting protein p29	-1.18	0.010
97253_at	1417106_at	Tpd52l2	tumor protein D52-like 2	-1.18	0.004
100339_at	1424898_at	Slc10a1	solute carrier family 10 (sodium/bile acid cotransporter family), member	-1.19	0.002
100500_at	1416888_at	Fadd	Fas (TNFRSF6)-associated via death domain	-1.19	0.002
101931_at	1416372_at	Ptdss1	phosphatidylserine synthase 1	-1.19	0.003
103033_at	1418021_at	C4	complement component 4 (within H-2S)	-1.19	0.001
103056_at	1454817_at	Wdr50	WD repeat domain 50	-1.19	0.006
103999_at	1455326_at	4932416N17Rik	RIKEN cDNA 4932416N17 gene	-1.19	0.000
160193_at	1424235_at	Ormdl2	ORM1-like 2 (S. cerevisiae)	-1.19	0.002
160949_at	1448725_at	Parg	poly (ADP-ribose) glycohydrolase	-1.19	0.003
92660_f_at	1416943_at	Ube2e1	ubiquitin-conjugating enzyme E2E 1, UBC4/5 homolog (yeast)	-1.19	0.000
92757_at	1427674_a_at	Sez6	seizure related gene 6	-1.19	0.003
94241_at	1423701_at	Coasy	Coenzyme A synthase	-1.19	0.001
94501_at	1420822_s_at	Sgpp1	sphingosine-1-phosphate phosphatase 1	-1.19	0.005
94957_at	1426345_at	Prepl	prolyl endopeptidase-like	-1.19	0.003
95469_at	1417987_at	Btd	biotinidase	-1.19	0.003
96603_at	1420832_at	Qscn6	quiescin Q6	-1.19	0.001
96616_at	1448763_at	Atad1	ATPase family, AAA domain containing 1	-1.19	0.000
97249_at	1417167_at	Exosc5	exosome component 5	-1.19	0.007
97734_at	1449569_at	Thpo	thrombopoietin	-1.19	0.003
98104_at	1416769_s_at	Atp6v0b	ATPase, H+ transporting, V0 subunit B	-1.19	0.009
98142_at	1434604_at	Eif5b	eukaryotic translation initiation factor 5B	-1.19	0.005
100912_at	1452839_at	2410012M04Rik	RIKEN cDNA 2410012M04 gene	-1.2	0.009
102206_at	1422787_at	Fkbpl	FK506 binding protein-like	-1.2	0.006
102895_at	1453576_at	Nipbl	Nipped-B homolog (Drosophila)	-1.2	0.010
160253_at	1423754_at	Ifitm3	interferon induced transmembrane protein 3	-1.2	0.003
160930_at	1416500_at	Sacm1l	SAC1 (supressor of actin mutations 1, homolog)-like (S. cerevisiae)	-1.2	0.000
95071_at	1449180_at	Kcmf1	potassium channel modulatory factor 1	-1.2	0.001
95591_at	1434703_at	Extl3	exostoses (multiple)-like 3	-1.2	0.003
96232_at	1451231_a_at	Cul2	cullin 2	-1.2	0.006
96259_at	1417386_at	Npepps	aminopeptidase puromycin sensitive	-1.2	0.008
98995_at	1460207_s_at	E2f5	E2F transcription factor 5	-1.2	0.002
101053_at	1424036_at	2610031L17Rik	RIKEN cDNA 2610031L17 gene	-1.21	0.002
101862_at	1419590_at	Cyp2b9	cytochrome P450, family 2, subfamily b, polypeptide 9	-1.21	0.001
102003_at	1415709_s_at	Gbf1	golgi-specific brefeldin A-resistance factor 1	-1.21	0.005
102070_at	1460734_at	Col9a3	procollagen, type IX, alpha 3	-1.21	0.002
102952_g_at	1427505_a_at	Cradd	CASP2 and RIPK1 domain containing adaptor with death domain	-1.21	0.005
104070_at	1434037_s_at	Pcaf	p300/CBP-associated factor	-1.21	0.000
104484_at	1419528_at	Sult2a2	sulfotransferase family 2A, dehydroepiandrosterone (DHEA)-preferring	-1.21	0.000
104583_at	1423300_at	Zdhhc6	zinc finger, DHHC domain containing 6	-1.21	0.010
104740_at	1424427_at	D1Ertd251e	DNA segment, Chr 1, ERATO Doi 251, expressed	-1.21	0.008
160217_at	1452159_at	2310001A20Rik	RIKEN cDNA 2310001A20 gene	-1.21	0.001
92411_at	1433843_at	Hs1bp3	HS1 binding protein 3	-1.21	0.002
92612_at	1449112_at	Slc27a5	solute carrier family 27 (fatty acid transporter), member 5	-1.21	0.000
95019_at	1418186_at	Gstt1	glutathione S-transferase, theta 1	-1.21	0.005
98029_at	1434658_at	3110056O03Rik	RIKEN cDNA 3110056O03 gene	-1.21	0.003
98962_at	1419560_at	Lipc	lipase, hepatic	-1.21	0.010

101067_at	1428709_a_at	Mrpl24	mitochondrial ribosomal protein L24	-1.22	0.001
102642_at	1417584_at	Slc11a2	solute carrier family 11 (proton-coupled divalent metal ion transporters)	-1.22	0.009
104381_at	1450444_a_at	Nr1h3	nuclear receptor subfamily 1, group H, member 3	-1.22	0.001
160267_at	1448882_at	0610009E20Rik	RIKEN cDNA 0610009E20 gene	-1.22	0.001
160362_at	1433576_at	Mat2a	methionine adenosyltransferase II, alpha	-1.22	0.000
160512_at	1423678_at	BC017643	cDNA sequence BC017643	-1.22	0.003
160738_at	1452835_a_at	Polrmt	polymerase (RNA) mitochondrial (DNA directed)	-1.22	0.001
160809_at	1423047_at	Tollip	toll interacting protein	-1.22	0.001
92195_at	1451639_at	Cebpg	CCAAT/enhancer binding protein (C/EBP), gamma	-1.22	0.006
93493_at	1423645_a_at	---	---	-1.22	0.001
93624_at	1428776_at	8430417G17Rik	RIKEN cDNA 8430417G17 gene	-1.22	0.001
96160_at	1417636_at	Slc6a9	solute carrier family 6 (neurotransmitter transporter, glycine), member 9	-1.22	0.000
96346_at	1448842_at	Cdo1	cysteine dioxygenase 1, cytosolic	-1.22	0.000
96732_at	1438669_at	Wdr40a	WD repeat domain 40A	-1.22	0.004
97418_at	1418914_s_at	Bhmt2	betaine-homocysteine methyltransferase 2	-1.22	0.003
97846_at	1460429_at	Cdc5l	cell division cycle 5-like (S. pombe)	-1.22	0.001
97866_at	1423217_a_at	2510049I19Rik	RIKEN cDNA 2510049I19 gene	-1.22	0.001
98767_at	1435824_at	Yy1	YY1 transcription factor	-1.22	0.001
99183_at	1433591_at	AI553587	expressed sequence AI553587	-1.22	0.000
100400_at	1416668_at	4921531G14Rik	RIKEN cDNA 4921531G14 gene	-1.23	0.004
100515_at	1418518_at	Furin	furin (paired basic amino acid cleaving enzyme)	-1.23	0.000
101127_at	1419628_at	Chx10	C. elegans ceh-10 homeo domain containing homolog	-1.23	0.003
102193_at	1435390_at	---	---	-1.23	0.001
103931_at	1418611_at	Grca	gene rich cluster, A gene	-1.23	0.004
104558_at	1454932_at	Rcor1	REST corepressor 1	-1.23	0.000
160302_at	1451238_at	1200003C05Rik	RIKEN cDNA 1200003C05 gene	-1.23	0.010
160525_f_at	1426730_a_at	Prlpk	prolactin-like protein K	-1.23	0.007
160536_at	1424132_at	Hras1	Harvey rat sarcoma virus oncogene 1	-1.23	0.005
93419_at	1425066_a_at	1110061O04Rik	RIKEN cDNA 1110061O04 gene	-1.23	0.001
93708_at	1421646_a_at	Pias3	protein inhibitor of activated STAT 3	-1.23	0.001
94292_at	1419912_s_at	Strap	Serine/threonine kinase receptor associated protein	-1.23	0.010
96088_at	1448154_at	Ndrg2	N-myc downstream regulated gene 2	-1.23	0.000
96325_at	1451078_at	2510039O18Rik	RIKEN cDNA 2510039O18 gene	-1.23	0.008
96891_at	1417082_at	Anp32b	acidic nuclear phosphoprotein 32 family, member B	-1.23	0.002
97492_at	1415738_at	0610040B21Rik	RIKEN cDNA 0610040B21 gene	-1.23	0.004
98506_r_at	1422492_at	Cpox	coproporphyrinogen oxidase	-1.23	0.001
99490_at	1423970_at	Thoc3	THO complex 3	-1.23	0.001
102210_at	1437651_a_at	Dtnb	dystrobrevin, beta	-1.24	0.001
103341_at	1416563_at	Ctps	cytidine 5'-triphosphate synthase	-1.24	0.008
104403_at	1434162_at	2700078E11Rik	RIKEN cDNA 2700078E11 gene	-1.24	0.000
104754_at	1448778_at	Sfrs4	splicing factor, arginine/serine-rich 4 (SRp75)	-1.24	0.003
160351_at	1451243_at	Rnpep	arginyl aminopeptidase (aminopeptidase B)	-1.24	0.005
160898_at	1424751_at	Abt1	activator of basal transcription	-1.24	0.003
92884_at	1435638_at	---	---	-1.24	0.003
93482_at	1425506_at	Mylk	myosin, light polypeptide kinase	-1.24	0.000
93579_at	1417064_at	Jagn1	jagunal homolog 1 (Drosophila)	-1.24	0.004
95479_at	1448505_at	MGI:1927354	nuclear DNA binding protein	-1.24	0.001
96712_at	1448321_at	Smoc1	SPARC related modular calcium binding 1	-1.24	0.002
96763_at	1448426_at	Sardh	Sarcosine dehydrogenase	-1.24	0.001
97933_at	1460695_a_at	2300006M17Rik	RIKEN cDNA 2300006M17 gene	-1.24	0.003
99121_at	1452247_at	Fxr1h	fragile X mental retardation gene 1, autosomal homolog	-1.24	0.001
100935_at	1418437_a_at	Tcfl4	transcription factor-like 4	-1.25	0.002
101926_at	1417216_at	---	---	-1.25	0.000

102824_g_at	1424631_a_at	Ighg	Immunoglobulin heavy chain (gamma polypeptide)	-1.25	0.004
103591_at	1418593_at	Taf6	TAF6 RNA polymerase II, TATA box binding protein (TBP)-associated	-1.25	0.003
104494_at	1454736_at	4921515A04Rik	RIKEN cDNA 4921515A04 gene	-1.25	0.006
160748_at	1448431_at	Asb6	ankyrin repeat and SOCS box-containing protein 6	-1.25	0.002
93512_f_at	1438292_x_at	Adk	adenosine kinase	-1.25	0.000
93806_at	1428107_at	Sh3bgrl	SH3-binding domain glutamic acid-rich protein like	-1.25	0.005
94471_r_at	1422497_at	Slc30a5	solute carrier family 30 (zinc transporter), member 5	-1.25	0.000
94832_at	1415963_at	Hnrph2	heterogeneous nuclear ribonucleoprotein H2	-1.25	0.001
94861_at	1449718_s_at	4930453N24Rik	RIKEN cDNA 4930453N24 gene	-1.25	0.003
96124_at	1428327_at	2310001H13Rik	RIKEN cDNA 2310001H13 gene	-1.25	0.006
96546_r_at	1435586_at	A730042J05Rik	RIKEN cDNA A730042J05 gene	-1.25	0.000
96787_at	1424758_s_at	Serpina10	serine (or cysteine) proteinase inhibitor, clade A (alpha-1 antiproteinase	-1.25	0.008
100570_at	1420535_a_at	6330412F12Rik	RIKEN cDNA 6330412F12 gene	-1.26	0.010
101905_at	1415769_at	Itch	itchy	-1.26	0.000
104157_at	1435369_at	C78212	expressed sequence C78212	-1.26	0.008
104174_at	1459546_s_at	Enpp1	ectonucleotide pyrophosphatase/phosphodiesterase 1	-1.26	0.003
104766_at	1418305_s_at	Nola1	nucleolar protein family A, member 1 (H/ACA small nucleolar RNPs)	-1.26	0.009
160151_i_at	1424462_at	1200009B18Rik	RIKEN cDNA 1200009B18 gene	-1.26	0.003
160228_at	1429555_at	1110019C08Rik	RIKEN cDNA 1110019C08 gene	-1.26	0.007
161129_r_at	1423948_at	Bag2	Bcl2-associated athanogene 2	-1.26	0.010
93095_at	1416176_at	Hmgb1	high mobility group box 1	-1.26	0.000
93845_at	1423863_at	Abcf2	ATP-binding cassette, sub-family F (GCN20), member 2	-1.26	0.006
94419_at	1448132_at	Slc19a1	solute carrier family 19 (sodium/hydrogen exchanger), member 1	-1.26	0.009
95609_at	1426798_a_at	---	---	-1.26	0.000
96949_at	1415767_at	Ythdf1	YTH domain family 1	-1.26	0.000
98144_f_at	1430029_a_at	Sas	sarcoma amplified sequence	-1.26	0.001
100099_at	1448621_a_at	Smpd1	sphingomyelin phosphodiesterase 1, acid lysosomal	-1.27	0.001
102291_at	1427393_at	F9	coagulation factor IX	-1.27	0.000
102555_at	1422417_at	X99300	epidymal sperm gene	-1.27	0.005
103044_g_at	1455710_x_at	Mtcp1	mature T-cell proliferation 1	-1.27	0.005
103220_at	1422554_at	Ndnl2	necdin-like 2	-1.27	0.001
103243_at	1425272_at	Emp2	epithelial membrane protein 2	-1.27	0.002
103752_r_at	1455156_at	Strn	striatin, calmodulin binding protein	-1.27	0.002
160262_at	1438843_x_at	Mtch2	mitochondrial carrier homolog 2 (C. elegans)	-1.27	0.000
92975_at	1448328_at	Sh3bp2	SH3-domain binding protein 2	-1.27	0.000
93009_at	1416411_at	Gstm2	glutathione S-transferase, mu 2	-1.27	0.003
93127_at	1426658_x_at	Phgdh	3-phosphoglycerate dehydrogenase	-1.27	0.001
93664_at	1422009_at	Atp1b2	ATPase, Na+/K+ transporting, beta 2 polypeptide	-1.27	0.000
94820_r_at	1448334_a_at	Ccni	cyclin I	-1.27	0.000
94977_at	1460203_at	Itpr1	inositol 1,4,5-triphosphate receptor 1	-1.27	0.004
95906_at	1459927_at	4833445I07Rik	RIKEN cDNA 4833445I07 gene	-1.27	0.008
95994_at	1447965_at	C80012	expressed sequence C80012	-1.27	0.003
96676_at	1454640_at	Chchd7	coiled-coil-helix-coiled-coil-helix domain containing 7	-1.27	0.006
97250_at	1423211_at	Nola3	nucleolar protein family A, member 3	-1.27	0.002
103476_at	1454796_at	D5Ertd40e	DNA segment, Chr 5, ERATO Doi 40, expressed	-1.28	0.004
160258_at	1416765_s_at	MGI:1913699	mitochondria-associated protein involved in granulocyte-macrophage c	-1.28	0.003
160717_at	1416841_at	1110059E24Rik	RIKEN cDNA 1110059E24 gene	-1.28	0.000
92637_at	1450269_a_at	Pfkl	phosphofructokinase, liver, B-type	-1.28	0.005
93046_at	1428328_at	Nup50	nucleoporin 50	-1.28	0.005
93204_r_at	1454831_at	3230402J05Rik	RIKEN cDNA 3230402J05 gene	-1.28	0.006
94956_at	1452434_s_at	Dgcr6	DiGeorge syndrome critical region gene 6	-1.28	0.006
96875_r_at	1420174_s_at	Tax1bp1	Tax1 (human T-cell leukemia virus type I) binding protein 1	-1.28	0.003
97740_at	1418401_a_at	Dusp16	dual specificity phosphatase 16	-1.28	0.001

99647_at	1416705_at	Rpe	ribulose-5-phosphate-3-epimerase	-1.28	0.005
100596_at	1450699_at	Selenbp1	selenium binding protein 1	-1.29	0.001
102863_at	1418801_at	Zkscan1	zinc finger with KRAB and SCAN domains 1	-1.29	0.003
103227_at	1427961_s_at	AI788959	expressed sequence AI788959	-1.29	0.006
103974_at	1419154_at	Tmprss2	transmembrane protease, serine 2	-1.29	0.001
104288_at	1451971_at	Cul4a	cullin 4A	-1.29	0.009
160275_at	1418888_a_at	Sepx1	selenoprotein X 1	-1.29	0.001
94928_at	1418099_at	Tnfrsf1b	tumor necrosis factor receptor superfamily, member 1b	-1.29	0.006
95914_at	1456405_at	6720461J16Rik	RIKEN cDNA 6720461J16 gene	-1.29	0.007
97331_at	1416265_at	Capn10	calpain 10	-1.29	0.003
97479_at	1417908_s_at	Ube2l3	ubiquitin-conjugating enzyme E2L 3	-1.29	0.000
97767_at	1420728_at	Krt1-2	keratin complex 1, acidic, gene 2	-1.29	0.004
98277_at	1460664_at	Zfpn1a4	zinc finger protein, subfamily 1A, 4 (Eos)	-1.29	0.004
99320_at	1421735_a_at	St8sia5	ST8 alpha-N-acetyl-neuraminide alpha-2,8-sialyltransferase 5	-1.29	0.006
99357_at	1447956_at	C76614	expressed sequence C76614	-1.29	0.004
100043_f_at	1429457_at	2310020A21Rik	RIKEN cDNA 2310020A21 gene	-1.3	0.001
100965_at	1455167_at	Cox8c	cytochrome c oxidase, subunit VIIIc	-1.3	0.004
100977_at	1423748_at	Pdk1	pyruvate dehydrogenase kinase, isoenzyme 1	-1.3	0.003
101366_f_at	1453018_at	Nvl	nuclear VCP-like	-1.3	0.004
102072_g_at	1434281_at	1500034J01Rik	RIKEN cDNA 1500034J01 gene	-1.3	0.001
103260_at	1430291_at	1110060D06Rik	RIKEN cDNA 1110060D06 gene	-1.3	0.004
94012_at	1417499_at	Timm13a	translocase of inner mitochondrial membrane 13 homolog a (yeast)	-1.3	0.010
94174_at	1420929_at	Catnal1	catenin alpha-like 1	-1.3	0.002
94212_at	1425557_x_at	Tsc22d3	TSC22 domain family 3	-1.3	0.002
95453_f_at	1419814_s_at	S100a1	S100 calcium binding protein A1	-1.3	0.008
96627_at	1416667_at	Ebp	phenylalkylamine Ca2+ antagonist (emopamil) binding protein	-1.3	0.006
96734_at	1417834_at	Synj2bp	synaptojanin 2 binding protein	-1.3	0.001
97868_at	1449935_a_at	Dnaja3	DnaJ (Hsp40) homolog, subfamily A, member 3	-1.3	0.000
98461_at	1417108_at	1200014P03Rik	RIKEN cDNA 1200014P03 gene	-1.3	0.001
99013_f_at	1423088_at	LOC434449	similar to Ubiquitous tropomodulin (U-Tmod) (Tropomodulin 3)	-1.3	0.006
101166_at	1450514_at	Zfp29	zinc finger protein 29	-1.31	0.005
102427_at	1451324_s_at	Fyttd1	forty-two-three domain containing 1	-1.31	0.002
104176_at	1452633_s_at	Aak1	AP2 associated kinase 1	-1.31	0.003
104726_at	1451625_a_at	1700013L23Rik	RIKEN cDNA 1700013L23 gene	-1.31	0.003
93827_at	1449337_at	Tdo2	tryptophan 2,3-dioxygenase	-1.31	0.001
94489_at	1438657_x_at	Ptp4a1	protein tyrosine phosphatase 4a1	-1.31	0.001
95594_at	1449697_s_at	Mfn1	mitofusin 1	-1.31	0.000
97430_at	1417042_at	Slc37a4	solute carrier family 37 (glycerol-6-phosphate transporter), member 4	-1.31	0.002
99326_at	1420584_at	Pla2g2c	phospholipase A2, group IIC	-1.31	0.000
102783_at	1452799_at	2310009E04Rik	RIKEN cDNA 2310009E04 gene	-1.32	0.004
103450_at	1428454_at	Bcas3	breast carcinoma amplified sequence 3	-1.32	0.003
104161_at	1420936_s_at	Cpsf2	cleavage and polyadenylation specific factor 2	-1.32	0.008
160139_at	1417013_at	Hspb8	heat shock 27kDa protein 8	-1.32	0.008
160220_at	1450998_at	Zfp110	zinc finger protein 110	-1.32	0.008
160990_r_at	1424243_at	BC016198	cDNA sequence BC016198	-1.32	0.009
93495_at	1435731_x_at	Stag1	Stromal antigen 1	-1.32	0.004
93975_at	1416129_at	1300002F13Rik	RIKEN cDNA 1300002F13 gene	-1.32	0.006
94361_at	1448270_at	Ddx21	DEAD (Asp-Glu-Ala-Asp) box polypeptide 21	-1.32	0.000
94522_at	1416247_at	Dctn3	dynactin 3	-1.32	0.005
97541_f_at	1427651_x_at	H2-D1	histocompatibility 2, D region locus 1	-1.32	0.002
98132_at	1422484_at	Cycs	cytochrome c, somatic	-1.32	0.001
99629_at	1416555_at	---	---	-1.32	0.004
102357_at	1454977_at	AU020772	expressed sequence AU020772	-1.33	0.001

104138_at	1448489_at	Pafah2	platelet-activating factor acetylhydrolase 2	-1.33	0.000
160666_at	1428502_at	Actr6	ARP6 actin-related protein 6 homolog (yeast)	-1.33	0.003
92826_at	1427173_a_at	Mrps33	mitochondrial ribosomal protein S33	-1.33	0.000
93779_at	1456635_at	---	---	-1.33	0.003
94343_at	1433887_at	Dnajc3	DnaJ (Hsp40) homolog, subfamily C, member 3	-1.33	0.000
95156_g_at	1424384_a_at	Znrf1	zinc and ring finger 1	-1.33	0.010
95486_at	1420056_s_at	Ptdsr	phosphatidylserine receptor	-1.33	0.003
96782_at	1427290_at	Krt2-19	keratin complex 2, basic, gene 19	-1.33	0.001
97302_at	1450084_s_at	Ivns1abp	influenza virus NS1A binding protein	-1.33	0.000
99037_at	1421416_at	Map2k7	mitogen activated protein kinase kinase 7	-1.33	0.004
99441_at	1426462_at	Gphn	gephyrin	-1.33	0.000
100713_at	1449546_a_at	Zfp617	zinc finger protein 617	-1.34	0.000
100715_at	1421687_at	Msmb	beta-microseminoprotein	-1.34	0.001
101088_f_at	1437850_a_at	Cnbp1	cellular nucleic acid binding protein 1	-1.34	0.001
102194_at	1427085_at	2810432D09Rik	RIKEN cDNA 2810432D09 gene	-1.34	0.000
103251_at	1455073_at	Cdadc1	cytidine and dCMP deaminase domain containing 1	-1.34	0.000
104021_at	1420414_at	Hoxa11	homeo box A11	-1.34	0.002
104033_at	1426445_at	Mgea6	meningioma expressed antigen 6 (coiled-coil proline-rich)	-1.34	0.000
160947_at	1451282_at	Centd3	centaurin, delta 3	-1.34	0.001
92490_at	1420395_a_at	Kif9	kinesin family member 9	-1.34	0.007
94225_at	1415684_at	Apg5l	autophagy 5-like (S. cerevisiae)	-1.34	0.005
96563_at	1448014_s_at	Usp24	ubiquitin specific protease 24	-1.34	0.003
98797_at	1422915_at	Gast	gastrin	-1.34	0.010
102819_at	1418046_at	Nap1l2	nucleosome assembly protein 1-like 2	-1.35	0.002
102852_at	1418815_at	Cdh2	cadherin 2	-1.35	0.002
103527_at	1436693_x_at	Slc35e4	solute carrier family 35, member E4	-1.35	0.001
160499_at	1415889_a_at	Tra1	tumor rejection antigen gp96	-1.35	0.000
92429_at	1422926_at	Mc2r	melanocortin 2 receptor	-1.35	0.005
94414_at	1436615_a_at	Otc	ornithine transcarbamylase	-1.35	0.001
95043_at	1424273_at	Cyp2c70	cytochrome P450, family 2, subfamily c, polypeptide 70	-1.35	0.000
95449_at	1451523_a_at	2310075G12Rik	RIKEN cDNA 2310075G12 gene	-1.35	0.000
96264_at	1416281_at	Wdr45l	Wdr45 like	-1.35	0.007
96644_at	1450742_at	Bysl	bystin-like	-1.35	0.000
99585_at	1449611_at	Kai1	kangai 1 (suppression of tumorigenicity 6, prostate)	-1.35	0.005
104036_at	1436479_a_at	Dpp7	dipeptidylpeptidase 7	-1.36	0.007
104259_at	1450416_at	Cbx5	chromobox homolog 5 (Drosophila HP1a)	-1.36	0.005
160624_at	1426796_at	Txln	Taxilin	-1.36	0.002
93195_at	1429005_at	Mfhas1	malignant fibrous histiocytoma amplified sequence 1	-1.36	0.004
93560_at	1450095_a_at	Acyp1	acylphosphatase 1, erythrocyte (common) type	-1.36	0.001
94036_at	1416511_a_at	Cdc42ep4	CDC42 effector protein (Rho GTPase binding) 4	-1.36	0.003
95395_at	1426840_at	Ythdf3	YTH domain family 3	-1.36	0.000
95522_i_at	1448760_at	Zfp68	zinc finger protein 68	-1.36	0.001
95949_at	1442492_at	DXErtd11e	DNA segment, Chr X, ERATO Doi 11, expressed	-1.36	0.004
96760_at	1449886_a_at	Timm10	translocase of inner mitochondrial membrane 10 homolog (yeast)	-1.36	0.000
96781_at	1415725_at	Rrn3	RRN3 RNA polymerase I transcription factor homolog (yeast)	-1.36	0.009
101318_at	1450527_at	Sstr1	somatostatin receptor 1	-1.37	0.005
104032_at	1435666_at	Mast3	microtubule associated serine/threonine kinase 3	-1.37	0.003
160248_at	1433482_a_at	Fubp1	far upstream element (FUSE) binding protein 1	-1.37	0.003
160449_at	1416018_at	Dr1	down-regulator of transcription 1	-1.37	0.001
93316_at	1460192_at	Osbpl1a	oxysterol binding protein-like 1A	-1.37	0.001
95596_at	1428353_at	1110054H05Rik	RIKEN cDNA 1110054H05 gene	-1.37	0.002
95889_at	1434787_at	AI854770	expressed sequence AI854770	-1.37	0.006
95954_at	1444292_at	D7Ertd143e	DNA segment, Chr 7, ERATO Doi 143, expressed	-1.37	0.007

96841_at	1451069_at	Pim3	proviral integration site 3	-1.37	0.008
97261_at	1460179_at	Dnaja1	DnaJ (Hsp40) homolog, subfamily A, member 1	-1.37	0.002
102385_at	1428389_s_at	Wdr43	WD repeat domain 43	-1.38	0.001
102816_at	1421921_at	Serpina3m	serine (or cysteine) proteinase inhibitor, clade A, member 3M	-1.38	0.000
103602_at	1420709_s_at	Dao1	D-amino acid oxidase 1	-1.38	0.001
103723_at	1454783_at	Il13ra1	interleukin 13 receptor, alpha 1	-1.38	0.000
160403_at	1423225_at	MGI:1931466	selenoprotein K	-1.38	0.000
92282_at	1427887_at	2610304G08Rik	RIKEN cDNA 2610304G08 gene	-1.38	0.000
92708_at	1424590_at	Ddx19b	DEAD (Asp-Glu-Ala-Asp) box polypeptide 19b	-1.38	0.000
93429_at	1449016_at	Zp2	zona pellucida glycoprotein 2	-1.38	0.000
94875_at	1416093_a_at	Mrpl20	mitochondrial ribosomal protein L20	-1.38	0.003
95783_g_at	1422345_s_at	Mageb1 /// Mageb2 /// Mag	melanoma antigen, family B, 1 /// melanoma antigen, family B, 2 /// mel	-1.38	0.005
96598_at	1455653_at	Ccnj	cyclin J	-1.38	0.000
97004_at	1422373_at	Olfr71	olfactory receptor 71	-1.38	0.006
99855_at	1421340_at	Map3k5	mitogen activated protein kinase kinase kinase 5	-1.38	0.009
100014_at	1416944_a_at	Tlk2	tousled-like kinase 2 (Arabidopsis)	-1.39	0.005
100015_at	1449090_a_at	Yes1	Yamaguchi sarcoma viral (v-yes) oncogene homolog 1	-1.39	0.001
100738_at	1426149_at	Spi15	serine protease inhibitor 15	-1.39	0.007
101853_f_at	1450876_at	Cfh	complement component factor h	-1.39	0.003
102766_at	1427940_s_at	Mycbp	c-myc binding protein	-1.39	0.003
103964_at	1460652_at	Esrra	Estrogen related receptor, alpha	-1.39	0.001
104215_at	1435444_at	Atf6	activating transcription factor 6	-1.39	0.001
160284_at	1418068_at	Ndufa10	NADH dehydrogenase (ubiquinone) 1 alpha subcomplex 10	-1.39	0.005
160475_at	1450418_a_at	2310034L04Rik	RIKEN cDNA 2310034L04 gene	-1.39	0.001
161034_at	1451502_at	Pla2g10	phospholipase A2, group X	-1.39	0.002
93165_at	1460438_at	2610022K04Rik	RIKEN cDNA 2610022K04 gene	-1.39	0.007
93485_at	1435537_at	Ptprd	Protein tyrosine phosphatase, receptor type, D	-1.39	0.002
93663_r_at	1439749_at	Zap70	zeta-chain (TCR) associated protein kinase	-1.39	0.000
94146_at	1421578_at	Ccl4	chemokine (C-C motif) ligand 4	-1.39	0.000
95054_at	1460323_at	Tars	threonyl-tRNA synthetase	-1.39	0.001
98459_at	1425179_at	Shmt1	serine hydroxymethyl transferase 1 (soluble)	-1.39	0.002
102166_g_at	1422380_at	V1rb5	vomeronasal 1 receptor, B5	-1.4	0.007
102969_at	1430417_s_at	0610025P10Rik	RIKEN cDNA 0610025P10 gene	-1.4	0.002
103313_r_at	1434941_s_at	2610101J03Rik	RIKEN cDNA 2610101J03 gene	-1.4	0.000
160256_at	1434732_x_at	Tomm7	Translocase of outer mitochondrial membrane 7 homolog (yeast)	-1.4	0.003
160998_at	1449677_s_at	Tmem38b	transmembrane protein 38B	-1.4	0.000
162058_f_at	1418767_at	Cyp4f13	cytochrome P450, family 4, subfamily f, polypeptide 13	-1.4	0.001
93269_at	1451240_a_at	Glo1	glyoxalase 1	-1.4	0.005
96423_at	1420555_at	Alx3	aristaless 3	-1.4	0.008
98073_at	1451435_at	Cutl1	cut-like 1 (Drosophila)	-1.4	0.000
99847_at	1418946_at	St3gal1	ST3 beta-galactoside alpha-2,3-sialyltransferase 1	-1.4	0.002
103249_at	1417304_at	Chrd	chordin	-1.41	0.002
94556_at	1431055_a_at	Snx10	sorting nexin 10	-1.41	0.001
95082_at	1423062_at	Igfbp3	insulin-like growth factor binding protein 3	-1.41	0.001
95694_at	1423474_at	Top1	topoisomerase (DNA) I	-1.41	0.000
95714_at	1436681_x_at	0610009D07Rik	RIKEN cDNA 0610009D07 gene	-1.41	0.000
95791_s_at	1427816_at	Sfrs2	splicing factor, arginine/serine-rich 2 (SC-35)	-1.41	0.004
99898_at	1417944_at	Gng4	guanine nucleotide binding protein (G protein), gamma 4 subunit	-1.41	0.001
101649_at	1421833_at	Pip5k1a	phosphatidylinositol-4-phosphate 5-kinase, type 1 alpha	-1.42	0.006
102574_at	1421793_at	Fgf11	fibroblast growth factor 11	-1.42	0.004
104179_at	1434312_at	AI788669	expressed sequence AI788669	-1.42	0.000
104298_at	1424915_s_at	---	---	-1.42	0.001
160710_at	1426709_a_at	Usp33	ubiquitin specific protease 33	-1.42	0.001

92261_at	1435231_at	Coq4	coenzyme Q4 homolog (yeast)	-1.42	0.000
94268_f_at	1416848_at	Ubl5	ubiquitin-like 5	-1.42	0.001
95733_at	1451782_a_at	Slc29a1	solute carrier family 29 (nucleoside transporters), member 1	-1.42	0.003
96794_at	1425030_at	Zfp622	zinc finger protein 622	-1.42	0.002
97317_at	1448136_at	Enpp2	ectonucleotide pyrophosphatase/phosphodiesterase 2	-1.42	0.010
98048_at	1423982_at	Fusip1	FUS interacting protein (serine-arginine rich) 1	-1.42	0.001
101527_at	1419258_at	Tcea1	transcription elongation factor A (SII) 1	-1.43	0.000
102776_at	1428592_s_at	Usp38	ubiquitin specific protease 38	-1.43	0.002
160518_at	1460706_s_at	Rer1	RER1 retention in endoplasmic reticulum 1 homolog (S. cerevisiae)	-1.43	0.004
98316_at	1427573_at	Chic1	cysteine-rich hydrophobic domain 1	-1.43	0.009
98447_at	1418982_at	Cebpa	CCAAT/enhancer binding protein (C/EBP), alpha	-1.43	0.002
99452_at	1451255_at	MGI:1927471	liver-specific bHLH-Zip transcription factor	-1.43	0.001
102725_at	1448468_a_at	Kcnab1	potassium voltage-gated channel, shaker-related subfamily, beta meml	-1.44	0.003
104406_at	1449450_at	Ptges	prostaglandin E synthase	-1.44	0.005
160740_at	1454921_at	Gm561	gene model 561, (NCBI)	-1.44	0.002
92919_at	1418268_at	Htr3a	5-hydroxytryptamine (serotonin) receptor 3A	-1.44	0.005
93528_s_at	1428289_at	Bteb1	basic transcription element binding protein 1	-1.44	0.009
94459_at	1455152_at	AI462493	Expressed sequence AI462493	-1.44	0.001
95926_at	1419778_at	---	---	-1.44	0.006
96984_at	1422384_at	Olfr1509	olfactory receptor 1509	-1.44	0.009
98794_at	1449287_at	Srms	src-related kinase lacking C-terminal regulatory tyrosine and N-termina	-1.44	0.000
101642_at	1427841_at	---	---	-1.45	0.000
160783_at	1426780_at	D14Ertd436e	DNA segment, Chr 14, ERATO Doi 436, expressed	-1.45	0.001
95066_at	1425129_a_at	Taldo1	transaldolase 1	-1.45	0.001
95142_s_at	1424168_a_at	---	---	-1.45	0.001
96229_at	1421946_at	Crp	C-reactive protein, petaxin related	-1.45	0.000
96487_at	1442976_at	C81072	expressed sequence C81072	-1.45	0.010
96803_at	1420654_a_at	Gbe1	glucan (1,4-alpha-), branching enzyme 1	-1.45	0.002
102799_at	1418037_at	C4bp	complement component 4 binding protein	-1.46	0.005
161947_f_at	1426039_a_at	Alox12e	arachidonate lipoxygenase, epidermal	-1.46	0.001
93117_at	1420365_a_at	Hnrpa2b1	heterogeneous nuclear ribonucleoprotein A2/B1	-1.46	0.003
94395_at	1433640_at	---	---	-1.46	0.005
98380_at	1418414_at	Kcnh1	potassium voltage-gated channel, subfamily H (eag-related), member 1	-1.46	0.001
98726_at	1421444_at	Pgr	progesterone receptor	-1.46	0.005
101150_at	1421773_at	Etsrp71	ets related protein 71	-1.47	0.009
101416_f_at	1423163_at	Bat4	HLA-B associated transcript 4	-1.47	0.002
102707_f_at	1421564_at	Serpina3c	serine (or cysteine) proteinase inhibitor, clade A, member 3C	-1.47	0.008
103516_at	1418925_at	Celsr1	cadherin EGF LAG seven-pass G-type receptor 1	-1.47	0.001
104704_at	1415986_at	Clcn4-2	chloride channel 4-2	-1.47	0.002
92469_at	1451031_at	Sfrp4	secreted frizzled-related sequence protein 4	-1.47	0.001
92802_s_at	1425467_a_at	Plp1	proteolipid protein (myelin) 1	-1.47	0.006
98423_at	1423271_at	Gjb2	gap junction membrane channel protein beta 2	-1.47	0.000
99851_at	1436308_at	Zfp292	zinc finger protein 292	-1.47	0.000
160417_at	1418429_at	Kif5b	kinesin family member 5B	-1.48	0.000
93623_at	1448665_at	Dmd	dystrophin, muscular dystrophy	-1.48	0.001
93692_f_at	1449417_at	Ambn	ameloblastin	-1.48	0.005
96924_at	1452189_at	9430077D24Rik	RIKEN cDNA 9430077D24 gene	-1.48	0.000
97526_at	1448308_at	Ap3m1	adaptor-related protein complex 3, mu 1 subunit	-1.48	0.000
99827_at	1429999_at	4933403M19Rik	RIKEN cDNA 4933403M19 gene	-1.48	0.007
101094_at	1416480_a_at	MGI:1930666	hypoxia induced gene 1	-1.49	0.003
104137_at	1449302_at	Abca2	ATP-binding cassette, sub-family A (ABC1), member 2	-1.49	0.000
96783_at	1426014_a_at	Mucdhl	mucin and cadherin like	-1.49	0.000
99180_at	1450873_at	Gtpbp4	GTP binding protein 4	-1.49	0.003

160392_at	1433448_at	B430110G05Rik	RIKEN cDNA B430110G05 gene	-1.5	0.000
104349_at	1428133_at	MGI:2156003	Smad nuclear interacting protein 1	-1.51	0.004
93290_at	1453299_a_at	Pnp	purine-nucleoside phosphorylase	-1.51	0.000
93978_at	1454842_a_at	B3galnt2	UDP-GalNAc:betaGlcNAc beta 1,3-galactosaminyltransferase, polypep	-1.51	0.002
95417_at	1426350_at	Mgat2	mannoside acetylglucosaminyltransferase 2	-1.51	0.000
101453_at	1419608_a_at	Mia1	melanoma inhibitory activity 1	-1.52	0.002
103612_at	1418903_at	Aqp2	aquaporin 2	-1.52	0.008
160850_at	1460673_at	Fpgs	folylpolyglutamyl synthetase	-1.52	0.000
95300_at	1449924_at	Prg3	proteoglycan 3	-1.52	0.005
97191_at	1449599_at	---	---	-1.52	0.001
98994_at	1416854_at	Slc34a2	solute carrier family 34 (sodium phosphate), member 2	-1.52	0.001
160376_at	1452646_at	Trp53inp2	tumor protein p53 inducible nuclear protein 2	-1.53	0.000
160582_at	1418551_at	Mybpc3	myosin binding protein C, cardiac	-1.53	0.001
92648_at	1435058_x_at	Stxbp3	syntaxin binding protein 3	-1.53	0.005
97420_at	1417290_at	Lrg1	leucine-rich alpha-2-glycoprotein 1	-1.53	0.000
104369_at	1422784_at	Krt2-6a	keratin complex 2, basic, gene 6a	-1.54	0.000
104745_at	1416793_at	Arl6ip2	ADP-ribosylation factor-like 6 interacting protein 2	-1.54	0.001
161077_f_at	1448400_a_at	Smarcd2	SWI/SNF related, matrix associated, actin dependent regulator of chror	-1.54	0.000
92634_at	1441342_at	Dpp4	Dipeptidylpeptidase 4	-1.54	0.003
94775_at	1420556_at	Oxt	oxytocin	-1.54	0.002
95870_at	1442974_at	D6Ertd160e	DNA segment, Chr 6, ERATO Doi 160, expressed	-1.54	0.006
97410_at	1454712_at	Mcart1	mitochondrial carrier triple repeat 1	-1.54	0.002
160617_at	1432543_a_at	Klf13	Kruppel-like factor 13	-1.55	0.002
95972_at	1459891_at	C78444	expressed sequence C78444	-1.55	0.009
96535_at	1435802_at	Gm157	gene model 157, (NCBI)	-1.55	0.009
101639_r_at	1421741_at	Cyp3a16	cytochrome P450, family 3, subfamily a, polypeptide 16	-1.56	0.001
104184_at	1450791_at	Nppb	natriuretic peptide precursor type B	-1.56	0.001
104206_at	1453287_at	5730557B15Rik	RIKEN cDNA 5730557B15 gene	-1.56	0.001
104525_at	1428351_at	Ppm1m	protein phosphatase 1M	-1.56	0.000
92735_at	1450128_at	Pla2g2a	phospholipase A2, group IIA (platelets, synovial fluid)	-1.56	0.002
95057_at	1448185_at	Herpud1	homocysteine-inducible, endoplasmic reticulum stress-inducible, ubiqui	-1.56	0.000
98137_at	1419525_at	Car5a	carbonic anhydrase 5a, mitochondrial	-1.56	0.000
103360_at	1417989_at	Tssk1	testis-specific serine kinase 1	-1.57	0.004
104199_at	1460743_at	Tigd5	tigger transposable element derived 5	-1.57	0.001
92728_at	1422873_at	Prg2	proteoglycan 2, bone marrow	-1.57	0.002
96227_at	1448506_at	Serpina6	serine (or cysteine) proteinase inhibitor, clade A, member 6	-1.57	0.005
101597_at	1446829_at	D12Ertd216e	DNA segment, Chr 12, ERATO Doi 216, expressed	-1.58	0.009
101822_at	1422237_at	Mc3r	melanocortin 3 receptor	-1.58	0.004
93926_at	1421382_at	Prlr	prolactin receptor	-1.58	0.002
103369_at	1436316_at	9430029L20Rik	RIKEN cDNA 9430029L20 gene	-1.59	0.000
161036_at	1426712_at	Slc6a15	solute carrier family 6 (neurotransmitter transporter), member 15	-1.59	0.009
101474_at	1419578_at	Mbl1	mannose binding lectin, liver (A)	-1.6	0.001
93058_at	1424343_a_at	Eif1a	eukaryotic translation initiation factor 1A	-1.6	0.000
96000_at	1459924_at	Atp6v0a1	ATPase, H+ transporting, lysosomal V0 subunit a isoform 1	-1.6	0.002
97710_f_at	1448028_at	C530046L02Rik	RIKEN cDNA C530046L02 gene	-1.6	0.000
96571_at	1449692_at	Prkcz	protein kinase C, zeta	-1.61	0.002
93915_at	1416957_at	Pou2af1	POU domain, class 2, associating factor 1	-1.62	0.009
98328_at	1421999_at	Tshr	thyroid stimulating hormone receptor	-1.62	0.006
100311_f_at	1421802_at	Ear1	eosinophil-associated, ribonuclease A family, member 1	-1.63	0.010
104273_at	1419002_s_at	Baat	bile acid-Coenzyme A: amino acid N-acyltransferase	-1.63	0.001
95122_g_at	1423371_at	Pole4	polymerase (DNA-directed), epsilon 4 (p12 subunit)	-1.63	0.001
100600_at	1448182_a_at	Cd24a	CD24a antigen	-1.64	0.003
102841_at	1435782_at	LOC216818	similar to ubiquitin A-52 residue ribosomal protein fusion product 1	-1.64	0.004

161033_at	1449208_at	Papolb	poly (A) polymerase beta (testis specific)	-1.64	0.005
103660_at	1449442_at	Pex11a	peroxisomal biogenesis factor 11a	-1.65	0.001
92531_at	1455698_at	Tloc1	translocation protein 1	-1.65	0.008
95343_at	1419323_at	Padi1	peptidyl arginine deiminase, type I	-1.65	0.004
99062_at	1417319_at	Pvrl3	poliovirus receptor-related 3	-1.65	0.001
103367_at	1418655_at	Galgt1	UDP-N-acetyl-alpha-D-galactosamine:(N-acetylneuraminyl)-galactosylg	-1.66	0.007
103084_at	1460318_at	Csrp3	cysteine and glycine-rich protein 3	-1.68	0.000
94297_at	1416125_at	Fkbp5	FK506 binding protein 5	-1.69	0.003
96985_at	1422385_at	Olfr1264	olfactory receptor 1264	-1.69	0.005
92925_at	1418901_at	---	---	-1.7	0.000
93500_at	1424126_at	Alas1	aminolevulinic acid synthase 1	-1.7	0.002
94627_at	1450369_at	Figla	factor in the germline alpha	-1.7	0.003
95546_g_at	1452014_a_at	Igf1	insulin-like growth factor 1	-1.7	0.005
96756_at	1435333_at	---	---	-1.7	0.000
92583_at	1423867_at	Serpina3k	serine (or cysteine) proteinase inhibitor, clade A, member 3K	-1.71	0.001
103636_at	1418746_at	MGI:1930773	brain protein 17	-1.72	0.006
92454_at	1422899_at	Slc6a20	solute carrier family 6 (neurotransmitter transporter), member 20	-1.72	0.008
94994_at	1425633_at	Cfh	complement component factor h	-1.72	0.001
102701_at	1425645_s_at	Cyp2b10	cytochrome P450, family 2, subfamily b, polypeptide 10	-1.73	0.003
97179_at	1460713_at	BC048355	CDNA sequence BC048355	-1.73	0.009
99411_at	1422288_at	Htr1b	5-hydroxytryptamine (serotonin) receptor 1B	-1.73	0.000
95981_at	1441401_at	C79329	expressed sequence C79329	-1.74	0.003
97125_f_at	1450534_x_at	LOC56628	MHC (A.CA/J(H-2K-f) class I antigen	-1.75	0.000
101539_f_at	1435371_x_at	Ces3	carboxylesterase 3	-1.76	0.006
101729_at	1423019_at	Gja9	gap junction membrane channel protein alpha 9	-1.78	0.009
103582_r_at	1452054_at	6130401J04Rik	RIKEN cDNA 6130401J04 gene	-1.81	0.002
93304_at	1448741_at	Slc3a1	solute carrier family 3, member 1	-1.81	0.000
99672_at	1448161_a_at	Clcn4-2	chloride channel 4-2	-1.82	0.000
97532_at	1451242_a_at	Ppp5c	protein phosphatase 5, catalytic subunit	-1.85	0.000
96269_at	1451122_at	Idi1	isopentenyl-diphosphate delta isomerase	-1.86	0.004
100755_at	1422928_at	---	---	-1.88	0.001
104285_at	1427229_at	Hmgcr	3-hydroxy-3-methylglutaryl-Coenzyme A reductase	-1.88	0.000
98370_at	1421781_at	Upk2	uroplakin 2	-1.89	0.000
92401_at	1419692_a_at	Ltc4s	leukotriene C4 synthase	-1.92	0.002
94185_at	1421419_at	Kcnk4	potassium channel, subfamily K, member 4	-1.92	0.000
98596_s_at	1449198_a_at	St3gal5	ST3 beta-galactoside alpha-2,3-sialyltransferase 5	-1.92	0.001
98111_at	1425993_a_at	Hsp105	heat shock protein 105	-1.94	0.001
92820_at	1417169_at	Usp2	ubiquitin specific protease 2	-1.98	0.000
93914_at	1448950_at	Il1r1	interleukin 1 receptor, type I	-1.98	0.001
98851_at	1421409_at	Msi1h	Musashi homolog 1(Drosophila)	-2.02	0.006
96643_at	1424351_at	Wfdc2	WAP four-disulfide core domain 2	-2.06	0.000
102204_at	1451715_at	Mafb	v-maf musculoaponeurotic fibrosarcoma oncogene family, protein B (av	-2.07	0.000
102998_at	1450715_at	Cyp1a2	cytochrome P450, family 1, subfamily a, polypeptide 2	-2.09	0.000
98892_at	1418288_at	Lpin1	lipin 1	-2.11	0.005
104659_g_at	1425107_a_at	Lifr	leukemia inhibitory factor receptor	-2.16	0.000
101647_at	1421767_at	---	---	-2.17	0.000
104008_at	1450220_a_at	Spdef	SAM pointed domain containing ets transcription factor	-2.18	0.001
101181_at	1422181_at	Cnga2	cyclic nucleotide gated channel alpha 2	-2.23	0.004
102847_s_at	1422230_s_at	Cyp2a4 /// Cyp2a5	cytochrome P450, family 2, subfamily a, polypeptide 4 /// cytochrome P	-2.27	0.004
93683_at	1450680_at	Rag1	recombination activating gene 1	-2.29	0.003
100069_at	1448792_a_at	Cyp2f2	cytochrome P450, family 2, subfamily f, polypeptide 2	-2.3	0.000
103887_at	1448756_at	S100a9	S100 calcium binding protein A9 (calgranulin B)	-2.3	0.000
97708_at	1459933_at	---	---	-2.31	0.005

95552_at	1417991_at	Dio1	deiodinase, iodothyronine, type I	-2.36	0.001
103599_at	1451053_a_at	Mdm1	transformed mouse 3T3 cell double minute 1	-2.43	0.000
97690_at	1449641_at	---	---	-2.43	0.005
94715_at	1422217_a_at	Cyp1a1	cytochrome P450, family 1, subfamily a, polypeptide 1	-2.46	0.010
92814_at	1417532_at	Cyp2j5	cytochrome P450, family 2, subfamily j, polypeptide 5	-2.48	0.001
92837_f_at	1417835_at	Mug1	murinoglobulin 1	-2.49	0.000
104421_at	1449525_at	Fmo3	flavin containing monooxygenase 3	-2.53	0.000
103448_at	1419394_s_at	S100a8	S100 calcium binding protein A8 (calgranulin A)	-2.6	0.000
92694_at	1425451_s_at	Chi3l3 /// Chi3l4	chitinase 3-like 3 /// chitinase 3-like 4	-2.61	0.000
101719_at	1450606_at	Pnmt	phenylethanolamine-N-methyltransferase	-2.62	0.007
101566_f_at	1420465_s_at	Mup1 /// Mup2	major urinary protein 1 /// major urinary protein 2	-2.63	0.001
92242_at	1419319_at	Saa4	serum amyloid A 4	-2.68	0.000
101473_at	1432517_a_at	Nnmt	nicotinamide N-methyltransferase	-2.69	0.000
102015_at	1435681_s_at	LOC234374	similar to hypothetical protein FLJ10432	-2.78	0.000
97168_at	1421456_at	P2ry1	purinergic receptor P2Y, G-protein coupled 1	-2.81	0.005
92821_at	1417168_a_at	Usp2	ubiquitin specific protease 2	-2.84	0.000
101115_at	1450009_at	Ltf	lactotransferrin	-2.88	0.000
100414_s_at	1415960_at	Mpo	myeloperoxidase	-2.97	0.000
97680_at	1448854_s_at	Mug-ps1 /// Mug1 /// Mug2	murinoglobulin, pseudogene 1 /// murinoglobulin 1 /// murinoglobulin 2 /	-2.97	0.000
104747_at	1448299_at	Slc1a1	solute carrier family 1 (neuronal/epithelial high affinity glutamate transp	-2.98	0.005
93386_at	1418958_at	Amac1	acyl-malonyl condensing enzyme 1	-3.07	0.001
99535_at	1425837_a_at	Ccrn4l	CCR4 carbon catabolite repression 4-like (S. cerevisiae)	-3.19	0.000
101912_at	1434484_at	1100001G20Rik	RIKEN cDNA 1100001G20 gene	-3.22	0.000
92644_s_at	1450194_a_at	Myb	myeloblastosis oncogene	-3.26	0.000
96672_at	1428662_a_at	MGI:1916782	homeobox only domain	-3.29	0.000
100717_at	1460667_at	U90926	cDNA sequence U90926	-3.59	0.008
96153_at	1418722_at	Ngp	neutrophilic granule protein	-4.14	0.000
102416_at	1417017_at	Cyp17a1	cytochrome P450, family 17, subfamily a, polypeptide 1	-4.58	0.000
102651_at	1425303_at	Gck	glucokinase	-7.45	0.000
92322_at	1419691_at	Camp	cathelicidin antimicrobial peptide	-10.12	0.000
160306_at	1424737_at	Thrsp	thyroid hormone responsive SPOT14 homolog (Rattus)	-11.55	0.000
