# Supplementary material for: Delayed and Accelerated Aging Share Common Longevity Assurance Mechanisms
Source: PLoS Genet. 2008 Aug 15;4(8):e1000161. doi: 10.1371/journal.pgen.1000161 (PMC2493043; doi:10.1371/journal.pgen.1000161)
Supplement: Table S3 — A list of all expression profiles of long-lived mice. FC: fold change, P: p-value. Expression profiles from these tables as well as all previously unpublished microarray data are available in public repository Array Express (www.ebi.ac.uk/arrayexpress/) and comply with the MIAME regulations. (0.78 MB PDF) [file pgen.1000161.s007.pdf]

Supplementary table S3. A list of all expression profiles of long-lived mice. FC: fold change, P: p-value

**Annotation data (from two independent Affymetrix platforms)**				**Ghr-/-**		**Ames**		**CR**		**Ames-CR**		**Snell**	
74code	430code	Gene Symbol	Gene Title	FC	P	FC	P	FC	P	FC	P	FC	P
100001_at	1419178_at	Cd3g	CD3 antigen, gamma polypeptide	1.08	0.88	1.17	0.5884	-1.12	0.714	1.42	0.3342	1.24	0.356
100002_at	1449123_at	Itih3	inter-alpha trypsin inhibitor, heavy chain 3	-1.17	0.27	1.12	0.3454	1.17	0.2541	1.3	0.1102	1.12	0.5721
100003_at	1427306_at	Ryr1	ryanodine receptor 1, skeletal muscle	1.27	0.36	-1.83	0.2348	-1.71	0.288	-1.77	0.2528	2.36	0.0251
100004_at	1428531_at	5930412E23Rik	RIKEN cDNA 5930412E23 gene	1.27	0.56	-1.54	0.0001	-1.31	0.0123	-1.62	0.0001	-1.12	0.337
100005_at	1460642_at	Traf4	Tnf receptor associated factor 4	-1.24	0.2	-1.17	0.112	-1	0.9845	-1.21	0.0334	-1.04	0.7293
100007_at	1451252_at	Irf2bp1	interferon regulatory factor 2 binding protein	-1.01	0.96	1.1	0.0893	-1.08	0.2386	-1.03	0.5977	1.23	0.1117
100009_r_at	1416967_at	Sox2	SRY-box containing gene 2	3.02	0.06	-1.47	0.147	-1.28	0.2911	-1.33	0.2314	1.58	0.2698
100010_at	1421604_a_at	Klf3	Kruppel-like factor 3 (basic)	4.46	0.07	1.06	0.3983	-1.14	0.1949	1.02	0.8764	1.38	0.4425
100011_at	1429360_at	Klf3	Kruppel-like factor 3 (basic)	1.05	0.87	-1.13	0.4086	-1.67	0.0012	-1.45	0.0279	1.61	0.0269
100012_at	1426025_s_at	Laptm5	lysosomal-associated protein transmembran	2.49	0.12	1.3	0.539	-1.4	0.1088	1.58	0.3913	2.32	0.1399
100013_at	1424617_at	Ifi35	interferon-induced protein 35	-1.1	0.62	-1.84	0.0087	-1.23	0.0694	-1.5	0.0372	-2.42	0.0157
100014_at	1416944_a_at	Tlk2	tousled-like kinase 2 (Arabidopsis)	-1.32	0.35	1.02	0.8719	1.16	0.1892	1.04	0.7064	-1.07	0.8055
100015_at	1449090_a_at	Yes1	Yamaguchi sarcoma viral (v-yes) oncogene	1.08	0.65	1	0.9994	1.01	0.9314	-1.03	0.5584	1.59	0.034
100016_at	1417234_at	Mmp11	matrix metalloproteinase 11	1.71	0.01	-1.34	0.0918	-1.06	0.7067	-1.15	0.3138	-1.02	0.9506
100017_at	1419487_at	Mybph	myosin binding protein H	-1.3	0.28	-1.28	0.0568	-1.19	0.1026	-1.24	0.0313	-1.08	0.6238
100018_at	1418333_at	Mtf1	metal response element binding transcriptio	-1.15	0.31	1.26	0.0556	1.13	0.39	1.06	0.7321	1.74	0.0558
100019_at	1427256_at	Cspg2	chondroitin sulfate proteoglycan 2	-2.8	0.37	-1.15	0.3047	1.3	0.5542	-1.27	0.1091	1.09	0.4982
100020_at	1416637_at	Slc4a2	solute carrier family 4 (anion exchanger), me	-1	0.99	1.02	0.6836	1.04	0.6444	-1.02	0.6993	1.13	0.0588
100021_at	1418852_at	Chrna1	cholinergic receptor, nicotinic, alpha polypep	-1.63	0.41	-1.01	0.9443	1.02	0.9351	1.28	0.3124	1.85	0.1162
100022_at	1448724_at	Cish	cytokine inducible SH2-containing protein	-2.88	0.03	-2.47	0.0001	-1.37	0.023	-2.5	0.0001	1	0.9967
100023_at	1417656_at	Mybl2	myeloblastosis oncogene-like 2	-1.75	0.41	1.42	0.0145	1.28	0.0453	1.16	0.1448	2.44	0.1583
100024_at	1422629_s_at	Shrm	shroom	-1.08	0.68	-1	0.9995	-1.26	0.0899	-1.08	0.5601	-1.12	0.4672
100026_at	1450871_a_at	Bcat1	branched chain aminotransferase 1, cytosoli	-1.2	0.8	-1.07	0.7478	-1.06	0.7933	1.11	0.6257	1.19	0.6729
100029_at	1419053_at	Pex14	peroxisomal biogenesis factor 14	-1.33	0.26	-1.04	0.6207	1.04	0.7261	-1.12	0.1134	-1.05	0.614
100030_at	1448562_at	Upp1	uridine phosphorylase 1	-1.5	0.43	1.5	0.0114	1.23	0.0585	1.43	0.0213	1.17	0.4779
100032_at	1418180_at	Sp1	Trans-acting transcription factor 1	1.03	0.97	-1	0.9812	-1.43	0.1824	-1.47	0.0954	1.42	0.1871
100033_at	1416988_at	Msh2	mutS homolog 2 (E. coli)	2.1	0.05	1.13	0.5904	1.1	0.4776	1.18	0.2821	1.63	0.2383
100034_at	1421752_a_at	Serpinb5	serine (or cysteine) proteinase inhibitor, clad	-2.59	0.04	-1.32	0.0901	1.1	0.5053	-1.08	0.607	1.32	0.5329
100035_at	1449160_at	Npr1 /// LOC435	natriuretic peptide receptor 1 /// similar to Tu	-5.03	0.35	1.05	0.8927	1.4	0.3748	1.04	0.9167	2.75	0.0784
100039_at	1448372_a_at	Tmem4	transmembrane protein 4	-1.01	0.92	-1.3	0.017	-1.02	0.7601	-1.37	0.0072	-1.15	0.0829
100040_at	1450866_a_at	Mrpl17	mitochondrial ribosomal protein L17	1.64	0.06	1.18	0.091	1.34	0.0041	1.28	0.0271	1.25	0.1266
100041_at	1456176_x_at	D11Ertd333e	DNA segment, Chr 11, ERATO Doi 333, exp	-1.18	0.11	-1.09	0.1684	1.04	0.4997	-1.07	0.3121	1.04	0.7279
100042_at	1424172_at	Hagh	hydroxyacyl glutathione hydrolase	-1.01	0.94	-1.15	0.0107	-1.05	0.1805	-1.25	0.0025	-1.47	0.007
100043_f_at	1429457_at	2310020A21Rik	RIKEN cDNA 2310020A21 gene	-1.06	0.85	-1.04	0.599	-1.09	0.3194	1.05	0.6008	1.59	0.0948
100044_at	1416003_at	Cldn11	claudin 11	-1.43	0.27	1.14	0.6121	-1.39	0.1646	1.26	0.3175	1.92	0.2518
100046_at	1419253_at	Mthfd2	methylenetetrahydrofolate dehydrogenase (I	-2.2	0.13	1.13	0.7688	1.18	0.5183	1.7	0.0629	1.17	0.6865
100047_at	1416828_at	Snap25	synaptosomal-associated protein 25	1.12	0.49	1.05	0.6373	1.1	0.4351	1.18	0.0649	-1.06	0.9263
100048_at	1424139_at	Rap1a	RAS-related protein-1a	1.85	0.19	-1.03	0.7524	-1.24	0.0258	-1.29	0.0109	-1.27	0.3927
100050_at	1425895_a_at	Id1	inhibitor of DNA binding 1	1.14	0.81	1.33	0.1777	1.12	0.5603	1.6	0.005	1.48	0.1685
100051_at	1419097_a_at	Stom	stomatin	-1.41	0.11	1.07	0.3876	1.02	0.738	1.09	0.2948	1.18	0.0167
100052_at	1452522_at	Stom	stomatin	-1.03	0.92	1.47	0.1417	1.18	0.3579	-1.02	0.8965	1.42	0.2221
100054_s_at	1434119_at	D2Wsu81e	DNA segment, Chr 2, Wayne State Universi	1.57	0.31	1.06	0.6857	1.36	0.1491	1.26	0.1837	2.11	0.0464
100056_at	1423442_a_at	Fbxw2	F-box and WD-40 domain protein 2	1.02	0.93	1.1	0.2408	-1.11	0.1749	-1.07	0.1758	1.07	0.172
100057_at	1436121_a_at	Nsmce1	non-SMC element 1 homolog (S. cerevisiae)	1.59	0.06	-1.03	0.5683	1.13	0.1543	1.14	0.0066	1.19	0.125
100058_at	1451975_at	2810453I06Rik	RIKEN cDNA 2810453I06 gene	-1.13	0.84	1.12	0.7645	-1.68	0.1001	1.58	0.231	3.61	0.0543
100059_at	1454268_a_at	Cyba	cytochrome b-245, alpha polypeptide	1.31	0.46	1.23	0.4371	-1.13	0.2391	1.61	0.3138	1.03	0.8729
100061_f_at	1455977_x_at	Klk6 /// Klk5	kallikrein 6 /// kallikrein 5	1.13	0.48	1.42	0.4442	2.29	0.0246	1.19	0.4989	1.49	0.0607
100064_f_at	1438973_x_at	Gja1	gap junction membrane channel protein alph	1.34	0.38	-1.05	0.6067	1	0.9693	-1.33	0.0066	1.68	0.0004
100065_r_at	1415800_at	Gja1	gap junction membrane channel protein alph	-1.2	0.29	1.39	0.0917	1.23	0.2158	1.19	0.4748	1.67	0.3229
100066_at	1416283_at	Gart	phosphoribosylglycinamide formyltransferas	1.12	0.59	-1.02	0.7392	1.01	0.8983	-1.01	0.8624	-1.12	0.1647

100068_at	1416468_at	Aldh1a1	aldehyde dehydrogenase family 1, subfamily	-1.01	0.95	1.03	0.5379	-1.11	0.1383	1.01	0.8081	-1.61	0.0066
100069_at	1448792_a_at	Cyp2f2	cytochrome P450, family 2, subfamily f, poly	-2.35	0.11	-2.4	0	-1.75	0.0017	-4.4	0	-6.62	0.0035
100072_at	1450953_at	Wdr39	WD repeat domain 39	1.6	0.08	1.03	0.6354	1.3	0.0204	1.04	0.5607	-1.11	0.4442
100073_at	1449072_a_at	2510005D08Rik	RIKEN cDNA 2510005D08 gene	1.29	0.36	-1.06	0.4808	-1.03	0.7734	-1.03	0.6922	1.01	0.8833
100074_at	1439448_x_at	Tmed9	Transmembrane emp24 protein transport dc	-1.03	0.89	-1.41	0.0008	-1.59	0.0019	-1.83	0	-1.73	0.0035
100078_at	1436504_x_at	---	---	-1.13	0.69	-3.96	0	1.1	0.6853	-3.3	0	-4.67	0.0001
100079_at	1452184_at	Ndufb9	NADH dehydrogenase (ubiquinone) 1 beta s	1.28	0.04	-1.04	0.565	1.02	0.7126	1.07	0.3868	-1.12	0.378
100080_at	1419348_at	Psp	parotid secretory protein	-2.2	0.2	1.12	0.6501	1.19	0.546	-1.52	0.0767	1.23	0.4043
100081_at	1415909_at	Stip1	stress-induced phosphoprotein 1	1.25	0.24	-1.42	0.0006	-1.37	0.013	-1.48	0.0003	1.01	0.944
100085_at	1448485_at	Ggt1	gamma-glutamyltransferase 1	1.78	0.24	-1.21	0.4145	-1.04	0.8604	-1.25	0.4191	2.37	0.0518
100086_at	1452148_at	Lrpap1	low density lipoprotein receptor-related prote	1.23	0.37	-1.09	0.2467	1.08	0.3384	1.04	0.6894	-1.08	0.6397
100088_at	1433540_x_at	Ppp1cb	protein phosphatase 1, catalytic subunit, bet	1.04	0.91	1.3	0.0168	1.16	0.1657	1.37	0.0052	-1	0.9917
100089_at	1416498_at	Ppic	peptidylprolyl isomerase C	1.47	0.08	1.31	0.0097	1.19	0.3706	1.27	0.0355	1.19	0.6134
100091_at	1448769_at	Slc35b1	solute carrier family 35, member B1	-1.31	0.04	-1.18	0.0528	-1.15	0.1995	-1.2	0.039	-2.18	0.0056
100093_at	1460169_a_at	Pctk1	PCTAIRE-motif protein kinase 1	-1.03	0.81	1.03	0.7074	1	0.9906	1.12	0.1077	1.09	0.3146
100094_at	1424255_at	Supt5h	suppressor of Ty 5 homolog (S. cerevisiae)	1.64	0.03	1.18	0.0259	1.16	0.1363	1.4	0	1.47	0.0022
100095_at	1416050_a_at	Scarb1	scavenger receptor class B, member 1	1.33	0.53	1.45	0.0004	1.06	0.6901	1.55	0.0001	1.23	0.1785
100099_at	1448621_a_at	Smpd1	sphingomyelin phosphodiesterase 1, acid ly:	-1.4	0.28	-1.17	0.0121	-1.15	0.26	-1.26	0.004	-1.34	0.0213
100101_at	1417274_at	Snrpa	small nuclear ribonucleoprotein polypeptide	1.43	0.14	1.01	0.8869	-1.12	0.1921	-1.08	0.2411	-1.25	0.3157
100104_r_at	1422435_at	2210010C04Rik	RIKEN cDNA 2210010C04 gene	-1.84	0.37	1.05	0.73	-1	0.9927	-1.09	0.6554	1.63	0.2242
100106_at	1417370_at	Tff3	trefoil factor 3, intestinal	1.8	0.46	2.51	0.1754	2.21	0.3423	8.28	0.0489	2.7	0.2278
100112_at	1417574_at	Cxcl12	chemokine (C-X-C motif) ligand 12	-1.02	0.93	-1.03	0.6108	-1.01	0.7813	-1.28	0.002	-1.14	0.1816
100113_s_at	1451783_a_at	Kifap3	kinesin-associated protein 3	1.28	0.27	1.04	0.8351	-1.06	0.7429	-1.3	0.2288	1.56	0.0766
100115_at	1426289_at	2610028H07Rik	RIKEN cDNA 2610028H07 gene	-1.24	0.29	-1.05	0.3118	-1.02	0.7375	1.04	0.639	1.18	0.0696
100116_at	1419153_at	2810417H13Rik	RIKEN cDNA 2810417H13 gene	1.01	0.98	1.54	0.0519	-1.05	0.7281	1.68	0.041	1.72	0.019
100120_at	1416808_at	Nid1	nidogen 1	-1.42	0.13	1.01	0.9548	-1.08	0.6358	1.27	0.1319	1.58	0.0542
100122_at	1422208_a_at	Gnb5	guanine nucleotide binding protein, beta 5	1.26	0.56	1.5	0.2876	-1.16	0.7197	-1.33	0.4765	1.45	0.4104
100125_at	1450854_at	Pa2g4	proliferation-associated 2G4	1.49	0.2	-1.02	0.8739	1.15	0.1117	1.13	0.171	-1.09	0.6973
100126_at	1422505_at	Chrac1	chromatin accessibility complex 1	1.62	0.01	1.2	0.0018	1.31	0	1.31	0	1.14	0.3369
100127_at	1451191_at	Crabp2	cellular retinoic acid binding protein II	1.15	0.85	-1.23	0.4247	1.2	0.6328	-1.22	0.5199	-1.38	0.5346
100128_at	1448314_at	Cdc2a	cell division cycle 2 homolog A (S. pombe)	2.37	0.38	2.45	0.2273	-1.03	0.8078	3.06	0.0661	2.01	0.1028
100130_at	1448694_at	Jun	Jun oncogene	1.86	0.16	1.86	0.1434	1.28	0.5023	1.35	0.4201	2.77	0.0052
100131_at	1423150_at	Sgne1	secretory granule neuroendocrine protein 1,	-2.45	0.38	1.05	0.8996	1.05	0.8964	1.36	0.3294	1.11	0.8231
100133_at	1448765_at	---	---	1.15	0.36	1.12	0.345	1.1	0.5668	1.03	0.8444	-1.04	0.8264
100134_at	1417271_a_at	Eng	endoglin	-1.27	0.16	-1.15	0.1841	1.09	0.2651	-1.22	0.0876	-1.11	0.3748
100136_at	1416344_at	Lamp2	lysosomal membrane glycoprotein 2	1.33	0.24	-1.21	0.0087	-1.24	0.0108	-1.13	0.086	-1.91	0
100138_f_at	1456566_x_at	Rbm14	RNA binding motif protein 14	1.84	0.06	-1.18	0.096	1.25	0.0764	1.02	0.8799	1.18	0.6764
100139_at	1416965_at	Pcsk1n	proprotein convertase subtilisin/kexin type 1	-1.3	0.14	-1.21	0.2481	-1.03	0.8246	-1.45	0.0171	1.64	0.0725
100142_at	1421810_at	---	---	1.13	0.65	-1.15	0.0356	-1.11	0.1083	-1.16	0.0163	1.17	0.4142
100144_at	1456528_x_at	---	---	2.18	0	1.22	0.0004	1.08	0.542	1.28	0.0027	1.62	0.0055
100147_at	1423644_at	Aco1	aconitase 1	-1.49	0.47	-1.6	0.0038	-1.07	0.538	-1.48	0.0114	-1.65	0.2683
100148_at	1418330_at	Ctcf	CCCTC-binding factor	-1.4	0.48	1.05	0.5301	1.08	0.4323	1.08	0.246	1.02	0.9757
100151_at	1455550_x_at	---	---	-1.19	0.13	1.12	0.0337	1.14	0.0378	1.14	0.0804	-1.09	0.4028
100152_at	1421966_at	Ncam1	neural cell adhesion molecule 1	3.21	0.06	-1.57	0.1074	1.35	0.381	-1.02	0.9415	1.79	0.1962
100153_at	1426864_a_at	Ncam1	neural cell adhesion molecule 1	-1.13	0.61	-1.08	0.4664	-1.04	0.7159	-1.21	0.1494	1.17	0.4529
100154_at	1421812_at	---	---	1.37	0.15	1.29	0.3905	-1.14	0.0798	1.47	0.1748	-1.38	0.0524
100155_at	1456226_x_at	Ddr1	discoidin domain receptor family, member 1	5.26	0	1.06	0.6871	1.04	0.8484	1.38	0.0977	-1.17	0.5823
100156_at	1415945_at	Mcm5	minichromosome maintenance deficient 5, c	2.07	0.23	1.2	0.4551	-1.18	0.2918	1.44	0.1416	-1.03	0.9249
100213_f_at	1455578_x_at	Rpl41	ribosomal protein L41	1.06	0.41	-1.06	0.3256	1.13	0.0071	-1.03	0.6541	1.31	0.0009
100272_at	1452563_a_at	Jarid1d	jumonji, AT rich interactive domain 1D (Rbp2	-1.95	0.32	1.11	0.6331	1.05	0.8267	-1.26	0.1731	-2.34	0.0507
100277_at	1422053_at	Inhba	inhibin beta-A	1.22	0.65	-1.47	0.0287	-1.59	0.0275	-1.6	0.0087	-2.46	0.0018
100278_at	1419497_at	Cdkn1b	cyclin-dependent kinase inhibitor 1B (P27)	-1.57	0.13	-1.31	0.286	-1.38	0.196	-1.65	0.0786	1.15	0.5703
100279_at	1421890_at	St3gal2	ST3 beta-galactoside alpha-2,3-sialyltransfe	-1.47	0.26	1.12	0.3346	1.01	0.968	1.02	0.8932	1.08	0.473

100280_at	1450608_at	Hist1h1t	histone 1, H1t	-1.79	0.02	1.01	0.9343	1.19	0.3728	1.11	0.6163	1.46	0.0964
100282_at	1422235_at	Htr7	5-hydroxytryptamine (serotonin) receptor 7	-1.42	0.23	-1	0.9639	1.11	0.3878	-1.04	0.7399	1.14	0.2956
100283_at	1424985_a_at	Sox10	SRY-box containing gene 10	1.23	0.56	-1.05	0.6227	-1.02	0.8613	-1.21	0.059	1.15	0.5108
100284_at	1421539_at	Zic4	zinc finger protein of the cerebellum 4	-3.03	0.19	-1.06	0.6871	1.09	0.5915	1.08	0.8051	1.41	0.4228
100286_at	1418267_at	Mst1	macrophage stimulating 1 (hepatocyte growt	-1.02	0.86	-1.03	0.5157	1.04	0.4432	1.16	0.0393	1.05	0.7447
100287_at	1430512_a_at	MGI:1354380	immunoglobulin (CD79A) binding protein 1b	-2.32	0.37	1.59	0.1257	1.61	0.1034	1.51	0.1159	1.4	0.4363
100290_f_at	1455488_at	6230416J20Rik	RIKEN cDNA 6230416J20 gene	7.14	0.05	1.08	0.7313	-1.35	0.466	1.35	0.1719	1.67	0.079
100291_at	1450457_at	Cbl	Casitas B-lineage lymphoma	1.06	0.91	1.23	0.3131	1.23	0.3483	1.2	0.4192	1.24	0.2108
100292_at	1449105_at	Sh2d2a	SH2 domain protein 2A	-1.18	0.57	1.23	0.0688	1.07	0.3621	1.02	0.8388	1.81	0.1682
100294_at	1421229_at	Dffb	DNA fragmentation factor, beta subunit	-1.36	0.68	1.26	0.5594	1.56	0.1793	1.27	0.4938	-1.01	0.9861
100297_at	1438234_at	Wdr26	WD repeat domain 26	-1.14	0.77	1.12	0.4548	-1.52	0.0209	1.01	0.9482	1.42	0.0613
100298_at	1450375_at	Pspn	persephin	-1.22	0.42	1.29	0.0223	1.22	0.1308	1.24	0.0529	1.39	0.2383
100300_at	1422978_at	Cybb	cytochrome b-245, beta polypeptide	-1.59	0.14	2.15	0.205	-1.16	0.4902	2.49	0.1998	1.17	0.5451
100301_at	1422986_at	Esrrb	estrogen related receptor, beta	1.1	0.52	1.59	0.02	1.65	0.0221	1.55	0.1288	-1.32	0.2687
100303_at	1450587_at	H2-M10.1	histocompatibility 2, M region locus 10.1	1.68	0.27	-1.02	0.9309	-1.36	0.2273	1.3	0.4594	1.09	0.8011
100306_at	1459902_at	2700007P21Rik	RIKEN cDNA 2700007P21 gene	3.39	0	1.17	0.3392	1.06	0.7588	1.32	0.1054	1.91	0.0882
100307_at	1459909_at	Nfix	Nuclear factor I/X	-1.51	0.03	-1.52	0.0004	-1.04	0.7957	-1.45	0.0044	-1.25	0.1693
100308_at	1418440_at	Col8a1	procollagen, type VIII, alpha 1	1.08	0.8	1.17	0.345	1.18	0.494	1.07	0.7173	2.31	0.1905
100309_at	1422990_at	Met	met proto-oncogene	-1.27	0.33	1.67	0.0591	1.24	0.4572	1.5	0.151	-2.21	0.3083
100311_f_at	1421802_at	Ear1	eosinophil-associated, ribonuclease A family	2.74	0.07	1.55	0.3989	-1.23	0.507	1.72	0.3232	1.66	0.0237
100317_at	1422159_at	Ppef2	protein phosphatase, EF hand calcium-bindi	-2.39	0.08	-1.32	0.2266	1.01	0.9607	-1.72	0.0389	1.47	0.4155
100318_at	1431119_at	1700014P03Rik	RIKEN cDNA 1700014P03 gene	1.14	0.6	1.04	0.7454	-1.01	0.8553	-1.02	0.8674	1.13	0.5261
100319_at	1450330_at	Il10	interleukin 10	1.07	0.9	-1.34	0.5385	-1.13	0.7918	1.88	0.1254	1.38	0.501
100321_f_at	1449910_at	2210418O10Rik	RIKEN cDNA 2210418O10 gene	-1.01	0.99	-1.16	0.1335	-1.35	0.011	-1.32	0.0254	-1.14	0.6066
100324_g_at	1416835_s_at	Amd1 /// Amd2	S-adenosylmethionine decarboxylase 1 /// S	1.12	0.78	1.12	0.4441	1.29	0.1693	1.06	0.6787	-1.02	0.9542
100326_f_at	1425417_x_at	---	---	-1.17	0.59	-1.21	0.1918	-1.22	0.1655	-1.01	0.929	1.11	0.4693
100328_s_at	1420464_s_at	Pira1 /// Pira2 //	paired-Ig-like receptor A1 /// paired-Ig-like re	-2.04	0.03	1.05	0.85	-1.22	0.1319	1.56	0.3727	1.08	0.6581
100331_g_at	1418506_a_at	Prdx2	peroxiredoxin 2	1.56	0.01	1.29	0	1.12	0.0336	1.39	0	1.35	0.0295
100332_s_at	1423223_a_at	Prdx6	peroxiredoxin 6	1.6	0.17	1.02	0.7389	-1.17	0.3489	1.1	0.4393	-1.3	0.3318
100334_f_at	1421788_x_at	Klk13	kallikrein 13	1.07	0.85	-1.08	0.6073	1.05	0.7362	1.03	0.8	1.31	0.2481
100335_at	1450283_at	Atp7b	ATPase, Cu++ transporting, beta polypeptid	-1.86	0.38	-1.01	0.9075	-1.33	0.1751	-1.09	0.6003	1.48	0.2657
100336_s_at	1449880_s_at	Bglap-rs1 /// Bgl	bone gamma-carboxyglutamate protein, rela	-1.03	0.65	-1.4	0.0179	1.02	0.886	-1.18	0.1819	1.45	0.0155
100337_at	1421692_at	Cacna1e	calcium channel, voltage-dependent, R type	-3.14	0.25	1.39	0.3353	-1.13	0.5593	1.29	0.4707	1.04	0.884
100339_at	1424898_at	Slc10a1	solute carrier family 10 (sodium/bile acid cot	-1.21	0.34	-1.48	0	-1.01	0.9294	-1.23	0.0135	-1.43	0.0533
100341_g_at	1450261_a_at	Slc10a1	solute carrier family 10 (sodium/bile acid cot	-1.42	0.26	-1.53	0	1.01	0.883	-1.36	0.0005	-1.59	0.0089
100343_f_at	1418884_x_at	Tuba1	tubulin, alpha 1	-1.24	0.12	-1.39	0.0307	-1.11	0.3268	-1.42	0.011	-1.88	0.0263
100344_at	1460696_at	BC026585	cDNA sequence BC026585	1.38	0.05	-1.13	0.4438	-1.09	0.4729	-1.39	0.0429	1.06	0.7801
100346_at	1420795_at	Fgf9	fibroblast growth factor 9	-1.82	0.26	-1.07	0.7747	1.06	0.8293	-1.25	0.332	1.64	0.3723
100347_at	1447986_at	D17892	expressed sequence D17892	1.07	0.15	1.06	0.5053	1.05	0.4636	1.1	0.2455	1.15	0.2491
100348_at	1456319_at	X83313	EST X83313	1.41	0.55	-1.01	0.9805	-1.13	0.7146	1.03	0.9213	-3.09	0.1928
100350_at	1441583_at	---	Transcribed locus, moderately similar to NP_	-1.01	0.95	1.01	0.9594	-1.17	0.6181	1.13	0.6718	1.59	0.18
100355_g_at	1422195_s_at	Tbx15	T-box 15	-1.04	0.95	1.5	0.1295	1.36	0.2495	1.46	0.2783	1.44	0.4123
100358_s_at	1422225_s_at	Tcp10a /// Tcp1	t-complex protein 10a /// t-complex protein 1	1.59	0.22	1	0.9979	-1.12	0.6612	-1.49	0.1842	1.05	0.9171
100359_at	1427421_at	Tcp10c	T-complex protein 10c	-1.85	0.24	-1.37	0.0835	1.07	0.7147	-2.2	0.0036	1.71	0.23
100364_at	1426106_a_at	Syt6	synaptotagmin 6	-1.01	0.97	1.21	0.4504	-1.24	0.3383	-1.07	0.7785	1.03	0.8653
100368_at	1426125_a_at	Casp9	caspase 9	-1.87	0.58	-1.09	0.4131	-1.57	0.0387	-1.71	0.0094	-1.96	0.0471
100374_at	1423582_at	---	---	-1.27	0.68	1.03	0.7675	1.33	0.0151	1.14	0.251	-1.17	0.6691
100378_at	1459701_x_at	---	---	1.41	0.56	-2.03	0.0018	-1.53	0.0937	-1.74	0.0344	1.46	0.3084
100380_at	1434127_a_at	H3f3a	H3 histone, family 3A	-1.18	0.5	1.29	0.0055	1.18	0.1626	1.34	0.0013	-1.1	0.4426
100381_at	1427735_a_at	Acta1	actin, alpha 1, skeletal muscle	1.18	0.78	-16.59	0.1812	-7.78	0.2144	-30.65	0.1693	1.46	0.2005
100382_at	1421517_at	St6galnac1	ST6 (alpha-N-acetyl-neuraminyl-2,3-beta-ga	1.4	0.41	1.22	0.1916	-1.01	0.9626	1.32	0.0851	1.48	0.3448
100383_at	1460385_a_at	Zfp179	zinc finger protein 179	1.27	0.59	1.04	0.8674	-1.24	0.3756	1.31	0.3141	2.58	0.052
100384_at	1425786_a_at	Hsf4	heat shock transcription factor 4	1.81	0.27	1.41	0.1764	1.18	0.592	1.34	0.4222	1.34	0.1109

100385_at	1422277_at	Glra1	glycine receptor, alpha 1 subunit	-1.59	0.41	-1.01	0.9576	1.02	0.8369	-1.1	0.5678	1.26	0.2845
100390_s_at	1450571_a_at	Bfsp1	beaded filament structural protein in lens-CF	1.45	0.49	-1.09	0.5713	-1	0.9875	-1.23	0.1418	2.61	0.0031
100391_at	1420931_at	Mapk8	mitogen activated protein kinase 8	-2.46	0.27	1.15	0.6624	1.09	0.801	-1.02	0.9485	1.66	0.1573
100394_at	1417396_at	Podxl	podocalyxin-like	-1.63	0.63	1.17	0.2385	1.17	0.239	1.25	0.173	1.33	0.2795
100397_at	1450792_at	Tyrobp	TYRO protein tyrosine kinase binding protein	1.43	0.14	1.69	0.1974	-1.03	0.835	1.96	0.1958	1.04	0.8709
100398_at	1449841_at	Kif3a	kinesin family member 3A	2.49	0.02	-1.11	0.4531	1.08	0.6999	-1.19	0.2307	-1.37	0.3321
100400_at	1416668_at	4921531G14Rik	RIKEN cDNA 4921531G14 gene	2.25	0	1.14	0.4169	1.09	0.6427	1.12	0.3426	-1.22	0.2401
100401_at	1420952_at	Son	Son cell proliferation protein	-1.07	0.6	1.22	0.0678	-1.07	0.588	1.11	0.4599	1.32	0.0932
100403_at	1449071_at	Myl7	myosin, light polypeptide 7, regulatory	-1.03	0.96	-1.09	0.5364	-1.17	0.163	-1.13	0.312	1.4	0.3473
100404_at	1448652_at	Ttc10	tetratricopeptide repeat domain 10	-1.28	0.18	1.09	0.1811	-1.15	0.1445	-1.07	0.4619	1.31	0.0202
100406_at	1423544_at	Ptpn5	protein tyrosine phosphatase, non-receptor t	-1.5	0.45	1.35	0.3225	1.06	0.862	1.09	0.7562	1.61	0.0264
100407_at	1460668_at	Gal	galanin	-1.03	0.83	1.25	0.0052	1.15	0.0316	1.1	0.1952	1.08	0.6849
100408_at	1421015_s_at	Pole3	polymerase (DNA directed), epsilon 3 (p17 s	1.24	0.24	-1.32	0.0703	-1.11	0.4127	-1.18	0.1347	1.27	0.3547
100409_at	1426673_at	Cdh3	cadherin 3	1.13	0.74	-1.22	0.1299	-1.08	0.4501	-1.16	0.1361	-1.01	0.8663
100410_at	1429558_a_at	C330027G06Rik	RIKEN cDNA C330027G06 gene	2.57	0.1	1.17	0.3248	-1.28	0.169	-1.02	0.8921	1.55	0.3236
100412_g_at	1450637_a_at	Aebp1	AE binding protein 1	2.14	0	-1.16	0.4493	-1.25	0.112	1.08	0.7472	2.02	0.2994
100413_at	1424532_at	Ylpm1	YLP motif containing 1	1.49	0.02	1.09	0.2753	1.03	0.7383	-1.01	0.8303	1.19	0.2116
100414_s_at	1415960_at	Mpo	myeloperoxidase	-1.22	0.72	1.22	0.3145	1.08	0.5554	1.31	0.2775	-1.09	0.6966
100416_at	1416558_at	Melk	maternal embryonic leucine zipper kinase	1.12	0.8	-1.58	0.1054	1.26	0.6601	-1.09	0.7739	2.58	0.027
100418_at	1418451_at	Gng2	guanine nucleotide binding protein (G protein	1.07	0.89	1.15	0.4866	-1.11	0.6446	1.19	0.4532	1.09	0.6599
100420_at	1427268_at	LOC433621	similar to Acidic ribosomal phosphoprotein P	-2.99	0.02	-1.13	0.4417	-1.31	0.0357	-1.33	0.0262	-1.19	0.5295
100421_at	1418735_at	Krt2-4	keratin complex 2, basic, gene 4	-1.57	0	-1.01	0.8767	-1.08	0.4589	-1.02	0.7926	1.23	0.043
100423_f_at	1421469_a_at	Stat5a	signal transducer and activator of transcriptio	-1.46	0.03	-1.13	0.2235	-1.15	0.2093	-1.11	0.2639	1.28	0.0621
100425_at	1418261_at	Syk	spleen tyrosine kinase	-1.41	0.13	1.02	0.8457	1.02	0.7981	1.1	0.5253	1.29	0.2298
100426_s_at	1425797_a_at	Syk	spleen tyrosine kinase	-1.13	0.15	1.06	0.5227	1.15	0.1463	1.1	0.3315	1.22	0.316
100427_at	1417676_a_at	Ptpro	protein tyrosine phosphatase, receptor type,	1.15	0.74	1.1	0.7074	-1.01	0.9708	1.15	0.6021	1.81	0.0972
100428_at	1421279_at	Lamc2	laminin, gamma 2	-2.58	0.07	-1.49	0.0368	-1.13	0.4091	-1.26	0.206	-1.13	0.5438
100429_at	1416618_at	Ppox	protoporphyrinogen oxidase	1.45	0.24	-1.16	0.1629	-1.17	0.1169	-1.13	0.1987	-1.31	0.1279
100430_at	1425873_a_at	Lepr	leptin receptor	-3.61	0.1	-1.14	0.5338	-1.11	0.767	-1.21	0.4231	1.56	0.2729
100431_at	1425644_at	Lepr	leptin receptor	3.23	0.23	2.59	0.1299	7.7	0.1662	7.89	0.0358	23.26	0.0855
100434_s_at	1420713_a_at	Mdfi	MyoD family inhibitor	1.11	0.74	1.06	0.4537	-1.16	0.2557	1.16	0.4009	-1.43	0.4362
100435_at	1417143_at	Edg2	endothelial differentiation, lysophosphatidic a	-1.38	0.21	-1.04	0.774	1.07	0.6092	-1	0.9748	1.18	0.5295
100437_g_at	1451054_at	Orm1	orosomucoid 1	-2.05	0.03	-1.21	0.2284	-1.12	0.1558	-1.08	0.6379	-13.45	0.0004
100438_at	1421756_a_at	Gpr19	G protein-coupled receptor 19	-1.66	0.24	1.56	0.0049	1.53	0.0304	1.45	0.0234	1.1	0.7897
100440_f_at	1419421_at	Ank1	ankyrin 1, erythroid	-1.31	0.26	1.11	0.1066	1.02	0.7857	1.09	0.1637	1.45	0.0077
100443_at	1425764_a_at	Bcat2	branched chain aminotransferase 2, mitocho	-1.22	0.31	1.08	0.5401	1.21	0.1701	-1	0.9927	1.35	0.1349
100444_at	1450674_at	Cdk5	cyclin-dependent kinase 5	1.21	0.25	1.01	0.8579	-1.09	0.4717	1.01	0.799	-1.2	0.5383
100447_at	1448679_at	Hyal2	hyaluronidase 2	-1.01	0.99	1.02	0.8263	-1.08	0.4987	-1.1	0.3483	-1.56	0.0833
100450_r_at	1451604_a_at	Acvrl1	activin A receptor, type II-like 1	1.76	0.08	-1.23	0.1729	-1.01	0.9209	-1.1	0.3169	-1.17	0.3148
100451_at	1424622_at	Hsf1	heat shock factor 1	1.57	0.18	1.31	0.162	1.65	0.027	1.35	0.1514	-1.26	0.3518
100452_at	1418600_at	Klf1	Kruppel-like factor 1 (erythroid)	1.64	0.13	1.34	0.0206	1.22	0.0958	1.29	0.0563	-1.18	0.3486
100453_at	1448676_at	Camk2b	calcium/calmodulin-dependent protein kinas	6.05	0.3	1.35	0.2876	-1.03	0.9247	2.31	0.0044	1.54	0.0143
100454_at	1416674_at	Ptpru	protein tyrosine phosphatase, receptor type,	2.48	0.01	1.03	0.8932	1.26	0.0488	1.11	0.4738	1.11	0.5869
100455_at	1416688_at	Snap91	synaptosomal-associated protein 91	-1.8	0.21	-1.35	0.4751	-1.89	0.1674	-1.17	0.6882	1.06	0.9087
100457_at	1448579_at	Glg1	golgi apparatus protein 1	1.32	0.41	-1.31	0.0987	-1.96	0.0055	-1.78	0.0043	-1.01	0.9379
100458_at	1423173_at	Napb	N-ethylmaleimide sensitive fusion protein att	-2.24	0.01	-1.17	0.1929	1.02	0.8603	-1.28	0.0758	1.47	0.3121
100459_at	1422630_at	Rad50	RAD50 homolog (S. cerevisiae)	1.36	0.36	1.15	0.209	1.21	0.2709	1.22	0.1114	1.14	0.4306
100460_at	1422679_s_at	Sh2bp1	SH2 domain binding protein 1 (tetratricopept	1.73	0.05	-1	0.9998	1.06	0.352	1.06	0.323	-1.11	0.4477
100461_at	1417720_at	Polr2j	polymerase (RNA) II (DNA directed) polypep	-1.31	0.32	-1.11	0.1087	-1.1	0.3768	1.01	0.875	1.02	0.924
100463_at	1436170_a_at	Csng	casein gamma	-1.28	0.14	-1.09	0.4178	1.08	0.4931	1	0.9789	2.88	0.317
100464_at	1428468_at	3110043O21Rik	RIKEN cDNA 3110043O21 gene	-1.19	0.06	1.11	0.4109	1.06	0.6205	1.35	0.0476	1.09	0.4892
100468_g_at	1419120_at	Lyl1	lymphoblastomic leukemia	1.92	0.11	1.43	0.2692	1.34	0.2486	2.08	0.0798	1.56	0.3693
100469_at	1452560_a_at	---	---	1.28	0.3	1.06	0.5265	1.19	0.1067	1.07	0.561	1.35	0.1158

100470_at	1448342_at	Mapk10	mitogen activated protein kinase 10	-1.82	0.12	-1.18	0.5009	-1.08	0.6917	1.1	0.6068	1.53	0.0163
100471_at	1455288_at	1110036O03Rik	RIKEN cDNA 1110036O03 gene	1.06	0.91	-1.07	0.8228	-1.14	0.6904	-1.04	0.9039	1.06	0.8405
100472_at	1424800_at	Enah	enabled homolog (Drosophila)	-1	0.99	-1.44	0.175	1.02	0.9415	-1.02	0.9386	-1.89	0.2411
100473_at	1460444_at	Arrb1	arrestin, beta 1	3.36	0.02	-1.01	0.962	1.01	0.9284	1.18	0.3393	1.59	0.0143
100474_at	1420377_at	St8sia2	ST8 alpha-N-acetyl-neuraminide alpha-2,8-s	-1.13	0.54	1.01	0.9285	-1.06	0.3814	-1.06	0.2171	1.26	0.3617
100475_at	1425974_a_at	Trim25	tripartite motif protein 25	-1.16	0.39	1.02	0.8321	1.15	0.1303	-1.1	0.32	-1.08	0.3776
100476_at	1417484_at	Ibsp	integrin binding sialoprotein	1.1	0.84	-1.61	0.1419	-1.3	0.3368	1.19	0.5982	1.51	0.3359
100477_at	1422587_at	Tmem45a	transmembrane protein 45a	-1.33	0.25	-1.39	0.2951	-1.01	0.979	-1.1	0.7362	1.33	0.5468
100481_at	1449154_at	Col11a1	procollagen, type XI, alpha 1	1.37	0.59	1.05	0.9082	1.66	0.1594	1.56	0.2675	4.62	0.039
100482_at	1460691_at	Zfp598	zinc finger protein 598	-1.41	0.2	1.06	0.3676	1.17	0.0283	1.11	0.0488	-1.05	0.5439
100483_at	1449026_at	Ifnar1	interferon (alpha and beta) receptor 1	1.72	0.16	1.12	0.1593	1.12	0.1023	1.19	0.042	-1.13	0.4495
100484_at	1417256_at	Mmp13	matrix metalloproteinase 13	2.11	0.09	1.34	0.1416	1.02	0.9091	1.43	0.035	4.15	0.034
100486_at	1449023_a_at	Ezh1	enhancer of zeste homolog 1 (Drosophila)	1.02	0.95	1.1	0.2133	1.07	0.4777	1.18	0.0399	1.41	0.0724
100488_at	1450099_a_at	Gba	glucosidase, beta, acid	-1.08	0.63	1	0.997	1.09	0.1288	1.06	0.4186	-1.1	0.4345
100489_at	1423313_at	Pde7a	phosphodiesterase 7A	2.2	0	1.89	0.0193	1.4	0.1223	2.57	0.0001	1.15	0.7792
100491_at	1418445_at	---	---	1.01	0.96	-1.29	0.004	-1.02	0.8403	-1.47	0.0002	-1.8	0.0001
100492_at	1455482_at	Ap2a2	adaptor protein complex AP-2, alpha 2 subu	1.11	0.69	1.01	0.9174	-1.14	0.1194	-1.2	0.0326	-1.38	0.029
100493_at	1416761_at	---	---	-2.36	0.28	1.04	0.6964	1.02	0.9014	-1.04	0.6698	1.68	0.004
100495_at	1417207_at	Dvl2	dishevelled 2, dsh homolog (Drosophila)	-2.22	0.2	1.21	0.3046	1.13	0.5277	1.13	0.5171	-1.05	0.8981
100496_at	1418908_at	Pam	peptidylglycine alpha-amidating monooxyger	1.56	0.49	-1.59	0.1563	-1.77	0.0846	-1.45	0.3744	1.46	0.5168
100498_g_at	1426151_a_at	Stx3	syntaxin 3	2.77	0.12	1.69	0.1502	1	0.9902	1.41	0.3736	-1.32	0.4687
100499_at	1425536_at	---	---	1.43	0.5	1.75	0.2506	1.39	0.3915	2.61	0.0412	1.59	0.5289
100500_at	1416888_at	Fadd	Fas (TNFRSF6)-associated via death domai	1.09	0.53	-1.03	0.5903	1.02	0.7616	-1.06	0.5848	-1.44	0.2672
100501_at	1448911_at	Atp4b	ATPase, H+/K+ transporting, beta polypeptic	-1.19	0.52	1.04	0.724	1.11	0.3905	1.25	0.275	1.41	0.3892
100502_at	1426036_a_at	Pde1c	phosphodiesterase 1C	-2.96	0.39	1.08	0.818	2.03	0.0387	1.65	0.0766	1.42	0.3141
100505_at	1448125_at	Rit2	Ras-like without CAAX 2	-2.17	0.43	-1.43	0.16	-1.06	0.807	-1.02	0.9241	1.82	0.2465
100507_at	1426852_x_at	Nov	nephroblastoma overexpressed gene	-3.33	0.06	-1.32	0.3074	-1.74	0.0414	-1.36	0.2676	1.1	0.8063
100508_at	1416992_at	Mfng	manic fringe homolog (Drosophila)	1.06	0.61	-1.21	0.015	-1.06	0.4711	-1.15	0.0937	1.04	0.7381
100509_at	1417508_at	Rnf19	ring finger protein (C3HC4 type) 19	1.18	0.33	1.15	0.0676	1.42	0.0084	1.17	0.0646	1.09	0.6143
100510_at	1418053_at	Sncb	synuclein, beta	-1.31	0.11	-1.24	0.0183	-1.02	0.8044	-1.1	0.4221	-1.05	0.7525
100511_at	1428242_at	6330406L22Rik	RIKEN cDNA 6330406L22 gene	1.08	0.8	1	0.9562	-1.12	0.1414	1.09	0.4791	1.07	0.4682
100512_at	1419452_at	Uchl5	ubiquitin carboxyl-terminal esterase L5	2.7	0.04	1.32	0.039	1.21	0.2535	1.28	0.0296	-1.02	0.8529
100513_at	1425572_a_at	Ddef1	development and differentiation enhancing	1.32	0.37	1	0.9686	-1.01	0.947	-1.05	0.7	-1.26	0.2498
100514_at	1450656_at	Gna13	guanine nucleotide binding protein, alpha 13	-1	0.99	1.19	0.379	-1.74	0.0087	-1.41	0.1056	1.27	0.0219
100515_at	1418518_at	Furin	furin (paired basic amino acid cleaving enzy	-1.47	0.01	1.08	0.2696	1.16	0.0219	1.22	0.003	1.06	0.4641
100516_at	1450264_a_at	Chka	choline kinase alpha	-1.85	0.11	-1.04	0.8368	-1.42	0.0271	1.04	0.7463	1.12	0.5522
100521_at	1426526_s_at	Ovgp1	oviductal glycoprotein 1	-2.12	0.07	1.1	0.3161	1.1	0.3065	1.14	0.1123	1.73	0.0034
100523_r_at	1451230_a_at	Wbp5	WW domain binding protein 5	1.36	0.57	1.01	0.9758	-1.02	0.954	1	0.9905	1.28	0.0294
100525_at	1448115_at	Htf9c	HpaII tiny fragments locus 9c	-1.27	0.35	1.03	0.6948	-1.02	0.8384	-1.04	0.6161	1.22	0.0811
100527_at	1419812_s_at	D11Ertd99e	DNA segment, Chr 11, ERATO Doi 99, expr	1.12	0.37	1.05	0.2699	-1.05	0.3512	-1.02	0.5818	1.05	0.7197
100529_at	1418632_at	Ube2h	ubiquitin-conjugating enzyme E2H	1.17	0.62	-1.25	0.0334	-1.24	0.1192	-1.41	0.008	-1.73	0.3347
100530_at	1460634_at	Ralgds	ral guanine nucleotide dissociation stimulato	-1.58	0.12	-1.19	0.0269	-1.21	0.0265	-1.16	0.0452	-1.29	0.0437
100534_at	1423395_at	Tsnax	translin-associated factor X	1.22	0.28	-1.04	0.4662	-1.07	0.3596	-1.04	0.5857	-1.06	0.6424
100535_at	1428362_at	E130105L11Rik	RIKEN cDNA E130105L11 gene	1.59	0.18	1.04	0.5609	1.14	0.2968	1.21	0.0283	-1.01	0.9603
100536_at	1433785_at	Mobp	myelin-associated oligodendrocytic basic prc	-1.01	0.96	-1.16	0.3935	-1.1	0.3349	-1.12	0.3536	1.36	0.144
100538_at	1451124_at	Sod1	superoxide dismutase 1, soluble	1.19	0.02	-1.02	0.7982	1.09	0.2349	1.03	0.6324	-1.12	0.7894
100539_at	1417094_at	MGI:1917275	brain acyl-CoA hydrolase	1.97	0.12	1.06	0.3598	1.15	0.1033	1.02	0.7929	1.05	0.8125
100540_at	1434790_a_at	Lta4h	leukotriene A4 hydrolase	1.52	0.24	-1.07	0.3197	-1.03	0.6563	-1.01	0.8816	-1.37	0.0337
100543_s_at	1448521_at	Brd7	bromodomain containing 7	1.16	0.4	-1.11	0.1199	1.05	0.6749	-1.04	0.5542	1	0.9852
100546_at	1416920_at	Rbm4	RNA binding motif protein 4	-1.28	0.7	-1.34	0.2516	1.15	0.6523	-1.28	0.2859	-2.11	0.4681
100548_at	1416406_at	Pea15	phosphoprotein enriched in astrocytes 15	1.43	0.59	1.28	0.387	1.42	0.3081	1.43	0.4022	1.06	0.7698
100549_at	1448716_at	Hba-x	hemoglobin X, alpha-like embryonic chain in	1.04	0.93	-1.19	0.0448	-1.16	0.1693	-1.23	0.046	1.46	0.0533
100550_f_at	1434491_a_at	---	Transcribed locus, strongly similar to NP_44	1.13	0.2	1.21	0.022	1.02	0.7843	1.24	0.0011	1.14	0.2218

100552_at	1448167_at	Ifngr1	interferon gamma receptor 1	1.17	0.13	1.45	0.0305	1.07	0.4094	1.6	0.0334	1.33	0.0785
100553_at	1448101_s_at	Trim27	tripartite motif protein 27	-1.48	0	1.08	0.0749	1.13	0.0357	1.08	0.1002	1.19	0.1896
100554_at	1416554_at	Pdlim1	PDZ and LIM domain 1 (elfin)	1.13	0.38	-1.49	0.0002	-1.28	0.0184	-1.6	0.0001	-1.59	0.0419
100555_at	1416600_a_at	Dscr1	Down syndrome critical region homolog 1 (h	-1.11	0.45	1.02	0.9081	1.06	0.7056	1.06	0.6536	1.04	0.8244
100557_g_at	1426378_at	Eif4b	eukaryotic translation initiation factor 4B	-1.05	0.8	1.13	0.4001	1.15	0.2694	1.09	0.3789	1.35	0.1433
100559_at	1423925_at	Dhx16	DEAH (Asp-Glu-Ala-His) box polypeptide 16	-1.05	0.81	-1.04	0.6308	1.05	0.6266	-1.02	0.7771	1.17	0.2308
100560_at	1448578_at	Pafah1b1	platelet-activating factor acetylhydrolase, isc	-1.22	0.12	-1.22	0.0077	-1.33	0.0118	-1.3	0.0004	-1.01	0.9391
100561_at	1417380_at	Iqgap1	IQ motif containing GTPase activating protei	-1.13	0.69	1.41	0.2282	-1.24	0.113	1.51	0.1583	1.34	0.2021
100562_at	1448740_at	2400006H24Rik	RIKEN cDNA 2400006H24 gene	2.38	0.36	1.51	0.3293	1.12	0.7988	1.11	0.8187	1.02	0.9368
100564_at	1417103_at	Ddt	D-dopachrome tautomerase	1.08	0.56	1.04	0.3643	1.15	0.0005	1.13	0.0348	1	0.9862
100565_at	1448163_at	---	---	-1.15	0.44	-1.23	0.0326	-1.14	0.0668	-1.54	0	1.05	0.5411
100566_at	1452114_s_at	Igfbp5	insulin-like growth factor binding protein 5	1.7	0.16	-2.56	0	-4.11	0	-2.75	0	1.68	0.1087
100567_at	1451263_a_at	Fabp4	fatty acid binding protein 4, adipocyte	1.29	0.47	-1.19	0.265	-1.06	0.6851	-1.11	0.402	1.54	0.1427
100568_at	1416015_s_at	Abce1	ATP-binding cassette, sub-family E (OABP),	-1.28	0.15	-1.11	0.0914	1.04	0.4956	1.06	0.5208	-1.26	0.0288
100569_at	1419091_a_at	Anxa2	annexin A2	1.37	0.04	1.38	0.0898	1.04	0.7318	1.15	0.4717	-1.02	0.9634
100570_at	1420535_a_at	6330412F12Rik	RIKEN cDNA 6330412F12 gene	1.07	0.6	-1.04	0.4443	-1.06	0.1357	1	0.9479	-1.05	0.6239
100571_at	1416148_at	Laptm4b	lysosomal-associated protein transmembran	-1.18	0.23	-1.05	0.549	1.02	0.7835	-1.12	0.1316	-1.14	0.1401
100574_f_at	1434814_x_at	Gpi1	glucose phosphate isomerase 1	1.05	0.68	1.04	0.5707	1.22	0.0307	1.28	0.0031	1.23	0.1362
100575_at	1417118_a_at	Ard1	N-acetyltransferase ARD1 homolog (S. cere	2.31	0.14	1.02	0.8836	1.15	0.1899	1.06	0.5168	-1.09	0.6509
100576_at	1416410_at	Pafah1b3	platelet-activating factor acetylhydrolase, isc	1.24	0.3	1.81	0.0009	1.42	0.1444	1.95	0.0001	1.56	0.0005
100578_at	1415852_at	Impdh2	inosine 5'-phosphate dehydrogenase 2	1.28	0.26	1.06	0.4026	1.03	0.7035	1.16	0.0779	1.26	0.2031
100579_s_at	1434540_a_at	Clta	clathrin, light polypeptide (Lca)	1.68	0.04	1.04	0.3512	1.18	0.001	1.11	0.1688	1.19	0.2362
100580_at	1421062_s_at	Clta	clathrin, light polypeptide (Lca)	-3.42	0	-1.17	0.6247	-1.32	0.2814	-1.63	0.1145	-1.09	0.7154
100581_at	1422507_at	Cstb	cystatin B	1.13	0.35	1.33	0.0534	1.07	0.32	1.56	0.0246	-1.09	0.5452
100583_at	1429381_x_at	Igh-VJ558	immunoglobulin heavy chain (J558 family)	1.88	0.09	1.84	0.0177	1.04	0.8565	1.55	0.0222	1.85	0.1242
100584_at	1421223_a_at	Anxa4	annexin A4	1.42	0.06	1.12	0.0879	1.01	0.8438	1.12	0.1604	-1.04	0.5924
100588_at	1417189_at	Psme2	proteasome (prosome, macropain) 28 subur	1.27	0.01	1.12	0.561	-1.26	0.0014	1.2	0.2615	-1.09	0.3637
100589_at	1433470_a_at	Immt	inner membrane protein, mitochondrial	1.58	0.51	-1.28	0.4033	-1.16	0.6515	1.22	0.5612	1.1	0.6983
100592_at	1415882_at	Ghitm	growth hormone inducible transmembrane p	-1.03	0.88	1.1	0.2452	1.06	0.5903	1.01	0.8733	-1.13	0.5927
100593_at	1424967_x_at	Tnnt2	troponin T2, cardiac	2.59	0.11	-1.02	0.9079	-1.21	0.3224	1.06	0.7197	1.75	0.2085
100594_at	1415946_at	Pigq	phosphatidylinositol glycan, class Q	1.2	0.17	1.02	0.6854	-1.01	0.8936	-1.03	0.5836	-1.38	0.0065
100595_at	1460707_at	Ptp4a2	protein tyrosine phosphatase 4a2	-1.01	0.94	1.23	0.0014	1.06	0.0839	1.28	0.0001	-1.19	0.0554
100596_at	1450699_at	Selenbp1	selenium binding protein 1	1.11	0.48	1.2	0.0008	1.1	0.1549	1.44	0	-1.06	0.7182
100597_at	1448429_at	Gyg1	glycogenin 1	1.76	0.15	1.19	0.3503	1.16	0.1717	1.23	0.1707	1.07	0.8855
100599_at	1448135_at	Atf4	activating transcription factor 4	-1.2	0.12	1.07	0.434	1.14	0.1107	1.13	0.1212	1.14	0.5605
100600_at	1448182_a_at	Cd24a	CD24a antigen	1.03	0.84	-1.1	0.4568	1.01	0.9076	-1.03	0.7861	1.68	0.002
100601_at	1417534_at	---	---	1.39	0.21	-1.08	0.1447	-1.15	0.025	-1.12	0.0473	-1.27	0.2339
100603_at	1415841_at	Dncic2	dynein, cytoplasmic, intermediate chain 2	2.22	0.01	1.25	0.0579	1.11	0.4259	1.32	0.0004	-1.39	0.1418
100605_at	1419739_at	Tpm2	tropomyosin 2, beta	2.01	0.21	-2.46	0.159	-1.91	0.2627	-2.06	0.2234	1.3	0.0129
100606_at	1448233_at	Prnp	prion protein	1.01	0.99	1.2	0.1391	-1.26	0.148	1.2	0.2163	-1.28	0.1687
100607_at	1416013_at	Pld3	phospholipase D3	1.35	0.37	-1.03	0.6192	-1.03	0.7817	-1.07	0.539	-1.18	0.7352
100610_at	1426400_a_at	Capns1	calpain, small subunit 1	1.45	0.14	1.05	0.5723	1.08	0.3751	1.16	0.1416	-1.2	0.0189
100611_at	1423547_at	Lyzs	lysozyme	2.05	0.08	2.31	0.2514	-1.04	0.8434	4.14	0.1337	-1.05	0.6837
100612_at	1415878_at	Rrm1	ribonucleotide reductase M1	1.28	0.55	2.23	0.0951	1.4	0.1755	2.53	0.0146	1.8	0.0791
100613_at	1416113_at	Fkbp8	FK506 binding protein 8	-1.31	0.4	-1.16	0.0177	-1.19	0.1258	-1.26	0.0278	-1.35	0.3023
100614_at	1451203_at	Mb	myoglobin	1.4	0.22	-6.61	0.1724	-4.94	0.2006	-10	0.149	-2.03	0.2815
100615_at	1422420_at	Mb	myoglobin	-2.38	0.17	1.54	0.0978	1.44	0.1336	1.1	0.6699	-1.01	0.9651
100617_at	1423772_x_at	Slc25a5	solute carrier family 25 (mitochondrial carrie	-3.87	0.06	1.14	0.6091	1.34	0.2424	-1.32	0.3137	2.28	0.1986
100621_at	1451201_s_at	Rnh1	ribonuclease/angiogenin inhibitor 1	1.42	0.13	-1.01	0.6892	-1.07	0.1575	1.04	0.2911	-1.62	0.0066
100626_at	1426040_a_at	Odf2	outer dense fiber of sperm tails 2	1.15	0.67	-1.37	0.4087	-1.37	0.326	-1.13	0.7093	2.09	0.136
100628_at	1448284_a_at	Ndufc1	NADH dehydrogenase (ubiquinone) 1, subc	1.28	0.6	-1.07	0.1079	1.06	0.1846	1.12	0.2527	-1.16	0.24
100629_at	1416842_at	Gstm5	glutathione S-transferase, mu 5	1.3	0.21	-1.03	0.6945	-1.01	0.944	-1.04	0.6139	1.33	0.0413
100632_at	1417690_at	Prkag1	protein kinase, AMP-activated, gamma 1 no	1.1	0.65	3.3	0	2.25	0.0139	2.99	0	2.97	0.0304

100633_at	1416766_at	2810484M10Rik	RIKEN cDNA 2810484M10 gene	1.84	0	1.01	0.7941	1.02	0.562	1.06	0.1989	1.4	0.0089
100634_at	1448723_at	Rdh7	retinol dehydrogenase 7	-1.19	0.28	-1.02	0.701	-1.06	0.2184	-1.06	0.2868	-1.01	0.8731
100635_at	1415696_at	Sara1	SAR1a gene homolog 1 (S. cerevisiae)	-1.28	0.32	-1.08	0.5178	-1.18	0.1304	-1.16	0.1444	1.11	0.3836
100636_at	1417562_at	Eif4ebp1	eukaryotic translation initiation factor 4E bind	1.15	0.43	-1.04	0.5375	1.14	0.184	1.01	0.8613	1.63	0.0013
100669_at	1426145_at	---	---	-2.13	0.02	-1	0.9944	1.25	0.3939	-1.02	0.9023	1.34	0.3962
100670_at	1421660_at	Scn9a /// LOC2(	sodium channel, voltage-gated, type IX, alph	-1	0.99	-1.89	0.0809	-1.23	0.5157	-2.26	0.0386	-1	0.9923
100671_at	1422332_at	Ifna11	interferon alpha family, gene 11	-2.75	0.21	1.76	0.0658	1.45	0.2319	1.02	0.9151	1.5	0.6031
100673_f_at	1420789_at	Klra5	killer cell lectin-like receptor, subfamily A, me	-1.8	0.01	-1.17	0.1385	-1.11	0.318	1.15	0.1815	1.49	0.1297
100675_at	1450300_at	Gabrr1	gamma-aminobutyric acid (GABA-C) recepto	-1.44	0.6	-1.15	0.6418	-1.31	0.3661	1.36	0.4676	1.7	0.3798
100678_s_at	1427681_s_at	V2r13 /// V2r3	vomeronasal 2, receptor, 13 /// vomeronasal	-2.16	0.22	1.15	0.7157	-1.11	0.7619	-1.06	0.8814	2.54	0.124
100679_at	1421701_at	V2r16	vomeronasal 2, receptor, 16	-1.24	0.51	1.32	0.4142	1.05	0.7554	1.26	0.2726	1.58	0.1024
100680_at	1420773_at	Dub1	deubiquitinating enzyme 1	1.2	0.71	1.43	0.2966	-1.19	0.2352	1.07	0.7182	-1.31	0.4055
100683_r_at	1451951_at	Igk-V8	Immunoglobulin kappa chain variable 8 (V8)	-3.35	0.01	1.1	0.668	1.75	0.1872	1.52	0.1069	1.31	0.4264
100684_at	1416339_a_at	Prkcsh	protein kinase C substrate 80K-H	1.31	0.29	-1.03	0.4359	-1.02	0.8617	-1.02	0.6514	-1.1	0.1309
100685_at	1422481_at	Krt2-1	keratin complex 2, basic, gene 1	-2.35	0.3	-1.36	0.3564	-1.92	0.0672	-1.73	0.1476	2.07	0.1951
100686_at	1431766_x_at	Rps2	ribosomal protein S2	1.06	0.69	1.04	0.3825	-1.02	0.7975	1.03	0.5487	1.27	0.0292
100688_at	1420536_at	Crybb2	crystallin, beta B2	-1.82	0.26	-1.28	0.1471	1.04	0.7891	-1.14	0.3815	1.29	0.2864
100690_at	1420546_at	Th	tyrosine hydroxylase	-1.89	0.01	-1.26	0.4516	-1.42	0.3558	1.02	0.9443	-1.02	0.9312
100691_at	1421705_at	Scn3a	sodium channel, voltage-gated, type III, alph	-3.08	0.24	-1.16	0.3164	-1.33	0.0597	-1.05	0.8761	-1.32	0.5193
100692_at	1427824_at	---	---	-1.52	0.21	1.19	0.229	1.14	0.0584	1.02	0.8617	2.06	0.252
100693_at	1422187_at	Gabrg3	gamma-aminobutyric acid (GABA-A) recepto	-1.3	0.45	1.08	0.5997	1.44	0.0081	1.15	0.4143	1.34	0.0962
100694_at	1416277_a_at	Rplp1	ribosomal protein, large, P1	-1.3	0.32	-1.08	0.1019	1.06	0.3178	1.05	0.5331	1.33	0.1894
100695_at	1449161_at	Edn2	endothelin 2	-1.08	0.74	1.05	0.6784	1.23	0.1439	1.15	0.3028	1.16	0.5351
100696_at	1450415_at	Pde6a	phosphodiesterase 6A, cGMP-specific, rod,	-1.18	0.57	-1.33	0.0306	-1.11	0.317	-1.17	0.238	1.45	0.1323
100697_at	1420389_at	Pax3	paired box gene 3	-1.47	0.18	1.81	0.0389	1.61	0.1318	1.55	0.2447	1.13	0.2793
100698_at	1450361_at	Prop1	paired like homeodomain factor 1	-1.75	0.04	1.31	0.1317	1.17	0.3732	1.05	0.7828	1.13	0.661
100699_at	1451781_at	Nfatc2ip	nuclear factor of activated T-cells, cytoplasm	1.35	0.29	1.05	0.8171	1.19	0.5078	-1.13	0.5795	1.84	0.2016
100702_at	1421602_at	Shbg	sex hormone binding globulin	-2	0.1	-1.17	0.1437	-1.02	0.7397	-1.19	0.0235	1.37	0.1015
100703_at	1417489_at	Npy2r	neuropeptide Y receptor Y2	1.04	0.91	-1.07	0.7569	-1.04	0.8595	1.13	0.5038	1.17	0.4844
100705_at	1450192_at	Lhcgr	luteinizing hormone/choriogonadotropin rece	-1.31	0.39	1.08	0.537	-1.07	0.6465	-1.09	0.4961	1.53	0.0921
100706_f_at	1434353_at	Sfmbt2	Scm-like with four mbt domains 2	-1.06	0.89	-2.12	0.027	-1.34	0.3612	-1.86	0.0589	1.8	0.2421
100707_at	1421270_at	Sh3md2	SH3 multiple domains 2	1.21	0.44	-1.32	0.1446	-1.03	0.8368	-1.15	0.3718	2.37	0.0758
100708_at	1420376_a_at	H3f3b	H3 histone, family 3B	-1.13	0.42	1.01	0.8952	-1.31	0.0005	-1.03	0.653	-1.14	0.2687
100709_at	1415808_at	Tpbpa	trophoblast specific protein alpha	1.47	0.47	1.8	0.327	2.3	0.1211	1.15	0.648	1.13	0.5625
100711_at	1431177_a_at	Rpl10a	ribosomal protein L10A	1.41	0.28	1.07	0.1779	1.17	0.0185	1.24	0.0031	1.2	0.2715
100712_at	1420801_at	Npas1	neuronal PAS domain protein 1	-1.06	0.82	-1.52	0.0686	-2.75	0.0006	-1.54	0.0977	1.86	0.0658
100713_at	1449546_a_at	Zfp617	zinc finger protein 617	1.21	0.71	1.1	0.4074	-1.08	0.4783	-1.01	0.9057	1.09	0.712
100714_at	1421464_at	Bapx1	bagpipe homeobox gene 1 homolog (Drosop	1.64	0.31	1.4	0.1809	-1.01	0.9487	1.47	0.1914	1.42	0.4906
100715_at	1421687_at	Msmb	beta-microseminoprotein	-5.81	0.12	-1.09	0.8025	-1.33	0.3086	-1.93	0.0179	1.28	0.6704
100716_at	1420590_at	Has1	hyaluronan synthase1	1.35	0.33	-1.5	0.0868	-1.23	0.4067	-1.59	0.0672	1.14	0.5315
100717_at	1460667_at	U90926	cDNA sequence U90926	-2.82	0.04	-1.31	0.0054	-1.31	0.0054	-1.24	0.0125	1.08	0.5615
100719_f_at	1420490_at	Klk16	kallikrein 16	1.46	0.42	-1.69	0.1256	-1.68	0.1597	-1.39	0.271	-1.55	0.3354
100720_at	1418883_a_at	Pabpc1	poly A binding protein, cytoplasmic 1	-1.11	0.75	1.11	0.2507	1.02	0.8672	1.18	0.0625	1.18	0.1048
100722_r_at	1450367_at	LOC56304	recombinant antineuraminidase single chain	-2.53	0.08	-1.31	0.55	-1.42	0.4475	-1.33	0.5637	1.17	0.626
100724_at	1427587_at	Zfp28	zinc finger protein 28	1.32	0.56	1.29	0.4516	1.15	0.6283	1.16	0.6314	1.34	0.523
100726_at	1421616_at	Grin2a	glutamate receptor, ionotropic, NMDA2A (ep	-5.01	0.15	1.31	0.273	1.27	0.2151	-1.11	0.4344	1.71	0.2443
100727_at	1416074_a_at	Rpl28	ribosomal protein L28	1.12	0.61	1.07	0.2737	1.23	0.0049	1.13	0.0383	1.54	0.0009
100728_at	1418663_at	Mpdz	multiple PDZ domain protein	-1.03	0.93	1.93	0.0035	1.94	0.0402	1.81	0.0202	1.14	0.3728
100729_at	1448109_a_at	Rpl26	ribosomal protein L26	1.21	0.28	1.05	0.4523	1.2	0.0812	1.21	0.0083	1.51	0.0112
100730_at	1421547_at	Ly78	lymphocyte antigen 78	1.03	0.88	-1.17	0.3217	-1.38	0.1223	-1.07	0.6808	1.44	0.0456
100731_at	1450373_at	Prp15	proline-rich protein 15	1.04	0.94	1.48	0.2274	1.81	0.0979	2.47	0.0001	-1.07	0.8967
100732_at	1436760_a_at	Rps8	ribosomal protein S8	1.33	0.21	1.01	0.7298	1.11	0.1057	1.12	0.0497	1.33	0.0858
100733_at	1448206_at	Psma2	proteasome (prosome, macropain) subunit, a	1.15	0.63	1.19	0.0037	1.18	0.0595	1.29	0.0005	-1.16	0.0738

100734_at	1449323_a_at	Rpl3	ribosomal protein L3	1.2	0.31	1.21	0.001	1.16	0.0005	1.23	0.0001	1.43	0.0187
100735_at	1427842_at	Krtap6-3	keratin associated protein 6-3	1.08	0.86	1.37	0.1877	1.22	0.3729	1.67	0.3275	1.69	0.1432
100736_at	1421761_a_at	Barx2	BarH-like homeobox 2	-1.14	0.46	-1.43	0.2449	-1.2	0.5122	-1.57	0.1191	-1.03	0.9044
100737_at	1450252_at	Onecut1	one cut domain, family member 1	-2.14	0	-3.02	0.0002	-2.09	0.0015	-3.34	0.0001	-1.1	0.8189
100738_at	1426149_at	Spi15	serine protease inhibitor 15	-2.14	0.18	-1.27	0.5487	-1.08	0.8334	-1.02	0.9694	-1.6	0.2968
100739_at	1427834_at	Spi16	serine protease inhibitor 16	-1.87	0.18	-1.16	0.51	-1.19	0.5404	-1.36	0.1478	1.67	0.2943
100740_at	1422817_at	Gp5	glycoprotein 5 (platelet)	1.37	0.26	-1.85	0.0913	-1.01	0.9911	-1.74	0.1092	1.2	0.4739
100741_at	1421661_at	Fprl1	formyl peptide receptor-like 1	1.31	0.61	1.09	0.6928	-1.11	0.6953	-1.04	0.8839	1.19	0.5165
100742_at	1420161_at	AA409749	expressed sequence AA409749	-1.08	0.89	1.2	0.3963	1.07	0.7705	1.37	0.2472	1.54	0.4031
100744_at	1441134_at	6820424L24Rik	RIKEN cDNA 6820424L24 gene	-1.05	0.92	1.09	0.5154	1.3	0.0165	1.08	0.6285	1.11	0.8176
100746_at	1457594_at	---	---	-1.74	0.44	-1.2	0.545	-1.64	0.0818	-1.24	0.3839	3.08	0.1842
100747_at	1420155_at	AA408954	expressed sequence AA408954	-2.24	0.09	-1.19	0.422	-1.18	0.3753	-1.18	0.3537	1.31	0.2101
100748_at	1446971_at	D10Wsu159e	DNA segment, Chr 10, Wayne State Univers	-1.01	0.98	-1.58	0.248	-1.21	0.6027	-1.97	0.1083	1.4	0.6053
100749_at	1457810_at	Zbtb37	zinc finger and BTB domain containing 37	-1.18	0.47	-1.14	0.099	-1.08	0.3355	-1.06	0.4108	1.22	0.3424
100750_at	1420301_at	AA414903	expressed sequence AA414903	-1.79	0.31	1.01	0.9501	1.07	0.7729	1.16	0.4407	1.35	0.3071
100751_at	1450104_at	Adam10	a disintegrin and metalloprotease domain 1(	-1.16	0.63	1.15	0.1235	-1.16	0.3927	-1.08	0.6091	1.24	0.489
100752_at	1450280_a_at	Rrh	retinal pigment epithelium derived rhodopsin	1.06	0.28	1.55	0.1171	-1.02	0.945	1.43	0.3669	1.25	0.455
100753_at	1449710_s_at	Atp5a1	ATP synthase, H+ transporting, mitochondri	1.22	0.15	1.1	0.019	1.08	0.1257	1.18	0.0009	-1.12	0.2548
100755_at	1422928_at	---	---	-1.79	0.06	-1.19	0.208	-1.07	0.6102	-1.44	0.0242	-1.08	0.6028
100756_r_at	1426119_at	Tyms-ps	thymidylate synthase, pseudogene	1.33	0.3	-1.1	0.3937	-1.03	0.8013	1.03	0.8459	2.83	0.0137
100757_at	1452476_at	Cacnb2	calcium channel, voltage-dependent, beta 2	-1.13	0.75	-1.17	0.2945	-1.17	0.3457	-1.18	0.3343	1.51	0.1679
100760_at	1421460_at	Dsc1	desmocollin 1	1.49	0.2	-2.09	0.1114	-1.41	0.3977	1	0.9926	1.38	0.6045
100761_at	1422036_at	Strn	striatin, calmodulin binding protein	1.19	0.69	-1	0.9883	-1.13	0.7289	-1.04	0.8982	1.78	0.1814
100762_at	1421414_a_at	Sema6a	sema domain, transmembrane domain (TM)	1.11	0.7	1.59	0.0911	1.77	0.087	1.61	0.1583	-1.13	0.7529
100763_at	1442374_at	D2Ertd127e	DNA segment, Chr 2, ERATO Doi 127, expr	1.59	0.12	1.15	0.5817	-1.32	0.3653	1.31	0.23	1.38	0.5593
100764_at	1417546_at	Il2rb	interleukin 2 receptor, beta chain	-1.32	0.34	-1.16	0.2047	-1.08	0.6926	1.1	0.4729	1.01	0.9619
100765_at	1448055_at	E230011A21Rik	RIKEN cDNA E230011A21 gene	-1.51	0.31	-1.69	0.0486	-1.13	0.6152	-1.7	0.0518	-1.72	0.2574
100766_at	1421136_at	Edn3	endothelin 3	-1.61	0.34	-1.1	0.5316	-1.01	0.9284	-1.04	0.733	1.65	0.1618
100767_at	1458196_at	---	---	-2.14	0.01	1.65	0.3004	1.66	0.1919	1.88	0.1407	1.44	0.6178
100772_g_at	1451780_at	Blnk	B-cell linker	-1.22	0.64	-1.93	0.0075	-1.17	0.4617	-1.61	0.0204	1.04	0.883
100773_at	1425454_a_at	Il12a	interleukin 12a	-1.46	0.1	-1.04	0.7608	-1.04	0.7756	1.02	0.8858	-1.16	0.7785
100774_at	1428015_at	D12Wsu118e	DNA segment, Chr 12, Wayne State Univers	-1.13	0.46	-1.17	0.1138	-1.1	0.4893	-1.11	0.3042	1.41	0.3749
100778_at	1450136_at	Cd38	CD38 antigen	-1.96	0.26	-1.28	0.1288	-1.24	0.1596	-1.32	0.1679	-1.16	0.7986
100779_at	1419530_at	Il12b	interleukin 12b	-1.68	0.23	-1.2	0.3191	1.12	0.7121	-1.09	0.7282	1.15	0.4821
100780_at	1416276_a_at	Rps4x	ribosomal protein S4, X-linked	1.25	0.31	1.03	0.4709	1.1	0.066	1.1	0.1601	1.48	0.0207
100828_at	1457126_at	Myl4	myosin, light polypeptide 4	-1.19	0.19	-1.28	0.0098	-1.15	0.097	-1.24	0.0075	-1.12	0.6425
100876_at	1454674_at	Fez1	fasciculation and elongation protein zeta 1 (z	1.28	0.33	1.24	0.0744	1.14	0.3254	1.27	0.1165	-1.33	0.299
100877_at	1436339_at	1810058I24Rik	RIKEN cDNA 1810058I24 gene	1.36	0.19	-1.03	0.6786	1.1	0.1987	-1.04	0.5297	-1.11	0.4373
100878_at	1423535_at	Strn3	striatin, calmodulin binding protein 3	1.4	0.13	1.82	0	1.54	0.0128	1.76	0	1.42	0.0653
100879_at	1418677_at	Actn3	actinin alpha 3	-1.2	0.67	1.15	0.3477	1.18	0.3357	1.16	0.2546	1.07	0.7473
100880_at	1434380_at	---	Diabetic nephropathy-like protein (Dnr12) m	-2.14	0.09	1.15	0.793	-1.81	0.0192	1.35	0.5889	-1.21	0.4561
100882_at	1419492_s_at	Defb1	defensin beta 1	2.62	0	2.82	0.0114	1.43	0.0601	4.61	0.0007	2.93	0.0062
100884_at	1448894_at	Akr1b8	aldo-keto reductase family 1, member B8	1.19	0.42	-1.25	0.0097	-1.16	0.2832	-1.19	0.2185	1.41	0.0033
100885_at	1417299_at	Nek2	NIMA (never in mitosis gene a)-related expr	-1.24	0.43	1.05	0.605	1.08	0.3747	-1	1	1.02	0.8763
100888_at	1460390_at	Sorl1	sortilin-related receptor, LDLR class A repea	1.15	0.72	-1.01	0.9828	-1.15	0.4324	1.08	0.7868	1.17	0.5006
100889_at	1460034_at	BC042901	cDNA sequence BC042901	-1.19	0.41	-1.12	0.2525	-1	0.9807	1	0.9847	1.16	0.1259
100890_at	1423877_at	Chaf1b	chromatin assembly factor 1, subunit B (p60	-1.04	0.79	1.65	0.045	1.7	0.0296	1.96	0.0019	1.19	0.4881
100891_at	1418358_at	Mcsp	mitochondrial capsule selenoprotein	1.36	0.49	1.15	0.4617	1.09	0.6353	-1.11	0.5525	-1.1	0.7516
100892_at	1423711_at	Ndufaf1	NADH dehydrogenase (ubiquinone) 1 alpha	1.09	0.5	-1.07	0.1448	-1.04	0.6107	-1.11	0.0915	-1.4	0.0057
100893_at	1460243_at	Sptlc2	serine palmitoyltransferase, long chain base	1.94	0.03	1.08	0.2936	-1.04	0.6974	1.19	0.0647	1.59	0.0049
100894_at	1415830_at	Orc5l	origin recognition complex, subunit 5-like (S.	1.19	0.28	1.01	0.8417	1.11	0.0989	1.03	0.6249	-1.09	0.3581
100895_at	1437630_at	D16Bwg1547e	DNA segment, Chr 16, Brigham & Women's	-1.24	0.27	1.01	0.799	1.04	0.5098	1.1	0.0703	-1.46	0.0035
100896_at	1422813_at	---	---	1.46	0.41	-2.05	0.1822	-1.09	0.851	-2.35	0.1285	1.45	0.397

100899_s_at	1448705_at	Zfp297	zinc finger protein 297	1.05	0.42	1.07	0.14	1	0.9357	1.07	0.279	1.15	0.1251
100900_at	1433736_at	Hcfc1	host cell factor C1	-1.1	0.14	1.18	0.0089	1.11	0.0153	1.19	0.0043	1.22	0.009
100901_at	1450439_at	Hcfc1	host cell factor C1	1.16	0.64	1.43	0.1172	1.37	0.3664	1.21	0.4219	1.49	0.0861
100902_at	1428357_at	2610019F03Rik	RIKEN cDNA 2610019F03 gene	1.08	0.74	-1.61	0.0113	1.07	0.5673	-1.37	0.0809	-1.24	0.1013
100903_at	1417800_at	Parp2	poly (ADP-ribose) polymerase family, memb	-1.04	0.91	1.06	0.2304	1.01	0.9387	1.09	0.1372	1.18	0.3407
100904_at	1452699_at	LOC386486	similar to FLJ20859 protein; false p73 target	-1.34	0.05	1.04	0.5653	-1.01	0.9186	1.03	0.606	1.23	0.043
100905_at	1427032_at	Herc4	hect domain and RLD 4	-1.1	0.75	1.21	0.1122	1.05	0.7078	1.33	0.0086	-1.48	0.0821
100906_at	1418741_at	Itgb7	integrin beta 7	-1.06	0.79	1.04	0.6257	-1.08	0.3717	1.24	0.0967	1.04	0.7612
100907_at	1416640_at	Kcne1l	potassium voltage-gated channel, Isk-relate	-1.58	0.31	1.09	0.3468	1.12	0.218	1.07	0.4261	1.16	0.4808
100910_at	1433609_s_at	---	---	1.35	0	1.13	0.3718	1.07	0.6488	1.13	0.2457	1.76	0.0747
100911_at	1448225_at	Gpaa1	GPI anchor attachment protein 1	1.04	0.95	1.22	0.1816	1.01	0.9763	1.03	0.8695	-1.49	0.4125
100912_at	1452839_at	2410012M04Ril	RIKEN cDNA 2410012M04 gene	2.55	0.12	1.16	0.0875	1.43	0.0854	1.32	0.0002	-1.06	0.8326
100913_at	1417443_at	BC026682	cDNA sequence BC026682	2.37	0.04	-1.03	0.9176	1.11	0.7623	-1.08	0.8257	-1	0.9953
100914_at	1437867_at	LOC385308	similar to protease	-1.93	0.04	-1.08	0.6205	-1.05	0.7185	-1.32	0.1109	-1.13	0.74
100915_at	1417472_at	Myh9	myosin, heavy polypeptide 9, non-muscle	-1.26	0.32	-1.13	0.4109	-1.74	0.0017	-1.55	0.0117	-1.29	0.0578
100916_at	1418118_at	Slc22a1	solute carrier family 22 (organic cation trans	-1.07	0.74	-1.45	0	-1.31	0.0018	-1.9	0	-3.6	0.0002
100921_at	1422536_at	Tnni3	troponin I, cardiac	-2.14	0.17	1.44	0.3218	1.36	0.3787	1.36	0.2945	-2.16	0.2021
100923_at	1450650_at	Myo10	myosin X	1.39	0.44	-1.2	0.0181	-1.08	0.5956	-1.36	0.0008	-1.26	0.3439
100924_at	1448886_at	Gata3	GATA binding protein 3	-1.39	0.12	-1.02	0.9371	1.44	0.1523	-1.24	0.417	1.43	0.262
100925_at	1453208_at	2700089E24Rik	RIKEN cDNA 2700089E24 gene	-1.4	0.24	1.01	0.9288	1.17	0.3323	-1.27	0.0898	-1.63	0.0735
100927_at	1456424_s_at	Pltp	phospholipid transfer protein	1.04	0.81	-1.93	0.0004	-1.17	0.3379	-1.87	0.001	-5.28	0.0025
100928_at	1423407_a_at	Fbln2	fibulin 2	-1.04	0.88	-1	0.9973	-1.13	0.35	-1.02	0.8827	2.65	0.0001
100929_at	1455058_at	Mtmr9	myotubularin related protein 9	1.29	0.15	-1.05	0.671	-1.04	0.8527	-1.36	0.0023	-1.18	0.3429
100931_at	1460346_at	Arsa	arylsulfatase A	-1.08	0.63	2.84	0	1.06	0.677	2.95	0	1.4	0.1511
100932_at	1417228_at	Capn1	calpain 1	-1.33	0.06	-2.67	0.0031	-1.7	0.0432	-2.18	0.0176	-1.14	0.4647
100933_at	1448366_at	Stx1a	syntaxin 1A (brain)	3.97	0.01	-1.43	0.26	1.02	0.9348	-1.6	0.0817	2.56	0.1376
100935_at	1418437_a_at	Tcfl4	transcription factor-like 4	-1.02	0.88	1.09	0.1711	1.08	0.2686	1.06	0.3393	1.25	0.0369
100939_at	1424298_at	Zfp282	zinc finger protein 282	-1.1	0.49	-1.13	0.6991	-1.12	0.7546	-1.06	0.8723	1.27	0.1494
100944_at	1434372_at	---	---	1.14	0.5	1.27	0.5933	-1.53	0.0193	1.32	0.5197	-2.93	0.0136
100946_at	1452318_a_at	Hspa1b	heat shock protein 1B	1.11	0.86	1.47	0.1228	2.32	0.0029	2.36	0.0116	1.64	0.0022
100949_at	1417688_at	BC004044	cDNA sequence BC004044	1.06	0.51	1.04	0.6976	1.04	0.6986	1.02	0.8491	1.1	0.0435
100951_at	1417753_at	Pkd2	polycystic kidney disease 2	-1.02	0.94	-1.13	0.293	1.04	0.8279	-1.01	0.9548	-1.61	0.1203
100953_at	1417586_at	Timeless	timeless homolog (Drosophila)	-1.09	0.74	-1.04	0.5557	1.02	0.7347	-1.12	0.1132	1.26	0.1452
100954_at	1426923_at	Hrb	HIV-1 Rev binding protein	-1.01	0.98	1.17	0.1024	1.15	0.3111	1.35	0.0065	1.2	0.1069
100955_at	1422462_at	2700084L22Rik	RIKEN cDNA 2700084L22 gene	-1.22	0.5	1.28	0.4055	-1.02	0.9297	1.53	0.0993	1.31	0.3477
100957_at	1452616_s_at	Ssbp1	single-stranded DNA binding protein 1	1.48	0.08	1.26	0.0046	1.09	0.5223	1.24	0.0003	-1.1	0.4671
100958_at	1454656_at	Spata13	spermatogenesis associated 13	-1.14	0.14	1.03	0.7695	1.15	0.2585	-1.04	0.6904	1.18	0.208
100960_g_at	1418704_at	S100a13	S100 calcium binding protein A13	-1.02	0.83	1.03	0.5477	1.04	0.2623	1.07	0.3197	1.34	0.0256
100961_at	1449544_a_at	Kcnh2	potassium voltage-gated channel, subfamily	-1.26	0.09	1.76	0.0002	1.49	0.0735	1.65	0.0124	1.33	0.3471
100962_at	1417930_at	Nab2	Ngfi-A binding protein 2	1.21	0.44	1.23	0.0096	-1.17	0.0732	1.29	0.0042	1.58	0.056
100963_at	1451399_at	MGI:1930773	brain protein 17	1.5	0.23	1.34	0.0372	1.18	0.1325	1.44	0.0015	1.32	0.1061
100964_at	1449003_a_at	Vti1b	vesicle transport through interaction with t-S	1.42	0.21	-1.04	0.4238	-1.15	0.1198	-1.14	0.0294	-1.1	0.4179
100965_at	1455167_at	Cox8c	cytochrome c oxidase, subunit VIIIc	-1.71	0	-1.07	0.2402	-1.06	0.3746	-1.03	0.6995	1.08	0.2655
100966_at	1418680_at	Serpind1	serine (or cysteine) proteinase inhibitor, clad	-1.2	0.24	1.05	0.1029	-1.01	0.7675	1.05	0.0436	1.01	0.8699
100967_at	1416316_at	---	---	1.03	0.83	1.07	0.4964	1.06	0.2704	1.08	0.3561	1.24	0.1845
100968_at	1424723_s_at	Cstf3	cleavage stimulation factor, 3' pre-RNA, sub	-1.2	0.6	-1.13	0.2674	-1.14	0.2607	-1.17	0.2135	1.25	0.4691
100970_at	1416657_at	Akt1	thymoma viral proto-oncogene 1	-1.02	0.86	-1.42	0.0006	-1.37	0.0043	-1.47	0.0004	1.08	0.5993
100972_s_at	1430375_a_at	Ccl27	chemokine (C-C motif) ligand 27	1.34	0.01	-1.06	0.6201	-1.11	0.2186	-1.08	0.5484	1.73	0.0119
100973_i_at	1434962_x_at	---	---	1.36	0.01	1.04	0.7615	1	0.9834	1.04	0.6837	1.47	0.4321
100974_at	1449511_a_at	Ssbp4	single stranded DNA binding protein 4	2.68	0.13	1.2	0.071	1.13	0.4195	1.33	0.0123	-1.38	0.4464
100976_at	1451037_at	---	---	-2.01	0.02	-1.32	0.0249	-1.04	0.669	-1.44	0.0056	1.12	0.5178
100977_at	1423748_at	Pdk1	pyruvate dehydrogenase kinase, isoenzyme	-1.1	0.76	-1.2	0.369	1.16	0.498	-1.37	0.1171	-1.25	0.1033
100978_at	1417253_at	Frg1	FSHD region gene 1	-1.01	0.97	1.1	0.4137	1.17	0.2503	1.07	0.6282	1.28	0.499

100979_at	1454064_a_at	Rnf138	ring finger protein 138	-1.11	0.74	1.35	0.0104	1.22	0.2842	1.34	0.0191	-1.04	0.7812
100981_at	1450783_at	Ifit1	interferon-induced protein with tetratricopept	-1.71	0.26	-1.71	0.0921	-2.47	0.0106	-1.88	0.0906	-4.14	0.0584
100982_at	1418399_at	Kctd9	potassium channel tetramerisation domain c	1.27	0.37	-1.04	0.8357	1.12	0.4726	-1.01	0.9504	1.03	0.8872
100983_at	1422796_at	Prep	prolyl endopeptidase	1.65	0.12	-1.03	0.8738	1.46	0.0184	1.53	0.0011	-1.11	0.7557
100984_at	1417296_at	Atf1	activating transcription factor 1	1.28	0.38	1.33	0.0019	1.07	0.5952	1.18	0.0528	1.07	0.7716
100985_at	1449733_s_at	Siah1a	seven in absentia 1A	1.29	0.49	1.08	0.4804	1.17	0.1499	1.1	0.4679	-1.02	0.8776
100986_at	1419184_a_at	Fhl2	four and a half LIM domains 2	-1.38	0.32	1	0.995	-1.13	0.4942	-1.11	0.456	1.98	0.0709
100988_at	1435448_at	Bcl2l11	BCL2-like 11 (apoptosis facilitator)	2.08	0.06	1.4	0.0256	1.49	0.0268	1.89	0.0003	1.41	0.4814
100990_g_at	1422157_a_at	Itgb1bp1	integrin beta 1 binding protein 1	1.75	0.14	-1.01	0.9272	1.2	0.0051	1.03	0.6433	1.03	0.8346
100994_at	1416936_at	Aatk	apoptosis-associated tyrosine kinase	-1.23	0.26	-1.13	0.2219	-1.15	0.0234	-1.16	0.0197	1.18	0.7363
100997_at	1426732_at	Des	desmin	1.28	0.79	-1.08	0.6645	1.02	0.9257	-1.08	0.6504	2.22	0.0835
100998_at	1451721_a_at	H2-Ab1	histocompatibility 2, class II antigen A, beta	2.08	0.04	2.97	0.1894	-1.35	0.2435	1.96	0.2851	2.39	0.0576
101000_at	1426763_at	Oaz2	ornithine decarboxylase antizyme 2	1.04	0.91	1.13	0.0846	-1.03	0.7688	1	0.9696	-1.05	0.6161
101001_at	1423824_at	5031439A09Rik	RIKEN cDNA 5031439A09 gene	2.79	0.03	1.11	0.2334	1.2	0.0714	1.14	0.1127	1.17	0.7876
101002_at	1450714_at	Oazin	ornithine decarboxylase antizyme inhibitor	1.43	0.11	-1	0.9187	-1.03	0.6926	-1.05	0.6764	1.04	0.7993
101003_at	1416151_at	Sfrs3	splicing factor, arginine/serine-rich 3 (SRp20	-1.08	0.73	-1.15	0.412	-1.21	0.2356	-1.13	0.4044	-1.07	0.8561
101004_f_at	1434512_x_at	Sfrs3 /// LOC43	splicing factor, arginine/serine-rich 3 (SRp20	1.44	0.03	1.31	0.1126	-1.06	0.6096	1.24	0.0367	1.13	0.5231
101007_at	1418300_a_at	Mknk2	MAP kinase-interacting serine/threonine kina	1.51	0.05	1.16	0.106	1.12	0.3175	1.34	0.0008	1.37	0.0379
101008_at	1434434_s_at	Tcerg1	transcription elongation regulator 1 (CA150)	-1.15	0.63	1.24	0.0262	1.26	0.1056	1.28	0.0961	-1.05	0.8025
101009_at	1435989_x_at	Krt2-8	keratin complex 2, basic, gene 8	1.32	0.46	1.01	0.9001	-1.09	0.3787	1.08	0.3337	-1.42	0.0768
101011_at	1415867_at	Cct4	chaperonin subunit 4 (delta)	1.68	0.06	-1.01	0.7613	1.14	0.3035	1.11	0.075	1.09	0.3349
101013_at	1436292_a_at	Oaz1	ornithine decarboxylase antizyme	-1.03	0.94	1.01	0.8979	1.06	0.3518	-1.03	0.3954	-1.12	0.1138
101014_at	1427691_a_at	Ifnar2	interferon (alpha and beta) receptor 2	-1.2	0.5	1.75	0.0703	1.01	0.9482	1.54	0.0399	1.22	0.4193
101015_s_at	1451462_a_at	Ifnar2	interferon (alpha and beta) receptor 2	1.39	0.11	1.46	0.0076	-1.01	0.9247	1.19	0.102	1.04	0.8735
101019_at	1416382_at	Ctsc	cathepsin C	-2.83	0.08	-1.28	0.4346	-1.09	0.4235	-1.18	0.6892	-4.75	0.0041
101023_f_at	1433936_at	0610010E21Rik	RIKEN cDNA 0610010E21 gene	1.3	0.35	1.11	0.1429	1.01	0.8293	1.1	0.053	1.01	0.9653
101025_f_at	1439016_x_at	Sprr2a	small proline-rich protein 2A	-1.34	0	-1.06	0.6921	1.14	0.293	-1.03	0.8254	1.03	0.8183
101026_at	1419620_at	Pttg1	pituitary tumor-transforming 1	1.96	0.21	-1.31	0.2691	-1.51	0.1091	-1.15	0.4964	1.61	0.197
101029_f_at	1415927_at	Actc1	actin, alpha, cardiac	-1.31	0.55	-1.4	0.3873	-2.09	0.1123	-2.1	0.1195	1.07	0.8046
101030_at	1449110_at	Rhob	ras homolog gene family, member B	-1.19	0.45	-1.08	0.1402	1.19	0.0021	1.06	0.2632	1.28	0.0195
101031_at	1450561_a_at	Surf1	surfeit gene 1	1.45	0.02	1.04	0.5039	1.05	0.3171	-1.01	0.9193	1.19	0.0958
101034_at	1418508_a_at	Grb2	growth factor receptor bound protein 2	-1.06	0.34	-1.15	0.0366	-1.05	0.5408	-1.26	0.0047	1.08	0.6222
101035_at	1415813_at	---	---	1.71	0.13	-1.01	0.7907	1.1	0.1817	1.1	0.0804	-1.2	0.1098
101036_at	1455357_x_at	Tomm20	translocase of outer mitochondrial membran	1.39	0.14	-1.13	0.1369	-1.14	0.1141	-1.11	0.312	-1.07	0.7793
101039_at	1424051_at	Col4a2	procollagen, type IV, alpha 2	-1.16	0.5	1.47	0.0004	1.22	0.0014	1.7	0	1.13	0.2138
101040_at	1416257_at	Capn2	calpain 2	1.16	0.55	-1.03	0.7153	-1	0.9922	-1.03	0.7007	1.05	0.8111
101043_f_at	1415954_at	Try4 /// Trygn16	trypsin 4 /// trypsinogen 16 /// RIKEN cDNA 1	1.31	0.54	1.1	0.6233	1.24	0.145	1.17	0.4041	-1.19	0.4762
101044_at	1424877_a_at	Alad	aminolevulinate, delta-, dehydratase	1.62	0.03	-1.09	0.1858	-1.14	0.1999	-1.22	0.0059	1.07	0.6948
101045_at	1448286_at	Hadh2	hydroxyacyl-Coenzyme A dehydrogenase ty	1.69	0.08	1.09	0.3151	1.03	0.7211	1.56	0	-1.35	0.08
101047_at	1453556_x_at	2410026K10Rik	RIKEN cDNA 2410026K10 gene	1.18	0.57	1.46	0.0047	1.07	0.7062	1.41	0.0786	-1.08	0.7299
101048_at	1422124_a_at	Ptprc	protein tyrosine phosphatase, receptor type,	1.28	0.36	1.88	0.1312	-1.28	0.1401	2.53	0.194	1.16	0.7582
101050_at	1415737_at	Rfk	riboflavin kinase	-1.02	0.94	-1.07	0.4281	-1.01	0.911	-1.09	0.361	-1.14	0.4142
101052_g_at	1421856_at	S100a3	S100 calcium binding protein A3	-1.16	0.75	-1.31	0.45	-1.38	0.394	-1.79	0.1221	1.08	0.8781
101053_at	1424036_at	2610031L17Rik	RIKEN cDNA 2610031L17 gene	-1.22	0.38	-1.09	0.163	1.01	0.8869	-1.01	0.8154	-1.12	0.2814
101054_at	1425519_a_at	Ii	Ia-associated invariant chain	1.35	0.45	2.38	0.1624	-1.43	0.0552	2.54	0.1286	1.89	0.0161
101055_at	1448128_at	Ppgb	protective protein for beta-galactosidase	1.38	0.17	1.13	0.026	1.09	0.2032	1.16	0.0054	1.34	0.0272
101056_at	1448236_at	Rdx	radixin	1.21	0.39	1.05	0.3717	1.04	0.6596	1.1	0.3089	1.13	0.3022
101057_at	1454682_at	A430005L14Rik	RIKEN cDNA A430005L14 gene	1.4	0	1.13	0.1691	1.08	0.2533	1.07	0.4211	1.18	0.1621
101058_at	1417765_a_at	Amy1	amylase 1, salivary	1.35	0.07	-1.3	0.0043	-1.12	0.0973	-1.27	0.0286	-1.65	0.0015
101059_at	1435383_x_at	Ndn	necdin	4.37	0.01	1.35	0.2599	1.62	0.2536	1.87	0.0664	1.16	0.5509
101060_at	1423423_at	Grp58	glucose regulated protein	1.72	0.1	1.01	0.9599	-1.45	0.0055	-1.18	0.131	-2.19	0.018
101061_at	1449930_a_at	Ssr2	signal sequence receptor, beta	-1.16	0.53	-1.2	0.0003	-1.11	0.2002	-1.27	0	-1.26	0.1306
101062_at	1416399_a_at	Hmox2	heme oxygenase (decycling) 2	1.41	0.05	1.27	0.0249	1.29	0.0166	1.46	0.0006	1.14	0.3611

101063_at	1418370_at	Tnnc1	troponin C, cardiac/slow skeletal	2.52	0.28	-2.87	0.0134	-2.79	0.0148	-2.35	0.0411	1.41	0.2144
101064_at	1448282_at	Plrg1	pleiotropic regulator 1, PRL1 homolog (Arab	1.68	0.16	1.12	0.0458	1.22	0.0316	1.19	0.0679	1.04	0.6226
101065_at	1417947_at	Pcna	proliferating cell nuclear antigen	1.19	0.4	1.11	0.4326	-1.13	0.1834	1.29	0.0158	1.15	0.1467
101067_at	1428709_a_at	Mrpl24	mitochondrial ribosomal protein L24	1.47	0.07	1.06	0.1541	1.11	0.2913	1.16	0.0056	1.13	0.2344
101069_g_at	1455504_a_at	Mkrn1	makorin, ring finger protein, 1	1.57	0.2	-1.06	0.5673	-1.17	0.0373	-1.01	0.8781	-1.04	0.7331
101070_at	1418434_at	Mkrn1	makorin, ring finger protein, 1	1.22	0.23	1.01	0.962	-1.12	0.4006	1.01	0.8733	1.03	0.8387
101071_at	1448826_at	Myh6	myosin, heavy polypeptide 6, cardiac muscle	1.51	0.44	-1.76	0.0662	-1.67	0.1602	-1.68	0.1329	1.96	0.3453
101072_at	1437733_at	Eif4ebp2	Eukaryotic translation initiation factor 4E bin	-1.29	0.05	-1.09	0.2456	1.06	0.1683	-1.14	0.0397	1.18	0.0281
101073_at	1448655_at	Lrp1	low density lipoprotein receptor-related prote	-1.08	0.74	-1.03	0.579	-1.19	0.0858	-1.09	0.1445	-1.13	0.6412
101075_f_at	1455802_x_at	Agr2	anterior gradient 2 (Xenopus laevis)	-1.6	0.18	1	0.9983	2.26	0.2757	1.09	0.5202	-1.05	0.9327
101077_at	1416464_at	Slc4a1	solute carrier family 4 (anion exchanger), me	-1.26	0.11	-1.14	0.3051	-1.26	0.1961	-1.06	0.6577	1.38	0.1407
101079_at	1416791_a_at	Nxf1	nuclear RNA export factor 1 homolog (S. cer	1.53	0.22	1.42	0.0001	1.44	0	1.64	0	1.13	0.5961
101080_at	1416740_at	Col5a1	procollagen, type V, alpha 1	2.3	0.18	2.17	0.0002	1.27	0.4203	1.71	0.0406	2.32	0.0707
101081_at	1415702_a_at	Ctbp1	C-terminal binding protein 1	1.1	0.45	1.02	0.6778	1.02	0.4342	1	0.9177	-1.05	0.6668
101082_at	1416632_at	Mod1	malic enzyme, supernatant	-1.3	0.25	-1.96	0.0031	-1.39	0.0907	-2.2	0.0019	-10.15	0
101085_at	1450865_s_at	Mrps24	mitochondrial ribosomal protein S24	1.45	0.01	1.04	0.5884	1.13	0.059	1.12	0.0581	1.1	0.3184
101086_f_at	1416244_a_at	Cnbp1	cellular nucleic acid binding protein 1	1.29	0.33	1.26	0.0038	1.19	0.0398	1.25	0.0271	1.46	0.036
101088_f_at	1437850_a_at	Cnbp1	cellular nucleic acid binding protein 1	2.08	0.02	1.2	0.0178	1.07	0.1217	1.11	0.0761	1.04	0.8372
101089_at	1449178_at	Pdlim3	PDZ and LIM domain 3	-1.47	0.42	-1.41	0.2643	-1.73	0.1344	-1.78	0.1047	1.41	0.4617
101090_at	1460208_at	Fbn1	fibrillin 1	1.6	0.31	1.42	0.0466	1.02	0.9071	1.6	0.0155	1.66	0.4723
101091_at	1451005_at	Sumo1	SMT3 suppressor of mif two 3 homolog 1 (ye	1.76	0.04	-1.05	0.5204	1	0.969	1.11	0.4049	1.16	0.4874
101093_at	1452035_at	Col4a1	procollagen, type IV, alpha 1	-1.16	0.64	1.87	0.0985	1.22	0.0817	2.02	0.0127	1.11	0.6798
101094_at	1416480_a_at	MGI:1930666	hypoxia induced gene 1	1.47	0.08	-1.06	0.564	-1.19	0.1616	-1.06	0.5481	-1.56	0.0012
101095_at	1417359_at	Mfap2	microfibrillar-associated protein 2	-1.94	0.26	-1.34	0.008	-1.16	0.1336	-1.27	0.0092	2.06	0.0857
101096_s_at	1426187_a_at	Hs1bp1	HS1 binding protein	-1	1	1.11	0.144	1.09	0.3624	1.08	0.2585	1.02	0.7102
101097_at	1452580_a_at	Mrpl21	mitochondrial ribosomal protein L21	1.52	0	1.06	0.4947	1.13	0.2889	1.11	0.2488	-1.33	0.0376
101099_at	1423056_at	Nsg1	neuron specific gene family member 1	-2.09	0.25	-1.05	0.7689	1.1	0.5809	1.1	0.5029	-1.13	0.761
101102_at	1460737_at	Igbp1	immunoglobulin (CD79A) binding protein 1	1.66	0.15	-1.04	0.5721	1.08	0.331	1.15	0.1485	1.12	0.1623
101103_at	1423257_at	---	---	1.87	0.05	11.87	0.0004	20.24	0	14.95	0.0015	6.79	0.0001
101104_at	1415745_a_at	Dscr3	Down syndrome critical region gene 3	-1.03	0.8	1.19	0.0006	1.22	0.0017	1.23	0.0006	1.15	0.2465
101105_at	1421082_s_at	Banf1	barrier to autointegration factor 1	1.03	0.89	-1.01	0.7784	1.11	0.1053	1.08	0.28	1.01	0.9663
101106_at	1423758_at	E430034L04Rik	RIKEN cDNA E430034L04 gene	1.11	0.61	-1.27	0.0426	-1.32	0.0262	-1.31	0.0167	-1.92	0.01
101107_at	1415870_at	---	---	1.27	0.24	1	0.9548	1.13	0.0244	1.01	0.9139	-1.03	0.5574
101108_at	1416042_s_at	Nasp	nuclear autoantigenic sperm protein (histone	5.09	0.01	1.7	0.0247	1.44	0.05	1.62	0.0149	-1.02	0.9521
101109_at	1426778_at	Dag1	dystroglycan 1	1	0.97	1.05	0.1749	-1.05	0.1746	1.06	0.1737	1.06	0.6765
101110_at	1424131_at	Col6a3	procollagen, type VI, alpha 3	1.07	0.52	-1.18	0.2886	-1.51	0.0138	-1.04	0.7965	1.11	0.5118
101112_g_at	1437628_s_at	Rhoa	ras homolog gene family, member A	1.22	0.24	1.1	0.1222	-1.06	0.2556	1.01	0.7922	-1.12	0.2283
101114_at	1421012_at	Srprb	signal recognition particle receptor, B subun	1.84	0.38	1.21	0.5022	1.45	0.147	-1.11	0.7208	-1.04	0.9099
101115_at	1450009_at	Ltf	lactotransferrin	-2.51	0.31	-1.28	0.3555	1.04	0.8562	1.32	0.3021	1.3	0.4343
101116_at	1420109_at	Pip5k2a	Phosphatidylinositol-4-phosphate 5-kinase, t	-1.33	0.46	1.25	0.2746	1.25	0.1419	1.18	0.408	1.89	0.1014
101117_at	1420163_at	AA511261	expressed sequence AA511261	1.01	0.96	-1.62	0.0621	-1.47	0.133	-1.67	0.058	1.32	0.41
101119_at	1449781_at	---	---	-2.33	0.15	1.12	0.7145	-1.03	0.9135	1.05	0.8516	1.49	0.2481
101121_at	1420236_at	Hat1	Histone aminotransferase 1	-1.78	0.33	1.07	0.6449	1.01	0.9198	-1.1	0.3048	-1.56	0.3852
101122_at	1421527_at	Epha6	Eph receptor A6	-2.27	0.12	-1.2	0.4255	1.01	0.9432	-1.19	0.3723	1.53	0.2604
101123_at	1417999_at	Itm2b	integral membrane protein 2B	-1.21	0.08	-1.04	0.1419	-1.15	0.0277	-1.16	0	-1.1	0.1666
101126_r_at	1420069_at	---	---	-1.04	0.95	-1.53	0.1977	1.02	0.967	-1.16	0.7243	-1.41	0.4768
101127_at	1419628_at	Chx10	C. elegans ceh-10 homeo domain containing	-7.47	0	1.06	0.7262	-1.36	0.1351	-1.01	0.9705	-1.14	0.7567
101128_at	1420442_at	Cacna1s /// LOC	calcium channel, voltage-dependent, L type,	1.11	0.84	-2.02	0.0901	-1.83	0.1314	-1.52	0.2881	1.99	0.1583
101129_at	1423665_a_at	Rpl5	ribosomal protein L5	1.12	0.15	1.22	0.0003	1.15	0.0111	1.19	0.0004	1.5	0.0491
101130_at	1450857_a_at	Col1a2	procollagen, type I, alpha 2	2.37	0.1	1.24	0.595	-1.13	0.7039	1.73	0.2415	1.51	0.0817
101131_at	1450299_at	Chrna7	cholinergic receptor, nicotinic, alpha polypep	-1.11	0.27	1	0.9728	-1.06	0.722	-1.11	0.4643	1.71	0.0771
101133_at	1422306_at	Lcn3	lipocalin 3	-1.22	0.35	1.17	0.4981	-1.17	0.2145	-1.24	0.0591	1.34	0.4416
101134_at	1452568_at	Gcap8	granule cell antiserum positive 8	-1.45	0.13	-1.16	0.3412	-1.19	0.2885	-1.44	0.0092	1.01	0.9811

101135_at	1418688_at	Calcr	calcitonin receptor	1.78	0.34	-1.82	0.0946	-1.11	0.7293	-1.22	0.5128	1.38	0.6203
101136_at	1450272_at	Tnfsf8	tumor necrosis factor (ligand) superfamily, m	-2.42	0.05	-1.12	0.4454	-1.07	0.6358	1.22	0.5222	-1.1	0.3148
101137_at	1435151_a_at	Rps3	ribosomal protein S3	1.23	0.34	1.19	0.0088	1.22	0.0309	1.16	0.1409	1.54	0.0336
101138_at	1450319_at	Gabrb2	gamma-aminobutyric acid (GABA-A) recepto	1.07	0.73	1.54	0.0586	1.53	0.2049	1.93	0.0976	2.41	0.023
101139_r_at	1449587_a_at	Muc10	mucin 10, submandibular gland salivary muc	1.32	0.54	1.12	0.7	1.39	0.3383	-1.06	0.8631	1.85	0.0269
101140_at	1450219_at	Htr1a	5-hydroxytryptamine (serotonin) receptor 1A	-1.82	0.04	1.06	0.7629	1.27	0.3101	-1.06	0.8395	1.7	0.1154
101141_at	1427806_at	---	---	1.8	0.13	-1.14	0.5897	1.05	0.8463	-1.18	0.497	1.52	0.5274
101142_at	1448662_at	Fzd6	frizzled homolog 6 (Drosophila)	-1.19	0.36	1.48	0.203	1.03	0.8767	1.21	0.3484	1.59	0.3507
101143_at	1450043_at	Fzd7	frizzled homolog 7 (Drosophila)	-1.67	0.04	-1.2	0.0296	-1.08	0.3915	-1.16	0.0249	1.11	0.6907
101144_at	1421628_at	Il18r1	interleukin 18 receptor 1	-1.07	0.85	1.31	0.375	1.35	0.1428	1.52	0.2215	1.46	0.2655
101145_at	1421512_at	Cep2	centrosomal protein 2	2.22	0.18	1.34	0.3672	1.01	0.9638	1.54	0.2204	-1	0.9959
101146_at	1450002_at	Gsh1	genomic screened homeo box 1	-1.81	0.05	-1.47	0.0331	-1.36	0.0349	-1.28	0.3878	1.24	0.5613
101147_at	1420544_at	Gcet2	germinal center expressed transcript 2	-1.69	0.09	-1.22	0.0366	-1.03	0.7539	-1.19	0.1887	1.05	0.8077
101148_at	1448695_at	Prkci	protein kinase C, iota	-1.01	0.99	-1.05	0.7128	-1.15	0.4026	-1.1	0.488	-1.09	0.5077
101149_at	1450326_at	Shc3	src homology 2 domain-containing transform	-5.25	0.24	1.22	0.2465	1.32	0.1256	1.24	0.1459	1.24	0.6437
101150_at	1421773_at	Etsrp71	ets related protein 71	2.68	0.01	-1.23	0.4012	-1.39	0.1539	-1.02	0.9036	-1.07	0.8444
101151_at	1450215_at	Rcvrn	recoverin	1.11	0.23	1.44	0.017	1.26	0.0282	1.18	0.0585	-1.02	0.9333
101152_at	1422207_at	Htr5a	5-hydroxytryptamine (serotonin) receptor 5A	-1.38	0.02	-1.21	0.0819	-1.05	0.4905	-1.15	0.1591	1.11	0.5269
101153_at	1452564_at	Ofa	oncofetal antigen	-1.09	0.91	-1.1	0.7513	-1.01	0.9616	1.16	0.6094	3.11	0.0379
101154_at	1420141_at	AA517023	expressed sequence AA517023	1.01	0.97	-1.46	0.2548	-1.56	0.3042	1.05	0.8943	3.64	0.1899
101155_at	1449306_at	Hsf2	heat shock factor 2	1.07	0.9	1.67	0.0821	-1.12	0.7986	-1.07	0.8233	1.8	0.0289
101156_at	1452552_at	Npn2	neoplastic progression 2	1.37	0.45	-1.19	0.7012	-1.47	0.3408	-1.15	0.745	1.42	0.3135
101158_at	1449913_at	Zfp2	zinc finger protein 2	2.29	0.07	-1.32	0.0237	-1.12	0.1949	-1.2	0.0401	-3.62	0.04
101159_at	1422025_at	Mitf	microphthalmia-associated transcription fact	-1.23	0.68	1.01	0.922	-1.43	0.1983	-1.07	0.7746	1.01	0.9886
101160_at	1449984_at	Cxcl2	chemokine (C-X-C motif) ligand 2	-1.1	0.88	2.69	0.3354	-1.09	0.7951	2.91	0.128	1.15	0.6845
101161_at	1422069_at	Mc1r	melanocortin 1 receptor	-1.51	0.26	-1.25	0.074	-1.09	0.3109	-1.32	0.0068	-1.26	0.3327
101162_at	1420757_at	Myf5	myogenic factor 5	-1.91	0.58	-1.03	0.9398	-1.21	0.6005	-1.76	0.1643	1.25	0.7718
101163_at	1418065_at	Rag2	recombination activating gene 2	-2.41	0.2	-1.08	0.612	1.04	0.7889	1.04	0.7954	1.51	0.0237
101164_at	1421744_at	Tnfsf4	tumor necrosis factor (ligand) superfamily, m	-1.05	0.89	1.01	0.9715	1.31	0.4236	1.2	0.4432	1.21	0.6853
101166_at	1450514_at	Zfp29	zinc finger protein 29	-1.37	0.24	1.28	0.0668	1.32	0.0906	1.27	0.0622	1.37	0.436
101167_at	1421598_at	---	---	1.97	0.05	-1.34	0.3198	-2.96	0.0017	-1.72	0.0241	1.11	0.8744
101169_at	1420511_at	Rds	retinal degeneration, slow (retinitis pigmento	-1	0.99	-1.06	0.8263	1.03	0.9289	-1.22	0.4977	-1.05	0.919
101170_at	1450548_at	Htr1f	5-hydroxytryptamine (serotonin) receptor 1F	1.04	0.94	-1.41	0.0625	-1.21	0.2764	1.1	0.686	2.06	0.1606
101171_at	1420457_at	Dvl3	dishevelled 3, dsh homolog (Drosophila)	1.61	0.14	-1.19	0.1731	-1.26	0.2078	1.07	0.6188	1.16	0.7275
101172_at	1421710_at	Zfp92	zinc finger protein 92	-1.92	0.35	1.42	0.164	1.04	0.8761	-1.37	0.3169	1.49	0.4084
101173_at	1420983_at	Pctp	phosphatidylcholine transfer protein	1.68	0.44	-1.58	0.0119	-1.59	0.0335	-2.45	0.0002	-1.63	0.5554
101175_at	1422250_at	Map3k2	mitogen activated protein kinase kinase kina	-1.23	0.13	-1.49	0.4023	-1.48	0.3411	-1.95	0.1638	-1.09	0.8654
101176_at	1422304_at	Lcn4	lipocalin 4	-1.13	0.72	1.27	0.0957	1.09	0.461	1.05	0.7337	1.02	0.9374
101177_at	1422098_at	Acvr1b	activin A receptor, type 1B	-1.5	0.65	1.01	0.9775	1.62	0.1007	-1.35	0.2681	1.21	0.2555
101178_at	1420696_at	Sema3c	sema domain, immunoglobulin domain (Ig),	1.35	0.44	1.04	0.747	1.11	0.2971	1.09	0.4601	1.36	0.2626
101180_at	1421205_at	Atm	ataxia telangiectasia mutated homolog (hum	-1.69	0.13	-1.07	0.5365	-1.48	0.0211	-1.16	0.4462	-1.17	0.1827
101181_at	1422181_at	Cnga2	cyclic nucleotide gated channel alpha 2	-1.31	0.57	1.04	0.8947	-1.01	0.9518	1.07	0.7848	-1.36	0.2482
101182_at	1450525_at	Gli3	GLI-Kruppel family member GLI3	-1.36	0.56	-1.14	0.492	-1.1	0.6419	-1.02	0.9276	-1.85	0.1518
101183_at	1449866_at	Syt2	synaptotagmin 2	-1.65	0.26	1.59	0.1201	1.85	0.0509	1.18	0.5901	1.34	0.4596
101184_at	1421671_at	Hsd17b3	hydroxysteroid (17-beta) dehydrogenase 3	-1.64	0.46	1.05	0.829	1.15	0.4582	-1.07	0.7498	1.28	0.4386
101185_at	1423027_at	Foxl1	Forkhead box L1	-1.63	0.01	-1.1	0.2016	-1.05	0.4713	-1.09	0.2083	1.29	0.1778
101186_at	1420747_at	MGI:1349458	per-pentamer repeat gene	-1.29	0.37	1.1	0.4548	-1.02	0.9048	-1.11	0.5733	1.28	0.3905
101187_at	1418813_at	Serpina5	serine (or cysteine) proteinase inhibitor, clad	-1.23	0.16	-1.94	0.0125	-1.25	0.3445	-1.5	0.1513	1.05	0.8917
101188_at	1421468_at	Kcnj3	potassium inwardly-rectifying channel, subfa	-1.37	0.24	1.15	0.5583	1.23	0.4077	1.2	0.4204	1.75	0.3045
101189_at	1450366_at	Bid3	BH3 interacting (with BCL2 family) domain, a	-1.36	0.05	1.18	0.4199	-1.04	0.8816	1.04	0.8813	1.34	0.6768
101190_at	1450352_at	Mtnr1a	melatonin receptor 1A	-1.36	0.58	1.22	0.5211	-1.22	0.537	1.75	0.1074	-1.82	0.1243
101191_at	1420666_at	Doc2b	double C2, beta	-2.58	0.31	1.03	0.9325	1.59	0.1762	-1.01	0.962	1	0.9925
101192_at	1420348_at	Lhx5	LIM homeobox protein 5	1.04	0.94	1.49	0.2022	1.43	0.078	1.27	0.1402	2.11	0.3451

101193_at	1421395_at	Zik1	zinc finger protein interacting with K protein	-1.04	0.87	-1.17	0.6631	1.31	0.4354	-1.16	0.6598	2.37	0.225
101194_at	1420568_at	Stra8	stimulated by retinoic acid gene 8	-1.9	0.09	-1.1	0.4844	-1.04	0.7937	1.03	0.8674	1.19	0.5615
101195_at	1420805_at	Mylc2pl	myosin light chain 2, precursor lymphocyte-s	-1.51	0.64	1	0.9903	-1.18	0.2765	-1.15	0.34	1.03	0.9287
101197_at	1421732_at	Glrp1	glutamine repeat protein 1	-1.69	0.11	1.05	0.6538	-1	0.9693	-1.03	0.7813	1.63	0.0346
101198_at	1417747_at	Cplx1	complexin 1	-1.74	0.09	-1.1	0.1618	1	0.9641	-1.02	0.7513	1.02	0.8015
101199_at	1426072_at	Cmklr1	chemokine-like receptor 1	-2.76	0.41	1.55	0.2556	-1	0.9962	1.23	0.4039	1.88	0.2687
101200_at	1449920_at	Cyp19a1	cytochrome P450, family 19, subfamily a, po	-1.72	0.27	1.51	0.2936	1.28	0.2412	1.28	0.1646	1.54	0.4891
101202_at	1420297_at	---	---	-1.04	0.91	1.1	0.5228	1.04	0.7583	1.11	0.4468	1.23	0.2508
101205_at	1449808_at	---	CDNA clone IMAGE:6405353	1.43	0.21	1.38	0.1961	1.19	0.5934	-1.02	0.8976	-1.57	0.4113
101206_at	1420289_at	T25656	expressed sequence T25656	1.59	0.39	-1.19	0.4365	-1.33	0.282	-1.09	0.608	1.01	0.9671
101207_at	1417451_a_at	Ppia	peptidylprolyl isomerase A	1.02	0.56	1.06	0.1398	1.09	0.0456	1.11	0.0225	1.05	0.5333
101208_at	1421533_at	Slc7a1	solute carrier family 7 (cationic amino acid tr	-1.01	0.96	-1	0.9723	1.13	0.5568	1.12	0.5244	1.25	0.1372
101209_at	1421775_at	Fcer1a	Fc receptor, IgE, high affinity I, alpha polype	-2.25	0.03	-1.77	0.007	1.1	0.8401	-1.42	0.1789	-1.04	0.9196
101211_at	1447494_at	D7Bwg0826e	DNA segment, Chr 7, Brigham & Women's G	2.41	0.01	-1.66	0.2197	-1.6	0.2533	-1.93	0.1377	-1.78	0.313
101212_at	1455364_a_at	Rps7	ribosomal protein S7	1.27	0.37	1.08	0.271	1.08	0.236	1.17	0.024	1.27	0.0393
101213_at	1419441_at	Arbp	acidic ribosomal phosphoprotein P0	1	0.98	-1.02	0.4962	1.15	0.0288	1.07	0.0958	1.13	0.3284
101215_at	1421738_at	Gabra2	gamma-aminobutyric acid (GABA-A) recepto	-1.6	0.62	-1.01	0.9441	1.33	0.4187	-1.17	0.5023	-1.82	0.0902
101216_at	1420287_at	---	Transcribed locus	1.73	0.35	-2.05	0.0318	-1.39	0.2857	-1.72	0.0507	1.14	0.7855
101220_at	1449765_at	---	---	-1.1	0.86	-1.62	0.031	-1.81	0.0363	-1.66	0.0705	1.58	0.1865
101221_at	1419992_x_at	---	---	1.02	0.9	-1.14	0.0158	1.16	0.0687	-1.15	0.017	1.39	0.133
101223_r_at	1449690_x_at	---	---	-1.61	0.18	-1.38	0.3343	-1.11	0.6937	-1.37	0.2876	1.48	0.5656
101224_at	1445125_at	D8Ertd107e	DNA segment, Chr 8, ERATO Doi 107, expr	-1.41	0.61	1.15	0.4047	-1.11	0.5492	1	0.9904	1.31	0.3542
101225_at	1448085_at	D5Ertd102e	DNA segment, Chr 5, ERATO Doi 102, expr	-1.44	0.1	-1.4	0.0001	-1.07	0.371	-1.38	0.0004	1.14	0.2444
101226_at	1444943_at	Sh3md2	SH3 multiple domains 2	-1.37	0.12	-1.08	0.1734	-1.08	0.3684	-1.19	0.0472	1.1	0.3493
101227_at	1448090_at	---	Transcribed locus	-2.05	0.13	-1.22	0.2553	1.12	0.4627	-1.15	0.3155	1.14	0.7038
101228_at	1459918_at	Bub3	budding uninhibited by benzimidazoles 3 hor	-2.92	0.26	-1.41	0.0809	1.03	0.9157	-1.35	0.1189	1.26	0.7052
101229_at	1448088_at	C78228	expressed sequence C78228	-1.12	0.67	-1.08	0.7263	-1.2	0.4896	-1.32	0.2774	1.03	0.8864
101231_at	1446695_at	Xrn1	RIKEN cDNA A130001C09 gene	-3.1	0.03	-1.44	0.032	-1.06	0.5015	-1.1	0.539	1.48	0.3552
101232_at	1444829_at	C78532	expressed sequence C78532	-1.19	0.72	-1.69	0.1995	-1.29	0.4869	-1.64	0.1578	1.01	0.9905
101233_at	1448082_at	Clcn1	Chloride channel 1	-1.46	0.43	1.22	0.3848	1.31	0.1999	1.25	0.3037	1.35	0.0084
101234_at	1458862_at	C78704	expressed sequence C78704	1.4	0.41	-1.38	0.4059	-1.68	0.2409	-1.55	0.231	1.65	0.4096
101254_at	1439270_x_at	Ran	RAN, member RAS oncogene family	1.01	0.92	-1.02	0.6756	1.05	0.474	-1.06	0.2618	-1.24	0.0016
101255_at	1453723_x_at	Ubb	ubiquitin B	1.15	0.26	1.13	0.0007	1.18	0.0047	1.13	0.0392	1.13	0.1448
101278_at	1432032_a_at	Artn	artemin	-2.64	0.07	1.03	0.8981	1.11	0.673	1.07	0.7938	1.2	0.5222
101280_at	1460313_at	Olfr2	olfactory receptor 2	-1.85	0.08	-1.05	0.675	1.38	0.0535	1.02	0.831	1.22	0.5684
101281_at	1422370_at	Olfr49 /// MOR1	olfactory receptor 49 /// olfactory receptor M(	-1.68	0.34	-1.08	0.7656	-1.15	0.5496	-1.22	0.3273	-2	0.0462
101282_at	1422275_at	Gpr44	G protein-coupled receptor 44	-1.11	0.63	-1.12	0.1552	-1.1	0.3184	-1.24	0.0099	1.2	0.1758
101286_at	1422274_at	Gja8	gap junction membrane channel protein alph	-1.64	0.4	1.32	0.2123	1.06	0.7975	1.07	0.7695	-1.6	0.0664
101288_at	1422319_at	Fv4	Friend virus susceptibility 4	-1.24	0.54	1.03	0.8806	1.35	0.1898	1.18	0.6392	2.07	0.2712
101289_f_at	1425182_x_at	Klk22 /// Klk9	kallikrein 22 /// kallikrein 9	1.02	0.92	-1.1	0.2155	-1.01	0.9372	-1.09	0.2729	-1.01	0.9446
101294_g_at	1422327_s_at	G6pd2 /// G6pd:	glucose-6-phosphate dehydrogenase 2 /// gl	-2.17	0.25	-1.12	0.7673	-1.16	0.5613	1.13	0.7661	-1.04	0.8934
101295_s_at	1427548_a_at	Clns1a	chloride channel, nucleotide-sensitive, 1A	-1.05	0.87	1.01	0.8943	1.1	0.3845	-1.02	0.8335	1.09	0.833
101302_at	1421538_at	Kcnd1	potassium voltage-gated channel, Shal-relat	1.2	0.51	-1.19	0.1652	-1.21	0.2287	1.02	0.9367	1.89	0.142
101303_at	1422372_at	Olfr15	olfactory receptor 15	-2.11	0.02	1.08	0.7246	-1.28	0.3033	-1.11	0.4296	1.49	0.0772
101305_at	1422331_at	Pou3f3	POU domain, class 3, transcription factor 3	1.47	0.31	-1.35	0.4205	-1.23	0.5656	-1.16	0.699	1.48	0.3747
101307_at	1424352_at	MGC25972	similar to cytochrome P450, 4a10	-4.55	0.06	2.23	0.0025	1.13	0.1555	2.15	0.0002	-5.69	0.0008
101309_at	1450577_at	Sstr3	somatostatin receptor 3	-1.26	0.43	1.16	0.262	-1.05	0.6596	1.04	0.724	1.2	0.3074
101310_at	1460309_at	Tal2	T-cell acute lymphocytic leukemia 2	1.28	0.63	-1.58	0.1232	-1.27	0.3779	-1.37	0.2669	-1.01	0.9874
101311_at	1427765_a_at	Tcrb-V13 /// LO(	T-cell receptor beta, variable 13 /// similar to	1.25	0.45	-2.5	0.0173	-1.28	0.4944	-2.77	0.0117	1.18	0.763
101312_at	1422223_at	Grin2b	glutamate receptor, ionotropic, NMDA2B (ep	1.55	0.24	-1.31	0.0527	1.18	0.4599	-1.09	0.6031	-1.2	0.6923
101315_at	1427832_at	Tuba-rs1	tubulin alpha, related sequence 1	1.22	0.66	1.1	0.3322	1.4	0.1372	1.09	0.4988	1.04	0.8247
101318_at	1450527_at	Sstr1	somatostatin receptor 1	1.05	0.92	1.43	0.0123	1.37	0.0105	1.17	0.1597	-1.09	0.7616
101325_r_at	1425276_at	Fbs1	fibrosin 1	-1.32	0.62	1.57	0.0121	1.39	0.104	1.5	0.0597	-1.07	0.7869

101329_f_at	1427837_at	Igk-V32	immunoglobulin kappa chain variable 32 (V3	1.66	0.26	1.55	0.2663	-1.02	0.9498	-1.05	0.8752	1.42	0.2488
101331_f_at	1427860_at	---	Similar to immunoglobulin light chain	2.08	0.19	2.58	0.0159	1.22	0.5476	2.06	0.0484	1.8	0.3685
101332_at	1426178_at	Igk-V8	Immunoglobulin kappa chain, constant regio	-1.74	0.29	-1.03	0.8874	-1.09	0.7452	1.01	0.9575	1.06	0.8876
101334_at	1452528_a_at	Nkx2-3	NK2 transcription factor related, locus 3 (Drc	-1.03	0.96	-1.37	0.2857	-1.14	0.7341	-1.16	0.5916	1.44	0.4471
101335_at	1450465_at	Cdk5r2	cyclin-dependent kinase 5, regulatory subun	-2	0.31	1.07	0.7444	1.2	0.2837	1.49	0.2167	-1.03	0.9579
101337_at	1426077_at	Moxd2	monooxygenase, DBH-like 2	-1.42	0.43	1.13	0.452	1.31	0.2728	-1.34	0.1211	1.68	0.0954
101341_at	1450529_at	H2-M9	histocompatibility 2, M region locus 9	1.36	0.51	1.07	0.6374	-1.04	0.8444	-1.07	0.6576	1.36	0.3469
101342_at	1451889_at	Notch2	Notch gene homolog 2 (Drosophila)	-2.12	0.29	1.11	0.7965	1.3	0.4391	1.02	0.9463	1.67	0.0779
101343_at	1423024_at	---	---	-1.76	0.1	-1.09	0.4969	-1.03	0.8508	-1.05	0.7406	1.22	0.4315
101344_at	1460663_at	Cckbr	cholecystokinin B receptor	-1.29	0.13	-1.1	0.1632	-1.05	0.3926	-1.2	0.0079	1.12	0.5311
101345_at	1447953_at	Egfl8	EGF-like domain 8	-1.13	0.32	-1.05	0.5009	-1.07	0.36	-1.11	0.173	1.26	0.2113
101346_at	1452535_at	---	Ig rearranged heavy chain (NC12-H7) mRN/	-3.06	0.03	1.17	0.5329	1.89	0.0715	1.15	0.6681	-1.8	0.0896
101347_at	1452536_s_at	Igk-V1	Immunoglobulin kappa chain variable 21 (V2	1.12	0.81	1.18	0.3197	1.06	0.7902	1.2	0.3677	1.59	0.0807
101348_at	1427753_at	---	Similar to Ig kappa chain precursor V region	-1.48	0.43	1.37	0.2004	1.14	0.5656	1.1	0.6634	2.83	0.0181
101354_at	1422203_at	Slc18a3	solute carrier family 18 (vesicular monoamin	-1.15	0.52	-1.05	0.6305	-1.05	0.5283	-1.15	0.1012	-1.07	0.7444
101355_at	1422396_s_at	Ascl2	achaete-scute complex homolog-like 2 (Dros	-1.81	0.22	-1.24	0.452	-1.39	0.3484	-1.86	0.0963	1.15	0.7288
101356_at	1426100_a_at	Tk2	thymidine kinase 2, mitochondrial	3.52	0.06	1.18	0.2279	-1.09	0.5735	1.12	0.4149	1.34	0.166
101357_at	1460724_at	Ap2a1	adaptor protein complex AP-2, alpha 1 subu	-1.27	0.55	1.12	0.342	1.1	0.4634	1.03	0.7155	-1.69	0.0239
101358_at	1448661_at	Plcb3	phospholipase C, beta 3	-1.07	0.88	1.04	0.705	1.09	0.2977	1.22	0.3897	-1.21	0.5167
101359_at	1416513_at	Lamb2	laminin, beta 2	1.98	0.03	1.17	0.2616	1.13	0.3505	-1.06	0.7682	1.07	0.8386
101363_at	1460710_at	---	---	-1.1	0.45	-1.01	0.937	-1.04	0.6311	-1.01	0.9314	1.23	0.265
101364_at	1427519_at	---	---	-2.87	0.01	-1.07	0.6472	1.01	0.9134	-1.13	0.2583	1.67	0.048
101366_f_at	1453018_at	Nvl	nuclear VCP-like	-1.17	0.5	-1.18	0.2869	1.06	0.6933	1.18	0.138	1.32	0.4099
101367_at	1435414_s_at	---	---	-1.15	0.73	-1.08	0.3741	-1.1	0.3632	-1.09	0.364	-1.04	0.8343
101368_at	1423429_at	Pem	placentae and embryos oncofetal gene	2.38	0.14	2.19	0.1136	1.11	0.6313	2.39	0.1343	-1.09	0.851
101369_at	1433705_at	6330415G19Rik	RIKEN cDNA 6330415G19 gene	1.02	0.96	1.09	0.6317	1.07	0.6937	-1.03	0.8769	1.88	0.1376
101370_at	1460260_s_at	Kpna1	karyopherin (importin) alpha 1	-1.06	0.86	-1.17	0.1048	1.09	0.223	-1.06	0.401	1.05	0.8454
101371_at	1424469_a_at	Cpsf4	cleavage and polyadenylation specific factor	1.89	0.07	1.05	0.6435	-1.01	0.9276	1.03	0.8033	-1.03	0.7735
101372_at	1429295_s_at	Trip13	thyroid hormone receptor interactor 13	-2.19	0.06	-1.21	0.6102	-1.33	0.2949	-1.38	0.3023	-2.56	0.2076
101374_at	1451859_at	Krtap6-1	keratin associated protein 6-1	-1.25	0.29	-1.69	0.0151	-1.26	0.1707	-1.39	0.0793	1.38	0.272
101375_at	1420751_at	Krtap6-1	keratin associated protein 6-1	-3.59	0.19	-2.37	0.0572	-2.71	0.0376	-2.27	0.068	-1.14	0.6948
101376_at	1425872_at	Krtap6-1	keratin associated protein 6-1	-1.56	0.05	-1.36	0.0235	-1.1	0.4824	-1.4	0.0396	1.19	0.324
101380_at	1424753_at	Nudt14	nudix (nucleoside diphosphate linked moiety	2.55	0.05	1.03	0.7862	1.12	0.3901	1.12	0.276	1.09	0.6306
101381_at	1421305_x_at	Rabep1	rabaptin, RAB GTPase binding effector prote	1.41	0.23	1.43	0.0001	1.47	0.007	1.33	0.0003	-1.28	0.5817
101382_at	1449261_at	Pbx2	pre B-cell leukemia transcription factor 2	1.51	0.28	-1.08	0.5519	-1.17	0.1663	1.05	0.6871	2.92	0.0005
101383_at	1419606_a_at	Tnnt1	troponin T1, skeletal, slow	-1.02	0.91	1.04	0.8348	1.1	0.68	1.09	0.6278	-1.4	0.2111
101384_at	1424549_at	Degs2	degenerative spermatocyte homolog 2 (Dros	-1.09	0.35	1.01	0.9369	1.28	0.1801	1.1	0.5926	1.42	0.1491
101386_at	1417735_at	1810030J14Rik	RIKEN cDNA 1810030J14 gene	-1.45	0.18	-1.17	0.4482	-1.26	0.2647	-1.17	0.4653	1.12	0.8407
101388_at	1416784_at	Pgk2	phosphoglycerate kinase 2	-2.62	0.26	-1.14	0.6913	1.32	0.4046	-1.42	0.3315	1.74	0.172
101389_at	1460235_at	Scarb2	scavenger receptor class B, member 2	1.22	0.37	1.3	0.0002	1.29	0.0009	1.22	0.0009	-1.01	0.9094
101390_at	1427374_at	Muc3	mucin 3, intestinal	-1.62	0.1	1.52	0.1941	1.48	0.563	-1.13	0.4555	1.7	0.3462
101392_at	1451300_a_at	Chmp7	CHMP family, member 7	-1.72	0.03	-1.12	0.0739	1.08	0.5216	-1.27	0.0021	-1.12	0.4506
101393_at	1460330_at	Anxa3	annexin A3	1.64	0.22	1.06	0.8444	1.03	0.9036	1.29	0.247	3.32	0.0099
101395_at	1436940_at	Purb	Purine rich element binding protein B	1.1	0.68	-1.04	0.6225	1	0.9995	-1.07	0.397	1.3	0.0056
101396_at	1451687_a_at	Tcf2	transcription factor 2	1.11	0.67	1.15	0.2486	-1.04	0.7422	1.04	0.7205	1.12	0.7645
101397_at	1450628_at	Slc2a8	solute carrier family 2, (facilitated glucose tra	-1.12	0.44	-1.03	0.4036	1.06	0.4131	1.01	0.8049	-1.05	0.5732
101398_at	1448341_a_at	Stxbp2	syntaxin binding protein 2	1.13	0.53	-1.1	0.0815	-1.02	0.8197	1	0.8961	-1.15	0.3609
101399_at	1418670_s_at	---	---	-1.21	0.81	1.02	0.9154	-1.02	0.9331	-1.19	0.374	1.35	0.1421
101401_at	1416867_at	Bet1	blocked early in transport 1 homolog (S. cer	-1.01	0.99	-1.17	0.1196	-1.11	0.4635	-1.22	0.0424	-1.47	0.1095
101402_at	1424180_a_at	Thrap4	thyroid hormone receptor associated protein	-1.47	0.01	-1.12	0.0692	-1.04	0.551	-1.12	0.0123	1.01	0.8239
101403_at	1418777_at	Ccl25	chemokine (C-C motif) ligand 25	1.84	0.1	2.34	0.0003	2.33	0.0209	1.98	0.0251	1.3	0.4199
101404_at	1451265_at	2310061I09Rik	RIKEN cDNA 2310061I09 gene	5.65	0.03	-1.02	0.8852	1.28	0.2368	1.3	0.0716	1.88	0.2708
101405_at	1448913_at	Smarcd1	SWI/SNF related, matrix associated, actin de	-1.23	0.59	1.19	0.0026	1.17	0.2014	1.01	0.8617	1.19	0.2899

101406_at	1417335_at	Sult2b1	sulfotransferase family, cytosolic, 2B, memb	-1.94	0.06	-1.5	0.0044	-1.17	0.1387	-1.19	0.0968	1.1	0.5358
101407_at	1427282_a_at	Fxn	frataxin	1.86	0.06	1.11	0.4238	1.11	0.4307	-1.09	0.3473	1.18	0.6402
101408_at	1422558_at	Gamt	guanidinoacetate methyltransferase	-1.01	0.97	-1.24	0.0017	-1.07	0.5126	-1.37	0.0011	-1.14	0.5427
101409_at	1451211_a_at	Lgtn	ligatin	1.07	0.83	-1.07	0.3049	1.17	0.0152	-1.04	0.5558	-1.44	0.0397
101410_at	1418283_at	Cldn4	claudin 4	-1.94	0.07	-1.54	0.0568	-1.49	0.116	-1.03	0.8609	1.12	0.7251
101412_at	1432103_a_at	Sh3gl3	SH3-domain GRB2-like 3	-1.06	0.81	1.07	0.7608	1.04	0.8161	-1.1	0.4845	2.67	0.1571
101414_at	1448531_at	Lmnb2	lamin B2	-1.3	0.28	1.11	0.4997	1.11	0.3854	1.17	0.3592	1.36	0.2961
101416_f_at	1423163_at	Bat4	HLA-B associated transcript 4	1.03	0.78	1.03	0.5078	1.04	0.677	1.02	0.769	1.21	0.1848
101417_at	1427252_at	Dmrtb1	DMRT-like family B with proline-rich C-termi	1.31	0.36	-1.23	0.5194	1.02	0.9391	-1.76	0.066	2.6	0.1022
101419_at	1423221_at	Tubb4	tubulin, beta 4	-2.07	0.07	-1.07	0.5387	1.06	0.5357	-1.01	0.9379	1.46	0.0617
101420_at	1422756_at	Slc32a1	solute carrier family 32 (GABA vesicular trar	-1.21	0.76	-1.12	0.5341	-1.18	0.4292	-1.15	0.3917	1.14	0.5929
101421_at	1422888_at	Rnf5	ring finger protein 5	1.03	0.9	-1.09	0.2199	-1.05	0.5107	-1.23	0.007	-1.37	0.0326
101424_at	1425719_a_at	Nmi	N-myc (and STAT) interactor	1.39	0.14	-1.32	0.2946	-1.16	0.4257	-1.16	0.3626	-1.76	0.2194
101425_at	1448273_at	Gss	glutathione synthetase	-1.04	0.83	1.21	0.0345	1.27	0.0471	1.3	0.0002	1.27	0.1039
101426_at	1434034_at	Cerk	ceramide kinase	5.58	0.15	-1.02	0.8937	-1.08	0.4476	2.14	0.2403	-1.22	0.6035
101429_at	1417516_at	Ddit3	DNA-damage inducible transcript 3	1.1	0.57	1.48	0.4259	-1.18	0.6459	1.51	0.3682	1.57	0.0012
101431_at	1418808_at	Rdh5	retinol dehydrogenase 5	5.39	0.04	-1	0.982	-1.05	0.5843	1.19	0.0551	-1.01	0.9623
101432_at	1455315_at	2410019G02Rik	RIKEN cDNA 2410019G02 gene	-1.11	0.63	-1.1	0.4024	-1.18	0.0514	-1.11	0.3207	1.41	0.1098
101436_at	1418652_at	Cxcl9	chemokine (C-X-C motif) ligand 9	-1.42	0.3	2.94	0.361	-1.86	0.1147	2.24	0.2979	1.04	0.9288
101437_at	1449336_a_at	Slk	STE20-like kinase (yeast)	1.21	0.73	-1.02	0.7853	1.02	0.8848	-1.11	0.2428	1.37	0.532
101439_at	1417331_a_at	Arl6	ADP-ribosylation factor-like 6	1.18	0.6	-1.05	0.7157	-1.03	0.7506	1.09	0.3932	-1.35	0.231
101440_at	1453025_at	2610107G07Rik	RIKEN cDNA 2610107G07 gene	-1.31	0.16	-1.4	0.0001	-1.14	0.0419	-1.55	0.0001	-1.26	0.1346
101442_f_at	1427287_s_at	Itpr2	inositol 1,4,5-triphosphate receptor 2	-1.15	0.49	1.1	0.389	1.25	0.0428	1.24	0.051	1.36	0.2718
101444_at	1417684_at	Thumpd3	THUMP domain containing 3	1.35	0.13	1.1	0.1041	1.13	0.0202	1.16	0.0385	-1.16	0.2822
101445_at	1422946_a_at	Dnmt1	DNA methyltransferase (cytosine-5) 1	-1.32	0.32	1.12	0.3617	1.04	0.7137	1.12	0.2921	2.1	0.1598
101446_at	1418412_at	Tpd52l1	tumor protein D52-like 1	1.55	0.06	1.23	0.0127	1.21	0.1038	1.34	0.0088	-1.06	0.5493
101447_at	1450056_at	Apc	adenomatosis polyposis coli	-1.25	0.68	-1	0.9822	1.01	0.8847	-1.09	0.2155	1.26	0.3547
101448_at	1416540_at	Hgs	HGF-regulated tyrosine kinase substrate	-1.2	0.09	-1.04	0.556	-1.01	0.8517	1.01	0.843	1.16	0.085
101449_at	1424275_s_at	Trim41	tripartite motif-containing 41	2.45	0.01	1.17	0.074	-1.05	0.6006	1.25	0.0078	-1.18	0.6907
101450_at	1460220_a_at	Csf1	colony stimulating factor 1 (macrophage)	1.15	0.67	-1.08	0.4199	-1.31	0.032	-1.08	0.627	1.16	0.7347
101451_at	1417355_at	Peg3	paternally expressed 3	1.51	0.27	-1.29	0.1746	1.07	0.647	-1.45	0.0132	-1.09	0.7769
101453_at	1419608_a_at	Mia1	melanoma inhibitory activity 1	-1.65	0.02	-1.19	0.2196	-1.06	0.6859	-1.12	0.3752	1.53	0.1632
101457_at	1421066_at	Jak2	Janus kinase 2	-1.1	0.54	1.23	0.0697	1.16	0.1217	1.22	0.0602	1.18	0.3135
101458_at	1416773_at	Wee1	wee 1 homolog (S. pombe)	1.62	0.4	1.47	0.006	1.32	0.015	1.28	0.0901	2.01	0.1319
101459_at	1450077_at	Chd1	chromodomain helicase DNA binding proteir	1.32	0.39	1.27	0.0087	1.53	0.0002	1.16	0.1484	1.32	0.0549
101462_r_at	1452083_a_at	Pja1	praja1, RING-H2 motif containing	-1.36	0.16	1.18	0.3022	1.32	0.1641	1.14	0.2694	-1.03	0.9293
101463_at	1418708_at	Apoc4	apolipoprotein C-IV	-1.19	0.62	1.05	0.3604	1.09	0.077	1.07	0.1926	1.21	0.0967
101464_at	1460227_at	Timp1	tissue inhibitor of metalloproteinase 1	-1.62	0.14	-1.36	0.09	-1.06	0.677	-1.11	0.4864	-1.33	0.1712
101465_at	1450034_at	Stat1	signal transducer and activator of transcripti	1.62	0.15	1.76	0.3209	-1.55	0.0811	1.78	0.3045	1.02	0.9371
101466_at	1448896_at	Pigf	phosphatidylinositol glycan, class F	-1.01	0.99	-1.08	0.4315	1.35	0.2097	-1.01	0.9204	1.26	0.1734
101467_at	1434342_at	---	---	-2.69	0.28	1.06	0.7345	-1.07	0.7132	-1.13	0.6006	1.83	0.212
101468_at	1452279_at	Pfc	properdin factor, complement	1.69	0.06	1.26	0.339	-1.03	0.756	1.39	0.3349	1.07	0.7697
101469_at	1422818_at	Nedd9	neural precursor cell expressed, developme	-1.43	0.32	1.33	0.1506	1.08	0.7469	1.57	0.03	1.6	0.2277
101470_at	1434793_at	BC028975	cDNA sequence BC028975	-2.03	0.05	-1.01	0.9227	-1.01	0.9134	-1.17	0.2019	1.39	0.1536
101471_at	1438711_at	---	---	-2.77	0.05	-1.7	0.0028	-1.26	0.1101	-2.47	0.0001	-2.43	0.0017
101472_s_at	1421259_at	Pklr	pyruvate kinase liver and red blood cell	-1.76	0.34	-1.55	0.0012	-1.38	0.0721	-1.97	0	-2.97	0.0692
101473_at	1432517_a_at	Nnmt	nicotinamide N-methyltransferase	2.64	0.25	-1.57	0.0006	-2.2	0.0017	-2.13	0	-1.22	0.5081
101474_at	1419578_at	Mbl1	mannose binding lectin, liver (A)	-1.27	0.29	-1.54	0	-1.32	0	-1.89	0	-2.87	0.0119
101475_at	1448733_at	Bmi1	B lymphoma Mo-MLV insertion region 1	1	0.99	1.19	0.1779	1.08	0.6332	1.14	0.2473	1.37	0.2425
101476_at	1415728_at	Pabpn1	poly(A) binding protein, nuclear 1	-1.58	0.07	1	0.9727	1.04	0.7094	1.04	0.6378	1.23	0.1168
101480_at	1416870_at	Dnalc4	dynein, axonemal, light chain 4	-1.11	0.78	1.08	0.6678	-1.17	0.348	1.08	0.532	-1.15	0.6737
101481_at	1415791_at	Rnf34	ring finger protein 34	-1.06	0.87	1.12	0.3824	1.13	0.3148	1.17	0.2194	-1.43	0.3624
101482_at	1450149_a_at	Ppp1cc	protein phosphatase 1, catalytic subunit, gar	1.24	0.05	1.15	0.006	-1.02	0.7081	1.08	0.0255	1.16	0.1833

101483_at	1420745_a_at	Ccndbp1	cyclin D-type binding-protein 1	-1.09	0.65	1.03	0.4814	1.07	0.1832	1.13	0.0016	-1.11	0.5513
101484_at	1448343_a_at	Nbr1	neighbor of Brca1 gene 1	1.8	0.04	1.16	0.0143	1.21	0.0032	1.27	0.0059	-1.02	0.8097
101485_at	1417242_at	Ddx48	DEAD (Asp-Glu-Ala-Asp) box polypeptide 48	1.53	0.03	1.01	0.8719	1.08	0.1618	1.08	0.1723	1.06	0.5559
101486_at	1448632_at	Psmb10	proteasome (prosome, macropain) subunit, I	1.14	0.49	1.22	0.492	-1.13	0.17	1.53	0.1555	-1.08	0.6707
101489_at	1448484_at	Amd1	S-adenosylmethionine decarboxylase 1	1.07	0.8	1.11	0.2207	1.33	0.0012	1.02	0.7779	-1.11	0.3676
101490_at	1423651_at	Hbld2	HESB like domain containing 2	1.35	0.18	1.02	0.8906	1.22	0.2195	1.13	0.3776	1.16	0.4208
101492_at	1416228_at	Pin1	protein (peptidyl-prolyl cis/trans isomerase) I	2.25	0.21	1.33	0.0774	1.12	0.5513	1.3	0.0376	-2.37	0.1928
101495_at	1416330_at	Cd81	CD 81 antigen	-1.03	0.72	-1.28	0.0043	-1.29	0.0017	-1.35	0	-1.15	0.1391
101498_at	1436848_x_at	Impa1	inositol (myo)-1(or 4)-monophosphatase 1	-1.11	0.5	-1.14	0.3338	-1.16	0.4072	-1.25	0.065	-2.58	0.0008
101499_at	1449942_a_at	Ilk	integrin linked kinase	1.33	0.02	-1.05	0.4039	1.04	0.4766	1.07	0.3364	-1.06	0.5887
101500_at	1433490_s_at	Epb4.1l2	erythrocyte protein band 4.1-like 2	-1.01	0.96	1.12	0.2548	1.18	0.1092	1.27	0.0243	2.33	0.0151
101501_r_at	1415911_at	Impact	imprinted and ancient	-1.06	0.9	1.59	0.0569	1.33	0.0387	1.34	0.1939	1.24	0.5919
101502_at	1422286_a_at	Tgif	TG interacting factor	-1.31	0.42	1.56	0.0397	1.09	0.5655	1.6	0.0632	1.07	0.6985
101505_at	1426644_at	Tbc1d20	TBC1 domain family, member 20	1.41	0.02	1.2	0.0056	1.28	0.086	1.4	0.0006	1.15	0.5472
101506_at	1417353_x_at	Snrpa1	small nuclear ribonucleoprotein polypeptide	1.42	0.27	1.07	0.5386	1.18	0.063	1.31	0.0145	-1.18	0.2242
101507_at	1428364_at	Scnm1	sodium channel modifier 1	6.86	0.04	1.31	0.0636	1.39	0.0263	1.47	0.0005	1.46	0.2326
101509_at	1421750_a_at	Vbp1	von Hippel-Lindau binding protein 1	1.4	0.22	1.28	0.0053	1.37	0.095	1.35	0.0051	1.23	0.37
101510_at	1417056_at	Psme1	proteasome (prosome, macropain) 28 subur	2.03	0.08	1.39	0.2485	1.1	0.4362	1.65	0.055	-1.23	0.1199
101513_at	1438358_x_at	Pfdn5 /// LOC43	prefoldin 5 /// similar to prefoldin 5; EIG-1; c-	-1.59	0.02	-1.2	0.161	1.01	0.9513	-1.39	0.0076	1.1	0.4317
101514_at	1416771_at	Trappc3	trafficking protein particle complex 3	-1.22	0.52	1.06	0.1942	-1.1	0.2634	-1.06	0.5132	-1.07	0.6086
101515_at	1416408_at	Acox1	acyl-Coenzyme A oxidase 1, palmitoyl	1.13	0.61	1.19	0.0457	-1.09	0.146	1.19	0.0913	-1.36	0.1097
101517_at	1450965_at	Tex261	testis expressed gene 261	-1.2	0.09	-1.06	0.5322	-1.37	0.0069	-1.26	0.0262	1.12	0.2572
101518_at	1451412_a_at	0610009H04Rik	RIKEN cDNA 0610009H04 gene	1.22	0.16	-1.04	0.4998	1.13	0.4207	1.06	0.5843	-1.01	0.9473
101519_at	1418568_x_at	Srp14	signal recognition particle 14	1.05	0.81	1.13	0.0641	1.02	0.7893	1.05	0.6104	-1.23	0.0106
101520_at	1418651_at	Spata6	spermatogenesis associated 6	-1.19	0.07	1.12	0.5616	1.09	0.3278	-1.03	0.7372	1.62	0.0094
101521_at	1424278_a_at	Birc5	baculoviral IAP repeat-containing 5	3.42	0.11	1.31	0.355	1.09	0.7925	1.6	0.088	2.21	0.0075
101522_at	1448069_at	Tm4sf1	transmembrane 4 superfamily member 1	-1.44	0.57	-1.2	0.2328	1	0.9858	-1.06	0.6584	1.72	0.0535
101523_at	1428262_s_at	Hnrpa3	heterogeneous nuclear ribonucleoprotein A3	1.19	0.26	1.08	0.4007	1.08	0.1591	1.1	0.1886	1.38	0.0886
101525_at	1428322_a_at	Ndufb10	NADH dehydrogenase (ubiquinone) 1 beta s	1.74	0.05	1.11	0.1295	1.17	0.0025	1.11	0.2142	1.12	0.2295
101526_at	1448601_s_at	Msx1	homeo box, msh-like 1	-2.14	0.03	-1.09	0.7365	-1.28	0.3885	1.05	0.8259	1.33	0.2169
101527_at	1419258_at	Tcea1	transcription elongation factor A (SII) 1	1.2	0.42	1.03	0.4598	-1	0.9528	-1.01	0.8634	-1.1	0.3939
101530_at	1438835_a_at	MGI:1336880	U5 small nuclear ribonucleoprotein	1.77	0.09	-1	0.9705	-1.07	0.3229	1.03	0.5699	-1.28	0.3451
101534_at	1422419_s_at	Tnp2	transition protein 2	-1.24	0.37	1.07	0.6877	-1.11	0.4577	-1.29	0.1013	2.29	0.0536
101536_at	1423200_at	Ncor1	nuclear receptor co-repressor 1	1.12	0.61	1.06	0.355	1.15	0.1383	1.07	0.3449	1.18	0.16
101537_at	1416913_at	Es1	esterase 1	-1.16	0.18	-1.19	0.0443	-1.05	0.5062	-1.48	0.0009	-1.1	0.5828
101539_f_at	1435371_x_at	Ces3	carboxylesterase 3	1.22	0.58	1.21	0.1284	1.1	0.1884	1.14	0.2948	1.61	0.0075
101540_at	1448462_at	Tdg	thymine DNA glycosylase	1.05	0.75	-1.05	0.4307	1.09	0.1387	-1.01	0.8726	-1.1	0.6176
101541_at	1456474_at	---	---	-1.19	0.58	1.05	0.785	1.4	0.0998	1.24	0.2841	1.71	0.1258
101542_f_at	1423043_s_at	Ddx3x /// Fin14	DEAD/H (Asp-Glu-Ala-Asp/His) box polypep	1.58	0.44	1.29	0.1051	-1.48	0.0623	-1.21	0.3118	1.05	0.6459
101551_s_at	1460378_a_at	Tes	testis derived transcript	-1.56	0.22	-1.25	0.5578	1.07	0.8565	1.37	0.3599	1.47	0.5291
101552_at	1423279_at	Slc34a1	solute carrier family 34 (sodium phosphate),	-1.21	0.08	1.1	0.3331	1.12	0.4361	-1.12	0.3839	-1.02	0.9544
101553_at	1424279_at	Fga	fibrinogen, alpha polypeptide	-2.01	0.04	1.11	0.5093	-1.05	0.7364	1.04	0.7373	1.25	0.0246
101554_at	1448306_at	Nfkbia	nuclear factor of kappa light chain gene enha	1.11	0.59	1.27	0.0026	1.21	0.0556	1.44	0.0021	1.24	0.3734
101555_at	1451086_s_at	Rac1	RAS-related C3 botulinum substrate 1	-1.38	0.05	1.1	0.0852	1.05	0.3808	1.08	0.1697	1.13	0.5069
101557_at	1460644_at	Bckdk	branched chain ketoacid dehydrogenase kin	-1.02	0.9	-1.04	0.6535	-1.01	0.9491	-1.05	0.4558	1.11	0.229
101558_s_at	1415676_a_at	Psmb5	proteasome (prosome, macropain) subunit, I	1.57	0.19	1.07	0.1196	1.09	0.1595	1.22	0.0002	-1.58	0.0121
101560_at	1415856_at	Emb	embigin	2.97	0.02	1.2	0.5841	1.14	0.583	1.49	0.2195	-1.04	0.911
101561_at	1428942_at	Mt2	metallothionein 2	6.83	0.03	1.49	0.0218	-1.93	0.0206	-1.1	0.4475	2.1	0.0401
101562_at	1448586_at	Hspa14	heat shock 70kDa protein 14	1.07	0.81	-1.09	0.5095	1.07	0.5673	1.14	0.1949	-1.87	0.1362
101564_at	1460665_a_at	Cnot7	CCR4-NOT transcription complex, subunit 7	1.37	0.45	-1.1	0.3449	-1	0.9739	-1.09	0.4446	-1.13	0.615
101565_f_at	1448680_at	Serpina1a	serine (or cysteine) proteinase inhibitor, clad	-1.4	0.2	-1.01	0.7965	-1.02	0.4818	-1.02	0.5619	1.37	0.0308
101566_f_at	1420465_s_at	Mup1 /// Mup2	major urinary protein 1 /// major urinary prote	-2.2	0.04	-4.59	0	-1.23	0.0016	-8.46	0	-33.45	0
101568_at	1423044_at	Prosc	proline synthetase co-transcribed	1.74	0.02	1.35	0	1.02	0.7147	1.27	0.0018	1.43	0.0094

101569_at	1448457_at	Krt2-6g	keratin complex 2, basic, gene 6g	-1.1	0.88	1.01	0.94	-1.01	0.9655	-1.15	0.3679	-1.04	0.8658
101571_g_at	1423756_s_at	Igfbp4	insulin-like growth factor binding protein 4	-1.16	0.61	-1.14	0.0609	-1.18	0.1662	-1.24	0.0022	1.01	0.9584
101572_f_at	1420553_x_at	Serpina1a	serine (or cysteine) proteinase inhibitor, clad	-1.17	0.27	1.01	0.7792	1	0.9932	-1.01	0.7504	1.13	0.1557
101576_f_at	1451513_x_at	Serpina1b	serine (or cysteine) proteinase inhibitor, clad	-1.2	0.44	-1.03	0.1713	-1.01	0.5804	-1.02	0.3605	1.33	0.0449
101577_at	1434377_x_at	Rps6	ribosomal protein S6	1.48	0.21	1.03	0.5688	1.16	0.0846	1.17	0.0053	1.43	0.0438
101579_at	1448753_at	---	---	1.21	0.35	1.01	0.9225	-1.04	0.7167	-1.14	0.1118	-1.19	0.2276
101581_at	1416680_at	Ube3a	ubiquitin protein ligase E3A	1.2	0.56	1.12	0.4125	-1.07	0.7107	1.11	0.5904	-1.16	0.4182
101582_at	1426579_at	Gnl2	guanine nucleotide binding protein-like 2 (nu	7.52	0.09	2.88	0.0049	2.59	0.0018	3.34	0	2.2	0.0672
101583_at	1448272_at	Btg2	B-cell translocation gene 2, anti-proliferative	1.17	0.6	1.56	0.0245	-1.28	0.2356	1.69	0.0129	3.89	0
101584_at	1419259_at	---	---	1.01	0.95	-1.02	0.6834	1	0.9602	1	0.9948	-1.18	0.231
101585_at	1423451_at	Pgrmc1	progesterone receptor membrane componer	1.16	0.65	1	0.9502	1.01	0.8072	-1.05	0.3226	-1.35	0.0046
101587_at	1422438_at	Ephx1	epoxide hydrolase 1, microsomal	1.28	0.54	1.48	0	1.43	0	1.51	0.0006	-1.57	0.0315
101588_at	1415802_at	Slc16a1	solute carrier family 16 (monocarboxylic acic	-1.1	0.39	-1.05	0.6974	1.45	0.0583	1.07	0.5956	1.17	0.6644
101589_at	1433507_a_at	Hmgn2	high mobility group nucleosomal binding dor	1.12	0.68	-1.02	0.831	-1.17	0.242	1.06	0.4632	1.01	0.9665
101590_at	1428094_at	Lamp2	lysosomal membrane glycoprotein 2	-1.37	0.3	-1.08	0.0717	-1.06	0.1994	-1.18	0.0067	-1.48	0.0039
101591_at	1422443_at	Xpnpep1	X-prolyl aminopeptidase (aminopeptidase P)	-1.13	0.55	-1.12	0.0704	-1.03	0.4274	-1.05	0.2387	-1.28	0.0132
101593_at	1417311_at	Crip2	cysteine rich protein 2	1.66	0.15	-1.06	0.6056	1.02	0.8032	-1.14	0.1708	-1.16	0.3934
101595_at	1448064_at	---	---	-1.06	0.83	-1.13	0.15	-1.04	0.7351	-1.21	0.0628	-1.1	0.622
101596_at	1420048_at	C78859	Expressed sequence C78859	-1.49	0.32	1.23	0.26	1.47	0.0791	1.95	0.0308	1.24	0.6013
101597_at	1446829_at	D12Ertd216e	DNA segment, Chr 12, ERATO Doi 216, exp	1.22	0.67	1.27	0.2359	1.09	0.6457	-1.13	0.5182	1.04	0.7437
101598_at	1458795_at	---	Transcribed locus	-1.33	0.33	-1.05	0.7563	1.04	0.8343	1.09	0.6465	1.41	0.3184
101599_at	1419940_at	C030018P15Ril	RIKEN cDNA C030018P15 gene	-1.37	0.25	1.06	0.5722	1.19	0.3922	1.08	0.5185	1.55	0.0112
101600_at	1444887_at	C79015	expressed sequence C79015	-1.39	0.3	1.12	0.3566	1.07	0.4409	-1.06	0.6116	1.97	0.0369
101601_at	1419885_at	DXErtd223e	DNA segment, Chr X, ERATO Doi 223, expr	1.77	0.14	-1.67	0.2691	1.23	0.6583	-1	0.9898	1.25	0.5651
101603_g_at	1457849_at	4933430F16Rik	RIKEN cDNA 4933430F16 gene	-1.35	0.12	-1.04	0.8062	1.04	0.79	-1.14	0.3367	1.03	0.9269
101604_at	1446379_at	C79296	expressed sequence C79296	-1.61	0.57	1.03	0.9301	-1.02	0.9598	-1.23	0.6156	-1.1	0.8914
101605_at	1446008_at	D3Ertd246e	DNA segment, Chr 3, ERATO Doi 246, expr	-1.62	0.23	1	0.9918	1.01	0.9807	1	0.9973	1.58	0.2967
101606_at	1459085_at	D1Ertd259e	DNA segment, Chr 1, ERATO Doi 259, expr	-1.38	0.01	1.15	0.516	1.09	0.5892	-1.3	0.1455	-1.01	0.9824
101607_at	1438755_at	C80068	expressed sequence C80068	-1.15	0.41	1.1	0.1993	1.08	0.3232	1.17	0.0294	1.41	0.0133
101608_at	1458041_at	C80360	expressed sequence C80360	3.18	0.16	1.29	0.2485	1.3	0.2708	1.4	0.0849	-1.54	0.2942
101609_at	1428752_at	Slc5a11	solute carrier family 5 (sodium/glucose cotra	-1.39	0.15	1	0.9769	1.07	0.4106	-1.07	0.4734	1.2	0.3094
101610_at	1449685_s_at	4933425A18Rik	RIKEN cDNA 4933425A18 gene	-1.61	0.36	-1.03	0.849	1.2	0.478	1.36	0.4002	1.76	0.046
101611_at	1420173_at	D11Ertd326e	DNA segment, Chr 11, ERATO Doi 326, exp	-1.24	0.48	-1.06	0.6798	1.02	0.9226	-1.04	0.8465	1.15	0.7595
101612_at	1460264_at	C81600	expressed sequence C81600	-1.52	0.2	1.34	0.3037	1.28	0.3703	1.12	0.6915	1.43	0.4948
101614_at	1419890_at	---	---	-1.62	0.22	-1.34	0.2139	-1.35	0.1116	-1.3	0.1155	1.68	0.1529
101615_at	1420149_at	---	---	1.05	0.88	-1.02	0.9033	1.3	0.3881	-1.22	0.0304	1.17	0.5912
101619_at	1450593_at	Ifna6	interferon alpha family, gene 6	-2.72	0.34	1.12	0.3385	-1.05	0.7008	1.09	0.4886	1.24	0.3832
101620_at	1422316_at	Gp1ba	glycoprotein 1b, alpha polypeptide	-3.76	0.37	1.02	0.8848	1	0.9863	-1.4	0.0249	1.12	0.8135
101621_at	1421803_at	Apbh	androgen-binding protein eta	-1.27	0.49	-1.05	0.4667	-1.18	0.0088	-1.13	0.0247	1.69	0.062
101622_at	1421737_at	Alx4	aristaless 4	-2.37	0.21	1.23	0.2697	1.46	0.0461	1.22	0.2918	1.09	0.5909
101623_at	1420445_at	Slc16a8	solute carrier family 16 (monocarboxylic acic	-1.36	0.53	-1.03	0.8886	1.26	0.5587	-1.1	0.7289	1.17	0.6
101624_at	1421518_at	Kcns1	K+ voltage-gated channel, subfamily S, 1	-1.23	0.83	1.76	0.1865	1.86	0.0406	2.15	0.0698	1.55	0.4928
101625_at	1421342_at	Kcns2	K+ voltage-gated channel, subfamily S, 2	-1.09	0.7	-1.25	0.0652	-1.12	0.2864	-1.21	0.2228	-1.21	0.5323
101626_at	1420742_at	Adam7	a disintegrin and metalloprotease domain 7	-2.42	0.02	1.08	0.7856	1.21	0.5012	-1.07	0.813	1.34	0.3834
101628_g_at	1425739_at	Pld1	phospholipase D1	-1.91	0.45	1.34	0.0603	1.26	0.1808	1.2	0.2989	1.06	0.9149
101629_s_at	1421673_s_at	Stx1b2 /// Stx1b	syntaxin 1B2 /// syntaxin 1B1	-1.16	0.8	-1.12	0.6956	2.38	0.1063	-1.04	0.8715	-2.04	0.311
101632_at	1419214_at	Tnfrsf11a	tumor necrosis factor receptor superfamily, r	-1.35	0.24	-1.06	0.4158	-1.1	0.3747	-1.13	0.108	1.68	0.0706
101634_at	1432416_a_at	Npm1	nucleophosmin 1	1.45	0.1	1.07	0.1171	1.16	0.1152	1.15	0.0191	1.38	0.0872
101635_f_at	1426166_at	Mup5	major urinary protein 5	-2.08	0.05	-5.06	0	-1.28	0.0141	-6.77	0	-10.01	0
101636_at	1420466_at	Spt2	salivary protein 2	1.85	0.03	1.31	0.362	1.47	0.1547	1.32	0.3431	1.19	0.6927
101637_at	1417074_at	Ceacam10	CEA-related cell adhesion molecule 10	-1.55	0.28	1.36	0.2641	1.26	0.4571	-1.1	0.7585	2.3	0.1931
101639_r_at	1421741_at	Cyp3a16	cytochrome P450, family 3, subfamily a, poly	-2.3	0.03	-3.76	0.0001	-1.62	0.0087	-3.56	0	1.27	0.3332
101642_at	1427841_at	---	---	1.1	0.31	1.18	0.0793	1.15	0.3121	1.05	0.3787	1.34	0.1277

101643_at	1450349_at	Stx1b1	syntaxin 1B1	1.13	0.84	1.21	0.1034	1.15	0.1893	1.37	0.2458	1.49	0.3609
101644_f_at	1450338_x_at	V2r8 /// V2r9	vomeronasal 2, receptor, 8 /// vomeronasal 2	2.16	0.26	1.15	0.5603	2.02	0.2046	-1.26	0.0434	1.46	0.5633
101646_at	1426056_at	Pigq	phosphatidylinositol glycan, class Q	1.63	0.19	-1.03	0.9418	1.29	0.2568	1.31	0.2972	4.47	0.0038
101647_at	1421767_at	---	---	-2.28	0.32	-1.34	0.3387	1.04	0.908	-1.14	0.6194	1.05	0.4812
101648_at	1422318_at	Foxd4	forkhead box D4	-3.67	0.18	-1.35	0.3058	-1.51	0.2173	-1.3	0.4484	1.59	0.5269
101649_at	1421833_at	Pip5k1a	phosphatidylinositol-4-phosphate 5-kinase, t	-1.54	0.15	1.18	0.3783	-1.23	0.1282	-1.17	0.1991	2.52	0.1418
101651_at	1419429_at	Cntfr	ciliary neurotrophic factor receptor	-1.66	0.38	-1.34	0.4404	-1.27	0.4669	-2.11	0.0527	1.73	0.0128
101653_f_at	1422405_at	H2-D4	histocompatibility 2, D region locus 4	1.77	0.58	1.16	0.4711	-1.38	0.0698	1.44	0.1363	-1.74	0.0475
101655_at	1419521_at	Zfp94	zinc finger protein 94	1.1	0.64	1	0.9735	1.05	0.7702	-1.12	0.5767	-1	1
101657_at	1450342_at	Bmp8b	bone morphogenetic protein 8b	-1.77	0.38	1.03	0.8475	-1.16	0.3475	1.03	0.8313	-1.03	0.8874
101659_at	1425127_at	Hsd3b2	hydroxysteroid dehydrogenase-2, delta<5>-3	-1.78	0.06	-1.23	0.1941	1.11	0.484	-1.1	0.4479	-1.77	0.0017
101661_r_at	1420290_at	---	Transcribed locus	-2.27	0.08	-1.01	0.9275	-1.12	0.5744	-1.37	0.1186	1.55	0.1071
101663_s_at	1420001_at	D7Ertd1e	DNA segment, Chr 7, ERATO Doi 1, express	2.14	0.24	1.25	0.4074	1.26	0.1038	-1.06	0.6999	1.16	0.5593
101664_at	1422475_a_at	Rps3a	ribosomal protein S3a	1.35	0.26	1.07	0.0103	1.11	0.0692	1.08	0.0559	1.32	0.0706
101665_at	1418315_at	Nr5a1	nuclear receptor subfamily 5, group A, meml	-1.4	0	-1.12	0.019	-1.17	0.0005	-1.21	0.0008	1.13	0.3763
101666_at	1421730_at	Nr5a1	nuclear receptor subfamily 5, group A, meml	-4.28	0.06	1.43	0.0076	1.3	0.0247	1.4	0.0333	1.28	0.4436
101668_at	1449761_at	AA589418	expressed sequence AA589418	-1.45	0.01	-1.03	0.8172	1.03	0.699	-1.09	0.5273	1.15	0.6006
101671_at	1420084_at	---	Transcribed locus	-1.53	0.66	1.2	0.5004	1.5	0.2081	1.32	0.1984	-1.04	0.9116
101676_at	1449106_at	Gpx3	glutathione peroxidase 3	-1.16	0.8	-1.55	0.0342	-1.15	0.2244	-1.32	0.0674	2.31	0.0381
101677_at	1453827_at	1110035H17Rik	RIKEN cDNA 1110035H17 gene	-1.23	0.23	-1.14	0.0164	-1.05	0.6056	-1.11	0.1406	1.11	0.3331
101679_at	1419723_at	Opn1mw	opsin 1 (cone pigments), medium-wave-sens	-1.77	0.03	1	0.9649	-1.04	0.6722	-1.12	0.354	1.26	0.1035
101680_at	1426661_at	Rpl27a	ribosomal protein L27a	-1.31	0.05	-1.04	0.5769	1.19	0.0177	1.08	0.1585	1.51	0.134
101681_f_at	1450531_at	H2-Bl	histocompatibility 2, blastocyst	-1.23	0.34	-1.13	0.1162	-1.18	0.0583	-1.18	0.0082	-1.1	0.3586
101682_f_at	1420793_at	Mup4	major urinary protein 4	-2.73	0.04	-3.7	0	-1.2	0.0936	-4.2	0	-9.72	0
101688_at	1448089_at	D4Ertd58e	DNA segment, Chr 4, ERATO Doi 58, expre	-1.05	0.89	-1.06	0.6646	-1.13	0.4226	-1.24	0.1406	1.85	0.2834
101689_at	1419831_at	AA416453	expressed sequence AA416453	-1.07	0.82	-1.47	0.308	-1.48	0.3056	-1.23	0.5464	1.15	0.7701
101691_s_at	1448093_s_at	C77405	expressed sequence C77405	-1.58	0.58	-1.7	0.0622	-1.16	0.6123	-1.74	0.0584	-2.11	0.1656
101693_f_at	1447997_s_at	LOC223262	similar to small zinc finger-like protein	-2.9	0.12	1.45	0.0052	1.52	0.2734	1.26	0.2484	1.75	0.0023
101694_f_at	1447631_at	Myst2	MYST histone acetyltransferase 2	-1.1	0.76	1.01	0.9236	-1.03	0.7568	-1.06	0.644	-1.01	0.966
101695_at	1419822_at	Eif3s6	Eukaryotic translation initiation factor 3, subu	-2.05	0.4	-1.43	0.128	1.05	0.8636	-1.24	0.3641	1.58	0.2972
101696_r_at	1419881_x_at	---	---	-1.34	0.23	1.02	0.8602	-1.02	0.8711	1.19	0.0063	1.58	0.4644
101699_at	1422287_at	Phxr2	per-hexamer repeat gene 2	-4.28	0.4	-1.01	0.9623	-1.63	0.0766	-1.17	0.5391	-1.32	0.62
101700_at	1422272_at	Phxr4	per-hexamer repeat gene 4	-1.32	0.36	-1.07	0.4254	-1.29	0.076	-1.17	0.171	1.16	0.4834
101701_at	1422052_at	Cdh8	cadherin 8	-1.77	0.57	-1.24	0.6319	-1.35	0.4954	-1.95	0.2064	-2.05	0.1622
101702_at	1421085_at	Rs1h	retinoschisis 1 homolog (human)	-1.23	0.2	1.34	0.01	1.24	0.0917	1.29	0.0375	1.36	0.5403
101704_at	1450518_at	Hnf4g	hepatocyte nuclear factor 4, gamma	-2.66	0.2	1.6	0.0598	1.7	0.0525	1.41	0.4381	1.14	0.8075
101705_at	1421800_at	Phxr1	per-hexamer repeat gene 1	-1.88	0.49	-1.18	0.631	-1.37	0.3545	-1.75	0.1404	1.56	0.1823
101708_at	1421618_at	Myo1f	myosin IF	-1.07	0.81	1.4	0.1787	1.06	0.7435	1.82	0.0801	1.44	0.2627
101709_at	1420796_at	Ahrr	aryl-hydrocarbon receptor repressor	1.41	0.52	-1	0.9919	1.02	0.9101	1.01	0.9452	1.56	0.1678
101710_at	1421351_at	Gria4	glutamate receptor, ionotropic, AMPA4 (alph	-1.15	0.55	1.1	0.7536	1.29	0.401	1.12	0.7698	1.56	0.4483
101711_at	1422295_at	Mds1	myelodysplasia syndrome 1 homolog (huma	-1.37	0.17	-1.01	0.9668	-2.01	0.0232	-1.39	0.2811	-3.81	0.0475
101712_at	1422218_at	P2rx7	purinergic receptor P2X, ligand-gated ion ch	-1.19	0.55	-1.12	0.2614	-1.1	0.4354	-1.15	0.2655	1.12	0.4958
101713_at	1420413_at	Slc7a11	solute carrier family 7 (cationic amino acid tr	-2.39	0.07	-1.35	0.3425	-1.31	0.4124	-1.41	0.2292	1.66	0.2418
101714_at	1421508_at	Odz1	odd Oz/ten-m homolog 1 (Drosophila)	1.32	0.48	1.09	0.8318	1.43	0.4349	1.37	0.5052	-1.07	0.7936
101715_at	1450615_at	Ear14	eosinophil-associated, ribonuclease A family	2.1	0.14	1.2	0.5571	2.42	0.1701	1.76	0.2039	1.31	0.266
101716_at	1450616_at	Ear5	eosinophil-associated, ribonuclease A family	-1.32	0.43	1.06	0.7514	-1.01	0.9644	-1.16	0.5203	1.14	0.6328
101717_at	1422281_at	Sstr4	somatostatin receptor 4	-1.28	0.04	-1	0.9967	1.03	0.6453	-1.01	0.7804	1.09	0.1772
101719_at	1450606_at	Pnmt	phenylethanolamine-N-methyltransferase	-1.08	0.36	1.25	0.1396	1.24	0.2245	1.02	0.8633	1.09	0.5376
101724_at	1423029_at	Hes2	hairy and enhancer of split 2 (Drosophila)	-1.31	0.58	1.03	0.8047	-1.14	0.3328	-1.41	0.0157	1.33	0.3248
101725_at	1450530_at	B3galt1	UDP-Gal:betaGlcNAc beta 1,3-galactosyltra	1.04	0.91	1.15	0.6958	-1.54	0.3257	-1.66	0.3243	1.14	0.7976
101726_at	1422342_at	Nmbr	neuromedin B receptor	1.3	0.68	-1.68	0.1388	-1.16	0.6476	-2.69	0.0116	2.31	0.0602
101728_at	1422190_at	C5r1	complement component 5, receptor 1	-1.56	0.09	-1.01	0.944	1.04	0.7298	1	0.9882	1.11	0.5393
101729_at	1423019_at	Gja9	gap junction membrane channel protein alph	-2.54	0.36	-1.26	0.2242	1.01	0.944	-1.09	0.6165	1.32	0.5439

101730_at	1450288_at	Cdh6	cadherin 6	2.87	0.12	-1.24	0.3532	-1.15	0.5394	-1.2	0.2709	2.19	0.0782
101732_at	1422320_x_at	Phxr5	per-hexamer repeat gene 5	-1.97	0.01	1.03	0.6948	-1.16	0.1298	-1.04	0.6079	1.1	0.3662
101733_at	1422349_at	Ccr1l1	chemokine (C-C motif) receptor 1-like 1	-1.84	0.32	1.25	0.4021	-1.01	0.9574	1.41	0.0442	2.13	0.0789
101734_at	1421435_at	Grid2	glutamate receptor, ionotropic, delta 2	-1.55	0.23	-1.03	0.9215	1.45	0.2378	-1.16	0.6741	2.81	0.142
101735_f_at	1422415_at	Ang2	angiogenin, ribonuclease A family, member	1.6	0.04	1.05	0.66	-1.12	0.1578	-1.07	0.4732	1.51	0.0057
101736_at	1422271_at	Ppyr1	pancreatic polypeptide receptor 1	-2.04	0.49	1.18	0.6636	1	0.9866	1.36	0.5016	4.42	0.0089
101738_at	1450795_at	Lhb	luteinizing hormone beta	-1.22	0.49	-1.04	0.4703	-1.12	0.1895	-1.18	0.0284	1.19	0.3053
101739_at	1450619_x_at	Cmar	cell matrix adhesion regulator	-1.09	0.45	-1.08	0.6281	1.23	0.2748	-1.06	0.7554	1.15	0.3818
101740_at	1421659_at	Adra1a	adrenergic receptor, alpha 1a	-2.55	0.03	1.13	0.2935	1.1	0.5918	-1.2	0.249	2.48	0.1276
101742_at	1421715_at	V2R2	tissue-type vomeronasal neurons putative pl	1.01	0.99	-1.08	0.8183	1.12	0.7393	-1.29	0.4874	5.22	0.0187
101745_f_at	1452574_x_at	Igh-VJ558	Similar to Ig H-chain V-JH3-region	-1.03	0.94	1.3	0.3372	1.14	0.7003	-1.01	0.9762	1.68	0.1896
101748_at	1450586_at	Bdkrb1	bradykinin receptor, beta 1	-1.35	0.64	1.09	0.5074	1.08	0.4571	-1.12	0.4829	1.55	0.3901
101755_f_at	1450811_at	Sprr2j	small proline-rich protein 2J	-1.8	0.01	1.31	0.1061	1.03	0.8946	1.12	0.5784	1.11	0.5981
101756_f_at	1422425_at	Sprr2k	small proline-rich protein 2K	-3.84	0	-1.6	0.1351	-1.33	0.3329	-1.37	0.3307	1.58	0.0694
101760_at	1450246_at	Fut2	fucosyltransferase 2	-1.18	0.45	-1.3	0.0875	-1.39	0.0448	-1.3	0.0551	1.04	0.8448
101762_at	1422401_at	Sprr3	small proline-rich protein 3	1.06	0.82	-1.58	0.0301	-1.06	0.6193	-1.57	0.0547	-1.42	0.4984
101763_at	1450346_at	Gpr50	G-protein-coupled receptor 50	-1.35	0.11	-1.01	0.9421	-1.03	0.5943	-1.04	0.5308	1.1	0.3566
101764_at	1422290_at	Htr1d	5-hydroxytryptamine (serotonin) receptor 1D	-1.08	0.68	-1.03	0.7902	-1.06	0.5968	-1.03	0.734	1.02	0.9092
101765_at	1450573_at	Amh	anti-Mullerian hormone	-1.16	0.23	-1.04	0.5864	1.04	0.5254	-1.06	0.4507	1.44	0.0626
101769_at	1427687_at	Pcdha10	protocadherin alpha 10	-1.29	0.6	-1.06	0.7425	1.04	0.8124	-1.09	0.5594	1.29	0.1761
101772_r_at	1425613_at	Pcdha11	protocadherin alpha 11	-1.98	0.1	-1.01	0.9718	1.36	0.2028	1.04	0.8161	1.69	0.0917
101775_at	1421769_at	Lmx1b	LIM homeobox transcription factor 1 beta	1.23	0.6	1.04	0.8458	-1.33	0.2793	-1.8	0.043	1.93	0.19
101777_at	1427783_at	Erbb4	v-erb-a erythroblastic leukemia viral oncoge	-1.59	0.25	-1.03	0.8601	1.08	0.7947	-1.23	0.0648	1.28	0.3869
101778_at	1422192_at	Gja5	gap junction membrane channel protein alph	-1.17	0.54	-1.06	0.6201	1.03	0.7905	-1.14	0.1966	1.22	0.1511
101779_at	1422278_at	Drd3	dopamine receptor 3	1.19	0.64	1.13	0.3691	1.32	0.0533	1.7	0.08	-1.36	0.5061
101786_at	1422255_at	Kcna4	potassium voltage-gated channel, shaker-re	-1.37	0.51	1.13	0.6809	-1.42	0.3533	1.13	0.6945	1.77	0.1609
101788_f_at	1450613_x_at	Ifnab	interferon alpha family, gene B	-1.06	0.84	-1.04	0.8638	-1	0.9988	1.07	0.8333	1.71	0.1313
101790_f_at	1422408_at	Ifna4	interferon alpha family, gene 4	-1.9	0.08	-1.44	0.2867	-1.17	0.6513	-1.4	0.3669	1.81	0.1436
101795_f_at	1422421_at	Defcr-rs12	defensin related cryptdin, related sequence	-1.33	0.2	-1.21	0.0459	-1.06	0.5237	-1.09	0.1483	1.38	0.221
101796_at	1427592_at	Pcdh7	protocadherin 7	-1.66	0	1.02	0.8937	1.16	0.1906	1.07	0.5176	1.2	0.3768
101800_at	1422953_at	Fpr-rs2	formyl peptide receptor, related sequence 2	-1.17	0.51	2.42	0.2987	-1.6	0.0604	4.29	0.2534	-1.6	0.2597
101801_at	1422350_at	Fpr-rs3	formyl peptide receptor, related sequence 3	-1.28	0.69	-1.67	0.0802	-1.34	0.2535	-1.05	0.8588	1.22	0.5703
101802_at	1422358_at	Fpr-rs4	formyl peptide receptor, related sequence 4	-1.26	0.39	1.27	0.4946	1.02	0.9605	1.25	0.3487	1.97	0.1112
101803_at	1430899_at	Muc5ac	mucin 5, subtypes A and C, tracheobronchia	1.67	0.29	1.24	0.1774	1.23	0.0848	-1	0.9813	1.21	0.4106
101806_at	1450581_at	Galr3	galanin receptor 3	-1.62	0.11	1.02	0.715	1.01	0.897	-1.09	0.1247	1.05	0.6322
101807_at	1450594_at	Magea4	melanoma antigen, family A, 4	-1.09	0.88	-2.22	0.0182	-1.35	0.3246	-1.03	0.9389	1.44	0.2795
101808_at	1422423_at	Magea7	melanoma antigen, family A, 7	-1.74	0.06	-1.13	0.3084	-1.11	0.3844	-1.27	0.0439	1.2	0.4609
101810_at	1450810_at	Fshr	follicle stimulating hormone receptor	-1.3	0.04	-1.39	0.0053	-1.23	0.0386	-1.1	0.496	1.15	0.5609
101811_at	1450796_at	Atoh7	atonal homolog 7 (Drosophila)	-2.52	0.22	1.24	0.0329	1.3	0.0144	1.19	0.2042	1.07	0.753
101815_at	1421763_at	Bmp10	bone morphogenetic protein 10	-1.75	0.27	-1.68	0.0332	1.01	0.9689	-2.06	0.0014	1.89	0.0464
101821_at	1450575_at	Chrm4	cholinergic receptor, muscarinic 4	-1.68	0.4	1.04	0.711	1.15	0.3999	1.1	0.6941	1.1	0.7521
101822_at	1422237_at	Mc3r	melanocortin 3 receptor	-1.92	0.31	1.51	0.1189	1.35	0.243	1.37	0.2944	2.5	0.0036
101825_at	1422961_at	Nat3	N-acetyltransferase 3	-1.86	0.48	-1.21	0.5882	1.08	0.8204	-1.32	0.4586	1.21	0.6066
101827_at	1422424_at	Hpvc2	human papillomavirus 18 E5 central sequen	-3.05	0.12	1	0.9912	-1.15	0.5793	-1.26	0.4288	1.08	0.7992
101828_at	1421359_at	Ret	ret proto-oncogene	1.13	0.58	1.2	0.0827	1.21	0.0587	1.12	0.2157	1.23	0.7247
101829_at	1450833_at	Chrm1	cholinergic receptor, muscarinic 1, CNS	1.38	0.56	-1.98	0.002	-1.16	0.3168	-1.64	0.0095	-1.01	0.9722
101830_at	1452533_at	Ryr3	ryanodine receptor 3	-1.13	0.64	-1.08	0.5481	-1.17	0.1535	-1.18	0.1593	1.62	0.0169
101831_at	1427571_at	Shh	sonic hedgehog	-1.33	0.43	-1.48	0.0644	-1.02	0.9426	-1.33	0.1334	1.02	0.9367
101834_at	1427060_at	Mapk3	mitogen activated protein kinase 3	-1.31	0.37	1.09	0.4953	1.05	0.81	1.12	0.4613	1.58	0.0611
101835_at	1448409_at	Lrmp	lymphoid-restricted membrane protein	-1.25	0.32	1.01	0.8948	1.03	0.6752	1.12	0.3326	1.32	0.2734
101838_r_at	1425330_a_at	Ppm1b	protein phosphatase 1B, magnesium depend	1.55	0.64	1	0.9826	1.03	0.7774	-1.31	0.0392	1.09	0.7034
101839_at	1432301_a_at	1700074P13Rik	RIKEN cDNA 1700074P13 gene	-1.15	0.79	-1.16	0.684	1.23	0.5067	1.13	0.6633	2.16	0.1564
101842_g_at	1460420_a_at	Egfr	epidermal growth factor receptor	-9.5	0	-2.82	0.0007	-1.14	0.4491	-2.67	0.001	-24.13	0

101843_at	1448874_a_at	Sh2bpsm1	SH2-B PH domain containing signaling med	1.43	0.35	1.15	0.2055	1.11	0.3286	1.14	0.1371	1.08	0.4429
101844_at	1449374_at	Pipox	pipecolic acid oxidase	1.14	0.51	1.25	0	1.07	0.0878	1.19	0.001	1.24	0.0148
101846_r_at	1460416_s_at	Csprs /// LOC43	component of Sp100-rs /// similar to homoge	-1.36	0.11	1.38	0.1347	1.25	0.3523	1.32	0.277	1.61	0.0036
101848_g_at	1451821_a_at	Sp100	nuclear antigen Sp100	1.79	0.24	1.66	0.1356	1.59	0.2091	1.84	0.1186	1.41	0.2997
101850_at	1417635_at	Spa17	sperm autoantigenic protein 17	1.17	0.66	-1.15	0.6444	-1.24	0.5815	-1.22	0.4567	1.03	0.9446
101851_at	1448788_at	Cd200	Cd200 antigen	-1.35	0.27	-1.41	0.0324	-1.2	0.356	-1.07	0.7083	1.04	0.846
101853_f_at	1450876_at	Cfh	complement component factor h	-1.84	0.1	-1.22	0.054	-1.2	0.086	-1.3	0.0042	-2.08	0.0003
101855_at	1422550_a_at	Mtap6	microtubule-associated protein 6	-1.03	0.93	-1.08	0.8749	-1.24	0.6317	-1.45	0.403	1.29	0.0341
101856_at	1435525_at	Kctd17	potassium channel tetramerisation domain c	-1.04	0.87	-1.09	0.618	-1.11	0.5755	-1.16	0.5025	-1.21	0.6921
101857_at	1448603_at	Srpk2	serine/arginine-rich protein specific kinase 2	3.34	0.08	-1.24	0.4205	1.04	0.8626	-1.11	0.6746	-1.36	0.6827
101858_at	1460704_at	Rfng	radical fringe gene homolog (Drosophila)	1	0.97	-1.12	0.021	-1.03	0.6223	-1.13	0.069	1.08	0.455
101859_at	1418848_at	Aqp7	aquaporin 7	-1.41	0.48	-1.35	0.1194	-1.06	0.7871	-1.06	0.7066	1.82	0.1908
101860_at	1448571_a_at	Gmfb	glia maturation factor, beta	1.26	0.45	1.36	0.1474	1.23	0.502	1.62	0.0027	1.45	0.5961
101861_at	1420688_a_at	Sgce	sarcoglycan, epsilon	-1.1	0.53	-1.19	0.061	-1.05	0.6627	1	0.9837	1.87	0.0027
101862_at	1419590_at	Cyp2b9	cytochrome P450, family 2, subfamily b, poly	12.19	0.07	-1.09	0.3779	-1.03	0.7263	-1.34	0.0234	4.22	0.0006
101864_at	1424678_at	Actl7b	actin-like 7b	-1.44	0.02	-2.18	0.0602	-1.62	0.1887	-1.85	0.1179	-1.12	0.2352
101865_at	1419279_at	Pip5k2a	phosphatidylinositol-4-phosphate 5-kinase, t	1.14	0.68	1.36	0.2587	-1.06	0.8115	1.49	0.0924	1.13	0.7511
101866_at	1425507_at	Arfrp1	ADP-ribosylation factor related protein 1	1.4	0.26	-1.01	0.8758	1.14	0.189	1.01	0.8837	-1.3	0.1283
101867_at	1419499_at	Gpam	Glycerol-3-phosphate acyltransferase, mitoc	-1.19	0.1	-1.71	0.0146	-1.38	0.1028	-1.65	0.0196	-2.01	0.0015
101868_i_at	1419744_at	H2-DMb2	histocompatibility 2, class II, locus Mb2	2.49	0.22	-1.21	0.6133	-1.75	0.1661	-1.3	0.483	-1.21	0.4719
101870_at	1427756_x_at	Igh-4	immunoglobulin heavy chain 4 (serum IgG1)	-1.42	0.31	1.03	0.7752	-1	0.9982	1.01	0.8993	1.24	0.536
101871_f_at	1452577_at	Igh-VJ558	Immunoglobulin heavy chain (J558 family)	-1.73	0.05	-1.01	0.9243	1.09	0.5674	1.17	0.2434	-1.07	0.4857
101872_at	1421041_s_at	Gsta2	glutathione S-transferase, alpha 2 (Yc2)	1.3	0.4	2.48	0	1.2	0.0378	2.68	0	1.85	0.0004
101873_at	1427092_at	---	---	-1.51	0.47	1.05	0.8041	-1.25	0.307	-1.11	0.5726	1.41	0.1468
101874_s_at	1449529_s_at	Prlpe	prolactin-like protein E	-3.81	0.03	1.7	0.1616	1.3	0.5281	-1.2	0.4315	1.4	0.3123
101875_at	1450560_a_at	Ppp2r5d	protein phosphatase 2, regulatory subunit B	1.94	0.35	-1.07	0.7913	1.64	0.097	-1.21	0.4337	1.18	0.517
101877_at	1455285_at	Slc31a1	solute carrier family 31, member 1	1.27	0.16	1.05	0.3935	-1.04	0.5381	-1.01	0.8605	-1.21	0.2351
101878_at	1426112_a_at	Cd72	CD72 antigen	1.14	0.85	-1.76	0.1981	-1.41	0.3654	-1.46	0.324	1.94	0.1401
101879_s_at	1422563_at	Crry	complement receptor related protein	1.71	0.01	1.07	0.3886	1.11	0.2976	1.06	0.3858	-1.18	0.0421
101882_s_at	1418237_s_at	Col18a1	procollagen, type XVIII, alpha 1	1.01	0.98	1.02	0.7895	-1.21	0.0902	1.04	0.6375	-1.09	0.7373
101883_s_at	1420357_s_at	Xlr3a /// Xlr3b	X-linked lymphocyte-regulated 3a /// X-linked	4.83	0.01	-1.51	0.2701	-1.17	0.744	-1.12	0.7182	1.01	0.9904
101884_at	1449347_a_at	Xlr4	X-linked lymphocyte-regulated 4	1.37	0.37	1.18	0.5343	1.35	0.3196	1.48	0.1677	1.62	0.314
101886_f_at	1451931_x_at	H2-D1	histocompatibility 2, D region locus 1	1.16	0.73	1.2	0.2692	-1.11	0.1789	1.25	0.2826	-1.48	0.1339
101887_at	1423396_at	Agt	angiotensinogen	1.26	0.27	1.26	0.0186	1.03	0.7811	1.13	0.1575	3.03	0.0003
101889_s_at	1420583_a_at	Rora	RAR-related orphan receptor alpha	-1.17	0.81	1.38	0.0111	1.26	0.0091	1.51	0.0013	1.21	0.4209
101891_at	1418352_at	Hsd17b2	hydroxysteroid (17-beta) dehydrogenase 2	-1.97	0.01	-2.81	0	-1.71	0	-3.9	0	-2.58	0.0004
101892_f_at	1437767_s_at	Fts	fused toes	1.88	0.08	1.11	0.5166	1.07	0.5832	1.04	0.7306	-1.17	0.4421
101894_s_at	1423364_a_at	Fts	fused toes	1.08	0.83	-1.04	0.8044	1.25	0.2196	1.08	0.5434	-1.15	0.6061
101897_g_at	1421647_at	Cd1d2	CD1d2 antigen	-1.3	0.51	-1.07	0.4885	-1.21	0.0127	-1.15	0.0957	-1.58	0.2037
101898_s_at	1425137_a_at	H2-Q10	histocompatibility 2, Q region locus 10	-1.1	0.41	-1.16	0.0001	-1.09	0.0551	-1.28	0	-1.15	0.165
101899_at	1418897_at	F2	coagulation factor II	-1.03	0.8	1.03	0.4633	-1.01	0.867	-1.05	0.2908	-1	0.9977
101900_at	1449152_at	Cdkn2b	cyclin-dependent kinase inhibitor 2B (p15, in	-2.14	0.3	-1.02	0.8973	1.01	0.9589	1.15	0.562	-1.48	0.5078
101902_at	1418114_at	Rbpsuh	recombining binding protein suppressor of h	1.1	0.19	1.03	0.7373	-1.03	0.699	1.15	0.0973	-1.18	0.6178
101904_at	1427714_at	Smyd1	SET and MYND domain containing 1	-1.36	0.64	-1.44	0.0155	1.02	0.8823	-1.12	0.3067	1.57	0.0979
101905_at	1415769_at	Itch	itchy	-1.4	0.05	-1.03	0.5614	1.07	0.3822	-1.01	0.9193	1.14	0.4018
101906_at	1452197_at	Smc4l1	SMC4 structural maintenance of chromosom	1.47	0.07	1.26	0.0504	1.08	0.5501	1.24	0.0048	1.23	0.239
101908_s_at	1422123_s_at	Ceacam1 /// Ce	CEA-related cell adhesion molecule 1 /// CE	-1.47	0.16	-1.18	0.1053	-1.08	0.647	-1.44	0.0079	-1.5	0.074
101910_f_at	1427631_x_at	LOC433826	similar to Mup3 protein	-2.21	0.06	-2.76	0	-1.06	0.3296	-3.98	0	-4.33	0
101912_at	1434484_at	1100001G20Rik	RIKEN cDNA 1100001G20 gene	-24.22	0	-57.99	0	-1.22	0.0017	-112.07	0	-136.16	0
101913_at	1429400_at	Clcn5	chloride channel 5	1.7	0.12	1.45	0.2154	1.48	0.2527	1.39	0.4733	1.85	0.1532
101914_at	1415731_at	D1Ertd396e	DNA segment, Chr 1, ERATO Doi 396, expr	1.37	0.1	1.13	0.2027	1.11	0.4626	1.23	0.041	1.14	0.0695
101916_at	1423938_at	Llglh2	lethal giant larvae homolog 2	1.27	0.5	1.02	0.8607	-1.2	0.1352	1.02	0.8704	1.15	0.2144
101917_at	1420692_at	Il2ra	interleukin 2 receptor, alpha chain	-2.95	0.26	1.01	0.9838	-1.36	0.5014	-1.17	0.7454	2.56	0.0723

101918_at	1420653_at	Tgfb1	transforming growth factor, beta 1	1.38	0.49	1.59	0.2863	-1.05	0.7118	2.29	0.1239	1.27	0.1982
101919_at	1425972_a_at	Zfx	zinc finger protein X-linked	1.2	0.4	1.12	0.3111	1.24	0.214	1.19	0.0395	-1.03	0.9095
101921_at	1449048_s_at	---	---	1.23	0.21	1	0.9681	1.19	0.0047	1.1	0.243	1.04	0.74
101922_at	1417204_at	Kdelr2	KDEL (Lys-Asp-Glu-Leu) endoplasmic reticu	1.08	0.81	1.06	0.3831	-1.06	0.451	1.01	0.8689	1.2	0.0872
101926_at	1417216_at	---	---	-1.11	0.68	-1.08	0.4681	1.01	0.9569	-1.11	0.418	2.15	0.0386
101927_at	1416754_at	Prkar1b	protein kinase, cAMP dependent regulatory,	-1.47	0.13	-1.11	0.4793	-1.07	0.7305	-1.04	0.8104	-1.02	0.9018
101928_at	1417498_at	Serpinf2	serine (or cysteine) proteinase inhibitor, clad	1.19	0.55	-1.02	0.8366	-1.01	0.9646	1	0.9948	-1.13	0.6224
101929_at	1418258_s_at	6720463E02Rik	RIKEN cDNA 6720463E02 gene	-1.39	0.56	-1.02	0.8294	-1.83	0.0003	-1.79	0.0003	-1.35	0.0912
101930_at	1436364_x_at	---	---	-1.14	0.83	-1.33	0.0198	-1.45	0.0811	-1.6	0.0208	-1.13	0.8472
101931_at	1416372_at	Ptdss1	phosphatidylserine synthase 1	-1.01	0.97	-1.1	0.0329	-1.11	0.0952	-1.08	0.071	-1.07	0.5084
101932_at	1418540_a_at	Ptpre	protein tyrosine phosphatase, receptor type,	1.36	0.26	1.58	0.0974	1.07	0.8183	1.17	0.6804	1.11	0.7239
101933_at	1422664_at	Rab10	RAB10, member RAS oncogene family	-1.53	0.42	-1.13	0.1835	-1.15	0.3052	-1.25	0.0123	1.21	0.4859
101934_at	1434348_at	Fez2	fasciculation and elongation protein zeta 2 (z	1.42	0.08	-1.02	0.8483	-1.06	0.5222	1.08	0.3594	-1.1	0.5622
101936_at	1422886_a_at	Clk4	CDC like kinase 4	1.97	0.05	1.38	0.0033	1.58	0.0539	1.58	0.0009	1.45	0.0788
101937_s_at	1427663_a_at	Clk4	CDC like kinase 4	1.78	0.34	1.22	0.078	1.16	0.2073	1.16	0.2062	1.44	0.4895
101938_at	1419500_at	Pabpc2	poly A binding protein, cytoplasmic 2	-1.53	0.03	1.01	0.9296	-1.04	0.6747	1.06	0.662	1.01	0.9081
101939_at	1433655_at	Rnf141	Ring finger protein 141	-1.38	0.02	1.1	0.3878	1.07	0.3992	1.13	0.2128	1.28	0.1218
101940_at	1416999_at	Smpd2	sphingomyelin phosphodiesterase 2, neutral	-1.08	0.59	-1.01	0.8624	-1.01	0.8712	-1.03	0.6491	-1.07	0.4576
101942_at	1435799_at	Sfrs14	splicing factor, arginine/serine-rich 14	-1.49	0.13	-1.27	0.0605	1.03	0.8205	-1.1	0.4737	1.02	0.9031
101943_at	1434117_at	Tceb3	transcription elongation factor B (SIII), polyp	-1.04	0.87	-1.07	0.4098	-1.03	0.6212	-1.07	0.2549	1.01	0.8732
101947_at	1417734_at	Akap8l	A kinase (PRKA) anchor protein 8-like	2.29	0.06	1.16	0.0956	1.25	0.0668	1.57	0.0004	1.5	0.3894
101948_at	1424114_s_at	Lamb1-1	laminin B1 subunit 1	1.17	0.56	1.35	0.2012	1.37	0.2248	1.4	0.0547	2.15	0.1863
101949_at	1431938_a_at	Pmm2	phosphomannomutase 2	1.43	0.27	-1.05	0.6953	1.08	0.2198	-1.04	0.6427	-1.67	0.1784
101950_at	1417178_at	MGI:1889209	semaF cytoplasmic domain associated prote	2.44	0.03	1.27	0.5472	1.06	0.8881	2.54	0.0288	1.66	0.3681
101952_at	1460373_a_at	ORF21	open reading frame 21	2.13	0.1	-3.46	0.0005	1.79	0.0219	-3.09	0.0017	1.35	0.3776
101954_at	1416415_a_at	H2afz	H2A histone family, member Z	1.3	0.22	1.27	0.038	1.06	0.6544	1.25	0.0787	-1.26	0.0923
101955_at	1427464_s_at	Hspa5	heat shock 70kD protein 5 (glucose-regulate	-1.11	0.59	-1.18	0.0925	-1.22	0.0671	-1.29	0.0126	-1.54	0.0107
101956_at	1452181_at	Ckap4	Cytoskeleton-associated protein 4	-1.22	0.64	1.75	0.2944	1.32	0.1965	-1.19	0.3881	1.42	0.2577
101957_f_at	1435368_a_at	Parp1	poly (ADP-ribose) polymerase family, memb	1.22	0.55	-1.25	0.3985	-1.3	0.3963	1.03	0.9121	-1.38	0.4835
101960_at	1423880_at	D10Wsu52e	DNA segment, Chr 10, Wayne State Univers	1.79	0.01	1.02	0.7125	-1.06	0.4552	1.1	0.0992	-1.07	0.5223
101961_at	1416815_s_at	Bub3	budding uninhibited by benzimidazoles 3 hor	1.11	0.27	1.02	0.7814	1.07	0.0551	1.05	0.2423	-1.12	0.3553
101962_at	1452155_a_at	Ddx17	DEAD (Asp-Glu-Ala-Asp) box polypeptide 17	-1.16	0.51	1.02	0.8427	-1.16	0.2308	1.05	0.5802	1.03	0.8936
101963_at	1451310_a_at	Ctsl	cathepsin L	1.17	0.6	1.16	0.0194	1.14	0.0957	1.19	0.0064	1.26	0.0446
101964_at	1451015_at	Tkt	transketolase	1.12	0.18	-1.31	0.0215	-1.2	0.2516	-1.65	0.0009	-2.68	0.0147
101965_at	1451074_at	Rnf13	ring finger protein 13	-1.06	0.77	1.03	0.7494	1.06	0.6091	-1.03	0.7741	1.12	0.3964
101966_s_at	1420620_a_at	Rnf13	ring finger protein 13	1.03	0.95	-1.12	0.2269	-1.06	0.5393	-1.24	0.0417	-1.04	0.8372
101967_at	1416857_at	Sdf2	stromal cell derived factor 2	-1.51	0.05	-1.46	0.0761	-1.19	0.2173	-1.27	0.211	1.54	0.0636
101968_at	1422585_at	Odf1	outer dense fiber of sperm tails 1	-1.01	0.98	-1.17	0.2802	1.12	0.2233	-1.16	0.0574	1.83	0.0204
101969_at	1448428_at	---	---	-1.44	0.02	1.27	0.4423	1.22	0.2344	-1.02	0.9015	-1.21	0.5446
101970_at	1419928_at	Lrig1	leucine-rich repeats and immunoglobulin-like	1.17	0.83	-1.31	0.4798	-1.11	0.8016	-1.28	0.5238	1.85	0.0003
101971_at	1423729_a_at	2500002L14Rik	RIKEN cDNA 2500002L14 gene	1.44	0.06	1.07	0.1712	-1.15	0.0853	1.15	0.0097	-1.21	0.0953
101972_at	1423590_at	Napsa	napsin A aspartic peptidase	1.46	0.25	1.25	0.2249	1.02	0.9094	3.11	0.2743	1.22	0.1496
101973_at	1452207_at	---	---	1.1	0.74	-1.07	0.4518	-1.19	0.2271	-1.25	0.02	-1.84	0.035
101975_at	1449939_s_at	Dlk1	delta-like 1 homolog (Drosophila)	-1.67	0.07	-1.02	0.8662	-1.04	0.7377	1.14	0.4757	1.72	0.0045
101976_at	1416163_at	Cops4	COP9 (constitutive photomorphogenic) hom	1.85	0.13	-1.01	0.8555	1.05	0.6277	1.01	0.8535	-1.26	0.2774
101977_at	1451575_a_at	Nudt3	nudix (nucleotide diphosphate linked moiety	1.05	0.77	-1.22	0.0343	-1.04	0.4902	-1.17	0.0077	1.27	0.0004
101978_at	1451624_a_at	1700048E23Rik	RIKEN cDNA 1700048E23 gene	1.46	0.47	1.13	0.3515	1.28	0.059	1.05	0.7191	-1.34	0.2453
101979_at	1453851_a_at	Gadd45g	growth arrest and DNA-damage-inducible 45	-1.66	0.07	-2.11	0.0317	-2.09	0.0393	-1.91	0.0503	-2.77	0.094
101982_at	1451097_at	---	---	1.24	0.53	1.16	0.3756	-1.1	0.4813	1.29	0.2687	1.07	0.7131
101984_at	1460639_a_at	Atox1	ATX1 (antioxidant protein 1) homolog 1 (yea	1.05	0.88	1.27	0.0044	1.06	0.3341	1.25	0.0086	1.04	0.8418
101985_at	1416729_at	Plg	plasminogen	1.19	0.73	-1.04	0.2587	-1.38	0.0537	-1.29	0.0758	-1.07	0.6301
101989_at	1428782_a_at	Uqcrc1	ubiquinol-cytochrome c reductase core prote	1.39	0.06	1.15	0.0634	1.13	0.1022	1.19	0.0064	1.12	0.4735
101990_at	1455235_x_at	Ldh2	lactate dehydrogenase 2, B chain	2.14	0.03	1.32	0.2298	1.33	0.1923	2.03	0.0035	2.38	0.0017

101991_at	1417429_at	Fmo1	flavin containing monooxygenase 1	-1.35	0.25	-1.28	0.0005	1	0.9901	-1.21	0.0103	1.01	0.9434
101992_at	1448822_at	Psmb6	proteasome (prosome, macropain) subunit, I	1.85	0.01	1.27	0.0225	1.35	0.0087	1.27	0.0382	-1.05	0.7856
101993_at	1416342_at	Tnc	tenascin C	-1.02	0.95	-1.33	0.3847	-1.24	0.4504	-1.09	0.7355	-1.08	0.8
101995_at	1450957_a_at	Sqstm1	sequestosome 1	-1.15	0.33	1.07	0.1364	-1.14	0.0298	-1.07	0.2073	1.11	0.3684
101996_at	1438562_a_at	Ptpn2	protein tyrosine phosphatase, non-receptor t	1.43	0.1	1.49	0.0002	1.27	0.3575	1.43	0.0102	1.18	0.3864
101997_at	1451747_a_at	Apg12l	autophagy 12-like (S. cerevisiae)	1.1	0.63	1.15	0.0455	1.18	0.046	1.09	0.0757	-1.16	0.4012
101998_at	1419636_at	4833420G17Rik	RIKEN cDNA 4833420G17 gene	1.59	0.08	1.27	0.2968	1.28	0.2306	1.25	0.0737	1.16	0.464
102000_f_at	1435757_a_at	Uqcrc2	ubiquinol cytochrome c reductase core prote	1.31	0.46	1.03	0.7268	-1.07	0.5853	1.11	0.2455	1.19	0.3824
102001_at	1448226_at	Rrm2	ribonucleotide reductase M2	-1.39	0.2	1.26	0.516	-1.4	0.0707	1.62	0.1888	1.25	0.392
102002_at	1450021_at	Ubqln2	ubiquilin 2	1.21	0.5	1.5	0.0068	1.34	0.1473	1.65	0.0008	1.3	0.1101
102003_at	1415709_s_at	Gbf1	golgi-specific brefeldin A-resistance factor 1	1.01	0.91	-1.13	0.0005	-1.11	0.0184	-1.21	0.0001	-1.11	0.2751
102009_at	1449273_at	Cyfip2	cytoplasmic FMR1 interacting protein 2	1.27	0.68	1.23	0.1342	1.01	0.9634	1.04	0.8419	-1.02	0.9523
102010_at	1427487_at	---	---	-1.27	0.75	-1.24	0.4774	-1.53	0.1417	-1.27	0.2574	1.49	0.5713
102011_at	1421948_a_at	2610507L03Rik	RIKEN cDNA 2610507L03 gene	1.76	0.03	1.18	0.1101	-1.26	0.1687	1.09	0.2919	1.97	0.096
102012_at	1418895_at	Scap2	src family associated phosphoprotein 2	1.37	0.1	1.2	0.057	1.1	0.0479	1.27	0.1197	-1.21	0.1557
102013_at	1420541_at	Rdh6	retinol dehydrogenase 6	2.99	0.14	-1.06	0.4865	-1.21	0.0953	-1.17	0.2603	-1.42	0.0377
102014_at	1424859_at	Homer3	homer homolog 3 (Drosophila)	1.33	0.29	1.07	0.8634	1.12	0.7293	1.04	0.9093	1.85	0.0381
102015_at	1435681_s_at	LOC234374	similar to hypothetical protein FLJ10432	1.09	0.74	-1.12	0.5045	-1.32	0.0918	1.09	0.772	1.79	0.0927
102016_at	1452260_at	Cidec	cell death-inducing DFFA-like effector c	1.24	0.69	-1.23	0.2501	-1.35	0.1152	-1.1	0.5572	-1.09	0.774
102017_at	1425498_at	Prpf4b	PRP4 pre-mRNA processing factor 4 homolo	2.14	0.21	1.24	0.0176	1.28	0.0102	1.31	0.0003	1.09	0.8758
102018_at	1435965_at	Cnot3	CCR4-NOT transcription complex, subunit 3	-1.29	0.18	-1.03	0.6426	-1.19	0.1669	-1.14	0.2489	1.21	0.2235
102019_at	1460354_a_at	Mrpl13	mitochondrial ribosomal protein L13	1.95	0.02	1.02	0.8384	1.2	0.229	1.14	0.0467	-1.16	0.266
102020_at	1426058_a_at	Kcnk3	potassium channel, subfamily K, member 3	3.21	0.31	-1.31	0.0673	-1.05	0.6807	-1.26	0.0784	-1.19	0.5889
102021_at	1421034_a_at	Il4ra	interleukin 4 receptor, alpha	1.2	0.45	1.11	0.2689	-1.01	0.8928	-1.06	0.5558	1.12	0.4028
102022_at	1438477_a_at	Mcee	methylmalonyl CoA epimerase	2.16	0.01	1.17	0.0198	-1.12	0.0159	1.05	0.469	-1.1	0.5883
102025_at	1417851_at	Cxcl13	chemokine (C-X-C motif) ligand 13	3.75	0.1	-1.63	0.2083	-1.11	0.8095	1.56	0.3134	2.84	0.0379
102026_s_at	1448448_a_at	Chkb	choline kinase beta	1.13	0.47	1.06	0.4867	-1.03	0.6593	1.07	0.3288	1.33	0.0769
102028_at	1422638_s_at	Rassf5	Ras association (RalGDS/AF-6) domain fam	1.07	0.74	-1.21	0.0679	1.02	0.8586	-1.15	0.1615	-1.38	0.0514
102029_at	1448686_at	Il16	interleukin 16	1.48	0.09	-1.02	0.7959	1.02	0.8048	-1.02	0.7795	1.44	0.1961
102030_at	1420948_s_at	4833408C14Rik	RIKEN cDNA 4833408C14 gene	2.88	0.07	1.31	0.1104	1.36	0.0898	1.21	0.2054	3.01	0.0748
102031_at	1453833_a_at	Rnaseh1	ribonuclease H1	3.17	0.06	1.18	0.6421	1.07	0.8434	1.52	0.1142	1.15	0.3933
102032_at	1450388_s_at	Twsg1 /// 18100	twisted gastrulation homolog 1 (Drosophila)	-1.11	0.51	1.12	0.1089	1.1	0.319	-1.03	0.6336	-1.01	0.963
102033_at	1450662_at	Tesk1	testis specific protein kinase 1	-1.13	0.7	-1.03	0.7773	-1.1	0.2115	-1.18	0.0697	-1.45	0.2075
102035_at	1430889_a_at	Tpmt	thiopurine methyltransferase	1.41	0.33	1.15	0.2294	-1.08	0.6154	1.42	0.0069	1.14	0.4802
102036_at	1452069_a_at	Thap7	THAP domain containing 7	1.07	0.87	1.17	0.0975	1.13	0.2019	1.21	0.0366	1.44	0.3665
102037_at	1451990_at	Mapre2	microtubule-associated protein, RP/EB famil	-1.06	0.93	-1.21	0.5364	-1.26	0.4778	1.08	0.726	1.1	0.8253
102038_at	1450251_a_at	Lnx1	ligand of numb-protein X 1	3.71	0.01	-2.43	0.0369	-1.11	0.7413	-1.94	0.1061	-2.42	0.2656
102039_at	1448585_at	Gtf2h4	general transcription factor II H, polypeptide	1.02	0.87	-1.08	0.2589	-1.07	0.4126	-1.08	0.3591	-1.06	0.6316
102040_at	1451672_at	Gprk6	G protein-coupled receptor kinase 6	2.32	0.29	1.2	0.3859	1.13	0.6053	1.21	0.4075	1.51	0.2843
102041_at	1450917_at	Myom2	myomesin 2	-2.42	0.13	-1.63	0.2329	-1.17	0.6766	-1.38	0.5751	-1.42	0.3666
102043_at	1416689_at	Tuft1	tuftelin 1	-1.53	0.03	-1.16	0.0908	-1.06	0.3037	-1.11	0.1042	1.05	0.8542
102044_at	1448594_at	Wisp1	WNT1 inducible signaling pathway protein 1	-1.35	0.16	1.08	0.5736	-1.18	0.4037	-1.31	0.0862	-1.31	0.2761
102046_at	1449121_at	Fusip1	FUS interacting protein (serine-arginine rich)	1.74	0.02	1.03	0.7034	-1.04	0.65	1.01	0.906	1.1	0.638
102047_at	1415683_at	Nmt1	N-myristoyltransferase 1	1.4	0.23	1.06	0.4894	1.04	0.5036	1.16	0.0061	-1.32	0.0306
102048_at	1420992_at	Ankrd1	ankyrin repeat domain 1 (cardiac muscle)	1.48	0.38	-1.44	0.2929	-1.24	0.5623	-1.25	0.541	1.41	0.3195
102049_at	1417273_at	Pdk4	pyruvate dehydrogenase kinase, isoenzyme	-1.53	0.09	-1.26	0.2198	-1.38	0.1446	-1.32	0.1545	1.1	0.5007
102052_at	1417566_at	Abhd5	abhydrolase domain containing 5	1.23	0.46	1.17	0.0994	-1.09	0.402	1.16	0.0403	-1.22	0.1749
102053_at	1448961_at	Plscr2	phospholipid scramblase 2	1.39	0.3	-1.48	0.0068	-1.08	0.5351	-1.5	0.0042	-6.87	0.0003
102054_at	1417685_at	Ankfy1	ankyrin repeat and FYVE domain containing	1.07	0.77	-1.06	0.4337	1.02	0.7683	-1.02	0.7556	-1.12	0.1829
102058_at	1427297_at	Mrpl9	mitochondrial ribosomal protein L9	1.48	0.06	1.08	0.2357	1.2	0.0656	1.13	0.1626	1.11	0.383
102059_at	1449377_at	Nicn1	nicolin 1	1.41	0.39	1.08	0.5154	1.01	0.9226	1.02	0.8472	-1.28	0.5603
102060_at	1460213_at	Golga4	golgi autoantigen, golgin subfamily a, 4	-1.22	0.19	-1.06	0.3694	-1.05	0.3287	-1.1	0.0602	1.01	0.915
102061_at	1418589_a_at	Mlf1	myeloid leukemia factor 1	1.96	0.4	-1.3	0.5241	1.1	0.8351	-1.43	0.4638	1.54	0.0796

102062_at	1423416_at	Smarcc1	SWI/SNF related, matrix associated, actin de	-1.36	0.44	-1.09	0.484	-1.42	0.0608	-1.42	0.1013	1.32	0.1178
102063_at	1416501_at	Pdpk1	3-phosphoinositide dependent protein kinase	-1.18	0.8	-1.05	0.7534	-2.02	0.0071	-1.67	0.0067	1.59	0.3662
102064_at	1449265_at	Casp1	caspase 1	1.15	0.43	1.16	0.4598	1.03	0.745	1.4	0.2126	-1.2	0.5628
102065_at	1418243_at	Fcna	ficolin A	1.2	0.28	-1.79	0	-1.15	0.0552	-1.99	0	-1.44	0.0437
102069_at	1418514_at	Mtf2	metal response element binding transcriptio	1.65	0.12	-1.21	0.0117	-1.32	0.0052	-1.16	0.0453	-1.03	0.896
102070_at	1460734_at	Col9a3	procollagen, type IX, alpha 3	-1.5	0.08	-1.26	0.07	-1.11	0.4316	-1.14	0.2381	1.37	0.0108
102071_at	1429039_s_at	1500034J01Rik	RIKEN cDNA 1500034J01 gene	-1.58	0.29	1.07	0.5204	-1.05	0.6672	-1.05	0.5162	1.33	0.0782
102072_g_at	1434281_at	1500034J01Rik	RIKEN cDNA 1500034J01 gene	1.22	0.07	-1.05	0.455	-1.06	0.3978	-1.03	0.6726	1.11	0.4259
102073_at	1420755_a_at	Park2	parkin	1.26	0.31	-1.11	0.7389	-1.31	0.3945	1.02	0.9306	1.61	0.1117
102074_at	1422285_at	Otp	orthopedia homolog (Drosophila)	1.23	0.47	-1.16	0.1306	-1.35	0.0118	-1.33	0.0402	-1.05	0.7868
102075_at	1427755_at	Mcpt-ps1	mast cell protease, pseudogene 1	-1.22	0.59	1.02	0.8906	-1.27	0.0573	-1.28	0.0212	1.07	0.8015
102077_at	1426173_at	Cyp51	Cytochrome P450, family 51	1.01	0.99	-1.27	0.2934	-2.25	0.0087	-1.47	0.0807	1.27	0.2615
102078_at	1419345_at	Pth	parathyroid hormone	-1.67	0.19	-1.14	0.72	-1.37	0.4374	-1.21	0.5579	4.29	0.0046
102079_at	1426190_at	Aym1	activator of yeast meiotic promoters 1	-1.43	0.54	1.29	0.2271	1.13	0.5764	-1.1	0.6961	1.48	0.1155
102080_at	1426038_at	---	---	3.64	0	-1.94	0.002	-1.14	0.5654	-1.73	0.0623	-1.95	0.3067
102081_at	1421565_at	Robo3	roundabout homolog 3 (Drosophila)	-4	0.11	-1.24	0.1867	1.13	0.3871	-1.1	0.3199	1.55	0.0919
102082_at	1421740_at	Gnas	GNAS (guanine nucleotide binding protein, a	-1.68	0.55	1.76	0.2851	1.05	0.8213	1.3	0.1727	1.62	0.2678
102083_at	1421779_at	Rit2	Ras-like without CAAX 2	1.08	0.38	-1.12	0.4048	1.03	0.7564	-1.11	0.2433	1.07	0.5505
102084_f_at	1452501_at	Cyp2c38	cytochrome P450, family 2, subfamily c, poly	-1.18	0.42	1.1	0.5509	1.02	0.874	-1.05	0.7131	2.83	0.0039
102085_at	1421399_at	Insm1	insulinoma-associated 1	-1.93	0.3	1.14	0.6357	-1.06	0.8027	-1.11	0.6816	-1.87	0.1677
102086_r_at	1448091_at	D15Ertd50e	DNA segment, Chr 15, ERATO Doi 50, expr	-6.14	0.07	1.54	0.0849	2.23	0.0376	1.28	0.2713	-1.06	0.8869
102087_at	1452421_at	Hoxa3	homeo box A3	3.05	0	-1.12	0.6881	1.44	0.2253	1.13	0.7133	1.31	0.3248
102088_at	1422227_at	Klf12 /// D53003	Kruppel-like factor 12 /// RIKEN cDNA D530	-1.73	0.39	-1.35	0.2773	1.2	0.4317	-1	0.9897	2.26	0.1304
102089_at	1422148_at	Matn3	matrilin 3	-2.53	0.15	1.24	0.2853	1.16	0.6158	1.28	0.5134	2.99	0.1076
102100_f_at	1449633_s_at	C330027I04Rik	RIKEN cDNA C330027I04 gene	4.22	0	1.09	0.8482	1.15	0.6713	1.6	0.1008	2.26	0.1959
102102_at	1447989_at	---	Transcribed locus, strongly similar to XP_34	-1.43	0.02	-1.12	0.4514	1.26	0.1709	-1.36	0.1385	-1.06	0.8519
102103_f_at	1449726_at	Txnl2	thioredoxin-like 2	2.17	0.09	-1.15	0.0512	1.08	0.5356	-1.2	0.0223	1.08	0.7696
102106_at	1459893_at	C76472	expressed sequence C76472	1.35	0.49	-1	0.9925	-1.86	0.0444	-1.46	0.1511	2.38	0.0539
102109_at	1460581_a_at	Rpl13 /// LOC28	ribosomal protein L13 /// similar to 60S ribos	1.08	0.72	1.1	0.0447	1.24	0.0138	1.2	0.0191	1.4	0.0557
102111_f_at	1447970_at	C81363	expressed sequence C81363	-1.32	0.51	-1.21	0.2198	1.1	0.7014	1.01	0.9563	1.45	0.027
102112_s_at	1419836_at	---	Transcribed locus	3.11	0	-1.11	0.2784	-1.01	0.8712	-1.21	0.0198	1.43	0.2165
102118_at	1459952_at	Ankrd17	DNA segment, Chr 5, Wayne State Universit	-1.66	0.27	1.1	0.5439	1.05	0.7876	-1.04	0.7842	-1.18	0.4561
102123_at	1423141_at	Lip1	lysosomal acid lipase 1	1.1	0.81	-1.29	0.0141	-1.58	0.0004	-1.74	0	-1.08	0.6647
102126_at	1448344_at	Rps12 /// LOC4	ribosomal protein S12 /// similar to ribosoma	1	1	1.02	0.6867	1.33	0.0044	1.22	0.0163	1.3	0.0641
102127_at	1445172_at	Lgtn	Ligatin	1.41	0.37	1.2	0.3933	1.4	0.2873	1.59	0.1442	1.86	0.0196
102130_f_at	1457841_at	---	---	-1.21	0.37	-1.03	0.6725	1.02	0.7017	-1.01	0.8242	1.04	0.8162
102133_at	1459929_at	C80731	expressed sequence C80731	-1.5	0.25	1.06	0.6576	1.11	0.3226	1.09	0.6813	1.36	0.1693
102135_at	1442211_at	C78893	expressed sequence C78893	-1.68	0.01	-1.39	0.0836	-1.09	0.5533	-1.19	0.2793	1.4	0.2077
102136_r_at	1445047_at	C79246	expressed sequence C79246	-1.44	0.1	-1.38	0.1011	1.1	0.5546	1.09	0.5421	1.58	0.2359
102138_at	1447276_at	D13Ertd37e	DNA segment, Chr 13, ERATO Doi 37, expr	-1.07	0.78	1.08	0.6451	-1.05	0.5811	-1.09	0.3849	1.77	0.2269
102139_at	1420105_at	D13Ertd205e	DNA segment, Chr 13, ERATO Doi 205, exp	-1.02	0.94	-1.08	0.5771	1.1	0.5122	-1.08	0.5611	1.12	0.6499
102140_at	1447287_at	C77137	expressed sequence C77137	-1.05	0.7	1.03	0.8845	1.07	0.6586	-1.08	0.7435	-1.05	0.8537
102142_r_at	1449597_at	LOC236874	similar to odorant binding protein Ib	-1.77	0.11	-1.03	0.8457	1.07	0.6029	-1.14	0.2614	1.85	0.1007
102143_at	1449631_at	Eno3	RIKEN cDNA 1700029K24 gene	-1.52	0.05	-1.21	0.0933	-1.09	0.4462	-1.32	0.1139	1.25	0.3647
102146_at	1450225_at	Insr	insulin receptor	-1.7	0.56	-1.72	0.0622	-2.02	0.0279	-1.34	0.3074	-1.19	0.5487
102147_at	1450572_at	Akp3	alkaline phosphatase 3, intestine, not Mn rec	-1.51	0.6	1.49	0.1614	2.13	0.386	1.09	0.746	1	0.9905
102148_f_at	1422406_at	Ifna9	interferon alpha family, gene 9	-1.68	0.44	-1.21	0.2661	-1.13	0.5425	-1.37	0.1105	1.05	0.7487
102149_f_at	1422404_x_at	Ifna1 /// Ifna2 ///	interferon alpha family, gene 1 /// interferon a	-3.09	0	-1.19	0.1328	1.09	0.4199	-1.16	0.1822	1.43	0.5812
102150_f_at	1450614_x_at	Ifna5	interferon alpha family, gene 5	1.93	0.28	1.85	0.079	-1.07	0.8341	1.34	0.5772	-1.17	0.4411
102157_f_at	1427799_x_at	Igk-C /// Igk-V21	immunoglobulin kappa chain, constant regio	-4.37	0.12	1.63	0.0853	1.2	0.1461	2.27	0.135	2.17	0.0448
102161_f_at	1422348_at	H2-Q2	histocompatibility 2, Q region locus 2	1.31	0.59	1.05	0.7345	-1.27	0.0066	1.14	0.4445	-1.52	0.2353
102164_at	1422328_at	Gja10	gap junction membrane channel protein alph	-1.37	0.36	1.07	0.4363	1.11	0.1736	1.07	0.3221	1.54	0.2643
102166_g_at	1422380_at	V1rb5	vomeronasal 1 receptor, B5	-1.01	0.99	-1.02	0.9336	1.25	0.3859	1.22	0.2893	-1.32	0.608

102167_at	1422383_at	V1rb6	vomeronasal 1 receptor, B6	-1.5	0.43	-1.07	0.4563	1.11	0.3646	-1.09	0.4706	1.28	0.3983
102193_at	1435390_at	---	---	-1.1	0.66	-1.28	0.0115	-1.05	0.5569	-1.19	0.0561	1.23	0.2532
102194_at	1427085_at	2810432D09Rik	RIKEN cDNA 2810432D09 gene	1.01	0.93	-1.13	0.0064	-1.03	0.3757	-1.19	0.0017	1.06	0.5636
102195_at	1448050_s_at	---	---	5.96	0	-1.06	0.7545	-1.1	0.6684	1.34	0.1803	-1.11	0.6657
102196_at	1449144_at	Gna11	guanine nucleotide binding protein, alpha 11	1.07	0.71	1	0.985	1.14	0.1866	1.17	0.0352	-1.41	0.2794
102197_at	1418355_at	Nucb2	nucleobindin 2	1.16	0.63	1.3	0.478	1.24	0.7291	1.69	0.1783	-1.1	0.7338
102198_at	1421038_a_at	Kcnn4	potassium intermediate/small conductance c	-1.76	0.28	-1.64	0.0023	-1.91	0.003	-1.71	0.0006	-1.07	0.7975
102199_at	1431125_a_at	Tarsl1	threonyl-tRNA synthetase-like 1	1.11	0.62	1.05	0.5693	1.08	0.3281	1.06	0.5011	1.05	0.8179
102200_at	1417828_at	Aqp8	aquaporin 8	-1.89	0.24	-2.44	0	-1.36	0.0579	-3.18	0	-4.33	0
102201_s_at	1421853_at	Psen1	presenilin 1	1.09	0.59	-1.07	0.2905	-1.11	0.0679	-1.09	0.1119	-1.02	0.8315
102202_s_at	1420387_at	Mpv17	Mpv17 transgene, kidney disease mutant	-1.32	0.44	-1.06	0.5033	-1.37	0.0083	-1.21	0.0712	1.08	0.7141
102203_at	1449937_at	Pp11r	placental protein 11 related	-1.47	0.08	1.43	0.2808	1.69	0.1031	1.37	0.2361	1.03	0.9269
102204_at	1451715_at	Mafb	v-maf musculoaponeurotic fibrosarcoma onc	1.05	0.93	-1.5	0.2329	-2.01	0.0482	-2.05	0.0448	-1.38	0.6324
102205_at	1452461_a_at	MGC79224	hypothetical LOC432486	-1.69	0.58	-1.09	0.6451	-1.68	0.0325	1.01	0.9805	1.1	0.8355
102206_at	1422787_at	Fkbpl	FK506 binding protein-like	1.15	0.75	1.1	0.3502	1.15	0.1732	1.31	0.0271	1.3	0.3673
102207_at	1424544_at	Nrbp2	nuclear receptor binding protein 2	1.21	0.05	1.5	0.0006	1.15	0.428	1.52	0.0041	3.06	0.0094
102208_at	1449078_at	St3gal6	ST3 beta-galactoside alpha-2,3-sialyltransfe	-1.02	0.95	-2.88	0	-1.29	0.1069	-2.65	0	2.89	0.0155
102209_at	1417621_at	Nfatc1	nuclear factor of activated T-cells, cytoplasm	-2.55	0.3	1.24	0.0977	1	0.995	1.1	0.4914	-1.25	0.5354
102210_at	1437651_a_at	Dtnb	dystrobrevin, beta	1	1	1.14	0.3534	1.15	0.3322	1.3	0.0608	-1.05	0.8307
102212_at	1419018_at	Psx1	placenta specific homeobox 1	-1.46	0.59	1.12	0.2809	1.06	0.5166	1.12	0.3921	1.43	0.1137
102213_at	1419220_at	---	---	-1.11	0.53	-1.19	0.361	1.02	0.9415	1.01	0.9763	1.22	0.3679
102214_at	1423292_a_at	Prx	periaxin	-1.54	0.42	1.5	0.2864	-1.16	0.5107	-1.2	0.4957	1.45	0.1991
102215_at	1449407_at	Cdv1	carnitine deficiency-associated gene expres	-2.06	0	-1.04	0.5221	-1.02	0.7776	-1.05	0.4994	1.29	0.4199
102216_at	1418266_at	Alox12b	arachidonate 12-lipoxygenase, 12R type	1.03	0.97	1.53	0.1561	1.76	0.0821	1.76	0.0878	-1.62	0.334
102217_at	1449514_at	Gprk5	G protein-coupled receptor kinase 5	1.07	0.76	1.05	0.6563	1.14	0.1772	1.15	0.1029	1.04	0.8105
102218_at	1450297_at	Il6	interleukin 6	-1.07	0.9	-1.96	0.1427	-1.38	0.417	1.21	0.5961	1.68	0.4014
102219_at	1417332_at	Rfx2	regulatory factor X, 2 (influences HLA class	-1.87	0.24	1.07	0.6989	1.39	0.0728	1.09	0.6406	1.32	0.4498
102220_at	1416899_at	Utf1	undifferentiated embryonic cell transcription	1.29	0.51	-1.07	0.462	-1.02	0.8236	-1.13	0.0638	1.05	0.8171
102221_at	1419289_a_at	Syngr1	synaptogyrin 1	1.17	0.71	1.3	0.3657	1.36	0.2375	1.94	0.0037	1.73	0.0784
102222_at	1427672_a_at	Utx	ubiquitously transcribed tetratricopeptide rep	2.27	0.44	1.03	0.8141	-1.06	0.4145	-1.03	0.6254	1.03	0.8166
102223_at	1460732_a_at	Ppl	periplakin	1.27	0.2	1.02	0.772	-1.04	0.6206	1.01	0.9371	1.27	0.1359
102225_at	1434062_at	Rabgap1l	RAB GTPase activating protein 1-like	1.12	0.39	1.19	0.0258	1.14	0.1785	1.11	0.0796	1.2	0.2191
102227_g_at	1460248_at	Cpxm2	carboxypeptidase X 2 (M14 family)	-1.23	0.75	-1.19	0.3435	1.12	0.5322	-1.06	0.7278	1.52	0.2296
102229_at	1423581_at	Nmt2	N-myristoyltransferase 2	2.5	0	1.52	0.1279	1.45	0.1446	1.42	0.1286	1.15	0.8502
102230_at	1449954_at	Hyal1 /// Nat6	hyaluronidase 1 /// N-acetyltransferase 6	-1.53	0.24	-1.18	0.2405	-1.49	0.012	-1.82	0.0012	-1.55	0.3034
102231_at	1419295_at	---	---	-1.9	0.04	-1.06	0.3806	-1.03	0.5708	-1.13	0.0754	1.08	0.5641
102232_at	1452765_at	Slc39a9	solute carrier family 39 (zinc transporter), me	1.18	0.14	-1.18	0.003	1.02	0.8383	-1.13	0.0093	-1.02	0.8617
102235_at	1434777_at	Lmyc1	lung carcinoma myc related oncogene 1	1.23	0.47	1.64	0.0044	1.45	0.1615	1.93	0.0106	2.84	0.0081
102237_at	1417597_at	Cd28	CD28 antigen	1.05	0.89	1.42	0.0491	1.27	0.254	1.2	0.444	1.25	0.4914
102238_at	1450164_at	Ascl1	achaete-scute complex homolog-like 1 (Dros	-1.04	0.94	-1.36	0.2448	-1.3	0.2731	-1.03	0.8911	1.47	0.4521
102239_at	1418133_at	Bcl3	B-cell leukemia/lymphoma 3	1.37	0.46	1.09	0.8046	1.02	0.9278	1.28	0.4824	-2.06	0.3102
102241_f_at	1425229_a_at	Tcf7l2	transcription factor 7-like 2, T-cell specific, H	1.53	0.27	-1.07	0.4718	-1.16	0.1589	-1.18	0.0321	-1.23	0.4763
102242_at	1460662_at	Per3	period homolog 3 (Drosophila)	-1.5	0.03	1.01	0.9188	1.13	0.3372	1.09	0.5585	-1.03	0.7098
102243_at	1419474_a_at	Ehf	ets homologous factor	1.56	0.36	1.01	0.9731	-1.04	0.8797	-1.55	0.2041	1.58	0.4085
102244_at	1418559_at	Tesp1	testicular serine protease 1	1.01	0.98	-1.04	0.7796	-1.07	0.7322	-1.14	0.508	1.14	0.7315
102247_at	1420971_at	Ubr1	ubiquitin protein ligase E3 component n-reco	-1.66	0.48	-1.06	0.5487	-1.06	0.6749	1.11	0.1761	-1.21	0.2494
102249_at	1419148_at	Avil	advillin	-1.19	0.39	-1.2	0.6468	-1.4	0.3593	-1.82	0.1224	1.95	0.1523
102250_at	1449508_at	Il27ra	interleukin 27 receptor, alpha	-1.11	0.51	-1.09	0.3151	-1.06	0.3974	-1.09	0.2501	1.31	0.1554
102251_at	1418935_at	Trpm1	transient receptor potential cation channel, s	-1.37	0.13	-1.12	0.4208	1.12	0.4214	-1.19	0.1982	1	0.997
102252_at	1421950_at	Pfdn2	prefoldin 2	2.16	0.11	1.19	0.0264	1.1	0.3655	1.06	0.505	1.21	0.2023
102253_at	1425160_at	AF067063	cDNA sequence AF067063	-1.14	0.38	-1.21	0.2175	-1	0.9772	-1.01	0.9316	1.04	0.8864
102255_at	1418674_at	Osmr	oncostatin M receptor	1.15	0.49	1.03	0.7854	1.09	0.5796	1.07	0.5511	1.53	0.0282
102256_at	1418755_at	Tbx15	T-box 15	1.18	0.38	-1.22	0.3405	-1.23	0.2522	-1.08	0.6608	1.29	0.2452

102257_at	1421233_at	Pknox1	Pbx/knotted 1 homeobox	-1.19	0.15	-1.2	0.083	-1.23	0.0711	-1.18	0.0734	1.58	0.0419
102258_at	1422723_at	Stra6	stimulated by retinoic acid gene 6	1.04	0.94	-1.5	0.3237	-1.05	0.9063	-1.47	0.3388	2.7	0.2451
102259_at	1420816_at	Ywhag	3-monooxygenase/tryptophan 5-monooxyge	1.11	0.88	-1.07	0.6787	-1.33	0.2489	-1.63	0.037	1.03	0.9489
102260_at	1420399_at	Gfi1b	growth factor independent 1B	-1.52	0.06	-1.38	0.4855	-1.24	0.5965	-1.13	0.7671	1.72	0.2552
102262_r_at	1422866_at	Col13a1	procollagen, type XIII, alpha 1	1.46	0.4	1.09	0.654	1.46	0.0242	-1.08	0.6807	1.69	0.0286
102264_at	1418612_at	Slfn1	schlafen 1	-1.52	0.21	1.25	0.3166	-1.03	0.7982	1.55	0.2395	-1.05	0.7895
102265_at	1419150_at	Myf6	myogenic factor 6	-4.72	0.01	-1.4	0.2679	1.39	0.1799	1.32	0.3645	2.37	0.0058
102266_at	1422728_at	Inha	inhibin alpha	1.33	0.49	-1.11	0.6258	1.07	0.8269	1.04	0.8865	1.06	0.739
102267_at	1419138_at	B3galt4	UDP-Gal:betaGalNAc beta 1,3-galactosyltra	1.08	0.77	1.03	0.795	1.13	0.1295	-1	0.9706	1.22	0.592
102268_at	1429124_s_at	Tceb3bp1	transcription elongation factor B polypeptide	-1.11	0.63	1.13	0.1073	1.04	0.5019	1.02	0.7305	1.33	0.0537
102271_at	1460739_at	D11Bwg0280e	DNA segment, Chr 11, Brigham & Women's	1.13	0.54	-1.11	0.018	-1.08	0.1978	-1.07	0.0881	1.03	0.6676
102272_at	1420066_s_at	Cd160	CD160 antigen	-1.78	0.37	1.09	0.7168	1.04	0.8888	-1.15	0.5592	1.64	0.4168
102273_at	1449870_a_at	Atp6v0a2	ATPase, H+ transporting, lysosomal V0 subu	1.3	0.17	1.21	0.0017	1.13	0.0283	1.16	0.0064	-1.19	0.231
102274_at	1419297_at	H2-Oa	histocompatibility 2, O region alpha locus	-1.09	0.87	1.22	0.3719	-1.1	0.6925	1.46	0.1006	1.24	0.7208
102275_at	1420943_at	Zfp185	zinc finger protein 185	-1.45	0.38	1.71	0.1193	1.11	0.5372	1.12	0.4538	3.27	0.2802
102278_at	1450657_at	Ppie	peptidylprolyl isomerase E (cyclophilin E)	2.55	0.22	1.16	0.4821	-1.1	0.7162	1.25	0.2895	1.54	0.3405
102279_at	1426971_at	Ube1l	ubiquitin-activating enzyme E1-like	-1.16	0.25	1.01	0.9541	-1.28	0.0078	1.04	0.8547	-1.49	0.0844
102280_at	1449249_at	Pcdh7	protocadherin 7	-1	1	-1.21	0.5281	1.01	0.9772	1.23	0.4915	2.44	0.2979
102282_g_at	1452389_at	Tnfrsf7	tumor necrosis factor receptor superfamily, r	-1.51	0.07	1.06	0.6481	-1	0.9997	-1.24	0.0671	1.44	0.0358
102283_at	1418057_at	Tiam1	T-cell lymphoma invasion and metastasis 1	-1.47	0.05	-1.12	0.2096	1.05	0.6861	1.07	0.5996	1.17	0.3754
102284_at	1419677_at	Masp1	mannan-binding lectin serine protease 1	-1.4	0.08	-1.57	0	-1.03	0.7665	-1.48	0	-1.75	0.0124
102285_at	1421309_at	Mgmt	O-6-methylguanine-DNA methyltransferase	1.02	0.94	-1.25	0.1282	-1.15	0.3127	-1.34	0.0743	-1.32	0.5077
102286_at	1426602_at	Araf	v-raf murine sarcoma 3611 viral oncogene h	1.19	0.61	-1.06	0.5734	-1.05	0.4941	-1.12	0.1334	1.02	0.9642
102287_at	1426396_at	Cd3z	CD3 antigen, zeta polypeptide	-1.14	0.77	1.4	0.1132	1.19	0.4315	1.59	0.0103	1.3	0.4187
102288_at	1426079_at	Cd3z	CD3 antigen, zeta polypeptide	-2.32	0.12	1.24	0.4792	-1.04	0.9089	1.52	0.165	-1.47	0.4917
102290_at	1422699_at	Alox12	arachidonate 12-lipoxygenase	-1.45	0.32	-1.01	0.9518	1.05	0.7267	-1.02	0.885	1.17	0.6753
102291_at	1427393_at	F9	coagulation factor IX	-1.26	0.28	-1.27	0.0003	-1.02	0.8366	-1.16	0.0094	-1.71	0.0005
102292_at	1449519_at	Gadd45a	growth arrest and DNA-damage-inducible 45	2.55	0.05	-1.23	0.1059	-1.32	0.1613	-1.54	0.0009	-4.5	0.0001
102293_at	1421303_at	Zfpn1a1	zinc finger protein, subfamily 1A, 1 (Ikaros)	-1.15	0.49	-1.04	0.8131	-1.31	0.3292	-1.01	0.9697	-1.41	0.2244
102295_at	1417680_at	Kcna5	potassium voltage-gated channel, shaker-re	1.22	0.59	1.37	0.1725	1.1	0.5401	1.23	0.2322	1.79	0.1003
102296_at	1448312_at	Pcsk2	proprotein convertase subtilisin/kexin type 2	1.09	0.83	1.35	0.3183	1.57	0.0762	1.58	0.0535	1.79	0.2814
102297_at	1448920_at	Zfp535	zinc finger protein 535	-1.4	0.63	-1.15	0.6192	-1.34	0.3213	1.35	0.2531	1.13	0.8025
102298_at	1419675_at	Ngfb	nerve growth factor, beta	2.02	0.14	1.34	0.2071	1.13	0.6317	1.64	0.0323	-1.81	0.1456
102300_at	1449331_a_at	Rapsn	receptor-associated protein of the synapse	-1.8	0.03	-1.21	0.0009	-1.15	0.0306	-1.22	0.0491	1.11	0.3682
102301_at	1418556_at	Capza3	capping protein (actin filament) muscle Z-line	-3.56	0.11	1.38	0.3571	1.7	0.0852	1.06	0.8682	1.29	0.588
102302_at	1427153_at	Bckdhb	branched chain ketoacid dehydrogenase E1	1.29	0.24	1.09	0.024	1.11	0.0053	1.26	0	-1.06	0.5058
102304_f_at	1417363_at	Zfp61	zinc finger protein 61	1.34	0.53	1.25	0.3577	1.15	0.6174	1.56	0.0334	-1	0.9967
102305_at	1424146_at	Gpr37l1	G protein-coupled receptor 37-like 1	-1.45	0.07	-1.09	0.278	1.1	0.3211	1.08	0.3117	2.55	0.006
102307_at	1418139_at	Dcx	doublecortin	-2.08	0.17	1.01	0.9432	1.06	0.7548	-1.18	0.2269	1.97	0.066
102308_at	1449008_at	Tulp3	tubby-like protein 3	-1.52	0.11	1.06	0.5542	1.12	0.2583	1.02	0.7671	1.44	0.0554
102309_at	1419181_at	Zfp326	zinc finger protein 326	-1	0.99	-1.05	0.5865	-1.15	0.2968	-1.03	0.707	1.01	0.9543
102311_at	1419720_at	Gp9	glycoprotein 9 (platelet)	-1.61	0.23	-1.3	0.1635	1.31	0.3064	-1.23	0.1535	1.02	0.9256
102313_at	1420499_at	Gch1	GTP cyclohydrolase 1	1.88	0	-1.05	0.43	1.45	0.0008	1.17	0.0022	-1.35	0.1188
102314_at	1415958_at	Slc2a4	solute carrier family 2 (facilitated glucose tra	2.53	0.11	1.08	0.8271	-1.24	0.5873	1.39	0.3069	1.1	0.3243
102315_at	1423884_at	Tex292	testis expressed gene 292	1.19	0.38	1.18	0.0129	1.35	0.0003	1.25	0.0003	1.28	0.0209
102316_at	1418671_at	Capn5	calpain 5	-1.2	0.32	1.09	0.203	-1.02	0.7885	1.05	0.527	1.43	0.2063
102317_at	1422895_at	Vamp4	vesicle-associated membrane protein 4	-1.03	0.72	1.28	0.0544	1.03	0.8069	1.12	0.3775	1.08	0.6789
102318_at	1419186_a_at	St8sia4	ST8 alpha-N-acetyl-neuraminide alpha-2,8-s	1.5	0.18	1.26	0.2384	1.02	0.8646	1.56	0.0599	1.65	0.3691
102319_at	1421797_a_at	Snx12	sorting nexin 12	1.98	0.17	-1.03	0.7309	-1.17	0.1336	-1.14	0.2188	1.29	0.6074
102320_at	1454807_a_at	Snx12	sorting nexin 12	-1.12	0.48	1.21	0.3692	1.27	0.3605	1.24	0.3733	1.65	0.1545
102321_at	1418128_at	Adcy6	adenylate cyclase 6	1.18	0.34	1.71	0.0002	1.37	0.0133	1.83	0	1.41	0.0418
102322_at	1416308_at	Ugdh	UDP-glucose dehydrogenase	1.08	0.67	1.1	0.2767	1.13	0.2232	1.25	0.0034	-1.58	0.0005
102323_at	1416997_a_at	Hap1	huntingtin-associated protein 1	-1.75	0.06	1.11	0.5173	-1.08	0.6238	-1.18	0.182	1.24	0.2208

102324_at	1417934_at	Dnajc4	DnaJ (Hsp40) homolog, subfamily C, membe	1.3	0.04	-1.04	0.5391	-1.02	0.7531	-1	0.9813	1.16	0.2524
102326_at	1448561_at	Ncf2	neutrophil cytosolic factor 2	1.36	0.31	1.74	0.2625	-1.06	0.7713	1.98	0.2288	2.27	0.0447
102327_at	1449396_at	Aoc3	amine oxidase, copper containing 3	-1.29	0.47	-1.27	0.0819	-1.13	0.2548	-1.36	0.0746	1.82	0.0319
102328_at	1424552_at	Casp8	caspase 8	-1.17	0.5	-1.09	0.3303	1.09	0.5794	1.1	0.3271	-1.12	0.5744
102329_at	1418976_s_at	Cideb	cell death-inducing DNA fragmentation facto	-1.2	0.1	-1.17	0.001	-1.05	0.2456	-1.19	0.0016	-1.38	0.0029
102330_at	1418204_s_at	Aif1	allograft inflammatory factor 1	2.45	0.02	1.31	0.5978	-1.05	0.8088	1.89	0.2493	1.38	0.0086
102331_at	1428372_at	St5	suppression of tumorigenicity 5	-1.38	0.24	-1.15	0.062	-2.14	0.001	-1.45	0.0233	1.2	0.5247
102332_at	1448370_at	Ulk1	Unc-51 like kinase 1 (C. elegans)	1.14	0.27	-1.07	0.2967	1.22	0.0458	1.02	0.7741	-1.17	0.038
102333_at	1427226_at	Epn2	epsin 2	-1.18	0.29	-1.03	0.488	1.01	0.9417	1.1	0.1302	1.24	0.0203
102334_at	1416333_at	Dok2	docking protein 2	1.54	0.07	1.22	0.0765	1.18	0.0834	1.31	0.0683	2.57	0.0236
102335_at	1455896_a_at	Kcnk1	potassium channel, subfamily K, member 1	-1.8	0.01	-1	0.9762	1.06	0.7129	-1.1	0.6059	1.31	0.5361
102336_at	1448396_at	D1Bwg0491e	DNA segment, Chr 1, Brigham & Women's (	-1.06	0.63	1.11	0.1734	1.19	0.143	1.32	0.0096	-1.02	0.7926
102337_s_at	1455332_x_at	Fcgr2b	Fc receptor, IgG, low affinity IIb	1.34	0.04	-1.1	0.3207	1.1	0.3449	1.02	0.9169	1.15	0.3005
102338_at	1449403_at	Pde9a	phosphodiesterase 9A	1.21	0.54	-1.37	0.2508	1.18	0.529	-3.43	0.0051	-1.93	0.0238
102340_at	1423595_at	Mina	myc induced nuclear antigen	1.19	0.35	1.06	0.5449	1.16	0.4146	1.07	0.435	1.16	0.4647
102341_at	1449006_at	Gla	galactosidase, alpha	-2.1	0.01	1.5	0.1853	1.27	0.4127	1.45	0.2166	-1.11	0.8665
102342_at	1422455_s_at	Nsf	N-ethylmaleimide sensitive fusion protein	-1.85	0.51	-1.08	0.6981	1.07	0.6799	1.14	0.4763	1.12	0.6641
102343_at	1417100_at	Cd320	CD320 antigen	-1.2	0.08	-1.09	0.3745	-1.06	0.4697	1.06	0.4929	1.77	0.1422
102344_s_at	1424531_a_at	Tcea3	transcription elongation factor A (SII), 3	3.21	0.03	1.2	0.033	-1.04	0.6737	1.16	0.1775	1.47	0.0098
102345_at	1417638_at	Lefty1	left right determination factor 1	-1.16	0.67	-1.18	0.2128	-1.14	0.2093	-1.41	0.0075	1.3	0.5248
102348_at	1424609_a_at	BC003993 /// L(	cDNA sequence BC003993 /// hypothetical L	1.11	0.86	1.75	0.048	-1.8	0.0178	1.15	0.6422	1.74	0.0673
102350_at	1427449_a_at	Adprhl2	ADP-ribosylhydrolase like 2	2.64	0.25	1.51	0.1587	1.24	0.4466	1.43	0.2163	2.42	0.076
102351_at	1448730_at	Cpa3	carboxypeptidase A3, mast cell	2.5	0.05	-1.22	0.5075	-1.14	0.6435	-1.25	0.4717	-1.19	0.6837
102352_at	1418195_at	Galnt10	UDP-N-acetyl-alpha-D-galactosamine:polyp	1.3	0.5	-1.13	0.1757	-1.41	0.0012	-1.58	0.002	-1.44	0.3147
102353_at	1450678_at	---	---	2.58	0.03	2.47	0.2174	1.08	0.7526	3.43	0.1936	1.25	0.6603
102354_at	1423809_at	Tcf19	transcription factor 19	1.65	0.3	-1.08	0.753	1.07	0.6994	1.21	0.3217	1.05	0.7106
102356_at	1448418_s_at	Wdr23	WD repeat domain 23	1.24	0.17	1.01	0.7742	-1.22	0.0073	-1.05	0.3909	-1.32	0.0022
102357_at	1454977_at	AU020772	expressed sequence AU020772	1.85	0.25	1.97	0.0344	2.15	0.0084	2.11	0.0068	-1.29	0.3946
102360_at	1434087_at	Mthfr	5,10-methylenetetrahydrofolate reductase	-1.35	0.57	1.3	0.4658	1.23	0.4206	1.6	0.2105	1.42	0.6311
102363_r_at	1415899_at	Junb	Jun-B oncogene	1.58	0.4	1.82	0.0335	-1.03	0.8678	1.59	0.0119	1.02	0.842
102364_at	1449117_at	Jund1	Jun proto-oncogene related gene d1	-1.13	0.62	1.06	0.4444	1.05	0.5425	1.03	0.7426	1.3	0.1443
102366_at	1449182_at	Retn	resistin	-1.46	0.28	-1.74	0.0835	-1.48	0.1826	-1.73	0.0876	1.29	0.0478
102368_at	1427934_at	2610208E05Rik	RIKEN cDNA 2610208E05 gene	1.55	0.12	-1.07	0.4041	-1.26	0.036	-1.06	0.3812	-1.26	0.0339
102370_at	1434642_at	Dhrs8	dehydrogenase/reductase (SDR family) mer	1.11	0.68	1.65	0	1.47	0	1.99	0	1.76	0.0009
102371_at	1416505_at	Nr4a1	nuclear receptor subfamily 4, group A, meml	6.56	0.1	3.78	0.0994	1.32	0.2358	2.3	0.0078	1.71	0.2797
102372_at	1424305_at	Igj	immunoglobulin joining chain	-1.18	0.18	1.52	0.0101	1.06	0.7117	1.17	0.1612	1.77	0.0326
102373_at	1448649_at	Enpep	glutamyl aminopeptidase	-1.84	0.06	-1.35	0.0573	-1.22	0.259	-1.78	0.0018	-1.49	0.0023
102374_at	1434027_at	Dscr1l2	Down syndrome critical region gene 1-like 2	3.27	0.16	1.11	0.4826	1.54	0.3239	1.26	0.1714	1.36	0.0899
102375_at	1451722_s_at	---	---	1.01	0.99	-1.09	0.5364	-1.03	0.8196	-1.05	0.7145	1.25	0.4891
102376_r_at	1419084_a_at	Pcp2	Purkinje cell protein 2 (L7)	-1.13	0.8	1.8	0.0055	1.49	0.1106	1.51	0.1334	2.14	0.0047
102378_at	1417644_at	Sspn	sarcospan	-1.38	0.21	1.2	0.6042	-1.15	0.6217	1.6	0.0645	2.95	0.0465
102379_at	1448855_at	Rassf1	Ras association (RalGDS/AF-6) domain fam	1.82	0.09	-1.06	0.8154	1.37	0.3141	2.51	0.0005	1.5	0.0458
102380_s_at	1450209_at	Hoxd4	homeo box D4	1.39	0.31	-1.33	0.1835	-1.45	0.1342	-1.08	0.7418	-1.24	0.4003
102381_at	1433531_at	Acsl4	acyl-CoA synthetase long-chain family mem	1.09	0.83	1.08	0.3972	-1.05	0.6541	1.46	0.0037	1.21	0.3563
102382_at	1425099_a_at	Arntl	aryl hydrocarbon receptor nuclear transloca	-2.4	0.04	-1.12	0.3887	-1.5	0.0215	-1.38	0.0454	-1.23	0.5821
102383_at	1433639_at	5730593F17Rik	RIKEN cDNA 5730593F17 gene	1.3	0.53	1.07	0.5466	1.11	0.5742	1.07	0.6168	1.6	0.1839
102384_at	1430526_a_at	Smarca2	SWI/SNF related, matrix associated, actin de	1.28	0.16	-1.01	0.8947	-1.18	0.0976	1.17	0.0815	1.2	0.3063
102385_at	1428389_s_at	Wdr43	WD repeat domain 43	-1.11	0.67	-1.08	0.3483	1.06	0.3362	-1.05	0.6486	1.15	0.3816
102387_at	1424377_at	BC003885	cDNA sequence BC003885	1.07	0.86	1.01	0.9195	1.07	0.3985	1.09	0.2155	-1.15	0.5374
102389_s_at	1423537_at	Gap43	growth associated protein 43	-2.11	0.03	-1.07	0.7096	1.02	0.9099	-1.24	0.25	1.16	0.7008
102395_at	1417133_at	Pmp22	peripheral myelin protein	1.28	0.17	-1.11	0.4022	-1.07	0.5718	-1.19	0.1638	1.22	0.1663
102396_at	1423068_at	4930553F24Rik	RIKEN cDNA 493055F24Rik	-1.06	0.84	1.03	0.7348	-1	0.9973	1.03	0.7383	1.1	0.5855
102397_at	1418582_at	Cbfa2t3h	core-binding factor, runt domain, alpha subu	1.29	0.7	1.35	0.1205	1.23	0.4725	-1.03	0.9229	1.26	0.4656

102398_at	1416990_at	Rxrb	retinoid X receptor beta	-1.26	0.4	-1.17	0.0686	-1.02	0.8065	-1.26	0.038	1.18	0.6094
102399_at	1429359_s_at	Rbpms	RNA binding protein gene with multiple splic	-1.08	0.82	1.08	0.2545	-1.26	0.0217	1.12	0.1263	1.25	0.5039
102400_at	1433663_s_at	LOC433702	nuclear cap binding protein subunit 1, 80kDa	-1.32	0.16	-1.23	0.0787	1.11	0.3456	-1.06	0.6044	-1.09	0.6695
102401_at	1448436_a_at	Irf1	interferon regulatory factor 1	-1.26	0.08	1.43	0.2624	-1.38	0.0123	1.58	0.2397	-1.14	0.5702
102403_at	1416575_at	Cdc45l	cell division cycle 45 homolog (S. cerevisiae	1.56	0.29	1.08	0.7172	-1	0.9965	1.21	0.2815	1.6	0.007
102405_at	1460219_at	Mag	myelin-associated glycoprotein	-1.76	0.03	1.4	0.0062	1.33	0.0113	1.2	0.0374	-1.05	0.8362
102408_g_at	1449456_a_at	Mcpt5	mast cell protease 5	1.04	0.82	1.18	0.1643	1.18	0.1504	1.04	0.6856	1.33	0.3027
102409_at	1448703_at	Lsm8	LSM8 homolog, U6 small nuclear RNA asso	1.57	0.18	1.36	0.0009	1.19	0.1224	1.45	0	1.1	0.575
102410_at	1423450_a_at	Hs3st1	heparan sulfate (glucosamine) 3-O-sulfotran	-1.14	0.65	1.14	0.3028	1.16	0.3895	1.15	0.1695	3.57	0.336
102411_at	1416586_at	Zfp239	zinc finger protein 239	-1.19	0.7	-1.65	0.2149	-1.28	0.5437	-1.71	0.1793	3.28	0.018
102412_at	1426751_s_at	Nup107	nucleoporin 107	1.02	0.9	1.09	0.5374	1.14	0.392	1.18	0.1855	-1.83	0.1829
102413_at	1418478_at	Lmo1	LIM domain only 1	-1.29	0.1	-1.04	0.6856	1.04	0.6041	-1.1	0.2535	1.23	0.28
102416_at	1417017_at	Cyp17a1	cytochrome P450, family 17, subfamily a, po	3.13	0.07	-5.03	0.0001	-2.39	0.0042	-9.22	0	2.1	0.1265
102418_at	1417482_at	Tex19	testis expressed gene 19	-1	0.99	-1.1	0.6912	1.11	0.6339	1.29	0.2153	-1.01	0.9709
102419_at	1419415_a_at	Rarg	retinoic acid receptor, gamma	1.28	0.26	-1.07	0.576	1.1	0.4548	1.11	0.3429	1.36	0.2181
102421_at	1424259_at	2400010G15Rik	RIKEN cDNA 2400010G15 gene	-1.16	0.2	-1.13	0.0257	-1.06	0.2975	-1.2	0.0034	-1.6	0.0281
102423_at	1452011_a_at	Uxs1	UDP-glucuronate decarboxylase 1	-1.74	0.33	-1.02	0.8806	-1.24	0.2648	-1.12	0.5016	-1.03	0.9264
102424_at	1419561_at	Ccl3	chemokine (C-C motif) ligand 3	2.22	0.05	1.78	0.042	1.21	0.514	2	0.0245	2.4	0.0213
102425_at	1422751_at	Tle1	transducin-like enhancer of split 1, homolog	1.6	0.3	-1.19	0.339	1.37	0.212	-1.32	0.138	1.26	0.1442
102426_at	1422598_at	Casq1	calsequestrin 1	-1.31	0.39	-1.1	0.5175	-1.06	0.613	-1.2	0.1087	1.07	0.4746
102427_at	1451324_s_at	Fyttd1	forty-two-three domain containing 1	1.18	0.61	1.23	0.1889	1.3	0.0193	1.15	0.2592	1.24	0.4403
102429_at	1422898_s_at	Slc22a12	solute carrier family 22 (organic anion/cation	-1.4	0.58	-1.37	0.0347	-1.24	0.1436	-1.32	0.0403	1.42	0.3466
102430_at	1419272_at	Myd88	myeloid differentiation primary response ger	1.08	0.69	1.19	0.0846	1.07	0.5021	1.2	0.1307	-1.05	0.8136
102552_at	1422339_at	Gja3	gap junction membrane channel protein alph	-2.74	0.3	-1.16	0.2437	-1.13	0.3351	-1.09	0.3713	1.26	0.4952
102554_at	1422382_at	Olfr16	olfactory receptor 16	-1.16	0.65	1.01	0.9449	1.27	0.1501	-1.07	0.724	1.34	0.2135
102555_at	1422417_at	X99300	epidymal sperm gene	-1.34	0.23	1.08	0.4095	1.13	0.4392	-1.01	0.905	1.51	0.31
102556_at	1423022_at	---	---	-1.6	0.25	1.36	0.0962	1.12	0.2252	1.2	0.088	1.82	0.1888
102560_at	1460321_at	Cntn4	contactin 4	1.04	0.88	-1.08	0.5045	2.35	0.2536	1.15	0.2626	1.27	0.5375
102566_at	1422162_at	Dcc	deleted in colorectal carcinoma	1.83	0.43	2.17	0.0421	1.43	0.1707	2.4	0.0964	-1.03	0.9032
102567_at	1422239_at	Hoxd13	homeo box D13	1.19	0.28	-1.14	0.1219	1.06	0.5526	-1.05	0.574	1.14	0.4364
102572_at	1421621_at	Rasgrf2	RAS protein-specific guanine nucleotide-rele	-2.31	0.28	-1.47	0.176	1.25	0.4032	-1.02	0.9467	3.14	0.0379
102574_at	1421793_at	Fgf11	fibroblast growth factor 11	-1.21	0.65	-1.29	0.012	-1.19	0.134	-1.24	0.0289	-1.33	0.311
102575_at	1450611_at	---	PREDICTED: Mus musculus similar to bM34	-1.49	0.19	-1.18	0.2998	-1.59	0.0002	-1.54	0.0014	-5.3	0.0358
102576_at	1419210_at	Hrh1	histamine receptor H 1	-1.91	0.12	1.22	0.3572	1.1	0.6912	-1.12	0.6553	1.14	0.6503
102577_at	1421790_a_at	Kcnab3	potassium voltage-gated channel, shaker-re	-1.68	0.25	1.39	0.0583	1.28	0.2578	1.29	0.2749	-1.09	0.8464
102578_at	1421765_at	Pax5	paired box gene 5	-1.31	0.52	-1.16	0.2062	1.26	0.0696	-1.05	0.7523	-2.07	0.2205
102581_at	1422266_at	Mycs	myc-like oncogene, s-myc protein	1.01	0.99	-1.28	0.1266	-1.1	0.6198	-1.51	0.0255	1.4	0.111
102582_at	1450497_at	Apc2	adenomatosis polyposis coli 2	-1.58	0.21	-1.06	0.6694	1.09	0.5776	1.09	0.5593	1.4	0.2232
102584_at	1422254_a_at	Dyrk1b	dual-specificity tyrosine-(Y)-phosphorylation	-1.12	0.31	-1.11	0.0771	-1.35	0.0053	-1.26	0.0068	1.39	0.0499
102586_at	1451014_at	Ror1	receptor tyrosine kinase-like orphan recepto	1.49	0.35	-1.02	0.8007	1.1	0.2228	-1.15	0.1711	1.36	0.3994
102599_at	1416642_a_at	Tpt1	tumor protein, translationally-controlled 1	-1.02	0.79	-1.02	0.4364	1.03	0.1921	1.01	0.674	1.19	0.0959
102613_at	1426071_at	Tiaf2	TGF-beta1-induced anti-apoptotic factor 2	1.21	0.68	-1.16	0.3505	1.08	0.6139	1.07	0.7173	2.52	0.3135
102614_at	1421336_at	Prox1	prospero-related homeobox 1	1.47	0.48	1.21	0.3242	-1.09	0.6652	-1.27	0.1419	-1.34	0.6353
102619_at	1450235_at	Fgd3	FYVE, RhoGEF and PH domain containing 3	1.46	0.62	1.01	0.9067	1.08	0.6412	1.35	0.1977	-1.33	0.1839
102620_at	1421961_a_at	Dnajb5	DnaJ (Hsp40) homolog, subfamily B, membe	-1.31	0.15	-1.03	0.7951	-1.08	0.4559	1.02	0.8317	1.02	0.9255
102621_at	1421452_at	Cdon	cell adhesion molecule-related/down-regulat	-1.36	0.5	-1.22	0.3375	-1.14	0.4803	-1.22	0.3086	1.3	0.0922
102623_at	1425840_a_at	Sema3f	sema domain, immunoglobulin domain (Ig),	-1.56	0.47	1.12	0.6178	1.57	0.0937	1.15	0.5867	1.1	0.7736
102624_at	1449484_at	Stc2	stanniocalcin 2	-1.18	0.38	-1.15	0.1759	-1.14	0.363	-1.08	0.4259	1.27	0.1706
102626_r_at	1419239_at	Zfp54	zinc finger protein 54	1.01	0.98	1.12	0.3955	1.44	0.0887	1.3	0.0445	-1.08	0.8294
102628_at	1425893_a_at	Fhit	fragile histidine triad gene	1.29	0.2	-1.18	0.1728	-1.26	0.142	-1.12	0.2883	-1.11	0.7587
102629_at	1419607_at	Tnf	tumor necrosis factor	-1.73	0.1	-1.12	0.6736	-1.13	0.7198	1.42	0.488	1.16	0.6141
102630_s_at	1420353_at	Lta	lymphotoxin A	-1.32	0.04	1.23	0.4304	1.14	0.2986	-1.03	0.7642	1.3	0.3727
102631_at	1448953_at	Blm	Bloom syndrome homolog (human)	-1.52	0.32	1.03	0.905	1.26	0.3572	-1.07	0.826	1.38	0.5171

102632_at	1422814_at	Calmbp1	calmodulin binding protein 1	-1.42	0.59	1.09	0.463	1.07	0.6457	1.12	0.2385	1.14	0.6504
102633_at	1460280_at	Grap2	GRB2-related adaptor protein 2	-1.21	0.5	-1.04	0.824	1.08	0.6643	-1	0.9989	1.89	0.1006
102635_at	1419190_at	Vti1a	vesicle transport through interaction with t-S	2.56	0.01	4.05	0.0014	2.25	0.0821	2.39	0.0261	1.43	0.0916
102636_at	1418214_at	Klc2	kinesin light chain 2	1.06	0.93	-1.27	0.4499	-1.14	0.6831	1.06	0.788	1.41	0.0451
102637_at	1425620_at	Tgfbr3	transforming growth factor, beta receptor III	-1.04	0.92	-1.15	0.3379	1.14	0.374	-1.04	0.8234	1.68	0.1726
102638_at	1419202_at	Cst7	cystatin F (leukocystatin)	1.11	0.69	-1.03	0.7277	1.05	0.7123	-1.06	0.4768	1.59	0.0103
102639_at	1422758_at	Chst2	carbohydrate sulfotransferase 2	2.83	0.01	1.27	0.5902	1.2	0.5823	1.3	0.4673	1.75	0.0624
102641_at	1418747_at	Sfpi1	SFFV proviral integration 1	-1.2	0.32	1.21	0.4151	-1.13	0.1306	1.21	0.3953	1.24	0.0758
102642_at	1417584_at	Slc11a2	solute carrier family 11 (proton-coupled diva	-1.19	0.34	1.16	0.1868	1.05	0.6049	1.18	0.1169	1.4	0.0807
102643_at	1419602_at	Hoxa2	homeo box A2	-1.66	0.05	-1.02	0.8764	1.12	0.2667	1.06	0.5621	1.15	0.4299
102644_at	1427040_at	Mdfic	MyoD family inhibitor domain containing	-1.08	0.71	-1.05	0.6075	-1.22	0.0844	-1.03	0.7892	-1.15	0.3949
102645_at	1438045_at	Eea1	Early endosome antigen 1	1.09	0.53	1.12	0.0329	1.07	0.3207	1.08	0.2894	1.19	0.1909
102647_g_at	1434392_at	Usp34	ubiquitin specific protease 34	-1.04	0.79	1.07	0.2782	-1.04	0.539	1.09	0.1705	1.02	0.7832
102649_s_at	1420603_s_at	Raet1a /// Raet1	retinoic acid early transcript 1, alpha /// retin	1.47	0.03	1.4	0.2672	-1.22	0.4767	1.65	0.0386	-1.54	0.0276
102650_at	1425245_a_at	Rgs11	regulator of G-protein signaling 11	-1.35	0.06	-1.26	0.0939	-1.17	0.1906	-1.24	0.1079	1.2	0.4215
102651_at	1425303_at	Gck	glucokinase	-1.83	0.12	-1.64	0.0015	-1.38	0.0175	-1.68	0.0001	-1.62	0.0354
102653_at	1450123_at	Ryr2	ryanodine receptor 2, cardiac	-4.47	0.34	-1.03	0.8462	2.64	0.0871	1.49	0.2221	1.96	0.1942
102654_at	1449232_at	Gata1	GATA binding protein 1	1.21	0.74	1.08	0.8544	-1.51	0.3159	1.38	0.3837	-1.26	0.6173
102656_at	1427615_at	Itga4	integrin alpha 4	-2.17	0.14	1.03	0.878	1.14	0.3319	-1.02	0.9191	-1.07	0.8664
102657_at	1418939_at	Hlx1	H2.0-like homeo box 1 (Drosophila)	2.26	0.03	1.82	0.0389	1.94	0.0037	1.39	0.3134	3.11	0.0058
102658_at	1419532_at	Il1r2	interleukin 1 receptor, type II	-1.7	0.03	-1.04	0.7795	1.02	0.8864	-1.28	0.1017	-1.06	0.8522
102659_at	1417551_at	Cln3	ceroid lipofuscinosis, neuronal 3, juvenile (B	-1.19	0.29	-1.13	0.1299	-1.08	0.2815	-1.1	0.1883	1.21	0.0156
102660_at	1422870_at	Hoxc4	homeo box C4	1.77	0.1	-1.13	0.4765	1.03	0.8194	-1.04	0.7266	1.32	0.1673
102661_at	1427683_at	Egr2	early growth response 2	1.48	0.29	1.08	0.5078	1.11	0.5084	1.02	0.8941	2.24	0.06
102662_at	1419747_at	Asgr2	asialoglycoprotein receptor 2	1.41	0.19	1.2	0.0048	1.02	0.8595	1.22	0.0347	1.66	0.0078
102663_at	1452521_a_at	Plaur	urokinase plasminogen activator receptor	-1.21	0.44	1.71	0.2039	1.12	0.195	1.95	0.2282	1.63	0.3998
102664_at	1421123_at	Cdk5r1	cyclin-dependent kinase 5, regulatory subun	1.77	0.23	-1.23	0.1851	-1.02	0.8854	1.01	0.9601	1.32	0.622
102665_at	1418756_at	Trh	thyrotropin releasing hormone	1.89	0.02	-1.2	0.1919	1.05	0.6367	-1.17	0.1389	1.79	0.0889
102666_at	1448821_at	Tyr	tyrosinase	-3.15	0.01	1.08	0.7291	1.27	0.3619	-1.34	0.2433	1.59	0.378
102667_at	1422093_at	Wnt3a	wingless-related MMTV integration site 3A	1.13	0.23	-1.39	0.1377	1.04	0.8187	-1.09	0.7088	1.17	0.3422
102668_at	1449051_at	Ppara	peroxisome proliferator activated receptor al	1.03	0.93	1.06	0.8295	-2.14	0.0153	-1.47	0.1983	-1.33	0.0063
102669_at	1422161_at	Sn	sialoadhesin	1.59	0.44	1.13	0.7272	-1.48	0.1466	-1.08	0.8341	1.18	0.6753
102670_at	1449095_at	Vps54	vacuolar protein sorting 54 (yeast)	-1.21	0.49	-1.13	0.0832	1.1	0.4705	-1.2	0.0436	-1.16	0.2396
102672_g_at	1452529_a_at	Creb1	cAMP responsive element binding protein 1	1.84	0.13	1	0.9932	1.2	0.4644	-1.02	0.9408	1.25	0.2825
102675_at	1419007_at	Zp3	zona pellucida glycoprotein 3	-1.08	0.82	-1.23	0.3564	1.02	0.9146	1.04	0.8372	1.65	0.209
102676_at	1421071_at	Vhlh	von Hippel-Lindau syndrome homolog	1.8	0.17	1.15	0.6741	-1.18	0.6676	-1.25	0.5415	1.5	0.551
102677_at	1448660_at	Arhgdig	Rho GDP dissociation inhibitor (GDI) gamma	-1.16	0.66	-1.12	0.6828	-1.24	0.4391	-1.18	0.5467	-1	0.9867
102678_at	1418077_at	Trim21	tripartite motif protein 21	-1.14	0.39	1.03	0.788	-1.03	0.7999	1.01	0.8953	1.05	0.7866
102680_g_at	1425253_a_at	Madcam1	mucosal vascular addressin cell adhesion m	1	0.99	1.08	0.7411	-1.31	0.1921	-1.31	0.2509	-1.04	0.8717
102681_at	1420351_at	Tnfrsf4	tumor necrosis factor receptor superfamily, r	-1.28	0.17	1.12	0.2524	1.34	0.0312	1.09	0.3087	1.44	0.1506
102682_at	1419341_at	Epha8	Eph receptor A8	1.31	0.35	-1.22	0.0213	-1.02	0.7944	-1.06	0.688	-1.03	0.8805
102683_at	1460654_at	---	---	-1.78	0.1	-1.13	0.1063	-1.07	0.433	-1.07	0.2709	1.01	0.9353
102684_at	1421782_a_at	Smr2	submaxillary gland androgen regulated prote	-2.34	0.04	-1.07	0.608	-1.29	0.1314	1.01	0.9287	1.67	0.257
102685_at	1451953_at	Smr2	submaxillary gland androgen regulated prote	-2.46	0.11	1.05	0.7727	1.01	0.9582	1.03	0.915	1.65	0.5364
102686_at	1424059_at	Suv420h2	suppressor of variegation 4-20 homolog 2 (D	-1.11	0.75	-1.12	0.0823	-1.13	0.2682	-1.28	0.0162	1.3	0.476
102687_at	1436561_at	Suv39h2	suppressor of variegation 3-9 homolog 2 (Dr	-3.57	0.05	-1.07	0.8349	1.08	0.8061	-1.38	0.3293	2.12	0.2539
102688_f_at	1420344_x_at	Gzmd	granzyme D	1.01	0.97	1.05	0.9026	1.1	0.7911	-1.08	0.833	1.23	0.6299
102689_at	1450378_at	Tapbp	TAP binding protein	-1.12	0.78	1.03	0.8031	-1.46	0.0069	-1.03	0.8203	-1.02	0.906
102690_at	1419731_at	Cyp2b19	cytochrome P450, family 2, subfamily b, poly	-1.34	0.27	1.09	0.2794	1.13	0.2206	1.06	0.4744	1.53	0.2623
102691_at	1418865_at	Zfp385	zinc finger protein 385	1.12	0.6	1.06	0.5493	-1.09	0.3152	-1.07	0.2732	-1.09	0.5512
102692_s_at	1452547_s_at	H2-D1 /// H2-T1	histocompatibility 2, D region locus 1 /// histo	-1.28	0.71	1.13	0.6241	1.18	0.6282	1.23	0.4102	2.21	0.0042
102693_f_at	1419090_x_at	Klk26	kallikrein 26	-2.26	0.13	-1.01	0.9497	1.05	0.7329	1	0.9655	1.64	0.0766
102694_at	1449238_at	Psg16	pregnancy specific glycoprotein 16	-1.44	0.05	-1.1	0.7123	1.55	0.3765	-1.09	0.6848	1.15	0.6851

102695_at	1427608_a_at	Tcrg-C	T-cell receptor gamma, constant region	-1.11	0.29	-1.03	0.6478	-1.17	0.0384	-1.1	0.3201	1.7	0.0257
102697_at	1421013_at	Pitpnb	phosphatidylinositol transfer protein, beta	-1.1	0.85	1	0.9675	-1.08	0.4396	-1.01	0.9548	-1.11	0.4839
102698_at	1449888_at	Epas1	endothelial PAS domain protein 1	-1.23	0.43	-1.06	0.6449	-1.03	0.859	-1.08	0.6042	1.4	0.1926
102699_at	1419676_at	Mx2	myxovirus (influenza virus) resistance 2	-1.13	0.71	-1.01	0.9171	-1.09	0.3489	1.06	0.6648	1.02	0.9102
102701_at	1425645_s_at	Cyp2b10	cytochrome P450, family 2, subfamily b, poly	3.8	0.08	1.01	0.8928	1.03	0.9171	1	0.9912	2.71	0.007
102702_at	1435358_at	Cuedc1	CUE domain containing 1	-1.36	0.38	-1.36	0.0911	1.03	0.8807	-1.37	0.132	1.54	0.0099
102703_s_at	1425382_a_at	Aqp4	aquaporin 4	1.33	0.61	2.19	0.0081	-1.19	0.5635	1.41	0.2738	-1.37	0.2138
102707_f_at	1421564_at	Serpina3c	serine (or cysteine) proteinase inhibitor, clad	-1.56	0.11	-1.69	0.0001	1.06	0.3924	-1.76	0	-1.93	0.0053
102708_at	1419175_a_at	Btn1a1	butyrophilin, subfamily 1, member A1	1.08	0.7	1.22	0.094	1.29	0.0709	1.12	0.2616	1.63	0.2827
102709_at	1436241_s_at	Hira	histone cell cycle regulation defective homol	-1.43	0.35	1.47	0.1156	1.83	0.0875	1.54	0.168	1.38	0.627
102711_at	1419221_a_at	Rgs14	regulator of G-protein signaling 14	-1.2	0.33	1.01	0.8532	-1.01	0.8794	1.01	0.8876	1.21	0.0486
102712_at	1450826_a_at	Saa3	serum amyloid A 3	2.49	0.09	14.01	0.1063	1.43	0.43	14.82	0.0447	-2.7	0.4419
102713_at	1418864_at	Gata4	GATA binding protein 4	-1.36	0.02	-1.14	0.2818	-1.02	0.8457	-1.08	0.4517	1.08	0.8167
102714_at	1419625_at	Hspa1l	heat shock protein 1-like	-1.89	0.13	1.06	0.7502	1.13	0.5639	-1.17	0.4222	1.86	0.0459
102715_at	1418157_at	Nr2f1	nuclear receptor subfamily 2, group F, meml	5.67	0	1.07	0.8061	1.25	0.4647	-1.14	0.6874	2.76	0.147
102716_at	1425989_a_at	Eya3	eyes absent 3 homolog (Drosophila)	1.18	0.63	-1.03	0.8794	1.25	0.3932	-1.16	0.4678	1.04	0.7653
102718_at	1424727_at	Ccr5	chemokine (C-C motif) receptor 5	2.37	0.05	1.62	0.1861	1	0.999	1.87	0.3208	-2.2	0.1365
102719_f_at	1422260_x_at	Ccr5	chemokine (C-C motif) receptor 5	1.23	0.67	1.52	0.4921	-3.47	0.0029	1.56	0.5995	1.68	0.4678
102720_at	1418788_at	Tek	endothelial-specific receptor tyrosine kinase	1.4	0.19	-1.25	0.0696	1.19	0.2069	-1.16	0.4021	1.18	0.2595
102722_g_at	1427503_at	AI324046	expressed sequence AI324046	-3.32	0.13	-3.39	0.2052	-1.3	0.6834	-3.05	0.2259	1.48	0.0386
102723_at	1447998_at	Igh-4	Expressed sequence AI324046	-1.41	0.01	-1.17	0.317	1.15	0.4292	-1.29	0.1492	2.55	0.2333
102724_at	1427448_at	Rabep1	rabaptin, RAB GTPase binding effector prote	-1.19	0.58	-1.06	0.5665	1.01	0.9184	-1.1	0.3918	-1.27	0.473
102725_at	1448468_a_at	Kcnab1	potassium voltage-gated channel, shaker-re	1.24	0.72	1.77	0.0418	-1.12	0.3571	1.78	0.1145	1.34	0.1942
102726_at	1416783_at	---	---	-1.55	0.1	1.01	0.9853	1.23	0.5225	1.23	0.552	1.98	0.0888
102728_f_at	1450171_x_at	Gzme	granzyme E	1.05	0.86	-1.05	0.7387	1.35	0.3496	1.44	0.2355	1.4	0.1004
102729_f_at	1460232_s_at	Hsd3b2 /// Hsd3	hydroxysteroid dehydrogenase-2, delta<5>-:	-1.26	0.1	1.65	0.0204	1.42	0.0924	2.15	0.0003	-1.54	0.0091
102731_g_at	1421358_at	H2-M3	histocompatibility 2, M region locus 3	-1.42	0.34	1.11	0.5718	-1.03	0.864	1.36	0.1725	-1.31	0.4128
102732_at	1436042_at	Tln1	Talin 1	-1.17	0.43	1.05	0.7079	-1.05	0.7932	1.02	0.813	1	0.9864
102733_at	1421256_at	Gzmc	granzyme C	-1.04	0.95	-1.39	0.3671	-1.68	0.1781	-1.36	0.4093	1.4	0.4251
102734_at	1418854_at	Birc2	baculoviral IAP repeat-containing 2	1.45	0.18	1.25	0.0164	1.16	0.2309	1.45	0.0013	1.17	0.2829
102735_at	1421356_at	Tex9	testis expressed gene 9	-1.24	0.21	-1.22	0.2706	1.17	0.4393	-1.19	0.2843	1.89	0.0226
102737_at	1451924_a_at	Edn1	endothelin 1	1.32	0.39	1.08	0.5314	-1	0.9884	1.15	0.2882	1.22	0.1327
102739_s_at	1420492_s_at	Smr1 /// Smr3	submaxillary gland androgen regulated prote	1.09	0.71	1.14	0.6742	-1.01	0.9722	-1.15	0.3002	1.26	0.5041
102740_at	1418160_at	Mkrn3	makorin, ring finger protein, 3	-1.32	0.75	1.01	0.9741	-1.32	0.2804	1.01	0.96	1.95	0.0566
102742_g_at	1424719_a_at	Mapt	microtubule-associated protein tau	1.23	0.29	-1.07	0.6851	-1.03	0.7963	-1.23	0.2066	1.43	0.2714
102743_at	1424718_at	Mapt	microtubule-associated protein tau	-1.45	0.17	-1.15	0.4782	-1.14	0.4595	1.01	0.9421	-1.12	0.7853
102745_at	1450521_a_at	Tcrg	T-cell receptor gamma chain	2.67	0.03	1.39	0.2121	1.15	0.6021	1.61	0.0164	1.31	0.7
102746_at	1456100_at	LOC280621	similar to selenoprotein W	1.16	0.54	-1.01	0.9213	1.01	0.9364	1.01	0.9477	1.15	0.4484
102748_at	1418907_at	F5	Coagulation factor V	-1.33	0.29	1.16	0.0188	1.23	0	1.28	0.0002	1.31	0.0334
102749_at	1418709_at	Cox7a1	cytochrome c oxidase, subunit VIIa 1	2.92	0.02	1.19	0.4773	1.29	0.3429	1.62	0.0498	2.55	0.0067
102750_at	1416182_at	Apba3	amyloid beta (A4) precursor protein-binding,	1.08	0.71	-1.35	0.0002	-1.16	0.0986	-1.46	0	-1.49	0.197
102751_at	1417455_at	Tgfb3	transforming growth factor, beta 3	-1.8	0	-1.15	0.139	1.01	0.9289	-1.08	0.4014	1.67	0.1101
102752_at	1416329_at	Cyfip1	cytoplasmic FMR1 interacting protein 1	-1.05	0.67	1.23	0.0202	1.07	0.4324	1.17	0.0293	1	0.9612
102753_at	1416347_at	Men1	multiple endocrine neoplasia 1	1.22	0.68	1.35	0.1544	1.12	0.6315	-1.36	0.2456	-1.07	0.7773
102754_at	1419563_at	Birc6	baculoviral IAP repeat-containing 6	1.15	0.51	1.18	0.1841	-1.07	0.6478	1.1	0.3977	1.21	0.1585
102755_at	1418165_at	Itlna	intelectin a	1.07	0.82	-1.08	0.5756	-1.03	0.8151	-1.13	0.5215	1.18	0.5442
102758_at	1452466_a_at	Rbm6	RNA binding motif protein 6	1.07	0.89	-1.11	0.5716	-1.18	0.3736	-1.09	0.6102	-1.23	0.4593
102759_at	1418463_at	Pik3r2	phosphatidylinositol 3-kinase, regulatory sub	-1.12	0.78	-1.16	0.6223	1.16	0.5338	1.39	0.2617	-1.14	0.5217
102761_at	1452262_at	Grpel2	GrpE-like 2, mitochondrial	2.04	0.02	1.17	0.3356	1.35	0.0017	1.13	0.2703	1.83	0.0311
102762_r_at	1419014_at	Rhag	Rhesus blood group-associated A glycoprot	-1.43	0.09	-1.4	0.0013	-1.07	0.3683	-1.19	0.0374	1.26	0.1515
102763_at	1418595_at	MGI:1929709	plasma membrane associated protein, S3-1:	1.58	0.39	1.97	0.2036	3.44	0.2391	2.42	0.044	3.63	0.003
102764_at	1460226_at	Trap1a	tumor rejection antigen P1A	1.01	0.99	-1.26	0.226	-1.26	0.2007	-1.26	0.2349	1.38	0.2112
102765_at	1418720_at	Cops7b	COP9 (constitutive photomorphogenic) hom	-1.18	0.21	1.19	0.0152	1.16	0.0397	1.15	0.0108	1.23	0.0034

102766_at	1427940_s_at	Mycbp	c-myc binding protein	1.89	0.18	1.62	0.018	1.13	0.4612	1.36	0.0379	1.21	0.6427
102767_at	1455089_at	Gng12	RIKEN cDNA 4930597O21 gene	-1	1	1.03	0.4327	1.09	0.1074	1.16	0.0045	-1.08	0.3259
102771_at	1451833_a_at	Setdb1	SET domain, bifurcated 1	1.23	0.11	1.09	0.3359	1.14	0.199	1.23	0.0322	1.03	0.8364
102772_at	1423999_at	Abl1	v-abl Abelson murine leukemia oncogene 1	1.03	0.86	1.09	0.0722	1.09	0.1117	1.13	0.0329	-1.02	0.9149
102773_at	1427482_a_at	Car8	carbonic anhydrase 8	1.71	0.05	1.26	0.1121	1.42	0.0409	1.26	0.1689	1.51	0.0269
102774_at	1418093_a_at	Egf	epidermal growth factor	1.17	0.21	-1.14	0.4579	1.01	0.9645	-1.22	0.1089	1.05	0.7025
102776_at	1428592_s_at	Usp38	ubiquitin specific protease 38	-1.03	0.66	-1.06	0.4037	-1.03	0.7145	-1.05	0.5579	-1.57	0.0117
102777_at	1422226_at	---	---	-1.01	0.98	1.5	0.0584	1.25	0.086	1.19	0.2648	-1.17	0.6633
102778_at	1418830_at	Cd79a	CD79A antigen (immunoglobulin-associated	-1.07	0.75	1.04	0.7614	1.03	0.8288	-1.05	0.6512	1.27	0.4379
102780_at	1426875_s_at	Npn3	neoplastic progression 3	2.15	0.08	1.85	0.0187	1.18	0.1279	2.29	0.007	1.19	0.4259
102781_at	1454149_a_at	Ccnl2	cyclin L2	-1.03	0.89	1.03	0.652	1.29	0.0236	1.17	0.0487	1.07	0.4656
102782_at	1449346_s_at	Riok1	RIO kinase 1 (yeast)	4.16	0.02	1.26	0.5142	1.5	0.2969	1.81	0.0938	1.23	0.4133
102783_at	1452799_at	2310009E04Rik	RIKEN cDNA 2310009E04 gene	1.18	0.06	-1.44	0.0003	-1.09	0.1897	-1.44	0.0018	-1.52	0.0023
102784_at	1426974_at	4632413K17Rik	RIKEN cDNA 4632413K17 gene	1.25	0.62	-1.12	0.1526	-1.29	0.0281	-1.29	0.0084	1.01	0.9741
102785_at	1418477_at	Matn1	matrilin 1, cartilage matrix protein 1	-2.15	0.04	1.08	0.6837	1	0.99	1.01	0.9557	1.57	0.3188
102786_at	1433486_at	Clcn3	chloride channel 3	-1.07	0.73	1.02	0.8578	-1.06	0.4859	-1.02	0.8091	-1.5	0.013
102787_at	1433485_x_at	Gpr56	G protein-coupled receptor 56	1.05	0.91	-1.24	0.1401	-1.23	0.198	1.39	0.2627	-1.11	0.7334
102788_s_at	1424797_a_at	Pitx2	paired-like homeodomain transcription factor	-1.45	0.14	1.07	0.5964	-1.21	0.1028	-1.01	0.9686	1.48	0.1738
102789_at	1450333_a_at	Gata2	GATA binding protein 2	2.46	0.03	-1.25	0.0177	-1.05	0.5454	-1.31	0.0084	1.18	0.074
102790_at	1424283_at	Jtb	jumping translocation breakpoint	1.1	0.41	-1.08	0.3051	1.02	0.788	-1.13	0.1431	-1.59	0.0108
102791_at	1422962_a_at	Psmb8	proteosome (prosome, macropain) subunit, I	1.2	0.09	1.71	0.323	-1.42	0.0117	2	0.1801	-1.59	0.198
102792_at	1425753_a_at	Ung	uracil-DNA glycosylase	-1.1	0.73	-1.22	0.0888	1.11	0.4376	-1.08	0.4807	1.08	0.6243
102793_at	1427211_at	Krtap8-1	keratin associated protein 8-1	-2.79	0.21	-1.45	0.0881	-1.22	0.4317	-1.09	0.5921	2.39	0.2585
102794_at	1448710_at	Cxcr4	chemokine (C-X-C motif) receptor 4	-1.17	0.81	1.5	0.2279	1.14	0.7287	2.13	0.0606	1.57	0.42
102795_at	1426557_at	Mesp1	mesoderm posterior 1	-1.88	0.32	-1.2	0.1707	1.17	0.3423	-1.15	0.2745	-1.05	0.9021
102796_at	1423522_at	Npm3	nucleoplasmin 3	1.52	0.63	-1.23	0.4526	-2.14	0.0072	-2.29	0.0082	1.64	0.0169
102797_at	1448390_a_at	Dhrs3	dehydrogenase/reductase (SDR family) mer	1.12	0.48	1.11	0.0847	-1.02	0.7257	-1.1	0.1192	-1.06	0.7719
102798_at	1416077_at	Adm	adrenomedullin	-1.31	0.31	-1.11	0.7273	-1.26	0.3542	1.21	0.5425	1.13	0.5725
102799_at	1418037_at	C4bp	complement component 4 binding protein	-1.02	0.92	1	0.9656	-1.14	0.1623	-1.09	0.4293	-1.91	0.0007
102800_at	1416693_at	Foxc2	forkhead box C2	-1.68	0.15	1.49	0.1246	1.92	0.0681	1.2	0.5031	-1.05	0.8875
102802_at	1417932_at	Il18	interleukin 18	1.68	0.12	1.09	0.6147	-1.15	0.3676	1.03	0.8152	1.08	0.6146
102804_at	1460681_at	Ceacam2	CEA-related cell adhesion molecule 2	-2.4	0	-1.09	0.1711	1.04	0.6504	-1.12	0.0763	-1.23	0.0338
102806_g_at	1425675_s_at	Ceacam1	CEA-related cell adhesion molecule 1	1.27	0.45	-1.05	0.5938	1.03	0.8622	-1.19	0.1509	-1.97	0.0149
102807_at	1434138_at	Prune	prune homolog (Drosophila)	1.01	0.93	1.03	0.579	-1.04	0.4391	-1.07	0.2233	1.12	0.2203
102808_at	1418738_at	Scn1b	sodium channel, voltage-gated, type I, beta	2.82	0.05	1.77	0.0012	1.55	0.0441	1.82	0.0016	1.91	0.123
102809_s_at	1425396_a_at	Lck	lymphocyte protein tyrosine kinase	1.01	0.97	1.41	0.0585	1.12	0.41	1.28	0.2061	2.08	0.0129
102811_at	1417730_at	Ext1	exostoses (multiple) 1	-1.13	0.63	-1.09	0.2746	-1.14	0.026	-1.19	0.0203	-1.07	0.5602
102813_f_at	1451973_at	Ube1dc1	ubiquitin-activating enzyme E1-domain conta	1.23	0.11	-1.05	0.4295	1.05	0.667	-1.08	0.1677	1.02	0.884
102815_at	1418468_at	---	---	1.03	0.87	-1	0.991	1.09	0.3883	1.15	0.0939	-1.63	0.0106
102816_at	1421921_at	Serpina3m	serine (or cysteine) proteinase inhibitor, clad	1.57	0.13	-1.11	0.0974	1.06	0.3819	-1.1	0.0589	1.06	0.5233
102817_at	1449354_at	U2af1-rs1	U2 small nuclear ribonucleoprotein auxiliary	1.77	0.16	1.03	0.7313	1.11	0.3726	1.1	0.3036	1.06	0.7685
102818_at	1422618_x_at	Xmr	Xlr-related, meiosis regulated	2	0.22	1.29	0.3589	1.86	0.0085	1.23	0.4267	2.45	0.0799
102819_at	1418046_at	Nap1l2	nucleosome assembly protein 1-like 2	-2.14	0.15	-1.28	0.2935	1	0.9903	-1.18	0.4915	-1.75	0.0493
102820_at	1449479_at	Cyp2b13	cytochrome P450, family 2, subfamily b, poly	42.04	0.15	2.87	0.0003	1.04	0.8646	2	0.0308	23.52	0
102822_at	1418679_at	Gzmf	granzyme F	-2.22	0.09	-1.53	0.2297	-1.11	0.7485	-1.12	0.8032	-1.58	0.2779
102824_g_at	1424631_a_at	Ighg	Immunoglobulin heavy chain (gamma polype	1.25	0.42	1.54	0.1518	-1.08	0.8185	1.74	0.0425	1.56	0.1355
102826_at	1423556_at	Akr1b7	aldo-keto reductase family 1, member B7	1.92	0.11	-1.06	0.5768	1.89	0.0582	1.1	0.2955	2.36	0.0048
102827_at	1416816_at	Nek7	NIMA (never in mitosis gene a)-related expre	-1.21	0.04	1.22	0.0065	1.25	0.0152	1.15	0.0088	1.16	0.1196
102828_at	1426850_a_at	Map2k6	mitogen activated protein kinase kinase 6	3.95	0.01	1.22	0.1768	1.51	0.0123	1.67	0.0047	1.27	0.4442
102831_s_at	1449858_at	---	---	-1.76	0.26	1.08	0.7666	-1.39	0.2351	-1.53	0.1723	1.65	0.1704
102832_at	1427790_at	Adam1a	a disintegrin and metalloproteinase domain 1	-1.67	0.14	-1.22	0.3489	-1.18	0.3724	-1.11	0.5858	-1.05	0.8825
102833_at	1434116_at	Cbx2	chromobox homolog 2 (Drosophila Pc class)	1.42	0.54	2.52	0.0012	1.75	0.0546	2.25	0.0009	1.88	0.006
102836_at	1416195_at	MGI:1194899	putative phosphatase	1.12	0.53	1.02	0.7565	1.01	0.921	1.03	0.5925	1.49	0.0255

102838_at	1419480_at	Sell	selectin, lymphocyte	1.1	0.64	1.17	0.104	1.34	0.0344	1.72	0.1655	1.17	0.7585
102839_at	1453181_x_at	Plscr1	phospholipid scramblase 1	-1.32	0.15	1.16	0.5746	1.07	0.5401	1.39	0.0886	1.17	0.3153
102840_at	1455105_at	Ptpn12	protein tyrosine phosphatase, non-receptor t	-1.76	0.01	1.26	0.3139	-1.14	0.6883	1.18	0.5087	1.29	0.402
102841_at	1435782_at	LOC216818	similar to ubiquitin A-52 residue ribosomal pr	1.05	0.8	-1.12	0.2582	-1.1	0.2445	-1.05	0.5319	-1.03	0.8767
102843_s_at	1451632_a_at	Igh-1a	immunoglobulin heavy chain 1a (serum IgG2	-1.63	0.48	-1.31	0.2789	-1.46	0.1637	-1.03	0.8618	1.25	0.3951
102846_at	1449489_at	Tssk2	testis-specific serine kinase 2	1.31	0.05	-1.28	0.4942	1.23	0.5552	1.04	0.9132	1.45	0.5802
102847_s_at	1422230_s_at	Cyp2a4 /// Cyp2	cytochrome P450, family 2, subfamily a, poly	5.52	0	1.03	0.2846	1.04	0.0205	1.12	0	4.3	0.0003
102848_f_at	1429244_at	2610524H06Rik	RIKEN cDNA 2610524H06 gene	1.87	0.15	-1.21	0.5208	1.03	0.9076	1.18	0.4623	1.05	0.9023
102849_at	1418142_at	Kcnj8	potassium inwardly-rectifying channel, subfa	3.21	0.04	1.15	0.6195	1.12	0.7963	1.5	0.2702	1.41	0.3025
102850_at	1448298_at	Tnk2	tyrosine kinase, non-receptor, 2	-1.17	0.6	-1.64	0.0016	-1.2	0.1038	-1.54	0.001	-1.52	0.3131
102851_s_at	1460188_at	Hcph	hemopoietic cell phosphatase	1.31	0.13	1.52	0.0313	-1.06	0.7011	1.69	0.0678	1.02	0.9632
102852_at	1418815_at	Cdh2	cadherin 2	-1.17	0.53	-1.12	0.3099	-1.17	0.1045	-1.22	0.0699	-1.52	0.0926
102853_at	1450950_at	Cspg6	chondroitin sulfate proteoglycan 6	1.92	0.35	1.27	0.0252	1.22	0.1584	1.25	0.0544	1.29	0.6444
102856_at	1451689_a_at	Sox10	SRY-box containing gene 10	-1.76	0.02	-1.07	0.5885	1.08	0.4589	-1.09	0.3712	1.34	0.0121
102857_at	1451662_x_at	Akap4	A kinase (PRKA) anchor protein 4	-2.54	0.32	1.15	0.6969	1.29	0.3945	-1.12	0.7348	-1.27	0.2704
102858_at	1421137_a_at	Pkib	protein kinase inhibitor beta, cAMP depende	-1.14	0.68	-1.01	0.9248	-1.14	0.2045	-1.06	0.7704	-1.08	0.7902
102859_at	1433509_s_at	D6Ertd253e	DNA segment, Chr 6, ERATO Doi 253, expr	-1.11	0.68	-1.2	0.0821	-1.32	0.0488	-1.4	0.0165	1.26	0.0988
102860_at	1424923_at	Serpina3g	serine (or cysteine) proteinase inhibitor, clad	1.09	0.34	1.42	0.348	-1.28	0.1471	1.74	0.2161	1.38	0.0301
102861_at	1417809_at	Slc22a18	solute carrier family 22 (organic cation trans	-1.04	0.68	1.05	0.552	-1.33	0.0106	-1.02	0.8303	-1.05	0.6859
102862_at	1436344_at	5830404H04Rik	RIKEN cDNA 5830404H04 gene	1.09	0.61	-1.03	0.5032	-1.09	0.073	-1.11	0.0497	-1.4	0.0016
102863_at	1418801_at	Zkscan1	zinc finger with KRAB and SCAN domains 1	1.07	0.64	1.26	0.1163	1.15	0.2075	1.15	0.1042	1.09	0.6452
102864_at	1449499_at	Hoxa7	homeo box A7	-1.49	0.17	1.16	0.1487	1.14	0.1701	1.11	0.1684	1.44	0.0179
102865_at	1421047_at	Smad5	MAD homolog 5 (Drosophila)	1.16	0.62	-1.17	0.239	-1.13	0.3949	-1.23	0.1973	-1.71	0.2352
102866_at	1460683_at	---	---	-1.61	0.03	1.05	0.5941	1.07	0.1574	-1.01	0.8402	1.16	0.3141
102868_g_at	1426337_a_at	Tead4	TEA domain family member 4	-1.1	0.78	1.21	0.1682	1.14	0.1302	1.01	0.9497	1.77	0.2251
102869_at	1417521_at	Efna2	ephrin A2	-1.14	0.83	1.35	0.1633	1.19	0.4138	-1.25	0.4366	1.63	0.0277
102871_at	1418051_at	Ephb6	Eph receptor B6	-1.16	0.78	-1.6	0.0194	-1.62	0.0052	-1.43	0.0309	1.28	0.5917
102872_f_at	1424706_at	Zfp51	zinc finger protein 51	1.16	0.41	1.14	0.3498	1.01	0.9514	1.23	0.2943	1.09	0.6507
102875_at	1434563_at	Rps6kc1	ribosomal protein S6 kinase polypeptide 1	1.86	0.22	1.2	0.2017	-1.09	0.6236	1.25	0.0717	-1.47	0.4461
102876_at	1422867_at	Gzmg	granzyme G	-1.81	0.02	1.05	0.5981	1.09	0.4215	-1.01	0.9332	1.07	0.7016
102877_at	1419060_at	Gzmb	granzyme B	1.14	0.83	2.03	0.0379	1.33	0.3254	1.75	0.1473	2.05	0.0355
102878_at	1416602_a_at	Rad52	RAD52 homolog (S. cerevisiae)	1.04	0.87	1.03	0.6877	1.04	0.5802	1.23	0.0137	1.33	0.0186
102879_s_at	1417876_at	Fcgr1	Fc receptor, IgG, high affinity I	1.35	0.24	1.24	0.4563	-1.08	0.3422	1.83	0.2711	-1.03	0.8655
102880_at	1427341_at	E130103I17Rik	RIKEN cDNA E130103I17 gene	1.33	0.05	-1.09	0.4506	-1.11	0.2366	-1.15	0.1514	1.1	0.2341
102882_at	1425231_a_at	Zfp46	zinc finger protein 46	1.86	0.18	1.16	0.7122	1.63	0.1554	1.86	0.1538	1.26	0.5265
102883_at	1426891_at	Rpap1	RNA polymerase II associated protein 1	2.2	0.08	-1.04	0.6403	1.08	0.3516	1.1	0.1817	1.12	0.3097
102884_at	1418110_a_at	Inpp5d	inositol polyphosphate-5-phosphatase D	-1.43	0.15	1.36	0.2851	-1.12	0.4783	1.96	0.3358	1.28	0.1979
102885_at	1452007_at	Sybl1	synaptobrevin like 1	-1.04	0.83	1.03	0.8456	-1.06	0.6182	1.02	0.8564	-2.58	0.001
102886_at	1421088_at	Gpc4	glypican 4	-1.62	0.05	-1.1	0.1771	-1.17	0.0779	-1.17	0.0297	-1	0.9954
102887_at	1449033_at	Tnfrsf11b	tumor necrosis factor receptor superfamily, r	-1.19	0.28	1.07	0.6375	1.11	0.4216	1.03	0.8665	1.2	0.4082
102888_s_at	1417627_a_at	Limk1	LIM-domain containing, protein kinase	-1.13	0.81	1.19	0.3752	-1.22	0.3634	1.28	0.2894	1.59	0.1563
102890_at	1416825_at	Snta1	syntrophin, acidic 1	-1.14	0.18	-1.03	0.5592	-1.04	0.4429	-1.03	0.3524	-1.18	0.2467
102891_at	1450163_a_at	Wrn	Werner syndrome homolog (human)	-1.39	0.08	1.03	0.8053	1.02	0.8664	1.01	0.9481	-1.61	0.3728
102892_at	1416956_at	Kcnab2	potassium voltage-gated channel, shaker-re	1.01	0.98	1.01	0.9706	-1.02	0.9079	1	0.9811	1.25	0.4034
102894_g_at	1419716_a_at	Pou2f1	POU domain, class 2, transcription factor 1	-1.04	0.77	1.29	0.1539	-1.38	0.1818	-1.04	0.892	1.31	0.0572
102895_at	1453576_at	Nipbl	Nipped-B homolog (Drosophila)	1.3	0.07	1.1	0.1201	1.09	0.3514	1.13	0.1106	-1.22	0.5835
102896_at	1417790_at	Dok1	docking protein 1	-1.62	0.05	1.03	0.8098	-1.03	0.7944	-1.04	0.7585	1.62	0.0055
102898_at	1425379_at	Hgf	hepatocyte growth factor	-1.25	0.82	1.18	0.2444	-1.22	0.3953	-1.03	0.8356	-1.74	0.3847
102899_at	1420902_at	St6galnac3	ST6 (alpha-N-acetyl-neuraminyl-2,3-beta-ga	-1.51	0.36	1.03	0.7782	1.31	0.0911	-1.06	0.5752	1.65	0.0303
102900_at	1427523_at	Six3	sine oculis-related homeobox 3 homolog (Dr	-3.67	0.25	1.25	0.662	-1.31	0.5883	-1.25	0.6517	2.52	0.0882
102901_at	1426638_at	Six3	sine oculis-related homeobox 3 homolog (Dr	-1.25	0.46	1.48	0.0444	1.64	0.0247	1.29	0.2478	1.16	0.2257
102902_at	1425041_at	Lhx3	LIM homeobox protein 3	-2.61	0.29	1.21	0.6544	1.74	0.1628	1.17	0.573	-1.15	0.5798
102904_at	1422892_s_at	---	---	2	0.07	1.22	0.6486	-1.65	0.0439	-1	0.9896	-1.14	0.8064

102905_at	1449591_at	Casp11	caspase 11, apoptosis-related cysteine prote	1.26	0.18	-1.03	0.8479	-1.04	0.801	1.28	0.2606	1.09	0.6286
102906_at	1449009_at	Tgtp	T-cell specific GTPase	-1.01	0.99	3.21	0.2298	-3.57	0.0675	3.61	0.1448	2.98	0.3568
102907_at	1454862_at	Phldb2	pleckstrin homology-like domain, family B, m	1.07	0.7	1.16	0.091	1.13	0.4914	1.16	0.0884	1.05	0.7943
102908_at	1417337_at	Epb4.2	erythrocyte protein band 4.2	1.37	0.55	-1.14	0.6654	-1.5	0.1985	1.24	0.4833	-1.67	0.2483
102910_at	1419759_at	Abcb1a	ATP-binding cassette, sub-family B (MDR/TA	7.22	0.02	1.51	0.003	1.24	0.1743	1.64	0.0017	1.98	0.0749
102911_at	1419076_a_at	Brca2	breast cancer 2	2.23	0.13	1.27	0.0549	1.27	0.0055	1.22	0.1411	-2.23	0.0477
102913_at	1437913_at	Bcl2a1a /// Bcl2	B-cell leukemia/lymphoma 2 related protein	-4.09	0.36	-1.35	0.4572	-1.5	0.3631	-1.19	0.7127	1.16	0.784
102915_at	1448931_at	F2rl1	coagulation factor II (thrombin) receptor-like	-1.83	0.45	-1.17	0.7073	-1.04	0.9167	-1.06	0.8809	1.43	0.5687
102916_s_at	1450798_at	Tnxb	tenascin XB	2.08	0.1	-1.09	0.7844	-1.45	0.2469	-2.2	0.0186	1.75	0.2298
102917_at	1421210_at	C2ta	class II transactivator	-1.92	0.31	1.16	0.5767	-1.1	0.3154	1.03	0.8563	-1.06	0.817
102918_at	1449199_at	Muc1	mucin 1, transmembrane	1.49	0.25	-1.22	0.5501	-1.09	0.769	-1.21	0.5682	1.32	0.1766
102919_at	1418616_at	Mafk	v-maf musculoaponeurotic fibrosarcoma onc	1.04	0.79	-1.01	0.854	-1.03	0.7018	1.12	0.1844	1.16	0.4794
102921_s_at	1460251_at	Fas	Fas (TNF receptor superfamily member)	1.98	0.23	1.22	0.5906	-1.25	0.2757	1.19	0.5734	-3.04	0.0202
102922_at	1435066_at	Pitpnc1	phosphatidylinositol transfer protein, cytopla	1.01	0.95	1.02	0.7917	1.17	0.0606	1.16	0.1547	1.41	0.062
102923_at	1425892_a_at	Pnoc	prepronociceptin	1.04	0.88	1.11	0.2413	1.19	0.1082	1.08	0.4213	1.04	0.8224
102924_at	1425822_a_at	Dtx1	deltex 1 homolog (Drosophila)	-1.83	0.02	1.02	0.8389	-1	0.9891	1.01	0.9236	1.54	0.0103
102925_at	1454737_at	Dusp9	dual specificity phosphatase 9	1.06	0.94	1.14	0.4236	1.03	0.8996	1.24	0.3642	-1.03	0.9004
102926_at	1418880_at	Gfra3	glial cell line derived neurotrophic factor fam	1.06	0.76	1.04	0.7654	1.09	0.2283	-1.04	0.695	1.3	0.058
102927_s_at	1425969_a_at	Hdh	Huntington disease gene homolog	1.55	0.58	-1.2	0.297	-1.23	0.3066	-1.23	0.2122	-1.43	0.0903
102930_at	1422523_at	Si	silver	1.45	0.19	1.39	0.0451	1.5	0.0037	1.46	0.0155	1.98	0.1097
102931_at	1448386_a_at	Wap	whey acidic protein	1.07	0.78	1.17	0.2846	1.06	0.7207	1.09	0.5509	1.45	0.3238
102932_at	1449706_s_at	Nr5a2	nuclear receptor subfamily 5, group A, meml	1.88	0.08	-1.01	0.9642	-1.24	0.162	-1.11	0.4135	1.11	0.7016
102933_at	1420995_at	Plxna3	plexin A3	-1.15	0.75	-1.52	0.0031	-1.42	0.0445	-1.4	0.0042	-1.24	0.5644
102935_at	1422252_a_at	Cdc25c	cell division cycle 25 homolog C (S. cerevisi	-2.71	0.15	-1.45	0.1677	-1.2	0.4967	-1.31	0.4159	1.37	0.4136
102936_at	1460329_at	B4galt6	UDP-Gal:betaGlcNAc beta 1,4-galactosyltra	1.67	0.28	-1.13	0.4719	1.05	0.8374	-1.06	0.7783	-1	0.9855
102938_at	1449492_a_at	Lect2	leukocyte cell-derived chemotaxin 2	1.7	0.1	-1.42	0.0004	-1.14	0.1363	-1.28	0.0008	-1.26	0.0277
102939_s_at	1419768_at	Cd22	CD22 antigen	1.03	0.9	1.59	0.2845	1	0.989	1.8	0.0409	-1.36	0.5238
102940_at	1419135_at	Ltb	lymphotoxin B	-1.04	0.9	1.81	0.0818	-1.46	0.0551	2.02	0.1858	1.98	0.0864
102941_at	1448009_at	Ugcgl1	UDP-glucose ceramide glucosyltransferase-	1.13	0.8	-1.09	0.3321	1.05	0.6694	1.02	0.8276	-1.56	0.0061
102942_at	1428794_at	2810012G08Rik	RIKEN cDNA 2810012G08 gene	3.9	0.07	1.32	0.2654	1.42	0.0089	1.84	0.0998	1.53	0.1585
102943_at	1428612_at	Apg7l	autophagy 7-like (S. cerevisiae)	1.08	0.72	-1.16	0.0502	-1.34	0.0006	-1.19	0.0156	-1.09	0.4599
102944_at	1455060_at	MGI:1351465	Ras-GTPase-activating protein SH3-domain	-1.09	0.61	-1.06	0.3783	1.07	0.2849	1.03	0.5778	-1.52	0.1527
102947_at	1419117_at	Slc22a2	solute carrier family 22 (organic cation trans	1.42	0.47	-1.21	0.2305	-1.04	0.778	-1.21	0.1665	1.45	0.1653
102950_at	1422400_a_at	Hemt1	hematopoietic cell transcript 1	2.05	0.02	1.33	0.258	1.24	0.396	-1.16	0.4698	1.07	0.876
102952_g_at	1427505_a_at	Cradd	CASP2 and RIPK1 domain containing adapt	1.32	0.2	1.13	0.239	-1.03	0.6796	1.04	0.6275	-1.03	0.8689
102954_at	1452511_at	Sox5	SRY-box containing gene 5	-1.79	0.45	-1.29	0.4284	-1.1	0.8143	-1.71	0.1141	1.99	0.23
102955_at	1418932_at	Nfil3	nuclear factor, interleukin 3, regulated	-1.83	0.18	-1.39	0.007	-1.76	0.001	-1.56	0.0068	-1.39	0.1097
102956_at	1449559_at	Msx2	homeo box, msh-like 2	-1.07	0.78	1.13	0.3272	1.3	0.0538	1.32	0.0184	1.14	0.4397
102957_at	1418641_at	Lcp2	lymphocyte cytosolic protein 2	1.59	0.19	1.4	0.3851	-1.42	0.0781	1.53	0.3493	-1.14	0.5383
102958_at	1418494_at	Ebf2	early B-cell factor 2	-1.66	0.17	-1.33	0.3934	1.16	0.614	-1.2	0.531	1.91	0.0457
102960_at	1448948_at	Rga	recombination activating gene 1 gene activa	1.3	0.03	1.05	0.3754	1.05	0.4864	1.23	0.0015	1.5	0.0029
102961_at	1449242_s_at	Hrg	histidine-rich glycoprotein	1.16	0.41	1.16	0.0228	-1.08	0.0518	1.32	0	1.56	0.0009
102962_at	1449898_at	1-Sep	septin 1	-2.15	0.04	-1.07	0.6672	1.09	0.4855	1.07	0.7311	-1.01	0.9808
102963_at	1417878_at	E2f1	E2F transcription factor 1	1.75	0.27	1.78	0.1498	-1.01	0.9633	1.36	0.3284	1.3	0.491
102964_at	1424237_at	Zfp639	zinc finger protein 639	1.13	0.63	1.16	0.1163	1.37	0.0208	1.14	0.1705	1.21	0.2379
102965_at	1427091_at	AI481105	expressed sequence AI481105	-1.4	0.26	-1.07	0.6139	-1.17	0.1335	1.15	0.3991	-1.61	0.0395
102966_at	1434311_at	Cnot6l	CCR4-NOT transcription complex, subunit 6	1.26	0.37	1.16	0.1498	1.35	0.0395	1.2	0.0415	1.04	0.8388
102967_at	1423328_at	Gdap1	ganglioside-induced differentiation-associate	2.39	0.15	1.37	0.0779	-1.02	0.8772	1.21	0.2893	1.77	0.0799
102968_at	1418216_at	Ggtla1	gamma-glutamyltransferase-like activity 1	2.29	0.14	1.01	0.9669	-1.02	0.8545	1.13	0.5176	1.45	0.4702
102969_at	1430417_s_at	0610025P10Rik	RIKEN cDNA 0610025P10 gene	-1.06	0.82	1.03	0.7623	1.22	0.0236	1.13	0.2349	1.8	0.0025
102970_at	1425271_at	Psmc3ip	proteasome (prosome, macropain) 26S subu	-1.51	0.35	1.13	0.4371	1.2	0.2286	1.11	0.2695	1.3	0.2601
102971_at	1422105_at	Cd3e	CD3 antigen, epsilon polypeptide	-1.54	0.02	1.74	0.1354	1.26	0.0164	1.26	0.1767	1.69	0.021
102972_s_at	1427307_a_at	Dab1	disabled homolog 1 (Drosophila)	-2.99	0.08	-1.37	0.1039	1.31	0.1123	1	0.9943	1.72	0.1017

102974_at	1449498_at	Marco	macrophage receptor with collagenous struc	3.86	0.05	1.44	0.4876	-1.02	0.9546	3.09	0.1438	1.18	0.2125
102975_at	1425335_at	Cd8a	CD8 antigen, alpha chain	-1.45	0.44	1.28	0.3549	1.36	0.4379	1.14	0.582	1.77	0.0775
102976_at	1424629_at	Brca1	breast cancer 1	2.02	0.03	1.32	0.1029	1.19	0.3078	1.67	0.0069	1.6	0.1709
102977_at	1420516_at	a	nonagouti	-1.23	0.4	-1.2	0.1935	1.12	0.3423	-1.16	0.2449	1.06	0.8102
102978_at	1448010_at	A430104N18Ril	RIKEN cDNA A430104N18 gene	-1.13	0.39	1.45	0.0179	1.13	0.5045	1.51	0.0251	1.08	0.315
102979_at	1436167_at	LOC435684	similar to Shb-like adapter protein, Shf - hum	4.25	0	1.13	0.4505	1.28	0.2448	1.2	0.2806	1.88	0.1881
102980_at	1454941_at	AW536594	expressed sequence AW536594	-1.58	0.02	-1.13	0.1431	-1.02	0.8202	-1.22	0.0016	1.06	0.5454
102982_at	1454872_at	B230308N11Ril	RIKEN cDNA B230308N11 gene	2.74	0.04	1.05	0.4183	1.08	0.5402	-1.02	0.7626	-1.22	0.1329
102984_g_at	1448208_at	Smad1	MAD homolog 1 (Drosophila)	-1.58	0.04	-1.09	0.2519	1.05	0.6	-1.13	0.0611	-1.07	0.6119
102985_at	1454042_a_at	Srpk1	serine/arginine-rich protein specific kinase 1	-1.47	0.23	1.04	0.7496	1.12	0.4137	1.14	0.3523	1.12	0.3137
102986_at	1418420_at	Myod1	myogenic differentiation 1	-1.51	0.03	-1.2	0.0288	-1.19	0.0425	-1.11	0.1912	1.32	0.2157
102988_at	1460394_a_at	Inppl1	inositol polyphosphate phosphatase-like 1	-1.02	0.77	-1.06	0.4902	-1.19	0.0957	-1.22	0.0465	1.54	0.2127
102989_at	1450645_at	---	---	1.06	0.85	-1.98	0.3941	-2.11	0.3638	-2.09	0.3669	-1.29	0.2411
102990_at	1427884_at	Col3a1	procollagen, type III, alpha 1	2.24	0.08	-1.37	0.1856	-1.53	0.05	-1.62	0.0255	1.19	0.7455
102991_s_at	1454987_a_at	H2-Ke6	H2-K region expressed gene 6	1.88	0	1.21	0.0261	1.13	0.0811	1.15	0.1106	-1.07	0.6169
102993_at	1418483_a_at	Ggta1	glycoprotein galactosyltransferase alpha 1, 3	-1.1	0.69	1.21	0.077	1.12	0.1763	1.19	0.1526	1.31	0.0299
102994_at	1448713_at	Stat4	signal transducer and activator of transcripti	-2.74	0.02	-1.26	0.4399	-1	0.9987	-1.09	0.8134	1.86	0.1799
102995_s_at	1417898_a_at	Gzma	granzyme A	-1.22	0.44	1.65	0.088	1.25	0.3058	1.65	0.1682	1.39	0.3195
102996_at	1460643_at	Ell	elongation factor RNA polymerase II	1.1	0.46	1.16	0.0019	1.09	0.1604	1.17	0.0211	1.26	0.0808
102998_at	1450715_at	Cyp1a2	cytochrome P450, family 1, subfamily a, poly	-1.26	0.18	1.24	0.0038	-1.16	0.0968	1.23	0.0116	1.78	0.0273
103001_at	1451803_a_at	Vegfb	vascular endothelial growth factor B	1.03	0.72	1.29	0.0297	1.24	0.0201	1.21	0.0213	1.85	0.0044
103002_at	1418014_a_at	B4galt1	UDP-Gal:betaGlcNAc beta 1,4- galactosyltra	-1.6	0.09	-1.25	0.0072	-1.21	0.0133	-1.29	0.0039	-1.25	0.0976
103005_s_at	1452483_a_at	Cd44	CD44 antigen	-1.25	0.05	1.53	0.3436	-1.33	0.1736	1.9	0.2521	1.87	0.3252
103006_at	1425927_a_at	Atf5	activating transcription factor 5	1.13	0.75	-1.25	0.0221	-1.2	0.2564	-1.63	0.0046	-1.68	0.062
103007_at	1416895_at	Efna1	ephrin A1	1.71	0.27	1.39	0.0654	-1.07	0.7173	1.46	0.0135	1.31	0.5947
103009_at	1450050_at	Hira	histone cell cycle regulation defective homol	3.48	0.02	1.19	0.1215	1.31	0.0498	1.17	0.3283	-1.54	0.0826
103011_at	1449343_s_at	Sin3a	transcriptional regulator, SIN3A (yeast)	-1.19	0.08	1.03	0.6248	-1.05	0.499	-1.02	0.722	1.43	0.0074
103012_at	1419426_s_at	Ccl21b /// Ccl21	chemokine (C-C motif) ligand 21b (serine) //.	1.99	0.33	1.08	0.722	-1.07	0.6766	1.04	0.7703	1.18	0.5897
103013_at	1460228_at	Usf2	upstream transcription factor 2	1.15	0.35	-1.3	0.0233	-1	0.9746	-1.19	0.0791	-1.06	0.6573
103015_at	1421818_at	Bcl6	B-cell leukemia/lymphoma 6	2.17	0.1	3.27	0	1.64	0.0565	3.84	0	-1.05	0.8474
103016_s_at	1449164_at	Cd68	CD68 antigen	1.23	0.49	-1.04	0.8203	-1.13	0.0532	1.31	0.3983	-1.09	0.6055
103017_at	1449670_x_at	Tm7sf1	transmembrane 7 superfamily member 1	2.1	0.05	1.4	0.1451	-1.24	0.4293	1.54	0.1466	1.53	0.2988
103018_at	1424274_at	MGI:1929095	vesicle docking protein	1.34	0.16	-1.28	0.0025	-1.09	0.1644	-1.16	0.0677	-1.81	0.0021
103020_s_at	1424850_at	Map3k1	mitogen activated protein kinase kinase kina	1.31	0.61	1.13	0.5964	-1.13	0.4681	1.47	0.0346	1.25	0.2609
103023_at	1417043_at	Lcat	lecithin cholesterol acyltransferase	-1.09	0.63	-1.07	0.3612	1.05	0.6948	-1.11	0.1067	1.14	0.3452
103024_at	1416871_at	Adam8	a disintegrin and metalloprotease domain 8	1.01	0.97	1.76	0.185	1.2	0.0984	2.64	0.3036	1.35	0.181
103025_at	1416380_at	Mov10	Moloney leukemia virus 10	-1.39	0.13	1.07	0.5345	-1.02	0.8902	1.31	0.0526	1.11	0.4991
103027_at	1433867_at	1810030O07Ril	RIKEN cDNA 1810030O07 gene	1.41	0.02	1.21	0.0381	1.15	0.3204	1.24	0.0073	1.07	0.757
103028_at	1417171_at	Itk	IL2-inducible T-cell kinase	-1.96	0.14	1.11	0.4405	1.05	0.7564	1.02	0.8766	1.43	0.2038
103029_at	1418840_at	Pdcd4	programmed cell death 4	-1.21	0.28	-1.04	0.5477	-1.03	0.8598	-1.12	0.2382	1.11	0.5429
103031_g_at	1460365_a_at	Dnm1	dynamin 1	-1.2	0.19	-1.09	0.2106	-1.03	0.6527	-1.13	0.07	1.3	0.1265
103032_at	1421733_a_at	Tpst1	protein-tyrosine sulfotransferase 1	1.02	0.87	-1.08	0.1194	1.02	0.7324	-1.03	0.5109	1.23	0.1798
103033_at	1418021_at	C4	complement component 4 (within H-2S)	-1.27	0.12	1.19	0.3386	1.09	0.6174	-1.54	0.1398	-2.64	0.0006
103035_at	1416016_at	---	---	1.25	0.08	1.48	0.289	-1.37	0.0825	1.57	0.2298	-1.44	0.1742
103036_at	1417437_at	G22p1	thyroid autoantigen	1.8	0.36	1.29	0.0571	1.26	0.2096	1.39	0.0225	-1.77	0.0165
103037_at	1449093_at	Ctf1	cardiotrophin 1	-1.28	0.46	1.03	0.7649	-1.05	0.5809	1.15	0.1778	1.25	0.0747
103038_at	1421061_at	Guca1a	guanylate cyclase activator 1a (retina)	2.93	0.03	-1.4	0.0872	-1.09	0.6056	-1.16	0.4252	-1.31	0.1457
103039_at	1423267_s_at	Itga5	integrin alpha 5 (fibronectin receptor alpha)	-2.79	0.34	-1.08	0.6555	1.03	0.8979	-1.11	0.5672	1.16	0.5608
103040_at	1416111_at	Cd83	CD83 antigen	5.23	0.01	1.32	0.4882	-1.39	0.3148	1.68	0.1297	4.95	0.0018
103044_g_at	1455710_x_at	Mtcp1	mature T-cell proliferation 1	-1.28	0.4	-1.09	0.1906	-1.1	0.1476	-1.17	0.0192	1.09	0.2291
103045_at	1425755_at	Mtcp1 /// LOC43	mature T-cell proliferation 1 /// similar to p13	-1.29	0.48	1.4	0.0325	-1.04	0.8273	1.46	0.0406	-2.05	0.0417
103046_at	1448949_at	Car4	carbonic anhydrase 4	-1.81	0.53	1.22	0.5584	1.42	0.325	1.31	0.3318	1.56	0.0944
103047_at	1420711_a_at	Pxmp3	peroxisomal membrane protein 3	1.39	0.3	-1.02	0.8292	1.15	0.4701	1.1	0.3787	-1.26	0.097

103048_at	1417155_at	Nmyc1	neuroblastoma myc-related oncogene 1	-1.15	0.84	-1.57	0.1468	-1.08	0.7845	-2.05	0.0158	-2.9	0.0661
103050_at	1417447_at	Tcf21	transcription factor 21	2	0.08	-1.02	0.7555	-1.04	0.7075	-1.05	0.5009	-1.05	0.8677
103051_at	1417160_s_at	Expi	extracellular proteinase inhibitor	1.45	0.55	-1.87	0.0309	-2.03	0.0265	-1.53	0.0712	-1.15	0.4903
103052_r_at	1416158_at	Nr2f2	nuclear receptor subfamily 2, group F, meml	-1.07	0.71	1.25	0.0503	1.26	0.0653	1.23	0.108	1.07	0.7732
103053_at	1419391_at	Myog	myogenin	-1.86	0.12	1.24	0.4616	1.45	0.0484	1.17	0.2079	1.12	0.7154
103054_at	1426242_at	---	---	-1.33	0.12	-1.14	0.0433	-1.03	0.7212	-1.04	0.6171	1.63	0.0434
103055_r_at	1422311_a_at	Polr2a	polymerase (RNA) II (DNA directed) polypep	-2.28	0	1.01	0.9426	-1.01	0.9055	-1	0.9901	1.54	0.0139
103056_at	1454817_at	Wdr50	WD repeat domain 50	1.7	0.2	1.08	0.1735	1.21	0.0191	1.15	0.1373	1.2	0.1913
103057_at	1448187_at	Pold1	polymerase (DNA directed), delta 1, catalytic	1.09	0.81	1.1	0.2634	-1.08	0.4832	1.2	0.0103	-1.03	0.8813
103058_f_at	1420527_s_at	Tcp10a /// Tcp1	t-complex protein 10a /// t-complex protein 1	1.66	0.42	1.48	0.0019	1.78	0.0404	1.51	0.1035	-1.04	0.8992
103059_at	1418374_at	Fxyd3	FXYD domain-containing ion transport regul	1.48	0.47	-1.32	0.4808	1.32	0.6126	1.33	0.4429	1.27	0.4889
103060_at	1434121_at	Lgi4	leucine-rich repeat LGI family, member 4	2.13	0.12	1.36	0.1133	1.28	0.1288	1.35	0.0803	-1.04	0.901
103061_at	1416561_at	Gad1	glutamic acid decarboxylase 1	1.49	0.28	1.57	0.3284	2	0.1148	1.13	0.7229	-1.17	0.8398
103063_at	1425495_at	Zfp62	zinc finger protein 62	1.47	0.23	1.18	0.0577	1.11	0.5272	1.15	0.2142	-1.19	0.3206
103064_at	1449708_s_at	Chek1	checkpoint kinase 1 homolog (S. pombe)	-1.1	0.78	-1.03	0.8052	-1.04	0.7685	-1.09	0.5164	-1.68	0.0613
103065_at	1448568_a_at	Slc20a1	solute carrier family 20, member 1	2.77	0.01	1.1	0.4013	2.23	0	1.54	0.0014	-1.08	0.8187
103066_at	1450484_a_at	Tyki	thymidylate kinase family LPS-inducible mer	-2.2	0.25	-1.06	0.8603	-1.34	0.2572	1.05	0.8568	1.81	0.0729
103067_at	1423137_at	Rala	v-ral simian leukemia viral oncogene homolo	1.81	0	1.13	0.2293	1.2	0.0624	1.1	0.2272	1.23	0.5331
103069_at	1428639_at	Lin9	lin-9 homolog (C. elegans)	1.13	0.59	1.16	0.3464	1.43	0.0042	1.18	0.1976	1.45	0.3676
103070_at	1416985_at	---	---	1.39	0.57	1.62	0.2598	-1.34	0.1667	2.72	0.1868	-1.34	0.6478
103071_at	1452241_at	Topbp1	topoisomerase (DNA) II beta binding protein	1.37	0.25	1.4	0.1554	-1.02	0.947	1.17	0.3168	1.22	0.7275
103072_at	1448453_at	Hsd3b1	hydroxysteroid dehydrogenase-1, delta<5>-3	-1.16	0.69	1.19	0.2122	1.26	0.2367	1.16	0.4937	-1.01	0.9702
103074_f_at	1438250_s_at	Taf9	TAF9 RNA polymerase II, TATA box binding	1.51	0.02	1.2	0.114	1.11	0.4279	1.37	0.0023	-1.06	0.7655
103075_at	1417945_at	Pou5f1	POU domain, class 5, transcription factor 1	-2.3	0.15	-1.62	0.1402	-1.94	0.049	-1.31	0.3095	1.43	0.1365
103076_at	1432270_a_at	Chmp5	chromatin modifying protein 5	-1.03	0.94	1.16	0.1082	1.12	0.4069	1.13	0.2484	1.01	0.985
103078_at	1424726_at	BC014685	cDNA sequence BC014685	-1.27	0.22	-1.05	0.5964	-1.22	0.1099	-1.05	0.6013	-1.36	0.1099
103079_at	1426525_at	Arid2	AT rich interactive domain 2 (Arid-rfx like)	1.12	0.09	1.16	0.0191	-1.01	0.7422	1.08	0.192	1.16	0.4168
103080_at	1418131_at	Samhd1	SAM domain and HD domain, 1	1.12	0.36	1.11	0.7851	-1.42	0.0058	1.09	0.7729	1.2	0.1455
103081_at	1434824_at	Baz1b	bromodomain adjacent to zinc finger domair	1.2	0.45	1.22	0.0149	1.49	0.0017	1.19	0.0064	1.49	0.0562
103084_at	1460318_at	Csrp3	cysteine and glycine-rich protein 3	-2.27	0.49	-16.46	0	-1.73	0.001	-20.32	0	-8.84	0.0009
103085_at	1418172_at	Hebp1	heme binding protein 1	1.23	0.35	1.14	0.1113	-1.11	0.34	1.03	0.8038	1.36	0.1869
103087_at	1427345_a_at	Sult1a1	sulfotransferase family 1A, phenol-preferring	2.08	0.03	-1.07	0.1984	-1.05	0.4706	1.06	0.4198	1.93	0.003
103088_at	1417795_at	Chl1	cell adhesion molecule with homology to L1(	1.47	0.18	1.24	0.0437	1.3	0.1297	1.04	0.6456	-1.1	0.7725
103089_at	1427301_at	Cd48	CD48 antigen	1.47	0.11	1.69	0.1772	1.06	0.7504	2.18	0.1475	2.48	0.0693
103090_at	1448337_at	2410003P15Rik	RIKEN cDNA 2410003P15 gene	1.35	0.33	1.02	0.8826	-1.05	0.7955	1.05	0.762	-1.02	0.9464
103091_at	1417856_at	Relb	avian reticuloendotheliosis viral (v-rel) oncog	1.09	0.77	1.16	0.2765	1.03	0.7645	1.49	0.0187	-1.08	0.6709
103092_at	1455012_s_at	Trim37	tripartite motif protein 37	1.34	0.42	1.15	0.1807	1.08	0.4585	1.42	0.0069	1.59	0.2853
103094_at	1449509_at	Serf1	small EDRK-rich factor 1	1.99	0.1	-1.21	0.4607	-1.15	0.5682	-1.34	0.2529	1.52	0.2592
103095_at	1448227_at	Grb7	growth factor receptor bound protein 7	-1.15	0.64	1.07	0.5982	-1.02	0.8668	1.04	0.7353	1.16	0.1904
103096_at	1419281_a_at	Zfp259	zinc finger protein 259	2.97	0.03	1.07	0.5412	1.21	0.0957	1.31	0.0703	2.31	0.0308
103097_at	1452941_at	0610038F07Rik	RIKEN cDNA 0610038F07 gene	1.35	0.33	1.12	0.0553	-1.08	0.3667	1.07	0.2315	-1.1	0.4123
103098_at	1425656_a_at	Baiap2	brain-specific angiogenesis inhibitor 1-assoc	-1.63	0.02	-1.11	0.0758	-1.08	0.5002	-1.38	0.0005	1.1	0.5747
103100_at	1417818_at	Wwtr1	WW domain containing transcription regulato	1.26	0.06	1.34	0.0008	1.16	0.2911	1.7	0.0004	-1.33	0.0352
103101_at	1423616_at	Tarbp2	TAR (HIV) RNA binding protein 2	-1.05	0.44	1.39	0.2027	1.87	0.0181	1.22	0.4113	1.27	0.2989
103132_at	1446790_at	C78948	expressed sequence C78948	-1.38	0.22	-1.26	0.2588	-1.34	0.1842	-1.05	0.7786	2.11	0.0094
103198_at	1455239_at	6330512M04Rik	RIKEN cDNA 6330512M04 gene	-1.31	0.23	1.06	0.486	1.09	0.2218	1.1	0.1502	-1.06	0.8532
103199_at	1419412_at	Xcl1	chemokine (C motif) ligand 1	-3.55	0	1.32	0.3646	1.03	0.9228	1.17	0.6112	1.26	0.3859
103200_at	1460555_at	6330500D04Rik	RIKEN cDNA 6330500D04 gene	1.24	0.38	-3.11	0	-1.71	0.0012	-2.47	0	-1.1	0.8263
103202_at	1418392_a_at	Gbp4	guanylate nucleotide binding protein 4	1.08	0.72	2.62	0.3114	-1.46	0.181	2.58	0.2248	-1.12	0.6925
103204_r_at	1436186_at	4432406C08Rik	RIKEN cDNA 4432406C08 gene	-3.05	0.01	-1.37	0.0789	-1.38	0.1276	-1.25	0.2107	-1.43	0.2413
103205_at	1420635_a_at	Tcirg1	T-cell, immune regulator 1	-1.24	0.14	-1.01	0.9359	-1.12	0.2334	1.07	0.6897	1.11	0.2766
103207_at	1419397_at	Pola1	polymerase (DNA directed), alpha 1	-1.11	0.75	-1.28	0.0421	-1.12	0.3192	-1.11	0.3374	1.07	0.6861
103210_at	1449360_at	Csf2rb2	colony stimulating factor 2 receptor, beta 2, l	-1.28	0.59	1.01	0.9791	-1.36	0.0239	1.22	0.6268	-1.09	0.5416

103211_at	1434517_at	Wdfy2	WD repeat and FYVE domain containing 2	1.32	0.11	1.12	0.4138	1.1	0.3874	1.03	0.7949	-1.48	0.2483
103212_at	1451306_at	BC006933	cDNA sequence BC006933	3.73	0	1.11	0.6241	-1.27	0.2985	1.09	0.7109	1.25	0.5672
103213_at	1418602_at	Cdh15	cadherin 15	1.86	0.03	-1.36	0.2852	-1.28	0.4093	-1.43	0.3769	1.47	0.0822
103215_g_at	1429888_a_at	Hspb2	heat shock protein 2	-1.04	0.74	-1.27	0.0346	-1.2	0.0738	-1.35	0.0339	-1.26	0.3278
103216_f_at	1454690_at	Ikbkg	inhibitor of kappaB kinase gamma	1.02	0.94	1.12	0.1239	1.16	0.2154	1.06	0.4584	-2.15	0.0074
103217_at	1425687_at	Cflar	CASP8 and FADD-like apoptosis regulator	-1.23	0.55	1.18	0.3958	-1.77	0.0051	-1.44	0.0298	-1.39	0.1654
103218_at	1451227_a_at	Slc10a3	solute carrier family 10 (sodium/bile acid cot	1.46	0.02	-1.18	0.2527	-1.04	0.7659	-1.11	0.4376	1.29	0.0598
103219_at	1419832_s_at	Acpp	acid phosphatase, prostate	-1.07	0.35	1.19	0.102	-1.09	0.4779	1.2	0.0918	1.3	0.502
103220_at	1422554_at	Ndnl2	necdin-like 2	1.05	0.8	1.03	0.6205	-1.01	0.9547	-1.06	0.3813	1.14	0.4158
103222_at	1422824_s_at	Eps8	epidermal growth factor receptor pathway su	-1.23	0.38	1.43	0.0352	1.25	0.4552	1.37	0.2489	3.66	0.0106
103223_at	1452878_at	Prkce	protein kinase C, epsilon	1.09	0.52	1.22	0.2361	1.1	0.5839	1.1	0.5834	1.49	0.1533
103224_at	1417529_at	Rab33a	RAB33A, member of RAS oncogene family	-1.41	0.46	-1.19	0.3701	1.09	0.7073	1.34	0.268	1.37	0.3948
103225_at	1429173_at	Dnase1l1	deoxyribonuclease 1-like 1	-1.01	0.9	1.05	0.5355	1.07	0.4325	1.08	0.4386	-1.09	0.8267
103226_at	1450430_at	Mrc1	mannose receptor, C type 1	1.72	0.07	1	0.9846	1.25	0.3129	-1.03	0.8695	1.06	0.7099
103227_at	1427961_s_at	AI788959	expressed sequence AI788959	-1.13	0.37	1.03	0.5389	-1.01	0.8833	1.06	0.1142	-1.11	0.4879
103228_at	1427025_at	Mtmr7	myotubularin related protein 7	1.21	0.67	1.24	0.1826	-1.23	0.0998	1.25	0.0411	1.35	0.1558
103229_at	1418594_a_at	Ncoa1	nuclear receptor coactivator 1	1.15	0.65	1.08	0.4731	1.22	0.0964	1.1	0.3867	1	0.9873
103231_at	1429319_at	Rhoh	ras homolog gene family, member H	-1.36	0.27	-1.08	0.6126	1.06	0.6781	-1.02	0.8951	1.66	0.3303
103232_at	1454732_at	Bpy2ip1	BPY2 interacting protein 1	-1.26	0.32	-1.69	0.105	-1.67	0.1088	-1.82	0.0752	-1.6	0.0588
103233_at	1419191_at	Hipk3	homeodomain interacting protein kinase 3	1.28	0.64	1.65	0.0065	1.37	0.112	1.31	0.0866	-1.34	0.3367
103234_at	1424847_at	Nefh	neurofilament, heavy polypeptide	-2.13	0.03	-1.29	0.2548	1.01	0.9805	-1.17	0.4421	2.18	0.1957
103235_at	1419127_at	Npy	neuropeptide Y	-2.37	0.28	-1.26	0.1673	-1.55	0.0212	1	0.979	1.53	0.2524
103236_at	1422647_at	Ring1	ring finger protein 1	-1.18	0.45	-1.05	0.7633	-1.08	0.6516	-1.32	0.1438	1.14	0.6999
103237_at	1448878_at	Mxd3	Max dimerization protein 3	-1.6	0.48	-1.01	0.9675	1.09	0.749	1.01	0.9744	1.04	0.6834
103238_at	1450782_at	Wnt4	wingless-related MMTV integration site 4	1.14	0.43	-1.05	0.5203	1.02	0.8033	-1.19	0.0191	1.14	0.4434
103239_at	1422074_at	Cdx2	caudal type homeo box 2	-1.02	0.88	1.7	0.0587	1.57	0.2484	1.06	0.8062	2.11	0.0946
103240_f_at	1422411_s_at	Ear1 /// Ear2 ///	eosinophil-associated, ribonuclease A family	1.36	0.37	1.38	0.2521	-1.07	0.5423	1.53	0.2385	1.09	0.6875
103241_at	1455462_at	Adcy2	adenylate cyclase 2	1.12	0.62	-1.1	0.6089	1.02	0.9089	1.03	0.8562	1.32	0.3313
103242_at	1460712_s_at	Ap1g1	adaptor protein complex AP-1, gamma 1 sul	-1.33	0.24	-1.13	0.201	1.01	0.877	-1.12	0.1233	-1.07	0.5227
103243_at	1425272_at	Emp2	epithelial membrane protein 2	-1.12	0.82	-1.03	0.7788	-1.07	0.6269	-1.17	0.2958	1.37	0.1663
103244_at	1418796_at	Clec11a	C-type lectin domain family 11, member a	1.34	0.47	1.06	0.5851	-1	0.9671	1.14	0.2533	-1.58	0.0797
103245_at	1424479_at	Cst8	cystatin 8 (cystatin-related epididymal sperm	-1.85	0.12	-1.34	0.294	1.22	0.4478	-1.27	0.3637	2.39	0.0308
103247_at	1419077_at	Mpp3	membrane protein, palmitoylated 3 (MAGUK	-1.16	0.63	1.16	0.4307	-1.09	0.6426	1.22	0.3092	1.88	0.1307
103248_at	1449429_at	Fkbp1b	FK506 binding protein 1b	-1.08	0.87	-1.11	0.4714	-1.01	0.9667	1.03	0.8599	1.5	0.0441
103249_at	1417304_at	Chrd	chordin	-1.67	0.38	1.25	0.1651	1.2	0.1719	1.14	0.2349	1.38	0.2595
103250_at	1417903_at	Dfna5h	deafness, autosomal dominant 5 homolog (h	1.23	0.77	1.21	0.4285	-1.41	0.2407	-1.3	0.3848	1.24	0.7015
103251_at	1455073_at	Cdadc1	cytidine and dCMP deaminase domain conta	1.35	0.24	-1.04	0.4481	-1.1	0.0924	-1.12	0.0304	-1.05	0.6505
103253_at	1449172_a_at	Lin7b	lin 7 homolog b (C. elegans)	-1.64	0.18	1.03	0.6799	1.12	0.2218	1.1	0.4091	-1.25	0.5438
103254_at	1428346_at	MGI:1923551	FLN29 gene product	-1.08	0.63	1.37	0.1462	1.02	0.8025	1.54	0.1418	-1.16	0.2935
103255_at	1448861_at	Traf5	Tnf receptor-associated factor 5	2.43	0.01	1.35	0.1176	1	0.9865	1.47	0.0198	1.3	0.4225
103256_at	1418990_at	Ms4a4d	membrane-spanning 4-domains, subfamily A	1.98	0.09	1.07	0.7607	1.24	0.1651	-1.09	0.6323	2.11	0.0122
103257_at	1434553_at	4930577M16Rik	RIKEN cDNA 4930577M16 gene	-1.04	0.84	1.06	0.3299	1.07	0.3252	1.1	0.0919	-1.27	0.0567
103258_at	1449328_at	Ly75	lymphocyte antigen 75	1.09	0.55	-1.21	0.4351	-1.36	0.0435	-1.05	0.8628	-1.12	0.7297
103259_at	1417679_at	Gfi1	growth factor independent 1	-1.01	0.99	1.01	0.9768	-1.09	0.7269	1.01	0.9709	1.18	0.679
103260_at	1430291_at	1110060D06Rik	RIKEN cDNA 1110060D06 gene	1.4	0.27	1.03	0.5504	1.14	0.0023	1.14	0.0354	1.06	0.6765
103261_at	1418109_at	Gspt2	G1 to phase transition 2	1.26	0.7	-1.16	0.2677	-1.17	0.4376	-1.01	0.9193	-1.32	0.5246
103262_at	1421298_a_at	Hipk1	homeodomain interacting protein kinase 1	1.15	0.81	1.56	0.1343	-1.36	0.2949	-1.05	0.8695	1.16	0.7168
103263_at	1418850_at	Epc1	enhancer of polycomb homolog 1 (Drosophil	1.09	0.5	1.1	0.2071	1.18	0.0288	1.07	0.3171	1.15	0.4426
103264_at	1421879_at	Mtmr1	myotubularin related protein 1	-1.16	0.75	-1.05	0.6444	-1.02	0.8837	1.17	0.1526	1.1	0.5227
103266_at	1420407_at	Ltb4r1	leukotriene B4 receptor 1	1.38	0.63	1.22	0.1483	1.27	0.1849	1.22	0.1161	-1.07	0.6327
103268_r_at	1427536_at	Zfp125	zinc finger protein 125	-2.98	0.02	-1.3	0.2395	-1.49	0.0896	-1.14	0.7284	1.44	0.4233
103270_at	1416969_at	Gtse1	G two S phase expressed protein 1	1.34	0.39	-1.35	0.1506	-1.52	0.0931	-1.27	0.1489	1.18	0.4675
103271_at	1451022_at	Lrp6	low density lipoprotein receptor-related prote	-1.14	0.84	-1.06	0.6154	-1.21	0.1214	-1.14	0.301	-1.71	0.2249

103273_s_at	1455765_a_at	Abcc8	ATP-binding cassette, sub-family C (CFTR/M	-1.48	0.24	1.01	0.9809	-1.03	0.9222	1	0.9921	-1.06	0.8681
103275_at	1460650_at	Atp6v0a1	ATPase, H+ transporting, lysosomal V0 subu	-1.44	0.11	-1.33	0.0135	-1.33	0.0552	-1.58	0.0012	-1.05	0.7886
103276_at	1417435_at	Large	like-glycosyltransferase	-1.18	0.25	1.11	0.4985	-1.01	0.9632	1.21	0.2411	1.67	0.048
103277_s_at	1425585_at	Tnrc11	trinucleotide repeat containing 11 (THR-asso	1.04	0.85	1.02	0.723	1.1	0.3487	1.03	0.6618	1.11	0.3432
103278_at	1422760_at	Padi4	peptidyl arginine deiminase, type IV	-2.71	0.07	-1.26	0.1482	1.21	0.4834	-1.09	0.7568	-1.71	0.4827
103279_at	1449393_at	Sh2d1a	SH2 domain protein 1A	-1.63	0.1	1.01	0.9439	-1.14	0.5008	1.34	0.0796	1.87	0.2925
103281_at	1420906_at	Cd2ap	CD2-associated protein	1.95	0.13	1.05	0.6975	-1.06	0.6948	1.24	0.2034	-1.36	0.4361
103282_at	1417804_at	LOC381240	similar to calcium and DAG-regulated guanin	-1.11	0.65	-1.05	0.6389	-1.09	0.2287	-1.18	0.0171	1.54	0.1268
103283_at	1419555_at	Elf5	E74-like factor 5	-2.28	0.37	-1.11	0.7505	1.54	0.2065	1.07	0.8194	-1.08	0.8697
103284_at	1449309_at	Cyp8b1	cytochrome P450, family 8, subfamily b, poly	-1.12	0.42	-1.32	0.0423	-1.19	0.1483	-1.16	0.24	-1.27	0.213
103285_at	1449490_at	Mbd4	methyl-CpG binding domain protein 4	1.46	0.31	1.05	0.5675	-1.08	0.4553	-1.01	0.9177	1.28	0.5811
103286_at	1416975_at	Stam2	signal transducing adaptor molecule (SH3 do	1.03	0.92	-1.01	0.9298	1.23	0.0456	1	0.9546	1.68	0.0117
103288_at	1449089_at	Nrip1	nuclear receptor interacting protein 1	-1.01	0.96	1.11	0.672	-2.02	0.0063	-1.77	0.0434	1.23	0.4144
103289_at	1419017_at	Corin	corin	-1.56	0.27	1.07	0.8518	1.6	0.2436	1.55	0.1654	1.45	0.1426
103291_at	1422325_at	Magea5	melanoma antigen, family A, 5	1.22	0.33	-1.02	0.9417	1.18	0.5348	1.11	0.6529	-1.2	0.6468
103292_at	1421984_at	Stc1	stanniocalcin 1	1.13	0.8	1.99	0.015	1.29	0.349	1.71	0.0249	-1.32	0.6794
103293_at	1448256_at	Gosr1	golgi SNAP receptor complex member 1	-1	0.99	-1.01	0.9142	-1.1	0.4293	-1.13	0.168	1.37	0.1499
103294_at	1420940_x_at	Rgs5	regulator of G-protein signaling 5	1.18	0.35	-1.01	0.9021	-1.36	0.137	-1.23	0.2553	1.2	0.6851
103295_at	1455066_s_at	9130229H14Rik	RIKEN cDNA 9130229H14 gene	-1.42	0.29	1.36	0.035	1.16	0.3091	1.53	0.0042	2.29	0.0056
103296_at	1449285_at	Cst9	cystatin 9	-7.34	0.08	-1.03	0.9282	1.1	0.7157	-1.18	0.5497	1.32	0.3443
103297_at	1427213_at	Pfkfb1	6-phosphofructo-2-kinase/fructose-2,6-bipho	2.31	0	-1.49	0.0322	1.01	0.9595	1.12	0.4055	-1.55	0.2507
103298_at	1448664_a_at	Apeg1	aortic preferentially expressed gene 1	-1.49	0.65	1.24	0.4474	-1.19	0.5884	1.19	0.5501	1.13	0.6362
103299_at	1433678_at	AI132321	expressed sequence AI132321	1.75	0.15	1.24	0.5141	-1.25	0.1162	1.62	0.2707	1.06	0.8843
103300_at	1435006_s_at	Abcb7	ATP-binding cassette, sub-family B (MDR/TA	-1.3	0.31	1.01	0.7794	1.17	0.0523	1.17	0.0059	-1.38	0.0198
103302_r_at	1435192_at	---	Transcribed locus	-1.84	0.05	-1.15	0.2935	-1.11	0.4363	-1.14	0.3731	-1.07	0.7306
103303_at	1448026_at	Chd7	Chromodomain helicase DNA binding protei	1.08	0.36	1.42	0.0048	1.34	0.029	1.67	0.0001	1.38	0.1441
103305_at	1427387_a_at	Itgb4	integrin beta 4	1.99	0.29	-1.03	0.6953	-1.02	0.8643	-1.01	0.8796	1.41	0.0797
103306_at	1451594_s_at	Serpinb6c	serine (or cysteine) proteinase inhibitor, clad	-1.15	0.72	1.06	0.8443	-1	0.9902	1.01	0.9805	1.09	0.8956
103308_at	1434767_at	C79407	expressed sequence C79407	-2.03	0.16	-1.05	0.8435	1.44	0.141	1.08	0.7309	2.06	0.2188
103309_at	1417592_at	Frap1	FK506 binding protein 12-rapamycin associa	1.86	0.23	1.05	0.3815	1.53	0.0003	1.22	0.0354	-1.23	0.3516
103310_at	1427142_s_at	Jarid1b	jumonji, AT rich interactive domain 1B (Rbp2	-1.01	0.93	-1.87	0.0001	-1.63	0.0054	-1.22	0.1203	1.1	0.7322
103311_at	1425391_a_at	Osbpl5	oxysterol binding protein-like 5	1.57	0.27	1.69	0.0079	1.47	0.018	1.79	0.0005	2.35	0.0025
103313_r_at	1434941_s_at	2610101J03Rik	RIKEN cDNA 2610101J03 gene	-1.31	0.63	-1.1	0.7043	1.3	0.3366	1.07	0.6801	-1.08	0.7906
103314_at	1451474_a_at	Parp8	poly (ADP-ribose) polymerase family, memb	2.53	0.16	2.42	0.0315	2.36	0.0829	4.02	0.0119	1.27	0.493
103315_at	1434898_at	Tnrc6a	trinucleotide repeat containing 6a	1.57	0.06	-1.1	0.1591	-1.05	0.3727	-1.01	0.8704	1.31	0.1396
103316_at	1435530_at	Camsap1	calmodulin regulated spectrin-associated pro	6.46	0.02	1.84	0.0896	2.47	0.0035	2.55	0.0001	-1.35	0.5533
103318_at	1436232_a_at	Gabpb1	GA repeat binding protein, beta 1	-1.05	0.86	-1.14	0.0657	1.06	0.6279	-1.12	0.13	-1.13	0.3299
103319_at	1436559_a_at	Psmd10	proteosome (prosome, macropain) 26S subu	2.78	0.04	1.43	0.008	1.14	0.4472	1.61	0.0003	-1.06	0.8638
103321_at	1455184_at	B230364F10	hypothetical protein B230364F10	1.66	0.09	1.15	0.0758	1.14	0.205	1.19	0.09	-1.76	0.0112
103322_at	1418337_at	Rpia	ribose 5-phosphate isomerase A	-1.16	0.26	-1.11	0.0152	1.02	0.5856	-1.17	0.0036	1.04	0.6324
103326_at	1434330_at	Lrrc35	leucine rich repeat containing 35	-1.05	0.79	-1.24	0.0011	1.3	0.0017	-1.04	0.4726	-2.66	0.0075
103327_at	1432331_a_at	Prrx2	paired related homeobox 2	-1.69	0.41	-1.01	0.9177	1.09	0.4367	-1.02	0.8762	1.02	0.9632
103328_at	1421640_a_at	Tank	TRAF family member-associated Nf-kappa B	-1.42	0.19	1.01	0.9538	-1.04	0.6966	1.12	0.2451	-1.77	0.0134
103329_at	1421125_at	Strbp	spermatid perinuclear RNA binding protein	-1.04	0.94	1.91	0.0011	1.24	0.5185	1.28	0.516	2.14	0.0739
103330_at	1452061_s_at	Strbp	spermatid perinuclear RNA binding protein	1.69	0.08	1.35	0.0004	1.27	0.0128	1.63	0	1.47	0.0029
103331_at	1451085_at	C030006K11Rik	RIKEN cDNA C030006K11 gene	1.18	0.53	1.01	0.93	-1.12	0.0688	-1.11	0.3057	1.06	0.8829
103332_at	1426782_at	Gpr125	G protein-coupled receptor 125	-1.15	0.7	-1.24	0.1726	-1.01	0.9524	-1.58	0.0024	-1.26	0.2736
103333_at	1417880_at	G6pc	glucose-6-phosphatase, catalytic	-2.94	0.04	-1.45	0.168	-1.2	0.4457	-1.35	0.2403	1.1	0.7714
103334_at	1418249_at	Crcp	calcitonin gene-related peptide-receptor com	1.26	0.33	1.23	0.0465	1.1	0.3848	1.18	0.0266	1.04	0.7954
103335_at	1421217_a_at	Lgals9	lectin, galactose binding, soluble 9	-1.28	0.29	-1.24	0.0025	1.05	0.4815	-1.32	0	-2.18	0.0002
103338_at	1428931_a_at	Parp6	poly (ADP-ribose) polymerase family, memb	1.64	0.11	1.04	0.6788	1	0.9898	-1.03	0.8002	1.03	0.8413
103340_at	1417049_at	Rhced	Rhesus blood group CE and D	-1.34	0.73	1.13	0.6683	1.38	0.2923	-1.07	0.8418	-2.56	0.0518
103341_at	1416563_at	Ctps	cytidine 5'-triphosphate synthase	-1.09	0.64	-1.14	0.1485	-1.03	0.5706	-1.02	0.7526	-1.3	0.099

103342_at	1448653_at	Eed	embryonic ectoderm development	1.56	0.14	1.26	0.0053	1.49	0.0173	1.3	0.0192	-1.14	0.2321
103343_at	1453768_a_at	5430432M24Ril	RIKEN cDNA 5430432M24 gene	1.13	0.59	1.09	0.6007	1	0.9987	1.03	0.8783	1.54	0.057
103344_at	1420501_at	Dnajc1	DnaJ (Hsp40) homolog, subfamily C, membe	1.27	0.25	1.11	0.2382	-1.27	0.0191	-1.02	0.8363	1.28	0.0315
103345_at	1427889_at	Spna2	spectrin alpha 2	1.71	0.09	1.28	0.042	1.07	0.5335	1.39	0.0001	1.64	0.0246
103346_at	1417743_at	---	---	1.09	0.61	1.14	0.0462	1.01	0.9129	1.11	0.1534	1.18	0.4065
103347_at	1420326_s_at	Tce4	T-complex expressed gene 4	-1.04	0.92	-1.1	0.3281	1.03	0.7582	-1.1	0.3308	1.64	0.1068
103348_at	1460350_at	---	---	-1.37	0.03	1.11	0.122	1.08	0.2708	1.02	0.7634	-1.24	0.0409
103352_at	1448549_a_at	Dpagt1	dolichyl-phosphate (UDP-N-acetylglucosami	-1.01	0.92	-1.08	0.1816	-1.02	0.839	-1.09	0.2032	-1.01	0.9451
103353_f_at	1416194_at	Cyp4b1	cytochrome P450, family 4, subfamily b, poly	1.11	0.52	1.19	0.1677	1.22	0.0058	1.21	0.1434	1.38	0.0205
103354_at	1417737_at	Mrps31	mitochondrial ribosomal protein S31	1.46	0.03	1.06	0.4282	1.06	0.5713	1.08	0.3494	1.26	0.0809
103355_at	1440213_a_at	2010001M06Ril	RIKEN cDNA 2010001M06 gene	4.13	0.15	-1.32	0.218	1.05	0.8563	-1.65	0.0435	1.28	0.1595
103356_at	1448892_at	Dock7	dedicator of cytokinesis 7	1.74	0.13	1.18	0.1666	1.04	0.8314	1.17	0.3557	2.32	0.1174
103357_at	1449067_at	Slc2a2	solute carrier family 2 (facilitated glucose tra	1.19	0.56	1.23	0.0056	1.33	0.0004	1.26	0.0119	1.31	0.0172
103359_at	1451141_at	BC004636	cDNA sequence BC004636	-1.28	0.1	-1.03	0.7403	1.03	0.7647	-1.21	0.0181	1.04	0.7121
103360_at	1417989_at	Tssk1	testis-specific serine kinase 1	1.07	0.88	1.42	0.2645	1.74	0.0288	1.43	0.3384	-1.24	0.4747
103362_at	1424208_at	---	---	1.56	0.5	1.78	0.4236	1.1	0.6286	2.23	0.2898	1.05	0.798
103363_at	1416451_s_at	Tbn	taube nuss	1.38	0.17	1.11	0.08	1.06	0.3129	1.21	0.0058	-2.46	0.0015
103364_f_at	1428189_at	5730494M16Ril	RIKEN cDNA 5730494M16 gene	1.06	0.81	-1.25	0.0034	-1.04	0.5026	-1.4	0.0001	-1.2	0.271
103365_s_at	1449619_s_at	Arhgap9	Rho GTPase activating protein 9	1.08	0.72	1.6	0.3723	1.02	0.74	2.53	0.2331	1.28	0.3526
103367_at	1418655_at	Galgt1	UDP-N-acetyl-alpha-D-galactosamine:(N-ac	-1.05	0.69	-1.15	0.3114	1.14	0.481	-1.08	0.5776	1.13	0.1678
103369_at	1436316_at	9430029L20Rik	RIKEN cDNA 9430029L20 gene	-1.18	0.54	-1.2	0.0727	1.29	0.2062	1	0.9728	1.07	0.8736
103371_at	1416949_s_at	Slc39a7	solute carrier family 39 (zinc transporter), me	1.03	0.79	-1.2	0.0342	1.02	0.8066	-1.16	0.0375	-1.84	0.0041
103375_at	1428401_at	Zcchc3	zinc finger, CCHC domain containing 3	-1.05	0.89	1.59	0.0388	1.17	0.4716	1.48	0.3042	1.24	0.3878
103376_s_at	1425760_a_at	Pitpnm1	phosphatidylinositol membrane-associated 1	1.32	0.41	2.59	0.0166	2.09	0.097	2.76	0.0368	1.86	0.1072
103377_at	1452320_at	LOC381369 /// I	similar to low density lipoprotein receptor-rel	-2.01	0.05	-1.02	0.8154	1.12	0.5244	1.03	0.7645	1.43	0.2939
103378_at	1448572_at	Prlpa	prolactin-like protein A	-2.46	0.03	2.09	0.0921	1.4	0.2303	1.8	0.108	2.15	0.2356
103379_at	1455047_at	Fbxo3	F-box only protein 3	1.29	0.08	1.41	0.0004	1.34	0.0028	1.45	0.0006	-1.09	0.5512
103381_at	1453014_a_at	Sec31l1	SEC31-like 1 (S. cerevisiae)	-1.08	0.71	-1.1	0.0089	-1.02	0.5346	-1.03	0.2212	-1.05	0.6601
103385_at	1448126_at	MGI:1929091	teratocarcinoma expressed, serine rich	1.43	0.08	1.24	0.4469	1.95	0.0156	1.18	0.5776	1.12	0.7955
103386_at	1417449_at	Pte1	peroxisomal acyl-CoA thioesterase 1	1.38	0.52	-1.09	0.4779	-1.15	0.4111	-1.22	0.1326	-1.59	0.2795
103387_at	1416542_at	Phf1	PHD finger protein 1	-1.74	0.06	1.09	0.1133	1.03	0.6441	1.13	0.0999	1.35	0.0803
103388_at	1451592_at	MGI:2446472	Myb protein P42POP	-1.18	0.15	-1.03	0.7624	-1.05	0.6665	-1.08	0.5199	1.03	0.8769
103389_at	1423523_at	Aass	aminoadipate-semialdehyde synthase	2.03	0.07	1.12	0.3019	1.03	0.7652	1.24	0.0636	1.39	0.0399
103391_at	1455991_at	Ccbl2	cysteine conjugate-beta lyase 2	-2.09	0.07	-1.24	0.092	1.14	0.2773	-1.34	0.0438	-1.31	0.0047
103392_at	1456307_s_at	Adcy7	Adenylate cyclase 7	1.09	0.84	1.11	0.7535	-1.45	0.0699	1.37	0.3038	-1.05	0.8873
103393_at	1423192_at	Pspc1	paraspeckle protein 1	-1.33	0.4	-1	0.9923	1.03	0.9106	-1.01	0.9616	-1.01	0.9815
103394_at	1418296_at	Fxyd5	FXYD domain-containing ion transport regul	1.3	0.6	1.68	0.205	-1.12	0.5658	2.02	0.0973	1.91	0.0516
103397_at	1426922_s_at	Hrb	HIV-1 Rev binding protein	1.62	0.28	1.19	0.1186	1.27	0.1698	1.37	0.0235	1.42	0.2025
103398_at	1424027_at	---	---	1.13	0.4	1.03	0.7487	-1.04	0.73	-1.03	0.8063	1.47	0
103399_at	1426241_a_at	Scmh1	sex comb on midleg homolog 1	-1.76	0.46	1.08	0.6076	1.06	0.7301	1.05	0.7784	2.23	0.0393
103400_at	1415708_at	AI316828	expressed sequence AI316828	1.32	0.21	1.22	0.011	1.21	0.0259	1.19	0.0014	1.32	0.04
103401_at	1460216_at	Acads	acyl-Coenzyme A dehydrogenase, short cha	1.62	0.18	-1.01	0.8309	-1.02	0.8678	1.02	0.8175	-1.25	0.5651
103402_at	1452664_a_at	Tm7sf3	transmembrane 7 superfamily member 3	-1.02	0.89	1.16	0.0492	1.1	0.5867	1.17	0.0239	-1.46	0.0498
103403_at	1452889_at	2310007H09Rik	RIKEN cDNA 2310007H09 gene	4.05	0.34	1.08	0.7911	-1.07	0.8102	-1.08	0.7934	1.17	0.8032
103404_at	1454670_at	Rere	arginine glutamic acid dipeptide (RE) repeat	1.45	0.04	1.16	0.0037	1.33	0.0001	1.31	0	1.7	0
103405_at	1428188_at	2610019A05Rik	RIKEN cDNA 2610019A05 gene	1.33	0.61	1.7	0.0202	1.15	0.6435	1.6	0.0355	1.04	0.9013
103406_at	1417390_at	Xab1	XPA binding protein 1	1.31	0.25	-1.06	0.1328	-1.01	0.9552	1.02	0.7648	-1.14	0.3175
103407_at	1424722_at	1300017J02Rik	RIKEN cDNA 1300017J02 gene	-1.55	0.01	-1.17	0.0062	1.11	0.173	-1.19	0.0032	-1.52	0.0003
103408_at	1436589_x_at	Prkd2	protein kinase D2	2.21	0.02	1.21	0.2645	1.11	0.6117	1.08	0.7151	1.25	0.5573
103409_at	1451539_at	Baiap2l1	BAI1-associated protein 2-like 1	1.2	0.07	1.25	0.0272	1.41	0.0002	1.33	0.0111	1.89	0.008
103411_at	1434254_at	Gna11	guanine nucleotide binding protein, alpha 11	1.63	0.07	-1.12	0.4246	1.01	0.9541	-1.17	0.335	1.24	0.1578
103412_at	1435321_at	3732412D22Rik	RIKEN cDNA 3732412D22 gene	-1.53	0.42	1.18	0.6389	-2.25	0.0263	-1.41	0.252	-1.16	0.8078
103413_at	1417873_at	2310058A11Rik	RIKEN cDNA 2310058A11 gene	-1.14	0.31	1.2	0.0047	1.11	0.31	-1	0.9794	1.33	0.1979

103414_at	1422584_at	Skiv2l	superkiller viralicidic activity 2-like (S. cerevis	-1.24	0.27	-1.06	0.1548	-1.02	0.6037	-1.11	0.0023	-1.23	0.1492
103415_at	1448376_at	Wrnip1	Werner helicase interacting protein 1	-1.14	0.46	-1.04	0.7115	1.03	0.847	-1.2	0.1149	1.03	0.7787
103416_at	1419169_at	Mapk6	mitogen-activated protein kinase 6	-1.05	0.82	-1.01	0.8036	1.13	0.1622	-1.05	0.3678	-1.3	0.1275
103418_at	1424321_at	Rfc4	replication factor C (activator 1) 4	2.36	0.16	-1.03	0.899	1.25	0.3513	1.54	0.0075	-2.44	0.0977
103420_at	1417357_at	Emd	emerin	1.05	0.74	1.01	0.8366	-1.2	0.0004	-1.09	0.0306	1.34	0.001
103421_at	1423465_at	Sdfr2	stromal cell derived factor receptor 2	1.15	0.59	-1.1	0.2015	1.3	0.0001	1.13	0.0422	-1.09	0.6853
103422_at	1449131_s_at	Cd1d1	CD1d1 antigen	-1.34	0.23	1.08	0.3397	-1.12	0.2691	-1.05	0.6312	-1.34	0.0054
103423_at	1417507_at	Cyb561	cytochrome b-561	1.39	0.53	-1.68	0.0583	-1.83	0.0592	-1.29	0.5633	-2.76	0.0329
103424_at	1454632_at	6330442E10Rik	RIKEN cDNA 6330442E10 gene	-1.18	0.44	1.16	0.4047	1.04	0.6943	1.28	0.1641	1.05	0.8269
103427_at	1454635_at	Fbxl3	F-box and leucine-rich repeat protein 3	1.36	0.35	1.04	0.616	1.07	0.6135	1.17	0.0052	1.25	0.1993
103428_at	1426838_at	Pold3	polymerase (DNA-directed), delta 3, access	-1.19	0.37	-1.05	0.3195	-1.07	0.3777	-1.1	0.1187	1.07	0.2846
103432_at	1419569_a_at	Isg20	interferon-stimulated protein	7.99	0.01	-1.32	0.4932	-1.54	0.2374	-1.23	0.5528	-1.05	0.7972
103433_at	1418758_a_at	Pscd3	pleckstrin homology, Sec7 and coiled-coil dc	2.03	0.1	1.22	0.1618	1.02	0.8977	1.28	0.1308	-1.08	0.8206
103434_at	1431707_a_at	Pscd3	pleckstrin homology, Sec7 and coiled-coil dc	1.63	0.14	1.11	0.3112	1.43	0.2784	1.12	0.2205	1	0.9905
103435_at	1434184_s_at	9430080K19Rik	RIKEN cDNA 9430080K19 gene	1.13	0.77	1.12	0.4932	1.1	0.5044	1.23	0.3716	2.95	0.0558
103436_at	1450023_at	---	---	-1.21	0.52	-1.15	0.3567	-1.03	0.8549	-1.11	0.3386	-1.17	0.7027
103437_at	1450929_at	Zfp57	zinc finger protein 57	-1.15	0.58	1.09	0.4548	1.3	0.015	1.01	0.8736	-1.03	0.8383
103438_at	1418937_at	Dio2	deiodinase, iodothyronine, type II	-1.83	0.03	-1.66	0.0213	-1.41	0.1103	-1.41	0.099	-1.07	0.8076
103439_at	1435780_at	Psd	pleckstrin and Sec7 domain containing	-1.65	0.03	-1.16	0.1771	-1.37	0.0367	-1.04	0.7783	-1.12	0.4051
103440_at	1450664_at	Gabpa	GA repeat binding protein, alpha	1.25	0.42	1.2	0.0485	1.23	0.1626	1.25	0.0077	1.05	0.6741
103441_at	1419036_at	Csnk2a1	casein kinase II, alpha 1 polypeptide	2.01	0.1	1.03	0.7001	1.12	0.1891	1.08	0.2984	1.02	0.9388
103442_at	1424869_at	BC003479	cDNA sequence BC003479	1.34	0.24	-1.17	0.0196	-1.27	0.0018	-1.24	0.0044	-1.78	0.0281
103443_at	1426942_at	Aim1	absent in melanoma 1	1.18	0.56	1.14	0.4338	1.13	0.3856	1.42	0.0394	1.01	0.9367
103444_at	1452210_at	Dna2l	DNA2 DNA replication helicase 2-like (yeast	-1.43	0.24	1.32	0.0589	2.03	0.0032	1.51	0.0004	1.18	0.5671
103445_at	1451660_a_at	Hoxb6	homeo box B6	-1.39	0.07	-1.24	0.2339	-1.18	0.4165	-1.17	0.3975	1.23	0.1419
103446_at	1426276_at	Ifih1	interferon induced with helicase C domain 1	-1.18	0.6	-1.06	0.6467	1.07	0.6434	1.05	0.6496	-1.67	0.0075
103447_at	1434445_at	D15Wsu169e	DNA segment, Chr 15, Wayne State Univers	-1.08	0.74	-1.13	0.0965	-1.06	0.4855	-1.14	0.036	-1.12	0.6657
103448_at	1419394_s_at	S100a8	S100 calcium binding protein A8 (calgranulir	1.45	0.43	1.5	0.2136	-1.27	0.1758	1.69	0.2724	1.74	0.0158
103449_at	1455155_at	BC040823	cDNA sequence BC040823	1.12	0.4	1.02	0.8264	1.41	0.0506	1.21	0.0946	1.16	0.5068
103450_at	1428454_at	Bcas3	breast carcinoma amplified sequence 3	1.35	0.21	-1.1	0.4662	1.06	0.7187	1.06	0.5789	1	0.9958
103451_at	1434653_at	Ptk2b	PTK2 protein tyrosine kinase 2 beta	-1.66	0.08	-1.3	0.0042	-1.3	0.0012	-1.39	0.0003	-1.04	0.4765
103452_at	1432099_a_at	Prodh2	proline dehydrogenase (oxidase) 2	1.28	0.32	-1.17	0.0139	-1.05	0.423	-1.2	0.0013	-1.16	0.3922
103456_at	1455741_a_at	Ece1	endothelin converting enzyme 1	1.21	0.18	1.07	0.5332	1.04	0.7373	1	0.9818	1.08	0.5844
103457_at	1424632_a_at	Rev3l	REV3-like, catalytic subunit of DNA polymer	-1.35	0.05	-1	0.9851	1.08	0.4369	-1.03	0.7299	-1.41	0.2096
103458_at	1419407_at	Hc	hemolytic complement	1.18	0.38	1.09	0.1146	1.03	0.5991	1.06	0.2472	-1.49	0.0121
103459_at	1424674_at	Slc39a6	solute carrier family 39 (metal ion transporte	1.3	0.53	-1.15	0.5137	-1.12	0.5658	1.09	0.5871	1.5	0.5211
103460_at	1428306_at	Ddit4	DNA-damage-inducible transcript 4	1.68	0.07	-1.1	0.6749	1.3	0.6807	-1.06	0.7912	-1.65	0.0961
103462_at	1422808_s_at	Dock2	dedicator of cyto-kinesis 2	-1.26	0.41	1.81	0.0351	-1.07	0.783	1.83	0.1979	1.69	0.2763
103463_at	1438468_at	Pramel4 /// Prar	preferentially expressed antigen in melanom	-2.08	0.26	-1.05	0.8399	-1.2	0.4427	1.14	0.5037	1.42	0.5596
103465_f_at	1449326_x_at	Saa2	serum amyloid A 2	3.32	0.02	1.57	0.4964	-3.87	0.0829	1.51	0.5144	-22.58	0.0776
103467_g_at	1435364_at	Cyhr1	Cysteine and histidine rich 1	1.39	0.23	1.39	0.0484	1.98	0.0008	1.6	0.0057	1.34	0.4013
103468_at	1419402_at	Mns1	meiosis-specific nuclear structural protein 1	-1.59	0.45	1.03	0.8859	-1.09	0.4736	-1.2	0.3505	1.28	0.2812
103470_at	1456270_s_at	Pramel6	preferentially expressed antigen in melanom	-2	0.41	-1.22	0.5236	-1.14	0.7222	-1.19	0.557	-1.05	0.8678
103471_at	1416061_at	Tbc1d15	TBC1 domain family, member 15	1.39	0.11	1.35	0.0115	1.45	0.0005	1.49	0.0009	1.17	0.1467
103472_at	1447604_at	BC053393	CDNA sequence BC053393	2.2	0.52	1.42	0.1251	1.24	0.1041	2.49	0.1555	1.5	0.2404
103476_at	1454796_at	D5Ertd40e	DNA segment, Chr 5, ERATO Doi 40, expre	-1.04	0.89	1.23	0.003	1.21	0.0164	1.2	0.0084	1.21	0.3507
103477_at	1449582_at	Cdx1	caudal type homeo box 1	-1.97	0.39	1.4	0.1103	1.38	0.1146	1.37	0.1374	2.47	0.1845
103478_at	1456869_at	2210018M03Rik	RIKEN cDNA 2210018M03 gene	-1.82	0.09	-1.17	0.2388	-1.11	0.4027	-1.43	0.0528	1.78	0.1102
103479_at	1435756_at	Samd10	sterile alpha motif domain containing 10	-1.07	0.74	1.78	0.0073	1.65	0.0015	1.5	0.0086	1.41	0.2458
103480_at	1419696_at	Cd4	CD4 antigen	-1.34	0.14	-1.19	0.2489	-1.09	0.5549	-1.02	0.9178	1.37	0.4055
103483_at	1450935_at	Ercc5	excision repair cross-complementing rodent	1.66	0.31	1.05	0.6412	1.26	0.0265	1.26	0.0795	1.7	0.2564
103484_at	1423856_at	Popdc3	popeye domain containing 3	-1.42	0.58	-1.1	0.7269	1.24	0.4671	-1.2	0.5291	2.03	0.2968
103485_at	1439064_at	BC030046	cDNA sequence BC030046	-1.32	0.15	-1.09	0.3351	1.07	0.4461	-1.16	0.0683	1.05	0.8115

103486_at	1449399_a_at	Il1b	interleukin 1 beta	2.23	0.14	4.85	0.203	1.77	0.3315	6.49	0.1646	1.1	0.779
103487_at	1422625_at	Ly6h	lymphocyte antigen 6 complex, locus H	-3.78	0.01	-1.09	0.7144	-1.01	0.9746	-1.29	0.3279	1.27	0.4721
103488_at	1449127_at	Selpl	selectin, platelet (p-selectin) ligand	-1.14	0.51	1.21	0.2056	-1	0.9985	1.33	0.1748	-1.02	0.843
103489_at	1426105_a_at	D4Bwg1540e	DNA segment, Chr 4, Brigham & Women's (	-2.12	0.05	1.07	0.8031	-1.2	0.5078	1.16	0.6511	1.05	0.8543
103490_at	1450772_at	Wnt11	wingless-related MMTV integration site 11	-1.11	0.74	-1.35	0.0711	-1.19	0.3084	-1.34	0.0367	1.27	0.1547
103492_at	1448901_at	Cpxm1	carboxypeptidase X 1 (M14 family)	2.1	0.1	1.63	0.0658	1.3	0.3914	1.2	0.4264	1.29	0.4363
103493_at	1419405_at	Nmb	neuromedin B	-1.45	0.5	1.13	0.7235	1.39	0.3886	1.56	0.268	-1.19	0.5875
103494_at	1424649_a_at	Tspan8	tetraspanin 8	1.72	0.46	-1.09	0.7378	1.5	0.3916	-1.01	0.9702	1.17	0.6083
103495_at	1449650_at	Etnk1	Ethanolamine kinase 1	-1.48	0.29	-1.16	0.6025	1.08	0.7786	-1.08	0.7839	2.02	0.2045
103496_at	1419396_at	Arid3a	AT rich interactive domain 3A (Bright like)	-1.51	0.27	1.21	0.3497	1.15	0.5865	1.35	0.2659	1.71	0.3206
103498_at	1450127_a_at	Gcgr	glucagon receptor	1.08	0.73	-1.08	0.2741	1.08	0.215	1.02	0.8583	-1.5	0.0282
103499_at	1435386_at	Vwf	Von Willebrand factor homolog	2.59	0.04	-1.32	0.4183	1.17	0.582	1.34	0.3218	2.06	0.0666
103500_at	1437382_at	Acvr2	activin receptor IIA	1.09	0.26	-1.05	0.5874	1.21	0.0567	-1.07	0.3698	-1.07	0.5354
103501_at	1449934_at	Pura	purine rich element binding protein A	-1.15	0.76	1.1	0.1966	-1.31	0.1058	-1.15	0.4021	1.38	0.157
103502_at	1436399_s_at	Nrk	Nik related kinase	-2.18	0.22	-2.15	0.0071	-1.5	0.0917	-1.17	0.6141	1.1	0.883
103503_at	1426926_at	Plcg2	phospholipase C, gamma 2	1.5	0.49	1.49	0.2696	-1.31	0.265	1.35	0.494	1.28	0.3521
103504_at	1449815_a_at	Ssbp2	single-stranded DNA binding protein 2	-1.29	0.51	1.08	0.4657	-1.03	0.7492	1.06	0.3903	-1.04	0.8271
103506_f_at	1426911_at	Dsc2	desmocollin 2	1.45	0.3	1.02	0.7478	1.01	0.9219	1.02	0.807	1.08	0.6596
103507_at	1451161_a_at	Emr1	EGF-like module containing, mucin-like, hor	1.07	0.69	-1.19	0.5058	-1.29	0.1266	1.29	0.5011	-1.71	0.0844
103509_at	1460469_at	Tnfrsf9	tumor necrosis factor receptor superfamily, r	-3.15	0.27	-1.14	0.5628	-1.08	0.7511	1.03	0.9093	2	0.1108
103510_at	1449382_at	Slc6a12	solute carrier family 6 (neurotransmitter tran	1.66	0.02	-1.12	0.1117	-1.09	0.2356	-1.17	0.0304	-1.29	0.1843
103512_at	1453047_at	Bzrpl1	benzodiazapine receptor, peripheral-like 1	-1.41	0.16	-1.2	0.0653	-1.22	0.042	-1.23	0.0312	1.07	0.615
103513_at	1422602_a_at	Wnt5b	wingless-related MMTV integration site 5B	-1.05	0.87	-1.48	0.0051	-1.09	0.3789	-1.49	0.0008	1.46	0.3822
103515_at	1433709_at	Cant1	calcium activated nucleotidase 1	1.08	0.74	1.01	0.9531	1.08	0.2394	1.06	0.4669	1.79	0.0113
103516_at	1418925_at	Celsr1	cadherin EGF LAG seven-pass G-type rece	-4.59	0	-7.23	0.002	-1.17	0.6238	-7.7	0.0019	-1.14	0.6416
103518_at	1452352_at	Ctla2b	cytotoxic T lymphocyte-associated protein 2	1.66	0	-2.8	0.0029	-1.16	0.5885	-1.18	0.6892	1.12	0.8006
103519_at	1449486_at	Ces1	carboxylesterase 1	2.11	0.1	1.32	0.0008	1.06	0.5654	1.34	0.0002	1.22	0.0431
103522_at	1450805_at	Sgcd	sarcoglycan, delta (dystrophin-associated gl	1.01	0.98	-1.48	0.1293	1.28	0.3939	-1.03	0.9259	1.05	0.9245
103523_at	1435152_at	Leng9	Leukocyte receptor cluster (LRC) member 9	-1.61	0.06	-1.04	0.7979	-1.03	0.8662	1.03	0.8436	1.53	0.581
103525_at	1425255_s_at	Hnrpll	heterogeneous nuclear ribonucleoprotein L-l	1.13	0.44	1.06	0.4736	1.25	0.1174	1.11	0.182	1.18	0.3925
103526_at	1418252_at	Padi2	peptidyl arginine deiminase, type II	-1.48	0.01	-1.03	0.7922	-1.04	0.819	1.08	0.6409	1.26	0.103
103527_at	1436693_x_at	Slc35e4	solute carrier family 35, member E4	-2.4	0.21	-1.99	0.0191	-1.3	0.4165	-1.02	0.9343	1.16	0.7433
103529_at	1449125_at	2600017J23Rik	RIKEN cDNA 2600017J23 gene	-1.02	0.93	-1.39	0.0005	1.06	0.5553	-1.55	0.0001	1.01	0.9481
103531_f_at	1434714_at	1300013B24Rik	RIKEN cDNA 1300013B24 gene	-1.7	0.1	-1.64	0	1.06	0.5301	-1.49	0	-2.51	0.0005
103532_at	1435172_at	Eomes	eomesodermin homolog (Xenopus laevis)	-1.47	0.63	1.1	0.8631	-2.28	0.2063	-1.28	0.6579	3.96	0.0049
103533_at	1450736_a_at	Hbb-bh1	hemoglobin Z, beta-like embryonic chain	-1.95	0.36	1.15	0.456	1.03	0.8673	1.62	0.1251	1.09	0.5727
103534_at	1417184_s_at	Hbb /// Hbb-b1 /	hemoglobin beta chain complex /// hemoglo	1.16	0.56	-1.16	0.2911	-1.97	0.0009	-1.2	0.2963	-1.47	0.247
103535_at	1450621_a_at	Hbb-y	hemoglobin Y, beta-like embryonic chain	1.06	0.81	1.1	0.3451	1.13	0.1612	-1.1	0.4202	-1.27	0.6127
103536_at	1419073_at	Tmeff2	transmembrane protein with EGF-like and tw	-1.44	0.42	1.1	0.7269	-1.28	0.47	1.2	0.6334	1.58	0.3199
103537_at	1449278_at	Eif2ak3	eukaryotic translation initiation factor 2 alph	-1.02	0.97	1.12	0.5859	1.31	0.1622	1.16	0.1749	1.77	0.0075
103538_at	1448029_at	Tbx3	T-box 3	-1.26	0.25	1.31	0.1036	-1.05	0.8136	-1.38	0.1529	2	0.0726
103539_at	1460204_at	Tec	cytoplasmic tyrosine kinase, Dscr28C relate	-1.07	0.48	-1.11	0.2472	-1.07	0.4492	-1.11	0.2079	1.18	0.7677
103540_at	1448038_at	---	---	-1.21	0.26	1.12	0.1263	-1.16	0.0653	1.03	0.6666	1.23	0.1725
103541_at	1425009_at	Tcte2	t-complex-associated testis expressed 2	-2.11	0.03	-1.33	0.1441	-1.05	0.7322	-1.19	0.2488	1.16	0.6069
103542_at	1427051_at	Tnks1bp1	tankyrase 1 binding protein 1	1.13	0.55	-1.05	0.6538	-1.15	0.1771	-1.26	0.0254	1.41	0.0916
103543_at	1424764_at	Sez6l	seizure related 6 homolog (mouse)-like	-1.65	0.3	-1.08	0.7487	-1.1	0.6969	1.02	0.9484	1.38	0.6177
103544_at	1418869_a_at	Pus1	pseudouridine synthase 1	2.46	0.01	1.27	0.1043	1.25	0.1131	1.09	0.5665	-1.28	0.3554
103546_at	1422931_at	Fosl2	fos-like antigen 2	-1.03	0.91	1.19	0.1811	-1.08	0.6208	-1.22	0.2281	1.24	0.6588
103547_at	1460565_at	Slc41a1	solute carrier family 41, member 1	2.44	0.07	1.2	0.5141	1.04	0.8783	1.73	0.0711	1.19	0.6524
103548_at	1419411_at	Tac2	tachykinin 2	-2.41	0.08	-1.12	0.5441	1.09	0.7173	-1.3	0.0915	1.13	0.7471
103549_at	1449022_at	Nes	nestin	1.26	0.2	-1.07	0.63	-1.08	0.5935	-1.11	0.4351	1.48	0.3333
103551_at	1425543_s_at	Plekha5	pleckstrin homology domain containing, fam	-1.04	0.78	-1.01	0.8588	-1.01	0.897	-1.06	0.4476	-1.03	0.8246
103552_at	1443856_at	Rabep1	rabaptin, RAB GTPase binding effector prot	1.18	0.59	1.04	0.7723	-1.08	0.6086	-1.14	0.1648	1.11	0.5948

103553_at	1433408_a_at	Mcm10	minichromosome maintenance deficient 10 (	-1.12	0.66	-1.16	0.2776	1.12	0.2534	-1.13	0.2542	-1.66	0.002
103554_at	1418402_at	Adam19	a disintegrin and metalloproteinase domain 1	1.67	0.42	1.5	0.3566	1.24	0.6042	1.37	0.3216	1.01	0.9803
103555_at	1451988_s_at	Chmp4b	chromatin modifying protein 4B	1.07	0.57	1.03	0.7165	-1.09	0.2659	-1.01	0.8249	-1.26	0.0258
103556_at	1455090_at	Ralgps1	Ral GEF with PH domain and SH3 binding m	1.16	0.12	-1.39	0.0001	1.04	0.4154	-1.3	0.0033	-3.7	0.002
103557_at	1426227_s_at	5730409F24Rik	RIKEN cDNA 5730409F24 gene	-1.05	0.78	1.11	0.1642	1.23	0.0256	1.29	0.0031	-1.03	0.8146
103558_at	1421589_at	Krt1-1	keratin complex 1, acidic, gene 1	1.4	0.42	-1.17	0.1518	-1.11	0.3209	-1.1	0.4528	1.58	0.2566
103559_at	1450519_a_at	Prkaca	protein kinase, cAMP dependent, catalytic, a	1.08	0.55	1.1	0.3507	-1.18	0.1275	-1.07	0.4556	-1.17	0.3483
103560_at	1428626_at	2210402C18Rik	RIKEN cDNA 2210402C18 gene	6.75	0.15	-1.12	0.6914	1.07	0.8217	1.09	0.7527	1.56	0.4152
103563_at	1435226_at	Ibrdc3	IBR domain containing 3	2.75	0.02	1.1	0.2833	1.06	0.4902	1.29	0.077	1.11	0.6466
103564_at	1454706_at	Uvrag	UV radiation resistance associated gene	1.25	0.31	-1.1	0.2171	-1.16	0.163	1.04	0.5861	1.04	0.9376
103565_at	1417886_at	1810009A15Rik	RIKEN cDNA 1810009A15 gene	1.46	0.08	-1.22	0.0537	-1.15	0.1832	-1.25	0.0865	-1.06	0.7998
103567_at	1415758_at	2510002A14Rik	RIKEN cDNA 2510002A14 gene	-1.26	0.09	-1.06	0.2995	1.02	0.6864	1	0.9975	1.14	0.3948
103568_at	1451939_a_at	Srpx	sushi-repeat-containing protein	-2.09	0.09	1.37	0.1897	1.17	0.5169	1.64	0.0448	1.33	0.2809
103570_at	1422606_at	C1qtnf3	C1q and tumor necrosis factor related protei	3.73	0	-1.74	0.0065	-1.67	0.0347	-1.44	0.2032	1.25	0.3795
103571_at	1425548_a_at	Lst1	leukocyte specific transcript 1	1.25	0.27	1.23	0.5223	-1.1	0.5911	1.12	0.7473	1.11	0.7439
103573_at	1450389_s_at	Pip5k1a	phosphatidylinositol-4-phosphate 5-kinase, t	-2.07	0.1	-1.16	0.4437	1.05	0.7007	-1.08	0.5085	1.15	0.4913
103574_at	1454708_at	Ablim1	actin-binding LIM protein 1	1.51	0.11	1.15	0.1594	1.18	0.1732	1.18	0.0558	1.73	0.053
103578_at	1431188_a_at	Tom1	target of myb1 homolog (chicken)	-1.2	0.08	-1.11	0.1211	-1.02	0.7735	-1.05	0.4233	-1.32	0.1017
103579_at	1417620_at	Rac2	RAS-related C3 botulinum substrate 2	-1.48	0.01	1	0.9761	-1.16	0.015	-1.01	0.9136	1.2	0.3224
103580_at	1424026_s_at	BC013529	cDNA sequence BC013529	1.47	0.1	-1.51	0.0007	-1.38	0.005	-1.37	0.0078	-2.51	0.0246
103581_at	1449065_at	Cte1	cytosolic acyl-CoA thioesterase 1	-1.58	0.02	-1.23	0.1905	1.6	0.0736	-1.21	0.353	-1.45	0.048
103582_r_at	1452054_at	6130401J04Rik	RIKEN cDNA 6130401J04 gene	1.44	0.46	1.3	0.0665	1.15	0.4597	1.39	0.0407	-1.13	0.6164
103584_at	1453015_at	5830471E12Rik	RIKEN cDNA 5830471E12 gene	-1.39	0.02	1.06	0.109	-1.01	0.8809	-1.08	0.0433	-1.02	0.9011
103585_at	1454794_at	---	---	-1.7	0	1.14	0.3055	1.15	0.4338	1.03	0.8116	1.42	0.0943
103588_at	1438760_x_at	Adam15	a disintegrin and metalloproteinase domain 1	-1.46	0.01	1.06	0.7505	-1.04	0.8254	1.07	0.7274	1.23	0.1413
103589_at	1448932_at	Krt1-16	keratin complex 1, acidic, gene 16	1.7	0.28	1.13	0.3083	1.01	0.9486	1.02	0.885	1.85	0.2183
103591_at	1418593_at	Taf6	TAF6 RNA polymerase II, TATA box binding	-1.44	0.16	1.03	0.8496	-1.1	0.593	-1.21	0.3302	-1.3	0.2589
103592_at	1417854_at	Map2k5	mitogen activated protein kinase kinase 5	1.25	0.33	1.03	0.5633	1.11	0.3857	-1.01	0.8583	-1.28	0.1838
103593_at	1456062_at	Nppa	natriuretic peptide precursor type A	-1.15	0.67	-1.03	0.749	-1.01	0.8767	-1.07	0.5135	-1.08	0.5585
103595_at	1448915_at	Zfp524	zinc finger protein 524	1.07	0.67	1.16	0.1888	1.1	0.2921	1.14	0.1733	1.31	0.359
103596_at	1418578_at	Dgka	diacylglycerol kinase, alpha	1.58	0.15	-1.2	0.1709	-1.18	0.087	-1.35	0.0398	1.08	0.8328
103597_at	1418028_at	Dct	dopachrome tautomerase	-1.6	0.31	-12.95	0	-1.88	0.0004	-8.51	0	-9.96	0
103598_at	1451770_s_at	---	---	1.29	0.42	1.1	0.0551	-1.15	0.0515	-1	0.9514	-1.02	0.914
103599_at	1451053_a_at	Mdm1	transformed mouse 3T3 cell double minute 1	-1.07	0.9	1.62	0.3119	1.08	0.8105	1.23	0.6686	-1.04	0.9389
103600_at	1460223_a_at	Epb4.9	erythrocyte protein band 4.9	-1.17	0.57	-1.02	0.737	1.02	0.7379	-1.21	0.0146	1.36	0.0111
103601_at	1436336_at	2210013M04Rik	RIKEN cDNA 2210013M04 gene	1.05	0.68	1.17	0.5265	-1.01	0.9333	1.25	0.3241	1.12	0.7238
103602_at	1420709_s_at	Dao1	D-amino acid oxidase 1	-2.02	0.29	-1.24	0.1972	-1.34	0.007	-1.25	0.1686	-1.03	0.8657
103603_at	1460562_at	Eftud1	elongation factor Tu GTP binding domain co	5.11	0	1.02	0.8928	-1.02	0.8668	1.15	0.1524	1.2	0.7084
103606_r_at	1417786_a_at	Rgs19	regulator of G-protein signaling 19	-1.23	0.61	1.09	0.521	1.03	0.7828	1.26	0.1304	1.36	0.0802
103607_at	1417836_at	Gpx7	glutathione peroxidase 7	1.71	0.25	1.03	0.934	1.18	0.5887	1.17	0.6521	1.21	0.5937
103608_at	1431354_a_at	Fars2	phenylalanine-tRNA synthetase 2 (mitochon	1.3	0.08	1.02	0.7577	1.12	0.0438	1.15	0.0462	-1.46	0.0006
103611_at	1419554_at	Cd47	CD47 antigen (Rh-related antigen, integrin-a	-1.24	0.45	1.2	0.0354	1.23	0.0537	1.38	0.0003	1.37	0.0191
103612_at	1418903_at	Aqp2	aquaporin 2	-1.31	0.53	1.01	0.9371	-1.01	0.9653	1.25	0.2942	1.05	0.8721
103613_at	1423498_at	Aldoa-ps1	aldolase 1, A isoform, pseudogene 1	2.67	0.05	1.72	0.0471	1.27	0.3243	1.15	0.6128	1.18	0.4023
103614_at	1425902_a_at	Nfkb2	nuclear factor of kappa light polypeptide gen	1.12	0.54	1.08	0.4557	-1.16	0.2562	1.14	0.3294	1.08	0.0747
103617_at	1460242_at	Daf1	decay accelerating factor 1	-1.02	0.96	1.28	0.1405	1.43	0.1115	1.3	0.1983	2.07	0.014
103618_at	1428722_at	Ckmt2	creatine kinase, mitochondrial 2	-2.5	0.08	-4.37	0.1317	-4.2	0.1377	-5.56	0.1101	2.91	0.0131
103619_at	1417766_at	1810044O22Rik	RIKEN cDNA 1810044O22 gene	-1.14	0.33	-1.17	0.0832	-1.2	0.0737	-1.38	0.0167	-2.06	0.0032
103622_at	1449964_a_at	Mlycd	malonyl-CoA decarboxylase	1.04	0.65	-1	0.9555	1.01	0.8959	1.1	0.2611	1.03	0.7192
103623_at	1422831_at	Fbn2	fibrillin 2	2.38	0.14	1.45	0.2233	1.3	0.3179	1.12	0.6463	1.44	0.2317
103624_at	1431753_x_at	2900073H19Rik	RIKEN cDNA 2900073H19 gene	-1.21	0.29	1.13	0.3119	-1.11	0.645	-1.08	0.7396	-1.27	0.5179
103625_at	1449045_at	Afg3l1	AFG3(ATPase family gene 3)-like 1 (yeast)	1.38	0.12	-1.09	0.3436	-1.02	0.859	-1.05	0.5698	-1.06	0.8361
103630_at	1448403_at	---	---	1.39	0.16	1.1	0.178	1.07	0.3665	1.17	0.0105	-1.07	0.5687

103631_at	1452588_at	2810407K09Rik	RIKEN cDNA 2810407K09 gene	1.64	0.13	1.35	0.0065	1.21	0.198	1.53	0.002	1.12	0.1679
103632_at	1429349_at	1700001F22Rik	RIKEN cDNA 1700001F22 gene	3.7	0.02	-1.5	0.1106	-1.34	0.2264	-1.79	0.0771	-1.01	0.9786
103635_at	1424526_a_at	Tgds	TDP-glucose 4,6-dehydratase	1.1	0.67	-1.09	0.1469	1.09	0.0784	-1.21	0.0149	-1.39	0.0313
103636_at	1418746_at	MGI:1930773	brain protein 17	-1.67	0.02	-1.15	0.0656	-1.34	0	-1.66	0	-1.57	0.0251
103637_at	1449043_at	Naga	N-acetyl galactosaminidase, alpha	-1.16	0.72	-1.09	0.1947	-1.13	0.1545	-1.19	0.0158	-1.07	0.8496
103638_at	1426983_at	Fnbp1	Formin binding protein 1	1.97	0.08	1.32	0.0721	1.04	0.782	1.27	0.2092	1.23	0.625
103639_at	1418293_at	Ifit2	interferon-induced protein with tetratricopept	1.71	0.24	6.21	0.228	1.05	0.8826	8.96	0.1531	1.2	0.5863
103641_at	1455587_at	BC030183	cDNA sequence BC030183	2.78	0.22	1.02	0.9257	1.69	0.0265	1.23	0.4971	4.03	0.0479
103643_at	1421115_a_at	Zdhhc16	zinc finger, DHHC domain containing 16	1.34	0.29	-1.09	0.3153	1.06	0.5366	1	0.9688	-1.26	0.0906
103644_at	1435943_at	Dpep1	Dipeptidase 1 (renal)	1.69	0.25	-2.61	0.052	-2.45	0.0614	-2.9	0.0408	-1.56	0.396
103645_at	1449223_at	Dnajb8	DnaJ (Hsp40) homolog, subfamily B, membe	1.24	0.6	-1.07	0.7815	-1.26	0.3804	-1.36	0.195	1.02	0.9313
103646_at	1417008_at	Crat	carnitine acetyltransferase	2.25	0.08	1.29	0.0478	1.17	0.2167	1.05	0.717	1.18	0.5344
103647_at	1416205_at	Glb1	galactosidase, beta 1	1.85	0.2	-1.12	0.6575	1.02	0.9379	1.05	0.8054	-1.33	0.1981
103649_at	1418654_at	Hao3	hydroxyacid oxidase (glycolate oxidase) 3	11.44	0.01	1.05	0.7231	-1.17	0.3749	-1.74	0.0131	6.22	0.0007
103650_at	1416989_at	3100002B05Rik	RIKEN cDNA 3100002B05 gene	1.38	0.4	-1.07	0.2775	-1.06	0.3855	-1	0.9462	-1.14	0.2052
103653_at	1449590_a_at	Mras	muscle and microspikes RAS	-1.12	0.69	1.23	0.4411	1.05	0.7632	1.13	0.5691	1.22	0.2804
103654_at	1418152_at	Nsbp1	nucleosome binding protein 1	1.2	0.5	1.2	0.0077	1.04	0.4374	1.15	0.0252	-1.1	0.4812
103655_at	1426437_s_at	Hdac3	histone deacetylase 3	2.29	0.33	1.02	0.9079	-1.43	0.1328	-1.25	0.31	-3.46	0.0167
103656_at	1427011_a_at	Lancl1	LanC (bacterial lantibiotic synthetase compo	-1.01	0.98	-1	0.9919	1.09	0.2113	1.13	0.0209	-1.13	0.4478
103658_r_at	1418515_at	Mtf2	metal response element binding transcriptio	1.09	0.85	1.26	0.34	1.16	0.423	1.19	0.1468	-1.12	0.596
103660_at	1449442_at	Pex11a	peroxisomal biogenesis factor 11a	-1	0.99	-1.17	0.2002	-1.19	0.3981	-1.48	0.0209	-1.51	0.0485
103662_at	1418465_at	Ncf4	neutrophil cytosolic factor 4	1.45	0.35	1.05	0.912	-3.54	0.0008	1.3	0.6243	1.59	0.3087
103663_at	1426618_a_at	4930467B06Rik	RIKEN cDNA 4930467B06 gene	1.12	0.78	1.05	0.6204	1	0.98	1.19	0.2175	1.15	0.7671
103664_r_at	1425830_a_at	2810452K22Rik	RIKEN cDNA 2810452K22 gene	1.33	0.33	1.15	0.0801	1.15	0.135	1.12	0.244	1.31	0.3103
103665_at	1417404_at	Elovl6	ELOVL family member 6, elongation of long	1.02	0.96	-5.3	0.002	-3.2	0.0082	-7.47	0.0012	-3.5	0.002
103666_at	1418415_at	Hoxb5	homeo box B5	-1.18	0.43	1.04	0.8215	1.27	0.0154	-1	0.99	1.07	0.8542
103667_at	1434513_at	Gm542	gene model 542, (NCBI)	-1.15	0.32	1.06	0.3213	1.05	0.4931	1.1	0.0718	-1.51	0.0034
103668_at	1417628_at	Supt6h	suppressor of Ty 6 homolog (S. cerevisiae)	1.3	0.25	1.14	0.047	1.18	0.0125	1.17	0.0298	1.65	0.0155
103669_at	1433798_a_at	E330034G19Ril	RIKEN cDNA E330034G19 gene	-2.58	0.02	-1.05	0.6902	1.07	0.529	-1.03	0.7476	1.43	0.0426
103670_at	1418821_at	Cyp2a12	cytochrome P450, family 2, subfamily a, poly	1.44	0.07	1.04	0.4151	-1.05	0.27	1.15	0.0026	1.05	0.7004
103671_at	1451814_a_at	Htatip2	HIV-1 tat interactive protein 2, homolog (hun	1.01	0.96	-1.11	0.2705	-1.13	0.0975	-1.19	0.0361	-1.15	0.4786
103672_at	1428997_at	2410141M05Ril	RIKEN cDNA 2410141M05 gene	-1.08	0.59	1.09	0.1883	1.17	0.0453	-1.08	0.2682	-1.08	0.5352
103673_at	1416051_at	C2	complement component 2 (within H-2S)	1.19	0.44	1.33	0.019	-1.02	0.7258	1.42	0.0075	1.25	0.0721
103674_f_at	1417210_at	Eif2s3y	eukaryotic translation initiation factor 2, subu	1.44	0.24	-2.64	0.0071	-1.81	0.03	-1.74	0.0442	-1.11	0.5388
103675_at	1427231_at	Robo1	roundabout homolog 1 (Drosophila)	1.15	0.83	1.78	0.0733	-2.15	0.1387	1.54	0.1474	2.8	0.003
103676_at	1448912_at	C1qtnf1	C1q and tumor necrosis factor related protei	1.67	0.46	1.16	0.5094	1.36	0.1615	1.04	0.8901	1.66	0.3345
103678_at	1434612_s_at	Sbno1	sno, strawberry notch homolog 1 (Drosophila	1.33	0.04	1.07	0.431	1.16	0.195	1.09	0.358	1.41	0.0872
103681_at	1434791_at	Atp6v0a2	ATPase, H+ transporting, lysosomal V0 subu	1.05	0.77	1.14	0.1286	1.04	0.5699	1.27	0.0189	1.08	0.5882
103682_at	1433913_at	C80913	expressed sequence C80913	1.73	0.04	1.33	0.0507	1.3	0.2366	1.56	0.0053	1.2	0.3207
103683_at	1417582_s_at	Dhodh	dihydroorotate dehydrogenase	1.65	0.19	-1.07	0.3114	-1.03	0.7176	-1.03	0.4795	1.11	0.7105
103684_at	1418484_at	Tekt2	tektin 2	-1.16	0.52	-1.72	0.2924	1.22	0.6677	-1.07	0.8803	1.12	0.5839
103685_at	1436570_at	---	Transcribed locus	1.03	0.89	-1.37	0.0107	-1.01	0.9397	-1.35	0.0111	-1.29	0.1735
103689_at	1428988_at	Abcc3	ATP-binding cassette, sub-family C (CFTR/M	1.3	0.41	1.13	0.1987	-1.08	0.3045	1.09	0.3188	1.33	0.0016
103691_at	1417888_at	Trim13	tripartite motif protein 13	-1.75	0.33	1.29	0.4986	1.05	0.8536	1.44	0.2605	1.43	0.4424
103692_at	1423085_at	Efnb3	ephrin B3	-1.49	0.23	1	0.994	-1.04	0.8512	1.07	0.7504	-1.13	0.6192
103693_at	1457676_at	C130027E04Ril	RIKEN cDNA C130027E04 gene	-1.4	0.17	-1.37	0.0221	-1.06	0.5872	-1.3	0.045	-1.37	0.2734
103694_at	1427047_at	Nup188	nucleoporin 188	1.07	0.68	-1.06	0.7501	-1.29	0.1739	1.08	0.7499	1.33	0.0877
103697_at	1454984_at	AW061234	expressed sequence AW061234	-1.42	0.1	-3.41	0.0001	-2.24	0.0009	-3.26	0.0001	-1.08	0.5843
103700_r_at	1455220_at	Frat2	frequently rearranged in advanced T-cell lym	-1.08	0.79	-1.05	0.7953	1.08	0.656	1.21	0.3145	1.26	0.1704
103701_at	1427031_s_at	D16Ertd480e	DNA segment, Chr 16, ERATO Doi 480, exp	-1.05	0.68	-1.04	0.7234	1.05	0.7196	1.03	0.797	1.49	0.1771
103703_f_at	1436162_at	C730048C13Ril	RIKEN cDNA C730048C13 gene	-2.44	0	-1.39	0.0001	-1.01	0.8861	-1.62	0	-3.01	0.0081
103704_at	1435376_at	Ddhd2	DDHD domain containing 2	1.31	0.29	1.15	0.1442	1.53	0.0036	1.31	0.0718	-1.44	0.1483
103706_at	1435896_at	Sfxn2	sideroflexin 2	1.4	0.02	1.35	0.0001	1.28	0.0082	1.43	0	1.66	0.0007

103707_at	1419483_at	C3ar1	complement component 3a receptor 1	-1.24	0.47	-1.06	0.6401	-1.11	0.1058	1.08	0.4969	1.15	0.5278
103708_at	1424344_s_at	Eif1a	eukaryotic translation initiation factor 1A	1.02	0.86	1.15	0.201	1.01	0.933	1.3	0.0218	-1.11	0.574
103709_at	1455494_at	---	Transcribed locus	-1.25	0.33	1.01	0.9773	-1.11	0.1758	1.21	0.2567	1.47	0.1822
103710_at	1455433_at	3110048L19Rik	RIKEN cDNA 3110048L19 gene	1.72	0.05	1.2	0.2488	1.4	0.0232	1.2	0.248	-1.03	0.8971
103711_at	1437537_at	Casp9	caspase 9	1.33	0.36	-1	0.9746	-1.14	0.1236	-1.06	0.446	-1.25	0.0158
103712_at	1433871_at	R3hdm	R3H domain (binds single-stranded nucleic a	-1.13	0.57	1.44	0.0234	1.7	0.0233	1.57	0.0022	-1.4	0.3534
103713_at	1428193_at	Usp9x	ubiquitin specific protease 9, X chromosome	-1.26	0.29	1.05	0.2999	1.03	0.5281	1.15	0.0345	1.07	0.6748
103714_at	1451547_at	---	---	1.2	0.25	1.01	0.9075	1.02	0.8994	-1	0.9917	-1.35	0.0245
103715_at	1450276_a_at	Scin	scinderin	1.34	0.15	-1.44	0.2112	1.05	0.8348	-1.05	0.8671	1.2	0.4361
103716_at	1455072_at	4933409L06Rik	RIKEN cDNA 4933409L06 gene	1.01	0.98	-1.05	0.6384	-1.01	0.9621	-1.04	0.6925	-1.14	0.3706
103717_at	1448146_at	Wwp2	WW domain containing E3 ubiquitin protein l	1.47	0.2	-1.14	0.1184	1.02	0.7899	-1.05	0.6524	1.28	0.2012
103718_at	1420645_at	Rnf110	ring finger protein 110	-1.9	0.41	1.35	0.0422	1.12	0.4996	1.53	0.0004	1.54	0.3216
103719_at	1449537_at	Msh5	mutS homolog 5 (E. coli)	-1.21	0.67	1.57	0.205	-1.24	0.595	1.17	0.6495	-1.05	0.8126
103720_at	1425565_at	Rest	RE1-silencing transcription factor	1.48	0.29	1.03	0.9016	-1.9	0.0094	-1.39	0.1337	1.38	0.0905
103721_at	1452107_s_at	Npnt	nephronectin	-1.22	0.58	-1.26	0.0109	-1.57	0.0007	-1.16	0.1409	-1.02	0.9598
103723_at	1454783_at	Il13ra1	interleukin 13 receptor, alpha 1	-1.65	0.08	-1.34	0.0025	-1.15	0.2662	-1.53	0	-2.79	0.0172
103727_at	1434840_at	Hrb	HIV-1 Rev binding protein	1.11	0.61	-1	0.9741	1.17	0.0723	1.18	0.019	1.28	0.0031
103728_at	1456521_at	---	Transcribed locus	1.37	0.16	1.13	0.0104	1.13	0.05	-1.01	0.8606	1.4	0.0094
103729_at	1418153_at	Lama1	laminin, alpha 1	1.08	0.81	-1.01	0.934	-1.06	0.767	-1.01	0.9412	-1.44	0.1692
103731_at	1455252_at	Tsc1	Tuberous sclerosis 1	1.14	0.45	1.23	0.0576	1.18	0.031	1.19	0.0382	1.49	0.0514
103732_at	1433941_at	Pib5pa	phosphatidylinositol (4,5) bisphosphate 5-ph	-1.01	0.99	1.07	0.8005	1	0.9953	1.01	0.9595	1.06	0.5544
103733_at	1448019_at	2900006A08Rik	RIKEN cDNA 2900006A08 gene	-1.05	0.83	-1.02	0.8032	1.25	0.0506	1.03	0.5822	1.73	0.0024
103734_at	1455177_at	Ahi1	Abelson helper integration site	1.67	0.34	-1.01	0.932	-1.09	0.571	-1.06	0.5906	1.21	0.558
103735_at	1419708_at	Wnt6	wingless-related MMTV integration site 6	-2.53	0.25	-1.06	0.7928	1.01	0.9455	1.11	0.6738	1.14	0.7363
103736_at	1448005_at	Sash1	SAM and SH3 domain containing 1	-1.19	0.37	1.09	0.3614	1.06	0.5886	1.17	0.0474	1.58	0.0115
103737_at	1418753_at	---	---	-4.51	0.15	1.87	0.1245	1.57	0.2681	1.15	0.7371	1.14	0.7802
103738_at	1454820_at	BC037034	cDNA sequence BC037034	-1.82	0.24	-1.18	0.0121	-1.15	0.0854	-1.13	0.0505	1.07	0.3877
103739_at	1428374_at	MGI:2136405	glucuronyl C5-epimerase	-1.28	0.1	1.01	0.9238	1.34	0.0731	-1.06	0.6939	1.2	0.2146
103742_at	1449522_at	Unc5c	unc-5 homolog C (C. elegans)	-1.5	0.45	1.21	0.5522	-1.28	0.3882	1.04	0.8893	2.78	0.1547
103743_at	1435106_at	---	Transcribed locus	2.4	0.02	1.28	0.1183	1.57	0.185	1.35	0.4355	-1.41	0.4093
103744_at	1434109_at	Sh3bgrl2	SH3 domain binding glutamic acid-rich prote	-1.14	0.49	-1.01	0.9154	1.16	0.3774	1.12	0.3566	-1.41	0.3112
103748_at	1427980_at	4933407C03Rik	RIKEN cDNA 4933407C03 gene	2.19	0.29	-1.01	0.9643	-4.62	0.0031	-3.36	0.0101	-1.05	0.8994
103751_at	1452273_at	AA409316	expressed sequence AA409316	-1.18	0.48	-1.2	0.0229	-1.2	0.0429	-1.3	0.0067	-1.1	0.4782
103752_r_at	1455156_at	Strn	striatin, calmodulin binding protein	-1.13	0.53	1.28	0.2464	1.13	0.6951	1.09	0.6991	1.14	0.7352
103753_at	1434332_at	Zzz3	zinc finger, ZZ domain containing 3	1.42	0.26	1.35	0.003	1.28	0.0254	1.27	0.0157	1.56	0.2062
103754_at	1450859_s_at	Ube2d3	ubiquitin-conjugating enzyme E2D 3 (UBC4/	1.21	0.33	-1.07	0.4019	-1.1	0.1416	-1.2	0.042	-1.36	0.0801
103755_at	1449084_s_at	Sh3d19	SH3 domain protein D19	-1.34	0.43	1.04	0.6828	-1.03	0.7361	-1.01	0.8582	-1.01	0.9346
103756_at	1435228_at	BC023829	cDNA sequence BC023829	1.24	0.09	1.09	0.1527	-1.03	0.629	1.1	0.1784	1.46	0.0011
103757_at	1440255_at	AA589481	expressed sequence AA589481	1.18	0.52	-1.35	0.0603	-1.28	0.1464	-1.26	0.1677	1.31	0.2578
103759_at	1449454_at	Bst1	bone marrow stromal cell antigen 1	1.53	0.43	1.17	0.5574	-1.08	0.7846	1.11	0.6211	1.91	0.412
103760_at	1428798_s_at	0610039J04Rik	RIKEN cDNA 0610039J04 gene	1.63	0.2	1.13	0.2887	1.28	0.1012	1.4	0.0055	-1.21	0.1285
103761_at	1418091_at	Tcfcp2l1	transcription factor CP2-like 1	-1.09	0.68	-1.34	0.0051	1.05	0.6275	-1.36	0.0044	1.07	0.6654
103762_at	1417698_at	Gtf2f1	general transcription factor IIF, polypeptide 1	1.05	0.76	1	0.9747	-1.01	0.9305	1	0.9545	1.03	0.786
103763_at	1450072_at	Ash1l	ash1 (absent, small, or homeotic)-like (Droso	1.27	0.07	1.23	0.0203	1.25	0.0062	1.21	0.0183	1.06	0.6127
103765_at	1418200_at	Hkr3	GLI-Kruppel family member HKR3	5.25	0.01	1.13	0.2355	1.13	0.171	1.23	0.1121	1.87	0.0821
103766_at	1422167_at	Sema5a	sema domain, seven thrombospondin repea	-1.69	0.46	-1.28	0.1374	1.29	0.0722	1.28	0.0411	2.49	0.0243
103767_f_at	1433773_at	Rrm2b	ribonucleotide reductase M2 B (TP53 induci	1.48	0.21	1.14	0.6112	-1.21	0.4966	1.14	0.6355	1.37	0.5953
103769_at	1421843_at	Il1rap	interleukin 1 receptor accessory protein	-2.31	0.15	1	0.9968	1.31	0.3148	-1.03	0.9259	1.07	0.8445
103770_at	1435572_at	2310014L17Rik	RIKEN cDNA 2310014L17 gene	-1.1	0.87	-1.67	0.0499	1.15	0.452	-1.11	0.5784	1.17	0.6644
103771_at	1435105_at	1110061N23Rik	RIKEN cDNA 1110061N23 gene	-1.67	0.11	1.05	0.3215	-1.02	0.7247	-1.07	0.2481	1.23	0.0492
103773_at	1439482_at	---	---	-1.23	0.36	1.14	0.0746	1.17	0.0407	1.39	0.0001	1.08	0.5862
103774_at	1419867_a_at	Ankhd1	ankyrin repeat and KH domain containing 1	-1.15	0.69	-1.03	0.8073	-1.52	0.0041	-1.22	0.1151	1.16	0.4698
103776_at	1442028_at	AI593864	expressed sequence AI593864	-3.08	0.24	-1.05	0.7819	1.44	0.4204	-1.12	0.5215	1.26	0.5252

103778_at	1448012_at	C76336	expressed sequence C76336	-2.32	0.35	-1.2	0.5337	-1.29	0.3936	-1.12	0.6979	-1	0.9948
103779_at	1434900_at	---	---	1.64	0.31	1.5	0.0413	1.43	0.1108	1.35	0.1044	1.65	0.089
103780_at	1421019_at	1700021F05Rik	RIKEN cDNA 1700021F05 gene	1.77	0.04	1.19	0.1042	1.26	0.0833	1.28	0.0676	-1	0.995
103781_at	1451573_a_at	Stx4a	syntaxin 4A (placental)	1.22	0.5	-1.1	0.2692	-1.08	0.3065	-1.06	0.3267	1.13	0.5657
103782_at	1455677_s_at	---	---	-1.54	0.22	-1.11	0.1782	-1.07	0.2937	-1.05	0.5987	1.42	0.1067
103783_at	1426992_at	Xpr1	xenotropic and polytropic retrovirus receptor	-1.11	0.52	-1.15	0.1954	1.12	0.429	-1.19	0.1031	-1.47	0.1328
103786_at	1435946_at	---	---	1.18	0.61	-1.12	0.6343	1.08	0.7647	-1.1	0.6858	-2.8	0.0023
103787_at	1417416_at	Kcna1	potassium voltage-gated channel, shaker-re	-1.72	0.04	1.14	0.1757	1.14	0.2337	1.03	0.7499	1.14	0.5705
103789_at	1424922_a_at	Brd4	bromodomain containing 4	-1.24	0.8	-1.25	0.5049	-2.95	0.013	-2.52	0.0404	1.13	0.6302
103790_at	1460742_at	Alg3	asparagine-linked glycosylation 3 homolog (	-1.19	0.21	-1.12	0.1326	-1.02	0.8005	-1.06	0.2145	1.1	0.385
103791_at	1418023_at	Narg1	NMDA receptor-regulated gene 1	1.78	0.27	1.26	0.019	1.39	0.0627	1.19	0.1817	-1.09	0.8615
103793_at	1448618_at	Mvp	major vault protein	1.25	0.07	-1.01	0.873	1.08	0.1411	1.06	0.5181	1.13	0.5117
103795_f_at	1418766_s_at	Timd2	T-cell immunoglobulin and mucin domain co	-1.34	0.05	-1.49	0.0009	-1.07	0.5903	-1.55	0.0006	-1.8	0.0251
103796_at	1452870_at	Apaf1	apoptotic protease activating factor 1	-1.34	0.4	1.14	0.3077	1.12	0.489	1.19	0.2643	1.08	0.761
103797_at	1426002_a_at	Cdc7	cell division cycle 7 (S. cerevisiae)	-1.33	0.58	1.44	0.3247	1.27	0.514	1.74	0.0295	2.72	0.0294
103799_at	1452254_at	Mtmr9	myotubularin related protein 9	1.13	0.64	-1.09	0.2942	-1.17	0.1413	-1.23	0.0125	1.19	0.5583
103800_at	1438056_x_at	Abcc5	ATP-binding cassette, sub-family C (CFTR/M	-1.48	0	-1.18	0.1174	-1.23	0.1122	-1.14	0.2751	1.26	0.1564
103801_at	1419310_s_at	Rfxank	regulatory factor X-associated ankyrin-conta	1.04	0.89	-1.13	0.0577	-1.07	0.3271	-1.14	0.0811	1.4	0.0035
103803_at	1419767_at	Padi3	peptidyl arginine deiminase, type III	1.15	0.71	1.12	0.6299	1.02	0.9641	1.17	0.5448	1.66	0.1259
103804_at	1450784_at	---	---	1.29	0.36	-1.72	0.1186	-1.29	0.3754	-1.8	0.0823	-2.21	0.0438
103805_at	1448746_at	Nbn	nibrin	1.72	0.21	-1.04	0.6557	1.38	0.0108	1.11	0.3095	-1.13	0.4003
103806_at	1449299_at	Lrp5	low density lipoprotein receptor-related prote	1.67	0.38	-1.16	0.1596	-1.13	0.1638	-1.38	0.0022	1.1	0.8302
103807_at	1418202_a_at	Wiz	widely-interspaced zinc finger motifs	-1.48	0.02	-1.07	0.3622	-1.04	0.4179	-1.14	0.0796	1.21	0.1953
103808_at	1418447_at	Golga5	golgi autoantigen, golgin subfamily a, 5	-1.09	0.71	1.15	0.1708	1.41	0.0531	1.35	0.0089	-1.27	0.4286
103809_r_at	1416361_a_at	Dncic1	dynein, cytoplasmic, intermediate chain 1	-1.07	0.64	1.02	0.8233	-1.03	0.7636	1.01	0.9545	1.33	0.0056
103810_at	1418158_at	Trp63	transformation related protein 63	-1.11	0.87	-1.1	0.2503	1.17	0.0133	-1.12	0.2045	1.32	0.0981
103811_at	1419308_at	Invs	inversin	-1.07	0.68	-1.18	0.0922	-1.2	0.2364	-1.35	0.0144	-1.37	0.3114
103812_at	1417852_x_at	Clca1	chloride channel calcium activated 1	1.25	0.31	1.45	0.0018	1.18	0.0705	1.43	0.0003	1.66	0.0434
103813_at	1449579_at	Sh3yl1	Sh3 domain YSC-like 1	1.06	0.8	-1.19	0.0627	-1.12	0.4754	-1.29	0.025	-1.33	0.4014
103814_at	1420460_a_at	Pex11b	peroxisomal biogenesis factor 11b	-1.2	0.42	1.03	0.6843	1.02	0.8532	-1.06	0.4145	-1.05	0.7806
103816_at	1424595_at	F11r	F11 receptor	1.23	0.22	1.1	0.4784	1.01	0.8891	1.16	0.1691	1.3	0.1957
103817_at	1448592_at	Crtap	cartilage associated protein	1.15	0.54	-1	0.96	1.02	0.7591	-1.06	0.4351	1.37	0.1039
103818_at	1417392_a_at	Slc7a7	solute carrier family 7 (cationic amino acid tr	3.12	0.01	1.38	0.2308	1.11	0.687	1.95	0.0949	-1.03	0.928
103819_at	1417486_at	Shd	src homology 2 domain-containing transform	-1.23	0.62	1.14	0.5678	1.17	0.4394	1.33	0.2024	2.21	0.1784
103821_at	1417019_a_at	Cdc6	cell division cycle 6 homolog (S. cerevisiae)	1.47	0.33	-1.13	0.0853	-1.16	0.2486	-1.1	0.3658	-1.2	0.4919
103822_at	1428961_a_at	Sfrs16	splicing factor, arginine/serine-rich 16 (suppr	-1.48	0.14	1.52	0.1122	2.09	0.0298	2.21	0.0151	1.83	0.1659
103823_at	1448173_a_at	Top3b	topoisomerase (DNA) III beta	-1.19	0.22	1	0.9959	1.07	0.4897	1.01	0.8714	-1	0.9916
103824_at	1448411_at	Wfs1	Wolfram syndrome 1 homolog (human)	1.19	0.69	1.36	0.3182	1.25	0.2741	1.24	0.6135	1.6	0.3292
103825_at	1422268_a_at	Rps6kb2	ribosomal protein S6 kinase, polypeptide 2	-2.18	0.21	1.16	0.6402	1.15	0.5982	-1.42	0.2231	1.02	0.9685
103826_at	1450093_s_at	Zbtb7	zinc finger and BTB domain containing 7	1.51	0.3	-1.15	0.1961	-1.3	0.0581	-1.28	0.0225	1.15	0.5553
103829_at	1426491_at	Herc2	hect (homologous to the E6-AP (UBE3A) ca	1.24	0.23	-1.28	0.0193	-1.19	0.1445	1.1	0.4813	-1.75	0.0974
103830_at	1448742_at	Snai1	snail homolog 1 (Drosophila)	-1.59	0.59	1.3	0.49	1.28	0.5367	-1.11	0.7951	1.71	0.3268
103831_at	1416734_at	Mkln1	muskelin 1, intracellular mediator containing	-1.29	0.61	1.02	0.9174	-1.2	0.3111	-1.2	0.284	1.25	0.5771
103832_at	1448976_at	Tfip11	tuftelin interacting protein 11	1.29	0.09	1.15	0.3021	1.19	0.1884	1.08	0.4416	1.29	0.065
103833_at	1448631_a_at	Hipk2	homeodomain interacting protein kinase 2	-1.32	0.68	-1.21	0.2209	-1.22	0.4289	-1.92	0.0162	-1.83	0.3125
103835_f_at	1448812_at	Hpcal1	hippocalcin-like 1	-1.04	0.76	1.2	0	1.12	0.021	1.2	0.0511	1.29	0.1462
103836_at	1419375_at	Wbp4	WW domain binding protein 4	-1.04	0.81	1.28	0.1036	1.16	0.5298	1.35	0.2192	1.07	0.6538
103837_at	1418748_at	Casp14	caspase 14	-2.09	0.21	-1.04	0.7273	-1.13	0.5265	-1.14	0.4843	1.74	0.0363
103838_at	1449206_at	Mg29	mitsugumin 29	-1.4	0.53	-1.08	0.6774	1.21	0.287	1.13	0.4488	1.52	0.0979
103839_at	1451596_a_at	Sphk1	sphingosine kinase 1	-1.14	0.69	1.03	0.7989	1.1	0.3069	1	0.9991	1.16	0.4554
103840_at	1448762_at	Rad17	RAD17 homolog (S. pombe)	1.4	0.27	1.26	0.0151	1.35	0.0787	1.38	0.0022	-1.2	0.3062
103841_at	1421030_at	Zfp64	zinc finger protein 64	-1.18	0.72	1.05	0.5499	1.09	0.6302	1.02	0.7986	-1.01	0.9893
103842_at	1452077_at	Ddx3y	DEAD (Asp-Glu-Ala-Asp) box polypeptide 3,	1.16	0.63	1.69	0.4251	-1.14	0.7993	1.57	0.3367	-1.14	0.517

103844_at	1460383_at	Gnao1	guanine nucleotide binding protein, alpha o	-1.22	0.3	1.1	0.4962	1.09	0.6068	1.03	0.7807	1.5	0.2963
103845_at	1433750_at	Slc31a1	solute carrier family 31, member 1	-1.01	0.98	-1.07	0.0509	1.04	0.3814	-1.17	0	-1.08	0.3352
103846_at	1422308_a_at	Lgals7	lectin, galactose binding, soluble 7	1.71	0.04	1.08	0.5706	1.06	0.5988	1.11	0.2686	1.12	0.6827
103849_at	1421953_at	Crkl	v-crk sarcoma virus CT10 oncogene homolo	1.36	0.45	-1.13	0.7348	1.11	0.7932	-1.76	0.1164	1.37	0.5838
103850_at	1451978_at	Loxl1	lysyl oxidase-like 1	-1.02	0.96	1.03	0.8173	-1.12	0.3476	-1.16	0.0929	1.23	0.3918
103852_at	1423958_a_at	2900001O04Rik	RIKEN cDNA 2900001O04 gene	1.39	0.15	1.13	0.2689	1.05	0.7456	1.16	0.1835	-1.41	0.0036
103853_at	1452171_at	Grwd1	glutamate-rich WD repeat containing 1	1.89	0.12	-1.12	0.3845	1.07	0.6391	1	0.9813	1.86	0.169
103854_at	1422549_at	Arl2	ADP-ribosylation factor-like 2	1.48	0.34	1.01	0.9501	1.05	0.5796	-1	0.9705	1.02	0.9597
103855_at	1419835_s_at	Plec1	plectin 1	1.13	0.52	1.09	0.34	1.24	0.041	1.21	0.1004	1.38	0.0032
103858_at	1460548_a_at	Eral1	Era (G-protein)-like 1 (E. coli)	1.06	0.68	1.04	0.3058	1.13	0.0353	1.08	0.107	-1.14	0.0182
103862_r_at	1448968_at	D7Wsu128e	DNA segment, Chr 7, Wayne State Universit	-1.1	0.16	-1.1	0.217	1.1	0.3251	-1.14	0.1078	1.08	0.6123
103863_at	1460656_a_at	5630401J11Rik	RIKEN cDNA 5630401J11 gene	-1.35	0.27	1.31	0.0068	1.13	0.4179	1.44	0.0152	1.07	0.7359
103865_at	1456598_at	LOC434451	similar to DNA segment, Chr 5, ERATO Doi	2.36	0.14	-1.53	0.0146	-1.18	0.3791	-1.27	0.1546	1.42	0.1023
103866_at	1453281_at	---	---	1.54	0.33	2.23	0.1348	1.12	0.1608	2.38	0.1546	1.23	0.2781
103868_at	1448802_at	Nufip1	nuclear fragile X mental retardation protein i	2.14	0.05	1.55	0.0235	1.55	0.0541	1.72	0.0031	-1.8	0.1905
103869_at	1416623_at	Thbs3	thrombospondin 3	1.23	0.67	1.07	0.8281	-1.1	0.7865	-1.21	0.5943	-1.31	0.4879
103871_at	1433627_at	Sec23ip	Sec23 interacting protein	2.88	0.05	1.54	0.0302	1.51	0.1275	1.63	0.0086	1.94	0.0817
103872_r_at	1450741_at	Stau1	staufen (RNA binding protein) homolog 1 (D	1.25	0.15	-1.04	0.7118	1.03	0.7788	-1.03	0.7875	1.68	0.051
103874_r_at	1428505_at	2310015N07Rik	RIKEN cDNA 2310015N07 gene	-1.26	0.18	1.57	0.1239	1.6	0.0948	1.59	0.156	-1.28	0.5261
103875_at	1416709_a_at	AW552001	expressed sequence AW552001	1.47	0.16	1.25	0.0002	1.3	0.0009	1.26	0.0063	1.08	0.4541
103876_at	1417583_a_at	Crlz1	charged amino acid rich leucine zipper 1	1.01	0.98	-1.03	0.5813	1.01	0.8558	-1.05	0.3189	-1.16	0.2847
103877_at	1460731_at	Slc35c2	solute carrier family 35, member C2	1.24	0.14	-1.02	0.7035	-1.01	0.9088	-1.04	0.4473	1.38	0.0021
103878_at	1423235_at	Ap3b1	adaptor-related protein complex 3, beta 1 su	1.06	0.79	-1.02	0.7465	1.05	0.5483	1.11	0.103	1.09	0.1541
103879_at	1451354_at	BC024806	cDNA sequence BC024806	-1.12	0.31	-1.01	0.9198	1.1	0.2369	1.13	0.1047	-1.02	0.9261
103881_at	1424488_a_at	1110013G13Rik	RIKEN cDNA 1110013G13 gene	1.48	0.03	-1	0.9454	1.16	0.183	1.05	0.4735	-1.59	0.0045
103882_at	1417745_at	Cpn1	carboxypeptidase N, polypeptide 1	1.1	0.74	-1.12	0.0305	-1.11	0.2553	-1.34	0.0001	-1.24	0.07
103884_at	1424682_at	D5Ertd708e	DNA segment, Chr 5, ERATO Doi 708, expr	2.32	0.08	1.2	0.0142	1.21	0.0656	1.17	0.1386	1.35	0.206
103885_at	1448633_at	Prpf31	PRP31 pre-mRNA processing factor 31 hom	1.64	0.25	1.18	0.3249	1.18	0.4297	1.3	0.1329	1.09	0.7661
103886_at	1426376_at	Dp1	deleted in polyposis 1	-1.01	0.97	-1.08	0.3547	1.08	0.3632	-1.23	0.1321	1.21	0.193
103887_at	1448756_at	S100a9	S100 calcium binding protein A9 (calgranulir	2.36	0.09	2.58	0.0266	1.12	0.7416	1.87	0.1571	3.94	0.0028
103888_at	1437161_x_at	Rbpms	RNA binding protein gene with multiple splic	1.19	0.5	1.2	0.0214	1.38	0.0225	1.29	0.0009	1.43	0.0209
103889_at	1434839_s_at	Tbl1xr1	transducin (beta)-like 1X-linked receptor 1	1.05	0.85	1.09	0.4124	-1.17	0.2159	1.09	0.3577	-1.12	0.5686
103890_at	1415866_at	AW538196	expressed sequence AW538196	1.74	0.19	-1.33	0.2625	1.02	0.9527	1.28	0.3477	-1.04	0.5947
103892_r_at	1450744_at	Ell2	elongation factor RNA polymerase II 2	1.38	0.14	1.09	0.4863	1.38	0.1184	-1	0.9973	-1.27	0.1357
103893_at	1452255_at	Fbxo38	F-box protein 38	-1.16	0.15	1.1	0.0786	1.09	0.1018	1.08	0.1391	-1.11	0.4921
103894_at	1424174_at	Shkbp1	Sh3kbp1 binding protein 1	1	0.99	-1.19	0.0535	-1.05	0.5858	-1.18	0.1092	-1.04	0.7503
103895_at	1426459_s_at	AW549877	expressed sequence AW549877	-1.24	0.21	-1.05	0.721	1.22	0.173	-1	0.9597	1.19	0.0917
103896_f_at	1418918_at	Igfbp1	insulin-like growth factor binding protein 1	3.25	0.06	11.33	0.0006	1.71	0.3508	18.87	0	36.97	0.011
103899_at	1456388_at	Atp11a	ATPase, class VI, type 11A	4.69	0.04	1.23	0.3428	-1.25	0.2314	1.11	0.4204	-3.12	0.063
103900_at	1423910_at	Centg3	centaurin, gamma 3	1.38	0.18	-1.32	0.0214	1.02	0.8662	-1.2	0.0577	1.23	0.4873
103901_at	1426899_at	4930451A13Rik	RIKEN cDNA 4930451A13 gene	1.04	0.84	1.05	0.4348	1.08	0.5609	1.04	0.513	1.58	0.0082
103903_at	1433881_at	Dnajc11	DnaJ (Hsp40) homolog, subfamily C, membe	1.38	0.51	1.48	0.2384	1.37	0.2201	1.11	0.7156	1.23	0.0232
103904_at	1417933_at	Igfbp6	insulin-like growth factor binding protein 6	-1.27	0.52	-1.11	0.4798	-1.12	0.2791	1.02	0.9415	1.11	0.2279
103905_at	1428485_at	Car12	carbonic anyhydrase 12	1.39	0.4	1.06	0.786	-1.26	0.4044	1.16	0.4225	1.35	0.2342
103907_at	1423269_a_at	Nedd4l	neural precursor cell expressed, developme	-1.57	0.32	-1	0.9967	-1.25	0.075	-1.03	0.7978	1.01	0.9683
103908_at	1453195_at	C330016H24Rik	RIKEN cDNA C330016H24 gene	-1.27	0.5	1.13	0.1491	1.05	0.5762	1.14	0.1055	-1.42	0.2804
103909_at	1448615_at	Ccs	copper chaperone for superoxide dismutase	1.11	0.84	-1.22	0.0008	-1.15	0.1245	-1.44	0	-1.72	0.0667
103910_at	1448784_at	Taf10	TAF10 RNA polymerase II, TATA box bindin	1.04	0.84	1.03	0.5661	1.17	0.0986	1.09	0.1763	-1.5	0.1223
103911_at	1424604_s_at	Sumf1	sulfatase modifying factor 1	1.01	0.99	-1.11	0.3524	1.11	0.5274	1.02	0.8675	-1.35	0.2568
103912_at	1428265_at	Ppp2r1b	protein phosphatase 2 (formerly 2A), regulat	-1.45	0.15	-1.38	0.0805	-1.11	0.5071	-1.29	0.0766	1.25	0.6185
103913_at	1449944_a_at	Sec61a2	Sec61, alpha subunit 2 (S. cerevisiae)	-1.13	0.23	1.22	0.0026	1.32	0.0047	1.2	0.0214	1.15	0.5391
103914_at	1420493_a_at	Pcyt2	phosphate cytidylyltransferase 2, ethanolam	-1.18	0.39	-1.14	0.0626	-1.01	0.9415	-1.15	0.0378	-1.22	0.3312
103916_at	1426436_at	8430420C20Rik	RIKEN cDNA 8430420C20 gene	1.76	0.13	1.08	0.5895	1.2	0.1868	1.18	0.3455	1.16	0.6611

103918_at	1424730_a_at	Slc15a2	solute carrier family 15 (H+/peptide transpor	-2.42	0.09	-1.31	0.3086	1.34	0.0922	-1.03	0.8915	1.36	0.3952
103922_f_at	1424048_a_at	Nqo3a2	NAD(P)H:quinone oxidoreductase type 3, pc	1.17	0.53	1.16	0.2597	1.68	0.0181	1.78	0.0004	1.52	0.1078
103924_at	1434287_at	Agpat5	1-acylglycerol-3-phosphate O-acyltransferas	-1.08	0.57	-1.15	0.0135	-1.11	0.0904	-1.17	0.0625	1.37	0.0662
103925_at	1431890_a_at	Mllt3	myeloid/lymphoid or mixed lineage-leukemia	1.13	0.75	-2.64	0.0001	1.12	0.4312	-3.66	0	1.17	0.5632
103927_at	1435091_at	C80731	expressed sequence C80731	1.3	0.36	1.08	0.3872	1.13	0.2494	1.07	0.3489	-1	0.9966
103931_at	1418611_at	Grca	gene rich cluster, A gene	-1.04	0.79	-1.17	0.5249	1.08	0.7661	-1.29	0.3612	1.58	0.1951
103932_at	1435442_at	Gm83	gene model 83, (NCBI)	1.94	0.05	1.42	0.0001	1.54	0.0049	1.56	0	1.36	0.0303
103934_at	1424338_at	Slc6a13	solute carrier family 6 (neurotransmitter tran:	-1.74	0.08	-1.15	0.0471	1.19	0.0818	-1.24	0.0006	-1.49	0.0095
103935_at	1421129_a_at	Atp2a3	ATPase, Ca++ transporting, ubiquitous	-1.19	0.32	1.19	0.0683	1.06	0.8112	1.07	0.5343	1.49	0.0972
103939_at	1451512_s_at	Hibch	3-hydroxyisobutyryl-Coenzyme A hydrolase	2.18	0.02	1.05	0.5866	1.24	0.213	1.06	0.506	-1.08	0.5659
103941_at	1421278_s_at	---	---	-1.75	0.06	1.02	0.9473	1.05	0.8312	-1.47	0.1379	2.31	0.2895
103942_at	1436370_at	Gucy2c	guanylate cyclase 2c	3.94	0.2	1.91	0.0523	1.93	0.0723	2.33	0.008	8.52	0.0009
103943_at	1452846_at	Ppfia4	protein tyrosine phosphatase, receptor type,	-2.26	0.01	-1.39	0.0148	-1.02	0.8634	1.07	0.8184	1.64	0.0843
103944_at	1421430_at	Rad51l1	RAD51-like 1 (S. cerevisiae)	1.02	0.96	1.09	0.4522	1.57	0.0003	-1.01	0.9468	1.41	0.2793
103945_at	1421524_at	Cfc1	cripto, FRL-1, cryptic family 1	1.06	0.89	-1.22	0.3789	1.26	0.3921	-1.11	0.6542	1.68	0.1354
103946_at	1424560_at	Pstpip1	proline-serine-threonine phosphatase-intera	1	0.99	2.03	0.0115	1.18	0.6163	1.77	0.1476	1.87	0.1069
103947_at	1424347_at	Ppp6c	protein phosphatase 6, catalytic subunit	1.19	0.25	1.07	0.2366	1.05	0.6018	1.13	0.1017	1.06	0.5811
103949_at	1450704_at	Ihh	Indian hedgehog	-1.01	0.94	1.06	0.4693	1.17	0.0679	-1.04	0.6098	-1.07	0.7843
103950_at	1452448_at	Aqr	aquarius	-2.31	0.4	-1.09	0.7431	-1.41	0.2571	1.03	0.9129	-1.79	0.2856
103952_at	1452317_at	Hoxb9	Homeo box B9	-2.47	0.17	-1.16	0.3371	-1.05	0.7777	-1.28	0.1815	1.21	0.1487
103953_at	1449063_at	Sec22l1	SEC22 vesicle trafficking protein-like 1 (S. c	1.16	0.71	-1.07	0.4951	1	0.994	-1.01	0.9206	-1.04	0.8676
103954_at	1449495_at	Reg3a	regenerating islet-derived 3 alpha	-1.66	0.14	-1.16	0.1573	1.14	0.176	1.02	0.8701	1.05	0.8392
103955_at	1416795_at	Cryl1	crystallin, lamda 1	-1.38	0.2	-1.16	0.0311	1.2	0.0247	-1.16	0.1911	-1.77	0.0141
103956_at	1419216_at	Azi1	5-azacytidine induced gene 1	-1.04	0.92	1.49	0.0619	1.56	0.0181	1.35	0.0544	-1.12	0.6457
103958_g_at	1422966_a_at	Tfrc	transferrin receptor	-1.33	0.13	-1.79	0.0149	-1.17	0.4271	-1.15	0.4889	-1.02	0.9542
103959_at	1455175_at	Phf13	PHD finger protein 13	1.06	0.77	1.54	0.0023	1.27	0.1332	1.46	0.0064	1.45	0.0389
103960_at	1449246_at	Rap2ip	Rap2 interacting protein	-1.01	0.97	-2.11	0.0006	1.06	0.6907	-1.77	0.0033	-1.47	0.2352
103962_at	1450545_a_at	Dntt	deoxynucleotidyltransferase, terminal	-1.1	0.86	-5.11	0.0026	-2.74	0.0151	-3.8	0.0053	1.11	0.772
103963_f_at	1419042_at	Iigp1	interferon inducible GTPase 1	-3.02	0	1.16	0.7637	-1.54	0.027	1.37	0.5366	-2.87	0.0101
103964_at	1460652_at	Esrra	Estrogen related receptor, alpha	-1.21	0.28	1.1	0.1475	1.07	0.5916	1.2	0.0575	1.2	0.2724
103965_at	1460433_at	Entpd6	ectonucleoside triphosphate diphosphohydro	-1.03	0.85	-1.47	0.0055	-1.19	0.0699	-1.26	0.0313	1.07	0.8273
103968_at	1422216_at	Mid2	midline 2	1.01	0.98	1.32	0.1677	-1	0.9795	1.29	0.1234	-1.05	0.9089
103969_at	1434704_at	---	---	1.07	0.45	1.18	0.0617	1.13	0.3255	1.16	0.0243	1.27	0.188
103970_at	1423526_at	Arid3b	AT rich interactive domain 3B (Bright like)	-1.17	0.57	1.05	0.6823	1.04	0.6704	-1.03	0.8518	1.41	0.339
103971_at	1460719_a_at	P2rx1	purinergic receptor P2X, ligand-gated ion ch	-1.26	0	-1.4	0.0199	-1.4	0.0069	-1.48	0.0034	1.08	0.6645
103972_at	1418614_at	Kcnj1	potassium inwardly-rectifying channel, subfa	-1.59	0.19	-1	0.9753	1.12	0.304	1.3	0.1782	1.28	0.3958
103973_at	1418613_at	Kcnj1	potassium inwardly-rectifying channel, subfa	1.06	0.92	-1.16	0.4857	-1.34	0.1591	-1.46	0.0715	1.57	0.457
103974_at	1419154_at	Tmprss2	transmembrane protease, serine 2	1.27	0.46	-1.37	0.0183	-1.09	0.5399	-1.52	0.0213	1.32	0.1052
103975_at	1418492_at	Grem2	gremlin 2 homolog, cysteine knot superfamil	2.89	0	2.63	0.0158	3.04	0.0011	5.66	0	9.29	0
103976_at	1449605_at	---	Transcribed locus	-1.07	0.85	1.8	0.0728	-1.26	0.3594	1.43	0.2734	2.26	0.3297
103977_at	1449305_at	F10	coagulation factor X	1.26	0.47	-1.27	0	-1.03	0.2106	-1.27	0	-1.2	0.0705
103978_at	1426361_at	5730454B08Rik	RIKEN cDNA 5730454B08 gene	-1.1	0.86	1.05	0.3356	1.03	0.7574	-1.04	0.6817	1.15	0.2084
103980_at	1421151_a_at	Epha2	Eph receptor A2	-1.26	0.13	1.1	0.5563	-1.04	0.6957	-1.05	0.6052	-1.09	0.7407
103981_at	1455727_at	U2af1-rs2	U2 small nuclear ribonucleoprotein auxiliary	-1.11	0.78	-1.09	0.5041	-1.04	0.8068	-1.12	0.3919	-1.16	0.5786
103983_at	1422070_at	Adh4	alcohol dehydrogenase 4 (class II), pi polype	1.33	0.33	1.29	0.0069	1.21	0.1313	1.4	0.001	-1.54	0.0471
103984_at	1419820_at	Pkhd1	polycystic kidney and hepatic disease 1	1.86	0.3	1.14	0.6169	-1.16	0.6481	-1.56	0.1272	-2.05	0.1782
103985_at	1450244_a_at	Map4k2	mitogen activated protein kinase kinase kina	1.01	0.98	1.5	0.0337	1.14	0.4077	1.27	0.0803	1.11	0.6141
103986_at	1422001_at	Inhbc	inhibin beta-C	-1.54	0.03	-1	0.9878	1.05	0.607	-1.14	0.1926	-1.12	0.3631
103987_at	1448768_at	Mog	myelin oligodendrocyte glycoprotein	-1.24	0.48	1.81	0.163	1.3	0.4641	-1.05	0.5909	1.11	0.6239
103988_at	1426843_at	BC023754	cDNA sequence BC023754	1.12	0.37	-1.04	0.6522	1.08	0.5952	1.11	0.0648	1.08	0.4995
103989_at	1432057_a_at	Prdm5	PR domain containing 5	1.26	0.73	-1.06	0.8235	-1.03	0.9324	-1.11	0.6772	1.81	0.2458
103990_at	1422134_at	Fosb	FBJ osteosarcoma oncogene B	-1.5	0.01	-1.09	0.4241	-1.09	0.4155	-1.18	0.186	1.28	0.0229
103991_at	1450636_s_at	Akp5	alkaline phosphatase 5	-1.36	0.17	1.01	0.9581	1.02	0.8096	-1.02	0.859	1.51	0.0442

103993_at	1451585_x_at	Grcc9	gene rich cluster, C9 gene	-1.23	0.22	-1.19	0.1999	1.06	0.5299	-1.19	0.1269	1.19	0.5982
103994_at	1426366_at	Eif2c2	eukaryotic translation initiation factor 2C, 2	1.47	0.22	1.19	0.4857	-1.11	0.6791	-1.03	0.8687	1.91	0.0086
103995_at	1419086_at	Fgfbp1	fibroblast growth factor binding protein 1	-1.99	0.2	1.11	0.4563	1.42	0.293	1.57	0.2544	1.33	0.3888
103997_at	1423344_at	Epor	erythropoietin receptor	1.66	0.11	-1.06	0.7978	1.24	0.2018	1.04	0.8139	1.75	0.1536
103998_at	1429063_s_at	Kif16b	kinesin family member 16B	1.49	0.02	1.21	0.0558	1.39	0.0099	1.17	0.1131	-1.44	0.3471
103999_at	1455326_at	4932416N17Rik	RIKEN cDNA 4932416N17 gene	1.11	0.63	-1.26	0.0009	-1.16	0.0279	-1.19	0.0247	-2	0.0231
104000_at	1424968_at	2210023G05Rik	RIKEN cDNA 2210023G05 gene	-1.65	0.11	-1.2	0.0504	1.17	0.2165	-1.04	0.6293	-1.23	0.3246
104002_at	1418397_at	Zfp275	Zinc finger protein 275	1.19	0.31	1.11	0.1096	1.07	0.3729	1.22	0.0163	1.58	0.1472
104003_at	1434518_at	Phka2	phosphorylase kinase alpha 2	2.37	0.16	1.01	0.9365	1.15	0.3182	1.4	0.0029	1.03	0.9248
104004_at	1428875_at	Golph4	golgi phosphoprotein 4	1.49	0.19	1.23	0.0909	1.15	0.4289	1.35	0.0061	1.27	0.146
104006_at	1419251_at	Eps15	epidermal growth factor receptor pathway su	-1.72	0.34	1.02	0.9324	1.53	0.0042	1.32	0.2736	-1.95	0.2452
104007_at	1420967_at	Slc25a15	solute carrier family 25 (mitochondrial carrie	1.4	0.25	-1.28	0.0001	-1.34	0.0002	-1.49	0	-1.79	0.0404
104008_at	1450220_a_at	Spdef	SAM pointed domain containing ets transcrip	-1.58	0.65	1.01	0.9532	1.52	0.3931	1.04	0.8919	1.9	0.3453
104010_at	1418607_at	Zfp99	zinc finger protein 99	-1.08	0.72	-1.1	0.5549	1.1	0.4174	1.27	0.0383	1.7	0.0834
104011_at	1419435_at	Aox1	aldehyde oxidase 1	-1.16	0.74	1.43	0.0147	1.21	0.2219	1.14	0.2821	-2.44	0.0001
104012_at	1427694_at	Gnrhr	gonadotropin releasing hormone receptor	-1.39	0.35	-1.3	0.1335	1.7	0.1701	-1.08	0.6882	-1.42	0.1355
104013_at	1427731_at	Gnrhr	Gonadotropin releasing hormone receptor	-1.69	0.08	-1.45	0.2137	-1.32	0.3198	-1.16	0.6706	1.17	0.4351
104014_at	1422645_at	Hfe	hemochromatosis	1.43	0.2	-1.09	0.0802	-1	0.9535	-1.12	0.0219	-2.31	0.0005
104015_at	1426836_s_at	Metap1	methionyl aminopeptidase 1	-1.06	0.6	1.06	0.3488	-1.01	0.9163	1.04	0.624	-1.06	0.5218
104016_at	1449204_at	Gjb5	gap junction membrane channel protein beta	-1.65	0.37	-1.25	0.0889	-1.06	0.6091	-1.29	0.0565	-1.35	0.2134
104017_at	1451828_a_at	Acsl4	acyl-CoA synthetase long-chain family mem	1.76	0.14	1.11	0.5515	1.12	0.468	1.41	0.1903	1.26	0.4074
104018_at	1439479_at	Lct	lactase	-1.43	0.14	-1.06	0.6674	1.07	0.7082	1.15	0.4603	1.52	0.1277
104019_at	1424539_at	---	---	2.3	0.13	-1.1	0.2307	-1.07	0.4877	-1.01	0.8755	1.36	0.4068
104020_at	1448885_at	Rap2b	RAP2B, member of RAS oncogene family	-1.89	0.07	-1.16	0.1936	-1.11	0.3758	-1.21	0.0971	1.2	0.3522
104021_at	1420414_at	Hoxa11	homeo box A11	1.22	0.82	1.52	0.3161	-1.22	0.5639	1.3	0.4733	2.61	0.0469
104022_at	1429080_at	Mphosph10	M-phase phosphoprotein 10 (U3 small nucle	4.47	0.05	2.24	0.1114	2.51	0.0523	2.73	0.0326	-1	0.9962
104023_at	1428018_a_at	Cd300c	Cd300C antigen	1.04	0.89	1.08	0.6056	-1.16	0.2303	1.19	0.528	1.6	0.0093
104024_at	1424973_at	Cyp3a25	cytochrome P450, family 3, subfamily a, poly	1.77	0.15	1.51	0.0019	1.12	0.1377	1.74	0	-1.05	0.599
104025_at	1448907_at	Thop1	thimet oligopeptidase 1	1.62	0.13	-1.47	0.1527	1.14	0.6585	1.22	0.3659	1.38	0.2583
104029_at	1430240_a_at	Clgn	calmegin	-1.43	0.22	-1.03	0.8898	-1.09	0.6663	1.2	0.179	-1.02	0.9512
104030_at	1428853_at	Ptch1	patched homolog 1	1.64	0	1.24	0.0681	1.17	0.083	1.31	0.0099	1.3	0.0545
104031_at	1450824_at	Ptch1	patched homolog 1	-1.05	0.63	-1.05	0.5747	-1.02	0.7834	-1.1	0.2564	1.23	0.1154
104032_at	1435666_at	Mast3	microtubule associated serine/threonine kina	1.99	0.43	1.4	0.3956	3.1	0.0136	2.71	0.0069	1.14	0.644
104033_at	1426445_at	Mgea6	meningioma expressed antigen 6 (coiled-coi	-1	0.99	1.3	0.0007	1.1	0.1451	1.36	0.0001	1.42	0.0061
104034_at	1435417_at	AI464131	expressed sequence AI464131	1.01	0.96	-1.22	0.0308	-1.01	0.9403	-1.4	0.0006	-1.19	0.2807
104035_at	1456492_at	---	---	-1.36	0.55	1.24	0.3991	-1.09	0.7641	-1.34	0.3583	1.35	0.272
104036_at	1436479_a_at	Dpp7	dipeptidylpeptidase 7	-1.68	0.05	-1.57	0	-1.08	0.313	-1.42	0.0004	-1.62	0.0132
104038_at	1448463_at	4933434E20Rik	RIKEN cDNA 4933434E20 gene	-1.24	0.01	-1.06	0.5158	1.14	0.2963	-1.03	0.7351	-1.08	0.6826
104041_at	1454668_at	1810009A16Rik	RIKEN cDNA 1810009A16 gene	-1.23	0.43	-1.04	0.3619	-1.06	0.5417	-1.1	0.0302	1.22	0.3416
104042_at	1423927_at	Slc35b2	solute carrier family 35, member B2	1.43	0.06	-1.1	0.1939	1.03	0.5803	1.08	0.2195	1.28	0.0951
104044_at	1430293_a_at	1300006N24Rik	RIKEN cDNA 1300006N24 gene	1.36	0.07	1.06	0.3092	-1	0.9937	1.06	0.2765	1.16	0.1872
104045_at	1424029_at	D10Bwg0791e	DNA segment, Chr 10, Brigham & Women's	-1.84	0.33	-2.02	0.0047	-1.46	0.0552	-1.78	0.0132	-2.99	0.0986
104047_at	1420932_at	Mapk8	mitogen activated protein kinase 8	-1.07	0.5	1.13	0.0845	1.15	0.0717	1.11	0.2408	1.2	0.1491
104049_at	1427898_at	Rnf6	ring finger protein (C3H2C3 type) 6	1.36	0.51	1.33	0.0222	1.18	0.3237	1.18	0.1465	-1.22	0.0213
104050_at	1419650_at	Zfr	zinc finger RNA binding protein	1.04	0.84	1.06	0.3163	-1.29	0.0031	-1.15	0.016	1.18	0.0939
104052_at	1433912_at	1810011K17Rik	RIKEN cDNA 1810011K17 gene	-1.09	0.71	-1.06	0.4662	1.04	0.5472	-1.07	0.3662	-1.06	0.621
104053_at	1454711_at	Trio	triple functional domain (PTPRF interacting)	-1.29	0.15	-1.07	0.1764	-1.02	0.6992	-1.14	0.0181	1.54	0.0429
104056_at	1433995_s_at	D16Bwg1543e	DNA segment, Chr 16, Brigham & Women's	1.01	0.91	1.29	0.0006	1.36	0.042	1.29	0.0069	1.31	0.0244
104057_at	1417320_at	Grpel1	GrpE-like 1, mitochondrial	2.02	0	-1.07	0.2881	-1.01	0.8715	-1.03	0.7435	-1.04	0.7264
104058_at	1428655_at	1110018J12Rik	RIKEN cDNA 1110018J12 gene	-1.16	0.26	-1.02	0.5087	-1.01	0.7899	-1.01	0.6802	1.01	0.8298
104059_at	1437297_at	Chd8	chromodomain helicase DNA binding proteir	-1.52	0.24	1.1	0.2749	1.19	0.1677	1.28	0.035	1.67	0.0309
104064_at	1452976_a_at	Slc9a3r2	solute carrier family 9 (sodium/hydrogen exc	1.25	0.41	-1.18	0.0918	1.02	0.8582	-1.28	0.0973	1.1	0.592
104065_at	1424065_at	Edem1	ER degradation enhancer, mannosidase alp	1.07	0.79	-1.14	0.0606	-1.05	0.6557	-1.14	0.1464	-1.47	0.0127

104066_at	1460411_s_at	AW548124	expressed sequence AW548124	1.19	0.81	1.11	0.5009	1.01	0.9403	1.14	0.1938	1.21	0.5813
104067_at	1426707_at	Tubgcp3	tubulin, gamma complex associated protein	1.45	0.01	1.25	0.0332	1.2	0.0673	1.37	0	1.23	0.3069
104069_at	1460677_at	---	---	-1.5	0.13	-1.14	0.1165	-1.03	0.6734	-1.07	0.187	1.02	0.7671
104070_at	1434037_s_at	Pcaf	p300/CBP-associated factor	1.08	0.6	1.15	0.0346	1.41	0.0057	1.35	0.0002	1.24	0.0999
104072_at	1419059_at	Apcs	serum amyloid P-component	-2.74	0.01	-2.14	0.0002	1.19	0.0208	-1.84	0.0018	-3.74	0.0031
104074_at	1436522_at	0610025L06Rik	RIKEN cDNA 0610025L06 gene	-1	0.96	1.21	0.0079	1.14	0.0667	1.2	0.0032	1.55	0.0088
104076_at	1417402_at	1190017O12Rik	RIKEN cDNA 1190017O12 gene	1.65	0.07	1.18	0.1726	1.25	0.2314	1.11	0.4147	1.46	0.0285
104078_g_at	1449213_at	1110049G11Rik	RIKEN cDNA 1110049G11 gene	1.48	0.07	-1.03	0.5079	1.03	0.7541	1.09	0.1327	-1.5	0.1032
104080_at	1434019_at	Pdap1	PDGFA associated protein 1	-1.23	0.47	-1.09	0.119	-1.12	0.1136	-1.1	0.1065	-1.03	0.7981
104082_at	1427992_a_at	Rab12	RAB12, member RAS oncogene family	-1.3	0.24	-1.08	0.0572	-1.12	0.0346	-1.19	0.0042	-1.15	0.2647
104083_at	1433956_at	Cdh5	cadherin 5	-1.23	0.19	-1.04	0.7705	-1.03	0.801	-1.06	0.6848	1.38	0.0483
104085_at	1453016_at	2900042B11Rik	RIKEN cDNA 2900042B11 gene	-1.02	0.95	-1.58	0.0115	-1.25	0.2404	-1.96	0.0039	-1.11	0.7911
104086_at	1452311_at	Dmgdh	dimethylglycine dehydrogenase precursor	1.43	0.11	-1.24	0.0246	-1.32	0.006	-1.23	0.0216	-1.14	0.3042
104087_at	1419994_s_at	D10Ertd641e	DNA segment, Chr 10, ERATO Doi 641, exp	1.77	0.15	1.09	0.3283	-1.07	0.4179	1.07	0.161	-1.64	0.0638
104088_at	1427908_at	Bnip1	BCL2/adenovirus E1B 19kDa-interacting pro	1.26	0.52	-1.05	0.1277	-1.09	0.3481	-1.12	0.0472	1.05	0.5476
104089_at	1428529_at	2810026P18Rik	RIKEN cDNA 2810026P18 gene	2.78	0.03	1.63	0.067	1.34	0.4406	2.06	0.0036	5.97	0.1107
104090_at	1433430_s_at	Cdc23	CDC23 (cell division cycle 23, yeast, homolo	1.94	0.06	1.02	0.8727	-1.1	0.5522	1.07	0.3913	-1.18	0.7497
104091_at	1428197_at	Tspan9	tetraspanin 9	-1.19	0.43	-1.36	0.0007	-1.07	0.503	-1.43	0.0004	-1.11	0.4298
104092_at	1424056_at	Usp48	Hypothetical gene supported by BC058410;	1.94	0.1	1.17	0.0917	1.25	0.0855	1.29	0.0048	1.28	0.4545
104093_at	1417756_a_at	Lsp1	lymphocyte specific 1	2.36	0.4	3.1	0.1814	-1.34	0.1924	3.78	0.1062	2.85	0.1546
104094_at	1417928_at	Pdlim4	PDZ and LIM domain 4	3.35	0.01	-1.35	0.1829	-1.07	0.7021	-1.17	0.3592	-1.1	0.7631
104095_at	1452150_at	AU040320	expressed sequence AU040320	-1.15	0.28	1.01	0.9431	-1.06	0.6052	-1.16	0.0938	1.09	0.4709
104096_at	1453804_a_at	Orc4l	origin recognition complex, subunit 4-like (S.	1.13	0.33	1.2	0.0899	1.37	0.0586	1.22	0.013	-1.23	0.2749
104097_at	1424046_at	Bub1	budding uninhibited by benzimidazoles 1 ho	1.07	0.85	-1.25	0.3625	-2.23	0.0028	-1.55	0.142	1.45	0.3144
104098_at	1417841_at	---	---	1.49	0.06	1.05	0.4442	-1.07	0.2176	1.03	0.6512	1.05	0.7858
104099_at	1449184_at	Pglyrp1	peptidoglycan recognition protein 1	1.01	0.98	1.74	0.0731	2.12	0.2536	1.89	0.0511	2.22	0.0913
104100_at	1424130_a_at	2310075E07Rik	RIKEN cDNA 2310075E07 gene	-2.07	0.13	-1.11	0.101	-1.06	0.2619	-1.1	0.1837	1.36	0.0729
104101_at	1454865_at	Slc9a8	solute carrier family 9 (sodium/hydrogen exc	1.19	0.12	1.07	0.0738	1.06	0.2741	1.17	0.0445	1.04	0.8009
104102_at	1460495_s_at	Prss25	protease, serine, 25	2.06	0.02	1.03	0.8044	1.03	0.846	1.28	0.008	1.26	0.6128
104104_at	1460605_at	AA606869	EST AA606869	-1.36	0.59	-1.14	0.6602	1.61	0.1012	-1.08	0.7795	2.31	0.3451
104105_at	1422759_a_at	Xpo6	exportin 6	-1.08	0.57	1.03	0.5604	1.11	0.0051	1.03	0.4385	1.03	0.7059
104106_at	1426559_at	Sbno1	sno, strawberry notch homolog 1 (Drosophila	-2.82	0.31	1.28	0.3851	-2.21	0.0095	-1.61	0.1166	-1.1	0.7673
104108_at	1424015_at	Rab6ip1	Rab6 interacting protein 1	1.01	0.96	1.45	0	1.29	0.011	1.56	0.0001	1.43	0.1755
104109_at	1452828_at	Fbxo21	F-box only protein 21	1.41	0.06	2.73	0.0001	2.34	0	3.71	0	3.65	0
104110_at	1451197_s_at	Gatad2a	GATA zinc finger domain containing 2A	-1.01	0.93	-1.04	0.3397	1.19	0.0521	1.02	0.6147	-1.16	0.4151
104112_at	1437742_at	Rab21	RAB21, member RAS oncogene family	1.04	0.89	-1.02	0.5917	-1.06	0.5403	-1.1	0.0545	-1.09	0.4301
104114_at	1460401_at	2310050N11Rik	RIKEN cDNA 2310050N11 gene	1.2	0.29	1.18	0.1391	1.25	0.1351	1.24	0.0522	-1.17	0.331
104115_at	1452211_at	Psme4	proteasome (prosome, macropain) activator	-1.18	0.16	1.09	0.2586	1.45	0.0019	1.2	0.007	-1.63	0.0353
104116_at	1434442_at	D5Ertd593e	DNA segment, Chr 5, ERATO Doi 593, expr	1.95	0.02	1.88	0.0001	1.98	0.0002	2.2	0	1.51	0.0001
104117_at	1451220_at	2310040A13Rik	RIKEN cDNA 2310040A13 gene	1.31	0.18	1.55	0.0026	1.71	0.0828	1.94	0.0075	1.16	0.5999
104118_at	1417062_at	2810037C14Rik	RIKEN cDNA 2810037C14 gene	1.21	0.27	1.09	0.3393	1.2	0.0826	1.24	0.0331	1.42	0.1058
104119_at	1434620_s_at	2610024E20Rik	RIKEN cDNA 2610024E20 gene	1.15	0.31	1.19	0.0243	-1.05	0.593	1.12	0.1621	1.45	0.0962
104121_at	1426873_s_at	Jup	junction plakoglobin	2.43	0.02	1.05	0.5973	-1.14	0.372	-1.03	0.7017	-1.22	0.6268
104122_at	1433808_at	D330001F17Rik	RIKEN cDNA D330001F17 gene	1.16	0.48	1.06	0.3178	1.16	0.0192	1.08	0.3089	1.28	0.0168
104123_at	1436078_at	Fcho1	FCH domain only 1	2.69	0.15	1.35	0.0901	1.26	0.031	1.32	0.0169	1.86	0.0071
104124_at	1428580_at	Blvra	biliverdin reductase A	2.01	0.11	-1.32	0.0194	-1.07	0.6041	-1.02	0.889	-1.05	0.6991
104125_at	1417250_at	Rnf12	ring finger protein 12	-1.1	0.72	1.01	0.9517	-1.01	0.9526	-1.04	0.7273	1.01	0.9423
104126_at	1415920_at	Cstf2t	cleavage stimulation factor, 3' pre-RNA sub	1.3	0.17	-1.06	0.3764	1.1	0.2359	-1.03	0.743	-1.04	0.763
104128_at	1416747_at	D15Mgi27	DNA Segment, Chr 15, Mouse Genome Info	-1.34	0.22	-1.02	0.7896	1.02	0.8335	-1.02	0.7867	1.17	0.0686
104131_at	1460276_a_at	Gpr175	G protein-coupled receptor 175	2.22	0.1	-1.26	0.096	-1.35	0.1602	1.05	0.6639	-1.01	0.9564
104132_at	1448677_at	Noc4	neighbor of Cox4	2.18	0.01	1.33	0.0023	1.15	0.1248	1.29	0.0012	1.59	0.0291
104134_at	1424057_at	Gdap2	ganglioside-induced differentiation-associate	-1.78	0.07	-1.49	0.0002	1.12	0.1602	-1.64	0	-1.12	0.4942
104135_at	1450706_a_at	Arl3	ADP-ribosylation factor-like 3	1.72	0.02	1.26	0.0145	1.05	0.2988	1.33	0.0005	1.31	0.03

104136_at	1426465_at	Dlgap4	discs, large homolog-associated protein 4 (D	1.32	0.57	-1.09	0.5439	-1.43	0.0426	1.02	0.8467	1.89	0.0751
104137_at	1449302_at	Abca2	ATP-binding cassette, sub-family A (ABC1),	-1.56	0.44	-1.6	0	-1.22	0.1579	-2.13	0	-1.34	0.411
104138_at	1448489_at	Pafah2	platelet-activating factor acetylhydrolase 2	1.47	0.47	-1.25	0.0049	-1.01	0.9072	-1.07	0.3216	-1.91	0.0279
104139_at	1452094_at	P4ha1	procollagen-proline, 2-oxoglutarate 4-dioxyg	1.58	0.37	1.1	0.5356	1.42	0.0321	1.11	0.6045	5.08	0.2011
104140_s_at	1451170_s_at	Nomo1	nodal modulator 1	-1.6	0.01	1.01	0.9166	-1.06	0.3523	-1.08	0.3248	1.05	0.6842
104141_at	1460689_at	D15Wsu75e	DNA segment, Chr 15, Wayne State Univers	-1.04	0.8	-1.03	0.5428	1.12	0.1616	1.11	0.0885	1.03	0.733
104143_at	1418455_at	Copz2	coatomer protein complex, subunit zeta 2	1.92	0.17	1.11	0.1465	-1.13	0.1405	1.06	0.6288	1.68	0.0905
104144_at	1448437_a_at	Gtpbp2	GTP binding protein 2	-1.07	0.74	1.16	0.2175	1.19	0.0751	1.19	0.055	1.38	0.051
104145_at	1424643_at	---	---	-1.05	0.62	1.11	0.0559	-1.08	0.341	1.1	0.0419	-1.19	0.3256
104147_at	1417773_at	Nans	N-acetylneuraminic acid synthase (sialic acid	1.55	0.26	-1.02	0.7962	-1.05	0.5939	-1.15	0.0697	-1.12	0.6144
104148_at	1452145_at	H6pd	hexose-6-phosphate dehydrogenase (glucos	-1.11	0.22	1.29	0.0014	1.21	0.0214	1.34	0	-1.01	0.9288
104149_at	1449731_s_at	---	---	1.38	0.32	1.25	0.1381	1.08	0.3695	1.52	0.0073	1.5	0.0128
104151_at	1451522_s_at	Lrch4	leucine-rich repeats and calponin homology	1.08	0.72	-1.09	0.3178	-1.04	0.656	-1.12	0.0737	-1.07	0.3748
104152_at	1429650_at	2310004N11Rik	RIKEN cDNA 2310004N11 gene	-1.35	0.04	1.03	0.6531	-1	0.9929	-1.13	0.0003	1.24	0.0019
104153_at	1418238_at	Ivd	isovaleryl coenzyme A dehydrogenase	2.12	0.01	1.25	0.0062	-1.13	0.0833	1.26	0.0011	1.2	0.2428
104156_r_at	1449363_at	Atf3	activating transcription factor 3	1.26	0.44	1.85	0.2924	-1.11	0.2965	1.48	0.3861	2.3	0.217
104157_at	1435369_at	C78212	expressed sequence C78212	2.41	0.1	1.38	0.1094	1.58	0.0344	1.5	0.0382	1.84	0.2728
104158_at	1429002_at	Skiip	SKI interacting protein	1.24	0.77	1.38	0.0321	-1.69	0.0308	-1.13	0.6606	3.24	0.0041
104159_at	1418218_at	Ceacam9	CEA-related cell adhesion molecule 9	-3.26	0.24	-2.63	0.0022	-1.08	0.8157	-1.46	0.2526	2.53	0.04
104160_at	1422717_at	Acp1	acid phosphatase 1, soluble	-1.45	0.18	-1.03	0.6874	-1.03	0.7458	-1.15	0.223	1.09	0.7121
104161_at	1420936_s_at	Cpsf2	cleavage and polyadenylation specific factor	-1.53	0.06	-1.08	0.4044	1.03	0.7606	-1.18	0.1411	-1.13	0.5389
104163_at	1426760_at	Ipo8	importin 8	-1.13	0.61	-1.29	0.0087	1.07	0.5385	-1.31	0.0016	-1.36	0.1573
104164_at	1428689_at	Tysnd1	trypsin domain containing 1	1.04	0.68	-1.15	0.0272	-1.16	0.0514	-1.34	0.0001	-1.35	0.1728
104165_at	1418486_at	Vnn1	vanin 1	1.68	0.32	-1.11	0.7006	1.24	0.2787	-1.59	0.0219	-2.28	0.1286
104166_at	1449476_at	Rage	renal tumor antigen	-2.02	0.21	1	0.984	-1.03	0.8635	-1.15	0.4562	1.45	0.0497
104169_at	1423477_at	Zic1	zinc finger protein of the cerebellum 1	2.6	0.36	-2.65	0.0397	-2.02	0.1122	-2.89	0.0311	2.14	0.1695
104171_f_at	1419141_at	---	---	1.41	0.17	1.06	0.6022	1.08	0.4677	-1.05	0.6061	-1.06	0.8358
104172_at	1451648_a_at	Folr2	folate receptor 2 (fetal)	1.17	0.57	-1.58	0.0003	-1.28	0.0124	-2.06	0	-1.14	0.825
104173_at	1450912_at	Ms4a1	membrane-spanning 4-domains, subfamily A	-1.31	0.18	-1.33	0.009	-1.28	0.0889	-1.63	0.0002	1.07	0.5411
104174_at	1459546_s_at	Enpp1	ectonucleotide pyrophosphatase/phosphodie	4.17	0.01	-1.11	0.3973	1.34	0.0091	-1.1	0.4516	2.17	0.1153
104175_at	1419580_at	---	---	-1.34	0.17	1.03	0.7117	1.06	0.4547	1.11	0.2049	1.46	0.2556
104176_at	1452633_s_at	Aak1	AP2 associated kinase 1	1.34	0.08	1.67	0.0025	1.27	0.044	1.92	0.0001	1.36	0.1248
104177_at	1436058_at	Rsad2	radical S-adenosyl methionine domain conta	-1.47	0.02	-1.18	0.5769	-2.1	0.0041	-1.13	0.6867	-1.7	0.2525
104179_at	1434312_at	AI788669	expressed sequence AI788669	1.08	0.75	-1.21	0.2243	-1.34	0.0074	-1.41	0.0042	-1.03	0.8851
104180_at	1420554_a_at	Rac3	RAS-related C3 botulinum substrate 3	1.21	0.23	1.16	0.3084	1.25	0.2363	1.12	0.3839	1.88	0.2822
104181_at	1420723_at	Vnn3	vanin 3	1.74	0.07	1.04	0.6381	-1.01	0.8551	1.05	0.6018	1.14	0.1253
104182_at	1418405_at	Hgfac	hepatocyte growth factor activator	-1.09	0.33	-1.06	0.405	-1.06	0.4232	-1.15	0.0062	1.36	0.0205
104184_at	1450791_at	Nppb	natriuretic peptide precursor type B	-2.5	0.1	1.03	0.8048	1.17	0.2088	-1.02	0.9023	1.86	0.0531
104185_at	1424689_at	Prss32	protease, serine, 32	-1.98	0.13	1.02	0.919	1.2	0.2729	1.14	0.4959	1.75	0.3971
104186_at	1429108_at	Rnf184	ring finger protein 184	-1.19	0.65	1.85	0.0449	1.24	0.5439	1.61	0.1219	2.75	0.0081
104187_at	1418375_at	D10Wsu93e	DNA segment, Chr 10, Wayne State Univers	-1.09	0.53	-1.09	0.3363	-1.04	0.7456	-1.02	0.749	1.63	0.0093
104188_at	1455556_at	Notch2	Notch gene homolog 2 (Drosophila)	1.34	0.16	1.31	0.1507	1.21	0.4064	1.24	0.0686	1.08	0.5107
104190_at	1435350_at	Traf6	Tnf receptor-associated factor 6	1.77	0.04	1.3	0.141	1.3	0.1947	1.17	0.4868	1.29	0.3453
104193_at	1452268_at	2810485I05Rik	RIKEN cDNA 2810485I05 gene	1.13	0.56	1.38	0.0012	1.53	0.0218	1.49	0.0001	1.52	0.0057
104194_at	1448696_at	Heph	hephaestin	-1.77	0.14	-1.27	0.1797	-1.08	0.7439	1.08	0.5317	2.47	0.2017
104195_at	1429207_at	5730408K05Rik	RIKEN cDNA 5730408K05 gene	-1.02	0.94	1.15	0.1879	1.05	0.6455	1.13	0.2299	1.4	0.2567
104196_at	1429253_at	Zfp262	zinc finger protein 262	1.24	0.11	-1.08	0.4678	-1.32	0.0103	-1.09	0.4206	-1.02	0.9604
104197_at	1434938_at	Rbm9	RNA binding motif protein 9	-1.41	0.42	1.07	0.7735	1.22	0.2343	1	0.9765	-1.11	0.3998
104198_at	1427095_at	Cdcp1	CUB domain containing protein 1	-1.13	0.69	-1.54	0.005	-1.99	0.0005	-2.11	0.0002	1.06	0.7121
104199_at	1460743_at	Tigd5	tigger transposable element derived 5	-1.05	0.57	1.08	0.2879	1.09	0.3052	1.04	0.683	1.16	0.208
104200_at	1435860_at	---	---	4.17	0.01	1.4	0.0008	1.7	0.0001	1.48	0.0001	1.81	0.0208
104201_at	1417553_at	Plac1	placental specific protein 1	-1.34	0.4	-1.09	0.23	-1.18	0.0101	-1.41	0.007	1.4	0.1133
104202_at	1435430_at	Tmem1	transmembrane protein 1	-1.26	0.31	-1.02	0.895	1.18	0.2499	1.11	0.5399	-1.07	0.8147

104205_at	1449827_at	Agc1	aggrecan 1	-1.24	0.79	-1.43	0.4112	1.14	0.7643	1.16	0.6904	-1.05	0.9347
104206_at	1453287_at	5730557B15Rik	RIKEN cDNA 5730557B15 gene	-1.14	0.39	-1.06	0.7511	-1.14	0.4034	-1.26	0.0825	-1.24	0.3229
104207_at	1439018_at	6330505N24Rik	RIKEN cDNA 6330505N24 gene	-1.61	0.32	-1.03	0.8366	1.11	0.5831	1.05	0.7955	1.83	0.1867
104208_at	1435003_at	Pik4ca	phosphatidylinositol 4-kinase, catalytic, alpha	3.05	0.07	1.14	0.2385	1.16	0.2455	1.27	0.0623	1.53	0.0006
104209_at	1449406_at	Cyhr1	cysteine and histidine rich 1	2.04	0.12	-1.16	0.0065	-1.09	0.146	-1.21	0.0018	-1.23	0.4577
104210_at	1455158_at	Itga3	integrin alpha 3	-1.42	0.62	-1.31	0.0191	-1.29	0.0335	-1.42	0.0003	1.35	0.1515
104211_at	1460305_at	Itga3	integrin alpha 3	-1.22	0.56	-1.07	0.2389	-1.09	0.1961	-1.21	0.0037	1.2	0.1216
104213_at	1451548_at	---	---	2.1	0.02	1.05	0.3592	1.1	0.1817	1.21	0.0137	1.12	0.6573
104214_at	1417929_at	Slc7a8	solute carrier family 7 (cationic amino acid tr	2.69	0.02	1.39	0.0671	1.03	0.9178	1.25	0.4581	1.47	0.4842
104215_at	1435444_at	Atf6	activating transcription factor 6	1.6	0.11	-1.04	0.6365	1.19	0.0787	-1.08	0.3401	-1.06	0.6266
104217_at	1449526_a_at	Gdpd3	glycerophosphodiester phosphodiesterase d	1.14	0.4	1.13	0.4945	1.32	0.0891	1.23	0.1504	1.2	0.2118
104220_at	1422771_at	---	---	-1	0.99	1.11	0.2055	1.05	0.5144	-1.03	0.7104	-1.06	0.6897
104221_at	1418326_at	Slc7a5	solute carrier family 7 (cationic amino acid tr	-1.12	0.11	-1.15	0.0382	1.09	0.4425	-1.08	0.3945	1.14	0.5011
104222_f_at	1419805_s_at	Ggps1	geranylgeranyl diphosphate synthase 1	1.37	0.6	1.04	0.8701	1.42	0.1449	1.4	0.0711	-1.07	0.8509
104225_at	1437074_at	---	---	1.11	0.52	1.08	0.62	-1.32	0.2158	-1	0.9931	-1.85	0.386
104227_at	1417894_at	Gpr97	G protein-coupled receptor 97	1.08	0.73	-1.1	0.4866	1.05	0.7059	-1.12	0.4464	1.64	0.1338
104228_at	1437534_at	---	---	-3.83	0.34	-1.01	0.9803	-1.04	0.866	-1.27	0.2902	2.29	0.197
104229_at	1449553_at	2610200G18Rik	RIKEN cDNA 2610200G18 gene	-1.78	0.17	1	0.9974	-1.17	0.2653	1.09	0.5033	-1.3	0.3127
104230_at	1417904_at	Dclre1a	DNA cross-link repair 1A, PSO2 homolog (S	4.77	0.05	1.99	0.0014	1.06	0.6305	1.92	0.0002	6.04	0.0051
104231_at	1455491_at	LOC432467	similar to heterogeneous nuclear ribonucleo	1.24	0.37	-1.14	0.2789	-1.1	0.2941	-1.06	0.5849	1.28	0.2561
104232_at	1416715_at	Gjb3	gap junction membrane channel protein beta	-1.48	0.41	-1.42	0.3822	1.15	0.7114	-1.4	0.3763	2.12	0.0871
104234_at	1449194_at	Mrps25	mitochondrial ribosomal protein S25	2.17	0.02	1.22	0.0946	1.06	0.6299	1.05	0.5852	-1.26	0.2286
104235_at	1433832_at	B230369L08Rik	RIKEN cDNA B230369L08 gene	1.36	0.2	1.49	0.0004	1.45	0.0011	1.72	0	2.22	0.0008
104237_at	1452621_at	Pcbd2	pterin 4 alpha carbinolamine dehydratase/di	1.5	0	-1.03	0.5656	1.07	0.5779	-1.12	0.1295	-1.03	0.7829
104238_at	1451553_at	Art5	ADP-ribosyltransferase 5	-1.93	0.02	-1.3	0.0007	-1.17	0.0173	-1.27	0.0013	-1.14	0.2093
104239_at	1418398_a_at	Phemx	pan hematopoietic expression	1.14	0.84	1.01	0.9488	-1.03	0.9092	1.25	0.2736	1.87	0.0093
104241_at	1434487_at	Mef2d	myocyte enhancer factor 2D	-1.46	0.05	-1.09	0.2855	1.04	0.5853	-1.05	0.508	1.25	0.1127
104244_at	1435889_at	Mark2	MAP/microtubule affinity-regulating kinase 2	-1.02	0.91	1.15	0.2495	1.18	0.1895	1.16	0.238	1.19	0.4631
104245_at	1452929_at	Rsn	restin (Reed-Steinberg cell-expressed intern	1.29	0.11	1.08	0.2844	1.08	0.3221	1.19	0.0815	1.35	0.1047
104246_at	1434561_at	Asxl1	additional sex combs like 1 (Drosophila)	-1.37	0.15	-1.01	0.9185	-1.14	0.174	-1.15	0.1567	1.39	0.0113
104247_at	1451380_at	Zfyve19	zinc finger, FYVE domain containing 19	1.09	0.61	-1.04	0.4467	-1.02	0.7188	1	0.997	1.04	0.7367
104250_at	1434694_at	Lrrc8	leucine rich repeat containing 8	1.02	0.93	-1.05	0.4126	1.12	0.1906	-1	0.9566	1.16	0.1546
104252_at	1433935_at	AU020206	expressed sequence AU020206	1.95	0.08	1.23	0.3814	-1.13	0.2833	1.38	0.2485	1.06	0.8599
104253_at	1434805_at	Mllt1	myeloid/lymphoid or mixed lineage-leukemia	-1.65	0.32	1.06	0.5505	-1.04	0.7228	-1.05	0.5591	1.45	0.1667
104254_at	1428789_at	Ralgps2	Ral GEF with PH domain and SH3 binding m	1.17	0.49	-1.2	0.0777	1.19	0.1339	-1.2	0.0831	1.92	0.0928
104255_at	1435551_at	FHOS2	formin-family protein FHOS2	-1.82	0.14	-1.15	0.5392	-1.15	0.5131	-1.03	0.8856	-1.03	0.9149
104257_g_at	1451206_s_at	Pscdbp	pleckstrin homology, Sec7 and coiled-coil do	-1.06	0.44	1.11	0.3549	-1.02	0.8294	1.15	0.2277	1.35	0.0221
104258_at	1427943_at	Acyp2	acylphosphatase 2, muscle type	1.3	0.55	1.08	0.7032	1.01	0.9568	-1.04	0.845	-2.42	0.0562
104259_at	1450416_at	Cbx5	chromobox homolog 5 (Drosophila HP1a)	-1.03	0.88	-1.06	0.5375	-1.2	0.0381	-1.11	0.2267	-1.64	0.0831
104260_at	1435326_at	AW112037	expressed sequence AW112037	-1.05	0.85	-1.19	0.0884	-1.22	0.0894	-1.25	0.0149	-1.69	0.0033
104261_at	1435077_at	Asxl1	additional sex combs like 1 (Drosophila)	1.87	0.2	1.28	0.0454	1.12	0.48	1.24	0.0279	1.38	0.3474
104262_at	1422324_a_at	Pthlh	parathyroid hormone-like peptide	-2.35	0.02	-1.18	0.4214	-1.17	0.5317	-1.12	0.5607	-1.08	0.8794
104264_at	1449099_at	Lrba	LPS-responsive beige-like anchor	1.02	0.96	1.02	0.7473	-1.03	0.6856	1.05	0.3124	-1.2	0.1033
104265_at	1449379_at	Kdr	kinase insert domain protein receptor	-1.49	0.31	-1.22	0.0404	-1.28	0.0598	-1.31	0.0914	1.71	0.0091
104266_at	1439506_at	Gm98	gene model 98, (NCBI)	1.42	0.23	1.09	0.2286	1.11	0.1794	1.16	0.0812	1.16	0.3813
104267_at	1417329_at	Slc23a2	solute carrier family 23 (nucleobase transpo	1.72	0.08	1.87	0.0051	1.31	0.0067	1.72	0	2.35	0.0012
104268_at	1452416_at	Il6ra	interleukin 6 receptor, alpha	1.39	0.31	-1.03	0.8998	-1.71	0.0213	-1.36	0.2182	1.44	0.1547
104269_at	1422531_at	Syt5	synaptotagmin 5	-1.08	0.49	1.03	0.7192	1.03	0.5737	-1.01	0.9363	1.15	0.1971
104270_at	1451992_at	Adrbk1	adrenergic receptor kinase, beta 1	2.9	0	-1.01	0.8946	1.2	0.0003	1.12	0.2466	1.39	0.439
104271_at	1418554_at	Admr	adrenomedullin receptor	-1.07	0.71	-1.26	0.0627	1.03	0.7641	-1.22	0.2214	1.13	0.1746
104272_s_at	1421450_a_at	Map3k4	mitogen activated protein kinase kinase kina	2.72	0.02	-1.09	0.361	-1.04	0.7947	-1.19	0.0186	-1.03	0.917
104273_at	1419002_s_at	Baat	bile acid-Coenzyme A: amino acid N-acyltra	1.01	0.97	-1	0.9989	-1.06	0.5711	-1.08	0.3703	-1.08	0.6554
104274_at	1435450_at	---	---	-1.36	0.21	1.1	0.2196	-1	0.9893	1.11	0.2251	1.12	0.5836

104275_g_at	1427739_a_at	Trp53	transformation related protein 53	-1.23	0.62	-1.12	0.4531	-1.09	0.6118	1.24	0.1688	1.03	0.9196
104276_at	1452803_at	Glipr2	GLI pathogenesis-related 2	1.21	0.45	1.84	0.2903	1.12	0.2755	1.53	0.1202	1.26	0.2961
104277_at	1421059_a_at	Alg2	asparagine-linked glycosylation 2 homolog (	1.2	0.41	1.24	0.0143	1.38	0.0065	1.15	0.0952	1.16	0.2911
104279_at	1415754_at	1810060D16Rik	RIKEN cDNA 1810060D16 gene	-1.31	0.53	-1.08	0.1663	-1.11	0.046	-1.09	0.0327	1.07	0.2979
104280_at	1417788_at	Sncg	synuclein, gamma	-1.03	0.96	-1.16	0.7034	-1.17	0.672	-1.28	0.5301	1.6	0.1637
104283_at	1416060_at	Tbc1d15	TBC1 domain family, member 15	-1.31	0.23	1.36	0.0022	1.43	0.0509	1.49	0.0003	1.93	0.0324
104284_at	1454786_at	5031439G07Rik	RIKEN cDNA 5031439G07 gene	1.17	0.41	1.21	0.0131	1.17	0.0381	1.28	0.0007	1.4	0.0008
104285_at	1427229_at	Hmgcr	3-hydroxy-3-methylglutaryl-Coenzyme A red	1.17	0.73	-1.41	0.0963	-1.33	0.2	-1.39	0.0896	-1.5	0.3829
104286_at	1448889_at	Slc38a4	solute carrier family 38, member 4	-1.01	0.94	1.23	0.0034	1.01	0.8748	1.19	0.0019	1.13	0.1262
104287_at	1448866_at	Senp3	SUMO/sentrin specific protease 3	1.41	0.23	1.05	0.6512	1.13	0.2455	1.01	0.9423	1.22	0.078
104288_at	1451971_at	Cul4a	cullin 4A	1.15	0.57	1.17	0.0643	1.24	0.0294	1.09	0.355	-1.06	0.6669
104289_at	1423713_at	Abcb8	ATP-binding cassette, sub-family B (MDR/T/	1.38	0.59	-1.21	0.2728	1.04	0.7223	1	0.9937	-1.5	0.005
104290_at	1449217_at	Casp8ap2	caspase 8 associated protein 2	1.89	0.03	1.08	0.3688	1.27	0.0642	1.23	0.0726	-1.04	0.7326
104292_at	1424127_at	Eya2	eyes absent 2 homolog (Drosophila)	-1.49	0.56	-1.24	0.4022	1.66	0.0488	1.23	0.421	1.71	0.3082
104293_at	1428081_at	Klhl21	kelch-like 21 (Drosophila)	1.14	0.37	1.19	0.0418	1.18	0.0753	1.26	0.0052	1.22	0.2132
104294_at	1454905_at	Ibtk	inhibitor of Bruton agammaglobulinemia tyro	1.34	0.23	1.27	0.0101	1.32	0.0528	1.21	0.029	-1.09	0.4905
104296_at	1434551_at	1110031M08Rik	RIKEN cDNA 1110031M08 gene /// similar t	1.24	0.45	-1.01	0.8952	-1.1	0.3393	-1.06	0.4014	-1.35	0.4267
104297_at	1428096_at	Ipo11	importin 11	1.18	0.2	-1.15	0.0321	-1.14	0.0677	-1.16	0.0018	-1.17	0.1754
104298_at	1424915_s_at	---	---	-1	1	-1.94	0	1.04	0.606	-1.97	0	-1.37	0.1406
104299_at	1423668_at	Zdhhc14	zinc finger, DHHC domain containing 14	1.12	0.72	-1.33	0.0564	1.39	0.0031	-1.21	0.2257	1.34	0.476
104300_at	1434998_at	Iqgap1	IQ motif containing GTPase activating protei	1.94	0	1.3	0.1614	1.05	0.7436	1.46	0.1577	1.61	0.2483
104301_at	1452591_a_at	2410018G20Rik	RIKEN cDNA 2410018G20 gene	-1.03	0.87	-1.04	0.5201	-1.05	0.4615	-1.11	0.1428	-1.24	0.0941
104302_f_at	1454781_x_at	Commd9	COMM domain containing 9	-1.08	0.59	-1.11	0.2841	-1.14	0.2097	-1.06	0.5863	1.05	0.713
104304_r_at	1439266_a_at	Polr3k	polymerase (RNA) III (DNA directed) polype	1.23	0.44	-1.25	0.0928	-1.07	0.5346	-1.16	0.1338	-2.07	0.0239
104306_at	1448215_a_at	Dpp3	dipeptidylpeptidase 3	-1.01	0.88	-1.14	0.0703	1.02	0.7972	-1.04	0.5684	-2.86	0.0279
104308_at	1419128_at	Itgax	integrin alpha X	-1.42	0	1.11	0.5751	1.04	0.5691	1.41	0.0663	1.39	0.0861
104310_at	1428245_at	G6pc3	glucose 6 phosphatase, catalytic, 3	1.06	0.68	-1.15	0.1073	-1.24	0.0231	-1.05	0.478	1.01	0.9869
104311_at	1426486_at	Ubxd2	UBX domain containing 2	1.65	0.29	1.03	0.8197	-1.22	0.0406	-1.11	0.2757	-1.13	0.1306
104312_at	1425699_a_at	Abhd14a	abhydrolase domain containing 14A	1	0.99	-1.03	0.8087	-1.05	0.6836	1	0.9993	1.49	0.0365
104313_at	1451149_at	Pgm2	phosphoglucomutase 2	1.09	0.61	1.19	0.0432	1.02	0.6306	1.26	0.0025	-1.1	0.45
104315_at	1451309_at	Arhgap1	Rho GTPase activating protein 1	1.32	0.17	1.02	0.7965	1	0.9711	1.18	0.0447	1.12	0.5602
104317_at	1448809_at	Cse1l	chromosome segregation 1-like (S. cerevisia	1.22	0.42	1.02	0.8719	-1.02	0.8972	1.02	0.846	1.08	0.3706
104318_at	1426770_at	Pex5	peroxisome biogenesis factor 5	-1.36	0.08	-1.02	0.781	-1.11	0.2518	-1.05	0.5101	-1.13	0.4216
104320_at	1427929_a_at	Pdxk	pyridoxal (pyridoxine, vitamin B6) kinase	1.26	0.17	1.15	0.1231	-1.02	0.8498	1.26	0.0112	1.43	0.0512
104322_at	1434748_at	Ckap2	cytoskeleton associated protein 2	-1.41	0.58	1.43	0.5013	-1.36	0.3541	1.97	0.209	1.41	0.3041
104324_at	1428467_at	Tardbp	TAR DNA binding protein	1.27	0.07	1.05	0.2588	1.02	0.8226	1.08	0.3803	1.2	0.5334
104325_at	1451371_at	Mrap	melanocortin 2 receptor accessory protein	1.16	0.45	-1.37	0.0001	-1.46	0	-1.56	0	-1.48	0.0207
104326_at	1451079_at	Adpgk	ADP-dependent glucokinase	1.06	0.71	1.08	0.4536	1.2	0.2462	1.07	0.4685	-1.07	0.6027
104327_at	1433784_at	AI265322	expressed sequence AI265322	1.04	0.91	1.64	0.0017	1.24	0.0301	1.67	0.0003	1.58	0.0024
104328_at	1424011_at	Aqp9	aquaporin 9	1.21	0.28	1.13	0.0288	1.24	0.0067	1.27	0.0012	1.11	0.2005
104331_at	1448709_at	Arid1a	AT rich interactive domain 1A (Swi1 like)	-1.04	0.92	1.25	0.0001	1.34	0.0165	1.21	0.0018	1.47	0.0064
104332_at	1423769_at	Ptcd2	pentatricopeptide repeat domain 2	1.32	0.15	1.05	0.3039	1.21	0.0599	1.03	0.4961	-1.22	0.3058
104333_at	1417822_at	D17H6S56E-5	DNA segment, Chr 17, human D6S56E 5	1.95	0.42	1.49	0.3012	-1.82	0.0056	3.92	0.0824	-1.1	0.6352
104334_at	1450987_a_at	2310004I24Rik	RIKEN cDNA 2310004I24 gene	1.15	0.11	1.2	0.0165	1.2	0.0292	1.26	0.0143	1.35	0.0207
104335_at	1416199_at	Kifc3	kinesin family member C3	-2.82	0.1	-1.59	0.1928	-1.19	0.5461	-1.18	0.6707	-1.33	0.4185
104336_at	1424841_s_at	Rbks	ribokinase	1.07	0.78	1.3	0.0328	1.29	0.0305	1.21	0.1469	-1.04	0.8411
104337_f_at	1449799_s_at	Pkp2	plakophilin 2	1.06	0.87	-1.21	0.1312	-1	0.977	-1.24	0.1055	-1.35	0.0955
104339_at	1428249_at	Pygo2	pygopus 2	1.05	0.84	-1.08	0.3409	1.05	0.5327	-1.05	0.4317	1.12	0.5486
104340_at	1417968_a_at	Mbd1	methyl-CpG binding domain protein 1	1.15	0.29	1.03	0.8026	1.34	0.078	1.17	0.1666	-1.28	0.1139
104341_at	1431299_a_at	2310014H01Rik	RIKEN cDNA 2310014H01 gene	-1.51	0.09	-1.03	0.7297	-1.15	0.1379	-1.15	0.0502	1.22	0.1971
104344_at	1449034_at	Klkb1	kallikrein B, plasma 1	1.35	0.24	-1.02	0.8038	1.18	0.0367	-1.07	0.3442	-1.27	0.0307
104345_at	1434865_a_at	Exoc7	exocyst complex component 7	1.83	0.13	-1.32	0.0008	-1.09	0.3271	-1.02	0.78	1.25	0.0094
104348_at	1426312_at	Bre	brain and reproductive organ-expressed pro	1.19	0.5	-1.06	0.346	-1.23	0.0174	-1.11	0.1451	-1.32	0.2304

104349_at	1428133_at	MGI:2156003	Smad nuclear interacting protein 1	-1.2	0.13	-1.03	0.65	1.11	0.0508	-1.03	0.6771	1.3	0.0549
104350_at	1422854_at	Shc1	src homology 2 domain-containing transform	-1.16	0.62	-1.13	0.0646	-1.01	0.9005	-1.04	0.5253	-1.29	0.1057
104351_at	1439030_at	Gmppb	GDP-mannose pyrophosphorylase B	-1.07	0.76	-1.78	0.0235	1.31	0.396	-1.82	0.019	-1.29	0.4339
104352_at	1450711_at	Brd4	bromodomain containing 4	-1.36	0.17	1.21	0.3321	1.23	0.2423	1.32	0.0855	1.31	0.2245
104354_at	1423593_a_at	Csf1r	colony stimulating factor 1 receptor	1.1	0.44	1.11	0.598	-1.1	0.3564	1.32	0.4279	1.09	0.6914
104356_at	1451509_at	Taf9	TAF9 RNA polymerase II, TATA box binding	1.89	0.15	-1.01	0.9313	1.3	0.0413	1.18	0.2743	1.55	0.066
104358_at	1426855_at	D10Ertd610e	DNA segment, Chr 10, ERATO Doi 610, exp	-1.15	0.76	-1.02	0.8623	1.12	0.2834	-1.01	0.941	1.26	0.28
104360_at	1460735_at	Svil	supervillin	-1.27	0.01	-1.26	0.0016	-1.15	0.0414	-1.29	0.0005	-1.22	0.0516
104362_at	1433765_at	B230113M03Ril	RIKEN cDNA B230113M03 gene	-1.58	0.04	-1.07	0.2641	-1.14	0.0609	-1.18	0.0338	1.18	0.2725
104363_at	1435191_at	Cdsn	corneodesmosin	-1.28	0.51	-1.03	0.874	1.1	0.5606	-1.27	0.2239	1.22	0.1591
104364_at	1417016_at	Mapkapk5	MAP kinase-activated protein kinase 5	1.34	0.34	-1.04	0.5279	1.05	0.5326	-1.07	0.3158	-1.34	0.2309
104365_at	1416611_at	Scamp2	secretory carrier membrane protein 2	1.51	0.17	1.13	0.125	-1.03	0.7685	1.18	0.0445	-1.26	0.1923
104366_at	1435087_at	BC039093	cDNA sequence BC039093	1.17	0.55	-1.06	0.3372	-1.19	0.0104	1.08	0.351	1.28	0.2264
104367_at	1419252_at	---	---	-1.61	0	-1.37	0.0137	1.15	0.3675	-1.19	0.0832	-1.95	0.0169
104368_at	1427079_at	Mapre3	microtubule-associated protein, RP/EB famil	-1.08	0.7	-1.04	0.7589	1.08	0.4267	1.12	0.0753	-1.11	0.5484
104369_at	1422784_at	Krt2-6a	keratin complex 2, basic, gene 6a	-1.53	0.01	-1	0.9813	-1	0.97	-1.01	0.922	1.11	0.356
104370_s_at	1427700_x_at	Krt2-6a	keratin complex 2, basic, gene 6a	-1.02	0.91	1.19	0.3583	-1.34	0.2556	1.17	0.3976	1.2	0.4167
104371_at	1418295_s_at	Dgat1	diacylglycerol O-acyltransferase 1	1.58	0.01	1.08	0.4353	-1.03	0.8069	1.12	0.2232	1.08	0.6193
104372_at	1416863_at	Abhd8	abhydrolase domain containing 8	1.31	0.6	1.79	0.122	1.54	0.1155	1.27	0.379	1.33	0.3384
104374_at	1419100_at	Serpina3n	serine (or cysteine) proteinase inhibitor, clad	1.2	0.34	1.26	0.011	-1.01	0.9103	1.15	0.1717	1.38	0.0184
104375_at	1435026_at	Spock2	sparc/osteonectin, cwcv and kazal-like doma	1.09	0.6	-1.08	0.5276	-1.06	0.6505	-1.09	0.4997	-1.46	0.3592
104376_at	1415743_at	Hdac5	histone deacetylase 5	-1.43	0.13	-1.26	0.0091	-1.2	0.0372	-1.21	0.0212	-1.08	0.3225
104378_at	1450686_at	---	---	1.19	0.19	1.09	0.1364	1.02	0.6349	1.11	0.0285	-1.42	0.1078
104380_at	1417538_at	Slc35a1	solute carrier family 35 (CMP-sialic acid tran	-1.04	0.68	-1.09	0.1589	-1.01	0.9512	-1.21	0.0252	-1.24	0.0714
104381_at	1450444_a_at	Nr1h3	nuclear receptor subfamily 1, group H, mem	1.08	0.64	-1.14	0.0256	-1.17	0.0101	-1.23	0.0003	1.06	0.5414
104383_at	1448289_at	Crmp1	collapsin response mediator protein 1	-1.45	0.42	-1.76	0.0785	-1.38	0.2601	-1.19	0.5617	-1.94	0.1792
104386_f_at	1452784_at	Itgav	integrin alpha V	1.19	0.33	1.04	0.4078	1.02	0.8209	1.08	0.2038	1.08	0.2106
104387_at	1450404_at	Slc23a1	solute carrier family 23 (nucleobase transpor	2.57	0.09	-1.9	0.0001	-1.25	0.1187	-1.91	0.001	-3.39	0.0186
104388_at	1417936_at	---	---	-1.1	0.84	1.06	0.5893	1.06	0.386	1.15	0.2988	1.62	0.0009
104389_at	1429758_at	1700017B05Rik	RIKEN cDNA 1700017B05 gene	1.37	0.04	1.02	0.8726	1.09	0.4514	1.21	0.138	1.77	0.0222
104390_at	1434555_at	Anp32a	acidic (leucine-rich) nuclear phosphoprotein	-1	0.99	1.04	0.6351	-1.13	0.0992	-1.1	0.193	-1.06	0.6096
104393_at	1418389_at	2810453I06Rik	RIKEN cDNA 2810453I06 gene	2.45	0.02	-1.53	0.1536	-1.4	0.3087	-1.04	0.8779	1.02	0.9727
104394_at	1416402_at	Abcb10	ATP-binding cassette, sub-family B (MDR/TA	-1.1	0.28	-1.09	0.281	-1.05	0.6822	-1.09	0.2908	-1.63	0.159
104395_at	1435272_at	Itpkb	inositol 1,4,5-trisphosphate 3-kinase B	-1.11	0.3	-1.12	0.2275	-1.03	0.6829	-1.11	0.2147	1.1	0.2627
104396_at	1434017_at	Znrf2	zinc and ring finger 2	-1.32	0.02	1.05	0.5017	1.14	0.0629	-1.14	0.0906	-1.34	0.1017
104398_at	1455618_x_at	1300010A20Rik	RIKEN cDNA 1300010A20 gene	-1.89	0	-1.2	0.0426	-1.14	0.2646	-1.18	0.0376	-1.21	0.0742
104399_at	1419645_at	Cstf2	cleavage stimulation factor, 3' pre-RNA subu	1.22	0.27	-1.06	0.4278	-1.01	0.9445	-1.1	0.1174	-1.25	0.1061
104401_at	1439803_at	6430526N21Rik	RIKEN cDNA 6430526N21 gene	-1.1	0.81	-1.07	0.5066	-1.1	0.3783	-1.22	0.0865	1.31	0.3588
104402_at	1434320_at	Gtf3c4	general transcription factor IIIC, polypeptide	1.83	0.13	-1.53	0.0238	-1.1	0.6351	-1.19	0.1534	-1.61	0.3317
104403_at	1434162_at	2700078E11Rik	RIKEN cDNA 2700078E11 gene	1.23	0.03	1.06	0.2754	1.06	0.3823	1.07	0.2229	-1.47	0.0109
104405_at	1434953_at	4633402D15Rik	RIKEN cDNA 4633402D15 gene	1.8	0.17	-1.06	0.6694	1.03	0.8306	-1.14	0.4147	-1.27	0.4398
104406_at	1449450_at	Ptges	prostaglandin E synthase	1.5	0.46	-1.35	0.0434	1.08	0.3175	-1.33	0.0515	1.74	0.0345
104407_at	1426301_at	Alcam	activated leukocyte cell adhesion molecule	1.41	0.27	1.49	0.0262	-1.32	0.0151	1.16	0.3234	1.52	0.0157
104409_at	1418784_at	Grik5	glutamate receptor, ionotropic, kainate 5 (ga	1.45	0.29	1.2	0.1673	-1.2	0.1979	1.03	0.8403	-1.02	0.9319
104410_at	1449188_at	Midn	midnolin	1.28	0.05	1.06	0.3842	-1.19	0.047	1	0.9868	1.27	0.0679
104411_at	1419893_at	D930048N14Ril	RIKEN cDNA D930048N14 gene	-1.89	0.52	-1.44	0.2274	-1.02	0.9355	-1.15	0.6017	-1.11	0.7004
104412_at	1454959_s_at	Gnai1	guanine nucleotide binding protein, alpha inh	-1.06	0.84	-1.19	0.5773	1.17	0.5423	-1.27	0.4449	1.4	0.1308
104413_at	1433595_at	---	---	-1.19	0.3	1.23	0.0001	1.18	0.0263	1.31	0	-1.45	0.0033
104414_at	1448031_at	AW050213	expressed sequence AW050213	-1.2	0.5	-1.11	0.6923	-1.16	0.3799	-1.04	0.8856	-1.27	0.6331
104415_at	1435221_at	Foxp1	Forkhead box P1	1.47	0.1	1.32	0.0538	-1.11	0.5611	1.51	0.0106	1.72	0.2796
104416_at	1421442_at	Flt4	FMS-like tyrosine kinase 4	1.2	0.69	-1.19	0.3569	1.15	0.394	-1.29	0.1828	1.08	0.7185
104417_at	1444052_at	Flt4	FMS-like tyrosine kinase 4	-1.04	0.77	-1.02	0.7405	1.26	0.0016	-1.11	0.3951	1.28	0.0504
104418_at	1455291_s_at	Znrf2	zinc and ring finger 2	1.01	0.96	1.05	0.6508	1.1	0.5078	-1.03	0.8235	-4.29	0.023

104419_at	1426903_at	Fndc3a	fibronectin type III domain containing 3a	1.08	0.67	1.29	0.017	1.1	0.4913	1.24	0.0101	-1.05	0.6592
104421_at	1449525_at	Fmo3	flavin containing monooxygenase 3	-2.84	0.23	-4.45	0	1.14	0.1421	-1.65	0.0008	45.85	0
104422_at	1416588_at	Ptprn	protein tyrosine phosphatase, receptor type,	-1.23	0.03	-1.05	0.6053	-1.24	0.0025	-1.2	0.0176	1.1	0.4318
104423_at	1434695_at	---	---	1.07	0.89	1.01	0.9747	-1.64	0.2975	-1.31	0.5208	1.5	0.3365
104424_at	1422815_at	---	---	-2.88	0.04	-1.29	0.1236	-1.52	0.0001	-1.32	0.1059	-6.32	0
104425_at	1418983_at	Inadl	InaD-like (Drosophila)	1.8	0.47	-1.32	0.4424	1.85	0.1816	2.04	0.051	-1.33	0.6035
104427_at	1443762_s_at	Mtmr13	myotubularin related protein 13	-1.05	0.76	1.08	0.2139	1.09	0.3936	1.13	0.036	1.28	0.0087
104428_s_at	1421739_a_at	Matk	megakaryocyte-associated tyrosine kinase	1.37	0.5	-1.49	0.0711	-1.47	0.1421	-1.26	0.2511	-1.11	0.756
104430_at	1419226_at	Cd96	CD96 antigen	-2.48	0.13	1.18	0.3966	-1.04	0.7597	1.03	0.8563	1.17	0.5109
104434_at	1460733_at	AA407659	expressed sequence AA407659	-1.3	0.09	1.1	0.1945	1.09	0.4813	-1.02	0.6524	-1.12	0.4327
104436_at	1421370_a_at	Il1f5	interleukin 1 family, member 5 (delta)	-1.28	0.63	1.54	0.2673	1.47	0.2448	1.25	0.512	1.72	0.2765
104437_at	1449813_at	Zfp30	zinc finger protein 30	1.33	0.46	1.24	0.0908	1.27	0.0403	1.12	0.3695	1.34	0.2384
104439_at	1419213_at	Nat6	N-acetyltransferase 6	-1.29	0	-1.08	0.2524	-1.1	0.3094	-1.19	0.0365	1.04	0.7877
104440_at	1420762_a_at	Ybx2	Y box protein 2	-1.33	0.12	1.17	0.3858	-1.05	0.6617	1.15	0.306	1.21	0.2399
104442_at	1441992_at	Rab14	RAB14, member RAS oncogene family	1.83	0.06	-1.01	0.9198	-2.02	0.0088	-1.56	0.0597	1.58	0.0997
104443_at	1423466_at	Ccr7	chemokine (C-C motif) receptor 7	2.02	0.08	1.66	0.1136	1.53	0.2413	1.12	0.7169	1.43	0.0769
104444_at	1425191_at	9430098E02Rik	RIKEN cDNA 9430098E02 gene	1.17	0.07	-1.03	0.6894	-1.11	0.1996	-1.06	0.4187	-1.14	0.4468
104445_at	1429159_at	4631408O11Rik	RIKEN cDNA 4631408O11 gene	-1.36	0.06	-1.03	0.6942	1.39	0.0512	1.15	0.3422	1.23	0.1941
104447_r_at	1426253_at	4933428G09Rik	RIKEN cDNA 4933428G09 gene	-1.02	0.95	1.07	0.6429	-1.18	0.3427	1.03	0.7643	-1.05	0.8742
104449_at	1422504_at	Glrb	glycine receptor, beta subunit	1.3	0.48	-1.29	0.2082	1.02	0.9313	-1.08	0.5855	1.44	0.196
104450_at	1425971_at	LOC277923	reverse transcriptase	-1.15	0.76	-1.25	0.5728	-1.47	0.3951	-1.03	0.9479	1.07	0.8187
104451_at	1426441_at	Slc11a2	solute carrier family 11 (proton-coupled diva	-1.41	0.25	-1.08	0.5876	1.07	0.5025	1.07	0.5884	-1.55	0.028
104453_at	1415718_at	2310079P12Rik	RIKEN cDNA 2310079P12 gene	1.1	0.65	-1.16	0.1242	1.02	0.845	-1.06	0.5989	1.29	0.0344
104454_at	1423539_at	Pms2	postmeiotic segregation increased 2 (S. cere	1.79	0.39	-1.12	0.4033	-1.18	0.3615	-1.25	0.0738	2.84	0.0977
104455_at	1421941_at	Camk4	calcium/calmodulin-dependent protein kinas	-1.43	0.58	1.15	0.4296	1.11	0.6734	-1.01	0.963	1.32	0.1734
104456_at	1436049_at	Mettl3	Methyltransferase-like 3	1.2	0.55	1.01	0.9428	1.16	0.2441	1.18	0.0829	-1.16	0.5318
104457_at	1448011_at	---	---	2.29	0.12	-1.46	0.2369	-1.59	0.1475	-1.44	0.3116	-1.05	0.8938
104458_at	1436207_at	Tcf3	Transcription factor 3	-1.02	0.93	1.7	0.0012	1.58	0.0059	1.54	0.0456	2.12	0.0076
104460_at	1423365_at	Cacna1g	calcium channel, voltage-dependent, T type,	-1.29	0.23	1.01	0.9162	1.27	0.1645	-1.13	0.1571	1.13	0.5144
104461_at	1460326_at	Pik3ca	phosphatidylinositol 3-kinase, catalytic, alpha	-1.04	0.85	1.17	0.0878	1.26	0.0063	1.29	0.0113	1.61	0.0101
104462_at	1449226_at	Hic1	hypermethylated in cancer 1	-1.39	0.68	-1.09	0.6487	-1.11	0.6259	-1.33	0.1152	-1.13	0.7926
104463_at	1452252_at	3830408P06Rik	RIKEN cDNA 3830408P06 gene	1.11	0.52	-1.24	0.0031	-1	0.9604	-1.28	0.0008	1.11	0.2869
104464_s_at	1418538_at	Kdelr3	KDEL (Lys-Asp-Glu-Leu) endoplasmic reticu	2	0.06	1.1	0.5334	1.12	0.4578	1.01	0.953	1.22	0.7907
104466_at	1448599_s_at	D4Wsu114e	DNA segment, Chr 4, Wayne State Universit	1.5	0.01	1.89	0.0298	1.83	0.0087	2.69	0	2.17	0.2631
104468_at	1423093_at	Incenp	inner centromere protein	-1.21	0.4	-1.04	0.7567	1.01	0.8801	-1.1	0.1912	1.46	0.1896
104469_at	1419309_at	Gp38	glycoprotein 38	-1.07	0.88	1.8	0.0013	1.76	0.0543	1.51	0.0171	1.64	0.2301
104471_at	1448928_at	Hdac6	histone deacetylase 6	1.3	0.21	1.1	0.2372	1.15	0.0735	1.06	0.3616	1.4	0.0002
104474_s_at	1426204_a_at	Oprl1	opioid receptor-like 1	-1.63	0.04	1.25	0.404	1.21	0.3192	1.25	0.3294	1.09	0.7058
104475_at	1434104_at	6030458H05	hypothetical protein 6030458H05	1.83	0.36	-1.07	0.5594	-1.17	0.2171	-1.13	0.2044	-1.5	0.1053
104476_at	1424156_at	Rbl1	retinoblastoma-like 1 (p107)	5.71	0.03	1.66	0.1908	1.43	0.0026	1.81	0.0889	1.89	0.0099
104477_at	1448021_at	---	Transcribed locus	1.22	0.54	-1.08	0.6776	1.09	0.5301	1.14	0.4823	-1.24	0.6482
104479_at	1450318_a_at	P2ry2	purinergic receptor P2Y, G-protein coupled 2	1.33	0.23	1.03	0.7396	1.66	0.0365	1.15	0.1763	-1.23	0.1727
104480_at	1449740_s_at	Dsg2	desmoglein 2	-2.03	0.05	-1.43	0.0002	-1.13	0.119	-1.47	0.0002	1.08	0.7491
104482_at	1418164_at	Epim	epimorphin	-1.24	0.62	1.11	0.2958	-1.33	0.0488	-1.36	0.0395	-1.29	0.3195
104483_at	1421381_a_at	Col9a1	procollagen, type IX, alpha 1	-1.22	0.66	1.04	0.8575	-1.17	0.6081	-1.14	0.6426	1.65	0.2967
104484_at	1419528_at	Sult2a2	sulfotransferase family 2A, dehydroepiandro	137.12	0	-1.04	0.6163	-1.02	0.7614	-1.32	0.0374	16.38	0.0027
104485_at	1452523_a_at	4930527D15Rik	RIKEN cDNA 4930527D15 gene	1.67	0.38	-1.23	0.4812	-1.43	0.3027	-1.48	0.2111	-1.03	0.867
104486_at	1434719_at	A2m	alpha-2-macroglobulin	-3.49	0.13	-1.15	0.6233	-1.18	0.6484	-1.65	0.1001	-1	0.9954
104487_at	1440252_at	E130307C13	Hypothetical protein E130307C13	1.34	0.54	1.06	0.8582	1.41	0.2995	1.52	0.1895	3.34	0.0915
104488_at	1427070_at	5730407K14Rik	RIKEN cDNA 5730407K14 gene	-1.03	0.89	1.18	0.3266	1.01	0.9379	1.03	0.867	1.61	0.1171
104489_at	1420371_at	Sntb2	syntrophin, basic 2	-2.78	0.08	1.21	0.3759	-1.02	0.9012	1.08	0.5811	1.57	0.0543
104491_at	1437067_at	Phtf2	putative homeodomain transcription factor 2	1.44	0.33	1.22	0.1361	1.25	0.1525	1.4	0.0445	1.23	0.388
104492_at	1460666_a_at	Ebf3	early B-cell factor 3	-1.41	0.28	-1.19	0.0745	-1.07	0.4853	-1.12	0.2062	1.09	0.3136

104493_at	1425433_a_at	Nrp2	neuropilin 2	-1.56	0.39	-1.52	0.1306	-1.91	0.0176	-1.63	0.0945	2.9	0.363
104494_at	1454736_at	4921515A04Rik	RIKEN cDNA 4921515A04 gene	1.05	0.54	1.13	0.4806	1.04	0.7736	1.28	0.0823	-1.13	0.1075
104495_f_at	1449313_at	Klk5	kallikrein 5	1.3	0.7	-1.24	0.3222	1.48	0.3173	1.02	0.9432	-1.35	0.5743
104497_f_at	1449463_at	Klk8	kallikrein 8	1.05	0.94	1.03	0.887	1.01	0.9711	-1.08	0.7215	1.48	0.1739
104498_at	1425671_at	Homer1	homer homolog 1 (Drosophila)	-1.08	0.88	1.09	0.8177	-1.12	0.7925	-1.48	0.4039	-1.89	0.4082
104499_at	1425710_a_at	Homer1	homer homolog 1 (Drosophila)	1.27	0.39	1.11	0.7678	-1.06	0.866	-1.11	0.7735	1.69	0.0361
104500_at	1418793_at	Idua	iduronidase, alpha-L-	1.38	0.41	1.4	0.0114	1.19	0.1902	1.57	0.0016	1.73	0.034
104501_at	1436079_s_at	Vapb	vesicle-associated membrane protein, assoc	-1.16	0.64	-1.08	0.4545	1.11	0.3316	-1.1	0.3021	1.33	0.085
104504_at	1451894_a_at	Scn8a	sodium channel, voltage-gated, type VIII, alp	1.04	0.93	-1.3	0.2884	1.19	0.4167	1.1	0.6855	1.02	0.9037
104507_g_at	1425392_a_at	Nr1i3	nuclear receptor subfamily 1, group I, memb	1.25	0.23	1.25	0.0351	1.19	0.0586	1.26	0.0012	2.21	0.0004
104508_at	1428787_at	4930568P13Rik	RIKEN cDNA 4930568P13 gene	-1.65	0.15	1.54	0.0049	1.19	0.1594	1.37	0.0937	1.46	0.0518
104509_at	1449227_at	Ch25h	cholesterol 25-hydroxylase	-1.14	0.85	1.08	0.7643	-1.19	0.3635	1.22	0.3769	1.57	0.1547
104510_at	1449999_a_at	Cacna2d1	calcium channel, voltage-dependent, alpha2	-3.44	0.16	1.09	0.3139	-1.01	0.9466	-1.04	0.7202	2.12	0.072
104512_at	1454881_s_at	Upk3b	uroplakin 3B	1.26	0.22	-1.19	0.1833	-1.37	0.0045	-1.26	0.0486	1.45	0.1579
104514_at	1427039_at	Epn1	epsin 1	-1.27	0.09	1.02	0.6766	1.03	0.4919	-1.05	0.2964	1.36	0.031
104516_at	1417839_at	Cldn5	claudin 5	2.39	0.01	1.21	0.0617	-1.09	0.6459	1.11	0.5135	1.75	0.0834
104518_at	1450671_at	Cryba1	crystallin, beta A1	-1.36	0.39	-1.12	0.4675	1.31	0.2028	-1.07	0.6358	1.85	0.1569
104519_at	1417618_at	Itih2	inter-alpha trypsin inhibitor, heavy chain 2	-1.09	0.68	-1.01	0.8814	1.03	0.4663	1	0.9106	1.26	0.0254
104522_at	1452338_s_at	Itsn1	intersectin 1 (SH3 domain protein 1A)	1.06	0.4	-1.06	0.167	-1.07	0.5178	-1.06	0.2981	1.08	0.4422
104523_at	1423614_at	E430036I04Rik	RIKEN cDNA E430036I04 gene	-2.48	0.01	1.44	0.0828	1.03	0.9118	1.49	0.1456	1.59	0.3482
104525_at	1428351_at	Ppm1m	protein phosphatase 1M	1.01	0.95	-1.09	0.2685	-1.01	0.9025	-1.14	0.0443	1.31	0.1532
104526_at	1422566_at	Tcfeb	transcription factor EB	-1.47	0.32	1.22	0.4856	-1.2	0.4306	-1.12	0.6564	2.08	0.1878
104527_at	1418281_at	Rad51	RAD51 homolog (S. cerevisiae)	1.18	0.72	-1.01	0.9428	1.01	0.937	-1.09	0.6816	-1.01	0.9768
104528_at	1416651_at	Znhit2	zinc finger, HIT domain containing 2	1.1	0.49	1.07	0.3779	1.02	0.7879	1.01	0.7677	1.01	0.9469
104529_at	1417842_at	Caml	calcium modulating ligand	-1.02	0.9	-1.02	0.8328	-1.04	0.7104	-1.08	0.4175	-1.42	0.1651
104532_at	1448345_at	Tomm34	translocase of outer mitochondrial membran	1.31	0.51	1.46	0.0022	1.33	0.0739	1.47	0.0309	1.14	0.0638
104533_at	1435458_at	---	---	-1.53	0.33	1.12	0.4547	-1.32	0.0034	1.05	0.6811	-1.81	0.0024
104535_at	1423360_at	Yme1l1	YME1-like 1 (S. cerevisiae)	-1.21	0.25	-1.15	0.0472	-1.14	0.1445	-1.12	0.1463	-1.46	0.1034
104536_at	1420634_a_at	Smad2	MAD homolog 2 (Drosophila)	1.59	0.08	1.04	0.6245	1.15	0.1072	1.05	0.527	-1.12	0.033
104537_at	1424505_at	0610042C05Rik	RIKEN cDNA 0610042C05 gene	2.59	0.12	1.07	0.4164	-1.08	0.4282	1.11	0.3257	1.19	0.1585
104538_at	1448816_at	Ptgis	prostaglandin I2 (prostacyclin) synthase	-1.14	0.69	-1.85	0.0081	-1.71	0.0282	-1.71	0.0068	1.75	0.1961
104539_at	1418940_at	Sult1b1	sulfotransferase family 1B, member 1	1.37	0.23	-1.21	0.0441	-1.11	0.5378	-1.19	0.2042	-1.53	0.0598
104541_at	1419669_at	Prtn3	proteinase 3	-1.69	0.4	-1.03	0.8922	-1.11	0.5105	1.36	0.3177	-1.69	0.5657
104544_at	1423327_at	4930517K11Rik	RIKEN cDNA 4930517K11 gene	-2.2	0.48	2	0.0587	1.3	0.3551	1.29	0.389	-1.07	0.8809
104546_g_at	1419035_s_at	Csnk2a1	casein kinase II, alpha 1 polypeptide	2.82	0.09	1.03	0.901	-1.42	0.1986	-1.38	0.2236	-1.29	0.4162
104547_at	1419172_at	Dhfr	dihydrofolate reductase	1.66	0.03	1.16	0.1175	-1.06	0.4706	1.23	0.0646	-1.04	0.6281
104548_at	1417837_at	Phlda2	pleckstrin homology-like domain, family A, m	2.4	0.05	1.23	0.2031	1.03	0.8715	1.31	0.0543	1.44	0.0153
104549_at	1433497_at	Aqr	aquarius	-1.09	0.2	1.06	0.6312	1.03	0.7211	1.17	0.058	1.13	0.3698
104550_at	1428283_at	Cyp2s1	cytochrome P450, family 2, subfamily s, poly	1.02	0.8	-1.01	0.9534	-1.24	0.2072	1.03	0.9067	-1.12	0.7192
104554_at	1460648_at	Nr2f6	nuclear receptor subfamily 2, group F, meml	-1.43	0.01	-1.17	0.007	-1.01	0.9352	-1.17	0.0046	-1.22	0.169
104555_at	1460202_at	Myoz1	myozenin 1	-1.43	0.62	-1.25	0.3928	1.01	0.9586	-1.02	0.9224	1.45	0.4199
104557_at	1420307_a_at	Pitpnb	phosphatidylinositol transfer protein, beta	1.15	0.68	1.03	0.3864	-1.02	0.877	-1	0.9996	-1.1	0.132
104558_at	1454932_at	Rcor1	REST corepressor 1	1.22	0.28	1.22	0.0134	1.47	0.0602	1.18	0.0503	-1.29	0.0936
104559_at	1460690_at	BC003940	cDNA sequence BC003940	-1.86	0.05	-1.07	0.24	-1.14	0.0695	-1.03	0.5189	1.17	0.0372
104560_at	1424776_a_at	Slc25a28	solute carrier family 25, member 28	1.48	0.02	-1.07	0.4666	1.08	0.4535	-1.03	0.7139	-1.07	0.6048
104561_at	1423998_at	Gtf3c5	general transcription factor IIIC, polypeptide	-1.05	0.79	1.58	0.0254	1.39	0.0936	1.37	0.1457	1.51	0.0228
104562_at	1434343_at	5730403M16Rik	RIKEN cDNA 5730403M16 gene	1.19	0.31	-1.18	0.0153	1	0.9474	-1.16	0.0076	1.27	0.0451
104564_at	1448628_at	Scg3	secretogranin III	-1.69	0.17	-1.14	0.5007	-1.15	0.4196	-1.01	0.9577	1.2	0.4239
104565_at	1451665_a_at	Ap4s1	adaptor-related protein complex AP-4, sigma	1.57	0.13	-1.1	0.288	1.18	0.2137	-1.04	0.5793	-1.32	0.0026
104567_at	1449004_at	Mrpl46	mitochondrial ribosomal protein L46	1.45	0.05	-1.03	0.7536	1.07	0.4225	-1.03	0.747	-1.02	0.8879
104568_at	1427283_at	Mll	myeloid/lymphoid or mixed-lineage leukemia	-1.18	0.5	-1.25	0.0215	-1.11	0.0952	-1.16	0.0149	1.37	0.0908
104572_at	1433910_at	Zcchc6	zinc finger, CCHC domain containing 6	-1.21	0.23	1.1	0.1779	1.14	0.1009	1.05	0.3664	-1.27	0.0785
104573_at	1434545_x_at	1110025L05Rik	RIKEN cDNA 1110025L05 gene	-1.1	0.78	1.13	0.0662	1.2	0.0185	1.23	0.0058	-1.08	0.7156

104574_at	1415730_at	5730453I16Rik	RIKEN cDNA 5730453I16 gene	-1.08	0.2	-1.09	0.2003	1.01	0.9034	1.01	0.8641	1.18	0.0973
104576_at	1426373_at	Ski	Sloan-Kettering viral oncogene homolog	-1.08	0.37	-1.26	0.0046	-1.16	0.0635	-1.36	0.0002	-1.24	0.0212
104577_at	1417360_at	Mlh1	mutL homolog 1 (E. coli)	1.18	0.53	1.07	0.4301	-1.1	0.1988	-1.03	0.6718	-1.22	0.2028
104579_r_at	1428585_at	Actn1	actinin, alpha 1	2.24	0.03	1.68	0.0015	1.3	0.0513	1.78	0.0003	1.55	0.036
104580_at	1448432_at	Plcd1	phospholipase C, delta 1	-1.15	0.64	1.26	0.4035	1.52	0.2337	1.15	0.7206	1.36	0.3922
104583_at	1423300_at	Zdhhc6	zinc finger, DHHC domain containing 6	1.34	0.08	-1.18	0.0405	-1.19	0.0443	-1.1	0.1807	-1.66	0.0075
104584_f_at	1434851_s_at	Crb3	crumbs homolog 3 (Drosophila)	1.28	0.46	-1.13	0.4368	1.18	0.2983	-1.07	0.7312	-1.06	0.9437
104585_at	1424809_at	Crb3	crumbs homolog 3 (Drosophila)	-1.29	0.09	1.06	0.5974	-1	0.9745	-1.04	0.6428	1.31	0.1018
104586_at	1428100_at	Sfrs1	splicing factor, arginine/serine-rich 1 (ASF/S	1.15	0.26	-1.02	0.7748	-1.09	0.3101	-1.09	0.1447	-1.35	0.004
104587_at	1424807_at	Lama4	laminin, alpha 4	1.41	0.04	-1.05	0.691	-1.05	0.6396	-1	0.9756	1.76	0.0829
104588_at	1419197_x_at	Hamp1	hepcidin antimicrobial peptide 1	1.52	0.32	1.07	0.2774	-1.16	0.0289	-1.03	0.7886	-2.01	0.0014
104589_at	1419273_at	C80913	expressed sequence C80913	-1.17	0.52	1.38	0.0114	-1.02	0.8957	1.36	0.0496	1.38	0.0587
104592_i_at	1421028_a_at	Mef2c	myocyte enhancer factor 2C	-2.13	0.22	1.38	0.1516	1.08	0.5848	-1.07	0.667	1.34	0.2334
104593_at	1451167_at	1700023O11Rik	RIKEN cDNA 1700023O11 gene	1.85	0.06	1.05	0.5947	1.08	0.5013	1.14	0.2626	1.23	0.6641
104595_at	1421849_at	Stag2	stromal antigen 2	1.17	0.62	1.3	0.0104	1.33	0.0615	1.61	0.001	1.51	0.0445
104597_at	1418240_at	Gbp2	guanylate nucleotide binding protein 2	1.77	0.03	4.98	0.1955	-1.7	0.0745	5.74	0.1072	6.31	0.0693
104598_at	1448830_at	Dusp1	dual specificity phosphatase 1	-1.27	0.44	1.36	0.2523	-1.26	0.1872	1.04	0.8201	-1.26	0.0644
104600_at	1452720_a_at	Fip1l1	FIP1 like 1 (S. cerevisiae)	1.55	0.14	1.29	0.0503	1.5	0.0004	1.41	0.002	1.5	0.3692
104601_at	1448529_at	Thbd	thrombomodulin	-1.44	0.62	-1.55	0.2332	-1.34	0.437	-1.31	0.3907	2.28	0.3598
104603_at	1417883_at	Gstt2	glutathione S-transferase, theta 2	2.93	0.03	1.22	0.0626	1.63	0.0001	1.54	0.0077	2.14	0.0192
104604_at	1451281_at	Zfp96	zinc finger protein 96	2.29	0.09	1.48	0.0026	1.18	0.1273	1.48	0.0087	1.05	0.7438
104605_at	1434329_s_at	Adipor2	adiponectin receptor 2	-1.41	0.14	-1.75	0	-1.3	0.0028	-2.43	0	-3.07	0
104606_at	1460218_at	Cd52	CD52 antigen	1.14	0.37	2.14	0.0914	-1.16	0.2806	2.58	0.1066	1.63	0.0166
104607_at	1419056_at	Rtn2	reticulon 2 (Z-band associated protein)	-1.21	0.38	-1.5	0.3228	-1.35	0.4274	-1.01	0.985	-1.03	0.9474
104608_at	1451996_at	Bbp	beta-amyloid binding protein precursor	1.23	0.64	1.04	0.6937	1.07	0.5038	-1.06	0.56	1.08	0.7281
104609_at	1415765_at	1110031M08Rik	RIKEN cDNA 1110031M08 gene /// similar t	1.19	0.02	1.19	0.0141	-1.12	0.0865	1.01	0.8489	1.57	0.0046
104610_at	1426381_at	Pprc1	peroxisome proliferative activated receptor,	1.15	0.54	-1.04	0.7739	1.3	0.0168	1.05	0.5881	2.2	0.0115
104612_g_at	1423962_at	Wdr26	WD repeat domain 26	1.2	0.43	1.2	0.0016	1.22	0.004	1.16	0.0259	-1.08	0.432
104614_at	1417389_at	Gpc1	glypican 1	-1.85	0	-1.25	0.0448	-1.15	0.1571	-1.47	0.0029	-2.36	0.0043
104616_g_at	1450187_a_at	Galt	galactose-1-phosphate uridyl transferase	1.28	0.16	1.05	0.6242	1.08	0.4769	1.11	0.1972	-1.34	0.0675
104617_at	1434906_at	0610005C13Rik	RIKEN cDNA 0610005C13 gene	1.49	0.21	1.02	0.9459	-1.14	0.6216	1.49	0.0018	1.1	0.8423
104618_at	1416174_at	Rbbp9	retinoblastoma binding protein 9	1.52	0.01	1.07	0.4469	-1.25	0.2091	1.06	0.4255	-1.22	0.2564
104619_at	1417431_a_at	Sphk2	sphingosine kinase 2	1.5	0.15	-1.16	0.1727	-1.15	0.2204	-1.15	0.1686	-1.02	0.9204
104620_at	1423649_at	2010300G19Rik	RIKEN cDNA 2010300G19 gene	-1.21	0.24	1.1	0.3893	1.28	0.0018	1.12	0.1473	1.33	0.078
104621_at	1433834_at	F830029L24Rik	Membrane-associated ring finger (C3HC4) 6	-1.09	0.46	-1.07	0.2034	-1.01	0.8698	-1.11	0.0868	-1.17	0.1575
104622_at	1418107_at	Tcea2	transcription elongation factor A (SII), 2	1.55	0.43	-1.02	0.9637	-1.23	0.5212	1.41	0.2936	-1.02	0.9515
104623_at	1419655_at	Tle3	transducin-like enhancer of split 3, homolog	1.4	0.22	1.2	0.0161	-1.26	0.0461	1.19	0.0375	-1.4	0.0548
104624_at	1426815_s_at	AU024582	expressed sequence AU024582	-1	0.99	-1.16	0.0257	-1.12	0.0422	-1.1	0.128	-1.03	0.8303
104625_at	1434035_at	Dnajb6	DnaJ (Hsp40) homolog, subfamily B, membe	1.03	0.79	-1	0.9936	1.24	0.0855	1.36	0.0057	1.42	0.0781
104626_at	1427964_at	Cklfsf8	chemokine-like factor super family 8	1.44	0.05	1.02	0.8412	-1.07	0.424	-1.03	0.7345	1.2	0.1295
104627_at	1434256_s_at	Cds2	CDP-diacylglycerol synthase (phosphatidate	1.93	0.15	1.33	0.0382	-1.13	0.4946	1.27	0.1304	1.06	0.8786
104628_at	1448647_at	Man2a1	mannosidase 2, alpha 1	1.24	0.4	1.43	0	1.19	0.0138	1.58	0	1.13	0.3841
104629_at	1417361_at	Asb3	ankyrin repeat and SOCS box-containing pro	-1.3	0.44	1	0.9925	1.06	0.4777	1.07	0.4356	1.19	0.2637
104632_at	1424651_at	BC021611	cDNA sequence BC021611	1.3	0.11	-1.11	0.0579	-1.09	0.1634	-1.1	0.1346	-1.68	0.0333
104633_at	1423805_at	Dab2	disabled homolog 2 (Drosophila)	1.04	0.89	-1.01	0.9148	1.14	0.1815	-1.07	0.5449	2.08	0.0002
104634_at	1418232_s_at	Lims1	LIM and senescent cell antigen-like domains	1.22	0.35	1.16	0.1078	1.02	0.8695	1.12	0.2705	1.41	0.066
104635_r_at	1449094_at	Gja7	gap junction membrane channel protein alph	-1.45	0.34	1.32	0.0442	1.4	0.0808	1.27	0.0325	1.13	0.4074
104637_at	1422072_a_at	Gstm6	glutathione S-transferase, mu 6	-1.28	0.05	1.16	0.0353	-1.03	0.6976	1.07	0.3913	-1.63	0.0355
104638_at	1451372_a_at	Art1	ADP-ribosyltransferase 1	-1.09	0.85	-1.2	0.2946	-1.06	0.7393	-1.04	0.7911	1.52	0.3515
104640_f_at	1436558_at	4930553M18Rik	RIKEN cDNA 4930553M18 gene	1.35	0.26	1.38	0.0003	1.42	0.0106	1.39	0.0063	1.75	0.0055
104641_f_at	1439113_at	Hpcal1 /// 2410(	hippocalcin-like 1 /// RIKEN cDNA 2410018L	-1.12	0.38	-1.11	0.5435	-1.24	0.3516	-1.08	0.6192	2.01	0.3851
104642_at	1449905_at	Clec4f	C-type lectin domain family 4, member f	1.12	0.65	-1.2	0.0557	-1.12	0.1075	-1.67	0.0002	-1.14	0.4939
104643_at	1427261_at	BC037006	cDNA sequence BC037006	-1.15	0.72	1.15	0.1141	-1.05	0.5041	-1.09	0.2229	-1.01	0.9552

104644_at	1450692_at	Kif4	kinesin family member 4	1.09	0.66	1.08	0.6307	-1.12	0.6993	1.05	0.8249	-1.28	0.4671
104645_at	1419354_at	Klf7	Kruppel-like factor 7 (ubiquitous)	1.15	0.83	-1.1	0.6603	-1.75	0.0148	-1.7	0.1016	1.33	0.2206
104646_at	1418949_at	Gdf15	growth differentiation factor 15	3.05	0.18	2.4	0.0218	1.04	0.7017	1.97	0.0419	1.33	0.1492
104647_at	1417262_at	Ptgs2	prostaglandin-endoperoxide synthase 2	-1.24	0.61	2.89	0.0463	1.29	0.5394	2.14	0.0877	-1.16	0.8082
104648_at	1435762_at	Pacs1	phosphofurin acidic cluster sorting protein 1	-1.02	0.96	1.4	0.0232	1.19	0.2904	1.47	0.0044	1.18	0.4183
104650_at	1422635_at	Ache	acetylcholinesterase	-1.26	0.29	1.04	0.7756	1.01	0.9649	-1.05	0.7256	1.07	0.8116
104651_at	1456039_at	Snx14	sorting nexin 14	1.72	0.06	1.13	0.0223	1.3	0.0417	1.2	0.0188	-1.08	0.6949
104652_at	1449158_at	Kcnk2	potassium channel, subfamily K, member 2	-1.41	0.14	-1.2	0.3545	-1.17	0.2343	-1.1	0.4832	1.23	0.6215
104654_at	1422564_at	Actl6b	actin-like 6B	-1.36	0.39	-1.49	0.1663	-1.44	0.2257	-1.29	0.4254	-1.04	0.923
104655_at	1422545_at	Tbx2	T-box 2	1.51	0.45	1.07	0.4351	1.15	0.3106	-1.12	0.273	1.81	0.021
104657_at	1450207_at	Lifr	leukemia inhibitory factor receptor	2.16	0.01	-2.56	0.0006	-1.63	0.0132	-2.33	0.0007	1.77	0.1728
104659_g_at	1425107_a_at	Lifr	leukemia inhibitory factor receptor	-3.22	0	-5.58	0	-2.11	0	-6.56	0	-3.09	0.0077
104660_at	1418751_at	MGI:1889342	SHP2 interacting transmembrane adaptor	-1.21	0.64	-1.38	0.325	-2.13	0.0192	-1.56	0.2168	1.73	0.0979
104661_at	1455134_at	D730040F13Rik	RIKEN cDNA A630051L19 gene	-1.11	0.39	1.3	0.0068	1.25	0.0209	1.25	0.1497	1.71	0.0313
104662_at	1422303_a_at	Tnfrsf18	tumor necrosis factor receptor superfamily, r	1.08	0.81	2.08	0.0017	2.06	0.0016	2.02	0.0031	2.12	0.0339
104663_at	1418144_a_at	Pip5k1b	phosphatidylinositol-4-phosphate 5-kinase, t	-1.36	0.24	-1.11	0.1539	-1.15	0.0116	-1.36	0.0011	1.34	0.0279
104667_at	1432083_a_at	Lrpb7	leucine rich protein, B7 gene	-1.25	0.1	-1.07	0.7413	1.01	0.9609	-1.13	0.5536	1.05	0.8582
104669_at	1417244_a_at	Irf7	interferon regulatory factor 7	-1.44	0.05	-1.12	0.6215	1.39	0.0176	1.19	0.6063	-1.13	0.7601
104670_at	1434846_at	1700065A05Rik	RIKEN cDNA 1700065A05 gene	1.04	0.82	1.29	0.0002	1.35	0.1712	1.19	0.055	-1.06	0.767
104671_at	1422573_at	---	---	1.19	0.59	1.76	0.0063	1.43	0.1395	1.38	0.3062	1.63	0.2154
104672_at	1416658_at	Frzb	frizzled-related protein	-1.22	0.49	1.35	0.0058	1.25	0.0718	1.14	0.3658	1.05	0.9016
104673_at	1421928_at	Epha4	Eph receptor A4	-2.38	0	-1.26	0.1734	1.01	0.9712	-1.29	0.1502	1.91	0.0018
104675_at	1425824_a_at	Pcsk4	proprotein convertase subtilisin/kexin type 4	2.01	0.25	2.02	0.0526	3.18	0	3.17	0.0021	1.01	0.9442
104677_at	1452235_at	Man1b1	mannosidase, alpha, class 1B, member 1	1.16	0.34	-1.02	0.8837	1.1	0.4146	-1.11	0.3251	1.11	0.3228
104678_at	1417859_at	Gas7	growth arrest specific 7	-1.07	0.87	-1.14	0.4959	-1.31	0.2526	-1.06	0.7536	1.71	0.0278
104680_at	1417481_at	Ramp1	receptor (calcitonin) activity modifying protei	1.02	0.95	1.05	0.5667	-1.04	0.5621	-1.03	0.504	-1.01	0.9684
104681_at	1421143_at	Diap1	diaphanous homolog 1 (Drosophila)	-1.6	0.02	-1.09	0.3852	-1.04	0.7113	-1.32	0.0097	1.02	0.9337
104682_at	1419518_at	Tuba8	tubulin, alpha 8	-1.3	0.03	-1.09	0.2216	-1.01	0.857	1.03	0.7205	1.26	0.0158
104684_at	1437968_at	Grin1	glutamate receptor, ionotropic, NMDA1 (zeta	-2.14	0.02	-1.07	0.3074	-1.06	0.4435	-1.17	0.0093	1.22	0.363
104686_at	1450202_at	Grin1	glutamate receptor, ionotropic, NMDA1 (zeta	-1.69	0.02	1.17	0.2468	1.67	0.0014	1.38	0.0448	-1.7	0.1621
104687_at	1451024_at	Edg6	endothelial differentiation, G-protein-couplec	-2.7	0.05	1.35	0.2569	-1.04	0.901	-1.02	0.943	-1.64	0.2454
104688_at	1439323_a_at	Map4k1	mitogen activated protein kinase kinase kina	1.54	0.06	-1.02	0.7362	-1.04	0.7584	-1.02	0.7819	1.19	0.3472
104689_at	1450464_at	E4f1	E4F transcription factor 1	1.77	0.32	1.98	0.0081	1.93	0.0003	1.95	0.0017	-1.08	0.8958
104690_at	1417249_at	Polm	polymerase (DNA directed), mu	-1.69	0.01	-1.11	0.0828	-1.05	0.4735	-1.06	0.2794	1.14	0.0637
104692_at	1420558_at	Selp	selectin, platelet	2.4	0.26	1.07	0.4062	1.24	0.0257	1.05	0.5663	1.23	0.2758
104693_at	1429126_at	2600001M11Rik	RIKEN cDNA 2600001M11 gene	1.91	0.05	1.54	0.0621	1.83	0.0141	1.6	0.0205	-1.01	0.9758
104694_at	1451902_at	BC021442	cDNA sequence BC021442	-1.07	0.58	1.24	0.1304	1.3	0.0394	1.06	0.6767	1.43	0.2017
104695_at	1453184_at	2310040C09Rik	RIKEN cDNA 2310040C09 gene	-1.38	0.09	-1.13	0.1051	-1.18	0.0884	-1.27	0.0657	1.01	0.9718
104696_at	1418989_at	Ctse	cathepsin E	-2.29	0.59	1.02	0.8761	1.63	0.0082	1.05	0.7303	1.76	0.2326
104698_at	1425463_at	Gata6	GATA binding protein 6	-1.15	0.7	-1.18	0.1113	-1.31	0.0945	-1.04	0.7032	1.43	0.0551
104700_at	1422198_a_at	Shmt1	serine hydroxymethyl transferase 1 (soluble)	-2.27	0.01	-1.58	0.2215	-1.51	0.2442	-1.75	0.0552	-1.04	0.9094
104701_at	1418025_at	Bhlhb2	basic helix-loop-helix domain containing, cla	-1.12	0.7	-1.14	0.3525	1.24	0.1718	-1.07	0.6412	-1.3	0.0471
104704_at	1415986_at	Clcn4-2	chloride channel 4-2	1.12	0.66	-1.17	0.0328	1.22	0.1642	-1.15	0.1736	-1.57	0.0151
104706_at	1418988_at	Pex7	peroxisome biogenesis factor 7	1.7	0.03	1.11	0.0424	1.04	0.6153	1.27	0.0017	-1.05	0.7095
104707_at	1424445_at	Tm4sf5	transmembrane 4 superfamily member 5	1.45	0.47	1.06	0.5794	3.03	0.241	1.06	0.4218	1.28	0.1849
104708_at	1455042_at	Tbl1x	Transducin (beta)-like 1 X-linked	-1.35	0.08	1.1	0.2223	1.13	0.2808	1.14	0.3097	1.27	0.1984
104709_at	1423347_at	---	---	-1.22	0.17	-1.02	0.6962	1.09	0.2617	-1.01	0.7941	-1.24	0.1818
104710_at	1418991_at	Bak1	BCL2-antagonist/killer 1	-1.25	0.2	-1.12	0.2436	-1.3	0.0077	-1.1	0.1843	-1.06	0.5271
104711_at	1417510_at	Vps4a	vacuolar protein sorting 4a (yeast)	-1.24	0.14	-1.06	0.3062	1.11	0.0191	-1.01	0.8084	1.04	0.7696
104712_at	1424942_a_at	Myc	myelocytomatosis oncogene	-1.06	0.92	-2.32	0.0025	-1.92	0.01	-2.05	0.0102	-1.17	0.7785
104713_at	1433565_at	2410002M20Rik	RIKEN cDNA 2410002M20 gene	-1.29	0.32	1.25	0.4292	1.48	0.0985	-1.01	0.9692	1.07	0.8436
104714_at	1450932_s_at	Dock9	dedicator of cytokinesis 9	1.75	0.01	-1.03	0.8127	-1.03	0.8285	-1.12	0.4108	-1.13	0.3342
104715_at	1424281_at	Ubap2	ubiquitin-associated protein 2	-1.21	0.02	-1.06	0.3136	-1.03	0.5959	-1.11	0.1632	1.4	0.0347

104716_at	1448754_at	Rbp1	retinol binding protein 1, cellular	1.87	0.35	1.57	0.0349	1.56	0.002	2.29	0.0206	2.51	0.0162
104717_at	1419984_s_at	Zfp644	zinc finger protein 644	1.59	0	1.2	0.0077	1.16	0.1667	1.15	0.0448	1.12	0.4669
104719_at	1418257_at	Slc12a7	solute carrier family 12, member 7	1.34	0.05	1.09	0.2423	-1.08	0.4993	1.06	0.3958	-1.25	0.2121
104720_at	1433780_at	Ubn1	ubinuclein 1	-1.08	0.48	1.02	0.7429	1.07	0.2494	1.03	0.5188	-1.03	0.8255
104722_at	1451165_at	D15Ertd735e	DNA segment, Chr 15, ERATO Doi 735, exp	1.46	0.23	-1.12	0.1323	-1.41	0.0156	-1.31	0.0125	1.12	0.6519
104725_at	1427918_a_at	Rhoq	ras homolog gene family, member Q	1.24	0.51	1.44	0.0093	1.51	0.0421	1.72	0.0018	1.21	0.4692
104726_at	1451625_a_at	1700013L23Rik	RIKEN cDNA 1700013L23 gene	-1.12	0.21	-1.46	0	-1.22	0.0001	-1.67	0	-2.59	0
104728_at	1426246_at	Pros1	protein S (alpha)	1.33	0.1	1.19	0.0489	-1.03	0.6911	1.2	0.0289	1.02	0.8635
104729_at	1416255_at	Gja4	gap junction membrane channel protein alph	-1.37	0.37	-1.46	0.0356	-1.12	0.6105	-1.11	0.6834	1.07	0.7009
104730_at	1417675_a_at	Mdn1	midasin homolog (yeast)	-1.49	0.65	1.2	0.3853	1.2	0.318	1.08	0.7132	1.4	0.2857
104731_at	1419564_at	Zfp467	zinc finger protein 467	1.06	0.84	1.28	0.0329	1.13	0.2447	1.36	0.0021	1.32	0.0636
104733_at	1418579_at	Cetn2	centrin 2	-1.21	0.59	-1.18	0.248	-1.07	0.7677	-1.19	0.1411	1.02	0.9796
104735_at	1434881_s_at	Kctd12	potassium channel tetramerisation domain c	1.33	0.31	1.61	0.0197	1.09	0.6128	1.98	0.0032	1.52	0.0033
104737_at	1435639_at	2610528A11Rik	RIKEN cDNA 2610528A11 gene	-1.38	0.2	1.3	0.1872	1.1	0.5449	1.03	0.829	1.94	0.234
104738_at	1417657_s_at	Dnajc2	DnaJ (Hsp40) homolog, subfamily C, memb	1.62	0.24	1.19	0.054	-1.03	0.7536	1.24	0.0223	-1.01	0.9513
104739_at	1424068_at	Tcta	T-cell leukemia translocation altered gene	1.33	0.48	-1.02	0.6299	-1.07	0.3577	-1.07	0.0846	1.19	0.1253
104740_at	1424427_at	D1Ertd251e	DNA segment, Chr 1, ERATO Doi 251, expr	1.04	0.86	-1.04	0.5227	-1.07	0.3478	-1.07	0.3604	-1.46	0.1497
104741_at	1454787_at	Zdhhc9	zinc finger, DHHC domain containing 9	1.08	0.58	-1.17	0.0383	-1.05	0.516	-1.14	0.0863	1.1	0.2358
104742_at	1452592_at	Mgst2	microsomal glutathione S-transferase 2	-1.02	0.95	1.27	0.2753	1.13	0.4735	2.07	0.0006	1.7	0.0813
104743_at	1423551_at	Cdh13	cadherin 13	1.14	0.75	-1.22	0.1413	-1.03	0.8472	-1.24	0.1213	1.8	0.0633
104744_at	1424293_s_at	2610319K07Rik	RIKEN cDNA 2610319K07 gene	1.41	0.15	1.05	0.4231	1.13	0.0932	1.12	0.008	-1.09	0.6411
104745_at	1416793_at	Arl6ip2	ADP-ribosylation factor-like 6 interacting pro	1.29	0.28	-1.2	0.0366	1.05	0.5849	-1.11	0.3546	-1.18	0.1432
104746_at	1416803_at	Fkbp7	FK506 binding protein 7	-1.04	0.77	-1.03	0.781	-1.08	0.5585	-1.12	0.2512	1.33	0.0275
104747_at	1448299_at	Slc1a1	solute carrier family 1 (neuronal/epithelial hig	-1.66	0.32	1.24	0.4449	1.51	0.2273	1.18	0.1867	1.86	0.1517
104749_at	1449664_s_at	Rnf20	ring finger protein 20	1.05	0.93	1.27	0.0972	1.3	0.2114	1.24	0.0848	1	0.9902
104750_at	1417292_at	Ifi47	interferon gamma inducible protein 47	-2.1	0.05	1.05	0.9233	-2.22	0.0013	1.11	0.8146	-2.45	0.0346
104751_at	1422530_at	Prph1	peripherin 1	-2.24	0.02	1.03	0.7573	-1.06	0.5817	-1.1	0.3584	1.26	0.4098
104752_at	1418254_at	Apip	APAF1 interacting protein	1.07	0.7	-1.12	0.016	-1.04	0.5969	-1.03	0.6712	-1.36	0.0233
104754_at	1448778_at	Sfrs4	splicing factor, arginine/serine-rich 4 (SRp75	1.04	0.82	1.07	0.4073	1.45	0.0001	1.41	0.038	1.42	0.0054
104755_at	1427689_a_at	Tnip1	TNFAIP3 interacting protein 1	1.04	0.73	-1.05	0.5037	-1.22	0.0011	1.1	0.4458	-1.06	0.5058
104756_at	1435431_at	2310047M15Rik	RIKEN cDNA 2310047M15 gene	-1.18	0.23	-1.02	0.8248	1.06	0.5021	1.08	0.2675	1.21	0.1967
104757_at	1424891_a_at	Zw10	ZW10 homolog (Drosophila), centromere/kir	1.41	0.09	-1.03	0.8489	1.03	0.8573	-1.09	0.5251	-1.04	0.8999
104758_at	1452710_at	Rpusd4	RNA pseudouridylate synthase domain cont	1.44	0.07	-1.32	0.0043	-1.08	0.1794	-1.14	0.3171	1.19	0.4114
104759_at	1454998_at	1200011I18Rik	RIKEN cDNA 1200011I18 gene	1.2	0.59	-1.12	0.09	1.02	0.8357	1.03	0.6011	-1.59	0.0846
104760_at	1451016_at	Ifrd2	interferon-related developmental regulator 2	1.23	0.06	1.18	0.017	1.09	0.2416	1.2	0.0002	1.13	0.2585
104761_at	1426708_at	Antxr2	anthrax toxin receptor 2	1.7	0.07	1.19	0.0925	-1.16	0.0234	1.07	0.2963	-1.79	0.088
104766_at	1418305_s_at	Nola1	nucleolar protein family A, member 1 (H/AC/	1.04	0.82	1	0.9964	1.01	0.9108	-1.05	0.3855	1.06	0.0786
160066_at	1422730_at	Limd1	LIM domains containing 1	1.62	0.02	1.14	0.1243	1.18	0.0678	1.12	0.1275	1.15	0.5021
160067_at	1423705_at	2310057D15Rik	RIKEN cDNA 2310057D15 gene	1.25	0.61	1.34	0.1554	1.04	0.8707	1.54	0.0091	1.6	0.1112
160068_at	1417719_at	Sap30	sin3 associated polypeptide	1.1	0.78	1.34	0.0519	1.36	0.0059	1.21	0.3136	1.28	0.3584
160069_at	1417506_at	Gmnn	geminin	3.31	0	1.58	0.0053	1.64	0.0011	1.8	0.0001	-1.42	0.1057
160070_at	1448186_at	Pnliprp2	pancreatic lipase-related protein 2	-1.73	0.21	1.41	0.042	1.11	0.6499	-1.15	0.6236	-1.1	0.8404
160071_at	1423373_at	Rpp30	ribonuclease P/MRP 30 subunit (human)	1.58	0	1.3	0.0118	1.32	0.0304	1.18	0.1173	1.3	0.0783
160074_at	1426215_at	---	---	1.11	0.83	-1.32	0.0073	-2.4	0	-2.1	0	-3.36	0.0021
160075_at	1417468_at	Nit1	nitrilase 1	1.31	0.11	1.23	0.0174	1.03	0.726	1.22	0.014	1.38	0.059
160076_at	1430500_s_at	Mtx2	metaxin 2	1.17	0.61	1.12	0.0345	1.31	0.0506	1.23	0.0184	-1.19	0.2429
160077_at	1431411_a_at	Rai12	retinoic acid induced 12	-3.7	0.4	1.36	0.0329	1.2	0.1814	1.3	0.3	2.61	0.0001
160078_at	1450914_at	Ppp1r14b	protein phosphatase 1, regulatory (inhibitor)	1.74	0.17	-1	0.9769	1.09	0.4098	1.03	0.6366	1.08	0.3886
160079_i_at	1451495_at	Wac	WW domain containing adaptor with coiled-c	1.02	0.95	1.08	0.5633	1.19	0.1822	1	0.9785	1.07	0.6619
160081_at	1416807_at	Rpl36a	ribosomal protein L36a	1.07	0.51	1.13	0.1965	1.19	0.06	1.26	0.0011	1.27	0.103
160082_s_at	1423052_at	Arf4	ADP-ribosylation factor 4	-1.51	0.24	-1.02	0.8124	-1.06	0.5297	-1.08	0.3163	-1.22	0.1341
160084_at	1438761_a_at	Odc1	ornithine decarboxylase, structural 1	-1.24	0.18	-1.28	0.0002	-1.1	0.1842	-1.35	0.0002	1.12	0.424
160085_at	1448609_at	Tst	thiosulfate sulfurtransferase, mitochondrial	-1.24	0.33	-1.34	0.0002	-1.2	0.0427	-1.45	0.0001	-1.19	0.4384

160088_at	1450332_s_at	Fmo5	flavin containing monooxygenase 5	1.33	0.26	1.3	0.0003	1.32	0.0551	1.75	0	-1.09	0.4017
160089_at	1415880_a_at	Lamp1	lysosomal membrane glycoprotein 1	-1.12	0.37	1.01	0.8709	1.05	0.3799	-1.01	0.9136	-1.29	0.0292
160090_f_at	1434799_x_at	Aldoa	aldolase 1, A isoform	1.45	0.01	1.19	0.3969	1.12	0.5108	1.5	0.0546	1.34	0.0976
160091_at	1426554_a_at	Pgam1	phosphoglycerate mutase 1	1.06	0.75	1.22	0.0042	1.18	0.0457	1.19	0.0018	-1.19	0.0841
160092_at	1416067_at	Ifrd1	interferon-related developmental regulator 1	1.7	0	1.06	0.6898	1.28	0.1172	1.17	0.1474	1.48	0.4267
160094_at	1423589_at	Arpc4	actin related protein 2/3 complex, subunit 4	-1.06	0.87	1.05	0.39	-1.01	0.8859	1.07	0.5186	1.29	0.5247
160095_at	1416121_at	Lox	lysyl oxidase	1.27	0.29	1.65	0.399	-1.55	0.1529	-1.28	0.5449	3.01	0.1074
160096_at	1416524_at	Spop	speckle-type POZ protein	-1.11	0.62	-1.21	0.0029	1.03	0.537	-1.23	0.0008	-1.17	0.045
160097_at	1427788_at	Nespas	neuroendocrine secretory protein antisense	1.11	0.65	-1.15	0.5636	1.27	0.246	1.16	0.5338	2.27	0.1251
160098_s_at	1434370_s_at	Faf1	Fas-associated factor 1	-1.27	0.53	-1.08	0.3105	1.08	0.2013	-1.15	0.1406	-1.03	0.6878
160099_at	1451336_at	Lgals4	lectin, galactose binding, soluble 4	1.59	0.01	-1.01	0.9121	1.53	0.0938	1.14	0.1561	1.32	0.1309
160101_at	1448239_at	Hmox1	heme oxygenase (decycling) 1	-1.46	0.06	1.19	0.3173	-1.27	0.0293	1.3	0.4118	1.05	0.7842
160102_at	1415785_a_at	Cct8	chaperonin subunit 8 (theta)	2.23	0.13	1.05	0.471	1.17	0.022	1.21	0.0292	-1.16	0.2105
160103_at	1420609_at	7-Mar	membrane-associated ring finger (C3HC4) 7	1.21	0.01	1.32	0.011	1.04	0.7369	1.23	0.031	1.14	0.4839
160104_at	1416968_a_at	Hsd3b7	hydroxy-delta-5-steroid dehydrogenase, 3 be	-1.31	0.02	-1.16	0.0636	-1.15	0.1884	-1.31	0.0045	-1.91	0.0055
160106_at	1450355_a_at	Capg	capping protein (actin filament), gelsolin-like	1.74	0.07	1.57	0.2351	-1.74	0.04	2.19	0.1853	1.2	0.2031
160107_at	1448736_a_at	Hprt1	hypoxanthine guanine phosphoribosyl transf	1.39	0.24	1.38	0.0003	1.22	0.0088	1.6	0	-1.44	0.0013
160108_at	1419666_x_at	Nupr1	nuclear protein 1	-1.52	0.39	-1.06	0.8548	-1.15	0.652	1.17	0.66	3.84	0.0557
160109_at	1419156_at	Sox4	SRY-box containing gene 4	-1.21	0.68	-1.01	0.946	-1.12	0.3574	-1.33	0.0778	-1.15	0.608
160110_at	1448145_at	Wwp2	WW domain containing E3 ubiquitin protein l	1.08	0.59	-1.19	0.0063	-1.06	0.4234	-1.1	0.1784	1.12	0.2323
160111_at	1449576_at	Eif1ay	eukaryotic translation initiation factor 1A, Y-l	-1.01	0.96	-1.07	0.4529	1.18	0.0571	-1.08	0.5076	-1.5	0.0384
160113_at	1416738_at	Brap	BRCA1 associated protein	-1.11	0.14	1.1	0.2107	-1.01	0.956	1.13	0.1529	1.34	0.0268
160114_at	1420815_at	Gdi3	guanosine diphosphate (GDP) dissociation i	-1.1	0.56	1.03	0.6884	1.08	0.3881	1.06	0.2137	-1.15	0.1672
160115_at	1455866_x_at	Txnl1	thioredoxin-like 1	1.08	0.7	1	0.9708	-1.02	0.8033	1.07	0.3057	-1.11	0.5224
160116_at	1454677_at	Timp2	tissue inhibitor of metalloproteinase 2	2.04	0.33	1.48	0.2601	1.81	0.1724	1.71	0.0986	1.11	0.8062
160117_at	1424175_at	Tef	thyrotroph embryonic factor	1.15	0.68	1.34	0.0383	1.23	0.0417	1.65	0.0002	1.86	0.0161
160118_at	1448383_at	---	---	-1.75	0.09	1.05	0.6651	-1.19	0.1391	-1.11	0.308	1.58	0.0868
160119_at	1452754_at	5730592L21Rik	RIKEN cDNA 5730592L21 gene	-1.52	0.41	-2.02	0.001	-1.6	0.0041	-2.1	0.0003	-1.32	0.1616
160120_i_at	1416626_at	Pla2g1b	phospholipase A2, group IB, pancreas	-3.69	0.08	-1.08	0.4164	-1.05	0.5573	-1.23	0.0287	1.3	0.0649
160121_at	1455798_at	Galk2	galactokinase 2	1.21	0.11	-1.03	0.6711	-1.09	0.301	-1.2	0.0326	1.04	0.7744
160123_at	1427903_at	Phpt1	phosphohistidine phosphatase 1	1.4	0.22	-1.03	0.5311	-1.09	0.0823	1.06	0.3083	-1.21	0.1967
160124_r_at	1419545_a_at	Atp6v1c1	ATPase, H+ transporting, V1 subunit C, isof	1.5	0.15	1.04	0.4573	1.09	0.349	1.19	0.0157	1.13	0.4518
160125_at	1452200_at	D11Ertd497e	DNA segment, Chr 11, ERATO Doi 497, exp	1.3	0.23	1.02	0.7622	1.09	0.2304	1.07	0.3413	-1.02	0.8553
160126_at	1416272_at	Map2k1ip1	mitogen-activated protein kinase kinase 1 in	2.05	0	-1.08	0.398	1.26	0.1756	1.08	0.5439	-1.25	0.553
160127_at	1420827_a_at	---	---	1.67	0.28	-1.05	0.6267	-1.07	0.6502	1.01	0.931	-1.14	0.224
160129_at	1449506_a_at	Eef1d	eukaryotic translation elongation factor 1 del	1.21	0.18	-1.03	0.7158	-1.04	0.6661	-1.05	0.4982	1.25	0.0456
160130_at	1451188_at	Wdr26	WD repeat domain 26	1.83	0.33	1.12	0.4468	-1.41	0.113	-1.19	0.3212	1.12	0.4562
160131_at	1452387_a_at	Amotl2	angiomotin like 2	1	0.99	1.16	0.162	-1.04	0.6272	1.24	0.0162	1.15	0.2877
160134_at	1451311_a_at	Adipor1	adiponectin receptor 1	-1.26	0.08	1	0.9991	1.13	0.3661	1	0.9687	1.58	0.1413
160135_at	1418763_at	Nit2	nitrilase family, member 2	-1.08	0.81	-1.36	0.0003	-1.13	0.1897	-1.65	0	-2.32	0.0002
160136_r_at	1433704_s_at	Tloc1	translocation protein 1	1.18	0.41	-1.06	0.6421	1.07	0.4965	1.04	0.7234	1.02	0.9375
160137_at	1450026_a_at	B3gnt1	UDP-GlcNAc:betaGal beta-1,3-N-acetylgluc	1.48	0.37	1.42	0.102	1.4	0.0585	1.55	0.1181	1.17	0.6043
160138_at	1450376_at	Mxi1	Max interacting protein 1	1.03	0.91	1.32	0.0298	1.24	0.0091	1.42	0.0107	1.25	0.2477
160139_at	1417013_at	Hspb8	heat shock 27kDa protein 8	1.3	0.28	1.12	0.1634	-1.24	0.0591	-1.01	0.9136	-1.15	0.1221
160140_at	1428282_at	Tbce	tubulin-specific chaperone e	1.6	0.25	1.09	0.4215	1.22	0.2019	1.43	0.0001	-1.11	0.6502
160142_at	1417593_at	Tusc2	tumor suppressor candidate 2	-1.21	0.23	-1.08	0.4675	-1.1	0.2061	-1.15	0.1298	-1.18	0.3617
160144_at	1426905_a_at	---	---	-1.48	0.13	1.21	0.017	1.24	0.0028	1.37	0.0035	1.29	0.0002
160145_at	1428359_s_at	1810010M01Rik	RIKEN cDNA 1810010M01 gene	1.19	0.81	1.75	0.1394	2.59	0.359	1.62	0.3364	1.66	0.2915
160150_f_at	1456380_x_at	Cnn3	calponin 3, acidic	1.33	0.13	1.45	0.0003	1.32	0.0139	1.46	0.0001	1.62	0.0345
160151_i_at	1424462_at	1200009B18Rik	RIKEN cDNA 1200009B18 gene	-1.15	0.46	-1	0.9813	1.22	0.1209	1.18	0.0607	1.11	0.615
160152_at	1416005_at	Psmc1	protease (prosome, macropain) 26S subunit	2.22	0.06	1.11	0.2406	-1.03	0.6869	1.19	0.0144	-1.21	0.0956
160153_at	1448362_at	Dnajc7	DnaJ (Hsp40) homolog, subfamily C, membe	1.78	0.1	1.01	0.9256	1.06	0.4466	1.08	0.2402	1.07	0.6123
160155_at	1433483_s_at	C86187	expressed sequence C86187	-1.35	0.23	1.02	0.8602	-1.17	0.2624	-1.04	0.7975	-1.61	0.3423

160156_at	1423829_at	0910001A06Rik	RIKEN cDNA 0910001A06 gene	1.2	0.41	1.62	0.07	1.2	0.257	1.76	0.0862	1.71	0.0966
160158_at	1450990_at	Gpc3	glypican 3	1.42	0.11	1.1	0.2504	-1.08	0.3945	1.08	0.4881	1.16	0.0297
160159_at	1448205_at	Ccnb1-rs1 /// Cc	cyclin B1, related sequence 1 /// cyclin B1	-1.06	0.92	1.4	0.2214	-1.58	0.2473	1.54	0.2296	-2.08	0.2934
160160_at	1434994_at	Dedd	death effector domain-containing	-1.54	0.62	-1.53	0.1072	1.18	0.4641	-1.34	0.2465	-1.6	0.2507
160161_at	1451981_at	Gtrgeo22	gene trap ROSA b-geo 22	1.26	0.69	-1.15	0.2027	-1.17	0.1903	-1.22	0.1018	-1.15	0.5794
160162_at	1426529_a_at	Tagln2	transgelin 2	1.27	0.23	1.12	0.1819	-1.06	0.1335	1.16	0.1577	1.17	0.0382
160164_at	1415755_a_at	Ube2v1	ubiquitin-conjugating enzyme E2 variant 1	1.24	0.09	-1.03	0.6216	-1.01	0.8949	1.05	0.344	-1.16	0.2732
160165_at	1424345_s_at	Ube2m	ubiquitin-conjugating enzyme E2M (UBC12	1.42	0.4	1.01	0.8523	1.06	0.4898	1.02	0.6909	-1.04	0.8095
160166_r_at	1460391_at	2810409H07Rik	RIKEN cDNA 2810409H07 gene	1.02	0.95	-1.06	0.6119	1.19	0.1876	-1.02	0.8398	-1.37	0.0712
160167_at	1438917_x_at	Nup62	nucleoporin 62	-1.28	0.06	-1.07	0.3714	-1.08	0.4071	1.01	0.8987	1.14	0.3937
160169_at	1429707_at	Plaa	phospholipase A2, activating protein	1.68	0.24	1.14	0.0552	1.16	0.1954	1.31	0.0028	-1.17	0.5176
160170_at	1460181_at	Stmn3	stathmin-like 3	-1.55	0.55	-1.04	0.7891	-1.1	0.3859	-1.09	0.4529	-1.1	0.849
160171_f_at	1418073_at	MGI:1928939	acyl-Coenzyme A thioesterase 2, mitochond	1.29	0.13	1.13	0.7306	1.05	0.8637	1.26	0.5196	-1.16	0.704
160172_at	1426758_s_at	Gtl2	GTL2, imprinted maternally expressed untra	1.33	0.05	1.05	0.5602	-1.11	0.1848	3.39	0.2936	1.21	0.3762
160173_at	1436713_s_at	---	---	1.21	0.38	1.12	0.6018	-1	0.9843	2.07	0.1602	-1.02	0.96
160174_at	1448278_at	Xab2	XPA binding protein 2	1.06	0.52	-1.23	0.0091	-1.16	0.0557	-1.26	0.002	-1.1	0.4672
160176_at	1418229_s_at	Hirip5	histone cell cycle regulation defective intera	1.68	0.03	1.16	0.2301	-1.08	0.5367	1.25	0.0947	1.3	0.1307
160177_at	1422442_at	Smu1	smu-1 suppressor of mec-8 and unc-52 hom	1.52	0.05	-1.15	0.0213	1.01	0.8198	-1.01	0.8917	-1.17	0.0333
160179_at	1448340_at	D9Wsu20e	DNA segment, Chr 9, Wayne State Universi	-1.83	0.03	-1.68	0	1.08	0.6008	-1.95	0	-1.4	0.1119
160180_at	1428935_at	Canx	calnexin	-1.59	0.08	-1.16	0.1081	-1.03	0.723	-1.15	0.1124	-1.07	0.7685
160181_at	1448280_at	Syp	synaptophysin	1.08	0.91	1.76	0.0093	1.41	0.2041	1.55	0.0935	-1.14	0.6002
160182_at	1448454_at	Sfrs6	splicing factor, arginine/serine-rich 6	-2.47	0.07	-1.14	0.1795	1	0.9996	-1.1	0.1924	1.71	0.0103
160184_at	1423334_at	1200007D18Rik	RIKEN cDNA 1200007D18 gene	1.04	0.88	-1.18	0.0894	-1.57	0.0008	-1.72	0	-1.42	0.0761
160185_at	1450683_at	Tagln3	transgelin 3	1.24	0.57	1.5	0.0504	1.46	0.0673	1.45	0.1177	2.77	0.0207
160186_at	1454236_a_at	C030004A17Rik	RIKEN cDNA C030004A17 gene	-1.53	0.22	-1.16	0.1001	1.04	0.8177	-1.01	0.9133	2.09	0.0058
160187_at	1450755_at	Pafah1b2	platelet-activating factor acetylhydrolase, isc	2.72	0.06	-1.09	0.3787	1.08	0.4861	-1.02	0.8736	-2.51	0.0087
160188_at	1418505_at	Nudt4	nudix (nucleoside diphosphate linked moiety	1.37	0.04	1.18	0.0247	1.18	0.1331	1.31	0.0032	1.2	0.1609
160190_at	1415844_at	Syt4	synaptotagmin 4	1.73	0.44	-1.08	0.8685	-1.33	0.5013	1.18	0.6792	-3.41	0.2269
160191_at	1452120_at	4931433E08Rik	RIKEN cDNA 4931433E08 gene	-1.6	0.27	1.35	0.2678	1.01	0.9718	1.56	0.0749	1.32	0.4262
160192_at	1416177_at	Rbmxrt	RNA binding motif protein, X chromosome re	1.3	0.25	1.09	0.3266	1.15	0.1574	1.25	0.0394	-1.34	0.2291
160193_at	1424235_at	Ormdl2	ORM1-like 2 (S. cerevisiae)	-1.07	0.69	-1.07	0.2363	-1.02	0.7555	-1.11	0.017	-1.41	0.0028
160194_at	1448717_at	Gcdh	glutaryl-Coenzyme A dehydrogenase	1.54	0.07	-1.04	0.4974	-1.13	0.0475	-1.01	0.8242	-1.16	0.2335
160195_at	1415721_a_at	1200013P24Rik	RIKEN cDNA 1200013P24 gene	-1.03	0.86	-1.06	0.4264	1.06	0.2957	-1.06	0.384	-1.12	0.2609
160196_at	1423956_at	Smap1	stromal membrane-associated protein 1	1.15	0.49	1.15	0.2474	1.26	0.2457	1.16	0.1586	-1.39	0.0342
160197_at	1416400_at	Pycrl	pyrroline-5-carboxylate reductase-like	2.02	0.07	-1.01	0.9506	-1.07	0.5822	-1.21	0.2076	1.29	0.1484
160198_at	1433442_at	Klhl9	Kelch-like 9 (Drosophila)	-1.1	0.8	1.23	0.0043	-1	0.9923	1.19	0.0143	1.12	0.2378
160199_at	1418693_at	Hnrpc	heterogeneous nuclear ribonucleoprotein C	1.47	0.12	1.1	0.3816	-1.18	0.3398	-1.11	0.2534	-1.59	0.0729
160200_at	1416856_at	3230401D17Rik	RIKEN cDNA 3230401D17 gene	1.34	0.26	1.38	0.0035	1.51	0.0125	1.33	0.0018	1.24	0.2459
160201_r_at	1423621_a_at	Slc33a1	solute carrier family 33 (acetyl-CoA transpor	-1.67	0.06	1	0.9926	1.03	0.8494	-1.04	0.7875	-2.05	0.0241
160202_at	1423662_at	Atp6ap2	ATPase, H+ transporting, lysosomal access	1.43	0.26	1.15	0.1143	1.19	0.0658	1.16	0.0499	1.09	0.4746
160203_at	1426473_at	Dnajc9	DnaJ (Hsp40) homolog, subfamily C, memb	1.28	0.22	1.22	0.0017	-1.03	0.6607	1.31	0.0158	-1.16	0.4203
160204_at	1428706_at	Prr6	proline-rich polypeptide 6	1.35	0.2	-1.03	0.6691	-1.01	0.8416	-1.06	0.4814	-1.02	0.8894
160205_f_at	1426405_at	Rnf11	ring finger protein 11	-1.96	0	1.26	0.0083	1.12	0.338	1.21	0.0155	-1.22	0.1524
160207_at	1451666_at	Acly	ATP citrate lyase	-1.65	0.1	-1.87	0.0016	-1.42	0.0481	-2.13	0.0005	-1.89	0.0166
160208_at	1415985_at	Sf3b3	splicing factor 3b, subunit 3	-1.01	0.96	-1.11	0.245	1.04	0.5072	1.01	0.8858	1	0.9792
160209_at	1423532_at	Rnf44	ring finger protein 44	1.26	0.06	1.38	0.0003	1.25	0.0561	1.45	0.0001	1.19	0.0355
160211_at	1451284_at	D17Wsu94e	DNA segment, Chr 17, Wayne State Univers	-1.53	0.09	-1.28	0.0109	-1.04	0.7134	-1.19	0.0222	-1.07	0.4404
160213_at	1415905_at	Reg1	regenerating islet-derived 1	2.18	0.12	1.32	0.0522	3.02	0.3372	1.23	0.2623	1.57	0.0681
160214_at	1450028_a_at	Lancl2	LanC (bacterial lantibiotic synthetase compo	1.55	0.13	1.43	0.0597	1.44	0.1175	1.32	0.1073	1.1	0.7649
160215_at	1420619_a_at	Aes	amino-terminal enhancer of split	-1.27	0.02	-1.28	0.0092	-1.34	0.0308	-1.41	0.0024	-1.48	0.0176
160216_f_at	1456017_x_at	Obox1	oocyte specific homeobox 1	1.22	0.32	-1.2	0.0929	1.01	0.9497	1.08	0.7395	1.03	0.9014
160217_at	1452159_at	2310001A20Rik	RIKEN cDNA 2310001A20 gene	1.34	0.4	-1.02	0.8484	1.16	0.0747	1.08	0.4146	-1.43	0.4011
160218_at	1451385_at	2310056P07Rik	RIKEN cDNA 2310056P07 gene	1.79	0.1	1.16	0.0582	1.23	0.107	1.38	0.0003	-1.13	0.3349

160220_at	1450998_at	Zfp110	zinc finger protein 110	1.19	0.55	-1	0.9624	1.22	0.2874	1.05	0.6078	-1.07	0.7718
160221_at	1417079_s_at	Lgals2	lectin, galactose-binding, soluble 2	1.01	0.98	-1.09	0.3912	-1	0.9845	-1.08	0.3948	1.23	0.4199
160223_at	1452665_at	2610511O17Rik	RIKEN cDNA 2610511O17 gene	3.73	0.16	1.39	0.3175	1.37	0.3283	1.3	0.4014	1.4	0.4228
160225_at	1451135_at	Gtf2b	general transcription factor IIB	1.18	0.53	1.36	0.0085	1.72	0.0076	1.67	0.0137	1.21	0.2052
160226_at	1416570_s_at	Gfm1	G elongation factor 1	1.47	0.17	1.04	0.6464	1.23	0.012	1.18	0.0092	-1.38	0.0072
160228_at	1429555_at	1110019C08Rik	RIKEN cDNA 1110019C08 gene	-1.09	0.69	1.2	0.0273	-1.06	0.5041	1.16	0.185	1.54	0.1016
160229_at	1420044_at	---	---	1.79	0.16	-1.34	0.177	1.01	0.9279	-1.21	0.3036	-1.04	0.9275
160230_at	1434435_s_at	Cox17	cytochrome c oxidase, subunit XVII assembl	1.53	0.12	1.08	0.1763	1.02	0.7875	1.08	0.0081	1.03	0.5822
160231_at	1424907_a_at	Farsla	phenylalanine-tRNA synthetase-like, alpha s	-1.07	0.92	1.17	0.3486	1.26	0.2451	1.58	0.0051	1.85	0.1181
160232_at	1418074_at	St6galnac4	ST6 (alpha-N-acetyl-neuraminyl-2,3-beta-ga	-2.41	0.42	1.31	0.1828	1.03	0.8964	1.07	0.7776	2.32	0.0962
160233_at	1423717_at	Ak3	adenylate kinase 3	-1.1	0.78	1.05	0.5638	1.06	0.661	1.04	0.7707	-1.12	0.5866
160234_at	1451080_at	Usp1	ubiquitin specific protease 1	1.4	0.14	1.21	0.1723	1.4	0.1403	1.48	0.0008	-1.23	0.3887
160235_at	1428465_at	5033425B17Rik	RIKEN cDNA 5033425B17 gene	1.39	0.13	-1.25	0.0001	-1.1	0.096	-1.33	0.0001	-2.89	0
160236_at	1424824_at	9630044O09Rik	RIKEN cDNA 9630044O09 gene	1.01	0.98	2.24	0.1974	1.62	0.324	1.17	0.6111	1.93	0.4332
160237_at	1448427_at	Ndufa6	NADH dehydrogenase (ubiquinone) 1 alpha	1.1	0.64	1.2	0.0165	1.27	0.0255	1.24	0.0142	1.3	0.1066
160239_at	1423697_at	2400006A19Rik	RIKEN cDNA 2400006A19 gene	1.33	0.28	1.01	0.9036	1.05	0.5122	1.05	0.2043	-1.46	0.005
160242_at	1423663_at	Flcn	folliculin	1.02	0.9	1.27	0.0528	1.39	0.0099	1.33	0.0002	1.58	0.0029
160243_r_at	1426650_at	Myh8 /// LOC38	myosin, heavy polypeptide 8, skeletal muscl	-2.62	0.32	-9.61	0.2851	-8.46	0.2924	-8.5	0.2922	2.79	0.0335
160245_at	1453013_at	1110034O07Rik	RIKEN cDNA 1110034O07 gene	1.41	0.23	1.1	0.1499	1.11	0.3475	1.16	0.021	2.26	0.3064
160246_at	1416950_at	Tnfaip8	tumor necrosis factor, alpha-induced protein	2.25	0.04	1.3	0.0606	1.35	0.1078	1.46	0.0451	1.47	0.2386
160247_at	1417983_a_at	Ube2v2	ubiquitin-conjugating enzyme E2 variant 2	2.1	0.11	1.33	0.0254	1.18	0.386	1.21	0.1053	1.1	0.4392
160248_at	1433482_a_at	Fubp1	far upstream element (FUSE) binding protein	3.91	0.23	1.52	0.2265	1.09	0.7598	2.1	0.0025	-2.35	0.0254
160249_at	1419493_a_at	Tpd52	tumor protein D52	-1.2	0.37	1.24	0.2194	1.15	0.4243	1.36	0.3495	-1.03	0.8351
160251_at	1427876_at	2610312B22Rik	RIKEN cDNA 2610312B22 gene	1.17	0.58	1.11	0.1211	1.28	0.0451	1.12	0.0433	1.07	0.603
160252_at	1454626_at	Cltc	clathrin, heavy polypeptide (Hc)	1.08	0.61	1.24	0.0002	1.17	0.0075	1.31	0.0005	1.12	0.0442
160253_at	1423754_at	Ifitm3	interferon induced transmembrane protein 3	-1.28	0.09	-1.02	0.855	-1.04	0.4526	1.01	0.9661	-1.43	0.0739
160254_at	1424254_at	Ifitm1	interferon induced transmembrane protein 1	-1.11	0.67	-1.05	0.7497	1.12	0.3662	-1.05	0.6672	1.06	0.7476
160255_at	1452217_at	Ahnak	AHNAK nucleoprotein (desmoyokin)	1.74	0	1.46	0.0595	1.24	0.3222	1.85	0.004	6.38	0.0051
160256_at	1434732_x_at	Tomm7	Translocase of outer mitochondrial membran	1.26	0.26	1.01	0.8562	-1.04	0.563	1.01	0.8163	-1.12	0.2451
160257_at	1448184_at	Fkbp1a	FK506 binding protein 1a	-1.16	0.44	1.12	0.0654	1.07	0.2609	1.09	0.1804	1.07	0.1786
160258_at	1416765_s_at	MGI:1913699	mitochondria-associated protein involved in	1.07	0.82	1.02	0.7766	1	0.9444	1.01	0.9105	1.06	0.8609
160260_at	1448461_a_at	1500006O09Rik	RIKEN cDNA 1500006O09 gene	1.22	0.33	1.08	0.3846	1.09	0.5588	1.22	0.0657	1.2	0.6283
160262_at	1438843_x_at	Mtch2	mitochondrial carrier homolog 2 (C. elegans)	-1.01	0.94	-1.03	0.6872	1.16	0.0397	1.07	0.3956	-1.22	0.0985
160265_at	1415723_at	Eif5	eukaryotic translation initiation factor 5	1.27	0.43	1.06	0.3972	1.31	0.0121	1.08	0.3085	-1.16	0.2147
160266_r_at	1451144_at	1110064N10Rik	RIKEN cDNA 1110064N10 gene	1.05	0.81	1.15	0.5149	1.17	0.5771	-1.01	0.9421	-1.41	0.0182
160267_at	1448882_at	0610009E20Rik	RIKEN cDNA 0610009E20 gene	1.36	0.03	-1.15	0.3766	-1.13	0.3871	-1.16	0.3175	-1.16	0.1276
160268_at	1424002_at	Pdcl3	phosducin-like 3	-1.01	0.97	1.08	0.4441	-1.04	0.6244	1.06	0.5648	1.35	0.052
160269_at	1426995_a_at	Gfer	growth factor, erv1 (S. cerevisiae)-like (augm	1.07	0.64	1.06	0.5969	-1.1	0.354	1.07	0.4362	-1.05	0.5326
160270_at	1452671_s_at	Lman1	lectin, mannose-binding, 1	-1.19	0.41	-1.15	0.0161	-1.01	0.9167	-1.1	0.1343	-1.46	0.0058
160271_at	1428380_at	0610007C21Rik	RIKEN cDNA 0610007C21 gene	1.37	0.01	1.02	0.7088	1.01	0.7971	1.09	0.2433	1.1	0.1864
160272_at	1448504_a_at	---	Mus musculus, Similar to heterochromatin-lik	1.56	0	1.12	0.1642	1.05	0.4031	1.16	0.0543	1.27	0.0727
160273_at	1437626_at	Zfp36l2	zinc finger protein 36, C3H type-like 2	1.65	0.06	1.8	0	1.5	0.0032	1.86	0	1.13	0.5637
160274_at	1418742_at	Krt1-4	keratin complex 1, acidic, gene 4	-1.1	0.09	1.08	0.5578	1.13	0.2172	1.06	0.4759	-2.45	0.1898
160275_at	1418888_a_at	Sepx1	selenoprotein X 1	-1.26	0.06	-1.05	0.4311	-1.06	0.237	-1.13	0.0247	-1.16	0.1283
160277_at	1448936_at	Stx12	syntaxin 12	1.05	0.81	-1.08	0.3536	1.01	0.9191	-1.06	0.4097	1.27	0.0267
160278_at	1418702_a_at	2810428I15Rik	RIKEN cDNA 2810428I15 gene	1.78	0.09	1.03	0.7237	1.08	0.264	1.12	0.1255	-1.43	0.1226
160279_at	1427881_at	4930588M11Rik	RIKEN cDNA 4930588M11 gene	2.36	0.06	1.17	0.1526	1.03	0.7645	1.19	0.0976	1.25	0.2345
160280_at	1449145_a_at	Cav1	caveolin, caveolae protein 1	1.33	0.34	-1.18	0.3162	-1.12	0.4441	-1.07	0.7763	3.01	0.0047
160282_at	1415717_at	4931406I20Rik	RIKEN cDNA 4931406I20 gene	-1.43	0.08	-1.13	0.1652	-1.24	0.0233	-1.16	0.1022	1.17	0.0539
160284_at	1418068_at	Ndufa10	NADH dehydrogenase (ubiquinone) 1 alpha	-1.29	0.01	1	0.9991	1	0.9386	-1	0.9538	1.02	0.7688
160285_at	1428267_at	Dhx40	DEAH (Asp-Glu-Ala-His) box polypeptide 40	1.48	0.21	1.08	0.0873	1.05	0.4387	1.12	0.0553	1.24	0.1011
160286_at	1452659_at	Dek	DEK oncogene (DNA binding)	1.86	0.01	1.31	0.0922	1.14	0.3158	1.44	0.0087	1.22	0.3966
160287_at	1415930_a_at	Map1lc3b /// LO	microtubule-associated protein 1 light chain	1.72	0.08	1.03	0.5535	1.4	0.0007	1.23	0.007	1.08	0.3392

160288_at	1415929_at	Map1lc3b	microtubule-associated protein 1 light chain	1.33	0.07	-1.18	0.0033	1.21	0.033	-1.01	0.8856	1.05	0.6308
160290_at	1423120_at	Ide	insulin degrading enzyme	1.64	0.29	1.01	0.9332	-1.35	0.0064	-1.28	0.0847	-1.54	0.0081
160291_at	1434986_a_at	Sec61a1	Sec61 alpha 1 subunit (S. cerevisiae)	1.01	0.97	-1.02	0.7801	1.12	0.1355	1.05	0.1952	-1.5	0.0293
160292_at	1432181_s_at	Ecgf1	endothelial cell growth factor 1 (platelet-deriv	-1.05	0.77	-1.08	0.5417	1.01	0.8889	-1.2	0.1037	-1.26	0.0337
160293_at	1459985_at	2700038L12Rik	RIKEN cDNA 2700038L12 gene	1.53	0.08	1.03	0.8576	1.08	0.5741	1.11	0.4214	-1.17	0.6017
160294_at	1448726_at	Snapc2	small nuclear RNA activating complex, polyp	1.42	0.4	1.02	0.7512	1.05	0.5213	1.08	0.1768	1.24	0.0017
160296_at	1421847_at	Wsb2	WD repeat and SOCS box-containing 2	1.42	0.12	1.09	0.0636	1.07	0.4225	1.01	0.8933	-1.01	0.9507
160297_at	1424465_at	AI413631	expressed sequence AI413631	1.46	0.25	1.17	0.0802	1.42	0.0122	1.33	0.0078	-1.14	0.3052
160298_at	1424186_at	2610001E17Rik	RIKEN cDNA 2610001E17 gene	1.32	0.16	1.23	0.1086	1.29	0.1609	1.35	0.0593	1.44	0.1291
160299_at	1427913_at	Rwdd1	RWD domain containing 1	1.18	0.22	-1.05	0.4005	-1.02	0.8103	-1.04	0.5084	1.06	0.5515
160300_at	1448156_at	Tff1	trefoil factor 1	-1.64	0.02	-1.06	0.4893	-1.09	0.2014	-1.09	0.2487	1.14	0.3648
160301_at	1460670_at	Riok3	RIO kinase 3 (yeast)	-1.83	0.12	1.04	0.293	1.06	0.1699	1.07	0.2663	-1.26	0.0644
160302_at	1451238_at	1200003C05Rik	RIKEN cDNA 1200003C05 gene	-1.04	0.89	1.05	0.4099	1.11	0.2415	1.06	0.5764	1.38	0.1575
160303_at	1453206_at	Acad9	acyl-Coenzyme A dehydrogenase family, me	1.17	0.51	-1.51	0.0001	-1.04	0.6494	-1.45	0.0001	-1.29	0.0793
160305_at	1437080_s_at	Psmd11	proteasome (prosome, macropain) 26S subu	1	1	1.08	0.3125	-1.09	0.382	-1.05	0.6469	-1.09	0.7225
160306_at	1424737_at	Thrsp	thyroid hormone responsive SPOT14 homol	-1.54	0.28	-2.18	0	-1.6	0.0008	-3.06	0	-24.42	0.0006
160307_at	1454605_a_at	Pi4k2a	phosphatidylinositol 4-kinase type 2 alpha	1.03	0.83	-1.18	0.0769	-1.24	0.0343	-1.32	0.0157	-1.12	0.6731
160308_at	1421814_at	---	---	1.16	0.36	1.33	0.2096	1.01	0.9402	1.48	0.1444	1.05	0.641
160309_at	1423462_at	Map3k7ip2	mitogen-activated protein kinase kinase kina	-1.23	0.41	1.27	0.0009	1.27	0.0852	1.31	0.0035	-1.22	0.3753
160310_at	1426931_s_at	D19Bwg1357e	DNA segment, Chr 19, Brigham & Women's	1.24	0.3	1.01	0.9326	1.11	0.2979	1.02	0.7394	1.32	0.0391
160311_at	1416683_at	Plxnb2	plexin B2	1.13	0.4	1.07	0.4995	-1.06	0.6113	1.1	0.3642	-1.21	0.1519
160312_at	1451114_at	Cklfsf6	chemokine-like factor super family 6	-2.47	0.03	-1.17	0.0722	1.03	0.7409	-1.29	0.0098	-2.47	0.002
160313_at	1426827_at	A730098D12Rik	RIKEN cDNA A730098D12 gene	1.05	0.77	1.18	0.1107	-1.03	0.6702	1.24	0.0109	1.39	0.0388
160314_at	1416939_at	Pyp	pyrophosphatase	1.61	0.2	1.17	0.0502	1.16	0.0546	1.28	0.0009	-1.43	0.0078
160316_at	1434390_at	Hnrpu	Heterogeneous nuclear ribonucleoprotein U	1.77	0.16	1.33	0.0096	1.14	0.2799	1.47	0.0019	1.2	0.1567
160317_at	1416591_at	Rab34	RAB34, member of RAS oncogene family	2.38	0.04	3.35	0.0048	3.78	0.0028	6.13	0	1.83	0.0322
160318_at	1416580_a_at	Stub1	STIP1 homology and U-Box containing prote	1.16	0.46	1.1	0.2876	1.16	0.2263	1.21	0.0375	1.13	0.1612
160319_at	1416114_at	Sparcl1	SPARC-like 1 (mast9, hevin)	1.52	0.06	1.07	0.6576	-1.3	0.0637	1.18	0.3403	3.69	0.0089
160320_at	1425826_a_at	Sorbs1	sorbin and SH3 domain containing 1	1.82	0.26	1.19	0.1297	1.14	0.3248	1.28	0.053	1.52	0.0054
160321_at	1416083_at	Za20d2	zinc finger, A20 domain containing 2	1.38	0.23	1.03	0.6916	-1.26	0.0686	-1.08	0.3669	1.02	0.7159
160322_at	1423759_a_at	4930403O06Rik	RIKEN cDNA 4930403O06 gene	1.19	0.38	1	0.9745	1.04	0.7373	-1.1	0.2728	-1.45	0.0106
160323_at	1437423_a_at	Sra1	steroid receptor RNA activator 1	1.37	0.2	-1.27	0.0059	-1.02	0.8509	-1.15	0.0575	-1.49	0.0043
160324_at	1448938_at	Rpa3	replication protein A3	1.46	0.2	1.05	0.6298	1.36	0.14	1.26	0.0247	-1.01	0.9189
160325_r_at	1451100_a_at	Cdv3	carnitine deficiency-associated gene expres	-1.07	0.79	1.09	0.5689	-1.09	0.6145	-1.14	0.5053	1.8	0.3725
160326_at	1415704_a_at	Cdv3	carnitine deficiency-associated gene expres	1.53	0.13	1.19	0.0764	1.39	0.0429	1.15	0.244	1.08	0.3945
160327_at	1448368_at	Dctn6	dynactin 6	2.05	0.02	1.2	0.1421	1.26	0.1039	1.31	0.0405	1.1	0.5557
160328_at	1428365_a_at	Prss15	protease, serine, 15	1.02	0.84	1.09	0.4021	1.08	0.3997	1.09	0.4603	1.28	0.0282
160329_at	1428675_at	1110049F12Rik	RIKEN cDNA 1110049F12 gene	-1.16	0.75	1.16	0.1345	1.15	0.2393	-1.03	0.6467	-1.13	0.5524
160330_at	1460645_at	Chordc1	cysteine and histidine-rich domain (CHORD)	-1.1	0.68	-1.06	0.6077	1.08	0.5009	-1.03	0.7744	1.14	0.4346
160331_at	1420663_at	Thpok	T helper-inducing POZ/Krueppel factor	1.29	0.63	-1.18	0.4435	-1.08	0.7776	-1.15	0.5206	1	0.9982
160333_at	1416699_at	1110008F13Rik	RIKEN cDNA 1110008F13 gene	1.07	0.79	-1.03	0.7063	1.23	0.057	1.01	0.9395	1.06	0.5473
160334_at	1421993_a_at	2410001H17Rik	RIKEN cDNA 2410001H17 gene	1.68	0.03	1.62	0.0508	1.12	0.5912	1.72	0.0122	-1.02	0.9602
160337_at	1415977_at	MGI:1919030	myo-inositol 1-phosphate synthase A1	1	0.99	1.09	0.2844	-1.05	0.4563	1.06	0.4085	-1.18	0.6091
160338_at	1460428_at	1100001D10Rik	RIKEN cDNA 1100001D10 gene	1.56	0.01	-1.03	0.7769	-1.04	0.7435	-1.14	0.2738	-1.27	0.1555
160339_at	1448315_a_at	Pycr2	pyrroline-5-carboxylate reductase family, me	1.63	0.26	1.08	0.6848	-1.16	0.3746	-1.12	0.3477	-1.24	0.4356
160341_at	1451262_a_at	MGI:2385237	JTV1 gene	1.33	0.08	1.02	0.7781	1.11	0.1546	1.02	0.8022	1.28	0.1698
160343_at	1450891_at	Srp19	signal recognition particle 19	1.06	0.8	-1.1	0.3391	1.14	0.333	-1.03	0.7045	-1.71	0.0677
160344_at	1416901_at	Npc2	Niemann Pick type C2	1.29	0.09	1.21	0.081	1.04	0.5423	1.21	0.1293	1.18	0.2242
160345_at	1416349_at	Mrpl34	mitochondrial ribosomal protein L34	1.18	0.56	-1.13	0.2097	-1.1	0.214	-1.18	0.0431	-1.65	0.0457
160346_at	1423894_a_at	Dalrd3	DALR anticodon binding domain containing	-1.14	0.37	1.02	0.7103	-1.01	0.8014	-1.05	0.1061	-1.01	0.929
160347_at	1423698_at	D15Ertd785e	DNA segment, Chr 15, ERATO Doi 785, exp	1.07	0.45	1.03	0.6241	-1.03	0.6977	1.01	0.9213	1.12	0.3349
160348_at	1452673_at	Ranbp3	RAN binding protein 3	1.12	0.58	1.02	0.7469	1.15	0.0528	1.03	0.6417	1.06	0.5549
160349_at	1460177_at	Cndp2	CNDP dipeptidase 2 (metallopeptidase M20	1.24	0.33	-1.06	0.312	1.06	0.2738	1.05	0.5289	1.01	0.9616

160351_at	1451243_at	Rnpep	arginyl aminopeptidase (aminopeptidase B)	2.19	0.05	-1.12	0.201	1.04	0.7099	-1.07	0.3491	-2.45	0.0519
160352_at	1449055_x_at	Pcbp4	poly(rC) binding protein 4	-1.12	0.64	1.48	0.0043	1.43	0.0463	1.37	0.0208	1.79	0.0214
160354_at	1416752_at	Ldb3	LIM domain binding 3	-2.99	0.07	-2.28	0.2908	-2.15	0.3135	-2.09	0.3241	2.05	0.1861
160355_at	1427099_at	Maz	MYC-associated zinc finger protein (purine-b	-2.04	0.4	1.02	0.8611	1.13	0.4515	1.02	0.8525	1.22	0.2478
160356_at	1423073_at	0610011D08Rik	RIKEN cDNA 0610011D08 gene	1.23	0.06	1.09	0.1844	1.05	0.332	1.06	0.3392	-1.38	0.019
160358_at	1416072_at	Cd34	CD34 antigen	-1.11	0.86	-1.25	0.4958	-1.5	0.2365	1.25	0.5577	2.35	0.2523
160359_at	1418003_at	1190002H23Rik	RIKEN cDNA 1190002H23 gene	-1.09	0.9	-1.43	0.3963	-1.1	0.7822	-1.26	0.5093	1.31	0.5296
160360_at	1448903_at	MGI:1927947	selenoprotein	-1.19	0.36	1.15	0.0236	1.12	0.136	1.1	0.0449	-1.86	0.0003
160361_at	1415674_a_at	Trappc4	trafficking protein particle complex 4	2.05	0.01	1.51	0	1.22	0.128	1.3	0.0156	1.15	0.3307
160362_at	1433576_at	Mat2a	methionine adenosyltransferase II, alpha	-1.04	0.77	1.02	0.8524	1.15	0.1306	1.18	0.1674	1.13	0.4876
160363_at	1418284_at	Tcfl1	transcription factor-like 1	2.05	0.07	1.08	0.408	-1.02	0.8352	1.02	0.8032	1.03	0.8962
160364_at	1427432_a_at	Sfrs10	splicing factor, arginine/serine-rich 10 (trans	1.65	0.05	1.26	0.0551	1.34	0.067	1.51	0.0012	1.3	0.2691
160365_at	1448819_at	Eif2s2	eukaryotic translation initiation factor 2, subu	-1.41	0.35	1.04	0.7213	1.11	0.604	-1.02	0.8927	-1.67	0.2197
160366_at	1415752_at	BC031181	cDNA sequence BC031181	1.16	0.17	1.06	0.4275	1.19	0.0682	1.12	0.1287	-1.24	0.0193
160367_at	1428319_at	Pdlim7	PDZ and LIM domain 7	-1.3	0.51	1.58	0.3033	1.81	0.1386	2.15	0.0378	-1.35	0.4281
160369_at	1451895_a_at	Dhcr24	24-dehydrocholesterol reductase	-1.15	0.35	-1.16	0.1223	-1.05	0.5206	-1.15	0.1807	-1.07	0.7908
160370_at	1416552_at	Dppa5	developmental pluripotency associated 5	1.19	0.6	1.5	0.2467	1.05	0.8694	1.4	0.3887	1.68	0.3187
160371_at	1423818_a_at	Arl6ip1	ADP-ribosylation factor-like 6 interacting pro	-1	1	1.39	0.0165	1.31	0.0682	1.52	0.0157	1.1	0.4921
160373_i_at	1416778_at	Sdpr	serum deprivation response	3.77	0.01	1.02	0.8376	1.22	0.3354	-1.11	0.39	1.66	0.0765
160375_at	1449434_at	---	---	-1.12	0.09	1.04	0.6436	-1.18	0.155	-1.18	0.0733	1.07	0.6248
160376_at	1452646_at	Trp53inp2	tumor protein p53 inducible nuclear protein 2	-1.56	0.03	-1.94	0	-1.23	0.0062	-1.9	0	-3.49	0.0003
160377_at	1423723_s_at	Tardbp	TAR DNA binding protein	1.22	0.31	1.46	0.0007	1.19	0.0253	1.47	0	-1.22	0.1296
160378_at	1416032_at	1110006I15Rik	RIKEN cDNA 1110006I15 gene	-1.15	0.74	1.04	0.4804	-1.04	0.6702	1.01	0.8918	-1.32	0.4271
160379_at	1424092_at	Epb4.1	erythrocyte protein band 4.1	1.54	0.21	-1.14	0.1678	-1.02	0.8685	-1.08	0.4576	-1.06	0.8552
160380_at	1455880_s_at	Becn1	beclin 1 (coiled-coil, myosin-like BCL2-intera	1.05	0.66	1.07	0.1627	1.08	0.3087	1.08	0.1854	-1.12	0.4701
160381_at	1451266_at	Mrpl50	mitochondrial ribosomal protein L50	2.6	0.06	1.15	0.2168	1.19	0.2086	1.2	0.207	1.19	0.3066
160382_at	1448141_at	1110014J01Rik	RIKEN cDNA 1110014J01 gene	1.54	0.09	-1.1	0.2089	-1.08	0.3662	-1.14	0.0848	1.86	0.059
160383_at	1432264_x_at	Cox7a2l	cytochrome c oxidase subunit VIIa polypepti	1.39	0.1	1.02	0.665	-1.01	0.8905	1.09	0.1368	1.03	0.6755
160384_at	1448221_at	Bat1a	HLA-B-associated transcript 1A	1.25	0.17	1.08	0.1717	1.04	0.4736	1.19	0.0032	1.09	0.343
160385_at	1432164_a_at	Gcsh	glycine cleavage system protein H (aminome	-1.24	0.03	-1.18	0.0001	-1.03	0.7729	-1.33	0	-1.28	0.0352
160386_at	1423730_at	C130052I12Rik	RIKEN cDNA C130052I12 gene	1.08	0.72	-1.17	0.122	-1.03	0.7166	-1.27	0.01	1.09	0.3123
160387_at	1448919_at	Cd302	CD302 antigen	1.17	0.24	-1.07	0.3117	-1.12	0.1836	-1.08	0.3341	-1.41	0.0004
160388_at	1423078_a_at	Sc4mol	sterol-C4-methyl oxidase-like	1.96	0.02	1.13	0.5342	-1.11	0.6943	-1.19	0.4761	-2.45	0.0031
160389_r_at	1450925_a_at	Rps27l	ribosomal protein S27-like	-6.58	0	1.3	0.2039	1.14	0.4058	1.08	0.5167	1.8	0.0782
160390_at	1448367_at	Sdf4	stromal cell derived factor 4	-3.89	0.18	1.23	0.1943	1.17	0.3136	1.22	0.5159	-1.36	0.4861
160391_at	1423680_at	Fads1	fatty acid desaturase 1	1.1	0.61	-1.41	0	-1.29	0.0037	-1.61	0	-1.58	0.0013
160392_at	1433448_at	B430110G05Ril	RIKEN cDNA B430110G05 gene	-1.23	0.39	-1.98	0.0001	-1.19	0.1026	-2.05	0	-1.31	0.2082
160393_at	1433514_at	Etnk1	ethanolamine kinase 1	-1.15	0.49	1.55	0	1.25	0.0182	1.47	0.0001	1.01	0.942
160394_at	1427244_at	Ttc15	tetratricopeptide repeat domain 15	1.21	0.14	1.02	0.6456	-1.04	0.7863	1.07	0.1504	-1.02	0.7385
160395_at	1449140_at	Nudcd2	NudC domain containing 2	1.22	0.4	1.16	0.3269	-1.11	0.5888	1.15	0.4657	-1.37	0.0008
160396_at	1423955_a_at	Lass2	longevity assurance homolog 2 (S. cerevisia	-1.16	0.12	-1.41	0.0004	-1.35	0.0007	-1.4	0.0015	-1.37	0.1248
160397_at	1451113_a_at	Ik	IK cytokine	2.46	0.05	1.32	0.0476	1.19	0.2729	1.41	0.0015	-1.08	0.6534
160398_at	1420486_at	Nol7	nucleolar protein 7	1.92	0.03	1.18	0.0676	1.2	0.2987	1.32	0.0045	-1.13	0.4502
160400_at	1428090_at	2810422B04Rik	RIKEN cDNA 2810422B04 gene	1.46	0.11	-1.09	0.2004	1.08	0.3975	1.06	0.436	1.01	0.9306
160401_r_at	1423641_s_at	Cnot7	CCR4-NOT transcription complex, subunit 7	-4.81	0.02	-1.22	0.5449	-1.19	0.6213	-1.07	0.7874	1.23	0.6484
160402_at	1428263_a_at	Tceb2	transcription elongation factor B (SIII), polyp	1.14	0.68	1.11	0.1351	1.2	0.0003	1.15	0.0133	-1.41	0.1143
160403_at	1423225_at	MGI:1931466	selenoprotein K	1.15	0.56	-1.11	0.0311	1.04	0.6396	-1.06	0.3365	-1.51	0.0018
160404_at	1448512_at	Hils1	histone H1-like protein in spermatids 1	1.72	0.44	-1.03	0.8859	-1.05	0.7761	-1.23	0.3405	1.45	0.1485
160406_at	1450652_at	Ctsk	cathepsin K	-1.73	0	1.4	0.221	1.37	0.1986	1.51	0.0644	1.28	0.0378
160409_at	1423282_at	Pitpna	phosphatidylinositol transfer protein, alpha	-1.08	0.51	-1.11	0.1547	1.13	0.0513	1.03	0.6954	-1.1	0.6055
160410_at	1416254_a_at	Vps16	vacuolar protein sorting 16 (yeast)	1.36	0.19	1.25	0.0678	1.21	0.1403	1.37	0.0025	1.4	0.165
160412_at	1423534_at	Pdcd2	programmed cell death 2	2.82	0.04	1.4	0.032	1.69	0.0502	1.41	0.0678	2.05	0.1123
160413_at	1416107_at	---	---	-1.03	0.92	1.15	0.2552	2.39	0.0582	1.18	0.0592	1.08	0.7897

160414_at	1427295_at	1810073N04Rik	RIKEN cDNA 1810073N04 gene	1.13	0.46	-1.07	0.0888	-1.08	0.4671	-1.09	0.2922	-1.61	0.0069
160415_at	1437932_a_at	Cldn1	claudin 1	-1.46	0.12	1.08	0.5896	-1.14	0.2387	-1.33	0.0473	1.01	0.9682
160416_at	1416859_at	Fkbp3	FK506 binding protein 3	-1.47	0.16	-1	0.9736	1.23	0.1128	1.05	0.5942	1.26	0.1555
160417_at	1418429_at	Kif5b	kinesin family member 5B	1.24	0.59	-1.07	0.5169	-1.37	0.0044	-1.15	0.2456	-1.05	0.7722
160418_at	1436391_s_at	MGI:2385186	Mid-1-related chloride channel 1	-1.39	0.14	1.42	0.0069	1.38	0.0439	1.33	0.024	1.18	0.5731
160419_r_at	1423928_at	Phgdhl1	phosphoglycerate dehydrogenase like 1	2.42	0.12	1.29	0.1556	-1.18	0.4113	-1.01	0.9419	-1.46	0.4468
160422_at	1422482_at	Ruvbl2	RuvB-like protein 2	1.18	0.36	-1.13	0.0523	-1.05	0.4645	-1.02	0.5714	1.32	0.0277
160423_at	1420846_at	Mrps2	mitochondrial ribosomal protein S2	1.29	0.15	1.12	0.161	1.12	0.2757	1.24	0.0034	-1.05	0.724
160425_at	1454116_a_at	2410017I18Rik	RIKEN cDNA 2410017I18 gene	2.31	0.02	1.18	0.1941	1.48	0.029	1.37	0.0592	1.21	0.3371
160426_at	1417041_at	Rpo1-1	RNA polymerase 1-1	1.07	0.62	-1.03	0.462	1.15	0.0019	-1.05	0.4041	-1.11	0.213
160427_at	1428084_at	Hrb2	HIV-1 Rev binding protein 2	1.1	0.58	1.11	0.1058	1.16	0.1033	1.06	0.3497	-1.02	0.942
160428_at	1427441_a_at	Suclg2	succinate-Coenzyme A ligase, GDP-forming	1.18	0.36	1.14	0.1118	1.07	0.4616	1.09	0.2958	-1.18	0.0826
160429_at	1422488_at	Nxt1	NTF2-related export protein 1	1.07	0.61	1.01	0.9316	1.16	0.0181	1.04	0.5553	-1.03	0.7812
160430_at	1420811_a_at	Catnb	catenin beta	1.12	0.48	-1.12	0.1735	-1.02	0.8577	-1.2	0.038	-1.53	0.0004
160431_at	1452048_at	Mrpl12	mitochondrial ribosomal protein L12	1.74	0	1	0.958	1.21	0.0396	1.17	0.1279	1.1	0.6059
160432_at	1434305_at	U2af1l4	U2 small nuclear RNA auxiliary factor 1-like	1.45	0.21	-1.05	0.1753	-1.06	0.3241	-1.02	0.7052	-1.03	0.6762
160433_at	1456716_s_at	3110002H16Rik	RIKEN cDNA 3110002H16 gene	-1.05	0.68	1.03	0.7251	1.09	0.3166	1.14	0.145	1.18	0.1208
160434_at	1448867_at	2310004K06Rik	RIKEN cDNA 2310004K06 gene	1.19	0.14	-1.09	0.0356	-1.07	0.2443	-1.05	0.2108	-1.19	0.2527
160435_at	1423965_at	Cd99l2	Cd99 antigen-like 2	1.1	0.83	-1.09	0.1841	1.01	0.8675	-1.18	0.077	1.34	0.0511
160437_at	1449097_at	Txnrd2	thioredoxin reductase 2	1.87	0.04	1.1	0.4545	1.06	0.6053	1.03	0.7869	1.36	0.1065
160439_at	1423273_at	Polg	polymerase (DNA directed), gamma	1.4	0.44	-1.34	0.0757	-1.27	0.2442	-1.4	0.0472	1.03	0.9141
160440_at	1422487_at	Smad4	MAD homolog 4 (Drosophila)	1.9	0.02	1.11	0.315	1.13	0.215	1.16	0.1893	1.38	0.0779
160441_at	1448197_at	Ceacam11	CEA-related cell adhesion molecule 11	-1.6	0.28	-1.52	0.0313	-1.11	0.5306	-1.4	0.0496	1.24	0.5554
160442_at	1433535_x_at	Cct2	chaperonin subunit 2 (beta)	1.13	0.49	-1.14	0.1274	-1.07	0.4553	1.01	0.838	1.01	0.9583
160444_at	1425134_a_at	Pigx	phosphatidylinositol glycan, class X	1.09	0.53	-1.19	0.0475	1.06	0.5479	-1.16	0.1174	-1.08	0.6723
160445_at	1453848_s_at	Zbed3	zinc finger, BED domain containing 3	-1.93	0.01	-1.22	0.0038	-1.19	0.0739	-1.28	0.0023	1.32	0.0385
160446_at	1423719_at	U46068	cDNA sequence U46068	-2.53	0.27	1.1	0.6912	-1.04	0.8291	-1.07	0.6694	2.2	0.2126
160447_at	1423195_at	Hiat1	hippocampus abundant gene transcript 1	1.13	0.54	1.13	0.0644	1.18	0.2884	1.13	0.1256	1.11	0.4584
160448_at	1426844_a_at	6030457N17Rik	RIKEN cDNA 6030457N17 gene	-1.01	0.96	-1.09	0.2289	-1.03	0.6218	-1.01	0.8837	-1	0.964
160449_at	1416018_at	Dr1	down-regulator of transcription 1	1.13	0.46	1.16	0.0133	1.24	0.0408	1.13	0.248	-1.03	0.8586
160450_at	1423031_at	---	---	1.33	0.17	1.13	0.0002	1.03	0.5829	1.08	0.1364	1.12	0.456
160451_at	1451208_at	Etf1	eukaryotic translation termination factor 1	1.33	0.39	1.1	0.0806	1.38	0.0005	1.16	0.0144	1.19	0.2043
160453_at	1426388_s_at	---	---	1.04	0.86	1.07	0.5788	1.2	0.2361	1.09	0.5947	1.34	0.071
160456_at	1424136_a_at	Ppih /// LOC433	peptidyl prolyl isomerase H /// similar to pept	3.56	0	2.02	0.041	1.78	0.235	2.35	0.0292	1.43	0.114
160457_at	1416496_at	Mrfap1	Morf4 family associated protein 1	1.5	0.2	1.12	0.0148	1.07	0.1556	1.08	0.0582	-1.2	0.1091
160458_at	1416357_a_at	Mcam	melanoma cell adhesion molecule	1.24	0.3	1.19	0.201	1.35	0.0076	1.26	0.0165	1.37	0.1685
160461_f_at	1416431_at	Tubb6	tubulin, beta 6	1.22	0.38	1.12	0.4913	1.04	0.6691	1.26	0.0263	-1.19	0.3432
160462_f_at	1415978_at	Tubb3	tubulin, beta 3	1.15	0.66	-1.22	0.067	-1.03	0.7401	-1.1	0.1742	-1.69	0.0224
160463_at	1448325_at	Myd116	myeloid differentiation primary response ger	1.13	0.59	1.37	0.0336	1.32	0.1979	1.34	0.0736	1.11	0.724
160466_at	1429528_at	Rae1	RAE1 RNA export 1 homolog (S. pombe)	1.73	0.02	1.19	0.0185	1.26	0.0061	1.32	0.0007	1.55	0.1276
160468_at	1420473_at	Mtpn	myotrophin	1.11	0.54	1.39	0.0143	1.02	0.9012	1.14	0.2667	-1.05	0.7568
160469_at	1421811_at	Thbs1	thrombospondin 1	1.2	0.78	1.55	0.1081	1.09	0.7433	1.21	0.5121	3.8	0.0602
160470_at	1448488_at	Mrps5	mitochondrial ribosomal protein S5	2.46	0.11	1.44	0.0144	1.33	0.0454	1.43	0.0211	-1.76	0.0426
160471_at	1460168_at	Slbp	stem-loop binding protein	1.88	0.02	1.25	0.1398	1.14	0.3959	1.35	0.0066	1.42	0.163
160472_r_at	1426649_at	Tmeff1	transmembrane protein with EGF-like and tw	-1.12	0.83	-1.12	0.7735	-1.24	0.6452	-1.76	0.3264	1.37	0.5003
160473_at	1416798_a_at	Nme4	expressed in non-metastatic cells 4, protein	1.93	0.14	1.16	0.3997	-1.03	0.8852	1.66	0.0036	-1.52	0.2707
160475_at	1450418_a_at	2310034L04Rik	RIKEN cDNA 2310034L04 gene	-1.28	0.4	-1.17	0.06	-1.15	0.2185	-1.36	0.0033	1.14	0.405
160476_f_at	1436699_x_at	Rpl18	ribosomal protein L18	1.24	0.27	1.03	0.5831	1.09	0.1661	1.11	0.0929	1.3	0.1078
160477_at	1424085_at	Ndufa4	NADH dehydrogenase (ubiquinone) 1 alpha	-1.4	0.45	1.21	0.2302	-1.02	0.9305	1.23	0.168	-1.16	0.698
160479_at	1416430_at	Cat	catalase	-1.36	0.08	1.09	0.1978	-1.09	0.2409	1.09	0.2767	-1.2	0.1374
160480_at	1426794_at	Ptprs	protein tyrosine phosphatase, receptor type,	1.26	0.41	1.42	0.0093	-1.01	0.9685	1.46	0.0086	1.84	0.0425
160481_at	1423439_at	Pck1	phosphoenolpyruvate carboxykinase 1, cyto	1.44	0.06	1.39	0.004	1.33	0.0087	1.65	0	1.46	0.1047
160482_at	1456011_x_at	Acaa1	acetyl-Coenzyme A acyltransferase 1	1.81	0.05	1.21	0.0034	1.14	0.0777	1.25	0.009	-1.04	0.8319

160483_at	1416723_at	Tcf4	transcription factor 4	1.54	0.23	-1.15	0.1095	-1.12	0.1412	-1.16	0.0823	1.19	0.367
160484_at	1452649_at	Rtn4	reticulon 4	1.38	0.13	1.01	0.9383	1.01	0.9594	-1.05	0.6199	-1.58	0.0128
160485_r_at	1435702_s_at	Ywhae	tyrosine 3-monooxygenase/tryptophan 5-mo	1.2	0.6	-1.14	0.0535	1.09	0.4493	1.03	0.669	1.27	0.3298
160487_at	1422580_at	Myl4	myosin, light polypeptide 4	-1.36	0.39	-1.02	0.8993	1.6	0.0025	1.23	0.2652	-1.2	0.5455
160488_at	1424166_at	Msh3	mutS homolog 3 (E. coli)	1.51	0.39	1.14	0.3522	1.3	0.0112	1.11	0.33	1.26	0.3919
160489_at	1438855_x_at	---	---	1.05	0.83	-1.07	0.6836	-1.19	0.0872	1.12	0.6321	-1.61	0.0872
160490_at	1415853_at	Def8	differentially expressed in FDCP 8	-1.02	0.8	-1.15	0.0149	-1.34	0.0014	-1.24	0.0005	-1.31	0.0792
160492_at	1416071_at	Ddx18	DEAD (Asp-Glu-Ala-Asp) box polypeptide 18	-1.01	0.95	-1.21	0.0648	-1.2	0.0241	-1.09	0.2263	1.06	0.6885
160494_at	1438317_a_at	Endog	endonuclease G	-1.14	0.25	1.03	0.7781	1.01	0.9374	-1.14	0.2231	1.3	0.1564
160495_at	1422631_at	Ahr	aryl-hydrocarbon receptor	1.41	0.31	1.15	0.0441	1.2	0.1648	1.13	0.1123	1.36	0.0902
160496_s_at	1449705_x_at	---	---	-1.42	0.23	1.01	0.9541	1.04	0.6222	1.12	0.2629	-1.15	0.3602
160497_at	1449842_at	1810059G22Rik	RIKEN cDNA 1810059G22 gene	-1.07	0.77	-1.06	0.258	-1.02	0.8011	-1.03	0.7046	-1.04	0.7064
160498_at	1452024_a_at	Ldb1	LIM domain binding 1	-1.39	0.18	1.36	0.0002	1.12	0.4187	1.63	0.0023	1.46	0.027
160499_at	1415889_a_at	Tra1	tumor rejection antigen gp96	-1.1	0.49	-1.21	0.0013	-1.15	0.0336	-1.32	0	-1.07	0.3181
160501_at	1449207_a_at	Kif20a	kinesin family member 20A	1.51	0.56	1.16	0.727	-1.02	0.9667	1.06	0.8971	1.49	0.2613
160502_at	1415948_at	Creg1	cellular repressor of E1A-stimulated genes 1	1.23	0.53	1.52	0	1.39	0.0012	1.6	0	-1.11	0.1811
160503_at	1416685_s_at	Fbl	fibrillarin	1.23	0.31	-1.03	0.5922	-1.02	0.7685	-1.01	0.8704	1.11	0.4132
160504_at	1416777_at	Ceacam12	CEA-related cell adhesion molecule 12	-4.55	0.1	2.42	0.0339	1.09	0.853	1.22	0.6294	1.97	0.3555
160505_at	1455808_at	4922502D21Rik	RIKEN cDNA 4922502D21 gene	-2.05	0.09	1.76	0.1234	-1.32	0.3047	2.06	0.2178	1.32	0.5858
160506_at	1423959_at	Ropn1l	ropporin 1-like	2.27	0.02	1.23	0.0799	-1.31	0.0979	1.16	0.182	1.1	0.6852
160507_at	1428078_at	0610013E23Rik	RIKEN cDNA 0610013E23 gene	-1.1	0.54	1.08	0.3253	-1.05	0.7201	-1.06	0.5659	-1.01	0.9327
160508_at	1451258_at	Psca	prostate stem cell antigen	-1.26	0.4	1.02	0.832	1.03	0.7016	1.01	0.9292	1.28	0.2636
160509_at	1416456_a_at	2200003E03Rik	RIKEN cDNA 2200003E03 gene	1.12	0.17	-1.04	0.7366	-1.01	0.9612	-1.06	0.5385	1.11	0.4604
160510_f_at	1422454_at	Krt1-13	keratin complex 1, acidic, gene 13	1.82	0.22	-1.4	0.3743	-1.55	0.2579	-1.48	0.3161	1.26	0.2688
160511_at	1448823_at	Cxcl12	chemokine (C-X-C motif) ligand 12	1.09	0.63	1.22	0.1253	1.21	0.2731	-1.11	0.3868	1.06	0.5503
160512_at	1423678_at	BC017643	cDNA sequence BC017643	-1.33	0.02	-1.34	0.0088	-1.33	0.0103	-1.4	0.001	1.07	0.6469
160513_at	1428331_at	2210016F16Rik	RIKEN cDNA 2210016F16 gene	1.19	0.38	1.01	0.9298	1.14	0.1634	1.06	0.3046	-1.02	0.8347
160514_at	1418277_at	Rp9h	retinitis pigmentosa 9 homolog (human)	1.56	0.14	-1.06	0.503	-1.06	0.4321	-1.08	0.3965	1.21	0.1405
160515_at	1425422_a_at	Parn	poly(A)-specific ribonuclease (deadenylation	1.04	0.73	1.08	0.1167	1.01	0.9282	1.07	0.2877	1.21	0.1376
160516_at	1419428_a_at	Gaa	glucosidase, alpha, acid	-1.44	0.06	-1.21	0.0285	1	0.9664	-1.2	0.0145	1.15	0.0359
160517_at	1423521_at	---	---	1.16	0.52	-1.12	0.1997	-1.36	0.0578	-1.46	0.0066	-1.56	0.0375
160518_at	1460706_s_at	Rer1	RER1 retention in endoplasmic reticulum 1 h	1.07	0.32	-1.06	0.2794	-1.02	0.8529	-1.13	0.0505	-1.22	0.048
160520_at	1448363_at	Yap1	yes-associated protein 1	-1.32	0.07	-1.04	0.3757	1.02	0.5536	-1.09	0.1059	-1.05	0.5823
160521_at	1426813_at	2610020N02Rik	RIKEN cDNA 2610020N02 gene	2.92	0.02	1.21	0.2427	1.16	0.4067	1.13	0.2338	1.32	0.6075
160522_at	1450839_at	D0H4S114	DNA segment, human D4S114	-3.65	0.05	-4.23	0	-1.48	0.0031	-4.15	0	-1.38	0.3899
160523_at	1421104_at	---	---	2.83	0.01	1.19	0.5866	1.37	0.3547	1.55	0.0934	2.11	0.008
160525_f_at	1426730_a_at	Prlpk	prolactin-like protein K	-1.89	0.45	-1.59	0.0219	-1.37	0.1117	-1.11	0.6622	-1.27	0.6823
160526_s_at	1418322_at	Crem	cAMP responsive element modulator	-1.66	0.02	-1.17	0.2666	-1.22	0.1634	-1.41	0.0166	1.27	0.2073
160528_at	1448105_at	Prm2	protamine 2	-5.16	0.1	1.29	0.5367	1.43	0.3182	-1.16	0.648	-1.01	0.9784
160529_r_at	1415990_at	Vdac2	voltage-dependent anion channel 2	1.49	0.14	1.25	0.0566	1.06	0.6006	1.35	0.0016	1.4	0.0893
160530_at	1415881_at	Ghitm	growth hormone inducible transmembrane p	1.39	0.09	1.25	0.0081	1.15	0.1571	1.25	0.0006	-1.11	0.1527
160531_at	1416393_at	Grcc2f	gene rich cluster, C2f gene	1.32	0.16	1.03	0.5534	-1.02	0.5148	1	0.995	1.22	0.0597
160532_at	1423721_at	Tpm1	tropomyosin 1, alpha	1.51	0.1	-1.04	0.7073	1.06	0.2636	1.06	0.3202	1.37	0.1231
160533_r_at	1415924_at	Tnp1	transition protein 1	-1.4	0.04	-1.08	0.1181	-1.11	0.0689	-1.11	0.1184	1.25	0.0685
160534_at	1415740_at	Psmc5	protease (prosome, macropain) 26S subunit	1.16	0.42	-1.01	0.8813	-1.06	0.096	1.02	0.7257	-1.09	0.4542
160535_at	1416331_a_at	Nfe2l1	nuclear factor, erythroid derived 2,-like 1	-1.17	0.26	-1.25	0.0023	-1.19	0.0234	-1.41	0	1.03	0.8121
160536_at	1424132_at	Hras1	Harvey rat sarcoma virus oncogene 1	-1.3	0.21	1.02	0.9006	1.08	0.5001	-1.2	0.144	-1.32	0.0259
160537_at	1418138_at	Sult1d1	sulfotransferase family 1D, member 1	2.17	0.06	-1.22	0.0603	-1.15	0.1769	-1.09	0.2294	2.3	0.0066
160538_at	1422441_x_at	Cdk4	cyclin-dependent kinase 4	-1.07	0.71	-1.12	0.0887	-1.1	0.1549	-1.03	0.4839	1.21	0.1701
160540_at	1416394_at	Bag1	Bcl2-associated athanogene 1	2	0.25	1.15	0.0378	1.15	0.1389	1.17	0.056	1.03	0.8318
160543_at	1422480_at	Snx3	sorting nexin 3	-1.23	0.13	1.02	0.6805	-1.07	0.3789	-1.12	0.0501	-1.13	0.0479
160544_at	1416022_at	Fabp5	fatty acid binding protein 5, epidermal	-3.81	0.06	-2.28	0.0006	-1.22	0.1115	-2.66	0.0004	-1.63	0.2046
160545_at	1415907_at	Ccnd3	cyclin D3	1.3	0.31	1.2	0.0026	1.05	0.6021	1.29	0.0011	1.34	0.0286

160546_at	1451461_a_at	Aldoc	aldolase 3, C isoform	1.12	0.69	1.21	0.1145	1.18	0.1088	1.49	0.0016	-1.14	0.3723
160547_s_at	1415996_at	Txnip	thioredoxin interacting protein	1.97	0.13	1.34	0.1271	2.69	0.0006	1.55	0.0025	2.58	0.0792
160549_at	1415741_at	Tparl	TPA regulated locus	-1.33	0.16	-1.01	0.8373	-1.07	0.1875	-1.07	0.1554	-1.03	0.8286
160550_i_at	1416212_at	Magoh	mago-nashi homolog, proliferation-associate	1.26	0.44	1.03	0.5164	1.04	0.5033	1.01	0.8234	1.04	0.6082
160551_at	1416175_a_at	Vdac3	voltage-dependent anion channel 3	1.02	0.84	1.09	0.1499	1.06	0.1556	1.16	0.0025	-1.23	0.1288
160552_at	1416087_at	---	---	-1.34	0.09	1.14	0.1103	-1.09	0.5336	-1.12	0.3585	-1.11	0.5816
160553_at	1416930_at	Ly6d	lymphocyte antigen 6 complex, locus D	-1.23	0.67	1.09	0.5536	-1.04	0.8523	1.02	0.9119	-2.31	0.1481
160554_at	1460432_a_at	Eif3s6	eukaryotic translation initiation factor 3, subu	1.7	0.05	-1.03	0.7973	-1.08	0.495	1.06	0.5233	1.58	0.041
160556_at	1450895_a_at	1810020G14Rik	RIKEN cDNA 1810020G14 gene	1.4	0.16	1.09	0.0848	1.1	0.4364	1.12	0.0241	-1.05	0.7623
160558_at	1424480_s_at	Akt2	thymoma viral proto-oncogene 2	1.35	0.07	-1.11	0.2226	-1.14	0.0889	-1.1	0.2553	1.08	0.7049
160561_at	1416006_at	Mdk	midkine	1.9	0.14	-1.1	0.5868	1.01	0.9462	-1.07	0.7387	1.17	0.602
160562_at	1415817_s_at	Cct7	chaperonin subunit 7 (eta)	1.51	0.03	1.1	0.2207	1.05	0.5565	1.06	0.3954	1.25	0.0802
160564_at	1427747_a_at	Lcn2	lipocalin 2	6.35	0.08	6.21	0.2286	-1.48	0.1302	9.82	0.1164	-10.78	0.3048
160565_at	1417089_a_at	Ckmt1	creatine kinase, mitochondrial 1, ubiquitous	1.17	0.64	1.37	0.1728	1.56	0.2207	1.07	0.8081	1.75	0.3212
160567_at	1448120_at	Gdf9	growth differentiation factor 9	-1.1	0.4	1.1	0.6096	1.13	0.5709	1.11	0.5981	1.86	0.1482
160568_at	1427404_x_at	Eno1	enolase 1, alpha non-neuron	1.78	0.04	-1.06	0.5675	1.24	0.1396	1.2	0.1789	1.09	0.1491
160569_at	1449139_at	2310008M10Rik	RIKEN cDNA 2310008M10 gene	1.01	0.98	-1.18	0.0622	-1.11	0.205	-1.33	0.0001	-1.48	0.0319
160570_at	1448327_at	Actn2	actinin alpha 2	-1.24	0.61	-1.28	0.021	-1.06	0.591	-1.16	0.0622	-1.2	0.5609
160571_at	1422433_s_at	Idh1	isocitrate dehydrogenase 1 (NADP+), solubl	1.07	0.46	-1.09	0.2284	-1.04	0.6991	-1.11	0.0537	-2.04	0.0003
160572_at	1416144_a_at	Dhx15	DEAH (Asp-Glu-Ala-His) box polypeptide 15	1.02	0.89	-1.06	0.5357	-1.02	0.701	-1.14	0.0277	1.15	0.4687
160573_at	1420890_at	Hccs	holocytochrome c synthetase	1.52	0.3	1.09	0.3901	1.24	0.1813	1.16	0.2318	-1.22	0.5309
160574_at	1416821_at	Es2el	expressed sequence 2 embryonic lethal	1.42	0.44	1.31	0.0167	1.02	0.8314	1.01	0.9561	-1.09	0.8756
160575_at	1454641_at	Cggbp1	CGG triplet repeat binding protein 1	-1.03	0.42	1.05	0.5304	1.04	0.7922	-1.02	0.8089	-1.05	0.5031
160577_at	1415932_x_at	Atp9a	ATPase, class II, type 9A	-1.55	0	-1.04	0.5512	-1.01	0.8466	-1.06	0.1874	-1.35	0.1803
160578_at	1415729_at	Pdpk1	3-phosphoinositide dependent protein kinase	-1.26	0.29	1.05	0.5245	1.04	0.4816	1.05	0.336	1.07	0.152
160579_at	1417111_at	Man1a	mannosidase 1, alpha	-1.49	0.25	1.02	0.8142	1.06	0.5192	-1.03	0.6314	-1.03	0.4192
160580_at	1417110_at	Man1a	mannosidase 1, alpha	1.41	0.15	1.23	0.0375	1.06	0.3737	1.16	0.0502	1.1	0.6197
160581_at	1428169_at	Apg16l	APG16 autophagy 16-like (S. cerevisiae)	-1.01	0.98	-1	0.9846	1.27	0.0139	-1.06	0.4765	-1.2	0.3177
160582_at	1418551_at	Mybpc3	myosin binding protein C, cardiac	1.13	0.73	-1.2	0.1601	-1.02	0.8628	-1.12	0.1787	-1.97	0.0606
160583_at	1429379_at	Xlkd1	extra cellular link domain-containing 1	1.48	0.15	1.63	0.0903	1.2	0.5705	1.43	0.2253	2.66	0.0333
160585_at	1450423_s_at	Bxdc1	brix domain containing 1	1.47	0.3	1.23	0.2269	1.04	0.8844	1.22	0.3856	2.54	0.0433
160587_at	1436955_at	Tssc1	tumor suppressing subtransferable candidat	2.49	0.09	1.44	0.1109	1.71	0.0443	1.37	0.1564	1.3	0.5261
160588_at	1428616_at	Zfp131	zinc finger protein 131	1.16	0.3	-1.13	0.2989	1.43	0.0019	-1.03	0.7798	1.02	0.8668
160590_r_at	1428635_at	Comtd1	catechol-O-methyltransferase domain contai	3.46	0.04	1.9	0.0279	1.96	0.0562	1.67	0.084	1.69	0.0504
160591_at	1418967_a_at	St7	Suppression of tumorigenicity 7	1.47	0.56	1	0.9747	1.18	0.0211	1.02	0.8268	-1.35	0.2264
160592_at	1423770_at	Tmc6	transmembrane channel-like gene family 6	1.14	0.33	-1.06	0.3866	-1.09	0.4319	-1.15	0.0416	1.14	0.3491
160593_at	1423076_at	Snx9	sorting nexin 9	-1.1	0.32	-1.04	0.0455	-1.13	0.1262	-1.09	0.0078	1.04	0.7126
160594_at	1418799_a_at	Col17a1	procollagen, type XVII, alpha 1	-2.03	0.15	-2.03	0.0128	-1.67	0.0752	-1.61	0.0956	-1.01	0.9799
160595_at	1448555_at	D15Ertd682e	DNA segment, Chr 15, ERATO Doi 682, exp	1.61	0.25	2.23	0.0234	1.29	0.2971	2.31	0.0012	1.26	0.5499
160596_at	1417739_at	1110030J09Rik	RIKEN cDNA 1110030J09 gene	1.47	0.28	1.19	0.0736	1.05	0.6486	1.33	0.0057	1.07	0.8324
160597_at	1448721_at	D1Ertd622e	DNA segment, Chr 1, ERATO Doi 622, expr	1.02	0.94	-1.04	0.6648	1.33	0.0765	1.15	0.1481	-1.17	0.4695
160601_at	1420643_at	Lfng	lunatic fringe gene homolog (Drosophila)	-1.21	0.74	1.79	0.0026	1.31	0.0921	1.78	0.0016	1.09	0.6718
160602_at	1416843_at	Pde6d	phosphodiesterase 6D, cGMP-specific, rod,	1.16	0.72	1.25	0.4017	1.22	0.4782	1.41	0.1335	-1.18	0.5367
160603_at	1421907_at	Pparbp	peroxisome proliferator activated receptor bi	-3.8	0.15	1.25	0.4749	-2.37	0.0086	-1.62	0.1158	2	0.0764
160607_at	1434775_at	Pard3	par-3 (partitioning defective 3) homolog (C. e	1.97	0	1.15	0.3094	-1.28	0.0661	1.22	0.0808	1.3	0.323
160608_at	1438097_at	Rab20	RAB20, member RAS oncogene family	1.13	0.48	1.26	0.4195	-1.69	0.0882	1.47	0.1172	1.15	0.495
160609_at	1418774_a_at	Atp7a	ATPase, Cu++ transporting, alpha polypepti	1.04	0.93	1.13	0.2272	-1.08	0.2872	1.09	0.3678	-1.07	0.8101
160610_at	1420798_s_at	Pcdha4 /// Pcdh	protocadherin alpha 4 /// protocadherin alpha	-1.37	0.74	-1.75	0.2037	1.03	0.9457	1.38	0.488	-1.73	0.1575
160612_at	1423570_at	Abcg1	ATP-binding cassette, sub-family G (WHITE	1.01	0.94	1.2	0.0881	-1.04	0.7291	1.12	0.3785	1.49	0.0075
160613_at	1417109_at	Tinagl	tubulointerstitial nephritis antigen-like	1.21	0.39	1.08	0.5408	1.23	0.1447	1.12	0.4154	1.5	0.0149
160614_at	1422553_at	Pten	phosphatase and tensin homolog	1.11	0.81	-1.07	0.8302	-2.82	0.0099	-2.17	0.0384	-1.61	0.0746
160615_at	1451115_at	Pias3	protein inhibitor of activated STAT 3	4.76	0	1.04	0.7457	1.12	0.184	1.19	0.0253	1.01	0.9835
160616_at	1427248_at	Whsc2	Wolf-Hirschhorn syndrome candidate 2 (hum	-1.41	0.04	-1.04	0.5854	-1.09	0.2947	-1.13	0.0458	-1.11	0.5405

160617_at	1432543_a_at	Klf13	Kruppel-like factor 13	2.08	0.22	-1.39	0.107	-1.97	0.0119	-2.13	0.0032	-1.15	0.7159
160618_at	1422661_at	Lgals8	lectin, galactose binding, soluble 8	-1.23	0.31	-1.25	0.0372	-1.41	0.0172	-1.56	0.007	-1.54	0.2122
160619_at	1452372_at	1110063F24Rik	RIKEN cDNA 1110063F24 gene	1.06	0.87	1.02	0.6826	1.01	0.9025	-1.03	0.5767	-1.1	0.5944
160620_at	1452239_at	Gt(ROSA)26So	gene trap ROSA 26, Philippe Soriano	1.29	0.44	-1.06	0.6118	1.32	0.1152	1.33	0.0881	-1.02	0.9564
160621_at	1416595_at	Mrps22	mitochondrial ribosomal protein S22	1.75	0.1	1.19	0.2047	1.18	0.3008	1.18	0.1645	-1.17	0.54
160622_at	1424123_at	BC011209	cDNA sequence BC011209	1.03	0.9	1.95	0.0002	1.39	0.0315	2.09	0	2.48	0.0006
160623_at	1449229_a_at	Cdkl2	cyclin-dependent kinase-like 2 (CDC2-relate	-1.15	0.8	-1.57	0.0302	-1.28	0.2302	-1.5	0.0743	-2.02	0.2178
160624_at	1426796_at	Txln	Taxilin	1.07	0.77	1.05	0.5587	-1.04	0.6747	-1.11	0.2048	-1.26	0.0759
160626_at	1425350_a_at	Myef2	myelin basic protein expression factor 2, rep	-1.53	0.2	1.14	0.2708	1.33	0.0249	1.28	0.0085	1.15	0.4542
160627_at	1434607_at	Ddx52	DEAD (Asp-Glu-Ala-Asp) box polypeptide 52	1.62	0.17	1.15	0.2734	1.17	0.3189	1.26	0.0655	-1.09	0.7571
160628_at	1417823_at	Gcat	glycine C-acetyltransferase (2-amino-3-ketol	1.35	0.18	-1.05	0.5257	1.06	0.3731	-1.09	0.3601	-1.03	0.9152
160630_at	1434190_at	Sms	spermine synthase	1.28	0.55	-1.19	0.19	1.04	0.7097	-1.31	0.0744	1.27	0.2283
160631_s_at	1422654_at	Sgca	sarcoglycan, alpha (dystrophin-associated g	-3.13	0.01	-1.18	0.4883	-1.3	0.3149	-1.41	0.2075	2.55	0.1844
160632_at	1428229_at	Prkcn	protein kinase C, nu	1.03	0.91	1.87	0.0003	1.86	0.0004	1.86	0	1.5	0.0194
160633_at	1417724_at	Thoc4	THO complex 4	1	0.99	1.05	0.5891	-1.04	0.6616	1.11	0.1608	-1.02	0.8954
160634_at	1428751_at	Pacrg	Park2 co-regulated	1.67	0.36	-1.01	0.969	1.54	0.1341	1.27	0.4125	1.02	0.9356
160635_at	1453819_x_at	Stx18	syntaxin 18	1.02	0.9	-1.14	0.1921	-1.12	0.2186	-1.13	0.1005	-1.3	0.5029
160636_at	1452599_s_at	AI413582	expressed sequence AI413582	1.66	0.21	1.12	0.0846	-1.04	0.5695	1.22	0.0761	1.39	0.3097
160637_at	1424309_a_at	Mocs2	molybdenum cofactor synthesis 2	1.54	0.22	1.02	0.5421	-1.01	0.8289	1.04	0.2599	-1.28	0.0382
160638_at	1416868_at	Cdkn2c	cyclin-dependent kinase inhibitor 2C (p18, ir	-1.27	0.19	-1.35	0.0007	1.07	0.4991	-1.17	0.0289	1.3	0.0234
160639_at	1420712_a_at	Hpn	hepsin	1.06	0.88	1.04	0.3281	-1.02	0.8406	1.08	0.1356	-1.27	0.2027
160640_at	1424748_at	Galnt11	UDP-N-acetyl-alpha-D-galactosamine:polype	-1.15	0.72	-1.03	0.7571	-1.01	0.9049	1.06	0.4542	-1.16	0.3415
160641_at	1416432_at	Pfkfb3	6-phosphofructo-2-kinase/fructose-2,6-bipho	1.65	0.11	-1.12	0.5239	1.54	0.0103	1.19	0.3345	-1.32	0.3184
160642_at	1424042_at	Tmem5	transmembrane protein 5	-1.1	0.79	-1.1	0.4799	1.05	0.8019	1.28	0.1001	1.49	0.0923
160643_at	1449080_at	Hdac2	histone deacetylase 2	-1.35	0.03	-1.16	0.1037	1.09	0.3563	1.18	0.33	-1	0.9857
160644_at	1433904_at	MGI:3055306	gametogenetin binding protein 1	-1.19	0.45	1.11	0.2749	1.11	0.1368	1.07	0.1927	1.33	0.0959
160645_at	1421392_a_at	Birc3	baculoviral IAP repeat-containing 3	-1.31	0.48	1.05	0.6486	1.16	0.3959	1.25	0.1587	1.13	0.5328
160646_at	1421817_at	Gsr	glutathione reductase 1	-1.2	0.6	1.23	0.0066	1.39	0.0062	1.52	0.0005	-1.23	0.0355
160647_at	1421001_a_at	Car6	carbonic anhydrase 6	-3.81	0.1	-1.29	0.2441	-1.16	0.4272	-1.27	0.2251	-1.75	0.2069
160648_at	1422430_at	Fignl1	fidgetin-like 1	-1.1	0.59	1.01	0.9586	-1.03	0.7295	-1.03	0.7382	1.33	0.2273
160649_at	1422977_at	Gp1bb	glycoprotein Ib, beta polypeptide	-1.25	0.39	1	0.9898	1.08	0.5048	-1.14	0.4035	1.26	0.1291
160651_at	1423323_at	Tacstd2	tumor-associated calcium signal transducer	-1.34	0	1.12	0.5216	1.28	0.1737	-1.04	0.7261	1.12	0.7627
160652_at	1448111_at	Ctps2	cytidine 5'-triphosphate synthase 2	3.81	0.01	1.21	0.1177	1.14	0.1678	1.32	0.0207	1.55	0.0068
160653_at	1426118_a_at	Tomm40	translocase of outer mitochondrial membran	5.9	0	1.39	0.0029	1.07	0.7097	1.29	0.045	-1.72	0.4807
160654_at	1428534_at	2310073E15Rik	RIKEN cDNA 2310073E15 gene	1.1	0.81	-1.06	0.3045	-1.02	0.7189	-1.14	0.0409	-1	0.9824
160655_at	1418018_at	Cpd	carboxypeptidase D	-1.27	0.35	-1.07	0.5737	-1.13	0.4246	-1.06	0.6805	1.73	0.1596
160657_at	1460669_at	Ilf3	interleukin enhancer binding factor 3	1.21	0.17	1.31	0.0019	1.16	0.0204	1.32	0.0029	1.44	0.1077
160658_at	1424257_at	Cdk7	cyclin-dependent kinase 7 (homolog of Xeno	-1.12	0.25	-1.02	0.862	1.03	0.8301	-1.19	0.2092	-1.05	0.6868
160659_at	1428823_at	2310057G13Rik	RIKEN cDNA 2310057G13 gene	2.09	0.3	1.56	0.0951	1.77	0.1565	1.84	0.2168	1.11	0.7427
160660_r_at	1423835_at	Zfp503	zinc finger protein 503	1.86	0.31	1.26	0.5635	1.45	0.2377	1.04	0.9063	1.87	0.1068
160661_at	1454621_s_at	5730472N09Rik	RIKEN cDNA 5730472N09 gene	-1.06	0.74	-1.28	0.0104	-1.52	0.0013	-1.53	0.0026	-2.24	0.0025
160663_at	1423814_at	Ddx41	DEAD (Asp-Glu-Ala-Asp) box polypeptide 41	2.08	0.14	1.12	0.3414	1.2	0.1372	1.02	0.8815	-1.43	0.5372
160666_at	1428502_at	Actr6	ARP6 actin-related protein 6 homolog (yeast	1.51	0.14	1.41	0.013	1.49	0.0786	1.58	0	1.25	0.2364
160667_at	1434920_a_at	Evl	Ena-vasodilator stimulated phosphoprotein	2.45	0.15	1.22	0.5213	-1.25	0.4299	1.76	0.14	-2.25	0.0845
160668_at	1422511_a_at	Ogfr	opioid growth factor receptor	-1.13	0.7	1.12	0.3243	1.04	0.7604	1.22	0.166	-1.02	0.9383
160669_at	1454760_at	Htatsf1	HIV TAT specific factor 1	1.14	0.33	1.23	0.0239	1.22	0.0472	1.33	0.0168	1.25	0.0492
160670_at	1448147_at	Tnfrsf19	tumor necrosis factor receptor superfamily, r	1.02	0.97	6.05	0	3.46	0.0303	4.56	0.0026	1.7	0.2917
160671_at	1459992_x_at	Cln8	ceroid-lipofuscinosis, neuronal 8	1.6	0.09	1.18	0.482	1.11	0.6868	1.07	0.8086	1.6	0.2963
160672_at	1423612_at	AI462438	expressed sequence AI462438	-1.22	0.41	-1.07	0.4839	1.11	0.2182	-1.16	0.1672	-1.05	0.7436
160673_at	1424803_at	BC020002	cDNA sequence BC020002	1.01	0.98	1.08	0.7217	1.03	0.911	1.09	0.6627	-1.33	0.3274
160674_at	1425381_a_at	Trfr2	transferrin receptor 2	1.06	0.8	-1.09	0.1444	-1.07	0.4984	-1.15	0.0056	-1.81	0.1409
160676_at	1427131_s_at	1810012N18Rik	RIKEN cDNA 1810012N18 gene	-1.63	0.18	1.16	0.0002	-1.05	0.4964	1.09	0.1412	1.18	0.1563
160677_at	1451198_at	Gatad2a	GATA zinc finger domain containing 2A	-1.38	0.3	-1.15	0.2772	1.02	0.8974	-1.17	0.3463	-1.01	0.9732

160678_at	1454604_s_at	Tspan12	tetraspanin 12	1.05	0.88	-1.04	0.7901	1.24	0.188	1.19	0.1523	-1.27	0.106
160679_at	1424746_at	Kif1c	Kinesin family member 1C	2.01	0.06	-1.09	0.2621	1.11	0.1212	-1.11	0.1645	1.06	0.6326
160682_at	1433685_a_at	6430706D22Rik	RIKEN cDNA 6430706D22 gene	1.91	0.04	1.01	0.8927	-1.07	0.6016	1.18	0.1847	-1.09	0.5549
160683_at	1419657_a_at	C330005L02Rik	RIKEN cDNA C330005L02 gene	1.02	0.89	1.14	0.3657	1.19	0.3067	1.21	0.159	1.18	0.3055
160684_at	1423786_at	8430410A17Rik	RIKEN cDNA 8430410A17 gene	1.37	0.62	1.5	0.1918	1.46	0.2007	1.53	0.2463	1.19	0.1196
160685_at	1452660_s_at	Klhl7	kelch-like 7 (Drosophila)	-1.08	0.58	-1.11	0.5685	-1.11	0.5773	-1.31	0.1869	-1.25	0.4739
160686_at	1424452_at	5730555F13Rik	RIKEN cDNA 5730555F13 gene	2.52	0.02	1.13	0.0523	1.22	0.0043	1.26	0.0018	1.34	0.153
160688_at	1450394_at	Golph3	golgi phosphoprotein 3	-1.09	0.52	1.08	0.1319	1.09	0.1571	1.12	0.0247	-1.03	0.7935
160690_at	1419037_at	Csnk2a1	casein kinase II, alpha 1 polypeptide	-1.16	0.4	-1.02	0.7395	-1.01	0.8144	-1.07	0.2829	-1.01	0.9489
160693_at	1416387_at	---	---	1.61	0.11	1.12	0.1139	-1.12	0.2181	1.01	0.8943	1.32	0.0122
160694_at	1423557_at	Ifngr2	interferon gamma receptor 2	1.94	0.11	1.08	0.6554	-1.08	0.6952	1.09	0.6834	-1.17	0.2814
160696_at	1416812_at	Tia1	cytotoxic granule-associated RNA binding pr	-1.07	0.82	1.1	0.2647	-1.38	0.0417	-1.15	0.3698	1.42	0.0304
160697_at	1433775_at	C77080	expressed sequence C77080	1.65	0.15	-1.1	0.4073	1.05	0.6367	-1.06	0.6309	-1.2	0.1484
160698_s_at	1422847_a_at	Prkcd	protein kinase C, delta	-1.12	0.18	1.22	0.0912	1.03	0.5371	1.22	0.2907	1.36	0.1872
160699_at	1448466_at	Cdca5	cell division cycle associated 5	-1.43	0.38	2.11	0.0343	1.31	0.2946	1.68	0.1827	1.15	0.7844
160701_at	1426966_at	Axin1	axin 1	-1.07	0.7	1.21	0.0155	1.1	0.2139	1.11	0.228	1.35	0.2804
160702_at	1454681_at	2210008M09Rik	RIKEN cDNA 2210008M09 gene	-2.85	0.43	-1.17	0.7107	1.24	0.5891	-1.28	0.5459	-1.11	0.8331
160703_at	1452709_at	Poldip3	polymerase (DNA-directed), delta interacting	-1.03	0.83	1.06	0.322	1.02	0.7489	1.07	0.3685	1.05	0.6961
160704_at	1451313_a_at	1110067D22Rik	RIKEN cDNA 1110067D22 gene	-1.52	0.08	-1.07	0.4715	-1.24	0.1302	-1.13	0.296	-1.36	0.0555
160705_at	1449031_at	Cited1	Cbp/p300-interacting transactivator with Glu	-3.22	0.01	-1.04	0.905	-1.05	0.9015	-1.28	0.5496	1.44	0.4154
160706_at	1453004_at	3110004L20Rik	RIKEN cDNA 3110004L20 gene	1.39	0.09	-1.19	0.1334	1.22	0.2091	-1.12	0.1459	-1.4	0.0003
160707_at	1454648_s_at	D10Wsu102e	DNA segment, Chr 10, Wayne State Univers	-1.2	0.3	1.26	0.1359	-1.15	0.5751	1.05	0.7668	1.02	0.8711
160708_at	1423025_a_at	Schip1	schwannomin interacting protein 1	-1.53	0.22	-1.15	0.3642	1.05	0.7025	-1.62	0.01	1.35	0.3957
160709_at	1435755_at	1110001A16Rik	RIKEN cDNA 1110001A16 gene	2.66	0	1.14	0.1516	1.03	0.8496	1.14	0.1787	-1.08	0.6398
160710_at	1426709_a_at	Usp33	ubiquitin specific protease 33	1.19	0.69	1.16	0.0502	1.12	0.3991	1.26	0.0157	-1.35	0.1822
160711_at	1419367_at	Decr1	2,4-dienoyl CoA reductase 1, mitochondrial	2.27	0.05	1.21	0.0799	1.3	0.0125	1.27	0.0164	-1.33	0.0159
160712_r_at	1426980_s_at	E130012A19Rik	RIKEN cDNA E130012A19 gene	1.24	0.34	1.42	0.3613	1	0.9786	1.96	0.0247	2.02	0.1214
160713_at	1415941_s_at	AA407930	expressed sequence AA407930	1.75	0.26	1.2	0.1195	-1.03	0.718	1.25	0.0384	-1.02	0.917
160714_at	1417694_at	Gab1	growth factor receptor bound protein 2-asso	4.02	0	1.24	0.1207	1.38	0.0289	1.44	0.0187	2.43	0.0006
160715_at	1451127_at	AW146242	expressed sequence AW146242	-1.36	0.3	-1.13	0.5014	-1.01	0.9586	-1.02	0.9425	-1.21	0.4463
160716_at	1451118_a_at	2410018C17Rik	RIKEN cDNA 2410018C17 gene	1.65	0.04	-1.03	0.8056	1.16	0.2056	1.11	0.2954	1.3	0.217
160717_at	1416841_at	1110059E24Rik	RIKEN cDNA 1110059E24 gene	1.08	0.61	1.01	0.8638	-1.06	0.4364	1.01	0.8458	1.01	0.906
160718_at	1423097_s_at	Capn7	calpain 7	1.06	0.78	1.23	0.0216	1.29	0.1826	1.13	0.0579	-1.25	0.0732
160719_at	1451845_a_at	A230072I16Rik	RIKEN cDNA A230072I16 gene	1.55	0.23	1.02	0.9215	-1.1	0.7287	1.15	0.4781	2.09	0.1347
160721_at	1417540_at	Elf1	E74-like factor 1	-1.6	0.48	-1.23	0.2958	-1.36	0.0809	-1.07	0.7083	1.23	0.7381
160722_at	1428766_at	Rnmtl1	RNA methyltransferase like 1	1.86	0.08	1.03	0.8242	1.13	0.2286	-1	0.9941	1.14	0.2357
160723_at	1428684_at	1500001M20Rik	RIKEN cDNA 1500001M20 gene	3.96	0.02	1.41	0.003	1.44	0.0917	1.56	0.014	1.69	0.3167
160724_at	1448478_at	Trfp	Trf (TATA binding protein-related factor)-pro	1.61	0.21	1.64	0.006	1.84	0.0004	1.67	0.0052	1.74	0.0492
160725_at	1434625_at	4930432O21Rik	RIKEN cDNA 4930432O21 gene	1.18	0.02	1.19	0.107	1.27	0.1462	-1.04	0.5948	-1.03	0.8709
160726_at	1417073_a_at	Qk	quaking	1.08	0.63	1.13	0.1416	1.06	0.5408	1.17	0.0769	1.05	0.7963
160727_at	1460672_at	2410002F23Rik	RIKEN cDNA 2410002F23 gene	-1.26	0.43	-1.04	0.5843	-1.12	0.1972	1.03	0.6272	1.07	0.7942
160729_f_at	1417076_at	Fabp9	fatty acid binding protein 9, testis	-1.25	0.68	-1.61	0.2112	-2.02	0.0754	-1.76	0.194	-2.36	0.0406
160732_at	1434507_at	Npepl1	aminopeptidase-like 1	-1.18	0.38	1.18	0.1381	1.16	0.1704	1.17	0.1376	1.16	0.372
160733_at	1451030_at	Akr1c21	aldo-keto reductase family 1, member C21	-1.31	0.68	1.14	0.7536	1.22	0.5838	-1.99	0.1246	1.27	0.6019
160734_at	1422593_at	Ap3s1	adaptor-related protein complex 3, sigma 1 s	-1.18	0.59	1.04	0.5376	1.05	0.5024	-1.09	0.1728	-1.31	0.083
160735_at	1452096_s_at	D230025D16Rik	RIKEN cDNA D230025D16 gene	-1.03	0.83	-1.15	0.0218	1.1	0.3138	-1.15	0.0505	1.09	0.2105
160736_at	1430805_s_at	4932432N11Rik	RIKEN cDNA 4932432N11 gene	1.22	0.64	1.14	0.4633	1.16	0.3688	1.2	0.1604	1.07	0.7915
160737_at	1420013_s_at	Lss	lanosterol synthase	1.25	0.33	-1.43	0.0836	-1.21	0.3939	-1.75	0.0187	-1.29	0.2222
160738_at	1452835_a_at	Polrmt	polymerase (RNA) mitochondrial (DNA direc	1.52	0.04	1.03	0.498	1.2	0.0055	1.09	0.1635	-1.3	0.3365
160739_at	1436746_at	Prkwnk1	protein kinase, lysine deficient 1	-1.08	0.83	1.02	0.8644	-1.41	0.1097	-1.64	0.0145	-1.49	0.3673
160740_at	1454921_at	Gm561	gene model 561, (NCBI)	1.72	0.08	1.01	0.9186	1.15	0.2719	-1.08	0.4808	-1.13	0.6896
160741_at	1439047_s_at	Recql	RecQ protein-like	1.51	0.17	-1.16	0.4839	-1.09	0.6219	1.03	0.8152	-1	0.9926
160742_at	1415901_at	Plod3	procollagen-lysine, 2-oxoglutarate 5-dioxyge	-1.29	0.28	-1.21	0.0106	-1.13	0.1456	-1.29	0.0089	-1.25	0.1892

160743_at	1452743_at	Pole3	polymerase (DNA directed), epsilon 3 (p17 s	-1.22	0.17	1.11	0.1411	-1.04	0.4838	1.02	0.573	1.1	0.4453
160744_r_at	1431763_a_at	Ctrl	chymotrypsin-like	1.08	0.76	-1.14	0.2284	1.09	0.6616	-1.06	0.5755	1.05	0.7014
160745_at	1426783_at	Gcn5l2	GCN5 general control of amino acid synthes	-2.39	0.29	-1.38	0.3284	1.06	0.8646	-1.62	0.1653	2.19	0.0423
160746_at	1450994_at	Rock1	Rho-associated coiled-coil forming kinase 1	1.7	0.12	1.31	0.1895	1.13	0.5054	1.38	0.0513	1.12	0.6842
160747_at	1425296_a_at	Rgs3	regulator of G-protein signaling 3	-1.41	0.02	-1.63	0	-1.29	0.0053	-1.53	0.0002	-1.23	0.1804
160748_at	1448431_at	Asb6	ankyrin repeat and SOCS box-containing pr	-1.09	0.85	1.15	0.3442	1.17	0.2109	1.05	0.5569	-1.02	0.9104
160749_at	1424192_at	1500011H22Rik	RIKEN cDNA 1500011H22 gene	-1.14	0.52	-1.15	0.3096	-1.04	0.7595	-1.04	0.7341	-1.11	0.738
160750_at	1452062_at	Prpsap2	phosphoribosyl pyrophosphate synthetase-a	-1.03	0.93	-1.08	0.3565	-1.14	0.2223	-1.09	0.3083	-1.25	0.4108
160752_at	1428312_at	2810002D13Rik	RIKEN cDNA 2810002D13 gene	-1.38	0.21	-1.05	0.7755	1.09	0.5783	1.17	0.2183	1.34	0.2339
160753_at	1427115_at	Myh3	myosin, heavy polypeptide 3, skeletal muscl	-2.46	0.35	1.21	0.5136	1.67	0.1354	1.91	0.1875	1.79	0.3168
160754_at	1448602_at	Pygm	muscle glycogen phosphorylase	-1.78	0.33	-3.38	0.282	-3.12	0.2984	-4.5	0.2353	1.38	0.5796
160756_at	1417744_a_at	Ralb	v-ral simian leukemia viral oncogene homolo	1.06	0.78	-1.08	0.2365	-1.19	0.0017	-1.09	0.2032	1.36	0.0735
160757_at	1428101_at	Rnf38	ring finger protein 38	1.37	0.12	1.08	0.4973	1.02	0.8984	1.08	0.51	1.2	0.4874
160758_at	1424200_s_at	Seh1l	SEH1-like (S. cerevisiae	1.4	0.26	1.06	0.4052	1.09	0.2904	1.08	0.3147	1.16	0.4373
160759_at	1417503_at	Rfc2	replication factor C (activator 1) 2	1.31	0.14	1.02	0.8105	-1.06	0.5188	-1.12	0.093	-1.2	0.1113
160761_at	1417626_at	Usmg4	upregulated during skeletal muscle growth 4	-1.1	0.3	-1.13	0.1259	1.1	0.1666	-1.06	0.3007	-1	0.9759
160762_at	1433477_at	Abr	active BCR-related gene	-2.95	0.12	1.35	0.5382	-1.71	0.0988	1.42	0.5931	-1.28	0.6888
160764_at	1437468_x_at	Fbxw11	F-box and WD-40 domain protein 11	1.34	0.31	1.09	0.1146	1.06	0.4094	1.15	0.0317	-1.03	0.7801
160765_at	1417591_at	Ptges2	prostaglandin E synthase 2	1.24	0.3	1.12	0.1584	-1.04	0.6287	1.08	0.2261	-1.05	0.7324
160767_at	1417696_at	Soat1	sterol O-acyltransferase 1	1.04	0.92	1.51	0.1505	1.14	0.663	1.27	0.4199	-1.34	0.1899
160768_at	1423879_at	D030056L22	hypothetical protein D030056L22	1.29	0.3	-1.05	0.7075	1.07	0.559	1.04	0.7624	1.32	0.1832
160769_at	1448438_at	Derl2	Der1-like domain family, member 2	-1.04	0.87	-1.39	0.007	-1.24	0.137	-1.59	0.0012	-2.83	0.0003
160770_at	1448663_s_at	Mvd	mevalonate (diphospho) decarboxylase	1.12	0.86	-1.87	0.0019	-1.27	0.2481	-2.21	0.0001	-2.17	0.1306
160772_i_at	1451727_at	D11Ertd730e	DNA segment, Chr 11, ERATO Doi 730, exp	1.17	0.4	1.22	0.0103	1.31	0.0333	1.21	0.0089	1.02	0.8516
160773_at	1417321_at	Zcchc7	zinc finger, CCHC domain containing 7	1.56	0.25	1.34	0.0037	1.24	0.1038	1.63	0.0007	1.58	0.0156
160774_at	1450939_at	Entpd1	ectonucleoside triphosphate diphosphohydro	1.09	0.85	1.17	0.6778	-1	0.9967	1.41	0.4514	2.27	0.1283
160775_at	1448548_at	Tulp4	tubby like protein 4	1.07	0.63	-1.06	0.4088	1.15	0.1199	1.04	0.6098	-1.12	0.5405
160776_at	1434133_s_at	---	---	1.03	0.82	-1.08	0.2303	1.02	0.6738	1.06	0.2945	1.04	0.6396
160777_at	1433460_at	Ttc7b	tetratricopeptide repeat domain 7B	1.46	0.35	-1.32	0.0031	-1.12	0.2378	-1.56	0.0002	-1.2	0.2793
160779_at	1423881_at	D19Ertd703e	DNA segment, Chr 19, ERATO Doi 703, exp	-1.16	0.18	-1.07	0.1485	-1.05	0.5001	-1.12	0.0195	1.02	0.7592
160780_at	1450117_at	Tcf3	transcription factor 3	-1.51	0.02	-1.06	0.5276	-1.12	0.173	-1.16	0.1137	-1.01	0.9487
160782_at	1454964_at	---	---	-1.25	0.3	-1.28	0.0194	-1.16	0.0154	-1.33	0.0008	-1.2	0.1417
160783_at	1426780_at	D14Ertd436e	DNA segment, Chr 14, ERATO Doi 436, exp	1.38	0.15	1.29	0.039	1.3	0.006	1.44	0.0169	1.49	0.0169
160784_r_at	1453314_x_at	2610039C10Rik	RIKEN cDNA 2610039C10 gene	1.14	0.31	1.06	0.6697	1.09	0.5366	1.17	0.3338	1.78	0.2188
160785_at	1428369_s_at	Arhgap21	Rho GTPase activating protein 21	-1.06	0.62	1.04	0.5457	1.21	0.0409	1.21	0.0021	1.42	0.0775
160787_at	1423656_x_at	1500010J02Rik	RIKEN cDNA 1500010J02 gene	1.2	0.69	-1.01	0.9649	1.04	0.8455	1.08	0.7077	1.02	0.8777
160788_at	1416172_at	Pes1	pescadillo homolog 1, containing BRCT dom	1.27	0.52	1.06	0.5715	1.19	0.1125	1.17	0.0737	1.2	0.3085
160789_at	1423695_at	9530090G24Rik	RIKEN cDNA 9530090G24 gene	-1.61	0.12	-1.23	0.0001	-1.18	0.0907	-1.23	0.0034	-1.16	0.2428
160791_at	1424802_a_at	3300001P08Rik	RIKEN cDNA 3300001P08 gene	1.49	0.25	1.29	0.0662	1.25	0.1917	1.58	0.0005	1.46	0.0703
160792_at	1415756_a_at	Snapap	SNAP-associated protein	-1.01	0.98	1.03	0.834	-1.17	0.2531	1.01	0.944	-1.05	0.8177
160793_at	1452844_at	Pou6f1	POU domain, class 6, transcription factor 1	1.22	0.54	1.33	0.0074	1.38	0.0651	1.36	0.0126	1.57	0.1269
160794_at	1424760_a_at	Smyd2	SET and MYND domain containing 2	-2.23	0.34	-1.46	0.3396	1.27	0.442	-2.15	0.0654	-1.32	0.3927
160795_at	1426775_s_at	Scamp1	secretory carrier membrane protein 1	1.15	0.6	1.58	0.0008	1.8	0.0019	2.06	0	1.27	0.2974
160797_r_at	1433718_a_at	E430007M08Ri	RIKEN cDNA E430007M08 gene	3.78	0.02	1.19	0.4999	-1.35	0.4119	-1.09	0.8128	-1.03	0.8677
160798_at	1417098_s_at	Nrbf1	nuclear receptor binding factor 1	1.31	0.07	-1.01	0.8131	-1	0.9669	-1.01	0.868	-1.23	0.0793
160799_at	1455869_at	Camk2b	Calcium/calmodulin-dependent protein kinas	3.71	0.35	1.4	0.2607	1.07	0.8198	2.11	0.0012	1.27	0.4969
160800_at	1415712_at	Zranb1	zinc finger, RAN-binding domain containing	-1.24	0.21	1.04	0.7562	-1.06	0.7087	1.02	0.907	1.47	0.07
160801_at	1430125_s_at	Pqlc1	PQ loop repeat containing 1	-1.31	0.15	-1.04	0.4535	-1.04	0.5614	-1.21	0.0001	-1.18	0.1814
160802_at	1423703_at	Ppan	peter pan homolog (Drosophila)	1.25	0.65	-1.22	0.0685	1.09	0.4947	-1.08	0.5778	-1.52	0.2777
160805_s_at	1416104_at	Mpdu1	mannose-P-dolichol utilization defect 1	1.25	0.36	-1.07	0.2085	-1.21	0.0766	-1.07	0.2511	-1.04	0.7359
160807_at	1450504_a_at	Agpat3	1-acylglycerol-3-phosphate O-acyltransferas	1.29	0.16	1	0.9417	1.12	0.1027	1.07	0.4565	-1.12	0.3467
160808_at	1424119_at	Prkab1	protein kinase, AMP-activated, beta 1 non-ca	1.36	0.25	1.06	0.2294	1.01	0.8973	-1.03	0.3744	1.17	0.3466
160809_at	1423047_at	Tollip	toll interacting protein	1.2	0.41	-1.04	0.5873	1.09	0.1785	-1.06	0.428	-1.16	0.2969

160811_at	1433554_at	AU022870	expressed sequence AU022870	2.01	0.04	1.21	0.0607	1.05	0.6745	1.33	0.011	-1.38	0.051
160812_at	1428141_at	Gga2	golgi associated, gamma adaptin ear contai	-1.05	0.9	-1.51	0.0053	-1.21	0.0586	-1.62	0.0012	1.1	0.7779
160814_at	1424052_at	Thap4	THAP domain containing 4	1.24	0.28	1.08	0.3334	1.05	0.3846	1.15	0.0056	1.75	0.0008
160815_at	1456014_s_at	BC032204	cDNA sequence BC032204	1.52	0.05	1.15	0.4549	-1.1	0.3647	1.29	0.2986	1.08	0.7375
160816_at	1450728_at	Fjx1	four jointed box 1 (Drosophila)	1.49	0.05	1.07	0.582	1.64	0.1859	1.24	0.1206	1.18	0.5825
160817_at	1451546_s_at	Tmem40	transmembrane protein 40	1.64	0.15	1.45	0.0552	1.16	0.2743	1.26	0.0844	-1.1	0.8491
160818_at	1436510_a_at	Lrrfip2	leucine rich repeat (in FLII) interacting protei	1.06	0.68	1.85	0.0011	1.84	0.0003	1.64	0.0024	1.31	0.5301
160819_at	1436188_a_at	---	---	-1.41	0.21	1.01	0.9663	1.35	0.3876	-1.58	0.1694	1.16	0.6759
160820_at	1460675_at	Igsf8	immunoglobulin superfamily, member 8	-1.13	0.68	-1.09	0.2899	1.04	0.612	1.08	0.3023	1.37	0.0114
160821_r_at	1424346_at	Ppp6c	protein phosphatase 6, catalytic subunit	1.37	0.23	1.03	0.9059	1.08	0.6196	1.07	0.7198	1.25	0.2941
160822_at	1428443_a_at	Rap1ga1	Rap1, GTPase-activating protein 1	1.25	0.56	1.02	0.8609	-1.01	0.8918	1.07	0.5282	1.36	0.1525
160823_at	1450899_at	Nedd1	neural precursor cell expressed, developme	1.09	0.68	-1.06	0.4065	1.05	0.6742	1.06	0.2274	-1.03	0.7913
160824_at	1428657_at	1110037N09Rik	RIKEN cDNA 1110037N09 gene	1.73	0.18	-1.03	0.7686	-1.01	0.9405	-1.12	0.2768	1.07	0.6266
160825_at	1433550_at	Chfr	checkpoint with forkhead and ring finger dom	1.1	0.74	1.06	0.2401	1.09	0.1239	1.04	0.417	-1.54	0.1788
160826_at	1416412_at	Nsmaf	neutral sphingomyelinase (N-SMase) activat	1.32	0.41	1.36	0.0202	1.28	0.0615	1.43	0.0013	2.53	0.0276
160828_at	1426858_at	Inhbb	inhibin beta-B	2.27	0.38	1.21	0.0909	1.1	0.3943	1.16	0.2194	1.19	0.55
160829_at	1418835_at	Phlda1	pleckstrin homology-like domain, family A, m	-1.91	0.28	-2.66	0	-2.34	0.0001	-3.67	0	-4.07	0.0448
160830_at	1417524_at	Cnih2	cornichon homolog 2 (Drosophila)	1.33	0.54	1.06	0.6214	1.18	0.1076	1.09	0.3136	1.15	0.4036
160831_at	1448836_s_at	AI838661	expressed sequence AI838661	2.2	0	1.05	0.6882	1.12	0.2638	1.08	0.4913	1.97	0.0774
160832_at	1421821_at	Ldlr	Low density lipoprotein receptor	1.08	0.13	-1.05	0.4001	-1.08	0.3748	-1.13	0.1548	-1.83	0.0006
160833_at	1417165_at	Mbd2	methyl-CpG binding domain protein 2	1.1	0.49	1.12	0.1281	1.19	0.3851	1.22	0.0453	-1.01	0.9547
160834_at	1417406_at	Sertad1	SERTA domain containing 1	1.93	0.08	1.14	0.4209	1.19	0.4035	1.17	0.3178	-1.41	0.5366
160835_i_at	1431359_a_at	1110007C09Rik	RIKEN cDNA 1110007C09 gene	1.72	0.24	1.14	0.3986	1.29	0.1658	1.22	0.1728	1.49	0.447
160836_at	1420824_at	Sema4d	sema domain, immunoglobulin domain (Ig),	-1.03	0.94	1.61	0.3183	-1.35	0.203	2	0.218	2.23	0.0225
160839_at	1416639_at	Slc2a5	solute carrier family 2 (facilitated glucose tra	-1.04	0.86	1.86	0.1138	-1.23	0.6589	1.67	0.26	-1.18	0.726
160840_at	1424250_a_at	Arhgef3	Rho guanine nucleotide exchange factor (GI	-1.01	0.99	1.49	0.0086	1.26	0.3664	1.56	0.0182	1.26	0.5833
160841_at	1438211_s_at	Dbp	D site albumin promoter binding protein	8.19	0.03	1.55	0.0059	1.59	0.0013	1.59	0.0199	1.27	0.4362
160842_at	1427317_at	Kin	antigenic determinant of rec-A protein	8.49	0.02	1.37	0.0136	1.32	0.1642	1.29	0.2348	1.49	0.2008
160843_at	1460164_at	Spin	spindlin	2.23	0.1	-1.32	0.2151	-1.39	0.1437	-1.58	0.0301	-2.66	0.0566
160844_at	1450660_at	Pts	6-pyruvoyl-tetrahydropterin synthase	1.54	0.08	1.08	0.264	-1.06	0.5244	-1.07	0.3167	-1.13	0.2599
160845_at	1424971_at	2600001J17Rik	RIKEN cDNA 2600001J17 gene	-2.58	0.28	-1.15	0.3267	-1.04	0.6071	-1.01	0.944	-1.2	0.7135
160846_at	1423596_at	Nek6	NIMA (never in mitosis gene a)-related expr	1.02	0.92	-1.13	0.1121	-1.1	0.0954	-1.15	0.0275	-1.44	0.0283
160847_at	1425562_s_at	Trnt1	tRNA nucleotidyl transferase, CCA-adding, 1	1.09	0.67	-1.07	0.5106	1	0.9948	1.01	0.9206	-1	0.9807
160848_at	1448875_at	Zhx1	zinc fingers and homeoboxes protein 1	1.07	0.83	1.01	0.866	1.12	0.2288	-1.05	0.3071	-1.08	0.5449
160849_at	1415691_at	Dlgh1	discs, large homolog 1 (Drosophila)	-1.11	0.34	1.23	0.0429	1.15	0.1555	1.13	0.0712	1.61	0.0002
160850_at	1460673_at	Fpgs	folylpolyglutamyl synthetase	-1.27	0.43	-1.26	0.0002	-1.58	0.0002	-1.3	0.0002	-1.89	0.0376
160851_r_at	1449218_at	Cox8b	cytochrome c oxidase, subunit VIIIb	-1.01	0.97	-1.07	0.5265	-1.14	0.2286	1.03	0.7107	1.09	0.6233
160853_at	1434587_x_at	Ptdss2	phosphatidylserine synthase 2	1.93	0.02	-1.05	0.7916	1.13	0.3582	1.21	0.1712	1.08	0.856
160855_at	1434345_at	AI649392	expressed sequence AI649392	-2.89	0.24	1.4	0.3485	1.43	0.3627	1.39	0.2787	1.02	0.9056
160856_at	1424538_at	Ubl4	ubiquitin-like 4	1.54	0.06	1.01	0.8139	1.01	0.8949	-1.07	0.3987	-1.12	0.6172
160857_at	1419639_at	---	---	3.48	0.01	2.23	0.0502	2.12	0.0478	2.36	0.078	1.2	0.3462
160858_at	1427921_s_at	2310061C15Rik	RIKEN cDNA 2310061C15 gene	1.04	0.85	-1.11	0.1976	-1.12	0.2285	-1.21	0.0471	1.6	0.0122
160860_at	1424007_at	Gdf10	growth differentiation factor 10	1.41	0.29	1.21	0.2268	1.06	0.8127	1.12	0.3818	3.51	0.0122
160861_s_at	1449298_a_at	Pde1a	phosphodiesterase 1A, calmodulin-depende	-1.72	0.21	-1.12	0.4393	-1.36	0.0268	-1.17	0.1229	1.41	0.3796
160862_at	1418181_at	Ptp4a3	protein tyrosine phosphatase 4a3	2.01	0.31	1.02	0.9213	1.11	0.6024	1.09	0.5034	1.3	0.63
160863_at	1417276_at	Tulp2	tubby-like protein 2	-2.57	0.14	-1.05	0.7697	-1.41	0.0392	-1.33	0.1726	-1.21	0.2872
160864_at	1425601_a_at	Rtkn	rhotekin	1.19	0.47	-1.08	0.4462	-1.08	0.4206	-1.04	0.6695	1.15	0.6719
160865_at	1451156_s_at	Vldlr	very low density lipoprotein receptor	-1.65	0.21	1.26	0.0759	-1.2	0.2459	1.17	0.2615	1.69	0.0327
160867_at	1417148_at	Pdgfrb	platelet derived growth factor receptor, beta	-1.44	0.1	1.11	0.41	-1.1	0.5725	1.33	0.0475	-2.29	0.1045
160868_at	1422583_at	Rab3b	RAB3B, member RAS oncogene family	1.19	0.35	-1.05	0.6924	1.01	0.8972	1.11	0.2277	1.27	0.2695
160869_at	1417892_a_at	Sirt3	sirtuin 3 (silent mating type information regul	1.84	0.04	1.19	0.2044	1.2	0.0711	1.29	0.0457	1.35	0.0135
160870_at	1436929_x_at	Adcy3	adenylate cyclase 3	1.35	0.67	1.22	0.1645	1.21	0.2332	1.15	0.3855	1.57	0.0609
160871_at	1451762_a_at	Kif1b	kinesin family member 1B	1.82	0.28	1.19	0.3195	1.33	0.1418	1.15	0.4496	1.05	0.8072

160876_at	1417077_at	Bcap29	B-cell receptor-associated protein 29	1.61	0.06	-1	0.961	-1.07	0.668	1.13	0.189	-1.07	0.6687
160877_at	1419663_at	Ogn	osteoglycin	-1.12	0.86	-1.31	0.6131	-1.27	0.63	-1.02	0.9709	2.05	0.2253
160878_at	1423264_at	Bop1	block of proliferation 1	-1.11	0.74	1.02	0.8038	-1.01	0.9607	-1.02	0.7807	-2.03	0.032
160880_at	1425975_a_at	Mapk8ip3	mitogen-activated protein kinase 8 interactin	-1.06	0.8	1.31	0.0176	1.25	0.0837	1.33	0.0017	1.39	0.0363
160881_at	1456565_s_at	Map3k12	mitogen activated protein kinase kinase kina	-1.16	0.71	1.45	0.1926	1.01	0.9613	1.15	0.5779	1.15	0.7013
160884_at	1429574_at	Clic3	chloride intracellular channel 3	1.16	0.45	-1.53	0.0745	-1.4	0.144	-1.56	0.0856	1.34	0.31
160885_at	1433519_at	8430423A01Rik	RIKEN cDNA 8430423A01 gene	-1.16	0.62	1.17	0.0015	1.07	0.269	1.23	0.0022	1.14	0.1184
160887_at	1418102_at	Hes1	hairy and enhancer of split 1 (Drosophila)	-1.07	0.79	-1.08	0.304	-1.13	0.1362	-1.21	0.0177	-1.01	0.923
160888_at	1416216_at	Reps1	RalBP1 associated Eps domain containing p	1.64	0.23	1.19	0.0464	1.26	0.0377	1.23	0.0261	2.07	0.1578
160889_at	1451133_s_at	8430437G11Rik	RIKEN cDNA 8430437G11 gene	1.21	0.34	1.21	0.0421	1.19	0.0681	1.21	0.0199	1.34	0.2976
160890_at	1415829_at	Lbr	lamin B receptor	1.04	0.81	1.02	0.7741	1.1	0.2553	1.09	0.1456	1.28	0.0728
160891_at	1437028_at	Sftpb	surfactant associated protein B	-1.55	0.24	1.48	0.1108	1.23	0.2076	1.14	0.4443	1.18	0.2871
160892_at	1416918_at	Dlgh3	discs, large homolog 3 (Drosophila)	1.32	0.44	1.31	0.3877	-1.12	0.6921	1.59	0.183	-2.32	0.2605
160894_at	1423233_at	Cebpd	CCAAT/enhancer binding protein (C/EBP), c	1.88	0.13	-1.1	0.4731	1.14	0.4825	1.49	0.0074	1.38	0.1485
160896_at	1417090_at	Rcn1	reticulocalbin 1	2.54	0.05	1.12	0.2532	1.23	0.1283	1.02	0.8665	-1.15	0.4895
160897_at	1451359_at	BC005662	cDNA sequence BC005662	1.86	0.08	1.25	0.0915	1.13	0.3686	1.3	0.0223	1.21	0.6857
160898_at	1424751_at	Abt1	activator of basal transcription	1.4	0.02	1.06	0.654	1.33	0.0711	1.31	0.0419	1.2	0.4183
160899_at	1460214_at	Pcp4	Purkinje cell protein 4	1.09	0.84	1.3	0.3455	1.02	0.9157	-1.14	0.365	1.21	0.3495
160900_at	1417594_at	Gkap1	G kinase anchoring protein 1	4.14	0.02	1.09	0.2928	1.32	0.0434	1.2	0.0978	2.5	0.0607
160901_at	1423100_at	Fos	FBJ osteosarcoma oncogene	3.34	0.15	2.15	0.0862	-1.29	0.1386	1.28	0.3636	-1.09	0.7888
160902_at	1417412_at	F8a	factor 8-associated gene A	1.21	0.59	-1.13	0.6217	-1.28	0.3055	-1.42	0.141	-1.25	0.2222
160903_at	1426719_at	Apbb2	amyloid beta (A4) precursor protein-binding,	-1.83	0.43	-1.03	0.7733	-1.14	0.3669	-1.05	0.7752	1.12	0.4183
160904_at	1417147_at	B230317C12Rik	RIKEN cDNA B230317C12 gene	1.11	0.72	1.05	0.5288	-1.19	0.0883	-1.1	0.3937	1.32	0.256
160905_s_at	1418100_at	A030009H04Rik	RIKEN cDNA A030009H04 gene	3.75	0.01	1.84	0.05	3.02	0.0561	2.38	0.0228	-1.32	0.329
160907_at	1454622_at	Slc38a5	solute carrier family 38, member 5	-1.17	0.73	-1.18	0.2583	-1.05	0.7273	-1.1	0.3766	1.15	0.3978
160908_r_at	1423672_at	2510042P03Rik	RIKEN cDNA 2510042P03 gene	-1.33	0.22	-1.22	0.0176	-1.15	0.3585	-1.21	0.0075	-1.26	0.3371
160909_at	1449133_at	Sprr1a	small proline-rich protein 1A	1.27	0.63	-1.27	0.2003	1.03	0.858	1.16	0.1269	1.06	0.8424
160910_at	1419634_a_at	Ghrh	growth hormone releasing hormone	1.33	0.5	-1.38	0.3417	-1.24	0.5337	-1.59	0.1593	-1.08	0.8414
160911_at	1421884_at	Sos1	Son of sevenless homolog 1 (Drosophila)	-1	0.99	-1.06	0.6706	1.09	0.4861	-1.11	0.2772	1.15	0.5622
160913_at	1421253_at	Nrap	nebulin-related anchoring protein	1.2	0.77	-1.36	0.0584	-1.18	0.1694	-1.37	0.05	1.37	0.4018
160914_at	1449191_at	Wfdc12	WAP four-disulfide core domain 12	-1.05	0.64	1.01	0.9224	-1.01	0.9291	-1.03	0.6569	1.02	0.9465
160915_at	1449435_at	B4galt3	UDP-Gal:betaGlcNAc beta 1,4-galactosyltra	-1.35	0.19	1	0.9033	1	0.9615	-1.05	0.1909	1.4	0.0276
160916_at	1417877_at	2310005P05Rik	RIKEN cDNA 2310005P05 gene	6.03	0.02	1.56	0.0281	1.27	0.2083	1.5	0.0345	-1.89	0.0431
160917_r_at	1422498_at	Mageh1	melanoma antigen, family H, 1	1.6	0.4	1.3	0.4471	1.44	0.2706	1.12	0.6754	-1.02	0.9801
160918_at	1448964_at	S100g	S100 calcium binding protein G	4.39	0.18	1.07	0.8184	1.91	0.3764	1.77	0.0019	1.95	0.141
160920_at	1423572_at	Bcl2l2	Bcl2-like 2	-1.04	0.88	1.02	0.7385	-1.01	0.938	-1	0.9969	1.52	0.0531
160921_at	1416617_at	Acas2l	acetyl-Coenzyme A synthetase 2 (AMP form	2.12	0.02	-1.35	0.2423	-1.15	0.6304	1.09	0.5669	1.75	0.1684
160923_at	1424600_at	Abp1	amiloride binding protein 1 (amine oxidase, (	-1.72	0.03	1.01	0.9378	1.01	0.8873	-1.12	0.102	1.03	0.9025
160924_at	1436896_at	D14Ertd231e	DNA segment, Chr 14, ERATO Doi 231, exp	3.28	0.12	1.49	0.0829	1.29	0.039	1.32	0.01	1.14	0.8199
160925_at	1422687_at	---	---	1.1	0.52	-1.04	0.584	-1.13	0.2326	1	0.977	1.14	0.5656
160927_at	1427034_at	Ace	angiotensin converting enzyme	-1.62	0.11	1.67	0.0754	1.68	0.143	2.03	0.0029	1.64	0.2922
160928_at	1448040_at	Tfpt	TCF3 (E2A) fusion partner	-1.58	0.23	-1.06	0.8043	1.18	0.4914	1.02	0.9371	2.05	0.0823
160929_at	1423099_a_at	Mettl3	methyltransferase-like 3	-1.21	0.32	1.54	0.0964	1.39	0.3271	1.23	0.5711	1.73	0.2751
160930_at	1416500_at	Sacm1l	SAC1 (supressor of actin mutations 1, homo	1.09	0.7	1.14	0.2088	-1.01	0.9091	1.02	0.8097	-1.41	0.0115
160931_at	1428086_at	Dnm1l	dynamin 1-like	1.28	0.24	1.14	0.0386	1.08	0.4807	1.22	0.0005	-1.01	0.9579
160933_at	1417141_at	Igtp	interferon gamma induced GTPase	-1.26	0.2	1.52	0.4916	-2.6	0.0194	1.28	0.6498	-1.75	0.1624
160937_at	1416776_at	Crym	crystallin, mu	1.86	0.08	1.71	0.0154	2.07	0.0119	2.06	0.0511	3.03	0.2249
160938_at	1417324_at	Mast2	microtubule associated serine/threonine kina	2.55	0.05	2.09	0.0432	1.39	0.39	2.75	0.0013	1.2	0.5599
160939_at	1448925_at	Twist2	twist homolog 2 (Drosophila)	-2.86	0.32	1.65	0.0535	1.13	0.6375	1.58	0.3172	1.26	0.4665
160941_at	1418406_at	Pde8a	phosphodiesterase 8A	-1.07	0.61	1.02	0.8434	1.22	0.0783	-1.04	0.5279	1.35	0.1032
160943_at	1460229_at	Stag3	stromal antigen 3	-1.07	0.79	-1.08	0.4698	-1.2	0.1128	-1.06	0.5614	1.28	0.548
160944_at	1418831_at	Pkp3	plakophilin 3	-1.36	0.38	1.26	0.0198	1.26	0.1407	1.22	0.0137	-1.02	0.9184
160945_at	1437786_at	C80008	expressed sequence C80008	-1.96	0.15	-1.23	0.649	-1.73	0.1794	-1.82	0.1363	1.43	0.5691

160946_at	1425805_a_at	Usp12	ubiquitin specific protease 12	1.19	0.79	-1.01	0.9738	-1.45	0.216	-1.07	0.8395	1.12	0.769
160947_at	1451282_at	Centd3	centaurin, delta 3	1.05	0.63	1.11	0.3368	1.11	0.0684	1.04	0.6761	1.12	0.295
160948_at	1420743_a_at	Ppp3cc	protein phosphatase 3, catalytic subunit, gar	2.4	0.02	-1.02	0.9401	-1.23	0.3668	1.37	0.0433	-1.96	0.1095
160949_at	1448725_at	Parg	poly (ADP-ribose) glycohydrolase	1.02	0.92	-1.01	0.9139	-1.34	0.1193	-1.22	0.2424	-1.37	0.0738
160951_at	1421092_at	Serpina12	serine (or cysteine) proteinase inhibitor, clad	-4.17	0.22	-1.14	0.4631	-1.08	0.6225	1.3	0.3151	-61.54	0.0001
160952_r_at	1420660_at	Lrrc6	leucine rich repeat containing 6 (testis)	-1.99	0.24	1.62	0.13	1.95	0.0488	-1.03	0.9486	2.58	0.0237
160955_at	1417075_at	2010309E21Rik	RIKEN cDNA 2010309E21 gene	1.47	0.15	-1.12	0.0758	-1.12	0.2237	-1.34	0.0017	-1.01	0.9339
160957_at	1460571_at	Dicer1	Dicer1, Dcr-1 homolog (Drosophila)	1.9	0.08	-1.01	0.9732	1.38	0.2439	1.22	0.2754	-1.23	0.7205
160958_at	1424075_at	9430016H08Rik	RIKEN cDNA 9430016H08 gene	1.68	0.06	1.32	0.0045	1.27	0.1591	1.29	0.0214	-1.65	0.0953
160961_at	1434261_at	Sipa1l2	signal-induced proliferation-associated 1 like	2.27	0.05	1.14	0.2809	1.66	0	1.37	0.0075	1.08	0.7681
160964_at	1452234_s_at	D16Bwg1494e	DNA segment, Chr 16, Brigham & Women's	-1.36	0.23	-1.04	0.3738	-1.07	0.0731	-1.08	0.1484	1.22	0.2148
160966_at	1457285_at	Zfp187	zinc finger protein 187	1.52	0.31	1.39	0.0219	1.36	0.0195	1.64	0.0001	1.53	0.0476
160968_at	1434528_at	Aard	alanine and arginine rich domain containing	1.29	0.36	-1.18	0.4529	1.16	0.2848	-1.15	0.4492	1.09	0.843
160969_at	1451752_at	Foxk1	forkhead box K1	1.87	0.04	1.05	0.7573	1.19	0.2996	1.16	0.348	-1.18	0.7297
160971_at	1436528_at	Kazald1	Kazal-type serine protease inhibitor domain	-1.26	0.18	-1.29	0.0118	-1.18	0.1256	1.04	0.7824	1.09	0.7535
160972_at	1418423_s_at	Serpinb9f /// Ser	serine (or cysteine) proteinase inhibitor, clad	-2.74	0.05	1.39	0.305	1.45	0.2886	-1.23	0.5378	-1.14	0.6457
160973_at	1449699_s_at	C330027C09Rik	RIKEN cDNA C330027C09 gene	-1.26	0.56	1.23	0.4764	-1.16	0.5607	1.3	0.3175	1.73	0.3176
160975_at	1435504_at	Rsnl2	Restin-like 2	-4.82	0.41	-2.25	0.079	-1.18	0.7322	-2.11	0.0959	-1.1	0.8947
160976_at	1454979_at	Diap1	diaphanous homolog 1 (Drosophila)	-1.01	0.92	1.14	0.0443	1.11	0.1766	-1	0.9515	-1.21	0.3459
160977_at	1452304_a_at	Arhgef5	Rho guanine nucleotide exchange factor (GI	1.59	0.45	-1.4	0.2077	1.16	0.4572	1.13	0.5198	-1.51	0.3497
160978_at	1451481_s_at	D630035O19Rik	RIKEN cDNA D630035O19 gene	-1.14	0.64	-1.08	0.4913	1.09	0.4151	-1.08	0.4372	-1.08	0.7333
160979_at	1434705_at	Ctbp2	C-terminal binding protein 2	1.83	0.1	1.1	0.5482	1	0.9988	1.07	0.6883	1.42	0.0972
160980_at	1428810_at	2700097O09Rik	RIKEN cDNA 2700097O09 gene	2.37	0.04	1.03	0.7925	-1.34	0.2033	1.2	0.1513	2.77	0.0431
160981_at	1450666_s_at	Sca10	spinocerebellar ataxia 10 homolog (human)	1.05	0.87	-1.19	0.0794	-1.01	0.939	-1.08	0.2728	-1.65	0.0222
160982_at	1428762_at	4921526G09Rik	RIKEN cDNA 4921526G09 gene	1.37	0.13	1.07	0.4731	1.09	0.5596	1.17	0.1062	1.25	0.1258
160983_at	1457964_at	1810044D09Rik	RIKEN cDNA 1810044D09 gene	1.13	0.58	-1.1	0.2542	-1.17	0.0627	-1.15	0.0167	1.36	0.229
160985_at	1453265_at	4930579C15Rik	RIKEN cDNA 4930579C15 gene	-2.14	0.35	-1.35	0.4599	-1.25	0.5524	-1.51	0.372	1.48	0.1179
160987_r_at	1423440_at	1110001A07Rik	RIKEN cDNA 1110001A07 gene	-2.19	0.36	-1.08	0.8524	1.84	0.0663	1.03	0.902	1.68	0.1432
160989_r_at	1435184_at	B430320C24Rik	RIKEN cDNA B430320C24 gene	1.67	0.3	1.11	0.51	1.14	0.5147	-1.13	0.3705	1.23	0.5259
160990_r_at	1424243_at	BC016198	cDNA sequence BC016198	1.46	0.27	1.13	0.4589	1.2	0.3643	1.32	0.1157	-1.58	0.0777
160991_at	1428503_a_at	Nkiras1	NFKB inhibitor interacting Ras-like protein 1	1.14	0.37	-1.07	0.5667	1.17	0.1179	1.13	0.1448	-1.19	0.0617
160992_at	1426928_at	4633402D15Rik	RIKEN cDNA 4633402D15 gene	1.6	0.46	1.03	0.929	-1.23	0.5607	1.21	0.5746	1.29	0.5756
160993_at	1459894_at	A630053O10	Hypothetical protein A630053O10	1.07	0.92	-1.24	0.1698	-1.77	0.0151	-1.81	0.0088	1.18	0.3849
160994_at	1433756_at	4930429A08Rik	RIKEN cDNA 4930429A08 gene	-1.14	0.64	1.04	0.7926	1.16	0.2847	1.41	0.0003	1.71	0.0247
160995_at	1434044_at	Repin1	replication initiator 1	-4	0	1.13	0.2637	-1.01	0.9205	1.12	0.0614	1.48	0.0109
160996_at	1418876_at	Foxd1	forkhead box D1	1.24	0.63	1.02	0.925	1.38	0.2211	-1.07	0.7926	1.17	0.6658
160997_at	1434981_at	E130303B06Rik	RIKEN cDNA E130303B06 gene	-1.04	0.86	1.13	0.1773	1.01	0.9038	-1.12	0.3605	-1.01	0.9628
160998_at	1449677_s_at	Tmem38b	transmembrane protein 38B	-1.36	0.06	1.29	0.0074	1.18	0.0078	1.32	0.0013	-1.06	0.4741
161000_i_at	1416309_at	Nusap1	nucleolar and spindle associated protein 1	1.19	0.75	1.18	0.5516	-1.03	0.9242	1.67	0.0854	-1.16	0.4727
161001_at	1455180_at	---	---	-2.51	0.18	1.02	0.847	1.18	0.3293	-1.12	0.3506	1.18	0.4099
161002_at	1434514_at	Rbm15	RNA binding motif protein 15	1.49	0.32	1.02	0.8303	1.08	0.5019	-1.04	0.5737	1.66	0.025
161003_at	1450073_at	Kif3b	kinesin family member 3B	-1.54	0.12	1.32	0.0231	1.12	0.4802	1.19	0.4226	-1.01	0.9817
161004_at	1455692_x_at	1700097N02Rik	RIKEN cDNA 1700097N02 gene	-1.56	0.12	1.11	0.1136	1.01	0.8999	1.02	0.7048	1.2	0.2921
161006_at	1434537_at	Slco3a1	Solute carrier organic anion transporter fami	1.19	0.57	1.11	0.6129	-1.04	0.7987	-1.14	0.4543	1.24	0.5104
161008_at	1427175_at	AI428936	expressed sequence AI428936	1.1	0.45	1.32	0.0711	-1.09	0.3405	1.08	0.4668	1.01	0.978
161009_at	1434436_at	Zcwcc2	zinc finger, CW-type with coiled-coil domain	3.08	0.01	-1.36	0.2844	1.38	0.2021	-1.16	0.4749	2.53	0.1502
161010_r_at	1434045_at	---	---	-1.24	0.54	1.46	0.0762	1.12	0.5877	1.53	0.0094	-1.63	0.268
161011_at	1454884_at	Btbd4	BTB (POZ) domain containing 4	-1.09	0.67	1.18	0.2525	1.22	0.2699	1.09	0.5647	-1.02	0.9734
161012_at	1417640_at	Cd79b	CD79B antigen	-1.13	0.6	1.67	0.0234	1.2	0.404	1.66	0.0085	1.91	0.013
161014_at	1426049_a_at	Terf2ip	telomeric repeat binding factor 2, interacting	1.02	0.9	1.07	0.5095	1.18	0.0688	1.11	0.2697	-1.01	0.9158
161015_at	1433906_at	4933402J24Rik	RIKEN cDNA 4933402J24 gene	-1.11	0.79	-1.27	0.4634	-1.31	0.3717	-1.39	0.3063	1.05	0.924
161016_at	1455915_at	Galnt4	UDP-N-acetyl-alpha-D-galactosamine:polype	-1.01	0.95	1	0.9782	-1.03	0.8495	-1.03	0.7847	-1.13	0.4226
161018_at	1439962_at	2310010J17Rik	RIKEN cDNA 2310010J17 gene	1.36	0.15	1.36	0.0052	1.09	0.4507	1.49	0.0017	1.41	0.0577

161019_at	1435015_at	2210018M03Rik	RIKEN cDNA 2210018M03 gene	1.4	0.01	1.05	0.5558	1.11	0.2403	1.06	0.3613	-1.45	0.3802
161021_at	1417923_at	Pak3	p21 (CDKN1A)-activated kinase 3	-2.27	0.31	-1.47	0.2025	-1.21	0.4783	-1.41	0.266	3.03	0.0036
161023_at	1448681_at	Il15ra	interleukin 15 receptor, alpha chain	-1.31	0.02	1.13	0.2867	-1.03	0.7859	1.08	0.471	1.09	0.7696
161024_at	1434889_at	Plekha7	Pleckstrin homology domain containing, fam	-1.31	0.41	1.17	0.2214	1.15	0.1099	1.01	0.9416	1.54	0.1119
161025_f_at	1449135_at	Sox18	SRY-box containing gene 18	1.13	0.69	1.1	0.3036	1.07	0.3263	-1.06	0.5157	1.34	0.0265
161028_at	1450759_at	Bmp6	bone morphogenetic protein 6	1.73	0.16	1.39	0.2572	2	0.0062	1.89	0.0473	1.45	0.253
161029_at	1426316_at	6330416G13Rik	RIKEN cDNA 6330416G13 gene	-1.68	0.06	-1.01	0.9645	-1.11	0.3119	1.09	0.6244	1.13	0.2474
161030_at	1428983_at	Scx	scleraxis	1.09	0.58	1.18	0.5792	-1.23	0.4853	1.28	0.3482	2.39	0.0343
161031_at	1453175_at	Zfp50	zinc finger protein 50	-1.02	0.97	-1.04	0.9034	1.3	0.3897	1.15	0.6075	-2.47	0.2345
161032_i_at	1419325_at	Nmu	neuromedin U	-1.25	0.61	-1.38	0.3093	-1.47	0.2741	-1.2	0.6002	1.52	0.1243
161033_at	1449208_at	Papolb	poly (A) polymerase beta (testis specific)	-1.06	0.79	1.01	0.9887	1.31	0.4724	-1.42	0.3345	1.36	0.2758
161034_at	1451502_at	Pla2g10	phospholipase A2, group X	-1.23	0.57	-1.19	0.3659	-1.29	0.1075	-1.65	0.0083	1.39	0.095
161036_at	1426712_at	Slc6a15	solute carrier family 6 (neurotransmitter trans	-2.1	0.08	-1.22	0.3165	-1.49	0.0439	-1.13	0.4359	-1.52	0.3478
161038_at	1420637_at	Prps2	phosphoribosyl pyrophosphate synthetase 2	-3.01	0.2	1.17	0.5424	1.28	0.3232	1.41	0.1772	1.79	0.0487
161039_at	1451812_at	Adam22	a disintegrin and metalloprotease domain 22	-1.31	0.24	-1.05	0.7072	-1.21	0.2873	-1.09	0.6329	-1.14	0.5125
161040_at	1422985_at	Fzd1	frizzled homolog 1 (Drosophila)	3.85	0.02	1.08	0.4173	-1.06	0.7359	-1.09	0.3821	-1.33	0.2282
161041_at	1418263_at	Ddx25	DEAD (Asp-Glu-Ala-Asp) box polypeptide 25	-1.37	0.28	-1.11	0.4762	1.02	0.8927	1.06	0.6766	-1.08	0.7772
161042_at	1427912_at	Cbr3	carbonyl reductase 3	2.93	0.4	-1.03	0.9122	-1.14	0.4935	-1.06	0.7883	1.34	0.1558
161044_at	1454985_at	D030051N19Rik	RIKEN cDNA D030051N19 gene	1.51	0.07	1.03	0.7383	-1.04	0.6831	1.05	0.6721	-1.04	0.5239
161045_at	1416053_at	Lrrn1	leucine rich repeat protein 1, neuronal	-1.8	0.24	-1.16	0.7088	1.01	0.9808	-1.39	0.3486	1.01	0.9753
161046_at	1418476_at	Crlf1	cytokine receptor-like factor 1	1.02	0.93	1.47	0.1653	1.63	0.0352	1.09	0.6353	1.43	0.2918
161049_at	1418357_at	Foxg1	forkhead box G1	1.06	0.73	1.04	0.8606	-1.07	0.7519	-1	0.9875	-1.42	0.5554
161050_at	1435981_at	---	10 days neonate cerebellum cDNA, RIKEN f	-1.44	0.07	1.11	0.2587	1.52	0.0037	-1.04	0.6631	1.26	0.4114
161051_at	1423146_at	Hes5	hairy and enhancer of split 5 (Drosophila)	-1.92	0.33	1.46	0.1326	2	0.0446	1.49	0.1642	1.08	0.6568
161054_at	1419672_at	Spock1	sparc/osteonectin, cwcv and kazal-like doma	-1.43	0.33	-1.32	0.3868	1.22	0.472	-1.32	0.2113	-1.23	0.5178
161056_at	1434901_at	---	Gene model 1103, (NCBI)	2.12	0	-1.29	0.3294	1.24	0.3295	-1.1	0.6835	1.24	0.7078
161057_at	1435788_at	2900086B20Rik	RIKEN cDNA 2900086B20 gene	1.35	0.65	-1.1	0.6472	-2.52	0.0029	-2.25	0.012	1.35	0.2453
161059_at	1452142_at	Slc6a1	solute carrier family 6 (neurotransmitter trans	-3.57	0.07	-1.41	0.3123	-1.5	0.2702	-2.31	0.0284	1.36	0.4168
161063_r_at	1436616_at	R74740	expressed sequence R74740	-1.75	0	1.1	0.0718	1.04	0.5573	1.01	0.8642	1.39	0.1573
161065_at	1429035_at	Dpep3	dipeptidase 3	-4.29	0.04	1.62	0.1023	1.9	0.047	-1.03	0.9087	1.32	0.2298
161067_at	1456225_x_at	Trib3	tribbles homolog 3 (Drosophila)	1.82	0.1	1.59	0.0098	1.6	0.0035	2.11	0.0002	1.73	0.0881
161069_at	1438551_at	Neurog1	neurogenin 1	1.4	0.52	-1.09	0.7968	-1.28	0.4493	-1.37	0.3589	1.61	0.255
161070_at	1434403_at	Spred2	sprouty-related, EVH1 domain containing 2	1.22	0.62	1.54	0.0006	1.05	0.7212	1.64	0	1.25	0.2709
161072_at	1429388_at	Nanog	Nanog homeobox	1.05	0.89	-1.18	0.567	-1.14	0.646	-1.49	0.2207	1.02	0.9203
161073_at	1427057_at	C630002B14Rik	RIKEN cDNA C630002B14 gene	2.35	0.06	1.08	0.5068	1.06	0.5752	-1.02	0.7978	1.69	0.091
161074_at	1438011_at	Pcyt1a	phosphate cytidylyltransferase 1, choline, alp	1.47	0.19	1.05	0.5822	1.01	0.8714	1.15	0.0218	1.03	0.6636
161075_at	1456481_at	D9Ertd280e	DNA segment, Chr 9, ERATO Doi 280, expr	-2.03	0.02	-1.09	0.5351	-1.01	0.9391	-1.26	0.048	-1.31	0.4455
161076_at	1423919_at	BC023882	cDNA sequence BC023882	1.37	0.22	1.39	0.0002	1.29	0.0377	1.25	0.0204	1.4	0.359
161077_f_at	1448400_a_at	Smarcd2	SWI/SNF related, matrix associated, actin de	1.03	0.89	-1.3	0.0023	-1.09	0.2222	-1.33	0.0001	-1.41	0.023
161078_at	1439625_at	---	Transcribed locus	-2.04	0.08	-1.19	0.3319	-1.24	0.2828	1.05	0.8226	-1.42	0.4064
161079_at	1422671_s_at	Naalad2	N-acetylated alpha-linked acidic dipeptidase	-1.42	0.39	-1.08	0.8167	1.53	0.052	-1.18	0.5954	2.4	0.1027
161081_at	1434272_at	Cpeb2	cytoplasmic polyadenylation element binding	-1.33	0.33	-1.04	0.5583	-1.2	0.231	-1.19	0.0828	-1.17	0.6576
161082_r_at	1428303_at	1500005I02Rik	RIKEN cDNA 1500005I02 gene	1.52	0.31	-1.26	0.2149	-1.18	0.4772	-1.28	0.3443	1.79	0.0369
161083_at	1455733_at	A130052D22	RIKEN cDNA A430105I05 gene	-1.01	0.99	-1.02	0.8657	-1.06	0.6917	-1.03	0.8479	1.22	0.1568
161084_at	1427104_at	Zfp612	zinc finger protein 612	1.31	0.54	1.24	0.1629	1.08	0.4878	1.34	0.0436	2.18	0.0839
161086_at	1418898_at	Lin7c	lin 7 homolog c (C. elegans)	-1.06	0.9	1.04	0.7858	1.02	0.9348	1.09	0.6003	1.12	0.7716
161092_at	1434859_at	Umps	uridine monophosphate synthetase	-1.47	0.04	-1.04	0.5449	1	0.9577	-1.18	0.018	1.12	0.1177
161097_at	1460480_at	1600014E20Rik	RIKEN cDNA 1600014E20 gene	-1.37	0.53	1.39	0.164	1.44	0.038	-1.18	0.4597	1.05	0.9272
161099_at	1457094_at	---	Transcribed locus	-1.02	0.75	-1.09	0.4898	1.16	0.1751	-1.19	0.1999	1.18	0.6336
161103_at	1435625_at	Entpd7	ectonucleoside triphosphate diphosphohydro	1.27	0.35	1.16	0.6471	1.55	0.2465	-1.13	0.7116	2.33	0.0895
161104_at	1437208_at	4921515A04Rik	RIKEN cDNA 4921515A04 gene	1.62	0.45	1.33	0.0552	1.25	0.1512	1.25	0.0983	1.12	0.2085
161110_at	1420064_s_at	Tktl1	transketolase-like 1	2.7	0.08	1.27	0.4697	1.01	0.9731	-1.51	0.2459	-2.65	0.28
161111_f_at	1434959_at	Dhh	desert hedgehog	-1.36	0.1	-1.01	0.8906	1.01	0.911	-1.18	0.0792	1.17	0.5318

161114_i_at	1453324_at	6330509M23Rik	RIKEN cDNA 6330509M23 gene	1.55	0.21	1.03	0.9233	-1.14	0.62	2.05	0.1418	2.56	0.3315
161117_at	1420512_at	Dkk2	dickkopf homolog 2 (Xenopus laevis)	-2.24	0.27	1.1	0.6807	-1.17	0.4167	-1.04	0.8544	1.26	0.2593
161119_at	1437920_at	Epha5	Eph receptor A5	-1.41	0.11	-1.41	0.071	-1.22	0.2311	-1.36	0.0702	1.35	0.2005
161122_f_at	1447919_x_at	Ndufab1	NADH dehydrogenase (ubiquinone) 1, alpha	1.63	0.07	-1.01	0.9451	1.08	0.4991	1.01	0.9511	-1.31	0.0007
161126_at	1435998_at	---	Gene model 288, (NCBI)	-2.77	0.01	1.6	0.1865	1.68	0.1561	1.57	0.2103	1.48	0.3933
161127_i_at	1447653_x_at	Rpl24 /// LOC27	ribosomal protein L24 /// similar to ribosomal	1.57	0.22	1.12	0.1644	1.04	0.6022	1.15	0.0282	1.38	0.0389
161129_r_at	1423948_at	Bag2	Bcl2-associated athanogene 2	-1.36	0.14	1.15	0.527	-1.16	0.4868	1.03	0.8807	1.1	0.3221
161132_at	1422837_at	Scel	sciellin	2.06	0.41	-2.38	0.0136	-1.12	0.8162	-1.18	0.7312	2.79	0.2205
161136_r_at	1448002_x_at	2610001J05Rik	RIKEN cDNA 2610001J05 gene	-1.23	0.68	1.03	0.9375	1.15	0.6124	1.15	0.5376	1.27	0.5699
161145_f_at	1456580_s_at	Atp5d	ATP synthase, H+ transporting, mitochondria	1.9	0.14	1.09	0.2819	1.06	0.4997	1.14	0.1095	-1.19	0.532
161147_f_at	1452597_at	2310061C15Rik	RIKEN cDNA 2310061C15 gene	1.14	0.11	-1.09	0.0816	-1.01	0.8855	-1.11	0.1241	1.09	0.3301
161351_r_at	1420251_at	---	---	-1.18	0.49	-1.2	0.4466	1.11	0.6813	-1.19	0.4769	2.97	0.3641
161362_at	1420229_at	---	---	1.33	0.51	-1.09	0.6256	-1.1	0.5476	-1.11	0.5116	1.29	0.4986
161450_r_at	1420261_at	Psen1	presenilin 1	-2.4	0.36	-1.1	0.8446	1.05	0.8909	-1.05	0.8788	1.37	0.5818
161493_at	1420224_at	---	---	2.32	0.1	1.12	0.7186	2.02	0.0413	1.37	0.3212	2.12	0.0995
161496_r_at	1420193_at	Krt1-17	keratin complex 1, acidic, gene 17	-3.02	0.08	1.21	0.1336	-1.16	0.3843	1.08	0.5742	2.1	0.0515
161511_f_at	1431591_s_at	G1p2	interferon, alpha-inducible protein	-1.02	0.92	1.09	0.8717	-1.17	0.4617	1.31	0.4723	-1.55	0.3328
161539_f_at	1434425_at	AI597080	expressed sequence AI597080	2.62	0.04	-1.42	0.1614	1.35	0.0904	-1.17	0.5296	1.59	0.4915
161543_at	1449783_at	---	---	3.18	0.04	-1.07	0.688	-1.16	0.4742	1.04	0.8089	1.65	0.1178
161595_at	1420243_at	Zfhx1a	zinc finger homeobox 1a	3.54	0.26	-1.17	0.3539	-1.44	0.0897	-2.05	0.0041	1.62	0.2793
161665_at	1449630_s_at	Mark1	MAP/microtubule affinity-regulating kinase 1	-1.11	0.62	-1.39	0.0886	-1.29	0.1328	-1.21	0.3009	1.77	0.1512
161666_f_at	1449773_s_at	Gadd45b	growth arrest and DNA-damage-inducible 45	2.56	0.22	3.15	0.1887	3.84	0.2414	4.05	0.06	13.24	0.034
161693_r_at	1420246_at	D1Ertd161e	DNA segment, Chr 1, ERATO Doi 161, expr	1.05	0.84	1.09	0.6617	1.2	0.3818	1.23	0.3417	1.9	0.1248
161705_r_at	1420202_at	---	---	-2.47	0.12	-1.05	0.8643	-1.17	0.3736	-1.33	0.1372	1.06	0.8801
161736_r_at	1449771_at	---	---	-2.81	0.13	1.12	0.7067	1.62	0.1229	-1.06	0.8256	1.87	0.031
161765_f_at	1416882_at	Rgs10	regulator of G-protein signalling 10	-1.18	0.05	1.12	0.3237	1.02	0.8763	1.12	0.4013	-1.23	0.6168
161769_r_at	1420235_at	Bphl	biphenyl hydrolase-like (serine hydrolase, br	-1.38	0.13	-1.33	0.0769	-1.36	0.0131	-1.4	0.0084	1.98	0.0812
161822_at	1427222_a_at	Svp2	seminal vesicle protein 2	-1.18	0.84	-1.1	0.6982	-1.44	0.1887	-1.17	0.5505	-1.76	0.3079
161825_f_at	1448573_a_at	Ceacam10	CEA-related cell adhesion molecule 10	1.29	0.18	-1.13	0.3712	-1.04	0.7725	-1.28	0.0861	-1.06	0.6468
161839_f_at	1422656_at	Rasl2-9	RAS-like, family 2, locus 9	2.51	0.01	1.26	0.0067	1.11	0.3443	1.42	0.0015	-1.1	0.652
161857_r_at	1427176_s_at	AI428936	expressed sequence AI428936	-1.05	0.92	-1.01	0.946	1.02	0.9021	1.04	0.7425	1.45	0.3382
161864_f_at	1441866_s_at	Ptdss1	phosphatidylserine synthase 1	1.01	0.92	-1.16	0.0132	-1.11	0.0453	1.04	0.7132	1.38	0.0039
161917_i_at	1449804_at	Pnmt	phenylethanolamine-N-methyltransferase	-1.18	0.47	-1.02	0.9568	1.49	0.1385	-1.09	0.8015	1.58	0.3858
161942_f_at	1439144_at	Cwf19l1	CWF19-like 1, cell cycle control (S. pombe)	-1.02	0.98	-1.78	0.0111	-1.47	0.0466	-2.44	0.0006	1.03	0.9456
161947_f_at	1426039_a_at	Alox12e	arachidonate lipoxygenase, epidermal	1.02	0.9	-1.22	0.0445	-1.04	0.5196	-1.25	0.0133	-1.05	0.7454
162049_f_at	1424150_at	Gdpd5	glycerophosphodiester phosphodiesterase d	1.77	0.11	-1.13	0.1835	-1.12	0.223	-1.17	0.1133	1.78	0.1811
162055_f_at	1433723_s_at	Serf2	Small EDRK-rich factor 2	1.17	0.57	-1.27	0.0465	-1.26	0.1147	-1.21	0.0819	1.08	0.5039
162058_f_at	1418767_at	Cyp4f13	cytochrome P450, family 4, subfamily f, poly	-1.67	0.27	-1.6	0.069	-1.05	0.8219	-1.57	0.117	-1.38	0.603
162061_f_at	1439393_x_at	Ppp2r4	protein phosphatase 2A, regulatory subunit I	1.09	0.92	1.19	0.2137	1.25	0.0674	-1.01	0.9702	1.48	0.1779
162084_i_at	1420240_at	---	---	-1.61	0.5	1.07	0.7334	1.03	0.8804	1.23	0.2218	1.7	0.0935
162099_f_at	1417224_a_at	Cd2bp2	CD2 antigen (cytoplasmic tail) binding protei	1.46	0.31	1.09	0.719	1.17	0.4645	1.04	0.8687	-1.48	0.168
162103_i_at	1420280_x_at	Col9a3	procollagen, type IX, alpha 3	1.34	0.6	-1.05	0.8516	-1.08	0.7542	-1.19	0.4708	-1.04	0.9339
162109_f_at	1455149_at	Sh3md2	SH3 multiple domains 2	-1.05	0.91	1.33	0.4838	1.05	0.9142	1.44	0.2782	1.99	0.2757
162114_f_at	1416826_a_at	Trfp	Trf (TATA binding protein-related factor)-pro	-1.61	0.17	1	0.9767	1.06	0.6895	-1.09	0.3493	-1.1	0.5067
162138_s_at	1424407_s_at	Cbx6	chromobox homolog 6	-1.06	0.87	1.22	0.0422	1.25	0.0202	1.27	0.0564	1.92	0.0572
162151_i_at	1420248_at	Tubg2	tubulin, gamma 2	1.1	0.73	-1.32	0.3497	-1.26	0.4564	-1.46	0.2226	-2.28	0.2682
162161_r_at	1420273_x_at	---	---	-1.25	0.54	-1.34	0.1916	-1.26	0.2023	-1.13	0.5493	1.3	0.5094
162174_at	1419349_a_at	Cyp2d9	cytochrome P450, family 2, subfamily d, poly	-1.3	0.36	1.76	0.0094	1.23	0.3105	1.82	0.0051	1	0.9967
162198_f_at	1420249_s_at	Ccl6	chemokine (C-C motif) ligand 6	1.61	0.46	-1.28	0.1189	-1.02	0.832	-1.35	0.0587	1.27	0.5636
162212_at	1420292_x_at	---	---	-2.94	0.49	-1.49	0.4076	-1.61	0.3739	1.03	0.9595	2.44	0.3629
162229_at	1420303_x_at	---	Transcribed locus, strongly similar to XP_34	-1.16	0.65	1.27	0.0597	1.16	0.105	1.24	0.0208	1.39	0.1414
162278_r_at	1449803_x_at	Tap1	Transporter 1, ATP-binding cassette, sub-fa	-1.33	0.49	-1.07	0.6804	-1.01	0.9506	-1.12	0.5404	1.18	0.3243
162330_f_at	1420270_at	---	---	-1.13	0.55	1.07	0.4964	1.05	0.6511	1.08	0.3214	1.24	0.3458

162453_at	1420189_at	---	---	2.31	0.08	1.08	0.6922	1.46	0.0271	1.34	0.0409	1.41	0.496
92180_at	1436517_at	H1fx	H1 histone family, member X	-1.05	0.9	1.32	0.0226	1.13	0.4794	1.26	0.1132	1.47	0.0805
92181_at	1420428_at	Ager	advanced glycosylation end product-specific	-1.53	0.56	1.63	0.0725	1.09	0.1317	1.36	0.198	1.72	0.12
92182_at	1434131_at	Rufy1	RUN and FYVE domain containing 1	-1.03	0.92	1.22	0.2021	1.31	0.094	1.09	0.601	-1.02	0.8832
92184_at	1427588_a_at	Dtna	dystrobrevin alpha	-1.44	0.28	-1.07	0.4461	-1.17	0.0742	-1.14	0.2491	-1.83	0.4473
92185_at	1436512_at	Arl7	ADP-ribosylation factor-like 7	-1.13	0.75	1.07	0.3614	1.03	0.7514	1.09	0.4706	-1.09	0.6997
92186_at	1420926_at	Arx	aristaless related homeobox gene (Drosophi	-1.23	0.68	1.94	0.032	1.51	0.0798	2	0.0027	1.4	0.4394
92188_s_at	1452410_a_at	Fes	feline sarcoma oncogene	2.65	0.01	1.13	0.4876	1.12	0.4114	1.15	0.5084	1.41	0.4345
92189_at	1449263_at	1810045K17Rik	RIKEN cDNA 1810045K17 gene	1.11	0.51	-1.12	0.088	-1.2	0.0892	-1.29	0.0041	-1.4	0.0134
92190_at	1418605_at	Nr2c1	nuclear receptor subfamily 2, group C, mem	1.09	0.83	-1.35	0.017	-1.04	0.5862	-1.3	0.0187	-1.42	0.1269
92192_s_at	1451406_a_at	Pcsk5	proprotein convertase subtilisin/kexin type 5	3.62	0.17	1.6	0.0657	1.5	0.272	-1.08	0.7474	3.79	0.0655
92194_at	1452475_at	Pcsk5	proprotein convertase subtilisin/kexin type 5	-1.31	0.1	-1.24	0.0107	-1.13	0.0197	-1.26	0.0009	1.29	0.1076
92195_at	1451639_at	Cebpg	CCAAT/enhancer binding protein (C/EBP), g	1.27	0.31	-1.02	0.872	1.26	0.1221	-1.09	0.4455	-1.33	0.2239
92197_r_at	1455546_s_at	Sf3a2	splicing factor 3a, subunit 2	-1.66	0.1	1.17	0.2221	1.07	0.6745	1.25	0.1294	1.59	0.2363
92198_s_at	1419727_at	Daf2	decay accelerating factor 2	1.88	0.11	-1.12	0.6191	-1.04	0.8645	-1.09	0.7468	1.26	0.5645
92199_at	1422102_a_at	Stat5b	signal transducer and activator of transcripti	2.18	0.24	-1.11	0.3107	-1.18	0.2624	-1.22	0.0596	-1.03	0.9258
92200_at	1423427_at	Adcyap1	adenylate cyclase activating polypeptide 1	-1.5	0.57	-1.39	0.2501	-1.22	0.4574	-1.12	0.7124	2	0.3249
92202_g_at	1442025_a_at	---	---	1.07	0.9	1.44	0.2341	3.12	0.0346	3.42	0.0013	3.17	0.0214
92204_at	1451910_a_at	Cd6	CD6 antigen	-1.03	0.83	1.17	0.2258	1.38	0.0874	1.25	0.1627	1.26	0.14
92205_at	1443969_at	LOC384783	similar to Insulin receptor substrate-2 (IRS-2	-2.5	0.01	1.79	0.1602	2.12	0.019	1.65	0.1093	3.3	0.0334
92206_at	1456870_at	A430107D22Rik	RIKEN cDNA A430107D22 gene	2.07	0.18	1.25	0.2947	1.16	0.4648	1.56	0.0344	1.59	0.131
92208_at	1427944_at	C1qdc1	C1q domain containing 1	1.04	0.79	1.53	0.0228	1.49	0.051	1.46	0.0453	1.94	0.012
92209_at	1416504_at	Ulk1	Unc-51 like kinase 1 (C. elegans)	1.18	0.85	-1.21	0.0255	1.06	0.6883	-1.14	0.1337	-3.05	0.1476
92210_at	1448831_at	Angpt2	angiopoietin 2	1.86	0	-1.13	0.3825	-1.04	0.728	1.03	0.8288	-1.47	0.1457
92213_at	1449201_at	Star	steroidogenic acute regulatory protein	1.11	0.81	1.57	0.0345	1.75	0.0224	1.82	0.0068	1.13	0.7087
92214_at	1422632_at	Ctsw	cathepsin W	-1.1	0.46	1.57	0.1374	1.07	0.546	1.18	0.2166	1.29	0.0354
92215_at	1450536_s_at	Krtap12-1	keratin associated protein 12-1	-1.22	0.03	-1.17	0.3749	-1.16	0.3608	-1.12	0.5132	1.3	0.3236
92216_at	1423389_at	Smad7	MAD homolog 7 (Drosophila)	-1.02	0.92	1.02	0.8727	1.17	0.3855	1.27	0.0331	1	0.9678
92217_s_at	1420394_s_at	Gp49a /// Lilrb4	glycoprotein 49 A /// leukocyte immunoglobu	2.25	0.04	2.08	0.2885	-1.12	0.6012	2.2	0.2825	1.44	0.031
92219_s_at	1422055_at	Mid1	midline 1	-1.57	0.08	1.4	0.2227	1.12	0.2455	1.03	0.7461	1.26	0.6947
92220_s_at	1425532_a_at	Bin1	bridging integrator 1	-1.32	0.38	-1.28	0.0452	-1.02	0.8719	-1.36	0.0195	-1.19	0.4918
92223_at	1449401_at	C1qg	complement component 1, q subcomponent	1.21	0.34	1.18	0.3338	-1.08	0.3111	1.35	0.3397	-1.03	0.8333
92224_at	1449466_at	Clec3b	C-type lectin domain family 3, member b	-1.39	0.63	-3.27	0.0368	-2.12	0.1154	-2.57	0.0719	-1.16	0.7808
92226_at	1417875_at	Ddx50	DEAD (Asp-Glu-Ala-Asp) box polypeptide 50	1.4	0.04	1.24	0.081	1.18	0.0618	1.27	0.0676	-1.1	0.6032
92228_at	1448895_a_at	Catna2	catenin alpha 2	-1.38	0.54	1.6	0.0893	1.48	0.0746	1.14	0.5798	1.42	0.6354
92229_at	1418790_at	Zfp312	zinc finger protein 312	1.5	0.08	-2.07	0.0096	-1.63	0.0304	-1.8	0.0085	1.88	0.0953
92230_at	1424981_at	Nln	neurolysin (metallopeptidase M3 family)	3.05	0	1.23	0.2138	1.27	0.1089	1.37	0.0163	1.4	0.253
92232_at	1416576_at	Socs3	suppressor of cytokine signaling 3	-4.3	0.14	1.81	0.3434	-1.21	0.3347	1.63	0.5042	-2.25	0.0493
92233_at	1418007_at	1810007M14Rik	RIKEN cDNA 1810007M14 gene	1.36	0.43	1.13	0.064	1.33	0.0264	1.46	0.0001	1.48	0.0562
92235_g_at	1425762_a_at	Rxra	retinoid X receptor alpha	-1.05	0.57	-1.01	0.8844	-1.02	0.8253	-1.17	0.1165	-1.11	0.6042
92237_at	1418782_at	Rxrg	retinoid X receptor gamma	-2.97	0.39	1.11	0.787	-1.58	0.3233	-1.55	0.3412	1.01	0.9717
92239_at	1452524_a_at	Elavl3	ELAV (embryonic lethal, abnormal vision, Dr	-1.15	0.83	-1.09	0.625	1.02	0.9346	-1.1	0.6311	-1.07	0.8566
92240_at	1422958_at	Krtap5-4	keratin associated protein 5-4	-2.92	0.08	-3.18	0.0001	-1.43	0.0915	-2.52	0.0007	-2.13	0.1993
92241_at	1435527_at	1500041O16Rik	RIKEN cDNA 1500041O16 gene	-1.5	0.18	-1.26	0.0412	-1.64	0.0132	-1.58	0.0068	-1.08	0.6663
92242_at	1419319_at	Saa4	serum amyloid A 4	-1.21	0.77	-1.13	0.5871	-1.44	0.1259	1.04	0.8311	-3.37	0.0917
92243_at	1434609_at	B930007L02Rik	RIKEN cDNA B930007L02 gene	-1.72	0.05	1.09	0.1288	1.25	0.0011	1.18	0.0396	1.26	0.0249
92244_at	1418026_at	Exo1	exonuclease 1	-1.43	0.56	-1.06	0.8619	-1.22	0.5815	-1.1	0.8022	1.04	0.8226
92246_at	1419707_at	Krtap14	keratin associated protein 14	-1.67	0.18	-1.3	0.0924	-1.15	0.4048	-1.23	0.1638	1.45	0.1318
92249_g_at	1450749_a_at	Nr4a2	nuclear receptor subfamily 4, group A, meml	-1.21	0.04	1.03	0.8349	1.1	0.4204	1.19	0.1784	1.38	0.4512
92250_s_at	1449250_at	Prcc	papillary renal cell carcinoma (translocation-	-1.18	0.32	-1.15	0.2257	-1.07	0.6235	-1.14	0.3184	-1.04	0.9027
92252_at	1421195_at	Cckar	cholecystokinin A receptor	1.77	0.34	-1.25	0.6213	-1.41	0.3846	1.26	0.6006	1.32	0.6618
92253_at	1449919_at	Krtap6-2	keratin associated protein 6-2	-1.99	0.1	-1.55	0.2827	-1.41	0.3621	-1.11	0.8071	-1.59	0.3434
92254_at	1452298_a_at	Myo5b	myosin Vb	1.41	0.28	-1.23	0.0004	-1.2	0.0017	-1.21	0.0055	-1.43	0.0789

92256_at	1417491_at	Ctsb	cathepsin B	1.82	0.13	1.08	0.453	1.19	0.0679	1.16	0.1903	1.03	0.8915
92257_at	1418659_at	Clock	circadian locomoter output cycles kaput	-1.3	0.42	-1.08	0.4083	-1.22	0.0149	-1.19	0.0375	1.07	0.7058
92258_at	1429597_at	Dppa4	developmental pluripotency associated 4	-3.66	0.09	-1.38	0.3458	1.31	0.4219	-1.27	0.5447	-2.33	0.0143
92260_at	1427804_at	---	MRNA containing B1 element, clone vario	-2.43	0.29	1.24	0.1903	1.11	0.4178	1.07	0.5325	1.6	0.4511
92261_at	1435231_at	Coq4	coenzyme Q4 homolog (yeast)	1.01	0.97	1.08	0.3873	-1.02	0.8379	1.17	0.0353	1.43	0.4683
92262_at	1449353_at	Wig1	wild-type p53-induced gene 1	1.03	0.92	-1.03	0.7799	-1.1	0.3325	1.09	0.5113	-1.16	0.5289
92263_at	1449531_at	Leprel2	leprecan-like 2	1.44	0.35	-1.04	0.6489	1.04	0.5623	-1.18	0.0141	1.59	0.302
92264_at	1450485_at	Sox3	SRY-box containing gene 3	-1.15	0.63	1.09	0.3133	1.09	0.3057	1.04	0.7553	1.17	0.5388
92266_at	1449982_at	Il11	interleukin 11	1.52	0	1.03	0.6805	1.23	0.0699	1.2	0.0536	1.52	0.2167
92268_at	1436859_at	2700007P21Rik	RIKEN cDNA 2700007P21 gene	-1.15	0.58	1.09	0.2748	1.3	0.1058	1.13	0.1151	-2.15	0.0056
92270_at	1426016_a_at	Tro	trophinin	-2.71	0.23	-1.23	0.1465	-1.28	0.0647	-1.34	0.0289	1.06	0.7929
92272_at	1455310_at	Rbm16	RNA binding motif protein 16	-1.08	0.69	1.12	0.4398	1.12	0.5748	1.07	0.6822	1.25	0.2239
92273_at	1419125_at	Ptpn18	protein tyrosine phosphatase, non-receptor t	-1.06	0.82	1.78	0	1.43	0	1.68	0	-1.03	0.9083
92274_at	1449830_at	Prlpi	prolactin-like protein I	-1.53	0.44	-1.33	0.2161	-1.95	0.0152	-1.42	0.2216	1.34	0.3884
92275_at	1436392_s_at	Tcfap2c	transcription factor AP-2, gamma	-1.7	0.04	1.21	0.1109	1.21	0.4168	1.09	0.5385	1.55	0.0875
92276_at	1449901_a_at	Map3k6	mitogen-activated protein kinase kinase kina	-1.33	0.24	1.11	0.4901	1.13	0.3278	1.07	0.5318	1.23	0.0619
92277_at	1420160_s_at	Myo1e	myosin IE	1.85	0.19	-1.07	0.8162	-1.17	0.5884	-1.14	0.6798	1.32	0.1183
92278_at	1460727_at	Ercc2	excision repair cross-complementing rodent	-1.02	0.92	-1.18	0.0936	-1.08	0.4651	-1.18	0.014	1.5	0.0186
92279_at	1460583_at	Golt1b	golgi transport 1 homolog B (S. cerevisiae)	-1.04	0.78	-1.25	0.046	1.17	0.1809	-1.19	0.2252	1.28	0.2868
92280_at	1427385_s_at	Actn1	actinin, alpha 1	-1.13	0.8	1.09	0.2543	-1.02	0.9305	-1.04	0.7795	1.5	0.0641
92282_at	1427887_at	2610304G08Rik	RIKEN cDNA 2610304G08 gene	1.52	0.15	1.11	0.2256	1.05	0.6409	1.07	0.4639	-1.15	0.0779
92286_g_at	1449864_at	Il4	interleukin 4	-1.02	0.96	-1.94	0.0011	-1.52	0.0464	-1.24	0.1976	2.13	0.2176
92289_at	1427486_at	Ptprb	protein tyrosine phosphatase, receptor type,	2.06	0.22	-1.24	0.424	-2.74	0.0019	-2.42	0.0073	2.09	0.0998
92290_at	1419404_s_at	Siah1a /// Siah1	seven in absentia 1A /// seven in absentia 1I	-1.37	0.23	-1.15	0.1458	-1.17	0.1058	-1.14	0.2036	1.06	0.4739
92291_f_at	1419436_at	Cfhl1	complement component factor h-like 1	-1.42	0.16	1.28	0.1355	1.29	0.1083	1.06	0.6172	-1.02	0.8469
92292_at	1421924_at	Slc2a3	solute carrier family 2 (facilitated glucose tra	1.86	0.11	1.25	0.2131	1.44	0.066	1.1	0.673	1.33	0.2505
92293_at	1434709_at	C130076O07Rik	RIKEN cDNA C130076O07 gene	-1.52	0.16	-1.12	0.4407	-1.04	0.8257	-1.18	0.2652	1.02	0.8685
92294_at	1447926_at	Arl5	ADP-ribosylation factor-like 5	1.41	0.21	-1.35	0.02	-1.17	0.2482	-1.66	0.0011	1.16	0.5075
92295_at	1419717_at	Sema3e	sema domain, immunoglobulin domain (Ig),	-1.73	0.5	-1.17	0.548	1.06	0.8001	-1.09	0.7281	-1.05	0.9136
92296_at	1421284_at	Pign	phosphatidylinositol glycan, class N	1.51	0.57	1.13	0.62	1.31	0.3783	-1.65	0.1469	-1.85	0.1846
92300_at	1418192_at	Mnt	max binding protein	2.87	0.14	2.71	0.0134	-1.28	0.4567	2.84	0.0219	1.25	0.2407
92301_at	1417813_at	Ikbke	inhibitor of kappaB kinase epsilon	1.91	0.38	1.47	0.3195	1.11	0.4718	2.46	0.003	1.05	0.6021
92302_at	1452281_at	Sos2	Son of sevenless homolog 2 (Drosophila)	1.63	0.15	1.54	0.0071	1.35	0.287	1.43	0.0047	1.01	0.9679
92305_s_at	1427725_a_at	Pou2f2	POU domain, class 2, transcription factor 2	-1.51	0.29	1.18	0.0602	-1.02	0.8875	1.35	0.0814	1.49	0.4222
92306_at	1427346_at	Ott /// LOC4348	ovary testis transcribed /// similar to Ott prote	1.01	0.94	1	0.9942	-1.16	0.6475	1.28	0.5445	2.36	0.219
92308_at	1422541_at	Ptprm	protein tyrosine phosphatase, receptor type,	-1.11	0.17	-1.09	0.7119	-1.23	0.422	1.12	0.5862	1.42	0.4587
92310_at	1427005_at	Plk2	polo-like kinase 2 (Drosophila)	-1.79	0.01	1.01	0.8427	1.11	0.1779	1.15	0.1673	1.08	0.5013
92311_s_at	1425862_a_at	Pik3c2a	phosphatidylinositol 3-kinase, C2 domain co	1.05	0.95	1.54	0.1771	2.22	0.0465	-1.13	0.5959	1.91	0.207
92314_at	1427391_a_at	Col12a1	procollagen, type XII, alpha 1	2.15	0.1	1.16	0.4307	1.19	0.3698	-1.41	0.2332	1.48	0.2175
92315_at	1427102_at	Slfn4	schlafen 4	-1.57	0.09	1.59	0.2285	1.01	0.9281	2.35	0.2025	1.29	0.1913
92317_at	1421881_a_at	Elavl2	ELAV (embryonic lethal, abnormal vision, Dr	-1.15	0.82	-1.34	0.0911	1.48	0.0825	1.15	0.6064	1.11	0.8417
92318_at	1419773_at	2010301N04Rik	RIKEN cDNA 2010301N04 gene	-2.17	0.35	1.32	0.4742	1.03	0.9292	1.06	0.8639	2.23	0.0335
92321_r_at	1447963_at	---	---	1.42	0.36	-2.01	0.0107	-1.34	0.232	-1.45	0.1344	1.32	0.1174
92322_at	1419691_at	Camp	cathelicidin antimicrobial peptide	-1.52	0.07	-1.3	0.1167	-1.38	0.0944	-1.8	0.0021	1.63	0.0288
92323_at	1449283_a_at	Mapk12	mitogen-activated protein kinase 12	-1.02	0.91	-1.13	0.3157	1.19	0.0896	1.07	0.6154	1.17	0.4246
92324_at	1429303_at	Zfp393	zinc finger protein 393	2	0.03	-1.04	0.858	1.05	0.8238	1.06	0.8213	-1.01	0.9736
92325_at	1452589_at	Ptk7	PTK7 protein tyrosine kinase 7	-1.6	0.28	1.57	0.223	1.5	0.1855	1.73	0.1333	1.67	0.1503
92328_at	1427655_a_at	A630038E17Rik	RIKEN cDNA A630038E17 gene	-2.6	0.01	2.8	0.0153	1.84	0.0759	2.55	0.0163	1.34	0.3094
92329_at	1450516_a_at	Rab17	RAB17, member RAS oncogene family	1.05	0.93	1.05	0.6744	1.04	0.7275	1.11	0.3033	-1.06	0.6064
92331_at	1422975_at	Mme	membrane metallo endopeptidase	-1.44	0.41	-6.12	0	-1.57	0.023	-9.85	0	-2.44	0.3673
92332_at	1448877_at	Dlx2	distal-less homeobox 2	-1.39	0.76	1.14	0.6531	-1.01	0.9789	1.17	0.6836	1.06	0.9323
92335_at	1418061_at	Ltbp2	latent transforming growth factor beta bindin	2.44	0.05	-1.32	0.2599	1.37	0.1807	-1.04	0.8555	-1.48	0.2511
92338_f_at	1424872_at	2310001H12Rik	RIKEN cDNA 2310001H12 gene	1.87	0.05	1.27	0.0931	-1.1	0.6242	1.13	0.297	-1.98	0.0625

92339_at	1419702_at	Taf1a	TATA box binding protein (Tbp)-associated f	2.14	0.22	1.19	0.216	1.48	0.0083	1.2	0.1452	1.38	0.4396
92342_at	1422205_at	Sox1	SRY-box containing gene 1	1.28	0.45	1.17	0.6125	1.08	0.8007	-1.11	0.7662	1.06	0.8681
92345_g_at	1451901_at	Smarca3	SWI/SNF related, matrix associated, actin de	-1.84	0.25	1.01	0.91	1.09	0.1009	1.02	0.753	1.55	0.0791
92347_at	1421980_at	Kcnc3	potassium voltage gated channel, Shaw-rela	-1.35	0.54	-1.6	0.07	-1.48	0.1422	-1.15	0.6305	1.6	0.2036
92348_at	1454675_at	Thra	thyroid hormone receptor alpha	1.07	0.62	1.07	0.2232	-1.07	0.4163	-1.01	0.9009	1.47	0.0139
92350_at	1428819_at	Mapre1	microtubule-associated protein, RP/EB famil	1.11	0.64	-1.36	0.004	-1.18	0.0522	-1.15	0.0607	1.01	0.9025
92352_at	1460661_at	Edg3	endothelial differentiation, sphingolipid G-pro	-1.51	0.03	-1.1	0.3288	-1.09	0.459	-1.14	0.1317	1.14	0.3797
92353_at	1448982_at	Prss18	protease, serine, 18	1.62	0.36	1.63	0.0127	1.06	0.7409	1.52	0.0149	-1.04	0.8685
92354_at	1447945_at	LOC436061	similar to Transcription factor Maf (Proto-onc	1.3	0.15	-1.15	0.5076	-1.21	0.381	-1.52	0.0863	-1.2	0.6843
92355_at	1419012_at	Zfpm2	zinc finger protein, multitype 2	-1.36	0.48	1.04	0.8219	-1.04	0.7988	1.1	0.4522	1.17	0.3523
92356_at	1417995_at	Ptpn22	protein tyrosine phosphatase, non-receptor t	3.62	0.26	2.35	0.2513	1.51	0.3832	2.64	0.2248	1.87	0.3261
92357_at	1423378_at	Adam23	a disintegrin and metalloprotease domain 23	1.44	0.26	1.11	0.2309	1.02	0.8787	1.11	0.3426	1.27	0.5328
92358_at	1423561_at	---	---	-1.33	0.39	-1.19	0.4093	-1.11	0.6516	-1.2	0.4816	2.05	0.2133
92360_at	1416861_at	Stam	signal transducing adaptor molecule (SH3 do	1.04	0.75	1.12	0.0416	1.22	0.0196	1.08	0.1546	1.22	0.1137
92361_at	1450258_a_at	Elavl4	ELAV (embryonic lethal, abnormal vision, Dr	-2.4	0.39	1.65	0.2511	1.53	0.1508	1.74	0.0786	1.19	0.6605
92362_at	1418714_at	Dusp8	dual specificity phosphatase 8	1.98	0.27	-1.11	0.7239	-1.27	0.3405	-1.43	0.169	1.16	0.5975
92364_at	1422073_a_at	Celsr2	cadherin EGF LAG seven-pass G-type recep	-1.76	0.23	1.42	0.1964	1.8	0.0309	1.38	0.1078	1.26	0.6125
92365_at	1449528_at	Figf	c-fos induced growth factor	1.81	0.09	1.25	0.0132	1.41	0.0033	1.31	0.0342	1.07	0.8342
92366_at	1426285_at	Lama2	laminin, alpha 2	1.1	0.8	-1.09	0.6789	1.1	0.6265	-1.21	0.3406	-1.1	0.8016
92367_at	1427707_a_at	---	---	-1.28	0.73	1.13	0.4402	1.17	0.1727	-1.22	0.1866	-1.35	0.398
92368_at	1420401_a_at	Ramp3	receptor (calcitonin) activity modifying protei	2.27	0.01	-1.61	0.169	-1.76	0.1058	-1.05	0.878	-1.02	0.931
92371_at	1419109_at	Hrc	histidine rich calcium binding protein	-1.88	0.02	-1.2	0.015	-1.09	0.2299	-1.22	0.0583	-1.14	0.5505
92374_at	1435495_at	Adora1	adenosine A1 receptor	2.8	0.02	3.84	0	2.69	0	4.64	0	1.73	0.0154
92375_at	1460703_at	Ascc1	activating signal cointegrator 1 complex sub	1.4	0.25	-1.11	0.0773	-1.05	0.4163	-1.04	0.2814	1.21	0.1163
92376_at	1420540_a_at	Rit1	Ras-like without CAAX 1	-1.05	0.76	-1.06	0.2747	-1.07	0.3102	1.02	0.7683	-1.07	0.5482
92377_at	1455279_at	Gm1060	gene model 1060, (NCBI)	-2.04	0.06	-1.18	0.564	1.07	0.8094	-1.31	0.3082	3	0.0096
92378_at	1427019_at	Ptprz1	protein tyrosine phosphatase, receptor type	1.17	0.76	-1.28	0.1643	-1.01	0.9622	-1.5	0.0438	-1.34	0.2911
92380_r_at	1418690_at	Ptprz1	protein tyrosine phosphatase, receptor type	-2.94	0.15	1.07	0.8545	-1.33	0.5232	-1.62	0.1379	1.25	0.5773
92381_at	1420975_at	Baz1b	bromodomain adjacent to zinc finger domain	-1.2	0.52	-1.11	0.3583	-1.04	0.8034	-1.3	0.0835	1.77	0.2636
92382_at	1421120_at	Myo6	myosin VI	1.31	0.63	1.08	0.2797	1.15	0.1303	1.1	0.2728	1.02	0.8764
92386_at	1448629_at	Hps4	Hermansky-Pudlak syndrome 4 homolog (hu	-1.01	0.98	-1.55	0.0089	-1.25	0.1217	-1.41	0.0038	1.45	0.2711
92387_at	1416827_at	Tbxas1	thromboxane A synthase 1, platelet	-1.09	0.45	1.08	0.6445	-1.08	0.5831	-1.03	0.8498	1.46	0.0364
92388_at	1415710_at	BC038311	cDNA sequence BC038311	1.11	0.38	-1.17	0.0372	1	0.9955	-1.06	0.5582	-1.2	0.137
92389_at	1420718_at	Odz2	odd Oz/ten-m homolog 2 (Drosophila)	-1.06	0.9	-1.38	0.0518	-1.26	0.0541	-1.33	0.0296	1.51	0.0684
92390_at	1422992_s_at	---	---	-2.84	0.2	1.28	0.3415	-1.04	0.8637	1.04	0.9036	1.32	0.2727
92392_at	1456064_at	AI504432	expressed sequence AI504432	-1.1	0.75	-1.21	0.6391	-1.17	0.6836	-1.48	0.3107	-1.17	0.8086
92393_at	1423018_at	Kcna3	potassium voltage-gated channel, shaker-re	-2.45	0.03	-1.08	0.6932	1.08	0.7273	-1.18	0.2173	-1.17	0.6973
92395_r_at	1417173_at	Crebl1	cAMP responsive element binding protein-lik	-1.19	0.54	-1.06	0.3584	-1.07	0.3439	-1.09	0.1871	1.31	0.2134
92398_at	1424380_at	BC026744	cDNA sequence BC026744	1.68	0.27	1.07	0.523	-1.23	0.2217	1.06	0.631	1.46	0.4637
92399_at	1422864_at	Runx1	runt related transcription factor 1	-1.52	0.18	1.11	0.7499	-1.09	0.7585	1.05	0.8543	1.31	0.4728
92400_at	1417931_at	Ndst2	N-deacetylase/N-sulfotransferase (heparan g	-1.34	0.68	-1.11	0.5895	1.06	0.7631	-1.13	0.5246	1.02	0.9522
92401_at	1419692_a_at	Ltc4s	leukotriene C4 synthase	-1.16	0.48	-1.2	0.0521	-1.21	0.06	-1.26	0.0268	1.47	0.1078
92403_at	1449468_at	St6galnac5	ST6 (alpha-N-acetyl-neuraminyl-2,3-beta-ga	-1.48	0.46	-1.12	0.5044	-1.08	0.6901	-1.12	0.5194	2.39	0.0301
92404_at	1420891_at	Wnt7b	wingless-related MMTV integration site 7B	-1.9	0.29	1.27	0.3592	1.54	0.231	1.37	0.4404	2.22	0.0229
92406_at	1419711_at	Cd7	CD7 antigen	-1.41	0.4	1.15	0.5434	-1.06	0.7905	1.3	0.2354	-1.27	0.5601
92407_at	1420693_at	Myom1	myomesin 1	1.07	0.79	-2.12	0.3333	-1.55	0.5342	-1.65	0.4767	-1.35	0.5236
92408_at	1438221_at	C130065N10Ril	RIKEN cDNA C130065N10 gene	1.48	0.2	1.23	0.0606	1.14	0.3535	1.23	0.1102	-1.02	0.8716
92409_at	1419164_at	Zfp260	zinc finger protein 260	1.23	0.13	1.13	0.2158	1.24	0.0205	1.13	0.1081	-1.32	0.136
92410_at	1422964_at	Rad23a	RAD23a homolog (S. cerevisiae)	1.25	0.68	-1.02	0.8397	-1.04	0.7642	-1.05	0.6264	-1.11	0.7772
92411_at	1433843_at	Hs1bp3	HS1 binding protein 3	-1.15	0.16	1	0.9723	1.11	0.0217	1.06	0.1035	1.26	0.0493
92412_s_at	1427498_a_at	Spag5	sperm associated antigen 5	-2.95	0.01	-1.19	0.6769	-1.63	0.2693	-1.76	0.2082	2.15	0.1995
92414_at	1421172_at	Adam12	a disintegrin and metalloproteinase domain 1	-1.12	0.85	-1.95	0.0513	-1.45	0.2574	-1.06	0.8727	-1.53	0.2548
92415_at	1422924_at	---	---	2.04	0.46	-1.1	0.6788	-1.17	0.4351	-1.22	0.3728	-1.67	0.3103

92416_at	1460293_at	Freq	frequenin homolog (Drosophila)	-2	0.06	-1.14	0.669	1.11	0.7297	-1.52	0.166	1.99	0.0279
92417_at	1417858_at	Rasal1	RAS protein activator like 1 (GAP1 like)	-1.17	0.29	1.1	0.2994	1.03	0.7258	1.18	0.1728	1.02	0.9085
92418_at	1416263_at	Abcb9	ATP-binding cassette, sub-family B (MDR/T/	1.04	0.91	1.04	0.6507	-1.01	0.8433	-1.07	0.2782	1.48	0.0429
92419_at	1419227_at	Cct6b	chaperonin subunit 6b (zeta)	-1.66	0.26	-1.05	0.5828	-1.05	0.5591	-1.09	0.3508	1.8	0.0499
92421_at	1453113_at	2610014F08Rik	RIKEN cDNA 2610014F08 gene	2.42	0	1.49	0.2266	2.39	0.0002	2.42	0.0087	1.67	0.1363
92422_at	1418149_at	Chga	chromogranin A	-1.3	0.45	-1.18	0.2458	-1.25	0.2285	-1.13	0.3032	-1.85	0.0404
92423_at	1449100_at	Pard6a	par-6 (partitioning defective 6,) homolog alpl	1.27	0.25	1.35	0.3566	1.4	0.2505	1.42	0.1841	1.2	0.6741
92426_at	1431530_a_at	Tspan5	tetraspanin 5	-1.11	0.57	1.14	0.2385	1.24	0.0712	1.18	0.1634	1.32	0.1745
92427_at	1420894_at	Tgfbr1	transforming growth factor, beta receptor I	-1.8	0.58	1.13	0.5299	-1.02	0.9187	1.66	0.1445	-1.66	0.087
92428_at	1418292_at	Asna1	arsA (bacterial) arsenite transporter, ATP-bi	1.16	0.74	-1.03	0.5857	-1.17	0.1939	-1.11	0.1912	-1.17	0.1758
92429_at	1422926_at	Mc2r	melanocortin 2 receptor	-2.02	0.26	-1.28	0.5654	1.03	0.9421	-1.14	0.6691	1.53	0.0932
92430_at	1424851_at	Chchd5	coiled-coil-helix-coiled-coil-helix domain con	-1.13	0.38	1.02	0.8269	1.07	0.4218	1.01	0.885	1.23	0.331
92432_at	1435202_at	Zfp574	zinc finger protein 574	-1.24	0.66	-1.83	0.0155	-1.04	0.8539	-1.54	0.095	-1.06	0.8832
92433_at	1450804_at	Kif5c	kinesin family member 5C	-3.82	0.22	1.04	0.8343	-1.1	0.471	-1.04	0.6629	1.38	0.0937
92434_at	1420707_a_at	Traip	TRAF-interacting protein	-2.47	0.35	1.31	0.2167	1.38	0.1814	1.26	0.1855	2.62	0.1404
92435_at	1418310_a_at	Rlbp1	retinaldehyde binding protein 1	-1.03	0.96	1.34	0.2162	1.59	0.0184	1.3	0.2945	1.95	0.2254
92436_at	1418798_s_at	Stk23	serine/threonine kinase 23	-1.17	0.54	1.17	0.1035	1.08	0.5893	1.07	0.4296	1.11	0.7734
92437_at	1460684_at	Tm7sf2	transmembrane 7 superfamily member 2	1.03	0.78	-1.55	0.0042	-1.64	0.0005	-2.1	0	-1.39	0.1391
92439_at	1452403_a_at	Oc90	otoconin 90	1.47	0.09	1.22	0.051	1.11	0.3458	1.25	0.0306	1.56	0.0882
92440_at	1418301_at	Irf6	interferon regulatory factor 6	-4.3	0	-6.39	0	-1.15	0.1897	-6.45	0	-3.34	0.0002
92441_at	1417552_at	Fap	fibroblast activation protein	-1.56	0.21	1.27	0.4712	1.72	0.2569	1.16	0.7072	1.42	0.3584
92444_f_at	1427702_at	Zfp1	zinc finger protein 1	1.85	0.2	1.21	0.0071	-1.15	0.0743	1.19	0.0037	1.19	0.3681
92449_at	1423007_a_at	Gfra2	glial cell line derived neurotrophic factor fam	-2.53	0.37	-1.26	0.5689	-1.12	0.8076	1.07	0.8774	1.28	0.3045
92450_at	1417446_at	Slc12a4	solute carrier family 12, member 4	-1.43	0.33	1.31	0.0314	1.46	0.0069	1.32	0.033	1.29	0.2678
92454_at	1422899_at	Slc6a20	solute carrier family 6 (neurotransmitter tran:	-1.6	0.07	-1.17	0.3442	-1.08	0.6513	-1.56	0.0427	1.12	0.349
92455_at	1450469_at	MGI:1929713	testis specific protein, Ddc8	-3.02	0.12	-1.75	0.0992	-1.95	0.0552	-2.05	0.0467	1.25	0.2617
92456_at	1423483_s_at	---	---	3.5	0.01	1.3	0.3537	1.15	0.6649	1.63	0.1448	-1.63	0.3919
92457_at	1452473_at	E130201N16Ril	RIKEN cDNA E130201N16 gene	1.62	0.02	-1.05	0.8063	1.32	0.2921	-1.3	0.181	1.05	0.8948
92458_at	1422663_at	Orc1l	origin recognition complex, subunit 1-like (S.	-1.41	0.35	-1.02	0.9243	-1.04	0.9119	1.04	0.8261	1.29	0.2354
92459_at	1419684_at	Ccl8	chemokine (C-C motif) ligand 8	-2.63	0.21	-1.16	0.2618	-1.13	0.3075	1.21	0.3281	1.37	0.1235
92460_at	1419328_at	Sema4f	sema domain, immunoglobulin domain (Ig),	-1.43	0.12	-1.03	0.666	-1.08	0.2225	-1.08	0.29	1.54	0.0304
92461_at	1448598_at	Mmp17	matrix metalloproteinase 17	-1.14	0.44	1.22	0.2821	1.16	0.2729	1.16	0.3727	-1.06	0.8645
92462_at	1460286_at	6-Sep	septin 6	2.15	0.16	1.24	0.5949	1.52	0.2646	1.44	0.2114	-1.34	0.4588
92465_at	1421170_a_at	Plcb1	phospholipase C, beta 1	-3.23	0.1	1.04	0.8335	1.02	0.8988	1.17	0.4032	-1.49	0.3213
92467_g_at	1425782_at	Plcb1	phospholipase C, beta 1	-1.62	0.5	-1.03	0.8352	-1.28	0.2351	-1.34	0.1073	-1.17	0.5936
92468_at	1423421_at	MGI:1930842	globin inducing factor, fetal	2.96	0.02	1.25	0.0359	-1	0.9696	1.37	0.0046	1.57	0.2804
92469_at	1451031_at	Sfrp4	secreted frizzled-related sequence protein 4	-1.11	0.81	-1.49	0.246	-1.04	0.8954	-1.31	0.3254	2.14	0.0625
92472_f_at	1450165_at	Slfn2	schlafen 2	2.03	0.22	1.9	0.1025	1.24	0.3432	1.9	0.1702	1.23	0.7554
92473_at	1449029_at	Mknk2	MAP kinase-interacting serine/threonine kin	-1.19	0.36	1.02	0.8395	-1.03	0.8749	-1.02	0.8987	1.09	0.7829
92476_at	1449288_at	Gdf3	growth differentiation factor 3	1.26	0.63	1.13	0.5871	1.04	0.5715	-1.16	0.1468	1.57	0.1549
92478_at	1421939_a_at	Stag1	stromal antigen 1	1.91	0.11	-1.05	0.7312	-1.08	0.5448	1.03	0.81	-1.01	0.9903
92480_f_at	1449414_at	Zfp53	zinc finger protein 53	1.12	0.64	1.45	0.044	1.36	0.157	1.53	0.0163	-1.14	0.5705
92481_at	1422747_at	Chek2	CHK2 checkpoint homolog (S. pombe)	-1.02	0.95	-1.06	0.4992	-1.1	0.1951	-1.17	0.0815	1.37	0.0111
92483_g_at	1450212_at	Fmnl1	formin-like 1	-2.62	0.29	-1.39	0.4038	-1.53	0.3103	1.42	0.3871	-1.03	0.9273
92484_at	1422018_at	Hivep2	human immunodeficiency virus type I enhan	-1.24	0.43	1.11	0.4713	-1.3	0.055	1.15	0.2964	-1.9	0.0593
92485_at	1450210_at	Bmp15	bone morphogenetic protein 15	-3.25	0.24	-1.12	0.6875	-1.27	0.4161	-1.51	0.0499	1.4	0.583
92486_at	1449215_at	Slc22a21	solute carrier family 22 (organic cation trans	1.17	0.73	-1.04	0.7155	1.06	0.6164	-1.08	0.4689	1.02	0.9341
92487_at	1416564_at	Sox7	SRY-box containing gene 7	1.77	0.13	-1.35	0.0009	-1.08	0.4175	-1.13	0.3336	-1.19	0.4183
92488_at	1449297_at	Casp12	caspase 12	-2.01	0.05	1.15	0.6136	1.1	0.5407	1.22	0.1081	-1.07	0.8829
92489_at	1449988_at	Gimap1	GTPase, IMAP family member 1	1.6	0.09	1.24	0.067	1.34	0.0514	1.09	0.5184	1.09	0.5955
92490_at	1420395_a_at	Kif9	kinesin family member 9	-1.21	0.3	-1.01	0.9662	1.2	0.583	-1.25	0.2827	1.05	0.8173
92493_at	1450433_at	Rbpsuhl	recombining binding protein suppressor of h	1.04	0.92	-1.19	0.182	-1.01	0.9386	1.01	0.9351	1.25	0.0408
92494_at	1449426_a_at	Anxa10	annexin A10	1.82	0.2	-1.08	0.8731	-1.17	0.6425	-1.77	0.143	1.15	0.6073

92495_at	1420999_at	Cnot4	CCR4-NOT transcription complex, subunit 4	1.01	0.91	1.08	0.3881	-1.07	0.4601	-1.1	0.2817	1.23	0.0096
92496_at	1430522_a_at	Vamp5	vesicle-associated membrane protein 5	2.52	0.05	1.28	0.131	1.58	0.0304	1.85	0.0077	1.92	0.2409
92497_at	1417639_at	Slc22a4	solute carrier family 22 (organic cation transp	2.54	0.23	1.77	0.0462	1.84	0.1941	1.96	0.0884	1.05	0.7493
92498_at	1417757_at	Unc13b	unc-13 homolog B (C. elegans)	-1.07	0.91	1.02	0.9083	-1.04	0.7256	1.09	0.5407	1.96	0.0856
92499_at	1419633_at	Uncx4.1	Unc4.1 homeobox (C. elegans)	-1.42	0.17	-1.55	0.1772	1.48	0.0966	-1.07	0.8344	2.29	0.0178
92500_at	1449315_at	Odz3	odd Oz/ten-m homolog 3 (Drosophila)	2.82	0.01	1.46	0.0052	1.29	0.0475	1.35	0.0327	1.12	0.4391
92501_s_at	1450533_a_at	Plagl1	pleiomorphic adenoma gene-like 1	2.05	0.11	1.38	0.0668	1.09	0.2504	1.87	0.1146	1.34	0.2142
92503_at	1418308_at	Hus1	Hus1 homolog (S. pombe)	1.14	0.57	-1.21	0.0217	-1.31	0.0131	-1.29	0.0372	-1.08	0.8004
92505_g_at	1425366_a_at	Hus1	Hus1 homolog (S. pombe)	-1.35	0.37	1.08	0.6925	1.08	0.6778	-1.11	0.6117	2.36	0.2463
92506_at	1426294_at	Hapln1	hyaluronan and proteoglycan link protein 1	-1.46	0.6	-1.7	0.0985	-1.83	0.03	-1.01	0.9662	3.41	0.1657
92508_s_at	1427569_a_at	Utrn	utrophin	-2.33	0.22	-1.37	0.1528	-1.44	0.0854	-1.37	0.1082	-1.29	0.4933
92509_at	1420496_at	F12	coagulation factor XII (Hageman factor)	-1.27	0.09	-1.2	0.0026	1.08	0.241	-1.25	0.0047	-2.01	0.0004
92510_at	1426431_at	Jag2	jagged 2	1.11	0.57	-1.1	0.3529	-1.06	0.5701	-1.14	0.2148	-1.1	0.6684
92512_g_at	1421666_a_at	Aanat	arylalkylamine N-acetyltransferase	-1.28	0.42	-1.01	0.9109	1.01	0.9043	-1.18	0.0736	1.24	0.1771
92514_at	1423342_at	Barx1	BarH-like homeobox 1	-2.6	0.01	-1.5	0.0565	-1.6	0.0405	-1.42	0.0862	1.85	0.1085
92515_at	1422720_at	Isl1	ISL1 transcription factor, LIM/homeodomain	-1.5	0.11	-1.07	0.4692	1.06	0.6498	1.06	0.7265	-1.03	0.9121
92516_at	1422044_at	Ndst1	N-deacetylase/N-sulfotransferase (heparan	-1.03	0.9	-1.41	0.1702	-2.43	0.0135	-3.41	0.0061	-2.16	0.425
92517_at	1423540_at	Rbms2	RNA binding motif, single stranded interactir	1.15	0.42	-1.31	0.0001	-1.16	0.0529	-1.42	0	-1.7	0.0875
92519_at	1422744_at	Phka1	phosphorylase kinase alpha 1	-1.1	0.83	-1.11	0.2169	-1.03	0.7557	-1.15	0.0836	1.08	0.5803
92520_at	1446914_at	C80425	expressed sequence C80425	1.64	0.25	1.1	0.741	1.73	0.0162	-1.14	0.5981	1.91	0.3204
92521_at	1420649_at	Atbf1	AT motif binding factor 1	1.02	0.9	1.17	0.0213	-1	0.9734	1.15	0.0579	1.47	0.0937
92522_at	1451808_at	Kcnj4	potassium inwardly-rectifying channel, subfa	-1.28	0.1	-1.11	0.3541	-1	0.989	-1.1	0.3834	1.38	0.0304
92523_at	1451868_at	Kcnj6	potassium inwardly-rectifying channel, subfa	-1.84	0.3	1.07	0.7553	-1.02	0.9265	-1.23	0.4179	1.38	0.1637
92524_at	1421581_at	Kcnj6	potassium inwardly-rectifying channel, subfa	1.95	0.28	-1.17	0.6452	1.01	0.9672	-1.35	0.3111	-1.44	0.525
92525_i_at	1429582_at	Btbd14a	BTB (POZ) domain containing 14A	1.42	0.49	1.32	0.1783	1.18	0.2985	1.31	0.0652	1.41	0.5479
92527_at	1418586_at	Adcy9	adenylate cyclase 9	1.26	0.1	1.47	0.0001	1.2	0.0923	1.56	0.0031	1.59	0.0018
92528_at	1455363_at	Bai1	brain-specific angiogenesis inhibitor 1	-1.11	0.86	1.26	0.032	1.14	0.086	1.1	0.4007	1.27	0.2638
92529_s_at	1421721_a_at	Arnt	aryl hydrocarbon receptor nuclear translocat	-1.36	0.46	-1	0.9857	-1.12	0.109	1.02	0.8291	-1.14	0.3818
92531_at	1455698_at	Tloc1	translocation protein 1	-1.61	0.05	-1.36	0.0526	-1.05	0.6908	-1.51	0.0122	-1.77	0.0874
92532_at	1418603_at	Avpr1a	arginine vasopressin receptor 1A	-1.32	0.29	-1.33	0.0068	-1.05	0.4984	-1.51	0.0005	1.35	0.0966
92533_at	1427355_at	Calca	calcitonin/calcitonin-related polypeptide, alpl	-2.2	0.12	-1.09	0.4816	1.3	0.1233	1.09	0.4637	-1.27	0.4757
92534_at	1426063_a_at	Gem	GTP binding protein (gene overexpressed in	-1.63	0.69	-1.12	0.7843	1.23	0.5599	-1.17	0.7086	-1.23	0.731
92535_at	1416302_at	Ebf1	early B-cell factor 1	-2.62	0.35	1.49	0.1393	-1.15	0.2742	-1.09	0.4902	2.13	0.054
92537_g_at	1455918_at	---	---	-1.11	0.85	1.06	0.7685	-1.65	0.0274	-1.03	0.8925	6.69	0.0011
92539_at	1416762_at	S100a10	S100 calcium binding protein A10 (calpactin	-2.25	0.14	-2.43	0.0001	-1.26	0.0695	-2.9	0	-6.67	0.001
92540_f_at	1421260_a_at	Srm	spermidine synthase	-1.12	0.13	-1.27	0.0046	-1.13	0.2072	-1.13	0.2037	1.01	0.9555
92541_at	1452651_a_at	Myl1	myosin, light polypeptide 1	-1.97	0.03	-2.93	0.1166	-2.55	0.1538	-3.25	0.0997	-1.04	0.7784
92543_at	1460636_at	Map2k2	Mitogen activated protein kinase kinase 2	1.17	0.1	1.06	0.3097	-1.06	0.3625	1.12	0.0659	1.33	0.0433
92544_f_at	1448442_a_at	Psma3	proteasome (prosome, macropain) subunit,	-1.11	0.53	1.05	0.5067	1.01	0.8488	1.02	0.6446	-1.11	0.4377
92546_r_at	1423860_at	Ptgds	prostaglandin D2 synthase (brain)	-1.2	0.06	-1.03	0.6972	1.34	0.0803	-1.17	0.028	1.12	0.4737
92547_at	1417186_at	Hip2	huntingtin interacting protein 2	1.23	0.2	1.14	0.0687	1.06	0.618	1.1	0.1329	-1.04	0.8257
92549_at	1423945_a_at	Pkig	protein kinase inhibitor, gamma	-1.15	0.27	-1.08	0.2069	-1.01	0.8619	1.01	0.9377	1.06	0.5765
92550_at	1417156_at	Krt1-19	keratin complex 1, acidic, gene 19	2.68	0.09	-1.85	0.1551	1.18	0.8377	-1.8	0.1741	1.31	0.3689
92551_at	1416641_at	Lig1	ligase I, DNA, ATP-dependent	3.47	0.06	2.74	0.0046	1.39	0.3933	3.3	0	1.62	0.2036
92553_at	1417825_at	Esd	esterase D/formylglutathione hydrolase	1.34	0.31	1.09	0.1039	1.03	0.732	1.14	0.0593	-1.15	0.1152
92554_at	1422887_a_at	Ctbp2	C-terminal binding protein 2	1.43	0.08	1.02	0.8058	-1.04	0.7295	1.05	0.5854	1.37	0.1411
92555_at	1448501_at	Tspan6	tetraspanin 6	2.21	0.11	1.94	0.2623	1.06	0.7186	1.52	0.1459	2.27	0.0799
92556_at	1417085_at	Akr1c6	aldo-keto reductase family 1, member C6	-1.19	0.21	1.04	0.3698	1.05	0.3922	-1.05	0.2977	-1.05	0.5082
92557_at	1449392_at	Hsd17b1	hydroxysteroid (17-beta) dehydrogenase 1	-2.54	0	1.59	0.0073	1.13	0.472	1.08	0.6558	1.02	0.9511
92558_at	1448162_at	Vcam1	vascular cell adhesion molecule 1	1.53	0.16	1.5	0.221	1.3	0.0197	1.9	0.114	2.13	0.0032
92560_g_at	1451314_a_at	Vcam1	vascular cell adhesion molecule 1	-2.37	0.13	1.28	0.42	-1.05	0.8349	1.61	0.292	1.74	0.2995
92561_at	1451765_a_at	Entpd5	ectonucleoside triphosphate diphosphohydro	1.97	0.17	1.08	0.4324	-1.08	0.3607	1.09	0.1881	-1.41	0.1735
92562_at	1416543_at	Nfe2l2	nuclear factor, erythroid derived 2, like 2	1.01	0.92	1.1	0.0766	1.16	0.1411	1.06	0.2543	-1.16	0.4279

92563_at	1448487_at	Lrrfip1	leucine rich repeat (in FLII) interacting protei	-1.3	0.62	1.02	0.8678	1.3	0.071	1.25	0.1079	1.07	0.8272
92564_at	1433842_at	Lrrfip1	leucine rich repeat (in FLII) interacting protei	1.65	0.05	1.09	0.5996	1.04	0.8103	1.21	0.0508	1.26	0.0626
92565_at	1416095_x_at	1110005A23Rik	RIKEN cDNA 1110005A23 gene	1.6	0.1	1.03	0.5746	1	0.9661	1.01	0.9169	-1.08	0.5463
92567_at	1422437_at	Col5a2	procollagen, type V, alpha 2	1.02	0.91	1.36	0.1331	1.33	0.1115	1.68	0.0966	-1.39	0.3688
92571_at	1416146_at	Hspa4	heat shock protein 4	2.28	0.12	1.12	0.1854	1.22	0.0727	1.26	0.0356	-1.18	0.2373
92573_at	1438174_x_at	Ppp2r1a	Protein phosphatase 2 (formerly 2A), regulat	-1.65	0.02	1.11	0.0371	1	0.9744	1.15	0.0472	-1.03	0.8225
92574_at	1428235_at	Sdhd	succinate dehydrogenase complex, subunit	1.48	0.07	1.05	0.5429	1.41	0.0034	1.16	0.214	1.41	0.0004
92575_at	1423128_at	Aip	aryl-hydrocarbon receptor-interacting proteir	1.06	0.64	1.01	0.8762	-1.01	0.8804	1.06	0.2814	1.39	0.0114
92577_f_at	1453729_a_at	Rpl37	ribosomal protein L37	1.29	0.06	1.22	0.0036	1.16	0.0005	1.29	0	1.34	0.0579
92578_at	1416486_at	Scye1	small inducible cytokine subfamily E, membe	1.4	0.08	-1.09	0.1272	1.09	0.2435	1.01	0.8608	-1.02	0.8744
92579_at	1416423_x_at	Ssb	Sjogren syndrome antigen B	1.35	0.28	1.37	0.0474	1.1	0.664	1.45	0.0021	-1.05	0.8148
92580_at	1417024_at	Hars	histidyl-tRNA synthetase	1.87	0.25	1.27	0.0272	1.11	0.3665	1.27	0.0158	1.19	0.4531
92581_at	1415984_at	Acadm	acetyl-Coenzyme A dehydrogenase, mediur	1.39	0.01	1.59	0.0009	1.27	0.0525	1.57	0.0007	1.42	0.0004
92582_at	1416629_at	Slc1a5	solute carrier family 1 (neutral amino acid tra	-1.19	0.17	1.12	0.4842	1.18	0.2859	1.22	0.1378	-1.21	0.6512
92583_at	1423867_at	Serpina3k	serine (or cysteine) proteinase inhibitor, clad	-1.65	0.35	-6.2	0	-1.19	0.4827	-6.85	0	-10.04	0.0118
92584_at	1424416_at	Nkiras2	NFKB inhibitor interacting Ras-like protein 2	-1.36	0.42	-1.08	0.1579	-1	0.9463	-1.11	0.1347	1.4	0.0543
92585_at	1416351_at	Map2k1	mitogen activated protein kinase kinase 1	1.01	0.98	1.11	0.1857	-1.07	0.3326	1.18	0.0245	-1.13	0.3428
92586_at	1448253_at	Glud1	glutamate dehydrogenase 1	-1.36	0.29	1.2	0.02	1.19	0.028	1.15	0.0168	-1.19	0.0664
92587_at	1449108_at	Fdx1	ferredoxin 1	1.15	0.31	-1.01	0.8576	-1.17	0.1251	1.01	0.92	1.05	0.7218
92589_at	1415673_at	Psph	phosphoserine phosphatase	-1.56	0.01	1.03	0.6433	1.03	0.6474	1.01	0.8453	1.07	0.7498
92590_at	1431833_a_at	Hmgcs2	3-hydroxy-3-methylglutaryl-Coenzyme A syr	1.15	0.45	-1.15	0.2526	1.12	0.0817	-1.32	0.0079	-1.24	0.0432
92593_at	1423606_at	Postn	periostin, osteoblast specific factor	2.46	0.02	1.3	0.0871	1.31	0.0603	1.27	0.0047	1.11	0.639
92595_r_at	1449181_at	Fech	ferrochelatase	-1.04	0.82	1.18	0.2363	1.15	0.5191	1.47	0.0147	1.4	0.2141
92596_at	1452047_at	Cacybp	calcyclin binding protein	1.48	0.06	-1.14	0.1406	-1.02	0.8975	-1.11	0.194	-1.31	0.1266
92597_s_at	1419883_s_at	Atp6v1b2	ATPase, H+ transporting, V1 subunit B, isof	-1.65	0.15	1.08	0.5699	1.1	0.352	1.17	0.21	1.4	0.095
92598_at	1415814_at	Atp6v1b2	ATPase, H+ transporting, V1 subunit B, isof	1.19	0.46	1.15	0.0593	1.16	0.0731	1.21	0.0115	1.19	0.0284
92599_at	1418373_at	Pgam2	phosphoglycerate mutase 2	-2.79	0.27	-3.34	0.2534	-4.13	0.218	-4.03	0.2214	1.38	0.1866
92600_f_at	1424853_s_at	Cyp4a10 /// BC(	cytochrome P450, family 4, subfamily a, poly	1.76	0.16	1.68	0.0217	2.08	0	2.01	0.0117	3.86	0.0011
92601_at	1415777_at	Pnliprp1	pancreatic lipase related protein 1	-1.06	0.76	1.35	0.0551	-1.04	0.8408	1.04	0.8458	1.56	0.3776
92602_at	1423145_a_at	Tcap	titin-cap	-1.33	0.36	-1.1	0.7979	-1.42	0.348	-1.71	0.1972	-1.15	0.7651
92603_at	1415671_at	Atp6v0d1	ATPase, H+ transporting, V0 subunit D isofc	1.37	0.07	-1.15	0.0194	1.02	0.7177	-1.04	0.4051	-1.3	0.067
92605_at	1456306_a_at	---	---	-2.16	0.3	-1.44	0.1484	1.05	0.8583	1.59	0.2758	1.34	0.4446
92607_at	1423294_at	Mest	mesoderm specific transcript	1.51	0.32	1.31	0.0311	1.38	0.0546	1.46	0.0233	2.6	0.0147
92608_at	1425811_a_at	Csrp1	cysteine and glycine-rich protein 1	1.58	0.1	1.34	0.165	-1.15	0.5155	1.49	0.0392	1.8	0.0276
92610_at	1450705_at	Rdbp	RD RNA-binding protein	1.19	0.48	1.03	0.7636	1.02	0.8906	1.25	0.082	1.44	0.0814
92611_at	1448347_a_at	Gpiap1	GPI-anchored membrane protein 1	1.12	0.81	-1.4	0.0003	-1.5	0.0001	-1.33	0.0022	-1.7	0.0923
92612_at	1449112_at	Slc27a5	solute carrier family 27 (fatty acid transporte	-1	0.99	1.07	0.2224	-1.07	0.1963	1.06	0.3727	-1.03	0.7885
92614_at	1416630_at	Id3	inhibitor of DNA binding 3	-1.7	0.23	-1.04	0.6296	-1.07	0.4504	-1.05	0.5376	-1.27	0.281
92615_at	1434057_at	Gm137	gene model 137, (NCBI)	-1.11	0.77	1.06	0.2798	1.12	0.0186	1.08	0.1252	-1.09	0.4337
92616_at	1448116_at	Ube1x	ubiquitin-activating enzyme E1, Chr X	-1.02	0.94	1.08	0.2123	1.03	0.6035	1.03	0.5473	-1.51	0.0097
92617_at	1450285_at	Ube1y1	ubiquitin-activating enzyme E1, Chr Y 1	2.07	0.13	1	0.9984	1.09	0.7796	1.28	0.333	1.49	0.062
92618_at	1455236_x_at	Serf2	small EDRK-rich factor 2	-2.55	0.17	1.1	0.43	-1.1	0.5191	-1.14	0.2853	1.37	0.5589
92619_at	1449441_a_at	Wbp1	WW domain binding protein 1	-1.65	0.07	-1.03	0.7269	-1.13	0.3269	-1.13	0.3171	-1.19	0.4918
92621_at	1421743_a_at	Pcbp2	poly(rC) binding protein 2	1.22	0.28	1.28	0.0023	1.16	0.0986	1.38	0	1.18	0.2912
92622_at	1431284_a_at	2210013K02Rik	RIKEN cDNA 2210013K02 gene	-1.16	0.42	-1.11	0.3219	-1.13	0.2055	-1.24	0.011	1.04	0.6897
92625_at	1448808_a_at	Nme2	expressed in non-metastatic cells 2, protein	1.07	0.48	1.1	0.0049	1.1	0.0388	1.03	0.6534	1.04	0.6932
92626_at	1415919_at	Npdc1	neural proliferation, differentiation and contro	-1.55	0.47	-1.01	0.9214	1.25	0.0279	1.02	0.8233	1.12	0.4142
92628_at	1416520_x_at	Rpl36	ribosomal protein L36	1.16	0.2	1.28	0.0001	1.37	0.0011	1.4	0.0001	1.9	0.005
92630_r_at	1419964_s_at	Hdgf	hepatoma-derived growth factor	-1.38	0.35	1.07	0.5654	1.03	0.812	-1.06	0.6044	1.21	0.4557
92632_at	1426710_at	Calm3	calmodulin 3	-1.19	0.7	-1.06	0.3942	-1.05	0.6322	-1.1	0.3025	-1.1	0.6146
92633_at	1417870_x_at	Ctsz	cathepsin Z	1.44	0.39	1.08	0.2528	1.41	0.0002	1.19	0.0244	1.36	0.2379
92634_at	1441342_at	Dpp4	Dipeptidylpeptidase 4	-1.34	0.15	-1.27	0.0288	1.1	0.2965	-1.3	0.0039	-1.12	0.2433
92636_f_at	1423090_x_at	Sec61g	SEC61, gamma subunit	-1.02	0.9	1.03	0.6755	1.21	0.0603	1.19	0.0052	-1.36	0.1729

92637_at	1450269_a_at	Pfkl	phosphofructokinase, liver, B-type	1.21	0.28	1.17	0.0063	-1.03	0.6579	1.01	0.8942	1.26	0.0503
92638_at	1456390_at	Ppp2ca	protein phosphatase 2a, catalytic subunit, al	-1.04	0.84	1.25	0.1105	1.16	0.5488	1.23	0.1462	1.24	0.0405
92639_at	1424511_at	Aurka	aurora kinase A	-1.05	0.87	-1.14	0.548	-1.36	0.2691	1.31	0.1444	1.37	0.4906
92640_at	1415783_at	Vps35	vacuolar protein sorting 35	1.22	0.02	1.07	0.4866	1.06	0.661	1.08	0.3028	1.04	0.8028
92642_at	1448752_at	Car2	carbonic anhydrase 2	-1.11	0.66	-1.77	0	1.69	0.0051	-1.03	0.7148	1.32	0.1237
92643_at	1421820_a_at	Nf2	neurofibromatosis 2	1.09	0.58	-1	0.961	-1.02	0.838	-1.05	0.5322	-1.1	0.539
92644_s_at	1450194_a_at	Myb	myeloblastosis oncogene	1.42	0.26	1.03	0.9382	1.01	0.9674	1.13	0.748	4.41	0.1279
92646_at	1416948_at	Mrpl23	mitochondrial ribosomal protein L23	1.64	0	1.08	0.2102	-1.02	0.759	1.18	0.0052	1.44	0.1098
92647_at	1434892_x_at	Rbbp4	retinoblastoma binding protein 4	-1.27	0.27	1.21	0.0642	1.11	0.3867	1.18	0.0345	1.06	0.7097
92648_at	1435058_x_at	Stxbp3	syntaxin binding protein 3	1.36	0.1	-1.02	0.8094	1.07	0.4438	1.08	0.3668	-1.39	0.0169
92649_at	1419516_at	D0HXS9928E	DNA segment, human DXS9928E	1.74	0.03	1.1	0.1019	1.16	0.1301	1.1	0.1338	1.23	0.1081
92650_at	1420976_at	Man1a2	mannosidase, alpha, class 1A, member 2	-3.73	0.15	1.34	0.5318	-1.03	0.9271	1.32	0.4084	-1.37	0.2016
92652_at	1449146_at	Notch4	Notch gene homolog 4 (Drosophila)	-1.04	0.91	-1.16	0.4594	1.09	0.607	-1.01	0.9724	-1.09	0.7906
92653_at	1437917_at	D530037H12Ril	RIKEN cDNA D530037H12 gene	-1.57	0.39	-1.09	0.4459	1.21	0.1707	-1.03	0.8237	2.86	0.0892
92654_at	1434453_at	Polr3a	polymerase (RNA) III (DNA directed) polype	2.91	0.02	-1.31	0.2569	-1.31	0.2669	1.03	0.9031	1.06	0.897
92655_at	1449538_a_at	Gcnt1	glucosaminyl (N-acetyl) transferase 1, core 2	-1.41	0.41	-1.17	0.0823	-1.25	0.1307	-1.25	0.0226	1.59	0.0691
92656_at	1436382_at	Zbtb12	zinc finger and BTB domain containing 12	1.09	0.78	1.25	0.2088	-1.11	0.4659	1.08	0.707	1.25	0.3523
92658_at	1422735_at	Foxq1	forkhead box Q1	1.2	0.63	-2.48	0.0013	-2.13	0.0033	-1.91	0.0053	-1.26	0.6123
92659_at	1421622_a_at	Rapgef4	Rap guanine nucleotide exchange factor (GI	-2.56	0.02	-1.24	0.1941	1.6	0.0418	-1.37	0.0206	1.07	0.9246
92660_f_at	1416943_at	Ube2e1	ubiquitin-conjugating enzyme E2E 1, UBC4/	1.37	0.19	-1.04	0.7294	-1.03	0.8764	-1.07	0.3064	-1.25	0.0887
92662_g_at	1449957_at	Ptprv	protein tyrosine phosphatase, receptor type,	-1.52	0.07	-1.07	0.5396	-1.03	0.7554	-1.02	0.7618	1.16	0.491
92665_f_at	1422011_s_at	Xlr /// 3830403N	X-linked lymphocyte-regulated complex /// R	2.07	0.17	-1.46	0.0722	-1.17	0.5528	-1.41	0.1015	1.15	0.8516
92666_at	1460222_at	Sh3bp1	SH3-domain binding protein 1	-1.03	0.96	1.84	0.0409	2.06	0.0269	2.22	0.0477	1.35	0.5417
92667_at	1422982_at	---	---	-1.58	0.01	-1.67	0.0004	-1.25	0.1178	-1.41	0.0369	-1.32	0.1752
92668_at	1422755_at	---	---	1.29	0.42	1.03	0.8707	-1.03	0.8481	1.24	0.1524	1.71	0.1265
92672_at	1453413_at	Gnas	GNAS (guanine nucleotide binding protein, a	-1.19	0.68	-1.38	0.2047	-1.16	0.5221	-1.58	0.0733	1.12	0.6505
92673_at	1418792_at	Sh3gl2	SH3-domain GRB2-like 2	-3.47	0.04	-1.42	0.0945	-1.33	0.107	-1.28	0.2377	1.01	0.9816
92674_at	1450508_at	Foxn1	forkhead box N1	-2.15	0.32	1.21	0.667	-1.12	0.7808	-1.45	0.3322	1.14	0.71
92676_at	1424704_at	Runx2	runt related transcription factor 2	1.81	0.31	1.94	0.1341	1.06	0.5894	1.99	0.1354	1.07	0.8255
92681_at	1417217_at	---	---	-1.35	0.59	-2.81	0.0424	-3.12	0.0306	-3.92	0.0198	-1.34	0.4831
92682_at	1450800_at	Syt8	synaptotagmin 8	1.8	0.15	1.64	0.1824	1.06	0.3684	1.59	0.1016	2.71	0.1663
92683_at	1422828_at	Cd3d	CD3 antigen, delta polypeptide	-1.06	0.86	1.5	0.2354	1.32	0.3008	1.54	0.1204	1.66	0.4079
92685_at	1419222_at	Tbxa2r	thromboxane A2 receptor	-2.06	0.22	1.17	0.4319	1.22	0.1767	-1.11	0.3112	1.8	0.1006
92686_at	1427180_at	Slc27a3	Solute carrier family 27 (fatty acid transporte	-1.03	0.9	1.04	0.7485	1.09	0.5091	-1.06	0.6616	1.43	0.2719
92688_at	1424655_at	Acp2	acid phosphatase 2, lysosomal	1.24	0.28	-1.15	0.1012	-1.29	0.0155	-1.16	0.1733	1	0.9962
92689_at	1450424_a_at	Il18bp	interleukin 18 binding protein	2.12	0.13	1.77	0.2017	-1.2	0.2349	1.82	0.2219	1.15	0.8002
92690_at	1419020_at	Gif	gastric intrinsic factor	-2.12	0.09	1.21	0.4975	1.07	0.8342	1.28	0.3502	-1.08	0.7474
92691_at	1426621_a_at	Ppp2r2b	protein phosphatase 2 (formerly 2A), regulat	-1.11	0.9	1.23	0.54	1.73	0.1013	1.18	0.5543	-1.16	0.6325
92692_at	1427291_at	Sycp1	synaptonemal complex protein 1	-1.05	0.9	1.39	0.4351	-1.34	0.3991	-1.49	0.1916	-1.67	0.1219
92693_at	1419078_at	Nin	ninein	1.01	0.98	1.46	0.3157	1.52	0.2343	1.8	0.1677	1.96	0.1147
92694_at	1425451_s_at	Chi3l3 /// Chi3l4	chitinase 3-like 3 /// chitinase 3-like 4	-1.39	0.55	1.14	0.6231	1.19	0.4654	1.31	0.2874	1.03	0.9334
92695_at	1449814_at	Frat1	frequently rearranged in advanced T-cell lym	1.07	0.65	1.54	0.0498	1.25	0.3962	1.67	0.004	1.56	0.3022
92696_at	1421515_at	Nr6a1	nuclear receptor subfamily 6, group A, meml	-2.31	0.34	-1.03	0.7611	-1.11	0.4425	-1.12	0.5116	1.47	0.4642
92697_at	1418496_at	Foxa1	forkhead box A1	1.22	0.06	-1.12	0.2788	-1	0.9617	-1.17	0.1019	1.09	0.7266
92698_at	1422869_at	Mertk	c-mer proto-oncogene tyrosine kinase	1.62	0.25	1.01	0.8054	1.03	0.7164	-1.04	0.5185	1.63	0.2278
92699_at	1448783_at	Slc7a9	solute carrier family 7 (cationic amino acid tr	-4.77	0.22	1.22	0.3706	1.58	0.1089	1.22	0.4707	-1.19	0.7054
92700_at	1416718_at	Bcan	brevican	-1.96	0.09	1.05	0.7368	1.19	0.023	1.12	0.1594	1.13	0.6159
92701_at	1427458_at	Bmp1	bone morphogenetic protein 1	-1.37	0.13	-1.45	0.0004	-1.19	0.0569	-1.46	0.0001	1.1	0.2585
92702_at	1418615_at	Astn1	astrotactin 1	-1.9	0.44	1.05	0.8598	1	0.9958	-1.87	0.0444	1.51	0.4075
92703_at	1427266_at	MGI:1923998	RIKEN cDNA 2310032M22 gene	1.27	0.61	1.01	0.9675	-1.53	0.0642	-1.5	0.1094	2.24	0.007
92706_at	1427140_at	Pvt1	plasmacytoma variant translocation 1	-1.49	0.42	-1.11	0.3379	1.07	0.5718	-1.1	0.2869	1.22	0.1683
92707_at	1450541_at	Pvt1	plasmacytoma variant translocation 1	-2.07	0.33	-1.05	0.8959	1.17	0.7499	1.05	0.896	-1.07	0.8997
92708_at	1424590_at	Ddx19b	DEAD (Asp-Glu-Ala-Asp) box polypeptide 19	-1.28	0.09	-1.01	0.9166	-1.03	0.6922	-1.03	0.7331	-1.31	0.1588

92710_at	1419457_at	Rgnef	Rho-guanine nucleotide exchange factor	2.53	0.01	1.21	0.5488	1.06	0.8637	1.16	0.6641	2.27	0.0176
92711_at	1451802_at	Mcpt6	mast cell protease 6	1.34	0.31	-1.45	0.0123	-1.34	0.0577	-1.23	0.0623	1.11	0.511
92712_at	1422918_at	1810009J06Rik	RIKEN cDNA 1810009J06 gene	-1.61	0.05	1.01	0.9488	1.02	0.8615	-1.06	0.6482	1.27	0.1649
92713_at	1450447_at	Hnf4a	hepatic nuclear factor 4, alpha	-4.59	0.11	-1.12	0.4657	1.04	0.8426	-1.42	0.0547	1.01	0.9847
92714_at	1418619_at	Icam5	intercellular adhesion molecule 5, telenceph	-1.12	0.85	-1.18	0.2319	-1.01	0.9727	1.07	0.7831	1.04	0.8366
92715_at	1419762_at	Ubd	ubiquitin D	1.48	0.35	48.33	0.1817	1.3	0.3218	88.77	0.0878	-1.65	0.5572
92717_at	1426413_at	Neurod1	neurogenic differentiation 1	-2.05	0.22	-1.59	0.0799	-1.73	0.0392	-2.04	0.0123	2.64	0.1994
92718_at	1426278_at	Ifi27	interferon, alpha-inducible protein 27	1.13	0.48	1.26	0.2533	1.25	0.1971	1.33	0.0843	-1.36	0.4719
92724_at	1455740_at	Hnrpa1	Heterogeneous nuclear ribonucleoprotein A1	1.09	0.64	1.29	0.0302	-1.01	0.932	1.42	0.0026	1.76	0.0014
92726_at	1427677_a_at	Sox6	SRY-box containing gene 6	1.36	0.49	-1.18	0.185	-1.28	0.0754	-1.11	0.5121	1.67	0.0205
92727_at	1427288_at	Apba2	amyloid beta (A4) precursor protein-binding,	1.18	0.8	-1.23	0.4084	-1.22	0.4757	-1.22	0.4322	1.72	0.0186
92728_at	1422873_at	Prg2	proteoglycan 2, bone marrow	-1.1	0.38	1.25	0.111	1.16	0.178	-1.07	0.311	1.31	0.209
92730_at	1418350_at	Hbegf	heparin-binding EGF-like growth factor	-1.51	0.05	-1.06	0.4845	-1.28	0.0197	1.01	0.9043	1.27	0.2882
92731_at	1418666_at	Ptx3	pentaxin related gene	1.34	0.65	1.49	0.0851	1.25	0.4578	1.11	0.7375	-1.22	0.597
92732_at	1460374_at	Adam2	a disintegrin and metalloprotease domain 2	-1.43	0.17	-2.25	0.1172	-1.27	0.6206	-2.17	0.1211	-1.01	0.9761
92734_at	1423415_at	Gpr83	G protein-coupled receptor 83	-2.22	0.13	-2.83	0.0069	-1.3	0.4728	-1.77	0.0981	-1.36	0.5243
92735_at	1450128_at	Pla2g2a	phospholipase A2, group IIA (platelets, syno	-1.52	0.18	1.32	0.0671	1.59	0.0167	1.24	0.221	1.19	0.614
92736_at	1422648_at	Slc7a2	solute carrier family 7 (cationic amino acid tr	1.63	0.23	1.58	0.0301	1.24	0.1752	1.77	0.0079	2.84	0.0766
92737_at	1421173_at	Irf4	interferon regulatory factor 4	-1.29	0.59	-1.06	0.5653	1.06	0.5299	-1.01	0.9151	1.2	0.5797
92738_at	1419080_at	Gdnf	glial cell line derived neurotrophic factor	-1.28	0.63	-1.21	0.0621	1.09	0.2955	1.01	0.9387	1.14	0.6975
92739_at	1422222_at	Ivl	involucrin	-1.67	0.44	1.7	0.0528	1.03	0.8834	1.05	0.8255	-1.31	0.4686
92742_at	1417789_at	Ccl11	small chemokine (C-C motif) ligand 11	2.29	0.2	-2.03	0.1729	-2.17	0.1815	-2.29	0.1328	1.09	0.868
92743_at	1419432_at	Spam1	sperm adhesion molecule 1	-1.14	0.65	-1.14	0.3912	-1.2	0.3121	-1.09	0.4592	1.53	0.0976
92744_at	1455231_s_at	Apc2	adenomatosis polyposis coli 2	1.47	0.52	-1.25	0.1325	-1.12	0.2693	-1.4	0.0196	-1.8	0.2673
92745_at	1421579_at	Hoxa9	homeo box A9	-1.39	0.46	1.05	0.8333	1.26	0.1641	1.03	0.8694	1.54	0.0821
92746_at	1449981_a_at	Nat2	N-acetyltransferase 2 (arylamine N-acetyltra	1.01	0.99	1.18	0.0655	1.14	0.2982	1.17	0.0356	-1.58	0.0343
92747_at	1421112_at	Nkx2-2	NK2 transcription factor related, locus 2 (Drc	1.65	0.47	-1.01	0.9632	1.09	0.7052	1.05	0.8132	1.12	0.8014
92749_at	1419719_at	Gabrb1	gamma-aminobutyric acid (GABA-A) recepto	-1.62	0.49	1.01	0.98	1.03	0.9529	1.36	0.5019	1.14	0.731
92753_at	1423984_a_at	Mrg2	myeloid ecotropic viral integration site-relate	2.85	0.13	1.2	0.5886	1.05	0.7602	-1.04	0.862	3.04	0.0501
92754_at	1416806_at	Fdxr	ferredoxin reductase	-1.04	0.8	1.15	0.1251	-1.09	0.1861	1.13	0.0913	1.19	0.1639
92756_r_at	1418805_at	Sct	secretin	-2.34	0.11	-1.06	0.6228	-1.06	0.4498	-1.18	0.0288	1.26	0.0875
92757_at	1427674_a_at	Sez6	seizure related gene 6	-1.37	0.34	1.38	0.1664	1.26	0.3444	1.15	0.4394	2.31	0.1831
92758_at	1450698_at	Dusp2	dual specificity phosphatase 2	3.08	0.01	1.16	0.712	-1.08	0.8185	1.04	0.9137	1.57	0.3906
92759_at	1417812_a_at	Lamb3	laminin, beta 3	3.56	0.12	1.38	0.112	1.19	0.5164	1.11	0.3975	1.56	0.1677
92760_at	1419631_at	Was	Wiskott-Aldrich syndrome homolog (human)	-1.51	0.5	1.36	0.4627	1.02	0.9458	1.64	0.2672	1.46	0.2702
92762_at	1422013_at	Clec4a2	C-type lectin domain family 4, member a2	1.03	0.96	1.48	0.4446	-1.29	0.4235	1.62	0.4598	2.81	0.2037
92763_at	1427490_at	Abcb7	ATP-binding cassette, sub-family B (MDR/TA	1.98	0.14	1.38	0.0541	1.2	0.3954	1.27	0.2287	-1.32	0.1476
92764_at	1450114_at	Ksr	kinase suppressor of ras	-1.13	0.38	-1.28	0.036	-1.16	0.1564	-1.25	0.0529	1.28	0.0128
92766_at	1417525_at	Hand1	heart and neural crest derivatives expressed	-1.27	0.63	-1.17	0.5261	1.03	0.8742	1.05	0.8145	1.23	0.4013
92767_at	1425493_at	Bmpr1a	bone morphogenetic protein receptor, type 1	1.83	0.38	1.26	0.2247	-1.13	0.7512	1.09	0.749	-1.6	0.4128
92768_s_at	1451675_a_at	Alas2	aminolevulinic acid synthase 2, erythroid	-1.99	0.07	1.78	0.0003	-1.28	0.0379	1.73	0	-5.43	0.0001
92769_at	1448479_at	Psmd3	proteasome (prosome, macropain) 26S subu	-1.15	0.34	-1.06	0.3263	1.09	0.5415	1.04	0.6008	1.18	0.3089
92770_at	1421375_a_at	S100a6	S100 calcium binding protein A6 (calcyclin)	2.06	0.17	1.68	0.2921	-1.38	0.3443	2.31	0.1059	1.46	0.2156
92773_at	1448943_at	Nrp1	neuropilin 1	-1.17	0.16	-1.26	0.0241	-1.05	0.691	-1.26	0.0622	-1.99	0.0081
92775_at	1451296_x_at	Pabpc4	poly A binding protein, cytoplasmic 4	1.15	0.25	1.03	0.6613	1.17	0.1692	1.23	0.0242	1.36	0.1028
92777_at	1438133_a_at	Cyr61	cysteine rich protein 61	1.26	0.26	1.09	0.5162	-1.02	0.8993	-1.28	0.1245	1.32	0.1141
92779_f_at	1425584_x_at	---	---	-1.36	0.71	1.11	0.7479	1.25	0.6215	-1.3	0.4675	2.52	0.075
92780_f_at	1425471_x_at	---	---	-3	0.22	1.34	0.4135	-1.32	0.3514	-1.05	0.8545	1.77	0.1826
92781_at	1426020_at	Tmpo	thymopoietin	-1.29	0.48	-1.45	0.009	-1.18	0.1039	-1.65	0.0005	1.19	0.2785
92783_at	1460310_a_at	Gh	growth hormone	1.45	0.07	-1.08	0.3722	1.03	0.7132	-1.01	0.9183	1.18	0.218
92784_at	1419980_at	Gba2	glucosidase beta 2	1.88	0.05	-1.31	0.436	-1.37	0.4396	1.12	0.7515	2.26	0.1522
92786_at	1448507_at	Efhd1	EF hand domain containing 1	1	0.99	-1.35	0.02	-1.01	0.9097	-1.19	0.1179	1.38	0.0481
92787_at	1448199_at	Ankrd10	ankyrin repeat domain 10	-1.19	0.14	1.15	0.0267	1.12	0.0902	1.06	0.4893	1.06	0.483

92789_r_at	1417239_at	Cetn3	centrin 3	1.11	0.13	1.13	0.3727	1.27	0.2523	1.26	0.1588	1.19	0.4096
92790_at	1415860_at	Kpna2	karyopherin (importin) alpha 2	2.24	0.09	-1.12	0.5369	1.1	0.5396	-1.03	0.7706	-1.84	0.0468
92793_at	1417291_at	Tnfrsf1a	tumor necrosis factor receptor superfamily, r	1.4	0.03	1.06	0.2803	1.03	0.6896	1.06	0.3063	1.18	0.0341
92795_at	1416091_at	Mtap4	microtubule-associated protein 4	2.07	0.17	1.14	0.2729	1.39	0.03	1.23	0.1054	1.11	0.1345
92796_at	1423611_at	Akp2	alkaline phosphatase 2, liver	-1.23	0.1	1.16	0.2416	1.41	0.0106	1.32	0.0055	1.37	0.125
92797_at	1422794_at	Cul3	cullin 3	2.2	0.11	1.31	0.0005	1.27	0.005	1.39	0.0007	1.23	0.1837
92800_i_at	1416058_s_at	Atp5c1	ATP synthase, H+ transporting, mitochondria	1.45	0.06	1.08	0.2293	1.12	0.1856	1.18	0.0455	1.09	0.5581
92801_at	1425468_at	Plp1	proteolipid protein (myelin) 1	-1.49	0.35	-1.6	0.094	-1.2	0.2259	-1.37	0.0931	1.31	0.5932
92802_s_at	1425467_a_at	Plp1	proteolipid protein (myelin) 1	-1.69	0.06	1.52	0.104	1.61	0.2267	1.49	0.1322	1.15	0.6991
92803_at	1429287_a_at	Prl	prolactin	-3.84	0.06	-1.04	0.9087	1.23	0.4957	1.08	0.8359	1.58	0.271
92804_at	1424473_at	LOC381040	similar to polymerase (RNA) II (DNA directed	1.16	0.47	1.03	0.7328	1.02	0.8396	-1.04	0.6329	1.76	0.0116
92805_s_at	1431429_a_at	Arl4	ADP-ribosylation factor-like 4	2.34	0.03	1.37	0.2125	-1.35	0.3164	1.51	0.0828	1.07	0.8985
92806_at	1428309_s_at	---	---	1.27	0.05	1.06	0.4117	1.05	0.4561	1.03	0.4747	1.47	0.0428
92807_at	1416119_at	Txn1	thioredoxin 1	-1.18	0.07	-1	0.9878	-1	0.9576	-1.08	0.4094	-1.21	0.1438
92811_at	1422846_at	Rbp2	retinol binding protein 2, cellular	-2.09	0.12	-1.76	0.0493	2.46	0.3732	-1.41	0.205	1.13	0.7682
92814_at	1417532_at	Cyp2j5	cytochrome P450, family 2, subfamily j, poly	-1.2	0.78	-1.5	0.1638	-3.66	0.0004	-3.99	0.0003	-2.21	0.0285
92816_r_at	1434985_a_at	Eif4a1	eukaryotic translation initiation factor 4A1	-1.08	0.44	1.1	0.3509	1.09	0.4864	1.23	0.1136	1.16	0.0267
92817_at	1416210_at	1190002L16Rik	RIKEN cDNA 1190002L16 gene	1.24	0.17	1.19	0.02	1.24	0.0061	1.23	0.0126	1.16	0.3107
92818_at	1417202_s_at	Ube1c	ubiquitin-activating enzyme E1C	1.43	0.02	1.09	0.235	1.18	0.1038	1.16	0.083	-1.1	0.4608
92820_at	1417169_at	Usp2	ubiquitin specific protease 2	9.81	0.17	1.22	0.0843	1.71	0.0001	1.46	0.0084	1.73	0.171
92821_at	1417168_a_at	Usp2	ubiquitin specific protease 2	3.35	0.31	1.15	0.2626	1.42	0.0189	1.33	0.0877	1.62	0.18
92824_at	1448574_at	Nme6	expressed in non-metastatic cells 6, protein	-1.17	0.62	-1.12	0.0512	1.14	0.0095	-1.04	0.5942	1.13	0.1979
92825_at	1416717_at	Crisp2	cysteine-rich secretory protein 2	-2.45	0.33	1.06	0.8265	-1.3	0.1983	-1.27	0.2396	-2.4	0.0664
92826_at	1427173_a_at	Mrps33	mitochondrial ribosomal protein S33	1.48	0.26	1	0.9839	1	0.9968	1.03	0.6556	-1.19	0.064
92827_at	1420881_at	Nsd1	nuclear receptor-binding SET-domain protein	-1.19	0.57	1.01	0.9637	-1.25	0.3783	-1.07	0.7719	1.11	0.6664
92828_at	1449438_at	Dpm1	dolichol-phosphate (beta-D) mannosyltransfe	1.73	0.03	1.11	0.108	1.07	0.5108	1.15	0.0629	-1.1	0.514
92829_at	1450668_s_at	Hspe1	heat shock protein 1 (chaperonin 10)	1.57	0.04	-1.01	0.7583	1.04	0.5254	-1.01	0.8561	-1.08	0.2579
92830_s_at	1452519_a_at	Zfp36	zinc finger protein 36	1.57	0.19	1.62	0.0033	1.08	0.4575	1.23	0.0429	1.17	0.4526
92831_at	1417560_at	Sfxn1	sideroflexin 1	1.3	0.34	1.31	0.0286	-1.29	0.0123	1.29	0.0274	1.17	0.3092
92832_at	1450446_a_at	Socs1	suppressor of cytokine signaling 1	1.19	0.54	1.78	0.2667	1.16	0.5252	2.72	0.0737	2.21	0.1558
92833_at	1418645_at	Hal	histidine ammonia lyase	1.1	0.69	1.21	0.0057	1.17	0.2223	1.19	0.027	1.03	0.7223
92834_at	1433928_a_at	Rpl13a	Ribosomal protein L13a	1.41	0.16	-1.12	0.1787	1.2	0.0222	-1.08	0.1802	-1.22	0.1371
92835_at	1418013_at	Cml1	camello-like 1	-1.2	0.21	-1.62	0.0003	-1.32	0.0158	-2	0	-3.99	0
92836_at	1420855_at	Eln	elastin	-1.66	0.32	-1.04	0.4877	1.02	0.7877	-1.01	0.9432	1.11	0.6632
92837_f_at	1417835_at	Mug1	murinoglobulin 1	-1.48	0.08	1.37	0.0026	1.14	0.3293	1.41	0.001	-1.19	0.2662
92838_at	1448378_at	Fscn1	fascin homolog 1, actin bundling protein (Str	1.03	0.67	1.31	0.0559	1.22	0.1012	1.18	0.2393	1.19	0.0883
92840_at	1433580_at	Nup54	nucleoporin 54	1.29	0.75	4.02	0.0005	4.22	0.0227	3.6	0.004	1.27	0.4493
92842_r_at	1415885_at	Chgb	chromogranin B	-1.27	0.56	1.03	0.946	-1.25	0.68	-1.22	0.7207	1.27	0.7908
92845_at	1428140_at	Oxct1	3-oxoacid CoA transferase 1	2.57	0.03	-1.32	0.3977	-1.43	0.1593	-1.05	0.8832	1.22	0.4518
92847_s_at	1416385_a_at	M6pr	mannose-6-phosphate receptor, cation depe	-1.27	0.12	1.11	0.3922	1.07	0.6351	1.05	0.5907	-1.24	0.1603
92848_at	1416452_at	Oat	ornithine aminotransferase	1.33	0.25	1.15	0.1977	-1.23	0.0468	1.13	0.3405	1.42	0.026
92849_at	1417266_at	Ccl6	chemokine (C-C motif) ligand 6	1.04	0.93	-1.17	0.1005	-1.1	0.3198	-1.12	0.296	1.61	0.0109
92850_at	1452767_at	Rrbp1	ribosome binding protein 1	-1.05	0.85	-1.07	0.3388	-1.25	0.0003	-1.24	0.0048	-1.23	0.0694
92851_at	1455393_at	---	---	-1.49	0.11	1.08	0.4514	-1.02	0.7258	1.07	0.5875	1.01	0.8861
92852_at	1426642_at	Fn1	fibronectin 1	-1.43	0	-1.01	0.9197	-1.01	0.8537	-1.06	0.2955	1.01	0.8393
92854_at	1449256_a_at	Rab11a	RAB11a, member RAS oncogene family	1.23	0.02	1.13	0.0119	1.17	0.0265	1.17	0.0158	-1.15	0.1814
92855_at	1423799_at	Sui1-rs1	suppressor of initiator codon mutations, relat	1.72	0	1.36	0.006	1.44	0	1.63	0	1.56	0.0317
92857_at	1448398_s_at	Rpl22	ribosomal protein L22	1.3	0.57	-1.11	0.2687	-1.02	0.7915	-1.16	0.1425	-1.04	0.8576
92858_at	1448377_at	Slpi	secretory leukocyte protease inhibitor	2.31	0.14	1.79	0.1425	-1.51	0.1329	2.13	0.1224	1.27	0.6727
92862_f_at	1423227_at	Krt1-17	keratin complex 1, acidic, gene 17	1.28	0.33	-1.18	0.2093	1.08	0.521	1.08	0.4218	1.12	0.6046
92863_at	1448121_at	Wbp2	WW domain binding protein 2	-1.26	0.17	-1	0.9891	1.04	0.6086	-1.01	0.8791	1.08	0.4991
92865_at	1424592_a_at	Dnase1	deoxyribonuclease I	-2.35	0.32	2.91	0.0138	1.24	0.464	2.69	0.0093	1.77	0.2408
92866_at	1452431_s_at	H2-Aa	histocompatibility 2, class II antigen A, alpha	1.92	0.16	3.78	0.1595	-1.46	0.1159	3.45	0.1966	8.43	0.0121

92867_at	1437239_x_at	Phc2	polyhomeotic-like 2 (Drosophila)	2.27	0.27	1.29	0.499	1.13	0.6999	1.3	0.4558	1.06	0.6239
92868_at	1416086_at	Tpst2	protein-tyrosine sulfotransferase 2	-1.28	0.44	-1.17	0.1857	1.04	0.6022	-1.06	0.2907	1.13	0.6022
92869_at	1417554_at	Hsd3b4	hydroxysteroid dehydrogenase-4, delta<5>-:	-1.44	0.28	-1.25	0.4422	-1.11	0.6933	-1.15	0.637	-1.08	0.655
92872_at	1448106_at	1200016B17Rik	RIKEN cDNA 1200016B17 gene	-1.36	0	-1.07	0.2434	1.18	0.0379	1	0.9736	1.16	0.1949
92873_f_at	1435507_x_at	Prss2	protease, serine, 2	-3.87	0.18	-1.6	0.0786	-1.51	0.1314	-1.29	0.3399	-1.1	0.8504
92874_f_at	1423245_at	Cops7a	COP9 (constitutive photomorphogenic) hom	1.43	0.05	1.12	0.2217	1.07	0.458	1.22	0.0154	1.08	0.6717
92875_s_at	1429078_a_at	Cops7a	COP9 (constitutive photomorphogenic) hom	1.79	0.48	1.12	0.06	-1.04	0.7856	1.06	0.5315	1.26	0.507
92876_at	1418117_at	Ndufs4	NADH dehydrogenase (ubiquinone) Fe-S pr	-1.05	0.89	1.02	0.8772	1.04	0.6806	1.17	0.1723	-1.07	0.8189
92877_at	1448123_s_at	---	---	1.74	0.04	1.3	0.0829	-1.02	0.7008	1.44	0.033	1.32	0.095
92879_at	1416792_at	Ppm1g	protein phosphatase 1G (formerly 2C), magr	-1.19	0.54	1.07	0.4493	1.24	0.0636	1.12	0.0837	1.08	0.5843
92880_at	1420911_a_at	Mfge8	milk fat globule-EGF factor 8 protein	-1.28	0.08	1.32	0.029	1.07	0.4314	1.37	0.0399	1.6	0.0055
92881_at	1448371_at	Mylpf	myosin light chain, phosphorylatable, fast sk	2.12	0.1	-1.59	0.0029	-1.3	0.0755	-1.54	0.0042	1.3	0.2886
92884_at	1435638_at	---	---	-1.23	0.09	-1.01	0.9393	1.18	0.0127	1.01	0.7926	1.28	0.0086
92885_at	1450118_a_at	Tnnt3	troponin T3, skeletal, fast	1.62	0.18	-4.75	0.1723	-3.85	0.2021	-6	0.1509	1.39	0.1711
92887_at	1416457_at	Ddah2	dimethylarginine dimethylaminohydrolase 2	1.12	0.72	1.03	0.6015	-1.04	0.7121	1.01	0.9244	2.33	0.0086
92888_s_at	1421664_a_at	Styx	phosphoserine/threonine/tyrosine interaction	2.09	0.35	1.86	0.0012	1.04	0.8849	1.4	0.2495	1.55	0.0034
92889_r_at	1422210_at	Foxd3	forkhead box D3	-1.14	0.71	1.16	0.6787	1.51	0.3147	1.08	0.7569	1.36	0.2997
92891_f_at	1449867_at	Hoxc9	homeo box C9	1.06	0.87	-1.34	0.1645	-1.25	0.3158	-1.2	0.4018	2.56	0.0048
92892_at	1418588_at	Vmp	vesicular membrane protein p24	1.03	0.94	-1.35	0.2888	1.09	0.7191	-1.34	0.3473	1.76	0.0681
92894_s_at	1421162_a_at	Nfia	nuclear factor I/A	2.89	0.03	-2.22	0.012	-1.55	0.1311	-2.55	0.0056	1.16	0.759
92895_at	1449967_at	Sim1	single-minded homolog 1 (Drosophila)	-2.46	0.04	-1.69	0.0745	-1.29	0.3188	-1.33	0.2276	1.37	0.1797
92896_s_at	1419437_at	Sim2	single-minded homolog 2 (Drosophila)	1.02	0.95	-1.34	0.4395	-1.22	0.5772	-1.32	0.4562	2.23	0.2506
92897_at	1418878_at	Acrv1	acrosomal vesicle protein 1	1.32	0.63	-1.22	0.5126	-1.12	0.6906	-1.34	0.3274	1.67	0.312
92898_at	1421075_s_at	Cyp7b1	cytochrome P450, family 7, subfamily b, poly	-2.32	0.22	-1.98	0.0014	-1.21	0.1431	-1.61	0.0015	-4.3	0.0019
92900_at	1448018_at	Rere	RIKEN cDNA E230012J19 gene	1.08	0.67	1.26	0.0725	1.07	0.6164	1.43	0.0014	1.23	0.3961
92901_at	1450180_a_at	Rara	retinoic acid receptor, alpha	1.89	0.24	1.07	0.8485	-2.28	0.0514	-1.17	0.6805	-1.99	0.2319
92902_at	1450481_at	Mybl1	myeloblastosis oncogene-like 1	-1.18	0.81	-1.65	0.1401	-1.29	0.473	-1.18	0.643	-1.82	0.0744
92903_at	1423340_at	Tcfap2b	transcription factor AP-2 beta	-1.6	0.43	-1.17	0.5433	-1.12	0.6988	-1.34	0.3293	1.42	0.4782
92904_at	1420425_at	Prdm1	PR domain containing 1, with ZNF domain	1.37	0.48	1.18	0.3236	1.36	0.0269	1.08	0.647	1.09	0.4198
92906_at	1452380_at	Epha7	Eph receptor A7	1.17	0.84	1.3	0.419	1.73	0.2676	1.02	0.9504	-1.28	0.7189
92907_at	1448873_at	Ocln	occludin	-3.64	0.12	1.37	0.031	1.23	0.3234	1.28	0.1977	1.42	0.1898
92908_at	1422742_at	Hivep1	human immunodeficiency virus type I enhan	1.33	0.35	1.32	0.0322	1.13	0.3416	1.44	0.003	-1.03	0.9226
92909_at	1418471_at	Pgf	placental growth factor	1.25	0.69	-1.07	0.4813	-1.06	0.5125	-1.25	0.0531	1.66	0.2291
92910_at	1420669_at	Arnt2	aryl hydrocarbon receptor nuclear translocat	-1.76	0.36	1.3	0.1206	1.47	0.0629	-1.03	0.8394	-1.04	0.9015
92911_at	1449177_at	Ccna1	cyclin A1	-3.09	0.27	-1.04	0.8481	1.02	0.9571	-1.5	0.0669	1.69	0.094
92912_at	1421597_a_at	Msx3	homeo box, msh-like 3	-1.02	0.96	-1.01	0.9242	-1.2	0.184	-1.08	0.6234	1.15	0.6414
92913_at	1419748_at	Abcd2	ATP-binding cassette, sub-family D (ALD), n	4.16	0.11	1.1	0.462	1.19	0.2414	1.19	0.0504	5.91	0.0717
92914_at	1427494_at	Hoxb7	homeo box B7	-2.01	0.01	-1.03	0.5735	1.07	0.3359	-1.03	0.6837	1	0.9883
92915_s_at	1452493_s_at	Hoxb7 /// Hoxb8	homeo box B7 /// homeo box B8	-1.22	0.12	-1.09	0.3483	-1.09	0.3197	-1.09	0.267	1.39	0.0263
92916_at	1417708_at	Syt3	synaptotagmin 3	-1.75	0.09	-1.07	0.4111	-1.1	0.2381	-1.18	0.0594	1.41	0.1788
92917_at	1449478_at	Mmp7	matrix metalloproteinase 7	-1.75	0.16	2.41	0.0764	1.15	0.4948	2.74	0.0768	1.76	0.1934
92918_at	1419321_at	F7	coagulation factor VII	1.14	0.3	-1.46	0	-1.17	0.1251	-1.53	0	-1.05	0.7664
92919_at	1418268_at	Htr3a	5-hydroxytryptamine (serotonin) receptor 3A	-1.23	0.31	-1.12	0.5758	-1.03	0.8028	-1.02	0.9086	2.96	0.0449
92920_at	1449488_at	Pitx1	paired-like homeodomain transcription factor	1.13	0.68	-1.14	0.6666	1.12	0.5998	-1.4	0.1863	1.34	0.2166
92923_g_at	1421996_at	Tcfap2a	transcription factor AP-2, alpha	-1.17	0.48	-1.03	0.8957	1.2	0.339	-1.13	0.5132	1.28	0.663
92925_at	1418901_at	---	---	1.24	0.3	-1.32	0.0682	-1.13	0.3119	-1.1	0.1905	-1.58	0.2539
92926_at	1421461_at	Mpl	myeloproliferative leukemia virus oncogene	-1.24	0.29	1.16	0.4381	1.14	0.315	1.05	0.7661	1.28	0.2979
92927_at	1422607_at	Etv1	ets variant gene 1	-1.01	0.98	-1.17	0.4322	-1.13	0.563	-1.13	0.6397	1.28	0.4476
92929_at	1450752_at	Cyct	cytochrome c, testis	-3.45	0.11	-1.46	0.3229	-1.68	0.1793	-2.05	0.0882	-1.72	0.2857
92930_at	1449863_a_at	Dlx5	distal-less homeobox 5	1.11	0.84	1.02	0.9319	1.29	0.3756	1.11	0.6692	-1.56	0.2818
92931_at	1419204_at	Dll1	delta-like 1 (Drosophila)	1.45	0.59	1.23	0.5246	1.32	0.3502	1.45	0.1338	1.45	0.1156
92932_at	1423288_s_at	Cbln1	cerebellin 1 precursor protein	-1.35	0.25	-1.13	0.4221	1.01	0.9287	-1.15	0.2541	1.32	0.0146
92934_at	1449126_at	Zfp90	zinc finger protein 90	-1.1	0.83	2.39	0.1043	1.53	0.0459	2.92	0.037	1.45	0.4734

92935_at	1448785_at	Cbfa2t1h	CBFA2T1 identified gene homolog (human)	-2.44	0.34	-1.35	0.3408	-1.07	0.8516	-1.17	0.6221	1.29	0.5243
92936_at	1449563_at	Cntn1	contactin 1	-1.06	0.92	1.21	0.3576	1.14	0.6569	1.07	0.822	1.61	0.0831
92937_at	1418596_at	---	---	-1.03	0.86	-1.3	0.0094	-1	0.974	-1.63	0.0001	-1.58	0.217
92938_at	1421281_at	Gabra1	gamma-aminobutyric acid (GABA-A) recepto	2	0.34	1.21	0.421	1.58	0.1096	1.12	0.6966	1.08	0.8898
92939_at	1417121_at	Gabra6	gamma-aminobutyric acid (GABA-A) recepto	-1.76	0.36	1.2	0.6096	1.51	0.4109	1.11	0.8146	-1.05	0.8637
92940_s_at	1451706_a_at	Gabra6	gamma-aminobutyric acid (GABA-A) recepto	1.04	0.93	1.06	0.7522	1.15	0.3667	1	0.9857	1.05	0.8687
92941_at	1436187_at	1110054M08Ril	RIKEN cDNA 1110054M08 gene	1.33	0.34	-1.23	0.0055	-1.02	0.8503	-1.39	0.062	-1.85	0.0529
92942_at	1448940_at	Trim21	tripartite motif protein 21	-1.05	0.81	-1.01	0.9304	-1.22	0.0476	1.01	0.9078	-1.05	0.7657
92943_at	1448972_at	Gria1	glutamate receptor, ionotropic, AMPA1 (alph	-1.9	0.06	1.44	0.0406	1.25	0.2272	1.39	0.3909	1.23	0.4716
92947_s_at	1421970_a_at	Gria2	glutamate receptor, ionotropic, AMPA2 (alph	-2.08	0.32	-1.15	0.439	1.1	0.7821	-1.12	0.345	1.22	0.5397
92948_at	1427429_at	Csf2	colony stimulating factor 2 (granulocyte-mac	-1.52	0.26	-1.28	0.2021	-1.07	0.7959	-1.13	0.5973	-1.08	0.8685
92949_at	1449380_at	Pacsin1	protein kinase C and casein kinase substrat	1.47	0.56	1.53	0.0459	1.58	0.0181	1.64	0.0064	1.32	0.3026
92950_at	1448257_at	Slc29a2	solute carrier family 29 (nucleoside transpor	1.14	0.42	1.43	0.4036	1.01	0.9219	-1.04	0.585	1.12	0.4795
92951_at	1450584_at	Hoxd11	homeo box D11	-2.06	0.23	-2.8	0.0226	-2.28	0.0513	-2.19	0.0633	1	0.9971
92952_f_at	1427470_s_at	Napb	N-ethylmaleimide sensitive fusion protein att	-1.09	0.61	1.51	0.0332	1.41	0.1417	1.32	0.1912	1.19	0.6449
92953_at	1427671_a_at	Fmn1	formin 1	-2.29	0.24	-1.86	0.0513	-1.32	0.354	-2.22	0.0171	-3.42	0.0116
92955_at	1419712_at	Il3ra	interleukin 3 receptor, alpha chain	2.25	0.14	1.27	0.1481	1.01	0.93	1.17	0.2789	1.78	0.1045
92956_at	1421965_s_at	Notch3	Notch gene homolog 3 (Drosophila)	1.31	0.48	1.19	0.1441	-1.19	0.3378	1.25	0.2261	-1.14	0.7099
92957_at	1441350_at	Fgf3	fibroblast growth factor 3	1.66	0.5	-1.25	0.4627	-1.32	0.3288	-2.04	0.032	1.06	0.8585
92958_at	1434832_at	Foxo3a	forkhead box O3a	1.18	0.04	-1.07	0.1594	-1.06	0.3591	-1.09	0.0505	-1.08	0.5454
92959_at	1426570_a_at	Frk	fyn-related kinase	-3.42	0.21	-1.18	0.6119	-1.29	0.5139	-1.28	0.4707	1.3	0.707
92960_at	1421206_at	Lif	leukemia inhibitory factor	-2.61	0.27	1.27	0.2561	2.19	0.1273	1.71	0.081	1.89	0.1549
92961_at	1421951_at	Lhx1	LIM homeobox protein 1	-1.5	0.5	-1.02	0.9533	1.12	0.7171	1.05	0.8298	-1.01	0.9796
92962_at	1449473_s_at	Tnfrsf5	tumor necrosis factor receptor superfamily, r	1.02	0.82	1.07	0.8616	-1.13	0.7988	1.22	0.6547	2.09	0.049
92963_at	1460234_at	BC054822	cDNA sequence BC054822	1.06	0.79	1.15	0.0552	-1.05	0.411	1.02	0.7917	1.11	0.3723
92967_r_at	1420560_at	Chrne	cholinergic receptor, nicotinic, epsilon polype	-2.4	0.06	-1.44	0.149	-1.21	0.403	-1.36	0.2102	1.54	0.0374
92968_at	1422807_at	Arf5	ADP-ribosylation factor 5	-1.02	0.88	-1.06	0.5004	-1.03	0.7319	-1.09	0.1199	-1.06	0.663
92969_at	1422738_at	Ddr2	discoidin domain receptor family, member 2	-1.83	0.16	-1.51	0.0069	-1.44	0.074	-1.38	0.0038	-1.18	0.6638
92970_at	1431475_a_at	Hoxa10	homeo box A10	-3.93	0.31	1.2	0.5532	-1.05	0.7096	-1.09	0.5162	1.02	0.9711
92971_at	1428891_at	9130213B05Rik	RIKEN cDNA 9130213B05 gene	1.4	0.51	-1.15	0.613	-1.06	0.8315	1.07	0.7541	2.95	0.0315
92972_at	1449869_at	Vpreb1	pre-B lymphocyte gene 1	-1.44	0.2	1.41	0.241	1.44	0.14	1.44	0.421	-1.26	0.4325
92974_at	1419207_at	Zfp37	zinc finger protein 37	-1.02	0.97	1.29	0.4181	1.22	0.5324	-1.19	0.6579	1.48	0.3363
92975_at	1448328_at	Sh3bp2	SH3-domain binding protein 2	2.08	0.08	1.08	0.3904	-1.34	0.0131	-1.03	0.7964	-2.04	0.0181
92976_at	1449251_at	Ndph	Norrie disease homolog	1.98	0.4	-1.14	0.6887	1.35	0.2268	-1.19	0.5746	1.54	0.2958
92977_s_at	1422150_at	Hmx3	H6 homeo box 3	-1.84	0.16	1.21	0.3641	-1.08	0.7701	1.03	0.9035	1.24	0.0603
92978_s_at	1419082_at	Serpinb2	serine (or cysteine) proteinase inhibitor, clad	-1.85	0.13	1.11	0.4044	-1.08	0.5422	1.05	0.7933	1.61	0.2492
92980_at	1418620_at	Phox2a	paired-like homeobox 2a	1.08	0.68	-1.03	0.825	1.53	0.2797	1.3	0.4065	1.02	0.9347
92981_at	1450708_at	Scg2	secretogranin II	-1.71	0.3	-1.3	0.262	-1.83	0.0733	-1.35	0.2974	1.1	0.8697
92982_at	1449873_at	Bmp8a	bone morphogenetic protein 8a	-1.06	0.87	-1.11	0.5775	-1.2	0.4586	-1.14	0.5826	1.26	0.6604
92984_g_at	1425587_a_at	Ptprj	protein tyrosine phosphatase, receptor type,	-1.01	0.99	-1.09	0.5528	-1.12	0.5776	-1.33	0.1266	-1.19	0.3486
92986_g_at	1427629_at	Ptprj	protein tyrosine phosphatase, receptor type,	-1.69	0.01	-1.01	0.9045	1.03	0.8166	1.02	0.8022	1.32	0.0052
92987_at	1418485_at	Slc4a3	solute carrier family 4 (anion exchanger), me	1.26	0.06	-1.21	0.4179	-1.13	0.6416	-1.42	0.1423	-1.23	0.6051
92989_f_at	1448955_s_at	Cadps	Ca<2+>dependent activator protein for secre	-2.26	0.17	1.02	0.944	-1.08	0.7246	-1.18	0.4525	-1.03	0.9505
92990_at	1418955_at	Zfp93	zinc finger protein 93	1.69	0.17	-1.1	0.6367	-1.28	0.2602	1.04	0.8755	1.19	0.4526
92991_at	1421504_at	Sp4	trans-acting transcription factor 4	1.48	0.46	-1.17	0.2286	-1.24	0.2164	-1.33	0.0483	1.15	0.7189
92993_r_at	1437508_at	5730497N03Rik	RIKEN cDNA 5730497N03 gene	-1.18	0.81	1.09	0.375	-1.67	0.0035	-1.06	0.709	1.2	0.4853
92994_at	1418363_at	Lalba	lactalbumin, alpha	-1.23	0.31	-1.05	0.7493	-1.09	0.5366	-1.23	0.2629	1.52	0.219
92996_at	1429177_x_at	Sox17	SRY-box containing gene 17	2.38	0.03	1.21	0.0618	1.21	0.0554	1.07	0.5455	1.28	0.4858
92997_g_at	1421657_a_at	Sox17	SRY-box containing gene 17	-3.01	0.21	1.19	0.1033	1.11	0.2585	1.34	0.0116	1.23	0.3513
92998_at	1421272_at	Vav2	Vav2 oncogene	-2.96	0.01	1.05	0.6232	-1.03	0.8609	-1.4	0.0351	-1.11	0.7976
93000_g_at	1425767_a_at	Six4	sine oculis-related homeobox 4 homolog (Dr	-1.3	0.57	1.19	0.4742	1.38	0.238	1.43	0.0881	1.54	0.3105
93002_r_at	1450989_at	Tdgf1	teratocarcinoma-derived growth factor	2.82	0.04	-1.05	0.8446	1.11	0.6052	-1.2	0.381	1.18	0.4143
93004_r_at	1424757_at	BC018242	cDNA sequence BC018242	-1.77	0.04	-1.01	0.9271	1.04	0.5957	1.07	0.4243	1.14	0.2851

93005_at	1421990_at	Syt1	synaptotagmin 1	1.16	0.75	-1.58	0.0072	1.09	0.6947	-1.43	0.0571	-1.09	0.8515
93006_at	1450661_x_at	Nfic	nuclear factor I/C	1.32	0.32	-1.41	0.2469	-3.18	0.0002	-2.6	0.0039	1.12	0.8548
93007_at	1421471_at	Npy1r	neuropeptide Y receptor Y1	-1.57	0.13	1.05	0.6437	-1.05	0.7317	1.09	0.4961	1.68	0.0754
93008_at	1448622_at	Lsm4	LSM4 homolog, U6 small nuclear RNA asso	1.49	0.47	-1.14	0.0616	1.09	0.3705	1.04	0.5266	1.19	0.0607
93009_at	1416411_at	Gstm2	glutathione S-transferase, mu 2	1.06	0.66	-1.02	0.9474	-1.38	0.0512	1.14	0.5206	-1.2	0.4637
93010_at	1417731_at	Pqbp1	polyglutamine binding protein 1	1.49	0.23	1.07	0.7889	-1.3	0.3398	1.34	0.2077	-2.73	0.1192
93011_at	1416419_s_at	Gabarapl1	gamma-aminobutyric acid (GABA(A)) recept	1.34	0.09	1.39	0.0032	1.17	0.0616	1.41	0.0003	2.12	0
93013_at	1435176_a_at	Id2	inhibitor of DNA binding 2	-1.49	0.32	1.2	0.0843	-1.07	0.6277	1.08	0.4119	-1.82	0.0047
93014_at	1448203_at	Atp5l	ATP synthase, H+ transporting, mitochondri	-1.18	0.56	1.14	0.0127	1.22	0.0037	1.23	0.0042	1	0.9697
93015_at	1423437_at	Gsta3	glutathione S-transferase, alpha 3	-1.19	0.18	1.13	0.0305	-1.14	0.189	1.05	0.3095	1.1	0.2158
93016_at	1460621_x_at	Ywhaq	tyrosine 3-monooxygenase/tryptophan 5-mo	-1.28	0.39	-1.07	0.718	-1.4	0.175	-1.65	0.0428	2.21	0.1282
93017_at	1450941_at	---	---	1.3	0.38	1.08	0.5506	1.17	0.395	1.22	0.2066	-1.58	0.149
93018_at	1417460_at	Ifitm2	interferon induced transmembrane protein 2	-1.16	0.78	-1.12	0.5911	-1.21	0.3526	-1.29	0.3269	-1.06	0.8633
93019_at	1416746_at	H2afx	H2A histone family, member X	1.04	0.8	1.11	0.1573	1.09	0.075	1.08	0.3179	1.12	0.5714
93020_at	1448595_a_at	Rex3	reduced expression 3	3.41	0.04	1.26	0.4777	-1.03	0.9111	-1.04	0.8845	1.3	0.5473
93021_at	1428209_at	LOC406217	RIKEN cDNA 2410004M13 gene	1.95	0.07	1.37	0.03	1.23	0.1868	1.71	0.0012	2.01	0.0038
93023_f_at	1460314_s_at	Hist2h3c1 /// His	histone 2, H3c1 /// histone 2, H3c2 /// histone	-1.55	0.37	-1.47	0.006	-1.54	0.011	-2	0.0002	1.47	0.3555
93025_at	1424821_at	Ndfip1	Nedd4 family interacting protein 1	-1.26	0.1	-1.14	0.0032	-1.01	0.8384	-1.23	0.0002	-1.39	0.0273
93026_at	1415897_a_at	Mgst1	microsomal glutathione S-transferase 1	-1.38	0.17	1.11	0.3574	-1.05	0.6457	1.1	0.2823	-1.14	0.3608
93028_at	1448194_a_at	---	---	11.99	0.1	1.14	0.8404	-2.58	0.2763	2.14	0.3001	25.04	0.0258
93029_at	1416789_at	Idh3g	isocitrate dehydrogenase 3 (NAD+), gamma	1.78	0.02	1.1	0.0919	1.11	0.0207	1.26	0.0011	1.05	0.5184
93030_at	1449196_a_at	Rps27a	ribosomal protein S27a	1.11	0.57	1.09	0.0352	1.18	0.0376	1.13	0.0031	1.19	0.3433
93033_at	1448671_at	Ube2e3	ubiquitin-conjugating enzyme E2E 3, UBC4/	-2.42	0.05	-1.42	0.0249	-1.34	0.1454	-1.82	0.0007	1.15	0.6671
93038_f_at	1448213_at	Anxa1	annexin A1	1.23	0.48	1.17	0.2725	-1.05	0.8379	1.4	0.1533	1.18	0.619
93039_at	1416441_at	MGI:1889205	plasma glutamate carboxypeptidase	1.05	0.84	-1.05	0.3388	1.03	0.8438	-1.25	0.0077	-1.18	0.1114
93040_at	1421374_a_at	Fxyd1	FXYD domain-containing ion transport regul	-1.36	0.28	-1.1	0.0668	-1.17	0.0867	-1.19	0.0273	-1.06	0.4573
93041_at	1416214_at	Mcm4	minichromosome maintenance deficient 4 ho	1.52	0.48	1.24	0.1955	-1.3	0.055	1.11	0.4298	1.15	0.3612
93042_at	1416695_at	Bzrp	benzodiazepine receptor, peripheral	1.01	0.97	-1.09	0.2936	1.11	0.3366	1.05	0.6388	-1.07	0.6519
93043_at	1415821_at	Sdfr1	stromal cell derived factor receptor 1	1.48	0	1.15	0.0623	1.14	0.2497	1.16	0.0111	1.03	0.8641
93045_at	1416679_at	Abcd3	ATP-binding cassette, sub-family D (ALD), m	1.1	0.46	-1.25	0.0013	-1.11	0.1793	-1.22	0.0698	-2.65	0.0001
93046_at	1428328_at	Nup50	nucleoporin 50	1.63	0.2	1.09	0.4742	1.31	0.1787	1.01	0.9133	-1.33	0.2876
93048_at	1416616_s_at	Clpp	caseinolytic protease, ATP-dependent, prote	1.36	0.02	1.08	0.334	1.12	0.1807	1.18	0.079	1.16	0.2491
93050_at	1448394_at	Myl2	myosin, light polypeptide 2, regulatory, cardi	1.01	0.99	-1.21	0.3465	1.1	0.672	-1.19	0.3455	1.38	0.3391
93051_at	1448499_a_at	Ephx2	epoxide hydrolase 2, cytoplasmic	1.23	0.12	1.71	0.0001	1.35	0.0152	1.83	0.0001	-1.39	0.0421
93053_at	1422529_s_at	Casq2	calsequestrin 2	1	1	-1.01	0.9696	-1.24	0.3461	-1.85	0.011	-1.08	0.7848
93054_at	1428063_at	1110054N06Rik	RIKEN cDNA 1110054N06 gene	-1.49	0.14	-1.14	0.2578	-1.1	0.2021	-1.13	0.1952	-1.3	0.2136
93057_at	1449643_s_at	Btf3	basic transcription factor 3	1.23	0.24	-1.06	0.1936	1.11	0.2769	1.05	0.2969	1.39	0.1593
93058_at	1424343_a_at	Eif1a	eukaryotic translation initiation factor 1A	1.13	0.56	1.49	0.0045	1.12	0.2795	1.58	0.0103	1.02	0.9164
93061_at	1418393_a_at	Itga7	integrin alpha 7	-1.52	0.45	-1.05	0.7116	-1.09	0.5426	1.12	0.3554	1.57	0.2081
93062_at	1448909_a_at	Mrpl39	mitochondrial ribosomal protein L39	2.14	0.02	-1.01	0.8334	1.01	0.8775	-1.07	0.1224	-1.23	0.107
93063_at	1427442_a_at	App	amyloid beta (A4) precursor protein	-1.38	0.26	1.48	0	1.16	0.0329	1.5	0.0002	2.24	0.0005
93064_at	1422490_at	Bnip2	BCL2/adenovirus E1B 19kDa-interacting pro	2.19	0.05	1.35	0.0006	1.12	0.1526	1.41	0.0001	2.11	0.0003
93066_at	1448148_at	Grn	granulin	-1.07	0.63	-1.33	0.004	1.02	0.7984	-1.26	0.066	-1.22	0.0993
93068_r_at	1418367_x_at	Hist2h2aa1 /// H	histone 2, H2aa1 /// histone 1, H2ad /// histo	1.64	0.12	-1.91	0.0187	-1.21	0.4566	-1.61	0.0619	1.14	0.0871
93069_at	1448356_at	Ube2d2	ubiquitin-conjugating enzyme E2D 2	1.06	0.75	-1.09	0.3165	-1.64	0.0008	-1.62	0.0001	-1.39	0.0807
93070_at	1426946_at	Ranbp5	RAN binding protein 5	1.61	0.23	-1.02	0.8546	-1.09	0.4865	1.13	0.03	-1.57	0.0604
93071_at	1415869_a_at	Trim28	tripartite motif protein 28	1.04	0.71	1.12	0.1196	1.02	0.834	1.2	0.0129	1.38	0.0331
93075_r_at	1425990_a_at	Nfatc2	nuclear factor of activated T-cells, cytoplasm	-2.57	0.01	1.22	0.5672	1.23	0.628	1.07	0.8395	1.96	0.3293
93076_at	1428537_at	Csnk1a1	casein kinase 1, alpha 1	1.12	0.49	1.01	0.766	-1.05	0.5544	-1.02	0.639	1.1	0.4352
93077_s_at	1421571_a_at	Ly6c	lymphocyte antigen 6 complex, locus C	-1.51	0.06	1.07	0.3822	-1.07	0.4127	1.12	0.4458	1.06	0.5606
93078_at	1417185_at	Ly6a	lymphocyte antigen 6 complex, locus A	1.06	0.62	1.54	0.4595	-1.49	0.0638	1.35	0.6424	-3.83	0.0005
93080_at	1420632_a_at	Bscl2	Bernardinelli-Seip congenital lipodystrophy 2	1.02	0.86	-1.03	0.5361	-1.02	0.7771	-1.04	0.4809	-1.16	0.1979
93081_at	1415775_at	Rbbp7	retinoblastoma binding protein 7	1.1	0.43	1.15	0.0066	1	0.9455	1.19	0.0039	1.07	0.3555

93082_at	1422032_a_at	Za20d3	zinc finger, A20 domain containing 3	-1.15	0.37	1.02	0.6872	1.3	0.0028	1.2	0.0029	-1.1	0.5352
93083_at	1425567_a_at	Anxa5	annexin A5	-1.22	0.53	1.39	0.1149	1.33	0.0019	1.74	0.0361	-1.06	0.5958
93084_at	1455069_x_at	Slc25a4	solute carrier family 25 (mitochondrial carrier	3.14	0.01	-1.29	0.3875	-1.35	0.3057	1.11	0.6745	1.63	0.179
93085_at	1450696_at	Psmb9	proteosome (prosome, macropain) subunit,	-1.09	0.6	1.49	0.4621	-2.08	0.0069	1.6	0.3379	-1.8	0.1486
93086_at	1452463_x_at	Igk-V8	Immunoglobulin kappa chain, constant regio	2.17	0.11	2.12	0.0481	-1.42	0.5907	1.53	0.3459	3.74	0.006
93088_at	1449289_a_at	B2m	beta-2 microglobulin	-1.37	0.05	1.02	0.7841	-1.09	0.2357	1.01	0.9323	-1.13	0.2438
93089_at	1450934_at	Eif4a2	eukaryotic translation initiation factor 4A2	-1.41	0.29	-1.23	0.0052	-1.14	0.0292	-1.3	0.0077	1.04	0.8057
93090_at	1433489_s_at	---	---	1.04	0.78	1.01	0.9429	-1.02	0.836	1.18	0.0843	1.05	0.8453
93091_s_at	1420847_a_at	Fgfr2	fibroblast growth factor receptor 2	1.61	0.31	1.12	0.3361	1.08	0.5821	1.09	0.4175	1.72	0.3202
93092_at	1422527_at	H2-DMa	histocompatibility 2, class II, locus DMa	1.23	0.7	2.31	0.1615	-1.56	0.045	2.2	0.2304	2.01	0.1
93093_at	1416880_at	Mcl1	myeloid cell leukemia sequence 1	2.48	0.01	1.2	0.0875	1.02	0.7978	1.35	0.0047	1.66	0.0064
93094_at	1417430_at	Cdr2	cerebellar degeneration-related 2	-1.05	0.88	-1.1	0.6824	1.13	0.5448	-1.23	0.1605	-1.44	0.3044
93095_at	1416176_at	Hmgb1	high mobility group box 1	-1.36	0.11	-1.03	0.6916	1.08	0.4415	-1.06	0.5409	-1.28	0.0896
93096_at	1416025_at	Fgg	fibrinogen, gamma polypeptide	-1.32	0.09	1.11	0.1701	1.1	0.2518	1.05	0.3722	-1.24	0.2303
93097_at	1419549_at	Arg1	arginase 1, liver	1.22	0.33	1.1	0.0355	-1.01	0.8354	1.16	0.0015	-1.31	0.0774
93099_f_at	1448191_at	Plk1	polo-like kinase 1 (Drosophila)	-1.74	0.34	1.17	0.4215	1.46	0.12	1.92	0.0086	1.52	0.1071
93100_at	1416454_s_at	Acta2	actin, alpha 2, smooth muscle, aorta	1.53	0.11	-1.07	0.8883	-1.1	0.8515	-1.06	0.9126	1.51	0.1748
93101_s_at	1450431_a_at	Nedd4	neural precursor cell expressed, developme	-1.34	0.13	1.14	0.0204	1.03	0.5194	1.2	0.0079	1.03	0.74
93102_f_at	1422340_a_at	Actg2	actin, gamma 2, smooth muscle, enteric	1.08	0.65	-1.18	0.0536	-1.37	0.0001	-1.28	0.0074	1.46	0.1477
93103_at	1415847_at	Ldh3	lactate dehydrogenase 3, C chain, sperm sp	1.23	0.36	1.02	0.8863	1.26	0.0788	1.14	0.302	1.03	0.918
93104_at	1426083_a_at	Btg1	B-cell translocation gene 1, anti-proliferative	1.4	0.01	1.7	0.0003	-1.01	0.9192	1.44	0.0395	1.67	0.0079
93105_s_at	1452205_x_at	Tcrb-V13	T-cell receptor beta, variable 13	1.07	0.65	1.29	0.0276	-1.14	0.1229	1.15	0.2562	1.48	0.0075
93107_r_at	1426772_x_at	LOC381765	similar to T cell antigen receptor	-1.83	0.55	-1.1	0.7738	-1.01	0.9875	1.19	0.6206	1.2	0.8264
93111_at	1448526_at	Kpnb1	karyopherin (importin) beta 1	1.33	0.14	-1.19	0.0196	-1.16	0.0518	-1.36	0.0004	-1.29	0.0035
93112_at	1448777_at	Mcm2	minichromosome maintenance deficient 2 m	-1.16	0.69	1.21	0.0511	-1.03	0.7735	1.05	0.4768	1.3	0.1976
93116_at	1420611_at	Prkacb	protein kinase, cAMP dependent, catalytic, b	-1.04	0.58	-1.04	0.4911	-1.07	0.223	-1.06	0.2756	1.1	0.5022
93117_at	1420365_a_at	Hnrpa2b1	heterogeneous nuclear ribonucleoprotein A2	-1.33	0.05	-1.03	0.5908	1.11	0.0825	1.05	0.1774	-1.3	0.1687
93118_at	1433829_a_at	Hnrpa2b1	heterogeneous nuclear ribonucleoprotein A2	1.37	0.08	1.41	0.0012	1.1	0.4518	1.48	0	1.34	0.0034
93119_at	1416902_a_at	Cox5b	cytochrome c oxidase, subunit Vb	1.56	0.01	1.04	0.3885	-1.02	0.7033	1.15	0.0221	1	0.9554
93120_f_at	1425336_x_at	H2-K1	histocompatibility 2, K1, K region	-1.08	0.15	1.14	0.3272	-1.12	0.0217	1.16	0.2911	-1.27	0.0937
93122_at	1416325_at	Crisp1	cysteine-rich secretory protein 1	1.9	0.35	1.62	0.2573	-1.11	0.4037	1.18	0.1118	1.14	0.7079
93126_at	1455106_a_at	Ckb	creatine kinase, brain	-1.14	0.38	-1.14	0.0776	-1.12	0.227	-1.04	0.6594	1.2	0.3668
93127_at	1426658_x_at	Phgdh	3-phosphoglycerate dehydrogenase	-1.18	0.68	-1.32	0.1579	-1.01	0.9746	-1.22	0.3213	1.28	0.4194
93128_at	1426054_at	Npy1r	neuropeptide Y receptor Y1	-1.4	0.28	-1.11	0.4453	-1.14	0.3384	-1.17	0.1938	1.04	0.8328
93129_at	1450181_at	Cutl2	cut-like 2 (Drosophila)	-1.08	0.74	-7.38	0.0007	1.66	0.1119	-8.34	0.0006	-1.13	0.6139
93130_at	1456117_at	2600005C20Rik	RIKEN cDNA 2600005C20 gene	-1.46	0.07	-1.02	0.7336	-1.28	0.0271	-1.03	0.5996	1.3	0.0626
93132_at	1427676_a_at	Grik1	Glutamate receptor, ionotropic, kainate 1	1.44	0.43	1.36	0.4387	2.03	0.1863	1.42	0.1343	1.07	0.915
93133_at	1417022_at	Slc7a3	solute carrier family 7 (cationic amino acid tr	-1.36	0.28	1.2	0.3376	1.07	0.7686	1.14	0.4981	-1.06	0.8896
93134_at	1422130_at	Nptx1	neuronal pentraxin 1	-2.45	0.07	1.4	0.0534	1.15	0.5009	1.12	0.6902	2.32	0.1186
93136_at	1421114_a_at	Dspg3	dermatan sulphate proteoglycan 3	-1.65	0.34	1.25	0.5195	1.73	0.0659	2.57	0.0655	1.37	0.2569
93137_at	1417151_a_at	Ntsr2	neurotensin receptor 2	2.6	0.11	1.33	0.3183	1.05	0.8386	1.14	0.5866	1.79	0.063
93138_at	1428515_at	2410012H22Rik	RIKEN cDNA 2410012H22 gene	1.46	0.07	1.01	0.8648	1.01	0.849	1.09	0.1476	1.19	0.0835
93139_at	1418490_at	Sdsl	serine dehydratase-like	-1.04	0.82	1.04	0.5992	1.12	0.4557	1.14	0.0259	-1.17	0.4799
93140_at	1420439_at	Tsx	testis specific X-linked gene	1.28	0.66	1.78	0.1138	1.55	0.2475	1.74	0.0987	1.03	0.9543
93141_at	1417760_at	Nr0b1	nuclear receptor subfamily 0, group B, mem	-2.08	0.17	-1.34	0.2018	-1.44	0.0977	-1.45	0.152	2.23	0.082
93142_at	1449311_at	Bach1	BTB and CNC homology 1	-1.07	0.49	2.39	0.1393	-1.36	0.2635	-1.42	0.2174	-1.3	0.6592
93143_at	1452609_at	1190005I06Rik	RIKEN cDNA 1190005I06 gene	1.14	0.62	1.24	0.0225	1.12	0.0271	1.23	0.0041	2.22	0.0013
93144_at	1434335_at	AI317237	expressed sequence AI317237	1.19	0.1	1.03	0.7095	1.25	0.0603	1.24	0.0658	1.26	0.0197
93145_at	1455505_at	---	Transcribed locus	1.15	0.48	1.04	0.7744	1.08	0.3946	1.14	0.2078	1.18	0.1747
93147_f_at	1452240_at	Brunol4	bruno-like 4, RNA binding protein (Drosophil	-1.25	0.2	-1.28	0.1579	-1.18	0.3696	-1.33	0.0626	1.52	0.3698
93148_at	1428862_at	Ttc17	tetratricopeptide repeat domain 17	1.05	0.86	1.23	0.1178	1.44	0.0139	1.28	0.0565	1.14	0.3869
93151_at	1449921_s_at	Cpne6	copine VI	-1.06	0.85	1.43	0.0996	1.12	0.6643	1.01	0.9641	1.14	0.675
93153_at	1434932_at	---	---	1.04	0.63	-1.18	0.0365	1.09	0.2433	-1.13	0.1262	1.14	0.5007

93155_at	1436243_at	A930004K21Rik	RIKEN cDNA A930004K21 gene	-1.17	0.8	1.19	0.161	1.14	0.5288	1.01	0.9719	1.58	0.0906
93158_at	1434992_at	9130206N08Rik	RIKEN cDNA 9130206N08 gene	-1.24	0.7	1.33	0.1106	1.07	0.6868	-1.32	0.1846	1.42	0.2704
93159_at	1440323_at	---	---	1.01	0.99	1.08	0.6794	1.25	0.1656	1.12	0.5717	-1.13	0.6994
93162_f_at	1425122_at	ORF9	open reading frame 9	-1.13	0.63	-1.09	0.6035	1.05	0.8073	1.12	0.6744	-1.24	0.5131
93165_at	1460438_at	2610022K04Rik	RIKEN cDNA 2610022K04 gene	1.44	0.17	1.06	0.582	1.25	0.0862	1.23	0.1035	2.35	0.134
93166_at	1447962_at	---	Transcribed locus	1.47	0.57	1.64	0.1687	-1.23	0.4819	-1.15	0.6879	1.4	0.314
93167_f_at	1419534_at	---	---	1.51	0.29	-1.53	0.0749	-1.69	0.0074	-1.54	0.0385	3.21	0.0181
93173_at	1417881_at	Slc39a3	solute carrier family 39 (zinc transporter), me	1.15	0.76	-1.05	0.8388	-1.3	0.2453	-1.15	0.5764	1.61	0.0501
93175_at	1454678_s_at	A130022J15Rik	RIKEN cDNA A130022J15 gene	-1.36	0.32	-1.12	0.1543	-1.01	0.9176	-1.05	0.5367	1.03	0.8828
93177_at	1436228_at	Zranb1	zinc finger, RAN-binding domain containing	1.46	0.26	1.09	0.5187	1.16	0.3803	1.31	0.0148	1.04	0.8508
93178_at	1448978_at	Ngef	neuronal guanine nucleotide exchange facto	1.44	0.01	-1.03	0.749	-1.3	0.0113	-1.24	0.0304	1.78	0.0008
93179_at	1455350_at	B830009D23Rik	RIKEN cDNA B830009D23 gene	-1.01	0.97	-1.2	0.188	-1.22	0.1093	-1.02	0.9113	-1.09	0.5391
93180_at	1439483_at	AI506816	expressed sequence AI506816	-1.08	0.83	1.01	0.903	1.17	0.0151	1.06	0.3982	1.17	0.6778
93183_at	1439350_s_at	Cdc91l1	CDC91 cell division cycle 91-like 1 (S. cerev	1.03	0.85	1.11	0.2401	1.03	0.7973	1.11	0.3632	1.14	0.5326
93184_at	1434224_at	Tbl2	transducin (beta)-like 2	-1.13	0.81	1.04	0.6601	1.1	0.2872	-1.03	0.7201	-1.2	0.263
93185_at	1437661_at	LOC239691	similar to SARG904	1.43	0.06	-1.12	0.3325	-1.17	0.2143	-1.08	0.6499	1.56	0.0158
93187_at	1426784_at	Trim47	tripartite motif protein 47	1.08	0.71	-1.02	0.8251	1.04	0.5517	1.03	0.601	1.19	0.1768
93188_at	1417312_at	Dkk3	dickkopf homolog 3 (Xenopus laevis)	-1.21	0.67	1.08	0.6398	-1.05	0.7328	-1.11	0.4959	1.55	0.3957
93190_at	1435013_at	MGC60818	similar to hypothetical protein FLJ20397	1.12	0.62	1.02	0.7531	1.11	0.1997	1.12	0.0334	1.1	0.525
93191_at	1434796_at	Vamp4	vesicle-associated membrane protein 4	1.56	0.02	1.16	0.0646	1.14	0.2979	1.16	0.2362	1.27	0.1363
93193_at	1437302_at	Adrb2	adrenergic receptor, beta 2	-1.24	0.6	-1.08	0.4708	1.08	0.693	1.24	0.1007	1.28	0.1813
93194_at	1449156_at	Ly9	lymphocyte antigen 9	1.43	0.7	1.32	0.0484	1.16	0.3631	1.63	0.1396	2.05	0.2279
93195_at	1429005_at	Mfhas1	malignant fibrous histiocytoma amplified seq	1.06	0.8	-1.22	0.2197	1.12	0.4816	-1.6	0.0177	1.04	0.8688
93198_at	1418806_at	Csf3r	colony stimulating factor 3 receptor (granulo	-4.65	0.12	-1.07	0.7916	-1.15	0.6353	1.48	0.4113	1.46	0.1445
93199_at	1452488_at	Dnajc8	DnaJ (Hsp40) homolog, subfamily C, membe	1.08	0.91	-1.23	0.2858	-1.31	0.1089	-1.46	0.0362	1.53	0.2829
93200_f_at	1422956_at	D1Pas1	DNA segment, Chr 1, Pasteur Institute 1	-3.88	0.09	1.49	0.2877	1.19	0.6581	-1.19	0.5781	-1.28	0.6064
93201_at	1437693_at	D1Pas1	DNA segment, Chr 1, Pasteur Institute 1	-2.22	0.2	-1.33	0.2825	-1.38	0.2625	-1.38	0.2183	2.32	0.3552
93202_at	1422974_at	Nt5e	5' nucleotidase, ecto	1.25	0.61	-2.16	0.0001	-1.34	0.033	-3.38	0	1.16	0.2109
93204_r_at	1454831_at	3230402J05Rik	RIKEN cDNA 3230402J05 gene	-2.19	0.44	1.18	0.648	-1.16	0.6825	1.01	0.9729	1.61	0.2547
93207_at	1449895_at	Acr	preproacrosin	-1.98	0.13	1.07	0.4779	1.02	0.8251	1.15	0.4364	1.62	0.0038
93208_at	1422682_s_at	Prss3 /// Trygn1	protease, serine, 3 /// trypsinogen 16	-1.38	0.2	-1.95	0.1314	-1.44	0.3653	-1.58	0.2319	2.36	0.0471
93210_g_at	1427639_a_at	Nek4	NIMA (never in mitosis gene a)-related expre	-1.25	0.66	-1.1	0.5725	-1.34	0.0499	-1.53	0.0126	-1.86	0.207
93213_at	1425871_a_at	LOC384413 /// l	similar to immunoglobulin light chain variable	-1	0.99	-1.35	0.1028	1.02	0.9384	-1.16	0.5141	2.13	0.0596
93214_at	1427763_a_at	Camk2d	calcium/calmodulin-dependent protein kinas	-2.57	0.39	1.23	0.5748	1.7	0.4158	1.22	0.5856	1.15	0.4904
93216_at	1449826_a_at	Fgf2	fibroblast growth factor 2	1.12	0.86	1.33	0.328	-1.28	0.0882	1.6	0.2277	1.46	0.3733
93217_at	1452895_at	Fbxo45	F-box protein 45	1.21	0.06	1.09	0.3814	1.2	0.2749	1.16	0.1891	1.31	0.1454
93220_at	1425475_at	Col4a5	procollagen, type IV, alpha 5	-1.32	0.32	3.16	0	1.7	0.0853	4.37	0	1.06	0.9141
93221_at	1431099_at	Hoxd8	homeo box D8	1.13	0.5	-1.58	0.2067	-1.14	0.7054	-1.92	0.1083	-1.12	0.7072
93224_at	1427766_at	---	Similar to T cell receptor V alpha 8.5	-2.28	0.08	1.4	0.1773	1.24	0.3905	1.34	0.2135	-1.17	0.7577
93225_s_at	1422333_at	Cyp21a1	cytochrome P450, family 21, subfamily a, po	-1.13	0.19	-1.14	0.0254	-1.19	0.0169	-1.29	0.0001	1.19	0.2096
93230_at	1450836_at	Neurog1	neurogenin 1	-1.63	0.06	1.23	0.2026	1.09	0.5861	1.09	0.3616	1.06	0.7648
93232_at	1435259_s_at	D2Ertd217e	DNA segment, Chr 2, ERATO Doi 217, expr	1.17	0.18	1.17	0.1171	1.15	0.2153	1.21	0.088	-1.45	0.0097
93234_at	1418417_at	Msc	musculin	-1.21	0.65	-1.22	0.6404	-1.76	0.1951	-1.33	0.4382	-1.33	0.4382
93241_r_at	1434842_s_at	Upf3b	UPF3 regulator of nonsense transcripts hom	1.18	0.65	1.29	0.0891	1.52	0.0738	1.46	0.0906	1.09	0.7753
93242_at	1422913_at	BC061127	cDNA sequence BC061127	-2.07	0.03	1.04	0.8653	1.19	0.6713	1.08	0.7466	-1.05	0.8492
93243_at	1418910_at	Bmp7	bone morphogenetic protein 7	2.37	0.32	1.29	0.0701	1.16	0.2713	1.13	0.3198	1.1	0.7409
93244_at	1425161_a_at	5730502D15Rik	RIKEN cDNA 5730502D15 gene	1.56	0.04	-1.48	0.0666	-1.17	0.4876	-1.26	0.3151	-1.63	0.4117
93245_at	1439810_s_at	Pramel7	preferentially expressed antigen in melanom	1.34	0.24	-1.23	0.0317	-1.21	0.2999	-1.33	0.0547	1.5	0.0623
93246_at	1418024_at	Narg1	NMDA receptor-regulated gene 1	1.09	0.86	1.1	0.3462	-1.41	0.0403	-1.35	0.0121	-1.25	0.0077
93250_r_at	1437313_x_at	Hmgb2	high mobility group box 2	-1.11	0.86	2.08	0.0206	-1.48	0.1862	1.34	0.2126	4.19	0.0809
93252_at	1456279_a_at	Bcap31	B-cell receptor-associated protein 31	1.07	0.68	-1.18	0.008	-1.16	0.0021	-1.24	0.0011	-1.35	0.04
93253_at	1419568_at	Mapk1	mitogen activated protein kinase 1	2.93	0.01	1.01	0.9637	-1.68	0.0071	-1.51	0.0283	-1.4	0.3432
93254_at	1426585_s_at	Mapk1	mitogen activated protein kinase 1	1	1	1.12	0.0002	1.12	0.0056	1.06	0.1422	1.05	0.485

93255_at	1448634_at	Ralbp1	ralA binding protein 1	-1.23	0.43	-1.02	0.6127	-1.08	0.1851	-1.1	0.0412	-1.2	0.14
93256_at	1415672_at	Golga7	golgi autoantigen, golgin subfamily a, 7	-1.37	0.14	1	0.9822	-1.1	0.3258	-1.15	0.1297	1.09	0.5423
93257_at	1415915_at	Ddx1	DEAD (Asp-Glu-Ala-Asp) box polypeptide 1	2.39	0.17	1.01	0.8933	1.11	0.1769	1.18	0.0244	-1.28	0.0831
93258_at	1426475_at	Hmbs	hydroxymethylbilane synthase	1.52	0.06	-1.05	0.3838	1.11	0.0831	-1.09	0.1883	-1.35	0.2407
93264_at	1426690_a_at	Srebf1	sterol regulatory element binding factor 1	-1.14	0.56	-1.18	0.0459	-1.18	0.0406	-1.42	0.0001	-2.72	0.0045
93266_at	1449997_at	Tpm3	tropomyosin 3, gamma	1.05	0.73	-1.03	0.4285	-1.05	0.5445	-1.07	0.2108	-1	0.98
93267_at	1426671_a_at	Rnpc2	RNA-binding region (RNP1, RRM) containin	1.56	0.07	1.19	0.0487	1.11	0.4513	1.21	0.0224	-1.09	0.3542
93269_at	1451240_a_at	Glo1	glyoxalase 1	-1.21	0.21	1.48	0.0094	1.37	0.0584	-1.24	0.1703	-1.84	0.0001
93270_at	1423784_at	Gars	glycyl-tRNA synthetase	1.68	0.24	-1.04	0.5887	1.08	0.2264	1.1	0.1983	-1.18	0.2151
93271_s_at	1450186_s_at	Gnas	GNAS (guanine nucleotide binding protein, a	-1.22	0.29	-1.02	0.5227	1.04	0.5905	-1.08	0.1148	-1.17	0.0448
93272_at	1426160_a_at	Stk16	serine/threonine kinase 16	1.22	0.26	-1.09	0.1899	1.06	0.2247	-1.14	0.0921	-1.21	0.0825
93274_at	1426124_a_at	Clk1	CDC-like kinase 1	1.48	0.05	1.25	0.0044	1.27	0.0693	1.4	0.0001	1.62	0.0015
93275_at	1416338_at	Sh3gl1	SH3-domain GRB2-like 1	-1.13	0.84	-1.02	0.8274	-1.05	0.5013	-1.09	0.2151	-1.02	0.9345
93276_at	1448180_a_at	Hn1	hematological and neurological expressed s	1.42	0.14	1.06	0.6813	-1.09	0.5569	1.13	0.5116	1.06	0.9042
93277_at	1426351_at	Hspd1	heat shock protein 1 (chaperonin)	-1.08	0.27	-1	0.9828	-1	0.989	-1.04	0.5154	1.25	0.2084
93278_at	1449686_s_at	Scp2	sterol carrier protein 2, liver	-1.7	0.14	1.01	0.8209	-1.09	0.388	-1.11	0.3161	-2.02	0.0333
93281_at	1422449_s_at	Rcn2	reticulocalbin 2	1.18	0.54	1.22	0.2203	1.14	0.4358	1.32	0.0899	1.24	0.0706
93283_at	1451703_s_at	---	---	-1.53	0.16	1.09	0.3359	1.16	0.372	1.17	0.0657	1.26	0.2234
93284_at	1416332_at	Cirbp	cold inducible RNA binding protein	-1.18	0.41	1.32	0.0284	1.14	0.1323	1.5	0.0001	1.12	0.3553
93285_at	1415834_at	Dusp6	dual specificity phosphatase 6	1.53	0.4	1.39	0.027	-1.32	0.2031	1.14	0.4374	1.62	0.0495
93287_at	1449836_x_at	Biklk	Bcl2-interacting killer-like	2.47	0.12	2.69	0.001	1.72	0.0596	2.77	0.0046	-1.17	0.6135
93288_at	1437148_at	Arpc2	actin related protein 2/3 complex, subunit 2	-1.03	0.9	1.13	0.2546	-1.03	0.6324	1.05	0.601	-1.2	0.3412
93290_at	1453299_a_at	Pnp	purine-nucleoside phosphorylase	1.34	0.3	1.14	0.0314	-1.04	0.3173	1.04	0.5413	1.07	0.4534
93293_at	1423807_a_at	Calm2	calmodulin 2	-1.06	0.85	1.08	0.2178	1.17	0.0458	1.12	0.0592	1.09	0.6033
93294_at	1416953_at	Ctgf	connective tissue growth factor	1.95	0.06	-1.18	0.3019	1.54	0.0167	1.84	0.0122	-1.04	0.8998
93295_at	1417258_at	Cct5	chaperonin subunit 5 (epsilon)	1.83	0.16	-1.11	0.0319	1.08	0.0301	-1.09	0.0791	-1	0.9782
93296_at	1422458_at	Tcl1	T-cell lymphoma breakpoint 1	-1.78	0.34	-1.06	0.8888	-1	0.9901	-1.02	0.9514	1.33	0.7394
93298_at	1415890_at	Papss1	3'-phosphoadenosine 5'-phosphosulfate syn	-1.36	0.11	-1.15	0.0084	-1.11	0.0536	-1.13	0.0237	1.12	0.1753
93300_at	1450923_at	Tgfb2	transforming growth factor, beta 2	-3.56	0.03	-1.16	0.597	-1.17	0.5774	-1.04	0.8603	1.05	0.9082
93301_at	1417199_at	1300007B12Rik	RIKEN cDNA 1300007B12 gene	1.31	0.15	-1.06	0.4949	-1.16	0.1035	-1.02	0.8735	-1.29	0.1731
93302_at	1422448_at	Tff2	trefoil factor 2 (spasmolytic protein 1)	-2.46	0.43	1.82	0.4783	57.68	0.3647	1.09	0.7914	1	0.9931
93304_at	1448741_at	Slc3a1	solute carrier family 3, member 1	1.51	0.28	-1.43	0.0018	-1.22	0.08	-1.56	0.0013	-1.1	0.7264
93305_f_at	1420624_a_at	Vamp8	vesicle-associated membrane protein 8	1.11	0.48	1.01	0.8965	1.14	0.1285	1.2	0.0185	1.17	0.4123
93306_at	1450740_a_at	Mapre1	microtubule-associated protein, RP/EB famil	-1.12	0.6	-1.04	0.6283	-1.17	0.014	-1.12	0.1627	1.09	0.5814
93308_s_at	1416383_a_at	Pcx	pyruvate carboxylase	1.52	0.09	1.15	0.0721	1.09	0.3542	1.2	0.0079	-1.18	0.2523
93310_at	1417771_a_at	Psmc6	proteasome (prosome, macropain) 26S subu	1.36	0.08	1.16	0.066	1.06	0.4825	1.13	0.1157	-1.24	0.1775
93311_at	1423849_a_at	Clk3	CDC-like kinase 3	-1.05	0.78	1.04	0.6646	1.17	0.1193	-1.02	0.8094	1.17	0.2852
93312_at	1415688_at	Ube2g1	ubiquitin-conjugating enzyme E2G 1	1.11	0.5	1.07	0.26	-1.06	0.2585	-1.04	0.6509	-1.07	0.6314
93315_at	1451714_a_at	Map2k3	mitogen activated protein kinase kinase 3	-1.09	0.34	-1.09	0.1493	-1.17	0.0008	-1.13	0.0062	-1.17	0.2026
93316_at	1460192_at	Osbpl1a	oxysterol binding protein-like 1A	-1.07	0.78	-1.04	0.5695	1.09	0.2642	1.09	0.1475	-1.36	0.0048
93318_at	1448417_at	Ninj1	ninjurin 1	1.82	0.31	-1.14	0.1093	-1.33	0.0207	-1.31	0.0065	-1.56	0.1125
93319_at	1415850_at	Rasa3	RAS p21 protein activator 3	2.53	0.01	1.26	0.098	-1.08	0.6052	1.26	0.1473	1.32	0.0431
93321_at	1448775_at	Ifi203	interferon activated gene 203	1.28	0.34	-1.05	0.8746	-1.26	0.4468	-1.15	0.6325	1.44	0.3782
93323_at	1453572_a_at	Plp2	proteolipid protein 2	-1.11	0.4	-1.06	0.3134	-1.12	0.1188	-1.05	0.4091	1.25	0.1309
93324_at	1450644_at	Zfp36l1	zinc finger protein 36, C3H type-like 1	1.14	0.44	1.1	0.2795	-1.2	0.0636	1.03	0.7496	1.07	0.6887
93326_at	1448737_at	Tspan7	tetraspanin 7	1.09	0.68	-1.21	0.0238	1.05	0.5298	-1.09	0.4149	1.32	0.1072
93327_at	1424169_at	Tax1bp3	Tax1 (human T-cell leukemia virus type I) bi	1.41	0.3	1.11	0.1546	1.06	0.4643	1.17	0.0253	-1.14	0.6188
93328_at	1454713_s_at	Hdc	histidine decarboxylase	3.58	0	1.01	0.982	-1.15	0.6281	-1.39	0.3091	-1.22	0.4949
93330_at	1416203_at	Aqp1	aquaporin 1	1.26	0.6	-1.15	0.3153	1.12	0.5199	-1.35	0.21	1.05	0.7371
93332_at	1450884_at	Cd36	CD36 antigen	2.72	0.01	1.51	0.0025	1.16	0.2974	1.28	0.0495	1.15	0.6274
93333_at	1417652_a_at	Tbca	tubulin cofactor a	1.28	0.45	1.09	0.1401	1.13	0.2816	1.05	0.4382	-1.14	0.4001
93336_at	1424708_at	1110014C03Rik	RIKEN cDNA 1110014C03 gene	1.1	0.71	-1.08	0.0275	1.11	0.063	1.06	0.343	-1.18	0.1811
93337_at	1417007_a_at	Vps4b	vacuolar protein sorting 4b (yeast)	1.47	0.05	1.11	0.0746	1.18	0.1691	1.07	0.2543	-1.15	0.2301

93338_at	1419558_at	Mdm4	transformed mouse 3T3 cell double minute 4	-1.66	0.28	1.07	0.8169	1.19	0.6701	1.93	0.104	2.11	0.198
93339_at	1460542_s_at	Mdm4	transformed mouse 3T3 cell double minute 4	1.51	0.19	1.34	0.0003	1.27	0.015	1.49	0.0001	1.57	0.0041
93341_r_at	1456175_a_at	Copb2	coatomer protein complex, subunit beta 2 (b	-1.38	0.03	-1.21	0.1085	-1.16	0.1348	-1.2	0.0105	-1.13	0.3915
93346_at	1417864_at	---	---	1.63	0.2	1.15	0.0995	1.23	0.0842	1.37	0.0051	-1.28	0.0636
93347_at	1421873_s_at	Rab24	RAB24, member RAS oncogene family	1.82	0.11	-1.05	0.5283	1.01	0.9515	1.16	0.0546	-1.09	0.5795
93348_at	1448517_at	Timm22	translocase of inner mitochondrial membran	2.42	0.02	1.09	0.3496	-1.06	0.4478	-1.08	0.1706	-1.35	0.2285
93349_at	1448433_a_at	Pcolce	procollagen C-proteinase enhancer protein	-1.01	0.95	1.13	0.0793	-1.01	0.8614	-1.04	0.6567	1.08	0.5803
93350_f_at	1451244_a_at	Zfp422	zinc finger protein 422	1.94	0.08	1.48	0.0061	-1.1	0.6672	1.18	0.2167	1.02	0.9505
93351_at	1419905_s_at	---	---	1.06	0.73	-1	0.9888	1.03	0.7743	-1.01	0.8962	1.06	0.7081
93352_at	1455900_x_at	Tgm2	transglutaminase 2, C polypeptide	-1.22	0.03	1.07	0.3063	-1.11	0.1197	1.04	0.3572	-1.17	0.477
93353_at	1423607_at	Lum	lumican	-1.23	0.47	-1.4	0.0024	-1.01	0.9093	-1.46	0.0006	1.07	0.7602
93354_at	1417561_at	Apoc1	apolipoprotein C-I	-1.1	0.82	1.07	0.0015	1.04	0.0784	1.07	0.0027	1.41	0.0024
93356_at	1416031_s_at	Mcm7	minichromosome maintenance deficient 7 (S	1.11	0.7	1.09	0.3768	-1	0.9948	1.24	0.1072	-1.21	0.5503
93358_at	1419736_a_at	Eif1ay	eukaryotic translation initiation factor 1A, Y-l	2.1	0.04	1.36	0.0003	1.33	0.0486	1.41	0	1.07	0.5693
93360_at	1424167_a_at	Pmm1	phosphomannomutase 1	-1.12	0.24	1.14	0.5725	-1.07	0.549	1.07	0.511	-1.26	0.2662
93362_at	1450894_a_at	Ap2m1	adaptor protein complex AP-2, mu1	1.1	0.59	1	0.9523	1.03	0.7575	1.02	0.733	-1	0.9889
93364_at	1448149_at	Catna1	catenin alpha 1	1.15	0.47	1.03	0.7308	1.18	0.1346	1.03	0.7082	-1.45	0.0368
93366_r_at	1426425_at	Sugt1	SGT1, suppressor of G2 allele of SKP1 (S. c	1.82	0.06	1.18	0.0242	1.11	0.451	1.26	0.0032	1.27	0.0381
93367_at	1418196_at	Tep1	telomerase associated protein 1	1.23	0.6	-1.01	0.9866	-1.04	0.9191	1.11	0.7456	1.03	0.8641
93369_at	1418745_at	Omd	osteomodulin	-1.32	0.55	-1.17	0.6185	-1.31	0.4234	2.06	0.0126	-2.02	0.1907
93371_at	1424890_at	Bnc1	basonuclin 1	2.36	0.11	-1.29	0.1008	-1.11	0.4164	-1.19	0.1288	1.02	0.9629
93372_at	1450407_a_at	Anp32a	acidic (leucine-rich) nuclear phosphoprotein	-1.1	0.93	-1.18	0.6708	-3.43	0.0021	-4.19	0.0009	1.39	0.0679
93373_at	1417706_at	Naglu	alpha-N-acetylglucosaminidase (Sanfilippo c	1.07	0.56	1.02	0.5952	-1.05	0.1909	1.01	0.7215	1.02	0.8475
93374_at	1418161_at	Jph3	junctophilin 3	-1.86	0.14	-1.21	0.3775	1.23	0.4651	-1.14	0.6035	1.18	0.7579
93375_at	1455893_at	2610028F08Rik	RIKEN cDNA 2610028F08 gene	4.07	0	-1.16	0.5663	-1.36	0.2946	1.03	0.8964	1.46	0.2184
93376_at	1437419_at	Bmp2k	BMP2 inducible kinase	8.79	0	1.25	0.3399	-1.14	0.6591	1.09	0.6758	1.26	0.6192
93379_at	1418298_s_at	Dpysl4	dihydropyrimidinase-like 4	-1.01	0.96	1.17	0.1857	1.21	0.1596	1.06	0.7677	1.44	0.1671
93380_at	1449570_at	Klrb1c	killer cell lectin-like receptor subfamily B mem	-1.04	0.93	1.06	0.8255	1.4	0.3049	1.36	0.2607	-1.46	0.4081
93381_at	1450794_at	Avp	arginine vasopressin	-1.35	0.23	1.14	0.2384	1.14	0.1482	1.09	0.1374	1.48	0.0654
93382_at	1449420_at	Pde1b	phosphodiesterase 1B, Ca2+-calmodulin de	-1.15	0.34	-1.04	0.7295	-1.24	0.1755	1.06	0.5304	1.68	0.1978
93383_at	1419613_at	Col7a1	procollagen, type VII, alpha 1	-1.3	0.27	-1.5	0.0836	1.05	0.8645	-1.36	0.1092	2.18	0.141
93384_at	1421455_at	Sntb1	syntrophin, basic 1	1.18	0.59	1.26	0.1326	-1.04	0.8298	1.05	0.7487	-1.71	0.3207
93385_at	1419433_at	Nthl1	nth (endonuclease III)-like 1 (E.coli)	3.13	0.02	1.36	0.1152	-1.83	0.0239	1.11	0.5049	1.51	0.4887
93386_at	1418958_at	Amac1	acyl-malonyl condensing enzyme 1	-1.38	0.55	1.14	0.5929	-1.15	0.6223	-1.02	0.9359	1.15	0.7681
93387_at	1418063_at	Kera	keratocan	-1.71	0.37	1.07	0.7467	1.04	0.8564	1.2	0.4183	2.05	0.2739
93390_g_at	1419700_a_at	Prom1	prominin 1	1.19	0.73	-3.13	0	-2.33	0.0001	-4.06	0	2.1	0.1353
93391_at	1420453_at	Crygs	crystallin, gamma S	-2.09	0.03	-1.31	0.0563	-1.05	0.7257	-1.31	0.0382	1.09	0.3917
93392_at	1420657_at	---	---	-1.11	0.69	-1.17	0.3439	-1.03	0.8667	-1.22	0.1679	1.01	0.9164
93393_at	1449524_at	Eda	ectodysplasin-A	-2.44	0.06	1.27	0.0456	1.07	0.6245	1.24	0.043	1.02	0.9075
93395_g_at	1419596_at	Eda	ectodysplasin-A	-1.88	0.07	-1.2	0.2636	-1.19	0.3838	-1.19	0.2193	1.18	0.7755
93396_at	1437758_a_at	Slc4a1ap	solute carrier family 4 (anion exchanger), me	1.71	0.03	1.21	0.0344	1.17	0.0296	1.26	0.0001	1.13	0.5388
93397_at	1421188_at	Ccr2	chemokine (C-C motif) receptor 2	2.67	0.01	4.47	0.2018	-1.07	0.7832	5.85	0.0902	1.32	0.4799
93398_at	1421128_at	U2af1-rs2	U2 small nuclear ribonucleoprotein auxiliary	-1.41	0.32	1.62	0.1464	-1.19	0.4187	-1.48	0.1794	-1.19	0.5914
93399_at	1452358_at	Rai2	retinoic acid induced 2	-1.3	0.53	-1.22	0.3232	-1.01	0.9749	-1.14	0.7018	1.33	0.2775
93400_at	1421394_a_at	---	---	1.2	0.58	-1.12	0.1475	-1.01	0.9167	1.04	0.6963	1.21	0.3895
93401_g_at	1450231_a_at	Birc4	baculoviral IAP repeat-containing 4	-8.67	0.24	-1.73	0.093	-1.36	0.3274	-1.02	0.9434	-1.4	0.374
93402_i_at	1426636_a_at	Birc4	baculoviral IAP repeat-containing 4	3.89	0.23	-1.03	0.7537	-1.09	0.3595	-1.22	0.0778	-3.02	0.0536
93404_g_at	1450124_a_at	Atp2a3	ATPase, Ca++ transporting, ubiquitous	-1.52	0.03	1.3	0.2118	1.13	0.1068	1.1	0.2587	2.46	0.0123
93405_at	1427462_at	E2f3	E2F transcription factor 3	2.14	0.23	-1.46	0.1273	-1.25	0.3117	-1.12	0.4984	1.56	0.4497
93407_at	1421212_at	Abcc6	ATP-binding cassette, sub-family C (CFTR/M	1.51	0.24	1.05	0.4737	1.16	0.1131	1.02	0.8333	-1.14	0.2162
93408_at	1436348_at	---	Transcribed locus	1.26	0.05	-1.13	0.1511	1.02	0.7834	-1.1	0.197	-1.02	0.9356
93409_at	1449593_at	---	Transcribed locus	-2.02	0.26	1.14	0.4285	1.1	0.5331	1.03	0.7984	1.65	0.0459
93410_at	1455424_at	1810073P09Rik	RIKEN cDNA 1810073P09 gene	-1.73	0.04	-1.02	0.8208	1.08	0.4268	-1.06	0.5136	1.28	0.1588

93411_at	1459903_at	Sema7a	sema domain, immunoglobulin domain (Ig),	-1.5	0.3	-1.12	0.7402	-1.04	0.9174	-1.17	0.5914	1.7	0.2963
93412_at	1432273_a_at	Dfy	Duffy blood group	-1.71	0.23	1.2	0.7181	-1.42	0.1587	-1	0.9913	1.17	0.7071
93413_at	1421147_at	Terf2	telomeric repeat binding factor 2	-1.05	0.89	-1.04	0.7547	-1.15	0.3442	1.01	0.9645	-1.02	0.9289
93414_at	1418872_at	Abcb1b	ATP-binding cassette, sub-family B (MDR/T/	-1.15	0.82	1.15	0.5271	1.24	0.4086	1.26	0.2186	1.13	0.3864
93416_at	1419083_at	Tnfsf11	tumor necrosis factor (ligand) superfamily, m	-1.32	0.63	1.1	0.5943	1.09	0.6524	-1.34	0.1244	1.6	0.2265
93418_g_at	1421541_a_at	Mef2b	myocyte enhancer factor 2B	1.05	0.85	-1.04	0.6366	-1.11	0.1366	-1.09	0.2174	-1.1	0.5366
93419_at	1425066_a_at	1110061O04Rik	RIKEN cDNA 1110061O04 gene	1.21	0.14	1.03	0.6453	1.04	0.5142	1.01	0.8472	1.01	0.9426
93421_at	1419249_at	Pftk1	PFTAIRE protein kinase 1	1.84	0.2	-1.32	0.266	-1.03	0.9293	-1.25	0.436	1.15	0.7736
93423_at	1423673_at	BC058638	cDNA sequence BC058638	-1.94	0.04	1.18	0.5075	1.26	0.3848	1.17	0.5135	1.21	0.4278
93424_at	1434937_at	Phr1	pam, highwire, rpm 1	1.39	0.27	1.19	0.148	1.06	0.5033	1.21	0.0326	-1.05	0.4777
93425_at	1460231_at	Irf5	interferon regulatory factor 5	-1.32	0.17	-1.14	0.4278	-1.41	0.0004	1.06	0.777	-1.27	0.1012
93426_at	1428982_at	---	---	-1.37	0.03	-1.18	0.1428	1.19	0.2626	-1.17	0.2558	-1.17	0.3569
93427_at	1435463_s_at	Myo1d	myosin ID	1.18	0.44	1.1	0.2443	-1.05	0.4488	-1.12	0.2061	-1.02	0.8752
93429_at	1449016_at	Zp2	zona pellucida glycoprotein 2	-2.06	0.46	1.99	0.0255	2.54	0.0526	1.7	0.1251	-1.17	0.6715
93430_at	1417625_s_at	Cmkor1	chemokine orphan receptor 1	2.76	0.01	-1.74	0.1237	-1.64	0.1817	-1.42	0.3131	-1.18	0.5112
93431_at	1434944_at	Dm15	dystrophia myotonica kinase, B15	-1.05	0.79	1.29	0.0135	-1.04	0.682	1.42	0.0027	1.74	0.0172
93433_s_at	1421944_a_at	Asgr1	asialoglycoprotein receptor 1	1.06	0.51	-1.2	0.0014	-1.06	0.1567	-1.26	0.0006	-1.6	0.012
93434_at	1449816_at	Sult5a1	sulfotransferase family 5A, member 1	-2.2	0.09	-5.66	0	-2.82	0.0006	-5.32	0	1.2	0.2967
93435_at	1418866_at	Cyp24a1	cytochrome P450, family 24, subfamily a, pc	-2.96	0.2	1	0.9977	1.21	0.5692	1.31	0.4359	-1.19	0.6901
93437_f_at	1433954_at	4632419I22Rik	RIKEN cDNA 4632419I22 gene	1.22	0.49	1.3	0.0008	1.08	0.5256	1.29	0.0028	-1.21	0.4362
93440_at	1415766_at	4930564D15Rik	RIKEN cDNA 4930564D15 gene	-1.05	0.69	-1.05	0.3091	1.02	0.8912	1.02	0.6477	-1.46	0.0234
93441_at	1427141_at	2700099C18Rik	RIKEN cDNA 2700099C18 gene	4.11	0.27	1.94	0.1974	1.21	0.7135	2.2	0.1303	-1.08	0.8194
93442_at	1450151_at	Zfp316	zinc finger protein 316	-1.2	0.38	1.1	0.2547	1.19	0.046	1.12	0.0875	1.44	0.0926
93443_at	1453321_at	Fndc1	fibronectin type III domain containing 1	1.07	0.88	-1.19	0.2363	-1.08	0.5399	-1.07	0.5403	1.15	0.5438
93444_at	1419410_at	Batf	basic leucine zipper transcription factor, ATF	-2.12	0.1	-1.21	0.0985	-1.26	0.0173	-1.21	0.1301	1.6	0.0291
93445_at	1449193_at	Cd5l	CD5 antigen-like	1.57	0.13	1.15	0.5967	-1.23	0.0109	1.12	0.7345	-1.22	0.2514
93446_at	1422954_at	Zfp60	zinc finger protein 60	-3.34	0.01	1.04	0.859	1.04	0.8683	-1.22	0.5409	-1.01	0.9892
93448_at	1451970_at	E330036I19Rik	RIKEN cDNA E330036I19 gene	1.23	0.16	1.06	0.7312	1.65	0.0643	1.43	0.0256	-3.54	0.0007
93449_at	1416650_at	Rfpl4	ret finger protein-like 4	-1.37	0.11	-1.72	0.0758	-1.05	0.8012	-1.01	0.9535	1.34	0.3732
93450_at	1439040_at	Cenpe	centromere protein E	1.15	0.49	-1.38	0.4443	-1.16	0.7409	1.19	0.6896	1.29	0.6224
93451_at	1455056_at	Lmo7	LIM domain only 7	1.36	0.41	1.5	0.0001	1.36	0.0067	1.64	0	1.84	0.0725
93452_at	1450256_at	Cer1	cerberus 1 homolog (Xenopus laevis)	-1.02	0.94	-1.38	0.1502	1.11	0.5149	-1.19	0.3861	1.16	0.2423
93453_at	1448996_at	Rom1	rod outer segment membrane protein 1	-1.4	0.25	1.21	0.0595	-1.13	0.6149	-1.08	0.663	1.64	0.1705
93454_at	1419589_at	C1qr1	complement component 1, q subcomponent	1.32	0.28	1.26	0.4142	1.07	0.5584	1.5	0.318	1.6	0.2258
93455_s_at	1422912_at	Bmp4	bone morphogenetic protein 4	-1.36	0.03	-1.06	0.3083	-1.04	0.4814	-1.03	0.5885	1.49	0.0086
93457_at	1427264_at	Crygb	crystallin, gamma B	-4.49	0.32	-1.52	0.2292	1.79	0.0852	1.36	0.5049	2.4	0.1193
93458_at	1419959_s_at	C330003B14Rik	RIKEN cDNA C330003B14 gene	1.02	0.94	-1.61	0.0009	1.06	0.7644	-1.75	0.0154	1.05	0.8757
93461_at	1425298_a_at	Birc1a	baculoviral IAP repeat-containing 1a	-1.71	0.48	-1.02	0.9614	-1.02	0.9445	1.19	0.5612	-1.2	0.8153
93462_at	1440192_at	1810054D07Rik	RIKEN cDNA 1810054D07 gene	1.1	0.89	1.12	0.7671	-1.07	0.8587	-2.02	0.1624	2.15	0.2486
93464_at	1455151_at	Akap9	A kinase (PRKA) anchor protein (yotiao) 9	1.45	0.55	-1.23	0.4768	-1.75	0.1087	-1.95	0.036	-1.38	0.2439
93465_at	1453076_at	9130211I03Rik	RIKEN cDNA 9130211I03 gene	-1.05	0.86	-1.02	0.7758	-1.07	0.1794	1.02	0.7815	1.07	0.5513
93467_at	1436030_at	B430218L07Rik	RIKEN cDNA B430218L07 gene	-1.31	0.26	-1.05	0.6239	1.11	0.3905	-1.08	0.4725	2.06	0.0208
93468_at	1425640_at	Mllt2h	homolog of human MLLT2 unidentified gene	-1.05	0.89	1.07	0.7119	-1.08	0.5965	-1.25	0.2236	1.25	0.2324
93469_at	1451550_at	Ephb3	Eph receptor B3	1.26	0.64	-1.44	0.2292	-1.48	0.1997	-1.4	0.249	-1.15	0.7949
93470_at	1419749_at	Dnmt2	DNA methyltransferase 2	1.58	0.07	1.12	0.3932	-1.22	0.3431	1.23	0.1685	-1.69	0.1663
93471_at	1438673_at	Slc4a7	solute carrier family 4, sodium bicarbonate c	-1.26	0.56	1.32	0.0278	1.76	0.0251	1.53	0.0073	1.08	0.6434
93472_at	1434479_at	AI413331	expressed sequence AI413331	-1.28	0.15	-1.09	0.1414	-1.09	0.1885	-1.09	0.1266	1.22	0.0321
93475_at	1424817_at	4931426K16Rik	RIKEN cDNA 4931426K16 gene	1.3	0.16	-1.27	0.0122	-1.03	0.7637	-1.13	0.158	1.06	0.7502
93476_at	1451576_at	Prkdc	protein kinase, DNA activated, catalytic poly	2.63	0.22	-1.12	0.4728	-1.06	0.7916	-1.33	0.097	-2.36	0.0299
93479_at	1455531_at	A930031D07Rik	RIKEN cDNA A930031D07 gene	-3.13	0.15	-1.01	0.9601	-1.23	0.2086	-1.32	0.1516	-1.02	0.9583
93480_at	1427029_at	Htra3	serine protease HTRA3	-1.9	0.26	-1.02	0.922	-1.01	0.9595	1.01	0.9773	-1.65	0.295
93481_at	1428835_at	Myh14	myosin, heavy polypeptide 14	1.17	0.69	-1.22	0.0674	-1.31	0.0707	-1.09	0.3378	1.19	0.436
93482_at	1425506_at	Mylk	myosin, light polypeptide kinase	-1.03	0.72	-1.1	0.0259	1	0.9432	-1.05	0.2397	1.07	0.4013

93483_at	1449455_at	Hck	hemopoietic cell kinase	1.57	0.15	1.33	0.3342	-1.1	0.2317	1.45	0.2895	-1.11	0.7858
93484_at	1428737_s_at	9130427A09Rik	RIKEN cDNA 9130427A09 gene	1.08	0.68	-1.05	0.4947	-1.02	0.8502	-1.2	0.1097	-1.54	0.0184
93485_at	1435537_at	Ptprd	Protein tyrosine phosphatase, receptor type,	-1.35	0.38	1.02	0.8715	-1.03	0.7651	1.15	0.172	-1.24	0.1775
93486_at	1422811_at	Slc27a1	solute carrier family 27 (fatty acid transporte	-1.13	0.5	-1.05	0.7321	1.08	0.5774	-1.02	0.8396	1.02	0.9221
93488_at	1433502_s_at	AW550801	expressed sequence AW550801	1.03	0.92	1	0.9889	1.13	0.2162	1.02	0.8167	1.44	0.0082
93490_at	1451126_at	1110068E11Rik	RIKEN cDNA 1110068E11 gene	1.12	0.13	1.18	0.1025	1.09	0.1727	1.12	0.0826	1.17	0.177
93491_f_at	1450858_a_at	Ube2d3	ubiquitin-conjugating enzyme E2D 3 (UBC4/	1.45	0.12	1.28	0.1075	1.26	0.1604	1.41	0.0087	-1.51	0.054
93492_at	1450103_a_at	Pscd2	pleckstrin homology, Sec7 and coiled-coil dc	1.03	0.85	-1.02	0.8424	1.06	0.5165	1.04	0.6734	1.61	0.0001
93493_at	1423645_a_at	---	---	1.5	0.15	-1.18	0.0113	-1.17	0.0042	-1.26	0.0011	1.01	0.9598
93495_at	1435731_x_at	Stag1	Stromal antigen 1	-1.3	0.14	-1.33	0.0004	1.01	0.9008	-1.52	0	-1.32	0.112
93496_at	1415840_at	Elovl5	ELOVL family member 5, elongation of long	-1.74	0.05	-1.53	0	-1.18	0.1317	-1.99	0	-3.85	0
93497_at	1423954_at	C3	complement component 3	-1.43	0.23	1.08	0.4321	1.09	0.226	1.06	0.4819	-1.16	0.0656
93498_s_at	1421888_x_at	Aplp2	amyloid beta (A4) precursor-like protein 2	-1.42	0.2	1.02	0.7793	-1.09	0.266	-1.05	0.4335	-1.31	0.3109
93499_at	1452038_at	Capza1	capping protein (actin filament) muscle Z-line	1.05	0.68	-1.08	0.4016	-1.03	0.8042	-1.14	0.1244	-1.19	0.2773
93500_at	1424126_at	Alas1	aminolevulinic acid synthase 1	1.21	0.75	-1.22	0.1575	1.16	0.1839	1.03	0.8112	1.68	0.1282
93503_at	1448201_at	Sfrp2	secreted frizzled-related sequence protein 2	-1.65	0.01	-1.06	0.7201	1.05	0.7308	-1.3	0.0871	1.33	0.171
93505_at	1451048_at	---	---	-1.16	0.52	-1.07	0.8382	1.04	0.9243	-1.05	0.8739	1.77	0.2175
93507_at	1420924_at	Timp2	tissue inhibitor of metalloproteinase 2	1.06	0.92	-1.14	0.1387	-1.25	0.1379	-1.22	0.0241	1.27	0.5438
93509_at	1423106_at	Ube2b	ubiquitin-conjugating enzyme E2B, RAD6 hc	1.11	0.22	-1.1	0.0451	-1.14	0.0824	-1.21	0.0228	-1.04	0.8137
93511_at	1423608_at	Itm2a	integral membrane protein 2A	1.47	0.2	1.59	0.028	1.36	0.076	1.54	0.0256	2.58	0.1244
93512_f_at	1438292_x_at	Adk	adenosine kinase	-1.09	0.69	1.12	0.0907	-1.13	0.0566	-1	0.9695	-1.24	0.0718
93514_at	1428266_at	Myl3	myosin, light polypeptide 3	-3.9	0.24	-17.55	0.2947	-19.02	0.2925	-19.14	0.2924	1.2	0.5427
93515_at	1448906_at	Cdh16	cadherin 16	-4.35	0.13	-1.45	0.2194	-1.42	0.237	-1.52	0.1711	1.83	0.2359
93517_at	1452250_a_at	Col6a2	procollagen, type VI, alpha 2	-1.02	0.92	-1.01	0.9311	-1.07	0.3616	1	0.981	1.11	0.5538
93518_at	1448324_at	Rnps1	ribonucleic acid binding protein S1	2.69	0.09	1.42	0.1632	1.66	0.0873	1.97	0.0184	1.04	0.8021
93519_s_at	1423715_a_at	Nedd8	neural precursor cell expressed, developmen	1.6	0.01	1.12	0.0012	1.2	0.0041	1.14	0.0524	-1.02	0.9043
93520_at	1450045_at	Srrm1	serine/arginine repetitive matrix 1	-1.37	0.33	-1.02	0.8032	-1.05	0.7252	-1.38	0.0784	1.45	0.2252
93521_at	1454689_at	Srrm1	serine/arginine repetitive matrix 1	1.01	0.98	1.09	0.0764	1.05	0.2255	1.07	0.1832	1.2	0.137
93523_at	1456170_x_at	Calr	calreticulin	-1.85	0.47	1.1	0.7835	-1.14	0.7202	1.07	0.8248	1.5	0.2859
93528_s_at	1428289_at	Bteb1	basic transcription element binding protein 1	-1.18	0.51	-1.07	0.5872	-1.29	0.1305	-1.35	0.0751	-1.51	0.0088
93529_at	1419041_at	D8Wsu49e	DNA segment, Chr 8, Wayne State Universit	1.34	0.27	-1.17	0.1139	-1.24	0.0475	-1.26	0.0383	-2.23	0.01
93530_at	1435637_at	D8Wsu49e	DNA segment, Chr 8, Wayne State Universit	1.14	0.16	-1.03	0.6525	-1.02	0.8727	-1.01	0.8615	-1.77	0.0076
93531_at	1423692_at	Ndufa8	NADH dehydrogenase (ubiquinone) 1 alpha	1.5	0.21	-1.08	0.151	1.11	0.1049	-1.02	0.7547	-1.5	0.026
93532_at	1416889_at	Tnni2	troponin I, skeletal, fast 2	1.63	0.05	-3.54	0.2506	-2.91	0.2929	-3.73	0.2417	1.14	0.4804
93533_at	1425674_a_at	1500011L16Rik	RIKEN cDNA 1500011L16 gene	1.43	0.15	1.02	0.7775	1.1	0.1286	1.09	0.1185	-1.44	0.1019
93535_at	1419866_s_at	Sca2	spinocerebellar ataxia 2 homolog (human)	1.25	0.43	1.21	0.0667	1.03	0.6814	1.14	0.1101	1.11	0.4905
93536_at	1416837_at	Bax	Bcl2-associated X protein	1.12	0.74	1.09	0.3865	1.05	0.5955	1.23	0.1693	-1.16	0.529
93538_at	1448706_at	Ttrap	Traf and Tnf receptor associated protein	1.43	0.21	1.29	0.0208	1.53	0.0065	1.14	0.262	-1.06	0.7021
93539_at	1452586_at	Anapc13	anaphase promoting complex subunit 13	1.44	0	-1.03	0.5645	1.08	0.4838	1.01	0.8582	1	0.9631
93540_at	1448333_at	Adprh	ADP-ribosylarginine hydrolase	-1.26	0.26	-1.06	0.3712	-1.02	0.7186	1.06	0.5237	1.06	0.3737
93541_at	1423505_at	Tagln	transgelin	1.52	0.05	1.08	0.5801	1.07	0.6368	1.12	0.3841	1.17	0.1195
93542_at	1417667_a_at	Pter	phosphotriesterase related	-1.44	0.11	-1.03	0.7919	1.24	0.217	-1.31	0.0222	-1.32	0.1443
93543_f_at	1448330_at	Gstm1	glutathione S-transferase, mu 1	1.04	0.77	1.21	0.0007	1.08	0.0804	1.24	0.0001	1.75	0.0003
93547_at	1460716_a_at	Cbfb	core binding factor beta	-1.47	0.01	-1.06	0.36	1.01	0.8976	-1.04	0.5988	1.04	0.5641
93548_at	1417083_at	Sec61b	Sec61 beta subunit	-1.31	0.53	-1.09	0.1027	1.05	0.4245	-1.01	0.8982	-1.48	0.1005
93550_at	1420731_a_at	Csrp2	cysteine and glycine-rich protein 2	1.3	0.38	1.09	0.2978	1.18	0.0244	1.11	0.1845	1.48	0.1757
93551_at	1428296_at	Polr2l	polymerase (RNA) II (DNA directed) polypep	1.11	0.63	-1.28	0.0403	1.08	0.5709	-1.23	0.0762	-1.12	0.2424
93555_at	1427118_at	Krt2-19	keratin complex 2, basic, gene 19	1.1	0.67	1.04	0.7506	1.24	0.0015	1.07	0.3112	1.12	0.3989
93556_at	1427365_at	Krt2-10	keratin complex 2, basic, gene 10	-1.21	0.24	-1.13	0.2543	-1.2	0.0937	-1.12	0.2248	1.13	0.1282
93557_at	1449040_a_at	Sephs2	selenophosphate synthetase 2	-1.06	0.6	1.22	0.0306	1.03	0.6804	1.33	0.0039	-1.01	0.9738
93559_at	1416135_at	Apex1	apurinic/apyrimidinic endonuclease 1	1.14	0.65	-1.11	0.0416	1.05	0.3763	-1.03	0.5125	-1.39	0.0292
93560_at	1450095_a_at	Acyp1	acylphosphatase 1, erythrocyte (common) ty	1.41	0.15	1.01	0.864	1.17	0.1312	1.01	0.8684	-1.23	0.125
93561_at	1423779_at	Chchd6	coiled-coil-helix-coiled-coil-helix domain con	3.14	0.04	1.34	0.2127	1.37	0.3046	1.33	0.2902	1.59	0.207

93562_at	1416547_at	Ndufb3	NADH dehydrogenase (ubiquinone) 1 beta s	1.74	0.02	1.07	0.5352	1.1	0.4489	1.05	0.625	-1.4	0.0038
93563_s_at	1423516_a_at	Nid2	nidogen 2	-1.26	0.43	-1.27	0.4126	-1.06	0.8827	-1.47	0.1693	1.06	0.8498
93564_at	1460638_at	Yars	tyrosyl-tRNA synthetase	1	1	-1.3	0.0015	1.02	0.7905	-1.23	0.0183	-1.07	0.4513
93565_at	1417005_at	Kns2	kinesin 2	-1.1	0.59	1.21	0.054	1.37	0.0028	1.12	0.3091	1	0.9917
93567_at	1418210_at	Pfn2	profilin 2	1.8	0.25	2.01	0.1123	1.7	0.1077	1.59	0.1411	-1.65	0.3319
93569_f_at	1452731_x_at	2610042L04Rik	RIKEN cDNA 2610042L04 gene	1.34	0.54	1.7	0.1954	2.25	0.0571	2.78	0.036	-2.4	0.222
93570_at	1422856_at	Slc12a3	solute carrier family 12, member 3	-1.55	0.01	1.18	0.5208	1.35	0.3389	1.14	0.6794	1.67	0.138
93571_at	1452143_at	Spnb2	spectrin beta 2	-1.07	0.17	-1.02	0.5536	1.02	0.647	1.01	0.9061	1.3	0.0254
93572_at	1425143_a_at	Ndufs1	NADH dehydrogenase (ubiquinone) Fe-S pr	1.48	0.03	1.09	0.2398	1.18	0.0426	1.24	0.0077	1.08	0.3049
93573_at	1422557_s_at	Mt1	metallothionein 1	8.98	0.03	1.6	0.0085	-1.44	0.1221	1.16	0.3193	3.02	0.0064
93574_at	1416168_at	Serpinf1	serine (or cysteine) proteinase inhibitor, clad	1.42	0.15	-1.02	0.7529	-1.29	0.0078	-1.13	0.1608	-1.22	0.1582
93575_at	1419595_a_at	Ggh	gamma-glutamyl hydrolase	1.28	0.21	1	0.9704	1.06	0.61	-1.16	0.1584	1.24	0.1379
93579_at	1417064_at	Jagn1	jagunal homolog 1 (Drosophila)	1	0.99	-1.06	0.5268	1.15	0.0577	-1.06	0.3656	-1.1	0.4641
93580_at	1418016_at	Pum2	pumilio 2 (Drosophila)	1.14	0.27	1.22	0.0002	1.16	0.0062	1.22	0.0008	1.17	0.0602
93581_at	1448198_a_at	Ndufb8	NADH dehydrogenase (ubiquinone) 1 beta s	2.75	0.01	-1	0.9299	-1.08	0.3599	1.02	0.7201	-1.32	0.0575
93582_at	1416665_at	Coq7	demethyl-Q 7	2.38	0.02	1.17	0.0681	1.19	0.1415	1.48	0.0014	1.29	0.0943
93583_s_at	1427329_a_at	Igh-6	immunoglobulin heavy chain 6 (heavy chain	-1.2	0.37	-1.29	0.0055	-1.57	0.0008	-1.27	0.0138	1.66	0.0009
93585_at	1417651_at	Cyp2c29	cytochrome P450, family 2, subfamily c, poly	1.61	0.16	1.2	0.1845	-1.09	0.256	1.3	0.0492	1.54	0.0008
93586_at	1448577_x_at	Syngr2	synaptogyrin 2	-1.19	0.08	-1.01	0.9243	-1.02	0.7353	1.04	0.5875	1.12	0.2926
93587_at	1416137_at	Anxa7	annexin A7	1.31	0.32	1.16	0.163	1.07	0.3061	1.29	0.005	1.07	0.7213
93588_at	1419462_s_at	Gtl3	gene trap locus 3	1.51	0.04	1.3	0.0449	1.45	0.0086	1.67	0.0001	1.26	0.3729
93589_at	1449190_a_at	Entpd4	ectonucleoside triphosphate diphosphohydro	-1.29	0.14	-1.02	0.7171	-1.15	0.0896	-1.02	0.7696	-1.13	0.3694
93590_at	1428367_at	Ndst1	N-deacetylase/N-sulfotransferase (heparan	1.09	0.7	-1.01	0.8649	-1.04	0.7521	-1.25	0.0584	-1.14	0.5344
93591_at	1448644_at	2600002E23Rik	RIKEN cDNA 2600002E23 gene	1.94	0.02	-1.08	0.3754	-1.1	0.3882	-1.09	0.2982	-1.31	0.4461
93592_at	1416371_at	Apod	apolipoprotein D	1.37	0.68	-1.03	0.89	-1.14	0.4929	1.16	0.6159	1.59	0.252
93594_r_at	1417104_at	Emp3	epithelial membrane protein 3	-2.08	0.04	1	0.9761	-1.05	0.4735	-1.15	0.0657	1.32	0.0079
93595_at	1448313_at	Cln2	ceroid-lipofuscinosis, neuronal 2	-1.74	0.03	-1.02	0.7492	-1.02	0.6745	-1.13	0.0276	1.07	0.6379
93596_i_at	1416567_s_at	Atp5e	ATP synthase, H+ transporting, mitochondria	1.18	0.63	1.17	0.0979	1.24	0.012	1.15	0.2178	-1.15	0.3591
93598_at	1417648_s_at	Snx5	sorting nexin 5	1.54	0.04	-1.24	0.0376	-1.35	0.0159	-1.14	0.0754	-1.02	0.9137
93599_at	1423408_a_at	2500003M10Rik	RIKEN cDNA 2500003M10 gene	1.77	0.04	-1.12	0.1032	-1.12	0.2303	-1.05	0.3253	-1.05	0.7349
93600_at	1424438_a_at	Leprot	leptin receptor overlapping transcript	1.06	0.78	1.15	0.2721	1.08	0.4546	1.14	0.2435	1.35	0.0807
93601_at	1450746_at	Keap1	kelch-like ECH-associated protein 1	2.82	0.01	-1.16	0.4558	1	0.9819	-1.43	0.0273	1.63	0.0301
93602_at	1448498_at	Rps6ka4	ribosomal protein S6 kinase, polypeptide 4	-1	0.99	1.05	0.5201	1.13	0.1818	1.07	0.6624	1.56	0.084
93603_at	1448849_at	Mrpl40	mitochondrial ribosomal protein L40	1.29	0.09	1.06	0.2832	1.04	0.316	1.08	0.053	-1.04	0.674
93605_r_at	1417376_a_at	Igsf4a	immunoglobulin superfamily, member 4A	1.22	0.52	1.07	0.5743	-1.04	0.7028	1.05	0.5976	1.38	0.2109
93608_at	1449222_at	Ebi3	Epstein-Barr virus induced gene 3	2.81	0.12	1.07	0.746	-1.04	0.7647	1.11	0.462	1.46	0.1892
93609_at	1424876_s_at	Spg20	spastic paraplegia 20, spartin (Troyer syndro	1.15	0.47	-1.02	0.8938	1.08	0.5377	-1.08	0.4876	1.19	0.6039
93611_at	1449868_at	Tbx6	T-box 6	1.44	0.1	1.16	0.2476	1.13	0.3424	1.04	0.7915	1.42	0.0141
93612_at	1422597_at	Mmp15	matrix metalloproteinase 15	-1.34	0.13	-1.13	0.1693	-1.18	0.282	-1.24	0.1167	1.1	0.7061
93618_at	1452269_at	Spnb3	spectrin beta 3	1.05	0.58	-1.08	0.4062	-1.05	0.7259	-1.04	0.69	-1.18	0.4444
93619_at	1449851_at	Per1	period homolog 1 (Drosophila)	1.32	0.55	1.04	0.8194	1.42	0.174	1.45	0.0165	2.33	0.1741
93620_at	1417775_at	Rpo1-4	RNA polymerase 1-4	1.27	0.05	-1.01	0.9053	1.13	0.1622	1.13	0.1835	1.33	0.1803
93621_at	1434400_at	Tgif2	TGFB-induced factor 2	-1.57	0.04	1.43	0.0045	1.23	0.0531	1.27	0.0337	1.59	0.0278
93623_at	1448665_at	Dmd	dystrophin, muscular dystrophy	-1.33	0.49	1.24	0.2578	1.28	0.2841	1.46	0.0279	1.07	0.8806
93624_at	1428776_at	8430417G17Rik	RIKEN cDNA 8430417G17 gene	1.12	0.8	1.24	0.1197	1.17	0.2859	1.15	0.4688	1.35	0.5184
93625_at	1418833_at	Agxt	alanine-glyoxylate aminotransferase	1.95	0.05	1.32	0.0015	1.01	0.859	1.31	0.0288	1.43	0.0184
93626_at	1422906_at	Abcg2	ATP-binding cassette, sub-family G (WHITE	-1.36	0.34	1.86	0.0011	1.1	0.4676	2.51	0	-2.68	0.0008
93627_at	1454801_at	---	---	1.24	0.46	1.02	0.7788	1.34	0.0565	1.16	0.193	1.05	0.7591
93628_at	1420571_at	Prlpb	prolactin-like protein B	-1.3	0.71	1.09	0.5432	1.3	0.0755	1.09	0.652	1.48	0.0463
93630_at	1425932_a_at	Cugbp1	CUG triplet repeat, RNA binding protein 1	-3.77	0.26	-1.24	0.3007	-1.27	0.313	-1.62	0.0608	-1.29	0.4355
93632_g_at	1427646_a_at	Arhgef2	rho/rac guanine nucleotide exchange factor	1.04	0.86	-1.03	0.8001	-1.11	0.4191	-1.12	0.2962	1.06	0.727
93633_at	1419217_at	MGI:1351630	guanine nucleotide exchange factor (RCC1	1.87	0.49	-1.25	0.5881	1.13	0.7662	-1.07	0.8743	-1.01	0.9589
93635_at	1448946_at	Kif3c	kinesin family member 3C	-1.28	0.13	-1.21	0.6533	1.27	0.5444	1.15	0.7308	3.48	0.0325

93636_at	1436588_at	Rttn	rotatin	-1.16	0.23	-1.08	0.3488	-1	0.9751	-1.15	0.1476	1.52	0.1351
93637_at	1418353_at	Cd5	CD5 antigen	-1.63	0.42	-1.06	0.6727	1.05	0.8131	1.46	0.195	-1.39	0.2805
93638_s_at	1424931_s_at	Igl-V1	immunoglobulin lambda chain, variable 1	1.57	0.08	1.06	0.6446	-1.2	0.1862	-1.16	0.3246	1.34	0.2923
93640_s_at	1453249_a_at	Tex21	testis expressed gene 21	-1.64	0.21	-1	0.9993	1.04	0.7238	1.78	0.2065	1.12	0.7627
93642_at	1416900_s_at	Gdf1 /// Lass1	growth differentiation factor 1 /// longevity as	-1.33	0.63	1.04	0.7151	1.17	0.01	-1.05	0.5449	1.34	0.1987
93643_at	1419324_at	Lhx9	LIM homeobox protein 9	-1.37	0.24	1.33	0.0248	1.11	0.346	1.01	0.9299	-1.02	0.9385
93645_at	1450659_at	Rgs7	regulator of G protein signaling 7	-1.5	0.53	-1.59	0.1188	-1.08	0.8243	-1.47	0.21	1.47	0.5027
93646_at	1420873_at	Ptk9	protein tyrosine kinase 9	2.18	0.24	1.03	0.8841	-1.19	0.4849	-1.1	0.6562	1.32	0.6921
93647_at	1428784_at	Gmip	Gem-interacting protein	-2.06	0.37	1.7	0.0457	1.06	0.8392	1.95	0.1366	2.23	0.1143
93648_at	1421446_at	Prkcc	protein kinase C, gamma	1.07	0.87	-1.24	0.0602	1.05	0.566	-1.23	0.0348	-1.03	0.7648
93651_r_at	1450837_at	Prh1	proline rich protein HaeIII subfamily 1	1.87	0.19	-1.62	0.0418	-1.49	0.0795	-1.3	0.2405	-1.42	0.4074
93654_at	1451693_a_at	Fgf12	fibroblast growth factor 12	-2.91	0.05	1.11	0.5481	1.38	0.2703	1.02	0.9216	1.07	0.8377
93657_at	1460407_at	Spib	Spi-B transcription factor (Spi-1/PU.1 relatec	1.05	0.94	-1.66	0.0693	-1.59	0.0448	-1.35	0.1805	1.78	0.2282
93658_at	1418759_at	Ptpn20	protein tyrosine phosphatase, non-receptor t	-1.92	0.53	1.67	0.0602	-1.01	0.9313	2.44	0.2238	4.76	0.1109
93660_at	1452453_a_at	Camk2a	calcium/calmodulin-dependent protein kinas	1.01	0.81	1.19	0.2143	1.18	0.2303	1.13	0.3315	1.18	0.1458
93661_at	1422701_at	Zap70	zeta-chain (TCR) associated protein kinase	-3.28	0.09	-7.81	0	-1.14	0.2619	-12.99	0	-3.6	0.0013
93663_r_at	1439749_at	Zap70	zeta-chain (TCR) associated protein kinase	-1.43	0.16	1.23	0.2799	1.09	0.3856	1.32	0.0578	1.19	0.4713
93664_at	1422009_at	Atp1b2	ATPase, Na+/K+ transporting, beta 2 polype	-1.41	0.12	1.01	0.962	1.01	0.9756	1.08	0.7507	1.32	0.3553
93667_at	1424986_s_at	Fbxw7	F-box and WD-40 domain protein 7, archipe	-1.91	0.42	1.03	0.7873	-1.1	0.3578	-1.06	0.6734	-1.13	0.5475
93668_at	1450823_at	Og9x	OG9 homeobox gene	1.3	0.02	-1.01	0.8587	1.09	0.5987	-1.06	0.3401	1.18	0.3322
93669_f_at	1436790_a_at	Sox11	SRY-box containing gene 11	-2.25	0.38	-1.44	0.2725	1.72	0.2944	-1.03	0.934	1.67	0.4702
93670_at	1435561_at	Erf	Ets2 repressor factor	1.33	0.4	1.35	0.0003	-1.03	0.7789	1.4	0.0079	1.5	0.1246
93671_at	1422114_at	Erf	Ets2 repressor factor	-1.74	0.03	-1.13	0.1635	-1.01	0.8774	-1.14	0.1861	-1.1	0.2849
93672_at	1422005_at	Prkr	protein kinase, interferon-inducible double st	-1.32	0.19	1	0.9685	-1.01	0.9437	-1.32	0.0601	-1.08	0.8025
93673_at	1449281_at	Nrtn	neurturin	-1.4	0	1.18	0.0692	1.28	0.0317	1.26	0.0244	1.16	0.2602
93674_at	1416865_at	Fgd1	FYVE, RhoGEF and PH domain containing 1	1.01	0.99	1.89	0.0674	1.24	0.3404	1.86	0.0486	3.13	0.0013
93676_at	1448899_s_at	Rad51ap1	RAD51 associated protein 1	1.02	0.95	-1.11	0.1173	-1.05	0.6219	-1.13	0.0604	1.03	0.8473
93677_at	1460245_at	Klrd1	killer cell lectin-like receptor, subfamily D, m	-1.19	0.43	1.48	0.1916	-1.23	0.5048	1.68	0.0924	-1.24	0.5974
93678_s_at	1450495_a_at	Klrk1	killer cell lectin-like receptor subfamily K, me	-1.14	0.62	1.12	0.6984	-1.23	0.4906	1.35	0.43	-1.84	0.0482
93680_at	1417751_at	Stk10	serine/threonine kinase 10	3.37	0.03	1.33	0.0215	1.06	0.5679	1.47	0.014	-1.22	0.364
93681_at	1418533_s_at	Fzd2	frizzled homolog 2 (Drosophila)	-3.45	0.03	1.31	0.3037	1.6	0.2586	1.54	0.3241	2.47	0.17
93682_at	1421101_a_at	Ldb2	LIM domain binding 2	-1.33	0.11	-1.12	0.1962	-1.14	0.0692	-1.09	0.2483	1.16	0.1797
93683_at	1450680_at	Rag1	recombination activating gene 1	-1.22	0.64	-1.7	0.1788	1.35	0.5187	-1.58	0.2493	2.08	0.0147
93687_r_at	1427669_a_at	Cit	citron	-1.77	0.03	-1.26	0.3064	-1.05	0.6961	-1.32	0.0158	-1.14	0.7667
93688_at	1428043_a_at	Cmah	cytidine monophospho-N-acetylneuraminic a	-1.59	0.28	-4.8	0.0036	-3.57	0.0085	-6.89	0.0021	-8.59	0.0734
93689_at	1449581_at	Emid1	EMI domain containing 1	1.4	0.53	1.08	0.4209	-1.06	0.6281	1.01	0.9217	1.21	0.4674
93691_s_at	1425457_a_at	Grb10	growth factor receptor bound protein 10	-1.78	0.17	-1.09	0.5397	-1.42	0.0759	-1.09	0.4901	-1.01	0.9671
93692_f_at	1449417_at	Ambn	ameloblastin	-1.26	0.74	1.18	0.6432	-1.27	0.4557	-1.55	0.1776	1.85	0.0396
93694_at	1417602_at	Per2	period homolog 2 (Drosophila)	2.22	0.35	1.27	0.0648	1.37	0.0962	1.46	0.0014	1.25	0.4715
93696_at	1425723_at	Nr1i2	nuclear receptor subfamily 1, group I, memb	1.58	0.05	-1.04	0.6564	-1.08	0.4079	1	0.9803	1.57	0.0182
93697_at	1419583_at	Cbx4	chromobox homolog 4 (Drosophila Pc class)	-1.19	0.49	1.15	0.3401	-1.03	0.761	1.26	0.0552	-1.12	0.2682
93698_at	1450191_a_at	Sox13	SRY-box containing gene 13	-1.58	0	-1.08	0.0243	-1.12	0.0108	-1.11	0.0223	1.02	0.8372
93699_at	1450816_at	Polg2	polymerase (DNA directed), gamma 2, acce	1.62	0.12	1.05	0.5893	1.02	0.9028	1.16	0.1057	-1.14	0.3841
93701_at	1424207_at	Smarca5	SWI/SNF related, matrix associated, actin de	-1.88	0.04	1.11	0.3012	1.08	0.4726	1.05	0.5411	1.23	0.4336
93702_at	1427128_at	Ptpn23	protein tyrosine phosphatase, non-receptor t	-1.19	0.7	1.13	0.4846	1	0.9976	1.25	0.1465	1.67	0.12
93703_at	1417845_at	Cldn6	claudin 6	1.28	0.52	1.24	0.2183	-1.44	0.192	-1.29	0.3147	1.22	0.6252
93704_at	1423013_at	Foxf1a	forkhead box F1a	1.14	0.81	1.04	0.9	1.04	0.9	1.36	0.3245	1.8	0.0734
93705_at	1420682_at	Chrnb1	cholinergic receptor, nicotinic, beta polypept	1.37	0.43	-1.01	0.888	1.03	0.7949	-1	0.9988	1.26	0.0908
93706_at	1426333_a_at	Ikbkb	inhibitor of kappaB kinase beta	1.41	0.34	-1.22	0.0259	-1.25	0.0515	-1.24	0.0073	1.03	0.7567
93708_at	1421646_a_at	Pias3	protein inhibitor of activated STAT 3	-1.62	0	1.01	0.9522	1.13	0.23	-1.05	0.6176	-1.01	0.9749
93709_at	1449952_s_at	Tmprss8	transmembrane protease, serine 8 (intestina	1.56	0.27	-1.34	0.0659	-1.08	0.6644	-1.52	0.0368	1.26	0.6549
93710_at	1450540_x_at	Krtap5-1	keratin associated protein 5-1	1.03	0.89	1.07	0.4853	1.03	0.656	1.07	0.2776	1.37	0.0911
93712_at	1419313_at	Ccnt1	cyclin T1	1.24	0.53	-1.09	0.487	1.01	0.9309	-1.17	0.0893	1.2	0.5932

93713_at	1422853_at	Shc1	src homology 2 domain-containing transforn	2.21	0.01	-1.34	0.1047	-1.47	0.0943	-1.9	0.0023	-2.73	0.1137
93717_at	1419282_at	Ccl12	chemokine (C-C motif) ligand 12	-1.11	0.68	-1.32	0.3652	-1.08	0.7912	-1.11	0.7561	-1.17	0.4311
93718_at	1422399_a_at	Rab23	RAB23, member RAS oncogene family	-1.69	0.41	-1.46	0.0116	-1.46	0.0278	-1.47	0.002	1.04	0.9409
93720_at	1421024_at	Agpat1	1-acylglycerol-3-phosphate O-acyltransferas	-2.09	0.12	-1.13	0.122	-1.22	0.0735	-1.32	0.0067	1.23	0.4142
93721_at	1417462_at	Cap1	CAP, adenylate cyclase-associated protein 1	-1.18	0.7	1.44	0.1372	-1.18	0.495	-1.03	0.8679	1.13	0.1834
93724_at	1423428_at	Ror2	receptor tyrosine kinase-like orphan recepto	1.03	0.93	-1.01	0.9712	-1.24	0.4667	1.01	0.9565	1.37	0.153
93727_at	1452168_x_at	Gspt1	G1 to S phase transition 1	-1.04	0.78	1.11	0.0671	1.28	0.084	1.1	0.3099	-1.38	0.205
93728_at	1454758_a_at	Tgfb1i4	transforming growth factor beta 1 induced tra	1.37	0.4	-1.35	0.1556	-1.48	0.0344	-1.46	0.0334	-6.12	0.0084
93729_at	1421836_at	---	---	-2.14	0.03	-1.22	0.0259	-1.05	0.4263	-1.08	0.4227	-1.45	0.0941
93730_at	1453467_s_at	Rps15a	ribosomal protein S15a	1.01	0.91	1.16	0.0086	1.4	0.0114	1.29	0.0058	1.98	0.0002
93731_at	1437687_x_at	Fkbp9	FK506 binding protein 9	1.24	0.02	1.03	0.6485	1.07	0.3633	1.07	0.3359	1.08	0.1963
93733_r_at	1422763_at	Rgs19ip1	regulator of G-protein signaling 19 interactin	-1.07	0.73	1.52	0.0321	1.3	0.2921	2.08	0.0625	1.27	0.1254
93735_f_at	1416282_at	Psmc3	proteasome (prosome, macropain) 26S subu	1.52	0.07	1.06	0.314	1.08	0.262	1.16	0.0154	-1.28	0.0299
93736_at	1448200_at	Tcn2	transcobalamin 2	1.03	0.93	-1.15	0.1963	-1.11	0.3577	-1.32	0.0054	1.86	0.0245
93737_at	1421901_at	Eif2ak1	eukaryotic translation initiation factor 2 alpha	-1.44	0.25	1.09	0.1693	1.08	0.2522	1.11	0.0649	-1.05	0.8791
93738_at	1434773_a_at	Slc2a1	solute carrier family 2 (facilitated glucose tra	4.11	0	1.25	0.3538	-3.75	0	1.25	0.4262	1.01	0.9822
93742_at	1449933_a_at	5730449L18Rik	RIKEN cDNA 5730449L18 gene	1.18	0.23	-1.13	0.2884	-1.23	0.0797	-1.26	0.0899	1.18	0.2945
93743_at	1423906_at	Hsbp1	heat shock factor binding protein 1	1.02	0.94	-1.11	0.2843	-1.19	0.1068	-1.11	0.3153	-1.57	0.0015
93744_at	1450633_at	Calm4	calmodulin 4	-1.46	0.02	1.01	0.9628	-1.02	0.8151	-1.11	0.4021	1.19	0.1135
93747_at	1454698_at	AW742319	expressed sequence AW742319	1.27	0.18	1.09	0.2548	1.17	0.1704	1.23	0.0229	1.11	0.2903
93748_at	1431390_a_at	Grinl1a	glutamate receptor, ionotropic, N-methyl D-a	-1.19	0.32	1.37	0.0111	1.14	0.1641	1.32	0.0128	1.48	0.0101
93749_at	1428667_at	Maoa	monoamine oxidase A	1.9	0.02	1.01	0.9411	1.06	0.5761	1.06	0.558	1.32	0.007
93750_at	1415812_at	Gsn	gelsolin	1.9	0.04	-1.59	0.2186	-1.69	0.1682	-1.32	0.4326	2.4	0.0416
93751_at	1455205_a_at	Usp19	ubiquitin specific protease 19	-1.3	0.18	1.13	0.1135	1.1	0.381	1.1	0.1865	1.18	0.2045
93752_at	1452154_at	Iars	isoleucine-tRNA synthetase	-1.2	0.07	-1.13	0.0539	1.12	0.0455	-1.07	0.2989	1.08	0.5266
93753_at	1416303_at	Litaf	LPS-induced TN factor	-1.25	0.26	1.12	0.3873	-1.28	0.0393	1.01	0.9552	-1.58	0.0155
93754_at	1448491_at	Ech1	enoyl coenzyme A hydratase 1, peroxisomal	1.42	0.01	1.01	0.8681	1.35	0	1.13	0.2385	1.03	0.7331
93755_at	1418368_at	Retnlb	resistin like beta	-1.22	0.56	1.01	0.867	1.02	0.745	1.02	0.7874	1.91	0.1187
93757_at	1423670_a_at	Srpr	signal recognition particle receptor ('docking	-1.59	0.13	-1.32	0.0041	1.21	0.0158	1.02	0.8182	-1.44	0.0107
93758_at	1423092_at	Incenp	inner centromere protein	1.05	0.92	1.09	0.3777	1.15	0.2161	1.41	0.0102	1.31	0.5905
93762_at	1448138_at	Ppp2r4	protein phosphatase 2A, regulatory subunit B	-1.54	0.04	-1.14	0.0776	-1.13	0.1098	-1.22	0.0135	-1.13	0.0398
93764_at	1430713_s_at	MGI:1914434	genes associated with retinoid-IFN-induced	1.31	0.47	1.41	0.0067	1.19	0.0194	1.44	0.0053	1.15	0.4944
93766_at	1448852_at	---	---	-1.38	0	-1.23	0.0005	-1.13	0.0448	-1.43	0	-1.45	0.0015
93768_f_at	1428237_at	MGC90611	similar to hypothetical protein FLJ10342	-1.16	0.53	1.27	0.0245	1.11	0.4676	1.2	0.0147	1.23	0.2269
93769_at	1418962_at	1110005F07Rik	RIKEN cDNA 1110005F07 gene	-1.4	0.52	1.21	0.0672	1.05	0.6767	1.26	0.0558	-1.04	0.8729
93770_at	1416809_at	Cyp3a11	cytochrome P450, family 3, subfamily a, poly	1.04	0.57	1.27	0.0004	1.2	0.0004	1.33	0	1.57	0.0036
93771_at	1448767_s_at	Gjb1	gap junction membrane channel protein beta	-1.42	0.39	-1.62	0	-1.36	0.0529	-1.83	0.0001	-1.66	0.0246
93773_f_at	1454652_at	Zfp265	zinc finger protein 265	1.21	0.39	1.25	0.0428	1.2	0.1925	1.32	0.0188	-1.05	0.8578
93774_at	1448202_x_at	2610524G07Rik	RIKEN cDNA 2610524G07 gene	-1.01	0.95	1.04	0.4309	1.01	0.749	1.1	0.0939	1.08	0.3246
93776_at	1438318_x_at	1500001L15Rik	RIKEN cDNA 1500001L15 gene	1.69	0.14	1.7	0.0077	1.47	0.1077	1.25	0.3391	1.54	0.2538
93777_at	1425173_s_at	Golph3l	golgi phosphoprotein 3-like	1.27	0.66	1.45	0.1622	1.16	0.5669	1.37	0.1578	1.61	0.3423
93779_at	1456635_at	---	---	1.24	0.37	1.25	0.5985	-1.54	0.128	1.6	0.4296	-1.05	0.8474
93780_at	1417316_at	Them2	thioesterase superfamily member 2	1.98	0	1.11	0.239	1.2	0.0942	1.14	0.3048	1.21	0.0947
93781_at	1416460_at	Aldrl6	aldehyde reductase (aldose reductase)-like 6	-1.55	0.2	-1.03	0.697	1.08	0.5517	1.06	0.6223	1.2	0.5598
93782_at	1423654_a_at	Rnf4	ring finger protein 4	-1.04	0.8	1.18	0.0108	1.22	0.0249	1.15	0.0859	1.17	0.1126
93783_at	1423693_at	Ela1	elastase 1, pancreatic	-1.92	0	-2.59	0	-1.49	0.0314	-2.63	0	-4.13	0.0006
93784_at	1416742_at	Cfdp1	craniofacial development protein 1	2.4	0.03	-1.13	0.2275	-1.08	0.4245	-1.03	0.7628	1.19	0.1481
93785_at	1450995_at	Folr1	folate receptor 1 (adult)	1.18	0.66	-2.16	0.0474	-1.88	0.0751	-2.07	0.0347	1.45	0.0565
93787_f_at	1448373_at	Mrpl18	mitochondrial ribosomal protein L18	-1.17	0.4	1.2	0.0375	1.21	0.097	1.3	0.0015	1.09	0.3099
93789_s_at	1424355_a_at	Sin3b	transcriptional regulator, SIN3B (yeast)	1.58	0.07	1.25	0.0014	1.33	0.0003	1.44	0	1.15	0.1904
93790_at	1425630_at	Sin3b	transcriptional regulator, SIN3B (yeast)	-1.33	0.17	1.16	0.589	-1.09	0.6914	1.13	0.6493	-1.54	0.3101
93793_at	1460173_at	Lasp1	LIM and SH3 protein 1	-1.11	0.66	-1.15	0.0747	-1.37	0.0001	-1.38	0.0022	-1.68	0.0004
93794_at	1423781_at	Appbp1	amyloid beta precursor protein binding prote	1.44	0.12	1.32	0.0102	1.5	0.0594	1.49	0.0025	-1.09	0.5895

93795_at	1416448_at	Itpa	inosine triphosphatase (nucleoside triphosph	1.84	0.08	-1.06	0.4875	-1.03	0.7051	-1.12	0.0535	-1.45	0.0699
93798_at	1423653_at	Atp1a1	ATPase, Na+/K+ transporting, alpha 1 polyp	1.82	0.01	-1.03	0.5799	-1.25	0.0292	-1.3	0.0003	1.35	0.015
93799_at	1433629_s_at	AI316787	expressed sequence AI316787	-1.13	0.39	1	0.9781	1.16	0.1909	-1	0.9968	1.01	0.935
93800_f_at	1449378_at	Krt1-c29	keratin complex-1, acidic, gene C29	1.04	0.93	-1.15	0.4251	1.29	0.4159	1.76	0.0471	2.03	0.0649
93801_at	1418173_at	4631426H08Rik	RIKEN cDNA 4631426H08 gene	-1.75	0.14	1.11	0.3189	-1.02	0.8465	-1.13	0.3388	1.26	0.6219
93802_at	1434882_at	Mtdh	Metadherin	1.15	0.36	1.11	0.228	1.28	0.1299	1.24	0.0672	-1.07	0.8254
93803_at	1418078_at	Psme3	proteaseome (prosome, macropain) 28 subu	-1.16	0.36	-1.12	0.1333	-1.11	0.1725	-1.15	0.0319	1.04	0.7616
93805_at	1448643_at	Ssna1	Sjogren's syndrome nuclear autoantigen 1	1.5	0.41	-1.02	0.7689	-1.04	0.3617	1.02	0.776	-1.03	0.8668
93806_at	1428107_at	Sh3bgrl	SH3-binding domain glutamic acid-rich prote	1.14	0.23	-1.07	0.0956	-1.01	0.9188	-1.02	0.5784	-1.5	0.0455
93807_at	1432322_at	2310016E02Rik	RIKEN cDNA 2310016E02 gene	1	1	-1.05	0.8889	1.02	0.9658	-1.1	0.7848	1.29	0.2585
93808_at	1418509_at	Cbr2	carbonyl reductase 2	-1.05	0.81	1.14	0.5807	-1.07	0.7621	-1.11	0.6254	2.39	0.0382
93809_at	1415742_at	Aup1	ancient ubiquitous protein	-1.12	0.28	-1.11	0.0476	-1.06	0.6457	-1.19	0.0141	-1.04	0.8767
93810_at	1448118_a_at	Ctsd	cathepsin D	1.01	0.98	-1.04	0.57	-1.12	0.359	-1.08	0.4915	-1.2	0.2549
93812_at	1423181_s_at	Clns1a	chloride channel, nucleotide-sensitive, 1A	1.21	0.48	1.02	0.75	1.19	0.0465	1.22	0.0638	-1.18	0.0697
93819_at	1431036_a_at	1110032D12Rik	RIKEN cDNA 1110032D12 gene	-1.27	0.37	-1.03	0.4868	1.09	0.142	1.07	0.2999	-1.41	0.006
93820_at	1416971_at	Cox7a2	cytochrome c oxidase, subunit VIIa 2	1.08	0.72	1.14	0.0069	1.18	0.0911	1.23	0.0006	1.14	0.3534
93821_at	1448611_at	D8Ertd594e	DNA segment, Chr 8, ERATO Doi 594, expr	1.48	0.58	-1.17	0.2555	-1.55	0.0292	-1.38	0.046	1.42	0.0481
93822_at	1416218_x_at	---	---	-1.66	0.01	1.03	0.7345	-1.15	0.2994	-1.09	0.4103	-1.04	0.6506
93823_at	1417948_s_at	Ilf2	interleukin enhancer binding factor 2	1.79	0.13	1.36	0.0167	1.16	0.1454	1.47	0.0135	1.1	0.552
93824_at	1455540_at	Cps1	carbamoyl-phosphate synthetase 1	-1.12	0.45	1	0.8178	1.06	0.0069	1.07	0.0031	1.5	0.0069
93826_at	1423911_at	Ppp2r5a	protein phosphatase 2, regulatory subunit B	-1.09	0.64	1.05	0.5355	-1.09	0.3934	-1.05	0.4865	1.14	0.1253
93827_at	1449337_at	Tdo2	tryptophan 2,3-dioxygenase	-1.31	0.61	1.49	0.0126	-1.19	0.1385	1.42	0.0186	1.7	0.0068
93830_at	1448103_s_at	Nono	non-POU-domain-containing, octamer bindir	-1.16	0.42	1.09	0.0829	-1.04	0.5496	1.05	0.2071	1.09	0.4091
93832_at	1433940_at	Spag7	sperm associated antigen 7	1.41	0.1	-1.08	0.4346	1	0.9732	1.07	0.337	1.08	0.736
93833_s_at	1418072_at	Hist1h2bc	histone 1, H2bc	-1.01	0.94	-1.19	0.0438	-1.24	0.0468	-1.41	0.0011	-1.92	0.0042
93835_at	1416109_at	Fuca1	fucosidase, alpha-L- 1, tissue	2.2	0.03	1.08	0.3603	-1.1	0.2018	1.09	0.2855	-1.24	0.1747
93837_at	1416676_at	Kng1	kininogen 1	-1.04	0.73	-1.1	0.066	-1.04	0.3985	-1.01	0.8132	1.16	0.0698
93838_at	1428381_a_at	2700038C09Rik	RIKEN cDNA 2700038C09 gene	-1.17	0.4	-1.23	0.0494	-1.2	0.0127	-1.49	0.0006	-1.62	0.1291
93839_at	1418101_a_at	Rtn3	reticulon 3	1.4	0.08	1.06	0.2441	-1.09	0.0745	1.02	0.6422	-1.05	0.5137
93840_at	1419096_at	Apom	apolipoprotein M	1.55	0.01	-1.4	0.0001	1.11	0.2499	-1.45	0.0001	1.74	0.0275
93842_at	1451112_s_at	Dap	death-associated protein	1.01	0.98	-1.19	0.0003	-1.14	0.0086	-1.35	0	-1.58	0.0072
93843_at	1415677_at	Dhrs1	dehydrogenase/reductase (SDR family) mer	-1.72	0	-1.2	0.0268	-1.11	0.1424	-1.41	0.0001	-1.68	0.0032
93844_at	1430326_s_at	1500040F11Rik	RIKEN cDNA 1500040F11 gene	-1.06	0.78	1.07	0.0698	1.08	0.0532	1.03	0.5887	1.12	0.354
93845_at	1423863_at	Abcf2	ATP-binding cassette, sub-family F (GCN20	1.12	0.89	-1.16	0.5226	1.87	0.3355	1.17	0.5539	1.06	0.802
93849_at	1425388_a_at	Tpk1	thiamin pyrophosphokinase	1.49	0.18	1.47	0.005	1.11	0.5266	1.36	0.0076	-1.21	0.4037
93851_at	1426046_a_at	Rabggta	Rab geranylgeranyl transferase, a subunit	1.1	0.72	-1.54	0.0013	-1.19	0.1803	-1.42	0.0072	-2.24	0.2206
93852_at	1452347_at	Mef2a	myocyte enhancer factor 2A	1.26	0.82	1.2	0.0085	1.12	0.0743	1.41	0.0009	2.02	0.0253
93853_at	1431734_a_at	Dnajb4	DnaJ (Hsp40) homolog, subfamily B, membe	1.59	0.26	1.28	0.0681	1.19	0.1186	1.06	0.6212	1.37	0.3577
93855_at	1450159_at	Rem1	rad and gem related GTP binding protein 1	2.48	0.09	1.33	0.0107	1.56	0.1852	1.15	0.2822	-1.15	0.7647
93856_at	1425995_s_at	Wt1	Wilms tumor homolog	-1.18	0.27	1.07	0.5658	1.1	0.3395	1.19	0.1037	1.63	0.0577
93858_at	1418930_at	Cxcl10	chemokine (C-X-C motif) ligand 10	1.18	0.08	3.34	0.1794	-1.11	0.6486	4.43	0.0704	1.05	0.9195
93859_at	1415685_at	Mtif2	mitochondrial translational initiation factor 2	1.24	0.35	-1.11	0.2905	-1.4	0.0015	-1.52	0.0005	-1.34	0.1203
93863_f_at	1450631_x_at	---	---	-1.78	0.04	-1.19	0.1872	1.06	0.6608	-1.09	0.5797	1.33	0.5481
93864_s_at	1421624_a_at	Enah	enabled homolog (Drosophila)	-1.88	0.18	-1.08	0.4341	-1.08	0.4168	-1.06	0.6142	1.11	0.7359
93865_s_at	1449875_s_at	H2-T10 /// H2-T	histocompatibility 2, T region locus 10 /// hist	-1.01	0.97	1.59	0.1912	-1.19	0.0841	1.56	0.2026	-1.38	0.0666
93866_s_at	1448416_at	Mglap	matrix gamma-carboxyglutamate (gla) protei	-1.24	0.31	-1.62	0.4495	-1.98	0.306	-1.16	0.774	-1.11	0.8807
93867_at	1419572_a_at	Abcd4	ATP-binding cassette, sub-family D (ALD), n	1.17	0.38	1.04	0.607	1.07	0.6175	-1.08	0.3305	1.5	0.3564
93869_s_at	1419004_s_at	Bcl2a1a /// Bcl2	B-cell leukemia/lymphoma 2 related protein	3.64	0.09	6.56	0.0283	2.03	0.0049	8.29	0.0448	4.86	0.0002
93870_at	1425693_at	Braf	Braf transforming gene	-1.06	0.85	-1.02	0.9095	1.02	0.9334	-1.01	0.971	1.26	0.1755
93871_at	1451798_at	Il1rn	interleukin 1 receptor antagonist	3.49	0.01	2.43	0.0026	1.09	0.6804	2.97	0.0009	16.08	0.005
93872_at	1450440_at	Gfra1	glial cell line derived neurotrophic factor fam	1.28	0.37	-1.24	0.1405	-1.06	0.7524	-1.18	0.237	-1.43	0.2283
93873_s_at	1452400_a_at	Hoxa11s	homeo box A11, opposite strand transcript	1.03	0.92	1.14	0.4513	1.09	0.586	1.13	0.3444	1.41	0.2108
93874_s_at	1417505_s_at	Il11ra1 /// Il11ra	interleukin 11 receptor, alpha chain 1 /// inter	1.3	0.33	-1.15	0.0043	-1.15	0.0038	-1.2	0.0042	-1.18	0.1747

93877_at	1428500_at	2210419D22Rik	RIKEN cDNA 2210419D22 gene	-1.04	0.86	1.05	0.6195	-1.05	0.6731	-1.01	0.9187	1.11	0.5322
93878_at	1420869_at	Mllt10	myeloid/lymphoid or mixed lineage-leukemia	-1.05	0.9	1.38	0.0154	1.35	0.0292	1.41	0.0046	1.29	0.4708
93880_at	1426001_at	Eomes	eomesodermin homolog (Xenopus laevis)	-1.62	0.58	1.13	0.3145	1.57	0.2728	1.41	0.3364	1.31	0.4867
93885_g_at	1425582_a_at	MGI:1891716	endomucin	1.68	0.28	-1.08	0.81	-1.02	0.9502	-1.33	0.3863	1.05	0.9159
93887_at	1418664_at	Mpdz	multiple PDZ domain protein	1.25	0.23	1.04	0.5397	1.48	0.0298	1.2	0.0462	-1.08	0.6499
93888_at	1453501_at	Hoxb1	homeo box B1	-1.01	0.98	-1.23	0.0765	-1.08	0.3459	-1.19	0.1711	1.43	0.081
93889_f_at	1452540_a_at	Hist1h2bc /// His	histone 1, H2bc /// histone 1, H2be /// histone	1.32	0.34	-1.23	0.0407	-1.3	0.0371	-1.31	0.0049	-1.31	0.0104
93891_at	1421312_a_at	Kifc2	kinesin family member C2	-2.13	0.39	-1.32	0.4302	1.19	0.6093	-1.24	0.4899	1.76	0.0173
93893_f_at	1425436_x_at	Klra3	killer cell lectin-like receptor, subfamily A, me	1.11	0.75	1.19	0.4708	1.02	0.9366	1.28	0.261	1.31	0.1656
93895_s_at	1417279_at	Itpr1	inositol 1,4,5-triphosphate receptor 1	1.03	0.93	-1.11	0.208	-1.03	0.7422	-1.17	0.0243	-2.22	0.1662
93896_at	1424886_at	Ptprd	protein tyrosine phosphatase, receptor type,	-1.07	0.84	-1.03	0.7622	-1.21	0.0471	-1.01	0.949	1.34	0.2477
93897_at	1425244_a_at	Theg	testicular haploid expressed gene	-1.23	0.76	1.01	0.9632	-1.28	0.5416	-1.2	0.6495	-1.21	0.7563
93898_at	1419667_at	Sgcb	sarcoglycan, beta (dystrophin-associated gly	-1.54	0.63	-1.41	0.1586	-1.39	0.1432	-1.24	0.2299	1.63	0.3762
93901_at	1448408_at	Hps1	Hermansky-Pudlak syndrome 1 homolog (hu	-1.16	0.43	1.01	0.9245	-1.04	0.4153	-1.03	0.6319	1.36	0.0005
93902_at	1448814_at	Gab1	growth factor receptor bound protein 2-asso	1.55	0.34	-1.2	0.1626	1.19	0.1323	1.02	0.8975	1.59	0.1617
93903_at	1419140_at	Acvr2b	activin receptor IIB	-1.1	0.73	-1.21	0.0181	-1.31	0.0028	-1.15	0.0681	1.45	0.0656
93905_at	1427717_at	Cd80	CD80 antigen	1.28	0.59	-1.07	0.7888	-2.16	0.0093	-1.22	0.5492	-1.85	0.2998
93906_s_at	1451950_a_at	Cd80	CD80 antigen	-1.87	0.26	-1.15	0.6386	1.23	0.4244	1.05	0.8725	1.18	0.3104
93910_at	1422711_a_at	Pnck	pregnancy upregulated non-ubiquitously exp	1.59	0.35	-1.05	0.8456	-1.03	0.8912	1.22	0.3131	1.51	0.2863
93911_at	1421904_at	Ncoa6ip	nuclear receptor coactivator 6 interacting pro	-1.02	0.88	1	0.9714	1.02	0.8843	-1.04	0.5494	1.03	0.8193
93913_at	1452751_at	Ebf3	early B-cell factor 3	-1.64	0.35	-1.24	0.3313	1.24	0.5636	-1.03	0.8714	1.05	0.8334
93914_at	1448950_at	Il1r1	interleukin 1 receptor, type I	-1.04	0.84	1.16	0.2377	1.31	0.1243	1.56	0.0044	-1.22	0.2347
93915_at	1416957_at	Pou2af1	POU domain, class 2, associating factor 1	-1.02	0.92	1.06	0.665	-1.18	0.2883	1.17	0.2273	1.6	0.0541
93917_at	1452440_at	Tnfsf12	tumor necrosis factor (ligand) superfamily, m	-1.18	0.45	-1.11	0.1803	1.02	0.7736	-1.04	0.5577	1.14	0.254
93919_at	1450078_at	Nrk	Nik related kinase	-1.49	0.34	1.12	0.3408	1.03	0.7831	1.03	0.8083	-1.25	0.4879
93923_at	1435019_at	Atxn7l3	ataxin 7-like 3	-1.47	0.14	1.08	0.3322	1.12	0.1838	1.01	0.8933	1.03	0.698
93924_f_at	1448296_x_at	---	---	1.34	0.38	-1.18	0.1087	1.08	0.5399	-1.12	0.3745	1.15	0.4898
93926_at	1421382_at	Prlr	prolactin receptor	-2.37	0.02	-3.09	0	-1.29	0.0245	-5.55	0	-2.83	0.0001
93929_s_at	1427760_s_at	Plf /// Plf2 /// Mrp	proliferin /// proliferin 2 /// mitogen regulated	-2.95	0.09	-1.02	0.945	1.63	0.0625	1.56	0.0577	-1.02	0.9524
93930_at	1448207_at	Lasp1	LIM and SH3 protein 1	-1.56	0.45	-1.12	0.2291	-1.44	0.0172	-1.6	0.0076	-1.81	0.454
93932_at	1450335_at	---	---	-1.32	0.03	1.04	0.7901	1.15	0.2618	1.07	0.6915	2.18	0.3373
93933_at	1425631_at	Ppp1r3c	protein phosphatase 1, regulatory (inhibitor)	-1.23	0.78	1.36	0.0407	-1.18	0.3566	1.05	0.6363	-1.07	0.9033
93934_at	1417232_at	Cldn2	claudin 2	-1.67	0.05	-1.66	0.063	-2.13	0.0423	-4.78	0.0009	-1.71	0.1965
93937_at	1418361_at	Gas8	growth arrest specific 8	1.35	0.56	1.06	0.7547	1.29	0.1115	1.16	0.4308	1.28	0.1316
93939_at	1450183_a_at	Lnk	linker of T-cell receptor pathways	1.3	0.11	-1.02	0.8085	-1.15	0.0935	-1.09	0.2246	-1.02	0.9385
93940_at	1419298_at	Pon3	paraoxonase 3	1.43	0.16	1.17	0.0675	1.07	0.3927	1.17	0.0594	1.08	0.4812
93941_at	1419304_at	T	brachyury	-1.15	0.6	1.01	0.9526	1.24	0.2992	1.23	0.159	1.33	0.1073
93942_at	1418045_at	Inpp1	inositol polyphosphate-1-phosphatase	-1.86	0.46	-1.08	0.3732	1.01	0.869	1.09	0.7607	1.2	0.408
93944_r_at	1451034_at	Zfp36l2	zinc finger protein 36, C3H type-like 2	1.67	0.11	1.06	0.6008	1.07	0.5986	-1.03	0.8066	1.52	0.0305
93945_at	1421244_at	Esr1	estrogen receptor 1 (alpha)	-1.61	0.09	-2.28	0.0342	-1.99	0.0587	-2.18	0.0395	-1.02	0.9498
93950_at	1422833_at	Foxa2	forkhead box A2	1.13	0.58	-1.44	0.0005	-1.46	0.0119	-1.81	0	-1.19	0.3563
93951_at	1419159_at	Golga3	golgi autoantigen, golgin subfamily a, 3	1.57	0.23	-1.01	0.9139	1.19	0.0467	1.06	0.385	1.17	0.7881
93953_at	1420388_at	Prss12	protease, serine, 12 neurotrypsin (motopsin)	-2.2	0.28	-1.2	0.464	-1.08	0.7803	-1.28	0.3575	1.57	0.0932
93954_at	1420872_at	Gucy1b3	guanylate cyclase 1, soluble, beta 3	-1.34	0.37	1.06	0.6967	1.21	0.2726	1.2	0.2646	1.78	0.1117
93955_at	1450751_at	Zpbp	zona pellucida binding protein	1.12	0.79	1.06	0.8614	-1.28	0.3898	-1.09	0.7918	2.73	0.2623
93956_at	1449025_at	Ifit3	interferon-induced protein with tetratricopept	1.23	0.37	-1.11	0.748	-1.93	0.0253	1.17	0.6774	1.11	0.8098
93957_at	1422921_at	Vpreb3	pre-B lymphocyte gene 3	1.12	0.78	1.51	0.2289	1.41	0.303	1.13	0.724	2.19	0.0347
93958_at	1425929_a_at	Rnf14	ring finger protein 14	1.32	0.33	1.12	0.1596	1.07	0.5843	1.26	0.0083	1.61	0.0666
93961_at	1449216_at	Itgae	integrin, alpha E, epithelial-associated	-1.13	0.64	1.64	0.0693	-1.11	0.7341	1.64	0.1064	-1.56	0.0098
93962_at	1448020_at	Rap1a	RAS-related protein-1a	1.45	0.19	-1.04	0.687	-1.21	0.1945	-1.15	0.1758	1.29	0.1035
93963_at	1436212_at	AI661017	expressed sequence AI661017	-1.07	0.83	1.33	0.3354	1.21	0.4801	2.01	0.0181	-1.02	0.9647
93965_r_at	1424598_at	Ddx6	DEAD (Asp-Glu-Ala-Asp) box polypeptide 6	-1.71	0.04	1.03	0.843	-1.38	0.0893	-1.22	0.285	-1.34	0.423
93966_at	1448410_at	Ube4b	ubiquitination factor E4B, UFD2 homolog (S	-1.06	0.79	-1.01	0.9625	1.03	0.6989	1.25	0.0391	1.19	0.401

93967_at	1417326_a_at	Anapc11	anaphase promoting complex subunit 11 hoı	1.16	0.55	1.01	0.9119	-1.27	0.0038	-1.07	0.4998	1.34	0.146
93968_at	1450638_at	Pdcd5	programmed cell death 5	1.18	0.38	1	0.9863	1.1	0.2047	1	0.9941	1.07	0.6744
93972_at	1451096_at	Ndufs2	NADH dehydrogenase (ubiquinone) Fe-S pr	1.94	0.12	-1.02	0.8207	1.05	0.5255	1.05	0.646	-1.13	0.6239
93973_at	1426674_at	Eif3s9	eukaryotic translation initiation factor 3, subu	1.01	0.94	-1.16	0.0187	-1.08	0.4273	-1.12	0.0058	-1.1	0.3442
93974_at	1419816_s_at	1300002F13Rik	RIKEN cDNA 1300002F13 gene	-1.81	0.49	-2.35	0	-1.15	0.353	-1.43	0.0046	-4.4	0.0198
93975_at	1416129_at	1300002F13Rik	RIKEN cDNA 1300002F13 gene	-1.94	0.18	-2.08	0	-1.07	0.4062	-1.3	0.0023	-2.69	0.01
93976_at	1418433_at	Cab39	calcium binding protein 39	1.14	0.73	1.2	0.0569	1.19	0.0522	1.21	0.039	1.12	0.3071
93978_at	1454842_a_at	B3galnt2	UDP-GalNAc:betaGlcNAc beta 1,3-galactos	-1.05	0.9	1.01	0.9538	1.03	0.862	-1.06	0.6233	-2.04	0.0987
93980_at	1426426_at	Rbm13	RNA binding motif protein 13	1.3	0.47	1.27	0.143	1.18	0.4331	1.28	0.0697	-1.21	0.4485
93981_at	1415806_at	---	---	1.08	0.87	1.28	0.3421	-1.01	0.9624	1.22	0.5069	-1.28	0.4931
93982_at	1415693_at	Derl1	Der1-like domain family, member 1	1	0.99	-1.02	0.7437	1.07	0.2838	-1.01	0.807	1.05	0.463
93983_at	1433563_s_at	Derl1	Der1-like domain family, member 1	-1.17	0.35	1.07	0.0978	1.16	0.0534	1.06	0.2879	1.08	0.4022
93984_at	1448770_a_at	Atpif1	ATPase inhibitory factor 1	1.76	0.09	1.08	0.4108	-1.12	0.08	-1	0.9875	1.42	0.0013
93985_at	1426721_s_at	Tiparp	TCDD-inducible poly(ADP-ribose) polymeras	-1	1	1.48	0.2075	1.75	0.2353	1.94	0.0108	1.33	0.436
93986_at	1428213_at	2410003A14Rik	RIKEN cDNA 2410003A14 gene	2.31	0.02	1.18	0.0214	1.22	0.0984	1.21	0.0329	-1.07	0.6044
93987_f_at	1417126_a_at	3110001N18Rik	RIKEN cDNA 3110001N18 gene	1.04	0.75	1.14	0.2368	1.39	0.0137	1.33	0.0021	-1.08	0.7187
93988_at	1423568_at	Psma7	proteasome (prosome, macropain) subunit, a	1.39	0.12	1.19	0.0128	1.06	0.1728	1.2	0.0029	-1.07	0.4627
93990_at	1415872_at	Hnrph1	heterogeneous nuclear ribonucleoprotein H1	1.13	0.63	1.05	0.4274	1.24	0.0124	1.15	0.0524	1.08	0.3724
93991_at	1416478_a_at	Mdh2	malate dehydrogenase 2, NAD (mitochondri	1.38	0.08	1.32	0	1.44	0.0002	1.56	0	1.14	0.3066
93992_at	1424073_at	5430437P03Rik	RIKEN cDNA 5430437P03 gene	1.11	0.47	1.03	0.4776	1.07	0.3653	1.03	0.5928	-1.13	0.2492
93994_at	1435446_a_at	Chpt1	choline phosphotransferase 1	1.24	0.27	2.38	0	1.24	0.036	2.41	0	-1.33	0.0315
93996_at	1415994_at	Cyp2e1	cytochrome P450, family 2, subfamily e, poly	-1.23	0.37	1.04	0.0671	1.03	0.2383	1.03	0.2604	1.54	0.0168
93997_at	1418116_at	MGI:1929890	interferon alpha responsive gene	-1.01	0.9	-1.15	0.0094	-1.06	0.5775	-1.16	0.0076	-1.26	0.068
93999_at	1448357_at	Snrpg	small nuclear ribonucleoprotein polypeptide	1.01	0.93	1.05	0.2786	1.08	0.3852	1.04	0.3894	1.21	0.2294
94000_at	1448569_at	Cd8b1	CD8 antigen, beta chain 1	-1.29	0.64	-1.21	0.076	-1.31	0.0038	-1.31	0.0029	-1.15	0.2984
94001_at	1448151_at	Elavl1	ELAV (embryonic lethal, abnormal vision, Dr	-1.03	0.9	1.01	0.8413	1.15	0.1179	1.06	0.3044	-1.21	0.1099
94002_at	1448174_at	Cul1	cullin 1	1.17	0.43	1.19	0	1.18	0.0006	1.25	0	1.02	0.7575
94003_at	1433676_at	Prkwnk1	protein kinase, lysine deficient 1	1.18	0.18	-1.04	0.4213	1.04	0.6212	-1.01	0.9171	1.12	0.2223
94004_at	1450981_at	Cnn2	calponin 2	1.48	0.08	1.53	0.0046	1.12	0.4078	1.44	0.0545	1.38	0.0926
94005_at	1433729_x_at	Pmpcb	peptidase (mitochondrial processing) beta	1.5	0.14	1.36	0.0157	1.22	0.1251	1.48	0.0002	-1.01	0.9363
94007_at	1456142_x_at	Morf4l1	mortality factor 4 like 1	-1.14	0.56	1.09	0.2732	1.05	0.3364	1.24	0.0283	-1.02	0.8796
94008_at	1427914_a_at	Tceb1	transcription elongation factor B (SIII), polyp	-1.08	0.48	1.04	0.5505	1.09	0.5136	1.04	0.4842	1.01	0.967
94009_at	1427915_s_at	Tceb1	transcription elongation factor B (SIII), polyp	1.24	0.65	1.11	0.2479	-1.02	0.8467	1.08	0.3559	-1.78	0.0241
94011_at	1415739_at	3100004P22Rik	RIKEN cDNA 3100004P22 gene	1.44	0.03	-1.17	0.0024	-1.14	0.0821	-1.07	0.1402	-1.11	0.3015
94012_at	1417499_at	Timm13a	translocase of inner mitochondrial membran	1.51	0.25	1.15	0.0859	1.25	0.0393	1.26	0.0441	1.28	0.4086
94014_at	1416979_at	2510048O06Rik	RIKEN cDNA 2510048O06 gene	1.3	0.3	-1.12	0.285	-1.11	0.4002	-1	0.9654	-1.37	0.0266
94015_at	1416198_at	Th1l	TH1-like homolog (Drosophila)	1.35	0.47	-1.01	0.8688	1.09	0.128	-1	0.9468	-1.52	0.045
94019_at	1423039_a_at	Bzw1	basic leucine zipper and W2 domains 1	-1.1	0.56	-1.03	0.6388	1.02	0.8891	-1.01	0.884	-1.55	0.002
94020_at	1431292_a_at	Ptk9l	protein tyrosine kinase 9-like (A6-related pro	1.4	0.5	1.45	0.3041	-1.14	0.553	1.81	0.1939	-1.05	0.9454
94021_at	1419630_a_at	Trim11	tripartite motif protein 11	1.51	0.02	-1.11	0.1459	-1.07	0.1526	1.05	0.4484	1.14	0.0561
94022_at	1452409_at	Gltscr2	glioma tumor suppressor candidate region g	1.18	0.14	1.1	0.2855	1.03	0.7653	1.03	0.7418	1.35	0.083
94024_at	1424144_at	Ris2	retroviral integration site 2	1.09	0.71	1.04	0.6351	1.02	0.8381	-1	0.9647	1.39	0.0267
94025_at	1460198_a_at	Psmb3	proteasome (prosome, macropain) subunit, I	-1.14	0.69	1.09	0.0241	1.11	0.1928	1.15	0.0043	1.06	0.5858
94026_at	1433800_a_at	Pomc1	pro-opiomelanocortin-alpha	-1.42	0.29	-2.32	0.0106	-2.41	0.0071	-1.77	0.0644	1.59	0.1825
94027_at	1434615_x_at	---	Transcribed locus, weakly similar to XP_484	1.17	0.64	-1.2	0.347	-1.29	0.158	-1.14	0.6424	1.13	0.8141
94030_at	1433594_at	Commd2	COMM domain containing 2	1.58	0.21	1.02	0.7476	1.17	0.0302	1.02	0.7623	-1.23	0.3111
94031_at	1419945_s_at	Rab2	RAB2, member RAS oncogene family	1.21	0.5	-1.12	0.1968	-1.14	0.1718	-1.06	0.4576	-1.32	0.0657
94032_at	1415727_at	Apoa1bp	apolipoprotein A-I binding protein	1.33	0.03	1.12	0.0727	-1.01	0.7973	1.03	0.5873	1.39	0.0002
94034_at	1451259_at	Smfn	small fragment nuclease	1.13	0.72	-1.14	0.0841	-1.12	0.0383	-1.21	0	-2.75	0.0061
94036_at	1416511_a_at	Cdc42ep4	CDC42 effector protein (Rho GTPase bindin	-1.1	0.65	1.18	0.0793	1.31	0.0025	1.23	0.0347	1.01	0.9361
94037_at	1448281_a_at	Ela2	elastase 2	-1.18	0.28	-1.14	0.0528	-1.02	0.7252	-1.02	0.8703	1.04	0.7021
94038_at	1448238_at	2700060E02Rik	RIKEN cDNA 2700060E02 gene	1.49	0.21	1.09	0.2002	1.09	0.3781	1.14	0.0194	-1.07	0.5767
94040_at	1430536_a_at	Erh	enhancer of rudimentary homolog (Drosophi	-1.48	0.36	-1	0.9747	-1.09	0.1201	1.02	0.7861	-1.39	0.0598

94041_at	1423684_at	Hnrpk	heterogeneous nuclear ribonucleoprotein K	1.47	0.03	1.18	0.0856	1.2	0.1494	1.27	0.0054	-1.07	0.4374
94042_f_at	1436946_s_at	Gng5	guanine nucleotide binding protein (G protei	-1.1	0.01	-1.06	0.3819	-1.08	0.3243	-1.08	0.2193	-1.02	0.889
94043_at	1449622_s_at	Atp6ap1	ATPase, H+ transporting, lysosomal access	1.13	0.47	1.15	0.0094	1.05	0.3575	1.1	0.117	1.04	0.764
94044_at	1420021_s_at	Suz12	suppressor of zeste 12 homolog (Drosophila	1.51	0.33	1.02	0.8884	1	0.998	1.07	0.4467	-1.12	0.4365
94045_at	1416649_at	Ambp	alpha 1 microglobulin/bikunin	1	0.97	1.06	0.0439	1.08	0.0028	1.09	0.0005	1	0.9306
94046_at	1423518_at	Csk	c-src tyrosine kinase	-1.53	0.01	1.01	0.8503	-1.07	0.3996	1.16	0.1839	1.01	0.9588
94047_at	1416830_at	0610031J06Rik	RIKEN cDNA 0610031J06 gene	-1.1	0.21	-1.04	0.3983	1.03	0.673	-1.07	0.1647	-1.19	0.0565
94048_at	1434879_at	Cdc34	cell division cycle 34 homolog (S. cerevisiae	-1.03	0.82	-1.16	0.0441	1.06	0.5014	-1.19	0.0278	-1.69	0.0048
94049_at	1450624_at	Bhmt	betaine-homocysteine methyltransferase	1.56	0.04	1.08	0.1553	-1.35	0.0083	1.08	0.2566	-1.24	0.1958
94051_at	1453095_at	Rab10	RAB10, member RAS oncogene family	-2.45	0.13	-1.45	0.2252	-1.83	0.0179	-1.63	0.0744	3.31	0.0637
94052_at	1415675_at	Dpm2	dolichol-phosphate (beta-D) mannosyltransf	-1.09	0.49	1.06	0.3149	-1.05	0.44	1.03	0.5113	1.04	0.7054
94054_at	1433908_a_at	Cttn	cortactin	-1.1	0.31	-1.05	0.5775	1.06	0.499	-1.05	0.5504	1.15	0.516
94055_at	1421313_s_at	Cttn	cortactin	2.34	0.13	-1.3	0.1543	1.03	0.8919	-1.06	0.6661	-2.07	0.474
94057_g_at	1415964_at	Scd1	stearoyl-Coenzyme A desaturase 1	-1.35	0.13	-1.13	0.2244	-1.47	0.0178	-1.38	0.03	-2.91	0.0003
94059_at	1423864_at	Nrbp	nuclear receptor binding protein	-1.02	0.9	1.02	0.6541	1.03	0.6427	1	0.9787	1.14	0.0996
94060_at	1423694_at	Kctd10	potassium channel tetramerisation domain c	1.49	0.06	1.08	0.3383	-1.01	0.88	1.08	0.4266	1.11	0.3987
94061_at	1416326_at	Crip1	cysteine-rich protein 1 (intestinal)	2.76	0.28	1.28	0.4714	-1.38	0.2912	1.52	0.1417	2.36	0.1902
94062_at	1452692_a_at	Ndufv2	NADH dehydrogenase (ubiquinone) flavopro	1.57	0.05	1.27	0	1.16	0.0115	1.34	0.0001	-1.18	0.1249
94063_at	1448110_at	Sema4a	sema domain, immunoglobulin domain (Ig),	-1.11	0.66	-1.24	0.0017	-1.2	0.0225	-1.4	0.0002	-1.18	0.2916
94064_at	1426326_at	Zfp91	zinc finger protein 91	1.09	0.77	1.1	0.3446	1.03	0.8209	-1.08	0.5032	-1.05	0.797
94065_at	1460176_at	Crk	v-crk sarcoma virus CT10 oncogene homolo	-1.25	0.55	-1.31	0.1119	-1.01	0.9416	-1.68	0.0074	-1.25	0.7051
94066_at	1422429_at	Rnf14	ring finger protein 14	1	0.99	1.09	0.0545	1.18	0.0036	1.26	0.0003	1.36	0.002
94067_at	1424080_at	Dcps	decapping enzyme, scavenger	1.05	0.83	-1.4	0.0029	-1.22	0.0561	-1.45	0.0037	1.19	0.1522
94068_at	1460442_at	Rps19	ribosomal protein S19	1.92	0.12	1.23	0.1273	1.32	0.0391	1.51	0.0061	1.14	0.334
94071_at	1419371_s_at	Gosr2	golgi SNAP receptor complex member 2	1.07	0.78	-1.09	0.1531	-1.02	0.6616	-1.07	0.0444	-1.35	0.0079
94073_at	1416270_at	Polr2g	polymerase (RNA) II (DNA directed) polype	1.43	0.32	1.23	0.0834	1.12	0.3513	1.3	0.0231	-1.01	0.9389
94075_at	1448764_a_at	Fabp1	fatty acid binding protein 1, liver	-1.28	0.29	-1.01	0.8649	1.01	0.8983	-1.04	0.4969	-1.71	0.0019
94077_f_at	1418896_a_at	Rpn2	ribophorin II	-1.11	0.38	-1.16	0.0086	-1.03	0.6753	-1.1	0.0294	-1.07	0.7517
94078_at	1434341_x_at	---	---	1.36	0.07	1.16	0.0159	1.24	0.0037	1.33	0.0011	1.11	0.4539
94079_at	1448729_a_at	4-Sep	septin 4	1.27	0.2	1.09	0.3586	1.14	0.3216	1.12	0.2957	-1.33	0.2477
94081_at	1452691_at	Rbm17	RNA binding motif protein 17	1.54	0.02	1.2	0.0888	1.24	0.0392	1.37	0.0001	1.59	0.0158
94084_at	1423987_at	Cdc26	cell division cycle 26	1.32	0.15	1.01	0.8923	1.09	0.3125	1.02	0.8143	-1.04	0.6809
94086_at	1450566_at	Il3	interleukin 3	1.57	0.27	-1.36	0.2664	1.05	0.8709	-1.36	0.2932	1.63	0.1013
94088_at	1423470_at	Ptbp2	polypyrimidine tract binding protein 2	-1.1	0.79	1.32	0.0974	1.08	0.5166	1.35	0.0254	-1.32	0.4286
94089_at	1444160_at	Pkd1l3	Polycystic kidney disease 1 like 3	1.11	0.8	1.01	0.9147	1.4	0.0844	1.04	0.8253	1.39	0.3153
94095_at	1420068_at	Hat1	Histone aminotransferase 1	-2.2	0.08	-1.2	0.1557	1.04	0.7952	-1.14	0.2833	1.27	0.3479
94097_at	1449058_at	Gli1	GLI-Kruppel family member GLI1	-2.47	0.22	1.22	0.1706	1.08	0.6507	1.1	0.3684	-1.09	0.7633
94098_at	1420471_at	Hcrt	hypocretin	-1.49	0.35	-1.15	0.7153	-1.03	0.9388	1.07	0.8372	1.05	0.8891
94099_at	1449960_at	Nptx2	neuronal pentraxin 2	-1.36	0.09	1.02	0.8367	-1.03	0.7854	-1.01	0.8856	1.39	0.1083
94100_s_at	1451033_a_at	Trpc4	transient receptor potential cation channel, s	-2.74	0.17	1.42	0.0796	1.11	0.6862	-1.03	0.8807	2.07	0.1371
94101_at	1420738_at	Zfp98	zinc finger protein 98	1.71	0.23	-1.98	0.0179	-1.64	0.1288	-2.12	0.0091	1.17	0.5186
94102_at	1422152_at	Hmx1	H6 homeo box 1	1.15	0.68	1.12	0.3466	-1	0.979	-1.01	0.8932	-1.66	0.2528
94104_at	1450254_at	Tert	telomerase reverse transcriptase	-1.05	0.89	-1.19	0.0499	-1.07	0.5003	-1.16	0.0613	1.31	0.2037
94105_at	1460708_s_at	Cdc42	cell division cycle 42 homolog (S. cerevisiae	1.12	0.65	1.11	0.0423	1.09	0.1051	1.21	0.0064	-1.26	0.1599
94106_at	1457272_at	---	Transcribed locus	1.05	0.54	1.05	0.2238	1.26	0.0008	1.09	0.0878	1.28	0.0895
94107_at	1421056_at	Dnase1l3	deoxyribonuclease 1-like 3	1.37	0.4	-1.13	0.7577	1.11	0.7555	1.17	0.619	-1.12	0.7934
94108_at	1421704_a_at	Pik3c2g	phosphatidylinositol 3-kinase, C2 domain co	1.43	0.1	-1.04	0.6581	1.01	0.9081	-1.04	0.6122	-1.26	0.1276
94109_at	1426377_at	Zfp281	zinc finger protein 281	1.1	0.39	1.33	0.0557	1.16	0.2325	1.15	0.3362	1.32	0.0801
94112_at	1420412_at	Tnfsf10	tumor necrosis factor (ligand) superfamily, m	-1.17	0.79	1.12	0.6853	1.11	0.5982	1.21	0.3409	1.17	0.2684
94113_at	1459920_at	---	Transcribed locus	-1.41	0.55	1	0.9917	-1.47	0.1286	1.12	0.498	1.09	0.8883
94115_at	1419008_at	Npy5r	neuropeptide Y receptor Y5	-1.07	0.69	-1.06	0.7032	-1.06	0.6736	-1.01	0.9052	1.21	0.6293
94116_at	1422265_at	Brs3	bombesin-like receptor 3	1.91	0.39	-1.49	0.1625	-1.19	0.5386	-1.96	0.0391	1.49	0.407
94119_at	1450228_a_at	Pip5k1c	phosphatidylinositol-4-phosphate 5-kinase, t	-1.06	0.69	1.05	0.6271	-1.07	0.3722	-1.05	0.6059	1.17	0.2633

94120_s_at	1449833_at	Sprr2f	small proline-rich protein 2F	-2	0.07	1.08	0.7081	1.06	0.7922	1.13	0.564	1.72	0.1079
94121_at	1422240_s_at	Sprr2h	small proline-rich protein 2H	-1.48	0.59	1.09	0.678	-1.09	0.6975	-1.05	0.8028	1.61	0.3607
94122_at	1450468_at	---	---	-1.59	0.16	1.14	0.322	1.42	0.0438	1.14	0.3272	2.84	0.0367
94123_at	1418054_at	Neurod4	neurogenic differentiation 4	-2.13	0.44	-1.74	0.2165	-1.49	0.3601	-1.73	0.2214	-1.02	0.9655
94124_at	1418597_at	Top3a	topoisomerase (DNA) III alpha	1.1	0.4	1.06	0.423	-1.03	0.7602	1.04	0.7201	1.28	0.4074
94125_at	1416237_at	Eva1	epithelial V-like antigen 1	1.28	0.53	-1.23	0.2738	-1.2	0.1939	-1.23	0.1034	-1.57	0.0832
94126_at	1421465_at	Wnt2b	wingless related MMTV integration site 2b	1.04	0.9	1.38	0.0571	1.61	0.0089	1.59	0.0172	2.94	0.0014
94127_at	1427564_at	Diap2	diaphanous homolog 2 (Drosophila)	-1.42	0.25	-1.06	0.7026	-1.3	0.1433	-1.53	0.0255	1.22	0.3178
94128_at	1420594_at	Bard1	BRCA1 associated RING domain 1	-3.11	0.01	-1.29	0.3593	1.08	0.8035	-1.21	0.4674	1.06	0.8369
94129_at	1419842_at	---	Transcribed locus, weakly similar to XP_486	-1.58	0.65	-1.33	0.3915	1.15	0.6807	1.17	0.6273	5.22	0.0112
94130_at	1420706_at	MGI:1933179	P140 gene	-1.79	0.34	-1.01	0.9713	1.09	0.6005	1.3	0.1726	1.85	0.1279
94131_at	1448070_at	Xpo1	exportin 1, CRM1 homolog (yeast)	1.02	0.96	1.08	0.8432	-1.27	0.4448	-1.04	0.8967	1.32	0.4743
94132_at	1460671_at	Gpx1	glutathione peroxidase 1	-1.19	0.48	-1.06	0.4643	-1.1	0.3002	-1.08	0.3042	1.12	0.1067
94134_at	1425377_at	Wnt1	wingless-related MMTV integration site 1	-1.68	0.14	-1.15	0.178	-1.03	0.7677	-1.2	0.0436	-1.08	0.6065
94135_at	1418159_at	Tcfcp2	transcription factor CP2	-1.3	0.08	-1.28	0.0013	-1.03	0.732	-1.23	0.0182	-1.09	0.2512
94137_at	1421734_at	Il8rb	interleukin 8 receptor, beta	-2.48	0.05	-1.12	0.6037	1.17	0.2859	-1.1	0.6714	1.32	0.1406
94138_s_at	1420756_at	Fut7	fucosyltransferase 7	-1.47	0.08	-1.04	0.7428	1.09	0.519	-1.03	0.8109	-1.07	0.5635
94139_at	1449179_at	Pdc	phosducin	1.41	0.38	-1.46	0.2389	1.07	0.859	-1.41	0.3068	1.41	0.2357
94141_at	1422122_at	Fcer2a	Fc receptor, IgE, low affinity II, alpha polype	-1.24	0.73	2.07	0.0185	1.43	0.2303	2.01	0.0328	2.3	0.1003
94142_at	1419427_at	Csf3	colony stimulating factor 3 (granulocyte)	-1.46	0.13	-1.16	0.4157	-1.09	0.5177	-1.25	0.1351	1.39	0.3617
94144_g_at	1426509_s_at	---	---	-1.09	0.69	-1.16	0.4396	-1.16	0.5067	-1.23	0.3225	-1.29	0.2005
94145_at	1422305_at	Ifnb1	interferon beta 1, fibroblast	-2.69	0.05	-1.12	0.7396	-1.44	0.3926	-1.29	0.5308	1.47	0.4815
94146_at	1421578_at	Ccl4	chemokine (C-C motif) ligand 4	-1.54	0.42	1.14	0.306	1.02	0.9348	-1.04	0.7756	-1.11	0.6651
94147_at	1419149_at	Serpine1	serine (or cysteine) proteinase inhibitor, clad	-1.47	0.32	2.52	0.2879	-1.36	0.1833	2.29	0.188	1.39	0.5949
94148_at	1420440_at	Ppy	pancreatic polypeptide	-1.46	0.45	1.14	0.4089	1.05	0.7217	1.13	0.3385	-1.07	0.8771
94149_at	1450132_at	Hivep3	human immunodeficiency virus type I enhan	-1.41	0.25	-1.1	0.6904	-1.05	0.8401	-1.17	0.5512	1.32	0.2399
94150_at	1419025_at	Sag	retinal S-antigen	2.14	0.26	1.03	0.9022	1.28	0.2807	1.12	0.542	1.05	0.9258
94151_at	1449212_at	Pip	prolactin induced protein	-1.53	0.62	-1.38	0.3591	1.3	0.4615	-1.47	0.325	1.79	0.4741
94153_g_at	1448168_a_at	Spt1	salivary protein 1	-1.16	0.45	1.04	0.79	-1.02	0.861	-1	0.981	1.12	0.6352
94154_at	1450790_at	Tgn	thyroglobulin	1.18	0.41	1.06	0.52	-1.11	0.3104	-1.07	0.4278	1.12	0.7082
94155_at	1448285_at	Rgs4	regulator of G-protein signaling 4	-1.68	0.4	1.06	0.7338	1.66	0.0452	1.07	0.7245	1.12	0.3749
94156_at	1422790_at	Nppc	natriuretic peptide precursor type C	-7.18	0.05	-1	0.9904	-1.19	0.1843	1.04	0.8712	2.45	0.3102
94158_f_at	1427872_at	Ptafr	platelet-activating factor receptor	-1.09	0.86	-1.2	0.1182	-1.28	0.0933	-1.03	0.8633	1.04	0.856
94161_at	1449956_at	Prkce	protein kinase C, epsilon	1.39	0.39	-1.03	0.8074	-1.09	0.6352	-1.56	0.0161	-1.08	0.6156
94163_at	1421286_a_at	Atp4a	ATPase, H+/K+ transporting, alpha polypept	-1.13	0.44	-1.12	0.0744	-1.02	0.7412	-1.23	0.0092	1.18	0.3207
94164_at	1419694_at	St8sia1	ST8 alpha-N-acetyl-neuraminide alpha-2,8-s	-3.47	0.28	-1.14	0.3385	1.51	0.1963	-1.07	0.6402	1.59	0.0901
94166_g_at	1421688_a_at	Ccl1 /// BF5343	chemokine (C-C motif) ligand 1 /// expressed	-1.71	0.37	-1.05	0.6388	-1.04	0.787	-1.17	0.2096	1.57	0.1311
94167_at	1422622_at	Nos3	nitric oxide synthase 3, endothelial cell	1.18	0.08	1.15	0.4434	1.33	0.0409	-1.05	0.7055	1.24	0.4733
94168_at	1420802_at	Il13	interleukin 13	1.04	0.91	1.1	0.5421	1.04	0.7026	1.16	0.2291	1.33	0.2636
94169_at	1450473_at	Pcdh12	protocadherin 12	-1.27	0.5	1.09	0.2207	1.06	0.5173	-1.05	0.6642	1.06	0.6077
94170_at	1460322_at	Chst3	carbohydrate (chondroitin 6/keratan) sulfotra	1.5	0.05	-1.02	0.9325	1.02	0.9097	-1.21	0.2763	-1.32	0.37
94171_at	1449829_at	Itgb2l	integrin beta 2-like	-1.32	0.38	1.26	0.3769	1.34	0.2519	1.31	0.2458	1.51	0.0009
94172_at	1453950_a_at	Xrcc2	X-ray repair complementing defective repair	-1	0.99	-1.23	0.4616	-1.01	0.9658	-1.23	0.5408	1.21	0.6587
94173_at	1449925_at	Cxcr3	chemokine (C-X-C motif) receptor 3	-2.45	0.27	1.27	0.1224	1.21	0.0853	1.11	0.4565	1.7	0.1669
94174_at	1420929_at	Catnal1	catenin alpha-like 1	-2.19	0.18	1.01	0.9577	1.35	0.0455	-1.04	0.8012	1.34	0.1286
94175_at	1421558_at	T2	brachyury 2	-1.53	0.28	1.19	0.5653	-1.06	0.8413	1.07	0.7749	-1.2	0.4608
94176_at	1425094_a_at	Lhx6	LIM homeobox protein 6	-1.44	0.06	1.11	0.3781	1.16	0.2532	-1.23	0.1302	-1.12	0.4938
94177_at	1417871_at	Hsd17b7	hydroxysteroid (17-beta) dehydrogenase 7	-1.08	0.9	-1.14	0.2813	-1.47	0.0252	-1.59	0.0026	-1.23	0.3766
94178_at	1421586_a_at	Mcp	membrane cofactor protein	2.29	0.03	1.3	0.2834	2.26	0.0505	1.45	0.1073	2.03	0.1502
94180_s_at	1420694_a_at	Dach1	dachshund 1 (Drosophila)	-3.14	0.13	-1	0.9876	1.23	0.5688	1.22	0.6859	1.99	0.0979
94181_at	1420764_at	Scrg1	scrapie responsive gene 1	-2.12	0.06	1.48	0.3046	1.07	0.586	1.06	0.6364	1.33	0.3265
94182_at	1420596_at	Cacng2	calcium channel, voltage-dependent, gamma	-4.84	0.09	1.22	0.3876	1.48	0.007	1.19	0.1038	1.32	0.1485
94183_at	1420358_at	Krtap13	keratin associated protein 13	-1.17	0.76	-1.06	0.5824	-1.05	0.5939	-1.13	0.2578	1.55	0.3059

94184_at	1451708_at	Gpr33	G protein-coupled receptor 33	-1.72	0.14	-1.11	0.4294	1.23	0.1421	1.01	0.8744	-1.42	0.0553
94185_at	1421419_at	Kcnk4	potassium channel, subfamily K, member 4	-1.09	0.86	1.4	0.1685	1.29	0.3327	1.23	0.3931	2.18	0.0202
94186_at	1423602_at	Traf1	Tnf receptor-associated factor 1	1.62	0.25	1.06	0.7437	-1.06	0.5602	1.24	0.1442	1.5	0.2114
94187_at	1421412_at	Gsc	goosecoid	-3.07	0.28	-1.2	0.1319	-1.02	0.8608	-1.07	0.443	-1.16	0.4966
94188_at	1451023_at	Hcn3	hyperpolarization-activated, cyclic nucleotide	-1.33	0.06	1.07	0.6887	1	0.9809	1.11	0.5139	1.08	0.4995
94189_at	1418421_at	Bcl6b	B-cell CLL/lymphoma 6, member B	-1.05	0.73	1.19	0.2862	-1.08	0.6214	-1.18	0.3007	1.11	0.7191
94190_at	1420782_at	Tnfrsf17	tumor necrosis factor receptor superfamily, r	-1.17	0.28	1.02	0.9268	-1.21	0.2188	-1.12	0.5356	1.56	0.1135
94192_at	1420342_at	Gdap10	Ganglioside-induced differentiation-associate	1.14	0.82	1.13	0.6835	-2.96	0.001	-1.87	0.0576	1.32	0.2152
94193_at	1450490_at	Kcna7	potassium voltage-gated channel, shaker-re	1.26	0.71	-1.02	0.9482	1.12	0.7121	-1.01	0.9577	1.04	0.8579
94195_r_at	1421154_at	Hcn2	hyperpolarization-activated, cyclic nucleotide	-1.45	0.03	1.03	0.6935	1.11	0.0833	1	0.9491	1.41	0.1506
94196_at	1450161_at	Ikbkg	inhibitor of kappaB kinase gamma	1.25	0.36	-1.01	0.9102	-1.07	0.2496	-1.11	0.1709	-1.47	0.1104
94197_at	1421268_at	Ugcg	UDP-glucose ceramide glucosyltransferase	1	1	-1.15	0.2774	-1.05	0.7687	-1.33	0.0502	-1.11	0.5961
94198_at	1425703_at	Ppard	peroxisome proliferator activator receptor de	1.24	0.71	-1.4	0.2657	-1.24	0.5252	-1.01	0.9846	1.6	0.0747
94199_at	1415969_s_at	Kap	kidney androgen regulated protein	-1.95	0.26	1.04	0.9079	-1.52	0.1326	-1.06	0.7609	-1.05	0.8992
94200_at	1420337_at	Gbx2	gastrulation brain homeobox 2	-1.23	0.47	1.12	0.3231	1.2	0.1253	1.23	0.3936	1.6	0.1091
94201_at	1450121_at	---	---	-2.36	0.25	1.13	0.4573	1.2	0.3405	1.57	0.0201	3.81	0.2063
94202_at	1427628_at	Tcrb-V8.2	T-cell receptor beta, variable 8.2	-1.02	0.97	1.08	0.5845	1.13	0.4341	1.05	0.7357	1.06	0.9077
94203_at	1459928_at	MGI:2443930	Mblk1-related protein-2	-1.81	0.14	-1.3	0.0234	-1.48	0.0187	-1.61	0.0007	1.73	0.1788
94205_at	1449136_at	---	---	-1.42	0.16	-1.16	0.2699	-1.07	0.6267	-1.11	0.4358	1.57	0.2396
94206_at	1416522_a_at	Grcc10	gene rich cluster, C10 gene	1.12	0.76	1.04	0.4992	1.01	0.8959	1.08	0.1088	1.17	0.2137
94210_at	1455211_a_at	Timm9	translocase of inner mitochondrial membran	-1.06	0.56	-1.02	0.6505	-1.06	0.5803	-1.18	0.0139	-1.2	0.4854
94211_at	1452073_at	6720460F02Rik	RIKEN cDNA 6720460F02 gene	-1.13	0.83	1.49	0.1583	-1.14	0.6939	1.17	0.6668	1.13	0.8026
94212_at	1425557_x_at	Tsc22d3	TSC22 domain family 3	1.14	0.48	-1.54	0.1645	-1.21	0.5294	1.08	0.8001	-1.2	0.4873
94214_at	1416023_at	Fabp3	fatty acid binding protein 3, muscle and hear	-1.19	0.51	-1.46	0.3699	-1.08	0.8411	-1.7	0.2697	-1.39	0.5249
94216_at	1448630_a_at	Sdhc	succinate dehydrogenase complex, subunit	1.1	0.43	-1.01	0.824	-1.04	0.5939	-1.04	0.5378	-1.09	0.4058
94218_at	1448122_at	Tcp1	t-complex protein 1	1.4	0.26	-1.17	0.038	-1.05	0.5021	-1.12	0.3774	1.65	0.0105
94219_at	1448262_at	Psmb2	proteasome (prosome, macropain) subunit, I	1.11	0.76	-1.07	0.382	-1.16	0.0238	1.08	0.3261	-1.26	0.051
94222_at	1437406_x_at	Igfbp4	insulin-like growth factor binding protein 4	-1.19	0.43	-1.02	0.8419	-1.01	0.8936	-1.09	0.46	1.26	0.0606
94224_s_at	1452349_x_at	Ifi205 /// Mnda	interferon activated gene 205 /// myeloid cell	1.68	0.1	4.68	0.2057	-1.53	0.3728	7.1	0.2212	1.39	0.1259
94225_at	1415684_at	Apg5l	autophagy 5-like (S. cerevisiae)	-1.2	0.16	-1.13	0.0121	-1.14	0.0614	-1.34	0	-1.78	0.0001
94226_at	1425068_a_at	Tex264	testis expressed gene 264	1.67	0.09	1.1	0.2466	-1	0.9696	1.01	0.8268	1.21	0.5623
94227_at	1422209_s_at	2310040M23Rik	RIKEN cDNA 2310040M23 gene	-1.2	0.61	1.13	0.253	1.18	0.1257	1.09	0.3684	1.19	0.1528
94228_at	1418442_at	Xpo1	exportin 1, CRM1 homolog (yeast)	1.23	0.52	-1.02	0.8876	-1.13	0.6436	-1.03	0.7887	1.12	0.7083
94229_at	1448412_a_at	Tsc22d4	TSC22 domain family 4	-1.12	0.37	1.04	0.4725	-1.06	0.3537	1.05	0.5706	1.21	0.1612
94231_at	1417419_at	Ccnd1	cyclin D1	-2.82	0.08	-1.76	0	-1.07	0.5394	-1.69	0.0024	2.11	0.0992
94232_at	1448698_at	Ccnd1	cyclin D1	-1.14	0.43	-1.66	0.0088	1.37	0.0486	-1.52	0.0217	1.4	0.0041
94233_at	1416885_at	1110038F14Rik	RIKEN cDNA 1110038F14 gene	1.46	0.15	1.32	0.0204	1.2	0.1299	1.22	0.1649	1.47	0.2269
94235_at	1423901_at	Trip12	thyroid hormone receptor interactor 12	1.19	0.7	-1.07	0.5102	1.04	0.6383	1.06	0.4732	-1.1	0.619
94236_at	1452156_a_at	Nisch	nischarin	-1.02	0.9	-1.05	0.4638	-1.03	0.6446	-1.05	0.4844	-1.08	0.5614
94237_at	1448353_x_at	Rpn1	ribophorin I	1.02	0.93	-1.01	0.8838	-1.18	0.0554	-1.06	0.3136	-1.49	0.1228
94238_at	1431057_a_at	2310046G15Rik	RIKEN cDNA 2310046G15 gene	1.3	0.53	1.51	0.0742	1.25	0.4136	1.63	0.0328	-2.33	0.0825
94239_at	1450938_at	---	---	-1.15	0.76	1.01	0.9515	1.03	0.7572	-1.09	0.391	1.1	0.6269
94240_i_at	1455348_x_at	Rpl29 /// LOC24	ribosomal protein L29 /// similar to ribosomal	-1.68	0.33	-1.75	0.2682	-1.49	0.414	-1.19	0.6596	1.2	0.5778
94241_at	1423701_at	Coasy	Coenzyme A synthase	1.49	0.04	-1.16	0.0068	-1.04	0.4227	-1.01	0.874	-1.26	0.0324
94242_at	1423036_at	Txnl5	thioredoxin-like 5	1.25	0.11	1.11	0.1249	1.15	0.3256	1.13	0.0262	-1.04	0.5535
94243_at	1437349_at	4930432B04Rik	RIKEN cDNA 4930432B04 gene	1.92	0.11	-1.11	0.2573	-1	0.987	-1.05	0.6536	1.01	0.9532
94244_at	1452694_at	Ihpk1	inositol hexaphosphate kinase 1	-1.66	0	1.03	0.6533	1.11	0.2536	1.13	0.1347	1.6	0.0269
94246_at	1416268_at	Ets2	E26 avian leukemia oncogene 2, 3' domain	1.05	0.57	1.09	0.4892	1.34	0	1.15	0.1586	1.77	0.0263
94247_at	1435494_s_at	Dsp	desmoplakin	-1.28	0.3	-1.04	0.8091	1.4	0.0216	-1.03	0.7496	-1.1	0.4531
94248_at	1416307_at	Ap1m1	adaptor-related protein complex AP-1, mu su	-1.21	0.41	-1.07	0.3474	-1.08	0.3815	-1.06	0.4613	1.22	0.0638
94249_at	1415679_at	Psenen	presenilin enhancer 2 homolog (C. elegans)	1.34	0.09	-1.01	0.8932	-1.01	0.8956	1.02	0.7955	1.15	0.1156
94250_at	1416659_at	Eif3s10	eukaryotic translation initiation factor 3, subu	1.28	0.32	1.23	0.1256	1.34	0.0123	1.2	0.1138	1.04	0.7067
94252_at	1451090_a_at	Eif2s3x	eukaryotic translation initiation factor 2, subu	-1.37	0.22	-1.06	0.2063	1	0.9208	-1.18	0.0031	-1.14	0.0319

94253_at	1452662_a_at	Eif2s1	eukaryotic translation initiation factor 2, subu	1.97	0.01	-1.03	0.5802	-1.03	0.6324	-1	0.9441	-1.1	0.5191
94255_g_at	1423393_at	Clic4	chloride intracellular channel 4 (mitochondria	-1.22	0.5	1.12	0.5277	-1.26	0.0082	1.04	0.7368	1.11	0.203
94256_at	1438606_a_at	Clic4	chloride intracellular channel 4 (mitochondria	-1.5	0.26	1.09	0.61	1.17	0.2212	1.23	0.153	1.31	0.2133
94257_at	1428905_at	Rraga	Ras-related GTP binding A	-1.18	0.61	-1.04	0.5337	-1.39	0.0126	-1.23	0.0219	-1.21	0.3138
94258_at	1426454_at	Arhgdib	Rho, GDP dissociation inhibitor (GDI) beta	-1.08	0.61	1.08	0.529	-1.1	0.1348	1.16	0.2967	1.37	0.0154
94259_at	1417998_at	MGI:1929282	telomerase binding protein, p23	1.79	0.09	1.07	0.2277	-1.09	0.2308	-1.02	0.8283	1.01	0.871
94260_at	1428244_at	3110040D16Rik	RIKEN cDNA 3110040D16 gene	-1	0.98	-1.13	0.0596	-1.09	0.0531	-1.07	0.1519	1.08	0.3801
94261_at	1460545_at	Thrap3	thyroid hormone receptor associated protein	-1.06	0.63	1.09	0.2407	1.23	0.0153	1.17	0.0183	1.58	0.0095
94263_f_at	1416240_at	Psmb7	proteasome (prosome, macropain) subunit, I	1.22	0.13	1.12	0.0427	1.1	0.1953	1.13	0.0058	-1.12	0.3394
94264_at	1425419_a_at	Raf1	v-raf-1 leukemia viral oncogene 1	-1.14	0.2	1.04	0.4513	1.17	0.04	1.12	0.1714	1.46	0.0053
94266_at	1428406_s_at	Hcfc1r1	host cell factor C1 regulator 1 (XPO1-depen	-1.01	0.88	-1.03	0.5768	-1.14	0.0062	-1.12	0.0244	-1.17	0.2159
94268_f_at	1416848_at	Ubl5	ubiquitin-like 5	-1	0.97	1.11	0.0019	1.08	0.3197	1.1	0.0047	-1.07	0.5878
94269_at	1427773_a_at	Rabac1	Rab acceptor 1 (prenylated)	1.27	0.6	1.14	0.0585	-1	0.9987	1.13	0.0492	1.23	0.2643
94270_at	1448169_at	Krt1-18	keratin complex 1, acidic, gene 18	1.66	0.13	1.13	0.5082	1.05	0.5277	1.28	0.013	1.03	0.8673
94273_at	1449142_a_at	2610311I19Rik	RIKEN cDNA 2610311I19 gene	1.01	0.95	-1.16	0.0521	-1.15	0.1073	-1.13	0.0264	-1.18	0.308
94274_at	1416726_s_at	Ube2s	ubiquitin-conjugating enzyme E2S	1.17	0.55	-1.06	0.5232	-1.02	0.8603	-1.02	0.7943	1.2	0.146
94275_at	1417206_at	Urod	uroporphyrinogen decarboxylase	1.13	0.4	1.06	0.3313	1.17	0.0668	1.08	0.3174	-1.07	0.4404
94276_at	1450011_at	Hsd17b12	hydroxysteroid (17-beta) dehydrogenase 12	-1.11	0.35	-1.15	0.0342	-1.1	0.2666	-1.18	0.0015	-2.95	0.0001
94277_at	1418522_at	Mtx1	metaxin 1	-1.21	0.15	-1.33	0.0003	-1.26	0.0517	-1.13	0.2191	1.25	0.1995
94278_at	1415983_at	Lcp1	lymphocyte cytosolic protein 1	-1.27	0.09	-1.06	0.5956	-1.2	0.0254	1.01	0.9626	-1.76	0.0035
94281_at	1454602_s_at	Cnot2	CCR4-NOT transcription complex, subunit 2	1.34	0.1	1.12	0.0199	1.09	0.1499	1.12	0.0264	1.19	0.011
94282_at	1416735_at	Asah1	N-acylsphingosine amidohydrolase 1	-1.24	0.21	-1.06	0.5201	1.11	0.1571	1.07	0.6362	1.04	0.6701
94284_at	1422186_s_at	Dia1	diaphorase 1 (NADH)	1.08	0.55	-1.45	0.0009	-1.56	0.0004	-1.52	0.0004	-1.67	0.02
94285_at	1417025_at	H2-Eb1	histocompatibility 2, class II antigen E beta	-1.02	0.95	2.65	0.1748	-1.2	0.2737	2.49	0.2006	1.8	0.0127
94286_at	1415705_at	9130011J15Rik	RIKEN cDNA 9130011J15 gene	1.33	0.09	1.07	0.3036	1.01	0.8141	1.11	0.048	-1.19	0.1923
94289_r_at	1426306_a_at	Maged2	melanoma antigen, family D, 2	-1.3	0.2	-1.05	0.5418	1.13	0.116	1.2	0.0704	1.26	0.4535
94291_at	1452543_a_at	Scgb1a1	secretoglobin, family 1A, member 1 (uterogl	-1.26	0.38	1.11	0.7163	1.15	0.6105	1.14	0.6233	1.37	0.5439
94292_at	1419912_s_at	Strap	Serine/threonine kinase receptor associated	-1.18	0.26	1.14	0.3472	1.32	0.1449	1.09	0.4806	-1.15	0.4222
94294_at	1450920_at	Ccnb2	cyclin B2	-1.89	0.04	1.2	0.0607	1.07	0.3955	1.42	0.0305	1.1	0.5447
94295_at	1425628_a_at	---	---	-1.13	0.54	-1.07	0.2155	-1.06	0.5654	-1.06	0.244	1.05	0.6195
94296_s_at	1460279_a_at	Gtf2i	general transcription factor II I	1.66	0.55	-1.12	0.071	-1.11	0.399	-1.31	0.0092	-1.58	0.2922
94297_at	1416125_at	Fkbp5	FK506 binding protein 5	-2.72	0.03	-2.04	0.0013	1.43	0.1411	-1.7	0.0057	-1.02	0.9349
94298_at	1448495_at	Tsta3	tissue specific transplantation antigen P35B	1.03	0.89	1.04	0.5907	1.13	0.206	1.17	0.0552	1.04	0.6431
94299_at	1424461_at	Dctn2	dynactin 2	1.93	0.15	1.12	0.202	1.21	0.2096	1.1	0.3918	-1.72	0.0531
94301_at	1416328_a_at	Atp6v0e	ATPase, H+ transporting, V0 subunit	1.47	0.11	1.07	0.4194	1.04	0.5953	1.18	0.1536	-1.07	0.4043
94302_at	1418874_a_at	Psmd4	proteasome (prosome, macropain) 26S subu	1.4	0.03	1.1	0.1641	1.07	0.4827	1.19	0.006	-1.19	0.0816
94303_at	1425142_a_at	Hnrpd	heterogeneous nuclear ribonucleoprotein D	-1.12	0.49	1.06	0.4254	1.08	0.2131	1.16	0.0745	1.12	0.4312
94304_at	1415818_at	Anxa6	annexin A6	1.26	0.37	-1.08	0.3616	-1.1	0.1063	-1.06	0.427	1.34	0.0766
94305_at	1423669_at	Col1a1	procollagen, type I, alpha 1	-2.13	0.35	1.83	0.2569	-1.29	0.443	2.32	0.2017	2.08	0.3254
94307_at	1422540_at	---	---	1.08	0.65	1.38	0.3886	1.28	0.4973	-1	0.9897	1.54	0.3036
94309_g_at	1451119_a_at	Fbln1	fibulin 1	-1.22	0.23	-1.17	0.0304	-1.17	0.0581	-1.06	0.454	1.5	0.0185
94312_at	1452705_at	AA415817	expressed sequence AA415817	1.03	0.84	1.16	0.0119	1.01	0.818	1.13	0.0947	2.32	0.0083
94313_at	1416197_at	Snrp1c	U1 small nuclear ribonucleoprotein 1C	1.13	0.63	-1.1	0.0542	-1.12	0.1386	-1.09	0.0809	1.08	0.7466
94318_at	1416677_at	Apoh	apolipoprotein H	1.26	0.19	1.1	0.016	1.12	0.0263	1.11	0.0041	1.34	0.0251
94321_at	1452166_a_at	Krt1-10	keratin complex 1, acidic, gene 10	1.66	0.15	1.05	0.7281	1.06	0.7407	1.07	0.6109	-2.27	0.0487
94322_at	1415993_at	Sqle	squalene epoxidase	1.03	0.93	-2.09	0.0085	-1.35	0.2279	-2.66	0.0007	-2.97	0.0055
94323_at	1434367_s_at	Nutf2	nuclear transport factor 2	-1.14	0.65	1	0.9931	-1.04	0.5101	-1.04	0.4607	-1.1	0.4501
94325_at	1433445_x_at	Hmgcs1	3-hydroxy-3-methylglutaryl-Coenzyme A syr	1.24	0.3	1.01	0.9417	1.12	0.6493	-1.34	0.0362	-2.39	0.0025
94327_at	1416984_at	Mrps18a	mitochondrial ribosomal protein S18A	1.61	0.13	-1.08	0.1103	-1	0.9826	-1.05	0.4537	-1.25	0.3705
94330_at	1424265_at	Npl	N-acetylneuraminate pyruvate lyase	1.44	0.4	1.57	0.1034	1.88	0.1119	1.76	0.2116	1.06	0.7276
94331_at	1426353_at	Stat6	signal transducer and activator of transcripti	1.95	0.02	-1.27	0.0324	-1.15	0.0373	-1.17	0.0221	-1.31	0.1585
94332_at	1452163_at	Ets1	E26 avian leukemia oncogene 1, 5' domain	1	1	1.21	0.1702	1.09	0.651	1.12	0.4763	1.38	0.3392
94335_r_at	1418178_at	Ina	internexin neuronal intermediate filament pro	1.43	0.28	1.5	0.1619	1.3	0.3888	1.33	0.4818	2.04	0.4034

94336_at	1448817_at	Otub1	OTU domain, ubiquitin aldehyde binding 1	-1.38	0.16	-1.04	0.7488	-1.15	0.1473	-1.08	0.4374	-1.21	0.6934
94338_g_at	1450112_a_at	Gas2	growth arrest specific 2	1.56	0.38	1.77	0.006	1.02	0.9115	1.73	0.0015	-1.47	0.0061
94339_at	1426715_s_at	D11Ertd18e	DNA segment, Chr 11, ERATO Doi 18, expr	-1.42	0.2	-1.52	0	-1.1	0.1681	-1.5	0.0001	-2.17	0.0066
94340_at	1428772_at	Xpot	exportin, tRNA (nuclear export receptor for t	1.28	0.26	-1.15	0.0191	1.1	0.2724	-1.06	0.2549	-1.24	0.1568
94341_at	1422698_s_at	Jarid2	jumonji, AT rich interactive domain 2	-1.32	0.34	-1.14	0.0636	-1.13	0.0383	-1.21	0.0075	1.02	0.8903
94343_at	1433887_at	Dnajc3	DnaJ (Hsp40) homolog, subfamily C, memb	-1.21	0.61	-1.3	0.0038	1	0.9997	-1.31	0.0038	-2.1	0.0017
94345_at	1452843_at	Il6st	interleukin 6 signal transducer	1.38	0.12	-1.06	0.5266	-1.08	0.3694	-1.05	0.6303	-1.26	0.2537
94346_at	1454805_at	Wtap	Wilms' tumour 1-associating protein	2.07	0.01	1.2	0.08	1.17	0.2618	1.31	0.0124	1.07	0.7678
94348_f_at	1422665_a_at	Pcmt1	protein-L-isoaspartate (D-aspartate) O-meth	1.13	0.33	1.09	0.0426	1	0.951	1.07	0.2317	1.21	0.0804
94349_at	1434164_s_at	LOC381251	similar to Nur77 downstream protein 1	-1.67	0.09	-1.22	0.1104	-1.04	0.6633	-1.18	0.1304	1.15	0.5181
94351_r_at	1423627_at	Nqo1	NAD(P)H dehydrogenase, quinone 1	3.69	0.03	1.57	0.0116	1.5	0.017	1.82	0.0096	1.74	0.2238
94353_at	1417084_at	Eif4ebp2	eukaryotic translation initiation factor 4E bin	-1.39	0.58	-1.27	0.0703	-1.29	0.0752	-1.81	0.0008	1.42	0.0203
94354_at	1421840_at	Abca1	ATP-binding cassette, sub-family A (ABC1),	-1.27	0.21	1.27	0.0187	-1.03	0.7302	1.21	0.0218	-1.07	0.5163
94355_at	1433659_at	---	---	3.13	0.05	1.13	0.6064	1.17	0.4852	1.09	0.6978	-3.84	0.0477
94356_at	1426957_at	Trp53bp1	transformation related protein 53 binding pro	1.26	0.42	1.52	0.2698	1.65	0.2546	1.81	0.0506	1.45	0.3529
94357_at	1419057_at	Slc5a1	solute carrier family 5 (sodium/glucose cotra	1.54	0.28	-1.24	0.3972	1.84	0.3369	-1.51	0.0594	-1.99	0.0895
94358_at	1427119_at	Spink4	serine protease inhibitor, Kazal type 4	1.01	0.95	1.11	0.4715	-1	0.9959	1.14	0.5016	1.04	0.9114
94359_at	1417965_at	Plekha1	pleckstrin homology domain containing, fam	-1.11	0.42	1.35	0.0094	1.08	0.3398	1.3	0.0505	-1.1	0.4209
94360_at	1452877_at	2700029M09Ril	RIKEN cDNA 2700029M09 gene	1.7	0.04	-1.1	0.2544	-1.06	0.6292	1.03	0.7766	-1.06	0.619
94361_at	1448270_at	Ddx21	DEAD (Asp-Glu-Ala-Asp) box polypeptide 2	-1.05	0.86	-1.13	0.1946	1.04	0.6375	-1.22	0.077	1.23	0.3359
94362_at	1454060_a_at	Nras	neuroblastoma ras oncogene	-1.37	0.36	1.02	0.637	-1	0.9575	-1.02	0.6388	-1.26	0.0728
94363_at	1433636_at	Bms1l	BMS1-like, ribosome assembly protein (yea	1.86	0.16	1.08	0.2968	1.34	0.006	1.19	0.006	1.57	0.153
94364_at	1417087_at	Glg1	golgi apparatus protein 1	-1.9	0.18	-1.15	0.4476	-1.83	0.0031	-1.63	0.0301	-1.12	0.3899
94365_at	1425196_a_at	Hint2	histidine triad nucleotide binding protein 2	-1.15	0.18	-1.21	0.0484	-1.2	0.0478	-1.13	0.2241	1.26	0.0255
94366_at	1417779_at	2310079N02Rik	RIKEN cDNA 2310079N02 gene	1.03	0.64	1.25	0.0121	-1.02	0.8482	1.37	0.0004	-1.17	0.4072
94367_at	1448604_at	Uck2	uridine-cytidine kinase 2	1.41	0.12	1.17	0.1764	1.12	0.3763	1.27	0.2164	1.46	0.101
94368_at	1460557_at	Supv3l1	suppressor of var1, 3-like 1 (S. cerevisiae)	1.56	0.01	1.02	0.8166	1.1	0.2968	1.06	0.3644	1.01	0.9725
94370_at	1424502_at	Oit1	oncoprotein induced transcript 1	-1.42	0.75	1.35	0.2299	1.51	0.4161	1.36	0.4893	-1.22	0.7172
94371_at	1417237_at	Pld2	phospholipase D2	-1.74	0.03	1.05	0.5624	-1.04	0.5989	-1.03	0.6538	1.18	0.5623
94372_at	1449349_at	Nudt1	nudix (nucleoside diphosphate linked moiety	1.38	0.1	-1.08	0.1989	-1.18	0.0334	-1.27	0.0005	-1.76	0.0012
94373_at	1428072_a_at	Dolpp1	dolichyl pyrophosphate phosphatase 1	-1.99	0.01	-1.08	0.1634	-1.18	0.0636	-1.22	0.0099	-1.06	0.5296
94374_at	1451728_at	Wdr13	WD repeat domain 13	-1.3	0.03	-1.24	0.0043	1.02	0.7627	-1.18	0.0015	-1.38	0.1682
94375_at	1422612_at	Hk2	hexokinase 2	-1.34	0.29	1.46	0.4567	-1.01	0.9716	1.55	0.4297	1.62	0.3059
94376_s_at	1416748_a_at	Mre11a	meiotic recombination 11 homolog A (S. cer	4.53	0.02	1.31	0.326	1.42	0.2015	1.66	0.0468	1.29	0.247
94377_at	1448942_at	Gng11	guanine nucleotide binding protein (G protei	1.4	0.14	1.06	0.7844	1.16	0.4831	1.2	0.2954	3.38	0.006
94378_at	1426037_a_at	---	---	2.33	0.05	1.98	0.0629	4.36	0.0014	3.13	0.0002	2.55	0.0217
94379_at	1425270_at	Kif1b	kinesin family member 1B	1.74	0.42	-1.55	0.008	-1.35	0.2402	-1.48	0.0194	1.22	0.3873
94380_at	1435140_at	Ide	insulin degrading enzyme	1.05	0.92	1.01	0.9141	1.11	0.2269	1.06	0.38	-4.42	0
94381_at	1424399_at	Uck1	uridine-cytidine kinase 1	-1.34	0.23	-1.78	0.0007	-1.53	0.0141	-2.48	0	-3.25	0.0003
94382_at	1428085_at	1110057K04Rik	RIKEN cDNA 1110057K04 gene	1.22	0.02	1.08	0.4004	1.06	0.3775	1.28	0.0008	-1.6	0.0351
94383_at	1418547_at	Tfpi2	tissue factor pathway inhibitor 2	-1.83	0.02	-1.56	0	-1.28	0.048	-2.43	0	-3.47	0.0001
94384_at	1419647_a_at	Ier3	immediate early response 3	1.15	0.65	1.62	0.318	1.14	0.4326	1.57	0.2574	2.76	0.0434
94385_at	1429763_at	E430023H19Rik	RIKEN cDNA E430023H19 gene	1.28	0.39	-1.23	0.0637	-1.34	0.0218	-1.18	0.0828	1.01	0.889
94386_at	1435862_at	Son	Son cell proliferation protein	1.32	0.04	1.14	0.004	1.26	0.024	1.25	0.005	1.23	0.1081
94387_at	1448639_a_at	Spata5	spermatogenesis associated 5	-1.24	0.76	1.2	0.2326	1.49	0.2572	1.46	0.2953	1.34	0.5658
94388_at	1422718_at	Ap3s2	adaptor-related protein complex 3, sigma 2 s	1.22	0.35	-1.16	0.1083	1.01	0.8357	-1.09	0.1986	-2.35	0.046
94389_at	1418656_at	Lsm5	LSM5 homolog, U6 small nuclear RNA asso	1.08	0.85	1.03	0.9352	1.13	0.6795	1.29	0.4283	1.12	0.7851
94390_at	1433669_at	Akap8	A kinase (PRKA) anchor protein 8	1.76	0.11	1.36	0.0042	1.51	0.0028	1.52	0.0002	1.1	0.6678
94391_at	1448397_at	Gjb6	gap junction membrane channel protein beta	1.28	0.61	-1.1	0.7376	-1.13	0.683	-1.3	0.4044	1.26	0.6872
94392_f_at	1450717_at	Ang1	angiogenin, ribonuclease A family, member	2.36	0.04	1.17	0.0043	1.01	0.9102	1.04	0.4402	2.17	0.0004
94393_r_at	1416444_at	Elovl2	elongation of very long chain fatty acids (FE	-1.59	0.17	-1	0.9875	1.16	0.0956	1.03	0.6612	-1.4	0.0286
94394_at	1418448_at	Rras	Harvey rat sarcoma oncogene, subgroup R	1.57	0.22	1.3	0.0054	-1.03	0.7772	1.35	0.0025	1.38	0.0494
94395_at	1433640_at	---	---	-1.03	0.83	1.23	0.0447	1.51	0.0281	1.34	0.0042	1.54	0.2844

94396_at	1448496_a_at	Ing1	inhibitor of growth family, member 1	1.25	0.47	1.16	0.0659	1.03	0.7868	1.17	0.1794	1.57	0.0057
94397_at	1426700_a_at	Usp52	ubiquitin specific protease 52	-1.03	0.94	1.02	0.8271	1.11	0.1704	1.07	0.3174	1.14	0.4986
94399_at	1451330_a_at	Inpp5b	inositol polyphosphate-5-phosphatase B	-1.16	0.1	1.08	0.4074	1.06	0.6367	1.06	0.6043	1.11	0.3286
94400_at	1428501_at	1110051M20Ril	RIKEN cDNA 1110051M20 gene	-1.22	0.77	1.1	0.6756	-1.11	0.4461	1.41	0.1081	1.94	0.3165
94402_r_at	1418199_at	Hemgn	hemogen	-4.21	0.35	-1.46	0.3964	-1.16	0.74	-1.25	0.573	2.03	0.0583
94403_at	1420196_s_at	Tbc1d14	TBC1 domain family, member 14	1.06	0.67	1.21	0.0202	1.2	0.0985	1.25	0.0233	1.03	0.8449
94404_at	1418143_at	Vps45	vacuolar protein sorting 45 (yeast)	1.64	0.12	-1	0.9788	-1.33	0.1399	-1.05	0.6682	-1.16	0.61
94405_at	1420148_at	Slc6a6	solute carrier family 6 (neurotransmitter trans	-1.5	0.1	-1.25	0.2192	-1	0.9861	-1.31	0.1219	1.41	0.0482
94406_at	1449148_a_at	Phtf1	putative homeodomain transcription factor 1	2.27	0.05	1.78	0.016	1.25	0.4261	1.94	0.0018	1.93	0.0372
94407_at	1416960_at	B3gat3	beta-1,3-glucuronyltransferase 3 (glucurono:	1.46	0.14	-1	0.9812	1.06	0.4013	-1.02	0.746	1.1	0.4937
94408_at	1417624_at	Nab1	Ngfi-A binding protein 1	1.01	0.95	-1.04	0.7411	1.18	0.0737	1.11	0.1986	1.09	0.5651
94409_at	1419802_at	Ccdc12	coiled-coil domain containing 12	-1.03	0.86	-1.04	0.7561	1.16	0.3536	1.06	0.5652	1.43	0.0064
94410_f_at	1419803_s_at	Ccdc12	coiled-coil domain containing 12	1.15	0.2	-1.1	0.0955	1.01	0.9166	-1.01	0.8974	-1.06	0.7361
94412_at	1416873_a_at	Cdk2	cyclin-dependent kinase 2	1.05	0.66	1.16	0.1886	1.36	0.0212	1.19	0.1286	1.23	0.173
94413_at	1454929_s_at	Safb	scaffold attachment factor B	1.17	0.38	1.17	0.0847	1.21	0.0614	1.15	0.0804	1.51	0.0537
94414_at	1436615_a_at	Otc	ornithine transcarbamylase	1.01	0.91	1.12	0.0656	-1.01	0.8789	-1	0.9999	-1.01	0.9095
94415_at	1440831_at	6230421P05Rik	RIKEN cDNA 6230421P05 gene	1.47	0.34	1.61	0.0117	1.42	0.1417	2.09	0.0011	2.08	0.0517
94416_at	1448714_at	Rngtt	RNA guanylyltransferase and 5'-phosphatas	-1.18	0.55	-1.13	0.2271	-1.09	0.3272	-1.13	0.2191	1.36	0.2154
94417_at	1452446_a_at	2010008E23Rik	RIKEN cDNA 2010008E23 gene	1.17	0.49	1.21	0.1058	1.1	0.456	1.19	0.1539	-1.01	0.942
94418_at	1417403_at	Elovl6	ELOVL family member 6, elongation of long	-1.37	0.2	-3.94	0.0055	-3.47	0.0083	-6.46	0.0025	-2.16	0.2222
94419_at	1448132_at	Slc19a1	solute carrier family 19 (sodium/hydrogen ex	1.12	0.82	1.64	0.2083	3.26	0.0081	3.28	0.0003	2.56	0.0636
94421_r_at	1433733_a_at	Cry1	cryptochrome 1 (photolyase-like)	2.2	0.19	-2.14	0.0892	1.32	0.4715	1.23	0.6079	3.25	0.1719
94422_at	1434038_at	Dnajc13	DnaJ (Hsp40) homolog, subfamily C, membe	1.4	0.35	1.15	0.1292	1.13	0.4105	1.2	0.0153	1.03	0.8685
94423_at	1425108_a_at	BC004728	cDNA sequence BC004728	-1.31	0.48	-1.1	0.3473	1.02	0.8603	-1.17	0.121	-1.11	0.4761
94424_at	1433521_at	LOC433667	similar to DKFZP566D1346 protein	1.34	0.25	1.08	0.4921	1.1	0.5313	1.01	0.9035	1.44	0.0371
94425_at	1422903_at	Ly86	lymphocyte antigen 86	1.6	0.04	1.77	0.1381	1.19	0.5131	2.11	0.1037	1.48	0.4255
94427_at	1416017_at	Copg	coatomer protein complex, subunit gamma	1.13	0.51	-1.04	0.4913	1.06	0.2231	1.08	0.2289	-1.35	0.1786
94429_at	1418062_at	Eef1a2	eukaryotic translation elongation factor 1 alp	-2.01	0.12	-1.44	0.0396	-1.29	0.1671	-1.61	0.0215	-1.27	0.006
94430_at	1451189_at	Zswim1	zinc finger, SWIM domain containing 1	-1.02	0.95	-1.04	0.7586	1.02	0.8703	-1.03	0.742	-1.21	0.6269
94431_at	1420927_at	St6gal1	beta galactoside alpha 2,6 sialyltransferase	-1.56	0.08	1.24	0.1118	-1.04	0.8369	-1.12	0.5392	-1.47	0.3928
94432_at	1420928_at	St6gal1	beta galactoside alpha 2,6 sialyltransferase	1.17	0.32	1.27	0.0007	1.12	0.2632	1.17	0.019	-1.1	0.5665
94433_at	1426722_at	Slc38a2	solute carrier family 38, member 2	-1.28	0.44	1.01	0.9484	1.44	0.0041	1.08	0.3354	1.48	0.0735
94434_at	1424892_at	Zfp95	zinc finger protein 95	1.35	0.4	-1.23	0.0335	-1.08	0.3243	-1.23	0.0241	-1.1	0.7655
94435_at	1419914_s_at	---	---	1.26	0.24	1.14	0.0896	1.14	0.3329	1.15	0.0764	-1.02	0.8073
94438_at	1416780_at	Pfkm	phosphofructokinase, muscle	1.03	0.89	1.08	0.1983	1.07	0.2524	1.15	0.0186	1.03	0.8184
94439_at	1455534_s_at	Osbpl11	oxysterol binding protein-like 11	1.2	0.48	1.04	0.6735	1.28	0.0522	1.12	0.2706	-1.02	0.8389
94440_at	1435007_s_at	AI132487	expressed sequence AI132487	1.15	0.52	1.26	0.1252	1.76	0.0022	1.45	0.0336	1.06	0.7388
94442_s_at	1418396_at	Gpsm3	G-protein signalling modulator 3 (AGS3-like,	-1.07	0.9	2.08	0.0059	-1.31	0.3573	1.82	0.1378	1.57	0.0535
94445_at	1423725_at	Pls3	plastin 3 (T-isoform)	1.19	0.45	1.25	0.0042	1.04	0.7374	1.22	0.0097	-1.59	0.0006
94447_at	1433923_at	Krt2-39	keratin complex 2, basic, gene 39	-1.19	0.42	-1.43	0.2211	1.09	0.6745	-1.25	0.4506	-1.67	0.322
94448_at	1418971_x_at	Bcl10	B-cell leukemia/lymphoma 10	1.03	0.88	1.15	0.1436	1.07	0.2331	1.2	0.0854	-1.03	0.9018
94449_at	1423628_s_at	Pcdhgb1 /// Pcd	protocadherin gamma subfamily B, 1 /// prot	-1.31	0.64	-1.04	0.7824	1.05	0.6995	-1.22	0.2517	1.08	0.826
94452_g_at	1423850_at	Nsun2	NOL1/NOP2/Sun domain family 2	1.12	0.6	-1.25	0.0021	-1.06	0.3809	-1.22	0.002	-1.44	0.0043
94454_at	1415796_at	Dazap2	DAZ associated protein 2	1.31	0.13	1.24	0.0019	1.3	0.0004	1.4	0	1.21	0.0904
94455_at	1448536_at	Lsm3	LSM3 homolog, U6 small nuclear RNA asso	1.66	0.02	1.08	0.3977	1.04	0.6299	-1.01	0.8459	-1.01	0.8863
94456_at	1426854_a_at	Set	SET translocation	1.07	0.85	-1.03	0.696	-1.55	0.0013	-1.23	0.0888	-1.22	0.3715
94457_at	1415792_at	AL033326	expressed sequence AL033326	1.02	0.91	-1.05	0.4188	-1.12	0.1921	-1.05	0.4311	1.02	0.8213
94458_at	1415995_at	Casp6	caspase 6	1.86	0.13	1.58	0.0041	1.6	0.0241	1.77	0.0002	2.6	0.0001
94459_at	1455152_at	AI462493	Expressed sequence AI462493	1.91	0.02	1.08	0.5747	1.26	0.2331	-1.04	0.7681	-1.02	0.9569
94460_at	1416252_at	Stk38	serine/threonine kinase 38	-1.01	0.92	1.2	0.0891	1.01	0.9223	-1.06	0.552	-1.1	0.5666
94465_g_at	1416610_a_at	Clcn3	chloride channel 3	1.45	0.44	1.17	0.2936	-1.16	0.3577	-1.07	0.6319	1.11	0.5672
94466_f_at	1420497_a_at	Cebpz	CCAAT/enhancer binding protein zeta	1.42	0.28	1.08	0.2439	1.19	0.0835	1.07	0.3461	1.28	0.147
94469_at	1448196_at	Mat2b	methionine adenosyltransferase II, beta	1.2	0.09	1.04	0.4873	1.06	0.3269	1.14	0.003	1.04	0.8172

94471_r_at	1422497_at	Slc30a5	solute carrier family 30 (zinc transporter), me	1.2	0.76	1.26	0.2191	1.11	0.5216	1.1	0.4823	-1.01	0.9381
94473_at	1420131_s_at	Pttg1ip	pituitary tumor-transforming 1 interacting pro	-1.12	0.23	-1.16	0.0155	-1.08	0.1569	-1.15	0.0754	-1.51	0.0004
94476_at	1430271_x_at	4930553M18Rik	RIKEN cDNA 4930553M18 gene	-1.08	0.87	1.58	0.033	1.69	0.0067	1.38	0.0579	1.25	0.4925
94478_at	1416426_at	Rab5a	RAB5A, member RAS oncogene family	-1.04	0.71	-1	0.9558	1.17	0.1189	1.08	0.1839	-1.42	0.0198
94480_at	1429014_at	D1Ertd161e	DNA segment, Chr 1, ERATO Doi 161, expr	-1.52	0.05	-1.08	0.3356	-1.01	0.898	-1.05	0.5569	1.87	0.0693
94481_at	1426461_at	Ugp2	UDP-glucose pyrophosphorylase 2	2.07	0.07	1.48	0.0004	1.08	0.6636	1.48	0.0001	2.85	0.0005
94482_at	1460646_at	Csnk2a2	casein kinase II, alpha 2, polypeptide	1.16	0.59	1.22	0.0878	1.37	0.0197	1.31	0.0009	1.24	0.0146
94483_at	1453099_at	Csnk2a2	casein kinase II, alpha 2, polypeptide	-1.24	0.3	1.37	0.1065	-1.24	0.0141	-1.19	0.2214	1.49	0.0621
94484_at	1421029_a_at	Hbs1l	Hbs1-like (S. cerevisiae)	1.35	0.35	1.01	0.8722	1.17	0.016	-1.1	0.1431	1.06	0.4247
94485_at	1431012_a_at	Peci	peroxisomal delta3, delta2-enoyl-Coenzyme	1.74	0.06	1.27	0.0445	1.34	0.003	1.55	0.0006	1.31	0.2071
94486_at	1416234_at	AA959742	expressed sequence AA959742	1.09	0.78	-1.12	0.1489	-1.25	0.001	-1.18	0.0123	-1.65	0.0001
94488_at	1415722_a_at	1110059P08Rik	RIKEN cDNA 1110059P08 gene	1.09	0.35	1.19	0.0425	1.14	0.173	1.23	0.0088	-1.15	0.2259
94489_at	1438657_x_at	Ptp4a1	protein tyrosine phosphatase 4a1	1.42	0.12	1.31	0.0013	-1.01	0.8519	1.3	0.0005	2.14	0.001
94490_at	1451102_at	Cnot8	CCR4-NOT transcription complex, subunit 8	1.21	0.35	-1.13	0.1807	-1.09	0.3152	-1.24	0.0124	-1.04	0.7874
94491_at	1422471_at	Pex13	peroxisomal biogenesis factor 13	-1.87	0.05	-1.09	0.3366	-1.22	0.0422	-1.23	0.0248	-1.13	0.3017
94493_at	1460569_x_at	Cldn3	claudin 3	1.26	0.21	-1.36	0.0013	-1.32	0.0074	-1.44	0.0002	-1.07	0.5824
94499_at	1433654_at	Mgea5	meningioma expressed antigen 5 (hyaluroni	1.12	0.56	1.19	0.116	1.11	0.341	1.09	0.2425	-1.12	0.1359
94501_at	1420822_s_at	Sgpp1	sphingosine-1-phosphate phosphatase 1	1.1	0.59	1.1	0.1208	1.03	0.7681	1.15	0.0988	-1.07	0.6884
94502_at	1448295_at	D13Wsu50e	DNA segment, Chr 13, Wayne State Univers	2.29	0.02	1.3	0.0043	1.17	0.1976	1.43	0	1.26	0.1686
94504_at	1450410_a_at	4930570C03Rik	RIKEN cDNA 4930570C03 gene	-1.22	0.27	-1.05	0.6177	-1.08	0.4837	-1.14	0.0958	1.23	0.4893
94505_at	1417371_at	Peli1	pellino 1	1.52	0.41	1.29	0.0476	1.18	0.1679	1.43	0.0001	1.26	0.0816
94506_at	1417681_at	Cpsf5	cleavage and polyadenylation specific factor	1.73	0.17	-1.03	0.7725	1.17	0.2187	1.18	0.0766	2.13	0.0935
94507_at	1423883_at	Acsl1	Acyl-CoA synthetase long-chain family mem	-1.34	0.09	1.26	0.1069	1.34	0.0223	1.24	0.135	-1.14	0.2003
94508_at	1451000_at	1810020E01Rik	RIKEN cDNA 1810020E01 gene	1.23	0.15	1.04	0.3375	1	0.9333	1.02	0.7551	-1.1	0.3715
94509_at	1423046_s_at	Ncbp2	nuclear cap binding protein subunit 2	1.35	0.16	-1.02	0.863	1	0.9933	-1.08	0.4166	-1.31	0.3487
94510_at	1422844_a_at	2610312E17Rik	RIKEN cDNA 2610312E17 gene	-1.05	0.82	1.25	0.0259	1.42	0.032	1.42	0.0085	-1.04	0.5957
94511_at	1448843_at	Ssr1	signal sequence receptor, alpha	-1.1	0.72	-1.52	0.0032	-1.36	0.0169	-1.66	0.0001	-2.9	0.1281
94514_s_at	1451089_a_at	Arcn1	archain 1	-1.26	0.03	1.05	0.4876	1.11	0.0466	1.16	0.0426	-1.06	0.3306
94515_at	1420641_a_at	Sqrdl	sulfide quinone reductase-like (yeast)	1.54	0.13	1.09	0.2423	-1.05	0.6935	-1.01	0.8993	-1.13	0.2683
94517_r_at	1427038_at	Penk1	preproenkephalin 1	1.2	0.63	-1.39	0.2357	-1.17	0.5523	-1.43	0.1814	1.47	0.3133
94518_at	1416245_at	0610033H09Rik	RIKEN cDNA 0610033H09 gene	1.71	0.06	-1.11	0.0657	-1.11	0.1512	-1.1	0.2031	-1.28	0.0824
94521_at	1416253_at	Cdkn2d	cyclin-dependent kinase inhibitor 2D (p19, in	1.66	0.17	1.29	0.3608	-1.31	0.0105	1.13	0.6145	1.11	0.8289
94522_at	1416247_at	Dctn3	dynactin 3	1.07	0.83	-1.07	0.077	-1.07	0.4099	-1.06	0.1126	-1.09	0.5054
94524_at	1450848_at	Dap3	death associated protein 3	2.29	0.01	1.08	0.3609	-1.01	0.964	1.16	0.1165	-1.31	0.4228
94526_at	1416850_s_at	D10Ertd214e	DNA segment, Chr 10, ERATO Doi 214, exp	1.25	0.18	1.05	0.5876	1.24	0.0528	1.1	0.4539	1.35	0.0375
94528_at	1418905_at	Nubp1	nucleotide binding protein 1	-1.07	0.59	-1.01	0.8957	1.13	0.0688	1.07	0.2468	-1.23	0.1401
94530_at	1451136_a_at	Eif2b2	eukaryotic translation initiation factor 2B, sub	-1.34	0.17	1	0.9633	1.02	0.8145	-1.02	0.8622	1.02	0.8676
94531_at	1428134_at	2310005O14Rik	RIKEN cDNA 2310005O14 gene	1.46	0.1	1.26	0.0126	1.21	0.0032	1.22	0.0072	1.06	0.5098
94534_at	1422501_s_at	Idh3a	isocitrate dehydrogenase 3 (NAD+) alpha	1.41	0.19	1.05	0.6002	1.06	0.4697	1.08	0.3941	-1.02	0.9275
94535_at	1450054_at	---	---	-1.8	0.49	1.19	0.0964	1.03	0.6743	1.15	0.1541	1.04	0.7368
94536_s_at	1420820_at	2900073G15Rik	RIKEN cDNA 2900073G15 gene	1.35	0.25	1.1	0.1112	1.05	0.314	1.22	0.0003	1.27	0.1748
94537_at	1428608_at	Mylc2b	myosin light chain, regulatory B	-1.18	0.26	-1.27	0.3467	1.07	0.7696	-1.2	0.4964	-1.41	0.1523
94540_at	1448683_at	Cyp2d26	cytochrome P450, family 2, subfamily d, poly	1.36	0.12	1.14	0.0086	1.08	0.0776	1.17	0.0003	1.34	0.0055
94543_at	1424087_at	1810042K04Rik	RIKEN cDNA 1810042K04 gene	-1.26	0.7	-1.22	0.3386	-1.11	0.5382	-1.55	0.0621	-1.53	0.2961
94548_at	1451247_at	Mfsd1	major facilitator superfamily domain containi	-1.1	0.59	-1.13	0.0764	-1.08	0.3737	-1.36	0.0001	-1.52	0.0049
94549_at	1424129_at	Mfsd1	major facilitator superfamily domain containi	1.16	0.49	-1.16	0.0289	-1.15	0.0421	-1.36	0.0005	1.04	0.8089
94550_at	1416260_a_at	Snx1	sorting nexin 1	-1.21	0.41	-1.02	0.8976	-1.08	0.2463	-1.08	0.4325	-1.19	0.5288
94551_at	1424102_at	Apg4b	APG4 (ATG4) autophagy-related homolog B	-1.12	0.45	-1.2	0.0853	-1.04	0.6181	-1.13	0.1605	1.12	0.3456
94552_at	1448642_at	Pcbp1	poly(rC) binding protein 1	-1.11	0.64	-1.05	0.3488	1.12	0.0596	1.01	0.8038	-1.24	0.0345
94554_at	1448999_at	Trappc5	trafficking protein particle complex 5	3.54	0.01	1.24	0.2436	1.09	0.699	1.39	0.1012	1.17	0.5659
94555_at	1430527_a_at	Rnf167	ring finger protein 167	1.15	0.55	-1.07	0.3115	-1.03	0.8028	-1.2	0.0028	1.14	0.5917
94556_at	1431055_a_at	Snx10	sorting nexin 10	-1.35	0.11	-1.16	0.3564	-1.12	0.2702	-1.09	0.6306	-1.3	0.0445
94558_g_at	1427027_a_at	Gtf3a	general transcription factor III A	1.21	0.29	1.01	0.9022	1.04	0.5138	1.03	0.6273	1.28	0.0975

94561_at	1422499_at	D15Ertd366e	DNA segment, Chr 15, ERATO Doi 366, exp	1.13	0.57	1.14	0.3453	1.09	0.4215	-1.25	0.141	1.13	0.3636
94562_at	1417456_at	Gnpat	glyceronephosphate O-acyltransferase	1.08	0.62	-1.07	0.218	-1	0.9403	-1.07	0.3224	-1.11	0.335
94563_at	1427440_a_at	Afm	afamin	-1.35	0.19	-1.25	0.0018	1.04	0.5976	-1.51	0	1.29	0.0614
94564_at	1421606_a_at	Sult4a1	sulfotransferase family 4A, member 1	2.26	0.01	-1.04	0.9123	1.24	0.442	-1.28	0.3993	1.54	0.148
94565_at	1421678_at	Itpr2	inositol 1,4,5-triphosphate receptor 2	-3.41	0.21	-1.13	0.6619	-1.03	0.9075	-1.24	0.4307	3.32	0.2574
94566_at	1421755_at	Gpr132	G protein-coupled receptor 132	-1.61	0.15	-1.07	0.7531	1.04	0.8574	-1.29	0.1859	-1.2	0.5953
94571_at	1450558_at	V1ra1	vomeronasal 1 receptor, A1	-1.71	0.52	-1.26	0.5048	-1.26	0.4883	-1.37	0.33	2.31	0.1102
94618_at	1422267_at	Foxb2	forkhead box B2	-1.18	0.54	-1.06	0.4767	1.23	0.0077	1	0.9876	1.09	0.6198
94619_at	1421569_at	Grid1	glutamate receptor, ionotropic, delta 1	1.35	0.38	1.02	0.8593	1.07	0.4929	-1.05	0.6611	1.09	0.4826
94621_at	1450289_at	Foxd2	forkhead box D2	-1.86	0.03	1.13	0.271	-1.02	0.8736	1.03	0.8261	1.55	0.0273
94622_at	1421530_a_at	Grm8	glutamate receptor, metabotropic 8	-1.21	0.57	-1.16	0.3619	-1.01	0.941	-1.1	0.6893	-1.2	0.378
94623_at	1421699_at	Enam	enamelin	-1.09	0.58	1.08	0.6882	1.18	0.3517	1.07	0.5959	1.28	0.371
94625_at	1420797_at	Otog	otogelin	1.15	0.43	1.26	0.3179	1.2	0.4937	1.4	0.1177	1.49	0.3761
94626_at	1421580_at	Cntnap1	contactin associated protein 1	-1.38	0.35	-1.25	0.0944	-1.09	0.4415	-1.13	0.3517	1.09	0.7535
94627_at	1450369_at	Figla	factor in the germline alpha	1.64	0.21	1.03	0.9127	-1.92	0.0512	-1.03	0.9119	1.47	0.2684
94628_r_at	1424063_at	Abpa	androgen binding protein alpha	-2.43	0.02	-1.34	0.3338	-1.08	0.7219	-1.4	0.2142	1.44	0.3722
94630_at	1421643_at	Zfpn1a2	zinc finger protein, subfamily 1A, 2 (Helios)	-1.86	0.34	-1.02	0.9705	1.29	0.4465	1.26	0.4456	-1.22	0.6877
94631_at	1450327_at	P2rxl1	purinergic receptor P2X-like 1, orphan recep	2.46	0.16	1.35	0.3631	-1.03	0.8906	1.04	0.8578	1.07	0.519
94632_at	1449320_at	Pbsn	probasin	1.28	0.68	-1.19	0.3771	1.08	0.6936	-1	0.9803	1.39	0.687
94634_at	1422234_at	Tacr2	tachykinin receptor 2	-1.75	0.15	-1.08	0.7728	-1.05	0.8429	-1.16	0.5699	-1.84	0.1435
94635_at	1422282_at	Tacr1	tachykinin receptor 1	-1.08	0.46	-1.35	0.0721	-1.1	0.5759	-1.48	0.0169	-1.27	0.5468
94636_at	1422336_at	Hoxa13	homeo box A13	-1.19	0.7	-1.06	0.7255	1.26	0.3441	1.12	0.6148	-1.44	0.5237
94637_at	1427586_at	Sema4b	sema domain, immunoglobulin domain (Ig), t	-1.33	0.13	1.26	0.2629	1.26	0.2058	1.28	0.1905	1.27	0.2876
94639_at	1422427_a_at	Svs2	seminal vesicle protein, secretion 2	-1.47	0.54	-1.01	0.9643	1.96	0.1494	1.28	0.5508	1.56	0.5257
94640_at	1427618_at	Cdh9	cadherin 9	-1.29	0.64	1.01	0.9704	1.78	0.057	1.03	0.9382	2.18	0.069
94642_at	1421742_at	Gdi2	guanosine diphosphate (GDP) dissociation i	-2.33	0.06	1.19	0.4739	-1.23	0.3092	1.2	0.4002	1.24	0.3027
94643_at	1450295_s_at	Pvr	poliovirus receptor	-1.69	0.35	-1.08	0.7575	-1.12	0.654	1.15	0.5899	1.81	0.0766
94644_at	1421218_at	Bche	butyrylcholinesterase	-2.44	0.04	1.19	0.3408	-1.01	0.9271	1.13	0.494	1.53	0.0525
94645_at	1421263_at	Gabra3	gamma-aminobutyric acid (GABA-A) recepto	-1.22	0.74	-1.71	0.178	-1.52	0.2755	-1.24	0.5408	-1.27	0.6797
94662_at	1456706_at	4833441D16Rik	RIKEN cDNA 4833441D16 gene	-1.72	0.19	1.36	0.0466	1.32	0.038	1.08	0.7026	1.84	0.3218
94663_at	1440874_at	Slco5a1	solute carrier organic anion transporter famil	-2.19	0.31	-1.39	0.1137	-1.25	0.2107	-1.35	0.1429	1.53	0.2358
94664_at	1437672_at	Irs3	insulin receptor substrate 3	-2.76	0.19	1.02	0.9526	1.35	0.4789	-1.56	0.2474	-1.15	0.8327
94667_at	1459478_at	AA408396	expressed sequence AA408396	-2.06	0.15	-1.14	0.5323	-1.16	0.4852	-1.35	0.1696	1.74	0.2957
94668_at	1446856_at	D3Wsu167e	DNA segment, Chr 3, Wayne State Universit	1.04	0.93	1.16	0.6761	1.09	0.7083	1.37	0.246	1.01	0.9751
94683_at	1419785_at	AA516738	expressed sequence AA516738	-1.08	0.76	-1.26	0.4632	1.11	0.7378	-1.2	0.5144	1.44	0.4856
94684_at	1449598_at	Denr	Density-regulated protein	-2.13	0.18	-1.22	0.3801	1.04	0.8457	1.17	0.4555	2	0.1756
94685_at	1419775_at	---	---	-2.39	0.27	1.11	0.4221	1.15	0.2096	1.03	0.7823	-1.74	0.3276
94686_at	1448078_at	C76533	expressed sequence C76533	1.14	0.74	-1.39	0.0781	-1.58	0.0253	-1.72	0.0243	1.93	0.2173
94688_at	1422002_at	Mad	Max dimerization protein	-1.22	0.34	1.33	0.1436	1.22	0.1474	1.3	0.042	1.45	0.2767
94689_at	1446148_x_at	C79248	expressed sequence C79248	1.79	0.44	1.14	0.4359	-1.81	0.0068	1.04	0.8682	1.65	0.1095
94690_at	1449595_at	C79490	expressed sequence C79490	-1.82	0.09	1.04	0.7452	1.06	0.712	-1.03	0.8007	-1.51	0.5484
94692_at	1420698_at	Gpx5	glutathione peroxidase 5	1.67	0.29	1.04	0.8616	1.07	0.8105	1.1	0.6906	1.41	0.2618
94693_at	1442529_at	Ogfrl1	Opioid growth factor receptor-like 1	-1.13	0.83	-1.22	0.5345	-1.36	0.3038	-1.66	0.1601	-1.47	0.2907
94694_at	1421396_at	Pcsk1	proprotein convertase subtilisin/kexin type 1	-3.07	0.18	-1.14	0.6887	1.14	0.6625	-1.34	0.4304	2.52	0.0989
94695_at	1441013_at	C81521	expressed sequence C81521	-1.26	0.2	2.02	0.0016	1.55	0.0177	2.29	0	1.19	0.3748
94696_at	1421655_a_at	Ccr4	chemokine (C-C motif) receptor 4	-2.98	0.19	-1.01	0.9249	1.18	0.294	1.15	0.6271	1.59	0.0176
94697_at	1419526_at	Fgr	Gardner-Rasheed feline sarcoma viral (Fgr)	-1.7	0.16	1.34	0.0686	1.34	0.0262	1.39	0.0801	1.65	0.0106
94698_at	1422024_at	Fli1	Friend leukemia integration 1	-2.86	0.04	1.04	0.8135	-1.28	0.2357	-1.25	0.2817	2.04	0.001
94699_at	1421771_a_at	Ipp	IAP promoted placental gene	-1.01	0.93	-1.12	0.2611	1.03	0.8061	-1.1	0.2188	-1.02	0.9425
94700_at	1418549_at	Cga	glycoprotein hormones, alpha subunit	1.02	0.97	-1.18	0.6484	1.21	0.5875	1.45	0.1062	1.08	0.8374
94701_at	1419740_at	Pde6b	phosphodiesterase 6B, cGMP, rod receptor,	1.26	0.76	-1.02	0.9159	1.16	0.6485	-1.11	0.5385	1.31	0.3625
94702_at	1422019_at	---	---	-1.54	0.65	1.25	0.353	1.26	0.5633	1.16	0.7745	3.2	0.0658
94703_at	1427154_at	Krt2-17	keratin complex 2, basic, gene 17	1.37	0.01	-1.06	0.857	-1.12	0.7137	1.21	0.6432	1.21	0.735

94704_at	1419015_at	Wisp2	WNT1 inducible signaling pathway protein 2	-2.73	0.04	1.27	0.4086	1.71	0.0916	1.39	0.282	1.04	0.8938
94705_at	1421727_at	Eya1	eyes absent 1 homolog (Drosophila)	-2	0.29	-1.95	0.0994	-1.43	0.3775	-2.67	0.0352	-1.99	0.1025
94707_s_at	1451686_x_at	Amelx	amelogenin X chromosome	-1.73	0.22	1.04	0.6257	-1.12	0.3832	1.01	0.9574	-1.17	0.704
94708_at	1425724_at	Ptprn2	protein tyrosine phosphatase, receptor type,	-1.25	0.55	-1.49	0.0752	1.09	0.6232	-1.3	0.2063	1.63	0.0861
94710_g_at	1420455_at	Gcm2	glial cells missing homolog 2 (Drosophila)	-1.09	0.78	1.32	0.4837	-1.13	0.7171	1.32	0.4903	-1.33	0.6207
94711_at	1420601_at	Gcm1	glial cells missing homolog 1 (Drosophila)	-3.94	0.28	2.52	0.0134	1.82	0.0746	2.05	0.1321	1.67	0.0988
94712_at	1419417_at	Vegfc	vascular endothelial growth factor C	2.31	0.09	-1	0.9958	1.03	0.9473	1.02	0.9459	-2.76	0.0336
94713_at	1421385_a_at	Myo7a	myosin VIIa	1.18	0.34	1.04	0.6747	1.15	0.1438	1.12	0.0958	1.28	0.1124
94714_at	1427380_at	Ngfg	nerve growth factor, gamma	1.54	0.31	1.21	0.5864	-1.25	0.373	-1.14	0.6135	1.67	0.1007
94715_at	1422217_a_at	Cyp1a1	cytochrome P450, family 1, subfamily a, poly	1.22	0.72	1.42	0.0173	1.17	0.3731	1.43	0.0437	1.31	0.5174
94717_f_at	1423028_at	Ifna2	interferon alpha family, gene 2	-1.79	0.04	-2.11	0.0003	-1.3	0.0517	-3.23	0	-1.08	0.8213
94718_at	1422398_at	Hist1h1e	histone 1, H1e	-1.26	0.36	1.06	0.3731	1.1	0.2116	-1.03	0.7531	1.36	0.2525
94719_at	1421568_at	Kcna6	potassium voltage-gated channel, shaker-re	-1.35	0.21	1.08	0.8259	1.26	0.2298	1.46	0.2173	1.18	0.3892
94720_at	1422027_a_at	Ets1	E26 avian leukemia oncogene 1, 5' domain	1.25	0.35	1	0.9905	-1.04	0.8381	-1.01	0.9522	-1.35	0.2619
94721_at	1422246_at	Dspp	dentin sialophosphoprotein	2.22	0.04	-1.04	0.9169	-1.05	0.8736	-1.18	0.6743	2.01	0.4209
94723_at	1421758_at	Nat1	N-acetyltransferase 1 (arylamine N-acetyltra	1.11	0.84	-1.18	0.2125	-1.38	0.1116	-1.21	0.2456	-1.31	0.1963
94724_at	1420450_at	Mmp10	matrix metalloproteinase 10	-2.5	0.22	-1.07	0.714	-1.32	0.205	-1.98	0.0143	1.32	0.5806
94727_f_at	1420729_at	MGI:1933437	2-cell-stage, variable group, member 1	1.32	0.42	-1.41	0.1261	-1.28	0.3104	-1.36	0.2027	1.17	0.1657
94729_r_at	1422352_at	Mcpt1	mast cell protease 1	-1.2	0.6	1.67	0.1241	1.94	0.0042	1.39	0.0655	-1.55	0.0864
94730_at	1449838_at	Crisp3	cysteine-rich secretory protein 3	-1.05	0.9	1.29	0.5184	1.02	0.9422	1.35	0.4633	2.36	0.1144
94731_at	1418957_at	Stac	src homology three (SH3) and cysteine rich	-1.39	0.5	-1.11	0.4882	1.2	0.2215	-1.2	0.3185	1.2	0.4661
94732_at	1420431_at	Rptn	repetin	1.1	0.72	-1.93	0.0139	-1.23	0.4759	-1.14	0.4199	-1.55	0.1898
94733_at	1449818_at	Abcb4	ATP-binding cassette, sub-family B (MDR/TA	-1.01	0.95	-1.43	0.0014	1.05	0.6104	-1.37	0.0017	-1.77	0.0081
94734_at	1420438_at	Orm2	orosomucoid 2	4.96	0.09	3.7	0.1175	2.83	0.002	5.89	0.0211	-16.98	0.2589
94736_at	1421384_at	Lyst	lysosomal trafficking regulator	-4.39	0	1.13	0.5912	1.2	0.3845	1.15	0.3857	1.04	0.9446
94737_at	1418754_at	Adcy8	adenylate cyclase 8	-1.61	0.23	1.17	0.3016	1.11	0.3961	-1.1	0.5485	1.21	0.4614
94738_s_at	1420598_x_at	Defcr-rs2	defensin related cryptdin, related sequence :	-1.69	0.07	1.12	0.3005	-1.05	0.5144	1.11	0.3993	2.35	0.1391
94740_g_at	1421095_a_at	Trpc1	transient receptor potential cation channel, s	-2.2	0.48	-1.42	0.0063	-1.27	0.0501	-1.58	0.0192	1.16	0.6819
94741_at	1432486_a_at	Tesp2	testicular serine protease 2	1	1	-1.73	0.1161	-1.22	0.5918	-1.17	0.688	1.36	0.4871
94743_f_at	1425999_at	Cfh	complement component factor h	-1.66	0.01	1.01	0.873	-1.02	0.8464	1.06	0.2684	-1.31	0.0327
94744_at	1422067_at	Klrb1d	killer cell lectin-like receptor subfamily B mer	-1.39	0.31	-1.2	0.3901	-1.09	0.6888	-1.11	0.5872	1.33	0.3924
94745_f_at	1427479_at	Eif1a /// LOC23(	eukaryotic translation initiation factor 1A /// s	-2.22	0.23	1.32	0.2125	1.53	0.0451	1.42	0.082	-1.11	0.8894
94746_at	1422160_at	H2-T24	histocompatibility 2, T region locus 24	-1.34	0.71	-1.03	0.8933	-1.16	0.436	-1.42	0.1225	-1	0.9844
94748_g_at	1450200_s_at	Csf2rb1 /// Csf2	colony stimulating factor 2 receptor, beta 1, l	-6.55	0	-1.17	0.3924	-1.27	0.1076	-1.59	0.0834	-1.61	0.0119
94750_at	1449502_at	Dazl	deleted in azoospermia-like	-1.19	0.34	1.04	0.8309	1.32	0.4598	-1.02	0.8712	2.24	0.0176
94752_s_at	1422054_a_at	Skil	SKI-like	-1.1	0.71	1.08	0.5783	1.24	0.2026	1.18	0.265	1.19	0.2608
94753_at	1421302_a_at	Gna15	guanine nucleotide binding protein, alpha 15	-1.37	0.24	1.24	0.297	1.23	0.2447	1.2	0.4206	-1.32	0.6537
94754_at	1427300_at	Lhx8	LIM homeobox protein 8	1.28	0.6	-1.51	0.1284	-1.36	0.119	-1.51	0.1055	1.01	0.9832
94755_at	1421473_at	Il1a	interleukin 1 alpha	-1.54	0.24	-1.26	0.1697	1	0.9968	-1.13	0.5515	-1.34	0.387
94757_at	1420579_s_at	Cftr	cystic fibrosis transmembrane conductance	-1.67	0.19	-1.08	0.4979	-1.03	0.8453	-1.13	0.1522	1.24	0.1451
94758_s_at	1427767_a_at	Cftr	cystic fibrosis transmembrane conductance	1.55	0.52	1.12	0.7551	1.37	0.4561	1.34	0.5052	-2.48	0.1475
94759_at	1459914_at	2810402A17Rik	RIKEN cDNA 2810402A17 gene	-1.82	0.5	1.11	0.7026	1.03	0.9058	-1.05	0.8706	2.5	0.0911
94760_at	1419770_at	---	---	1.15	0.55	-1.34	0.496	-1.66	0.2434	-1.27	0.6069	-1.53	0.2144
94761_at	1421228_at	Ccl7	chemokine (C-C motif) ligand 7	1.39	0.43	1.08	0.8001	-1.58	0.1758	1.01	0.9851	1.89	0.1939
94762_at	1421407_at	F2rl2	coagulation factor II (thrombin) receptor-like	1.21	0.58	-1.12	0.2872	1.1	0.414	1.1	0.3555	1.19	0.2498
94763_at	1450511_at	Musk	muscle, skeletal, receptor tyrosine kinase	-1.63	0.14	-1.2	0.4628	-1.07	0.8215	-1.19	0.4444	-1.13	0.6677
94764_at	1425367_at	Itgal	integrin alpha L	1.19	0.77	1.4	0.1513	1.03	0.8666	1.46	0.2661	-1.12	0.7016
94765_at	1433799_at	Rdh13	retinol dehydrogenase 13 (all-trans and 9-cis	1.79	0.43	1.87	0.0117	2.58	0.0055	2.52	0.0327	-1.01	0.9844
94766_at	1424635_at	Eef1a1	eukaryotic translation elongation factor 1 alp	1.06	0.53	1.02	0.2089	1.08	0.0003	1.06	0.0128	1.29	0.0259
94767_at	1424000_a_at	Rps11	ribosomal protein S11	-1	0.98	1.02	0.8951	-1.06	0.6248	1.25	0.0611	1.38	0.0888
94768_at	1416161_at	Rad21	RAD21 homolog (S. pombe)	1.06	0.76	1.12	0.0544	1.22	0.0139	1.14	0.0403	1.25	0.1133
94769_at	1449366_at	---	---	-1.43	0.19	1.06	0.7586	1.18	0.4415	1.23	0.3056	1.2	0.5715
94770_at	1435573_at	DXImx46e	DNA segment, Chr X, Immunex 46, express	1.15	0.4	-1.11	0.0967	1.02	0.7174	-1.2	0.0053	-1.03	0.7753

94772_at	1450296_at	Klrb1a	killer cell lectin-like receptor subfamily B mer	-2.74	0.22	-2.17	0.0712	-1.09	0.8056	-1.73	0.1551	1.09	0.8059
94773_at	1450222_x_at	Ngfa	nerve growth factor, alpha	-1.29	0.77	-1.41	0.4701	-1.51	0.4248	-1.66	0.2851	-2.18	0.2701
94774_at	1421551_s_at	Ifi202b	interferon activated gene 202B	-8.26	0.1	2.26	0.2854	-1.17	0.4392	2.75	0.2968	-1.04	0.92
94775_at	1420556_at	Oxt	oxytocin	-1.35	0.13	1.41	0.0307	1.13	0.4395	1.46	0.0057	-1.11	0.5004
94777_at	1425260_at	Alb1	albumin 1	-1.28	0.36	-1.03	0.036	-1.02	0.089	-1.01	0.1966	1.37	0.0249
94778_at	1418601_at	---	---	1.43	0.3	-1.07	0.5918	-1.12	0.4629	1.02	0.9017	-2.39	0.0007
94781_at	1452757_s_at	Hba-a1	hemoglobin alpha, adult chain 1	1.05	0.91	-1.12	0.3843	-1.7	0.0007	-1.2	0.2025	1	0.9899
94782_at	1449596_at	C78142	expressed sequence C78142	-3.1	0.01	1.23	0.6258	2.14	0.2187	-1.46	0.1964	1.92	0.1724
94783_at	1442948_at	C79452	expressed sequence C79452	-1.15	0.75	1.22	0.3049	1.32	0.2025	1.37	0.2072	1.21	0.4067
94784_at	1434850_at	D030034H08	Hypothetical protein D030034H08	-1.44	0.32	1.08	0.4863	1.03	0.8237	1.05	0.6029	-1.15	0.3989
94785_at	1447957_at	D7Ertd128e	DNA segment, Chr 7, ERATO Doi 128, expr	-3.32	0.18	-1.52	0.1895	-1.28	0.4042	-1.16	0.6146	1.49	0.2543
94786_at	1443605_at	C81272	expressed sequence C81272	-1.13	0.7	-1.47	0.1124	1	0.9842	-1.3	0.2476	1.42	0.1199
94787_at	1447952_at	C77140	expressed sequence C77140	1.8	0.17	-1.09	0.2496	-1.17	0.1075	-1.15	0.1662	1.64	0.1091
94789_r_at	1416256_a_at	Tubb5	tubulin, beta 5	-1.01	0.96	1.3	0.0344	1.26	0.1099	1.45	0.0099	1.33	0.3643
94791_s_at	1460300_a_at	Ltk	leukocyte tyrosine kinase	-1.49	0.02	-1.14	0.0982	1.05	0.3988	-1.11	0.1079	-1.07	0.6251
94792_at	1448061_at	Msr1	Macrophage scavenger receptor 1	1.18	0.22	1.09	0.6946	-1.06	0.6448	1.34	0.1584	1.1	0.1769
94793_at	1455511_at	Sephs1	selenophosphate synthetase 1	1.49	0.49	1.03	0.8948	1.54	0.3021	1.13	0.4926	1.31	0.4544
94794_at	1448771_a_at	Fth1	ferritin heavy chain 1	1.43	0.1	1.2	0.0003	1.09	0.0296	1.21	0.0008	1.06	0.4715
94795_at	1420531_at	Hsd3b5	hydroxysteroid dehydrogenase-5, delta<5>-3	-19.65	0.01	-1.61	0.0073	-1.02	0.8818	-1.68	0.0162	-67.67	0.0036
94796_at	1440253_at	---	---	-1.21	0.35	1.02	0.718	1.07	0.289	-1.06	0.2605	-1.4	0.1453
94797_at	1451239_a_at	Slc26a1	solute carrier family 26 (sulfate transporter),	1.76	0.13	-1.54	0	-1.25	0.0914	-1.4	0.0001	-1.6	0.0446
94798_at	1449594_at	Abcb1b	ATP-binding cassette, sub-family B (MDR/TA	2.25	0.22	1.21	0.5971	1.13	0.7554	1.33	0.4207	1.24	0.4161
94799_at	1449558_at	F8	coagulation factor VIII	1.56	0.15	1.02	0.8518	1.23	0.0769	-1.11	0.5422	1.34	0.1889
94800_at	1419772_at	Mllt10	Myeloid/lymphoid or mixed lineage-leukemia	-1.23	0.71	1.37	0.1159	1.3	0.278	1.14	0.632	1.59	0.1545
94801_at	1452882_at	Pgrmc2	progesterone receptor membrane componen	1.37	0.46	-1.41	0	-1.02	0.8294	-1.4	0.0002	-2.17	0.0185
94803_at	1449542_at	Pbx1	pre B-cell leukemia transcription factor 1	-1.18	0.19	1.12	0.3955	-1.04	0.758	1.04	0.6969	2.71	0.0237
94804_at	1425383_a_at	Pbx1	pre B-cell leukemia transcription factor 1	1.33	0.35	-1.09	0.3827	-1.14	0.0629	-1.16	0.0887	1.23	0.2228
94806_at	1416090_at	Pdhb	pyruvate dehydrogenase (lipoamide) beta	1.62	0.01	-1.26	0.0029	1.12	0.1416	-1.17	0.077	-1.65	0.0032
94810_at	1436884_x_at	---	---	-1.12	0.46	1.17	0.0268	1.17	0.1749	1.29	0.0054	1.26	0.0684
94813_at	1416855_at	Gas1	growth arrest specific 1	1.12	0.57	-1.5	0.1463	-1.11	0.5718	-1.56	0.0327	1.03	0.8386
94814_at	1428645_at	Gnai3	guanine nucleotide binding protein, alpha inh	-1.96	0.07	-1.04	0.7073	1.19	0.0243	-1.18	0.0667	-1.14	0.502
94815_at	1415865_s_at	Bpgm	2,3-bisphosphoglycerate mutase	1.29	0.27	-1.23	0.2798	-1.06	0.6884	-1.17	0.2567	-3.13	0.0008
94817_at	1450843_a_at	Serpinh1	serine (or cysteine) proteinase inhibitor, clad	-1.03	0.91	1.05	0.7447	-1.17	0.4077	-1.18	0.319	1.57	0.3249
94818_at	1460631_at	Ogt	O-linked N-acetylglucosamine (GlcNAc) tran	-1.03	0.84	1.5	0.0035	1.3	0.2051	1.86	0	1.62	0.0142
94820_r_at	1448334_a_at	Ccni	cyclin I	-1.16	0.46	1.16	0.4646	-1.12	0.5112	1	0.9925	-1.42	0.0835
94821_at	1420886_a_at	Xbp1	X-box binding protein 1	-1.19	0.49	-1.14	0.1617	-1.2	0.0789	-1.09	0.3113	-1.44	0.014
94823_at	1426660_x_at	Rpl23a	ribosomal protein L23a	1.14	0.32	1.1	0.1439	1.07	0.4018	1.14	0.0093	1.39	0.0544
94825_at	1426019_at	Plaa	phospholipase A2, activating protein	-1.44	0.09	-1.52	0.0674	-1.31	0.1371	-1.18	0.3932	1.12	0.8281
94827_at	1456601_x_at	Fxyd2	FXYD domain-containing ion transport regul	-1.18	0.8	1.06	0.7019	1.51	0.0321	1.37	0.123	-1.09	0.7716
94828_at	1416750_at	Oprs1	opioid receptor, sigma 1	-1.3	0.14	-1.41	0.0013	-1.24	0.0356	-1.61	0	-1.91	0.0004
94831_at	1417490_at	Ctsb	cathepsin B	1.05	0.77	1.06	0.1487	-1.05	0.0671	-1.06	0.2855	1.06	0.4361
94832_at	1415963_at	Hnrph2	heterogeneous nuclear ribonucleoprotein H2	1.04	0.93	1.1	0.3266	1.29	0.2531	1.24	0.1452	-1.54	0.0468
94833_at	1448259_at	Fstl1	follistatin-like 1	1	0.95	-1.1	0.5543	-1.37	0.1219	1.11	0.4257	2.1	0.0519
94834_at	1418365_at	Ctsh	cathepsin H	-1.08	0.24	1.09	0.0387	1.12	0.0112	1.07	0.0579	-1.11	0.1126
94835_f_at	1427347_s_at	---	---	-1.44	0.19	-1.14	0.443	1.04	0.8233	-1.17	0.2627	-1.86	0.0704
94836_at	1419965_at	C79445	expressed sequence C79445	-1.75	0.48	-1.11	0.7535	-1.07	0.8304	1.13	0.754	-1.46	0.4178
94837_at	1415858_at	Eif3s8	eukaryotic translation initiation factor 3, subu	1.41	0.18	-1.08	0.0114	1.04	0.3918	1.02	0.6132	-1.3	0.0806
94839_at	1416903_at	Nucb1	nucleobindin 1	1.03	0.81	-1.05	0.3574	-1.04	0.395	-1.17	0.0009	1.1	0.3608
94840_at	1449024_a_at	Hexa	hexosaminidase A	1.2	0.25	1.44	0	1.11	0.1378	1.39	0.0001	1.6	0.0077
94841_at	1424681_a_at	Psma5	proteasome (prosome, macropain) subunit, a	1	0.99	1.17	0.1878	1.08	0.3459	1.15	0.121	-1.47	0.006
94843_at	1427885_at	Pold4	polymerase (DNA-directed), delta 4	2.98	0.03	1.06	0.2568	-1.1	0.121	1.05	0.4396	1.21	0.387
94845_at	1415764_at	5730454B08Rik	RIKEN cDNA 5730454B08 gene	-1.08	0.62	1.28	0.0105	1.29	0.0107	1.22	0.0295	1.17	0.0985
94850_at	1449968_s_at	MGI:1928939 //.	acyl-Coenzyme A thioesterase 2, mitochond	1.11	0.51	1.13	0.4519	1.09	0.2861	1.14	0.4858	1.07	0.6827

94852_at	1426236_a_at	Glul	glutamate-ammonia ligase (glutamine syntha	-1.51	0.4	-1.43	0.0001	1.04	0.5016	-1.63	0	-1.57	0.2493
94853_at	1417432_a_at	Gnb1	guanine nucleotide binding protein, beta 1	1.63	0.01	1.25	0.0972	1.23	0.0424	1.44	0.0001	1.29	0.062
94855_at	1448563_at	Phb	prohibitin	3.01	0	-1.12	0.1532	1.09	0.41	-1.06	0.5704	-1.59	0.0634
94857_at	1417572_at	Mpg	N-methylpurine-DNA glycosylase	1.13	0.11	-1.05	0.4938	-1.03	0.5063	1.06	0.3478	-1.18	0.6058
94860_at	1426256_at	Timm17a	translocator of inner mitochondrial membran	1.42	0.06	-1	0.9845	1.19	0.1262	1.17	0.1036	-1.08	0.5044
94861_at	1449718_s_at	4930453N24Rik	RIKEN cDNA 4930453N24 gene	-1.18	0.4	-1.01	0.9077	-1.12	0.1483	-1	0.9776	-1.95	0.0072
94865_at	1415788_at	BC002236	cDNA sequence BC002236	1	0.99	1.04	0.6524	1.3	0.0443	1.18	0.153	-1.11	0.6149
94866_at	1448869_a_at	Mrps16	mitochondrial ribosomal protein S16	-1.49	0.43	1.39	0.0406	1.31	0.27	1.38	0.1623	1.05	0.8808
94868_at	1423712_a_at	Qars	glutaminyl-tRNA synthetase	1.27	0.21	1.04	0.451	1.13	0.1206	1.16	0.024	-1.16	0.6434
94871_r_at	1428163_at	Sara2	SAR1a gene homolog 2 (S. cerevisiae)	1.08	0.59	1.19	0.1975	1.03	0.8225	1.18	0.2464	-1.38	0.0235
94872_at	1416635_at	Smpdl3a	sphingomyelin phosphodiesterase, acid-like	1.44	0.29	-1.14	0.106	1.11	0.4917	-1.11	0.3043	-1.42	0.0149
94875_at	1416093_a_at	Mrpl20	mitochondrial ribosomal protein L20	1.34	0.1	-1.07	0.1526	1.11	0.1313	1.04	0.5849	-1.66	0.0706
94876_f_at	1424710_a_at	Gorasp2	golgi reassembly stacking protein 2	-1.53	0.02	-1.13	0.0565	1.17	0.2314	-1.06	0.3585	-1.49	0.0047
94878_at	1455286_at	Btbd1	BTB (POZ) domain containing 1	1.3	0.1	-1.15	0.0911	-1.1	0.2573	-1.21	0.0142	1.13	0.1668
94881_at	1424638_at	Cdkn1a	cyclin-dependent kinase inhibitor 1A (P21)	1.32	0.12	1.21	0.2996	-1.33	0.0734	1.41	0.1191	-1.05	0.9313
94882_at	1416446_at	D9Wsu20e	DNA segment, Chr 9, Wayne State Universit	-1.11	0.51	-1.11	0.1996	1.06	0.5408	-1.08	0.2344	-1.29	0.1853
94885_at	1426666_a_at	Unc84a	unc-84 homolog A (C. elegans)	1.78	0.06	-1.01	0.8985	1	0.9915	1.01	0.929	1.05	0.8717
94886_at	1415692_s_at	Canx	calnexin	-1.34	0.09	1.1	0.3118	1.03	0.7452	-1.12	0.1526	1.1	0.4247
94889_at	1416466_at	Vapa	vesicle-associated membrane protein, assoc	-1.03	0.91	-1.17	0.0024	1.03	0.4614	-1.07	0.0118	-1.09	0.5901
94892_r_at	1416810_at	Mea1	male enhanced antigen 1	1.12	0.3	1.03	0.7186	1.07	0.1325	1.02	0.8097	1.16	0.2897
94893_at	1429451_at	2610301B20Rik	RIKEN cDNA 2610301B20 gene	-1.43	0.29	-1.06	0.6733	1.22	0.0675	1.01	0.8984	-1.42	0.0012
94895_at	1460357_at	Ythdf2	YTH domain family 2	1.33	0.5	1.13	0.1102	1.09	0.337	1.15	0.0425	-1.03	0.5236
94896_at	1448144_at	Hnrpab	heterogeneous nuclear ribonucleoprotein A/	-1.22	0.04	-1.06	0.3254	-1.09	0.2375	-1.04	0.5904	1.1	0.4654
94897_at	1451695_a_at	Gpx4	glutathione peroxidase 4	1.17	0.24	1.22	0.0099	1.05	0.5647	1.43	0	1.05	0.6333
94898_at	1420608_at	Rbm18	RNA binding motif protein 18	1.29	0.2	1.04	0.5541	1.15	0.1316	1.04	0.6312	1.07	0.7214
94899_at	1460342_s_at	AA536749	expressed sequence AA536749	-1.01	0.95	1.27	0.0164	1.23	0.0707	1.46	0	1.13	0.5257
94900_at	1418639_at	Sftpc	surfactant associated protein C	-1.15	0.76	-1.03	0.859	-1.1	0.608	1.2	0.2935	-1.3	0.5595
94902_at	1417633_at	Sod3	superoxide dismutase 3, extracellular	-1.87	0.01	-1.21	0.0646	-1.08	0.4297	-1.56	0.0001	1.11	0.3519
94903_at	1419917_s_at	Tmed7	transmembrane emp24 protein transport dor	-1.58	0.02	-1.02	0.7812	1.04	0.5722	1.05	0.3476	-1.4	0.0085
94906_at	1416225_at	Adh1	alcohol dehydrogenase 1 (class I)	1.15	0.16	1.04	0.4194	1.03	0.3288	1.03	0.3631	1.31	0.0466
94908_r_at	1416367_at	1110001J03Rik	RIKEN cDNA 1110001J03 gene	1.12	0.83	1.11	0.2475	1.16	0.0575	1.14	0.2099	-1.02	0.8536
94909_at	1453728_a_at	Mrps17	mitochondrial ribosomal protein S17	1.5	0.17	-1.09	0.1641	1.08	0.0591	1.04	0.5741	-1.17	0.1017
94910_at	1435737_a_at	Nde1	nuclear distribution gene E homolog 1 (A nic	1.06	0.48	-1.04	0.4616	1.04	0.472	-1.02	0.7731	-1.4	0.0142
94912_at	1422451_at	Mrps21	mitochondrial ribosomal protein S21	1.5	0.12	-1.14	0.0918	1.02	0.8733	-1.1	0.2058	-1.07	0.6525
94913_at	1415697_at	E430034L04Rik	RIKEN cDNA E430034L04 gene	-1.11	0.63	-1.21	0.1115	-1.2	0.0205	-1.12	0.1816	-1.17	0.3374
94914_at	1451441_at	2210415F13Rik	RIKEN cDNA 2210415F13 gene	1.34	0.55	-1.22	0.2452	-1.14	0.2607	-1.33	0.0121	-1.27	0.1392
94915_at	1450911_at	Ppib	peptidylprolyl isomerase B	-1.01	0.99	-1.13	0.1692	-1.04	0.6694	-1.07	0.2751	-1.27	0.0506
94918_at	1451083_s_at	Aars	alanyl-tRNA synthetase	-1.3	0.1	-1.4	0.0005	-1.13	0.0486	-1.46	0.0001	-1.39	0.0192
94920_at	1415793_at	Pnpo	pyridoxine 5'-phosphate oxidase	1.53	0.03	-1.11	0.1413	-1.28	0.0183	-1.24	0.0116	-1.28	0.0002
94923_f_at	1434468_at	D8Ertd69e	DNA segment, Chr 8, ERATO Doi 69, expre	-1.01	0.98	-1.36	0.021	-1.25	0.1932	-1.31	0.1669	1.18	0.0999
94924_at	1424329_a_at	Prrg2	proline-rich Gla (G-carboxyglutamic acid) po	1.06	0.8	-1.12	0.2312	-1.1	0.374	-1.25	0.0307	-1.07	0.7582
94925_at	1423625_a_at	1810055D05Rik	RIKEN cDNA 1810055D05 gene	1.4	0.1	1.01	0.8636	1.11	0.2611	1.06	0.4935	-1.45	0.1056
94927_at	1421804_at	Fabp9	fatty acid binding protein 9, testis	-2.19	0.46	1.12	0.5004	1.12	0.4777	-1.01	0.9553	-1.48	0.0267
94928_at	1418099_at	Tnfrsf1b	tumor necrosis factor receptor superfamily, r	1.03	0.77	-1.22	0.0303	-1	0.9816	1.12	0.358	1.03	0.7092
94929_at	1417068_a_at	Ptpn1	protein tyrosine phosphatase, non-receptor t	1.66	0.43	1.29	0.2229	-1.16	0.3939	1.25	0.2533	-1.04	0.791
94930_at	1450663_at	Thbs2	thrombospondin 2	-1.59	0.37	1.18	0.522	1.21	0.3681	1.31	0.2154	2.45	0.0332
94931_at	1418899_at	1810045K17Rik	RIKEN cDNA 1810045K17 gene	1.29	0.26	-1.12	0.1004	-1.11	0.1275	-1.2	0.0027	-1.54	0.0317
94932_at	1418711_at	Pdgfa	platelet derived growth factor, alpha	-1.29	0.33	-1.12	0.3189	-1.33	0.0436	-1.37	0.0231	1.34	0.0402
94933_at	1425323_a_at	BC008155	cDNA sequence BC008155	1.01	0.95	1.07	0.1677	-1.11	0.3596	-1.01	0.8503	-1.48	0.3888
94934_at	1415832_at	Agtr2	angiotensin II receptor, type 2	-2.49	0.03	1.45	0.2928	-1.1	0.6486	1.17	0.4984	1.14	0.5756
94936_at	1418215_at	Mep1b	meprin 1 beta	-2.34	0.37	-1.11	0.4475	1.24	0.3097	1.06	0.7496	-1.21	0.6577
94937_at	1433953_at	Zfp277	zinc finger protein 277	1.74	0.22	1.24	0.0586	1.16	0.4529	1.2	0.0896	1.87	0.1615
94939_at	1448617_at	Cd53	CD53 antigen	1.88	0.1	2.49	0.2609	1.1	0.5515	3.37	0.169	-1.15	0.7528

94940_at	1417227_at	Mccc1	methylcrotonoyl-Coenzyme A carboxylase 1	1.5	0.2	-1.05	0.5117	-1.07	0.6023	-1.17	0.0851	-1.17	0.2002
94941_at	1421389_a_at	Eif2ak4	eukaryotic translation initiation factor 2 alpha	2.39	0.1	-1.04	0.8221	-1.05	0.8123	-1.19	0.2797	1.03	0.833
94942_at	1448597_at	Cstf1	cleavage stimulation factor, 3' pre-RNA, sub	1.09	0.57	1.07	0.2777	1.09	0.1733	-1.02	0.7226	-1.09	0.4252
94944_at	1416753_at	Prkar1b	protein kinase, cAMP dependent regulatory,	-1.77	0.36	1.53	0.0063	1.09	0.6337	1.1	0.6365	2.4	0.2693
94945_at	1449694_s_at	Commd5	COMM domain containing 5	2.67	0	-1.27	0.1037	1.02	0.9051	-1.04	0.7044	-1.85	0.2357
94946_at	1426686_s_at	Map3k3	mitogen activated protein kinase kinase kina	-1.01	0.94	1.24	0.0809	-1.07	0.6531	1.11	0.3413	1.47	0.0043
94948_at	1449041_a_at	Trip6	thyroid hormone receptor interactor 6	1.21	0.31	1.18	0.1107	1.28	0.1174	1.39	0.0018	-1.05	0.7644
94951_at	1452266_at	1810030A06Rik	RIKEN cDNA 1810030A06 gene	1.73	0.01	1.17	0.0304	1.16	0.2736	1.35	0.006	1.45	0.0023
94952_at	1437103_at	C330012H03Ril	RIKEN cDNA C330012H03 gene	-1.56	0.13	-1.16	0.0879	-1.16	0.1155	-1.22	0.0543	1.1	0.7068
94953_at	1421546_a_at	Racgap1	Rac GTPase-activating protein 1	1.93	0.28	2.26	0.007	1.46	0.2727	2.3	0.026	-1.38	0.4897
94954_at	1423931_s_at	Anapc4	anaphase promoting complex subunit 4	3.72	0.03	1.1	0.4748	1.04	0.8511	1.13	0.2488	-2.15	0.0612
94956_at	1452434_s_at	Dgcr6	DiGeorge syndrome critical region gene 6	1.27	0.23	-1.24	0.0103	-1.11	0.0317	-1.29	0.0094	-1.61	0.0559
94957_at	1426345_at	Prepl	prolyl endopeptidase-like	1.31	0.17	-1.2	0.0181	-1.09	0.1629	-1.15	0.0404	-1.86	0.0024
94958_at	1454893_at	1110013L07Rik	RIKEN cDNA 1110013L07 gene	-1.29	0.28	1.23	0.1401	1.07	0.6017	1.55	0.0039	1.35	0.3801
94963_at	1416156_at	---	---	1.27	0.39	1.43	0.0008	1.12	0.4128	1.69	0	-1.32	0.0791
94964_at	1416157_at	Vcl	vinculin	1.32	0.71	1.54	0.0221	1.07	0.8036	1.35	0.1091	2.05	0.1316
94966_at	1448354_at	G6pdx	glucose-6-phosphate dehydrogenase X-linke	-1.17	0.2	1.06	0.784	-1.37	0.0276	1.05	0.8602	2.14	0.0175
94967_at	1433702_at	D19Wsu12e	DNA segment, Chr 19, Wayne State Univers	1.21	0.37	1.15	0.1204	-1.06	0.4499	1.23	0.0004	-1.28	0.0887
94968_at	1448963_at	Nfyc	nuclear transcription factor-Y gamma	1.05	0.78	1.2	0.016	1.21	0.0849	1.31	0.0035	1.37	0.0932
94973_at	1428320_at	Jmjd1b	jumonji domain containing 1B	1.06	0.71	-1.07	0.442	-1.02	0.8468	1	0.978	-1.05	0.6338
94975_at	1433527_at	---	---	-1	1	1.02	0.8359	-1.02	0.782	-1.06	0.565	-1.28	0.0846
94976_at	1423975_s_at	Numa1	nuclear mitotic apparatus protein 1	1.01	0.91	1.16	0.0131	1.09	0.3222	-1.05	0.5175	1.27	0.0721
94977_at	1460203_at	Itpr1	inositol 1,4,5-triphosphate receptor 1	-1.13	0.46	-1.15	0.2164	1.08	0.6479	-1.28	0.0483	-1.6	0.0157
94978_at	1416171_at	2310037I24Rik	RIKEN cDNA 2310037I24 gene	1.43	0.33	1.08	0.3553	1.25	0.0228	1.19	0.1667	-1.1	0.4258
94979_at	1424071_s_at	BC018507	cDNA sequence BC018507	1.15	0.39	1.11	0.3325	1.36	0.0677	1.17	0.1769	1.03	0.7884
94980_at	1452594_at	Dusp11	dual specificity phosphatase 11 (RNA/RNP c	1.33	0.2	-1.01	0.8933	1.01	0.9035	1.01	0.8861	1.43	0.4038
94983_at	1451283_at	1810073G14Rik	RIKEN cDNA 1810073G14 gene	1.38	0.58	1.11	0.2854	1.04	0.7856	1.09	0.4106	1.14	0.4735
94985_at	1426402_at	Syncrip	synaptotagmin binding, cytoplasmic RNA int	1.49	0.13	1.08	0.494	1.26	0.0972	1.11	0.1878	-1.36	0.229
94986_at	1417428_at	Gng3	guanine nucleotide binding protein (G protei	-1.32	0.2	-1.07	0.767	1.09	0.6811	-1.01	0.9563	1.05	0.8624
94987_at	1450612_a_at	Pemt	phosphatidylethanolamine N-methyltransfera	-1.02	0.93	-1.2	0.0018	1.1	0.193	-1.27	0	-1.11	0.4431
94989_at	1454722_at	Herc1	hect (homologous to the E6-AP (UBE3A) ca	1.04	0.89	-1.1	0.0231	-1.1	0.3106	-1.19	0.0002	-1.2	0.019
94990_at	1448567_at	C78915	expressed sequence C78915	1.82	0.05	-1.07	0.3513	-1.03	0.7146	-1.1	0.0589	1.17	0.5294
94991_at	1434089_at	Synpo	synaptopodin	1.21	0.48	-1.41	0.0147	-1.11	0.2802	-1.24	0.102	1.27	0.5424
94992_at	1423301_at	Copb1	coatomer protein complex, subunit beta 1	1.11	0.62	-1.12	0.1858	1.07	0.6177	-1.07	0.2345	-1.36	0.1188
94994_at	1425633_at	Cfh	complement component factor h	1.14	0.68	-1.42	0.0112	-1.07	0.414	-1.55	0.0005	-1.52	0.3913
94995_at	1435695_a_at	A030007L17Rik	RIKEN cDNA A030007L17 gene	3.25	0.02	-1.3	0.0044	-1.12	0.1345	-1.36	0.0012	-2.27	0.0204
94998_at	1450870_at	Rala	v-ral simian leukemia viral oncogene homolo	1.73	0.16	-1.18	0.1669	1.02	0.8745	-1.01	0.9382	-1.05	0.6135
95000_g_at	1452270_s_at	Cubn	cubilin (intrinsic factor-cobalamin receptor)	-1.17	0.56	-1.27	0.0228	-1.22	0.119	-1.12	0.088	-1.13	0.8336
95001_at	1450983_at	Akap8	A kinase (PRKA) anchor protein 8	1.88	0.14	1.47	0.1605	1.87	0.0345	1.71	0.036	-1.03	0.869
95002_at	1460741_x_at	D17Wsu92e	DNA segment, Chr 17, Wayne State Univers	1.24	0.18	1.03	0.6617	1.07	0.5115	-1.11	0.1333	1.04	0.7418
95003_at	1452596_at	Polr2k	polymerase (RNA) II (DNA directed) polypep	1.17	0.26	1.02	0.5247	1.16	0.1489	1.08	0.1258	-1.09	0.6276
95004_at	1428255_at	Luc7l	Luc7 homolog (S. cerevisiae)-like	-1.26	0.18	1.18	0.0219	1.08	0.2408	1.18	0.1276	1.52	0.0016
95007_at	1426949_s_at	Tpr	translocated promoter region	2.52	0.09	1.5	0.0372	-1.06	0.4896	1.33	0.0081	-1.32	0.3018
95010_at	1418587_at	Traf3	Tnf receptor-associated factor 3	1.16	0.59	-1.01	0.886	-1.04	0.5481	1.04	0.7105	-1.16	0.2984
95011_at	1427905_at	1810063B07Rik	RIKEN cDNA 1810063B07 gene	1.2	0.13	-1.03	0.5751	1.11	0.1825	1.07	0.1924	-1.18	0.4807
95012_at	1448209_a_at	Slc22a17	solute carrier family 22 (organic cation trans	-1.09	0.82	-1.39	0.0157	-1.25	0.1174	-1.46	0.0219	1.13	0.757
95014_at	1417501_at	Fbxo6b	F-box only protein 6b	-1.1	0.58	-1.2	0.1072	1.12	0.0762	-1.07	0.322	-1.34	0.0089
95015_at	1450455_s_at	Akr1c12	aldo-keto reductase family 1, member C12	1.29	0.28	1.14	0.188	-1	0.975	1.25	0.0338	-1.08	0.1523
95016_at	1418084_at	Nrp1	neuropilin 1	-1.38	0.31	-1.45	0.0016	-1.22	0.046	-1.63	0.0001	-1.78	0.0851
95018_r_at	1433903_at	AU021838	expressed sequence AU021838	-1.52	0.07	-1.06	0.6847	-1.13	0.4351	-1.02	0.8487	1.01	0.9238
95019_at	1418186_at	Gstt1	glutathione S-transferase, theta 1	1.8	0.02	1.09	0.1236	-1.13	0.0255	-1	0.9917	1.47	0.0372
95020_at	1428187_at	Cd47	CD47 antigen (Rh-related antigen, integrin-a	-1.03	0.93	1.25	0.0394	1.29	0.0415	1.49	0.0002	1.05	0.573
95021_at	1433597_at	9430010O03Rik	RIKEN cDNA 9430010O03 gene	1.33	0.09	1.1	0.1845	1.04	0.6281	-1.05	0.5873	-1.05	0.7465

95022_at	1419706_a_at	Akap12	A kinase (PRKA) anchor protein (gravin) 12	3.88	0.01	-2.14	0.0157	-1.56	0.138	-1.58	0.1359	2.93	0.0325
95023_at	1426940_at	Sidt2	SID1 transmembrane family, member 2	-1.29	0.05	-1.35	0	-1.12	0.0612	-1.37	0	-1.32	0.0031
95024_at	1418191_at	Usp18	ubiquitin specific protease 18	1.3	0.51	2.26	0.05	1.61	0.059	2.86	0.0244	5.77	0
95025_at	1448900_at	D16H22S680E	DNA segment, Chr 16, human D22S680E, e	1.16	0.38	-1.04	0.5278	1.24	0.116	1.33	0.0008	-1.14	0.0578
95027_at	1454034_a_at	Usp21	ubiquitin specific protease 21	-1.14	0.83	-1.01	0.9386	-1.02	0.8696	1.04	0.6752	1.55	0.2819
95029_at	1417475_at	Atp13a1	ATPase type 13A1	1.41	0.31	1.13	0.2924	1.22	0.0367	1.14	0.1408	-1.12	0.3833
95030_at	1448556_at	Prlr	prolactin receptor	-5.06	0.01	-1.9	0.0008	-1.28	0.1572	-4.13	0	-2.44	0.0234
95032_at	1423775_s_at	Prc1	protein regulator of cytokinesis 1	1.63	0.36	1	0.9842	-1.06	0.7472	1.22	0.3818	1.78	0.3156
95033_at	1426810_at	Jmjd1a	jumonji domain containing 1A	-1.23	0.21	1.34	0.0102	1.35	0.0171	1.4	0.0014	1.23	0.113
95034_f_at	1436420_a_at	Ipo4	importin 4	-1.19	0.25	-1.45	0.0015	-1.12	0.2773	-1.23	0.0316	-1.14	0.1801
95035_at	1450814_a_at	Ipo4	importin 4	-1.04	0.89	1.11	0.0425	1.01	0.8781	1.07	0.1946	-1.01	0.9461
95036_at	1451129_at	Calb2	calbindin 2	-1.23	0.62	1.08	0.4296	1.07	0.2901	1.16	0.1403	1.1	0.454
95037_at	1417269_at	Cdk9	cyclin-dependent kinase 9 (CDC2-related kir	1.26	0.12	1.16	0.0769	1.04	0.699	1.23	0.0037	-1.03	0.745
95040_at	1415937_s_at	Pdcd6ip	programmed cell death 6 interacting protein	-1.12	0.58	1.05	0.5831	-1.09	0.4907	-1.1	0.4312	-1.33	0.0217
95041_at	1449046_a_at	1110007C05Rik	RIKEN cDNA 1110007C05 gene	-1.52	0.13	-1.28	0.0037	-1.17	0.1511	-1.39	0.0036	1.32	0.4646
95042_at	1424196_at	C030002N13Ril	RIKEN cDNA C030002N13 gene	1.23	0.23	-1.13	0.0454	-1.01	0.8228	-1.1	0.1383	-1.27	0.0396
95043_at	1424273_at	Cyp2c70	cytochrome P450, family 2, subfamily c, poly	-1.32	0.41	-1.48	0.0004	-1.4	0.0007	-1.71	0.0001	-2.07	0.0233
95044_at	1416508_at	Med28	mediator of RNA polymerase II transcription	2.3	0.01	1.11	0.3888	-1.21	0.0195	1.01	0.8608	-1.58	0.0895
95045_at	1417710_at	0610012D09Rik	RIKEN cDNA 0610012D09 gene	1.02	0.89	1.14	0.0459	1.03	0.7102	1.11	0.0667	-1.53	0.0019
95046_s_at	1449940_a_at	Eif2b4	eukaryotic translation initiation factor 2B, sul	1.31	0.13	-1.01	0.8233	-1.05	0.4586	-1.06	0.2596	1.01	0.9736
95049_at	1452680_at	Snrpd2	small nuclear ribonucleoprotein D2	1.35	0.2	1.03	0.6319	1.04	0.4942	1.06	0.4446	1.04	0.8253
95053_s_at	1418005_at	Sdhb	succinate dehydrogenase complex, subunit	1.62	0.09	1.08	0.0421	-1.02	0.4769	1.1	0.103	1.08	0.3926
95054_at	1460323_at	Tars	threonyl-tRNA synthetase	-1.63	0.02	-1.73	0	-1.04	0.5028	-1.66	0	-2.52	0
95057_at	1448185_at	Herpud1	homocysteine-inducible, endoplasmic reticul	1.7	0.15	1.44	0.003	1.56	0.0001	1.75	0	1.35	0.0754
95058_f_at	1423833_a_at	2610205H19Rik	RIKEN cDNA 2610205H19 gene	1.18	0.33	1.19	0.0364	1.07	0.4725	1.14	0.0713	1.38	0.0061
95059_at	1416187_s_at	Pnrc2	proline-rich nuclear receptor coactivator 2	-1.5	0.14	1.06	0.3307	-1.16	0.1547	-1.07	0.4283	-1.35	0.1466
95060_at	1448502_at	Slc16a7	solute carrier family 16 (monocarboxylic acic	1.77	0.09	1.84	0.0001	1.27	0.1009	2	0.0001	2.49	0
95061_at	1418234_s_at	Bcas2	breast carcinoma amplified sequence 2	1.76	0.16	1.23	0.142	1.37	0.0958	1.46	0.0083	1.51	0.3765
95062_at	1451413_at	Cast	calpastatin	1.14	0.13	-1.11	0.2589	-1.06	0.5032	-1.01	0.8752	-1.14	0.38
95063_at	1428069_at	Cdca7	cell division cycle associated 7	-1.19	0.29	-1.26	0.0377	1.1	0.3228	-1.43	0.0233	-1.64	0.3113
95064_at	1428146_s_at	Acaa2	acetyl-Coenzyme A acyltransferase 2 (mitoc	-1.03	0.88	-1.03	0.3509	1.06	0.0228	1.01	0.7982	1.17	0.0705
95065_at	1416373_at	Nfs1	nitrogen fixation gene 1 (S. cerevisiae)	-1.17	0.04	-1.03	0.5763	1.09	0.134	-1.09	0.0387	-1.53	0.0077
95066_at	1425129_a_at	Taldo1	transaldolase 1	1.5	0.07	-1.19	0.0134	-1.14	0.0261	-1.19	0.0031	-2.11	0.0004
95067_at	1448811_at	Mrpl2	mitochondrial ribosomal protein L2	1.51	0.11	-1.07	0.2828	1.12	0.0968	1.06	0.4013	-1.37	0.097
95068_at	1417182_at	Dnaja2	DnaJ (Hsp40) homolog, subfamily A, membe	-1.06	0.75	-1.16	0.0166	1.13	0.0663	-1.08	0.2038	-1.32	0.0222
95069_at	1426789_s_at	Ssrp1	structure specific recognition protein 1	-1.01	0.98	1.04	0.7389	1.19	0.2612	1.09	0.4871	-1.05	0.8422
95070_at	1452866_at	Nars	asparaginyl-tRNA synthetase	1.03	0.88	1.15	0.0592	1.35	0.0094	1.13	0.0768	-1.57	0.0061
95071_at	1449180_at	Kcmf1	potassium channel modulatory factor 1	-1.08	0.49	1.03	0.7232	1.02	0.6799	-1.02	0.6497	1.22	0.2172
95072_at	1416604_at	Cyc1	cytochrome c-1	1.28	0.02	1.08	0.0241	1.11	0.0438	1.11	0.0396	-1.05	0.6585
95074_at	1416425_at	Pex19	peroxisome biogenesis factor 19	1.31	0.24	-1.11	0.1767	1.2	0.1592	-1.02	0.8144	-1.48	0.0402
95075_at	1426447_at	Nup35	nucleoporin 35	-1.02	0.79	1.03	0.8499	-1.03	0.8607	1.06	0.7328	1.01	0.9803
95076_at	1452584_at	1500032L24Rik	RIKEN cDNA 1500032L24 gene	-1.09	0.67	-1.04	0.4912	1.09	0.0459	-1.01	0.8331	1.02	0.8391
95077_at	1419553_a_at	Rabggtb	RAB geranylgeranyl transferase, b subunit	1.22	0.11	1.1	0.3404	1.32	0.0367	1.22	0.0502	-1.07	0.637
95078_at	1452704_at	1200015F23Rik	RIKEN cDNA 1200015F23 gene	-1.19	0.15	1.03	0.7891	-1.08	0.1908	-1.08	0.5264	-1.81	0.0065
95079_at	1421917_at	Pdgfra	platelet derived growth factor receptor, alpha	1.98	0	1.09	0.2877	-1.05	0.7603	1.29	0.0046	3.23	0.0105
95081_at	1420115_at	Exosc8	exosome component 8	-1.16	0.43	-1.04	0.6485	-1.18	0.102	-1.12	0.3311	1.18	0.1022
95082_at	1423062_at	Igfbp3	insulin-like growth factor binding protein 3	-1.17	0.57	1.23	0.2473	1.35	0.1459	1.47	0.0408	2.17	0.0126
95086_at	1428201_at	2310036O22Rik	RIKEN cDNA 2310036O22 gene	1.46	0.1	-1.08	0.1036	1.15	0.0142	1.03	0.415	-1.11	0.5759
95090_at	1455752_a_at	2410001H17Rik	RIKEN cDNA 2410001H17 gene	1.09	0.29	1.15	0.0375	-1.11	0.1957	1.14	0.0043	1.18	0.0312
95091_at	1416241_at	Sec13l1	SEC13-like 1 (S. cerevisiae)	-1.28	0.31	-1.13	0.0981	1	0.8931	-1.06	0.4141	-1.61	0.0001
95095_at	1448559_at	Flot1	flotillin 1	1.4	0.01	1.33	0.0148	1.73	0.0008	1.65	0.0002	1.88	0.0096
95096_at	1451179_a_at	Qk	quaking	-1.01	0.97	1.18	0.0081	1.2	0.0152	1.25	0.0025	-1.04	0.8336
95097_at	1417157_at	Actr10	ARP10 actin-related protein 10 homolog (S.	1.41	0.13	1.12	0.0197	1.25	0.0867	1.2	0.0069	-1.17	0.2353

95098_at	1423650_at	Rnf26	ring finger protein 26	-1.35	0.16	1.05	0.5937	1.12	0.1206	1.08	0.3741	1.28	0.1614
95100_at	1456695_x_at	Anapc5	anaphase-promoting complex subunit 5	-1.01	0.95	-1.09	0.1579	1.02	0.7855	-1.08	0.1939	1.23	0.1932
95101_at	1448108_at	Tde2	tumor differentially expressed 2	1.27	0.36	1.18	0.1248	1.02	0.8982	1.18	0.098	1.02	0.8729
95102_at	1423986_a_at	MGI:1915044	scotin gene	1.09	0.31	1.16	0.4435	-1.04	0.6612	1.32	0.3031	1.01	0.9516
95103_at	1417243_at	2310065K24Rik	RIKEN cDNA 2310065K24 gene	-1.12	0.41	-1.1	0.1245	1.12	0.1753	1.01	0.8901	-1.23	0.2113
95104_at	1417011_at	Sdc2	syndecan 2	-1.03	0.88	-1.03	0.6846	-1.06	0.0342	-1.21	0.0007	-1.08	0.488
95105_at	1418002_at	2010110M21Ril	RIKEN cDNA 2010110M21 gene	-1.06	0.72	1.06	0.2131	-1.03	0.3976	1.05	0.2462	-1.13	0.3178
95109_at	1455035_s_at	Nol5a	nucleolar protein 5A	1.71	0.18	1.05	0.5968	1.36	0.0258	1.25	0.0257	1.37	0.0466
95110_at	1452920_a_at	Ppil2	peptidylprolyl isomerase (cyclophilin)-like 2	1.1	0.66	-1.05	0.4386	-1.05	0.641	-1.03	0.6122	1.46	0.1564
95114_s_at	1451387_s_at	0610039D01Rik	RIKEN cDNA 0610039D01 gene	1.13	0.57	1.01	0.9212	-1.03	0.7556	-1.02	0.7423	-1.07	0.6042
95117_at	1424111_at	Igf2r	insulin-like growth factor 2 receptor	-1.04	0.77	1.17	0.0086	1.03	0.5303	1.28	0.0047	1.18	0.0226
95118_r_at	1451128_s_at	Kif22	kinesin family member 22	-1.21	0.52	1.15	0.5929	-1.08	0.5012	1.25	0.3424	-1.02	0.8279
95119_at	1428071_at	1110038D17Rik	RIKEN cDNA 1110038D17 gene	-1.03	0.87	1.09	0.0858	1.17	0.0001	1.15	0.0082	1.01	0.916
95120_at	1418643_at	Tspan13	tetraspanin 13	-1.12	0.87	1.1	0.6852	-1.53	0.0904	1.31	0.1979	3.63	0.0617
95122_g_at	1423371_at	Pole4	polymerase (DNA-directed), epsilon 4 (p12 s	-1.27	0.35	-1.33	0.0026	-1.4	0.0075	-1.9	0	-1.21	0.4506
95123_at	1426889_at	4930566A11Rik	RIKEN cDNA 4930566A11 gene	2.2	0.16	-1.2	0.174	1	0.9782	1.07	0.6076	1.28	0.5395
95125_f_at	1416578_at	Rbx1	ring-box 1	1.33	0.05	1.11	0.2284	1.12	0.2141	1.06	0.489	-1.11	0.5223
95128_at	1415707_at	Anapc2	anaphase promoting complex subunit 2	1.32	0.33	-1.04	0.4495	-1.11	0.2209	-1.07	0.1317	-1.01	0.9237
95129_at	1448893_at	Ncor2	nuclear receptor co-repressor 2	-1.25	0.39	-1.08	0.3142	-1.08	0.4864	1.05	0.4404	1.03	0.8673
95132_r_at	1453806_at	Ndufb2	NADH dehydrogenase (ubiquinone) 1 beta s	-1.21	0.61	-1.07	0.1923	-1.05	0.5276	-1.11	0.1366	1.02	0.9387
95133_at	1451095_at	Asns	asparagine synthetase	-1.97	0.11	1.1	0.1349	1.16	0.0233	1.13	0.0707	1.14	0.2281
95135_at	1416840_at	Mid1ip1	Mid1 interacting protein 1 (gastrulation speci	-1.19	0.43	-1.03	0.8365	-1.02	0.8986	-1.32	0.0187	-2.55	0.0038
95136_at	1424285_s_at	Arl6ip4	ADP-ribosylation factor-like 6 interacting pro	1.64	0	1.16	0.0293	1.31	0.0106	1.33	0.0041	1	0.9755
95137_at	1416376_at	1810014L12Rik	RIKEN cDNA 1810014L12 gene	1.57	0.03	1.02	0.7411	1.11	0.1001	1.11	0.0453	-1.11	0.5989
95138_at	1429367_at	2510001I10Rik	RIKEN cDNA 2510001I10 gene	1.67	0.02	-1.15	0.0327	-1.2	0.0102	-1.1	0.0741	-2.09	0.0258
95140_at	1456736_x_at	5230400G24Ril	RIKEN cDNA 5230400G24 gene	-1.04	0.57	1.12	0.0957	1.23	0.0591	1.15	0.0378	-1.15	0.276
95142_s_at	1424168_a_at	---	---	1.06	0.8	1	0.9887	-1.09	0.4466	1.05	0.6651	-1.26	0.1679
95144_at	1416079_a_at	Arpc1a	actin related protein 2/3 complex, subunit 1A	1.16	0.56	1.07	0.1293	1.09	0.2123	1.01	0.8229	-1.55	0.0505
95146_at	1424039_at	1810045K07Rik	RIKEN cDNA 1810045K07 gene	1.32	0.14	-1.02	0.5938	-1.14	0.0227	1.01	0.8861	-1.15	0.1453
95147_at	1417722_at	Pgls	6-phosphogluconolactonase	-1.03	0.91	-1	0.9511	-1.04	0.725	-1.1	0.1207	-1.04	0.8115
95148_at	1448450_at	---	---	1.25	0.14	1.19	0.0517	1.15	0.0661	1.24	0.0039	1.1	0.5364
95149_at	1451825_a_at	Copz1	coatomer protein complex, subunit zeta 1	1.13	0.66	-1.15	0.0013	-1.12	0.0269	-1.24	0.0013	-1.31	0.0184
95150_at	1417128_at	2810052M02Ril	RIKEN cDNA 2810052M02 gene	1.14	0.59	1.47	0.3281	-1.29	0.1405	1.67	0.3492	1.2	0.3134
95153_at	1435220_s_at	Cdc42se2	CDC42 small effector 2	-1.25	0.42	1.18	0.0191	1.08	0.3511	1.23	0.0521	1.28	0.4689
95156_g_at	1424384_a_at	Znrf1	zinc and ring finger 1	1.6	0.27	3.2	0.0024	1.95	0.1319	3.03	0.0037	-1.05	0.9108
95157_at	1420950_at	Znrf1	zinc and ring finger 1	2.34	0.14	1.45	0.3389	1.3	0.2156	1.09	0.6783	1.6	0.1252
95158_at	1415744_at	---	---	1.44	0.1	1.05	0.4567	1.11	0.1936	-1.04	0.539	-1.03	0.7249
95159_at	1451164_a_at	Mrps18b	mitochondrial ribosomal protein S18B	1.4	0.07	-1.09	0.1564	-1.01	0.914	-1.02	0.8317	-1.17	0.071
95161_at	1451075_s_at	Ctdsp2	CTD (carboxy-terminal domain, RNA polyme	-1.19	0.49	1.15	0.022	-1	0.9968	1.1	0.1073	1.63	0.0002
95165_at	1448060_at	Sema6d	sema domain, transmembrane domain (TM)	1.19	0.08	-1.03	0.7832	1.07	0.5422	-1.05	0.5811	1.41	0.1919
95177_at	1449785_at	AA414993	expressed sequence AA414993	-1.49	0.57	-1	0.9923	1.01	0.9758	-1.27	0.4828	4.73	0.0745
95182_at	1449624_at	---	---	-1.67	0.26	1.4	0.1649	1.4	0.2034	-1.37	0.105	1.66	0.3387
95215_f_at	1437666_x_at	Ubc	ubiquitin C	1.14	0.56	1.06	0.6121	-1.16	0.3106	1.05	0.652	1.05	0.7679
95228_f_at	1421791_at	Ang3	angiogenin, ribonuclease A family, member	1.11	0.78	1.01	0.9655	1.09	0.3488	1.09	0.3856	1.74	0.0414
95232_at	1424101_at	Hnrpl	heterogeneous nuclear ribonucleoprotein L	-1.37	0.26	1.01	0.9402	1.04	0.8322	-1.02	0.8306	-1.15	0.3226
95244_at	1421251_at	Zfp40	zinc finger protein 40	1.49	0.3	1.04	0.6991	1.12	0.2401	-1.04	0.7163	1.01	0.9722
95248_at	1420168_at	AA517841	expressed sequence AA517841	-2.28	0.26	1.21	0.0877	1.25	0.2057	1.08	0.5484	-1.21	0.5937
95249_at	1420121_at	AA517858	expressed sequence AA517858	-1.36	0.17	-1.09	0.6283	1.23	0.5883	1.47	0.4181	-1.67	0.4642
95275_at	1421570_at	Il9r	interleukin 9 receptor	-1.16	0.51	1.06	0.6681	-1.11	0.2374	-1.12	0.2558	1.6	0.0597
95282_at	1438902_a_at	Hspca	heat shock protein 1, alpha	1.35	0.31	-1.34	0.0162	-1.11	0.261	-1.55	0.0001	-1.19	0.1724
95283_at	1450109_s_at	Abcc2	ATP-binding cassette, sub-family C (CFTR/M	1.53	0.14	1.27	0.0357	1.09	0.5992	1.65	0.0006	-1.34	0.3529
95284_at	1419907_s_at	BB219290	expressed sequence BB219290	-1.62	0.03	-1.07	0.6925	-1.01	0.9227	-1.03	0.8101	2.01	0.0068
95285_at	1425058_at	Zfp472	zinc finger protein 472	-1.35	0.13	-1.24	0.2817	-1.35	0.1757	-1.1	0.5299	-1.38	0.2584

95286_at	1418626_a_at	Clu	clusterin	1.14	0.62	1.22	0.0003	1	0.9943	1.14	0.1428	-1.22	0.2211
95290_at	1418810_at	Crhr1	corticotropin releasing hormone receptor 1	2.31	0.01	-1.07	0.7278	1.08	0.6758	-1.24	0.3017	1.53	0.245
95291_r_at	1419846_at	---	---	-3.85	0.19	1.46	0.2502	1.11	0.7455	-1.07	0.8295	1.22	0.659
95292_at	1421194_at	Itga4	integrin alpha 4	1.01	0.96	1.04	0.8606	1.16	0.3332	1.26	0.2459	-1.09	0.6928
95293_at	1419843_at	---	---	-1.09	0.73	-1.08	0.8025	-1.2	0.5433	-1.2	0.4629	1.27	0.2508
95294_at	1419789_at	---	MKIAA0167 protein	1.33	0.58	1.13	0.6302	-1.01	0.932	1.07	0.6467	1.18	0.4893
95295_s_at	1419538_at	Flt3	FMS-like tyrosine kinase 3	-1.31	0.19	1.03	0.7261	-1.07	0.3483	-1	0.9622	1.29	0.0814
95297_at	1420565_at	Hoxa1	homeo box A1	1.42	0.06	1.1	0.519	-1.01	0.9333	1.02	0.8862	1.37	0.2179
95298_at	1425574_at	Epha3	Eph receptor A3	-1.14	0.86	-1.23	0.3948	-1.12	0.5793	-1.46	0.2331	1.05	0.9295
95300_at	1449924_at	Prg3	proteoglycan 3	-1.95	0.11	1.36	0.3847	1.48	0.1817	1.09	0.7903	1.48	0.2199
95301_at	1449927_at	S100a5	S100 calcium binding protein A5	-1.01	0.98	-1.52	0.0794	-1.13	0.6069	-1.26	0.434	1.3	0.4297
95302_at	1436267_a_at	Frap1	FK506 binding protein 12-rapamycin associa	-1.19	0.08	-1.04	0.5282	1.07	0.5148	-1.02	0.8129	1.13	0.5707
95303_at	1440865_at	Ifitm6	interferon induced transmembrane protein 6	-1.12	0.67	1.18	0.17	-1.02	0.7433	1.2	0.2492	1.18	0.4502
95304_at	1421785_at	Attp	signaling molecule ATTP	-2.54	0.17	-1.19	0.4536	1.3	0.4169	1.18	0.5404	2.51	0.0856
95305_at	1460745_at	---	---	-1.85	0.11	-1.08	0.5046	-1.03	0.786	-1.07	0.5798	1.2	0.2922
95306_at	1451863_at	Uts2r	urotensin 2 receptor	1.21	0.38	-1.05	0.5406	1.05	0.491	-1.03	0.639	1.25	0.1026
95308_at	1418098_at	Adcy4	adenylate cyclase 4	-1.36	0.01	-1.09	0.2605	-1.08	0.4132	-1.26	0.0292	1.57	0.1602
95310_at	1421161_at	Btc	betacellulin, epidermal growth factor family r	-2.01	0.23	-1.36	0.454	-1.87	0.1561	1.44	0.3672	-1.74	0.5342
95312_at	1450832_at	Hoxc5	homeo box C5	-1.63	0.05	-1.21	0.477	1.19	0.4471	-1.33	0.3743	1.69	0.1595
95316_at	1449545_at	Fgf18	fibroblast growth factor 18	1.52	0.36	-1.09	0.8451	1.07	0.873	1.61	0.1946	1.07	0.8788
95317_at	1422023_at	Bsn	bassoon	-1.42	0.62	1	0.9947	1.08	0.6277	1.04	0.8133	1.25	0.2997
95318_at	1460252_s_at	Zfp105	zinc finger protein 105	-1.86	0	-1.2	0.0171	-1.05	0.4844	-1.11	0.144	1.22	0.2686
95319_at	1420799_at	Ntsr1	neurotensin receptor 1	-1.31	0.18	1.15	0.1262	1.22	0.03	1.01	0.9081	1.06	0.746
95320_at	1420788_at	Klrg1	killer cell lectin-like receptor subfamily G, me	-1.85	0.07	1.87	0.1355	1.13	0.6963	1.7	0.2248	-1.26	0.6473
95322_g_at	1427654_a_at	Htr4	5 hydroxytryptamine (serotonin) receptor 4	-1.73	0.02	-1.13	0.2072	-1.05	0.5477	-1.13	0.2739	1.38	0.0242
95324_at	1420402_at	Atp2b2	ATPase, Ca++ transporting, plasma membra	1.15	0.02	1.09	0.7686	1.53	0.0852	-1.02	0.9474	1.34	0.0863
95325_at	1448997_at	Pscd1	pleckstrin homology, Sec7 and coiled-coil do	-1.59	0.42	-1.07	0.4875	-1.25	0.32	-1.49	0.0418	-1.18	0.7137
95326_at	1460717_at	Tspyl1	testis-specific protein, Y-encoded-like 1	-1.86	0.12	-1.05	0.7533	1.04	0.8125	-1.22	0.1397	1.46	0.064
95327_at	1422163_at	Sh3md1	SH3 multiple domains 1	-1.1	0.54	1.15	0.2178	1.15	0.1982	1.05	0.5162	1.35	0.0927
95328_at	1450771_at	Fut9	fucosyltransferase 9	-1.28	0.75	-1.09	0.8092	1.52	0.3316	2.38	0.1572	-1.1	0.8934
95329_at	1450139_at	Ern2	endoplasmic reticulum (ER) to nucleus signa	-2.5	0.14	1.08	0.4155	1.21	0.1811	-1.01	0.8695	1.53	0.1613
95330_at	1423529_at	G6pc2	glucose-6-phosphatase, catalytic, 2	-1.43	0.42	1.27	0.1917	1.26	0.1415	1.26	0.1418	1.03	0.9354
95331_at	1422832_at	Rgr	retinal G protein coupled receptor	-4.68	0.26	1.37	0.3197	1.42	0.0674	2.42	0.0495	1.52	0.3622
95332_at	1422125_at	Htr2b	5-hydroxytryptamine (serotonin) receptor 2B	-1.89	0.49	-1.09	0.699	1.01	0.9805	-1.06	0.7689	-1.43	0.2148
95333_at	1421291_at	Il18rap	interleukin 18 receptor accessory protein	-1.19	0.46	1.42	0.306	1.85	0.037	1.85	0.0569	1.25	0.3832
95334_at	1422329_a_at	Ntrk3	neurotrophic tyrosine kinase, receptor, type	2.2	0.02	1.29	0.3922	-1.11	0.6977	1.21	0.3864	1.03	0.9085
95335_at	1450019_at	Cx3cr1	chemokine (C-X3-C) receptor 1	-1.19	0.45	-1.06	0.53	-1.03	0.7084	-1.22	0.1244	-1.25	0.3646
95336_at	1421572_at	Hif3a	hypoxia inducible factor 3, alpha subunit	-2.34	0.09	1.04	0.5909	1.03	0.7173	-1	0.9987	1.15	0.5098
95339_r_at	1449153_at	Mmp12	matrix metalloproteinase 12	-1.15	0.62	1.8	0.3735	-1.71	0	1.84	0.2715	1.5	0.3784
95340_at	1420575_at	Mt3	metallothionein 3	1.43	0.15	-1.13	0.4274	-1.06	0.665	-1.1	0.5136	1.64	0.0245
95342_at	1427675_at	V1ra2	vomeronasal 1 receptor, A2	-5.09	0	-1.35	0.4902	-1.36	0.5261	-1.62	0.332	-1.02	0.9816
95343_at	1419323_at	Padi1	peptidyl arginine deiminase, type I	-1.68	0.42	1.09	0.656	-1.06	0.5308	-1.11	0.28	1.8	0.2436
95344_at	1422177_at	Il13ra2	interleukin 13 receptor, alpha 2	1.81	0.04	-2.14	0.0286	-1.04	0.889	-1.25	0.4949	1.29	0.5083
95345_at	1423311_s_at	Tpbg	trophoblast glycoprotein	1.22	0.57	1.05	0.7215	1.1	0.6098	-1.44	0.0591	1.12	0.7565
95346_at	1426137_at	Rex2	reduced expression 2	-7.1	0.13	-1.26	0.4866	1.36	0.4508	-1.32	0.4219	-1.4	0.377
95347_at	1422773_at	Myt1	myelin transcription factor 1	-1.18	0.46	1.04	0.8255	-1.12	0.5685	1.63	0.4052	1.64	0.1133
95349_g_at	1419209_at	---	---	2.7	0.02	4.09	0	-1.07	0.6797	4.22	0.002	1.17	0.6301
95352_at	1459932_at	D8Ertd28e	DNA segment, Chr 8, ERATO Doi 28, expre	-1.55	0.31	-1.02	0.8518	1.36	0.3886	1.43	0.1609	1.56	0.2576
95353_at	1439973_at	LOC434427	LOC434427	-1.35	0.01	-1.13	0.5399	1.09	0.6993	-1.16	0.5077	-1.65	0.2601
95356_at	1432466_a_at	Apoe	apolipoprotein E	-1.16	0.57	1.03	0.1011	1.02	0.3182	1.01	0.6502	1.32	0.0477
95358_at	1449404_at	Pip5k2a	phosphatidylinositol-4-phosphate 5-kinase, t	1.11	0.74	-1.05	0.6485	-1.18	0.0121	1.02	0.8792	1.25	0.2983
95359_at	1416364_at	Hspcb	heat shock protein 1, beta	-1.41	0.01	-1.29	0.0028	-1.09	0.3212	-1.45	0.0001	-1.15	0.1729
95360_at	1441986_at	Zcchc6	zinc finger, CCHC domain containing 6	-1.15	0.71	-1.26	0.2326	-1.28	0.1592	-1.34	0.1174	1.07	0.9335

95363_at	1449501_a_at	Gzmm	granzyme M (lymphocyte met-ase 1)	3.31	0.01	1.22	0.4631	-1.17	0.2734	1.01	0.967	1.25	0.6464
95364_at	1449848_at	Gna14	guanine nucleotide binding protein, alpha 14	-1.18	0.6	-5.94	0	-1.75	0.073	-6.66	0	-5.49	0.0009
95368_at	1451753_at	Plxna2	plexin A2	1.37	0.45	-1.46	0.0759	1.02	0.9273	-1.1	0.6516	-1.24	0.2849
95371_g_at	1449637_at	Cdh4	Cadherin 4	-1.97	0.33	1.06	0.8214	-1.21	0.4741	-1.02	0.9425	1.38	0.354
95372_at	1419847_at	AW539964	Protein serine kinase H1	-1.52	0.31	1.17	0.6024	1.68	0.1403	1.18	0.574	1.51	0.436
95373_at	1418770_at	Cd2	CD2 antigen	-1.33	0.16	1.23	0.1944	1.23	0.2765	1.3	0.0747	-1.24	0.6457
95376_at	1427591_at	Clcn1	Chloride channel 1	-1.86	0.24	-1.74	0.1394	1.1	0.8198	1.22	0.6028	1.23	0.1589
95377_at	1439959_at	Fgf11	Fibroblast growth factor 11	1.04	0.92	1.06	0.6742	1.17	0.1866	-1.04	0.7417	1.34	0.4372
95378_at	1448034_at	AI842396	expressed sequence AI842396	1.16	0.68	-1.03	0.6805	1.14	0.2491	1.09	0.4906	-1.15	0.4915
95379_at	1418934_at	Mab21l2	mab-21-like 2 (C. elegans)	-1.39	0.29	-1.26	0.0817	1.02	0.8709	-1.24	0.1353	1.2	0.6756
95380_at	1449991_at	Cd244	CD244 natural killer cell receptor 2B4	-1.52	0.24	1.13	0.4945	1.31	0.1285	1.44	0.1374	1.09	0.7815
95381_at	1425437_a_at	Kcnk7	potassium channel, subfamily K, member 7	1.41	0.36	-1.09	0.3571	-1	0.999	-1.15	0.1608	-1.07	0.6483
95382_at	1459922_at	AI646383	expressed sequence AI646383	1.05	0.92	-1.13	0.4014	1.12	0.3633	1.28	0.1276	-1.17	0.7401
95385_at	1456672_at	AA408556	Expressed sequence AA408556	-1.77	0.01	-1.11	0.1861	1.04	0.4692	-1.08	0.2552	-1.01	0.9578
95386_at	1434690_at	Lycat	lysocardiolipin acyltransferase	1.21	0.48	1.14	0.273	1.08	0.517	1.06	0.6509	1.27	0.5035
95389_at	1451763_at	Cnga1	cyclic nucleotide gated channel alpha 1	-1.48	0.21	-1.01	0.9358	1.02	0.9192	1.34	0.0383	1.48	0.2813
95390_at	1421036_at	Npas2	neuronal PAS domain protein 2	-1.09	0.7	-1.11	0.3671	-1.25	0.095	-1.01	0.9542	-1	1
95391_at	1421774_at	Vax1	ventral anterior homeobox containing gene 1	-1.14	0.72	-1.05	0.6327	-1.09	0.3033	-1.09	0.3555	1.02	0.9644
95392_at	1450238_at	Gcnt2	glucosaminyl (N-acetyl) transferase 2, I-bran	-2.95	0.35	-1.1	0.6937	1.21	0.2955	-1.71	0.053	-2.27	0.1045
95393_at	1425660_at	Btbd3	BTB (POZ) domain containing 3	-1.15	0.29	-1.07	0.2755	-1.09	0.3066	-1.19	0.0397	-1.04	0.7698
95395_at	1426840_at	Ythdf3	YTH domain family 3	1.1	0.44	1.19	0.051	1.15	0.1618	1.15	0.0815	1.12	0.5635
95396_at	1427585_at	---	---	-2.62	0.08	1	0.9919	1.28	0.6686	1.02	0.9575	2.36	0.2795
95397_at	1455447_at	D430019H16Ril	RIKEN cDNA D430019H16 gene	1.03	0.89	-1.3	0.0048	1.08	0.3913	-1.08	0.3755	1.5	0.3764
95398_at	1419849_at	E130014H08Ril	RIKEN cDNA E130014H08 gene	1.1	0.86	1.03	0.8017	1.1	0.2061	1.08	0.5626	1.43	0.2558
95399_at	1419771_at	Dchs2	Dachsous 2 (Drosophila)	1.19	0.75	-2.74	0.017	-1.24	0.5455	-1.46	0.2615	-1.08	0.7734
95401_at	1452164_at	BC038286	cDNA sequence BC038286	-1.3	0.09	1.07	0.4042	-1.01	0.9282	-1.02	0.6995	1.11	0.4013
95404_at	1422792_at	Pafah1b2	platelet-activating factor acetylhydrolase, isc	-1.02	0.92	1.13	0.2723	1.18	0.0805	1.19	0.0323	1.41	0.1971
95405_at	1416181_at	Mesdc2	mesoderm development candiate 2	1.11	0.74	-1.1	0.2294	-1.03	0.8196	-1.21	0.0245	1.13	0.288
95406_at	1424365_at	1810037I17Rik	RIKEN cDNA 1810037I17 gene	-1.13	0.63	-1.12	0.0941	1.08	0.4515	-1.17	0.0484	-1.1	0.359
95407_at	1454638_a_at	Pah	phenylalanine hydroxylase	-1.04	0.47	1.03	0.6609	-1.04	0.5374	1.05	0.3737	1.28	0.068
95408_at	1423206_s_at	2310003F16Rik	RIKEN cDNA 2310003F16 gene	1.16	0.32	1.01	0.8316	1.07	0.3963	1.05	0.284	-1.1	0.3994
95409_at	1415733_a_at	1110019J04Rik	RIKEN cDNA 1110019J04 gene	1.18	0.58	-1.03	0.4511	-1.04	0.5468	-1.07	0.0954	-1	0.9912
95412_at	1416027_at	Pdcd6	programmed cell death 6	1.55	0.04	1.08	0.1236	-1.04	0.738	1.05	0.3592	-1.29	0.0231
95413_at	1449889_a_at	Ociad1	OCIA domain containing 1	-1.12	0.11	-1.02	0.6647	-1.11	0.0833	-1.05	0.2937	1.16	0.2269
95417_at	1426350_at	Mgat2	mannoside acetylglucosaminyltransferase 2	1.28	0.1	-1.12	0.1257	1.25	0.0765	-1.07	0.3952	-1.29	0.3067
95418_at	1423854_a_at	Rasl11b	RAS-like, family 11, member B	-1.05	0.85	1.18	0.1195	-1.02	0.8785	-1.09	0.5278	1.34	0.0605
95419_at	1423702_at	H1f0	H1 histone family, member 0	1.11	0.52	-1.03	0.7036	1	0.9879	-1.06	0.3983	-1.31	0.0054
95420_at	1423706_a_at	---	---	-1.08	0.65	-1.2	0.0836	-1.16	0.0444	-1.14	0.2302	-1.44	0.2121
95423_at	1416497_at	Cai	calcium binding protein, intestinal	-2.75	0.03	-1.21	0.0613	-1.37	0.0561	-1.41	0.0106	-1.76	0.0514
95424_at	1422457_s_at	Sumo3	SMT3 suppressor of mif two 3 homolog 3 (ye	-1.19	0.27	-1.08	0.1651	-1.08	0.1299	-1.17	0.039	-1.36	0.0166
95427_at	1423293_at	Rpa1	replication protein A1	1.53	0.02	1.02	0.8059	-1.23	0.0499	1.17	0.1295	-1.09	0.3994
95428_at	1426213_at	Imp4	IMP4, U3 small nucleolar ribonucleoprotein,	-1.17	0.55	1.1	0.4742	1.04	0.7754	1.1	0.4768	1.05	0.8316
95431_at	1426676_s_at	Tomm70a	translocase of outer mitochondrial membran	-1.06	0.84	-1.01	0.9537	-1.03	0.6889	-1.09	0.3373	-1.42	0.0075
95432_f_at	1426675_at	Tomm70a	translocase of outer mitochondrial membran	1.05	0.86	1.02	0.6652	-1	0.9933	-1.02	0.645	1.04	0.6639
95433_at	1460396_at	Ddx54	DEAD (Asp-Glu-Ala-Asp) box polypeptide 54	1.17	0.24	-1.16	0.0199	1.07	0.3762	-1.05	0.4722	1.04	0.8477
95434_at	1448279_at	Arpc3	actin related protein 2/3 complex, subunit 3	1.55	0.08	1.01	0.8593	-1.59	0.0032	-1.34	0.0227	-1.13	0.3256
95436_at	1417954_at	Sst	somatostatin	2.17	0.14	1.52	0.0032	1.71	0.0296	1.75	0.1481	2.44	0.1105
95437_at	1426912_at	Rfwd2	ring finger and WD repeat domain 2	1.54	0.16	-1.08	0.443	-1.08	0.497	-1.13	0.3454	-1.07	0.5992
95438_at	1423533_a_at	Rhot1	ras homolog gene family, member T1	1.91	0.02	-1.08	0.3246	1.12	0.0669	1.09	0.352	1.14	0.5037
95439_at	1448813_at	Aadac	arylacetamide deacetylase (esterase)	1.1	0.72	-1.28	0.0103	-1.11	0.4165	-1.2	0.0203	-1.47	0.013
95440_at	1423679_at	2810432L12Rik	RIKEN cDNA 2810432L12 gene	-1.46	0.17	-1.03	0.898	-1.28	0.1634	-1.14	0.4303	1.12	0.7579
95441_at	1416485_at	Timm23	translocase of inner mitochondrial membran	1.39	0.13	-1.1	0.0188	-1.06	0.2119	-1.05	0.5673	-1.43	0.0005
95444_at	1423722_at	4930579A11Rik	RIKEN cDNA 4930579A11 gene	-1.33	0.11	1.23	0.0095	1.18	0.1174	1.49	0.0017	-1.27	0.0086

95445_at	1448100_at	4833439L19Rik	RIKEN cDNA 4833439L19 gene	1.18	0.23	-1.18	0.0401	1.03	0.6554	-1.14	0.0895	-1.27	0.0581
95446_at	1425194_a_at	6330577E15Rik	RIKEN cDNA 6330577E15 gene	1.12	0.61	-1.02	0.5962	1.24	0.0001	1.1	0.0719	-1.2	0.1338
95447_at	1450067_a_at	1810034K20Rik	RIKEN cDNA 1810034K20 gene	1.74	0.03	-1.05	0.4989	-1.03	0.7352	-1.1	0.1898	-1.15	0.1799
95448_at	1426611_at	Psmc2	proteasome (prosome, macropain) 26S subu	1.32	0.35	-1	0.9557	1.02	0.7699	1.03	0.5284	-1.84	0.001
95449_at	1451523_a_at	2310075G12Rik	RIKEN cDNA 2310075G12 gene	-1.3	0.25	-1.28	0.0029	-1.28	0.0037	-1.38	0.0006	-1.29	0.1117
95451_at	1416445_at	2810405J04Rik	RIKEN cDNA 2810405J04 gene	1.52	0.23	-1.14	0.1309	1.04	0.6595	-1.13	0.1508	-1.28	0.121
95453_f_at	1419814_s_at	S100a1	S100 calcium binding protein A1	1.56	0.25	1.32	0.0001	1.06	0.3154	1.38	0.0013	-1.11	0.5107
95456_r_at	1418574_a_at	Shfdg1	split hand/foot deleted gene 1	1.05	0.88	-1.1	0.1515	1.02	0.7835	-1.12	0.0293	-1.27	0.141
95457_at	1437290_at	1110001C20Rik	RIKEN cDNA 1110001C20 gene	1.14	0.52	1.15	0.0002	1.18	0.0581	1.25	0.0031	-1.19	0.1769
95458_s_at	1437288_at	1110001C20Rik	RIKEN cDNA 1110001C20 gene	-1	1	1.01	0.9552	-1.59	0.0025	-1.35	0.0504	-1.23	0.032
95460_at	1460171_at	Cops5	COP9 (constitutive photomorphogenic) hom	1.43	0.01	1.07	0.304	-1.09	0.3031	1.05	0.5321	-1.28	0.0567
95465_s_at	1417611_at	Tmem37	transmembrane protein 37	-1.36	0.42	-1.51	0.0002	-1.38	0.0059	-1.7	0	-1.59	0.0329
95467_at	1416267_at	Scoc	short coiled-coil protein	1.28	0.25	-1.1	0.2467	-1.03	0.7412	1.14	0.1388	1.53	0.0542
95468_at	1423785_at	Egln1	EGL nine homolog 1 (C. elegans)	1.43	0.18	1.04	0.5837	-1.04	0.6733	-1.05	0.6098	-1.37	0.1381
95469_at	1417987_at	Btd	biotinidase	-1.38	0.04	-1.38	0.0078	-1.12	0.3266	-1.58	0.0001	-1.22	0.082
95470_at	1423766_at	Pak1ip1	PAK1 interacting protein 1	2.26	0.05	1.31	0.005	1.19	0.2407	1.31	0.0064	-1.04	0.894
95471_at	1417649_at	Cdkn1c	cyclin-dependent kinase inhibitor 1C (P57)	3.04	0	1.51	0.0162	1.41	0.0224	1.78	0.0028	2.49	0.001
95472_f_at	1416337_at	Uqcrb	ubiquinol-cytochrome c reductase binding pr	1.45	0.04	-1.01	0.8942	1.15	0.2188	1.02	0.7322	1.01	0.9631
95474_at	1437308_s_at	F2r	coagulation factor II (thrombin) receptor	1.25	0.58	1.31	0.0012	-1.11	0.0053	1.44	0.0138	1.12	0.4272
95478_at	1427955_a_at	Deb1	differentially expressed in B16F10 1	1.51	0.08	-1.03	0.6146	1.14	0.2343	1.01	0.8677	-1.13	0.4461
95479_at	1448505_at	MGI:1927354	nuclear DNA binding protein	1.68	0.35	1.12	0.5159	-1.06	0.7308	1.14	0.391	-1.54	0.0289
95480_at	1428621_a_at	D11Wsu68e	DNA segment, Chr 11, Wayne State Univers	-1.04	0.72	-1.07	0.189	-1.02	0.7233	-1.02	0.7355	-1.29	0.4075
95482_at	1419920_s_at	Usp7	ubiquitin specific protease 7	1.27	0.18	1.18	0.0085	1.13	0.041	1.14	0.0066	1.19	0.1301
95485_at	1460184_at	Hadhsc	L-3-hydroxyacyl-Coenzyme A dehydrogenas	1.4	0.08	1.01	0.8472	1	0.9937	-1.04	0.4084	-1.01	0.8607
95486_at	1420056_s_at	Ptdsr	phosphatidylserine receptor	1.48	0.05	-1.26	0.0013	-1.04	0.6139	-1.24	0.0042	1.06	0.6191
95489_at	1416044_at	Fliih	flightless I homolog (Drosophila)	1.6	0.11	1.15	0.0041	1.16	0.0061	1.15	0.0237	1.04	0.8339
95490_at	1460211_a_at	Kdelr1	KDEL (Lys-Asp-Glu-Leu) endoplasmic reticu	-1.14	0.35	-1.04	0.3357	-1.11	0.0452	-1.26	0	-1.12	0.1128
95491_at	1456194_a_at	Park7	Parkinson disease (autosomal recessive, ea	1.68	0.05	-1.04	0.4228	-1.09	0.2132	-1.02	0.804	-1.12	0.5817
95493_at	1448590_at	Col6a1	procollagen, type VI, alpha 1	-1.27	0.11	-1.1	0.3033	-1.01	0.8811	-1	0.9885	1.43	0.0075
95496_at	1428182_at	Prpsap1	phosphoribosyl pyrophosphate synthetase-a	1.4	0.1	1.03	0.6763	1.21	0.0907	1.41	0	-1.72	0.0743
95497_at	1426612_at	MGI:1921571	timeless interacting protein	4.51	0.03	1.42	0.0288	1.4	0.14	1.4	0.0464	1.12	0.6352
95501_at	1424564_at	2410001C21Rik	RIKEN cDNA 2410001C21 gene	1.3	0.14	1.1	0.3144	1	0.9902	1.18	0.0286	-1.37	0.0073
95502_at	1423507_a_at	Sirt2	sirtuin 2 (silent mating type information regul	1.29	0.44	-1.1	0.2116	-1.15	0.1501	-1.09	0.1978	-1.04	0.7535
95503_at	1416313_at	AI839562	expressed sequence AI839562	-2.66	0.15	-1.03	0.897	-1.03	0.8645	-1.22	0.3187	1.2	0.7576
95505_at	1448848_at	---	---	1.54	0.15	-1	0.9334	1.07	0.1792	1.05	0.5124	1.14	0.2609
95506_at	1419649_s_at	Myo1c	myosin IC	1.69	0.35	1.39	0.3162	2.14	0.0104	1.93	0.0081	2.59	0.121
95507_at	1416052_at	Prps1	phosphoribosyl pyrophosphate synthetase 1	1.19	0.46	1.04	0.5421	1.01	0.823	-1.04	0.4377	-1.04	0.4904
95508_at	1452196_a_at	Nckap1	NCK-associated protein 1	1.12	0.54	1	0.9748	1.07	0.3108	1.04	0.6181	-1.12	0.306
95509_at	1451228_a_at	Sycn	syncollin	-1.09	0.89	1.52	0.2139	1.2	0.227	1.28	0.1965	1.11	0.7837
95511_at	1422445_at	Itga6	integrin alpha 6	1.5	0.6	-1.23	0.5666	1.07	0.8237	-1.05	0.873	1.24	0.68
95513_at	1415774_at	Statip1	signal transducer and activator of transcriptio	1.89	0.13	1	0.949	1.1	0.4325	1.15	0.1263	1.04	0.8453
95514_at	1426897_at	2610510H01Rik	RIKEN cDNA 2610510H01 gene	1.86	0.29	-1.01	0.9501	1.14	0.4908	1.02	0.9224	-1.24	0.3068
95516_at	1448391_at	Rab9	RAB9, member RAS oncogene family	-1.09	0.53	1.09	0.2148	1.29	0.002	1.1	0.195	-1.29	0.0864
95518_at	1424683_at	1810015C04Rik	RIKEN cDNA 1810015C04 gene	1.61	0.07	1.3	0.0047	1.33	0.0889	1.63	0	2.9	0.0002
95520_at	1417162_at	2310061B02Rik	RIKEN cDNA 2310061B02 gene	1.55	0.02	1.1	0.327	1.02	0.8172	1.03	0.7598	1.01	0.9245
95522_i_at	1448760_at	Zfp68	zinc finger protein 68	1.5	0.16	1.33	0.0086	1.28	0.1165	1.4	0.0037	1.41	0.0784
95523_at	1428333_at	6530401D17Rik	RIKEN cDNA 6530401D17 gene	2.73	0	-1.42	0.0069	-1.14	0.4045	-1.11	0.316	-1.11	0.691
95525_at	1423374_at	Ncoa6	nuclear receptor coactivator 6	1.21	0.16	1.25	0.0031	1.22	0.126	1.22	0.0173	1.57	0.2365
95526_at	1415682_at	Xpo7	exportin 7	1.12	0.17	1.2	0.0082	1.08	0.2832	1.11	0.0207	-1.26	0.0352
95528_at	1419840_at	AI507495	expressed sequence AI507495	-1.77	0	-1.09	0.4661	-1.01	0.9231	-1.04	0.755	1.59	0.3341
95530_at	1454631_at	6330549H03Rik	RIKEN cDNA 6330549H03 gene	-1.1	0.68	1.09	0.2204	1	0.9964	1.08	0.1183	1.05	0.4932
95531_at	1454890_at	Amot	angiomotin	1.13	0.5	1.17	0.1851	1.28	0.0307	1.64	0.0004	1.34	0.0248
95532_at	1439771_s_at	LOC236374	similar to 2-cell-stage, variable group, memb	1.02	0.88	-1.32	0.0956	-1.36	0.0541	-1.31	0.0746	2.61	0.1165

95533_at	1425332_at	Zfp106	zinc finger protein 106	1.93	0.08	1.01	0.9347	1.07	0.7038	1.05	0.7559	1.27	0.2048
95536_at	1422591_at	Tceb3	transcription elongation factor B (SIII), polyp	1.25	0.16	-1.09	0.4064	-1.01	0.9169	-1.46	0.0018	1.42	0.0142
95537_at	1417846_at	Ulk2	Unc-51 like kinase 2 (C. elegans)	1.2	0.32	-1.2	0.0007	-1.01	0.933	-1.29	0.0016	-1.53	0.069
95538_at	1452044_at	Arpc5l	actin related protein 2/3 complex, subunit 5-l	1.07	0.43	-1.08	0.2183	-1.07	0.3016	-1.11	0.2377	1.15	0.1188
95539_at	1434306_at	Rab3ip	RAB3A interacting protein	1.33	0.11	1.1	0.2955	1.07	0.3883	1.22	0.0359	1.25	0.0084
95541_at	1417953_at	D6Wsu176e	DNA segment, Chr 6, Wayne State Universit	-1.29	0.13	-1.1	0.3148	-1.12	0.4233	-1.07	0.4024	-1.37	0.1434
95542_at	1433883_at	Tpm4	tropomyosin 4	1	1	1.06	0.7261	1.04	0.6311	1.05	0.7676	1.55	0.043
95544_at	1438017_at	Rusc1	RUN and SH3 domain containing 1	1.41	0.23	1.15	0.1889	1.19	0.1107	1.36	0.0066	1.51	0.1792
95546_g_at	1452014_a_at	Igf1	insulin-like growth factor 1	-15.55	0	-9.09	0	-1.07	0.3507	-17.36	0	-14.75	0
95547_at	1419061_at	Rhod	ras homolog gene family, member D	1.11	0.56	1.05	0.5314	-1.05	0.6335	1.04	0.5716	1.16	0.0625
95549_at	1418036_at	Prim2	DNA primase, p58 subunit	1.06	0.84	2.62	0.0336	2.32	0.0268	2.62	0.0271	1.42	0.2712
95550_at	1427467_a_at	Rpgr	retinitis pigmentosa GTPase regulator	1.23	0.62	-1.06	0.9009	-1.94	0.0977	1.34	0.4541	-1.78	0.0757
95551_at	1452736_at	1700020M16Rik	RIKEN cDNA 1700020M16 gene	1.24	0.49	-1.11	0.3341	-1.15	0.1558	-1.07	0.5325	1.05	0.7019
95552_at	1417991_at	Dio1	deiodinase, iodothyronine, type I	1.65	0.33	-11.22	0	-6.26	0	-11.55	0	-10.51	0.0005
95555_at	1418123_at	Unc119	unc-119 homolog (C. elegans)	-1.22	0.37	-1.03	0.7276	1.14	0.0407	1.07	0.3312	1.13	0.5367
95556_at	1423492_at	Mrpl45	mitochondrial ribosomal protein L45	4.52	0.05	-1.04	0.7969	-1.29	0.1913	-1.24	0.2084	2.15	0.047
95557_at	1426238_at	Bmp1	bone morphogenetic protein 1	-1.34	0.19	-1.25	0.055	-1.09	0.3726	-1.36	0.0045	-1.34	0.1428
95559_at	1426766_at	6330403K07Rik	RIKEN cDNA 6330403K07 gene	1.74	0.27	1.17	0.1772	1.09	0.524	1.03	0.6998	1.02	0.9277
95561_at	1429270_a_at	1700013H19Rik	RIKEN cDNA 1700013H19 gene	1.89	0.07	1.57	0.1301	1.44	0.2146	1.64	0.0235	1.04	0.9276
95562_at	1429483_at	Ndp52	nuclear domain 10 protein 52	1.01	0.92	1.28	0.1724	1.04	0.7584	1.15	0.2016	1.44	0.2157
95563_at	1427188_at	Arih1	ariadne ubiquitin-conjugating enzyme E2 bir	1.54	0.3	1.01	0.9052	-1.07	0.3283	-1.1	0.2238	-1.16	0.102
95565_at	1415720_s_at	Mad2l1bp	MAD2L1 binding protein	-1.03	0.8	1.07	0.5552	1.13	0.3207	-1.02	0.8808	1.13	0.2975
95566_at	1418050_at	Gpld1	glycosylphosphatidylinositol specific phosph	1.01	0.97	1.07	0.1905	1	0.9693	1.08	0.1355	1.38	0.0426
95567_at	1452884_at	Sfrs2ip	splicing factor, arginine/serine-rich 2, interac	2.2	0.36	1.57	0.0804	1.71	0.0383	1.76	0.0387	1.04	0.8248
95568_at	1454074_a_at	1500011J06Rik	RIKEN cDNA 1500011J06 gene	1.19	0.43	1.39	0.0574	1.33	0.2082	1.38	0.0695	1.11	0.8223
95569_at	1460233_at	Guca2b	guanylate cyclase activator 2b (retina)	1.01	0.91	2.14	0.3412	1.74	0.2598	4.54	0.145	1.11	0.4747
95571_at	1418843_at	Slc30a4	solute carrier family 30 (zinc transporter), me	1.57	0.09	1.05	0.632	1.06	0.5438	1.06	0.5067	1.07	0.8155
95573_at	1438192_s_at	Baz2a	bromodomain adjacent to zinc finger domain	-1.08	0.69	1.11	0.1734	1.06	0.3656	1.24	0.0003	1.18	0.2512
95577_at	1452122_at	AI314180	expressed sequence AI314180	1.46	0.14	1.18	0.036	1.2	0.0421	1.3	0.0005	1.04	0.7063
95580_at	1424385_at	5830417I10Rik	RIKEN cDNA 5830417I10 gene	1.68	0.14	1.05	0.5964	1.18	0.0746	1.28	0.0121	1.41	0.2138
95584_at	1453223_s_at	Dppa2	developmental pluripotency associated 2	5.41	0	1.04	0.8858	-1.16	0.6631	1.11	0.7154	1.11	0.8243
95585_at	1418845_at	Proc	protein C	1.04	0.8	1.02	0.7298	1.01	0.7966	-1.01	0.7499	-1.08	0.6523
95587_at	1433628_at	Gm1	gene model 1, (NCBI)	-1.07	0.63	-1.2	0.0141	1.02	0.81	-1.09	0.2815	1	0.9693
95588_at	1417208_at	Amacr	alpha-methylacyl-CoA racemase	-1.95	0	1.35	0.0589	1.34	0.0423	1.65	0.0022	-1.41	0.0832
95590_at	1424160_at	Alg5	asparagine-linked glycosylation 5 homolog (	1.55	0.13	1.07	0.2396	1.1	0.2622	1.13	0.2375	1.08	0.4957
95591_at	1434703_at	Extl3	exostoses (multiple)-like 3	1.71	0.24	1.06	0.2531	1.01	0.9366	-1.04	0.448	1.02	0.9521
95592_at	1428874_at	1110019N10Rik	RIKEN cDNA 1110019N10 gene	-1.07	0.68	1.03	0.6272	1.16	0.0775	-1.05	0.5618	1.13	0.2376
95593_at	1415698_at	Golph2	golgi phosphoprotein 2	-1.07	0.73	-1.06	0.4965	1.12	0.2935	1.04	0.7575	1.28	0.227
95594_at	1449697_s_at	Mfn1	mitofusin 1	1.64	0.09	1.05	0.5194	1.19	0.0655	1.27	0.0003	1.14	0.3411
95596_at	1428353_at	1110054H05Rik	RIKEN cDNA 1110054H05 gene	1.46	0.3	1.01	0.9591	1.27	0.0894	-1.08	0.5929	-1.25	0.5578
95597_at	1436448_a_at	Ptgs1	prostaglandin-endoperoxide synthase 1	1.13	0.57	1.22	0.4633	1.13	0.4589	1.3	0.1847	1.28	0.1341
95600_at	1449119_at	Arih2	ariadne homolog 2 (Drosophila)	-1.4	0.52	1.18	0.0169	1.12	0.3699	1.16	0.0637	-1.56	0.5359
95601_at	1419385_a_at	Ubqln1	ubiquilin 1	-1.08	0.46	-1.05	0.6156	-1.01	0.9246	-1.26	0.0239	-1.4	0.0982
95602_at	1460720_at	Trpc4ap	transient receptor potential cation channel, s	-1.27	0.21	-1.04	0.4378	-1.11	0.0844	-1.07	0.1606	-1.02	0.6944
95603_at	1416049_at	Gldc	glycine decarboxylase	1.11	0.77	1.27	0.005	1.07	0.4087	1.25	0.0063	-1.27	0.2242
95604_at	1433618_at	C330006A16Rik	RIKEN cDNA C330006A16 gene	-1.24	0.15	-1.25	0.0021	-1.26	0.0035	-1.26	0.0179	1.07	0.6398
95606_at	1422768_at	Syncrip	synaptotagmin binding, cytoplasmic RNA int	-1.45	0.21	-1.12	0.7325	1.26	0.4001	1.33	0.2193	-3	0.0032
95607_at	1417405_at	Stard3	START domain containing 3	-1.11	0.59	-1.06	0.1553	-1.02	0.6959	-1.09	0.041	1.14	0.1126
95608_at	1417492_at	Ctsb	cathepsin B	1.89	0.02	1.35	0.0077	1.07	0.6483	1.41	0.0065	1.23	0.3259
95609_at	1426798_a_at	---	---	1.36	0.37	1.04	0.8011	1.17	0.1977	-1.04	0.7027	1.05	0.7874
95610_at	1428092_at	Cdc5l	cell division cycle 5-like (S. pombe)	-2.94	0.21	1.11	0.2664	-1.02	0.8764	-1.03	0.8626	1.06	0.7787
95611_at	1415904_at	Lpl	lipoprotein lipase	2.85	0.08	3.79	0.0014	-1.5	0.0992	7.05	0	11.79	0.0002
95612_at	1452917_at	Rfc5	replication factor C (activator 1) 5	1.12	0.58	1.08	0.3453	-1.04	0.6061	1.03	0.6169	-1.35	0.0812

95613_at	1448339_at	D9Wsu20e	DNA segment, Chr 9, Wayne State Universi	-1.46	0.04	1.05	0.4497	-1.02	0.7242	-1.08	0.1686	-1.57	0.0006
95614_at	1454636_at	Cbx5	chromobox homolog 5 (Drosophila HP1a)	-1.15	0.76	1.24	0.0018	-1.12	0.2048	1.2	0.0779	1.26	0.5871
95616_at	1452064_at	Crsp3	cofactor required for Sp1 transcriptional acti	1.17	0.25	1.19	0.074	1.17	0.3079	1.2	0.0502	1.2	0.2207
95617_at	1425981_a_at	Rbl2	retinoblastoma-like 2	1.76	0.16	1.2	0.112	1.11	0.5441	1.28	0.0411	1.16	0.441
95619_at	1429122_a_at	1700040I03Rik	RIKEN cDNA 1700040I03 gene	1.44	0.03	1.08	0.4921	1.09	0.4393	1.05	0.7017	-1.02	0.8553
95620_at	1426440_at	Dhrs7	dehydrogenase/reductase (SDR family) mer	2.13	0.02	-1.16	0.004	-1.24	0.0016	-1.17	0.0594	1.13	0.1568
95621_at	1426284_at	Krt20	keratin 20	4.56	0.04	1.35	0.3148	1.62	0.3191	1.17	0.5857	1.49	0.1546
95622_at	1460678_at	Klhdc2	kelch domain containing 2	1.47	0.01	-1.05	0.2805	1.09	0.1777	1.14	0.0419	1.16	0.0605
95625_at	1417116_at	Slc6a8	solute carrier family 6 (neurotransmitter tran	3.94	0.05	-1	0.9987	1.34	0.1158	1.47	0.1461	2.08	0.0044
95626_at	1422532_at	---	---	1.24	0.02	1.05	0.567	1.11	0.2779	1.1	0.2438	-1.34	0.3447
95627_at	1437155_a_at	Wwtr1	WW domain containing transcription regulat	2.04	0.25	1.03	0.798	-1.15	0.324	1.09	0.5442	-1.08	0.7379
95628_at	1422944_a_at	Diap3	diaphanous homolog 3 (Drosophila)	-1.32	0.71	1.02	0.9288	1.06	0.7689	1.51	0.0118	-1.52	0.4979
95629_at	1436019_a_at	---	---	1.21	0.05	-1.21	0.0004	-1.17	0.001	-1.15	0.0127	-1.55	0.0391
95630_at	1448522_at	Sars2	seryl-aminoacyl-tRNA synthetase 2	-1.2	0.36	-1.1	0.3637	-1.03	0.7438	-1.1	0.2061	1.2	0.0331
95631_at	1460288_a_at	Ppp4c	protein phosphatase 4, catalytic subunit	-1.11	0.77	-1.09	0.276	-1.16	0.1498	-1.12	0.1737	1.22	0.095
95635_g_at	1429252_at	0610010K14Rik	RIKEN cDNA 0610010K14 gene	1.1	0.57	-1.04	0.7562	-1.18	0.1859	-1.02	0.8772	3.93	0.0615
95636_at	1428679_s_at	0610010K14Rik	RIKEN cDNA 0610010K14 gene	1.29	0.26	-1.11	0.2273	-1.15	0.2341	-1.02	0.7989	-1.26	0.5061
95637_at	1426750_at	Flnb	filamin, beta	1.43	0.15	1.04	0.7171	1.14	0.2277	1.17	0.0544	1.53	0.1084
95639_at	1449532_at	Chrng	cholinergic receptor, nicotinic, gamma polyp	-1.03	0.85	1.64	0.1763	1.29	0.3631	1.81	0.0866	1.61	0.2541
95641_at	1452590_a_at	MGC41689	hypothetical LOC211623	1.92	0.04	-1.01	0.9766	1.09	0.7888	-1.2	0.5918	1.8	0.3726
95643_at	1415770_at	Wdr6	WD repeat domain 6	2.58	0.01	1.05	0.6934	1.67	0.0005	1.3	0.0587	2.05	0.0468
95646_at	1416772_at	Cpt2	carnitine palmitoyltransferase 2	1.17	0.41	-1.05	0.4781	-1.14	0.0765	-1.22	0.001	-1.6	0.0031
95648_at	1423782_at	Mobk1b	MOB1, Mps One Binder kinase activator-like	-1.28	0.35	1.21	0.0027	1.08	0.1328	1.3	0.0036	1.13	0.1906
95649_at	1424170_at	Phf5a	PHD finger protein 5A	1.22	0.12	1.04	0.5968	1.19	0.0603	1.1	0.2136	-1.06	0.4227
95650_at	1416972_at	Nhp2l1	NHP2 non-histone chromosome protein 2-lik	-1.29	0.27	-1.08	0.2393	-1.13	0.0231	-1.15	0.0246	1.13	0.2104
95652_at	1455749_x_at	---	---	-1.12	0.61	-1	0.9431	-1.04	0.2392	-1.06	0.2091	1.2	0.1045
95653_at	1423764_s_at	Mrpl37	mitochondrial ribosomal protein L37	1.4	0.05	-1.02	0.7522	1.09	0.19	-1.03	0.6212	1.03	0.783
95655_at	1452203_at	5830411E10Rik	RIKEN cDNA 5830411E10 gene	1.38	0.28	1.14	0.1958	1.13	0.4639	1.26	0.1064	-1.11	0.7875
95658_at	1424121_at	Commd1	COMM domain containing 1	1.8	0.07	1.05	0.6319	1.05	0.6169	1.01	0.8612	-1.01	0.9546
95659_at	1416569_at	Actl6a	actin-like 6A	-1.1	0.63	1.02	0.7744	1.05	0.5004	1.09	0.1629	-1.33	0.0742
95660_at	1417203_at	Ethe1	ethylmalonic encephalopathy 1	-1.11	0.68	-1.1	0.4272	-1.02	0.79	-1.09	0.4111	-1.07	0.8023
95661_at	1416066_at	Cd9	CD9 antigen	-1.1	0.39	-1.17	0.0693	-1.05	0.6194	-1.12	0.0836	-1.27	0.2391
95662_at	1417241_at	X83328	EST X83328	1.45	0.19	-1.05	0.4234	1.03	0.7315	-1.04	0.5984	1.04	0.8428
95665_at	1453412_a_at	Sec14l1	SEC14-like 1 (S. cerevisiae)	1.19	0.76	1.08	0.6162	1.01	0.9722	1.06	0.4213	1.04	0.9387
95666_at	1415934_at	Cops8	COP9 (constitutive photomorphogenic) hom	1.25	0.16	-1.01	0.9199	1.03	0.711	-1.06	0.3681	-1.02	0.7436
95670_at	1423280_at	Stmn2	stathmin-like 2	-1.17	0.64	1.01	0.9656	1.07	0.6794	-1.05	0.7923	1.71	0.3641
95671_at	1415999_at	Hey1	hairy/enhancer-of-split related with YRPW m	-1.14	0.55	-1.12	0.661	-1.12	0.6867	-1.32	0.2977	1.22	0.4229
95674_r_at	1428572_at	Basp1	brain abundant, membrane attached signal p	-1.52	0.01	1.02	0.7021	-1.17	0.0303	-1.11	0.107	1.15	0.1669
95675_at	1426759_at	Map4k3	mitogen-activated protein kinase kinase kina	1.25	0.15	1.15	0.0165	1.24	0.0283	1.17	0.0309	1.05	0.6611
95677_at	1452713_a_at	0610009C03Rik	RIKEN cDNA 0610009C03 gene	1.25	0.46	1.16	0.0932	1.06	0.3545	1.07	0.4082	1.26	0.3828
95679_at	1417161_at	D19Ertd144e	DNA segment, Chr 19, ERATO Doi 144, exp	1.27	0.47	1.17	0.0085	-1.02	0.775	1.08	0.2524	1.18	0.2958
95680_at	1417342_at	---	---	1.38	0.51	-1.02	0.8837	-1.1	0.3604	-1.17	0.0882	-1.32	0.2458
95681_f_at	1417341_a_at	Ppp1r2	protein phosphatase 1, regulatory (inhibitor)	1.35	0.34	1.07	0.8137	1.45	0.1757	1.58	0.0412	1.29	0.6951
95683_g_at	1415735_at	Ddb1	damage specific DNA binding protein 1	-1.01	0.97	1	0.9809	1.02	0.9015	1.11	0.1442	-1.04	0.8309
95685_at	1416890_at	5730436H21Rik	RIKEN cDNA 5730436H21 gene	2.64	0.28	1.25	0.14	1.25	0.0511	1.18	0.264	1.77	0.1156
95688_at	1423346_at	Degs1	degenerative spermatocyte homolog 1 (Dros	1.35	0.2	1.14	0.056	1.11	0.3206	1.14	0.0033	1.01	0.9423
95690_at	1428068_at	1110030L07Rik	RIKEN cDNA 1110030L07 gene	1.27	0.11	1.02	0.5974	1.22	0.0143	1.25	0.0038	-1.02	0.7939
95692_at	1433708_at	Srp68	signal recognition particle 68	1.16	0.37	-1.12	0.069	-1.11	0.2546	-1.1	0.1151	-1.06	0.6223
95693_at	1450048_a_at	Idh2	isocitrate dehydrogenase 2 (NADP+), mitoch	1.97	0	1.61	0.0018	1.43	0.0065	1.69	0.0002	3.05	0
95694_at	1423474_at	Top1	topoisomerase (DNA) I	-1.22	0.26	-1.05	0.6763	1.03	0.8319	-1.12	0.3957	-1.13	0.5762
95695_at	1423109_s_at	Slc25a20	solute carrier family 25 (mitochondrial carniti	1.11	0.47	1.05	0.6178	1.13	0.1258	1.11	0.1577	-1.4	0.0169
95697_at	1451470_s_at	Eif5a	eukaryotic translation initiation factor 5A	-1	0.99	-1.22	0.3234	1.14	0.315	-1.16	0.3235	-1.09	0.814
95698_at	1448331_at	Ndufb7	NADH dehydrogenase (ubiquinone) 1 beta s	1.08	0.33	-1.04	0.3666	-1	0.9715	1.03	0.5052	-1.02	0.948

95700_r_at	1452683_at	Dnajc8	DnaJ (Hsp40) homolog, subfamily C, membe	1.03	0.87	1.54	0.0012	1.4	0.0151	1.73	0.0007	-1.03	0.8896
95701_at	1448960_at	Cxxc5	CXXC finger 5	1.47	0.27	1.24	0.0027	1.07	0.5044	1.16	0.1364	1.15	0.3657
95702_at	1426342_at	1300006C19Rik	RIKEN cDNA 1300006C19 gene	1.08	0.65	1.18	0.076	1.11	0.3118	1.13	0.1104	-1.21	0.3893
95703_at	1416443_a_at	Uble1a	ubiquitin-like 1 (sentrin) activating enzyme E	1.94	0.01	-1.03	0.5427	-1.03	0.7374	-1.08	0.083	-1.17	0.1507
95704_at	1416279_at	Ap1b1	adaptor protein complex AP-1, beta 1 subun	-1.11	0.24	-1.09	0.2815	-1.09	0.2473	-1.07	0.2888	1.05	0.5299
95705_s_at	1419734_at	Actb	actin, beta, cytoplasmic	-1.14	0.49	1.1	0.4477	-1.33	0.0244	-1.02	0.8649	1.15	0.7102
95706_at	1426808_at	Lgals3	lectin, galactose binding, soluble 3	2.57	0.1	2.96	0.2998	-1.11	0.6411	4.42	0.2007	-1.14	0.8003
95707_at	1448685_at	2900010M23Rik	RIKEN cDNA 2900010M23 gene	1.24	0.34	-1.13	0.169	-1.11	0.2119	-1.16	0.0925	-1	0.9571
95708_at	1415827_a_at	D3Ucla1	DNA segment, Chr 3, University of California	-1.75	0	-1.18	0.0089	-1.08	0.0517	-1.08	0.0394	-1.98	0
95709_at	1452770_at	Vkorc1	vitamin K epoxide reductase complex, subur	-1.11	0.18	-1.16	0.0074	1.11	0.0438	-1.19	0.0102	-1.01	0.9406
95712_at	1417037_at	Orc6l	origin recognition complex, subunit 6-like (S.	1.95	0.08	1.15	0.3257	1.05	0.7836	-1.05	0.7558	1.24	0.5439
95713_at	1416883_at	Clptm1	cleft lip and palate associated transmembrar	-1.34	0.12	1.04	0.5605	1.15	0.1488	1.12	0.1106	-1.15	0.2533
95714_at	1436681_x_at	0610009D07Rik	RIKEN cDNA 0610009D07 gene	1.01	0.91	1.04	0.5333	-1.01	0.9629	1.03	0.5965	-1.34	0.0209
95715_at	1452674_a_at	Eif3s12	eukaryotic translation initiation factor 3, subu	1.19	0.16	-1.04	0.2888	1.13	0.0538	1.03	0.4918	1.16	0.0697
95716_at	1450012_x_at	Ywhag	3-monooxygenase/tryptophan 5-monooxyge	1.23	0.02	-1.21	0.0836	-1.32	0.0174	-1.02	0.8564	1.1	0.5684
95717_at	1426643_at	Elp3	elongation protein 3 homolog (S. cerevisiae)	1.34	0.18	-1.1	0.257	-1.01	0.9197	1.05	0.4405	-1.78	0.1142
95718_f_at	1448179_at	Usmg5	upregulated during skeletal muscle growth 5	1.03	0.87	-1.01	0.8339	1.14	0.1636	1.11	0.275	-1.33	0.0745
95721_at	1426648_at	Mapkapk2	MAP kinase-activated protein kinase 2	-1.09	0.64	1.29	0.0055	-1.08	0.5537	1.21	0.1936	1.2	0.0841
95722_at	1416592_at	Glrx1	glutaredoxin 1 (thioltransferase)	1.07	0.61	1.01	0.8451	1.23	0.0068	1.32	0	1.02	0.8813
95723_r_at	1428708_x_at	2610009E16Rik	RIKEN cDNA 2610009E16 gene	-1.63	0.09	-1.12	0.0447	-1.16	0.1102	-1.25	0	-1.08	0.3616
95725_at	1448539_a_at	Acy3	aspartoacylase (aminoacylase) 3	1.23	0.27	-1.32	0.0003	-1.28	0	-1.34	0	-2.16	0.0007
95726_at	1423916_s_at	Mlf2	myeloid leukemia factor 2	1.16	0.7	1.19	0.0166	1.05	0.6697	1.16	0.0131	-1.53	0.1097
95728_g_at	1417610_at	Apoa5	apolipoprotein A-V	-1.42	0.03	-1.01	0.8423	1.12	0.0278	-1.08	0.215	1.07	0.7348
95730_at	1421971_a_at	Mrps34	mitochondrial ribosomal protein S34	1.9	0.02	1.08	0.2006	1.2	0.0441	1.3	0.0006	-1.19	0.45
95731_at	1454699_at	Sesn1	sestrin 1	1.3	0.19	1	0.9686	1.05	0.6609	-1.05	0.5282	-1.43	0.1011
95732_at	1451185_at	Sf3b5	splicing factor 3b, subunit 5	1.05	0.78	1.01	0.9305	1.03	0.6966	1.01	0.9083	-1.13	0.0524
95733_at	1451782_a_at	Slc29a1	solute carrier family 29 (nucleoside transpor	-1.4	0.04	-1.67	0	-1.12	0.1949	-1.82	0	-1.52	0.0467
95734_at	1422464_at	Mrpl3	mitochondrial ribosomal protein L3	-1.57	0.01	-1.14	0.0899	-1.09	0.2743	-1.04	0.6179	1.27	0.0292
95736_at	1416510_at	Mrpl4	mitochondrial ribosomal protein L4	1.01	0.98	-1.09	0.1952	-1.1	0.3686	-1.15	0.0621	-1.59	0.0099
95737_at	1451172_at	1200015A19Rik	RIKEN cDNA 1200015A19 gene	1.72	0	1.04	0.4638	1.11	0.3688	1.14	0.0653	-1.25	0.0211
95738_at	1415836_at	Aldh18a1	aldehyde dehydrogenase 18 family, member	-1.08	0.89	-1.24	0.401	-1.6	0.096	-1.4	0.2319	-1.06	0.8895
95740_at	1452732_at	2300003P22Rik	RIKEN cDNA 2300003P22 gene	-1.74	0.34	1.59	0.0113	1.47	0.1033	1.05	0.7929	1.54	0.0197
95742_at	1437220_x_at	Psmd13	proteasome (prosome, macropain) 26S subu	1.54	0.17	1.02	0.7881	1.14	0.2712	1.14	0.0928	-1.53	0.0631
95746_at	1422508_at	Atp6v1a1	ATPase, H+ transporting, V1 subunit A, isofo	1.07	0.02	1.24	0.0012	1.14	0.275	1.24	0	1.01	0.8992
95749_at	1428112_at	Armet	arginine-rich, mutated in early stage tumors	-1.29	0.42	-1.34	0.0649	-1.22	0.0322	-1.22	0.0364	-2.26	0.0004
95750_at	1426654_at	Zc3hc1	zinc finger, C3HC type 1	2.43	0.1	-1.09	0.7062	1	0.9949	-1.12	0.627	1.59	0.2108
95752_at	1426480_at	Sbds	Shwachman-Bodian-Diamond syndrome hor	-1.07	0.8	1.13	0.219	1.06	0.5643	1.11	0.189	1.12	0.599
95753_at	1423920_at	Brrn1	barren homolog (Drosophila)	-1.28	0.77	1.05	0.9177	-1.42	0.4657	-1.47	0.4288	-1.01	0.9667
95754_at	1448240_at	Mbtps1	membrane-bound transcription factor protea	-1.35	0.09	-1.06	0.2049	1.09	0.0179	-1.01	0.6316	-1.07	0.2668
95755_at	1435800_a_at	Csda	cold shock domain protein A	-1.02	0.89	-1.01	0.8558	1.05	0.6497	-1.1	0.28	-1.01	0.9693
95756_at	1451026_at	Ftsj3	FtsJ homolog 3 (E. coli)	1.82	0.07	1	0.9556	1.15	0.1628	1.15	0.0991	1.12	0.4805
95758_at	1415822_at	Scd2	stearoyl-Coenzyme A desaturase 2	10.43	0.08	3.85	0.0014	-1.25	0.2302	2.51	0.0001	1.58	0.0521
95759_at	1424014_at	2900092E17Rik	RIKEN cDNA 2900092E17 gene	1.41	0.1	1.11	0.247	-1.05	0.4181	1	0.9588	-1	0.9814
95760_at	1423820_at	Elof1	elongation factor 1 homolog (ELF1, S. cerev	1.27	0.52	1.01	0.8794	1.09	0.2952	1.07	0.3916	1.19	0.3123
95770_s_at	1450583_s_at	Fprl1	formyl peptide receptor-like 1	-1.17	0.61	-1.26	0.1236	-1.13	0.4969	-1.32	0.1397	1.76	0.0509
95772_r_at	1419301_at	Fzd4	frizzled homolog 4 (Drosophila)	2.88	0.14	-1.51	0.3336	-1.44	0.3373	-1.33	0.4621	1.99	0.267
95775_f_at	1420701_at	Klk1	kallikrein 1	-1.26	0.18	-1.07	0.6528	-1.04	0.7463	-1.13	0.2325	-1.05	0.4164
95783_g_at	1422345_s_at	Mageb1 /// Mag	melanoma antigen, family B, 1 /// melanoma	2.24	0.18	-1.49	0.278	-1.31	0.4467	-1.07	0.8634	1.25	0.5618
95784_at	1418809_at	Pira1	paired-Ig-like receptor A1	-1.96	0.21	1.4	0.4848	-1.18	0.5359	1.45	0.2962	2.29	0.0779
95786_at	1416139_at	Reg2	regenerating islet-derived 2	-3.1	0.37	-1.04	0.8787	-1.29	0.4032	-1.19	0.5598	1.79	0.4933
95787_s_at	1426219_at	Scp2	sterol carrier protein 2, liver	-1.22	0.65	1.03	0.6413	-1.04	0.7561	-1.05	0.4848	-2.17	0.0094
95791_s_at	1427816_at	Sfrs2	splicing factor, arginine/serine-rich 2 (SC-35	1.16	0.65	1.12	0.2075	1.18	0.0875	1.06	0.5359	-1.01	0.973
95792_at	1450190_at	Zfp106	zinc finger protein 106	-1.66	0.59	-1.5	0.2643	-1.1	0.8125	-2.3	0.0396	1.87	0.4671

95793_at	1420771_at	Sprr2d	small proline-rich protein 2D	1.15	0.74	1.16	0.5717	-1.17	0.5874	-1.15	0.6137	1.19	0.6384
95796_g_at	1422418_s_at	Supt4h1 /// Sup	suppressor of Ty 4 homolog 1 (S. cerevisiae	1.4	0.34	-1.01	0.9002	1.03	0.4601	1.07	0.2653	1.08	0.4742
95799_s_at	1450331_s_at	V2r4	vomeronasal 2, receptor, 4	-2.37	0.43	-1.01	0.904	-1.08	0.4157	-1.01	0.9086	1.17	0.6133
95800_s_at	1422249_s_at	Zfa /// Zfx	zinc finger protein, autosomal /// zinc finger p	-1.23	0.19	1.13	0.3348	-1	0.9915	1.07	0.5379	1.63	0.2162
95801_s_at	1427831_s_at	Zfp260	zinc finger protein 260	-1.33	0.42	1.12	0.2921	1.08	0.647	1.18	0.2334	-1	0.9926
95804_g_at	1448534_at	Ptpns1	protein tyrosine phosphatase, non-receptor t	1.02	0.77	2.43	0.0461	-1.02	0.9271	3.75	0.1319	-1.04	0.8561
95806_f_at	1450538_s_at	Cma2 /// Mcpt9	chymase 2, mast cell /// mast cell protease 9	-2.44	0.07	-1.25	0.2608	1.17	0.1861	-1.1	0.3549	-1.2	0.4274
95852_at	1445367_at	---	Transcribed locus	1.04	0.9	-1.28	0.4037	-1.01	0.9776	-1.23	0.499	1.01	0.9429
95853_at	1446828_at	---	---	1.63	0.1	1.05	0.8283	1.54	0.2506	1.46	0.3916	2.36	0.0645
95854_at	1447990_at	C76332	expressed sequence C76332	-1.75	0.39	-1.41	0.0938	-1.13	0.4986	-1.09	0.6228	1.35	0.4937
95855_at	1448059_at	Mipol1	mirror-image polydactyly gene 1 homolog (h	1.01	0.98	-1.16	0.4224	-1.1	0.7548	1.24	0.193	1.83	0.0095
95856_at	1447983_at	Zfml	Zinc finger, matrin-like	-1.8	0.2	-1.4	0.0873	-1.02	0.9378	-1.33	0.1009	1.24	0.5158
95857_at	1444866_at	E230006M18Ril	RIKEN cDNA E230006M18 gene	-1.3	0.28	1.23	0.1806	1.71	0.1416	1.14	0.4193	1.86	0.0983
95858_at	1445116_at	Usp25	Ubiquitin specific protease 25	-1.62	0.38	-1.15	0.4173	-2.12	0.0015	-1.61	0.038	-1.37	0.454
95861_at	1419929_at	D15Ertd55e	DNA segment, Chr 15, ERATO Doi 55, expr	-1.1	0.62	-1.26	0.0487	-1.29	0.033	-1.25	0.0526	1	0.9837
95862_at	1420253_at	D2Ertd63e	DNA segment, Chr 2, ERATO Doi 63, expre	-1.77	0.18	-1.01	0.955	-1.03	0.8141	-1.06	0.5501	1.38	0.0261
95863_at	1443428_at	---	---	1.47	0.57	-2.2	0.0169	-1.04	0.8739	-1.83	0.0707	-1.03	0.956
95864_at	1447182_at	C77815	expressed sequence C77815	-2.59	0.17	-1.53	0.1641	1.03	0.9086	-1.48	0.2105	1.07	0.8563
95869_at	1420072_s_at	---	---	1.27	0.68	1.44	0.1281	-1.1	0.7442	-1.64	0.1571	1.23	0.6929
95870_at	1442974_at	D6Ertd160e	DNA segment, Chr 6, ERATO Doi 160, expr	-1.07	0.93	1.42	0.2102	2.02	0.2162	1.41	0.184	2.63	0.0423
95871_at	1448086_at	D1Ertd164e	DNA segment, Chr 1, ERATO Doi 164, expr	-1.41	0.47	-1.15	0.6253	-1.29	0.4699	1.01	0.9847	1.51	0.1878
95872_at	1449755_at	D7Ertd183e	DNA segment, Chr 7, ERATO Doi 183, expr	1.25	0.54	1.02	0.8921	-1.07	0.5456	1.3	0.0373	1.27	0.6305
95875_at	1459220_at	C78651	expressed sequence C78651	-1.11	0.56	-1.01	0.9212	-1.07	0.4741	-1.03	0.753	1.11	0.6286
95876_at	1441659_at	Dpf3	D4, zinc and double PHD fingers, family 3	-2.11	0.01	1.56	0.0743	1.21	0.3029	1.35	0.0516	-1.01	0.9416
95877_at	1420101_at	2610020H08Rik	RIKEN cDNA 2610020H08 gene	-1.43	0.45	-1.81	0.0673	-1.22	0.5851	-2.19	0.0219	1.79	0.397
95878_at	1459410_at	LOC493582	hypothetical LOC493582	-1.02	0.96	1.42	0.2545	1.35	0.3709	1.12	0.7555	1.73	0.3272
95880_s_at	1443461_at	C79130	expressed sequence C79130	-2.02	0.17	1.14	0.5601	1.04	0.8195	-1.06	0.7714	1.68	0.1901
95881_f_at	1419235_s_at	Helb	helicase (DNA) B	1.23	0.34	1.07	0.6105	1.2	0.1713	1.07	0.6283	1.12	0.3821
95882_at	1449647_at	---	---	2.59	0.15	-1.61	0.2456	-2.15	0.0725	-1.13	0.7298	2.96	0.1749
95883_at	1452180_at	Phf17	PHD finger protein 17	1.21	0.71	1.49	0.0172	1.38	0.0264	1.36	0.0265	1.59	0.0425
95884_at	1449640_at	---	---	-1.29	0.74	-1.06	0.6187	1.4	0.2768	1.53	0.2359	-1.27	0.5469
95886_g_at	1434633_at	AW558298	expressed sequence AW558298	-1.08	0.6	1.41	0.0395	1.44	0.1482	1.58	0.0083	1.11	0.3996
95887_at	1448068_at	8430426K15Rik	RIKEN cDNA 8430426K15 gene	-1.2	0.7	-1.11	0.7801	-1.43	0.2229	1	0.9989	1.98	0.1628
95889_at	1434787_at	AI854770	expressed sequence AI854770	-1.14	0.51	1.04	0.6242	1.24	0.0073	-1.06	0.3792	1.45	0.0023
95890_r_at	1459916_at	5730488B01Rik	RIKEN cDNA 5730488B01 gene	1.51	0.21	-1.13	0.6133	1.08	0.788	1.06	0.8343	-1.27	0.5602
95891_at	1459923_at	LOC328660	similar to RIKEN cDNA 2410004M13 gene; s	-1.09	0.68	-1.19	0.1482	-1.04	0.7757	-1.05	0.6689	1.39	0.1261
95892_at	1449609_at	LOC433755	LOC433755	2.35	0.23	-2.41	0.001	-1.7	0.0617	-1.97	0.0093	1.47	0.4029
95893_at	1422775_at	Blk	B lymphoid kinase	-1.46	0.01	-1.06	0.6897	-1.02	0.8742	-1.13	0.3753	-1.11	0.6912
95894_at	1447935_at	C730036B14Rik	RIKEN cDNA C730036B14 gene	1.36	0.54	-1.1	0.8206	-1.37	0.3057	-1.21	0.5283	1.08	0.8462
95895_at	1448046_at	9530020D24Rik	RIKEN cDNA 9530020D24 gene	-1.29	0.63	-1.36	0.3162	-1.6	0.0892	-1.59	0.0856	1.3	0.3915
95896_at	1459885_s_at	Cox7c	cytochrome c oxidase, subunit VIIc	1.29	0.38	1.17	0.1007	1.43	0.0184	1.46	0.0028	1.37	0.1353
95897_at	1434386_at	Atp2c1	ATPase, Ca++-sequestering	1.45	0.01	1.03	0.6888	1.09	0.5171	-1.03	0.7345	-1.08	0.7035
95898_at	1449422_at	Cdh4	cadherin 4	1.23	0.15	1.15	0.1231	1.19	0.0517	1.09	0.3213	1.36	0.0775
95901_f_at	1419857_at	Apoa1	Apolipoprotein A-I	-2.68	0.02	-2.14	0	-1.63	0.0012	-2.47	0	-2.23	0.0781
95902_at	1440964_s_at	AI465270	expressed sequence AI465270	1.89	0.16	1.15	0.5762	1.59	0.0786	-1.3	0.1037	1.37	0.4384
95903_at	1447959_at	Zfp398	zinc finger protein 398	1.05	0.92	-1.11	0.5579	-1.08	0.6811	-1.02	0.8944	1.42	0.3699
95904_at	1455628_at	6430543G08Rik	RIKEN cDNA 6430543G08 gene	-1.34	0.28	-1.15	0.2561	-1.1	0.3966	-1.09	0.3958	-1.07	0.7052
95905_at	1436112_at	AI118078	expressed sequence AI118078	-1.22	0.66	1.01	0.9637	1.45	0.0173	1.26	0.3843	2.67	0.0604
95906_at	1459927_at	4833445I07Rik	RIKEN cDNA 4833445I07 gene	1.16	0.87	-1.17	0.4452	-1.08	0.7583	1.25	0.2065	-1.53	0.1853
95908_at	1420790_x_at	Klra16	killer cell lectin-like receptor, subfamily A, m	1.92	0.29	-1.14	0.521	-1.45	0.1911	-1	0.9973	-2.71	0.1982
95909_at	1419859_at	BC057079	CDNA sequence BC057079	1.02	0.91	1.34	0.0084	1.2	0.017	1.24	0.0277	1.5	0.4337
95912_at	1448043_x_at	Rnf2	ring finger protein 2	1.61	0.04	1.08	0.7682	1.14	0.501	1.06	0.8197	-1.06	0.925
95913_at	1457265_at	B230333C21Ril	RIKEN cDNA B230333C21 gene	1.17	0.69	-1.07	0.7514	1.07	0.7086	-1.1	0.6109	-2.17	0.1454

95914_at	1456405_at	6720461J16Rik	RIKEN cDNA 6720461J16 gene	1.83	0.26	-1.16	0.3013	1.19	0.2592	1.02	0.8766	1.99	0.136
95917_at	1445535_at	AA407107	expressed sequence AA407107	-2.95	0.25	1.11	0.6815	-1.3	0.1783	1.01	0.9517	1.71	0.0237
95919_at	1443482_at	LOC240677 /// I	similar to protease /// similar to protease	-1.18	0.47	-1.31	0.1851	1.1	0.7396	1.2	0.6642	-1.1	0.758
95920_at	1448048_at	BC016495	cDNA sequence BC016495	-1.44	0.55	1.1	0.5246	1.02	0.8715	-1.16	0.4182	1.31	0.4746
95923_at	1440959_s_at	Mynn	myoneurin	-1.36	0.36	-1.16	0.5533	1.32	0.3976	-1.59	0.063	2.14	0.038
95925_at	1419795_at	AA517631	expressed sequence AA517631	-1.67	0.2	1.06	0.8835	-1.3	0.3879	-1.52	0.2116	-1.04	0.8866
95926_at	1419778_at	---	---	-1.82	0.6	1.22	0.4993	-1.29	0.446	-1.12	0.6733	1.22	0.5865
95927_f_at	1439516_at	2610201A13Rik	RIKEN cDNA 2610201A13 gene	1.51	0.39	-1.17	0.3699	1.24	0.2649	-1.15	0.2563	1.39	0.5916
95929_at	1419533_at	Nhlh1	nescient helix loop helix 1	-1.89	0.21	-1.04	0.8937	-1.03	0.926	1.15	0.6369	1.07	0.859
95930_at	1419796_at	Popdc3	Popeye domain containing 3	-1.08	0.92	-1.21	0.481	-1.24	0.4124	1.3	0.3865	1.4	0.4307
95931_at	1439116_at	---	---	-1.16	0.49	-1.3	0.3948	1.11	0.707	-1.73	0.0742	-1.02	0.9403
95932_at	1450306_at	Zp1	zona pellucida glycoprotein 1	-1.68	0.04	-1.18	0.4261	-1.02	0.9057	1.13	0.3217	1.36	0.2684
95933_f_at	1448037_at	Zcchc6	Zinc finger, CCHC domain containing 6	-1.01	0.98	-1.32	0.0922	1.15	0.2506	-1.04	0.7391	-1.6	0.4281
95934_at	1445485_at	D7Ertd187e	DNA segment, Chr 7, ERATO Doi 187, expr	-2	0.28	1.99	0.2422	2.26	0.0457	2.14	0.1012	1.02	0.9673
95935_at	1421145_at	Slc26a2	solute carrier family 26 (sulfate transporter),	-1.76	0.13	1.15	0.1574	1.02	0.85	1.19	0.1686	2.45	0.215
95936_at	1448022_at	---	---	2.17	0.05	1.31	0.4031	1.45	0.3203	1.78	0.1842	-1.03	0.9581
95937_at	1449639_at	0610040J01Rik	RIKEN cDNA 0610040J01 gene	-1.15	0.47	-1.08	0.5532	-1.24	0.1373	-1.27	0.1187	1.26	0.3736
95938_at	1448056_at	Huwe1	HECT, UBA and WWE domain containing 1	-1.05	0.91	-1.14	0.576	-2.01	0.0335	-1.63	0.0488	1.62	0.0907
95940_f_at	1435375_at	Trio	triple functional domain (PTPRF interacting)	1.01	0.97	2.87	0.0222	1.48	0.1423	2.9	0.0562	1.7	0.2235
95941_at	1420060_s_at	AA675344	expressed sequence AA675344	1.16	0.54	1.13	0.6357	1.19	0.5116	-1.15	0.6506	1.84	0.1457
95943_at	1448051_at	---	---	-1.03	0.9	1.1	0.7293	1.06	0.8279	-1.18	0.4545	1.27	0.2184
95944_at	1424398_at	Dhx36	DEAH (Asp-Glu-Ala-His) box polypeptide 36	1.05	0.96	1.07	0.6748	-1.97	0.0016	-1.57	0.0375	1.21	0.3939
95945_at	1442900_at	Prdm5	EST AI197291	-1.63	0.44	-1.55	0.1363	-1.07	0.7711	-1.25	0.412	2.16	0.2484
95946_at	1458173_at	---	Transcribed locus, moderately similar to NP_	2.63	0.17	-1.77	0.0359	-1.51	0.1097	-2.65	0.0005	1.58	0.4352
95947_at	1443431_at	---	---	-1.41	0.11	1.75	0.0039	1.34	0.0041	1.41	0.0143	1.96	0.1275
95948_at	1457520_at	Tcfcp2l3	Transcription factor CP2-like 3	-1.42	0.17	-1.08	0.5741	-1.11	0.5232	1.05	0.738	1.8	0.0228
95949_at	1442492_at	DXErtd11e	DNA segment, Chr X, ERATO Doi 11, expre	1.36	0.44	1.07	0.8302	-1.05	0.8912	1.35	0.3227	1.49	0.193
95950_at	1443326_at	---	---	-1.09	0.83	1.52	0.1408	2.4	0.0943	1.65	0.1578	2.46	0.0322
95951_at	1420804_s_at	Clec4d	C-type lectin domain family 4, member d	-1.69	0.52	1.3	0.6479	1.22	0.6845	2.59	0.366	-1.03	0.9226
95952_at	1453355_at	1810073P09Rik	RIKEN cDNA 1810073P09 gene	1.12	0.73	-1.08	0.5152	-1.19	0.3048	-1.17	0.3387	1.12	0.7527
95953_at	1442531_at	D12Ertd123e	DNA segment, Chr 12, ERATO Doi 123, exp	-4.13	0.25	1.28	0.4992	1.07	0.813	-1.16	0.605	1.73	0.0744
95954_at	1444292_at	D7Ertd143e	DNA segment, Chr 7, ERATO Doi 143, expr	-1.22	0.54	-1.01	0.9761	-1.09	0.7371	-1.03	0.8904	-1.98	0.2251
95955_at	1443949_at	Ppp2r5e	Protein phosphatase 2, regulatory subunit B	1.15	0.86	-1.06	0.7809	1.11	0.7369	1.08	0.7769	3.05	0.0228
95956_at	1459926_at	C77068	expressed sequence C77068	-1.47	0.23	1.08	0.6041	1.28	0.107	1.03	0.8684	-1.06	0.5366
95957_at	1458215_at	Setdb1	SET domain, bifurcated 1	-4.09	0	1.22	0.3937	-1.01	0.9652	-1.09	0.7369	-1.01	0.9456
95959_at	1443427_at	---	---	-5.34	0.05	1.23	0.5306	1.7	0.1635	1.3	0.55	-1.02	0.9709
95960_at	1458011_s_at	D4Ertd117e	DNA segment, Chr 4, ERATO Doi 117, expr	1.74	0.33	1.14	0.7095	1.09	0.7717	-1.25	0.4212	1.4	0.538
95961_at	1455694_at	Nbeal2	neurobeachin-like 2	-1.17	0.55	1.01	0.9862	-1.46	0.1661	1.06	0.8572	1.16	0.4582
95962_at	1448076_at	Sh2bp1	SH2 domain binding protein 1 (tetratricopept	2.04	0.19	-1.07	0.777	-1.16	0.5617	-1.65	0.1518	1.09	0.8829
95963_at	1419968_at	C77370	expressed sequence C77370	-1.44	0.31	-1	0.9873	1.19	0.5422	-1.07	0.8001	2.39	0.0685
95965_at	1419891_s_at	C77545	expressed sequence C77545	-1.32	0.52	-1.22	0.6669	1.04	0.9311	-1.48	0.3852	1.45	0.5539
95966_at	1442540_at	C77609	expressed sequence C77609	-6.44	0.33	1.05	0.8392	-1.01	0.9469	1.12	0.8095	-1.57	0.208
95967_at	1442526_at	C77681	expressed sequence C77681	-1.37	0.51	-1.67	0.126	-1.1	0.7487	-1.26	0.4553	1.2	0.6058
95968_at	1443472_at	C77691	expressed sequence C77691	-3.31	0.23	1.33	0.5113	1.58	0.1747	1.14	0.696	1.22	0.5728
95970_at	1442665_at	C77874	expressed sequence C77874	-1.67	0.38	-1.18	0.635	-1.35	0.4306	1.01	0.9863	1.48	0.4449
95971_at	1459925_at	C78282	expressed sequence C78282	-1.2	0.73	1.12	0.6623	-1.28	0.1977	-1.03	0.8801	1.84	0.4644
95972_at	1459891_at	C78444	expressed sequence C78444	-1.16	0.76	-1.2	0.6825	-1.55	0.2445	-1.98	0.0935	-1.11	0.691
95973_at	1442566_at	C78878	expressed sequence C78878	-2.72	0.22	-1.54	0.1661	-1.47	0.2038	1.4	0.3555	4.25	0.2035
95974_at	1420549_at	Gbp1	guanylate nucleotide binding protein 1	-1.31	0.66	3.37	0.335	-4.86	0.179	3.21	0.3827	3.69	0.3183
95975_at	1443385_at	A930012N16Ril	RIKEN cDNA A930012N16 gene	1.24	0.57	-1.29	0.2612	1.02	0.9144	-1.5	0.0232	1.13	0.7363
95976_at	1420690_at	Fgf10	fibroblast growth factor 10	-1.09	0.64	1.12	0.1831	1.2	0.018	1.09	0.2346	1.35	0.2127
95977_at	1419774_at	D2Ertd239e	DNA segment, Chr 2, ERATO Doi 239, expr	1.29	0.69	1.28	0.3813	1.25	0.5065	1.28	0.6129	1.4	0.4475
95978_at	1442145_at	Gm542	gene model 542, (NCBI)	1.2	0.54	-1.29	0.0088	1.21	0.0122	-1.21	0.0081	-2.08	0.0119

95979_at	1457860_at	---	---	-2.29	0.35	1.1	0.6494	1.25	0.3017	1.1	0.7195	2.06	0.1627
95981_at	1441401_at	C79329	expressed sequence C79329	1.12	0.56	-1.02	0.9096	1.06	0.6013	-1	0.9965	-1.42	0.4843
95982_at	1449638_at	---	---	1.19	0.55	1.16	0.2644	1.16	0.2624	1.34	0.0857	2.22	0.0279
95983_at	1458846_at	---	---	-1.49	0.47	1.18	0.5625	1.25	0.5359	1.02	0.9466	1.12	0.8319
95984_at	1459900_at	C79468	expressed sequence C79468	1.1	0.66	1.02	0.782	1.21	0.1337	1.06	0.6137	-1.64	0.3055
95985_at	1441182_at	Brwd3	bromodomain and WD repeat domain contai	-1.22	0.17	1.1	0.3135	1.09	0.4685	-1.07	0.5851	1.38	0.1917
95986_at	1457887_at	Scospondin	Subcommissural organ spondin	-1.67	0.01	-1.14	0.0809	-1.08	0.2476	-1.03	0.6212	1.33	0.0484
95987_at	1442694_at	---	---	-1.11	0.87	-1.32	0.1814	1.15	0.4836	-1.09	0.7127	-1.96	0.1575
95988_r_at	1447968_at	---	Transcribed locus	-1.19	0.35	-1.28	0.43	-1.54	0.3133	-1.49	0.3315	1.28	0.6127
95990_at	1419780_at	---	---	1.77	0	-1.19	0.0167	-1.11	0.2606	-1.13	0.2202	1.1	0.7543
95991_at	1419776_at	---	---	1.72	0.39	-1.13	0.7138	-1.47	0.3007	-1.54	0.2335	1.17	0.8258
95992_at	1443416_at	C79741	expressed sequence C79741	-2.14	0.02	1.19	0.2723	1.26	0.1147	1.01	0.9531	1.2	0.5434
95994_at	1447965_at	C80012	expressed sequence C80012	-1.12	0.47	-1.09	0.0553	1	0.9458	-1.17	0.0013	-1.06	0.5246
95996_at	1456972_at	C80256	expressed sequence C80256	-1.33	0.55	-1.2	0.4206	1.32	0.23	-1.21	0.5026	3.08	0.0078
95997_at	1442669_at	---	13 days embryo heart cDNA, RIKEN full-len	1.06	0.94	1.09	0.6553	1.26	0.3204	1.22	0.4106	1.51	0.0449
95998_at	1457872_at	---	---	1.01	0.99	-1.81	0.0193	-1.23	0.3138	-1.41	0.1293	1.25	0.5126
95999_at	1440591_at	---	---	-1.22	0.69	1.18	0.3419	1.22	0.2288	1.11	0.5124	-2.72	0.3685
96000_at	1459924_at	Atp6v0a1	ATPase, H+ transporting, lysosomal V0 sub	-1.38	0.32	-1.73	0.1177	-1	0.9872	-1.46	0.2959	-1.66	0.2497
96002_at	1428165_at	Vps24	vacuolar protein sorting 24 (yeast)	-1.08	0.72	-1.17	0.0097	-1.05	0.4941	-1.2	0.0035	-1.25	0.3207
96004_at	1450878_at	Sri	sorcin	-1.08	0.55	-1.27	0.0017	-1.08	0.1799	-1.24	0.0001	-1.39	0.2525
96007_at	1415700_a_at	Ssr3	signal sequence receptor, gamma	-1.34	0.03	1.08	0.0534	1.12	0.1049	1.09	0.1109	-1.44	0.0004
96008_at	1418528_a_at	Dad1	defender against cell death 1	-1.12	0.38	-1.13	0.1336	1.22	0.0251	-1.05	0.6351	-1.34	0.0453
96009_s_at	1448290_at	Pap	pancreatitis-associated protein	1.02	0.93	-1.21	0.066	1.13	0.5685	-1.19	0.1638	1.3	0.2726
96010_at	1450386_at	Kpna3	karyopherin (importin) alpha 3	-1.07	0.39	1.04	0.4008	1.12	0.2779	1.18	0.0912	1.07	0.1497
96012_f_at	1438368_a_at	Matr3	matrin 3	1.08	0.72	1.13	0.0939	1.07	0.4266	1.07	0.1495	1.37	0.0509
96013_r_at	1434888_a_at	Matr3	matrin 3	1.29	0.41	1.17	0.3085	1.07	0.668	1.16	0.1263	1.75	0.0181
96014_at	1452686_s_at	D4Ertd196e	DNA segment, Chr 4, ERATO Doi 196, expr	1.66	0.01	1.17	0.0812	-1.02	0.7864	1.13	0.1933	1.33	0.1878
96016_at	1436349_at	2700094K13Rik	RIKEN cDNA 2700094K13 gene	-1.01	0.97	-1.19	0.1224	-1.17	0.1421	-1.06	0.5483	-1.16	0.4147
96017_at	1416628_at	0610006I08Rik	RIKEN cDNA 0610006I08 gene	1.08	0.42	-1.16	0.0741	-1.12	0.2604	-1.1	0.2984	-1.23	0.1932
96019_at	1422881_s_at	Sypl	synaptophysin-like protein	-1.25	0.31	1.13	0.0976	1.23	0.0643	1.06	0.3761	-1.02	0.8359
96020_at	1417063_at	C1qb	complement component 1, q subcomponent	1.24	0.4	1.05	0.8183	-1.16	0.0715	1.27	0.5287	1.04	0.7514
96021_at	1424154_a_at	Hbld1	HESB like domain containing 1	1.83	0.01	-1.04	0.572	1.06	0.3781	1.08	0.305	1.05	0.6695
96023_at	1416231_at	Vac14	Vac14 homolog (S. cerevisiae)	1.6	0.09	1.02	0.8816	-1.11	0.3085	1.25	0.0155	1.01	0.9797
96025_g_at	1417125_at	Ahcy	S-adenosylhomocysteine hydrolase	-1.1	0.38	1.07	0.0207	1	0.8657	1.06	0.0575	1.46	0.0027
96027_at	1449333_at	Sf3a1	splicing factor 3a, subunit 1	1.39	0.17	-1.04	0.6324	-1.01	0.8484	-1.04	0.5412	1.01	0.9254
96029_at	1423811_at	Sf3a3	splicing factor 3a, subunit 3	1.62	0.02	1.07	0.404	1.08	0.2296	1.08	0.1658	1.07	0.5095
96030_at	1420627_a_at	Csna	casein alpha	-1.67	0.43	1.29	0.3424	1.22	0.3483	1.19	0.381	3.26	0.3129
96033_at	1415944_at	Sdc1	syndecan 1	-1.28	0.28	-1.14	0.1232	-1.28	0.0071	-1.48	0	-1.12	0.4117
96035_at	1416647_at	Bckdha	branched chain ketoacid dehydrogenase E1	1.32	0.38	1.14	0.0835	1.1	0.3794	1.24	0.0159	-1.12	0.6415
96037_at	1416924_at	Bri3	brain protein I3	1.21	0.65	1.06	0.5104	1.32	0.0065	1.04	0.6567	1.08	0.5972
96038_at	1422603_at	Rnase4	ribonuclease, RNase A family 4	1.48	0.03	1.28	0.0149	-1.02	0.7524	1.21	0.0146	1.76	0.0067
96041_at	1422660_at	Rbm3	RNA binding motif protein 3	1.37	0.14	2.26	0.0054	1.91	0.0011	3.53	0	2.19	0.0018
96042_at	1448610_a_at	Sod2	superoxide dismutase 2, mitochondrial	1.38	0	1.22	0.0273	1.15	0.0952	1.34	0.0005	-1.36	0.116
96044_at	1416434_at	Bcl2l10	Bcl2-like 10	-1.86	0.02	-1.02	0.8944	-1.12	0.1034	-1.12	0.0589	1.24	0.2077
96046_at	1448246_at	Hdac1	histone deacetylase 1	1.37	0.57	-1.01	0.7448	-1.06	0.4501	-1.07	0.1608	-1.08	0.6425
96047_at	1426225_at	Rbp4	retinol binding protein 4, plasma	-1.04	0.77	1.05	0.2107	1.04	0.0241	1.08	0.0275	1.58	0.007
96048_at	1428326_s_at	Hrsp12	heat-responsive protein 12	-1.08	0.7	-1.05	0.491	-1.1	0.3155	-1.14	0.0538	-1.53	0.0033
96049_at	1448323_a_at	Bgn	biglycan	-1.05	0.76	-1.1	0.3935	1.04	0.6838	-1.13	0.1527	1.18	0.1623
96050_at	1435856_x_at	Smarcb1	SWI/SNF related, matrix associated, actin de	1.31	0.12	1.1	0.2179	1.04	0.5185	1.19	0.0218	1.04	0.3022
96052_at	1450721_at	Acp1	acid phosphatase 1, soluble	1.28	0.23	-1.14	0.0156	1.03	0.6746	-1.15	0.0417	1.04	0.4616
96054_f_at	1422715_s_at	Acp1	acid phosphatase 1, soluble	1.19	0.72	-1.05	0.3329	1.04	0.6546	-1.12	0.0972	-1.22	0.406
96055_at	1419473_a_at	Cck	cholecystokinin	-2.23	0.25	1.07	0.649	-1.2	0.196	-1.33	0.0672	1.03	0.9457
96056_at	1448605_at	Rhoc	ras homolog gene family, member C	-1.08	0.77	1.09	0.6107	1.08	0.5082	1.14	0.3582	1.41	0.1545

96058_s_at	1448143_at	Aldh2	aldehyde dehydrogenase 2, mitochondrial	1.14	0.44	-1.01	0.7675	-1.03	0.5924	-1.08	0.1807	-1.21	0.0969
96059_at	1428302_at	Mrpl48	mitochondrial ribosomal protein L48	1.96	0.02	1.11	0.1428	1.14	0.1341	1.18	0.0129	-1.05	0.647
96060_at	1450138_a_at	Serpinb6a	serine (or cysteine) proteinase inhibitor, clad	1.34	0.16	-1.14	0.2883	-1.08	0.5631	1.03	0.8413	-1.23	0.326
96063_at	1451968_at	Xrcc5	X-ray repair complementing defective repair	2.53	0.22	-1.14	0.3084	-1.06	0.7131	-1.03	0.8282	-1.12	0.7274
96064_at	1448872_at	Reg3g	regenerating islet-derived 3 gamma	-1.64	0.14	1.01	0.929	1.08	0.6316	-1.13	0.0444	1.63	0.0723
96065_at	1416503_at	Lxn	latexin	2.96	0.03	1.23	0.5601	1.34	0.2896	1.9	0.0199	-1.33	0.3494
96066_s_at	1417308_at	Pkm2	pyruvate kinase, muscle	1.18	0.64	1.85	0.2017	-1.08	0.7268	2.58	0.1622	1.6	0.1094
96068_at	1451217_a_at	1500034J20Rik	RIKEN cDNA 1500034J20 gene	1.63	0.03	-1.08	0.5423	1.03	0.8511	-1	0.9805	1.15	0.5422
96069_at	1417294_at	Akr7a5	aldo-keto reductase family 7, member A5 (a	1.28	0.28	1.01	0.8546	1.04	0.7323	-1.06	0.4805	1.23	0.2299
96070_at	1449018_at	Pfn1	profilin 1	-1.35	0.64	-1.13	0.4278	-1.16	0.4523	1.01	0.9519	1.43	0.3507
96072_at	1419737_a_at	Ldh1	lactate dehydrogenase 1, A chain	-1.02	0.84	1.23	0.0236	1.3	0.006	1.23	0.0327	-1.42	0.0174
96073_at	1416534_at	Dpf2	D4, zinc and double PHD fingers family 2	-1.16	0.16	1.02	0.6672	1.03	0.6334	-1.03	0.5982	-1.07	0.2048
96074_at	1418239_at	Apof	apolipoprotein F	-1.05	0.61	-1.35	0.0185	-1.1	0.1398	-1.38	0.0001	-3.03	0.0005
96075_at	1450851_at	Wdr1	WD repeat domain 1	-1.13	0.77	1.1	0.4261	1.01	0.855	1.09	0.3412	-1.52	0.0045
96076_at	1449679_s_at	Stx5a	syntaxin 5A	1.1	0.48	1.04	0.1914	1.16	0.0129	1.13	0.0924	-1.25	0.2536
96078_g_at	1417280_at	Slc17a1	solute carrier family 17 (sodium phosphate),	-1.17	0.44	1.03	0.7688	1.25	0.0245	-1.53	0.0002	-1.21	0.3165
96079_at	1433463_at	0610010K06Rik	RIKEN cDNA 0610010K06 gene	1.41	0.14	1.09	0.1298	1.23	0.0075	1.2	0.017	-1.06	0.7967
96081_at	1416258_at	Tk1	thymidine kinase 1	1.54	0.12	-1.35	0.0002	-1.11	0.311	-1.15	0.0397	-1.03	0.9346
96082_at	1423857_at	Mrpl30	mitochondrial ribosomal protein L30	-1.24	0.64	-1.13	0.1258	-1.09	0.3936	-1.24	0.0081	1.08	0.4172
96083_s_at	1428225_s_at	Hnrpdl	heterogeneous nuclear ribonucleoprotein D-	-1.21	0.4	1.34	0.001	1.23	0.0069	1.32	0	1.41	0.0447
96084_at	1428224_at	Hnrpdl	heterogeneous nuclear ribonucleoprotein D-	1.23	0.08	-1.03	0.6865	1.01	0.965	1.02	0.795	1.2	0.1685
96085_at	1416368_at	Gsta4	glutathione S-transferase, alpha 4	4.48	0.05	2.11	0.0001	1.32	0.0791	2.34	0	1.74	0.0019
96086_at	1423745_at	1110031B06Rik	RIKEN cDNA 1110031B06 gene	-1.07	0.69	-1.04	0.5363	-1.25	0.0044	1.03	0.4919	-1.23	0.2239
96087_at	1423609_a_at	Mgat1	mannoside acetylglucosaminyltransferase 1	-1.04	0.7	-1.18	0.003	-1.15	0.0867	-1.2	0.0007	-1.11	0.5395
96088_at	1448154_at	Ndrg2	N-myc downstream regulated gene 2	-1	0.99	-1.07	0.1632	1.04	0.3809	-1.19	0.0024	-1.69	0
96090_g_at	1416607_at	4931406C07Rik	RIKEN cDNA 4931406C07 gene	1.22	0.06	-1.01	0.7968	-1.09	0.172	-1.02	0.8297	-1.56	0.0023
96092_at	1448881_at	Hp	haptoglobin	1.09	0.24	1.25	0.0029	1.05	0.4499	1.26	0.002	1.12	0.3145
96093_at	1454161_s_at	0610007P14Rik	RIKEN cDNA 0610007P14 gene	-1.16	0.16	-1.16	0.0606	1.05	0.5211	-1.09	0.3041	-1.2	0.0374
96094_at	1455201_x_at	Apoa1	apolipoprotein A-I	-1.38	0.19	1.02	0.5071	1.04	0.162	1.01	0.841	1.39	0.0221
96096_f_at	1426856_at	Hsdl2	hydroxysteroid dehydrogenase like 2	1.16	0.46	-1.11	0.2374	1.07	0.2972	-1.15	0.055	-1.01	0.9789
96097_at	1433684_at	---	Similar to hypothetical protein FLJ11749	-1.03	0.86	1.01	0.7939	1.03	0.6211	1.02	0.6027	1.03	0.6484
96098_at	1422819_at	Mrpl36	mitochondrial ribosomal protein L36	1.15	0.15	1.03	0.6946	-1.08	0.1425	-1	0.9871	-1.3	0.0336
96104_at	1452769_at	3732413I11Rik	RIKEN cDNA 3732413I11 gene	3.83	0.03	2.65	0	2.5	0	3.42	0	2.83	0.0223
96109_at	1448890_at	Klf2	Kruppel-like factor 2 (lung)	-1.66	0.4	1.11	0.6416	1.36	0.1885	1.21	0.472	-2.49	0.0004
96112_at	1423972_at	Etfa	electron transferring flavoprotein, alpha poly	1.21	0.07	1.11	0.1028	1.06	0.3204	1.17	0.0373	1.06	0.5981
96113_at	1433698_a_at	D18Wsu98e	DNA segment, Chr 18, Wayne State Univers	-1.01	0.94	1.12	0.1967	1.48	0.0023	1.11	0.4383	1.02	0.8861
96115_at	1438001_x_at	Dp1	deleted in polyposis 1	-1.95	0.07	-1.39	0.0387	1.22	0.0128	-1.11	0.5065	-1.17	0.1227
96117_r_at	1417287_at	H13	histocompatibility 13	1.13	0.34	-1.09	0.18	1.14	0.0459	-1.06	0.1566	-1.54	0.0121
96119_s_at	1417130_s_at	Angptl4	angiopoietin-like 4	1.15	0.72	-1.15	0.351	1.48	0.0461	-1.03	0.7373	1.35	0.2716
96120_at	1448234_at	Dnajb6	DnaJ (Hsp40) homolog, subfamily B, membe	1.36	0.1	1.01	0.9156	1.05	0.5264	1.07	0.2393	1.25	0.2651
96121_at	1434012_at	1110055N21Rik	RIKEN cDNA 1110055N21 gene	-1.26	0.27	1.25	0.0025	1.28	0.0078	1.22	0.006	-1.5	0.1609
96122_at	1451322_at	2310016A09Rik	RIKEN cDNA 2310016A09 gene	1.45	0.09	-1.22	0.0149	-1.12	0.0647	-1.43	0.0009	-1	0.9405
96123_at	1448550_at	Lbp	lipopolysaccharide binding protein	-1.53	0.11	1.19	0.1469	1.17	0.2726	1.24	0.1896	1.38	0.0322
96124_at	1428327_at	2310001H13Rik	RIKEN cDNA 2310001H13 gene	1.32	0.33	1	0.9504	1.03	0.5798	1.11	0.1327	-1.07	0.609
96125_at	1419026_at	Daxx	Fas death domain-associated protein	-1.52	0	1.73	0.0014	1.55	0.0122	1.75	0.0034	1.18	0.3714
96127_at	1415892_at	Sgpl1	sphingosine phosphate lyase 1	1.11	0.69	1.24	0.0002	-1.09	0.3685	1.16	0.0458	1.37	0.0291
96128_at	1449151_at	Pctk3	PCTAIRE-motif protein kinase 3	1.32	0.11	1.13	0.1423	-1.02	0.6084	1.22	0.0239	-1.08	0.4029
96130_at	1433999_at	Slk	STE20-like kinase (yeast)	-1.12	0.16	1	0.9418	1.04	0.3795	1.05	0.2746	1.18	0.0453
96131_at	1448587_at	Tbc1d10a	TBC1 domain family, member 10a	-1.02	0.93	1.35	0.0241	-1.05	0.8023	-1.07	0.718	1.54	0.3749
96132_at	1449070_x_at	AB023957	cDNA sequence AB023957	-1.43	0.39	1.01	0.9569	-1.79	0.0167	-1.14	0.5348	1.76	0.2086
96134_at	1430128_a_at	Dp1l1	deleted in polyposis 1-like 1	1.24	0.35	1.05	0.5432	-1.06	0.3174	-1.03	0.7969	-1.16	0.1936
96135_at	1426964_at	3110003A17Rik	RIKEN cDNA 3110003A17 gene	1.37	0.16	1.03	0.866	-1.03	0.8152	1.04	0.8195	-1.55	0.023
96136_at	1423587_a_at	Exosc10	exosome component 10	3.32	0.01	-1.12	0.3841	1.01	0.9004	-1.12	0.4245	1.04	0.9301

96139_at	1422489_at	Gcs1	glucosidase 1	-1.25	0.59	-1.08	0.3424	-1.02	0.6998	-1.15	0.0456	1.16	0.2442
96143_at	1418294_at	Epb4.1l4b	erythrocyte protein band 4.1-like 4b	-2.09	0.02	-1.21	0.1347	1.09	0.411	-1.24	0.0729	1.38	0.0699
96145_at	1428740_a_at	Pigt	phosphatidylinositol glycan, class T	-2.26	0.16	-1.21	0.0297	-1.33	0.026	-1.36	0.0005	1.04	0.8773
96146_at	1449007_at	Btg3	B-cell translocation gene 3	1.24	0.24	1.06	0.6072	1.55	0.0039	1.49	0.0098	2.21	0.0122
96147_at	1448916_at	Mafg	v-maf musculoaponeurotic fibrosarcoma onc	-1.04	0.87	1.23	0.0029	1	0.9719	1.19	0.014	1.16	0.5338
96148_at	1452627_at	Senp6	SUMO/sentrin specific protease 6	2.3	0.28	1.14	0.2516	1.09	0.3983	1.23	0.1141	1.34	0.5056
96151_at	1429352_at	Mocos	molybdenum cofactor sulfurase	-1.31	0.14	-1.28	0.0338	-1.27	0.0109	-1.29	0.014	-1.57	0.0179
96152_at	1418022_at	Narg1	NMDA receptor-regulated gene 1	1.49	0.44	1.14	0.0563	1.33	0.1209	1.24	0.0051	1	0.989
96153_at	1418722_at	Ngp	neutrophilic granule protein	-2.14	0.14	1.01	0.9369	1.04	0.737	-1.08	0.5419	1.2	0.25
96154_at	1450107_a_at	Renbp	renin binding protein	-1.09	0.22	1	0.9643	1.11	0.1576	-1.01	0.9091	1.15	0.5315
96155_at	1423067_at	Cdk5rap3	CDK5 regulatory subunit associated protein	1.14	0.59	-1.08	0.5261	1.27	0.0145	1.12	0.2484	-1.79	0.072
96156_at	1436506_a_at	1110008H02Rik	RIKEN cDNA 1110008H02 gene	1.17	0.59	-1.01	0.7854	-1.11	0.039	-1.06	0.1101	1.18	0.1766
96157_at	1429615_at	Zfp91	zinc finger protein 91	1.15	0.55	-1.03	0.6482	1.15	0.2442	1.05	0.5521	-1.23	0.2261
96158_at	1451125_at	BC017133	cDNA sequence BC017133	1.49	0.27	-1.19	0.054	-1.04	0.6769	-1.26	0.0013	1	0.9687
96160_at	1417636_at	Slc6a9	solute carrier family 6 (neurotransmitter tran	-1.39	0.06	-1.47	0.0002	-1.1	0.1977	-1.5	0	-1.46	0.0508
96162_at	1427238_at	Fbxo15	F-box protein 15	1.14	0.7	-1.16	0.5992	-1.19	0.6257	-1.16	0.6581	-1.08	0.8844
96165_at	1433737_at	Uhmk1	U2AF homology motif (UHM) kinase 1	1.32	0.25	1.26	0.002	1.06	0.6446	1.07	0.3393	-1.02	0.8814
96166_at	1452768_at	Tex261	testis expressed gene 261	1.11	0.26	-1.07	0.4923	-1.13	0.1881	1.07	0.4143	-1.25	0.0723
96167_at	1422452_at	Bag3	Bcl2-associated athanogene 3	-1.06	0.87	1.02	0.8682	1.24	0.1131	-1.16	0.1433	-1.73	0.0515
96168_at	1455990_at	Kif23	kinesin family member 23	2.46	0.02	1.47	0.0644	-1.2	0.4943	1.21	0.4849	2.63	0.0384
96169_at	1424152_at	Sall4	sal-like 4 (Drosophila)	-3.07	0.38	1.01	0.9199	-1.13	0.4757	-1.07	0.5057	-1.03	0.9064
96171_at	1448446_at	Deaf1	deformed epidermal autoregulatory factor 1	1.23	0.35	-1.31	0.0108	1.18	0.317	-1.07	0.4927	-1.4	0.1718
96172_at	1424375_s_at	Gimap4	GTPase, IMAP family member 4	-1.11	0.53	1.25	0.0986	-1	0.9896	1.17	0.1929	1.56	0.0068
96174_at	1427886_at	Pom121	nuclear pore membrane protein 121	-1.06	0.78	1.01	0.8549	1.12	0.2655	1.04	0.432	1.46	0.0557
96176_at	1455013_at	Arih2	ariadne homolog 2 (Drosophila)	-1.1	0.56	1.11	0.2046	1.21	0.0126	1.05	0.4543	1.01	0.9642
96177_at	1417967_at	Mms19l	MMS19 (MET18 S. cerevisiae)-like	1.63	0.2	1.07	0.3891	1.11	0.3023	1.22	0.0222	-2.1	0.0122
96178_at	1433433_at	Myst2	MYST histone acetyltransferase 2	1.07	0.51	1.08	0.0743	1.1	0.0887	1.1	0.1808	1.26	0.0584
96183_at	1421142_s_at	Foxp1	forkhead box P1	-1.25	0.56	1.18	0.0803	1.07	0.6451	1.28	0.14	2.13	0.0013
96184_at	1454753_at	Rnpepl1	arginyl aminopeptidase (aminopeptidase B)-	1.31	0.26	-1.3	0.0021	1.16	0.1889	-1.18	0.1657	-1.42	0.0063
96185_at	1417964_at	Ap3d1	adaptor-related protein complex 3, delta 1 su	1.17	0.38	1.12	0.083	1.16	0.1249	1.12	0.0144	-1.04	0.6736
96186_at	1416836_at	Lrp10	low-density lipoprotein receptor-related prote	1.18	0.28	-1.21	0.017	-1.05	0.6366	-1.1	0.2722	-1.41	0.0208
96187_at	1452209_at	Pkp4	plakophilin 4	1.56	0.34	1.04	0.3585	1.17	0.1348	1	0.9171	1.03	0.8235
96188_at	1425405_a_at	Adar	adenosine deaminase, RNA-specific	-1.32	0.36	1.01	0.9474	-1.24	0.1585	-1.22	0.1381	-1.06	0.8619
96189_at	1418820_s_at	Zcchc10	zinc finger, CCHC domain containing 10	1.4	0.17	1.02	0.8567	1.03	0.8138	1.03	0.8004	1	0.9954
96191_at	1415711_at	Arfgef1	ADP-ribosylation factor guanine nucleotide-e	-1.29	0.14	-1.03	0.5368	-1.01	0.8557	-1.04	0.4176	-1.2	0.0277
96195_at	1416716_at	Efs	embryonal Fyn-associated substrate	-1.46	0.26	-1.3	0.03	1.09	0.4328	-1.24	0.0623	1.48	0.0281
96197_f_at	1416574_at	5730589K01Rik	RIKEN cDNA 5730589K01 gene	1.24	0.12	1.02	0.7875	1.02	0.8723	1.04	0.6243	-1.09	0.2375
96198_at	1418085_at	Prkcz	protein kinase C, zeta	1.96	0.06	-1.08	0.4582	-1.02	0.904	-1.17	0.2417	1.84	0.1413
96199_at	1416934_at	Mtm1	X-linked myotubular myopathy gene 1	1.08	0.58	1.06	0.333	1.36	0.0021	1.23	0.0147	-1.3	0.14
96200_at	1423683_at	Cdca4	cell division cycle associated 4	-1.04	0.89	1.09	0.3601	1.11	0.2509	1.2	0.0621	1.38	0.0221
96203_at	1424713_at	Calml4	calmodulin-like 4	1.37	0.23	-1.19	0.3308	1.32	0.1097	1.36	0.3623	1.14	0.7051
96205_at	1422644_at	Sh3bgr	SH3-binding domain glutamic acid-rich prote	1.48	0.65	1.1	0.7885	-1.48	0.2426	-1.21	0.5887	1.98	0.206
96206_at	1424468_s_at	Phldb1	pleckstrin homology-like domain, family B, m	2.04	0.1	-1.38	0.0886	-1.01	0.9534	-1.13	0.4454	1.09	0.7888
96207_at	1434005_at	Rbms1	RIKEN cDNA 6030432P03 gene	1.61	0.05	1.73	0.0011	1.44	0.0432	1.9	0	2.52	0.0111
96208_at	1434009_at	6430596G11Rik	RIKEN cDNA 6430596G11 gene	1.05	0.85	1.27	0.0709	1.36	0.0234	1.17	0.2099	-1.16	0.213
96211_at	1428698_at	2310004I03Rik	RIKEN cDNA 2310004I03 gene	-1.19	0.34	-1.21	0.0881	-1.05	0.6431	-1.09	0.3515	1.13	0.3348
96212_at	1437985_a_at	2310061I04Rik	RIKEN cDNA 2310061I04 gene	1.74	0.04	1.11	0.1401	-1.1	0.2256	1.09	0.1432	1.01	0.8386
96215_f_at	1427820_at	---	Mus musculus, clone IMAGE:3983821, mRN	-2.84	0.17	1.21	0.1922	1.5	0.0157	1.82	0.0029	-1.03	0.8695
96216_at	1451219_at	Ormdl1	ORM1-like 1 (S. cerevisiae)	-1.3	0.42	-1.02	0.8256	-1.04	0.8958	-1.08	0.5507	-1.41	0.2409
96217_at	1434230_at	---	---	1.26	0.11	1.09	0.3893	-1.17	0.0758	1.09	0.3429	-1.05	0.7655
96218_at	1434309_at	Fntb	farnesyltransferase, CAAX box, beta	-1.08	0.48	-1.07	0.601	-1.05	0.6738	-1.19	0.154	1.07	0.7157
96219_at	1450675_at	1810031K02Rik	RIKEN cDNA 1810031K02 gene	1.24	0.2	1.07	0.3913	-1.01	0.9108	1.13	0.1001	1.33	0.0099
96220_at	1437198_at	---	---	-1.19	0.54	1.14	0.2529	1.14	0.3032	1.06	0.5329	-1.62	0.0161

96221_at	1448508_at	Traf3ip2	Traf3 interacting protein 2	-1.4	0.43	-1.1	0.4711	-1.27	0.0568	-1.28	0.078	1.32	0.2677
96222_at	1438278_a_at	BC003993	cDNA sequence BC003993	1.32	0.33	1.45	0.0309	1.45	0.0374	1.37	0.0806	1.06	0.8103
96223_at	1433606_at	Mitc1	MAD homolog 4 interacting transcription coa	-1.67	0.38	-1.04	0.8773	-1.18	0.4633	1.18	0.4177	1.6	0.2763
96224_at	1425680_a_at	Btrc	beta-transducin repeat containing protein	1.25	0.3	1.07	0.4544	-1.01	0.876	1.11	0.2345	1.14	0.285
96226_at	1451180_a_at	C330027I04Rik	RIKEN cDNA C330027I04 gene	-1.11	0.65	1.03	0.5923	-1.07	0.1934	1	0.9552	1.53	0.0484
96227_at	1448506_at	Serpina6	serine (or cysteine) proteinase inhibitor, clad	2.95	0	1.12	0.023	-1	0.9727	1.11	0.0045	2.92	0.0004
96228_at	1448804_at	Cyp11a1	cytochrome P450, family 11, subfamily a, po	-1.74	0.39	1.16	0.5091	1.07	0.698	1.02	0.9211	1.5	0.4004
96229_at	1421946_at	Crp	C-reactive protein, petaxin related	-1.69	0.01	-1.3	0.0007	-1.15	0.0387	-1.27	0.0367	1.22	0.1449
96230_at	1433958_at	9830165K03Rik	RIKEN cDNA 9830165K03 gene	1.6	0.47	1.52	0.0332	1.32	0.2315	1.04	0.7823	2.08	0.0579
96231_at	1424242_at	Bphl	biphenyl hydrolase-like (serine hydrolase, br	1.11	0.6	-1.21	0.0136	-1.19	0.024	-1.4	0.0034	-1.5	0.0016
96232_at	1451231_a_at	Cul2	cullin 2	1.01	0.98	-1.11	0.4333	1.02	0.8466	-1.16	0.1534	-1.48	0.0029
96234_at	1422855_at	Cpsf3	cleavage and polyadenylation specificity fact	1.54	0.03	1.02	0.8201	1.17	0.1926	1.21	0.0225	1	0.9781
96236_at	1425554_a_at	Cdc16	CDC16 cell division cycle 16 homolog (S. ce	1.08	0.78	-1.03	0.6485	1.07	0.4633	1.03	0.6319	1.09	0.6587
96237_at	1424729_at	BC054059	cDNA sequence BC054059	1.47	0.37	-1.11	0.7136	1.04	0.9197	1.03	0.9195	1.88	0.1795
96238_at	1434549_at	Rab11a	RAB11a, member RAS oncogene family	1.46	0.07	1.06	0.5496	1.05	0.7992	1.16	0.2528	-1.35	0.1673
96239_at	1418121_at	Vrk3	vaccinia related kinase 3	1.32	0.1	1.1	0.1808	1.18	0.0504	1.26	0.0186	-1.48	0.0134
96240_at	1434784_s_at	D15Ertd405e	DNA segment, Chr 15, ERATO Doi 405, exp	-1.01	0.89	-1.15	0.0393	-1.12	0.0165	-1.08	0.2091	1.03	0.8792
96243_f_at	1436689_a_at	Aldh9a1	aldehyde dehydrogenase 9, subfamily A1	1.14	0.59	-1.16	0.0162	1.02	0.8641	-1.09	0.2806	-1.5	0.0157
96244_at	1448260_at	Uchl1	ubiquitin carboxy-terminal hydrolase L1	1.1	0.65	-1.43	0.245	1.54	0.4045	-1.58	0.1506	-1.05	0.9031
96245_at	1423815_at	Ddx56	DEAD (Asp-Glu-Ala-Asp) box polypeptide 56	1.12	0.43	-1.15	0.0023	-1.01	0.8988	-1.12	0.0336	1.15	0.1066
96252_at	1449674_s_at	---	---	-1.17	0.15	-1.07	0.0582	1.01	0.8207	-1	0.9446	1.04	0.7775
96254_at	1416755_at	Dnajb1	DnaJ (Hsp40) homolog, subfamily B, membe	1.22	0.46	-1.01	0.9458	1.16	0.2699	-1.2	0.204	1.24	0.3843
96255_at	1416923_a_at	Bnip3l	BCL2/adenovirus E1B 19kDa-interacting pro	1.31	0.17	-1.15	0.008	1.12	0.3399	-1.02	0.8061	-1.16	0.2958
96256_at	1416292_at	---	---	-1.02	0.93	-1.06	0.5175	-1.04	0.6121	-1.12	0.1367	-1.48	0.0039
96257_at	1415751_at	Hp1bp3	heterochromatin protein 1, binding protein 3	1.05	0.67	1.18	0.0503	1.03	0.7205	1.24	0.0065	1.3	0.0721
96259_at	1417386_at	Npepps	aminopeptidase puromycin sensitive	1.3	0.22	1.14	0.0087	1.17	0.0039	1.16	0.0017	1.32	0.0551
96260_at	1416038_at	AL033314	expressed sequence AL033314	-1.14	0.77	1.15	0.0317	1.1	0.3926	1.2	0.0133	1.39	0.1443
96261_at	1433616_a_at	2310028O11Rik	RIKEN cDNA 2310028O11 gene /// similar to	1.4	0.08	-1.01	0.7409	1.04	0.3987	1.06	0.2468	-1.23	0.1816
96262_at	1424684_at	Rab5c	RAB5C, member RAS oncogene family	1.11	0.63	1.02	0.861	-1.01	0.876	-1.01	0.8391	-1.11	0.3324
96263_at	1423119_at	Rshl2	radial spokehead-like 2	1.5	0.39	1.15	0.3909	-1.08	0.6245	1.01	0.9308	-1.9	0.1878
96264_at	1416281_at	Wdr45l	Wdr45 like	-1.01	0.92	-1.22	0.0378	-1.06	0.3909	-1.33	0.0071	-1.29	0.0192
96266_at	1426698_a_at	Hnrpm	heterogeneous nuclear ribonucleoprotein M	1.14	0.57	1.11	0.3501	1.05	0.589	1.14	0.2782	1.17	0.1529
96267_at	1415967_at	Ndufv1	NADH dehydrogenase (ubiquinone) flavopro	1.19	0.1	1.08	0.3317	1.13	0.268	1.16	0.0353	1.21	0.1285
96268_at	1415891_at	Suclg1	succinate-CoA ligase, GDP-forming, alpha s	1.6	0	1.25	0.0095	1.14	0.005	1.3	0.002	1.15	0.1704
96269_at	1451122_at	Idi1	isopentenyl-diphosphate delta isomerase	-1.08	0.73	-1.5	0.0368	1.07	0.8064	-2.01	0.0015	-4.02	0.0094
96271_at	1448623_at	2310075C12Rik	RIKEN cDNA 2310075C12 gene	1.13	0.65	1.52	0	1.09	0.46	1.44	0.002	-1.22	0.0605
96272_at	1420842_at	Ptprf	protein tyrosine phosphatase, receptor type,	-1.02	0.7	-1.1	0.1104	-1.02	0.7437	-1.11	0.1082	-1.28	0.058
96273_at	1423231_at	Nrgn	neurogranin	-1.8	0.16	-1.15	0.2381	-1.17	0.1232	1.01	0.9424	1.66	0.1778
96276_r_at	1416509_at	MGI:1914262	SM-11044 binding protein	1.44	0.23	1.04	0.7918	-1.14	0.2017	1.03	0.7981	-1.14	0.3668
96277_at	1448129_at	Arpc5	actin related protein 2/3 complex, subunit 5	1.19	0.51	-1.08	0.0605	-1.08	0.3324	1.01	0.8998	1.13	0.121
96278_at	1423686_a_at	1110020C13Rik	RIKEN cDNA 1110020C13 gene	1.27	0.4	-1.07	0.4433	-1.21	0.1007	-1.18	0.0756	-1.16	0.1
96280_at	1417368_s_at	Ndufa2	NADH dehydrogenase (ubiquinone) 1 alpha	1.53	0.02	1.23	0.0022	1.04	0.3963	1.2	0.0151	1.25	0.4203
96281_at	1423256_a_at	Atp6v1g1	ATPase, H+ transporting, V1 subunit G isofo	-1.41	0.14	1.04	0.6802	1.12	0.3114	-1.03	0.5954	-1.35	0.0012
96283_at	1415961_at	Itm2c	integral membrane protein 2C	1.1	0.65	1	0.9767	-1.09	0.156	1.11	0.5278	1.1	0.507
96284_at	1423370_a_at	Csnk1g2	casein kinase 1, gamma 2	-1.1	0.7	1.06	0.2931	-1.02	0.8726	1.05	0.3579	1.09	0.7326
96285_at	1439389_s_at	Myadm	myeloid-associated differentiation marker	-1.54	0.04	1.15	0.1052	-1.06	0.4257	1.2	0.0704	1.18	0.2703
96286_at	1423185_a_at	Ubap1	ubiquitin-associated protein 1	-1.55	0.03	-1.12	0.0434	-1.05	0.2602	-1.11	0.1764	-1.44	0.0109
96287_at	1419269_at	Dut	deoxyuridine triphosphatase	1.35	0.35	1.07	0.8108	-1.15	0.5546	-1.55	0.1582	-1.24	0.2539
96288_at	1424058_at	1190002C06Rik	RIKEN cDNA 1190002C06 gene	1.26	0.01	-1.04	0.5609	1.07	0.3463	-1.02	0.7447	-1.37	0.1309
96289_at	1448774_at	Stoml2	stomatin (Epb7.2)-like 2	-1.11	0.69	1.01	0.8864	1.05	0.6283	-1.06	0.3208	-1.12	0.0249
96292_r_at	1433603_at	Ndufs6	NADH dehydrogenase (ubiquinone) Fe-S pr	1	0.99	1.04	0.2788	1.05	0.309	1.03	0.5288	-1.07	0.5021
96294_s_at	1416439_at	2410015N17Rik	RIKEN cDNA 2410015N17 gene	1.37	0.17	1.03	0.5516	1.11	0.143	1.15	0.0974	1.54	0.0021
96295_at	1451064_a_at	Psat1	phosphoserine aminotransferase 1	1.76	0.2	1.49	0.1594	1.03	0.9265	1.32	0.3971	1.44	0.1684

96296_at	1435232_x_at	---	---	1.37	0.36	-1.09	0.2199	-1.02	0.6473	-1.07	0.513	1.03	0.643
96298_f_at	1456125_a_at	Dnclc1	dynein, cytoplasmic, light chain 1	-1.02	0.81	-1.07	0.673	1.3	0.0617	1.22	0.185	1.16	0.3164
96299_at	1428286_at	2900097C17Rik	RIKEN cDNA 2900097C17 gene	1.54	0.02	1.36	0.0328	1.05	0.5624	1.25	0.0396	1.3	0.0308
96301_at	1460699_at	Rps27	ribosomal protein S27	-1.16	0.36	-1.07	0.2402	-1.07	0.2827	-1.08	0.2119	1.16	0.064
96305_at	1433611_s_at	---	---	1.64	0.2	-1.06	0.6333	1.02	0.8676	-1.06	0.5955	-1.36	0.0152
96306_at	1428494_a_at	Polr2i	polymerase (RNA) II (DNA directed) polypep	-1.34	0.71	-1.13	0.2831	1.4	0.2078	-1.01	0.9321	1.18	0.3632
96307_s_at	1427875_a_at	1100001I22Rik	RIKEN cDNA 1100001I22 gene	-1.16	0.33	1.04	0.2797	1.2	0.0126	1.09	0.2063	1.38	0.0439
96310_at	1425264_s_at	Mbp	myelin basic protein	-1.28	0.42	1.28	0.0625	1	0.971	1.16	0.2763	2.24	0.0247
96311_at	1433532_a_at	Mbp	myelin basic protein	-1.69	0.39	-1.2	0.3509	-1.05	0.7617	-1.14	0.5334	1.09	0.7149
96313_at	1422600_at	Rasgrf1	RAS protein-specific guanine nucleotide-rele	2.45	0.07	1.6	0.3933	1.01	0.976	1.17	0.6965	1.55	0.2512
96318_at	1416696_at	D17Wsu104e	DNA segment, Chr 17, Wayne State Univers	1.11	0.7	-1.27	0	-1.05	0.5701	-1.33	0	-1.42	0.0028
96319_at	1416664_at	Cdc20	cell division cycle 20 homolog (S. cerevisiae	-1.47	0.21	1.1	0.4624	-1.1	0.3701	1.14	0.3165	1.16	0.1305
96320_at	1424038_a_at	2310044H10Rik	RIKEN cDNA 2310044H10 gene	-1.02	0.83	-1.21	0.0075	1.01	0.8967	-1.33	0.0003	-1.13	0.3515
96321_at	1416663_at	Ndufa9	NADH dehydrogenase (ubiquinone) 1 alpha	1.51	0.02	1	0.9709	1.08	0.3263	1.07	0.3506	1.22	0.0277
96322_at	1448266_at	Edf1	endothelial differentiation-related factor 1	1.18	0.49	-1.08	0.1334	1.11	0.0271	-1.01	0.8935	-1.59	0.0984
96325_at	1451078_at	2510039O18Rik	RIKEN cDNA 2510039O18 gene	1.11	0.55	1.13	0.1091	1.09	0.1617	1.13	0.0289	1.05	0.6316
96326_at	1451557_at	Tat	tyrosine aminotransferase	-1.46	0.08	-1.03	0.7747	1.03	0.8321	1.01	0.8846	1.28	0.2142
96327_at	1415689_s_at	Zfp306	zinc finger protein 306	-1.06	0.42	1.44	0.0002	1.29	0.0453	1.56	0	-1.03	0.8822
96329_at	1416438_at	2410104I19Rik	RIKEN cDNA 2410104I19 gene	1.23	0.3	1.03	0.5281	1.08	0.1532	1.1	0.0584	-1.07	0.5367
96331_at	1460224_at	Snx2	sorting nexin 2	1.5	0.21	1.41	0.0013	1.42	0.0102	1.58	0	1.64	0.0047
96334_f_at	1423244_at	LOC433247	similar to Cytochrome P450, family 2, subfar	-1.22	0.14	-1.63	0	-1.05	0.3257	-1.69	0	-1.05	0.752
96335_at	1447961_s_at	Mrpl38	mitochondrial ribosomal protein L38	1.02	0.8	1.03	0.5531	1.09	0.2906	1.03	0.481	1.04	0.6869
96337_at	1452357_at	Gp1bb /// Sept5	glycoprotein Ib, beta polypeptide /// septin 5	-1.93	0.14	-1.08	0.7532	1.22	0.3947	-1.12	0.6539	2.57	0.0095
96338_at	1416533_at	Egln2	EGL nine homolog 2 (C. elegans)	1.44	0.15	1.11	0.0287	1.15	0.0864	1.19	0.0003	1.28	0.1876
96340_at	1423707_at	B230114J08Rik	RIKEN cDNA B230114J08 gene	1.4	0.2	-1.04	0.7399	1.15	0.0524	-1.03	0.7896	-1.13	0.5094
96341_at	1424104_at	MGI:1915842	GCIP-interacting protein p29	1.24	0.2	1.22	0.0004	1.22	0.0034	1.2	0.0002	-1.06	0.6301
96342_at	1419287_at	MGI:1913570	HSPC171 protein	1.09	0.74	-1.04	0.4148	1.11	0.1405	-1.05	0.3596	-1.28	0.0707
96344_at	1417951_at	Eno3	enolase 3, beta muscle	1.15	0.53	-1.4	0.2622	-1.43	0.2348	-1.38	0.2903	1.22	0.0832
96345_at	1423548_s_at	Sdbcag84	serologically defined breast cancer antigen 8	1.37	0.23	-1.01	0.9053	1.07	0.4309	-1.4	0.0009	-1.45	0.0471
96346_at	1448842_at	Cdo1	cysteine dioxygenase 1, cytosolic	-1.06	0.3	1.16	0.0098	1.11	0.069	1.22	0.0006	1.33	0.0349
96348_at	1428143_a_at	Pnpla2	patatin-like phospholipase domain containin	1.32	0.22	1.27	0.0004	1.32	0.1523	1.6	0	1.29	0.0991
96352_at	1448718_at	2400001E08Rik	RIKEN cDNA 2400001E08 gene	-1.14	0.52	-1.1	0.1734	-1.18	0.0169	-1.18	0.0008	-1.13	0.4759
96353_at	1416479_a_at	Tmem14c	transmembrane protein 14C	1.35	0.05	-1.05	0.2653	-1.05	0.2692	-1.06	0.273	-1.34	0.1081
96354_at	1416904_at	Mbnl1	muscleblind-like 1 (Drosophila)	1.36	0.03	1.2	0.021	1.05	0.3587	1.16	0.0634	1.12	0.5713
96358_at	1460175_at	Rps23	ribosomal protein S23	-1.17	0.46	1.14	0.0281	1.18	0.0555	1.21	0.0291	1.47	0.0124
96359_at	1419869_s_at	Hdlbp	high density lipoprotein (HDL) binding protei	-1.24	0.13	-1	0.9933	1.1	0.0842	1.03	0.5482	-1.09	0.1929
96360_at	1451168_a_at	Arhgdia	Rho GDP dissociation inhibitor (GDI) alpha	1.18	0.49	1.09	0.3583	-1.1	0.3704	1.13	0.3317	1.62	0.0327
96365_at	1459092_at	Slco6c1	solute carrier organic anion transporter famil	1.14	0.83	1.32	0.5234	-1.55	0.2203	1.11	0.7603	-1.83	0.3063
96367_at	1442494_at	C79242	expressed sequence C79242	-1.01	0.99	-1.24	0.0538	1.25	0.0797	-1.15	0.4346	1.19	0.7336
96373_at	1459930_at	---	---	1	0.98	-1.19	0.0879	-1.11	0.1929	-1.19	0.056	1.35	0.2238
96374_at	1449704_at	C80171	expressed sequence C80171	-1.65	0.08	-1.39	0.1881	-1.11	0.6145	-1.54	0.1338	1.13	0.7194
96375_at	1442484_at	D9Ertd306e	DNA segment, Chr 9, ERATO Doi 306, expr	-1.59	0.48	-1.17	0.1902	-1.19	0.0919	-1.06	0.5563	1.58	0.0463
96388_at	1419953_at	C80678	expressed sequence C80678	1.06	0.93	-1.07	0.825	1.06	0.8327	1.29	0.1663	1.29	0.6641
96389_at	1447978_at	C80719	expressed sequence C80719	-1.27	0.43	-1.18	0.4223	1.14	0.5413	-1.16	0.5239	1.57	0.3888
96390_at	1446572_at	Usp53	Ubiquitin specific protease 53	1.55	0.59	-1.41	0.0953	-1.3	0.2073	-1.61	0.0617	-1.11	0.8097
96391_at	1436092_at	---	Transcribed locus, moderately similar to XP_	-1.31	0.16	-1.07	0.5564	-1.02	0.8298	-1.18	0.0937	-1.16	0.5549
96392_at	1419807_at	D4Ertd335e	DNA segment, Chr 4, ERATO Doi 335, expr	-1.23	0.06	-1.11	0.3381	-1.05	0.5618	-1.17	0.114	-1.01	0.948
96393_at	1419962_at	Depdc6	DEP domain containing 6	1.25	0.43	1.98	0.0142	1.36	0.0541	1.34	0.0333	1.54	0.1866
96394_at	1446378_at	D2Ertd357e	DNA segment, Chr 2, ERATO Doi 357, expr	-1.27	0.57	1.14	0.6427	-1.03	0.9317	1.2	0.6006	1.37	0.3568
96400_at	1421722_at	MGI:1930074	interferon response element binding factor 1	4.61	0.09	-1.46	0.325	1.33	0.4264	-1.39	0.3558	1.25	0.6646
96408_at	1420041_at	---	---	-1.98	0.12	1.17	0.5353	1.17	0.3624	1.05	0.8405	-1.15	0.2332
96413_at	1422143_at	Akap7	A kinase (PRKA) anchor protein 7	1.45	0.38	-1.19	0.7075	-1.39	0.4961	-1.08	0.8679	1.11	0.841
96414_at	1450336_at	---	---	-1.54	0.21	1.11	0.5302	1.16	0.6225	1.12	0.4728	1.49	0.289

96416_f_at	1427864_at	Hist2h3c1 /// His	histone 2, H3c1 /// histone1, H3d /// histone	-1.05	0.29	-1.16	0.1094	1.02	0.7791	-1.33	0.0019	1.2	0.2073
96420_at	1427693_at	Itpr2	Inositol 1,4,5-triphosphate receptor 2	-1.03	0.89	1.12	0.7053	1.92	0.0438	1.33	0.3283	1.75	0.206
96421_at	1420470_at	Sult1c1	sulfotransferase family, cytosolic, 1C, memb	1.65	0.42	1.07	0.7555	1.06	0.7873	1.13	0.3885	1.38	0.5087
96422_at	1422284_at	Nkx2-9	NK2 transcription factor related, locus 9 (Dro	1.17	0.45	1.45	0.2003	1.22	0.5534	-1.05	0.7706	1.93	0.002
96423_at	1420555_at	Alx3	aristaless 3	-1.01	0.98	-1.05	0.9064	1.32	0.4069	-1.14	0.7035	1.18	0.4819
96426_at	1415906_at	Tmsb4x	thymosin, beta 4, X chromosome	1.3	0.25	1.59	0.2151	-1.12	0.3272	1.85	0.1758	1.03	0.8777
96431_at	1444959_at	D1Ertd83e	DNA segment, Chr 1, ERATO Doi 83, expre	-2.13	0.46	-1.08	0.7929	1.48	0.1742	1.77	0.0436	3.05	0.245
96435_at	1420754_at	Ttf1	transcription termination factor 1	-1.07	0.49	-1.06	0.4228	-1.11	0.0945	-1.15	0.0808	-1.11	0.6375
96474_at	1420864_at	Zfp161	zinc finger protein 161	1.1	0.68	1.83	0.0259	1.64	0.1071	1.53	0.0334	1.53	0.0626
96481_at	1439995_at	C80638	expressed sequence C80638	-1.02	0.96	-1.54	0.1411	-1.12	0.741	-2.61	0.0076	1.68	0.2899
96482_at	1448071_at	Lmtk2	Lemur tyrosine kinase 2	3.27	0.27	1.14	0.6958	1.52	0.3408	1.28	0.2029	1.77	0.2731
96483_at	1442661_at	C80113	expressed sequence C80113	-1.58	0.41	1.01	0.928	1.04	0.7352	-1.06	0.5712	-1.63	0.4972
96484_at	1442984_at	C80060	expressed sequence C80060	-1.27	0.58	1.18	0.4862	1.23	0.4289	-1	0.9844	1.76	0.2804
96485_at	1442975_at	---	---	-1.01	0.98	1.54	0.0639	1.88	0.0723	1.27	0.1013	1.22	0.4816
96486_at	1443464_at	Sntb1	Syntrophin, basic 1	-1.81	0.45	1.5	0.1539	-1.47	0.126	-1.08	0.768	6.99	0.01
96487_at	1442976_at	C81072	expressed sequence C81072	-1.54	0.43	1.41	0.2328	1.54	0.0664	1.08	0.7583	1.51	0.452
96490_at	1435941_at	Rhbdl4	rhomboid, veinlet-like 4 (Drosophila)	-1.71	0.01	1.05	0.7779	-1.65	0.0011	-1.05	0.7627	1.08	0.7702
96492_at	1449600_at	Gm1683	gene model 1683, (NCBI)	1.08	0.87	-1.11	0.7952	1.23	0.5113	1.05	0.9013	1.16	0.3398
96493_at	1460744_at	2810002I04Rik	RIKEN cDNA 2810002I04 gene	-1.3	0.02	-1.03	0.7935	-1.04	0.6403	-1.07	0.4304	1.4	0.0342
96494_at	1451793_at	4930429H24Rik	RIKEN cDNA 4930429H24 gene	4.15	0.1	1.2	0.1847	-1.08	0.6455	-1.07	0.7472	1.14	0.7321
96496_g_at	1421175_at	Myt1l	myelin transcription factor 1-like	-4.87	0.1	1.18	0.1746	1.13	0.4776	-1.15	0.2837	1.69	0.0416
96498_at	1420335_at	Dmc1h	disrupted meiotic cDNA 1 homolog	-2.74	0.01	1.18	0.269	1.51	0.0272	1.16	0.3474	-1.21	0.5008
96499_at	1449603_at	AI594671	expressed sequence AI594671	-4.04	0.32	-1.13	0.6909	-1.22	0.6013	-1.07	0.8083	1.01	0.9902
96500_at	1450125_at	Gata5	GATA binding protein 5	1.16	0.49	-1.03	0.8552	1.08	0.4969	1.24	0.3006	1.47	0.0637
96501_at	1418993_s_at	F10	coagulation factor X	1.32	0.17	1.11	0.1294	1.12	0.0756	1.15	0.0319	-1.06	0.7532
96502_at	1450445_at	Phex	phosphate regulating gene with homologies	-1.96	0.2	-1.06	0.7776	1.31	0.343	1.2	0.323	1.49	0.339
96503_at	1451551_at	Krt2-16	keratin complex 2, basic, gene 16	-1.42	0.15	1.11	0.5459	1.09	0.4219	1.1	0.2139	1.04	0.8088
96504_at	1418208_at	Pax8	paired box gene 8	-1.29	0.39	1.1	0.1438	1.13	0.0899	-1.02	0.7507	-1.1	0.7713
96505_at	1420605_at	MGI:1860766	metastasis associated gene 2	-2.15	0.26	1.09	0.6498	-1	0.9869	1.02	0.8992	1.57	0.0928
96506_at	1449987_at	Alk	anaplastic lymphoma kinase	-1.39	0.12	-1.31	0.0012	-1.16	0.1565	-1.33	0.0011	1.09	0.6267
96507_at	1421577_at	Evx2	even skipped homeotic gene 2 homolog	-1.02	0.94	-1.06	0.6526	-1	0.9915	1.11	0.5011	-1.24	0.3862
96508_at	1450458_at	Ncoa2	nuclear receptor coactivator 2	-1.1	0.88	-1.05	0.8095	1.08	0.6144	1.11	0.5397	1.5	0.4674
96509_at	1421475_at	Ms4a2	membrane-spanning 4-domains, subfamily A	-2.25	0.25	-1.02	0.9463	1.11	0.7303	-1.46	0.2837	1.07	0.8901
96510_at	1421441_at	Angpt1	angiopoietin 1	-1.33	0.56	-1.16	0.387	-1.11	0.5581	-1.65	0.0206	1.07	0.9014
96513_at	1459917_at	AI451896	expressed sequence AI451896	-1.5	0.18	-1.03	0.89	-2.3	0.0007	-1.79	0.0107	1.1	0.7227
96515_at	1419192_at	Il4i1	interleukin 4 induced 1	2.05	0.32	1.03	0.806	1.33	0.241	1.38	0.1515	1.6	0.0705
96516_at	1429884_at	---	---	3.04	0.08	1.14	0.5011	1.09	0.6993	1.2	0.3618	1.11	0.7534
96517_at	1443696_s_at	Habp2	hyaluronic acid binding protein 2	-2.46	0.4	-1.21	0.1045	-1.13	0.4801	-1.52	0.0058	-1.52	0.5427
96518_at	1420008_s_at	BC037006	cDNA sequence BC037006	1.28	0.37	1.44	0.0223	1.31	0.0047	1.18	0.2	1.31	0.2419
96519_at	1427931_s_at	Pdxk	pyridoxal (pyridoxine, vitamin B6) kinase	4.94	0.03	1.6	0.0877	2.06	0.0032	1.59	0.0947	1.87	0.1507
96520_at	1457776_at	D9Ertd720e	DNA segment, Chr 9, ERATO Doi 720, expr	1.12	0.85	1.81	0.0096	1.65	0.1127	1.08	0.7568	1.48	0.3814
96521_at	1450775_at	Mos	Moloney sarcoma oncogene	-1.09	0.86	1.41	0.2853	1.42	0.3427	-1.17	0.5184	-1.09	0.7586
96522_at	1417365_a_at	Calm1	calmodulin 1	1.04	0.89	1.04	0.4933	-1.02	0.6602	1.01	0.8246	-1.58	0.0223
96523_at	1418618_at	En1	engrailed 1	1.45	0.36	1.35	0.3034	1.29	0.3721	1.09	0.735	1.34	0.4601
96524_at	1459911_at	D030068L24Rik	RIKEN cDNA D030068L24 gene	1.8	0.17	1.37	0.0864	1.4	0.0639	1.1	0.644	-1.14	0.6994
96525_at	1448731_at	Il10ra	interleukin 10 receptor, alpha	-2.44	0.43	2.6	0.1776	1.12	0.5879	3.75	0.048	-1.24	0.4153
96526_at	1437885_at	D030029J20Rik	RIKEN cDNA D030029J20 gene	-1.29	0.13	1.53	0.0023	1.24	0.0992	1.48	0.0006	1.54	0.0308
96527_at	1419877_x_at	---	---	-1.23	0.22	-1.24	0.0405	-1.47	0.0311	-1.38	0.0046	1.3	0.4236
96529_at	1457695_at	Ap1gbp1	AP1 gamma subunit binding protein 1	-1.12	0.83	1.05	0.8782	-1.04	0.9118	-1.25	0.5534	-1.19	0.6082
96530_at	1442006_at	---	Transcribed locus	1.02	0.86	1.15	0.0998	1.26	0.0039	1.33	0.0016	1.17	0.3816
96533_at	1421998_at	Tor3a	torsin family 3, member A	-1.38	0.08	-1.16	0.1799	1.1	0.4272	1.02	0.861	1.07	0.7392
96534_at	1442169_at	Vldlr	very low density lipoprotein receptor	3.26	0.07	2.32	0.0034	1.31	0.1732	2.36	0.003	5.56	0.0004
96535_at	1435802_at	Gm157	gene model 157, (NCBI)	-1.23	0.43	-1.07	0.5018	-1.06	0.5509	-1.06	0.5068	1.01	0.9534

96536_at	1444274_at	---	Transcribed locus, moderately similar to XP_	4.15	0	-1.12	0.6918	-1.29	0.4404	-1.02	0.9486	-1.11	0.7308
96537_at	1420562_at	Slurp1	secreted Ly6/Plaur domain containing 1	1.13	0.61	-1.18	0.4831	-1.14	0.6039	-1.46	0.1235	-1.14	0.5201
96539_at	1440195_at	1200009K13Rik	RIKEN cDNA 1200009K13 gene	2.4	0.12	-1.16	0.154	-1.1	0.3282	-1.25	0.0787	-1.37	0.2301
96540_at	1449896_at	Mlph	melanophilin	-1.35	0.05	-1.07	0.5236	1.17	0.2568	-1.03	0.8194	1.76	0.0249
96541_at	1434964_at	AA408420	expressed sequence AA408420	1.22	0.41	-1.46	0.0019	-1.14	0.264	-1.21	0.0667	-1.06	0.7184
96542_at	1448255_a_at	Surf4	surfeit gene 4	-1.4	0.04	-1.05	0.463	-1.09	0.1952	-1.1	0.0412	1.07	0.5943
96543_at	1455601_at	---	LOC434299	-1.45	0.69	-1.48	0.2153	1.2	0.6174	-2.04	0.0768	1.58	0.3683
96544_at	1419797_at	AA672641	expressed sequence AA672641	1.93	0.28	1.85	0.0858	1.29	0.2377	1.38	0.2045	-1.41	0.4271
96546_r_at	1435586_at	A730042J05Rik	RIKEN cDNA A730042J05 gene	-1.18	0.04	-1.03	0.7256	-1.09	0.1933	-1.06	0.2871	1.18	0.1709
96547_at	1419863_at	Tmod1	Tropomodulin 1	1.05	0.91	-1.25	0.0391	-1.11	0.3092	-1.25	0.0556	1.7	0.1159
96548_at	1421522_at	Galgt2	UDP-N-acetyl-alpha-D-galactosamine:(N-ac	1.91	0.31	2.01	0.0586	1.92	0.1728	1.28	0.4772	1.04	0.9072
96549_at	1420761_at	Chrnd	cholinergic receptor, nicotinic, delta polypept	-1.24	0.71	-1.08	0.8419	1.04	0.8965	-1.03	0.9371	1.9	0.032
96550_at	1443706_at	1500002I11Rik	C1GALT1-specific chaperone 1	1.23	0.61	-1.5	0.0126	-1.35	0.1063	-1.44	0.0337	1.53	0.1523
96551_at	1420331_at	Clec4e	C-type lectin domain family 4, member e	1.07	0.92	-1.09	0.7236	1.48	0.2935	1.36	0.3069	1.02	0.9495
96552_at	1442296_at	2410118I19Rik	RIKEN cDNA 2410118I19 gene	-1.72	0.18	-1.12	0.0949	-1.15	0.0085	-1.13	0.0717	1.28	0.1914
96553_at	1449175_at	Gpr65	G-protein coupled receptor 65	2.04	0.06	2.09	0.2095	1.79	0.2182	3.2	0.1217	-1.24	0.6949
96556_at	1457899_at	Kalrn	kalirin, RhoGEF kinase	1.5	0.38	1.34	0.2148	1.23	0.1958	1.1	0.6102	-1.18	0.5284
96557_at	1422655_at	Ptch2	Patched homolog 2	2.11	0.11	1.01	0.9356	1.08	0.755	1.01	0.9785	2.18	0.2144
96558_at	1422816_a_at	Mutyh	mutY homolog (E. coli)	3.55	0	-1.17	0.1705	-1.3	0.0248	-1.32	0.0557	1.01	0.9691
96560_at	1435559_at	Myo6	myosin VI	-1.4	0.2	-1.42	0	1.02	0.7645	-1.44	0.0001	-1.21	0.3161
96561_at	1434836_at	Nfatc2ip	nuclear factor of activated T-cells, cytoplasm	1.67	0.18	1.42	0.0121	1.4	0.0671	1.04	0.8049	1.28	0.5172
96562_at	1420361_at	Slc11a1	solute carrier family 11 (proton-coupled diva	-1.01	0.91	1.12	0.653	-1.17	0.2569	1.14	0.5945	-1.09	0.8775
96563_at	1448014_s_at	Usp24	ubiquitin specific protease 24	1.3	0.31	-1.04	0.5643	1.12	0.0385	-1.16	0.064	-1.45	0.0531
96564_at	1420623_x_at	Hspa8	heat shock protein 8	1.43	0.21	-1.07	0.5302	-1.15	0.1465	-1.02	0.8366	1.37	0.0482
96566_at	1421355_at	Tgm3	transglutaminase 3, E polypeptide	-2.15	0.2	1.33	0.3047	1.35	0.2325	1.21	0.5709	1.06	0.8758
96567_at	1425171_at	Rho	rhodopsin	-1.09	0.72	-1.16	0.1795	1.45	0.312	1.32	0.4366	-1.46	0.3077
96568_at	1420739_at	Cntn3	contactin 3	-1	0.98	1.48	0.1872	1.09	0.5424	1.15	0.2808	1.5	0.2499
96569_at	1460746_at	---	---	-1.57	0.07	-1.13	0.1118	-1.08	0.2	-1.11	0.0763	1.3	0.2311
96570_at	1439103_at	Hrpt2	hyperparathyroidism 2 homolog (human)	3.85	0.01	-1.02	0.906	-1.03	0.8698	-1.05	0.6973	-1.01	0.9778
96571_at	1449692_at	Prkcz	protein kinase C, zeta	-1.81	0.38	1.38	0.181	1.71	0.1944	1.42	0.1059	1.5	0.409
96572_at	1448032_at	Azi2	5-azacytidine induced gene 2	1.29	0.38	1.14	0.5357	1.5	0.0759	1.21	0.1833	1.04	0.9485
96573_at	1415779_s_at	Actg1	actin, gamma, cytoplasmic 1	1.81	0.17	1.29	0.0085	-1.27	0.0011	1.09	0.3944	1.06	0.5252
96574_at	1450565_at	Il9	interleukin 9	-1.87	0.4	1.23	0.3104	1.22	0.1882	-1.04	0.8679	1.07	0.7394
96575_at	1417762_a_at	Rpl8	ribosomal protein L8	-1.03	0.85	1.05	0.3482	1.12	0.1704	1.08	0.1107	1.41	0.0359
96576_at	1449642_at	Ebi2	Epstein-Barr virus induced gene 2	-1.43	0.02	1.14	0.2521	1.14	0.3915	1.37	0.0131	3.2	0.0045
96579_at	1448016_at	---	Transcribed locus	-1.63	0.6	1.27	0.3214	-1.12	0.7034	-1.51	0.024	-1.05	0.8785
96580_at	1421193_a_at	Pbx3	pre B-cell leukemia transcription factor 3	-1.24	0.51	1.05	0.7757	1.02	0.8432	-1.11	0.5744	1.09	0.8346
96581_at	1421593_at	Ncam2	neural cell adhesion molecule 2	-2.13	0.42	1.12	0.4615	1.48	0.1574	1.25	0.1042	1.43	0.1549
96582_at	1425301_at	Ncam2	neural cell adhesion molecule 2	-1.71	0.42	1.18	0.6867	1.15	0.7023	1.18	0.5928	-2.32	0.0633
96583_s_at	1450249_s_at	Kif5a /// Kif5c	kinesin family member 5A /// kinesin family r	-1.21	0.61	1.61	0.1735	1.24	0.5695	1.45	0.2983	1.69	0.2124
96584_f_at	1451852_at	MGI:2675349	2-cell-stage, variable group, member 3	1.14	0.85	-1.47	0.1468	-1.75	0.0432	-1.9	0.0433	5.46	0.001
96585_at	1450277_at	Avpr2	arginine vasopressin receptor 2	-1.66	0.25	-1.4	0.1132	1.02	0.9179	-1.56	0.0649	1.18	0.4596
96586_at	1422674_s_at	Crygb /// Crygc	crystallin, gamma B /// crystallin, gamma C	-1.18	0.42	1.18	0.1377	1.19	0.171	1.05	0.5275	-1.03	0.8962
96587_at	1421789_s_at	Arf3	ADP-ribosylation factor 3	-1.74	0.01	-1.23	0.0939	-1.53	0.0179	-1.3	0.0509	-1	0.9866
96588_at	1450344_a_at	Ptger3	prostaglandin E receptor 3 (subtype EP3)	-1.21	0.75	-1.41	0.4114	-1.09	0.8638	1.09	0.8168	-1.27	0.5229
96589_at	1425251_at	Ptger3	prostaglandin E receptor 3 (subtype EP3)	2.43	0.12	-1.23	0.4691	-1.26	0.4441	-1.08	0.8501	-2.1	0.1582
96590_f_at	1429139_at	2900060B22Rik	RIKEN cDNA 2900060B22 gene	1.15	0.63	1.13	0.4506	1.12	0.6088	1.14	0.4236	3.37	0.0224
96591_at	1449465_at	Reln	reelin	-1.2	0.32	-1.07	0.4401	1.18	0.1006	-1.02	0.8427	1.79	0.0076
96592_at	1451737_at	Pik3r1	phosphatidylinositol 3-kinase, regulatory sub	3.27	0.01	-1.31	0.1618	-2.22	0.0031	-2.18	0.0034	-2.19	0.2257
96593_at	1421896_at	Elk1	ELK1, member of ETS oncogene family	-3.87	0	-1.2	0.2276	1.13	0.2839	-1.22	0.1935	1.46	0.4191
96595_at	1449359_at	Pax1	paired box gene 1	-3.34	0.13	-1.11	0.421	-1.08	0.4423	-1.13	0.2461	1.28	0.2509
96596_at	1420760_s_at	Ndrl	N-myc downstream regulated-like	1.08	0.76	1.38	0.0275	1.06	0.7089	1.23	0.1371	1.9	0.0038
96597_at	1421566_at	Pet2	plasmacytoma expressed transcript 2	-2.09	0.28	-1.74	0.072	-1.57	0.1215	-1.74	0.036	-2.39	0.0152

96598_at	1455653_at	Ccnj	cyclin J	1.22	0.52	-1.57	0.0046	-1.07	0.5351	-1.16	0.1992	-1.18	0.5998
96599_at	1435420_at	C330016K18Rik	RIKEN cDNA C330016K18 gene	-1.32	0.53	1.36	0.1044	1.33	0.0648	1.16	0.3821	1.48	0.1964
96600_at	1441618_at	B130017I01Rik	RIKEN cDNA B130017I01 gene	-1.33	0.17	-1.02	0.9355	-1.81	0.0182	-1.91	0.0159	-2.42	0.372
96603_at	1420832_at	Qscn6	quiescin Q6	-1.38	0.31	-2.05	0	-1.41	0.027	-2.35	0	-3.37	0.008
96604_at	1423943_at	1110032N12Rik	RIKEN cDNA 1110032N12 gene	1.46	0.38	1.08	0.4021	1.54	0.0055	1.05	0.6322	-1.47	0.0404
96605_at	1423909_at	0610011I04Rik	RIKEN cDNA 0610011I04 gene	1.49	0.25	1.66	0.0067	-1.14	0.1898	1.67	0.0344	-1.75	0.0766
96606_at	1450007_at	1500003O03Rik	RIKEN cDNA 1500003O03 gene	1.35	0.49	-1.25	0.159	-1.06	0.7535	-1.39	0.0356	-1.42	0.5199
96607_at	1420810_at	1500003O03Rik	RIKEN cDNA 1500003O03 gene	-1.22	0.42	-1.19	0.0211	1.08	0.2126	-1.11	0.2472	-1.1	0.4314
96608_at	1460194_at	Phyh	phytanoyl-CoA hydroxylase	1.44	0.03	1.02	0.7036	1.08	0.1369	1.14	0.0286	1.1	0.4586
96609_at	1452371_at	Sfrs11	splicing factor, arginine/serine-rich 11	-1.04	0.71	1.23	0.0298	1.33	0.128	1.42	0.0016	1.62	0.0025
96610_at	1415826_at	Atp6v1h	ATPase, H+ transporting, lysosomal 50/57kD	-1.02	0.91	1.02	0.6646	1.03	0.5488	1.03	0.2393	1.16	0.162
96611_at	1429708_at	Ndufa11	NADH dehydrogenase (ubiquinone) 1 alpha	1.87	0.13	-1.03	0.6233	1.14	0.023	1.26	0.0119	-1.07	0.7365
96613_at	1416634_at	5730536A07Rik	RIKEN cDNA 5730536A07 gene	1.14	0.73	-1.04	0.3949	-1.02	0.6117	-1.08	0.1052	-1.26	0.0049
96614_at	1454606_at	4933426M11Rik	RIKEN cDNA 4933426M11 gene	1.24	0.27	1.08	0.1639	1.18	0.0947	1.3	0.0017	1.06	0.6055
96615_at	1426624_a_at	Ypel3	yippee-like 3 (Drosophila)	-1.17	0.21	-1.12	0.1696	1.04	0.7772	-1.14	0.0407	1.01	0.9472
96616_at	1448763_at	Atad1	ATPase family, AAA domain containing 1	-1.15	0.38	-1.11	0.0404	-1.03	0.776	-1.08	0.2166	-1.03	0.7557
96617_at	1451676_at	Drap1	Dr1 associated protein 1 (negative cofactor 2	1.39	0.03	1.16	0.08	-1.05	0.6252	1.1	0.268	1.24	0.2595
96618_at	1449021_at	Rpp21	ribonuclease P 21 subunit (human)	1.26	0.13	-1.1	0.0643	-1.09	0.1227	-1.1	0.0835	-1.19	0.348
96619_at	1452177_at	Abcf3	ATP-binding cassette, sub-family F (GCN20	1.78	0.13	-1.09	0.3264	-1.19	0.1	-1.32	0.0174	-1.11	0.7716
96620_at	1417550_a_at	Gsg1	germ cell-specific gene 1	1.1	0.57	-1	0.935	1.05	0.3262	-1.03	0.6845	1.47	0.0582
96621_at	1452726_a_at	1110061L23Rik	RIKEN cDNA 1110061L23 gene	1.47	0.2	1.04	0.6995	-1.05	0.6007	1.04	0.6134	1.04	0.7434
96623_at	1435133_at	Ugcg	UDP-glucose ceramide glucosyltransferase	1.18	0.58	1.19	0.1136	1.2	0.1978	1.2	0.1094	1.34	0.1319
96625_at	1435114_at	Wdhd1	WD repeat and HMG-box DNA binding prote	-1.11	0.77	-1.27	0.0536	-1.26	0.0597	-1.18	0.1869	-1.08	0.6429
96626_at	1449672_s_at	Tufm	Tu translation elongation factor, mitochondri	1.63	0.13	-1.08	0.2456	-1.08	0.4686	-1.1	0.3095	-1.22	0.1087
96627_at	1416667_at	Ebp	phenylalkylamine Ca2+ antagonist (emopam	1.29	0.36	-1.42	0	-1.2	0.0167	-1.72	0	-1.68	0.0004
96628_at	1452157_at	Eprs	glutamyl-prolyl-tRNA synthetase	-1.3	0.29	-1.06	0.3921	-1.04	0.6054	1.03	0.4413	-1.17	0.2988
96629_at	1434216_a_at	D7Rp2e	DNA segment, Chr 7, Roswell Park 2 compl	1.75	0.02	1.52	0.0001	1.16	0.0599	1.53	0	1.64	0.0024
96630_at	1415938_at	Spink3	serine protease inhibitor, Kazal type 3	12.28	0.01	2.43	0.1062	1.06	0.9116	2.95	0.0046	4.26	0.0161
96633_s_at	1415778_at	Morf4l2	mortality factor 4 like 2	-1.35	0.47	-1.15	0.1029	-1.08	0.3826	-1.15	0.1404	-1.45	0.0007
96634_at	1452716_at	5730469M10Rik	RIKEN cDNA 5730469M10 gene	1.69	0.09	1.53	0.0039	1.14	0.4056	1.67	0.0009	1.31	0.1891
96635_at	1426344_at	Gle1l	GLE1 RNA export mediator-like (yeast	2.04	0.15	-1.01	0.813	1.13	0.1209	-1.01	0.9369	-1.5	0.0601
96636_at	1415748_a_at	Dctn5	dynactin 5	1.26	0.29	-1.03	0.5787	-1.01	0.904	1.03	0.6156	-1.06	0.7282
96637_at	1419447_s_at	---	---	-1.01	0.97	1.03	0.6736	1.08	0.2828	1.02	0.8395	-1.3	0.497
96640_at	1448509_at	3110001A13Rik	RIKEN cDNA 3110001A13 gene	1.42	0.09	1.16	0.0346	1.03	0.653	1.08	0.2948	-1.11	0.336
96641_at	1433478_at	Psarl	presenilin associated, rhomboid-like	1.75	0.06	1.03	0.7554	1.19	0.0493	1.13	0.1827	1.11	0.2439
96643_at	1424351_at	Wfdc2	WAP four-disulfide core domain 2	1.53	0.09	1.04	0.8413	-1.09	0.5272	-1.73	0.0033	-3.5	0.0645
96644_at	1450742_at	Bysl	bystin-like	1.37	0.49	-1.77	0.0806	-2.05	0.0379	-1.73	0.0802	1.17	0.7341
96646_at	1460209_at	Usp39	ubiquitin specific protease 39	1.44	0.13	-1.12	0.1767	-1.01	0.8932	-1.06	0.4556	-1.62	0.0362
96647_at	1432271_a_at	4833420K19Rik	RIKEN cDNA 4833420K19 gene	-1.28	0.42	1.09	0.2604	1.25	0.0532	1.14	0.2403	-1.16	0.4225
96648_at	1455269_a_at	Coro1a	coronin, actin binding protein 1A	1.13	0.71	2.15	0.007	-1.29	0.3026	2.03	0.0621	1.43	0.201
96649_at	1452130_at	2310042M24Rik	RIKEN cDNA 2310042M24 gene	1.93	0.14	-1.01	0.8647	-1.17	0.0045	-1.06	0.3237	-1.25	0.0146
96650_at	1420776_a_at	Auh	AU RNA binding protein/enoyl-coenzyme A h	1.09	0.21	1.21	0.0062	1.24	0.0543	1.32	0.0001	1.16	0.2067
96651_at	1422676_at	Smarce1	SWI/SNF related, matrix associated, actin de	1.41	0.11	1.13	0.3223	1.34	0.0435	1.13	0.333	-1	0.9892
96652_at	1456313_x_at	Mrpl28	mitochondrial ribosomal protein L28	1.03	0.74	-1.04	0.6124	-1.04	0.3851	-1.02	0.8087	-1.11	0.0371
96653_at	1452734_at	Rnaset2	ribonuclease T2	1.2	0.42	1.06	0.6626	-1.16	0.0547	1.01	0.9521	-1.09	0.078
96656_at	1426723_at	Wdr48	WD repeat domain 48	2.05	0.03	1.01	0.8397	1.11	0.1564	1.07	0.177	1.46	0.0086
96657_at	1420502_at	Sat1	spermidine/spermine N1-acetyl transferase 1	1.25	0.44	1.38	0.0081	1.28	0.0646	1.61	0.0164	1.04	0.8525
96658_at	1433480_at	2900010J23Rik	RIKEN cDNA 2900010J23 gene	1.73	0.02	-1.09	0.1147	-1.35	0.0141	-1.18	0.0309	-1.15	0.4758
96661_at	1424391_at	Nrd1	nardilysin, N-arginine dibasic convertase, NF	1.99	0.24	1.19	0.0009	1.23	0.0001	1.29	0.0003	1.03	0.776
96662_at	1429514_at	Ppap2b	phosphatidic acid phosphatase type 2B	1.01	0.97	-1.02	0.7829	1.18	0.1893	-1.16	0.1118	-1.13	0.239
96663_at	1416864_at	Surf6	surfeit gene 6	2.47	0.26	1.25	0.1322	1.44	0.0151	1.36	0.0403	-2.17	0.0664
96665_at	1420495_a_at	Vps26	vacuolar protein sorting 26 (yeast)	-1.09	0.7	1.08	0.7164	1.02	0.9058	1.08	0.6742	1.46	0.2645
96666_at	1423538_at	Ntan1	N-terminal Asn amidase	2.45	0.01	1.13	0.3427	-1.08	0.6453	1.1	0.3655	1.39	0.2321

96667_at	1433666_s_at	Vps41	vacuolar protein sorting 41 (yeast)	-1.04	0.86	1.13	0.0713	1.09	0.2095	1.17	0.0111	-1.27	0.072
96668_at	1460685_at	Timm17b	translocator of inner mitochondrial membran	-1.12	0.5	-1.07	0.4195	-1.08	0.3679	-1.07	0.4488	1.02	0.7581
96669_at	1423838_s_at	2400003C14Rik	RIKEN cDNA 2400003C14 gene	1.61	0.02	1.14	0.0132	1.16	0.205	1.3	0.0003	-1.31	0.1789
96670_at	1452823_at	Gstk1	glutathione S-transferase kappa 1	1.53	0.02	-1.01	0.9397	-1.16	0.1502	-1.07	0.5951	-1.19	0.2054
96672_at	1428662_a_at	MGI:1916782	homeobox only domain	-1.58	0.08	-1.25	0.2448	-2.04	0.0315	-1.06	0.8135	-1.68	0.0279
96674_at	1419950_s_at	Tnpo3	transportin 3	1.53	0.46	1.15	0.3184	1	0.994	1.01	0.9532	1.68	0.0329
96675_at	1435811_a_at	Unc50	unc-50 homolog (C. elegans)	1.17	0.4	1.09	0.0697	1.18	0.0027	1.18	0.017	-1.12	0.3315
96676_at	1454640_at	Chchd7	coiled-coil-helix-coiled-coil-helix domain con	1.24	0.51	1.08	0.3508	1.2	0.0469	1.22	0.0682	1.07	0.7268
96677_at	1460687_at	2410195B05Rik	RIKEN cDNA 2410195B05 gene	1.18	0.49	1.1	0.3641	1.03	0.8034	1.16	0.2663	1.89	0
96678_at	1451559_a_at	Dhrs4	dehydrogenase/reductase (SDR family) mer	1.53	0.05	1.14	0.1355	1.15	0.0614	1.25	0.0349	-1.03	0.8259
96680_at	1417191_at	Dnajb9	DnaJ (Hsp40) homolog, subfamily B, memb	1.6	0.3	1.95	0.0038	1.97	0.0026	1.89	0.0005	1.01	0.9589
96682_at	1418075_at	St6galnac4	ST6 (alpha-N-acetyl-neuraminyl-2,3-beta-ga	-1.13	0.61	-1.04	0.8815	-1.2	0.4804	1.12	0.7718	-1.43	0.5607
96684_at	1433457_s_at	Grsf1	G-rich RNA sequence binding factor 1	1.04	0.84	1.32	0.0026	1.15	0.0961	1.43	0.0002	1.26	0.0228
96687_f_at	1420642_a_at	2010100O12Rik	RIKEN cDNA 2010100O12 gene	1.68	0.03	1.2	0.0073	1.03	0.7103	1.21	0.0001	-1.34	0.1163
96691_at	1451995_at	Taf11	TAF11 RNA polymerase II, TATA box bindin	-1.21	0	1.06	0.4331	1.17	0.077	-1.01	0.9197	-1.34	0.1671
96692_at	1423591_at	Fgfr1op2	FGFR1 oncogene partner 2	-1.67	0.01	-1.14	0.2984	1.02	0.8947	1.03	0.7874	1.44	0.0619
96693_at	1416312_at	Rars	arginyl-tRNA synthetase	1.32	0.3	1.05	0.3674	1.04	0.4649	1.09	0.1071	-1.1	0.5224
96695_at	1417609_at	Ube2a	ubiquitin-conjugating enzyme E2A, RAD6 hc	-1.07	0.76	-1.05	0.6465	-1.08	0.6298	-1.27	0.1203	-1.91	0.0048
96696_at	1452787_a_at	Hrmt1l2	heterogeneous nuclear ribonucleoproteins r	1.69	0.25	1.41	0.0029	1.05	0.7234	1.42	0.0017	1.37	0.0798
96698_at	1423234_at	Psmd5	proteasome (prosome, macropain) 26S subu	-1.31	0.29	1.16	0.1659	1.24	0.1446	1.16	0.3422	-1.23	0.308
96699_at	1422495_a_at	Hmgn1	high mobility group nucleosomal binding dor	1.27	0.2	-1.03	0.7351	1.06	0.5641	-1.01	0.9542	1.3	0.0575
96700_r_at	1455349_at	Rap1b	RAS related protein 1b	1.2	0.44	1.01	0.9062	1.13	0.559	1.15	0.1669	1.24	0.1775
96701_at	1448283_a_at	Uble1b	ubiquitin-like 1 (sentrin) activating enzyme E	1.58	0.03	1.29	0.0035	1.22	0.0814	1.23	0.0867	-1.23	0.3013
96703_at	1450062_a_at	Maged1	melanoma antigen, family D, 1	-1.15	0.14	-1.11	0.0893	1.14	0.0966	-1.06	0.3147	-1.16	0.281
96704_at	1448612_at	Sfn	stratifin	-1.45	0.6	-1.07	0.7494	-1.97	0.009	1.04	0.8469	-1.47	0.3467
96707_at	1416370_at	Zipro1	zinc finger proliferation 1	1.01	0.95	1.07	0.1896	1.2	0.0666	1.05	0.3431	1.25	0.0216
96708_at	1416108_a_at	Tmed3	transmembrane emp24 domain containing 3	1.36	0.12	-1.09	0.2888	1.09	0.1535	1	0.9745	-1.12	0.3339
96709_at	1459890_s_at	1110008P14Rik	RIKEN cDNA 1110008P14 gene	-1.04	0.83	-1.24	0.0019	-1.1	0.2004	-1.15	0.0301	-2.48	0.0109
96710_at	1428029_a_at	H2afv	H2A histone family, member V	1.49	0.02	1.36	0.0026	1.32	0.0305	1.42	0	1.22	0.1792
96711_at	1422517_a_at	Znrd1	zinc ribbon domain containing, 1	-1	0.99	1.1	0.2563	1.03	0.7314	1.02	0.7306	1.12	0.4761
96712_at	1448321_at	Smoc1	SPARC related modular calcium binding 1	1.11	0.68	1.09	0.3922	1.2	0.0111	1.04	0.5738	1.48	0.1328
96713_at	1434510_at	Papss2	3'-phosphoadenosine 5'-phosphosulfate syn	2.4	0.08	-1.23	0.0103	-1.29	0.0075	-1.11	0.1878	-1.1	0.5206
96716_at	1416768_at	1110003E01Rik	RIKEN cDNA 1110003E01 gene	-1.18	0.41	1.12	0.0061	1.03	0.5721	1.19	0.0057	-1	0.9941
96717_at	1423752_at	Ddx47	DEAD (Asp-Glu-Ala-Asp) box polypeptide 47	1.32	0.19	1.02	0.8211	1.08	0.1238	1.04	0.542	1.1	0.7099
96718_at	1416914_s_at	Mtvr2 /// Sssca1	mammary tumor virus receptor 2 /// Sjogren'	-1.17	0.15	1.09	0.102	1.05	0.5317	1.22	0.0083	1.11	0.2451
96719_i_at	1417653_at	Pvalb	parvalbumin	-1.68	0.05	1.1	0.3511	-1.13	0.1107	-1.07	0.4617	1.49	0.0185
96724_r_at	1448743_at	Ssx2ip	synovial sarcoma, X breakpoint 2 interacting	1.02	0.9	-1.15	0.1364	1.07	0.4409	-1.06	0.5135	1.07	0.5752
96725_at	1415746_at	Cic	capicua homolog (Drosophila)	-1.44	0.06	1.07	0.3427	-1.08	0.1744	-1.09	0.2703	1.12	0.1748
96726_at	1460389_at	Cdk8	cyclin-dependent kinase 8	1.19	0.23	1.21	0.0001	1.19	0.0015	1.13	0.0013	-1.01	0.9704
96728_at	1424786_s_at	Wdr45	WD repeat domain 45	1.24	0.11	1.05	0.5326	1.34	0.0003	1.15	0.1257	1	0.9941
96729_at	1455575_at	Eif4ebp2	eukaryotic translation initiation factor 4E bin	1.04	0.92	-1.05	0.774	1.04	0.8837	-1.32	0.1868	-1.88	0.2688
96730_at	1421893_a_at	Tpp2	tripeptidyl peptidase II	-1.33	0.55	1.21	0.2827	1.26	0.1569	1.08	0.6729	1.22	0.6938
96731_at	1434697_at	1110001P04Rik	RIKEN cDNA 1110001P04 gene	-1.14	0.58	1.12	0.2338	-1.12	0.0543	-1.02	0.703	1.44	0.0143
96732_at	1438669_at	Wdr40a	WD repeat domain 40A	1.01	0.98	1.45	0.0053	1.41	0.0088	1.5	0.0003	1.05	0.4098
96733_at	1425266_a_at	Rap1gds1	RAP1, GTP-GDP dissociation stimulator 1	-1.06	0.75	1.01	0.8536	1.14	0.1139	1.01	0.8707	1.15	0.3473
96734_at	1417834_at	Synj2bp	synaptojanin 2 binding protein	-1.31	0.21	-1.09	0.1571	-1.01	0.8009	-1.14	0.108	-1.26	0.0006
96735_at	1448956_at	Stard10	START domain containing 10	1.09	0.47	1.01	0.8608	-1.11	0.4296	-1.14	0.186	1.08	0.7041
96736_at	1454899_at	Lpp	LIM domain containing preferred translocatic	-1.67	0.14	1.06	0.2346	1.12	0.2574	-1.03	0.6853	1.23	0.1411
96737_at	1451107_at	Tbc1d22a	TBC1 domain family, member 22a	1.19	0.31	-1.04	0.4885	-1.02	0.8107	-1.1	0.1454	-1.15	0.6317
96738_at	1416094_at	Adam9	a disintegrin and metalloproteinase domain !	-1.15	0.55	-1.09	0.1908	1.08	0.4572	-1.14	0.1972	-1.7	0.0079
96739_at	1439028_at	1810045K17Rik	RIKEN cDNA 1810045K17 gene	1.25	0.49	-1.13	0.3455	-1.28	0.0281	-1.24	0.1393	-1	0.9862
96742_at	1418511_at	Dpt	dermatopontin	1.37	0.03	-1.16	0.4724	-1.37	0.3024	-1.24	0.3839	1.17	0.7385
96743_at	1428554_a_at	1810035L17Rik	RIKEN cDNA 1810035L17 gene	1.76	0.12	-1.01	0.9254	-1.03	0.7895	1.11	0.1255	-1.13	0.6066

96744_at	1448445_at	Acp6	acid phosphatase 6, lysophosphatidic	1.06	0.8	1.09	0.0755	1.07	0.1282	-1.04	0.5179	1.14	0.2175
96746_at	1426265_x_at	Dlat	dihydrolipoamide S-acetyltransferase (E2 cc	1.25	0.07	1.16	0.0407	1.38	0.0156	1.17	0.1436	1.01	0.9584
96747_at	1449027_at	Rhou	ras homolog gene family, member U	1.19	0.53	-1.01	0.7991	1.02	0.7066	-1.19	0.0118	-1.68	0.0401
96749_f_at	1433579_at	Tmem30b	transmembrane protein 30B	1.03	0.93	1.15	0.3603	1.14	0.3842	1.37	0.086	1.47	0.0325
96751_at	1433951_at	Arl5	ADP-ribosylation factor-like 5	-1.19	0.17	-1.04	0.5591	-1.05	0.5428	-1.23	0	-1.09	0.3176
96752_at	1424067_at	Icam1	intercellular adhesion molecule	1.98	0.04	2.71	0.1008	1.26	0.0389	4.04	0.046	1.79	0.0065
96753_at	1449523_at	Bcl7c	B-cell CLL/lymphoma 7C	1.3	0.57	1	0.9908	-1.13	0.203	-1.05	0.5893	1.04	0.9252
96755_at	1424013_at	Etf1	eukaryotic translation termination factor 1	1.66	0.1	1.38	0.0035	1.31	0.005	1.44	0	1.73	0.0018
96756_at	1435333_at	---	---	2.32	0.06	-1.03	0.824	1.26	0.0059	1.05	0.6274	1.02	0.9175
96757_at	1449000_at	D10Jhu81e	DNA segment, Chr 10, Johns Hopkins Unive	1.64	0.04	1.08	0.2468	-1.07	0.2676	1.02	0.743	1.01	0.9307
96759_r_at	1424530_at	Sec14l2	SEC14-like 2 (S. cerevisiae)	1.08	0.73	-1.15	0.3164	-1.13	0.3007	-1.33	0.0537	-1.38	0.0144
96760_at	1449886_a_at	Timm10	translocase of inner mitochondrial membran	1.66	0.1	1.3	0.013	1.35	0.0013	1.6	0.0022	1.16	0.4636
96761_at	1416596_at	2210409B01Rik	RIKEN cDNA 2210409B01 gene	-1.68	0.01	-1.05	0.504	-1.1	0.1918	-1.05	0.4591	1.01	0.9515
96762_at	1433585_at	Tnpo1	Transportin 1	-1.67	0.09	1.1	0.3603	1.06	0.5165	1	0.9986	1.19	0.2906
96763_at	1448426_at	Sardh	Sarcosine dehydrogenase	2.33	0.03	1.13	0.0906	-1.44	0.0013	1.07	0.3328	-1.18	0.3152
96764_at	1419043_a_at	Iigp1	interferon inducible GTPase 1	-3.93	0.04	-1.01	0.9804	-1.8	0.0021	1.03	0.9285	-2.87	0.0267
96765_at	1433924_at	Peg3	Paternally expressed 3	1.31	0.06	1.03	0.8384	1.53	0.0063	-1.13	0.4457	2.33	0.0281
96766_s_at	1425248_a_at	Tyro3	TYRO3 protein tyrosine kinase 3	1.45	0.1	1.04	0.802	1.07	0.7471	-1.09	0.6207	1.31	0.1554
96767_at	1451099_at	Mbc2	membrane bound C2 domain containing pro	-1.28	0.02	1.17	0.0194	1.03	0.5609	1.31	0.0038	-1.44	0.1755
96768_at	1417226_at	Fbxw4	F-box and WD-40 domain protein 4	-1.53	0.07	1.09	0.3713	1.03	0.5985	-1.07	0.3559	1.14	0.1023
96770_at	1450936_a_at	Dnase1l2	deoxyribonuclease 1-like 2	2.44	0.12	-1.21	0.3367	1.06	0.8102	-1.35	0.1888	-1.62	0.567
96771_at	1434606_at	Erbb3	V-erb-b2 erythroblastic leukemia viral oncog	-1.03	0.91	1.05	0.5316	1.09	0.2946	1.09	0.2682	-1.03	0.9087
96772_at	1449061_a_at	Prim1	DNA primase, p49 subunit	1.94	0.26	1.32	0.4043	1.18	0.5852	1.51	0.1773	1.78	0.0601
96773_at	1423247_at	Txndc4	thioredoxin domain containing 4 (endoplasm	1.05	0.84	-1.02	0.8232	1.05	0.6354	-1.01	0.8461	-1.2	0.078
96774_at	1434180_at	Plekhc1	pleckstrin homology domain containing, fam	1.09	0.69	1.05	0.5594	-1.05	0.652	-1.05	0.5928	-1.33	0.0521
96777_at	1449138_at	Sf3b1	splicing factor 3b, subunit 1	1.66	0.04	1.17	0.1475	1.22	0.113	1.36	0.0012	1.03	0.7379
96778_at	1416998_at	Rrs1	RRS1 ribosome biogenesis regulator homolo	-1.38	0.4	1.01	0.9196	1.39	0.0262	-1.08	0.5853	1.02	0.8116
96779_f_at	1420113_s_at	2410022L05Rik	RIKEN cDNA 2410022L05 gene	2.07	0.01	-1.09	0.3693	1.14	0.1602	-1.02	0.7762	1.3	0.0621
96780_at	1448971_at	2410022L05Rik	RIKEN cDNA 2410022L05 gene	2.02	0.19	1.4	0.2311	1.41	0.2304	1.29	0.3157	-1.01	0.9491
96781_at	1415725_at	Rrn3	RRN3 RNA polymerase I transcription factor	1.33	0.21	1.17	0.1196	1.33	0.0131	1.34	0.0095	1.44	0.1632
96782_at	1427290_at	Krt2-19	keratin complex 2, basic, gene 19	-1.7	0.24	-1.11	0.2988	-1.17	0.1848	-1.3	0.0548	-1.3	0.1708
96783_at	1426014_a_at	Mucdhl	mucin and cadherin like	-1.15	0.52	-1.04	0.7237	1.07	0.6824	1.02	0.868	-1.23	0.2389
96784_at	1433543_at	Anln	anillin, actin binding protein (scraps homolog	2.54	0.14	1.04	0.8981	1.37	0.2403	1.25	0.4732	1.53	0.3662
96785_at	1448839_at	D17Ertd288e	DNA segment, Chr 17, ERATO Doi 288, exp	1.36	0.22	1.02	0.8746	1.22	0.1381	1.14	0.3588	2.2	0.0463
96787_at	1424758_s_at	Serpina10	serine (or cysteine) proteinase inhibitor, clad	-1.17	0.26	-1.18	0.0159	-1.26	0.0025	-1.44	0.0001	-1.35	0.1795
96790_f_at	1452583_s_at	Galm	galactose mutarotase	1.19	0.36	1.07	0.3062	1	0.9617	1.1	0.0858	1.16	0.0527
96791_at	1456603_at	1500005K14Rik	RIKEN cDNA 1500005K14 gene	-1.2	0.16	1.06	0.4901	1.11	0.1792	1.05	0.42	1.79	0.0004
96792_at	1455593_at	Apob	apolipoprotein B	1.42	0.14	1.18	0.5005	-2.41	0.0033	-1.47	0.1274	-1.06	0.8427
96794_at	1425030_at	Zfp622	zinc finger protein 622	1.13	0.53	-1.03	0.7356	-1.01	0.8747	1.04	0.6631	1.24	0.28
96796_f_at	1419622_at	Ugt2b5	UDP-glucuronosyltransferase 2 family, mem	-1.47	0.11	-1.14	0.0002	1.06	0.0611	-1.17	0.0055	-1.94	0.0001
96799_at	1417476_at	Fbxw5	F-box and WD-40 domain protein 5	1.41	0.03	1.06	0.5593	-1.02	0.8429	1.16	0.1403	-1.61	0.385
96801_at	1422184_a_at	Ak1	adenylate kinase 1	-1.57	0.38	-1.13	0.823	-1.01	0.9905	-1.33	0.6002	1.13	0.6008
96802_at	1416377_at	Pdcd7	programmed cell death protein 7	-1.07	0.72	-1.06	0.6125	-1.01	0.8967	-1.06	0.6191	1.3	0.3008
96803_at	1420654_a_at	Gbe1	glucan (1,4-alpha-), branching enzyme 1	2.02	0.1	-1.01	0.8855	1.15	0.4385	1.11	0.4805	-1.78	0.0013
96804_at	1424019_at	Nol1	nucleolar protein 1	-1.08	0.58	1.39	0.0066	1.38	0.0096	1.4	0.0012	1.07	0.7275
96806_at	1452836_at	Lpin2	lipin 2	1.95	0.01	1.24	0.0877	1.35	0.0198	1.8	0.0001	1.92	0.0239
96807_at	1416296_at	---	---	1.99	0.18	1	0.9956	-1.32	0.1686	1.21	0.1742	-1.42	0.4926
96808_at	1423563_at	ORF31	open reading frame 31	-1.37	0.17	-1.05	0.7994	1.08	0.7855	1.39	0.257	1.06	0.7034
96810_at	1454086_a_at	Lmo2	LIM domain only 2	2.2	0.12	1.5	0.0035	-1.01	0.9376	1.45	0.0259	1.53	0.0336
96811_at	1416165_at	1700093E07Rik	RIKEN cDNA 1700093E07 gene	2.96	0.02	1.29	0.3363	1.04	0.8689	1.49	0.0924	1.06	0.8935
96812_at	1427049_s_at	Smo	smoothened homolog (Drosophila)	1.38	0.57	1.05	0.6796	-1.02	0.9088	1.02	0.8584	1.86	0.1921
96817_at	1457303_at	2700067D09Rik	RIKEN cDNA 2700067D09 gene	1.5	0.31	1.26	0.1167	1.2	0.3514	1.22	0.2221	4.17	0
96818_at	1421720_a_at	Dtx2	deltex 2 homolog (Drosophila)	1	1	1.2	0.0838	1.18	0.3248	1.15	0.3257	1.07	0.8372

96819_at	1428241_at	2310035K24Rik	RIKEN cDNA 2310035K24 gene	1.51	0.52	-1.06	0.4895	-1.04	0.6701	-1.14	0.1962	-1.19	0.4714
96822_at	1433886_at	Eif2b5	eukaryotic translation initiation factor 2B, sub	1.74	0.11	1.03	0.7449	1.07	0.4796	1.14	0.1526	-1.02	0.9199
96824_at	1452017_at	Sox15	SRY-box containing gene 15	-1.61	0.15	-1.12	0.4605	1.08	0.5576	1.01	0.9509	1.47	0.0779
96825_at	1452264_at	Tenc1	tensin like C1 domain-containing phosphata	2.48	0.05	1.54	0.0008	1.21	0.2569	1.53	0.005	1.46	0.1035
96827_at	1452830_s_at	Cad	carbamoyl-phosphate synthetase 2, asparta	1.58	0.46	1.17	0.2725	1.35	0.275	1.68	0.0625	1.36	0.0512
96828_at	1417422_at	Gnmt	glycine N-methyltransferase	-1.06	0.67	1.07	0.1366	-1.13	0.0041	-1.01	0.7635	1.19	0.1611
96829_at	1433717_at	D19Wsu162e	DNA segment, Chr 19, Wayne State Univers	1.3	0.34	1.02	0.8149	1.07	0.5536	-1.03	0.506	-1.05	0.5255
96831_at	1424650_at	Pdia5	protein disulfide isomerase-associated 5	1.06	0.74	-1.05	0.5368	1.09	0.4334	-1.05	0.5847	1.11	0.3508
96832_at	1424424_at	Slc39a1	solute carrier family 39 (zinc transporter), me	1.39	0.56	-1	0.9754	1.06	0.633	-1.02	0.856	1.05	0.8309
96833_at	1447924_at	8430423A01Rik	RIKEN cDNA 8430423A01 gene	1.38	0.36	-1.08	0.3544	-1.04	0.6902	-1.12	0.143	-1.02	0.9637
96834_at	1417727_at	Sfrs9	splicing factor, arginine/serine rich 9	-1.05	0.88	1.07	0.0553	1.11	0.0093	1.12	0.0366	-1.24	0.139
96835_at	1418464_at	Matn4	matrilin 4	1.48	0.6	-1.16	0.3594	-1.76	0.0178	-1.28	0.0923	-1.21	0.5409
96836_r_at	1437585_x_at	Zfp161	zinc finger protein 161	3.1	0.04	1.47	0.0517	1.41	0.2418	1.81	0.0003	-1.02	0.9743
96837_at	1427109_at	ORF61	open reading frame 61	-1.42	0.04	-1.03	0.6423	-1.04	0.6923	-1.14	0.0375	1.08	0.5104
96838_at	1418779_at	Rce1	Ras and a-factor-converting enzyme 1 homo	-1.51	0.2	-1.39	0.0257	-1.22	0.1234	-1.57	0.0065	1.62	0.0318
96840_at	1423187_at	Gabarapl2	gamma-aminobutyric acid (GABA-A) recepto	1.03	0.87	-1.05	0.5407	1.02	0.7116	-1.1	0.2088	-1.34	0.0011
96841_at	1451069_at	Pim3	proviral integration site 3	1.23	0.35	-1.04	0.5796	-1.05	0.7575	1.06	0.6313	1.38	0.6179
96845_at	1426668_at	Slc30a9	solute carrier family 30 (zinc transporter), me	1.35	0.42	1.4	0.0127	1.28	0.1142	1.53	0.0017	-1.28	0.1167
96846_at	1417909_at	Serpinc1	serine (or cysteine) proteinase inhibitor, clad	-1.05	0.49	-1.07	0.019	-1.07	0.1969	-1.2	0	-1.12	0.1908
96847_at	1448447_at	Vps28	vacuolar protein sorting 28 (yeast)	1.54	0.04	1	0.9144	1.07	0.2928	1.08	0.3094	1.06	0.3877
96849_at	1416345_at	Timm8a	translocase of inner mitochondrial membran	1.93	0.08	1.27	0.0078	1.34	0.0391	1.52	0.0008	1.39	0.161
96852_at	1452032_at	Prkar1a	protein kinase, cAMP dependent regulatory,	-1.08	0.7	1.19	0.0004	1.01	0.9394	1.08	0.0305	1.23	0.0939
96854_at	1415706_at	Copa	coatomer protein complex subunit alpha	-1.16	0.38	-1.06	0.4289	1.08	0.1852	1.05	0.3666	-1.04	0.7014
96855_at	1423215_at	Spcs2	signal peptidase complex subunit 2 homolog	-1.12	0.54	-1.2	0.0123	-1.28	0	-1.24	0.0002	-1.19	0.0214
96856_at	1455821_x_at	C1qbp	complement component 1, q subcomponent	-1.11	0.6	-1.22	0.3497	1.02	0.8929	1.09	0.6939	2.31	0.2068
96858_at	1418127_a_at	Pdcd8	programmed cell death 8	-1.08	0.57	1.04	0.5334	1.12	0.3364	1.1	0.2444	-1.39	0.0368
96859_at	1423102_a_at	Rnf10	ring finger protein 10	1.34	0.46	-1.13	0.0939	-1.08	0.564	-1.18	0.0015	1.28	0.321
96861_at	1424164_at	Mrpl50	mitochondrial ribosomal protein L50	1.26	0.28	1.37	0.0051	1.4	0.0412	1.42	0.0206	1.16	0.1842
96862_at	1448388_a_at	1110002B05Rik	RIKEN cDNA 1110002B05 gene	1.4	0.37	1.38	0.0001	1.24	0.0139	1.71	0	1.38	0.0207
96864_at	1433878_at	Mrps26	mitochondrial ribosomal protein S26	1.49	0.08	1.21	0.0052	1.21	0.0649	1.24	0.0282	1.11	0.1369
96865_at	1415973_at	Marcks	Myristoylated alanine rich protein kinase C s	1.4	0.47	1.11	0.4625	-1.04	0.7842	1.34	0.0735	1.58	0.0959
96866_at	1423122_at	Avpi1	arginine vasopressin-induced 1	2.96	0.18	1.21	0.605	1.59	0.1791	1.99	0.0348	-2.09	0.2403
96867_at	1417776_at	Azgp1	alpha-2-glycoprotein 1, zinc	1.05	0.62	1.15	0.1138	1.01	0.9261	1.25	0.0107	-1.18	0.0736
96868_at	1428079_at	Fgb	fibrinogen, B beta polypeptide	-1.27	0.2	1.22	0.0896	1.03	0.7735	1.26	0.0337	-1.44	0.0445
96869_at	1416937_at	Gabarap	gamma-aminobutyric acid receptor associate	1	0.98	-1.04	0.3572	-1.14	0.2099	-1.12	0.0299	-1.05	0.5345
96870_at	1451002_at	Aco2	aconitase 2, mitochondrial	1.37	0.03	1.11	0.0692	1.02	0.6052	1.14	0.1103	1.58	0.0045
96871_at	1448543_at	2310042G06Rik	RIKEN cDNA 2310042G06 gene	-1	0.98	-1.02	0.8667	1.24	0.0813	1.15	0.269	-1.77	0.0217
96872_at	1451067_at	Sgta	small glutamine-rich tetratricopeptide repeat	-1.01	0.95	1.04	0.4749	-1.01	0.9111	-1.01	0.8976	1.4	0.0184
96875_r_at	1420174_s_at	Tax1bp1	Tax1 (human T-cell leukemia virus type I) bi	-1.04	0.81	1.08	0.7077	-1.14	0.4706	1.17	0.3186	1.01	0.9751
96879_at	1451274_at	Ogdh	oxoglutarate dehydrogenase (lipoamide)	1.39	0.13	1.21	0	1.2	0.0363	1.33	0.0002	1.62	0.0198
96881_at	1435863_at	Commd6	COMM domain containing 6	1.06	0.63	-1.13	0.0439	-1.01	0.915	-1.14	0.0103	-1.71	0.0036
96883_at	1417718_at	Eif3s4	eukaryotic translation initiation factor 3, subu	1.02	0.91	-1.04	0.7132	-1	0.9711	-1.03	0.8303	1.16	0.3035
96884_at	1415975_at	Carhsp1	calcium regulated heat stable protein 1	1.11	0.63	-1.11	0.057	-1.14	0.0283	-1.18	0.003	-1.35	0.0264
96885_at	1424882_a_at	2510015F01Rik	RIKEN cDNA 2510015F01 gene	2.02	0.18	1.17	0.2581	1.61	0.0007	1.3	0.1239	2.35	0.0501
96886_at	1450199_a_at	Stab1	stabilin 1	1.45	0.11	1.05	0.5369	1.19	0.0234	1.25	0.0174	1.94	0.0041
96887_at	1416057_at	Np15	nuclear protein 15.6	1.39	0.1	1.09	0.0678	1.08	0.0168	1.14	0.1371	1.1	0.3697
96888_at	1451035_a_at	Akr1a4	aldo-keto reductase family 1, member A4 (al	1.15	0.47	1.14	0.0253	1.15	0.0069	1.14	0.0308	-1.13	0.1851
96890_at	1460178_at	1300002A08Rik	RIKEN cDNA 1300002A08 gene	1.08	0.64	1.01	0.8325	1.22	0.1103	-1.03	0.6017	-3.21	0
96891_at	1417082_at	Anp32b	acidic nuclear phosphoprotein 32 family, me	1.35	0.09	1.12	0.1843	1.05	0.6157	1.1	0.2158	1.04	0.7876
96892_at	1415695_at	Psma1	proteasome (prosome, macropain) subunit, a	1.52	0.08	1.27	0.0384	1.18	0.1726	1.27	0.0039	-1.02	0.8633
96894_at	1448422_at	Tmed4	transmembrane emp24 protein transport dor	1.31	0.36	1.08	0.4401	1.27	0.0978	1.18	0.1244	1.04	0.8177
96895_at	1418190_at	Pon1	paraoxonase 1	1.17	0.65	-1.04	0.2721	-1.08	0.0564	-1.12	0.0683	1.23	0.0749
96896_at	1452587_at	Actr2	ARP2 actin-related protein 2 homolog (yeast	1.02	0.84	1.08	0.5571	1.15	0.1269	1.05	0.5712	-1.67	0.0531

96898_at	1433562_s_at	Atp5f1	ATP synthase, H+ transporting, mitochondria	-1.25	0.01	-1.08	0.4653	-1.09	0.4137	-1.01	0.9267	1.01	0.9591
96899_at	1423737_at	Ndufs3	NADH dehydrogenase (ubiquinone) Fe-S pr	1.63	0.04	1.11	0.0181	1.12	0.0719	1.18	0.006	-1.06	0.4765
96900_at	1436990_s_at	MGI:2143558	Nur77 downstream gene 2	1.09	0.6	1.12	0.0449	1.23	0.0001	1.21	0.0083	1.46	0.001
96902_at	1452721_a_at	2900091E11Rik	RIKEN cDNA 2900091E11 gene	1.44	0.05	-1.06	0.5872	-1.16	0.0971	-1	0.9784	-1.31	0.1139
96904_at	1453725_a_at	Mrps7	mitchondrial ribosomal protein S7	1.59	0.25	1.03	0.7328	1.08	0.6346	1.05	0.5899	-1.05	0.524
96907_at	1451896_a_at	Cherp	calcium homeostasis endoplasmic reticulum	1.78	0.17	1.18	0.0562	1.16	0.0715	1.25	0.025	1.21	0.0825
96909_at	1428159_s_at	Ndufab1	NADH dehydrogenase (ubiquinone) 1, alpha	1.54	0.03	-1.04	0.5384	1.16	0.2028	1.05	0.5413	-1.07	0.6913
96910_at	1456190_a_at	BC031140	cDNA sequence BC031140	-2.92	0.14	-1.14	0.6947	-1.39	0.2757	-1.74	0.0917	-1.92	0.118
96911_at	1450623_at	Gnb2	guanine nucleotide binding protein, beta 2	1.05	0.85	1.07	0.2237	-1.04	0.7189	1.01	0.9132	-1.06	0.8457
96912_s_at	1448471_a_at	Ctla2a	cytotoxic T lymphocyte-associated protein 2	1.99	0.02	-1.03	0.7778	1.13	0.2071	1.21	0.4807	1.12	0.6601
96913_at	1426522_at	Hadhb	hydroxyacyl-Coenzyme A dehydrogenase/3-	1.36	0.11	1.06	0.3552	1.43	0	1.2	0.0417	-1.03	0.7324
96915_f_at	1452790_x_at	Ndufa3	NADH dehydrogenase (ubiquinone) 1 alpha	1.25	0.14	1.06	0.2124	-1.06	0.4154	1.02	0.7131	-1.08	0.636
96916_at	1448190_at	Mrpl33	Mitochondrial ribosomal protein L33	1.42	0.06	1	0.9639	-1.04	0.6165	1.05	0.4609	-1.21	0.4848
96917_at	1420058_s_at	2410166I05Rik	RIKEN cDNA 2410166I05 gene	1.03	0.77	1.08	0.1732	1	0.882	1.06	0.2983	-1.29	0.1665
96918_at	1448470_at	Fbp1	fructose bisphosphatase 1	1.01	0.93	1.04	0.4269	-1.04	0.4176	-1.04	0.558	1.03	0.7654
96919_at	1435732_x_at	Atp6v0c	ATPase, H+ transporting, V0 subunit C	-1.13	0.3	1.06	0.3414	1.12	0.1403	1.08	0.2311	1.06	0.6893
96920_at	1416749_at	Prss11	protease, serine, 11 (Igf binding)	-1.39	0.34	-1.23	0.1282	-1.19	0.0934	-1.28	0.043	-1.4	0.12
96921_at	1448537_at	Ttc1	tetratricopeptide repeat domain 1	1.4	0.1	1.05	0.6478	1.17	0.1663	1.28	0.0102	2.23	0.1435
96924_at	1452189_at	9430077D24Rik	RIKEN cDNA 9430077D24 gene	1.18	0.37	1.05	0.3722	1.13	0.1392	1.1	0.0667	1.36	0.1449
96925_at	1433495_at	Glt25d1	glycosyltransferase 25 domain containing 1	1.81	0.02	-1.02	0.8233	1.02	0.7527	1.1	0.1043	-1.24	0.0146
96926_at	1415935_at	Smoc2	SPARC related modular calcium binding 2	2.41	0.01	1.05	0.6176	-1.01	0.9415	1.09	0.4256	-1.12	0.6746
96930_at	1416012_at	Ehd1	EH-domain containing 1	1.15	0.53	-1.37	0.018	-1.27	0.0509	-1.26	0.0645	-1.55	0.1387
96934_at	1434823_x_at	1110002M09Rik	RIKEN cDNA 1110002M09 gene	1.14	0.2	1.15	0.123	1.1	0.152	1.09	0.3308	1.04	0.8456
96935_at	1455477_s_at	Pdzk1ip1	PDZK1 interacting protein 1	-1.57	0.2	-1.14	0.3368	-1.13	0.4401	-1.35	0.0448	-1	0.9875
96936_at	1415670_at	Copg	coatomer protein complex, subunit gamma	-1.25	0.14	-1.04	0.5677	-1.06	0.209	-1.03	0.5844	-1.34	0.0161
96938_at	1416833_at	Keg1	kidney expressed gene 1	-8.74	0	-3.01	0	-1.44	0.0082	-3.48	0	-8.47	0.0004
96940_at	1448519_at	Tead2	TEA domain family member 2	-2.82	0.17	1.47	0.186	2.25	0.0084	1.17	0.6284	1.43	0.3394
96941_at	1424648_at	Rabl4	RAB, member of RAS oncogene family-like 4	1.27	0.15	-1.16	0.0285	-1.19	0.0173	-1.23	0.0025	-1.06	0.5352
96942_at	1423728_at	Eif3s6ip	eukaryotic translation initiation factor 3, subu	-1.04	0.84	-1.16	0.0394	-1.05	0.4789	-1.06	0.3867	-1.2	0.0723
96943_at	1415699_a_at	Gps1	G protein pathway suppressor 1	1.22	0.16	-1.04	0.3166	-1.01	0.827	-1.07	0.2451	-1.21	0.0086
96945_at	1420898_at	Snap23	synaptosomal-associated protein 23	-1.73	0.07	1.62	0.012	1.33	0.0124	1.18	0.1375	1.59	0.161
96946_at	1423873_at	Lsm1	LSM1 homolog, U6 small nuclear RNA asso	-1.06	0.79	1.09	0.1259	1.14	0.2888	1.23	0.003	-1.65	0.0319
96947_at	1428181_at	Etfb	electron transferring flavoprotein, beta polyp	1.55	0.04	1.02	0.6083	1.17	0.0001	1.08	0.2195	1.01	0.9196
96948_at	1423664_at	Qdpr	quininoid dihydropteridine reductase	1.13	0.51	-1.17	0.0273	-1.14	0.0189	-1.33	0.0031	-1.36	0.011
96949_at	1415767_at	Ythdf1	YTH domain family 1	1.33	0.26	1.09	0.1537	1.14	0.029	1.12	0.11	-1.04	0.6488
96950_at	1449277_at	Ccl19	chemokine (C-C motif) ligand 19	-1.24	0.33	1.08	0.3848	1.19	0.0511	1.19	0.0439	1.18	0.1116
96951_at	1438993_a_at	Atp6v1d	ATPase, H+ transporting, V1 subunit D	1.01	0.9	1.19	0.0068	1.15	0.1925	1.28	0.001	-1.05	0.7769
96952_at	1416506_at	Psma6	proteasome (prosome, macropain) subunit, a	1.37	0.25	1.27	0.0049	1.23	0.1902	1.45	0.0002	1.14	0.5021
96953_at	1418456_a_at	Cxcl14	chemokine (C-X-C motif) ligand 14	-2	0.41	-1.37	0.2656	-1.42	0.1156	-2.09	0.0104	1.81	0.3193
96956_at	1416381_a_at	Prdx5	peroxiredoxin 5	1.21	0.19	-1.01	0.7966	1.01	0.922	1.09	0.2865	-1.38	0.0439
96959_at	1422559_at	Ube2n	ubiquitin-conjugating enzyme E2N	1.12	0.46	1.06	0.4124	-1.01	0.8795	1.08	0.1544	-1.09	0.296
96962_at	1416546_a_at	Rpl6	ribosomal protein L6	1.1	0.66	1.12	0.0006	1.17	0.0112	1.19	0.0012	1.26	0.0784
96968_at	1425322_at	Ifnz	interferon zeta	-1.68	0.24	-1.19	0.4243	-1.03	0.8984	-1.42	0.2663	1.05	0.7605
96978_at	1417336_a_at	Sytl4	synaptotagmin-like 4	1.49	0.2	-1.39	0.0156	-1.1	0.4074	-1.12	0.3589	-1.43	0.2945
96979_at	1450347_at	Syt10	synaptotagmin 10	-1.28	0.36	1.26	0.3073	1.21	0.3632	1.06	0.7841	1.36	0.4412
96982_g_at	1422371_at	Olfr1507	olfactory receptor 1507	-1.41	0.14	1.03	0.6636	1.02	0.7873	1.09	0.3846	1.58	0.0161
96983_at	1450600_at	Olfr1508	olfactory receptor 1508	-1.07	0.62	1.27	0.3597	1.21	0.032	-1	0.9922	1.27	0.0496
96984_at	1422384_at	Olfr1509	olfactory receptor 1509	-2.26	0.09	-1.14	0.3555	-1.18	0.1101	-1.26	0.1178	1.07	0.755
96985_at	1422385_at	Olfr1264	olfactory receptor 1264	-1.09	0.83	-1.57	0.0127	-1.25	0.0631	-1.14	0.3431	1.43	0.0074
96986_at	1450604_at	Olfr140	olfactory receptor 140	-1.12	0.1	1.32	0.0726	1.02	0.8886	1.07	0.7027	1.38	0.392
96987_at	1422149_at	Mllt7	myeloid/lymphoid or mixed lineage-leukemia	-1.41	0.08	1.07	0.3848	1.03	0.6403	1.03	0.7354	1.28	0.1268
96996_at	1450426_at	Chrna6	cholinergic receptor, nicotinic, alpha polypep	1.14	0.77	-1.06	0.8054	1.19	0.3485	1.08	0.6843	1.38	0.1134
96997_at	1420451_at	Accn5	amiloride-sensitive cation channel 5, intestin	-1.4	0.13	-1.2	0.2735	-1.16	0.4301	1.03	0.8801	-1.39	0.1072

96998_at	1427424_at	Galnt6	UDP-N-acetyl-alpha-D-galactosamine:polype	-1.11	0.73	1.19	0.5743	-1.03	0.8601	1.08	0.8097	1.38	0.4056
96999_at	1422343_at	Olfr155	olfactory receptor 155	1.25	0.71	1.04	0.9201	1.07	0.8485	1.13	0.6796	2.34	0.3007
97003_at	1450590_at	Olfr159	olfactory receptor 159	-2.73	0.18	1.21	0.4134	1.49	0.1788	1.28	0.371	-1.52	0.3397
97004_at	1422373_at	Olfr71	olfactory receptor 71	-1.13	0.13	1.01	0.898	-1.03	0.7077	1.09	0.2446	-1.05	0.6741
97005_at	1422367_at	Olfr70	olfactory receptor 70	-1.19	0.17	-1.09	0.4007	-1.05	0.5552	-1.76	0.0007	-1.33	0.177
97007_at	1452516_at	Smok2	sperm motility kinase 2	-1.83	0.41	1.07	0.8725	1.29	0.5055	1.23	0.6343	-1.5	0.2598
97053_at	1422294_at	Xcr1	chemokine (C motif) receptor 1	-1.75	0.05	-1.15	0.1429	-1.06	0.5149	-1.29	0.0183	1.4	0.0711
97061_g_at	1460590_s_at	Ywhaq	tyrosine 3-monooxygenase/tryptophan 5-mo	1.2	0.35	1.1	0.1969	1.04	0.5834	1.14	0.1328	-1.28	0.0171
97073_at	1451954_at	---	---	-2.39	0.13	1.63	0.175	-1.28	0.5031	1.26	0.6867	1.49	0.5036
97080_at	1443473_at	C79562	expressed sequence C79562	-1.44	0.53	-1.02	0.8999	1.21	0.1166	-1.06	0.5569	1.03	0.9042
97082_at	1418211_at	p	pink-eyed dilution	-1.5	0.41	-1.48	0.2885	-1.13	0.7629	-1.6	0.1883	-1.74	0.2035
97083_at	1441023_at	Eif2s2	eukaryotic translation initiation factor 2, subu	-3.05	0.26	1.33	0.3648	-1.81	0.0321	-1.5	0.1777	1.44	0.3524
97084_at	1457475_at	C80993	expressed sequence C80993	-4.14	0.05	1.04	0.8511	1.19	0.6378	1	0.9924	1.31	0.1628
97085_at	1457476_at	D8Ertd362e	DNA segment, Chr 8, ERATO Doi 362, expr	-1.72	0.26	-1.4	0.3702	-1.95	0.1131	-1.61	0.1855	1.57	0.0555
97087_f_at	1451616_at	Psg-ps1	pregnancy specific glycoprotein pseudogene	-1.22	0.33	-1.1	0.5987	-1.19	0.3098	1.06	0.8124	-3.02	0.1131
97088_at	1448072_at	---	---	-1.28	0.47	1.05	0.6814	-1.02	0.8513	1.01	0.9419	-1.03	0.907
97089_at	1450154_at	Folh1	folate hydrolase	-1.51	0.25	4.94	0.1876	1.09	0.4646	22.82	0.1514	1.03	0.8972
97091_at	1419508_at	Ripk1	receptor (TNFRSF)-interacting serine-threor	-1.3	0.45	1.08	0.4052	-1.26	0.1203	1.1	0.3154	-1.18	0.7195
97092_at	1419629_at	Mesp2	mesoderm posterior 2	-2.07	0.35	1.19	0.0027	1.18	0.0011	1.15	0.0502	1.77	0.1994
97094_at	1425164_a_at	Phkg1	phosphorylase kinase gamma 1	-1.15	0.71	1.6	0.1264	1.38	0.2174	1.51	0.1946	2.92	0.2571
97095_at	1438571_at	Bub1	Budding uninhibited by benzimidazoles 1 ho	1.48	0.5	-1.15	0.2459	1.17	0.3524	1.06	0.6785	1.03	0.9207
97097_at	1436307_at	Myo9a	myosin IXa	1.71	0.02	1.23	0.2111	1.34	0.0262	1.16	0.3004	1	0.9658
97098_at	1428780_at	1300017K07Rik	RIKEN cDNA 1300017K07 gene	1.45	0.14	-1.18	0.1454	1.04	0.8322	-1.09	0.4959	1.05	0.7115
97099_at	1419851_at	---	---	1.65	0.27	1.46	0.2038	1.67	0.3123	1.11	0.2319	-1.06	0.8044
97100_at	1449536_at	Kcnn1	potassium intermediate/small conductance c	-1	0.99	-1.86	0.029	-1.39	0.1914	-1.38	0.227	1.05	0.8878
97101_at	1430661_at	BC037527	cDNA sequence BC037527	2.18	0.12	-1.35	0.1795	-1.26	0.3359	-2.05	0.0112	1.61	0.283
97104_g_at	1416229_at	Rfk	riboflavin kinase	-1.11	0.75	-1.98	0	-1.46	0.0023	-2.47	0	-2.32	0.0434
97105_at	1437111_at	C230027N18Rik	RIKEN cDNA C230027N18 gene	1.37	0.17	1.08	0.2884	1.36	0.004	1.01	0.8685	1.37	0.1039
97106_at	1419208_at	Map3k8	mitogen activated protein kinase kinase kina	3.51	0.05	2.54	0.1693	2.78	0.065	2.87	0.0951	1.12	0.6116
97107_at	1455700_at	1700007D05Rik	RIKEN cDNA 1700007D05 gene	1.66	0.1	1.24	0.0748	-1.13	0.5612	1.17	0.1707	1.18	0.6407
97109_at	1426175_a_at	Mcpt7	mast cell protease 7	-1.93	0.02	-1.04	0.7945	-1.07	0.6105	-1.15	0.2909	1.26	0.3629
97113_at	1449235_at	Fasl	Fas ligand (TNF superfamily, member 6)	1.12	0.76	1.08	0.5592	1.13	0.555	-1.06	0.5373	-1.16	0.7437
97114_at	1415687_a_at	Psap	prosaposin	1.05	0.59	1.09	0.4701	-1.26	0.0446	-1.01	0.9668	-1.11	0.3809
97115_at	1459931_a_at	2810021G02Rik	RIKEN cDNA 2810021G02 gene	-1.14	0.77	-1.05	0.6118	-1.04	0.6794	-1.18	0.1478	1.3	0.1294
97116_at	1424930_s_at	MGC27770	hypothetical protein MGC27770	1.01	0.95	1.12	0.1195	-1.5	0.0014	1.15	0.183	-1.31	0.251
97117_at	1419845_at	Dlx1	Distal-less homeobox 1	-1.59	0.04	1.48	0.1473	1.21	0.2841	1.03	0.8748	1.85	0.1123
97118_at	1436073_at	A630007B06Rik	RIKEN cDNA A630007B06 gene	1.38	0.2	-1.42	0.1151	-1.16	0.3231	-1.53	0.0304	3.27	0.0021
97119_at	1447976_at	AI596198	expressed sequence AI596198	-3.58	0.12	-1.07	0.7737	1.29	0.3156	1.27	0.4258	1.02	0.9844
97120_at	1457905_at	D7Ertd59e	DNA segment, Chr 7, ERATO Doi 59, expre	-1.3	0.74	1.42	0.2307	1.77	0.0079	1.15	0.5581	-1.08	0.7736
97121_at	1427582_at	Fgf6	fibroblast growth factor 6	-1.27	0.26	1.06	0.5967	-1.48	0.0541	-1.05	0.7474	1.47	0.5513
97122_at	1427560_at	Six5	sine oculis-related homeobox 5 homolog (Dr	-1.51	0.33	-1.14	0.3291	1	0.9825	-1.11	0.2796	1.26	0.1136
97123_at	1449854_at	Nr0b2	nuclear receptor subfamily 0, group B, meml	1.35	0.62	1.17	0.18	-1.15	0.3503	1.21	0.0779	-1.04	0.8478
97124_at	1421534_at	---	---	4.15	0.21	1.13	0.2245	1	0.9766	1.17	0.1027	1.48	0.2827
97125_f_at	1450534_x_at	LOC56628	MHC (A.CA/J(H-2K-f) class I antigen	-1.15	0.41	1.5	0.056	-1.05	0.444	1.46	0.1314	-1.37	0.0174
97126_at	1427427_at	Ryr3	ryanodine receptor 3	-1.28	0.63	-1.29	0.3534	-1.2	0.5744	-1.33	0.2966	-1.04	0.9137
97128_at	1421620_at	Il5ra	interleukin 5 receptor, alpha	-1.86	0.35	-1.91	0.2314	1.23	0.7457	-1.69	0.3112	1.18	0.3309
97130_at	1420651_at	Ate1	arginine-tRNA-protein transferase 1	2.95	0.13	1.04	0.8253	-1.1	0.7521	-1.2	0.3743	-1.45	0.4002
97131_at	1431792_a_at	Stk11ip	serine/threonine kinase 11 interacting protei	2.02	0.24	-1.03	0.8123	-1.38	0.0231	-1.02	0.8396	1.18	0.6673
97134_at	1419782_at	AA517864	expressed sequence AA517864	-3.2	0.3	-1.04	0.911	-1.14	0.7663	-1.41	0.4005	1.01	0.9911
97135_at	1443312_at	---	---	1.63	0.52	-3.72	0.0021	-2.87	0.0048	-3.37	0.0028	-1.53	0.4017
97137_at	1442941_at	C77027	expressed sequence C77027	-2.84	0.07	1.41	0.0809	1.34	0.2996	1.21	0.5376	1.64	0.3817
97138_at	1457147_at	BC026657	cDNA sequence BC026657	-1.44	0.26	-1.1	0.3522	-1.42	0.0151	-1.3	0.0171	1.24	0.0158
97139_at	1443352_at	C77614	EST C77614	-1.45	0.13	-1.12	0.4825	-1.1	0.2511	-1.1	0.582	1.12	0.7002

97140_at	1447984_at	D1Ertd75e	DNA segment, Chr 1, ERATO Doi 75, expre	-1.07	0.85	1.03	0.9241	-1.31	0.3529	-1.32	0.3299	1.09	0.8519
97141_s_at	1441147_at	D3Ertd229e	DNA segment, Chr 3, ERATO Doi 229, expr	-3.09	0.21	-1.08	0.8032	1.02	0.9234	-1.1	0.692	1.11	0.7389
97142_at	1443589_at	DXErtd242e	DNA segment, Chr X, ERATO Doi 242, expr	-1.69	0.37	-1.37	0.3188	-1.24	0.4691	-1.35	0.2629	1.05	0.8838
97143_at	1448054_at	---	---	-1.88	0.01	-1.15	0.1241	-1.07	0.4511	-1.26	0.0283	1.04	0.7836
97144_at	1447981_at	C78441	expressed sequence C78441	1.07	0.93	1.08	0.8231	-1.11	0.7423	1.26	0.4528	1.15	0.5813
97146_g_at	1441344_at	Spfh1	SPFH domain family, member 1	1.1	0.85	-1.04	0.8458	1.07	0.7724	-1.04	0.8996	-1.15	0.6325
97147_at	1441421_at	C530030P08Rik	RIKEN cDNA C530030P08 gene	-2.47	0.01	1.01	0.9814	1.29	0.4861	-1.43	0.3127	-1.01	0.9806
97148_at	1456136_at	---	---	1.04	0.95	1.18	0.5697	-1.16	0.5941	1.09	0.7589	1.41	0.304
97149_at	1418558_at	Rax	retina and anterior neural fold homeobox	-1.41	0.52	1.25	0.5887	1.22	0.525	-1.22	0.6215	1.55	0.1901
97150_at	1450245_at	Slc10a2	solute carrier family 10, member 2	-2.43	0.27	-2.24	0.0043	-3.63	0.0002	-2.33	0.0072	-1.64	0.2465
97151_at	1422154_at	Gpr27	G protein-coupled receptor 27	-1.03	0.81	1.02	0.8497	1.01	0.9425	-1.13	0.312	1.07	0.7716
97152_at	1421689_at	Krtap8-2	keratin associated protein 8-2	-1.08	0.8	1.12	0.4452	1.06	0.5905	1.1	0.3072	1.11	0.6232
97153_at	1422144_at	Inhbe	inhibin beta E	-2.94	0.01	-1.03	0.887	1.33	0.1842	-1.44	0.0636	-2.07	0.0338
97154_f_at	1439065_x_at	---	Similar to RIKEN cDNA 6330416L07 gene	-3	0.26	1.66	0.1492	1.41	0.1154	2.21	0.0746	-1.22	0.7528
97156_at	1422409_at	Hes3	hairy and enhancer of split 3 (Drosophila)	-1.8	0.01	-1.06	0.8206	1.53	0.145	1.11	0.6701	1.75	0.0254
97157_at	1449998_at	Nkx3-1	NK-3 transcription factor, locus 1 (Drosophila	-1.49	0.39	-1.31	0.3095	-1.35	0.2711	-1.21	0.4492	2.52	0.0255
97158_at	1435360_at	BC030045	cDNA sequence BC030045	-1.2	0.74	-1.17	0.2621	-1.11	0.4714	-1.39	0.0179	1.26	0.6044
97159_at	1419241_a_at	Aire	autoimmune regulator (autoimmune polyend	-2.21	0.13	-1.13	0.1847	-1.1	0.4021	-1.12	0.2756	-1.12	0.628
97160_at	1448392_at	Sparc	secreted acidic cysteine rich glycoprotein	1.33	0.2	1.03	0.7312	1.29	0.0942	1.16	0.2621	1.51	0.0491
97162_at	1419858_at	---	Transcribed locus	1.07	0.94	1.15	0.3865	-1.07	0.6384	1.28	0.0776	1.98	0.2738
97163_g_at	1437909_at	D030022P06Rik	RIKEN cDNA D030022P06 gene	-1.32	0.15	1.05	0.2503	1.04	0.672	1.01	0.7903	1.13	0.4929
97165_r_at	1458414_at	D2Ertd93e	DNA segment, Chr 2, ERATO Doi 93, expre	-1.73	0.13	1.3	0.2052	-1	0.9943	1.37	0.1508	1.28	0.5321
97166_at	1428476_a_at	Elac2	elaC homolog 2 (E. coli)	1.56	0.01	-1.03	0.7452	1.03	0.6764	-1.02	0.804	-1.18	0.6912
97168_at	1421456_at	P2ry1	purinergic receptor P2Y, G-protein coupled 1	-1.35	0.23	1.98	0.1564	1.53	0.3345	2.43	0.0001	1.51	0.19
97169_f_at	1425427_at	AF067061	CDNA sequence AF067061	1.08	0.82	1.16	0.3577	-1.05	0.8516	-1.34	0.2408	1.47	0.1309
97170_at	1443001_at	C78344	expressed sequence C78344	1.2	0.68	-1.62	0.1135	1.11	0.7163	1.78	0.037	1.78	0.4642
97172_s_at	1420408_a_at	Abcc9	ATP-binding cassette, sub-family C (CFTR/M	1.45	0.28	-1.06	0.5921	1.02	0.898	-1.04	0.7163	1.09	0.5315
97176_at	1420075_at	Atp8b1	ATPase, class I, type 8B, member 1	-1.81	0.01	1.06	0.6477	-1.02	0.7745	-1.14	0.1422	1.13	0.0676
97177_at	1426101_at	Klhl2	Kelch-like 2, Mayven (Drosophila)	1.26	0.73	-1.59	0.0378	-1.26	0.3079	-1.58	0.0269	1.3	0.6555
97179_at	1460713_at	BC048355	CDNA sequence BC048355	-1.24	0	-1.4	0.0004	-1.2	0.0002	-1.5	0	-1.13	0.2958
97186_s_at	1419777_at	---	---	1.31	0.28	-1.55	0.0288	-1.49	0.1111	-1.39	0.1201	-1.19	0.5115
97187_at	1447462_at	D7Wsu130e	DNA segment, Chr 7, Wayne State Universit	-1.04	0.91	-1.09	0.4176	1.08	0.492	1.26	0.0772	1.36	0.4997
97188_at	1457372_at	AW121686	expressed sequence AW121686	-1.75	0.29	-1.21	0.3629	1.04	0.9021	-1.55	0.0189	1.94	0.1532
97189_at	1448097_at	Ube3c	Ubiquitin protein ligase E3C	-1.2	0.75	-1.13	0.7792	-1.09	0.8514	1.1	0.855	-1.15	0.8528
97190_f_at	1459935_at	AA517562	EST AA517562	1.08	0.82	1.35	0.3053	1.48	0.1714	-1.24	0.5545	-1.01	0.9878
97191_at	1449599_at	---	---	-1.12	0.8	-1.12	0.6706	-1.26	0.4246	-1.03	0.8931	1.06	0.8435
97192_at	1419779_at	---	---	-1.95	0.16	-1.19	0.3999	-1.1	0.6205	-1.12	0.6304	1.67	0.2143
97194_at	1448075_at	AA408251	expressed sequence AA408251	-1.52	0.37	-1.17	0.2891	-1.06	0.6574	-1.07	0.6306	-1.28	0.2795
97195_at	1427510_at	Gnai1	guanine nucleotide binding protein, alpha inh	-1.17	0.72	1.13	0.4859	1.09	0.5895	1.11	0.5642	2.37	0.0071
97196_at	1427727_x_at	Psg19	pregnancy specific glycoprotein 19	-1.17	0.83	-1.01	0.9869	1.32	0.4438	-1.16	0.6242	2.04	0.3535
97198_at	1421839_at	Abca1	ATP-binding cassette, sub-family A (ABC1),	1.45	0.58	1.12	0.3184	-1.03	0.7936	1.01	0.9173	-1.93	0.3029
97199_at	1433439_at	Cpne1	copine I	-1.12	0.68	1.35	0.0066	1.28	0.2581	1.39	0.0004	1.66	0.0109
97200_f_at	1451294_s_at	Snrpe	small nuclear ribonucleoprotein E	1.52	0.11	1.15	0.0436	1.07	0.4455	1.17	0.0135	-1.16	0.4813
97201_s_at	1417285_a_at	Ndufa5	NADH dehydrogenase (ubiquinone) 1 alpha	1.74	0.06	1.12	0.0059	1.15	0.1874	1.15	0.1324	1.17	0.1551
97203_at	1415922_s_at	Mlp	MARCKS-like protein	-1.88	0.16	1.4	0.044	1.13	0.2824	1.57	0.1217	1.36	0.1258
97204_s_at	1416910_at	Dnajd1	DnaJ (Hsp40) homolog, subfamily D, membe	1.39	0.12	1.03	0.6408	1.1	0.158	-1.03	0.7228	-1.1	0.4948
97205_at	1426395_s_at	Eif3s1	eukaryotic translation initiation factor 3, subu	1.31	0.05	1.07	0.2435	1.08	0.144	1.19	0.0326	-1.19	0.3084
97206_at	1416627_at	Spint1	serine protease inhibitor, Kunitz type 1	-2.59	0.36	1	0.9948	1.15	0.1248	1.09	0.141	-1.51	0.3477
97207_f_at	1448244_at	Lypla1	lysophospholipase 1	1.1	0.39	1.08	0.6061	1.05	0.7567	1.11	0.4293	-1.31	0.0205
97210_at	1460262_a_at	1700037H04Rik	RIKEN cDNA 1700037H04 gene	-1.33	0.56	1.11	0.1889	-1.05	0.5348	-1.1	0.1815	-1	0.9952
97211_at	1415780_a_at	Armcx2	armadillo repeat containing, X-linked 2	-1.09	0.62	1.3	0.124	1.22	0.2339	1.19	0.3129	-1.03	0.9498
97213_at	1423583_at	Fem1a	feminization 1 homolog a (C. elegans)	1.07	0.66	-1.07	0.8437	1.51	0.3384	1.05	0.9191	-1.03	0.8748
97216_at	1417246_at	Pzp	pregnancy zone protein	-1.12	0.37	-1.04	0.0796	-1.01	0.7279	-1.04	0.2518	1.07	0.182

97217_at	1426830_a_at	Ahcyl1	S-adenosylhomocysteine hydrolase-like 1	1.16	0.47	-1.06	0.3324	1.04	0.5778	-1.01	0.8663	-1.2	0.1696
97220_at	1448307_at	Dscr2	Down syndrome critical region homolog 2 (h	1.58	0.06	-1.03	0.6827	1.05	0.5369	-1.03	0.6351	-1.02	0.9011
97226_at	1455008_at	6530401C20Rik	RIKEN cDNA 6530401C20 gene	1.54	0.01	1.47	0.0031	1.35	0.0023	1.59	0.0001	1.14	0.4519
97227_at	1421026_at	Gna12	guanine nucleotide binding protein, alpha 12	3.19	0	1.5	0.0078	1.13	0.5917	1.27	0.2239	-1.11	0.8399
97228_at	1451421_a_at	MGI:1913299	leucine zipper domain protein	1.24	0.67	1.08	0.3247	-1.2	0.0985	1.11	0.441	-1.02	0.956
97237_at	1450735_at	1810003N24Rik	RIKEN cDNA 1810003N24 gene	1.05	0.58	1.03	0.5838	-1.02	0.8432	-1.01	0.843	-1.29	0.0369
97238_at	1417450_a_at	Tacc3	transforming, acidic coiled-coil containing pr	1.08	0.89	-1.17	0.7507	-1.25	0.536	1.21	0.6319	-1.73	0.0158
97241_at	1436342_a_at	D19Ertd721e	DNA segment, Chr 19, ERATO Doi 721, exp	2.12	0.06	-1.07	0.3274	1.08	0.5407	-1.14	0.0292	-1.15	0.4345
97242_at	1427878_at	0610010O12Rik	RIKEN cDNA 0610010O12 gene	-1.04	0.86	1.18	0.0223	1.15	0.0427	1.08	0.1223	1.14	0.4558
97243_at	1450982_at	Slc9a3r1	solute carrier family 9 (sodium/hydrogen exc	1.37	0.3	-1.08	0.077	-1.14	0.0002	1.07	0.0537	-1.01	0.9172
97247_at	1425052_at	2610034N03Rik	Isochorismatase domain containing 1	1.94	0.23	-1.02	0.8478	1.14	0.5115	-1.04	0.844	-1.29	0.3699
97248_at	1422432_at	Dbi	diazepam binding inhibitor	1.06	0.5	-1.29	0.0006	-1.17	0.0643	-1.45	0.0001	-1.57	0.0558
97249_at	1417167_at	Exosc5	exosome component 5	1.31	0.48	-1.05	0.4732	-1.02	0.8274	1.08	0.1184	-1.04	0.7937
97250_at	1423211_at	Nola3	nucleolar protein family A, member 3	-1.49	0.18	1.1	0.4033	1.06	0.5183	1.09	0.2813	-1.1	0.5729
97252_at	1423823_at	2610012O22Rik	RIKEN cDNA 2610012O22 gene	1.83	0.13	-1.06	0.7499	1.13	0.5011	1.08	0.6548	-1.19	0.4844
97253_at	1417106_at	Tpd52l2	tumor protein D52-like 2	1.59	0.24	1.12	0.1822	1.14	0.4151	1.21	0.0588	-1.37	0.2406
97254_at	1418119_at	Rbm8a	RNA binding motif protein 8a	1.36	0.13	1.21	0.0076	1.18	0.0428	1.18	0.023	1.26	0.0846
97255_at	1451154_a_at	Cugbp2	CUG triplet repeat,RNA binding protein 2	-1.06	0.85	1.57	0.0036	1.17	0.449	1.88	0.0019	1.12	0.5517
97257_at	1425141_at	---	---	-1.58	0.07	-1.08	0.4252	-1.2	0.1419	-1.11	0.4561	-2.47	0
97259_at	1422608_at	Arpp19	cAMP-regulated phosphoprotein 19	-1.06	0.6	1.13	0.3107	1.1	0.2999	1.11	0.2898	1.03	0.9178
97260_at	1450685_at	Arpp19	cAMP-regulated phosphoprotein 19	-1.28	0.12	-1.05	0.4292	-1.04	0.7487	-1.08	0.1294	-1.62	0.1494
97261_at	1460179_at	Dnaja1	DnaJ (Hsp40) homolog, subfamily A, membe	-1.02	0.92	-1.32	0.0054	-1.44	0.002	-1.43	0.0016	-1.68	0.002
97262_at	1415825_s_at	---	---	1.07	0.51	-1.03	0.5475	-1.07	0.4082	-1.03	0.457	1.09	0.5086
97264_r_at	1418889_a_at	Csnk1d	casein kinase 1, delta	2.63	0.16	-1.04	0.7476	1.03	0.8164	-1.1	0.3808	-1.21	0.3894
97267_at	1416912_at	6330407G11Rik	RIKEN cDNA 6330407G11 gene	-1.06	0.7	-1.13	0.3754	1.12	0.349	-1.21	0.1782	-1.5	0.1678
97269_f_at	1417059_at	Krtcap2	keratinocyte associated protein 2	1.05	0.79	-1.01	0.9276	1.19	0.2805	1.01	0.9062	-1.2	0.311
97270_at	1422466_at	---	---	-2.77	0.21	-1.24	0.3434	-1.31	0.2611	-1.23	0.3781	1.37	0.2108
97271_at	1428526_at	1500034E06Rik	RIKEN cDNA 1500034E06 gene	1.06	0.77	-1.01	0.7951	1.1	0.1337	1.01	0.9366	1.11	0.535
97273_at	1417655_a_at	---	---	-1.05	0.73	1.3	0.089	1.18	0.3311	1.44	0.0141	1.1	0.5219
97274_at	1421751_a_at	Psmd14	proteasome (prosome, macropain) 26S subu	1.87	0.05	1.22	0.0785	-1.04	0.7902	1.17	0.0475	-1.44	0.0188
97276_at	1448533_at	Ckap1	cytoskeleton-associated protein 1	1.19	0.45	1.18	0.1021	1.02	0.8972	1.21	0.1747	1.2	0.6124
97277_at	1460702_at	1810015M01Rik	RIKEN cDNA 1810015M01 gene	1.22	0.35	-1.15	0.1079	-1.02	0.8426	-1.12	0.1254	-1.66	0.0118
97279_at	1435967_s_at	Hibadh	3-hydroxyisobutyrate dehydrogenase	-1.09	0.63	1.04	0.5478	-1.04	0.5404	-1.07	0.2663	-1.33	0.0093
97281_at	1416745_x_at	Uap1	UDP-N-acetylglucosamine pyrophosphoryla:	-1.55	0.24	1.11	0.457	1.24	0.0719	1.06	0.7073	1.08	0.7751
97282_at	1456456_x_at	Mela	Melanoma antigen	1.73	0.38	-1.31	0.0119	-1.23	0.3153	-1.29	0.0076	-1.58	0.2077
97283_at	1424295_at	Dppa3	developmental pluripotency-associated 3	1.38	0.52	-1.53	0.2627	-1.47	0.3575	-1.31	0.4661	2.26	0.1358
97284_at	1424406_at	Bcl2l13	BCL2-like 13 (apoptosis facilitator)	-1	1	1.04	0.5527	1.09	0.1691	1.1	0.2005	-1.15	0.3246
97292_at	1452167_at	2810407C02Rik	RIKEN cDNA 2810407C02 gene	-1.37	0.02	1.07	0.1109	1.02	0.8193	-1.02	0.6654	-1.11	0.006
97293_at	1423741_at	Rbm10	RNA binding motif protein 10	7.94	0.08	1.27	0.2015	1.69	0.0056	1.65	0.0275	-1.17	0.6946
97296_at	1452144_a_at	Mrpl44	mitochondrial ribosomal protein L44	1.9	0.01	1.37	0.0042	1.17	0.1139	1.27	0.0168	1.07	0.5201
97297_at	1452913_at	Pcp4l1	Purkinje cell protein 4-like 1	12.41	0.1	3.69	0.0001	1.56	0.0022	2.58	0.0004	19.6	0
97301_at	1419246_s_at	Rab14	RAB14, member RAS oncogene family	1.08	0.33	1.18	0.0007	1.11	0.2481	1.11	0.0814	-1.06	0.3231
97302_at	1450084_s_at	Ivns1abp	influenza virus NS1A binding protein	2.03	0.07	1.88	0.0009	1.44	0.0338	2.19	0	2.04	0.0107
97304_at	1460655_a_at	Ubp1	upstream binding protein 1	-1.13	0.63	-1.02	0.8244	1.17	0.1737	1.06	0.5894	-1.15	0.2418
97305_at	1448480_at	1110017C15Rik	RIKEN cDNA 1110017C15 gene	1.43	0.04	1.15	0.0719	1.01	0.9264	1.09	0.3658	1.07	0.7675
97307_f_at	1417102_a_at	Ndufb5	NADH dehydrogenase (ubiquinone) 1 beta s	1.25	0.19	1.04	0.4754	1.11	0.1816	1.07	0.3452	1.14	0.2359
97308_at	1454669_at	5730466P16Rik	RIKEN cDNA 5730466P16 gene	1.02	0.84	1.09	0.21	1.14	0.2094	1.06	0.4067	1.21	0.1654
97309_at	1460193_at	St13	suppression of tumorigenicity 13	1.01	0.88	1.13	0.0769	1.07	0.4191	-1	0.9721	1.26	0.2576
97311_at	1423189_at	6720456B07Rik	RIKEN cDNA 6720456B07 gene	1.05	0.49	1.05	0.5086	1.07	0.2978	1.01	0.9396	-1.14	0.2714
97312_at	1416440_at	Cd164	CD164 antigen	-1.67	0.11	1.04	0.7117	1.07	0.6361	-1.11	0.2005	-1.73	0.0004
97313_at	1451070_at	Gdi1	guanosine diphosphate (GDP) dissociation i	-1.1	0.41	-1.13	0.2699	1.08	0.4627	-1.09	0.4754	1.03	0.8591
97315_at	1424258_at	Polr2d	polymerase (RNA) II (DNA directed) polypep	1.13	0.73	-1.06	0.4854	-1.26	0.0114	-1.1	0.1222	-1.2	0.0534
97316_at	1448382_at	Ehhadh	enoyl-Coenzyme A, hydratase/3-hydroxyacy	1.92	0.01	1.26	0.0239	1.26	0.0376	1.46	0.0107	-1.33	0.0723

97317_at	1448136_at	Enpp2	ectonucleotide pyrophosphatase/phosphodie	2.51	0.01	3.75	0	1.66	0.001	4.14	0	5.34	0.0016
97318_at	1451040_at	Hars2	histidyl tRNA synthetase 2	1.31	0.15	1.3	0.0012	1.19	0.1015	1.26	0.0209	1.01	0.9673
97319_at	1422562_at	Rrad	Ras-related associated with diabetes	1.05	0.91	1.2	0.3943	-1.49	0.1039	1.11	0.6627	1.04	0.8903
97320_at	1451139_at	Slc39a4	solute carrier family 39 (zinc transporter), me	-1.22	0.01	1.1	0.1323	1.01	0.9104	1.17	0.1528	-1.59	0.0451
97322_at	1418826_at	Ms4a6b	membrane-spanning 4-domains, subfamily A	-1.63	0.22	2.4	0.4019	-1.98	0.1164	3.21	0.1916	1.04	0.9282
97324_at	1424496_at	5133401N09Rik	RIKEN cDNA 5133401N09 gene	1.23	0.49	1.14	0.3482	-1.14	0.2325	1.21	0.165	-1.07	0.8186
97325_at	1451253_at	---	---	1.16	0.28	-1.01	0.8061	1.13	0.1719	1.2	0.1134	1.28	0.139
97327_at	1421731_a_at	Fen1	flap structure specific endonuclease 1	-1.6	0.09	1.55	0.0131	1.39	0.0068	1.57	0.0197	1.04	0.842
97328_at	1456618_at	---	---	-1.43	0.43	-1.35	0.4065	-1.97	0.0907	-1.27	0.5067	1.88	0.2351
97329_at	1423922_s_at	C77668	expressed sequence C77668	2.53	0.01	-1.22	0.0026	-1.21	0.0465	-1.18	0.0121	-1.52	0.2693
97330_at	1420157_s_at	Abcf1	ATP-binding cassette, sub-family F (GCN20	-1.06	0.49	1.04	0.584	-1.03	0.7556	-1.01	0.9487	1.14	0.2985
97331_at	1416265_at	Capn10	calpain 10	-1.01	0.98	-1.02	0.8075	-1.03	0.7861	-1.21	0.0056	-1.44	0.0518
97332_at	1416378_at	Pnkp	polynucleotide kinase 3'- phosphatase	1.18	0.43	1.02	0.7668	1.2	0.0291	1.21	0.0037	-1.48	0.0457
97333_at	1416648_at	Dnchc1	dynein, cytoplasmic, heavy chain 1	1.22	0.48	-1.11	0.2285	1.09	0.3	-1.01	0.8938	1.32	0.0205
97334_at	1452021_a_at	Hes6	hairy and enhancer of split 6 (Drosophila)	-1.4	0.02	-2.37	0	-1.61	0.0007	-2.65	0	-3.42	0.0313
97335_at	1418765_at	Timd2	T-cell immunoglobulin and mucin domain co	1.06	0.77	-1.29	0.001	-1.03	0.7563	-1.56	0	-2.51	0
97336_at	1451019_at	Ctsf	cathepsin F	1.47	0.04	1.2	0.0645	1.11	0.3117	1.22	0.0068	1.41	0.1742
97339_at	1454759_at	Git1	G protein-coupled receptor kinase-interactor	1.41	0.15	1.21	0.0305	1.21	0.0171	1.16	0.0285	1.42	0.0691
97340_at	1417547_at	Sart3	squamous cell carcinoma antigen recognize	2.39	0.2	1.05	0.6554	1.33	0.0121	1.24	0.0671	1.41	0.0057
97341_at	1416782_s_at	Praf2	PRA1 domain family 2	1.5	0.41	1.45	0.2582	-1.12	0.7226	1.42	0.3899	1.49	0.1389
97342_at	1420489_at	Mrps14	mitochondrial ribosomal protein S14	1.38	0.02	-1.03	0.5467	1.07	0.1826	1.06	0.3114	-1.21	0.1037
97343_at	1424288_at	D5Wsu46e	DNA segment, Chr 5, Wayne State Universit	-1.15	0.44	-1.01	0.901	1.13	0.1099	1.05	0.329	1.47	0.0179
97345_at	1420592_a_at	Anp32e	acidic (leucine-rich) nuclear phosphoprotein	-1.2	0.44	-1.03	0.6943	-1.32	0.0237	-1.35	0.0051	1.08	0.6724
97346_at	1428552_at	2610001J05Rik	RIKEN cDNA 2610001J05 gene	-1.1	0.8	1.23	0.1004	1.08	0.6596	1.11	0.3231	1.18	0.5739
97347_at	1436665_a_at	Ltbp4	latent transforming growth factor beta bindin	1.3	0.32	-1.15	0.195	1.06	0.6001	-1.22	0.0894	-1.3	0.1709
97349_at	1451264_at	4930488L10Rik	RIKEN cDNA 4930488L10 gene	-2.11	0.24	1.22	0.3357	-1.01	0.9713	1.21	0.3246	2.59	0.0191
97352_f_at	1435275_at	Cox6b2	cytochrome c oxidase subunit VIb polypeptic	3.37	0.14	1.35	0.2623	2.36	0.0145	2.76	0.0134	1.76	0.0562
97353_at	1433558_at	Dab2ip	disabled homolog 2 (Drosophila) interacting	1.82	0.37	1.3	0.2425	-1.12	0.7303	1.53	0.0372	1.96	0.0232
97354_at	1434066_at	Gtf3c1	general transcription factor III C 1	-1.24	0.34	1.02	0.7926	1.05	0.6156	1.03	0.769	1.14	0.3318
97355_at	1437728_at	AW050020	expressed sequence AW050020	2.16	0.01	-1.31	0.123	-1.36	0.1005	1.11	0.3959	-2.05	0.0402
97356_at	1460424_at	1810008O21Rik	RIKEN cDNA 1810008O21 gene	-1.16	0.75	1.14	0.0309	1.1	0.3477	1.11	0.0735	1.02	0.9318
97357_at	1451507_at	Mef2c	myocyte enhancer factor 2C	1.24	0.7	-1.36	0.3933	-1.32	0.4208	1.05	0.8559	2.03	0.0849
97358_at	1428510_at	Lphn1	Latrophilin 1	-1.12	0.6	1.14	0.2461	1.38	0.0141	1.26	0.0354	1.4	0.0097
97359_at	1418198_a_at	Tm9sf1	transmembrane 9 superfamily member 1	-1.56	0.13	1.03	0.5631	1.01	0.8376	-1.06	0.2065	-1.18	0.1562
97360_at	1448316_at	Cklfsf3	chemokine-like factor super family 3	-1.34	0.64	2.2	0.0415	1.84	0.1831	1.94	0.1513	4.25	0.0007
97363_at	1423059_at	Ptk2	PTK2 protein tyrosine kinase 2	1.15	0.33	-1.05	0.6759	1.07	0.5705	-1.08	0.4695	1.05	0.5428
97364_at	1423714_at	Asf1b	ASF1 anti-silencing function 1 homolog B (S	-1.45	0.07	-1.14	0.7094	-1.71	0.1157	1.19	0.6006	1.07	0.8398
97365_at	1434755_at	Coro2b	coronin, actin binding protein, 2B	-1.63	0.42	-1.08	0.6186	1.15	0.1794	-1.19	0.2435	2.01	0.2365
97366_at	1424326_at	Lemd2	LEM domain containing 2	-1.33	0.08	1.04	0.5663	1.22	0.0054	1.1	0.0515	1.09	0.2687
97367_at	1449019_at	Akap1	A kinase (PRKA) anchor protein 1	-1.21	0.62	1.16	0.0819	1.06	0.3838	1.12	0.1931	-1.06	0.6021
97369_g_at	1418279_a_at	Akap1	A kinase (PRKA) anchor protein 1	-1.48	0.52	1.04	0.73	-1.04	0.7629	-1.17	0.1041	-1.05	0.9021
97370_at	1452076_at	4633402N23Rik	RIKEN cDNA 4633402N23 gene	1.06	0.8	1.01	0.7464	1.09	0.1312	1.09	0.1183	-1	0.929
97371_at	1452715_at	2310022K01Rik	RIKEN cDNA 2310022K01 gene	-1.43	0.21	1.39	0.129	-1.01	0.9568	1.49	0.1558	1.25	0.3396
97373_at	1424316_at	Slc25a19	solute carrier family 25 (mitochondrial deoxy	-1.14	0.77	-1.05	0.5329	-1.07	0.4619	-1.19	0.054	1.11	0.2664
97374_at	1428452_at	2810025M15Rik	RIKEN cDNA 2810025M15 gene	2.01	0.07	-1.35	0.0751	-1.17	0.2725	-1.3	0.0966	1.1	0.8532
97375_at	1460210_at	Pkd1	polycystic kidney disease 1 homolog	-1.23	0	1.09	0.4831	-1.02	0.8306	-1.02	0.8274	1.38	0.1035
97377_at	1416692_at	Coil	coilin	1.06	0.9	1.47	0.1577	1.37	0.273	1.28	0.353	-1.26	0.4285
97379_at	1449088_at	Fbp2	fructose bisphosphatase 2	-2.17	0.24	-1.15	0.4188	1.44	0.3479	1.1	0.7433	-1.9	0.2369
97380_at	1431745_a_at	2700069A02Rik	RIKEN cDNA 2700069A02 gene	1.3	0.49	1.2	0.0164	1.26	0.0024	1.12	0.1678	-1.03	0.893
97381_s_at	1420730_a_at	Tcp11	t-complex protein 11	-1.16	0.7	-1.35	0.2942	-1.02	0.9424	-1.53	0.1639	1.06	0.8156
97385_at	1419509_a_at	Nagk	N-acetylglucosamine kinase	1.28	0.52	1.01	0.8712	-1.06	0.4649	-1.03	0.6096	1.47	0.0308
97386_at	1436026_at	MGI:2662729	ES neuronal differentiation 2	-1.12	0.53	-1.26	0.0372	1.17	0.1555	-1.18	0.142	-1.09	0.6893
97390_at	1417132_at	Cdc25a	cell division cycle 25 homolog A (S. cerevisia	-1.35	0.17	1.1	0.1593	1.18	0.0519	1.04	0.5426	1.73	0.0563

97392_at	1415715_at	2410015J15Rik	RIKEN cDNA 2410015J15 gene	-1.39	0.11	1.16	0.1528	1.09	0.2945	1.09	0.3314	1.12	0.3467
97393_at	1425006_a_at	Vrk1	vaccinia related kinase 1	1.71	0.48	1.89	0.1011	1.66	0.183	2.43	0.0169	1.2	0.4307
97395_at	1460633_at	Prp19	PRP19/PSO4 homolog (S. cerevisiae)	1.22	0.26	-1.08	0.3197	-1.27	0.0119	-1.26	0.0183	1.02	0.899
97397_at	1417265_s_at	D5Ertd33e	DNA segment, Chr 5, ERATO Doi 33, expre	1.07	0.82	1.03	0.6058	-1.09	0.3847	1.01	0.9226	1.28	0.0465
97398_at	1425615_a_at	Pck2	phosphoenolpyruvate carboxykinase 2 (mito	-1.2	0.62	1.36	0.2183	1.45	0.2	1.51	0.1324	1.35	0.2705
97400_at	1424806_s_at	1110039B18Rik	RIKEN cDNA 1110039B18 gene	1.16	0.38	-1.31	0.1566	-1.01	0.9496	-1.01	0.926	-1.7	0.0403
97401_at	1460676_at	1300006C06Rik	RIKEN cDNA 1300006C06 gene	1.3	0.01	1.23	0.0017	1.13	0.0585	1.22	0.002	1.43	0.0094
97402_at	1418697_at	Inmt	indolethylamine N-methyltransferase	1.14	0.23	1.44	0.0006	1.19	0.0462	1.62	0	1.4	0.0083
97404_at	1429038_at	1500034J01Rik	RIKEN cDNA 1500034J01 gene	1.85	0.19	1.23	0.016	1.03	0.808	1.25	0.0084	1.02	0.9589
97405_at	1416896_at	Rps6ka1	ribosomal protein S6 kinase polypeptide 1	-1.43	0.04	1	0.9169	1.02	0.6608	1.03	0.5698	-1.13	0.5101
97406_at	1434757_at	Cbfa2t2h	core-binding factor, runt domain, alpha subu	1.23	0.06	1.08	0.3488	-1	0.9661	1.04	0.5501	1.05	0.7331
97407_at	1416963_at	---	---	1.37	0.08	1.28	0.0602	1.28	0.0267	1.17	0.2069	-1.39	0.0524
97409_at	1418825_at	Ifi1	interferon inducible protein 1	1.1	0.68	1.58	0.391	-1.64	0.0267	1.41	0.4398	-1.75	0.009
97410_at	1454712_at	Mcart1	mitochondrial carrier triple repeat 1	1.26	0.49	-1.26	0.0608	-1.49	0.0096	-1.38	0.0121	-1.16	0.4763
97411_at	1419513_a_at	Ect2	ect2 oncogene	-1.13	0.66	-2.12	0.002	-2	0.0039	-2.4	0.0006	-1.47	0.2304
97412_at	1428004_at	3300001G02Rik	RIKEN cDNA 3300001G02 gene	1.29	0.03	-1.06	0.3223	1.06	0.1835	-1.08	0.1134	-1.2	0.2218
97413_at	1423933_a_at	1600029D21Rik	RIKEN cDNA 1600029D21 gene	-1.54	0.02	-1.95	0.0027	-1.18	0.3102	-3.04	0.0001	1.1	0.6698
97414_at	1423827_s_at	AI326906	expressed sequence AI326906	1.24	0.19	1.15	0.1142	-1.05	0.453	-1.01	0.8712	-1.11	0.4159
97415_at	1418890_a_at	Rab3d	RAB3D, member RAS oncogene family	4.79	0.01	1.39	0.0156	1.71	0.0064	1.8	0.0017	1.32	0.5017
97418_at	1418914_s_at	Bhmt2	betaine-homocysteine methyltransferase 2	1.36	0.21	-1.03	0.5906	-1.1	0.1304	-1.04	0.3564	-1.24	0.0111
97419_at	1426765_at	Commd7	COMM domain containing 7	-1.09	0.44	-1.11	0.1139	-1.19	0.0261	-1.13	0.1132	1.05	0.5381
97420_at	1417290_at	Lrg1	leucine-rich alpha-2-glycoprotein 1	-1.87	0.04	-1.04	0.8491	1.09	0.6104	1.23	0.3116	-5.73	0.0193
97421_at	1448635_at	Smc2l1	SMC2 structural maintenance of chromosom	1.54	0.43	1.16	0.3923	-1.03	0.8891	1.2	0.2489	1.05	0.8784
97422_at	1426735_at	2010002H18Rik	RIKEN cDNA 2010002H18 gene	1.56	0.3	1.54	0	1.46	0.0076	1.72	0	1.11	0.2737
97423_at	1417474_at	1500035H01Rik	RIKEN cDNA 1500035H01 gene	-1	0.99	1.09	0.1698	1.25	0.0005	1.19	0.0083	1.44	0.0269
97424_at	1417225_at	Arl6ip5	ADP-ribosylation factor-like 6 interacting pro	2.16	0	-1.12	0.0719	-1.18	0.0076	-1.15	0.0076	-1.42	0.0458
97425_at	1433645_at	2210409B22Rik	RIKEN cDNA 2210409B22 gene	-1.17	0.6	-1.01	0.9473	-1.02	0.9225	-1.06	0.5256	-1.21	0.2994
97426_at	1416529_at	Emp1	epithelial membrane protein 1	1.73	0.01	-1.07	0.6732	-1.32	0.2113	-1.09	0.6841	2.28	0.0259
97427_at	1418787_at	Mbl2	mannose binding lectin, serum (C)	-1.26	0.22	-1.85	0	-1.37	0.0014	-2.22	0	-1.66	0.0004
97428_at	1423116_at	Dom3z	DOM-3 homolog Z (C. elegans)	-1.08	0.1	1.07	0.3539	-1.07	0.4616	1.01	0.8941	-1.12	0.7001
97429_at	1425678_a_at	Snrk	SNF related kinase	1.05	0.79	-1.54	0.0038	-1.09	0.4045	-1.46	0.0089	-1.03	0.9252
97430_at	1417042_at	Slc37a4	solute carrier family 37 (glycerol-6-phosphat	1.44	0.12	1.04	0.6093	1.51	0.001	1.34	0.0084	-1.12	0.4705
97431_at	1417072_at	Slc22a6	solute carrier family 22 (organic anion transp	1.54	0.17	-1.01	0.9502	1.07	0.5592	-1.11	0.3442	1.42	0.1676
97433_at	1448711_at	---	---	1.14	0.52	1.11	0.2337	1.15	0.0272	1.12	0.1922	-1.07	0.6567
97434_at	1424839_a_at	2810405F18Rik	RIKEN cDNA 2810405F18 gene	1.25	0.62	-1.02	0.6976	1.17	0.0681	1.13	0.0479	-1.46	0.1319
97435_at	1419131_at	F13b	coagulation factor XIII, beta subunit	-1.19	0.37	1.11	0.1159	1.1	0.3493	-1	0.9611	-1.12	0.2458
97436_at	1423687_a_at	Man2c1	mannosidase, alpha, class 2C, member 1	1.51	0.1	-1.09	0.146	-1.03	0.4314	1.04	0.2915	-1.03	0.7999
97437_f_at	1447985_s_at	---	---	-1.09	0.74	-1.08	0.7645	-1.14	0.5144	-1.01	0.9745	1.98	0.0011
97438_r_at	1429193_at	---	---	1.09	0.81	1.22	0.3988	1.35	0.2844	1.38	0.1385	-3.09	0.1182
97441_at	1421197_a_at	Acin1	apoptotic chromatin condensation inducer 1	1.03	0.96	1.4	0.0457	1.26	0.3052	1.28	0.18	1.89	0.039
97442_at	1416832_at	Slc39a8	solute carrier family 39 (metal ion transporte	-1.08	0.59	-1.04	0.621	-1.04	0.6001	-1.17	0.0835	-1.04	0.767
97443_at	1460701_a_at	Mrpl52	mitochondrial ribosomal protein L52	1.46	0.04	-1.06	0.1122	1.03	0.6183	1.04	0.1791	1.05	0.719
97444_at	1422476_at	Ifi30	interferon gamma inducible protein 30	2.15	0.14	1.55	0.1543	1.06	0.6907	1.54	0.2876	-1.36	0.1474
97446_at	1416140_a_at	Dhx30	DEAH (Asp-Glu-Ala-His) box polypeptide 30	1.09	0.5	-1.05	0.7787	1.11	0.5218	1.09	0.6632	-1.03	0.9073
97447_at	1451290_at	Map1lc3a	microtubule-associated protein 1 light chain	1.21	0.28	-1.08	0.2511	1.02	0.8499	-1.21	0.0072	-1.09	0.5732
97450_s_at	1460167_at	Aldh7a1	aldehyde dehydrogenase family 7, member	1.02	0.94	1.07	0.2893	-1.02	0.5542	1.14	0.0161	-1.42	0.0061
97451_at	1424024_at	Mcfd2	multiple coagulation factor deficiency 2	-1.18	0.42	-1.21	0.0005	-1.11	0.061	-1.42	0	-1.6	0.0004
97452_at	1424572_a_at	H2afy	H2A histone family, member Y	-1.54	0.37	1.01	0.9149	-1.18	0.0564	-1.2	0.0219	1.27	0.1623
97456_at	1428082_at	Acsl5	acyl-CoA synthetase long-chain family mem	1.39	0.3	-1.13	0.1157	1.04	0.7913	-1.1	0.1856	-1.64	0.0013
97458_at	1454696_at	LOC433812	LOC433812	1.12	0.49	-1.05	0.5769	1.04	0.6175	1.08	0.3588	-1.74	0.1615
97459_at	1460339_at	Psma4	proteasome (prosome, macropain) subunit,	1.08	0.76	-1.1	0.0901	-1.01	0.7141	1.05	0.3246	-1.47	0.0212
97463_g_at	1436687_x_at	3110006P09Rik	RIKEN cDNA 3110006P09 gene	-1.02	0.92	1.01	0.8539	1.04	0.4926	-1.04	0.5164	-1.29	0.1341
97464_at	1426678_at	3110006P09Rik	RIKEN cDNA 3110006P09 gene	-1.2	0.37	1	0.9686	-1.14	0.1311	-1.25	0.0031	-1.81	0.0002

97465_at	1451879_a_at	Slc6a18	solute carrier family 6 (neurotransmitter trans	1.18	0.82	1.41	0.2037	1.79	0.0671	1.46	0.1379	1.5	0.0551
97468_at	1448441_at	Cks1b	CDC28 protein kinase 1b	1.75	0.17	1.48	0.0073	1.4	0.0546	1.53	0.0006	-1.3	0.1466
97469_at	1452957_at	Krtap3-3	keratin associated protein 3-3	1.1	0.75	1.28	0.0471	1.29	0.0599	-1.03	0.8309	1.21	0.3203
97470_at	1427366_at	Krtap3-1	keratin associated protein 3-1	1.19	0.26	1.1	0.4176	1.04	0.5045	1.09	0.5705	1.18	0.0144
97471_at	1415719_s_at	Armc1	armadillo repeat containing 1	1.11	0.59	1.08	0.1326	1.09	0.1682	1.15	0.0949	1.07	0.2396
97472_at	1424912_at	Slc25a17	solute carrier family 25 (mitochondrial carrier	1.03	0.89	1.03	0.6619	-1.07	0.2339	-1.08	0.3881	-1.33	0.0415
97473_at	1448276_at	Tspan4	tetraspanin 4	-1.07	0.61	-1.23	0.0502	-1.1	0.4708	-1.17	0.1605	-1.45	0.0304
97474_r_at	1448254_at	Ptn	pleiotrophin	1.25	0.58	1.42	0.4011	1.67	0.2665	2.07	0.0497	-3	0.2144
97475_at	1422843_at	---	---	1.43	0.55	1.15	0.3851	1.51	0.1199	1.38	0.043	-1.32	0.558
97477_at	1431665_a_at	Timm8b	translocase of inner mitochondrial membran	1.12	0.39	-1.02	0.465	1.09	0.1482	1.01	0.7792	-1.07	0.5673
97478_at	1460544_at	Mak10	MAK10 homolog, amino-acid N-acetyltransfe	1.37	0.15	1.04	0.6954	1.13	0.2135	1.11	0.2608	1.22	0.2125
97479_at	1417908_s_at	Ube2l3	ubiquitin-conjugating enzyme E2L 3	-1.3	0.04	-1.2	0.0105	-1.18	0.0281	-1.3	0.0002	-1.35	0.0012
97483_at	1416219_at	Rpl19	ribosomal protein L19	1.15	0.37	1.12	0.024	1.15	0.0204	1.14	0.0106	1.52	0.026
97484_at	1426531_at	Zmynd11	zinc finger, MYND domain containing 11	-1.03	0.62	1.08	0.321	1.02	0.8219	1.07	0.4393	1.42	0.0364
97486_at	1422509_at	U2af1	U2 small nuclear ribonucleoprotein auxiliary	1.7	0.14	1.06	0.4624	-1.06	0.6072	-1.02	0.791	1.17	0.3656
97487_at	1416666_at	Serpine2	serine (or cysteine) proteinase inhibitor, clad	2.27	0.01	-1.47	0.3854	-1.88	0.1907	-1.87	0.1838	-3.18	0.0069
97488_at	1428144_at	1200011O22Rik	RIKEN cDNA 1200011O22 gene	-1.27	0.24	1.12	0.3552	1.07	0.503	1.12	0.1997	1.18	0.1176
97489_at	1433504_at	Pygb	brain glycogen phosphorylase	2.05	0.14	-1.08	0.7852	-1.46	0.1955	-1.19	0.5355	-1.13	0.5505
97490_at	1448247_at	Bcl7b	B-cell CLL/lymphoma 7B	-1.29	0.25	1.29	0.0138	1.47	0.0018	1.29	0.0153	1.2	0.4611
97491_at	1427129_a_at	Hnrpr	heterogeneous nuclear ribonucleoprotein R	1.25	0.36	-1.07	0.3342	-1.16	0.2002	-1.05	0.5476	-1.4	0.0745
97492_at	1415738_at	0610040B21Rik	RIKEN cDNA 0610040B21 gene	-1.18	0.48	-1.04	0.5015	1.13	0.1975	-1.06	0.3832	-1.04	0.7494
97496_f_at	1423771_at	Prkcdbp	protein kinase C, delta binding protein	4.24	0	1.04	0.893	-1.79	0.1009	1.13	0.6913	2.31	0.0167
97497_at	1418634_at	Notch1	Notch gene homolog 1 (Drosophila)	2.41	0.18	1.64	0.0218	1.25	0.2857	1.41	0.0517	1.95	0.1621
97498_at	1417872_at	Fhl1	four and a half LIM domains 1	-1.14	0.67	-1.51	0.3593	-1.44	0.4019	-1.43	0.4148	1.86	0.0329
97504_at	1448229_s_at	Ccnd2	cyclin D2	-1.33	0.55	-1.52	0.1341	-1.8	0.0319	-1.99	0.0194	1.22	0.6779
97505_at	1451025_at	Arl1	ADP-ribosylation factor-like 1	1.14	0.49	-1.01	0.868	1.06	0.5355	1.14	0.0572	-1.52	0.004
97506_at	1424873_at	Rnf2	ring finger protein 2	1.39	0.35	1.16	0.4576	1.12	0.5607	1.24	0.2229	-1.37	0.5425
97507_at	1448380_at	Lgals3bp	lectin, galactoside-binding, soluble, 3 binding	-1.14	0.23	1.06	0.6783	-1.1	0.2254	1.22	0.3668	-1.38	0.0454
97509_f_at	1424050_s_at	Fgfr1	fibroblast growth factor receptor 1	-1.24	0.42	1.19	0.3465	1.19	0.3194	1.28	0.3472	1.83	0.069
97511_at	1450391_a_at	Mgll	monoglyceride lipase	-1.02	0.94	-1.58	0	-1.22	0.0047	-1.91	0	-2.6	0.0077
97512_at	1428258_at	2010107E04Rik	RIKEN cDNA 2010107E04 gene	1.59	0.22	1.06	0.3908	1.11	0.1377	1.08	0.3883	1.08	0.6662
97514_at	1435864_a_at	1810063B05Rik	RIKEN cDNA 1810063B05 gene	1.54	0.05	-1.05	0.5083	-1.03	0.7728	1.24	0.0021	-1.02	0.8026
97515_at	1417369_at	Hsd17b4	hydroxysteroid (17-beta) dehydrogenase 4	1.39	0.17	1.02	0.7994	1.17	0.0501	-1	0.9739	-2.21	0.0001
97516_at	1415787_at	Ganab	alpha glucosidase 2 alpha neutral subunit	-1.09	0.58	-1.21	0.0176	-1.31	0.0068	-1.42	0	-1.12	0.0333
97517_at	1460679_at	Exosc4	exosome component 4	1.02	0.85	1.31	0.001	1.05	0.4711	1.2	0.0171	1.22	0.2057
97518_at	1448130_at	Fdft1	farnesyl diphosphate farnesyl transferase 1	-1.12	0.73	-1.04	0.6876	1.12	0.3326	-1.12	0.183	1.41	0.0259
97519_at	1449254_at	Spp1	secreted phosphoprotein 1	1.59	0	1.09	0.5274	1.23	0.2845	1.14	0.2634	-1.57	0.0867
97520_s_at	1423506_a_at	Nnat	neuronatin	-2.12	0.42	1.22	0.3385	1.21	0.1643	1.21	0.1208	1.48	0.0865
97524_f_at	1416055_at	Amy2	amylase 2, pancreatic	1.27	0.5	-1.47	0.0175	-1.18	0.3557	-1.35	0.0754	-1.15	0.536
97525_at	1422704_at	Gyk	glycerol kinase	-1.39	0.03	1.32	0.0003	1.27	0.0223	1.37	0	-1.05	0.668
97526_at	1448308_at	Ap3m1	adaptor-related protein complex 3, mu 1 sub	1.07	0.71	-1.07	0.4254	1.05	0.7006	-1.02	0.8336	-1.28	0.1095
97527_at	1417458_s_at	Cks2	CDC28 protein kinase regulatory subunit 2	1.26	0.25	1.13	0.2424	-1.09	0.5281	1.1	0.3833	-1.02	0.9257
97528_at	1423727_at	Cnih	cornichon homolog (Drosophila)	1.31	0.13	-1.13	0.1352	-1.11	0.4015	-1.28	0.0092	-1.92	0
97529_at	1417732_at	Anxa8	annexin A8	-1.36	0.68	1.23	0.5485	1.34	0.3627	1.04	0.8905	-1.93	0.1006
97530_at	1425478_x_at	Ube2i	ubiquitin-conjugating enzyme E2I	1.52	0.09	-1.15	0.1413	-1.09	0.329	-1.16	0.0056	-1.62	0.0902
97531_at	1448700_at	---	---	-1.31	0.56	-1.04	0.883	-2.26	0.0033	-2.19	0.0042	-1.51	0.1441
97532_at	1451242_a_at	Ppp5c	protein phosphatase 5, catalytic subunit	1.95	0.04	1.23	0.0987	1.12	0.4612	1.1	0.4022	-1.32	0.4979
97535_at	1416004_at	Ywhah	tyrosine 3-monooxygenase/tryptophan 5-mo	1.2	0.36	1.19	0.0381	1.22	0.1772	1.27	0.0349	1.01	0.9236
97536_at	1434560_at	Wdtc1	WD and tetratricopeptide repeats 1	-1.11	0.74	-1.09	0.3386	-1.04	0.6854	-1.04	0.6451	1.01	0.8981
97538_at	1448124_at	Gusb	glucuronidase, beta	1.05	0.72	1.22	0.0504	1.18	0.1563	1.37	0.0089	-1.28	0.2387
97539_at	1426749_at	Hrmt1l3	heterogeneous nuclear ribonucleoprotein me	-1.01	0.99	-1.1	0.7369	-1.43	0.2487	1.99	0.0019	4.83	0.0886
97541_f_at	1427651_x_at	H2-D1	histocompatibility 2, D region locus 1	-1.43	0.11	1.32	0.1923	-1.05	0.5159	1.36	0.111	-1.61	0.0017
97543_at	1423472_at	2-Sep	septin 2	1.44	0.01	1.02	0.7903	-1.12	0.3678	1.03	0.7302	-1.15	0.2851

97546_at	1450014_at	Cldn1	claudin 1	1.03	0.93	-1.02	0.8975	-1.05	0.7707	-1.34	0.1463	1.55	0.1459
97548_at	1428172_at	Prpf39	PRP39 pre-mRNA processing factor 39 hom	3.41	0.13	-1.18	0.3123	1.25	0.1564	1.16	0.2947	-2.97	0.0887
97549_at	1418066_at	Cfl2	cofilin 2, muscle	-1.11	0.81	-1.16	0.1927	1.08	0.5684	-1.37	0.0095	-2.5	0.0008
97550_at	1420813_at	Hdac7a	histone deacetylase 7A	1.08	0.85	1.07	0.2848	-1	0.967	1.1	0.254	1.13	0.4177
97551_at	1425553_s_at	Hip1r	huntingtin interacting protein 1 related	-1.28	0.63	-1.32	0.0609	-1.14	0.3998	-1.21	0.29	-1.03	0.955
97553_at	1452182_at	Galnt2	UDP-N-acetyl-alpha-D-galactosamine:polyp	-1.28	0.03	-1.24	0.0004	-1.02	0.7452	-1.28	0.0001	-1.25	0.0891
97554_at	1456016_x_at	BC005624	CDNA sequence BC005624	1.18	0.29	1.08	0.1954	1.05	0.5181	-1.04	0.591	1.18	0.2041
97556_at	1451356_at	Anp32e	acidic (leucine-rich) nuclear phosphoprotein	-1.07	0.71	-1.32	0.0265	-1.41	0.0205	-1.32	0.0258	-1.68	0.022
97557_at	1438547_x_at	Tor2a	torsin family 2, member A	1.53	0.05	-1.04	0.7211	1.03	0.703	-1.12	0.238	-1.52	0.0735
97559_at	1424736_at	Eef2	eukaryotic translation elongation factor 2	1.08	0.78	-1.05	0.2225	-1.01	0.91	-1.09	0.0475	1.14	0.4069
97564_f_at	1460423_x_at	IgM	Ig kappa chain	-1.99	0.19	1.54	0.1429	1.11	0.7258	1.65	0.149	1.03	0.9161
97580_at	1422048_at	Trpc5	transient receptor potential cation channel, s	-1.51	0.53	-1.26	0.6232	-2.19	0.039	-1.33	0.434	1.64	0.176
97594_r_at	1448189_a_at	Fliih	flightless I homolog (Drosophila)	-1.27	0.21	1.03	0.5656	1.01	0.8824	-1.01	0.9137	1.2	0.2592
97645_f_at	1447377_at	LOC433721	similar to hypothetical protein 4930503F14	-1.75	0.24	1.17	0.4186	1.08	0.5805	-1.04	0.7761	1.3	0.3865
97647_at	1416404_s_at	Rps16	ribosomal protein S16	-1.04	0.87	1.09	0.0042	1.2	0.0014	1.23	0.0008	1.36	0.1985
97651_at	1419501_at	Polk	polymerase (DNA directed), kappa	1.44	0.54	-1.64	0.1152	-1.71	0.0957	1.05	0.873	3.01	0.0724
97652_at	1427611_at	Ghrhr	growth hormone releasing hormone recepto	-1.46	0.1	-1.01	0.8965	1.03	0.7506	-1.06	0.5506	1.31	0.0973
97653_at	1427622_at	Mem1	maternal embryonic message 1	-1.18	0.42	-1.57	0.3116	-1.34	0.4706	-1.67	0.2655	1.17	0.2568
97654_at	1427563_at	Dlx6as	distal-less homeobox 6, antisense	1.93	0.42	1.88	0.0339	2	0.0773	1.4	0.0592	1.52	0.3647
97655_at	1427637_a_at	Dsc3	desmocollin 3	-3.52	0.26	-1.27	0.1727	-1.18	0.3892	-1.26	0.128	-1.21	0.5523
97659_r_at	1422447_at	Ins1	insulin I	-1.48	0	1.07	0.2647	1.01	0.863	-1	0.9651	1.18	0.2894
97661_at	1418682_at	Tenr	testis nuclear RNA binding protein	1.25	0.35	1	0.9998	1.23	0.1626	-1.04	0.6831	-1.43	0.2101
97666_r_at	1457588_at	C76213	expressed sequence C76213	2.07	0	-1.29	0.4551	-1.22	0.5369	1.24	0.5279	2.04	0.0665
97679_at	1421770_a_at	Tlx2	T-cell leukemia, homeobox 2	1.35	0.44	1.07	0.7422	-1.04	0.8109	-1.28	0.1694	-1.09	0.7643
97680_at	1448854_s_at	Mug-ps1 /// Mug	murinoglobulin, pseudogene 1 /// murinoglob	-1.62	0.03	1.16	0.068	1.11	0.2512	1.24	0.0173	1.04	0.7472
97681_f_at	1427474_s_at	Gstm3	glutathione S-transferase, mu 3	1.12	0.74	1.64	0.0073	1.3	0.0016	1.9	0.0001	1.75	0.0002
97683_at	1421481_at	Tnfrsf9	tumor necrosis factor receptor superfamily, r	-2.25	0.43	1.24	0.4517	1.78	0.0091	1.72	0.0043	-1.1	0.7474
97684_at	1419384_at	Prkcabp	protein kinase C, alpha binding protein	-1.15	0.55	1.02	0.8899	-1.04	0.8394	1	0.9734	2.09	0.0121
97688_at	1447958_at	---	16 days neonate thymus cDNA, RIKEN full-l	-1.58	0.18	1.11	0.3078	1.18	0.0629	1.07	0.427	1.5	0.0028
97689_at	1417408_at	F3	coagulation factor III	1.04	0.9	1.15	0.3498	1.16	0.3968	1.33	0.0675	-1.05	0.8754
97690_at	1449641_at	---	---	1.32	0.51	-1.27	0.0892	-1.45	0.0097	-1.22	0.0524	-1.33	0.4572
97691_at	1419799_at	---	Transcribed locus, strongly similar to XP_52	-1.27	0.42	-1.15	0.2042	-1.19	0.2515	-1.3	0.0441	1.29	0.1263
97692_at	1450477_at	Htr2c	5-hydroxytryptamine (serotonin) receptor 2C	-2.78	0.14	-1.26	0.4893	-1.05	0.869	-1.15	0.6519	1.94	0.0491
97693_at	1443414_at	C78513	EST C78513	-3.11	0.2	-1.35	0.3484	1.72	0.0727	1.38	0.399	-1.36	0.327
97695_s_at	1426163_x_at	Rpl7	ribosomal protein L7	1.25	0.31	1.11	0.0374	1.09	0.1467	1.17	0.0017	1.35	0.0272
97697_at	1427452_at	Rpl7	ribosomal protein L7	-1.81	0.23	-1.12	0.7656	-1.4	0.4039	-1.5	0.2956	1.33	0.6342
97698_at	1418548_at	---	---	-1.39	0.55	-1.48	0.1561	1.22	0.6485	-1.1	0.808	1.28	0.589
97699_at	1421232_at	Plxna1	plexin A1	1.4	0.53	-1.18	0.0444	-1.01	0.8529	-1.11	0.3489	1.96	0.0156
97700_at	1451593_at	H2-Q1	Histocompatibility 2, D region locus 1	1.07	0.85	1.81	0.0386	-1.18	0.4989	2.07	0.1349	1.02	0.9701
97701_at	1458219_at	Bcas2	breast carcinoma amplified sequence 2	1.03	0.9	-1.06	0.5789	-1	0.9647	1.01	0.9219	1.19	0.6653
97703_at	1421945_a_at	Bxdc1	brix domain containing 1	-1.15	0.6	-1.24	0.0972	-1.06	0.6625	-1.12	0.3424	-1.08	0.8089
97705_at	1453077_a_at	Snapc3	small nuclear RNA activating complex, polyp	2.05	0.18	1.19	0.3786	-1.15	0.5634	-1.16	0.574	1.41	0.2024
97707_at	1448073_at	LOC541456	hypothetical LOC541456	-1.08	0.69	1.1	0.6484	-1.07	0.809	-1.47	0.1598	1.66	0.2355
97708_at	1459933_at	---	---	-1.43	0.29	1.23	0.5472	1.15	0.7169	1.13	0.688	1.32	0.606
97709_at	1422080_at	Il7	interleukin 7	-1.36	0.65	-1.36	0.4308	-1.34	0.439	-1.3	0.4637	-1.55	0.552
97710_f_at	1448028_at	C530046L02Rik	RIKEN cDNA C530046L02 gene	2.45	0.04	1.61	0.0025	2.26	0.0012	1.97	0.0004	3.13	0.0065
97711_at	1448024_at	B430320C24Rik	RIKEN cDNA B430320C24 gene	1.97	0.22	1.09	0.5996	1.07	0.6335	1.11	0.5239	1.47	0.327
97712_at	1422994_at	---	---	-1.43	0	1.42	0.2859	1.22	0.563	1.6	0.1699	3.7	0.1141
97713_at	1419327_at	AA415817	expressed sequence AA415817	-1.37	0.28	-1.18	0.5285	-1.38	0.1876	-1.48	0.0698	-1.04	0.6927
97714_r_at	1457970_at	Actr1a	ARP1 actin-related protein 1 homolog A (yea	2.08	0.06	1.2	0.5139	1.5	0.1167	-1.13	0.7133	1.35	0.5348
97717_at	1449592_at	Tcf15	transcription factor 15	-1.83	0.17	1.06	0.8428	1.03	0.9079	1.08	0.7394	1.07	0.6403
97718_at	1419334_at	Ctla4	cytotoxic T-lymphocyte-associated protein 4	-1.57	0.57	-1.19	0.6683	1.45	0.2363	-1.06	0.8678	1.25	0.7271
97719_at	1420461_at	Mst1r	macrophage stimulating 1 receptor (c-met-re	-1.66	0.01	1.05	0.7223	-1.01	0.9553	-1.06	0.7135	2.89	0.1053

97721_at	1418376_at	Fgf15	fibroblast growth factor 15	1.09	0.56	-1.18	0.3244	-1.16	0.4151	-1.02	0.8979	1.14	0.6914
97722_at	1417764_at	Ssr1	signal sequence receptor, alpha	-1.25	0.38	-1.18	0.3096	-1.35	0.1447	-1.92	0.0004	-1.25	0.3053
97723_at	1422059_at	Cbx2	chromobox homolog 2 (Drosophila Pc class)	-1.8	0.07	-1.13	0.3731	-1.26	0.248	-1.31	0.0707	1.36	0.1203
97725_at	1422872_at	Bmpr1b	bone morphogenetic protein receptor, type 1	-1.18	0.68	-1.14	0.6197	1.36	0.1667	1	0.9917	1.4	0.2748
97726_at	1449245_at	Grin2c	glutamate receptor, ionotropic, NMDA2C (ep	-2.11	0	-1.03	0.7929	-1.1	0.3579	-1.18	0.2346	1.14	0.5643
97727_at	1422300_at	Nog	noggin	-1.2	0.33	-1.12	0.5651	1.04	0.7829	-1.21	0.2805	-1.18	0.7324
97728_at	1422987_at	Ntn1	netrin 1	2.25	0.13	-1.05	0.6032	1.05	0.6518	-1.03	0.8162	-1.16	0.701
97729_at	1420964_at	Enc1	ectodermal-neural cortex 1	-3.74	0.01	1.44	0.1829	1.17	0.6095	1.24	0.4059	1.01	0.979
97730_at	1449588_at	Abca4	ATP-binding cassette, sub-family A (ABC1),	-1.55	0.29	-1.27	0.4262	-1.09	0.8228	-1.53	0.2192	1.65	0.0663
97731_at	1420437_at	Indo	indoleamine-pyrrole 2,3 dioxygenase	-1.41	0.45	-1.98	0.1725	-1.24	0.5885	-1.38	0.3818	1.39	0.5719
97732_at	1450453_a_at	Pde6g	phosphodiesterase 6G, cGMP-specific, rod,	1.13	0.8	-1.04	0.8371	1.08	0.6107	-1.06	0.7823	-1.24	0.5076
97734_at	1449569_at	Thpo	thrombopoietin	-1.38	0.11	-1.19	0.081	-1.41	0.002	-1.03	0.7721	1.23	0.5196
97735_at	1449571_at	Trhr	thyrotropin releasing hormone receptor	-1.02	0.97	1.03	0.9157	-1.22	0.4118	1.39	0.3177	-1.44	0.326
97736_at	1442384_at	---	Transcribed locus	-1.4	0.64	1.1	0.7257	1.04	0.9015	-1.04	0.8894	2.5	0.0861
97738_r_at	1433661_at	BC034204	cDNA sequence BC034204	-1.1	0.62	-1.03	0.6622	-1	0.985	1.11	0.0968	1.01	0.8434
97740_at	1418401_a_at	Dusp16	dual specificity phosphatase 16	1.4	0.26	1.08	0.2086	-1.03	0.7165	1.07	0.2903	1.18	0.1611
97741_at	1450371_at	Tshb	thyroid stimulating hormone, beta subunit	1.55	0.55	-1.39	0.315	-1	0.9937	1.22	0.5214	1.11	0.8557
97742_s_at	1451882_a_at	Fgf8	fibroblast growth factor 8	-1.07	0.73	1.1	0.3678	1.36	0.0064	1.01	0.9025	1.17	0.6462
97743_at	1435949_at	Zc3hdc3	zinc finger CCCH type domain containing 3	-1.76	0.07	1.81	0.0009	1.66	0.0666	1.54	0.1275	1.39	0.2235
97745_at	1427354_at	Hoxa4	homeo box A4	-1.58	0.1	-1.35	0.0262	-1.29	0.0495	-1.64	0.0004	1.28	0.0541
97750_at	1448245_at	Lamr1	laminin receptor 1 (ribosomal protein SA)	1.1	0.33	-1.02	0.6805	1.07	0.4331	1.11	0.0224	1.21	0.3031
97751_f_at	1447999_x_at	LOC380690 /// I	similar to glyceraldehyde-3-phosphate dehy	1.19	0.32	1.16	0.1676	1.15	0.0147	1.2	0.0172	-1.16	0.0679
97752_at	1434292_at	E130013N09Ril	RIKEN cDNA E130013N09 gene	2.67	0.28	2.35	0.0005	1.62	0.0832	2.93	0	3.3	0.0227
97753_at	1419392_at	Pclo	piccolo (presynaptic cytomatrix protein)	-1.29	0.35	1.1	0.4967	1.26	0.1348	1.17	0.2909	1.52	0.3005
97754_at	1456757_at	G630024C07Ril	RIKEN cDNA G630024C07 gene	-2.45	0.18	-1.22	0.3702	1.01	0.9741	-1.07	0.7359	-1.28	0.3944
97755_at	1422830_s_at	Drd4	dopamine receptor 4	-2.17	0.01	-1.02	0.8742	1.08	0.6557	1.01	0.9602	1.89	0.0267
97756_s_at	1451769_s_at	Pcdha4 /// Pcdh	protocadherin alpha 4 /// protocadherin alpha	-1.31	0.59	1.07	0.7047	1.27	0.1188	1.01	0.9246	-1.06	0.7637
97757_at	1420359_at	Sva	seminal vesicle antigen	-1.16	0.81	-1.21	0.4891	1.35	0.4388	-1.84	0.0175	-1.01	0.988
97758_at	1416000_a_at	Prdx1	peroxiredoxin 1	1.05	0.74	-1.07	0.1101	-1.03	0.5351	-1.09	0.0171	-1.01	0.7539
97760_at	1421327_at	Mtap2	microtubule-associated protein 2	1.14	0.59	1.08	0.8269	-1.08	0.59	-1.03	0.8987	1.12	0.6005
97762_f_at	1426171_x_at	Klra7	killer cell lectin-like receptor, subfamily A, m	-1.16	0.56	-1.06	0.7779	1.01	0.9613	-1.07	0.7418	1.44	0.1751
97763_at	1451767_at	Ncf1	neutrophil cytosolic factor 1	-1.19	0.06	2.23	0.0925	-1.43	0.2114	2.35	0.2827	1.84	0.0044
97765_g_at	1425970_a_at	Ros1	Ros1 proto-oncogene	-1.5	0.67	-1.37	0.5275	1.59	0.4116	1.47	0.4211	-1.12	0.7523
97767_at	1420728_at	Krt1-2	keratin complex 1, acidic, gene 2	1.15	0.44	-1.3	0.0393	-1.09	0.5195	-1.11	0.338	1.53	0.0728
97768_at	1421156_a_at	Dsc2	desmocollin 2	1.58	0.37	1.41	0.0211	1.68	0.0269	1.33	0.0476	-1.26	0.6451
97769_at	1420349_at	Ptgfr	prostaglandin F receptor	-1.25	0.21	1.59	0.1981	1.76	0.0607	1.68	0.2012	1.59	0.3796
97771_r_at	1448904_at	D6Wsu176e	DNA segment, Chr 6, Wayne State Universit	-4.05	0.27	-1.25	0.2394	1.28	0.3778	-1.6	0.0582	1.18	0.7906
97772_at	1422139_at	Plau	plasminogen activator, urokinase	-1.08	0.62	-1.02	0.8681	1.05	0.4841	-1.02	0.9068	-1.1	0.7901
97774_at	1425432_at	Oprm1	opioid receptor, mu 1	-1.47	0.14	1.93	0.0005	1.3	0.1111	1.47	0.0195	1.18	0.5353
97775_at	1442778_at	AA511254	expressed sequence AA511254	-1.17	0.62	-1.33	0.0016	-1.12	0.1148	-1.13	0.1592	1.39	0.4152
97776_at	1418950_at	Drd2	dopamine receptor 2	-1.95	0.01	-1.05	0.6872	-1.03	0.8031	-1.03	0.7953	1.69	0.0157
97777_at	1449566_at	Nkx2-5	NK2 transcription factor related, locus 5 (Dro	-1.63	0.22	1.1	0.4098	1.07	0.4323	-1.03	0.7529	1.39	0.0533
97778_at	1421915_a_at	St3gal3	ST3 beta-galactoside alpha-2,3-sialyltransfe	5.28	0.19	-1.41	0.0225	-2.04	0.0018	-2.06	0.0005	-2.31	0.0082
97779_at	1449965_at	Mcpt8	mast cell protease 8	-1.46	0.25	1.09	0.7721	1.34	0.3349	-1.29	0.5026	1.14	0.7638
97780_at	1459934_at	Usp42	ubiquitin specific protease 42	-2.09	0.3	-1.16	0.5063	-1.42	0.0949	-1.07	0.6987	2.05	0.0797
97781_at	1422089_at	Ncr1	natural cytotoxicity triggering receptor 1	-1.16	0.85	1.9	0.0787	1.31	0.4835	2.45	0.0709	1.34	0.5902
97782_at	1450554_at	Defb2	defensin beta 2	1.24	0.54	1.71	0.1013	1.35	0.2337	1.7	0.1056	3.41	0.0584
97783_at	1419413_at	Ccl17	chemokine (C-C motif) ligand 17	1.29	0.64	1.05	0.4209	1.22	0.1038	1.1	0.2146	1.48	0.1519
97784_at	1449237_at	Aloxe3	arachidonate lipoxygenase 3	-1.24	0.25	-1.05	0.6495	-1.01	0.9228	-1.08	0.3683	1.23	0.1877
97785_at	1419419_at	D0H6S2654E	DNA segment, human D6S2654E	1.11	0.8	-1.03	0.805	-1.06	0.5196	-1.22	0.1124	1.21	0.399
97786_at	1419621_at	Ankrd2	ankyrin repeat domain 2 (stretch responsive	1.55	0.16	-1.27	0.1688	-1.22	0.2059	-1.3	0.1093	2.16	0.0074
97788_at	1419231_s_at	---	---	-1.41	0.6	-1.3	0.3228	-1.22	0.4708	-1.38	0.1671	-1.04	0.8097
97789_at	1422213_s_at	---	---	-1	0.96	1.03	0.6286	1.01	0.8999	-1.03	0.662	1.2	0.2931

97790_s_at	1427512_a_at	Lama3	laminin, alpha 3	-1.45	0.11	1.28	0.2376	1.13	0.5437	1.56	0.0436	-1.99	0.1672
97791_at	1422146_at	Sema5b	sema domain, seven thrombospondin repea	-2.31	0.21	-1.14	0.7386	1.19	0.6105	-1.09	0.8378	1.09	0.8363
97793_at	1420563_at	Gria3	glutamate receptor, ionotropic, AMPA3 (alph	1.11	0.67	-1.19	0.501	-1.1	0.6989	1.36	0.4472	-1.01	0.9871
97794_at	1422040_at	Sema7a	sema domain, immunoglobulin domain (Ig),	-1.38	0.52	-1.15	0.3779	-1.01	0.9239	-1.44	0.0281	-1.1	0.6309
97796_at	1450229_at	Crsp2	cofactor required for Sp1 transcriptional activ	-1.45	0.41	-1.25	0.3704	1.09	0.722	-1.04	0.8353	-1.88	0.1355
97797_at	1430656_a_at	2210409M21Rik	RIKEN cDNA 2210409M21 gene	1.55	0.19	-1.01	0.8539	1.05	0.4354	1.08	0.205	-1.06	0.6175
97798_at	1426656_at	4930504E06Rik	RIKEN cDNA 4930504E06 gene	-1.4	0.05	-1.53	0	-1.14	0.1453	-1.64	0	-1.49	0.0047
97800_at	1460635_at	Fastk	Fas-activated serine/threonine kinase	1.24	0.55	1.08	0.216	1.01	0.9591	1.14	0.2226	1.13	0.5764
97803_at	1450919_at	Mpp1	membrane protein, palmitoylated	1.98	0.06	1.22	0.0361	1.22	0.0233	1.8	0	1.82	0.0106
97807_at	1416327_at	1110021H02Rik	RIKEN cDNA 1110021H02 gene	1.34	0.14	1.05	0.459	-1.06	0.3724	1.09	0.11	-1.17	0.0702
97808_at	1418562_at	Sf3b1	splicing factor 3b, subunit 1	1.73	0.36	1.15	0.5579	-2.62	0.001	-1.8	0.0234	1.13	0.2841
97809_at	1460692_at	Bat8	HLA-B associated transcript 8	1.17	0.28	1.16	0.0219	1.12	0.3373	1.2	0.0525	1.03	0.752
97811_at	1426534_a_at	Arfgap3	ADP-ribosylation factor GTPase activating p	1.37	0.53	2.28	0.0221	2.31	0.02	2.88	0.0116	1.31	0.5774
97813_at	1419536_a_at	Rela	v-rel reticuloendotheliosis viral oncogene ho	-1.49	0.12	-1.16	0.019	-1.08	0.3632	-1.14	0.1315	-1.03	0.8025
97814_at	1427179_at	Krt1-3	keratin complex 1, acidic, gene 3	1.81	0.08	-1.07	0.508	-1.04	0.684	1	0.9932	1.16	0.3556
97816_at	1428907_at	2600011C06Rik	RIKEN cDNA 2600011C06 gene	-1.04	0.8	-1.01	0.9656	-1.08	0.7007	1.17	0.2776	-1.2	0.5759
97817_at	1428131_a_at	Cdc42se1	CDC42 small effector 1	-1.01	0.94	1.03	0.7139	-1.02	0.7249	1.12	0.205	1.26	0.0787
97818_at	1460182_at	Snx4	sorting nexin 4	1.06	0.79	1.09	0.1241	1.09	0.4128	1.12	0.0236	-1.2	0.3403
97819_at	1416531_at	Gsto1	glutathione S-transferase omega 1	1.34	0.16	-1.07	0.1401	1.13	0.0934	1.01	0.9238	1.32	0.0324
97820_at	1417177_at	Galk1	galactokinase 1	1.61	0.06	1.84	0.0086	1.57	0.0018	1.86	0	1.85	0.0112
97823_g_at	1454887_at	Pak2	p21 (CDKN1A)-activated kinase 2	-1.12	0.08	1	0.9906	1.06	0.3374	1.04	0.4765	1.24	0.057
97824_at	1416606_s_at	Nola2	nucleolar protein family A, member 2	1.38	0.1	-1.25	0.0369	-1.1	0.4818	-1.24	0.0578	-1.03	0.7608
97825_at	1416271_at	Perp	PERP, TP53 apoptosis effector	1.24	0.46	-1.1	0.1667	1.11	0.2446	-1.08	0.288	-2.24	0.0009
97826_at	1426872_at	A430096B05Rik	RIKEN cDNA A430096B05 gene	-1.14	0.74	-1.18	0.2176	1.21	0.3125	-1.15	0.2843	-1.08	0.1651
97828_at	1418377_a_at	MGI:1353606	Cd27 binding protein (Hindu God of destruct	1.61	0.04	1.35	0	-1.01	0.7934	1.2	0.003	2.32	0.0006
97829_at	1423657_at	Cdipt	CDP-diacylglycerol--inositol 3-phosphatidyltr	-1	0.96	-1.14	0.1286	1.05	0.6247	-1.05	0.4911	-1.08	0.3
97834_g_at	1416069_at	Pfkp	phosphofructokinase, platelet	2.99	0.03	3.15	0.1048	1.61	0.2289	3.3	0.0705	-1.6	0.423
97835_at	1437902_s_at	Rarres2	retinoic acid receptor responder (tazarotene	1.62	0.03	1.31	0	1	0.9355	1.36	0	1.63	0.0011
97836_at	1456600_a_at	Rnf7	ring finger protein 7	1.5	0.04	1	0.9799	-1.01	0.946	1	0.9945	-1.15	0.576
97838_at	1454703_x_at	Rnu22	RNA, U22 small nucleolar	1.8	0.01	1.22	0.0252	1.17	0.3224	1.37	0.0031	1.64	0.0396
97839_at	1429497_s_at	Snx6	sorting nexin 6	1.05	0.45	1.05	0.2819	1.07	0.2944	1.02	0.7634	1.13	0.0823
97841_at	1425591_a_at	Chmp2a	chromatin modifying protein 2A	1.65	0.12	1.2	0.1949	1.1	0.5291	-1.03	0.785	-1.02	0.8793
97843_at	1420808_at	Ncoa4	nuclear receptor coactivator 4	-1.13	0.1	1.07	0.2208	1.08	0.3597	1.22	0.0008	-1.16	0.2042
97844_at	1419248_at	Rgs2	regulator of G-protein signaling 2	1.39	0.37	1.81	0.2084	-1.02	0.9393	1.88	0.1628	1.41	0.2062
97845_at	1416581_at	Wdr5	WD repeat domain 5	-1.48	0.28	-1.23	0.0313	1.15	0.0364	-1.31	0.0062	-1.67	0.1247
97846_at	1460429_at	Cdc5l	cell division cycle 5-like (S. pombe)	1.45	0.32	-1.09	0.4082	1.22	0.0139	-1.06	0.5414	1.23	0.1102
97847_at	1416354_at	Rbmx	RNA binding motif protein, X chromosome	1.3	0.3	1.01	0.9042	-1.01	0.8511	-1.04	0.706	1.51	0.0081
97848_at	1426863_at	Rbmx	RNA binding motif protein, X chromosome	1.68	0.12	1.38	0.0151	1.43	0.1706	1.61	0.0009	1.03	0.9536
97853_at	1417166_at	Psip1	PC4 and SFRS1 interacting protein 1	1.49	0.24	1.14	0.1589	1.16	0.0615	1.06	0.6356	-1.13	0.4131
97857_at	1423646_at	Zdhhc3	zinc finger, DHHC domain containing 3	1.51	0.21	-1.23	0.0878	1.08	0.4851	-1.08	0.4704	-1.34	0.188
97859_at	1433605_at	Inpp5a	inositol polyphosphate-5-phosphatase A	1.37	0.04	-1.11	0.2958	1.15	0.0385	-1.14	0.3149	-1.32	0.3251
97862_s_at	1451145_s_at	0610039A15Rik	RIKEN cDNA 0610039A15 gene	1.42	0.07	1.01	0.9301	1.02	0.7429	-1	0.9612	-1.19	0.4469
97863_at	1450868_at	D8Ertd354e	DNA segment, Chr 8, ERATO Doi 354, expr	1.06	0.69	1.16	0.0758	1.24	0.0049	1.29	0	1.36	0.0125
97866_at	1423217_a_at	2510049I19Rik	RIKEN cDNA 2510049I19 gene	-1.02	0.92	-1.1	0.1943	-1.06	0.6781	-1.11	0.0656	1.09	0.5204
97867_at	1449038_at	---	---	1.16	0.21	1.16	0.0156	1.11	0.0373	1.08	0.1876	1.28	0.0193
97868_at	1449935_a_at	Dnaja3	DnaJ (Hsp40) homolog, subfamily A, membe	-1.02	0.81	1.34	0.0002	1.2	0.0365	1.36	0	-1.04	0.6843
97869_at	1451084_at	Etfdh	electron transferring flavoprotein, dehydroge	1.45	0.11	1.19	0.0481	1.24	0.0881	1.28	0.0246	-1.22	0.3148
97871_at	1449324_at	Ero1l /// LOC43	ERO1-like (S. cerevisiae) /// hypothetical ge	-1.12	0.43	1.09	0.2489	1.14	0.0119	1.16	0.0221	1.22	0.0419
97875_at	1419263_a_at	Adrm1	adhesion regulating molecule 1	1.39	0.06	-1.08	0.2455	1.08	0.1703	1.06	0.4316	-1.1	0.5735
97876_at	1417660_s_at	Vps29	vacuolar protein sorting 29 (S. pombe)	1.25	0.15	1.14	0.066	1.04	0.7476	1.09	0.1004	1.09	0.3866
97880_at	1423710_at	Dlst	dihydrolipoamide S-succinyltransferase (E2	1.62	0.02	1.16	0.0969	-1.01	0.9461	1	0.9869	-1.71	0.0096
97882_at	1448242_at	Sec61a1	Sec61 alpha 1 subunit (S. cerevisiae)	-1.04	0.81	-1.13	0.0302	-1.02	0.84	-1.1	0.0347	-1.19	0.1471
97884_at	1460195_at	Mrps11	mitochondrial ribosomal protein S11	1.53	0.23	1.07	0.2764	1.02	0.8116	1.04	0.6307	-1.3	0.1216

97885_at	1418004_a_at	1810009M01Rik	RIKEN cDNA 1810009M01 gene	1.28	0.3	1.48	0.0009	1.03	0.3168	1.49	0.0136	1.07	0.6181
97886_at	1432372_a_at	Spr	sepiapterin reductase	1.56	0.09	-1.04	0.4629	-1.21	0.1731	-1.1	0.3864	-1.05	0.6239
97887_at	1418069_at	Apoc2	apolipoprotein C-II	1.37	0.18	1.45	0	1.32	0.0279	1.9	0	-1.27	0.1601
97888_at	1424616_s_at	---	---	2.67	0.07	1.15	0.2064	-1.47	0.0043	1.28	0.0072	-1.23	0.2196
97889_at	1418438_at	Fabp2	fatty acid binding protein 2, intestinal	-2.76	0.03	-1.92	0	-1.18	0.2222	-2.06	0	-14.57	0
97890_at	1416041_at	Sgk	serum/glucocorticoid regulated kinase	2.32	0.08	1.3	0.0633	1.33	0.0494	1.57	0.0023	2.69	0.0316
97891_at	1417035_at	Sac3d1	SAC3 domain containing 1	1.24	0.5	1.03	0.7587	-1.08	0.3865	-1.05	0.5909	1.56	0.2213
97892_at	1451137_a_at	Brd8	bromodomain containing 8	1.35	0.34	1.28	0.1033	1.48	0.0007	1.36	0.0061	1.75	0.0027
97893_at	1431415_a_at	Tbpl1	TATA box binding protein-like 1	1.39	0.53	1.49	0.0009	1.7	0.1007	1.41	0.0001	1.23	0.6521
97894_at	1448472_at	Vars2	valyl-tRNA synthetase 2	-1.02	0.94	-1.32	0.0045	-1.01	0.9331	1.13	0.3945	-1.16	0.4573
97896_r_at	1428061_at	Hat1	histone aminotransferase 1	1.76	0.01	1.3	0.1228	1.07	0.6499	1.49	0.0148	1.62	0.1849
97897_at	1433476_at	C78339	expressed sequence C78339	1.16	0.56	1.02	0.5984	-1.06	0.3966	1.05	0.3871	1.05	0.6886
97901_at	1453097_a_at	Ubtf	upstream binding transcription factor, RNA p	1.06	0.84	1.29	0	1.23	0	1.27	0.0183	1.1	0.7987
97903_at	1431593_a_at	Leng5	leukocyte receptor cluster (LRC) member 5	1.05	0.88	-1.12	0.123	-1.15	0.1518	-1.17	0.0306	-1.24	0.3299
97904_at	1452051_at	Actr3	ARP3 actin-related protein 3 homolog (yeast	-1.24	0.26	1.08	0.4016	1.08	0.492	1.25	0.0893	1.09	0.3718
97906_at	1448170_at	Siah2	seven in absentia 2	-1.57	0.04	1.01	0.934	1.16	0.0441	-1.13	0.0766	1.27	0.0045
97907_at	1417313_at	Lsm7	LSM7 homolog, U6 small nuclear RNA asso	2.29	0.03	1.09	0.1978	-1.03	0.8259	1.02	0.7493	-1.09	0.6987
97908_at	1426884_at	1110007A06Rik	RIKEN cDNA 1110007A06 gene	-1.08	0.6	-1.12	0.0958	1.06	0.4713	-1.11	0.1624	-1.38	0.0196
97909_at	1415849_s_at	Stmn1	stathmin 1	1.85	0.25	1.75	0.0188	1.25	0.1526	2.18	0.0023	1.65	0.2248
97911_at	1434214_at	0910001L09Rik	RIKEN cDNA 0910001L09 gene	1.13	0.75	-1.03	0.5837	1.08	0.1589	-1.06	0.3813	-1.41	0.0399
97912_at	1416009_at	Tspan3	tetraspanin 3	-1.03	0.96	-1	0.9512	1.12	0.2013	1.18	0.1777	1.12	0.7158
97914_at	1418503_at	Hspa9a	heat shock protein 9A	1.55	0.08	1.19	0.0011	1.48	0.0003	1.36	0.0003	1.46	0.0291
97915_at	1460341_at	Plekhb2	pleckstrin homology domain containing, fam	1.4	0.13	-1.22	0.2326	-1.04	0.7679	-1.15	0.3779	1.1	0.8165
97916_at	1452773_at	5730494N06Rik	RIKEN cDNA 5730494N06 gene	1.24	0	1.04	0.6268	-1.13	0.1842	-1.16	0.0507	1.08	0.4575
97917_at	1422614_s_at	Bloc1s1	biogenesis of lysosome-related organelles c	1.36	0.2	1.08	0.0634	-1.12	0.0242	1.07	0.2574	1.07	0.3713
97918_at	1427243_at	AA536743	expressed sequence AA536743	1.31	0.06	1.15	0.0903	-1.16	0.0606	1.2	0.002	1.44	0.0439
97919_at	1452869_at	1110021E09Rik	RIKEN cDNA 1110021E09 gene	-1.26	0	1	0.971	1.04	0.689	-1.01	0.8279	-1.16	0.213
97920_at	1423952_a_at	Krt2-7	keratin complex 2, basic, gene 7	2.31	0.18	1.05	0.756	-1.06	0.7155	1.05	0.7034	1.19	0.5507
97921_at	1426670_at	Agrn	agrin	1.12	0.47	-1.08	0.2786	-1.07	0.4874	-1.03	0.7014	1.1	0.5247
97922_at	1426307_at	---	---	1.17	0.38	-1.14	0.0829	-1.1	0.139	-1.12	0.0309	-1.12	0.1252
97923_at	1434060_at	Herc1	hect (homologous to the E6-AP (UBE3A) ca	1.05	0.84	1.08	0.3864	1.26	0.0121	1.1	0.31	1.17	0.359
97924_at	1448810_at	Gne	glucosamine	1.15	0.08	-1.17	0.0082	1.36	0.0318	1.1	0.1501	-1.19	0.3954
97925_at	1417176_at	Csnk1e	casein kinase 1, epsilon	1.02	0.93	-1.35	0.4305	-2.99	0.0076	-1.27	0.508	1.55	0.0957
97926_s_at	1420715_a_at	Pparg	peroxisome proliferator activated receptor ga	6.19	0.01	1.65	0.0014	1.39	0.052	1.72	0.0002	2.68	0.0181
97927_at	1422572_at	Rhog	ras homolog gene family, member G	-1.07	0.93	1.05	0.751	-1.17	0.3947	-1.05	0.7321	1.71	0.2187
97928_at	1454837_at	Cln6	ceroid-lipofuscinosis, neuronal 6	2.33	0.07	1.98	0.0005	2.47	0.0058	2.1	0.0019	5.66	0.0018
97930_f_at	1451232_at	Cd151	CD151 antigen	-1.37	0.03	-1.05	0.5069	-1.16	0.0507	-1.1	0.1711	-1.46	0.011
97932_f_at	1420921_at	Cd151	CD151 antigen	-1.21	0.03	1.07	0.038	-1.05	0.2424	-1	0.9695	-1.16	0.1456
97933_at	1460695_a_at	2300006M17Rik	RIKEN cDNA 2300006M17 gene	1.05	0.81	-1.12	0.2522	1.06	0.6846	1.01	0.9667	1.27	0.0139
97934_at	1423236_at	Galnt1	UDP-N-acetyl-alpha-D-galactosamine:polype	-1.15	0.34	-1.01	0.7975	1.12	0.0246	1.11	0.1778	-1.11	0.4926
97935_at	1428871_at	4121402D02Rik	RIKEN cDNA 4121402D02 gene	1.08	0.46	1.22	0.0004	1.14	0.1106	1.2	0.0174	1.17	0.0166
97936_at	1435243_at	2810407L07Rik	RIKEN cDNA 2810407L07 gene	-1.06	0.88	1.06	0.4663	1.01	0.8701	1.11	0.2316	2.15	0.0631
97937_at	1451021_a_at	Klf5	Kruppel-like factor 5	1.97	0.22	-1.32	0.2663	1.41	0.4419	-1.12	0.6124	1.49	0.4531
97939_at	1451195_a_at	Txndc1	thioredoxin domain containing 1	1.25	0.21	1.23	0.0016	1.12	0.1461	1.33	0	1.36	0.1053
97940_at	1415848_at	Csh1	chorionic somatomammotropin hormone 1	-2.07	0.28	1.56	0.196	-1.05	0.8841	1.72	0.1387	1.34	0.0284
97943_at	1450429_at	Capn6	calpain 6	-1.17	0.39	1.11	0.2839	1	0.9837	-1	0.9518	1.41	0.1028
97944_f_at	1452405_x_at	Tcra	T-cell receptor alpha chain	-2.52	0	-1.05	0.6092	1.1	0.2904	-1.07	0.5021	1.18	0.1069
97945_at	1426168_a_at	Tcra	T-cell receptor alpha chain	-1.61	0.41	3.43	0.0089	1.79	0.1397	2.01	0.1152	1.4	0.2316
97946_at	1417905_at	Prlpf	prolactin-like protein F	-1.59	0.18	-1.42	0.2912	-1.33	0.4592	-1.22	0.5573	-1.55	0.3398
97947_at	1428103_at	---	---	1.16	0.71	1.26	0.1041	1.24	0.1021	1.36	0.0603	-1.16	0.5532
97948_at	1417850_at	Rb1	retinoblastoma 1	1.8	0.16	-1.22	0.0452	-1.2	0.357	-1.03	0.8242	-2.12	0.0677
97949_at	1421855_at	Fgl2	fibrinogen-like protein 2	1.51	0.35	2.27	0.1662	-1.38	0.1382	2.92	0.1355	1.34	0.2451
97950_at	1451006_at	---	---	1.45	0.2	1.31	0.1836	-1.05	0.6623	1.3	0.3716	1.17	0.1623

97954_at	1415861_at	Tyrp1	tyrosinase-related protein 1	-2.35	0.26	1.12	0.762	-1.05	0.8177	1.04	0.8892	-1.44	0.3966
97957_at	1424441_at	Slc27a4	solute carrier family 27 (fatty acid transporte	1.27	0.52	1.06	0.6324	-1.15	0.4863	-1.63	0.0074	-1.96	0.0574
97958_at	1426614_at	Prkcbp1	protein kinase C binding protein 1	1.46	0	-1.11	0.3409	-1.05	0.771	-1.23	0.1928	-2.28	0.3713
97960_at	1456043_at	Usp22	ubiquitin specific protease 22	-1.08	0.62	1.11	0.3923	1.18	0.2259	-1.02	0.8114	1.17	0.5772
97962_at	1452344_at	Synj2	synaptojanin 2	1.24	0.6	1.01	0.9542	-1.09	0.5784	-1.11	0.5794	-1.2	0.4928
97963_at	1416206_at	Sipa1	signal-induced proliferation associated gene	3.69	0.09	1	0.9992	1.08	0.7439	1.09	0.7682	2.31	0.1909
97964_at	1417267_s_at	Fkbp11	FK506 binding protein 11	-1.09	0.69	-1.05	0.8474	1.42	0.0469	1.18	0.4341	-2.06	0.0339
97965_at	1422147_a_at	Pla2g6	phospholipase A2, group VI	4.14	0.01	1.05	0.6432	-1.47	0.0338	1.04	0.7321	-1.7	0.4926
97966_at	1424055_at	Ncoa5	nuclear receptor coactivator 5	-1.4	0.1	-1.12	0.1611	-1.01	0.8961	-1.09	0.1089	1.13	0.6389
97970_at	1419755_at	Mfi2	antigen p97 (melanoma associated) identifie	-1.96	0.01	1.34	0.1836	1.5	0.0594	1.29	0.092	1.79	0.1311
97971_at	1428511_at	Phkg2	phosphorylase kinase, gamma 2 (testis)	1.37	0.13	1.08	0.241	-1	0.9693	1.15	0.1038	1.04	0.8214
97972_at	1448434_at	Rnf103	ring finger protein 103	-1.11	0.41	-1.28	0.0344	-1.11	0.4341	-1.28	0.0302	-1.6	0.0016
97973_at	1449389_at	Tal1	T-cell acute lymphocytic leukemia 1	1.27	0.68	1.38	0.0179	1.51	0.0901	1.06	0.7103	1.1	0.3982
97974_at	1451046_at	Zfpm1	zinc finger protein, multitype 1	-1.4	0.07	1.06	0.4188	1.13	0.1623	-1.04	0.4831	1.49	0.0269
97975_at	1452497_a_at	Nfatc3	nuclear factor of activated T-cells, cytoplasm	-1.12	0.77	1.07	0.552	-1.3	0.0662	-1.36	0.0194	-1	0.9854
97976_at	1455434_a_at	Ktn1	kinectin 1	-1.39	0.3	-1.02	0.8478	1.02	0.889	-1.1	0.2155	-1.18	0.2932
97977_at	1454974_at	Ntn1	netrin 1	-1.11	0.55	1.01	0.9464	1.09	0.1336	-1.14	0.1543	1.21	0.0285
97979_at	1417919_at	Ppp1r7	protein phosphatase 1, regulatory (inhibitor)	2.24	0.01	1.07	0.4174	1.03	0.6914	1.13	0.0335	1.03	0.9426
97980_at	1416435_at	Ltbr	lymphotoxin B receptor	-2.01	0.01	-1.84	0	-1.37	0.0012	-1.72	0	-2.71	0.0059
97982_at	1425310_a_at	Emid2	EMI domain containing 2	-1.85	0	-1.12	0.2683	-1	0.9637	-1.08	0.5185	1.31	0.1018
97983_s_at	1420505_a_at	Stxbp1	syntaxin binding protein 1	1.41	0.45	1.17	0.1794	-1.04	0.6216	1.18	0.1824	1.39	0.032
97985_f_at	1428881_at	Kns2	kinesin 2	-1.53	0.03	1.08	0.3659	1.1	0.1652	1.04	0.531	1.12	0.4122
97986_at	1418259_a_at	Entpd2	ectonucleoside triphosphate diphosphohydro	-1.81	0.05	1.03	0.885	1.22	0.329	-1	0.9918	1.09	0.5993
97987_at	1422826_at	Igfals	insulin-like growth factor binding protein, aci	-21.66	0	-11.92	0	-1.66	0	-61.92	0	-35.03	0
97990_at	1448962_at	Myh11	myosin, heavy polypeptide 11, smooth musc	1.29	0.42	-1.25	0.6125	1.06	0.9002	-1.05	0.9152	-2.85	0.14
97991_at	1451979_at	Kras2	V-Ki-ras2 Kirsten rat sarcoma viral oncogen	1.47	0.34	1.15	0.0139	1.09	0.3121	1.28	0.0021	-1.25	0.3203
97992_at	1434546_at	BC024683	cDNA sequence BC024683	-1.06	0.43	1.07	0.7795	1.46	0.2594	-1.17	0.2485	1.14	0.0748
97993_at	1423482_at	Uros	uroporphyrinogen III synthase	1.07	0.68	-1.44	0	-1.3	0	-1.49	0	-1.39	0.0141
97994_at	1433471_at	Tcf7	transcription factor 7, T-cell specific	1.72	0.12	-1.07	0.4035	1.27	0.0375	1.05	0.5012	1.8	0.0304
97995_at	1450461_at	Tcf7	transcription factor 7, T-cell specific	-1.6	0.11	-1.16	0.0998	1.1	0.2341	-1.13	0.2221	1.12	0.5069
97996_at	1424217_at	Papola	poly (A) polymerase alpha	-1.25	0.56	-1.06	0.5729	-1.18	0.2777	-1.26	0.0404	1	0.9872
97997_at	1448395_at	Sfrp1	secreted frizzled-related sequence protein 1	-1.34	0.33	-1.13	0.4872	1.02	0.8812	1.02	0.871	1.89	0.0257
97998_at	1421149_a_at	Drpla	dentatorubral pallidoluysian atrophy	1	1	1.05	0.6467	1.11	0.3531	1.09	0.2659	-1.17	0.4809
98000_at	1419387_s_at	Ly64	lymphocyte antigen 64	-2.47	0.21	1.13	0.6354	-1.03	0.8977	-1.25	0.3336	1.9	0.2011
98001_at	1421164_a_at	Arhgef1	Rho guanine nucleotide exchange factor (GI	1.8	0.25	1.14	0.2706	1.06	0.7578	1.13	0.3813	1.11	0.7643
98002_at	1416714_at	Icsbp1	interferon consensus sequence binding prot	1.25	0.19	1.37	0.2444	-1.1	0.2053	1.51	0.1563	1.28	0.1884
98003_at	1424302_at	Pirb	paired-Ig-like receptor B	1.35	0.23	1.18	0.4837	-1.02	0.8129	1.56	0.2161	1.33	0.0246
98004_at	1420858_at	Pkia	protein kinase inhibitor, alpha	-1.25	0.43	-1.75	0.0435	-1.61	0.0954	-1.45	0.1082	1.49	0.4697
98005_at	1420859_at	Pkia	protein kinase inhibitor, alpha	-1.16	0.77	-1.07	0.7497	1.5	0.124	-1.15	0.4283	1.79	0.2412
98006_at	1448369_at	Pola2	polymerase (DNA directed), alpha 2	2.24	0.08	1.19	0.0328	1.01	0.9401	1.11	0.1231	1.17	0.6841
98007_at	1417542_at	Rps6ka2	ribosomal protein S6 kinase, polypeptide 2	3.5	0.02	1.53	0.0827	1.94	0.1214	2.3	0.0126	-1.1	0.7731
98008_at	1415803_at	Cx3cl1	chemokine (C-X3-C motif) ligand 1	-1.68	0.3	-1	0.9888	-1.18	0.5117	-1.04	0.839	-1.02	0.9593
98010_at	1452001_at	Nfe2	nuclear factor, erythroid derived 2	1.14	0.33	-1.02	0.9247	1.05	0.6532	-1.1	0.3668	1.16	0.5564
98011_at	1455021_at	Gabbr1	gamma-aminobutyric acid (GABA-B) recepto	-1.38	0.13	1.59	0.0859	1.16	0.5317	1.21	0.3827	1.38	0.2977
98013_at	1454705_at	D15Ertd621e	DNA segment, Chr 15, ERATO Doi 621, exp	1.45	0.17	1.18	0.1226	1.24	0.0287	1.46	0.0054	1.09	0.5262
98014_at	1417973_at	Itih1	inter-alpha trypsin inhibitor, heavy chain 1	-1.52	0.08	-1.48	0.0001	-1.02	0.7925	-1.79	0	-2.57	0
98015_at	1416294_at	Scamp3	secretory carrier membrane protein 3	-1.19	0.47	1.15	0.0391	1.03	0.7368	1.15	0.0433	-1.26	0.2784
98016_at	1428310_at	D3Wsu161e	DNA segment, Chr 3, Wayne State Universit	1.96	0.07	1.04	0.7475	-1.11	0.6493	-1.08	0.6573	-1.26	0.3299
98017_at	1434071_a_at	Pelo	pelota homolog (Drosophila)	2.1	0	1.01	0.9128	1.09	0.4968	-1.3	0.0259	-1.58	0.1201
98018_at	1420664_s_at	Procr	protein C receptor, endothelial	-1.02	0.9	1.02	0.9517	-1.04	0.8089	1.12	0.7639	-1.13	0.835
98019_at	1418136_at	Tgfb1i1	transforming growth factor beta 1 induced tra	-1.01	0.98	1.19	0.108	-1.07	0.5705	1.02	0.8805	1.2	0.5709
98020_at	1448336_at	Drg1	developmentally regulated GTP binding prot	1.14	0.59	-1.1	0.0123	-1.1	0.0426	-1.06	0.0555	1.23	0.2752
98021_at	1435057_x_at	MGI:1929022	RNA polymerase I associated factor	-1.37	0.17	-1.15	0.1226	1.18	0.1858	1.02	0.8812	-1.24	0.5169

98022_at	1417180_at	Pcsk7	proprotein convertase subtilisin/kexin type 7	-1.31	0.23	-1.16	0.2096	1.11	0.2524	-1.06	0.5625	1.23	0.3964
98023_r_at	1460217_at	Plfr	proliferin related protein	-3.85	0.22	1.02	0.8708	1.93	0.0725	1.29	0.1907	2.54	0.0006
98024_at	1419266_at	Nfyb	nuclear transcription factor-Y beta	1.18	0.22	1.18	0.0241	1.07	0.4547	1.09	0.1157	-1.16	0.198
98026_g_at	1450241_a_at	Evi2a	ecotropic viral integration site 2a	1.67	0.16	1.26	0.5003	-1.17	0.3446	1.37	0.4163	-1.13	0.6978
98027_at	1450673_at	Col9a2	procollagen, type IX, alpha 2	-1.17	0.3	-1.09	0.174	-1.02	0.833	-1.13	0.2695	1.58	0.0279
98028_at	1418733_at	Twist1	twist gene homolog 1 (Drosophila)	-1.08	0.76	1.56	0.3098	1.33	0.499	1.04	0.8719	1.68	0.4121
98029_at	1434658_at	3110056O03Rik	RIKEN cDNA 3110056O03 gene	1.31	0.19	-1.13	0.1906	-1.21	0.0797	-1.32	0.0206	1.26	0.6787
98030_at	1451860_a_at	Trim30	tripartite motif protein 30	1.81	0.09	1.78	0.0677	1.5	0.2094	2.35	0.0399	1.15	0.7122
98031_at	1417040_a_at	Bok	Bcl-2-related ovarian killer protein	-1.34	0.1	1.14	0.2048	1.05	0.6791	1.25	0.0907	1.31	0.0303
98032_at	1417778_at	Zfp35	zinc finger protein 35	2.19	0.26	1.31	0.2374	1.35	0.1676	1.26	0.3127	-1.17	0.4679
98033_at	1448786_at	1100001H23Rik	RIKEN cDNA 1100001H23 gene	1.31	0.01	-1.7	0.0001	1.08	0.1909	-1.46	0.0085	-2.97	0.0006
98035_g_at	1449580_s_at	H2-DMb1 /// H2	histocompatibility 2, class II, locus Mb1 /// his	1.22	0.52	2.46	0.199	-1.54	0.0775	2.37	0.2408	1.28	0.4892
98036_at	1433538_at	Marveld1	MARVEL (membrane-associating) domain c	1.11	0.57	-1.16	0.2086	1.04	0.5812	-1.1	0.193	-1.12	0.653
98037_at	1415759_a_at	Hbxip	hepatitis B virus x interacting protein	1.09	0.71	1.01	0.8668	1.13	0.1232	1.09	0.1482	-1.11	0.2867
98038_at	1416155_at	Hmgb3	high mobility group box 3	1.1	0.55	-1.16	0.0855	1.07	0.2421	-1.13	0.0574	-1.76	0.0014
98039_at	1427909_at	2410015M20Rik	RIKEN cDNA 2410015M20 gene	1.28	0.07	1.14	0.0565	1.04	0.5739	1.25	0.0107	-1.07	0.3407
98042_at	1426555_at	Scpep1	serine carboxypeptidase 1	1.17	0.63	1.45	0.037	1.04	0.8434	1.02	0.8979	-1.08	0.6837
98045_s_at	1420498_a_at	Dab2	disabled homolog 2 (Drosophila)	-1.02	0.96	-1.05	0.7347	1.22	0.216	-1.06	0.6205	1.63	0.1756
98047_at	1454616_at	5730410I19Rik	RIKEN cDNA 5730410I19 gene	1.05	0.85	1.12	0.0524	1.18	0.0054	1.09	0.3278	-1.13	0.2376
98048_at	1423982_at	Fusip1	FUS interacting protein (serine-arginine rich)	1.02	0.97	1.84	0.0058	1.48	0.2051	2.16	0.0006	-1.76	0.27
98049_at	1423681_at	1300018I05Rik	RIKEN cDNA 1300018I05 gene /// mKIAA06	-1.13	0.54	-1.11	0.1662	1.01	0.8378	-1.06	0.4543	-1.17	0.3406
98051_at	1424819_a_at	AI114950	expressed sequence AI114950	1.27	0.26	1.05	0.5077	1.13	0.0572	1.14	0.0668	1.13	0.0938
98052_at	1453570_x_at	Bet1l	blocked early in transport 1 homolog (S. cere	1.6	0.05	1.11	0.2081	1.04	0.6336	1.09	0.297	-1.23	0.3235
98053_at	1455815_a_at	Ywhab	tyrosine 3-monooxygenase/tryptophan 5-mo	-1.25	0.12	-1.74	0	-1.47	0.0083	-1.75	0.0001	1.01	0.9487
98055_at	1420631_a_at	Blcap	bladder cancer associated protein homolog (	-1.53	0.03	1.01	0.9656	-1.03	0.8511	-1.11	0.4621	-1.42	0.0799
98056_at	1449002_at	Phlda3	pleckstrin homology-like domain, family A, m	-1.28	0.15	1.03	0.8513	-1.03	0.8622	1.24	0.3033	1.28	0.1052
98057_at	1436906_at	Rnf166	ring finger protein 166	1.25	0.5	1.13	0.1257	1.31	0.0022	1.15	0.1136	1.19	0.1781
98059_s_at	1421654_a_at	Lmna	lamin A	1.31	0.19	1	0.9474	-1.05	0.6983	1.07	0.5016	-1.31	0.3433
98060_at	1425472_a_at	Lmna	lamin A	1.05	0.73	-1.02	0.8531	1.09	0.1679	1	0.9664	1.03	0.826
98061_at	1428049_a_at	1110001K21Rik	RIKEN cDNA 1110001K21 gene	-1.05	0.85	-1.2	0.0378	-1.06	0.449	-1.2	0.033	-1.04	0.6768
98063_at	1424825_a_at	Glycam1	glycosylation dependent cell adhesion molec	-2.59	0.13	2.38	0.4402	-1.15	0.6357	-1.19	0.5053	2.32	0.0862
98064_at	1454754_a_at	Aamp	angio-associated migratory protein	-1.25	0.61	1.07	0.1752	1.07	0.6233	1.02	0.7115	-1.06	0.7996
98066_r_at	1423502_at	Brd2	bromodomain containing 2	-1.6	0	-1.03	0.8772	1.17	0.3125	1.21	0.2857	-1.14	0.7648
98067_at	1421679_a_at	Cdkn1a	cyclin-dependent kinase inhibitor 1A (P21)	1.11	0.7	1.3	0.2024	-1.14	0.3744	1.36	0.2031	1.1	0.5365
98069_s_at	1427897_s_at	2400003N08Rik	RIKEN cDNA 2400003N08 gene	-1.11	0.4	-1.01	0.8885	1.05	0.3626	-1.15	0.0863	-1.06	0.341
98070_at	1427896_at	2400003N08Rik	RIKEN cDNA 2400003N08 gene	-1.16	0.08	-1.15	0.0344	-1.08	0.1707	-1.25	0.0003	-1.15	0.0467
98072_r_at	1449176_a_at	Dck	deoxycytidine kinase	-1.11	0.48	1.2	0.4608	-1.06	0.8329	1.4	0.1451	-1.08	0.855
98073_at	1451435_at	Cutl1	cut-like 1 (Drosophila)	-1.18	0.45	-1.1	0.0679	1.1	0.3027	-1.08	0.2922	-1.46	0.0812
98075_at	1416763_at	G431001I09Rik	RIKEN cDNA G431001I09 gene	1.48	0.01	1.21	0.0007	1.03	0.5978	1.13	0.0424	-1.08	0.5102
98076_at	1453634_a_at	Erp29	endoplasmic reticulum protein 29	1.09	0.52	-1.04	0.7268	-1.12	0.1489	-1.01	0.9531	-1.17	0.1347
98077_at	1422884_at	Snrpd3	small nuclear ribonucleoprotein D3	1.31	0.57	1.25	0.0926	1.02	0.8838	1.16	0.2952	-1.11	0.7396
98078_at	1424979_at	Aph1a	anterior pharynx defective 1a homolog (C. e	-1.77	0.14	-1.19	0.0239	-1.19	0.0054	-1.3	0.0011	1.15	0.4473
98079_at	1450725_s_at	Car14	carbonic anhydrase 14	-1.61	0.21	-1.62	0.0008	-1.44	0.0003	-1.98	0	-1.01	0.9369
98081_at	1451120_at	Rpo1-3	RNA polymerase 1-3	1.76	0.12	1.06	0.5445	-1.07	0.5512	-1.02	0.7952	1.06	0.8571
98082_at	1448287_at	Rpo1-3	RNA polymerase 1-3	-1.91	0.23	-1.23	0.5038	-1.31	0.3869	-1.13	0.6905	1.17	0.6602
98083_at	1433508_at	Copeb	Kruppel-like factor 6	2.07	0.14	1.48	0.1008	1.46	0.0013	1.89	0.0251	2.06	0.0404
98084_at	1417112_at	Arl2bp	ADP-ribosylation factor-like 2 binding protein	-1.12	0.57	-1.16	0.0064	-1.05	0.394	-1.12	0.0583	-1.08	0.6254
98085_f_at	1454778_x_at	Rps28	Ribosomal protein S28	-1.05	0.74	-1.04	0.652	-1	0.9789	1.17	0.1058	1.6	0.0053
98087_at	1460315_s_at	Tbk1	TANK-binding kinase 1	-1.06	0.75	1.21	0.1116	1.18	0.1177	1.23	0.0506	-1.34	0.0086
98088_at	1417268_at	Cd14	CD14 antigen	-1.42	0.03	1.2	0.3278	-1.07	0.381	1.45	0.1965	1.37	0.06
98092_at	1451335_at	Plac8	placenta-specific 8	1.09	0.81	1.46	0.3843	-1.06	0.7684	2.23	0.2197	1.06	0.7376
98097_r_at	1451052_at	2610019N19Rik	RIKEN cDNA 2610019N19 gene	1.19	0.16	-1	0.9791	1.06	0.454	-1.06	0.4998	-1.06	0.6577
98098_at	1416193_at	Car1	carbonic anhydrase 1	1.25	0.33	1.39	0.1346	1.14	0.6143	1.12	0.6421	1.36	0.3309

98102_at	1418560_at	Pdha1	pyruvate dehydrogenase E1 alpha 1	1.45	0.03	1.26	0.0264	1.31	0.0004	1.31	0.0043	-1.44	0.1801
98104_at	1416769_s_at	Atp6v0b	ATPase, H+ transporting, V0 subunit B	1.39	0.47	-1.01	0.9321	1.12	0.3158	-1.03	0.5654	-1.14	0.4296
98106_at	1448801_a_at	Timm44	translocator of inner mitochondrial membran	1.56	0	1.06	0.3495	1.09	0.5027	1.05	0.467	1.21	0.1025
98107_at	1449660_s_at	Coro1c	coronin, actin binding protein 1C	1.23	0.14	1.06	0.4687	1.03	0.7612	1.03	0.6705	-1.45	0.5083
98108_at	1448326_a_at	Crabp1	cellular retinoic acid binding protein I	1.08	0.68	1.26	0.1398	1.19	0.269	1.27	0.0761	1.19	0.7069
98109_at	1429453_a_at	Mrpl55	mitochondrial ribosomal protein L55	1.15	0.23	1.03	0.7647	1.01	0.9366	1.12	0.1746	-1.28	0.3032
98111_at	1425993_a_at	Hsp105	heat shock protein 105	1.24	0.4	-1.63	0.0088	-1.09	0.556	-1.98	0.0013	1.04	0.8509
98113_at	1448166_a_at	Psmb1	proteasome (prosome, macropain) subunit, I	1.41	0.12	1.16	0.0522	1.2	0.0466	1.24	0.0089	-1.03	0.769
98116_at	1423944_at	Hpxn	hemopexin	-1.44	0.11	1.05	0.3992	1.05	0.3996	1.09	0.2544	-1.15	0.0492
98118_at	1422241_a_at	Ndufa1	NADH dehydrogenase (ubiquinone) 1 alpha	1.57	0.56	-1.98	0.0278	-1.13	0.6654	-1.95	0.0287	-1.12	0.812
98119_at	1418273_a_at	Rpl30	ribosomal protein L30	1.27	0.18	1.07	0.0076	1.14	0.0666	1.13	0.0002	1.5	0.0001
98120_at	1415690_at	Mrpl27	mitochondrial ribosomal protein L27	1.5	0	1.05	0.5386	1.01	0.9319	1.28	0.0054	-1.11	0.3447
98121_at	1417465_at	Fnta	farnesyltransferase, CAAX box, alpha	1.44	0.06	1.06	0.5656	1.11	0.3025	1.15	0.1892	1.2	0.2685
98123_at	1418519_at	Aadat	aminoadipate aminotransferase	1.32	0.13	1.03	0.6574	-1.04	0.7365	1.11	0.2225	-1.22	0.2645
98125_at	1423951_at	1110025I09Rik	RIKEN cDNA 1110025I09 gene	1.27	0.27	-1.25	0.0025	-1.12	0.0682	-1.14	0.0323	-1.2	0.4092
98126_s_at	1419312_at	Atp2a1	ATPase, Ca++ transporting, cardiac muscle,	-1.02	0.94	-2.95	0.1089	-1.76	0.2998	-2.66	0.1321	1.14	0.6002
98127_at	1423057_at	Capza2	capping protein (actin filament) muscle Z-line	-1.23	0.51	1.19	0.0022	1.16	0.2844	1.25	0.0284	1.07	0.6695
98128_at	1416143_at	Atp5j	ATP synthase, H+ transporting, mitochondria	1.09	0.69	-1.03	0.6928	-1	0.9959	1.02	0.8209	-1.03	0.801
98129_at	1417219_s_at	Tmsb10	thymosin, beta 10	1.82	0.03	1.96	0.085	1.13	0.4308	1.98	0.0975	1.75	0.077
98130_at	1452782_a_at	Txn2	thioredoxin 2	1.14	0.71	1.03	0.6408	1.09	0.2835	1.03	0.7	1.09	0.5789
98131_at	1451848_a_at	Cryz	crystallin, zeta	1.08	0.66	1.01	0.9229	1.01	0.8859	-1.01	0.8583	-1.36	0.0184
98132_at	1422484_at	Cycs	cytochrome c, somatic	1.8	0.02	1.28	0.0862	1.38	0.0378	1.45	0.0011	-1.86	0.002
98133_at	1417504_at	Calb1	calbindin-28K	-1.03	0.93	1.02	0.8319	1.18	0.2508	1.5	0.1152	-1.04	0.7986
98136_at	1421052_a_at	Sms	spermine synthase	1.91	0.09	1.07	0.3673	-1.02	0.8927	-1.03	0.8635	1.03	0.5906
98137_at	1419525_at	Car5a	carbonic anhydrase 5a, mitochondrial	1.44	0.12	-1.33	0.0021	-1.28	0.012	-1.58	0.0002	-1.44	0.2462
98138_at	1452049_at	Rpl7l1	ribosomal protein L7-like 1	1.2	0.31	1.1	0.2824	1.09	0.4026	1.06	0.5494	1.1	0.4235
98140_at	1448261_at	Cdh1	cadherin 1	-1.14	0.65	-1.07	0.347	-1.14	0.0281	-1.11	0.0824	1.28	0.1017
98142_at	1434604_at	Eif5b	eukaryotic translation initiation factor 5B	1.41	0.41	1.36	0.0146	1.39	0.0521	1.37	0.0051	1.07	0.5378
98143_at	1460319_at	Fut8	fucosyltransferase 8	1.11	0.76	-1.61	0.0024	-1.07	0.6151	-2.1	0.0002	-1.55	0.1389
98144_f_at	1430029_a_at	Sas	sarcoma amplified sequence	-1.45	0.04	-1.04	0.6483	-1.04	0.7963	-1.13	0.2419	-1.74	0.0021
98146_at	1428073_a_at	Nup88	nucleoporin 88	1.89	0.05	-1.02	0.7986	1.08	0.22	1.05	0.533	1.1	0.7047
98147_at	1448311_at	Usp5	ubiquitin specific protease 5 (isopeptidase T	-1.2	0.3	-1.04	0.5493	-1.05	0.7454	1.01	0.9019	1.09	0.7562
98149_s_at	1452730_at	1110033J19Rik	RIKEN cDNA 1110033J19 gene	-1.25	0.43	-1.26	0.1491	-1.02	0.8481	1.07	0.5474	-1.83	0.0964
98150_at	1423448_at	Rab11b	RAB11B, member RAS oncogene family	1.01	0.97	1.15	0.0111	1.08	0.0995	1.2	0.0153	1.14	0.1941
98152_at	1422450_at	Catns	catenin src	-1.4	0.02	-1.06	0.3811	-1.12	0.1547	-1.12	0.0648	1.11	0.2298
98153_at	1459987_s_at	Cct3	chaperonin subunit 3 (gamma)	1.16	0.43	-1.13	0.0929	-1.02	0.7358	-1.19	0.0111	-1.16	0.1746
98154_at	1452781_a_at	Gtf3c2	general transcription factor IIIC, polypeptide	1.3	0.15	1.02	0.8259	-1.15	0.1114	1.12	0.2298	1.2	0.3704
98155_r_at	1448277_at	Pold2	polymerase (DNA directed), delta 2, regulato	1.37	0.47	-1.34	0.2497	1.08	0.6855	-1.61	0.0232	1.08	0.7792
98168_at	1422613_a_at	Rpl7a	ribosomal protein L7a	1.02	0.83	1.05	0.2863	1.1	0.0006	1.12	0.0132	1.33	0.0245
98169_s_at	1449730_s_at	Fzd3	frizzled homolog 3 (Drosophila)	1.22	0.18	1.18	0.1144	1.08	0.4957	1.09	0.4324	1.1	0.4495
98240_at	1418166_at	---	---	1.04	0.9	1.04	0.8663	1.07	0.7902	1.29	0.2947	1.25	0.4141
98245_at	1419935_s_at	Csnk2a2	casein kinase II, alpha 2, polypeptide	-3.02	0.12	-1.09	0.2952	-1.12	0.2855	-1.34	0.0299	1.37	0.2355
98247_at	1420025_s_at	Aak1	AP2 associated kinase 1	-1.37	0.35	1.16	0.2497	1.36	0.0236	1.31	0.0066	1.76	0.0353
98254_f_at	1455316_x_at	Ccrn4l	CCR4 carbon catabolite repression 4-like (S	1.11	0.64	1.63	0.0002	1.28	0.1272	1.71	0.0002	1.89	0.0015
98276_at	1420785_at	Gab2	growth factor receptor bound protein 2-asso	-1.2	0.68	1.17	0.1749	1.23	0.0419	1.19	0.1942	1.26	0.4633
98277_at	1460664_at	Zfpn1a4	zinc finger protein, subfamily 1A, 4 (Eos)	-1.6	0.03	-1.4	0.0074	-1.16	0.2235	-1.33	0.0298	1.23	0.2179
98278_at	1422020_at	Spry4	sprouty homolog 4 (Drosophila)	-1.45	0.01	-1.04	0.7588	-1.16	0.1931	-1.16	0.2387	1.31	0.1222
98279_at	1420564_at	Insrr	insulin receptor-related receptor	-2	0.08	1.34	0.2439	-1.21	0.3934	1.03	0.9183	1.47	0.1761
98280_at	1422626_at	Mmp16	matrix metalloproteinase 16	-1.55	0.03	1.07	0.3879	1.18	0.1352	-1	0.997	1.39	0.216
98281_at	1427794_at	AJ242955	hypothetical protein, P4(21)n gene	-2.21	0.25	1.22	0.5052	1.62	0.2818	-1.03	0.8651	1.47	0.4396
98283_at	1450308_a_at	Xrn1	5'-3' exoribonuclease 1	-1.24	0.15	1.01	0.9331	-1.29	0.1026	-1.11	0.516	4.53	0.0193
98284_f_at	1460418_x_at	H2-T18	histocompatibility 2, T region locus 18	-1.54	0.08	1.12	0.4779	-1.2	0.2582	-1.05	0.7693	-1.04	0.785
98285_at	1422135_at	Zfp146	zinc finger protein 146	2.52	0.28	-1.2	0.3382	-1.2	0.4555	-1.32	0.2465	-1.15	0.7705

98286_at	1450774_at	Ly6g6d	lymphocyte antigen 6 complex, locus G6D	-1.42	0.65	-1.92	0.1008	-1.11	0.7643	-1.18	0.6183	-1.29	0.5054
98287_at	1451394_at	Dpp6	dipeptidylpeptidase 6	1.49	0.24	1.17	0.5348	1.39	0.0888	1.3	0.1853	1.22	0.6637
98288_at	1420763_at	Klk4	kallikrein 4 (prostase, enamel matrix, prostat	-1.22	0.26	-1.24	0.0539	-1.16	0.1884	-1.25	0.1053	1.68	0.1059
98289_at	1421337_at	Elf4	E74-like factor 4 (ets domain transcription fa	1.06	0.84	1.07	0.5915	1.04	0.7992	-1.04	0.7432	1.53	0.0675
98290_at	1450360_at	Plcg1	phospholipase C, gamma 1	-1.14	0.54	-1.18	0.0454	-1.06	0.482	-1.14	0.2379	1.09	0.441
98291_at	1421155_at	B3galt6	UDP-Gal:betaGal beta 1,3-galactosyltransfe	1.1	0.52	-1.01	0.932	1.1	0.3732	1.01	0.9453	-1.15	0.1699
98294_at	1419790_at	---	---	-3.07	0.04	1.06	0.6145	1.17	0.1606	-1.03	0.8364	-1.29	0.2723
98295_at	1421363_at	Cyp2c39	cytochrome P450, family 2, subfamily c, poly	-1.02	0.95	1.16	0.7376	-1.32	0.5931	1.13	0.7762	3.17	0.0024
98296_at	1451582_at	Tulp1	tubby like protein 1	-1.73	0.21	-1.11	0.2306	1.04	0.6112	-1.18	0.02	1.27	0.2392
98297_at	1450988_at	Gpr49	G protein-coupled receptor 49	-1.22	0.69	2.47	0.0473	-1.21	0.3837	1.49	0.388	1.25	0.4722
98298_at	1450502_at	Dpysl2	dihydropyrimidinase-like 2	-1.55	0.12	-1.13	0.224	-1.02	0.6827	-1.15	0.0145	-1.14	0.3986
98299_s_at	1450322_s_at	Slfn3 /// Slfn4	schlafen 3 /// schlafen 4	-1.15	0.89	1.89	0.297	1.29	0.4779	2.17	0.2636	-1.7	0.1735
98300_at	1419225_at	Cacna2d3	calcium channel, voltage-dependent, alpha2	-1.72	0.04	-1.06	0.481	2.61	0.1845	1.07	0.5756	1.11	0.5981
98301_at	1430912_a_at	Tectb	tectorin beta	-1.15	0.75	-1.84	0.1078	-1.61	0.2053	-1.99	0.0983	1.47	0.3526
98302_at	1450266_at	Scn10a	sodium channel, voltage-gated, type X, alph	-1.45	0.55	1.16	0.5834	1.6	0.3224	1.3	0.345	3.44	0.1146
98303_at	1421004_at	Cep1	centrosomal protein 1	-2.38	0.19	-1.14	0.4297	-1.19	0.2462	1.06	0.5431	-1.06	0.8718
98304_at	1421352_at	Tlr6	toll-like receptor 6	-1.14	0.56	1.29	0.3598	1.17	0.4613	1.76	0.1299	1.4	0.4572
98306_g_at	1448834_at	Foxm1	forkhead box M1	-1.63	0.01	-1.16	0.1412	1.04	0.7353	-1.29	0.0234	1.42	0.0992
98307_at	1421176_at	Rasgrp1	RAS guanyl releasing protein 1	-1.51	0.36	1.33	0.3113	1.04	0.8964	-1.07	0.8482	1.6	0.1835
98308_at	1427868_x_at	Myh1	myosin, heavy polypeptide 1, skeletal muscl	-1.74	0.41	-12.67	0.1847	-6.42	0.222	-8.91	0.2002	-2.04	0.2111
98309_at	1422112_at	Ccbp2	chemokine binding protein 2	1.05	0.75	-1.75	0.0056	-1.61	0.0199	-1.65	0.0022	1.48	0.2137
98310_at	1449535_at	Znrf4	zinc and ring finger 4	-1.58	0.37	-1.49	0.1376	-1.22	0.4456	1.08	0.7712	1.41	0.4784
98311_at	1420737_at	Pmfbp1	polyamine modulated factor 1 binding protei	-2.13	0.15	1.43	0.4474	1.54	0.3341	1.61	0.3204	-1.56	0.4613
98312_at	1449319_at	MGI:2183426	thrombospondin type 1 domain containing g	-1.06	0.91	-1.11	0.5312	1.04	0.84	1.57	0.1525	2.63	0.259
98314_g_at	1451888_a_at	Odz4	odd Oz/ten-m homolog 4 (Drosophila)	-1.3	0.57	-1.06	0.7638	1.16	0.4393	1.16	0.4622	1.45	0.1902
98315_at	1419632_at	Tecta	tectorin alpha	-3.16	0.05	-1.26	0.0884	-1.14	0.2763	-1.3	0.0311	1.2	0.5081
98316_at	1427573_at	Chic1	cysteine-rich hydrophobic domain 1	-1.95	0.01	-1.07	0.8396	-1.04	0.9293	-1.93	0.1071	1.22	0.3013
98317_at	1422232_at	Phox2b	paired-like homeobox 2b	-1.48	0.45	-1.41	0.2222	-1.66	0.079	-1.69	0.0802	1.11	0.794
98318_at	1449926_at	Tnfsf7	tumor necrosis factor (ligand) superfamily, m	1.37	0.3	-1.87	0.0266	-1.46	0.1102	-1.08	0.7854	1.32	0.1513
98319_at	1460380_at	Dsg2	desmoglein 2	-1.96	0.02	-1.39	0.0324	-1.36	0.0609	-1.47	0.0086	-1	0.9958
98320_at	1419430_at	Cyp26a1	cytochrome P450, family 26, subfamily a, po	1.23	0.26	1.68	0.0705	-3.38	0.0008	1.36	0.1583	-1.41	0.255
98322_at	1421848_at	Slc22a5	solute carrier family 22 (organic cation trans	1.55	0.33	-1.24	0.0856	-1.18	0.2045	-1.19	0.2419	1.75	0.0575
98323_at	1421919_a_at	Ccr9	chemokine (C-C motif) receptor 9	-1.47	0.33	1.4	0.3198	1.14	0.7211	1.31	0.3949	-1.01	0.9669
98324_at	1431900_a_at	Foxa3	forkhead box A3	1.04	0.86	1.53	0.0033	1.64	0.0064	1.94	0	1.63	0.004
98325_at	1448098_at	AA517545	expressed sequence AA517545	-1.4	0.6	1.31	0.2229	1.46	0.1605	1.33	0.3781	1.85	0.2059
98326_f_at	1419306_at	4921513E08Rik	RIKEN cDNA 4921513E08 gene	-1.64	0.02	-1.31	0.355	-1.21	0.5648	-1.59	0.14	1.24	0.4751
98327_at	1457308_at	---	---	-1.29	0.27	1.06	0.6996	1.07	0.5623	-1.11	0.3719	2.01	0.1872
98328_at	1421999_at	Tshr	thyroid stimulating hormone receptor	-2.25	0.47	-1.07	0.8551	1.17	0.6823	-1.11	0.79	1.63	0.3314
98329_at	1422091_at	Pfkfb2	6-phosphofructo-2-kinase/fructose-2,6-biphc	-3.63	0.2	1.22	0.3912	1.03	0.8972	1.08	0.7464	2.16	0.1277
98330_at	1423424_at	Zic3	zinc finger protein of the cerebellum 3	-1.26	0.58	-1.11	0.4166	1.04	0.7141	-1.33	0.0522	-1.22	0.4166
98331_at	1427883_a_at	Col3a1	procollagen, type III, alpha 1	-1.05	0.86	1.29	0.2918	1.03	0.8666	1.45	0.1888	1.12	0.3558
98333_at	1448739_x_at	Rps18	ribosomal protein S18	-1.04	0.79	-1.01	0.7941	1.08	0.0839	1.18	0.0082	1.54	0.0046
98334_at	1449903_at	Crtam	cytotoxic and regulatory T cell molecule	1.12	0.88	1.05	0.8691	1.25	0.3736	1.18	0.453	1.34	0.3386
98335_at	1418342_at	Recc1	replication factor C 1	1.98	0.11	1.16	0.1919	1.11	0.2797	1.14	0.0401	-1.14	0.3453
98336_s_at	1451920_a_at	Recc1	replication factor C 1	-1.41	0.1	-1.02	0.8753	-1	0.9984	-1.01	0.972	-1.13	0.6016
98337_at	1422049_at	Nkx1-2	NK1 transcription factor related, locus 2 (Drc	-1.4	0.53	-1.67	0.0486	-1.28	0.2876	-1.45	0.0594	-1.09	0.6442
98338_at	1418868_at	En2	engrailed 2	-1.42	0.36	-1.12	0.3519	-1.2	0.1909	1.11	0.5932	1.25	0.039
98339_at	1449264_at	Syt11	synaptotagmin 11	-2.11	0.18	-1.25	0.4843	-1.44	0.2699	-1.63	0.0916	-1.27	0.38
98342_at	1448157_s_at	Rpl10	ribosomal protein 10	1.13	0.43	1.01	0.6911	1.08	0.0206	1.03	0.2561	1.38	0.0008
98343_s_at	1449978_at	Zfy2	zinc finger protein 2, Y linked	-1.17	0.87	-1.03	0.9305	-1.23	0.6051	-1.26	0.486	2.02	0.3872
98345_at	1419895_at	AA536748	expressed sequence AA536748	1.25	0.57	1.1	0.8293	-1.26	0.5117	1.05	0.9203	4.76	0.0408
98346_at	1440230_at	9530051K01Rik	RIKEN cDNA 9530051K01 gene	1.58	0.38	-1.39	0.0312	-1.16	0.2316	-1.4	0.0204	-12.39	0.0014
98347_at	1421552_at	Cdx4	caudal type homeo box 4	-1.05	0.93	-1.22	0.5932	-1.52	0.1971	-1.96	0.0393	3.05	0.0792

98348_at	1421157_at	Fzd3	frizzled homolog 3 (Drosophila)	1.15	0.73	-1.33	0.0138	-1.2	0.0894	-1.21	0.1423	-1.2	0.5736
98349_at	1421239_at	Il6st	interleukin 6 signal transducer	-1.13	0.74	-1.37	0.0208	-1.27	0.1124	-1.36	0.0385	-1.04	0.8815
98351_g_at	1422256_at	Sstr2	somatostatin receptor 2	-1.1	0.78	-1.42	0.2417	-1.4	0.3318	-1.03	0.92	2.25	0.0356
98354_at	1421760_at	Ptcra	pre T-cell antigen receptor alpha	-1.76	0.4	1.05	0.8216	1.22	0.2345	1.09	0.6316	1.7	0.1157
98355_at	1455466_at	Gpr133	G protein-coupled receptor 133	-1.21	0.32	-1.05	0.4574	-1.26	0.0076	-1.17	0.0405	1.39	0.173
98356_at	1449620_s_at	D16Wsu65e	DNA segment, Chr 16, Wayne State Univers	-1.1	0.41	1.35	0.0036	1.28	0.0092	1.52	0	1.21	0.1343
98357_at	1438297_at	LOC433991	LOC433991	1.28	0.2	-1.06	0.6991	1.24	0.157	-1.11	0.4537	1.07	0.7401
98360_at	1425812_a_at	Cacna1b	calcium channel, voltage-dependent, N type	-2.05	0.03	-1.15	0.1633	-1.06	0.4646	-1.24	0.0549	1.8	0.0666
98361_at	1450799_at	Adcyap1r1	adenylate cyclase activating polypeptide 1 re	1.42	0.64	1.86	0.0326	2.14	0.0474	2.28	0.0235	-1.17	0.6749
98365_at	1422949_at	Nos1	nitric oxide synthase 1, neuronal	-1.88	0.3	-1.37	0.1363	1.09	0.7077	1.06	0.7921	1.29	0.3486
98366_at	1421198_at	Itgav	integrin alpha V	-1.07	0.83	-1.04	0.7599	-1.08	0.5313	1.08	0.5916	1.05	0.9215
98367_at	1421537_at	Hoxd3	homeo box D3	-1.07	0.84	-1.14	0.4902	1.21	0.3442	-1.12	0.4863	-1.28	0.4602
98368_at	1421354_at	Prkg2	protein kinase, cGMP-dependent, type II	-1.04	0.85	-1.01	0.9079	-1.35	0.0086	-1.15	0.1422	-1.05	0.8807
98370_at	1421781_at	Upk2	uroplakin 2	-3.05	0.13	-1.2	0.41	-1.3	0.2218	-1.42	0.1298	1.25	0.5926
98372_at	1448789_at	Aldh1a3	aldehyde dehydrogenase family 1, subfamily	-1.75	0.16	1.17	0.5459	1.42	0.035	1.51	0.0277	2.74	0.0065
98373_at	1450291_s_at	Ms4a4c	membrane-spanning 4-domains, subfamily A	1.1	0.78	2	0.2746	1.03	0.9263	2.83	0.2008	1.27	0.4374
98374_at	1450550_at	Il5	interleukin 5	-1.51	0.32	1.06	0.7722	1.12	0.6924	1.19	0.4244	1.76	0.0707
98376_at	1420557_at	Epha5	Eph receptor A5	-2.01	0.11	1.48	0.2632	1.65	0.318	1.32	0.2124	1.54	0.2261
98380_at	1418414_at	Kcnh1	potassium voltage-gated channel, subfamily	-1.24	0.66	1.21	0.24	-1.04	0.6883	1.13	0.4679	2.05	0.1776
98383_r_at	1427745_x_at	MP4 /// Prb1	proline rich protein MP4 /// proline-rich prote	-1.18	0.77	1.28	0.477	1.44	0.1329	1.18	0.6363	1.59	0.2211
98384_at	1450271_at	Ptk6	PTK6 protein tyrosine kinase 6	-1.95	0.16	-1.51	0.004	-1.36	0.0395	-2.05	0.0007	1.08	0.818
98385_at	1421499_a_at	Ptpn14	protein tyrosine phosphatase, non-receptor t	-1.74	0.05	-1.24	0.0697	-1.29	0.0245	-1.17	0.0952	1.35	0.1645
98386_s_at	1421297_a_at	Cacna1c	calcium channel, voltage-dependent, L type,	2.2	0.07	-1.08	0.7065	-1.15	0.3939	-1.17	0.4124	1.03	0.9235
98387_at	1422263_at	Bdkrb2	bradykinin receptor, beta 2	-5.07	0.09	1.13	0.287	1.18	0.3038	1.08	0.5457	1.64	0.2375
98391_at	1425144_at	Klk11	kallikrein 11	1.98	0.16	1.24	0.2754	-1.13	0.2566	1.22	0.2599	1.27	0.5886
98392_at	1449911_at	Lag3	lymphocyte-activation gene 3	-1.2	0.76	-1.91	0.0115	-1.33	0.2068	-1.57	0.0448	1.78	0.2345
98394_at	1449470_at	Dlx1	distal-less homeobox 1	-1.35	0.34	1	0.9488	1.15	0.0504	1.05	0.4698	1.02	0.9343
98395_at	1422012_at	Crhr2	corticotropin releasing hormone receptor 2	-1.53	0.15	-1.51	0.2235	-1.33	0.3632	-1.55	0.1891	1.82	0.0439
98397_at	1418973_at	Blzf1	basic leucine zipper nuclear factor 1	2.08	0.06	1.08	0.2913	1.1	0.4317	1.15	0.1756	-1.22	0.4283
98398_s_at	1451755_a_at	Apobec1	apolipoprotein B editing complex 1	-1.21	0.74	-1.07	0.7298	-1.41	0.0056	1.09	0.6777	1.05	0.7315
98400_at	1418264_at	Solt	SoxLZ/Sox6 leucine zipper binding protein ir	1.7	0.21	-1.06	0.7071	1.11	0.5442	1.21	0.1319	1.45	0.3874
98401_at	1427377_x_at	Hsd3b3	hydroxysteroid dehydrogenase-3, delta<5>-:	1.04	0.5	1.39	0.0199	1.11	0.4005	2.14	0.0007	-1.38	0.0136
98402_at	1428847_a_at	Macf1	microtubule-actin crosslinking factor 1	1.06	0.65	1.41	0.0004	1.25	0.1455	1.45	0.0081	1.68	0.0106
98403_at	1418646_at	Gna-rs1	guanine nucleotide binding protein, related s	-1.2	0.29	1.2	0.0412	1.06	0.5824	-1.01	0.8527	1.05	0.8739
98404_at	1417260_at	U2af2	U2 small nuclear ribonucleoprotein auxiliary	-1.15	0.2	1.06	0.1459	1.04	0.2959	-1	0.8464	1.29	0.0004
98405_at	1422601_at	Serpinb9	serine (or cysteine) proteinase inhibitor, clad	-1.12	0.85	1.04	0.8238	-1.23	0.0656	1.12	0.5647	-1.16	0.3985
98406_at	1418126_at	Ccl5	chemokine (C-C motif) ligand 5	1.86	0.1	8.35	0.1606	-1.03	0.935	15.59	0.0989	1.24	0.3613
98407_at	1418285_at	Efnb1	ephrin B1	-1.65	0.05	-1.11	0.1324	-1.28	0.0289	-1.22	0.0356	1.43	0.2124
98408_at	1423319_at	Hhex	hematopoietically expressed homeobox	-1.37	0.23	-1.02	0.8334	1.25	0.0429	-1.26	0.048	1.03	0.9309
98410_at	1417793_at	Iigp2	interferon inducible GTPase 2	-1.09	0.79	1.25	0.54	-1.81	0.0265	1.2	0.6274	-1.53	0.2164
98414_at	1418362_at	Zfp42	zinc finger protein 42	1.01	0.98	1.15	0.6326	1.08	0.5644	-1.14	0.2497	1.09	0.6849
98416_at	1451233_at	Traf2	Tnf receptor-associated factor 2	-1.36	0.34	1.14	0.1813	1.09	0.3445	1.05	0.5233	1.25	0.0704
98417_at	1451905_a_at	Mx1	myxovirus (influenza virus) resistance 1	-1.21	0.73	2.16	0.374	1.09	0.7735	3.87	0.1187	-1.15	0.7784
98418_at	1450978_at	Dvl1	dishevelled, dsh homolog 1 (Drosophila)	-1.1	0.41	1.03	0.7354	-1.07	0.621	-1.07	0.4844	1.42	0.0006
98419_at	1417595_at	Meox1	mesenchyme homeobox 1	-1.45	0.35	-1.1	0.6229	-1.21	0.1614	1.03	0.8718	1.14	0.6985
98420_at	1459983_at	Impa2	inositol (myo)-1(or 4)-monophosphatase 2	-1.16	0.78	1.81	0.3288	1.47	0.0027	1.06	0.6703	1.71	0.0275
98421_at	1456612_at	Il17d	Expressed sequence AU020094	-1.83	0.34	1.03	0.9033	-1.1	0.645	1.39	0.3359	1.69	0.0142
98423_at	1423271_at	Gjb2	gap junction membrane channel protein beta	-1.22	0.32	-1.51	0	-1.32	0.0061	-2.46	0	-2.62	0
98424_at	1452127_a_at	Ptpn13	protein tyrosine phosphatase, non-receptor t	1.32	0.44	-1.06	0.7277	-1.18	0.4352	-1.2	0.3224	1.38	0.2165
98426_at	1418302_at	Ppt2	palmitoyl-protein thioesterase 2	-1.18	0.15	1.02	0.8034	-1.05	0.5622	-1.01	0.8849	-1.13	0.4765
98427_s_at	1427705_a_at	Nfkb1	nuclear factor of kappa light chain gene enha	-1.27	0.24	1.08	0.3855	-1.07	0.3138	1.12	0.1132	-1.02	0.7821
98428_at	1460400_at	Spg4	spastic paraplegia 4 homolog (human)	1.96	0.08	-1.08	0.3946	1.22	0.0584	-1.01	0.8794	1.19	0.4044
98429_at	1417433_at	Lypla2	lysophospholipase 2	-1.27	0.14	-1.02	0.8028	-1	0.9864	-1.1	0.3542	-1.03	0.668

98430_at	1421528_a_at	Surf5	surfeit gene 5	-1.33	0.09	-1.04	0.5945	-1.08	0.2992	-1.05	0.4336	-1.2	0.5984
98431_at	1420636_a_at	Dusp12	dual specificity phosphatase 12	1.12	0.67	-1.03	0.7608	-1.01	0.932	-1.11	0.2834	1.32	0.2665
98432_at	1448134_at	X99384	cDNA sequence X99384	1.09	0.49	-1.26	0.0217	1.08	0.2532	-1.24	0.0166	1.03	0.8275
98433_at	1417045_at	Bid	BH3 interacting domain death agonist	1.5	0.31	1.15	0.0753	1.1	0.334	1.13	0.2045	-1.05	0.8964
98434_at	1449066_a_at	Arhgef7	Rho guanine nucleotide exchange factor (GI	-1.3	0.12	-1.03	0.7918	1.09	0.3327	-1	0.9952	1.28	0.3185
98435_at	1449383_at	Adssl1	adenylosuccinate synthetase like 1	3.23	0.01	1.91	0	1.2	0.1308	2.01	0	2.3	0.0006
98437_at	1449839_at	Casp3	caspase 3, apoptosis related cysteine protea	-1.31	0.03	-1.18	0.0519	-1.05	0.5009	-1.15	0.0568	-1.1	0.3772
98438_f_at	1418536_at	H2-Q7	Histocompatibility 2, Q region locus 6	-1.12	0.48	1.34	0.2054	-1.12	0.0692	1.38	0.196	-1.41	0.0756
98440_at	1417777_at	Ltb4dh	leukotriene B4 12-hydroxydehydrogenase	2.27	0.33	1.15	0.3602	1.69	0.0043	1.36	0.1038	-1.2	0.4145
98441_at	1423369_at	Fmr1	fragile X mental retardation syndrome 1 hom	1.15	0.56	1.26	0.0076	1.11	0.5575	1.25	0.0024	1.04	0.7712
98444_g_at	1422582_at	Lep	leptin	-1.1	0.75	1.29	0.191	1.39	0.0983	1.37	0.4167	2.37	0.1125
98445_at	1423834_s_at	Gga1	golgi associated, gamma adaptin ear contai	1.33	0.46	1.09	0.3356	1.14	0.3165	1.12	0.2145	1.65	0.2193
98447_at	1418982_at	Cebpa	CCAAT/enhancer binding protein (C/EBP), a	-1.26	0.33	-1.03	0.6038	-1	0.9955	-1.14	0.0227	-1.11	0.4739
98449_at	1448658_at	Sart1	squamous cell carcinoma antigen recognize	1.05	0.88	1.05	0.5673	1.14	0.155	1.12	0.1487	1.59	0.056
98451_at	1448657_a_at	Dnajb10	DnaJ (Hsp40) homolog, subfamily B, membe	1.92	0.01	-1.08	0.3861	-1.13	0.1118	-1.17	0.1024	1.02	0.9303
98452_at	1419300_at	Flt1	FMS-like tyrosine kinase 1	-1.09	0.19	-1.15	0.1573	-1.22	0.0747	-1.16	0.2749	1.39	0.0506
98453_at	1451756_at	Flt1	FMS-like tyrosine kinase 1	-1.54	0.08	1.27	0.2248	-1.01	0.9152	1.04	0.7099	1.46	0.0393
98454_at	1423967_at	Palm	paralemmin	-1.09	0.62	-1.13	0.1194	1.02	0.7196	-1.12	0.1825	-1.17	0.4858
98455_at	1452116_s_at	Atf2	activating transcription factor 2	-1.07	0.57	1.18	0.0433	1.24	0.1262	1.2	0.0126	-1.11	0.5174
98456_at	1417334_at	Stk19	serine/threonine kinase 19	1.61	0.36	-1	0.9953	-1.1	0.1938	-1.07	0.32	-1.16	0.6998
98457_at	1421225_a_at	Slc4a4	solute carrier family 4 (anion exchanger), me	2.51	0.07	1.35	0.0309	-1.47	0.0795	-1.15	0.5249	1.42	0.3253
98459_at	1425179_at	Shmt1	serine hydroxymethyl transferase 1 (soluble)	1.18	0.29	-1.48	0	-1.19	0.0054	-1.65	0	-1.55	0.0039
98460_at	1448223_at	---	---	-1.2	0.18	-1.13	0.1961	-1.09	0.3289	-1.02	0.8519	1.08	0.3687
98461_at	1417108_at	1200014P03Rik	RIKEN cDNA 1200014P03 gene	1.03	0.75	-1.24	0.0116	-1.11	0.3097	-1.3	0.0015	-1.2	0.1498
98462_s_at	1425547_a_at	1200014P03Rik	RIKEN cDNA 1200014P03 gene	1.16	0.73	-1.1	0.3976	-1.44	0.0762	-1.35	0.0437	1.3	0.5791
98463_at	1426804_at	Smarca4	SWI/SNF related, matrix associated, actin de	1.24	0.35	-1.07	0.2883	-1.14	0.0297	-1.17	0.0131	-1.54	0.0323
98466_r_at	1419603_at	---	---	1.06	0.87	1.46	0.1554	1.2	0.3712	1.44	0.3814	1.83	0.0639
98467_at	1431808_a_at	Itih4	inter alpha-trypsin inhibitor, heavy chain 4	1.13	0.36	-1.01	0.7676	-1.08	0.1479	-1.16	0.0218	-1.69	0.0194
98468_r_at	1434821_at	Brd1	bromodomain containing 1	1.85	0.06	1.05	0.7218	1.2	0.5136	1.34	0.0698	1.09	0.5847
98469_at	1451246_s_at	Aurkb	aurora kinase B	3.3	0.33	1.08	0.3357	1.11	0.2529	1.47	0.1518	1.58	0.1628
98470_at	1417154_at	Slc25a14	solute carrier family 25 (mitochondrial carrie	1.48	0.33	-1.03	0.7801	1.48	0.2379	-1.01	0.9451	-1.11	0.6466
98471_f_at	1449877_s_at	Kifc1	kinesin family member C1	-1.24	0.29	1.04	0.7841	1.08	0.4702	1	0.9952	1.03	0.7748
98472_at	1449556_at	H2-T23 /// C920	histocompatibility 2, T region locus 23 /// RIK	1.01	0.92	1.16	0.4386	-1.15	0.0303	1.2	0.3582	1.17	0.1341
98473_at	1438841_s_at	Arg2	arginase type II	-1.4	0.01	-1.33	0.4194	1.38	0.5897	1.04	0.9066	2.05	0.0289
98474_r_at	1418424_at	Tnfaip6	tumor necrosis factor alpha induced protein	-6.72	0.07	-1.04	0.9222	-1.11	0.7774	-1.05	0.9015	1.85	0.3122
98475_at	1419442_at	Matn2	matrilin 2	1.42	0.39	1.08	0.6929	-1.08	0.6847	-1.03	0.8879	-1.46	0.287
98476_at	1452124_at	Ank3 /// 290005	ankyrin 3, epithelial /// RIKEN cDNA 290005	1.5	0.01	1.28	0.0216	1.17	0.2278	1.35	0.0106	1.87	0.0373
98478_at	1416488_at	Ccng2	cyclin G2	-1.46	0.15	1.13	0.4764	-1.15	0.4747	1.12	0.4704	1.07	0.8279
98480_s_at	1448975_s_at	Ren1 /// Ren2	renin 1 structural /// renin 2 tandem duplicati	1.3	0.67	1.23	0.0786	1.32	0.0475	1.1	0.4723	1.56	0.127
98482_at	1417092_at	Pthr1	parathyroid hormone receptor 1	1.6	0.43	-1.08	0.5245	1.07	0.5749	1.16	0.1913	1.1	0.7808
98483_at	1448656_at	Cacnb3	Calcium channel, voltage-dependent, beta 3	1.42	0.57	1.01	0.9385	-1.66	0.0268	-1.1	0.6735	1.4	0.3501
98484_at	1417901_a_at	Ica1	islet cell autoantigen 1	1.17	0.35	1.21	0.2052	1.07	0.6918	1.17	0.4487	-1.05	0.7686
98485_at	1431805_a_at	Rhpn2	rhophilin, Rho GTPase binding protein 2	1.57	0.16	-1.08	0.5763	1.05	0.7499	-1.05	0.717	-1.52	0.2931
98486_at	1418105_at	Stmn4	stathmin-like 4	-1.09	0.58	-1.17	0.1236	-1.03	0.7917	-1.05	0.5166	1.53	0.0165
98488_at	1427026_at	Myh4	myosin, heavy polypeptide 4, skeletal muscl	-1.53	0.46	-6.92	0.2879	-2.04	0.5354	-4.05	0.3491	1.39	0.6843
98489_at	1455730_at	Dlg7	discs, large homolog 7 (Drosophila)	1.26	0.49	1.02	0.9403	1.16	0.603	-1	0.997	2.13	0.0529
98492_at	1460253_at	Cklfsf7	chemokine-like factor super family 7	1.13	0.49	1.04	0.6326	1.08	0.4889	1.03	0.7779	1.03	0.8151
98493_at	1424328_s_at	3200002M19Rik	RIKEN cDNA 3200002M19 gene	1.93	0.02	1.12	0.0507	-1.03	0.6101	1.06	0.2044	-1.16	0.6092
98495_at	1460361_at	5033414D02Rik	RIKEN cDNA 5033414D02 gene	1.27	0.32	1.15	0.4285	-1.27	0.1985	1.25	0.2497	1.33	0.2221
98496_at	1416737_at	Gys3	glycogen synthase 3, brain	1	1	1.16	0.1973	1.08	0.2469	1.06	0.4525	1.53	0.0689
98497_at	1449355_a_at	Eps15-rs	epidermal growth factor receptor pathway su	1.07	0.61	1.23	0.1168	1.16	0.1883	1.22	0.1003	1.17	0.328
98498_at	1448659_at	Casp7	caspase 7	1	0.99	-1.08	0.3839	1.01	0.8776	1.02	0.824	-1.71	0.0048
98499_s_at	1426062_a_at	Casp7	caspase 7	-1.47	0.68	-1.21	0.0852	-1.31	0.0011	-1.23	0.0133	-4.23	0.0042

98500_at	1425145_at	Il1rl1	interleukin 1 receptor-like 1	-2.01	0.33	-1.08	0.7775	-1.12	0.6287	1.1	0.6595	1.97	0.0278
98501_at	1422317_a_at	Il1rl1	interleukin 1 receptor-like 1	-2.76	0.22	-1.01	0.9644	1.46	0.1138	-1.2	0.4549	-1.21	0.6302
98502_at	1448922_at	Dusp19	dual specificity phosphatase 19	1.07	0.69	-1.18	0.049	-1.19	0.1281	-1.27	0.0024	-1.81	0.0031
98503_at	1417512_at	---	---	1.1	0.48	1.11	0.164	1.07	0.4281	1.17	0.0239	-1.08	0.4093
98506_r_at	1422492_at	Cpox	coproporphyrinogen oxidase	-1.12	0.63	1.06	0.6426	-1.08	0.3711	-1	0.9728	-1.14	0.4057
98508_s_at	1422620_s_at	Ppap2a	phosphatidic acid phosphatase 2a	1.7	0.08	1.4	0.0026	1.23	0.0744	1.37	0.0361	1.49	0.0358
98509_at	1426234_s_at	BC002199	cDNA sequence BC002199	1.54	0.02	1.57	0	1.31	0.008	1.45	0	1.33	0.0169
98511_at	1418573_a_at	Raly	hnRNP-associated with lethal yellow	1.45	0.4	1.05	0.7495	1.04	0.8206	-1.04	0.7961	1.19	0.5284
98512_at	1452462_a_at	Banp	Btg3 associated nuclear protein	-1.1	0.75	1.2	0.0572	1.18	0.1511	1.11	0.3935	1.89	0.0347
98513_at	1415842_at	MGI:1929514	G protein beta subunit-like	-1.31	0.01	1.02	0.8241	1	0.9605	-1.04	0.5797	1.05	0.7441
98514_at	1451791_at	Tfpi	tissue factor pathway inhibitor	2.73	0.02	1.3	0.3447	1.21	0.609	1.28	0.3386	3.78	0.0037
98515_at	1448884_at	Gtf2e2	general transcription factor II E, polypeptide	1.36	0.09	-1.15	0.1791	1.07	0.447	-1.05	0.6617	1.13	0.6342
98516_at	1424117_at	BC056474	cDNA sequence BC056474	-1.34	0.11	-1.12	0.0151	-1.08	0.153	-1.12	0.0714	1.03	0.7977
98519_r_at	1453092_at	2300002G24Rik	RIKEN cDNA 2300002G24 gene	-1.92	0.07	1.1	0.4803	1.33	0.0149	1.28	0.2116	1.66	0.2035
98521_at	1433916_at	Vamp3	vesicle-associated membrane protein 3	1.08	0.71	1.04	0.7442	1	0.9854	1.08	0.3911	1.15	0.2982
98524_f_at	1434278_at	---	---	1.72	0.14	1.57	0.0009	-1.05	0.7472	1.79	0.0007	1.65	0.015
98525_f_at	1452406_x_at	MGI:2384747	erythroid differentiation regulator 1	-8.41	0.06	1.06	0.6692	1.5	0.0017	1.44	0.0032	1	0.9897
98527_at	1418321_at	Dci	dodecenoyl-Coenzyme A delta isomerase (3	1.41	0.04	1.19	0.0543	1.25	0.0161	1.28	0.0484	1.09	0.4006
98528_at	1415763_a_at	---	---	1.18	0.6	-1.04	0.6315	-1.03	0.7076	-1.07	0.399	-1.1	0.3421
98529_at	1448608_at	Dtprp	decidual/trophoblast prolactin-related protein	-2.48	0.11	1.43	0.2811	1.23	0.6006	-1.17	0.6123	-1.17	0.6904
98531_g_at	1436222_at	Gas5	Growth arrest specific 5	1.74	0.01	1.21	0.0301	1.14	0.185	1.22	0.0011	1.53	0.0076
98532_at	1456314_x_at	Cdk2ap1	CDK2 (cyclin-dependent kinase 2)-associate	-1.29	0.36	1.03	0.7146	1.05	0.5652	-1.09	0.2484	-1.34	0.1667
98533_at	1416727_a_at	Cyb5	cytochrome b-5	1.06	0.66	1.04	0.4564	-1.02	0.7295	1.07	0.2465	1.46	0.0012
98534_at	1449480_at	Sap18	Sin3-associated polypeptide 18	1.04	0.87	1.08	0.5283	1.16	0.203	-1.04	0.6248	1.42	0.2395
98535_at	1449183_at	Comt	catechol-O-methyltransferase	-2.39	0	-1.24	0.0004	-1.1	0.2269	-1.33	0.0017	-1.54	0.0013
98538_at	1426820_at	2610507B11Rik	RIKEN cDNA 2610507B11 gene	1.17	0.33	-1.18	0.0201	-1.06	0.263	-1.12	0.0382	-1.25	0.1184
98540_g_at	1437039_at	---	---	-1.25	0.64	1.31	0.0025	1.33	0.0982	1.44	0.0001	1.07	0.8252
98543_at	1448591_at	Ctss	cathepsin S	1.25	0.35	1.55	0.3448	-1.18	0.1409	2	0.2564	1.16	0.4053
98544_at	1416395_at	Guk1	guanylate kinase 1	1.23	0.2	-1.09	0.3264	-1.1	0.4094	-1.04	0.6614	-1.06	0.3487
98545_at	1416202_at	Bcap37	B-cell receptor-associated protein 37	1.43	0.05	1.22	0.0013	1.08	0.5208	1.16	0.0253	1.51	0.0964
98547_at	1448799_s_at	Mrps12	mitochondrial ribosomal protein S12	1.06	0.67	-1.17	0.0876	-1.01	0.9517	-1.29	0.0094	-1.18	0.5872
98549_at	1420484_a_at	Vtn	vitronectin	-1.42	0.05	-1.03	0.4695	1.01	0.8462	-1.05	0.289	-1.28	0.0374
98550_at	1426853_at	Set	SET translocation	1.34	0.02	1.11	0.1304	1.17	0.1593	1.12	0.055	1.11	0.5512
98552_at	1416707_a_at	Pmf1	polyamine-modulated factor 1	1.76	0.19	-1.22	0.0239	-1.28	0.0678	-1.28	0.0462	1.19	0.343
98553_at	1426457_at	Slmap	sarcolemma associated protein	1.25	0.09	-1.06	0.2968	1.07	0.5313	1.08	0.3507	-1.13	0.4286
98554_at	1417663_a_at	Ndrg3	N-myc downstream regulated gene 3	4.25	0.02	1.06	0.8199	1.34	0.2543	1.31	0.2563	-2.35	0.1511
98555_at	1448361_at	Ttc3	tetratricopeptide repeat domain 3	2.28	0.03	1.47	0.0007	1.55	0.0056	1.54	0.0172	2.75	0.0124
98558_r_at	1451205_at	Psmb4	proteasome (prosome, macropain) subunit, I	-5.36	0.2	1.28	0.3169	2.66	0.1215	1.57	0.3825	-1.31	0.5841
98559_at	1452469_a_at	Smtn	smoothelin	-4.02	0.29	1.09	0.1972	1.17	0.0145	-1.03	0.6705	1.26	0.4496
98560_at	1460740_at	Cltb	clathrin, light polypeptide (Lcb)	1.26	0.43	-1.15	0.0655	1.02	0.8477	-1.13	0.0222	-1.18	0.4255
98561_at	1450813_a_at	Tnni1	troponin I, skeletal, slow 1	-1.69	0.09	1.45	0.2014	1.32	0.3846	1.42	0.1717	1.05	0.9121
98562_at	1417381_at	C1qa	complement component 1, q subcomponent	1.39	0.51	-1.05	0.8	-1.4	0.0428	-1.04	0.8908	-1.13	0.6299
98564_f_at	1415876_a_at	Rps26	ribosomal protein S26	-1.08	0.58	1.17	0.0001	1.28	0.0057	1.16	0.0003	1.54	0.0159
98568_at	1415835_at	Csh2	chorionic somatomammotropin hormone 2	-1.12	0.27	-1.23	0.1352	-1.02	0.9177	-1.18	0.1986	1.19	0.7133
98571_s_at	1416669_s_at	Naca	nascent polypeptide-associated complex alp	1.73	0.11	1.05	0.5265	1.05	0.6759	1.08	0.301	1.06	0.6638
98574_at	1422453_at	Prpf8	pre-mRNA processing factor 8	1.4	0.15	-1.03	0.6488	-1.05	0.3744	-1.07	0.3618	1.14	0.1649
98575_at	1423828_at	Fasn	fatty acid synthase	-1.61	0.28	-3.12	0.0009	-1.9	0.016	-3.97	0.0003	-5.51	0.001
98578_at	1455041_at	Mosg	mosg protein	-1.17	0.42	1.03	0.7855	1.05	0.7392	-1.12	0.3733	1.17	0.6023
98579_at	1417065_at	Egr1	early growth response 1	1.53	0.08	1.65	0.0975	-1.08	0.7335	1.95	0.0045	2.51	0.0034
98580_at	1415678_at	Ppm1a	protein phosphatase 1A, magnesium depend	1.4	0.25	-1	0.9606	1.17	0.0412	1.03	0.8017	-1.07	0.561
98582_at	1452986_at	Hgd	homogentisate 1, 2-dioxygenase	1.34	0.26	-1.03	0.6171	1.03	0.7657	-1.02	0.7889	-1.1	0.43
98583_at	1426520_at	Btg4	B-cell translocation gene 4	-2.4	0.39	1.32	0.4396	1.19	0.6775	-1.09	0.7407	1.01	0.9773
98586_at	1420477_at	Nap1l1	nucleosome assembly protein 1-like 1	1.04	0.92	-1.08	0.5999	1.02	0.8867	-1.12	0.3369	1.45	0.3577

98587_at	1420478_at	Nap1l1	nucleosome assembly protein 1-like 1	-1.21	0.68	1.02	0.7424	1.09	0.3204	1	0.9514	-1.19	0.4296
98588_at	1417220_at	Fah	fumarylacetoacetate hydrolase	1	0.95	-1.07	0.0071	-1.02	0.2903	-1.07	0.0534	-1.01	0.9122
98589_at	1448318_at	Adfp	adipose differentiation related protein	-1.03	0.89	-1.52	0.0051	-1.31	0.1351	-1.82	0.0004	-3.16	0.0003
98590_at	1417654_at	Sdc4	syndecan 4	1.03	0.95	1.04	0.5119	1.17	0.3911	1.29	0.2677	1.07	0.6066
98593_at	1426662_at	Cmas	cytidine monophospho-N-acetylneuraminic a	-1.03	0.92	-1.21	0.0055	-1.15	0.0671	-1.15	0.0281	-1.94	0.0065
98594_at	1433581_at	1190002N15Rik	RIKEN cDNA 1190002N15 gene	1.46	0.36	1.61	0	1.14	0.4249	1.86	0.0001	-1.25	0.4462
98596_s_at	1449198_a_at	St3gal5	ST3 beta-galactoside alpha-2,3-sialyltransfe	-1.18	0.58	-1.99	0.0027	1.41	0.2064	-1.61	0.0132	1.27	0.4719
98597_at	1417172_at	Ube2l6	ubiquitin-conjugating enzyme E2L 6	-1.25	0.49	1.15	0.4113	-1.01	0.9377	1.09	0.5823	-1.05	0.8193
98598_at	1458854_at	C78891	expressed sequence C78891	1.92	0.12	-1.77	0.1674	-1.25	0.5347	-1.63	0.2365	-1.8	0.2842
98599_at	1416597_at	Hdgfrp2	hepatoma-derived growth factor, related pro	1.5	0.13	-1.09	0.2311	-1.03	0.751	1	0.9463	-1.02	0.9457
98600_at	1460351_at	S100a11	S100 calcium binding protein A11 (calizzarir	1.46	0.1	2.02	0.2158	1.2	0.4975	2.44	0.1533	1.44	0.0987
98602_at	1423749_s_at	Rangap1	RAN GTPase activating protein 1	-1.64	0.05	-1.76	0	-1.04	0.7205	-1.78	0	-1.58	0.0105
98604_at	1427720_a_at	Nnp1	novel nuclear protein 1	1.87	0.05	1.03	0.7274	-1.01	0.9284	1.17	0.0137	1.16	0.27
98605_at	1415694_at	Wars	tryptophanyl-tRNA synthetase	-1.21	0.44	-1.08	0.561	-1.09	0.4695	-1.12	0.3458	1.18	0.1096
98606_s_at	1437832_x_at	Wars	tryptophanyl-tRNA synthetase	1.08	0.8	1.01	0.9799	-1.23	0.0869	-1.07	0.6927	-1.08	0.3599
98608_at	1420024_s_at	Etf1	eukaryotic translation termination factor 1	-1.54	0.14	-1.1	0.265	1.13	0.3348	-1.07	0.3899	1.29	0.0166
98609_at	1417038_at	9-Sep	septin 9	-1.06	0.84	-1.21	0.0002	-1.45	0.0001	-1.42	0	-1.35	0.1475
98610_at	1452585_at	Mrps28	mitochondrial ribosomal protein S28	1.22	0.51	1.08	0.2442	1.3	0.0008	1.12	0.0645	-1.05	0.7592
98612_at	1418113_at	Cyp2d10	cytochrome P450, family 2, subfamily d, poly	-1.07	0.76	-1.03	0.6091	1.11	0.0608	1.01	0.832	-1.3	0.059
98613_at	1428421_a_at	2700085E05Rik	RIKEN cDNA 2700085E05 gene	1.65	0.14	1.14	0.0835	1.28	0.0027	1.05	0.5191	-1.16	0.1503
98614_at	1453686_x_at	Nphp1	nephronophthisis 1 (juvenile) homolog (hum	1.13	0.7	-1.01	0.9187	-1.15	0.4596	1.13	0.4167	1.44	0.2672
98615_at	1425780_a_at	0610041E09Rik	RIKEN cDNA 0610041E09 gene	1.28	0.01	1.12	0.4536	-1.05	0.7335	1.14	0.172	-1.63	0.0011
98616_f_at	1448554_s_at	Myh6	myosin, heavy polypeptide 6, cardiac muscle	-1.15	0.5	1.14	0.2894	1.18	0.4193	1.1	0.4219	1.59	0.0406
98617_at	1415799_at	Wbp11	WW domain binding protein 11	-1.01	0.96	1.2	0.0796	1.27	0.0275	1.22	0.0364	1.02	0.839
98618_at	1449116_a_at	Dtymk	deoxythymidylate kinase	-1.15	0.27	-1.1	0.2632	1.06	0.5647	1.06	0.5933	-1.15	0.2269
98619_at	1452681_at	Dtymk	deoxythymidylate kinase	1.18	0.44	-1.1	0.319	-1.16	0.1034	-1.04	0.6875	-1.44	0.2975
98624_at	1421265_a_at	Rnpc1	RNA-binding region (RNP1, RRM) containin	-1.08	0.7	1.13	0.3015	1.09	0.4955	-1	0.9967	1.18	0.4939
98625_s_at	1416185_a_at	Adh5	alcohol dehydrogenase 5 (class III), chi poly	1.13	0.46	1.14	0.0045	1.08	0.1992	1.21	0	1	0.9439
98626_at	1417105_at	1810017G16Rik	RIKEN cDNA 1810017G16 gene	1.69	0.19	1.03	0.5531	1.25	0.0055	1.17	0.0971	1.38	0.1168
98627_at	1454159_a_at	Igfbp2	insulin-like growth factor binding protein 2	2.43	0.19	1.93	0.0003	1.91	0.0027	2.68	0	7.6	0.0007
98629_f_at	1427418_a_at	Hif1a	hypoxia inducible factor 1, alpha subunit	-1.3	0.29	-1.07	0.5737	1.07	0.712	-1.03	0.7983	-1.25	0.1301
98632_at	1417976_at	Ada	adenosine deaminase	-1.88	0.1	-1.07	0.7956	1.05	0.8595	-1.24	0.2986	2.06	0.3049
98635_at	1423949_at	D11Moh35	DNA segment, Chr 11, KL Mohlke 35	1.22	0.18	1.01	0.9694	1.08	0.5303	-1.07	0.5982	1.36	0.0005
98726_at	1421444_at	Pgr	progesterone receptor	-4.27	0.11	-1.3	0.4564	1.08	0.8246	-1.01	0.983	-1.25	0.4793
98727_at	1422193_at	Gucy2e	guanylate cyclase 2e	-1.53	0.05	-1.24	0.336	1.26	0.0945	1.01	0.9751	1.13	0.5342
98728_at	1421649_at	Tnfrsf8	tumor necrosis factor receptor superfamily, r	1.15	0.77	-1.06	0.8219	-1.2	0.4066	-1.46	0.0744	1.17	0.8059
98730_at	1421523_at	Fgf17	fibroblast growth factor 17	-1.56	0.54	-1.16	0.651	1.13	0.7421	1.29	0.5511	1.35	0.4882
98731_at	1422119_at	Rab5b	RAB5B, member RAS oncogene family	1.05	0.69	-1.21	0.0026	-1.26	0.1424	-1.71	0	1.38	0.2453
98732_at	1450549_s_at	Elk4	ELK4, member of ETS oncogene family	-1.96	0.34	1.19	0.3647	1.03	0.8909	-1.07	0.7452	1.63	0.0786
98756_at	1430195_at	2810043O03Rik	RIKEN cDNA 2810043O03 gene	1.12	0.87	1.76	0.1795	2.09	0.129	1.62	0.1994	2.7	0.2077
98758_at	1420338_at	---	---	-3.02	0.29	1.14	0.6833	-1.3	0.5013	-1.63	0.2077	-1.52	0.3008
98759_f_at	1423846_x_at	Tuba2	tubulin, alpha 2	-1.07	0.71	-1.27	0.1337	-1.05	0.6684	-1.22	0.1558	-1.97	0.0011
98762_f_at	1449972_s_at	Zfp97 /// BC018	zinc finger protein 97 /// cDNA sequence BC	2.16	0.01	1.78	0.0132	1.56	0.137	2.06	0.0008	1.53	0.0543
98764_at	1427738_at	D0Kist2	DNA segment, KIST 2	-1.02	0.95	1.02	0.9643	1.34	0.3859	1.21	0.6685	-1.94	0.4161
98766_at	1421922_at	---	---	2.09	0.16	-1.06	0.4567	-1.25	0.1141	-1.34	0.0097	-1.36	0.6343
98767_at	1435824_at	Yy1	YY1 transcription factor	1.05	0.89	-1.03	0.7386	1.43	0.0019	1.01	0.9028	-1.26	0.1978
98768_at	1449310_at	Ptger2	prostaglandin E receptor 2 (subtype EP2)	-1.03	0.87	-1.04	0.8698	1.28	0.2171	1.09	0.6896	1.3	0.6557
98770_at	1420441_at	Cenpc1	centromere autoantigen C1	1.06	0.89	-1.39	0.1265	-1.11	0.7017	-1.36	0.1918	1.04	0.9203
98772_at	1419728_at	Cxcl5	chemokine (C-X-C motif) ligand 5	-1.86	0.17	-1.14	0.5755	-1.98	0.0221	-1.41	0.1418	-1.66	0.2917
98774_at	1427381_at	---	---	-1.2	0.64	1.26	0.6016	1.05	0.8631	1.41	0.3437	1.43	0.4954
98775_at	1418513_at	Stk3	serine/threonine kinase 3 (Ste20, yeast hom	3.24	0.09	-1.31	0.1471	-1.38	0.2305	-1.23	0.1757	-1.07	0.9019
98777_at	1423004_at	Vipr1	vasoactive intestinal peptide receptor 1	-1.55	0.27	1.16	0.5922	-1.15	0.4441	-1.35	0.1114	2.04	0.1437
98778_at	1460626_at	D5Ertd606e	DNA segment, Chr 5, ERATO Doi 606, expr	-1.34	0.01	-1.1	0.2822	-1.13	0.2323	-1.07	0.5043	1.52	0.0239

98780_at	1427605_at	Hoxb3	homeo box B3	-2.02	0.05	-1.16	0.276	1.1	0.5247	-1.02	0.8832	1.22	0.5717
98781_at	1421470_at	Grpr	gastrin releasing peptide receptor	-1.05	0.93	-1.38	0.1586	-1.16	0.488	-1.15	0.5574	1.54	0.4644
98782_at	1421477_at	Cplx2	complexin 2	-1.85	0	-1.23	0.0734	-1.26	0.0793	-1.32	0.0121	1.05	0.8498
98783_at	1420786_a_at	Rbmy1a1	RNA binding motif protein, Y chromosome, f	1.12	0.63	1.23	0.0576	1.42	0.0206	1.2	0.3967	-1.62	0.1619
98784_at	1449472_at	Gpr12	G-protein coupled receptor 12	-3.3	0	-1.05	0.589	-1.06	0.3903	-1.19	0.0116	1.2	0.3701
98786_at	1460275_at	Gpr3	G-protein coupled receptor 3	1.81	0.17	-1.44	0.3128	-1.19	0.5989	-1.28	0.4729	1.46	0.0964
98787_at	1450515_at	Kcnj11	potassium inwardly rectifying channel, subfa	-2.42	0.2	1.35	0.2947	1.1	0.6046	1.07	0.7769	1.06	0.8877
98788_at	1422220_at	Pit1	pituitary specific transcription factor 1	-2.08	0.17	-1.15	0.5689	-1.13	0.6901	-1.34	0.3042	1.05	0.9092
98789_at	1450140_a_at	Cdkn2a	cyclin-dependent kinase inhibitor 2A	-1.37	0.54	-1.1	0.7077	-1.34	0.2465	-1.25	0.3994	1.74	0.2314
98790_s_at	1450992_a_at	Meis1	myeloid ecotropic viral integration site 1	4.75	0.03	1.54	0.1466	1.45	0.3542	2.88	0	3.55	0.0989
98791_at	1420454_at	Rai1	retinoic acid induced 1	-1.51	0.13	-1	0.9847	1.1	0.262	-1.03	0.7159	-1.03	0.7662
98793_at	1419139_at	Gdf5	growth differentiation factor 5	1.58	0.53	-1	0.9903	-1.04	0.8207	-1.08	0.645	1.39	0.3751
98794_at	1449287_at	Srms	src-related kinase lacking C-terminal regulat	1.37	0.67	-1.16	0.6261	1.09	0.8083	-1.32	0.2996	2.93	0.0242
98795_at	1422202_at	Thrb	thyroid hormone receptor beta	-1.11	0.39	-1.02	0.8438	-1.24	0.1994	-1.19	0.2461	1.19	0.6291
98796_at	1421039_at	Mip	major intrinsic protein of eye lens fiber	-1.61	0.43	-1.27	0.5035	1.22	0.5813	-1.45	0.3294	1.29	0.3371
98797_at	1422915_at	Gast	gastrin	-1.47	0.61	-1.13	0.5264	1.23	0.2752	-1.44	0.1167	1.84	0.2376
98799_g_at	1423604_at	Cspg3	chondroitin sulfate proteoglycan 3	-1.39	0.28	-1.05	0.8846	-1.44	0.1956	-1.45	0.1782	2.39	0.0701
98800_at	1460042_at	Slc23a3	solute carrier family 23 (nucleobase transpo	-1.49	0.14	-1.38	0.0204	-1.05	0.6821	-1.36	0.012	1.29	0.1085
98801_at	1421304_at	Klra2	killer cell lectin-like receptor, subfamily A, m	-1.8	0.54	1.33	0.5904	1.02	0.9608	1.44	0.5439	-1.18	0.7262
98802_at	1419431_at	Ereg	epiregulin	1.98	0.31	1.04	0.879	-1.07	0.8313	-1.18	0.5564	4.16	0.22
98803_at	1420390_s_at	Zfp354a	zinc finger protein 354A	-1.39	0.65	1.78	0.0514	1.44	0.2365	1.38	0.3283	1.99	0.0789
98804_at	1420604_at	Hesx1	homeo box gene expressed in ES cells	-1.43	0.15	1.43	0.2823	2.15	0.1378	1.63	0.2012	3.07	0.0224
98806_s_at	1450041_a_at	Tub	tubby candidate gene	-1.06	0.85	1.16	0.1527	1.19	0.1279	-1.02	0.8842	-1.12	0.6352
98807_at	1422907_at	Gnat2	guanine nucleotide binding protein, alpha tra	-1.32	0.29	-1.11	0.6237	-1.02	0.912	-1.13	0.5448	2.43	0.1605
98808_at	1418995_at	Neurod2	neurogenic differentiation 2	-1.83	0.06	-1.2	0.3991	-1.13	0.462	-1.01	0.9286	-1.15	0.6263
98809_s_at	1452525_a_at	Nf1	neurofibromatosis 1	-2.36	0.17	-1.09	0.701	-1.03	0.9154	1.59	0.2051	1.42	0.5662
98810_at	1422196_at	Htr5b	5-hydroxytryptamine (serotonin) receptor 5B	-1.05	0.75	1.03	0.8317	1.04	0.7745	-1.17	0.3401	1.8	0.0687
98811_at	1427313_at	Ptgir	prostaglandin I receptor (IP)	1.11	0.83	-1.01	0.9695	-1.29	0.1949	-1.24	0.2213	1.17	0.7304
98812_at	1422283_at	Tnfsf5	tumor necrosis factor (ligand) superfamily, m	1.12	0.83	1.32	0.2007	1.41	0.1106	1.41	0.052	1.25	0.5514
98814_at	1418221_at	Csnd	casein delta	-2.42	0.19	-1.1	0.7528	1.24	0.4899	-1.13	0.7113	-2.19	0.1802
98815_at	1421627_at	Evx1	even skipped homeotic gene 1 homolog	-1.6	0.03	1.11	0.2527	1.23	0.0235	1.06	0.4902	1.04	0.8466
98817_at	1421365_at	Fst	follistatin	1.4	0.31	1.96	0.1098	1.27	0.4027	2.19	0.0328	1.29	0.3739
98818_at	1421866_at	Nr3c1	nuclear receptor subfamily 3, group C, mem	3.17	0	-1.02	0.8676	-1.22	0.338	-1.24	0.1319	-1.41	0.5567
98820_g_at	1420573_at	Hoxd1	homeo box D1	1.13	0.81	1.83	0.1369	-1.11	0.8117	1.16	0.6944	1.68	0.2451
98821_at	1422173_at	Ipf1	insulin promoter factor 1, homeodomain tran	-3.02	0.16	-1.19	0.5193	1.68	0.2392	-1.09	0.8062	1.23	0.5895
98823_at	1450503_at	Kcnj2	potassium inwardly-rectifying channel, subfa	-1.44	0.62	-1.15	0.381	1.09	0.5059	1.39	0.2655	-1.79	0.3391
98825_at	1420672_at	Kcne1	potassium voltage-gated channel, Isk-relate	-2.76	0.01	-1.18	0.5611	1.04	0.8637	-1.33	0.3096	-1.04	0.911
98826_at	1420086_x_at	---	---	-2.85	0.16	1.25	0.4455	1.65	0.0781	1.07	0.8224	-1.32	0.4295
98827_i_at	1427635_at	Kif5c	kinesin family member 5C	1.35	0.49	-1.83	0.1561	1.08	0.8316	-1.5	0.3135	-1.48	0.5531
98828_at	1422046_at	Itgam	integrin alpha M	-1.07	0.81	1.94	0.0605	1.09	0.5936	1.99	0.1496	1.12	0.8143
98829_at	1422871_at	Kcnj12	potassium inwardly-rectifying channel, subfa	-1.06	0.92	1.47	0.1309	-1.09	0.7151	-1.37	0.2154	-1.15	0.6981
98830_at	1418307_a_at	Sbp	spermine binding protein	-1.79	0.38	1.74	0.0139	1.75	0.123	1.67	0.0485	1.07	0.8043
98831_at	1425291_at	Foxj1	forkhead box J1	-2.37	0.06	-1.55	0.0657	-1.09	0.6211	-1.35	0.147	1.28	0.4997
98832_at	1420600_at	Tpo	thyroid peroxidase	1.21	0.69	-1.14	0.5171	-1.32	0.1265	-1.32	0.276	-1.44	0.318
98833_at	1418945_at	Mmp3	matrix metalloproteinase 3	-2.61	0.29	-1.28	0.3246	-1.41	0.2711	-1.85	0.0663	1.26	0.7328
98834_at	1450501_at	Itga2	integrin alpha 2	-1.74	0.14	1.19	0.3834	1.13	0.6459	-1.04	0.8442	1.35	0.067
98835_at	1449865_at	Sema3a	sema domain, immunoglobulin domain (Ig),	-1.79	0.36	1.35	0.1881	1.29	0.3386	1.19	0.6303	-1.23	0.5315
98836_at	1449835_at	Pdcd1	programmed cell death 1	1.14	0.72	-1.13	0.711	1.41	0.2456	1.21	0.5738	1.44	0.3499
98837_at	1448074_at	Rln1	Relaxin 1	-1.46	0.21	-1.04	0.8107	1.16	0.372	1.19	0.1943	1.05	0.906
98838_at	1421246_at	Pax9	paired box gene 9	1.01	0.99	1.08	0.7345	1.23	0.4319	-1.11	0.7081	1.2	0.6117
98839_at	1427436_at	Six2	sine oculis-related homeobox 2 homolog (Dr	1.23	0.61	-1.33	0.0195	-1.19	0.2399	-1.37	0.0038	1	0.9791
98840_at	1422084_at	Bmx	BMX non-receptor tyrosine kinase	-1.15	0.64	-1.05	0.8388	1.14	0.5015	1.06	0.7871	1.25	0.6772
98842_at	1456124_x_at	Svs5	seminal vesicle secretion 5	-5.26	0.33	1.29	0.4257	1.4	0.3284	-1.02	0.8827	1.79	0.1961

98843_at	1421301_at	Zic2	Zic finger protein of the cerebellum 2	-1.25	0.59	1.08	0.5161	2.36	0.0947	1.37	0.446	1.47	0.3934
98845_at	1426186_a_at	Fgf5	fibroblast growth factor 5	1.57	0.43	1.13	0.5431	1.14	0.3255	1.17	0.3302	2.34	0.2154
98846_f_at	1421638_at	Psg17	pregnancy specific glycoprotein 17	-1.19	0.58	1.3	0.0076	1.09	0.4868	1.14	0.2168	1.26	0.4058
98847_at	1420853_at	Sdc3	syndecan 3	-1.56	0.23	-1.04	0.8981	1.02	0.928	-1.01	0.9651	1.11	0.5566
98848_at	1419329_at	Sh3d4	SH3 domain protein 4	1.05	0.93	2.6	0.0345	1.46	0.4275	2.09	0.0785	1.15	0.4028
98849_at	1439964_at	D8Bwg1414e	DNA segment, Chr 8, Brigham & Women's (	-1.25	0.41	1.13	0.18	1.32	0.0053	1	0.9935	1.29	0.1693
98850_at	1420753_at	Tll1	tolloid-like	-1.2	0.58	1.11	0.474	1.22	0.119	-1.02	0.8851	-1.17	0.6036
98851_at	1421409_at	Msi1h	Musashi homolog 1(Drosophila)	1.15	0.74	1	0.9961	1.04	0.8641	1.01	0.9642	1.28	0.5908
98852_at	1452386_at	Sall3	sal-like 3 (Drosophila)	-1.26	0.36	-1.03	0.8663	1.1	0.6671	-1.1	0.7604	-1.33	0.211
98853_at	1450144_at	Pla2g1br	phospholipase A2, group IB, pancreas, rece	-1.4	0.39	1.24	0.2401	1.22	0.356	1.19	0.4399	2.02	0.1391
98854_at	1450499_at	Sca1	spinocerebellar ataxia 1 homolog (human)	-1.5	0.26	-1.12	0.4527	1.11	0.5076	-1.12	0.3333	1.87	0.0677
98856_at	1422171_at	Ptgdr	prostaglandin D receptor	1.37	0.5	-1.12	0.686	-1.17	0.7126	1.06	0.8491	-1.26	0.5797
98857_at	1418047_at	Neurod6	neurogenic differentiation 6	3.26	0.13	-1.34	0.4845	-1.69	0.1181	-1.94	0.0494	1.07	0.8607
98858_at	1449908_at	Gip	gastric inhibitory polypeptide	-1.49	0.32	1.23	0.2311	1.63	0.0021	1.29	0.0868	2.15	0.1699
98859_at	1431609_a_at	Acp5	acid phosphatase 5, tartrate resistant	1.06	0.77	-1.09	0.1411	1.03	0.5695	-1.14	0.0153	-1.13	0.322
98860_at	1421500_at	Sts	steroid sulfatase	-4.54	0.14	2.04	0.0227	1.57	0.2425	1.68	0.166	-1.52	0.2879
98862_at	1460657_at	Wnt10a	wingless related MMTV integration site 10a	-1.02	0.96	-1.26	0.0075	-1.09	0.1896	-1.25	0.0149	1.26	0.1007
98864_s_at	1425790_a_at	Grik2	glutamate receptor, ionotropic, kainate 2 (be	-1.79	0.03	-1.25	0.4503	1.25	0.4062	-1.04	0.8976	1.22	0.4496
98865_at	1418678_at	Has2	hyaluronan synthase 2	-1.15	0.48	-1.3	0.4597	-1.14	0.6562	-1.6	0.2197	1.09	0.7624
98866_at	1452507_at	Dlx6	distal-less homeobox 6	-1.53	0.37	-1.55	0.1725	-1.55	0.1866	-1.53	0.18	1.54	0.3528
98870_at	1422938_at	Bcl2	B-cell leukemia/lymphoma 2	-1.19	0.11	1.09	0.1992	1.04	0.3992	1.08	0.0131	1.3	0.0405
98871_at	1420581_at	Gpr143	G protein-coupled receptor 143	1.33	0.47	-1.34	0.2676	-1.83	0.0246	-1.17	0.53	-1.79	0.1962
98872_at	1419064_a_at	Ugt8	UDP-glucuronosyltransferase 8	-2.59	0.31	1.21	0.567	-1.03	0.941	-1.51	0.2803	1.23	0.7598
98873_at	1420595_at	Dlx4	distal-less homeobox 4	1.93	0.1	-1.29	0.4398	1.22	0.5604	1.25	0.4511	-1.23	0.6557
98874_at	1421376_at	Traf6	Tnf receptor-associated factor 6	1.44	0.02	1.09	0.8266	-1.03	0.9267	1.22	0.5962	-1.2	0.597
98875_at	1423793_at	D2Ertd391e	DNA segment, Chr 2, ERATO Doi 391, expr	1.19	0.35	1.1	0.0926	1.13	0.1443	1.18	0.0252	-1.19	0.2857
98876_at	1417918_at	Mrpl11	mitochondrial ribosomal protein L11	1.62	0.18	1.15	0.0222	1.21	0.0355	1.14	0.0512	1.16	0.4439
98880_at	1433588_at	D6Wsu116e	DNA segment, Chr 6, Wayne State Universi	-1.14	0.42	-1.14	0.1061	-1.01	0.8845	-1.06	0.418	-1.17	0.2135
98881_at	1428507_at	Hdhd2	haloacid dehalogenase-like hydrolase doma	1.05	0.69	1.1	0.2775	1.17	0.1096	1.08	0.2121	1.03	0.8547
98884_r_at	1422568_at	Ndel1	nuclear distribution gene E-like homolog 1 (/	1.54	0.18	1.82	0.0044	1.4	0.0196	1.96	0.0051	1.37	0.243
98886_at	1423767_at	2810410M20Ril	RIKEN cDNA 2810410M20 gene	-1.3	0.07	1.05	0.6163	1.02	0.8677	-1.07	0.4676	1.4	0.0523
98887_at	1448243_at	Napa	N-ethylmaleimide sensitive fusion protein att	1.06	0.85	1.2	0.0212	1.11	0.2152	1.34	0	1.25	0.0362
98891_at	1428217_at	1600012H06Rik	RIKEN cDNA 1600012H06 gene	1.67	0.39	-1.12	0.2753	-1.3	0.046	-1.32	0.0189	-1.88	0.0669
98892_at	1418288_at	Lpin1	lipin 1	1.39	0.12	-1.87	0.0015	1.75	0.0143	1.08	0.5811	2.04	0.0517
98894_at	1426878_at	2610016F04Rik	RIKEN cDNA 2610016F04 gene	-1.12	0.72	1.12	0.2672	-1.14	0.2248	1.06	0.5882	1.75	0.0926
98896_at	1424028_at	5830457O10Ril	RIKEN cDNA 5830457O10 gene	1.19	0.51	1.01	0.8447	-1.02	0.7959	-1.06	0.284	1.43	0.0241
98905_at	1454610_at	7-Sep	septin 7	-1.27	0.5	-1.08	0.2203	-1.05	0.6061	-1.1	0.1462	-1.09	0.5915
98906_at	1417480_at	Fbxo9	f-box only protein 9	1.1	0.58	-1	0.963	1.16	0.0323	1.13	0.2912	-1.03	0.7141
98908_at	1451073_at	Sppl3	Signal peptide peptidase 3	-1.17	0.17	1.04	0.3299	-1.11	0.1653	-1.1	0.1251	-1.07	0.5338
98909_at	1418700_at	Lias	lipoic acid synthetase	1.35	0.23	1.16	0.1127	-1.02	0.804	1.1	0.3265	-1.07	0.7357
98910_at	1436747_at	1110014K08Rik	RIKEN cDNA 1110014K08 gene	-1.29	0.24	1.01	0.943	1.07	0.1362	1.09	0.1443	-1.15	0.0885
98911_at	1433803_at	Jak1	Janus kinase 1	1.48	0.09	1.03	0.5936	-1.03	0.3496	1.14	0.0022	-1.04	0.7359
98912_at	1434597_at	D13Wsu64e	DNA segment, Chr 13, Wayne State Univers	1.33	0.15	1.1	0.0566	1.22	0.0775	1.13	0.0683	1.13	0.5023
98914_at	1423511_at	Asf1a	ASF1 anti-silencing function 1 homolog A (S	1.51	0.01	1.37	0.0009	1.23	0.0885	1.36	0.0128	-1.15	0.265
98915_at	1429321_at	Rnf149	ring finger protein 149	-1.39	0.34	1.04	0.6996	1.14	0.0768	1.04	0.5362	1.36	0.2167
98917_at	1416369_at	5730414C17Rik	RIKEN cDNA 5730414C17 gene	1.04	0.93	1.02	0.7764	1.07	0.5102	-1.03	0.62	-1.17	0.2098
98918_at	1451091_at	Txndc5	thioredoxin domain containing 5	-1.31	0.06	-1.07	0.4657	1.07	0.4347	-1.05	0.594	-1.19	0.0332
98921_at	1451134_a_at	2410018G23Rik	RIKEN cDNA 2410018G23 gene	-1.51	0.19	-1.15	0.051	1.04	0.4842	-1.11	0.2459	-1.19	0.0601
98923_at	1416730_at	Rcl1	RNA terminal phosphate cyclase-like 1	1.42	0.05	1.3	0.0015	1.33	0	1.48	0	1.33	0.0739
98924_at	1452474_a_at	Art3	ADP-ribosyltransferase 3	1.66	0.21	-1.52	0.0122	-1.24	0.2008	-1.35	0.165	-1.64	0.4254
98926_at	1420834_at	Vamp2	vesicle-associated membrane protein 2	1.21	0.53	1.31	0.0006	1.07	0.4689	1.33	0.0034	1.12	0.2872
98927_at	1448304_a_at	Rab6	RAB6, member RAS oncogene family	-1.02	0.97	1	0.9781	-1.64	0.0176	-1.24	0.1583	1.17	0.3656
98928_at	1448351_at	Coro1b	coronin, actin binding protein 1B	1.53	0.03	1.29	0.0004	1.17	0.0734	1.45	0	1.05	0.7224

98929_at	1423242_at	Mrps36	mitochondrial ribosomal protein S36	1.77	0.05	-1.13	0.08	1	0.9578	-1.04	0.4816	1.08	0.536
98930_at	1416384_a_at	Cope	coatomer protein complex, subunit epsilon	1.23	0.05	1.04	0.4024	1.1	0.1061	1.16	0.0011	-1.08	0.3956
98931_at	1433546_at	Gns	glucosamine (N-acetyl)-6-sulfatase	1.76	0.1	1.03	0.7174	1.06	0.4719	1.05	0.469	-1.17	0.1283
98934_at	1419352_at	0610007P06Rik	RIKEN cDNA 0610007P06 gene	-1.04	0.77	-1.18	0.1115	-1.02	0.8198	-1.21	0.0418	-1.39	0.2581
98936_at	1452000_s_at	Sars1	seryl-aminoacyl-tRNA synthetase 1	1.59	0.1	1.09	0.3832	1.1	0.378	1.14	0.1644	1.86	0.0569
98937_at	1452648_at	Tbrg1	transforming growth factor beta regulated ge	1.42	0.03	-1.12	0.1009	-1.17	0.0384	-1.04	0.5188	-1.12	0.3899
98938_at	1417427_at	1500026D16Rik	RIKEN cDNA 1500026D16 gene	1.31	0.53	1.01	0.8844	-1.07	0.5757	-1.12	0.147	1.05	0.7554
98944_at	1416059_at	Sec23b	SEC23B (S. cerevisiae)	-1.17	0.52	-1.26	0.0003	-1.19	0.0039	-1.2	0.0056	-1.35	0.002
98945_at	1418010_a_at	Sh3glb1	SH3-domain GRB2-like B1 (endophilin)	1.55	0.17	1.26	0.1667	1.17	0.3596	1.37	0.0256	-1.02	0.9373
98946_at	1425241_a_at	Wsb1	WD repeat and SOCS box-containing 1	-2.6	0.02	-1.63	0.0086	1.07	0.7326	-1.51	0.0454	-1.89	0.0591
98948_at	1433656_a_at	Gnl3	guanine nucleotide binding protein-like 3 (nu	1.77	0.25	1.05	0.5277	1.33	0.0038	1.08	0.3657	1.22	0.1388
98950_at	1415749_a_at	Rragc	Ras-related GTP binding C	1.13	0.42	1.23	0.0003	1.21	0.0004	1.34	0	1.39	0.015
98951_at	1437205_at	---	---	-1.21	0.76	-1.06	0.3401	-1.14	0.3231	-1.22	0.1145	-1.19	0.6501
98952_at	1451081_a_at	D8Ertd325e	DNA segment, Chr 8, ERATO Doi 325, expr	1.21	0.71	1.02	0.7683	1.01	0.9247	1.09	0.4009	1.49	0.1598
98953_at	1428272_at	1500010M16Rik	RIKEN cDNA 1500010M16 gene	-1.02	0.88	-1.05	0.5569	1.11	0.3448	-1.16	0.0674	1.11	0.3327
98954_f_at	1427977_x_at	Oog1	oogenesin 1	-2.21	0.1	-1.99	0.0462	-1.35	0.363	-1.55	0.1485	1.02	0.9552
98956_at	1423732_at	Tram1	translocating chain-associating membrane p	-1.31	0.1	-1.16	0.0057	-1.06	0.2868	-1.12	0.0255	-2.08	0.0048
98959_at	1449821_a_at	0610016J10Rik	RIKEN cDNA 0610016J10 gene	1.13	0.57	-1	0.9802	1.15	0.2413	1.15	0.2691	-1.34	0.2019
98961_at	1418736_at	B3galt3	UDP-Gal:betaGlcNAc beta 1,3-galactosyltra	-1.24	0.53	-1.15	0.6536	1.42	0.2775	1.34	0.2611	1.03	0.9453
98962_at	1419560_at	Lipc	lipase, hepatic	-1.26	0.25	-1.72	0	-1.36	0.0043	-2.85	0	-1.76	0.0219
98963_at	1416935_at	Trpv2	transient receptor potential cation channel, s	-1.01	0.99	1.43	0.0931	1.25	0.258	1.55	0.1142	1.45	0.2902
98965_at	1423960_at	Grcc3f	gene rich cluster, C3f gene	-1.14	0.5	1.01	0.8795	1.1	0.198	1.09	0.3685	-1.18	0.1328
98966_at	1449118_at	Dbt	dihydrolipoamide branched chain transacyla	1.11	0.77	1.44	0.0163	-1.25	0.1488	1.34	0.0918	1.36	0.1918
98967_at	1450779_at	Fabp7	fatty acid binding protein 7, brain	1.54	0.26	-1.66	0.0381	-1.51	0.1217	-2.75	0.0007	-2.24	0.3131
98968_at	1419754_at	Myo5a	myosin Va	1.1	0.66	-1.78	0.3091	-1.84	0.2916	-1.33	0.6045	-3.5	0.2344
98969_at	1418838_at	Abcd1	ATP-binding cassette, sub-family D (ALD), n	1.1	0.69	-1.24	0.0848	-1.27	0.0201	-1.27	0.0117	-1.94	0.0316
98970_at	1419505_a_at	Ggps1	geranylgeranyl diphosphate synthase 1	-1.1	0.23	-1.02	0.9203	-1.17	0.4629	1.2	0.2498	1.94	0.0664
98973_at	1428390_at	Wdr43	WD repeat domain 43	-1.41	0.41	1.19	0.1926	1.19	0.2226	1.08	0.4794	1.08	0.8346
98974_at	1455530_at	Igh-5	immunoglobulin heavy chain 5 (delta-like he	-1.83	0.51	2.78	0.0306	1.15	0.6537	2.93	0.0209	3.75	0.1475
98975_at	1424291_at	Nup93	nucleoporin 93	-1.4	0.64	1.09	0.6449	1.06	0.6779	1.16	0.2548	1.2	0.6399
98976_at	1419476_at	Adamdec1	ADAM-like, decysin 1	-1.86	0.01	2.25	0.0822	1.3	0.4802	3.05	0.0679	2.97	0.0018
98977_at	1451163_at	Tinf2	Terf1 (TRF1)-interacting nuclear factor 2	1.16	0.38	-1.1	0.2651	1.1	0.2796	-1.03	0.7324	1.11	0.4634
98979_at	1432177_a_at	Mnat1	menage a trois 1	1.71	0.09	1.19	0.188	1.11	0.5125	1.3	0.0589	1.66	0.0166
98980_at	1419206_at	Cd37	CD37 antigen	-1.26	0.2	1.44	0.1317	1.32	0.1918	1.58	0.0454	1.47	0.3354
98981_s_at	1427670_a_at	Tcf12	transcription factor 12	1.74	0	1.18	0.0742	1.09	0.4616	1.2	0.0585	1.44	0.0782
98982_at	1426349_s_at	Tmpo	thymopoietin	-1.19	0.42	1.05	0.3972	-1.12	0.1371	-1.05	0.4319	1.24	0.0978
98983_at	1417149_at	P4ha2	procollagen-proline, 2-oxoglutarate 4-dioxyg	-1.26	0.19	-1.07	0.2846	1.1	0.2452	-1.22	0.0202	1.91	0.0335
98987_at	1418242_at	Faf1	Fas-associated factor 1	1.35	0.35	-1.38	0.0392	-1.1	0.5137	-1.23	0.1069	1.3	0.4467
98988_at	1417483_at	Nfkbiz	nuclear factor of kappa light polypeptide gen	1.2	0.43	1.31	0.0769	1.04	0.8151	1.47	0.0233	1.33	0.418
98989_at	1448619_at	Dhcr7	7-dehydrocholesterol reductase	1.22	0.58	-1.93	0.0009	-1.15	0.3811	-1.68	0.0006	-1.42	0.0391
98990_at	1450622_at	Bcar1	breast cancer anti-estrogen resistance 1	1.39	0.36	1.05	0.7832	1.15	0.3232	1.08	0.5183	-1.31	0.5056
98992_at	1417596_at	Eppb9	endothelial precursor protein B9	1	0.99	-1.29	0.3556	-1.19	0.5008	-1.22	0.4894	1.5	0.3886
98993_at	1425542_a_at	Ppp2r5c	protein phosphatase 2, regulatory subunit B	-1.5	0.05	-1.07	0.3363	-1.07	0.4204	-1.13	0.1368	-1.51	0.1851
98994_at	1416854_at	Slc34a2	solute carrier family 34 (sodium phosphate),	-4.75	0	-1.73	0.1498	-1.31	0.4825	-1.53	0.2472	-1.09	0.7873
98995_at	1460207_s_at	E2f5	E2F transcription factor 5	-1.41	0.29	1.01	0.9445	1.14	0.2915	-1	0.9839	1.1	0.7207
98996_at	1419838_s_at	Plk4	polo-like kinase 4 (Drosophila)	-1.25	0.68	1.36	0.0845	1.21	0.3937	1.01	0.96	1.73	0.0073
98998_r_at	1420409_at	Krt1-24	keratin complex 1, acidic, gene 24	-1.74	0.06	1.12	0.2544	1.09	0.225	-1.09	0.3563	1.61	0.016
98999_at	1418372_at	---	---	2.03	0.04	1.05	0.5249	1.04	0.7085	1.13	0.1591	-1.17	0.2348
99000_at	1418060_a_at	Mapk7	mitogen activated protein kinase 7	1.32	0.32	1.14	0.1854	1.47	0.0008	1.06	0.5518	1.25	0.0656
99001_at	1449515_at	Zfp292	zinc finger protein 292	-2.16	0.33	-1.23	0.0988	-1.55	0.0013	-1.75	0.0001	1.17	0.4664
99005_at	1448414_at	Rad1	RAD1 homolog (S. pombe)	1.92	0.17	1.12	0.5237	1.14	0.4455	1.11	0.5087	-1.01	0.9799
99006_at	1430749_at	2810040C05Rik	RIKEN cDNA 2810040C05 gene	-1.67	0.03	1.32	0.025	1.39	0.006	1.13	0.2114	-1.09	0.7978
99007_at	1417544_a_at	Flot2	flotillin 2	-1.2	0.25	-1.1	0.1012	-1.07	0.3921	-1.11	0.0881	1.05	0.6648

99009_at	1416105_at	Nnt	nicotinamide nucleotide transhydrogenase	2.63	0.05	-1.09	0.2352	1.08	0.3773	1	0.9804	1.21	0.2336
99010_at	1418450_at	Islr	immunoglobulin superfamily containing leuci	-1.25	0.17	-1.11	0.2427	-1.13	0.1815	-1.11	0.2502	1.52	0.0759
99011_at	1417588_at	Galnt3	UDP-N-acetyl-alpha-D-galactosamine:polyp	2.77	0.03	-1.06	0.8604	1.4	0.1234	1.18	0.6241	1.46	0.2945
99013_f_at	1423088_at	LOC434449	similar to Ubiquitous tropomodulin (U-Tmod)	-1.46	0.07	1.05	0.2025	-1.02	0.5619	1.01	0.8732	-1	0.979
99014_at	1423893_x_at	Apbb1	amyloid beta (A4) precursor protein-binding,	-1.85	0.07	-1.41	0.0167	-1.24	0.1214	-1.61	0.0055	-1.87	0.02
99015_at	1448757_at	Pml	promyelocytic leukemia	-1.37	0.02	-1.13	0.0875	-1.12	0.0472	-1.15	0.0325	-1.19	0.3264
99016_at	1420103_at	Sertad2	SERTA domain containing 2	-1.19	0.22	-1.12	0.2664	-1.06	0.46	-1.09	0.3712	1.16	0.3309
99018_at	1428844_a_at	Bclaf1	BCL2-associated transcription factor 1	-1.11	0.85	1.28	0.0164	1.17	0.0652	1.19	0.1061	1.92	0.0602
99019_at	1416933_at	Por	P450 (cytochrome) oxidoreductase	2.1	0.02	-1.15	0.0346	-1.02	0.9247	-1.17	0.0929	1.82	0.163
99020_at	1457834_at	Yy1	YY1 transcription factor	2.82	0.06	1.36	0.223	1.45	0.2471	1.27	0.3231	1.48	0.1034
99021_at	1425527_at	Prrx1	paired related homeobox 1	-1.17	0.77	-1.19	0.3105	-1.01	0.9504	-1.36	0.0221	1.26	0.5066
99023_at	1422791_at	Pafah1b2	platelet-activating factor acetylhydrolase, isc	-1.38	0.12	1.09	0.4778	-1.03	0.83	1.03	0.8294	1.01	0.9684
99025_at	1417927_at	Ddx19a	DEAD (Asp-Glu-Ala-Asp) box polypeptide 1!	1.15	0	1.12	0.1158	1.14	0.1909	1.29	0.0003	1.14	0.2191
99026_at	1426050_at	Bcl2l1	Bcl2-like 1	-2.69	0.34	-1.3	0.3477	-1.12	0.6829	-1.31	0.3754	2.01	0.1551
99027_at	1420888_at	Bcl2l1	Bcl2-like 1	-1.1	0.33	-1.02	0.814	-1.07	0.5302	1.02	0.8344	-1.09	0.4731
99028_at	1424120_at	Rnf8	ring finger protein 8	1.57	0.15	1.04	0.6087	1.07	0.3092	1.07	0.4562	-1.03	0.8536
99029_at	1422581_at	Pias1	protein inhibitor of activated STAT 1	-1.13	0.67	1.25	0.0299	1.13	0.133	1.2	0.1335	1.44	0.1528
99030_at	1448575_at	Il7r	interleukin 7 receptor	1.29	0.58	-1.11	0.3419	1.03	0.8054	1.1	0.4763	2.46	0.0225
99031_at	1454763_at	Ankrd17	ankyrin repeat domain 17	-1.12	0.37	1.39	0.0245	1.34	0.0978	1.38	0.0119	1.14	0.3434
99033_at	1426733_at	Itpk1	inositol 1,3,4-triphosphate 5/6 kinase	-1.07	0.34	1.05	0.2289	-1.13	0.1182	-1.05	0.5462	-1.06	0.5686
99034_at	1418517_at	Irx3	Iroquois related homeobox 3 (Drosophila)	-2.41	0.03	-1.56	0.0097	-1.34	0.0692	-1.54	0.0094	1.23	0.4519
99036_s_at	1425393_a_at	Map2k7	mitogen activated protein kinase kinase 7	1.06	0.93	1.04	0.8238	-1.33	0.155	1	0.9775	1.14	0.7407
99037_at	1421416_at	Map2k7	mitogen activated protein kinase kinase 7	-1.39	0.29	1.36	0.1044	1.12	0.398	1.42	0.2563	1.04	0.9124
99039_g_at	1460726_at	Adss	adenylosuccinate synthetase, non muscle	-1.11	0.48	-1.1	0.0148	-1.14	0.0006	-1.15	0.0158	-1.35	0.0319
99040_at	1417150_at	Slc6a4	solute carrier family 6 (neurotransmitter trans	1.63	0.35	-1.2	0.1541	1.48	0.2743	-1.17	0.0708	1.1	0.5194
99043_s_at	1420559_a_at	Shox2	short stature homeobox 2	-1.48	0.06	-1.17	0.1073	-1.05	0.7104	-1.08	0.5087	1.43	0.2125
99044_at	1451011_at	Zfp358	zinc finger protein 358	-1.64	0.58	-1.05	0.7985	1.15	0.5281	1.06	0.7532	-1.12	0.7992
99045_at	1418829_a_at	Eno2	enolase 2, gamma neuronal	-2.3	0.12	-1.28	0.3066	-1.22	0.2306	-1.29	0.3178	-1.05	0.8551
99046_at	1421010_at	Mobp	myelin-associated oligodendrocytic basic pro	-1.03	0.97	-2.17	0.225	-2	0.2632	-2.14	0.2315	-1.66	0.234
99048_g_at	1450088_a_at	Mobp	myelin-associated oligodendrocytic basic pro	-1.44	0.09	-1.03	0.9378	-1.56	0.213	-1.61	0.1889	1.93	0.2695
99049_at	1448165_at	Casp2	caspase 2	-1.16	0.31	1.02	0.8001	-1.03	0.6642	1.08	0.3552	1.41	0.0103
99050_at	1418413_at	Cav3	caveolin 3	1.25	0.7	-1.45	0.0816	1.12	0.5869	1.13	0.6164	-1.05	0.8754
99051_at	1424542_at	S100a4	S100 calcium binding protein A4	1.2	0.73	2.24	0.1208	1.33	0.4945	3.25	0.0522	1.57	0.2688
99052_at	1418926_at	Zfhx1a	zinc finger homeobox 1a	-1.05	0.83	-1.11	0.124	1.01	0.8862	-1.1	0.2364	1.22	0.1774
99053_at	1448862_at	Icam2	intercellular adhesion molecule 2	-1.09	0.8	1.13	0.5157	-1.04	0.7001	1.33	0.2469	2	0.069
99054_at	1416621_at	Llglh	lethal giant larvae homolog	-1.49	0.11	1.01	0.8622	-1.06	0.4811	-1.01	0.9434	1.2	0.0122
99055_at	1417616_at	St6galnac2	ST6 (alpha-N-acetyl-neuraminyl-2,3-beta-ga	-2.16	0.01	-1.11	0.5071	-1	0.9938	-1.3	0.0401	-1.27	0.5347
99056_at	1418713_at	Pcbd1	pterin 4 alpha carbinolamine dehydratase/di	1.09	0.32	1.01	0.7785	-1	0.9645	-1.04	0.5023	-1.16	0.123
99057_at	1423135_at	Thy1	thymus cell antigen 1, theta	3.5	0	-1.31	0.4547	-1.67	0.171	-1.6	0.2076	1.52	0.0892
99058_at	1450780_s_at	Hmga2	high mobility group AT-hook 2	-1.49	0.61	-1.59	0.234	-1.72	0.1794	1.03	0.9497	2.8	0.1121
99059_at	1416916_at	Elf3	E74-like factor 3	-1.36	0.52	1.32	0.246	1.31	0.0227	1.53	0.0758	1.2	0.567
99062_at	1417319_at	Pvrl3	poliovirus receptor-related 3	-1.06	0.87	-1.23	0.0395	1.11	0.3932	-1.33	0.0334	-1.48	0.2069
99063_at	1418089_at	Stx8	syntaxin 8	2.17	0.05	-1.04	0.5676	1.07	0.3761	1.01	0.8529	-1.37	0.403
99065_at	1419735_at	Csnk	casein kappa	-1.88	0.3	1.09	0.8441	1.38	0.4573	-1.14	0.6687	1.03	0.9539
99067_at	1417399_at	Gas6	growth arrest specific 6	1.15	0.59	-2.15	0	-1.87	0	-2.45	0	-1.77	0.0706
99068_at	1415680_at	Anapc1	anaphase promoting complex subunit 1	1.09	0.62	1.11	0.022	1.09	0.084	1.18	0.0024	1.02	0.8409
99069_at	1420837_at	Ntrk2	neurotrophic tyrosine kinase, receptor, type	-1.17	0.57	-1.14	0.2058	-1.09	0.3649	-1.33	0.005	-1.16	0.6568
99070_at	1417091_at	Chuk	conserved helix-loop-helix ubiquitous kinase	-1.08	0.75	-1	0.9964	1.03	0.7152	-1.06	0.4229	-1.78	0.0011
99071_at	1427076_at	Mpeg1	macrophage expressed gene 1	1.18	0.65	1.99	0.1774	-1.33	0.0675	2.54	0.1003	-1.44	0.1679
99073_at	1422513_at	Ccnf	cyclin F	-2.17	0	-1	0.9951	-1.05	0.2707	-1.02	0.7808	-1.16	0.1022
99074_at	1448837_at	Vil1	villin 1	-2.04	0.44	1.18	0.3103	4.25	0.3672	1.6	0.2122	2.73	0.0641
99077_at	1426997_at	Thra	thyroid hormone receptor alpha	-1.44	0.65	-1.06	0.8294	-1.6	0.1253	1.15	0.5508	2.26	0.0295
99078_at	1417322_at	1110033C18Rik	RIKEN cDNA 1110033C18 gene	1.99	0.12	1.11	0.235	1.08	0.5575	1.02	0.8104	1.2	0.7202

99080_at	1428311_at	2810012H18Rik	RIKEN cDNA 2810012H18 gene	2.7	0.12	-1.07	0.4886	1.01	0.9311	1.08	0.4468	-1.3	0.4108
99081_at	1416625_at	Serping1	serine (or cysteine) proteinase inhibitor, clad	-2.12	0	1.02	0.8342	-1.14	0.0058	-1.06	0.604	-1.66	0.002
99082_at	1415951_at	Fkbp10	FK506 binding protein 10	1.2	0.63	-1.2	0.2651	-1.01	0.9382	-1.21	0.1508	1.44	0.3356
99084_s_at	1425023_at	Usp3	ubiquitin specific protease 3	-1.21	0.09	-1.08	0.1628	-1.11	0.0371	-1.08	0.2789	1.06	0.5231
99086_g_at	1425022_at	Usp3	ubiquitin specific protease 3	1.28	0.38	1.02	0.8329	1.15	0.0067	1.09	0.1747	1.2	0.2385
99087_at	1423724_at	Zwint	ZW10 interactor	-1.31	0.12	-1.06	0.4833	-1.12	0.1286	-1.08	0.4024	1.11	0.1165
99089_at	1417275_at	Mal	myelin and lymphocyte protein, T-cell differe	-1.12	0.61	-1.01	0.9111	-1	0.981	-1.11	0.4438	-1.18	0.5341
99093_at	1456497_x_at	Rps10	ribosomal protein S10	1.13	0.31	1.1	0.122	1.17	0.026	1.25	0.0019	1.67	0.0054
99094_at	1424260_at	---	---	1.48	0.18	-1.19	0.2496	-1.14	0.3778	-1.19	0.1392	1.45	0.0932
99096_at	1415713_a_at	Ddx24	DEAD (Asp-Glu-Ala-Asp) box polypeptide 24	1.53	0.41	1.1	0.3179	1.12	0.1595	1.19	0.0336	-1.03	0.7028
99097_at	1450909_at	Eif4e	eukaryotic translation initiation factor 4E	1.29	0.6	-1.13	0.5566	1.05	0.82	-1.08	0.6988	1.15	0.4707
99098_at	1423418_at	Fdps	farnesyl diphosphate synthetase	-1.21	0.45	-2.03	0.0001	-1.28	0.2294	-2.7	0	-2.69	0.0051
99100_at	1460700_at	Stat3	signal transducer and activator of transcriptio	1.21	0.31	1.11	0.1195	1.01	0.8334	1.12	0.3307	1.14	0.4847
99101_at	1416100_at	Eif3s7	eukaryotic translation initiation factor 3, subu	1.11	0.46	-1	0.9639	1.1	0.2407	1.07	0.2267	-1.07	0.639
99102_at	1428194_at	Usp9x	ubiquitin specific protease 9, X chromosome	-1.15	0.34	1.42	0.0057	1.27	0.0941	1.39	0.0098	-1.01	0.9763
99103_at	1426111_x_at	Irf3	interferon regulatory factor 3	1.12	0.04	1.06	0.4402	1.02	0.7324	1.18	0.0277	1.34	0.0048
99104_at	1422651_at	Adipoq	adiponectin, C1Q and collagen domain conta	-1.23	0.68	-1.8	0.0771	-1.36	0.3329	-1.73	0.0895	1.27	0.3512
99106_at	1451366_at	Cops6	COP9 (constitutive photomorphogenic) hom	1.47	0.02	-1.04	0.2623	1	0.9501	-1.1	0.0157	-1.19	0.3299
99107_at	1451871_a_at	Ghr	Growth hormone receptor	-2.37	0.02	-1	0.9895	-1.18	0.2599	-1.2	0.1984	-2.06	0.1827
99108_s_at	1451501_a_at	Ghr	Growth hormone receptor	-6.32	0	1.09	0.1611	-1.24	0.1102	-1.11	0.196	-1.48	0.0028
99109_at	1416442_at	Ier2	immediate early response 2	1.53	0.23	-1.23	0.0176	-1.42	0.0004	-1.2	0.0269	-1.6	0.0898
99111_at	1416541_at	Skd3	suppressor of K+ transport defect 3	1.77	0.13	1.08	0.1917	1.06	0.2388	-1.04	0.6951	-1.51	0.0236
99112_at	1416954_at	Slc25a10	solute carrier family 25 (mitochondrial carrier	1.53	0.02	1.3	0.0115	-1.15	0.2676	1.15	0.1233	-1.44	0.3
99113_at	1416678_at	Cops3	COP9 (constitutive photomorphogenic) hom	1.78	0.14	-1.14	0.0131	1.01	0.9415	-1.14	0.0785	1.04	0.7301
99115_at	1452133_at	Uqcrh	ubiquinol-cytochrome c reductase hinge prot	1.4	0.23	1.06	0.1155	1.23	0.005	1.16	0.0367	1.14	0.4824
99119_at	1448346_at	Cfl1	cofilin 1, non-muscle	-1.04	0.84	1.07	0.351	1.02	0.866	1.07	0.5029	-1.34	0.2109
99120_f_at	1436343_at	Chd4	chromodomain helicase DNA binding protein	-1.26	0.28	1.04	0.7229	-1.01	0.919	-1.06	0.5906	1.17	0.2322
99121_at	1452247_at	Fxr1h	fragile X mental retardation gene 1, autosom	-1.34	0.46	1.01	0.8777	1.17	0.1825	1.02	0.7817	1.83	0.0693
99123_s_at	1448887_x_at	Fxc1	fractured callus expressed transcript 1	1.16	0.59	-1.25	0.0051	1.07	0.486	-1.12	0.1858	-1.16	0.1673
99126_at	1427262_at	---	---	1.17	0.74	-1.02	0.9333	-2.28	0.0048	-1.56	0.1151	1.13	0.5452
99128_at	1416278_a_at	Atp5o	ATP synthase, H+ transporting, mitochondria	1.6	0.09	1.22	0.0502	1.24	0.0204	1.31	0.0168	1.27	0.0467
99129_at	1448212_at	Tnfsf5ip1	tumor necrosis factor superfamily, member 5	1.84	0.16	1.12	0.0844	1.21	0.308	1.32	0.0027	-1.48	0.2135
99130_at	1420370_s_at	Csnb	casein beta	1.25	0.65	-1.28	0.1051	1.14	0.3545	-1.26	0.3222	1.39	0.2757
99133_at	1425364_a_at	Slc3a2	solute carrier family 3 (activators of dibasic a	-1.03	0.87	-1.23	0.0067	-1.18	0.0536	-1.25	0.0074	-1.01	0.9598
99134_at	1421682_a_at	Tcte3	t-complex-associated testis expressed 3	1.18	0.62	1.5	0.113	1	0.9999	2.26	0.0006	1.45	0.4109
99135_at	1416819_at	Cdc37	cell division cycle 37 homolog (S. cerevisiae	1.31	0.44	-1.01	0.9103	-1.04	0.6885	-1.07	0.1001	-1.01	0.9496
99139_at	1428246_at	2310075A12Rik	RIKEN cDNA 2310075A12 gene	-1.24	0.49	1	0.9575	1.07	0.709	-1.06	0.4353	-1.13	0.3157
99140_at	1450880_at	Mrpl16	mitochondrial ribosomal protein L16	1.5	0.05	-1.03	0.6739	-1.09	0.1087	-1.05	0.3999	-1.11	0.4275
99141_at	1448241_at	Gm2a	GM2 ganglioside activator protein	-1.54	0.05	-1.28	0.0183	-1.31	0.0104	-1.24	0.0335	-1.09	0.6388
99142_at	1460674_at	MGI:1919154	membrane progestin receptor alpha	-1.03	0.9	-1.08	0.5191	-1.32	0.0171	-1.23	0.0428	-1.11	0.3965
99144_s_at	1423307_s_at	Tgoln1 /// Tgoln	trans-golgi network protein /// trans-golgi net	-1.28	0.14	-1.13	0.1947	-1.07	0.465	-1.26	0.0242	-1.12	0.426
99146_at	1423038_at	Stx6	syntaxin 6	1.34	0.65	-1.21	0.0404	-1.16	0.1759	-1.47	0.006	-1.57	0.4903
99147_at	1451745_a_at	Znhit1	zinc finger, HIT domain containing 1	1.41	0.26	-1.15	0.0207	-1.04	0.4882	-1.07	0.1702	1.04	0.7465
99148_at	1424828_a_at	---	---	1.82	0.06	1.32	0.0001	1.13	0.1775	1.26	0.0265	1.47	0.0671
99149_at	1416118_at	---	---	1.53	0.58	1.22	0.5568	-1.26	0.4899	1.32	0.5803	-1.32	0.4812
99150_at	1460308_a_at	Ict1	immature colon carcinoma transcript 1	1.8	0.01	-1.05	0.2313	1.06	0.3946	-1	0.9193	1.07	0.7121
99151_at	1448365_at	Exosc7	exosome component 7	1.16	0.52	-1.02	0.5149	-1.07	0.2811	-1.03	0.5878	-1.43	0.0911
99152_at	1415939_at	Fmod	fibromodulin	1.41	0.26	1.33	0.0236	1.18	0.218	1.47	0.0179	-1	0.9864
99153_at	1451178_at	Mrpl53	mitochondrial ribosomal protein L53	1.27	0.17	-1.01	0.784	1.22	0.0491	1.12	0.1937	-1.19	0.1224
99154_s_at	1451381_at	1810020D17Rik	RIKEN cDNA 1810020D17 gene	1.65	0.08	1.01	0.819	1.13	0.0471	1.07	0.525	-1.15	0.2864
99156_at	1451269_at	Pdzk11	PDZ domain containing 11	1.49	0.26	-1.17	0.0026	-1.22	0.0069	-1.11	0.1552	-1.9	0.3669
99157_at	1427895_at	2310004N24Rik	RIKEN cDNA 2310004N24 gene	1.87	0.26	1.3	0.0287	1.07	0.427	1.22	0.089	1.46	0.0391
99159_at	1416940_at	Ppif	peptidylprolyl isomerase F (cyclophilin F)	1.46	0.06	-1.13	0.1505	-1.07	0.5768	-1.01	0.9135	1.49	0.0246

99161_at	1417423_at	Grina	glutamate receptor, ionotropic, N-methyl D-a	-1.36	0.08	-1.11	0.1515	-1	0.9839	-1.11	0.3558	-1.23	0.0697
99162_at	1429043_at	Smndc1	survival motor neuron domain containing 1	-1.03	0.9	1.19	0.05	1.03	0.776	1.12	0.1275	1.11	0.3475
99164_at	1422797_at	MGI:1932697	mitogen activated protein binding protein inte	1.38	0.25	1.03	0.6585	-1.16	0.0047	-1.11	0.1209	-1.06	0.736
99166_at	1448540_a_at	0610012G03Rik	RIKEN cDNA 0610012G03 gene	1.24	0.12	1.15	0.0854	1.13	0.0046	1.19	0.0296	1.35	0.0429
99167_at	1417006_at	Commd4	COMM domain containing 4	1.57	0.4	1.48	0.0058	1.16	0.3244	1.41	0.0511	-1.26	0.2018
99168_at	1433429_at	Pigs	phosphatidylinositol glycan, class S	-1.37	0.04	-1.02	0.7734	-1.01	0.8088	1.03	0.6643	-1.2	0.0943
99169_at	1419743_s_at	Carm1	coactivator-associated arginine methyltransf	1.07	0.73	-1.1	0.2541	-1.1	0.2374	-1.13	0.1619	1.05	0.7843
99175_at	1450649_at	Gng10	guanine nucleotide binding protein (G protein	-1.12	0.67	-1.29	0.0761	-1.17	0.3166	-1.24	0.036	-1.52	0.0045
99176_at	1422516_a_at	Fibp	fibroblast growth factor (acidic) intracellular b	1.33	0.31	1.27	0.0002	1.27	0.0006	1.32	0.0003	1.44	0.0845
99178_at	1423091_a_at	Gpm6b	glycoprotein m6b	-1.18	0.81	1.37	0.0757	1.39	0.0588	1.11	0.6089	2.21	0.1322
99179_at	1431145_a_at	Cuedc2	CUE domain containing 2	1.47	0.06	1.14	0.1872	1.02	0.8386	1.22	0.049	1.42	0.5587
99180_at	1450873_at	Gtpbp4	GTP binding protein 4	-1.15	0.54	1.09	0.3683	1.08	0.347	1.12	0.1706	-1.01	0.9261
99182_at	1419461_at	Rpp14	ribonuclease P 14 subunit (human)	-1.7	0	1.05	0.4719	1.12	0.135	1.01	0.8585	1.03	0.7455
99183_at	1433591_at	AI553587	expressed sequence AI553587	1.05	0.87	1.07	0.3878	1.03	0.7223	1.05	0.3913	1.19	0.082
99184_at	1427981_a_at	Csad	cysteine sulfinic acid decarboxylase	-5.05	0	-1.13	0.5787	2.57	0.0238	-1.38	0.1799	-2.1	0.0057
99185_at	1428589_at	2810443J12Rik	RIKEN cDNA 2810443J12 gene	1.3	0.03	1.11	0.2202	1.2	0.0335	1.01	0.9114	1.05	0.7778
99186_at	1417910_at	Ccna2	cyclin A2	-1.17	0.55	1.17	0.3257	1.03	0.8463	1.15	0.3493	-1.16	0.651
99188_at	1451130_at	2010315L10Rik	RIKEN cDNA 2010315L10 gene	1.53	0.06	1.17	0.015	1.03	0.6123	1.15	0.0836	1.31	0.0999
99190_at	1434516_at	Pstk	phosphoseryl-tRNA kinase	1.51	0.13	1.11	0.2035	1.23	0.1076	1.04	0.7229	1.05	0.918
99191_at	1448405_a_at	Cri1	CREBBP/EP300 inhibitory protein 1	1.12	0.47	1.61	0.0014	1.52	0.003	1.8	0.0007	1.97	0.0085
99194_at	1452676_a_at	Pnpt1	polyribonucleotide nucleotidyltransferase 1	1.55	0.02	1.02	0.8026	1.25	0.0425	1.2	0.0132	-1.07	0.5017
99196_at	1428314_at	Pcnp	PEST-containing nuclear protein	1.21	0.35	-1.02	0.8672	-1	0.9887	-1.2	0.0353	-1.11	0.3
99197_at	1426547_at	Gc	group specific component	-1.18	0.24	1.03	0.3183	-1.02	0.5535	-1.03	0.3611	1.18	0.0391
99236_at	1419336_at	Lcn5	lipocalin 5	-3.24	0.19	1.11	0.7681	-1.45	0.2772	1.67	0.2132	-2	0.1667
99237_at	1450362_at	U55872	cDNA sequence U55872	-1.13	0.83	-1.41	0.1607	1.1	0.7426	1.73	0.3301	1.87	0.4654
99238_at	1449159_at	Gnb3	guanine nucleotide binding protein, beta 3	-1.02	0.96	1.16	0.432	-1.04	0.8737	-1.19	0.4914	1.55	0.319
99239_at	1421341_at	Axin2	axin2	-1.26	0.66	-1.13	0.4249	1.07	0.7565	-1.05	0.7434	1.16	0.6548
99320_at	1421735_a_at	St8sia5	ST8 alpha-N-acetyl-neuraminide alpha-2,8-s	-1.57	0.22	-1.5	0.0353	-1.2	0.4042	-1.56	0.0358	-1.35	0.2198
99321_at	1421729_a_at	Fert2	fer (fms/fps related) protein kinase, testis spe	1.45	0.26	1.1	0.813	1.05	0.8869	-1.13	0.7452	-1.2	0.4743
99322_at	1449464_at	Kcnq1	potassium voltage-gated channel, subfamily	1.12	0.86	-1.54	0.0107	-1.54	0.039	-1.12	0.6433	1.87	0.3519
99323_at	1421623_at	Il12rb2	interleukin 12 receptor, beta 2	-1.6	0.53	-1.2	0.5481	-1.1	0.7471	-1.01	0.9793	1.64	0.2041
99324_at	1421510_at	Prss7	protease, serine, 7 (enterokinase)	-1.42	0.45	1.16	0.3612	1.29	0.1425	-1.09	0.7497	1.51	0.3044
99326_at	1420584_at	Pla2g2c	phospholipase A2, group IIC	1.3	0.5	-1.26	0.0073	-1.2	0.0232	-1.18	0.0363	1.24	0.173
99327_at	1419722_at	Prss19	protease, serine, 19 (neuropsin)	-1.16	0.79	1.2	0.3471	-1.26	0.2507	1.02	0.8952	1.17	0.5322
99328_at	1450475_at	Dlx3	distal-less homeobox 3	1.3	0.59	-1.3	0.3564	1.18	0.4782	-1.1	0.6731	-1.49	0.1287
99329_at	1421378_s_at	Abcc1	ATP-binding cassette, sub-family C (CFTR/M	-1.12	0.71	1.04	0.9027	-1.06	0.8115	1.2	0.5952	1.63	0.1401
99330_at	1420703_at	Csf2ra	colony stimulating factor 2 receptor, alpha, lo	1.17	0.61	1.27	0.3332	1.01	0.9261	1.9	0.1642	1.02	0.9506
99331_at	1425968_s_at	Apeg1	aortic preferentially expressed gene 1	-1.97	0.3	-1.13	0.5832	1.04	0.9211	-1.15	0.5483	3.47	0.0212
99332_at	1422121_at	Oprd1	opioid receptor, delta 1	-2.31	0.32	1.3	0.3022	1.12	0.7306	1.18	0.5421	1.37	0.524
99333_at	1421712_at	Sele	selectin, endothelial cell	-1.54	0.64	1.37	0.1633	-1.05	0.8281	1.48	0.054	2.23	0.4116
99334_at	1425947_at	Ifng	interferon gamma	-6.87	0.13	2.44	0.4049	1.1	0.6577	2.07	0.3387	-1.58	0.2988
99335_at	1423855_x_at	Rpl17	ribosomal protein L17	1.31	0.47	1.18	0.2255	1.3	0.0227	1.19	0.0678	1.32	0.0476
99336_at	1416054_at	Rps5	ribosomal protein S5	1.19	0.05	1.19	0.0015	1.2	0.0115	1.31	0	1.73	0.0018
99337_at	1435128_at	Baiap2	brain-specific angiogenesis inhibitor 1-assoc	1.26	0.75	-1.19	0.4702	1.06	0.7251	1.06	0.7521	1.54	0.4646
99339_r_at	1422835_at	Kcnd2	potassium voltage-gated channel, Shal-relat	-2.21	0.37	1.79	0.2784	1.15	0.7435	1.4	0.59	2.07	0.1665
99340_at	1455168_a_at	Gnb2-rs1	guanine nucleotide binding protein, beta 2, r	1.52	0.06	1.07	0.1919	1.21	0.0203	1.16	0.006	1.26	0.285
99342_at	1449980_a_at	Gabrd	gamma-aminobutyric acid (GABA-A) recepto	-1.62	0	1.01	0.9006	1.03	0.7208	1.02	0.8036	1.23	0.3059
99345_at	1449659_s_at	Mphosph9	M-phase phosphoprotein 9	1.46	0.39	1.19	0.5864	1.26	0.4128	1.15	0.5499	-1.02	0.9377
99347_f_at	1419801_x_at	2700069A02Rik	Echinoderm microtubule associated protein	1.08	0.87	1.09	0.4268	1.17	0.1139	1.12	0.2552	-1.04	0.8693
99349_at	1421672_at	Il17	interleukin 17	-1.5	0.54	1.08	0.8388	1	0.9973	1.25	0.5277	1.02	0.9561
99350_at	1424926_at	Sec63	SEC63-like (S. cerevisiae)	-2.22	0.27	1.01	0.8975	-1.14	0.3741	-1.08	0.4563	-1.28	0.1156
99351_at	1441146_at	C76132	expressed sequence C76132	-1.38	0.28	-1.15	0.4841	-1.11	0.6648	-1.49	0.1024	-1.12	0.6445
99354_s_at	1441394_at	C76554	expressed sequence C76554	-1.42	0.41	1.11	0.711	1.01	0.9762	1.1	0.7456	1.39	0.3992

99356_r_at	1419864_x_at	Tnpo1	Transportin 1	1.07	0.77	-1.21	0.4975	1.06	0.7802	-1.18	0.4829	1.47	0.094
99357_at	1447956_at	C76614	expressed sequence C76614	1.36	0.56	-1.34	0.386	1.31	0.4767	1.16	0.6113	1.31	0.4131
99358_at	1442833_at	D15Ertd30e	DNA segment, Chr 15, ERATO Doi 30, expr	-1.34	0.35	1.22	0.1317	1.1	0.4542	1.19	0.0917	1.42	0.5708
99359_at	1440499_at	D9Ertd26e	DNA segment, Chr 9, ERATO Doi 26, expre	1.31	0.29	1.35	0.0179	1.42	0.1125	1.79	0.1025	1.34	0.2187
99360_at	1440936_at	D17Ertd141e	DNA segment, Chr 17, ERATO Doi 141, exp	1.42	0.48	1.07	0.7238	1.1	0.6213	1.08	0.6072	-1.61	0.1078
99361_at	1422228_at	Wnt8a	wingless-related MMTV integration site 8A	-1.02	0.94	1.14	0.6836	1.23	0.4477	1.31	0.4244	1.46	0.1117
99363_at	1443317_at	D6Ertd47e	DNA segment, Chr 6, ERATO Doi 47, expre	-3.01	0.2	-1.95	0.1133	-1.41	0.4483	-2.22	0.0467	1.32	0.3679
99364_at	1425114_at	Rbbp6	retinoblastoma binding protein 6	1.41	0.38	1.06	0.5424	-1.15	0.4079	1.1	0.3335	1.83	0.3365
99365_at	1436351_at	Coq3	coenzyme Q3 homolog, methyltransferase (y	1.37	0.07	-1.22	0.0036	-1.15	0.019	-1.28	0.0009	-1.39	0.0031
99370_at	1426182_a_at	Klrc1	killer cell lectin-like receptor subfamily C, me	-1.14	0.44	-1.29	0.0287	-1.15	0.2168	-1.08	0.4583	1.47	0.0523
99371_at	1426070_a_at	Kcnd3	potassium voltage-gated channel, Shal-relat	-3.01	0.26	1.3	0.4133	-1.03	0.9316	1.53	0.1482	-1.44	0.5093
99373_at	1422179_at	Gjb4	gap junction membrane channel protein beta	-1.2	0.15	-1.11	0.7576	1.02	0.9333	-1.1	0.7715	-1.6	0.4532
99375_at	1423009_at	MGI:1928893	secretory blood group 1	1.43	0.27	-1.5	0.0886	-1.22	0.2969	-1.11	0.5519	1.23	0.6628
99376_at	1421762_at	Kcnj5	potassium inwardly-rectifying channel, subfa	-1.58	0.02	-1.14	0.3281	-1.09	0.3541	-1.08	0.4756	1.46	0.1285
99377_at	1453644_at	Obp1a	odorant binding protein Ia	-4.37	0.37	-1.52	0.4251	-1.15	0.7936	-1.05	0.9136	1.36	0.7362
99380_at	1422197_at	Kcna2	potassium voltage-gated channel, shaker-re	1.02	0.97	-1.24	0.4026	-1.14	0.6334	1.18	0.5493	2.67	0.0835
99381_at	1450359_at	Fut1	fucosyltransferase 1	2.39	0.24	1.33	0.0358	1.09	0.6895	1.31	0.0536	1.34	0.1433
99382_at	1421393_at	Grin2d	glutamate receptor, ionotropic, NMDA2D (ep	1.1	0.57	-1.21	0.189	-1.25	0.1009	-1.21	0.1043	1.09	0.6547
99384_at	1423006_at	Pim1	proviral integration site 1	-1.09	0.78	1.04	0.711	-1.28	0.0596	-1.41	0.0486	1.22	0.5625
99385_at	1450831_at	Pou3f2	POU domain, class 3, transcription factor 2	-1.74	0.42	1.01	0.9206	1.31	0.3282	-1.07	0.3763	2.25	0.0513
99386_at	1422165_at	Pou3f4	POU domain, class 3, transcription factor 4	-2.7	0.39	1.04	0.7311	1.1	0.6722	-1.1	0.5395	-1.15	0.4903
99387_at	1450808_at	Fpr1	formyl peptide receptor 1	1.11	0.77	-1.08	0.7201	-1.41	0.0873	1.09	0.7447	1.32	0.6073
99393_at	1421282_at	Bmp5	bone morphogenetic protein 5	-1.39	0.2	-1.07	0.4213	-1.08	0.4664	-1.36	0.0059	1.05	0.7762
99398_at	1422280_at	Gzmk	granzyme K	1.94	0.34	-1.12	0.4441	1.18	0.5851	1.08	0.7437	-1.3	0.4618
99399_at	1421690_s_at	Agrp	agouti related protein	-1.4	0.09	1.18	0.0849	1.14	0.1576	1.2	0.0652	1.24	0.2469
99401_at	1422136_at	Uhmk1	U2AF homology motif (UHM) kinase 1	1.49	0.49	-2.44	0.0333	-1.65	0.2645	-1.59	0.2065	5.71	0.018
99402_at	1420794_at	Art2b	ADP-ribosyltransferase 2b	-1.43	0.66	-1.59	0.2765	-1.34	0.4738	-2.04	0.0743	2.53	0.1936
99403_at	1423008_at	Art2a	ADP-ribosyltransferase 2a	-8.75	0.24	-1.33	0.4905	-1.52	0.3729	-2.09	0.1212	2.62	0.2627
99404_at	1422100_at	Cyp7a1	cytochrome P450, family 7, subfamily a, poly	1.74	0.26	-1.18	0.3601	1.28	0.2664	-1.87	0.0074	-1.21	0.7353
99405_at	1427577_x_at	Igk-V8	Immunoglobulin kappa chain, constant regio	-1.05	0.74	-1	0.9716	-1.09	0.4588	-1.02	0.8212	1.41	0.2577
99406_at	1450834_at	Fut4	fucosyltransferase 4	-1.42	0.13	-1.36	0.2853	-1.46	0.2284	-1.01	0.9764	1.25	0.6322
99407_at	1422199_at	Omp	olfactory marker protein	-1.25	0.15	-1.4	0.3541	-1.08	0.8343	-1.42	0.3343	1.07	0.7968
99408_at	1421641_at	Slc6a2	solute carrier family 6 (neurotransmitter trans	-2.58	0.02	1.12	0.5744	-1.43	0.1473	-1.29	0.2788	1.27	0.144
99410_at	1422838_at	Kcnma3	potassium large conductance pH-sensitive c	-1.26	0.64	-1.48	0.1489	-1.96	0.0389	-1.51	0.1429	1.46	0.3222
99411_at	1422288_at	Htr1b	5-hydroxytryptamine (serotonin) receptor 1B	-1.96	0.01	-1.05	0.767	1.09	0.5258	-1.11	0.408	1.21	0.5962
99413_at	1419609_at	Ccr1	chemokine (C-C motif) receptor 1	1.79	0.2	1.71	0.3839	-1.56	0.1811	2.49	0.1248	2.65	0.099
99414_at	1450789_at	Rhpn1	rhophilin, Rho GTPase binding protein 1	-1.35	0.61	1.09	0.5196	1.47	0.089	1.08	0.5639	1.07	0.8539
99415_at	1423348_at	Fzd8	frizzled homolog 8 (Drosophila)	1.15	0.41	-1.01	0.8622	-1.35	0.0059	-1.15	0.0709	-1.06	0.8214
99416_at	1439283_at	Osbpl9	Oxysterol binding protein-like 9	2.74	0.28	2.42	0.0076	-1.51	0.3022	2.14	0.08	3.85	0.0174
99417_at	1421667_at	Nmur1	neuromedin U receptor 1	-1.83	0.34	2.19	0.0143	1.52	0.1886	1.75	0.0958	-1.12	0.7702
99419_g_at	1426334_a_at	Bcl2l11	BCL2-like 11 (apoptosis facilitator)	1.87	0.27	2.59	0.0759	2.01	0.0476	1.87	0.0584	-1.3	0.1915
99423_at	1422145_at	Mgat3	mannoside acetylglucosaminyltransferase 3	-1.29	0.11	1.29	0.1686	1.13	0.2021	-1.01	0.9008	1.16	0.6442
99424_at	1449958_a_at	Fgf14	fibroblast growth factor 14	-1.47	0.55	-1.09	0.3486	-1.03	0.8437	-1.06	0.709	1.27	0.4379
99427_at	1450553_at	Hoxd12	homeo box D12	-2.08	0.18	1.14	0.2024	1.53	0.0083	1.92	0.0703	1.14	0.6516
99430_at	1422942_at	Galr2	galanin receptor 2	-1.09	0.74	1.08	0.4354	1.04	0.722	1.01	0.9021	1.36	0.0279
99432_at	1422984_at	---	---	1.27	0.6	1.09	0.7104	1.05	0.8869	1.89	0.0553	1.42	0.6596
99433_at	1421411_at	Pstpip2	proline-serine-threonine phosphatase-intera	1.18	0.81	-1.4	0.0568	-1.48	0.0508	-1.65	0.006	-2.3	0.0732
99435_at	1422243_at	Fgf7	fibroblast growth factor 7	-1.34	0.25	-1.01	0.9474	1.08	0.7008	-1.03	0.8733	2.07	0.1043
99437_at	1450562_at	Ly6f	lymphocyte antigen 6 complex, locus F	-1.43	0.37	-1.2	0.6172	1.14	0.7288	1.18	0.7322	1.53	0.3362
99438_at	1457379_at	C78549	expressed sequence C78549	-1.45	0.04	1.65	0.0291	1.16	0.5478	1.54	0.168	1.49	0.3824
99440_at	1427680_a_at	Nfib	nuclear factor I/B	1.26	0.36	1.09	0.4035	-1.08	0.3902	-1.02	0.8591	1.1	0.7503
99441_at	1426462_at	Gphn	gephyrin	1.74	0	1.01	0.8289	-1.01	0.9198	1.11	0.1054	-1.38	0.0363
99442_at	1417988_at	Resp18	regulated endocrine-specific protein 18	-1.47	0.57	-1.26	0.0357	-1.24	0.1558	-1.28	0.0862	-1.08	0.8626

99444_at	1418187_at	Ramp2	receptor (calcitonin) activity modifying protei	1.02	0.8	-1.19	0.2302	1.1	0.3993	-1.3	0.0759	1.88	0.0088
99445_at	1428065_at	1110028E10Rik	RIKEN cDNA 1110028E10 gene	1.19	0.34	1.1	0.5507	1.19	0.2253	1.11	0.5557	-1.13	0.2658
99446_at	1423226_at	Ms4a1	membrane-spanning 4-domains, subfamily A	1.47	0.59	1.67	0.0437	1.18	0.6289	1.27	0.4005	2.86	0.0277
99447_at	1428528_at	1110007L15Rik	RIKEN cDNA 1110007L15 gene	-1.9	0.24	1.13	0.2006	1.07	0.5076	1.04	0.6933	1.4	0.1679
99448_at	1448402_at	Tln1	talin 1	-1.29	0.04	1.09	0.1502	-1.09	0.4215	1.06	0.45	1.13	0.4918
99449_at	1451595_a_at	Kcnq2	potassium voltage-gated channel, subfamily	1.93	0.05	-1.14	0.5631	1.19	0.42	-1.08	0.6992	1.17	0.6438
99450_at	1420800_a_at	Kcnq2	potassium voltage-gated channel, subfamily	-1.71	0.03	-1	0.9781	-1.13	0.4445	-1.63	0.0144	1.51	0.0786
99451_at	1426894_s_at	C230093N12Rik	RIKEN cDNA C230093N12 gene	-1.31	0.22	-1.61	0.0007	-1.16	0.1173	-1.38	0.0047	-1.32	0.1747
99452_at	1451255_at	MGI:1927471	liver-specific bHLH-Zip transcription factor	-1.36	0.03	-1.75	0.0003	-1.24	0.0963	-1.97	0.0001	-1.26	0.0331
99455_s_at	1451333_a_at	Acrbp	proacrosin binding protein	-1.18	0.62	1.01	0.9645	1.71	0.1021	1.14	0.6971	2.06	0.0221
99456_at	1422874_at	Acrbp	proacrosin binding protein	-1.03	0.9	1.03	0.8563	1.02	0.8507	1.02	0.8717	1.46	0.0037
99457_at	1426817_at	Mki67	antigen identified by monoclonal antibody Ki	11.02	0.08	2.45	0.2058	1.11	0.7759	3.37	0.103	1.41	0.5389
99460_at	1448415_a_at	Sema3b	sema domain, immunoglobulin domain (Ig),	-1.35	0.25	-1.09	0.4585	-1.03	0.7979	1.2	0.2806	2.46	0.3513
99461_at	1418842_at	Hcls1	hematopoietic cell specific Lyn substrate 1	3.5	0.05	1.24	0.2995	-1.53	0.0244	1.2	0.5585	2.51	0.1292
99462_at	1416731_at	Top2b	topoisomerase (DNA) II beta	1.69	0.11	-1.07	0.3922	1.09	0.4829	1.24	0.021	-1.03	0.8493
99463_at	1419523_at	Cyp3a13	cytochrome P450, family 3, subfamily a, poly	1.54	0.11	-1.14	0.1144	1	0.9644	1.04	0.6522	-1.25	0.3317
99464_at	1426829_at	Rxrip110	retinoid X receptor interacting protein 110	1.22	0.36	1.23	0.1646	1.29	0.0397	1.44	0.0031	1.08	0.5284
99465_at	1460246_at	Mecp2	methyl CpG binding protein 2	1.42	0.3	1.07	0.3737	-1.01	0.9346	1.09	0.2928	1.74	0.0111
99466_at	1452376_at	6230401O10Rik	RIKEN cDNA 6230401O10 gene	1.07	0.75	-1.34	0.0001	-1.25	0.0285	-1.43	0.0001	-1.16	0.7079
99467_at	1426476_at	Rasa1	RAS p21 protein activator 1	-1.05	0.78	1.19	0.0761	1.19	0.2968	1.13	0.1232	1.05	0.7772
99469_at	1451226_at	Pex6	peroxisomal biogenesis factor 6	1.12	0.64	-1.13	0.1673	1.02	0.826	-1.23	0.0636	1.14	0.3311
99471_at	1449628_s_at	Stard7	START domain containing 7	1.33	0.37	1.12	0.0793	1.14	0.1318	1.19	0.0661	-1.05	0.6316
99474_at	1424798_a_at	Adam5	a disintegrin and metalloprotease domain 5	-3.18	0.27	-1.02	0.9748	-1.22	0.4731	-1.5	0.1033	1.47	0.6053
99475_at	1449109_at	Socs2	suppressor of cytokine signaling 2	-10.57	0.05	-6.98	0.0003	1.03	0.8795	-7.86	0.0003	-1.98	0.0805
99476_at	1427168_a_at	Col14a1	procollagen, type XIV, alpha 1	-1.24	0.37	1.15	0.2501	1.11	0.381	1.08	0.5371	-1.03	0.9199
99478_at	1448511_at	Ptprcap	protein tyrosine phosphatase, receptor type,	-1.36	0.02	1.31	0.0015	1.1	0.2952	1.18	0.0626	1.21	0.1095
99479_at	1418287_a_at	Dmbt1	deleted in malignant brain tumors 1	-2.22	0.38	-2.2	0.3996	-1.23	0.7903	-2.49	0.3457	1.96	0.0913
99481_at	1427465_at	Atp1a2	ATPase, Na+/K+ transporting, alpha 2 polyp	1.44	0.13	1.17	0.1916	1.02	0.8673	1.01	0.931	1.14	0.6999
99485_at	1450885_at	---	---	1.31	0.37	1.34	0.0036	1.25	0.0225	1.27	0.002	1.14	0.3686
99488_at	1423510_at	Iapp	islet amyloid polypeptide	-1.45	0.31	1.28	0.1422	1.25	0.1137	1.28	0.1497	-1.17	0.2074
99489_at	1418253_a_at	Hspa4l	heat shock 70kDa protein 4 like	1.57	0.33	-1.64	0.0001	-1.43	0.0023	-1.83	0	-1.21	0.2264
99490_at	1423970_at	Thoc3	THO complex 3	1.35	0.35	1.03	0.7714	-1.16	0.2566	1.01	0.9155	1.99	0.1569
99491_at	1419455_at	Il10rb	interleukin 10 receptor, beta	1.07	0.74	1.14	0.2799	1.19	0.1081	1.29	0.2454	1.35	0.0483
99493_at	1453406_a_at	Rab28	RAB28, member RAS oncogene family	1.03	0.89	1.18	0.0028	1.27	0.0239	1.17	0.0477	1.13	0.5424
99494_at	1448443_at	Serpini1	serine (or cysteine) proteinase inhibitor, clad	-1.68	0.35	1.16	0.5427	-1.27	0.3683	1.4	0.1562	1.86	0.0675
99497_at	1424240_at	Arfip2	ADP-ribosylation factor interacting protein 2	-1.16	0.54	1.03	0.8459	1.08	0.4921	1.15	0.3005	1.06	0.8277
99499_at	1419122_at	Mettl1	methyltransferase-like 1	1.2	0.26	-1.06	0.5278	-1.17	0.2998	-1.12	0.4379	1.02	0.9328
99500_at	1417622_at	Slc12a2	solute carrier family 12, member 2	1.22	0.44	1.14	0.1497	-1.13	0.4103	-1.13	0.3533	-1.02	0.9259
99502_at	1449069_at	Zfp148	zinc finger protein 148	-1.47	0.59	1.1	0.5801	-1.3	0.1253	-1.24	0.218	1.29	0.2434
99503_at	1455958_s_at	9130017A15Rik	RIKEN cDNA 9130017A15 gene	1.66	0.25	-1.04	0.7022	1.59	0.0302	1.25	0.164	1.93	0.0855
99504_at	1451008_at	St8sia3	ST8 alpha-N-acetyl-neuraminide alpha-2,8-s	1.1	0.52	-1.46	0.1153	1.05	0.8044	-1.03	0.8867	-1.11	0.6653
99505_at	1451643_a_at	Rab4b	RAB4B, member RAS oncogene family	1.12	0.78	-1.05	0.3477	-1.08	0.4863	-1.08	0.3504	1.14	0.5408
99506_at	1451453_at	Dapk2	death-associated kinase 2	-1.42	0.08	-1.27	0.0024	1.04	0.3906	-1.29	0.0003	1.11	0.3747
99507_at	1418197_at	Ucp1	uncoupling protein 1 (mitochondrial, proton c	-1.11	0.53	-1.41	0.4923	-1.34	0.5731	-1.6	0.3753	1.27	0.6291
99508_at	1419200_at	Fxyd7	FXYD domain-containing ion transport regul	-1.24	0.4	-1.21	0.2002	-1.04	0.8339	-1.11	0.446	2.06	0.0586
99509_s_at	1425750_a_at	Jak3	Janus kinase 3	-1.97	0.11	1.08	0.1199	-1.14	0.0637	-1.01	0.8007	-1.37	0.0221
99510_at	1460419_a_at	Prkcb1	protein kinase C, beta 1	1.05	0.73	-1.09	0.5313	-1.09	0.2798	-1.12	0.2655	1.5	0.1228
99512_at	1434251_at	Cnot1	CCR4-NOT transcription complex, subunit 1	1.1	0.5	1.15	0.1267	1.39	0.0019	1.22	0.0594	-1.1	0.5289
99513_at	1448558_a_at	Pla2g4a	phospholipase A2, group IVA (cytosolic, calc	1.98	0.11	1.48	0.2024	1.01	0.9564	1.31	0.4462	-1.56	0.2831
99514_at	1428669_at	Bmyc	brain expressed myelocytomatosis oncogen	-1.72	0.36	-1.65	0.0071	-1.11	0.3757	-1.84	0.0005	-4.94	0.0002
99516_at	1422522_at	---	---	1.01	0.99	-1.01	0.9546	1.04	0.7494	1.08	0.7141	-1.67	0.1914
99518_at	1418454_at	Mfap5	microfibrillar associated protein 5	-1.16	0.76	1.09	0.6763	-1.19	0.5207	-1.72	0.0596	1.07	0.9283
99521_at	1421830_at	Ak3l1	adenylate kinase 3 alpha-like 1	2.34	0.19	1.42	0.0227	1.35	0.0143	1.67	0.0015	1.78	0.0132

99522_at	1450886_at	Gsg2	germ cell-specific gene 2	-1.55	0.56	-1.23	0.4572	-1.18	0.6781	1.26	0.4713	1.78	0.353
99525_at	1425817_a_at	Slc8a1	solute carrier family 8 (sodium/calcium exch	-3.46	0.15	-1.5	0.1301	-1.45	0.2196	-1.33	0.405	1.42	0.4079
99527_at	1417520_at	Nfe2l3	nuclear factor, erythroid derived 2, like 3	-2.29	0.4	1.17	0.7186	-1.38	0.3649	1.31	0.5652	2.65	0.0378
99528_at	1415795_at	Spin	spindlin	1.35	0.32	-1.03	0.7765	1.03	0.7754	-1.08	0.4326	-1.25	0.1201
99529_f_at	1419368_a_at	Rnf138	ring finger protein 138	1.77	0.11	1.33	0.0682	1.45	0.0048	1.58	0.0086	1.04	0.878
99530_at	1418418_a_at	Prlpc1	prolactin-like protein C 1	-1.78	0.46	-1.33	0.27	-1.26	0.3546	-1.13	0.6859	2.56	0.2613
99531_at	1418875_at	Syngr4	synaptogyrin 4	1.05	0.74	-1.06	0.4152	-1.06	0.3409	-1.25	0.0161	1.25	0.1929
99532_at	1423176_at	Tob1	transducer of ErbB-2.1	1.05	0.88	-1.02	0.8887	-1.35	0.0358	-1.09	0.4667	1.06	0.4753
99535_at	1425837_a_at	Ccrn4l	CCR4 carbon catabolite repression 4-like (S	3.87	0.27	-1.62	0.2195	-1.05	0.856	-1.33	0.387	2.17	0.1386
99536_at	1422852_at	Cib2	calcium and integrin binding family member	-1.25	0.19	-1.3	0.0183	1.05	0.7412	-1.11	0.2031	-1.02	0.9304
99537_at	1416585_at	Ruvbl1	RuvB-like protein 1	1.62	0.23	1.08	0.6274	1.33	0.0836	1.12	0.424	1.65	0.0335
99541_at	1435306_a_at	Kif11	kinesin family member 11	1.69	0.49	-1.03	0.9464	-1.11	0.8163	1.28	0.4458	1.33	0.2128
99542_at	1450962_at	Pdha2	pyruvate dehydrogenase E1 alpha 2	-1.98	0.29	1.13	0.4846	1.07	0.7015	-1.18	0.2703	1.12	0.7711
99544_at	1425228_a_at	Dguok	deoxyguanosine kinase	1.5	0.39	-1.01	0.9265	1.11	0.1015	1.06	0.6112	1.57	0.1821
99545_at	1432075_a_at	Tekt1	tektin 1	1.9	0.28	-1.58	0.2291	-1.34	0.47	-1.34	0.4504	1.35	0.2922
99546_at	1450694_at	Fkbp2	FK506 binding protein 2	1.25	0.4	-1.01	0.82	-1.02	0.7542	-1.01	0.853	1.2	0.0741
99548_at	1418752_at	Aldh3a1	aldehyde dehydrogenase family 3, subfamily	-1.83	0.12	1.54	0.0127	1.65	0.0181	1.37	0.1261	-1.28	0.3556
99549_at	1419662_at	Ogn	osteoglycin	1.5	0.08	-2.29	0	-2.09	0.0024	-1.5	0.0306	1.98	0.288
99551_f_at	1450709_at	Defcr5	defensin related cryptdin 5	-2.45	0.1	-1.04	0.9184	-1.2	0.4757	-1.2	0.528	1.13	0.7892
99552_at	1418673_at	Snai2	snail homolog 2 (Drosophila)	1.13	0.53	1.15	0.2021	1.28	0.027	1.18	0.0827	1.35	0.0465
99553_f_at	1453060_at	6530413N01Rik	RIKEN cDNA 6530413N01 gene	-1.21	0.11	-1.15	0.2509	-1.11	0.4449	-1.07	0.5706	-1.02	0.9229
99555_at	1449742_at	AA522020	expressed sequence AA522020	1.4	0.54	-1.34	0.3206	1.13	0.6575	1.22	0.5345	1.21	0.7201
99556_s_at	1450579_x_at	Sry	sex determining region of Chr Y	1.11	0.82	-1.23	0.1657	-1.33	0.0304	-1.39	0.053	1.83	0.0945
99558_at	1417861_at	---	---	-1.05	0.86	-1.05	0.3014	-1.04	0.691	-1.1	0.1804	-1.5	0.1237
99559_at	1415776_at	---	---	1.74	0.04	1.08	0.436	1.07	0.2081	1.03	0.8124	1.15	0.4263
99561_f_at	1448393_at	Cldn7	claudin 7	1.18	0.55	1.34	0.0994	1.3	0.6374	-1.11	0.5989	-1.11	0.6809
99562_at	1416340_a_at	Man2b1	mannosidase 2, alpha B1	1.13	0.54	1.2	0.0167	1.07	0.3853	1.31	0.0032	1.26	0.061
99563_at	1415794_a_at	Spin	spindlin	-1.1	0.62	-1.16	0.0165	1.22	0.1545	1.01	0.76	-1.04	0.8099
99564_at	1415810_at	---	---	-1.15	0.61	1.37	0.3314	1.51	0.0283	1.15	0.5804	1.2	0.5892
99566_at	1452927_x_at	Tpi1	triosephosphate isomerase 1	1.54	0.1	1.05	0.4096	1.11	0.1397	1.14	0.0015	-1.06	0.6505
99569_at	1460185_at	Krt2-18	keratin complex 2, basic, gene 18	-6.02	0	1.39	0.2706	1.11	0.7835	1.37	0.3816	1.86	0.1496
99570_s_at	1452363_a_at	Atp2a2	ATPase, Ca++ transporting, cardiac muscle,	1.23	0.62	-1.25	0.0199	-1.06	0.6243	-1.17	0.054	-1.1	0.7547
99571_at	1424451_at	MGC29978	3-ketoacyl-CoA thiolase B	1.16	0.26	1.15	0.0644	1.03	0.5266	1.08	0.3429	-1.98	0.0227
99574_at	1434016_at	Znrf2	zinc and ring finger 2	-1.15	0.18	1.09	0.0496	1.07	0.1568	1.04	0.379	-1.13	0.377
99577_at	1415855_at	Kitl	kit ligand	2.3	0.06	-1.07	0.6382	1.17	0.3395	-1.04	0.8327	1.73	0.2898
99578_at	1454694_a_at	Top2a	topoisomerase (DNA) II alpha	1.37	0.36	1.94	0.1813	1.07	0.826	2.06	0.2791	2.62	0.1861
99580_s_at	1424783_a_at	Ugt1a2 /// Ugt1a	UDP-glucuronosyltransferase 1 family, mem	-1.09	0.47	-1	0.941	1.02	0.6423	-1.01	0.8102	1.35	0.0037
99581_at	1424018_at	Hint1	histidine triad nucleotide binding protein 1	1.21	0.24	1.05	0.1647	1.18	0.0637	1.22	0.0005	1.11	0.1616
99583_at	1449575_a_at	Gstp1	glutathione S-transferase, pi 1	-1.24	0.17	2	0.0001	1.8	0	2.47	0	1.1	0.3753
99584_at	1416401_at	Kai1	kangai 1 (suppression of tumorigenicity 6, pr	1.13	0.71	1.28	0.0025	1.12	0.3567	1.2	0.1275	1.07	0.7603
99585_at	1449611_at	Kai1	kangai 1 (suppression of tumorigenicity 6, pr	-1.44	0.17	-1.06	0.6481	-1.16	0.2466	-1.15	0.2688	1.19	0.5665
99586_at	1426195_a_at	Cst3	cystatin C	1.3	0.06	-1.01	0.8784	-1.19	0.0001	-1.06	0.481	-1.31	0.0899
99587_at	1415734_at	Rab7	RAB7, member RAS oncogene family	-1.18	0.4	1.15	0.1142	1.21	0.0937	1.27	0.029	1.33	0.0129
99589_f_at	1439379_x_at	Prm1	protamine 1	-2.51	0.05	1.2	0.569	-1	0.9841	-1.24	0.1853	2.59	0.0361
99590_at	1459986_a_at	Rps17	ribosomal protein S17	1.24	0.15	1.22	0.0026	1.26	0.0315	1.28	0.0006	1.5	0.0218
99593_at	1416495_s_at	Ndufs5	NADH dehydrogenase (ubiquinone) Fe-S pr	1.97	0.02	1.25	0.0027	1.34	0.0132	1.2	0.054	1.05	0.5112
99594_at	1416877_a_at	Mrpl51	mitochondrial ribosomal protein L51	1.46	0.02	1.01	0.8486	1.16	0.0669	1.11	0.2282	1.06	0.5906
99596_f_at	1435652_a_at	Gnai2	guanine nucleotide binding protein, alpha inl	-1.32	0.12	1.08	0.1907	-1.11	0.0005	1.04	0.4611	1.34	0.0022
99599_s_at	1438058_s_at	Ptov1	prostate tumor over expressed gene 1	-1.16	0.45	1.07	0.4165	-1.01	0.952	1.02	0.7742	-1.01	0.9488
99600_at	1416945_at	Ptov1	prostate tumor over expressed gene 1	1.24	0.37	1.18	0.0704	1.07	0.5814	1.22	0.0873	-1.12	0.407
99603_g_at	1416029_at	Tieg1	TGFB inducible early growth response 1	-1.56	0.16	-1.12	0.5153	1.8	0.0021	1.2	0.3154	-1.49	0.1867
99604_at	1428843_at	5-Mar	membrane-associated ring finger (C3HC4) 5	1.39	0.12	1.31	0.0018	1.14	0.0477	1.37	0	1.02	0.8454
99606_at	1452060_a_at	Limk2	LIM motif-containing protein kinase 2	1.4	0.12	1.05	0.681	-1.05	0.6006	1.02	0.8425	1.25	0.6293

99607_at	1423149_at	Skp1a	S-phase kinase-associated protein 1A	1.06	0.8	1.05	0.646	-1.57	0.0015	-1.34	0.0658	-1.41	0.0003
99608_at	1430979_a_at	Prdx2	peroxiredoxin 2	1.94	0.01	1.58	0.0427	1.27	0.2759	1.58	0.0265	1.36	0.3783
99610_at	1419360_a_at	Ss18	synovial sarcoma translocation, Chromosom	1.38	0.07	1.14	0.1948	1.09	0.4581	1.08	0.4111	-1.07	0.4455
99613_at	1448486_at	Mut	methylmalonyl-Coenzyme A mutase	1.15	0.12	1.32	0.0016	1.16	0.063	1.32	0.0192	-1.2	0.2289
99616_s_at	1423133_at	0610040D20Rik	RIKEN cDNA 0610040D20 gene	-1.1	0.82	1.39	0.0021	1.29	0.121	1.38	0.0036	1.17	0.4103
99617_at	1452147_at	Sec24c	SEC24 related gene family, member C (S. c	-1.04	0.78	1.08	0.0928	1.03	0.7011	1.09	0.038	1	0.9459
99618_at	1448292_at	Uqcr	ubiquinol-cytochrome c reductase (6.4kD) su	-1.01	0.99	1.17	0.0289	1.08	0.1089	1.16	0.0568	-1.21	0.3237
99619_at	1438790_x_at	Tmem41b	transmembrane protein 41B	-1	0.98	-1.31	0.0276	-1.15	0.2221	-1.41	0.0077	-1.37	0.0107
99620_at	1423796_at	Sfpq	splicing factor proline/glutamine rich (polypy	1.06	0.89	1.1	0.7244	1.11	0.7039	1.6	0.0087	1.22	0.6788
99622_at	1417394_at	Klf4	Kruppel-like factor 4 (gut)	-1.26	0.58	1.07	0.6801	-1.01	0.9634	-1.09	0.6269	1.51	0.324
99623_s_at	1426562_a_at	Olfm1	olfactomedin 1	1.07	0.84	1.38	0.2401	1.05	0.9073	1.28	0.3493	1.2	0.3654
99624_at	1427108_at	9530068E07Rik	RIKEN cDNA 9530068E07 gene	-1.2	0.09	-1.25	0.0002	-1.16	0.0696	-1.21	0.0022	-1.19	0.1091
99629_at	1416555_at	---	---	1.41	0.19	-1	0.9978	-1.27	0.0089	-1.23	0.0137	-1.81	0.0223
99630_at	1448699_at	Mrpl54	mitochondrial ribosomal protein L54	1.68	0.24	1.3	0.0393	1.22	0.1319	1.46	0.0153	-1.18	0.6123
99633_at	1416853_at	Ncdn	neurochondrin	1.05	0.8	1.09	0.4991	1.16	0.1951	1.16	0.2889	-1.05	0.8771
99635_at	1460728_s_at	Ing4	inhibitor of growth family, member 4	-1.45	0.01	-1.07	0.4574	-1.1	0.3045	-1.29	0.0009	1.22	0.13
99636_at	1422732_at	Poldip2	polymerase (DNA-directed), delta interacting	1.24	0.12	1.1	0.0608	1.14	0.0112	1.2	0.0049	1.05	0.3647
99637_at	1448755_at	Col15a1	procollagen, type XV	1.12	0.75	-2.28	0	-2.74	0	-5.98	0	-6.9	0.0001
99638_at	1426955_at	Col18a1	procollagen, type XVIII, alpha 1	-1.1	0.6	1.12	0.0878	1.04	0.5143	1.07	0.0758	-1.08	0.1917
99639_at	1448230_at	Usp10	ubiquitin specific protease 10	-1.16	0.22	1.41	0.0011	1.34	0.0085	1.45	0.0003	-1.32	0.0154
99640_at	1423265_at	Minpp1	multiple inositol polyphosphate histidine pho	1.08	0.88	1.27	0.0218	1.29	0.0051	1.42	0.0107	1.02	0.9266
99641_at	1451431_a_at	D2Bwg0891e	DNA segment, Chr 2, Brigham & Women's G	4.01	0.01	-1.09	0.4178	1.06	0.4399	-1.11	0.3294	1.05	0.8945
99643_f_at	1415949_at	Cpe	carboxypeptidase E	10.62	0.02	-1.05	0.9146	-2.09	0.1814	1.1	0.8155	1.84	0.3277
99644_at	1448672_a_at	Zfp289	zinc finger protein 289	-1.09	0.66	1.02	0.7552	1.12	0.066	1.11	0.1459	1.38	0.0159
99645_at	1428125_at	4921506J03Rik	RIKEN cDNA 4921506J03 gene	-1.03	0.93	-1.03	0.6241	-1.11	0.303	-1.17	0.0475	-1.29	0.2414
99647_at	1416705_at	Rpe	ribulose-5-phosphate-3-epimerase	-1.51	0.19	1.15	0.3632	1.13	0.4351	-1.07	0.7186	-1.08	0.7635
99649_at	1455959_s_at	D9Wsu168e	DNA segment, Chr 9, Wayne State Universit	-1.54	0.05	1.53	0	1.48	0.0024	1.72	0.0028	-1.24	0.0864
99650_at	1424827_a_at	Csnk1a1	casein kinase 1, alpha 1	1.01	0.93	-1.02	0.7454	-1.07	0.3691	-1.1	0.0817	-1.07	0.3968
99651_at	1460697_s_at	2610209M04Rik	RIKEN cDNA 2610209M04 gene	1.26	0.56	1.47	0.014	1.23	0.1864	1.82	0	1.11	0.3209
99652_at	1460709_a_at	Bat5	HLA-B associated transcript 5	-1.05	0.84	-1.02	0.7144	-1.07	0.4506	1.02	0.7578	1.11	0.5387
99653_at	1438626_x_at	Rpl14	ribosomal protein L14	-1.12	0.49	-1.13	0.0536	-1.02	0.6642	-1.12	0.0946	-1.57	0.2139
99654_s_at	1434227_at	---	---	-2.14	0.21	-1.65	0.0691	-1.39	0.1627	-1.35	0.1165	1.18	0.3984
99655_at	1418223_at	Sec11l1	Sec11-like 1 (S. cerevisiae)	1.16	0.25	-1.31	0.0001	-1.07	0.1772	-1.18	0.03	-1.44	0.0172
99656_at	1451249_at	D8Ertd812e	DNA segment, Chr 8, ERATO Doi 812, expr	-1.12	0.79	1.04	0.3273	1.08	0.0766	1.15	0.0308	-1.01	0.9842
99659_r_at	1419547_at	Fahd1	fumarylacetoacetate hydrolase domain conta	-1.1	0.85	1.68	0.0152	1.71	0.0408	1.58	0.0326	1.16	0.5401
99661_r_at	1448112_at	Cox7c	cytochrome c oxidase, subunit VIIc	-1.22	0.33	-1.02	0.7908	1.06	0.4285	-1.04	0.5263	1.22	0.031
99664_at	1416073_a_at	Pcnt1	pericentrin 1	-1.88	0.42	1.97	0.138	1.87	0.1932	1.87	0.1991	2.14	0.215
99665_at	1416007_at	Satb1	special AT-rich sequence binding protein 1	1.01	0.96	1.05	0.7009	-1.09	0.5199	1.16	0.2224	1.47	0.1249
99666_at	1450667_a_at	Cs	citrate synthase	-1.16	0.3	-1.28	0.0007	-1.03	0.616	-1.22	0.0058	-1.54	0.0004
99667_at	1417607_at	Cox6a2	cytochrome c oxidase, subunit VI a, polypep	-2.72	0.06	-3.39	0.125	-2.47	0.2019	-3.39	0.1234	1.51	0.2742
99669_at	1419573_a_at	Lgals1	lectin, galactose binding, soluble 1	2.97	0.01	1.83	0.0012	1.02	0.8461	1.83	0.0002	1.5	0.0565
99670_at	1416583_at	Bad	Bcl-associated death promoter	1.42	0.62	-1.18	0.2981	-1.05	0.806	-1.09	0.4728	1.5	0.5281
99671_at	1417867_at	Adn	adipsin	-2.12	0.12	-5.95	0.15	-4.04	0.1914	-5.52	0.1558	-1.47	0.5171
99672_at	1448161_a_at	Clcn4-2	chloride channel 4-2	-1.09	0.77	-1.16	0.0443	-1.07	0.438	-1.35	0.01	-1.42	0.4428
99674_at	1415681_at	Mrpl43	mitochondrial ribosomal protein L43	1.28	0.17	1.03	0.7213	1.04	0.4897	1.02	0.7686	-1.06	0.755
99675_at	1423940_at	Yif1	Yip1 interacting factor homolog (S. cerevisia	1.44	0.18	-1.08	0.5385	-1.01	0.8995	-1.26	0.0498	1.06	0.6734
99677_at	1450057_at	2610042O14Rik	RIKEN cDNA 2610042O14 gene	1.01	0.94	-1.01	0.8486	-1.02	0.6855	-1.05	0.2678	-1.04	0.7994
99681_at	1420223_at	---	---	1.13	0.74	1.37	0.2096	1.82	0.054	-1.14	0.5624	1.13	0.726
99699_at	1427828_at	---	Murine retrovirus readthrough RNA sequenc	1.28	0.57	1.31	0.4041	1.12	0.3774	1.37	0.1965	1.23	0.7818
99700_at	1421719_at	V2r1b	vomeronasal 2, receptor, 1b	-1.46	0.52	-1.46	0.0468	-1.3	0.206	-1.74	0.0125	1.67	0.1568
99702_at	1450617_at	---	---	-1.74	0.05	-1.08	0.7735	1.09	0.7542	1.13	0.6296	1.44	0.0746
99708_at	1449805_at	Isp2	Implantation serine protease 2	-3.44	0.07	-1.68	0.1603	1.14	0.7623	-1.42	0.3086	-1.13	0.7837
99779_at	1446244_at	D6Bwg1452e	DNA segment, Chr 6, Brigham & Women's G	-1.06	0.55	-1.05	0.7117	-1.1	0.4219	1.06	0.5657	1.33	0.4251

99799_at	1422932_a_at	Vav1	vav 1 oncogene	-1.02	0.95	1.87	0.1248	-1.08	0.7982	2.56	0.1872	1.45	0.1092
99800_at	1421958_at	L1cam	L1 cell adhesion molecule	-1.01	0.98	1.05	0.8652	1.13	0.6555	1.3	0.3863	2.16	0.0376
99801_at	1422058_at	Nodal	nodal	1.1	0.81	1.09	0.5166	1.07	0.5242	1.04	0.6966	-1.06	0.8403
99802_at	1450003_at	Adra2b	adrenergic receptor, alpha 2b	-1.36	0.62	1.98	0.048	1.14	0.6047	1.15	0.5588	1.03	0.9015
99804_at	1422335_at	Adra2c	adrenergic receptor, alpha 2c	-1.48	0.45	-1.09	0.3201	-1.05	0.5981	-1.15	0.3105	1.96	0.0375
99806_at	1450286_at	Npr3	natriuretic peptide receptor 3	-2.77	0.23	-1.34	0.3908	-1.02	0.9659	1.12	0.7752	-3.59	0.2316
99807_r_at	1422279_at	Fv1	Friend virus susceptibility 1	-1.29	0.62	-1.16	0.6852	-1.76	0.0619	1.05	0.8589	1.14	0.7807
99808_at	1419576_at	Hoxb13	homeo box B13	-1.26	0.66	-1.17	0.1494	-1.07	0.5414	-1.05	0.6487	-1.72	0.1383
99810_at	1449279_at	Gpx2	glutathione peroxidase 2	-1.05	0.85	-1.12	0.1602	-1.08	0.3747	-1.2	0.031	1.4	0.1433
99811_at	1422347_at	Npy6r	neuropeptide Y receptor Y6	-1.83	0.07	-1.2	0.141	1.07	0.5042	-1.29	0.0446	-1.24	0.468
99813_g_at	1426043_a_at	Capn3	calpain 3	1.48	0.38	-1.28	0.3147	1.26	0.3151	1.17	0.3471	1.39	0.3698
99814_at	1422312_a_at	Neurog3	neurogenin 3	-2.48	0.06	-1.01	0.9636	1.13	0.3067	-1.1	0.3377	1.43	0.1277
99816_at	1417101_at	Hspa2	heat shock protein 2	-1.13	0.8	1.81	0.0562	1.5	0.1393	1.98	0.0127	1.96	0.2623
99817_at	1450303_at	Vax2	ventral anterior homeobox containing gene 2	-1.95	0.23	1.03	0.9342	-1.01	0.98	-1.05	0.8803	1.06	0.9166
99818_at	1442597_at	C79122	expressed sequence C79122	-2.17	0.37	1.11	0.5058	-1.12	0.4424	-1.14	0.2988	-1.27	0.596
99819_at	1442932_at	C79709	expressed sequence C79709	-3.77	0.12	-1.66	0.0152	-1.04	0.8043	-1.27	0.2584	1.59	0.1233
99821_at	1443711_at	Chd8	Chromodomain helicase DNA binding protei	-1.28	0.63	-1.01	0.9256	-1.01	0.9529	-1.05	0.72	1.22	0.3132
99824_at	1457521_at	---	---	-1.76	0.17	1.5	0.1647	1.19	0.5748	1.19	0.4382	1.09	0.7501
99826_at	1419861_at	---	Transcribed locus	-3.81	0.15	1.3	0.684	1.03	0.9432	1.27	0.5496	-1.06	0.9129
99827_at	1429999_at	4933403M19Ril	RIKEN cDNA 4933403M19 gene	-3.58	0.02	-1.43	0.2274	-1.02	0.9531	-1.57	0.1319	-1.39	0.4282
99828_at	1456430_at	D3Ertd789e	DNA segment, Chr 3, ERATO Doi 789, expr	1.12	0.77	1.07	0.6958	1.68	0.0288	1.44	0.0144	1.61	0.3056
99830_at	1436066_at	Kalrn	kalirin, RhoGEF kinase	-1.05	0.93	1.04	0.9153	-1.29	0.4737	1.09	0.7753	-1.43	0.3605
99831_at	1449820_at	Cort	cortistatin	-1.07	0.93	1.02	0.9501	1.03	0.9305	-1.13	0.6143	-1.03	0.9097
99832_at	1421400_at	Kcnmb1	potassium large conductance calcium-activa	1.2	0.58	-1.57	0.0036	-1.49	0.0115	-1.8	0.0007	1.72	0.2236
99833_at	1422876_at	Capn9	calpain 9 (nCL-4)	-1.07	0.67	-1.38	0.0927	-1.01	0.9192	-1.45	0.0683	1.39	0.2027
99834_at	1460289_at	Nrg3	neuregulin 3	-1.49	0.56	-1.18	0.6846	1.1	0.7914	-2	0.066	-1.05	0.9068
99835_at	1417487_at	Fosl1	fos-like antigen 1	-2.36	0.15	1.9	0.0487	1.24	0.2182	1.09	0.3785	1.62	0.4196
99836_at	1427372_at	Cyp27b1	cytochrome P450, family 27, subfamily b, po	-2.84	0.05	-1.64	0.1077	1.1	0.7656	1.2	0.5267	-1.05	0.9259
99837_at	1450568_at	Galr1	galanin receptor 1	-1.44	0.5	1.07	0.6178	1.07	0.707	-1.02	0.9118	1.73	0.142
99839_at	1420735_at	Gabrr2	gamma-aminobutyric acid (GABA-C) recepto	1.23	0.41	1.33	0.0761	1.12	0.3501	1.01	0.9538	1.21	0.7164
99840_at	1416266_at	Pdyn	prodynorphin	-1.09	0.73	1.15	0.1076	1.34	0.022	1.09	0.3202	-1.03	0.9315
99841_at	1422003_at	Blr1	Burkitt lymphoma receptor 1	1.71	0.02	-1.09	0.8341	1.16	0.7397	-1.37	0.4094	1.73	0.1675
99842_at	1421698_a_at	Col19a1	procollagen, type XIX, alpha 1	1.39	0.51	-1.06	0.7219	1.1	0.4513	1.06	0.7036	1.96	0.0866
99843_at	1449917_at	Pitx3	paired-like homeodomain transcription facto	-2.38	0.15	-1.1	0.2823	1.02	0.8573	-1.01	0.8905	-1.03	0.8791
99844_at	1427529_at	Fzd9	frizzled homolog 9 (Drosophila)	1.22	0.72	1.66	0.0227	1.48	0.0895	1.39	0.2295	-1.2	0.6818
99845_at	1418933_at	Slc1a6	solute carrier family 1 (high affinity aspartate	-1.07	0.88	-1.02	0.9346	-1.69	0.0358	-1.35	0.2674	1.75	0.0861
99846_at	1418220_at	Foxf2	forkhead box F2	-1.22	0.48	1.17	0.3117	1.33	0.103	1.34	0.0392	-1.24	0.5053
99847_at	1418946_at	St3gal1	ST3 beta-galactoside alpha-2,3-sialyltransfe	-1.18	0.12	-1.32	0	-1.39	0	-1.88	0	-1.03	0.8406
99848_at	1425376_at	Alox15b	arachidonate 15-lipoxygenase, second type	-1.68	0.36	-1.5	0.021	-1.23	0.0453	-1.32	0.0145	-1.05	0.8909
99851_at	1436308_at	Zfp292	zinc finger protein 292	-1.11	0.31	-1.19	0.0213	-1.18	0.0699	-1.25	0.007	1.27	0.0322
99854_at	1421669_at	Sult3a1	sulfotransferase family 3A, member 1	-2.82	0.38	-165.01	0	-4.28	0.0001	-205.69	0	2.32	0.0193
99855_at	1421340_at	Map3k5	mitogen activated protein kinase kinase kina	-1.18	0.52	1.15	0.1859	1.32	0.06	1.19	0.1527	1.68	0.0924
99856_r_at	1422592_at	Catnd2	catenin delta 2	-1.74	0.03	-1.21	0.2288	-1.26	0.1312	-1.27	0.1016	1.22	0.2662
99861_at	1422127_at	Dhh	desert hedgehog	-1.72	0.14	-1.12	0.4569	-1.06	0.7006	-1.65	0.0037	1.03	0.9382
99862_at	1455093_a_at	Ahsg	alpha-2-HS-glycoprotein	-1.04	0.81	1.04	0.2382	1.02	0.237	1.01	0.6327	1.38	0.0083
99863_at	1433467_at	Slc7a6	solute carrier family 7 (cationic amino acid tr	-1.37	0.42	1.07	0.4455	1.08	0.3003	1.16	0.3	1.32	0.4863
99864_at	1434772_at	Adora2b	adenosine A2b receptor	-1.2	0.59	-1.06	0.8195	-1.07	0.8074	-1.55	0.129	-1.32	0.6191
99865_at	1419616_at	Bmpr2	bone morphogenic protein receptor, type II (	1.62	0.39	-1.24	0.3675	-1.02	0.9429	-1.18	0.4986	1.65	0.0459
99866_at	1445689_at	Rbms1	RIKEN cDNA 6030432P03 gene	-2.54	0.03	1.22	0.3957	-1.52	0.0218	-1.24	0.3126	1.67	0.1879
99867_at	1419800_at	---	---	-1.06	0.88	1.59	0.0596	-1.02	0.851	-1.03	0.7614	-1.09	0.8147
99868_at	1419875_at	---	Transcribed locus	-1.22	0.77	1.15	0.6002	1.37	0.226	1.11	0.7561	3.35	0.0412
99869_at	1421293_at	Hdgfl1	hepatoma derived growth factor-like 1	-1.43	0.2	-1.15	0.681	-1.15	0.5801	-1.7	0.0728	1.58	0.1863
99872_s_at	1422302_s_at	Ftl1 /// Ftl2 /// Gı	ferritin light chain 1 /// ferritin light chain 2 ///	-1.14	0.56	-1.06	0.1367	-1.11	0.3581	-1.07	0.1603	1.14	0.0829

99873_at	1452018_at	Nkx2-6	NK2 transcription factor related, locus 6 (Drc	1.17	0.61	1.26	0.0686	1.11	0.3256	1.29	0.0218	1.41	0.0655
99874_at	1439548_at	Rap2b	RAP2B, member of RAS oncogene family	-2.02	0.33	-1.15	0.5738	-1.1	0.628	-1.09	0.768	2.03	0.2183
99875_at	1422110_at	Hr	hairless	-2.06	0.05	1.16	0.1848	1.12	0.3052	1.1	0.2922	-1.35	0.2696
99876_at	1420818_at	Sla	src-like adaptor	-1.68	0.13	-1.5	0.172	-1.48	0.1947	-1.08	0.8449	-1.12	0.6843
99880_at	1422109_at	Rfx1	regulatory factor X, 1 (influences HLA class	1.03	0.79	1.31	0.1014	1.23	0.2945	1.45	0.2139	-1.23	0.4044
99881_at	1420360_at	Dkk1	dickkopf homolog 1 (Xenopus laevis)	-2.98	0.41	1.28	0.5327	-1.53	0.2172	-1.34	0.3902	-2.33	0.0585
99883_g_at	1450166_at	Ids	iduronate 2-sulfatase	-2.31	0.01	-1.06	0.4792	-1.09	0.4389	-1.13	0.1693	1.29	0.2459
99884_at	1427813_at	Ids	iduronate 2-sulfatase	-1.32	0.28	1.1	0.6709	1.44	0.3461	1.24	0.308	1.14	0.71
99885_at	1451958_at	Igh-6	Immunoglobulin heavy chain 6 (heavy chain	2.38	0.05	-1.07	0.8525	-1.11	0.7927	-1.26	0.5353	2.74	0.2731
99886_at	1451813_at	Oprk1	opioid receptor, kappa 1	-1.56	0.32	-1.44	0.1205	1.16	0.4609	1.18	0.5039	2.82	0.0467
99887_at	1421796_a_at	Efna5	ephrin A5	-1.42	0.67	-1.52	0.1348	-1.05	0.8632	-1.27	0.4138	1.02	0.9824
99888_at	1421614_at	Zan	zonadhesin	-1.93	0.01	1.06	0.5695	1.17	0.096	-1.08	0.4662	1.38	0.1647
99891_at	1420749_a_at	Pou6f1	POU domain, class 6, transcription factor 1	1.83	0.1	-1.02	0.9264	1.29	0.3046	-1.82	0.021	-1.08	0.8781
99892_at	1419425_at	Cnr1	cannabinoid receptor 1 (brain)	-1.63	0.08	-1.42	0.0327	-1.29	0.1094	-1.44	0.0406	-1.17	0.1705
99893_at	1418498_at	Fgf13	fibroblast growth factor 13	-1.48	0.54	1.03	0.8425	1.11	0.5429	1.07	0.6422	1.43	0.0721
99895_at	1422291_at	Ccr8	chemokine (C-C motif) receptor 8	-1.11	0.64	-1.07	0.6552	-1.01	0.956	-1.11	0.4925	1.07	0.643
99896_at	1460723_at	Mc5r	melanocortin 5 receptor	-1.87	0.06	1.1	0.4924	-1.02	0.8877	-1.04	0.7559	1.43	0.0188
99897_at	1421189_at	Gabrb3	gamma-aminobutyric acid (GABA-A) recepto	-1.29	0.57	-1.47	0.2846	1.11	0.789	1.03	0.9272	1	1
99898_at	1417944_at	Gng4	guanine nucleotide binding protein (G protei	-1.88	0.19	-1.08	0.5597	1.06	0.7725	-1.14	0.1928	1.1	0.7941
99899_at	1450357_a_at	Ccr6	chemokine (C-C motif) receptor 6	-1.1	0.49	1.25	0.2226	2.68	0.0669	2.01	0.1718	1.07	0.773
99900_at	1449185_at	Crx	cone-rod homeobox containing gene	-2.12	0.02	-1.02	0.825	-1.1	0.339	-1.1	0.5603	1.28	0.3043
99901_at	1421431_at	Ptrf	polymerase I and transcript release factor	-1.43	0.21	-1.05	0.5595	-1.01	0.8718	-1.02	0.8805	1.37	0.0688
99902_at	1450193_at	Hcn1	hyperpolarization-activated, cyclic nucleotide	-1.2	0.56	1.04	0.6787	1.1	0.4188	1.03	0.7655	1.12	0.724
99903_at	1426005_at	Dmp1	dentin matrix protein 1	-3.4	0.12	1.21	0.5697	1.01	0.9648	1.05	0.8673	1.35	0.4029
99904_at	1421511_at	Itgb3	integrin beta 3	-3.24	0.01	1.2	0.461	-1.02	0.9487	-1.21	0.4311	1.33	0.2594
99905_at	1450547_x_at	Dub2	deubiquitinating enzyme 2	-1.84	0.2	1.08	0.7049	-1.13	0.4272	-1.25	0.0564	1.57	0.338
99906_at	1420602_a_at	Esx1	extraembryonic, spermatogenesis, homeobo	-1.55	0.45	-2.91	0.001	-2.34	0.0099	-2.19	0.0123	1.22	0.3053
99907_at	1426033_at	Rgs9	regulator of G-protein signaling 9	-2.15	0.22	-1.21	0.5119	-1.28	0.271	-1.54	0.1201	1.71	0.3207
99908_at	1451598_at	Pax4	paired box gene 4	-1.93	0.04	1.11	0.6668	1.14	0.6279	1.29	0.3445	1.7	0.0407
99909_at	1449431_at	Trpc6	transient receptor potential cation channel, s	-4.56	0.35	1.91	0.2245	1.61	0.2701	1.36	0.5337	2.73	0.2007
99910_at	1417994_a_at	Accn1	amiloride-sensitive cation channel 1, neuron	1.32	0.51	1.34	0.3649	-1.15	0.2455	-1.15	0.3256	1.61	0.1026
99911_at	1448790_at	Sema6b	sema domain, transmembrane domain (TM)	-2.45	0.26	1.46	0.0713	1.35	0.0971	1.34	0.0299	-1.01	0.9731
99912_at	1422116_at	Fmr2	fragile X mental retardation 2 homolog	1.56	0.45	1.06	0.8467	-1.12	0.6872	-1.1	0.8148	-1.06	0.9061
99913_at	1420569_at	Chad	chondroadherin	1.04	0.84	-1.15	0.2892	-1.27	0.0972	-1.17	0.2241	1.25	0.2559
99914_at	1422221_at	Hand2	heart and neural crest derivatives expressec	1.38	0.37	-1.14	0.6937	1.25	0.4541	-1.09	0.787	1.44	0.1326
99915_at	1421134_at	Areg	amphiregulin	-1.51	0.26	-1.28	0.373	-1.03	0.905	-1.08	0.7509	1.55	0.3967
99916_at	1422079_at	Prkch	protein kinase C, eta	1.09	0.88	2.23	0.0128	1.71	0.1735	2.79	0.0347	-1.13	0.7899
99917_at	1416544_at	Ezh2	enhancer of zeste homolog 2 (Drosophila)	1.42	0.15	1.42	0.0138	1.22	0.0365	1.34	0.0018	1.41	0.0605
99920_at	1418380_at	Terf1	telomeric repeat binding factor 1	1.83	0.31	1.26	0.2125	1.37	0.021	1.31	0.0386	1.01	0.9868
99922_at	1417397_at	Slc9a1	solute carrier family 9 (sodium/hydrogen exc	-1.61	0.14	1.06	0.527	-1.04	0.5333	-1.03	0.5705	1.01	0.9479
99923_at	1427160_at	2500001H09Rik	RIKEN cDNA 2500001H09 gene	1.29	0.22	1.15	0.2532	1.21	0.3026	1.04	0.7642	1.02	0.8723
99924_at	1417144_at	Tubg1	tubulin, gamma 1	-1.69	0.02	-1.01	0.8993	-1.09	0.4462	1.11	0.3566	-2.33	0.0764
99926_at	1455490_at	Pigr	polymeric immunoglobulin receptor	1.07	0.69	-1.04	0.5582	1.02	0.6478	-1.09	0.1252	1.3	0.0807
99927_at	1418724_at	Cfi	complement component factor i	-1.18	0.12	-1.05	0.3072	-1.08	0.2941	-1.14	0.0471	-1.19	0.0434
99929_at	1460170_at	Ext2	exostoses (multiple) 2	-1.44	0.09	-1.1	0.2807	-1.2	0.0321	-1.07	0.3993	-1.07	0.6164
99931_at	1427010_s_at	Lama5	laminin, alpha 5	1.48	0.31	1.71	0.041	1.5	0.0693	2.13	0.0022	1.59	0.1904
99932_at	1416224_at	Zbtb17	zinc finger and BTB domain containing 17	1.42	0.26	1.07	0.4777	1.19	0.0589	1.02	0.8276	-1.16	0.4621
99933_at	1417703_at	Pvrl2	poliovirus receptor-related 2	-1.51	0.24	-1.2	0.0583	-1.12	0.2625	1.06	0.5237	1.06	0.8165
99934_at	1424456_at	Pvrl2	poliovirus receptor-related 2	-1.21	0.14	-1.08	0.1148	-1.1	0.2021	-1.03	0.6303	-1.2	0.4284
99935_at	1417749_a_at	Tjp1	tight junction protein 1	-1.2	0.41	-1.23	0.0487	-1.04	0.7844	-1.2	0.0756	1.02	0.8416
99936_at	1416238_at	Tie1	tyrosine kinase receptor 1	2.4	0.05	-1.07	0.6273	-1.03	0.8212	-1.26	0.1125	-1.01	0.9728
99937_at	1424234_s_at	Meox2	mesenchyme homeobox 2	-1.42	0.3	1.05	0.7859	1.05	0.7678	-1	0.9654	1.14	0.5265
99938_at	1416587_a_at	Xrcc1	X-ray repair complementing defective repair	2.04	0.08	1.09	0.5076	1.12	0.1965	1.16	0.1416	1.3	0.4049

99939_at	1417257_at	Cel	carboxyl ester lipase	1.37	0.32	1.01	0.8586	-1.01	0.9382	-1.02	0.7445	-1.29	0.3208
99940_at	1416324_s_at	2410004N11Rik	RIKEN cDNA 2410004N11 gene	2.75	0.03	1.06	0.41	1.08	0.3251	1.08	0.2873	1.02	0.908
99941_at	1451600_s_at	LOC13909 /// Es	esterase 31-like /// esterase 31	-6.7	0	-4.54	0	-1.23	0.0666	-4.79	0	-11.48	0
99942_s_at	1417917_at	Cnn1	calponin 1	-4.29	0.17	-1.62	0.3295	-1.13	0.7764	-1.12	0.7947	1.42	0.4501
99944_at	1450930_at	Hpca	hippocalcin	-1.41	0.49	1.34	0.0434	1.09	0.6499	1.17	0.3371	2.01	0.096
99945_at	1450570_a_at	Cd19	CD19 antigen	1.18	0.45	1.15	0.0388	-1.03	0.6063	1.03	0.6784	1.53	0.0018
99947_at	1455676_x_at	Tial1	Tial1 cytotoxic granule-associated RNA bind	1.73	0.11	1.12	0.0025	1.32	0.0007	1.22	0.0012	1.15	0.3897
99948_at	1451347_at	AI225782	expressed sequence AI225782	1.74	0.14	1.13	0.5328	1.09	0.7455	1.53	0.0224	1.34	0.2338
99949_at	1424428_at	AI225782	expressed sequence AI225782	2.29	0.1	-1.14	0.3427	-1.31	0.0941	-1.23	0.1	-1.52	0.0986
99950_at	1426469_a_at	Tbp	TATA box binding protein	1.64	0.03	1.71	0.0628	2.33	0.0015	1.73	0.0292	1.93	0.1413
99951_at	1425793_a_at	Rorc	RAR-related orphan receptor gamma	1.61	0.29	1.15	0.4533	-1.23	0.1971	1.07	0.6421	1.4	0.0593
99952_at	1451537_at	Chi3l1	chitinase 3-like 1	8.26	0.07	22.92	0.1169	1.48	0.1575	21.74	0.0337	-3.63	0.0018
99953_at	1450688_at	Rgl2	ral guanine nucleotide dissociation stimulato	-1.01	0.97	1.28	0.0125	1.15	0.1937	1.36	0.0035	1.07	0.6641
99954_at	1451293_at	Rnu3ip2	RNA, U3 small nucleolar interacting protein :	-1.28	0.11	-1.17	0.0211	-1.06	0.5722	-1.17	0.0168	1.08	0.511
99955_at	1416891_at	Numb	numb gene homolog (Drosophila)	1.47	0.07	1.06	0.3617	1.15	0.1875	1.11	0.076	-1.32	0.0244
99956_at	1452514_a_at	Kit	kit oncogene	-1.36	0.13	-1.04	0.7448	1.02	0.9196	-1.16	0.3518	1.06	0.8027
99957_at	1416298_at	Mmp9	matrix metalloproteinase 9	1.13	0.61	1.27	0.0598	1.11	0.2297	1.39	0.0629	-1.1	0.7195
99958_at	1449989_at	Mcpt2	mast cell protease 2	-1.99	0.32	-1.81	0.0065	-1.52	0.1436	-1.66	0.0128	1.4	0.5614
99959_at	1450387_s_at	Ak3l1	adenylate kinase 3 alpha-like 1	2.16	0.16	1.51	0.0005	1.3	0.0249	1.6	0.0002	1.49	0.0909
99960_at	1426233_at	Map2k4	mitogen activated protein kinase kinase 4	-1.59	0.05	1.02	0.8092	1.05	0.5375	1.03	0.6285	-1.03	0.9402
99961_s_at	1418841_s_at	Cdc2l1	cell division cycle 2-like 1	1.85	0.13	-1.02	0.8251	1.04	0.6384	1.17	0.0432	-3.06	0.0013
99962_at	1450053_at	Kif2a	RIKEN cDNA D930043N17 gene	1.08	0.88	1.35	0.0052	1.12	0.4009	1.43	0.0003	-1.15	0.302
99963_at	1450090_at	Zfp101	zinc finger protein 101	2.97	0.21	-1.49	0.0977	-2.6	0.0043	-1.49	0.1095	1.35	0.3967
99964_at	1418176_at	Vdr	vitamin D receptor	-1.55	0.22	1.15	0.2973	1.73	0.3284	1.02	0.9027	1.77	0.3546
99970_at	1419054_a_at	Ptpn21	protein tyrosine phosphatase, non-receptor t	-1.17	0.64	1.31	0.0383	1.38	0.021	1.36	0.0222	1.75	0.0177
99972_at	1419524_at	Tph1	tryptophan hydroxylase 1	1.28	0.41	-1.04	0.7553	-1.05	0.6678	-1.11	0.4259	1.72	0.1638
99973_s_at	1450185_a_at	Kcnj15	potassium inwardly-rectifying channel, subfa	-1.11	0.76	-1	0.9965	-1.13	0.7253	-1.64	0.0754	1.98	0.3614
99975_at	1426483_at	Prkrir	protein-kinase, interferon-inducible double st	1	0.99	1.11	0.2339	-1.06	0.5267	1.21	0.0363	1.43	0.0295
99977_at	1450682_at	---	---	-1.81	0.09	-1.11	0.4239	1.26	0.1207	1.04	0.702	1.16	0.6717
99978_s_at	1416703_at	Mapk14	mitogen activated protein kinase 14	-1.06	0.82	-1.21	0.0054	-1.02	0.8303	-1.19	0.0169	-1.24	0.0269
99979_at	1416612_at	Cyp1b1	cytochrome P450, family 1, subfamily b, poly	1.73	0.36	-1.08	0.7889	-1.02	0.9435	1.17	0.649	1.25	0.4352
99980_at	1427454_at	Hoxc6	homeo box C6	-1.33	0.36	-1.08	0.7146	-1.18	0.336	-1.27	0.1648	1.25	0.597
99981_at	1450115_at	Gnaq	guanine nucleotide binding protein, alpha q p	-2.79	0.01	-1.1	0.5892	1.15	0.4013	-1.17	0.4256	1.14	0.3805
99982_at	1421266_s_at	Nfkbib	nuclear factor of kappa light chain gene enha	1.31	0.22	1.09	0.3266	-1.02	0.7821	1.19	0.07	-1.16	0.7415
99984_at	1417662_at	Elk3	ELK3, member of ETS oncogene family	-2.34	0.36	-1.11	0.5257	1.13	0.4064	-1.17	0.3446	3.1	0.0019
99985_at	1421529_a_at	Txnrd1	thioredoxin reductase 1	1.31	0.45	1.21	0.0054	1.1	0.1618	1.18	0.0403	-1.03	0.788
99986_at	1419372_at	Gosr2	golgi SNAP receptor complex member 2	-1.04	0.76	1.02	0.8526	1.04	0.7721	-1.08	0.3915	-1.04	0.841
99987_at	1456486_at	Zfp574	zinc finger protein 574	-1.25	0.4	1.74	0.0762	1.92	0.0581	1.66	0.1632	-1.2	0.5145
99988_at	1423736_a_at	Dym	dymeclin	1.42	0.22	-1.17	0.0209	-1.11	0.2549	-1.02	0.7821	-1.2	0.0505
99990_at	1426487_a_at	Rbbp6	retinoblastoma binding protein 6	-1.34	0.03	1.02	0.7598	1.27	0.0467	-1	0.9844	1.17	0.173
99991_at	1420904_at	Il17r	interleukin 17 receptor	1.24	0.65	1.14	0.2136	1.09	0.4842	1.3	0.0288	2.15	0.0027
99992_at	1420905_at	Il17r	Interleukin 17 receptor	1.22	0.34	1.05	0.5485	1.03	0.7725	1.11	0.2426	1.65	0.0043
99993_at	1421424_a_at	Anpep	alanyl (membrane) aminopeptidase	-1.13	0.5	1.07	0.6568	1.01	0.9463	-1	0.9871	1.07	0.7213
99994_at	1417956_at	Cidea	cell death-inducing DNA fragmentation facto	-1.98	0.22	-1.1	0.7564	-1.68	0.1998	-1.43	0.3399	1.36	0.2861
99995_at	1423203_a_at	Cetn1	centrin 1	2.39	0.04	-1.15	0.5886	-1.91	0.038	-1.51	0.1448	-1.48	0.131
99996_at	1449586_at	Pkp1	plakophilin 1	-1.62	0.23	-1.27	0.4122	-1.15	0.583	-1.39	0.2516	-1.01	0.9621
99999_at	1417791_a_at	Zfml	zinc finger, matrin-like	-1.19	0.4	1.08	0.347	1.15	0.2208	1.22	0.0258	1.08	0.6771
