# Supplementary material for: Delayed and Accelerated Aging Share Common Longevity Assurance Mechanisms
Source: PLoS Genet. 2008 Aug 15;4(8):e1000161. doi: 10.1371/journal.pgen.1000161 (PMC2493043; doi:10.1371/journal.pgen.1000161)
Supplement: Table S4 — Expression changes in the kidney, liver, lung and spleen of 130-week old as compared to 13-week old naturally aged mice. Expression profiles from these tables as well as all previously unpublished microarray data are available in public repository Array Express (www.ebi.ac.uk/arrayexpress/) and comply with the MIAME regulations. (3.7 MB PDF) [file pgen.1000161.s008.pdf]

**Supplementary table S4. Expression changes in the kidney, liver, lung and spleen of 130-week old as compared to 13-week old naturally aging wt mice**

| code | Gene Symbol | Gene Title | Kidney | p1 | Liver | p2 | Lung | p3 | Spleen | p4 | av. FC |
|---|---|---|---|---|---|---|---|---|---|---|---|
| 1433190_at | 4933433N18Rik | RIKEN cDNA 4933433N18 gene | -2.49 | 0.333 | -4.93 | 0.104 | -3.63 | 0.25 | -1.59 | 0.443 | -3.16 |
| 1447050_at | --- | --- | -3.47 | 0.057 | -3.95 | 0.051 | -2.33 | 0.244 | -1.32 | 0.503 | -2.77 |
| 1459319_at | Pola1 | Polymerase (DNA directed), alpha 1 | -4.66 | 0.176 | -3.66 | 0.003 | -2.05 | 0.241 | -1.43 | 0.584 | -2.95 |
| 1443451_at | --- | --- | -5.77 | 0.099 | -2.72 | 0.183 | -1.85 | 0.247 | -3.14 | 0.002 | -3.37 |
| 1460418_x_at | H2-T18 | histocompatibility 2, T region locus 18 | -2.51 | 0.488 | -3.16 | 0.095 | -2.68 | 0.216 | -1.57 | 0.524 | -2.48 |
| 1427977_x_at | Oog1 | oogenesin 1 | -2.26 | 0.393 | -2.97 | 0.146 | -3.15 | 0.123 | -1.2 | 0.672 | -2.39 |
| 1458907_at | Slco6b1 | Solute carrier organic anion transporter family, member 6b1 | -2.78 | 0.284 | -2.54 | 0.02 | -2.8 | 0.014 | -1.56 | 0.34 | -2.42 |
| 1420357_s_at | Xlr3a /// Xlr3b /// MC | X-linked lymphocyte-regulated 3A /// X-linked lymphocyte-regulated 3B /// hypothe | -4.4 | 0.036 | -1.81 | 0.272 | -2.99 | 0.057 | -3.56 | 0.076 | -3.19 |
| 1459139_at | --- | --- | -2.88 | 0.032 | -1.78 | 0.079 | -3.62 | 0.001 | -1.19 | 0.663 | -2.37 |
| 1419539_at | Irx4 | Iroquois related homeobox 4 (Drosophila) | -3.88 | 0.318 | -3.3 | 0.163 | -1.57 | 0.364 | -2.3 | 0.05 | -2.76 |
| 1443076_at | D030041N04Rik | RIKEN cDNA D030041N04 gene | -2.24 | 0.153 | -3.33 | 0.033 | -2.07 | 0.315 | -1.6 | 0.32 | -2.31 |
| 1416640_at | Kcne1l | potassium voltage-gated channel, Isk-related family, member 1-like | -4.63 | 0.096 | -1.78 | 0.196 | -2.17 | 0.206 | -1.15 | 0.848 | -2.43 |
| 1441853_at | Dpp10 | Dipeptidylpeptidase 10 | -1.83 | 0.213 | -2.9 | 0.058 | -2.87 | 0.352 | -1.37 | 0.539 | -2.24 |
| 1443070_at | Auts2 | Autism susceptibility candidate 2 | -1.31 | 0.593 | -3.74 | 0.054 | -4.67 | 0.057 | -1.36 | 0.711 | -2.77 |
| 1427800_at | Krtap16-2 | keratin associated protein 16-2 | -2.61 | 0.101 | -2.03 | 0.077 | -2.65 | 0.169 | -1.59 | 0.301 | -2.22 |
| 1436736_x_at | D0H4S114 | DNA segment, human D4S114 | -1.36 | 0.451 | -3.85 | 0.013 | -3.89 | 0.019 | -1.19 | 0.304 | -2.57 |
| 1443714_at | C530044N13Rik | RIKEN cDNA C530044N13 gene | -2.31 | 0.154 | -3.2 | 0.048 | -1.94 | 0.089 | -1.78 | 0.381 | -2.31 |
| 1437954_at | --- | --- | -2.36 | 0.224 | -2.79 | 0.331 | -2.04 | 0.02 | -1.05 | 0.936 | -2.06 |
| 1432440_at | 1700111E14Rik | RIKEN cDNA 1700111E14 gene | -2.06 | 0.302 | -3.02 | 0.076 | -2.16 | 0.34 | -1.17 | 0.208 | -2.1 |
| 1430938_at | Zfp64 | zinc finger protein 64 | -3.88 | 0.004 | -1.43 | 0.556 | -2.98 | 0.331 | -1.85 | 0.241 | -2.53 |
| 1448964_at | S100g | S100 calcium binding protein G | -2.46 | 0.048 | -2.14 | 0.029 | -2.32 | 0.093 | -1.63 | 0.511 | -2.14 |
| 1440001_at | Rian | RNA imprinted and accumulated in nucleus | -2.29 | 0.146 | -3.38 | 0.111 | -1.73 | 0.106 | -1.06 | 0.689 | -2.11 |
| 1444919_at | --- | --- | -2.11 | 0.142 | -3.43 | 0.053 | -1.82 | 0.422 | -1.16 | 0.828 | -2.13 |
| 1456139_at | Air | antisense Igf2r RNA | -1.68 | 0.057 | -2.84 | 0.208 | -2.68 | 0.01 | -1.17 | 0.708 | -2.09 |
| 1453507_at | Mbd5 | methyl-CpG binding domain protein 5 | -1.69 | 0.243 | -1.92 | 0.43 | -4.65 | 0 | -1.44 | 0.401 | -2.42 |
| 1432627_at | 5730507A11Rik | RIKEN cDNA 5730507A11 gene | -2.65 | 0.182 | -2.57 | 0.029 | -1.75 | 0.212 | -1.71 | 0.407 | -2.17 |
| 1427686_at | V2r13 /// LOC6326( | vomeronasal 2, receptor, 13 /// similar to vomeronasal 2, receptor, 8 | -2.05 | 0.434 | -2.21 | 0.272 | -2.49 | 0.299 | -1.27 | 0.809 | -2 |
| 1445745_at | Rbm17 /// LOC545( | RNA binding motif protein 17 /// similar to RNA binding motif protein 17 | -2.82 | 0.182 | -1.81 | 0.237 | -2.29 | 0.026 | -2.46 | 0.043 | -2.34 |
| 1458225_at | Fkbp14 | FK506 binding protein 14 | -3.17 | 0.113 | -2.12 | 0.43 | -1.79 | 0.273 | -1.44 | 0.566 | -2.13 |
| 1458967_at | --- | Transcribed locus | -1.48 | 0.685 | -3.42 | 0.004 | -2.58 | 0.009 | -1.07 | 0.865 | -2.14 |
| 1434672_at | Gpr22 | G protein-coupled receptor 22 | -2.55 | 0.367 | -2.53 | 0.198 | -1.75 | 0.293 | -2.15 | 0.319 | -2.24 |
| 1450806_at | Tlx3 | T-cell leukemia, homeobox 3 | -1.8 | 0.003 | -3.9 | 0.149 | -1.82 | 0.104 | -1.21 | 0.634 | -2.18 |
| 1459025_at | 1700001J04Rik | RIKEN cDNA 1700001J04 gene | -1.43 | 0.677 | -3.7 | 0.039 | -2.54 | 0.122 | -1.78 | 0.284 | -2.36 |
| 1453621_at | Thoc7 /// LOC6708: | THO complex 7 homolog (Drosophila) /// similar to Ngg1 interacting factor 3 like 1 | -2.45 | 0.487 | -2.42 | 0.25 | -1.84 | 0.375 | -1.68 | 0.274 | -2.1 |
| 1437258_at | Sprr2a | small proline-rich protein 2A | -3.62 | 0.053 | -1.99 | 0.163 | -1.69 | 0.227 | -1.38 | 0.66 | -2.17 |
| 1433023_at | 2310068G24Rik | RIKEN cDNA 2310068G24 gene | -2.52 | 0.198 | -2.26 | 0.014 | -1.88 | 0.028 | -1.42 | 0.332 | -2.02 |
| 1432950_at | 5330421F21Rik | RIKEN cDNA 5330421F21 gene | -3.67 | 0.206 | -3.31 | 0.144 | -1.25 | 0.347 | -1.42 | 0.622 | -2.41 |
| 1433847_at | D330017J20Rik | RIKEN cDNA D330017J20 gene | -2.14 | 0.144 | -3.2 | 0.194 | -1.65 | 0.058 | -1.39 | 0.178 | -2.09 |
| 1443270_at | --- | Adult male hypothalamus cDNA, RIKEN full-length enriched library, clone:A23002 | -2 | 0.093 | -1.79 | 0.039 | -3.03 | 0.009 | -1.06 | 0.913 | -1.97 |
| 1438483_at | Nos1 | nitric oxide synthase 1, neuronal | -4.83 | 0.108 | -1.55 | 0.411 | -1.85 | 0.271 | -1.93 | 0.262 | -2.54 |
| 1458569_at | Ell2 | elongation factor RNA polymerase II 2 | -1.71 | 0.088 | -3.05 | 0.249 | -2.09 | 0.307 | -1.39 | 0.524 | -2.06 |
| 1446261_at | D1Ertd507e | DNA segment, Chr 1, ERATO Doi 507, expressed | -2.81 | 0.402 | -3.81 | 0.004 | -1.29 | 0.638 | -1.04 | 0.786 | -2.24 |
| 1453453_at | 1700080O16Rik /// | RIKEN cDNA 1700080O16 gene /// similar to melanoma antigen family A, 5 /// sir | -3.29 | 0.05 | -3.29 | 0.102 | -1.27 | 0.698 | -1.03 | 0.961 | -2.22 |
| 1459795_at | Gpr85 | G protein-coupled receptor 85 | -2.12 | 0.359 | -2.14 | 0.463 | -2.17 | 0.084 | -1.14 | 0.746 | -1.89 |
| 1447329_at | --- | --- | -1.77 | 0.293 | -2.21 | 0.129 | -2.61 | 0.299 | -2.14 | 0.373 | -2.18 |
| 1447288_at | --- | Transcribed locus | -3.95 | 0.049 | -1.32 | 0.569 | -2.55 | 0.101 | -1.14 | 0.854 | -2.24 |
| 1457911_at | A930002I21Rik | RIKEN cDNA A930002I21 gene | -3.61 | 0.081 | -1.51 | 0.367 | -2.12 | 0.113 | -1.18 | 0.626 | -2.1 |
| 1417925_at | Ccl22 | chemokine (C-C motif) ligand 22 | -1.18 | 0.588 | -2.84 | 0.029 | -4.61 | 0.072 | -2.31 | 0.043 | -2.74 |
| 1441560_at | Ctnna3 | catenin (cadherin associated protein), alpha 3 | -2.22 | 0.054 | -2.13 | 0.226 | -2.01 | 0.138 | -1.51 | 0.425 | -1.97 |
| 1419740_at | Pde6b | phosphodiesterase 6B, cGMP, rod receptor, beta polypeptide | -4.62 | 0.037 | -1.77 | 0.336 | -1.56 | 0.306 | -1.26 | 0.252 | -2.3 |
| 1450825_at | Asah3 | N-acylsphingosine amidohydrolase (alkaline ceramidase) 3 | -1.44 | 0.611 | -3.76 | 0.038 | -2.17 | 0.275 | -1.52 | 0.628 | -2.22 |

| | | | | | | | | | | | |
|---|---|---|---|---|---|---|---|---|---|---|---|
| 1422588_at | Krt6b | keratin 6B | -2.16 | 0.301 | -3.29 | 0.285 | -1.52 | 0.326 | -1.04 | 0.953 | -2 |
| 1446251_at | 4930565A17 | hypothetical protein 4930565A17 | -2.74 | 0.126 | -2.04 | 0.238 | -1.75 | 0.066 | -1.07 | 0.906 | -1.9 |
| 1429424_at | Cst13 | cystatin 13 | -4.95 | 0.083 | -2.71 | 0.075 | -1.17 | 0.801 | -1.23 | 0.729 | -2.52 |
| 1421623_at | Il12rb2 | interleukin 12 receptor, beta 2 | -1.97 | 0.293 | -2.66 | 0.022 | -1.84 | 0.029 | -1.6 | 0.07 | -2.02 |
| 1459037_at | Gm1614 | gene model 1614, (NCBI) | -2.4 | 0.172 | -1.31 | 0.129 | -4.01 | 0.092 | -1.56 | 0.529 | -2.32 |
| 1438901_at | BC024814 | cDNA sequence BC024814 | -2.34 | 0.106 | -3.04 | 0.045 | -1.48 | 0.331 | -1.95 | 0.292 | -2.2 |
| 1459171_at | Emp1 | Epithelial membrane protein 1 | -1.79 | 0.593 | -2.74 | 0.164 | -1.97 | 0.38 | -1.08 | 0.903 | -1.89 |
| 1443209_at | Hist1h1e | Histone 1, H1e | -2.02 | 0.385 | -2.07 | 0.285 | -2.19 | 0.193 | -1.12 | 0.829 | -1.85 |
| 1425290_at | Stx19 | syntaxin 19 | -3.1 | 0.232 | -2.25 | 0.061 | -1.47 | 0.143 | -1.2 | 0.772 | -2.01 |
| 1458702_at | 2610316D01Rik | RIKEN cDNA 2610316D01 gene | -1.62 | 0.474 | -2.9 | 0.037 | -2.06 | 0.21 | -2.5 | 0.299 | -2.27 |
| 1447183_at | Odz3 | Odd Oz/ten-m homolog 3 (Drosophila) | -2.12 | 0.269 | -1.94 | 0.341 | -2.18 | 0.334 | -1.52 | 0.667 | -1.94 |
| 1459275_at | Rnf17 | ring finger protein 17 | -2.41 | 0.28 | -2.01 | 0.296 | -1.87 | 0.179 | -1.18 | 0.728 | -1.87 |
| 1415808_at | Tpbpa | trophoblast specific protein alpha | -2.55 | 0.085 | -1.96 | 0.329 | -1.83 | 0.259 | -1.45 | 0.459 | -1.95 |
| 1422196_at | Htr5b | 5-hydroxytryptamine (serotonin) receptor 5B | -1.41 | 0.219 | -2.46 | 0.078 | -2.96 | 0.128 | -1.71 | 0.489 | -2.14 |
| 1439607_at | --- | --- | -2.25 | 0.245 | -2.71 | 0.016 | -1.55 | 0.421 | -1.06 | 0.54 | -1.89 |
| 1420213_x_at | --- | --- | -2.5 | 0.095 | -1.72 | 0.504 | -2.09 | 0.083 | -3.31 | 0.252 | -2.4 |
| 1419019_a_at | Akap4 | A kinase (PRKA) anchor protein 4 | -2.32 | 0.28 | -2.14 | 0.331 | -1.77 | 0.392 | -1.4 | 0.039 | -1.91 |
| 1443618_at | Pdzd2 | PDZ domain containing 2 | -1.43 | 0.292 | -2.67 | 0.153 | -2.53 | 0.057 | -1.78 | 0.225 | -2.1 |
| 1420343_at | Gzmd | granzyme D | -2.16 | 0.319 | -2.57 | 0.08 | -1.61 | 0.347 | -1.08 | 0.473 | -1.86 |
| 1444192_at | 9630020C08Rik | RIKEN cDNA 9630020C08 gene | -2.9 | 0.283 | -2.67 | 0.083 | -1.33 | 0.307 | -1.95 | 0.404 | -2.21 |
| 1445666_at | D15Wsu169e | DNA segment, Chr 15, Wayne State University 169, expressed | -1.54 | 0.356 | -2.97 | 0.015 | -2.06 | 0.06 | -1.01 | 0.976 | -1.89 |
| 1457808_at | LOC432842 | hypothetical LOC432842 | -1.93 | 0.549 | -2.43 | 0.129 | -1.83 | 0.181 | -1.06 | 0.261 | -1.81 |
| 1453079_at | 5730494M16Rik | RIKEN cDNA 5730494M16 gene | -1.92 | 0.168 | -2.56 | 0.03 | -1.76 | 0.165 | -3.49 | 0.02 | -2.43 |
| 1419328_at | Sema4f | sema domain, immunoglobulin domain (Ig), TM domain, and short cytoplasmic dc | -3.99 | 0.172 | -2.92 | 0.236 | -1.13 | 0.079 | -1.45 | 0.415 | -2.37 |
| 1453486_a_at | Scube2 | signal peptide, CUB domain, EGF-like 2 | -2.61 | 0.159 | -1.91 | 0.152 | -1.74 | 0.171 | -1.03 | 0.881 | -1.82 |
| 1416905_at | Guca2a | guanylate cyclase activator 2a (guanylin) | -2 | 0.205 | -2.58 | 0.028 | -1.67 | 0.201 | -1.98 | 0.377 | -2.06 |
| 1449838_at | Crisp3 | cysteine-rich secretory protein 3 | -1.4 | 0.388 | -2.34 | 0.215 | -2.9 | 0.209 | -2.89 | 0.156 | -2.38 |
| 1454173_at | Sh2d4b | SH2 domain containing 4B | -2.1 | 0.02 | -1.71 | 0.527 | -2.33 | 0.097 | -1.83 | 0.28 | -1.99 |
| 1447067_at | --- | --- | -3.51 | 0.073 | -2.33 | 0.118 | -1.29 | 0.609 | -1.48 | 0.384 | -2.15 |
| 1427215_at | Acsm2 | acyl-CoA synthetase medium-chain family member 2 | -1.31 | 0.15 | -4.37 | 0.003 | -2.01 | 0.184 | -2.39 | 0.345 | -2.52 |
| 1429840_at | 4933439G12Rik | RIKEN cDNA 4933439G12 gene | -3.08 | 0.175 | -1.3 | 0.425 | -2.49 | 0.056 | -1.47 | 0.427 | -2.09 |
| 1457558_at | A330050F15Rik | RIKEN cDNA A330050F15 gene | -2.37 | 0.077 | -1.73 | 0.312 | -2.02 | 0.166 | -1.36 | 0.62 | -1.87 |
| 1458593_at | Fbn1 | Fibrillin 1 | -1.79 | 0.12 | -2.61 | 0.191 | -1.8 | 0.361 | -1.36 | 0.527 | -1.89 |
| 1426171_x_at | Klra7 | killer cell lectin-like receptor, subfamily A, member 7 | -1.73 | 0.046 | -3.8 | 0.256 | -1.52 | 0.324 | -2.6 | 0.038 | -2.41 |
| 1455925_at | Prdm8 | PR domain containing 8 | -3.25 | 0.016 | -2.09 | 0.116 | -1.41 | 0.229 | -2.13 | 0.018 | -2.22 |
| 1445054_at | Foxn2 | Forkhead box N2 | -1.65 | 0.258 | -2.87 | 0.164 | -1.84 | 0.114 | -1.87 | 0.278 | -2.06 |
| 1448107_x_at | Klk1 | kallikrein 1 | -1.34 | 0.01 | -10.89 | 0.127 | -1.51 | 0.239 | -2.45 | 0.082 | -4.05 |
| 1443646_at | Igsf10 | immunoglobulin superfamily, member 10 | -2.18 | 0.494 | -1.97 | 0.283 | -1.87 | 0.235 | -1.48 | 0.626 | -1.87 |
| 1450331_s_at | V2r4 /// V2r5 /// LO( | vomeronasal 2, receptor, 4 /// vomeronasal 2, receptor, 5 /// similar to vomeronas | -1.85 | 0.098 | -2.71 | 0.289 | -1.68 | 0.331 | -1.58 | 0.35 | -1.95 |
| 1445580_at | Matn2 | Matrilin 2 | -2.41 | 0.508 | -2.21 | 0.324 | -1.56 | 0.515 | -2.27 | 0.149 | -2.11 |
| 1429636_at | 1700010D01Rik /// | RIKEN cDNA 1700010D01 gene /// similar to hypothetical protein LOC76386 | -2.43 | 0.476 | -1.56 | 0.126 | -2.17 | 0.299 | -1.09 | 0.784 | -1.81 |
| 1446128_at | Ebf2 | Early B-cell factor 2 | -1.7 | 0.374 | -1.76 | 0.312 | -2.79 | 0.037 | -1.64 | 0.359 | -1.98 |
| 1439661_at | Slc16a14 | solute carrier family 16 (monocarboxylic acid transporters), member 14 | -1.98 | 0.414 | -2.63 | 0.176 | -1.59 | 0.357 | -1.76 | 0.018 | -1.99 |
| 1444427_at | D930038D03Rik | RIKEN cDNA D930038D03 gene | -2.81 | 0.432 | -1.66 | 0.238 | -1.8 | 0.21 | -1.68 | 0.339 | -1.99 |
| 1430842_at | 1500002F19Rik | RIKEN cDNA 1500002F19 gene | -3.11 | 0.018 | -1.55 | 0.442 | -1.81 | 0.071 | -1.23 | 0.325 | -1.93 |
| 1459988_at | Mbd3l2 | methyl-CpG binding domain protein 3-like 2 | -2.68 | 0.03 | -3.4 | 0 | -1.18 | 0.593 | -1.12 | 0.513 | -2.1 |
| 1456684_at | Tmem74 | transmembrane protein 74 | -2.26 | 0.412 | -2.43 | 0.092 | -1.5 | 0.057 | -1.12 | 0.589 | -1.83 |
| 1447552_s_at | Ctnnd2 | Catenin (cadherin associated protein), delta 2 | -1.82 | 0.371 | -1.87 | 0.372 | -2.28 | 0.19 | -1.6 | 0.3 | -1.89 |
| 1459841_x_at | Laptm5 | lysosomal-associated protein transmembrane 5 | -2.97 | 0.104 | -2.25 | 0.044 | -1.34 | 0.467 | -1.27 | 0.554 | -1.96 |
| 1444492_at | Ptprd | protein tyrosine phosphatase, receptor type, D | -2.17 | 0.131 | -2.5 | 0.034 | -1.51 | 0.514 | -3.16 | 0.02 | -2.33 |
| 1429703_at | 2900072G11Rik | RIKEN cDNA 2900072G11 gene | -1.41 | 0.142 | -2.39 | 0.088 | -2.52 | 0.02 | -1.2 | 0.603 | -1.88 |
| 1420448_at | Rhox2 | reproductive homeobox 2 | -2.46 | 0.046 | -2.53 | 0.012 | -1.38 | 0.36 | -1.15 | 0.66 | -1.88 |
| 1446132_at | --- | --- | -2.42 | 0.211 | -1.35 | 0.522 | -2.61 | 0.036 | -1.01 | 0.983 | -1.85 |

| | | | | | | | | | | | |
|---|---|---|---|---|---|---|---|---|---|---|---|
| 1433163_at | 6230414M07Rik | RIKEN cDNA 6230414M07 gene | -2.25 | 0.015 | -3.91 | 0.015 | -1.19 | 0.159 | -1.52 | 0.14 | -2.22 |
| 1455977_x_at | Klk1 /// Klk1b5 | kallikrein 1 /// kallikrein 1-related peptidase b5 | -1.42 | 0.048 | -8.54 | 0.101 | -1.4 | 0.424 | -3.26 | 0.203 | -3.65 |
| 1458854_at | C78891 | expressed sequence C78891 | -1.9 | 0.394 | -4.27 | 0.029 | -1.28 | 0.608 | -1.43 | 0.522 | -2.22 |
| 1422956_at | D1Pas1 | DNA segment, Chr 1, Pasteur Institute 1 | -3.32 | 0.328 | -3.59 | 0.047 | -1.04 | 0.844 | -1.31 | 0.452 | -2.31 |
| 1454585_at | 1110065P19Rik | RIKEN cDNA 1110065P19 gene | -3.72 | 0.008 | -1.9 | 0.427 | -1.34 | 0.491 | -1.02 | 0.983 | -2 |
| 1432992_at | 4930403L11Rik | RIKEN cDNA 4930403L11 gene | -2.43 | 0.332 | -1.56 | 0.308 | -2.04 | 0.064 | -1.21 | 0.763 | -1.81 |
| 1444882_at | --- | --- | -2.3 | 0.113 | -2.53 | 0.19 | -1.39 | 0.56 | -2.29 | 0.244 | -2.13 |
| 1457589_at | Fat3 | FAT tumor suppressor homolog 3 (Drosophila) | -2.72 | 0.039 | -2.35 | 0.147 | -1.32 | 0.045 | -1.31 | 0.613 | -1.93 |
| 1430732_at | 4921525D07Rik | RIKEN cDNA 4921525D07 gene | -1.08 | 0.91 | -3.08 | 0.006 | -3.35 | 0.113 | -1.09 | 0.917 | -2.15 |
| 1454821_at | B3gat1 | beta-1,3-glucuronyltransferase 1 (glucuronosyltransferase P) | -2.08 | 0.487 | -1.29 | 0.332 | -3.37 | 0.003 | -1.33 | 0.195 | -2.02 |
| 1443469_at | --- | Transcribed locus | -2.13 | 0.482 | -2.63 | 0.018 | -1.42 | 0.645 | -1.18 | 0.758 | -1.84 |
| 1442728_at | Nasp | Nuclear autoantigenic sperm protein (histone-binding) | -2.66 | 0.452 | -1.91 | 0.19 | -1.52 | 0.193 | -1.61 | 0.617 | -1.92 |
| 1430785_at | Sdro | orphan short chain dehydrogenase/reductase | -3.07 | 0.069 | -1.33 | 0.232 | -2.07 | 0.082 | -1.15 | 0.717 | -1.9 |
| 1418218_at | Ceacam9 | CEA-related cell adhesion molecule 9 | -3.53 | 0.004 | -1.16 | 0.592 | -2.39 | 0.05 | -1.31 | 0.383 | -2.1 |
| 1433435_at | AW551984 | expressed sequence AW551984 | -2.88 | 0.05 | -1.37 | 0.039 | -2.04 | 0.148 | -1.55 | 0.236 | -1.96 |
| 1448906_at | Cdh16 | cadherin 16 | -1.15 | 0.13 | -2.52 | 0.265 | -3.34 | 0.016 | -1.11 | 0.9 | -2.03 |
| 1444733_at | --- | Transcribed locus | -1.98 | 0.252 | -1.42 | 0.365 | -2.78 | 0.178 | -2.72 | 0.117 | -2.22 |
| 1420084_at | --- | Transcribed locus | -1.98 | 0.003 | -1.92 | 0.227 | -1.83 | 0.33 | -1.18 | 0.192 | -1.73 |
| 1424972_at | --- | --- | -2.21 | 0.129 | -1.59 | 0.447 | -2.03 | 0.056 | -1.19 | 0.571 | -1.76 |
| 1422089_at | Ncr1 | natural cytotoxicity triggering receptor 1 | -2.45 | 0.057 | -1.63 | 0.368 | -1.82 | 0.142 | -1.44 | 0.072 | -1.83 |
| 1430226_at | Xlr4c | X-linked lymphocyte-regulated 4C | -2.27 | 0.497 | -1.96 | 0.29 | -1.61 | 0.311 | -2.9 | 0.031 | -2.18 |
| 1439725_at | Ptprt | protein tyrosine phosphatase, receptor type, T | -2.19 | 0.133 | -3.35 | 0 | -1.22 | 0.358 | -1.32 | 0.617 | -2.02 |
| 1421080_at | Nr4a3 | nuclear receptor subfamily 4, group A, member 3 | -2.17 | 0.299 | -2.3 | 0.033 | -1.47 | 0.182 | -1.19 | 0.413 | -1.78 |
| 1458525_at | App | Amyloid beta (A4) precursor protein | -1.27 | 0.525 | -3.63 | 0.141 | -1.95 | 0.024 | -1.34 | 0.076 | -2.05 |
| 1458068_at | 4932417H02Rik | RIKEN cDNA 4932417H02 gene | -1.88 | 0.515 | -2.66 | 0.075 | -1.49 | 0.624 | -1.05 | 0.894 | -1.77 |
| 1444081_at | 5033428A16Rik | RIKEN cDNA 5033428A16 gene | -2.12 | 0.11 | -3.76 | 0.139 | -1.19 | 0.827 | -1.05 | 0.933 | -2.03 |
| 1451062_a_at | Pex2 | peroxin 2 | -1.67 | 0.478 | -2.43 | 0.427 | -1.75 | 0.33 | -1.8 | 0.242 | -1.91 |
| 1434877_at | Nptx1 | neuronal pentraxin 1 | -1.44 | 0.591 | -4.49 | 0.034 | -1.5 | 0.339 | -1.82 | 0.351 | -2.31 |
| 1445929_at | Kcnk2 | Potassium channel, subfamily K, member 2 | -1.25 | 0.653 | -2.62 | 0.072 | -2.51 | 0.024 | -1.11 | 0.894 | -1.87 |
| 1421774_at | Vax1 | ventral anterior homeobox containing gene 1 | -2.6 | 0.396 | -1.19 | 0.718 | -2.79 | 0.107 | -1.47 | 0.389 | -2.01 |
| 1455891_at | 4933425K02Rik | RIKEN cDNA 4933425K02 gene | -1.75 | 0.324 | -1.96 | 0.494 | -1.98 | 0.139 | -1.03 | 0.957 | -1.68 |
| 1448006_at | Zc3h7b | zinc finger CCCH type containing 7B | -1.43 | 0.396 | -2.21 | 0.052 | -2.29 | 0.164 | -1.62 | 0.018 | -1.89 |
| 1459778_at | Rpn1 | Ribophorin I | -2.18 | 0.178 | -1.53 | 0.244 | -2.11 | 0.159 | -2.17 | 0.156 | -1.99 |
| 1427825_at | --- | --- | -4.13 | 0 | -1.23 | 0.667 | -1.86 | 0.382 | -1.04 | 0.943 | -2.07 |
| 1430405_at | --- | --- | -2.36 | 0.024 | -1.46 | 0.386 | -2.05 | 0.014 | -1.02 | 0.977 | -1.72 |
| 1444253_at | Adamts18 | a disintegrin-like and metallopeptidase (reprolysin type) with thrombospondin type | -2.2 | 0.202 | -2.02 | 0.466 | -1.54 | 0.445 | -1.24 | 0.69 | -1.75 |
| 1433036_at | 4930564B12Rik | RIKEN cDNA 4930564B12 gene | -1.79 | 0.303 | -2.4 | 0.044 | -1.61 | 0.378 | -1.15 | 0.828 | -1.74 |
| 1446903_at | Angpt2 | Angiopoietin 2 | -2.26 | 0.243 | -1.77 | 0.061 | -1.68 | 0.315 | -3.13 | 0.087 | -2.21 |
| 1430714_at | 4930451C15Rik | RIKEN cDNA 4930451C15 gene | -2.7 | 0.316 | -2.93 | 0.004 | -1.12 | 0.685 | -1.06 | 0.906 | -1.95 |
| 1454435_at | 5530400N10Rik | RIKEN cDNA 5530400N10 gene | -3.34 | 0.396 | -3.07 | 0.225 | -1.02 | 0.899 | -1.07 | 0.666 | -2.13 |
| 1457918_at | Tnfaip2 | Tumor necrosis factor, alpha-induced protein 2 | -2.47 | 0.022 | -1.86 | 0.098 | -1.51 | 0.095 | -1.11 | 0.585 | -1.74 |
| 1432399_a_at | Epha1 | Eph receptor A1 | -2.14 | 0.337 | -2.52 | 0.379 | -1.35 | 0.405 | -1.53 | 0.657 | -1.89 |
| 1437326_x_at | Ela3 /// LOC63841ε | elastase 3, pancreatic /// similar to elastase 3B, pancreatic | -2.6 | 0.409 | -2.08 | 0.222 | -1.35 | 0.346 | -2.77 | 0.157 | -2.2 |
| 1427545_at | Scml4 | sex comb on midleg-like 4 (Drosophila) | -1.74 | 0.183 | -1.97 | 0.363 | -1.91 | 0.154 | -3.18 | 0.018 | -2.2 |
| 1447385_at | --- | Adult male corpora quadrigemina cDNA, RIKEN full-length enriched library, clone | -2.17 | 0.146 | -2.93 | 0.023 | -1.24 | 0.126 | -1.72 | 0.225 | -2.02 |
| 1452028_a_at | Cdh23 | cadherin 23 (otocadherin) | -1.41 | 0.161 | -3.08 | 0.215 | -1.73 | 0.255 | -1.14 | 0.858 | -1.84 |
| 1422282_at | Tacr1 | tachykinin receptor 1 | -2.11 | 0.231 | -1.46 | 0.581 | -2.21 | 0.208 | -1.39 | 0.057 | -1.79 |
| 1457077_at | --- | 2 days neonate sympathetic ganglion cDNA, RIKEN full-length enriched library, c | -2.69 | 0.292 | -2.12 | 0.058 | -1.3 | 0.204 | -1.21 | 0.441 | -1.83 |
| 1450524_at | Cldn9 | claudin 9 | -1.54 | 0.592 | -2.92 | 0.004 | -1.61 | 0.117 | -1.39 | 0.008 | -1.86 |
| 1457604_x_at | Cyp11a1 | cytochrome P450, family 11, subfamily a, polypeptide 1 | -1.45 | 0.239 | -3.21 | 0.058 | -1.63 | 0.354 | -2 | 0.244 | -2.07 |
| 1453423_at | 4933409L14Rik | RIKEN cDNA 4933409L14 gene | -3.08 | 0.368 | -1.88 | 0.235 | -1.32 | 0.274 | -1.01 | 0.99 | -1.82 |
| 1447305_at | Tcba1 | T-cell lymphoma breakpoint associated target 1 | -2.73 | 0.021 | -2.14 | 0.112 | -1.28 | 0.357 | -1.16 | 0.687 | -1.83 |
| 1450532_at | 2310002J15Rik | RIKEN cDNA 2310002J15 gene | -3.03 | 0.169 | -1.78 | 0.254 | -1.38 | 0.216 | -1.21 | 0.171 | -1.85 |

| | | | | | | | | | | | |
|---|---|---|---|---|---|---|---|---|---|---|---|
| 1447081_at | --- | --- | -2.14 | 0.451 | -3.53 | 0.01 | -1.15 | 0.535 | -2.25 | 0.064 | -2.27 |
| 1456185_at | Nalp9a | NACHT, LRR and PYD containing protein 9a | -3.01 | 0.264 | -1.44 | 0.604 | -1.68 | 0.183 | -1.67 | 0.397 | -1.95 |
| 1455148_at | Tmem130 | transmembrane protein 130 | -3.88 | 0.013 | -1.39 | 0.415 | -1.55 | 0.51 | -2.32 | 0.393 | -2.29 |
| 1431357_a_at | Rpgrip1 | retinitis pigmentosa GTPase regulator interacting protein 1 | -1.81 | 0.202 | -2.98 | 0.034 | -1.36 | 0.31 | -1.32 | 0.714 | -1.87 |
| 1446841_at | --- | 12 days embryo spinal ganglion cDNA, RIKEN full-length enriched library, clone:[ | -1.7 | 0.024 | -2.04 | 0.151 | -1.84 | 0.088 | -1.51 | 0.425 | -1.77 |
| 1432258_at | 9430014N10Rik | RIKEN cDNA 9430014N10 gene | -1.76 | 0.017 | -2.08 | 0.385 | -1.74 | 0.363 | -1.54 | 0.17 | -1.78 |
| 1441316_at | LOC677060 | similar to wingless related MMTV integration site 8b | -2.53 | 0.378 | -1.91 | 0.106 | -1.42 | 0.171 | -2.52 | 0.243 | -2.1 |
| 1445131_at | Fbxl13 | F-box and leucine-rich repeat protein 13 | -3.25 | 0.372 | -1.3 | 0.598 | -1.83 | 0.298 | -1.34 | 0.677 | -1.93 |
| 1430779_at | Ntng1 | netrin G1 | -1.6 | 0.346 | -3.38 | 0.001 | -1.42 | 0.031 | -1.89 | 0.128 | -2.07 |
| 1437970_at | BC053401 | CDNA sequence BC053401 | -3.22 | 0.188 | -1.19 | 0.672 | -2.11 | 0.188 | -2.33 | 0.31 | -2.21 |
| 1459056_at | Rasgrf1 | RAS protein-specific guanine nucleotide-releasing factor 1 | -3.31 | 0.062 | -1.67 | 0.477 | -1.38 | 0.064 | -1.01 | 0.979 | -1.84 |
| 1444529_at | LOC666806 | similar to X-linked eukaryotic translation initiation factor 1A | -4.85 | 0.005 | -1.12 | 0.901 | -1.9 | 0.192 | -1.46 | 0.69 | -2.33 |
| 1432682_at | 5530400K19Rik | RIKEN cDNA 5530400K19 gene | -2.3 | 0.163 | -2.17 | 0.306 | -1.37 | 0.376 | -1.89 | 0.111 | -1.93 |
| 1435974_at | Arhgef9 | Cdc42 guanine nucleotide exchange factor (GEF) 9 | -2.72 | 0.321 | -2.51 | 0.462 | -1.16 | 0.737 | -1.26 | 0.35 | -1.91 |
| 1443533_at | Ppp2r5e | Protein phosphatase 2, regulatory subunit B (B56), epsilon isoform | -1.87 | 0.018 | -1.44 | 0.042 | -2.5 | 0.053 | -1.36 | 0.087 | -1.79 |
| 1446607_at | Mtap7 | Microtubule-associated protein 7 | -2.89 | 0.466 | -1.31 | 0.59 | -1.91 | 0.071 | -2.57 | 0.434 | -2.17 |
| 1443802_at | Tnik | TRAF2 and NCK interacting kinase | -1.91 | 0.04 | -2.47 | 0.082 | -1.42 | 0.323 | -1.69 | 0.076 | -1.87 |
| 1444652_at | B830004H01Rik | RIKEN cDNA B830004H01 gene | -1.52 | 0.515 | -3.64 | 0.058 | -1.42 | 0.587 | -1.55 | 0.592 | -2.03 |
| 1426167_a_at | Camk4 | calcium/calmodulin-dependent protein kinase IV | -2.61 | 0.062 | -2.05 | 0.198 | -1.31 | 0.483 | -1.55 | 0.087 | -1.88 |
| 1438237_at | Rex2 | reduced expression 2 | -4 | 0.349 | -1.36 | 0.197 | -1.54 | 0.387 | -1.19 | 0.439 | -2.02 |
| 1447988_at | --- | --- | -1.6 | 0.117 | -1.5 | 0.573 | -2.9 | 0.001 | -1.12 | 0.681 | -1.78 |
| 1457619_at | BC015286 | cDNA sequence BC015286 | -1.99 | 0.311 | -1.75 | 0.425 | -1.77 | 0.019 | -1.61 | 0.114 | -1.78 |
| 1433399_at | 2900078I11Rik | RIKEN cDNA 2900078I11 gene | -1.25 | 0.499 | -2.4 | 0.396 | -2.36 | 0.042 | -1.16 | 0.444 | -1.79 |
| 1460508_at | Mrpl1 | mitochondrial ribosomal protein L1 | -3.42 | 0.018 | -2.13 | 0.211 | -1.14 | 0.758 | -1.26 | 0.711 | -1.99 |
| 1444568_at | Arpc5 | Actin related protein 2/3 complex, subunit 5 | -2.81 | 0.005 | -1.55 | 0.437 | -1.56 | 0.171 | -2.04 | 0.452 | -1.99 |
| 1422396_s_at | Ascl2 | achaete-scute complex homolog-like 2 (Drosophila) | -2.52 | 0.395 | -1.14 | 0.815 | -2.72 | 0.089 | -2.93 | 0.369 | -2.33 |
| 1443242_at | D5Ertd121e | DNA segment, Chr 5, ERATO Doi 121, expressed | -2.53 | 0.181 | -1.68 | 0.062 | -1.53 | 0.051 | -1.55 | 0.173 | -1.82 |
| 1420605_at | Mtag2 | metastasis associated gene 2 | -2.05 | 0.397 | -1.99 | 0.228 | -1.52 | 0.286 | -1.82 | 0.115 | -1.85 |
| 1458166_at | Phf21a | PHD finger protein 21A | -1.81 | 0.281 | -1.64 | 0.228 | -2.05 | 0.009 | -1.23 | 0.696 | -1.68 |
| 1460219_at | Mag | myelin-associated glycoprotein | -2.7 | 0.154 | -2.33 | 0.247 | -1.17 | 0.368 | -1.07 | 0.515 | -1.82 |
| 1446334_at | --- | --- | -3.94 | 0.013 | -1.84 | 0.45 | -1.17 | 0.775 | -1.03 | 0.903 | -1.99 |
| 1456654_at | Paqr5 | progestin and adipoQ receptor family member V | -1.19 | 0.074 | -3.35 | 0.043 | -1.94 | 0.137 | -1.34 | 0.324 | -1.95 |
| 1436398_at | Lef1 | Lymphoid enhancer binding factor 1 | -1.36 | 0.269 | -2.01 | 0.236 | -2.36 | 0.022 | -6.05 | 0.025 | -2.95 |
| 1442734_at | --- | --- | -1.89 | 0.185 | -1.46 | 0.607 | -2.26 | 0.084 | -1.44 | 0.591 | -1.76 |
| 1459739_at | E130308A19Rik | RIKEN cDNA E130308A19 gene | -2.44 | 0.208 | -1.41 | 0.587 | -1.86 | 0.216 | -1.27 | 0.667 | -1.74 |
| 1420230_at | AA414993 | expressed sequence AA414993 | -2.76 | 0.043 | -2.01 | 0.028 | -1.25 | 0.502 | -1.95 | 0.039 | -1.99 |
| 1457654_at | --- | Transcribed locus, moderately similar to NP_598437.1 O-sialoglycoprotein endop | -2.07 | 0.018 | -1.29 | 0.659 | -2.5 | 0.007 | -1.25 | 0.648 | -1.78 |
| 1443417_at | C87122 | expressed sequence C87122 | -1.59 | 0.134 | -2.07 | 0.221 | -1.83 | 0.219 | -1.14 | 0.269 | -1.66 |
| 1454083_at | 4930440C22Rik | RIKEN cDNA 4930440C22 gene | -1.27 | 0.767 | -2.29 | 0.064 | -2.31 | 0.376 | -1.26 | 0.673 | -1.78 |
| 1419788_at | --- | Transcribed locus | -1.47 | 0.224 | -2.79 | 0.02 | -1.61 | 0.107 | -2.04 | 0.31 | -1.98 |
| 1440753_at | --- | Transcribed locus | -1.34 | 0.198 | -2.33 | 0.066 | -2.06 | 0.241 | -1.58 | 0.328 | -1.83 |
| 1427402_at | Polr2f | polymerase (RNA) II (DNA directed) polypeptide F | -2.12 | 0.094 | -2.69 | 0.283 | -1.22 | 0.105 | -1.07 | 0.704 | -1.78 |
| 1437501_at | AF529169 | cDNA sequence AF529169 | -3.7 | 0.108 | -1.51 | 0.417 | -1.37 | 0.58 | -1.02 | 0.968 | -1.9 |
| 1459498_at | --- | --- | -2.95 | 0.137 | -1.6 | 0.506 | -1.43 | 0.498 | -1.34 | 0.435 | -1.83 |
| 1444887_at | C79015 | expressed sequence C79015 | -1.25 | 0.779 | -2.54 | 0.144 | -2.12 | 0.241 | -1.41 | 0.509 | -1.83 |
| 1447319_at | --- | --- | -2.77 | 0.222 | -1.17 | 0.689 | -2.22 | 0.016 | -1.74 | 0.413 | -1.98 |
| 1427126_at | Hspa1b | heat shock protein 1B | -1.45 | 0.279 | -1.7 | 0.141 | -2.58 | 0.198 | -1.83 | 0.537 | -1.89 |
| 1422523_at | Si | silver | -1.46 | 0.013 | -3.07 | 0.022 | -1.53 | 0.141 | -1.82 | 0.334 | -1.97 |
| 1446984_at | Prtg | Protogenin homolog (Gallus gallus) | -1.22 | 0.811 | -4.74 | 0.179 | -1.56 | 0.387 | -1.82 | 0.454 | -2.34 |
| 1447236_at | Kif21a | kinesin family member 21A | -3.11 | 0.053 | -2.12 | 0.271 | -1.14 | 0.769 | -2.37 | 0.284 | -2.18 |
| 1446746_at | Osbpl3 | Oxysterol binding protein-like 3 | -1.92 | 0.292 | -2.7 | 0.191 | -1.28 | 0.504 | -2.33 | 0.4 | -2.06 |
| 1419514_at | Pitx1 | paired-like homeodomain transcription factor 1 | -2.95 | 0.087 | -1.39 | 0.489 | -1.64 | 0.108 | -1.06 | 0.909 | -1.76 |
| 1432132_at | 4933401D09Rik | RIKEN cDNA 4933401D09 gene | -1.29 | 0.764 | -1.76 | 0.475 | -3 | 0.108 | -2.08 | 0.292 | -2.03 |

| | | | | | | | | | | | |
|---|---|---|---|---|---|---|---|---|---|---|---|
| 1458110_at | D430030G11Rik | RIKEN cDNA D430030G11 gene | -1.87 | 0.052 | -2.05 | 0.06 | -1.53 | 0.254 | -1.16 | 0.628 | -1.65 |
| 1442605_at | Bach2 | BTB and CNC homology 2 | -1.9 | 0.023 | -1.74 | 0.107 | -1.74 | 0.107 | -2.51 | 0.116 | -1.97 |
| 1419011_at | Cryba2 | crystallin, beta A2 | -1.88 | 0.357 | -2.33 | 0.119 | -1.39 | 0.469 | -2.14 | 0.335 | -1.93 |
| 1422037_at | Dlx3 | distal-less homeobox 3 | -1.18 | 0.57 | -2.4 | 0.137 | -2.41 | 0.022 | -2.24 | 0.107 | -2.06 |
| 1455277_at | Hhip | Hedgehog-interacting protein | -1.85 | 0.334 | -1.31 | 0.452 | -2.65 | 0.008 | -1.54 | 0.512 | -1.84 |
| 1439529_at | A430110N23Rik | RIKEN cDNA A430110N23 gene | -4.17 | 0.208 | -2 | 0.146 | -1.06 | 0.872 | -1.28 | 0.636 | -2.13 |
| 1455561_at | Cndp1 | carnosine dipeptidase 1 (metallopeptidase M20 family) | -1.28 | 0.265 | -2.23 | 0.057 | -2.22 | 0.1 | -1.18 | 0.651 | -1.73 |
| 1443277_at | Ndst3 | N-deacetylase/N-sulfotransferase (heparan glucosaminyl) 3 | -1.42 | 0.52 | -6.15 | 0.075 | -1.22 | 0.669 | -1.47 | 0.251 | -2.57 |
| 1457667_x_at | Dyrk2 | dual-specificity tyrosine-(Y)-phosphorylation regulated kinase 2 | -2.19 | 0.236 | -2.98 | 0.293 | -1.12 | 0.776 | -1.29 | 0.48 | -1.89 |
| 1432022_at | Cdgap | Cdc42 GTPase-activating protein | -1.3 | 0.048 | -2.41 | 0.053 | -2 | 0.019 | -1.07 | 0.624 | -1.69 |
| 1448030_at | 1700023D09Rik | RIKEN cDNA 1700023D09 gene | -3.97 | 0.067 | -2.19 | 0.083 | -1.02 | 0.921 | -2.92 | 0.181 | -2.53 |
| 1432895_at | 9030409K20Rik | RIKEN cDNA 9030409K20 gene | -2.43 | 0.189 | -1.91 | 0.076 | -1.33 | 0.506 | -1.43 | 0.46 | -1.77 |
| 1432015_a_at | 4933428M03Rik | RIKEN cDNA 4933428M03 gene | -2.25 | 0.002 | -1.12 | 0.876 | -2.88 | 0.015 | -1.71 | 0.306 | -1.99 |
| 1433061_at | 4933423L19Rik | RIKEN cDNA 4933423L19 gene | -2.13 | 0.326 | -1.84 | 0.328 | -1.48 | 0.061 | -2.1 | 0.301 | -1.89 |
| 1458231_at | --- | PREDICTED: Mus musculus similar to Zinc finger protein 195 (LOC625519), mRI | -1.29 | 0.406 | -2.55 | 0.185 | -1.93 | 0.348 | -1.04 | 0.931 | -1.7 |
| 1460071_at | Sap30l | SAP30-like | -1.57 | 0.629 | -1.71 | 0.085 | -2.14 | 0.048 | -4.07 | 0.022 | -2.37 |
| 1427952_at | Aipl1 | aryl hydrocarbon receptor-interacting protein-like 1 | -1.59 | 0.289 | -2.43 | 0.435 | -1.54 | 0.209 | -1.9 | 0.211 | -1.87 |
| 1432145_at | 2310026I22Rik | RIKEN cDNA 2310026I22 gene | -1.47 | 0.595 | -2.5 | 0.025 | -1.64 | 0.432 | -1.83 | 0.39 | -1.86 |
| 1457982_at | 1700052M18Rik | RIKEN cDNA 1700052M18 gene | -1.94 | 0.044 | -1.47 | 0.329 | -2.02 | 0.134 | -1.22 | 0.632 | -1.66 |
| 1431240_at | Clec2h | C-type lectin domain family 2, member h | -1.13 | 0.736 | -4.18 | 0.2 | -1.76 | 0.381 | -1.09 | 0.814 | -2.04 |
| 1442893_at | Ctnna2 | Catenin (cadherin associated protein), alpha 2 | -1.8 | 0.263 | -1.84 | 0.187 | -1.69 | 0.311 | -1.98 | 0.366 | -1.83 |
| 1445279_at | --- | Transcribed locus | -1.88 | 0.303 | -3.24 | 0.033 | -1.17 | 0.566 | -2.09 | 0.345 | -2.1 |
| 1459640_at | --- | --- | -1.32 | 0.559 | -2.26 | 0.227 | -2.02 | 0.12 | -1.18 | 0.736 | -1.7 |
| 1422391_at | V1rc3 | vomeronasal 1 receptor, C3 | -1.08 | 0.721 | -3.12 | 0.344 | -2.25 | 0.162 | -2.65 | 0.139 | -2.27 |
| 1453291_at | Hmgb2l1 | high mobility group box 2-like 1 | -1.93 | 0.01 | -2.27 | 0.138 | -1.36 | 0.102 | -1.11 | 0.524 | -1.66 |
| 1456370_s_at | 0610037L13Rik | RIKEN cDNA 0610037L13 gene | -2.14 | 0.14 | -1.75 | 0.326 | -1.52 | 0.136 | -1.68 | 0.168 | -1.77 |
| 1430689_at | 2810019C22Rik | RIKEN cDNA 2810019C22 gene | -1.41 | 0.203 | -2.76 | 0.009 | -1.6 | 0.054 | -1.2 | 0.286 | -1.74 |
| 1456166_at | --- | --- | -2.67 | 0.116 | -1.73 | 0.168 | -1.34 | 0.466 | -1.46 | 0.523 | -1.8 |
| 1422117_s_at | Khdrbs2 | KH domain containing, RNA binding, signal transduction associated 2 | -2.28 | 0.22 | -1.22 | 0.729 | -2.26 | 0.043 | -1.27 | 0.604 | -1.76 |
| 1444780_at | 5330421F07Rik | RIKEN cDNA 5330421F07 gene | -5.33 | 0.004 | -1.17 | 0.602 | -1.52 | 0.031 | -1.13 | 0.635 | -2.29 |
| 1450989_at | Tdgf1 | teratocarcinoma-derived growth factor | -2.15 | 0.411 | -1.21 | 0.467 | -2.47 | 0.142 | -1.2 | 0.773 | -1.76 |
| 1434163_at | AU024076 | expressed sequence AU024076 | -1.82 | 0.364 | -2.81 | 0.323 | -1.26 | 0.408 | -1.68 | 0.095 | -1.89 |
| 1460502_at | 2010007E15Rik | RIKEN cDNA 2010007E15 gene | -1.57 | 0.513 | -2.86 | 0.141 | -1.4 | 0.293 | -1.49 | 0.454 | -1.83 |
| 1459606_at | C79130 | expressed sequence C79130 | -1.35 | 0.58 | -2.51 | 0.043 | -1.78 | 0.319 | -1.91 | 0.309 | -1.89 |
| 1426005_at | Dmp1 | dentin matrix protein 1 | -2.16 | 0.331 | -1.18 | 0.807 | -2.57 | 0.214 | -1.01 | 0.992 | -1.73 |
| 1442955_at | --- | --- | -1.35 | 0.579 | -2.76 | 0.126 | -1.67 | 0.291 | -1.11 | 0.848 | -1.72 |
| 1446487_at | --- | --- | -3.98 | 0.289 | -1.17 | 0.819 | -1.67 | 0.276 | -1.03 | 0.936 | -1.96 |
| 1442563_at | Tmem8 | Transmembrane protein 8 (five membrane-spanning domains) | -2.49 | 0.283 | -1.46 | 0.527 | -1.62 | 0.383 | -1.1 | 0.836 | -1.67 |
| 1445177_at | --- | 4 days neonate male adipose cDNA, RIKEN full-length enriched library, clone:B43 | -2.33 | 0.428 | -2.6 | 0.327 | -1.12 | 0.617 | -1.18 | 0.819 | -1.81 |
| 1445108_at | Lphn3 | Latrophilin 3 | -2.25 | 0.453 | -1.47 | 0.417 | -1.73 | 0.198 | -1.51 | 0.218 | -1.74 |
| 1444823_at | --- | --- | -1.86 | 0.169 | -2.22 | 0.076 | -1.39 | 0.194 | -2 | 0.118 | -1.87 |
| 1456002_at | Xpa | xeroderma pigmentosum, complementation group A | -1.94 | 0.316 | -2.79 | 0.04 | -1.2 | 0.692 | -2.51 | 0.114 | -2.11 |
| 1458657_at | Antxr1 | Anthrax toxin receptor 1 | -1.1 | 0.045 | -4.35 | 0.032 | -1.75 | 0.189 | -1.84 | 0.128 | -2.26 |
| 1436927_at | Cypt4 /// Cypt9 /// C | cysteine-rich perinuclear theca 4 /// cysteine-rich perinuclear theca 9 /// cysteine-r | -2.31 | 0.097 | -2.79 | 0.024 | -1.09 | 0.863 | -1.38 | 0.46 | -1.89 |
| 1459826_at | Kcnq2 | Potassium voltage-gated channel, subfamily Q, member 2 | -2.77 | 0.314 | -1.5 | 0.025 | -1.47 | 0.09 | -1.58 | 0.594 | -1.83 |
| 1450871_a_at | Bcat1 | branched chain aminotransferase 1, cytosolic | -1.69 | 0.388 | -2.83 | 0.126 | -1.31 | 0.572 | -1.19 | 0.695 | -1.75 |
| 1422122_at | Fcer2a | Fc receptor, IgE, low affinity II, alpha polypeptide | -3.01 | 0.138 | -1.42 | 0.678 | -1.49 | 0.31 | -3.51 | 0.07 | -2.36 |
| 1417923_at | Pak3 | p21 (CDKN1A)-activated kinase 3 | -1.56 | 0.35 | -2.49 | 0.019 | -1.5 | 0.037 | -1.46 | 0.038 | -1.75 |
| 1444436_at | Hsf2 /// 9630030I15 | heat shock factor 2 /// RIKEN cDNA 9630030I15 gene | -1.65 | 0.094 | -1.76 | 0.025 | -1.86 | 0.005 | -1.2 | 0.453 | -1.62 |
| 1457507_at | Nrg3 | Neuregulin 3 | -1.34 | 0.713 | -2.5 | 0.142 | -1.77 | 0.512 | -1.78 | 0.372 | -1.85 |
| 1441875_at | 1700063J08Rik | RIKEN cDNA 1700063J08 gene | -1.77 | 0.442 | -1.86 | 0.483 | -1.65 | 0.221 | -1.48 | 0.523 | -1.69 |
| 1434728_at | Gria3 | glutamate receptor, ionotropic, AMPA3 (alpha 3) | -1.32 | 0.255 | -2.61 | 0.037 | -1.75 | 0.074 | -1.2 | 0.338 | -1.72 |
| 1443927_at | Ube1l2 | ubiquitin-activating enzyme E1-like 2 | -1.17 | 0.771 | -2.39 | 0.237 | -2.29 | 0.215 | -2.57 | 0.036 | -2.1 |

| | | | | | | | | | | | |
|---|---|---|---|---|---|---|---|---|---|---|---|
| 1450795_at | Lhb | luteinizing hormone beta | -2.82 | 0.008 | -2.46 | 0.189 | -1.05 | 0.878 | -1.6 | 0.62 | -1.98 |
| 1451915_at | Cct3 | chaperonin subunit 3 (gamma) | -4.08 | 0.005 | -1.65 | 0.424 | -1.16 | 0.772 | -1.02 | 0.973 | -1.98 |
| 1445288_at | --- | --- | -2.13 | 0.161 | -1.22 | 0.662 | -2.38 | 0.144 | -1.91 | 0.085 | -1.91 |
| 1446882_at | --- | --- | -1.61 | 0.493 | -2.04 | 0.01 | -1.66 | 0.349 | -3.29 | 0.034 | -2.15 |
| 1448062_at | Stk11 | serine/threonine kinase 11 | -3.13 | 0.024 | -1.6 | 0.498 | -1.3 | 0.5 | -1.62 | 0.424 | -1.91 |
| 1454054_at | 4930459I23Rik | RIKEN cDNA 4930459I23 gene | -1.98 | 0.469 | -2.05 | 0.282 | -1.39 | 0.475 | -1.1 | 0.873 | -1.63 |
| 1442924_at | Clstn2 | Calsyntenin 2 | -1.19 | 0.317 | -1.75 | 0.175 | -3.27 | 0.03 | -1.89 | 0.295 | -2.03 |
| 1457244_at | D6Ertd474e | DNA segment, Chr 6, ERATO Doi 474, expressed | -3.26 | 0.381 | -1.54 | 0.254 | -1.31 | 0.415 | -2.59 | 0.045 | -2.18 |
| 1453839_a_at | Pi16 | peptidase inhibitor 16 | -1.91 | 0.367 | -3.41 | 0.05 | -1.11 | 0.702 | -1.38 | 0.615 | -1.95 |
| 1456629_at | Ankrd47 | ankyrin repeat domain 47 | -1.78 | 0.345 | -1.81 | 0.134 | -1.65 | 0.048 | -1.27 | 0.518 | -1.63 |
| 1445703_at | AI848149 | expressed sequence AI848149 | -3.46 | 0.098 | -1.19 | 0.776 | -1.69 | 0.335 | -1.47 | 0.392 | -1.95 |
| 1459012_at | A730041O05Rik | RIKEN cDNA A730041O05 gene | -3.36 | 0.022 | -1.6 | 0.086 | -1.25 | 0.692 | -1.81 | 0.215 | -2 |
| 1457208_at | Nfxl1 | nuclear transcription factor, X-box binding-like 1 | -1.64 | 0.612 | -2.15 | 0.309 | -1.54 | 0.203 | -1.01 | 0.991 | -1.58 |
| 1442445_at | 2610027H17Rik | RIKEN cDNA 2610027H17 gene | -1.17 | 0.654 | -2.15 | 0.066 | -2.46 | 0.026 | -1.13 | 0.592 | -1.73 |
| 1441732_at | Tshz1 | Teashirt zinc finger family member 1 | -1.58 | 0.076 | -1.7 | 0.159 | -1.98 | 0.081 | -1.41 | 0.227 | -1.67 |
| 1432077_at | Pot1 | protection of telomeres 1 | -4.67 | 0.137 | -1.41 | 0.415 | -1.24 | 0.489 | -2.63 | 0.247 | -2.49 |
| 1446046_at | BC023818 | CDNA sequence BC023818 | -2 | 0.099 | -1.38 | 0.376 | -1.99 | 0.022 | -1.77 | 0.328 | -1.79 |
| 1457685_at | D330034E10Rik | RIKEN cDNA D330034E10 gene | -1.24 | 0.801 | -1.96 | 0.085 | -2.44 | 0.003 | -1.21 | 0.727 | -1.71 |
| 1451308_at | Elovl4 | elongation of very long chain fatty acids (FEN1/Elo2, SUR4/Elo3, yeast)-like 4 | -2.79 | 0.009 | -1.25 | 0.707 | -1.74 | 0.093 | -2.02 | 0.263 | -1.95 |
| 1433474_at | Edil3 | EGF-like repeats and discoidin I-like domains 3 | -1.11 | 0.579 | -1.82 | 0.193 | -3.61 | 0.004 | -1.37 | 0.26 | -1.98 |
| 1433184_at | 6720477C19Rik | RIKEN cDNA 6720477C19 gene | -1.29 | 0.222 | -1.47 | 0.425 | -3.66 | 0.062 | -1.13 | 0.158 | -1.88 |
| 1446729_at | Disp2 | dispatched homolog 2 (Drosophila) | -1.64 | 0.354 | -1.97 | 0.169 | -1.62 | 0.31 | -1.67 | 0.494 | -1.73 |
| 1438399_at | Pex2 | peroxin 2 | -3.56 | 0.18 | -2.21 | 0.266 | -1 | 0.997 | -6.27 | 0.051 | -3.26 |
| 1459506_at | --- | --- | -1.67 | 0.38 | -1.79 | 0.28 | -1.75 | 0.181 | -1.89 | 0.049 | -1.77 |
| 1419314_at | Tinag | tubulointerstitial nephritis antigen | -1.18 | 0.11 | -2.9 | 0.067 | -1.85 | 0.002 | -1.3 | 0.646 | -1.81 |
| 1458713_at | --- | --- | -1.82 | 0.019 | -1.47 | 0.481 | -1.98 | 0.115 | -1.31 | 0.313 | -1.65 |
| 1453907_at | C030041B07Rik | RIKEN cDNA C030041B07 gene | -1.33 | 0.712 | -2.09 | 0.233 | -1.99 | 0.302 | -1.8 | 0.536 | -1.8 |
| 1457187_at | A630071L07Rik | RIKEN cDNA A630071L07 gene | -2.3 | 0.223 | -1.61 | 0.481 | -1.47 | 0.267 | -1.37 | 0.567 | -1.69 |
| 1433279_at | 5830440H09Rik | RIKEN cDNA 5830440H09 gene | -2.24 | 0.063 | -1.85 | 0.133 | -1.33 | 0.191 | -1.58 | 0.181 | -1.75 |
| 1446664_at | D15Ertd154e | DNA segment, Chr 15, ERATO Doi 154, expressed | -2.12 | 0.264 | -1.7 | 0.32 | -1.48 | 0.487 | -1.62 | 0.448 | -1.73 |
| 1415837_at | Klk1 | kallikrein 1 | -1.36 | 0.036 | -10.34 | 0.111 | -1.1 | 0.643 | -1.79 | 0.121 | -3.65 |
| 1433404_at | 4930423O20Rik | RIKEN cDNA 4930423O20 gene | -1.38 | 0.677 | -1.79 | 0.248 | -2.2 | 0.018 | -1.66 | 0.049 | -1.76 |
| 1420135_at | BC021438 | CDNA sequence BC021438 | -1.38 | 0.483 | -1.8 | 0.272 | -2.19 | 0.01 | -1.18 | 0.838 | -1.64 |
| 1444316_at | --- | --- | -2.91 | 0.119 | -1.41 | 0.28 | -1.46 | 0.325 | -1.06 | 0.852 | -1.71 |
| 1445924_at | LOC385211 | similar to RIKEN cDNA 1700029I01 | -1.57 | 0.1 | -2.44 | 0.018 | -1.44 | 0.523 | -1.76 | 0.464 | -1.8 |
| 1445749_at | Rnf4 | Ring finger protein 4 | -2.16 | 0.193 | -1.94 | 0.359 | -1.31 | 0.087 | -1.64 | 0.588 | -1.76 |
| 1423415_at | Gpr83 | G protein-coupled receptor 83 | -2 | 0.03 | -1.46 | 0.3 | -1.79 | 0.242 | -1.9 | 0.304 | -1.79 |
| 1459310_at | 5330434G04Rik | RIKEN cDNA 5330434G04 gene | -1.17 | 0.569 | -2.91 | 0.333 | -1.83 | 0.158 | -1.37 | 0.752 | -1.82 |
| 1440724_at | AI854703 | expressed sequence AI854703 | -1.61 | 0.016 | -1.44 | 0.382 | -2.35 | 0.073 | -2.28 | 0.336 | -1.92 |
| 1442335_at | --- | Transcribed locus | -1.94 | 0.275 | -2.52 | 0.018 | -1.2 | 0.436 | -1.43 | 0.275 | -1.77 |
| 1432901_at | BC026657 | cDNA sequence BC026657 | -2.07 | 0.038 | -1.52 | 0.104 | -1.66 | 0.011 | -1.24 | 0.579 | -1.62 |
| 1451711_at | Wnt9b | wingless-type MMTV integration site 9B | -4.76 | 0.03 | -1.87 | 0.187 | -1 | 0.999 | -1.13 | 0.712 | -2.19 |
| 1457012_at | Dbx1 | developing brain homeobox 1 | -2.63 | 0.269 | -2.24 | 0.053 | -1.09 | 0.836 | -1.42 | 0.387 | -1.85 |
| 1431910_at | 5730460C07Rik | RIKEN cDNA 5730460C07 gene | -1.22 | 0.558 | -2.99 | 0.006 | -1.68 | 0.185 | -1.51 | 0.537 | -1.85 |
| 1441373_at | Msi2 | Musashi homolog 2 (Drosophila) | -1.56 | 0.159 | -1.6 | 0.471 | -2.1 | 0.074 | -1.5 | 0.192 | -1.69 |
| 1444605_at | 1700061F12Rik | RIKEN cDNA 1700061F12 gene | -1.27 | 0.779 | -1.98 | 0.514 | -2.19 | 0.055 | -2.4 | 0.224 | -1.96 |
| 1422365_at | Olfr17 | olfactory receptor 17 | -3.21 | 0.004 | -1.03 | 0.965 | -2.17 | 0.013 | -1.18 | 0.801 | -1.9 |
| 1439299_at | Spnb2 | Spectrin beta 2 | -1.58 | 0.023 | -1.54 | 0.043 | -2.15 | 0.051 | -1.22 | 0.501 | -1.62 |
| 1447419_at | --- | Transcribed locus | -2.49 | 0.246 | -1.59 | 0.246 | -1.39 | 0.104 | -1.19 | 0.151 | -1.67 |
| 1425038_at | Slc22a19 | solute carrier family 22 (organic anion transporter), member 19 | -1.1 | 0.611 | -2.96 | 0.447 | -2.01 | 0.296 | -1.08 | 0.834 | -1.79 |
| 1440780_x_at | 1500015O10Rik | RIKEN cDNA 1500015O10 gene | -2.07 | 0.086 | -1.86 | 0.093 | -1.37 | 0.205 | -1.57 | 0.294 | -1.72 |
| 1456926_at | Rims4 | regulating synaptic membrane exocytosis 4 | -4.33 | 0.038 | -1.16 | 0.631 | -1.52 | 0.145 | -1.44 | 0.378 | -2.12 |
| 1459532_at | Grm7 | glutamate receptor, metabotropic 7 | -1.2 | 0.851 | -2.37 | 0.214 | -2.02 | 0.269 | -1.99 | 0.484 | -1.9 |

| | | | | | | | | | | | |
|---|---|---|---|---|---|---|---|---|---|---|---|
| 1449735_at | --- | --- | -3.71 | 0.161 | -1.64 | 0.437 | -1.15 | 0.716 | -1.45 | 0.491 | -1.99 |
| 1429635_at | Ccdc89 | coiled-coil domain containing 89 | -1.6 | 0.492 | -1.68 | 0.064 | -1.9 | 0.411 | -1.39 | 0.423 | -1.64 |
| 1443328_at | 4921505C17Rik | RIKEN cDNA 4921505C17 gene | -1.92 | 0.071 | -1.81 | 0.266 | -1.48 | 0.165 | -1.64 | 0.223 | -1.71 |
| 1421602_at | Shbg | sex hormone binding globulin | -1.89 | 0.09 | -2.33 | 0.019 | -1.26 | 0.475 | -1.46 | 0.243 | -1.73 |
| 1451061_at | 1700018B24Rik | RIKEN cDNA 1700018B24 gene | -1.29 | 0.752 | -1.44 | 0.571 | -3.5 | 0.006 | -1.44 | 0.04 | -1.92 |
| 1430821_at | 1700024C24Rik | RIKEN cDNA 1700024C24 gene | -2.19 | 0.064 | -2.48 | 0.216 | -1.12 | 0.602 | -1.35 | 0.386 | -1.79 |
| 1440587_at | --- | --- | -1.41 | 0.038 | -1.77 | 0.135 | -2.08 | 0.019 | -1.96 | 0.162 | -1.8 |
| 1447161_at | --- | Transcribed locus | -2.22 | 0.365 | -1.33 | 0.55 | -1.8 | 0.138 | -2.75 | 0.186 | -2.03 |
| 1445562_at | Zbtb16 | Zinc finger and BTB domain containing 16 | -1.66 | 0.394 | -1.37 | 0.322 | -2.38 | 0.154 | -1.21 | 0.59 | -1.65 |
| 1440304_at | Bach2 | BTB and CNC homology 2 | -1.46 | 0.106 | -1.59 | 0.173 | -2.27 | 0.027 | -3.23 | 0.045 | -2.14 |
| 1433262_at | 9430052A13Rik | RIKEN cDNA 9430052A13 gene | -1.95 | 0.106 | -1.54 | 0.367 | -1.68 | 0.137 | -1 | 0.991 | -1.54 |
| 1430649_at | Baalc | brain and acute leukemia, cytoplasmic | -2.01 | 0.21 | -2.81 | 0.043 | -1.1 | 0.797 | -1.36 | 0.675 | -1.82 |
| 1440678_at | Ltbp1 | Latent transforming growth factor beta binding protein 1 | -1.77 | 0.023 | -1.81 | 0.079 | -1.56 | 0.169 | -1.1 | 0.842 | -1.56 |
| 1455233_at | Mrps11 | mitochondrial ribosomal protein S11 | -1.28 | 0.552 | -2.06 | 0.164 | -2.01 | 0.044 | -1.04 | 0.873 | -1.6 |
| 1455557_at | LOC553095 | hypothetical LOC553095 | -1.66 | 0.002 | -2.13 | 0.345 | -1.45 | 0.577 | -1.16 | 0.825 | -1.6 |
| 1431044_at | Thoc1 | THO complex 1 | -1.42 | 0.733 | -2.14 | 0.205 | -1.69 | 0.128 | -1.15 | 0.57 | -1.6 |
| 1431901_a_at | Pfkfb2 | 6-phosphofructo-2-kinase/fructose-2,6-biphosphatase 2 | -4.26 | 0.147 | -1.44 | 0.647 | -1.2 | 0.683 | -1.55 | 0.188 | -2.11 |
| 1442789_at | --- | Transcribed locus | -1.58 | 0.157 | -1.49 | 0.143 | -2.17 | 0.068 | -1.6 | 0.431 | -1.71 |
| 1439823_at | Ctbp2 | C-terminal binding protein 2 | -2.11 | 0.117 | -2.29 | 0.237 | -1.17 | 0.755 | -4.08 | 0.228 | -2.41 |
| 1458383_at | Idh2 | Isocitrate dehydrogenase 2 (NADP+), mitochondrial | -1.61 | 0.347 | -1.3 | 0.617 | -2.69 | 0.022 | -1.07 | 0.864 | -1.67 |
| 1446885_at | --- | --- | -1.63 | 0.557 | -2.1 | 0.285 | -1.47 | 0.321 | -1.3 | 0.316 | -1.63 |
| 1431852_at | Prdm8 | PR domain containing 8 | -2.5 | 0.153 | -2.19 | 0.12 | -1.1 | 0.859 | -1.32 | 0.605 | -1.78 |
| 1459395_at | --- | Transcribed locus | -1.21 | 0.537 | -1.88 | 0.064 | -2.43 | 0.172 | -1.3 | 0.29 | -1.7 |
| 1420263_at | --- | --- | -3.24 | 0.052 | -1.08 | 0.868 | -1.87 | 0.072 | -1.1 | 0.811 | -1.82 |
| 1443156_at | --- | --- | -1.29 | 0.579 | -1.57 | 0.147 | -2.8 | 0.005 | -1.72 | 0.02 | -1.84 |
| 1445819_at | A830018L16Rik | RIKEN cDNA A830018L16 gene | -3.07 | 0.013 | -1.9 | 0.023 | -1.09 | 0.561 | -1.55 | 0.098 | -1.9 |
| 1445664_at | Igf1r | Insulin-like growth factor I receptor | -1.35 | 0.224 | -1.36 | 0.716 | -3.35 | 0.052 | -1.07 | 0.626 | -1.78 |
| 1458883_at | --- | --- | -1.32 | 0.724 | -1.95 | 0.302 | -2 | 0.202 | -1.43 | 0.381 | -1.67 |
| 1459721_at | --- | CDNA clone IMAGE:6406267 | -1.79 | 0.228 | -1.69 | 0.372 | -1.6 | 0.488 | -1.94 | 0.609 | -1.76 |
| 1416643_at | Tpt1 | tumor protein, translationally-controlled 1 | -1.53 | 0.704 | -2.75 | 0.092 | -1.32 | 0.117 | -1.24 | 0.606 | -1.71 |
| 1442492_at | DXErtd11e | DNA segment, Chr X, ERATO Doi 11, expressed | -3.23 | 0.061 | -1.16 | 0.704 | -1.66 | 0.106 | -1.14 | 0.574 | -1.8 |
| 1422014_at | Foxp2 | forkhead box P2 | -2 | 0.255 | -1.57 | 0.22 | -1.57 | 0.32 | -1 | 0.998 | -1.54 |
| 1459259_at | A030007L22 | hypothetical protein A030007L22 | -1.76 | 0.263 | -2.66 | 0.079 | -1.21 | 0.44 | -1.58 | 0.244 | -1.8 |
| 1445533_at | A630047E20Rik | RIKEN cDNA A630047E20 gene | -2.78 | 0.061 | -1.89 | 0.303 | -1.13 | 0.76 | -3.44 | 0.025 | -2.31 |
| 1443112_at | Api5 | apoptosis inhibitor 5 | -1.41 | 0.263 | -2.47 | 0.026 | -1.51 | 0.055 | -1.31 | 0.388 | -1.67 |
| 1445625_at | --- | Transcribed locus | -1.5 | 0.46 | -1.72 | 0.512 | -1.89 | 0.225 | -1.33 | 0.584 | -1.61 |
| 1437755_at | Slc5a12 | solute carrier family 5 (sodium/glucose cotransporter), member 12 | -1.02 | 0.945 | -2.46 | 0.268 | -2.57 | 0.004 | -1.22 | 0.577 | -1.82 |
| 1420783_at | Trpd52l3 | tumor protein D52-like 3 | -1.41 | 0.395 | -2.15 | 0.213 | -1.65 | 0.104 | -1.38 | 0.139 | -1.65 |
| 1416966_at | Slc22a8 | solute carrier family 22 (organic anion transporter), member 8 | -1.29 | 0.253 | -2.59 | 0.126 | -1.62 | 0.293 | -1.68 | 0.194 | -1.79 |
| 1452318_a_at | Hspa1b | heat shock protein 1B | -1.45 | 0.164 | -1.62 | 0.192 | -2.12 | 0.188 | -2.19 | 0.534 | -1.84 |
| 1460264_at | Lats1 | large tumor suppressor | -2.01 | 0.036 | -1.35 | 0.693 | -1.84 | 0.113 | -2.6 | 0.066 | -1.95 |
| 1454877_at | Sertad4 | SERTA domain containing 4 | -1.33 | 0.235 | -2.35 | 0.133 | -1.66 | 0.161 | -1.36 | 0.567 | -1.68 |
| 1426042_at | Fgd4 | FYVE, RhoGEF and PH domain containing 4 | -1.53 | 0.67 | -1.9 | 0.379 | -1.67 | 0.074 | -1.16 | 0.756 | -1.56 |
| 1436118_at | Vangl2 | vang-like 2 (van gogh, Drosophila) | -1.28 | 0.111 | -2.78 | 0.06 | -1.57 | 0.044 | -1.55 | 0.153 | -1.79 |
| 1459507_at | --- | --- | -2.68 | 0.499 | -1.35 | 0.535 | -1.5 | 0.355 | -3.94 | 0.001 | -2.37 |
| 1420072_s_at | --- | --- | -1.22 | 0.638 | -2.13 | 0.034 | -2.02 | 0.039 | -1.63 | 0.255 | -1.75 |
| 1454202_a_at | 1700061J05Rik | RIKEN cDNA 1700061J05 gene | -1.62 | 0.449 | -1.47 | 0.528 | -2.04 | 0.096 | -1.2 | 0.543 | -1.58 |
| 1420627_a_at | Csn1s1 | casein alpha s1 | -2.24 | 0.113 | -1.5 | 0.537 | -1.48 | 0.393 | -1.08 | 0.894 | -1.58 |
| 1447134_at | Setd5 | SET domain containing 5 | -1.54 | 0.572 | -2.17 | 0.098 | -1.48 | 0.452 | -2.02 | 0.036 | -1.8 |
| 1443635_at | Gm128 | gene model 128, (NCBI) | -1.23 | 0.13 | -2.29 | 0.268 | -1.86 | 0.19 | -2.48 | 0.308 | -1.97 |
| 1433779_at | Casc4 | cancer susceptibility candidate 4 | -1.12 | 0.301 | -2.24 | 0.03 | -2.24 | 0.016 | -1.23 | 0.359 | -1.71 |
| 1420299_at | Gabra2 | gamma-aminobutyric acid (GABA-A) receptor, subunit alpha 2 | -1.46 | 0.652 | -2.89 | 0.003 | -1.32 | 0.608 | -1.29 | 0.392 | -1.74 |
| 1432643_at | 4933411E06Rik | RIKEN cDNA 4933411E06 gene | -1.24 | 0.593 | -2.01 | 0.183 | -2.05 | 0.108 | -1.22 | 0.721 | -1.63 |

| | | | | | | | | | | | |
|---|---|---|---|---|---|---|---|---|---|---|---|
| 1457446_at | Opcml | opioid binding protein/cell adhesion molecule-like | -1.46 | 0.173 | -1.67 | 0.527 | -1.96 | 0.094 | -2.59 | 0.338 | -1.92 |
| 1458004_at | Prrx1 | paired related homeobox 1 | -1.52 | 0.021 | -2.58 | 0.269 | -1.34 | 0.528 | -1.1 | 0.476 | -1.63 |
| 1440276_at | Gm62 | gene model 62, (NCBI) | -1.33 | 0.686 | -2.05 | 0.198 | -1.8 | 0.17 | -1.79 | 0.487 | -1.75 |
| 1459825_x_at | Igf2bp1 | insulin-like growth factor 2 mRNA binding protein 1 | -2.4 | 0.026 | -1.42 | 0.289 | -1.49 | 0.208 | -1.22 | 0.769 | -1.63 |
| 1440711_at | C630001G18Rik | RIKEN cDNA C630001G18 gene | -2.12 | 0.239 | -1.74 | 0.393 | -1.34 | 0.241 | -1.69 | 0.371 | -1.72 |
| 1440582_at | Itch /// 8030492O04 | itchy /// RIKEN cDNA 8030492O04 gene | -1.8 | 0.176 | -1.52 | 0.023 | -1.72 | 0.062 | -1.58 | 0.078 | -1.65 |
| 1442040_at | 6330530A05Rik | RIKEN cDNA 6330530A05 gene | -1.55 | 0.311 | -1.65 | 0.189 | -1.84 | 0.121 | -1.7 | 0.064 | -1.69 |
| 1446762_at | --- | Transcribed locus | -1.22 | 0.131 | -2.04 | 0.143 | -2.07 | 0.002 | -1.1 | 0.596 | -1.61 |
| 1427127_x_at | Hspa1b | heat shock protein 1B | -1.4 | 0.308 | -1.75 | 0.075 | -1.96 | 0.242 | -2.15 | 0.51 | -1.82 |
| 1439480_at | AU015850 | Expressed sequence AU015850 | -1.91 | 0.244 | -1.34 | 0.594 | -1.89 | 0.372 | -2.45 | 0.157 | -1.9 |
| 1458934_at | D5Ertd505e | DNA segment, Chr 5, ERATO Doi 505 , expressed | -1.51 | 0.584 | -1.49 | 0.156 | -2.16 | 0.031 | -1.92 | 0.146 | -1.77 |
| 1421405_at | Zim1 | zinc finger, imprinted 1 | -1.42 | 0.288 | -1.35 | 0.56 | -2.85 | 0.097 | -1.04 | 0.942 | -1.67 |
| 1439086_at | A930009L07Rik | RIKEN cDNA A930009L07 gene | -1.16 | 0.512 | -4.37 | 0.088 | -1.42 | 0.214 | -1.53 | 0.596 | -2.12 |
| 1432330_at | LOC381294 | complement factor H-like | -2.38 | 0.116 | -1.06 | 0.662 | -2.33 | 0.166 | -2.18 | 0.277 | -1.98 |
| 1421716_at | V1rd6 | vomeronasal 1 receptor, D6 | -2.26 | 0.228 | -1.71 | 0.209 | -1.3 | 0.257 | -1.87 | 0.039 | -1.78 |
| 1432768_at | 6430710M23Rik | RIKEN cDNA 6430710M23 gene | -2.09 | 0.057 | -1.42 | 0.232 | -1.63 | 0.274 | -1.12 | 0.67 | -1.56 |
| 1429632_at | Trappc4 | trafficking protein particle complex 4 | -2.26 | 0.049 | -1.47 | 0.231 | -1.48 | 0.077 | -1.18 | 0.585 | -1.6 |
| 1444044_at | Nedd4 | Neural precursor cell expressed, developmentally down-regulted gene 4 | -2.33 | 0.02 | -1.36 | 0.581 | -1.57 | 0.167 | -1.28 | 0.725 | -1.64 |
| 1415832_at | Agtr2 | angiotensin II receptor, type 2 | -1.67 | 0.532 | -1.24 | 0.698 | -2.51 | 0.082 | -1.17 | 0.663 | -1.65 |
| 1455542_at | C630043F03Rik | RIKEN cDNA C630043F03 gene | -1.19 | 0.705 | -2.49 | 0.024 | -1.78 | 0.117 | -1.07 | 0.896 | -1.63 |
| 1447380_at | Amdhd1 | amidohydrolase domain containing 1 | -1.73 | 0.565 | -1.2 | 0.144 | -2.57 | 0.015 | -1.27 | 0.636 | -1.69 |
| 1456474_at | --- | --- | -1.37 | 0.607 | -1.81 | 0.3 | -1.91 | 0.155 | -2.33 | 0.011 | -1.85 |
| 1425648_at | Trim60 /// Trim61 | tripartite motif-containing 60 /// tripartite motif-containing 61 | -2.25 | 0.126 | -1.38 | 0.44 | -1.57 | 0.183 | -1.2 | 0.826 | -1.6 |
| 1451774_at | LOC435285 | similar to keratin associated protein 4-10; keratin associated protein 4.10 | -1.67 | 0.098 | -1.77 | 0.311 | -1.56 | 0.438 | -1.58 | 0.156 | -1.65 |
| 1425683_at | --- | --- | -1.77 | 0.454 | -2.5 | 0.267 | -1.19 | 0.567 | -1.16 | 0.853 | -1.66 |
| 1428741_at | Elavl4 | ELAV (embryonic lethal, abnormal vision, Drosophila)-like 4 (Hu antigen D) | -1.27 | 0.741 | -2.25 | 0.305 | -1.73 | 0.244 | -1.11 | 0.799 | -1.59 |
| 1437558_at | B130021B11Rik | RIKEN cDNA B130021B11 gene | -1.12 | 0.084 | -2.45 | 0.029 | -1.96 | 0.027 | -1.98 | 0.028 | -1.88 |
| 1447458_at | St3gal4 | ST3 beta-galactoside alpha-2,3-sialyltransferase 4 | -3.4 | 0.033 | -1.18 | 0.488 | -1.49 | 0.034 | -1.22 | 0.744 | -1.82 |
| 1439710_at | Ncoa6ip | nuclear receptor coactivator 6 interacting protein | -1.3 | 0.414 | -2.72 | 0.006 | -1.49 | 0.112 | -1.31 | 0.387 | -1.7 |
| 1427293_a_at | Auts2 | autism susceptibility candidate 2 | -1.79 | 0.048 | -1.49 | 0.592 | -1.73 | 0.294 | -1.53 | 0.608 | -1.63 |
| 1419779_at | --- | --- | -1.39 | 0.436 | -1.35 | 0.442 | -2.85 | 0.006 | -1.31 | 0.618 | -1.72 |
| 1440910_at | --- | CDNA clone IMAGE:5252333 | -1.89 | 0.189 | -1.45 | 0.117 | -1.68 | 0.008 | -2.41 | 0.267 | -1.86 |
| 1433313_at | 6720454L07Rik | RIKEN cDNA 6720454L07 gene | -1.66 | 0.609 | -1.87 | 0.149 | -1.48 | 0.444 | -1.59 | 0.515 | -1.65 |
| 1439384_at | Slc13a3 | solute carrier family 13 (sodium-dependent dicarboxylate transporter), member 3 | -1.28 | 0.764 | -3.89 | 0.026 | -1.29 | 0.467 | -1.47 | 0.594 | -1.98 |
| 1453883_at | 4933425B07Rik | RIKEN cDNA 4933425B07 gene | -1.41 | 0.607 | -1.6 | 0.64 | -2.1 | 0.104 | -1.22 | 0.571 | -1.58 |
| 1444959_at | D1Ertd83e | DNA segment, Chr 1, ERATO Doi 83, expressed | -2.64 | 0.353 | -1.81 | 0.435 | -1.13 | 0.54 | -1.11 | 0.83 | -1.67 |
| 1446497_at | Nav1 | Neuron navigator 1 | -1.2 | 0.556 | -1.65 | 0.223 | -2.68 | 0.011 | -1.25 | 0.479 | -1.7 |
| 1442814_at | Glcci1 | Glucocorticoid induced transcript 1 | -2.26 | 0.03 | -1.63 | 0.446 | -1.32 | 0.424 | -1.16 | 0.798 | -1.59 |
| 1449678_at | --- | --- | -1.58 | 0.249 | -1.73 | 0.086 | -1.66 | 0.134 | -1.03 | 0.84 | -1.5 |
| 1419200_at | Fxyd7 | FXYD domain-containing ion transport regulator 7 | -1.43 | 0.39 | -1.55 | 0.243 | -2.13 | 0.209 | -1.55 | 0.233 | -1.67 |
| 1437826_at | Pfpl | Pore forming protein-like | -1.32 | 0.489 | -2.14 | 0.163 | -1.7 | 0.338 | -1.64 | 0.417 | -1.7 |
| 1435307_at | 6430502M16Rik | RIKEN cDNA 6430502M16 gene | -1.24 | 0.225 | -1.71 | 0.389 | -2.38 | 0.223 | -1.37 | 0.506 | -1.67 |
| 1440069_at | Efnb2 | Ephrin B2 | -1.46 | 0.419 | -1.69 | 0.428 | -1.86 | 0.003 | -1.57 | 0.572 | -1.64 |
| 1439522_at | --- | --- | -1.6 | 0.08 | -2 | 0.089 | -1.45 | 0.253 | -3.16 | 0.063 | -2.05 |
| 1420771_at | Sprr2d | small proline-rich protein 2D | -1.63 | 0.381 | -2.44 | 0.028 | -1.26 | 0.544 | -1.59 | 0.409 | -1.73 |
| 1442803_at | Rad51l3 | RAD51-like 3 (S. cerevisiae) | -1.73 | 0.131 | -1.51 | 0.355 | -1.73 | 0.532 | -3.47 | 0.254 | -2.11 |
| 1436889_at | Gabra1 | gamma-aminobutyric acid (GABA-A) receptor, subunit alpha 1 | -3.83 | 0.015 | -1.19 | 0.745 | -1.4 | 0.202 | -1 | 0.995 | -1.85 |
| 1427421_at | Tcp10b | t-complex protein 10b | -1.88 | 0.242 | -1.59 | 0.353 | -1.52 | 0.514 | -1.67 | 0.397 | -1.67 |
| 1433924_at | Peg3 | paternally expressed 3 | -1.49 | 0.464 | -1.16 | 0.708 | -3.5 | 0.008 | -2.4 | 0.151 | -2.14 |
| 1454176_at | Ercc8 | excision repaiross-complementing rodent repair deficiency, complementation grou | -1.18 | 0.821 | -2.3 | 0.253 | -1.88 | 0.142 | -1.32 | 0.49 | -1.67 |
| 1431268_at | 1700047I17Rik | RIKEN cDNA 1700047I17 gene | -1.16 | 0.804 | -2.71 | 0.148 | -1.69 | 0.145 | -1.9 | 0.124 | -1.87 |
| 1460214_at | Pcp4 | Purkinje cell protein 4 | -1.21 | 0.304 | -2.01 | 0.353 | -2.03 | 0.1 | -2.23 | 0.194 | -1.87 |
| 1440644_at | Parp6 | poly (ADP-ribose) polymerase family, member 6 | -1.44 | 0.474 | -1.77 | 0.169 | -1.79 | 0.049 | -1.87 | 0.004 | -1.72 |

| | | | | | | | | | | | |
|---|---|---|---|---|---|---|---|---|---|---|---|
| 1442516_at | D2Ertd623e | DNA segment, Chr 2, ERATO Doi 623, expressed | -1.56 | 0.285 | -1.46 | 0.505 | -2.04 | 0.197 | -1.22 | 0.699 | -1.57 |
| 1456988_at | AI848258 | expressed sequence AI848258 | -2.41 | 0.359 | -1.68 | 0.366 | -1.23 | 0.711 | -1.05 | 0.776 | -1.59 |
| 1447149_at | --- | --- | -3.66 | 0.026 | -1.53 | 0.14 | -1.12 | 0.482 | -1.06 | 0.764 | -1.84 |
| 1440064_at | BC026657 | cDNA sequence BC026657 | -4.41 | 0.102 | -1.08 | 0.846 | -1.5 | 0.254 | -1.31 | 0.087 | -2.07 |
| 1440258_at | Kcnq2 | Potassium voltage-gated channel, subfamily Q, member 2 | -2.25 | 0.052 | -1.25 | 0.678 | -1.73 | 0.152 | -1.08 | 0.931 | -1.58 |
| 1426077_at | Moxd2 | monooxygenase, DBH-like 2 | -1.2 | 0.555 | -1.34 | 0.675 | -4.13 | 0.038 | -1.7 | 0.563 | -2.09 |
| 1417356_at | Peg3 | paternally expressed 3 | -1.23 | 0.769 | -1.41 | 0.608 | -3.35 | 0.166 | -1.93 | 0.23 | -1.98 |
| 1447791_s_at | Gna14 | guanine nucleotide binding protein, alpha 14 | -1.54 | 0.108 | -1.65 | 0.179 | -1.77 | 0.002 | -1.06 | 0.664 | -1.5 |
| 1431459_at | 9430013L14Rik | RIKEN cDNA 9430013L14 gene | -1.13 | 0.865 | -1.62 | 0.244 | -3.14 | 0.111 | -2.06 | 0.044 | -1.99 |
| 1426070_a_at | Kcnd3 | potassium voltage-gated channel, Shal-related family, member 3 | -1.32 | 0.29 | -5.42 | 0.028 | -1.13 | 0.815 | -1.26 | 0.762 | -2.28 |
| 1456946_at | 4831416G18Rik | RIKEN cDNA 4831416G18 gene | -1.74 | 0.071 | -1.61 | 0.125 | -1.59 | 0.143 | -1.09 | 0.643 | -1.51 |
| 1441537_at | Recql5 | RecQ protein-like 5 | -2.13 | 0.044 | -1.26 | 0.526 | -1.77 | 0.147 | -1.05 | 0.935 | -1.55 |
| 1450820_a_at | Ntng2 | netrin G2 | -1.91 | 0.287 | -2.23 | 0.219 | -1.16 | 0.543 | -1.23 | 0.439 | -1.64 |
| 1432078_at | 4930453L07Rik | RIKEN cDNA 4930453L07 gene | -1.82 | 0.48 | -1.25 | 0.524 | -2.09 | 0.123 | -1.34 | 0.363 | -1.63 |
| 1445169_at | --- | --- | -2.56 | 0.031 | -1.26 | 0.677 | -1.54 | 0.041 | -1.1 | 0.616 | -1.62 |
| 1429856_at | Tspan18 | tetraspanin 18 | -4.07 | 0.073 | -1.44 | 0.425 | -1.12 | 0.739 | -1.12 | 0.771 | -1.94 |
| 1459083_at | A330084C13Rik | RIKEN cDNA A330084C13 gene | -1.87 | 0.197 | -2.34 | 0.131 | -1.15 | 0.757 | -2.38 | 0.415 | -1.94 |
| 1458867_at | Edil3 | EGF-like repeats and discoidin I-like domains 3 | -1.36 | 0.704 | -2.59 | 0.028 | -1.41 | 0.308 | -1.12 | 0.861 | -1.62 |
| 1456306_a_at | Umod | uromodulin | -1.18 | 0.001 | -9.02 | 0.127 | -1.14 | 0.568 | -3.46 | 0.193 | -3.7 |
| 1459051_at | 6530418L21Rik | RIKEN cDNA 6530418L21 gene | -1.69 | 0.21 | -2.71 | 0.035 | -1.15 | 0.8 | -1.24 | 0.149 | -1.7 |
| 1425182_x_at | Klk1b22 /// Klk1b9 | kallikrein 1-related peptidase b22 /// kallikrein 1-related peptidase b9 | -1.66 | 0.086 | -1.68 | 0.338 | -1.57 | 0.421 | -1.56 | 0.293 | -1.62 |
| 1433395_at | 4921504P13Rik | RIKEN cDNA 4921504P13 gene | -1.85 | 0.041 | -1.22 | 0.685 | -2.13 | 0.135 | -1.22 | 0.774 | -1.6 |
| 1425922_a_at | Mycn | v-myc myelocytomatosis viral related oncogene, neuroblastoma derived (avian) | -1.91 | 0.223 | -2.27 | 0.154 | -1.15 | 0.707 | -1.14 | 0.804 | -1.62 |
| 1428077_at | Tmem163 | transmembrane protein 163 | -3.4 | 0.007 | -1.68 | 0.261 | -1.06 | 0.823 | -1.12 | 0.623 | -1.81 |
| 1458805_at | --- | --- | -1.28 | 0.094 | -1.64 | 0.166 | -2.25 | 0.041 | -1.21 | 0.539 | -1.6 |
| 1459550_at | Syn3 | Synapsin III | -2.39 | 0.46 | -1.15 | 0.64 | -1.82 | 0.133 | -1.77 | 0.388 | -1.78 |
| 1421740_at | Gnas | GNAS (guanine nucleotide binding protein, alpha stimulating) complex locus | -2.11 | 0.302 | -2.53 | 0.109 | -1.04 | 0.706 | -1.51 | 0.339 | -1.8 |
| 1439693_a_at | Fbxw16 | F-box and WD-40 domain protein 16 | -1.89 | 0.191 | -1.61 | 0.211 | -1.46 | 0.443 | -1.29 | 0.238 | -1.56 |
| 1442695_at | C030007I01Rik | RIKEN cDNA C030007I01 gene | -1.33 | 0.449 | -1.69 | 0.187 | -2.03 | 0.053 | -2.29 | 0.012 | -1.84 |
| 1443915_at | Mrpl47 | mitochondrial ribosomal protein L47 | -1.45 | 0.15 | -2.14 | 0.085 | -1.47 | 0.275 | -1.09 | 0.853 | -1.54 |
| 1447098_at | --- | --- | -1.38 | 0.616 | -1.67 | 0.284 | -1.93 | 0.179 | -2.64 | 0.184 | -1.91 |
| 1439261_x_at | Mitd1 | MIT, microtubule interacting and transport, domain containing 1 | -2.19 | 0.122 | -1.1 | 0.691 | -2.11 | 0.125 | -1.23 | 0.41 | -1.66 |
| 1445561_at | Grip1 | Glutamate receptor interacting protein 1 | -1.57 | 0.355 | -1.32 | 0.03 | -2.25 | 0 | -1.51 | 0.023 | -1.66 |
| 1447844_at | --- | Transcribed locus | -1.84 | 0.205 | -1.93 | 0.133 | -1.29 | 0.336 | -1.52 | 0.248 | -1.64 |
| 1442226_at | 6430702L12 | hypothetical protein 6430702L12 | -1.22 | 0.539 | -2.56 | 0.272 | -1.59 | 0.013 | -3.9 | 0.148 | -2.32 |
| 1457482_at | Jarid1b | jumonji, AT rich interactive domain 1B (Rbp2 like) | -1.46 | 0.153 | -2.33 | 0.123 | -1.38 | 0.369 | -2.29 | 0.047 | -1.87 |
| 1422839_at | Neurog2 | neurogenin 2 | -1.26 | 0.453 | -2.15 | 0.156 | -1.72 | 0.37 | -1.73 | 0.543 | -1.72 |
| 1458354_x_at | Krt28 | keratin 28 | -1.2 | 0.415 | -1.48 | 0.516 | -3 | 0.041 | -2.24 | 0.497 | -1.98 |
| 1442018_at | AI426953 | expressed sequence AI426953 | -1.27 | 0.605 | -2.42 | 0.091 | -1.57 | 0.184 | -2.84 | 0.219 | -2.02 |
| 1458087_at | Stac3 | SH3 and cysteine rich domain 3 | -1.46 | 0.183 | -1.82 | 0.29 | -1.65 | 0.422 | -1.71 | 0.557 | -1.66 |
| 1429523_a_at | Slc39a5 | solute carrier family 39 (metal ion transporter), member 5 | -1.02 | 0.955 | -2.25 | 0.029 | -2.38 | 0.086 | -1.22 | 0.776 | -1.72 |
| 1455423_at | Ndg1 /// LOC62318 | Nur77 downstream gene 1 /// similar to chromosome 6 open reading frame 148 | -2.19 | 0.437 | -1.12 | 0.732 | -2.01 | 0.434 | -1.6 | 0.466 | -1.73 |
| 1432252_a_at | 4933406K04Rik | RIKEN cDNA 4933406K04 gene | -1.91 | 0.224 | -1.78 | 0.376 | -1.32 | 0.275 | -1.36 | 0.295 | -1.59 |
| 1457950_at | Lphn3 | Latrophilin 3 | -2.4 | 0.223 | -1.09 | 0.815 | -1.97 | 0.277 | -1.53 | 0.516 | -1.75 |
| 1438918_at | Fdft1 /// LOC62771 | farnesyl diphosphate farnesyl transferase 1 /// similar to farnesyl diphosphate farr | -2.33 | 0.245 | -1.04 | 0.92 | -2.2 | 0.203 | -1.4 | 0.246 | -1.74 |
| 1435214_at | Gja12 | gap junction membrane channel protein alpha 12 | -1.6 | 0.596 | -4.52 | 0.027 | -1 | 0.998 | -1.12 | 0.827 | -2.06 |
| 1426990_at | Cubn | cubilin (intrinsic factor-cobalamin receptor) | -1.03 | 0.708 | -4.84 | 0.108 | -1.51 | 0.386 | -1.03 | 0.971 | -2.1 |
| 1453459_at | 1700028I16Rik | RIKEN cDNA 1700028I16 gene | -1.42 | 0.588 | -1.64 | 0.292 | -1.88 | 0.194 | -1.4 | 0.52 | -1.58 |
| 1445653_at | BC031361 | cDNA sequence BC031361 | -1.61 | 0.484 | -2.13 | 0.17 | -1.32 | 0.131 | -1.35 | 0.284 | -1.6 |
| 1454049_at | 4933426G20Rik | RIKEN cDNA 4933426G20 gene | -1.28 | 0.237 | -1.68 | 0.312 | -2.14 | 0.4 | -2.08 | 0.179 | -1.79 |
| 1457543_at | Nsun7 | NOL1/NOP2/Sun domain family, member 7 | -5.89 | 0.08 | -1.36 | 0.589 | -1.06 | 0.636 | -1.76 | 0.455 | -2.52 |
| 1419968_at | C77370 | expressed sequence C77370 | -2.77 | 0.267 | -1.98 | 0.148 | -1.02 | 0.941 | -1.42 | 0.582 | -1.8 |
| 1443510_at | AW822216 | expressed sequence AW822216 | -1.17 | 0.509 | -2.16 | 0.322 | -1.88 | 0.377 | -3.42 | 0.23 | -2.16 |

| | | | | | | | | | | | |
|---|---|---|---|---|---|---|---|---|---|---|---|
| 1444567_at | 1700066J03Rik | RIKEN cDNA 1700066J03 gene | -1.18 | 0.711 | -1.81 | 0.377 | -2.22 | 0.004 | -1.63 | 0.353 | -1.71 |
| 1431650_at | 1110054P19Rik | RIKEN cDNA 1110054P19 gene | -3.26 | 0.291 | -1.63 | 0.129 | -1.07 | 0.875 | -1.3 | 0.574 | -1.82 |
| 1447620_at | Cno | cappuccino | -1.22 | 0.381 | -2.64 | 0.232 | -1.53 | 0.185 | -1.96 | 0.389 | -1.84 |
| 1441441_at | 2010007L08Rik | RIKEN cDNA 2010007L08 gene | -1.79 | 0.002 | -1.39 | 0.494 | -1.74 | 0.012 | -1.2 | 0.493 | -1.53 |
| 1460265_at | Pa2g4 | Proliferation-associated 2G4 | -1.39 | 0.451 | -1.85 | 0.333 | -1.69 | 0.052 | -1.86 | 0.205 | -1.7 |
| 1421017_at | Nrg3 | neuregulin 3 | -1.4 | 0.735 | -2.12 | 0.115 | -1.5 | 0.471 | -1.63 | 0.289 | -1.66 |
| 1436931_at | Rfx4 | regulatory factor X, 4 (influences HLA class II expression) | -2.43 | 0.038 | -1.21 | 0.487 | -1.62 | 0.436 | -2.4 | 0.153 | -1.91 |
| 1429037_at | 1700019A02Rik | RIKEN cDNA 1700019A02 gene | -2.37 | 0.317 | -1.03 | 0.962 | -2.19 | 0.043 | -1.15 | 0.753 | -1.68 |
| 1432065_at | 3100003L13Rik | RIKEN cDNA 3100003L13 gene | -1.69 | 0.072 | -2.69 | 0.028 | -1.12 | 0.745 | -1.67 | 0.121 | -1.79 |
| 1419629_at | Mesp2 | mesoderm posterior 2 | -3.45 | 0.134 | -1.19 | 0.838 | -1.38 | 0.031 | -1.66 | 0.256 | -1.92 |
| 1450547_x_at | Dub2 | deubiquitinating enzyme 2 | -2.72 | 0.227 | -1.13 | 0.86 | -1.66 | 0.321 | -1.74 | 0.273 | -1.81 |
| 1444023_at | Ank2 | ankyrin 2, brain | -1.86 | 0.149 | -1.61 | 0.178 | -1.43 | 0.188 | -1.19 | 0.759 | -1.52 |
| 1426338_a_at | Ntng1 | netrin G1 | -1.15 | 0.791 | -2.7 | 0.023 | -1.62 | 0.277 | -1.24 | 0.429 | -1.68 |
| 1422481_at | Krt1 | keratin 1 | -1.47 | 0.423 | -2.75 | 0.107 | -1.23 | 0.669 | -1.28 | 0.201 | -1.68 |
| 1446849_at | Auts2 | Autism susceptibility candidate 2 | -1.16 | 0.727 | -2.04 | 0.123 | -1.98 | 0.048 | -1.24 | 0.218 | -1.61 |
| 1433082_at | 4930448K20Rik | RIKEN cDNA 4930448K20 gene | -2.09 | 0.346 | -2.04 | 0.285 | -1.13 | 0.809 | -1.41 | 0.427 | -1.66 |
| 1420273_x_at | --- | --- | -1.39 | 0.449 | -1.56 | 0.289 | -2.01 | 0.177 | -1.26 | 0.517 | -1.55 |
| 1449447_at | Cst10 | cystatin 10 (chondrocytes) | -1.31 | 0.515 | -2.02 | 0.26 | -1.67 | 0.358 | -1.16 | 0.577 | -1.54 |
| 1457344_at | Neto2 | neuropilin (NRP) and tolloid (TLL)-like 2 | -1.34 | 0.624 | -2.69 | 0.118 | -1.35 | 0.627 | -1.29 | 0.642 | -1.67 |
| 1457786_at | Siglech | Sialic acid binding Ig-like lectin H | -1.29 | 0.581 | -1.62 | 0.263 | -2.13 | 0.06 | -1.15 | 0.695 | -1.55 |
| 1446892_at | Lrrc16 | Leucine rich repeat containing 16 | -1.15 | 0.718 | -2.24 | 0.16 | -1.83 | 0.012 | -3.47 | 0.044 | -2.17 |
| 1422236_at | 4930560E09Rik | RIKEN cDNA 4930560E09 gene | -1.75 | 0.553 | -1.41 | 0.365 | -1.72 | 0.16 | -1.45 | 0.451 | -1.58 |
| 1430286_s_at | Ppp1r14c | protein phosphatase 1, regulatory (inhibitor) subunit 14c | -1.39 | 0.069 | -1.35 | 0.228 | -2.49 | 0.003 | -1.01 | 0.985 | -1.56 |
| 1453888_at | Cpne4 | copine IV | -1.23 | 0.693 | -1.79 | 0.164 | -2.03 | 0.223 | -1.2 | 0.764 | -1.56 |
| 1453698_at | 6030451C04Rik | RIKEN cDNA 6030451C04 gene | -1.03 | 0.943 | -1.64 | 0.229 | -3.6 | 0.004 | -1.54 | 0.002 | -1.95 |
| 1459774_at | Tspan5 | Tetraspanin 5 | -1.28 | 0.256 | -1.62 | 0.479 | -2.17 | 0.057 | -2.03 | 0.173 | -1.77 |
| 1444754_at | Asxl2 | additional sex combs like 2 (Drosophila) | -2.02 | 0.361 | -1.77 | 0.047 | -1.24 | 0.596 | -2.02 | 0.174 | -1.77 |
| 1431259_at | --- | --- | -2.23 | 0.135 | -1.8 | 0.346 | -1.16 | 0.564 | -1.24 | 0.507 | -1.61 |
| 1444846_at | D15Ertd529e | DNA segment, Chr 15, ERATO Doi 529, expressed | -2.94 | 0.162 | -1.33 | 0.731 | -1.29 | 0.459 | -1.14 | 0.786 | -1.68 |
| 1430478_at | 3110043A19Rik | RIKEN cDNA 3110043A19 gene | -1.57 | 0.148 | -2.07 | 0.143 | -1.34 | 0.367 | -1.13 | 0.836 | -1.53 |
| 1457844_a_at | 8430427H17Rik | RIKEN cDNA 8430427H17 gene | -1.92 | 0.513 | -1.25 | 0.641 | -1.85 | 0.268 | -2.2 | 0.194 | -1.8 |
| 1459876_at | Npr1 | Natriuretic peptide receptor 1 | -1.4 | 0.08 | -1.6 | 0.145 | -1.9 | 0.063 | -1.3 | 0.145 | -1.55 |
| 1447428_at | --- | --- | -1.73 | 0.557 | -2.05 | 0.154 | -1.25 | 0.481 | -2.29 | 0.227 | -1.83 |
| 1443134_at | A930005G22Rik | RIKEN cDNA A930005G22 gene | -3.33 | 0.003 | -1.28 | 0.744 | -1.27 | 0.559 | -1.4 | 0.641 | -1.82 |
| 1425604_at | Crkl | v-crk sarcoma virus CT10 oncogene homolog (avian)-like | -1.85 | 0.538 | -1.24 | 0.741 | -1.92 | 0.281 | -1 | 0.998 | -1.5 |
| 1432497_at | Ccdc93 | coiled-coil domain containing 93 | -1.3 | 0.723 | -2 | 0.389 | -1.67 | 0.298 | -1.09 | 0.422 | -1.52 |
| 1440935_at | Grb10 | Growth factor receptor bound protein 10 | -1.26 | 0.261 | -1.48 | 0.483 | -2.48 | 0.035 | -1.11 | 0.699 | -1.58 |
| 1421532_at | Rxfp2 | relaxin/insulin-like family peptide receptor 2 | -1.22 | 0.527 | -1.99 | 0.092 | -1.82 | 0.018 | -1.6 | 0.305 | -1.66 |
| 1431685_at | 4930549O18Rik | RIKEN cDNA 4930549O18 gene | -1.75 | 0.136 | -2.13 | 0.345 | -1.21 | 0.785 | -1.3 | 0.333 | -1.6 |
| 1459191_at | B130020M22Rik | RIKEN cDNA B130020M22 gene | -1.6 | 0.506 | -2.51 | 0.254 | -1.18 | 0.765 | -1.65 | 0.224 | -1.73 |
| 1442576_at | Creb5 | CAMP responsive element binding protein 5 | -1.92 | 0.391 | -1.57 | 0.253 | -1.4 | 0.469 | -1.62 | 0.605 | -1.63 |
| 1456517_at | Tmem44 | transmembrane protein 44 | -2.03 | 0.113 | -2.16 | 0.184 | -1.09 | 0.719 | -1.02 | 0.956 | -1.58 |
| 1456591_x_at | Akap3 | A kinase (PRKA) anchor protein 3 | -1.49 | 0.698 | -1.92 | 0.367 | -1.47 | 0.409 | -1.21 | 0.76 | -1.52 |
| 1443154_at | 6030438J01 | hypothetical protein 6030438J01 | -1.92 | 0.042 | -1.61 | 0.349 | -1.37 | 0.115 | -1.42 | 0.184 | -1.58 |
| 1459000_at | --- | --- | -1.58 | 0.328 | -1.3 | 0.522 | -2.1 | 0.148 | -1.31 | 0.469 | -1.58 |
| 1443772_at | --- | --- | -1.39 | 0.472 | -3.24 | 0.206 | -1.18 | 0.079 | -2.6 | 0.048 | -2.1 |
| 1448608_at | Dtprp | decidual/trophoblast prolactin-related protein | -2.6 | 0.063 | -1.15 | 0.623 | -1.61 | 0.036 | -1.02 | 0.939 | -1.59 |
| 1442262_at | --- | --- | -1.94 | 0.124 | -1.72 | 0.021 | -1.28 | 0.399 | -3.65 | 0.079 | -2.15 |
| 1439468_at | Bach2 | BTB and CNC homology 2 | -1.64 | 0.029 | -1.31 | 0.287 | -1.98 | 0.048 | -2.26 | 0.122 | -1.8 |
| 1458742_at | Lphn3 | latrophilin 3 | -1.27 | 0.777 | -2.96 | 0.051 | -1.33 | 0.297 | -1.69 | 0.405 | -1.81 |
| 1439044_at | Zfp354c | zinc finger protein 354C | -1.15 | 0.491 | -2.1 | 0.043 | -1.87 | 0.064 | -2.02 | 0.216 | -1.79 |
| 1443228_at | --- | --- | -1.57 | 0.227 | -1.8 | 0.229 | -1.46 | 0.191 | -1.2 | 0.72 | -1.51 |
| 1440484_at | Unc5d | unc-5 homolog D (C. elegans) | -1.12 | 0.693 | -2.36 | 0.016 | -1.78 | 0.182 | -1.38 | 0.637 | -1.66 |

| | | | | | | | | | | | |
|---|---|---|---|---|---|---|---|---|---|---|---|
| 1458804_at | Slco4c1 | Solute carrier organic anion transporter family, member 4C1 | -4.56 | 0.025 | -1.03 | 0.962 | -1.46 | 0.493 | -1.54 | 0.489 | -2.14 |
| 1432854_at | 1700018P08Rik | RIKEN cDNA 1700018P08 gene | -1.36 | 0.281 | -1.61 | 0.154 | -1.92 | 0.378 | -1.06 | 0.819 | -1.49 |
| 1445318_at | Klhl4 | Kelch-like 4 (Drosophila) | -1.22 | 0.84 | -2.05 | 0.034 | -1.75 | 0.329 | -1.57 | 0.074 | -1.65 |
| 1433192_at | A430105P17Rik | RIKEN cDNA A430105P17 gene | -1.44 | 0.296 | -3.53 | 0.043 | -1.11 | 0.704 | -1.35 | 0.557 | -1.86 |
| 1453218_at | 1110014K05Rik | RIKEN cDNA 1110014K05 gene | -1.55 | 0.091 | -2.25 | 0.233 | -1.26 | 0.35 | -1.68 | 0.026 | -1.69 |
| 1432878_at | 4930544N03Rik | RIKEN cDNA 4930544N03 gene | -1.76 | 0.216 | -2.31 | 0.232 | -1.14 | 0.494 | -3.67 | 0.033 | -2.22 |
| 1458238_at | Arid5b | AT rich interactive domain 5B (Mrf1 like) | -1.77 | 0.086 | -2.5 | 0.075 | -1.09 | 0.729 | -1.32 | 0.373 | -1.67 |
| 1431475_a_at | Hoxa10 | homeo box A10 | -1.23 | 0.318 | -2.36 | 0.052 | -1.55 | 0.002 | -1.09 | 0.875 | -1.56 |
| 1421562_at | Cd209c | CD209c antigen | -3.06 | 0.023 | -1.15 | 0.68 | -1.45 | 0.208 | -4.73 | 0 | -2.6 |
| 1457517_at | A730046G19Rik | RIKEN cDNA A730046G19 gene | -1.4 | 0.312 | -1.85 | 0.239 | -1.59 | 0.154 | -1.1 | 0.633 | -1.49 |
| 1431195_at | Rnf170 | ring finger protein 170 | -1.25 | 0.669 | -2 | 0.285 | -1.72 | 0.077 | -1.35 | 0.157 | -1.58 |
| 1459654_at | --- | --- | -1.06 | 0.535 | -1.92 | 0.069 | -2.41 | 0.152 | -1.9 | 0.062 | -1.82 |
| 1430460_at | 5830410F13Rik | RIKEN cDNA 5830410F13 gene | -1.45 | 0.201 | -1.97 | 0.034 | -1.45 | 0.003 | -1.52 | 0.151 | -1.6 |
| 1433416_at | 5430432H19Rik | RIKEN cDNA 5430432H19 gene | -1.26 | 0.711 | -2.2 | 0.148 | -1.57 | 0.415 | -1.16 | 0.79 | -1.55 |
| 1431376_at | Wdr62 | WD repeat domain 62 | -1.64 | 0.609 | -1.83 | 0.246 | -1.37 | 0.422 | -1.84 | 0.288 | -1.67 |
| 1442541_at | Oaz2 | Ornithine decarboxylase antizyme 2 | -1.46 | 0.272 | -1.8 | 0.053 | -1.54 | 0.238 | -1.38 | 0.001 | -1.55 |
| 1443593_at | --- | --- | -1.79 | 0.189 | -1.12 | 0.834 | -2.29 | 0.067 | -1.35 | 0.632 | -1.64 |
| 1457898_at | Ga17 | Dendritic cell protein GA17 | -1.13 | 0.635 | -1.41 | 0.362 | -3.45 | 0.008 | -1.89 | 0.123 | -1.97 |
| 1456152_at | Paqr5 | progestin and adipoQ receptor family member V | -1.02 | 0.744 | -1.82 | 0.203 | -2.81 | 0.092 | -2.13 | 0.008 | -1.94 |
| 1442992_at | 130004C03 | hypothetical LOC403343 | -1.3 | 0.199 | -1.58 | 0.026 | -2.05 | 0.094 | -1.68 | 0.058 | -1.65 |
| 1421919_a_at | Ccr9 | chemokine (C-C motif) receptor 9 | -1.69 | 0.303 | -2.48 | 0.166 | -1.12 | 0.718 | -1.63 | 0.12 | -1.73 |
| 1437796_at | --- | Transcribed locus | -1.58 | 0.006 | -1.21 | 0.584 | -2.33 | 0.055 | -1.74 | 0.087 | -1.71 |
| 1459055_at | Foxj3 | forkhead box J3 | -2.59 | 0.167 | -1.16 | 0.763 | -1.56 | 0.248 | -1.29 | 0.563 | -1.65 |
| 1442373_at | 2610203C20Rik | RIKEN cDNA 2610203C20 gene | -1.5 | 0.43 | -1.19 | 0.734 | -2.63 | 0.023 | -1.17 | 0.769 | -1.62 |
| 1447743_x_at | Ext2 | exostoses (multiple) 2 | -2.27 | 0.037 | -1.67 | 0.018 | -1.18 | 0.49 | -1.28 | 0.093 | -1.6 |
| 1447729_s_at | 1700036D21Rik | RIKEN cDNA 1700036D21 gene | -1.84 | 0.208 | -1.75 | 0.344 | -1.29 | 0.246 | -1.56 | 0.028 | -1.61 |
| 1454360_at | 4930418C01Rik | RIKEN cDNA 4930418C01 gene | -1.09 | 0.749 | -2.15 | 0.171 | -1.95 | 0.391 | -2.06 | 0.028 | -1.81 |
| 1443456_at | 6330416L07Rik | RIKEN cDNA 6330416L07 gene | -1.45 | 0.082 | -1.62 | 0.055 | -1.72 | 0.081 | -1.08 | 0.683 | -1.46 |
| 1458872_at | --- | --- | -1.24 | 0.112 | -2.23 | 0.035 | -1.57 | 0.329 | -1.17 | 0.766 | -1.55 |
| 1423379_at | Nfatc4 /// LOC6720 | nuclear factor of activated T-cells, cytoplasmic, calcineurin-dependent 4 /// similar | -1.6 | 0.096 | -1.97 | 0.056 | -1.31 | 0.245 | -1.63 | 0.54 | -1.63 |
| 1441369_at | C030017B01Rik | RIKEN cDNA C030017B01 gene | -2.47 | 0.447 | -1.08 | 0.878 | -1.78 | 0.274 | -1.07 | 0.721 | -1.6 |
| 1442667_at | Cdc40 | cell division cycle 40 homolog (yeast) | -1.45 | 0.696 | -1.24 | 0.559 | -2.52 | 0.032 | -1.28 | 0.544 | -1.62 |
| 1440624_at | Ptprd | Protein tyrosine phosphatase, receptor type, D | -1.48 | 0.094 | -1.48 | 0.09 | -1.83 | 0.087 | -1.33 | 0.519 | -1.53 |
| 1420450_at | Mmp10 | matrix metallopeptidase 10 | -1.56 | 0.059 | -2.57 | 0.29 | -1.16 | 0.603 | -1.3 | 0.381 | -1.65 |
| 1422247_a_at | Uty | ubiquitously transcribed tetratricopeptide repeat gene, Y chromosome | -1.2 | 0.698 | -4.13 | 0.033 | -1.22 | 0.682 | -1.4 | 0.395 | -1.99 |
| 1454691_at | Nrxn1 | neurexin I | -1.43 | 0.607 | -1.15 | 0.5 | -3.06 | 0.091 | -1.24 | 0.516 | -1.72 |
| 1423279_at | Slc34a1 | solute carrier family 34 (sodium phosphate), member 1 | -1.23 | 0.16 | -4.31 | 0.153 | -1.18 | 0.622 | -1.11 | 0.764 | -1.96 |
| 1431407_at | 2310024H09Rik | RIKEN cDNA 2310024H09 gene | -1.94 | 0.424 | -1.19 | 0.696 | -1.85 | 0.222 | -1.23 | 0.709 | -1.55 |
| 1435460_at | Prkg2 | protein kinase, cGMP-dependent, type II | -1.46 | 0.033 | -1.88 | 0.156 | -1.47 | 0.077 | -1.46 | 0.425 | -1.57 |
| 1458901_at | Mef2d | Myocyte enhancer factor 2D | -1.46 | 0.005 | -3.15 | 0.014 | -1.12 | 0.591 | -1.21 | 0.371 | -1.73 |
| 1441718_at | Edd1 | E3 ubiquitin protein ligase, HECT domain containing, 1 | -1.37 | 0.202 | -1.42 | 0.072 | -2.15 | 0.01 | -1.22 | 0.121 | -1.54 |
| 1418726_a_at | Tnnt2 | troponin T2, cardiac | -3.58 | 0.191 | -1.61 | 0.154 | -1 | 0.991 | -1.23 | 0.077 | -1.86 |
| 1449967_at | Sim1 | single-minded homolog 1 (Drosophila) | -1.09 | 0.513 | -3.21 | 0.005 | -1.5 | 0.143 | -1.62 | 0.407 | -1.86 |
| 1454315_at | 3300002A11Rik | RIKEN cDNA 3300002A11 gene | -1.93 | 0.336 | -2.31 | 0.177 | -1.06 | 0.821 | -1.09 | 0.44 | -1.59 |
| 1441498_at | Ptprd | Protein tyrosine phosphatase, receptor type, D | -1.29 | 0.312 | -1.74 | 0.069 | -1.82 | 0.052 | -1.19 | 0.593 | -1.51 |
| 1446967_at | Fbxl7 | F-box and leucine-rich repeat protein 7 | -1.21 | 0.093 | -1.69 | 0.027 | -2.08 | 0.007 | -1.78 | 0.038 | -1.69 |
| 1440549_at | B230334L07Rik | RIKEN cDNA B230334L07 gene | -1.24 | 0.303 | -1.73 | 0.124 | -1.95 | 0.001 | -2.11 | 0.041 | -1.76 |
| 1454394_at | 4932432N04Rik | RIKEN cDNA 4932432N04 gene | -2.11 | 0.501 | -1.18 | 0.706 | -1.73 | 0.385 | -1.68 | 0.496 | -1.68 |
| 1458054_at | Ext1 | Exostoses (multiple) 1 | -1.35 | 0.457 | -1.68 | 0.121 | -1.76 | 0.046 | -1 | 0.985 | -1.45 |
| 1445682_at | 2700099C18Rik | RIKEN cDNA 2700099C18 gene | -2.14 | 0.253 | -1.36 | 0.194 | -1.43 | 0.446 | -1.24 | 0.304 | -1.54 |
| 1449963_at | 2310040M23Rik | RIKEN cDNA 2310040M23 gene | -2.1 | 0.16 | -1.22 | 0.782 | -1.65 | 0.338 | -2.23 | 0.543 | -1.8 |
| 1445791_at | --- | --- | -4.13 | 0.032 | -1.01 | 0.963 | -1.48 | 0.203 | -1.06 | 0.924 | -1.92 |
| 1456564_at | C030046I01Rik | RIKEN cDNA C030046I01 gene | -1.36 | 0.455 | -2.27 | 0.163 | -1.38 | 0.131 | -1.07 | 0.831 | -1.52 |

| | | | | | | | | | | | |
|---|---|---|---|---|---|---|---|---|---|---|---|
| 1423037_at | Agtrl1 | angiotensin receptor-like 1 | -1.08 | 0.752 | -1.67 | 0.085 | -2.64 | 0.048 | -1.68 | 0.143 | -1.77 |
| 1441393_at | --- | --- | -1.15 | 0.848 | -1.92 | 0.122 | -1.93 | 0.133 | -1.25 | 0.517 | -1.56 |
| 1438896_at | Dnajc6 | DnaJ (Hsp40) homolog, subfamily C, member 6 | -1.29 | 0.49 | -2.72 | 0 | -1.31 | 0.585 | -1.08 | 0.8 | -1.6 |
| 1430646_at | 5830407P18Rik | RIKEN cDNA 5830407P18 gene | -1.56 | 0.083 | -1.27 | 0.495 | -2.09 | 0.001 | -1.21 | 0.71 | -1.53 |
| 1445842_at | --- | --- | -1.96 | 0.287 | -1.3 | 0.679 | -1.59 | 0.122 | -1.2 | 0.44 | -1.51 |
| 1439591_at | Ulk2 | Unc-51 like kinase 2 (C. elegans) | -1.18 | 0.025 | -2.08 | 0.08 | -1.73 | 0.049 | -1.32 | 0.101 | -1.58 |
| 1459344_at | 9630019E01Rik | RIKEN cDNA 9630019E01 gene | -1.47 | 0.005 | -1.33 | 0.53 | -2.1 | 0.027 | -1.56 | 0.165 | -1.62 |
| 1443480_at | --- | --- | -1.15 | 0.808 | -1.8 | 0.136 | -2.07 | 0.036 | -1.29 | 0.253 | -1.58 |
| 1444479_at | --- | --- | -1.04 | 0.853 | -4.31 | 0.066 | -1.39 | 0.149 | -1.09 | 0.674 | -1.96 |
| 1445594_at | Hnrpl | Heterogeneous nuclear ribonucleoprotein L | -1.9 | 0.212 | -1.14 | 0.847 | -1.98 | 0.029 | -1.16 | 0.791 | -1.55 |
| 1453214_at | Lrrc15 | leucine rich repeat containing 15 | -1.74 | 0.504 | -1.39 | 0.48 | -1.63 | 0.436 | -1.11 | 0.849 | -1.47 |
| 1432485_at | 3110057O12Rik | RIKEN cDNA 3110057O12 gene | -1.02 | 0.962 | -2.82 | 0.185 | -1.74 | 0.255 | -1.06 | 0.814 | -1.66 |
| 1425661_at | Cdadc1 | cytidine and dCMP deaminase domain containing 1 | -1.32 | 0.228 | -1.14 | 0.816 | -3.65 | 0.01 | -1.42 | 0.659 | -1.88 |
| 1443284_at | Satb1 | Special AT-rich sequence binding protein 1 | -1.17 | 0.552 | -1.44 | 0.281 | -2.77 | 0.073 | -4.26 | 0.04 | -2.41 |
| 1439321_at | Arid1b | AT rich interactive domain 1B (Swi1 like) | -1.48 | 0.199 | -1.41 | 0.146 | -1.89 | 0.062 | -1.67 | 0.161 | -1.62 |
| 1441446_at | --- | --- | -1.13 | 0.837 | -2.97 | 0.128 | -1.44 | 0.197 | -1.63 | 0.276 | -1.8 |
| 1431590_at | 4930521C21Rik | RIKEN cDNA 4930521C21 gene | -1.77 | 0.117 | -1.99 | 0.269 | -1.18 | 0.068 | -1.26 | 0.26 | -1.55 |
| 1438697_at | 4632425D07Rik | RIKEN cDNA 4632425D07 gene | -1.37 | 0.471 | -1.94 | 0.116 | -1.49 | 0.02 | -1.35 | 0.113 | -1.54 |
| 1440309_at | 4833430A08Rik | RIKEN cDNA 4833430A08 gene | -1.87 | 0.135 | -1.13 | 0.727 | -2.01 | 0.006 | -1.5 | 0.051 | -1.63 |
| 1442107_at | Flnb | filamin, beta | -1.4 | 0.211 | -1.48 | 0.109 | -1.89 | 0.155 | -1.21 | 0.507 | -1.5 |
| 1442854_at | --- | Transcribed locus | -2.12 | 0.081 | -2.11 | 0.408 | -1.03 | 0.961 | -1 | 0.995 | -1.57 |
| 1440097_at | Mirg | miRNA containing gene | -1.75 | 0.035 | -1.29 | 0.269 | -1.76 | 0.129 | -1.01 | 0.992 | -1.45 |
| 1431853_at | 4933413C19Rik | RIKEN cDNA 4933413C19 gene | -3.02 | 0.274 | -1.26 | 0.377 | -1.27 | 0.526 | -1.56 | 0.509 | -1.78 |
| 1457922_at | --- | --- | -2.31 | 0.502 | -1.03 | 0.967 | -1.96 | 0.218 | -1.12 | 0.79 | -1.6 |
| 1429932_at | 4930566F21Rik | RIKEN cDNA 4930566F21 gene | -1.34 | 0.509 | -1.76 | 0.159 | -1.65 | 0.013 | -2.07 | 0.127 | -1.71 |
| 1460003_at | AI956758 | expressed sequence AI956758 | -1.17 | 0.435 | -1.9 | 0.238 | -1.87 | 0.067 | -1.03 | 0.922 | -1.49 |
| 1422275_at | Gpr44 | G protein-coupled receptor 44 | -1.78 | 0.469 | -1.35 | 0.174 | -1.63 | 0.465 | -1.76 | 0.324 | -1.63 |
| 1423555_a_at | Ifi44 | interferon-induced protein 44 | -1.57 | 0.019 | -1.07 | 0.704 | -2.88 | 0.021 | -1.18 | 0.713 | -1.67 |
| 1432969_at | 4933423K11Rik | RIKEN cDNA 4933423K11 gene | -1.67 | 0.572 | -1.24 | 0.284 | -1.94 | 0.038 | -1.38 | 0.282 | -1.56 |
| 1432660_at | 5830420C07Rik | RIKEN cDNA 5830420C07 gene | -1.17 | 0.741 | -1.61 | 0.454 | -2.25 | 0.037 | -1.45 | 0.556 | -1.62 |
| 1432684_at | 4921521C08Rik | RIKEN cDNA 4921521C08 gene | -1.37 | 0.566 | -1.2 | 0.722 | -2.84 | 0.119 | -2.21 | 0.493 | -1.9 |
| 1422281_at | Sstr4 | somatostatin receptor 4 | -1.22 | 0.741 | -2.44 | 0.085 | -1.44 | 0.437 | -1.38 | 0.578 | -1.62 |
| 1431756_at | --- | Adult male testis cDNA, RIKEN full-length enriched library, clone:4930516B21 pro | -2.05 | 0.446 | -1.81 | 0.451 | -1.14 | 0.788 | -1.52 | 0.474 | -1.63 |
| 1420555_at | Alx3 | aristaless 3 | -2.67 | 0.39 | -1.09 | 0.694 | -1.59 | 0.451 | -1.44 | 0.641 | -1.7 |
| 1443204_at | Rbms3 | RNA binding motif, single stranded interacting protein | -1.18 | 0.604 | -1.74 | 0.068 | -2 | 0.044 | -1.1 | 0.739 | -1.51 |
| 1442069_at | D5Wsu178e | DNA segment, Chr 5, Wayne State University 178, expressed | -1.4 | 0.735 | -2.56 | 0.004 | -1.22 | 0.731 | -1.17 | 0.419 | -1.59 |
| 1440963_at | AI465270 | expressed sequence AI465270 | -1.2 | 0.732 | -2.45 | 0.1 | -1.47 | 0.147 | -1.17 | 0.547 | -1.57 |
| 1422587_at | Tmem45a | transmembrane protein 45a | -1.03 | 0.896 | -2.14 | 0.038 | -2.05 | 0.006 | -2.6 | 0.521 | -1.96 |
| 1446210_at | --- | --- | -1.8 | 0.574 | -1.42 | 0.072 | -1.51 | 0.063 | -1.2 | 0.477 | -1.48 |
| 1440119_at | Sipa1l1 | signal-induced proliferation-associated 1 like 1 | -1.16 | 0.602 | -3.87 | 0.099 | -1.25 | 0.38 | -1.27 | 0.415 | -1.89 |
| 1453785_at | Taz | tafazzin | -1.53 | 0.454 | -1.62 | 0.32 | -1.54 | 0.056 | -2 | 0.555 | -1.67 |
| 1459118_at | D230038C21 | hypothetical protein D230038C21 | -1.5 | 0.36 | -1.91 | 0.147 | -1.36 | 0.188 | -1.1 | 0.495 | -1.47 |
| 1425987_a_at | Kcnma1 | potassium large conductance calcium-activated channel, subfamily M, alpha men | -1.82 | 0.062 | -2.15 | 0.299 | -1.1 | 0.578 | -1.14 | 0.447 | -1.55 |
| 1458431_at | Eif4g2 | eukaryotic translation initiation factor 4, gamma 2 | -2.01 | 0.05 | -1.54 | 0.203 | -1.29 | 0.064 | -1.14 | 0.4 | -1.49 |
| 1446767_at | Bcl9 | B-cell CLL/lymphoma 9 | -1.36 | 0.643 | -1.93 | 0.085 | -1.5 | 0.477 | -1.91 | 0.147 | -1.67 |
| 1441869_x_at | Auts2 | Autism susceptibility candidate 2 | -1.34 | 0.07 | -1.52 | 0.106 | -1.92 | 0.08 | -1.5 | 0.286 | -1.57 |
| 1441744_at | Cacna1c | Calcium channel, voltage-dependent, L type, alpha 1C subunit | -1.71 | 0.354 | -1.22 | 0.647 | -1.92 | 0.078 | -1.45 | 0.524 | -1.57 |
| 1438398_at | Rnpc2 | RNA-binding region (RNP1, RRM) containing 2 | -2.2 | 0.423 | -1.76 | 0.192 | -1.11 | 0.755 | -2.35 | 0.22 | -1.85 |
| 1454253_at | 2810452K22Rik | RIKEN cDNA 2810452K22 gene | -2.29 | 0.249 | -1.74 | 0.054 | -1.09 | 0.671 | -1.28 | 0.522 | -1.6 |
| 1435848_at | D430041D05Rik | RIKEN cDNA D430041D05 gene | -1.48 | 0.481 | -1.41 | 0.491 | -1.86 | 0.205 | -1.66 | 0.164 | -1.6 |
| 1457572_at | Magi1 | Membrane associated guanylate kinase, WW and PDZ domain containing 1 | -2.45 | 0.222 | -1.19 | 0.089 | -1.48 | 0.234 | -1.27 | 0.741 | -1.6 |
| 1445706_x_at | --- | --- | -1.71 | 0.152 | -1.34 | 0.687 | -1.69 | 0.058 | -1.06 | 0.851 | -1.45 |
| 1441337_at | --- | Transcribed locus | -1.29 | 0.413 | -1.14 | 0.826 | -3.7 | 0.006 | -1.9 | 0.25 | -2.01 |

| | | | | | | | | | | | |
|---|---|---|---|---|---|---|---|---|---|---|---|
| 1433015_at | 6330436F06Rik | RIKEN cDNA 6330436F06 gene | -1.17 | 0.343 | -1.58 | 0.222 | -2.27 | 0.033 | -1.11 | 0.818 | -1.53 |
| 1453905_at | 5033415L01Rik | RIKEN cDNA 5033415L01 gene | -1.41 | 0.031 | -1.24 | 0.763 | -2.41 | 0.021 | -1.15 | 0.558 | -1.55 |
| 1445033_at | Abca16 | ATP-binding cassette, sub-family A (ABC1), member 16 | -1.25 | 0.388 | -1.84 | 0.454 | -1.71 | 0.129 | -1.02 | 0.893 | -1.46 |
| 1457203_at | --- | Transcribed locus | -1.39 | 0.678 | -1.79 | 0.174 | -1.54 | 0.088 | -1.27 | 0.768 | -1.5 |
| 1446007_at | Bmp1 | Bone morphogenetic protein 1 | -1.23 | 0.786 | -1.2 | 0.438 | -3.5 | 0.089 | -1.79 | 0.448 | -1.93 |
| 1444339_at | --- | 0 day neonate kidney cDNA, RIKEN full-length enriched library, clone:D630003P1 | -1.27 | 0.168 | -1.49 | 0.234 | -2.13 | 0.009 | -1.06 | 0.855 | -1.49 |
| 1452561_at | Lphn3 | Latrophilin 3 | -1.42 | 0.338 | -1.19 | 0.791 | -2.59 | 0.042 | -1.87 | 0.156 | -1.77 |
| 1450289_at | Foxd2 | forkhead box D2 | -1.81 | 0.617 | -1.21 | 0.475 | -1.82 | 0.31 | -1.62 | 0.078 | -1.61 |
| 1430083_at | 2610307P16Rik | RIKEN cDNA 2610307P16 gene | -1.77 | 0.503 | -1.44 | 0.492 | -1.49 | 0.001 | -1.18 | 0.789 | -1.47 |
| 1430616_at | 4930528A17Rik | RIKEN cDNA 4930528A17 gene | -1.49 | 0.043 | -2.55 | 0.406 | -1.16 | 0.728 | -1.06 | 0.819 | -1.57 |
| 1459829_x_at | BC031781 | CDNA sequence BC031781 | -1.74 | 0.209 | -1.49 | 0.135 | -1.46 | 0.116 | -1.19 | 0.504 | -1.47 |
| 1431670_at | Ndufc2 | NADH dehydrogenase (ubiquinone) 1, subcomplex unknown, 2 | -2.5 | 0.171 | -1.33 | 0.624 | -1.29 | 0.625 | -2.2 | 0.316 | -1.83 |
| 1440537_at | Kcnv2 | potassium channel, subfamily V, member 2 | -3.01 | 0.003 | -1.27 | 0.393 | -1.24 | 0.386 | -1.55 | 0.019 | -1.77 |
| 1452730_at | 1110033J19Rik | RIKEN cDNA 1110033J19 gene | -1.19 | 0.194 | -2.65 | 0.003 | -1.41 | 0.157 | -1.83 | 0.138 | -1.77 |
| 1447997_s_at | Timm8a2 | translocase of inner mitochondrial membrane 8 homolog a2 (yeast) | -2.72 | 0.133 | -1.26 | 0.73 | -1.3 | 0.555 | -1.23 | 0.762 | -1.63 |
| 1436646_at | --- | Transcribed locus | -1.36 | 0.224 | -3.75 | 0.103 | -1.08 | 0.803 | -1.33 | 0.067 | -1.88 |
| 1433303_at | 4930483C01Rik | RIKEN cDNA 4930483C01 gene | -1.63 | 0.634 | -3.05 | 0.253 | -1.01 | 0.975 | -1.15 | 0.643 | -1.71 |
| 1459595_at | AA516738 | Expressed sequence AA516738 | -1.31 | 0.142 | -1.5 | 0.364 | -2 | 0.041 | -1.77 | 0.046 | -1.64 |
| 1435201_at | 0610009B10Rik | RIKEN cDNA 0610009B10 gene | -1.01 | 0.915 | -5.12 | 0.134 | -1.34 | 0.596 | -1.51 | 0.568 | -2.25 |
| 1436406_at | A930007I19Rik | RIKEN cDNA A930007I19 gene | -2.11 | 0.249 | -1.3 | 0.042 | -1.46 | 0.298 | -1.37 | 0.34 | -1.56 |
| 1457334_at | C130057M05Rik | RIKEN cDNA C130057M05 gene | -1.29 | 0.044 | -1.86 | 0.114 | -1.61 | 0.047 | -1.07 | 0.804 | -1.46 |
| 1441242_at | Dpp4 | Dipeptidylpeptidase 4 | -1.44 | 0.173 | -1.48 | 0.149 | -1.77 | 0.061 | -2.72 | 0.058 | -1.85 |
| 1420086_x_at | Fgf4 | fibroblast growth factor 4 | -2.39 | 0.302 | -1.09 | 0.822 | -1.67 | 0.232 | -1.48 | 0.294 | -1.66 |
| 1442752_at | Opcml | Opioid binding protein/cell adhesion molecule-like | -1.56 | 0.283 | -2.09 | 0.259 | -1.23 | 0.753 | -1.06 | 0.886 | -1.49 |
| 1458743_at | --- | --- | -1.02 | 0.97 | -1.67 | 0.39 | -2.86 | 0.128 | -1.48 | 0.571 | -1.75 |
| 1446187_at | 4831416G18Rik | RIKEN cDNA 4831416G18 gene | -2.92 | 0.011 | -1.29 | 0.42 | -1.22 | 0.658 | -1.31 | 0.557 | -1.69 |
| 1440954_at | Pbx1 | Pre B-cell leukemia transcription factor 1 | -1.43 | 0.107 | -1.26 | 0.157 | -2.26 | 0.001 | -1.12 | 0.524 | -1.52 |
| 1459689_at | Gpd2 | Glycerol phosphate dehydrogenase 2, mitochondrial | -1.76 | 0.131 | -1.57 | 0.134 | -1.37 | 0.065 | -1.63 | 0.198 | -1.58 |
| 1437307_at | Senp8 | SUMO/sentrin specific peptidase 8 | -1.15 | 0.205 | -2.71 | 0.396 | -1.43 | 0.161 | -1.19 | 0.305 | -1.62 |
| 1443151_at | Synj2bp | Synaptojanin 2 binding protein | -1.41 | 0.027 | -1.51 | 0.075 | -1.77 | 0.027 | -1.72 | 0.122 | -1.6 |
| 1420195_at | --- | --- | -1.66 | 0.316 | -1.94 | 0.138 | -1.22 | 0.541 | -1.11 | 0.556 | -1.48 |
| 1444185_at | Stim2 | Stromal interaction molecule 2 | -1.28 | 0.121 | -1.68 | 0.16 | -1.79 | 0.009 | -1.63 | 0.132 | -1.6 |
| 1440643_at | --- | --- | -1.18 | 0.418 | -2.22 | 0.344 | -1.57 | 0.157 | -1.32 | 0.502 | -1.57 |
| 1459157_at | Stox2 | Storkhead box 2 | -1.35 | 0.358 | -1.7 | 0.11 | -1.64 | 0.494 | -1.14 | 0.601 | -1.46 |
| 1441325_at | Nkrf | NF-kappaB repressing factor | -1.28 | 0.055 | -1.31 | 0.532 | -2.55 | 0.113 | -1.2 | 0.55 | -1.59 |
| 1444939_at | --- | --- | -1.45 | 0.291 | -1.29 | 0.694 | -2.13 | 0.044 | -2.29 | 0.258 | -1.79 |
| 1443129_at | --- | --- | -1.74 | 0.431 | -1.53 | 0.251 | -1.42 | 0.188 | -1.22 | 0.595 | -1.48 |
| 1454115_at | 4930572J10Rik | RIKEN cDNA 4930572J10 gene | -1.13 | 0.816 | -1.93 | 0.319 | -1.87 | 0.081 | -1.45 | 0.321 | -1.6 |
| 1453143_at | 4921509F24Rik | RIKEN cDNA 4921509F24 gene | -3.28 | 0.158 | -1.33 | 0.689 | -1.14 | 0.607 | -1.34 | 0.478 | -1.77 |
| 1436035_at | 3830431G21Rik | RIKEN cDNA 3830431G21 gene | -1.28 | 0.047 | -1.91 | 0.268 | -1.59 | 0.02 | -1.29 | 0.637 | -1.52 |
| 1435775_at | Clock | circadian locomoter output cycles kaput | -1.32 | 0.123 | -1.66 | 0.031 | -1.74 | 0.151 | -1.01 | 0.975 | -1.43 |
| 1429977_at | 9030425L15Rik | RIKEN cDNA 9030425L15 gene | -1.28 | 0.124 | -1.25 | 0.363 | -2.81 | 0.007 | -1.23 | 0.492 | -1.64 |
| 1443302_at | 6720403M19Rik | RIKEN cDNA 6720403M19 gene | -1.75 | 0.043 | -1.26 | 0.293 | -1.76 | 0.109 | -1.6 | 0.041 | -1.59 |
| 1444684_at | 8030475D13Rik | RIKEN cDNA 8030475D13 gene | -1.36 | 0.498 | -1.67 | 0.16 | -1.66 | 0.461 | -1.24 | 0.603 | -1.48 |
| 1459809_x_at | 1700063D05Rik | RIKEN cDNA 1700063D05 gene | -1.76 | 0.454 | -1.05 | 0.888 | -2.38 | 0.1 | -1.37 | 0.664 | -1.64 |
| 1439681_at | Farp2 | FERM, RhoGEF and pleckstrin domain protein 2 | -1.8 | 0.462 | -1.49 | 0.427 | -1.4 | 0.484 | -3.04 | 0.067 | -1.93 |
| 1433247_at | C530007A02Rik | RIKEN cDNA C530007A02 gene | -1.51 | 0.647 | -2.03 | 0.164 | -1.28 | 0.689 | -1.28 | 0.768 | -1.52 |
| 1424798_a_at | Adam5 | a disintegrin and metallopeptidase domain 5 | -1.1 | 0.832 | -1.78 | 0.075 | -2.13 | 0.034 | -1.05 | 0.912 | -1.51 |
| 1446545_at | Spnb2 | Spectrin beta 2 | -1.57 | 0.331 | -1.36 | 0.213 | -1.75 | 0.181 | -1.06 | 0.92 | -1.44 |
| 1440023_at | Pcdh12 | protocadherin 12 | -1.36 | 0.524 | -1.44 | 0.158 | -1.97 | 0.327 | -1.37 | 0.528 | -1.53 |
| 1446947_at | --- | --- | -1.57 | 0.431 | -1.23 | 0.676 | -2.03 | 0.085 | -1.18 | 0.801 | -1.5 |
| 1445893_at | Plod1 | procollagen-lysine, 2-oxoglutarate 5-dioxygenase 1 | -2.13 | 0.018 | -1.58 | 0.314 | -1.19 | 0.455 | -1.32 | 0.541 | -1.55 |
| 1433317_at | 4930477O15Rik | RIKEN cDNA 4930477O15 gene | -3 | 0.002 | -1.35 | 0.553 | -1.16 | 0.738 | -1.07 | 0.935 | -1.64 |

| | | | | | | | | | | | |
|---|---|---|---|---|---|---|---|---|---|---|---|
| 1439640_at | --- | 16 days neonate thymus cDNA, RIKEN full-length enriched library, clone:A13004 | -1.58 | 0.099 | -1.46 | 0.223 | -1.6 | 0.145 | -1.19 | 0.508 | -1.46 |
| 1442180_at | Dleu7 | deleted in lymphocytic leukemia, 7 | -1.32 | 0.288 | -2.04 | 0.073 | -1.44 | 0.281 | -1.46 | 0.137 | -1.57 |
| 1432744_at | 4930443G03Rik | RIKEN cDNA 4930443G03 gene | -4.41 | 0.379 | -1.38 | 0.569 | -1.01 | 0.977 | -1.94 | 0.36 | -2.18 |
| 1420701_at | Klk1b1 | kallikrein 1-related peptidase b1 | -1.5 | 0.644 | -1.48 | 0.119 | -1.66 | 0.152 | -1.11 | 0.531 | -1.44 |
| 1430243_at | 1700058C01Rik | RIKEN cDNA 1700058C01 gene | -1.71 | 0.145 | -1.54 | 0.351 | -1.41 | 0.525 | -1.36 | 0.591 | -1.5 |
| 1441562_at | A230048O21Rik | RIKEN cDNA A230048O21 gene | -1.23 | 0.151 | -2.3 | 0.193 | -1.44 | 0.469 | -1.26 | 0.61 | -1.56 |
| 1429894_a_at | Mtap7 | microtubule-associated protein 7 | -1.14 | 0.73 | -2.98 | 0.213 | -1.37 | 0.471 | -1.23 | 0.749 | -1.68 |
| 1449428_at | Cldn18 | claudin 18 | -2.65 | 0.165 | -1 | 0.992 | -1.76 | 0.065 | -1.82 | 0.442 | -1.81 |
| 1440107_at | Smg7 | Smg-7 homolog, nonsense mediated mRNA decay factor (C. elegans) | -1.25 | 0.099 | -1.55 | 0.018 | -2.01 | 0.009 | -1.63 | 0.032 | -1.61 |
| 1430380_at | Hemk2 | HemK methyltransferase family member 2 | -1.15 | 0.749 | -1.58 | 0.457 | -2.27 | 0.011 | -2.1 | 0.113 | -1.77 |
| 1447697_at | Ccdc40 | Coiled-coil domain containing 40 | -1.32 | 0.373 | -1.59 | 0.516 | -1.79 | 0.066 | -1.2 | 0.713 | -1.47 |
| 1439354_at | LOC668373 /// LOC | region containing RIKEN cDNA 2310008H09 gene; RIKEN cDNA A230094G09 g | -1.73 | 0.291 | -2.31 | 0.222 | -1.07 | 0.896 | -1.04 | 0.872 | -1.54 |
| 1440598_at | Sntg1 | syntrophin, gamma 1 | -1.1 | 0.769 | -1.73 | 0.538 | -2.19 | 0.269 | -1.63 | 0.329 | -1.66 |
| 1442829_at | Rin2 | Ras and Rab interactor 2 | -1.72 | 0.202 | -1.93 | 0.305 | -1.18 | 0.216 | -1.02 | 0.857 | -1.46 |
| 1442863_at | Cacna2d4 | calcium channel, voltage-dependent, alpha 2/delta subunit 4 | -1.71 | 0.505 | -1.42 | 0.361 | -1.53 | 0.396 | -1.23 | 0.725 | -1.47 |
| 1435730_at | D930026N18Rik | RIKEN cDNA D930026N18 gene | -1.94 | 0.028 | -1.19 | 0.431 | -1.7 | 0.134 | -1.41 | 0.362 | -1.56 |
| 1446223_at | Cox10 | COX10 homolog, cytochrome c oxidase assembly protein, heme A: farnesyltrans | -1.57 | 0.231 | -1.64 | 0.283 | -1.43 | 0.299 | -1.19 | 0.215 | -1.46 |
| 1419776_at | --- | --- | -1.24 | 0.216 | -2.36 | 0.214 | -1.39 | 0.536 | -1.52 | 0.573 | -1.63 |
| 1433245_at | 6720475M21Rik | RIKEN cDNA 6720475M21 gene | -1.94 | 0.12 | -1.22 | 0.311 | -1.62 | 0.167 | -1.45 | 0.028 | -1.56 |
| 1441051_at | Trim27 | tripartite motif protein 27 | -2 | 0.163 | -1.6 | 0.153 | -1.22 | 0.26 | -1.32 | 0.221 | -1.53 |
| 1433092_at | 3110062G12Rik | RIKEN cDNA 3110062G12 gene | -3.11 | 0.095 | -1.17 | 0.79 | -1.29 | 0.722 | -1.39 | 0.522 | -1.74 |
| 1457366_at | Cdc40 | cell division cycle 40 homolog (yeast) | -1.58 | 0.204 | -1.44 | 0.07 | -1.61 | 0.005 | -1.43 | 0.139 | -1.51 |
| 1439943_at | Vps54 | vacuolar protein sorting 54 (yeast) | -1.29 | 0.446 | -1.82 | 0.248 | -1.6 | 0.008 | -1.61 | 0.062 | -1.58 |
| 1446221_at | Eln | Elastin | -2.36 | 0.509 | -1.06 | 0.9 | -1.72 | 0.4 | -1.15 | 0.8 | -1.57 |
| 1439355_at | Plekha5 | Pleckstrin homology domain containing, family A member 5 | -1.61 | 0.031 | -1.2 | 0.092 | -2.01 | 0.009 | -1.08 | 0.699 | -1.48 |
| 1446335_at | BC013481 | CDNA sequence BC013481 | -1.86 | 0.089 | -1.61 | 0.207 | -1.26 | 0.081 | -1.04 | 0.558 | -1.44 |
| 1444810_at | Acaca | Acetyl-Coenzyme A carboxylase alpha | -1.8 | 0.154 | -1.18 | 0.416 | -1.81 | 0 | -1.06 | 0.527 | -1.47 |
| 1450838_x_at | Rpl37 /// LOC3834; | ribosomal protein L37 /// similar to ribosomal protein L37 | -1.49 | 0.191 | -1.98 | 0.026 | -1.29 | 0.063 | -1.07 | 0.699 | -1.46 |
| 1440306_at | Nr3c2 | Nuclear receptor subfamily 3, group C, member 2 | -2.12 | 0.47 | -1.35 | 0.496 | -1.35 | 0.66 | -1.83 | 0.517 | -1.66 |
| 1444212_at | Hip2 | Huntingtin interacting protein 2 | -1.32 | 0.684 | -2.06 | 0.128 | -1.42 | 0.023 | -1.62 | 0.027 | -1.6 |
| 1441506_at | Dcn | decorin | -1.83 | 0.197 | -1.26 | 0.179 | -1.64 | 0.294 | -1.08 | 0.875 | -1.45 |
| 1447139_at | Bcl7c | B-cell CLL/lymphoma 7C | -1.14 | 0.501 | -1.89 | 0.128 | -1.83 | 0.063 | -1.02 | 0.965 | -1.47 |
| 1430357_at | H3f3b | H3 histone, family 3B | -1.89 | 0.422 | -1.13 | 0.792 | -1.87 | 0.185 | -1.38 | 0.518 | -1.57 |
| 1432757_at | 2900011L18Rik | RIKEN cDNA 2900011L18 gene | -1.13 | 0.413 | -1.61 | 0.007 | -2.26 | 0.001 | -1.3 | 0.429 | -1.57 |
| 1457453_at | 1700057G04Rik | RIKEN cDNA 1700057G04 gene | -1.64 | 0.013 | -1.59 | 0.152 | -1.41 | 0.364 | -1.45 | 0.307 | -1.52 |
| 1446249_at | Antxr2 | Anthrax toxin receptor 2 | -1.57 | 0.643 | -2.08 | 0.337 | -1.2 | 0.665 | -1.15 | 0.601 | -1.5 |
| 1460268_at | --- | --- | -1.66 | 0.081 | -1.27 | 0.389 | -1.78 | 0.189 | -1.82 | 0.153 | -1.63 |
| 1459632_at | Crim1 | Cysteine rich transmembrane BMP regulator 1 (chordin like) | -1.14 | 0.206 | -1.69 | 0.121 | -2.07 | 0.013 | -1.28 | 0.378 | -1.54 |
| 1454236_a_at | C030004A17Rik | RIKEN cDNA C030004A17 gene | -1.48 | 0.214 | -1.47 | 0.07 | -1.67 | 0.019 | -1.73 | 0.145 | -1.59 |
| 1428349_s_at | Ebf3 | early B-cell factor 3 | -3.66 | 0.001 | -1.3 | 0.635 | -1.1 | 0.843 | -1.85 | 0.457 | -1.98 |
| 1417607_at | Cox6a2 | cytochrome c oxidase, subunit VI a, polypeptide 2 | -1.84 | 0.044 | -2.15 | 0.075 | -1.06 | 0.811 | -1.47 | 0.078 | -1.63 |
| 1419304_at | T | brachyury | -2.07 | 0.306 | -1.16 | 0.745 | -1.64 | 0.313 | -1.88 | 0.259 | -1.69 |
| 1453233_s_at | Calr3 | calreticulin 3 | -1.74 | 0.508 | -1.65 | 0.359 | -1.29 | 0.534 | -1.36 | 0.028 | -1.51 |
| 1455785_at | --- | Transcribed locus | -2.55 | 0 | -1.34 | 0.479 | -1.23 | 0.538 | -1.61 | 0.276 | -1.68 |
| 1444135_at | 6332401O19Rik | RIKEN cDNA 6332401O19 gene | -1.27 | 0.261 | -1.24 | 0.53 | -2.77 | 0.135 | -1.5 | 0.361 | -1.69 |
| 1444313_at | Dym | Dymeclin | -2.7 | 0.178 | -1.43 | 0.517 | -1.13 | 0.737 | -1.57 | 0.257 | -1.71 |
| 1423019_at | Gja9 | gap junction membrane channel protein alpha 9 | -1.47 | 0.568 | -2.4 | 0.172 | -1.16 | 0.475 | -1.28 | 0.699 | -1.58 |
| 1418933_at | Slc1a6 | solute carrier family 1 (high affinity aspartate/glutamate transporter), member 6 | -1.11 | 0.804 | -2.23 | 0.081 | -1.64 | 0.145 | -1.19 | 0.333 | -1.54 |
| 1459194_at | 2210408I21Rik | RIKEN cDNA 2210408I21 gene | -1.42 | 0.376 | -1.77 | 0.09 | -1.45 | 0.128 | -1.09 | 0.798 | -1.43 |
| 1437713_x_at | 1500010J02Rik | RIKEN cDNA 1500010J02 gene | -1.45 | 0.592 | -2.34 | 0.117 | -1.19 | 0.676 | -1.54 | 0.364 | -1.63 |
| 1444869_at | Gphn | Gephyrin | -1.47 | 0.135 | -1.56 | 0.054 | -1.58 | 0.057 | -1.04 | 0.837 | -1.41 |
| 1438585_at | Syne2 | synaptic nuclear envelope 2 | -1.32 | 0.562 | -2.59 | 0.192 | -1.23 | 0.611 | -1.17 | 0.626 | -1.58 |
| 1440702_at | AU022297 | expressed sequence AU022297 | -1.7 | 0.049 | -1.61 | 0.404 | -1.33 | 0.387 | -1.18 | 0.45 | -1.46 |

| | | | | | | | | | | | |
|---|---|---|---|---|---|---|---|---|---|---|---|
| 1439941_at | --- | --- | -1.11 | 0.859 | -1.5 | 0.561 | -2.54 | 0.016 | -1.01 | 0.987 | -1.54 |
| 1423859_a_at | Ptgds | prostaglandin D2 synthase (brain) | -1.36 | 0.397 | -3.35 | 0.056 | -1.08 | 0.744 | -1.23 | 0.283 | -1.76 |
| 1440485_at | --- | --- | -1.68 | 0.384 | -1.14 | 0.74 | -2.06 | 0.12 | -1.09 | 0.881 | -1.49 |
| 1443321_at | Tmtc2 | Transmembrane and tetratricopeptide repeat containing 2 | -1.38 | 0.634 | -1.71 | 0.11 | -1.55 | 0.307 | -1.79 | 0.374 | -1.6 |
| 1433129_at | 2900040J22Rik | RIKEN cDNA 2900040J22 gene | -2.05 | 0.14 | -1.09 | 0.338 | -1.8 | 0.433 | -1.26 | 0.546 | -1.55 |
| 1459515_at | 2610037D02Rik | RIKEN cDNA 2610037D02 gene | -1.94 | 0.311 | -1.48 | 0.534 | -1.31 | 0.568 | -1.41 | 0.527 | -1.53 |
| 1447600_at | Tspan5 | Tetraspanin 5 | -1.27 | 0.362 | -1.59 | 0.228 | -1.85 | 0.144 | -1.35 | 0.175 | -1.51 |
| 1440290_at | LOC434089 | hypothetical gene supported by AK039686 | -1.24 | 0.66 | -1.72 | 0.472 | -1.74 | 0.369 | -1.47 | 0.607 | -1.55 |
| 1438114_x_at | Efs | Embryonal Fyn-associated substrate | -1.86 | 0.314 | -1.92 | 0.29 | -1.11 | 0.263 | -1.04 | 0.844 | -1.48 |
| 1433845_x_at | Dusp9 | dual specificity phosphatase 9 | -1.21 | 0.66 | -1.69 | 0.421 | -1.84 | 0.088 | -1.92 | 0.118 | -1.67 |
| 1459708_at | Ube4a | Ubiquitination factor E4A, UFD2 homolog (S. cerevisiae) | -3.02 | 0.204 | -1.58 | 0.224 | -1 | 0.957 | -1.52 | 0.453 | -1.78 |
| 1445080_at | Wwc1 | WW, C2 and coiled-coil domain containing 1 | -1.32 | 0.319 | -1.78 | 0.189 | -1.56 | 0.004 | -1.95 | 0.21 | -1.65 |
| 1458094_at | Zfp407 | Zinc finger protein 407 | -3.5 | 0.035 | -1.01 | 0.962 | -1.47 | 0.14 | -1.16 | 0.259 | -1.78 |
| 1457994_at | 4930563J15Rik | RIKEN cDNA 4930563J15 gene | -1.41 | 0.086 | -1.44 | 0.25 | -1.8 | 0.119 | -2.66 | 0.47 | -1.83 |
| 1419642_at | Purb | purine rich element binding protein B | -1.6 | 0.282 | -1.42 | 0.175 | -1.59 | 0.213 | -1.45 | 0.249 | -1.51 |
| 1457022_at | Dmd | Dystrophin, muscular dystrophy | -2.54 | 0.412 | -1.11 | 0.793 | -1.51 | 0.105 | -1.79 | 0.246 | -1.73 |
| 1423608_at | Itm2a | integral membrane protein 2A | -1.69 | 0.037 | -1.1 | 0.747 | -2.17 | 0.004 | -2.99 | 0.046 | -1.99 |
| 1447991_at | Pcsk2 | proprotein convertase subtilisin/kexin type 2 | -2.05 | 0.508 | -1.6 | 0.31 | -1.18 | 0.811 | -3.01 | 0.471 | -1.96 |
| 1421524_at | Cfc1 | cripto, FRL-1, cryptic family 1 | -3.84 | 0.118 | -1.24 | 0.666 | -1.11 | 0.515 | -1.2 | 0.585 | -1.85 |
| 1422518_at | Cask | calcium/calmodulin-dependent serine protein kinase (MAGUK family) | -1.26 | 0.506 | -1.59 | 0.046 | -1.85 | 0.01 | -1.14 | 0.598 | -1.46 |
| 1446184_at | --- | --- | -3.67 | 0.121 | -1.25 | 0.711 | -1.12 | 0.802 | -1.73 | 0.393 | -1.94 |
| 1430625_at | Krtap3-2 | keratin associated protein 3-2 | -1.39 | 0.515 | -1.59 | 0.654 | -1.62 | 0.364 | -3.28 | 0.007 | -1.97 |
| 1440333_at | Mamdc4 | MAM domain containing 4 | -1.19 | 0.049 | -1.87 | 0.052 | -1.69 | 0.089 | -1.5 | 0.163 | -1.56 |
| 1457794_at | Whsc1l1 | Wolf-Hirschhorn syndrome candidate 1-like 1 (human) | -1.5 | 0.104 | -1.39 | 0.42 | -1.73 | 0.005 | -1.12 | 0.571 | -1.44 |
| 1442422_at | Tmem55a | Transmembrane protein 55A | -1.27 | 0.175 | -1.62 | 0.076 | -1.8 | 0.006 | -1.57 | 0.014 | -1.56 |
| 1443093_at | Ajap1 | Adherens junction associated protein 1 | -2 | 0.401 | -1.25 | 0.771 | -1.5 | 0.237 | -3.06 | 0.025 | -1.95 |
| 1430042_at | A930012M21Rik | RIKEN cDNA A930012M21 gene | -1.18 | 0.28 | -1.71 | 0.049 | -1.87 | 0.014 | -1.08 | 0.8 | -1.46 |
| 1446806_at | --- | --- | -1.48 | 0.023 | -3.4 | 0.293 | -1 | 0.966 | -1.44 | 0.284 | -1.83 |
| 1459196_at | Dnmbp | Dynamin binding protein | -1.49 | 0.498 | -1.66 | 0.124 | -1.45 | 0.398 | -2.91 | 0.095 | -1.88 |
| 1446156_at | Dmd | Dystrophin, muscular dystrophy | -2.02 | 0.322 | -1.71 | 0.315 | -1.13 | 0.477 | -2.72 | 0.034 | -1.9 |
| 1435933_at | Scn2a1 | sodium channel, voltage-gated, type II, alpha 1 | -1.08 | 0.929 | -1.78 | 0.36 | -2.09 | 0.021 | -1.27 | 0.671 | -1.56 |
| 1443027_at | 4930523C07Rik | RIKEN cDNA 4930523C07 gene | -1.36 | 0.49 | -1.48 | 0.034 | -1.79 | 0.019 | -2.08 | 0.028 | -1.68 |
| 1445395_at | Prkca | Protein kinase C, alpha | -1.22 | 0.206 | -2 | 0.132 | -1.55 | 0.165 | -1.54 | 0.233 | -1.58 |
| 1442340_x_at | Cyr61 | cysteine rich protein 61 | -1.51 | 0.302 | -2.28 | 0.198 | -1.15 | 0.67 | -1.12 | 0.736 | -1.52 |
| 1445355_at | Spata6 | spermatogenesis associated 6 | -2.05 | 0.061 | -1.14 | 0.766 | -1.65 | 0.279 | -2 | 0.356 | -1.71 |
| 1453124_at | Tnpo3 | transportin 3 | -2.14 | 0.453 | -1.62 | 0.3 | -1.14 | 0.831 | -2.7 | 0.202 | -1.9 |
| 1458113_at | 9530019H20Rik | RIKEN cDNA 9530019H20 gene | -1.23 | 0.749 | -1.73 | 0.238 | -1.74 | 0.003 | -1.35 | 0.494 | -1.51 |
| 1458020_at | Reln | Reelin | -1.87 | 0.271 | -1.91 | 0.087 | -1.1 | 0.732 | -2.92 | 0.221 | -1.95 |
| 1457105_at | Pkd2l1 | polycystic kidney disease 2-like 1 | -1.25 | 0.591 | -1.83 | 0.271 | -1.61 | 0.223 | -2.73 | 0.057 | -1.86 |
| 1434722_at | Ampd1 | adenosine monophosphate deaminase 1 (isoform M) | -1.13 | 0.786 | -5.41 | 0.028 | -1.11 | 0.771 | -2.61 | 0.355 | -2.57 |
| 1420070_a_at | Slc9a5 | solute carrier family 9 (sodium/hydrogen exchanger), member 5 | -2.19 | 0.207 | -1.88 | 0.28 | -1.02 | 0.956 | -1.5 | 0.545 | -1.65 |
| 1445792_at | Ppme1 | Protein phosphatase methylesterase 1 | -1.74 | 0.59 | -1.03 | 0.958 | -2.34 | 0.114 | -1.41 | 0.633 | -1.63 |
| 1453798_at | Ccdc93 | coiled-coil domain containing 93 | -1.06 | 0.844 | -2.81 | 0.326 | -1.49 | 0.532 | -1.62 | 0.37 | -1.74 |
| 1420248_at | Tubg2 | tubulin, gamma 2 | -1.28 | 0.363 | -1.84 | 0.251 | -1.55 | 0.208 | -1.25 | 0.375 | -1.48 |
| 1443235_at | Eif2ak4 | Eukaryotic translation initiation factor 2 alpha kinase 4 | -1.77 | 0.196 | -1.55 | 0.138 | -1.32 | 0.117 | -1.49 | 0.226 | -1.53 |
| 1454482_at | 5830474E16Rik | RIKEN cDNA 5830474E16 gene | -1.33 | 0.729 | -2.26 | 0.241 | -1.29 | 0.58 | -3.19 | 0.215 | -2.02 |
| 1437798_at | 6720422M22Rik | RIKEN cDNA 6720422M22 gene | -1.17 | 0.465 | -1.34 | 0.086 | -2.7 | 0.04 | -1.14 | 0.469 | -1.59 |
| 1436683_at | 1700011M02Rik | RIKEN cDNA 1700011M02 gene | -1.99 | 0.304 | -1.22 | 0.692 | -1.54 | 0.242 | -1.19 | 0.837 | -1.49 |
| 1437934_at | Tia1 | cytotoxic granule-associated RNA binding protein 1 | -1.47 | 0.04 | -1.68 | 0.075 | -1.43 | 0.014 | -1.34 | 0.261 | -1.48 |
| 1457505_at | Utrn | Utrophin | -1.38 | 0.587 | -1.22 | 0.718 | -2.32 | 0.013 | -1.22 | 0.505 | -1.54 |
| 1444622_at | Ptprd | Protein tyrosine phosphatase, receptor type, D | -1.3 | 0.224 | -2.08 | 0.021 | -1.39 | 0.121 | -4.71 | 0.023 | -2.37 |
| 1425836_a_at | Limk1 | LIM-domain containing, protein kinase | -1.73 | 0.603 | -1.67 | 0.173 | -1.26 | 0.646 | -3.29 | 0.254 | -1.99 |
| 1427470_s_at | Napb | N-ethylmaleimide sensitive fusion protein attachment protein beta | -1.27 | 0.287 | -1.46 | 0.514 | -1.99 | 0.017 | -1.32 | 0.306 | -1.51 |

| | | | | | | | | | | | |
|---|---|---|---|---|---|---|---|---|---|---|---|
| 1442785_at | Smyd3 | SET and MYND domain containing 3 | -1.6 | 0.259 | -1.27 | 0.19 | -1.78 | 0.245 | -2.87 | 0.025 | -1.88 |
| 1457823_at | Cyr61 | cysteine rich protein 61 | -1.68 | 0.133 | -2.17 | 0.138 | -1.09 | 0.794 | -1.18 | 0.722 | -1.53 |
| 1447362_at | Bub1b | budding uninhibited by benzimidazoles 1 homolog, beta (S. cerevisiae) | -3.31 | 0.167 | -1.45 | 0.191 | -1.02 | 0.919 | -1.22 | 0.446 | -1.75 |
| 1424162_at | Trim29 | tripartite motif protein 29 | -2.54 | 0.035 | -1.51 | 0.256 | -1.09 | 0.669 | -1.98 | 0.22 | -1.78 |
| 1459397_at | Arih1 | ariadne ubiquitin-conjugating enzyme E2 binding protein homolog 1 (Drosophila) | -2.01 | 0.208 | -1.14 | 0.722 | -1.66 | 0.238 | -1.36 | 0.603 | -1.54 |
| 1439933_at | B430316J06Rik | RIKEN cDNA B430316J06 gene | -1.62 | 0.208 | -1.12 | 0.515 | -2.14 | 0.011 | -1.88 | 0.223 | -1.69 |
| 1439311_at | B830012L14Rik | RIKEN cDNA B830012L14 gene | -1.31 | 0.728 | -1.37 | 0.394 | -2.09 | 0.02 | -4.26 | 0.194 | -2.26 |
| 1439362_at | --- | Adult male urinary bladder cDNA, RIKEN full-length enriched library, clone:95300 | -1.11 | 0.646 | -2.75 | 0.062 | -1.41 | 0.438 | -1.27 | 0.518 | -1.63 |
| 1442388_at | 2410137F16Rik | RIKEN cDNA 2410137F16 gene | -1.63 | 0.455 | -2.26 | 0.171 | -1.08 | 0.829 | -1.51 | 0.555 | -1.62 |
| 1432962_at | 2610024D14Rik | RIKEN cDNA 2610024D14 gene | -1.59 | 0.348 | -1.62 | 0.152 | -1.37 | 0.239 | -1.21 | 0.499 | -1.45 |
| 1456910_at | --- | Transcribed locus | -1.17 | 0.78 | -2.88 | 0.115 | -1.29 | 0.414 | -1.45 | 0.61 | -1.7 |
| 1430642_at | 2900001G08Rik | RIKEN cDNA 2900001G08 gene | -1.48 | 0.072 | -1.62 | 0.35 | -1.46 | 0.291 | -1.96 | 0.331 | -1.63 |
| 1445460_at | --- | Transcribed locus | -1.56 | 0.332 | -1.86 | 0.127 | -1.25 | 0.416 | -1.12 | 0.378 | -1.45 |
| 1435833_at | --- | --- | -2.43 | 0.448 | -1.74 | 0.313 | -1.01 | 0.988 | -1.49 | 0.507 | -1.67 |
| 1425409_at | Chrna2 | cholinergic receptor, nicotinic, alpha polypeptide 2 (neuronal) | -1.13 | 0.072 | -3.15 | 0.238 | -1.29 | 0.194 | -1.5 | 0.126 | -1.77 |
| 1456188_at | Svs2 | seminal vesicle protein, secretion 2 | -1.08 | 0.392 | -2.69 | 0.216 | -1.47 | 0.015 | -1.37 | 0.295 | -1.65 |
| 1435166_at | Cntn2 | contactin 2 | -1.37 | 0.18 | -1.69 | 0.119 | -1.53 | 0.304 | -1.07 | 0.79 | -1.41 |
| 1429342_s_at | 2310021H06Rik | RIKEN cDNA 2310021H06 gene | -1.45 | 0.451 | -1.8 | 0.33 | -1.37 | 0.185 | -4.05 | 0.003 | -2.17 |
| 1420800_a_at | Kcnq2 | potassium voltage-gated channel, subfamily Q, member 2 | -2.74 | 0.073 | -1.21 | 0.584 | -1.27 | 0.563 | -1.35 | 0.182 | -1.64 |
| 1459588_at | Kit | Kit oncogene | -1.22 | 0.234 | -1.89 | 0.412 | -1.59 | 0.23 | -1.05 | 0.756 | -1.44 |
| 1439566_at | C030038J10Rik | RIKEN cDNA C030038J10 gene | -1.02 | 0.963 | -2.18 | 0.108 | -1.86 | 0.328 | -1.23 | 0.568 | -1.57 |
| 1457870_at | --- | Transcribed locus | -1.1 | 0.822 | -2.76 | 0.106 | -1.41 | 0.519 | -1.23 | 0.292 | -1.63 |
| 1459412_at | Mfhas1 | Malignant fibrous histiocytoma amplified sequence 1 | -1.42 | 0.134 | -1.8 | 0.061 | -1.4 | 0.049 | -1.01 | 0.97 | -1.4 |
| 1458546_at | D18Ertd653e | DNA segment, Chr 18, ERATO Doi 653, expressed | -1.41 | 0.374 | -1.98 | 0.115 | -1.31 | 0.34 | -1.57 | 0.371 | -1.57 |
| 1432612_at | 9030405F24Rik | RIKEN cDNA 9030405F24 gene | -1.94 | 0.154 | -1.32 | 0.522 | -1.41 | 0.397 | -1.3 | 0.56 | -1.49 |
| 1444337_at | Numb | Numb gene homolog (Drosophila) | -1.4 | 0.227 | -1.42 | 0.092 | -1.77 | 0.015 | -1.2 | 0.334 | -1.45 |
| 1458588_at | Schip1 | Schwannomin interacting protein 1 | -1.33 | 0.067 | -1.52 | 0.429 | -1.75 | 0.039 | -1.02 | 0.949 | -1.41 |
| 1460117_at | --- | --- | -1.12 | 0.317 | -1.49 | 0.175 | -2.43 | 0.008 | -1.14 | 0.356 | -1.54 |
| 1431894_at | 4833424O12Rik | RIKEN cDNA 4833424O12 gene | -1.18 | 0.423 | -1.66 | 0.163 | -1.89 | 0.085 | -1.89 | 0.086 | -1.65 |
| 1421766_at | Uchl4 | ubiquitin carboxyl-terminal esterase L4 | -1.17 | 0.797 | -1.28 | 0.301 | -2.91 | 0.18 | -1.62 | 0.464 | -1.74 |
| 1443536_at | Slc7a11 | solute carrier family 7 (cationic amino acid transporter, y+ system), member 11 | -1.13 | 0.88 | -3.2 | 0.021 | -1.28 | 0.489 | -1.62 | 0.208 | -1.81 |
| 1447193_at | --- | --- | -1.79 | 0.286 | -1.27 | 0.584 | -1.57 | 0.033 | -1.17 | 0.453 | -1.45 |
| 1446946_at | Tgfbr1 | Transforming growth factor, beta receptor I | -1.1 | 0.679 | -1.56 | 0.167 | -2.3 | 0.022 | -1.28 | 0.136 | -1.56 |
| 1444070_at | --- | --- | -1.28 | 0.51 | -1.57 | 0.229 | -1.78 | 0.188 | -1.49 | 0.394 | -1.53 |
| 1443588_at | --- | Transcribed locus | -1.31 | 0.391 | -1.36 | 0.369 | -2.07 | 0.065 | -1.8 | 0.331 | -1.64 |
| 1433411_at | --- | --- | -1.6 | 0.216 | -1.13 | 0.791 | -2.12 | 0.221 | -1.31 | 0.786 | -1.54 |
| 1438464_at | Arid4a | AT rich interactive domain 4A (Rbp1 like) | -1.68 | 0.352 | -1.22 | 0.559 | -1.76 | 0.199 | -1.3 | 0.083 | -1.49 |
| 1445024_at | Stard7 | START domain containing 7 | -1.66 | 0.027 | -1.44 | 0.388 | -1.46 | 0.041 | -1.44 | 0.368 | -1.5 |
| 1428991_at | Hrasls | HRAS-like suppressor | -1.23 | 0.392 | -2.38 | 0.05 | -1.33 | 0.296 | -1.3 | 0.523 | -1.56 |
| 1431981_at | Hif1a | hypoxia inducible factor 1, alpha subunit | -1.53 | 0.339 | -1.78 | 0.463 | -1.3 | 0.438 | -1.05 | 0.843 | -1.41 |
| 1443607_at | --- | --- | -1.76 | 0.487 | -2.13 | 0.348 | -1.06 | 0.865 | -1.06 | 0.873 | -1.5 |
| 1446484_at | Mef2c | Myocyte enhancer factor 2C | -1.44 | 0.176 | -1.86 | 0.205 | -1.33 | 0.399 | -2.34 | 0.085 | -1.74 |
| 1459947_at | Bmp6 | Bone morphogenetic protein 6 | -1.38 | 0.199 | -1.4 | 0.144 | -1.83 | 0.027 | -1.01 | 0.929 | -1.41 |
| 1459784_x_at | 1300002A08Rik | RIKEN cDNA 1300002A08 gene | -1.68 | 0.042 | -2 | 0.011 | -1.12 | 0.74 | -2.11 | 0.058 | -1.73 |
| 1421094_at | Zbtb33 | zinc finger and BTB domain containing 33 | -1.77 | 0.419 | -2.08 | 0.054 | -1.06 | 0.918 | -1.08 | 0.901 | -1.5 |
| 1459605_at | Apba1 | amyloid beta (A4) precursor protein binding, family A, member 1 | -1.19 | 0.851 | -2.27 | 0.165 | -1.42 | 0.299 | -1.03 | 0.955 | -1.48 |
| 1456400_at | Terf2ip | telomeric repeat binding factor 2, interacting protein | -2.36 | 0.473 | -1.01 | 0.982 | -1.75 | 0.402 | -4.67 | 0.209 | -2.45 |
| 1459442_at | Gnb1 | Guanine nucleotide binding protein, beta 1 | -1.06 | 0.658 | -1.48 | 0.41 | -2.69 | 0.005 | -1.23 | 0.374 | -1.62 |
| 1445171_at | 1700067C01Rik /// | RIKEN cDNA 1700067C01 gene /// RIKEN cDNA A230107C01 gene | -1.22 | 0.615 | -1.46 | 0.501 | -2.07 | 0.127 | -1.52 | 0.31 | -1.57 |
| 1419215_at | Aox4 | aldehyde oxidase 4 | -1.28 | 0.621 | -1.68 | 0.387 | -1.63 | 0.455 | -1.1 | 0.846 | -1.42 |
| 1453236_at | 1700054F22Rik | RIKEN cDNA 1700054F22 gene | -2.06 | 0.485 | -1.47 | 0.431 | -1.22 | 0.635 | -2.58 | 0.317 | -1.83 |
| 1457169_at | Ppp1r12b | Protein phosphatase 1, regulatory (inhibitor) subunit 12B | -1.09 | 0.821 | -2.06 | 0.065 | -1.72 | 0.174 | -1.38 | 0.39 | -1.56 |
| 1443535_at | Rabgap1 | RAB GTPase activating protein 1 | -1.27 | 0.149 | -1.46 | 0.149 | -1.94 | 0.012 | -1.32 | 0.059 | -1.5 |

| | | | | | | | | | | | |
|---|---|---|---|---|---|---|---|---|---|---|---|
| 1439056_at | LOC636791 | hypothetical protein LOC636791 | -1.61 | 0.628 | -2.42 | 0.115 | -1.05 | 0.818 | -1.09 | 0.822 | -1.54 |
| 1458029_at | --- | --- | -1.19 | 0.679 | -3.59 | 0.006 | -1.15 | 0.806 | -2.08 | 0.316 | -2 |
| 1458003_at | Zfp398 | zinc finger protein 398 | -1.61 | 0.492 | -2.26 | 0.044 | -1.08 | 0.679 | -1.42 | 0.286 | -1.59 |
| 1450810_at | Fshr | follicle stimulating hormone receptor | -1.92 | 0.207 | -1.21 | 0.657 | -1.56 | 0.157 | -1.16 | 0.756 | -1.46 |
| 1421745_at | Plag1 | pleiomorphic adenoma gene 1 | -1.1 | 0.823 | -3.94 | 0.037 | -1.21 | 0.74 | -1.11 | 0.768 | -1.84 |
| 1458093_at | Mtrr | 5-methyltetrahydrofolate-homocysteine methyltransferase reductase | -1.35 | 0.631 | -1.54 | 0.244 | -1.68 | 0.326 | -2.23 | 0.28 | -1.7 |
| 1450215_at | Rcvrn | recoverin | -1.62 | 0.421 | -1.53 | 0.625 | -1.39 | 0.276 | -1.36 | 0.519 | -1.48 |
| 1444693_at | Cacnb2 | Calcium channel, voltage-dependent, beta 2 subunit | -1.37 | 0.276 | -1.13 | 0.762 | -2.71 | 0.004 | -1.05 | 0.82 | -1.56 |
| 1453626_at | 3930402G23Rik | RIKEN cDNA 3930402G23 gene | -4.04 | 0.001 | -1.3 | 0.233 | -1.03 | 0.871 | -1.22 | 0.478 | -1.9 |
| 1460060_at | --- | --- | -1.64 | 0.288 | -1.31 | 0.657 | -1.63 | 0.326 | -1.68 | 0.378 | -1.56 |
| 1440848_at | 1110028C15Rik | RIKEN cDNA 1110028C15 gene | -1.34 | 0.519 | -1.54 | 0.298 | -1.69 | 0.031 | -1.48 | 0.038 | -1.51 |
| 1443961_at | LOC627488 /// LOC | similar to THO complex subunit 4 (Tho4) (RNA and export factor binding protein 1 | -1.34 | 0.462 | -1.57 | 0.2 | -1.65 | 0.145 | -1.1 | 0.798 | -1.42 |
| 1444381_at | A830005F24Rik | RIKEN cDNA A830005F24 gene | -2.2 | 0.171 | -1.02 | 0.97 | -1.81 | 0.128 | -1.48 | 0.198 | -1.63 |
| 1420155_at | AA408954 | expressed sequence AA408954 | -1.39 | 0.541 | -1.41 | 0.6 | -1.77 | 0.28 | -1.48 | 0.552 | -1.51 |
| 1457353_at | Tomm70a | Translocase of outer mitochondrial membrane 70 homolog A (yeast) | -1.63 | 0.242 | -1.48 | 0.271 | -1.42 | 0.536 | -1.69 | 0.07 | -1.56 |
| 1447296_at | Fbxo27 | F-box protein 27 | -1.61 | 0.217 | -1.43 | 0.207 | -1.5 | 0.502 | -1.46 | 0.162 | -1.5 |
| 1427744_at | Ccnb3 | cyclin B3 | -1.08 | 0.832 | -2.3 | 0.237 | -1.58 | 0.608 | -1.01 | 0.978 | -1.49 |
| 1457809_at | Pcaf /// LOC330129 | p300/CBP-associated factor /// similar to p300/CBP-associated factor | -1.57 | 0.064 | -1.28 | 0.296 | -1.75 | 0.011 | -1.62 | 0.011 | -1.55 |
| 1436408_at | Rprml | reprimo-like | -1.67 | 0.211 | -1.41 | 0.715 | -1.46 | 0.039 | -1.07 | 0.918 | -1.4 |
| 1456979_at | Zhx3 | Zinc fingers and homeoboxes 3 | -1.24 | 0.246 | -1.58 | 0.016 | -1.8 | 0.032 | -1.3 | 0.16 | -1.48 |
| 1440528_at | --- | Transcribed locus | -1.21 | 0.791 | -2.02 | 0.218 | -1.49 | 0.253 | -1.65 | 0.416 | -1.59 |
| 1432587_at | 5730596P11Rik | RIKEN cDNA 5730596P11 gene | -1.1 | 0.666 | -2.02 | 0.083 | -1.69 | 0.195 | -1.48 | 0.451 | -1.57 |
| 1421752_a_at | Serpinb5 | serine (or cysteine) peptidase inhibitor, clade B, member 5 | -1.39 | 0.741 | -2.01 | 0.29 | -1.29 | 0.343 | -1.09 | 0.751 | -1.44 |
| 1436267_a_at | Frap1 | FK506 binding protein 12-rapamycin associated protein 1 | -1.3 | 0.308 | -1.68 | 0.046 | -1.58 | 0.099 | -1.13 | 0.649 | -1.42 |
| 1425920_at | Cuedc1 | CUE domain containing 1 | -1.45 | 0.254 | -2.78 | 0.063 | -1.06 | 0.732 | -1.18 | 0.373 | -1.62 |
| 1442447_at | --- | --- | -1.26 | 0.135 | -1.84 | 0.119 | -1.52 | 0.19 | -1.05 | 0.302 | -1.42 |
| 1447705_at | 4833432M17Rik | RIKEN cDNA 4833432M17 gene | -2.22 | 0.056 | -1.44 | 0.015 | -1.18 | 0.666 | -1.56 | 0.158 | -1.6 |
| 1438470_at | Socs2 | suppressor of cytokine signaling 2 | -1.07 | 0.808 | -2.39 | 0.017 | -1.54 | 0.06 | -1.44 | 0.258 | -1.61 |
| 1458578_at | Rbms1 | RNA binding motif, single stranded interacting protein 1 | -1.31 | 0.242 | -1.26 | 0.313 | -2.26 | 0.009 | -1.31 | 0.021 | -1.53 |
| 1432971_at | 4921518B13Rik | RIKEN cDNA 4921518B13 gene | -2.03 | 0.117 | -1.27 | 0.614 | -1.4 | 0.092 | -1.02 | 0.922 | -1.43 |
| 1448459_at | Kcnip1 | Kv channel-interacting protein 1 | -1.57 | 0.518 | -2.67 | 0.026 | -1.01 | 0.967 | -4.31 | 0.029 | -2.39 |
| 1432118_at | 2410012E07Rik | RIKEN cDNA 2410012E07 gene | -1.13 | 0.724 | -2.98 | 0.049 | -1.29 | 0.36 | -1.11 | 0.461 | -1.63 |
| 1431148_at | --- | --- | -1.85 | 0.121 | -1.47 | 0.114 | -1.28 | 0.637 | -1.15 | 0.759 | -1.44 |
| 1435354_at | Kcnj15 | potassium inwardly-rectifying channel, subfamily J, member 15 | -1.01 | 0.925 | -2.7 | 0.132 | -1.57 | 0.017 | -1.24 | 0.711 | -1.63 |
| 1425945_at | 4933426I21Rik | RIKEN cDNA 4933426I21 gene | -3.91 | 0.371 | -1.24 | 0.682 | -1.07 | 0.881 | -2 | 0.455 | -2.05 |
| 1441665_at | Iars2 | isoleucine-tRNA synthetase 2, mitochondrial | -1.82 | 0.153 | -1.08 | 0.906 | -1.92 | 0.106 | -1.07 | 0.905 | -1.47 |
| 1448097_at | Ube3c | Ubiquitin protein ligase E3C | -1 | 0.988 | -2.78 | 0.089 | -1.56 | 0.449 | -2.57 | 0.404 | -1.98 |
| 1419982_s_at | --- | --- | -1.89 | 0.097 | -1.77 | 0.179 | -1.1 | 0.501 | -1.85 | 0.269 | -1.65 |
| 1433091_at | 6430514K02Rik | RIKEN cDNA 6430514K02 gene | -1.39 | 0.413 | -1.84 | 0.171 | -1.36 | 0.577 | -1.01 | 0.972 | -1.4 |
| 1438186_at | Pdlim5 | PDZ and LIM domain 5 | -1.16 | 0.762 | -1.66 | 0.19 | -1.87 | 0.309 | -1.67 | 0.328 | -1.59 |
| 1421776_at | Olfr74 | olfactory receptor 74 | -1.29 | 0.606 | -1.46 | 0.405 | -1.86 | 0.356 | -1.17 | 0.782 | -1.44 |
| 1440599_at | Olfr658 | olfactory receptor 658 | -1.09 | 0.922 | -1.5 | 0.471 | -2.43 | 0.201 | -2.74 | 0.095 | -1.94 |
| 1437785_at | Adamts9 | a disintegrin-like and metallopeptidase (reprolysin type) with thrombospondin type | -1.38 | 0.287 | -1.53 | 0.034 | -1.61 | 0.022 | -1.38 | 0.247 | -1.47 |
| 1448421_s_at | Aspn | asporin | -1.71 | 0.441 | -1.39 | 0.661 | -1.44 | 0.188 | -1.04 | 0.914 | -1.39 |
| 1432530_s_at | Boll | bol, boule-like (Drosophila) | -1.75 | 0.211 | -1.1 | 0.886 | -1.92 | 0.04 | -1.14 | 0.777 | -1.48 |
| 1447765_at | Wdr48 | WD repeat domain 48 | -1.99 | 0.274 | -1.34 | 0.627 | -1.33 | 0.246 | -1.78 | 0.495 | -1.61 |
| 1445934_at | Arih2 /// LOC43256 | ariadne homolog 2 (Drosophila) /// similar to ariadne homolog 2 | -1.57 | 0.115 | -1.35 | 0.183 | -1.6 | 0.112 | -1.05 | 0.824 | -1.39 |
| 1447706_at | --- | --- | -1.41 | 0.441 | -1.55 | 0.03 | -1.54 | 0.313 | -1.59 | 0.32 | -1.52 |
| 1445773_at | Meis1 | Myeloid ecotropic viral integration site 1 | -1.12 | 0.836 | -1.5 | 0.123 | -2.26 | 0.01 | -1.19 | 0.17 | -1.52 |
| 1444500_at | Ahsa1 | AHA1, activator of heat shock protein ATPase homolog 1 (yeast) | -1.66 | 0.018 | -1.73 | 0.033 | -1.22 | 0.188 | -1.12 | 0.582 | -1.43 |
| 1445807_at | Prkg1 | Protein kinase, cGMP-dependent, type I | -1.58 | 0.514 | -1.38 | 0.42 | -1.55 | 0.056 | -1.06 | 0.871 | -1.39 |
| 1454111_at | 4930404A10Rik | RIKEN cDNA 4930404A10 gene | -1.75 | 0.524 | -1.24 | 0.625 | -1.6 | 0.184 | -1.62 | 0.393 | -1.55 |
| 1447103_at | Cdk5rap2 | CDK5 regulatory subunit associated protein 2 | -1.04 | 0.959 | -3.79 | 0.149 | -1.28 | 0.676 | -1.1 | 0.892 | -1.8 |

| | | | | | | | | | | | |
|---|---|---|---|---|---|---|---|---|---|---|---|
| 1458346_x_at | Atp2b1 | ATPase, Ca++ transporting, plasma membrane 1 | -1.58 | 0.242 | -1.23 | 0.357 | -1.8 | 0.177 | -2.45 | 0.09 | -1.76 |
| 1436474_at | Mrcl | mannose receptor-like precursor | -1.48 | 0.146 | -1.43 | 0.204 | -1.58 | 0.404 | -1.38 | 0.442 | -1.47 |
| 1427871_at | Ptafr | platelet-activating factor receptor | -1.79 | 0.444 | -1.59 | 0.568 | -1.22 | 0.766 | -1.56 | 0.483 | -1.54 |
| 1440126_at | BC037704 | cDNA sequence BC037704 | -1.72 | 0.378 | -1.54 | 0.379 | -1.28 | 0.551 | -1.56 | 0.327 | -1.53 |
| 1417355_at | Peg3 | paternally expressed 3 | -1.25 | 0.558 | -1.22 | 0.512 | -2.58 | 0.006 | -2.35 | 0.126 | -1.85 |
| 1459644_at | AU020772 | expressed sequence AU020772 | -1.23 | 0.294 | -1.86 | 0.148 | -1.53 | 0.363 | -1.03 | 0.848 | -1.41 |
| 1437531_at | Trpm1 | transient receptor potential cation channel, subfamily M, member 1 | -1.07 | 0.823 | -1.88 | 0.127 | -1.84 | 0.26 | -1.8 | 0.296 | -1.65 |
| 1436613_at | Coro6 | Coronin, actin binding protein 6 | -2.78 | 0.028 | -1.19 | 0.768 | -1.24 | 0.608 | -2.17 | 0.528 | -1.84 |
| 1437599_at | Chchd3 | Coiled-coil-helix-coiled-coil-helix domain containing 3 | -1.41 | 0.056 | -1.38 | 0.127 | -1.74 | 0.012 | -1.13 | 0.421 | -1.41 |
| 1450559_at | 5730559C18Rik | RIKEN cDNA 5730559C18 gene | -1.72 | 0.107 | -1.46 | 0.432 | -1.35 | 0.405 | -3.22 | 0.105 | -1.94 |
| 1421319_at | Ptgfrn | prostaglandin F2 receptor negative regulator | -2.28 | 0.108 | -1.72 | 0.108 | -1.01 | 0.958 | -1.72 | 0.147 | -1.68 |
| 1442399_at | Xrn1 | 5'-3' exoribonuclease 1 | -1.13 | 0.443 | -1.58 | 0.162 | -2.03 | 0.08 | -1.03 | 0.857 | -1.44 |
| 1445433_at | Glt8d2 | glycosyltransferase 8 domain containing 2 | -3.48 | 0.085 | -1.06 | 0.919 | -1.29 | 0.268 | -1.07 | 0.925 | -1.72 |
| 1439310_at | A630012P03Rik | RIKEN cDNA A630012P03 gene | -1.77 | 0.451 | -1.23 | 0.601 | -1.58 | 0.35 | -1.53 | 0.569 | -1.53 |
| 1431855_at | 4930570E03Rik | RIKEN cDNA 4930570E03 gene | -1.14 | 0.825 | -1.91 | 0.125 | -1.63 | 0.273 | -1.14 | 0.646 | -1.46 |
| 1453012_at | Tsc22d2 | TSC22 domain family 2 | -1.43 | 0.403 | -1.47 | 0.284 | -1.59 | 0.049 | -1.14 | 0.414 | -1.41 |
| 1421190_at | Gabrb3 | gamma-aminobutyric acid (GABA-A) receptor, subunit beta 3 | -1.32 | 0.487 | -1.26 | 0.602 | -2.19 | 0.203 | -1.46 | 0.316 | -1.56 |
| 1435592_at | Eif5b | eukaryotic translation initiation factor 5B | -1.48 | 0.127 | -1.29 | 0.44 | -1.79 | 0.068 | -1.17 | 0.516 | -1.43 |
| 1442491_at | Dpp10 | Dipeptidylpeptidase 10 | -1.66 | 0.232 | -1.74 | 0.166 | -1.2 | 0.681 | -2.59 | 0.074 | -1.8 |
| 1432293_at | 4930579G18Rik | RIKEN cDNA 4930579G18 gene | -1.77 | 0.305 | -1.5 | 0.329 | -1.28 | 0.637 | -1.03 | 0.954 | -1.4 |
| 1433121_at | 5830490A04Rik | RIKEN cDNA 5830490A04 gene | -1.16 | 0.745 | -1.71 | 0.462 | -1.76 | 0.258 | -1.33 | 0.691 | -1.49 |
| 1451801_at | Trdn | triadin | -1.65 | 0.028 | -2.05 | 0.438 | -1.09 | 0.8 | -1.44 | 0.745 | -1.56 |
| 1440201_at | Mapk10 | mitogen activated protein kinase 10 | -1.22 | 0.019 | -2.41 | 0.014 | -1.29 | 0.4 | -1.38 | 0.367 | -1.57 |
| 1448606_at | Edg2 | endothelial differentiation, lysophosphatidic acid G-protein-coupled receptor, 2 | -1.36 | 0.257 | -1.64 | 0.013 | -1.5 | 0.109 | -1.47 | 0.174 | -1.49 |
| 1454370_at | 4930557B21Rik | RIKEN cDNA 4930557B21 gene | -1.6 | 0.553 | -1.72 | 0.404 | -1.24 | 0.681 | -2.52 | 0.15 | -1.77 |
| 1436309_at | Neto2 | neuropilin (NRP) and tolloid (TLL)-like 2 | -2.06 | 0.356 | -1.41 | 0.52 | -1.22 | 0.446 | -1.33 | 0.479 | -1.51 |
| 1446174_at | LOC245440 | similar to melanoma antigen family A, 10 | -1.61 | 0.324 | -2.33 | 0.13 | -1.04 | 0.947 | -1.17 | 0.837 | -1.54 |
| 1458000_at | Dsg1a | desmoglein 1 alpha | -1.85 | 0.364 | -1.63 | 0.356 | -1.16 | 0.717 | -1.95 | 0.318 | -1.65 |
| 1445850_at | 9530009M10Rik | RIKEN cDNA 9530009M10 gene | -1.31 | 0.481 | -1.23 | 0.559 | -2.27 | 0.015 | -1.33 | 0.292 | -1.54 |
| 1436138_at | --- | --- | -1.48 | 0.182 | -1.47 | 0.233 | -1.52 | 0.117 | -1.22 | 0.557 | -1.42 |
| 1440116_at | D630045J12Rik | RIKEN cDNA D630045J12 gene | -1.13 | 0.203 | -2.62 | 0.221 | -1.34 | 0.338 | -1.2 | 0.685 | -1.57 |
| 1445966_at | Igf2r | Insulin-like growth factor 2 receptor | -1.29 | 0.105 | -1.26 | 0.417 | -2.25 | 0 | -1.07 | 0.638 | -1.47 |
| 1444857_at | C87312 | expressed sequence C87312 | -1.03 | 0.751 | -1.45 | 0.549 | -2.87 | 0.133 | -1.54 | 0.374 | -1.72 |
| 1425476_at | Col4a5 | procollagen, type IV, alpha 5 | -1.37 | 0.163 | -1.96 | 0.044 | -1.29 | 0.353 | -1.18 | 0.479 | -1.45 |
| 1446025_at | AI462493 | Expressed sequence AI462493 | -2.3 | 0.197 | -1.12 | 0.758 | -1.45 | 0.277 | -1.04 | 0.91 | -1.48 |
| 1432938_at | 5430439C14Rik | RIKEN cDNA 5430439C14 gene | -2.35 | 0.479 | -1.03 | 0.966 | -1.61 | 0.299 | -4.79 | 0.153 | -2.45 |
| 1457410_at | Arhgap5 | Rho GTPase activating protein 5 | -1.41 | 0.005 | -1.32 | 0.429 | -1.83 | 0.016 | -1.74 | 0.007 | -1.57 |
| 1441874_x_at | Prlpe | prolactin-like protein E | -5.5 | 0.004 | -1.12 | 0.78 | -1.06 | 0.895 | -1.8 | 0.453 | -2.37 |
| 1432582_at | 3110054G05Rik | RIKEN cDNA 3110054G05 gene | -1.15 | 0.759 | -1.36 | 0.669 | -2.4 | 0.037 | -1.11 | 0.86 | -1.51 |
| 1441798_at | --- | --- | -1.26 | 0.782 | -2.94 | 0.184 | -1.13 | 0.661 | -3.1 | 0.224 | -2.11 |
| 1446581_at | Pcdh11x | Protocadherin 11 X-linked | -1.91 | 0.378 | -1.15 | 0.254 | -1.6 | 0.525 | -3.08 | 0.324 | -1.94 |
| 1423400_at | Kl | klotho | -1.57 | 0.091 | -1.3 | 0.387 | -1.62 | 0.147 | -1.21 | 0.763 | -1.43 |
| 1421691_at | Krtap16-7 | keratin associated protein 16-7 | -1.48 | 0.408 | -1.3 | 0.673 | -1.73 | 0.241 | -2.47 | 0.201 | -1.75 |
| 1454496_at | 5031415H12Rik | RIKEN cDNA 5031415H12 gene | -2.26 | 0.017 | -1.25 | 0.289 | -1.29 | 0.598 | -1.2 | 0.758 | -1.5 |
| 1422305_at | Ifnb1 | interferon beta 1, fibroblast | -1.5 | 0.143 | -1.93 | 0.444 | -1.19 | 0.705 | -1.11 | 0.681 | -1.44 |
| 1439250_at | Slitrk3 | SLIT and NTRK-like family, member 3 | -1.75 | 0.029 | -1.07 | 0.762 | -1.94 | 0.463 | -1.61 | 0.474 | -1.59 |
| 1424633_at | Camk1g | calcium/calmodulin-dependent protein kinase I gamma | -1.49 | 0.438 | -1.64 | 0.071 | -1.35 | 0.505 | -1.44 | 0.573 | -1.48 |
| 1460064_at | BC028789 | cDNA sequence BC028789 | -3.01 | 0.082 | -1.19 | 0.454 | -1.18 | 0.798 | -1.18 | 0.678 | -1.64 |
| 1429836_at | Ugcgl2 | UDP-glucose ceramide glucosyltransferase-like 2 | -1.26 | 0.345 | -1.51 | 0.189 | -1.76 | 0.053 | -1.97 | 0.147 | -1.63 |
| 1436459_at | Gpr161 | G protein-coupled receptor 161 | -1.11 | 0.831 | -1.95 | 0.202 | -1.63 | 0.338 | -2.03 | 0.503 | -1.68 |
| 1447712_x_at | Ep400 | E1A binding protein p400 | -1.58 | 0.224 | -1.47 | 0.48 | -1.4 | 0.09 | -1.26 | 0.439 | -1.43 |
| 1422378_at | V1rb7 | vomeronasal 1 receptor, B7 | -1.14 | 0.853 | -2.7 | 0.034 | -1.29 | 0.662 | -1.36 | 0.684 | -1.62 |
| 1443430_at | Cradd | CASP2 and RIPK1 domain containing adaptor with death domain | -1.34 | 0.021 | -1.42 | 0.025 | -1.73 | 0.031 | -1.21 | 0.512 | -1.43 |

| | | | | | | | | | | | |
|---|---|---|---|---|---|---|---|---|---|---|---|
| 1441748_at | Zzz3 | Zinc finger, ZZ domain containing 3 | -1.57 | 0.234 | -1.45 | 0.283 | -1.43 | 0.23 | -1.19 | 0.07 | -1.41 |
| 1430267_at | 4833422M21Rik | RIKEN cDNA 4833422M21 gene | -2.19 | 0.224 | -1.08 | 0.825 | -1.55 | 0.481 | -3.38 | 0.013 | -2.05 |
| 1416039_x_at | Cyr61 | cysteine rich protein 61 | -1.29 | 0.492 | -1.57 | 0.085 | -1.63 | 0.281 | -1.42 | 0.329 | -1.48 |
| 1450490_at | Kcna7 | potassium voltage-gated channel, shaker-related subfamily, member 7 | -1.39 | 0.674 | -2.42 | 0.148 | -1.12 | 0.787 | -2.45 | 0.046 | -1.85 |
| 1441634_at | Ntng1 | netrin G1 | -1.55 | 0.173 | -2 | 0.248 | -1.14 | 0.809 | -1.02 | 0.976 | -1.43 |
| 1453938_at | 9330198N18Rik | RIKEN cDNA 9330198N18 gene | -1.48 | 0.135 | -1.44 | 0.621 | -1.52 | 0.3 | -1.04 | 0.781 | -1.37 |
| 1446118_at | D17Ertd165e | DNA segment, Chr 17, ERATO Doi 165, expressed | -1.04 | 0.675 | -1.44 | 0.058 | -2.69 | 0.01 | -1.17 | 0.652 | -1.59 |
| 1430673_a_at | 2210009G21Rik | RIKEN cDNA 2210009G21 gene | -1.68 | 0.478 | -1.85 | 0.196 | -1.12 | 0.748 | -1 | 0.997 | -1.41 |
| 1429646_at | 1700112C13Rik | RIKEN cDNA 1700112C13 gene | -2.07 | 0.317 | -1.16 | 0.81 | -1.46 | 0.239 | -1.45 | 0.565 | -1.54 |
| 1431613_a_at | 1700024D23Rik | RIKEN cDNA 1700024D23 gene | -1.7 | 0.008 | -2.24 | 0.369 | -1.01 | 0.984 | -2.15 | 0.19 | -1.78 |
| 1450758_at | Cntnap2 | contactin associated protein-like 2 | -1.43 | 0.604 | -1.95 | 0.439 | -1.23 | 0.21 | -1.18 | 0.719 | -1.45 |
| 1445151_at | --- | --- | -1.59 | 0.029 | -1.76 | 0.19 | -1.2 | 0.391 | -1.46 | 0.14 | -1.5 |
| 1423802_at | Camkv | CaM kinase-like vesicle-associated | -2.33 | 0.438 | -1.03 | 0.869 | -1.59 | 0.049 | -1.83 | 0.519 | -1.7 |
| 1453221_at | Gopc | golgi associated PDZ and coiled-coil motif containing | -1.55 | 0.1 | -1.54 | 0.007 | -1.36 | 0.106 | -2.14 | 0.076 | -1.65 |
| 1427618_at | Cdh9 | cadherin 9 | -1.25 | 0.799 | -1.79 | 0.068 | -1.49 | 0.475 | -1.73 | 0.293 | -1.57 |
| 1446723_at | --- | --- | -2.03 | 0.444 | -1.24 | 0.338 | -1.37 | 0.569 | -2.31 | 0.363 | -1.74 |
| 1420144_x_at | Mnab | membrane associated DNA binding protein | -1.04 | 0.972 | -3.04 | 0.01 | -1.35 | 0.557 | -1.13 | 0.873 | -1.64 |
| 1447523_at | Zfp294 | Zinc finger protein 294 | -1.5 | 0.311 | -2.04 | 0.042 | -1.15 | 0.651 | -1.24 | 0.246 | -1.48 |
| 1430471_at | 9230110K08Rik | RIKEN cDNA 9230110K08 gene | -1.9 | 0.182 | -1.76 | 0.327 | -1.07 | 0.788 | -1.02 | 0.945 | -1.44 |
| 1443949_at | Ppp2r5e | Protein phosphatase 2, regulatory subunit B (B56), epsilon isoform | -1.2 | 0.347 | -1.4 | 0.089 | -2.07 | 0.004 | -1.19 | 0.478 | -1.46 |
| 1439536_at | AI846133 | Expressed sequence AI846133 | -1.22 | 0.102 | -1.99 | 0.296 | -1.41 | 0.103 | -1.51 | 0.342 | -1.53 |
| 1456644_at | 5730528L13Rik | RIKEN cDNA 5730528L13 gene | -1.89 | 0.128 | -1.37 | 0.662 | -1.3 | 0.428 | -1.17 | 0.805 | -1.43 |
| 1457332_at | Prmt3 | protein arginine N-methyltransferase 3 | -1.74 | 0.208 | -1.92 | 0.416 | -1.07 | 0.587 | -1.2 | 0.443 | -1.48 |
| 1444826_at | --- | --- | -2.92 | 0.018 | -1.12 | 0.543 | -1.26 | 0.397 | -1.16 | 0.562 | -1.61 |
| 1427133_s_at | Lrp2 | low density lipoprotein receptor-related protein 2 | -1.12 | 0.196 | -3.89 | 0.125 | -1.13 | 0.534 | -1.46 | 0.457 | -1.9 |
| 1421800_at | Phxr1 | per-hexamer repeat gene 1 | -1.3 | 0.485 | -1.82 | 0.063 | -1.4 | 0.16 | -1.19 | 0.009 | -1.43 |
| 1440401_at | --- | --- | -1.99 | 0.592 | -1.24 | 0.735 | -1.38 | 0.527 | -1.04 | 0.914 | -1.41 |
| 1421598_at | --- | --- | -1.13 | 0.529 | -2.34 | 0.058 | -1.39 | 0.233 | -2.2 | 0.089 | -1.77 |
| 1451582_at | Tulp1 | tubby like protein 1 | -1.54 | 0.055 | -1.23 | 0.172 | -1.76 | 0.227 | -1.77 | 0.509 | -1.57 |
| 1441684_at | Ttc3 | tetratricopeptide repeat domain 3 | -1.08 | 0.845 | -1.79 | 0.304 | -1.83 | 0.053 | -1.77 | 0.189 | -1.62 |
| 1433344_at | 4930448K20Rik | RIKEN cDNA 4930448K20 gene | -1.34 | 0.502 | -1.5 | 0.274 | -1.61 | 0.235 | -1.08 | 0.864 | -1.38 |
| 1457991_at | Abcd3 | ATP-binding cassette, sub-family D (ALD), member 3 | -1.23 | 0.673 | -1.77 | 0.111 | -1.52 | 0.152 | -1.95 | 0.406 | -1.62 |
| 1459768_x_at | Rap1gds1 | RAP1, GTP-GDP dissociation stimulator 1 | -1.09 | 0.776 | -1.85 | 0.12 | -1.75 | 0.158 | -1.04 | 0.909 | -1.43 |
| 1430754_at | 4930511H11Rik | RIKEN cDNA 4930511H11 gene | -1.82 | 0.111 | -1.68 | 0.337 | -1.13 | 0.794 | -1.28 | 0.104 | -1.48 |
| 1447142_at | Cts7 | cathepsin 7 | -1.21 | 0.436 | -1.54 | 0.52 | -1.79 | 0.494 | -1.05 | 0.807 | -1.4 |
| 1450625_at | Col5a2 | procollagen, type V, alpha 2 | -1.41 | 0.132 | -1.32 | 0.652 | -1.76 | 0.02 | -1.26 | 0.389 | -1.44 |
| 1455235_x_at | Ldhb | lactate dehydrogenase B | -1.26 | 0.154 | -2.13 | 0.107 | -1.29 | 0.132 | -1.49 | 0.281 | -1.54 |
| 1432067_at | 4930473O22Rik | RIKEN cDNA 4930473O22 gene | -2.36 | 0.015 | -1.19 | 0.77 | -1.3 | 0.586 | -1.24 | 0.772 | -1.52 |
| 1446201_at | 9030420J04Rik | RIKEN cDNA 9030420J04 gene | -1.16 | 0.479 | -1.58 | 0.147 | -1.86 | 0.114 | -1.24 | 0.771 | -1.46 |
| 1450760_a_at | Ing3 | inhibitor of growth family, member 3 | -1.86 | 0.373 | -1.3 | 0.397 | -1.37 | 0.294 | -2.07 | 0.161 | -1.65 |
| 1438279_at | Dpp4 | Dipeptidylpeptidase 4 | -1.31 | 0.135 | -1.38 | 0.233 | -1.82 | 0.081 | -2.15 | 0.113 | -1.67 |
| 1428948_at | 5730414M22Rik | RIKEN cDNA 5730414M22 gene | -1.24 | 0.045 | -3.46 | 0.048 | -1.06 | 0.855 | -1.09 | 0.885 | -1.71 |
| 1443817_x_at | Nkx2-6 | NK2 transcription factor related, locus 6 (Drosophila) | -1.26 | 0.799 | -2.49 | 0.052 | -1.19 | 0.557 | -1.49 | 0.325 | -1.61 |
| 1433213_at | 2700008E08Rik | RIKEN cDNA 2700008E08 gene | -1.51 | 0.34 | -1.27 | 0.481 | -1.69 | 0.101 | -1.31 | 0.569 | -1.45 |
| 1440111_at | Fmnl2 | Formin-like 2 | -1.52 | 0.173 | -1.25 | 0.435 | -1.74 | 0.086 | -1.82 | 0.005 | -1.58 |
| 1454762_at | Xkrx | X Kell blood group precursor related X linked | -1.83 | 0.356 | -1.68 | 0.61 | -1.11 | 0.61 | -2.3 | 0.128 | -1.73 |
| 1445083_at | --- | Transcribed locus | -2.6 | 0.141 | -1.15 | 0.653 | -1.28 | 0.619 | -1.21 | 0.61 | -1.56 |
| 1442592_at | Ppapdc3 | Phosphatidic acid phosphatase type 2 domain containing 3 | -1.33 | 0.62 | -1.48 | 0.153 | -1.63 | 0.388 | -1.53 | 0.39 | -1.49 |
| 1437676_at | Spag9 | sperm associated antigen 9 | -1.49 | 0.024 | -1.19 | 0.285 | -1.89 | 0.02 | -1.52 | 0.232 | -1.52 |
| 1444677_at | C77673 | expressed sequence C77673 | -1.54 | 0.145 | -1.28 | 0.182 | -1.64 | 0.098 | -1.2 | 0.141 | -1.42 |
| 1447741_x_at | Acad10 | acyl-Coenzyme A dehydrogenase family, member 10 | -1.95 | 0.29 | -1.44 | 0.563 | -1.2 | 0.724 | -1.52 | 0.601 | -1.53 |
| 1459434_at | Pdhx | Pyruvate dehydrogenase complex, component X | -1.2 | 0.555 | -1.64 | 0.139 | -1.68 | 0.217 | -1.31 | 0.439 | -1.46 |
| 1452270_s_at | Cubn | cubilin (intrinsic factor-cobalamin receptor) | -1.1 | 0.085 | -3.97 | 0.174 | -1.13 | 0.859 | -1.1 | 0.857 | -1.83 |

| | | | | | | | | | | | |
|---|---|---|---|---|---|---|---|---|---|---|---|
| 1422134_at | Fosb | FBJ osteosarcoma oncogene B | -2.33 | 0.241 | -1.54 | 0.231 | -1.04 | 0.927 | -1.43 | 0.12 | -1.58 |
| 1441479_at | Ppm1b | Protein phosphatase 1B, magnesium dependent, beta isoform | -1.35 | 0.399 | -1.36 | 0.298 | -1.77 | 0.022 | -1.18 | 0.29 | -1.41 |
| 1439679_at | Cct3 | chaperonin subunit 3 (gamma) | -1.58 | 0.113 | -1.38 | 0.615 | -1.46 | 0.385 | -1.35 | 0.685 | -1.44 |
| 1457712_at | Chd8 | chromodomain helicase DNA binding protein 8 | -1.16 | 0.125 | -1.58 | 0.022 | -1.84 | 0.002 | -1.43 | 0.098 | -1.5 |
| 1445895_at | Tcrb-V8.2 | T-cell receptor beta, variable 8.2 | -2.22 | 0.279 | -1.18 | 0.723 | -1.34 | 0.259 | -1.43 | 0.029 | -1.54 |
| 1435298_at | Cog5 | component of oligomeric golgi complex 5 | -1.33 | 0.556 | -1.63 | 0.027 | -1.48 | 0.017 | -1.9 | 0.2 | -1.58 |
| 1435673_at | AI448005 | expressed sequence AI448005 | -1.48 | 0.036 | -1.39 | 0.402 | -1.54 | 0.145 | -1.11 | 0.636 | -1.38 |
| 1441167_at | --- | Transcribed locus | -1.45 | 0.48 | -1.53 | 0.249 | -1.43 | 0.441 | -1.5 | 0.165 | -1.48 |
| 1438109_at | Clca5 | chloride channel calcium activated 5 | -1.3 | 0.671 | -1.58 | 0.486 | -1.55 | 0.356 | -1.15 | 0.783 | -1.4 |
| 1447313_at | --- | --- | -1.36 | 0.169 | -1.45 | 0.172 | -1.61 | 0.087 | -1.01 | 0.976 | -1.36 |
| 1443299_at | Pdlim3 | PDZ and LIM domain 3 | -1.63 | 0.029 | -1.47 | 0.202 | -1.33 | 0.287 | -1.69 | 0.153 | -1.53 |
| 1440638_at | A230107N01Rik | RIKEN cDNA A230107N01 gene | -1 | 0.996 | -1.75 | 0.119 | -2.1 | 0.084 | -2.17 | 0.401 | -1.75 |
| 1428030_at | Adam34 | a disintegrin and metallopeptidase domain 34 | -2.24 | 0.038 | -1.06 | 0.903 | -1.53 | 0.494 | -1.78 | 0.075 | -1.65 |
| 1453494_at | 4921513H07Rik | RIKEN cDNA 4921513H07 gene | -1.48 | 0.591 | -1.32 | 0.662 | -1.64 | 0.026 | -1.02 | 0.97 | -1.36 |
| 1438674_a_at | Sfrs8 | splicing factor, arginine/serine-rich 8 | -1.48 | 0.074 | -1.31 | 0.037 | -1.64 | 0.207 | -1.36 | 0.129 | -1.45 |
| 1427648_at | Rgs3 | Regulator of G-protein signaling 3 | -1.34 | 0.516 | -1.71 | 0.253 | -1.39 | 0.685 | -1.67 | 0.53 | -1.53 |
| 1445914_at | Nrf1 | Nuclear respiratory factor 1 | -1.34 | 0.119 | -1.32 | 0.327 | -1.85 | 0.04 | -1.01 | 0.88 | -1.38 |
| 1419439_at | Stk22s1 /// LOC672 | serine/threonine kinase 22 substrate 1 /// similar to serine/threonine kinase 22 sul | -1.71 | 0.205 | -1.23 | 0.171 | -1.56 | 0.155 | -1.84 | 0.055 | -1.58 |
| 1458240_at | Magi1 | Membrane associated guanylate kinase, WW and PDZ domain containing 1 | -1.22 | 0.229 | -1.4 | 0.191 | -1.97 | 0.033 | -1.56 | 0.224 | -1.54 |
| 1436629_at | A830041P22Rik | RIKEN cDNA A830041P22 gene | -1.23 | 0.325 | -2.27 | 0.106 | -1.26 | 0.644 | -4.88 | 0.022 | -2.41 |
| 1443416_at | C79741 | expressed sequence C79741 | -1.48 | 0.266 | -1.42 | 0.113 | -1.5 | 0.002 | -1.06 | 0.673 | -1.36 |
| 1427791_a_at | Adam1a | a disintegrin and metallopeptidase domain 1a | -1.1 | 0.616 | -1.43 | 0.422 | -2.29 | 0.013 | -1.8 | 0.067 | -1.65 |
| 1441342_at | Dpp4 | Dipeptidylpeptidase 4 | -1.15 | 0.185 | -1.46 | 0.015 | -2.02 | 0.01 | -2.74 | 0.066 | -1.84 |
| 1421437_x_at | Pcdhb14 | protocadherin beta 14 | -1.48 | 0.6 | -1.19 | 0.471 | -1.87 | 0.196 | -2.39 | 0.229 | -1.73 |
| 1458326_at | Polr2e | polymerase (RNA) II (DNA directed) polypeptide E | -1.55 | 0.322 | -1.43 | 0.539 | -1.43 | 0.456 | -1.29 | 0.155 | -1.42 |
| 1440675_at | Grlf1 | Glucocorticoid receptor DNA binding factor 1 | -1.34 | 0.039 | -1.43 | 0.02 | -1.67 | 0.143 | -1.53 | 0.395 | -1.49 |
| 1448831_at | Angpt2 | angiopoietin 2 | -1.19 | 0.2 | -1.35 | 0.386 | -2.13 | 0.018 | -1.76 | 0.073 | -1.61 |
| 1442067_at | Ror1 | Receptor tyrosine kinase-like orphan receptor 1 | -1.15 | 0.4 | -1.32 | 0.219 | -2.39 | 0.033 | -1.55 | 0.166 | -1.6 |
| 1457544_at | Ube2w | ubiquitin-conjugating enzyme E2W (putative) | -1.12 | 0.888 | -3.04 | 0.024 | -1.2 | 0.583 | -1.24 | 0.749 | -1.65 |
| 1446943_at | Dst | Dystonin | -1.05 | 0.873 | -1.47 | 0.497 | -2.39 | 0.015 | -1.13 | 0.864 | -1.51 |
| 1437919_at | Bdp1 | B double prime 1, subunit of RNA polymerase III transcription initiation factor IIIB | -1.16 | 0.593 | -1.38 | 0.318 | -2.14 | 0.001 | -1.29 | 0.365 | -1.5 |
| 1459512_at | Meis1 | Myeloid ecotropic viral integration site 1 | -1.03 | 0.889 | -1.68 | 0.215 | -2.05 | 0.057 | -1.13 | 0.629 | -1.47 |
| 1437493_at | 5230400M03Rik | RIKEN cDNA 5230400M03 gene | -1.21 | 0.704 | -1.41 | 0.218 | -1.93 | 0.031 | -2.03 | 0.013 | -1.65 |
| 1441362_at | Ptprg | Protein tyrosine phosphatase, receptor type, G | -1.55 | 0.29 | -1.45 | 0.126 | -1.4 | 0.013 | -1.29 | 0.404 | -1.42 |
| 1430185_at | 5830460E08Rik | RIKEN cDNA 5830460E08 gene | -1.43 | 0.087 | -1.28 | 0.177 | -1.76 | 0.009 | -1.22 | 0.555 | -1.42 |
| 1456723_at | Prr14 | Proline rich 14 | -1.27 | 0.693 | -1.93 | 0.127 | -1.34 | 0.3 | -1.08 | 0.769 | -1.41 |
| 1446400_at | Olfr558 | olfactory receptor 558 | -1.18 | 0.274 | -1.43 | 0.1 | -1.97 | 0.24 | -2.92 | 0.341 | -1.88 |
| 1445710_x_at | 1110051B16Rik | RIKEN cDNA 1110051B16 gene | -1.29 | 0.754 | -1.42 | 0.337 | -1.75 | 0.032 | -1.17 | 0.752 | -1.41 |
| 1444378_at | Csnk1d | Casein kinase 1, delta | -1.55 | 0.161 | -1.58 | 0.014 | -1.29 | 0.366 | -1.11 | 0.033 | -1.38 |
| 1444591_at | Pik3r1 | Phosphatidylinositol 3-kinase, regulatory subunit, polypeptide 1 (p85 alpha) | -1.15 | 0.61 | -1.47 | 0.069 | -1.99 | 0.038 | -2.14 | 0.035 | -1.69 |
| 1440432_at | Lztfl1 | Leucine zipper transcription factor-like 1 | -1.19 | 0.733 | -2.39 | 0.081 | -1.26 | 0.437 | -2.6 | 0.054 | -1.86 |
| 1423014_at | Pcdhb6 | protocadherin beta 6 | -1.41 | 0.68 | -1.67 | 0.226 | -1.34 | 0.171 | -1.03 | 0.937 | -1.36 |
| 1456692_at | Brd1 | Bromodomain containing 1 | -1.35 | 0.411 | -1.12 | 0.352 | -2.39 | 0.374 | -1.21 | 0.569 | -1.52 |
| 1457742_at | D9Wsu74e | DNA segment, Chr 9, Wayne State University 74, expressed | -1.04 | 0.581 | -2.52 | 0.219 | -1.43 | 0.123 | -1.06 | 0.913 | -1.51 |
| 1458426_at | Kif1b | Kinesin family member 1B | -1.33 | 0.123 | -1.32 | 0.285 | -1.85 | 0.012 | -1.38 | 0.021 | -1.47 |
| 1420281_at | --- | --- | -1.07 | 0.937 | -1.75 | 0.323 | -1.83 | 0.255 | -1.35 | 0.655 | -1.5 |
| 1441657_at | Bach2 | BTB and CNC homology 2 | -2.05 | 0.012 | -1.48 | 0.35 | -1.12 | 0.703 | -1.89 | 0.142 | -1.64 |
| 1438538_at | D630021H01Rik | RIKEN cDNA D630021H01 gene | -1.31 | 0.291 | -1.32 | 0.081 | -1.88 | 0.05 | -1.17 | 0.562 | -1.42 |
| 1446818_at | Mib1 | Mindbomb homolog 1 (Drosophila) | -1.27 | 0.439 | -2.06 | 0.062 | -1.28 | 0.203 | -1.3 | 0.476 | -1.48 |
| 1454413_at | 4933424H11Rik | RIKEN cDNA 4933424H11 gene | -1.09 | 0.495 | -2.02 | 0.334 | -1.55 | 0.346 | -1.33 | 0.651 | -1.5 |
| 1434760_at | Lrrtm3 | leucine rich repeat transmembrane neuronal 3 | -1.19 | 0.758 | -1.72 | 0.332 | -1.59 | 0.36 | -1.97 | 0.09 | -1.62 |
| 1439348_at | S100a10 | S100 calcium binding protein A10 (calpactin) | -1.3 | 0.553 | -1.48 | 0.077 | -1.65 | 0.157 | -1.5 | 0.214 | -1.48 |
| 1429903_at | Gja10 /// Dpf2 | gap junction membrane channel protein alpha 10 /// D4, zinc and double PHD fing | -1.53 | 0.194 | -1.31 | 0.246 | -1.58 | 0.09 | -1.24 | 0.181 | -1.41 |

| | | | | | | | | | | | |
|---|---|---|---|---|---|---|---|---|---|---|---|
| 1446110_at | D3Ertd711e | DNA segment, Chr 3, ERATO Doi 711, expressed | -2.06 | 0.32 | -1.25 | 0.329 | -1.31 | 0.548 | -1.23 | 0.477 | -1.46 |
| 1426129_at | Brms1 | breast cancer metastasis-suppressor 1 | -1.82 | 0.197 | -1.15 | 0.574 | -1.57 | 0.057 | -1.19 | 0.492 | -1.43 |
| 1447010_at | Zfp609 | Zinc finger protein 609 | -1.06 | 0.692 | -1.36 | 0.242 | -2.62 | 0.028 | -1.48 | 0.176 | -1.63 |
| 1441452_at | Lsm1 | LSM1 homolog, U6 small nuclear RNA associated (S. cerevisiae) | -1.07 | 0.235 | -2.17 | 0.121 | -1.51 | 0.095 | -1.43 | 0.061 | -1.55 |
| 1431880_at | 3100003M19Rik | RIKEN cDNA 3100003M19 gene | -1.14 | 0.805 | -1.48 | 0.318 | -1.99 | 0.103 | -1.06 | 0.886 | -1.42 |
| 1441357_at | Kirrel3 | kin of IRRE like 3 (Drosophila) | -1.22 | 0.395 | -3.16 | 0.169 | -1.09 | 0.696 | -1.06 | 0.876 | -1.63 |
| 1460152_at | --- | --- | -1.7 | 0.194 | -1.54 | 0.556 | -1.22 | 0.675 | -1.64 | 0.417 | -1.53 |
| 1456067_at | Gli3 | GLI-Kruppel family member GLI3 | -1.09 | 0.737 | -2.44 | 0.236 | -1.37 | 0.121 | -1.23 | 0.475 | -1.53 |
| 1453672_at | 2810038L03Rik | RIKEN cDNA 2810038L03 gene | -2.14 | 0.239 | -1 | 0.995 | -1.68 | 0.224 | -1.05 | 0.933 | -1.47 |
| 1441135_at | Zfp672 | Zinc finger protein 672 | -1.5 | 0.227 | -1.24 | 0.539 | -1.7 | 0.224 | -1.13 | 0.585 | -1.39 |
| 1432285_at | 1700128F08Rik | RIKEN cDNA 1700128F08 gene | -1.49 | 0.63 | -1.12 | 0.748 | -2.02 | 0.036 | -2.1 | 0.554 | -1.68 |
| 1434862_at | Fut2 | fucosyltransferase 2 | -1.44 | 0.563 | -1.56 | 0.614 | -1.38 | 0.476 | -1.43 | 0.382 | -1.45 |
| 1442740_at | --- | --- | -1.62 | 0.407 | -1.04 | 0.868 | -2.09 | 0.081 | -1.16 | 0.321 | -1.48 |
| 1453881_x_at | Cetn1 | centrin 1 | -1.51 | 0.184 | -1.79 | 0.482 | -1.2 | 0.605 | -1.1 | 0.792 | -1.4 |
| 1441614_at | --- | --- | -1.66 | 0.352 | -1.08 | 0.903 | -1.89 | 0.103 | -1.69 | 0.206 | -1.58 |
| 1440615_at | Dusp16 | dual specificity phosphatase 16 | -1.13 | 0.489 | -1.32 | 0.465 | -2.38 | 0.105 | -2.56 | 0.021 | -1.85 |
| 1430774_at | A430106A12Rik | RIKEN cDNA A430106A12 gene | -1.27 | 0.56 | -1.56 | 0.04 | -1.58 | 0.164 | -1.41 | 0.231 | -1.46 |
| 1453387_at | 4833432E10Rik | RIKEN cDNA 4833432E10 gene | -1.36 | 0.716 | -1.29 | 0.605 | -1.82 | 0.103 | -1.01 | 0.988 | -1.37 |
| 1416687_at | Plod2 | procollagen lysine, 2-oxoglutarate 5-dioxygenase 2 | -1.15 | 0.656 | -1.44 | 0.442 | -2.01 | 0.008 | -1.16 | 0.095 | -1.44 |
| 1458826_at | C78809 | expressed sequence C78809 | -1.11 | 0.866 | -2.08 | 0.325 | -1.47 | 0.335 | -1.75 | 0.223 | -1.6 |
| 1442017_at | Nfs1 | nitrogen fixation gene 1 (S. cerevisiae) | -1.32 | 0.442 | -1.6 | 0.072 | -1.48 | 0.031 | -1.37 | 0.03 | -1.44 |
| 1445958_at | --- | Transcribed locus | -2.27 | 0.142 | -1.57 | 0.273 | -1.02 | 0.966 | -1.47 | 0.472 | -1.58 |
| 1442653_at | --- | --- | -1.62 | 0.451 | -1.49 | 0.284 | -1.29 | 0.284 | -1.25 | 0.544 | -1.41 |
| 1442916_at | 4931420C21Rik | RIKEN cDNA 4931420C21 gene | -1.37 | 0.414 | -1.39 | 0.074 | -1.63 | 0.02 | -2.62 | 0.064 | -1.75 |
| 1437294_at | Exosc10 | exosome component 10 | -1.06 | 0.051 | -2.03 | 0.027 | -1.6 | 0.136 | -1.42 | 0.037 | -1.53 |
| 1440989_at | --- | --- | -1.78 | 0.198 | -1.41 | 0.045 | -1.27 | 0.386 | -1.2 | 0.441 | -1.41 |
| 1443424_at | 2210018M11Rik | RIKEN cDNA 2210018M11 gene | -1.78 | 0.245 | -1.82 | 0.007 | -1.06 | 0.871 | -1.14 | 0.514 | -1.45 |
| 1438133_a_at | Cyr61 | cysteine rich protein 61 | -1.38 | 0.362 | -1.81 | 0.103 | -1.28 | 0.646 | -1.27 | 0.425 | -1.43 |
| 1460593_at | Susd4 | sushi domain containing 4 | -1.12 | 0.582 | -2.32 | 0.015 | -1.36 | 0.094 | -2.11 | 0.191 | -1.73 |
| 1432306_at | A930023M06Rik | RIKEN cDNA A930023M06 gene | -1.29 | 0.084 | -1.84 | 0.282 | -1.35 | 0.385 | -1.48 | 0.136 | -1.49 |
| 1430818_at | Tmc1 | transmembrane channel-like gene family 1 | -1.47 | 0.136 | -1.4 | 0.335 | -1.5 | 0.245 | -1.7 | 0.08 | -1.52 |
| 1440012_at | --- | --- | -1.17 | 0.385 | -1.66 | 0.028 | -1.65 | 0.088 | -1.19 | 0.599 | -1.42 |
| 1432509_at | 5033430I15Rik | RIKEN cDNA 5033430I15 gene | -1.27 | 0.357 | -1.78 | 0.112 | -1.4 | 0.188 | -1.59 | 0.293 | -1.51 |
| 1457282_x_at | Tubgcp5 | Tubulin, gamma complex associated protein 5 | -1.59 | 0.275 | -1.26 | 0.556 | -1.57 | 0.068 | -1.16 | 0.391 | -1.39 |
| 1459049_at | Rasgrf1 | RAS protein-specific guanine nucleotide-releasing factor 1 | -2.43 | 0.093 | -1.44 | 0.331 | -1.04 | 0.924 | -1.84 | 0.107 | -1.69 |
| 1445996_at | Plac9 | Placenta specific 9 | -1.17 | 0.63 | -1.54 | 0.095 | -1.78 | 0.084 | -1.11 | 0.817 | -1.4 |
| 1430121_at | 1700006E09Rik | RIKEN cDNA 1700006E09 gene | -1.27 | 0.556 | -1.62 | 0.353 | -1.53 | 0.417 | -1.21 | 0.541 | -1.4 |
| 1458337_at | Tcf12 | Transcription factor 12 | -1.74 | 0.256 | -1.2 | 0.774 | -1.52 | 0.467 | -1.29 | 0.572 | -1.44 |
| 1446850_at | Ppap2b | Phosphatidic acid phosphatase type 2B | -1.36 | 0.04 | -1.23 | 0.323 | -1.94 | 0.03 | -1.32 | 0.478 | -1.46 |
| 1430685_at | 6330503C03Rik | RIKEN cDNA 6330503C03 gene | -2.05 | 0.295 | -1.14 | 0.748 | -1.43 | 0.405 | -1.59 | 0.156 | -1.55 |
| 1442755_at | --- | Transcribed locus | -1.12 | 0.693 | -2 | 0.051 | -1.49 | 0.26 | -1.26 | 0.235 | -1.47 |
| 1420747_at | Ppnr | per-pentamer repeat gene | -1.52 | 0.332 | -1.13 | 0.689 | -1.94 | 0.135 | -1.05 | 0.859 | -1.41 |
| 1433197_at | 4930445B03Rik | RIKEN cDNA 4930445B03 gene | -1.4 | 0.296 | -1.56 | 0.298 | -1.42 | 0.35 | -1.13 | 0.471 | -1.37 |
| 1455419_at | D630045J12Rik | RIKEN cDNA D630045J12 gene | -1.44 | 0.084 | -2.07 | 0.313 | -1.12 | 0.327 | -1.14 | 0.823 | -1.44 |
| 1438879_at | Ddah1 | Dimethylarginine dimethylaminohydrolase 1 | -1.32 | 0.083 | -2.08 | 0.072 | -1.21 | 0.521 | -2.39 | 0.251 | -1.75 |
| 1456708_at | 2610028E06Rik | RIKEN cDNA 2610028E06 gene | -2.45 | 0.129 | -1.42 | 0.255 | -1.05 | 0.586 | -1.06 | 0.87 | -1.49 |
| 1442214_at | Nfib | Nuclear factor I/B | -1.17 | 0.346 | -1.4 | 0.132 | -2.01 | 0.005 | -1.03 | 0.746 | -1.4 |
| 1446477_at | Zfp622 | zinc finger protein 622 | -1.61 | 0.484 | -1.94 | 0.102 | -1.07 | 0.786 | -1.59 | 0.108 | -1.55 |
| 1425688_a_at | Dpys | dihydropyrimidinase | -1.33 | 0.496 | -1.28 | 0.035 | -1.88 | 0.2 | -1.88 | 0.221 | -1.59 |
| 1432888_at | 4930455M05Rik | RIKEN cDNA 4930455M05 gene | -1.35 | 0.112 | -1.86 | 0 | -1.27 | 0.312 | -1.4 | 0.339 | -1.47 |
| 1439131_at | 4932441K18Rik | RIKEN cDNA 4932441K18 gene | -1.88 | 0.208 | -1.47 | 0.255 | -1.17 | 0.382 | -1.22 | 0.199 | -1.44 |
| 1429597_at | Dppa4 | developmental pluripotency associated 4 | -1.79 | 0.027 | -1.32 | 0.499 | -1.33 | 0.448 | -1.16 | 0.309 | -1.4 |
| 1442405_at | Csrp2bp | Cysteine and glycine-rich protein 2 binding protein | -1.29 | 0.619 | -1.6 | 0.039 | -1.5 | 0.232 | -1.53 | 0.063 | -1.48 |

| | | | | | | | | | | | |
|---|---|---|---|---|---|---|---|---|---|---|---|
| 1440794_x_at | 1700029M03Rik | RIKEN cDNA 1700029M03 gene | -1.39 | 0.583 | -2.28 | 0.247 | -1.1 | 0.818 | -1.3 | 0.556 | -1.52 |
| 1427790_at | Mapkapk5 /// Adam | MAP kinase-activated protein kinase 5 /// a disintegrin and metallopeptidase dom | -1.75 | 0.136 | -1.56 | 0.188 | -1.16 | 0.676 | -1.29 | 0.385 | -1.44 |
| 1441596_at | 5730601F06Rik | RIKEN cDNA 5730601F06 gene | -1.13 | 0.777 | -1.39 | 0.387 | -2.15 | 0.271 | -1.76 | 0.145 | -1.61 |
| 1441620_at | Grb10 | Growth factor receptor bound protein 10 | -1.44 | 0.282 | -1.31 | 0.494 | -1.63 | 0.082 | -1.28 | 0.686 | -1.42 |
| 1442184_at | Slc24a3 | Solute carrier family 24 (sodium/potassium/calcium exchanger), member 3 | -1.87 | 0.43 | -1.69 | 0.269 | -1.06 | 0.893 | -1.56 | 0.528 | -1.55 |
| 1458648_at | AU042950 | expressed sequence AU042950 | -1.3 | 0.2 | -1.21 | 0.638 | -2.1 | 0.002 | -1.21 | 0.569 | -1.46 |
| 1425124_at | Rnf183 | ring finger protein 183 | -1.33 | 0.33 | -1.45 | 0.273 | -1.59 | 0.168 | -1.38 | 0.694 | -1.44 |
| 1441148_at | Fcmd | Fukuyama type congenital muscular dystrophy homolog (human) | -1.32 | 0.047 | -1.73 | 0.113 | -1.37 | 0.241 | -1.22 | 0.543 | -1.41 |
| 1431399_at | Adamts9 | a disintegrin-like and metallopeptidase (reprolysin type) with thrombospondin type | -1.34 | 0.353 | -1.4 | 0.375 | -1.63 | 0.237 | -1.59 | 0.312 | -1.49 |
| 1445225_at | 2410001C21Rik | RIKEN cDNA 2410001C21 gene | -1.32 | 0.437 | -2.33 | 0.053 | -1.13 | 0.352 | -1.2 | 0.09 | -1.5 |
| 1429813_at | Pank1 | pantothenate kinase 1 | -1.17 | 0.662 | -1.48 | 0.261 | -1.85 | 0.327 | -1.05 | 0.934 | -1.39 |
| 1446220_at | Gm484 | gene model 484, (NCBI) | -1.36 | 0.499 | -1.64 | 0.368 | -1.38 | 0.626 | -1.08 | 0.899 | -1.37 |
| 1428662_a_at | Hod | homeobox only domain | -1.49 | 0.024 | -1.39 | 0.157 | -1.47 | 0.005 | -1.15 | 0.754 | -1.37 |
| 1430846_at | 1700061G19Rik | RIKEN cDNA 1700061G19 gene | -1.35 | 0.436 | -1.76 | 0.234 | -1.31 | 0.415 | -1.39 | 0.079 | -1.45 |
| 1460421_at | Zfp133 | zinc finger protein 133 | -1.29 | 0.363 | -2.58 | 0.062 | -1.1 | 0.792 | -1.03 | 0.532 | -1.5 |
| 1445610_at | BC039093 | CDNA sequence BC039093 | -1.92 | 0.39 | -1.1 | 0.859 | -1.56 | 0.293 | -1.32 | 0.392 | -1.48 |
| 1429068_at | 2810488G03Rik | RIKEN cDNA 2810488G03 gene | -1.12 | 0.675 | -2.19 | 0.111 | -1.39 | 0.142 | -1.2 | 0.558 | -1.48 |
| 1457245_at | Dirc2 | disrupted in renal carcinoma 2 (human) | -1.08 | 0.66 | -2.13 | 0.132 | -1.48 | 0.151 | -1.17 | 0.143 | -1.46 |
| 1437884_at | Arl5b | ADP-ribosylation factor-like 5B | -1.21 | 0.048 | -1.3 | 0.136 | -2.09 | 0.011 | -1.09 | 0.791 | -1.42 |
| 1458502_at | Ktn1 | Kinectin 1 | -1.32 | 0.153 | -3.16 | 0.129 | -1 | 0.994 | -2.16 | 0.039 | -1.91 |
| 1441038_at | Utrn | Utrophin | -1.12 | 0.501 | -1.19 | 0.194 | -2.97 | 0.028 | -1.71 | 0.135 | -1.75 |
| 1459033_at | Plxna2 | Plexin A2 | -1.33 | 0.305 | -1.11 | 0.877 | -2.42 | 0.123 | -1.09 | 0.865 | -1.48 |
| 1430103_at | 9030607L20Rik | RIKEN cDNA 9030607L20 gene | -1.36 | 0.075 | -1.67 | 0.265 | -1.36 | 0.011 | -1.14 | 0.588 | -1.38 |
| 1435603_at | Sned1 | sushi, nidogen and EGF-like domains 1 | -1.14 | 0.034 | -2.11 | 0.061 | -1.38 | 0.278 | -1.3 | 0.609 | -1.48 |
| 1435411_at | --- | Transcribed locus | -1.35 | 0.59 | -1.36 | 0.098 | -1.68 | 0.26 | -2.14 | 0.181 | -1.63 |
| 1447409_at | --- | --- | -1.75 | 0.028 | -1.41 | 0.1 | -1.27 | 0.169 | -1.64 | 0.101 | -1.52 |
| 1444208_at | Dnahc1 | Dynein, axonemal, heavy chain 1 | -1.45 | 0.498 | -1.29 | 0.382 | -1.65 | 0.109 | -1.04 | 0.931 | -1.36 |
| 1442901_at | Tax1bp1 | Tax1 (human T-cell leukemia virus type I) binding protein 1 | -1.46 | 0.314 | -1.89 | 0.192 | -1.17 | 0.6 | -1.03 | 0.896 | -1.38 |
| 1456101_at | LOC640072 /// LOC | hypothetical protein LOC640072 /// hypothetical protein LOC677194 | -1.34 | 0.647 | -1.53 | 0.334 | -1.48 | 0.507 | -1.61 | 0.305 | -1.49 |
| 1446386_at | Tcf4 | Transcription factor 4 | -1.2 | 0.622 | -1.64 | 0.006 | -1.58 | 0.002 | -1.58 | 0.245 | -1.5 |
| 1444917_at | Ube2w | Ubiquitin-conjugating enzyme E2W (putative) | -2.16 | 0.012 | -1.19 | 0.823 | -1.3 | 0.072 | -1.86 | 0.163 | -1.63 |
| 1420149_at | Pcnt | Pericentrin (kendrin) | -1.58 | 0.242 | -1.15 | 0.755 | -1.75 | 0.431 | -1.7 | 0.478 | -1.55 |
| 1459142_at | Spag17 | sperm associated antigen 17 | -1.79 | 0.477 | -1.86 | 0.473 | -1.02 | 0.653 | -1.99 | 0.441 | -1.67 |
| 1441763_at | Zdhhc6 | zinc finger, DHHC domain containing 6 | -1.24 | 0.681 | -1.59 | 0.189 | -1.56 | 0.106 | -1.43 | 0.181 | -1.46 |
| 1430175_at | 4930588G05Rik | RIKEN cDNA 4930588G05 gene | -1.1 | 0.891 | -1.85 | 0.017 | -1.59 | 0.134 | -1.54 | 0.154 | -1.52 |
| 1453847_at | A930037O16Rik | RIKEN cDNA A930037O16 gene | -1.12 | 0.866 | -2.76 | 0.219 | -1.22 | 0.477 | -1.07 | 0.935 | -1.54 |
| 1445720_at | Sltm | SAFB-like, transcription modulator | -1.73 | 0.025 | -1.67 | 0.1 | -1.11 | 0.422 | -1.52 | 0.274 | -1.51 |
| 1421084_at | Rs1h | retinoschisis 1 homolog (human) | -1.14 | 0.771 | -1.69 | 0.287 | -1.65 | 0.093 | -1.46 | 0.204 | -1.49 |
| 1425709_at | Rnf17 | ring finger protein 17 | -1.53 | 0.638 | -1.53 | 0.575 | -1.3 | 0.583 | -3.87 | 0.122 | -2.06 |
| 1443661_at | --- | --- | -1.51 | 0.269 | -2.01 | 0.264 | -1.1 | 0.821 | -1.07 | 0.908 | -1.42 |
| 1418188_a_at | Malat1 | Metastasis associated lung adenocarcinoma transcript 1 (non-coding RNA) | -1.18 | 0.656 | -1.54 | 0.123 | -1.73 | 0.02 | -1.69 | 0.34 | -1.54 |
| 1443376_at | --- | Transcribed locus | -1.61 | 0.317 | -1.32 | 0.595 | -1.44 | 0.401 | -1.06 | 0.876 | -1.36 |
| 1445834_at | --- | Transcribed locus | -2.31 | 0.397 | -1.08 | 0.669 | -1.4 | 0.464 | -1.25 | 0.391 | -1.51 |
| 1444318_at | Chchd7 | Coiled-coil-helix-coiled-coil-helix domain containing 7 | -1.11 | 0.314 | -2.41 | 0.021 | -1.31 | 0.251 | -1.81 | 0 | -1.66 |
| 1441449_at | Jarid1c | jumonji, AT rich interactive domain 1C (Rbp2 like) | -1.05 | 0.949 | -1.44 | 0.25 | -2.33 | 0.01 | -1.02 | 0.92 | -1.46 |
| 1440155_at | Fstl4 | follistatin-like 4 | -1.71 | 0.298 | -1.85 | 0.336 | -1.05 | 0.858 | -2.51 | 0.116 | -1.78 |
| 1418660_at | Clock /// LOC62072 | circadian locomoter output cycles kaput /// hypothetical protein LOC620729 | -1.38 | 0.022 | -1.24 | 0.159 | -1.83 | 0.014 | -1.39 | 0.292 | -1.46 |
| 1433226_at | 6330403N20Rik | RIKEN cDNA 6330403N20 gene | -1.54 | 0.566 | -1.49 | 0.598 | -1.32 | 0.302 | -1.28 | 0.25 | -1.41 |
| 1429302_at | Csnk2a2 | casein kinase 2, alpha prime polypeptide | -1.29 | 0.193 | -1.38 | 0.079 | -1.73 | 0.086 | -1.16 | 0.045 | -1.39 |
| 1440271_at | --- | Transcribed locus | -1.51 | 0.446 | -1.55 | 0.17 | -1.3 | 0.052 | -1.52 | 0.147 | -1.47 |
| 1459078_at | Rbpms | RNA binding protein gene with multiple splicing | -1.36 | 0.357 | -1.25 | 0.43 | -1.83 | 0.013 | -1.68 | 0.462 | -1.53 |
| 1447038_x_at | Phip | pleckstrin homology domain interacting protein | -1.37 | 0.283 | -1.89 | 0.202 | -1.22 | 0.222 | -1.67 | 0.308 | -1.54 |
| 1447542_at | AY702102 | cDNA sequence AY702102 | -1.36 | 0.456 | -2.22 | 0.448 | -1.12 | 0.447 | -1.99 | 0.211 | -1.67 |

| | | | | | | | | | | | |
|---|---|---|---|---|---|---|---|---|---|---|---|
| 1431940_at | LOC74457 | hypothetical protein LOC74457 | -1.3 | 0.29 | -1.55 | 0.305 | -1.51 | 0.121 | -1.22 | 0.048 | -1.39 |
| 1419065_at | 5730521E12Rik | RIKEN cDNA 5730521E12 gene | -1.27 | 0.353 | -1.03 | 0.847 | -3.15 | 0.05 | -1.44 | 0.515 | -1.72 |
| 1445135_at | --- | --- | -1.02 | 0.988 | -2.9 | 0.153 | -1.34 | 0.428 | -1.5 | 0.635 | -1.69 |
| 1457386_at | 9530010C24Rik | RIKEN cDNA 9530010C24 gene | -1.12 | 0.607 | -1.23 | 0.305 | -2.69 | 0.014 | -3.37 | 0.13 | -2.1 |
| 1451529_at | Sgtb | small glutamine-rich tetratricopeptide repeat (TPR)-containing, beta | -1.67 | 0.094 | -1.39 | 0.311 | -1.31 | 0.026 | -3.08 | 0.243 | -1.86 |
| 1438900_at | Sacm1l | SAC1 (suppressor of actin mutations 1, homolog)-like (S. cerevisiae) | -1.27 | 0.553 | -1.37 | 0.197 | -1.77 | 0.016 | -1.09 | 0.781 | -1.38 |
| 1433152_at | 2900022B07Rik | RIKEN cDNA 2900022B07 gene | -1.77 | 0.472 | -1.24 | 0.634 | -1.42 | 0.701 | -1.42 | 0.59 | -1.46 |
| 1454649_at | Srd5a1 | steroid 5 alpha-reductase 1 | -1.18 | 0.438 | -2.27 | 0.008 | -1.26 | 0.53 | -1.63 | 0.21 | -1.59 |
| 1441787_at | Zc3h12c | Zinc finger CCCH-type containing 12C | -1.19 | 0.56 | -2.55 | 0.114 | -1.18 | 0.591 | -1.3 | 0.56 | -1.56 |
| 1441267_at | Rsrc1 | Arginine/serine-rich coiled-coil 1 | -1.74 | 0.33 | -1.19 | 0.192 | -1.51 | 0.4 | -2.74 | 0.102 | -1.79 |
| 1456132_x_at | Tsc22d1 | TSC22 domain family, member 1 | -1.28 | 0.741 | -1.37 | 0.258 | -1.75 | 0.278 | -1.01 | 0.987 | -1.35 |
| 1458836_at | Calb1 | calbindin-28K | -1.5 | 0.227 | -1.47 | 0.622 | -1.36 | 0.264 | -1.26 | 0.698 | -1.4 |
| 1440947_at | Bat2d | BAT2 domain containing 1 | -1.38 | 0.307 | -1.56 | 0.294 | -1.4 | 0.128 | -1.52 | 0.141 | -1.46 |
| 1449599_at | --- | --- | -1.77 | 0.544 | -1.1 | 0.844 | -1.64 | 0.115 | -1.81 | 0.362 | -1.58 |
| 1436413_at | Frk | fyn-related kinase | -1.29 | 0.093 | -1.53 | 0.124 | -1.54 | 0.171 | -1.03 | 0.948 | -1.35 |
| 1432182_at | 4922502B01Rik | RIKEN cDNA 4922502B01 gene | -1.25 | 0.781 | -1.47 | 0.156 | -1.67 | 0.546 | -1.14 | 0.768 | -1.38 |
| 1456341_a_at | Klf9 | Kruppel-like factor 9 | -1.42 | 0.479 | -1.65 | 0.102 | -1.3 | 0.302 | -1.12 | 0.788 | -1.37 |
| 1432686_at | 4833406M21Rik | RIKEN cDNA 4833406M21 gene | -1.43 | 0.097 | -1.1 | 0.806 | -2.11 | 0.016 | -1.2 | 0.232 | -1.46 |
| 1447328_at | Itpr2 | Inositol 1,4,5-triphosphate receptor 2 | -1.12 | 0.692 | -1.74 | 0.164 | -1.63 | 0.184 | -1.35 | 0.436 | -1.46 |
| 1458691_at | Synpo2l | Synaptopodin 2-like | -1.41 | 0.488 | -1.73 | 0.378 | -1.26 | 0.623 | -1.83 | 0.068 | -1.56 |
| 1430955_at | 2810403A07Rik | RIKEN cDNA 2810403A07 gene | -1.58 | 0.146 | -1.55 | 0.012 | -1.24 | 0.375 | -1.35 | 0.335 | -1.43 |
| 1451725_a_at | Psmd4 | proteasome (prosome, macropain) 26S subunit, non-ATPase, 4 | -1.38 | 0.153 | -2.27 | 0.103 | -1.09 | 0.864 | -1.07 | 0.761 | -1.45 |
| 1435269_at | 2510005D08Rik | RIKEN cDNA 2510005D08 gene | -1.05 | 0.871 | -2.19 | 0.223 | -1.47 | 0.593 | -1.16 | 0.829 | -1.47 |
| 1447595_x_at | 1810012K16Rik | RIKEN cDNA 1810012K16 gene | -1.68 | 0.262 | -1.18 | 0.719 | -1.57 | 0.429 | -1.61 | 0.508 | -1.51 |
| 1420212_at | --- | --- | -1.16 | 0.796 | -1.55 | 0.372 | -1.73 | 0.28 | -3.22 | 0.15 | -1.92 |
| 1456973_at | Arid5b | AT rich interactive domain 5B (Mrf1 like) | -1.21 | 0.202 | -1.34 | 0.219 | -1.94 | 0.001 | -1.79 | 0.114 | -1.57 |
| 1446127_at | Zfhx1a | Zinc finger homeobox 1a | -1.1 | 0.576 | -1.51 | 0.199 | -1.97 | 0.047 | -1.37 | 0.444 | -1.49 |
| 1453331_at | 1700013H16Rik | RIKEN cDNA 1700013H16 gene | -1.44 | 0.515 | -1.44 | 0.397 | -1.44 | 0.461 | -1.38 | 0.663 | -1.42 |
| 1437652_at | Sema6c | sema domain, transmembrane domain (TM), and cytoplasmic domain, (semapho | -2.78 | 0.004 | -1.03 | 0.942 | -1.32 | 0.497 | -3.05 | 0.1 | -2.05 |
| 1440250_at | Col4a4 | procollagen, type IV, alpha 4 | -1.24 | 0.256 | -1.6 | 0.326 | -1.52 | 0.063 | -2.71 | 0.031 | -1.77 |
| 1427806_at | --- | --- | -1.42 | 0.141 | -1.65 | 0.031 | -1.29 | 0.526 | -2.48 | 0.021 | -1.71 |
| 1444763_at | Ptprk | protein tyrosine phosphatase, receptor type, K | -1.11 | 0.773 | -1.81 | 0.079 | -1.58 | 0.01 | -1.17 | 0.656 | -1.42 |
| 1448024_at | Npr3 | natriuretic peptide receptor 3 | -1.25 | 0.497 | -2.38 | 0.007 | -1.16 | 0.462 | -2.08 | 0.166 | -1.72 |
| 1442778_at | AA511254 | expressed sequence AA511254 | -1.79 | 0.005 | -1.43 | 0.098 | -1.21 | 0.422 | -1.11 | 0.84 | -1.38 |
| 1454560_at | 4930519N06Rik | RIKEN cDNA 4930519N06 gene | -1.02 | 0.958 | -2.69 | 0.175 | -1.35 | 0.441 | -1.28 | 0.722 | -1.59 |
| 1422048_at | Trpc5 | transient receptor potential cation channel, subfamily C, member 5 | -1.8 | 0.212 | -1.43 | 0.303 | -1.21 | 0.471 | -1.13 | 0.694 | -1.39 |
| 1441799_at | 6030422H21Rik | RIKEN cDNA 6030422H21 gene | -1.16 | 0.844 | -1.56 | 0.477 | -1.7 | 0.375 | -1.38 | 0.395 | -1.45 |
| 1442607_a_at | --- | --- | -1.36 | 0.196 | -1.19 | 0.424 | -1.95 | 0.075 | -1.54 | 0.392 | -1.51 |
| 1442821_at | Tm9sf3 | Transmembrane 9 superfamily member 3 | -1.3 | 0.372 | -1.48 | 0.027 | -1.56 | 0.143 | -1.49 | 0.27 | -1.46 |
| 1445802_at | AU017455 | Expressed sequence AU017455 | -1.95 | 0.229 | -1.38 | 0.381 | -1.18 | 0.482 | -1.07 | 0.664 | -1.39 |
| 1435184_at | Npr3 | natriuretic peptide receptor 3 | -1.28 | 0.408 | -1.5 | 0.262 | -1.57 | 0.253 | -1.59 | 0.278 | -1.48 |
| 1446006_at | Immp1l | IMP1 inner mitochondrial membrane peptidase-like (S. cerevisiae) | -1.3 | 0.155 | -1.45 | 0.043 | -1.59 | 0.027 | -1.34 | 0.349 | -1.42 |
| 1431819_at | --- | --- | -1.34 | 0.527 | -1.59 | 0.305 | -1.4 | 0.015 | -1.56 | 0.495 | -1.47 |
| 1438338_at | Mdh1 | malate dehydrogenase 1, NAD (soluble) | -1.23 | 0.305 | -1.84 | 0.022 | -1.36 | 0.047 | -1.72 | 0.147 | -1.54 |
| 1430415_at | Phf6 | PHD finger protein 6 | -1.22 | 0.591 | -1.62 | 0.051 | -1.53 | 0.009 | -1.5 | 0.154 | -1.47 |
| 1437071_at | Eif1ay | eukaryotic translation initiation factor 1A, Y-linked | -1.18 | 0.62 | -1.44 | 0.286 | -1.83 | 0.024 | -1.08 | 0.777 | -1.38 |
| 1430792_at | --- | --- | -1.16 | 0.617 | -1.47 | 0.313 | -1.84 | 0.028 | -1.32 | 0.626 | -1.45 |
| 1438152_at | Gpaa1 | GPI anchor attachment protein 1 | -3.13 | 0.021 | -1.12 | 0.557 | -1.15 | 0.491 | -1.14 | 0.806 | -1.63 |
| 1447268_at | Nol12 | nucleolar protein 12 | -1.8 | 0.123 | -1.35 | 0.515 | -1.26 | 0.396 | -1.45 | 0.551 | -1.47 |
| 1436650_at | Filip1 | filamin A interacting protein 1 | -1.23 | 0.633 | -2.38 | 0.395 | -1.17 | 0.399 | -1.15 | 0.664 | -1.48 |
| 1434788_at | D930050A07Rik | RIKEN cDNA D930050A07 gene | -1.32 | 0.013 | -1.53 | 0.513 | -1.48 | 0.017 | -1.13 | 0.544 | -1.36 |
| 1441738_at | --- | --- | -2.03 | 0.062 | -1.46 | 0.237 | -1.1 | 0.737 | -1.01 | 0.946 | -1.4 |
| 1445598_at | Galnt7 | UDP-N-acetyl-alpha-D-galactosamine: polypeptide N-acetylgalactosaminyltransfe | -1.06 | 0.658 | -2.01 | 0.123 | -1.55 | 0.457 | -1.35 | 0.489 | -1.49 |

| | | | | | | | | | | | |
|---|---|---|---|---|---|---|---|---|---|---|---|
| 1446672_at | --- | --- | -1.33 | 0.798 | -1.39 | 0.505 | -1.62 | 0.303 | -1.34 | 0.588 | -1.42 |
| 1450784_at | Reck | reversion-inducing-cysteine-rich protein with kazal motifs | -1.11 | 0.384 | -1.81 | 0.054 | -1.58 | 0.015 | -1 | 0.993 | -1.37 |
| 1452989_at | 2900009J20Rik | RIKEN cDNA 2900009J20 gene | -1.23 | 0.151 | -1.28 | 0.355 | -2.02 | 0.016 | -1.69 | 0.167 | -1.56 |
| 1447062_at | Vps24 | Vacuolar protein sorting 24 (yeast) | -1.15 | 0.302 | -1.42 | 0.024 | -1.93 | 0.131 | -1.12 | 0.484 | -1.4 |
| 1458134_at | Gstz1 | Glutathione transferase zeta 1 (maleylacetoacetate isomerase) | -1.47 | 0.237 | -1.63 | 0.157 | -1.26 | 0.024 | -1.27 | 0.412 | -1.41 |
| 1446069_at | Ablim3 | Actin binding LIM protein family, member 3 | -1.06 | 0.855 | -2.55 | 0.002 | -1.33 | 0.109 | -1.06 | 0.591 | -1.5 |
| 1446464_at | Psme4 | Proteasome (prosome, macropain) activator subunit 4 | -1.09 | 0.617 | -1.3 | 0.182 | -2.46 | 0.033 | -1.14 | 0.438 | -1.5 |
| 1446609_at | --- | --- | -1.07 | 0.832 | -1.51 | 0.124 | -2.01 | 0.049 | -1.37 | 0.421 | -1.49 |
| 1441287_at | --- | --- | -1.03 | 0.897 | -3.71 | 0.019 | -1.17 | 0.599 | -1.15 | 0.136 | -1.77 |
| 1443094_at | BB031773 | expressed sequence BB031773 | -1.21 | 0.109 | -2.58 | 0.253 | -1.14 | 0.238 | -2.28 | 0.471 | -1.8 |
| 1430008_x_at | Speer5-ps1 | spermatogenesis associated glutamate (E)-rich protein 5, pseudogene 1 | -1.01 | 0.99 | -2.81 | 0.104 | -1.34 | 0.542 | -1.31 | 0.671 | -1.62 |
| 1437971_at | Skiv2l2 | Superkiller viralicidic activity 2-like 2 (S. cerevisiae) | -2.7 | 0.347 | -1.09 | 0.767 | -1.25 | 0.388 | -1.14 | 0.799 | -1.54 |
| 1438831_at | Crkrs | Cdc2-related kinase, arginine/serine-rich | -1.15 | 0.138 | -1.47 | 0.105 | -1.83 | 0.013 | -1.23 | 0.178 | -1.42 |
| 1442613_at | --- | --- | -1.3 | 0.15 | -1.99 | 0.183 | -1.22 | 0.304 | -1.38 | 0.128 | -1.47 |
| 1421538_at | Kcnd1 | potassium voltage-gated channel, Shal-related family, member 1 | -1.25 | 0.629 | -1.46 | 0.427 | -1.64 | 0.244 | -1.39 | 0.086 | -1.43 |
| 1442168_at | A230071A22Rik | RIKEN cDNA A230071A22 gene | -1.45 | 0.46 | -1.22 | 0.473 | -1.71 | 0.031 | -1.45 | 0.149 | -1.46 |
| 1441633_at | Nudt9 | Nudix (nucleoside diphosphate linked moiety X)-type motif 9 | -1.07 | 0.869 | -1.44 | 0.512 | -2.13 | 0.018 | -1.43 | 0.464 | -1.52 |
| 1433431_at | Pnlip | pancreatic lipase | -1.35 | 0.585 | -1.95 | 0.237 | -1.19 | 0.694 | -2.39 | 0.152 | -1.72 |
| 1421393_at | Grin2d | glutamate receptor, ionotropic, NMDA2D (epsilon 4) | -1.98 | 0.3 | -1.05 | 0.83 | -1.56 | 0.236 | -1.48 | 0.52 | -1.52 |
| 1431492_at | 2210418G03Rik | RIKEN cDNA 2210418G03 gene | -2.03 | 0.101 | -1.16 | 0.63 | -1.35 | 0.513 | -1.05 | 0.905 | -1.4 |
| 1418745_at | Omd | osteomodulin | -1.58 | 0.532 | -1.33 | 0.634 | -1.41 | 0.237 | -2.22 | 0.303 | -1.63 |
| 1432105_at | Sirt5 | sirtuin 5 (silent mating type information regulation 2 homolog) 5 (S. cerevisiae) | -1.61 | 0.514 | -1.93 | 0.357 | -1.04 | 0.953 | -1.3 | 0.175 | -1.47 |
| 1459338_at | Auts2 | Autism susceptibility candidate 2 | -1.18 | 0.613 | -1.56 | 0.187 | -1.65 | 0.113 | -1.73 | 0.112 | -1.53 |
| 1459170_at | Foxo1 | Forkhead box O1 | -1.2 | 0.659 | -1.31 | 0.387 | -1.99 | 0.018 | -2.39 | 0.093 | -1.72 |
| 1450349_at | Stx1b1 | syntaxin 1B1 | -1.36 | 0.434 | -1.27 | 0.402 | -1.74 | 0.446 | -1.29 | 0.577 | -1.41 |
| 1453217_at | LOC625508 /// LOC | similar to Xlr-related, meiosis regulated /// similar to Xlr-related, meiosis regulated | -2.13 | 0.179 | -1.58 | 0.479 | -1.01 | 0.991 | -2.11 | 0.169 | -1.71 |
| 1431960_at | Wwox | WW domain-containing oxidoreductase | -1.18 | 0.117 | -1.45 | 0.056 | -1.79 | 0.022 | -1.04 | 0.791 | -1.36 |
| 1458386_at | C030009J22Rik | RIKEN cDNA C030009J22 gene | -1.5 | 0.446 | -1.68 | 0.106 | -1.2 | 0.057 | -1.3 | 0.657 | -1.42 |
| 1445820_at | --- | --- | -1.37 | 0.238 | -1.51 | 0.039 | -1.41 | 0.528 | -1.77 | 0.023 | -1.52 |
| 1458065_at | --- | --- | -1.38 | 0.147 | -1.25 | 0.512 | -1.75 | 0.145 | -1.68 | 0.1 | -1.51 |
| 1446105_at | Exoc1 | exocyst complex component 1 | -1.69 | 0.539 | -1.37 | 0.01 | -1.29 | 0.588 | -1.88 | 0.345 | -1.56 |
| 1421599_at | Hs6st3 | heparan sulfate 6-O-sulfotransferase 3 | -1.69 | 0.457 | -1.48 | 0.018 | -1.2 | 0.448 | -1.01 | 0.934 | -1.35 |
| 1449109_at | Socs2 | suppressor of cytokine signaling 2 | -1.03 | 0.955 | -2.56 | 0.182 | -1.35 | 0.007 | -1.28 | 0.197 | -1.56 |
| 1442991_at | B3gat2 | beta-1,3-glucuronyltransferase 2 (glucuronosyltransferase S) | -1.04 | 0.929 | -1.69 | 0.498 | -1.84 | 0.308 | -1.13 | 0.732 | -1.42 |
| 1426215_at | Ddc | dopa decarboxylase | -1.11 | 0.624 | -1.26 | 0.374 | -2.45 | 0 | -1.53 | 0.216 | -1.59 |
| 1430890_at | 2210010C17Rik | RIKEN cDNA 2210010C17 gene | -2.01 | 0.322 | -1.52 | 0.089 | -1.06 | 0.781 | -3.77 | 0.014 | -2.09 |
| 1442090_at | 8030463A06Rik | RIKEN cDNA 8030463A06 gene | -1.97 | 0.502 | -1.23 | 0.623 | -1.28 | 0.617 | -1.14 | 0.625 | -1.4 |
| 1437880_at | Lbxcor1 | ladybird homeobox 1 homolog (Drosophila) corepressor 1 | -1.18 | 0.821 | -1.48 | 0.52 | -1.73 | 0.2 | -1.02 | 0.942 | -1.35 |
| 1446348_at | Map4k5 | Mitogen-activated protein kinase kinase kinase kinase 5 | -1.21 | 0.329 | -2.08 | 0.08 | -1.26 | 0.433 | -1.35 | 0.113 | -1.48 |
| 1441217_at | Rabgap1 | RAB GTPase activating protein 1 | -1.31 | 0.13 | -1.28 | 0.597 | -1.79 | 0.022 | -1.06 | 0.772 | -1.36 |
| 1447051_at | Rnf43 | Ring finger protein 43 | -1.17 | 0.836 | -1.53 | 0.199 | -1.68 | 0.462 | -1.03 | 0.954 | -1.35 |
| 1440513_at | C80258 | expressed sequence C80258 | -1.29 | 0.033 | -1.28 | 0.166 | -1.83 | 0.047 | -1.29 | 0.057 | -1.42 |
| 1454884_at | Btbd4 | BTB (POZ) domain containing 4 | -1.91 | 0.038 | -1.24 | 0.277 | -1.29 | 0.335 | -1.5 | 0.038 | -1.49 |
| 1444453_at | Trp53rk | Trp53 regulating kinase | -1.08 | 0.724 | -1.68 | 0.049 | -1.71 | 0.323 | -3.09 | 0.069 | -1.89 |
| 1459314_at | Cdkal1 | CDK5 regulatory subunit associated protein 1-like 1 | -1.64 | 0.248 | -1.12 | 0.694 | -1.67 | 0.143 | -1.11 | 0.841 | -1.38 |
| 1457360_at | Cfdp1 | Craniofacial development protein 1 | -1.04 | 0.689 | -1.58 | 0.051 | -1.98 | 0.02 | -1.12 | 0.763 | -1.43 |
| 1417595_at | Meox1 | mesenchyme homeobox 1 | -1.38 | 0.16 | -1.12 | 0.861 | -2.07 | 0.029 | -1.25 | 0.743 | -1.45 |
| 1423004_at | Vipr1 | vasoactive intestinal peptide receptor 1 | -1.08 | 0.449 | -2.61 | 0.358 | -1.26 | 0.579 | -1.96 | 0.528 | -1.73 |
| 1459572_at | C86371 | expressed sequence C86371 | -1.32 | 0.735 | -1.11 | 0.766 | -2.25 | 0.195 | -1.34 | 0.562 | -1.5 |
| 1457230_at | Gnpda2 | glucosamine-6-phosphate deaminase 2 | -1.34 | 0.135 | -1.82 | 0.319 | -1.23 | 0.457 | -1.29 | 0.248 | -1.42 |
| 1454069_at | 9030409C19Rik | RIKEN cDNA 9030409C19 gene | -2.07 | 0.053 | -1.03 | 0.933 | -1.54 | 0.189 | -1.27 | 0.606 | -1.48 |
| 1416710_at | Tmem35 | transmembrane protein 35 | -1.35 | 0.013 | -1.77 | 0.358 | -1.25 | 0.634 | -1.14 | 0.62 | -1.38 |
| 1432687_at | 4833406M21Rik | RIKEN cDNA 4833406M21 gene | -1.49 | 0.532 | -1.37 | 0.354 | -1.42 | 0.244 | -1.51 | 0.148 | -1.45 |

| | | | | | | | | | | | |
|---|---|---|---|---|---|---|---|---|---|---|---|
| 1456451_at | Ceacam15 | CEA-related cell adhesion molecule 15 | -1.43 | 0.253 | -1.37 | 0.502 | -1.48 | 0.426 | -1.14 | 0.482 | -1.35 |
| 1453606_at | 4931403G20Rik | RIKEN cDNA 4931403G20 gene | -1.19 | 0.069 | -1.31 | 0.253 | -1.99 | 0.359 | -1.48 | 0.193 | -1.49 |
| 1455635_at | --- | Transcribed locus | -1.2 | 0.626 | -1.96 | 0.071 | -1.31 | 0.268 | -1.27 | 0.639 | -1.44 |
| 1443231_at | Magi1 | Membrane associated guanylate kinase, WW and PDZ domain containing 1 | -1.19 | 0.464 | -1.27 | 0.017 | -2.09 | 0.015 | -1.23 | 0.203 | -1.45 |
| 1457752_at | AU014876 | expressed sequence AU014876 | -1.15 | 0.663 | -2.03 | 0.247 | -1.35 | 0.018 | -2.17 | 0.352 | -1.67 |
| 1457548_at | Adamts6 | A disintegrin-like and metallopeptidase (reprolysin type) with thrombospondin typ | -1.76 | 0.152 | -1.41 | 0.542 | -1.2 | 0.497 | -1.63 | 0.207 | -1.5 |
| 1441231_at | --- | PREDICTED: Mus musculus hypothetical protein LOC626097 (LOC626097), mR | -1.24 | 0.069 | -1.28 | 0.519 | -1.93 | 0.074 | -1.53 | 0.226 | -1.49 |
| 1424211_at | 5730438N18Rik | RIKEN cDNA 5730438N18 gene | -1.75 | 0.424 | -1.44 | 0.283 | -1.19 | 0.454 | -1.21 | 0.065 | -1.4 |
| 1419698_at | Cxcl11 | chemokine (C-X-C motif) ligand 11 | -1.83 | 0.273 | -1.51 | 0.11 | -1.11 | 0.546 | -1.19 | 0.468 | -1.41 |
| 1445022_at | --- | Transcribed locus | -1.19 | 0.653 | -1.85 | 0.311 | -1.38 | 0.591 | -1.02 | 0.954 | -1.36 |
| 1458814_at | AU022855 | expressed sequence AU022855 | -1.1 | 0.475 | -2.01 | 0.037 | -1.42 | 0.103 | -1.24 | 0.483 | -1.44 |
| 1439596_at | --- | --- | -1.28 | 0.399 | -1.63 | 0.113 | -1.4 | 0.026 | -1.91 | 0.175 | -1.55 |
| 1458124_at | Iqgap1 | IQ motif containing GTPase activating protein 1 | -1.39 | 0.215 | -1.29 | 0.544 | -1.64 | 0.25 | -1.44 | 0.032 | -1.44 |
| 1427029_at | Htra3 | HtrA serine peptidase 3 | -2.4 | 0.217 | -1.1 | 0.847 | -1.28 | 0.468 | -1.49 | 0.083 | -1.57 |
| 1435832_at | Lrrc4 | leucine rich repeat containing 4 | -1.34 | 0.588 | -1.86 | 0.141 | -1.21 | 0.528 | -1.31 | 0.661 | -1.43 |
| 1425077_at | Dnajc18 | DnaJ (Hsp40) homolog, subfamily C, member 18 | -1.32 | 0.088 | -1.46 | 0.138 | -1.5 | 0.13 | -1.02 | 0.949 | -1.33 |
| 1420218_at | --- | --- | -1.13 | 0.597 | -1.39 | 0.371 | -1.99 | 0.015 | -2.13 | 0.218 | -1.66 |
| 1430306_a_at | Atp6v1c2 | ATPase, H+ transporting, lysosomal V1 subunit C2 | -1.42 | 0.078 | -1.49 | 0.555 | -1.36 | 0.135 | -1.91 | 0.086 | -1.55 |
| 1443595_at | Cnot6l | CCR4-NOT transcription complex, subunit 6-like | -1.08 | 0.773 | -1.79 | 0.02 | -1.6 | 0.215 | -1.12 | 0.856 | -1.4 |
| 1446290_at | 2410129H14Rik | RIKEN cDNA 2410129H14 gene | -1.19 | 0.588 | -1.38 | 0.431 | -1.85 | 0.115 | -1.18 | 0.317 | -1.4 |
| 1452875_at | Hddc3 | HD domain containing 3 | -1.26 | 0.607 | -2.33 | 0.145 | -1.13 | 0.639 | -1.49 | 0.123 | -1.55 |
| 1430923_at | 2610300M13Rik | RIKEN cDNA 2610300M13 gene | -1.14 | 0.71 | -2.13 | 0.237 | -1.31 | 0.569 | -1.13 | 0.837 | -1.43 |
| 1458748_at | Rbm27 | RNA binding motif protein 27 | -1.05 | 0.745 | -2.1 | 0.006 | -1.47 | 0.101 | -1.17 | 0.449 | -1.45 |
| 1445370_at | Vapb | Vesicle-associated membrane protein, associated protein B and C | -1.24 | 0.196 | -1.41 | 0.572 | -1.68 | 0.057 | -2 | 0.078 | -1.58 |
| 1445770_at | --- | --- | -2.1 | 0.2 | -1.18 | 0.735 | -1.26 | 0.214 | -1.75 | 0.392 | -1.57 |
| 1432107_at | 2310010M20Rik | RIKEN cDNA 2310010M20 gene | -1.83 | 0.169 | -1.42 | 0.635 | -1.16 | 0.596 | -1.33 | 0.589 | -1.44 |
| 1454212_x_at | Gsdmdc2 /// 49334 | gasdermin domain containing 2 /// RIKEN cDNA 4933426G20 gene /// RIKEN cD | -1.4 | 0.674 | -1.74 | 0.517 | -1.21 | 0.151 | -1.9 | 0.155 | -1.56 |
| 1419391_at | Myog | myogenin | -1.44 | 0.441 | -1.8 | 0.52 | -1.16 | 0.778 | -1.36 | 0.414 | -1.44 |
| 1442845_at | C130075A20Rik | RIKEN cDNA C130075A20 gene | -1.21 | 0.117 | -1.03 | 0.82 | -3.16 | 0.009 | -1.4 | 0.077 | -1.7 |
| 1420067_at | Cd160 | CD160 antigen | -1.91 | 0.41 | -1.04 | 0.89 | -1.59 | 0.038 | -1.46 | 0.51 | -1.5 |
| 1456124_x_at | Svs5 | seminal vesicle secretion 5 | -3.17 | 0.001 | -1.17 | 0.762 | -1.06 | 0.894 | -1.38 | 0.609 | -1.7 |
| 1458050_at | --- | --- | -1.12 | 0.844 | -2.88 | 0.01 | -1.14 | 0.475 | -1.41 | 0.165 | -1.64 |
| 1436392_s_at | Tcfap2c | transcription factor AP-2, gamma | -1.48 | 0.281 | -1.46 | 0.167 | -1.33 | 0.308 | -1.34 | 0.596 | -1.4 |
| 1440540_at | Gle1l | GLE1 RNA export mediator-like (yeast | -1.21 | 0.589 | -1.47 | 0.342 | -1.65 | 0.052 | -1.22 | 0.387 | -1.39 |
| 1456816_at | Rai17 | Retinoic acid induced 17 | -1.49 | 0.28 | -1.43 | 0.241 | -1.35 | 0.287 | -1.5 | 0.232 | -1.44 |
| 1425935_at | 2900042B11Rik | RIKEN cDNA 2900042B11 gene | -1.14 | 0.714 | -3.01 | 0.114 | -1.1 | 0.531 | -1.27 | 0.284 | -1.63 |
| 1430277_at | 9430034F23Rik | RIKEN cDNA 9430034F23 gene | -1.17 | 0.493 | -1.6 | 0.405 | -1.58 | 0.093 | -1.21 | 0.479 | -1.39 |
| 1456050_at | C80998 | expressed sequence C80998 | -1.22 | 0.427 | -1.27 | 0.385 | -1.99 | 0.058 | -1.06 | 0.523 | -1.38 |
| 1435656_at | Gmps | guanine monphosphate synthetase | -1.25 | 0.425 | -1.38 | 0.019 | -1.7 | 0.12 | -1.17 | 0.113 | -1.37 |
| 1416505_at | Nr4a1 | nuclear receptor subfamily 4, group A, member 1 | -1.92 | 0.057 | -1.17 | 0.409 | -1.36 | 0.506 | -1.93 | 0.154 | -1.59 |
| 1453841_at | 2310050P20Rik | RIKEN cDNA 2310050P20 gene | -1.34 | 0.688 | -1.1 | 0.782 | -2.17 | 0.095 | -2.01 | 0.11 | -1.66 |
| 1432375_a_at | Spata3 | spermatogenesis associated 3 | -1.05 | 0.961 | -1.12 | 0.601 | -3.74 | 0.119 | -1.6 | 0.153 | -1.88 |
| 1440693_at | Palld | palladin, cytoskeletal associated protein | -1.01 | 0.963 | -1.65 | 0.254 | -1.92 | 0.083 | -1.05 | 0.923 | -1.41 |
| 1447464_at | Anks1b | ankyrin repeat and sterile alpha motif domain containing 1B | -1.19 | 0.697 | -2.71 | 0.01 | -1.1 | 0.805 | -1.07 | 0.721 | -1.52 |
| 1443770_x_at | Auts2 | Autism susceptibility candidate 2 | -1.3 | 0.243 | -1.21 | 0.789 | -1.92 | 0.177 | -1.07 | 0.617 | -1.38 |
| 1453598_at | 3110082D06Rik | RIKEN cDNA 3110082D06 gene | -2.13 | 0.438 | -1.22 | 0.449 | -1.21 | 0.731 | -1.08 | 0.595 | -1.41 |
| 1454348_at | 4933407C09Rik | RIKEN cDNA 4933407C09 gene | -2.16 | 0.117 | -1.24 | 0.058 | -1.18 | 0.714 | -1.3 | 0.756 | -1.47 |
| 1433035_at | 5430430K15Rik | RIKEN cDNA 5430430K15 gene | -1.29 | 0.564 | -1.74 | 0.181 | -1.3 | 0.677 | -1.59 | 0.236 | -1.48 |
| 1433410_at | C030014O09Rik | RIKEN cDNA C030014O09 gene | -1.57 | 0.412 | -1.14 | 0.841 | -1.66 | 0.209 | -1.67 | 0.418 | -1.51 |
| 1441082_at | Dscam | Down syndrome cell adhesion molecule | -1.36 | 0.116 | -1.86 | 0.086 | -1.18 | 0.674 | -1.91 | 0.39 | -1.58 |
| 1447202_at | 1200009F10Rik | RIKEN cDNA 1200009F10 gene | -1.6 | 0.359 | -1.4 | 0.438 | -1.28 | 0.162 | -1.19 | 0.462 | -1.37 |
| 1447167_at | AL033314 | Expressed sequence AL033314 | -1.33 | 0.327 | -1.2 | 0.363 | -1.87 | 0.031 | -1.36 | 0.237 | -1.44 |
| 1447820_x_at | Cpt2 | carnitine palmitoyltransferase 2 | -1.59 | 0.156 | -1.24 | 0.52 | -1.46 | 0.118 | -1.08 | 0.661 | -1.34 |

| | | | | | | | | | | | |
|---|---|---|---|---|---|---|---|---|---|---|---|
| 1437957_at | 7030407O06Rik | RIKEN cDNA 7030407O06 gene | -1.29 | 0.053 | -1.5 | 0.24 | -1.48 | 0.178 | -1.1 | 0.645 | -1.34 |
| 1430828_at | Trmu | tRNA 5-methylaminomethyl-2-thiouridylate methyltransferase | -1.26 | 0.478 | -1.79 | 0.483 | -1.31 | 0.526 | -1.67 | 0.155 | -1.51 |
| 1456782_at | Tada1l | transcriptional adaptor 1 (HFI1 homolog, yeast) like | -1.32 | 0.146 | -2.07 | 0.018 | -1.14 | 0.426 | -1 | 0.989 | -1.38 |
| 1455610_at | Dmn | desmuslin | -1.09 | 0.765 | -1.87 | 0.139 | -1.51 | 0.087 | -1.22 | 0.105 | -1.42 |
| 1452477_at | 3110057O12Rik | RIKEN cDNA 3110057O12 gene | -1.52 | 0.14 | -1.19 | 0.682 | -1.61 | 0.192 | -1 | 0.998 | -1.33 |
| 1460108_at | Trit1 | TRNA isopentenyltransferase 1 | -1.3 | 0.597 | -1.35 | 0.357 | -1.64 | 0.191 | -1.04 | 0.931 | -1.33 |
| 1438590_at | Rapgef3 | Rap guanine nucleotide exchange factor (GEF) 3 | -1.24 | 0.258 | -1.63 | 0.139 | -1.42 | 0.325 | -1.34 | 0.289 | -1.41 |
| 1440802_at | Clasp2 | CLIP associating protein 2 | -1.29 | 0.503 | -1.31 | 0.327 | -1.71 | 0.019 | -1.39 | 0.24 | -1.43 |
| 1457567_at | Nagk | N-acetylglucosamine kinase | -1.5 | 0.041 | -1.53 | 0.008 | -1.25 | 0.26 | -1.03 | 0.887 | -1.33 |
| 1437744_at | Slitrk4 | SLIT and NTRK-like family, member 4 | -1.99 | 0.203 | -1.54 | 0.2 | -1.03 | 0.921 | -1.25 | 0.754 | -1.45 |
| 1445637_at | Dnmt1 | DNA methyltransferase (cytosine-5) 1 | -2.73 | 0.357 | -1.23 | 0.772 | -1.06 | 0.931 | -1.28 | 0.733 | -1.57 |
| 1431697_at | Synj2 | synaptojanin 2 | -2.64 | 0.03 | -1.2 | 0.516 | -1.1 | 0.698 | -1.03 | 0.891 | -1.49 |
| 1443020_at | Hmbox1 | Homeobox containing 1 | -1.37 | 0.012 | -1.15 | 0.585 | -1.91 | 0.052 | -1.15 | 0.362 | -1.4 |
| 1455281_at | Wdr33 | WD repeat domain 33 | -1.2 | 0.338 | -1.51 | 0.153 | -1.59 | 0.025 | -1.29 | 0.352 | -1.4 |
| 1454257_at | Sptlc2 | serine palmitoyltransferase, long chain base subunit 2 | -1.33 | 0.413 | -1.25 | 0.512 | -1.77 | 0.083 | -1.11 | 0.31 | -1.36 |
| 1453456_at | 2900084O13Rik | RIKEN cDNA 2900084O13 gene | -1.18 | 0.759 | -1.23 | 0.606 | -2.16 | 0.01 | -2.38 | 0.167 | -1.74 |
| 1459074_at | --- | --- | -1.67 | 0.352 | -1 | 0.99 | -1.9 | 0.316 | -2.6 | 0.094 | -1.79 |
| 1444377_at | Psmb2 | Proteasome (prosome, macropain) subunit, beta type 2 | -1.41 | 0.258 | -1.42 | 0.103 | -1.42 | 0.405 | -1.51 | 0.124 | -1.44 |
| 1456783_at | 9330107J05Rik | RIKEN cDNA 9330107J05 gene | -1.22 | 0.044 | -1.28 | 0.119 | -1.93 | 0.06 | -3.19 | 0.085 | -1.9 |
| 1459444_at | Zfp91 | zinc finger protein 91 | -1.52 | 0.029 | -1.14 | 0.785 | -1.71 | 0.017 | -1.6 | 0.012 | -1.49 |
| 1439833_at | 3-Sep | septin 3 | -1.51 | 0.336 | -1.01 | 0.977 | -2.11 | 0.013 | -1.02 | 0.884 | -1.41 |
| 1457247_at | AI465495 | expressed sequence AI465495 | -1.74 | 0.147 | -1.32 | 0.568 | -1.26 | 0.119 | -1.18 | 0.687 | -1.38 |
| 1436067_at | Zbtb10 /// LOC6696 | zinc finger and BTB domain containing 10 /// similar to zinc finger and BTB domai | -1.2 | 0.173 | -1.66 | 0.065 | -1.46 | 0.078 | -1.99 | 0.079 | -1.57 |
| 1447736_at | Fkbp5 | FK506 binding protein 5 | -1.04 | 0.82 | -1.59 | 0.526 | -1.88 | 0.298 | -1.22 | 0.838 | -1.43 |
| 1441771_at | Atrx | Alpha thalassemia/mental retardation syndrome X-linked homolog (human) | -1.2 | 0.53 | -1.38 | 0.171 | -1.77 | 0.065 | -1.11 | 0.727 | -1.36 |
| 1441608_at | Cacna2d1 | Calcium channel, voltage-dependent, alpha2/delta subunit 1 | -1.11 | 0.903 | -2.24 | 0.129 | -1.28 | 0.383 | -1.09 | 0.804 | -1.43 |
| 1454138_a_at | Stk31 | serine threonine kinase 31 | -1.14 | 0.757 | -2.9 | 0.04 | -1.11 | 0.471 | -2.04 | 0.416 | -1.8 |
| 1418189_s_at | Malat1 | Metastasis associated lung adenocarcinoma transcript 1 (non-coding RNA) | -1.17 | 0.582 | -1.63 | 0.15 | -1.51 | 0.057 | -1.41 | 0.248 | -1.43 |
| 1417803_at | 1110032A04Rik | RIKEN cDNA 1110032A04 gene | -1.52 | 0.066 | -1.26 | 0.639 | -1.48 | 0.151 | -2.24 | 0.099 | -1.63 |
| 1457199_at | --- | --- | -1.12 | 0.554 | -1.68 | 0.102 | -1.57 | 0.014 | -1.19 | 0.285 | -1.39 |
| 1446485_at | 4930553C11Rik | RIKEN cDNA 4930553C11 gene | -1.12 | 0.821 | -1.83 | 0.18 | -1.46 | 0.513 | -1.22 | 0.51 | -1.41 |
| 1458497_at | Crim1 | Cysteine rich transmembrane BMP regulator 1 (chordin like) | -1.2 | 0.023 | -1.31 | 0.24 | -1.89 | 0.014 | -1.36 | 0.058 | -1.44 |
| 1442401_at | Sorbs1 | Sorbin and SH3 domain containing 1 | -1.62 | 0.464 | -1.29 | 0.454 | -1.37 | 0.517 | -1.73 | 0.199 | -1.5 |
| 1445347_at | --- | --- | -1.03 | 0.93 | -1.65 | 0.411 | -1.82 | 0.28 | -4.26 | 0.212 | -2.19 |
| 1455846_at | Aqp11 | aquaporin 11 | -3.32 | 0.08 | -1.09 | 0.756 | -1.1 | 0.741 | -1.27 | 0.672 | -1.7 |
| 1443622_at | Dscr3 | Down syndrome critical region gene 3 | -1.93 | 0.575 | -1.15 | 0.76 | -1.35 | 0.173 | -1.19 | 0.678 | -1.41 |
| 1447426_at | Dip3b | Dip3 beta | -1.21 | 0.422 | -1.24 | 0.418 | -2.03 | 0.054 | -1.25 | 0.343 | -1.43 |
| 1427142_s_at | Jarid1b | jumonji, AT rich interactive domain 1B (Rbp2 like) | -1.39 | 0.155 | -1.44 | 0.02 | -1.41 | 0.051 | -1.07 | 0.524 | -1.33 |
| 1454377_at | 2700080J24Rik | RIKEN cDNA 2700080J24 gene | -1.92 | 0.031 | -1.18 | 0.485 | -1.31 | 0.024 | -1.06 | 0.801 | -1.37 |
| 1427403_at | Slco1a5 | solute carrier organic anion transporter family, member 1a5 | -1.19 | 0.775 | -2.03 | 0.455 | -1.26 | 0.63 | -1.28 | 0.69 | -1.44 |
| 1439801_at | Sunc1 | Sad1 and UNC84 domain containing 1 | -1.16 | 0.674 | -1.81 | 0.189 | -1.41 | 0.5 | -1.73 | 0.361 | -1.52 |
| 1429333_at | Kif2b | kinesin family member 2B | -4.06 | 0.084 | -1.08 | 0.798 | -1.05 | 0.883 | -1.16 | 0.872 | -1.84 |
| 1419881_x_at | --- | --- | -1.18 | 0.57 | -1.19 | 0.825 | -2.25 | 0.134 | -1.27 | 0.761 | -1.47 |
| 1440683_at | A930004D18Rik | RIKEN cDNA A930004D18 gene | -1.27 | 0.689 | -1.54 | 0.279 | -1.44 | 0.368 | -1.05 | 0.838 | -1.33 |
| 1430341_at | Nudt5 | nudix (nucleoside diphosphate linked moiety X)-type motif 5 | -1.3 | 0.073 | -1.75 | 0.006 | -1.27 | 0.275 | -1.11 | 0.395 | -1.36 |
| 1455361_at | Dgkb | diacylglycerol kinase, beta | -1.25 | 0.247 | -1.3 | 0.36 | -1.78 | 0.039 | -1.85 | 0.385 | -1.55 |
| 1446703_at | Rfwd2 | Ring finger and WD repeat domain 2 | -1.53 | 0.531 | -1.26 | 0.637 | -1.47 | 0.406 | -1.05 | 0.935 | -1.33 |
| 1456413_at | Pde4dip | phosphodiesterase 4D interacting protein (myomegalin) | -1.32 | 0.277 | -1.35 | 0.152 | -1.58 | 0.163 | -1.72 | 0.117 | -1.49 |
| 1447116_at | Parl | Presenilin associated, rhomboid-like | -1.42 | 0.439 | -1.95 | 0.076 | -1.09 | 0.758 | -3.27 | 0.029 | -1.94 |
| 1436373_at | Map3k10 | mitogen activated protein kinase kinase kinase 10 | -1.6 | 0.591 | -1.4 | 0.441 | -1.26 | 0.475 | -2.08 | 0.113 | -1.59 |
| 1458096_at | Nhs | Nance-Horan syndrome (human) | -1.02 | 0.885 | -1.59 | 0.003 | -1.91 | 0.009 | -1.12 | 0.621 | -1.41 |
| 1457588_at | C76213 | expressed sequence C76213 | -1.18 | 0.176 | -1.41 | 0.036 | -1.75 | 0.02 | -1.21 | 0.28 | -1.39 |
| 1454105_at | --- | --- | -1.43 | 0.586 | -2.03 | 0.264 | -1.07 | 0.911 | -1.85 | 0.594 | -1.59 |

| | | | | | | | | | | | |
|---|---|---|---|---|---|---|---|---|---|---|---|
| 1421647_at | Cd1d2 | CD1d2 antigen | -1.03 | 0.98 | -2.8 | 0.022 | -1.25 | 0.575 | -1.24 | 0.628 | -1.58 |
| 1419498_at | Tmigd1 | transmembrane and immunoglobulin domain containing 1 | -1.09 | 0.32 | -2.05 | 0.092 | -1.38 | 0.334 | -1.15 | 0.566 | -1.42 |
| 1445337_at | Dnajc13 | DnaJ (Hsp40) homolog, subfamily C, member 13 | -1.2 | 0.136 | -1.32 | 0.041 | -1.84 | 0.02 | -1.35 | 0.386 | -1.43 |
| 1442698_at | --- | 7 days neonate cerebellum cDNA, RIKEN full-length enriched library, clone:A730 | -1.44 | 0.494 | -2.18 | 0.356 | -1.03 | 0.892 | -2.88 | 0.356 | -1.88 |
| 1442364_at | Mapk14 | Mitogen activated protein kinase 14 | -1.08 | 0.909 | -1.83 | 0.175 | -1.53 | 0.29 | -2.69 | 0.026 | -1.78 |
| 1457928_at | 4930435E12Rik | RIKEN cDNA 4930435E12 gene | -1.42 | 0.443 | -1.84 | 0.336 | -1.13 | 0.527 | -1.07 | 0.911 | -1.37 |
| 1439928_at | Spnb2 | Spectrin beta 2 | -1.2 | 0.1 | -1.15 | 0.624 | -2.35 | 0.014 | -1.59 | 0.222 | -1.57 |
| 1459540_at | Cdc26 | Cell division cycle 26 | -1.11 | 0.623 | -1.65 | 0.165 | -1.61 | 0.293 | -1.34 | 0.424 | -1.43 |
| 1442012_at | AU015791 | expressed sequence AU015791 | -2.56 | 0.518 | -1.18 | 0.195 | -1.12 | 0.655 | -1.44 | 0.425 | -1.57 |
| 1419567_at | Fank1 | fibronectin type 3 and ankyrin repeat domains 1 | -1.68 | 0.056 | -1.61 | 0.208 | -1.09 | 0.784 | -1.59 | 0.542 | -1.49 |
| 1430503_at | 6330522J23Rik | RIKEN cDNA 6330522J23 gene | -1.65 | 0.236 | -1.3 | 0.497 | -1.33 | 0.436 | -1.49 | 0.67 | -1.44 |
| 1439828_at | Mapkapk5 | MAP kinase-activated protein kinase 5 | -1.7 | 0.033 | -1.32 | 0.293 | -1.27 | 0.486 | -1.61 | 0.083 | -1.48 |
| 1432196_a_at | Dscaml1 | Down syndrome cell adhesion molecule-like 1 | -1.26 | 0.754 | -1.51 | 0.448 | -1.48 | 0.117 | -1.7 | 0.385 | -1.49 |
| 1444933_at | --- | Transcribed locus, moderately similar to XP_231006.3 PREDICTED: similar to for | -1.14 | 0.685 | -1.26 | 0.623 | -2.16 | 0.184 | -2.08 | 0.297 | -1.66 |
| 1444538_at | AL033314 | Expressed sequence AL033314 | -1.22 | 0.445 | -1.43 | 0.111 | -1.63 | 0.011 | -1.34 | 0.154 | -1.41 |
| 1422279_at | Fv1 | Friend virus susceptibility 1 | -2.02 | 0.267 | -1.37 | 0.507 | -1.1 | 0.743 | -2.13 | 0.557 | -1.65 |
| 1452388_at | Hspa1a | heat shock protein 1A | -1.51 | 0.014 | -1.45 | 0.14 | -1.28 | 0.639 | -1.79 | 0.418 | -1.51 |
| 1430433_at | 4933406J08Rik /// L | RIKEN cDNA 4933406J08 gene /// hypothetical protein LOC640572 | -3.65 | 0.134 | -1.06 | 0.865 | -1.09 | 0.428 | -1.06 | 0.895 | -1.72 |
| 1420446_at | Odf3 | outer dense fiber of sperm tails 3 | -1.63 | 0.044 | -1.33 | 0.138 | -1.3 | 0.095 | -1.39 | 0.312 | -1.41 |
| 1457223_at | Elmod1 | ELMO domain containing 1 | -1.1 | 0.487 | -1.02 | 0.951 | -4.12 | 0.005 | -1.03 | 0.847 | -1.82 |
| 1430749_at | 2810040C05Rik | RIKEN cDNA 2810040C05 gene | -1.27 | 0.338 | -1.26 | 0.392 | -1.81 | 0.1 | -1.46 | 0.385 | -1.45 |
| 1431732_at | Spag16 | sperm associated antigen 16 | -1.68 | 0.109 | -1.09 | 0.901 | -1.62 | 0.241 | -2.69 | 0.24 | -1.77 |
| 1458694_at | AI789011 | expressed sequence AI789011 | -2.43 | 0.113 | -1.14 | 0.796 | -1.18 | 0.263 | -1.03 | 0.813 | -1.45 |
| 1458191_at | Foxp2 | forkhead box P2 | -1.27 | 0.454 | -1.54 | 0.023 | -1.43 | 0.088 | -1.08 | 0.821 | -1.33 |
| 1416129_at | Errfi1 | ERBB receptor feedback inhibitor 1 | -1.47 | 0.162 | -1.19 | 0.229 | -1.63 | 0.004 | -2.22 | 0.215 | -1.63 |
| 1450432_s_at | Mus81 | MUS81 endonuclease homolog (yeast) | -1.45 | 0.133 | -2 | 0.011 | -1.06 | 0.819 | -1.34 | 0.271 | -1.46 |
| 1437190_at | Styk1 | serine/threonine/tyrosine kinase 1 | -1.23 | 0.584 | -2.12 | 0.389 | -1.18 | 0.5 | -1.05 | 0.923 | -1.39 |
| 1449392_at | Hsd17b1 | hydroxysteroid (17-beta) dehydrogenase 1 | -1.11 | 0.89 | -2.89 | 0.054 | -1.13 | 0.857 | -1.14 | 0.539 | -1.57 |
| 1439800_at | Rsn | restin (Reed-Steinberg cell-expressed intermediate filament-associated protein) | -1.15 | 0.101 | -1.32 | 0.251 | -1.96 | 0.04 | -1.33 | 0.298 | -1.44 |
| 1458763_at | C78024 | expressed sequence C78024 | -1 | 0.999 | -1.2 | 0.746 | -3.26 | 0.002 | -1.13 | 0.748 | -1.65 |
| 1437367_at | H2-K1 | Histocompatibility 2, K1, K region | -1.04 | #DIV/0! | -2.72 | 0.099 | -1.24 | 0.413 | -1.35 | 0.268 | -1.59 |
| 1441493_at | Erc1 | ELKS/RAB6-interacting/CAST family member 1 | -1.18 | 0.452 | -1.63 | 0.042 | -1.47 | 0.006 | -1.07 | 0.74 | -1.34 |
| 1456659_at | LOC552902 | hypothetical LOC552902 | -1.39 | 0.238 | -1.04 | 0.588 | -2.2 | 0.023 | -2.2 | 0.053 | -1.71 |
| 1450936_a_at | Dnase1l2 | deoxyribonuclease 1-like 2 | -1.19 | 0.417 | -2.79 | 0.12 | -1.06 | 0.9 | -1.29 | 0.222 | -1.58 |
| 1440143_at | Pigz | phosphatidylinositol glycan anchor biosynthesis, class Z | -1.14 | 0.533 | -1.75 | 0.259 | -1.45 | 0.022 | -1.09 | 0.38 | -1.36 |
| 1442772_at | Ehbp1 | EH domain binding protein 1 | -1.05 | 0.91 | -2.23 | 0.009 | -1.36 | 0.095 | -1.81 | 0.443 | -1.61 |
| 1458542_at | Msi2 | Musashi homolog 2 (Drosophila) | -1.08 | 0.912 | -1.71 | 0.319 | -1.59 | 0.155 | -1.42 | 0.475 | -1.45 |
| 1443257_at | 9630050E16Rik | RIKEN cDNA 9630050E16 gene | -1.12 | 0.414 | -1.19 | 0.174 | -2.47 | 0.014 | -1.47 | 0.064 | -1.56 |
| 1425107_a_at | Lifr | leukemia inhibitory factor receptor | -1.03 | 0.778 | -2.68 | 0.005 | -1.26 | 0.11 | -1.92 | 0.025 | -1.72 |
| 1440977_at | Akap13 | A kinase (PRKA) anchor protein 13 | -1.35 | 0.182 | -1.33 | 0.23 | -1.55 | 0.023 | -1.11 | 0.436 | -1.33 |
| 1433164_at | 4930570E01Rik | RIKEN cDNA 4930570E01 gene | -1.37 | 0.612 | -1.95 | 0.103 | -1.12 | 0.776 | -1.1 | 0.693 | -1.39 |
| 1444130_at | 1700081L11Rik | RIKEN cDNA 1700081L11 gene | -1.27 | 0.076 | -1.34 | 0.017 | -1.65 | 0.042 | -1.79 | 0.069 | -1.51 |
| 1454338_at | Bat2d | BAT2 domain containing 1 | -1.35 | 0.313 | -1.32 | 0.672 | -1.55 | 0.22 | -1.26 | 0.578 | -1.37 |
| 1458467_at | 1110032D16Rik | RIKEN cDNA 1110032D16 gene | -2.19 | 0.224 | -1.18 | 0.741 | -1.2 | 0.694 | -1.14 | 0.547 | -1.43 |
| 1446182_at | Zmynd11 | Zinc finger, MYND domain containing 11 | -1.21 | 0.262 | -1.51 | 0.036 | -1.53 | 0.142 | -1.92 | 0.009 | -1.54 |
| 1442052_at | C330019G07Rik | RIKEN cDNA C330019G07 gene | -1.47 | 0.037 | -1.32 | 0.082 | -1.43 | 0.178 | -1.27 | 0.45 | -1.37 |
| 1432887_at | 3110048L19Rik | RIKEN cDNA 3110048L19 gene | -1.19 | 0.386 | -1.41 | 0.14 | -1.69 | 0.112 | -1.47 | 0.537 | -1.44 |
| 1449673_s_at | --- | --- | -1.14 | 0.731 | -1.35 | 0.492 | -1.9 | 0.353 | -1.46 | 0.621 | -1.46 |
| 1459026_at | Snw1 | SNW domain containing 1 | -1.05 | 0.909 | -1.52 | 0.201 | -1.89 | 0.065 | -1.67 | 0.027 | -1.53 |
| 1431919_at | Rttn | rotatin | -1.27 | 0.744 | -1.82 | 0.176 | -1.24 | 0.649 | -1.03 | 0.905 | -1.34 |
| 1438682_at | Pik3r1 | phosphatidylinositol 3-kinase, regulatory subunit, polypeptide 1 (p85 alpha) | -1.69 | 0.406 | -1.71 | 0.092 | -1.04 | 0.915 | -1.54 | 0.352 | -1.49 |
| 1458482_at | Tnni3k | TNNI3 interacting kinase | -1.01 | 0.973 | -2.52 | 0.123 | -1.32 | 0.646 | -1.26 | 0.661 | -1.53 |
| 1444961_at | --- | --- | -1.14 | 0.485 | -2.62 | 0.099 | -1.13 | 0.605 | -1.07 | 0.918 | -1.49 |

| | | | | | | | | | | | |
|---|---|---|---|---|---|---|---|---|---|---|---|
| 1437587_at | --- | Transcribed locus | -1.76 | 0.075 | -1.41 | 0.255 | -1.15 | 0.382 | -1.13 | 0.523 | -1.37 |
| 1421804_at | Fabp9 | fatty acid binding protein 9, testis | -1.87 | 0.415 | -1.45 | 0.44 | -1.09 | 0.503 | -1 | 0.986 | -1.35 |
| 1459427_at | Stam | signal transducing adaptor molecule (SH3 domain and ITAM motif) 1 | -1.32 | 0.703 | -1.73 | 0.148 | -1.24 | 0.648 | -1.62 | 0.604 | -1.48 |
| 1457417_at | --- | --- | -1.12 | 0.643 | -1.8 | 0.02 | -1.44 | 0.179 | -2.17 | 0.095 | -1.63 |
| 1457738_at | Lsm14a | LSM14 homolog A (SCD6, S. cerevisiae) | -1.71 | 0.609 | -1.01 | 0.924 | -1.75 | 0.432 | -1.86 | 0.41 | -1.58 |
| 1444514_at | B930096F20Rik | RIKEN cDNA B930096F20 gene | -1.23 | 0.668 | -1.29 | 0.695 | -1.81 | 0.079 | -1.17 | 0.39 | -1.37 |
| 1438064_at | Ybx1 | Y box protein 1 | -1.43 | 0.165 | -1.24 | 0.621 | -1.57 | 0.126 | -1.28 | 0.225 | -1.38 |
| 1458159_at | Gnaq | Guanine nucleotide binding protein, alpha q polypeptide | -1.16 | 0.629 | -1.29 | 0.033 | -1.98 | 0.048 | -1.71 | 0.009 | -1.53 |
| 1457636_x_at | --- | --- | -1.36 | 0.503 | -1.41 | 0.043 | -1.44 | 0.068 | -1.94 | 0.162 | -1.53 |
| 1430488_at | 1700066B19Rik | RIKEN cDNA 1700066B19 gene | -1.44 | 0.1 | -1.86 | 0.14 | -1.09 | 0.463 | -1.35 | 0.243 | -1.44 |
| 1431121_at | Amotl1 | angiomotin-like 1 | -1.61 | 0.223 | -1.22 | 0.774 | -1.42 | 0.415 | -1.15 | 0.763 | -1.35 |
| 1457524_at | Tcf12 | Transcription factor 12 | -1.07 | 0.834 | -2.59 | 0.085 | -1.21 | 0.314 | -1.6 | 0.259 | -1.62 |
| 1441015_at | Fut8 | Fucosyltransferase 8 | -1.61 | 0.219 | -1.55 | 0.604 | -1.14 | 0.363 | -2.34 | 0.183 | -1.66 |
| 1420062_at | --- | Transcribed locus | -1.69 | 0.185 | -1.33 | 0.093 | -1.25 | 0.428 | -1.14 | 0.429 | -1.35 |
| 1447120_at | Btrc | Beta-transducin repeat containing protein | -1.47 | 0.095 | -1.19 | 0.25 | -1.62 | 0.087 | -1.17 | 0.413 | -1.36 |
| 1421342_at | Kcns2 | K+ voltage-gated channel, subfamily S, 2 | -1.85 | 0.018 | -1.03 | 0.88 | -1.58 | 0.319 | -1.2 | 0.452 | -1.41 |
| 1427819_at | --- | --- | -1.52 | 0.113 | -1.68 | 0.355 | -1.12 | 0.834 | -1.29 | 0.526 | -1.4 |
| 1447740_at | 1700034J05Rik /// L | RIKEN cDNA 1700034J05 gene /// hypothetical protein LOC635035 | -1.97 | 0.371 | -1.31 | 0.671 | -1.14 | 0.633 | -1.07 | 0.935 | -1.37 |
| 1443653_at | --- | --- | -1.69 | 0.059 | -1.18 | 0.776 | -1.42 | 0.134 | -1.79 | 0.065 | -1.52 |
| 1444364_at | Rcor3 | REST corepressor 3 | -1.29 | 0.549 | -1.34 | 0.103 | -1.61 | 0.139 | -1.19 | 0.514 | -1.36 |
| 1434201_at | Chrdl1 | Chordin-like 1 | -1.24 | 0.694 | -1.52 | 0.037 | -1.46 | 0.319 | -1.18 | 0.683 | -1.35 |
| 1440892_at | BC017647 | CDNA sequence BC017647 | -1.19 | 0.291 | -1.42 | 0.007 | -1.67 | 0.104 | -1.26 | 0.346 | -1.38 |
| 1437833_at | Ltbp3 | latent transforming growth factor beta binding protein 3 | -2.8 | 0.238 | -1.08 | 0.792 | -1.15 | 0.738 | -1.02 | 0.972 | -1.51 |
| 1447209_at | Foxp1 | Forkhead box P1 | -1.37 | 0.183 | -1.22 | 0.263 | -1.67 | 0.147 | -2.59 | 0.114 | -1.71 |
| 1430363_at | 4933407O12Rik | RIKEN cDNA 4933407O12 gene | -3.51 | 0.145 | -1.1 | 0.795 | -1.05 | 0.851 | -1.07 | 0.628 | -1.68 |
| 1441552_at | A430107P09Rik | RIKEN cDNA A430107P09 gene | -1.13 | 0.876 | -1.38 | 0.528 | -1.87 | 0.058 | -1.92 | 0.176 | -1.57 |
| 1439328_at | Nfat5 | nuclear factor of activated T-cells 5 | -1.23 | 0.464 | -1.64 | 0.016 | -1.39 | 0.086 | -1.41 | 0.159 | -1.42 |
| 1451432_x_at | Alkbh4 | alkB, alkylation repair homolog 4 (E. coli) | -1.42 | 0.061 | -1.53 | 0.33 | -1.26 | 0.279 | -1.22 | 0.258 | -1.36 |
| 1456843_at | Yes1 | Yamaguchi sarcoma viral (v-yes) oncogene homolog 1 | -1.24 | 0.49 | -1.5 | 0.128 | -1.48 | 0.161 | -1.1 | 0.858 | -1.33 |
| 1447571_at | Elk4 | ELK4, member of ETS oncogene family | -1.54 | 0.017 | -1.37 | 0.005 | -1.31 | 0.414 | -1.05 | 0.815 | -1.32 |
| 1430345_at | 5530402H23Rik | RIKEN cDNA 5530402H23 gene | -1.25 | 0.11 | -1.48 | 0.086 | -1.5 | 0.022 | -1.78 | 0.158 | -1.5 |
| 1445686_at | --- | 12 days embryo spinal ganglion cDNA, RIKEN full-length enriched library, clone:[ | -1.2 | 0.588 | -1.22 | 0.583 | -2.04 | 0.17 | -2.53 | 0.285 | -1.75 |
| 1445689_at | --- | Transcribed locus | -1.14 | 0.628 | -1.26 | 0.484 | -2.11 | 0.075 | -1.08 | 0.818 | -1.4 |
| 1444045_at | --- | Transcribed locus | -1.15 | 0.66 | -1.11 | 0.585 | -2.64 | 0.035 | -1.09 | 0.851 | -1.5 |
| 1439243_x_at | Cops5 | COP9 (constitutive photomorphogenic) homolog, subunit 5 (Arabidopsis thaliana) | -1.12 | 0.818 | -1.46 | 0.61 | -1.75 | 0.077 | -1.88 | 0.013 | -1.55 |
| 1452547_s_at | H2-T18 /// LOC633 | histocompatibility 2, T region locus 18 /// similar to histocompatibility 2, T region lo | -1.52 | 0.657 | -1.23 | 0.681 | -1.48 | 0.426 | -1.02 | 0.962 | -1.31 |
| 1439673_at | E230008O15Rik | RIKEN cDNA E230008O15 gene | -1.54 | 0.162 | -1.23 | 0.35 | -1.46 | 0.086 | -1.02 | 0.906 | -1.31 |
| 1455063_at | 4921513D11Rik /// | RIKEN cDNA 4921513D11 gene /// expressed sequence AW061290 | -1.46 | 0.329 | -1.42 | 0.179 | -1.32 | 0.095 | -1.47 | 0.226 | -1.42 |
| 1445535_at | AA407107 | expressed sequence AA407107 | -1.23 | 0.077 | -1.37 | 0.282 | -1.66 | 0.205 | -2.84 | 0.045 | -1.77 |
| 1446636_at | Tns3 | tensin 3 | -1.3 | 0.065 | -1.21 | 0.326 | -1.81 | 0.005 | -1.09 | 0.215 | -1.35 |
| 1427303_at | Enpp3 | ectonucleotide pyrophosphatase/phosphodiesterase 3 | -1.64 | 0.154 | -1.03 | 0.921 | -1.77 | 0.024 | -2.67 | 0.013 | -1.77 |
| 1458821_at | Sec24d | SEC24 related gene family, member D (S. cerevisiae) | -2.22 | 0.231 | -1.24 | 0.777 | -1.12 | 0.816 | -1.34 | 0.279 | -1.48 |
| 1433248_at | 2310075M01Rik | RIKEN cDNA 2310075M01 gene | -1.13 | 0.816 | -2.73 | 0.046 | -1.11 | 0.719 | -1.41 | 0.385 | -1.6 |
| 1430093_at | 4933406P04Rik | RIKEN cDNA 4933406P04 gene | -1.25 | 0.612 | -1.46 | 0.326 | -1.51 | 0.156 | -1.15 | 0.419 | -1.34 |
| 1421053_at | Kif1a | kinesin family member 1A | -2.13 | 0.395 | -1.3 | 0.314 | -1.1 | 0.696 | -1.08 | 0.777 | -1.4 |
| 1437822_at | Yme1l1 | YME1-like 1 (S. cerevisiae) | -1.34 | 0.176 | -1.22 | 0.637 | -1.71 | 0.172 | -1 | 0.993 | -1.32 |
| 1439233_at | Tloc1 | Translocation protein 1 | -1.23 | 0.6 | -1.16 | 0.389 | -2.12 | 0.047 | -1.03 | 0.662 | -1.39 |
| 1420994_at | B3gnt5 | UDP-GlcNAc:betaGal beta-1,3-N-acetylglucosaminyltransferase 5 | -1.09 | 0.783 | -2.86 | 0.098 | -1.13 | 0.587 | -2.39 | 0.127 | -1.87 |
| 1422335_at | Adra2c | adrenergic receptor, alpha 2c | -1.52 | 0.544 | -1.64 | 0.339 | -1.13 | 0.861 | -1.03 | 0.968 | -1.33 |
| 1439752_at | --- | PREDICTED: Mus musculus similar to O-acyltransferase (membrane bound) don | -1.01 | 0.981 | -1.22 | 0.602 | -2.95 | 0.047 | -1.65 | 0.454 | -1.71 |
| 1440937_at | Rbm41 | RNA binding motif protein 41 | -1.23 | 0.473 | -1.59 | 0.415 | -1.41 | 0.527 | -1.06 | 0.901 | -1.32 |
| 1432100_a_at | 4930478P22Rik | RIKEN cDNA 4930478P22 gene | -1.42 | 0.266 | -1.39 | 0.361 | -1.38 | 0.106 | -1.13 | 0.472 | -1.33 |
| 1434671_at | B230337E12Rik | RIKEN cDNA B230337E12 gene | -1.15 | 0.32 | -1.46 | 0.025 | -1.68 | 0.003 | -1.31 | 0.275 | -1.4 |

| | | | | | | | | | | | |
|---|---|---|---|---|---|---|---|---|---|---|---|
| 1432834_at | Cpb2 | carboxypeptidase B2 (plasma) | -1.39 | 0.329 | -1.69 | 0.307 | -1.19 | 0.693 | -1.46 | 0.664 | -1.43 |
| 1451705_a_at | Oprm1 | opioid receptor, mu 1 | -2.18 | 0.219 | -1.01 | 0.955 | -1.41 | 0.302 | -1.77 | 0.413 | -1.6 |
| 1433731_at | Igf2bp3 | insulin-like growth factor 2 mRNA binding protein 3 | -1.59 | 0.005 | -1.38 | 0.095 | -1.25 | 0.431 | -1.08 | 0.603 | -1.33 |
| 1439216_at | 2900075B16Rik | RIKEN cDNA 2900075B16 gene | -1.24 | 0.563 | -1.95 | 0.01 | -1.2 | 0.413 | -1.5 | 0.128 | -1.47 |
| 1447168_at | --- | Transcribed locus | -1.73 | 0.106 | -1.08 | 0.868 | -1.54 | 0.319 | -2.07 | 0.194 | -1.61 |
| 1430263_at | 1700109F18Rik | RIKEN cDNA 1700109F18 gene | -1.04 | 0.957 | -2.72 | 0.089 | -1.22 | 0.482 | -1.48 | 0.56 | -1.61 |
| 1444019_at | Lrrc19 | leucine rich repeat containing 19 | -1.04 | 0.387 | -1.51 | 0.144 | -1.89 | 0.211 | -1.17 | 0.591 | -1.4 |
| 1440121_at | St6galnac3 | ST6 (alpha-N-acetyl-neuraminyl-2,3-beta-galactosyl-1,3)-N-acetylgalactosaminide | -1.33 | 0.191 | -1.08 | 0.891 | -2.1 | 0.007 | -1.48 | 0.257 | -1.5 |
| 1416288_at | Dnaja1 | DnaJ (Hsp40) homolog, subfamily A, member 1 | -1.2 | 0.106 | -1.58 | 0.014 | -1.46 | 0.026 | -1.6 | 0.292 | -1.46 |
| 1442424_at | Ctdspl2 | CTD (carboxy-terminal domain, RNA polymerase II, polypeptide A) small phospha | -1.4 | 0.201 | -1.26 | 0.501 | -1.56 | 0.011 | -1.1 | 0.346 | -1.33 |
| 1428978_at | 2900009J20Rik | RIKEN cDNA 2900009J20 gene | -1.24 | 0.577 | -1.45 | 0.186 | -1.52 | 0.212 | -1.21 | 0.055 | -1.36 |
| 1446433_at | Acbd5 | acyl-Coenzyme A binding domain containing 5 | -1.66 | 0.034 | -1.38 | 0.105 | -1.21 | 0.548 | -1.32 | 0.168 | -1.39 |
| 1451406_a_at | Pcsk5 | proprotein convertase subtilisin/kexin type 5 | -1.21 | 0.374 | -1.59 | 0.035 | -1.44 | 0.299 | -1.53 | 0.485 | -1.44 |
| 1440041_at | Tgfbr3 | Transforming growth factor, beta receptor III | -1.15 | 0.216 | -1.28 | 0.645 | -2 | 0.074 | -1.49 | 0.057 | -1.48 |
| 1431854_a_at | 4930452B06Rik | RIKEN cDNA 4930452B06 gene | -2.31 | 0.087 | -1.16 | 0.813 | -1.17 | 0.344 | -1.22 | 0.699 | -1.46 |
| 1455932_at | Mtdh | Metadherin | -2.99 | 0.332 | -1.03 | 0.952 | -1.18 | 0.662 | -1.03 | 0.952 | -1.56 |
| 1441352_at | 9430034N14Rik | RIKEN cDNA 9430034N14 gene | -1.07 | 0.405 | -1.98 | 0.026 | -1.41 | 0.12 | -1.69 | 0.184 | -1.54 |
| 1446437_at | 9630028H03Rik | RIKEN cDNA 9630028H03 gene | -2.41 | 0.268 | -1.04 | 0.956 | -1.28 | 0.6 | -2.19 | 0.257 | -1.73 |
| 1440854_at | 2810403A07Rik | RIKEN cDNA 2810403A07 gene | -1.06 | 0.826 | -1.49 | 0.05 | -1.85 | 0.033 | -1.49 | 0.193 | -1.47 |
| 1460139_at | C79256 | expressed sequence C79256 | -1.08 | 0.207 | -2.86 | 0.303 | -1.14 | 0.734 | -1.71 | 0.195 | -1.7 |
| 1441750_x_at | 4930447F24Rik | RIKEN cDNA 4930447F24 gene | -2.31 | 0.378 | -1.27 | 0.729 | -1.07 | 0.891 | -1.61 | 0.368 | -1.57 |
| 1430146_at | 4933402N03Rik | RIKEN cDNA 4933402N03 gene | -1.62 | 0.223 | -1.66 | 0.369 | -1.07 | 0.89 | -1.6 | 0.486 | -1.49 |
| 1454551_at | 9530034D02Rik | RIKEN cDNA 9530034D02 gene | -1.18 | 0.664 | -1.58 | 0.031 | -1.49 | 0.058 | -1.13 | 0.829 | -1.34 |
| 1442981_at | --- | --- | -1.51 | 0.192 | -1.97 | 0.123 | -1.01 | 0.917 | -1.13 | 0.731 | -1.41 |
| 1430330_at | Wiz | widely-interspaced zinc finger motifs | -1.33 | 0.314 | -1.77 | 0.194 | -1.19 | 0.642 | -1.07 | 0.809 | -1.34 |
| 1418690_at | Ptprz1 | protein tyrosine phosphatase, receptor type Z, polypeptide 1 | -2.22 | 0.159 | -1.11 | 0.835 | -1.24 | 0.554 | -1 | 1 | -1.39 |
| 1436049_at | 5730589K01Rik | RIKEN cDNA 5730589K01 gene | -1.25 | 0.491 | -1.48 | 0.029 | -1.48 | 0.017 | -1.32 | 0.461 | -1.38 |
| 1438105_at | LOC632687 | hypothetical protein LOC632686 | -1.38 | 0.35 | -1.58 | 0.066 | -1.25 | 0.601 | -1.34 | 0.495 | -1.39 |
| 1424928_at | 2210018M11Rik | RIKEN cDNA 2210018M11 gene | -1.13 | 0.324 | -1.71 | 0.036 | -1.46 | 0.006 | -1.07 | 0.633 | -1.34 |
| 1455280_at | Frem1 | Fras1 related extracellular matrix protein 1 | -1.38 | 0.044 | -1.08 | 0.91 | -1.96 | 0.008 | -1.25 | 0.394 | -1.42 |
| 1430479_at | 2010007H06Rik | RIKEN cDNA 2010007H06 gene | -2.01 | 0.215 | -1.2 | 0.794 | -1.22 | 0.333 | -1.37 | 0.519 | -1.45 |
| 1455260_at | Lcorl | ligand dependent nuclear receptor corepressor-like | -1.52 | 0.131 | -1.4 | 0.18 | -1.27 | 0.059 | -1.33 | 0.268 | -1.38 |
| 1433131_at | 5033425B01Rik | RIKEN cDNA 5033425B01 gene | -1.18 | 0.458 | -1.6 | 0.266 | -1.46 | 0.313 | -1.46 | 0.587 | -1.42 |
| 1445038_at | --- | --- | -1.26 | 0.768 | -1.24 | 0.555 | -1.78 | 0.312 | -1.42 | 0.313 | -1.43 |
| 1459242_at | Elovl5 | ELOVL family member 5, elongation of long chain fatty acids (yeast) | -1.62 | 0.136 | -1.12 | 0.864 | -1.55 | 0.093 | -1.21 | 0.411 | -1.38 |
| 1460101_at | Nrxn3 | Neurexin III | -1.27 | 0.598 | -2.21 | 0.219 | -1.09 | 0.88 | -2.52 | 0.021 | -1.77 |
| 1420278_at | BC048599 | cDNA sequence BC048599 | -1.83 | 0.462 | -1.04 | 0.909 | -1.54 | 0.516 | -1.1 | 0.76 | -1.38 |
| 1424886_at | Ptprd | protein tyrosine phosphatase, receptor type, D | -1.02 | 0.754 | -1.45 | 0.11 | -2.04 | 0.035 | -1.03 | 0.921 | -1.39 |
| 1432997_at | 5830462P14Rik | RIKEN cDNA 5830462P14 gene | -1.36 | 0.164 | -1.33 | 0.335 | -1.49 | 0.098 | -2.14 | 0.07 | -1.58 |
| 1441939_x_at | 2410003I16Rik | RIKEN cDNA 2410003I16 gene | -1.08 | 0.924 | -1.35 | 0.534 | -2.04 | 0.116 | -1.32 | 0.663 | -1.45 |
| 1437570_at | AI503301 | expressed sequence AI503301 | -1.36 | 0.324 | -1.48 | 0.362 | -1.34 | 0.353 | -1.01 | 0.919 | -1.3 |
| 1429902_at | 5830443J22Rik | RIKEN cDNA 5830443J22 gene | -1.42 | 0.022 | -1.19 | 0.161 | -1.63 | 0.002 | -1.55 | 0.038 | -1.45 |
| 1458618_at | Ireb2 | Iron responsive element binding protein 2 | -1.46 | 0.407 | -1.07 | 0.635 | -1.87 | 0.034 | -1.76 | 0.014 | -1.54 |
| 1442775_at | St13 | Suppression of tumorigenicity 13 | -1.28 | 0.481 | -1.63 | 0.022 | -1.31 | 0.329 | -1.71 | 0.238 | -1.48 |
| 1440680_at | --- | Transcribed locus | -1.37 | 0.168 | -1.54 | 0.429 | -1.29 | 0.219 | -1.01 | 0.993 | -1.3 |
| 1439717_at | B230362M20Rik | RIKEN cDNA B230362M20 gene | -1.46 | 0.464 | -1.4 | 0.161 | -1.32 | 0.051 | -1.04 | 0.804 | -1.3 |
| 1460073_at | Prkar1b | Protein kinase, cAMP dependent regulatory, type I beta | -1.29 | 0.453 | -1.5 | 0.509 | -1.39 | 0.029 | -1.21 | 0.651 | -1.35 |
| 1429526_at | Brd8 | bromodomain containing 8 | -1.05 | 0.324 | -1.89 | 0.056 | -1.48 | 0.061 | -1.01 | 0.978 | -1.36 |
| 1443384_at | Ptk2 | PTK2 protein tyrosine kinase 2 | -1.39 | 0.405 | -1.12 | 0.74 | -1.82 | 0.006 | -1.86 | 0.008 | -1.55 |
| 1447592_at | Dbh | dopamine beta hydroxylase | -1.67 | 0.396 | -1.06 | 0.773 | -1.62 | 0.476 | -2.99 | 0.23 | -1.84 |
| 1430039_at | Cdkal1 | CDK5 regulatory subunit associated protein 1-like 1 | -1.23 | 0.507 | -1.36 | 0.237 | -1.62 | 0.028 | -1.45 | 0.011 | -1.42 |
| 1456906_at | 4833432P19Rik | RIKEN cDNA 4833432P19 gene | -1.32 | 0.187 | -1.22 | 0.376 | -1.72 | 0.125 | -1.06 | 0.787 | -1.33 |
| 1447201_at | --- | --- | -1.25 | 0.724 | -1.57 | 0.229 | -1.38 | 0.441 | -2.85 | 0.117 | -1.76 |

| | | | | | | | | | | | |
|---|---|---|---|---|---|---|---|---|---|---|---|
| 1431902_at | 4930401A09Rik | RIKEN cDNA 4930401A09 gene | -1.09 | 0.812 | -1.36 | 0.595 | -1.96 | 0.36 | -1.54 | 0.026 | -1.49 |
| 1456209_x_at | --- | --- | -1.4 | 0.547 | -1.17 | 0.563 | -1.69 | 0.025 | -1.46 | 0.009 | -1.43 |
| 1458019_at | --- | --- | -2.35 | 0.338 | -1.08 | 0.897 | -1.23 | 0.689 | -1.15 | 0.864 | -1.45 |
| 1433450_at | Cdk5r1 | cyclin-dependent kinase 5, regulatory subunit (p35) 1 | -1.33 | 0.51 | -2.39 | 0.024 | -1.01 | 0.97 | -1.58 | 0.044 | -1.58 |
| 1444682_at | BC037032 | cDNA Sequence BC037032 | -1.09 | 0.404 | -1.91 | 0.233 | -1.38 | 0.052 | -1.07 | 0.878 | -1.37 |
| 1427480_at | Leap2 | liver-expressed antimicrobial peptide 2 | -1.51 | 0.375 | -1.04 | 0.839 | -1.87 | 0.281 | -1.66 | 0.249 | -1.52 |
| 1449682_s_at | Tubb2b | tubulin, beta 2b | -1.16 | 0.842 | -1.38 | 0.646 | -1.76 | 0.244 | -1.46 | 0.506 | -1.44 |
| 1445055_at | --- | Adult male hypothalamus cDNA, RIKEN full-length enriched library, clone:A23001 | -1.34 | 0.6 | -1.26 | 0.703 | -1.61 | 0.223 | -2.67 | 0.237 | -1.72 |
| 1430418_at | Tmem57 | transmembrane protein 57 | -1.35 | 0.089 | -1.46 | 0.107 | -1.36 | 0.132 | -1.24 | 0.504 | -1.35 |
| 1459956_at | --- | --- | -1.1 | 0.314 | -2.83 | 0.078 | -1.11 | 0.21 | -2.49 | 0.058 | -1.88 |
| 1430350_at | 2610035F20Rik | RIKEN cDNA 2610035F20 gene | -1.72 | 0.531 | -1.06 | 0.721 | -1.58 | 0.414 | -1.02 | 0.974 | -1.34 |
| 1455092_at | Zfp207 | zinc finger protein 207 | -1.32 | 0.63 | -1.57 | 0.053 | -1.31 | 0.266 | -1.08 | 0.869 | -1.32 |
| 1417836_at | Gpx7 | glutathione peroxidase 7 | -1.09 | 0.711 | -1.59 | 0.007 | -1.62 | 0.007 | -1.02 | 0.811 | -1.33 |
| 1425282_at | Ibrdc2 | IBR domain containing 2 | -2.17 | 0.085 | -1.15 | 0.674 | -1.2 | 0.491 | -1.01 | 0.948 | -1.38 |
| 1432935_at | 5330433J24Rik | RIKEN cDNA 5330433J24 gene | -1.8 | 0.24 | -1.04 | 0.935 | -1.55 | 0.457 | -1.51 | 0.141 | -1.48 |
| 1419633_at | Uncx4.1 | Unc4.1 homeobox (C. elegans) | -1.09 | 0.846 | -1.48 | 0.623 | -1.77 | 0.003 | -1.02 | 0.967 | -1.34 |
| 1419469_at | Gnb4 | guanine nucleotide binding protein, beta 4 | -1.58 | 0.177 | -1.37 | 0.193 | -1.25 | 0.009 | -1.2 | 0.315 | -1.35 |
| 1436154_at | --- | 0 day neonate thymus cDNA, RIKEN full-length enriched library, clone:A430066P | -1.31 | 0.693 | -1.85 | 0.248 | -1.16 | 0.74 | -1.2 | 0.767 | -1.38 |
| 1442812_at | LOC433844 | similar to Anapc5 protein | -1.66 | 0.44 | -1.29 | 0.515 | -1.28 | 0.036 | -1.51 | 0.185 | -1.43 |
| 1457021_x_at | Amhr2 | anti-Mullerian hormone type 2 receptor | -1.15 | 0.79 | -2.51 | 0.33 | -1.12 | 0.297 | -1.08 | 0.897 | -1.46 |
| 1440642_at | D630042P16Rik | RIKEN cDNA D630042P16 gene | -1.36 | 0.205 | -1.52 | 0.084 | -1.3 | 0.247 | -1.14 | 0.813 | -1.33 |
| 1437504_at | Sla2 | Src-like-adaptor 2 | -2 | 0.554 | -1.14 | 0.709 | -1.28 | 0.214 | -1.39 | 0.078 | -1.45 |
| 1443592_at | AA617406 | expressed sequence AA617406 | -1.31 | 0.261 | -1.19 | 0.671 | -1.79 | 0.155 | -1.71 | 0.212 | -1.5 |
| 1446694_at | Dnm3 | Dynamin 3 | -1.19 | 0.506 | -1.81 | 0.433 | -1.3 | 0.552 | -1.06 | 0.512 | -1.34 |
| 1442296_at | Wdr75 | WD repeat domain 75 | -1.49 | 0.043 | -1.36 | 0.156 | -1.32 | 0.03 | -1.7 | 0.043 | -1.47 |
| 1457813_at | Mrps18a | Mitochondrial ribosomal protein S18A | -1.38 | 0.437 | -1.41 | 0.317 | -1.37 | 0.312 | -1.46 | 0.195 | -1.41 |
| 1427193_at | Brd8 | bromodomain containing 8 | -1.66 | 0.053 | -1.13 | 0.724 | -1.48 | 0.05 | -1.05 | 0.879 | -1.33 |
| 1453967_at | 1700093J21Rik | RIKEN cDNA 1700093J21 gene | -1.32 | 0.385 | -1.21 | 0.812 | -1.72 | 0.06 | -3.53 | 0.002 | -1.95 |
| 1433058_at | 4933411O13Rik | RIKEN cDNA 4933411O13 gene | -1.29 | 0.741 | -1.18 | 0.789 | -1.85 | 0.117 | -1.76 | 0.439 | -1.52 |
| 1437617_x_at | 1110034G24Rik | RIKEN cDNA 1110034G24 gene | -1.37 | 0.274 | -1.16 | 0.491 | -1.76 | 0.034 | -1.01 | 0.735 | -1.32 |
| 1442470_at | --- | --- | -1.08 | 0.568 | -1.41 | 0.11 | -1.88 | 0.058 | -1.12 | 0.157 | -1.37 |
| 1420077_at | AI452102 | expressed sequence AI452102 | -1.08 | 0.619 | -1.62 | 0.46 | -1.61 | 0.213 | -1.17 | 0.797 | -1.37 |
| 1420092_at | Morc3 | microrchidia 3 | -1.54 | 0.415 | -1.09 | 0.776 | -1.67 | 0.039 | -1.27 | 0.012 | -1.39 |
| 1451585_x_at | Spsb2 | splA/ryanodine receptor domain and SOCS box containing 2 | -1.44 | 0.114 | -1.69 | 0.003 | -1.14 | 0.318 | -1.05 | 0.694 | -1.33 |
| 1459451_at | --- | --- | -2.62 | 0.329 | -1.14 | 0.769 | -1.1 | 0.789 | -1.44 | 0.122 | -1.58 |
| 1421377_at | Traf6 | Tnf receptor-associated factor 6 | -1.65 | 0.043 | -1.25 | 0.73 | -1.32 | 0.652 | -1.54 | 0.571 | -1.44 |
| 1446700_at | --- | --- | -1.2 | 0.646 | -1.39 | 0.589 | -1.63 | 0.119 | -1.64 | 0.533 | -1.46 |
| 1422144_at | Inhbe | inhibin beta E | -1.15 | 0.549 | -1.36 | 0.327 | -1.79 | 0.021 | -1.69 | 0.209 | -1.5 |
| 1432875_at | 4632409D06Rik | RIKEN cDNA 4632409D06 gene | -1.94 | 0.128 | -1.53 | 0.451 | -1 | 0.998 | -2.31 | 0.221 | -1.7 |
| 1440227_at | BF642829 | expressed sequence BF642829 | -1 | 0.968 | -1.8 | 0.107 | -1.63 | 0.033 | -1 | 0.993 | -1.36 |
| 1423377_at | Igfbpl1 | insulin-like growth factor binding protein-like 1 | -1.55 | 0.061 | -1.55 | 0.288 | -1.14 | 0.454 | -1.24 | 0.481 | -1.37 |
| 1457146_at | Dock4 | dedicator of cytokinesis 4 | -1.2 | 0.077 | -1.1 | 0.247 | -2.35 | 0.012 | -1.17 | 0.333 | -1.45 |
| 1455526_at | Diras1 | DIRAS family, GTP-binding RAS-like 1 | -1.22 | 0.378 | -1.79 | 0.149 | -1.27 | 0.111 | -1.54 | 0.034 | -1.46 |
| 1424387_at | Prlpc3 | prolactin-like protein C 3 | -1 | 0.992 | -1.52 | 0.493 | -1.95 | 0.246 | -1.32 | 0.57 | -1.45 |
| 1442620_at | 4931420C21Rik | RIKEN cDNA 4931420C21 gene | -1.21 | 0.252 | -1.22 | 0.259 | -1.93 | 0 | -2.25 | 0.088 | -1.65 |
| 1451642_at | Kif1b | kinesin family member 1B | -1.09 | 0.261 | -1.76 | 0 | -1.46 | 0.115 | -1.33 | 0.227 | -1.41 |
| 1459206_at | --- | --- | -1.02 | 0.934 | -1.44 | 0.132 | -2.05 | 0.146 | -4.61 | 0 | -2.28 |
| 1421479_at | Zfp318 | zinc finger protein 318 | -1.58 | 0.389 | -1.48 | 0.004 | -1.16 | 0.367 | -3.12 | 0.087 | -1.84 |
| 1439546_at | 4933417O08Rik | RIKEN cDNA 4933417O08 gene | -1.27 | 0.426 | -1.22 | 0.213 | -1.79 | 0.437 | -1.47 | 0.316 | -1.44 |
| 1444106_at | D330012F22Rik | RIKEN cDNA D330012F22 gene | -1.38 | 0.242 | -1.51 | 0.168 | -1.28 | 0.149 | -1.07 | 0.76 | -1.31 |
| 1440158_x_at | Chchd7 | Coiled-coil-helix-coiled-coil-helix domain containing 7 | -1.1 | 0.554 | -3.25 | 0.014 | -1.05 | 0.9 | -1.11 | 0.81 | -1.63 |
| 1424529_s_at | Cgref1 | cell growth regulator with EF hand domain 1 | -1.01 | 0.775 | -1.82 | 0.168 | -1.59 | 0.015 | -1.04 | 0.886 | -1.36 |
| 1447711_x_at | 4933412E12Rik | RIKEN cDNA 4933412E12 gene | -1.52 | 0.412 | -1.8 | 0.127 | -1.05 | 0.73 | -1.36 | 0.455 | -1.43 |

| | | | | | | | | | | | |
|---|---|---|---|---|---|---|---|---|---|---|---|
| 1446871_at | D7Ertd481e | DNA segment, Chr 7, ERATO Doi 481, expressed | -1.01 | 0.987 | -2.35 | 0.141 | -1.33 | 0.361 | -1.4 | 0.192 | -1.52 |
| 1431147_at | Rint1 | RAD50 interactor 1 | -2.08 | 0.244 | -1.14 | 0.681 | -1.23 | 0.2 | -1.01 | 0.952 | -1.37 |
| 1422177_at | Il13ra2 | interleukin 13 receptor, alpha 2 | -1.24 | 0.642 | -1.69 | 0.198 | -1.3 | 0.145 | -1.36 | 0.322 | -1.4 |
| 1457821_at | 2610511M17Rik | RIKEN cDNA 2610511M17 gene | -1.08 | 0.319 | -1.56 | 0.127 | -1.66 | 0.071 | -2.79 | 0.27 | -1.77 |
| 1430512_a_at | Igbp1b | immunoglobulin (CD79A) binding protein 1b | -1.76 | 0.037 | -1.52 | 0.442 | -1.06 | 0.674 | -1.16 | 0.642 | -1.38 |
| 1434499_a_at | Ldhb | lactate dehydrogenase B | -1.07 | 0.361 | -2.23 | 0.172 | -1.27 | 0.188 | -1.91 | 0.032 | -1.62 |
| 1443025_at | --- | 10 days neonate medulla oblongata cDNA, RIKEN full-length enriched library, clo | -2.43 | 0.083 | -1.03 | 0.85 | -1.26 | 0.711 | -2 | 0.383 | -1.68 |
| 1432811_at | --- | --- | -1.3 | 0.27 | -1.35 | 0.572 | -1.5 | 0.115 | -1.5 | 0.333 | -1.42 |
| 1459885_s_at | Cox7c | cytochrome c oxidase, subunit VIIc | -1.4 | 0.215 | -1.4 | 0.123 | -1.35 | 0.122 | -1.16 | 0.41 | -1.33 |
| 1448040_at | Tfpt | TCF3 (E2A) fusion partner | -1.55 | 0.114 | -1.64 | 0.405 | -1.09 | 0.751 | -1.29 | 0.177 | -1.39 |
| 1438999_a_at | Nfat5 | nuclear factor of activated T-cells 5 | -1.23 | 0.158 | -1.52 | 0.001 | -1.43 | 0.143 | -1.46 | 0.356 | -1.41 |
| 1421416_at | Map2k7 | mitogen activated protein kinase kinase 7 | -1.63 | 0.026 | -1.11 | 0.839 | -1.51 | 0.377 | -1.15 | 0.358 | -1.35 |
| 1454318_at | 2810403D21Rik | RIKEN cDNA 2810403D21 gene | -1.37 | 0.267 | -1.51 | 0.172 | -1.28 | 0.553 | -1.47 | 0.279 | -1.41 |
| 1427657_at | 2310042L06Rik | RIKEN cDNA 2310042L06 gene | -2.5 | 0.098 | -1.01 | 0.951 | -1.27 | 0.317 | -1.19 | 0.191 | -1.49 |
| 1432002_at | 2810471M01Rik | RIKEN cDNA 2810471M01 gene | -1.68 | 0.572 | -1.62 | 0.364 | -1.04 | 0.917 | -1.54 | 0.313 | -1.47 |
| 1435926_at | E030003F13Rik | RIKEN cDNA E030003F13 gene | -1.2 | 0.165 | -1.29 | 0.182 | -1.77 | 0.005 | -1.39 | 0.132 | -1.41 |
| 1457748_at | Pogz | pogo transposable element with ZNF domain | -2.15 | 0.118 | -1.25 | 0.638 | -1.1 | 0.762 | -1.51 | 0.194 | -1.5 |
| 1432715_at | Lrrn1 | leucine rich repeat protein 1, neuronal | -1.77 | 0.483 | -1.18 | 0.732 | -1.32 | 0.43 | -1.07 | 0.896 | -1.34 |
| 1445552_at | A830039H10Rik | RIKEN cDNA A830039H10 gene | -1.48 | 0.244 | -1.42 | 0.365 | -1.26 | 0.126 | -1.5 | 0.048 | -1.42 |
| 1459959_at | Gldc | Glycine decarboxylase | -1.04 | 0.925 | -1.86 | 0.238 | -1.48 | 0.307 | -1.05 | 0.95 | -1.36 |
| 1440430_at | A130004G07Rik | RIKEN cDNA A130004G07 gene | -1.18 | 0.418 | -1.27 | 0.158 | -1.86 | 0.049 | -1.37 | 0.065 | -1.42 |
| 1457182_at | Map2k7 | mitogen activated protein kinase kinase 7 | -1.91 | 0.172 | -1.1 | 0.839 | -1.35 | 0.281 | -2.01 | 0.434 | -1.59 |
| 1441292_at | RP23-14F5.7 | Hypothetical gene Rp23-14f5.7 | -1.55 | 0.086 | -1.12 | 0.874 | -1.57 | 0.462 | -5.35 | 0.129 | -2.4 |
| 1448340_at | Tmem30a | transmembrane protein 30A | -1.18 | 0.088 | -1.55 | 0.059 | -1.47 | 0.043 | -1.21 | 0.268 | -1.35 |
| 1454401_at | 5730488B01Rik | RIKEN cDNA 5730488B01 gene | -2.46 | 0.253 | -1.22 | 0.691 | -1.06 | 0.845 | -1.76 | 0.155 | -1.62 |
| 1453540_at | 5430404G13Rik | RIKEN cDNA 5430404G13 gene | -1.3 | 0.245 | -1.03 | 0.877 | -2.33 | 0.021 | -1.43 | 0.243 | -1.52 |
| 1443300_at | Notch3 | Notch gene homolog 3 (Drosophila) | -1.18 | 0.566 | -1.36 | 0.732 | -1.69 | 0.015 | -2.3 | 0.349 | -1.63 |
| 1419974_at | Scp2 | sterol carrier protein 2, liver | -1.8 | 0.082 | -1.46 | 0.111 | -1.07 | 0.776 | -1.1 | 0.523 | -1.36 |
| 1446098_at | Zfp407 | Zinc finger protein 407 | -1.47 | 0.001 | -1.02 | 0.951 | -1.97 | 0.005 | -1.33 | 0.2 | -1.45 |
| 1457653_at | A630042L21Rik | RIKEN cDNA A630042L21 gene | -1.12 | 0.811 | -1.72 | 0.158 | -1.43 | 0.053 | -1.55 | 0.172 | -1.45 |
| 1459709_at | --- | --- | -1.15 | 0.486 | -1.07 | 0.895 | -2.74 | 0.098 | -1.71 | 0.502 | -1.67 |
| 1416552_at | Dppa5 | developmental pluripotency associated 5 | -1.5 | 0.092 | -1.44 | 0.303 | -1.23 | 0.251 | -1.14 | 0.51 | -1.33 |
| 1447982_at | 1110008P14Rik | RIKEN cDNA 1110008P14 gene | -1.69 | 0.003 | -1.22 | 0.369 | -1.31 | 0.282 | -1.04 | 0.938 | -1.32 |
| 1433401_at | 5033405D04Rik | RIKEN cDNA 5033405D04 gene | -1.1 | 0.674 | -1.35 | 0.24 | -1.92 | 0.379 | -1.26 | 0.696 | -1.41 |
| 1430404_at | 4833416J08Rik | RIKEN cDNA 4833416J08 gene | -1.02 | 0.931 | -1.63 | 0.108 | -1.73 | 0.009 | -1.13 | 0.325 | -1.38 |
| 1446403_at | AU022899 | expressed sequence AU022899 | -1.38 | 0.5 | -1.63 | 0.534 | -1.2 | 0.643 | -1.03 | 0.964 | -1.31 |
| 1420758_at | 5830411J07Rik | RIKEN cDNA 5830411J07 gene | -1.28 | 0.442 | -1.11 | 0.83 | -2.03 | 0.155 | -1.35 | 0.7 | -1.44 |
| 1447147_at | Atg7 | Autophagy-related 7 (yeast) | -1.59 | 0.165 | -1.1 | 0.769 | -1.57 | 0.176 | -1.55 | 0.236 | -1.45 |
| 1427743_at | Gm672 | Gene model 672, (NCBI) | -1.41 | 0.298 | -1.31 | 0.18 | -1.42 | 0.126 | -1.12 | 0.564 | -1.32 |
| 1437958_at | Xpr1 | xenotropic and polytropic retrovirus receptor 1 | -1.48 | 0.015 | -1.37 | 0.45 | -1.3 | 0.048 | -1.06 | 0.749 | -1.3 |
| 1439493_at | D630040G17Rik /// | RIKEN cDNA D630040G17 gene /// similar to zinc finger protein 64 (predicted) | -1.36 | 0.075 | -1.42 | 0.456 | -1.36 | 0.161 | -1.91 | 0.044 | -1.51 |
| 1442684_x_at | Hmg20a | high mobility group 20A | -1.61 | 0.064 | -1.11 | 0.657 | -1.52 | 0.106 | -1.75 | 0.298 | -1.5 |
| 1437793_at | Tnks2 | Tankyrase, TRF1-interacting ankyrin-related ADP-ribose polymerase 2 | -1.06 | 0.436 | -1.5 | 0.309 | -1.76 | 0.008 | -1.34 | 0.159 | -1.42 |
| 1440398_at | --- | --- | -1.53 | 0.072 | -1.51 | 0.08 | -1.16 | 0.586 | -1.22 | 0.015 | -1.36 |
| 1440976_at | 1110035M17Rik | RIKEN cDNA 1110035M17 gene | -1.58 | 0.171 | -1.53 | 0.146 | -1.12 | 0.141 | -1.01 | 0.928 | -1.31 |
| 1439475_at | AI929863 | expressed sequence AI929863 | -1.58 | 0.072 | -1.69 | 0.055 | -1.05 | 0.75 | -2.13 | 0.016 | -1.61 |
| 1434943_at | BC023055 | cDNA sequence BC023055 | -1.21 | 0.438 | -2.34 | 0.132 | -1.08 | 0.683 | -1.62 | 0.432 | -1.56 |
| 1439275_s_at | 9530010C24Rik | RIKEN cDNA 9530010C24 gene | -1.23 | 0.061 | -1.13 | 0.816 | -2.07 | 0.025 | -2.54 | 0.175 | -1.75 |
| 1441840_x_at | Stno | Strawberry notch homolog (Drosophila) | -1.66 | 0.466 | -1.05 | 0.791 | -1.59 | 0.002 | -1.47 | 0.206 | -1.44 |
| 1428298_at | 1700029G01Rik | RIKEN cDNA 1700029G01 gene | -1.64 | 0.026 | -1.07 | 0.88 | -1.58 | 0.015 | -1 | 0.998 | -1.32 |
| 1450174_at | Ptprt | protein tyrosine phosphatase, receptor type, T | -2.43 | 0.239 | -1.24 | 0.305 | -1.04 | 0.876 | -1.27 | 0.595 | -1.5 |
| 1458182_at | Rbm18 | RNA binding motif protein 18 | -1.45 | 0.015 | -1.29 | 0.012 | -1.41 | 0.217 | -1.21 | 0.373 | -1.34 |
| 1443128_at | Ints6 | integrator complex subunit 6 | -1.45 | 0.033 | -1.71 | 0.031 | -1.1 | 0.593 | -1.03 | 0.897 | -1.32 |

| | | | | | | | | | | | |
|---|---|---|---|---|---|---|---|---|---|---|---|
| 1444522_at | --- | Transcribed locus | -1.29 | 0.481 | -1.93 | 0.182 | -1.13 | 0.801 | -1.32 | 0.607 | -1.42 |
| 1433266_at | 2810416A17Rik | RIKEN cDNA 2810416A17 gene | -1.15 | 0.463 | -1.36 | 0.177 | -1.74 | 0.009 | -1.69 | 0.015 | -1.49 |
| 1439316_at | Rbm39 | RNA binding motif protein 39 | -1.21 | 0.268 | -1.25 | 0.144 | -1.8 | 0.056 | -1.32 | 0.104 | -1.4 |
| 1440010_at | C230066K19Rik | RIKEN cDNA C230066K19 gene | -1.57 | 0.243 | -1.36 | 0.289 | -1.24 | 0.172 | -1.62 | 0 | -1.45 |
| 1455492_at | B330016D10Rik | RIKEN cDNA B330016D10 gene | -1.19 | 0.043 | -1.96 | 0.016 | -1.2 | 0.245 | -1.41 | 0.149 | -1.44 |
| 1459559_at | --- | --- | -1.01 | 0.967 | -1.73 | 0.454 | -1.63 | 0.06 | -1.52 | 0.621 | -1.47 |
| 1446200_at | Dcc | Deleted in colorectal carcinoma | -1.98 | 0.233 | -1.05 | 0.942 | -1.38 | 0.477 | -1.12 | 0.86 | -1.38 |
| 1440066_at | Smarcad1 | SWI/SNF-related, matrix-associated actin-dependent regulator of chromatin, subf | -1.41 | 0.068 | -1.26 | 0.142 | -1.46 | 0.058 | -1.03 | 0.867 | -1.29 |
| 1450591_at | Olfr154 | olfactory receptor 154 | -1.05 | 0.823 | -1.86 | 0.534 | -1.44 | 0.129 | -1.58 | 0.092 | -1.48 |
| 1439046_at | Ccdc55 | coiled-coil domain containing 55 | -1.08 | 0.545 | -1.66 | 0.031 | -1.53 | 0.123 | -1.25 | 0.366 | -1.38 |
| 1444400_at | Rb1 | retinoblastoma 1 | -1 | 0.999 | -2.96 | 0.243 | -1.19 | 0.779 | -1.8 | 0.449 | -1.74 |
| 1422096_at | 4933434I20Rik | RIKEN cDNA 4933434I20 gene | -1.4 | 0.035 | -1.78 | 0.363 | -1.1 | 0.702 | -3.04 | 0.203 | -1.83 |
| 1435119_at | --- | Adult male medulla oblongata cDNA, RIKEN full-length enriched library, clone:63 | -1.71 | 0.487 | -1.1 | 0.729 | -1.45 | 0.107 | -1.42 | 0.494 | -1.42 |
| 1457449_at | Lass6 | Longevity assurance homolog 6 (S. cerevisiae) | -2.18 | 0.002 | -1.04 | 0.891 | -1.32 | 0.449 | -1.07 | 0.852 | -1.4 |
| 1421467_at | Runx3 | runt related transcription factor 3 | -1.04 | 0.971 | -3.42 | 0.181 | -1.08 | 0.907 | -1.36 | 0.694 | -1.73 |
| 1443399_at | AU021001 | expressed sequence AU021001 | -1.31 | 0.249 | -1.45 | 0.47 | -1.38 | 0.297 | -1.57 | 0.006 | -1.43 |
| 1421270_at | Sh3rf1 | SH3 domain containing ring finger 1 | -1.58 | 0.286 | -1.25 | 0.754 | -1.34 | 0.444 | -2.6 | 0.315 | -1.69 |
| 1444188_at | --- | --- | -1.16 | 0.357 | -1.34 | 0.435 | -1.74 | 0.006 | -2.17 | 0.026 | -1.6 |
| 1452572_at | Camk4 | calcium/calmodulin-dependent protein kinase IV | -1.39 | 0.236 | -1.27 | 0.532 | -1.47 | 0.169 | -1.09 | 0.849 | -1.31 |
| 1434294_at | --- | Transcribed locus | -1.24 | 0.572 | -1.21 | 0.349 | -1.83 | 0.019 | -1.5 | 0.187 | -1.44 |
| 1449727_x_at | --- | --- | -1.33 | 0.62 | -1.56 | 0.279 | -1.27 | 0.628 | -1.03 | 0.942 | -1.3 |
| 1457399_at | Elavl4 | ELAV (embryonic lethal, abnormal vision, Drosophila)-like 4 (Hu antigen D) | -1.37 | 0.1 | -1.17 | 0.488 | -1.68 | 0.135 | -1.06 | 0.715 | -1.32 |
| 1438871_at | Son | Son cell proliferation protein | -1.09 | 0.663 | -1.49 | 0.085 | -1.69 | 0.078 | -1.5 | 0.19 | -1.44 |
| 1441463_at | --- | --- | -1.48 | 0.46 | -1.19 | 0.414 | -1.49 | 0.076 | -1.21 | 0.378 | -1.34 |
| 1433325_at | 9330154F10Rik | RIKEN cDNA 9330154F10 gene | -1.31 | 0.297 | -1.98 | 0.338 | -1.09 | 0.858 | -1.61 | 0.433 | -1.5 |
| 1445814_at | --- | Adult male testis cDNA, RIKEN full-length enriched library, clone:4930402N10 pr | -1.95 | 0.365 | -1.39 | 0.631 | -1.05 | 0.898 | -1.37 | 0.653 | -1.44 |
| 1426104_at | Mapk14 | mitogen activated protein kinase 14 | -1.15 | 0.817 | -2.77 | 0.021 | -1.05 | 0.909 | -1.02 | 0.96 | -1.5 |
| 1425883_at | Smg6 | Smg-6 homolog, nonsense mediated mRNA decay factor (C. elegans) | -1.71 | 0.248 | -1.66 | 0.237 | -1 | 0.991 | -1.34 | 0.465 | -1.43 |
| 1440959_s_at | Mynn | myoneurin | -1.25 | 0.519 | -1.26 | 0.163 | -1.69 | 0.013 | -1.15 | 0.455 | -1.34 |
| 1439639_at | Phc2 | Polyhomeotic-like 2 (Drosophila) | -1.21 | 0.642 | -1.32 | 0.687 | -1.65 | 0.177 | -1.48 | 0.328 | -1.42 |
| 1442375_at | Ubap2 | Ubiquitin-associated protein 2 | -1.2 | 0.219 | -1.2 | 0.64 | -1.91 | 0.003 | -1.02 | 0.958 | -1.33 |
| 1457608_at | Gimap8 | GTPase, IMAP family member 8 | -1.42 | 0.411 | -1.18 | 0.667 | -1.58 | 0.142 | -1.92 | 0.346 | -1.52 |
| 1429542_at | 1700007B14Rik | RIKEN cDNA 1700007B14 gene | -1.04 | 0.95 | -1.94 | 0.042 | -1.4 | 0.51 | -1.3 | 0.584 | -1.42 |
| 1455421_x_at | 6330503C03Rik | RIKEN cDNA 6330503C03 gene | -1.21 | 0.786 | -2.06 | 0.031 | -1.15 | 0.718 | -1.94 | 0.334 | -1.59 |
| 1433221_at | 2610311E24Rik | RIKEN cDNA 2610311E24 gene | -1.82 | 0.036 | -1.32 | 0.632 | -1.13 | 0.456 | -1.13 | 0.565 | -1.35 |
| 1456812_at | Abcd2 | ATP-binding cassette, sub-family D (ALD), member 2 | -1.16 | 0.462 | -1.71 | 0.194 | -1.35 | 0.336 | -1.06 | 0.897 | -1.32 |
| 1427789_s_at | Gnas | GNAS (guanine nucleotide binding protein, alpha stimulating) complex locus | -1.55 | 0.088 | -1.46 | 0.44 | -1.17 | 0.734 | -2.09 | 0.115 | -1.57 |
| 1441844_at | Eps15l1 | Epidermal growth factor receptor pathway substrate 15-like 1 | -1.42 | 0.19 | -1.29 | 0.219 | -1.41 | 0.223 | -1.19 | 0.64 | -1.33 |
| 1420059_at | 2410166I05Rik | RIKEN cDNA 2410166I05 gene | -1.26 | 0.016 | -1.37 | 0.134 | -1.51 | 0.27 | -1.05 | 0.833 | -1.3 |
| 1439810_s_at | Pramel7 | preferentially expressed antigen in melanoma like 7 | -1.1 | 0.504 | -1.09 | 0.87 | -2.78 | 0.293 | -3.11 | 0.015 | -2.02 |
| 1436223_at | Itgb8 | integrin beta 8 | -1.05 | 0.639 | -2.38 | 0.079 | -1.23 | 0.134 | -1.17 | 0.691 | -1.46 |
| 1447506_at | --- | --- | -1.35 | 0.246 | -1.37 | 0.156 | -1.39 | 0.254 | -1.04 | 0.908 | -1.29 |
| 1454951_at | Zfp606 | zinc finger protein 606 | -1.24 | 0.168 | -1.45 | 0.139 | -1.44 | 0.022 | -1.52 | 0.162 | -1.41 |
| 1446066_at | Zcchc17 | zinc finger, CCHC domain containing 17 | -1.47 | 0.204 | -1.09 | 0.746 | -1.69 | 0.118 | -1.3 | 0.42 | -1.39 |
| 1419245_at | Rab14 | RAB14, member RAS oncogene family | -1.3 | 0.251 | -1.64 | 0.051 | -1.23 | 0.376 | -1.1 | 0.763 | -1.32 |
| 1449500_at | Serpinb7 | serine (or cysteine) peptidase inhibitor, clade B, member 7 | -1.32 | 0.666 | -1.79 | 0.269 | -1.14 | 0.814 | -1.43 | 0.384 | -1.42 |
| 1436818_a_at | Msi2 | Musashi homolog 2 (Drosophila) | -1.31 | 0.18 | -1.16 | 0.406 | -1.75 | 0.045 | -2.03 | 0.002 | -1.56 |
| 1439818_at | AI931714 | expressed sequence AI931714 | -1.44 | 0.186 | -1.14 | 0.668 | -1.61 | 0.157 | -1.36 | 0.194 | -1.39 |
| 1432106_at | 6530403M18Rik | RIKEN cDNA 6530403M18 gene | -1.31 | 0.624 | -1.42 | 0.598 | -1.38 | 0.51 | -1.34 | 0.39 | -1.36 |
| 1445794_at | Spag16 | Sperm associated antigen 16 | -1.11 | 0.816 | -1.34 | 0.617 | -1.83 | 0.194 | -2.46 | 0.16 | -1.68 |
| 1450836_at | Neurog1 | neurogenin 1 | -1.65 | 0.061 | -1.2 | 0.765 | -1.33 | 0.655 | -1.27 | 0.695 | -1.36 |
| 1440092_at | Ext1 | Exostoses (multiple) 1 | -1.16 | 0.346 | -1.16 | 0.494 | -2.17 | 0.006 | -1.01 | 0.939 | -1.37 |
| 1455203_at | A930003A15Rik | RIKEN cDNA A930003A15 gene | -2.05 | 0.323 | -1.19 | 0.793 | -1.16 | 0.764 | -1.27 | 0.438 | -1.42 |

| | | | | | | | | | | | |
|---|---|---|---|---|---|---|---|---|---|---|---|
| 1443649_at | Huwe1 | HECT, UBA and WWE domain containing 1 | -1.3 | 0.19 | -1.34 | 0.23 | -1.48 | 0.107 | -1.3 | 0.371 | -1.35 |
| 1441588_at | Kcnq1ot1 | KCNQ1 overlapping transcript 1 | -2.13 | 0.141 | -1.09 | 0.903 | -1.25 | 0.484 | -1.29 | 0.649 | -1.44 |
| 1445432_at | --- | --- | -1.18 | 0.684 | -1.06 | 0.796 | -2.51 | 0.209 | -1.53 | 0.158 | -1.57 |
| 1441859_x_at | Ccdc64 | Coiled-coil domain containing 64 | -1.25 | 0.707 | -1.46 | 0.349 | -1.41 | 0.384 | -3.48 | 0.007 | -1.9 |
| 1450079_at | Nrk | Nik related kinase | -1.82 | 0.253 | -1.11 | 0.63 | -1.35 | 0.53 | -1.47 | 0.507 | -1.44 |
| 1443011_at | Prmt7 | protein arginine N-methyltransferase 7 | -3.14 | 0.14 | -1.06 | 0.853 | -1.07 | 0.857 | -1.41 | 0.397 | -1.67 |
| 1419950_s_at | Tnpo3 | transportin 3 | -2.09 | 0.342 | -1.25 | 0.535 | -1.09 | 0.827 | -1.84 | 0.292 | -1.57 |
| 1430043_at | Ttc19 | tetratricopeptide repeat domain 19 | -1.3 | 0.483 | -1.23 | 0.178 | -1.65 | 0.015 | -1.19 | 0.516 | -1.34 |
| 1444936_at | D5Ertd566e | DNA segment, Chr 5, ERATO Doi 566, expressed | -2.37 | 0.125 | -1.26 | 0.365 | -1.02 | 0.923 | -1.03 | 0.92 | -1.42 |
| 1452958_at | Asphd2 | aspartate beta-hydroxylase domain containing 2 | -1.28 | 0.703 | -1.82 | 0.166 | -1.16 | 0.468 | -1.26 | 0.476 | -1.38 |
| 1438802_at | Foxp1 | Forkhead box P1 | -1.37 | 0.366 | -1.23 | 0.384 | -1.54 | 0.166 | -2.58 | 0.091 | -1.68 |
| 1432387_at | Pgcp | plasma glutamate carboxypeptidase | -2.63 | 0.044 | -1.17 | 0.456 | -1.04 | 0.943 | -1.91 | 0.342 | -1.69 |
| 1445178_at | Sh3rf1 | SH3 domain containing ring finger 1 | -1.01 | 0.969 | -1.3 | 0.33 | -2.27 | 0.061 | -1.82 | 0.144 | -1.6 |
| 1458879_at | C76798 | expressed sequence C76798 | -1.42 | 0.105 | -1.66 | 0.202 | -1.12 | 0.56 | -1.43 | 0.161 | -1.41 |
| 1445838_at | Galnt5 | UDP-N-acetyl-alpha-D-galactosamine:polypeptide N-acetylgalactosaminyltransfer | -1.08 | 0.237 | -2.52 | 0.076 | -1.15 | 0.689 | -1.9 | 0.379 | -1.66 |
| 1434494_at | Zar1 | zygote arrest 1 | -1.26 | 0.416 | -1.48 | 0.089 | -1.37 | 0.348 | -1.4 | 0.623 | -1.38 |
| 1458716_at | Dusp27 | dual specificity phosphatase 27 (putative) | -1.4 | 0.635 | -1.38 | 0.352 | -1.33 | 0.23 | -2.25 | 0.108 | -1.59 |
| 1431528_at | 5830427D02Rik | RIKEN cDNA 5830427D02 gene | -1.31 | 0.355 | -1.25 | 0.066 | -1.59 | 0.032 | -1.61 | 0.123 | -1.44 |
| 1455648_at | LOC278757 /// LOC | similar to reduced expression 2 /// similar to reduced expression 2 | -1.47 | 0.268 | -1.19 | 0.466 | -1.49 | 0.049 | -1.45 | 0.233 | -1.4 |
| 1441243_at | Zfp532 | Zinc finger protein 532 | -1.28 | 0.429 | -1.6 | 0.046 | -1.27 | 0.263 | -1.14 | 0.511 | -1.32 |
| 1455680_at | 9630025H16Rik | RIKEN cDNA 9630025H16 gene | -1.19 | 0.136 | -1.48 | 0.057 | -1.48 | 0.073 | -1.51 | 0.013 | -1.41 |
| 1447838_x_at | Eml4 | echinoderm microtubule associated protein like 4 | -1.77 | 0.116 | -1.22 | 0.665 | -1.23 | 0.332 | -1.36 | 0.274 | -1.4 |
| 1460616_at | Slco4c1 | solute carrier organic anion transporter family, member 4C1 | -1.09 | 0.858 | -1.57 | 0.204 | -1.57 | 0.203 | -1.16 | 0.889 | -1.35 |
| 1453676_at | A930041D05Rik | RIKEN cDNA A930041D05 gene | -1.31 | 0.157 | -1.27 | 0.481 | -1.56 | 0.2 | -1.26 | 0.347 | -1.35 |
| 1437170_x_at | Pdlim1 /// LOC5457 | PDZ and LIM domain 1 (elfin) /// similar to PDZ and LIM domain protein 1 (Elfin) ( | -1.25 | 0.623 | -1.59 | 0.381 | -1.3 | 0.653 | -2.93 | 0.014 | -1.77 |
| 1453844_at | Chit1 | chitinase 1 (chitotriosidase) | -2.13 | 0.45 | -1.3 | 0.426 | -1.04 | 0.891 | -2.75 | 0.009 | -1.81 |
| 1420514_at | Tmem47 | Transmembrane protein 47 | -1.26 | 0.551 | -1.05 | 0.324 | -2.21 | 0.001 | -2.18 | 0.129 | -1.67 |
| 1451855_at | Bcl7a | B-cell CLL/lymphoma 7A | -1.38 | 0.581 | -1.97 | 0.258 | -1.04 | 0.958 | -1.53 | 0.474 | -1.48 |
| 1436876_at | D13Bwg1146e | DNA segment, Chr 13, Brigham & Women's Genetics 1146 expressed | -1.6 | 0.047 | -1.4 | 0.058 | -1.17 | 0.126 | -1.78 | 0.179 | -1.49 |
| 1458221_at | G630014P10Rik | RIKEN cDNA G630014P10 gene | -1.35 | 0.234 | -1.52 | 0.068 | -1.25 | 0.04 | -1.38 | 0.244 | -1.38 |
| 1457592_x_at | 2210018M03Rik | RIKEN cDNA 2210018M03 gene | -1.39 | 0.287 | -2.08 | 0.002 | -1.01 | 0.978 | -1.21 | 0.299 | -1.42 |
| 1456638_at | Wdr59 | WD repeat domain 59 | -1.52 | 0.046 | -1.27 | 0.063 | -1.32 | 0.238 | -1.15 | 0.398 | -1.32 |
| 1422226_at | --- | --- | -1.06 | 0.838 | -1.7 | 0.444 | -1.5 | 0.097 | -1.16 | 0.798 | -1.35 |
| 1440219_at | Cspp1 | Centrosome and spindle pole associated protein 1 | -1.32 | 0.214 | -1.15 | 0.371 | -1.75 | 0.022 | -1.22 | 0.068 | -1.36 |
| 1455877_a_at | Nanos1 | nanos homolog 1 (Drosophila) | -1.5 | 0.662 | -1.16 | 0.71 | -1.49 | 0.155 | -1.04 | 0.844 | -1.3 |
| 1440651_at | Dusp16 | Dual specificity phosphatase 16 | -1.02 | 0.888 | -1.67 | 0.03 | -1.61 | 0.132 | -1.69 | 0.038 | -1.5 |
| 1430910_at | 4930544L04Rik | RIKEN cDNA 4930544L04 gene | -2.14 | 0.512 | -1.22 | 0.667 | -1.1 | 0.512 | -1.93 | 0.378 | -1.6 |
| 1422646_at | Mga | MAX gene associated | -1.69 | 0.221 | -1.23 | 0.404 | -1.26 | 0.212 | -1 | 0.987 | -1.3 |
| 1441155_at | 2810446P07Rik | RIKEN cDNA 2810446P07 gene | -1.37 | 0.054 | -1.14 | 0.46 | -1.69 | 0.043 | -1.23 | 0.325 | -1.36 |
| 1444551_at | Itpr2 | Inositol 1,4,5-triphosphate receptor 2 | -1.32 | 0.193 | -1.16 | 0.601 | -1.72 | 0.376 | -1.47 | 0.263 | -1.42 |
| 1449796_at | Prph1 | Peripherin 1 | -1.2 | 0.69 | -1.71 | 0.339 | -1.29 | 0.724 | -1.09 | 0.847 | -1.32 |
| 1421703_at | Zfp59 | zinc finger protein 59 | -1.15 | 0.477 | -2.18 | 0.075 | -1.15 | 0.581 | -1.05 | 0.865 | -1.38 |
| 1451933_a_at | Cts7 | cathepsin 7 | -1.37 | 0.412 | -1.93 | 0.34 | -1.05 | 0.786 | -1.02 | 0.931 | -1.34 |
| 1447555_at | Utrn | Utrophin | -1.08 | 0.862 | -1.15 | 0.564 | -2.47 | 0.003 | -1.51 | 0.135 | -1.55 |
| 1454456_at | 6530413G14Rik | RIKEN cDNA 6530413G14 gene | -1.48 | 0.415 | -1.28 | 0.165 | -1.35 | 0.425 | -1.25 | 0.547 | -1.34 |
| 1432352_at | 5730405I09Rik | RIKEN cDNA 5730405I09 gene | -1.16 | 0.055 | -1.12 | 0.203 | -2.24 | 0.008 | -1.59 | 0.151 | -1.53 |
| 1421420_at | Ccr10 | chemokine (C-C motif) receptor 10 | -1.54 | 0.254 | -1.06 | 0.932 | -1.65 | 0.452 | -1.77 | 0.212 | -1.51 |
| 1437420_at | Baz1b | bromodomain adjacent to zinc finger domain, 1B | -1.37 | 0.275 | -1.1 | 0.863 | -1.79 | 0.156 | -1.12 | 0.879 | -1.34 |
| 1454919_at | AU044698 | Expressed sequence AU044698 | -1.19 | 0.152 | -1.46 | 0.079 | -1.48 | 0.007 | -1.06 | 0.695 | -1.3 |
| 1435639_at | 2610528A11Rik | RIKEN cDNA 2610528A11 gene | -1.01 | 0.965 | -3.14 | 0.037 | -1.12 | 0.131 | -1.53 | 0.48 | -1.7 |
| 1427724_at | Top2a | topoisomerase (DNA) II alpha | -1.33 | 0.477 | -1.56 | 0.017 | -1.23 | 0.315 | -1.13 | 0.229 | -1.32 |
| 1457591_at | Nol10 | Nucleolar protein 10 | -1.15 | 0.537 | -1.75 | 0.042 | -1.31 | 0.379 | -1.51 | 0.131 | -1.43 |
| 1422091_at | Pfkfb2 | 6-phosphofructo-2-kinase/fructose-2,6-biphosphatase 2 | -1.56 | 0.298 | -1.64 | 0.395 | -1.05 | 0.871 | -1.64 | 0.275 | -1.47 |

| | | | | | | | | | | | |
|---|---|---|---|---|---|---|---|---|---|---|---|
| 1446014_at | --- | --- | -1.17 | 0.216 | -1.49 | 0.056 | -1.48 | 0.024 | -1.12 | 0.596 | -1.32 |
| 1426073_at | Twsg1 | twisted gastrulation homolog 1 (Drosophila) | -1.19 | 0.321 | -1.89 | 0.223 | -1.2 | 0.352 | -1.07 | 0.837 | -1.34 |
| 1458564_at | --- | --- | -1.4 | 0.485 | -1.04 | 0.874 | -1.9 | 0.033 | -1.6 | 0.505 | -1.49 |
| 1419313_at | Ccnt1 | cyclin T1 | -1.19 | 0.017 | -1.25 | 0.333 | -1.79 | 0.05 | -1.06 | 0.837 | -1.32 |
| 1447505_at | 1700020G03Rik | RIKEN cDNA 1700020G03 gene | -1.71 | 0.221 | -1.11 | 0.731 | -1.39 | 0.175 | -1.45 | 0.408 | -1.42 |
| 1440563_at | 2010111I01Rik | RIKEN cDNA 2010111I01 gene | -1.09 | 0.831 | -1.3 | 0.468 | -1.94 | 0.028 | -1.21 | 0.362 | -1.39 |
| 1433140_a_at | Btbd14b | BTB (POZ) domain containing 14B | -1.76 | 0.291 | -1.02 | 0.96 | -1.52 | 0.571 | -1 | 0.982 | -1.33 |
| 1458220_at | Dlc1 | deleted in liver cancer 1 | -1.16 | 0.59 | -1.07 | 0.847 | -2.48 | 0.063 | -1.18 | 0.555 | -1.47 |
| 1422357_at | Magea6 | melanoma antigen, family A, 6 | -1.06 | 0.948 | -2.09 | 0.29 | -1.28 | 0.631 | -1.66 | 0.527 | -1.52 |
| 1429898_at | 2310008M10Rik | RIKEN cDNA 2310008M10 gene | -1.19 | 0.616 | -1.51 | 0.087 | -1.44 | 0.139 | -1.12 | 0.246 | -1.31 |
| 1443468_at | --- | --- | -1.38 | 0.165 | -1.15 | 0.559 | -1.66 | 0.113 | -1.5 | 0.184 | -1.42 |
| 1438814_at | --- | --- | -1.43 | 0.111 | -1.28 | 0.178 | -1.38 | 0.453 | -1.37 | 0.292 | -1.37 |
| 1456924_at | AW492981 | expressed sequence AW492981 | -1.28 | 0.498 | -1.89 | 0.285 | -1.12 | 0.557 | -1.21 | 0.59 | -1.37 |
| 1456550_at | --- | --- | -1.23 | 0.209 | -1.4 | 0.481 | -1.49 | 0.195 | -1.01 | 0.986 | -1.28 |
| 1448329_at | Adam3 | a disintegrin and metallopeptidase domain 3 (cyritestin) | -1.26 | 0.528 | -1.33 | 0.35 | -1.52 | 0.291 | -1.08 | 0.826 | -1.3 |
| 1459343_at | 2700008B19Rik | RIKEN cDNA 2700008B19 gene | -2.12 | 0.197 | -1.3 | 0.445 | -1.04 | 0.887 | -1.17 | 0.713 | -1.41 |
| 1435392_at | Wdr17 | WD repeat domain 17 | -1.26 | 0.691 | -1.99 | 0.253 | -1.1 | 0.876 | -3.07 | 0.375 | -1.86 |
| 1437916_at | --- | --- | -1.49 | 0.414 | -1.14 | 0.458 | -1.53 | 0.067 | -1.42 | 0.018 | -1.39 |
| 1421079_at | Nr4a3 | nuclear receptor subfamily 4, group A, member 3 | -1.33 | 0.568 | -1.32 | 0.684 | -1.44 | 0.478 | -1.81 | 0.24 | -1.48 |
| 1447653_x_at | Rpl24 /// LOC62499 | ribosomal protein L24 /// similar to ribosomal protein L24 /// similar to ribosomal pr | -1.17 | 0.514 | -1.53 | 0.005 | -1.45 | 0.234 | -1.24 | 0.169 | -1.34 |
| 1422354_at | Olfr544 | olfactory receptor 544 | -1.48 | 0.38 | -1.78 | 0.153 | -1.03 | 0.918 | -2.01 | 0.09 | -1.58 |
| 1458704_at | --- | Transcribed locus, moderately similar to NP_663461.2 membrane-associated ring | -1.23 | 0.269 | -1.23 | 0.628 | -1.73 | 0.206 | -2.23 | 0.172 | -1.6 |
| 1457228_x_at | Gle1l | GLE1 RNA export mediator-like (yeast | -1.3 | 0.165 | -1.16 | 0.493 | -1.75 | 0.081 | -1.79 | 0.151 | -1.5 |
| 1427522_at | Arhgap20 | Rho GTPase activating protein 20 | -1.46 | 0.65 | -1.05 | 0.94 | -1.75 | 0.002 | -1.47 | 0.508 | -1.43 |
| 1445204_at | Ccdc85a | Coiled-coil domain containing 85A | -1.12 | 0.452 | -1.27 | 0.57 | -1.89 | 0.149 | -1.55 | 0.263 | -1.46 |
| 1457781_at | Kcnq1ot1 | KCNQ1 overlapping transcript 1 | -1.28 | 0.15 | -1.08 | 0.57 | -2.02 | 0.014 | -1.46 | 0.004 | -1.46 |
| 1430902_at | Ankrd24 | ankyrin repeat domain 24 | -1.02 | 0.929 | -1.23 | 0.631 | -2.43 | 0.041 | -1.83 | 0.051 | -1.63 |
| 1447951_at | 5730403B10Rik | RIKEN cDNA 5730403B10 gene | -1.07 | 0.88 | -1.38 | 0.318 | -1.84 | 0.087 | -1.43 | 0.264 | -1.43 |
| 1422960_at | Srd5a2 | steroid 5 alpha-reductase 2 | -1.22 | 0.032 | -1 | 0.988 | -2.59 | 0.066 | -1.18 | 0.782 | -1.5 |
| 1445026_at | Acvr2a | Activin receptor IIA | -1.92 | 0.125 | -1.06 | 0.902 | -1.34 | 0.6 | -1.14 | 0.729 | -1.37 |
| 1447339_at | --- | Transcribed locus | -1.16 | 0.625 | -1.68 | 0.447 | -1.34 | 0.227 | -1.7 | 0.263 | -1.47 |
| 1421021_at | --- | --- | -1.01 | 0.926 | -1.58 | 0.184 | -1.7 | 0.052 | -1.72 | 0.177 | -1.51 |
| 1437426_at | Wac | WW domain containing adaptor with coiled-coil | -1.25 | 0.129 | -1.25 | 0.127 | -1.66 | 0.009 | -1.08 | 0.137 | -1.31 |
| 1448125_at | Rit2 | Ras-like without CAAX 2 | -1.3 | 0.787 | -1.72 | 0.046 | -1.17 | 0.75 | -1.96 | 0.221 | -1.54 |
| 1432010_at | 4933408N05Rik | RIKEN cDNA 4933408N05 gene | -1.3 | 0.762 | -1.69 | 0.146 | -1.19 | 0.337 | -1.72 | 0.305 | -1.47 |
| 1422402_a_at | Asz1 | ankyrin repeat, SAM and basic leucine zipper domain containing 1 | -1.49 | 0.29 | -1.47 | 0.395 | -1.17 | 0.57 | -1.22 | 0.67 | -1.34 |
| 1418091_at | Tcfcp2l1 | transcription factor CP2-like 1 | -1.31 | 0.143 | -1.16 | 0.66 | -1.72 | 0.061 | -1.27 | 0.615 | -1.37 |
| 1446815_at | Zcsl3 | Zinc finger, CSL-type containing 3 | -1.21 | 0.658 | -1.3 | 0.21 | -1.62 | 0.048 | -1.65 | 0.254 | -1.45 |
| 1441618_at | Arhgap29 | Rho GTPase activating protein 29 | -1.39 | 0.247 | -1.03 | 0.911 | -1.94 | 0.005 | -1.1 | 0.748 | -1.36 |
| 1446840_at | Thrap1 | Thyroid hormone receptor associated protein 1 | -1.27 | 0.142 | -1.32 | 0.484 | -1.51 | 0.071 | -1.29 | 0.289 | -1.35 |
| 1450824_at | Ptch1 | patched homolog 1 | -1.28 | 0.742 | -1.6 | 0.018 | -1.25 | 0.579 | -1.94 | 0.249 | -1.52 |
| 1440162_x_at | A630043P06 | hypothetical protein A630043P06 | -1.46 | 0.147 | -1.45 | 0.407 | -1.21 | 0.654 | -3.38 | 0.04 | -1.87 |
| 1453917_at | Ifitm7 | interferon induced transmembrane protein 7 | -1.39 | 0.328 | -1.37 | 0.589 | -1.32 | 0.074 | -1.3 | 0.461 | -1.34 |
| 1456661_at | Jarid2 | Jumonji, AT rich interactive domain 2 | -1.47 | 0.042 | -1.17 | 0.333 | -1.49 | 0.068 | -1.69 | 0.044 | -1.46 |
| 1443263_at | Bach2 | BTB and CNC homology 2 | -1.08 | 0.669 | -1.4 | 0.369 | -1.77 | 0.062 | -3.87 | 0.039 | -2.03 |
| 1422682_s_at | Prss3 /// Prss1 /// L( | protease, serine, 3 /// protease, serine, 1 (trypsin 1) /// trypsinogen 12 | -1.16 | 0.728 | -1.66 | 0.562 | -1.36 | 0.585 | -3.1 | 0.422 | -1.82 |
| 1456787_at | --- | --- | -1.15 | 0.423 | -1.18 | 0.495 | -2.05 | 0.053 | -1.92 | 0.199 | -1.57 |
| 1454426_at | 4930519E07Rik | RIKEN cDNA 4930519E07 gene | -1.03 | 0.973 | -1.89 | 0.358 | -1.42 | 0.527 | -1.18 | 0.549 | -1.38 |
| 1457489_at | Eif4e | Eukaryotic translation initiation factor 4E | -1.4 | 0.33 | -1.14 | 0.689 | -1.63 | 0.028 | -1.31 | 0.033 | -1.37 |
| 1416117_at | Orc3l | origin recognition complex, subunit 3-like (S. cerevisiae) | -1.09 | 0.837 | -1.88 | 0.045 | -1.31 | 0.184 | -1.46 | 0.139 | -1.44 |
| 1422644_at | Sh3bgr | SH3-binding domain glutamic acid-rich protein | -1.07 | 0.864 | -1.66 | 0.281 | -1.5 | 0.296 | -1.06 | 0.753 | -1.32 |
| 1449590_a_at | Mras | muscle and microspikes RAS | -1.01 | 0.951 | -1.95 | 0.129 | -1.42 | 0.015 | -1.75 | 0.043 | -1.53 |
| 1446365_at | Vti1a | vesicle transport through interaction with t-SNAREs homolog 1A (yeast) | -1.36 | 0.127 | -1.23 | 0.182 | -1.51 | 0.002 | -1.15 | 0.391 | -1.31 |

| | | | | | | | | | | | |
|---|---|---|---|---|---|---|---|---|---|---|---|
| 1430200_at | 4930579D07Rik | RIKEN cDNA 4930579D07 gene | -1.97 | 0.543 | -1.21 | 0.698 | -1.14 | 0.717 | -1.25 | 0.744 | -1.39 |
| 1440137_at | Bcl11b | B-cell leukemia/lymphoma 11B | -1.97 | 0.553 | -1.13 | 0.24 | -1.23 | 0.333 | -2.11 | 0.235 | -1.61 |
| 1441839_s_at | BC024814 | cDNA sequence BC024814 | -1.87 | 0.12 | -1.34 | 0.405 | -1.08 | 0.664 | -1.45 | 0.165 | -1.43 |
| 1440685_at | Calcrl | Calcitonin receptor-like | -1.36 | 0.182 | -1.32 | 0.431 | -1.4 | 0.478 | -1.32 | 0.091 | -1.35 |
| 1419166_at | Slc5a2 | solute carrier family 5 (sodium/glucose cotransporter), member 2 | -1.21 | 0.053 | -2.05 | 0.269 | -1.12 | 0.278 | -1.89 | 0.496 | -1.57 |
| 1453397_at | 9130016M20Rik | RIKEN cDNA 9130016M20 gene | -1.31 | 0.021 | -1.1 | 0.498 | -1.87 | 0.122 | -1.36 | 0.541 | -1.41 |
| 1430827_a_at | Ptk2 | PTK2 protein tyrosine kinase 2 | -1.37 | 0.612 | -1.6 | 0.065 | -1.17 | 0.783 | -1.1 | 0.831 | -1.31 |
| 1423380_s_at | Nfatc4 | nuclear factor of activated T-cells, cytoplasmic, calcineurin-dependent 4 | -1.21 | 0.138 | -1.58 | 0.057 | -1.33 | 0.253 | -1.43 | 0.099 | -1.39 |
| 1445835_at | --- | Transcribed locus | -1.95 | 0.052 | -1.18 | 0.737 | -1.18 | 0.684 | -1.01 | 0.984 | -1.33 |
| 1438595_at | Gm546 | gene model 546, (NCBI) | -1.85 | 0.008 | -1.43 | 0.236 | -1.03 | 0.842 | -1.34 | 0.342 | -1.41 |
| 1423253_at | Mpz | myelin protein zero | -1.93 | 0.361 | -1.15 | 0.687 | -1.22 | 0.603 | -1.95 | 0.532 | -1.56 |
| 1450374_at | Tifp39 | tuberoinfundibular peptide of 39 residues (TIP39) preprohormone | -2.46 | 0.026 | -1.01 | 0.991 | -1.23 | 0.614 | -3.81 | 0.255 | -2.13 |
| 1444343_at | A130064L14Rik | RIKEN cDNA A130064L14 gene | -1.24 | 0.221 | -1.07 | 0.794 | -2.13 | 0.001 | -1.94 | 0.06 | -1.59 |
| 1426357_at | Taok1 | TAO kinase 1 | -1.37 | 0.022 | -1.31 | 0.061 | -1.39 | 0.059 | -1.06 | 0.779 | -1.28 |
| 1459624_at | --- | --- | -1.14 | 0.543 | -1.4 | 0.702 | -1.62 | 0.508 | -1.17 | 0.735 | -1.33 |
| 1447213_at | Sp100 | Nuclear antigen Sp100 | -1.15 | 0.401 | -1.63 | 0.06 | -1.37 | 0.283 | -1.9 | 0.08 | -1.51 |
| 1427317_at | Kin | antigenic determinant of rec-A protein | -1.7 | 0.141 | -1.22 | 0.305 | -1.24 | 0.034 | -1.01 | 0.955 | -1.29 |
| 1456832_at | Atrx | Alpha thalassemia/mental retardation syndrome X-linked homolog (human) | -1.49 | 0.049 | -1.11 | 0.749 | -1.56 | 0.024 | -1.31 | 0.211 | -1.37 |
| 1453611_at | Rbbp6 | retinoblastoma binding protein 6 | -1.73 | 0.14 | -1.5 | 0.222 | -1.04 | 0.899 | -2.41 | 0.278 | -1.67 |
| 1458928_at | --- | --- | -1.32 | 0.056 | -1.16 | 0.739 | -1.7 | 0.324 | -1.63 | 0.427 | -1.45 |
| 1460622_x_at | Wdr18 | WD repeat domain 18 | -1.79 | 0.053 | -1.37 | 0.314 | -1.09 | 0.76 | -1.34 | 0.129 | -1.4 |
| 1432370_at | 4933411E02Rik | RIKEN cDNA 4933411E02 gene | -1.22 | 0.639 | -1.83 | 0.277 | -1.19 | 0.609 | -2.47 | 0.239 | -1.68 |
| 1436918_at | Dyrk2 | dual-specificity tyrosine-(Y)-phosphorylation regulated kinase 2 | -1.37 | 0.037 | -1.31 | 0.109 | -1.39 | 0.056 | -1.72 | 0.108 | -1.45 |
| 1431040_at | 6530401N04Rik /// | RIKEN cDNA 6530401N04 gene /// hypothetical protein LOC638977 | -1.27 | 0.645 | -1.14 | 0.797 | -1.84 | 0.307 | -1.03 | 0.917 | -1.32 |
| 1442706_at | Nelf | nasal embryonic LHRH factor | -1.6 | 0.156 | -1.02 | 0.972 | -1.64 | 0.395 | -2.31 | 0.373 | -1.64 |
| 1438336_at | Fbxw11 | F-box and WD-40 domain protein 11 | -1.35 | 0.361 | -1.38 | 0.411 | -1.34 | 0.229 | -1.23 | 0.134 | -1.32 |
| 1416515_at | Fscn1 | fascin homolog 1, actin bundling protein (Strongylocentrotus purpuratus) | -1.95 | 0.315 | -1.01 | 0.992 | -1.41 | 0.4 | -1.27 | 0.359 | -1.41 |
| 1420702_at | 1700093K21Rik | RIKEN cDNA 1700093K21 gene | -1.22 | 0.858 | -1.07 | 0.911 | -2.2 | 0.132 | -1.28 | 0.318 | -1.44 |
| 1457056_at | Ftcd | Formiminotransferase cyclodeaminase | -1.07 | 0.767 | -1.47 | 0.534 | -1.67 | 0.066 | -1.67 | 0.499 | -1.47 |
| 1421706_at | Mmp20 | matrix metallopeptidase 20 (enamelysin) | -1.59 | 0.073 | -1.5 | 0.064 | -1.09 | 0.832 | -1.09 | 0.863 | -1.32 |
| 1451779_at | BC027061 | cDNA sequence BC027061 | -1.47 | 0.381 | -1.02 | 0.832 | -1.81 | 0.061 | -2.22 | 0.151 | -1.63 |
| 1457605_at | Atxn1 | Ataxin 1 | -1.13 | 0.691 | -1.37 | 0.257 | -1.67 | 0.018 | -1.51 | 0.144 | -1.42 |
| 1449652_at | Nudcd1 | NudC domain containing 1 | -1.29 | 0.539 | -1.48 | 0.187 | -1.3 | 0.443 | -1.2 | 0.749 | -1.32 |
| 1452321_at | Brwd1 | bromodomain and WD repeat domain containing 1 | -1.05 | 0.801 | -1.28 | 0.057 | -2.07 | 0.011 | -2.5 | 0.058 | -1.73 |
| 1434368_at | 1700008K24Rik | RIKEN cDNA 1700008K24 gene | -2.47 | 0.214 | -1.23 | 0.489 | -1.01 | 0.982 | -1.18 | 0.775 | -1.47 |
| 1446697_at | --- | --- | -1.07 | 0.84 | -1.47 | 0.493 | -1.67 | 0.154 | -1.08 | 0.84 | -1.32 |
| 1460326_at | Pik3ca | phosphatidylinositol 3-kinase, catalytic, alpha polypeptide | -1.22 | 0.01 | -1.26 | 0.144 | -1.66 | 0.05 | -1.21 | 0.296 | -1.34 |
| 1425905_at | --- | --- | -1.16 | 0.868 | -1.29 | 0.684 | -1.72 | 0.274 | -1.99 | 0.144 | -1.54 |
| 1439687_at | Rab14 | RAB14, member RAS oncogene family | -1.22 | 0.302 | -1.3 | 0 | -1.6 | 0.112 | -1.38 | 0.234 | -1.37 |
| 1439949_at | Gsk3b | Glycogen synthase kinase 3 beta | -1.11 | 0.702 | -1.29 | 0.438 | -1.85 | 0.005 | -1.04 | 0.273 | -1.32 |
| 1455032_at | 9630037P07Rik | RIKEN cDNA 9630037P07 gene | -1.14 | 0.148 | -1.51 | 0.074 | -1.47 | 0.031 | -1.11 | 0.383 | -1.31 |
| 1423104_at | Irs1 | insulin receptor substrate 1 | -1.16 | 0.153 | -1.34 | 0.228 | -1.64 | 0.156 | -1.71 | 0.142 | -1.46 |
| 1425673_at | Lpp | LIM domain containing preferred translocation partner in lipoma | -1.11 | 0.435 | -1.18 | 0.261 | -2.15 | 0.015 | -1.69 | 0.184 | -1.53 |
| 1445965_at | --- | --- | -1.18 | 0.339 | -1.07 | 0.797 | -2.3 | 0.116 | -1.09 | 0.796 | -1.41 |
| 1430683_at | Dock6 /// LOC6700 | dedicator of cytokinesis 6 /// similar to Dedicator of cytokinesis protein 6 | -1.23 | 0.289 | -1.28 | 0.387 | -1.61 | 0.097 | -1.5 | 0.266 | -1.4 |
| 1443736_at | Ttll10 | Tubulin tyrosine ligase-like family, member 10 | -1.27 | 0.362 | -1.13 | 0.878 | -1.84 | 0.153 | -1.72 | 0.519 | -1.49 |
| 1436084_at | Scrt1 | scratch homolog 1, zinc finger protein (Drosophila) | -1.52 | 0.151 | -1.45 | 0.299 | -1.15 | 0.797 | -1.57 | 0.474 | -1.42 |
| 1458116_at | 9530079D04Rik | RIKEN cDNA 9530079D04 gene | -1.19 | 0.825 | -1.8 | 0.275 | -1.21 | 0.474 | -2.09 | 0.065 | -1.57 |
| 1458335_x_at | 2900073H19Rik | RIKEN cDNA 2900073H19 gene | -1.26 | 0.254 | -1.57 | 0.301 | -1.28 | 0.586 | -2.01 | 0.161 | -1.53 |
| 1430665_at | 5730480H06Rik | RIKEN cDNA 5730480H06 gene | -1.47 | 0.008 | -1.43 | 0.104 | -1.2 | 0.191 | -1.43 | 0.183 | -1.38 |
| 1455258_at | Kcnc2 | potassium voltage gated channel, Shaw-related subfamily, member 2 | -1.12 | 0.513 | -1.13 | 0.824 | -2.24 | 0.34 | -1.71 | 0.297 | -1.55 |
| 1450510_a_at | Cacna1a | calcium channel, voltage-dependent, P/Q type, alpha 1A subunit | -1.06 | 0.569 | -2.44 | 0.099 | -1.16 | 0.485 | -1.22 | 0.253 | -1.47 |
| 1457776_at | D9Ertd720e | DNA segment, Chr 9, ERATO Doi 720, expressed | -1.48 | 0.463 | -1.15 | 0.623 | -1.48 | 0.038 | -1.35 | 0.329 | -1.37 |

| | | | | | | | | | | | |
|---|---|---|---|---|---|---|---|---|---|---|---|
| 1456667_at | Hdh | Huntington disease gene homolog | -1.5 | 0.048 | -1.51 | 0.087 | -1.12 | 0.378 | -1.09 | 0.293 | -1.31 |
| 1447371_at | --- | --- | -1.67 | 0.161 | -1.42 | 0.191 | -1.1 | 0.784 | -1.23 | 0.617 | -1.35 |
| 1434122_at | Fbxo27 | F-box protein 27 | -1.39 | 0.51 | -1.69 | 0.048 | -1.1 | 0.493 | -1.53 | 0.046 | -1.43 |
| 1435670_at | Tcfap2b | transcription factor AP-2 beta | -1.2 | 0.044 | -1.12 | 0.802 | -2.04 | 0.078 | -1.15 | 0.64 | -1.38 |
| 1459567_at | C77905 | expressed sequence C77905 | -1.25 | 0.622 | -1.6 | 0.303 | -1.25 | 0.583 | -1.49 | 0.387 | -1.4 |
| 1428815_at | 4930583C14Rik | RIKEN cDNA 4930583C14 gene | -1.03 | 0.962 | -1.83 | 0.343 | -1.42 | 0.117 | -1.74 | 0.386 | -1.51 |
| 1444033_at | Anapc10 | Anaphase promoting complex subunit 10 | -1.14 | 0.113 | -1.24 | 0.557 | -1.85 | 0.023 | -1.56 | 0.261 | -1.45 |
| 1433801_at | 9930012K11Rik | RIKEN cDNA 9930012K11 gene | -1.1 | 0.733 | -1.83 | 0.052 | -1.3 | 0.307 | -1.71 | 0.111 | -1.49 |
| 1457722_at | Klrb1f | killer cell lectin-like receptor subfamily B member 1F | -1.72 | 0.337 | -1.27 | 0.336 | -1.17 | 0.72 | -1.43 | 0.145 | -1.4 |
| 1432906_at | 1700126A01Rik | RIKEN cDNA 1700126A01 gene | -1.29 | 0.707 | -1.56 | 0.421 | -1.24 | 0.47 | -2.39 | 0.204 | -1.62 |
| 1459409_at | D130073L02Rik | RIKEN cDNA D130073L02 gene | -1.14 | 0.036 | -1.52 | 0.222 | -1.46 | 0.062 | -1.22 | 0.558 | -1.34 |
| 1450687_at | Igf2bp3 | insulin-like growth factor 2 mRNA binding protein 3 | -1.21 | 0.634 | -1 | 0.996 | -2.53 | 0.236 | -1.25 | 0.452 | -1.5 |
| 1419255_at | Spnb2 | spectrin beta 2 | -1.13 | 0.013 | -1.24 | 0.383 | -1.89 | 0.065 | -1.47 | 0.078 | -1.43 |
| 1459756_at | Cnot10 | CCR4-NOT transcription complex, subunit 10 | -1.6 | 0.05 | -1.27 | 0.109 | -1.24 | 0.233 | -1.25 | 0.102 | -1.34 |
| 1457806_at | Dock1 | dedicator of cyto-kinesis 1 | -1.14 | 0.476 | -1.16 | 0.372 | -2.08 | 0.071 | -1.1 | 0.713 | -1.37 |
| 1441739_at | Ebf4 | Early B-cell factor 4 | -1.65 | 0.282 | -1.23 | 0.61 | -1.25 | 0.745 | -1.65 | 0.27 | -1.44 |
| 1446100_at | Trp53bp1 | Transformation related protein 53 binding protein 1 | -2 | 0.033 | -1.26 | 0.382 | -1.08 | 0.871 | -1.12 | 0.155 | -1.36 |
| 1458400_at | 9630050P21Rik | RIKEN cDNA 9630050P21 gene | -1.03 | 0.714 | -1.63 | 0.052 | -1.57 | 0.033 | -1.46 | 0.224 | -1.42 |
| 1452320_at | Lrp2 | low density lipoprotein receptor-related protein 2 | -1 | 0.992 | -4.28 | 0.243 | -1.01 | 0.955 | -1.18 | 0.263 | -1.87 |
| 1455126_x_at | 2310028O11Rik | RIKEN cDNA 2310028O11 gene | -1.2 | 0.065 | -1.3 | 0.091 | -1.6 | 0.027 | -1.44 | 0.305 | -1.39 |
| 1440225_at | Gpr116 | G protein-coupled receptor 116 | -1.32 | 0.431 | -1.49 | 0.088 | -1.26 | 0.371 | -1.67 | 0.076 | -1.43 |
| 1418881_at | Efcbp2 | EF hand calcium binding protein 2 | -1.78 | 0.138 | -1.07 | 0.721 | -1.38 | 0.107 | -1.18 | 0.616 | -1.35 |
| 1435880_at | Ankrd50 | ankrin repeat domain 50 | -1.26 | 0.481 | -1.26 | 0.003 | -1.57 | 0.029 | -1.19 | 0.395 | -1.32 |
| 1423327_at | 4930517K11Rik | RIKEN cDNA 4930517K11 gene | -1.19 | 0.807 | -1.84 | 0.162 | -1.19 | 0.65 | -2.16 | 0.341 | -1.6 |
| 1444872_at | D3Ertd740e | DNA segment, Chr 3, ERATO Doi 740, expressed | -1.09 | 0.791 | -1.51 | 0.137 | -1.55 | 0.037 | -1.11 | 0.625 | -1.32 |
| 1430069_at | 4921517L17Rik | RIKEN cDNA 4921517L17 gene | -1.58 | 0.325 | -1.15 | 0.671 | -1.38 | 0.254 | -1.24 | 0.569 | -1.34 |
| 1421305_x_at | Rabep1 | rabaptin, RAB GTPase binding effector protein 1 | -1.27 | 0.217 | -1.46 | 0.001 | -1.33 | 0.18 | -1.5 | 0.078 | -1.39 |
| 1458615_at | Depdc5 | DEP domain containing 5 | -1.47 | 0.2 | -1.49 | 0.07 | -1.15 | 0.769 | -1.24 | 0.564 | -1.34 |
| 1454536_at | 4933401B01Rik | RIKEN cDNA 4933401B01 gene | -1.29 | 0.05 | -1.53 | 0.498 | -1.26 | 0.144 | -1.52 | 0.032 | -1.4 |
| 1432828_at | --- | --- | -1.62 | 0.187 | -1.45 | 0.148 | -1.09 | 0.864 | -1.35 | 0.512 | -1.38 |
| 1421658_x_at | Psg18 | pregnancy specific glycoprotein 18 | -1.65 | 0.233 | -1.07 | 0.886 | -1.47 | 0.455 | -1.82 | 0.321 | -1.5 |
| 1431393_at | Six6os1 | Six6 opposite strand transcript 1 | -1.66 | 0.198 | -1.01 | 0.992 | -1.59 | 0.441 | -1.29 | 0.554 | -1.39 |
| 1453858_at | Slc35a2 | solute carrier family 35 (UDP-galactose transporter), member 2 | -1.06 | 0.778 | -1.51 | 0.224 | -1.62 | 0.082 | -1.01 | 0.954 | -1.3 |
| 1444475_at | --- | --- | -1.71 | 0.085 | -1.03 | 0.781 | -1.5 | 0.525 | -1.35 | 0.638 | -1.4 |
| 1447902_at | 1810013A23Rik | RIKEN cDNA 1810013A23 gene | -1.16 | 0.686 | -1.66 | 0.292 | -1.31 | 0.274 | -1 | 0.993 | -1.28 |
| 1458441_at | Slc6a17 | solute carrier family 6 (neurotransmitter transporter), member 17 | -1.17 | 0.648 | -1.98 | 0.164 | -1.16 | 0.712 | -2.28 | 0.06 | -1.65 |
| 1456938_at | Smarcb1 | SWI/SNF related, matrix associated, actin dependent regulator of chromatin, subf | -1.28 | 0.568 | -1.03 | 0.912 | -2.09 | 0.197 | -1.4 | 0.554 | -1.45 |
| 1441233_at | Srp54 | Signal recognition particle 54 | -1.03 | 0.796 | -1.38 | 0.241 | -1.91 | 0.002 | -1.46 | 0.094 | -1.44 |
| 1430728_at | Krtap5-5 | keratin associated protein 5-5 | -1.12 | 0.765 | -1.12 | 0.838 | -2.29 | 0.061 | -1.61 | 0.263 | -1.54 |
| 1443426_at | Sltm | SAFB-like, transcription modulator | -1.11 | 0.309 | -1.54 | 0.069 | -1.48 | 0.012 | -1.5 | 0.173 | -1.41 |
| 1442525_at | --- | Transcribed locus | -1.13 | 0.683 | -1.56 | 0.088 | -1.43 | 0.272 | -1.33 | 0.374 | -1.36 |
| 1448081_at | Colq | Collagen-like tail subunit (single strand of homotrimer) of asymmetric acetylcholin | -1.16 | 0.368 | -1.94 | 0.184 | -1.18 | 0.659 | -1.67 | 0.183 | -1.49 |
| 1453806_at | Ndufb2 | NADH dehydrogenase (ubiquinone) 1 beta subcomplex, 2 | -1.24 | 0.707 | -1.59 | 0.273 | -1.26 | 0.588 | -1.05 | 0.646 | -1.29 |
| 1450252_at | Onecut1 | one cut domain, family member 1 | -1.07 | 0.742 | -2.14 | 0.265 | -1.21 | 0.065 | -1.25 | 0.426 | -1.42 |
| 1454493_at | 5033425B01Rik | RIKEN cDNA 5033425B01 gene | -1.22 | 0.345 | -1.31 | 0.534 | -1.54 | 0.479 | -1.45 | 0.394 | -1.38 |
| 1455638_at | Zfp319 | zinc finger protein 319 | -1 | 0.999 | -1.76 | 0.476 | -1.52 | 0.046 | -2.4 | 0.027 | -1.67 |
| 1453580_at | 5630401D24Rik | RIKEN cDNA 5630401D24 gene | -1.29 | 0.662 | -1.53 | 0.225 | -1.25 | 0.175 | -1.05 | 0.846 | -1.28 |
| 1444653_at | --- | 10 days neonate skin cDNA, RIKEN full-length enriched library, clone:4732412F1 | -1.22 | 0.731 | -1.4 | 0.058 | -1.44 | 0.627 | -2.26 | 0.09 | -1.58 |
| 1447314_at | Tgfbr3 | Transforming growth factor, beta receptor III | -1.24 | 0.384 | -1.1 | 0.752 | -1.95 | 0.071 | -1.35 | 0.247 | -1.41 |
| 1453237_at | Qars | glutaminyl-tRNA synthetase | -1.18 | 0.653 | -1.83 | 0.052 | -1.21 | 0.53 | -2.33 | 0.006 | -1.64 |
| 1455327_at | Senp2 | SUMO/sentrin specific peptidase 2 | -1.31 | 0.402 | -1.32 | 0.281 | -1.42 | 0 | -1.02 | 0.929 | -1.27 |
| 1437665_at | Il22ra2 | Interleukin 22 receptor, alpha 2 | -1.94 | 0.178 | -1.04 | 0.932 | -1.34 | 0.487 | -1.84 | 0.249 | -1.54 |
| 1446079_at | Zmat2 | zinc finger, matrin type 2 | -1.14 | 0.335 | -1.71 | 0.158 | -1.32 | 0.091 | -1.22 | 0.323 | -1.35 |

| | | | | | | | | | | | |
|---|---|---|---|---|---|---|---|---|---|---|---|
| 1443355_at | --- | --- | -1.03 | 0.925 | -1.25 | 0.346 | -2.21 | 0.006 | -1.28 | 0.444 | -1.44 |
| 1432781_at | Pdzd6 | PDZ domain containing 6 | -1.01 | 0.984 | -1.31 | 0.413 | -2.11 | 0.34 | -1.24 | 0.443 | -1.42 |
| 1431569_a_at | Lypd1 | Ly6/Plaur domain containing 1 | -1.25 | 0.61 | -1.35 | 0.651 | -1.46 | 0.584 | -1.33 | 0.71 | -1.35 |
| 1440394_at | --- | --- | -1.64 | 0.229 | -1.32 | 0.166 | -1.17 | 0.494 | -1.31 | 0.458 | -1.36 |
| 1440467_at | BC028799 /// 49225 | cDNA sequence BC028799 /// RIKEN cDNA 4922501C03 gene | -1.39 | 0.546 | -1.58 | 0.411 | -1.14 | 0.509 | -1.11 | 0.663 | -1.3 |
| 1419095_a_at | Apom | apolipoprotein M | -1.15 | 0.455 | -1.21 | 0.096 | -1.9 | 0.356 | -1.25 | 0.448 | -1.38 |
| 1428512_at | Bhlhb9 | basic helix-loop-helix domain containing, class B9 | -1.12 | 0.196 | -1.71 | 0.086 | -1.34 | 0.035 | -1.36 | 0.189 | -1.38 |
| 1457908_at | Zfp407 | Zinc finger protein 407 | -1.02 | 0.956 | -1.4 | 0.18 | -1.89 | 0.043 | -1.32 | 0.147 | -1.41 |
| 1456683_at | Sltm | SAFB-like, transcription modulator | -1.33 | 0.093 | -1.24 | 0.14 | -1.48 | 0.001 | -1.43 | 0.22 | -1.37 |
| 1431865_a_at | 4933405K07Rik | RIKEN cDNA 4933405K07 gene | -1.07 | 0.829 | -1.42 | 0.211 | -1.7 | 0.408 | -1.89 | 0.199 | -1.52 |
| 1442983_at | Slc23a2 | Solute carrier family 23 (nucleobase transporters), member 2 | -1.24 | 0.356 | -1.1 | 0.64 | -1.92 | 0.027 | -1.19 | 0.144 | -1.37 |
| 1450514_at | Zscan2 | zinc finger and SCAN domain containing 2 | -1.13 | 0.16 | -1.38 | 0.656 | -1.62 | 0.44 | -1.25 | 0.715 | -1.34 |
| 1424491_at | --- | --- | -1.02 | 0.943 | -1.61 | 0.338 | -1.6 | 0.208 | -1.19 | 0.391 | -1.35 |
| 1444621_at | Iqsec2 | IQ motif and Sec7 domain 2 | -1.55 | 0.44 | -1.08 | 0.852 | -1.51 | 0.557 | -1.85 | 0.485 | -1.5 |
| 1437775_at | Dlst | dihydrolipoamide S-succinyltransferase (E2 component of 2-oxo-glutarate comple | -1.22 | 0.209 | -1.34 | 0.214 | -1.51 | 0.086 | -1.1 | 0.549 | -1.29 |
| 1441378_at | --- | --- | -1.1 | 0.163 | -1.49 | 0.179 | -1.54 | 0.2 | -1.22 | 0.649 | -1.34 |
| 1442111_at | D430033H22Rik | RIKEN cDNA D430033H22 gene | -1.06 | 0.527 | -1.37 | 0.004 | -1.79 | 0.032 | -1.24 | 0.335 | -1.36 |
| 1446353_at | Tubb6 | Tubulin, beta 6 | -1.14 | 0.652 | -1.4 | 0.477 | -1.56 | 0.063 | -1.42 | 0.153 | -1.38 |
| 1432676_at | --- | --- | -1.29 | 0.394 | -1.84 | 0.073 | -1.1 | 0.648 | -1.71 | 0.018 | -1.48 |
| 1451590_at | Cplx4 | complexin 4 | -1.99 | 0.073 | -1.19 | 0.735 | -1.13 | 0.783 | -1.6 | 0.532 | -1.48 |
| 1420275_at | --- | --- | -2.21 | 0.048 | -1.24 | 0.572 | -1.03 | 0.93 | -1.19 | 0.522 | -1.42 |
| 1419167_at | Prap1 | proline-rich acidic protein 1 | -1.22 | 0.595 | -1.79 | 0.15 | -1.17 | 0.5 | -1.23 | 0.199 | -1.35 |
| 1435904_at | Eif2c3 | eukaryotic translation initiation factor 2C, 3 | -1.31 | 0.015 | -1.3 | 0.037 | -1.44 | 0.187 | -1.17 | 0.564 | -1.3 |
| 1430068_at | C030011L09Rik | RIKEN cDNA C030011L09 gene | -1.4 | 0.411 | -1.4 | 0.555 | -1.25 | 0.4 | -2.08 | 0.33 | -1.53 |
| 1453083_at | 6430701C03Rik | RIKEN cDNA 6430701C03 gene | -1.48 | 0.071 | -1.24 | 0.292 | -1.33 | 0.175 | -1.17 | 0.34 | -1.31 |
| 1452108_at | Igf1r | insulin-like growth factor I receptor | -1.74 | 0.01 | -1.5 | 0.344 | -1.01 | 0.934 | -1.02 | 0.979 | -1.32 |
| 1419363_a_at | Mrpl35 | mitochondrial ribosomal protein L35 | -1.3 | 0.024 | -1.43 | 0.013 | -1.3 | 0.039 | -1.07 | 0.712 | -1.28 |
| 1449921_s_at | Cpne6 | copine VI | -1.45 | 0.128 | -1.21 | 0.448 | -1.4 | 0.468 | -1.12 | 0.488 | -1.29 |
| 1442181_at | Grlf1 | Glucocorticoid receptor DNA binding factor 1 | -1.17 | 0.505 | -1.21 | 0.242 | -1.79 | 0.059 | -1.52 | 0.013 | -1.43 |
| 1443538_at | --- | --- | -1.09 | 0.806 | -1.19 | 0.75 | -2.08 | 0.174 | -1.06 | 0.8 | -1.36 |
| 1432897_at | 4930435N07Rik | RIKEN cDNA 4930435N07 gene | -1.05 | 0.716 | -2.32 | 0.047 | -1.17 | 0.407 | -1.79 | 0.37 | -1.58 |
| 1420662_at | 4933439F18Rik | RIKEN cDNA 4933439F18 gene | -1.28 | 0.073 | -1.5 | 0.59 | -1.27 | 0.492 | -1.19 | 0.442 | -1.31 |
| 1418207_at | Fxyd4 | FXYD domain-containing ion transport regulator 4 | -1.2 | 0.359 | -1.63 | 0.169 | -1.27 | 0.214 | -1.31 | 0.568 | -1.35 |
| 1432656_at | 3222402N08Rik | RIKEN cDNA 3222402N08 gene | -2.16 | 0.391 | -1.27 | 0.548 | -1.02 | 0.711 | -1.02 | 0.971 | -1.37 |
| 1439283_at | Osbpl9 | Oxysterol binding protein-like 9 | -1.34 | 0.351 | -1.05 | 0.579 | -1.86 | 0.038 | -1.57 | 0.153 | -1.46 |
| 1454215_at | 2410007B07Rik | RIKEN cDNA 2410007B07 gene | -1.2 | 0.283 | -1.62 | 0.223 | -1.28 | 0.069 | -1.63 | 0.622 | -1.43 |
| 1442206_at | Mamdc1 | MAM domain containing 1 | -1.83 | 0.343 | -1.11 | 0.639 | -1.27 | 0.024 | -1.43 | 0.066 | -1.41 |
| 1440353_at | Ntf5 | neurotrophin 5 | -1.47 | 0.308 | -1.38 | 0.206 | -1.21 | 0.222 | -1.33 | 0.663 | -1.35 |
| 1431302_a_at | Nudt7 | nudix (nucleoside diphosphate linked moiety X)-type motif 7 | -1.15 | 0.282 | -1.92 | 0.103 | -1.18 | 0.138 | -1.18 | 0.438 | -1.36 |
| 1456712_at | Lcorl | ligand dependent nuclear receptor corepressor-like | -1.31 | 0.056 | -1.41 | 0.017 | -1.31 | 0.115 | -1.16 | 0.527 | -1.3 |
| 1459322_at | Syne2 | Synaptic nuclear envelope 2 | -1.21 | 0.405 | -1.32 | 0.432 | -1.55 | 0.119 | -1.05 | 0.741 | -1.28 |
| 1425662_at | Cdadc1 | cytidine and dCMP deaminase domain containing 1 | -1.09 | 0.689 | -1.68 | 0.123 | -1.38 | 0.096 | -1.42 | 0.427 | -1.39 |
| 1433337_at | 6720460K10Rik | RIKEN cDNA 6720460K10 gene | -1.63 | 0.411 | -1.05 | 0.812 | -1.5 | 0.288 | -1.22 | 0.73 | -1.35 |
| 1439163_at | Zbtb16 | zinc finger and BTB domain containing 16 | -1.48 | 0.758 | -1.46 | 0.45 | -1.15 | 0.785 | -1.98 | 0.381 | -1.52 |
| 1440274_at | Rapgef4 | Rap guanine nucleotide exchange factor (GEF) 4 | -1.61 | 0.022 | -1 | 0.989 | -1.62 | 0.1 | -2.88 | 0.13 | -1.78 |
| 1445001_at | --- | --- | -1.1 | 0.536 | -1.29 | 0.185 | -1.81 | 0.03 | -1.15 | 0.547 | -1.34 |
| 1455789_x_at | LOC621284 | similar to heat shock protein 8 | -1.35 | 0.114 | -1.31 | 0.266 | -1.36 | 0.056 | -1.17 | 0.139 | -1.3 |
| 1458226_at | Flnb | filamin, beta | -1.16 | 0.459 | -2.43 | 0.281 | -1.04 | 0.877 | -1.18 | 0.813 | -1.45 |
| 1446790_at | C78948 | expressed sequence C78948 | -1.55 | 0.469 | -1.13 | 0.737 | -1.42 | 0.125 | -4.34 | 0.063 | -2.11 |
| 1438273_at | --- | --- | -1.11 | 0.752 | -1.03 | 0.951 | -2.71 | 0.027 | -1.36 | 0.287 | -1.55 |
| 1425083_at | Otor | otoraplin | -1.5 | 0.494 | -1.29 | 0.609 | -1.26 | 0.572 | -1.73 | 0.405 | -1.45 |
| 1430196_at | 8430408J09Rik | RIKEN cDNA 8430408J09 gene | -1.37 | 0.118 | -1.14 | 0.626 | -1.59 | 0.061 | -1.75 | 0.096 | -1.46 |
| 1437963_at | C530045E16Rik | RIKEN cDNA C530045E16 gene | -1.35 | 0.497 | -1.98 | 0.255 | -1.01 | 0.973 | -1.67 | 0.146 | -1.5 |

| | | | | | | | | | | | |
|---|---|---|---|---|---|---|---|---|---|---|---|
| 1458185_at | Thap4 | THAP domain containing 4 | -1.38 | 0.111 | -1.3 | 0.338 | -1.35 | 0.121 | -1.25 | 0.082 | -1.32 |
| 1456121_at | Lrriq2 | leucine-rich repeats and IQ motif containing 2 | -1.49 | 0.276 | -1.12 | 0.785 | -1.49 | 0.068 | -1.31 | 0.371 | -1.35 |
| 1452549_at | Gm1549 | gene model 1549, (NCBI) | -3.51 | 0.039 | -1.05 | 0.939 | -1 | 0.994 | -1.8 | 0.007 | -1.84 |
| 1458186_at | --- | Transcribed locus | -1.42 | 0.036 | -1.09 | 0.729 | -1.63 | 0.064 | -1.29 | 0.513 | -1.36 |
| 1458977_at | A530021J07 | hypothetical protein A530021J07 | -1.6 | 0.3 | -1.06 | 0.923 | -1.49 | 0.431 | -2.33 | 0.151 | -1.62 |
| 1426320_at | 4933437K13Rik | RIKEN cDNA 4933437K13 gene | -1.22 | 0.368 | -1.6 | 0.139 | -1.26 | 0.266 | -2.15 | 0.033 | -1.56 |
| 1420577_at | Aicda | activation-induced cytidine deaminase | -1.97 | 0.099 | -1.02 | 0.946 | -1.33 | 0.422 | -1.69 | 0.331 | -1.5 |
| 1422800_at | Bat2 | HLA-B associated transcript 2 | -1.74 | 0.255 | -1.5 | 0.37 | -1 | 0.993 | -1.54 | 0.252 | -1.45 |
| 1442485_at | --- | --- | -1.71 | 0.183 | -1.13 | 0.616 | -1.31 | 0.519 | -1.26 | 0.523 | -1.35 |
| 1439945_at | Zfp449 | zinc finger protein 449 | -1.27 | 0.358 | -1.35 | 0.299 | -1.42 | 0.134 | -1.29 | 0.353 | -1.33 |
| 1451614_a_at | Amelx | amelogenin X chromosome | -1.63 | 0.259 | -1.57 | 0.019 | -1.01 | 0.955 | -1.68 | 0.543 | -1.47 |
| 1449793_at | --- | --- | -1.2 | 0.525 | -1.31 | 0.581 | -1.55 | 0.101 | -1.94 | 0.155 | -1.5 |
| 1435559_at | Myo6 | myosin VI | -1.23 | 0.288 | -1.38 | 0.07 | -1.43 | 0.024 | -1.26 | 0.682 | -1.32 |
| 1460081_at | Syt7 | Synaptotagmin VII | -1.84 | 0.051 | -1.14 | 0.321 | -1.22 | 0.337 | -1.83 | 0.253 | -1.51 |
| 1460103_at | --- | --- | -2.11 | 0.116 | -1.11 | 0.685 | -1.15 | 0.742 | -1.22 | 0.512 | -1.4 |
| 1431403_a_at | 2900002G04Rik | RIKEN cDNA 2900002G04 gene | -1.19 | 0.2 | -1.58 | 0.254 | -1.31 | 0.417 | -1.07 | 0.897 | -1.29 |
| 1446138_at | Kifap3 | Kinesin-associated protein 3 | -1.02 | 0.939 | -1.52 | 0.484 | -1.67 | 0.111 | -1.64 | 0.135 | -1.46 |
| 1438285_at | 2210015D19Rik | RIKEN cDNA 2210015D19 gene | -1.26 | 0.16 | -1.38 | 0.155 | -1.39 | 0.152 | -1.29 | 0.219 | -1.33 |
| 1458881_at | 9530001P21Rik | RIKEN cDNA 9530001P21 gene | -2.43 | 0.116 | -1.18 | 0.655 | -1.02 | 0.941 | -1.36 | 0.484 | -1.5 |
| 1438127_at | LOC552906 | hypothetical LOC552906 | -1.1 | 0.698 | -1.34 | 0.184 | -1.71 | 0.114 | -1.18 | 0.33 | -1.33 |
| 1427944_at | C1qdc1 | C1q domain containing 1 | -1.38 | 0.036 | -1.19 | 0.551 | -1.49 | 0.065 | -1.2 | 0.271 | -1.31 |
| 1441046_at | --- | --- | -1.26 | 0.429 | -1.3 | 0.243 | -1.49 | 0.144 | -1.27 | 0.244 | -1.33 |
| 1438457_at | --- | Transcribed locus, strongly similar to XP_574375.1 PREDICTED: similar to MGC | -1.82 | 0.178 | -1.38 | 0.386 | -1.04 | 0.83 | -1.03 | 0.965 | -1.31 |
| 1433800_a_at | Pomc1 | pro-opiomelanocortin-alpha | -1.42 | 0.019 | -1.36 | 0.545 | -1.25 | 0.395 | -1.73 | 0.021 | -1.44 |
| 1459481_at | Pcdh11x | Protocadherin 11 X-linked | -2.1 | 0.028 | -1.12 | 0.871 | -1.14 | 0.762 | -1.79 | 0.345 | -1.54 |
| 1434490_at | Scarf1 | scavenger receptor class F, member 1 | -1.28 | 0.398 | -1.49 | 0.303 | -1.27 | 0.42 | -1.1 | 0.715 | -1.28 |
| 1459276_at | A530088E08Rik | RIKEN cDNA A530088E08 gene | -2.44 | 0.441 | -1.04 | 0.943 | -1.15 | 0.767 | -1.38 | 0.395 | -1.5 |
| 1455425_at | BB001228 | expressed sequence BB001228 | -1.29 | 0.087 | -1.24 | 0.717 | -1.52 | 0.083 | -2.25 | 0.032 | -1.58 |
| 1441779_at | 9530006C21Rik | RIKEN cDNA 9530006C21 gene | -1.29 | 0.191 | -1.09 | 0.694 | -1.84 | 0.055 | -1.92 | 0.124 | -1.53 |
| 1459398_at | --- | --- | -1.26 | 0.449 | -1.45 | 0.502 | -1.32 | 0.273 | -1.97 | 0.192 | -1.5 |
| 1445543_at | 9830124H08Rik | RIKEN cDNA 9830124H08 gene | -1.09 | 0.268 | -1.44 | 0.16 | -1.6 | 0.027 | -1.31 | 0.442 | -1.36 |
| 1439484_at | 4930431H11Rik | RIKEN cDNA 4930431H11 gene | -1.1 | 0.745 | -1.91 | 0.101 | -1.24 | 0.197 | -1.62 | 0.22 | -1.46 |
| 1423595_at | Mina | myc induced nuclear antigen | -1.32 | 0.087 | -1.47 | 0.05 | -1.25 | 0.084 | -1.22 | 0.118 | -1.31 |
| 1443086_at | Alcam | activated leukocyte cell adhesion molecule | -1.17 | 0.411 | -1.18 | 0.158 | -1.85 | 0.001 | -1.37 | 0.313 | -1.39 |
| 1433863_at | Btf3 /// LOC640665 | basic transcription factor 3 /// similar to basic transcription factor 3 /// similar to ba | -1.32 | 0.115 | -1.18 | 0.493 | -1.58 | 0.07 | -1.18 | 0.502 | -1.31 |
| 1440247_at | Phf14 | PHD finger protein 14 | -1.58 | 0.196 | -1.09 | 0.757 | -1.46 | 0.35 | -2.15 | 0.033 | -1.57 |
| 1435480_at | Braf | Braf transforming gene | -1.15 | 0.279 | -1.29 | 0.001 | -1.67 | 0.03 | -1.17 | 0.07 | -1.32 |
| 1443862_at | --- | --- | -1.02 | 0.892 | -1.39 | 0.049 | -1.84 | 0.025 | -1.58 | 0.169 | -1.46 |
| 1416602_a_at | Rad52 | RAD52 homolog (S. cerevisiae) | -1.56 | 0.004 | -1.33 | 0.002 | -1.18 | 0.65 | -1.35 | 0.118 | -1.35 |
| 1432222_at | A930015G24Rik | RIKEN cDNA A930015G24 gene | -1.4 | 0.066 | -1.11 | 0.864 | -1.6 | 0.205 | -1.14 | 0.78 | -1.31 |
| 1456257_at | C130065N10Rik | RIKEN cDNA C130065N10 gene | -1.09 | 0.628 | -1.45 | 0.346 | -1.58 | 0.047 | -1.24 | 0.38 | -1.34 |
| 1456321_at | Npal1 | NIPA-like domain containing 1 | -1.73 | 0.673 | -1.04 | 0.933 | -1.43 | 0.247 | -1.98 | 0.019 | -1.54 |
| 1442749_at | Braf | Braf transforming gene | -1.01 | 0.958 | -1.49 | 0.281 | -1.71 | 0.032 | -1.49 | 0.333 | -1.43 |
| 1444909_at | --- | Transcribed locus | -2.56 | 0.187 | -1.15 | 0.522 | -1.02 | 0.94 | -1.18 | 0.785 | -1.48 |
| 1445231_at | B230112C05Rik | RIKEN cDNA B230112C05 gene | -1.58 | 0.114 | -1.07 | 0.642 | -1.48 | 0.42 | -1.71 | 0.229 | -1.46 |
| 1425223_at | Birc3 | baculoviral IAP repeat-containing 3 | -1.28 | 0.602 | -1.23 | 0.361 | -1.54 | 0.244 | -2.17 | 0.174 | -1.55 |
| 1444345_at | --- | Transcribed locus | -1.02 | 0.938 | -1.55 | 0.443 | -1.62 | 0.313 | -2.05 | 0.239 | -1.56 |
| 1440365_at | Actb | Actin, beta, cytoplasmic | -1.24 | 0.292 | -1.19 | 0.557 | -1.68 | 0.016 | -1.14 | 0.347 | -1.31 |
| 1447983_at | Zfml | Zinc finger, matrin-like | -1.84 | 0.28 | -1.13 | 0.853 | -1.23 | 0.55 | -1.46 | 0.354 | -1.41 |
| 1460546_at | Lgi3 | leucine-rich repeat LGI family, member 3 | -1.14 | 0.672 | -1.91 | 0.007 | -1.19 | 0.209 | -2.19 | 0.214 | -1.61 |
| 1447387_at | --- | --- | -1.23 | 0.464 | -1.26 | 0.573 | -1.58 | 0.261 | -1.41 | 0.674 | -1.37 |
| 1436093_at | Epha10 | Eph receptor A10 | -1.12 | 0.598 | -1.23 | 0.453 | -1.85 | 0.202 | -1.22 | 0.175 | -1.36 |
| 1446504_at | --- | --- | -1.01 | 0.933 | -1.49 | 0.043 | -1.71 | 0.056 | -1.3 | 0.268 | -1.38 |

| | | | | | | | | | | | |
|---|---|---|---|---|---|---|---|---|---|---|---|
| 1451980_at | Casd1 | CAS1 domain containing 1 | -1.3 | 0.351 | -1.24 | 0.105 | -1.5 | 0.002 | -1.47 | 0.09 | -1.38 |
| 1432813_at | 2900064F13Rik | RIKEN cDNA 2900064F13 gene | -1.33 | 0.621 | -1.64 | 0.402 | -1.13 | 0.574 | -1.38 | 0.275 | -1.37 |
| 1434311_at | Cnot6l | CCR4-NOT transcription complex, subunit 6-like | -1.22 | 0.176 | -1.26 | 0.289 | -1.59 | 0.049 | -1.28 | 0.289 | -1.34 |
| 1457398_at | Gdi2 | Guanosine diphosphate (GDP) dissociation inhibitor 2 | -1.18 | 0.071 | -1.3 | 0.13 | -1.59 | 0.008 | -1.81 | 0.12 | -1.47 |
| 1438842_at | Mtch2 | mitochondrial carrier homolog 2 (C. elegans) | -1.19 | 0.399 | -1.59 | 0.348 | -1.3 | 0.431 | -1.34 | 0.415 | -1.35 |
| 1441100_at | Mbtd1 | mbt domain containing 1 | -1.15 | 0.195 | -1.38 | 0.04 | -1.54 | 0.049 | -1.75 | 0.054 | -1.45 |
| 1446940_at | Traf6 | Tnf receptor-associated factor 6 | -2.01 | 0.343 | -1.02 | 0.982 | -1.31 | 0.435 | -1.09 | 0.902 | -1.36 |
| 1452646_at | Trp53inp2 | tumor protein p53 inducible nuclear protein 2 | -1.25 | 0.021 | -1.58 | 0.213 | -1.23 | 0.458 | -1.29 | 0.279 | -1.34 |
| 1429721_s_at | 4930579C15Rik | RIKEN cDNA 4930579C15 gene | -1.19 | 0.484 | -1.06 | 0.794 | -2.15 | 0.164 | -1.77 | 0.291 | -1.54 |
| 1439689_at | 9030224M15Rik | RIKEN cDNA 9030224M15 gene | -1.29 | 0.255 | -1.12 | 0.839 | -1.74 | 0.042 | -1.29 | 0.464 | -1.36 |
| 1429820_at | Rutbc2 | RUN and TBC1 domain containing 2 | -1.05 | 0.935 | -2.27 | 0.008 | -1.18 | 0.781 | -2.5 | 0.097 | -1.75 |
| 1438036_x_at | AW061290 | expressed sequence AW061290 | -1.32 | 0.27 | -1.44 | 0.029 | -1.26 | 0.226 | -1.09 | 0.707 | -1.28 |
| 1432915_at | 5730407I07Rik | RIKEN cDNA 5730407I07 gene | -1.31 | 0.742 | -1.25 | 0.591 | -1.47 | 0.4 | -1.89 | 0.317 | -1.48 |
| 1434467_at | Atcay | ataxia, cerebellar, Cayman type homolog (human) | -1.16 | 0.563 | -1.71 | 0.17 | -1.26 | 0.619 | -1.52 | 0.468 | -1.41 |
| 1440698_at | Unc5c | Unc-5 homolog C (C. elegans) | -1.27 | 0.109 | -1.35 | 0.621 | -1.39 | 0.309 | -1.01 | 0.988 | -1.25 |
| 1444647_at | Plaa | phospholipase A2, activating protein | -1.05 | 0.248 | -1.34 | 0.327 | -1.83 | 0.046 | -1.18 | 0.296 | -1.35 |
| 1441925_at | Slc15a4 | solute carrier family 15, member 4 | -1.77 | 0.518 | -1.25 | 0.091 | -1.13 | 0.631 | -2.13 | 0.401 | -1.57 |
| 1445589_at | Slc23a2 | Solute carrier family 23 (nucleobase transporters), member 2 | -1.01 | 0.979 | -1.23 | 0.221 | -2.29 | 0.002 | -1.44 | 0.152 | -1.49 |
| 1440653_at | Phip | Pleckstrin homology domain interacting protein | -1 | 0.992 | -2.61 | 0.064 | -1.15 | 0.707 | -1.17 | 0.317 | -1.49 |
| 1445733_at | 2610206G21Rik | RIKEN cDNA 2610206G21 gene | -1.29 | 0.115 | -1.1 | 0.82 | -1.77 | 0.269 | -1.22 | 0.697 | -1.35 |
| 1457775_at | Sacs | sacsin | -2.05 | 0.168 | -1.03 | 0.954 | -1.28 | 0.56 | -1.7 | 0.473 | -1.51 |
| 1447948_at | A430107O13Rik | RIKEN cDNA A430107O13 gene | -1.19 | 0.192 | -1.36 | 0.097 | -1.48 | 0.217 | -1.22 | 0.142 | -1.31 |
| 1437431_at | --- | --- | -1.44 | 0.328 | -1.03 | 0.903 | -1.73 | 0.096 | -1.51 | 0.181 | -1.43 |
| 1458790_at | --- | --- | -1.45 | 0.484 | -1.22 | 0.691 | -1.35 | 0.642 | -2.33 | 0.36 | -1.59 |
| 1442535_at | Zfp609 | Zinc finger protein 609 | -1.18 | 0.04 | -1.18 | 0.52 | -1.8 | 0.008 | -1.26 | 0.443 | -1.36 |
| 1440223_at | Rbm6 | RNA binding motif protein 6 | -1.07 | 0.499 | -1.29 | 0.001 | -1.85 | 0.006 | -1.34 | 0.085 | -1.39 |
| 1418559_at | Tesp1 | testicular serine protease 1 | -1.3 | 0.728 | -1.61 | 0.445 | -1.17 | 0.756 | -1.31 | 0.701 | -1.35 |
| 1440649_at | Tes | Testis derived transcript | -1.21 | 0.492 | -1.83 | 0.202 | -1.14 | 0.765 | -1.75 | 0.081 | -1.48 |
| 1456771_at | Zyg11bl | zyg-11 homolog B (C. elegans)-like | -1.13 | 0.322 | -2.2 | 0.026 | -1.1 | 0.656 | -1.73 | 0.261 | -1.54 |
| 1456453_at | Evl | Ena-vasodilator stimulated phosphoprotein | -2.03 | 0.166 | -1.13 | 0.504 | -1.15 | 0.456 | -2.58 | 0.03 | -1.72 |
| 1444496_at | 2600011C06Rik | RIKEN cDNA 2600011C06 gene | -1.48 | 0.117 | -1.52 | 0.16 | -1.09 | 0.754 | -1.69 | 0.197 | -1.45 |
| 1425292_at | Dtna | dystrobrevin alpha | -1.17 | 0.048 | -1.21 | 0.643 | -1.75 | 0.043 | -1.47 | 0.6 | -1.4 |
| 1437073_x_at | AV025504 | expressed sequence AV025504 | -1.02 | 0.707 | -2.78 | 0.004 | -1.1 | 0.43 | -1.08 | 0.367 | -1.49 |
| 1447016_at | Tbc1d1 | TBC1 domain family, member 1 | -1.48 | 0.073 | -1.51 | 0.289 | -1.1 | 0.745 | -2.69 | 0.076 | -1.69 |
| 1460119_at | --- | 16 days embryo lung cDNA, RIKEN full-length enriched library, clone:8430415C1 | -1.12 | 0.547 | -1.26 | 0.357 | -1.78 | 0.079 | -1.3 | 0.111 | -1.36 |
| 1449068_at | Zfp148 | zinc finger protein 148 | -1.16 | 0.212 | -1.32 | 0.022 | -1.59 | 0.054 | -1.36 | 0.114 | -1.36 |
| 1417757_at | Unc13b | unc-13 homolog B (C. elegans) | -1.63 | 0.285 | -1.04 | 0.91 | -1.49 | 0.132 | -1.16 | 0.711 | -1.33 |
| 1424272_at | Stat3 | signal transducer and activator of transcription 3 | -1.57 | 0.302 | -1.52 | 0.36 | -1.05 | 0.757 | -1.32 | 0.54 | -1.36 |
| 1449542_at | Pbx1 | pre B-cell leukemia transcription factor 1 | -1.13 | 0.589 | -1.4 | 0.315 | -1.53 | 0.083 | -1.99 | 0.015 | -1.52 |
| 1440931_at | Scrt2 | scratch homolog 2, zinc finger protein (Drosophila) | -1.48 | 0.595 | -1.06 | 0.632 | -1.58 | 0.467 | -1.09 | 0.708 | -1.3 |
| 1441490_at | Tmem39a | transmembrane protein 39a | -1.93 | 0.008 | -1.05 | 0.736 | -1.28 | 0.109 | -1.26 | 0.317 | -1.38 |
| 1454229_a_at | Exod1 | exonuclease domain containing 1 | -1.52 | 0.117 | -1.21 | 0.66 | -1.31 | 0.578 | -1.06 | 0.922 | -1.28 |
| 1439958_at | LOC546257 /// LOC | similar to transcription elongation factor B polypeptide 3 binding protein 1 isoform | -1.69 | 0.464 | -1.08 | 0.828 | -1.37 | 0.285 | -1.26 | 0.609 | -1.35 |
| 1443169_at | Cnot4 | CCR4-NOT transcription complex, subunit 4 | -1.81 | 0.121 | -1.41 | 0.587 | -1.01 | 0.977 | -1.22 | 0.289 | -1.36 |
| 1429131_at | Ube2v2 | ubiquitin-conjugating enzyme E2 variant 2 | -1.45 | 0.089 | -1.13 | 0.517 | -1.49 | 0.112 | -1.1 | 0.253 | -1.29 |
| 1448700_at | G0s2 | G0/G1 switch gene 2 | -1.18 | 0.462 | -1.33 | 0.56 | -1.53 | 0.244 | -1.46 | 0.016 | -1.38 |
| 1449125_at | Tnfaip8l1 | tumor necrosis factor, alpha-induced protein 8-like 1 | -1.71 | 0.394 | -1.44 | 0.091 | -1.03 | 0.81 | -1.3 | 0.089 | -1.37 |
| 1442750_at | --- | Transcribed locus | -1.29 | 0.673 | -1.02 | 0.923 | -2 | 0.068 | -1.03 | 0.905 | -1.34 |
| 1442392_at | --- | 0 day neonate thymus cDNA, RIKEN full-length enriched library, clone:A430024M | -1.32 | 0.216 | -1.64 | 0.523 | -1.13 | 0.699 | -1.08 | 0.6 | -1.29 |
| 1459906_at | Dgkh | Diacylglycerol kinase, eta | -1.3 | 0.422 | -1.52 | 0.119 | -1.21 | 0.248 | -1.42 | 0 | -1.36 |
| 1456656_at | Lin7a | lin-7 homolog A (C. elegans) | -1.35 | 0.517 | -1.4 | 0.112 | -1.25 | 0.591 | -1.05 | 0.924 | -1.26 |
| 1460128_at | Tmed2 | Transmembrane emp24 domain trafficking protein 2 | -1.74 | 0.165 | -1.04 | 0.938 | -1.4 | 0.15 | -1.39 | 0.645 | -1.39 |
| 1441964_at | 1110003F05Rik | RIKEN cDNA 1110003F05 gene | -1.24 | 0.265 | -1.29 | 0.116 | -1.5 | 0.103 | -1.17 | 0.771 | -1.3 |

| | | | | | | | | | | | |
|---|---|---|---|---|---|---|---|---|---|---|---|
| 1441002_at | Pop1 | processing of precursor 1, ribonuclease P/MRP family, (S. cerevisiae) | -1.48 | 0.423 | -1.04 | 0.926 | -1.62 | 0.101 | -1.61 | 0.068 | -1.44 |
| 1442907_at | --- | Adult male aorta and vein cDNA, RIKEN full-length enriched library, clone:A5300 | -1.4 | 0.429 | -1.73 | 0.112 | -1.04 | 0.871 | -1.09 | 0.696 | -1.32 |
| 1422148_at | Matn3 | matrilin 3 | -1.81 | 0.532 | -1.02 | 0.974 | -1.39 | 0.398 | -1.77 | 0.389 | -1.5 |
| 1428940_at | Gnaq | guanine nucleotide binding protein, alpha q polypeptide | -1.29 | 0.022 | -1.51 | 0.142 | -1.23 | 0.078 | -1.08 | 0.821 | -1.28 |
| 1447719_at | Apoh | Apolipoprotein H | -2.01 | 0.185 | -1.14 | 0.486 | -1.14 | 0.733 | -1.33 | 0.471 | -1.4 |
| 1454839_a_at | Ccdc84 | coiled-coil domain containing 84 | -1.31 | 0.287 | -1.41 | 0.029 | -1.29 | 0.02 | -1.44 | 0.087 | -1.36 |
| 1444902_at | Fbxl10 | F-box and leucine-rich repeat protein 10 | -1.36 | 0.037 | -1.23 | 0.243 | -1.42 | 0.179 | -1.25 | 0.245 | -1.32 |
| 1421652_at | Htr3b | 5-hydroxytryptamine (serotonin) receptor 3B | -1.65 | 0.334 | -1.21 | 0.799 | -1.22 | 0.708 | -2.5 | 0.164 | -1.65 |
| 1438034_at | 2410005O16Rik | RIKEN cDNA 2410005O16 gene | -1.29 | 0.316 | -1.23 | 0.028 | -1.52 | 0.13 | -1.19 | 0.351 | -1.31 |
| 1431024_a_at | Arid4b | AT rich interactive domain 4B (Rbp1 like) | -1.41 | 0.298 | -1.36 | 0.109 | -1.24 | 0.345 | -1.18 | 0.543 | -1.3 |
| 1431660_at | 4930564D02Rik | RIKEN cDNA 4930564D02 gene | -1.08 | 0.819 | -1.41 | 0.278 | -1.62 | 0.483 | -1.57 | 0.333 | -1.42 |
| 1430397_at | Got2 /// LOC64084 | glutamate oxaloacetate transaminase 2, mitochondrial /// similar to Aspartate ami | -1.03 | 0.741 | -1.93 | 0.166 | -1.31 | 0.456 | -1.52 | 0.166 | -1.45 |
| 1437037_x_at | AL033314 | expressed sequence AL033314 | -1.46 | 0.341 | -1.37 | 0.406 | -1.19 | 0.711 | -1.51 | 0.163 | -1.38 |
| 1418863_at | Gata4 | GATA binding protein 4 | -1.51 | 0.304 | -1.27 | 0.361 | -1.25 | 0.662 | -4.28 | 0 | -2.08 |
| 1454367_at | 4930413G21Rik | RIKEN cDNA 4930413G21 gene | -1.09 | 0.868 | -1.46 | 0.202 | -1.53 | 0.191 | -1.1 | 0.678 | -1.3 |
| 1422419_s_at | Tnp2 | transition protein 2 | -1.38 | 0.004 | -1.12 | 0.825 | -1.58 | 0.141 | -1.23 | 0.165 | -1.33 |
| 1458507_at | 2810055G22Rik | RIKEN cDNA 2810055G22 gene | -1.94 | 0.315 | -1.06 | 0.91 | -1.25 | 0.275 | -1.34 | 0.425 | -1.4 |
| 1449995_at | 4933421I07Rik | RIKEN cDNA 4933421I07 gene | -1.36 | 0.162 | -1.5 | 0.273 | -1.17 | 0.723 | -1.47 | 0.547 | -1.38 |
| 1430014_at | 5430419D17Rik | RIKEN cDNA 5430419D17 gene | -1.5 | 0.506 | -1.12 | 0.715 | -1.44 | 0.216 | -1.63 | 0.236 | -1.42 |
| 1455687_at | Ick | intestinal cell kinase | -1.22 | 0.591 | -1.42 | 0.175 | -1.36 | 0.213 | -1.4 | 0.448 | -1.35 |
| 1454984_at | AW061234 | expressed sequence AW061234 | -1.12 | 0.656 | -1.63 | 0.093 | -1.34 | 0.163 | -1.49 | 0.126 | -1.4 |
| 1442497_at | Smchd1 | SMC hinge domain containing 1 | -1.32 | 0.562 | -1.84 | 0.043 | -1.05 | 0.853 | -1.21 | 0.306 | -1.35 |
| 1454233_at | 2310006M14Rik | RIKEN cDNA 2310006M14 gene | -1.45 | 0.381 | -1.39 | 0.273 | -1.18 | 0.548 | -2.69 | 0.06 | -1.68 |
| 1433212_at | 2700008E08Rik | RIKEN cDNA 2700008E08 gene | -1.17 | 0.738 | -1.47 | 0.217 | -1.39 | 0.152 | -1.12 | 0.74 | -1.29 |
| 1438035_at | AW061290 | expressed sequence AW061290 | -1.32 | 0.237 | -1.43 | 0.019 | -1.26 | 0.289 | -1.18 | 0.235 | -1.3 |
| 1440028_at | Atg7 | Autophagy-related 7 (yeast) | -1.19 | 0.294 | -1.16 | 0.669 | -1.83 | 0.135 | -1.77 | 0.08 | -1.48 |
| 1442079_at | Tmem23 | transmembrane protein 23 | -1.12 | 0.037 | -1.22 | 0.616 | -1.83 | 0.024 | -1.24 | 0.517 | -1.35 |
| 1423934_at | --- | --- | -1.39 | 0.163 | -1.38 | 0.422 | -1.23 | 0.518 | -1.92 | 0.22 | -1.48 |
| 1443286_at | AU019754 | expressed sequence AU019754 | -1.24 | 0.564 | -1.23 | 0.648 | -1.57 | 0.163 | -1.06 | 0.893 | -1.28 |
| 1459680_at | Tomm40 | Translocase of outer mitochondrial membrane 40 homolog (yeast) | -1.2 | 0.326 | -1.57 | 0.228 | -1.27 | 0.586 | -1.1 | 0.619 | -1.28 |
| 1417319_at | Pvrl3 | poliovirus receptor-related 3 | -1.22 | 0.056 | -1.07 | 0.582 | -1.98 | 0.009 | -1.55 | 0.172 | -1.46 |
| 1427714_at | Smyd1 | SET and MYND domain containing 1 | -1.56 | 0.542 | -1.17 | 0.31 | -1.32 | 0.588 | -1.11 | 0.725 | -1.29 |
| 1429811_at | 4933424B01Rik | RIKEN cDNA 4933424B01 gene | -1.48 | 0.178 | -1.42 | 0.114 | -1.14 | 0.665 | -1.37 | 0.099 | -1.35 |
| 1455878_at | 2700023E23Rik | RIKEN cDNA 2700023E23 gene | -1.27 | 0.164 | -1.44 | 0.141 | -1.29 | 0.191 | -1.24 | 0.528 | -1.31 |
| 1453390_at | 4930428F12Rik | RIKEN cDNA 4930428F12 gene | -1.22 | 0.542 | -1.49 | 0.225 | -1.3 | 0.386 | -2.34 | 0.301 | -1.59 |
| 1431911_at | 4931423N10Rik | RIKEN cDNA 4931423N10 gene | -1.27 | 0.479 | -1.61 | 0.561 | -1.18 | 0.334 | -1 | 0.997 | -1.27 |
| 1430075_at | Sf3b3 | splicing factor 3b, subunit 3 | -1.25 | 0.254 | -1.16 | 0.563 | -1.67 | 0.022 | -1.38 | 0.2 | -1.37 |
| 1450523_at | Cntn2 | contactin 2 | -1.39 | 0.412 | -1.26 | 0.202 | -1.35 | 0.255 | -1.45 | 0.718 | -1.36 |
| 1458643_at | Dtx1 | deltex 1 homolog (Drosophila) | -1.92 | 0.424 | -1.33 | 0.513 | -1.02 | 0.847 | -1.25 | 0.698 | -1.38 |
| 1457896_at | Esrrg | estrogen-related receptor gamma | -1.31 | 0.058 | -1.57 | 0.333 | -1.17 | 0.208 | -1.99 | 0.196 | -1.51 |
| 1458464_at | Hecw2 | HECT, C2 and WW domain containing E3 ubiquitin protein ligase 2 | -1.07 | 0.91 | -1.23 | 0.538 | -1.95 | 0.073 | -1.84 | 0.082 | -1.52 |
| 1418721_at | Cops7b | COP9 (constitutive photomorphogenic) homolog, subunit 7b (Arabidopsis thaliana | -1.09 | 0.476 | -1.49 | 0.036 | -1.5 | 0.01 | -1.3 | 0.04 | -1.34 |
| 1452602_a_at | 1700001C19Rik | RIKEN cDNA 1700001C19 gene | -2.1 | 0.248 | -1.01 | 0.964 | -1.26 | 0.517 | -1.24 | 0.691 | -1.4 |
| 1439192_at | Nova2 | neuro-oncological ventral antigen 2 | -1.44 | 0.247 | -1.22 | 0.178 | -1.34 | 0.391 | -1.23 | 0.338 | -1.31 |
| 1458632_at | Dpysl2 | Dihydropyrimidinase-like 2 | -1.55 | 0.335 | -1.2 | 0.776 | -1.28 | 0.328 | -1.38 | 0.029 | -1.35 |
| 1423633_at | 6530403A03Rik | RIKEN cDNA 6530403A03 gene | -1.01 | 0.975 | -1.48 | 0.101 | -1.67 | 0.152 | -1.2 | 0.08 | -1.34 |
| 1459821_x_at | A630082K20Rik | RIKEN cDNA A630082K20 gene | -1.31 | 0.257 | -1.58 | 0.605 | -1.16 | 0.643 | -1.63 | 0.496 | -1.42 |
| 1458144_at | BC031353 | CDNA sequence BC031353 | -1 | 0.997 | -1.81 | 0.105 | -1.42 | 0.341 | -1.31 | 0.57 | -1.38 |
| 1459923_at | Bex6 | brain expressed gene 6 | -1 | 0.998 | -1.7 | 0.077 | -1.49 | 0.316 | -2.24 | 0.021 | -1.61 |
| 1459743_at | --- | Transcribed locus | -1.68 | 0.342 | -1.11 | 0.607 | -1.31 | 0.343 | -1.41 | 0.475 | -1.38 |
| 1451375_at | Ehf | ets homologous factor | -1.27 | 0.38 | -1.36 | 0.55 | -1.36 | 0.037 | -1.48 | 0.22 | -1.37 |
| 1456955_at | --- | --- | -1.21 | 0.383 | -1.43 | 0.026 | -1.36 | 0.227 | -1.25 | 0.553 | -1.31 |
| 1432752_at | 4930403O18Rik | RIKEN cDNA 4930403O18 gene | -1.63 | 0.429 | -1.27 | 0.711 | -1.17 | 0.844 | -1.02 | 0.976 | -1.27 |

| | | | | | | | | | | | |
|---|---|---|---|---|---|---|---|---|---|---|---|
| 1438828_at | Rapgef6 | Rap guanine nucleotide exchange factor (GEF) 6 | -1.23 | 0.39 | -1.5 | 0.314 | -1.28 | 0.121 | -2.05 | 0.053 | -1.51 |
| 1441880_x_at | MGC30332 | hypothetical protein MGC30332 | -1.3 | 0.42 | -1.24 | 0.288 | -1.46 | 0.069 | -1.65 | 0.104 | -1.41 |
| 1420974_at | Setdb1 | SET domain, bifurcated 1 | -1.2 | 0.5 | -1.36 | 0.087 | -1.45 | 0.096 | -1.05 | 0.84 | -1.26 |
| 1432852_at | Phactr1 | phosphatase and actin regulator 1 | -1.28 | 0.621 | -1.22 | 0.189 | -1.51 | 0.162 | -3.66 | 0.13 | -1.92 |
| 1456868_at | 2900073G15Rik | RIKEN cDNA 2900073G15 gene | -1.13 | 0.613 | -1.13 | 0.792 | -2.04 | 0.028 | -1.28 | 0.333 | -1.4 |
| 1420220_x_at | 4930461P20Rik | RIKEN cDNA 4930461P20 gene | -1.28 | 0.531 | -1.19 | 0.681 | -1.56 | 0.162 | -1.1 | 0.877 | -1.28 |
| 1420147_at | AA407331 | expressed sequence AA407331 | -2.18 | 0.42 | -1.12 | 0.805 | -1.1 | 0.851 | -1.79 | 0.386 | -1.55 |
| 1432465_at | 4933414I06Rik | RIKEN cDNA 4933414I06 gene | -1.86 | 0.066 | -1.22 | 0.396 | -1.1 | 0.566 | -1.09 | 0.737 | -1.32 |
| 1450083_at | Cnot4 | CCR4-NOT transcription complex, subunit 4 | -1.18 | 0.396 | -1.39 | 0.214 | -1.45 | 0.312 | -1.02 | 0.939 | -1.26 |
| 1455751_at | Cand1 | cullin associated and neddylation disassociated 1 | -1.28 | 0.736 | -1.84 | 0.298 | -1.07 | 0.662 | -1.08 | 0.78 | -1.32 |
| 1457388_at | A830080L01Rik | RIKEN cDNA A830080L01 gene | -1.15 | 0.829 | -2.26 | 0.252 | -1.05 | 0.914 | -1.19 | 0.656 | -1.41 |
| 1459009_at | Utrn | Utrophin | -1.13 | 0.611 | -1.09 | 0.76 | -2.18 | 0.027 | -1.88 | 0.118 | -1.57 |
| 1437440_at | --- | Transcribed locus | -1.3 | 0.691 | -1.2 | 0.738 | -1.52 | 0.357 | -1.11 | 0.589 | -1.28 |
| 1430114_at | 5430420C16Rik | RIKEN cDNA 5430420C16 gene | -1.04 | 0.952 | -1.6 | 0.563 | -1.47 | 0.493 | -1.54 | 0.355 | -1.41 |
| 1428923_at | Ppp1r3g | protein phosphatase 1, regulatory (inhibitor) subunit 3G | -1.5 | 0.494 | -1.14 | 0.847 | -1.39 | 0.183 | -1.57 | 0.196 | -1.4 |
| 1420724_at | 1700067P10Rik | RIKEN cDNA 1700067P10 gene | -1.19 | 0.688 | -1.18 | 0.434 | -1.73 | 0.482 | -2.78 | 0.212 | -1.72 |
| 1424464_s_at | 2210010L05Rik | RIKEN cDNA 2210010L05 gene | -1.27 | 0.243 | -1.1 | 0.777 | -1.76 | 0.058 | -1.11 | 0.687 | -1.31 |
| 1438721_a_at | Irf3 | interferon regulatory factor 3 | -1.59 | 0.24 | -1.14 | 0.723 | -1.32 | 0.044 | -1.55 | 0.281 | -1.4 |
| 1442456_at | Spata5 | spermatogenesis associated 5 | -1.56 | 0.022 | -1.12 | 0.69 | -1.37 | 0.424 | -1.42 | 0.564 | -1.37 |
| 1442151_at | D230040A04Rik | RIKEN cDNA D230040A04 gene | -1.04 | 0.643 | -1.17 | 0.358 | -2.27 | 0.026 | -1.42 | 0.034 | -1.47 |
| 1438197_at | Vezt | vezatin, adherens junctions transmembrane protein | -1.24 | 0.215 | -1.23 | 0.032 | -1.55 | 0.013 | -1.02 | 0.936 | -1.26 |
| 1429114_at | Sestd1 | SEC14 and spectrin domains 1 | -1.26 | 0.098 | -1.34 | 0.312 | -1.38 | 0.135 | -1.36 | 0.122 | -1.34 |
| 1425817_a_at | Slc8a1 | solute carrier family 8 (sodium/calcium exchanger), member 1 | -1.33 | 0.208 | -1.34 | 0.269 | -1.31 | 0.109 | -1.12 | 0.812 | -1.27 |
| 1427549_s_at | Krtap16-10 /// Krtap | keratin associated protein 16-10 /// keratin associated protein 16-3 /// keratin assoc | -1.33 | 0.488 | -1.48 | 0.354 | -1.2 | 0.706 | -1.5 | 0.668 | -1.38 |
| 1445975_at | --- | --- | -1.17 | 0.186 | -1.39 | 0.163 | -1.45 | 0.074 | -1.2 | 0.239 | -1.3 |
| 1447344_at | --- | Transcribed locus | -1.02 | 0.646 | -1.59 | 0.036 | -1.53 | 0.034 | -1.13 | 0.499 | -1.32 |
| 1456821_at | 4932439K10Rik | RIKEN cDNA 4932439K10 gene | -1.27 | 0.619 | -1.33 | 0.351 | -1.38 | 0.163 | -1.6 | 0.099 | -1.4 |
| 1444248_at | Rcn2 | reticulocalbin 2 | -1.23 | 0.468 | -1.33 | 0.511 | -1.43 | 0.263 | -1.76 | 0.237 | -1.44 |
| 1459862_at | --- | Transcribed locus | -1.18 | 0.604 | -1.15 | 0.789 | -1.82 | 0.012 | -1.06 | 0.897 | -1.3 |
| 1430195_at | 2810043O03Rik | RIKEN cDNA 2810043O03 gene | -1.16 | 0.466 | -1.28 | 0.152 | -1.61 | 0.011 | -1.45 | 0.295 | -1.37 |
| 1446584_at | Txndc4 | Thioredoxin domain containing 4 (endoplasmic reticulum) | -1.25 | 0.25 | -1.25 | 0.658 | -1.51 | 0.465 | -2.1 | 0.12 | -1.53 |
| 1424860_at | D930016D06Rik | RIKEN cDNA D930016D06 gene | -1.33 | 0.273 | -1.15 | 0.279 | -1.55 | 0.004 | -1.13 | 0.634 | -1.29 |
| 1459408_at | Sec22a | SEC22 vesicle trafficking protein-like A (S. cerevisiae) | -2.4 | 0.215 | -1.1 | 0.771 | -1.06 | 0.946 | -1.11 | 0.788 | -1.42 |
| 1445259_at | Scn2a1 | sodium channel, voltage-gated, type II, alpha 1 | -1.4 | 0.582 | -1.18 | 0.698 | -1.41 | 0.297 | -1.47 | 0.374 | -1.37 |
| 1430737_at | 2810409K11Rik | RIKEN cDNA 2810409K11 gene | -1.12 | 0.861 | -1.2 | 0.797 | -1.83 | 0.053 | -1.86 | 0.324 | -1.5 |
| 1444079_at | Defb8 | defensin beta 8 | -1.59 | 0.371 | -1.33 | 0.259 | -1.13 | 0.677 | -1.86 | 0.011 | -1.48 |
| 1453356_at | 5730453I16Rik | RIKEN cDNA 5730453I16 gene | -1.01 | 0.967 | -1.29 | 0.435 | -1.99 | 0.069 | -1.64 | 0.213 | -1.48 |
| 1425903_at | Sema6a | sema domain, transmembrane domain (TM), and cytoplasmic domain, (semapho | -1.07 | 0.904 | -1.55 | 0.573 | -1.45 | 0.361 | -1.22 | 0.676 | -1.32 |
| 1436925_at | Ches1 | checkpoint suppressor 1 | -1.38 | 0.071 | -1.25 | 0.002 | -1.34 | 0.223 | -1.6 | 0.117 | -1.39 |
| 1455166_at | AW146430 | expressed sequence AW146430 | -1.18 | 0.156 | -1.27 | 0.036 | -1.58 | 0.009 | -1.38 | 0.019 | -1.35 |
| 1428010_at | Timm9 | translocase of inner mitochondrial membrane 9 homolog (yeast) | -1.11 | 0.489 | -1.51 | 0.142 | -1.41 | 0.238 | -1.59 | 0.023 | -1.41 |
| 1435466_at | A830091I15Rik | RIKEN cDNA A830091I15 gene | -1.39 | 0.071 | -1.63 | 0.457 | -1.07 | 0.89 | -1.63 | 0.347 | -1.43 |
| 1443192_at | --- | --- | -2.14 | 0.098 | -1.12 | 0.794 | -1.1 | 0.506 | -2.37 | 0.054 | -1.68 |
| 1420606_at | Npff | neuropeptide FF-amide peptide precursor | -1.48 | 0.529 | -1.03 | 0.954 | -1.61 | 0.093 | -1.39 | 0.292 | -1.38 |
| 1440961_at | 9130604C24Rik | RIKEN cDNA 9130604C24 gene | -1.58 | 0.373 | -1.55 | 0.276 | -1.01 | 0.973 | -1.43 | 0.095 | -1.39 |
| 1458660_at | AI414330 | expressed sequence AI414330 | -1.28 | 0.559 | -1.75 | 0.207 | -1.09 | 0.725 | -1.12 | 0.723 | -1.31 |
| 1439846_at | Klf12 | Kruppel-like factor 12 | -1.22 | 0.43 | -1.35 | 0.281 | -1.41 | 0.216 | -1.16 | 0.614 | -1.29 |
| 1443891_at | Rad51l3 | RAD51-like 3 (S. cerevisiae) | -1.18 | 0.654 | -1.54 | 0.078 | -1.29 | 0.489 | -1.21 | 0.406 | -1.3 |
| 1425355_at | BC018371 /// LOC4 | cDNA sequence BC018371 /// hypothetical LOC433604 | -1.33 | 0.553 | -1.22 | 0.498 | -1.43 | 0.414 | -1.12 | 0.86 | -1.28 |
| 1441185_at | Msi2 | Musashi homolog 2 (Drosophila) | -1.25 | 0.181 | -1.08 | 0.84 | -1.83 | 0.075 | -1.43 | 0.111 | -1.4 |
| 1456991_at | Cobll1 | Cobl-like 1 | -1.14 | 0.603 | -1.18 | 0.218 | -1.83 | 0.009 | -1.97 | 0.119 | -1.53 |
| 1439887_at | --- | 16 days embryo head cDNA, RIKEN full-length enriched library, clone:C130025B | -1.41 | 0.1 | -1.22 | 0.521 | -1.35 | 0.36 | -1.03 | 0.932 | -1.25 |
| 1432880_at | 6820402A03Rik | RIKEN cDNA 6820402A03 gene | -1.09 | 0.678 | -1.65 | 0.007 | -1.34 | 0.228 | -2.45 | 0.002 | -1.63 |

| | | | | | | | | | | | |
|---|---|---|---|---|---|---|---|---|---|---|---|
| 1429057_at | Narg1l | NMDA receptor regulated 1-like | -1.2 | 0.347 | -1.3 | 0.046 | -1.5 | 0.057 | -1.01 | 0.984 | -1.25 |
| 1435194_at | --- | --- | -1.41 | 0.119 | -1.34 | 0.543 | -1.23 | 0.509 | -1.03 | 0.879 | -1.25 |
| 1448096_at | Ogfod1 | 2-oxoglutarate and iron-dependent oxygenase domain containing 1 | -1.39 | 0.045 | -1.21 | 0.124 | -1.38 | 0.162 | -1.34 | 0.167 | -1.33 |
| 1444742_at | Asb14 | Ankyrin repeat and SOCS box-containing protein 14 | -1.67 | 0.328 | -1.1 | 0.872 | -1.31 | 0.53 | -1.98 | 0.19 | -1.51 |
| 1447408_at | Nipbl | Nipped-B homolog (Drosophila) | -1.1 | 0.313 | -1.37 | 0.001 | -1.57 | 0.035 | -1.41 | 0.045 | -1.36 |
| 1430555_s_at | Lrig3 | leucine-rich repeats and immunoglobulin-like domains 3 | -1.2 | 0.283 | -1.44 | 0.088 | -1.34 | 0.303 | -1.11 | 0.889 | -1.27 |
| 1423249_at | Nktr | natural killer tumor recognition sequence | -1.51 | 0.298 | -1.36 | 0.216 | -1.14 | 0.565 | -1.53 | 0.127 | -1.38 |
| 1440377_at | Tigd5 | tigger transposable element derived 5 | -1.18 | 0.851 | -1.66 | 0.513 | -1.21 | 0.678 | -1.2 | 0.699 | -1.31 |
| 1438771_at | Brd1 | bromodomain containing 1 | -1.11 | 0.674 | -1.63 | 0.007 | -1.32 | 0.21 | -1.36 | 0.231 | -1.35 |
| 1443253_at | Glis3 | GLIS family zinc finger 3 | -1 | 0.993 | -1.14 | 0.567 | -2.49 | 0.008 | -1.4 | 0.272 | -1.51 |
| 1460082_at | Ing3 | inhibitor of growth family, member 3 | -1.33 | 0.563 | -1.56 | 0.101 | -1.14 | 0.316 | -1.55 | 0.267 | -1.39 |
| 1447023_at | --- | --- | -1.12 | 0.619 | -1.94 | 0.009 | -1.16 | 0.261 | -1.09 | 0.485 | -1.33 |
| 1418777_at | Ccl25 | chemokine (C-C motif) ligand 25 | -1.33 | 0.097 | -1.56 | 0.21 | -1.14 | 0.551 | -1.02 | 0.954 | -1.26 |
| 1447400_at | Ryk | receptor-like tyrosine kinase | -1.08 | 0.855 | -2.29 | 0.191 | -1.09 | 0.765 | -1.61 | 0.542 | -1.52 |
| 1434511_at | Phkb | phosphorylase kinase beta | -1.18 | 0.546 | -1.48 | 0.011 | -1.33 | 0.186 | -1.22 | 0.365 | -1.3 |
| 1460147_at | A730009E18Rik | RIKEN cDNA A730009E18 gene | -1.08 | 0.827 | -1.16 | 0.67 | -2.05 | 0.032 | -1.13 | 0.856 | -1.35 |
| 1438363_at | LOC434128 | similar to KIAA1183 protein | -1.5 | 0.431 | -1.47 | 0.396 | -1.08 | 0.707 | -2.27 | 0.14 | -1.58 |
| 1459570_at | Gpr143 | G protein-coupled receptor 143 | -1.38 | 0.567 | -1.49 | 0.596 | -1.14 | 0.823 | -1.05 | 0.886 | -1.27 |
| 1460200_s_at | Lztfl1 | leucine zipper transcription factor-like 1 | -1.37 | 0.151 | -1.21 | 0.112 | -1.39 | 0.29 | -1.29 | 0.368 | -1.32 |
| 1437387_at | Susd5 | sushi domain containing 5 | -1.2 | 0.712 | -1.54 | 0.238 | -1.27 | 0.584 | -1.68 | 0.384 | -1.42 |
| 1456526_at | C130034I24Rik | RIKEN cDNA C130034I24 gene | -1.07 | 0.349 | -1.24 | 0.385 | -1.86 | 0.007 | -1.43 | 0.124 | -1.4 |
| 1441550_at | 9330184L24Rik | RIKEN cDNA 9330184L24 gene | -2.41 | 0.465 | -1 | 1 | -1.16 | 0.832 | -1.84 | 0.322 | -1.6 |
| 1451160_s_at | Pvr | poliovirus receptor | -1.55 | 0.095 | -1.17 | 0.586 | -1.29 | 0.346 | -1.19 | 0.317 | -1.3 |
| 1456916_at | Nsd1 | Nuclear receptor-binding SET-domain protein 1 | -1.22 | 0.418 | -1.15 | 0.243 | -1.69 | 0.03 | -1.44 | 0.208 | -1.38 |
| 1454617_at | Arrdc3 | arrestin domain containing 3 | -1.25 | 0.234 | -1.22 | 0.507 | -1.54 | 0.039 | -1.06 | 0.831 | -1.27 |
| 1446598_at | Prkca | Protein kinase C, alpha | -1.38 | 0.488 | -1.11 | 0.712 | -1.53 | 0.16 | -1.33 | 0.313 | -1.34 |
| 1430220_at | 4833420G17Rik | RIKEN cDNA 4833420G17 gene | -1.23 | 0.446 | -1.21 | 0.474 | -1.56 | 0.143 | -1.36 | 0.126 | -1.34 |
| 1445182_at | Zfp672 | Zinc finger protein 672 | -1.08 | 0.768 | -1.11 | 0.698 | -2.2 | 0.211 | -2.38 | 0.004 | -1.69 |
| 1445941_at | Atbf1 | AT motif binding factor 1 | -1.18 | 0.489 | -1.3 | 0.257 | -1.51 | 0.103 | -1.3 | 0.456 | -1.32 |
| 1442710_at | Pdlim5 | PDZ and LIM domain 5 | -1.22 | 0.447 | -1.19 | 0.226 | -1.62 | 0.076 | -1.4 | 0.23 | -1.36 |
| 1445758_at | BC049816 | CDNA sequence BC049816 | -1.34 | 0.063 | -1.06 | 0.209 | -1.7 | 0.019 | -1.44 | 0.065 | -1.39 |
| 1443126_at | Slc12a1 | Solute carrier family 12, member 1 | -1.25 | 0.027 | -1.08 | 0.838 | -1.83 | 0.249 | -1.64 | 0.444 | -1.45 |
| 1422216_at | Mid2 | midline 2 | -1.14 | 0.489 | -1.62 | 0.06 | -1.28 | 0.364 | -1.05 | 0.67 | -1.27 |
| 1459682_at | LOC434249 | hypothetical LOC434249 | -1.12 | 0.637 | -1.6 | 0.12 | -1.32 | 0.428 | -1.64 | 0.218 | -1.42 |
| 1448673_at | Pvrl3 | poliovirus receptor-related 3 | -1.23 | 0.179 | -1.14 | 0.01 | -1.7 | 0.007 | -1.41 | 0.108 | -1.37 |
| 1458048_at | 6720406K03 | hypothetical protein 6720406K03 | -1.26 | 0.536 | -1.2 | 0.664 | -1.53 | 0.247 | -1.61 | 0.633 | -1.4 |
| 1457346_at | --- | Transcribed locus | -1.02 | 0.984 | -1.85 | 0.307 | -1.33 | 0.533 | -2.32 | 0.383 | -1.63 |
| 1455987_at | Sec61a1 | Sec61 alpha 1 subunit (S. cerevisiae) | -1.11 | 0.632 | -1.5 | 0.044 | -1.4 | 0.177 | -1.47 | 0.049 | -1.37 |
| 1442738_at | Crebl2 | CAMP responsive element binding protein-like 2 | -1.15 | 0.438 | -1.1 | 0.871 | -1.99 | 0.286 | -1.95 | 0.062 | -1.55 |
| 1446293_at | Bcl11a | B-cell CLL/lymphoma 11A (zinc finger protein) | -1.39 | 0.077 | -1.52 | 0.274 | -1.11 | 0.667 | -1.25 | 0.404 | -1.32 |
| 1438310_at | --- | 10 days neonate cerebellum cDNA, RIKEN full-length enriched library, clone:B93 | -1.1 | 0.812 | -1.45 | 0.493 | -1.48 | 0.152 | -1.45 | 0.043 | -1.37 |
| 1445421_at | --- | Transcribed locus | -1.14 | 0.487 | -1.17 | 0.734 | -1.83 | 0.098 | -1.03 | 0.871 | -1.29 |
| 1427818_at | Bcl2 | B-cell leukemia/lymphoma 2 | -1.71 | 0.411 | -1.13 | 0.764 | -1.24 | 0.577 | -1.14 | 0.589 | -1.3 |
| 1457381_at | Angpt1 | Angiopoietin 1 | -1.08 | 0.856 | -1.92 | 0.001 | -1.21 | 0.405 | -4.76 | 0.05 | -2.24 |
| 1428536_at | Kcng4 | potassium voltage-gated channel, subfamily G, member 4 | -2.03 | 0.143 | -1.17 | 0.8 | -1.07 | 0.806 | -1.1 | 0.269 | -1.34 |
| 1430247_at | Daam2 | dishevelled associated activator of morphogenesis 2 | -1.27 | 0.154 | -1.91 | 0.127 | -1.03 | 0.942 | -1.47 | 0.649 | -1.42 |
| 1455549_at | Sestd1 | SEC14 and spectrin domains 1 | -1.21 | 0.17 | -1.19 | 0.198 | -1.64 | 0.022 | -1.49 | 0.158 | -1.38 |
| 1445737_at | 1700057H21Rik | RIKEN cDNA 1700057H21 gene | -1.41 | 0.617 | -1.35 | 0.107 | -1.2 | 0.359 | -1.12 | 0.793 | -1.27 |
| 1458266_at | Mad1l1 | Mitotic arrest deficient 1-like 1 | -1.18 | 0.701 | -2.01 | 0.314 | -1.07 | 0.866 | -2.26 | 0.352 | -1.63 |
| 1441956_s_at | --- | --- | -1.3 | 0.447 | -1.12 | 0.634 | -1.62 | 0.126 | -1.68 | 0.086 | -1.43 |
| 1436273_at | Pms2 | postmeiotic segregation increased 2 (S. cerevisiae) | -1.54 | 0.043 | -1.54 | 0.184 | -1.02 | 0.941 | -1.04 | 0.863 | -1.28 |
| 1449241_at | Klhl1 | kelch-like 1 (Drosophila) | -1.08 | 0.366 | -1.64 | 0.178 | -1.34 | 0.356 | -1.3 | 0.229 | -1.34 |
| 1431205_at | Slc9a8 | Solute carrier family 9 (sodium/hydrogen exchanger), member 8 | -1.4 | 0.58 | -1.04 | 0.957 | -1.65 | 0.08 | -2.77 | 0.056 | -1.71 |

| | | | | | | | | | | | |
|---|---|---|---|---|---|---|---|---|---|---|---|
| 1442198_at | Nek7 | NIMA (never in mitosis gene a)-related expressed kinase 7 | -1.1 | 0.757 | -1.77 | 0.144 | -1.24 | 0.413 | -1.23 | 0.426 | -1.33 |
| 1442241_at | Srpk2 | Serine/arginine-rich protein specific kinase 2 | -1.28 | 0.487 | -1.11 | 0.612 | -1.68 | 0.032 | -1.4 | 0.156 | -1.37 |
| 1447203_at | --- | --- | -1.35 | 0.541 | -1.44 | 0.069 | -1.18 | 0.363 | -1.05 | 0.931 | -1.25 |
| 1429054_at | Mrpl47 | mitochondrial ribosomal protein L47 | -1.19 | 0.203 | -1.4 | 0.02 | -1.38 | 0.028 | -1.13 | 0.489 | -1.27 |
| 1419457_at | Rgnef | Rho-guanine nucleotide exchange factor | -1.1 | 0.202 | -2.44 | 0.001 | -1.04 | 0.813 | -3.45 | 0.054 | -2.01 |
| 1429933_at | Agtpbp1 | ATP/GTP binding protein 1 | -1.13 | 0.836 | -1.27 | 0.557 | -1.64 | 0.029 | -1.38 | 0.308 | -1.35 |
| 1440046_at | BC031748 | cDNA sequence BC031748 | -1.03 | 0.969 | -2.9 | 0.211 | -1.03 | 0.939 | -1.13 | 0.75 | -1.52 |
| 1448359_a_at | Higd1a | HIG1 domain family, member 1A | -1.31 | 0.059 | -1.28 | 0.136 | -1.35 | 0.172 | -1.23 | 0.616 | -1.29 |
| 1432688_at | 4631422I05Rik | RIKEN cDNA 4631422I05 gene | -1.05 | 0.918 | -2.18 | 0.103 | -1.15 | 0.803 | -1.44 | 0.601 | -1.45 |
| 1425162_at | Rorb | RAR-related orphan receptor beta | -1.28 | 0.805 | -1.98 | 0.093 | -1 | 1 | -1.15 | 0.773 | -1.35 |
| 1445105_at | Pcaf | P300/CBP-associated factor | -1.05 | 0.899 | -1.44 | 0.299 | -1.58 | 0.082 | -1.41 | 0.351 | -1.37 |
| 1443730_at | 9130404D14Rik | RIKEN cDNA 9130404D14 gene | -1.1 | 0.885 | -1.26 | 0.631 | -1.72 | 0.383 | -1.34 | 0.575 | -1.35 |
| 1459224_at | Ppp1r13b | Protein phosphatase 1, regulatory (inhibitor) subunit 13B | -1.66 | 0.417 | -1.08 | 0.774 | -1.32 | 0.095 | -1.33 | 0.363 | -1.35 |
| 1417740_at | Cdc37l1 | cell division cycle 37 homolog (S. cerevisiae)-like 1 | -1.41 | 0.263 | -1.3 | 0.006 | -1.24 | 0.136 | -1.07 | 0.481 | -1.26 |
| 1418659_at | Clock | circadian locomoter output cycles kaput | -1.07 | 0.716 | -1.45 | 0.081 | -1.52 | 0.01 | -1.25 | 0.409 | -1.32 |
| 1416183_a_at | Ldhb | lactate dehydrogenase B | -1.32 | 0.121 | -1.45 | 0.21 | -1.2 | 0.327 | -1.14 | 0.319 | -1.27 |
| 1457468_at | Tnks2 | Tankyrase, TRF1-interacting ankyrin-related ADP-ribose polymerase 2 | -1.4 | 0.173 | -1.05 | 0.896 | -1.62 | 0.129 | -2.03 | 0.087 | -1.53 |
| 1433062_at | Wdr66 | WD repeat domain 66 | -1.44 | 0.17 | -1.22 | 0.465 | -1.3 | 0.075 | -1.31 | 0.414 | -1.32 |
| 1442416_at | D930001B02 | hypothetical protein D930001B02 | -1.14 | 0.38 | -1.24 | 0.024 | -1.65 | 0.089 | -1.41 | 0.286 | -1.36 |
| 1444876_at | 2700049A03Rik | RIKEN cDNA 2700049A03 gene | -1.21 | 0.389 | -1.36 | 0.078 | -1.38 | 0.169 | -1.07 | 0.672 | -1.26 |
| 1418835_at | Phlda1 | pleckstrin homology-like domain, family A, member 1 | -1.15 | 0.457 | -1.7 | 0.138 | -1.21 | 0.328 | -1.04 | 0.86 | -1.27 |
| 1432861_at | 2900046F13Rik | RIKEN cDNA 2900046F13 gene | -1.56 | 0.472 | -1.31 | 0.451 | -1.14 | 0.548 | -1.08 | 0.764 | -1.27 |
| 1431181_a_at | Luc7l | Luc7 homolog (S. cerevisiae)-like | -1.49 | 0.038 | -1.5 | 0.095 | -1.06 | 0.735 | -1.3 | 0.003 | -1.34 |
| 1440331_at | 9430079B08Rik | RIKEN cDNA 9430079B08 gene | -1.12 | 0.522 | -1.38 | 0.309 | -1.49 | 0.141 | -1.12 | 0.402 | -1.28 |
| 1457587_at | Ga17 | Dendritic cell protein GA17 | -1.02 | 0.745 | -1.22 | 0.699 | -2.07 | 0.013 | -1.51 | 0.207 | -1.45 |
| 1446875_at | Il1rapl2 | Interleukin 1 receptor accessory protein-like 2 | -1.52 | 0.264 | -1.22 | 0.514 | -1.24 | 0.143 | -1.77 | 0.173 | -1.44 |
| 1441685_at | Prosc | proline synthetase co-transcribed | -1.24 | 0.03 | -1.54 | 0.309 | -1.21 | 0.607 | -1.09 | 0.844 | -1.27 |
| 1417485_at | Ibsp | integrin binding sialoprotein | -1.04 | 0.92 | -1.16 | 0.13 | -2.15 | 0.046 | -1.16 | 0.095 | -1.38 |
| 1434449_at | Aqp4 | aquaporin 4 | -1.11 | 0.506 | -1.54 | 0.373 | -1.36 | 0.164 | -1.8 | 0.121 | -1.45 |
| 1440679_at | --- | Transcribed locus | -1.26 | 0.182 | -1.42 | 0.156 | -1.27 | 0.164 | -1.37 | 0.226 | -1.33 |
| 1440717_at | AA407881 | expressed sequence AA407881 | -1.21 | 0.379 | -1.21 | 0.014 | -1.58 | 0.087 | -1.41 | 0.238 | -1.35 |
| 1442732_at | --- | --- | -1.43 | 0.17 | -1.16 | 0.52 | -1.38 | 0.435 | -1.5 | 0.038 | -1.37 |
| 1458148_at | D230007K08Rik | RIKEN cDNA D230007K08 gene | -1.33 | 0.015 | -1.77 | 0.217 | -1.03 | 0.922 | -2.47 | 0.048 | -1.65 |
| 1439569_at | Gpr83 | G protein-coupled receptor 83 | -1.02 | 0.955 | -1.73 | 0.289 | -1.38 | 0.593 | -4.41 | 0.006 | -2.13 |
| 1437667_a_at | Bach2 | BTB and CNC homology 2 | -1.29 | 0.322 | -1.51 | 0.186 | -1.18 | 0.588 | -2.6 | 0.19 | -1.64 |
| 1430776_s_at | Ankrd24 | ankyrin repeat domain 24 | -1.36 | 0.32 | -1.39 | 0.247 | -1.21 | 0.034 | -1.17 | 0.538 | -1.28 |
| 1459237_at | Atf2 | Activating transcription factor 2 | -1.26 | 0.497 | -1.22 | 0.424 | -1.47 | 0.037 | -1.15 | 0.715 | -1.28 |
| 1440896_at | Sod1 | Superoxide dismutase 1, soluble | -1.55 | 0.157 | -1.1 | 0.688 | -1.36 | 0.319 | -1.34 | 0.317 | -1.34 |
| 1418042_a_at | Abcc5 | ATP-binding cassette, sub-family C (CFTR/MRP), member 5 | -1.17 | 0.496 | -1.95 | 0.099 | -1.08 | 0.254 | -1.13 | 0.669 | -1.33 |
| 1421715_at | V2rx | pheromone receptor V2Rx | -1.13 | 0.237 | -1.95 | 0.285 | -1.12 | 0.741 | -1.38 | 0.568 | -1.4 |
| 1441157_at | C230006B20 | hypothetical LOC403344 | -1.36 | 0.061 | -1.78 | 0.099 | -1.01 | 0.965 | -1.19 | 0.416 | -1.33 |
| 1435629_at | --- | Transcribed locus | -1.17 | 0.62 | -1.37 | 0.382 | -1.43 | 0.006 | -1.07 | 0.908 | -1.26 |
| 1436096_at | --- | Adult male corpora quadrigemina cDNA, RIKEN full-length enriched library, clone | -1.36 | 0.127 | -1.09 | 0.844 | -1.57 | 0.148 | -1.15 | 0.703 | -1.29 |
| 1422207_at | Htr5a | 5-hydroxytryptamine (serotonin) receptor 5A | -1.08 | 0.89 | -2.11 | 0.135 | -1.13 | 0.842 | -2.57 | 0.222 | -1.72 |
| 1419413_at | Ccl17 | chemokine (C-C motif) ligand 17 | -1.08 | 0.705 | -1.07 | 0.843 | -2.35 | 0.116 | -2.46 | 0.063 | -1.74 |
| 1459734_at | Psmd14 | Proteasome (prosome, macropain) 26S subunit, non-ATPase, 14 | -1.23 | 0.071 | -1.3 | 0.27 | -1.42 | 0.271 | -1.04 | 0.84 | -1.25 |
| 1459710_at | --- | Transcribed locus | -1.16 | 0.588 | -1.89 | 0.006 | -1.11 | 0.652 | -1.1 | 0.272 | -1.32 |
| 1458541_at | Dctn4 | dynactin 4 | -1.88 | 0.096 | -1.11 | 0.613 | -1.17 | 0.207 | -1.03 | 0.859 | -1.3 |
| 1460035_at | Phb2 | prohibitin 2 | -1.04 | 0.832 | -1.37 | 0.2 | -1.67 | 0.109 | -1.21 | 0.013 | -1.32 |
| 1427643_at | 1200009O22Rik | RIKEN cDNA 1200009O22 gene | -1.56 | 0.044 | -1.14 | 0.752 | -1.29 | 0.414 | -1.2 | 0.66 | -1.3 |
| 1451834_at | Cacnb1 | calcium channel, voltage-dependent, beta 1 subunit | -1.18 | 0.644 | -1.07 | 0.791 | -1.96 | 0.011 | -1.16 | 0.841 | -1.34 |
| 1427702_at | Zfp1 | zinc finger protein 1 | -1.22 | 0.564 | -1.78 | 0.31 | -1.1 | 0.475 | -1.68 | 0.248 | -1.45 |
| 1455472_at | A630071D13Rik | RIKEN cDNA A630071D13 gene | -1.28 | 0.173 | -1.31 | 0.286 | -1.34 | 0.31 | -1.22 | 0.139 | -1.29 |

| | | | | | | | | | | | |
|---|---|---|---|---|---|---|---|---|---|---|---|
| 1430928_at | 2900002J02Rik | RIKEN cDNA 2900002J02 gene | -1.15 | 0.758 | -1.24 | 0.68 | -1.64 | 0.317 | -1.62 | 0.498 | -1.41 |
| 1439683_at | --- | In vitro fertilized eggs cDNA, RIKEN full-length enriched library, clone:7420434E( | -1.25 | 0.784 | -1.67 | 0.456 | -1.12 | 0.883 | -1.19 | 0.774 | -1.31 |
| 1432076_at | 4933430H16Rik | RIKEN cDNA 4933430H16 gene | -1.11 | 0.882 | -2.34 | 0.245 | -1.04 | 0.87 | -1.12 | 0.826 | -1.4 |
| 1458547_at | --- | Transcribed locus | -1.46 | 0.608 | -1.28 | 0.486 | -1.22 | 0.594 | -1.57 | 0.209 | -1.38 |
| 1420040_at | 5730472N09Rik | RIKEN cDNA 5730472N09 gene | -1.07 | 0.918 | -1.63 | 0.227 | -1.34 | 0.26 | -1.46 | 0.421 | -1.38 |
| 1453268_at | 5730409G07Rik | RIKEN cDNA 5730409G07 gene | -1.19 | 0.616 | -1.94 | 0.115 | -1.07 | 0.468 | -1.16 | 0.458 | -1.34 |
| 1418916_a_at | Spp2 | secreted phosphoprotein 2 | -1.22 | 0.165 | -1.38 | 0.047 | -1.34 | 0.557 | -1.14 | 0.835 | -1.27 |
| 1438488_at | Esd | esterase D/formylglutathione hydrolase | -1.65 | 0.191 | -1.36 | 0.594 | -1.05 | 0.883 | -2.14 | 0.046 | -1.55 |
| 1418590_at | Kpna6 | karyopherin (importin) alpha 6 | -1.38 | 0.144 | -1.4 | 0.107 | -1.17 | 0.57 | -1.27 | 0.425 | -1.31 |
| 1418452_at | Gng2 | guanine nucleotide binding protein (G protein), gamma 2 subunit | -1.33 | 0.671 | -1.82 | 0.506 | -1.01 | 0.985 | -1.4 | 0.306 | -1.39 |
| 1438231_at | Foxp2 | forkhead box P2 | -1.06 | 0.802 | -1.22 | 0.341 | -1.89 | 0.022 | -1.38 | 0.382 | -1.39 |
| 1441022_at | Arih1 | ariadne ubiquitin-conjugating enzyme E2 binding protein homolog 1 (Drosophila) | -1.15 | 0.74 | -1.43 | 0.349 | -1.39 | 0.081 | -1.18 | 0.629 | -1.29 |
| 1429846_at | 9030411K21Rik | RIKEN cDNA 9030411K21 gene | -1.58 | 0.047 | -1.04 | 0.906 | -1.44 | 0.136 | -1.47 | 0.286 | -1.38 |
| 1457832_at | --- | 7 days neonate cerebellum cDNA, RIKEN full-length enriched library, clone:A730 | -1.2 | 0.485 | -1.64 | 0.064 | -1.17 | 0.137 | -1.1 | 0.697 | -1.28 |
| 1441522_at | AW495713 | expressed sequence AW495713 | -1.27 | 0.472 | -1.71 | 0.004 | -1.08 | 0.532 | -1.1 | 0.506 | -1.29 |
| 1443943_at | Plag1 | pleiomorphic adenoma gene 1 | -1.11 | 0.857 | -2.07 | 0.172 | -1.1 | 0.889 | -2.23 | 0.298 | -1.63 |
| 1460667_at | U90926 | cDNA sequence U90926 | -1.06 | 0.882 | -2.49 | 0.32 | -1.06 | 0.232 | -1.17 | 0.327 | -1.44 |
| 1431783_at | 1700010L04Rik | RIKEN cDNA 1700010L04 gene | -1.58 | 0.276 | -1.28 | 0.697 | -1.14 | 0.58 | -2.02 | 0.313 | -1.5 |
| 1446326_at | Col1a2 | procollagen, type I, alpha 2 | -1.04 | 0.901 | -1.11 | 0.844 | -2.35 | 0.005 | -1.6 | 0.072 | -1.52 |
| 1421152_a_at | Gnao1 | guanine nucleotide binding protein, alpha o | -1.3 | 0.257 | -1.12 | 0.445 | -1.6 | 0.206 | -1.21 | 0.454 | -1.3 |
| 1445933_at | Slc12a5 | solute carrier family 12, member 5 | -1.12 | 0.252 | -1.22 | 0.364 | -1.74 | 0.016 | -1.19 | 0.581 | -1.31 |
| 1457488_at | B230339M05Rik | RIKEN cDNA B230339M05 gene | -1.11 | 0.89 | -1.67 | 0.111 | -1.26 | 0.305 | -3.03 | 0.09 | -1.77 |
| 1445255_at | Rapgef5 | Rap guanine nucleotide exchange factor (GEF) 5 | -1.03 | 0.842 | -1.73 | 0.344 | -1.34 | 0.623 | -1.05 | 0.94 | -1.29 |
| 1441997_at | Zfp184 | zinc finger protein 184 (Kruppel-like) | -1.14 | 0.794 | -1.92 | 0.342 | -1.12 | 0.611 | -1.47 | 0.487 | -1.41 |
| 1458719_at | --- | Transcribed locus | -1.3 | 0.038 | -1.33 | 0.374 | -1.28 | 0.335 | -1.08 | 0.296 | -1.25 |
| 1430145_at | Lrrc28 | leucine rich repeat containing 28 | -1.59 | 0.024 | -1.22 | 0.592 | -1.18 | 0.59 | -1.08 | 0.799 | -1.27 |
| 1420801_at | Npas1 | neuronal PAS domain protein 1 | -1.18 | 0.012 | -1.52 | 0.473 | -1.27 | 0.16 | -1.58 | 0.011 | -1.39 |
| 1431281_at | Dysfip1 | dysferlin interacting protein 1 | -1.49 | 0.29 | -1.51 | 0.427 | -1.04 | 0.911 | -2.62 | 0.114 | -1.67 |
| 1444246_at | Chd2 | Chromodomain helicase DNA binding protein 2 | -1.53 | 0.177 | -1.23 | 0.416 | -1.21 | 0.561 | -1.52 | 0.188 | -1.37 |
| 1445280_at | Ap3d1 | adaptor-related protein complex 3, delta 1 subunit | -1.05 | 0.923 | -1.45 | 0.322 | -1.54 | 0.009 | -1.02 | 0.899 | -1.26 |
| 1420492_s_at | Smr1 /// Smr3 | submaxillary gland androgen regulated protein 1 /// submaxillary gland androgen | -1.35 | 0.004 | -1.13 | 0.832 | -1.49 | 0.257 | -2.49 | 0.492 | -1.62 |
| 1444018_at | B930098A02Rik | RIKEN cDNA B930098A02 gene | -1.16 | 0.628 | -1.37 | 0.165 | -1.42 | 0.02 | -1.18 | 0.51 | -1.28 |
| 1447249_at | --- | Transcribed locus | -1.8 | 0.076 | -1.03 | 0.864 | -1.29 | 0.515 | -1.51 | 0.105 | -1.41 |
| 1454380_at | Phactr4 | phosphatase and actin regulator 4 | -1.32 | 0.655 | -1.54 | 0.093 | -1.12 | 0.615 | -1.31 | 0.74 | -1.32 |
| 1447589_at | Ppp4r1 | Protein phosphatase 4, regulatory subunit 1 | -1.44 | 0.488 | -1.34 | 0.655 | -1.17 | 0.171 | -1.16 | 0.446 | -1.28 |
| 1421012_at | Srprb | signal recognition particle receptor, B subunit | -1.32 | 0.392 | -1.67 | 0.016 | -1.07 | 0.708 | -1.16 | 0.509 | -1.3 |
| 1440616_at | AU042410 | expressed sequence AU042410 | -1.2 | 0.611 | -1.33 | 0.485 | -1.4 | 0.596 | -1.04 | 0.924 | -1.24 |
| 1446835_at | Ftl1 | Ferritin light chain 1 | -1.18 | 0.143 | -1.22 | 0.128 | -1.58 | 0.165 | -1.95 | 0.091 | -1.48 |
| 1430320_at | Dmd | dystrophin, muscular dystrophy | -1.06 | 0.656 | -1.41 | 0.004 | -1.54 | 0.354 | -1.6 | 0.373 | -1.41 |
| 1441426_at | BC002230 | CDNA sequence BC002230 | -1.54 | 0.426 | -1.18 | 0.341 | -1.25 | 0.287 | -1.05 | 0.909 | -1.25 |
| 1419640_at | Purb | purine rich element binding protein B | -1.27 | 0.32 | -1.18 | 0.485 | -1.51 | 0.096 | -1.14 | 0.188 | -1.28 |
| 1460109_at | Tcf25 | Transcription factor 25 (basic helix-loop-helix) | -1.29 | 0.576 | -1.61 | 0.002 | -1.12 | 0.366 | -1.1 | 0.283 | -1.28 |
| 1430152_at | Eps15 | epidermal growth factor receptor pathway substrate 15 | -1.19 | 0.38 | -1.21 | 0.428 | -1.59 | 0.003 | -1.07 | 0.809 | -1.26 |
| 1445566_at | Centb2 | Centaurin, beta 2 | -1.14 | 0.652 | -1.66 | 0.353 | -1.22 | 0.581 | -1.15 | 0.544 | -1.29 |
| 1441769_at | Arl15 | ADP-ribosylation factor-like 15 | -1.16 | 0.25 | -1.04 | 0.817 | -2.11 | 0.053 | -1.28 | 0.35 | -1.4 |
| 1419218_at | Vangl2 | vang-like 2 (van gogh, Drosophila) | -1.89 | 0.033 | -1.13 | 0.322 | -1.13 | 0.698 | -1.39 | 0.031 | -1.38 |
| 1435543_at | Apc | adenomatosis polyposis coli | -1.24 | 0.069 | -1.13 | 0.353 | -1.64 | 0.011 | -1.35 | 0.288 | -1.34 |
| 1417623_at | Slc12a2 | solute carrier family 12, member 2 | -1.1 | 0.731 | -1.16 | 0.799 | -1.9 | 0.041 | -1.08 | 0.854 | -1.31 |
| 1419224_at | Cecr6 | cat eye syndrome chromosome region, candidate 6 homolog (human) | -1.35 | 0.615 | -1.15 | 0.764 | -1.45 | 0.299 | -1.13 | 0.865 | -1.27 |
| 1446058_at | --- | PREDICTED: Mus musculus hypothetical protein LOC628925 (LOC628925), mR | -1.18 | 0.448 | -1.41 | 0.46 | -1.36 | 0.222 | -1.12 | 0.566 | -1.26 |
| 1453193_s_at | Kif12 | kinesin family member 12 | -1.08 | 0.816 | -1.56 | 0.355 | -1.37 | 0.446 | -1.5 | 0.51 | -1.38 |
| 1455329_at | --- | --- | -1.5 | 0.359 | -1.27 | 0.131 | -1.18 | 0.314 | -1.4 | 0.137 | -1.34 |
| 1438735_at | Rsf1 | remodeling and spacing factor 1 | -1.62 | 0.034 | -1.05 | 0.737 | -1.37 | 0.122 | -1.17 | 0.137 | -1.3 |

| | | | | | | | | | | | |
|---|---|---|---|---|---|---|---|---|---|---|---|
| 1432622_a_at | 4930507D05Rik | RIKEN cDNA 4930507D05 gene | -1.07 | 0.94 | -1.29 | 0.341 | -1.71 | 0.213 | -1.21 | 0.306 | -1.32 |
| 1419115_at | Alg14 | asparagine-linked glycosylation 14 homolog (yeast) | -1.14 | 0.489 | -1.37 | 0.036 | -1.44 | 0.059 | -1.06 | 0.87 | -1.25 |
| 1429634_at | Zfp580 | zinc finger protein 580 | -1.19 | 0.392 | -1.53 | 0.006 | -1.24 | 0.177 | -1.02 | 0.965 | -1.24 |
| 1450176_at | Ern1 | endoplasmic reticulum (ER) to nucleus signalling 1 | -1.16 | 0.529 | -1.64 | 0.075 | -1.2 | 0.65 | -1.31 | 0.626 | -1.33 |
| 1439169_at | Eif4enif1 | Eukaryotic translation initiation factor 4E nuclear import factor 1 | -1.16 | 0.519 | -1.11 | 0.23 | -1.86 | 0.047 | -1.17 | 0.308 | -1.32 |
| 1437705_at | Zfp551 | zinc fingr protein 551 | -1.09 | 0.723 | -1.39 | 0.562 | -1.5 | 0.043 | -1.06 | 0.9 | -1.26 |
| 1445492_at | Mapre2 | Microtubule-associated protein, RP/EB family, member 2 | -1.12 | 0.413 | -1.13 | 0.739 | -1.93 | 0.04 | -1.24 | 0.501 | -1.35 |
| 1438221_at | C130065N10Rik | RIKEN cDNA C130065N10 gene | -1.24 | 0.459 | -1.33 | 0.253 | -1.35 | 0.116 | -1.36 | 0.033 | -1.32 |
| 1446705_at | --- | --- | -1.11 | 0.713 | -1.39 | 0.617 | -1.46 | 0.23 | -1.57 | 0.354 | -1.38 |
| 1442616_at | Grasp | GRP1 (general receptor for phosphoinositides 1)-associated scaffold protein | -1.17 | 0.585 | -1.59 | 0.186 | -1.23 | 0.213 | -1.05 | 0.485 | -1.26 |
| 1443233_at | Gtf2f2 | General transcription factor IIF, polypeptide 2 | -1.14 | 0.205 | -1.36 | 0.107 | -1.45 | 0.04 | -1.04 | 0.731 | -1.25 |
| 1434212_at | Ndufs8 | NADH dehydrogenase (ubiquinone) Fe-S protein 8 | -1.14 | 0.551 | -1.5 | 0.135 | -1.32 | 0.348 | -1.07 | 0.813 | -1.26 |
| 1446876_at | Wdr22 | WD repeat domain 22 | -1.06 | 0.877 | -1.23 | 0.509 | -1.82 | 0.183 | -1.86 | 0.162 | -1.49 |
| 1423499_at | Sncaip | synuclein, alpha interacting protein (synphilin) | -1.77 | 0.018 | -1.06 | 0.843 | -1.25 | 0.197 | -1.73 | 0.307 | -1.45 |
| 1421189_at | Gabrb3 | gamma-aminobutyric acid (GABA-A) receptor, subunit beta 3 | -1.4 | 0.067 | -1.28 | 0.107 | -1.24 | 0.559 | -1.16 | 0.815 | -1.27 |
| 1419722_at | Klk8 | kallikrein 8 | -1.16 | 0.282 | -1.22 | 0.708 | -1.61 | 0.033 | -2.76 | 0.236 | -1.69 |
| 1427794_at | AJ242955 | hypothetical protein, P4(21)n gene | -1.16 | 0.623 | -1.73 | 0.274 | -1.16 | 0.703 | -2.18 | 0.207 | -1.56 |
| 1435574_at | Chordc1 | Cysteine and histidine-rich domain (CHORD)-containing, zinc-binding protein 1 | -1.09 | 0.766 | -1.41 | 0.145 | -1.48 | 0.034 | -1.53 | 0.116 | -1.38 |
| 1456216_at | Csnk1a1 | Casein kinase 1, alpha 1 | -1.24 | 0.328 | -1.09 | 0.513 | -1.72 | 0.014 | -1.12 | 0.031 | -1.29 |
| 1453635_at | 4932416N17Rik | RIKEN cDNA 4932416N17 gene | -1.41 | 0.398 | -1.43 | 0.261 | -1.11 | 0.393 | -1.02 | 0.843 | -1.25 |
| 1425229_a_at | Tcf7l2 | transcription factor 7-like 2, T-cell specific, HMG-box | -1.09 | 0.76 | -1.41 | 0.212 | -1.47 | 0.082 | -1.34 | 0.145 | -1.33 |
| 1459324_at | --- | --- | -1.07 | 0.55 | -1.13 | 0.42 | -2.06 | 0.013 | -1.84 | 0.317 | -1.53 |
| 1440163_at | 6030490B17Rik | RIKEN cDNA 6030490B17 gene | -1.36 | 0.274 | -1.08 | 0.619 | -1.57 | 0.044 | -1.12 | 0.709 | -1.28 |
| 1444506_at | Tram1 | Translocating chain-associating membrane protein 1 | -1.15 | 0.193 | -1.23 | 0.447 | -1.62 | 0.122 | -1.26 | 0.236 | -1.31 |
| 1440552_at | Ubr1 | Ubiquitin protein ligase E3 component n-recognin 1 | -2.95 | 0.02 | -1.03 | 0.939 | -1 | 0.979 | -2.13 | 0.369 | -1.78 |
| 1421368_at | Scrt1 | scratch homolog 1, zinc finger protein (Drosophila) | -1.37 | 0.56 | -1.4 | 0.488 | -1.17 | 0.659 | -1.43 | 0.335 | -1.34 |
| 1426302_at | Tmprss4 | transmembrane protease, serine 4 | -1.21 | 0.415 | -1.31 | 0.624 | -1.39 | 0.019 | -1.35 | 0.57 | -1.32 |
| 1459312_at | --- | Transcribed locus | -1.12 | 0.668 | -1.09 | 0.849 | -2.01 | 0.241 | -1.48 | 0.654 | -1.43 |
| 1451004_at | Acvr2a | activin receptor IIA | -1.05 | 0.897 | -1.61 | 0.103 | -1.37 | 0.073 | -1.09 | 0.883 | -1.28 |
| 1429435_x_at | Pik3ca | phosphatidylinositol 3-kinase, catalytic, alpha polypeptide | -1.26 | 0.248 | -1.18 | 0.363 | -1.5 | 0.065 | -1.09 | 0.469 | -1.26 |
| 1430319_at | 4833411C07Rik | RIKEN cDNA 4833411C07 gene | -1.51 | 0.335 | -1.16 | 0.589 | -1.28 | 0.018 | -1.11 | 0.773 | -1.27 |
| 1459109_at | Sema5a | sema domain, seven thrombospondin repeats (type 1 and type 1-like), transmeml | -1.65 | 0.65 | -1.29 | 0.635 | -1.08 | 0.841 | -1.46 | 0.464 | -1.37 |
| 1436045_at | Tsga10 | testis specific 10 | -1.22 | 0.583 | -1.22 | 0.525 | -1.5 | 0.168 | -1.1 | 0.669 | -1.26 |
| 1456302_at | Pex6 | peroxisomal biogenesis factor 6 | -1.33 | 0.125 | -1.56 | 0.033 | -1.1 | 0.811 | -1.42 | 0.3 | -1.35 |
| 1420074_at | D8Ertd738e | DNA segment, Chr 8, ERATO Doi 738, expressed | -1.28 | 0.315 | -1.06 | 0.686 | -1.72 | 0.142 | -1.18 | 0.771 | -1.31 |
| 1441327_a_at | Ssr1 | signal sequence receptor, alpha | -1.14 | 0.169 | -1.2 | 0.116 | -1.67 | 0.06 | -1.02 | 0.951 | -1.26 |
| 1440950_at | Akt1 | Thymoma viral proto-oncogene 1 | -1.39 | 0.328 | -1.25 | 0.311 | -1.27 | 0.569 | -1.25 | 0.135 | -1.29 |
| 1429413_at | Cpm | carboxypeptidase M | -1.25 | 0.091 | -1.53 | 0.123 | -1.18 | 0.461 | -3 | 0.066 | -1.74 |
| 1455659_at | --- | --- | -1.41 | 0.222 | -1.19 | 0.315 | -1.33 | 0.072 | -1.01 | 0.982 | -1.23 |
| 1455134_at | A630051L19Rik | RIKEN cDNA A630051L19 gene | -1.12 | 0.095 | -1.38 | 0.126 | -1.45 | 0.117 | -1.67 | 0.215 | -1.4 |
| 1436679_at | 2210010N04Rik | RIKEN cDNA 2210010N04 gene | -1.18 | 0.59 | -1.19 | 0.704 | -1.61 | 0.488 | -2.15 | 0.21 | -1.53 |
| 1433107_at | --- | --- | -1.46 | 0.169 | -1.03 | 0.944 | -1.53 | 0.023 | -1.11 | 0.804 | -1.28 |
| 1459746_at | R3hdm1 | R3H domain 1 (binds single-stranded nucleic acids) | -1.27 | 0.369 | -1.28 | 0.34 | -1.36 | 0.017 | -1.08 | 0.825 | -1.25 |
| 1444817_at | Plekhh2 | Pleckstrin homology domain containing, family H (with MyTH4 domain) member 2 | -1.05 | 0.77 | -1.34 | 0.662 | -1.65 | 0.217 | -1.07 | 0.837 | -1.28 |
| 1436261_at | Dysf | Dysferlin | -1.46 | 0.565 | -1.05 | 0.898 | -1.5 | 0.476 | -1.35 | 0.675 | -1.34 |
| 1425339_at | Plcb4 | phospholipase C, beta 4 | -1.41 | 0.497 | -1.43 | 0.356 | -1.11 | 0.562 | -2.25 | 0.314 | -1.55 |
| 1423312_at | Tpbg | trophoblast glycoprotein | -1.05 | 0.595 | -1.52 | 0.551 | -1.43 | 0.353 | -2.76 | 0.169 | -1.69 |
| 1445138_at | A530083I20Rik | RIKEN cDNA A530083I20 gene | -1.09 | 0.84 | -1.75 | 0.217 | -1.21 | 0.406 | -2.32 | 0.066 | -1.6 |
| 1440511_at | Psme4 | Proteasome (prosome, macropain) activator subunit 4 | -1.19 | 0.546 | -1.41 | 0.074 | -1.31 | 0.28 | -1.13 | 0.588 | -1.26 |
| 1435484_at | BF642829 | expressed sequence BF642829 | -1.14 | 0.694 | -1.82 | 0.133 | -1.14 | 0.477 | -1.6 | 0.026 | -1.42 |
| 1433729_x_at | Pmpcb | peptidase (mitochondrial processing) beta | -1.43 | 0.166 | -1.44 | 0.024 | -1.1 | 0.314 | -1.16 | 0.195 | -1.28 |
| 1429345_at | D2Ertd435e | DNA segment, Chr 2, ERATO Doi 435, expressed | -1.33 | 0.266 | -1.2 | 0.114 | -1.39 | 0.151 | -1.3 | 0.063 | -1.3 |
| 1442240_at | Ctr9 | Ctr9, Paf1/RNA polymerase II complex component, homolog (S. cerevisiae) | -1.16 | 0.579 | -1.49 | 0 | -1.29 | 0.384 | -1.35 | 0.144 | -1.32 |

| | | | | | | | | | | | |
|---|---|---|---|---|---|---|---|---|---|---|---|
| 1447560_at | --- | --- | -1.39 | 0.696 | -1.31 | 0.166 | -1.21 | 0.306 | -1.15 | 0.649 | -1.27 |
| 1432843_at | 5730405O12Rik | RIKEN cDNA 5730405O12 gene | -1.11 | 0.608 | -1.52 | 0.313 | -1.33 | 0.143 | -1.28 | 0.274 | -1.31 |
| 1447516_at | --- | Transcribed locus | -1.37 | 0.445 | -1.3 | 0.621 | -1.24 | 0.396 | -1.31 | 0.068 | -1.3 |
| 1446101_at | --- | --- | -1.19 | 0.724 | -1.11 | 0.702 | -1.78 | 0.065 | -1.6 | 0.077 | -1.42 |
| 1441354_at | AU018740 | expressed sequence AU018740 | -1.14 | 0.673 | -1.23 | 0.462 | -1.62 | 0.208 | -1.41 | 0.394 | -1.35 |
| 1430261_at | --- | --- | -1.5 | 0.265 | -1 | 0.997 | -1.55 | 0.008 | -1.32 | 0.701 | -1.34 |
| 1456221_at | Cdh8 | Cadherin 8 | -2.04 | 0.44 | -1.05 | 0.876 | -1.16 | 0.747 | -1.07 | 0.885 | -1.33 |
| 1443795_at | Tcf19 | transcription factor 19 | -1.01 | 0.992 | -1.66 | 0.473 | -1.41 | 0.579 | -1.4 | 0.437 | -1.37 |
| 1457154_at | 6-Mar | Membrane-associated ring finger (C3HC4) 6 | -1.02 | 0.942 | -1.49 | 0.146 | -1.53 | 0.24 | -1.17 | 0.595 | -1.3 |
| 1460179_at | Dnaja1 | DnaJ (Hsp40) homolog, subfamily A, member 1 | -1.15 | 0.193 | -1.31 | 0.053 | -1.48 | 0.014 | -1.43 | 0.274 | -1.34 |
| 1459696_at | Fry | Furry homolog (Drosophila) | -1.78 | 0.134 | -1.14 | 0.574 | -1.15 | 0.807 | -1.93 | 0.218 | -1.5 |
| 1445148_at | Plcl1 | Phospholipase C-like 1 | -1.04 | 0.885 | -1.03 | 0.919 | -2.66 | 0.056 | -1.44 | 0.247 | -1.54 |
| 1454210_at | 4933428M09Rik | RIKEN cDNA 4933428M09 gene | -1.94 | 0.307 | -1.25 | 0.603 | -1.01 | 0.978 | -1.66 | 0.319 | -1.46 |
| 1460063_at | D5Ertd798e | DNA segment, Chr 5, ERATO Doi 798, expressed | -1.07 | 0.675 | -1.33 | 0.073 | -1.59 | 0.015 | -1.32 | 0.357 | -1.33 |
| 1429715_at | Ppp2r2a | protein phosphatase 2 (formerly 2A), regulatory subunit B (PR 52), alpha isoform | -1.02 | 0.856 | -1.27 | 0.438 | -1.83 | 0.005 | -1 | 0.987 | -1.28 |
| 1458033_at | Ubr1 | Ubiquitin protein ligase E3 component n-recognin 1 | -1.03 | 0.898 | -1.39 | 0.074 | -1.61 | 0.001 | -1.16 | 0.53 | -1.3 |
| 1453565_at | Ndufab1 | NADH dehydrogenase (ubiquinone) 1, alpha/beta subcomplex, 1 | -1.29 | 0.112 | -1.33 | 0.525 | -1.27 | 0.159 | -1.22 | 0.299 | -1.28 |
| 1430386_at | E030024N20Rik | RIKEN cDNA E030024N20 gene | -1.14 | 0.313 | -1.13 | 0.414 | -1.83 | 0.03 | -1.07 | 0.623 | -1.29 |
| 1423561_at | Nell2 | NEL-like 2 (chicken) | -1.11 | 0.873 | -1.84 | 0.48 | -1.15 | 0.757 | -4.24 | 0.348 | -2.09 |
| 1444195_at | Rmnd5a | Required for meiotic nuclear division 5 homolog A (S. cerevisiae) | -1.04 | 0.76 | -1.13 | 0.368 | -2.17 | 0.079 | -1.1 | 0.667 | -1.36 |
| 1445345_at | Plekha5 | Pleckstrin homology domain containing, family A member 5 | -1.51 | 0.024 | -1.08 | 0.648 | -1.39 | 0.058 | -1.38 | 0.639 | -1.34 |
| 1420728_at | Krt32 | keratin 32 | -1.28 | 0.17 | -1.48 | 0.332 | -1.17 | 0.472 | -1.01 | 0.964 | -1.23 |
| 1427157_at | Ccdc85a | coiled-coil domain containing 85A | -1.26 | 0.397 | -1.33 | 0.669 | -1.3 | 0.229 | -1.31 | 0.573 | -1.3 |
| 1439408_a_at | Pparbp | peroxisome proliferator activated receptor binding protein | -1.42 | 0.362 | -1.37 | 0.147 | -1.14 | 0.689 | -1.09 | 0.784 | -1.25 |
| 1430115_at | 4833413E03Rik | RIKEN cDNA 4833413E03 gene | -1.32 | 0.441 | -1 | 0.992 | -1.79 | 0.143 | -1.07 | 0.72 | -1.29 |
| 1420175_at | Tax1bp1 | Tax1 (human T-cell leukemia virus type I) binding protein 1 | -1.18 | 0.773 | -1.45 | 0.293 | -1.29 | 0.289 | -1.03 | 0.89 | -1.24 |
| 1453451_at | 6820402I19Rik | RIKEN cDNA 6820402I19 gene | -1.24 | 0.251 | -1.09 | 0.494 | -1.7 | 0.027 | -1.16 | 0.374 | -1.3 |
| 1449425_at | Wnt2 | wingless-related MMTV integration site 2 | -1.6 | 0.572 | -1.39 | 0.027 | -1.03 | 0.92 | -1.94 | 0.106 | -1.49 |
| 1459873_x_at | Maged2 | Melanoma antigen, family D, 2 | -1.02 | 0.944 | -1.74 | 0.165 | -1.31 | 0.357 | -2.03 | 0.238 | -1.53 |
| 1449571_at | Trhr | thyrotropin releasing hormone receptor | -2.02 | 0.291 | -1.08 | 0.727 | -1.12 | 0.702 | -2.59 | 0.001 | -1.7 |
| 1416718_at | Bcan | brevican | -1.46 | 0.707 | -1.34 | 0.591 | -1.13 | 0.602 | -1.07 | 0.906 | -1.25 |
| 1427656_at | Tcrb-V13 | T-cell receptor beta, variable 13 | -1.52 | 0.635 | -1.06 | 0.892 | -1.41 | 0.367 | -3.71 | 0.148 | -1.92 |
| 1440295_at | 1110057K04Rik | RIKEN cDNA 1110057K04 gene | -1.61 | 0.147 | -1.21 | 0.336 | -1.16 | 0.522 | -1.09 | 0.74 | -1.27 |
| 1457356_at | Igf2r | Insulin-like growth factor 2 receptor | -1.11 | 0.68 | -1.06 | 0.86 | -2.14 | 0.038 | -1.27 | 0.41 | -1.39 |
| 1422597_at | Mmp15 | matrix metallopeptidase 15 | -1.24 | 0.593 | -1.51 | 0.12 | -1.19 | 0.235 | -1.52 | 0.366 | -1.36 |
| 1447824_x_at | Hspa5 | heat shock 70kD protein 5 (glucose-regulated protein) | -1.61 | 0.121 | -1.27 | 0.172 | -1.1 | 0.545 | -1.07 | 0.319 | -1.26 |
| 1425489_at | D11Ertd730e | DNA segment, Chr 11, ERATO Doi 730, expressed | -1.39 | 0.093 | -1.28 | 0.394 | -1.23 | 0.12 | -1.08 | 0.882 | -1.25 |
| 1416679_at | Abcd3 | ATP-binding cassette, sub-family D (ALD), member 3 | -1.14 | 0.458 | -1.52 | 0.064 | -1.29 | 0.047 | -1.06 | 0.788 | -1.25 |
| 1422574_at | Mxd4 | Max dimerization protein 4 | -1.02 | 0.489 | -1.46 | 0.057 | -1.54 | 0.079 | -1.36 | 0.14 | -1.35 |
| 1417261_at | Mbtd1 | mbt domain containing 1 | -1.24 | 0.211 | -1.38 | 0.088 | -1.28 | 0.142 | -1.11 | 0.647 | -1.25 |
| 1455519_at | Dsg1b | desmoglein 1 beta | -1.29 | 0.7 | -1.5 | 0.548 | -1.15 | 0.501 | -2.79 | 0.289 | -1.68 |
| 1425771_at | Akr1d1 | aldo-keto reductase family 1, member D1 | -1.6 | 0.047 | -1.31 | 0.042 | -1.08 | 0.856 | -1.16 | 0.718 | -1.29 |
| 1453863_at | 3300002P09Rik | RIKEN cDNA 3300002P09 gene | -1.25 | 0.745 | -1.63 | 0.43 | -1.11 | 0.394 | -1.04 | 0.907 | -1.26 |
| 1421330_at | Ptpn4 | protein tyrosine phosphatase, non-receptor type 4 | -1.17 | 0.841 | -1.54 | 0.041 | -1.23 | 0.411 | -1.27 | 0.291 | -1.3 |
| 1439537_at | --- | --- | -1.15 | 0.439 | -1.23 | 0.12 | -1.58 | 0.182 | -1.64 | 0.26 | -1.4 |
| 1442306_at | --- | Transcribed locus | -1.36 | 0.635 | -1.23 | 0.55 | -1.31 | 0.473 | -2.55 | 0.331 | -1.61 |
| 1445128_at | --- | CDNA clone IMAGE:30463708 | -1.4 | 0.33 | -1.12 | 0.855 | -1.4 | 0.041 | -1.17 | 0.458 | -1.27 |
| 1432310_at | 6530401F13Rik | RIKEN cDNA 6530401F13 gene | -1.45 | 0.579 | -1.24 | 0.68 | -1.22 | 0.723 | -1.82 | 0.428 | -1.43 |
| 1454232_at | 9430027B09Rik | RIKEN cDNA 9430027B09 gene | -1.01 | 0.963 | -1.86 | 0.28 | -1.26 | 0.562 | -1.79 | 0.023 | -1.48 |
| 1437619_x_at | Ddr1 | discoidin domain receptor family, member 1 | -1.65 | 0.041 | -1.22 | 0.274 | -1.12 | 0.434 | -1.57 | 0.045 | -1.39 |
| 1418446_at | Slc16a2 | solute carrier family 16 (monocarboxylic acid transporters), member 2 | -1.32 | 0.15 | -1.21 | 0.357 | -1.37 | 0.069 | -1.51 | 0.243 | -1.35 |
| 1436427_at | Prpf4b | PRP4 pre-mRNA processing factor 4 homolog B (yeast) | -1.22 | 0.14 | -1.15 | 0.492 | -1.59 | 0.074 | -1.16 | 0.662 | -1.28 |
| 1436857_at | Defcr21 /// Defcr22 | defensin related cryptdin 21 /// defensin related cryptdin 22 | -1.1 | 0.254 | -1.53 | 0.532 | -1.33 | 0.336 | -1.1 | 0.568 | -1.26 |

| | | | | | | | | | | | |
|---|---|---|---|---|---|---|---|---|---|---|---|
| 1459270_at | Abcc1 | ATP-binding cassette, sub-family C (CFTR/MRP), member 1 | -1.07 | 0.71 | -1.46 | 0.139 | -1.43 | 0.185 | -1.07 | 0.791 | -1.26 |
| 1430659_at | 4930548H24Rik | RIKEN cDNA 4930548H24 gene | -1.28 | 0.142 | -1.06 | 0.867 | -1.69 | 0.162 | -1.22 | 0.271 | -1.31 |
| 1436318_at | Tardbp | TAR DNA binding protein | -1.26 | 0.355 | -1.38 | 0.076 | -1.25 | 0.158 | -1.18 | 0.493 | -1.27 |
| 1442022_at | E430004N04Rik | RIKEN cDNA E430004N04 gene | -1.38 | 0.772 | -1.68 | 0.431 | -1 | 0.996 | -2.07 | 0.331 | -1.53 |
| 1446299_at | 6720456B07Rik | RIKEN cDNA 6720456B07 gene | -1.03 | 0.881 | -1.42 | 0 | -1.56 | 0.118 | -1.2 | 0.518 | -1.3 |
| 1428540_at | 3321401G04Rik | RIKEN cDNA 3321401G04 gene | -1.44 | 0.302 | -1.21 | 0.468 | -1.25 | 0.26 | -1.47 | 0.019 | -1.34 |
| 1419176_at | Vps37a | vacuolar protein sorting 37A (yeast) | -1.12 | 0.022 | -1.43 | 0.039 | -1.38 | 0.069 | -1.11 | 0.564 | -1.26 |
| 1445952_at | Magi1 | Membrane associated guanylate kinase, WW and PDZ domain containing 1 | -1.28 | 0.612 | -1.11 | 0.742 | -1.56 | 0.072 | -2.36 | 0.015 | -1.58 |
| 1457649_x_at | AI848149 | expressed sequence AI848149 | -1.46 | 0.345 | -1.16 | 0.684 | -1.29 | 0.397 | -1.86 | 0.047 | -1.44 |
| 1421274_at | Socs4 | suppressor of cytokine signaling 4 | -1.39 | 0.464 | -1.08 | 0.79 | -1.48 | 0.416 | -1.07 | 0.784 | -1.26 |
| 1424921_at | Brd4 /// Bst2 | bromodomain containing 4 /// bone marrow stromal cell antigen 2 | -1.54 | 0.116 | -1.1 | 0.17 | -1.32 | 0.035 | -2.27 | 0.086 | -1.56 |
| 1457062_at | 1700081L11Rik | RIKEN cDNA 1700081L11 gene | -1.15 | 0.129 | -1.18 | 0 | -1.66 | 0.018 | -1.29 | 0.165 | -1.32 |
| 1430272_at | Gtpbp5 | GTP binding protein 5 | -1.45 | 0.029 | -1.4 | 0.281 | -1.09 | 0.632 | -1.19 | 0.248 | -1.28 |
| 1441225_at | Gsn | Gelsolin | -1.08 | 0.619 | -1.21 | 0.504 | -1.78 | 0.361 | -1.1 | 0.827 | -1.29 |
| 1431165_at | A030009A09Rik | RIKEN cDNA A030009A09 gene | -1.6 | 0.207 | -1.15 | 0.539 | -1.22 | 0.096 | -1.2 | 0.748 | -1.29 |
| 1429741_at | Kcnv1 | potassium channel, subfamily V, member 1 | -1 | 0.99 | -1.49 | 0.47 | -1.53 | 0.294 | -1.35 | 0.613 | -1.34 |
| 1447693_s_at | Neo1 | neogenin | -1.22 | 0.032 | -1.21 | 0.066 | -1.49 | 0.036 | -1.18 | 0.495 | -1.27 |
| 1440764_at | Araf | v-raf murine sarcoma 3611 viral oncogene homolog | -1.04 | 0.701 | -1.22 | 0.046 | -1.86 | 0.016 | -1.44 | 0.36 | -1.39 |
| 1423752_at | Ddx47 | DEAD (Asp-Glu-Ala-Asp) box polypeptide 47 | -1.43 | 0.118 | -1.24 | 0.005 | -1.23 | 0.007 | -1.1 | 0.413 | -1.25 |
| 1433010_at | 5530400N10Rik | RIKEN cDNA 5530400N10 gene | -1.77 | 0.058 | -1.13 | 0.503 | -1.15 | 0.595 | -1.06 | 0.64 | -1.28 |
| 1438121_at | Sec24a | SEC24 related gene family, member A (S. cerevisiae) | -1.17 | 0.524 | -1.26 | 0.101 | -1.49 | 0.339 | -1 | 0.998 | -1.23 |
| 1459265_at | Myohd1 | myosin head domain containing 1 | -1.17 | 0.586 | -1.59 | 0.43 | -1.19 | 0.561 | -1.07 | 0.827 | -1.26 |
| 1446298_at | C630016I17Rik | RIKEN cDNA C630016I17 gene | -1.07 | 0.484 | -1.3 | 0.096 | -1.63 | 0.028 | -1.81 | 0.184 | -1.45 |
| 1418291_at | Zfp87 | zinc finger protein 87 | -1.25 | 0.143 | -1.12 | 0.662 | -1.59 | 0.007 | -1.77 | 0.057 | -1.43 |
| 1453897_at | C030014A21Rik | RIKEN cDNA C030014A21 gene | -1.04 | 0.915 | -2 | 0.036 | -1.16 | 0.622 | -1.02 | 0.963 | -1.31 |
| 1442568_at | C330002I19Rik | RIKEN cDNA C330002I19 gene | -1.46 | 0.02 | -1.16 | 0.398 | -1.28 | 0.186 | -1.6 | 0.04 | -1.38 |
| 1435917_at | --- | Transcribed locus | -1.36 | 0.093 | -1 | 0.969 | -1.69 | 0.033 | -1.05 | 0.872 | -1.28 |
| 1430958_at | 4933430N04Rik | RIKEN cDNA 4933430N04 gene | -1.64 | 0.527 | -1.11 | 0.862 | -1.24 | 0.669 | -1.35 | 0.716 | -1.33 |
| 1429983_at | 2010002M09Rik | RIKEN cDNA 2010002M09 gene | -1.31 | 0.477 | -1.2 | 0.121 | -1.37 | 0.071 | -1.42 | 0.313 | -1.33 |
| 1424462_at | Ergic2 | ERGIC and golgi 2 | -1.09 | 0.369 | -1.38 | 0.011 | -1.46 | 0.021 | -1.07 | 0.431 | -1.25 |
| 1446276_at | 2310008H04Rik | RIKEN cDNA 2310008H04 gene | -1.08 | 0.722 | -1.33 | 0.107 | -1.55 | 0.051 | -1.18 | 0.46 | -1.28 |
| 1455144_s_at | AU040829 | expressed sequence AU040829 | -1.01 | 0.936 | -1.96 | 0.035 | -1.22 | 0.075 | -1.17 | 0.56 | -1.34 |
| 1451271_a_at | Acat1 | acetyl-Coenzyme A acetyltransferase 1 | -1.23 | 0.116 | -1.48 | 0.157 | -1.2 | 0.191 | -1.09 | 0.113 | -1.25 |
| 1434951_at | Armc8 | armadillo repeat containing 8 | -1.36 | 0.047 | -1.44 | 0.065 | -1.12 | 0.247 | -1.41 | 0.133 | -1.33 |
| 1424671_at | Plekhf1 | pleckstrin homology domain containing, family F (with FYVE domain) member 1 | -1.11 | 0.443 | -1.63 | 0.215 | -1.24 | 0.178 | -1.21 | 0.244 | -1.3 |
| 1442939_at | Rif1 /// LOC671598 | Rap1 interacting factor 1 homolog (yeast) /// similar to Telomere-associated prote | -1.37 | 0.669 | -1.57 | 0.157 | -1.05 | 0.745 | -1.09 | 0.763 | -1.27 |
| 1436632_at | D130059P03Rik | RIKEN cDNA D130059P03 gene | -1.19 | 0.11 | -1.35 | 0.214 | -1.35 | 0.15 | -1.07 | 0.78 | -1.24 |
| 1417549_at | Zfp68 | zinc finger protein 68 | -1.17 | 0.091 | -1.2 | 0.581 | -1.56 | 0.102 | -1.22 | 0.311 | -1.29 |
| 1445455_at | D3Ertd270e | DNA segment, Chr 3, ERATO Doi 270, expressed | -1.1 | 0.89 | -1.96 | 0.08 | -1.1 | 0.847 | -1.02 | 0.93 | -1.3 |
| 1458084_at | Zdhhc17 | Zinc finger, DHHC domain containing 17 | -1.31 | 0.02 | -1.25 | 0.07 | -1.31 | 0.277 | -1.26 | 0.288 | -1.28 |
| 1428254_at | Purb | Purine rich element binding protein B | -1.15 | 0.64 | -1.19 | 0.143 | -1.61 | 0.025 | -1.08 | 0.397 | -1.26 |
| 1437872_at | AB112350 | cDNA sequence AB112350 | -1.18 | 0.446 | -1.76 | 0.374 | -1.1 | 0.776 | -1.21 | 0.555 | -1.31 |
| 1457138_x_at | Optc | Opticin | -1.28 | 0.193 | -1.31 | 0.51 | -1.28 | 0.4 | -1.59 | 0.474 | -1.36 |
| 1456156_at | Lepr | leptin receptor | -1.03 | 0.704 | -1.53 | 0.359 | -1.42 | 0.199 | -1.14 | 0.682 | -1.28 |
| 1435899_at | 9430079B08Rik | RIKEN cDNA 9430079B08 gene | -1.17 | 0.356 | -1.44 | 0.332 | -1.29 | 0.411 | -1.21 | 0.273 | -1.28 |
| 1435691_at | C630028N24Rik | RIKEN cDNA C630028N24 gene | -1.12 | 0.36 | -1.34 | 0.366 | -1.47 | 0.005 | -1.23 | 0.5 | -1.29 |
| 1458921_at | LOC434348 | hypothetical gene supported by AK043371 | -1.18 | 0.571 | -1.33 | 0.483 | -1.38 | 0.047 | -1.03 | 0.946 | -1.23 |
| 1430270_at | 4933411K05Rik | RIKEN cDNA 4933411K05 gene | -1.11 | 0.691 | -1.41 | 0.139 | -1.39 | 0.294 | -1.22 | 0.325 | -1.28 |
| 1439676_at | Aatf | apoptosis antagonizing transcription factor | -1.21 | 0.55 | -1.21 | 0.653 | -1.48 | 0.031 | -1.34 | 0.455 | -1.31 |
| 1419888_at | 1110033M05Rik | RIKEN cDNA 1110033M05 gene | -1.19 | 0.168 | -1.28 | 0.549 | -1.41 | 0.128 | -1.59 | 0.27 | -1.37 |
| 1440356_at | Ciapin1 | cytokine induced apoptosis inhibitor 1 | -1.51 | 0.493 | -1 | 0.995 | -1.49 | 0.218 | -1.47 | 0.327 | -1.37 |
| 1442956_at | Ppp1r13b | Protein phosphatase 1, regulatory (inhibitor) subunit 13B | -1.17 | 0.787 | -1.67 | 0.271 | -1.14 | 0.399 | -1.54 | 0.127 | -1.38 |
| 1436487_x_at | Fbxw2 | F-box and WD-40 domain protein 2 | -1.11 | 0.644 | -1.65 | 0.246 | -1.21 | 0.519 | -1.57 | 0.041 | -1.39 |

| | | | | | | | | | | | |
|---|---|---|---|---|---|---|---|---|---|---|---|
| 1431316_at | Itch | itchy | -1.08 | 0.587 | -1.18 | 0.273 | -1.81 | 0.005 | -1.33 | 0.482 | -1.35 |
| 1447204_at | Map4k5 | Mitogen-activated protein kinase kinase kinase kinase 5 | -1.04 | 0.889 | -2.46 | 0.067 | -1.04 | 0.911 | -1.58 | 0.007 | -1.53 |
| 1443755_at | --- | --- | -1.12 | 0.865 | -1.16 | 0.759 | -1.74 | 0.098 | -1.96 | 0.122 | -1.49 |
| 1448008_at | Ankhd1 | ankyrin repeat and KH domain containing 1 | -1.2 | 0.409 | -1.41 | 0.023 | -1.27 | 0.339 | -1.28 | 0.456 | -1.29 |
| 1441140_at | --- | --- | -1.09 | 0.511 | -1.34 | 0.057 | -1.51 | 0.048 | -1.37 | 0.188 | -1.33 |
| 1442307_at | --- | Transcribed locus | -1.13 | 0.676 | -1.68 | 0.176 | -1.18 | 0.504 | -1.01 | 0.978 | -1.25 |
| 1439775_at | Brwd3 | bromodomain and WD repeat domain containing 3 | -1.4 | 0.237 | -1.11 | 0.449 | -1.39 | 0.008 | -1.1 | 0.25 | -1.25 |
| 1441385_at | Rhoh | ras homolog gene family, member H | -1.34 | 0.265 | -1.42 | 0.304 | -1.14 | 0.667 | -5.48 | 0.002 | -2.34 |
| 1422218_at | P2rx7 | purinergic receptor P2X, ligand-gated ion channel, 7 | -1.04 | 0.877 | -1.61 | 0.247 | -1.34 | 0.289 | -1.46 | 0.057 | -1.36 |
| 1437819_s_at | 9530020G05Rik | RIKEN cDNA 9530020G05 gene | -1.19 | 0.675 | -1.63 | 0.072 | -1.15 | 0.272 | -1.21 | 0.323 | -1.29 |
| 1457107_at | Nmt2 | N-myristoyltransferase 2 | -1.39 | 0.017 | -1.19 | 0.624 | -1.31 | 0.306 | -1.09 | 0.699 | -1.24 |
| 1444444_at | --- | Adult male medulla oblongata cDNA, RIKEN full-length enriched library, clone:63 | -1.36 | 0.42 | -1.2 | 0.332 | -1.31 | 0.554 | -1.75 | 0.25 | -1.41 |
| 1432331_a_at | Prrx2 | paired related homeobox 2 | -1.39 | 0.492 | -1.3 | 0.626 | -1.19 | 0.313 | -1.42 | 0.023 | -1.32 |
| 1420098_s_at | D13Ertd787e | DNA segment, Chr 13, ERATO Doi 787, expressed | -1.14 | 0.418 | -1.18 | 0.404 | -1.65 | 0.022 | -1.76 | 0.139 | -1.43 |
| 1445107_at | A530064N14Rik | RIKEN cDNA A530064N14 gene | -1.64 | 0.031 | -1.11 | 0.767 | -1.22 | 0.575 | -1.2 | 0.384 | -1.29 |
| 1443495_at | Zfp99 | Zinc finger protein 99 | -1.4 | 0.158 | -1.45 | 0.111 | -1.08 | 0.777 | -1.33 | 0.225 | -1.31 |
| 1422850_at | Pabpn1 | poly(A) binding protein, nuclear 1 | -1.3 | 0.135 | -1.27 | 0.049 | -1.3 | 0.292 | -1.16 | 0.511 | -1.26 |
| 1421350_a_at | Grip1 | glutamate receptor interacting protein 1 | -1.47 | 0.33 | -1.34 | 0.441 | -1.1 | 0.69 | -1.05 | 0.951 | -1.24 |
| 1457593_at | 2610202C22Rik | RIKEN cDNA 2610202C22 gene | -1.11 | 0.799 | -1.15 | 0.289 | -1.79 | 0.065 | -1.18 | 0.583 | -1.31 |
| 1442385_at | Msra | Methionine sulfoxide reductase A | -1.28 | 0.813 | -1.77 | 0.076 | -1.01 | 0.965 | -1.27 | 0.51 | -1.33 |
| 1443023_at | --- | Transcribed locus | -1.24 | 0.677 | -1.53 | 0.162 | -1.14 | 0.714 | -1.34 | 0.123 | -1.32 |
| 1427177_at | Fyco1 | FYVE and coiled-coil domain containing 1 | -1.03 | 0.917 | -1.36 | 0.103 | -1.61 | 0.017 | -1.28 | 0.431 | -1.32 |
| 1420939_at | Hs6st2 | heparan sulfate 6-O-sulfotransferase 2 | -1.12 | 0.696 | -1.96 | 0.277 | -1.07 | 0.912 | -1.23 | 0.722 | -1.35 |
| 1459323_at | 1700106N22Rik | RIKEN cDNA 1700106N22 gene | -1.55 | 0.183 | -1.27 | 0.183 | -1.11 | 0.676 | -1.07 | 0.848 | -1.25 |
| 1455927_x_at | --- | --- | -1.16 | 0.112 | -1.29 | 0.059 | -1.44 | 0.036 | -1.13 | 0.58 | -1.26 |
| 1438960_at | --- | --- | -1.41 | 0.632 | -1.15 | 0.836 | -1.32 | 0.561 | -4.11 | 0.024 | -2 |
| 1456272_at | Setd5 | SET domain containing 5 | -1.04 | 0.887 | -1.22 | 0.321 | -1.81 | 0.001 | -1.11 | 0.709 | -1.3 |
| 1425993_a_at | Hsp110 | heat shock protein 110 | -1.11 | 0.628 | -1.54 | 0.106 | -1.28 | 0.285 | -1.09 | 0.824 | -1.25 |
| 1441644_at | Ptprk | Protein tyrosine phosphatase, receptor type, K | -1.05 | 0.862 | -1.31 | 0.535 | -1.63 | 0.428 | -3.82 | 0.095 | -1.95 |
| 1419915_at | D10Ertd438e | DNA segment, Chr 10, ERATO Doi 438, expressed | -1.36 | 0.36 | -1.26 | 0.267 | -1.24 | 0.116 | -1.22 | 0.525 | -1.27 |
| 1433016_s_at | 4933412F11Rik /// 4 | RIKEN cDNA 4933412F11 gene /// RIKEN cDNA 4930529H12 gene /// hypothetic | -1.51 | 0.013 | -1.24 | 0.38 | -1.16 | 0.483 | -1.02 | 0.864 | -1.23 |
| 1429434_at | Pik3ca | phosphatidylinositol 3-kinase, catalytic, alpha polypeptide | -1.11 | 0.57 | -1.25 | 0.437 | -1.57 | 0.101 | -1.14 | 0.488 | -1.27 |
| 1425848_a_at | Dusp26 | dual specificity phosphatase 26 (putative) | -1.15 | 0.706 | -1.67 | 0.36 | -1.16 | 0.587 | -1.17 | 0.635 | -1.29 |
| 1440647_at | Sipa1l1 | signal-induced proliferation-associated 1 like 1 | -1.1 | 0.812 | -1.38 | 0.369 | -1.43 | 0.039 | -1.3 | 0.235 | -1.3 |
| 1459552_at | --- | Transcribed locus | -1.45 | 0.592 | -1.07 | 0.829 | -1.4 | 0.157 | -1.19 | 0.755 | -1.28 |
| 1447021_at | --- | CDNA clone IMAGE:5686051 | -1.08 | 0.877 | -1.31 | 0.679 | -1.54 | 0.379 | -1.2 | 0.833 | -1.28 |
| 1434843_at | A430041B07Rik | RIKEN cDNA A430041B07 gene | -1.3 | 0.173 | -1.19 | 0.033 | -1.38 | 0.073 | -1.34 | 0.064 | -1.3 |
| 1433764_at | Clec2l /// LOC6651 | C-type lectin domain family, member l /// similar to CD69 antigen (p60, early T-ce | -1.33 | 0.246 | -1.41 | 0.053 | -1.14 | 0.036 | -1.12 | 0.269 | -1.25 |
| 1435779_at | Cep110 | centrosomal protein 110 | -1.19 | 0.29 | -1.71 | 0.001 | -1.1 | 0.363 | -1.57 | 0.017 | -1.39 |
| 1417568_at | Ncald | neurocalcin delta | -1.15 | 0.219 | -1.21 | 0.3 | -1.57 | 0.028 | -1.21 | 0.569 | -1.28 |
| 1453434_at | 1110019D14Rik | RIKEN cDNA 1110019D14 gene | -1.25 | 0.105 | -1.19 | 0.104 | -1.43 | 0.092 | -1.1 | 0.515 | -1.25 |
| 1424665_at | 5430405G24Rik | RIKEN cDNA 5430405G24 gene | -1.34 | 0.177 | -1.12 | 0.57 | -1.44 | 0.057 | -1.01 | 0.897 | -1.23 |
| 1432601_at | Mll5 | myeloid/lymphoid or mixed-lineage leukemia 5 | -1.22 | 0.395 | -1.64 | 0.071 | -1.11 | 0.716 | -1.5 | 0.223 | -1.37 |
| 1460527_at | 4933407C09Rik | RIKEN cDNA 4933407C09 gene | -1.05 | 0.767 | -1.33 | 0.658 | -1.6 | 0.129 | -1.14 | 0.423 | -1.28 |
| 1447034_at | Tnpo1 | Transportin 1 | -1.05 | 0.748 | -1.13 | 0.492 | -2.02 | 0.052 | -1.53 | 0.036 | -1.43 |
| 1443425_at | Adamts6 | A disintegrin-like and metallopeptidase (reprolysin type) with thrombospondin type | -1.3 | 0.209 | -1.73 | 0.068 | -1.01 | 0.979 | -1.4 | 0.127 | -1.36 |
| 1437788_at | Sp6 | trans-acting transcription factor 6 | -1.54 | 0.647 | -1.08 | 0.784 | -1.32 | 0.372 | -1.39 | 0.001 | -1.33 |
| 1430917_at | 9130401L11Rik | RIKEN cDNA 9130401L11 gene | -1.03 | 0.929 | -1.48 | 0.468 | -1.45 | 0.477 | -1.52 | 0.413 | -1.37 |
| 1456899_at | 1500004F05Rik | RIKEN cDNA 1500004F05 gene | -1.23 | 0.078 | -1.65 | 0.609 | -1.09 | 0.744 | -1.5 | 0.302 | -1.37 |
| 1441597_at | Ankhd1 | Ankyrin repeat and KH domain containing 1 | -1.12 | 0.511 | -1.14 | 0.591 | -1.77 | 0.01 | -1.12 | 0.288 | -1.29 |
| 1445570_at | Nsun4 | NOL1/NOP2/Sun domain family, member 4 | -1.36 | 0.277 | -1.1 | 0.74 | -1.43 | 0.001 | -1.62 | 0.113 | -1.38 |
| 1438245_at | --- | --- | -1.03 | 0.893 | -1.07 | 0.724 | -2.32 | 0.036 | -1.17 | 0.6 | -1.4 |
| 1444387_at | Nmt2 | N-myristoyltransferase 2 | -1.14 | 0.333 | -1.16 | 0.513 | -1.66 | 0.076 | -1.41 | 0.013 | -1.34 |

| | | | | | | | | | | | |
|---|---|---|---|---|---|---|---|---|---|---|---|
| 1438708_x_at | Ywhab | tyrosine 3-monooxygenase/tryptophan 5-monooxygenase activation protein, beta | -1.25 | 0.303 | -1.05 | 0.774 | -1.7 | 0.108 | -1.71 | 0.099 | -1.43 |
| 1436493_at | LOC626281 | similar to Cortexin-1 | -1.35 | 0.084 | -1.36 | 0.399 | -1.16 | 0.561 | -1.79 | 0.222 | -1.42 |
| 1453591_at | 5730437N04Rik | RIKEN cDNA 5730437N04 gene | -1.74 | 0.107 | -1.14 | 0.48 | -1.13 | 0.059 | -1.09 | 0.607 | -1.28 |
| 1423314_s_at | Pde7a | phosphodiesterase 7A | -1.12 | 0.136 | -1.45 | 0.255 | -1.32 | 0.059 | -1.75 | 0.137 | -1.41 |
| 1429937_at | D530033C11Rik | RIKEN cDNA D530033C11 gene | -2.14 | 0.063 | -1.06 | 0.833 | -1.08 | 0.675 | -1.04 | 0.883 | -1.33 |
| 1455459_at | Prdm15 | PR domain containing 15 | -1.17 | 0.564 | -1.35 | 0.158 | -1.34 | 0.075 | -1.36 | 0.121 | -1.31 |
| 1449306_at | Hsf2 | heat shock factor 2 | -1.16 | 0.835 | -1.37 | 0.297 | -1.34 | 0.585 | -1.18 | 0.799 | -1.26 |
| 1457358_at | --- | --- | -1.23 | 0.156 | -1.2 | 0.566 | -1.43 | 0.264 | -1.04 | 0.809 | -1.23 |
| 1439992_at | 2610206G21Rik | RIKEN cDNA 2610206G21 gene | -1.15 | 0.81 | -1.63 | 0.154 | -1.17 | 0.62 | -2.14 | 0.512 | -1.52 |
| 1419582_at | Cyp2c55 | cytochrome P450, family 2, subfamily c, polypeptide 55 | -1.21 | 0.405 | -1.24 | 0.706 | -1.42 | 0.489 | -1.1 | 0.899 | -1.24 |
| 1451859_at | Krtap6-1 | keratin associated protein 6-1 | -1.16 | 0.799 | -1.46 | 0.393 | -1.27 | 0.451 | -1.66 | 0.302 | -1.38 |
| 1417221_at | --- | --- | -1.08 | 0.733 | -1.44 | 0.298 | -1.38 | 0.207 | -1.78 | 0.467 | -1.42 |
| 1440429_at | Arpp21 | cyclic AMP-regulated phosphoprotein, 21 | -1.38 | 0.692 | -1.03 | 0.962 | -1.56 | 0.309 | -2.17 | 0.122 | -1.53 |
| 1421093_at | Slc7a10 | solute carrier family 7 (cationic amino acid transporter, y+ system), member 10 | -1.49 | 0.171 | -1.18 | 0.709 | -1.22 | 0.498 | -1.2 | 0.253 | -1.27 |
| 1437129_at | E330018D03Rik | RIKEN cDNA E330018D03 gene | -1.31 | 0.11 | -1.1 | 0.68 | -1.49 | 0.111 | -1.54 | 0.375 | -1.36 |
| 1457174_at | AU015680 | expressed sequence AU015680 | -1.16 | 0.685 | -1.11 | 0.81 | -1.74 | 0.046 | -3.57 | 0.093 | -1.89 |
| 1450896_at | Arhgap5 | Rho GTPase activating protein 5 | -1.41 | 0.416 | -1.07 | 0.763 | -1.45 | 0.076 | -1.51 | 0.223 | -1.36 |
| 1447086_at | Smc6l1 | SMC6 structural maintenance of chromosomes 6-like 1 (yeast) | -1.29 | 0.56 | -1.04 | 0.895 | -1.65 | 0.051 | -1.13 | 0.798 | -1.28 |
| 1427564_at | Diap2 | diaphanous homolog 2 (Drosophila) | -1.08 | 0.708 | -1.31 | 0.037 | -1.53 | 0.04 | -1.3 | 0.583 | -1.31 |
| 1444229_at | Nr2f2 | nuclear receptor subfamily 2, group F, member 2 | -1.15 | 0.391 | -1.57 | 0.174 | -1.2 | 0.507 | -1.1 | 0.806 | -1.26 |
| 1447537_at | 1500032P08Rik | RIKEN cDNA 1500032P08 gene | -1.02 | 0.838 | -1.63 | 0.058 | -1.34 | 0.268 | -1.21 | 0.525 | -1.3 |
| 1422289_a_at | Ctsq | cathepsin Q | -1.4 | 0.142 | -1.22 | 0.107 | -1.25 | 0.235 | -1.05 | 0.85 | -1.23 |
| 1431568_at | 4933427E13Rik | RIKEN cDNA 4933427E13 gene | -1.03 | 0.734 | -2.37 | 0.377 | -1.05 | 0.825 | -1.22 | 0.507 | -1.42 |
| 1459864_at | Gpr146 | G protein-coupled receptor 146 | -1.24 | 0.529 | -1.6 | 0.2 | -1.1 | 0.793 | -1.13 | 0.659 | -1.27 |
| 1439167_at | Pecr | peroxisomal trans-2-enoyl-CoA reductase | -1.65 | 0.036 | -1.28 | 0.319 | -1.05 | 0.838 | -1.06 | 0.897 | -1.26 |
| 1458097_at | Cobll1 | Cobl-like 1 | -1.17 | 0.061 | -1.18 | 0.185 | -1.56 | 0.021 | -1.68 | 0.104 | -1.4 |
| 1432822_at | 4930557B21Rik | RIKEN cDNA 4930557B21 gene | -1.85 | 0.43 | -1 | 0.987 | -1.24 | 0.356 | -1.35 | 0.582 | -1.36 |
| 1439980_at | D14Ertd725e | DNA segment, Chr 14, ERATO Doi 725, expressed | -1.31 | 0.219 | -1.03 | 0.762 | -1.64 | 0.089 | -1.28 | 0.226 | -1.31 |
| 1453534_at | 2810004I08Rik | RIKEN cDNA 2810004I08 gene | -1.37 | 0.396 | -1.01 | 0.969 | -1.6 | 0.06 | -1.11 | 0.693 | -1.27 |
| 1417179_at | Tspan5 | tetraspanin 5 | -1.01 | 0.984 | -1.51 | 0.159 | -1.46 | 0.156 | -1.14 | 0.408 | -1.28 |
| 1458284_at | Ptbp1 | polypyrimidine tract binding protein 1 | -1.49 | 0.006 | -1.33 | 0.221 | -1.09 | 0.693 | -1.08 | 0.788 | -1.25 |
| 1429358_at | 4921533L14Rik | RIKEN cDNA 4921533L14 gene | -1.16 | 0.822 | -1.46 | 0.381 | -1.26 | 0.637 | -3.55 | 0.025 | -1.86 |
| 1448156_at | Tff1 | trefoil factor 1 | -1.29 | 0.568 | -1.51 | 0.235 | -1.11 | 0.676 | -1.01 | 0.951 | -1.23 |
| 1418008_at | 1810007M14Rik | RIKEN cDNA 1810007M14 gene | -1.25 | 0.143 | -1.43 | 0.084 | -1.19 | 0.386 | -1.22 | 0.564 | -1.27 |
| 1444890_at | AA536749 | Expressed sequence AA536749 | -1.17 | 0.453 | -1.11 | 0.711 | -1.7 | 0.069 | -1.3 | 0.319 | -1.32 |
| 1453353_at | 1700047I16Rik | RIKEN cDNA 1700047I16 gene | -2.24 | 0.478 | -1.03 | 0.901 | -1.08 | 0.877 | -1.08 | 0.585 | -1.36 |
| 1436540_at | 6720427I07Rik | RIKEN cDNA 6720427I07 gene | -1.39 | 0.334 | -1.17 | 0.56 | -1.29 | 0.254 | -1.35 | 0.219 | -1.3 |
| 1437388_at | Fut10 | fucosyltransferase 10 | -1.12 | 0.619 | -1.62 | 0.359 | -1.19 | 0.494 | -1.5 | 0.143 | -1.36 |
| 1441462_at | Dock4 | dedicator of cytokinesis 4 | -1.12 | 0.429 | -1.1 | 0.083 | -1.84 | 0.031 | -1.12 | 0.558 | -1.29 |
| 1437942_x_at | Tube1 | epsilon-tubulin 1 | -1.27 | 0.439 | -1.43 | 0.38 | -1.17 | 0.347 | -1.07 | 0.799 | -1.23 |
| 1442759_at | Akt1 | thymoma viral proto-oncogene 1 | -1.2 | 0.52 | -1.12 | 0.839 | -1.62 | 0.151 | -1.02 | 0.905 | -1.24 |
| 1457639_at | Atp6v1h | ATPase, H+ transporting, lysosomal V1 subunit H | -1.18 | 0.402 | -1 | 0.99 | -2 | 0.039 | -1.99 | 0.186 | -1.54 |
| 1421101_a_at | Ldb2 | LIM domain binding 2 | -1.34 | 0.11 | -1.21 | 0.407 | -1.3 | 0.263 | -1.62 | 0.071 | -1.37 |
| 1420187_at | C76628 | expressed sequence C76628 | -1.95 | 0.056 | -1.19 | 0.815 | -1.01 | 0.979 | -1.26 | 0.665 | -1.35 |
| 1429784_at | C130032J12Rik | RIKEN cDNA C130032J12 gene | -1.15 | 0.392 | -1.22 | 0.16 | -1.53 | 0.006 | -1.23 | 0.245 | -1.28 |
| 1442671_at | Hip2 | Huntingtin interacting protein 2 | -1.41 | 0.214 | -1.05 | 0.795 | -1.46 | 0.075 | -1.02 | 0.872 | -1.24 |
| 1450666_s_at | Atxn10 | ataxin 10 | -1.5 | 0.066 | -1.09 | 0.679 | -1.31 | 0.362 | -1.28 | 0.277 | -1.3 |
| 1425644_at | Lepr | leptin receptor | -1.21 | 0.359 | -1.44 | 0.599 | -1.22 | 0.35 | -1.28 | 0.251 | -1.28 |
| 1443362_at | Zfp277 | zinc finger protein 277 | -1.23 | 0.261 | -1.28 | 0.183 | -1.33 | 0.012 | -1.67 | 0.281 | -1.38 |
| 1440068_at | Rfxdc2 | Regulatory factor X domain containing 2 homolog (human) | -1.38 | 0.183 | -1.47 | 0.092 | -1.07 | 0.607 | -2.08 | 0.13 | -1.5 |
| 1422223_at | Grin2b | glutamate receptor, ionotropic, NMDA2B (epsilon 2) | -1.43 | 0.476 | -1.06 | 0.906 | -1.42 | 0.572 | -2.74 | 0.2 | -1.66 |
| 1421364_at | Lrfn1 | leucine rich repeat and fibronectin type III domain containing 1 | -1.6 | 0.055 | -1.25 | 0.279 | -1.09 | 0.663 | -1.22 | 0.259 | -1.29 |
| 1447859_at | Adck2 | aarF domain containing kinase 2 | -1.06 | 0.861 | -1.28 | 0.71 | -1.6 | 0.277 | -1.28 | 0.167 | -1.31 |

| | | | | | | | | | | | |
|---|---|---|---|---|---|---|---|---|---|---|---|
| 1459086_at | A430107P09Rik | RIKEN cDNA A430107P09 gene | -1.09 | 0.867 | -2.06 | 0.373 | -1.06 | 0.882 | -1.35 | 0.474 | -1.39 |
| 1434172_at | Cnr1 | cannabinoid receptor 1 (brain) | -1.15 | 0.844 | -1.08 | 0.915 | -1.82 | 0.067 | -2.46 | 0.046 | -1.63 |
| 1456514_at | --- | --- | -1.11 | 0.109 | -1.34 | 0.057 | -1.42 | 0.116 | -1.28 | 0.245 | -1.29 |
| 1419261_at | Acad8 | acyl-Coenzyme A dehydrogenase family, member 8 | -1.14 | 0.765 | -1.62 | 0.037 | -1.18 | 0.273 | -1.02 | 0.932 | -1.24 |
| 1459722_at | Zswim6 | Zinc finger, SWIM domain containing 6 | -1.13 | 0.316 | -1.12 | 0.27 | -1.77 | 0.041 | -1.74 | 0.077 | -1.44 |
| 1456822_at | Rad23b | RAD23b homolog (S. cerevisiae) | -1.37 | 0.485 | -1.17 | 0.069 | -1.32 | 0.182 | -1.52 | 0.339 | -1.34 |
| 1442703_at | AK220484 | cDNA sequence AK220484 | -1.59 | 0.312 | -1.31 | 0.552 | -1.05 | 0.913 | -1.23 | 0.594 | -1.29 |
| 1421312_a_at | Kifc2 | kinesin family member C2 | -1.31 | 0.647 | -1.4 | 0.237 | -1.15 | 0.723 | -1.91 | 0.361 | -1.44 |
| 1442494_at | Ubr2 | Ubiquitin protein ligase E3 component n-recognin 2 | -1.38 | 0.161 | -1.09 | 0.553 | -1.42 | 0.016 | -1.37 | 0.036 | -1.31 |
| 1437994_x_at | Mier2 | mesoderm induction early response 1, family member 2 | -1.41 | 0.026 | -1.23 | 0.245 | -1.21 | 0.055 | -1.81 | 0.024 | -1.42 |
| 1450242_at | Tlr5 | toll-like receptor 5 | -1.22 | 0.684 | -1.83 | 0.162 | -1.02 | 0.91 | -1.17 | 0.435 | -1.31 |
| 1420378_at | Sftpd | surfactant associated protein D | -1.34 | 0.418 | -1.09 | 0.884 | -1.46 | 0.003 | -1.08 | 0.801 | -1.24 |
| 1459573_at | Bxdc5 | brix domain containing 5 | -1.19 | 0.394 | -1.21 | 0.1 | -1.48 | 0.023 | -1.31 | 0.252 | -1.29 |
| 1457197_at | Prkacb | protein kinase, cAMP dependent, catalytic, beta | -1.09 | 0.933 | -1.14 | 0.733 | -1.81 | 0.064 | -1.02 | 0.945 | -1.27 |
| 1432504_at | 1700106N22Rik | RIKEN cDNA 1700106N22 gene | -1.32 | 0.505 | -1.45 | 0.204 | -1.11 | 0.788 | -2.05 | 0.038 | -1.48 |
| 1426061_x_at | --- | --- | -1.79 | 0.373 | -1.21 | 0.473 | -1.04 | 0.831 | -1.59 | 0.182 | -1.41 |
| 1424690_at | 5930434B04Rik | RIKEN cDNA 5930434B04 gene | -1.04 | 0.918 | -1.45 | 0.091 | -1.43 | 0.254 | -1.72 | 0.073 | -1.41 |
| 1419581_at | Dlgh4 | discs, large homolog 4 (Drosophila) | -1.11 | 0.787 | -1.81 | 0.213 | -1.12 | 0.697 | -1.43 | 0.516 | -1.37 |
| 1436873_at | 4732454E20Rik | RIKEN cDNA 4732454E20 gene | -1.15 | 0.691 | -1.6 | 0.284 | -1.18 | 0.731 | -1.46 | 0.514 | -1.35 |
| 1426082_a_at | Slc16a4 | solute carrier family 16 (monocarboxylic acid transporters), member 4 | -1.06 | 0.265 | -1.95 | 0.093 | -1.12 | 0.079 | -1.02 | 0.934 | -1.29 |
| 1452227_at | 2310045A20Rik | RIKEN cDNA 2310045A20 gene | -1.28 | 0.231 | -1.68 | 0.171 | -1.03 | 0.858 | -1.27 | 0.394 | -1.31 |
| 1440872_at | BC010304 | CDNA sequence BC010304 | -1.68 | 0.165 | -1.31 | 0.511 | -1.01 | 0.964 | -1.27 | 0.487 | -1.32 |
| 1446244_at | 2810482G21Rik | RIKEN cDNA 2810482G21 gene | -1.33 | 0.303 | -1.25 | 0.078 | -1.25 | 0.29 | -1.88 | 0.122 | -1.43 |
| 1444406_at | Map4k3 | Mitogen-activated protein kinase kinase kinase kinase 3 | -1.02 | 0.893 | -1.15 | 0.479 | -2.01 | 0.014 | -1.2 | 0.107 | -1.34 |
| 1428412_at | Tm9sf3 | transmembrane 9 superfamily member 3 | -1.14 | 0.385 | -1.26 | 0.055 | -1.48 | 0.033 | -1.26 | 0.162 | -1.28 |
| 1452728_at | Kirrel3 | kin of IRRE like 3 (Drosophila) | -1.24 | 0.627 | -1.42 | 0.174 | -1.19 | 0.416 | -1.49 | 0.581 | -1.34 |
| 1440374_at | Pde1c | Phosphodiesterase 1C | -1.31 | 0.428 | -1.05 | 0.891 | -1.57 | 0.089 | -1 | 0.996 | -1.23 |
| 1436052_at | 1700020O03Rik | RIKEN cDNA 1700020O03 gene | -1.18 | 0.634 | -1.15 | 0.457 | -1.59 | 0.01 | -1.42 | 0.11 | -1.33 |
| 1456171_at | Rnf190 | ring finger protein 190 | -1.4 | 0.266 | -1.37 | 0.444 | -1.1 | 0.489 | -1.1 | 0.822 | -1.24 |
| 1429900_at | 5330406M23Rik | RIKEN cDNA 5330406M23 gene | -1.12 | 0.576 | -1.16 | 0.39 | -1.68 | 0.073 | -1.51 | 0.184 | -1.37 |
| 1437045_at | Mapk8 | mitogen activated protein kinase 8 | -1.18 | 0.126 | -1.21 | 0.298 | -1.47 | 0.057 | -1.33 | 0.292 | -1.3 |
| 1430017_at | 1600016N20Rik | RIKEN cDNA 1600016N20 gene | -1.38 | 0.015 | -1.6 | 0.43 | -1 | 0.997 | -1.3 | 0.152 | -1.32 |
| 1446521_at | Psmd14 | Proteasome (prosome, macropain) 26S subunit, non-ATPase, 14 | -1.17 | 0.534 | -1.46 | 0.013 | -1.23 | 0.195 | -1.24 | 0.103 | -1.28 |
| 1437339_s_at | Pcsk5 | proprotein convertase subtilisin/kexin type 5 | -1.1 | 0.393 | -1.13 | 0.68 | -1.8 | 0.012 | -1.58 | 0.333 | -1.4 |
| 1456879_at | C130022K22Rik | RIKEN cDNA C130022K22 gene | -1.15 | 0.62 | -1.51 | 0.208 | -1.22 | 0.166 | -1.09 | 0.407 | -1.24 |
| 1446773_at | E230025N22 | Hypothetical protein E230025N22 | -1.12 | 0.897 | -1.07 | 0.874 | -1.93 | 0.045 | -1.29 | 0.449 | -1.35 |
| 1442026_at | --- | --- | -1.2 | 0.846 | -1.18 | 0.645 | -1.5 | 0.476 | -1.46 | 0.15 | -1.33 |
| 1417406_at | Sertad1 | SERTA domain containing 1 | -1.1 | 0.575 | -1.3 | 0.004 | -1.48 | 0.106 | -1.16 | 0.338 | -1.26 |
| 1444302_at | BC049816 | cDNA sequence BC049816 | -1.66 | 0.427 | -1.17 | 0.743 | -1.12 | 0.761 | -2.15 | 0.151 | -1.52 |
| 1440022_at | Poldip3 | polymerase (DNA-directed), delta interacting protein 3 | -1.06 | 0.852 | -1.48 | 0.323 | -1.37 | 0.14 | -1.35 | 0.244 | -1.31 |
| 1443260_at | Meis1 | myeloid ecotropic viral integration site 1 | -1.09 | 0.5 | -1.17 | 0.692 | -1.74 | 0.116 | -1.17 | 0.575 | -1.29 |
| 1458510_at | AU019823 | expressed sequence AU019823 | -1.42 | 0.187 | -1.07 | 0.808 | -1.39 | 0.069 | -1.51 | 0.301 | -1.35 |
| 1442979_at | --- | PREDICTED: Mus musculus similar to zinc finger protein 709 (LOC629191), mRI | -1.17 | 0.864 | -1.95 | 0.408 | -1.02 | 0.889 | -1.19 | 0.679 | -1.33 |
| 1459942_at | ORF34 | Open reading frame 34 | -1.17 | 0.544 | -1.14 | 0.738 | -1.61 | 0.163 | -1 | 0.997 | -1.23 |
| 1447450_at | Pparbp | Peroxisome proliferator activated receptor binding protein | -1.25 | 0.603 | -1.26 | 0.068 | -1.33 | 0.088 | -1.3 | 0.328 | -1.28 |
| 1427200_at | Zranb1 | zinc finger, RAN-binding domain containing 1 | -1.11 | 0.492 | -1.34 | 0.019 | -1.41 | 0.031 | -1.01 | 0.943 | -1.22 |
| 1428583_at | Nufip2 | nuclear fragile X mental retardation protein interacting protein 2 | -1.06 | 0.734 | -1.42 | 0.088 | -1.42 | 0.093 | -1.09 | 0.67 | -1.25 |
| 1441408_at | AU044856 | expressed sequence AU044856 | -1.11 | 0.782 | -1.34 | 0.58 | -1.41 | 0.31 | -1.16 | 0.778 | -1.26 |
| 1429963_at | Mapk6 | mitogen-activated protein kinase 6 | -1.24 | 0.722 | -1.2 | 0.74 | -1.41 | 0.03 | -1.23 | 0.319 | -1.27 |
| 1426361_at | Zc3h11a | zinc finger CCCH type containing 11A | -1.14 | 0.433 | -1.32 | 0.121 | -1.4 | 0.025 | -1.23 | 0.348 | -1.27 |
| 1444473_at | Pcaf | P300/CBP-associated factor | -1.2 | 0.418 | -1.19 | 0.087 | -1.47 | 0.099 | -1.26 | 0.185 | -1.28 |
| 1446889_at | D2Ertd105e | DNA segment, Chr 2, ERATO Doi 105, expressed | -1.12 | 0.911 | -1.32 | 0.468 | -1.43 | 0.418 | -1.4 | 0.442 | -1.32 |
| 1441169_at | B4galt6 | UDP-Gal:betaGlcNAc beta 1,4-galactosyltransferase, polypeptide 6 | -1.38 | 0.268 | -1.19 | 0.797 | -1.27 | 0.449 | -1.01 | 0.977 | -1.21 |

| | | | | | | | | | | | |
|---|---|---|---|---|---|---|---|---|---|---|---|
| 1434081_at | Ap1g1 | adaptor protein complex AP-1, gamma 1 subunit | -1.16 | 0.567 | -1.3 | 0.143 | -1.39 | 0.061 | -1.31 | 0.399 | -1.29 |
| 1443993_at | Zc3h12a | zinc finger CCCH type containing 12A | -1.56 | 0.468 | -1.11 | 0.743 | -1.23 | 0.507 | -1.29 | 0.266 | -1.3 |
| 1438033_at | Tef | thyrotroph embryonic factor | -1.54 | 0.405 | -1.28 | 0.498 | -1.08 | 0.769 | -1.2 | 0.23 | -1.27 |
| 1427512_a_at | Lama3 /// LOC6698 | laminin, alpha 3 /// similar to Laminin alpha-3 chain precursor (Nicein alpha subur | -1.41 | 0.062 | -1.24 | 0.799 | -1.19 | 0.636 | -1.59 | 0.334 | -1.36 |
| 1457218_at | 6430510M02Rik | RIKEN cDNA 6430510M02 gene | -1.02 | 0.837 | -1.47 | 0.162 | -1.44 | 0.233 | -2.02 | 0.193 | -1.49 |
| 1436854_at | Trpc2 | transient receptor potential cation channel, subfamily C, member 2 | -1.26 | 0.301 | -1.44 | 0.186 | -1.16 | 0.518 | -1.41 | 0.151 | -1.32 |
| 1459173_at | Ptprd | protein tyrosine phosphatase, receptor type, D | -2.17 | 0.018 | -1.06 | 0.865 | -1.05 | 0.924 | -1.8 | 0.015 | -1.52 |
| 1454230_a_at | Slc25a27 | solute carrier family 25, member 27 | -1.04 | 0.921 | -1.59 | 0.392 | -1.3 | 0.44 | -1.66 | 0.389 | -1.4 |
| 1442351_a_at | BC029214 | cDNA sequence BC029214 | -1.03 | 0.934 | -2.05 | 0.097 | -1.12 | 0.554 | -2.52 | 0.081 | -1.68 |
| 1446571_at | A830039H10Rik | RIKEN cDNA A830039H10 gene | -1.03 | 0.724 | -1.28 | 0.118 | -1.64 | 0.007 | -1.16 | 0.237 | -1.28 |
| 1420721_at | 4921536K21Rik | RIKEN cDNA 4921536K21 gene | -1.14 | 0.696 | -1.18 | 0.696 | -1.59 | 0.256 | -1.7 | 0.232 | -1.4 |
| 1431560_at | Dcakd | dephospho-CoA kinase domain containing | -1.21 | 0.507 | -1.58 | 0.231 | -1.11 | 0.696 | -1.4 | 0.036 | -1.33 |
| 1417425_at | Prkrip1 | Prkr interacting protein 1 (IL11 inducible) | -1.02 | 0.932 | -1.41 | 0.027 | -1.49 | 0.015 | -1.15 | 0.667 | -1.27 |
| 1418416_x_at | Psg23 | pregnancy-specific glycoprotein 23 | -1.8 | 0.144 | -1.21 | 0.533 | -1.03 | 0.913 | -1.66 | 0.33 | -1.42 |
| 1446722_at | Gna13 | Guanine nucleotide binding protein, alpha 13 | -1.06 | 0.715 | -1.2 | 0.35 | -1.73 | 0.072 | -1.33 | 0.181 | -1.33 |
| 1450935_at | Ercc5 | excision repair cross-complementing rodent repair deficiency, complementation g | -1.21 | 0.43 | -1.36 | 0.002 | -1.26 | 0.256 | -1.39 | 0.069 | -1.31 |
| 1457478_at | Strbp | Spermatid perinuclear RNA binding protein | -1.17 | 0.68 | -1.35 | 0.561 | -1.32 | 0.119 | -1.98 | 0.253 | -1.45 |
| 1442683_at | Hmg20a | high mobility group 20A | -1.33 | 0.45 | -1.07 | 0.867 | -1.5 | 0.107 | -2.26 | 0.107 | -1.54 |
| 1440873_at | --- | --- | -1.71 | 0.033 | -1.08 | 0.582 | -1.18 | 0.359 | -1.04 | 0.942 | -1.25 |
| 1438987_at | --- | --- | -1.63 | 0.042 | -1.24 | 0.618 | -1.06 | 0.802 | -1.15 | 0.675 | -1.27 |
| 1440579_at | --- | --- | -1.16 | 0.189 | -1.13 | 0.333 | -1.65 | 0.007 | -1.13 | 0.633 | -1.27 |
| 1457642_at | 5730507N06Rik | RIKEN cDNA 5730507N06 gene | -1.26 | 0.225 | -1.17 | 0.258 | -1.41 | 0.321 | -1.08 | 0.881 | -1.23 |
| 1426899_at | Tbc1d23 | TBC1 domain family, member 23 | -1.53 | 0.123 | -1.23 | 0.191 | -1.12 | 0.35 | -1.24 | 0.633 | -1.28 |
| 1428288_at | Klf9 | Kruppel-like factor 9 | -1.2 | 0.651 | -1.25 | 0.229 | -1.38 | 0.166 | -1.05 | 0.883 | -1.22 |
| 1423473_at | 2-Sep | septin 2 | -1.27 | 0.525 | -1.15 | 0.672 | -1.42 | 0.299 | -1 | 0.995 | -1.21 |
| 1457454_at | Usp47 | ubiquitin specific peptidase 47 | -1.01 | 0.966 | -1.35 | 0.313 | -1.59 | 0.043 | -1.3 | 0.234 | -1.31 |
| 1451568_at | A630054L15Rik | RIKEN cDNA A630054L15 gene | -1.11 | 0.506 | -1.3 | 0.426 | -1.45 | 0.13 | -1.04 | 0.887 | -1.23 |
| 1428651_at | Klhl24 | kelch-like 24 (Drosophila) | -1.16 | 0.313 | -1.29 | 0.232 | -1.39 | 0.37 | -1.86 | 0.155 | -1.42 |
| 1453874_at | 4933401B06Rik | RIKEN cDNA 4933401B06 gene | -1.26 | 0.531 | -1 | 0.994 | -1.75 | 0.052 | -1.33 | 0.303 | -1.34 |
| 1459332_at | Itch | itchy | -1.16 | 0.227 | -1.18 | 0.328 | -1.54 | 0.091 | -1.46 | 0.045 | -1.34 |
| 1429538_a_at | 5730406M06Rik | RIKEN cDNA 5730406M06 gene | -1.26 | 0.016 | -1.15 | 0.377 | -1.44 | 0.14 | -1.29 | 0.371 | -1.28 |
| 1448112_at | Cox7c | cytochrome c oxidase, subunit VIIc | -1.18 | 0.027 | -1.33 | 0.013 | -1.31 | 0.019 | -1.12 | 0.16 | -1.24 |
| 1429914_at | Epc1 | enhancer of polycomb homolog 1 (Drosophila) | -1.08 | 0.313 | -1.62 | 0.134 | -1.22 | 0.098 | -1.13 | 0.66 | -1.26 |
| 1433063_at | Wdr66 | WD repeat domain 66 | -1.24 | 0.712 | -1.36 | 0.528 | -1.22 | 0.392 | -1.55 | 0.041 | -1.34 |
| 1428637_at | Dyrk2 | dual-specificity tyrosine-(Y)-phosphorylation regulated kinase 2 | -1.04 | 0.803 | -1.25 | 0.062 | -1.67 | 0.024 | -1.72 | 0.122 | -1.42 |
| 1419730_at | 4631427C17Rik | RIKEN cDNA 4631427C17 gene | -1.26 | 0.206 | -1.15 | 0.437 | -1.44 | 0.04 | -1.09 | 0.527 | -1.23 |
| 1441364_at | Gata4 | GATA binding protein 4 | -1.02 | 0.97 | -1.14 | 0.409 | -1.99 | 0.166 | -1.48 | 0.594 | -1.41 |
| 1431391_at | Ralgps1 | Ral GEF with PH domain and SH3 binding motif 1 | -1.37 | 0.262 | -1.06 | 0.855 | -1.46 | 0.012 | -1.03 | 0.851 | -1.23 |
| 1460487_at | 1110014L15Rik | RIKEN cDNA 1110014L15 gene | -1.44 | 0.599 | -1.13 | 0.871 | -1.28 | 0.164 | -1.16 | 0.538 | -1.25 |
| 1440734_at | Ttbk2 | tau tubulin kinase 2 | -1.13 | 0.69 | -1.4 | 0.208 | -1.32 | 0.477 | -1.3 | 0.73 | -1.29 |
| 1459008_at | --- | --- | -1.12 | 0.32 | -1.13 | 0.177 | -1.72 | 0.021 | -1.11 | 0.098 | -1.27 |
| 1443279_at | Nlk | Nemo like kinase | -1.3 | 0.36 | -1.04 | 0.903 | -1.59 | 0.059 | -1.33 | 0.289 | -1.32 |
| 1445605_s_at | 4921533L14Rik | RIKEN cDNA 4921533L14 gene | -1.39 | 0.213 | -1.14 | 0.789 | -1.32 | 0.435 | -2.44 | 0.238 | -1.57 |
| 1421829_at | Ak3l1 | adenylate kinase 3 alpha-like 1 | -1.04 | 0.848 | -2.26 | 0.042 | -1.04 | 0.945 | -1.1 | 0.654 | -1.36 |
| 1446802_at | Atox1 | ATX1 (antioxidant protein 1) homolog 1 (yeast) | -1.09 | 0.205 | -1.1 | 0.639 | -1.86 | 0.021 | -1.08 | 0.717 | -1.28 |
| 1443312_at | Rfx4 | Regulatory factor X, 4 (influences HLA class II expression) | -1.18 | 0.534 | -1.41 | 0.288 | -1.25 | 0.784 | -1.06 | 0.903 | -1.22 |
| 1426192_at | Smarcd2 | SWI/SNF related, matrix associated, actin dependent regulator of chromatin, subf | -1.39 | 0.029 | -1.16 | 0.564 | -1.28 | 0.268 | -1.32 | 0.298 | -1.29 |
| 1429565_s_at | Lce5a | late cornified envelope 5A | -1.18 | 0.872 | -1.69 | 0.253 | -1.09 | 0.807 | -2.19 | 0.019 | -1.54 |
| 1421951_at | Lhx1 | LIM homeobox protein 1 | -1.15 | 0.134 | -1.56 | 0.141 | -1.18 | 0.822 | -2.89 | 0.054 | -1.69 |
| 1444396_at | Trp53inp2 | Tumor protein p53 inducible nuclear protein 2 | -1.22 | 0.251 | -1.59 | 0.112 | -1.1 | 0.748 | -1.39 | 0.471 | -1.32 |
| 1428467_at | Tardbp | TAR DNA binding protein | -1.21 | 0.057 | -1.31 | 0.135 | -1.29 | 0.249 | -1.31 | 0.4 | -1.28 |
| 1457895_at | Kif18a | kinesin family member 18A | -1.34 | 0.545 | -1.57 | 0.629 | -1.02 | 0.95 | -1.17 | 0.809 | -1.27 |
| 1449329_at | Zfp235 | zinc finger protein 235 | -1.14 | 0.769 | -1.7 | 0.079 | -1.12 | 0.723 | -2 | 0.268 | -1.49 |

| | | | | | | | | | | | |
|---|---|---|---|---|---|---|---|---|---|---|---|
| 1444184_at | Alkbh3 | alkB, alkylation repair homolog 3 (E. coli) | -1.4 | 0.04 | -1.13 | 0.433 | -1.3 | 0.303 | -1.01 | 0.818 | -1.21 |
| 1434770_at | Iqcb1 | IQ calmodulin-binding motif containing 1 | -1.25 | 0.321 | -1.39 | 0.105 | -1.19 | 0.092 | -1.13 | 0.601 | -1.24 |
| 1455170_at | 2810001G20Rik | RIKEN cDNA 2810001G20 gene | -1.07 | 0.522 | -1.58 | 0.003 | -1.25 | 0.418 | -1.28 | 0.524 | -1.3 |
| 1438429_at | 2610319H10Rik | RIKEN cDNA 2610319H10 gene | -1.1 | 0.523 | -1.25 | 0.295 | -1.51 | 0.208 | -1.55 | 0.16 | -1.36 |
| 1454770_at | Cckbr | cholecystokinin B receptor | -1.67 | 0.599 | -1 | 0.993 | -1.3 | 0.167 | -1.08 | 0.606 | -1.26 |
| 1431135_at | --- | --- | -1.21 | 0.405 | -1.45 | 0.18 | -1.18 | 0.546 | -1.14 | 0.841 | -1.24 |
| 1444934_at | Zfp276 | zinc finger protein (C2H2 type) 276 | -1.15 | 0.414 | -1.38 | 0.103 | -1.3 | 0.192 | -1.25 | 0.063 | -1.27 |
| 1432716_at | Lrrn1 | leucine rich repeat protein 1, neuronal | -1.23 | 0.772 | -1.07 | 0.829 | -1.63 | 0.212 | -1.44 | 0.567 | -1.34 |
| 1418066_at | Cfl2 | cofilin 2, muscle | -1.28 | 0.082 | -1.18 | 0.335 | -1.36 | 0.062 | -1.21 | 0.529 | -1.26 |
| 1455449_at | Gm468 | gene model 468, (NCBI) | -1.07 | 0.662 | -1.17 | 0.684 | -1.72 | 0.343 | -1.4 | 0.358 | -1.34 |
| 1445759_at | Smpd3 | Sphingomyelin phosphodiesterase 3, neutral | -1.21 | 0.336 | -1.18 | 0.705 | -1.44 | 0.044 | -1.48 | 0.024 | -1.33 |
| 1439089_at | Zbtb41 | zinc finger and BTB domain containing 41 homolog | -1.16 | 0.21 | -1.48 | 0.009 | -1.21 | 0.037 | -1.01 | 0.914 | -1.22 |
| 1447690_at | Lsr | lipolysis stimulated lipoprotein receptor | -2.36 | 0.415 | -1.05 | 0.863 | -1.01 | 0.971 | -2.27 | 0.181 | -1.67 |
| 1437205_at | Tcf25 | transcription factor 25 (basic helix-loop-helix) | -1.18 | 0.182 | -1.28 | 0.005 | -1.35 | 0.108 | -1.24 | 0.311 | -1.26 |
| 1447442_at | Helz | Helicase with zinc finger domain | -1.31 | 0.221 | -1.18 | 0.194 | -1.33 | 0.179 | -1.52 | 0.204 | -1.34 |
| 1442766_at | --- | Transcribed locus | -1.63 | 0.309 | -1.03 | 0.904 | -1.27 | 0.453 | -1.51 | 0.047 | -1.36 |
| 1426568_at | Slc2a9 | solute carrier family 2 (facilitated glucose transporter), member 9 | -1.44 | 0.349 | -1.44 | 0.247 | -1.02 | 0.895 | -1.77 | 0.268 | -1.42 |
| 1420056_s_at | Ptdsr | phosphatidylserine receptor | -1.06 | 0.714 | -1.55 | 0.031 | -1.29 | 0.132 | -1.12 | 0.067 | -1.25 |
| 1456575_at | Ndn | Necdin | -2.23 | 0.166 | -1.07 | 0.909 | -1.02 | 0.961 | -1.07 | 0.923 | -1.35 |
| 1429284_at | 8430436F23Rik | RIKEN cDNA 8430436F23 gene | -1.13 | 0.006 | -1.22 | 0.297 | -1.51 | 0.104 | -1.55 | 0.13 | -1.35 |
| 1455366_at | Mrpl52 | mitochondrial ribosomal protein L52 | -1.59 | 0.348 | -1.29 | 0.177 | -1.04 | 0.88 | -1.25 | 0.478 | -1.29 |
| 1442864_at | Esrra | estrogen related receptor, alpha | -1.33 | 0.316 | -1.18 | 0.682 | -1.3 | 0.353 | -1.51 | 0.32 | -1.33 |
| 1454970_at | --- | --- | -1.35 | 0.364 | -1.59 | 0.207 | -1.01 | 0.981 | -1.02 | 0.879 | -1.24 |
| 1445021_at | 6030465E24Rik | RIKEN cDNA 6030465E24 gene | -1.18 | 0.688 | -1.41 | 0.547 | -1.24 | 0.647 | -2.13 | 0.011 | -1.49 |
| 1427666_a_at | Tcrb-J | T-cell receptor beta, joining region | -1.19 | 0.544 | -1.28 | 0.374 | -1.35 | 0.092 | -3.86 | 0.06 | -1.92 |
| 1448975_s_at | Ren1 /// Ren2 | renin 1 structural /// renin 2 tandem duplication of Ren1 | -1.69 | 0.307 | -1.09 | 0.777 | -1.16 | 0.239 | -1.42 | 0.502 | -1.34 |
| 1434420_x_at | Tomm22 | translocase of outer mitochondrial membrane 22 homolog (yeast) | -1.17 | 0.234 | -1.21 | 0.244 | -1.46 | 0.039 | -1.14 | 0.547 | -1.25 |
| 1453384_at | 4632404N19Rik | RIKEN cDNA 4632404N19 gene | -1.05 | 0.87 | -1.47 | 0.021 | -1.37 | 0.081 | -1.7 | 0.2 | -1.4 |
| 1454604_s_at | Tspan12 | tetraspanin 12 | -1.31 | 0.315 | -1.09 | 0.516 | -1.47 | 0.101 | -1.08 | 0.599 | -1.24 |
| 1458156_at | E230012J19Rik | RIKEN cDNA E230012J19 gene | -1.21 | 0.059 | -1.23 | 0.289 | -1.38 | 0.237 | -1.22 | 0.421 | -1.26 |
| 1453710_at | Tmem116 | transmembrane protein 116 | -1.04 | 0.507 | -1.31 | 0.658 | -1.56 | 0.229 | -2.27 | 0.145 | -1.54 |
| 1434656_at | B230339M05Rik | RIKEN cDNA B230339M05 gene | -1.14 | 0.231 | -1.39 | 0.043 | -1.3 | 0.089 | -1.2 | 0.12 | -1.26 |
| 1447913_x_at | Akap9 | A kinase (PRKA) anchor protein (yotiao) 9 | -1.18 | 0.144 | -1.6 | 0.165 | -1.12 | 0.714 | -1.42 | 0.333 | -1.33 |
| 1441253_at | Rfx3 | Regulatory factor X, 3 (influences HLA class II expression) | -1.13 | 0.698 | -1.31 | 0.092 | -1.39 | 0.197 | -1.43 | 0.047 | -1.32 |
| 1442692_at | Mapkap1 | Mitogen-activated protein kinase associated protein 1 | -1.15 | 0.662 | -1.28 | 0.357 | -1.4 | 0.271 | -1.26 | 0.338 | -1.27 |
| 1434283_at | Arid5b | AT rich interactive domain 5B (Mrf1 like) | -1.14 | 0.315 | -1.57 | 0.166 | -1.18 | 0.254 | -1.28 | 0.293 | -1.29 |
| 1439317_at | Krit1 | KRIT1, ankyrin repeat containing | -1.18 | 0.603 | -1.3 | 0.24 | -1.33 | 0.164 | -1.62 | 0.024 | -1.36 |
| 1439209_at | Tcf12 | Transcription factor 12 | -1.29 | 0.416 | -1.04 | 0.91 | -1.58 | 0.004 | -1.38 | 0.084 | -1.32 |
| 1434642_at | Dhrs8 | dehydrogenase/reductase (SDR family) member 8 | -1.05 | 0.531 | -1.78 | 0.032 | -1.17 | 0.21 | -1.31 | 0.459 | -1.33 |
| 1421433_at | Zfhx4 | zinc finger homeodomain 4 | -1.69 | 0.368 | -1.09 | 0.626 | -1.16 | 0.789 | -1.97 | 0.268 | -1.48 |
| 1442358_at | AA409587 | expressed sequence AA409587 | -1.23 | 0.001 | -1.11 | 0.239 | -1.53 | 0.032 | -1.23 | 0.267 | -1.27 |
| 1454284_at | Slc25a25 | solute carrier family 25 (mitochondrial carrier, phosphate carrier), member 25 | -1.02 | 0.966 | -1.38 | 0.556 | -1.51 | 0.314 | -1.08 | 0.88 | -1.25 |
| 1445866_at | Mast4 | microtubule associated serine/threonine kinase family member 4 | -1.15 | 0.371 | -1.12 | 0.514 | -1.63 | 0.076 | -1.6 | 0.021 | -1.38 |
| 1436673_at | 2610020H08Rik | RIKEN cDNA 2610020H08 gene | -1.18 | 0.509 | -1.14 | 0.653 | -1.55 | 0.016 | -1.64 | 0.035 | -1.38 |
| 1454819_at | --- | --- | -1.04 | 0.814 | -1.49 | 0.101 | -1.36 | 0.041 | -1.03 | 0.875 | -1.23 |
| 1458919_at | Mkln1 | muskelin 1, intracellular mediator containing kelch motifs | -1.14 | 0.194 | -1.08 | 0.503 | -1.77 | 0.024 | -1.03 | 0.862 | -1.26 |
| 1434692_at | 1110034B05Rik | RIKEN cDNA 1110034B05 gene | -1.21 | 0.045 | -1.39 | 0.025 | -1.22 | 0.146 | -1.37 | 0.258 | -1.3 |
| 1444877_at | --- | --- | -1.8 | 0.261 | -1.01 | 0.979 | -1.22 | 0.576 | -2.56 | 0.048 | -1.65 |
| 1431222_at | Tiam2 | T-cell lymphoma invasion and metastasis 2 | -1.17 | 0.494 | -1.31 | 0.099 | -1.33 | 0.241 | -1.04 | 0.819 | -1.21 |
| 1415769_at | Itch | itchy | -1.28 | 0.249 | -1.23 | 0.23 | -1.29 | 0.049 | -1.57 | 0.012 | -1.34 |
| 1432160_at | Git2 | G protein-coupled receptor kinase-interactor 2 | -1.18 | 0.676 | -1.53 | 0.47 | -1.15 | 0.568 | -1.92 | 0.118 | -1.44 |
| 1454635_at | Fbxl3 | F-box and leucine-rich repeat protein 3 | -1.33 | 0.103 | -1.06 | 0.334 | -1.47 | 0.113 | -1.41 | 0.162 | -1.32 |
| 1440622_at | Sec15l2 | SEC15-like 2 (S. cerevisiae) | -1.2 | 0.654 | -1.03 | 0.885 | -1.77 | 0.04 | -1.52 | 0.503 | -1.38 |

| | | | | | | | | | | | |
|---|---|---|---|---|---|---|---|---|---|---|---|
| 1453321_at | Fndc1 | fibronectin type III domain containing 1 | -1.18 | 0.372 | -1.36 | 0.205 | -1.27 | 0.478 | -1.03 | 0.838 | -1.21 |
| 1430681_at | Cryl1 | crystallin, lamda 1 | -1.34 | 0.057 | -1.27 | 0.428 | -1.2 | 0.278 | -1.46 | 0.378 | -1.32 |
| 1432351_at | 9030625G05Rik | RIKEN cDNA 9030625G05 gene | -1.11 | 0.856 | -1.5 | 0.136 | -1.25 | 0.76 | -2.07 | 0.314 | -1.48 |
| 1431961_at | Dock8 | dedicator of cytokinesis 8 | -1.09 | 0.486 | -1.25 | 0.193 | -1.54 | 0.223 | -2.24 | 0.06 | -1.53 |
| 1429650_at | Stk40 | serine/threonine kinase 40 | -1.28 | 0.166 | -1.3 | 0.02 | -1.22 | 0.431 | -1.06 | 0.767 | -1.21 |
| 1447153_x_at | Ulk2 | Unc-51 like kinase 2 (C. elegans) | -1.03 | 0.926 | -1.44 | 0.158 | -1.42 | 0.235 | -1.49 | 0.248 | -1.35 |
| 1430856_at | Pex11c | peroxisomal biogenesis factor 11c | -1.46 | 0.282 | -1.37 | 0.006 | -1.04 | 0.857 | -1.7 | 0.183 | -1.39 |
| 1415731_at | Angel2 | angel homolog 2 (Drosophila) | -1.35 | 0.194 | -1.2 | 0.494 | -1.25 | 0.133 | -1.24 | 0.179 | -1.26 |
| 1459143_at | Chchd3 | coiled-coil-helix-coiled-coil-helix domain containing 3 | -1.07 | 0.777 | -1.64 | 0.038 | -1.21 | 0.579 | -1.59 | 0.169 | -1.38 |
| 1459150_at | Lrch1 | Leucine-rich repeats and calponin homology (CH) domain containing 1 | -1.29 | 0.008 | -1.11 | 0.612 | -1.43 | 0.12 | -1.73 | 0.074 | -1.39 |
| 1423533_a_at | Rhot1 | ras homolog gene family, member T1 | -1.17 | 0.192 | -1.38 | 0.03 | -1.26 | 0.105 | -1.08 | 0.478 | -1.22 |
| 1456439_x_at | Mical1 | microtubule associated monoxygenase, calponin and LIM domain containing 1 | -1.12 | 0.688 | -1.68 | 0.36 | -1.13 | 0.355 | -2.13 | 0.07 | -1.51 |
| 1418563_at | Serbp1 | Serpine1 mRNA binding protein 1 | -1.15 | 0.099 | -1.26 | 0.032 | -1.4 | 0.02 | -1.06 | 0.38 | -1.22 |
| 1449647_at | --- | Transcribed locus | -1.65 | 0.226 | -1.28 | 0.626 | -1.02 | 0.978 | -1.03 | 0.86 | -1.25 |
| 1456997_at | Ncoa5 | Nuclear receptor coactivator 5 | -1.08 | 0.702 | -1.17 | 0.548 | -1.7 | 0.027 | -1.16 | 0.273 | -1.28 |
| 1443068_at | D130084N16Rik | RIKEN cDNA D130084N16 gene | -1.18 | 0.273 | -1.33 | 0.295 | -1.29 | 0.207 | -1.42 | 0.522 | -1.31 |
| 1446269_at | Hbp1 | high mobility group box transcription factor 1 | -1.33 | 0.284 | -1.34 | 0.512 | -1.14 | 0.686 | -1.41 | 0.208 | -1.31 |
| 1437839_x_at | Mrpl11 | mitochondrial ribosomal protein L11 | -1.34 | 0.141 | -1.03 | 0.888 | -1.52 | 0.003 | -1.35 | 0.296 | -1.31 |
| 1433252_at | 4932703K07Rik | RIKEN cDNA 4932703K07 gene | -1.77 | 0.417 | -1.04 | 0.935 | -1.19 | 0.633 | -1.34 | 0.381 | -1.33 |
| 1418374_at | Fxyd3 | FXYD domain-containing ion transport regulator 3 | -1.12 | 0.783 | -1.06 | 0.734 | -1.85 | 0.018 | -1.28 | 0.459 | -1.33 |
| 1454177_at | 4930597G03Rik | RIKEN cDNA 4930597G03 gene | -1.4 | 0.599 | -1.4 | 0.408 | -1.06 | 0.8 | -1.07 | 0.676 | -1.23 |
| 1428996_at | 4833426J09Rik | RIKEN cDNA 4833426J09 gene | -1.09 | 0.78 | -1.64 | 0.217 | -1.19 | 0.542 | -1.07 | 0.746 | -1.25 |
| 1447504_at | Ghitm | Growth hormone inducible transmembrane protein | -1.17 | 0.438 | -1.13 | 0.555 | -1.58 | 0.169 | -1.11 | 0.577 | -1.25 |
| 1430833_at | Itk | IL2-inducible T-cell kinase | -1.04 | 0.926 | -1.31 | 0.683 | -1.54 | 0.207 | -1.72 | 0.342 | -1.4 |
| 1422980_a_at | Bet1l | blocked early in transport 1 homolog (S. cerevisiae)-like | -1.24 | 0.265 | -1.22 | 0.029 | -1.35 | 0.058 | -1.45 | 0.016 | -1.31 |
| 1447821_at | 1700051K22Rik | RIKEN cDNA 1700051K22 gene | -1.61 | 0.072 | -1.27 | 0.065 | -1.04 | 0.824 | -1.5 | 0.051 | -1.35 |
| 1441823_at | Rai17 | retinoic acid induced 17 | -1.18 | 0.475 | -1.16 | 0.464 | -1.5 | 0.101 | -1.62 | 0.088 | -1.37 |
| 1436316_at | 9430029L20Rik | RIKEN cDNA 9430029L20 gene | -1.33 | 0.642 | -1.19 | 0.573 | -1.28 | 0.393 | -1.28 | 0.498 | -1.27 |
| 1435350_at | Traf6 | Tnf receptor-associated factor 6 | -1.17 | 0.339 | -1.38 | 0.41 | -1.27 | 0.116 | -1.36 | 0.323 | -1.29 |
| 1422575_at | Mxd4 /// LOC63515 | Max dimerization protein 4 /// similar to Max dimerization protein 4 | -1.54 | 0.05 | -1.27 | 0.176 | -1.07 | 0.848 | -1.17 | 0.731 | -1.26 |
| 1429356_s_at | Ggps1 | geranylgeranyl diphosphate synthase 1 | -1.11 | 0.408 | -1.99 | 0.038 | -1.04 | 0.746 | -1.07 | 0.629 | -1.3 |
| 1417866_at | Tnfaip1 | tumor necrosis factor, alpha-induced protein 1 (endothelial) | -1.23 | 0.2 | -1.13 | 0.514 | -1.47 | 0.04 | -1.38 | 0.064 | -1.3 |
| 1444726_at | Krr1 | KRR1, small subunit (SSU) processome component, homolog (yeast) | -1.19 | 0.315 | -1.49 | 0.101 | -1.16 | 0.534 | -1.4 | 0.209 | -1.31 |
| 1432719_at | 4833412K13Rik | RIKEN cDNA 4833412K13 gene | -1.19 | 0.019 | -1.2 | 0.031 | -1.43 | 0.124 | -1.17 | 0.594 | -1.25 |
| 1436798_at | Rpl9 /// LOC432768 | ribosomal protein L9 /// similar to ribosomal protein L9 /// similar to ribosomal prot | -1.25 | 0.018 | -1.34 | 0.066 | -1.2 | 0.233 | -1.36 | 0.141 | -1.29 |
| 1457455_at | Suhw4 | suppressor of hairy wing homolog 4 (Drosophila) | -1.13 | 0.403 | -1.17 | 0.135 | -1.58 | 0.133 | -1 | 0.972 | -1.22 |
| 1455086_at | Uchl5 | Ubiquitin carboxyl-terminal esterase L5 | -1.12 | 0.724 | -1.36 | 0.437 | -1.34 | 0.338 | -1.83 | 0.174 | -1.41 |
| 1417622_at | Slc12a2 | solute carrier family 12, member 2 | -1.16 | 0.498 | -1.08 | 0.29 | -1.7 | 0.026 | -1.02 | 0.963 | -1.24 |
| 1441716_at | --- | 12 days embryo eyeball cDNA, RIKEN full-length enriched library, clone:D230024 | -1.49 | 0.295 | -1.15 | 0.659 | -1.2 | 0.103 | -1.12 | 0.16 | -1.24 |
| 1431566_at | --- | --- | -1.24 | 0.519 | -1.37 | 0.029 | -1.19 | 0.284 | -1.14 | 0.635 | -1.24 |
| 1429463_at | Prkaa2 | protein kinase, AMP-activated, alpha 2 catalytic subunit | -1 | 0.998 | -1.42 | 0.302 | -1.49 | 0.193 | -1.21 | 0.745 | -1.28 |
| 1449775_x_at | Slc35a4 /// LOC545 | solute carrier family 35, member A4 /// similar to solute carrier family 35, member | -1.56 | 0.536 | -1.28 | 0.686 | -1.05 | 0.918 | -1.02 | 0.952 | -1.23 |
| 1428364_at | Scnm1 | sodium channel modifier 1 | -1.23 | 0.172 | -1.36 | 0.103 | -1.21 | 0.156 | -1.13 | 0.55 | -1.23 |
| 1437557_at | D3Ertd254e | DNA segment, Chr 3, ERATO Doi 254, expressed | -1.26 | 0.101 | -1.28 | 0.154 | -1.24 | 0.078 | -1.47 | 0.068 | -1.31 |
| 1425289_a_at | Cr2 | complement receptor 2 | -1.18 | 0.527 | -1.5 | 0.63 | -1.16 | 0.586 | -4.2 | 0.027 | -2.01 |
| 1449581_at | Emid1 | EMI domain containing 1 | -1.05 | 0.779 | -1.21 | 0.679 | -1.68 | 0.162 | -2.73 | 0.059 | -1.67 |
| 1443350_at | Mta3 | metastasis associated 3 | -1.31 | 0.328 | -1.25 | 0.484 | -1.23 | 0.202 | -1.47 | 0.169 | -1.32 |
| 1439262_x_at | 2310001A20Rik | RIKEN cDNA 2310001A20 gene | -1.21 | 0.584 | -1.31 | 0.232 | -1.27 | 0.066 | -1.43 | 0.204 | -1.31 |
| 1442704_at | Baz2b | Bromodomain adjacent to zinc finger domain, 2B | -1.04 | 0.702 | -1.23 | 0.097 | -1.66 | 0.057 | -1.76 | 0.063 | -1.42 |
| 1417525_at | Hand1 | heart and neural crest derivatives expressed transcript 1 | -2.01 | 0.227 | -1.1 | 0.793 | -1.03 | 0.921 | -1.35 | 0.537 | -1.37 |
| 1446470_at | --- | --- | -1.5 | 0.088 | -1.21 | 0.543 | -1.13 | 0.613 | -1.21 | 0.496 | -1.26 |
| 1432174_a_at | 4933425K02Rik | RIKEN cDNA 4933425K02 gene | -1.26 | 0.64 | -1.36 | 0.553 | -1.18 | 0.628 | -1.28 | 0.679 | -1.27 |
| 1431552_at | 1810006J02Rik | RIKEN cDNA 1810006J02 gene | -1.51 | 0.2 | -1.1 | 0.883 | -1.25 | 0.58 | -1.43 | 0.483 | -1.32 |

| | | | | | | | | | | | |
|---|---|---|---|---|---|---|---|---|---|---|---|
| 1459995_at | 1700015G11Rik | RIKEN cDNA 1700015G11 gene | -1.21 | 0.831 | -1.01 | 0.925 | -1.79 | 0.154 | -1.61 | 0.507 | -1.4 |
| 1456001_at | LOC545548 | similar to late cornified envelope protein | -1.27 | 0.362 | -1.38 | 0.409 | -1.16 | 0.471 | -1.51 | 0.496 | -1.33 |
| 1418720_at | Cops7b | COP9 (constitutive photomorphogenic) homolog, subunit 7b (Arabidopsis thaliana | -1.34 | 0.086 | -1.57 | 0.01 | -1 | 0.979 | -1.07 | 0.722 | -1.25 |
| 1449187_at | Pdgfa | platelet derived growth factor, alpha | -1 | 0.96 | -1.54 | 0.088 | -1.37 | 0.163 | -1.19 | 0.396 | -1.28 |
| 1445695_at | Atxn1 | Ataxin 1 | -1.1 | 0.723 | -1.13 | 0.344 | -1.71 | 0.017 | -1.25 | 0.364 | -1.3 |
| 1460085_at | Zfp281 | Zinc finger protein 281 | -1.73 | 0.049 | -1.14 | 0.418 | -1.08 | 0.462 | -1.3 | 0.224 | -1.31 |
| 1440051_at | Ppp3ca | Protein phosphatase 3, catalytic subunit, alpha isoform | -1.1 | 0.783 | -1.06 | 0.791 | -1.91 | 0.004 | -1.87 | 0.186 | -1.49 |
| 1457598_at | Txnl2 | Thioredoxin-like 2 | -1.09 | 0.665 | -1.75 | 0.099 | -1.13 | 0.678 | -1.16 | 0.317 | -1.28 |
| 1440853_at | Rhot1 | ras homolog gene family, member T1 | -1.12 | 0.829 | -1.61 | 0.046 | -1.16 | 0.239 | -1.06 | 0.82 | -1.24 |
| 1435074_at | Tmem106b | transmembrane protein 106B | -1.23 | 0.294 | -1.24 | 0.106 | -1.31 | 0.231 | -1.6 | 0.238 | -1.35 |
| 1446304_at | --- | --- | -1.25 | 0.294 | -1.18 | 0.409 | -1.37 | 0.273 | -1.17 | 0.588 | -1.24 |
| 1443719_x_at | Ddx42 | DEAD (Asp-Glu-Ala-Asp) box polypeptide 42 | -1.06 | 0.68 | -1.22 | 0.302 | -1.63 | 0.038 | -1.2 | 0.169 | -1.28 |
| 1434584_a_at | Pramel5 /// LOC384 | preferentially expressed antigen in melanoma like 5 /// Unknown (protein for IMAC | -1.34 | 0.685 | -1.4 | 0.397 | -1.1 | 0.73 | -2.5 | 0.094 | -1.58 |
| 1455322_at | Ttc21b | tetratricopeptide repeat domain 21B | -1.34 | 0.187 | -1.27 | 0.14 | -1.19 | 0.226 | -1.2 | 0.326 | -1.25 |
| 1433766_at | C330023M02Rik | RIKEN cDNA C330023M02 gene | -1.22 | 0.242 | -1.26 | 0.017 | -1.31 | 0.009 | -1.15 | 0.011 | -1.24 |
| 1451058_at | Mcts2 | malignant T cell amplified sequence 2 | -1.16 | 0.104 | -1.17 | 0.112 | -1.52 | 0.023 | -1.34 | 0.062 | -1.3 |
| 1447002_at | --- | --- | -1.75 | 0.618 | -1.23 | 0.666 | -1.01 | 0.991 | -1.12 | 0.79 | -1.28 |
| 1452899_at | Rian | RNA imprinted and accumulated in nucleus | -1.26 | 0.398 | -1.02 | 0.925 | -1.65 | 0.329 | -1.11 | 0.882 | -1.26 |
| 1435218_at | Rasgef1a | RasGEF domain family, member 1A | -1.39 | 0.513 | -1.05 | 0.776 | -1.42 | 0.125 | -1.53 | 0.192 | -1.35 |
| 1418623_at | Rab2 | RAB2, member RAS oncogene family | -1.44 | 0.194 | -1.38 | 0.457 | -1.04 | 0.837 | -1.64 | 0.221 | -1.38 |
| 1458994_at | Csnk1g3 | Casein kinase 1, gamma 3 | -1.16 | 0.101 | -1.13 | 0.487 | -1.58 | 0.016 | -1.98 | 0.089 | -1.46 |
| 1423278_at | Ptprk | protein tyrosine phosphatase, receptor type, K | -1.16 | 0.27 | -1.09 | 0.226 | -1.66 | 0.016 | -1.27 | 0.153 | -1.3 |
| 1429996_at | 6330417A16Rik | RIKEN cDNA 6330417A16 gene | -1.14 | 0.405 | -1.27 | 0.184 | -1.4 | 0.179 | -1.26 | 0.299 | -1.27 |
| 1453270_a_at | Phf14 | PHD finger protein 14 | -1.08 | 0.881 | -1.35 | 0.413 | -1.4 | 0.22 | -1.37 | 0.271 | -1.3 |
| 1442823_at | --- | 0 day neonate eyeball cDNA, RIKEN full-length enriched library, clone:E130001F | -1.04 | 0.588 | -1.8 | 0.203 | -1.16 | 0.515 | -1.29 | 0.711 | -1.32 |
| 1437264_at | BC051142 | cDNA sequence BC051142 | -1.81 | 0.44 | -1.06 | 0.893 | -1.13 | 0.468 | -1.12 | 0.721 | -1.28 |
| 1457064_at | D030002E05Rik | RIKEN cDNA D030002E05 gene | -1.08 | 0.867 | -1.36 | 0.315 | -1.39 | 0.126 | -1.59 | 0.061 | -1.36 |
| 1429518_at | Faim2 | Fas apoptotic inhibitory molecule 2 | -1.43 | 0.226 | -1.37 | 0.117 | -1.05 | 0.861 | -1.02 | 0.955 | -1.22 |
| 1435300_at | --- | --- | -1.45 | 0.021 | -1.36 | 0.142 | -1.05 | 0.78 | -1.78 | 0.009 | -1.41 |
| 1456913_at | Tmod3 | Tropomodulin 3 | -1.09 | 0.776 | -1.18 | 0.373 | -1.63 | 0.02 | -1.59 | 0.132 | -1.37 |
| 1437227_at | 2900073H19Rik | RIKEN cDNA 2900073H19 gene | -1.12 | 0.749 | -1.73 | 0.01 | -1.1 | 0.797 | -1.9 | 0.222 | -1.46 |
| 1446608_at | Cbl | Casitas B-lineage lymphoma | -1.46 | 0.063 | -1.28 | 0.506 | -1.09 | 0.765 | -1.12 | 0.714 | -1.24 |
| 1421569_at | Grid1 | glutamate receptor, ionotropic, delta 1 | -1.32 | 0.586 | -1.23 | 0.734 | -1.24 | 0.618 | -1.25 | 0.198 | -1.26 |
| 1424664_at | BC017647 | cDNA sequence BC017647 | -1.6 | 0.179 | -1.27 | 0.415 | -1.03 | 0.899 | -1.19 | 0.784 | -1.27 |
| 1443338_at | Ap2m1 | Adaptor protein complex AP-2, mu1 | -1.06 | 0.826 | -1.27 | 0.118 | -1.54 | 0.027 | -1.19 | 0.029 | -1.26 |
| 1424377_at | LOC670726 | similar to ribosomal protein L24-like | -1.14 | 0.17 | -1.39 | 0.061 | -1.28 | 0.128 | -1.01 | 0.885 | -1.2 |
| 1440167_s_at | Lpp | LIM domain containing preferred translocation partner in lipoma | -1.05 | 0.558 | -1.18 | 0.396 | -1.72 | 0.03 | -1.33 | 0.37 | -1.32 |
| 1435788_at | 2900086B20Rik | RIKEN cDNA 2900086B20 gene | -1.13 | 0.035 | -1.33 | 0.135 | -1.34 | 0.129 | -1.13 | 0.394 | -1.23 |
| 1444371_at | A630007B06Rik | RIKEN cDNA A630007B06 gene | -1.11 | 0.564 | -1.22 | 0.558 | -1.51 | 0.07 | -1.54 | 0.017 | -1.35 |
| 1457051_at | Trim27 | tripartite motif protein 27 | -1.43 | 0.539 | -1.25 | 0.268 | -1.13 | 0.68 | -1.17 | 0.476 | -1.25 |
| 1456952_at | Arid1b | AT rich interactive domain 1B (Swi1 like) | -1.2 | 0.38 | -1.14 | 0.255 | -1.49 | 0.071 | -1.79 | 0.042 | -1.4 |
| 1432665_at | 2210416J07Rik | RIKEN cDNA 2210416J07 gene | -1.28 | 0.34 | -1.26 | 0.748 | -1.24 | 0.3 | -1.04 | 0.779 | -1.21 |
| 1423637_at | Galnt4 | UDP-N-acetyl-alpha-D-galactosamine:polypeptide N-acetylgalactosaminyltransfer | -1.32 | 0.267 | -1.14 | 0.766 | -1.35 | 0.253 | -1.7 | 0.024 | -1.37 |
| 1452041_at | Klhl26 | kelch-like 26 (Drosophila) | -1.44 | 0.201 | -1.25 | 0.058 | -1.13 | 0.58 | -1.45 | 0.01 | -1.32 |
| 1455617_at | Lmbrd1 | LMBR1 domain containing 1 | -1.13 | 0.143 | -1.33 | 0.254 | -1.34 | 0.204 | -1.41 | 0.02 | -1.3 |
| 1453790_at | 5730403B10Rik | RIKEN cDNA 5730403B10 gene | -1.03 | 0.899 | -1.86 | 0.012 | -1.15 | 0.541 | -1.32 | 0.441 | -1.34 |
| 1439769_at | Zfp644 | Zinc finger protein 644 | -1.02 | 0.925 | -1.69 | 0.162 | -1.23 | 0.139 | -1.09 | 0.565 | -1.26 |
| 1432536_at | 1700055M20Rik | RIKEN cDNA 1700055M20 gene | -1.43 | 0.682 | -1.04 | 0.947 | -1.39 | 0.292 | -1.06 | 0.75 | -1.23 |
| 1453106_a_at | Rnmt | RNA (guanine-7-) methyltransferase | -1.26 | 0.091 | -1.16 | 0.149 | -1.36 | 0.025 | -1.01 | 0.954 | -1.2 |
| 1425895_a_at | Id1 | inhibitor of DNA binding 1 | -1.87 | 0.317 | -1.11 | 0.837 | -1.06 | 0.777 | -1.51 | 0.082 | -1.39 |
| 1455115_a_at | Crb3 | crumbs homolog 3 (Drosophila) | -1.22 | 0.51 | -1.16 | 0.736 | -1.41 | 0.149 | -1.88 | 0.414 | -1.42 |
| 1439221_s_at | Cd40 | CD40 antigen | -1.27 | 0.628 | -1.03 | 0.948 | -1.6 | 0.006 | -2.76 | 0.147 | -1.66 |
| 1448773_at | Rpl11 /// LOC1941 | ribosomal protein L11 /// similar to 60S ribosomal protein L11 /// similar to 60S rib | -1.39 | 0.325 | -1.14 | 0.314 | -1.26 | 0.334 | -1.22 | 0.201 | -1.25 |

| | | | | | | | | | | | |
|---|---|---|---|---|---|---|---|---|---|---|---|
| 1418749_at | Psd3 | pleckstrin and Sec7 domain containing 3 | -1.08 | 0.48 | -1.2 | 0.12 | -1.6 | 0.016 | -1.08 | 0.659 | -1.24 |
| 1439583_x_at | D130029J02Rik | RIKEN cDNA D130029J02 gene | -1.55 | 0.32 | -1.02 | 0.863 | -1.32 | 0.045 | -1.78 | 0.011 | -1.42 |
| 1443148_at | Hip2 | huntingtin interacting protein 2 | -1.2 | 0.518 | -1.09 | 0.505 | -1.58 | 0.061 | -1.55 | 0.031 | -1.36 |
| 1450809_at | Sval2 | seminal vesicle antigen-like 2 | -1.64 | 0.413 | -1.11 | 0.839 | -1.14 | 0.844 | -1.05 | 0.953 | -1.24 |
| 1440013_at | Trim44 | Tripartite motif-containing 44 | -1.06 | 0.437 | -1.09 | 0.522 | -1.91 | 0.004 | -1.27 | 0.256 | -1.33 |
| 1439010_at | D330037H05Rik /// | RIKEN cDNA D330037H05 gene /// similar to La-related protein 4 (La ribonucleop | -1.18 | 0.06 | -1.17 | 0.156 | -1.46 | 0.07 | -1.04 | 0.659 | -1.21 |
| 1453286_at | Plxna2 | plexin A2 | -1.09 | 0.75 | -1.27 | 0.3 | -1.49 | 0.161 | -1.03 | 0.879 | -1.22 |
| 1458556_at | Tnrc6c | Trinucleotide repeat containing 6C | -1.01 | 0.938 | -1.31 | 0.477 | -1.6 | 0.17 | -2.11 | 0.297 | -1.51 |
| 1458023_at | DXErtd697e | DNA segment, Chr X, ERATO Doi 697, expressed | -1.19 | 0.121 | -1.15 | 0.627 | -1.47 | 0.035 | -1.53 | 0.033 | -1.34 |
| 1423342_at | Barx1 | BarH-like homeobox 1 | -1.59 | 0.064 | -1.23 | 0.371 | -1.06 | 0.796 | -2.6 | 0.002 | -1.62 |
| 1418116_at | Ifrg15 | interferon alpha responsive gene | -1.14 | 0.502 | -1.34 | 0.103 | -1.32 | 0.03 | -1.08 | 0.533 | -1.22 |
| 1442415_at | 5830454E08Rik | RIKEN cDNA 5830454E08 gene | -1.01 | 0.973 | -1.91 | 0.019 | -1.16 | 0.408 | -1.38 | 0.269 | -1.36 |
| 1434954_at | --- | --- | -1.17 | 0.717 | -1.14 | 0.365 | -1.53 | 0.134 | -1.18 | 0.312 | -1.26 |
| 1456430_at | Ccdc39 | coiled-coil domain containing 39 | -1.15 | 0.266 | -1.08 | 0.699 | -1.7 | 0.125 | -1.37 | 0.152 | -1.33 |
| 1416483_at | Ttc3 | tetratricopeptide repeat domain 3 | -1.17 | 0.252 | -1.09 | 0.4 | -1.64 | 0.098 | -1.6 | 0.101 | -1.37 |
| 1445199_at | --- | Transcribed locus | -1.68 | 0.439 | -1.13 | 0.814 | -1.11 | 0.598 | -1.03 | 0.902 | -1.24 |
| 1436145_at | Igf2bp3 | Insulin-like growth factor 2, binding protein 3 | -1.11 | 0.55 | -1.2 | 0.308 | -1.54 | 0.055 | -1.23 | 0.493 | -1.27 |
| 1435321_at | 3732412D22Rik | RIKEN cDNA 3732412D22 gene | -1.14 | 0.199 | -1.31 | 0.463 | -1.34 | 0.153 | -1.67 | 0.065 | -1.37 |
| 1460151_at | --- | --- | -1.04 | 0.912 | -1.2 | 0.331 | -1.68 | 0.066 | -1.21 | 0.081 | -1.29 |
| 1422692_at | Sub1 | SUB1 homolog (S. cerevisia | -1.12 | 0.136 | -1.4 | 0.003 | -1.29 | 0.114 | -1.16 | 0.609 | -1.24 |
| 1451552_at | MGC28431 | Similar to lipoyltransferase | -1.28 | 0.071 | -1.3 | 0.048 | -1.2 | 0.295 | -1.18 | 0.32 | -1.24 |
| 1419977_s_at | Rbm35b | RNA binding motif protein 35b | -1.45 | 0.107 | -1.01 | 0.959 | -1.42 | 0.12 | -1.17 | 0.217 | -1.26 |
| 1455014_at | AV009015 | expressed sequence AV009015 | -1.21 | 0.157 | -1.12 | 0.067 | -1.5 | 0.013 | -1.28 | 0.103 | -1.28 |
| 1442660_at | Ypel1 | Yippee-like 1 (Drosophila) | -1.12 | 0.61 | -1.39 | 0.065 | -1.29 | 0.359 | -1.29 | 0.306 | -1.27 |
| 1441324_at | Zfp395 | Zinc finger protein 395 | -1.24 | 0.642 | -1.28 | 0.408 | -1.25 | 0.436 | -1.4 | 0.287 | -1.29 |
| 1450657_at | Ppie | peptidylprolyl isomerase E (cyclophilin E) | -1.25 | 0.404 | -1 | 0.969 | -1.7 | 0.156 | -1.19 | 0.163 | -1.28 |
| 1439467_at | Mtap4 | Microtubule-associated protein 4 | -1.11 | 0.651 | -1.09 | 0.117 | -1.77 | 0.022 | -1.49 | 0.133 | -1.36 |
| 1430064_at | 4933424A20Rik | RIKEN cDNA 4933424A20 gene | -1.23 | 0.328 | -1.73 | 0.294 | -1 | 0.988 | -1.2 | 0.201 | -1.29 |
| 1439276_at | --- | --- | -1.47 | 0.061 | -1.3 | 0.241 | -1.07 | 0.84 | -1.5 | 0.092 | -1.33 |
| 1427849_a_at | Tcrb-V13 | T-cell receptor beta, variable 13 | -1.07 | 0.923 | -1.23 | 0.604 | -1.58 | 0.41 | -2.15 | 0.041 | -1.5 |
| 1439447_x_at | Rpl37a /// LOC629 | ribosomal protein L37a /// similar to 60S ribosomal protein L37a /// similar to 60S | -1.53 | 0.126 | -1.04 | 0.778 | -1.3 | 0.315 | -1.47 | 0.131 | -1.33 |
| 1423873_at | Lsm1 | LSM1 homolog, U6 small nuclear RNA associated (S. cerevisiae) | -1.12 | 0.085 | -1.05 | 0.716 | -1.86 | 0.024 | -1.49 | 0.072 | -1.38 |
| 1429215_at | 2310058N22Rik | RIKEN cDNA 2310058N22 gene | -1.15 | 0.649 | -1.15 | 0.489 | -1.54 | 0.018 | -1.12 | 0.664 | -1.24 |
| 1440650_at | Slit2 | Slit homolog 2 (Drosophila) | -1.33 | 0.119 | -1.01 | 0.964 | -1.56 | 0.17 | -1.46 | 0.653 | -1.34 |
| 1419177_at | Vps37a | vacuolar protein sorting 37A (yeast) | -1.06 | 0.669 | -1.23 | 0.2 | -1.58 | 0.045 | -1.27 | 0.442 | -1.29 |
| 1446209_at | Usp40 | ubiquitin specific peptidase 40 | -1.26 | 0.047 | -1.02 | 0.727 | -1.63 | 0.008 | -1.53 | 0.149 | -1.36 |
| 1420295_x_at | Clcn5 | chloride channel 5 | -1.12 | 0.359 | -1.4 | 0.216 | -1.27 | 0.43 | -1.24 | 0.694 | -1.26 |
| 1444224_at | Tnfaip8 | Tumor necrosis factor, alpha-induced protein 8 | -1.4 | 0.557 | -1.41 | 0.349 | -1.04 | 0.941 | -1.4 | 0.36 | -1.31 |
| 1437238_x_at | Nmd3 | NMD3 homolog (S. cerevisiae) | -1.11 | 0.402 | -1.41 | 0.079 | -1.29 | 0.129 | -1.45 | 0.031 | -1.31 |
| 1428658_at | Pin4 /// LOC62816 | protein (peptidyl-prolyl cis/trans isomerase) NIMA-interacting, 4 (parvulin) /// simil | -1.1 | 0.423 | -1.16 | 0.154 | -1.63 | 0.006 | -1.04 | 0.808 | -1.23 |
| 1455146_at | C230082I21Rik | RIKEN cDNA C230082I21 gene | -1.3 | 0.17 | -1.11 | 0.725 | -1.39 | 0.107 | -1.93 | 0.281 | -1.43 |
| 1442258_at | --- | --- | -1.1 | 0.621 | -1.65 | 0 | -1.15 | 0.468 | -1.49 | 0.111 | -1.34 |
| 1459063_at | --- | Transcribed locus | -1.11 | 0.786 | -1.66 | 0.052 | -1.13 | 0.796 | -1 | 0.998 | -1.23 |
| 1429611_at | 1700034E13Rik | RIKEN cDNA 1700034E13 gene | -1.86 | 0.344 | -1.01 | 0.972 | -1.16 | 0.629 | -1.17 | 0.879 | -1.3 |
| 1453023_at | Ankhd1 | ankyrin repeat and KH domain containing 1 | -1.22 | 0.518 | -1.26 | 0.589 | -1.28 | 0.064 | -1.2 | 0.222 | -1.24 |
| 1444818_at | --- | --- | -1.37 | 0.631 | -1.21 | 0.491 | -1.2 | 0.72 | -1.1 | 0.666 | -1.22 |
| 1454439_at | 4833419G08Rik | RIKEN cDNA 4833419G08 gene | -1.14 | 0.615 | -1.58 | 0.181 | -1.13 | 0.745 | -1.48 | 0.035 | -1.34 |
| 1445985_at | Traf3ip2 | Traf3 interacting protein 2 | -1.02 | 0.981 | -1.28 | 0.716 | -1.59 | 0.158 | -2.06 | 0.236 | -1.49 |
| 1440906_at | Bcl7c | B-cell CLL/lymphoma 7C | -1.17 | 0.635 | -1.3 | 0.36 | -1.3 | 0.439 | -1.54 | 0.583 | -1.33 |
| 1432933_at | 4930429N05Rik | RIKEN cDNA 4930429N05 gene | -1.08 | 0.502 | -2.14 | 0.158 | -1.01 | 0.993 | -1.69 | 0.43 | -1.48 |
| 1447860_x_at | Cog8 | component of oligomeric golgi complex 8 | -1.06 | 0.696 | -1.25 | 0.169 | -1.55 | 0.146 | -1.91 | 0.173 | -1.44 |
| 1460548_a_at | Eral1 | Era (G-protein)-like 1 (E. coli) | -1.08 | 0.661 | -1.26 | 0.186 | -1.49 | 0.014 | -1.69 | 0.052 | -1.38 |
| 1454678_s_at | A130022J15Rik | RIKEN cDNA A130022J15 gene | -1.15 | 0.099 | -1.34 | 0.047 | -1.29 | 0.118 | -1.15 | 0.571 | -1.23 |

| | | | | | | | | | | | |
|---|---|---|---|---|---|---|---|---|---|---|---|
| 1417984_at | Ube2v2 | ubiquitin-conjugating enzyme E2 variant 2 | -1.34 | 0.326 | -1.34 | 0.109 | -1.11 | 0.319 | -1 | 0.982 | -1.2 |
| 1425087_at | 2310003F16Rik | RIKEN cDNA 2310003F16 gene | -1.22 | 0.58 | -1.71 | 0.164 | -1.01 | 0.962 | -1.32 | 0.267 | -1.32 |
| 1436515_at | E030004N02Rik | RIKEN cDNA E030004N02 gene | -1.21 | 0.017 | -1.33 | 0.485 | -1.23 | 0.428 | -2.55 | 0.085 | -1.58 |
| 1437647_at | Dido1 | death inducer-obliterator 1 | -1.4 | 0.081 | -1.43 | 0.008 | -1.02 | 0.928 | -1.12 | 0.622 | -1.24 |
| 1445513_at | Vezt | Vezatin, adherens junctions transmembrane protein | -1.21 | 0.519 | -1.62 | 0.027 | -1.05 | 0.671 | -1.15 | 0.802 | -1.26 |
| 1430216_at | Zfp292 | zinc finger protein 292 | -1.42 | 0.374 | -1.26 | 0.561 | -1.12 | 0.628 | -1.53 | 0.387 | -1.33 |
| 1453109_at | Arsk | arylsulfatase K | -1.33 | 0.105 | -1.12 | 0.187 | -1.34 | 0.122 | -1.15 | 0.412 | -1.24 |
| 1423592_at | Rock2 | Rho-associated coiled-coil containing protein kinase 2 | -1.12 | 0.465 | -1.13 | 0.565 | -1.63 | 0.105 | -1.3 | 0.352 | -1.3 |
| 1429264_at | C030044B11Rik | RIKEN cDNA C030044B11 gene | -1.04 | 0.182 | -1.36 | 0.109 | -1.44 | 0.088 | -1.12 | 0.383 | -1.24 |
| 1445211_at | --- | PREDICTED: Mus musculus hypothetical protein LOC626411 (LOC626411), mR | -1.19 | 0.438 | -1.38 | 0.08 | -1.2 | 0.401 | -1.52 | 0.026 | -1.32 |
| 1422074_at | Cdx2 | caudal type homeo box 2 | -1.03 | 0.957 | -1.25 | 0.326 | -1.61 | 0.015 | -1.17 | 0.522 | -1.27 |
| 1423666_s_at | Rpl5 /// LOC38274( | ribosomal protein L5 /// similar to 60S ribosomal protein L5 /// similar to 60S ribos | -1.03 | 0.783 | -1.17 | 0.216 | -1.75 | 0.015 | -1.15 | 0.322 | -1.28 |
| 1446615_at | --- | --- | -1.25 | 0.208 | -1.17 | 0.704 | -1.36 | 0.141 | -1.51 | 0.153 | -1.32 |
| 1445585_at | Zfp313 | zinc finger protein 313 | -1.19 | 0.259 | -1.23 | 0.609 | -1.35 | 0.721 | -1.2 | 0.765 | -1.24 |
| 1440658_at | E230022H04Rik | RIKEN cDNA E230022H04 gene | -1.02 | 0.96 | -1.25 | 0.454 | -1.62 | 0.131 | -1.28 | 0.45 | -1.29 |
| 1444432_at | D330040H18Rik | RIKEN cDNA D330040H18 gene | -1.16 | 0.654 | -1.03 | 0.872 | -1.77 | 0.005 | -1.44 | 0.363 | -1.35 |
| 1439097_at | D10Wsu52e | DNA segment, Chr 10, Wayne State University 52, expressed | -1.23 | 0.219 | -1.31 | 0.152 | -1.23 | 0.435 | -1.8 | 0.064 | -1.39 |
| 1460272_at | Ubl4b | ubiquitin-like 4B | -1.71 | 0.134 | -1.17 | 0.142 | -1.05 | 0.827 | -1.14 | 0.541 | -1.27 |
| 1428373_at | Ihpk2 | inositol hexaphosphate kinase 2 | -1.42 | 0.199 | -1.14 | 0.554 | -1.23 | 0.281 | -1.69 | 0.186 | -1.37 |
| 1444021_at | --- | --- | -1.23 | 0.513 | -1.33 | 0.617 | -1.2 | 0.115 | -1.35 | 0.285 | -1.28 |
| 1434029_at | 2410025L10Rik | RIKEN cDNA 2410025L10 gene | -1.08 | 0.459 | -1.51 | 0.083 | -1.24 | 0.423 | -1.01 | 0.959 | -1.21 |
| 1429874_at | Spata17 | spermatogenesis associated 17 | -1.18 | 0.777 | -1.7 | 0.149 | -1.05 | 0.583 | -1.93 | 0.1 | -1.46 |
| 1424813_at | AA536717 | expressed sequence AA536717 | -1.24 | 0.208 | -1.24 | 0.02 | -1.28 | 0.138 | -1.16 | 0.273 | -1.23 |
| 1446955_at | Nr2c2 | Nuclear receptor subfamily 2, group C, member 2 | -1.1 | 0.652 | -1.3 | 0.024 | -1.39 | 0.244 | -1.04 | 0.86 | -1.21 |
| 1424861_at | D930016D06Rik | RIKEN cDNA D930016D06 gene | -1.11 | 0.622 | -1.14 | 0.153 | -1.62 | 0.002 | -1.45 | 0.028 | -1.33 |
| 1439398_x_at | Nelf | nasal embryonic LHRH factor | -1.4 | 0.329 | -1.23 | 0.088 | -1.15 | 0.385 | -1.31 | 0.163 | -1.27 |
| 1427057_at | C630002B14Rik | RIKEN cDNA C630002B14 gene | -1.57 | 0.301 | -1.25 | 0.014 | -1.05 | 0.611 | -1.1 | 0.629 | -1.24 |
| 1438065_at | --- | --- | -1.43 | 0.251 | -1.21 | 0.438 | -1.15 | 0.535 | -1.17 | 0.455 | -1.24 |
| 1435109_at | 0710007G10Rik /// | RIKEN cDNA 0710007G10 gene /// RIKEN cDNA 3010001K23 gene | -1.14 | 0.411 | -1.25 | 0.317 | -1.39 | 0.122 | -1.27 | 0.154 | -1.26 |
| 1436263_at | Mobp | myelin-associated oligodendrocytic basic protein | -1.85 | 0.009 | -1.09 | 0.68 | -1.07 | 0.817 | -1.62 | 0.045 | -1.41 |
| 1460408_at | Gabrg1 | gamma-aminobutyric acid (GABA-A) receptor, subunit gamma 1 | -1.37 | 0.627 | -1.26 | 0.739 | -1.15 | 0.739 | -1 | 0.995 | -1.2 |
| 1433409_at | C030014O09Rik | RIKEN cDNA C030014O09 gene | -1.15 | 0.415 | -1.35 | 0.184 | -1.27 | 0.01 | -1.34 | 0.512 | -1.28 |
| 1451195_a_at | Txndc1 | thioredoxin domain containing 1 | -1.94 | 0.368 | -1.1 | 0.831 | -1.03 | 0.937 | -1.13 | 0.76 | -1.3 |
| 1446033_at | C87882 | expressed sequence C87882 | -1.21 | 0.754 | -1.09 | 0.847 | -1.52 | 0.202 | -1.56 | 0.227 | -1.35 |
| 1451360_at | Ergic2 | ERGIC and golgi 2 | -1.21 | 0.091 | -1.09 | 0.404 | -1.53 | 0.007 | -1.14 | 0.447 | -1.24 |
| 1440994_at | Shprh | SNF2 histone linker PHD RING helicase | -1.35 | 0.184 | -1.26 | 0.254 | -1.16 | 0.303 | -1.19 | 0.388 | -1.24 |
| 1450232_at | Birc4 | baculoviral IAP repeat-containing 4 | -1.07 | 0.854 | -1.28 | 0.067 | -1.47 | 0.091 | -1.11 | 0.687 | -1.23 |
| 1432011_at | 2900052L18Rik | RIKEN cDNA 2900052L18 gene | -1.16 | 0.257 | -1.34 | 0.103 | -1.26 | 0.332 | -1.82 | 0.023 | -1.4 |
| 1431823_at | Moap1 | modulator of apoptosis 1 | -1.17 | 0.099 | -1.39 | 0.153 | -1.21 | 0.067 | -1.33 | 0.053 | -1.28 |
| 1458490_at | 9230116N13Rik | RIKEN cDNA 9230116N13 gene | -1.09 | 0.798 | -1.38 | 0.175 | -1.33 | 0.177 | -1.42 | 0.243 | -1.31 |
| 1447322_at | --- | --- | -1.11 | 0.561 | -1.13 | 0.58 | -1.64 | 0.019 | -1.39 | 0.132 | -1.32 |
| 1436217_at | Zfp148 | zinc finger protein 148 | -1.15 | 0.041 | -1.08 | 0.547 | -1.67 | 0.079 | -1.32 | 0.08 | -1.3 |
| 1444271_at | Anapc1 | anaphase promoting complex subunit 1 | -1.02 | 0.929 | -1.21 | 0.575 | -1.68 | 0.068 | -1.28 | 0.297 | -1.3 |
| 1431158_at | 8430406H22Rik | RIKEN cDNA 8430406H22 gene | -1.04 | 0.94 | -1.31 | 0.213 | -1.49 | 0.018 | -1 | 0.991 | -1.21 |
| 1443039_at | --- | --- | -1.14 | 0.812 | -1.05 | 0.924 | -1.77 | 0.337 | -4.09 | 0.03 | -2.01 |
| 1430077_at | Sfrs11 | splicing factor, arginine/serine-rich 11 | -1.24 | 0.343 | -1.17 | 0.09 | -1.36 | 0.115 | -1.18 | 0.555 | -1.24 |
| 1443798_at | Pik3cd | phosphatidylinositol 3-kinase catalytic delta polypeptide | -1.61 | 0.095 | -1.03 | 0.794 | -1.24 | 0.358 | -1.02 | 0.902 | -1.23 |
| 1426359_at | Zc3h11a | zinc finger CCCH type containing 11A | -1.06 | 0.552 | -1.33 | 0.132 | -1.41 | 0.067 | -1.31 | 0.071 | -1.28 |
| 1440062_at | 2510009E07Rik | RIKEN cDNA 2510009E07 gene | -1.53 | 0.568 | -1.07 | 0.902 | -1.23 | 0.584 | -1.4 | 0.248 | -1.31 |
| 1455605_at | Rufy3 | RUN and FYVE domain containing 3 | -1.2 | 0.04 | -1.15 | 0.209 | -1.44 | 0.007 | -1.7 | 0.031 | -1.37 |
| 1453951_a_at | D19Ertd652e /// LO | DNA segment, Chr 19, ERATO Doi 652, expressed /// similar to DNA segment, C | -1.02 | 0.972 | -1.68 | 0.031 | -1.21 | 0.537 | -1.3 | 0.322 | -1.31 |
| 1457161_at | 9530029O12Rik | RIKEN cDNA 9530029O12 gene | -1.02 | 0.904 | -1.22 | 0.143 | -1.69 | 0.005 | -1.16 | 0.373 | -1.27 |
| 1447486_at | Ppp1r9b | protein phosphatase 1, regulatory subunit 9B | -1.48 | 0.131 | -1.03 | 0.931 | -1.33 | 0.161 | -1.05 | 0.875 | -1.22 |

| | | | | | | | | | | | |
|---|---|---|---|---|---|---|---|---|---|---|---|
| 1452042_a_at | Tmem144 | transmembrane protein 144 | -1.25 | 0.324 | -1.32 | 0.373 | -1.19 | 0.243 | -1.17 | 0.776 | -1.23 |
| 1457480_at | Pcgf3 | Polycomb group ring finger 3 | -1.03 | 0.911 | -1.32 | 0.083 | -1.49 | 0.041 | -1.22 | 0.336 | -1.27 |
| 1444465_at | Ube4b | Ubiquitination factor E4B, UFD2 homolog (S. cerevisiae) | -1.11 | 0.828 | -1.45 | 0.531 | -1.24 | 0.491 | -1.63 | 0.163 | -1.36 |
| 1437392_at | LOC433485 | hypothetical LOC433485 | -1.95 | 0.332 | -1.07 | 0.892 | -1.05 | 0.944 | -1.09 | 0.742 | -1.29 |
| 1419672_at | Spock1 | sparc/osteonectin, cwcv and kazal-like domains proteoglycan 1 | -1.1 | 0.742 | -1.19 | 0.599 | -1.53 | 0.38 | -1.74 | 0.271 | -1.39 |
| 1446242_at | 4930535B03Rik | RIKEN cDNA 4930535B03 gene | -1.33 | 0.051 | -1.25 | 0.516 | -1.18 | 0.589 | -1.13 | 0.68 | -1.22 |
| 1443856_at | --- | --- | -1.12 | 0.123 | -1.22 | 0.059 | -1.46 | 0.191 | -1.3 | 0.306 | -1.27 |
| 1418684_at | 2310012P17Rik | RIKEN cDNA 2310012P17 gene | -1.34 | 0.23 | -1.15 | 0.497 | -1.27 | 0.329 | -1.09 | 0.672 | -1.21 |
| 1439392_x_at | Psmc1 /// LOC3860 | protease (prosome, macropain) 26S subunit, ATPase 1 /// hypothetical gene supp | -1.51 | 0.336 | -1.02 | 0.917 | -1.33 | 0.668 | -2.29 | 0.055 | -1.54 |
| 1455728_at | Pten | phosphatase and tensin homolog | -1.25 | 0.069 | -1.14 | 0.143 | -1.39 | 0.135 | -1.56 | 0.122 | -1.33 |
| 1459483_at | Tmem28 | Transmembrane protein 28 | -1.39 | 0.426 | -1.01 | 0.985 | -1.46 | 0.072 | -1 | 0.928 | -1.21 |
| 1421897_at | Elk1 | ELK1, member of ETS oncogene family | -2.09 | 0.134 | -1.02 | 0.956 | -1.06 | 0.85 | -1.17 | 0.528 | -1.34 |
| 1457956_at | 2310058O09Rik | RIKEN cDNA 2310058O09 gene | -1.67 | 0.148 | -1.23 | 0.663 | -1.01 | 0.98 | -1.42 | 0.257 | -1.33 |
| 1430219_at | Fts | fused toes | -1.78 | 0.33 | -1.16 | 0.864 | -1.03 | 0.961 | -1.32 | 0.743 | -1.32 |
| 1459362_at | --- | Transcribed locus | -1.67 | 0.316 | -1.03 | 0.957 | -1.2 | 0.608 | -2.09 | 0.088 | -1.5 |
| 1451776_s_at | Hod | homeobox only domain | -1.2 | 0.385 | -1.29 | 0.306 | -1.27 | 0.186 | -1.15 | 0.687 | -1.22 |
| 1445909_at | --- | --- | -1.03 | 0.885 | -1.8 | 0.133 | -1.14 | 0.323 | -1.03 | 0.965 | -1.25 |
| 1459098_at | Dst | Dystonin | -1.21 | 0.601 | -1.2 | 0.439 | -1.35 | 0.227 | -1 | 0.994 | -1.19 |
| 1445290_at | Hunk | Hormonally upregulated Neu-associated kinase | -1.54 | 0.554 | -1.1 | 0.88 | -1.18 | 0.63 | -1.62 | 0.296 | -1.36 |
| 1448049_at | Jmjd1c | jumonji domain containing 1C | -1.15 | 0.377 | -1.2 | 0.233 | -1.43 | 0.08 | -1.7 | 0.07 | -1.37 |
| 1440590_at | --- | --- | -1.07 | 0.736 | -1.16 | 0.678 | -1.66 | 0.033 | -1.07 | 0.723 | -1.24 |
| 1427322_at | Brwd1 | bromodomain and WD repeat domain containing 1 | -1.24 | 0.502 | -1.27 | 0.306 | -1.23 | 0.299 | -2.12 | 0.194 | -1.47 |
| 1444096_at | Zfr | Zinc finger RNA binding protein | -1.12 | 0.653 | -1.2 | 0.181 | -1.48 | 0.065 | -1.09 | 0.729 | -1.22 |
| 1443095_at | --- | --- | -1.12 | 0.16 | -1.13 | 0.425 | -1.61 | 0.034 | -1.17 | 0.417 | -1.26 |
| 1441794_at | 4933437K13Rik | RIKEN cDNA 4933437K13 gene | -1.34 | 0.264 | -1.21 | 0.566 | -1.21 | 0.574 | -2.89 | 0.067 | -1.66 |
| 1445242_at | Gyk | glycerol kinase | -1.09 | 0.17 | -1.29 | 0.017 | -1.42 | 0.27 | -1.53 | 0.332 | -1.33 |
| 1460605_at | Crxos1 | Crx opposite strand transcript 1 | -1.3 | 0.704 | -1.25 | 0.586 | -1.2 | 0.813 | -1.22 | 0.738 | -1.24 |
| 1419475_a_at | Ehf | ets homologous factor | -1.03 | 0.882 | -2.17 | 0.251 | -1.03 | 0.9 | -1.07 | 0.824 | -1.33 |
| 1447908_x_at | Ttc3 | tetratricopeptide repeat domain 3 | -1.57 | 0.106 | -1.07 | 0.354 | -1.21 | 0.323 | -1.11 | 0.442 | -1.24 |
| 1424867_a_at | Glyat | glycine-N-acyltransferase | -1.37 | 0.097 | -1.13 | 0.291 | -1.27 | 0.55 | -1.13 | 0.852 | -1.23 |
| 1456576_x_at | Cnot2 | CCR4-NOT transcription complex, subunit 2 | -1.14 | 0.477 | -1.18 | 0.226 | -1.47 | 0.09 | -1.54 | 0.123 | -1.33 |
| 1447092_at | Pecam1 | Platelet/endothelial cell adhesion molecule 1 | -1.19 | 0.51 | -1.03 | 0.878 | -1.7 | 0.128 | -1.64 | 0.231 | -1.39 |
| 1433988_s_at | C230098O21Rik | RIKEN cDNA C230098O21 gene | -1.29 | 0.032 | -1.32 | 0.147 | -1.15 | 0.424 | -1.62 | 0.023 | -1.34 |
| 1450190_at | Zfp106 | zinc finger protein 106 | -1.24 | 0.635 | -1.11 | 0.841 | -1.44 | 0.552 | -1.13 | 0.805 | -1.23 |
| 1442679_at | Map2k4 | Mitogen activated protein kinase kinase 4 | -1.33 | 0.378 | -1.1 | 0.724 | -1.36 | 0.477 | -1.35 | 0.2 | -1.28 |
| 1456880_at | Sfrs8 | Splicing factor, arginine/serine-rich 8 | -1.14 | 0.567 | -1.35 | 0.001 | -1.27 | 0.379 | -1.25 | 0.08 | -1.25 |
| 1419506_at | Ggps1 | geranylgeranyl diphosphate synthase 1 | -1.02 | 0.88 | -1.38 | 0.029 | -1.44 | 0.02 | -1.29 | 0.212 | -1.28 |
| 1446709_at | --- | --- | -1.3 | 0.712 | -1.13 | 0.614 | -1.34 | 0.469 | -1.14 | 0.828 | -1.23 |
| 1453036_at | 5430439M09Rik | RIKEN cDNA 5430439M09 gene | -1.31 | 0.268 | -1.2 | 0.426 | -1.24 | 0.077 | -1.12 | 0.526 | -1.22 |
| 1455035_s_at | Nol5a | nucleolar protein 5A | -1.08 | 0.723 | -1.53 | 0.001 | -1.21 | 0.422 | -1.31 | 0.118 | -1.28 |
| 1434471_at | BC003331 | cDNA sequence BC003331 | -1.09 | 0.616 | -1.15 | 0.398 | -1.63 | 0.062 | -1.12 | 0.588 | -1.25 |
| 1456734_at | Txn1 | thioredoxin 1 | -1.09 | 0.791 | -1.63 | 0.243 | -1.14 | 0.701 | -1.19 | 0.758 | -1.26 |
| 1432216_s_at | Mpp7 | membrane protein, palmitoylated 7 (MAGUK p55 subfamily member 7) | -1.2 | 0.543 | -1.12 | 0.675 | -1.47 | 0.019 | -1.45 | 0.098 | -1.31 |
| 1448094_at | LOC546100 | hypothetical LOC546100 | -1.39 | 0.728 | -1.45 | 0.474 | -1 | 0.992 | -1.26 | 0.563 | -1.28 |
| 1428654_at | 1200016B10Rik | RIKEN cDNA 1200016B10 gene | -1.05 | 0.766 | -1.22 | 0.13 | -1.6 | 0.034 | -1 | 0.991 | -1.22 |
| 1436756_x_at | Hadhsc | L-3-hydroxyacyl-Coenzyme A dehydrogenase, short chain | -1.85 | 0.05 | -1.05 | 0.847 | -1.09 | 0.531 | -2.34 | 0.153 | -1.58 |
| 1457188_at | Arhgef11 | Rho guanine nucleotide exchange factor (GEF) 11 | -1.24 | 0.188 | -1.02 | 0.958 | -1.62 | 0.07 | -1.67 | 0.279 | -1.39 |
| 1437498_at | --- | --- | -1.1 | 0.629 | -1.27 | 0.265 | -1.4 | 0.168 | -1.35 | 0.513 | -1.28 |
| 1459005_at | D030051N19Rik | RIKEN cDNA D030051N19 gene | -1.23 | 0.317 | -1.01 | 0.972 | -1.66 | 0.006 | -1.59 | 0.028 | -1.37 |
| 1455442_at | Slc6a19 | solute carrier family 6 (neurotransmitter transporter), member 19 | -1.17 | 0.395 | -1.59 | 0.305 | -1.08 | 0.736 | -2.23 | 0.052 | -1.52 |
| 1424034_at | Rora | RAR-related orphan receptor alpha | -1.1 | 0.683 | -1.25 | 0.041 | -1.44 | 0.146 | -1.61 | 0.128 | -1.35 |
| 1420543_at | ORF28 | open reading frame 28 | -1.23 | 0.217 | -1.13 | 0.506 | -1.4 | 0.138 | -1.05 | 0.701 | -1.21 |
| 1453311_at | 2310008B10Rik | RIKEN cDNA 2310008B10 gene | -1.21 | 0.246 | -1.22 | 0.524 | -1.31 | 0.311 | -1.64 | 0.177 | -1.35 |

| | | | | | | | | | | | |
|---|---|---|---|---|---|---|---|---|---|---|---|
| 1424811_at | Cml5 | camello-like 5 | -1.03 | 0.975 | -1.8 | 0.059 | -1.14 | 0.845 | -3.03 | 0.182 | -1.75 |
| 1442186_at | Atxn7 | ataxin 7 | -1.17 | 0.486 | -1.08 | 0.779 | -1.62 | 0.131 | -1.26 | 0.258 | -1.28 |
| 1438072_at | Nfib | Nuclear factor I/B | -1.03 | 0.85 | -1.08 | 0.549 | -1.94 | 0.016 | -1.22 | 0.561 | -1.32 |
| 1423560_at | Nell2 | NEL-like 2 (chicken) | -1.07 | 0.597 | -1.74 | 0.014 | -1.12 | 0.385 | -1.56 | 0.093 | -1.37 |
| 1430328_at | Polr3f | polymerase (RNA) III (DNA directed) polypeptide F | -1.09 | 0.339 | -1.42 | 0.239 | -1.28 | 0.22 | -1.13 | 0.707 | -1.23 |
| 1460645_at | Chordc1 | cysteine and histidine-rich domain (CHORD)-containing, zinc-binding protein 1 | -1.06 | 0.742 | -1.32 | 0.176 | -1.41 | 0.012 | -1.29 | 0.279 | -1.27 |
| 1442569_at | Cct4 | Chaperonin subunit 4 (delta) | -1.18 | 0.549 | -1.19 | 0.412 | -1.4 | 0.104 | -1.49 | 0.425 | -1.31 |
| 1417814_at | Pla2g5 | phospholipase A2, group V | -1.14 | 0.566 | -1.31 | 0.701 | -1.31 | 0.549 | -2.2 | 0.088 | -1.49 |
| 1452984_at | 5730405I09Rik | RIKEN cDNA 5730405I09 gene | -1.16 | 0.202 | -1.34 | 0.096 | -1.26 | 0.334 | -1.15 | 0.38 | -1.23 |
| 1442387_at | Hars | Histidyl-tRNA synthetase | -1.36 | 0.578 | -1.33 | 0.522 | -1.08 | 0.737 | -1.08 | 0.726 | -1.22 |
| 1444557_at | Sumo1 | SMT3 suppressor of mif two 3 homolog 1 (yeast) | -1.17 | 0.119 | -1.06 | 0.763 | -1.64 | 0.027 | -1.36 | 0.032 | -1.31 |
| 1429041_at | 2610005L07Rik | RIKEN cDNA 2610005L07 gene | -1.5 | 0.016 | -1.18 | 0.302 | -1.12 | 0.453 | -1.23 | 0.272 | -1.26 |
| 1457193_at | Mll3 | myeloid/lymphoid or mixed-lineage leukemia 3 | -1.12 | 0.434 | -1.13 | 0.53 | -1.59 | 0.106 | -1.15 | 0.321 | -1.25 |
| 1432053_at | Rfx4 | regulatory factor X, 4 (influences HLA class II expression) | -1.54 | 0.262 | -1.08 | 0.8 | -1.21 | 0.449 | -1 | 0.971 | -1.21 |
| 1445786_at | Braf | Braf transforming gene | -1.03 | 0.83 | -1.26 | 0.364 | -1.54 | 0.004 | -1.03 | 0.898 | -1.22 |
| 1426455_at | Sdccag10 | serologically defined colon cancer antigen 10 | -1.15 | 0.338 | -1.48 | 0.189 | -1.17 | 0.427 | -1.35 | 0.188 | -1.29 |
| 1419473_a_at | Cck | cholecystokinin | -1.12 | 0.356 | -1.66 | 0.37 | -1.09 | 0.501 | -2.74 | 0.078 | -1.65 |
| 1421426_at | Hhip | Hedgehog-interacting protein | -1.11 | 0.893 | -1.83 | 0.355 | -1.04 | 0.905 | -1.01 | 0.973 | -1.25 |
| 1445907_at | --- | --- | -1.05 | 0.941 | -1.65 | 0.228 | -1.17 | 0.611 | -1.26 | 0.254 | -1.29 |
| 1437990_x_at | Hbb-bh1 | hemoglobin Z, beta-like embryonic chain | -1.03 | 0.937 | -1.31 | 0.687 | -1.49 | 0.376 | -1.53 | 0.526 | -1.34 |
| 1416480_a_at | Higd1a | HIG1 domain family, member 1A | -1.06 | 0.608 | -1.46 | 0.005 | -1.29 | 0.02 | -1.06 | 0.742 | -1.22 |
| 1431189_a_at | Fahd2a | fumarylacetoacetate hydrolase domain containing 2A | -1.12 | 0.267 | -1.7 | 0.032 | -1.08 | 0.402 | -1.7 | 0.156 | -1.4 |
| 1429328_at | Nsfl1c | NSFL1 (p97) cofactor (p47) | -1.25 | 0.593 | -1.58 | 0.064 | -1.03 | 0.859 | -1.01 | 0.973 | -1.22 |
| 1459067_at | 1190002N15Rik | RIKEN cDNA 1190002N15 gene | -1.1 | 0.603 | -1.3 | 0.45 | -1.37 | 0.691 | -2.76 | 0.02 | -1.63 |
| 1451693_a_at | Fgf12 | fibroblast growth factor 12 | -1.62 | 0.349 | -1.08 | 0.783 | -1.16 | 0.326 | -1.75 | 0.448 | -1.4 |
| 1445034_at | Coro2a | Coronin, actin binding protein 2A | -1.38 | 0.041 | -1 | 0.998 | -1.46 | 0.246 | -1.66 | 0.136 | -1.37 |
| 1458126_at | D030041H20Rik | RIKEN cDNA D030041H20 gene | -1.02 | 0.927 | -1.14 | 0.585 | -1.82 | 0.124 | -1.29 | 0.415 | -1.32 |
| 1441296_at | --- | --- | -1.33 | 0.471 | -1.33 | 0.172 | -1.1 | 0.85 | -1.19 | 0.433 | -1.24 |
| 1434394_at | B3bp | Bcl3 binding protein | -1.2 | 0.547 | -1.47 | 0.009 | -1.12 | 0.467 | -1.45 | 0.196 | -1.31 |
| 1429735_at | 1110003F05Rik | RIKEN cDNA 1110003F05 gene | -1.06 | 0.236 | -1.1 | 0.013 | -1.8 | 0.003 | -1.06 | 0.131 | -1.25 |
| 1434002_at | Ches1 | checkpoint suppressor 1 | -1.14 | 0.228 | -1.2 | 0.136 | -1.44 | 0.052 | -1.51 | 0.129 | -1.32 |
| 1426224_x_at | Cmtm2a | CKLF-like MARVEL transmembrane domain containing 2A | -1.51 | 0.41 | -1.29 | 0.592 | -1.03 | 0.96 | -1.02 | 0.975 | -1.21 |
| 1441905_x_at | Snrpn | Small nuclear ribonucleoprotein N | -1.34 | 0.312 | -1.46 | 0.571 | -1.02 | 0.967 | -1.96 | 0.229 | -1.45 |
| 1420490_at | Klk1b16 | kallikrein 1-related peptidase b16 | -1.14 | 0.789 | -1.71 | 0.304 | -1.05 | 0.938 | -1.49 | 0.338 | -1.35 |
| 1427370_at | Amdhd1 | amidohydrolase domain containing 1 | -1.31 | 0.526 | -1.15 | 0.202 | -1.29 | 0.541 | -1.6 | 0.548 | -1.34 |
| 1450395_at | Slc22a5 | solute carrier family 22 (organic cation transporter), member 5 | -1.16 | 0.511 | -1.44 | 0.319 | -1.18 | 0.486 | -1.18 | 0.584 | -1.24 |
| 1420614_at | Dynlt3 | dynein light chain Tctex-type 3 | -1.3 | 0.053 | -1.13 | 0.211 | -1.33 | 0.191 | -1.07 | 0.833 | -1.21 |
| 1450873_at | Gtpbp4 | GTP binding protein 4 | -1.39 | 0.249 | -1.18 | 0.106 | -1.19 | 0.144 | -1.19 | 0.263 | -1.24 |
| 1442150_at | --- | --- | -1.02 | 0.813 | -1.21 | 0.492 | -1.67 | 0.042 | -1.23 | 0.372 | -1.28 |
| 1447032_at | Clybl | Citrate lyase beta like | -1.1 | 0.36 | -1.04 | 0.91 | -1.83 | 0.03 | -1.48 | 0.606 | -1.37 |
| 1444589_at | LOC240038 | similar to reduced expression 2 | -1.34 | 0.112 | -1.07 | 0.748 | -1.37 | 0.029 | -1.42 | 0.192 | -1.3 |
| 1450356_at | Trhr2 | thyrotropin releasing hormone receptor 2 | -1.26 | 0.333 | -1.05 | 0.95 | -1.51 | 0.068 | -1.01 | 0.986 | -1.21 |
| 1440267_at | Pex1 | peroxisome biogenesis factor 1 | -1.1 | 0.686 | -1.24 | 0.275 | -1.45 | 0.102 | -1.44 | 0.405 | -1.31 |
| 1449923_at | 1700018F24Rik | RIKEN cDNA 1700018F24 gene | -1.5 | 0.131 | -1.12 | 0.683 | -1.17 | 0.102 | -1.15 | 0.125 | -1.24 |
| 1459575_at | D1Ertd704e | DNA segment, Chr 1, ERATO Doi 704, expressed | -1.5 | 0.388 | -1.23 | 0.612 | -1.07 | 0.875 | -1.81 | 0.39 | -1.4 |
| 1434901_at | Gm1103 | gene model 1103, (NCBI) | -1.21 | 0.268 | -1.46 | 0.013 | -1.11 | 0.146 | -1.22 | 0.011 | -1.25 |
| 1455091_at | 3222402P14Rik | RIKEN cDNA 3222402P14 gene | -1.11 | 0.394 | -1.27 | 0.125 | -1.38 | 0.131 | -1.43 | 0.178 | -1.3 |
| 1437543_at | Fubp1 | far upstream element (FUSE) binding protein 1 | -1.13 | 0.218 | -1.26 | 0.275 | -1.37 | 0.088 | -1.17 | 0.372 | -1.23 |
| 1447491_at | Lrrc8d | Leucine rich repeat containing 8D | -1.39 | 0.016 | -1.28 | 0.191 | -1.1 | 0.722 | -1.26 | 0.39 | -1.26 |
| 1434807_s_at | Mtx3 | metaxin 3 | -1.28 | 0.092 | -1.08 | 0.724 | -1.42 | 0.058 | -1.19 | 0.578 | -1.24 |
| 1426850_a_at | Map2k6 | mitogen activated protein kinase kinase 6 | -1.17 | 0.17 | -1.44 | 0.396 | -1.16 | 0.519 | -1.36 | 0.127 | -1.28 |
| 1442361_at | Itga2b | Integrin alpha 2b | -1.14 | 0.618 | -1.65 | 0.38 | -1.07 | 0.903 | -1.02 | 0.964 | -1.22 |
| 1448095_at | --- | Transcribed locus | -1.15 | 0.232 | -1.46 | 0.447 | -1.16 | 0.37 | -1.42 | 0.281 | -1.3 |

| | | | | | | | | | | | |
|---|---|---|---|---|---|---|---|---|---|---|---|
| 1436581_at | Ccdc39 | coiled-coil domain containing 39 | -1.16 | 0.238 | -1.55 | 0.119 | -1.11 | 0.671 | -1.18 | 0.309 | -1.25 |
| 1435982_at | Stx12 | syntaxin 12 | -1.12 | 0.463 | -1.08 | 0.612 | -1.68 | 0.064 | -1.05 | 0.523 | -1.23 |
| 1429819_at | Nmnat1 | nicotinamide nucleotide adenylyltransferase 1 | -1.09 | 0.57 | -1.33 | 0.246 | -1.35 | 0.166 | -2.21 | 0.037 | -1.49 |
| 1439837_at | Tnrc15 | trinucleotide repeat containing 15 | -1.26 | 0.232 | -1.23 | 0.42 | -1.24 | 0.016 | -1.46 | 0.034 | -1.3 |
| 1428214_at | Tomm7 | translocase of outer mitochondrial membrane 7 homolog (yeast) | -1.14 | 0.264 | -1.27 | 0.182 | -1.34 | 0.18 | -1.22 | 0.229 | -1.24 |
| 1457112_at | A230106N23 | hypothetical protein A230106N23 | -1.1 | 0.805 | -1.45 | 0.162 | -1.23 | 0.226 | -1.18 | 0.366 | -1.24 |
| 1419205_x_at | Gpatc4 | G patch domain containing 4 | -1.02 | 0.704 | -1.27 | 0.332 | -1.55 | 0.11 | -1.19 | 0.234 | -1.26 |
| 1454402_at | 3110048L19Rik | RIKEN cDNA 3110048L19 gene | -1.32 | 0.162 | -1.03 | 0.867 | -1.45 | 0.009 | -1.71 | 0.065 | -1.38 |
| 1421762_at | Kcnj5 | potassium inwardly-rectifying channel, subfamily J, member 5 | -1.14 | 0.71 | -1.24 | 0.079 | -1.36 | 0.246 | -1.38 | 0.541 | -1.28 |
| 1456825_at | Wdr33 | WD repeat domain 33 | -1.1 | 0.574 | -1.14 | 0.807 | -1.59 | 0.15 | -1.58 | 0.026 | -1.35 |
| 1447522_s_at | Tnks2 | tankyrase, TRF1-interacting ankyrin-related ADP-ribose polymerase 2 | -1.19 | 0.027 | -1.1 | 0.541 | -1.51 | 0 | -1.29 | 0.123 | -1.27 |
| 1430713_s_at | Ndufa13 | NADH dehydrogenase (ubiquinone) 1 alpha subcomplex, 13 | -1.29 | 0.222 | -1.31 | 0.12 | -1.14 | 0.318 | -1.29 | 0.176 | -1.26 |
| 1456142_x_at | Morf4l1 /// LOC433 | mortality factor 4 like 1 /// similar to mortality factor 4 like 1 isoform b /// RIKEN cD | -1.2 | 0.133 | -1.26 | 0.047 | -1.27 | 0.042 | -1.2 | 0.019 | -1.23 |
| 1430476_at | Srp54 | signal recognition particle 54 | -1.03 | 0.972 | -2.11 | 0.065 | -1.03 | 0.931 | -1.27 | 0.271 | -1.36 |
| 1434062_at | Rabgap1l | RAB GTPase activating protein 1-like | -1.24 | 0.098 | -1.16 | 0.336 | -1.35 | 0.011 | -1.39 | 0.374 | -1.28 |
| 1425358_at | Riok1 | RIO kinase 1 (yeast) | -1.12 | 0.665 | -1.36 | 0.081 | -1.27 | 0.315 | -1.21 | 0.507 | -1.24 |
| 1455630_at | 1700010H15Rik | RIKEN cDNA 1700010H15 gene | -1.23 | 0.216 | -1.26 | 0.601 | -1.23 | 0.092 | -1.41 | 0.256 | -1.29 |
| 1437359_at | Rnps1 | Ribonucleic acid binding protein S1 | -1.53 | 0.239 | -1.22 | 0.19 | -1.07 | 0.799 | -1.07 | 0.517 | -1.22 |
| 1456928_at | --- | --- | -1.06 | 0.887 | -1.44 | 0.066 | -1.29 | 0.119 | -1.08 | 0.846 | -1.22 |
| 1458414_at | D2Ertd93e | DNA segment, Chr 2, ERATO Doi 93, expressed | -1.06 | 0.756 | -1.32 | 0.409 | -1.41 | 0.108 | -1.11 | 0.402 | -1.22 |
| 1430180_at | Map2k5 | mitogen activated protein kinase kinase 5 | -1.16 | 0.644 | -1.32 | 0.29 | -1.26 | 0.223 | -1.73 | 0.039 | -1.37 |
| 1457111_at | AA415038 | expressed sequence AA415038 | -1.04 | 0.753 | -1.09 | 0.552 | -1.84 | 0.102 | -1.31 | 0.501 | -1.32 |
| 1441063_at | Eif2c3 | eukaryotic translation initiation factor 2C, 3 | -1.11 | 0.541 | -1.1 | 0.552 | -1.67 | 0.087 | -1.14 | 0.318 | -1.25 |
| 1450640_x_at | Atp5k | ATP synthase, H+ transporting, mitochondrial F1F0 complex, subunit e | -1.25 | 0.012 | -1.35 | 0.029 | -1.14 | 0.09 | -1.04 | 0.488 | -1.2 |
| 1434343_at | 5730403M16Rik | RIKEN cDNA 5730403M16 gene | -1.18 | 0.601 | -1.1 | 0.659 | -1.51 | 0.018 | -1.5 | 0.148 | -1.32 |
| 1457510_at | Phf14 | PHD finger protein 14 | -1 | 0.998 | -1.19 | 0.325 | -1.74 | 0.081 | -1.56 | 0.195 | -1.37 |
| 1437721_at | Coro1c | coronin, actin binding protein 1C | -1.15 | 0.896 | -1.25 | 0.631 | -1.34 | 0.479 | -1.1 | 0.64 | -1.21 |
| 1429283_at | 1500009M05Rik | RIKEN cDNA 1500009M05 gene | -1.09 | 0.785 | -1.15 | 0.228 | -1.6 | 0.13 | -1.36 | 0.297 | -1.3 |
| 1429940_at | Cdc73 | Vcell division cycle 73, Paf1/RNA polymerase II complex component, homolog (S | -1.12 | 0.687 | -1.23 | 0.232 | -1.41 | 0.052 | -1.27 | 0.239 | -1.26 |
| 1439931_at | Gsk3b | glycogen synthase kinase 3 beta | -1.15 | 0.006 | -1.05 | 0.837 | -1.69 | 0.022 | -1.22 | 0.03 | -1.28 |
| 1420982_at | Rbm39 | RNA binding motif protein 39 | -1.04 | 0.887 | -1.5 | 0.194 | -1.28 | 0.064 | -1.13 | 0.214 | -1.23 |
| 1442510_at | LOC665792 /// LOC | hypothetical protein LOC665792 /// hypothetical protein LOC669957 /// hypothetic | -1.07 | 0.757 | -1.32 | 0.278 | -1.38 | 0.562 | -1.47 | 0.196 | -1.31 |
| 1421867_at | Nr3c1 | nuclear receptor subfamily 3, group C, member 1 | -1.25 | 0.104 | -1.1 | 0.122 | -1.41 | 0.202 | -1.55 | 0.109 | -1.33 |
| 1454810_s_at | AI315068 | expressed sequence AI315068 | -1.45 | 0.13 | -1.16 | 0.278 | -1.16 | 0.351 | -1.13 | 0.208 | -1.22 |
| 1419365_at | Pex11a | peroxisomal biogenesis factor 11a | -1.15 | 0.656 | -1.56 | 0.309 | -1.11 | 0.656 | -1.21 | 0.586 | -1.26 |
| 1419886_at | DXErtd223e | DNA segment, Chr X, ERATO Doi 223, expressed | -1.1 | 0.721 | -1.27 | 0.158 | -1.4 | 0.148 | -1.01 | 0.973 | -1.19 |
| 1428844_a_at | Bclaf1 | BCL2-associated transcription factor 1 | -1.16 | 0.642 | -1.26 | 0.129 | -1.32 | 0.062 | -1.4 | 0.058 | -1.28 |
| 1425319_s_at | 6530403A03Rik | RIKEN cDNA 6530403A03 gene | -1.05 | 0.833 | -1.28 | 0.32 | -1.45 | 0.042 | -1.65 | 0.029 | -1.36 |
| 1444408_at | B130040O20Rik | RIKEN cDNA B130040O20 gene | -1.27 | 0.208 | -1.19 | 0.408 | -1.27 | 0.102 | -1.77 | 0.143 | -1.38 |
| 1428951_at | Nol8 | nucleolar protein 8 | -1.07 | 0.8 | -1.3 | 0.115 | -1.4 | 0.042 | -1.09 | 0.57 | -1.22 |
| 1425728_at | RP23-14F5.7 | hypothetical gene Rp23-14f5.7 | -1.17 | 0.715 | -1.01 | 0.971 | -1.73 | 0.024 | -2.12 | 0.002 | -1.51 |
| 1438675_at | Sfrs8 | splicing factor, arginine/serine-rich 8 | -1.04 | 0.867 | -1.21 | 0.135 | -1.6 | 0.306 | -1.52 | 0.066 | -1.34 |
| 1445884_at | 1110007A13Rik | RIKEN cDNA 1110007A13 gene | -1.35 | 0.014 | -1.08 | 0.828 | -1.33 | 0.094 | -1.38 | 0.097 | -1.29 |
| 1440260_at | A930007B11Rik | RIKEN cDNA A930007B11 gene | -1.1 | 0.628 | -1.09 | 0.447 | -1.69 | 0.031 | -1.71 | 0.081 | -1.4 |
| 1445091_at | Atr | ataxia telangiectasia and Rad3 related | -1.22 | 0.315 | -1.32 | 0.407 | -1.19 | 0.196 | -1.64 | 0.089 | -1.34 |
| 1424423_at | Lenep /// Flad1 | lens epithelial protein /// RFad1, flavin adenine dinucleotide synthetase, homolog | -1.08 | 0.883 | -1.27 | 0.224 | -1.41 | 0.142 | -1.11 | 0.474 | -1.22 |
| 1424276_at | Snx16 | sorting nexin 16 | -1.11 | 0.55 | -1.35 | 0.127 | -1.29 | 0.018 | -1.26 | 0.315 | -1.25 |
| 1441901_x_at | Fahd2a | fumarylacetoacetate hydrolase domain containing 2A | -1.44 | 0.019 | -1.14 | 0.177 | -1.18 | 0.473 | -1.26 | 0.192 | -1.25 |
| 1445484_at | Cycs | cytochrome c, somatic | -1.05 | 0.826 | -1.63 | 0.491 | -1.18 | 0.536 | -1.13 | 0.839 | -1.25 |
| 1439582_at | Macf1 | Microtubule-actin crosslinking factor 1 | -1.06 | 0.636 | -1.05 | 0.698 | -1.93 | 0.024 | -1.81 | 0.243 | -1.46 |
| 1437699_at | E430014B02Rik | RIKEN cDNA E430014B02 gene | -1.06 | 0.882 | -1.04 | 0.919 | -1.96 | 0.031 | -3.88 | 0.055 | -1.98 |
| 1452491_at | 2900060N18Rik | RIKEN cDNA 2900060N18 gene | -1.04 | 0.073 | -1.27 | 0.647 | -1.49 | 0.343 | -1.03 | 0.965 | -1.21 |
| 1448047_at | --- | Adult male medulla oblongata cDNA, RIKEN full-length enriched library, clone:63 | -1.33 | 0.045 | -1.42 | 0.203 | -1.04 | 0.843 | -1.08 | 0.699 | -1.22 |

| | | | | | | | | | | | |
|---|---|---|---|---|---|---|---|---|---|---|---|
| 1452642_at | Tmem16f | Transmembrane protein 16F | -1.2 | 0.381 | -1.44 | 0.319 | -1.12 | 0.328 | -1.31 | 0.299 | -1.27 |
| 1420623_x_at | Hspa8 /// LOC6212 | heat shock protein 8 /// similar to heat shock protein 8 /// heat shock protein 8 pse | -1.25 | 0.265 | -1.3 | 0.088 | -1.18 | 0.018 | -1.25 | 0.042 | -1.24 |
| 1433941_at | Pib5pa | phosphatidylinositol (4,5) bisphosphate 5-phosphatase, A | -1.17 | 0.539 | -1.27 | 0.044 | -1.29 | 0.554 | -1.03 | 0.865 | -1.19 |
| 1450340_a_at | Clcnkb | chloride channel Kb | -1.03 | 0.781 | -1.03 | 0.961 | -2.11 | 0.08 | -1.61 | 0.565 | -1.44 |
| 1425043_s_at | 0610037D15Rik | RIKEN cDNA 0610037D15 gene | -1.66 | 0.004 | -1.02 | 0.977 | -1.2 | 0.456 | -1.7 | 0.042 | -1.39 |
| 1420068_at | --- | --- | -1.12 | 0.848 | -1.14 | 0.705 | -1.53 | 0.178 | -1.47 | 0.577 | -1.32 |
| 1437224_at | Rtn4 | reticulon 4 | -1.21 | 0.331 | -1.05 | 0.818 | -1.55 | 0.011 | -1.07 | 0.691 | -1.22 |
| 1450966_at | Crot | carnitine O-octanoyltransferase | -1.18 | 0.201 | -1.72 | 0.006 | -1.01 | 0.98 | -1.33 | 0.216 | -1.31 |
| 1444728_at | Exoc4 | Exocyst complex component 4 | -1.02 | 0.947 | -1.21 | 0.132 | -1.63 | 0.061 | -1.17 | 0.389 | -1.26 |
| 1445556_at | --- | Transcribed locus | -1.36 | 0.442 | -1.13 | 0.773 | -1.25 | 0.7 | -1.67 | 0.408 | -1.35 |
| 1446205_at | --- | 0 day neonate thymus cDNA, RIKEN full-length enriched library, clone:A430057L | -1.05 | 0.729 | -1.51 | 0.335 | -1.25 | 0.41 | -1.34 | 0.196 | -1.28 |
| 1440690_at | Nipbl | Nipped-B homolog (Drosophila) | -1.06 | 0.68 | -1.16 | 0.175 | -1.63 | 0.011 | -1.29 | 0.377 | -1.28 |
| 1433574_at | Cdc37l1 | cell division cycle 37 homolog (S. cerevisiae)-like 1 | -1.11 | 0.591 | -1.11 | 0.146 | -1.63 | 0.006 | -1.41 | 0.061 | -1.31 |
| 1441624_at | Sorbs2 | sorbin and SH3 domain containing 2 | -1.25 | 0.444 | -1.34 | 0.279 | -1.14 | 0.221 | -1.34 | 0.224 | -1.27 |
| 1417980_a_at | Insig2 | insulin induced gene 2 | -1.03 | 0.861 | -1.46 | 0.071 | -1.32 | 0.016 | -1.46 | 0.054 | -1.31 |
| 1437290_at | Impad1 | inositol monophosphatase domain containing 1 | -1.31 | 0.296 | -1.1 | 0.331 | -1.34 | 0.059 | -1.23 | 0.543 | -1.25 |
| 1420932_at | Mapk8 | mitogen activated protein kinase 8 | -1.41 | 0.027 | -1.08 | 0.084 | -1.27 | 0 | -1.3 | 0.146 | -1.27 |
| 1428175_at | Tmem161b | transmembrane protein 161B | -1.19 | 0.342 | -1.18 | 0.434 | -1.36 | 0.049 | -1 | 0.989 | -1.18 |
| 1432995_at | 2900057E15Rik | RIKEN cDNA 2900057E15 gene | -1.21 | 0.389 | -1.26 | 0.42 | -1.25 | 0.225 | -1.08 | 0.767 | -1.2 |
| 1447150_at | Phr1 | pam, highwire, rpm 1 | -1.23 | 0.173 | -1.41 | 0.125 | -1.11 | 0.462 | -1.85 | 0.075 | -1.4 |
| 1420586_at | 2900054J07Rik | RIKEN cDNA 2900054J07 gene | -1.43 | 0.313 | -1.24 | 0.65 | -1.1 | 0.559 | -1.07 | 0.848 | -1.21 |
| 1416977_at | Stam2 | signal transducing adaptor molecule (SH3 domain and ITAM motif) 2 | -1.73 | 0.082 | -1.05 | 0.888 | -1.12 | 0.477 | -1.57 | 0.012 | -1.37 |
| 1459305_at | Rai17 | Retinoic acid induced 17 | -1.21 | 0.188 | -1.34 | 0.271 | -1.18 | 0.279 | -1.01 | 0.965 | -1.19 |
| 1457679_at | Dclre1c | DNA cross-link repair 1C, PSO2 homolog (S. cerevisiae) | -1 | 0.995 | -1.41 | 0.454 | -1.4 | 0.03 | -1.4 | 0.409 | -1.3 |
| 1454703_x_at | Snord22 | small nucleolar RNA, C/D box 22 | -1.41 | 0.394 | -1.33 | 0.177 | -1.04 | 0.874 | -1.06 | 0.84 | -1.21 |
| 1434166_at | 9330151L19Rik | RIKEN cDNA 9330151L19 gene | -1.2 | 0.389 | -1.41 | 0.186 | -1.13 | 0.535 | -1.09 | 0.704 | -1.21 |
| 1429595_at | 2700049A03Rik | RIKEN cDNA 2700049A03 gene | -1.38 | 0.03 | -1.23 | 0.513 | -1.13 | 0.549 | -1.04 | 0.835 | -1.2 |
| 1441753_at | 4921505C17Rik | RIKEN cDNA 4921505C17 gene | -1.11 | 0.481 | -1.09 | 0.805 | -1.65 | 0.127 | -1.35 | 0.154 | -1.3 |
| 1428755_at | Creb1 | cAMP responsive element binding protein 1 | -1.34 | 0.022 | -1.13 | 0.254 | -1.26 | 0.007 | -1.39 | 0.13 | -1.28 |
| 1459928_at | Lcor | ligand dependent nuclear receptor corepressor | -1.37 | 0.574 | -1.2 | 0.718 | -1.16 | 0.269 | -1.01 | 0.967 | -1.19 |
| 1435565_at | 1500011H22Rik | RIKEN cDNA 1500011H22 gene | -1.2 | 0.323 | -1.09 | 0.559 | -1.49 | 0.052 | -1.41 | 0.015 | -1.3 |
| 1456806_at | A130010C12Rik | RIKEN cDNA A130010C12 gene | -1.55 | 0.283 | -1.14 | 0.77 | -1.11 | 0.671 | -1.22 | 0.183 | -1.26 |
| 1419608_a_at | Mia1 | melanoma inhibitory activity 1 | -1.18 | 0.148 | -1.16 | 0.79 | -1.41 | 0.251 | -1.46 | 0.475 | -1.3 |
| 1458508_at | Matr3 | matrin 3 | -1.01 | 0.942 | -1.18 | 0.379 | -1.69 | 0.004 | -1.56 | 0.076 | -1.36 |
| 1437384_at | 1700015E13Rik | RIKEN cDNA 1700015E13 gene | -1.47 | 0.147 | -1.25 | 0.378 | -1.06 | 0.848 | -1.77 | 0.086 | -1.39 |
| 1435274_at | 4921517N04Rik | RIKEN cDNA 4921517N04 gene | -1.14 | 0.142 | -1.17 | 0.097 | -1.44 | 0.036 | -1.16 | 0.478 | -1.23 |
| 1442213_at | LOC552908 | hypothetical LOC552908 | -1.01 | 0.974 | -1.14 | 0.769 | -1.79 | 0.035 | -1.07 | 0.879 | -1.25 |
| 1430680_a_at | 5830417I10Rik /// L | RIKEN cDNA 5830417I10 gene /// similar to Dingo protein isoform 2 /// similar to I | -1.19 | 0.111 | -1.69 | 0.08 | -1.01 | 0.914 | -1.35 | 0.291 | -1.31 |
| 1440861_a_at | --- | --- | -1.16 | 0.261 | -1.03 | 0.863 | -1.68 | 0.004 | -1.29 | 0.06 | -1.29 |
| 1427208_at | Zfp451 | zinc finger protein 451 | -1.27 | 0.282 | -1.4 | 0.033 | -1.09 | 0.662 | -1.15 | 0.621 | -1.23 |
| 1454640_at | Chchd7 | coiled-coil-helix-coiled-coil-helix domain containing 7 | -1.07 | 0.462 | -1.54 | 0.003 | -1.2 | 0.093 | -1.05 | 0.622 | -1.21 |
| 1443579_s_at | Depdc6 | DEP domain containing 6 | -1.04 | 0.581 | -1.35 | 0.053 | -1.39 | 0.329 | -1.02 | 0.965 | -1.2 |
| 1452322_a_at | Brwd1 | bromodomain and WD repeat domain containing 1 | -1.39 | 0.028 | -1.12 | 0.677 | -1.23 | 0.208 | -1.73 | 0.029 | -1.37 |
| 1428920_at | --- | --- | -1.25 | 0.345 | -1.37 | 0.198 | -1.11 | 0.482 | -1.38 | 0.265 | -1.28 |
| 1459011_at | Uchl5 | Ubiquitin carboxyl-terminal esterase L5 | -1.25 | 0.045 | -1.09 | 0.636 | -1.41 | 0.008 | -1.36 | 0.419 | -1.28 |
| 1435980_x_at | Wnt6 | wingless-related MMTV integration site 6 | -1.2 | 0.453 | -1.47 | 0.309 | -1.1 | 0.544 | -1.08 | 0.785 | -1.21 |
| 1418211_at | p | pink-eyed dilution | -1.49 | 0.284 | -1.3 | 0.102 | -1.01 | 0.946 | -1.05 | 0.85 | -1.21 |
| 1458369_at | 2310050B05Rik | RIKEN cDNA 2310050B05 gene | -1.22 | 0.249 | -1.27 | 0.536 | -1.22 | 0.323 | -1.01 | 0.944 | -1.18 |
| 1420107_at | Bbs4 | Bardet-Biedl syndrome 4 homolog (human) | -1.01 | 0.971 | -1.55 | 0.416 | -1.26 | 0.089 | -1.19 | 0.348 | -1.25 |
| 1443088_at | 9930031P18Rik | RIKEN cDNA 9930031P18 gene | -1.01 | 0.972 | -1.08 | 0.602 | -1.98 | 0.034 | -1.54 | 0.156 | -1.4 |
| 1424252_at | Hnrpdl | heterogeneous nuclear ribonucleoprotein D-like | -1.31 | 0.194 | -1.21 | 0.104 | -1.2 | 0.213 | -1.34 | 0.225 | -1.26 |
| 1445870_at | Cdc42ep2 | CDC42 effector protein (Rho GTPase binding) 2 | -1.12 | 0.426 | -1.34 | 0.395 | -1.26 | 0.473 | -1.29 | 0.388 | -1.26 |
| 1451464_at | Mfap3 | microfibrillar-associated protein 3 | -1.16 | 0.716 | -1.42 | 0.386 | -1.17 | 0.444 | -1.11 | 0.507 | -1.21 |

| | | | | | | | | | | | |
|---|---|---|---|---|---|---|---|---|---|---|---|
| 1432099_a_at | Prodh2 | proline dehydrogenase (oxidase) 2 | -1.27 | 0.125 | -1.2 | 0.128 | -1.24 | 0.203 | -1.24 | 0.178 | -1.24 |
| 1428084_at | Krr1 | KRR1, small subunit (SSU) processome component, homolog (yeast) | -1.14 | 0.052 | -1.2 | 0.025 | -1.39 | 0.034 | -1.11 | 0.333 | -1.21 |
| 1426232_at | BC024479 | cDNA sequence BC024479 | -1.19 | 0.535 | -1.07 | 0.793 | -1.53 | 0.113 | -1.42 | 0.062 | -1.3 |
| 1425788_a_at | Echdc2 | enoyl Coenzyme A hydratase domain containing 2 | -1.05 | 0.781 | -1.1 | 0.07 | -1.79 | 0.032 | -2.25 | 0.081 | -1.55 |
| 1454969_at | Lypd6 | LY6/PLAUR domain containing 6 | -1.13 | 0.017 | -1.39 | 0.1 | -1.22 | 0.287 | -1.85 | 0.222 | -1.4 |
| 1454921_at | Gm561 | gene model 561, (NCBI) | -1.05 | 0.321 | -1.4 | 0.046 | -1.32 | 0.121 | -1.25 | 0.18 | -1.25 |
| 1437461_s_at | Rnpc3 | RNA-binding region (RNP1, RRM) containing 3 | -1.3 | 0.458 | -1.16 | 0.425 | -1.26 | 0.164 | -1.05 | 0.707 | -1.19 |
| 1429128_x_at | Nfkb2 | nuclear factor of kappa light polypeptide gene enhancer in B-cells 2, p49/p100 | -1.09 | 0.558 | -1.35 | 0.714 | -1.3 | 0.199 | -1.78 | 0.213 | -1.38 |
| 1431239_at | Nono | non-POU-domain-containing, octamer binding protein | -1.13 | 0.512 | -1.29 | 0.157 | -1.3 | 0.155 | -1.12 | 0.551 | -1.21 |
| 1437022_at | D130059P03Rik | RIKEN cDNA D130059P03 gene | -1.05 | 0.114 | -1.16 | 0.394 | -1.63 | 0.018 | -1.18 | 0.263 | -1.25 |
| 1444753_at | Nek7 | NIMA (never in mitosis gene a)-related expressed kinase 7 | -1 | 0.981 | -1.18 | 0.549 | -1.72 | 0.042 | -1.38 | 0.431 | -1.32 |
| 1438497_at | 2810423E13Rik | RIKEN cDNA 2810423E13 gene | -1.1 | 0.44 | -1.44 | 0.161 | -1.21 | 0.337 | -1.32 | 0.218 | -1.27 |
| 1432073_at | 1700113I22Rik | RIKEN cDNA 1700113I22 gene | -1.65 | 0.049 | -1.2 | 0.402 | -1.01 | 0.927 | -1.3 | 0.429 | -1.29 |
| 1440254_at | --- | Transcribed locus, weakly similar to XP_417295.1 PREDICTED: similar to TAF3 | -1.08 | 0.685 | -1.22 | 0.013 | -1.45 | 0.196 | -1.21 | 0.249 | -1.24 |
| 1426013_s_at | Plekha4 | pleckstrin homology domain containing, family A (phosphoinositide binding specif | -1.18 | 0.258 | -1.12 | 0.767 | -1.44 | 0.104 | -1.17 | 0.576 | -1.23 |
| 1435041_at | Myl6 | myosin, light polypeptide 6, alkali, smooth muscle and non-muscle | -1.77 | 0.276 | -1.11 | 0.489 | -1.04 | 0.868 | -1.68 | 0.161 | -1.4 |
| 1433756_at | S100pbp | S100P binding protein | -1.2 | 0.573 | -1.21 | 0.226 | -1.3 | 0.297 | -1.46 | 0.357 | -1.29 |
| 1437717_x_at | 2610005L07Rik /// | RIKEN cDNA 2610005L07 gene /// RIKEN cDNA A430108E01 gene /// similar to | -1.17 | 0.669 | -1.13 | 0.669 | -1.45 | 0.3 | -1.31 | 0.593 | -1.27 |
| 1460564_at | Suhw2 | suppressor of hairy wing homolog 2 (Drosophila) | -1.19 | 0.641 | -1.32 | 0.126 | -1.21 | 0.231 | -1.11 | 0.302 | -1.21 |
| 1458419_at | E130215H24Rik | RIKEN cDNA E130215H24 gene | -1.06 | 0.736 | -1.93 | 0.153 | -1.03 | 0.855 | -1.2 | 0.571 | -1.31 |
| 1455406_at | --- | 0 day neonate head cDNA, RIKEN full-length enriched library, clone:4833431M1 | -1.04 | 0.744 | -1.48 | 0.062 | -1.26 | 0.129 | -1.18 | 0.688 | -1.24 |
| 1458677_at | Entpd5 | ectonucleoside triphosphate diphosphohydrolase 5 | -1.2 | 0.426 | -1.17 | 0.73 | -1.36 | 0.154 | -1.63 | 0.065 | -1.34 |
| 1429760_at | Rps6ka6 | ribosomal protein S6 kinase polypeptide 6 | -1.27 | 0.497 | -1.53 | 0.461 | -1.01 | 0.957 | -2.39 | 0.058 | -1.55 |
| 1459736_at | Stk10 | Serine/threonine kinase 10 | -1.65 | 0.594 | -1.04 | 0.928 | -1.16 | 0.684 | -1.69 | 0.087 | -1.39 |
| 1448959_at | Ndufs4 | NADH dehydrogenase (ubiquinone) Fe-S protein 4 | -1.19 | 0.061 | -1.34 | 0.028 | -1.18 | 0.118 | -1.3 | 0.154 | -1.25 |
| 1440886_at | Cdc37l1 | cell division cycle 37 homolog (S. cerevisiae)-like 1 | -1.29 | 0.463 | -1.17 | 0.578 | -1.25 | 0.248 | -1.97 | 0.054 | -1.42 |
| 1438597_x_at | Morf4l1 /// LOC433 | mortality factor 4 like 1 /// similar to mortality factor 4 like 1 isoform b /// similar to | -1.34 | 0.122 | -1.09 | 0.779 | -1.31 | 0.01 | -1.16 | 0.076 | -1.22 |
| 1421372_at | Klk1b4 | kallikrein 1-related pepidase b4 | -1.05 | 0.869 | -1.74 | 0.031 | -1.1 | 0.665 | -1.36 | 0.509 | -1.31 |
| 1436455_at | Asph | aspartate-beta-hydroxylase | -1.02 | 0.937 | -1.72 | 0.475 | -1.14 | 0.757 | -1.51 | 0.359 | -1.35 |
| 1440636_at | Mrpl3 | Mitochondrial ribosomal protein L3 | -1.38 | 0.115 | -1.09 | 0.759 | -1.27 | 0.036 | -1.4 | 0.385 | -1.28 |
| 1420177_at | Igll1 | immunoglobulin lambda-like polypeptide 1 | -1.06 | 0.842 | -1.32 | 0.367 | -1.37 | 0.516 | -1.42 | 0.641 | -1.29 |
| 1439642_at | 5930403N24Rik | RIKEN cDNA 5930403N24 gene | -1.12 | 0.857 | -1.08 | 0.837 | -1.63 | 0.259 | -1.07 | 0.922 | -1.22 |
| 1454375_at | 5730458M16Rik | RIKEN cDNA 5730458M16 gene | -1.41 | 0.084 | -1.08 | 0.137 | -1.25 | 0.021 | -1.31 | 0.365 | -1.26 |
| 1452613_at | Uqcrq | ubiquinol-cytochrome c reductase, complex III subunit VII | -1.3 | 0.284 | -1.18 | 0.557 | -1.22 | 0.748 | -1.08 | 0.536 | -1.2 |
| 1438888_at | Gmcl1 | germ cell-less homolog 1 (Drosophila) | -1.31 | 0.482 | -1.01 | 0.979 | -1.47 | 0.074 | -1.53 | 0.156 | -1.33 |
| 1430444_at | 0610006L08Rik | RIKEN cDNA 0610006L08 gene | -1.34 | 0.064 | -1.35 | 0.506 | -1.06 | 0.903 | -1.43 | 0.458 | -1.29 |
| 1432084_at | 4921515G04Rik | RIKEN cDNA 4921515G04 gene | -1.06 | 0.923 | -1.66 | 0.36 | -1.13 | 0.763 | -2.98 | 0.091 | -1.71 |
| 1447956_at | C76614 | expressed sequence C76614 | -1.05 | 0.937 | -1.21 | 0.421 | -1.53 | 0.321 | -1.69 | 0.323 | -1.37 |
| 1457205_at | Nsf | N-ethylmaleimide sensitive fusion protein | -1.11 | 0.769 | -1.16 | 0.161 | -1.49 | 0.179 | -1.49 | 0.087 | -1.31 |
| 1426420_at | Rbm26 | RNA binding motif protein 26 | -1.34 | 0.125 | -1.1 | 0.763 | -1.3 | 0.148 | -1.15 | 0.459 | -1.22 |
| 1425001_at | Rnf146 | ring finger protein 146 | -1.22 | 0.494 | -1.23 | 0.183 | -1.25 | 0.176 | -1.01 | 0.931 | -1.18 |
| 1430375_a_at | Ccl27 | chemokine (C-C motif) ligand 27 | -1.17 | 0.094 | -1.15 | 0.478 | -1.41 | 0.222 | -1.15 | 0.642 | -1.22 |
| 1422537_a_at | Id2 | inhibitor of DNA binding 2 | -1.33 | 0.57 | -1.09 | 0.728 | -1.31 | 0.051 | -1.16 | 0.614 | -1.22 |
| 1459791_at | Dnajc1 | DnaJ (Hsp40) homolog, subfamily C, member 1 | -1.03 | 0.942 | -1.53 | 0.244 | -1.24 | 0.373 | -1.99 | 0.193 | -1.45 |
| 1451347_at | Ccdc95 | coiled-coil domain containing 95 | -1.37 | 0.109 | -1.4 | 0.351 | -1.01 | 0.958 | -1.07 | 0.515 | -1.21 |
| 1438762_at | Tcf12 | Transcription factor 12 | -1.02 | 0.917 | -1.11 | 0.318 | -1.84 | 0.005 | -1.73 | 0.105 | -1.42 |
| 1424658_at | Taok1 | TAO kinase 1 | -1.02 | 0.931 | -1.29 | 0.05 | -1.47 | 0.128 | -1.1 | 0.655 | -1.22 |
| 1438898_at | --- | Adult male corpora quadrigemina cDNA, RIKEN full-length enriched library, clone | -1.26 | 0.669 | -1.45 | 0.392 | -1.05 | 0.936 | -3.56 | 0.016 | -1.83 |
| 1419772_at | Mllt10 | Myeloid/lymphoid or mixed lineage-leukemia translocation to 10 homolog (Drosop | -1.3 | 0.441 | -1.23 | 0.406 | -1.18 | 0.621 | -1.28 | 0.635 | -1.25 |
| 1419815_at | Mett11d1 | methyltransferase 11 domain containing 1 | -1.49 | 0.084 | -1.26 | 0.201 | -1.03 | 0.717 | -2.09 | 0.102 | -1.47 |
| 1446693_at | --- | --- | -1.13 | 0.52 | -1.02 | 0.938 | -1.76 | 0.274 | -1.56 | 0.438 | -1.37 |
| 1427352_at | BC031593 | cDNA sequence BC031593 | -1.24 | 0.284 | -1.31 | 0.456 | -1.15 | 0.545 | -2.65 | 0.105 | -1.59 |
| 1439466_s_at | Bud31 | BUD31 homolog (yeast) | -1.14 | 0.358 | -1.15 | 0.016 | -1.46 | 0.018 | -1.08 | 0.268 | -1.21 |

| | | | | | | | | | | | |
|---|---|---|---|---|---|---|---|---|---|---|---|
| 1460728_s_at | Ing4 | inhibitor of growth family, member 4 | -1.3 | 0.186 | -1.11 | 0.525 | -1.31 | 0.343 | -1.17 | 0.286 | -1.22 |
| 1449535_at | Znrf4 | zinc and ring finger 4 | -1.02 | 0.945 | -1.14 | 0.742 | -1.73 | 0.086 | -1.64 | 0.087 | -1.38 |
| 1450226_at | Prlr | prolactin receptor | -1.46 | 0.266 | -1.03 | 0.881 | -1.29 | 0.434 | -2.02 | 0.506 | -1.45 |
| 1444351_at | Cyfip2 | Cytoplasmic FMR1 interacting protein 2 | -1.23 | 0.451 | -1.57 | 0.414 | -1.02 | 0.959 | -1.42 | 0.235 | -1.31 |
| 1416817_at | Nek7 | NIMA (never in mitosis gene a)-related expressed kinase 7 | -1.18 | 0.239 | -1.15 | 0.377 | -1.39 | 0.063 | -1.61 | 0.052 | -1.33 |
| 1443654_at | Ankfy1 | Ankyrin repeat and FYVE domain containing 1 | -1.19 | 0.285 | -1.28 | 0.351 | -1.23 | 0.16 | -1.54 | 0.382 | -1.31 |
| 1446489_at | Sufu | Suppressor of fused homolog (Drosophila) | -1.29 | 0.232 | -1.22 | 0.359 | -1.19 | 0.454 | -1.76 | 0.06 | -1.36 |
| 1446097_at | Rbbp8 | Retinoblastoma binding protein 8 | -1.24 | 0.505 | -1.13 | 0.735 | -1.34 | 0.37 | -1 | 0.995 | -1.18 |
| 1438532_at | Hmcn1 | hemicentin 1 | -1.19 | 0.323 | -1.11 | 0.697 | -1.45 | 0.24 | -1 | 0.992 | -1.19 |
| 1457690_at | Kalrn | kalirin, RhoGEF kinase | -1.05 | 0.942 | -1.22 | 0.139 | -1.51 | 0.182 | -1.46 | 0.616 | -1.31 |
| 1434307_at | Tmem64 | transmembrane protein 64 | -1.49 | 0.075 | -1.18 | 0.091 | -1.09 | 0.599 | -1.03 | 0.842 | -1.2 |
| 1430585_at | 5930436O19Rik | RIKEN cDNA 5930436O19 gene | -1.21 | 0.278 | -1.1 | 0.64 | -1.41 | 0.165 | -1.68 | 0.117 | -1.35 |
| 1445651_at | Atp11b | ATPase, Class VI, type 11B | -1.36 | 0.654 | -1.15 | 0.182 | -1.21 | 0.459 | -1.07 | 0.629 | -1.2 |
| 1436602_x_at | Cacna1b | calcium channel, voltage-dependent, N type, alpha 1B subunit | -1.57 | 0.467 | -1.24 | 0.719 | -1 | 0.993 | -2.06 | 0.183 | -1.47 |
| 1443386_at | C86727 | expressed sequence C86727 | -1.01 | 0.983 | -1.6 | 0.596 | -1.22 | 0.695 | -1.74 | 0.33 | -1.39 |
| 1456583_x_at | Tmem144 | transmembrane protein 144 | -1.34 | 0.392 | -1.08 | 0.809 | -1.31 | 0.6 | -1.02 | 0.959 | -1.19 |
| 1455530_at | Igh-1a | Immunoglobulin heavy chain 1a (serum IgG2a) | -1.29 | 0.719 | -1.12 | 0.675 | -1.3 | 0.483 | -2.91 | 0.106 | -1.66 |
| 1438326_at | 3300001M20Rik | RIKEN cDNA 3300001M20 gene | -1.23 | 0.189 | -1.26 | 0.334 | -1.2 | 0.315 | -1.08 | 0.567 | -1.19 |
| 1416665_at | Coq7 | demethyl-Q 7 | -1.22 | 0.044 | -1.23 | 0.045 | -1.24 | 0.028 | -1.1 | 0.587 | -1.2 |
| 1449985_at | 1700072E05Rik | RIKEN cDNA 1700072E05 gene | -1.32 | 0.777 | -1.15 | 0.777 | -1.23 | 0.635 | -1.78 | 0.539 | -1.37 |
| 1438887_a_at | Gmcl1 | germ cell-less homolog 1 (Drosophila) | -1.37 | 0.189 | -1.28 | 0.402 | -1.08 | 0.742 | -1.85 | 0.133 | -1.39 |
| 1429108_at | Msl2l1 | male-specific lethal 2-like 1 (Drosophila) | -1.05 | 0.697 | -1.4 | 0.019 | -1.3 | 0.045 | -1.12 | 0.437 | -1.22 |
| 1460506_s_at | Ndufc2 /// LOC675 | NADH dehydrogenase (ubiquinone) 1, subcomplex unknown, 2 /// similar to NAD | -1.21 | 0.067 | -1.14 | 0.12 | -1.36 | 0.038 | -1.12 | 0.167 | -1.21 |
| 1445534_at | Flnb | Filamin, beta | -1.08 | 0.762 | -1.3 | 0.245 | -1.36 | 0.321 | -1.42 | 0.318 | -1.29 |
| 1438046_at | AU019823 | expressed sequence AU019823 | -1.15 | 0.495 | -1.26 | 0.329 | -1.29 | 0.166 | -1.16 | 0.364 | -1.21 |
| 1434942_at | 2610101J03Rik | RIKEN cDNA 2610101J03 gene | -1.18 | 0.144 | -1.11 | 0.311 | -1.45 | 0.003 | -1.12 | 0.577 | -1.22 |
| 1457552_at | Zfp295 | zinc finger protein 295 | -1.17 | 0.4 | -1.24 | 0.232 | -1.28 | 0.154 | -1.45 | 0.142 | -1.29 |
| 1438247_at | Klhl15 | kelch-like 15 (Drosophila) | -1.24 | 0.381 | -1.21 | 0.293 | -1.24 | 0.438 | -1.22 | 0.42 | -1.23 |
| 1459465_at | 9630020C08Rik | RIKEN cDNA 9630020C08 gene | -1.18 | 0.756 | -1.33 | 0.681 | -1.19 | 0.709 | -1.31 | 0.432 | -1.25 |
| 1439341_at | AK220484 | cDNA sequence AK220484 | -1.21 | 0.646 | -1.55 | 0.105 | -1.03 | 0.707 | -1.72 | 0.15 | -1.38 |
| 1457904_at | Car8 | carbonic anhydrase 8 | -1 | 0.982 | -1.23 | 0.297 | -1.58 | 0.019 | -1.09 | 0.841 | -1.23 |
| 1423063_at | Dnmt3a | DNA methyltransferase 3A | -1.44 | 0.067 | -1.14 | 0.286 | -1.15 | 0.595 | -1.22 | 0.567 | -1.24 |
| 1451466_at | D16Ertd472e | DNA segment, Chr 16, ERATO Doi 472, expressed | -1.43 | 0.059 | -1.08 | 0.382 | -1.23 | 0.092 | -1.43 | 0.078 | -1.29 |
| 1457309_at | AU015228 | expressed sequence AU015228 | -1.09 | 0.885 | -1.13 | 0.804 | -1.58 | 0.369 | -1 | 0.997 | -1.2 |
| 1458032_at | Tpd52l2 | Tumor protein D52-like 2 | -1.2 | 0.779 | -1.31 | 0.687 | -1.19 | 0.737 | -1.11 | 0.775 | -1.2 |
| 1453303_at | 4833417J20Rik | RIKEN cDNA 4833417J20 gene | -1.11 | 0.663 | -1.41 | 0.379 | -1.21 | 0.516 | -1.26 | 0.45 | -1.25 |
| 1448519_at | Tead2 | TEA domain family member 2 | -1.11 | 0.92 | -1.35 | 0.714 | -1.25 | 0.185 | -1.18 | 0.693 | -1.22 |
| 1444955_at | D6Ertd469e | DNA segment, Chr 6, ERATO Doi 469, expressed | -1.05 | 0.931 | -1.23 | 0.556 | -1.48 | 0.213 | -1.18 | 0.698 | -1.24 |
| 1440640_at | 2010111I01Rik | RIKEN cDNA 2010111I01 gene | -1.08 | 0.579 | -1.14 | 0.482 | -1.58 | 0.052 | -1.52 | 0.365 | -1.33 |
| 1428280_at | Fip1l1 | FIP1 like 1 (S. cerevisiae) | -1.03 | 0.909 | -1.39 | 0.112 | -1.34 | 0.025 | -1.29 | 0.101 | -1.26 |
| 1447818_x_at | Rhebl1 | Ras homolog enriched in brain like 1 | -1.3 | 0.22 | -1.11 | 0.69 | -1.31 | 0.388 | -1.54 | 0.221 | -1.31 |
| 1416791_a_at | Nxf1 | nuclear RNA export factor 1 homolog (S. cerevisiae) | -1.04 | 0.737 | -1.28 | 0.034 | -1.44 | 0.072 | -1.39 | 0.175 | -1.29 |
| 1455783_at | --- | --- | -1.15 | 0.066 | -1.23 | 0.763 | -1.32 | 0.556 | -1.67 | 0.242 | -1.34 |
| 1443516_at | Atxn2 | Ataxin 2 | -1.03 | 0.933 | -1.08 | 0.67 | -1.85 | 0.032 | -1.01 | 0.984 | -1.24 |
| 1434395_at | Man1a2 | mannosidase, alpha, class 1A, member 2 | -1.17 | 0.488 | -1.08 | 0.635 | -1.51 | 0.119 | -1.15 | 0.552 | -1.23 |
| 1452988_at | 2610306M01Rik | RIKEN cDNA 2610306M01 gene | -1.15 | 0.486 | -1.29 | 0.351 | -1.26 | 0.383 | -1.23 | 0.265 | -1.23 |
| 1436484_at | C030019I05Rik | RIKEN cDNA C030019I05 gene | -1.03 | 0.972 | -1.07 | 0.801 | -1.89 | 0.382 | -2.59 | 0.174 | -1.65 |
| 1440303_at | Slc7a6os | Solute carrier family 7, member 6 opposite strand | -1.55 | 0.261 | -1.14 | 0.745 | -1.09 | 0.216 | -1.39 | 0.39 | -1.29 |
| 1423246_at | Txndc4 | thioredoxin domain containing 4 (endoplasmic reticulum) | -1.25 | 0.022 | -1.23 | 0.172 | -1.2 | 0.332 | -1.18 | 0.174 | -1.22 |
| 1455997_a_at | Uqcrb | ubiquinol-cytochrome c reductase binding protein | -1.21 | 0.018 | -1.22 | 0.457 | -1.26 | 0.008 | -1.05 | 0.107 | -1.19 |
| 1444232_at | Prkg1 | protein kinase, cGMP-dependent, type I | -1.15 | 0.225 | -1.02 | 0.931 | -1.69 | 0.028 | -1 | 0.996 | -1.22 |
| 1455696_a_at | Prpf4b | PRP4 pre-mRNA processing factor 4 homolog B (yeast) | -1.15 | 0.602 | -1.14 | 0.506 | -1.45 | 0.071 | -1.31 | 0.399 | -1.26 |
| 1437274_at | Copa | coatomer protein complex subunit alpha | -1.05 | 0.817 | -1.04 | 0.85 | -1.9 | 0 | -2.02 | 0.065 | -1.5 |

| | | | | | | | | | | | |
|---|---|---|---|---|---|---|---|---|---|---|---|
| 1434613_at | 1810013L24Rik | RIKEN cDNA 1810013L24 gene | -1.08 | 0.662 | -1.14 | 0.136 | -1.58 | 0.034 | -1.42 | 0.047 | -1.3 |
| 1454532_at | C030043A13Rik | RIKEN cDNA C030043A13 gene | -1 | 0.999 | -1.47 | 0.01 | -1.32 | 0.163 | -1.25 | 0.182 | -1.26 |
| 1454149_a_at | Ccnl2 | cyclin L2 | -1.32 | 0.452 | -1.18 | 0.399 | -1.19 | 0.222 | -1.3 | 0.142 | -1.25 |
| 1456148_a_at | 1110014K08Rik | RIKEN cDNA 1110014K08 gene | -1.22 | 0.55 | -1.19 | 0.231 | -1.28 | 0.368 | -2.22 | 0.167 | -1.48 |
| 1447679_s_at | Bms1l | BMS1-like, ribosome assembly protein (yeast) | -1.02 | 0.832 | -1.29 | 0.03 | -1.46 | 0.094 | -1.75 | 0.249 | -1.38 |
| 1427935_at | 2610208E05Rik | RIKEN cDNA 2610208E05 gene | -1.03 | 0.837 | -1.32 | 0.044 | -1.4 | 0.188 | -1.3 | 0.508 | -1.26 |
| 1440144_x_at | C330046E03 | hypothetical protein C330046E03 | -1.81 | 0.181 | -1.03 | 0.919 | -1.09 | 0.663 | -1.36 | 0.018 | -1.32 |
| 1455165_at | Rora | RAR-related orphan receptor alpha | -1.05 | 0.9 | -1.13 | 0.308 | -1.67 | 0.12 | -1.64 | 0.103 | -1.37 |
| 1442078_at | Ctdspl2 | CTD (carboxy-terminal domain, RNA polymerase II, polypeptide A) small phospha | -1.33 | 0.409 | -1.12 | 0.589 | -1.26 | 0.294 | -1.26 | 0.207 | -1.24 |
| 1428213_at | 2410003A14Rik | RIKEN cDNA 2410003A14 gene | -1.24 | 0.019 | -1.38 | 0.018 | -1.1 | 0.354 | -1.07 | 0.501 | -1.2 |
| 1432806_at | 9430099H24Rik | RIKEN cDNA 9430099H24 gene | -1.46 | 0.08 | -1.09 | 0.643 | -1.19 | 0.484 | -1.47 | 0.14 | -1.3 |
| 1434633_at | Crebbp | CREB binding protein | -1.07 | 0.395 | -1.26 | 0.073 | -1.39 | 0.098 | -1.31 | 0.141 | -1.26 |
| 1460113_at | B930093H17Rik | RIKEN cDNA B930093H17 gene | -1.09 | 0.789 | -1.23 | 0.464 | -1.4 | 0.065 | -1.32 | 0.454 | -1.26 |
| 1441262_at | Slk | STE20-like kinase (yeast) | -1.02 | 0.89 | -1.03 | 0.914 | -2.04 | 0.007 | -1.4 | 0.1 | -1.37 |
| 1435499_at | D030041N04Rik | RIKEN cDNA D030041N04 gene | -1.42 | 0.118 | -1.18 | 0.628 | -1.12 | 0.582 | -1.51 | 0.057 | -1.31 |
| 1437338_x_at | Elp3 | Elongation protein 3 homolog (S. cerevisiae) | -1.18 | 0.15 | -1.26 | 0.152 | -1.25 | 0.136 | -1.47 | 0.075 | -1.29 |
| 1447970_at | --- | --- | -1.33 | 0.37 | -1.28 | 0.355 | -1.1 | 0.685 | -1.11 | 0.661 | -1.21 |
| 1460430_at | Rap2c | RAP2C, member of RAS oncogene family | -1.18 | 0.301 | -1.41 | 0.067 | -1.13 | 0.515 | -1.15 | 0.508 | -1.22 |
| 1439123_at | Phf21a | PHD finger protein 21A | -1.18 | 0.232 | -1.02 | 0.844 | -1.63 | 0.015 | -2.01 | 0.077 | -1.46 |
| 1457214_at | Ncoa3 | Nuclear receptor coactivator 3 | -1.31 | 0.547 | -1.17 | 0.57 | -1.21 | 0.526 | -1.77 | 0.223 | -1.37 |
| 1439080_at | Erbb2ip | Erbb2 interacting protein | -1.14 | 0.36 | -1.15 | 0.588 | -1.42 | 0.142 | -1.55 | 0.203 | -1.32 |
| 1441285_at | 4833426J09Rik | RIKEN cDNA 4833426J09 gene | -1.06 | 0.872 | -1.73 | 0.019 | -1.09 | 0.701 | -1.89 | 0.025 | -1.44 |
| 1458974_at | Tox | Thymocyte selection-associated HMG box gene | -1.06 | 0.793 | -1.64 | 0.058 | -1.12 | 0.276 | -1.81 | 0.142 | -1.41 |
| 1428546_at | Syncrip | synaptotagmin binding, cytoplasmic RNA interacting protein | -1.13 | 0.528 | -1.22 | 0.03 | -1.35 | 0 | -1.03 | 0.876 | -1.18 |
| 1437111_at | Zc3h12c | zinc finger CCCH type containing 12C | -1.06 | 0.52 | -1.21 | 0.247 | -1.49 | 0.058 | -1.03 | 0.768 | -1.2 |
| 1438198_at | Bri3bp | Bri3 binding protein | -1.08 | 0.663 | -1.45 | 0.008 | -1.21 | 0.108 | -1.08 | 0.721 | -1.2 |
| 1430603_at | 4930579K19Rik | RIKEN cDNA 4930579K19 gene | -1.05 | 0.717 | -1.65 | 0.295 | -1.13 | 0.706 | -1.27 | 0.213 | -1.27 |
| 1424834_s_at | Itpr2 | inositol 1,4,5-triphosphate receptor 2 | -1.39 | 0.052 | -1.29 | 0.095 | -1.05 | 0.791 | -1.59 | 0.31 | -1.33 |
| 1423336_at | Orc4l | origin recognition complex, subunit 4-like (S. cerevisiae) | -1.12 | 0.545 | -1.23 | 0.134 | -1.36 | 0.024 | -1.23 | 0.055 | -1.24 |
| 1435995_at | Mrpl22 | mitochondrial ribosomal protein L22 | -1.12 | 0.237 | -1.21 | 0.071 | -1.38 | 0.049 | -1.12 | 0.214 | -1.21 |
| 1455644_at | Vps53 | vacuolar protein sorting 53 (yeast) | -1.25 | 0.032 | -1.24 | 0.197 | -1.19 | 0.251 | -1.28 | 0.117 | -1.24 |
| 1430694_at | --- | --- | -1.36 | 0.377 | -1.15 | 0.482 | -1.2 | 0.564 | -1.32 | 0.265 | -1.26 |
| 1434931_at | Neo1 | neogenin | -1.24 | 0.056 | -1.4 | 0.06 | -1.08 | 0.709 | -1.63 | 0.52 | -1.34 |
| 1449939_s_at | Dlk1 | delta-like 1 homolog (Drosophila) | -1.48 | 0.229 | -1.01 | 0.978 | -1.29 | 0.664 | -1.04 | 0.759 | -1.2 |
| 1453198_at | Zfp422-rs1 | zinc finger protein 422, related sequence 1 | -1.19 | 0.591 | -1.23 | 0.277 | -1.26 | 0.314 | -1.24 | 0.298 | -1.23 |
| 1434842_s_at | Upf3b | UPF3 regulator of nonsense transcripts homolog B (yeast) | -1.1 | 0.226 | -1.35 | 0.098 | -1.26 | 0.145 | -1.09 | 0.633 | -1.2 |
| 1439329_a_at | Brsk2 | BR serine/threonine kinase 2 | -1.37 | 0.563 | -1.18 | 0.787 | -1.15 | 0.82 | -1.62 | 0.313 | -1.33 |
| 1455103_at | Ddx46 | DEAD (Asp-Glu-Ala-Asp) box polypeptide 46 | -1.32 | 0.121 | -1.21 | 0.249 | -1.17 | 0.235 | -1.29 | 0.137 | -1.24 |
| 1448974_at | Dcx | doublecortin | -1.04 | 0.95 | -1.51 | 0.099 | -1.22 | 0.297 | -2.84 | 0.174 | -1.65 |
| 1453306_at | 6330531I01Rik | RIKEN cDNA 6330531I01 gene | -1.17 | 0.12 | -1.23 | 0.574 | -1.29 | 0.246 | -1.54 | 0.158 | -1.31 |
| 1441006_at | Dus4l | dihydrouridine synthase 4-like (S. cerevisiae) | -1.01 | 0.972 | -1.19 | 0.549 | -1.64 | 0.11 | -1.14 | 0.459 | -1.24 |
| 1429016_at | D1Ertd161e /// Glb1 | DNA segment, Chr 1, ERATO Doi 161, expressed /// galactosidase, beta 1-like | -1 | 0.997 | -1.64 | 0.194 | -1.2 | 0.505 | -1.02 | 0.926 | -1.21 |
| 1436660_at | Rrbp1 | ribosome binding protein 1 | -1.1 | 0.371 | -1.18 | 0.474 | -1.45 | 0.289 | -1.39 | 0.277 | -1.28 |
| 1455441_at | --- | --- | -1.09 | 0.395 | -1.26 | 0.127 | -1.36 | 0.05 | -1.02 | 0.891 | -1.18 |
| 1455986_at | Zdhhc17 | zinc finger, DHHC domain containing 17 | -1.05 | 0.556 | -1.39 | 0.535 | -1.29 | 0.482 | -1.53 | 0.242 | -1.32 |
| 1428273_at | Abhd13 | abhydrolase domain containing 13 | -1.17 | 0.198 | -1.24 | 0.328 | -1.28 | 0.124 | -1.22 | 0.391 | -1.23 |
| 1419222_at | Tbxa2r | thromboxane A2 receptor | -1.34 | 0.398 | -1.32 | 0.135 | -1.06 | 0.767 | -1.58 | 0 | -1.32 |
| 1439170_at | --- | --- | -1.05 | 0.776 | -1.26 | 0.408 | -1.43 | 0.05 | -1 | 0.97 | -1.19 |
| 1451997_at | Zfp426 | zinc finger protein 426 | -1.07 | 0.677 | -1.15 | 0.156 | -1.54 | 0.038 | -1.34 | 0.045 | -1.28 |
| 1443037_at | Nptn | Neuroplastin | -1.02 | 0.923 | -1.15 | 0.479 | -1.69 | 0.031 | -1.16 | 0.189 | -1.25 |
| 1436429_at | Zfp606 | zinc finger protein 606 | -1.17 | 0.571 | -1.42 | 0.451 | -1.13 | 0.499 | -2.18 | 0.029 | -1.47 |
| 1434629_at | Zbtb26 | zinc finger and BTB domain containing 26 | -1.14 | 0.719 | -1.19 | 0.42 | -1.38 | 0.057 | -1.3 | 0.364 | -1.25 |
| 1454406_at | 4930453J04Rik | RIKEN cDNA 4930453J04 gene | -1.17 | 0.757 | -1.36 | 0.617 | -1.17 | 0.515 | -2.48 | 0.079 | -1.54 |

| | | | | | | | | | | | |
|---|---|---|---|---|---|---|---|---|---|---|---|
| 1421023_at | Pik3c2a | phosphatidylinositol 3-kinase, C2 domain containing, alpha polypeptide | -1.12 | 0.191 | -1.19 | 0.012 | -1.41 | 0.1 | -1.6 | 0.144 | -1.33 |
| 1434451_at | --- | NOD-derived CD11c +ve dendritic cells cDNA, RIKEN full-length enriched library, | -1.26 | 0.125 | -1.26 | 0.128 | -1.16 | 0.331 | -1.21 | 0.46 | -1.22 |
| 1449750_at | AA407331 | expressed sequence AA407331 | -1.1 | 0.727 | -1.08 | 0.213 | -1.64 | 0.005 | -1.26 | 0.448 | -1.27 |
| 1446804_at | Nxn | nucleoredoxin | -1.11 | 0.759 | -1.21 | 0.54 | -1.39 | 0.31 | -1.71 | 0.01 | -1.36 |
| 1425082_s_at | 9030416H16Rik | RIKEN cDNA 9030416H16 gene | -1.13 | 0.514 | -1.1 | 0.333 | -1.52 | 0.014 | -1.12 | 0.396 | -1.22 |
| 1457005_at | Mtfmt | mitochondrial methionyl-tRNA formyltransferase | -1.13 | 0.203 | -1.38 | 0.231 | -1.19 | 0.357 | -1.28 | 0.493 | -1.25 |
| 1452762_at | 8430436O14Rik | RIKEN cDNA 8430436O14 gene | -1.07 | 0.705 | -1.12 | 0.59 | -1.61 | 0.076 | -1.41 | 0.224 | -1.3 |
| 1452977_at | Zhx3 | zinc fingers and homeoboxes 3 | -1.16 | 0.53 | -1.27 | 0.109 | -1.26 | 0.259 | -1.62 | 0.086 | -1.33 |
| 1437719_x_at | A230046K03Rik | RIKEN cDNA A230046K03 gene | -1.07 | 0.606 | -1.16 | 0.287 | -1.53 | 0.003 | -1.98 | 0.192 | -1.44 |
| 1455653_at | Ccnj | cyclin J | -1.13 | 0.637 | -1.27 | 0.427 | -1.29 | 0.252 | -1.65 | 0.098 | -1.33 |
| 1438580_at | Zcchc7 | zinc finger, CCHC domain containing 7 | -1.2 | 0.531 | -1.1 | 0.424 | -1.42 | 0.026 | -1.79 | 0.111 | -1.38 |
| 1431126_a_at | 0610011F06Rik | RIKEN cDNA 0610011F06 gene | -1.1 | 0.149 | -1.46 | 0.021 | -1.18 | 0.402 | -1.06 | 0.813 | -1.2 |
| 1455589_at | 1700100M05Rik | RIKEN cDNA 1700100M05 gene | -1.32 | 0.098 | -1.18 | 0.213 | -1.19 | 0.349 | -1.43 | 0.096 | -1.28 |
| 1457020_at | Gpbp1l1 | GC-rich promoter binding protein 1-like 1 | -1.06 | 0.781 | -1.18 | 0.366 | -1.53 | 0.159 | -1.16 | 0.465 | -1.23 |
| 1430366_at | 5430405H02Rik | RIKEN cDNA 5430405H02 gene | -1.21 | 0.342 | -1.08 | 0.864 | -1.45 | 0.249 | -1.01 | 0.984 | -1.18 |
| 1455314_at | Lpp | LIM domain containing preferred translocation partner in lipoma | -1.1 | 0.578 | -1.07 | 0.504 | -1.65 | 0.016 | -1.58 | 0.088 | -1.35 |
| 1431161_at | Rgnef | Rho-guanine nucleotide exchange factor | -1.03 | 0.866 | -1.93 | 0.376 | -1.04 | 0.911 | -2.59 | 0.428 | -1.65 |
| 1434390_at | Hnrpu | Heterogeneous nuclear ribonucleoprotein U | -1.2 | 0.535 | -1.28 | 0.349 | -1.2 | 0.455 | -1.13 | 0.702 | -1.2 |
| 1448391_at | Rab9 | RAB9, member RAS oncogene family | -1.12 | 0.151 | -1.16 | 0.016 | -1.44 | 0.006 | -1.02 | 0.772 | -1.19 |
| 1440533_at | Bfar | Bifunctional apoptosis regulator | -1.03 | 0.895 | -1.13 | 0.482 | -1.69 | 0.088 | -1.28 | 0.457 | -1.28 |
| 1439705_at | Ptpn12 | Protein tyrosine phosphatase, non-receptor type 12 | -1.14 | 0.469 | -1.02 | 0.911 | -1.69 | 0.007 | -1.08 | 0.355 | -1.23 |
| 1425820_x_at | Gpatc4 | G patch domain containing 4 | -1.44 | 0.216 | -1.12 | 0.702 | -1.16 | 0.287 | -1.26 | 0.282 | -1.25 |
| 1450328_at | --- | Adult male testis cDNA, RIKEN full-length enriched library, clone:1700129L04 pro | -1.01 | 0.983 | -1.85 | 0.39 | -1.09 | 0.455 | -2.92 | 0.215 | -1.72 |
| 1419793_at | D5Ertd615e | DNA segment, Chr 5, ERATO Doi 615, expressed | -1.47 | 0.073 | -1.06 | 0.875 | -1.21 | 0.384 | -1.23 | 0.714 | -1.24 |
| 1446302_at | Ccbl1 | Cysteine conjugate-beta lyase 1 | -1.15 | 0.54 | -1.15 | 0.625 | -1.41 | 0.214 | -1.55 | 0.523 | -1.31 |
| 1458712_at | --- | --- | -1.02 | 0.957 | -1.42 | 0.345 | -1.31 | 0.38 | -2.55 | 0.129 | -1.57 |
| 1442465_s_at | Strbp | spermatid perinuclear RNA binding protein | -1.04 | 0.438 | -1.32 | 0.104 | -1.37 | 0.124 | -1.79 | 0.055 | -1.38 |
| 1453934_at | 4930535C22Rik | RIKEN cDNA 4930535C22 gene | -1.86 | 0.247 | -1.06 | 0.799 | -1.03 | 0.827 | -1.51 | 0.256 | -1.37 |
| 1446245_at | Fmnl2 | Formin-like 2 | -1.04 | 0.904 | -1.06 | 0.826 | -1.83 | 0.014 | -1.4 | 0.098 | -1.33 |
| 1453024_at | Wdr37 | WD repeat domain 37 | -1.47 | 0.187 | -1.05 | 0.808 | -1.23 | 0.296 | -1.37 | 0.426 | -1.28 |
| 1424590_at | Ddx19b | DEAD (Asp-Glu-Ala-Asp) box polypeptide 19b | -1.36 | 0.059 | -1.32 | 0.147 | -1.04 | 0.875 | -1.52 | 0.136 | -1.31 |
| 1455776_x_at | Bola2 | bolA-like 2 (E. coli) | -1.39 | 0.059 | -1.13 | 0.409 | -1.18 | 0.179 | -1.03 | 0.843 | -1.18 |
| 1439820_at | Ebf1 | Early B-cell factor 1 | -1.11 | 0.64 | -1.48 | 0.403 | -1.14 | 0.568 | -2.18 | 0.124 | -1.48 |
| 1439293_at | C130047D21Rik | RIKEN cDNA C130047D21 gene | -1.21 | 0.7 | -1.49 | 0.108 | -1.05 | 0.884 | -1.82 | 0.224 | -1.39 |
| 1456320_at | BC049806 | cDNA sequence BC049806 | -1.04 | 0.824 | -1.5 | 0.022 | -1.21 | 0.349 | -1.12 | 0.537 | -1.22 |
| 1437635_at | Dcbld2 | discoidin, CUB and LCCL domain containing 2 | -1.09 | 0.571 | -1.23 | 0.073 | -1.39 | 0.02 | -1.03 | 0.87 | -1.18 |
| 1415826_at | Atp6v1h | ATPase, H+ transporting, lysosomal V1 subunit H | -1.27 | 0.173 | -1.15 | 0.264 | -1.25 | 0.054 | -1.22 | 0.36 | -1.23 |
| 1421318_at | Ndst4 | N-deacetylase/N-sulfotransferase (heparin glucosaminyl) 4 | -1.01 | 0.929 | -1.2 | 0.792 | -1.6 | 0.269 | -1.71 | 0.293 | -1.38 |
| 1448237_x_at | Ldhb | lactate dehydrogenase B | -1.2 | 0.108 | -1.43 | 0.169 | -1.09 | 0.575 | -1.42 | 0.088 | -1.28 |
| 1416141_a_at | Rps6 /// LOC66657 | ribosomal protein S6 /// similar to 40S ribosomal protein S6 /// similar to 40S ribos | -1.06 | 0.526 | -1.37 | 0.118 | -1.29 | 0.012 | -1.18 | 0.004 | -1.22 |
| 1441549_at | --- | --- | -1.32 | 0.111 | -1.19 | 0.283 | -1.18 | 0.377 | -1.36 | 0.367 | -1.26 |
| 1434478_at | Heca | headcase homolog (Drosophila) | -1.17 | 0.383 | -1.3 | 0.098 | -1.2 | 0.358 | -1.6 | 0.236 | -1.32 |
| 1429080_at | Mphosph10 | M-phase phosphoprotein 10 (U3 small nucleolar ribonucleoprotein) | -1.35 | 0.224 | -1.11 | 0.461 | -1.22 | 0.141 | -1.01 | 0.956 | -1.18 |
| 1457033_at | Gm397 /// XM_486 | gene model 397, (NCBI) /// Mus musculus sequence XM_486399 /// similar to zin | -1.14 | 0.55 | -1.54 | 0.475 | -1.08 | 0.839 | -1.14 | 0.491 | -1.22 |
| 1456715_at | Phr1 | Pam, highwire, rpm 1 | -1.52 | 0.488 | -1.23 | 0.378 | -1.02 | 0.965 | -2.25 | 0.155 | -1.51 |
| 1448018_at | --- | --- | -1.1 | 0.704 | -1.11 | 0.593 | -1.56 | 0.142 | -1.3 | 0.571 | -1.27 |
| 1438781_at | E130014J05Rik | RIKEN cDNA E130014J05 gene | -1.21 | 0.12 | -1.07 | 0.669 | -1.44 | 0.08 | -1.38 | 0.239 | -1.27 |
| 1460155_at | 2410005O16Rik | RIKEN cDNA 2410005O16 gene | -1.3 | 0.332 | -1.36 | 0.12 | -1.05 | 0.847 | -1.6 | 0.237 | -1.33 |
| 1423930_at | Anapc4 | anaphase promoting complex subunit 4 | -1.2 | 0.171 | -1.06 | 0.498 | -1.48 | 0.154 | -1.1 | 0.598 | -1.21 |
| 1453254_at | 1700034O15Rik | RIKEN cDNA 1700034O15 gene | -1.13 | 0.72 | -1.62 | 0.377 | -1.05 | 0.81 | -2.79 | 0.238 | -1.65 |
| 1426148_at | Gbgt1 | globoside alpha-1,3-N-acetylgalactosaminyltransferase 1 | -1.39 | 0.479 | -1.2 | 0.27 | -1.11 | 0.797 | -1.29 | 0.212 | -1.25 |
| 1442178_at | Gmds | GDP-mannose 4, 6-dehydratase | -1.21 | 0.476 | -1.11 | 0.705 | -1.37 | 0.226 | -1.39 | 0.01 | -1.27 |
| 1428914_at | 2310014D11Rik | RIKEN cDNA 2310014D11 gene | -1.05 | 0.759 | -1.32 | 0.059 | -1.34 | 0.191 | -1.72 | 0.085 | -1.36 |

| | | | | | | | | | | | |
|---|---|---|---|---|---|---|---|---|---|---|---|
| 1444558_at | Wdr33 | WD repeat domain 33 | -1.26 | 0.502 | -1.46 | 0.343 | -1.02 | 0.938 | -1.3 | 0.396 | -1.26 |
| 1417886_at | 1810009A15Rik | RIKEN cDNA 1810009A15 gene | -1.14 | 0.023 | -1.23 | 0.011 | -1.3 | 0.213 | -1.15 | 0.234 | -1.21 |
| 1436133_at | 0610011N22Rik | RIKEN cDNA 0610011N22 gene | -1.32 | 0.317 | -1.33 | 0.532 | -1.05 | 0.787 | -1.2 | 0.613 | -1.23 |
| 1428231_at | Cpsf6 | cleavage and polyadenylation specific factor 6 | -1.09 | 0.5 | -1.33 | 0.171 | -1.27 | 0.075 | -1.43 | 0.132 | -1.28 |
| 1446412_at | Wwox | WW domain-containing oxidoreductase | -1 | 0.999 | -1.59 | 0.34 | -1.21 | 0.34 | -1.97 | 0.047 | -1.44 |
| 1432800_at | C030011G24Rik | RIKEN cDNA C030011G24 gene | -1.21 | 0.606 | -1.35 | 0.16 | -1.12 | 0.317 | -1.45 | 0.172 | -1.28 |
| 1441843_s_at | 5230400M03Rik | RIKEN cDNA 5230400M03 gene | -1.05 | 0.197 | -1.1 | 0.521 | -1.69 | 0.058 | -1.2 | 0.438 | -1.26 |
| 1429640_at | 1700025F22Rik | RIKEN cDNA 1700025F22 gene | -1.44 | 0.705 | -1.2 | 0.429 | -1.08 | 0.698 | -1.03 | 0.822 | -1.18 |
| 1450366_at | Hrk | harakiri, BCL2 interacting protein (contains only BH3 domain) | -1.44 | 0.589 | -1.02 | 0.918 | -1.27 | 0.205 | -1.53 | 0.553 | -1.32 |
| 1442663_at | --- | --- | -1.12 | 0.098 | -1.27 | 0.388 | -1.29 | 0.334 | -1.27 | 0.27 | -1.24 |
| 1423903_at | Pvr | poliovirus receptor | -1.32 | 0.006 | -1.42 | 0.003 | -1 | 0.985 | -1.16 | 0.265 | -1.23 |
| 1457717_at | AI987986 | expressed sequence AI987986 | -1.27 | 0.09 | -1.04 | 0.802 | -1.41 | 0.067 | -1.63 | 0.26 | -1.34 |
| 1441346_at | 2810421E14Rik | RIKEN cDNA 2810421E14 gene | -1.15 | 0.688 | -1.46 | 0.387 | -1.1 | 0.574 | -1.57 | 0.031 | -1.32 |
| 1423470_at | Ptbp2 | polypyrimidine tract binding protein 2 | -1.13 | 0.644 | -1.37 | 0.009 | -1.18 | 0.338 | -1.39 | 0.336 | -1.27 |
| 1458136_at | Msi2 | Musashi homolog 2 (Drosophila) | -1.5 | 0.11 | -1.1 | 0.723 | -1.13 | 0.537 | -1.09 | 0.692 | -1.21 |
| 1454980_at | 4930402E16Rik | RIKEN cDNA 4930402E16 gene | -1.16 | 0.184 | -1.32 | 0.144 | -1.19 | 0.24 | -1.37 | 0.235 | -1.26 |
| 1441402_at | LOC674229 | similar to cysteine and glycine-rich protein 2 binding protein | -1.21 | 0.489 | -1.48 | 0.081 | -1.04 | 0.916 | -1.25 | 0.187 | -1.25 |
| 1455564_at | Bcr | breakpoint cluster region homolog | -1.29 | 0.071 | -1.1 | 0.303 | -1.29 | 0.312 | -1.12 | 0.064 | -1.2 |
| 1423432_at | Phip | pleckstrin homology domain interacting protein | -1.17 | 0.398 | -1.14 | 0.407 | -1.38 | 0.148 | -1.42 | 0.174 | -1.28 |
| 1460579_at | Dnpep | aspartyl aminopeptidase | -1.32 | 0.586 | -1.1 | 0.397 | -1.26 | 0.422 | -1.31 | 0.125 | -1.25 |
| 1451732_at | Prpf4b | PRP4 pre-mRNA processing factor 4 homolog B (yeast) | -1.12 | 0.47 | -1.44 | 0.049 | -1.14 | 0.3 | -1.26 | 0.363 | -1.24 |
| 1460425_at | 1700001C19Rik | RIKEN cDNA 1700001C19 gene | -1.66 | 0.003 | -1.12 | 0.062 | -1.04 | 0.871 | -1.22 | 0.152 | -1.26 |
| 1443052_at | C330019L16Rik | RIKEN cDNA C330019L16 gene | -1.58 | 0.15 | -1.12 | 0.73 | -1.07 | 0.697 | -1.35 | 0.472 | -1.28 |
| 1416320_at | Sec22a | SEC22 vesicle trafficking protein-like A (S. cerevisiae) | -1.19 | 0.001 | -1.11 | 0.464 | -1.39 | 0.031 | -1.16 | 0.257 | -1.21 |
| 1438895_at | A430102J17Rik | RIKEN cDNA A430102J17 gene | -1.18 | 0.217 | -1.19 | 0.193 | -1.3 | 0.212 | -1.15 | 0.068 | -1.2 |
| 1435595_at | 1810011O10Rik | RIKEN cDNA 1810011O10 gene | -1.26 | 0.425 | -1.19 | 0.43 | -1.21 | 0.165 | -1.58 | 0.205 | -1.31 |
| 1436001_at | 2310028O11Rik | RIKEN cDNA 2310028O11 gene | -1.35 | 0.11 | -1.07 | 0.774 | -1.27 | 0.043 | -1.34 | 0.119 | -1.26 |
| 1428593_at | 1700029F09Rik | RIKEN cDNA 1700029F09 gene | -1.22 | 0.126 | -1.26 | 0.018 | -1.18 | 0.166 | -1 | 0.986 | -1.17 |
| 1457464_at | C130015C19 | hypothetical LOC403342 | -1.05 | 0.855 | -1.36 | 0.01 | -1.3 | 0.299 | -1.71 | 0.041 | -1.35 |
| 1434632_at | --- | Transcribed locus | -1.19 | 0.325 | -1.18 | 0.524 | -1.29 | 0.364 | -1.43 | 0.233 | -1.27 |
| 1418582_at | Cbfa2t3h | core-binding factor, runt domain, alpha subunit 2, translocated to, 3 homolog (hur | -1.23 | 0.106 | -1.4 | 0.121 | -1.07 | 0.784 | -1.15 | 0.275 | -1.21 |
| 1453171_s_at | Ppm1a | protein phosphatase 1A, magnesium dependent, alpha isoform | -1.03 | 0.566 | -1.22 | 0.064 | -1.49 | 0.034 | -1.04 | 0.476 | -1.2 |
| 1441077_at | AW124847 | expressed sequence AW124847 | -1.5 | 0.179 | -1.14 | 0.455 | -1.09 | 0.855 | -1.47 | 0.097 | -1.3 |
| 1454166_at | 1700040N02Rik | RIKEN cDNA 1700040N02 gene | -1.23 | 0.335 | -1.24 | 0.071 | -1.19 | 0.464 | -1.61 | 0.415 | -1.32 |
| 1441318_at | Jarid1a | jumonji, AT rich interactive domain 1A (Rbp2 like) | -1.46 | 0.037 | -1.04 | 0.924 | -1.22 | 0.253 | -1.25 | 0.377 | -1.25 |
| 1456663_x_at | Tm2d2 | TM2 domain containing 2 | -1 | 0.97 | -1.34 | 0.126 | -1.39 | 0.021 | -1.36 | 0.091 | -1.27 |
| 1457528_at | Slc4a7 | solute carrier family 4, sodium bicarbonate cotransporter, member 7 | -1.15 | 0.705 | -1.18 | 0.217 | -1.35 | 0.148 | -2.49 | 0.129 | -1.54 |
| 1431594_at | Dpp3 | dipeptidylpeptidase 3 | -1.02 | 0.969 | -1.38 | 0.652 | -1.32 | 0.566 | -1.7 | 0.004 | -1.36 |
| 1422109_at | Rfx1 | regulatory factor X, 1 (influences HLA class II expression) | -1.36 | 0.125 | -1.25 | 0.56 | -1.08 | 0.735 | -1.73 | 0.087 | -1.35 |
| 1435695_a_at | A030007L17Rik | RIKEN cDNA A030007L17 gene | -1.37 | 0.13 | -1.21 | 0.3 | -1.11 | 0.103 | -2.23 | 0.064 | -1.48 |
| 1453596_at | Id2 | inhibitor of DNA binding 2 | -1.23 | 0.597 | -1.45 | 0.371 | -1.04 | 0.747 | -1.22 | 0.338 | -1.24 |
| 1418801_at | Zkscan1 | zinc finger with KRAB and SCAN domains 1 | -1.15 | 0.184 | -1.21 | 0.146 | -1.3 | 0.208 | -1.06 | 0.76 | -1.18 |
| 1443339_at | 2310056P07Rik | RIKEN cDNA 2310056P07 gene | -1.04 | 0.917 | -1.76 | 0.037 | -1.07 | 0.882 | -1.63 | 0.262 | -1.38 |
| 1459731_at | --- | --- | -1.19 | 0.826 | -1.21 | 0.608 | -1.26 | 0.463 | -1.58 | 0.539 | -1.31 |
| 1422322_at | Chml | choroideremia-like | -1.38 | 0.548 | -1.13 | 0.705 | -1.17 | 0.655 | -1.6 | 0.192 | -1.32 |
| 1441371_at | Plxna4 | plexin A4 | -1.08 | 0.769 | -1.33 | 0.01 | -1.28 | 0.09 | -1.07 | 0.566 | -1.19 |
| 1438405_at | Fgf7 | fibroblast growth factor 7 | -1.12 | 0.505 | -1.37 | 0.538 | -1.19 | 0.014 | -1.07 | 0.686 | -1.19 |
| 1449472_at | Gpr12 | G-protein coupled receptor 12 | -1.08 | 0.881 | -1.81 | 0.236 | -1.02 | 0.867 | -1.26 | 0.181 | -1.29 |
| 1448521_at | Brd7 /// LOC63432 | bromodomain containing 7 /// similar to bromodomain containing 7 | -1.13 | 0.496 | -1.32 | 0.087 | -1.23 | 0.18 | -1.36 | 0.069 | -1.26 |
| 1432224_at | 4831407H17Rik | RIKEN cDNA 4831407H17 gene | -1.06 | 0.852 | -1.4 | 0.045 | -1.24 | 0.29 | -1.21 | 0.517 | -1.23 |
| 1452759_s_at | Ppfibp1 | PTPRF interacting protein, binding protein 1 (liprin beta 1) | -1.05 | 0.36 | -1.16 | 0.251 | -1.55 | 0.03 | -1.6 | 0.056 | -1.34 |
| 1446511_at | Msh3 | mutS homolog 3 (E. coli) | -1.24 | 0.426 | -1.06 | 0.631 | -1.41 | 0.194 | -1.37 | 0.379 | -1.27 |
| 1455113_at | Armc8 | armadillo repeat containing 8 | -1.21 | 0.149 | -1.18 | 0.12 | -1.26 | 0.175 | -1.06 | 0.514 | -1.18 |

| | | | | | | | | | | | |
|---|---|---|---|---|---|---|---|---|---|---|---|
| 1420209_at | Dus4l | Dihydrouridine synthase 4-like (S. cerevisiae) | -1.34 | 0.423 | -1.28 | 0.404 | -1.07 | 0.728 | -2.21 | 0.487 | -1.47 |
| 1448203_at | Atp5l | ATP synthase, H+ transporting, mitochondrial F0 complex, subunit g | -1.24 | 0.02 | -1.23 | 0.043 | -1.18 | 0.013 | -1.14 | 0.352 | -1.2 |
| 1459835_s_at | Dnaja1 | DnaJ (Hsp40) homolog, subfamily A, member 1 | -1.24 | 0.294 | -1.13 | 0.302 | -1.29 | 0.001 | -1.68 | 0.327 | -1.34 |
| 1419765_at | Cul2 | cullin 2 | -1.01 | 0.924 | -1.24 | 0.515 | -1.51 | 0.302 | -1.59 | 0.125 | -1.34 |
| 1435608_at | Znrf3 /// LOC63180 | zinc and ring finger 3 /// similar to Goliath homolog precursor (Ring finger protein | -1.12 | 0.351 | -1.4 | 0.204 | -1.17 | 0.492 | -1.02 | 0.609 | -1.18 |
| 1424333_at | Rg9mtd1 | RNA (guanine-9-) methyltransferase domain containing 1 | -1.13 | 0.455 | -1.3 | 0.112 | -1.24 | 0.026 | -1.22 | 0.356 | -1.22 |
| 1456234_at | Limk1 | LIM-domain containing, protein kinase | -1.22 | 0.153 | -1.34 | 0.237 | -1.12 | 0.543 | -1.29 | 0.268 | -1.24 |
| 1442164_at | Blzf1 | basic leucine zipper nuclear factor 1 | -1.06 | 0.772 | -1.07 | 0.87 | -1.7 | 0.032 | -1.29 | 0.238 | -1.28 |
| 1441946_at | Itih5 | inter-alpha (globulin) inhibitor H5 | -1.23 | 0.118 | -1.44 | 0.298 | -1.05 | 0.808 | -3.69 | 0.057 | -1.85 |
| 1426259_at | Pank3 | pantothenate kinase 3 | -1.01 | 0.937 | -1.15 | 0.389 | -1.66 | 0.044 | -1.05 | 0.827 | -1.22 |
| 1455831_at | Fus | fusion, derived from t(12;16) malignant liposarcoma (human) | -1.47 | 0.497 | -1.08 | 0.815 | -1.17 | 0.178 | -1.35 | 0.136 | -1.27 |
| 1449075_at | Exdl2 | exonuclease 3''-5'' domain-like 2 | -1.16 | 0.303 | -1.33 | 0.089 | -1.18 | 0.396 | -1 | 0.987 | -1.17 |
| 1438678_at | 1500011K16Rik | RIKEN cDNA 1500011K16 gene | -1.3 | 0.142 | -1.17 | 0.187 | -1.19 | 0.053 | -1.36 | 0.245 | -1.25 |
| 1458281_at | Arsb | Arylsulfatase B | -1.22 | 0.277 | -1.28 | 0.579 | -1.16 | 0.121 | -1.96 | 0.043 | -1.4 |
| 1452596_at | Polr2k | polymerase (RNA) II (DNA directed) polypeptide K | -1.08 | 0.733 | -1.25 | 0.342 | -1.36 | 0.001 | -1.25 | 0.314 | -1.24 |
| 1441099_at | Plekhg3 | Pleckstrin homology domain containing, family G (with RhoGef domain) member | -1.22 | 0.761 | -1.2 | 0.232 | -1.23 | 0.308 | -1.03 | 0.922 | -1.17 |
| 1428474_at | Ppp3cb | protein phosphatase 3, catalytic subunit, beta isoform | -1.12 | 0.777 | -1.24 | 0.202 | -1.31 | 0.014 | -1.14 | 0.251 | -1.2 |
| 1434834_at | Socs7 | suppressor of cytokine signaling 7 | -1.23 | 0.166 | -1.53 | 0.05 | -1 | 0.989 | -1.26 | 0.11 | -1.25 |
| 1419204_at | Dll1 | delta-like 1 (Drosophila) | -1.47 | 0.171 | -1.25 | 0.334 | -1.01 | 0.97 | -1.48 | 0.155 | -1.3 |
| 1453583_at | Zzef1 | zinc finger, ZZ-type with EF hand domain 1 | -1.27 | 0.26 | -1.03 | 0.794 | -1.4 | 0.089 | -1.32 | 0.184 | -1.26 |
| 1454132_at | 7630402F16Rik | RIKEN cDNA 7630402F16 gene | -1.11 | 0.873 | -1.06 | 0.802 | -1.6 | 0.433 | -1.24 | 0.776 | -1.25 |
| 1434194_at | Mtap2 | microtubule-associated protein 2 | -1.44 | 0.007 | -1.08 | 0.911 | -1.19 | 0.418 | -1.97 | 0.036 | -1.42 |
| 1456282_at | --- | CDNA clone IMAGE:30014192 | -1.02 | 0.88 | -1.44 | 0.256 | -1.26 | 0.408 | -1.42 | 0.113 | -1.29 |
| 1435239_at | Gria1 | glutamate receptor, ionotropic, AMPA1 (alpha 1) | -1.13 | 0.803 | -1.64 | 0.458 | -1.03 | 0.861 | -1.04 | 0.834 | -1.21 |
| 1440719_at | Gle1l | GLE1 RNA export mediator-like (yeast | -1.07 | 0.803 | -1.2 | 0.444 | -1.43 | 0.249 | -1.79 | 0.036 | -1.37 |
| 1454554_at | 9330159N22Rik | RIKEN cDNA 9330159N22 gene | -1.32 | 0.62 | -1.29 | 0.603 | -1.07 | 0.733 | -1.22 | 0.768 | -1.22 |
| 1439817_at | 2900064A13Rik | RIKEN cDNA 2900064A13 gene | -1.21 | 0.034 | -1.1 | 0.381 | -1.37 | 0.143 | -1.34 | 0.388 | -1.25 |
| 1429624_at | Sltm | SAFB-like, transcription modulator | -1.23 | 0.288 | -1.14 | 0.506 | -1.29 | 0.093 | -1.34 | 0.291 | -1.25 |
| 1437182_at | Dido1 | death inducer-obliterator 1 | -1.13 | 0.375 | -1.19 | 0.358 | -1.34 | 0.098 | -1.58 | 0.101 | -1.31 |
| 1436157_at | Ccar1 | cell division cycle and apoptosis regulator 1 | -1.19 | 0.487 | -1.1 | 0.188 | -1.39 | 0.043 | -1.02 | 0.909 | -1.17 |
| 1419575_s_at | Zfp292 | zinc finger protein 292 | -1.21 | 0.201 | -1.01 | 0.939 | -1.53 | 0.101 | -1.39 | 0.205 | -1.29 |
| 1434105_at | Epm2aip1 | EPM2A (laforin) interacting protein 1 | -1.1 | 0.228 | -1.2 | 0.116 | -1.37 | 0.172 | -1.19 | 0.469 | -1.22 |
| 1442656_at | --- | --- | -1.13 | 0.771 | -1.24 | 0.231 | -1.29 | 0.588 | -1.01 | 0.959 | -1.17 |
| 1424546_at | BC003965 | cDNA sequence BC003965 | -1.14 | 0.631 | -1.49 | 0.14 | -1.09 | 0.275 | -1.02 | 0.9 | -1.18 |
| 1418664_at | Mpdz | multiple PDZ domain protein | -1.2 | 0.595 | -1.26 | 0.106 | -1.19 | 0.524 | -1.61 | 0.032 | -1.31 |
| 1416630_at | Id3 | inhibitor of DNA binding 3 | -1.46 | 0.132 | -1.06 | 0.806 | -1.19 | 0.436 | -2.42 | 0.047 | -1.53 |
| 1445862_at | 4921513D23Rik | RIKEN cDNA 4921513D23 gene | -1.15 | 0.119 | -1.02 | 0.755 | -1.6 | 0.072 | -1.32 | 0.237 | -1.28 |
| 1459517_at | Cdc14a | CDC14 cell division cycle 14 homolog A (S. cerevisiae) | -1.32 | 0.56 | -1.02 | 0.961 | -1.37 | 0.137 | -1.13 | 0.678 | -1.21 |
| 1452799_at | 2310009E04Rik | RIKEN cDNA 2310009E04 gene | -1.2 | 0.138 | -1.15 | 0.015 | -1.3 | 0.042 | -1.17 | 0.668 | -1.2 |
| 1428257_s_at | Dynlrb1 | dynein light chain roadblock-type 1 | -1.14 | 0.194 | -1.2 | 0.003 | -1.31 | 0.044 | -1.39 | 0.15 | -1.26 |
| 1457303_at | Anks3 | Ankyrin repeat and sterile alpha motif domain containing 3 | -1.24 | 0.274 | -1.17 | 0.592 | -1.24 | 0.128 | -1.5 | 0.299 | -1.29 |
| 1456855_at | --- | --- | -1.32 | 0.485 | -1.06 | 0.226 | -1.3 | 0.235 | -1.2 | 0.422 | -1.22 |
| 1437490_x_at | Uap1 /// LOC64050 | UDP-N-acetylglucosamine pyrophosphorylase 1 /// similar to UDP-N-acetylhexos | -1.17 | 0.492 | -1.21 | 0.124 | -1.27 | 0.042 | -1.21 | 0.349 | -1.21 |
| 1435283_s_at | Gm967 | gene model 967, (NCBI) | -1.03 | 0.854 | -1.45 | 0.158 | -1.24 | 0.198 | -1.09 | 0.637 | -1.2 |
| 1456662_at | LOC546058 | Hypothetical protein LOC546058 | -1.15 | 0.55 | -1.04 | 0.865 | -1.56 | 0.039 | -1.61 | 0.083 | -1.34 |
| 1444237_at | C330019G07Rik | RIKEN cDNA C330019G07 gene | -1.19 | 0.542 | -1.11 | 0.382 | -1.37 | 0.011 | -1.15 | 0.357 | -1.2 |
| 1449108_at | Fdx1 | ferredoxin 1 | -1.07 | 0.429 | -1.2 | 0.208 | -1.43 | 0.065 | -1.43 | 0.144 | -1.28 |
| 1457785_at | Zswim3 | Zinc finger, SWIM domain containing 3 | -1.31 | 0.619 | -1.12 | 0.832 | -1.23 | 0.549 | -1.29 | 0.685 | -1.24 |
| 1446166_at | --- | --- | -1.03 | 0.755 | -1.86 | 0.193 | -1.04 | 0.812 | -1.14 | 0.851 | -1.27 |
| 1457129_at | Zfp458 | Zinc finger protein 458 | -1.45 | 0.348 | -1.02 | 0.926 | -1.25 | 0.019 | -1.19 | 0.61 | -1.23 |
| 1443115_at | Tgfbr2 | Transforming growth factor, beta receptor II | -1.12 | 0.737 | -1.13 | 0.798 | -1.46 | 0.252 | -1.88 | 0.146 | -1.39 |
| 1443464_at | Sntb1 | Syntrophin, basic 1 | -1.11 | 0.742 | -1.18 | 0.693 | -1.38 | 0.258 | -1.67 | 0.374 | -1.34 |
| 1443099_at | BC087945 | CDNA sequence BC087945 | -1.22 | 0.666 | -1.36 | 0.367 | -1.09 | 0.644 | -1.11 | 0.462 | -1.2 |

| | | | | | | | | | | | |
|---|---|---|---|---|---|---|---|---|---|---|---|
| 1455196_s_at | AA987161 | expressed sequence AA987161 | -1.3 | 0.445 | -1.11 | 0.599 | -1.25 | 0.205 | -1.41 | 0.2 | -1.27 |
| 1417254_at | Spata5 | spermatogenesis associated 5 | -1.35 | 0.073 | -1.21 | 0.287 | -1.11 | 0.407 | -1.5 | 0.186 | -1.29 |
| 1438110_at | Zbtb1 | Zinc finger and BTB domain containing 1 | -1.05 | 0.893 | -1.39 | 0.352 | -1.25 | 0.364 | -1.23 | 0.355 | -1.23 |
| 1453134_at | Pik3ca | phosphatidylinositol 3-kinase, catalytic, alpha polypeptide | -1.04 | 0.915 | -1.1 | 0.585 | -1.66 | 0.007 | -1.67 | 0.039 | -1.37 |
| 1439940_at | 2900019G14Rik | RIKEN cDNA 2900019G14 gene | -1.63 | 0.468 | -1.16 | 0.522 | -1.01 | 0.985 | -1.2 | 0.706 | -1.25 |
| 1427158_at | Mrps30 | mitochondrial ribosomal protein S30 | -1.3 | 0.059 | -1.27 | 0.009 | -1.09 | 0.527 | -1.11 | 0.382 | -1.19 |
| 1445959_at | --- | --- | -1.05 | 0.782 | -1.27 | 0.274 | -1.36 | 0.668 | -1.75 | 0.616 | -1.36 |
| 1443643_at | --- | --- | -1.45 | 0.467 | -1.09 | 0.838 | -1.16 | 0.459 | -1.13 | 0.802 | -1.21 |
| 1452854_at | --- | --- | -1.08 | 0.224 | -1.2 | 0.029 | -1.41 | 0.128 | -1.65 | 0.259 | -1.33 |
| 1440736_at | AI131651 | expressed sequence AI131651 | -1.22 | 0.021 | -1.03 | 0.884 | -1.47 | 0.057 | -1.61 | 0.112 | -1.33 |
| 1459154_at | --- | Transcribed locus | -1.21 | 0.568 | -1.06 | 0.852 | -1.43 | 0.35 | -1.47 | 0.153 | -1.29 |
| 1420593_a_at | Tead3 | TEA domain family member 3 | -1.39 | 0.172 | -1.07 | 0.762 | -1.23 | 0.458 | -1.06 | 0.841 | -1.19 |
| 1429625_at | 2900054C01Rik | RIKEN cDNA 2900054C01 gene | -1.1 | 0.28 | -1.04 | 0.77 | -1.65 | 0.011 | -1.28 | 0.389 | -1.27 |
| 1452774_at | Hnrpa3 | heterogeneous nuclear ribonucleoprotein A3 | -1.07 | 0.741 | -1.31 | 0.084 | -1.29 | 0.19 | -1.39 | 0.136 | -1.26 |
| 1445529_at | --- | --- | -1.03 | 0.857 | -1.51 | 0.373 | -1.19 | 0.689 | -1.26 | 0.667 | -1.25 |
| 1434207_at | 2900057K09Rik | RIKEN cDNA 2900057K09 gene | -1.19 | 0.579 | -1.15 | 0.447 | -1.31 | 0.223 | -1.55 | 0.097 | -1.3 |
| 1440607_at | Ches1 | Checkpoint suppressor 1 | -1.06 | 0.63 | -1.3 | 0.169 | -1.31 | 0.511 | -1.72 | 0.162 | -1.35 |
| 1453302_at | Tmem81 | transmembrane protein 81 | -1.01 | 0.965 | -1.99 | 0.16 | -1.02 | 0.928 | -1.34 | 0.12 | -1.34 |
| 1446195_at | LOC632689 | similar to WD repeat and SOCS box containing protein 2 (WSB-2) (SOCS box-co | -1.45 | 0.636 | -1.02 | 0.954 | -1.25 | 0.693 | -1.06 | 0.234 | -1.19 |
| 1418301_at | Irf6 | interferon regulatory factor 6 | -1.15 | 0.317 | -1.18 | 0.299 | -1.31 | 0.19 | -1.76 | 0.216 | -1.35 |
| 1460017_at | LOC434179 | hypothetical LOC434179 | -1.29 | 0.246 | -1.02 | 0.919 | -1.39 | 0.207 | -1.54 | 0.164 | -1.31 |
| 1448136_at | Enpp2 | ectonucleotide pyrophosphatase/phosphodiesterase 2 | -1.24 | 0.229 | -1.13 | 0.22 | -1.28 | 0.237 | -1.51 | 0.425 | -1.29 |
| 1432974_at | 4933421H12Rik | RIKEN cDNA 4933421H12 gene | -1.09 | 0.706 | -1.21 | 0.77 | -1.37 | 0.579 | -1.7 | 0.393 | -1.34 |
| 1438961_s_at | Blmh | bleomycin hydrolase | -1.19 | 0.322 | -1.19 | 0.047 | -1.27 | 0.044 | -1.49 | 0.034 | -1.28 |
| 1439090_at | D030022P07Rik | RIKEN cDNA D030022P07 gene | -1.15 | 0.003 | -1.11 | 0.436 | -1.42 | 0.149 | -1.63 | 0.082 | -1.33 |
| 1458228_at | Opcml | Opioid binding protein/cell adhesion molecule-like | -1.48 | 0.295 | -1.14 | 0.25 | -1.08 | 0.82 | -1.58 | 0.508 | -1.32 |
| 1452158_at | Eprs /// LOC633677 | glutamyl-prolyl-tRNA synthetase /// similar to Bifunctional aminoacyl-tRNA synthe | -1.28 | 0.161 | -1.03 | 0.802 | -1.39 | 0.08 | -1.05 | 0.853 | -1.18 |
| 1452107_s_at | Npnt | Nephronectin | -1.09 | 0.535 | -1.13 | 0.85 | -1.48 | 0.253 | -2.1 | 0.157 | -1.45 |
| 1454449_at | Rabepk | Rab9 effector protein with kelch motifs | -1.11 | 0.592 | -1.19 | 0.699 | -1.37 | 0.029 | -1.07 | 0.839 | -1.18 |
| 1416766_at | Mosc2 | MOCO sulphurase C-terminal domain containing 2 | -1.04 | 0.702 | -1.36 | 0.126 | -1.28 | 0.059 | -1.17 | 0.228 | -1.21 |
| 1446284_at | Mtss1 | metastasis suppressor 1 | -1.09 | 0.804 | -1.09 | 0.777 | -1.55 | 0.39 | -2.17 | 0.132 | -1.48 |
| 1445490_at | C77805 | expressed sequence C77805 | -1.19 | 0.59 | -1.03 | 0.915 | -1.5 | 0.154 | -1.77 | 0.053 | -1.37 |
| 1446621_at | Chd7 | Chromodomain helicase DNA binding protein 7 | -1.03 | 0.941 | -1.36 | 0.441 | -1.31 | 0.077 | -1.88 | 0.097 | -1.39 |
| 1435614_s_at | Rasgrf1 | RAS protein-specific guanine nucleotide-releasing factor 1 | -1.56 | 0.479 | -1.02 | 0.895 | -1.17 | 0.474 | -1 | 0.993 | -1.19 |
| 1439388_s_at | Bcar1 | breast cancer anti-estrogen resistance 1 | -1.09 | 0.42 | -1.22 | 0.23 | -1.35 | 0.121 | -1.35 | 0.379 | -1.25 |
| 1457522_at | 1110034C04Rik | RIKEN cDNA 1110034C04 gene | -1.09 | 0.286 | -1.25 | 0.271 | -1.33 | 0.36 | -1.33 | 0.223 | -1.25 |
| 1456856_at | Ppfia2 | protein tyrosine phosphatase, receptor type, f polypeptide (PTPRF), interacting pı | -1.2 | 0.685 | -1.34 | 0.353 | -1.12 | 0.751 | -1.22 | 0.535 | -1.22 |
| 1457503_at | 2310035C23Rik | RIKEN cDNA 2310035C23 gene | -1.26 | 0.124 | -1.15 | 0.542 | -1.22 | 0.17 | -1.68 | 0.057 | -1.33 |
| 1441010_at | --- | Adult male vesicular gland cDNA, RIKEN full-length enriched library, clone:G630( | -1.3 | 0.039 | -1.02 | 0.966 | -1.37 | 0.328 | -1.1 | 0.76 | -1.2 |
| 1430335_a_at | Pax3 | paired box gene 3 | -1.09 | 0.867 | -1.68 | 0.378 | -1.04 | 0.946 | -1.02 | 0.946 | -1.21 |
| 1432312_a_at | 4931440B09Rik | RIKEN cDNA 4931440B09 gene | -1.26 | 0.051 | -1.18 | 0.75 | -1.2 | 0.705 | -1.29 | 0.215 | -1.23 |
| 1433951_at | Arl5a | ADP-ribosylation factor-like 5A | -1.06 | 0.474 | -1.2 | 0.004 | -1.42 | 0.042 | -1.47 | 0.148 | -1.29 |
| 1456516_x_at | Uap1 /// LOC64050 | UDP-N-acetylglucosamine pyrophosphorylase 1 /// similar to UDP-N-acetylhexosa | -1.25 | 0.468 | -1.15 | 0.167 | -1.24 | 0.007 | -1.34 | 0.186 | -1.24 |
| 1426147_s_at | Cldn10 | claudin 10 | -1.25 | 0.112 | -1.32 | 0.693 | -1.09 | 0.748 | -1.47 | 0.005 | -1.28 |
| 1431023_at | C030046E11Rik | RIKEN cDNA C030046E11 gene | -1.06 | 0.861 | -1.36 | 0.634 | -1.25 | 0.447 | -1.72 | 0.302 | -1.35 |
| 1444806_at | 6720456B07Rik | RIKEN cDNA 6720456B07 gene | -1.22 | 0.563 | -1.07 | 0.785 | -1.39 | 0.1 | -1.42 | 0.13 | -1.27 |
| 1426807_at | Lta4h | Leukotriene A4 hydrolase | -1.12 | 0.129 | -1.16 | 0.519 | -1.39 | 0.131 | -1.11 | 0.694 | -1.19 |
| 1429480_at | 1700011N24Rik | RIKEN cDNA 1700011N24 gene | -1.09 | 0.845 | -1.5 | 0.071 | -1.12 | 0.435 | -2.22 | 0.301 | -1.48 |
| 1457938_at | Ncor1 | Nuclear receptor co-repressor 1 | -1.44 | 0.39 | -1.02 | 0.92 | -1.25 | 0.356 | -1.47 | 0.211 | -1.29 |
| 1431913_a_at | Pde3a | phosphodiesterase 3A, cGMP inhibited | -1.02 | 0.971 | -1.64 | 0.456 | -1.13 | 0.801 | -1.26 | 0.027 | -1.26 |
| 1435157_at | 5830454D03Rik | RIKEN cDNA 5830454D03 gene | -1.29 | 0.183 | -1.16 | 0.211 | -1.19 | 0.293 | -1.06 | 0.85 | -1.17 |
| 1420996_at | Plxna3 | plexin A3 | -1.61 | 0.048 | -1.07 | 0.725 | -1.08 | 0.339 | -1.15 | 0.048 | -1.23 |
| 1424632_a_at | Rev3l | REV3-like, catalytic subunit of DNA polymerase zeta RAD54 like (S. cerevisiae) | -1.22 | 0.393 | -1.15 | 0.189 | -1.27 | 0.023 | -1.18 | 0.228 | -1.21 |

| | | | | | | | | | | | |
|---|---|---|---|---|---|---|---|---|---|---|---|
| 1440317_at | C130068B02Rik | RIKEN cDNA C130068B02 gene | -1.15 | 0.355 | -1.01 | 0.941 | -1.63 | 0.113 | -1.34 | 0.157 | -1.28 |
| 1430808_at | Tbc1d5 | TBC1 domain family, member 5 | -1.02 | 0.943 | -1.45 | 0.013 | -1.24 | 0.266 | -1.53 | 0.248 | -1.31 |
| 1436775_a_at | Ankrd17 | ankyrin repeat domain 17 | -1.16 | 0.412 | -1.16 | 0.097 | -1.33 | 0.092 | -1.45 | 0.028 | -1.27 |
| 1455676_x_at | Tial1 | Tial1 cytotoxic granule-associated RNA binding protein-like 1 | -1.36 | 0.081 | -1.08 | 0.588 | -1.22 | 0.367 | -1.01 | 0.921 | -1.17 |
| 1458292_at | Psma1 | proteasome (prosome, macropain) subunit, alpha type 1 | -1.08 | 0.759 | -1.47 | 0.183 | -1.15 | 0.123 | -1.16 | 0.271 | -1.21 |
| 1459981_s_at | Rsbn1 | rosbin, round spermatid basic protein 1 | -1.24 | 0.451 | -1.14 | 0.504 | -1.26 | 0.126 | -1.28 | 0.058 | -1.23 |
| 1415785_a_at | Cct8 | chaperonin subunit 8 (theta) | -1.27 | 0.167 | -1.14 | 0.086 | -1.23 | 0.053 | -1.11 | 0.324 | -1.19 |
| 1445042_at | Aass | Aminoadipate-semialdehyde synthase | -1.09 | 0.008 | -1.59 | 0.386 | -1.07 | 0.853 | -1.22 | 0.668 | -1.24 |
| 1428729_at | Krit1 | KRIT1, ankyrin repeat containing | -1.21 | 0.004 | -1.15 | 0.275 | -1.27 | 0.333 | -1.23 | 0.347 | -1.22 |
| 1434966_at | Sfrs8 | splicing factor, arginine/serine-rich 8 | -1.16 | 0.431 | -1.46 | 0.063 | -1.08 | 0.221 | -1.31 | 0.256 | -1.25 |
| 1440856_at | Mapk8 | mitogen activated protein kinase 8 | -1.16 | 0.087 | -1.12 | 0.289 | -1.38 | 0.058 | -1.32 | 0.256 | -1.25 |
| 1433937_at | Trp53bp2 | transformation related protein 53 binding protein 2 | -1.38 | 0.352 | -1.26 | 0.247 | -1.04 | 0.597 | -1.4 | 0.229 | -1.27 |
| 1428210_s_at | Chuk | conserved helix-loop-helix ubiquitous kinase | -1.3 | 0.069 | -1.15 | 0.308 | -1.18 | 0.313 | -1.63 | 0.06 | -1.32 |
| 1442585_at | B230220N19Rik | RIKEN cDNA B230220N19 gene | -1.42 | 0.326 | -1.12 | 0.843 | -1.14 | 0.61 | -1 | 0.989 | -1.17 |
| 1419972_at | Slc35a5 | Solute carrier family 35, member A5 | -1.02 | 0.914 | -1.31 | 0.55 | -1.35 | 0.213 | -1.58 | 0.399 | -1.32 |
| 1416547_at | Ndufb3 | NADH dehydrogenase (ubiquinone) 1 beta subcomplex 3 | -1.14 | 0.257 | -1.22 | 0.065 | -1.28 | 0.042 | -1.23 | 0.072 | -1.22 |
| 1428226_at | Ngdn | neuroguidin, EIF4E binding protein | -1.07 | 0.14 | -1.35 | 0.065 | -1.24 | 0.071 | -1.38 | 0.017 | -1.26 |
| 1419778_at | --- | --- | -1.06 | 0.935 | -1.22 | 0.586 | -1.4 | 0.139 | -2.89 | 0.165 | -1.64 |
| 1429779_at | Eif2c4 | eukaryotic translation initiation factor 2C, 4 | -1.32 | 0.453 | -1.11 | 0.258 | -1.22 | 0.349 | -1.37 | 0.385 | -1.25 |
| 1423248_at | Nktr | natural killer tumor recognition sequence | -1.18 | 0.366 | -1.1 | 0.368 | -1.38 | 0.216 | -1.25 | 0.446 | -1.23 |
| 1444273_at | AW555355 | expressed sequence AW555355 | -1.03 | 0.901 | -1.33 | 0.006 | -1.32 | 0.428 | -1.73 | 0.175 | -1.35 |
| 1443065_at | Rhbdd1 | Rhomboid domain containing 1 | -1.49 | 0.624 | -1.15 | 0.603 | -1.06 | 0.726 | -1.04 | 0.839 | -1.19 |
| 1448803_at | Golga4 | golgi autoantigen, golgin subfamily a, 4 | -1.02 | 0.823 | -1.34 | 0.063 | -1.32 | 0.191 | -1.25 | 0.562 | -1.23 |
| 1441705_at | AU015680 | expressed sequence AU015680 | -1.17 | 0.693 | -1.25 | 0.619 | -1.21 | 0.596 | -1.91 | 0.116 | -1.39 |
| 1435632_at | Nufip2 | nuclear fragile X mental retardation protein interacting protein 2 | -1.11 | 0.31 | -1.15 | 0.224 | -1.39 | 0.043 | -1.2 | 0.379 | -1.21 |
| 1428616_at | Zfp131 | zinc finger protein 131 | -1.03 | 0.833 | -1.21 | 0.072 | -1.45 | 0.004 | -1.12 | 0.104 | -1.2 |
| 1425346_at | Zfp318 | zinc finger protein 318 | -1.02 | 0.806 | -1.21 | 0.025 | -1.48 | 0.005 | -2.12 | 0.123 | -1.46 |
| 1451234_at | BC021381 | cDNA sequence BC021381 | -1.09 | 0.416 | -1.18 | 0.275 | -1.38 | 0.159 | -1.29 | 0.457 | -1.24 |
| 1418982_at | Cebpa | CCAAT/enhancer binding protein (C/EBP), alpha | -1.4 | 0.29 | -1.09 | 0.747 | -1.18 | 0.424 | -1.21 | 0.427 | -1.22 |
| 1441208_at | Hdhd2 | Haloacid dehalogenase-like hydrolase domain containing 2 | -1.13 | 0.491 | -1.26 | 0.255 | -1.25 | 0.228 | -1.28 | 0.249 | -1.23 |
| 1451953_at | Smr2 | submaxillary gland androgen regulated protein 2 | -1.4 | 0.294 | -1.06 | 0.93 | -1.22 | 0.736 | -1.13 | 0.772 | -1.2 |
| 1428831_at | 6230429P13Rik | RIKEN cDNA 6230429P13 gene | -1.03 | 0.84 | -1.32 | 0.04 | -1.33 | 0.058 | -1.12 | 0.472 | -1.2 |
| 1434999_at | Suv420h1 | suppressor of variegation 4-20 homolog 1 (Drosophila) | -1.11 | 0.536 | -1.15 | 0.38 | -1.41 | 0.036 | -1.49 | 0.086 | -1.29 |
| 1433640_at | Fubp1 | Far upstream element (FUSE) binding protein 1 | -1.37 | 0.312 | -1.2 | 0.563 | -1.09 | 0.6 | -1.26 | 0.37 | -1.23 |
| 1417488_at | Fosl1 | fos-like antigen 1 | -1.15 | 0.728 | -1.4 | 0.167 | -1.11 | 0.674 | -1.67 | 0.365 | -1.33 |
| 1433888_at | Atp2b2 | ATPase, Ca++ transporting, plasma membrane 2 | -1.46 | 0.305 | -1.18 | 0.711 | -1.06 | 0.805 | -1 | 0.931 | -1.17 |
| 1442089_at | --- | --- | -1.37 | 0.584 | -1.1 | 0.855 | -1.19 | 0.759 | -2.39 | 0.064 | -1.51 |
| 1454826_at | Zbtb11 | zinc finger and BTB domain containing 11 | -1.27 | 0.28 | -1.21 | 0.152 | -1.15 | 0.14 | -1.32 | 0.07 | -1.24 |
| 1443450_at | --- | 7 days neonate cerebellum cDNA, RIKEN full-length enriched library, clone:A730 | -1.29 | 0.621 | -1.22 | 0.668 | -1.12 | 0.761 | -1.2 | 0.498 | -1.21 |
| 1437763_at | Dcun1d3 | DCN1, defective in cullin neddylation 1, domain containing 3 (S. cerevisiae) | -1.2 | 0.44 | -1.24 | 0.432 | -1.18 | 0.281 | -1.02 | 0.882 | -1.16 |
| 1419114_at | Alg14 | asparagine-linked glycosylation 14 homolog (yeast) | -1.08 | 0.511 | -1.31 | 0.014 | -1.25 | 0.104 | -1.04 | 0.723 | -1.17 |
| 1430317_at | Ube2j2 | ubiquitin-conjugating enzyme E2, J2 homolog (yeast) | -1.09 | 0.818 | -1.17 | 0.392 | -1.4 | 0.208 | -1.46 | 0.054 | -1.28 |
| 1441604_at | Esd | Esterase D/formylglutathione hydrolase | -1.09 | 0.602 | -1.2 | 0.403 | -1.36 | 0.083 | -1.28 | 0.214 | -1.23 |
| 1460625_at | Gm1568 | gene model 1568, (NCBI) | -1.24 | 0.799 | -1.27 | 0.607 | -1.12 | 0.78 | -1.42 | 0.251 | -1.26 |
| 1451391_at | 2700050L05Rik | RIKEN cDNA 2700050L05 gene | -1.17 | 0.389 | -1.14 | 0.475 | -1.33 | 0.192 | -1.08 | 0.648 | -1.18 |
| 1439826_at | Hspa14 | heat shock protein 14 | -1.07 | 0.708 | -1.73 | 0.012 | -1.02 | 0.929 | -1.36 | 0.304 | -1.3 |
| 1444560_at | --- | Transcribed locus | -1.13 | 0.497 | -1.39 | 0.154 | -1.14 | 0.287 | -1.27 | 0.372 | -1.23 |
| 1416694_at | Gk2 | glycerol kinase 2 | -1.64 | 0.216 | -1.12 | 0.679 | -1.02 | 0.974 | -1.45 | 0.405 | -1.31 |
| 1434749_at | BC067068 | cDNA sequence BC067068 | -1.5 | 0.002 | -1.03 | 0.875 | -1.18 | 0.206 | -1.21 | 0.441 | -1.23 |
| 1445831_at | --- | --- | -1.12 | 0.429 | -1.04 | 0.73 | -1.58 | 0.008 | -1.1 | 0.685 | -1.21 |
| 1427044_a_at | Amph | amphiphysin | -1.36 | 0.293 | -1.15 | 0.762 | -1.13 | 0.48 | -2.17 | 0.115 | -1.45 |
| 1436085_at | Zbtb34 | zinc finger and BTB domain containing 34 | -1.57 | 0.209 | -1.18 | 0.458 | -1 | 0.997 | -1.23 | 0.522 | -1.24 |
| 1422366_at | V1ra9 | vomeronasal 1 receptor, A9 | -1.23 | 0.119 | -1.21 | 0.611 | -1.18 | 0.588 | -1.28 | 0.628 | -1.23 |

| | | | | | | | | | | | |
|---|---|---|---|---|---|---|---|---|---|---|---|
| 1459966_at | Osbpl7 | Oxysterol binding protein-like 7 | -1.17 | 0.677 | -1.05 | 0.905 | -1.48 | 0.018 | -1.54 | 0.314 | -1.31 |
| 1434284_at | Bdp1 | B double prime 1, subunit of RNA polymerase III transcription initiation factor IIIB | -1.13 | 0.632 | -1.32 | 0.022 | -1.19 | 0.091 | -1.07 | 0.642 | -1.18 |
| 1449217_at | Casp8ap2 | caspase 8 associated protein 2 | -1.17 | 0.371 | -1.04 | 0.762 | -1.48 | 0.011 | -1.07 | 0.734 | -1.19 |
| 1433417_at | 8030497O21Rik | RIKEN cDNA 8030497O21 gene | -1.23 | 0.24 | -1.23 | 0.312 | -1.16 | 0.713 | -1.51 | 0.405 | -1.28 |
| 1436980_x_at | Cnot2 | CCR4-NOT transcription complex, subunit 2 | -1.03 | 0.734 | -1.16 | 0.363 | -1.53 | 0.042 | -1.4 | 0.085 | -1.28 |
| 1450486_a_at | Oprl1 | opioid receptor-like 1 | -1.29 | 0.583 | -1.25 | 0.498 | -1.1 | 0.874 | -2.75 | 0.085 | -1.6 |
| 1437643_at | Cenpj | centromere protein J | -1.29 | 0.149 | -1.11 | 0.608 | -1.23 | 0.433 | -1.07 | 0.752 | -1.18 |
| 1433740_at | 2610301K12Rik | RIKEN cDNA 2610301K12 gene | -1.38 | 0.115 | -1.07 | 0.588 | -1.2 | 0.141 | -1.27 | 0.263 | -1.23 |
| AFFX-MURINE_B2_ | --- | --- | -1.11 | 0.039 | -1.2 | 0.457 | -1.33 | 0.001 | -1.2 | 0.046 | -1.21 |
| 1436032_at | --- | Bone marrow macrophage cDNA, RIKEN full-length enriched library, clone:I8300 | -1.19 | 0.509 | -1.28 | 0.26 | -1.15 | 0.359 | -2.01 | 0.099 | -1.41 |
| 1446235_at | BC002199 | cDNA sequence BC002199 | -1.16 | 0.066 | -1.1 | 0.536 | -1.39 | 0.031 | -1.22 | 0.52 | -1.22 |
| 1442381_at | Mkln1 | Muskelin 1, intracellular mediator containing kelch motifs | -1.12 | 0.271 | -1.08 | 0.605 | -1.51 | 0.024 | -1.16 | 0.544 | -1.21 |
| 1416275_at | Slc26a6 | solute carrier family 26, member 6 | -1.56 | 0.02 | -1.02 | 0.929 | -1.15 | 0.373 | -1.53 | 0.094 | -1.32 |
| 1446183_at | Als2 | Amyotrophic lateral sclerosis 2 (juvenile) homolog (human) | -1.31 | 0.183 | -1.03 | 0.961 | -1.32 | 0.386 | -1.21 | 0.054 | -1.22 |
| 1416006_at | Mdk | midkine | -1.15 | 0.281 | -1.05 | 0.814 | -1.51 | 0.001 | -1.43 | 0.311 | -1.28 |
| 1454521_at | 2810421E14Rik | RIKEN cDNA 2810421E14 gene | -1.05 | 0.87 | -1.34 | 0.409 | -1.27 | 0.384 | -1.35 | 0.48 | -1.25 |
| 1453421_at | Srr | serine racemase | -1.05 | 0.78 | -1.23 | 0.347 | -1.38 | 0.138 | -1.2 | 0.575 | -1.22 |
| 1447406_at | --- | PREDICTED: Mus musculus similar to Hemoglobin alpha subunit (Hemoglobin al | -1.45 | 0.331 | -1.22 | 0.696 | -1.02 | 0.955 | -1.14 | 0.625 | -1.21 |
| 1447947_at | Zfyve16 | zinc finger, FYVE domain containing 16 | -1.19 | 0.393 | -1.13 | 0.67 | -1.32 | 0.241 | -1.18 | 0.585 | -1.2 |
| 1440604_at | 8030494B02Rik | Riken cDNA 8030494B02 gene | -1.01 | 0.916 | -1.1 | 0.41 | -1.68 | 0.224 | -1.27 | 0.25 | -1.27 |
| 1419555_at | Elf5 | E74-like factor 5 | -1.07 | 0.771 | -1.24 | 0.103 | -1.33 | 0.194 | -1.12 | 0.826 | -1.19 |
| 1439059_at | BC031748 | CDNA sequence BC031748 | -1.06 | 0.735 | -1.12 | 0.505 | -1.52 | 0.065 | -1.56 | 0.111 | -1.32 |
| 1446830_at | AU015892 | expressed sequence AU015892 | -1.22 | 0.739 | -1.25 | 0.478 | -1.15 | 0.783 | -1.43 | 0.208 | -1.26 |
| 1440369_at | Ptger3 | Prostaglandin E receptor 3 (subtype EP3) | -1.21 | 0.637 | -1.06 | 0.857 | -1.4 | 0.018 | -1.36 | 0.131 | -1.26 |
| 1440551_at | Dnajc1 | DnaJ (Hsp40) homolog, subfamily C, member 1 | -1.1 | 0.199 | -1.07 | 0.442 | -1.54 | 0.003 | -1.3 | 0.095 | -1.25 |
| 1429823_at | 5430420E18Rik | RIKEN cDNA 5430420E18 gene | -1.08 | 0.876 | -1.33 | 0.685 | -1.23 | 0.605 | -2.39 | 0.128 | -1.51 |
| 1439771_s_at | D13Ertd608e | DNA segment, Chr 13, ERATO Doi 608, expressed | -1.33 | 0.462 | -1.21 | 0.481 | -1.1 | 0.692 | -2.62 | 0.025 | -1.56 |
| 1459238_at | --- | --- | -1.19 | 0.017 | -1.11 | 0.416 | -1.33 | 0.039 | -1.3 | 0.309 | -1.23 |
| 1433961_at | BC023814 | cDNA sequence BC023814 | -1.54 | 0.028 | -1.01 | 0.93 | -1.17 | 0.143 | -1.44 | 0.012 | -1.29 |
| 1453824_at | A930028L21Rik | RIKEN cDNA A930028L21 gene | -1.27 | 0.095 | -1.26 | 0.252 | -1.1 | 0.648 | -1.36 | 0.373 | -1.25 |
| 1429226_at | 2310014F06Rik | RIKEN cDNA 2310014F06 gene | -1.19 | 0.489 | -1.29 | 0.099 | -1.15 | 0.301 | -1.07 | 0.808 | -1.17 |
| 1457677_at | BC057593 | CDNA sequence BC057593 | -1.2 | 0.135 | -1.18 | 0.153 | -1.24 | 0.435 | -1.41 | 0.065 | -1.26 |
| 1417154_at | Slc25a14 | solute carrier family 25 (mitochondrial carrier, brain), member 14 | -1.41 | 0.419 | -1.09 | 0.84 | -1.15 | 0.606 | -1.29 | 0.318 | -1.24 |
| 1426088_at | --- | --- | -1.04 | 0.746 | -1.24 | 0.364 | -1.37 | 0.144 | -1.21 | 0.487 | -1.22 |
| 1433757_a_at | Nisch | nischarin | -1.25 | 0.473 | -1.01 | 0.933 | -1.43 | 0.125 | -1.65 | 0.143 | -1.33 |
| 1428409_at | Nat13 | N-acetyltransferase 13 | -1.16 | 0.408 | -1.12 | 0.057 | -1.36 | 0.1 | -1.29 | 0.193 | -1.23 |
| 1416375_at | Ap3m1 | adaptor-related protein complex 3, mu 1 subunit | -1.02 | 0.906 | -1.41 | 0.066 | -1.25 | 0.125 | -1.38 | 0.002 | -1.26 |
| 1420861_at | Dctn4 | dynactin 4 | -1.07 | 0.638 | -1.04 | 0.741 | -1.68 | 0.036 | -1.05 | 0.536 | -1.21 |
| 1451847_s_at | Arid4b | AT rich interactive domain 4B (Rbp1 like) | -1.31 | 0.706 | -1.11 | 0.734 | -1.2 | 0.335 | -1.79 | 0.215 | -1.36 |
| 1439161_at | Saps3 | SAPS domain family, member 3 | -1.13 | 0.356 | -1.06 | 0.337 | -1.51 | 0.029 | -1.31 | 0.056 | -1.25 |
| 1449374_at | Pipox | pipecolic acid oxidase | -1.06 | 0.726 | -1.17 | 0.108 | -1.44 | 0.161 | -1.9 | 0.378 | -1.39 |
| 1434631_at | D3Ertd751e | DNA segment, Chr 3, ERATO Doi 751, expressed | -1.2 | 0.554 | -1.45 | 0.008 | -1.03 | 0.854 | -1.42 | 0.205 | -1.28 |
| 1426719_at | Apbb2 | amyloid beta (A4) precursor protein-binding, family B, member 2 | -1.13 | 0.065 | -1.2 | 0.111 | -1.3 | 0.33 | -1.33 | 0.058 | -1.24 |
| 1453381_at | C030033M12Rik | RIKEN cDNA C030033M12 gene | -1.04 | 0.845 | -1.26 | 0.067 | -1.35 | 0.229 | -1.34 | 0.277 | -1.25 |
| 1432792_at | 4930401B11Rik | RIKEN cDNA 4930401B11 gene | -1.16 | 0.871 | -1.3 | 0.631 | -1.16 | 0.792 | -1.15 | 0.826 | -1.19 |
| 1458747_at | Fbxo45 | F-box protein 45 | -1.37 | 0.696 | -1.05 | 0.946 | -1.23 | 0.641 | -1.11 | 0.638 | -1.19 |
| 1433992_at | Shroom2 /// LOC67 | shroom family member 2 /// similar to Apical-like protein (APXL protein) | -1.04 | 0.682 | -1.33 | 0.027 | -1.29 | 0.226 | -1.19 | 0.665 | -1.21 |
| 1451757_at | BC003883 | cDNA sequence BC003883 | -1.42 | 0.072 | -1.12 | 0.748 | -1.12 | 0.681 | -1.92 | 0.019 | -1.39 |
| 1434708_at | Vhlh | von Hippel-Lindau syndrome homolog | -1.24 | 0.097 | -1.24 | 0.235 | -1.14 | 0.422 | -1.32 | 0.054 | -1.23 |
| 1432548_at | Parp14 | poly (ADP-ribose) polymerase family, member 14 | -1.05 | 0.115 | -1.16 | 0.757 | -1.47 | 0.181 | -1.7 | 0.117 | -1.35 |
| 1451211_a_at | Lgtn | ligatin | -1.14 | 0.407 | -1.21 | 0.028 | -1.27 | 0.179 | -1.11 | 0.347 | -1.18 |
| 1446062_at | B830028B13Rik | RIKEN cDNA B830028B13 gene | -1.13 | 0.851 | -1.2 | 0.65 | -1.29 | 0.201 | -1.46 | 0.389 | -1.27 |
| 1417943_at | Gng4 | guanine nucleotide binding protein (G protein), gamma 4 subunit | -1.92 | 0.283 | -1.03 | 0.962 | -1 | 0.998 | -1.78 | 0.41 | -1.43 |

| | | | | | | | | | | | |
|---|---|---|---|---|---|---|---|---|---|---|---|
| 1422435_at | 2210010C04Rik | RIKEN cDNA 2210010C04 gene | -1.06 | 0.823 | -1.02 | 0.946 | -1.75 | 0.26 | -1.58 | 0.4 | -1.35 |
| 1419445_s_at | Sap18 | Sin3-associated polypeptide 18 | -1.14 | 0.144 | -1.41 | 0.015 | -1.1 | 0.572 | -1.26 | 0.504 | -1.23 |
| 1427055_at | Them4 | thioesterase superfamily member 4 | -1.05 | 0.803 | -1.33 | 0.136 | -1.27 | 0.179 | -1.9 | 0.222 | -1.39 |
| 1458311_at | Usp36 | ubiquitin specific peptidase 36 | -1.2 | 0.499 | -1.06 | 0.8 | -1.4 | 0.105 | -1.08 | 0.838 | -1.19 |
| 1428219_at | Rybp /// LOC62874 | RING1 and YY1 binding protein /// similar to RING1 and YY1 binding protein | -1.12 | 0.234 | -1.22 | 0.093 | -1.28 | 0.04 | -1.11 | 0.069 | -1.18 |
| 1445827_at | Prkcbp1 | Protein kinase C binding protein 1 | -1.22 | 0.306 | -1.17 | 0.279 | -1.22 | 0.374 | -1.05 | 0.788 | -1.17 |
| 1455121_at | Lcor | ligand dependent nuclear receptor corepressor | -1.2 | 0.039 | -1.13 | 0.193 | -1.29 | 0.009 | -1.01 | 0.956 | -1.16 |
| 1439005_x_at | Ywhaz | tyrosine 3-monooxygenase/tryptophan 5-monooxygenase activation protein, zeta | -1.25 | 0.511 | -1.22 | 0.485 | -1.14 | 0.29 | -1.08 | 0.456 | -1.17 |
| 1423551_at | Cdh13 | cadherin 13 | -1.22 | 0.06 | -1.11 | 0.644 | -1.28 | 0.495 | -1.72 | 0.309 | -1.33 |
| 1434957_at | Cdon | cell adhesion molecule-related/down-regulated by oncogenes | -1.3 | 0.046 | -1.17 | 0.605 | -1.16 | 0.713 | -1.97 | 0.078 | -1.4 |
| 1455668_at | Whsc1l1 | Wolf-Hirschhorn syndrome candidate 1-like 1 (human) | -1.16 | 0.252 | -1.33 | 0.073 | -1.14 | 0.248 | -1.15 | 0.201 | -1.19 |
| 1430376_at | Lrrc9 | leucine rich repeat containing 9 | -1.04 | 0.95 | -1.45 | 0.524 | -1.19 | 0.772 | -1.33 | 0.556 | -1.25 |
| 1426695_at | 9030624J02Rik | RIKEN cDNA 9030624J02 gene | -1.39 | 0.159 | -1.06 | 0.845 | -1.2 | 0.371 | -1.28 | 0.351 | -1.23 |
| 1443526_at | --- | --- | -1.12 | 0.414 | -1.05 | 0.593 | -1.53 | 0.101 | -1.76 | 0.117 | -1.37 |
| 1428507_at | Hdhd2 | haloacid dehalogenase-like hydrolase domain containing 2 | -1.24 | 0.014 | -1.25 | 0.003 | -1.12 | 0.425 | -1.07 | 0.674 | -1.17 |
| 1454697_at | Tloc1 | translocation protein 1 | -1.05 | 0.731 | -1.14 | 0.111 | -1.5 | 0.03 | -1.05 | 0.595 | -1.19 |
| 1434890_at | Opa1 | optic atrophy 1 homolog (human) | -1.19 | 0.192 | -1.15 | 0.066 | -1.28 | 0.03 | -1.32 | 0.121 | -1.23 |
| 1420451_at | Accn5 | amiloride-sensitive cation channel 5, intestinal | -1.22 | 0.693 | -1.36 | 0.249 | -1.06 | 0.909 | -1.28 | 0.755 | -1.23 |
| 1429675_at | 1700023A16Rik | RIKEN cDNA 1700023A16 gene | -1.2 | 0.747 | -1.29 | 0.641 | -1.13 | 0.862 | -2.11 | 0.449 | -1.43 |
| 1428453_at | Nat12 | N-acetyltransferase 12 | -1.12 | 0.353 | -1.15 | 0.033 | -1.36 | 0.005 | -1.21 | 0.152 | -1.21 |
| 1450901_a_at | AW011752 | expressed sequence AW011752 | -1.16 | 0.31 | -1.33 | 0.043 | -1.14 | 0.26 | -1.24 | 0.227 | -1.21 |
| 1445374_at | Vti1a | Vesicle transport through interaction with t-SNAREs homolog 1A (yeast) | -1.14 | 0.08 | -1.13 | 0.59 | -1.35 | 0.141 | -1.2 | 0.329 | -1.21 |
| 1446219_at | D930015E06Rik | RIKEN cDNA D930015E06 gene | -1.41 | 0.096 | -1.15 | 0.736 | -1.09 | 0.72 | -1.06 | 0.607 | -1.18 |
| 1454612_at | Rkhd2 | ring finger and KH domain containing 2 | -1.21 | 0.268 | -1.03 | 0.82 | -1.43 | 0.13 | -1.13 | 0.444 | -1.2 |
| 1424693_at | 4933407N01Rik | RIKEN cDNA 4933407N01 gene | -1.19 | 0.038 | -1.17 | 0.179 | -1.24 | 0.388 | -1.1 | 0.53 | -1.18 |
| 1449630_s_at | Mark1 | MAP/microtubule affinity-regulating kinase 1 | -1.1 | 0.028 | -1.47 | 0.219 | -1.11 | 0.534 | -1.31 | 0.117 | -1.25 |
| 1420188_at | Syt6 | synaptotagmin VI | -1.31 | 0.736 | -1.34 | 0.52 | -1.01 | 0.978 | -1.1 | 0.728 | -1.19 |
| 1440364_a_at | A230062G08Rik | RIKEN cDNA A230062G08 gene | -1.04 | 0.943 | -1.59 | 0.035 | -1.11 | 0.632 | -1.73 | 0.029 | -1.37 |
| 1422621_at | Ranbp2 | RAN binding protein 2 | -1.19 | 0.537 | -1.21 | 0.109 | -1.21 | 0.209 | -1.04 | 0.839 | -1.16 |
| 1454195_at | 4933433G19Rik | RIKEN cDNA 4933433G19 gene | -1.12 | 0.674 | -1.51 | 0.328 | -1.06 | 0.819 | -1.02 | 0.899 | -1.18 |
| 1429060_at | --- | --- | -1.26 | 0.325 | -1.34 | 0.061 | -1.04 | 0.724 | -1.29 | 0.372 | -1.23 |
| 1423286_at | Cbln1 | cerebellin 1 precursor protein | -1.63 | 0.089 | -1.11 | 0.754 | -1.02 | 0.942 | -1.22 | 0.611 | -1.24 |
| 1416947_s_at | Acaa1a /// Acaa1b | acetyl-Coenzyme A acyltransferase 1A /// acetyl-Coenzyme A acyltransferase 1B | -1.05 | 0.867 | -1.73 | 0.235 | -1.03 | 0.739 | -1.38 | 0.241 | -1.3 |
| 1443610_at | --- | Transcribed locus | -1.73 | 0.015 | -1.05 | 0.881 | -1.04 | 0.876 | -1.61 | 0.012 | -1.36 |
| 1421664_a_at | Styx | phosphoserine/threonine/tyrosine interaction protein | -1.14 | 0.145 | -1.1 | 0.619 | -1.4 | 0.007 | -1.21 | 0.041 | -1.21 |
| 1425217_a_at | Synj2 | synaptojanin 2 | -1.38 | 0.751 | -1.14 | 0.841 | -1.12 | 0.811 | -1.55 | 0.515 | -1.3 |
| 1435198_at | LOC545228 | hypothetical protein LOC545228 | -1.27 | 0.128 | -1.29 | 0.314 | -1.07 | 0.712 | -1.25 | 0.328 | -1.22 |
| 1419637_s_at | 4833420G17Rik | RIKEN cDNA 4833420G17 gene | -1.12 | 0.57 | -1.41 | 0.069 | -1.12 | 0.516 | -1.37 | 0.204 | -1.25 |
| 1419845_at | Dlx1as | distal-less homeobox 1, antisense | -1.55 | 0.136 | -1.02 | 0.952 | -1.15 | 0.792 | -1.03 | 0.937 | -1.19 |
| 1438783_at | LOC637870 | similar to Nedd4 WW binding protein 4 | -1.1 | 0.859 | -1.3 | 0.527 | -1.23 | 0.64 | -1.93 | 0.184 | -1.39 |
| 1437869_at | 3222402P14Rik | RIKEN cDNA 3222402P14 gene | -1.04 | 0.84 | -1.4 | 0.279 | -1.22 | 0.438 | -1.22 | 0.545 | -1.22 |
| 1433331_at | C030046M01Rik | RIKEN cDNA C030046M01 gene | -1.15 | 0.821 | -1.45 | 0.547 | -1.07 | 0.937 | -1.5 | 0.456 | -1.29 |
| 1430131_at | Crry | complement receptor related protein | -1.07 | 0.777 | -1.17 | 0.551 | -1.41 | 0.222 | -1.7 | 0.003 | -1.34 |
| 1434787_at | Arf3 | ADP-ribosylation factor 3 | -1.11 | 0.555 | -1.26 | 0.055 | -1.25 | 0.375 | -1.38 | 0.13 | -1.25 |
| 1443758_at | Wdr18 | WD repeat domain 18 | -1.29 | 0.776 | -1.25 | 0.623 | -1.09 | 0.865 | -1.58 | 0.331 | -1.3 |
| 1416143_at | Atp5j | ATP synthase, H+ transporting, mitochondrial F0 complex, subunit F | -1.13 | 0.177 | -1.23 | 0.097 | -1.25 | 0.028 | -1.03 | 0.809 | -1.16 |
| 1443444_at | Mgat5 | Mannoside acetylglucosaminyltransferase 5 | -1.1 | 0.829 | -1.19 | 0.683 | -1.33 | 0.39 | -1.48 | 0.127 | -1.28 |
| 1452122_at | AI314180 | expressed sequence AI314180 | -1.14 | 0 | -1.33 | 0.036 | -1.15 | 0.583 | -1.18 | 0.44 | -1.2 |
| 1425287_at | Zfp189 | zinc finger protein 189 | -1.37 | 0.213 | -1.15 | 0.334 | -1.11 | 0.526 | -1.4 | 0.11 | -1.26 |
| 1459704_at | Dnajc7 | DnaJ (Hsp40) homolog, subfamily C, member 7 | -1.25 | 0.128 | -1.32 | 0.414 | -1.06 | 0.859 | -1.18 | 0.226 | -1.2 |
| 1431381_at | 3110005L24Rik | RIKEN cDNA 3110005L24 gene | -1.01 | 0.857 | -1.45 | 0.115 | -1.21 | 0.422 | -1.51 | 0.011 | -1.3 |
| 1460311_at | Srst | simple repeat sequence-containing transcript | -1.52 | 0.176 | -1.12 | 0.855 | -1.05 | 0.893 | -2.78 | 0.111 | -1.62 |
| 1444662_at | Pla2g4c | phospholipase A2, group IVC (cytosolic, calcium-independent) | -1.37 | 0.158 | -1.16 | 0.257 | -1.1 | 0.69 | -1.14 | 0.402 | -1.19 |

| | | | | | | | | | | | |
|---|---|---|---|---|---|---|---|---|---|---|---|
| 1434010_at | Als2cr13 | amyotrophic lateral sclerosis 2 (juvenile) chromosome region, candidate 13 (huma | -1.1 | 0.599 | -1.22 | 0.095 | -1.29 | 0.292 | -1.21 | 0.28 | -1.21 |
| 1439847_s_at | Klf12 | Kruppel-like factor 12 | -1.05 | 0.861 | -1.01 | 0.967 | -1.8 | 0.036 | -1.63 | 0.292 | -1.37 |
| 1421075_s_at | Cyp7b1 | cytochrome P450, family 7, subfamily b, polypeptide 1 | -1.13 | 0.496 | -1.54 | 0.075 | -1.03 | 0.705 | -1.43 | 0.158 | -1.29 |
| 1434389_at | AI449023 | expressed sequence AI449023 | -1.18 | 0.299 | -1.15 | 0.303 | -1.28 | 0.31 | -1.19 | 0.176 | -1.2 |
| 1451383_a_at | Chuk | conserved helix-loop-helix ubiquitous kinase | -1.15 | 0.057 | -1.24 | 0.001 | -1.21 | 0.03 | -1.44 | 0.021 | -1.26 |
| 1453191_at | Col27a1 | procollagen, type XXVII, alpha 1 | -1.19 | 0.195 | -1.04 | 0.828 | -1.44 | 0.035 | -1.25 | 0.182 | -1.23 |
| 1434100_x_at | Ppargc1a | Peroxisome proliferative activated receptor, gamma, coactivator 1 alpha | -1.19 | 0.536 | -1.25 | 0.514 | -1.17 | 0.37 | -1.13 | 0.856 | -1.18 |
| 1428374_at | Glce | glucuronyl C5-epimerase | -1.03 | 0.85 | -1.17 | 0.269 | -1.49 | 0.046 | -1.16 | 0.382 | -1.21 |
| 1434933_at | 5730557L09Rik | RIKEN cDNA 5730557L09 gene | -1.13 | 0.105 | -1.06 | 0.394 | -1.49 | 0.048 | -1.12 | 0.338 | -1.2 |
| 1440952_at | Smad7 | MAD homolog 7 (Drosophila) | -1.41 | 0.236 | -1.07 | 0.867 | -1.17 | 0.658 | -2.13 | 0.037 | -1.45 |
| 1418322_at | Crem | cAMP responsive element modulator | -1.1 | 0.654 | -1.21 | 0.305 | -1.31 | 0.08 | -1.17 | 0.409 | -1.2 |
| 1436757_a_at | Cox6b1 | cytochrome c oxidase, subunit VIb polypeptide 1 | -1.12 | 0.213 | -1.24 | 0.258 | -1.25 | 0 | -1.01 | 0.857 | -1.15 |
| 1444995_at | Cdyl2 | Chromodomain protein, Y chromosome-like 2 | -1.05 | 0.959 | -1.66 | 0.311 | -1.06 | 0.838 | -1.12 | 0.603 | -1.22 |
| 1453108_at | Arsk | arylsulfatase K | -1.19 | 0.114 | -1.13 | 0.244 | -1.3 | 0.164 | -1.17 | 0.011 | -1.19 |
| 1435333_at | 1110007M04Rik | RIKEN cDNA 1110007M04 gene | -1.25 | 0.425 | -1.09 | 0.597 | -1.28 | 0.165 | -1.04 | 0.884 | -1.16 |
| 1444284_at | A130050O07Rik | RIKEN cDNA A130050O07 gene | -1.03 | 0.967 | -1.85 | 0.316 | -1.01 | 0.955 | -3.92 | 0.327 | -1.95 |
| 1446516_at | Bcl7c | B-cell CLL/lymphoma 7C | -1.42 | 0.413 | -1.25 | 0.766 | -1 | 0.98 | -1.04 | 0.891 | -1.18 |
| 1439121_at | H2-T22 | histocompatibility 2, T region locus 22 | -1.51 | 0.419 | -1.05 | 0.925 | -1.12 | 0.737 | -1.93 | 0.097 | -1.4 |
| 1459625_at | Frap1 | FK506 binding protein 12-rapamycin associated protein 1 | -1.01 | 0.955 | -1.29 | 0.211 | -1.36 | 0.224 | -1.12 | 0.156 | -1.19 |
| 1424361_at | BC019943 | cDNA sequence BC019943 | -1.24 | 0.245 | -1.17 | 0.248 | -1.19 | 0.319 | -1.25 | 0.568 | -1.21 |
| 1445844_at | --- | Adult male testis cDNA, RIKEN full-length enriched library, clone:4930547H19 pro | -1.24 | 0.683 | -1.19 | 0.658 | -1.16 | 0.636 | -1.05 | 0.908 | -1.16 |
| 1423869_s_at | Txnrd3 | thioredoxin reductase 3 | -1.12 | 0.579 | -1.32 | 0.329 | -1.18 | 0.458 | -1.18 | 0.501 | -1.2 |
| 1433342_at | 5730416F02Rik | RIKEN cDNA 5730416F02 gene | -1.17 | 0.795 | -1.48 | 0.394 | -1.03 | 0.833 | -1.82 | 0.23 | -1.37 |
| 1417194_at | Sod2 | superoxide dismutase 2, mitochondrial | -1.1 | 0.741 | -1.04 | 0.846 | -1.58 | 0.056 | -1.75 | 0.017 | -1.37 |
| 1440042_at | Ank2 | ankyrin 2, brain | -1.12 | 0.492 | -1.3 | 0.691 | -1.19 | 0.14 | -1.28 | 0.273 | -1.22 |
| 1434723_at | AI426748 | expressed sequence AI426748 | -1.1 | 0.696 | -1.45 | 0.378 | -1.11 | 0.262 | -1.34 | 0.074 | -1.25 |
| 1416201_at | Crk | v-crk sarcoma virus CT10 oncogene homolog (avian) | -1.21 | 0.314 | -1.21 | 0.151 | -1.18 | 0.068 | -1.09 | 0.693 | -1.17 |
| 1446324_at | Cacna2d1 | Calcium channel, voltage-dependent, alpha2/delta subunit 1 | -1.04 | 0.715 | -1.11 | 0.785 | -1.57 | 0.029 | -1.28 | 0.282 | -1.25 |
| 1421765_at | Pax5 | paired box gene 5 | -1.35 | 0.328 | -1.23 | 0.598 | -1.05 | 0.909 | -5.14 | 0.051 | -2.19 |
| 1449867_at | Hoxc9 | homeo box C9 | -1.07 | 0.504 | -1.35 | 0.161 | -1.21 | 0.384 | -2.47 | 0.415 | -1.53 |
| 1459875_x_at | 5730494M16Rik | RIKEN cDNA 5730494M16 gene | -1.36 | 0.129 | -1.17 | 0.373 | -1.1 | 0.369 | -1.21 | 0.398 | -1.21 |
| 1448830_at | Dusp1 | dual specificity phosphatase 1 | -1.18 | 0.674 | -1.32 | 0.28 | -1.11 | 0.631 | -1.13 | 0.726 | -1.18 |
| 1459460_at | --- | --- | -1.05 | 0.905 | -1.19 | 0.519 | -1.41 | 0.418 | -1.16 | 0.72 | -1.2 |
| 1427224_at | Acsm2 | Acyl-CoA synthetase medium-chain family member 2 | -1.12 | 0.863 | -1.25 | 0.741 | -1.23 | 0.611 | -1.89 | 0.056 | -1.37 |
| 1441970_at | E430010N07Rik | RIKEN cDNA E430010N07 gene | -1.05 | 0.746 | -1.02 | 0.93 | -1.78 | 0.035 | -1.13 | 0.606 | -1.24 |
| 1446188_at | Lrriq2 | leucine-rich repeats and IQ motif containing 2 | -1.54 | 0.381 | -1.12 | 0.467 | -1.04 | 0.853 | -1.3 | 0.299 | -1.25 |
| 1457715_at | 1010001B22Rik | RIKEN cDNA 1010001B22 gene | -1.25 | 0.778 | -1.21 | 0.432 | -1.14 | 0.49 | -1.16 | 0.756 | -1.19 |
| 1435461_at | Magi3 | membrane associated guanylate kinase, WW and PDZ domain containing 3 | -1.07 | 0.271 | -1.08 | 0.301 | -1.57 | 0.08 | -1.37 | 0.216 | -1.27 |
| 1424372_at | Mrpl32 | mitochondrial ribosomal protein L32 | -1.21 | 0.017 | -1.09 | 0.137 | -1.32 | 0.128 | -1.11 | 0.639 | -1.18 |
| 1427043_s_at | Cova1 | cytosolic ovarian carcinoma antigen 1 | -1.2 | 0.019 | -1.1 | 0.514 | -1.32 | 0.014 | -1.25 | 0.426 | -1.22 |
| 1456397_at | Cdh4 | cadherin 4 | -1.27 | 0.09 | -1.09 | 0.716 | -1.25 | 0.243 | -1.62 | 0.004 | -1.31 |
| 1419183_at | Papd4 | PAP associated domain containing 4 | -1.28 | 0.263 | -1.11 | 0.323 | -1.22 | 0.061 | -1.03 | 0.828 | -1.16 |
| 1439721_at | C85627 | expressed sequence C85627 | -1.18 | 0.641 | -1.19 | 0.702 | -1.23 | 0.542 | -1 | 0.999 | -1.15 |
| 1456274_at | C230071H18Rik | RIKEN cDNA C230071H18 gene | -1.58 | 0.319 | -1.11 | 0.684 | -1.03 | 0.888 | -2.53 | 0.053 | -1.56 |
| 1442195_at | BB283564 | expressed sequence BB283564 | -1.05 | 0.93 | -1.2 | 0.474 | -1.39 | 0.049 | -1.27 | 0.263 | -1.23 |
| 1454369_a_at | Nfatc4 | nuclear factor of activated T-cells, cytoplasmic, calcineurin-dependent 4 | -1.25 | 0.34 | -1.26 | 0.695 | -1.1 | 0.667 | -1.08 | 0.763 | -1.17 |
| 1442053_at | Phkb | Phosphorylase kinase beta | -1.15 | 0.713 | -1.3 | 0.206 | -1.15 | 0.527 | -1.56 | 0.365 | -1.29 |
| 1455160_at | 2610203C20Rik | RIKEN cDNA 2610203C20 gene | -1.28 | 0.32 | -1.13 | 0.472 | -1.19 | 0.479 | -1.08 | 0.793 | -1.17 |
| 1422442_at | Smu1 | smu-1 suppressor of mec-8 and unc-52 homolog (C. elegans) | -1.18 | 0.014 | -1.21 | 0.003 | -1.2 | 0.033 | -1.2 | 0.213 | -1.2 |
| 1428563_at | Ddx10 | DEAD (Asp-Glu-Ala-Asp) box polypeptide 10 | -1.01 | 0.907 | -1.68 | 0.044 | -1.09 | 0.716 | -1.38 | 0.207 | -1.29 |
| 1433749_at | Gna13 | guanine nucleotide binding protein, alpha 13 | -1.08 | 0.597 | -1.26 | 0.084 | -1.27 | 0.101 | -1.1 | 0.598 | -1.18 |
| 1420952_at | Son | Son cell proliferation protein | -1.06 | 0.578 | -1.18 | 0.221 | -1.41 | 0.09 | -1.29 | 0.198 | -1.23 |
| 1439292_at | 2210408F21Rik | RIKEN cDNA 2210408F21 gene | -1.04 | 0.865 | -1.17 | 0.556 | -1.45 | 0.139 | -1.41 | 0.21 | -1.27 |

| | | | | | | | | | | | |
|---|---|---|---|---|---|---|---|---|---|---|---|
| 1438805_at | 9230106B05Rik | RIKEN cDNA 9230106B05 gene | -1 | 0.991 | -1.76 | 0.262 | -1.06 | 0.865 | -2.32 | 0.273 | -1.54 |
| 1425309_at | Catsper2 | cation channel, sperm associated 2 | -1.29 | 0.096 | -1.28 | 0.088 | -1.05 | 0.833 | -1.09 | 0.437 | -1.18 |
| 1455637_x_at | LOC245305 | Similar to hypothetical protein FLJ38281 | -1.05 | 0.867 | -1.23 | 0.165 | -1.34 | 0.22 | -1.37 | 0.222 | -1.25 |
| 1455070_at | Dcp2 | DCP2 decapping enzyme homolog (S. cerevisiae) | -1.19 | 0.023 | -1.14 | 0.061 | -1.26 | 0.129 | -1.28 | 0.056 | -1.22 |
| 1457298_at | --- | --- | -1.03 | 0.944 | -1.27 | 0.646 | -1.34 | 0.127 | -1.39 | 0.385 | -1.26 |
| 1442281_at | D4Ertd796e | DNA segment, Chr 4, ERATO Doi 796, expressed | -1.14 | 0.084 | -1.49 | 0.404 | -1.04 | 0.858 | -1.94 | 0.127 | -1.4 |
| 1443187_at | --- | --- | -1.89 | 0.383 | -1.02 | 0.971 | -1.01 | 0.987 | -1.97 | 0.094 | -1.47 |
| 1434609_at | B930007L02Rik | RIKEN cDNA B930007L02 gene | -1.23 | 0.156 | -1.25 | 0.004 | -1.11 | 0.447 | -1.04 | 0.349 | -1.16 |
| 1446720_at | Alcam | Activated leukocyte cell adhesion molecule | -1.07 | 0.87 | -1.12 | 0.576 | -1.47 | 0.144 | -1.32 | 0.144 | -1.25 |
| 1435251_at | Snx13 | sorting nexin 13 | -1.21 | 0.142 | -1.14 | 0.048 | -1.25 | 0.179 | -1.35 | 0.258 | -1.24 |
| 1455702_at | --- | --- | -1.03 | 0.925 | -1.38 | 0.257 | -1.23 | 0.543 | -1.29 | 0.317 | -1.23 |
| 1452502_at | Serf1 | small EDRK-rich factor 1 | -1.27 | 0.438 | -1.07 | 0.454 | -1.28 | 0.341 | -1.11 | 0.774 | -1.18 |
| 1419180_at | Bcl9l | B-cell CLL/lymphoma 9-like | -1.15 | 0.202 | -1.2 | 0.251 | -1.25 | 0.304 | -1.17 | 0.557 | -1.19 |
| 1435511_at | Syn2 | synapsin II | -1.2 | 0.111 | -1.16 | 0.702 | -1.23 | 0.501 | -1.53 | 0.097 | -1.28 |
| 1459119_at | Cenpo | centromere protein O | -1.21 | 0.566 | -1 | 0.998 | -1.47 | 0.148 | -1.17 | 0.547 | -1.21 |
| 1455681_at | Zfp369 | zinc finger protein 369 | -1.22 | 0.066 | -1.14 | 0.294 | -1.23 | 0.293 | -1.27 | 0.455 | -1.22 |
| 1429004_at | Phip | pleckstrin homology domain interacting protein | -1.16 | 0.245 | -1.1 | 0.367 | -1.36 | 0.063 | -1.38 | 0.104 | -1.25 |
| 1456162_x_at | Add3 | adducin 3 (gamma) | -1.26 | 0.394 | -1.07 | 0.527 | -1.29 | 0.281 | -1.7 | 0.234 | -1.33 |
| 1442643_at | Jmjd3 | jumonji domain containing 3 | -1.29 | 0.692 | -1.36 | 0.038 | -1 | 0.99 | -1.77 | 0.231 | -1.36 |
| 1429137_at | 2810422O20Rik | RIKEN cDNA 2810422O20 gene | -1.13 | 0.696 | -1.22 | 0.036 | -1.25 | 0.22 | -1.76 | 0.049 | -1.34 |
| 1429868_at | --- | --- | -1.11 | 0.605 | -1.34 | 0.235 | -1.16 | 0.29 | -1.4 | 0.545 | -1.25 |
| 1416642_a_at | Tpt1 | tumor protein, translationally-controlled 1 | -1.13 | 0.015 | -1.16 | 0.481 | -1.31 | 0.01 | -1.2 | 0.013 | -1.2 |
| 1444149_at | Zfp96 | zinc finger protein 96 | -1.02 | 0.925 | -1.07 | 0.869 | -1.69 | 0.073 | -1.15 | 0.39 | -1.23 |
| 1439486_at | Kcnt1 | potassium channel, subfamily T, member 1 | -1.18 | 0.451 | -1.18 | 0.698 | -1.22 | 0.369 | -1.57 | 0.352 | -1.29 |
| 1456116_at | Ctnnd2 | catenin (cadherin associated protein), delta 2 | -1.02 | 0.924 | -1.14 | 0.751 | -1.52 | 0.08 | -1.28 | 0.476 | -1.24 |
| 1447575_at | --- | --- | -1.01 | 0.808 | -1.12 | 0.738 | -1.59 | 0.268 | -1.13 | 0.383 | -1.21 |
| 1443779_s_at | Lcor | ligand dependent nuclear receptor corepressor | -1.02 | 0.826 | -1.17 | 0.038 | -1.49 | 0.012 | -1.26 | 0.003 | -1.23 |
| 1455158_at | Itga3 | integrin alpha 3 | -1.25 | 0.052 | -1.17 | 0.124 | -1.17 | 0.421 | -1.07 | 0.819 | -1.16 |
| 1449410_a_at | Gas5 | growth arrest specific 5 | -1.14 | 0.102 | -1.14 | 0.337 | -1.33 | 0.179 | -1.46 | 0.004 | -1.27 |
| 1419916_at | Rnf20 | Ring finger protein 20 | -1.43 | 0.496 | -1.05 | 0.897 | -1.16 | 0.412 | -1.08 | 0.668 | -1.18 |
| 1431425_a_at | 4930535B03Rik | RIKEN cDNA 4930535B03 gene | -1.18 | 0.245 | -1.27 | 0.697 | -1.13 | 0.44 | -1.18 | 0.537 | -1.19 |
| 1443899_at | BC028799 /// 49225 | cDNA sequence BC028799 /// RIKEN cDNA 4922501C03 gene | -1.15 | 0.327 | -1.08 | 0.628 | -1.4 | 0.069 | -1.15 | 0.409 | -1.19 |
| 1444772_at | Smyd3 | SET and MYND domain containing 3 | -1.33 | 0.31 | -1.13 | 0.522 | -1.15 | 0.328 | -1.4 | 0.119 | -1.25 |
| 1441359_at | 9230115F04Rik | RIKEN cDNA 9230115F04 gene | -1.1 | 0.641 | -1.28 | 0.503 | -1.22 | 0.102 | -2.71 | 0.062 | -1.58 |
| 1429806_at | Eral1 | Era (G-protein)-like 1 (E. coli) | -1.01 | 0.968 | -1.27 | 0.292 | -1.37 | 0.363 | -1.24 | 0.36 | -1.22 |
| 1421742_at | Gdi2 | guanosine diphosphate (GDP) dissociation inhibitor 2 | -1.16 | 0.194 | -1.12 | 0.725 | -1.33 | 0.513 | -1.89 | 0.099 | -1.37 |
| 1416064_a_at | Hspa5 | heat shock 70kD protein 5 (glucose-regulated protein) | -1.12 | 0.591 | -1.09 | 0.647 | -1.43 | 0.088 | -1.28 | 0.063 | -1.23 |
| 1436344_at | --- | --- | -1.2 | 0.324 | -1.3 | 0.084 | -1.1 | 0.646 | -1.29 | 0.063 | -1.22 |
| 1460317_s_at | Gna13 | guanine nucleotide binding protein, alpha 13 | -1.33 | 0.221 | -1.12 | 0.362 | -1.16 | 0.592 | -1.22 | 0.15 | -1.21 |
| 1447700_x_at | Ss18l1 | synovial sarcoma translocation gene on chromosome 18-like 1 | -1.15 | 0.323 | -1.18 | 0.775 | -1.26 | 0.247 | -1.51 | 0.269 | -1.27 |
| 1417747_at | Cplx1 | complexin 1 | -1.16 | 0.39 | -1.39 | 0.101 | -1.07 | 0.679 | -1.57 | 0.011 | -1.3 |
| 1438876_at | A130014A01Rik | RIKEN cDNA A130014A01 gene | -1.43 | 0.081 | -1.12 | 0.686 | -1.09 | 0.725 | -1.24 | 0.584 | -1.22 |
| 1437706_x_at | Rps14 /// LOC6728 | ribosomal protein S14 /// similar to ribosomal protein S14 | -1.13 | 0.057 | -1.17 | 0.186 | -1.3 | 0.007 | -1.19 | 0.039 | -1.2 |
| 1433732_x_at | Igf2bp3 | insulin-like growth factor 2 mRNA binding protein 3 | -1.09 | 0.655 | -1.29 | 0.216 | -1.22 | 0.336 | -1.23 | 0.352 | -1.21 |
| 1417388_at | Bex2 | brain expressed X-linked 2 | -1.01 | 0.968 | -1.11 | 0.806 | -1.61 | 0.163 | -1.14 | 0.709 | -1.22 |
| 1447432_s_at | Zfp263 | zinc finger protein 263 | -1.16 | 0.37 | -1.07 | 0.651 | -1.39 | 0.057 | -1.71 | 0.117 | -1.33 |
| 1452061_s_at | Strbp | spermatid perinuclear RNA binding protein | -1.28 | 0.123 | -1.22 | 0.104 | -1.1 | 0.39 | -1.94 | 0.152 | -1.38 |
| 1443843_x_at | LOC433546 | similar to ribosomal protein L9 | -1.15 | 0.061 | -1.14 | 0.536 | -1.31 | 0.001 | -1.25 | 0.004 | -1.21 |
| 1418433_at | Cab39 | calcium binding protein 39 | -1.14 | 0.388 | -1.19 | 0.112 | -1.26 | 0.116 | -1.07 | 0.688 | -1.16 |
| 1452141_a_at | Sepp1 | selenoprotein P, plasma, 1 | -1.07 | 0.045 | -1.17 | 0.435 | -1.38 | 0.001 | -1.13 | 0.338 | -1.19 |
| 1429503_at | 2900024C23Rik | RIKEN cDNA 2900024C23 gene | -1.36 | 0.111 | -1.15 | 0.347 | -1.1 | 0.505 | -1.68 | 0.134 | -1.32 |
| 1458079_at | Usp40 | ubiquitin specific peptidase 40 | -1.11 | 0.096 | -1.16 | 0.388 | -1.33 | 0.315 | -1.7 | 0.065 | -1.33 |
| 1439804_at | 2310015A10Rik | RIKEN cDNA 2310015A10 gene | -1.69 | 0.204 | -1.05 | 0.814 | -1.03 | 0.902 | -1.24 | 0.53 | -1.25 |

| | | | | | | | | | | | |
|---|---|---|---|---|---|---|---|---|---|---|---|
| 1422626_at | Mmp16 | matrix metallopeptidase 16 | -1.46 | 0.436 | -1.13 | 0.72 | -1.05 | 0.878 | -1.28 | 0.136 | -1.23 |
| 1436506_a_at | 1110008H02Rik | RIKEN cDNA 1110008H02 gene | -1.3 | 0.026 | -1.05 | 0.534 | -1.26 | 0.154 | -1.42 | 0.003 | -1.26 |
| 1430245_at | --- | --- | -1.23 | 0.262 | -1.07 | 0.64 | -1.3 | 0.116 | -1.16 | 0.632 | -1.19 |
| 1428495_at | 2410003K15Rik | RIKEN cDNA 2410003K15 gene | -1.13 | 0.126 | -1.23 | 0.023 | -1.23 | 0.153 | -1.17 | 0.368 | -1.19 |
| 1456702_x_at | Mat2a | methionine adenosyltransferase II, alpha | -1.24 | 0.523 | -1.13 | 0.479 | -1.22 | 0.189 | -1.21 | 0.11 | -1.2 |
| 1455073_at | Cdadc1 | cytidine and dCMP deaminase domain containing 1 | -1.1 | 0.503 | -1.08 | 0.349 | -1.48 | 0.099 | -1.21 | 0.301 | -1.22 |
| 1418607_at | Zfp99 | zinc finger protein 99 | -1.12 | 0.205 | -1.27 | 0.152 | -1.19 | 0.349 | -1.23 | 0.201 | -1.2 |
| 1431190_x_at | Fahd2a | fumarylacetoacetate hydrolase domain containing 2A | -1.17 | 0.156 | -1.28 | 0.107 | -1.14 | 0.262 | -1.16 | 0.379 | -1.19 |
| 1442715_at | --- | --- | -1.48 | 0.124 | -1.14 | 0.859 | -1.03 | 0.887 | -1.04 | 0.907 | -1.17 |
| 1434596_at | --- | PREDICTED: Mus musculus hypothetical protein LOC625568 (LOC625568), mR | -1.09 | 0.429 | -1.08 | 0.907 | -1.49 | 0.273 | -1.88 | 0.22 | -1.38 |
| 1452501_at | Cyp2c38 | cytochrome P450, family 2, subfamily c, polypeptide 38 | -1.13 | 0.439 | -1.11 | 0.54 | -1.37 | 0.109 | -1.92 | 0.169 | -1.38 |
| 1445998_at | Prkg1 | Protein kinase, cGMP-dependent, type I | -1.01 | 0.907 | -1.1 | 0.886 | -1.61 | 0.06 | -1.36 | 0.578 | -1.27 |
| 1430065_at | A930005N03Rik | RIKEN cDNA A930005N03 gene | -1.25 | 0.743 | -1.19 | 0.759 | -1.14 | 0.588 | -2.3 | 0.122 | -1.47 |
| 1453158_at | Zfp622 | zinc finger protein 622 | -1.19 | 0.814 | -1.22 | 0.749 | -1.17 | 0.579 | -1.25 | 0.658 | -1.21 |
| 1416841_at | 1110059E24Rik | RIKEN cDNA 1110059E24 gene | -1.27 | 0.113 | -1.19 | 0.001 | -1.12 | 0.374 | -1.77 | 0.11 | -1.34 |
| 1453804_a_at | Orc4l | origin recognition complex, subunit 4-like (S. cerevisiae) | -1.09 | 0.361 | -1.29 | 0.038 | -1.21 | 0.094 | -1.21 | 0.18 | -1.2 |
| 1416795_at | Cryl1 | crystallin, lamda 1 | -1.32 | 0.077 | -1.31 | 0.497 | -1 | 0.985 | -1.02 | 0.925 | -1.16 |
| 1415970_at | Cox6c | cytochrome c oxidase, subunit VIc | -1.22 | 0.057 | -1.19 | 0.08 | -1.16 | 0.133 | -1.08 | 0.156 | -1.17 |
| 1438555_x_at | 4933405I11Rik /// M | RIKEN cDNA 4933405I11 gene /// mucin 4 | -1.32 | 0.468 | -1.11 | 0.849 | -1.16 | 0.157 | -3.29 | 0.292 | -1.72 |
| 1443528_at | Ttbk1 | tau tubulin kinase 1 | -1.22 | 0.54 | -1.41 | 0.266 | -1.01 | 0.951 | -1.02 | 0.775 | -1.17 |
| 1436960_at | Brd3 | bromodomain containing 3 | -1.1 | 0.104 | -1.18 | 0.514 | -1.32 | 0.202 | -1.09 | 0.022 | -1.17 |
| 1433856_at | Hisppd1 | histidine acid phosphatase domain containing 1 | -1.3 | 0.067 | -1.19 | 0.434 | -1.1 | 0.185 | -1.07 | 0.328 | -1.17 |
| 1452961_at | 1200009O22Rik | RIKEN cDNA 1200009O22 gene | -1.29 | 0.278 | -1.2 | 0.434 | -1.1 | 0.541 | -1.17 | 0.218 | -1.19 |
| 1416446_at | Tmem30a | transmembrane protein 30A | -1.02 | 0.749 | -1.28 | 0.036 | -1.32 | 0.073 | -1.38 | 0.239 | -1.25 |
| 1451133_s_at | Tmem168 | transmembrane protein 168 | -1.07 | 0.779 | -1.11 | 0.529 | -1.45 | 0.034 | -1.48 | 0.181 | -1.28 |
| 1452206_at | Sucla2 | succinate-Coenzyme A ligase, ADP-forming, beta subunit | -1.15 | 0.24 | -1.24 | 0.026 | -1.19 | 0.181 | -1.13 | 0.08 | -1.18 |
| 1434485_a_at | Ugp2 | UDP-glucose pyrophosphorylase 2 | -1.19 | 0.077 | -1.34 | 0.206 | -1.08 | 0.527 | -1.16 | 0.556 | -1.19 |
| 1451199_at | Qtrtd1 | queuine tRNA-ribosyltransferase domain containing 1 | -1.29 | 0.094 | -1.27 | 0.487 | -1.05 | 0.724 | -1.35 | 0.117 | -1.24 |
| 1438736_at | Thoc2 | THO complex 2 | -1.1 | 0.557 | -1.13 | 0.125 | -1.38 | 0.045 | -1.07 | 0.815 | -1.17 |
| 1433942_at | Myo6 | myosin VI | -1.1 | 0.299 | -1.34 | 0.01 | -1.16 | 0.491 | -1.17 | 0.015 | -1.19 |
| 1453776_at | 5730407K14Rik | RIKEN cDNA 5730407K14 gene | -1.1 | 0.399 | -1.18 | 0.449 | -1.32 | 0.176 | -1.38 | 0.002 | -1.24 |
| 1447423_at | --- | --- | -1.24 | 0.46 | -1.01 | 0.98 | -1.39 | 0.145 | -1.06 | 0.906 | -1.17 |
| 1453701_at | 4921515E04Rik | RIKEN cDNA 4921515E04 gene | -1.33 | 0.693 | -1.17 | 0.789 | -1.09 | 0.667 | -3.04 | 0.02 | -1.66 |
| 1456761_at | D630030B22Rik | RIKEN cDNA D630030B22 gene | -1.37 | 0.433 | -1.16 | 0.674 | -1.08 | 0.807 | -1.06 | 0.892 | -1.17 |
| 1450043_at | Fzd7 | frizzled homolog 7 (Drosophila) | -1.04 | 0.889 | -1.51 | 0.502 | -1.12 | 0.557 | -1.5 | 0.195 | -1.29 |
| 1441331_at | A230061C15Rik | RIKEN cDNA A230061C15 gene | -1.13 | 0.256 | -1.06 | 0.595 | -1.46 | 0.077 | -1.84 | 0.172 | -1.37 |
| 1454822_x_at | Apcdd1 | adenomatosis polyposis coli down-regulated 1 | -1.35 | 0.313 | -1.01 | 0.968 | -1.27 | 0.587 | -1.24 | 0.385 | -1.22 |
| 1458607_at | Tgfbr2 | Transforming growth factor, beta receptor II | -1.05 | 0.933 | -1.18 | 0.754 | -1.39 | 0.088 | -1.61 | 0.136 | -1.31 |
| 1452708_a_at | Luc7l | Luc7 homolog (S. cerevisiae)-like | -1.16 | 0.723 | -1.13 | 0.509 | -1.29 | 0.083 | -1.13 | 0.668 | -1.18 |
| 1427269_at | Sfrs11 | splicing factor, arginine/serine-rich 11 | -1.21 | 0.451 | -1.24 | 0.088 | -1.13 | 0.222 | -1.14 | 0.35 | -1.18 |
| 1424519_at | Mtg1 | mitochondrial GTPase 1 homolog (S. cerevisiae) | -1.12 | 0.354 | -1.38 | 0.009 | -1.11 | 0.462 | -1.02 | 0.788 | -1.16 |
| 1446483_at | Akr1e1 | aldo-keto reductase family 1, member E1 | -1.08 | 0.657 | -1.32 | 0.195 | -1.2 | 0.491 | -1.8 | 0.344 | -1.35 |
| 1417007_a_at | Vps4b | vacuolar protein sorting 4b (yeast) | -1.2 | 0.438 | -1.25 | 0.05 | -1.13 | 0.252 | -1.48 | 0.114 | -1.26 |
| 1416978_at | Fcgrt | Fc receptor, IgG, alpha chain transporter | -1.1 | 0.437 | -1.15 | 0.096 | -1.36 | 0.204 | -1.03 | 0.932 | -1.16 |
| 1449055_x_at | Pcbp4 | poly(rC) binding protein 4 | -1.29 | 0.119 | -1.08 | 0.645 | -1.22 | 0.002 | -1.59 | 0.121 | -1.3 |
| 1427484_at | Eml5 | echinoderm microtubule associated protein like 5 | -1.26 | 0.004 | -1.15 | 0.476 | -1.17 | 0.726 | -1.11 | 0.594 | -1.17 |
| 1445163_at | --- | --- | -1.25 | 0.808 | -1.27 | 0.416 | -1.07 | 0.655 | -1.04 | 0.964 | -1.16 |
| 1457082_at | Foxp3 | forkhead box P3 | -1.38 | 0.688 | -1.05 | 0.929 | -1.19 | 0.552 | -2.4 | 0.068 | -1.5 |
| 1457176_at | Garnl1 | GTPase activating RANGAP domain-like 1 | -1.07 | 0.521 | -1.23 | 0.382 | -1.29 | 0.238 | -1.44 | 0.18 | -1.26 |
| 1431638_at | 4930592A05Rik | RIKEN cDNA 4930592A05 gene | -1.01 | 0.824 | -1.35 | 0.194 | -1.26 | 0.552 | -1.77 | 0.247 | -1.35 |
| 1460411_s_at | AW548124 | expressed sequence AW548124 | -1.24 | 0.232 | -1.35 | 0.163 | -1.02 | 0.904 | -1.35 | 0.424 | -1.24 |
| 1438283_at | 3110057O12Rik | RIKEN cDNA 3110057O12 gene | -1.39 | 0.301 | -1.05 | 0.89 | -1.18 | 0.429 | -1 | 0.99 | -1.16 |
| 1422595_s_at | 5730470L24Rik | RIKEN cDNA 5730470L24 gene | -1.06 | 0.736 | -1.13 | 0.163 | -1.44 | 0.009 | -1.52 | 0.207 | -1.29 |

| | | | | | | | | | | | |
|---|---|---|---|---|---|---|---|---|---|---|---|
| 1460663_at | Cckbr | cholecystokinin B receptor | -1.15 | 0.109 | -1.05 | 0.677 | -1.43 | 0.554 | -1.24 | 0.487 | -1.22 |
| 1456548_at | Mtdh | Metadherin | -1.04 | 0.951 | -1.28 | 0.166 | -1.28 | 0.285 | -1.15 | 0.528 | -1.19 |
| 1448271_a_at | Ddx21 | DEAD (Asp-Glu-Ala-Asp) box polypeptide 21 | -1.36 | 0.043 | -1.18 | 0.01 | -1.07 | 0.659 | -1.09 | 0.719 | -1.17 |
| 1438868_at | D14Ertd668e | DNA segment, Chr 14, ERATO Doi 668, expressed | -1.43 | 0.052 | -1.08 | 0.317 | -1.12 | 0.497 | -1.79 | 0.053 | -1.35 |
| 1416056_a_at | Ndufb11 | NADH dehydrogenase (ubiquinone) 1 beta subcomplex, 11 | -1.1 | 0.135 | -1.17 | 0.069 | -1.31 | 0.025 | -1.26 | 0.265 | -1.21 |
| 1460391_at | 2810409H07Rik | RIKEN cDNA 2810409H07 gene | -1.18 | 0.436 | -1.19 | 0.028 | -1.19 | 0.51 | -1.07 | 0.809 | -1.16 |
| 1457889_at | AU014972 | expressed sequence AU014972 | -1.01 | 0.974 | -1.18 | 0.687 | -1.47 | 0.124 | -1.14 | 0.469 | -1.2 |
| 1441003_at | Ercc4 | excision repair cross-complementing rodent repair deficiency, complementation g | -1.26 | 0.437 | -1.08 | 0.775 | -1.24 | 0.257 | -1.22 | 0.569 | -1.2 |
| 1455729_at | Gnaq | guanine nucleotide binding protein, alpha q polypeptide | -1.11 | 0.535 | -1.08 | 0.573 | -1.44 | 0.011 | -1.2 | 0.582 | -1.21 |
| 1455717_s_at | Daam2 | dishevelled associated activator of morphogenesis 2 | -1.11 | 0.546 | -1.07 | 0.635 | -1.45 | 0.333 | -1.06 | 0.636 | -1.17 |
| 1450937_at | Lin7c | lin-7 homolog C (C. elegans) | -1.29 | 0.12 | -1.05 | 0.659 | -1.26 | 0.128 | -1.4 | 0.132 | -1.25 |
| 1421852_at | Kcnk5 | potassium channel, subfamily K, member 5 | -1.31 | 0.379 | -1.31 | 0.484 | -1 | 0.983 | -1.48 | 0.26 | -1.28 |
| 1437240_at | Pgm2 | phosphoglucomutase 2 | -1.16 | 0.542 | -1.38 | 0.211 | -1.06 | 0.833 | -1.67 | 0.272 | -1.32 |
| 1456383_at | Rsl1d1 | ribosomal L1 domain containing 1 | -1.29 | 0.146 | -1.27 | 0.004 | -1.05 | 0.813 | -1.1 | 0.763 | -1.17 |
| 1438506_s_at | Pdss1 | Prenyl (solanesyl) diphosphate synthase, subunit 1 | -1.25 | 0.49 | -1.06 | 0.708 | -1.29 | 0.224 | -1.77 | 0.113 | -1.34 |
| 1428921_at | 2810021B07Rik | RIKEN cDNA 2810021B07 gene | -1.05 | 0.625 | -1.2 | 0.348 | -1.36 | 0.023 | -1.24 | 0.382 | -1.21 |
| 1417766_at | Cyb5b | cytochrome b5 type B | -1.2 | 0.12 | -1.1 | 0.402 | -1.28 | 0.041 | -1.3 | 0.238 | -1.22 |
| 1458077_at | Baz2b | Bromodomain adjacent to zinc finger domain, 2B | -1.11 | 0.678 | -1.25 | 0.134 | -1.22 | 0.353 | -1.74 | 0.192 | -1.33 |
| 1427097_at | Wwp1 | WW domain containing E3 ubiquitin protein ligase 1 | -1.21 | 0.135 | -1.17 | 0.044 | -1.18 | 0.294 | -1.17 | 0.44 | -1.18 |
| 1418007_at | 1810007M14Rik | RIKEN cDNA 1810007M14 gene | -1.33 | 0.309 | -1.15 | 0.328 | -1.11 | 0.59 | -1.23 | 0.432 | -1.2 |
| 1447964_at | Ttl | tubulin tyrosine ligase | -1.25 | 0.436 | -1.28 | 0.541 | -1.06 | 0.873 | -1.34 | 0.582 | -1.23 |
| 1435106_at | 3732412D22Rik | RIKEN cDNA 3732412D22 gene | -1.19 | 0.19 | -1.02 | 0.912 | -1.42 | 0.102 | -1.67 | 0.168 | -1.33 |
| 1451495_at | Wac | WW domain containing adaptor with coiled-coil | -1.18 | 0.292 | -1.14 | 0.242 | -1.25 | 0.125 | -1.12 | 0.106 | -1.17 |
| 1431467_at | 4931402G19Rik | RIKEN cDNA 4931402G19 gene | -1.43 | 0 | -1.16 | 0.656 | -1.04 | 0.684 | -1.73 | 0.533 | -1.34 |
| 1435164_s_at | Ube1c | ubiquitin-activating enzyme E1C | -1.32 | 0.171 | -1.08 | 0.507 | -1.2 | 0.229 | -1.19 | 0.413 | -1.19 |
| 1438390_s_at | Pttg1 | pituitary tumor-transforming 1 | -1.09 | 0.342 | -1.28 | 0.147 | -1.21 | 0.037 | -1.17 | 0.33 | -1.19 |
| 1427542_at | 5330439J01Rik | RIKEN cDNA 5330439J01 gene | -1.08 | 0.663 | -1.4 | 0.224 | -1.14 | 0.655 | -1.24 | 0.22 | -1.21 |
| 1448702_at | Ier3ip1 | immediate early response 3 interacting protein 1 | -1.18 | 0.087 | -1.03 | 0.642 | -1.42 | 0.001 | -1.08 | 0.618 | -1.18 |
| 1430318_at | Sat2 | spermidine/spermine N1-acetyl transferase 2 | -1.24 | 0.299 | -1.11 | 0.009 | -1.22 | 0.269 | -1.23 | 0.277 | -1.2 |
| 1418515_at | Mtf2 | metal response element binding transcription factor 2 | -1.07 | 0.636 | -1.28 | 0.091 | -1.24 | 0.299 | -1.02 | 0.884 | -1.15 |
| 1455387_at | Nufip2 | nuclear fragile X mental retardation protein interacting protein 2 | -1.14 | 0.352 | -1.04 | 0.695 | -1.46 | 0.078 | -1.25 | 0.576 | -1.22 |
| 1428826_at | Nr6a1 | nuclear receptor subfamily 6, group A, member 1 | -1.02 | 0.705 | -1.51 | 0.051 | -1.13 | 0.375 | -1.1 | 0.843 | -1.19 |
| 1456729_x_at | Rtel1 | regulator of telomere elongation helicase 1 | -1.14 | 0.698 | -1.42 | 0.094 | -1.06 | 0.773 | -1.23 | 0.196 | -1.21 |
| 1441981_at | Tmprss11f | transmembrane protease, serine 11f | -1.08 | 0.922 | -1.36 | 0.646 | -1.16 | 0.721 | -2.24 | 0.117 | -1.46 |
| 1421089_a_at | 2610028A01Rik | RIKEN cDNA 2610028A01 gene | -1.11 | 0.431 | -1.24 | 0.509 | -1.22 | 0.506 | -1.26 | 0.116 | -1.21 |
| 1455077_a_at | Morf4l1 /// LOC433! | mortality factor 4 like 1 /// similar to mortality factor 4 like 1 isoform b /// RIKEN cD | -1.14 | 0.043 | -1.07 | 0.154 | -1.4 | 0.005 | -1.24 | 0.05 | -1.21 |
| 1423504_at | Jam3 | junction adhesion molecule 3 | -1.05 | 0.782 | -1.01 | 0.977 | -1.72 | 0.109 | -1.35 | 0.531 | -1.28 |
| 1434481_at | 4121402D02Rik | RIKEN cDNA 4121402D02 gene | -1.09 | 0.606 | -1.31 | 0.084 | -1.19 | 0.039 | -1.14 | 0.323 | -1.18 |
| 1421993_a_at | Tmem134 | transmembrane protein 134 | -1.23 | 0.459 | -1.25 | 0.36 | -1.09 | 0.789 | -1.24 | 0.605 | -1.2 |
| 1418313_at | Zfp276 | zinc finger protein (C2H2 type) 276 | -1.31 | 0.375 | -1.25 | 0.344 | -1.04 | 0.729 | -1.14 | 0.704 | -1.19 |
| 1428282_at | Tbce | tubulin-specific chaperone e | -1.18 | 0.384 | -1.11 | 0.316 | -1.29 | 0.069 | -1.23 | 0.216 | -1.2 |
| 1431726_a_at | Tmem80 | transmembrane protein 80 | -1.12 | 0.645 | -1.25 | 0.021 | -1.19 | 0.237 | -1.06 | 0.25 | -1.16 |
| 1445321_at | E430025E21Rik | RIKEN cDNA E430025E21 gene | -1.03 | 0.907 | -1.49 | 0.01 | -1.13 | 0.47 | -1.33 | 0.426 | -1.25 |
| 1437322_at | Rbm4 | RNA binding motif protein 4 | -1.08 | 0.582 | -1.21 | 0.394 | -1.29 | 0.304 | -1.09 | 0.183 | -1.17 |
| 1428149_at | Coro7 | coronin 7 | -1.03 | 0.93 | -1.29 | 0.04 | -1.29 | 0.137 | -1.71 | 0.121 | -1.33 |
| 1453518_at | Pdzd11 | PDZ domain containing 11 | -1.57 | 0.056 | -1.01 | 0.988 | -1.12 | 0.778 | -1.32 | 0.441 | -1.25 |
| 1435302_at | Taf4b | TAF4B RNA polymerase II, TATA box binding protein (TBP)-associated factor | -1.23 | 0.607 | -1.07 | 0.775 | -1.29 | 0.101 | -1.14 | 0.303 | -1.18 |
| 1417285_a_at | Ndufa5 | NADH dehydrogenase (ubiquinone) 1 alpha subcomplex, 5 | -1.11 | 0.082 | -1.15 | 0.234 | -1.32 | 0.009 | -1.11 | 0.434 | -1.17 |
| 1443205_at | D5Buc30e | DNA segment, Chr 5, Bucan 30 expressed | -1.5 | 0.291 | -1.06 | 0.858 | -1.09 | 0.846 | -1.18 | 0.714 | -1.21 |
| 1438591_at | 2610019A05Rik | RIKEN cDNA 2610019A05 gene | -1.33 | 0.278 | -1.11 | 0.76 | -1.14 | 0.63 | -1.32 | 0.11 | -1.23 |
| 1436667_at | Slc6a20 | solute carrier family 6 (neurotransmitter transporter), member 20 | -1.09 | 0.502 | -1.06 | 0.853 | -1.51 | 0.397 | -1.23 | 0.166 | -1.22 |
| 1435070_at | Aebp2 | AE binding protein 2 | -1.1 | 0.602 | -1.1 | 0.328 | -1.41 | 0.068 | -1.28 | 0.162 | -1.22 |
| 1443520_at | AU022870 | Expressed sequence AU022870 | -1.02 | 0.936 | -1.23 | 0.469 | -1.36 | 0.32 | -1.15 | 0.669 | -1.19 |

| | | | | | | | | | | | |
|---|---|---|---|---|---|---|---|---|---|---|---|
| 1460744_at | 2810002I04Rik | RIKEN cDNA 2810002I04 gene | -1.11 | 0.61 | -1.02 | 0.968 | -1.55 | 0.184 | -1.53 | 0.307 | -1.3 |
| 1438296_at | C430010C01 | hypothetical protein C430010C01 | -1.2 | 0.576 | -1.41 | 0.501 | -1.02 | 0.963 | -2.11 | 0.165 | -1.43 |
| 1420023_at | Etf1 | eukaryotic translation termination factor 1 | -1.39 | 0.03 | -1.09 | 0.739 | -1.12 | 0.566 | -1.01 | 0.958 | -1.15 |
| 1434941_s_at | 2610101J03Rik | RIKEN cDNA 2610101J03 gene | -1.06 | 0.766 | -1.09 | 0.531 | -1.5 | 0.021 | -1.06 | 0.716 | -1.18 |
| 1442355_at | LOC546100 | hypothetical LOC546100 | -1.05 | 0.653 | -1.59 | 0.535 | -1.06 | 0.823 | -1.26 | 0.053 | -1.24 |
| 1440909_at | Irgc1 | immunity-related GTPase family, cinema 1 | -1.07 | 0.826 | -1.47 | 0.476 | -1.1 | 0.542 | -1.01 | 0.965 | -1.16 |
| 1416107_at | Nsg2 | neuron specific gene family member 2 | -1.29 | 0.255 | -1.11 | 0.857 | -1.18 | 0.685 | -2.66 | 0.037 | -1.56 |
| 1435689_at | 9030025P20Rik | RIKEN cDNA 9030025P20 gene | -1.21 | 0.269 | -1.28 | 0.059 | -1.08 | 0.632 | -1.65 | 0.075 | -1.31 |
| 1441613_at | 1110033F14Rik | RIKEN cDNA 1110033F14 gene | -1.23 | 0.458 | -1.24 | 0.724 | -1.1 | 0.714 | -1.57 | 0.304 | -1.28 |
| 1454945_at | Lrriq2 | leucine-rich repeats and IQ motif containing 2 | -1.07 | 0.425 | -1.34 | 0.367 | -1.18 | 0.382 | -1.37 | 0.183 | -1.24 |
| 1456220_at | Fbxl7 | F-box and leucine-rich repeat protein 7 | -1.27 | 0.407 | -1.14 | 0.767 | -1.16 | 0.347 | -1.6 | 0.07 | -1.29 |
| 1446053_at | Gpkow | G patch domain and KOW motifs | -1.3 | 0.177 | -1.2 | 0.678 | -1.08 | 0.69 | -1.25 | 0.173 | -1.21 |
| 1438668_x_at | Atxn2l | ataxin 2-like | -1.35 | 0.022 | -1.19 | 0.145 | -1.06 | 0.389 | -1.04 | 0.794 | -1.16 |
| 1449515_at | Zfp292 | zinc finger protein 292 | -1.18 | 0.275 | -1.05 | 0.752 | -1.37 | 0.038 | -1.69 | 0.136 | -1.32 |
| 1428038_at | Gm568 | gene model 568, (NCBI) | -1.16 | 0.872 | -1.18 | 0.745 | -1.22 | 0.651 | -1.31 | 0.313 | -1.22 |
| 1437131_x_at | Mrpl11 | mitochondrial ribosomal protein L11 | -1.01 | 0.892 | -1.44 | 0.089 | -1.18 | 0.716 | -1.27 | 0.228 | -1.22 |
| 1447689_at | Gprasp1 | G protein-coupled receptor associated sorting protein 1 | -1.5 | 0.696 | -1.15 | 0.6 | -1.01 | 0.977 | -1.76 | 0.399 | -1.36 |
| 1416971_at | Cox7a2 | cytochrome c oxidase, subunit VIIa 2 | -1.13 | 0.06 | -1.25 | 0.012 | -1.18 | 0.012 | -1.1 | 0.529 | -1.16 |
| 1453702_at | Ccdc105 | coiled-coil domain containing 105 | -1.03 | 0.971 | -1.51 | 0.482 | -1.11 | 0.865 | -1.6 | 0.465 | -1.31 |
| 1418390_at | Phf21a | PHD finger protein 21A | -1.07 | 0.444 | -1.29 | 0.268 | -1.22 | 0.129 | -1.81 | 0.121 | -1.35 |
| 1443038_at | --- | --- | -1.31 | 0.547 | -1.05 | 0.873 | -1.22 | 0.109 | -1.35 | 0.108 | -1.23 |
| 1453420_at | 1700013G10Rik | RIKEN cDNA 1700013G10 gene | -1.15 | 0.454 | -1.06 | 0.869 | -1.4 | 0.078 | -1.53 | 0.34 | -1.28 |
| 1448063_at | Iqsec2 | IQ motif and Sec7 domain 2 | -1.1 | 0.542 | -1.22 | 0.367 | -1.24 | 0.486 | -2.12 | 0.123 | -1.42 |
| 1421295_at | Chrdl1 | chordin-like 1 | -1.27 | 0.475 | -1.34 | 0.509 | -1.01 | 0.985 | -1.42 | 0.157 | -1.26 |
| 1431494_at | A930008B05Rik | RIKEN cDNA A930008B05 gene | -1.06 | 0.885 | -1.68 | 0.366 | -1.01 | 0.953 | -1.83 | 0.122 | -1.39 |
| 1444638_at | Ttn | titin | -1.59 | 0.116 | -1.02 | 0.941 | -1.09 | 0.692 | -1.21 | 0.444 | -1.23 |
| 1435541_at | Btc | betacellulin, epidermal growth factor family member | -1.37 | 0.295 | -1.17 | 0.661 | -1.06 | 0.714 | -1.08 | 0.845 | -1.17 |
| 1452867_at | Col4a3bp | procollagen, type IV, alpha 3 (Goodpasture antigen) binding protein | -1.24 | 0.237 | -1.11 | 0.25 | -1.22 | 0.074 | -1.19 | 0.348 | -1.19 |
| 1452335_at | 2810423E13Rik | RIKEN cDNA 2810423E13 gene | -1.22 | 0.278 | -1.37 | 0.166 | -1.02 | 0.935 | -1.42 | 0.347 | -1.26 |
| 1417102_a_at | Ndufb5 | NADH dehydrogenase (ubiquinone) 1 beta subcomplex, 5 | -1.08 | 0.221 | -1.2 | 0.144 | -1.29 | 0.033 | -1.38 | 0.221 | -1.24 |
| 1449745_at | --- | Transcribed locus | -1.17 | 0.598 | -1.2 | 0.401 | -1.19 | 0.453 | -1.24 | 0.354 | -1.2 |
| 1427984_at | Senp6 | SUMO/sentrin specific peptidase 6 | -1.11 | 0.774 | -1.47 | 0.499 | -1.06 | 0.794 | -1.01 | 0.965 | -1.16 |
| 1420608_at | Rbm18 | RNA binding motif protein 18 | -1.27 | 0.258 | -1.06 | 0.453 | -1.24 | 0.005 | -1.19 | 0.381 | -1.19 |
| 1426829_at | Rxrip110 | retinoid X receptor interacting protein 110 | -1.16 | 0.514 | -1.17 | 0.058 | -1.22 | 0.099 | -1.28 | 0.184 | -1.21 |
| 1459080_at | --- | --- | -1.26 | 0.611 | -1.08 | 0.761 | -1.23 | 0.646 | -1.05 | 0.901 | -1.16 |
| 1457746_at | 2610028H07Rik | RIKEN cDNA 2610028H07 gene | -1.08 | 0.6 | -1.26 | 0.022 | -1.23 | 0.406 | -1.57 | 0.044 | -1.29 |
| 1437158_at | Nipbl | Nipped-B homolog (Drosophila) | -1.11 | 0.409 | -1.17 | 0.324 | -1.29 | 0.232 | -1.16 | 0.4 | -1.18 |
| 1451275_at | E030041M21Rik | RIKEN cDNA E030041M21 gene | -1.25 | 0.124 | -1.12 | 0.385 | -1.19 | 0.103 | -1.27 | 0.358 | -1.21 |
| 1434106_at | Epm2aip1 | EPM2A (laforin) interacting protein 1 | -1.13 | 0.232 | -1.14 | 0.462 | -1.3 | 0.114 | -1.31 | 0.211 | -1.22 |
| 1451153_a_at | Cyhr1 | cysteine and histidine rich 1 | -1.17 | 0.442 | -1.19 | 0.296 | -1.19 | 0.267 | -1.31 | 0.08 | -1.22 |
| 1416979_at | Pomp | proteasome maturation protein | -1.23 | 0.031 | -1.16 | 0.019 | -1.17 | 0.278 | -1.29 | 0.147 | -1.21 |
| 1443942_at | Gabpb2 | GA repeat binding protein, beta 2 | -1.36 | 0.306 | -1.19 | 0.663 | -1.05 | 0.841 | -1.38 | 0.25 | -1.24 |
| 1434796_at | Vamp4 | vesicle-associated membrane protein 4 | -1.19 | 0.26 | -1.12 | 0.31 | -1.25 | 0.335 | -1.72 | 0.105 | -1.32 |
| 1416379_at | Panx1 | pannexin 1 | -1.33 | 0.045 | -1.23 | 0.766 | -1.04 | 0.86 | -2.11 | 0.142 | -1.43 |
| 1433654_at | Mgea5 | meningioma expressed antigen 5 (hyaluronidase) | -1.13 | 0.557 | -1.32 | 0.14 | -1.12 | 0.391 | -1.33 | 0.285 | -1.23 |
| 1421022_x_at | Acyp1 | acylphosphatase 1, erythrocyte (common) type | -1.39 | 0.097 | -1.14 | 0.2 | -1.07 | 0.534 | -1.2 | 0.531 | -1.2 |
| 1459117_at | --- | Transcribed locus | -1.11 | 0.591 | -1.35 | 0.512 | -1.12 | 0.685 | -2.14 | 0.077 | -1.43 |
| 1444173_at | --- | 8 days neonate male testis cDNA, RIKEN full-length enriched library, clone:G630 | -1.26 | 0.752 | -1.22 | 0.655 | -1.09 | 0.818 | -1.18 | 0.709 | -1.19 |
| 1459623_at | --- | --- | -1.12 | 0.777 | -1.53 | 0.439 | -1.01 | 0.986 | -1.23 | 0.784 | -1.22 |
| 1451403_at | BC024868 | cDNA sequence BC024868 | -1.03 | 0.905 | -1.4 | 0.01 | -1.18 | 0.443 | -1.18 | 0.592 | -1.2 |
| 1417555_at | Atad1 | ATPase family, AAA domain containing 1 | -1.15 | 0.555 | -1.17 | 0.453 | -1.23 | 0.237 | -1.19 | 0.404 | -1.19 |
| 1434806_at | Mtx3 | metaxin 3 | -1.4 | 0.238 | -1.1 | 0.561 | -1.1 | 0.627 | -1.34 | 0.57 | -1.24 |
| 1420532_at | Magi2 | membrane associated guanylate kinase, WW and PDZ domain containing 2 | -1.21 | 0.422 | -1.2 | 0.725 | -1.14 | 0.33 | -1.48 | 0.2 | -1.26 |

| | | | | | | | | | | | |
|---|---|---|---|---|---|---|---|---|---|---|---|
| 1437981_x_at | 1110057K04Rik | RIKEN cDNA 1110057K04 gene | -1.14 | 0.581 | -1.3 | 0.61 | -1.13 | 0.254 | -1.03 | 0.94 | -1.15 |
| 1455502_at | Madd | MAP-kinase activating death domain | -1.14 | 0.596 | -1.46 | 0.1 | -1.03 | 0.887 | -1.67 | 0.059 | -1.32 |
| 1433957_at | C030048B08Rik | RIKEN cDNA C030048B08 gene | -1.08 | 0.573 | -1.1 | 0.597 | -1.42 | 0.237 | -1.46 | 0.061 | -1.27 |
| 1438971_x_at | Ube2h | ubiquitin-conjugating enzyme E2H | -1.31 | 0.004 | -1.21 | 0.25 | -1.06 | 0.725 | -1.06 | 0.562 | -1.16 |
| 1424398_at | Dhx36 | DEAH (Asp-Glu-Ala-His) box polypeptide 36 | -1.01 | 0.988 | -1.36 | 0.038 | -1.24 | 0.391 | -1.14 | 0.682 | -1.19 |
| 1456303_at | Phf14 | PHD finger protein 14 | -1.01 | 0.954 | -1.11 | 0.311 | -1.55 | 0.026 | -1.22 | 0.071 | -1.22 |
| 1452986_at | Hgd | homogentisate 1, 2-dioxygenase | -1.16 | 0.309 | -1.24 | 0.242 | -1.15 | 0.683 | -1.13 | 0.819 | -1.17 |
| 1455483_at | Zfp148 | zinc finger protein 148 | -1.23 | 0.184 | -1.08 | 0.334 | -1.26 | 0.215 | -1.07 | 0.367 | -1.16 |
| 1428240_at | Nrxn1 | neurexin I | -1.17 | 0.77 | -1.17 | 0.517 | -1.21 | 0.259 | -1.16 | 0.091 | -1.18 |
| 1459062_x_at | AU041133 | Expressed sequence AU041133 | -1.1 | 0.906 | -1.18 | 0.699 | -1.29 | 0.107 | -1.35 | 0.312 | -1.23 |
| 1440183_x_at | Coq9 | Coenzyme Q9 homolog (yeast) | -1.19 | 0.254 | -1.18 | 0.154 | -1.18 | 0.452 | -1.35 | 0.263 | -1.23 |
| 1432784_at | 4930556A17Rik | RIKEN cDNA 4930556A17 gene | -1.33 | 0.456 | -1.21 | 0.745 | -1.05 | 0.935 | -1.34 | 0.536 | -1.23 |
| 1450954_at | Yme1l1 | YME1-like 1 (S. cerevisiae) | -1.15 | 0.256 | -1.2 | 0.185 | -1.2 | 0.117 | -1 | 0.966 | -1.14 |
| 1436656_at | BC062109 | cDNA sequence BC062109 | -1.04 | 0.848 | -1.37 | 0.646 | -1.19 | 0.377 | -1.48 | 0.312 | -1.27 |
| 1416000_a_at | Prdx1 | peroxiredoxin 1 | -1.22 | 0.13 | -1.13 | 0.44 | -1.21 | 0.018 | -1.2 | 0.147 | -1.19 |
| 1427075_s_at | Pcmtd2 | protein-L-isoaspartate (D-aspartate) O-methyltransferase domain containing 2 | -1.34 | 0.223 | -1.04 | 0.775 | -1.21 | 0.193 | -1.28 | 0.061 | -1.22 |
| 1436284_s_at | Zfp319 | zinc finger protein 319 | -1.11 | 0.377 | -1.15 | 0.502 | -1.3 | 0.111 | -1.17 | 0.466 | -1.18 |
| 1432403_at | 4933402C06Rik | RIKEN cDNA 4933402C06 gene | -1 | 1 | -1.38 | 0.675 | -1.23 | 0.249 | -1.42 | 0.458 | -1.26 |
| 1430222_at | 9130007G19Rik | RIKEN cDNA 9130007G19 gene | -1.27 | 0.385 | -1 | 0.998 | -1.34 | 0.153 | -2.76 | 0.001 | -1.59 |
| 1451442_at | Ccdc104 | coiled-coil domain containing 104 | -1.1 | 0.502 | -1.34 | 0.09 | -1.14 | 0.301 | -1.05 | 0.54 | -1.16 |
| 1433643_at | Cacna2d1 | calcium channel, voltage-dependent, alpha2/delta subunit 1 | -1.1 | 0.603 | -1.08 | 0.665 | -1.43 | 0.219 | -1.02 | 0.952 | -1.16 |
| 1456204_at | 2010107H07Rik | RIKEN cDNA 2010107H07 gene | -1.02 | 0.527 | -1.17 | 0.385 | -1.41 | 0.077 | -1.03 | 0.892 | -1.16 |
| 1446481_at | Apbb2 | Amyloid beta (A4) precursor protein-binding, family B, member 2 | -1.04 | 0.907 | -1.04 | 0.912 | -1.62 | 0.165 | -1.58 | 0.211 | -1.32 |
| 1447853_x_at | Kif13a | Kinesin family member 13A | -1.03 | 0.303 | -1.35 | 0.554 | -1.21 | 0.168 | -1.02 | 0.955 | -1.15 |
| 1438756_at | Ankrd29 | ankyrin repeat domain 29 | -1.08 | 0.749 | -1.15 | 0.605 | -1.35 | 0.278 | -1.66 | 0.178 | -1.31 |
| 1431701_a_at | Pdzk1 | PDZ domain containing 1 | -1.12 | 0.339 | -1.26 | 0.06 | -1.18 | 0.436 | -2.05 | 0.306 | -1.4 |
| 1448518_at | Timm22 | translocase of inner mitochondrial membrane 22 homolog (yeast) | -1.27 | 0.388 | -1.28 | 0.423 | -1.03 | 0.766 | -1.05 | 0.741 | -1.16 |
| 1441796_at | 2010309E21Rik | RIKEN cDNA 2010309E21 gene | -1.27 | 0.576 | -1.31 | 0.004 | -1.02 | 0.959 | -1.11 | 0.76 | -1.18 |
| 1449664_s_at | Rnf20 | ring finger protein 20 | -1.33 | 0.088 | -1.04 | 0.688 | -1.21 | 0.206 | -1.1 | 0.398 | -1.17 |
| 1457472_at | Tnrc15 | trinucleotide repeat containing 15 | -1.14 | 0.476 | -1.11 | 0.536 | -1.32 | 0.155 | -1.1 | 0.61 | -1.17 |
| 1438080_at | Mrpl11 | mitochondrial ribosomal protein L11 | -1.13 | 0.858 | -1.53 | 0.029 | -1 | 1 | -1.29 | 0.354 | -1.24 |
| 1434732_x_at | Tomm7 | Translocase of outer mitochondrial membrane 7 homolog (yeast) | -1.26 | 0.196 | -1.08 | 0.654 | -1.23 | 0.358 | -1.17 | 0.499 | -1.18 |
| 1443335_at | Pdzrn3 | PDZ domain containing RING finger 3 | -1.06 | 0.792 | -1.13 | 0.589 | -1.41 | 0.088 | -1.2 | 0.549 | -1.2 |
| 1437924_at | --- | --- | -1.24 | 0.143 | -1.33 | 0.045 | -1.02 | 0.856 | -1.03 | 0.653 | -1.16 |
| 1456120_at | 3110001I20Rik | RIKEN cDNA 3110001I20 gene | -1.05 | 0.757 | -1.38 | 0.452 | -1.17 | 0.724 | -1.37 | 0.495 | -1.24 |
| 1433597_at | 9430010O03Rik | RIKEN cDNA 9430010O03 gene | -1.23 | 0.386 | -1.11 | 0.525 | -1.21 | 0.401 | -1.08 | 0.724 | -1.16 |
| 1439210_at | Mrps9 | mitochondrial ribosomal protein S9 | -1.21 | 0.062 | -1.02 | 0.861 | -1.36 | 0.335 | -1.27 | 0.318 | -1.22 |
| 1436115_at | Gm266 | gene model 266, (NCBI) | -1.45 | 0.262 | -1.05 | 0.905 | -1.11 | 0.808 | -3.02 | 0.042 | -1.66 |
| 1444679_at | Phf21a | PHD finger protein 21A | -1.04 | 0.901 | -1.05 | 0.739 | -1.6 | 0.07 | -1.19 | 0.439 | -1.22 |
| 1455664_at | Rtn4rl1 | reticulon 4 receptor-like 1 | -1.17 | 0.445 | -1.37 | 0.352 | -1.05 | 0.823 | -1.77 | 0.121 | -1.34 |
| 1442528_at | Xpo4 | exportin 4 | -1.28 | 0.112 | -1.13 | 0.665 | -1.14 | 0.733 | -1.16 | 0.686 | -1.18 |
| 1457696_at | Rilp | Rab interacting lysosomal protein | -1.17 | 0.152 | -1.24 | 0.15 | -1.14 | 0.387 | -1.43 | 0.364 | -1.24 |
| 1450066_at | Ubr1 | ubiquitin protein ligase E3 component n-recognin 1 | -1.18 | 0.368 | -1.06 | 0.577 | -1.33 | 0.126 | -1.3 | 0.217 | -1.22 |
| 1431009_at | B230219D22Rik | RIKEN cDNA B230219D22 gene | -1.03 | 0.652 | -1.21 | 0.687 | -1.35 | 0.11 | -1.1 | 0.781 | -1.17 |
| 1419702_at | Taf1a | TATA box binding protein (Tbp)-associated factor, RNA polymerase I, A | -1.21 | 0.183 | -1.26 | 0.167 | -1.09 | 0.532 | -1.48 | 0.012 | -1.26 |
| 1419006_s_at | Peli2 | pellino 2 | -1.46 | 0.474 | -1.03 | 0.933 | -1.14 | 0.299 | -1.74 | 0.2 | -1.34 |
| 1422026_at | Pi16 | peptidase inhibitor 16 | -1.09 | 0.755 | -1.39 | 0.412 | -1.1 | 0.776 | -1.78 | 0.591 | -1.34 |
| 1429027_at | 0610007N19Rik | RIKEN cDNA 0610007N19 gene | -1.07 | 0.692 | -1.12 | 0.759 | -1.4 | 0.042 | -1.34 | 0.211 | -1.23 |
| 1444004_at | Thoc2 | THO complex 2 | -1.03 | 0.898 | -1.1 | 0.118 | -1.52 | 0.081 | -1.04 | 0.867 | -1.17 |
| 1436516_at | Abhd13 | abhydrolase domain containing 13 | -1.07 | 0.818 | -1.28 | 0.013 | -1.22 | 0.108 | -1.31 | 0.094 | -1.22 |
| 1421052_a_at | Sms /// LOC67187[illegible] | spermine synthase /// similar to Spermine synthase (Spermidine aminopropyltrans[illegible] | -1.26 | 0.231 | -1.15 | 0.505 | -1.15 | 0.152 | -1.28 | 0.004 | -1.21 |
| 1447112_s_at | Cryl1 | crystallin, lamda 1 | -1.29 | 0.188 | -1.16 | 0.525 | -1.1 | 0.377 | -1.11 | 0.729 | -1.17 |
| 1454890_at | Amot | angiomotin | -1.02 | 0.803 | -1.17 | 0.099 | -1.42 | 0.078 | -1.28 | 0.53 | -1.22 |

| | | | | | | | | | | | |
|---|---|---|---|---|---|---|---|---|---|---|---|
| 1419158_a_at | Harsl | histidyl-tRNA synthetase-like | -1.14 | 0.313 | -1.34 | 0.01 | -1.09 | 0.55 | -1.19 | 0.383 | -1.19 |
| 1440835_at | Zfp27 | zinc finger protein 27 | -1.03 | 0.813 | -1.34 | 0.3 | -1.21 | 0.251 | -1.37 | 0.024 | -1.24 |
| 1416810_at | Mea1 | male enhanced antigen 1 | -1.08 | 0.102 | -1.15 | 0.264 | -1.34 | 0.062 | -1.28 | 0.13 | -1.21 |
| 1449655_a_at | --- | CDNA clone IMAGE:5690974 | -1.03 | 0.532 | -1.6 | 0.171 | -1.05 | 0.867 | -1.87 | 0.155 | -1.39 |
| 1444366_at | Taok3 | TAO kinase 3 | -1.11 | 0.758 | -1.17 | 0.617 | -1.28 | 0.432 | -1.26 | 0.449 | -1.2 |
| 1457944_at | Etv6 | Ets variant gene 6 (TEL oncogene) | -1.03 | 0.864 | -1.06 | 0.439 | -1.59 | 0.234 | -1.51 | 0.082 | -1.3 |
| 1421256_at | Gzmc | granzyme C | -1 | 0.999 | -1.02 | 0.975 | -1.79 | 0.155 | -3 | 0.351 | -1.7 |
| 1415776_at | Aldh3a2 | aldehyde dehydrogenase family 3, subfamily A2 | -1.01 | 0.848 | -1.41 | 0.386 | -1.19 | 0.204 | -1.51 | 0.178 | -1.28 |
| 1459856_at | Mrpl13 | Mitochondrial ribosomal protein L13 | -1.32 | 0.278 | -1.01 | 0.971 | -1.26 | 0.523 | -1.11 | 0.101 | -1.18 |
| 1417842_at | Caml | calcium modulating ligand | -1.29 | 0.119 | -1.09 | 0.162 | -1.18 | 0.168 | -1.14 | 0.444 | -1.17 |
| 1456896_at | 6720462K09Rik | RIKEN cDNA 6720462K09 gene | -1.02 | 0.657 | -1.02 | 0.933 | -1.73 | 0.019 | -1.04 | 0.722 | -1.2 |
| 1424456_at | Pvrl2 | poliovirus receptor-related 2 | -1.09 | 0.647 | -1.45 | 0.011 | -1.07 | 0.392 | -1.28 | 0.316 | -1.22 |
| 1439345_at | Gpnmb | glycoprotein (transmembrane) nmb | -1.26 | 0.53 | -1.32 | 0.519 | -1 | 0.983 | -1.07 | 0.804 | -1.17 |
| 1440840_at | D630004K10Rik | RIKEN cDNA D630004K10 gene | -1.12 | 0.008 | -1.15 | 0.563 | -1.28 | 0.289 | -1 | 0.993 | -1.14 |
| 1442288_at | Anxa6 | Annexin A6 | -1.04 | 0.861 | -1.11 | 0.674 | -1.46 | 0.068 | -1.33 | 0.588 | -1.24 |
| 1418966_a_at | Dcbld1 | discoidin, CUB and LCCL domain containing 1 | -1.13 | 0.61 | -1.13 | 0.368 | -1.3 | 0.149 | -1.28 | 0.071 | -1.21 |
| 1416471_at | Cept1 | choline/ethanolaminephosphotransferase 1 | -1.35 | 0.064 | -1.15 | 0.176 | -1.07 | 0.463 | -1.59 | 0.078 | -1.29 |
| 1442166_at | Cpne5 | copine V | -1.33 | 0.097 | -1.2 | 0.508 | -1.04 | 0.819 | -1.14 | 0.228 | -1.18 |
| 1417736_at | Smc6 | structural maintenance of chromosomes 6 | -1.24 | 0.134 | -1.11 | 0.593 | -1.19 | 0.076 | -1.01 | 0.968 | -1.14 |
| 1442784_at | Zfp608 | Zinc finger protein 608 | -1.03 | 0.732 | -1.19 | 0.775 | -1.37 | 0.397 | -2.78 | 0.024 | -1.59 |
| 1416370_at | Zipro1 | zinc finger proliferation 1 | -1.21 | 0.201 | -1.05 | 0.719 | -1.31 | 0.093 | -1.24 | 0.493 | -1.2 |
| 1428919_at | Fgfr1op | Fgfr1 oncogene partner | -1.17 | 0.081 | -1.1 | 0.464 | -1.28 | 0.122 | -1.38 | 0.251 | -1.23 |
| 1427978_at | 4732418C07Rik | RIKEN cDNA 4732418C07 gene | -1.12 | 0.501 | -1.14 | 0.509 | -1.29 | 0.274 | -1.17 | 0.533 | -1.18 |
| 1420590_at | Has1 | hyaluronan synthase1 | -1.23 | 0.605 | -1.13 | 0.653 | -1.17 | 0.565 | -1.01 | 0.991 | -1.14 |
| 1428067_at | Rasl12 | RAS-like, family 12 | -1.22 | 0.449 | -1.07 | 0.598 | -1.27 | 0.214 | -1 | 0.994 | -1.14 |
| 1436222_at | Gas5 | growth arrest specific 5 | -1.32 | 0.416 | -1.24 | 0.45 | -1.02 | 0.929 | -1.2 | 0.467 | -1.19 |
| 1446550_at | Gspt1 | G1 to S phase transition 1 | -1.06 | 0.423 | -1.07 | 0.748 | -1.5 | 0.019 | -1.12 | 0.519 | -1.19 |
| 1456851_at | Foxp1 | Forkhead box P1 | -1.02 | 0.974 | -1.28 | 0.354 | -1.27 | 0.376 | -2.66 | 0.1 | -1.56 |
| 1434175_s_at | 2210010N04Rik | RIKEN cDNA 2210010N04 gene | -1.28 | 0.294 | -1.22 | 0.669 | -1.06 | 0.772 | -1.7 | 0.314 | -1.31 |
| 1416874_a_at | Paf1 | Paf1, RNA polymerase II associated factor, homolog (S. cerevisiae) | -1.39 | 0.3 | -1.07 | 0.546 | -1.13 | 0.654 | -1.64 | 0.175 | -1.3 |
| 1433672_at | 4732479N06Rik | RIKEN cDNA 4732479N06 gene | -1.15 | 0.066 | -1.33 | 0.027 | -1.09 | 0.301 | -1.2 | 0.271 | -1.19 |
| 1456763_at | AA536749 | expressed sequence AA536749 | -1.01 | 0.912 | -1.15 | 0.339 | -1.45 | 0.094 | -1.22 | 0.342 | -1.21 |
| 1420964_at | Enc1 | ectodermal-neural cortex 1 | -1.27 | 0.019 | -1.2 | 0.8 | -1.09 | 0.886 | -1.22 | 0.848 | -1.19 |
| 1426559_at | Sbno1 | sno, strawberry notch homolog 1 (Drosophila) | -1.16 | 0.319 | -1.2 | 0.01 | -1.17 | 0.04 | -1.15 | 0.149 | -1.17 |
| 1449303_at | Sesn3 | sestrin 3 | -1.32 | 0.044 | -1.15 | 0.378 | -1.09 | 0.779 | -2.24 | 0.068 | -1.45 |
| 1417673_at | Grb14 | growth factor receptor bound protein 14 | -1.13 | 0.342 | -1.31 | 0.013 | -1.12 | 0.475 | -1.66 | 0.518 | -1.3 |
| 1457508_at | C430003N24Rik | RIKEN cDNA C430003N24 gene | -1.03 | 0.908 | -1.08 | 0.678 | -1.54 | 0.071 | -1.36 | 0.367 | -1.25 |
| 1450482_a_at | Pitx2 | paired-like homeodomain transcription factor 2 | -1.14 | 0.717 | -1.15 | 0.699 | -1.25 | 0.477 | -1.04 | 0.942 | -1.15 |
| 1452765_at | LOC674308 | similar to solute carrier family 39 (zinc transporter), member 9 | -1.18 | 0.249 | -1.16 | 0.226 | -1.19 | 0.077 | -1.12 | 0.453 | -1.16 |
| 1421478_a_at | Zfp318 | zinc finger protein 318 | -1.07 | 0.599 | -1.36 | 0.043 | -1.14 | 0.052 | -2.25 | 0.131 | -1.46 |
| 1447990_at | C76332 | expressed sequence C76332 | -1.27 | 0.487 | -1.03 | 0.954 | -1.27 | 0.622 | -3.94 | 0.023 | -1.88 |
| 1452333_at | Smarca2 | SWI/SNF related, matrix associated, actin dependent regulator of chromatin, subf | -1.02 | 0.952 | -1.26 | 0.436 | -1.3 | 0.092 | -1.8 | 0.273 | -1.34 |
| 1433752_s_at | D030016E14Rik | RIKEN cDNA D030016E14 gene | -1.14 | 0.315 | -1.35 | 0.007 | -1.07 | 0.649 | -1.27 | 0.094 | -1.21 |
| 1427475_a_at | LOC669660 | similar to PDZ and LIM domain protein 5 (Enigma homolog) (Enigma-like PDZ an | -1.17 | 0.203 | -1.23 | 0.078 | -1.13 | 0.335 | -1.07 | 0.679 | -1.15 |
| 1436993_x_at | Pfn2 | profilin 2 | -1.28 | 0.137 | -1.22 | 0.507 | -1.05 | 0.754 | -1.52 | 0.175 | -1.27 |
| 1417390_at | Xab1 | XPA binding protein 1 | -1.41 | 0.04 | -1.16 | 0.124 | -1.02 | 0.838 | -1.43 | 0.114 | -1.26 |
| 1428388_at | Tnks2 | tankyrase, TRF1-interacting ankyrin-related ADP-ribose polymerase 2 | -1.1 | 0.133 | -1.03 | 0.818 | -1.48 | 0.297 | -1.53 | 0.226 | -1.29 |
| 1446520_at | Il1rapl2 | interleukin 1 receptor accessory protein-like 2 | -1.05 | 0.937 | -1.12 | 0.777 | -1.43 | 0.373 | -1.07 | 0.898 | -1.17 |
| 1453636_at | Pcgf5 | polycomb group ring finger 5 | -1.09 | 0.467 | -1.12 | 0.633 | -1.36 | 0.232 | -1.91 | 0.156 | -1.37 |
| 1451556_a_at | 2700078E11Rik | RIKEN cDNA 2700078E11 gene | -1.28 | 0.044 | -1.11 | 0.704 | -1.16 | 0.094 | -1.16 | 0.055 | -1.18 |
| 1438104_at | --- | --- | -1.01 | 0.898 | -1.06 | 0.678 | -1.63 | 0.275 | -1.16 | 0.03 | -1.21 |
| 1416281_at | Wdr45l | Wdr45 like | -1.11 | 0.639 | -1.09 | 0.352 | -1.37 | 0.037 | -1.24 | 0.177 | -1.2 |
| 1444310_at | Polr3a | polymerase (RNA) III (DNA directed) polypeptide A | -1.13 | 0.799 | -1.23 | 0.661 | -1.18 | 0.748 | -1.01 | 0.894 | -1.14 |

| | | | | | | | | | | | |
|---|---|---|---|---|---|---|---|---|---|---|---|
| 1434379_at | Mxd4 | Max dimerization protein 4 | -1.01 | 0.988 | -1.13 | 0.505 | -1.49 | 0.058 | -1.56 | 0.169 | -1.3 |
| 1417834_at | Synj2bp | synaptojanin 2 binding protein | -1.1 | 0.358 | -1.19 | 0.058 | -1.24 | 0.054 | -1.18 | 0.11 | -1.18 |
| 1436226_at | Tceb1 | transcription elongation factor B (SIII), polypeptide 1 | -1.06 | 0.388 | -1.07 | 0.33 | -1.48 | 0 | -1.11 | 0.602 | -1.18 |
| 1456460_at | BC068281 | cDNA sequence BC068281 | -1.39 | 0.431 | -1.17 | 0.492 | -1.02 | 0.949 | -1.31 | 0.412 | -1.22 |
| AFFX-MUR_b2_at | --- | --- | -1.13 | 0.334 | -1.09 | 0.255 | -1.35 | 0.111 | -1.08 | 0.706 | -1.16 |
| 1428180_at | 2810422J05Rik | RIKEN cDNA 2810422J05 gene | -1.15 | 0.452 | -1.15 | 0.311 | -1.23 | 0.181 | -1.36 | 0.051 | -1.22 |
| 1442911_at | Riok2 | RIO kinase 2 (yeast) | -1.05 | 0.809 | -1.09 | 0.801 | -1.47 | 0.073 | -1.44 | 0.098 | -1.26 |
| 1425892_a_at | Pnoc | prepronociceptin | -1.28 | 0.716 | -1.11 | 0.744 | -1.15 | 0.699 | -1.2 | 0.552 | -1.18 |
| 1424873_at | Rnf2 | ring finger protein 2 | -1.16 | 0.391 | -1.1 | 0.154 | -1.28 | 0.004 | -1.59 | 0.069 | -1.28 |
| 1434823_x_at | Myeov2 | myeloma overexpressed 2 | -1.16 | 0.209 | -1.1 | 0.026 | -1.28 | 0.176 | -1.49 | 0.117 | -1.26 |
| 1426861_at | Aftph | aftiphilin | -1.23 | 0.3 | -1.05 | 0.64 | -1.27 | 0.072 | -1.16 | 0.553 | -1.18 |
| 1428500_at | 2210419D22Rik | RIKEN cDNA 2210419D22 gene | -1.14 | 0.358 | -1.04 | 0.659 | -1.41 | 0.112 | -1.1 | 0.729 | -1.17 |
| 1428248_at | Nfx1 | nuclear transcription factor, X-box binding 1 | -1.13 | 0.639 | -1.15 | 0.442 | -1.26 | 0.178 | -1.34 | 0.149 | -1.22 |
| 1439901_at | 2610208M17Rik | RIKEN cDNA 2610208M17 gene | -1.12 | 0.681 | -1.05 | 0.833 | -1.42 | 0.008 | -1.34 | 0.127 | -1.23 |
| 1460420_a_at | Egfr | epidermal growth factor receptor | -1.28 | 0.225 | -1.16 | 0.685 | -1.1 | 0.663 | -1.1 | 0.64 | -1.16 |
| 1446938_at | AA408213 | expressed sequence AA408213 | -1.07 | 0.791 | -1.06 | 0.845 | -1.48 | 0.19 | -1.44 | 0.36 | -1.26 |
| 1423331_a_at | Pvrl3 | poliovirus receptor-related 3 | -1.11 | 0.508 | -1.13 | 0.427 | -1.32 | 0.18 | -1.4 | 0.005 | -1.24 |
| 1428825_at | Nr6a1 | nuclear receptor subfamily 6, group A, member 1 | -1.29 | 0.167 | -1.18 | 0.61 | -1.08 | 0.572 | -1.69 | 0.323 | -1.31 |
| 1428853_at | Ptch1 | patched homolog 1 | -1.01 | 0.933 | -1.19 | 0.143 | -1.39 | 0.226 | -1.29 | 0.056 | -1.22 |
| 1449025_at | Ifit3 | interferon-induced protein with tetratricopeptide repeats 3 | -1.18 | 0.43 | -1.13 | 0.681 | -1.22 | 0.444 | -1.55 | 0.3 | -1.27 |
| 1419660_at | 1600012F09Rik | RIKEN cDNA 1600012F09 gene | -1.17 | 0.038 | -1.17 | 0.205 | -1.19 | 0.122 | -1.07 | 0.748 | -1.15 |
| 1419007_at | Zp3 | zona pellucida glycoprotein 3 | -1.05 | 0.95 | -1.25 | 0.647 | -1.25 | 0.308 | -2.79 | 0.496 | -1.59 |
| 1450780_s_at | Hmga2 | high mobility group AT-hook 2 | -1.02 | 0.939 | -1.46 | 0.053 | -1.12 | 0.595 | -1.6 | 0.219 | -1.3 |
| 1425577_at | 9830124H08Rik | RIKEN cDNA 9830124H08 gene | -1.07 | 0.437 | -1.16 | 0.112 | -1.33 | 0.067 | -1.55 | 0.119 | -1.28 |
| 1443001_at | C78344 | expressed sequence C78344 | -1.07 | 0.883 | -1.16 | 0.821 | -1.32 | 0.585 | -1.03 | 0.961 | -1.15 |
| 1432171_at | 4933409F18Rik | RIKEN cDNA 4933409F18 gene | -1.15 | 0.603 | -1.09 | 0.859 | -1.31 | 0.48 | -1.27 | 0.577 | -1.2 |
| 1440846_at | A930041I02Rik | RIKEN cDNA A930041I02 gene | -1.38 | 0.406 | -1.12 | 0.481 | -1.07 | 0.797 | -1.42 | 0.027 | -1.25 |
| 1454422_at | 4930456J16Rik | RIKEN cDNA 4930456J16 gene | -1.02 | 0.842 | -1.22 | 0.573 | -1.34 | 0.011 | -1.01 | 0.975 | -1.14 |
| 1415872_at | Hnrph1 | heterogeneous nuclear ribonucleoprotein H1 | -1.07 | 0.325 | -1.17 | 0.142 | -1.3 | 0.118 | -1.15 | 0.571 | -1.17 |
| 1427020_at | Scara3 | scavenger receptor class A, member 3 | -1.03 | 0.838 | -1.43 | 0.191 | -1.14 | 0.64 | -1.42 | 0.45 | -1.25 |
| 1426803_at | Rbm26 | RNA binding motif protein 26 | -1.29 | 0.315 | -1.22 | 0.102 | -1.04 | 0.754 | -1.17 | 0.588 | -1.18 |
| 1422532_at | Xpc | | -1.21 | 0.095 | -1.22 | 0.069 | -1.11 | 0.508 | -1.38 | 0.151 | -1.23 |
| 1438766_at | Pnrc2 | proline-rich nuclear receptor coactivator 2 | -1.03 | 0.858 | -1.35 | 0.139 | -1.19 | 0.494 | -1.46 | 0.079 | -1.26 |
| 1442002_at | 7030402D04Rik | RIKEN cDNA 7030402D04 gene | -1.08 | 0.643 | -1.13 | 0.356 | -1.35 | 0.153 | -1.1 | 0.405 | -1.16 |
| 1433810_x_at | Ddx5 | DEAD (Asp-Glu-Ala-Asp) box polypeptide 5 | -1.21 | 0.215 | -1.13 | 0.397 | -1.19 | 0.507 | -1.18 | 0.709 | -1.18 |
| 1424047_at | Dera | 2-deoxyribose-5-phosphate aldolase homolog (C. elegans) | -1.28 | 0.106 | -1.22 | 0.103 | -1.05 | 0.717 | -1.17 | 0.323 | -1.18 |
| 1460544_at | Mak10 | MAK10 homolog, amino-acid N-acetyltransferase subunit, (S. cerevisiae) | -1.23 | 0.102 | -1.29 | 0.277 | -1.04 | 0.873 | -1.65 | 0.003 | -1.3 |
| 1419105_at | Nr1h4 | nuclear receptor subfamily 1, group H, member 4 | -1.32 | 0.382 | -1.18 | 0.048 | -1.06 | 0.89 | -1.93 | 0.06 | -1.37 |
| 1444231_at | Zdhhc6 | Zinc finger, DHHC domain containing 6 | -1.09 | 0.3 | -1 | 0.994 | -1.56 | 0.053 | -1.15 | 0.51 | -1.2 |
| 1416067_at | Ifrd1 | interferon-related developmental regulator 1 | -1.05 | 0.878 | -1.29 | 0.156 | -1.21 | 0.116 | -1.03 | 0.696 | -1.14 |
| 1453123_at | Sf3b2 | splicing factor 3b, subunit 2 | -1.21 | 0.072 | -1.28 | 0.207 | -1.06 | 0.67 | -1.16 | 0.234 | -1.18 |
| 1436248_at | --- | Adult male spinal cord cDNA, RIKEN full-length enriched library, clone:A330033L | -1.11 | 0.572 | -1.2 | 0.016 | -1.23 | 0.22 | -1.04 | 0.792 | -1.14 |
| 1441144_at | Arr3 | arrestin 3, retinal | -1.44 | 0.316 | -1.04 | 0.884 | -1.11 | 0.76 | -1.53 | 0.156 | -1.28 |
| 1440893_at | --- | --- | -1.04 | 0.88 | -1.09 | 0.735 | -1.48 | 0.155 | -1.11 | 0.673 | -1.18 |
| 1427876_at | 2610312B22Rik | RIKEN cDNA 2610312B22 gene | -1.11 | 0.483 | -1.2 | 0.154 | -1.22 | 0.054 | -1.16 | 0.044 | -1.17 |
| 1438843_x_at | Mtch2 | mitochondrial carrier homolog 2 (C. elegans) | -1.28 | 0.117 | -1.19 | 0.041 | -1.07 | 0.758 | -1.16 | 0.318 | -1.18 |
| 1426429_at | Thap7 | THAP domain containing 7 | -1.26 | 0.729 | -1.12 | 0.629 | -1.16 | 0.712 | -1.24 | 0.091 | -1.19 |
| 1429556_at | 2610024B07Rik | RIKEN cDNA 2610024B07 gene | -1.22 | 0.424 | -1 | 0.986 | -1.36 | 0.411 | -1.17 | 0.546 | -1.19 |
| 1422806_x_at | Ing3 | inhibitor of growth family, member 3 | -1.28 | 0.49 | -1.15 | 0.307 | -1.1 | 0.503 | -1.79 | 0.13 | -1.33 |
| 1417321_at | D4Wsu132e | DNA segment, Chr 4, Wayne State University 132, expressed | -1.21 | 0.385 | -1.1 | 0.324 | -1.22 | 0.32 | -1.63 | 0.204 | -1.29 |
| 1428321_at | Eml1 /// LOC63410 | echinoderm microtubule associated protein like 1 /// similar to echinoderm microtu | -1.02 | 0.955 | -1.45 | 0.618 | -1.13 | 0.602 | -1.7 | 0.144 | -1.32 |
| 1416154_at | Srp54 /// LOC6651 | signal recognition particle 54 /// similar to signal recognition particle 54 | -1.32 | 0.732 | -1.18 | 0.698 | -1.05 | 0.806 | -1.1 | 0.342 | -1.16 |
| 1429733_at | Ccdc94 | coiled-coil domain containing 94 | -1.2 | 0.269 | -1.11 | 0.758 | -1.21 | 0.08 | -1.32 | 0.156 | -1.21 |

| | | | | | | | | | | | |
|---|---|---|---|---|---|---|---|---|---|---|---|
| 1438695_at | C230091D08Rik | RIKEN cDNA C230091D08 gene | -1.06 | 0.725 | -1.1 | 0.132 | -1.42 | 0.068 | -1.13 | 0.327 | -1.18 |
| 1418117_at | Ndufs4 | NADH dehydrogenase (ubiquinone) Fe-S protein 4 | -1.09 | 0.564 | -1.54 | 0.005 | -1.01 | 0.942 | -1.13 | 0.49 | -1.19 |
| 1417451_a_at | Ppia | peptidylprolyl isomerase A | -1.17 | 0.151 | -1.13 | 0.342 | -1.22 | 0.006 | -1.23 | 0.016 | -1.19 |
| 1434550_at | 3830406C13Rik | RIKEN cDNA 3830406C13 gene | -1.12 | 0.059 | -1.1 | 0.528 | -1.32 | 0.108 | -1.46 | 0.157 | -1.25 |
| 1429000_at | Phf3 | PHD finger protein 3 | -1.1 | 0.454 | -1.18 | 0.313 | -1.25 | 0.157 | -1.01 | 0.968 | -1.13 |
| 1420626_at | 2410016F19Rik | RIKEN cDNA 2410016F19 gene | -1.1 | 0.276 | -1.1 | 0.386 | -1.35 | 0.017 | -1.07 | 0.653 | -1.16 |
| 1442231_at | AI616248 | expressed sequence AI616248 | -1.38 | 0.562 | -1.1 | 0.837 | -1.08 | 0.669 | -1.6 | 0.424 | -1.29 |
| 1455958_s_at | Pptc7 | PTC7 protein phosphatase homolog (S. cerevisiae) | -1.17 | 0.345 | -1.2 | 0.449 | -1.15 | 0.304 | -1.62 | 0.124 | -1.28 |
| 1428556_at | Pigy | phosphatidylinositol glycan anchor biosynthesis, class Y | -1.14 | 0.293 | -1.23 | 0.034 | -1.16 | 0.061 | -1.03 | 0.75 | -1.14 |
| 1437538_at | 2610002F03Rik | RIKEN cDNA 2610002F03 gene | -1.12 | 0.526 | -1.45 | 0.083 | -1.03 | 0.814 | -1.05 | 0.914 | -1.16 |
| 1449800_x_at | Phf7 | PHD finger protein 7 | -1.13 | 0.403 | -1.1 | 0.582 | -1.32 | 0.134 | -1.48 | 0.256 | -1.26 |
| 1435781_at | Cand1 | cullin associated and neddylation disassociated 1 | -1.03 | 0.873 | -1.15 | 0.31 | -1.38 | 0.117 | -1.19 | 0.05 | -1.19 |
| 1439071_at | 5430416N02Rik | RIKEN cDNA 5430416N02 gene | -1.09 | 0.743 | -1.12 | 0.661 | -1.33 | 0.074 | -1.34 | 0.117 | -1.22 |
| 1417250_at | Rnf12 | ring finger protein 12 | -1.05 | 0.716 | -1 | 0.976 | -1.65 | 0.05 | -1.06 | 0.787 | -1.19 |
| 1426489_s_at | Bfar | bifunctional apoptosis regulator | -1.25 | 0.077 | -1.13 | 0.264 | -1.15 | 0.148 | -1.48 | 0.112 | -1.25 |
| 1424489_a_at | Trit1 | tRNA isopentenyltransferase 1 | -1.26 | 0.284 | -1.19 | 0.188 | -1.09 | 0.627 | -1.86 | 0.065 | -1.35 |
| 1423451_at | Pgrmc1 | progesterone receptor membrane component 1 | -1.19 | 0.337 | -1.22 | 0.223 | -1.11 | 0.315 | -1.18 | 0.385 | -1.18 |
| 1443212_at | Large | Like-glycosyltransferase | -1.07 | 0.579 | -1.06 | 0.875 | -1.46 | 0.249 | -1.11 | 0.825 | -1.17 |
| 1428022_at | Lcn13 | lipocalin 13 | -1.11 | 0.735 | -1.26 | 0.599 | -1.15 | 0.456 | -1.37 | 0.17 | -1.22 |
| 1428785_at | Amotl1 | angiomotin-like 1 | -1.09 | 0.29 | -1.23 | 0.34 | -1.2 | 0.391 | -1.12 | 0.464 | -1.16 |
| 1452047_at | Cacybp | calcyclin binding protein | -1.12 | 0.285 | -1.18 | 0.134 | -1.22 | 0.026 | -1.16 | 0.645 | -1.17 |
| 1434851_s_at | Crb3 | crumbs homolog 3 (Drosophila) | -1.03 | 0.794 | -1.18 | 0.103 | -1.34 | 0.088 | -1.46 | 0.134 | -1.25 |
| 1438041_at | --- | --- | -1.01 | 0.973 | -1.14 | 0.598 | -1.45 | 0.115 | -1.93 | 0.133 | -1.38 |
| 1445843_at | Chd2 | chromodomain helicase DNA binding protein 2 | -1.08 | 0.695 | -1.06 | 0.228 | -1.44 | 0.01 | -1.6 | 0.185 | -1.3 |
| 1434555_at | Anp32a | acidic (leucine-rich) nuclear phosphoprotein 32 family, member A | -1.06 | 0.46 | -1.17 | 0.298 | -1.31 | 0.031 | -1.37 | 0.035 | -1.23 |
| 1458870_x_at | Phr1 | pam, highwire, rpm 1 | -1.29 | 0.496 | -1.09 | 0.809 | -1.15 | 0.476 | -1.02 | 0.96 | -1.14 |
| 1428725_at | Pias2 | protein inhibitor of activated STAT 2 | -1.12 | 0.553 | -1.19 | 0.182 | -1.21 | 0.09 | -1.26 | 0.151 | -1.2 |
| 1449291_a_at | Dcbld1 | discoidin, CUB and LCCL domain containing 1 | -1.09 | 0.272 | -1.07 | 0.778 | -1.42 | 0.105 | -1.78 | 0.107 | -1.34 |
| 1433482_a_at | Fubp1 | far upstream element (FUSE) binding protein 1 | -1.09 | 0.684 | -1.5 | 0.13 | -1.03 | 0.871 | -1.67 | 0.2 | -1.32 |
| 1434180_at | Plekhc1 | pleckstrin homology domain containing, family C (with FERM domain) member 1 | -1.02 | 0.608 | -1.15 | 0.351 | -1.41 | 0.062 | -1.48 | 0.216 | -1.26 |
| 1416278_a_at | Atp5o | ATP synthase, H+ transporting, mitochondrial F1 complex, O subunit | -1.15 | 0.191 | -1.23 | 0.081 | -1.15 | 0.389 | -1.06 | 0.525 | -1.14 |
| 1437041_at | 5730406M06Rik | RIKEN cDNA 5730406M06 gene | -1.19 | 0.375 | -1.14 | 0.518 | -1.18 | 0.347 | -1.37 | 0.355 | -1.22 |
| 1418578_at | Dgka | diacylglycerol kinase, alpha | -1.1 | 0.14 | -1.36 | 0.047 | -1.09 | 0.592 | -3.33 | 0.023 | -1.72 |
| 1459537_at | 4631427C17Rik | RIKEN cDNA 4631427C17 gene | -1.54 | 0.004 | -1.03 | 0.921 | -1.06 | 0.718 | -1.44 | 0.112 | -1.27 |
| 1435221_at | --- | Adult male corpora quadrigemina cDNA, RIKEN full-length enriched library, clone | -1.15 | 0.428 | -1.27 | 0.112 | -1.11 | 0.6 | -1.98 | 0.141 | -1.38 |
| 1445056_at | 9230102K24Rik | RIKEN cDNA 9230102K24 gene | -1.36 | 0.595 | -1.06 | 0.889 | -1.14 | 0.78 | -1.18 | 0.475 | -1.18 |
| 1436505_at | Ppig | peptidyl-prolyl isomerase G (cyclophilin G) | -1 | 0.997 | -1.22 | 0.135 | -1.35 | 0.165 | -1.13 | 0.615 | -1.17 |
| 1426365_at | 2810403A07Rik | RIKEN cDNA 2810403A07 gene | -1.22 | 0.416 | -1.27 | 0.057 | -1.04 | 0.833 | -1.24 | 0.214 | -1.19 |
| 1416058_s_at | Atp5c1 | ATP synthase, H+ transporting, mitochondrial F1 complex, gamma polypeptide 1 | -1.25 | 0.008 | -1.3 | 0.011 | -1.01 | 0.849 | -1.05 | 0.675 | -1.15 |
| 1454888_at | Pfdn4 | prefoldin 4 | -1.02 | 0.689 | -1.14 | 0.019 | -1.42 | 0.034 | -1.23 | 0.219 | -1.2 |
| 1438478_a_at | Ppp3ca | protein phosphatase 3, catalytic subunit, alpha isoform | -1.15 | 0.019 | -1.21 | 0.277 | -1.15 | 0.447 | -1.48 | 0.176 | -1.25 |
| 1420456_at | Gcm2 | glial cells missing homolog 2 (Drosophila) | -1.34 | 0.341 | -1.13 | 0.712 | -1.08 | 0.723 | -1.16 | 0.79 | -1.18 |
| 1420628_at | Pura | purine rich element binding protein A | -1.05 | 0.166 | -1.2 | 0.026 | -1.3 | 0.054 | -1.4 | 0.169 | -1.24 |
| 1452611_at | Zfp294 | zinc finger protein 294 | -1.09 | 0.46 | -1.33 | 0.12 | -1.12 | 0.561 | -1.09 | 0.655 | -1.16 |
| 1423781_at | Appbp1 | amyloid beta precursor protein binding protein 1 | -1.17 | 0.446 | -1.03 | 0.663 | -1.36 | 0.039 | -1.43 | 0.016 | -1.25 |
| 1442666_at | D9Ertd12e | DNA segment, Chr 9, ERATO Doi 12, expressed | -1.08 | 0.632 | -1.24 | 0.758 | -1.21 | 0.597 | -1.43 | 0.52 | -1.24 |
| 1423442_a_at | Fbxw2 | F-box and WD-40 domain protein 2 | -1.2 | 0.497 | -1.22 | 0.106 | -1.1 | 0.61 | -1.04 | 0.881 | -1.14 |
| 1415894_at | Enpp2 | ectonucleotide pyrophosphatase/phosphodiesterase 2 | -1.25 | 0.011 | -1.02 | 0.835 | -1.27 | 0.168 | -1.14 | 0.335 | -1.17 |
| 1458900_at | Slc24a3 | Solute carrier family 24 (sodium/potassium/calcium exchanger), member 3 | -1.04 | 0.907 | -1.13 | 0.764 | -1.41 | 0.044 | -1.19 | 0.588 | -1.19 |
| 1429202_at | 2610019N06Rik | RIKEN cDNA 2610019N06 gene | -1.12 | 0.135 | -1.37 | 0.374 | -1.07 | 0.722 | -1.36 | 0.095 | -1.23 |
| 1440348_at | Zfyve9 | zinc finger, FYVE domain containing 9 | -1.16 | 0.439 | -1.13 | 0.284 | -1.23 | 0.183 | -1.27 | 0.263 | -1.2 |
| 1434306_at | Rab3ip | RAB3A interacting protein | -1.07 | 0.304 | -1.13 | 0.449 | -1.35 | 0.007 | -1.65 | 0.023 | -1.3 |
| 1457635_s_at | Nr3c1 | nuclear receptor subfamily 3, group C, member 1 | -1.16 | 0.314 | -1.01 | 0.958 | -1.41 | 0.022 | -1.28 | 0.301 | -1.22 |

| | | | | | | | | | | | |
|---|---|---|---|---|---|---|---|---|---|---|---|
| 1424016_at | 2310007F21Rik | RIKEN cDNA 2310007F21 gene | -1.11 | 0.21 | -1.29 | 0.183 | -1.13 | 0.378 | -1.16 | 0.586 | -1.17 |
| 1439524_at | Dnajb4 | DnaJ (Hsp40) homolog, subfamily B, member 4 | -1.09 | 0.807 | -1.15 | 0.667 | -1.3 | 0.445 | -1.24 | 0.253 | -1.19 |
| 1418407_at | Pde8a | phosphodiesterase 8A | -1.07 | 0.717 | -1.5 | 0.315 | -1.04 | 0.896 | -1.36 | 0.478 | -1.24 |
| 1437288_at | Impad1 | inositol monophosphatase domain containing 1 | -1.03 | 0.611 | -1.07 | 0.577 | -1.53 | 0.058 | -1.08 | 0.577 | -1.18 |
| 1453886_a_at | Slc25a26 | solute carrier family 25 (mitochondrial carrier, phosphate carrier), member 26 | -1.23 | 0.009 | -1.22 | 0.357 | -1.08 | 0.76 | -1.3 | 0.358 | -1.21 |
| 1422525_at | Atp5k | ATP synthase, H+ transporting, mitochondrial F1F0 complex, subunit e | -1.18 | 0.007 | -1.31 | 0.024 | -1.05 | 0.593 | -1.05 | 0.599 | -1.15 |
| 1452153_at | Fbxo18 | F-box protein 18 | -1.12 | 0.291 | -1.2 | 0.083 | -1.19 | 0.129 | -1.22 | 0.383 | -1.18 |
| 1442519_at | Ga17 | Dendritic cell protein GA17 | -1.07 | 0.687 | -1.35 | 0.079 | -1.12 | 0.427 | -1.05 | 0.776 | -1.15 |
| 1454852_at | Sp1 | trans-acting transcription factor 1 | -1.13 | 0.173 | -1.06 | 0.618 | -1.36 | 0.024 | -1.13 | 0.54 | -1.17 |
| 1460052_at | Acsm1 | Acyl-CoA synthetase medium-chain family member 1 | -1.02 | 0.896 | -1.22 | 0.297 | -1.31 | 0.632 | -1.58 | 0.345 | -1.28 |
| 1423298_at | Add3 | adducin 3 (gamma) | -1.23 | 0.134 | -1.1 | 0.688 | -1.19 | 0.327 | -1.78 | 0.169 | -1.33 |
| 1431996_at | --- | --- | -1.13 | 0.413 | -1.14 | 0.548 | -1.25 | 0.517 | -1.03 | 0.932 | -1.14 |
| 1453419_at | 2900078C09Rik | RIKEN cDNA 2900078C09 gene | -1.15 | 0.64 | -1.44 | 0.129 | -1 | 0.972 | -1.55 | 0.201 | -1.28 |
| 1432519_at | 1810059H22Rik | RIKEN cDNA 1810059H22 gene | -1.34 | 0.207 | -1.15 | 0.746 | -1.05 | 0.919 | -1.52 | 0.118 | -1.27 |
| 1417280_at | Slc17a1 | solute carrier family 17 (sodium phosphate), member 1 | -1.05 | 0.261 | -1.2 | 0.192 | -1.28 | 0.674 | -1.28 | 0.327 | -1.2 |
| 1441190_at | Arpc5l | actin related protein 2/3 complex, subunit 5-like | -1.01 | 0.981 | -1.53 | 0.131 | -1.09 | 0.776 | -1.11 | 0.784 | -1.19 |
| 1441944_s_at | Gpr135 | G protein-coupled receptor 135 | -1.31 | 0.448 | -1.15 | 0.789 | -1.08 | 0.896 | -1.36 | 0.332 | -1.22 |
| 1451287_s_at | 2810003C17Rik | RIKEN cDNA 2810003C17 gene | -1.21 | 0.239 | -1.17 | 0.755 | -1.13 | 0.562 | -1.54 | 0.501 | -1.26 |
| 1456594_at | --- | --- | -1.29 | 0.216 | -1.13 | 0.466 | -1.11 | 0.788 | -1.69 | 0.425 | -1.3 |
| 1428681_at | Gm608 | gene model 608, (NCBI) | -1.08 | 0.064 | -1.13 | 0.186 | -1.33 | 0.012 | -1.19 | 0.117 | -1.18 |
| 1460575_at | Eif2a | eukaryotic translation initiation factor 2a | -1.18 | 0.486 | -1.16 | 0.286 | -1.16 | 0 | -1.14 | 0.297 | -1.16 |
| 1439979_at | Zfp398 | zinc finger protein 398 | -1.35 | 0.185 | -1.18 | 0.587 | -1.02 | 0.891 | -1.42 | 0.351 | -1.24 |
| 1416624_a_at | Uba52 /// LOC6665 | ubiquitin A-52 residue ribosomal protein fusion product 1 /// similar to ubiquitin A-! | -1.06 | 0.271 | -1.19 | 0.395 | -1.28 | 0.007 | -1.23 | 0.058 | -1.19 |
| 1416482_at | Ttc3 | tetratricopeptide repeat domain 3 | -1.37 | 0.327 | -1.19 | 0.367 | -1.01 | 0.981 | -1.92 | 0.067 | -1.37 |
| 1426760_at | Ipo8 | importin 8 | -1.02 | 0.757 | -1.31 | 0.055 | -1.21 | 0.095 | -1.06 | 0.798 | -1.15 |
| 1429165_at | 3110001I22Rik | RIKEN cDNA 3110001I22 gene | -1.06 | 0.737 | -1.37 | 0.092 | -1.12 | 0.309 | -1.34 | 0.183 | -1.22 |
| 1424493_s_at | Ugt3a1 | UDP glycosyltransferases 3 family, polypeptide A1 | -1.03 | 0.827 | -1.22 | 0.152 | -1.29 | 0.503 | -1.02 | 0.943 | -1.14 |
| 1458242_at | Gphn | Gephyrin | -1.43 | 0.521 | -1.04 | 0.943 | -1.1 | 0.786 | -1.31 | 0.759 | -1.22 |
| 1450897_at | Arhgap5 | Rho GTPase activating protein 5 | -1.18 | 0.503 | -1.14 | 0.39 | -1.18 | 0.065 | -1.37 | 0.314 | -1.22 |
| 1430546_at | Cryzl1 | crystallin, zeta (quinone reductase)-like 1 | -1.3 | 0.297 | -1.02 | 0.934 | -1.22 | 0.34 | -1.21 | 0.588 | -1.19 |
| 1434178_at | Mll3 | myeloid/lymphoid or mixed-lineage leukemia 3 | -1.08 | 0.509 | -1.1 | 0.498 | -1.36 | 0.114 | -1.06 | 0.576 | -1.15 |
| 1435088_at | Nsd1 | nuclear receptor-binding SET-domain protein 1 | -1.06 | 0.588 | -1.1 | 0.304 | -1.39 | 0.069 | -1.69 | 0.065 | -1.31 |
| 1456154_at | Zfp444 | Zinc finger protein 444 | -1.27 | 0.773 | -1.07 | 0.924 | -1.18 | 0.526 | -1.05 | 0.862 | -1.14 |
| 1426709_a_at | Usp33 | ubiquitin specific peptidase 33 | -1.23 | 0.458 | -1.07 | 0.473 | -1.21 | 0.185 | -1.19 | 0.281 | -1.18 |
| 1455646_at | 2010004M13Rik | RIKEN cDNA 2010004M13 gene | -1.07 | 0.485 | -1.34 | 0.525 | -1.12 | 0.501 | -1.78 | 0.066 | -1.33 |
| 1438986_x_at | Rps17 | ribosomal protein S17 | -1.11 | 0.027 | -1.06 | 0.567 | -1.38 | 0 | -1.36 | 0.008 | -1.23 |
| 1437271_at | Clcf1 | cardiotrophin-like cytokine factor 1 | -1.06 | 0.572 | -1.16 | 0.451 | -1.31 | 0.242 | -2.04 | 0.078 | -1.39 |
| AFFX-r2-P1-cre-3_a | --- | --- | -1.01 | 0.9 | -1.17 | 0.553 | -1.38 | 0.043 | -1.23 | 0.082 | -1.2 |
| 1428819_at | Mapre1 | microtubule-associated protein, RP/EB family, member 1 | -1.14 | 0.358 | -1.08 | 0.769 | -1.3 | 0.289 | -1.11 | 0.815 | -1.16 |
| 1430703_at | --- | --- | -1.05 | 0.869 | -1.52 | 0.378 | -1.05 | 0.806 | -1.11 | 0.877 | -1.18 |
| 1424324_at | Esco1 | establishment of cohesion 1 homolog 1 (S. cerevisiae) | -1.15 | 0.065 | -1.06 | 0.438 | -1.33 | 0.026 | -1.5 | 0.04 | -1.26 |
| 1420967_at | Slc25a15 | solute carrier family 25 (mitochondrial carrier ornithine transporter), member 15 | -1.19 | 0.027 | -1.09 | 0.289 | -1.24 | 0.203 | -1.14 | 0.349 | -1.16 |
| 1424912_at | Slc25a17 | solute carrier family 25 (mitochondrial carrier, peroxisomal membrane protein), m | -1.24 | 0.003 | -1.27 | 0.047 | -1.03 | 0.857 | -1.03 | 0.576 | -1.14 |
| 1428845_at | Bclaf1 | BCL2-associated transcription factor 1 | -1.19 | 0.318 | -1.14 | 0.246 | -1.17 | 0.353 | -1.18 | 0.233 | -1.17 |
| 1424526_a_at | Tgds | TDP-glucose 4,6-dehydratase | -1.07 | 0.149 | -1.05 | 0.654 | -1.49 | 0.026 | -1.69 | 0.106 | -1.32 |
| 1417720_at | Polr2j | polymerase (RNA) II (DNA directed) polypeptide J | -1.15 | 0.39 | -1.14 | 0.009 | -1.21 | 0.106 | -1.01 | 0.952 | -1.13 |
| 1452261_at | Shprh | SNF2 histone linker PHD RING helicase | -1.1 | 0.417 | -1.23 | 0.229 | -1.18 | 0.034 | -1.29 | 0.108 | -1.2 |
| 1433649_at | Aof1 | amine oxidase, flavin containing 1 | -1.18 | 0.328 | -1.07 | 0.601 | -1.27 | 0.145 | -1.77 | 0.177 | -1.32 |
| 1425102_a_at | Ace2 | angiotensin I converting enzyme (peptidyl-dipeptidase A) 2 | -1.08 | 0.692 | -1.22 | 0.746 | -1.22 | 0.469 | -2.01 | 0.224 | -1.38 |
| 1424635_at | Eef1a1 | eukaryotic translation elongation factor 1 alpha 1 | -1.13 | 0.034 | -1.18 | 0.447 | -1.2 | 0.01 | -1.23 | 0.052 | -1.19 |
| 1426477_at | Rasa1 | RAS p21 protein activator 1 | -1.04 | 0.05 | -1.02 | 0.703 | -1.58 | 0 | -1.47 | 0.132 | -1.28 |
| 1431689_at | 5830400J07Rik | RIKEN cDNA 5830400J07 gene | -1.28 | 0.169 | -1.06 | 0.864 | -1.18 | 0.604 | -1.2 | 0.167 | -1.18 |
| 1451649_a_at | Wdr75 | WD repeat domain 75 | -1.18 | 0.411 | -1.05 | 0.296 | -1.31 | 0.05 | -1.4 | 0.131 | -1.23 |

| | | | | | | | | | | | |
|---|---|---|---|---|---|---|---|---|---|---|---|
| 1427254_at | Zfp445 | zinc finger protein 445 | -1.07 | 0.718 | -1.06 | 0.795 | -1.45 | 0.13 | -1.39 | 0.322 | -1.24 |
| 1441062_at | Ddx11 | DEAD/H (Asp-Glu-Ala-Asp/His) box polypeptide 11 (CHL1-like helicase homolog, | -1.1 | 0.747 | -1.08 | 0.891 | -1.37 | 0.461 | -1.12 | 0.72 | -1.17 |
| 1452101_at | Blmh | bleomycin hydrolase | -1.35 | 0.113 | -1.14 | 0.297 | -1.05 | 0.749 | -1.03 | 0.919 | -1.14 |
| 1423112_at | Ube2d3 | ubiquitin-conjugating enzyme E2D 3 (UBC4/5 homolog, yeast) | -1.23 | 0.524 | -1.27 | 0.079 | -1.03 | 0.909 | -1.09 | 0.698 | -1.15 |
| 1455491_at | Hnrph3 /// LOC669 | heterogeneous nuclear ribonucleoprotein H3 /// similar to heterogeneous nuclear | -1.25 | 0.295 | -1.18 | 0.363 | -1.08 | 0.531 | -1.38 | 0.189 | -1.22 |
| 1457743_at | D13Bwg1146e | DNA segment, Chr 13, Brigham & Women's Genetics 1146 expressed | -1.07 | 0.928 | -1.34 | 0.718 | -1.13 | 0.674 | -1.24 | 0.534 | -1.19 |
| 1417981_at | Insig2 | insulin induced gene 2 | -1.04 | 0.562 | -1.22 | 0.367 | -1.27 | 0.07 | -1.31 | 0.119 | -1.21 |
| 1424603_at | Sumf1 | sulfatase modifying factor 1 | -1.25 | 0.488 | -1.01 | 0.971 | -1.29 | 0.464 | -1.72 | 0.319 | -1.32 |
| 1429882_at | 2610005L07Rik | RIKEN cDNA 2610005L07 gene | -1.1 | 0.679 | -1.16 | 0.429 | -1.25 | 0.268 | -1.74 | 0.159 | -1.31 |
| 1428998_at | Phf3 | PHD finger protein 3 | -1.1 | 0.135 | -1.17 | 0.145 | -1.25 | 0.11 | -1.04 | 0.77 | -1.14 |
| 1457751_at | 4832420A03Rik | RIKEN cDNA 4832420A03 gene | -1.01 | 0.54 | -1.11 | 0.553 | -1.47 | 0.019 | -1.12 | 0.474 | -1.18 |
| 1460207_s_at | E2f5 | E2F transcription factor 5 | -1.24 | 0.079 | -1.12 | 0.361 | -1.15 | 0.092 | -1.16 | 0.47 | -1.17 |
| 1451554_a_at | Aph1a | anterior pharynx defective 1a homolog (C. elegans) | -1.05 | 0.627 | -1.29 | 0.043 | -1.19 | 0.329 | -1.54 | 0.093 | -1.27 |
| 1437050_s_at | Angel2 | angel homolog 2 (Drosophila) | -1.05 | 0.494 | -1.01 | 0.939 | -1.58 | 0.043 | -1.53 | 0.003 | -1.29 |
| 1424564_at | 2410001C21Rik | RIKEN cDNA 2410001C21 gene | -1.06 | 0.537 | -1.41 | 0.113 | -1.08 | 0.608 | -1.05 | 0.221 | -1.15 |
| 1448248_at | Crk | v-crk sarcoma virus CT10 oncogene homolog (avian) | -1.07 | 0.664 | -1.14 | 0.004 | -1.33 | 0.064 | -1.39 | 0.005 | -1.23 |
| 1452992_at | LOC668450 /// LOC | similar to cell division cycle 26 /// similar to cell division cycle 26 /// similar to cell c | -1.35 | 0.256 | -1.11 | 0.393 | -1.08 | 0.446 | -1.21 | 0.391 | -1.19 |
| 1419141_at | Crygd | crystallin, gamma D | -1.04 | 0.946 | -1.18 | 0.272 | -1.3 | 0.106 | -1.15 | 0.778 | -1.17 |
| 1436740_at | A430108E01Rik | RIKEN cDNA A430108E01 gene | -1.09 | 0.357 | -1.16 | 0.03 | -1.27 | 0.182 | -1.53 | 0.097 | -1.26 |
| 1417465_at | Fnta | farnesyltransferase, CAAX box, alpha | -1.11 | 0.113 | -1.09 | 0.432 | -1.32 | 0 | -1.25 | 0.108 | -1.19 |
| 1452422_a_at | Snrpb2 | U2 small nuclear ribonucleoprotein B | -1.16 | 0.15 | -1.07 | 0.497 | -1.29 | 0.008 | -1.28 | 0.21 | -1.2 |
| 1430398_at | 4921517O11Rik | RIKEN cDNA 4921517O11 gene | -1.1 | 0.733 | -1.47 | 0.45 | -1.02 | 0.895 | -1.13 | 0.869 | -1.18 |
| 1455273_at | --- | --- | -1.13 | 0.732 | -1.07 | 0.736 | -1.32 | 0.142 | -1.1 | 0.727 | -1.16 |
| 1415788_at | Ublcp1 | ubiquitin-like domain containing CTD phosphatase 1 | -1.16 | 0.197 | -1.15 | 0.154 | -1.19 | 0.14 | -1.18 | 0.307 | -1.17 |
| 1437456_x_at | Ythdf1 | YTH domain family 1 | -1.11 | 0.2 | -1.22 | 0.039 | -1.18 | 0.079 | -1.05 | 0.509 | -1.14 |
| 1455290_at | Znrf2 | zinc and ring finger 2 | -1.22 | 0.244 | -1.19 | 0.309 | -1.1 | 0.561 | -1.4 | 0.212 | -1.23 |
| 1448828_at | Smc6 | structural maintenance of chromosomes 6 | -1.18 | 0.231 | -1.26 | 0.135 | -1.07 | 0.636 | -1.05 | 0.766 | -1.14 |
| 1445440_at | A430106J12Rik | RIKEN cDNA A430106J12 gene | -1.34 | 0.023 | -1.05 | 0.81 | -1.14 | 0.467 | -1.02 | 0.852 | -1.14 |
| 1424033_at | Sfrs7 | splicing factor, arginine/serine-rich 7 | -1.2 | 0.372 | -1.15 | 0.106 | -1.15 | 0.248 | -1.32 | 0.124 | -1.21 |
| 1431465_s_at | Fyttd1 | forty-two-three domain containing 1 | -1.27 | 0.509 | -1.18 | 0.599 | -1.07 | 0.802 | -1.19 | 0.294 | -1.18 |
| 1428302_at | Mrpl48 | mitochondrial ribosomal protein L48 | -1.22 | 0.016 | -1.13 | 0.122 | -1.15 | 0.25 | -1.09 | 0.683 | -1.15 |
| 1454989_at | Cspp1 | centrosome and spindle pole associated protein 1 | -1.35 | 0.164 | -1.14 | 0.319 | -1.04 | 0.785 | -1.1 | 0.734 | -1.16 |
| 1431610_at | 5330439A09Rik | RIKEN cDNA 5330439A09 gene | -1.06 | 0.926 | -1.1 | 0.851 | -1.39 | 0.206 | -1.07 | 0.869 | -1.16 |
| 1434328_at | Rpl15 | ribosomal protein L15 | -1.15 | 0.295 | -1.05 | 0.351 | -1.34 | 0.099 | -1.58 | 0.078 | -1.28 |
| 1435341_at | Ppig | peptidyl-prolyl isomerase G (cyclophilin G) | -1.13 | 0.452 | -1.03 | 0.832 | -1.39 | 0.065 | -1.15 | 0.325 | -1.18 |
| 1451325_at | Fyttd1 | forty-two-three domain containing 1 | -1.21 | 0.496 | -1.06 | 0.854 | -1.25 | 0.131 | -1.25 | 0.36 | -1.19 |
| 1423149_at | Skp1a | S-phase kinase-associated protein 1A | -1.02 | 0.865 | -1.11 | 0.419 | -1.43 | 0.002 | -1 | 0.989 | -1.14 |
| 1423108_at | Slc25a20 | solute carrier family 25 (mitochondrial carnitine/acylcarnitine translocase), membe | -1.04 | 0.921 | -1.45 | 0.186 | -1.09 | 0.568 | -1.4 | 0.271 | -1.24 |
| 1426954_at | Trim33 /// 8030451 | tripartite motif protein 33 /// RIKEN cDNA 8030451N04 gene | -1.3 | 0.101 | -1.12 | 0.264 | -1.1 | 0.283 | -1.28 | 0.011 | -1.2 |
| 1445239_at | Gatad2a | GATA zinc finger domain containing 2A | -1.13 | 0.682 | -1.17 | 0.442 | -1.2 | 0.417 | -1.22 | 0.109 | -1.18 |
| 1428589_at | Mrpl41 | mitochondrial ribosomal protein L41 | -1.13 | 0.201 | -1.16 | 0.042 | -1.21 | 0.153 | -1.12 | 0.323 | -1.15 |
| 1436371_at | Recql | RecQ protein-like | -1.19 | 0.576 | -1.1 | 0.733 | -1.22 | 0.436 | -1.5 | 0.16 | -1.25 |
| 1452825_at | 5330410G16Rik | RIKEN cDNA 5330410G16 gene | -1.2 | 0.61 | -1.29 | 0.595 | -1.03 | 0.796 | -1.02 | 0.869 | -1.14 |
| 1427406_at | Trip11 | thyroid hormone receptor interactor 11 | -1.15 | 0.707 | -1.02 | 0.942 | -1.39 | 0.232 | -1.6 | 0.26 | -1.29 |
| 1417557_at | Ubxd1 | UBX domain containing 1 | -1.09 | 0.286 | -1.18 | 0.138 | -1.23 | 0.021 | -1.6 | 0.082 | -1.28 |
| 1435001_at | Plaa | phospholipase A2, activating protein | -1.24 | 0.167 | -1.13 | 0.508 | -1.14 | 0.037 | -1.07 | 0.793 | -1.14 |
| 1429633_at | Lcor | ligand dependent nuclear receptor corepressor | -1.05 | 0.467 | -1.16 | 0.078 | -1.31 | 0.07 | -1.17 | 0.235 | -1.17 |
| 1454894_at | Smurf2 | SMAD specific E3 ubiquitin protein ligase 2 | -1.17 | 0.087 | -1.13 | 0.021 | -1.21 | 0.094 | -1.42 | 0.182 | -1.23 |
| 1417846_at | Ulk2 | Unc-51 like kinase 2 (C. elegans) | -1.08 | 0.575 | -1.17 | 0.089 | -1.26 | 0.111 | -1.4 | 0.287 | -1.23 |
| 1436995_a_at | Rpl26 | ribosomal protein L26 | -1.17 | 0.094 | -1.04 | 0.635 | -1.31 | 0.004 | -1.32 | 0.013 | -1.21 |
| 1444049_at | Adat1 | adenosine deaminase, tRNA-specific 1 | -1.48 | 0.34 | -1.09 | 0.733 | -1.02 | 0.959 | -1.08 | 0.541 | -1.17 |
| 1418934_at | Mab21l2 | mab-21-like 2 (C. elegans) | -1.04 | 0.846 | -1.26 | 0.388 | -1.22 | 0.637 | -1.06 | 0.818 | -1.14 |
| 1437406_x_at | Igfbp4 | insulin-like growth factor binding protein 4 | -1.08 | 0.437 | -1.13 | 0.439 | -1.31 | 0.55 | -1.17 | 0.765 | -1.17 |

| | | | | | | | | | | | |
|---|---|---|---|---|---|---|---|---|---|---|---|
| 1416210_at | Imp3 | IMP3, U3 small nucleolar ribonucleoprotein, homolog (yeast) | -1.09 | 0.512 | -1.26 | 0.034 | -1.16 | 0.254 | -1.36 | 0.077 | -1.22 |
| 1457241_at | Lrrc48 | Leucine rich repeat containing 48 | -1.16 | 0.406 | -1.03 | 0.898 | -1.34 | 0.207 | -1.47 | 0.223 | -1.25 |
| 1446306_at | --- | --- | -1.14 | 0.531 | -1.08 | 0.861 | -1.29 | 0.56 | -1.08 | 0.819 | -1.15 |
| 1427088_at | Ccnt2 | cyclin T2 | -1.17 | 0.348 | -1.18 | 0.3 | -1.15 | 0.458 | -1.21 | 0.34 | -1.18 |
| 1446562_at | Sh3kbp1 | SH3-domain kinase binding protein 1 | -1.41 | 0.2 | -1.09 | 0.88 | -1.05 | 0.879 | -1.5 | 0.028 | -1.26 |
| 1429698_at | Mterf | mitochondrial transcription termination factor | -1.31 | 0.492 | -1.03 | 0.838 | -1.19 | 0.277 | -1.37 | 0.139 | -1.22 |
| 1457349_at | LOC639426 | similar to Tetratricopeptide repeat protein 6 (TPR repeat protein 6) | -1.03 | 0.953 | -1.28 | 0.387 | -1.21 | 0.12 | -3.33 | 0.036 | -1.71 |
| 1455538_at | 6330403M23Rik | RIKEN cDNA 6330403M23 gene | -1.13 | 0.2 | -1.06 | 0.525 | -1.34 | 0.024 | -1.36 | 0.182 | -1.22 |
| 1427949_at | Zfp294 | zinc finger protein 294 | -1.15 | 0.096 | -1.14 | 0.049 | -1.21 | 0.109 | -1.05 | 0.593 | -1.14 |
| 1435531_at | Usp3 | ubiquitin specific peptidase 3 | -1.11 | 0.446 | -1.28 | 0.011 | -1.12 | 0.247 | -1.71 | 0.012 | -1.3 |
| 1454740_at | Mib1 | mindbomb homolog 1 (Drosophila) | -1.14 | 0.637 | -1.01 | 0.925 | -1.41 | 0.044 | -1.13 | 0.676 | -1.17 |
| 1444585_at | Adc | arginine decarboxylase | -1.17 | 0.285 | -1.06 | 0.639 | -1.28 | 0.175 | -1.1 | 0.36 | -1.15 |
| 1436698_x_at | BC054438 | cDNA sequence BC054438 | -1.07 | 0.81 | -1.01 | 0.935 | -1.54 | 0.026 | -1.77 | 0.252 | -1.35 |
| 1429159_at | 4631408O11Rik | RIKEN cDNA 4631408O11 gene | -1.16 | 0.61 | -1.26 | 0.054 | -1.09 | 0.821 | -2.06 | 0.103 | -1.39 |
| 1424282_at | Pet112l | PET112-like (yeast) | -1.08 | 0.696 | -1.23 | 0.416 | -1.2 | 0.382 | -1.22 | 0.088 | -1.18 |
| 1429890_at | A930007D18Rik | RIKEN cDNA A930007D18 gene | -1.12 | 0.615 | -1.07 | 0.849 | -1.32 | 0.51 | -1.7 | 0.112 | -1.3 |
| 1435843_x_at | Mrps9 | mitochondrial ribosomal protein S9 | -1.12 | 0.186 | -1.07 | 0.28 | -1.34 | 0.247 | -1.25 | 0.014 | -1.19 |
| 1424197_s_at | Fance | Fanconi anemia, complementation group E | -1.16 | 0.57 | -1.25 | 0.308 | -1.09 | 0.691 | -1.6 | 0.271 | -1.27 |
| 1454987_a_at | H2-Ke6 | H2-K region expressed gene 6 | -1.02 | 0.904 | -1.25 | 0.247 | -1.25 | 0.053 | -1.77 | 0.215 | -1.32 |
| 1452692_a_at | Ndufv2 | NADH dehydrogenase (ubiquinone) flavoprotein 2 | -1.04 | 0.649 | -1.21 | 0.114 | -1.27 | 0.064 | -1.08 | 0.499 | -1.15 |
| 1426841_at | Ythdf3 | YTH domain family 3 | -1.05 | 0.013 | -1.25 | 0.008 | -1.2 | 0.04 | -1.15 | 0.342 | -1.16 |
| 1456803_at | Polr3c | Polymerase (RNA) III (DNA directed) polypeptide C | -1.01 | 0.977 | -1.16 | 0.535 | -1.37 | 0.287 | -1.29 | 0.211 | -1.21 |
| 1432458_at | 1700011F14Rik | RIKEN cDNA 1700011F14 gene | -1.07 | 0.178 | -1.08 | 0.654 | -1.39 | 0.111 | -1.29 | 0.185 | -1.21 |
| 1437055_x_at | Mfsd1 | Major facilitator superfamily domain containing 1 | -1.52 | 0.06 | -1.04 | 0.757 | -1.04 | 0.845 | -1.26 | 0.203 | -1.21 |
| 1418316_a_at | Mark3 | MAP/microtubule affinity-regulating kinase 3 | -1.07 | 0.783 | -1.22 | 0.112 | -1.22 | 0.361 | -1.01 | 0.96 | -1.13 |
| 1416655_at | C1galt1c1 | C1GALT1-specific chaperone 1 | -1.06 | 0.095 | -1.12 | 0.248 | -1.35 | 0.008 | -1.01 | 0.889 | -1.13 |
| 1425482_s_at | Ankmy2 | ankyrin repeat and MYND domain containing 2 | -1.16 | 0.033 | -1.11 | 0.459 | -1.23 | 0.191 | -1.37 | 0.164 | -1.22 |
| 1434718_at | Cul3 | cullin 3 | -1.19 | 0.57 | -1.21 | 0.3 | -1.1 | 0.591 | -1.15 | 0.417 | -1.16 |
| 1418704_at | S100a13 | S100 calcium binding protein A13 | -1.03 | 0.714 | -1.07 | 0.638 | -1.49 | 0.025 | -1.11 | 0.677 | -1.17 |
| 1418101_a_at | Rtn3 | reticulon 3 | -1.09 | 0.383 | -1.25 | 0.094 | -1.16 | 0.075 | -1.04 | 0.77 | -1.14 |
| 1432423_a_at | C530008M17Rik | RIKEN cDNA C530008M17 gene | -1.27 | 0.468 | -1.2 | 0.349 | -1.04 | 0.778 | -1.17 | 0.382 | -1.17 |
| 1451901_at | Smarca3 | SWI/SNF related, matrix associated, actin dependent regulator of chromatin, subf | -1.25 | 0.023 | -1.03 | 0.937 | -1.25 | 0.15 | -1.31 | 0.331 | -1.21 |
| 1429709_at | Pmpcb | peptidase (mitochondrial processing) beta | -1.1 | 0.233 | -1.26 | 0.042 | -1.14 | 0.039 | -1.07 | 0.577 | -1.14 |
| 1435139_at | Narg1 | NMDA receptor-regulated gene 1 | -1.19 | 0.26 | -1.15 | 0.122 | -1.15 | 0.258 | -1.02 | 0.948 | -1.13 |
| 1448998_at | Lpo | lactoperoxidase | -1.33 | 0.588 | -1.13 | 0.499 | -1.06 | 0.635 | -2 | 0.12 | -1.38 |
| 1451902_at | Zfp758 | zinc finger protein 758 | -1.15 | 0.011 | -1.19 | 0.019 | -1.14 | 0.066 | -1.39 | 0.061 | -1.22 |
| 1435901_at | Usp40 | ubiquitin specific peptidase 40 | -1.06 | 0.693 | -1.14 | 0.336 | -1.32 | 0.063 | -1.34 | 0.324 | -1.21 |
| 1423065_at | Dnmt3a | DNA methyltransferase 3A | -1.07 | 0.575 | -1.18 | 0.344 | -1.24 | 0.386 | -1.19 | 0.274 | -1.17 |
| 1434099_at | Ppargc1a | Peroxisome proliferative activated receptor, gamma, coactivator 1 alpha | -1.23 | 0.456 | -1.26 | 0.499 | -1.03 | 0.918 | -2.55 | 0.422 | -1.52 |
| 1436691_x_at | Prdx1 | peroxiredoxin 1 | -1.16 | 0.07 | -1.11 | 0.411 | -1.22 | 0.011 | -1.32 | 0.053 | -1.2 |
| 1449623_at | Txnrd3 | thioredoxin reductase 3 | -1.11 | 0.092 | -1.06 | 0.741 | -1.35 | 0.069 | -1.27 | 0.218 | -1.2 |
| 1434065_at | Cwf19l1 | CWF19-like 1, cell cycle control (S. pombe) | -1.27 | 0.01 | -1.17 | 0.386 | -1.07 | 0.388 | -1.17 | 0.003 | -1.17 |
| 1418038_s_at | Dusp19 | dual specificity phosphatase 19 | -1.08 | 0.021 | -1.16 | 0.186 | -1.26 | 0.063 | -1.18 | 0.385 | -1.17 |
| 1451968_at | Xrcc5 | X-ray repair complementing defective repair in Chinese hamster cells 5 | -1.32 | 0.017 | -1.05 | 0.692 | -1.15 | 0.339 | -1.28 | 0.049 | -1.2 |
| 1452234_s_at | D16Bwg1494e | DNA segment, Chr 16, Brigham & Women's Genetics 1494 expressed | -1.1 | 0.695 | -1.24 | 0.416 | -1.16 | 0.637 | -1.35 | 0.102 | -1.21 |
| 1434618_at | --- | --- | -1.21 | 0.075 | -1.18 | 0.083 | -1.1 | 0.573 | -1.3 | 0.094 | -1.2 |
| 1447066_at | --- | Adult inner ear cDNA, RIKEN full-length enriched library, clone:F930039H20 proc | -1.35 | 0.745 | -1.07 | 0.891 | -1.11 | 0.734 | -2.69 | 0.434 | -1.55 |
| 1455682_at | AA536808 | expressed sequence AA536808 | -1.17 | 0.027 | -1.16 | 0.288 | -1.16 | 0.322 | -1.35 | 0.082 | -1.21 |
| 1433302_at | Cdh10 | cadherin 10 | -1.23 | 0.504 | -1.1 | 0.812 | -1.17 | 0.586 | -1.48 | 0.209 | -1.24 |
| 1428236_at | Acbd5 | acyl-Coenzyme A binding domain containing 5 | -1.07 | 0.671 | -1.16 | 0.067 | -1.27 | 0.025 | -1.21 | 0.144 | -1.18 |
| 1457302_at | Slc20a2 | Solute carrier family 20, member 2 | -1.23 | 0.107 | -1.17 | 0.351 | -1.09 | 0.592 | -1.39 | 0.246 | -1.22 |
| 1457735_at | --- | Transcribed locus | -1.16 | 0.168 | -1.03 | 0.965 | -1.34 | 0.538 | -1.02 | 0.708 | -1.14 |
| 1444402_at | Zc3h12c | zinc finger CCCH type containing 12C | -1.12 | 0.125 | -1.32 | 0.029 | -1.07 | 0.698 | -1.31 | 0.149 | -1.21 |

| | | | | | | | | | | | |
|---|---|---|---|---|---|---|---|---|---|---|---|
| 1448488_at | Mrps5 | mitochondrial ribosomal protein S5 | -1.06 | 0.738 | -1.13 | 0.132 | -1.32 | 0.019 | -1.11 | 0.515 | -1.15 |
| 1427031_s_at | Ccdc52 | coiled-coil domain containing 52 | -1.19 | 0.44 | -1.29 | 0.213 | -1.03 | 0.53 | -1.33 | 0.054 | -1.21 |
| 1420870_at | Mllt10 | myeloid/lymphoid or mixed lineage-leukemia translocation to 10 homolog (Drosop | -1.21 | 0.401 | -1.29 | 0.048 | -1.02 | 0.918 | -1.12 | 0.41 | -1.16 |
| 1457070_at | Uhrf2 | Ubiquitin-like, containing PHD and RING finger domains 2 | -1.1 | 0.628 | -1.18 | 0.62 | -1.21 | 0.313 | -1.2 | 0.689 | -1.17 |
| 1423622_a_at | Ccnl1 | cyclin L1 | -1.04 | 0.849 | -1.13 | 0.188 | -1.37 | 0.097 | -1.31 | 0.199 | -1.21 |
| 1453475_at | 4930445K14Rik | RIKEN cDNA 4930445K14 gene | -1.18 | 0.045 | -1.08 | 0.622 | -1.23 | 0.382 | -1.26 | 0.004 | -1.19 |
| 1424634_at | Tceal1 | transcription elongation factor A (SII)-like 1 | -1.06 | 0.808 | -1.15 | 0.623 | -1.31 | 0.115 | -1.15 | 0.346 | -1.17 |
| 1453774_at | 2810002O09Rik | RIKEN cDNA 2810002O09 gene | -1.06 | 0.63 | -1.12 | 0.597 | -1.34 | 0.209 | -1.32 | 0.371 | -1.21 |
| 1448858_at | Ulk2 | Unc-51 like kinase 2 (C. elegans) | -1.09 | 0.262 | -1.29 | 0.191 | -1.12 | 0.56 | -1.13 | 0.217 | -1.16 |
| 1459359_at | --- | 0 day neonate cerebellum cDNA, RIKEN full-length enriched library, clone:C2300 | -1.09 | 0.552 | -1.26 | 0.662 | -1.14 | 0.75 | -1.76 | 0.315 | -1.31 |
| 1434949_at | Armc8 | armadillo repeat containing 8 | -1.16 | 0.529 | -1.08 | 0.501 | -1.25 | 0.103 | -1.24 | 0.413 | -1.18 |
| 1416668_at | 4921531G14Rik | RIKEN cDNA 4921531G14 gene | -1.1 | 0.254 | -1.02 | 0.591 | -1.44 | 0.001 | -1.38 | 0.247 | -1.24 |
| 1426152_a_at | Kitl | kit ligand | -1.03 | 0.862 | -1.26 | 0.473 | -1.22 | 0.454 | -1.01 | 0.924 | -1.13 |
| 1435096_at | Ric8b | resistance to inhibitors of cholinesterase 8 homolog B (C. elegans) | -1.13 | 0.424 | -1.28 | 0.067 | -1.09 | 0.499 | -1.37 | 0.054 | -1.22 |
| 1449056_at | E330009J07Rik | RIKEN cDNA E330009J07 gene | -1.03 | 0.846 | -1.16 | 0.236 | -1.32 | 0.145 | -1.83 | 0.013 | -1.34 |
| 1438821_at | Rfwd2 | Ring finger and WD repeat domain 2 | -1.22 | 0.678 | -1.07 | 0.801 | -1.2 | 0.379 | -1.26 | 0.251 | -1.19 |
| 1425480_at | Cnot6l | CCR4-NOT transcription complex, subunit 6-like | -1 | 0.995 | -1.25 | 0.04 | -1.27 | 0.041 | -1.26 | 0.512 | -1.2 |
| 1423324_at | Pnn | pinin | -1.16 | 0.453 | -1.11 | 0.543 | -1.21 | 0.468 | -1.33 | 0.414 | -1.2 |
| 1450973_s_at | Mapkbp1 | mitogen activated protein kinase binding protein 1 | -1.06 | 0.738 | -1.16 | 0.256 | -1.29 | 0.207 | -1.27 | 0.29 | -1.19 |
| 1436436_at | Cnih4 | cornichon homolog 4 (Drosophila) | -1.03 | 0.748 | -1.26 | 0.129 | -1.21 | 0.086 | -1.12 | 0.28 | -1.16 |
| 1439962_at | 2310010J17Rik | RIKEN cDNA 2310010J17 gene | -1.26 | 0.256 | -1.03 | 0.862 | -1.22 | 0.424 | -1.18 | 0.767 | -1.17 |
| 1416926_at | Trp53inp1 | transformation related protein 53 inducible nuclear protein 1 | -1.06 | 0.784 | -1.12 | 0.478 | -1.33 | 0.287 | -1.06 | 0.883 | -1.14 |
| 1443534_at | --- | --- | -1.03 | 0.629 | -1.04 | 0.831 | -1.54 | 0.099 | -1.81 | 0.129 | -1.35 |
| 1422126_a_at | Nudt13 | nudix (nucleoside diphosphate linked moiety X)-type motif 13 | -1.28 | 0.328 | -1.01 | 0.958 | -1.23 | 0.119 | -1.35 | 0.214 | -1.22 |
| 1430578_at | Eif4g3 | eukaryotic translation initiation factor 4 gamma, 3 | -1.05 | 0.943 | -1.13 | 0.598 | -1.34 | 0.076 | -1.08 | 0.804 | -1.15 |
| 1450840_a_at | Rpl39 | ribosomal protein L39 | -1.04 | 0.753 | -1.08 | 0.638 | -1.43 | 0.017 | -1.24 | 0.008 | -1.2 |
| 1452133_at | Uqcrh | ubiquinol-cytochrome c reductase hinge protein | -1.13 | 0.094 | -1.2 | 0.029 | -1.15 | 0.381 | -1.15 | 0.39 | -1.16 |
| 1430344_at | 2900064P18Rik | RIKEN cDNA 2900064P18 gene | -1.01 | 0.949 | -1.3 | 0.474 | -1.21 | 0.72 | -1.6 | 0.385 | -1.28 |
| 1448032_at | Azi2 | 5-azacytidine induced gene 2 | -1.04 | 0.851 | -1.19 | 0.104 | -1.27 | 0.234 | -1.17 | 0.185 | -1.17 |
| 1453160_at | Thrap1 | thyroid hormone receptor associated protein 1 | -1.06 | 0.351 | -1.06 | 0.699 | -1.43 | 0.06 | -1.12 | 0.353 | -1.17 |
| 1441642_at | Large | Like-glycosyltransferase | -1.36 | 0.328 | -1.01 | 0.976 | -1.16 | 0.669 | -1.2 | 0.412 | -1.18 |
| 1431198_x_at | 9430038I01Rik | RIKEN cDNA 9430038I01 gene | -1.09 | 0.61 | -1.43 | 0.272 | -1.03 | 0.871 | -1.3 | 0.284 | -1.21 |
| 1435977_at | Hdgfrp3 | hepatoma-derived growth factor, related protein 3 | -1.1 | 0.467 | -1.11 | 0.802 | -1.28 | 0.3 | -1.22 | 0.594 | -1.18 |
| 1453186_at | 5730406M06Rik | RIKEN cDNA 5730406M06 gene | -1.09 | 0.53 | -1.12 | 0.619 | -1.29 | 0.181 | -1.19 | 0.645 | -1.17 |
| 1437751_at | Ppargc1a | peroxisome proliferative activated receptor, gamma, coactivator 1 alpha | -1.11 | 0.362 | -1.24 | 0.36 | -1.13 | 0.195 | -1.95 | 0.146 | -1.36 |
| 1435462_at | LOC433022 | hypothetical LOC433022 | -1.05 | 0.549 | -1.27 | 0.304 | -1.17 | 0.499 | -1.62 | 0.166 | -1.28 |
| 1442093_at | Adc | Arginine decarboxylase | -1.27 | 0.68 | -1.02 | 0.962 | -1.22 | 0.364 | -1.34 | 0.529 | -1.21 |
| 1421560_at | Snai3 | snail homolog 3 (Drosophila) | -1.23 | 0.153 | -1.04 | 0.89 | -1.24 | 0.304 | -1.29 | 0.491 | -1.2 |
| 1438859_x_at | Rps29 /// LOC6299 | ribosomal protein S29 /// similar to 40S ribosomal protein S29 /// similar to 40S rib | -1.18 | 0.068 | -1.03 | 0.856 | -1.29 | 0.013 | -1.22 | 0.051 | -1.18 |
| 1448598_at | Mmp17 | matrix metallopeptidase 17 | -1.24 | 0.337 | -1.17 | 0.754 | -1.08 | 0.767 | -1.13 | 0.049 | -1.15 |
| 1426675_at | Tomm70a | translocase of outer mitochondrial membrane 70 homolog A (yeast) | -1.08 | 0.694 | -1.07 | 0.101 | -1.37 | 0.074 | -1.04 | 0.688 | -1.14 |
| 1433738_at | 5730445M16Rik | RIKEN cDNA 5730445M16 gene | -1.02 | 0.752 | -1.16 | 0.201 | -1.33 | 0.036 | -1.29 | 0.089 | -1.2 |
| 1429050_at | Chic2 | cysteine-rich hydrophobic domain 2 | -1.03 | 0.709 | -1.23 | 0.25 | -1.25 | 0.077 | -1.06 | 0.509 | -1.14 |
| 1428161_a_at | Chchd2 | coiled-coil-helix-coiled-coil-helix domain containing 2 | -1.13 | 0.045 | -1.19 | 0.163 | -1.15 | 0.033 | -1.13 | 0.027 | -1.15 |
| 1453229_s_at | Uqcrh | ubiquinol-cytochrome c reductase hinge protein | -1.21 | 0.046 | -1.11 | 0.191 | -1.16 | 0.207 | -1.59 | 0.083 | -1.27 |
| 1434041_at | Appbp2 | amyloid beta precursor protein (cytoplasmic tail) binding protein 2 | -1.12 | 0.327 | -1.06 | 0.67 | -1.34 | 0.134 | -1.28 | 0.135 | -1.2 |
| 1441973_at | Zfp295 | zinc finger protein 295 | -1.04 | 0.178 | -1.17 | 0.596 | -1.3 | 0.413 | -1.16 | 0.157 | -1.17 |
| 1434562_at | Mfap3 | microfibrillar-associated protein 3 | -1.15 | 0.305 | -1.05 | 0.639 | -1.31 | 0.03 | -1.1 | 0.505 | -1.15 |
| 1418544_at | Kcnip3 | Kv channel interacting protein 3, calsenilin | -1.26 | 0.375 | -1.12 | 0.142 | -1.11 | 0.516 | -1.24 | 0.446 | -1.18 |
| 1457355_at | --- | --- | -1.23 | 0.329 | -1.19 | 0.384 | -1.07 | 0.555 | -1.19 | 0.518 | -1.17 |
| 1441339_at | Chd9 | chromodomain helicase DNA binding protein 9 | -1.26 | 0.346 | -1.1 | 0.288 | -1.13 | 0.443 | -1.38 | 0.101 | -1.22 |
| 1453399_at | Ccnt2 | Cyclin T2 | -1.1 | 0.577 | -1.09 | 0.494 | -1.31 | 0.24 | -1.55 | 0.004 | -1.26 |
| 1426776_at | Wasl | Wiskott-Aldrich syndrome-like (human) | -1.07 | 0.264 | -1.08 | 0.097 | -1.36 | 0.182 | -1 | 0.995 | -1.13 |

| | | | | | | | | | | | |
|---|---|---|---|---|---|---|---|---|---|---|---|
| 1419945_s_at | Rab2 | RAB2, member RAS oncogene family | -1.14 | 0.194 | -1.05 | 0.207 | -1.3 | 0.019 | -1.18 | 0.383 | -1.17 |
| 1445317_at | Sdha | Succinate dehydrogenase complex, subunit A, flavoprotein (Fp) | -1 | 0.909 | -1.09 | 0.547 | -1.49 | 0.065 | -1.36 | 0.224 | -1.23 |
| 1455036_s_at | Ndufc2 /// LOC675 | NADH dehydrogenase (ubiquinone) 1, subcomplex unknown, 2 /// similar to NAD | -1.17 | 0.017 | -1.1 | 0.219 | -1.21 | 0.039 | -1.06 | 0.212 | -1.14 |
| 1459773_x_at | Snip1 | Smad nuclear interacting protein 1 | -1.1 | 0.228 | -1.23 | 0.107 | -1.15 | 0.373 | -1.29 | 0.146 | -1.19 |
| 1426531_at | Zmynd11 | zinc finger, MYND domain containing 11 | -1.08 | 0.258 | -1.14 | 0.329 | -1.27 | 0.075 | -1.52 | 0.242 | -1.25 |
| 1423696_a_at | Psmd6 | proteasome (prosome, macropain) 26S subunit, non-ATPase, 6 | -1.18 | 0.104 | -1.04 | 0.764 | -1.27 | 0.02 | -1.19 | 0.362 | -1.17 |
| 1435207_at | Dixdc1 | DIX domain containing 1 | -1.23 | 0.421 | -1.11 | 0.564 | -1.14 | 0.055 | -1.64 | 0.039 | -1.28 |
| 1455543_at | Klhl18 | kelch-like 18 (Drosophila) | -1.24 | 0.423 | -1.25 | 0.348 | -1.01 | 0.942 | -1.47 | 0.234 | -1.24 |
| 1423211_at | Nola3 | nucleolar protein family A, member 3 | -1.22 | 0.226 | -1.09 | 0.151 | -1.17 | 0.276 | -1.16 | 0.271 | -1.16 |
| 1424577_at | Msto1 | misato homolog 1 (Drosophila) | -1.13 | 0.222 | -1.13 | 0.661 | -1.22 | 0 | -1.14 | 0.477 | -1.15 |
| 1431745_a_at | Zc3h14 | zinc finger CCCH type containing 14 | -1.21 | 0.133 | -1.27 | 0.005 | -1.02 | 0.824 | -1.02 | 0.861 | -1.13 |
| 1428505_at | 2310015N07Rik | RIKEN cDNA 2310015N07 gene | -1.17 | 0.107 | -1.07 | 0.19 | -1.25 | 0.002 | -1.18 | 0.071 | -1.17 |
| 1422581_at | Pias1 | protein inhibitor of activated STAT 1 | -1.18 | 0.223 | -1.05 | 0.648 | -1.26 | 0.152 | -1.43 | 0.048 | -1.23 |
| 1435803_a_at | Eif4e2 | eukaryotic translation initiation factor 4E member 2 | -1.16 | 0.223 | -1.18 | 0.246 | -1.14 | 0.243 | -1.1 | 0.381 | -1.14 |
| 1428594_at | Garnl1 | GTPase activating RANGAP domain-like 1 | -1.21 | 0.08 | -1.09 | 0.452 | -1.17 | 0.247 | -1.2 | 0.353 | -1.17 |
| 1448689_at | Rras2 | related RAS viral (r-ras) oncogene homolog 2 | -1.05 | 0.52 | -1.07 | 0.013 | -1.42 | 0.038 | -2.29 | 0.097 | -1.46 |
| 1428760_at | Snapc3 | small nuclear RNA activating complex, polypeptide 3 | -1.3 | 0.016 | -1.09 | 0.571 | -1.1 | 0.379 | -1.24 | 0.437 | -1.18 |
| 1443167_at | Rnf12 | Ring finger protein 12 | -1 | 0.999 | -1.05 | 0.739 | -1.56 | 0 | -1.21 | 0.071 | -1.21 |
| 1429512_at | Zpbp2 | zona pellucida binding protein 2 | -1.11 | 0.821 | -1.37 | 0.533 | -1.04 | 0.877 | -1.24 | 0.489 | -1.19 |
| 1428230_at | Prkcn | protein kinase C, nu | -1.12 | 0.457 | -1.24 | 0.213 | -1.12 | 0.295 | -1.89 | 0.082 | -1.34 |
| 1424430_at | Mterfd2 | MTERF domain containing 2 | -1.19 | 0.215 | -1.19 | 0.171 | -1.1 | 0.615 | -1.14 | 0.438 | -1.15 |
| 1420139_s_at | Krr1 | KRR1, small subunit (SSU) processome component, homolog (yeast) | -1.15 | 0.38 | -1.14 | 0.133 | -1.17 | 0.059 | -1.12 | 0.169 | -1.15 |
| 1419188_s_at | Ccl27 | chemokine (C-C motif) ligand 27 | -1.11 | 0.188 | -1.17 | 0.343 | -1.2 | 0.317 | -1.31 | 0.511 | -1.2 |
| 1429087_at | 1110054O05Rik | RIKEN cDNA 1110054O05 gene | -1.3 | 0.625 | -1.21 | 0.329 | -1 | 0.997 | -1.06 | 0.589 | -1.14 |
| 1458656_at | AW050198 | expressed sequence AW050198 | -1.13 | 0.489 | -1.08 | 0.478 | -1.28 | 0.141 | -1.32 | 0.061 | -1.2 |
| 1446616_at | Gltscr1 | Glioma tumor suppressor candidate region gene 1 | -1.09 | 0.872 | -1.2 | 0.512 | -1.19 | 0.272 | -1.08 | 0.831 | -1.14 |
| 1444315_at | D630024D03Rik | RIKEN cDNA D630024D03 gene | -1.14 | 0.316 | -1.28 | 0.499 | -1.07 | 0.701 | -1.68 | 0.42 | -1.29 |
| 1417264_at | Coq5 | coenzyme Q5 homolog, methyltransferase (yeast) | -1.07 | 0.312 | -1.15 | 0.335 | -1.27 | 0.283 | -1 | 0.999 | -1.12 |
| 1425534_at | Stau2 | staufen (RNA binding protein) homolog 2 (Drosophila) | -1.06 | 0.292 | -1.22 | 0.347 | -1.2 | 0.407 | -1.61 | 0.114 | -1.27 |
| 1453322_at | Wdr33 | WD repeat domain 33 | -1.14 | 0.089 | -1.04 | 0.797 | -1.32 | 0.161 | -1.18 | 0.205 | -1.17 |
| 1449702_at | Zfand2a | zinc finger, AN1-type domain 2A | -1.07 | 0.629 | -1.2 | 0.364 | -1.2 | 0.118 | -1.36 | 0.105 | -1.21 |
| 1428086_at | Dnm1l | dynamin 1-like | -1.2 | 0.443 | -1.13 | 0.205 | -1.14 | 0.324 | -1.24 | 0.153 | -1.18 |
| 1439738_at | --- | --- | -1.21 | 0.488 | -1.04 | 0.84 | -1.23 | 0.571 | -1.23 | 0.623 | -1.18 |
| 1419489_at | AW049604 | expressed sequence AW049604 | -1.11 | 0.04 | -1.05 | 0.897 | -1.35 | 0.42 | -1.61 | 0.368 | -1.28 |
| 1440384_at | Tmcc1 | transmembrane and coiled coil domains 1 | -1.32 | 0.13 | -1.04 | 0.49 | -1.15 | 0.136 | -1.01 | 0.973 | -1.13 |
| 1422508_at | Atp6v1a | ATPase, H+ transporting, lysosomal V1 subunit A | -1.2 | 0.314 | -1.09 | 0.424 | -1.19 | 0.213 | -1.15 | 0.277 | -1.16 |
| 1424893_at | Ndel1 | nuclear distribution gene E-like homolog 1 (A. nidulans) | -1.06 | 0.796 | -1.03 | 0.724 | -1.46 | 0.209 | -1.14 | 0.456 | -1.17 |
| 1452632_at | LOC637741 | hypothetical protein LOC637741 | -1.08 | 0.531 | -1.36 | 0.141 | -1.07 | 0.786 | -1.43 | 0.103 | -1.24 |
| 1452056_s_at | Ppp3ca | protein phosphatase 3, catalytic subunit, alpha isoform | -1.09 | 0.274 | -1.1 | 0.255 | -1.3 | 0.019 | -1.5 | 0.107 | -1.25 |
| 1436051_at | 9630007J19Rik | RIKEN cDNA 9630007J19 gene | -1.31 | 0.05 | -1.14 | 0.493 | -1.05 | 0.848 | -1.41 | 0.311 | -1.23 |
| 1434401_at | Zcchc2 | zinc finger, CCHC domain containing 2 | -1.08 | 0.492 | -1.3 | 0.094 | -1.11 | 0.459 | -1.65 | 0.058 | -1.29 |
| 1418312_at | Zfp276 | zinc finger protein (C2H2 type) 276 | -1.13 | 0.485 | -1.27 | 0.178 | -1.08 | 0.334 | -1.02 | 0.891 | -1.13 |
| 1436317_at | D230012E17Rik | RIKEN cDNA D230012E17 gene | -1.1 | 0.67 | -1.15 | 0.258 | -1.22 | 0.271 | -2.35 | 0.054 | -1.46 |
| 1432749_at | --- | --- | -1.51 | 0.154 | -1.06 | 0.543 | -1.01 | 0.97 | -1.47 | 0.654 | -1.26 |
| 1441536_at | Hmgcs1 | 3-hydroxy-3-methylglutaryl-Coenzyme A synthase 1 | -1.26 | 0.136 | -1.03 | 0.927 | -1.2 | 0.065 | -1.56 | 0.43 | -1.26 |
| 1417553_at | Plac1 | placental specific protein 1 | -1.25 | 0.663 | -1.01 | 0.964 | -1.23 | 0.401 | -1.04 | 0.844 | -1.14 |
| 1453145_at | 4933439C20Rik | RIKEN cDNA 4933439C20 gene | -1.08 | 0.161 | -1.03 | 0.916 | -1.44 | 0.071 | -1.55 | 0.261 | -1.27 |
| 1446375_at | Exoc6 | Exocyst complex component 6 | -1.06 | 0.91 | -1.1 | 0.149 | -1.34 | 0.628 | -1.09 | 0.871 | -1.15 |
| 1429923_x_at | Spata3 | spermatogenesis associated 3 | -1.22 | 0.715 | -1.19 | 0.329 | -1.06 | 0.525 | -2.37 | 0.124 | -1.46 |
| 1448337_at | 2410003P15Rik | RIKEN cDNA 2410003P15 gene | -1.05 | 0.717 | -1.39 | 0.13 | -1.08 | 0.576 | -1.06 | 0.532 | -1.15 |
| 1433478_at | Parl | presenilin associated, rhomboid-like | -1.24 | 0.161 | -1.21 | 0.024 | -1.04 | 0.745 | -1.53 | 0.056 | -1.25 |
| 1452256_at | 1110002N22Rik | RIKEN cDNA 1110002N22 gene | -1.18 | 0.146 | -1.1 | 0.21 | -1.19 | 0.238 | -1.17 | 0.253 | -1.16 |
| 1441203_at | --- | CDNA clone IMAGE:30543871 | -1.32 | 0.04 | -1.13 | 0.731 | -1.04 | 0.806 | -1.07 | 0.767 | -1.14 |

| | | | | | | | | | | | |
|---|---|---|---|---|---|---|---|---|---|---|---|
| 1420487_at | Nol7 | nucleolar protein 7 | -1.25 | 0.473 | -1.13 | 0.628 | -1.1 | 0.508 | -1.37 | 0.26 | -1.21 |
| 1447158_at | --- | --- | -1.23 | 0.547 | -1.1 | 0.69 | -1.15 | 0.407 | -1.55 | 0.506 | -1.25 |
| 1437070_at | Cdc14b | CDC14 cell division cycle 14 homolog B (S. cerevisiae) | -1.21 | 0.317 | -1.23 | 0.056 | -1.04 | 0.832 | -1.34 | 0.294 | -1.21 |
| 1456820_at | --- | 9 days embryo whole body cDNA, RIKEN full-length enriched library, clone:D030( | -1.09 | 0.701 | -1.04 | 0.933 | -1.4 | 0.47 | -1.19 | 0.592 | -1.18 |
| 1424001_at | Mki67ip | Mki67 (FHA domain) interacting nucleolar phosphoprotein | -1.07 | 0.733 | -1.12 | 0.123 | -1.3 | 0.096 | -1.03 | 0.761 | -1.13 |
| 1417157_at | Actr10 | ARP10 actin-related protein 10 homolog (S. cerevisiae) | -1.16 | 0.093 | -1.15 | 0.245 | -1.16 | 0.102 | -1.18 | 0.375 | -1.16 |
| 1437446_at | Rab5b | RAB5B, member RAS oncogene family | -1.06 | 0.565 | -1.07 | 0.802 | -1.38 | 0.11 | -1.3 | 0.059 | -1.2 |
| 1435557_at | Fhod1 | formin homology 2 domain containing 1 | -1.42 | 0.208 | -1.06 | 0.862 | -1.05 | 0.879 | -1.14 | 0.714 | -1.17 |
| 1443677_at | E030011O05Rik | RIKEN cDNA E030011O05 gene | -1.35 | 0.441 | -1.02 | 0.962 | -1.14 | 0.623 | -1.42 | 0.081 | -1.23 |
| 1454705_at | D15Ertd621e | DNA segment, Chr 15, ERATO Doi 621, expressed | -1.21 | 0.031 | -1.08 | 0.065 | -1.18 | 0.172 | -1.15 | 0.519 | -1.16 |
| 1456798_at | 9330118A15Rik | RIKEN cDNA 9330118A15 gene | -1.02 | 0.944 | -1.05 | 0.947 | -1.5 | 0.222 | -1.45 | 0.674 | -1.26 |
| 1425178_s_at | Shmt1 | serine hydroxymethyl transferase 1 (soluble) | -1.3 | 0.093 | -1.06 | 0.725 | -1.13 | 0.487 | -1.1 | 0.358 | -1.15 |
| 1436117_at | A830010M20Rik | RIKEN cDNA A830010M20 gene | -1.31 | 0.006 | -1.06 | 0.082 | -1.12 | 0.382 | -1.28 | 0.524 | -1.19 |
| 1424188_at | Rabgap1 | RAB GTPase activating protein 1 | -1.04 | 0.089 | -1.13 | 0.04 | -1.33 | 0.122 | -1.22 | 0.319 | -1.18 |
| 1436767_at | Luc7l2 | LUC7-like 2 (S. cerevisiae) | -1.1 | 0.536 | -1.12 | 0.305 | -1.26 | 0.19 | -1.21 | 0.246 | -1.17 |
| 1433562_s_at | Atp5f1 | ATP synthase, H+ transporting, mitochondrial F0 complex, subunit b, isoform 1 | -1.16 | 0.267 | -1.19 | 0.071 | -1.12 | 0.121 | -1.03 | 0.745 | -1.12 |
| 1456975_at | Taok1 | TAO kinase 1 | -1.08 | 0.837 | -1.14 | 0.638 | -1.26 | 0.307 | -1.33 | 0.265 | -1.2 |
| 1421643_at | Zfpn1a2 | zinc finger protein, subfamily 1A, 2 (Helios) | -1.38 | 0.28 | -1.02 | 0.955 | -1.12 | 0.448 | -1.57 | 0.604 | -1.27 |
| 1455418_at | --- | Transcribed locus | -1.04 | 0.752 | -1.05 | 0.872 | -1.47 | 0.11 | -1.29 | 0.145 | -1.21 |
| 1417663_a_at | Ndrg3 | N-myc downstream regulated gene 3 | -1.1 | 0.31 | -1.09 | 0.414 | -1.31 | 0.104 | -1.24 | 0.038 | -1.18 |
| 1416212_at | Magoh | mago-nashi homolog, proliferation-associated (Drosophila) | -1.14 | 0.058 | -1.13 | 0.023 | -1.2 | 0.095 | -1.16 | 0.326 | -1.16 |
| 1435295_at | Dopey1 | dopey family member 1 | -1.28 | 0.152 | -1.05 | 0.748 | -1.16 | 0.515 | -1.28 | 0.196 | -1.19 |
| 1460123_at | Gpr1 | G protein-coupled receptor 1 | -1.21 | 0.561 | -1.22 | 0.699 | -1.05 | 0.837 | -1.02 | 0.915 | -1.13 |
| 1436040_at | 2310005L22Rik | RIKEN cDNA 2310005L22 gene | -1.3 | 0.298 | -1.2 | 0.265 | -1.01 | 0.954 | -1.23 | 0.152 | -1.18 |
| 1442086_at | Mta3 | Metastasis associated 3 | -1.32 | 0.065 | -1.17 | 0.793 | -1.02 | 0.952 | -1.31 | 0.253 | -1.2 |
| 1444010_at | LOC668879 /// LOC | hypothetical protein LOC668879 /// hypothetical protein LOC669500 | -1.05 | 0.853 | -1.36 | 0.413 | -1.1 | 0.315 | -1.62 | 0.144 | -1.28 |
| 1458887_at | Tcf21 | transcription factor 21 | -1.09 | 0.718 | -1.23 | 0.658 | -1.15 | 0.529 | -1.45 | 0.172 | -1.23 |
| 1428258_at | 2010107E04Rik | RIKEN cDNA 2010107E04 gene | -1.19 | 0.079 | -1.15 | 0.123 | -1.13 | 0.473 | -1.02 | 0.91 | -1.12 |
| 1434839_s_at | Tbl1xr1 | transducin (beta)-like 1X-linked receptor 1 | -1.27 | 0.103 | -1.13 | 0.221 | -1.08 | 0.496 | -1.24 | 0.371 | -1.18 |
| 1422893_at | Sfmbt1 | Scm-like with four mbt domains 1 | -1.07 | 0.589 | -1.35 | 0.032 | -1.08 | 0.61 | -1.11 | 0.644 | -1.15 |
| 1436191_at | Arid4a | AT rich interactive domain 4A (Rbp1 like) | -1.16 | 0.338 | -1.04 | 0.746 | -1.3 | 0.138 | -1.29 | 0.354 | -1.19 |
| 1428798_s_at | 0610039J04Rik | RIKEN cDNA 0610039J04 gene | -1.19 | 0.361 | -1.16 | 0.438 | -1.12 | 0.497 | -1.17 | 0.376 | -1.16 |
| 1418983_at | Inadl | InaD-like (Drosophila) | -1.05 | 0.76 | -1.37 | 0.153 | -1.09 | 0.626 | -1.86 | 0.052 | -1.34 |
| 1452342_at | Apbb2 | amyloid beta (A4) precursor protein-binding, family B, member 2 | -1.04 | 0.705 | -1 | 0.986 | -1.55 | 0.082 | -2.02 | 0.036 | -1.4 |
| 1416057_at | Ndufb11 | NADH dehydrogenase (ubiquinone) 1 beta subcomplex, 11 | -1.15 | 0.296 | -1.24 | 0.104 | -1.09 | 0.427 | -1 | 0.956 | -1.12 |
| 1433641_at | Smad5 | MAD homolog 5 (Drosophila) | -1.04 | 0.111 | -1.05 | 0.541 | -1.46 | 0.03 | -1.25 | 0.039 | -1.2 |
| 1449446_at | Ccdc59 | coiled-coil domain containing 59 | -1.16 | 0.349 | -1.16 | 0.177 | -1.14 | 0.407 | -1.3 | 0.241 | -1.19 |
| 1435161_at | D5Ertd135e | DNA segment, Chr 5, ERATO Doi 135, expressed | -1.08 | 0.68 | -1.24 | 0.202 | -1.15 | 0.394 | -1.03 | 0.751 | -1.13 |
| 1434181_at | Plekhc1 | pleckstrin homology domain containing, family C (with FERM domain) member 1 | -1.05 | 0.415 | -1.19 | 0.577 | -1.23 | 0.343 | -1.77 | 0.209 | -1.31 |
| 1418495_at | Zc3h8 | zinc finger CCCH type containing 8 | -1.45 | 0.043 | -1.06 | 0.818 | -1.03 | 0.852 | -1.39 | 0.111 | -1.23 |
| 1436128_at | Plekha8 | pleckstrin homology domain containing, family A (phosphoinositide binding specif | -1.05 | 0.783 | -1.07 | 0.621 | -1.4 | 0.18 | -1.03 | 0.928 | -1.14 |
| 1422486_a_at | Smad4 | MAD homolog 4 (Drosophila) | -1.04 | 0.752 | -1.21 | 0.002 | -1.24 | 0.014 | -2.01 | 0.057 | -1.37 |
| 1426879_at | 1190005F20Rik | RIKEN cDNA 1190005F20 gene | -1.06 | 0.394 | -1.28 | 0.095 | -1.14 | 0.458 | -1.44 | 0.023 | -1.23 |
| 1417733_at | Rnf146 | ring finger protein 146 | -1.11 | 0.436 | -1.09 | 0.099 | -1.27 | 0.014 | -1.27 | 0.053 | -1.19 |
| 1419914_s_at | D10Ertd438e | DNA segment, Chr 10, ERATO Doi 438, expressed | -1.13 | 0.525 | -1.1 | 0.197 | -1.23 | 0.12 | -1.2 | 0.2 | -1.17 |
| 1433784_at | AI265322 | expressed sequence AI265322 | -1.09 | 0.161 | -1.1 | 0.397 | -1.29 | 0.092 | -1.19 | 0.346 | -1.17 |
| 1452113_a_at | Rab23 | RAB23, member RAS oncogene family | -1.3 | 0.241 | -1.18 | 0.436 | -1.01 | 0.934 | -1.01 | 0.903 | -1.13 |
| 1454574_at | C430049A07Rik | RIKEN cDNA C430049A07 gene | -1.47 | 0.635 | -1.06 | 0.927 | -1.02 | 0.883 | -1.19 | 0.64 | -1.18 |
| 1427416_x_at | LOC674944 | similar to dual specificity phosphatase 7 | -1.17 | 0.572 | -1.12 | 0.692 | -1.16 | 0.458 | -1.48 | 0.244 | -1.24 |
| 1429504_at | Rnpc3 | RNA-binding region (RNP1, RRM) containing 3 | -1.3 | 0.218 | -1.1 | 0.693 | -1.08 | 0.8 | -1.32 | 0.259 | -1.2 |
| 1420999_at | Cnot4 | CCR4-NOT transcription complex, subunit 4 | -1.01 | 0.739 | -1.32 | 0.075 | -1.17 | 0.157 | -1.08 | 0.277 | -1.14 |
| 1435329_at | Fbxl11 | F-box and leucine-rich repeat protein 11 | -1.11 | 0.464 | -1.37 | 0.07 | -1.03 | 0.9 | -1.23 | 0.162 | -1.19 |
| 1452633_s_at | LOC637741 | hypothetical protein LOC637741 | -1.19 | 0.258 | -1.05 | 0.833 | -1.24 | 0.272 | -1.23 | 0.236 | -1.18 |

| | | | | | | | | | | | |
|---|---|---|---|---|---|---|---|---|---|---|---|
| 1452309_at | Cgnl1 /// LOC67748 | cingulin-like 1 /// similar to cingulin-like 1 isoform 1 | -1.09 | 0.137 | -1.03 | 0.782 | -1.39 | 0.109 | -1.22 | 0.385 | -1.18 |
| 1448020_at | Rap1a | RAS-related protein-1a | -1.2 | 0.107 | -1.07 | 0.575 | -1.2 | 0.233 | -1.1 | 0.703 | -1.14 |
| 1437987_at | --- | --- | -1.09 | 0.47 | -1.14 | 0.748 | -1.23 | 0.315 | -1.67 | 0.097 | -1.28 |
| 1454880_s_at | Bmf | Bcl2 modifying factor | -1.19 | 0.497 | -1.27 | 0.322 | -1.03 | 0.899 | -2 | 0.136 | -1.37 |
| 1434468_at | Otud4 | OTU domain containing 4 | -1.11 | 0.544 | -1.12 | 0.229 | -1.22 | 0.029 | -1.27 | 0.296 | -1.18 |
| 1435927_at | Dennd3 | DENN/MADD domain containing 3 | -1.28 | 0.006 | -1 | 0.997 | -1.21 | 0.16 | -2.07 | 0.082 | -1.39 |
| 1419454_x_at | Pias2 | protein inhibitor of activated STAT 2 | -1.18 | 0.341 | -1.08 | 0.634 | -1.21 | 0.059 | -1.02 | 0.909 | -1.12 |
| 1452627_at | Senp6 | SUMO/sentrin specific peptidase 6 | -1.08 | 0.576 | -1.09 | 0.377 | -1.32 | 0.078 | -1.19 | 0.26 | -1.17 |
| 1443760_at | Dock1 | Dedicator of cyto-kinesis 1 | -1.24 | 0.729 | -1.08 | 0.827 | -1.15 | 0.734 | -2.01 | 0.202 | -1.37 |
| 1418016_at | Pum2 | pumilio 2 (Drosophila) | -1.16 | 0.054 | -1 | 0.97 | -1.34 | 0.086 | -1.15 | 0.175 | -1.16 |
| 1435470_at | LOC547150 | similar to p47 protein isoform a | -1.35 | 0.034 | -1.01 | 0.977 | -1.15 | 0.25 | -1.2 | 0.487 | -1.18 |
| 1448753_at | Srp9 | signal recognition particle 9 | -1.12 | 0.342 | -1.03 | 0.518 | -1.34 | 0.121 | -1.38 | 0.196 | -1.22 |
| 1423715_a_at | Nedd8 | neural precursor cell expressed, developmentally down-regulated gene 8 | -1.07 | 0.627 | -1.14 | 0.001 | -1.25 | 0.021 | -1.36 | 0.213 | -1.21 |
| 1439278_at | Zbtb20 | zinc finger and BTB domain containing 20 | -1.26 | 0.317 | -1 | 0.95 | -1.23 | 0.392 | -1.12 | 0.782 | -1.15 |
| 1428631_a_at | Uqcrc2 | ubiquinol cytochrome c reductase core protein 2 | -1.18 | 0.017 | -1.29 | 0.079 | -1.02 | 0.901 | -1.2 | 0.289 | -1.17 |
| 1433527_at | Ireb2 | iron responsive element binding protein 2 | -1.17 | 0.367 | -1.05 | 0.354 | -1.26 | 0.075 | -1.13 | 0.496 | -1.15 |
| 1422925_s_at | Acot3 | acyl-CoA thioesterase 3 | -1.21 | 0.583 | -1.22 | 0.788 | -1.04 | 0.827 | -1.11 | 0.833 | -1.15 |
| 1435966_x_at | Mrpl13 | mitochondrial ribosomal protein L13 | -1.13 | 0.51 | -1.08 | 0.642 | -1.27 | 0.066 | -1.17 | 0.437 | -1.16 |
| 1459677_at | --- | --- | -1.17 | 0.67 | -1.03 | 0.892 | -1.29 | 0.246 | -1.39 | 0.246 | -1.22 |
| 1420374_at | Foxj2 | forkhead box J2 | -1.09 | 0.188 | -1.06 | 0.783 | -1.33 | 0.059 | -1.3 | 0.146 | -1.2 |
| 1423868_at | Txnrd3 | thioredoxin reductase 3 | -1.07 | 0.591 | -1.23 | 0.086 | -1.17 | 0.417 | -1.47 | 0.54 | -1.23 |
| 1422068_at | Pou3f1 | POU domain, class 3, transcription factor 1 | -1.09 | 0.481 | -1.1 | 0.822 | -1.29 | 0.29 | -1.38 | 0.558 | -1.21 |
| 1455911_x_at | Ndufb11 | NADH dehydrogenase (ubiquinone) 1 beta subcomplex, 11 | -1.07 | 0.294 | -1.14 | 0.152 | -1.26 | 0.036 | -1.19 | 0.259 | -1.17 |
| 1426485_at | Ubxd2 | UBX domain containing 2 | -1.08 | 0.638 | -1.27 | 0.254 | -1.12 | 0.457 | -1.08 | 0.78 | -1.14 |
| 1447919_x_at | Ndufab1 | NADH dehydrogenase (ubiquinone) 1, alpha/beta subcomplex, 1 | -1.07 | 0.52 | -1.39 | 0.07 | -1.05 | 0.655 | -1.27 | 0.435 | -1.2 |
| 1438069_a_at | Rbm5 | RNA binding motif protein 5 | -1.06 | 0.863 | -1.04 | 0.803 | -1.43 | 0.273 | -1.1 | 0.702 | -1.16 |
| 1434397_at | Zdhhc17 | zinc finger, DHHC domain containing 17 | -1.08 | 0.254 | -1.05 | 0.476 | -1.37 | 0.034 | -1.32 | 0.429 | -1.21 |
| 1442995_at | Ndst1 | N-deacetylase/N-sulfotransferase (heparan glucosaminyl) 1 | -1.23 | 0.088 | -1.11 | 0.617 | -1.12 | 0.719 | -1.4 | 0.278 | -1.22 |
| 1436681_x_at | 0610009D07Rik | RIKEN cDNA 0610009D07 gene | -1.44 | 0.069 | -1.01 | 0.957 | -1.08 | 0.634 | -1.02 | 0.801 | -1.14 |
| 1455574_at | Smg7 | Smg-7 homolog, nonsense mediated mRNA decay factor (C. elegans) | -1.13 | 0.652 | -1.01 | 0.97 | -1.37 | 0.087 | -1.09 | 0.526 | -1.15 |
| 1436122_at | Zfp667 | zinc finger protein 667 | -1.15 | 0.442 | -1.14 | 0.622 | -1.16 | 0.26 | -1.34 | 0.129 | -1.2 |
| 1425134_a_at | Pigx | phosphatidylinositol glycan anchor biosynthesis, class X | -1.11 | 0.369 | -1.2 | 0.109 | -1.14 | 0.365 | -1.66 | 0.046 | -1.28 |
| 1434125_at | Utp15 | UTP15, U3 small nucleolar ribonucleoprotein, homolog (yeast) | -1.07 | 0.444 | -1.24 | 0.016 | -1.15 | 0.367 | -1.07 | 0.719 | -1.13 |
| 1450505_a_at | 1810015C04Rik | RIKEN cDNA 1810015C04 gene | -1.2 | 0.565 | -1.15 | 0.364 | -1.11 | 0.657 | -1.86 | 0.136 | -1.33 |
| 1453524_at | 5530401D11Rik | RIKEN cDNA 5530401D11 gene | -1.15 | 0.52 | -1.09 | 0.806 | -1.22 | 0.164 | -1.46 | 0.217 | -1.23 |
| 1459836_x_at | Sf3b2 | splicing factor 3b, subunit 2 | -1.21 | 0.815 | -1.04 | 0.915 | -1.22 | 0.022 | -1.24 | 0.438 | -1.18 |
| 1423273_at | Polg | polymerase (DNA directed), gamma | -1.16 | 0.542 | -1.12 | 0.498 | -1.17 | 0.01 | -1.34 | 0.004 | -1.2 |
| 1455554_at | A830039N20Rik | RIKEN cDNA A830039N20 gene | -1.15 | 0.705 | -1.25 | 0.491 | -1.06 | 0.86 | -1.5 | 0.446 | -1.24 |
| 1434994_at | Dedd | death effector domain-containing | -1.13 | 0.332 | -1.21 | 0.213 | -1.12 | 0.661 | -1.11 | 0.575 | -1.14 |
| 1428877_at | Srp72 | signal recognition particle 72 | -1.14 | 0.33 | -1.2 | 0.004 | -1.11 | 0.137 | -1.05 | 0.53 | -1.13 |
| 1418244_at | Nat5 | N-acetyltransferase 5 (ARD1 homolog, S. cerevisiae) | -1.19 | 0.042 | -1.06 | 0.46 | -1.21 | 0.178 | -1.01 | 0.792 | -1.12 |
| 1427342_at | Fastkd1 | FAST kinase domains 1 | -1.17 | 0.32 | -1.14 | 0.455 | -1.14 | 0.415 | -1.27 | 0.267 | -1.18 |
| 1457681_at | 2610301F02Rik | RIKEN cDNA 2610301F02 gene | -1.04 | 0.908 | -1.29 | 0.11 | -1.15 | 0.439 | -1.01 | 0.873 | -1.12 |
| 1418520_at | Tgoln1 | trans-golgi network protein | -1.02 | 0.822 | -1.45 | 0.018 | -1.07 | 0.791 | -1.06 | 0.784 | -1.15 |
| 1456343_at | Slc35f1 | solute carrier family 35, member F1 | -1.19 | 0.493 | -1.07 | 0.872 | -1.2 | 0.433 | -1.36 | 0.351 | -1.2 |
| 1435280_at | AI452195 | expressed sequence AI452195 | -1.07 | 0.848 | -1.05 | 0.853 | -1.38 | 0.046 | -1.23 | 0.329 | -1.18 |
| 1441491_at | A330068G13Rik | RIKEN cDNA A330068G13 gene | -1.06 | 0.865 | -1.09 | 0.854 | -1.34 | 0.435 | -1.29 | 0.185 | -1.2 |
| 1430251_at | D330022H12Rik | RIKEN cDNA D330022H12 gene | -1.01 | 0.846 | -1.05 | 0.9 | -1.49 | 0.089 | -1.08 | 0.894 | -1.16 |
| 1438501_at | Rps17 | Ribosomal protein S17 | -1.17 | 0.029 | -1.24 | 0.509 | -1.06 | 0.804 | -1.53 | 0.045 | -1.25 |
| 1434604_at | Eif5b | eukaryotic translation initiation factor 5B | -1.16 | 0.452 | -1.07 | 0.444 | -1.23 | 0.182 | -1.16 | 0.259 | -1.16 |
| 1444057_at | --- | Transcribed locus | -1.28 | 0.131 | -1.09 | 0.142 | -1.1 | 0.704 | -1.32 | 0.163 | -1.2 |
| 1460303_at | Nr3c1 | nuclear receptor subfamily 3, group C, member 1 | -1.09 | 0.6 | -1.03 | 0.687 | -1.38 | 0.103 | -1.23 | 0.375 | -1.18 |
| 1436256_at | Grpel2 | GrpE-like 2, mitochondrial | -1.08 | 0.531 | -1.21 | 0.452 | -1.17 | 0.446 | -1.28 | 0.162 | -1.18 |

| | | | | | | | | | | | |
|---|---|---|---|---|---|---|---|---|---|---|---|
| 1428360_x_at | Ndufa7 | NADH dehydrogenase (ubiquinone) 1 alpha subcomplex, 7 (B14.5a) | -1.09 | 0.19 | -1.16 | 0.291 | -1.2 | 0.098 | -1.12 | 0.341 | -1.14 |
| 1439780_at | Rpl7l1 | ribosomal protein L7-like 1 | -1.08 | 0.649 | -1.14 | 0.217 | -1.24 | 0.22 | -1.26 | 0.133 | -1.18 |
| 1434126_at | 4930402H24Rik | RIKEN cDNA 4930402H24 gene | -1.25 | 0.429 | -1.2 | 0.221 | -1.02 | 0.937 | -1.25 | 0.542 | -1.18 |
| 1417674_s_at | Golga4 | golgi autoantigen, golgin subfamily a, 4 | -1.15 | 0.266 | -1.17 | 0.148 | -1.13 | 0.261 | -1.12 | 0.404 | -1.14 |
| 1422475_a_at | Rps3a | ribosomal protein S3a | -1.11 | 0.156 | -1.05 | 0.202 | -1.32 | 0.001 | -1.36 | 0.024 | -1.21 |
| 1433502_s_at | Tsr1 | TSR1, 20S rRNA accumulation, homolog (yeast) | -1.03 | 0.884 | -1.17 | 0.421 | -1.28 | 0.062 | -1.38 | 0.533 | -1.21 |
| 1447936_at | 2410006H16Rik | RIKEN cDNA 2410006H16 gene | -1.07 | 0.591 | -1.26 | 0.111 | -1.13 | 0.66 | -2.45 | 0.034 | -1.48 |
| 1431972_a_at | Gcap14 | granule cell antiserum positive 14 | -1.09 | 0.761 | -1.3 | 0.361 | -1.08 | 0.746 | -1.64 | 0.175 | -1.28 |
| 1458979_at | --- | --- | -1.04 | 0.968 | -1.41 | 0.22 | -1.06 | 0.901 | -1.5 | 0.497 | -1.25 |
| 1460096_at | Lamp1 | Lysosomal membrane glycoprotein 1 | -1.1 | 0.387 | -1.08 | 0.355 | -1.29 | 0.261 | -1.04 | 0.895 | -1.13 |
| 1449371_at | Harsl | histidyl-tRNA synthetase-like | -1.35 | 0.171 | -1.07 | 0.595 | -1.07 | 0.775 | -1.21 | 0.183 | -1.18 |
| 1418118_at | Slc22a1 | solute carrier family 22 (organic cation transporter), member 1 | -1.18 | 0.33 | -1.26 | 0.002 | -1.03 | 0.861 | -1.39 | 0.283 | -1.22 |
| 1424455_at | Gprasp1 | G protein-coupled receptor associated sorting protein 1 | -1.07 | 0.605 | -1.12 | 0.376 | -1.27 | 0.178 | -1.39 | 0.322 | -1.21 |
| 1433895_at | Tmem127 | transmembrane protein 127 | -1.05 | 0.825 | -1.13 | 0.334 | -1.3 | 0.222 | -1.41 | 0.066 | -1.22 |
| 1438560_x_at | Cct4 | chaperonin subunit 4 (delta) | -1.2 | 0.326 | -1.03 | 0.82 | -1.24 | 0.072 | -1.22 | 0.078 | -1.17 |
| 1419244_a_at | Rab14 | RAB14, member RAS oncogene family | -1.06 | 0.621 | -1.21 | 0.2 | -1.19 | 0.266 | -1.62 | 0.085 | -1.27 |
| 1428309_s_at | Pdrg1 | p53 and DNA damage regulated 1 | -1.02 | 0.806 | -1.2 | 0.385 | -1.25 | 0.19 | -1.86 | 0.084 | -1.33 |
| 1427078_at | Snx19 | sorting nexin 19 | -1.15 | 0.387 | -1.26 | 0.096 | -1.05 | 0.652 | -1.13 | 0.387 | -1.15 |
| 1451324_s_at | Fyttd1 | forty-two-three domain containing 1 | -1.28 | 0.424 | -1.08 | 0.437 | -1.1 | 0.068 | -1.39 | 0.152 | -1.21 |
| 1454781_x_at | Commd9 | COMM domain containing 9 | -1.05 | 0.375 | -1.09 | 0.435 | -1.36 | 0.267 | -1.11 | 0.541 | -1.15 |
| 1457011_at | --- | Transcribed locus | -1.15 | 0.771 | -1.18 | 0.492 | -1.12 | 0.778 | -3.13 | 0.072 | -1.64 |
| 1429666_at | Kctd16 | potassium channel tetramerisation domain containing 16 | -1.16 | 0.771 | -1.09 | 0.812 | -1.2 | 0.576 | -1.01 | 0.963 | -1.11 |
| 1417847_at | Ulk2 | Unc-51 like kinase 2 (C. elegans) | -1.06 | 0.752 | -1.29 | 0.007 | -1.12 | 0.362 | -1.14 | 0.609 | -1.15 |
| 1427997_at | 1110007M04Rik | RIKEN cDNA 1110007M04 gene | -1.06 | 0.778 | -1.34 | 0.035 | -1.08 | 0.347 | -1.03 | 0.879 | -1.13 |
| 1444403_at | Cbfa2t2h | Core-binding factor, runt domain, alpha subunit 2, translocated to, 2 homolog (hu | -1.15 | 0.725 | -1.15 | 0.396 | -1.14 | 0.681 | -1.31 | 0.218 | -1.19 |
| 1452901_at | Creb1 | cAMP responsive element binding protein 1 | -1.04 | 0.815 | -1.06 | 0.353 | -1.4 | 0.018 | -1.36 | 0.24 | -1.22 |
| 1428633_at | Twistnb /// LOC627 | TWIST neighbor /// similar to TWIST neighbor /// similar to TWIST neighbor | -1.18 | 0.31 | -1.03 | 0.935 | -1.26 | 0.228 | -1.23 | 0.375 | -1.17 |
| 1441139_at | B330003H21 | hypothetical protein B330003H21 | -1.45 | 0.227 | -1.03 | 0.92 | -1.05 | 0.841 | -1.09 | 0.74 | -1.15 |
| 1440332_at | Cdv3 | carnitine deficiency-associated gene expressed in ventricle 3 | -1.01 | 0.907 | -1.13 | 0.386 | -1.36 | 0.138 | -1.32 | 0.396 | -1.2 |
| 1436636_at | --- | --- | -1.03 | 0.806 | -1.37 | 0.068 | -1.1 | 0.682 | -1.07 | 0.679 | -1.14 |
| 1422490_at | Bnip2 | BCL2/adenovirus E1B interacting protein 1, NIP2 | -1.1 | 0.287 | -1.02 | 0.81 | -1.37 | 0.032 | -1.32 | 0.141 | -1.21 |
| 1456758_at | 9930017N22Rik | RIKEN cDNA 9930017N22 gene | -1.05 | 0.199 | -1.14 | 0.215 | -1.26 | 0.167 | -1.46 | 0.095 | -1.23 |
| 1436924_x_at | Ero1l /// Rpl31 /// L( | ERO1-like (S. cerevisiae) /// ribosomal protein L31 /// similar to ribosomal protein | -1.1 | 0.186 | -1.07 | 0.449 | -1.3 | 0.003 | -1.31 | 0 | -1.19 |
| 1424637_s_at | 2610204L23Rik | RIKEN cDNA 2610204L23 gene | -1.03 | 0.919 | -1.08 | 0.254 | -1.39 | 0.043 | -1.1 | 0.741 | -1.15 |
| 1453741_x_at | Angel2 | angel homolog 2 (Drosophila) | -1.16 | 0.7 | -1.1 | 0.758 | -1.19 | 0.714 | -2.14 | 0.111 | -1.4 |
| 1416669_s_at | Naca | nascent polypeptide-associated complex alpha polypeptide | -1.07 | 0.258 | -1.22 | 0.131 | -1.16 | 0.098 | -1.32 | 0.038 | -1.19 |
| 1451399_at | Pnkd | paroxysmal nonkinesiogenic dyskinesia | -1.03 | 0.785 | -1.2 | 0.096 | -1.22 | 0.134 | -1.34 | 0.23 | -1.2 |
| 1415734_at | Rab7 | RAB7, member RAS oncogene family | -1.15 | 0.052 | -1.09 | 0.014 | -1.21 | 0.186 | -1.28 | 0.293 | -1.18 |
| 1437100_x_at | Pim3 | proviral integration site 3 | -1.11 | 0.64 | -1.32 | 0.289 | -1.04 | 0.765 | -1.2 | 0.194 | -1.17 |
| 1438025_at | Mtrf1l | mitochondrial translational release factor 1-like | -1.11 | 0.082 | -1.12 | 0.459 | -1.21 | 0.239 | -1.01 | 0.96 | -1.11 |
| 1456633_at | Trpm3 | transient receptor potential cation channel, subfamily M, member 3 | -1.08 | 0.729 | -1.3 | 0.591 | -1.08 | 0.775 | -1.48 | 0.227 | -1.24 |
| 1454859_a_at | Rpl23 | ribosomal protein L23 | -1.08 | 0.476 | -1.06 | 0.646 | -1.35 | 0.001 | -1.38 | 0.026 | -1.21 |
| 1442886_at | Tra2a | transformer 2 alpha homolog (Drosophila) | -1.09 | 0.689 | -1.06 | 0.726 | -1.32 | 0.19 | -1.17 | 0.474 | -1.16 |
| 1451570_a_at | 6720467C03Rik | RIKEN cDNA 6720467C03 gene | -1.34 | 0.032 | -1.06 | 0.43 | -1.08 | 0.427 | -1.07 | 0.385 | -1.14 |
| 1423142_a_at | Gtpbp4 | GTP binding protein 4 | -1.26 | 0.516 | -1.11 | 0.433 | -1.08 | 0.569 | -1.32 | 0.166 | -1.19 |
| 1451113_a_at | Ik | IK cytokine | -1.15 | 0.362 | -1.03 | 0.254 | -1.28 | 0.048 | -1.32 | 0.254 | -1.2 |
| 1456018_at | AL022779 | expressed sequence AL022779 | -1.15 | 0.218 | -1.15 | 0.116 | -1.14 | 0.216 | -1.14 | 0.227 | -1.14 |
| 1448909_a_at | Mrpl39 | mitochondrial ribosomal protein L39 | -1.03 | 0.85 | -1.35 | 0.104 | -1.1 | 0.383 | -1.37 | 0.1 | -1.21 |
| 1441069_at | Zdhhc23 | zinc finger, DHHC domain containing 23 | -1.15 | 0.603 | -1.2 | 0.8 | -1.09 | 0.698 | -1.28 | 0.385 | -1.18 |
| 1443199_at | Lrch3 | Leucine-rich repeats and calponin homology (CH) domain containing 3 | -1.14 | 0.555 | -1.02 | 0.949 | -1.32 | 0.247 | -1.01 | 0.973 | -1.12 |
| 1416577_a_at | Rbx1 | ring-box 1 | -1.11 | 0.495 | -1.1 | 0.052 | -1.23 | 0.262 | -1.02 | 0.841 | -1.12 |
| 1452594_at | Dusp11 | dual specificity phosphatase 11 (RNA/RNP complex 1-interacting) | -1.1 | 0.738 | -1.05 | 0.661 | -1.32 | 0.098 | -1.76 | 0.025 | -1.31 |
| 1437375_at | --- | 0 day neonate thymus cDNA, RIKEN full-length enriched library, clone:A430010E | -1.13 | 0.686 | -1.15 | 0.645 | -1.16 | 0.307 | -1.32 | 0.358 | -1.19 |

| | | | | | | | | | | | |
|---|---|---|---|---|---|---|---|---|---|---|---|
| 1419805_s_at | Ggps1 | geranylgeranyl diphosphate synthase 1 | -1.2 | 0.003 | -1.11 | 0.48 | -1.13 | 0.303 | -1.56 | 0.022 | -1.25 |
| 1430123_a_at | Akr1a4 | aldo-keto reductase family 1, member A4 (aldehyde reductase) | -1.22 | 0.053 | -1.13 | 0.576 | -1.09 | 0.16 | -1.16 | 0.212 | -1.15 |
| 1434681_at | 4932441K18Rik | RIKEN cDNA 4932441K18 gene | -1.28 | 0.169 | -1.02 | 0.878 | -1.17 | 0.359 | -1.16 | 0.54 | -1.16 |
| 1434793_at | Wdr78 | WD repeat domain 78 | -1.25 | 0.263 | -1.21 | 0.572 | -1.01 | 0.968 | -1.19 | 0.579 | -1.16 |
| 1448050_s_at | Map4k4 | mitogen-activated protein kinase kinase kinase kinase 4 | -1.25 | 0.561 | -1.13 | 0.838 | -1.07 | 0.36 | -1.94 | 0.023 | -1.35 |
| 1454568_at | 6030460B20Rik | RIKEN cDNA 6030460B20 gene | -1.14 | 0.703 | -1.13 | 0.739 | -1.17 | 0.277 | -1.47 | 0.01 | -1.23 |
| 1449929_at | Dynlt3 | dynein light chain Tctex-type 3 | -1.07 | 0.375 | -1.06 | 0.127 | -1.35 | 0.029 | -1.17 | 0.37 | -1.16 |
| 1450084_s_at | Ivns1abp | influenza virus NS1A binding protein | -1.19 | 0.052 | -1.11 | 0.603 | -1.14 | 0.402 | -1.24 | 0.438 | -1.17 |
| 1428033_at | LOC245174 | hypothetical protein LOC245174 | -1.08 | 0.55 | -1.23 | 0.654 | -1.14 | 0.447 | -1.17 | 0.5 | -1.15 |
| 1443074_at | --- | --- | -1.09 | 0.075 | -1.32 | 0.444 | -1.06 | 0.768 | -1.36 | 0.626 | -1.21 |
| 1451494_at | Wac | WW domain containing adaptor with coiled-coil | -1.05 | 0.436 | -1.07 | 0.327 | -1.37 | 0.016 | -1.33 | 0.052 | -1.21 |
| 1437413_x_at | Rps29 /// LOC6299 | ribosomal protein S29 /// similar to 40S ribosomal protein S29 /// similar to 40S rib | -1.15 | 0.011 | -1.03 | 0.78 | -1.29 | 0.009 | -1.25 | 0.025 | -1.18 |
| 1429608_at | Adh6a | alcohol dehydrogenase 6A (class V) | -1.13 | 0.866 | -1.19 | 0.784 | -1.12 | 0.876 | -1.81 | 0.391 | -1.31 |
| 1457537_at | Rab1 | RAB1, member RAS oncogene family | -1.02 | 0.945 | -1.16 | 0.677 | -1.29 | 0.227 | -1.19 | 0.233 | -1.16 |
| 1455749_x_at | Ndufa7 | NADH dehydrogenase (ubiquinone) 1 alpha subcomplex, 7 (B14.5a) | -1.05 | 0.606 | -1.13 | 0.128 | -1.27 | 0.169 | -1.03 | 0.911 | -1.12 |
| 1428339_at | Nudt21 | nudix (nucleoside diphosphate linked moiety X)-type motif 21 | -1.09 | 0.537 | -1.15 | 0.161 | -1.2 | 0.247 | -1.03 | 0.881 | -1.12 |
| 1455949_at | Atp1b1 | ATPase, Na+/K+ transporting, beta 1 polypeptide | -1.28 | 0.002 | -1.14 | 0.829 | -1.04 | 0.795 | -1.01 | 0.988 | -1.12 |
| 1460543_x_at | Rpl37a | ribosomal protein L37a | -1.11 | 0.077 | -1.04 | 0.69 | -1.33 | 0.001 | -1.32 | 0.023 | -1.2 |
| 1423898_a_at | Trip12 | thyroid hormone receptor interactor 12 | -1.2 | 0.265 | -1.09 | 0.141 | -1.15 | 0.304 | -1.3 | 0.06 | -1.19 |
| 1435696_s_at | 3110031B13Rik | RIKEN cDNA 3110031B13 gene | -1.24 | 0.014 | -1.04 | 0.661 | -1.17 | 0.347 | -1.02 | 0.848 | -1.12 |
| 1426577_a_at | 1810054G18Rik | RIKEN cDNA 1810054G18 gene | -1.15 | 0.539 | -1.12 | 0.189 | -1.16 | 0.137 | -1.28 | 0.324 | -1.18 |
| 1431202_at | Herc3 | hect domain and RLD 3 | -1.35 | 0.346 | -1.13 | 0.838 | -1.01 | 0.979 | -1.13 | 0.483 | -1.15 |
| 1457658_x_at | Anxa4 | annexin A4 | -1.22 | 0.32 | -1.15 | 0.382 | -1.07 | 0.769 | -1.4 | 0.429 | -1.21 |
| 1455021_at | Gabbr1 | gamma-aminobutyric acid (GABA-B) receptor, 1 | -1.01 | 0.901 | -1.4 | 0.322 | -1.09 | 0.682 | -1.03 | 0.948 | -1.13 |
| 1427916_at | St7l | suppression of tumorigenicity 7-like | -1.19 | 0.281 | -1.06 | 0.661 | -1.19 | 0.06 | -1.08 | 0.497 | -1.13 |
| 1426486_at | Ubxd2 | UBX domain containing 2 | -1.13 | 0.168 | -1.1 | 0.214 | -1.21 | 0.067 | -1.06 | 0.674 | -1.13 |
| 1423874_at | Wdr33 | WD repeat domain 33 | -1.28 | 0.199 | -1.1 | 0.684 | -1.08 | 0.538 | -1.42 | 0.45 | -1.22 |
| 1430526_a_at | Smarca2 | SWI/SNF related, matrix associated, actin dependent regulator of chromatin, subf | -1.13 | 0.475 | -1.03 | 0.881 | -1.32 | 0.19 | -1.4 | 0.342 | -1.22 |
| 1451276_at | E030041M21Rik | RIKEN cDNA E030041M21 gene | -1.08 | 0.188 | -1.12 | 0.145 | -1.25 | 0.119 | -1.35 | 0.295 | -1.2 |
| 1416938_at | Chchd1 | coiled-coil-helix-coiled-coil-helix domain containing 1 | -1.06 | 0.124 | -1.08 | 0.087 | -1.34 | 0.01 | -1.29 | 0.324 | -1.19 |
| 1441351_at | Ankib1 | Ankyrin repeat and IBR domain containing 1 | -1.28 | 0.446 | -1.02 | 0.963 | -1.17 | 0.36 | -1.56 | 0.136 | -1.26 |
| 1424488_a_at | Ppa2 | pyrophosphatase (inorganic) 2 | -1.08 | 0.017 | -1.14 | 0.085 | -1.21 | 0.046 | -1.03 | 0.868 | -1.12 |
| 1453545_at | 5730492I20Rik | RIKEN cDNA 5730492I20 gene | -1.08 | 0.892 | -1.1 | 0.425 | -1.28 | 0.679 | -1.68 | 0.074 | -1.28 |
| 1438456_at | H13 | histocompatibility 13 | -1 | 0.981 | -1.41 | 0.138 | -1.09 | 0.549 | -1.23 | 0.484 | -1.18 |
| 1439859_at | 9630033F20Rik | RIKEN cDNA 9630033F20 gene | -1.18 | 0.086 | -1.11 | 0.576 | -1.14 | 0.437 | -1.14 | 0.464 | -1.14 |
| 1436103_at | Rab3ip | RAB3A interacting protein | -1.07 | 0.563 | -1.16 | 0.417 | -1.2 | 0.118 | -1.9 | 0.1 | -1.34 |
| 1443496_at | Elmod1 | ELMO domain containing 1 | -1.53 | 0.104 | -1.01 | 0.985 | -1.02 | 0.955 | -1.46 | 0.58 | -1.26 |
| 1436234_at | 4732471D19Rik | RIKEN cDNA 4732471D19 gene | -1.12 | 0.52 | -1.36 | 0.074 | -1 | 0.98 | -1.2 | 0.475 | -1.17 |
| 1430978_at | Rps25 | ribosomal protein S25 | -1.14 | 0.021 | -1.2 | 0.34 | -1.1 | 0.728 | -1.47 | 0.073 | -1.23 |
| 1425207_at | BC026439 | cDNA sequence BC026439 | -1.2 | 0.292 | -1.22 | 0.636 | -1.04 | 0.803 | -2.37 | 0.289 | -1.45 |
| 1437770_at | AW557046 | expressed sequence AW557046 | -1.02 | 0.489 | -1.36 | 0.545 | -1.11 | 0.8 | -1 | 0.986 | -1.12 |
| 1428503_a_at | Nkiras1 | NFKB inhibitor interacting Ras-like protein 1 | -1.12 | 0.381 | -1.16 | 0.099 | -1.15 | 0.399 | -1.4 | 0.17 | -1.21 |
| 1448584_at | Rsrc1 | arginine/serine-rich coiled-coil 1 | -1.08 | 0.549 | -1.03 | 0.651 | -1.37 | 0.064 | -1.31 | 0.062 | -1.2 |
| 1434919_at | --- | Transcribed locus | -1.18 | 0.597 | -1.1 | 0.767 | -1.15 | 0.484 | -1.13 | 0.707 | -1.14 |
| 1457323_at | LOC632191 | hypothetical protein LOC632191 | -1.05 | 0.109 | -1.05 | 0.726 | -1.4 | 0.466 | -1.16 | 0.644 | -1.16 |
| 1444268_at | BC004004 | cDNA sequence BC004004 | -1.14 | 0.683 | -1.28 | 0.27 | -1.04 | 0.892 | -1.24 | 0.47 | -1.17 |
| 1452586_at | Anapc13 | anaphase promoting complex subunit 13 | -1.14 | 0.148 | -1.08 | 0.084 | -1.21 | 0.172 | -1.14 | 0.318 | -1.14 |
| 1419081_at | Atg10 | autophagy-related 10 (yeast) | -1.1 | 0.469 | -1.18 | 0.319 | -1.15 | 0.039 | -1.33 | 0.23 | -1.19 |
| 1428830_at | Atm | ataxia telangiectasia mutated homolog (human) | -1.14 | 0.201 | -1.18 | 0.093 | -1.11 | 0.636 | -1.89 | 0.143 | -1.33 |
| 1424578_at | Arrdc1 | arrestin domain containing 1 | -1.14 | 0.516 | -1.06 | 0.651 | -1.24 | 0.096 | -1.72 | 0.038 | -1.29 |
| 1433068_at | 6330582A15Rik | RIKEN cDNA 6330582A15 gene | -1.11 | 0.691 | -1.25 | 0.238 | -1.09 | 0.667 | -1.1 | 0.804 | -1.14 |
| 1426863_at | Rbmx | RNA binding motif protein, X chromosome | -1.16 | 0.586 | -1 | 0.964 | -1.3 | 0.385 | -1.33 | 0.37 | -1.2 |
| 1417704_a_at | Arhgap6 | Rho GTPase activating protein 6 | -1.2 | 0.4 | -1.15 | 0.249 | -1.09 | 0.526 | -1.07 | 0.818 | -1.13 |

| | | | | | | | | | | | |
|---|---|---|---|---|---|---|---|---|---|---|---|
| 1429163_at | Dchs1 | dachsous 1 (Drosophila) | -1.11 | 0.725 | -1.13 | 0.661 | -1.2 | 0.395 | -1.74 | 0.032 | -1.29 |
| 1434070_at | Jag1 | jagged 1 | -1.22 | 0.056 | -1.19 | 0.541 | -1.04 | 0.769 | -1.66 | 0.103 | -1.28 |
| 1451903_at | Kynu | kynureninase (L-kynurenine hydrolase) | -1.18 | 0.811 | -1.08 | 0.801 | -1.17 | 0.576 | -1.34 | 0.223 | -1.19 |
| 1418092_s_at | Trip10 | thyroid hormone receptor interactor 10 | -1.07 | 0.672 | -1.28 | 0.105 | -1.1 | 0.512 | -1.38 | 0.046 | -1.21 |
| 1419291_x_at | Gas5 | growth arrest specific 5 | -1.02 | 0.715 | -1.11 | 0.426 | -1.34 | 0.158 | -1.52 | 0.008 | -1.25 |
| 1433594_at | Commd2 | COMM domain containing 2 | -1.12 | 0.535 | -1.03 | 0.552 | -1.32 | 0.168 | -1.58 | 0.164 | -1.26 |
| 1429456_a_at | Polr3e | polymerase (RNA) III (DNA directed) polypeptide E | -1.21 | 0.722 | -1.19 | 0.322 | -1.04 | 0.784 | -1.2 | 0.13 | -1.16 |
| 1451217_a_at | Immp1l | IMP1 inner mitochondrial membrane peptidase-like (S. cerevisiae) | -1.06 | 0.231 | -1.27 | 0.031 | -1.12 | 0.377 | -1.17 | 0.457 | -1.15 |
| 1459630_at | --- | Transcribed locus | -1.14 | 0.658 | -1.06 | 0.8 | -1.25 | 0.218 | -1.09 | 0.52 | -1.13 |
| 1434474_at | Abca5 | ATP-binding cassette, sub-family A (ABC1), member 5 | -1.09 | 0.529 | -1.06 | 0.613 | -1.31 | 0.177 | -1.5 | 0.344 | -1.24 |
| 1438673_at | --- | --- | -1.13 | 0.588 | -1.17 | 0.068 | -1.13 | 0.087 | -1.51 | 0.133 | -1.24 |
| 1446060_at | 9230109A22Rik | RIKEN cDNA 9230109A22 gene | -1.2 | 0.817 | -1.06 | 0.915 | -1.18 | 0.575 | -1.38 | 0.23 | -1.2 |
| 1460572_a_at | Zfp511 | zinc finger protein 511 | -1.07 | 0.763 | -1.11 | 0.571 | -1.27 | 0.315 | -1.48 | 0.034 | -1.23 |
| 1425159_at | Golt1a | golgi transport 1 homolog A (S. cerevisiae) | -1.42 | 0.217 | -1.04 | 0.636 | -1.04 | 0.815 | -1.35 | 0.536 | -1.21 |
| 1418622_at | Rab2 | RAB2, member RAS oncogene family | -1.1 | 0.238 | -1.1 | 0.225 | -1.24 | 0.088 | -1.13 | 0.596 | -1.14 |
| 1457086_at | LOC434147 | hypothetical LOC434147 | -1.13 | 0.509 | -1.09 | 0.788 | -1.22 | 0.622 | -1.3 | 0.597 | -1.18 |
| 1439049_at | Dph5 | DPH5 homolog (S. cerevisiae) | -1.14 | 0.579 | -1.03 | 0.837 | -1.29 | 0.256 | -2.48 | 0.056 | -1.49 |
| 1448497_at | Ercc3 | excision repair cross-complementing rodent repair deficiency, complementation g | -1.04 | 0.791 | -1.22 | 0.122 | -1.19 | 0.289 | -1.18 | 0.339 | -1.16 |
| 1422561_at | Adamts5 | a disintegrin-like and metallopeptidase (reprolysin type) with thrombospondin type | -1.05 | 0.216 | -1.42 | 0.153 | -1.03 | 0.739 | -1.39 | 0.269 | -1.22 |
| 1434410_at | BC043118 | cDNA sequence BC043118 | -1.18 | 0.49 | -1.18 | 0.153 | -1.08 | 0.643 | -1 | 0.989 | -1.11 |
| 1436175_at | A430107N12Rik | RIKEN cDNA A430107N12 gene | -1.09 | 0.536 | -1.02 | 0.858 | -1.36 | 0.044 | -1.43 | 0.212 | -1.23 |
| 1423876_at | AI450540 | expressed sequence AI450540 | -1.1 | 0.52 | -1.08 | 0.689 | -1.26 | 0.183 | -1.18 | 0.605 | -1.16 |
| 1416015_s_at | Abce1 | ATP-binding cassette, sub-family E (OABP), member 1 | -1.1 | 0.47 | -1.11 | 0.222 | -1.23 | 0.019 | -1.02 | 0.883 | -1.11 |
| 1456349_x_at | Sumo1 | SMT3 suppressor of mif two 3 homolog 1 (yeast) | -1.13 | 0.435 | -1.03 | 0.805 | -1.3 | 0.007 | -1.35 | 0.107 | -1.2 |
| 1430300_at | Sco1 | SCO cytochrome oxidase deficient homolog 1 (yeast) | -1.15 | 0.386 | -1.16 | 0.292 | -1.12 | 0.257 | -1.12 | 0.469 | -1.14 |
| 1434392_at | Usp34 | ubiquitin specific peptidase 34 | -1.08 | 0.734 | -1.09 | 0.436 | -1.28 | 0.059 | -1.13 | 0.545 | -1.14 |
| 1419351_a_at | 0610007P06Rik | RIKEN cDNA 0610007P06 gene | -1.13 | 0.041 | -1.23 | 0.204 | -1.07 | 0.639 | -1.12 | 0.41 | -1.14 |
| 1428115_a_at | Rab2b | RAB2B, member RAS oncogene family | -1.16 | 0.397 | -1.2 | 0.522 | -1.07 | 0.586 | -1.65 | 0.152 | -1.27 |
| 1418000_a_at | Itm2b | integral membrane protein 2B | -1.08 | 0.066 | -1.23 | 0.336 | -1.13 | 0.084 | -1.04 | 0.531 | -1.12 |
| 1435128_at | Baiap2 | brain-specific angiogenesis inhibitor 1-associated protein 2 | -1 | 0.994 | -1.5 | 0.249 | -1.04 | 0.913 | -1.09 | 0.761 | -1.16 |
| 1441837_at | Chrnb2 | cholinergic receptor, nicotinic, beta polypeptide 2 (neuronal) | -1.16 | 0.637 | -1.04 | 0.899 | -1.24 | 0.597 | -4.29 | 0.002 | -1.93 |
| 1449436_s_at | Ubb /// LOC218963 | ubiquitin B /// ubiquitin pseudogene /// gene model 1821, (NCBI) /// similar to poly | -1.08 | 0.351 | -1.15 | 0.22 | -1.2 | 0.031 | -1.11 | 0.132 | -1.14 |
| 1417340_at | Txnl2 | thioredoxin-like 2 | -1.18 | 0.123 | -1.1 | 0.1 | -1.15 | 0.142 | -1.34 | 0.26 | -1.19 |
| 1455403_at | Manea | mannosidase, endo-alpha | -1.23 | 0.041 | -1.09 | 0.165 | -1.11 | 0.163 | -1.04 | 0.877 | -1.12 |
| 1430575_a_at | Tpp2 | tripeptidyl peptidase II | -1.02 | 0.84 | -1.14 | 0.531 | -1.3 | 0.07 | -1.49 | 0.083 | -1.24 |
| 1428461_at | Ppp2r5e | protein phosphatase 2, regulatory subunit B (B56), epsilon isoform | -1.19 | 0.587 | -1.14 | 0.35 | -1.09 | 0.224 | -1.03 | 0.774 | -1.12 |
| 1452970_at | Zmym2 | zinc finger, MYM-type 2 | -1.17 | 0.16 | -1.09 | 0.337 | -1.16 | 0.134 | -1.2 | 0.089 | -1.16 |
| 1446313_at | Zfp30 | zinc finger protein 30 | -1.05 | 0.707 | -1.35 | 0.513 | -1.07 | 0.87 | -1.71 | 0.183 | -1.3 |
| 1428898_at | Mon1a | MON1 homolog A (yeast) | -1.1 | 0.657 | -1.17 | 0.298 | -1.16 | 0.203 | -1.2 | 0.298 | -1.16 |
| 1443262_at | Mrps14 | mitochondrial ribosomal protein S14 | -1.18 | 0.196 | -1.22 | 0.23 | -1.04 | 0.827 | -1.65 | 0.206 | -1.27 |
| 1434568_at | --- | Transcribed locus | -1.01 | 0.924 | -1.13 | 0.643 | -1.32 | 0.003 | -1.21 | 0.509 | -1.17 |
| 1460448_s_at | Ttc14 | tetratricopeptide repeat domain 14 | -1.06 | 0.85 | -1.18 | 0.365 | -1.2 | 0.361 | -1.23 | 0.091 | -1.17 |
| 1439788_at | Ccdc111 | coiled-coil domain containing 111 | -1.31 | 0.404 | -1.12 | 0.193 | -1.03 | 0.904 | -1.2 | 0.2 | -1.16 |
| 1416472_at | Syap1 | synapse associated protein 1 | -1.1 | 0.05 | -1.12 | 0.211 | -1.22 | 0.077 | -1.08 | 0.708 | -1.13 |
| 1454955_at | Ipo7 | importin 7 | -1.13 | 0.515 | -1.18 | 0.21 | -1.11 | 0.263 | -1.06 | 0.829 | -1.12 |
| 1454874_at | Btbd7 | BTB (POZ) domain containing 7 | -1.14 | 0.075 | -1.1 | 0.548 | -1.18 | 0.132 | -1.21 | 0.323 | -1.16 |
| 1427913_at | Rwdd1 | RWD domain containing 1 | -1.07 | 0.56 | -1.09 | 0.272 | -1.29 | 0.048 | -1.3 | 0.113 | -1.19 |
| 1430287_s_at | Hemk1 | HemK methyltransferase family member 1 | -1.09 | 0.758 | -1.13 | 0.453 | -1.21 | 0.524 | -1.33 | 0.355 | -1.19 |
| 1424604_s_at | Sumf1 | sulfatase modifying factor 1 | -1.04 | 0.742 | -1.1 | 0.375 | -1.32 | 0.085 | -1.11 | 0.667 | -1.14 |
| 1455742_x_at | Morf4l1 /// A730098 | mortality factor 4 like 1 /// RIKEN cDNA A730098P11 gene /// similar to mortality f | -1.08 | 0.057 | -1.07 | 0.08 | -1.3 | 0.001 | -1.18 | 0.034 | -1.16 |
| 1428172_at | Prpf39 | PRP39 pre-mRNA processing factor 39 homolog (yeast) | -1.03 | 0.725 | -1.12 | 0.633 | -1.31 | 0.147 | -1.45 | 0.314 | -1.23 |
| 1453520_at | Ttc21b | tetratricopeptide repeat domain 21B | -1.02 | 0.987 | -1.09 | 0.918 | -1.37 | 0.258 | -2.47 | 0.099 | -1.49 |
| 1437489_x_at | Sdhd | succinate dehydrogenase complex, subunit D, integral membrane protein | -1.3 | 0.086 | -1.04 | 0.753 | -1.11 | 0.388 | -1.16 | 0.328 | -1.15 |

| | | | | | | | | | | | |
|---|---|---|---|---|---|---|---|---|---|---|---|
| 1437993_x_at | Qdpr | quininoid dihydropteridine reductase | -1.16 | 0.035 | -1.13 | 0.44 | -1.13 | 0.191 | -1.2 | 0.461 | -1.15 |
| 1441980_at | C030007I09Rik | RIKEN cDNA C030007I09 gene | -1.19 | 0.326 | -1.01 | 0.87 | -1.24 | 0.179 | -1.23 | 0.377 | -1.17 |
| 1457814_at | 1110014D18Rik | RIKEN cDNA 1110014D18 gene | -1.01 | 0.974 | -1.45 | 0.056 | -1.05 | 0.797 | -1.18 | 0.369 | -1.17 |
| 1455072_at | Cep350 | centrosomal protein 350 | -1.2 | 0.101 | -1.07 | 0.499 | -1.16 | 0.335 | -1.12 | 0.467 | -1.14 |
| 1435501_at | C030004M05Rik | RIKEN cDNA C030004M05 gene | -1.17 | 0.597 | -1.24 | 0.222 | -1.03 | 0.909 | -1.24 | 0.417 | -1.17 |
| 1429116_at | Slc17a5 | solute carrier family 17 (anion/sugar transporter), member 5 | -1.13 | 0.401 | -1.03 | 0.873 | -1.29 | 0.231 | -1.04 | 0.91 | -1.12 |
| 1458453_at | Lmo7 | LIM domain only 7 | -1.11 | 0.164 | -1.31 | 0.651 | -1.03 | 0.883 | -1.91 | 0.044 | -1.34 |
| 1428431_at | 2310047A01Rik | RIKEN cDNA 2310047A01 gene | -1.17 | 0.264 | -1.02 | 0.82 | -1.26 | 0.235 | -1.43 | 0.088 | -1.22 |
| 1416943_at | Ube2e1 /// LOC631 | ubiquitin-conjugating enzyme E2E 1, UBC4/5 homolog (yeast) /// similar to Ubiqui | -1.26 | 0 | -1.1 | 0.021 | -1.07 | 0.407 | -1.45 | 0.088 | -1.22 |
| 1434396_a_at | Myl6 /// LOC43329 | myosin, light polypeptide 6, alkali, smooth muscle and non-muscle /// similar to m | -1.01 | 0.922 | -1.02 | 0.881 | -1.53 | 0.003 | -1.27 | 0.05 | -1.21 |
| 1437405_a_at | Igfbp4 | insulin-like growth factor binding protein 4 | -1.13 | 0.39 | -1.17 | 0.382 | -1.12 | 0.599 | -1.21 | 0.41 | -1.16 |
| 1426124_a_at | Clk1 | CDC-like kinase 1 | -1.13 | 0.333 | -1.16 | 0.377 | -1.13 | 0.411 | -1.44 | 0.182 | -1.22 |
| 1438348_x_at | Ccbl1 | cysteine conjugate-beta lyase 1 | -1.06 | 0.891 | -1.03 | 0.928 | -1.39 | 0.396 | -1.37 | 0.236 | -1.21 |
| 1431333_at | Hist1h4h | histone 1, H4h | -1.23 | 0.806 | -1.15 | 0.471 | -1.06 | 0.814 | -1.01 | 0.977 | -1.11 |
| 1444883_at | Tmem19 | transmembrane protein 19 | -1 | 0.989 | -1.01 | 0.977 | -1.55 | 0.043 | -1.59 | 0.23 | -1.29 |
| 1434659_at | 5830411G16Rik | RIKEN cDNA 5830411G16 gene | -1.05 | 0.535 | -1.11 | 0.083 | -1.27 | 0.065 | -1.44 | 0.17 | -1.22 |
| 1417935_at | Mkrn2 | makorin, ring finger protein, 2 | -1.22 | 0.054 | -1.1 | 0.374 | -1.11 | 0.421 | -1.2 | 0.027 | -1.16 |
| 1452239_at | Gt(ROSA)26Sor | gene trap ROSA 26, Philippe Soriano | -1.25 | 0.106 | -1.06 | 0.746 | -1.13 | 0.407 | -1.28 | 0.062 | -1.18 |
| 1429988_at | Zfp235 | zinc finger protein 235 | -1.15 | 0.46 | -1.18 | 0.219 | -1.09 | 0.526 | -1 | 0.998 | -1.11 |
| 1424681_a_at | Psma5 | proteasome (prosome, macropain) subunit, alpha type 5 | -1.17 | 0.025 | -1.13 | 0.137 | -1.12 | 0.299 | -1.04 | 0.879 | -1.11 |
| 1434162_at | 2700078E11Rik | RIKEN cDNA 2700078E11 gene | -1.07 | 0.525 | -1.13 | 0.155 | -1.23 | 0.07 | -1.19 | 0.231 | -1.16 |
| 1434756_at | 5430421B17 | hypothetical protein 5430421B17 | -1.05 | 0.632 | -1.01 | 0.954 | -1.43 | 0.132 | -1.27 | 0.229 | -1.19 |
| 1456540_s_at | Mtmr6 | myotubularin related protein 6 | -1.17 | 0.528 | -1.06 | 0.704 | -1.19 | 0.089 | -1.42 | 0.103 | -1.21 |
| 1459013_at | --- | --- | -1.16 | 0.61 | -1.15 | 0.78 | -1.11 | 0.823 | -1.01 | 0.989 | -1.11 |
| 1435662_at | 2610020O08Rik | RIKEN cDNA 2610020O08 gene | -1.08 | 0.501 | -1.1 | 0.464 | -1.25 | 0.016 | -1.28 | 0.307 | -1.18 |
| 1434520_at | Sc5d | sterol-C5-desaturase (fungal ERG3, delta-5-desaturase) homolog (S. cerevisae) | -1.08 | 0.46 | -1.17 | 0.528 | -1.17 | 0.358 | -1.27 | 0.006 | -1.17 |
| 1441119_at | Garnl1 | GTPase activating RANGAP domain-like 1 | -1.21 | 0.034 | -1.06 | 0.84 | -1.15 | 0.396 | -1.09 | 0.667 | -1.13 |
| 1426598_at | Uty | ubiquitously transcribed tetratricopeptide repeat gene, Y chromosome | -1.14 | 0.861 | -1.19 | 0.838 | -1.09 | 0.723 | -1.36 | 0.639 | -1.2 |
| 1427089_at | Ccnt2 | cyclin T2 | -1.1 | 0.564 | -1.23 | 0.155 | -1.09 | 0.59 | -1.15 | 0.551 | -1.14 |
| 1433669_at | Akap8 | A kinase (PRKA) anchor protein 8 | -1.12 | 0.229 | -1.13 | 0.413 | -1.17 | 0.205 | -1.28 | 0.228 | -1.17 |
| 1429549_at | Col27a1 | procollagen, type XXVII, alpha 1 | -1.06 | 0.607 | -1.4 | 0.307 | -1.03 | 0.952 | -1.33 | 0.158 | -1.2 |
| 1446548_at | Gltscr1 | Glioma tumor suppressor candidate region gene 1 | -1.04 | 0.927 | -1.37 | 0.143 | -1.06 | 0.874 | -1.2 | 0.512 | -1.17 |
| 1425533_a_at | Stau2 | staufen (RNA binding protein) homolog 2 (Drosophila) | -1.07 | 0.639 | -1.35 | 0.099 | -1.04 | 0.718 | -1.03 | 0.894 | -1.12 |
| 1446194_at | Nr2c1 | nuclear receptor subfamily 2, group C, member 1 | -1.24 | 0.419 | -1.04 | 0.857 | -1.15 | 0.625 | -1.17 | 0.336 | -1.15 |
| 1447703_x_at | Zfp593 | zinc finger protein 593 | -1.21 | 0.083 | -1.22 | 0.144 | -1.02 | 0.953 | -1.68 | 0.236 | -1.28 |
| 1427319_at | A230046K03Rik | RIKEN cDNA A230046K03 gene | -1.16 | 0.49 | -1.28 | 0.091 | -1 | 0.987 | -1.48 | 0.271 | -1.23 |
| 1415833_x_at | Rps29 /// LOC6299 | ribosomal protein S29 /// similar to 40S ribosomal protein S29 /// similar to 40S rib | -1.12 | 0.101 | -1.02 | 0.824 | -1.32 | 0.007 | -1.33 | 0.05 | -1.2 |
| 1451219_at | Ormdl1 | ORM1-like 1 (S. cerevisiae) | -1.03 | 0.861 | -1.38 | 0.135 | -1.07 | 0.719 | -1.24 | 0.171 | -1.18 |
| 1448137_at | Aldh7a1 | aldehyde dehydrogenase family 7, member A1 | -1.16 | 0.553 | -1.15 | 0.664 | -1.1 | 0.621 | -1.24 | 0.29 | -1.16 |
| 1455613_at | E130308A19Rik | RIKEN cDNA E130308A19 gene | -1.22 | 0.225 | -1.21 | 0.323 | -1 | 0.966 | -1.02 | 0.857 | -1.11 |
| 1439704_at | Hdac2 | histone deacetylase 2 | -1.08 | 0.814 | -1.24 | 0.62 | -1.1 | 0.61 | -1.31 | 0.4 | -1.18 |
| 1434078_at | D7Wsu128e | DNA segment, Chr 7, Wayne State University 128, expressed | -1.09 | 0.621 | -1.15 | 0.235 | -1.18 | 0.52 | -1.11 | 0.114 | -1.13 |
| 1456871_a_at | Phf20l1 | PHD finger protein 20-like 1 | -1.02 | 0.792 | -1 | 0.979 | -1.51 | 0.02 | -1.12 | 0.446 | -1.16 |
| 1417918_at | Mrpl11 | mitochondrial ribosomal protein L11 | -1.22 | 0.062 | -1.16 | 0.023 | -1.05 | 0.827 | -1.39 | 0.052 | -1.2 |
| 1434404_at | C030011O14Rik | RIKEN cDNA C030011O14 gene | -1.11 | 0.547 | -1.19 | 0.583 | -1.11 | 0.491 | -1.26 | 0.447 | -1.17 |
| 1456038_at | Fbxl4 | F-box and leucine-rich repeat protein 4 | -1.24 | 0.146 | -1.08 | 0.154 | -1.11 | 0.385 | -1.11 | 0.724 | -1.13 |
| 1436276_at | --- | Transcribed locus | -1.01 | 0.94 | -1.52 | 0.176 | -1.01 | 0.959 | -1.13 | 0.435 | -1.17 |
| 1426518_at | Tubgcp5 | tubulin, gamma complex associated protein 5 | -1.12 | 0.43 | -1.16 | 0.33 | -1.13 | 0.5 | -1.26 | 0.451 | -1.17 |
| 1428152_a_at | Rpl18a | Ribosomal protein L18A | -1.02 | 0.887 | -1.12 | 0.065 | -1.31 | 0.007 | -1.26 | 0.034 | -1.18 |
| 1437704_at | 2900024O10Rik | RIKEN cDNA 2900024O10 gene | -1.15 | 0.301 | -1.07 | 0.685 | -1.2 | 0.013 | -1.5 | 0.072 | -1.23 |
| 1438430_at | Hbp1 | high mobility group box transcription factor 1 | -1.05 | 0.094 | -1.1 | 0.317 | -1.29 | 0.007 | -1.36 | 0.151 | -1.2 |
| 1426844_a_at | Pdcd2l | programmed cell death 2-like | -1.29 | 0.139 | -1.14 | 0.371 | -1.02 | 0.915 | -1.78 | 0.012 | -1.3 |
| 1456429_at | Malt1 | mucosa associated lymphoid tissue lymphoma translocation gene 1 | -1.23 | 0.384 | -1.13 | 0.518 | -1.06 | 0.842 | -1.84 | 0.143 | -1.32 |

| | | | | | | | | | | | |
|---|---|---|---|---|---|---|---|---|---|---|---|
| 1426216_at | Cog6 | component of oligomeric golgi complex 6 | -1.22 | 0.009 | -1.21 | 0.169 | -1.01 | 0.9 | -1.07 | 0.649 | -1.13 |
| 1457606_x_at | AU015228 | expressed sequence AU015228 | -1.02 | 0.947 | -1.07 | 0.432 | -1.38 | 0.005 | -1.42 | 0.289 | -1.22 |
| 1442858_at | Phr1 | Pam, highwire, rpm 1 | -1.17 | 0.469 | -1.05 | 0.895 | -1.2 | 0.294 | -1.53 | 0.148 | -1.24 |
| 1459638_at | Ptpre | Protein tyrosine phosphatase, receptor type, E | -1.12 | 0.673 | -1.14 | 0.649 | -1.15 | 0.535 | -1.21 | 0.532 | -1.16 |
| 1441982_at | Taf7 | TAF7 RNA polymerase II, TATA box binding protein (TBP)-associated factor | -1.1 | 0.549 | -1.27 | 0.516 | -1.07 | 0.871 | -1.28 | 0.749 | -1.18 |
| 1425343_at | Hdhd3 | haloacid dehalogenase-like hydrolase domain containing 3 | -1.24 | 0.379 | -1.04 | 0.77 | -1.15 | 0.418 | -1.27 | 0.69 | -1.17 |
| 1425523_at | 2600011C06Rik | RIKEN cDNA 2600011C06 gene | -1.2 | 0.12 | -1.15 | 0.095 | -1.06 | 0.5 | -1.03 | 0.857 | -1.11 |
| 1446239_at | 4930444F02Rik | RIKEN cDNA 4930444F02 gene | -1.09 | 0.878 | -1.18 | 0.472 | -1.15 | 0.702 | -1.27 | 0.348 | -1.17 |
| 1445796_at | AI315068 | Expressed sequence AI315068 | -1.32 | 0.251 | -1.02 | 0.954 | -1.11 | 0.544 | -1.08 | 0.653 | -1.13 |
| 1447320_x_at | Rpo1-3 | RNA polymerase 1-3 | -1.08 | 0.735 | -1.1 | 0.451 | -1.25 | 0.174 | -1.2 | 0.179 | -1.16 |
| 1423444_at | Rock1 | Rho-associated coiled-coil containing protein kinase 1 | -1.1 | 0.561 | -1.09 | 0.207 | -1.23 | 0.062 | -1.02 | 0.866 | -1.11 |
| 1431150_at | 1700110M21Rik | RIKEN cDNA 1700110M21 gene | -1.05 | 0.889 | -1.19 | 0.729 | -1.18 | 0.696 | -1.61 | 0.494 | -1.26 |
| 1422886_a_at | Clk4 | CDC like kinase 4 | -1.14 | 0.434 | -1.08 | 0.606 | -1.2 | 0.443 | -1.12 | 0.633 | -1.13 |
| 1451035_a_at | Akr1a4 | aldo-keto reductase family 1, member A4 (aldehyde reductase) | -1.24 | 0.021 | -1.09 | 0.224 | -1.09 | 0.323 | -1.19 | 0.291 | -1.15 |
| 1450047_at | Hs6st2 | heparan sulfate 6-O-sulfotransferase 2 | -1.11 | 0.725 | -1.19 | 0.675 | -1.12 | 0.538 | -1.24 | 0.501 | -1.16 |
| 1448853_at | Synj2bp | synaptojanin 2 binding protein | -1.05 | 0.616 | -1.12 | 0.15 | -1.25 | 0.135 | -1.58 | 0.068 | -1.25 |
| 1454750_a_at | BC057552 | cDNA sequence BC057552 | -1.03 | 0.803 | -1.36 | 0.231 | -1.07 | 0.725 | -1.2 | 0.135 | -1.17 |
| 1437180_at | 6530403A03Rik | RIKEN cDNA 6530403A03 gene | -1.26 | 0.075 | -1 | 0.991 | -1.18 | 0.328 | -1.38 | 0.185 | -1.2 |
| 1431784_a_at | Bxdc5 | brix domain containing 5 | -1.13 | 0.369 | -1.26 | 0.195 | -1.04 | 0.764 | -1.33 | 0.229 | -1.19 |
| 1423322_at | Lin7c | lin-7 homolog C (C. elegans) | -1.09 | 0.026 | -1.15 | 0.097 | -1.17 | 0.009 | -1.16 | 0.393 | -1.14 |
| 1457219_at | Arhgef18 | rho/rac guanine nucleotide exchange factor (GEF) 18 | -1.04 | 0.69 | -1.21 | 0.712 | -1.16 | 0.306 | -2.12 | 0.084 | -1.39 |
| 1445609_at | --- | Transcribed locus | -1.02 | 0.889 | -1.15 | 0.699 | -1.26 | 0.69 | -1.63 | 0.451 | -1.27 |
| 1428777_at | Spred1 | sprouty protein with EVH-1 domain 1, related sequence | -1.05 | 0.564 | -1.37 | 0.149 | -1.05 | 0.829 | -1.03 | 0.86 | -1.12 |
| 1455916_at | AI931714 | expressed sequence AI931714 | -1.13 | 0.517 | -1.01 | 0.887 | -1.3 | 0.197 | -1.22 | 0.257 | -1.17 |
| 1416174_at | Rbbp9 | retinoblastoma binding protein 9 | -1.1 | 0.374 | -1.06 | 0.141 | -1.26 | 0.147 | -1.54 | 0.104 | -1.24 |
| 1443693_at | AU022332 | expressed sequence AU022332 | -1.08 | 0.912 | -1.28 | 0.651 | -1.07 | 0.851 | -1.03 | 0.921 | -1.12 |
| 1429107_at | 1110059H15Rik | RIKEN cDNA 1110059H15 gene | -1.12 | 0.357 | -1.19 | 0.086 | -1.1 | 0.459 | -1.02 | 0.933 | -1.11 |
| 1445905_at | C79206 | expressed sequence C79206 | -1.3 | 0.693 | -1.04 | 0.928 | -1.1 | 0.77 | -1.04 | 0.935 | -1.12 |
| 1422485_at | Smad4 | MAD homolog 4 (Drosophila) | -1.11 | 0.057 | -1.19 | 0.121 | -1.12 | 0.451 | -1.75 | 0.152 | -1.29 |
| 1429193_at | Ankib1 | ankyrin repeat and IBR domain containing 1 | -1.15 | 0.144 | -1.1 | 0.39 | -1.16 | 0.313 | -1.3 | 0.076 | -1.18 |
| 1423756_s_at | Igfbp4 | insulin-like growth factor binding protein 4 | -1.21 | 0.453 | -1.16 | 0.392 | -1.05 | 0.87 | -1.29 | 0.441 | -1.18 |
| 1439100_s_at | --- | --- | -1.02 | 0.962 | -1.11 | 0.796 | -1.32 | 0.105 | -2.17 | 0.432 | -1.41 |
| 1423577_at | Ankrd32 | ankyrin repeat domain 32 | -1.14 | 0.591 | -1.11 | 0.306 | -1.15 | 0.176 | -1 | 0.998 | -1.1 |
| 1448109_a_at | Rpl26 | ribosomal protein L26 | -1.06 | 0.167 | -1.07 | 0.499 | -1.32 | 0.025 | -1.26 | 0.001 | -1.18 |
| 1427599_at | Boll | bol, boule-like (Drosophila) | -1.14 | 0.749 | -1.3 | 0.416 | -1 | 0.993 | -1.1 | 0.729 | -1.14 |
| 1451231_a_at | Cul2 | cullin 2 | -1.1 | 0.405 | -1.14 | 0.195 | -1.17 | 0.033 | -1.02 | 0.879 | -1.11 |
| 1433457_s_at | Grsf1 | G-rich RNA sequence binding factor 1 | -1.1 | 0.405 | -1.22 | 0.102 | -1.09 | 0.585 | -1.04 | 0.851 | -1.11 |
| 1430028_at | 2210018M11Rik | RIKEN cDNA 2210018M11 gene | -1.01 | 0.966 | -1.35 | 0.239 | -1.1 | 0.121 | -1.05 | 0.603 | -1.13 |
| 1434935_at | LOC637741 | hypothetical protein LOC637741 | -1.12 | 0.237 | -1.1 | 0.099 | -1.18 | 0.183 | -1.72 | 0.037 | -1.28 |
| 1417974_at | Kpna4 | karyopherin (importin) alpha 4 | -1.07 | 0.662 | -1.06 | 0.598 | -1.31 | 0.053 | -1.46 | 0.131 | -1.22 |
| 1446868_at | Hcfc1 | host cell factor C1 | -1.12 | 0.477 | -1.17 | 0.721 | -1.11 | 0.731 | -1.97 | 0.249 | -1.35 |
| 1452039_a_at | Bap1 | Brca1 associated protein 1 | -1.13 | 0.322 | -1.05 | 0.713 | -1.24 | 0.087 | -1.15 | 0.097 | -1.14 |
| 1428007_at | Krtap13-1 | keratin associated protein 13-1 | -1.09 | 0.893 | -1.2 | 0.617 | -1.11 | 0.768 | -1.07 | 0.846 | -1.12 |
| 1422266_at | Mycs | myc-like oncogene, s-myc protein | -1.23 | 0.704 | -1.08 | 0.883 | -1.11 | 0.771 | -1.57 | 0.08 | -1.24 |
| 1423450_a_at | Hs3st1 | heparan sulfate (glucosamine) 3-O-sulfotransferase 1 | -1.23 | 0.422 | -1.05 | 0.908 | -1.14 | 0.279 | -2.76 | 0.048 | -1.54 |
| 1417670_at | Timm44 | translocase of inner mitochondrial membrane 44 | -1.16 | 0.405 | -1.2 | 0.205 | -1.06 | 0.149 | -1.06 | 0.706 | -1.12 |
| 1442161_at | Igbp1 | immunoglobulin (CD79A) binding protein 1 | -1.21 | 0.672 | -1.21 | 0.218 | -1.01 | 0.987 | -1.26 | 0.329 | -1.17 |
| 1445443_at | --- | --- | -1.05 | 0.527 | -1.05 | 0.843 | -1.35 | 0.147 | -1.11 | 0.553 | -1.14 |
| 1456109_a_at | Mrps15 | mitochondrial ribosomal protein S15 | -1.07 | 0.499 | -1.18 | 0.014 | -1.16 | 0.232 | -1.39 | 0.059 | -1.2 |
| 1416270_at | Polr2g | polymerase (RNA) II (DNA directed) polypeptide G | -1.18 | 0.051 | -1.11 | 0.055 | -1.11 | 0.142 | -1.31 | 0.064 | -1.18 |
| 1425972_a_at | Zfx | zinc finger protein X-linked | -1.08 | 0.386 | -1 | 0.982 | -1.38 | 0.074 | -1.24 | 0.249 | -1.18 |
| 1423757_x_at | Igfbp4 | insulin-like growth factor binding protein 4 | -1.13 | 0.759 | -1.2 | 0.401 | -1.08 | 0.684 | -1.24 | 0.45 | -1.16 |
| 1426457_at | Slmap | sarcolemma associated protein | -1.09 | 0.595 | -1.13 | 0.07 | -1.19 | 0.12 | -1.09 | 0.589 | -1.13 |

| | | | | | | | | | | | |
|---|---|---|---|---|---|---|---|---|---|---|---|
| 1428911_at | Ttll4 | tubulin tyrosine ligase-like family, member 4 | -1.15 | 0.601 | -1.18 | 0.188 | -1.08 | 0.581 | -1.2 | 0.341 | -1.15 |
| 1436219_at | 4933403F05Rik | RIKEN cDNA 4933403F05 gene | -1.17 | 0.421 | -1.02 | 0.897 | -1.23 | 0.054 | -1.01 | 0.925 | -1.11 |
| 1436165_at | Luc7l2 | LUC7-like 2 (S. cerevisiae) | -1.11 | 0.031 | -1.06 | 0.459 | -1.26 | 0.15 | -1.03 | 0.867 | -1.11 |
| 1433781_a_at | Cldn12 | claudin 12 | -1 | 0.97 | -1.22 | 0.163 | -1.21 | 0.126 | -1.37 | 0.42 | -1.2 |
| 1456600_a_at | Rnf7 | ring finger protein 7 | -1.07 | 0.659 | -1.01 | 0.939 | -1.38 | 0.02 | -1.1 | 0.706 | -1.14 |
| 1446508_at | --- | --- | -1.07 | 0.553 | -1.08 | 0.749 | -1.28 | 0.446 | -1.01 | 0.937 | -1.11 |
| 1431507_a_at | Synj2bp /// 181002( | synaptojanin 2 binding protein /// RIKEN cDNA 1810020G14 gene | -1.18 | 0.382 | -1.22 | 0.143 | -1.03 | 0.757 | -1.29 | 0.488 | -1.18 |
| 1434476_at | Crtc1 | CREB regulated transcription coactivator 1 | -1.19 | 0.246 | -1.04 | 0.652 | -1.19 | 0.1 | -1.22 | 0.417 | -1.16 |
| 1423195_at | Hiat1 | hippocampus abundant gene transcript 1 | -1.21 | 0.243 | -1.1 | 0.42 | -1.09 | 0.259 | -1.33 | 0.204 | -1.18 |
| 1424311_at | 0710008K08Rik | RIKEN cDNA 0710008K08 gene | -1.13 | 0.498 | -1.1 | 0.105 | -1.17 | 0.533 | -1.56 | 0.094 | -1.24 |
| 1424800_at | Enah | enabled homolog (Drosophila) | -1.04 | 0.855 | -1.15 | 0.752 | -1.23 | 0.169 | -1.35 | 0.247 | -1.19 |
| 1449099_at | Lrba | LPS-responsive beige-like anchor | -1.03 | 0.802 | -1.33 | 0.015 | -1.08 | 0.399 | -1.16 | 0.028 | -1.15 |
| 1424517_at | Ccdc12 | coiled-coil domain containing 12 | -1.25 | 0.269 | -1.16 | 0.036 | -1.02 | 0.935 | -1.43 | 0.094 | -1.21 |
| 1418185_at | 4733401H18Rik | RIKEN cDNA 4733401H18 gene | -1.16 | 0.066 | -1.2 | 0.002 | -1.06 | 0.736 | -1.09 | 0.671 | -1.13 |
| 1425063_at | --- | --- | -1.42 | 0.643 | -1.04 | 0.859 | -1.02 | 0.972 | -2.92 | 0.098 | -1.6 |
| 1456722_at | Chrdl1 | chordin-like 1 | -1.32 | 0.198 | -1.05 | 0.9 | -1.07 | 0.835 | -1.58 | 0.319 | -1.26 |
| 1427894_at | Vasn | vasorin | -1.12 | 0.563 | -1.17 | 0.313 | -1.11 | 0.674 | -1.48 | 0.335 | -1.22 |
| 1439722_at | --- | --- | -1.22 | 0.377 | -1.02 | 0.924 | -1.18 | 0.358 | -1.59 | 0.086 | -1.25 |
| 1417594_at | Gkap1 | G kinase anchoring protein 1 | -1.08 | 0.69 | -1.08 | 0.681 | -1.27 | 0.246 | -1.12 | 0.765 | -1.13 |
| 1416217_a_at | Rpl37a | ribosomal protein L37a | -1.01 | 0.953 | -1.14 | 0.202 | -1.29 | 0.001 | -1.25 | 0.027 | -1.17 |
| 1434757_at | Cbfa2t2h | core-binding factor, runt domain, alpha subunit 2, translocated to, 2 homolog (hur | -1.11 | 0.384 | -1.02 | 0.91 | -1.31 | 0.089 | -1.13 | 0.113 | -1.14 |
| 1443385_at | A930012N16Rik | RIKEN cDNA A930012N16 gene | -1.09 | 0.916 | -1.23 | 0.582 | -1.09 | 0.865 | -1.08 | 0.914 | -1.12 |
| 1455596_a_at | BC029214 | cDNA sequence BC029214 | -1.03 | 0.81 | -1.32 | 0.108 | -1.09 | 0.374 | -1.24 | 0.313 | -1.17 |
| 1416765_s_at | Magmas | mitochondria-associated protein involved in granulocyte-macrophage colony-stim | -1.03 | 0.375 | -1.13 | 0.226 | -1.26 | 0.157 | -1.2 | 0.237 | -1.15 |
| 1453794_at | Fer1l4 | fer-1-like 4 (C. elegans) | -1.14 | 0.826 | -1.01 | 0.975 | -1.28 | 0.416 | -1.09 | 0.652 | -1.13 |
| 1448866_at | Senp3 | SUMO/sentrin specific peptidase 3 | -1.24 | 0.213 | -1 | 0.994 | -1.19 | 0.032 | -1.21 | 0.248 | -1.16 |
| 1441440_at | Atg4c | autophagy-related 4C (yeast) | -1.06 | 0.627 | -1.01 | 0.966 | -1.4 | 0.006 | -1.57 | 0.097 | -1.26 |
| 1456403_at | Pag1 | phosphoprotein associated with glycosphingolipid microdomains 1 | -1.17 | 0.643 | -1.24 | 0.654 | -1.02 | 0.951 | -1.39 | 0.28 | -1.2 |
| 1442567_at | Zbtb24 | zinc finger and BTB domain containing 24 | -1.25 | 0.542 | -1.13 | 0.758 | -1.04 | 0.811 | -1.02 | 0.944 | -1.11 |
| 1440150_at | Tgm3 | Transglutaminase 3, E polypeptide | -1.17 | 0.63 | -1.11 | 0.777 | -1.12 | 0.803 | -1.28 | 0.737 | -1.17 |
| 1436555_at | Slc7a2 | solute carrier family 7 (cationic amino acid transporter, y+ system), member 2 | -1.1 | 0.089 | -1.2 | 0.464 | -1.11 | 0.608 | -1.11 | 0.703 | -1.13 |
| 1456201_at | 4632427E13Rik | RIKEN cDNA 4632427E13 gene | -1.04 | 0.865 | -1.07 | 0.403 | -1.32 | 0.161 | -1.04 | 0.674 | -1.12 |
| 1429707_at | Plaa | phospholipase A2, activating protein | -1.18 | 0.223 | -1.14 | 0.211 | -1.08 | 0.576 | -1.06 | 0.294 | -1.12 |
| 1437977_at | Sgtb | Small glutamine-rich tetratricopeptide repeat (TPR)-containing, beta | -1.23 | 0.29 | -1.14 | 0.506 | -1.05 | 0.852 | -1.04 | 0.852 | -1.11 |
| 1422130_at | Nptx1 | neuronal pentraxin 1 | -1.16 | 0.421 | -1.08 | 0.615 | -1.16 | 0.27 | -1.67 | 0.091 | -1.27 |
| 1450730_at | Hs2st1 | heparan sulfate 2-O-sulfotransferase 1 | -1.08 | 0.462 | -1.1 | 0.571 | -1.23 | 0.45 | -1.08 | 0.801 | -1.12 |
| 1436056_at | Kif13b | kinesin family member 13B | -1.13 | 0.266 | -1.12 | 0.267 | -1.16 | 0.328 | -1.03 | 0.874 | -1.11 |
| 1431515_at | 4932414N04Rik | RIKEN cDNA 4932414N04 gene | -1.04 | 0.947 | -1.41 | 0.488 | -1.02 | 0.964 | -1.83 | 0.337 | -1.32 |
| 1418503_at | Hspa9a | heat shock protein 9A | -1.11 | 0.373 | -1.15 | 0.206 | -1.14 | 0.169 | -1.31 | 0.267 | -1.18 |
| 1435250_at | Ints8 | integrator complex subunit 8 | -1.09 | 0.593 | -1.13 | 0.282 | -1.18 | 0.268 | -1.13 | 0.572 | -1.13 |
| 1452770_at | Vkorc1 | vitamin K epoxide reductase complex, subunit 1 | -1.04 | 0.625 | -1.15 | 0.004 | -1.22 | 0.03 | -1.12 | 0.387 | -1.13 |
| 1436008_at | Tpd52 | Tumor protein D52 | -1.19 | 0.354 | -1.12 | 0.697 | -1.09 | 0.766 | -1.21 | 0.132 | -1.15 |
| 1436848_x_at | Impa1 | inositol (myo)-1(or 4)-monophosphatase 1 | -1.07 | 0.625 | -1.17 | 0.251 | -1.16 | 0.108 | -1.06 | 0.374 | -1.12 |
| 1452580_a_at | Mrpl21 | mitochondrial ribosomal protein L21 | -1.16 | 0.008 | -1.18 | 0.018 | -1.06 | 0.63 | -1.26 | 0.205 | -1.17 |
| 1438330_at | BC065397 | cDNA sequence BC065397 | -1.19 | 0.375 | -1.15 | 0.307 | -1.06 | 0.812 | -1.17 | 0.554 | -1.14 |
| 1417510_at | Vps4a | vacuolar protein sorting 4a (yeast) | -1.05 | 0.573 | -1.06 | 0.2 | -1.31 | 0.046 | -1.28 | 0.201 | -1.18 |
| 1417538_at | Slc35a1 | solute carrier family 35 (CMP-sialic acid transporter), member 1 | -1.06 | 0.503 | -1.04 | 0.718 | -1.34 | 0.009 | -1.05 | 0.778 | -1.12 |
| 1460008_x_at | Ero1l /// Rpl31 /// L( | ERO1-like (S. cerevisiae) /// ribosomal protein L31 /// similar to ribosomal protein | -1.13 | 0.025 | -1.02 | 0.73 | -1.26 | 0.021 | -1.27 | 0.001 | -1.17 |
| 1428829_at | 6820401H01Rik | RIKEN cDNA 6820401H01 gene | -1.1 | 0.624 | -1.21 | 0.11 | -1.1 | 0.361 | -1.55 | 0.214 | -1.24 |
| 1445729_at | Dnaja1 | DnaJ (Hsp40) homolog, subfamily A, member 1 | -1.27 | 0.072 | -1.15 | 0.316 | -1 | 0.975 | -1.05 | 0.838 | -1.12 |
| 1445396_at | Lrch1 | leucine-rich repeats and calponin homology (CH) domain containing 1 | -1.12 | 0.792 | -1.03 | 0.934 | -1.26 | 0.427 | -1.61 | 0.287 | -1.26 |
| 1437508_at | Sp4 | trans-acting transcription factor 4 | -1.08 | 0.766 | -1.05 | 0.858 | -1.3 | 0.067 | -1.01 | 0.963 | -1.11 |
| 1434921_at | Nr2e1 | nuclear receptor subfamily 2, group E, member 1 | -1.11 | 0.887 | -1.08 | 0.838 | -1.21 | 0.772 | -1.06 | 0.815 | -1.11 |

| | | | | | | | | | | | |
|---|---|---|---|---|---|---|---|---|---|---|---|
| 1443830_x_at | Rnf103 | ring finger protein 103 | -1.08 | 0.708 | -1.1 | 0.392 | -1.22 | 0.245 | -1.17 | 0.748 | -1.14 |
| 1429017_at | Smcr8 | Smith-Magenis syndrome chromosome region, candidate 8 homolog (human) | -1.07 | 0.634 | -1.2 | 0.245 | -1.13 | 0.404 | -1.19 | 0.119 | -1.15 |
| 1428421_a_at | 2700085E05Rik | RIKEN cDNA 2700085E05 gene | -1.1 | 0.018 | -1.23 | 0.099 | -1.07 | 0.557 | -1.05 | 0.686 | -1.11 |
| 1442937_at | C77190 | expressed sequence C77190 | -1.12 | 0.889 | -1.18 | 0.779 | -1.1 | 0.836 | -1.17 | 0.844 | -1.14 |
| 1456358_at | Etv3 | Ets variant gene 3 | -1.06 | 0.625 | -1.16 | 0.377 | -1.18 | 0.624 | -1.93 | 0.102 | -1.33 |
| 1420855_at | Eln | elastin | -1.06 | 0.514 | -1.08 | 0.534 | -1.27 | 0.138 | -1.39 | 0.291 | -1.2 |
| 1423654_a_at | Rnf4 | ring finger protein 4 | -1.17 | 0.489 | -1.09 | 0.02 | -1.14 | 0.152 | -1.07 | 0.372 | -1.12 |
| 1445181_at | Eml5 | echinoderm microtubule associated protein like 5 | -1.08 | 0.659 | -1.02 | 0.95 | -1.34 | 0.009 | -1.15 | 0.15 | -1.15 |
| 1450543_at | Myo1h | myosin 1H | -1.19 | 0.807 | -1.11 | 0.818 | -1.09 | 0.731 | -1.25 | 0.471 | -1.16 |
| 1455420_at | Rad23b | RAD23b homolog (S. cerevisiae) | -1.2 | 0.071 | -1.02 | 0.912 | -1.19 | 0.247 | -1.25 | 0.305 | -1.17 |
| 1446410_at | Cept1 | Choline/ethanolaminephosphotransferase 1 | -1.05 | 0.594 | -1.07 | 0.777 | -1.31 | 0.123 | -1.57 | 0.083 | -1.25 |
| 1450859_s_at | Ube2d3 | ubiquitin-conjugating enzyme E2D 3 (UBC4/5 homolog, yeast) | -1.1 | 0.504 | -1.06 | 0.563 | -1.25 | 0.364 | -1.31 | 0.072 | -1.18 |
| 1419174_at | 2410004B18Rik | RIKEN cDNA 2410004B18 gene | -1.09 | 0.093 | -1.11 | 0.242 | -1.2 | 0.021 | -1.27 | 0.028 | -1.17 |
| 1434274_at | Phr1 | pam, highwire, rpm 1 | -1.15 | 0.194 | -1.04 | 0.76 | -1.22 | 0.134 | -1.63 | 0.09 | -1.26 |
| 1439271_x_at | Ik | IK cytokine | -1.12 | 0.482 | -1.09 | 0.288 | -1.19 | 0.067 | -1.18 | 0.292 | -1.14 |
| 1433101_at | 9030419F21Rik | RIKEN cDNA 9030419F21 gene | -1.19 | 0.193 | -1.05 | 0.735 | -1.16 | 0.44 | -1.29 | 0.389 | -1.17 |
| 1458095_at | Gtl2 | GTL2, imprinted maternally expressed untranslated mRNA | -1.09 | 0.484 | -1.27 | 0.752 | -1.06 | 0.869 | -1.95 | 0.184 | -1.34 |
| 1431951_a_at | Usp16 | ubiquitin specific peptidase 16 | -1.13 | 0.585 | -1.04 | 0.682 | -1.24 | 0.109 | -1.21 | 0.216 | -1.16 |
| 1436580_at | --- | --- | -1.14 | 0.327 | -1.17 | 0.627 | -1.09 | 0.393 | -1.02 | 0.959 | -1.1 |
| 1429485_a_at | Utp11l | UTP11-like, U3 small nucleolar ribonucleoprotein, (yeast) | -1.13 | 0.177 | -1.07 | 0.167 | -1.2 | 0.036 | -1.21 | 0.069 | -1.15 |
| 1447333_at | Slc11a2 | Solute carrier family 11 (proton-coupled divalent metal ion transporters), member | -1.23 | 0.695 | -1.04 | 0.94 | -1.13 | 0.805 | -1.09 | 0.876 | -1.13 |
| 1423890_x_at | Atp1b1 | ATPase, Na+/K+ transporting, beta 1 polypeptide | -1.07 | 0.065 | -1.28 | 0.126 | -1.07 | 0.698 | -2.21 | 0.073 | -1.41 |
| 1423046_s_at | Ncbp2 | nuclear cap binding protein subunit 2 | -1.04 | 0.691 | -1.11 | 0.466 | -1.26 | 0.052 | -1.3 | 0.114 | -1.18 |
| 1424295_at | Dppa3 | developmental pluripotency-associated 3 | -1.35 | 0.614 | -1 | 0.994 | -1.09 | 0.808 | -1.09 | 0.787 | -1.13 |
| 1427134_at | Sfrs12 | splicing factor, arginine/serine-rich 12 | -1.02 | 0.883 | -1.13 | 0.317 | -1.27 | 0.019 | -1.02 | 0.937 | -1.11 |
| 1456153_at | Ssh2 | slingshot homolog 2 (Drosophila) | -1.05 | 0.866 | -1.16 | 0.334 | -1.19 | 0.068 | -1.46 | 0.118 | -1.21 |
| 1426556_at | Suhw4 | suppressor of hairy wing homolog 4 (Drosophila) | -1.07 | 0.629 | -1.15 | 0.264 | -1.18 | 0.143 | -1.27 | 0.006 | -1.17 |
| 1417982_at | Insig2 | insulin induced gene 2 | -1.02 | 0.867 | -1.35 | 0.207 | -1.07 | 0.693 | -1.17 | 0.37 | -1.15 |
| 1425068_a_at | Tex264 | testis expressed gene 264 | -1.01 | 0.931 | -1.19 | 0.272 | -1.22 | 0.249 | -1.48 | 0.185 | -1.22 |
| 1449004_at | Mrpl46 | mitochondrial ribosomal protein L46 | -1.06 | 0.577 | -1.11 | 0.284 | -1.25 | 0.047 | -1.07 | 0.738 | -1.12 |
| 1454851_at | Nr2c2 | nuclear receptor subfamily 2, group C, member 2 | -1.1 | 0.219 | -1.06 | 0.598 | -1.24 | 0.282 | -1.1 | 0.65 | -1.13 |
| 1452721_a_at | Ccdc53 | coiled-coil domain containing 53 | -1.12 | 0.366 | -1.2 | 0.18 | -1.07 | 0.568 | -1.46 | 0.077 | -1.22 |
| 1454602_s_at | Cnot2 | CCR4-NOT transcription complex, subunit 2 | -1.06 | 0.188 | -1.17 | 0.07 | -1.17 | 0.268 | -1.12 | 0.315 | -1.13 |
| 1421881_a_at | Elavl2 | ELAV (embryonic lethal, abnormal vision, Drosophila)-like 2 (Hu antigen B) | -1.21 | 0.808 | -1.09 | 0.858 | -1.1 | 0.73 | -1.01 | 0.971 | -1.1 |
| 1424052_at | Thap4 | THAP domain containing 4 | -1.08 | 0.008 | -1.18 | 0.51 | -1.14 | 0.4 | -1.15 | 0.047 | -1.14 |
| 1455565_at | Bcl9 | B-cell CLL/lymphoma 9 | -1.23 | 0.777 | -1.04 | 0.93 | -1.14 | 0.667 | -1.94 | 0.065 | -1.34 |
| 1460213_at | Golga4 | golgi autoantigen, golgin subfamily a, 4 | -1.05 | 0.718 | -1.25 | 0.125 | -1.11 | 0.744 | -1.06 | 0.828 | -1.12 |
| 1417405_at | Stard3 | START domain containing 3 | -1.11 | 0.157 | -1.08 | 0.435 | -1.21 | 0.127 | -1.35 | 0.206 | -1.19 |
| 1444456_at | 9030425P06Rik | RIKEN cDNA 9030425P06 gene | -1.06 | 0.661 | -1.04 | 0.761 | -1.33 | 0.149 | -1.44 | 0.146 | -1.22 |
| 1434192_at | Zzef1 | zinc finger, ZZ-type with EF hand domain 1 | -1.12 | 0.3 | -1.15 | 0.297 | -1.13 | 0.462 | -1.25 | 0.305 | -1.16 |
| 1433882_at | Cnot10 | CCR4-NOT transcription complex, subunit 10 | -1.12 | 0.439 | -1.09 | 0.101 | -1.18 | 0.107 | -1.04 | 0.677 | -1.11 |
| 1426840_at | Ythdf3 | YTH domain family 3 | -1.15 | 0.578 | -1.03 | 0.739 | -1.23 | 0.072 | -1.38 | 0.124 | -1.2 |
| 1419287_at | Hspc171 | HSPC171 protein | -1.05 | 0.041 | -1.1 | 0.176 | -1.27 | 0.139 | -1.01 | 0.925 | -1.11 |
| 1428448_a_at | Gtf3c2 | general transcription factor IIIC, polypeptide 2, beta | -1.19 | 0.099 | -1.13 | 0.39 | -1.07 | 0.563 | -1.08 | 0.108 | -1.12 |
| 1430293_a_at | B230118G17Rik | RIKEN cDNA B230118G17 gene | -1.16 | 0.294 | -1.12 | 0.183 | -1.11 | 0.508 | -1.11 | 0.251 | -1.12 |
| 1448589_at | Ndufb5 | NADH dehydrogenase (ubiquinone) 1 beta subcomplex, 5 | -1.1 | 0.158 | -1.28 | 0.043 | -1.03 | 0.788 | -1.48 | 0.144 | -1.23 |
| 1447507_at | C80406 | expressed sequence C80406 | -1.2 | 0.113 | -1.1 | 0.499 | -1.1 | 0.433 | -1.38 | 0.176 | -1.19 |
| 1416996_at | Tbc1d8 | TBC1 domain family, member 8 | -1.07 | 0.8 | -1.31 | 0.39 | -1.05 | 0.595 | -1.32 | 0.091 | -1.19 |
| 1450785_at | Trip4 | thyroid hormone receptor interactor 4 | -1.12 | 0.348 | -1.15 | 0.57 | -1.12 | 0.68 | -1.19 | 0.457 | -1.14 |
| 1448483_a_at | Ndufb2 | NADH dehydrogenase (ubiquinone) 1 beta subcomplex, 2 | -1.09 | 0.26 | -1.08 | 0.224 | -1.23 | 0.203 | -1.01 | 0.959 | -1.1 |
| 1451385_at | 2310056P07Rik | RIKEN cDNA 2310056P07 gene | -1.1 | 0.197 | -1.12 | 0.054 | -1.17 | 0.271 | -1.32 | 0.112 | -1.18 |
| 1419111_at | Ing2 | inhibitor of growth family, member 2 | -1.18 | 0.249 | -1.03 | 0.711 | -1.19 | 0.105 | -1.17 | 0.575 | -1.14 |
| 1434340_at | --- | --- | -1.08 | 0.335 | -1.34 | 0.018 | -1.02 | 0.953 | -1.04 | 0.677 | -1.12 |

| | | | | | | | | | | | |
|---|---|---|---|---|---|---|---|---|---|---|---|
| 1435769_at | Akap9 | A kinase (PRKA) anchor protein (yotiao) 9 | -1.08 | 0.506 | -1.12 | 0.106 | -1.19 | 0.023 | -1.24 | 0.447 | -1.16 |
| 1455762_at | C330002I19Rik | RIKEN cDNA C330002I19 gene | -1.1 | 0.287 | -1.13 | 0.423 | -1.16 | 0.503 | -1.31 | 0.504 | -1.18 |
| 1455189_at | Trim33 /// 8030451I | tripartite motif protein 33 /// RIKEN cDNA 8030451N04 gene | -1.2 | 0.082 | -1.02 | 0.916 | -1.18 | 0.196 | -1.07 | 0.456 | -1.12 |
| 1438409_at | Cep63 | centrosomal protein 63 | -1.01 | 0.884 | -1.02 | 0.915 | -1.45 | 0.024 | -1.38 | 0.119 | -1.22 |
| 1416096_at | AI413782 | expressed sequence AI413782 | -1.19 | 0.313 | -1.05 | 0.573 | -1.16 | 0.251 | -1.19 | 0.35 | -1.15 |
| 1427519_at | Adora2a | adenosine A2a receptor | -1.31 | 0.411 | -1.04 | 0.92 | -1.08 | 0.719 | -1.04 | 0.928 | -1.12 |
| 1417410_s_at | Prkci | protein kinase C, iota | -1.05 | 0.629 | -1.1 | 0.289 | -1.26 | 0.196 | -1.48 | 0.17 | -1.22 |
| 1458404_at | Ndufb8 | NADH dehydrogenase (ubiquinone) 1 beta subcomplex 8 | -1.15 | 0.183 | -1.26 | 0.43 | -1.01 | 0.978 | -1.18 | 0.034 | -1.15 |
| 1447195_at | Elp4 | Elongation protein 4 homolog (S. cerevisiae) | -1.11 | 0.5 | -1 | 0.997 | -1.32 | 0.282 | -1.34 | 0.092 | -1.19 |
| 1416721_s_at | Sfrs6 | splicing factor, arginine/serine-rich 6 | -1 | 0.983 | -1.25 | 0.018 | -1.16 | 0.162 | -1.01 | 0.967 | -1.11 |
| 1423070_at | Rpl21 | ribosomal protein L21 | -1.1 | 0.694 | -1.03 | 0.879 | -1.29 | 0.245 | -1.14 | 0.532 | -1.14 |
| 1424017_a_at | Hint1 | histidine triad nucleotide binding protein 1 | -1.11 | 0.133 | -1.13 | 0.556 | -1.15 | 0.065 | -1.18 | 0.153 | -1.14 |
| 1458202_at | 6330500D04Rik | RIKEN cDNA 6330500D04 gene | -1.06 | 0.885 | -1.3 | 0.195 | -1.06 | 0.844 | -2.68 | 0.038 | -1.53 |
| 1420500_at | Dnajc1 | DnaJ (Hsp40) homolog, subfamily C, member 1 | -1.04 | 0.795 | -1.05 | 0.714 | -1.35 | 0.091 | -1.5 | 0.166 | -1.24 |
| 1416488_at | Ccng2 | cyclin G2 | -1.11 | 0.667 | -1.12 | 0.512 | -1.15 | 0.255 | -1.57 | 0.084 | -1.24 |
| 1454774_at | Zfp445 | zinc finger protein 445 | -1.18 | 0.531 | -1.05 | 0.292 | -1.16 | 0.235 | -1.44 | 0.118 | -1.21 |
| 1416420_a_at | Rpl9 /// LOC637379 | ribosomal protein L9 /// similar to ribosomal protein L9 | -1.11 | 0.082 | -1.07 | 0.281 | -1.22 | 0.003 | -1.25 | 0.003 | -1.16 |
| 1438391_x_at | Hadh2 | hydroxyacyl-Coenzyme A dehydrogenase type II | -1.04 | 0.698 | -1.16 | 0.175 | -1.2 | 0.052 | -1.1 | 0.708 | -1.13 |
| 1424025_at | BC013529 | cDNA sequence BC013529 | -1.17 | 0.014 | -1.11 | 0.491 | -1.1 | 0.251 | -1.08 | 0.556 | -1.12 |
| 1456063_at | ORF34 | open reading frame 34 | -1.09 | 0.338 | -1.13 | 0.519 | -1.17 | 0.316 | -1.07 | 0.837 | -1.11 |
| 1448442_a_at | Psma3 | proteasome (prosome, macropain) subunit, alpha type 3 | -1.04 | 0.51 | -1.16 | 0.007 | -1.19 | 0.027 | -1.19 | 0.049 | -1.15 |
| 1426924_at | Mnab | membrane associated DNA binding protein | -1.07 | 0.8 | -1.02 | 0.813 | -1.34 | 0.023 | -1.01 | 0.824 | -1.11 |
| 1416463_at | Gpiap1 | GPI-anchored membrane protein 1 | -1.01 | 0.941 | -1.48 | 0.346 | -1 | 0.993 | -1.06 | 0.709 | -1.14 |
| 1450357_a_at | Ccr6 | chemokine (C-C motif) receptor 6 | -1.08 | 0.814 | -1.32 | 0.575 | -1.02 | 0.795 | -3.17 | 0.046 | -1.65 |
| 1440020_at | Rlf | Rearranged L-myc fusion sequence | -1.09 | 0.775 | -1.07 | 0.761 | -1.24 | 0.12 | -1.08 | 0.45 | -1.12 |
| 1450895_a_at | 1810020G14Rik | RIKEN cDNA 1810020G14 gene | -1.25 | 0.062 | -1.06 | 0.406 | -1.09 | 0.519 | -1.63 | 0.202 | -1.26 |
| 1438018_at | Hook1 | hook homolog 1 (Drosophila) | -1.08 | 0.586 | -1.1 | 0.203 | -1.22 | 0.122 | -1.86 | 0.087 | -1.31 |
| 1424251_a_at | Hnrpdl | heterogeneous nuclear ribonucleoprotein D-like | -1.07 | 0.705 | -1.25 | 0.074 | -1.08 | 0.426 | -1.11 | 0.509 | -1.13 |
| 1425018_at | Mcts1 | malignant T cell amplified sequence 1 | -1.18 | 0.007 | -1.17 | 0.041 | -1.04 | 0.669 | -1.23 | 0.25 | -1.16 |
| 1457248_x_at | Hsd17b7 | hydroxysteroid (17-beta) dehydrogenase 7 | -1.06 | 0.631 | -1.03 | 0.872 | -1.34 | 0.001 | -1.36 | 0.307 | -1.2 |
| 1427073_at | Lace1 | lactation elevated 1 | -1.12 | 0.103 | -1.22 | 0.278 | -1.05 | 0.698 | -1.21 | 0.125 | -1.15 |
| 1459986_a_at | Rps17 | ribosomal protein S17 | -1.1 | 0.211 | -1.06 | 0.576 | -1.23 | 0.005 | -1.24 | 0.003 | -1.16 |
| 1432195_s_at | Ccnl2 | cyclin L2 | -1.33 | 0.003 | -1.01 | 0.913 | -1.08 | 0.545 | -1.26 | 0.226 | -1.17 |
| 1439470_at | 2410025L10Rik | RIKEN cDNA 2410025L10 gene | -1.19 | 0.534 | -1.06 | 0.73 | -1.14 | 0.421 | -1.15 | 0.266 | -1.14 |
| 1434406_at | Srgap2 | SLIT-ROBO Rho GTPase activating protein 2 | -1.23 | 0.057 | -1.13 | 0.368 | -1.04 | 0.827 | -1.07 | 0.157 | -1.12 |
| 1428908_at | 2600011C06Rik | RIKEN cDNA 2600011C06 gene | -1.16 | 0.415 | -1.12 | 0.143 | -1.1 | 0.374 | -1.1 | 0.783 | -1.12 |
| 1430875_a_at | Pak1ip1 | PAK1 interacting protein 1 | -1.02 | 0.896 | -1.36 | 0.043 | -1.06 | 0.68 | -1.11 | 0.732 | -1.14 |
| 1452286_at | 5033405K12Rik | RIKEN cDNA 5033405K12 gene | -1.13 | 0.668 | -1.19 | 0.076 | -1.06 | 0.664 | -1.16 | 0.078 | -1.14 |
| 1460182_at | Snx4 | sorting nexin 4 | -1.09 | 0.347 | -1.04 | 0.267 | -1.27 | 0.028 | -1.33 | 0.204 | -1.18 |
| 1434009_at | Grlf1 | glucocorticoid receptor DNA binding factor 1 | -1.04 | 0.524 | -1.21 | 0.013 | -1.14 | 0.389 | -1.56 | 0.018 | -1.24 |
| 1437435_at | 1700061G19Rik /// | RIKEN cDNA 1700061G19 gene /// similar to gonadotropin-regulated long chain a | -1.08 | 0.508 | -1.24 | 0.562 | -1.07 | 0.601 | -1.73 | 0.206 | -1.28 |
| 1454657_s_at | Mak10 | MAK10 homolog, amino-acid N-acetyltransferase subunit, (S. cerevisiae) | -1.13 | 0.12 | -1.16 | 0.218 | -1.09 | 0.082 | -1.19 | 0.218 | -1.14 |
| 1418205_at | --- | --- | -1.2 | 0.358 | -1.17 | 0.647 | -1.02 | 0.872 | -1.39 | 0.143 | -1.2 |
| 1453928_a_at | Ssb | Sjogren syndrome antigen B | -1.05 | 0.725 | -1.04 | 0.544 | -1.34 | 0.088 | -1.31 | 0.205 | -1.18 |
| 1433472_x_at | Rpl38 /// LOC62564 | ribosomal protein L38 /// similar to ribosomal protein L38 /// similar to ribosomal pr | -1.11 | 0.048 | -1.02 | 0.832 | -1.28 | 0.02 | -1.29 | 0.015 | -1.18 |
| 1418150_at | Mtmr4 | myotubularin related protein 4 | -1.07 | 0.781 | -1.26 | 0.044 | -1.07 | 0.851 | -1.03 | 0.946 | -1.11 |
| 1419818_x_at | D1Ertd161e | DNA segment, Chr 1, ERATO Doi 161, expressed | -1.3 | 0.288 | -1.05 | 0.674 | -1.06 | 0.684 | -1.03 | 0.893 | -1.11 |
| 1448308_at | Ap3m1 | adaptor-related protein complex 3, mu 1 subunit | -1.07 | 0.279 | -1.15 | 0.481 | -1.17 | 0.139 | -1.01 | 0.917 | -1.1 |
| 1455152_at | AI462493 | expressed sequence AI462493 | -1.02 | 0.854 | -1.15 | 0.072 | -1.22 | 0.092 | -1.5 | 0.128 | -1.22 |
| 1452749_at | Papd1 | PAP associated domain containing 1 | -1.17 | 0.049 | -1.1 | 0.267 | -1.12 | 0.348 | -1.37 | 0.009 | -1.19 |
| 1443438_at | Ncoa1 | Nuclear receptor coactivator 1 | -1.02 | 0.915 | -1.11 | 0.75 | -1.28 | 0.038 | -1.83 | 0.068 | -1.31 |
| 1449083_at | Ccdc91 | coiled-coil domain containing 91 | -1.2 | 0.014 | -1.02 | 0.92 | -1.18 | 0.155 | -1.22 | 0.41 | -1.15 |
| 1426915_at | Dapk1 | death associated protein kinase 1 | -1.15 | 0.046 | -1 | 0.999 | -1.25 | 0.014 | -1.16 | 0.486 | -1.14 |

| | | | | | | | | | | | |
|---|---|---|---|---|---|---|---|---|---|---|---|
| 1451283_at | 1810073G14Rik | RIKEN cDNA 1810073G14 gene | -1.08 | 0.169 | -1.24 | 0.017 | -1.07 | 0.647 | -1.32 | 0.003 | -1.18 |
| 1425956_a_at | Cdadc1 | cytidine and dCMP deaminase domain containing 1 | -1.11 | 0.636 | -1.15 | 0.045 | -1.11 | 0.336 | -1.13 | 0.364 | -1.13 |
| 1442432_x_at | Atxn7l1 | ataxin 7-like 1 | -1.13 | 0.432 | -1.22 | 0.568 | -1.04 | 0.92 | -1.12 | 0.665 | -1.13 |
| 1435247_at | Ube1dc1 | ubiquitin-activating enzyme E1-domain containing 1 | -1.09 | 0.273 | -1 | 0.962 | -1.33 | 0.106 | -1.36 | 0.055 | -1.2 |
| 1450804_at | Kif5c | kinesin family member 5C | -1.23 | 0.049 | -1.07 | 0.853 | -1.09 | 0.811 | -1 | 0.986 | -1.1 |
| 1435347_at | Stau1 | staufen (RNA binding protein) homolog 1 (Drosophila) | -1.08 | 0.425 | -1.17 | 0.123 | -1.12 | 0.475 | -1.09 | 0.732 | -1.12 |
| 1438082_at | 2310028N02Rik | RIKEN cDNA 2310028N02 gene | -1.05 | 0.789 | -1.25 | 0.206 | -1.1 | 0.755 | -1.29 | 0.214 | -1.17 |
| 1453723_x_at | Ubb /// Gm1821 | ubiquitin B /// gene model 1821, (NCBI) | -1.06 | 0.065 | -1.15 | 0.222 | -1.18 | 0.031 | -1.12 | 0.035 | -1.12 |
| 1444028_s_at | Dock9 /// LOC6703 | dedicator of cytokinesis 9 /// similar to Dedicator of cytokinesis protein 9 (Cdc42 g | -1.16 | 0.449 | -1.19 | 0.283 | -1.03 | 0.866 | -1.38 | 0.158 | -1.19 |
| 1445375_at | Itsn2 | intersectin 2 | -1.09 | 0.611 | -1.04 | 0.524 | -1.27 | 0.154 | -1.48 | 0.166 | -1.22 |
| 1417402_at | 1190017O12Rik | RIKEN cDNA 1190017O12 gene | -1.11 | 0.669 | -1.05 | 0.453 | -1.23 | 0.229 | -1.29 | 0.262 | -1.17 |
| 1455740_at | Hnrpa1 | heterogeneous nuclear ribonucleoprotein A1 | -1.16 | 0.808 | -1.09 | 0.711 | -1.12 | 0.648 | -1.34 | 0.595 | -1.18 |
| 1456467_s_at | Nlk | nemo like kinase | -1.15 | 0.608 | -1.06 | 0.805 | -1.18 | 0.153 | -1.47 | 0.027 | -1.21 |
| 1429418_at | Cdc14b | CDC14 cell division cycle 14 homolog B (S. cerevisiae) | -1.1 | 0.652 | -1.14 | 0.213 | -1.14 | 0.47 | -1.29 | 0.446 | -1.17 |
| 1452116_s_at | Atf2 | activating transcription factor 2 | -1.07 | 0.477 | -1.08 | 0.256 | -1.23 | 0.091 | -1.01 | 0.811 | -1.1 |
| 1439973_at | Crxos1 | Crx opposite strand transcript 1 | -1.29 | 0.085 | -1.01 | 0.965 | -1.11 | 0.55 | -1.45 | 0.104 | -1.21 |
| 1417369_at | Hsd17b4 | hydroxysteroid (17-beta) dehydrogenase 4 | -1.05 | 0.813 | -1.36 | 0.098 | -1.03 | 0.868 | -1.13 | 0.692 | -1.14 |
| 1443970_at | Ntrk3 | neurotrophic tyrosine kinase, receptor, type 3 | -1.02 | 0.965 | -1.35 | 0.145 | -1.06 | 0.875 | -1.17 | 0.602 | -1.15 |
| 1415684_at | Atg5 | autophagy-related 5 (yeast) | -1.12 | 0.436 | -1.06 | 0.721 | -1.21 | 0.491 | -1.08 | 0.231 | -1.12 |
| 1435099_at | Utp14a | UTP14, U3 small nucleolar ribonucleoprotein, homolog A (yeast) | -1.23 | 0.268 | -1.05 | 0.478 | -1.1 | 0.353 | -1.26 | 0.073 | -1.16 |
| 1415738_at | Txndc12 | thioredoxin domain containing 12 (endoplasmic reticulum) | -1.29 | 0.076 | -1.03 | 0.701 | -1.09 | 0.596 | -1.45 | 0.101 | -1.21 |
| 1455252_at | Tsc1 | tuberous sclerosis 1 | -1.06 | 0.251 | -1.08 | 0.561 | -1.26 | 0.175 | -1.16 | 0.013 | -1.14 |
| 1430702_at | 5830427D03Rik | RIKEN cDNA 5830427D03 gene | -1.08 | 0.504 | -1.01 | 0.971 | -1.34 | 0.349 | -1.35 | 0.34 | -1.19 |
| 1457559_at | Wac | WW domain containing adaptor with coiled-coil | -1.05 | 0.746 | -1.08 | 0.219 | -1.27 | 0.116 | -1.04 | 0.755 | -1.11 |
| 1423033_at | Stt3a | STT3, subunit of the oligosaccharyltransferase complex, homolog A (S. cerevisia | -1.13 | 0.528 | -1.13 | 0.578 | -1.12 | 0.542 | -1.12 | 0.615 | -1.12 |
| 1425554_a_at | Cdc16 | CDC16 cell division cycle 16 homolog (S. cerevisiae) | -1.17 | 0.128 | -1.07 | 0.627 | -1.14 | 0.145 | -1.1 | 0.458 | -1.12 |
| 1427050_at | 5730420B22Rik | RIKEN cDNA 5730420B22 gene | -1.09 | 0.673 | -1.24 | 0.315 | -1.06 | 0.281 | -1.87 | 0.077 | -1.32 |
| 1420709_s_at | Dao1 | D-amino acid oxidase 1 | -1.09 | 0.805 | -1.21 | 0.616 | -1.09 | 0.761 | -1.15 | 0.596 | -1.13 |
| 1450642_at | 3110001I20Rik | RIKEN cDNA 3110001I20 gene | -1.1 | 0.679 | -1.14 | 0.19 | -1.13 | 0.257 | -1.17 | 0.468 | -1.14 |
| 1444159_at | Pip5k2a | Phosphatidylinositol-4-phosphate 5-kinase, type II, alpha | -1.09 | 0.315 | -1.04 | 0.863 | -1.27 | 0.433 | -1.13 | 0.732 | -1.13 |
| 1428188_at | 2610019A05Rik | RIKEN cDNA 2610019A05 gene | -1.09 | 0.74 | -1.12 | 0.791 | -1.16 | 0.64 | -1.43 | 0.275 | -1.2 |
| 1459874_s_at | Mtmr4 | myotubularin related protein 4 | -1.02 | 0.934 | -1.13 | 0.51 | -1.24 | 0.013 | -1.33 | 0.07 | -1.18 |
| 1448958_at | Ifrg15 | interferon alpha responsive gene | -1.2 | 0.205 | -1.08 | 0.729 | -1.1 | 0.676 | -1.15 | 0.57 | -1.13 |
| 1434215_at | B230308N11Rik | RIKEN cDNA B230308N11 gene | -1.11 | 0.281 | -1.1 | 0.387 | -1.16 | 0.1 | -1.04 | 0.402 | -1.1 |
| 1432984_at | 1700026H06Rik | RIKEN cDNA 1700026H06 gene | -1.09 | 0.665 | -1.07 | 0.609 | -1.23 | 0.129 | -1.14 | 0.612 | -1.13 |
| 1438993_a_at | Atp6v1d | ATPase, H+ transporting, lysosomal V1 subunit D | -1.06 | 0.711 | -1.15 | 0.1 | -1.16 | 0.1 | -1.25 | 0.083 | -1.16 |
| 1437470_at | Pknox1 | Pbx/knotted 1 homeobox | -1.1 | 0.696 | -1.19 | 0.338 | -1.09 | 0.732 | -1.06 | 0.596 | -1.11 |
| 1456395_at | Ppargc1a | peroxisome proliferative activated receptor, gamma, coactivator 1 alpha | -1.11 | 0.599 | -1.29 | 0.358 | -1.01 | 0.921 | -1.68 | 0.346 | -1.27 |
| 1451576_at | Prkdc | protein kinase, DNA activated, catalytic polypeptide | -1.14 | 0.549 | -1.18 | 0.288 | -1.05 | 0.681 | -1.07 | 0.708 | -1.11 |
| 1426527_at | Toe1 | target of EGR1, member 1 (nuclear) | -1.12 | 0.541 | -1.04 | 0.859 | -1.23 | 0.103 | -1.29 | 0.046 | -1.17 |
| 1454620_x_at | Rps6 /// LOC43440 | ribosomal protein S6 /// similar to 40S ribosomal protein S6 /// similar to 40S ribos | -1.01 | 0.88 | -1.09 | 0.368 | -1.31 | 0.002 | -1.32 | 0.006 | -1.18 |
| 1444031_at | --- | --- | -1.12 | 0.756 | -1.13 | 0.525 | -1.12 | 0.682 | -1.34 | 0.522 | -1.18 |
| 1433768_at | Palld | palladin, cytoskeletal associated protein | -1.06 | 0.681 | -1.31 | 0.064 | -1.04 | 0.854 | -1.27 | 0.463 | -1.17 |
| 1460571_at | Dicer1 | Dicer1, Dcr-1 homolog (Drosophila) | -1.08 | 0.03 | -1.1 | 0.124 | -1.2 | 0.31 | -1.54 | 0.153 | -1.23 |
| 1444858_at | Inoc1 | INO80 complex homolog 1 (S. cerevisiae) | -1.11 | 0.136 | -1.11 | 0.645 | -1.15 | 0.442 | -1.5 | 0.1 | -1.22 |
| 1420883_at | Sln | sarcolipin | -1.03 | 0.896 | -1.17 | 0.665 | -1.18 | 0.669 | -1.1 | 0.84 | -1.12 |
| 1416312_at | Rars | arginyl-tRNA synthetase | -1.1 | 0.593 | -1.17 | 0.053 | -1.11 | 0.045 | -1.08 | 0.553 | -1.11 |
| 1448990_a_at | Myo1b | myosin IB | -1.13 | 0.769 | -1.18 | 0.301 | -1.07 | 0.739 | -1.75 | 0.057 | -1.28 |
| 1438051_at | Ttc14 | tetratricopeptide repeat domain 14 | -1.16 | 0.659 | -1.11 | 0.736 | -1.1 | 0.331 | -1.2 | 0.38 | -1.14 |
| 1459854_s_at | Dynlt3 | dynein light chain Tctex-type 3 | -1.14 | 0.374 | -1.05 | 0.608 | -1.19 | 0.032 | -1.14 | 0.57 | -1.13 |
| 1446107_at | Stk24 | Serine/threonine kinase 24 (STE20 homolog, yeast) | -1.19 | 0.621 | -1.08 | 0.578 | -1.1 | 0.755 | -1.33 | 0.247 | -1.18 |
| 1434214_at | 0910001L09Rik | RIKEN cDNA 0910001L09 gene | -1.06 | 0.696 | -1.14 | 0.115 | -1.18 | 0.339 | -1.52 | 0.081 | -1.22 |
| 1455434_a_at | Ktn1 | kinectin 1 | -1.1 | 0.672 | -1.16 | 0.199 | -1.11 | 0.196 | -1.47 | 0.247 | -1.21 |

| | | | | | | | | | | | |
|---|---|---|---|---|---|---|---|---|---|---|---|
| 1418209_a_at | Pfn2 | profilin 2 | -1.09 | 0.303 | -1.03 | 0.921 | -1.28 | 0.202 | -1.38 | 0.237 | -1.2 |
| 1439795_at | Gpr64 | G protein-coupled receptor 64 | -1.02 | 0.974 | -1.28 | 0.695 | -1.1 | 0.605 | -1.42 | 0.371 | -1.21 |
| 1424721_at | Mfap3 | microfibrillar-associated protein 3 | -1.07 | 0.759 | -1.05 | 0.769 | -1.28 | 0.061 | -1.36 | 0.037 | -1.19 |
| 1437791_s_at | Eml5 | echinoderm microtubule associated protein like 5 | -1.05 | 0.786 | -1.08 | 0.713 | -1.27 | 0.246 | -1.26 | 0.121 | -1.16 |
| 1449533_at | Tmem100 | transmembrane protein 100 | -1.06 | 0.873 | -1.3 | 0.567 | -1.04 | 0.608 | -1.37 | 0.42 | -1.19 |
| 1431665_a_at | Timm8b | translocase of inner mitochondrial membrane 8 homolog b (yeast) | -1.06 | 0.593 | -1.05 | 0.596 | -1.29 | 0.012 | -1.02 | 0.946 | -1.1 |
| 1459457_at | Camk2d | calcium/calmodulin-dependent protein kinase II, delta | -1.09 | 0.254 | -1.13 | 0.648 | -1.15 | 0.543 | -1 | 0.989 | -1.09 |
| 1417791_a_at | Zfml | zinc finger, matrin-like | -1.09 | 0.599 | -1.09 | 0.623 | -1.19 | 0.369 | -1.4 | 0.174 | -1.19 |
| 1436539_at | Clmn | calmin | -1.3 | 0.094 | -1.05 | 0.862 | -1.05 | 0.932 | -1.18 | 0.147 | -1.15 |
| 1433846_s_at | C430003P19Rik | RIKEN cDNA C430003P19 gene | -1.15 | 0.019 | -1.04 | 0.477 | -1.19 | 0.09 | -1.31 | 0.103 | -1.17 |
| 1435011_x_at | Akr1a4 | aldo-keto reductase family 1, member A4 (aldehyde reductase) | -1.23 | 0.026 | -1.07 | 0.246 | -1.08 | 0.209 | -1.2 | 0.341 | -1.15 |
| 1428394_at | Phyhd1 /// Lrrc8a | phytanoyl-CoA dioxygenase domain containing 1 /// leucine rich repeat containing | -1.08 | 0.301 | -1.17 | 0.054 | -1.12 | 0.629 | -1.35 | 0.215 | -1.18 |
| 1457672_at | Chd9 | chromodomain helicase DNA binding protein 9 | -1 | 0.972 | -1.23 | 0.126 | -1.16 | 0.237 | -1.41 | 0.23 | -1.2 |
| 1423272_at | Polg | polymerase (DNA directed), gamma | -1.1 | 0.236 | -1.21 | 0.011 | -1.07 | 0.727 | -1.32 | 0.011 | -1.17 |
| 1435158_at | Rbm12b | RNA binding motif protein 12B | -1.3 | 0.475 | -1.01 | 0.953 | -1.09 | 0.649 | -1.77 | 0.039 | -1.29 |
| 1419046_at | Brp16 | brain protein 16 | -1.06 | 0.685 | -1.23 | 0.143 | -1.09 | 0.643 | -1.32 | 0.242 | -1.18 |
| 1455139_at | AI851716 | expressed sequence AI851716 | -1.08 | 0.69 | -1.3 | 0.294 | -1.02 | 0.823 | -1.1 | 0.647 | -1.13 |
| 1448794_s_at | Dnajc2 | DnaJ (Hsp40) homolog, subfamily C, member 2 | -1.02 | 0.896 | -1.18 | 0.326 | -1.18 | 0.344 | -1.12 | 0.71 | -1.13 |
| 1427407_s_at | Trip11 | thyroid hormone receptor interactor 11 | -1.13 | 0.333 | -1.01 | 0.894 | -1.25 | 0.075 | -1.3 | 0.263 | -1.17 |
| 1440966_at | 7-Mar | membrane-associated ring finger (C3HC4) 7 | -1.01 | 0.966 | -1.08 | 0.659 | -1.33 | 0.129 | -1.58 | 0.131 | -1.25 |
| 1434232_a_at | 2610030H06Rik | RIKEN cDNA 2610030H06 gene | -1.07 | 0.675 | -1.25 | 0.151 | -1.07 | 0.703 | -1.06 | 0.847 | -1.11 |
| 1448448_a_at | Chkb | choline kinase beta | -1.1 | 0.526 | -1.27 | 0.045 | -1.02 | 0.775 | -1.44 | 0.134 | -1.21 |
| 1424299_at | Oma1 | OMA1 homolog, zinc metallopeptidase (S. cerevisiae) | -1.13 | 0.246 | -1.1 | 0.242 | -1.14 | 0.262 | -1.39 | 0.076 | -1.19 |
| 1438418_at | 4932432K03Rik | RIKEN cDNA 4932432K03 gene | -1.12 | 0.086 | -1.06 | 0.797 | -1.19 | 0.345 | -1.32 | 0.307 | -1.17 |
| 1426862_at | Aftph | aftiphilin | -1.12 | 0.308 | -1.08 | 0.333 | -1.17 | 0.205 | -1.07 | 0.642 | -1.11 |
| 1427881_at | Dnttip2 | deoxynucleotidyltransferase, terminal, interacting protein 2 | -1.08 | 0.745 | -1.19 | 0.118 | -1.11 | 0.586 | -1.21 | 0.206 | -1.15 |
| 1441229_at | D230019N24Rik | RIKEN cDNA D230019N24 gene | -1.03 | 0.614 | -1.32 | 0.113 | -1.06 | 0.708 | -1.59 | 0.123 | -1.25 |
| 1434952_at | Cox4nb | COX4 neighbor | -1.03 | 0.853 | -1.24 | 0.426 | -1.12 | 0.585 | -1.08 | 0.884 | -1.12 |
| 1446096_at | 2310001H17Rik | RIKEN cDNA 2310001H17 gene | -1.14 | 0.499 | -1.19 | 0.117 | -1.04 | 0.916 | -1.43 | 0.124 | -1.2 |
| 1416616_s_at | Clpp | caseinolytic peptidase, ATP-dependent, proteolytic subunit homolog (E. coli) | -1.1 | 0.401 | -1.09 | 0.523 | -1.18 | 0.201 | -1.03 | 0.739 | -1.1 |
| 1434179_at | Mll3 | myeloid/lymphoid or mixed-lineage leukemia 3 | -1.06 | 0.374 | -1.16 | 0.093 | -1.16 | 0.431 | -1.24 | 0.273 | -1.15 |
| 1435568_at | AK129128 | cDNA sequence AK129128 | -1.16 | 0.275 | -1.09 | 0.483 | -1.11 | 0.334 | -1.03 | 0.848 | -1.1 |
| 1428406_s_at | Hcfc1r1 | host cell factor C1 regulator 1 (XPO1-dependent) | -1.12 | 0.578 | -1.18 | 0.456 | -1.07 | 0.633 | -1.04 | 0.747 | -1.1 |
| 1423301_at | Copb1 | coatomer protein complex, subunit beta 1 | -1.11 | 0.524 | -1.16 | 0.015 | -1.09 | 0.371 | -1.24 | 0.072 | -1.15 |
| 1433852_at | C330002I19Rik | RIKEN cDNA C330002I19 gene | -1.15 | 0.164 | -1.03 | 0.596 | -1.2 | 0.304 | -1.25 | 0.419 | -1.16 |
| 1459715_at | 1500011J06Rik | RIKEN cDNA 1500011J06 gene | -1.12 | 0.71 | -1.09 | 0.48 | -1.16 | 0.597 | -1.23 | 0.494 | -1.15 |
| 1434884_at | Mtdh | Metadherin | -1.18 | 0.483 | -1.09 | 0.575 | -1.1 | 0.415 | -1.01 | 0.969 | -1.09 |
| 1426536_at | Narg2 | NMDA receptor-regulated gene 2 | -1.12 | 0.479 | -1.03 | 0.718 | -1.23 | 0.118 | -1.42 | 0.121 | -1.2 |
| 1445244_at | Tm2d1 | TM2 domain containing 1 | -1.11 | 0.808 | -1.07 | 0.914 | -1.19 | 0.484 | -1.13 | 0.778 | -1.12 |
| 1426741_a_at | Fastkd2 | FAST kinase domains 2 | -1.23 | 0.352 | -1.12 | 0.137 | -1.03 | 0.411 | -1.04 | 0.47 | -1.1 |
| 1422888_at | Rnf5 | ring finger protein 5 | -1.36 | 0.207 | -1.06 | 0.709 | -1 | 0.995 | -1.03 | 0.766 | -1.11 |
| 1428177_at | Trappc6b /// LOC43 | trafficking protein particle complex 6B /// similar to Trafficking protein particle com | -1.18 | 0.066 | -1.17 | 0.017 | -1.03 | 0.764 | -1.03 | 0.847 | -1.1 |
| 1430678_at | 5730457N03Rik | RIKEN cDNA 5730457N03 gene | -1.17 | 0.843 | -1.17 | 0.604 | -1.03 | 0.945 | -2.37 | 0.284 | -1.44 |
| 1439558_at | Zfp75 | zinc finger protein 75 | -1.23 | 0.735 | -1.08 | 0.784 | -1.06 | 0.489 | -1.2 | 0.568 | -1.14 |
| 1457787_at | --- | --- | -1.24 | 0.341 | -1.02 | 0.917 | -1.12 | 0.531 | -1.18 | 0.238 | -1.14 |
| 1456058_at | Rbm27 | RNA binding motif protein 27 | -1.02 | 0.883 | -1.02 | 0.846 | -1.38 | 0.014 | -1.12 | 0.045 | -1.14 |
| 1433778_at | Tnks | tankyrase, TRF1-interacting ankyrin-related ADP-ribose polymerase | -1.01 | 0.916 | -1.21 | 0.059 | -1.16 | 0.42 | -1 | 0.989 | -1.1 |
| 1454794_at | Spast | spastin | -1.04 | 0.77 | -1.17 | 0.234 | -1.16 | 0.152 | -1.03 | 0.609 | -1.1 |
| 1428170_at | Zfp180 | zinc finger protein 180 | -1.02 | 0.914 | -1.11 | 0.503 | -1.27 | 0.06 | -1.06 | 0.769 | -1.11 |
| 1454116_a_at | Mterfd1 | MTERF domain containing 1 | -1.19 | 0.412 | -1.01 | 0.898 | -1.18 | 0.074 | -1.64 | 0.062 | -1.25 |
| 1438972_x_at | 2810410L24Rik | RIKEN cDNA 2810410L24 gene | -1.15 | 0.414 | -1.07 | 0.77 | -1.15 | 0.502 | -1.26 | 0.462 | -1.16 |
| 1447883_x_at | Map1lc3a | microtubule-associated protein 1 light chain 3 alpha | -1.09 | 0.38 | -1.11 | 0.525 | -1.17 | 0.206 | -1.01 | 0.931 | -1.09 |
| 1420895_at | Tgfbr1 | transforming growth factor, beta receptor I | -1.03 | 0.751 | -1.04 | 0.698 | -1.35 | 0.014 | -1.36 | 0.192 | -1.19 |

| | | | | | | | | | | | |
|---|---|---|---|---|---|---|---|---|---|---|---|
| 1448641_at | Mbtd1 | mbt domain containing 1 | -1.05 | 0.513 | -1.08 | 0.311 | -1.25 | 0.141 | -1.26 | 0.041 | -1.16 |
| 1456553_at | Zcchc11 | Zinc finger, CCHC domain containing 11 | -1.02 | 0.922 | -1.15 | 0.484 | -1.21 | 0.264 | -2.37 | 0.112 | -1.44 |
| 1448403_at | Lars | leucyl-tRNA synthetase | -1.15 | 0.467 | -1.04 | 0.32 | -1.18 | 0.256 | -1.01 | 0.953 | -1.09 |
| 1426432_a_at | Slc4a4 | solute carrier family 4 (anion exchanger), member 4 | -1.08 | 0.496 | -1.03 | 0.696 | -1.28 | 0.111 | -1.09 | 0.896 | -1.12 |
| 1448707_at | Taf13 | TAF13 RNA polymerase II, TATA box binding protein (TBP)-associated factor | -1.05 | 0.712 | -1.03 | 0.661 | -1.31 | 0.042 | -1.28 | 0.167 | -1.17 |
| 1418479_at | Vps54 | vacuolar protein sorting 54 (yeast) | -1.16 | 0.375 | -1.2 | 0.043 | -1.02 | 0.749 | -1.1 | 0.341 | -1.12 |
| 1432119_at | 4933405I11Rik | RIKEN cDNA 4933405I11 gene | -1.21 | 0.701 | -1.03 | 0.911 | -1.13 | 0.405 | -1.32 | 0.62 | -1.17 |
| 1426763_at | Oaz2 | ornithine decarboxylase antizyme 2 | -1.22 | 0.081 | -1 | 0.976 | -1.16 | 0.218 | -1.26 | 0.104 | -1.16 |
| 1448179_at | Usmg5 | upregulated during skeletal muscle growth 5 | -1.22 | 0.005 | -1.13 | 0.692 | -1.03 | 0.923 | -1.33 | 0.21 | -1.18 |
| 1428155_at | Commd9 | COMM domain containing 9 | -1.12 | 0.697 | -1.09 | 0.457 | -1.16 | 0.255 | -1.05 | 0.687 | -1.1 |
| 1422627_a_at | Mkks | McKusick-Kaufman syndrome protein | -1.08 | 0.55 | -1.05 | 0.63 | -1.24 | 0.016 | -1.2 | 0.345 | -1.14 |
| 1429473_at | 1200003I07Rik | RIKEN cDNA 1200003I07 gene | -1.17 | 0.499 | -1.08 | 0.057 | -1.11 | 0.172 | -1.58 | 0.063 | -1.23 |
| 1443932_at | Klhdc1 | kelch domain containing 1 | -1.27 | 0.059 | -1.03 | 0.966 | -1.09 | 0.481 | -1.66 | 0.089 | -1.26 |
| 1437020_at | Ep400 | E1A binding protein p400 | -1.08 | 0.665 | -1.16 | 0.716 | -1.13 | 0.433 | -1.09 | 0.75 | -1.11 |
| 1435637_at | Itfg1 | integrin alpha FG-GAP repeat containing 1 | -1.11 | 0.085 | -1.12 | 0.081 | -1.13 | 0.144 | -1.16 | 0.427 | -1.13 |
| 1437441_at | AA388235 | expressed sequence AA388235 | -1.3 | 0.161 | -1.03 | 0.954 | -1.07 | 0.716 | -1.51 | 0.239 | -1.23 |
| 1428337_at | 1810034K20Rik | RIKEN cDNA 1810034K20 gene | -1.15 | 0.038 | -1.06 | 0.612 | -1.16 | 0.223 | -1.53 | 0.123 | -1.22 |
| 1427733_a_at | Nfia | nuclear factor I/A | -1.17 | 0.415 | -1.01 | 0.966 | -1.2 | 0.385 | -1.23 | 0.309 | -1.15 |
| 1433953_at | Zfp277 | zinc finger protein 277 | -1.07 | 0.424 | -1.14 | 0.15 | -1.15 | 0.221 | -1.93 | 0.071 | -1.32 |
| 1441998_at | --- | Transcribed locus | -1.14 | 0.517 | -1.18 | 0.485 | -1.05 | 0.616 | -1.23 | 0.437 | -1.15 |
| 1424384_a_at | Znrf1 | zinc and ring finger 1 | -1.01 | 0.939 | -1.08 | 0.648 | -1.31 | 0.044 | -2.09 | 0.053 | -1.37 |
| 1437212_at | B230312I18Rik | RIKEN cDNA B230312I18 gene | -1.14 | 0.388 | -1 | 0.996 | -1.25 | 0.169 | -1.85 | 0.095 | -1.31 |
| 1422698_s_at | Jarid2 | jumonji, AT rich interactive domain 2 | -1.11 | 0.562 | -1.12 | 0.521 | -1.12 | 0.248 | -1.29 | 0.024 | -1.16 |
| 1426241_a_at | Scmh1 | sex comb on midleg homolog 1 | -1.22 | 0.298 | -1.15 | 0.358 | -1.01 | 0.948 | -1.13 | 0.185 | -1.13 |
| 1460429_at | Cdc5l | cell division cycle 5-like (S. pombe) | -1.25 | 0.132 | -1.04 | 0.446 | -1.09 | 0.306 | -1.21 | 0.129 | -1.15 |
| 1433648_at | Spag9 | sperm associated antigen 9 | -1.09 | 0.64 | -1.03 | 0.76 | -1.25 | 0.139 | -1.38 | 0.404 | -1.19 |
| 1417598_a_at | Fxr1h | fragile X mental retardation gene 1, autosomal homolog | -1.12 | 0.544 | -1.27 | 0.006 | -1 | 0.983 | -1.13 | 0.399 | -1.13 |
| 1437447_s_at | Ercc1 | excision repair cross-complementing rodent repair deficiency, complementation g | -1.11 | 0.537 | -1.07 | 0.775 | -1.19 | 0.067 | -1.37 | 0.272 | -1.18 |
| 1450775_at | Mos | Moloney sarcoma oncogene | -1.25 | 0.597 | -1.11 | 0.502 | -1.02 | 0.926 | -1.51 | 0.018 | -1.22 |
| 1415729_at | Pdpk1 | 3-phosphoinositide dependent protein kinase-1 | -1.1 | 0.482 | -1.13 | 0.302 | -1.12 | 0.37 | -1.06 | 0.672 | -1.1 |
| 1420731_a_at | Csrp2 | cysteine and glycine-rich protein 2 | -1.03 | 0.394 | -1.11 | 0.209 | -1.23 | 0.284 | -1.64 | 0.078 | -1.25 |
| 1429699_at | Oxsm | 3-oxoacyl-ACP synthase, mitochondrial | -1.01 | 0.966 | -1.33 | 0.025 | -1.07 | 0.823 | -1.04 | 0.845 | -1.11 |
| 1452897_at | Cdc2l5 | cell division cycle 2-like 5 (cholinesterase-related cell division controller) | -1.18 | 0.002 | -1.13 | 0.278 | -1.05 | 0.643 | -1.35 | 0.056 | -1.18 |
| 1432902_at | 9130414P19Rik | RIKEN cDNA 9130414P19 gene | -1.24 | 0.669 | -1.02 | 0.901 | -1.11 | 0.549 | -1.4 | 0.653 | -1.19 |
| 1452710_at | Rpusd4 | RNA pseudouridylate synthase domain containing 4 | -1.12 | 0.224 | -1.11 | 0.603 | -1.12 | 0.434 | -1.3 | 0.08 | -1.16 |
| 1441052_at | 2310035C23Rik | RIKEN cDNA 2310035C23 gene | -1.06 | 0.329 | -1.09 | 0.614 | -1.22 | 0.082 | -1.37 | 0.055 | -1.19 |
| 1435746_at | Srpk2 | serine/arginine-rich protein specific kinase 2 | -1 | 0.999 | -1.03 | 0.768 | -1.41 | 0.098 | -1.69 | 0.099 | -1.28 |
| 1418440_at | Col8a1 | procollagen, type VIII, alpha 1 | -1.22 | 0.543 | -1.11 | 0.776 | -1.04 | 0.855 | -1.01 | 0.966 | -1.1 |
| 1425201_a_at | Hyi | hydroxypyruvate isomerase homolog (E. coli) | -1.02 | 0.927 | -1.28 | 0.453 | -1.09 | 0.67 | -1.7 | 0.004 | -1.27 |
| 1426494_at | Rg9mtd3 | RNA (guanine-9-) methyltransferase domain containing 3 | -1.08 | 0.593 | -1.08 | 0.355 | -1.2 | 0.134 | -1.28 | 0.211 | -1.16 |
| 1437196_x_at | Rps16 | ribosomal protein S16 | -1 | 0.974 | -1.05 | 0.694 | -1.36 | 0.006 | -1.32 | 0.007 | -1.18 |
| 1424792_at | Rpp40 | ribonuclease P 40 subunit (human) | -1.25 | 0.184 | -1.06 | 0.765 | -1.07 | 0.538 | -1.27 | 0.369 | -1.16 |
| 1426424_at | Sugt1 | SGT1, suppressor of G2 allele of SKP1 (S. cerevisiae) | -1.07 | 0.434 | -1.15 | 0.536 | -1.14 | 0.034 | -1.15 | 0.308 | -1.13 |
| 1453846_at | 2810013C04Rik | RIKEN cDNA 2810013C04 gene | -1.19 | 0.718 | -1.03 | 0.937 | -1.15 | 0.16 | -1.78 | 0.035 | -1.29 |
| 1459952_at | Ankrd17 | Ankyrin repeat domain 17 | -1.04 | 0.618 | -1.04 | 0.833 | -1.32 | 0.223 | -1.5 | 0.073 | -1.22 |
| 1446923_at | Ptplb | Protein tyrosine phosphatase-like (proline instead of catalytic arginine), member b | -1.09 | 0.484 | -1.11 | 0.758 | -1.16 | 0.549 | -1.41 | 0.403 | -1.19 |
| 1455180_at | AA407270 | expressed sequence AA407270 | -1.01 | 0.959 | -1.16 | 0.716 | -1.21 | 0.197 | -1.48 | 0.261 | -1.21 |
| 1449118_at | Dbt | dihydrolipoamide branched chain transacylase E2 | -1.14 | 0.027 | -1.17 | 0.133 | -1.06 | 0.575 | -1.14 | 0.444 | -1.13 |
| 1437203_at | Cbll1 | Casitas B-lineage lymphoma-like 1 | -1.12 | 0.146 | -1.07 | 0.562 | -1.16 | 0.078 | -1.3 | 0.267 | -1.16 |
| 1417683_at | Diablo | diablo homolog (Drosophila) | -1.14 | 0.124 | -1.09 | 0.057 | -1.12 | 0.109 | -1.09 | 0.291 | -1.11 |
| 1455006_at | 2310016M24Rik | RIKEN cDNA 2310016M24 gene | -1.06 | 0.432 | -1.17 | 0.163 | -1.13 | 0.311 | -1.27 | 0.298 | -1.16 |
| 1426490_at | Bfar | bifunctional apoptosis regulator | -1.03 | 0.503 | -1.19 | 0.118 | -1.16 | 0.266 | -1.37 | 0.153 | -1.18 |
| 1423641_s_at | Cnot7 | CCR4-NOT transcription complex, subunit 7 | -1.13 | 0.249 | -1.06 | 0.436 | -1.17 | 0.049 | -1.12 | 0.449 | -1.12 |

| | | | | | | | | | | | |
|---|---|---|---|---|---|---|---|---|---|---|---|
| 1419979_s_at | Creb3 | cAMP responsive element binding protein 3 | -1.05 | 0.033 | -1.03 | 0.68 | -1.3 | 0.012 | -1.06 | 0.626 | -1.11 |
| 1422805_a_at | Ing3 | inhibitor of growth family, member 3 | -1.2 | 0.018 | -1.07 | 0.547 | -1.09 | 0.641 | -1.52 | 0.177 | -1.22 |
| 1422697_s_at | --- | --- | -1.14 | 0.339 | -1.07 | 0.165 | -1.14 | 0.121 | -1.27 | 0.048 | -1.16 |
| 1415774_at | Statip1 | signal transducer and activator of transcription interacting protein 1 | -1.15 | 0.69 | -1.05 | 0.746 | -1.16 | 0.279 | -1.22 | 0.418 | -1.15 |
| 1433516_a_at | Myeov2 | myeloma overexpressed 2 | -1.06 | 0.524 | -1.01 | 0.879 | -1.34 | 0.097 | -1.62 | 0.106 | -1.26 |
| 1428083_at | 2310043N10Rik | RIKEN cDNA 2310043N10 gene | -1.11 | 0.597 | -1.24 | 0.386 | -1.02 | 0.918 | -1.18 | 0.48 | -1.14 |
| 1433726_at | --- | --- | -1.11 | 0.397 | -1.08 | 0.548 | -1.17 | 0.004 | -1.24 | 0.368 | -1.15 |
| 1454785_at | Dusp11 | dual specificity phosphatase 11 (RNA/RNP complex 1-interacting) | -1.07 | 0.689 | -1.17 | 0.08 | -1.12 | 0.31 | -1.24 | 0.238 | -1.15 |
| 1435174_at | Rsbn1 | rosbin, round spermatid basic protein 1 | -1.14 | 0.464 | -1.01 | 0.934 | -1.21 | 0.009 | -1.23 | 0.276 | -1.15 |
| 1444051_at | 5830405N20Rik | RIKEN cDNA 5830405N20 gene | -1.21 | 0.507 | -1.14 | 0.423 | -1.02 | 0.93 | -2.05 | 0.087 | -1.35 |
| 1423665_a_at | Rpl5 | ribosomal protein L5 | -1.11 | 0.212 | -1.05 | 0.372 | -1.19 | 0.01 | -1.21 | 0.005 | -1.14 |
| 1448157_s_at | Rpl10 | ribosomal protein 10 | -1.04 | 0.239 | -1.06 | 0.65 | -1.28 | 0 | -1.24 | 0.032 | -1.15 |
| 1429400_at | Clcn5 | chloride channel 5 | -1.18 | 0.33 | -1.08 | 0.277 | -1.09 | 0.186 | -1.11 | 0.721 | -1.12 |
| 1452604_at | Stard13 | serologically defined colon cancer antigen 13 | -1.3 | 0.026 | -1.04 | 0.859 | -1.04 | 0.869 | -1.68 | 0.081 | -1.27 |
| 1454747_a_at | Klhdc3 | kelch domain containing 3 | -1.22 | 0.001 | -1.12 | 0.218 | -1.03 | 0.832 | -1.07 | 0.684 | -1.11 |
| 1424358_at | Ube2e2 | ubiquitin-conjugating enzyme E2E 2 (UBC4/5 homolog, yeast) | -1 | 0.975 | -1.05 | 0.536 | -1.35 | 0.119 | -2.41 | 0.024 | -1.45 |
| 1452083_a_at | Pja1 | praja1, RING-H2 motif containing | -1.12 | 0.594 | -1.11 | 0.537 | -1.12 | 0.387 | -1.25 | 0.545 | -1.15 |
| 1448801_a_at | Timm44 | translocase of inner mitochondrial membrane 44 | -1.19 | 0.141 | -1.12 | 0.107 | -1.05 | 0.775 | -1.09 | 0.462 | -1.11 |
| 1450664_at | Gabpa | GA repeat binding protein, alpha | -1.27 | 0.077 | -1.03 | 0.524 | -1.08 | 0.302 | -1.07 | 0.423 | -1.11 |
| 1420489_at | Mrps14 | mitochondrial ribosomal protein S14 | -1.13 | 0.267 | -1.15 | 0.008 | -1.08 | 0.224 | -1.14 | 0.419 | -1.12 |
| 1449686_s_at | Scp2 | sterol carrier protein 2, liver | -1.09 | 0.476 | -1.09 | 0.363 | -1.18 | 0.181 | -1.53 | 0.164 | -1.22 |
| 1434742_s_at | 2810401C16Rik | RIKEN cDNA 2810401C16 gene | -1.17 | 0.599 | -1.09 | 0.719 | -1.09 | 0.788 | -1.05 | 0.931 | -1.1 |
| 1458501_at | Vapb | vesicle-associated membrane protein, associated protein B and C | -1.04 | 0.897 | -1.06 | 0.781 | -1.28 | 0.052 | -1.04 | 0.853 | -1.1 |
| 1426870_at | Fbxo33 | F-box only protein 33 | -1.15 | 0.252 | -1.08 | 0.544 | -1.12 | 0.33 | -1.06 | 0.733 | -1.1 |
| 1448356_at | Ube2d2 | ubiquitin-conjugating enzyme E2D 2 | -1.01 | 0.818 | -1.16 | 0.02 | -1.2 | 0.002 | -1.31 | 0.132 | -1.17 |
| 1423893_x_at | Apbb1 | amyloid beta (A4) precursor protein-binding, family B, member 1 | -1.07 | 0.74 | -1.26 | 0.584 | -1.04 | 0.556 | -1.34 | 0.163 | -1.18 |
| 1437696_at | BC049807 | cDNA sequence BC049807 | -1.05 | 0.906 | -1.07 | 0.786 | -1.25 | 0.344 | -1.07 | 0.765 | -1.11 |
| 1436597_at | Ankhd1 | ankyrin repeat and KH domain containing 1 | -1.15 | 0.645 | -1.09 | 0.417 | -1.12 | 0.367 | -1.43 | 0.073 | -1.2 |
| 1454722_at | Pten | phosphatase and tensin homolog | -1.06 | 0.635 | -1.02 | 0.085 | -1.3 | 0.132 | -1.44 | 0.278 | -1.21 |
| 1435317_x_at | Psma6 | proteasome (prosome, macropain) subunit, alpha type 6 | -1.16 | 0.258 | -1.01 | 0.938 | -1.19 | 0.379 | -1.33 | 0.252 | -1.17 |
| 1452878_at | Prkce | protein kinase C, epsilon | -1.26 | 0.12 | -1 | 0.986 | -1.12 | 0.492 | -1.2 | 0.448 | -1.14 |
| 1443573_at | Parp1 | poly (ADP-ribose) polymerase family, member 1 | -1.08 | 0.817 | -1.1 | 0.748 | -1.17 | 0.468 | -1.39 | 0.289 | -1.19 |
| 1445960_at | --- | --- | -1.13 | 0.813 | -1.13 | 0.768 | -1.09 | 0.627 | -1.64 | 0.303 | -1.25 |
| 1430060_at | 1700034G24Rik | RIKEN cDNA 1700034G24 gene | -1.02 | 0.922 | -1.2 | 0.384 | -1.14 | 0.339 | -1.25 | 0.262 | -1.15 |
| 1426558_x_at | 3100002L24Rik /// I | RIKEN cDNA 3100002L24 gene /// similar to zinc finger protein 14 /// RIKEN cDN | -1.22 | 0.218 | -1.14 | 0.11 | -1.01 | 0.946 | -1.13 | 0.468 | -1.12 |
| 1439108_at | Mll5 | myeloid/lymphoid or mixed-lineage leukemia 5 | -1.05 | 0.859 | -1.33 | 0.022 | -1.02 | 0.946 | -1.32 | 0.243 | -1.18 |
| 1431500_at | 4933406J10Rik | RIKEN cDNA 4933406J10 gene | -1.01 | 0.962 | -1.08 | 0.708 | -1.3 | 0.345 | -1.23 | 0.202 | -1.15 |
| 1416244_a_at | Cnbp | cellular nucleic acid binding protein | -1.06 | 0.224 | -1.19 | 0.108 | -1.1 | 0.228 | -1.15 | 0.074 | -1.13 |
| 1423911_at | Ppp2r5a | protein phosphatase 2, regulatory subunit B (B56), alpha isoform | -1.17 | 0.199 | -1.1 | 0.432 | -1.08 | 0.154 | -1.29 | 0.29 | -1.16 |
| 1438626_x_at | Rpl14 | ribosomal protein L14 | -1.19 | 0.264 | -1.05 | 0.633 | -1.12 | 0.411 | -1.31 | 0.024 | -1.17 |
| 1430045_at | Tsnax | translin-associated factor X | -1.03 | 0.884 | -1.02 | 0.828 | -1.35 | 0.139 | -1.23 | 0.247 | -1.16 |
| 1434332_at | Zzz3 | zinc finger, ZZ domain containing 3 | -1.05 | 0.314 | -1.17 | 0.067 | -1.12 | 0.026 | -1.1 | 0.298 | -1.11 |
| 1437256_at | --- | --- | -1.1 | 0.821 | -1.26 | 0.648 | -1.02 | 0.957 | -2.06 | 0.184 | -1.36 |
| 1449679_s_at | Stx5a | syntaxin 5A | -1.06 | 0.092 | -1.06 | 0.615 | -1.24 | 0.076 | -1.14 | 0.199 | -1.13 |
| 1452790_x_at | Ndufa3 | NADH dehydrogenase (ubiquinone) 1 alpha subcomplex, 3 | -1.04 | 0.755 | -1.21 | 0.028 | -1.11 | 0.448 | -1.13 | 0.353 | -1.12 |
| 1426578_s_at | Snapap | SNAP-associated protein | -1.01 | 0.932 | -1.22 | 0.344 | -1.13 | 0.639 | -1.1 | 0.596 | -1.12 |
| 1449585_at | Il1rap | interleukin 1 receptor accessory protein | -1.07 | 0.903 | -1.29 | 0.104 | -1.01 | 0.961 | -1 | 0.993 | -1.1 |
| 1454595_at | 4930451E06Rik | RIKEN cDNA 4930451E06 gene | -1.1 | 0.876 | -1.13 | 0.699 | -1.11 | 0.82 | -1.39 | 0.194 | -1.18 |
| 1434486_x_at | Ugp2 | UDP-glucose pyrophosphorylase 2 | -1.09 | 0.263 | -1.15 | 0.404 | -1.1 | 0.334 | -1.13 | 0.681 | -1.12 |
| 1434402_at | Samd8 | sterile alpha motif domain containing 8 | -1.19 | 0.265 | -1.09 | 0.434 | -1.07 | 0.666 | -1.22 | 0.307 | -1.14 |
| 1438563_s_at | Mrps24 | mitochondrial ribosomal protein S24 | -1.01 | 0.944 | -1.06 | 0.646 | -1.32 | 0.131 | -1.13 | 0.532 | -1.13 |
| 1424862_s_at | Mib2 | mindbomb homolog 2 (Drosophila) | -1.04 | 0.894 | -1.19 | 0.354 | -1.13 | 0.46 | -1.21 | 0.437 | -1.14 |
| 1435440_at | Pdzd8 | PDZ domain containing 8 | -1.01 | 0.932 | -1.18 | 0.017 | -1.17 | 0.186 | -1.07 | 0.255 | -1.11 |

| | | | | | | | | | | | |
|---|---|---|---|---|---|---|---|---|---|---|---|
| 1420583_a_at | Rora | RAR-related orphan receptor alpha | -1.06 | 0.746 | -1.11 | 0.46 | -1.18 | 0.181 | -1.26 | 0.114 | -1.15 |
| 1439998_at | Jmjd1c | jumonji domain containing 1C | -1.02 | 0.943 | -1.04 | 0.773 | -1.33 | 0.214 | -1.6 | 0.11 | -1.25 |
| 1422517_a_at | Znrd1 | zinc ribbon domain containing, 1 | -1.03 | 0.498 | -1.01 | 0.726 | -1.36 | 0.017 | -1.58 | 0.134 | -1.25 |
| 1452266_at | Las1l | LAS1-like (S. cerevisiae) | -1.05 | 0.335 | -1.17 | 0.056 | -1.13 | 0.541 | -1.13 | 0.27 | -1.12 |
| 1454064_a_at | Rnf138 | ring finger protein 138 | -1.16 | 0.408 | -1.11 | 0.269 | -1.08 | 0.479 | -1.55 | 0.073 | -1.22 |
| 1452188_at | Maml1 | mastermind like 1 (Drosophila) | -1.19 | 0.005 | -1.14 | 0.311 | -1.02 | 0.859 | -1.38 | 0.087 | -1.18 |
| 1423338_at | Ccdc16 | coiled-coil domain containing 16 | -1.14 | 0.308 | -1.19 | 0.067 | -1.03 | 0.855 | -1.15 | 0.311 | -1.13 |
| 1416153_at | Srp54 /// LOC6651 | signal recognition particle 54 /// similar to signal recognition particle 54 | -1.19 | 0.032 | -1.05 | 0.348 | -1.11 | 0.396 | -1.08 | 0.37 | -1.11 |
| 1456726_x_at | Qars | glutaminyl-tRNA synthetase | -1.13 | 0.158 | -1.17 | 0.16 | -1.05 | 0.688 | -1.38 | 0.043 | -1.18 |
| 1429351_at | Klhl24 | kelch-like 24 (Drosophila) | -1.16 | 0.477 | -1.11 | 0.13 | -1.07 | 0.65 | -1.78 | 0.229 | -1.28 |
| 1415942_at | Rpl10 /// LOC2347 | ribosomal protein 10 /// similar to ribosomal protein L10 /// similar to ribosomal pro | -1.02 | 0.871 | -1.14 | 0.328 | -1.2 | 0.004 | -1.18 | 0.013 | -1.13 |
| 1448427_at | Ndufa6 | NADH dehydrogenase (ubiquinone) 1 alpha subcomplex, 6 (B14) | -1.06 | 0.394 | -1.17 | 0.099 | -1.11 | 0.181 | -1.4 | 0.128 | -1.19 |
| 1418815_at | Cdh2 | cadherin 2 | -1.02 | 0.795 | -1 | 0.974 | -1.39 | 0.377 | -1.2 | 0.72 | -1.15 |
| 1424545_at | BC003965 | cDNA sequence BC003965 | -1.05 | 0.391 | -1.14 | 0.047 | -1.16 | 0.154 | -1.07 | 0.466 | -1.11 |
| 1425085_at | 6330416L07Rik | RIKEN cDNA 6330416L07 gene | -1.35 | 0.338 | -1.03 | 0.81 | -1.02 | 0.951 | -1 | 0.992 | -1.1 |
| 1448760_at | Zfp68 | zinc finger protein 68 | -1.02 | 0.885 | -1.06 | 0.143 | -1.3 | 0.067 | -1.41 | 0.214 | -1.2 |
| 1419374_at | Wbp4 | WW domain binding protein 4 | -1.08 | 0.602 | -1.08 | 0.216 | -1.18 | 0.187 | -1.02 | 0.929 | -1.09 |
| 1419112_at | Nlk | nemo like kinase | -1.16 | 0.175 | -1.06 | 0.643 | -1.12 | 0.302 | -1.74 | 0.045 | -1.27 |
| 1417934_at | Dnajc4 | DnaJ (Hsp40) homolog, subfamily C, member 4 | -1.19 | 0.052 | -1.17 | 0.184 | -1 | 0.965 | -1.34 | 0.044 | -1.17 |
| 1419352_at | 0610007P06Rik | RIKEN cDNA 0610007P06 gene | -1.17 | 0.247 | -1.15 | 0.175 | -1.03 | 0.847 | -1.05 | 0.742 | -1.1 |
| 1451804_a_at | Lrrc16 | leucine rich repeat containing 16 | -1 | 0.983 | -1.39 | 0.265 | -1.02 | 0.953 | -2.18 | 0.073 | -1.4 |
| 1434683_at | Cutl1 | Cut-like 1 (Drosophila) | -1.19 | 0.144 | -1.03 | 0.887 | -1.13 | 0.376 | -1.18 | 0.565 | -1.13 |
| 1430264_at | 2610030P05Rik | RIKEN cDNA 2610030P05 gene | -1.1 | 0.789 | -1.04 | 0.815 | -1.22 | 0.13 | -1.19 | 0.191 | -1.14 |
| 1423840_at | Ccdc56 | coiled-coil domain containing 56 | -1.19 | 0.325 | -1.08 | 0.52 | -1.08 | 0.782 | -1.53 | 0.209 | -1.22 |
| 1453491_at | 4833409A17Rik | RIKEN cDNA 4833409A17 gene | -1.14 | 0.689 | -1.21 | 0.646 | -1.01 | 0.984 | -2.33 | 0.048 | -1.42 |
| 1433831_at | 4833418A01Rik | RIKEN cDNA 4833418A01 gene | -1.04 | 0.718 | -1.01 | 0.928 | -1.35 | 0.044 | -1.66 | 0.021 | -1.26 |
| 1422858_at | Trip4 | thyroid hormone receptor interactor 4 | -1.12 | 0.188 | -1.05 | 0.584 | -1.17 | 0.418 | -1.17 | 0.154 | -1.13 |
| 1424607_a_at | BC003993 /// LOC5 | cDNA sequence BC003993 /// hypothetical LOC544818 | -1.04 | 0.859 | -1.14 | 0.013 | -1.17 | 0.322 | -1.05 | 0.526 | -1.1 |
| 1416751_a_at | Ddx20 | DEAD (Asp-Glu-Ala-Asp) box polypeptide 20 | -1.12 | 0.535 | -1.23 | 0.22 | -1.01 | 0.859 | -1.01 | 0.955 | -1.09 |
| 1419360_a_at | Ss18 | synovial sarcoma translocation, Chromosome 18 | -1.05 | 0.69 | -1.09 | 0.128 | -1.2 | 0.11 | -1.24 | 0.341 | -1.15 |
| 1438288_x_at | 1110059G02Rik | RIKEN cDNA 1110059G02 gene | -1.24 | 0.178 | -1.11 | 0.664 | -1.01 | 0.951 | -1.27 | 0.244 | -1.16 |
| 1460716_a_at | Cbfb | core binding factor beta | -1.04 | 0.429 | -1.11 | 0.491 | -1.2 | 0.124 | -1.53 | 0.168 | -1.22 |
| 1439504_s_at | Zfp28 | zinc finger protein 28 | -1.18 | 0.562 | -1.17 | 0.803 | -1.01 | 0.986 | -1.78 | 0.303 | -1.28 |
| 1415722_a_at | 1110059P08Rik | RIKEN cDNA 1110059P08 gene | -1.11 | 0.232 | -1.11 | 0.176 | -1.11 | 0.351 | -1.19 | 0.268 | -1.13 |
| 1455402_at | Socs7 | suppressor of cytokine signaling 7 | -1.25 | 0.102 | -1.05 | 0.659 | -1.06 | 0.637 | -1.17 | 0.183 | -1.13 |
| 1434551_at | Hnrpul2 | heterogeneous nuclear ribonucleoprotein U-like 2 | -1.15 | 0.457 | -1.15 | 0.395 | -1.04 | 0.831 | -1.02 | 0.928 | -1.09 |
| 1423923_a_at | Wdr8 | WD repeat domain 8 | -1 | 0.986 | -1.25 | 0.222 | -1.11 | 0.095 | -1.1 | 0.528 | -1.12 |
| 1452048_at | Mrpl12 | mitochondrial ribosomal protein L12 | -1.06 | 0.455 | -1.1 | 0.1 | -1.18 | 0.029 | -1.14 | 0.472 | -1.12 |
| 1435442_at | Wdsof1 | WD repeats and SOF domain containing 1 | -1.09 | 0.437 | -1.16 | 0.187 | -1.09 | 0.589 | -1.17 | 0.205 | -1.13 |
| 1433934_at | Sec24a | SEC24 related gene family, member A (S. cerevisiae) | -1.22 | 0.287 | -1.04 | 0.678 | -1.09 | 0.493 | -1.08 | 0.781 | -1.11 |
| 1425672_a_at | Trpc2 | transient receptor potential cation channel, subfamily C, member 2 | -1.06 | 0.746 | -1.1 | 0.54 | -1.18 | 0.582 | -1.35 | 0.648 | -1.17 |
| 1429160_at | 2810012L14Rik | RIKEN cDNA 2810012L14 gene | -1 | 0.982 | -1.27 | 0.137 | -1.09 | 0.657 | -1.85 | 0.112 | -1.3 |
| 1424309_a_at | Mocs2 | molybdenum cofactor synthesis 2 | -1.06 | 0.372 | -1.18 | 0.095 | -1.09 | 0.233 | -1.12 | 0.052 | -1.12 |
| 1424209_at | Rarsl | arginyl-tRNA synthetase-like | -1.26 | 0.167 | -1.09 | 0.293 | -1.01 | 0.922 | -1.04 | 0.713 | -1.1 |
| 1454641_at | Cggbp1 | CGG triplet repeat binding protein 1 | -1.1 | 0.02 | -1.03 | 0.546 | -1.22 | 0.016 | -1.04 | 0.042 | -1.1 |
| 1435590_at | D430047L21Rik | RIKEN cDNA D430047L21 gene | -1.02 | 0.953 | -1.09 | 0.7 | -1.26 | 0.353 | -1.18 | 0.446 | -1.14 |
| 1448965_at | Inoc1 | INO80 complex homolog 1 (S. cerevisiae) | -1.1 | 0.722 | -1.22 | 0.24 | -1.03 | 0.926 | -1.12 | 0.74 | -1.12 |
| 1454756_at | Lrch3 | leucine-rich repeats and calponin homology (CH) domain containing 3 | -1.19 | 0.357 | -1.05 | 0.629 | -1.1 | 0.652 | -1.62 | 0.081 | -1.24 |
| 1424703_at | Hemk1 | HemK methyltransferase family member 1 | -1.07 | 0.65 | -1.26 | 0.152 | -1.03 | 0.941 | -1.62 | 0.123 | -1.24 |
| 1460339_at | Psma4 | proteasome (prosome, macropain) subunit, alpha type 4 | -1.12 | 0.098 | -1.12 | 0.246 | -1.1 | 0.272 | -1.17 | 0.483 | -1.13 |
| 1457046_s_at | C77370 | expressed sequence C77370 | -1.15 | 0.779 | -1.12 | 0.717 | -1.06 | 0.888 | -1.93 | 0.435 | -1.32 |
| 1420265_x_at | Cog8 | component of oligomeric golgi complex 8 | -1.23 | 0.268 | -1.09 | 0.478 | -1.04 | 0.898 | -1.73 | 0.02 | -1.27 |
| 1422797_at | Mapbpip | mitogen activated protein binding protein interacting protein | -1.11 | 0.203 | -1.18 | 0.141 | -1.05 | 0.209 | -1.63 | 0.063 | -1.24 |

| | | | | | | | | | | | |
|---|---|---|---|---|---|---|---|---|---|---|---|
| 1452166_a_at | Krt10 | keratin 10 | -1.04 | 0.027 | -1.06 | 0.67 | -1.26 | 0.071 | -1.21 | 0.503 | -1.14 |
| 1431028_a_at | Pank1 | pantothenate kinase 1 | -1.06 | 0.849 | -1.09 | 0.55 | -1.19 | 0.26 | -1.24 | 0.354 | -1.15 |
| 1433830_at | Hnrpa2b1 | heterogeneous nuclear ribonucleoprotein A2/B1 | -1.22 | 0.152 | -1.04 | 0.562 | -1.09 | 0.488 | -1.15 | 0.407 | -1.12 |
| 1416983_s_at | Foxo1 | forkhead box O1 | -1.07 | 0.801 | -1.17 | 0.495 | -1.1 | 0.587 | -2.06 | 0.101 | -1.35 |
| 1434841_at | BC010584 | CDNA sequence BC010584 | -1.14 | 0.259 | -1.02 | 0.905 | -1.2 | 0.255 | -1.41 | 0.143 | -1.19 |
| 1456253_s_at | Plekhn1 /// Klhl17 | pleckstrin homology domain containing, family N member 1 /// kelch-like 17 (Dros | -1.02 | 0.919 | -1.19 | 0.17 | -1.13 | 0.287 | -1.05 | 0.676 | -1.1 |
| 1435149_at | Plcg1 | phospholipase C, gamma 1 | -1.02 | 0.895 | -1.14 | 0.332 | -1.18 | 0.437 | -1.55 | 0.278 | -1.22 |
| 1454217_at | 4930556N09Rik | RIKEN cDNA 4930556N09 gene | -1.24 | 0.776 | -1.04 | 0.794 | -1.07 | 0.875 | -1.4 | 0.079 | -1.19 |
| 1433397_at | 9430012M22Rik | RIKEN cDNA 9430012M22 gene | -1.19 | 0.534 | -1.14 | 0.769 | -1.01 | 0.972 | -2.13 | 0.051 | -1.37 |
| 1440130_at | BB116930 | expressed sequence BB116930 | -1.36 | 0.133 | -1.03 | 0.932 | -1 | 0.994 | -1.31 | 0.346 | -1.18 |
| 1424839_a_at | Nsun4 | NOL1/NOP2/Sun domain family, member 4 | -1.21 | 0.269 | -1.11 | 0.512 | -1.03 | 0.827 | -1.11 | 0.302 | -1.11 |
| 1460680_a_at | Rpl23 | ribosomal protein L23 | -1.15 | 0.237 | -1.05 | 0.381 | -1.15 | 0.087 | -1.34 | 0.011 | -1.17 |
| 1433824_x_at | Grsf1 | G-rich RNA sequence binding factor 1 | -1.18 | 0.298 | -1.16 | 0.057 | -1.01 | 0.857 | -1.25 | 0.127 | -1.15 |
| 1436121_a_at | Nsmce1 | non-SMC element 1 homolog (S. cerevisiae) | -1.08 | 0.154 | -1.09 | 0.009 | -1.16 | 0.201 | -1.31 | 0.277 | -1.16 |
| 1454022_at | Ephb2 | Eph receptor B2 | -1.22 | 0.434 | -1.05 | 0.722 | -1.07 | 0.534 | -1.07 | 0.719 | -1.1 |
| 1419636_at | 4833420G17Rik | RIKEN cDNA 4833420G17 gene | -1.05 | 0.813 | -1.11 | 0.475 | -1.17 | 0.457 | -1.65 | 0.052 | -1.25 |
| 1432271_a_at | Dcun1d5 | DCN1, defective in cullin neddylation 1, domain containing 5 (S. cerevisiae) | -1.12 | 0.321 | -1.11 | 0.204 | -1.1 | 0.442 | -1.19 | 0.274 | -1.13 |
| 1417792_at | Zfml | zinc finger, matrin-like | -1.02 | 0.829 | -1.26 | 0.272 | -1.08 | 0.679 | -1.08 | 0.749 | -1.11 |
| 1447923_at | 2810013C04Rik | RIKEN cDNA 2810013C04 gene | -1.17 | 0.543 | -1.03 | 0.744 | -1.14 | 0.206 | -2.02 | 0.063 | -1.34 |
| 1423625_a_at | Dnajc19 | DnaJ (Hsp40) homolog, subfamily C, member 19 | -1.08 | 0.63 | -1.11 | 0.368 | -1.14 | 0.459 | -1.23 | 0.492 | -1.14 |
| 1436982_at | Tnrc6b | trinucleotide repeat containing 6b | -1.12 | 0.334 | -1.07 | 0.529 | -1.14 | 0.464 | -1.03 | 0.606 | -1.09 |
| 1416857_at | Sdf2 | stromal cell derived factor 2 | -1.06 | 0.144 | -1.09 | 0.018 | -1.19 | 0.031 | -1.24 | 0.337 | -1.14 |
| 1416764_at | Fis1 | fission 1 (mitochondrial outer membrane) homolog (yeast) | -1.09 | 0.532 | -1.02 | 0.698 | -1.24 | 0.08 | -1.19 | 0.375 | -1.14 |
| 1455195_at | Rps24 | ribosomal protein S24 | -1.16 | 0.055 | -1.07 | 0.502 | -1.1 | 0.641 | -1.49 | 0.038 | -1.21 |
| 1437776_at | Tmcc1 | transmembrane and coiled coil domains 1 | -1.07 | 0.688 | -1.11 | 0.619 | -1.16 | 0.089 | -1.29 | 0.023 | -1.16 |
| 1427095_at | Cdcp1 | CUB domain containing protein 1 | -1.18 | 0.245 | -1.01 | 0.938 | -1.15 | 0.203 | -1.21 | 0.507 | -1.14 |
| 1429949_at | 6530415H11Rik | RIKEN cDNA 6530415H11 gene | -1.07 | 0.747 | -1.29 | 0.302 | -1.01 | 0.977 | -1.01 | 0.909 | -1.1 |
| 1428357_at | 2610019F03Rik | RIKEN cDNA 2610019F03 gene | -1.06 | 0.642 | -1.17 | 0.385 | -1.1 | 0.723 | -2.74 | 0.126 | -1.52 |
| 1427544_a_at | Papola | poly (A) polymerase alpha | -1.03 | 0.937 | -1.23 | 0.27 | -1.1 | 0.684 | -1.06 | 0.797 | -1.1 |
| 1434377_x_at | Rps6 /// LOC66773 | ribosomal protein S6 /// similar to 40S ribosomal protein S6 /// similar to 40S ribos | -1.07 | 0.14 | -1.04 | 0.574 | -1.24 | 0.003 | -1.25 | 0.004 | -1.15 |
| 1416981_at | Foxo1 | forkhead box O1 | -1.03 | 0.848 | -1.2 | 0.294 | -1.1 | 0.493 | -2.31 | 0.052 | -1.41 |
| 1450949_at | Katna1 | katanin p60 (ATPase-containing) subunit A1 | -1.09 | 0.568 | -1.23 | 0.019 | -1.03 | 0.82 | -1.06 | 0.693 | -1.1 |
| 1429711_at | Styx | phosphoserine/threonine/tyrosine interaction protein | -1.04 | 0.547 | -1.02 | 0.838 | -1.3 | 0.099 | -1.15 | 0.122 | -1.13 |
| 1426810_at | Jmjd1a | jumonji domain containing 1A | -1.15 | 0.388 | -1.09 | 0.443 | -1.09 | 0.39 | -1.15 | 0.002 | -1.12 |
| 1420862_at | Dctn4 | dynactin 4 | -1.07 | 0.444 | -1.2 | 0.129 | -1.07 | 0.527 | -1.11 | 0.559 | -1.11 |
| 1428163_at | Sar1b | SAR1 gene homolog B (S. cerevisiae) | -1.05 | 0.588 | -1.14 | 0.415 | -1.14 | 0.135 | -1.03 | 0.778 | -1.09 |
| 1448347_a_at | Gpiap1 | GPI-anchored membrane protein 1 | -1.07 | 0.679 | -1.05 | 0.708 | -1.22 | 0.085 | -1.02 | 0.742 | -1.09 |
| 1442243_at | Per3 | period homolog 3 (Drosophila) | -1.05 | 0.903 | -1.09 | 0.865 | -1.19 | 0.733 | -1.06 | 0.376 | -1.1 |
| 1448286_at | Hadh2 | hydroxyacyl-Coenzyme A dehydrogenase type II | -1.13 | 0.238 | -1.12 | 0.062 | -1.08 | 0.655 | -1.11 | 0.666 | -1.11 |
| 1417474_at | 1500035H01Rik | RIKEN cDNA 1500035H01 gene | -1 | 0.88 | -1.14 | 0.259 | -1.2 | 0.277 | -1.3 | 0.032 | -1.16 |
| 1460581_a_at | Rpl13 /// LOC28004 | ribosomal protein L13 /// similar to 60S ribosomal protein L13 /// similar to 60S rib | -1.05 | 0.412 | -1.06 | 0.663 | -1.23 | 0.042 | -1.3 | 0.07 | -1.16 |
| 1443762_s_at | Sbf2 | SET binding factor 2 | -1.08 | 0.526 | -1.09 | 0.535 | -1.16 | 0.253 | -1.11 | 0.527 | -1.11 |
| 1426845_at | Pdcd2l | programmed cell death 2-like | -1.13 | 0.273 | -1.19 | 0.028 | -1.02 | 0.866 | -1.28 | 0.095 | -1.15 |
| 1424105_a_at | Pttg1 | pituitary tumor-transforming 1 | -1.11 | 0.229 | -1.23 | 0.035 | -1.01 | 0.94 | -1.01 | 0.946 | -1.09 |
| 1433927_at | Uspl1 | ubiquitin specific peptidase like 1 | -1.03 | 0.731 | -1.08 | 0.401 | -1.24 | 0.178 | -1.29 | 0.019 | -1.16 |
| 1453929_at | Rnf24 | ring finger protein 24 | -1.27 | 0.417 | -1.07 | 0.708 | -1.01 | 0.807 | -1.03 | 0.616 | -1.1 |
| 1423793_at | D2Ertd391e | DNA segment, Chr 2, ERATO Doi 391, expressed | -1.05 | 0.564 | -1.31 | 0.183 | -1.01 | 0.964 | -1.17 | 0.481 | -1.13 |
| 1451076_s_at | Ga17 | dendritic cell protein GA17 | -1.07 | 0.604 | -1.12 | 0.017 | -1.14 | 0.068 | -1.38 | 0.112 | -1.18 |
| 1415699_a_at | Gps1 | G protein pathway suppressor 1 | -1.01 | 0.934 | -1.08 | 0.013 | -1.26 | 0.047 | -1.13 | 0.148 | -1.12 |
| 1442098_at | AU022434 | expressed sequence AU022434 | -1.17 | 0.364 | -1.14 | 0.225 | -1.02 | 0.863 | -1.83 | 0.089 | -1.29 |
| 1426084_a_at | Tor1aip1 | torsin A interacting protein 1 | -1.11 | 0.061 | -1.19 | 0.122 | -1.03 | 0.633 | -1.29 | 0.25 | -1.16 |
| 1419308_at | Invs | inversin | -1.09 | 0.264 | -1.25 | 0.061 | -1.01 | 0.966 | -1.62 | 0.13 | -1.24 |
| 1437210_a_at | Brd2 | bromodomain containing 2 | -1 | 0.987 | -1.23 | 0.136 | -1.11 | 0.685 | -1.14 | 0.443 | -1.12 |

| | | | | | | | | | | | |
|---|---|---|---|---|---|---|---|---|---|---|---|
| 1445747_at | --- | 15 days embryo male testis cDNA, RIKEN full-length enriched library, clone:8030 | -1.18 | 0.5 | -1 | 0.998 | -1.16 | 0.47 | -1.36 | 0.307 | -1.17 |
| 1457432_at | Prox1 | prospero-related homeobox 1 | -1.09 | 0.796 | -1.05 | 0.588 | -1.2 | 0.189 | -1.33 | 0.237 | -1.16 |
| 1422451_at | Mrps21 | mitochondrial ribosomal protein S21 | -1.04 | 0.534 | -1.12 | 0.143 | -1.17 | 0.168 | -1.25 | 0.275 | -1.14 |
| 1427878_at | 0610010O12Rik | RIKEN cDNA 0610010O12 gene | -1.15 | 0.189 | -1.02 | 0.881 | -1.15 | 0.254 | -1.21 | 0.52 | -1.14 |
| 1432263_a_at | Cox7a2l | cytochrome c oxidase subunit VIIa polypeptide 2-like | -1.12 | 0.048 | -1.12 | 0.093 | -1.08 | 0.645 | -1.96 | 0.131 | -1.32 |
| 1440830_at | Gpr116 | G protein-coupled receptor 116 | -1.01 | 0.948 | -1.01 | 0.864 | -1.37 | 0.104 | -1.32 | 0.382 | -1.18 |
| 1451146_at | Zfp386 /// LOC6769 | zinc finger protein 386 (Kruppel-like) /// similar to zinc finger protein 386 (Kruppel- | -1.14 | 0.389 | -1.14 | 0.33 | -1.04 | 0.835 | -1.93 | 0.041 | -1.31 |
| 1423989_at | 2210010N04Rik | RIKEN cDNA 2210010N04 gene | -1.02 | 0.916 | -1.25 | 0.417 | -1.08 | 0.687 | -2.4 | 0.071 | -1.44 |
| 1455227_at | Aadacl1 | arylacetamide deacetylase-like 1 | -1.04 | 0.486 | -1.29 | 0.178 | -1.02 | 0.889 | -1.13 | 0.668 | -1.12 |
| 1448398_s_at | Rpl22 | ribosomal protein L22 | -1.14 | 0.57 | -1.04 | 0.793 | -1.15 | 0.306 | -1.38 | 0.159 | -1.18 |
| 1448493_at | Paip2 | polyadenylate-binding protein-interacting protein 2 | -1.04 | 0.695 | -1.08 | 0.184 | -1.21 | 0.093 | -1.25 | 0.181 | -1.15 |
| 1452075_at | 4933435A13Rik | RIKEN cDNA 4933435A13 gene | -1.17 | 0.295 | -1.11 | 0.532 | -1.05 | 0.773 | -1.02 | 0.823 | -1.09 |
| 1422471_at | Pex13 | peroxisomal biogenesis factor 13 | -1.1 | 0.01 | -1.14 | 0.135 | -1.09 | 0.484 | -1.17 | 0.347 | -1.12 |
| 1424005_at | B230219D22Rik | RIKEN cDNA B230219D22 gene | -1.02 | 0.644 | -1.14 | 0.109 | -1.17 | 0.388 | -1.04 | 0.76 | -1.09 |
| 1426830_a_at | Ahcyl1 | S-adenosylhomocysteine hydrolase-like 1 | -1.02 | 0.875 | -1.04 | 0.176 | -1.3 | 0.071 | -1.1 | 0.493 | -1.11 |
| 1417885_at | Mapt | microtubule-associated protein tau | -1.07 | 0.781 | -1.15 | 0.751 | -1.1 | 0.264 | -2.25 | 0.478 | -1.39 |
| 1439841_at | Zfyve27 | zinc finger, FYVE domain containing 27 | -1.22 | 0.288 | -1.08 | 0.805 | -1.03 | 0.879 | -1.08 | 0.729 | -1.1 |
| 1421212_at | Abcc6 | ATP-binding cassette, sub-family C (CFTR/MRP), member 6 | -1.01 | 0.954 | -1.13 | 0.416 | -1.2 | 0.111 | -1.15 | 0.686 | -1.12 |
| 1451294_s_at | Snrpe | small nuclear ribonucleoprotein E | -1.03 | 0.728 | -1.1 | 0.175 | -1.2 | 0.214 | -1.2 | 0.17 | -1.13 |
| 1418516_at | Mtf2 | metal response element binding transcription factor 2 | -1.05 | 0.767 | -1.17 | 0.227 | -1.11 | 0.362 | -1.29 | 0.227 | -1.15 |
| 1417113_at | Gmcl1 | germ cell-less homolog 1 (Drosophila) | -1.1 | 0.061 | -1.07 | 0.412 | -1.16 | 0.039 | -1.27 | 0.205 | -1.15 |
| 1428215_x_at | Tomm7 | translocase of outer mitochondrial membrane 7 homolog (yeast) | -1.03 | 0.713 | -1.08 | 0.351 | -1.22 | 0.137 | -1.29 | 0.304 | -1.16 |
| 1457674_at | Vps13a | vacuolar protein sorting 13A (yeast) | -1.25 | 0.289 | -1.02 | 0.947 | -1.08 | 0.582 | -1.43 | 0.004 | -1.19 |
| 1424463_at | 2210010L05Rik | RIKEN cDNA 2210010L05 gene | -1.03 | 0.554 | -1.11 | 0.699 | -1.2 | 0.016 | -1.24 | 0.186 | -1.14 |
| 1459433_at | C130051F05Rik | RIKEN cDNA C130051F05 gene | -1.06 | 0.847 | -1.02 | 0.891 | -1.27 | 0.159 | -1.68 | 0.056 | -1.26 |
| 1416439_at | 2410015N17Rik | RIKEN cDNA 2410015N17 gene | -1.1 | 0.682 | -1.15 | 0.333 | -1.07 | 0.741 | -1.28 | 0.5 | -1.15 |
| 1449386_at | Hsd17b9 | hydroxysteroid (17-beta) dehydrogenase 9 | -1.07 | 0.938 | -1.15 | 0.703 | -1.1 | 0.504 | -2.08 | 0.25 | -1.35 |
| 1430417_s_at | 0610025P10Rik | RIKEN cDNA 0610025P10 gene | -1.02 | 0.782 | -1.14 | 0.318 | -1.17 | 0.267 | -1.1 | 0.616 | -1.11 |
| 1433570_s_at | Mak10 | MAK10 homolog, amino-acid N-acetyltransferase subunit, (S. cerevisiae) | -1.06 | 0.751 | -1.27 | 0.03 | -1.02 | 0.904 | -1.14 | 0.384 | -1.12 |
| 1416762_at | S100a10 | S100 calcium binding protein A10 (calpactin) | -1.02 | 0.638 | -1.09 | 0.425 | -1.24 | 0.046 | -1.57 | 0.302 | -1.23 |
| 1434344_at | Gpkow | G patch domain and KOW motifs | -1.13 | 0.268 | -1.17 | 0.01 | -1.03 | 0.473 | -1.07 | 0.557 | -1.1 |
| 1426820_at | 2610507B11Rik | RIKEN cDNA 2610507B11 gene | -1.01 | 0.728 | -1.23 | 0.08 | -1.1 | 0.406 | -1.23 | 0.138 | -1.14 |
| 1455153_at | Zfp236 | zinc finger protein 236 | -1.11 | 0.435 | -1.21 | 0.117 | -1.02 | 0.859 | -1.31 | 0.092 | -1.16 |
| 1416465_a_at | Vapa | vesicle-associated membrane protein, associated protein A | -1.04 | 0.801 | -1.29 | 0.037 | -1.03 | 0.791 | -1.13 | 0.291 | -1.12 |
| 1422613_a_at | Rpl7a | ribosomal protein L7a | -1.08 | 0.146 | -1.1 | 0.204 | -1.14 | 0.088 | -1.29 | 0.002 | -1.15 |
| 1433464_at | Ipo13 | importin 13 | -1.12 | 0.248 | -1.13 | 0.224 | -1.07 | 0.662 | -1.17 | 0.2 | -1.12 |
| 1426724_at | Cnn3 | calponin 3, acidic | -1.03 | 0.606 | -1.08 | 0.531 | -1.22 | 0.225 | -2.32 | 0.04 | -1.41 |
| 1453032_at | 2610109B12Rik | RIKEN cDNA 2610109B12 gene | -1.04 | 0.412 | -1.02 | 0.744 | -1.29 | 0.012 | -1.18 | 0.024 | -1.13 |
| 1416171_at | 2310037I24Rik | RIKEN cDNA 2310037I24 gene | -1.03 | 0.759 | -1.15 | 0.224 | -1.14 | 0.377 | -1.26 | 0.013 | -1.15 |
| 1447483_s_at | 2610002F03Rik | RIKEN cDNA 2610002F03 gene | -1.02 | 0.898 | -1.27 | 0.392 | -1.05 | 0.642 | -1 | 0.993 | -1.09 |
| 1427898_at | Rnf6 | ring finger protein (C3H2C3 type) 6 | -1.05 | 0.413 | -1.08 | 0.452 | -1.2 | 0.156 | -1.17 | 0.034 | -1.12 |
| 1437657_at | Zfp291 | zinc finger protein 291 | -1.03 | 0.917 | -1.15 | 0.675 | -1.14 | 0.598 | -1.48 | 0.283 | -1.2 |
| 1417590_at | Cyp27a1 | cytochrome P450, family 27, subfamily a, polypeptide 1 | -1.01 | 0.946 | -1.21 | 0.174 | -1.11 | 0.637 | -2.13 | 0.123 | -1.36 |
| 1424706_at | Zfp51 | zinc finger protein 51 | -1.11 | 0.683 | -1.1 | 0.476 | -1.1 | 0.469 | -1.73 | 0.083 | -1.26 |
| 1453729_a_at | Rpl37 | ribosomal protein L37 | -1.06 | 0.076 | -1 | 0.906 | -1.29 | 0.018 | -1.22 | 0.012 | -1.14 |
| 1428449_at | Gtf3c2 | general transcription factor IIIC, polypeptide 2, beta | -1.14 | 0.312 | -1.13 | 0.417 | -1.05 | 0.806 | -1.03 | 0.93 | -1.09 |
| 1448921_a_at | Mrps9 | mitochondrial ribosomal protein S9 | -1.1 | 0.643 | -1.11 | 0.526 | -1.1 | 0.031 | -1.14 | 0.191 | -1.11 |
| 1451388_a_at | Atp11b | ATPase, Class VI, type 11B | -1.06 | 0.574 | -1.22 | 0.276 | -1.05 | 0.696 | -1.02 | 0.909 | -1.09 |
| 1443089_at | 4930506C02Rik | RIKEN cDNA 4930506C02 gene | -1.02 | 0.919 | -1.32 | 0.27 | -1.02 | 0.974 | -1.46 | 0.365 | -1.21 |
| 1443453_at | Zfp651 | zinc finger protein 651 | -1 | 0.994 | -1.13 | 0.876 | -1.19 | 0.649 | -1.56 | 0.396 | -1.22 |
| 1451096_at | Ndufs2 | NADH dehydrogenase (ubiquinone) Fe-S protein 2 | -1.11 | 0.401 | -1.16 | 0.234 | -1.05 | 0.734 | -1.43 | 0.068 | -1.19 |
| 1436936_s_at | Xist | inactive X specific transcripts | -1.05 | 0.792 | -1.15 | 0.26 | -1.12 | 0.425 | -1.07 | 0.615 | -1.1 |
| 1424782_at | Tmem77 | transmembrane protein 77 | -1.13 | 0.364 | -1.03 | 0.613 | -1.16 | 0.036 | -1.28 | 0.102 | -1.15 |

| | | | | | | | | | | | |
|---|---|---|---|---|---|---|---|---|---|---|---|
| 1451905_a_at | Mx1 | myxovirus (influenza virus) resistance 1 | -1.13 | 0.737 | -1.02 | 0.965 | -1.18 | 0.523 | -2.34 | 0.147 | -1.42 |
| 1452701_x_at | Uba52 /// LOC6665 | ubiquitin A-52 residue ribosomal protein fusion product 1 /// similar to ubiquitin A-5 | -1.07 | 0.049 | -1.04 | 0.723 | -1.21 | 0.024 | -1.28 | 0.017 | -1.15 |
| 1450601_at | V1rb8 | vomeronasal 1 receptor, B8 | -1.14 | 0.519 | -1.05 | 0.902 | -1.13 | 0.854 | -1.19 | 0.766 | -1.13 |
| 1424218_a_at | Creb3l4 | cAMP responsive element binding protein 3-like 4 | -1.02 | 0.967 | -1.24 | 0.56 | -1.07 | 0.895 | -1.95 | 0.443 | -1.32 |
| 1433550_at | Chfr | checkpoint with forkhead and ring finger domains | -1.27 | 0.149 | -1.04 | 0.481 | -1.03 | 0.726 | -1.14 | 0.125 | -1.12 |
| 1429043_at | Smndc1 | survival motor neuron domain containing 1 | -1.17 | 0.275 | -1.08 | 0.453 | -1.06 | 0.554 | -1.11 | 0.422 | -1.11 |
| 1422608_at | Arpp19 /// 2700024 | cAMP-regulated phosphoprotein 19 /// RIKEN cDNA 2700024H10 gene | -1.03 | 0.753 | -1.01 | 0.902 | -1.33 | 0.205 | -1.04 | 0.804 | -1.1 |
| 1428709_a_at | Mrpl24 | mitochondrial ribosomal protein L24 | -1.12 | 0.133 | -1.16 | 0.002 | -1.04 | 0.598 | -1.63 | 0.073 | -1.24 |
| 1435171_at | 2810416G20Rik | RIKEN cDNA 2810416G20 gene | -1.16 | 0.413 | -1.08 | 0.751 | -1.07 | 0.76 | -1.27 | 0.113 | -1.15 |
| 1422601_at | Serpinb9 | serine (or cysteine) peptidase inhibitor, clade B, member 9 | -1.01 | 0.956 | -1.1 | 0.418 | -1.22 | 0.006 | -1.05 | 0.428 | -1.1 |
| 1415841_at | Dync1i2 | dynein cytoplasmic 1 intermediate chain 2 | -1.03 | 0.817 | -1.08 | 0.355 | -1.21 | 0.012 | -1.12 | 0.074 | -1.11 |
| 1451302_at | 1110012L19Rik | RIKEN cDNA 1110012L19 gene | -1.07 | 0.666 | -1.04 | 0.711 | -1.22 | 0.234 | -1.12 | 0.568 | -1.11 |
| 1415711_at | Arfgef1 | ADP-ribosylation factor guanine nucleotide-exchange factor 1(brefeldin A-inhibite | -1.11 | 0.342 | -1.09 | 0.195 | -1.1 | 0.406 | -1.28 | 0.119 | -1.15 |
| 1416447_at | Tmem30a | transmembrane protein 30A | -1.09 | 0.499 | -1.16 | 0.446 | -1.07 | 0.655 | -1.37 | 0.149 | -1.17 |
| 1430171_at | Fibp | fibroblast growth factor (acidic) intracellular binding protein | -1.15 | 0.478 | -1.05 | 0.66 | -1.12 | 0.52 | -1.4 | 0.22 | -1.18 |
| 1457304_at | D13Ertd787e | DNA segment, Chr 13, ERATO Doi 787, expressed | -1.06 | 0.902 | -1.02 | 0.932 | -1.26 | 0.468 | -1.52 | 0.122 | -1.21 |
| 1428327_at | Trak1 | trafficking protein, kinesin binding 1 | -1.11 | 0.672 | -1.18 | 0.345 | -1.03 | 0.859 | -1.14 | 0.392 | -1.12 |
| 1434127_a_at | H3f3a /// LOC5449 | H3 histone, family 3A /// similar to H3 histone, family 3B /// similar to H3 histone, f | -1.06 | 0.701 | -1.05 | 0.334 | -1.21 | 0.043 | -1.12 | 0.152 | -1.11 |
| 1426290_at | Dimt1 | DIM1 dimethyladenosine transferase 1-like (S. cerevisiae) | -1.18 | 0.422 | -1.07 | 0.829 | -1.06 | 0.532 | -1.13 | 0.498 | -1.11 |
| 1436079_s_at | Vapb | vesicle-associated membrane protein, associated protein B and C | -1.04 | 0.881 | -1.11 | 0.235 | -1.16 | 0.292 | -1.33 | 0.389 | -1.16 |
| 1425781_a_at | Plcb1 | phospholipase C, beta 1 | -1.15 | 0.476 | -1.13 | 0.372 | -1.04 | 0.875 | -1.21 | 0.323 | -1.13 |
| 1455662_x_at | Rps17 | ribosomal protein S17 | -1.11 | 0.123 | -1.01 | 0.802 | -1.2 | 0.017 | -1.31 | 0.003 | -1.16 |
| 1444167_at | --- | Transcribed locus | -1.15 | 0.432 | -1.16 | 0.64 | -1.01 | 0.945 | -1.07 | 0.678 | -1.1 |
| 1455429_at | AI450540 | expressed sequence AI450540 | -1.01 | 0.939 | -1.15 | 0.407 | -1.16 | 0.199 | -1.26 | 0.021 | -1.15 |
| 1444507_at | Usp53 | ubiquitin specific peptidase 53 | -1.09 | 0.553 | -1.04 | 0.814 | -1.18 | 0.204 | -1.82 | 0.112 | -1.28 |
| 1418564_s_at | Serbp1 | Serpine1 mRNA binding protein 1 | -1.06 | 0.276 | -1.05 | 0.262 | -1.22 | 0.104 | -1.1 | 0.356 | -1.1 |
| 1429436_at | Prpf40a | PRP40 pre-mRNA processing factor 40 homolog A (yeast) | -1.02 | 0.712 | -1.03 | 0.795 | -1.29 | 0.082 | -1.08 | 0.718 | -1.11 |
| 1418501_a_at | Oxr1 | oxidation resistance 1 | -1.05 | 0.604 | -1.08 | 0.106 | -1.19 | 0.129 | -1.14 | 0.456 | -1.11 |
| 1433723_s_at | Serf2 | small EDRK-rich factor 2 | -1.15 | 0.236 | -1.09 | 0.02 | -1.06 | 0.542 | -1.37 | 0.04 | -1.17 |
| 1453207_at | 2900053A13Rik | RIKEN cDNA 2900053A13 gene | -1.08 | 0.462 | -1.02 | 0.786 | -1.23 | 0.331 | -1.05 | 0.802 | -1.09 |
| 1433603_at | Ndufs6 | NADH dehydrogenase (ubiquinone) Fe-S protein 6 | -1.08 | 0.376 | -1.1 | 0.211 | -1.13 | 0.328 | -1.05 | 0.816 | -1.09 |
| 1416372_at | Ptdss1 | phosphatidylserine synthase 1 | -1.02 | 0.391 | -1.09 | 0.48 | -1.2 | 0.033 | -1.06 | 0.343 | -1.1 |
| 1419013_at | Gpatc1 | G patch domain containing 1 | -1.18 | 0.293 | -1.05 | 0.445 | -1.08 | 0.629 | -1.04 | 0.777 | -1.09 |
| 1438386_x_at | Mat2a | methionine adenosyltransferase II, alpha | -1.02 | 0.94 | -1.07 | 0.681 | -1.23 | 0.125 | -1.18 | 0.136 | -1.13 |
| 1416344_at | Lamp2 | lysosomal membrane glycoprotein 2 | -1.07 | 0.43 | -1.03 | 0.484 | -1.22 | 0.086 | -1.03 | 0.829 | -1.09 |
| 1450444_a_at | Nr1h3 | nuclear receptor subfamily 1, group H, member 3 | -1.08 | 0.523 | -1.01 | 0.884 | -1.23 | 0.127 | -1.04 | 0.891 | -1.09 |
| 1451135_at | Gtf2b | general transcription factor IIB | -1.2 | 0.015 | -1.03 | 0.708 | -1.09 | 0.426 | -1.27 | 0.075 | -1.15 |
| 1436967_at | Ankrd11 | ankyrin repeat domain 11 | -1.07 | 0.454 | -1.07 | 0.597 | -1.17 | 0.55 | -1.43 | 0.299 | -1.18 |
| 1449973_a_at | Pelp1 | proline, glutamic acid and leucine rich protein 1 | -1.31 | 0.463 | -1.04 | 0.948 | -1 | 0.991 | -1.77 | 0.311 | -1.28 |
| 1431544_at | 4930524B17Rik | RIKEN cDNA 4930524B17 gene | -1.15 | 0.837 | -1.04 | 0.932 | -1.12 | 0.664 | -1.26 | 0.232 | -1.14 |
| 1455500_at | D11Ertd759e /// LO | DNA segment, Chr 11, ERATO Doi 759, expressed /// similar to chromosome 17 | -1.09 | 0.629 | -1.11 | 0.28 | -1.11 | 0.59 | -1.27 | 0.318 | -1.14 |
| 1431428_a_at | Nosip | nitric oxide synthase interacting protein | -1.01 | 0.934 | -1.15 | 0.282 | -1.16 | 0.213 | -1.11 | 0.66 | -1.11 |
| 1425354_a_at | Aggf1 | angiogenic factor with G patch and FHA domains 1 | -1.04 | 0.776 | -1.03 | 0.723 | -1.26 | 0.016 | -1.04 | 0.775 | -1.09 |
| 1418715_at | Pank1 | pantothenate kinase 1 | -1.05 | 0.87 | -1.04 | 0.752 | -1.24 | 0.091 | -1.06 | 0.412 | -1.1 |
| 1432444_a_at | 1810011O16Rik | RIKEN cDNA 1810011O16 gene | -1.01 | 0.916 | -1.21 | 0.026 | -1.09 | 0.477 | -1.33 | 0.281 | -1.16 |
| 1417075_at | 2010309E21Rik | RIKEN cDNA 2010309E21 gene | -1.08 | 0.545 | -1.04 | 0.493 | -1.2 | 0.312 | -1.04 | 0.815 | -1.09 |
| 1416356_at | Gmpr2 | guanosine monophosphate reductase 2 | -1.07 | 0.245 | -1.11 | 0.141 | -1.12 | 0.317 | -1.21 | 0.028 | -1.13 |
| 1433537_at | 4833408C14Rik | RIKEN cDNA 4833408C14 gene | -1.02 | 0.94 | -1.01 | 0.881 | -1.33 | 0.024 | -1.12 | 0.661 | -1.12 |
| 1425266_a_at | Rap1gds1 | RAP1, GTP-GDP dissociation stimulator 1 | -1.05 | 0.477 | -1.16 | 0.285 | -1.1 | 0.488 | -1.43 | 0.248 | -1.18 |
| 1454301_at | 2900073C17Rik | RIKEN cDNA 2900073C17 gene | -1.03 | 0.964 | -1.13 | 0.811 | -1.16 | 0.803 | -2.17 | 0.074 | -1.37 |
| 1447388_at | 5830484A20Rik | RIKEN cDNA 5830484A20 gene | -1.15 | 0.804 | -1.1 | 0.864 | -1.05 | 0.893 | -1.01 | 0.972 | -1.08 |
| 1422994_at | Pip5k3 | phosphatidylinositol-3-phosphate/phosphatidylinositol 5-kinase, type III | -1.21 | 0.55 | -1.08 | 0.881 | -1.03 | 0.919 | -2.24 | 0.032 | -1.39 |
| 1418319_at | 1810047C23Rik | RIKEN cDNA 1810047C23 gene | -1.09 | 0.446 | -1.08 | 0.04 | -1.14 | 0.366 | -1.21 | 0.066 | -1.13 |

| | | | | | | | | | | | |
|---|---|---|---|---|---|---|---|---|---|---|---|
| 1429421_at | 2610203E10Rik | RIKEN cDNA 2610203E10 gene | -1.14 | 0.588 | -1.17 | 0.525 | -1.01 | 0.957 | -1.05 | 0.809 | -1.09 |
| 1460578_at | Fgd5 | FYVE, RhoGEF and PH domain containing 5 | -1.18 | 0.433 | -1.05 | 0.827 | -1.08 | 0.494 | -1.07 | 0.592 | -1.1 |
| 1455693_x_at | Rps6 /// LOC43440 | ribosomal protein S6 /// similar to 40S ribosomal protein S6 /// similar to 40S ribos | -1.04 | 0.753 | -1.07 | 0.561 | -1.21 | 0.014 | -1.28 | 0.006 | -1.15 |
| 1415688_at | Ube2g1 | ubiquitin-conjugating enzyme E2G 1 (UBC7 homolog, C. elegans) | -1.12 | 0.297 | -1.14 | 0.076 | -1.05 | 0.343 | -1.02 | 0.781 | -1.08 |
| 1427074_at | Pcmtd2 | protein-L-isoaspartate (D-aspartate) O-methyltransferase domain containing 2 | -1.18 | 0.416 | -1.07 | 0.345 | -1.06 | 0.644 | -1.03 | 0.821 | -1.09 |
| 1452583_s_at | Galm | galactose mutarotase | -1.13 | 0.132 | -1.17 | 0.43 | -1.02 | 0.92 | -1.61 | 0.196 | -1.23 |
| 1452249_at | Prickle1 | prickle like 1 (Drosophila) | -1.06 | 0.171 | -1.05 | 0.725 | -1.2 | 0.546 | -1.2 | 0.247 | -1.13 |
| 1450858_a_at | Ube2d3 | ubiquitin-conjugating enzyme E2D 3 (UBC4/5 homolog, yeast) | -1.06 | 0.438 | -1.15 | 0.001 | -1.09 | 0.321 | -1.16 | 0.201 | -1.11 |
| 1426513_at | Rbm28 | RNA binding motif protein 28 | -1 | 0.972 | -1.13 | 0.326 | -1.18 | 0.556 | -1.2 | 0.493 | -1.13 |
| 1436187_at | 1110054M08Rik | RIKEN cDNA 1110054M08 gene | -1.11 | 0.37 | -1.18 | 0.101 | -1.03 | 0.844 | -1.45 | 0.305 | -1.19 |
| 1451645_at | Fbs1 | fibrosin 1 | -1 | 0.989 | -1.37 | 0.017 | -1 | 0.968 | -1.38 | 0.017 | -1.19 |
| 1450921_at | Aptx | aprataxin | -1.11 | 0.262 | -1.08 | 0.529 | -1.11 | 0.372 | -1.29 | 0.254 | -1.15 |
| 1448373_at | Mrpl18 | mitochondrial ribosomal protein L18 | -1.09 | 0.251 | -1.12 | 0.278 | -1.09 | 0.24 | -1.01 | 0.945 | -1.08 |
| 1432158_a_at | Trappc2 | trafficking protein particle complex 2 | -1.1 | 0.112 | -1.04 | 0.505 | -1.17 | 0.01 | -1.21 | 0.027 | -1.13 |
| 1417999_at | Itm2b | integral membrane protein 2B | -1.1 | 0.588 | -1.08 | 0.513 | -1.13 | 0.05 | -1.08 | 0.368 | -1.09 |
| 1419803_s_at | Ccdc12 | coiled-coil domain containing 12 | -1.14 | 0.402 | -1.03 | 0.779 | -1.14 | 0.174 | -1.62 | 0.019 | -1.23 |
| 1451238_at | 1200003C05Rik | RIKEN cDNA 1200003C05 gene | -1.1 | 0.549 | -1.11 | 0.238 | -1.09 | 0.436 | -1.13 | 0.18 | -1.11 |
| 1436163_at | Kcnj16 | potassium inwardly-rectifying channel, subfamily J, member 16 | -1.03 | 0.74 | -1.26 | 0.255 | -1.04 | 0.777 | -1.29 | 0.564 | -1.15 |
| 1440044_at | Ttf1 | Transcription termination factor 1 | -1.06 | 0.657 | -1.28 | 0.432 | -1 | 0.988 | -1.36 | 0.486 | -1.17 |
| 1420668_a_at | Yipf2 | Yip1 domain family, member 2 | -1.06 | 0.797 | -1.15 | 0.49 | -1.1 | 0.771 | -1.02 | 0.937 | -1.08 |
| 1422996_at | Acot2 | acyl-CoA thioesterase 2 | -1 | 0.995 | -1.14 | 0.809 | -1.17 | 0.538 | -1.12 | 0.672 | -1.11 |
| 1455283_x_at | Ndufs8 | NADH dehydrogenase (ubiquinone) Fe-S protein 8 | -1.05 | 0.636 | -1.07 | 0.23 | -1.2 | 0.056 | -1.04 | 0.691 | -1.09 |
| 1437013_x_at | Atp6v0b | ATPase, H+ transporting, lysosomal V0 subunit B | -1.02 | 0.853 | -1.19 | 0.052 | -1.1 | 0.477 | -1.29 | 0.025 | -1.15 |
| 1436153_a_at | Zmynd11 | zinc finger, MYND domain containing 11 | -1.13 | 0.518 | -1.08 | 0.421 | -1.09 | 0.674 | -2.18 | 0.161 | -1.37 |
| 1418255_s_at | Srf | serum response factor | -1.2 | 0.496 | -1.06 | 0.803 | -1.05 | 0.833 | -1.66 | 0.09 | -1.24 |
| 1455312_at | Phc3 | polyhomeotic-like 3 (Drosophila) | -1.01 | 0.793 | -1.01 | 0.957 | -1.33 | 0.119 | -1.28 | 0.257 | -1.16 |
| 1452312_at | 2810002D19Rik | RIKEN cDNA 2810002D19 gene | -1.07 | 0.376 | -1.02 | 0.802 | -1.22 | 0.016 | -1.42 | 0.126 | -1.18 |
| 1455700_at | Mterfd3 | MTERF domain containing 3 | -1.12 | 0.717 | -1.05 | 0.856 | -1.14 | 0.428 | -2.11 | 0.11 | -1.35 |
| 1416095_x_at | 1110005A23Rik | RIKEN cDNA 1110005A23 gene | -1.1 | 0.032 | -1.01 | 0.908 | -1.2 | 0.094 | -1.03 | 0.795 | -1.09 |
| 1419077_at | Mpp3 | membrane protein, palmitoylated 3 (MAGUK p55 subfamily member 3) | -1.16 | 0.316 | -1.07 | 0.891 | -1.07 | 0.728 | -1.23 | 0.611 | -1.13 |
| 1423127_at | Impa1 | inositol (myo)-1(or 4)-monophosphatase 1 | -1.03 | 0.713 | -1.13 | 0.028 | -1.15 | 0.155 | -1.15 | 0.251 | -1.11 |
| 1449014_at | Lactb | lactamase, beta | -1.02 | 0.832 | -1.15 | 0.237 | -1.15 | 0.397 | -1.7 | 0.049 | -1.25 |
| 1433463_at | 0610010K06Rik | RIKEN cDNA 0610010K06 gene | -1.06 | 0.287 | -1.01 | 0.744 | -1.26 | 0.119 | -1.1 | 0.386 | -1.11 |
| 1424458_at | Jmjd2c | jumonji domain containing 2C | -1.16 | 0.48 | -1.01 | 0.91 | -1.15 | 0.291 | -1.27 | 0.192 | -1.15 |
| 1417002_at | 0610012G03Rik | RIKEN cDNA 0610012G03 gene | -1.08 | 0.452 | -1.2 | 0.046 | -1.03 | 0.708 | -1.06 | 0.503 | -1.09 |
| 1449444_a_at | Mfap1 | microfibrillar-associated protein 1 | -1 | 0.978 | -1.2 | 0.181 | -1.11 | 0.113 | -1.13 | 0.45 | -1.11 |
| 1428954_at | Slc9a3r2 | solute carrier family 9 (sodium/hydrogen exchanger), isoform 3 regulator 2 | -1.09 | 0.802 | -1.09 | 0.789 | -1.12 | 0.653 | -1.41 | 0.204 | -1.18 |
| 1424496_at | 5133401N09Rik | RIKEN cDNA 5133401N09 gene | -1.06 | 0.694 | -1.19 | 0.034 | -1.06 | 0.545 | -1.5 | 0.048 | -1.2 |
| 1435717_at | 4833428C12Rik | RIKEN cDNA 4833428C12 gene | -1.06 | 0.83 | -1.03 | 0.779 | -1.23 | 0.36 | -1.44 | 0.303 | -1.19 |
| 1451783_a_at | Kifap3 | kinesin-associated protein 3 | -1.15 | 0.4 | -1.02 | 0.837 | -1.13 | 0.238 | -1.07 | 0.827 | -1.09 |
| 1437306_at | C130092O11Rik | RIKEN cDNA C130092O11 gene | -1.01 | 0.913 | -1.14 | 0.805 | -1.15 | 0.19 | -1.37 | 0.356 | -1.17 |
| 1428708_x_at | Ptms | parathymosin | -1.07 | 0.86 | -1.07 | 0.76 | -1.17 | 0.246 | -1.05 | 0.896 | -1.09 |
| 1427173_a_at | Mrps33 | mitochondrial ribosomal protein S33 | -1.23 | 0.079 | -1.05 | 0.532 | -1.03 | 0.737 | -1.28 | 0.103 | -1.15 |
| 1450900_at | AW011752 | expressed sequence AW011752 | -1.01 | 0.884 | -1.09 | 0.491 | -1.21 | 0.182 | -1.34 | 0.056 | -1.16 |
| 1448762_at | Rad17 | RAD17 homolog (S. pombe) | -1.27 | 0.311 | -1 | 0.96 | -1.06 | 0.695 | -1.25 | 0.104 | -1.15 |
| 1417344_at | 2900064A13Rik | RIKEN cDNA 2900064A13 gene | -1.06 | 0.121 | -1.03 | 0.634 | -1.23 | 0.006 | -1 | 0.976 | -1.08 |
| 1424104_at | Syf2 | SYF2 homolog, RNA splicing factor (S. cerevisiae) | -1.13 | 0.207 | -1.07 | 0.34 | -1.09 | 0.26 | -1.12 | 0.151 | -1.1 |
| 1442446_at | Zfp523 | Zinc finger protein 523 | -1.04 | 0.911 | -1.09 | 0.756 | -1.17 | 0.525 | -1.29 | 0.603 | -1.15 |
| 1451244_a_at | Zfp422 | zinc finger protein 422 | -1.16 | 0.372 | -1.11 | 0.238 | -1.03 | 0.807 | -1.19 | 0.35 | -1.12 |
| 1424821_at | Ndfip1 | Nedd4 family interacting protein 1 | -1.07 | 0.414 | -1.14 | 0.123 | -1.09 | 0.343 | -1.34 | 0.344 | -1.16 |
| 1443203_at | Zfhx1a | Zinc finger homeobox 1a | -1.05 | 0.949 | -1.05 | 0.796 | -1.22 | 0.053 | -1.07 | 0.701 | -1.1 |
| 1435757_a_at | Uqcrc2 | ubiquinol cytochrome c reductase core protein 2 | -1.05 | 0.245 | -1.11 | 0.357 | -1.14 | 0.152 | -1.43 | 0.216 | -1.18 |
| 1418100_at | A030009H04Rik | RIKEN cDNA A030009H04 gene | -1.11 | 0.378 | -1.16 | 0.847 | -1.03 | 0.898 | -1.13 | 0.657 | -1.11 |

| | | | | | | | | | | | |
|---|---|---|---|---|---|---|---|---|---|---|---|
| 1448425_at | Eif3s10 | eukaryotic translation initiation factor 3, subunit 10 (theta) | -1.02 | 0.925 | -1.1 | 0.121 | -1.19 | 0.297 | -1.02 | 0.782 | -1.08 |
| 1416277_a_at | Rplp1 | ribosomal protein, large, P1 | -1.08 | 0.231 | -1.03 | 0.714 | -1.2 | 0.095 | -1.3 | 0.029 | -1.15 |
| 1455727_at | U2af1-rs2 | U2 small nuclear ribonucleoprotein auxiliary factor (U2AF) 1, related sequence 2 | -1.08 | 0.525 | -1.05 | 0.715 | -1.17 | 0.238 | -1 | 0.998 | -1.08 |
| 1416280_at | Uble1b | ubiquitin-like 1 (sentrin) activating enzyme E1B | -1.13 | 0.182 | -1.13 | 0.464 | -1.04 | 0.524 | -1.04 | 0.752 | -1.08 |
| 1423773_at | Gpbp1 | GC-rich promoter binding protein 1 | -1.02 | 0.84 | -1.03 | 0.461 | -1.29 | 0.074 | -1.09 | 0.394 | -1.1 |
| 1419512_at | Prpf40b | PRP40 pre-mRNA processing factor 40 homolog B (yeast) | -1.06 | 0.781 | -1.04 | 0.693 | -1.21 | 0.34 | -1.51 | 0.173 | -1.2 |
| 1448799_s_at | Mrps12 | mitochondrial ribosomal protein S12 | -1.1 | 0.141 | -1.03 | 0.687 | -1.17 | 0.165 | -1.15 | 0.484 | -1.11 |
| 1427941_at | Dicer1 | Dicer1, Dcr-1 homolog (Drosophila) | -1.11 | 0.798 | -1.11 | 0.642 | -1.08 | 0.662 | -1.16 | 0.628 | -1.11 |
| 1424241_at | Slc30a6 | solute carrier family 30 (zinc transporter), member 6 | -1.05 | 0.676 | -1.16 | 0.011 | -1.09 | 0.433 | -1.03 | 0.369 | -1.08 |
| 1420113_s_at | 2410022L05Rik | RIKEN cDNA 2410022L05 gene | -1 | 0.959 | -1 | 0.989 | -1.36 | 0.003 | -1.28 | 0.044 | -1.16 |
| 1435571_at | A530065I17Rik | RIKEN cDNA A530065I17 gene | -1.04 | 0.921 | -1.2 | 0.265 | -1.07 | 0.745 | -1.02 | 0.872 | -1.08 |
| 1455291_s_at | Znrf2 | zinc and ring finger 2 | -1.1 | 0.542 | -1.04 | 0.734 | -1.16 | 0.268 | -1.68 | 0.163 | -1.24 |
| 1434493_at | 1810022K09Rik | RIKEN cDNA 1810022K09 gene | -1.01 | 0.964 | -1.28 | 0.063 | -1.04 | 0.572 | -1.63 | 0.146 | -1.24 |
| 1430992_s_at | 1500009M05Rik | RIKEN cDNA 1500009M05 gene | -1.16 | 0.189 | -1.04 | 0.684 | -1.1 | 0.755 | -1.1 | 0.689 | -1.1 |
| 1435680_a_at | Dpp7 | dipeptidylpeptidase 7 | -1.17 | 0.228 | -1.02 | 0.932 | -1.12 | 0.374 | -1.27 | 0.13 | -1.14 |
| 1423707_at | Tmem50b | transmembrane protein 50B | -1.07 | 0.469 | -1.06 | 0.201 | -1.17 | 0.072 | -1.1 | 0.549 | -1.1 |
| 1455941_s_at | Map2k5 | mitogen activated protein kinase kinase 5 | -1 | 0.995 | -1.3 | 0.198 | -1.03 | 0.871 | -1.39 | 0.257 | -1.18 |
| 1429255_at | 2010007H12Rik | RIKEN cDNA 2010007H12 gene | -1.07 | 0.682 | -1.05 | 0.708 | -1.18 | 0.312 | -1.24 | 0.441 | -1.14 |
| 1452451_at | Bean | brain expressed, associated with Nedd4 | -1.01 | 0.989 | -1.14 | 0.684 | -1.16 | 0.519 | -3.12 | 0.091 | -1.61 |
| 1427108_at | 9530068E07Rik | RIKEN cDNA 9530068E07 gene | -1 | 0.946 | -1.26 | 0.008 | -1.06 | 0.661 | -1.18 | 0.226 | -1.13 |
| 1416858_a_at | Fkbp3 | FK506 binding protein 3 | -1.11 | 0.284 | -1.04 | 0.735 | -1.15 | 0.136 | -1.65 | 0.065 | -1.24 |
| 1428619_at | 2310005N03Rik | RIKEN cDNA 2310005N03 gene | -1.1 | 0.156 | -1 | 0.999 | -1.21 | 0.019 | -1.14 | 0.263 | -1.11 |
| 1423197_a_at | AW011752 | expressed sequence AW011752 | -1 | 0.965 | -1.01 | 0.913 | -1.34 | 0.175 | -1.39 | 0.084 | -1.19 |
| 1452775_at | Csrp2bp | cysteine and glycine-rich protein 2 binding protein | -1.18 | 0.368 | -1.11 | 0.145 | -1.01 | 0.957 | -1.07 | 0.575 | -1.09 |
| 1452186_at | Rbm5 | RNA binding motif protein 5 | -1.23 | 0.154 | -1.05 | 0.628 | -1.03 | 0.852 | -1.41 | 0.171 | -1.18 |
| 1423131_at | LOC433230 | similar to TGF beta-inducible nuclear protein 1 (L-name-related protein 42) (LNR4 | -1.05 | 0.33 | -1.11 | 0.509 | -1.12 | 0.222 | -1.45 | 0.157 | -1.19 |
| 1454863_at | Ankrd11 | ankyrin repeat domain 11 | -1.02 | 0.937 | -1.17 | 0.135 | -1.11 | 0.19 | -1.18 | 0.4 | -1.12 |
| 1455507_s_at | D8Ertd587e | DNA segment, Chr 8, ERATO Doi 587, expressed | -1.01 | 0.929 | -1.03 | 0.635 | -1.28 | 0.152 | -1.34 | 0.329 | -1.17 |
| 1428539_at | 2610207I05Rik | RIKEN cDNA 2610207I05 gene | -1.06 | 0.776 | -1.06 | 0.824 | -1.19 | 0.579 | -1.26 | 0.607 | -1.14 |
| 1425486_s_at | Mtmr6 | myotubularin related protein 6 | -1.11 | 0.522 | -1.01 | 0.834 | -1.18 | 0.243 | -1.29 | 0.193 | -1.15 |
| 1424189_at | Pigc | phosphatidylinositol glycan anchor biosynthesis, class C | -1.07 | 0.416 | -1.1 | 0.384 | -1.12 | 0.202 | -1.28 | 0.037 | -1.14 |
| 1456194_a_at | Park7 | Parkinson disease (autosomal recessive, early onset) 7 | -1.1 | 0.097 | -1.07 | 0.564 | -1.12 | 0.024 | -1.2 | 0.315 | -1.12 |
| 1456332_at | Tmem17 | transmembrane protein 17 | -1.03 | 0.963 | -1.28 | 0.136 | -1.02 | 0.929 | -1.57 | 0.492 | -1.22 |
| 1426087_at | Icosl | icos ligand | -1.05 | 0.661 | -1.15 | 0.662 | -1.09 | 0.459 | -1.15 | 0.59 | -1.11 |
| 1436545_at | Dtx4 | deltex 4 homolog (Drosophila) | -1.08 | 0.844 | -1.07 | 0.696 | -1.14 | 0.432 | -1.05 | 0.706 | -1.09 |
| 1445499_at | Zc3h13 | zinc finger CCCH type containing 13 | -1.1 | 0.75 | -1.13 | 0.656 | -1.06 | 0.776 | -1.23 | 0.409 | -1.13 |
| 1448505_at | C1d | nuclear DNA binding protein | -1.15 | 0.099 | -1.07 | 0.338 | -1.07 | 0.645 | -1.05 | 0.444 | -1.08 |
| 1454772_at | Ascc3l1 | activating signal cointegrator 1 complex subunit 3-like 1 | -1.04 | 0.614 | -1.19 | 0.327 | -1.07 | 0.488 | -1.42 | 0.139 | -1.18 |
| 1428466_at | Chd3 | chromodomain helicase DNA binding protein 3 | -1.13 | 0.441 | -1.07 | 0.688 | -1.09 | 0.669 | -1.41 | 0.162 | -1.17 |
| 1420631_a_at | Blcap | bladder cancer associated protein homolog (human) | -1.04 | 0.846 | -1.1 | 0.177 | -1.15 | 0.261 | -1.36 | 0.406 | -1.16 |
| 1450934_at | Eif4a2 | eukaryotic translation initiation factor 4A2 | -1.04 | 0.415 | -1.14 | 0.017 | -1.11 | 0.307 | -1.45 | 0.22 | -1.19 |
| 1451223_a_at | Btf3l4 | basic transcription factor 3-like 4 | -1.06 | 0.698 | -1.06 | 0.325 | -1.17 | 0.374 | -1.18 | 0.194 | -1.12 |
| 1448251_at | 9030425E11Rik | RIKEN cDNA 9030425E11 gene | -1.05 | 0.772 | -1.07 | 0.923 | -1.17 | 0.264 | -1.23 | 0.219 | -1.13 |
| 1460621_x_at | Ywhaq | tyrosine 3-monooxygenase/tryptophan 5-monooxygenase activation protein, theta | -1.03 | 0.656 | -1.08 | 0.163 | -1.18 | 0.044 | -1.18 | 0.316 | -1.12 |
| 1416911_a_at | 6330407G11Rik | RIKEN cDNA 6330407G11 gene | -1.21 | 0.526 | -1.03 | 0.874 | -1.06 | 0.664 | -1.32 | 0.094 | -1.16 |
| 1427212_at | Mapkap1 | mitogen-activated protein kinase associated protein 1 | -1.02 | 0.917 | -1.2 | 0.137 | -1.09 | 0.379 | -1.06 | 0.307 | -1.09 |
| 1419635_at | 4833420G17Rik | RIKEN cDNA 4833420G17 gene | -1 | 0.974 | -1.25 | 0.331 | -1.06 | 0.539 | -1.57 | 0.003 | -1.22 |
| 1428218_a_at | 1600012H06Rik | RIKEN cDNA 1600012H06 gene | -1.03 | 0.829 | -1.13 | 0.391 | -1.12 | 0.453 | -1.21 | 0.213 | -1.12 |
| 1420530_at | Neud4 | neuronal d4 domain family member | -1.07 | 0.908 | -1.01 | 0.984 | -1.23 | 0.516 | -1.15 | 0.016 | -1.11 |
| 1418223_at | Sec11l1 | Sec11-like 1 (S. cerevisiae) | -1.13 | 0.501 | -1.05 | 0.468 | -1.1 | 0.329 | -1.14 | 0.098 | -1.11 |
| 1456873_at | Clic5 | chloride intracellular channel 5 | -1.04 | 0.728 | -1.12 | 0.858 | -1.13 | 0.531 | -1.63 | 0.13 | -1.23 |
| 1427132_at | Sbf2 | SET binding factor 2 | -1.09 | 0.477 | -1.05 | 0.487 | -1.15 | 0.639 | -1.01 | 0.947 | -1.08 |
| 1448697_s_at | Rpl36al | ribosomal protein L36a-like | -1.03 | 0.606 | -1.04 | 0.611 | -1.24 | 0.02 | -1.44 | 0.028 | -1.19 |

| | | | | | | | | | | | |
|---|---|---|---|---|---|---|---|---|---|---|---|
| 1423043_s_at | Ddx3x | DEAD/H (Asp-Glu-Ala-Asp/His) box polypeptide 3, X-linked | -1.04 | 0.395 | -1.04 | 0.642 | -1.22 | 0.008 | -1.07 | 0.314 | -1.09 |
| 1433039_at | 4930555B11Rik | RIKEN cDNA 4930555B11 gene | -1.11 | 0.543 | -1.03 | 0.924 | -1.15 | 0.815 | -1.05 | 0.942 | -1.08 |
| 1450778_a_at | Rnuxa | RNA U, small nuclear RNA export adaptor | -1.1 | 0.711 | -1.07 | 0.435 | -1.11 | 0.551 | -1.41 | 0.032 | -1.17 |
| 1428441_at | 1500009M05Rik | RIKEN cDNA 1500009M05 gene | -1.03 | 0.761 | -1.05 | 0.496 | -1.22 | 0 | -1.21 | 0.288 | -1.13 |
| 1417098_s_at | Mecr | mitochondrial trans-2-enoyl-CoA reductase | -1.15 | 0.597 | -1.1 | 0.083 | -1.03 | 0.845 | -1.03 | 0.863 | -1.08 |
| 1435900_at | Zfp297b | zinc finger protein 297B | -1.11 | 0.338 | -1.03 | 0.82 | -1.15 | 0.134 | -1.2 | 0.021 | -1.12 |
| 1415701_x_at | Rpl23 | ribosomal protein L23 | -1.08 | 0.444 | -1.02 | 0.591 | -1.2 | 0.019 | -1.25 | 0.009 | -1.14 |
| 1425462_at | Fbxw11 | F-box and WD-40 domain protein 11 | -1.14 | 0.156 | -1.03 | 0.78 | -1.12 | 0.371 | -1.13 | 0.188 | -1.11 |
| 1438491_x_at | A530054K11Rik | RIKEN cDNA A530054K11 gene | -1.11 | 0.326 | -1.03 | 0.93 | -1.15 | 0.688 | -1.73 | 0.267 | -1.25 |
| 1439452_x_at | Dnpep | aspartyl aminopeptidase | -1.21 | 0.19 | -1.08 | 0.19 | -1.01 | 0.962 | -1.09 | 0.262 | -1.1 |
| 1438983_x_at | AI413782 | expressed sequence AI413782 | -1.11 | 0.481 | -1.08 | 0.076 | -1.08 | 0.15 | -1.46 | 0.133 | -1.19 |
| 1450818_a_at | Ndufa7 | NADH dehydrogenase (ubiquinone) 1 alpha subcomplex, 7 (B14.5a) | -1.07 | 0.474 | -1.09 | 0.287 | -1.12 | 0.46 | -1.16 | 0.34 | -1.11 |
| 1426219_at | Scp2 | sterol carrier protein 2, liver | -1.03 | 0.64 | -1.27 | 0.221 | -1.02 | 0.9 | -1.33 | 0.076 | -1.16 |
| 1415736_at | Pfdn5 | prefoldin 5 | -1.06 | 0.691 | -1.01 | 0.827 | -1.23 | 0.058 | -2.28 | 0.205 | -1.4 |
| 1448712_at | Chm | choroidermia | -1.11 | 0.241 | -1.08 | 0.033 | -1.09 | 0.033 | -1.19 | 0.604 | -1.12 |
| 1436949_a_at | Tceb2 /// LOC6685 | transcription elongation factor B (SIII), polypeptide 2 /// similar to transcription elo | -1.01 | 0.877 | -1.05 | 0.13 | -1.25 | 0.056 | -1.09 | 0.638 | -1.1 |
| 1437005_a_at | Rpl18 | ribosomal protein L18 | -1 | 0.977 | -1.06 | 0.446 | -1.25 | 0.012 | -1.26 | 0.008 | -1.14 |
| 1418077_at | Trim21 | tripartite motif protein 21 | -1.14 | 0.528 | -1.09 | 0.552 | -1.05 | 0.628 | -1.43 | 0.209 | -1.18 |
| 1446371_at | --- | --- | -1.02 | 0.957 | -1.04 | 0.868 | -1.25 | 0.146 | -1.23 | 0.309 | -1.14 |
| 1448540_a_at | 0610012G03Rik /// | RIKEN cDNA 0610012G03 gene /// hypothetical protein LOC638521 | -1.06 | 0.503 | -1.14 | 0.056 | -1.08 | 0.542 | -1.38 | 0.331 | -1.17 |
| 1436034_at | Cep68 | centrosomal protein 68 | -1.09 | 0.428 | -1.09 | 0.571 | -1.1 | 0.087 | -1.43 | 0.097 | -1.18 |
| 1440500_at | Map3k10 | Mitogen activated protein kinase kinase kinase 10 | -1.01 | 0.923 | -1.2 | 0.542 | -1.09 | 0.585 | -1.07 | 0.653 | -1.09 |
| 1445693_at | Araf | v-raf murine sarcoma 3611 viral oncogene homolog | -1.09 | 0.713 | -1.02 | 0.917 | -1.19 | 0.397 | -1.13 | 0.632 | -1.11 |
| 1449956_at | Prkce | protein kinase C, epsilon | -1.17 | 0.37 | -1.04 | 0.783 | -1.07 | 0.479 | -1.3 | 0.119 | -1.15 |
| 1448763_at | Atad1 | ATPase family, AAA domain containing 1 | -1.11 | 0.038 | -1.08 | 0.23 | -1.09 | 0.242 | -1.22 | 0.147 | -1.13 |
| 1455976_x_at | Dbi | diazepam binding inhibitor | -1.16 | 0.243 | -1.03 | 0.798 | -1.1 | 0.405 | -1.06 | 0.621 | -1.09 |
| 1434703_at | Extl3 | exostoses (multiple)-like 3 | -1.02 | 0.887 | -1.14 | 0.177 | -1.13 | 0.392 | -1.02 | 0.847 | -1.08 |
| 1459749_s_at | Fat4 | FAT tumor suppressor homolog 4 (Drosophila) | -1.05 | 0.926 | -1.06 | 0.672 | -1.18 | 0.396 | -1.26 | 0.463 | -1.14 |
| 1434219_at | Stim2 | stromal interaction molecule 2 | -1.06 | 0.77 | -1.12 | 0.12 | -1.1 | 0.258 | -1.4 | 0.108 | -1.17 |
| 1425321_a_at | Clmn | calmin | -1.08 | 0.603 | -1.16 | 0.411 | -1.05 | 0.772 | -1.57 | 0.164 | -1.21 |
| 1424355_a_at | Sin3b | transcriptional regulator, SIN3B (yeast) | -1.11 | 0.341 | -1.17 | 0.192 | -1.01 | 0.926 | -1.22 | 0.124 | -1.13 |
| 1435862_at | Son | Son cell proliferation protein | -1.02 | 0.869 | -1.07 | 0.509 | -1.21 | 0.387 | -1.43 | 0.107 | -1.18 |
| 1417684_at | Thumpd3 | THUMP domain containing 3 | -1.1 | 0.17 | -1.09 | 0.354 | -1.09 | 0.53 | -1.21 | 0.141 | -1.12 |
| 1424277_at | 1110020L19Rik | RIKEN cDNA 1110020L19 gene | -1.14 | 0.66 | -1.13 | 0.661 | -1.02 | 0.749 | -1.08 | 0.673 | -1.09 |
| 1428303_at | 1500005I02Rik | RIKEN cDNA 1500005I02 gene | -1 | 0.997 | -1.14 | 0.865 | -1.15 | 0.73 | -1.32 | 0.728 | -1.15 |
| 1426978_at | Klhl2 | kelch-like 2, Mayven (Drosophila) | -1.07 | 0.643 | -1.08 | 0.402 | -1.13 | 0.586 | -1.09 | 0.818 | -1.09 |
| 1449272_at | Igsf4b | immunoglobulin superfamily, member 4B | -1.03 | 0.969 | -1.06 | 0.913 | -1.2 | 0.609 | -1.18 | 0.686 | -1.12 |
| 1459227_at | --- | Transcribed locus | -1.02 | 0.895 | -1.07 | 0.734 | -1.19 | 0.558 | -1.23 | 0.293 | -1.13 |
| 1417317_s_at | Rpl35a | ribosomal protein L35a | -1.03 | 0.304 | -1.01 | 0.83 | -1.27 | 0.001 | -1.42 | 0.016 | -1.18 |
| 1428465_at | Tmem147 | transmembrane protein 147 | -1.06 | 0.668 | -1.13 | 0.122 | -1.1 | 0.452 | -1.01 | 0.928 | -1.07 |
| 1439455_x_at | Capza1 | capping protein (actin filament) muscle Z-line, alpha 1 | -1.06 | 0.588 | -1.16 | 0.16 | -1.07 | 0.621 | -1.34 | 0.091 | -1.16 |
| 1415761_at | Mrpl52 | mitochondrial ribosomal protein L52 | -1.03 | 0.652 | -1.01 | 0.865 | -1.26 | 0.162 | -1.22 | 0.445 | -1.13 |
| 1426660_x_at | Rpl23a | ribosomal protein L23a | -1.02 | 0.679 | -1 | 0.914 | -1.3 | 0.006 | -1.28 | 0.005 | -1.15 |
| 1450889_at | Smarca3 | SWI/SNF related, matrix associated, actin dependent regulator of chromatin, subf | -1.1 | 0.48 | -1.13 | 0.292 | -1.05 | 0.671 | -1.75 | 0.064 | -1.26 |
| 1437773_x_at | Ddx17 | DEAD (Asp-Glu-Ala-Asp) box polypeptide 17 | -1.14 | 0.348 | -1.02 | 0.852 | -1.12 | 0.273 | -1.28 | 0.117 | -1.14 |
| 1437016_x_at | Rap2c | RAP2C, member of RAS oncogene family | -1.12 | 0.703 | -1.15 | 0.344 | -1.02 | 0.78 | -1.09 | 0.711 | -1.09 |
| 1436272_at | Rab3gap2 | RAB3 GTPase activating protein subunit 2 | -1.15 | 0.062 | -1.01 | 0.979 | -1.13 | 0.384 | -1.02 | 0.769 | -1.08 |
| 1431614_at | 2810049E08Rik | RIKEN cDNA 2810049E08 gene | -1.06 | 0.835 | -1.03 | 0.87 | -1.2 | 0.32 | -1.2 | 0.36 | -1.12 |
| 1450031_at | Aff4 | AF4/FMR2 family, member 4 | -1.12 | 0.176 | -1.04 | 0.733 | -1.12 | 0.066 | -1.35 | 0.096 | -1.16 |
| 1426484_at | Ubxd2 | UBX domain containing 2 | -1.1 | 0.315 | -1.07 | 0.426 | -1.11 | 0.098 | -1.14 | 0.342 | -1.1 |
| 1426487_a_at | Rbbp6 | retinoblastoma binding protein 6 | -1.08 | 0.689 | -1 | 0.985 | -1.21 | 0.144 | -1.33 | 0.041 | -1.16 |
| 1418134_at | 1200007B05Rik | RIKEN cDNA 1200007B05 gene | -1.08 | 0.648 | -1.16 | 0.047 | -1.04 | 0.736 | -1.02 | 0.889 | -1.08 |
| 1444433_at | Auh | AU RNA binding protein/enoyl-coenzyme A hydratase | -1.07 | 0.742 | -1.12 | 0.564 | -1.09 | 0.529 | -1.02 | 0.913 | -1.07 |

| | | | | | | | | | | | |
|---|---|---|---|---|---|---|---|---|---|---|---|
| 1426968_a_at | Rdh10 | retinol dehydrogenase 10 (all-trans) | -1.13 | 0.513 | -1.11 | 0.078 | -1.04 | 0.808 | -1.03 | 0.898 | -1.08 |
| 1422340_a_at | Actg2 | actin, gamma 2, smooth muscle, enteric | -1.24 | 0.606 | -1.01 | 0.958 | -1.04 | 0.87 | -1.46 | 0.057 | -1.19 |
| 1418419_at | Fbxl20 | F-box and leucine-rich repeat protein 20 | -1.15 | 0.189 | -1.1 | 0.012 | -1.03 | 0.58 | -1.16 | 0.393 | -1.11 |
| 1425262_at | Cebpg | CCAAT/enhancer binding protein (C/EBP), gamma | -1.05 | 0.753 | -1.01 | 0.846 | -1.23 | 0.228 | -1.06 | 0.389 | -1.09 |
| 1451005_at | Sumo1 | SMT3 suppressor of mif two 3 homolog 1 (yeast) | -1.06 | 0.554 | -1.01 | 0.905 | -1.23 | 0.105 | -1.21 | 0.15 | -1.13 |
| 1452985_at | Uaca | uveal autoantigen with coiled-coil domains and ankyrin repeats | -1.05 | 0.75 | -1.11 | 0.572 | -1.11 | 0.722 | -1.05 | 0.912 | -1.08 |
| 1417526_at | Pcbp3 | poly(rC) binding protein 3 | -1.08 | 0.635 | -1.03 | 0.933 | -1.17 | 0.536 | -1.19 | 0.561 | -1.12 |
| 1434516_at | Pstk | phosphoseryl-tRNA kinase | -1.17 | 0.018 | -1.1 | 0.467 | -1.02 | 0.909 | -1.07 | 0.337 | -1.09 |
| 1438684_at | Nuak1 | NUAK family, SNF1-like kinase, 1 | -1.18 | 0.041 | -1.02 | 0.936 | -1.08 | 0.774 | -1.25 | 0.334 | -1.13 |
| 1433879_a_at | C130032J12Rik | RIKEN cDNA C130032J12 gene | -1.1 | 0.512 | -1.15 | 0.124 | -1.03 | 0.895 | -1.03 | 0.576 | -1.08 |
| 1441878_s_at | 1810049H13Rik | RIKEN cDNA 1810049H13 gene | -1.02 | 0.887 | -1.14 | 0.441 | -1.12 | 0.316 | -1.41 | 0.035 | -1.17 |
| 1437253_at | A630054L15Rik | RIKEN cDNA A630054L15 gene | -1.14 | 0.353 | -1.02 | 0.793 | -1.11 | 0.4 | -1.08 | 0.499 | -1.09 |
| 1457594_at | --- | Transcribed locus | -1.07 | 0.843 | -1.07 | 0.791 | -1.13 | 0.191 | -1.27 | 0.478 | -1.14 |
| 1428985_at | Ints12 | integrator complex subunit 12 | -1.06 | 0.567 | -1.08 | 0.516 | -1.13 | 0.127 | -1.31 | 0.045 | -1.15 |
| 1418040_at | 4432406C05Rik | RIKEN cDNA 4432406C05 gene | -1.04 | 0.885 | -1.18 | 0.47 | -1.06 | 0.506 | -1.13 | 0.622 | -1.1 |
| 1455323_at | Rbak | RB-associated KRAB repressor | -1.07 | 0.814 | -1.02 | 0.848 | -1.2 | 0.389 | -1.08 | 0.768 | -1.09 |
| 1418401_a_at | Dusp16 | dual specificity phosphatase 16 | -1.06 | 0.87 | -1 | 0.976 | -1.23 | 0.166 | -1.72 | 0.021 | -1.26 |
| 1448430_a_at | Naca /// LOC62827 | nascent polypeptide-associated complex alpha polypeptide /// similar to nascent p | -1.09 | 0.476 | -1.11 | 0.068 | -1.07 | 0.091 | -1.41 | 0.027 | -1.17 |
| 1416103_at | Ywhaz | tyrosine 3-monooxygenase/tryptophan 5-monooxygenase activation protein, zeta | -1.01 | 0.816 | -1.29 | 0.247 | -1.02 | 0.911 | -1.05 | 0.644 | -1.09 |
| 1434521_at | Rfxdc2 | regulatory factor X domain containing 2 homolog (human) | -1.15 | 0.296 | -1.11 | 0.201 | -1.02 | 0.927 | -1.53 | 0.163 | -1.2 |
| 1434301_at | D330050I23Rik | RIKEN cDNA D330050I23 gene | -1.09 | 0.513 | -1.19 | 0.321 | -1 | 0.965 | -2.04 | 0.056 | -1.33 |
| 1420129_s_at | D10Wsu52e | DNA segment, Chr 10, Wayne State University 52, expressed | -1.1 | 0.475 | -1.15 | 0.16 | -1.03 | 0.791 | -1.53 | 0.041 | -1.2 |
| 1450668_s_at | Hspe1 | heat shock protein 1 (chaperonin 10) | -1.11 | 0.065 | -1.13 | 0.186 | -1.03 | 0.816 | -1.47 | 0.005 | -1.19 |
| 1446865_at | Sema5a | Sema domain, seven thrombospondin repeats (type 1 and type 1-like), transmem | -1.1 | 0.659 | -1 | 0.997 | -1.18 | 0.753 | -2.66 | 0.043 | -1.49 |
| 1449951_at | Nfkbil1 | nuclear factor of kappa light polypeptide gene enhancer in B-cells inhibitor-like 1 | -1.14 | 0.268 | -1.09 | 0.713 | -1.05 | 0.738 | -1.68 | 0.127 | -1.24 |
| 1415867_at | Cct4 | chaperonin subunit 4 (delta) | -1.12 | 0.097 | -1.09 | 0.275 | -1.06 | 0.736 | -1.12 | 0.352 | -1.1 |
| 1427236_a_at | Mll5 | myeloid/lymphoid or mixed-lineage leukemia 5 | -1.06 | 0.51 | -1.18 | 0.087 | -1.04 | 0.773 | -1.43 | 0.206 | -1.18 |
| 1422059_at | Cbx2 | chromobox homolog 2 (Drosophila Pc class) | -1.01 | 0.99 | -1.16 | 0.799 | -1.11 | 0.65 | -1.52 | 0.736 | -1.2 |
| 1427321_s_at | Cxadr | coxsackievirus and adenovirus receptor | -1.06 | 0.718 | -1.1 | 0.794 | -1.11 | 0.6 | -1.14 | 0.663 | -1.1 |
| 1437521_s_at | E230022H04Rik | RIKEN cDNA E230022H04 gene | -1.02 | 0.905 | -1.01 | 0.777 | -1.28 | 0.132 | -1.34 | 0.044 | -1.16 |
| 1428293_at | 2310022M17Rik | RIKEN cDNA 2310022M17 gene | -1.05 | 0.295 | -1.03 | 0.664 | -1.2 | 0.056 | -1.6 | 0.113 | -1.22 |
| 1433690_at | 2210016L21Rik | RIKEN cDNA 2210016L21 gene | -1.17 | 0.292 | -1.08 | 0.565 | -1.03 | 0.754 | -1.48 | 0.112 | -1.19 |
| 1448645_at | Msl31 | male-specific lethal-3 homolog 1 (Drosophila) | -1.01 | 0.943 | -1.06 | 0.585 | -1.22 | 0.063 | -1.12 | 0.452 | -1.1 |
| 1429141_at | Neurl2 | neuralized-like 2 (Drosophila) | -1.27 | 0.068 | -1.01 | 0.953 | -1.02 | 0.882 | -1.66 | 0.028 | -1.24 |
| 1428944_at | Ube1l2 | ubiquitin-activating enzyme E1-like 2 | -1.02 | 0.843 | -1.05 | 0.715 | -1.22 | 0.247 | -1.17 | 0.344 | -1.11 |
| 1455320_at | --- | --- | -1.04 | 0.871 | -1.04 | 0.817 | -1.21 | 0.341 | -1.02 | 0.88 | -1.08 |
| 1451200_at | Kif1b | kinesin family member 1B | -1.05 | 0.292 | -1.19 | 0.109 | -1.03 | 0.819 | -1.32 | 0.162 | -1.15 |
| 1450892_a_at | Usp4 | ubiquitin specific peptidase 4 (proto-oncogene) | -1.05 | 0.586 | -1.09 | 0.079 | -1.13 | 0.293 | -1.07 | 0.647 | -1.09 |
| 1423568_at | Psma7 | proteasome (prosome, macropain) subunit, alpha type 7 | -1.07 | 0.358 | -1.15 | 0.014 | -1.05 | 0.701 | -1.34 | 0.196 | -1.15 |
| 1415818_at | Anxa6 | annexin A6 | -1.08 | 0.753 | -1.09 | 0.375 | -1.1 | 0.457 | -1.59 | 0.255 | -1.21 |
| 1416207_at | Taz | tafazzin | -1.14 | 0.085 | -1.09 | 0.411 | -1.04 | 0.328 | -1.41 | 0.22 | -1.17 |
| 1427162_a_at | Elk4 | ELK4, member of ETS oncogene family | -1.07 | 0.64 | -1.01 | 0.917 | -1.2 | 0.256 | -1.37 | 0.144 | -1.16 |
| 1425058_at | Zfp472 | zinc finger protein 472 | -1.17 | 0.305 | -1.02 | 0.932 | -1.09 | 0.592 | -1.9 | 0.088 | -1.29 |
| 1416393_at | Emg1 | EMG1 nucleolar protein homolog (S. cerevisiae) | -1.04 | 0.51 | -1.04 | 0.387 | -1.2 | 0.308 | -1.05 | 0.604 | -1.08 |
| 1446661_at | --- | --- | -1.06 | 0.923 | -1.08 | 0.834 | -1.13 | 0.624 | -1.61 | 0.32 | -1.22 |
| 1453833_a_at | Rnaseh1 | ribonuclease H1 | -1.2 | 0.625 | -1.06 | 0.706 | -1.02 | 0.917 | -1.28 | 0.114 | -1.14 |
| 1423202_a_at | Ncor1 | nuclear receptor co-repressor 1 | -1.05 | 0.482 | -1.12 | 0.054 | -1.09 | 0.501 | -1.14 | 0.231 | -1.1 |
| 1419760_a_at | ORF5 | open reading frame 5 | -1.03 | 0.811 | -1.11 | 0.193 | -1.14 | 0.503 | -1.04 | 0.643 | -1.08 |
| 1419041_at | Itfg1 | integrin alpha FG-GAP repeat containing 1 | -1.02 | 0.766 | -1.08 | 0.124 | -1.17 | 0.134 | -1.09 | 0.606 | -1.09 |
| 1422569_at | Yy1 | YY1 transcription factor | -1.15 | 0.186 | -1.02 | 0.514 | -1.1 | 0.169 | -1.09 | 0.277 | -1.09 |
| 1434018_at | BC043098 | cDNA sequence BC043098 | -1.02 | 0.951 | -1.12 | 0.463 | -1.14 | 0.667 | -1.72 | 0.058 | -1.25 |
| 1416245_at | Aurkaip1 | aurora kinase A interacting protein 1 | -1.07 | 0.451 | -1.11 | 0.194 | -1.09 | 0.454 | -1.04 | 0.77 | -1.08 |
| 1451089_a_at | Arcn1 | archain 1 | -1.1 | 0.107 | -1 | 0.946 | -1.18 | 0.009 | -1.21 | 0.048 | -1.12 |

| | | | | | | | | | | | |
|---|---|---|---|---|---|---|---|---|---|---|---|
| 1428428_at | Abhd11 | abhydrolase domain containing 11 | -1.1 | 0.123 | -1.09 | 0.209 | -1.07 | 0.672 | -1.31 | 0.043 | -1.14 |
| 1427877_at | 2610312B22Rik | RIKEN cDNA 2610312B22 gene | -1.05 | 0.557 | -1.07 | 0.667 | -1.14 | 0.432 | -1.23 | 0.417 | -1.12 |
| 1440281_at | 0610042C05Rik | RIKEN cDNA 0610042C05 gene | -1.03 | 0.853 | -1.2 | 0.236 | -1.04 | 0.855 | -1.68 | 0.081 | -1.24 |
| 1454853_s_at | AW146154 /// AI98 | expressed sequence AW146154 /// expressed sequence AI987944 | -1.13 | 0.432 | -1.08 | 0.491 | -1.06 | 0.82 | -1.51 | 0.152 | -1.19 |
| 1452621_at | Pcbd2 | pterin 4 alpha carbinolamine dehydratase/dimerization cofactor of hepatocyte nuc | -1.04 | 0.72 | -1.18 | 0.252 | -1.06 | 0.472 | -1.06 | 0.054 | -1.08 |
| 1418125_at | Inoc1 | INO80 complex homolog 1 (S. cerevisiae) | -1.15 | 0.514 | -1.08 | 0.613 | -1.03 | 0.77 | -1.15 | 0.259 | -1.1 |
| 1428470_at | Exoc2 | exocyst complex component 2 | -1.05 | 0.516 | -1.16 | 0.264 | -1.05 | 0.651 | -1.08 | 0.409 | -1.09 |
| 1438208_at | Taok2 | TAO kinase 2 | -1.04 | 0.829 | -1.23 | 0.239 | -1.01 | 0.941 | -1.07 | 0.852 | -1.09 |
| 1439235_x_at | Tm2d2 | TM2 domain containing 2 | -1.07 | 0.676 | -1.06 | 0.607 | -1.13 | 0.037 | -1.06 | 0.316 | -1.08 |
| 1416821_at | Es2el | expressed sequence 2 embryonic lethal | -1.08 | 0.666 | -1.19 | 0.69 | -1 | 0.989 | -1.21 | 0.198 | -1.12 |
| 1424095_at | Rtcd1 | RNA terminal phosphate cyclase domain 1 | -1.04 | 0.742 | -1.18 | 0.001 | -1.06 | 0.564 | -1.05 | 0.67 | -1.08 |
| 1422498_at | Mageh1 | melanoma antigen, family H, 1 | -1.12 | 0.518 | -1.05 | 0.725 | -1.09 | 0.761 | -1.12 | 0.748 | -1.1 |
| 1448808_a_at | Nme2 | expressed in non-metastatic cells 2, protein | -1.17 | 0.035 | -1.04 | 0.687 | -1.06 | 0.427 | -1.54 | 0.139 | -1.2 |
| 1421914_s_at | Mrpl19 | mitochondrial ribosomal protein L19 | -1.06 | 0.506 | -1.13 | 0.15 | -1.07 | 0.542 | -1.06 | 0.67 | -1.08 |
| 1457002_at | Zfp408 | zinc finger protein 408 | -1.18 | 0.24 | -1.06 | 0.716 | -1.03 | 0.818 | -1.25 | 0.288 | -1.13 |
| 1426748_s_at | Abcf3 | ATP-binding cassette, sub-family F (GCN20), member 3 | -1.14 | 0.484 | -1.02 | 0.929 | -1.11 | 0.062 | -1.07 | 0.47 | -1.09 |
| 1424319_at | Oraov1 | oral cancer overexpressed 1 | -1.05 | 0.627 | -1.17 | 0.242 | -1.06 | 0.786 | -1.35 | 0.017 | -1.15 |
| 1452798_s_at | Mapk1ip1 | mitogen activated protein kinase 1 interacting protein 1 | -1.1 | 0.454 | -1.11 | 0.18 | -1.05 | 0.732 | -1.6 | 0.071 | -1.22 |
| 1416775_at | 2310004L02Rik | RIKEN cDNA 2310004L02 gene | -1.16 | 0.622 | -1.09 | 0.669 | -1.02 | 0.898 | -2.04 | 0.078 | -1.33 |
| 1435232_x_at | --- | --- | -1.11 | 0.069 | -1.05 | 0.41 | -1.09 | 0.543 | -1.07 | 0.615 | -1.08 |
| 1437976_x_at | Rpl23a | ribosomal protein L23a | -1.03 | 0.306 | -1 | 0.957 | -1.26 | 0.017 | -1.23 | 0 | -1.13 |
| 1418737_at | Nudt2 | nudix (nucleoside diphosphate linked moiety X)-type motif 2 | -1.23 | 0.385 | -1.03 | 0.842 | -1.02 | 0.781 | -1.26 | 0.143 | -1.14 |
| 1454690_at | Ikbkg | inhibitor of kappaB kinase gamma | -1.01 | 0.923 | -1.2 | 0.072 | -1.07 | 0.401 | -1.08 | 0.755 | -1.09 |
| 1435437_at | Setd7 | SET domain containing (lysine methyltransferase) 7 | -1.18 | 0.249 | -1.05 | 0.81 | -1.04 | 0.855 | -1.4 | 0.195 | -1.17 |
| 1419002_s_at | Baat | bile acid-Coenzyme A: amino acid N-acyltransferase | -1.03 | 0.108 | -1.22 | 0.098 | -1.03 | 0.893 | -1.77 | 0.321 | -1.26 |
| 1418462_at | Exosc9 | exosome component 9 | -1.18 | 0.338 | -1.03 | 0.785 | -1.06 | 0.45 | -1.11 | 0.467 | -1.1 |
| 1422356_at | V1rb10 | vomeronasal 1 receptor, B10 | -1.18 | 0.462 | -1.02 | 0.863 | -1.07 | 0.338 | -1.18 | 0.507 | -1.11 |
| 1426515_a_at | Tor1a | torsin family 1, member A (torsin A) | -1.09 | 0.524 | -1.14 | 0.336 | -1.03 | 0.745 | -1.46 | 0.002 | -1.18 |
| 1455110_at | Gabpb2 | GA repeat binding protein, beta 2 | -1.01 | 0.926 | -1.09 | 0.152 | -1.17 | 0.254 | -1.06 | 0.724 | -1.08 |
| 1455169_at | Rab11fip2 | RAB11 family interacting protein 2 (class I) | -1.05 | 0.739 | -1.13 | 0.034 | -1.08 | 0.309 | -1.2 | 0.34 | -1.11 |
| 1452462_a_at | Banp | Btg3 associated nuclear protein | -1.12 | 0.244 | -1.06 | 0.697 | -1.08 | 0.655 | -1.22 | 0.46 | -1.12 |
| 1441239_at | Kctd18 | potassium channel tetramerisation domain containing 18 | -1 | 0.999 | -1.14 | 0.417 | -1.13 | 0.581 | -1.44 | 0.22 | -1.18 |
| 1455284_x_at | Pigx | phosphatidylinositol glycan anchor biosynthesis, class X | -1.02 | 0.958 | -1.03 | 0.938 | -1.23 | 0.368 | -1.09 | 0.781 | -1.09 |
| 1437181_at | Peli2 | pellino 2 | -1.17 | 0.578 | -1.05 | 0.454 | -1.05 | 0.825 | -1.57 | 0.288 | -1.21 |
| 1435002_at | zfp507 | zinc finger protein 507 | -1.09 | 0.641 | -1.06 | 0.531 | -1.12 | 0.384 | -1.26 | 0.092 | -1.13 |
| 1426853_at | Set | SET translocation | -1.05 | 0.775 | -1.01 | 0.881 | -1.22 | 0.106 | -1.19 | 0.313 | -1.12 |
| 1420497_a_at | Cebpz | CCAAT/enhancer binding protein zeta | -1.03 | 0.885 | -1.07 | 0.24 | -1.17 | 0.334 | -1.37 | 0.191 | -1.16 |
| 1415759_a_at | Hbxip | hepatitis B virus x interacting protein | -1.12 | 0.359 | -1.08 | 0.383 | -1.06 | 0.631 | -1.38 | 0.081 | -1.16 |
| 1419976_s_at | Nfatc3 | nuclear factor of activated T-cells, cytoplasmic, calcineurin-dependent 3 | -1.1 | 0.246 | -1.01 | 0.901 | -1.16 | 0.113 | -1.62 | 0.06 | -1.22 |
| 1451448_a_at | 1110005A03Rik | RIKEN cDNA 1110005A03 gene | -1.08 | 0.323 | -1.03 | 0.657 | -1.16 | 0.397 | -1.96 | 0.101 | -1.31 |
| 1433666_s_at | Vps41 | vacuolar protein sorting 41 (yeast) | -1.08 | 0.497 | -1.08 | 0.039 | -1.09 | 0.153 | -1.19 | 0.43 | -1.11 |
| 1438568_at | Mrgpre | MAS-related GPR, member E | -1.02 | 0.985 | -1.06 | 0.818 | -1.2 | 0.368 | -1.21 | 0.037 | -1.12 |
| 1448480_at | Nip7 | nuclear import 7 homolog (S. cerevisiae) | -1.13 | 0.241 | -1.04 | 0.759 | -1.09 | 0.497 | -1.21 | 0.441 | -1.12 |
| 1448387_at | Rbx1 | ring-box 1 | -1.1 | 0.149 | -1.1 | 0.043 | -1.05 | 0.553 | -1.1 | 0.331 | -1.09 |
| 1436804_s_at | Scyl1 | SCY1-like 1 (S. cerevisiae) | -1.04 | 0.827 | -1.14 | 0.273 | -1.08 | 0.701 | -1.28 | 0.045 | -1.14 |
| 1428746_a_at | Trspap1 | tRNA selenocysteine associated protein 1 | -1.04 | 0.758 | -1.01 | 0.813 | -1.23 | 0.24 | -1.32 | 0.248 | -1.15 |
| 1434254_at | Gna11 | guanine nucleotide binding protein, alpha 11 | -1 | 0.978 | -1.02 | 0.77 | -1.26 | 0.028 | -1.38 | 0.139 | -1.17 |
| 1438610_a_at | Cryz | Crystallin, zeta | -1.04 | 0.491 | -1.08 | 0.631 | -1.14 | 0.386 | -1.02 | 0.829 | -1.07 |
| 1424106_at | 1200003C05Rik | RIKEN cDNA 1200003C05 gene | -1.03 | 0.634 | -1.03 | 0.55 | -1.22 | 0.071 | -1.14 | 0.359 | -1.1 |
| 1444998_at | --- | Transcribed locus | -1.04 | 0.234 | -1.22 | 0.146 | -1.02 | 0.893 | -1.29 | 0.494 | -1.14 |
| 1433794_at | Setx | senataxin | -1.09 | 0.585 | -1.01 | 0.811 | -1.17 | 0.173 | -1.51 | 0.082 | -1.19 |
| 1455474_at | D6Wsu116e | DNA segment, Chr 6, Wayne State University 116, expressed | -1.19 | 0.22 | -1.02 | 0.925 | -1.05 | 0.789 | -1.46 | 0.455 | -1.18 |
| 1417823_at | Gcat | glycine C-acetyltransferase (2-amino-3-ketobutyrate-coenzyme A ligase) | -1.23 | 0.53 | -1.05 | 0.875 | -1 | 0.985 | -1.59 | 0.415 | -1.22 |

| | | | | | | | | | | | |
|---|---|---|---|---|---|---|---|---|---|---|---|
| 1452756_at | 0610009J22Rik | RIKEN cDNA 0610009J22 gene | -1.07 | 0.71 | -1.14 | 0.396 | -1.04 | 0.758 | -1.16 | 0.569 | -1.1 |
| 1457285_at | Zfp187 | zinc finger protein 187 | -1.02 | 0.613 | -1.09 | 0.337 | -1.15 | 0.23 | -1.28 | 0.474 | -1.13 |
| 1433976_at | D10Ucla1 | DNA segment, Chr 10, University of California at Los Angeles 1 | -1.04 | 0.241 | -1.13 | 0.208 | -1.09 | 0.35 | -1.19 | 0.487 | -1.11 |
| 1436899_at | 2700019D07Rik | RIKEN cDNA 2700019D07 gene | -1.1 | 0.747 | -1.01 | 0.881 | -1.15 | 0.115 | -1.81 | 0.027 | -1.27 |
| 1435734_x_at | Dus1l | dihydrouridine synthase 1-like (S. cerevisiae) | -1.02 | 0.467 | -1.17 | 0.086 | -1.07 | 0.667 | -1.07 | 0.628 | -1.08 |
| 1428667_at | Maoa | monoamine oxidase A | -1.03 | 0.846 | -1.07 | 0.004 | -1.16 | 0.341 | -1.01 | 0.953 | -1.07 |
| 1434213_x_at | Ndufs8 | NADH dehydrogenase (ubiquinone) Fe-S protein 8 | -1.11 | 0.501 | -1.03 | 0.721 | -1.11 | 0.267 | -1.35 | 0.175 | -1.15 |
| 1416486_at | Scye1 | small inducible cytokine subfamily E, member 1 | -1.01 | 0.967 | -1.14 | 0.154 | -1.12 | 0.24 | -1.28 | 0.053 | -1.14 |
| 1417372_a_at | Peli1 | pellino 1 | -1.14 | 0.555 | -1.01 | 0.922 | -1.11 | 0.354 | -2.02 | 0.073 | -1.32 |
| 1455042_at | Tbl1x | Transducin (beta)-like 1 X-linked | -1.09 | 0.524 | -1.13 | 0.241 | -1.04 | 0.723 | -1.14 | 0.388 | -1.1 |
| 1434617_x_at | 1810073N04Rik | RIKEN cDNA 1810073N04 gene | -1.07 | 0.661 | -1 | 0.97 | -1.2 | 0.643 | -1.26 | 0.229 | -1.13 |
| 1434145_s_at | Serhl | serine hydrolase-like | -1.02 | 0.906 | -1.17 | 0.41 | -1.07 | 0.767 | -1.31 | 0.117 | -1.14 |
| 1420850_at | Crnkl1 | Crn, crooked neck-like 1 (Drosophila) | -1.05 | 0.347 | -1.18 | 0.293 | -1.04 | 0.814 | -1.15 | 0.615 | -1.1 |
| 1435205_at | Tcfap2e | transcription factor AP-2, epsilon | -1.01 | 0.942 | -1.18 | 0.532 | -1.07 | 0.304 | -1.29 | 0.233 | -1.14 |
| 1427136_s_at | Sfrs12 | splicing factor, arginine/serine-rich 12 | -1.08 | 0.819 | -1.02 | 0.924 | -1.16 | 0.463 | -1.19 | 0.404 | -1.11 |
| 1433677_at | Sfrs8 | splicing factor, arginine/serine-rich 8 | -1.15 | 0.206 | -1.04 | 0.678 | -1.06 | 0.432 | -1.03 | 0.796 | -1.07 |
| 1435377_at | --- | --- | -1.03 | 0.882 | -1.08 | 0.348 | -1.15 | 0.317 | -1.11 | 0.279 | -1.09 |
| 1459396_at | 6030446N20Rik | RIKEN cDNA 6030446N20 gene | -1.07 | 0.897 | -1.17 | 0.69 | -1.02 | 0.972 | -2.11 | 0.114 | -1.34 |
| 1452155_a_at | Ddx17 | DEAD (Asp-Glu-Ala-Asp) box polypeptide 17 | -1.06 | 0.712 | -1.03 | 0.519 | -1.16 | 0.386 | -1.33 | 0.19 | -1.15 |
| 1427192_a_at | Brd8 | bromodomain containing 8 | -1.12 | 0.476 | -1.01 | 0.927 | -1.13 | 0.274 | -1.15 | 0.232 | -1.1 |
| 1440689_at | Foxk2 | forkhead box K2 | -1.1 | 0.665 | -1.04 | 0.573 | -1.12 | 0.422 | -1.25 | 0.155 | -1.12 |
| 1430401_at | 3110045C21Rik | RIKEN cDNA 3110045C21 gene | -1.14 | 0.51 | -1.02 | 0.975 | -1.09 | 0.857 | -1.37 | 0.616 | -1.16 |
| 1425183_a_at | Rpl4 | ribosomal protein L4 | -1.06 | 0.476 | -1.05 | 0.171 | -1.14 | 0.013 | -1.31 | 0.004 | -1.14 |
| 1455268_at | Zcsl2 | zinc finger, CSL domain containing 2 | -1.02 | 0.905 | -1.01 | 0.954 | -1.24 | 0.065 | -1.14 | 0.676 | -1.11 |
| 1437984_x_at | Bat1a | HLA-B-associated transcript 1A | -1.13 | 0.016 | -1.09 | 0.537 | -1.04 | 0.805 | -1.3 | 0.087 | -1.14 |
| 1434571_at | Vps13b /// LOC666 | vacuolar protein sorting 13B (yeast) /// similar to vacuolar protein sorting 13B isof | -1.21 | 0.339 | -1.04 | 0.689 | -1.02 | 0.695 | -1.12 | 0.309 | -1.1 |
| 1460338_a_at | Crlf3 | cytokine receptor-like factor 3 | -1.16 | 0.025 | -1.08 | 0.314 | -1.02 | 0.888 | -1.68 | 0.017 | -1.24 |
| 1436315_at | Myst3 | MYST histone acetyltransferase (monocytic leukemia) 3 | -1.04 | 0.482 | -1 | 0.983 | -1.23 | 0.106 | -1.09 | 0.311 | -1.09 |
| 1453379_at | 4930519P11Rik | RIKEN cDNA 4930519P11 gene | -1.18 | 0.846 | -1.04 | 0.921 | -1.04 | 0.926 | -2.25 | 0.291 | -1.38 |
| 1428464_at | Ndufa3 | NADH dehydrogenase (ubiquinone) 1 alpha subcomplex, 3 | -1.01 | 0.97 | -1.17 | 0.121 | -1.09 | 0.525 | -1.14 | 0.38 | -1.1 |
| 1456581_x_at | Gdi2 | guanosine diphosphate (GDP) dissociation inhibitor 2 | -1.03 | 0.529 | -1.07 | 0.01 | -1.16 | 0.027 | -1.3 | 0.161 | -1.14 |
| 1420611_at | Prkacb | protein kinase, cAMP dependent, catalytic, beta | -1.11 | 0.3 | -1.09 | 0.461 | -1.05 | 0.73 | -1.43 | 0.224 | -1.17 |
| 1426949_s_at | Tpr | translocated promoter region | -1.08 | 0.485 | -1.03 | 0.615 | -1.13 | 0.349 | -1 | 0.974 | -1.06 |
| 1433591_at | Ppp3r1 | protein phosphatase 3, regulatory subunit B, alpha isoform (calcineurin B, type I) | -1.06 | 0.823 | -1.05 | 0.674 | -1.13 | 0.359 | -1.02 | 0.904 | -1.07 |
| 1423099_a_at | Mettl3 | methyltransferase-like 3 | -1.09 | 0.309 | -1.01 | 0.81 | -1.15 | 0.113 | -1.41 | 0.1 | -1.17 |
| 1418850_at | Epc1 | enhancer of polycomb homolog 1 (Drosophila) | -1.05 | 0.57 | -1.06 | 0.33 | -1.14 | 0.468 | -1.29 | 0.253 | -1.13 |
| 1449445_x_at | Mfap1 | microfibrillar-associated protein 1 | -1.06 | 0.552 | -1.07 | 0.323 | -1.12 | 0.007 | -1.2 | 0.127 | -1.11 |
| 1452169_a_at | Dgkz | diacylglycerol kinase zeta | -1.05 | 0.804 | -1.13 | 0.399 | -1.07 | 0.755 | -2.36 | 0.026 | -1.4 |
| 1435186_s_at | Lrrcc1 | leucine rich repeat and coiled-coil domain containing 1 | -1.03 | 0.817 | -1.16 | 0.532 | -1.06 | 0.499 | -1.19 | 0.165 | -1.11 |
| 1436113_a_at | St13 | suppression of tumorigenicity 13 | -1.07 | 0.408 | -1.16 | 0.337 | -1.02 | 0.881 | -1.13 | 0.435 | -1.09 |
| 1424080_at | Dcps | decapping enzyme, scavenger | -1.11 | 0.541 | -1.12 | 0.293 | -1.02 | 0.922 | -1.07 | 0.592 | -1.08 |
| 1424388_at | Cluap1 | clusterin associated protein 1 | -1.02 | 0.936 | -1.16 | 0.16 | -1.07 | 0.689 | -1.36 | 0.19 | -1.15 |
| 1441940_x_at | Klhdc8a | kelch domain containing 8A | -1.1 | 0.658 | -1.08 | 0.88 | -1.06 | 0.774 | -1.37 | 0.552 | -1.15 |
| 1417794_at | Zmym3 | zinc finger, MYM-type 3 | -1.18 | 0.199 | -1 | 0.997 | -1.07 | 0.623 | -1.13 | 0.375 | -1.1 |
| 1423763_x_at | Tia1 | Cytotoxic granule-associated RNA binding protein 1 | -1.02 | 0.685 | -1.01 | 0.89 | -1.23 | 0.06 | -1.21 | 0.044 | -1.12 |
| 1416327_at | Ufc1 | ubiquitin-fold modifier conjugating enzyme 1 | -1.11 | 0.369 | -1.09 | 0.064 | -1.05 | 0.713 | -1.09 | 0.373 | -1.08 |
| 1440151_s_at | Edf1 | endothelial differentiation-related factor 1 | -1.05 | 0.045 | -1.13 | 0.268 | -1.07 | 0.169 | -1.29 | 0.19 | -1.13 |
| 1416506_at | Psma6 | proteasome (prosome, macropain) subunit, alpha type 6 | -1.09 | 0.04 | -1.09 | 0.041 | -1.06 | 0.644 | -1.11 | 0.507 | -1.09 |
| 1436546_at | Lix1l | Lix1-like | -1.21 | 0.064 | -1.03 | 0.862 | -1.03 | 0.892 | -1.12 | 0.62 | -1.09 |
| 1431289_at | Tspan32 | tetraspanin 32 | -1.22 | 0.726 | -1 | 0.999 | -1.04 | 0.906 | -1.1 | 0.501 | -1.09 |
| 1448722_s_at | Ppcs | phosphopantothenoylcysteine synthetase | -1.05 | 0.428 | -1.19 | 0.118 | -1.01 | 0.881 | -1.56 | 0.122 | -1.2 |
| 1430605_at | Ccdc101 | coiled-coil domain containing 101 | -1.08 | 0.724 | -1.05 | 0.796 | -1.12 | 0.355 | -1.02 | 0.946 | -1.07 |
| 1452625_at | Kctd2 | potassium channel tetramerisation domain containing 2 | -1.1 | 0.319 | -1.08 | 0.639 | -1.06 | 0.738 | -1.35 | 0.268 | -1.15 |

| | | | | | | | | | | | |
|---|---|---|---|---|---|---|---|---|---|---|---|
| 1434085_at | Zfp523 | zinc finger protein 523 | -1.14 | 0.463 | -1.04 | 0.645 | -1.06 | 0.556 | -1.25 | 0.321 | -1.12 |
| 1449323_a_at | Rpl3 /// LOC66821 | ribosomal protein L3 /// similar to ribosomal protein L3 isoform b /// similar to 60S | -1.03 | 0.606 | -1.04 | 0.573 | -1.18 | 0.057 | -1.26 | 0.015 | -1.13 |
| 1440235_at | Itga10 | integrin, alpha 10 | -1.09 | 0.596 | -1.15 | 0.752 | -1.01 | 0.977 | -1.09 | 0.621 | -1.09 |
| 1460352_s_at | Pik3r4 | phosphatidylinositol 3 kinase, regulatory subunit, polypeptide 4, p150 | -1.05 | 0.573 | -1.09 | 0.125 | -1.1 | 0.59 | -2.89 | 0.021 | -1.53 |
| 1420089_at | Nfkbia | Nuclear factor of kappa light chain gene enhancer in B-cells inhibitor, alpha | -1.06 | 0.679 | -1.01 | 0.923 | -1.18 | 0.275 | -1.34 | 0.157 | -1.15 |
| 1427966_at | BC087945 | cDNA sequence BC087945 | -1.03 | 0.779 | -1.15 | 0.307 | -1.06 | 0.67 | -1.73 | 0.145 | -1.24 |
| 1427442_a_at | App | amyloid beta (A4) precursor protein | -1.05 | 0.207 | -1.08 | 0.345 | -1.11 | 0.345 | -1.07 | 0.673 | -1.08 |
| 1424342_at | Fyttd1 | forty-two-three domain containing 1 | -1.05 | 0.768 | -1.08 | 0.384 | -1.1 | 0.703 | -1.31 | 0.216 | -1.14 |
| 1439799_at | --- | Transcribed locus | -1.13 | 0.211 | -1.07 | 0.78 | -1.04 | 0.88 | -1.54 | 0.306 | -1.2 |
| 1422669_at | Ebag9 | estrogen receptor-binding fragment-associated gene 9 | -1.13 | 0.021 | -1.11 | 0.046 | -1.01 | 0.918 | -1.14 | 0.2 | -1.1 |
| 1435739_at | AW208599 | expressed sequence AW208599 | -1.03 | 0.555 | -1.11 | 0.321 | -1.1 | 0.412 | -1.04 | 0.687 | -1.07 |
| 1451550_at | Ephb3 | Eph receptor B3 | -1.22 | 0.661 | -1.03 | 0.94 | -1.01 | 0.932 | -1.2 | 0.709 | -1.12 |
| 1416883_at | Clptm1 | cleft lip and palate associated transmembrane protein 1 | -1.09 | 0.543 | -1.06 | 0.434 | -1.09 | 0.563 | -1.06 | 0.468 | -1.07 |
| 1418628_at | Khdrbs1 | KH domain containing, RNA binding, signal transduction associated 1 | -1.01 | 0.959 | -1.18 | 0.134 | -1.06 | 0.722 | -1.02 | 0.922 | -1.07 |
| 1438318_x_at | Ngdn | neuroguidin, EIF4E binding protein | -1.01 | 0.895 | -1.04 | 0.601 | -1.21 | 0.212 | -1.39 | 0.019 | -1.16 |
| 1426296_at | Rad52 | RAD52 homolog (S. cerevisiae) | -1.04 | 0.828 | -1.03 | 0.85 | -1.18 | 0.022 | -1.11 | 0.658 | -1.09 |
| 1420119_s_at | Phf3 | PHD finger protein 3 | -1.12 | 0.732 | -1.11 | 0.683 | -1.01 | 0.894 | -1.13 | 0.606 | -1.09 |
| 1439531_at | E130311K13Rik | RIKEN cDNA E130311K13 gene | -1.02 | 0.841 | -1.04 | 0.638 | -1.19 | 0.306 | -1.45 | 0.211 | -1.18 |
| 1438032_at | Lrch1 | leucine-rich repeats and calponin homology (CH) domain containing 1 | -1.02 | 0.949 | -1.04 | 0.757 | -1.19 | 0.181 | -1.43 | 0.02 | -1.17 |
| 1439563_at | Zfp3 | zinc finger protein 3 | -1.08 | 0.85 | -1.11 | 0.638 | -1.05 | 0.563 | -1.23 | 0.471 | -1.12 |
| 1418960_at | Phf20l1 | PHD finger protein 20-like 1 | -1.11 | 0.517 | -1.02 | 0.931 | -1.12 | 0.416 | -1.45 | 0.031 | -1.17 |
| 1416647_at | Bckdha | branched chain ketoacid dehydrogenase E1, alpha polypeptide | -1.05 | 0.798 | -1.2 | 0.23 | -1.01 | 0.96 | -1.29 | 0.309 | -1.14 |
| 1454350_at | Pdzd6 | PDZ domain containing 6 | -1.04 | 0.894 | -1.14 | 0.378 | -1.06 | 0.753 | -1.28 | 0.225 | -1.13 |
| 1426789_s_at | Ssrp1 | structure specific recognition protein 1 | -1.21 | 0.04 | -1.03 | 0.657 | -1.01 | 0.938 | -1 | 0.987 | -1.06 |
| 1423130_a_at | Sfrs5 /// LOC62246 | splicing factor, arginine/serine-rich 5 (SRp40, HRS) /// similar to Splicing factor, a | -1.1 | 0.747 | -1.05 | 0.746 | -1.08 | 0.556 | -1.17 | 0.581 | -1.1 |
| 1429103_at | Tomm22 | translocase of outer mitochondrial membrane 22 homolog (yeast) | -1.01 | 0.956 | -1.22 | 0.049 | -1.03 | 0.809 | -1.01 | 0.966 | -1.07 |
| 1434251_at | Cnot1 | CCR4-NOT transcription complex, subunit 1 | -1.06 | 0.363 | -1.03 | 0.798 | -1.15 | 0.079 | -1.19 | 0.111 | -1.11 |
| 1423045_at | Ncbp2 | nuclear cap binding protein subunit 2 | -1.05 | 0.134 | -1.04 | 0.694 | -1.15 | 0.203 | -1.17 | 0.199 | -1.1 |
| 1432629_at | --- | --- | -1.08 | 0.92 | -1.14 | 0.232 | -1.02 | 0.953 | -1.32 | 0.258 | -1.14 |
| 1416708_a_at | Gramd1a | GRAM domain containing 1A | -1.18 | 0.156 | -1.03 | 0.855 | -1.04 | 0.757 | -2.39 | 0.023 | -1.41 |
| 1440076_at | Sqstm1 | Sequestosome 1 | -1.06 | 0.132 | -1.09 | 0.496 | -1.08 | 0.522 | -1.12 | 0.699 | -1.09 |
| 1422555_s_at | Gna13 | guanine nucleotide binding protein, alpha 13 | -1.02 | 0.744 | -1 | 0.96 | -1.23 | 0.076 | -1.28 | 0.435 | -1.13 |
| 1416291_at | Psmc4 | proteasome (prosome, macropain) 26S subunit, ATPase, 4 | -1.07 | 0.33 | -1.11 | 0.02 | -1.05 | 0.659 | -1.17 | 0.372 | -1.1 |
| 1428950_s_at | Nol8 | nucleolar protein 8 | -1.05 | 0.912 | -1.07 | 0.565 | -1.12 | 0.63 | -1.05 | 0.77 | -1.07 |
| 1434019_at | Pdap1 | PDGFA associated protein 1 | -1.09 | 0.124 | -1.1 | 0.401 | -1.05 | 0.768 | -1.02 | 0.904 | -1.06 |
| 1451134_a_at | Tm2d2 | TM2 domain containing 2 | -1.12 | 0.437 | -1.05 | 0.598 | -1.06 | 0.557 | -1.2 | 0.301 | -1.11 |
| 1436595_at | Rbm34 | RNA binding motif protein 34 | -1.08 | 0.603 | -1.07 | 0.329 | -1.08 | 0.554 | -1.12 | 0.122 | -1.09 |
| 1449294_at | Mrps15 | mitochondrial ribosomal protein S15 | -1.04 | 0.644 | -1.02 | 0.887 | -1.19 | 0.077 | -1.32 | 0.384 | -1.14 |
| 1428367_at | Ndst1 | N-deacetylase/N-sulfotransferase (heparan glucosaminyl) 1 | -1.13 | 0.678 | -1.01 | 0.954 | -1.09 | 0.825 | -1.37 | 0.47 | -1.15 |
| 1460726_at | Adss | adenylosuccinate synthetase, non muscle | -1.01 | 0.926 | -1.22 | 0.022 | -1.02 | 0.881 | -1.04 | 0.683 | -1.07 |
| 1415751_at | Hp1bp3 | heterochromatin protein 1, binding protein 3 | -1.04 | 0.666 | -1.16 | 0.079 | -1.04 | 0.709 | -1.08 | 0.522 | -1.08 |
| 1423361_at | Yme1l1 | YME1-like 1 (S. cerevisiae) | -1.08 | 0.718 | -1.07 | 0.313 | -1.08 | 0.484 | -1.12 | 0.539 | -1.09 |
| 1433761_at | Pde4dip | phosphodiesterase 4D interacting protein (myomegalin) | -1.21 | 0.521 | -1.02 | 0.902 | -1.02 | 0.946 | -1.63 | 0.161 | -1.22 |
| 1428855_at | H13 | histocompatibility 13 | -1.12 | 0.638 | -1.09 | 0.885 | -1.02 | 0.929 | -3.5 | 0.096 | -1.68 |
| 1429194_at | Tigd2 | tigger transposable element derived 2 | -1.04 | 0.751 | -1 | 0.971 | -1.21 | 0.183 | -1.9 | 0.095 | -1.29 |
| 1417979_at | Tnmd | tenomodulin | -1.04 | 0.936 | -1.02 | 0.947 | -1.18 | 0.697 | -1.22 | 0.699 | -1.12 |
| 1441089_at | Eif2c3 | eukaryotic translation initiation factor 2C, 3 | -1.05 | 0.915 | -1.04 | 0.863 | -1.14 | 0.416 | -1.31 | 0.438 | -1.14 |
| 1418568_x_at | Srp14 | signal recognition particle 14 | -1.02 | 0.702 | -1.04 | 0.216 | -1.18 | 0.026 | -1.28 | 0.081 | -1.13 |
| 1428520_at | 1110032A13Rik | RIKEN cDNA 1110032A13 gene | -1.05 | 0.556 | -1.13 | 0.088 | -1.05 | 0.764 | -1.08 | 0.315 | -1.08 |
| 1454947_a_at | Ublcp1 /// LOC665 | ubiquitin-like domain containing CTD phosphatase 1 /// similar to ubiquitin-like do | -1.07 | 0.784 | -1.04 | 0.845 | -1.12 | 0.543 | -1.19 | 0.253 | -1.11 |
| 1435278_at | Sft2d3 | SFT2 domain containing 3 | -1.02 | 0.902 | -1.15 | 0.263 | -1.06 | 0.705 | -1.27 | 0.284 | -1.13 |
| 1416500_at | Sacm1l | SAC1 (suppressor of actin mutations 1, homolog)-like (S. cerevisiae) | -1.04 | 0.545 | -1.03 | 0.653 | -1.17 | 0.125 | -1.08 | 0.177 | -1.08 |
| 1417812_a_at | Lamb3 | laminin, beta 3 | -1.06 | 0.925 | -1.15 | 0.881 | -1.03 | 0.944 | -1.47 | 0.295 | -1.18 |

| | | | | | | | | | | | |
|---|---|---|---|---|---|---|---|---|---|---|---|
| 1451185_at | Sf3b5 | splicing factor 3b, subunit 5 | -1.04 | 0.444 | -1.09 | 0.144 | -1.1 | 0.501 | -1.06 | 0.667 | -1.07 |
| 1423441_at | Tfb2m | transcription factor B2, mitochondrial | -1.07 | 0.066 | -1.06 | 0.409 | -1.1 | 0.355 | -1.11 | 0.574 | -1.08 |
| 1426890_a_at | Rpap1 | RNA polymerase II associated protein 1 | -1.1 | 0.496 | -1.09 | 0.598 | -1.03 | 0.9 | -1.19 | 0.209 | -1.1 |
| 1451487_at | Rabepk | Rab9 effector protein with kelch motifs | -1.03 | 0.698 | -1.02 | 0.878 | -1.19 | 0.406 | -1.25 | 0.362 | -1.12 |
| 1423567_a_at | Psma7 | proteasome (prosome, macropain) subunit, alpha type 7 | -1.16 | 0.022 | -1.05 | 0.438 | -1.03 | 0.778 | -1.16 | 0.442 | -1.1 |
| 1443955_at | --- | --- | -1.13 | 0.467 | -1.01 | 0.835 | -1.1 | 0.723 | -1.18 | 0.333 | -1.1 |
| 1416495_s_at | Ndufs5 | NADH dehydrogenase (ubiquinone) Fe-S protein 5 | -1.02 | 0.716 | -1.12 | 0.03 | -1.09 | 0.189 | -1.12 | 0.554 | -1.09 |
| 1457501_at | LOC622665 | hypothetical protein LOC622665 | -1.05 | 0.896 | -1.15 | 0.738 | -1.03 | 0.907 | -1.55 | 0.177 | -1.2 |
| 1424078_s_at | Pex6 | peroxisomal biogenesis factor 6 | -1 | 0.986 | -1.21 | 0.168 | -1.03 | 0.599 | -1.33 | 0.152 | -1.14 |
| 1452953_at | Fam18b | family with sequence similarity 18, member B | -1.08 | 0.22 | -1.01 | 0.873 | -1.15 | 0.192 | -1.04 | 0.794 | -1.07 |
| 1417827_at | Ngly1 | N-glycanase 1 | -1.21 | 0.076 | -1.03 | 0.66 | -1 | 0.959 | -1.1 | 0.278 | -1.09 |
| 1434057_at | Ndufb6 | NADH dehydrogenase (ubiquinone) 1 beta subcomplex, 6 | -1.03 | 0.802 | -1.17 | 0.12 | -1.03 | 0.435 | -1.02 | 0.909 | -1.06 |
| 1435017_at | Mel13 | melanoma nuclear protein 13 | -1.04 | 0.483 | -1.05 | 0.492 | -1.14 | 0.141 | -1.53 | 0.142 | -1.19 |
| 1437391_x_at | Mrpl44 | mitochondrial ribosomal protein L44 | -1.01 | 0.837 | -1.19 | 0.063 | -1.04 | 0.782 | -1.17 | 0.195 | -1.1 |
| 1448027_at | 2010305B15Rik | RIKEN cDNA 2010305B15 gene | -1.02 | 0.904 | -1.13 | 0.454 | -1.08 | 0.632 | -1.42 | 0.372 | -1.16 |
| 1455175_at | Phf13 | PHD finger protein 13 | -1.06 | 0.363 | -1.1 | 0.658 | -1.07 | 0.476 | -1.52 | 0.246 | -1.19 |
| 1460436_at | Ndst1 | N-deacetylase/N-sulfotransferase (heparan glucosaminyl) 1 | -1.07 | 0.464 | -1.13 | 0.148 | -1.02 | 0.945 | -1.93 | 0.085 | -1.29 |
| 1420088_at | Nfkbia | nuclear factor of kappa light chain gene enhancer in B-cells inhibitor, alpha | -1.07 | 0.845 | -1.03 | 0.783 | -1.13 | 0.069 | -1.51 | 0.096 | -1.18 |
| 1423791_at | Ik | IK cytokine | -1.1 | 0.6 | -1.03 | 0.576 | -1.1 | 0.425 | -1.13 | 0.605 | -1.09 |
| 1419490_at | AW049604 | expressed sequence AW049604 | -1.22 | 0.338 | -1.01 | 0.974 | -1.02 | 0.914 | -1.4 | 0.651 | -1.16 |
| 1430969_at | Mtch2 | mitochondrial carrier homolog 2 (C. elegans) | -1.05 | 0.926 | -1.09 | 0.843 | -1.09 | 0.77 | -1.05 | 0.892 | -1.07 |
| 1426716_at | Tdrd7 | tudor domain containing 7 | -1.05 | 0.735 | -1.15 | 0.224 | -1.03 | 0.804 | -1.36 | 0.049 | -1.15 |
| 1425023_at | Usp3 | ubiquitin specific peptidase 3 | -1.08 | 0.552 | -1.15 | 0.357 | -1 | 0.99 | -1.7 | 0.041 | -1.23 |
| 1442593_at | --- | --- | -1.05 | 0.857 | -1.15 | 0.184 | -1.02 | 0.949 | -2.13 | 0.109 | -1.34 |
| 1448241_at | Gm2a | GM2 ganglioside activator protein | -1.1 | 0.022 | -1.01 | 0.92 | -1.12 | 0.352 | -1.38 | 0.322 | -1.15 |
| 1434946_at | C330021A05Rik | RIKEN cDNA C330021A05 gene | -1.22 | 0.448 | -1.01 | 0.983 | -1.02 | 0.925 | -1.15 | 0.521 | -1.1 |
| 1452066_a_at | Ndfip2 | Nedd4 family interacting protein 2 | -1.07 | 0.318 | -1.1 | 0.12 | -1.05 | 0.649 | -1.36 | 0.155 | -1.15 |
| 1435617_at | AI480743 | expressed sequence AI480743 | -1 | 0.999 | -1.19 | 0.226 | -1.05 | 0.765 | -1.44 | 0.111 | -1.17 |
| 1433447_x_at | Cct4 | chaperonin subunit 4 (delta) | -1.05 | 0.594 | -1.07 | 0.552 | -1.1 | 0.405 | -1.22 | 0.022 | -1.11 |
| 1447805_s_at | D11Ertd730e | DNA segment, Chr 11, ERATO Doi 730, expressed | -1.02 | 0.664 | -1.08 | 0.469 | -1.12 | 0.496 | -1.16 | 0.244 | -1.1 |
| 1448639_a_at | Spata5 | spermatogenesis associated 5 | -1 | 0.968 | -1.2 | 0.406 | -1.03 | 0.648 | -1.1 | 0.611 | -1.09 |
| 1436178_at | Leprel1 | leprecan-like 1 | -1.05 | 0.677 | -1.02 | 0.975 | -1.16 | 0.607 | -1.27 | 0.172 | -1.12 |
| 1454704_at | Scarb2 | scavenger receptor class B, member 2 | -1.01 | 0.95 | -1.02 | 0.849 | -1.2 | 0.46 | -1.25 | 0.481 | -1.12 |
| 1423335_at | 1110004F10Rik | RIKEN cDNA 1110004F10 gene | -1.11 | 0.256 | -1.07 | 0.564 | -1.03 | 0.711 | -1.01 | 0.946 | -1.06 |
| 1448867_at | Tmem9b | TMEM9 domain family, member B | -1 | 0.971 | -1.02 | 0.805 | -1.22 | 0.106 | -1.15 | 0.274 | -1.1 |
| 1424616_s_at | Frag1 | FGF receptor activating protein 1 | -1.13 | 0.13 | -1.01 | 0.865 | -1.08 | 0.426 | -1.57 | 0.083 | -1.2 |
| 1451049_at | Bcap31 | B-cell receptor-associated protein 31 | -1.03 | 0.544 | -1.08 | 0.266 | -1.11 | 0.153 | -1.03 | 0.862 | -1.06 |
| 1448178_a_at | Cct3 | chaperonin subunit 3 (gamma) | -1.13 | 0.243 | -1.08 | 0.437 | -1.01 | 0.91 | -1.13 | 0.1 | -1.09 |
| 1438289_a_at | Sumo1 | SMT3 suppressor of mif two 3 homolog 1 (yeast) | -1.01 | 0.938 | -1.02 | 0.858 | -1.22 | 0.028 | -1.39 | 0.092 | -1.16 |
| 1454776_at | Ehmt1 | euchromatic histone methyltransferase 1 | -1.08 | 0.392 | -1.14 | 0.129 | -1.01 | 0.915 | -1.05 | 0.615 | -1.07 |
| 1436004_at | Usp27x | ubiquitin specific peptidase 27, X chromosome | -1.19 | 0.546 | -1.02 | 0.947 | -1.03 | 0.849 | -1.08 | 0.714 | -1.08 |
| 1434056_a_at | Ndufb6 | NADH dehydrogenase (ubiquinone) 1 beta subcomplex, 6 | -1.1 | 0.11 | -1.07 | 0.277 | -1.04 | 0.605 | -1.16 | 0.228 | -1.1 |
| 1454480_at | 4933439N06Rik | RIKEN cDNA 4933439N06 gene | -1.02 | 0.949 | -1.16 | 0.787 | -1.05 | 0.845 | -1.26 | 0.653 | -1.12 |
| 1451051_a_at | Scyl1 | SCY1-like 1 (S. cerevisiae) | -1.11 | 0.161 | -1.09 | 0.234 | -1.02 | 0.901 | -1.18 | 0.179 | -1.1 |
| 1460235_at | Scarb2 | scavenger receptor class B, member 2 | -1.04 | 0.884 | -1.07 | 0.626 | -1.12 | 0.346 | -1.16 | 0.535 | -1.09 |
| 1430594_at | Rab11fip1 | RAB11 family interacting protein 1 (class I) | -1.11 | 0.474 | -1.09 | 0.618 | -1.02 | 0.944 | -1.33 | 0.533 | -1.14 |
| 1416179_a_at | Rdx | radixin | -1.06 | 0.489 | -1.04 | 0.561 | -1.12 | 0.151 | -1.08 | 0.168 | -1.07 |
| 1428078_at | 0610013E23Rik | RIKEN cDNA 0610013E23 gene | -1.18 | 0.459 | -1.05 | 0.727 | -1 | 1 | -1.33 | 0.171 | -1.14 |
| 1439268_x_at | Eif3s6 | eukaryotic translation initiation factor 3, subunit 6 | -1.03 | 0.763 | -1.05 | 0.491 | -1.15 | 0.381 | -1.36 | 0.073 | -1.15 |
| 1421446_at | Prkcc | protein kinase C, gamma | -1 | 0.996 | -1.16 | 0.67 | -1.06 | 0.798 | -1.22 | 0.693 | -1.11 |
| 1433712_at | AW555464 | expressed sequence AW555464 | -1.05 | 0.787 | -1.09 | 0.348 | -1.07 | 0.342 | -1.04 | 0.79 | -1.06 |
| 1416522_a_at | Grcc10 | gene rich cluster, C10 gene | -1 | 0.977 | -1.1 | 0.234 | -1.12 | 0.46 | -1.7 | 0.194 | -1.23 |
| 1426307_at | Cyb5r4 | cytochrome b5 reductase 4 | -1.15 | 0.35 | -1.04 | 0.48 | -1.03 | 0.786 | -1.29 | 0.012 | -1.13 |

| | | | | | | | | | | | |
|---|---|---|---|---|---|---|---|---|---|---|---|
| 1460135_at | A930005H10Rik | RIKEN cDNA A930005H10 gene | -1.01 | 0.977 | -1.23 | 0.294 | -1 | 0.998 | -2.65 | 0.06 | -1.47 |
| 1424313_a_at | Ndufs7 | NADH dehydrogenase (ubiquinone) Fe-S protein 7 | -1.02 | 0.764 | -1.14 | 0.3 | -1.06 | 0.52 | -1.12 | 0.089 | -1.09 |
| 1442278_at | Jarid1b | Jumonji, AT rich interactive domain 1B (Rbp2 like) | -1.12 | 0.811 | -1.01 | 0.968 | -1.09 | 0.861 | -1.04 | 0.885 | -1.07 |
| 1460432_a_at | Eif3s6 | eukaryotic translation initiation factor 3, subunit 6 | -1.1 | 0.002 | -1.01 | 0.847 | -1.11 | 0.446 | -1.29 | 0.155 | -1.13 |
| 1415920_at | Cstf2t | cleavage stimulation factor, 3' pre-RNA subunit 2, tau | -1.1 | 0.18 | -1.01 | 0.86 | -1.1 | 0.024 | -1.56 | 0.048 | -1.19 |
| 1418521_a_at | Mtx1 | metaxin 1 | -1.14 | 0.127 | -1.07 | 0.411 | -1.01 | 0.937 | -1.11 | 0.218 | -1.08 |
| 1432487_at | 4930405H06Rik | RIKEN cDNA 4930405H06 gene | -1.02 | 0.958 | -1.06 | 0.873 | -1.13 | 0.576 | -1.31 | 0.317 | -1.13 |
| 1456324_at | Zfp748 | zinc finger protein 748 | -1.03 | 0.847 | -1.05 | 0.793 | -1.13 | 0.087 | -1.63 | 0.059 | -1.21 |
| 1460308_a_at | Ict1 | immature colon carcinoma transcript 1 | -1.03 | 0.826 | -1.11 | 0.02 | -1.07 | 0.67 | -1.37 | 0.146 | -1.14 |
| 1448363_at | Yap1 | yes-associated protein 1 | -1.02 | 0.494 | -1.18 | 0.068 | -1.02 | 0.859 | -1.15 | 0.643 | -1.09 |
| 1420473_at | Mtpn | myotrophin | -1.02 | 0.902 | -1.03 | 0.7 | -1.17 | 0.135 | -1.37 | 0.195 | -1.15 |
| 1426111_x_at | Irf3 | interferon regulatory factor 3 | -1.09 | 0.556 | -1.12 | 0.05 | -1.01 | 0.931 | -1.55 | 0.167 | -1.19 |
| 1457259_at | --- | --- | -1.12 | 0.452 | -1.07 | 0.76 | -1.02 | 0.85 | -1.22 | 0.399 | -1.11 |
| 1436400_at | --- | --- | -1.04 | 0.845 | -1.08 | 0.717 | -1.08 | 0.707 | -1.11 | 0.388 | -1.08 |
| 1433753_x_at | Eral1 | Era (G-protein)-like 1 (E. coli) | -1.03 | 0.831 | -1.09 | 0.502 | -1.09 | 0.609 | -1.38 | 0.147 | -1.15 |
| 1452852_at | Twistnb | TWIST neighbor | -1.06 | 0.213 | -1.04 | 0.422 | -1.11 | 0.414 | -1.24 | 0.325 | -1.11 |
| 1437734_at | Ppp1r12a | protein phosphatase 1, regulatory (inhibitor) subunit 12A | -1.16 | 0.186 | -1 | 0.992 | -1.06 | 0.566 | -1.08 | 0.509 | -1.07 |
| 1426289_at | 2610028H07Rik | RIKEN cDNA 2610028H07 gene | -1.07 | 0.492 | -1.1 | 0.348 | -1.04 | 0.82 | -1.1 | 0.567 | -1.08 |
| 1420834_at | Vamp2 | vesicle-associated membrane protein 2 | -1.03 | 0.894 | -1.13 | 0.573 | -1.05 | 0.849 | -1.11 | 0.629 | -1.08 |
| 1454074_a_at | 1500011J06Rik | RIKEN cDNA 1500011J06 gene | -1.03 | 0.885 | -1.03 | 0.715 | -1.15 | 0.087 | -1.14 | 0.162 | -1.09 |
| 1418841_s_at | Cdc2l1 | cell division cycle 2-like 1 | -1.06 | 0.73 | -1.12 | 0.03 | -1.03 | 0.753 | -1.29 | 0.089 | -1.12 |
| 1417844_at | Med4 | mediator of RNA polymerase II transcription, subunit 4 homolog (yeast) | -1.01 | 0.797 | -1.08 | 0.402 | -1.11 | 0.424 | -1.15 | 0.134 | -1.09 |
| 1455942_at | Fbxl11 | F-box and leucine-rich repeat protein 11 | -1.01 | 0.927 | -1.09 | 0.6 | -1.11 | 0.552 | -1.13 | 0.221 | -1.09 |
| 1434844_at | Hexdc | hexosaminidase (glycosyl hydrolase family 20, catalytic domain) containing | -1.13 | 0.551 | -1.08 | 0.515 | -1 | 0.982 | -1.54 | 0.188 | -1.19 |
| 1426537_at | Narg2 | NMDA receptor-regulated gene 2 | -1.04 | 0.918 | -1.09 | 0.617 | -1.07 | 0.712 | -1.75 | 0.085 | -1.24 |
| 1423096_at | Capn7 | calpain 7 | -1.05 | 0.792 | -1.09 | 0.274 | -1.06 | 0.608 | -1.21 | 0.197 | -1.1 |
| 1456011_x_at | Acaa1a | acetyl-Coenzyme A acyltransferase 1A | -1.07 | 0.878 | -1.05 | 0.874 | -1.08 | 0.709 | -1.8 | 0.088 | -1.25 |
| 1454765_at | Gtf3c3 | general transcription factor IIIC, polypeptide 3 | -1.11 | 0.546 | -1.09 | 0.591 | -1.01 | 0.984 | -1.05 | 0.352 | -1.06 |
| 1444607_at | Cdsn | Corneodesmosin | -1 | 0.985 | -1.1 | 0.657 | -1.1 | 0.661 | -1.43 | 0.315 | -1.16 |
| 1424196_at | Yipf1 | Yip1 domain family, member 1 | -1.01 | 0.858 | -1.04 | 0.393 | -1.16 | 0.35 | -1.18 | 0.24 | -1.1 |
| 1436179_a_at | Dnajc5 | DnaJ (Hsp40) homolog, subfamily C, member 5 | -1.1 | 0.579 | -1.03 | 0.688 | -1.08 | 0.775 | -1.27 | 0.214 | -1.12 |
| 1455211_a_at | Timm13 | translocase of inner mitochondrial membrane 13 homolog (yeast) | -1 | 0.963 | -1.17 | 0.065 | -1.04 | 0.653 | -1.18 | 0.45 | -1.1 |
| 1451573_a_at | Stx4a | syntaxin 4A (placental) | -1.1 | 0.641 | -1.01 | 0.947 | -1.1 | 0.486 | -1.3 | 0.222 | -1.12 |
| 1449842_at | 1810059G22Rik | RIKEN cDNA 1810059G22 gene | -1.09 | 0.41 | -1.1 | 0.499 | -1.01 | 0.879 | -1.02 | 0.797 | -1.05 |
| 1416867_at | Bet1 | blocked early in transport 1 homolog (S. cerevisiae) | -1.06 | 0.597 | -1.06 | 0.742 | -1.07 | 0.724 | -1.07 | 0.756 | -1.07 |
| 1435271_at | Irf3 | interferon regulatory factor 3 | -1.04 | 0.753 | -1.04 | 0.891 | -1.13 | 0.773 | -1.7 | 0.259 | -1.23 |
| 1452973_at | Ppm1k | protein phosphatase 1K (PP2C domain containing) | -1.05 | 0.616 | -1.05 | 0.523 | -1.1 | 0.558 | -1.85 | 0.154 | -1.26 |
| 1455022_at | Strn | striatin, calmodulin binding protein | -1.03 | 0.667 | -1.01 | 0.872 | -1.17 | 0.243 | -1.03 | 0.889 | -1.06 |
| 1418577_at | Trim8 | tripartite motif protein 8 | -1.1 | 0.68 | -1.07 | 0.767 | -1.03 | 0.856 | -1.3 | 0.23 | -1.12 |
| 1434441_at | 1110018J18Rik | RIKEN cDNA 1110018J18 gene | -1.04 | 0.598 | -1.01 | 0.919 | -1.15 | 0.035 | -1.35 | 0.127 | -1.14 |
| 1428405_at | Hcfc1r1 | host cell factor C1 regulator 1 (XPO1-dependent) | -1.07 | 0.269 | -1.06 | 0.479 | -1.07 | 0.238 | -1.18 | 0.434 | -1.09 |
| 1430292_a_at | 1810030N24Rik | RIKEN cDNA 1810030N24 gene | -1.13 | 0.054 | -1.01 | 0.896 | -1.07 | 0.554 | -1.01 | 0.935 | -1.05 |
| 1423958_a_at | 2900001O04Rik | RIKEN cDNA 2900001O04 gene | -1.07 | 0.516 | -1.03 | 0.674 | -1.1 | 0.426 | -1.16 | 0.139 | -1.09 |
| 1436069_at | Ing5 | inhibitor of growth family, member 5 | -1.16 | 0.416 | -1.04 | 0.775 | -1.01 | 0.91 | -1.03 | 0.839 | -1.06 |
| 1438631_x_at | Ttc13 | tetratricopeptide repeat domain 13 | -1.01 | 0.965 | -1 | 0.998 | -1.21 | 0.285 | -1.53 | 0.02 | -1.19 |
| 1418502_a_at | Oxr1 | oxidation resistance 1 | -1.14 | 0.372 | -1.05 | 0.629 | -1.01 | 0.9 | -1.12 | 0.272 | -1.08 |
| 1434791_at | Atp6v0a2 | ATPase, H+ transporting, lysosomal V0 subunit A2 | -1.08 | 0.53 | -1.11 | 0.404 | -1.02 | 0.932 | -1.03 | 0.901 | -1.06 |
| 1445059_at | C80435 | expressed sequence C80435 | -1.13 | 0.811 | -1.01 | 0.987 | -1.07 | 0.86 | -2.04 | 0.072 | -1.31 |
| 1460637_s_at | Pfdn5 | prefoldin 5 | -1.03 | 0.745 | -1.06 | 0.352 | -1.12 | 0.279 | -1.75 | 0.129 | -1.24 |
| 1437297_at | Chd8 | chromodomain helicase DNA binding protein 8 | -1.02 | 0.873 | -1.03 | 0.769 | -1.16 | 0.237 | -1.39 | 0.159 | -1.15 |
| 1437730_at | Ppp2r2a | protein phosphatase 2 (formerly 2A), regulatory subunit B (PR 52), alpha isoform | -1.05 | 0.287 | -1.05 | 0.637 | -1.09 | 0.137 | -1.17 | 0.219 | -1.09 |
| 1433511_at | 6330549H03Rik | RIKEN cDNA 6330549H03 gene | -1.08 | 0.726 | -1.11 | 0.757 | -1.01 | 0.922 | -1.16 | 0.326 | -1.09 |
| 1434022_at | Zbtb33 | zinc finger and BTB domain containing 33 | -1.02 | 0.807 | -1.05 | 0.47 | -1.13 | 0.045 | -1.24 | 0.331 | -1.11 |

| | | | | | | | | | | | |
|---|---|---|---|---|---|---|---|---|---|---|---|
| 1423486_at | Cript | cysteine-rich PDZ-binding protein | -1.07 | 0.231 | -1.11 | 0.214 | -1.02 | 0.891 | -1.17 | 0.447 | -1.09 |
| 1428634_at | Twistnb | TWIST neighbor | -1.05 | 0.563 | -1.08 | 0.551 | -1.07 | 0.687 | -1.12 | 0.426 | -1.08 |
| 1450904_at | Tmem167 | transmembrane protein 167 | -1.09 | 0.619 | -1.01 | 0.975 | -1.11 | 0.626 | -1.2 | 0.525 | -1.1 |
| 1418742_at | Krt34 | keratin 34 | -1.01 | 0.959 | -1.16 | 0.669 | -1.03 | 0.835 | -1.16 | 0.549 | -1.09 |
| 1438071_at | Pms1 | postmeiotic segregation increased 1 (S. cerevisiae) | -1.03 | 0.038 | -1.17 | 0.638 | -1 | 0.992 | -1.4 | 0.062 | -1.15 |
| 1421870_at | Trim44 | tripartite motif-containing 44 | -1.04 | 0.507 | -1.13 | 0.085 | -1.03 | 0.809 | -1.02 | 0.901 | -1.05 |
| 1423167_at | Prei3 | preimplantation protein 3 | -1.05 | 0.765 | -1.04 | 0.833 | -1.1 | 0.634 | -1.2 | 0.492 | -1.1 |
| 1444113_at | --- | Transcribed locus | -1.06 | 0.796 | -1.05 | 0.734 | -1.09 | 0.587 | -1.57 | 0.044 | -1.19 |
| 1440268_at | Trim41 | tripartite motif-containing 41 | -1.1 | 0.49 | -1.01 | 0.97 | -1.09 | 0.602 | -1.03 | 0.892 | -1.06 |
| 1416813_at | Tia1 | cytotoxic granule-associated RNA binding protein 1 | -1.06 | 0.604 | -1.08 | 0.599 | -1.05 | 0.661 | -1.49 | 0.208 | -1.17 |
| 1443382_s_at | Btbd14b | BTB (POZ) domain containing 14B | -1.03 | 0.846 | -1.17 | 0.757 | -1.01 | 0.966 | -1 | 0.984 | -1.05 |
| 1415980_at | Atp5g2 | ATP synthase, H+ transporting, mitochondrial F0 complex, subunit c (subunit 9), i | -1.03 | 0.899 | -1.05 | 0.689 | -1.11 | 0.55 | -1.29 | 0.271 | -1.12 |
| 1426995_a_at | Gfer | growth factor, erv1 (S. cerevisiae)-like (augmenter of liver regeneration) | -1.02 | 0.907 | -1.1 | 0.183 | -1.07 | 0.653 | -1.01 | 0.908 | -1.05 |
| 1436027_at | Osbpl11 | oxysterol binding protein-like 11 | -1.1 | 0.705 | -1.01 | 0.942 | -1.09 | 0.583 | -1.37 | 0.054 | -1.14 |
| 1451020_at | Gsk3b | glycogen synthase kinase 3 beta | -1.11 | 0.581 | -1.05 | 0.649 | -1.03 | 0.779 | -1 | 0.992 | -1.05 |
| 1454614_at | 1810013D10Rik | RIKEN cDNA 1810013D10 gene | -1.09 | 0.271 | -1.01 | 0.921 | -1.09 | 0.471 | -1.19 | 0.169 | -1.09 |
| 1424883_s_at | Sfrs7 | splicing factor, arginine/serine-rich 7 | -1.05 | 0.827 | -1.07 | 0.55 | -1.07 | 0.636 | -1.19 | 0.123 | -1.09 |
| 1428178_s_at | Trappc6b | trafficking protein particle complex 6B | -1.15 | 0.086 | -1.01 | 0.815 | -1.04 | 0.672 | -1.21 | 0.209 | -1.1 |
| 1426602_at | Araf | v-raf murine sarcoma 3611 viral oncogene homolog | -1.01 | 0.96 | -1.07 | 0.575 | -1.11 | 0.348 | -1.08 | 0.671 | -1.07 |
| 1416538_at | Siae | sialic acid acetylesterase | -1.1 | 0.232 | -1.08 | 0.521 | -1.02 | 0.932 | -1.05 | 0.798 | -1.06 |
| 1421859_at | Adam17 | a disintegrin and metallopeptidase domain 17 | -1.11 | 0.188 | -1.03 | 0.841 | -1.05 | 0.819 | -1.36 | 0.107 | -1.14 |
| 1422849_a_at | Pabpn1 | poly(A) binding protein, nuclear 1 | -1.03 | 0.862 | -1.03 | 0.859 | -1.13 | 0.297 | -1.16 | 0.236 | -1.09 |
| 1417010_at | Zfp238 | zinc finger protein 238 | -1.03 | 0.491 | -1.02 | 0.862 | -1.15 | 0.049 | -1.75 | 0.051 | -1.24 |
| 1416526_a_at | Park7 | Parkinson disease (autosomal recessive, early onset) 7 | -1.05 | 0.36 | -1.13 | 0.252 | -1.01 | 0.803 | -1.12 | 0.407 | -1.08 |
| 1417054_a_at | 0610009D07Rik | RIKEN cDNA 0610009D07 gene | -1.09 | 0.036 | -1.07 | 0.187 | -1.03 | 0.854 | -1.11 | 0.35 | -1.07 |
| 1451525_at | Arhgap12 | Rho GTPase activating protein 12 | -1.06 | 0.42 | -1.04 | 0.711 | -1.09 | 0.516 | -1.21 | 0.603 | -1.1 |
| 1450865_s_at | Mrps24 | mitochondrial ribosomal protein S24 | -1.08 | 0.5 | -1.01 | 0.77 | -1.1 | 0.618 | -1.04 | 0.788 | -1.06 |
| 1417029_a_at | Trim2 | tripartite motif protein 2 | -1.16 | 0.603 | -1.01 | 0.981 | -1.02 | 0.918 | -1.08 | 0.818 | -1.07 |
| 1440799_s_at | Farp2 | FERM, RhoGEF and pleckstrin domain protein 2 | -1.03 | 0.89 | -1.05 | 0.741 | -1.11 | 0.662 | -1.09 | 0.815 | -1.07 |
| 1449343_s_at | Sin3a | transcriptional regulator, SIN3A (yeast) | -1.02 | 0.828 | -1.09 | 0.489 | -1.07 | 0.636 | -1.17 | 0.25 | -1.09 |
| 1450811_at | Sprr2j | small proline-rich protein 2J | -1.03 | 0.704 | -1.04 | 0.906 | -1.12 | 0.536 | -1.3 | 0.496 | -1.12 |
| 1421261_at | Lipg | lipase, endothelial | -1.02 | 0.961 | -1.04 | 0.847 | -1.12 | 0.697 | -1.27 | 0.231 | -1.11 |
| 1455141_at | Tnrc6a | trinucleotide repeat containing 6a | -1.11 | 0.094 | -1.01 | 0.913 | -1.06 | 0.605 | -1.59 | 0.063 | -1.19 |
| 1447960_at | 9630017O17 | hypothetical protein 9630017O17 | -1.09 | 0.418 | -1.04 | 0.887 | -1.05 | 0.829 | -1.17 | 0.276 | -1.09 |
| 1448993_at | Atg3 | autophagy-related 3 (yeast) | -1.07 | 0.243 | -1.11 | 0.262 | -1 | 0.979 | -1.29 | 0.262 | -1.12 |
| 1426999_at | Zc3h14 | zinc finger CCCH type containing 14 | -1.06 | 0.529 | -1.08 | 0.081 | -1.04 | 0.81 | -1.11 | 0.439 | -1.07 |
| 1428874_at | 1110019N10Rik | RIKEN cDNA 1110019N10 gene | -1.05 | 0.835 | -1.13 | 0.312 | -1 | 0.98 | -1.11 | 0.507 | -1.07 |
| 1451220_at | 2310040A13Rik | RIKEN cDNA 2310040A13 gene | -1.08 | 0.575 | -1.09 | 0.258 | -1.01 | 0.835 | -1.34 | 0.095 | -1.13 |
| 1428645_at | Gnai3 | guanine nucleotide binding protein, alpha inhibiting 3 | -1.06 | 0.164 | -1.07 | 0.235 | -1.05 | 0.658 | -1.45 | 0.074 | -1.16 |
| 1417452_a_at | Fau | Finkel-Biskis-Reilly murine sarcoma virus (FBR-MuSV) ubiquitously expressed (fo | -1.04 | 0.703 | -1.03 | 0.756 | -1.11 | 0.417 | -1.46 | 0.052 | -1.16 |
| 1426749_at | Prmt3 | protein arginine N-methyltransferase 3 | -1.03 | 0.709 | -1.06 | 0.38 | -1.09 | 0.306 | -1.34 | 0.146 | -1.13 |
| 1418017_at | Pum2 | pumilio 2 (Drosophila) | -1.12 | 0.146 | -1.01 | 0.952 | -1.05 | 0.681 | -1.05 | 0.597 | -1.06 |
| 1451520_at | --- | --- | -1.02 | 0.869 | -1.09 | 0.394 | -1.07 | 0.836 | -1.32 | 0.435 | -1.12 |
| 1442830_at | Nusap1 | Nucleolar and spindle associated protein 1 | -1.02 | 0.893 | -1.06 | 0.937 | -1.1 | 0.801 | -1.97 | 0.171 | -1.29 |
| 1427449_a_at | Adprhl2 | ADP-ribosylhydrolase like 2 | -1.03 | 0.849 | -1.11 | 0.574 | -1.03 | 0.846 | -1.15 | 0.135 | -1.08 |
| 1455534_s_at | Osbpl11 | oxysterol binding protein-like 11 | -1.08 | 0.542 | -1.05 | 0.676 | -1.05 | 0.747 | -1.31 | 0.042 | -1.12 |
| 1417348_at | 2310039H08Rik | RIKEN cDNA 2310039H08 gene | -1.05 | 0.516 | -1.04 | 0.537 | -1.08 | 0.567 | -1.17 | 0.391 | -1.09 |
| 1452044_at | Arpc5l | actin related protein 2/3 complex, subunit 5-like | -1.01 | 0.924 | -1.13 | 0.087 | -1.04 | 0.715 | -1.81 | 0.104 | -1.25 |
| 1436016_x_at | Gdi2 | guanosine diphosphate (GDP) dissociation inhibitor 2 | -1.04 | 0.163 | -1 | 0.934 | -1.15 | 0.129 | -1.3 | 0.122 | -1.12 |
| 1449642_at | Phgdhl1 | Phosphoglycerate dehydrogenase like 1 | -1.06 | 0.708 | -1.03 | 0.936 | -1.08 | 0.837 | -1.3 | 0.529 | -1.12 |
| 1424541_at | Tmem70 | transmembrane protein 70 | -1.1 | 0.168 | -1.02 | 0.805 | -1.05 | 0.592 | -1.01 | 0.929 | -1.05 |
| 1448224_at | Tfam | transcription factor A, mitochondrial | -1.04 | 0.887 | -1.04 | 0.772 | -1.1 | 0.004 | -1.78 | 0.069 | -1.24 |
| 1417798_at | 1810019J16Rik | RIKEN cDNA 1810019J16 gene | -1.01 | 0.966 | -1.02 | 0.951 | -1.16 | 0.278 | -1.45 | 0.535 | -1.16 |

| | | | | | | | | | | | |
|---|---|---|---|---|---|---|---|---|---|---|---|
| 1436783_x_at | Ywhab | tyrosine 3-monooxygenase/tryptophan 5-monooxygenase activation protein, beta | -1.05 | 0.428 | -1 | 0.958 | -1.12 | 0.429 | -1.07 | 0.512 | -1.06 |
| 1442372_at | --- | Transcribed locus | -1.06 | 0.758 | -1.03 | 0.825 | -1.08 | 0.547 | -1.05 | 0.831 | -1.05 |
| 1436652_at | 5830418K08Rik | RIKEN cDNA 5830418K08 gene | -1.03 | 0.669 | -1.12 | 0.717 | -1.02 | 0.959 | -1.01 | 0.942 | -1.05 |
| 1426643_at | Elp3 | elongation protein 3 homolog (S. cerevisiae) | -1.11 | 0.13 | -1.02 | 0.701 | -1.04 | 0.525 | -1.28 | 0.04 | -1.11 |
| 1435865_at | Hist3h2a | histone 3, H2a | -1.15 | 0.463 | -1.02 | 0.882 | -1.01 | 0.866 | -1.06 | 0.711 | -1.06 |
| 1428526_at | Lsmd1 | LSM domain containing 1 | -1.1 | 0.582 | -1.01 | 0.884 | -1.06 | 0.634 | -1.03 | 0.87 | -1.05 |
| 1424018_at | Hint1 | histidine triad nucleotide binding protein 1 | -1.03 | 0.825 | -1.03 | 0.686 | -1.1 | 0.482 | -1.12 | 0.37 | -1.07 |
| 1450726_at | Asah2 | N-acylsphingosine amidohydrolase 2 | -1.03 | 0.777 | -1.06 | 0.593 | -1.08 | 0.626 | -1.04 | 0.775 | -1.05 |
| 1448240_at | Mbtps1 | membrane-bound transcription factor peptidase, site 1 | -1.07 | 0.351 | -1.04 | 0.318 | -1.06 | 0.66 | -1.35 | 0.046 | -1.13 |
| 1454964_at | BC021395 | cDNA sequence BC021395 | -1.1 | 0.413 | -1.01 | 0.908 | -1.06 | 0.641 | -1.04 | 0.894 | -1.05 |
| 1435085_at | Crebl2 | cAMP responsive element binding protein-like 2 | -1.04 | 0.867 | -1.08 | 0.646 | -1.05 | 0.622 | -1.23 | 0.501 | -1.1 |
| 1417032_at | Ube2g2 | ubiquitin-conjugating enzyme E2G 2 | -1.01 | 0.862 | -1.08 | 0.508 | -1.07 | 0.166 | -1.02 | 0.859 | -1.05 |
| 1426762_s_at | Aof2 | amine oxidase (flavin containing) domain 2 | -1.13 | 0.169 | -1.02 | 0.601 | -1.02 | 0.7 | -1.2 | 0.184 | -1.09 |
| 1426979_at | Mlxip | MLX interacting protein | -1.09 | 0.792 | -1.06 | 0.584 | -1.01 | 0.953 | -1.58 | 0.247 | -1.19 |
| 1448917_at | Thrap6 | thyroid hormone receptor associated protein 6 | -1.04 | 0.648 | -1.08 | 0.57 | -1.05 | 0.698 | -1.06 | 0.263 | -1.06 |
| 1425719_a_at | Nmi | N-myc (and STAT) interactor | -1.07 | 0.499 | -1.02 | 0.839 | -1.08 | 0.349 | -1.52 | 0.19 | -1.17 |
| 1417136_s_at | Srpk2 | serine/arginine-rich protein specific kinase 2 | -1.06 | 0.511 | -1.06 | 0.747 | -1.04 | 0.657 | -1.47 | 0.397 | -1.16 |
| 1432094_a_at | 1700034M03Rik | RIKEN cDNA 1700034M03 gene | -1.12 | 0.379 | -1 | 0.939 | -1.04 | 0.828 | -1.07 | 0.665 | -1.06 |
| 1423645_a_at | Ddx5 | DEAD (Asp-Glu-Ala-Asp) box polypeptide 5 | -1.07 | 0.782 | -1.01 | 0.908 | -1.08 | 0.353 | -1.33 | 0.253 | -1.12 |
| 1455058_at | Mtmr9 | myotubularin related protein 9 | -1.05 | 0.649 | -1.05 | 0.605 | -1.06 | 0.541 | -1.26 | 0.288 | -1.11 |
| 1420609_at | 7-Mar | membrane-associated ring finger (C3HC4) 7 | -1.08 | 0.741 | -1.08 | 0.336 | -1 | 0.989 | -1.37 | 0.21 | -1.13 |
| 1451184_at | Hnrpa3 /// LOC545 | heterogeneous nuclear ribonucleoprotein A3 /// similar to heterogeneous nuclear | -1.05 | 0.259 | -1.03 | 0.677 | -1.09 | 0.336 | -1.2 | 0.038 | -1.09 |
| 1453342_at | Cdc40 | cell division cycle 40 homolog (yeast) | -1.06 | 0.076 | -1.09 | 0.267 | -1.02 | 0.865 | -1.34 | 0.068 | -1.13 |
| 1417468_at | Nit1 | nitrilase 1 | -1.06 | 0.412 | -1.07 | 0.403 | -1.03 | 0.748 | -1.11 | 0.404 | -1.07 |
| 1428769_at | Tatdn3 | TatD DNase domain containing 3 | -1.12 | 0.268 | -1.02 | 0.899 | -1.02 | 0.875 | -1.03 | 0.88 | -1.05 |
| 1451025_at | Arl1 | ADP-ribosylation factor-like 1 | -1.08 | 0.225 | -1.03 | 0.424 | -1.05 | 0.485 | -1.31 | 0.16 | -1.12 |
| 1426481_at | Klhl22 | kelch-like 22 (Drosophila) | -1.1 | 0.022 | -1.02 | 0.803 | -1.05 | 0.595 | -1.06 | 0.571 | -1.05 |
| 1429429_s_at | Pcmtd1 | protein-L-isoaspartate (D-aspartate) O-methyltransferase domain containing 1 | -1 | 0.99 | -1.09 | 0.098 | -1.07 | 0.615 | -1.17 | 0.491 | -1.08 |
| 1438030_at | Rasgrp3 | RAS, guanyl releasing protein 3 | -1.07 | 0.607 | -1.03 | 0.904 | -1.06 | 0.847 | -1.5 | 0.294 | -1.16 |
| 1415727_at | Apoa1bp | apolipoprotein A-I binding protein | -1 | 0.996 | -1.08 | 0.61 | -1.08 | 0.492 | -1.14 | 0.573 | -1.08 |
| 1452023_at | Asb16 | ankyrin repeat and SOCS box-containing 16 | -1.02 | 0.839 | -1.04 | 0.927 | -1.1 | 0.634 | -1.54 | 0.202 | -1.17 |
| 1435153_at | Btbd6 | BTB (POZ) domain containing 6 | -1.12 | 0.358 | -1.01 | 0.936 | -1.04 | 0.682 | -1.18 | 0.519 | -1.09 |
| 1434680_at | Plekhg3 | pleckstrin homology domain containing, family G (with RhoGef domain) member : | -1.06 | 0.463 | -1.02 | 0.886 | -1.08 | 0.565 | -1.2 | 0.22 | -1.09 |
| 1455340_at | AI852444 | Expressed sequence AI852444 | -1.09 | 0.616 | -1.06 | 0.522 | -1.01 | 0.92 | -1.12 | 0.748 | -1.07 |
| 1437284_at | Fzd1 | frizzled homolog 1 (Drosophila) | -1.09 | 0.257 | -1.07 | 0.691 | -1 | 0.995 | -1.1 | 0.769 | -1.06 |
| 1448287_at | Rpo1-3 | RNA polymerase 1-3 | -1.12 | 0.444 | -1.02 | 0.716 | -1.02 | 0.907 | -1.15 | 0.468 | -1.08 |
| 1431766_x_at | Rps2 | ribosomal protein S2 | -1.1 | 0.218 | -1.03 | 0.859 | -1.03 | 0.486 | -1.2 | 0.003 | -1.09 |
| 1433475_a_at | C78339 | expressed sequence C78339 | -1.01 | 0.703 | -1.01 | 0.941 | -1.15 | 0.146 | -1.38 | 0.013 | -1.14 |
| 1453206_at | Acad9 | acyl-Coenzyme A dehydrogenase family, member 9 | -1.08 | 0.428 | -1.04 | 0.756 | -1.03 | 0.666 | -1.2 | 0.461 | -1.09 |
| 1431825_at | Srpk3 | serine/arginine-rich protein specific kinase 3 | -1.04 | 0.73 | -1.04 | 0.91 | -1.07 | 0.746 | -1.93 | 0.151 | -1.27 |
| 1423789_at | BC005624 | cDNA sequence BC005624 | -1.06 | 0.65 | -1.03 | 0.642 | -1.06 | 0.667 | -1.41 | 0.031 | -1.14 |
| 1455348_x_at | Rpl29 /// LOC4333 | ribosomal protein L29 /// similar to 60S ribosomal protein L29 /// similar to 60S rib | -1 | 0.968 | -1.01 | 0.87 | -1.15 | 0.063 | -1.31 | 0.044 | -1.12 |
| 1425459_at | Mtmr2 | myotubularin related protein 2 | -1.08 | 0.715 | -1.02 | 0.885 | -1.05 | 0.776 | -1.08 | 0.695 | -1.06 |
| 1428080_at | Pgam5 | phosphoglycerate mutase family member 5 | -1.04 | 0.686 | -1.04 | 0.48 | -1.06 | 0.607 | -1.14 | 0.294 | -1.07 |
| 1429596_at | Slc7a6os | solute carrier family 7, member 6 opposite strand | -1.03 | 0.848 | -1.03 | 0.805 | -1.09 | 0.687 | -1.72 | 0.04 | -1.22 |
| 1454922_at | AI553587 | expressed sequence AI553587 | -1.01 | 0.848 | -1.11 | 0.294 | -1.04 | 0.855 | -1.03 | 0.802 | -1.05 |
| 1424275_s_at | Trim41 | tripartite motif-containing 41 | -1.11 | 0.57 | -1.01 | 0.935 | -1.03 | 0.292 | -1.18 | 0.481 | -1.08 |
| 1421773_at | Etv2 | ets variant gene 2 | -1.03 | 0.934 | -1.1 | 0.801 | -1.02 | 0.92 | -1.81 | 0.298 | -1.24 |
| 1418695_a_at | Kcmf1 | potassium channel modulatory factor 1 | -1.02 | 0.817 | -1 | 0.858 | -1.13 | 0.054 | -1.04 | 0.48 | -1.05 |
| 1452971_at | Upf3a | UPF3 regulator of nonsense transcripts homolog A (yeast) | -1.02 | 0.929 | -1.1 | 0.706 | -1.03 | 0.867 | -1.14 | 0.269 | -1.07 |
| 1427022_at | Ddx42 | DEAD (Asp-Glu-Ala-Asp) box polypeptide 42 | -1.01 | 0.944 | -1.02 | 0.898 | -1.12 | 0.387 | -1.04 | 0.825 | -1.05 |
| 1448295_at | D13Wsu50e | DNA segment, Chr 13, Wayne State University 50, expressed | -1.06 | 0.332 | -1.01 | 0.945 | -1.08 | 0.025 | -1.37 | 0.123 | -1.13 |
| 1421810_at | Dgcr2 | DiGeorge syndrome critical region gene 2 | -1.15 | 0.264 | -1.01 | 0.936 | -1 | 0.994 | -1.21 | 0.292 | -1.09 |

| | | | | | | | | | | | |
|---|---|---|---|---|---|---|---|---|---|---|---|
| 1423838_s_at | 2400003C14Rik | RIKEN cDNA 2400003C14 gene | -1.01 | 0.728 | -1.02 | 0.755 | -1.12 | 0.161 | -1.32 | 0.203 | -1.12 |
| 1420829_a_at | Ywhaq | tyrosine 3-monooxygenase/tryptophan 5-monooxygenase activation protein, theta | -1.04 | 0.685 | -1.02 | 0.84 | -1.09 | 0.204 | -1.08 | 0.621 | -1.06 |
| 1427185_at | Mef2a | myocyte enhancer factor 2A | -1.05 | 0.791 | -1.02 | 0.659 | -1.07 | 0.703 | -1.31 | 0.292 | -1.11 |
| 1437852_x_at | Cpsf3 | cleavage and polyadenylation specificity factor 3 | -1.02 | 0.928 | -1.1 | 0.202 | -1.02 | 0.846 | -1.68 | 0.133 | -1.21 |
| 1424473_at | Polr2h | polymerase (RNA) II (DNA directed) polypeptide H | -1.09 | 0.626 | -1.01 | 0.952 | -1.05 | 0.817 | -1.03 | 0.896 | -1.04 |
| 1456538_at | Sdccag8 | serologically defined colon cancer antigen 8 | -1.01 | 0.933 | -1.06 | 0.534 | -1.08 | 0.568 | -1.5 | 0.088 | -1.16 |
| 1456748_a_at | Nipsnap1 | 4-nitrophenylphosphatase domain and non-neuronal SNAP25-like protein homolo | -1.01 | 0.454 | -1.13 | 0.377 | -1.01 | 0.875 | -1.24 | 0.039 | -1.1 |
| 1433823_at | AW456874 | expressed sequence AW456874 | -1.02 | 0.872 | -1 | 1 | -1.13 | 0.726 | -1.13 | 0.708 | -1.07 |
| 1453380_a_at | Xrcc6bp1 | XRCC6 binding protein 1 | -1.05 | 0.702 | -1.08 | 0.454 | -1.01 | 0.957 | -1.34 | 0.045 | -1.12 |
| 1437225_x_at | Gnai3 | guanine nucleotide binding protein, alpha inhibiting 3 | -1.13 | 0.619 | -1 | 0.976 | -1.02 | 0.871 | -1.34 | 0.039 | -1.12 |
| 1460547_a_at | Hnrpk | heterogeneous nuclear ribonucleoprotein K | -1.02 | 0.747 | -1.01 | 0.896 | -1.12 | 0.014 | -1.05 | 0.128 | -1.05 |
| 1448115_at | Htf9c | HpaII tiny fragments locus 9c | -1.02 | 0.381 | -1.01 | 0.926 | -1.12 | 0.498 | -1.18 | 0.086 | -1.08 |
| 1435230_at | Ankrd12 | ankyrin repeat domain 12 | -1.07 | 0.681 | -1.01 | 0.964 | -1.05 | 0.849 | -1.18 | 0.402 | -1.08 |
| 1441121_at | Pcid2 | PCI domain containing 2 | -1.01 | 0.985 | -1.01 | 0.902 | -1.13 | 0.505 | -1.1 | 0.562 | -1.06 |
| 1417174_at | 1810021J13Rik | RIKEN cDNA 1810021J13 gene | -1.01 | 0.893 | -1.03 | 0.795 | -1.09 | 0.626 | -1.46 | 0.168 | -1.15 |
| 1450816_at | Polg2 | polymerase (DNA directed), gamma 2, accessory subunit | -1.01 | 0.976 | -1.04 | 0.812 | -1.1 | 0.492 | -1.57 | 0.02 | -1.18 |
| 1460363_at | Tnrc6c | trinucleotide repeat containing 6C | -1.04 | 0.729 | -1 | 0.987 | -1.1 | 0.622 | -1.56 | 0.082 | -1.18 |
| 1448495_at | Tsta3 | tissue specific transplantation antigen P35B | -1.02 | 0.87 | -1.02 | 0.803 | -1.09 | 0.479 | -1.33 | 0.086 | -1.12 |
| 1426903_at | Fndc3a | fibronectin type III domain containing 3a | -1.01 | 0.952 | -1.05 | 0.569 | -1.08 | 0.559 | -1.43 | 0.383 | -1.14 |
| 1425323_a_at | BC008155 | cDNA sequence BC008155 | -1.02 | 0.666 | -1.02 | 0.73 | -1.1 | 0.375 | -1.29 | 0.129 | -1.11 |
| 1426347_at | 2010321M09Rik | RIKEN cDNA 2010321M09 gene | -1.03 | 0.87 | -1.08 | 0.155 | -1.03 | 0.708 | -1.15 | 0.178 | -1.07 |
| 1448345_at | Tomm34 | translocase of outer mitochondrial membrane 34 | -1.12 | 0.022 | -1 | 0.992 | -1.02 | 0.705 | -1.37 | 0.249 | -1.13 |
| 1451728_at | Wdr13 | WD repeat domain 13 | -1.08 | 0.427 | -1.01 | 0.908 | -1.04 | 0.795 | -1.14 | 0.546 | -1.07 |
| 1454998_at | 1200011I18Rik | RIKEN cDNA 1200011I18 gene | -1.06 | 0.568 | -1 | 0.999 | -1.07 | 0.645 | -1.07 | 0.496 | -1.05 |
| 1431704_a_at | Ralgps2 | Ral GEF with PH domain and SH3 binding motif 2 | -1.07 | 0.768 | -1.06 | 0.557 | -1 | 0.988 | -2.48 | 0.143 | -1.4 |
| 1429476_s_at | Dnaja2 | DnaJ (Hsp40) homolog, subfamily A, member 2 | -1.08 | 0.227 | -1.04 | 0.465 | -1.01 | 0.874 | -1.1 | 0.223 | -1.06 |
| 1452587_at | Actr2 | ARP2 actin-related protein 2 homolog (yeast) | -1.06 | 0.484 | -1.04 | 0.515 | -1.03 | 0.726 | -1.43 | 0.15 | -1.14 |
| 1420633_a_at | Csn1s2a | casein alpha s2-like A | -1 | 0.985 | -1.03 | 0.943 | -1.1 | 0.561 | -1.08 | 0.671 | -1.05 |
| 1439105_at | Cdadc1 | cytidine and dCMP deaminase domain containing 1 | -1.08 | 0.609 | -1.04 | 0.91 | -1.01 | 0.964 | -1.18 | 0.382 | -1.08 |
| 1437838_x_at | Grsf1 | G-rich RNA sequence binding factor 1 | -1.06 | 0.684 | -1.05 | 0.309 | -1.01 | 0.738 | -1.04 | 0.568 | -1.04 |
| 1424652_at | Tmem166 | transmembrane protein 166 | -1.1 | 0.549 | -1 | 0.992 | -1.03 | 0.843 | -1.18 | 0.761 | -1.08 |
| 1422184_a_at | Ak1 | adenylate kinase 1 | -1.06 | 0.818 | -1.03 | 0.901 | -1.03 | 0.846 | -1.59 | 0.053 | -1.18 |
| 1452084_at | Zcchc17 | zinc finger, CCHC domain containing 17 | -1.05 | 0.381 | -1.02 | 0.799 | -1.05 | 0.798 | -1.08 | 0.54 | -1.05 |
| 1429155_at | 4933411K20Rik | RIKEN cDNA 4933411K20 gene | -1 | 0.976 | -1.03 | 0.429 | -1.09 | 0.541 | -1.09 | 0.644 | -1.05 |
| 1448418_s_at | Wdr23 | WD repeat domain 23 | -1.06 | 0.5 | -1.06 | 0.611 | -1 | 0.966 | -1.27 | 0.01 | -1.1 |
| 1426700_a_at | Usp52 | ubiquitin specific peptidase 52 | -1.04 | 0.015 | -1.04 | 0.623 | -1.04 | 0.9 | -1.11 | 0.544 | -1.06 |
| 1425936_a_at | Ankmy2 | ankyrin repeat and MYND domain containing 2 | -1.01 | 0.977 | -1.06 | 0.867 | -1.05 | 0.737 | -1.21 | 0.309 | -1.08 |
| 1434082_at | --- | --- | -1.07 | 0.631 | -1.04 | 0.371 | -1 | 0.979 | -1.05 | 0.578 | -1.04 |
| 1455357_x_at | Tomm20 | translocase of outer mitochondrial membrane 20 homolog (yeast) | -1.01 | 0.939 | -1.01 | 0.818 | -1.1 | 0.52 | -1.32 | 0.232 | -1.11 |
| 1431112_at | Ulk3 | unc-51-like kinase 3 (C. elegans) | -1.01 | 0.979 | -1.09 | 0.697 | -1.01 | 0.944 | -1.18 | 0.2 | -1.08 |
| 1433476_at | C78339 | expressed sequence C78339 | -1 | 0.999 | -1.04 | 0.135 | -1.08 | 0.478 | -1.38 | 0.033 | -1.12 |
| 1423455_at | Ptma | prothymosin alpha | -1.02 | 0.878 | -1.02 | 0.816 | -1.07 | 0.417 | -1.07 | 0.013 | -1.05 |
| 1441276_at | Ptprk | protein tyrosine phosphatase, receptor type, K | -1.06 | 0.865 | -1.04 | 0.881 | -1.02 | 0.953 | -1.61 | 0.402 | -1.18 |
| 1419435_at | Aox1 | aldehyde oxidase 1 | -1.04 | 0.911 | -1.07 | 0.688 | -1.01 | 0.941 | -1.51 | 0.437 | -1.15 |
| 1426426_at | Rbm13 | RNA binding motif protein 13 | -1.02 | 0.533 | -1.03 | 0.779 | -1.06 | 0.635 | -1.25 | 0.125 | -1.09 |
| 1432430_a_at | 1700081L11Rik | RIKEN cDNA 1700081L11 gene | -1.03 | 0.947 | -1.02 | 0.95 | -1.06 | 0.89 | -1.44 | 0.025 | -1.14 |
| 1424753_at | Nudt14 | nudix (nucleoside diphosphate linked moiety X)-type motif 14 | -1.01 | 0.957 | -1.09 | 0.329 | -1.01 | 0.962 | -1.55 | 0.177 | -1.16 |
| 1437423_a_at | Sra1 | steroid receptor RNA activator 1 | -1.04 | 0.059 | -1.02 | 0.862 | -1.05 | 0.664 | -1.18 | 0.26 | -1.07 |
| 1423181_s_at | Clns1a | chloride channel, nucleotide-sensitive, 1A | -1.05 | 0.671 | -1.01 | 0.903 | -1.05 | 0.155 | -1.12 | 0.045 | -1.06 |
| 1436710_at | Zswim4 | zinc finger, SWIM domain containing 4 | -1.05 | 0.864 | -1.03 | 0.823 | -1.02 | 0.904 | -1.04 | 0.723 | -1.04 |
| 1423114_at | Ube2d3 | ubiquitin-conjugating enzyme E2D 3 (UBC4/5 homolog, yeast) | -1.04 | 0.697 | -1.06 | 0.533 | -1.01 | 0.959 | -1.08 | 0.292 | -1.05 |
| 1433040_at | 2900018E21Rik | RIKEN cDNA 2900018E21 gene | -1.01 | 0.971 | -1.04 | 0.852 | -1.05 | 0.756 | -1.01 | 0.963 | -1.03 |
| 1418583_at | Hint3 | histidine triad nucleotide binding protein 3 | -1.05 | 0.56 | -1.05 | 0.706 | -1.01 | 0.93 | -1.51 | 0.174 | -1.15 |

| | | | | | | | | | | | |
|---|---|---|---|---|---|---|---|---|---|---|---|
| 1447302_at | --- | Transcribed locus | -1.01 | 0.929 | -1.03 | 0.939 | -1.06 | 0.889 | -1.77 | 0.394 | -1.22 |
| 1425528_at | Prrx1 | paired related homeobox 1 | -1.05 | 0.875 | -1 | 0.993 | -1.05 | 0.86 | -1.46 | 0.34 | -1.14 |
| 1425674_a_at | Ssu72 | Ssu72 RNA polymerase II CTD phosphatase homolog (yeast) | -1.03 | 0.515 | -1.07 | 0.411 | -1 | 1 | -1.48 | 0.135 | -1.15 |
| 1458181_at | 9030624J02Rik | RIKEN cDNA 9030624J02 gene | -1.09 | 0.151 | -1 | 0.99 | -1.01 | 0.969 | -1.16 | 0.235 | -1.07 |
| 1454669_at | Tmem11 | transmembrane protein 11 | -1.04 | 0.735 | -1.05 | 0.513 | -1 | 0.988 | -1.13 | 0.315 | -1.06 |
| 1416440_at | Cd164 | CD164 antigen | -1.08 | 0.422 | -1.01 | 0.945 | -1.01 | 0.748 | -1.06 | 0.779 | -1.04 |
| 1452734_at | Rnaset2 | ribonuclease T2 | -1.05 | 0.445 | -1.04 | 0.696 | -1.01 | 0.898 | -1.37 | 0.149 | -1.12 |
| 1450988_at | Lgr5 | leucine rich repeat containing G protein coupled receptor 5 | -1.05 | 0.101 | -1.01 | 0.966 | -1.04 | 0.825 | -1.16 | 0.709 | -1.06 |
| 1422449_s_at | Rcn2 | reticulocalbin 2 | -1.06 | 0.572 | -1.03 | 0.684 | -1 | 0.973 | -1.42 | 0.143 | -1.13 |
| 1449138_at | Sf3b1 | splicing factor 3b, subunit 1 | -1.07 | 0.254 | -1.01 | 0.939 | -1.02 | 0.931 | -1.28 | 0.307 | -1.09 |
| 1455581_x_at | 9530028C05 | hypothetical protein 9530028C05 | -1.06 | 0.735 | -1.02 | 0.894 | -1.01 | 0.885 | -2.56 | 0.057 | -1.41 |
| 1452009_at | 9130422G05Rik | RIKEN cDNA 9130422G05 gene | -1 | 0.999 | -1.02 | 0.742 | -1.06 | 0.688 | -1.28 | 0.372 | -1.09 |
| 1419317_x_at | 2310002A05Rik /// | RIKEN cDNA 2310002A05 gene /// small proline rich-like 1 /// similar to late cornif | -1 | 0.998 | -1.07 | 0.621 | -1.01 | 0.906 | -2.2 | 0.47 | -1.32 |
| 1429690_at | 1300003B13Rik | RIKEN cDNA 1300003B13 gene | -1.06 | 0.69 | -1.01 | 0.964 | -1.01 | 0.933 | -1.99 | 0.144 | -1.27 |
| 1448353_x_at | Rpn1 | ribophorin I | -1.03 | 0.845 | -1.03 | 0.805 | -1.02 | 0.774 | -1.04 | 0.725 | -1.03 |
| 1423880_at | D10Wsu52e | DNA segment, Chr 10, Wayne State University 52, expressed | -1.02 | 0.821 | -1.06 | 0.307 | -1 | 0.977 | -1.65 | 0.057 | -1.18 |
| 1423132_a_at | 5730427N09Rik /// | RIKEN cDNA 5730427N09 gene /// similar to TGF beta-inducible nuclear protein | -1.07 | 0.688 | -1.01 | 0.852 | -1 | 0.987 | -1.97 | 0.108 | -1.26 |
| 1429311_at | Ube2q | ubiquitin-conjugating enzyme E2Q (putative) | -1.02 | 0.795 | -1.03 | 0.614 | -1.03 | 0.663 | -1.48 | 0.057 | -1.14 |
| 1430995_at | --- | --- | -1.06 | 0.775 | -1 | 0.994 | -1.02 | 0.922 | -1.26 | 0.141 | -1.09 |
| 1416466_at | Vapa | vesicle-associated membrane protein, associated protein A | -1.06 | 0.282 | -1 | 0.975 | -1.02 | 0.873 | -1.03 | 0.787 | -1.03 |
| 1451216_at | Zfp330 | zinc finger protein 330 | -1 | 0.965 | -1.06 | 0.57 | -1.02 | 0.849 | -1.33 | 0.079 | -1.1 |
| 1435223_at | Spfh2 | SPFH domain family, member 2 | -1.02 | 0.795 | -1.02 | 0.782 | -1.04 | 0.61 | -1.03 | 0.841 | -1.03 |
| 1416796_at | Nck2 | non-catalytic region of tyrosine kinase adaptor protein 2 | -1.01 | 0.889 | -1.04 | 0.836 | -1.03 | 0.752 | -1.29 | 0.116 | -1.09 |
| 1424139_at | Rap1a | RAS-related protein-1a | -1.04 | 0.746 | -1.03 | 0.559 | -1.01 | 0.93 | -1 | 0.962 | -1.02 |
| 1420321_at | Rsad1 | Radical S-adenosyl methionine domain containing 1 | -1.07 | 0.84 | -1 | 0.994 | -1.01 | 0.986 | -1.24 | 0.266 | -1.08 |
| 1435273_at | Wars2 | tryptophanyl tRNA synthetase 2 (mitochondrial) | -1.01 | 0.973 | -1.01 | 0.925 | -1.05 | 0.718 | -1.05 | 0.662 | -1.03 |
| 1433542_at | Inpp5f | inositol polyphosphate-5-phosphatase F | -1.01 | 0.869 | -1.04 | 0.692 | -1.02 | 0.771 | -2 | 0.027 | -1.27 |
| 1436956_at | Bace1 | Beta-site APP cleaving enzyme 1 | -1.02 | 0.672 | -1.04 | 0.69 | -1.01 | 0.95 | -1.08 | 0.494 | -1.04 |
| 1415737_at | Rfk | riboflavin kinase | -1.02 | 0.745 | -1.02 | 0.718 | -1.03 | 0.738 | -1.01 | 0.969 | -1.02 |
| 1415796_at | Dazap2 | DAZ associated protein 2 | -1.02 | 0.783 | -1.02 | 0.776 | -1.01 | 0.885 | -1.29 | 0.071 | -1.09 |
| 1451036_at | Spg21 | spastic paraplegia 21 homolog (human) | -1.01 | 0.758 | -1.02 | 0.856 | -1.01 | 0.941 | -1.27 | 0.408 | -1.08 |
| 1455435_s_at | --- | --- | -1 | 0.995 | -1.03 | 0.562 | -1 | 0.997 | -1.37 | 0.199 | -1.1 |
| 1423262_a_at | H3f3a /// LOC6233 | H3 histone, family 3A /// similar to H3 histone, family 3B /// similar to H3 histone, f | -1.01 | 0.946 | -1 | 0.886 | -1.02 | 0.822 | -1.03 | 0.834 | -1.02 |
| 1416094_at | Adam9 | a disintegrin and metallopeptidase domain 9 (meltrin gamma) | -1.01 | 0.95 | -1.01 | 0.778 | -1 | 0.997 | -1.22 | 0.248 | -1.06 |
| 1437952_at | AU040096 | expressed sequence AU040096 | -3.88 | 0.026 | -2.67 | 0.016 | -2.28 | 0.229 | 1.21 | 0.676 | -1.9 |
| 1441825_x_at | Ighmbp2 | Immunoglobulin mu binding protein 2 | -2.78 | 0.041 | -3.42 | 0.02 | -2.36 | 0.079 | 1.32 | 0.594 | -1.81 |
| 1444636_at | 4930592I03Rik | RIKEN cDNA 4930592I03 gene | -3.53 | 0.386 | -3.32 | 0.354 | -1.92 | 0.098 | 1.44 | 0.46 | -1.83 |
| 1454342_at | C030007D22Rik | RIKEN cDNA C030007D22 gene | -3.06 | 0.064 | -3.29 | 0.111 | -2.08 | 0.142 | 1.56 | 0.489 | -1.72 |
| 1458434_at | A730009L09Rik | RIKEN cDNA A730009L09 gene | -2.39 | 0.347 | -2.49 | 0.246 | -3.36 | 0.094 | 1.26 | 0.348 | -1.75 |
| 1421554_at | Lmx1a | LIM homeobox transcription factor 1 alpha | -2.52 | 0.339 | -3.31 | 0.365 | -2.08 | 0.2 | 2.04 | 0.214 | -1.47 |
| 1432648_at | 4930466F19Rik | RIKEN cDNA 4930466F19 gene | -4.79 | 0.161 | -1.85 | 0.233 | -2.26 | 0.29 | 1.29 | 0.745 | -1.9 |
| 1446030_at | --- | Transcribed locus | -1.98 | 0.162 | -2.62 | 0.019 | -2.92 | 0.001 | 3.54 | 0 | -1 |
| 1459633_at | --- | --- | -2.29 | 0.394 | -3.99 | 0.072 | -1.81 | 0.229 | 2.03 | 0.255 | -1.51 |
| 1433153_at | 9030201C23Rik | RIKEN cDNA 9030201C23 gene | -3.07 | 0.034 | -1.59 | 0.16 | -3.44 | 0.009 | 1.2 | 0.794 | -1.72 |
| 1450839_at | D0H4S114 | DNA segment, human D4S114 | -1.16 | 0.748 | -4.7 | 0.021 | -5.42 | 0.005 | 1.06 | 0.729 | -2.56 |
| 1440705_at | AU021720 | expressed sequence AU021720 | -3.05 | 0.029 | -4.24 | 0.009 | -1.44 | 0.499 | 1.73 | 0.464 | -1.75 |
| 1445454_at | D2Ertd282e | DNA segment, Chr 2, ERATO Doi 282, expressed | -2.22 | 0.107 | -2.94 | 0.17 | -2.14 | 0.114 | 1.68 | 0.147 | -1.4 |
| 1432680_at | 9130009M17Rik | RIKEN cDNA 9130009M17 gene | -2.48 | 0.008 | -2 | 0.336 | -2.78 | 0.064 | 2.83 | 0.339 | -1.11 |
| 1457261_at | A930025H08Rik | RIKEN cDNA A930025H08 gene | -2.49 | 0.413 | -2.52 | 0.046 | -2.07 | 0.226 | 3.84 | 0.002 | -0.81 |
| 1458612_at | A930006A01Rik | RIKEN cDNA A930006A01 gene | -4.1 | 0.009 | -2.92 | 0.021 | -1.4 | 0.493 | 1.23 | 0.566 | -1.8 |
| 1445496_at | Auh | AU RNA binding protein/enoyl-coenzyme A hydratase | -2.12 | 0.015 | -2.36 | 0.031 | -2.39 | 0.032 | 1.19 | 0.254 | -1.42 |
| 1457104_at | D930036F22Rik | RIKEN cDNA D930036F22 gene | -2.66 | 0.154 | -2.38 | 0.028 | -1.92 | 0.149 | 1.04 | 0.918 | -1.48 |
| 1422328_at | Gja10 | gap junction membrane channel protein alpha 10 | -3.43 | 0.01 | -1.6 | 0.507 | -2.38 | 0.12 | 1.65 | 0.165 | -1.44 |

| | | | | | | | | | | | |
|---|---|---|---|---|---|---|---|---|---|---|---|
| 1454228_a_at | Ccdc77 | coiled-coil domain containing 77 | -2.51 | 0.009 | -3.06 | 0.137 | -1.63 | 0.485 | 1.31 | 0.441 | -1.47 |
| 1441012_at | Hip2 | Huntingtin interacting protein 2 | -4.03 | 0.092 | -2.13 | 0.519 | -1.6 | 0.197 | 2.54 | 0.326 | -1.3 |
| 1453503_at | 9230117E20Rik | RIKEN cDNA 9230117E20 gene | -3.09 | 0.052 | -2.84 | 0.097 | -1.48 | 0.149 | 1.14 | 0.572 | -1.57 |
| 1417899_at | Zp3r | zona pellucida 3 receptor | -2.52 | 0.065 | -4.09 | 0.087 | -1.4 | 0.43 | 3.65 | 0.004 | -1.09 |
| 1442589_at | Trpc5 | Transient receptor potential cation channel, subfamily C, member 5 | -3.2 | 0.096 | -3.74 | 0.024 | -1.28 | 0.474 | 1.38 | 0.419 | -1.71 |
| 1458699_at | Ccrn4l | CCR4 carbon catabolite repression 4-like (S. cerevisiae) | -2.98 | 0.38 | -2 | 0.221 | -1.89 | 0.123 | 4.28 | 0.168 | -0.65 |
| 1442097_at | --- | 0 day neonate kidney cDNA, RIKEN full-length enriched library, clone:D630011L( | -2.29 | 0.128 | -3.07 | 0.002 | -1.65 | 0.5 | 1.15 | 0.814 | -1.46 |
| 1446492_at | Tmem132d | transmembrane protein 132D | -3.38 | 0.095 | -1.86 | 0.344 | -1.86 | 0.03 | 1.29 | 0.211 | -1.45 |
| 1421984_at | Stc1 | stanniocalcin 1 | -2.07 | 0.102 | -2.55 | 0.012 | -1.99 | 0.101 | 3.82 | 0.008 | -0.7 |
| 1444327_at | Edd1 | E3 ubiquitin protein ligase, HECT domain containing, 1 | -2.41 | 0.43 | -3.91 | 0.001 | -1.4 | 0.278 | 1.27 | 0.712 | -1.61 |
| 1430973_at | Zfp558 | zinc finger protein 558 | -3.82 | 0.002 | -1.66 | 0.331 | -1.89 | 0.246 | 1.37 | 0.664 | -1.5 |
| 1453935_a_at | Ccdc7 | coiled-coil domain containing 7 | -2.07 | 0.156 | -2.1 | 0.413 | -2.32 | 0.017 | 2.02 | 0.121 | -1.12 |
| 1432980_at | 5730420F10Rik | RIKEN cDNA 5730420F10 gene | -1.39 | 0.526 | -2.37 | 0.242 | -3.97 | 0.055 | 2.22 | 0.265 | -1.38 |
| 1451898_a_at | Sema6c | sema domain, transmembrane domain (TM), and cytoplasmic domain, (semapho | -4.47 | 0.002 | -1.58 | 0.233 | -1.87 | 0.169 | 2.39 | 0.064 | -1.38 |
| 1449493_at | Insl5 | insulin-like 5 | -1.96 | 0.126 | -2.49 | 0.004 | -2.05 | 0.395 | 1.29 | 0.58 | -1.3 |
| 1447769_x_at | Amigo2 | adhesion molecule with Ig like domain 2 | -1.37 | 0.282 | -1.89 | 0.348 | -7.1 | 0.067 | 1.12 | 0.759 | -2.31 |
| 1419229_at | Rhox4b /// Rhox4e | reproductive homeobox 4B /// reproductive homeobox 4E /// reproductive homeob | -3.61 | 0.054 | -2.39 | 0.039 | -1.41 | 0.119 | 1.15 | 0.796 | -1.56 |
| 1433276_at | 4930556A12Rik | RIKEN cDNA 4930556A12 gene | -4.14 | 0.341 | -2.09 | 0.099 | -1.44 | 0.461 | 1.06 | 0.904 | -1.65 |
| 1421591_at | Cylc1 | cylicin, basic protein of sperm head cytoskeleton 1 | -2.27 | 0.471 | -2.19 | 0.178 | -1.93 | 0.297 | 1.38 | 0.567 | -1.25 |
| 1433346_at | 5830432F11Rik | RIKEN cDNA 5830432F11 gene | -2.24 | 0.449 | -1.87 | 0.067 | -2.26 | 0.388 | 1.72 | 0.23 | -1.16 |
| 1438909_at | Sccpdh | saccharopine dehydrogenase (putative) | -1.79 | 0.269 | -2.44 | 0.171 | -2.19 | 0.037 | 1.78 | 0.101 | -1.16 |
| 1422248_at | Irs4 /// LOC669982 | insulin receptor substrate 4 /// similar to insulin receptor substrate 4 | -2.35 | 0.344 | -2.05 | 0.088 | -1.95 | 0.172 | 2.78 | 0.375 | -0.89 |
| 1439720_at | Ralgps1 | Ral GEF with PH domain and SH3 binding motif 1 | -3.83 | 0.393 | -2.39 | 0.2 | -1.33 | 0.563 | 1.56 | 0.373 | -1.5 |
| 1454190_at | 9630013K17Rik | RIKEN cDNA 9630013K17 gene | -1.66 | 0.055 | -2.79 | 0.026 | -2.13 | 0.258 | 1.03 | 0.943 | -1.39 |
| 1460368_at | Mpp4 | membrane protein, palmitoylated 4 (MAGUK p55 subfamily member 4) | -2.42 | 0.171 | -2.2 | 0.187 | -1.77 | 0.389 | 1.84 | 0.282 | -1.14 |
| 1442559_at | --- | 10 days neonate cerebellum cDNA, RIKEN full-length enriched library, clone:B93 | -2.78 | 0.26 | -1.4 | 0.299 | -2.78 | 0 | 1.12 | 0.828 | -1.46 |
| 1431373_at | Pop5 | processing of precursor 5, ribonuclease P/MRP family (S. cerevisiae) | -5.01 | 0.005 | -1.57 | 0.608 | -1.67 | 0.172 | 2.73 | 0.287 | -1.38 |
| 1436643_x_at | Hamp2 | hepcidin antimicrobial peptide 2 | -2.77 | 0.027 | -2.13 | 0.321 | -1.65 | 0.034 | 16.19 | 0.142 | 2.41 |
| 1445262_at | --- | --- | -2.97 | 0.064 | -1.45 | 0.572 | -2.43 | 0.03 | 1.64 | 0.573 | -1.3 |
| 1442565_at | Pcdh10 | protocadherin 10 | -1.97 | 0.312 | -2.25 | 0.113 | -2.03 | 0.298 | 2.56 | 0.223 | -0.92 |
| 1417746_at | Cplx1 | complexin 1 | -1.34 | 0.532 | -4.87 | 0.001 | -2 | 0.392 | 1.09 | 0.726 | -1.78 |
| 1439409_x_at | Tyrp1 | tyrosinase-related protein 1 | -1.44 | 0.316 | -3.14 | 0.035 | -2.28 | 0.267 | 1.27 | 0.336 | -1.4 |
| 1419904_at | --- | Adult male corpora quadrigemina cDNA, RIKEN full-length enriched library, clone | -2.07 | 0.24 | -1.93 | 0.174 | -2.18 | 0.016 | 1.9 | 0.13 | -1.07 |
| 1447044_at | --- | --- | -2.2 | 0.123 | -2.11 | 0.38 | -1.87 | 0.371 | 2.25 | 0.345 | -0.98 |
| 1440118_at | D11Ertd333e | DNA segment, Chr 11, ERATO Doi 333, expressed | -3.82 | 0.443 | -2.72 | 0.138 | -1.2 | 0.134 | 1.2 | 0.524 | -1.63 |
| 1459081_at | 1700007B14Rik | RIKEN cDNA 1700007B14 gene | -3.23 | 0.068 | -3.54 | 0.024 | -1.15 | 0.764 | 2.49 | 0.294 | -1.36 |
| 1431438_at | C530001K22Rik | RIKEN cDNA C530001K22 gene | -1.72 | 0.191 | -2.52 | 0.057 | -2.05 | 0.231 | 1.18 | 0.281 | -1.28 |
| 1440232_at | LOC622645 /// LOC | similar to KIAA1913 /// similar to KIAA1913 | -3.39 | 0.014 | -1.82 | 0.072 | -1.6 | 0.226 | 1.64 | 0.041 | -1.29 |
| 1446263_at | AU015619 | expressed sequence AU015619 | -2.77 | 0.38 | -1.29 | 0.66 | -2.91 | 0.151 | 1.27 | 0.61 | -1.43 |
| 1460470_at | Acoxl | acyl-Coenzyme A oxidase-like | -1.56 | 0.569 | -2.68 | 0.031 | -2.15 | 0.005 | 1.07 | 0.918 | -1.33 |
| 1446686_at | Dnajc11 | DnaJ (Hsp40) homolog, subfamily C, member 11 | -1.75 | 0.374 | -2.45 | 0.161 | -1.98 | 0.074 | 1.54 | 0.339 | -1.16 |
| 1454308_at | LOC385154 /// LOC | similar to development- and differentiation-enhancing factor 2; PYK2 C terminus-a | -3.63 | 0.136 | -1.19 | 0.627 | -2.7 | 0.048 | 1.32 | 0.411 | -1.55 |
| 1431581_at | 4922502B01Rik | RIKEN cDNA 4922502B01 gene | -1.59 | 0.12 | -2.7 | 0.08 | -2.03 | 0.279 | 1.92 | 0.069 | -1.1 |
| 1428055_at | Rian | RNA imprinted and accumulated in nucleus | -1.85 | 0.504 | -4.59 | 0.145 | -1.35 | 0.627 | 1.51 | 0.615 | -1.57 |
| 1442081_at | AU017455 | Expressed sequence AU017455 | -2.27 | 0.116 | -2.04 | 0.239 | -1.74 | 0.201 | 2.03 | 0.097 | -1 |
| 1456268_at | Cks1b | CDC28 protein kinase 1b | -2.68 | 0.054 | -1.39 | 0.419 | -2.42 | 0.225 | 1.12 | 0.776 | -1.34 |
| 1429997_at | 4832441B07Rik | RIKEN cDNA 4832441B07 gene | -2.1 | 0.101 | -1.96 | 0.479 | -1.93 | 0.042 | 1.48 | 0.507 | -1.13 |
| 1420137_at | --- | --- | -2.56 | 0.17 | -2.14 | 0.047 | -1.53 | 0.365 | 2.04 | 0.249 | -1.05 |
| 1420759_s_at | Zfy1 /// Zfy2 | zinc finger protein 1, Y linked /// zinc finger protein 2, Y linked | -2.35 | 0.488 | -2.72 | 0.205 | -1.39 | 0.275 | 1.18 | 0.631 | -1.32 |
| 1442091_at | --- | Transcribed locus | -3.53 | 0.028 | -1.42 | 0.467 | -1.9 | 0.032 | 1.36 | 0.424 | -1.37 |
| 1442318_at | --- | Transcribed locus | -2.39 | 0.293 | -2.73 | 0.085 | -1.37 | 0.381 | 1.35 | 0.62 | -1.28 |
| 1439854_at | Hrk | harakiri, BCL2 interacting protein (contains only BH3 domain) | -2.6 | 0.473 | -1.9 | 0.143 | -1.65 | 0.415 | 1.85 | 0.521 | -1.07 |
| 1449703_at | Zfand2a | Zinc finger, AN1-type domain 2A | -1.93 | 0.512 | -1.54 | 0.471 | -2.85 | 0.138 | 2.08 | 0.032 | -1.06 |

| | | | | | | | | | | | |
|---|---|---|---|---|---|---|---|---|---|---|---|
| 1433323_at | 4930529F21Rik | RIKEN cDNA 4930529F21 gene | -1.48 | 0.108 | -3.04 | 0.166 | -1.94 | 0.164 | 1.45 | 0.46 | -1.25 |
| 1453637_at | 5430410E06Rik | RIKEN cDNA 5430410E06 gene | -1.74 | 0.255 | -2.53 | 0.048 | -1.79 | 0.183 | 1.7 | 0.267 | -1.09 |
| 1440949_at | --- | --- | -2.51 | 0.2 | -1.85 | 0.161 | -1.69 | 0.088 | 1.02 | 0.974 | -1.26 |
| 1442600_at | --- | --- | -2.81 | 0.069 | -2.19 | 0.125 | -1.39 | 0.33 | 1.07 | 0.615 | -1.33 |
| 1458687_at | LOC668172 /// LOC | hypothetical protein LOC668172 /// hypothetical protein LOC672498 /// hypothetic | -3.63 | 0.299 | -1.84 | 0.406 | -1.4 | 0.347 | 1.05 | 0.871 | -1.46 |
| 1454600_at | 9530086P17Rik | RIKEN cDNA 9530086P17 gene | -3.06 | 0.026 | -2.71 | 0.18 | -1.19 | 0.665 | 1.18 | 0.776 | -1.44 |
| 1441602_at | 1110005A03Rik | RIKEN cDNA 1110005A03 gene | -4.24 | 0.022 | -3.05 | 0.187 | -1.03 | 0.943 | 1.13 | 0.841 | -1.8 |
| 1446009_at | LOC240444 | Similar to Potassium voltage-gated channel subfamily G member 2 (Voltage-gate | -2.58 | 0.163 | -1.91 | 0.028 | -1.59 | 0.334 | 1.27 | 0.423 | -1.2 |
| 1432175_at | 5430434I15Rik | RIKEN cDNA 5430434I15 gene | -1.43 | 0.467 | -3.21 | 0.006 | -1.89 | 0.133 | 1.83 | 0.028 | -1.17 |
| 1432364_at | 4930556N13Rik | RIKEN cDNA 4930556N13 gene | -1.35 | 0.75 | -2.2 | 0.002 | -2.84 | 0.148 | 1.36 | 0.642 | -1.26 |
| 1444964_at | Csnk1g1 | casein kinase 1, gamma 1 | -5.44 | 0.054 | -1.24 | 0.738 | -1.79 | 0.043 | 1.55 | 0.157 | -1.73 |
| 1444957_at | Pspc1 | Paraspeckle protein 1 | -3.47 | 0.123 | -1.08 | 0.908 | -2.95 | 0.077 | 1.3 | 0.618 | -1.55 |
| 1445851_at | --- | --- | -2.23 | 0.092 | -1.88 | 0.103 | -1.75 | 0.258 | 1.27 | 0.679 | -1.15 |
| 1456982_at | 6430562O15Rik | RIKEN cDNA 6430562O15 gene | -2.01 | 0.219 | -1.5 | 0.031 | -2.53 | 0.085 | 1.08 | 0.896 | -1.24 |
| 1444146_at | Rnf20 | ring finger protein 20 | -2.26 | 0.182 | -2.42 | 0.005 | -1.42 | 0.503 | 1.1 | 0.75 | -1.25 |
| 1437011_x_at | Oaz3 | ornithine decarboxylase antizyme 3 | -1.7 | 0.446 | -2.27 | 0.174 | -1.88 | 0.271 | 1.71 | 0.513 | -1.03 |
| 1457778_at | Stox2 | storkhead box 2 | -2.68 | 0.062 | -2.16 | 0.302 | -1.38 | 0.607 | 1.01 | 0.989 | -1.3 |
| 1431483_at | 4930544M13Rik | RIKEN cDNA 4930544M13 gene | -1.72 | 0.574 | -1.7 | 0.351 | -2.54 | 0.273 | 1.41 | 0.614 | -1.14 |
| 1435215_at | Ap1gbp1 | AP1 gamma subunit binding protein 1 | -4.08 | 0.067 | -1.54 | 0.396 | -1.49 | 0.388 | 1.46 | 0.331 | -1.41 |
| 1451808_at | Kcnj4 | potassium inwardly-rectifying channel, subfamily J, member 4 | -3.12 | 0.35 | -1.6 | 0.472 | -1.6 | 0.075 | 1.56 | 0.435 | -1.19 |
| 1442345_at | 1700031L13Rik | RIKEN cDNA 1700031L13 gene | -2.07 | 0.531 | -2.01 | 0.431 | -1.7 | 0.371 | 1.26 | 0.692 | -1.13 |
| 1440749_at | Map3k13 | Mitogen-activated protein kinase kinase kinase 13 | -2.44 | 0.085 | -1.96 | 0.019 | -1.53 | 0.428 | 1.12 | 0.876 | -1.2 |
| 1429388_at | Nanog | Nanog homeobox | -1.51 | 0.562 | -1.72 | 0.231 | -3.01 | 0 | 1.06 | 0.758 | -1.29 |
| 1419411_at | Tac2 | tachykinin 2 | -2.12 | 0.358 | -1.59 | 0.071 | -2.08 | 0.24 | 11.54 | 0.237 | 1.43 |
| 1456725_x_at | Vil2 | villin 2 | -6.99 | 0.019 | -1.65 | 0.147 | -1.2 | 0.579 | 2.07 | 0.077 | -1.94 |
| 1430760_a_at | Usp50 | ubiquitin specific peptidase 50 | -2.42 | 0.046 | -2.12 | 0.172 | -1.43 | 0.538 | 1.47 | 0.598 | -1.13 |
| 1420805_at | Mylc2pl | myosin light chain 2, precursor lymphocyte-specific | -3.46 | 0.407 | -1.09 | 0.486 | -2.68 | 0.015 | 1.65 | 0.101 | -1.39 |
| 1445754_at | Acacb | Acetyl-Coenzyme A carboxylase beta | -1.45 | 0.08 | -1.89 | 0.149 | -2.73 | 0.044 | 1.35 | 0.512 | -1.18 |
| 1432702_at | 2810403G07Rik | RIKEN cDNA 2810403G07 gene | -2.76 | 0.078 | -1.52 | 0.507 | -1.76 | 0.127 | 1.32 | 0.592 | -1.18 |
| 1428525_at | 4930488B01Rik | RIKEN cDNA 4930488B01 gene | -4.63 | 0.004 | -1.11 | 0.734 | -2.11 | 0.156 | 1.18 | 0.803 | -1.67 |
| 1440109_at | D7Ertd413e | DNA segment, Chr 7, ERATO Doi 413, expressed | -1.43 | 0.642 | -3.41 | 0.071 | -1.67 | 0.24 | 1.71 | 0.202 | -1.2 |
| 1449540_at | Rhox9 | reproductive homeobox 9 | -3.32 | 0.023 | -1.44 | 0.24 | -1.69 | 0.301 | 1.03 | 0.958 | -1.35 |
| 1451772_at | Tpcn1 | two pore channel 1 | -3.08 | 0.271 | -1.76 | 0.444 | -1.42 | 0.378 | 2.5 | 0.036 | -0.94 |
| 1449873_at | Bmp8a | bone morphogenetic protein 8a | -1.45 | 0.29 | -1.6 | 0.597 | -3.6 | 0.099 | 1.07 | 0.93 | -1.39 |
| 1446777_at | Spp2 | Secreted phosphoprotein 2 | -2.24 | 0.313 | -1.96 | 0.309 | -1.55 | 0.231 | 2.8 | 0.001 | -0.74 |
| 1437081_at | Timp2 | Tissue inhibitor of metalloproteinase 2 | -1.78 | 0.26 | -2.61 | 0.361 | -1.52 | 0.325 | 2.04 | 0.012 | -0.97 |
| 1432979_at | Gatad2b | GATA zinc finger domain containing 2B | -2.48 | 0.457 | -2.6 | 0.112 | -1.23 | 0.748 | 1.51 | 0.61 | -1.2 |
| 1460059_at | Upp2 | uridine phosphorylase 2 | -1.36 | 0.018 | -2.46 | 0.27 | -2.17 | 0.288 | 1.16 | 0.088 | -1.21 |
| 1447482_at | 5830457H20Rik | RIKEN cDNA 5830457H20 gene | -2.23 | 0.046 | -2.63 | 0.139 | -1.29 | 0.615 | 1.06 | 0.926 | -1.27 |
| 1446401_at | Atrnl1 | Attractin like 1 | -2 | 0.459 | -1.77 | 0.175 | -1.85 | 0.42 | 2.78 | 0.037 | -0.71 |
| 1460504_at | --- | --- | -2.19 | 0.463 | -1.6 | 0.523 | -1.91 | 0.161 | 1.12 | 0.732 | -1.15 |
| 1447607_at | --- | --- | -2.41 | 0.228 | -1.57 | 0.163 | -1.79 | 0.086 | 1.39 | 0.373 | -1.1 |
| 1439756_at | 4933406J04Rik | RIKEN cDNA 4933406J04 gene | -1.71 | 0.425 | -1.79 | 0.421 | -2.16 | 0.217 | 1.72 | 0.377 | -0.98 |
| 1446629_at | C85938 | expressed sequence C85938 | -1.29 | 0.63 | -2.52 | 0.181 | -2.27 | 0.068 | 1.5 | 0.629 | -1.14 |
| 1441000_at | --- | PREDICTED: Mus musculus similar to T-cell immunoglobulin and mucin domain | -2.06 | 0.158 | -1.81 | 0.227 | -1.72 | 0.181 | 1.03 | 0.843 | -1.14 |
| 1442670_at | 6430517E21Rik | RIKEN cDNA 6430517E21 gene | -2.09 | 0.033 | -3.82 | 0.262 | -1.14 | 0.815 | 1.73 | 0.25 | -1.33 |
| 1420955_at | Vsnl1 | visinin-like 1 | -1.73 | 0.472 | -1.47 | 0.05 | -2.74 | 0.012 | 1.55 | 0.195 | -1.1 |
| 1450352_at | Mtnr1a | melatonin receptor 1A | -2.13 | 0.45 | -1.98 | 0.237 | -1.53 | 0.343 | 1.46 | 0.527 | -1.05 |
| 1443548_at | Ank2 | Ankyrin 2, brain | -2.1 | 0.138 | -1.81 | 0.415 | -1.67 | 0.018 | 1.51 | 0.526 | -1.02 |
| 1420145_at | --- | Transcribed locus | -2.92 | 0.106 | -1.51 | 0.254 | -1.6 | 0.097 | 1.04 | 0.969 | -1.25 |
| 1430240_a_at | Clgn | calmegin | -3.03 | 0.013 | -2.11 | 0.314 | -1.21 | 0.201 | 1.68 | 0.535 | -1.17 |
| 1457103_at | AU046084 | expressed sequence AU046084 | -1.87 | 0.351 | -1.7 | 0.194 | -1.97 | 0.193 | 1.41 | 0.219 | -1.03 |
| 1460038_at | Pou3f1 | POU domain, class 3, transcription factor 1 | -1.82 | 0.278 | -3.27 | 0.022 | -1.29 | 0.717 | 1.45 | 0.485 | -1.23 |

| | | | | | | | | | | | | |
|---|---|---|---|---|---|---|---|---|---|---|---|---|
| 1459183_at | --- | PREDICTED: Mus musculus similar to C05G5.5 (LOC230628), mRNA | -1.41 | 0.38 | -2.43 | 0.109 | -1.97 | 0.032 | 1.14 | 0.666 | -1.16 |
| 1443364_at | --- | Transcribed locus | -1.19 | 0.011 | -2.35 | 0.158 | -2.71 | 0.013 | 1.41 | 0.235 | -1.21 |
| 1459565_at | Uty | ubiquitously transcribed tetratricopeptide repeat gene, Y chromosome | -3.21 | 0.008 | -1.76 | 0.245 | -1.33 | 0.065 | 1.65 | 0.415 | -1.16 |
| 1441777_at | Emx1 | empty spiracles homolog 1 (Drosophila) | -1.43 | 0.027 | -1.8 | 0.147 | -2.64 | 0.116 | 1.17 | 0.785 | -1.18 |
| 1445472_at | 2210412E05Rik /// | RIKEN cDNA 2210412E05 gene /// similar to Carcinoembryonic antigen-related c | -3.69 | 0.005 | -1.9 | 0.135 | -1.19 | 0.641 | 1.01 | 0.985 | -1.44 |
| 1441961_at | Mtap9 | microtubule-associated protein 9 | -1.92 | 0.252 | -1.95 | 0.096 | -1.65 | 0.205 | 1.66 | 0.067 | -0.97 |
| 1442515_at | --- | --- | -2.54 | 0.234 | -1.67 | 0.438 | -1.55 | 0.368 | 1.29 | 0.501 | -1.12 |
| 1441421_at | C530030P08Rik | RIKEN cDNA C530030P08 gene | -1.97 | 0.421 | -1.72 | 0.331 | -1.83 | 0.078 | 1.74 | 0.495 | -0.94 |
| 1419958_at | C330003B14Rik | RIKEN cDNA C330003B14 gene | -3.01 | 0.295 | -1.84 | 0.494 | -1.31 | 0.4 | 1.04 | 0.915 | -1.28 |
| 1420432_at | Tas1r1 | taste receptor, type 1, member 1 | -1.47 | 0.241 | -3.26 | 0.284 | -1.52 | 0.355 | 1.39 | 0.384 | -1.22 |
| 1454508_at | 4930485E13Rik | RIKEN cDNA 4930485E13 gene | -2.74 | 0.102 | -1.35 | 0.646 | -1.87 | 0.246 | 1.15 | 0.774 | -1.2 |
| 1441628_at | Diap3 | Diaphanous homolog 3 (Drosophila) | -3.56 | 0.05 | -1.64 | 0.476 | -1.33 | 0.491 | 3.57 | 0.197 | -0.74 |
| 1454293_at | Rbl1 | retinoblastoma-like 1 (p107) | -1.27 | 0.802 | -2.6 | 0.24 | -2.11 | 0.186 | 1.12 | 0.86 | -1.22 |
| 1433286_at | 4833417J16Rik | RIKEN cDNA 4833417J16 gene | -1.43 | 0.166 | -2.01 | 0.099 | -2.22 | 0.028 | 1.49 | 0.236 | -1.04 |
| 1418306_at | Crybb1 | crystallin, beta B1 | -1.96 | 0.531 | -1.64 | 0.457 | -1.9 | 0.155 | 1.34 | 0.665 | -1.04 |
| 1420468_at | Asb17 | ankyrin repeat and SOCS box-containing protein 17 | -1.65 | 0.176 | -2.38 | 0.022 | -1.61 | 0.204 | 5.42 | 0.158 | -0.05 |
| 1437700_at | Schip1 | Schwannomin interacting protein 1 | -1.63 | 0.03 | -2.09 | 0.132 | -1.79 | 0.044 | 1.35 | 0.093 | -1.04 |
| 1435308_at | Fut9 | fucosyltransferase 9 | -1.28 | 0.025 | -4.33 | 0.01 | -1.57 | 0.291 | 1.21 | 0.494 | -1.49 |
| 1459620_at | 9130004C02Rik | RIKEN cDNA 9130004C02 gene | -2.59 | 0.457 | -1.76 | 0.286 | -1.43 | 0.397 | 1.86 | 0.144 | -0.98 |
| 1443211_at | --- | --- | -1.87 | 0.634 | -4.69 | 0 | -1.1 | 0.377 | 1.84 | 0.401 | -1.45 |
| 1431297_a_at | 4933436C20Rik | RIKEN cDNA 4933436C20 gene | -1.58 | 0.219 | -3.04 | 0.083 | -1.44 | 0.072 | 1.58 | 0.238 | -1.12 |
| 1444069_at | Auts2 | Autism susceptibility candidate 2 | -1.19 | 0.709 | -4.63 | 0.191 | -1.68 | 0.464 | 1.28 | 0.493 | -1.55 |
| 1442713_at | Osbpl10 | Oxysterol binding protein-like 10 | -2.28 | 0.27 | -1.6 | 0.043 | -1.69 | 0.246 | 1.01 | 0.972 | -1.14 |
| 1454520_at | 5830415B17Rik | RIKEN cDNA 5830415B17 gene | -2.49 | 0.141 | -2.38 | 0.06 | -1.2 | 0.469 | 1.11 | 0.764 | -1.24 |
| 1432441_at | 4933413J09Rik | RIKEN cDNA 4933413J09 gene | -2.13 | 0.092 | -1.62 | 0.114 | -1.76 | 0.104 | 1 | 0.999 | -1.13 |
| 1419930_at | D15Ertd55e | DNA segment, Chr 15, ERATO Doi 55, expressed | -1.92 | 0.523 | -2.49 | 0.04 | -1.36 | 0.581 | 1.96 | 0.22 | -0.95 |
| 1444470_x_at | Bmp5 | Bone morphogenetic protein 5 | -4.18 | 0.07 | 1.1 | 0.838 | -3.1 | 0.003 | -3.45 | 0.091 | -2.41 |
| 1456838_at | BC072620 | cDNA sequence BC072620 | -1.88 | 0.319 | -1.57 | 0.434 | -2.03 | 0.432 | 2.62 | 0.057 | -0.72 |
| 1444649_at | Prkce | Protein kinase C, epsilon | -1.62 | 0.646 | -1.63 | 0.438 | -2.32 | 0.117 | 1.51 | 0.559 | -1.02 |
| 1460232_s_at | Hsd3b2 /// Hsd3b3 | hydroxy-delta-5-steroid dehydrogenase, 3 beta- and steroid delta-isomerase 2 /// | -1.19 | 0.127 | -2.2 | 0.004 | -2.71 | 0.064 | 1.29 | 0.71 | -1.2 |
| 1431379_a_at | Slc13a1 | solute carrier family 13 (sodium/sulphate symporters), member 1 | -1.03 | 0.833 | -5.14 | 0.115 | -2.04 | 0.254 | 1.49 | 0.461 | -1.68 |
| 1449064_at | Tdh | L-threonine dehydrogenase | -1.65 | 0.372 | -2.49 | 0.08 | -1.53 | 0.469 | 1.42 | 0.419 | -1.06 |
| 1430709_at | 4833405L11Rik | RIKEN cDNA 4833405L11 gene | -2.42 | 0.002 | -2.68 | 0.188 | -1.14 | 0.822 | 2.86 | 0.223 | -0.84 |
| 1431695_at | Rph3al | rabphilin 3A-like (without C2 domains) | -1.7 | 0.11 | -1.63 | 0.407 | -2.16 | 0.245 | 1.25 | 0.622 | -1.06 |
| 1433123_at | 2900069G24Rik | RIKEN cDNA 2900069G24 gene | -1.82 | 0.291 | -1.76 | 0.382 | -1.82 | 0.334 | 1.85 | 0.216 | -0.89 |
| 1432690_at | 9030407C09Rik | RIKEN cDNA 9030407C09 gene | -2.16 | 0.451 | -2.15 | 0.083 | -1.35 | 0.445 | 1.9 | 0.047 | -0.94 |
| 1429231_at | --- | --- | -1.82 | 0.041 | -1.27 | 0.542 | -3.01 | 0.155 | 1.11 | 0.858 | -1.24 |
| 1450302_at | 4930470P17Rik | RIKEN cDNA 4930470P17 gene | -1.31 | 0.105 | -3.32 | 0.028 | -1.64 | 0.115 | 1.81 | 0.165 | -1.12 |
| 1446026_at | Kif13a | Kinesin family member 13A | -1.66 | 0.439 | -1.18 | 0.767 | -4.5 | 0.012 | 1.79 | 0.078 | -1.39 |
| 1460383_at | Gnao1 | guanine nucleotide binding protein, alpha o | -3.17 | 0.466 | -1.12 | 0.875 | -2.12 | 0.183 | 1.18 | 0.208 | -1.31 |
| 1426028_a_at | Cit | citron | -1.91 | 0.405 | -2.34 | 0.287 | -1.37 | 0.471 | 2.53 | 0.307 | -0.77 |
| 1426203_at | Krtap16-4 | keratin associated protein 16-4 | -4.42 | 0.12 | -1.23 | 0.699 | -1.56 | 0.3 | 1.84 | 0.202 | -1.34 |
| 1457221_at | Igsf11 | Immunoglobulin superfamily, member 11 | -1.22 | 0.51 | -3.05 | 0.013 | -1.88 | 0.141 | 1.23 | 0.633 | -1.23 |
| 1449559_at | Msx2 | homeo box, msh-like 2 | -2.5 | 0.212 | -1.28 | 0.679 | -2 | 0.031 | 1.07 | 0.895 | -1.18 |
| 1444540_at | --- | --- | -1.46 | 0.468 | -2.82 | 0.252 | -1.56 | 0.31 | 1.02 | 0.931 | -1.2 |
| 1432005_at | Dnm2 | dynamin 2 | -2.11 | 0.156 | -2.3 | 0 | -1.29 | 0.651 | 1.57 | 0.515 | -1.03 |
| 1441256_at | Wdr45l | Wdr45 like | -2.32 | 0.031 | -1.52 | 0.534 | -1.68 | 0.383 | 1.49 | 0.45 | -1.01 |
| 1441775_at | Wdr7 | WD repeat domain 7 | -2.14 | 0.149 | -1.26 | 0.485 | -2.34 | 0.013 | 1.25 | 0.362 | -1.12 |
| 1443705_at | D14Ertd171e | DNA segment, Chr 14, ERATO Doi 171, expressed | -1.76 | 0.276 | -2.2 | 0.006 | -1.51 | 0.318 | 1.81 | 0.382 | -0.91 |
| 1458728_at | --- | --- | -1.55 | 0.482 | -2.45 | 0.35 | -1.57 | 0.106 | 1.19 | 0.531 | -1.1 |
| 1420672_at | Kcne1 | potassium voltage-gated channel, Isk-related subfamily, member 1 | -2.79 | 0.065 | -2.06 | 0.387 | -1.18 | 0.415 | 1.01 | 0.986 | -1.26 |
| 1424561_at | Ece2 | endothelin converting enzyme 2 | -1.97 | 0.267 | -1.43 | 0.503 | -2.06 | 0.171 | 1.24 | 0.738 | -1.06 |
| 1442650_at | --- | Transcribed locus | -3.08 | 0.014 | -1.32 | 0.487 | -1.63 | 0.137 | 1.1 | 0.701 | -1.23 |

| | | | | | | | | | | | |
|---|---|---|---|---|---|---|---|---|---|---|---|
| 1458539_at | R3hdm1 | R3H domain 1 (binds single-stranded nucleic acids) | -1.18 | 0.485 | -2.56 | 0.048 | -2.2 | 0.096 | 2.06 | 0.201 | -0.97 |
| 1439990_at | --- | 12 days embryo spinal ganglion cDNA, RIKEN full-length enriched library, clone:[illegible] | -1.45 | 0.348 | -1.87 | 0.356 | -2.12 | 0.313 | 1.01 | 0.98 | -1.11 |
| 1453088_at | 6430628N08Rik | RIKEN cDNA 6430628N08 gene | -1.9 | 0.066 | -1.59 | 0.202 | -1.84 | 0.182 | 1.06 | 0.86 | -1.07 |
| 1427669_a_at | Cit | citron | -2.11 | 0.113 | -2.47 | 0.208 | -1.22 | 0.504 | 3.26 | 0.077 | -0.64 |
| 1426139_a_at | Ccrl1 | chemokine (C-C motif) receptor-like 1 | -1.27 | 0.723 | -1.69 | 0.111 | -3.1 | 0.237 | 1.13 | 0.804 | -1.23 |
| 1433240_at | 9430013L17Rik | RIKEN cDNA 9430013L17 gene | -3.79 | 0.063 | 1.05 | 0.92 | -2.62 | 0.017 | -3.01 | 0.06 | -2.09 |
| 1456035_at | Nxf3 | nuclear RNA export factor 3 | -1.43 | 0.608 | -2.25 | 0.256 | -1.81 | 0.107 | 1.31 | 0.7 | -1.05 |
| 1439265_at | Xkr6 | X Kell blood group precursor related family member 6 homolog | -1.54 | 0.137 | -1.34 | 0.72 | -3.3 | 0.049 | 1.16 | 0.832 | -1.26 |
| 1427751_a_at | Krt36 | keratin 36 | -1.82 | 0.206 | -1.5 | 0.411 | -2.07 | 0.002 | 1.12 | 0.829 | -1.07 |
| 1424606_at | Cplx3 | complexin 3 | -3.58 | 0.166 | -3.58 | 0.007 | 1.14 | 0.652 | -1.53 | 0.235 | -1.89 |
| 1446065_at | Kcnd2 | Potassium voltage-gated channel, Shal-related family, member 2 | -1.02 | 0.953 | -2.84 | 0.053 | -2.7 | 0.056 | 1.69 | 0.503 | -1.22 |
| 1446287_at | 4931440B09Rik | RIKEN cDNA 4931440B09 gene | -1.2 | 0.237 | -3.64 | 0.001 | -1.69 | 0.211 | 1.6 | 0.38 | -1.23 |
| 1436202_at | --- | --- | -1.76 | 0.129 | -1.79 | 0.053 | -1.73 | 0.025 | 1.16 | 0.382 | -1.03 |
| 1436437_at | Gm97 | gene model 97, (NCBI) | -4.09 | 0.047 | -1.85 | 0.099 | -1.09 | 0.754 | 1.38 | 0.52 | -1.41 |
| 1419576_at | Hoxb13 | homeo box B13 | -1.57 | 0.486 | -1.73 | 0.271 | -2.04 | 0.071 | 1.01 | 0.989 | -1.08 |
| 1452570_at | D0Kist5 | DNA segment, KIST 5 | -2.18 | 0.225 | -1.82 | 0.227 | -1.43 | 0.384 | 1.2 | 0.791 | -1.06 |
| 1454100_at | 4930480E11Rik | RIKEN cDNA 4930480E11 gene | -1.73 | 0.411 | -1.63 | 0.4 | -1.93 | 0.136 | 1.59 | 0.304 | -0.93 |
| 1434074_x_at | Arf4 | ADP-ribosylation factor 4 | -3.04 | 0.145 | -1.62 | 0.63 | -1.32 | 0.278 | 1.28 | 0.666 | -1.17 |
| 1456123_at | AI481121 | expressed sequence AI481121 | -1.35 | 0.232 | -1.52 | 0.491 | -3.26 | 0.004 | 2.01 | 0.1 | -1.03 |
| 1429020_at | Pon2 | paraoxonase 2 | -2.41 | 0.259 | -1.36 | 0.404 | -1.8 | 0.005 | 1.56 | 0.362 | -1 |
| 1446528_at | Scyl2 | SCY1-like 2 (S. cerevisiae) | -1.59 | 0.272 | -2.01 | 0.073 | -1.72 | 0.226 | 1.12 | 0.493 | -1.05 |
| 1422108_at | Ppp1r3a | protein phosphatase 1, regulatory (inhibitor) subunit 3A | -3.23 | 0.143 | -1.48 | 0.29 | -1.38 | 0.27 | 1.55 | 0.446 | -1.13 |
| 1419432_at | Spam1 | sperm adhesion molecule 1 | -2.29 | 0.059 | -1.38 | 0.366 | -1.82 | 0.252 | 1.1 | 0.836 | -1.1 |
| 1460512_a_at | 4930558O21Rik | RIKEN cDNA 4930558O21 gene | -3.06 | 0.347 | 1.03 | 0.919 | -2.85 | 0.025 | -1.08 | 0.893 | -1.49 |
| 1453900_at | 1700018A14Rik | RIKEN cDNA 1700018A14 gene | -2.41 | 0.098 | -1.8 | 0.458 | -1.35 | 0.668 | 2.67 | 0.046 | -0.72 |
| 1430493_at | 2310061G22Rik | RIKEN cDNA 2310061G22 gene | -2.27 | 0.146 | -1.2 | 0.539 | -2.29 | 0.035 | 1.16 | 0.524 | -1.15 |
| 1430420_at | Alg9 | asparagine-linked glycosylation 9 homolog (yeast, alpha 1,2 mannosyltransferase | -2.73 | 0.06 | -2.19 | 0.293 | -1.12 | 0.807 | 1.77 | 0.302 | -1.07 |
| 1440618_at | 9130416B15 | hypothetical protein 9130416B15 | -1.57 | 0.541 | -1.33 | 0.467 | -3.13 | 0.07 | 1.11 | 0.832 | -1.23 |
| 1443317_at | D6Ertd47e | DNA segment, Chr 6, ERATO Doi 47, expressed | -4.07 | 0.259 | -1.12 | 0.885 | -1.74 | 0.121 | 1.47 | 0.182 | -1.37 |
| 1432890_at | 2610011E03Rik | RIKEN cDNA 2610011E03 gene | -2.1 | 0.365 | -1.29 | 0.586 | -2.17 | 0.28 | 1.02 | 0.973 | -1.13 |
| 1439003_s_at | 1700008F21Rik /// [illegible] | RIKEN cDNA 1700008F21 gene /// similar to coiled-coil domain containing 7 | -1.47 | 0.579 | -1.81 | 0.376 | -2.09 | 0.372 | 1.33 | 0.53 | -1.01 |
| 1427547_a_at | Slc26a3 | solute carrier family 26, member 3 | -1.6 | 0.15 | -1.78 | 0.348 | -1.9 | 0.064 | 1.71 | 0.287 | -0.89 |
| 1456391_at | Tdrd5 | tudor domain containing 5 | -1.07 | 0.836 | -3.55 | 0.091 | -1.97 | 0.129 | 1.12 | 0.767 | -1.37 |
| 1436612_at | 2410004I01Rik | RIKEN cDNA 2410004I01 gene | -4.42 | 0.011 | -1.3 | 0.762 | -1.37 | 0.233 | 1.25 | 0.763 | -1.46 |
| 1442545_at | --- | --- | -3.05 | 0.064 | -2.32 | 0.307 | -1.04 | 0.943 | 1.38 | 0.373 | -1.26 |
| 1458498_at | --- | --- | -1.52 | 0.519 | -1.75 | 0.409 | -2.02 | 0.169 | 1.06 | 0.896 | -1.06 |
| 1442552_at | Thsd4 | thrombospondin, type I, domain containing 4 | -2.63 | 0.359 | -1.25 | 0.368 | -1.84 | 0.077 | 2.8 | 0.282 | -0.73 |
| 1431451_at | Glt6d1 | glycosyltransferase 6 domain containing 1 | -3.42 | 0.04 | -2.15 | 0.123 | -1.03 | 0.934 | 1.05 | 0.895 | -1.39 |
| 1438086_at | Npy6r | neuropeptide Y receptor Y6 | -1.82 | 0.187 | -3.63 | 0.071 | -1.11 | 0.889 | 2.94 | 0.021 | -0.9 |
| 1447768_at | Got2 /// LOC64084[illegible] | glutamate oxaloacetate transaminase 2, mitochondrial /// similar to Aspartate ami | -1.67 | 0.058 | -2.12 | 0.074 | -1.53 | 0.059 | 1.39 | 0.58 | -0.98 |
| 1440945_at | --- | --- | -1.26 | 0.694 | -2.72 | 0.023 | -1.74 | 0.15 | 1.41 | 0.299 | -1.08 |
| 1459421_at | Hkdc1 | hexokinase domain containing 1 | -1.68 | 0.539 | -1.92 | 0.298 | -1.62 | 0.263 | 1.63 | 0.437 | -0.9 |
| 1450636_s_at | Akp5 | alkaline phosphatase 5 | -2.07 | 0.146 | -1.81 | 0.166 | -1.43 | 0.421 | 1.65 | 0.221 | -0.92 |
| 1443812_x_at | Prkg1 | Protein kinase, cGMP-dependent, type I | -1.91 | 0.193 | -1.67 | 0.157 | -1.63 | 0.15 | 1.5 | 0.324 | -0.93 |
| 1442729_at | D12Ertd553e | DNA segment, Chr 12, ERATO Doi 553, expressed | -5.96 | 0.003 | -1.76 | 0.468 | 1 | 0.996 | -1.2 | 0.364 | -1.98 |
| 1457853_at | BC021785 | CDNA sequence BC021785 | -1.25 | 0.123 | -2.27 | 0.383 | -2.02 | 0.291 | 1.23 | 0.317 | -1.08 |
| 1459577_at | --- | --- | -2.07 | 0.011 | -1.67 | 0.288 | -1.52 | 0.329 | 1.16 | 0.809 | -1.03 |
| 1450188_s_at | Lipg | lipase, endothelial | -1.29 | 0.704 | -2.07 | 0.198 | -2.07 | 0.005 | 5 | 0.276 | -0.11 |
| 1446753_at | --- | --- | -2.28 | 0.169 | -1.81 | 0.167 | -1.34 | 0.686 | 1.09 | 0.891 | -1.08 |
| 1440453_at | 1700012C15Rik | RIKEN cDNA 1700012C15 gene | -1.83 | 0.453 | -2.52 | 0.348 | -1.25 | 0.064 | 1.19 | 0.401 | -1.1 |
| 1432675_at | Mdn1 | midasin homolog (yeast) | -1.92 | 0.048 | -1.56 | 0.556 | -1.72 | 0.213 | 1.54 | 0.142 | -0.92 |
| 1450162_at | Dpf3 | D4, zinc and double PHD fingers, family 3 | -3.23 | 0.082 | -1.26 | 0.596 | -1.56 | 0.282 | 2.39 | 0.126 | -0.92 |
| 1438997_at | Ern1 | Endoplasmic reticulum (ER) to nucleus signalling 1 | -1.88 | 0.179 | -1.99 | 0.189 | -1.41 | 0.485 | 1.47 | 0.121 | -0.95 |

| | | | | | | | | | | | |
|---|---|---|---|---|---|---|---|---|---|---|---|
| 1432354_at | 1700003H04Rik | RIKEN cDNA 1700003H04 gene | -1.52 | 0.346 | -2.27 | 0.203 | -1.55 | 0.259 | 1.52 | 0.464 | -0.96 |
| 1453383_at | 1700022F17Rik | RIKEN cDNA 1700022F17 gene | -1.19 | 0.677 | -2.91 | 0.097 | -1.77 | 0.501 | 1.35 | 0.593 | -1.13 |
| 1445287_at | --- | Adult male spinal cord cDNA, RIKEN full-length enriched library, clone:A330010P | -1.14 | 0.882 | -1.98 | 0.228 | -2.71 | 0.149 | 1.59 | 0.439 | -1.06 |
| 1432148_at | 4930533K18Rik | RIKEN cDNA 4930533K18 gene | -2.23 | 0.364 | -2.09 | 0.36 | -1.22 | 0.609 | 1.27 | 0.09 | -1.07 |
| 1439139_at | D2Ertd640e | DNA segment, Chr 2, ERATO Doi 640, expressed | -1.33 | 0.37 | -1.58 | 0.202 | -2.76 | 0.007 | 1.72 | 0.034 | -0.99 |
| 1444423_at | --- | Transcribed locus | -2.77 | 0.472 | -1.67 | 0.229 | -1.27 | 0.602 | 1.88 | 0.338 | -0.96 |
| 1453856_at | Btbd4 | BTB (POZ) domain containing 4 | -2.07 | 0.369 | -3.36 | 0.004 | -1.03 | 0.959 | 2.75 | 0.02 | -0.93 |
| 1442794_at | Col8a1 | procollagen, type VIII, alpha 1 | -1.35 | 0.569 | -1.89 | 0.311 | -2.09 | 0.057 | 1.16 | 0.839 | -1.04 |
| 1458954_at | --- | --- | -2.38 | 0.059 | -2.31 | 0.266 | -1.12 | 0.802 | 1.02 | 0.977 | -1.2 |
| 1437753_at | AW742931 | Expressed sequence AW742931 | -1.72 | 0.395 | -1.6 | 0.041 | -1.85 | 0.121 | 1.21 | 0.647 | -0.99 |
| 1421483_at | Lhx4 | LIM homeobox protein 4 | -2.48 | 0.348 | -1.29 | 0.426 | -1.74 | 0.284 | 1.05 | 0.947 | -1.12 |
| 1447129_at | --- | --- | -1.25 | 0.425 | -2.13 | 0.261 | -2.06 | 0.143 | 1.02 | 0.936 | -1.11 |
| 1438613_at | Kcna4 | potassium voltage-gated channel, shaker-related subfamily, member 4 | -1.49 | 0.541 | -1.62 | 0.312 | -2.16 | 0.122 | 1.41 | 0.624 | -0.96 |
| 1438341_at | Tgm4 | transglutaminase 4 (prostate) | -2.67 | 0.083 | -1.33 | 0.644 | -1.6 | 0.423 | 1.04 | 0.946 | -1.14 |
| 1441189_at | --- | Transcribed locus | -1.39 | 0.679 | -1.83 | 0.181 | -2.05 | 0.114 | 1.29 | 0.751 | -0.99 |
| 1426704_at | Gak | cyclin G associated kinase | -2.17 | 0.173 | -1.3 | 0.454 | -1.91 | 0.012 | 1.32 | 0.736 | -1.01 |
| 1431122_at | Armc9 | armadillo repeat containing 9 | -1.74 | 0.376 | -2.49 | 0.173 | -1.28 | 0.435 | 1.84 | 0.3 | -0.92 |
| 1456508_at | A030001H23Rik | RIKEN cDNA A030001H23 gene | -1.21 | 0.594 | -2.22 | 0.1 | -2.08 | 0.379 | 3.93 | 0.315 | -0.4 |
| 1444142_at | LOC432591 /// LOC | hypothetical LOC432591 /// hypothetical protein LOC639155 | -1.26 | 0.543 | -1.81 | 0.24 | -2.45 | 0.082 | 1.6 | 0.398 | -0.98 |
| 1433254_at | 5033423K11Rik | RIKEN cDNA 5033423K11 gene | -2.24 | 0.396 | -1.05 | 0.946 | -2.76 | 0.181 | 2.49 | 0.045 | -0.89 |
| 1446734_at | C77438 | expressed sequence C77438 | -1.97 | 0.071 | -1.4 | 0.494 | -1.86 | 0.151 | 2.11 | 0.102 | -0.78 |
| 1432529_at | Boll | bol, boule-like (Drosophila) | -1.95 | 0.029 | -1.29 | 0.603 | -2.14 | 0.016 | 1.35 | 0.535 | -1.01 |
| 1442211_at | C78893 | expressed sequence C78893 | -1.92 | 0.166 | -2.49 | 0.169 | -1.19 | 0.674 | 1.1 | 0.809 | -1.13 |
| 1418093_a_at | Egf | epidermal growth factor | -1.36 | 0.12 | -4.43 | 0.077 | -1.25 | 0.263 | 1.16 | 0.564 | -1.47 |
| 1430203_at | Usp16 | ubiquitin specific peptidase 16 | -2.09 | 0.054 | -2.71 | 0.03 | -1.1 | 0.757 | 1.12 | 0.703 | -1.19 |
| 1446170_at | Abcc12 | ATP-binding cassette, sub-family C (CFTR/MRP), member 12 | -1.35 | 0.168 | -2.26 | 0.377 | -1.72 | 0.264 | 1.05 | 0.956 | -1.07 |
| 1456570_at | --- | --- | -1.92 | 0.247 | -1.82 | 0.265 | -1.44 | 0.502 | 1.32 | 0.734 | -0.97 |
| 1437575_at | Mcmdc1 | minichromosome maintenance deficient domain containing 1 | -1.35 | 0.695 | -1.42 | 0.426 | -3.09 | 0.027 | 1.14 | 0.803 | -1.18 |
| 1442285_at | Syne2 | synaptic nuclear envelope 2 | 1.01 | 0.917 | -1.79 | 0.095 | -5.07 | 0.085 | -1.17 | 0.622 | -1.76 |
| 1421443_at | Gpr110 | G protein-coupled receptor 110 | -1.03 | 0.965 | -2.99 | 0.156 | -2.18 | 0.112 | 1.81 | 0.076 | -1.1 |
| 1430174_at | A930010G16Rik | RIKEN cDNA A930010G16 gene | -1.68 | 0.37 | -1.68 | 0.616 | -1.74 | 0.049 | 2.86 | 0.094 | -0.56 |
| 1428053_at | Gprc6a | G protein-coupled receptor, family C, group 6, member A | -1.25 | 0.666 | -1.41 | 0.575 | -3.88 | 0.014 | 1.53 | 0.182 | -1.25 |
| 1447875_x_at | AA407452 | EST AA407452 | -1.32 | 0.698 | -1.42 | 0.507 | -3.29 | 0.011 | 1.42 | 0.509 | -1.15 |
| 1445409_at | Lrba | LPS-responsive beige-like anchor | -1.42 | 0.277 | -3.91 | 0.03 | -1.24 | 0.709 | 1.4 | 0.653 | -1.29 |
| 1428363_at | Eif4g2 | eukaryotic translation initiation factor 4, gamma 2 | -1.27 | 0.632 | -3.94 | 0.168 | -1.38 | 0.489 | 1.2 | 0.591 | -1.34 |
| 1430904_at | Arfgap3 | ADP-ribosylation factor GTPase activating protein 3 | -1.34 | 0.137 | -2.09 | 0.291 | -1.84 | 0.016 | 1.34 | 0.108 | -0.98 |
| 1445908_at | Eif2b5 | Eukaryotic translation initiation factor 2B, subunit 5 epsilon | -1.18 | 0.219 | -2.44 | 0.194 | -1.96 | 0.038 | 1.3 | 0.588 | -1.07 |
| 1445202_at | --- | 0 day neonate cerebellum cDNA, RIKEN full-length enriched library, clone:C2300 | -3.04 | 0.266 | -1.7 | 0.267 | -1.17 | 0.83 | 2.03 | 0.023 | -0.97 |
| 1453705_at | B230110C06Rik | RIKEN cDNA B230110C06 gene | -2.15 | 0.457 | -1.35 | 0.57 | -1.78 | 0.004 | 1.12 | 0.706 | -1.04 |
| 1449739_at | Ptdss1 | Phosphatidylserine synthase 1 | 1.23 | 0.638 | -2.71 | 0.058 | -5.7 | 0.012 | -2.11 | 0.098 | -2.32 |
| 1418697_at | Inmt | indolethylamine N-methyltransferase | -2.02 | 0.225 | -1.95 | 0.161 | -1.31 | 0.003 | 2.6 | 0.314 | -0.67 |
| 1458942_at | C230037E05Rik | RIKEN cDNA C230037E05 gene | -1.47 | 0.168 | -1.85 | 0.318 | -1.81 | 0.057 | 1.43 | 0.462 | -0.93 |
| 1445887_at | --- | --- | -1.7 | 0.08 | -1.28 | 0.587 | -2.49 | 0.008 | 1.65 | 0.114 | -0.95 |
| 1421428_at | Slc5a7 | solute carrier family 5 (choline transporter), member 7 | -3.19 | 0.302 | -1.41 | 0.394 | -1.34 | 0.105 | 1.27 | 0.622 | -1.17 |
| 1453697_at | 6530402F18Rik | RIKEN cDNA 6530402F18 gene | -3.28 | 0.059 | -2.18 | 0.014 | 1.01 | 0.981 | -1.74 | 0.177 | -1.55 |
| 1419895_at | AA536748 | expressed sequence AA536748 | -1.55 | 0.445 | -2.89 | 0.11 | -1.27 | 0.499 | 1.23 | 0.671 | -1.12 |
| 1436010_at | BC036313 | cDNA sequence BC036313 | -1.77 | 0.17 | -1.89 | 0.085 | -1.47 | 0.144 | 1.14 | 0.663 | -1 |
| 1445352_at | --- | Transcribed locus | -1.35 | 0.18 | -6.02 | 0.024 | -1.15 | 0.579 | 2.56 | 0.047 | -1.49 |
| 1420169_at | AA517841 | expressed sequence AA517841 | -2.51 | 0.352 | -1.34 | 0.214 | -1.58 | 0.276 | 1.18 | 0.27 | -1.06 |
| 1442220_at | --- | --- | -2.46 | 0.153 | -1.16 | 0.619 | -1.96 | 0.306 | 1.69 | 0.257 | -0.97 |
| 1419919_at | 4833422M21Rik | RIKEN cDNA 4833422M21 gene | -2.66 | 0.091 | -1.9 | 0.141 | -1.14 | 0.789 | 1.82 | 0.155 | -0.97 |
| 1440621_at | Kitl | Kit ligand | -1.02 | 0.851 | -2.2 | 0.127 | -2.94 | 0.002 | 1.16 | 0.522 | -1.25 |
| 1459470_at | Runx1 | Runt related transcription factor 1 | -1.74 | 0.076 | -1.78 | 0.087 | -1.56 | 0.137 | 1.16 | 0.252 | -0.98 |

| | | | | | | | | | | | |
|---|---|---|---|---|---|---|---|---|---|---|---|
| 1457651_x_at | Rem2 | rad and gem related GTP binding protein 2 | -2.04 | 0.532 | -1.23 | 0.562 | -2.11 | 0.227 | 1.33 | 0.547 | -1.01 |
| 1423562_at | Prrt1 | proline-rich transmembrane protein 1 | -3.27 | 0.097 | -1.49 | 0.015 | -1.25 | 0.604 | 1.19 | 0.757 | -1.21 |
| 1439877_at | Ext1 | Exostoses (multiple) 1 | -1.65 | 0.188 | -1.46 | 0.082 | -2.05 | 0.001 | 1.24 | 0.398 | -0.98 |
| 1441425_at | --- | Transcribed locus | -1.34 | 0.219 | -2.05 | 0.106 | -1.84 | 0.141 | 1.13 | 0.247 | -1.02 |
| 1441283_at | 5730405I09Rik | RIKEN cDNA 5730405I09 gene | 1.04 | 0.723 | -4.38 | 0.089 | -1.95 | 0.108 | -2.16 | 0.129 | -1.86 |
| 1421979_at | Phex | phosphate regulating gene with homologies to endopeptidases on the X chromos | -1.35 | 0.711 | -1.23 | 0.748 | -4.44 | 0.002 | 1 | 0.996 | -1.5 |
| 1417429_at | Fmo1 | flavin containing monooxygenase 1 | -1.58 | 0.124 | -1.6 | 0.01 | -1.92 | 0.006 | 1.38 | 0.257 | -0.93 |
| 1445328_at | Col4a4 | procollagen, type IV, alpha 4 | -1.23 | 0.419 | -2.18 | 0.452 | -1.96 | 0.039 | 1.02 | 0.961 | -1.09 |
| 1447819_x_at | Col8a1 | procollagen, type VIII, alpha 1 | -1.52 | 0.135 | -4.58 | 0.042 | -1.11 | 0.625 | 1.86 | 0.208 | -1.34 |
| 1449792_at | --- | --- | -1.12 | 0.858 | -2.27 | 0.282 | -2.23 | 0.208 | 1.2 | 0.19 | -1.11 |
| 1425758_a_at | Impg1 | interphotoreceptor matrix proteoglycan 1 | -1.42 | 0.184 | -1.44 | 0.014 | -2.58 | 0.113 | 1.14 | 0.403 | -1.08 |
| 1431918_at | 1700036A12Rik | RIKEN cDNA 1700036A12 gene | -2.08 | 0.137 | -1.11 | 0.856 | -2.47 | 0.055 | 1.48 | 0.395 | -1.05 |
| 1443075_at | Ptprd | Protein tyrosine phosphatase, receptor type, D | -1.28 | 0.319 | -2.27 | 0.105 | -1.78 | 0.223 | 1.2 | 0.808 | -1.03 |
| 1455034_at | Nr4a2 | Nuclear receptor subfamily 4, group A, member 2 | -1.53 | 0.055 | -2.2 | 0.103 | -1.47 | 0.367 | 2.35 | 0.053 | -0.71 |
| 1447684_at | Lzic | Leucine zipper and CTNNBIP1 domain containing | -1.67 | 0.403 | -1.62 | 0.379 | -1.74 | 0.052 | 1.49 | 0.301 | -0.89 |
| 1446264_at | --- | --- | -1.44 | 0.256 | -2.47 | 0.01 | -1.45 | 0.095 | 1.55 | 0.32 | -0.95 |
| 1432071_at | 6330437I11Rik | RIKEN cDNA 6330437I11 gene | -1.15 | 0.452 | -2.83 | 0.259 | -1.77 | 0.226 | 1.44 | 0.319 | -1.08 |
| 1449654_s_at | C77545 | expressed sequence C77545 | -1.61 | 0.003 | -1.82 | 0.412 | -1.61 | 0.215 | 1.08 | 0.859 | -0.99 |
| 1459468_at | C79743 | expressed sequence C79743 | -2.08 | 0.189 | -1.88 | 0.101 | -1.28 | 0.582 | 1.26 | 0.434 | -0.99 |
| 1432956_at | 4930444K16Rik | RIKEN cDNA 4930444K16 gene | -1.53 | 0.636 | -2.66 | 0.306 | -1.31 | 0.178 | 1.1 | 0.923 | -1.1 |
| 1453465_x_at | Ppp1r14c | protein phosphatase 1, regulatory (inhibitor) subunit 14c | -1.44 | 0.153 | -2.46 | 0.004 | -1.44 | 0.202 | 1.26 | 0.572 | -1.02 |
| 1446929_at | Bach2 | BTB and CNC homology 2 | 1.05 | 0.942 | -3.44 | 0.019 | -2.21 | 0.012 | -3.06 | 0.05 | -1.91 |
| 1416193_at | Car1 | carbonic anhydrase 1 | -2.32 | 0.173 | -1.53 | 0.394 | -1.41 | 0.602 | 4.83 | 0.089 | -0.11 |
| 1444901_at | --- | --- | 1.01 | 0.991 | -2.26 | 0.199 | -2.94 | 0.122 | -1.79 | 0.258 | -1.49 |
| 1432412_at | 9530004P13Rik | RIKEN cDNA 9530004P13 gene | -2.55 | 0.089 | -1.58 | 0.363 | -1.3 | 0.359 | 1.58 | 0.215 | -0.96 |
| 1443239_at | Mtap2 | Microtubule-associated protein 2 | -2.14 | 0.317 | -2.75 | 0.141 | -1.04 | 0.934 | 1.38 | 0.494 | -1.14 |
| 1453644_at | Obp1a | odorant binding protein Ia | -1.71 | 0.506 | -2.83 | 0.135 | -1.17 | 0.292 | 1.96 | 0.083 | -0.94 |
| 1453624_at | C430046K18Rik | RIKEN cDNA C430046K18 gene | -1.48 | 0.169 | -1.39 | 0.544 | -2.5 | 0.051 | 1.68 | 0.302 | -0.92 |
| 1435849_at | 6330417G02Rik | RIKEN cDNA 6330417G02 gene | -1.56 | 0.513 | -1.3 | 0.696 | -2.59 | 0.252 | 1.12 | 0.829 | -1.08 |
| 1444975_at | Chchd6 | Coiled-coil-helix-coiled-coil-helix domain containing 6 | -1.77 | 0.18 | -1.77 | 0.321 | -1.5 | 0.164 | 1.16 | 0.626 | -0.97 |
| 1430941_at | Bbs7 | Bardet-Biedl syndrome 7 | -1.93 | 0.387 | -1.19 | 0.747 | -2.3 | 0.195 | 1.3 | 0.742 | -1.03 |
| 1446469_at | A030008J09 | hypothetical protein A030008J09 | -2.56 | 0.028 | -1.2 | 0.754 | -1.74 | 0.358 | 1.49 | 0.574 | -1 |
| 1430717_at | 4930403C10Rik | RIKEN cDNA 4930403C10 gene | -2.19 | 0.103 | -1.9 | 0.19 | -1.23 | 0.312 | 1.34 | 0.707 | -0.99 |
| 1435310_at | Fbxo7 | F-box only protein 7 | -1.78 | 0.312 | -1.56 | 0.286 | -1.68 | 0.067 | 1.14 | 0.781 | -0.97 |
| 1453334_at | B230216N24Rik | RIKEN cDNA B230216N24 gene | -1.71 | 0.186 | -1.46 | 0.439 | -1.88 | 0.001 | 1.36 | 0.601 | -0.93 |
| 1440541_at | 2810442I21Rik | RIKEN cDNA 2810442I21 gene | -1.27 | 0.524 | -2.12 | 0.24 | -1.84 | 0.264 | 1.6 | 0.322 | -0.91 |
| 1420536_at | Crybb2 | crystallin, beta B2 | -1.92 | 0.294 | -1.86 | 0.152 | -1.35 | 0.389 | 1.36 | 0.462 | -0.94 |
| 1453573_at | Hist1h3d | histone1, H3d | -1.96 | 0.003 | -1.84 | 0.123 | -1.34 | 0.173 | 1.57 | 0.223 | -0.89 |
| 1432393_a_at | 5730409G07Rik | RIKEN cDNA 5730409G07 gene | -1.2 | 0.608 | -2.69 | 0.018 | -1.68 | 0.014 | 1.07 | 0.9 | -1.12 |
| 1432408_a_at | D0Kist2 | DNA segment, KIST 2 | -2.25 | 0.155 | -1.5 | 0.192 | -1.45 | 0.555 | 1.33 | 0.452 | -0.97 |
| 1439996_at | Tatdn1 | TatD DNase domain containing 1 | -2 | 0.013 | -1.52 | 0.194 | -1.54 | 0.181 | 1.4 | 0.004 | -0.92 |
| 1438210_at | Gpr149 | G protein-coupled receptor 149 | -3.9 | 0.007 | -1.88 | 0.063 | 1.02 | 0.979 | -1.17 | 0.336 | -1.49 |
| 1425952_a_at | Gcg | glucagon | -3.72 | 0.348 | -1.91 | 0.321 | 1.01 | 0.983 | -2.78 | 0.155 | -1.85 |
| 1425056_s_at | Saps2 | SAPS domain family, member 2 | -1.66 | 0.021 | -1.73 | 0.156 | -1.59 | 0.145 | 1.72 | 0.281 | -0.82 |
| 1432928_at | 4921507K24Rik | RIKEN cDNA 4921507K24 gene | -3.35 | 0.043 | -1.5 | 0.467 | -1.18 | 0.684 | 1.32 | 0.127 | -1.18 |
| 1443443_at | --- | --- | -1.51 | 0.281 | -3.16 | 0.033 | -1.2 | 0.139 | 1.37 | 0.61 | -1.13 |
| 1449458_at | Foxi1 | forkhead box I1 | -1.63 | 0.182 | -2.16 | 0.375 | -1.37 | 0.454 | 2.14 | 0.004 | -0.75 |
| 1431300_at | Sgip1 | SH3-domain GRB2-like (endophilin) interacting protein 1 | -1.36 | 0.215 | -1.67 | 0.21 | -2.11 | 0.018 | 1.91 | 0.158 | -0.81 |
| 1446884_at | AU022554 | expressed sequence AU022554 | -2.57 | 0.067 | -1.16 | 0.68 | -1.8 | 0.15 | 1.12 | 0.836 | -1.1 |
| 1431489_at | 4933432K03Rik | RIKEN cDNA 4933432K03 gene | -2.71 | 0.094 | -1.79 | 0.438 | -1.13 | 0.815 | 2.22 | 0.14 | -0.85 |
| 1419995_at | D10Ertd641e | DNA segment, Chr 10, ERATO Doi 641, expressed | -1.4 | 0.283 | -2.04 | 0.411 | -1.64 | 0.11 | 1.16 | 0.773 | -0.98 |
| 1433119_at | 4930456G14Rik | RIKEN cDNA 4930456G14 gene | -3.13 | 0.013 | -1.38 | 0.462 | -1.3 | 0.562 | 1.85 | 0.114 | -0.99 |
| 1420689_at | 4933415F23Rik | RIKEN cDNA 4933415F23 gene | -1.96 | 0.43 | -1.53 | 0.581 | -1.54 | 0.335 | 1.09 | 0.887 | -0.99 |

| | | | | | | | | | | | |
|---|---|---|---|---|---|---|---|---|---|---|---|
| 1426150_at | Gipc3 | GIPC PDZ domain containing family, member 3 | -1.43 | 0.564 | -1.66 | 0.078 | -1.95 | 0.173 | 1.37 | 0.373 | -0.92 |
| 1420252_at | --- | --- | -1.56 | 0.442 | -1.64 | 0.208 | -1.78 | 0.136 | 1.65 | 0.12 | -0.83 |
| 1431877_a_at | Grhl2 | grainyhead-like 2 (Drosophila) | -1.79 | 0.375 | -1.35 | 0.275 | -1.93 | 0 | 2.3 | 0.286 | -0.69 |
| 1458356_at | --- | --- | -1.24 | 0.033 | -4.28 | 0.095 | -1.29 | 0.596 | 1.03 | 0.676 | -1.44 |
| 1425013_at | Slc25a2 | solute carrier family 25 (mitochondrial carrier, ornithine transporter) member 2 | -1.67 | 0.216 | -2.06 | 0.024 | -1.36 | 0.29 | 1.09 | 0.798 | -1 |
| 1444778_at | 3-Sep | septin 3 | -2.06 | 0.238 | -1.02 | 0.96 | -2.83 | 0.016 | 1.02 | 0.966 | -1.22 |
| 1450584_at | Hoxd11 | homeo box D11 | -1.83 | 0.01 | -2.05 | 0.085 | -1.28 | 0.639 | 1.03 | 0.972 | -1.03 |
| 1455907_x_at | Phox2b | paired-like homeobox 2b | -2.34 | 0.249 | -1.21 | 0.51 | -1.76 | 0.265 | 1.45 | 0.492 | -0.97 |
| 1440425_at | --- | --- | -2.72 | 0.027 | -1.62 | 0.031 | -1.2 | 0.741 | 1.84 | 0.443 | -0.92 |
| 1432808_at | 5830426K05Rik | RIKEN cDNA 5830426K05 gene | -1.43 | 0.634 | -2.39 | 0.18 | -1.42 | 0.464 | 4.19 | 0.357 | -0.26 |
| 1430666_at | LOC665270 | similar to phospholipase B | -2.01 | 0.351 | -1.46 | 0.494 | -1.57 | 0.204 | 1.32 | 0.199 | -0.93 |
| 1440009_at | Olfr78 | olfactory receptor 78 | -2.96 | 0.342 | 1.13 | 0.814 | -2.85 | 0.297 | -1.65 | 0.566 | -1.58 |
| 1433175_at | --- | --- | -1.84 | 0.184 | -1.16 | 0.823 | -2.39 | 0.236 | 1.92 | 0.179 | -0.87 |
| 1442904_at | LOC622118 | similar to RNA (guanine-9-) methyltransferase domain containing 2 | -1.82 | 0.496 | -2.37 | 0.406 | -1.17 | 0.339 | 1.43 | 0.526 | -0.98 |
| 1444639_at | Nphp4 | Nephronophthisis 4 (juvenile) homolog (human) | -4.13 | 0.012 | -2.34 | 0.406 | 1.15 | 0.776 | -1.52 | 0.529 | -1.71 |
| 1418917_at | Hebp2 | heme binding protein 2 | -1.28 | 0.659 | -2.47 | 0.23 | -1.57 | 0.152 | 1.15 | 0.692 | -1.04 |
| 1421218_at | Bche | butyrylcholinesterase | -1.51 | 0.382 | -1.96 | 0.188 | -1.53 | 0.344 | 1.16 | 0.681 | -0.96 |
| 1449605_at | --- | Transcribed locus | -1.93 | 0.248 | -1.45 | 0.224 | -1.62 | 0.51 | 1.03 | 0.86 | -0.99 |
| 1456364_at | C230057M02Rik | RIKEN cDNA C230057M02 gene | -2.75 | 0.118 | -2.56 | 0.117 | 1.07 | 0.87 | -2.51 | 0.067 | -1.69 |
| 1446742_at | Nfia | Nuclear factor I/A | -2.24 | 0.539 | -2.42 | 0.034 | -1.04 | 0.907 | 1.62 | 0.423 | -1.02 |
| 1456417_at | Zic4 | zinc finger protein of the cerebellum 4 | -1.73 | 0.173 | -1.73 | 0.475 | -1.5 | 0.508 | 4.62 | 0.011 | -0.08 |
| 1424437_s_at | Abcg4 | ATP-binding cassette, sub-family G (WHITE), member 4 | -2.96 | 0.037 | -1.2 | 0.49 | -1.52 | 0.377 | 7 | 0.162 | 0.33 |
| 1450979_at | Ceacam14 | CEA-related cell adhesion molecule 14 | -1.7 | 0.169 | -2.63 | 0.118 | -1.16 | 0.801 | 1 | 0.995 | -1.12 |
| 1457798_at | C130039O16Rik | RIKEN cDNA C130039O16 gene | -1.5 | 0.275 | -2.55 | 0.002 | -1.29 | 0.594 | 1.22 | 0.716 | -1.03 |
| 1438779_at | Col4a3 | procollagen, type IV, alpha 3 | -1.37 | 0.284 | -1.82 | 0.009 | -1.8 | 0.095 | 1.28 | 0.294 | -0.93 |
| 1458775_at | 1700041E20Rik | RIKEN cDNA 1700041E20 gene | -1.75 | 0.44 | -1.18 | 0.791 | -2.39 | 0.149 | 2.29 | 0.31 | -0.76 |
| 1437778_at | Rbm15b | RNA binding motif protein 15B | -3.42 | 0.029 | -1.29 | 0.58 | -1.3 | 0.393 | 1.67 | 0.406 | -1.08 |
| 1445675_at | --- | Transcribed locus | -2.09 | 0.448 | -1.81 | 0.197 | -1.24 | 0.29 | 1.46 | 0.471 | -0.92 |
| 1446181_at | C85699 | expressed sequence C85699 | -3.7 | 0.462 | -1.07 | 0.733 | -1.58 | 0.084 | 1.52 | 0.49 | -1.21 |
| 1420988_at | Polh | polymerase (DNA directed), eta (RAD 30 related) | -1.86 | 0.311 | -1.42 | 0.606 | -1.68 | 0.166 | 2.65 | 0.134 | -0.58 |
| 1444481_at | --- | --- | -1.8 | 0.569 | -1.62 | 0.182 | -1.5 | 0.188 | 1.45 | 0.357 | -0.87 |
| 1450794_at | Avp | arginine vasopressin | -2.43 | 0.13 | -1.14 | 0.758 | -1.82 | 0.164 | 1.3 | 0.505 | -1.02 |
| 1433217_at | 4930566N20Rik | RIKEN cDNA 4930566N20 gene | -1.79 | 0.549 | -2.11 | 0.515 | -1.24 | 0.696 | 1.22 | 0.636 | -0.98 |
| 1453610_at | 4931406B18Rik | RIKEN cDNA 4931406B18 gene | -1.43 | 0.085 | -1.82 | 0.114 | -1.69 | 0.239 | 1.39 | 0.091 | -0.89 |
| 1445633_at | Rex2 | Reduced expression 2 | -1.81 | 0.315 | -2.45 | 0.05 | -1.13 | 0.797 | 1.51 | 0.226 | -0.97 |
| 1448000_at | Cdca3 | cell division cycle associated 3 | -1.7 | 0.202 | -1.97 | 0.223 | -1.34 | 0.154 | 1.81 | 0.188 | -0.8 |
| 1446665_at | C80918 | expressed sequence C80918 | -1.12 | 0.517 | -2.12 | 0.102 | -2.09 | 0.122 | 1.02 | 0.969 | -1.08 |
| 1432166_at | E130018N17Rik | RIKEN cDNA E130018N17 gene | -4.48 | 0.066 | -1.15 | 0.672 | -1.32 | 0.468 | 3.2 | 0.219 | -0.94 |
| 1420358_at | Krtap13 | keratin associated protein 13 | -4.46 | 0.008 | -3.2 | 0.003 | 1.31 | 0.701 | -2.05 | 0.109 | -2.1 |
| 1417518_at | Plagl2 | pleiomorphic adenoma gene-like 2 | -2.87 | 0.015 | -1.31 | 0.524 | -1.35 | 0.502 | 1.08 | 0.782 | -1.11 |
| 1445873_at | Tfdp2 | Transcription factor Dp 2 | -2.07 | 0.132 | -1.34 | 0.269 | -1.62 | 0.097 | 3.8 | 0.158 | -0.31 |
| 1443332_at | Slc12a2 | Solute carrier family 12, member 2 | -1.33 | 0.225 | -1.95 | 0.482 | -1.71 | 0.179 | 1.33 | 0.523 | -0.91 |
| 1425741_at | Srgap3 | SLIT-ROBO Rho GTPase activating protein 3 | -2.05 | 0.191 | -1.02 | 0.958 | -2.63 | 0.182 | 1.01 | 0.99 | -1.17 |
| 1425563_s_at | Pcdh10 | protocadherin 10 | -2.8 | 0.031 | -3.63 | 0.277 | 1.22 | 0.659 | -1.33 | 0.297 | -1.63 |
| 1422100_at | Cyp7a1 | cytochrome P450, family 7, subfamily a, polypeptide 1 | -3.16 | 0.414 | -1.34 | 0.473 | -1.27 | 0.702 | 2.46 | 0.098 | -0.83 |
| 1441205_at | --- | --- | -1.56 | 0.298 | -1.02 | 0.942 | -4.25 | 0.027 | 1.19 | 0.683 | -1.41 |
| 1423001_at | Spred2 | sprouty-related, EVH1 domain containing 2 | -1.28 | 0.65 | -1.93 | 0.408 | -1.8 | 0.445 | 2.03 | 0.191 | -0.75 |
| 1459075_at | AU022479 | expressed sequence AU022479 | -1.14 | 0.676 | -1.42 | 0.312 | -3.69 | 0.031 | 1.38 | 0.678 | -1.22 |
| 1418754_at | Adcy8 | adenylate cyclase 8 | -1.37 | 0.375 | -1.49 | 0.439 | -2.2 | 0.025 | 2.92 | 0.134 | -0.54 |
| 1444874_at | Atp5g1 | ATP synthase, H+ transporting, mitochondrial F0 complex, subunit c (subunit 9), i | -1.27 | 0.122 | -2.47 | 0.137 | -1.51 | 0.109 | 2.09 | 0.084 | -0.79 |
| 1444047_at | Kctd8 | Potassium channel tetramerisation domain containing 8 | -1.81 | 0.284 | -1.11 | 0.644 | -2.48 | 0.023 | 1.04 | 0.94 | -1.09 |
| 1458725_at | --- | --- | -1.89 | 0.498 | -2.09 | 0.192 | -1.18 | 0.753 | 2.89 | 0 | -0.57 |
| 1423434_at | Tead1 | TEA domain family member 1 | -3.22 | 0.085 | -1.69 | 0.343 | -1.05 | 0.912 | 1.93 | 0.362 | -1.01 |

| | | | | | | | | | | | |
|---|---|---|---|---|---|---|---|---|---|---|---|
| 1433284_at | 4921522E08Rik | RIKEN cDNA 4921522E08 gene | -3.29 | 0.002 | -2.04 | 0.027 | 1.06 | 0.886 | -1.32 | 0.564 | -1.4 |
| 1429744_at | 4931407G18Rik | RIKEN cDNA 4931407G18 gene | -1.48 | 0.297 | -2.49 | 0.337 | -1.29 | 0.396 | 1.1 | 0.715 | -1.04 |
| 1418094_s_at | Car4 | carbonic anhydrase 4 | -1.27 | 0.078 | -1.92 | 0.09 | -1.82 | 0.006 | 1.41 | 0.213 | -0.9 |
| 1418139_at | Dcx | doublecortin | -1.52 | 0.607 | -1.64 | 0.421 | -1.7 | 0.525 | 1.04 | 0.936 | -0.95 |
| 1432319_at | 1700085C21Rik | RIKEN cDNA 1700085C21 gene | -4.18 | 0.002 | 1.34 | 0.523 | -3.54 | 0.07 | -1.02 | 0.953 | -1.85 |
| 1457979_at | --- | Adult male hypothalamus cDNA, RIKEN full-length enriched library, clone:A23001 | -2.39 | 0.503 | -3.36 | 0.198 | 1.14 | 0.741 | -1.06 | 0.922 | -1.42 |
| 1426603_at | Rnasel | ribonuclease L (2', 5'-oligoisoadenylate synthetase-dependent) | -1.94 | 0.302 | -1.63 | 0.428 | -1.36 | 0.331 | 1.55 | 0.134 | -0.85 |
| 1431486_at | 4930503E24Rik | RIKEN cDNA 4930503E24 gene | -1.37 | 0.681 | -2.46 | 0.247 | -1.37 | 0.59 | 1.07 | 0.928 | -1.03 |
| 1422361_at | Tlm | T lymphoma oncogene | -1.44 | 0.588 | -1.5 | 0.389 | -1.97 | 0.01 | 1.5 | 0.139 | -0.85 |
| 1457804_at | Stx8 | Syntaxin 8 | -1.34 | 0.004 | -1.19 | 0.722 | -3.61 | 0.008 | 1.71 | 0.43 | -1.11 |
| 1445089_at | D16Ertd778e | DNA segment, Chr 16, ERATO Doi 778, expressed | -2.03 | 0.334 | -1.45 | 0.05 | -1.47 | 0.185 | 2.04 | 0.045 | -0.73 |
| 1432812_at | 4930554P06Rik | RIKEN cDNA 4930554P06 gene | -1.96 | 0.46 | -1.13 | 0.441 | -2.12 | 0.213 | 1.41 | 0.433 | -0.95 |
| 1445548_at | Rdx | Radixin | -2.69 | 0.034 | -1.67 | 0.492 | -1.12 | 0.552 | 1.72 | 0.269 | -0.94 |
| 1440601_at | --- | Transcribed locus | -1.19 | 0.337 | -1.72 | 0.144 | -2.23 | 0.216 | 1.14 | 0.831 | -1 |
| 1449717_at | --- | --- | -1.38 | 0.57 | -1.41 | 0.069 | -2.31 | 0.226 | 1.16 | 0.331 | -0.98 |
| 1456190_a_at | Acsm2 | Acyl-CoA synthetase medium-chain family member 2 | -1.16 | 0.043 | -3.01 | 0.059 | -1.48 | 0.104 | 1.16 | 0.566 | -1.12 |
| 1436238_at | Lgi3 | leucine-rich repeat LGI family, member 3 | -2.34 | 0.484 | -1.39 | 0.518 | -1.39 | 0.332 | 1.01 | 0.97 | -1.03 |
| 1445139_at | AV312086 | Expressed sequence AV312086 | -4.17 | 0.231 | -1.37 | 0.256 | -1.11 | 0.731 | 1.08 | 0.9 | -1.39 |
| 1452229_at | Camta2 | Calmodulin binding transcription activator 2 | -1.62 | 0.346 | -1.61 | 0.384 | -1.59 | 0.233 | 1.08 | 0.913 | -0.93 |
| 1420437_at | Indo | indoleamine-pyrrole 2,3 dioxygenase | -1.36 | 0.384 | -2.63 | 0.007 | -1.33 | 0.34 | 1.03 | 0.932 | -1.07 |
| 1426034_a_at | Runx2 | runt related transcription factor 2 | -1.14 | 0.863 | -3.42 | 0.21 | -1.43 | 0.306 | 1.31 | 0.483 | -1.17 |
| 1443828_x_at | Herpud2 | HERPUD family member 2 | -1.67 | 0.207 | -1.99 | 0.138 | -1.3 | 0.667 | 1.6 | 0.53 | -0.84 |
| 1428209_at | Bex4 | brain expressed gene 4 | -1.45 | 0.068 | -1.36 | 0.277 | -2.23 | 0.013 | 5.13 | 0.218 | 0.02 |
| 1444515_at | Myo1d | Myosin ID | -1.71 | 0.159 | -1.45 | 0.185 | -1.67 | 0.036 | 3.16 | 0.14 | -0.42 |
| 1457640_x_at | --- | Transcribed locus | -1.8 | 0.153 | -1.73 | 0.267 | -1.35 | 0.265 | 1.41 | 0.433 | -0.87 |
| 1438077_at | Nalp4a | NACHT, leucine rich repeat and PYD containing 4A | -1.95 | 0.402 | -1.12 | 0.863 | -2.13 | 0.088 | 1.18 | 0.792 | -1 |
| 1443374_at | --- | Transcribed locus | -1.21 | 0.739 | -2.4 | 0.207 | -1.59 | 0.012 | 1.18 | 0.158 | -1 |
| 1417504_at | Calb1 | calbindin-28K | -1.43 | 0.039 | -6.69 | 0.123 | 1.03 | 0.944 | -1.21 | 0.619 | -2.07 |
| 1457931_at | --- | --- | -1.84 | 0.367 | -1.27 | 0.361 | -1.85 | 0.192 | 1.77 | 0.242 | -0.79 |
| 1442696_at | Dhcr7 | 7-dehydrocholesterol reductase | 1.17 | 0.773 | -2.89 | 0.028 | -2.76 | 0.12 | -1.03 | 0.948 | -1.38 |
| 1428002_at | Saal1 | serum amyloid A-like 1 | -2.1 | 0.078 | -1.26 | 0.651 | -1.65 | 0.227 | 1.39 | 0.419 | -0.9 |
| 1458670_at | Adarb2 | Adenosine deaminase, RNA-specific, B2 | -2.03 | 0.064 | 1.09 | 0.919 | -3.46 | 0 | -1.56 | 0.593 | -1.49 |
| 1421696_at | Pkhd1l1 | polycystic kidney and hepatic disease 1-like 1 | -1.16 | 0.668 | -1.72 | 0.426 | -2.3 | 0.196 | 4.78 | 0.157 | -0.1 |
| 1460531_at | 2900069M18Rik | RIKEN cDNA 2900069M18 gene | -1.19 | 0.54 | -2.07 | 0.248 | -1.8 | 0.019 | 1.4 | 0.384 | -0.92 |
| 1446675_at | Adk | adenosine kinase | -1.68 | 0.51 | -1.42 | 0.314 | -1.73 | 0.383 | 1.19 | 0.771 | -0.91 |
| 1418875_at | Syngr4 | synaptogyrin 4 | -2.35 | 0.187 | -1.04 | 0.94 | -2.02 | 0.014 | 1.22 | 0.627 | -1.05 |
| 1430859_at | 5330427O13Rik | RIKEN cDNA 5330427O13 gene | -1.57 | 0.339 | -1.41 | 0.495 | -1.89 | 0.176 | 1.32 | 0.492 | -0.89 |
| 1441114_at | 9330156P08Rik | RIKEN cDNA 9330156P08 gene | -1.1 | 0.385 | -1.8 | 0.011 | -2.39 | 0.031 | 2.43 | 0.252 | -0.72 |
| 1438332_at | Slc22a6 | solute carrier family 22 (organic anion transporter), member 6 | -1.19 | 0.445 | -1.62 | 0.482 | -2.37 | 0.219 | 2.14 | 0.19 | -0.76 |
| 1450550_at | Il5 | interleukin 5 | -1.83 | 0.229 | -1.39 | 0.23 | -1.62 | 0.344 | 1.89 | 0.188 | -0.74 |
| 1447216_at | Nrxn1 | neurexin I | -1.51 | 0.722 | -1.42 | 0.419 | -1.95 | 0.009 | 1.23 | 0.393 | -0.91 |
| 1458341_x_at | --- | --- | 1.04 | 0.732 | -1.85 | 0.047 | -3.38 | 0.033 | -2.18 | 0.153 | -1.59 |
| 1444694_at | Tuft1 | Tuftelin 1 | -1.37 | 0.705 | -2.46 | 0.174 | -1.35 | 0.615 | 1.38 | 0.413 | -0.95 |
| 1458348_at | --- | Transcribed locus | -1.42 | 0.15 | -1.5 | 0.326 | -1.97 | 0.167 | 1.09 | 0.823 | -0.95 |
| 1458272_at | --- | Transcribed locus | -1.53 | 0.448 | -5.25 | 0.002 | 1.04 | 0.945 | -1.35 | 0.342 | -1.77 |
| 1420019_at | Tspan8 | Tetraspanin 8 | -1.12 | 0.717 | -2.06 | 0.208 | -1.97 | 0.017 | 2.14 | 0.219 | -0.75 |
| 1454158_at | Mpp7 | membrane protein, palmitoylated 7 (MAGUK p55 subfamily member 7) | -1.46 | 0.526 | -1.64 | 0.011 | -1.7 | 0.033 | 1.83 | 0.314 | -0.74 |
| 1456823_at | Gm70 | gene model 70, (NCBI) | -2.22 | 0.259 | -2.4 | 0.146 | 1.02 | 0.961 | -1.49 | 0.124 | -1.27 |
| 1439809_at | Pramel7 | preferentially expressed antigen in melanoma like 7 | -1.7 | 0.305 | -2.43 | 0.08 | -1.13 | 0.85 | 1.65 | 0.218 | -0.9 |
| 1441715_at | B130055D15Rik | RIKEN cDNA B130055D15 gene | -1.85 | 0.209 | -2.06 | 0.341 | -1.16 | 0.413 | 1.07 | 0.829 | -1 |
| 1449989_at | Mcpt2 | mast cell protease 2 | -3.39 | 0.001 | -1.05 | 0.954 | -1.58 | 0.483 | 1.25 | 0.69 | -1.19 |
| 1420211_at | --- | --- | -1.64 | 0.475 | -1.81 | 0.255 | -1.38 | 0.441 | 1.35 | 0.246 | -0.87 |
| 1444295_at | Neo1 | neogenin | -1.15 | 0.739 | -2.07 | 0.229 | -1.88 | 0.132 | 1.6 | 0.036 | -0.87 |

| | | | | | | | | | | | |
|---|---|---|---|---|---|---|---|---|---|---|---|
| 1445811_at | Nedd8 | Neural precursor cell expressed, developmentally down-regulated gene 8 | -1.37 | 0.369 | -1.77 | 0.086 | -1.69 | 0.388 | 1.21 | 0.553 | -0.91 |
| 1441179_at | 9530020O07Rik | RIKEN cDNA 9530020O07 gene | -1.36 | 0.538 | -1.58 | 0.287 | -1.93 | 0.205 | 1.41 | 0.648 | -0.86 |
| 1460296_a_at | Fgf22 | fibroblast growth factor 22 | -1.54 | 0.674 | -1.36 | 0.658 | -1.99 | 0.165 | 1.02 | 0.935 | -0.97 |
| 1444225_at | Sgip1 | SH3-domain GRB2-like (endophilin) interacting protein 1 | -1.22 | 0.457 | -2.92 | 0.21 | -1.38 | 0.438 | 1.18 | 0.734 | -1.08 |
| 1418882_at | Nalp5 | NACHT, leucine rich repeat and PYD containing 5 | -3.05 | 0.329 | -2.17 | 0.249 | 1.1 | 0.685 | -1.1 | 0.816 | -1.3 |
| 1429263_at | 4933425D22Rik | RIKEN cDNA 4933425D22 gene | -1.88 | 0.108 | -2.05 | 0.12 | -1.15 | 0.135 | 1.34 | 0.608 | -0.94 |
| 1429975_at | 4931432M23Rik /// | RIKEN cDNA 4931432M23 gene /// similar to plasma kallikrein-like protein 4 | -1.18 | 0.605 | -3.7 | 0.001 | -1.3 | 0.626 | 1.48 | 0.07 | -1.17 |
| 1430803_at | Lin28b | lin-28 homolog B (C. elegans) | -1.11 | 0.901 | -2.39 | 0.123 | -1.76 | 0.228 | 2.45 | 0.098 | -0.7 |
| 1420146_at | --- | --- | -2.03 | 0.348 | -1.55 | 0.392 | -1.33 | 0.141 | 1.34 | 0.565 | -0.89 |
| 1447661_at | --- | Transcribed locus | -1.37 | 0.704 | -1.94 | 0.337 | -1.56 | 0.251 | 2.23 | 0.012 | -0.66 |
| 1438851_x_at | Cldn1 | claudin 1 | -1.39 | 0.632 | -3.07 | 0.018 | -1.18 | 0.725 | 2.32 | 0.023 | -0.83 |
| 1420698_at | Gpx5 | glutathione peroxidase 5 | -1.76 | 0.455 | -3.73 | 0.297 | 1.05 | 0.663 | -1.22 | 0.185 | -1.42 |
| 1422890_at | Pcdh18 | protocadherin 18 | -1.51 | 0.026 | -1.39 | 0.266 | -1.97 | 0.278 | 1.11 | 0.802 | -0.94 |
| 1434136_at | 6332401O19Rik | RIKEN cDNA 6332401O19 gene | -1.27 | 0.31 | -1.47 | 0.448 | -2.35 | 0.139 | 1.78 | 0.255 | -0.83 |
| 1443652_x_at | Spred1 | sprouty protein with EVH-1 domain 1, related sequence | -2.47 | 0.244 | -1.56 | 0.179 | -1.18 | 0.816 | 2.32 | 0.344 | -0.72 |
| 1444630_at | D2Ertd127e | DNA segment, Chr 2, ERATO Doi 127, expressed | -1.52 | 0.334 | -2.3 | 0.246 | -1.25 | 0.511 | 1.51 | 0.058 | -0.89 |
| 1446197_at | Lphn3 | Latrophilin 3 | -1.16 | 0.532 | -4.48 | 0.034 | -1.24 | 0.688 | 1.29 | 0.776 | -1.4 |
| 1446763_at | Kcnh1 | Potassium voltage-gated channel, subfamily H (eag-related), member 1 | -1.99 | 0.348 | -1.49 | 0.467 | -1.39 | 0.117 | 1.1 | 0.647 | -0.94 |
| 1446104_at | Srgap3 | SLIT-ROBO Rho GTPase activating protein 3 | -1.58 | 0.205 | -1.4 | 0.121 | -1.84 | 0.198 | 1.03 | 0.78 | -0.95 |
| 1445190_at | Golph4 | golgi phosphoprotein 4 | -1.79 | 0.563 | -2.11 | 0.117 | -1.16 | 0.661 | 1.06 | 0.939 | -1 |
| 1445309_at | --- | --- | -2.4 | 0.015 | -2.16 | 0.013 | 1.01 | 0.966 | -1.51 | 0.417 | -1.26 |
| 1444953_at | Nucks1 | Nuclear casein kinase and cyclin-dependent kinase substrate 1 | -2.72 | 0.098 | -1.21 | 0.728 | -1.43 | 0.337 | 1.1 | 0.847 | -1.07 |
| 1458492_x_at | Hnt | neurotrimin | -1.58 | 0.543 | -1.83 | 0.084 | -1.4 | 0.242 | 1.29 | 0.569 | -0.88 |
| 1458244_at | Tmco5 | transmembrane and coiled-coil domains 5 | -1.25 | 0.716 | -1.91 | 0.069 | -1.75 | 0.174 | 1.78 | 0.23 | -0.78 |
| 1444168_at | Xpr1 | xenotropic and polytropic retrovirus receptor 1 | -1.1 | 0.876 | -2.68 | 0.051 | -1.63 | 0.168 | 1.01 | 0.97 | -1.1 |
| 1429697_at | 1700023D19Rik | RIKEN cDNA 1700023D19 gene | -1.51 | 0.434 | -1.45 | 0.188 | -1.85 | 0.213 | 1.7 | 0.465 | -0.78 |
| 1446419_at | --- | --- | 1.16 | 0.548 | -3.41 | 0.168 | -2.28 | 0.367 | -1.57 | 0.187 | -1.52 |
| 1443912_at | --- | --- | -2.4 | 0.089 | -1.3 | 0.606 | -1.41 | 0.41 | 2.81 | 0.188 | -0.58 |
| 1433483_s_at | C86187 | expressed sequence C86187 | -1.07 | 0.866 | -1.79 | 0.268 | -2.46 | 0.035 | 1.11 | 0.343 | -1.05 |
| 1459149_at | --- | --- | -1.37 | 0.141 | -1.84 | 0.23 | -1.6 | 0.15 | 1.65 | 0.355 | -0.79 |
| 1447572_at | 1700052I22Rik | RIKEN cDNA 1700052I22 gene | -3.33 | 0.027 | -1.01 | 0.981 | -1.63 | 0.325 | 1.51 | 0.61 | -1.12 |
| 1442632_at | Centg2 | Centaurin, gamma 2 | -1.66 | 0.166 | -1.44 | 0.189 | -1.66 | 0.148 | 1.25 | 0.375 | -0.88 |
| 1460746_at | Fignl1 | fidgetin-like 1 | -1.49 | 0.002 | -2.01 | 0.049 | -1.37 | 0.156 | 1.73 | 0.335 | -0.79 |
| 1454513_at | 9430031J08Rik | RIKEN cDNA 9430031J08 gene | -2.39 | 0.174 | -1.29 | 0.616 | -1.41 | 0.123 | 1.41 | 0.49 | -0.92 |
| 1422722_at | 1700001K19Rik | RIKEN cDNA 1700001K19 gene | -1.76 | 0.466 | -1.57 | 0.386 | -1.44 | 0.452 | 1.07 | 0.836 | -0.92 |
| 1458930_at | A4gnt | alpha-1,4-N-acetylglucosaminyltransferase | -1.31 | 0.094 | -1.83 | 0.168 | -1.7 | 0.184 | 1.41 | 0.14 | -0.86 |
| 1425770_at | A730028G07Rik | RIKEN cDNA A730028G07 gene | -4.91 | 0.359 | -1.86 | 0.248 | 1.16 | 0.283 | -1.06 | 0.777 | -1.67 |
| 1442742_at | Atp2c1 | ATPase, Ca++-sequestering | -1.27 | 0.222 | -3.17 | 0.113 | -1.26 | 0.249 | 1.25 | 0.325 | -1.11 |
| 1441752_at | Art3 | ADP-ribosyltransferase 3 | -1.7 | 0.481 | -1.52 | 0.437 | -1.53 | 0.182 | 1.74 | 0.259 | -0.75 |
| 1445795_at | Chka | choline kinase alpha | -3.08 | 0.246 | 1.15 | 0.646 | -2.33 | 0.034 | -1.12 | 0.857 | -1.34 |
| 1443725_at | --- | --- | -2.31 | 0.12 | -1.22 | 0.606 | -1.54 | 0.35 | 2.56 | 0.154 | -0.62 |
| 1421764_at | V1rd9 | vomeronasal 1 receptor, D9 | -2.02 | 0.472 | -1.7 | 0.122 | -1.22 | 0.239 | 1.76 | 0.244 | -0.79 |
| 1425218_a_at | Scgb3a2 | secretoglobin, family 3A, member 2 | -1.94 | 0.006 | -1.53 | 0.344 | -1.36 | 0.005 | 1.2 | 0.303 | -0.91 |
| 1429663_at | Katnal2 | katanin p60 subunit A-like 2 | -2.43 | 0.209 | -1.72 | 0.161 | -1.1 | 0.593 | 1.15 | 0.764 | -1.02 |
| 1458982_at | AU014965 | expressed sequence AU014965 | -2.24 | 0.034 | -2.13 | 0.081 | -1.01 | 0.985 | 2.85 | 0.324 | -0.63 |
| 1456549_at | Gm550 | gene model 550, (NCBI) | -1.27 | 0.614 | -1.47 | 0.33 | -2.28 | 0.013 | 1.42 | 0.313 | -0.9 |
| 1443865_at | Gabra2 | gamma-aminobutyric acid (GABA-A) receptor, subunit alpha 2 | 1.07 | 0.931 | -3.79 | 0.016 | -1.76 | 0.043 | -3.58 | 0.011 | -2.01 |
| 1447899_x_at | Tacstd1 | tumor-associated calcium signal transducer 1 | -1.5 | 0.136 | -1.85 | 0.178 | -1.43 | 0.095 | 1.11 | 0.743 | -0.92 |
| 1446656_at | Angpt1 | Angiopoietin 1 | -1.64 | 0.05 | -1.44 | 0.035 | -1.66 | 0.05 | 1.02 | 0.961 | -0.93 |
| 1429200_at | Saal1 | serum amyloid A-like 1 | -1.09 | 0.927 | -2.55 | 0.151 | -1.67 | 0.012 | 1.04 | 0.931 | -1.07 |
| 1419822_at | Eif3s6 | Eukaryotic translation initiation factor 3, subunit 6 | -1.55 | 0.161 | -1.33 | 0.587 | -1.96 | 0.043 | 1.11 | 0.886 | -0.93 |
| 1430765_at | 5730521K06Rik | RIKEN cDNA 5730521K06 gene | -1.69 | 0.535 | -2.13 | 0.052 | -1.18 | 0.613 | 1.01 | 0.948 | -1 |
| 1443194_at | --- | --- | -1.09 | 0.919 | -1.56 | 0.514 | -2.84 | 0.015 | 1.04 | 0.929 | -1.11 |

| | | | | | | | | | | | |
|---|---|---|---|---|---|---|---|---|---|---|---|
| 1421293_at | Hdgfl1 | hepatoma derived growth factor-like 1 | 1.02 | 0.976 | -2.29 | 0.034 | -2.19 | 0.045 | -1.05 | 0.944 | -1.13 |
| 1430877_at | 8030425K09Rik | RIKEN cDNA 8030425K09 gene | -2.25 | 0.477 | -1.33 | 0.052 | -1.4 | 0.471 | 1.3 | 0.069 | -0.92 |
| 1443876_at | Camk2a | Calcium/calmodulin-dependent protein kinase II alpha | -1.45 | 0.528 | -3.95 | 0.027 | -1.03 | 0.921 | 1.82 | 0.28 | -1.15 |
| 1444990_at | Hivep2 | Human immunodeficiency virus type I enhancer binding protein 2 | -1.72 | 0.255 | -1.38 | 0.347 | -1.65 | 0.051 | 1.41 | 0.462 | -0.83 |
| 1442936_at | Dsg3 | Desmoglein 3 | -3.46 | 0.028 | -1.8 | 0.069 | 1.07 | 0.743 | -1.68 | 0.481 | -1.47 |
| 1429791_at | A930004D18Rik | RIKEN cDNA A930004D18 gene | -1.86 | 0.299 | -1.01 | 0.947 | -2.56 | 0.038 | 1.05 | 0.932 | -1.1 |
| 1450094_at | Ranbp17 | RAN binding protein 17 | -3.13 | 0.447 | -1.37 | 0.401 | -1.16 | 0.785 | 1.69 | 0.447 | -0.99 |
| 1460111_at | Myt1l | myelin transcription factor 1-like | -2.87 | 0.002 | 1.03 | 0.948 | -1.86 | 0.051 | -2.73 | 0.069 | -1.61 |
| 1442457_at | --- | --- | -2.98 | 0.334 | -1.1 | 0.803 | -1.48 | 0.397 | 1.57 | 0.413 | -1 |
| 1435054_at | Eme1 | essential meiotic endonuclease 1 homolog 1 (S. pombe) | -1.6 | 0.606 | -1.48 | 0.488 | -1.62 | 0.223 | 2.47 | 0.276 | -0.56 |
| 1426309_at | Asb9 | ankyrin repeat and SOCS box-containing protein 9 | -1.47 | 0.033 | -1.38 | 0.102 | -1.97 | 0.297 | 1.1 | 0.846 | -0.93 |
| 1435895_at | D930023J12Rik | RIKEN cDNA D930023J12 gene | -2.57 | 0.165 | -1.35 | 0.556 | -1.27 | 0.376 | 1.14 | 0.691 | -1.01 |
| 1440212_at | --- | --- | -1.23 | 0.287 | -2.27 | 0.339 | -1.5 | 0.483 | 1.04 | 0.961 | -0.99 |
| 1439598_at | Foxo3a | Forkhead box O3a | -1.61 | 0.181 | -1.28 | 0.031 | -1.94 | 0.05 | 1.64 | 0.254 | -0.8 |
| 1458635_at | 4832428D23Rik | RIKEN cDNA 4832428D23 gene | -1.92 | 0.516 | 1 | 0.999 | -2.53 | 0.148 | -1.39 | 0.678 | -1.21 |
| 1454417_at | 4933408M05Rik | RIKEN cDNA 4933408M05 gene | -1.79 | 0.495 | -2 | 0.262 | -1.17 | 0.638 | 1.01 | 0.983 | -0.98 |
| 1429657_at | Zfand5 | zinc finger, AN1-type domain 5 | -2.75 | 0.051 | -1.16 | 0.832 | -1.45 | 0.188 | 1.76 | 0.317 | -0.9 |
| 1430824_at | Arhgap23 | Rho GTPase activating protein 23 | -1.76 | 0.055 | -1.09 | 0.749 | -2.32 | 0.042 | 1.85 | 0.396 | -0.83 |
| 1430874_at | 9430085M18Rik | RIKEN cDNA 9430085M18 gene | -1.14 | 0.663 | -2.03 | 0.504 | -1.82 | 0.19 | 1.49 | 0.191 | -0.87 |
| 1449479_at | Cyp2b13 | cytochrome P450, family 2, subfamily b, polypeptide 13 | -2.74 | 0.07 | -1.55 | 0.471 | -1.1 | 0.681 | 1.93 | 0.017 | -0.87 |
| 1443658_at | D8Ertd457e | DNA segment, Chr 8, ERATO Doi 457, expressed | 1.17 | 0.741 | -2.54 | 0.016 | -2.85 | 0.047 | -1.05 | 0.163 | -1.32 |
| 1459692_at | AI661323 | expressed sequence AI661323 | -2.27 | 0.025 | -1.26 | 0.757 | -1.45 | 0.115 | 8.85 | 0.156 | 0.97 |
| 1444631_at | Zfp410 | Zinc finger protein 410 | -1.54 | 0.575 | -1.59 | 0.443 | -1.56 | 0.403 | 2.31 | 0.327 | -0.59 |
| 1429992_at | Speer4b | spermatogenesis associated glutamate (E)-rich protein 4b | -1.88 | 0.538 | -2.09 | 0.233 | -1.1 | 0.653 | 1.11 | 0.859 | -0.99 |
| 1446202_at | --- | Transcribed locus | -2.56 | 0.155 | -1.07 | 0.931 | -1.69 | 0.357 | 1.23 | 0.656 | -1.02 |
| 1423915_at | Olfml2b | olfactomedin-like 2B | -2.36 | 0.266 | -1.33 | 0.376 | -1.34 | 0.154 | 1.8 | 0.284 | -0.81 |
| 1456456_x_at | Mela | Melanoma antigen | -1.84 | 0.329 | -1.25 | 0.635 | -1.73 | 0.388 | 1.97 | 0.499 | -0.71 |
| 1424146_at | Gpr37l1 | G protein-coupled receptor 37-like 1 | -1.18 | 0.528 | -2.14 | 0.152 | -1.64 | 0.381 | 1.15 | 0.678 | -0.95 |
| 1448911_at | Atp4b | ATPase, H+/K+ exchanging, beta polypeptide | -5.87 | 0.006 | -1.68 | 0.074 | 1.16 | 0.427 | -1.7 | 0.41 | -2.02 |
| 1420354_at | Cnnm1 | cyclin M1 | -1.43 | 0.462 | -1.94 | 0.342 | -1.41 | 0.566 | 1.21 | 0.803 | -0.89 |
| 1420418_at | Syt2 | synaptotagmin II | -1.79 | 0.425 | -1.89 | 0.337 | -1.2 | 0.604 | 1.42 | 0.57 | -0.86 |
| 1428460_at | Syn2 | synapsin II | -1.45 | 0.224 | -3.47 | 0.327 | -1.06 | 0.885 | 1.12 | 0.836 | -1.21 |
| 1444262_at | 1110017F19Rik | RIKEN cDNA 1110017F19 gene | -1.21 | 0.286 | -1.9 | 0.072 | -1.74 | 0.021 | 3.08 | 0.295 | -0.44 |
| 1441398_at | --- | --- | -1.97 | 0.339 | -1.04 | 0.93 | -2.19 | 0.05 | 1.28 | 0.364 | -0.98 |
| 1440256_at | Rgs9bp | regulator of G-protein signalling 9 binding protein | -1.22 | 0.727 | -1.82 | 0.15 | -1.8 | 0.368 | 1.21 | 0.106 | -0.91 |
| 1433186_at | 2610019A05Rik | RIKEN cDNA 2610019A05 gene | -2.17 | 0.182 | -1.35 | 0.357 | -1.38 | 0.335 | 1.41 | 0.229 | -0.87 |
| 1456289_at | Hamp2 /// Hamp1 | hepcidin antimicrobial peptide 2 /// hepcidin antimicrobial peptide 1 | -1.86 | 0.315 | -1.32 | 0.355 | -1.58 | 0.21 | 2.61 | 0.376 | -0.54 |
| 1460480_at | 1600014E20Rik | RIKEN cDNA 1600014E20 gene | -1.26 | 0.743 | -1.73 | 0.018 | -1.79 | 0.037 | 1.34 | 0.657 | -0.86 |
| 1447110_at | 4921513D23Rik | RIKEN cDNA 4921513D23 gene | -2.55 | 0.011 | -1.03 | 0.933 | -1.77 | 0.181 | 1.15 | 0.493 | -1.05 |
| 1419214_at | Tnfrsf11a | tumor necrosis factor receptor superfamily, member 11a | -1.83 | 0.32 | -1.49 | 0.204 | -1.41 | 0.189 | 1.83 | 0.12 | -0.72 |
| 1447714_x_at | --- | Transcribed locus | -2.35 | 0.127 | -2.1 | 0.298 | 1.03 | 0.958 | -2.17 | 0.097 | -1.4 |
| 1460683_at | Tead4 | TEA domain family member 4 | -1.26 | 0.32 | -1.37 | 0.281 | -2.46 | 0.232 | 1.44 | 0.135 | -0.91 |
| 1434324_x_at | Eif3s3 /// LOC6250 | eukaryotic translation initiation factor 3, subunit 3 (gamma) /// similar to eukaryotic | -2.08 | 0.219 | -1.18 | 0.42 | -1.66 | 0.38 | 1.03 | 0.93 | -0.97 |
| 1448039_at | LOC622175 | Similar to gonadotropin inducible ovarian transcription factor 2 | -3.06 | 0.444 | 1.07 | 0.928 | -1.87 | 0.292 | -1.04 | 0.878 | -1.23 |
| 1445679_at | Atp2b3 | ATPase, Ca++ transporting, plasma membrane 3 | -2.48 | 0.124 | 1.11 | 0.527 | -2.41 | 0.015 | -2.09 | 0.255 | -1.47 |
| 1446668_at | --- | PREDICTED: Mus musculus similar to sidekick homolog 1 (LOC620969), mRNA | -3.6 | 0.255 | -2.02 | 0.335 | 1.16 | 0.801 | -1.41 | 0.366 | -1.47 |
| 1443580_at | Stxbp6 | Syntaxin binding protein 6 (amisyn) | -1.98 | 0.467 | -1.33 | 0.662 | -1.49 | 0.546 | 2.22 | 0.055 | -0.65 |
| 1441847_at | Mast2 | Microtubule associated serine/threonine kinase 2 | -1.2 | 0.831 | -1.96 | 0.047 | -1.72 | 0.337 | 2.81 | 0.313 | -0.51 |
| 1439865_at | Kbtbd7 | kelch repeat and BTB (POZ) domain containing 7 | -2.72 | 0.337 | -1.4 | 0.005 | -1.18 | 0.063 | 1.46 | 0.43 | -0.96 |
| 1421975_a_at | Add2 | adducin 2 (beta) | -1.74 | 0.269 | -1.11 | 0.863 | -2.18 | 0.331 | 5.63 | 0.172 | 0.15 |
| 1446355_at | 9530085L11Rik | RIKEN cDNA 9530085L11 gene | -2.01 | 0.319 | -1.82 | 0.25 | -1.13 | 0.606 | 1.55 | 0.447 | -0.85 |
| 1457830_at | Net1 | Neuroepithelial cell transforming gene 1 | -1.74 | 0.058 | -1.3 | 0.287 | -1.71 | 0.039 | 1.75 | 0.322 | -0.75 |
| 1436786_at | 1110069O07Rik | RIKEN cDNA 1110069O07 gene | -1.95 | 0.243 | -1.61 | 0.324 | -1.26 | 0.007 | 1.16 | 0.794 | -0.91 |

| | | | | | | | | | | | |
|---|---|---|---|---|---|---|---|---|---|---|---|
| 1441776_at | Tspan11 | tetraspanin 11 | -1.79 | 0.381 | -1.54 | 0.056 | -1.38 | 0.401 | 1.1 | 0.836 | -0.9 |
| 1456931_at | C85363 | expressed sequence C85363 | -1.22 | 0.061 | -1.71 | 0.264 | -1.89 | 0.093 | 1.02 | 0.942 | -0.95 |
| 1424679_at | Mab21l1 | mab-21-like 1 (C. elegans) | -1.59 | 0.496 | -3.21 | 0.137 | -1.01 | 0.987 | 1.59 | 0.118 | -1.05 |
| 1420807_a_at | Egfl9 | EGF-like-domain, multiple 9 | -1.54 | 0.088 | -2.09 | 0.196 | -1.25 | 0.625 | 1.64 | 0.563 | -0.81 |
| 1421349_x_at | Cend1 | cell cycle exit and neuronal differentiation 1 | -1.08 | 0.829 | -2.36 | 0.217 | -1.7 | 0.357 | 1.01 | 0.99 | -1.04 |
| 1457409_at | Fut9 | fucosyltransferase 9 | -1.05 | 0.912 | -3.3 | 0.152 | -1.47 | 0.576 | 1.04 | 0.902 | -1.2 |
| 1444784_at | 4930564K09Rik | RIKEN cDNA 4930564K09 gene | -1.81 | 0.141 | -1.18 | 0.805 | -1.88 | 0.139 | 1.24 | 0.467 | -0.91 |
| 1444190_at | E030049G20Rik | RIKEN cDNA E030049G20 gene | -1.79 | 0.012 | -1.26 | 0.491 | -1.73 | 0.088 | 1.03 | 0.953 | -0.94 |
| 1446920_at | E130308A19Rik | RIKEN cDNA E130308A19 gene | -1.49 | 0.096 | -1.37 | 0.324 | -1.88 | 0.005 | 1.75 | 0.271 | -0.75 |
| 1446641_at | Syt7 | Synaptotagmin VII | -2.22 | 0.139 | -1.67 | 0.033 | -1.13 | 0.778 | 1.16 | 0.722 | -0.97 |
| 1445614_at | --- | --- | -1.81 | 0.177 | -1.28 | 0.678 | -1.67 | 0.23 | 1.29 | 0.692 | -0.87 |
| 1427122_at | Copg2as2 | coatomer protein complex, subunit gamma 2, antisense 2 | -2.43 | 0.454 | -1.17 | 0.766 | -1.49 | 0.116 | 1.28 | 0.762 | -0.95 |
| 1447269_at | 9630031F12Rik | RIKEN cDNA 9630031F12 gene | -1.09 | 0.727 | -2.88 | 0.087 | -1.49 | 0.139 | 1.04 | 0.934 | -1.11 |
| 1445977_at | AU022793 | expressed sequence AU022793 | -1.21 | 0.798 | -1.7 | 0.202 | -1.9 | 0.403 | 1.64 | 0.227 | -0.79 |
| 1426252_a_at | Umod | uromodulin | -1.13 | 0.049 | -11.04 | 0.133 | -1.04 | 0.95 | 1.73 | 0.23 | -2.87 |
| 1446926_at | Pycard | PYD and CARD domain containing | -1.27 | 0.445 | -2.56 | 0.01 | -1.31 | 0.103 | 1.11 | 0.609 | -1.01 |
| 1432045_at | Tssk5 | testis-specific serine kinase 5 | -1.83 | 0.323 | -2.09 | 0.366 | -1.1 | 0.765 | 2.34 | 0.005 | -0.67 |
| 1449522_at | Unc5c | unc-5 homolog C (C. elegans) | -1.59 | 0.004 | -1.49 | 0.428 | -1.57 | 0.054 | 1.05 | 0.786 | -0.9 |
| 1426166_at | Mup5 | major urinary protein 5 | -1.44 | 0.496 | -1.56 | 0.087 | -1.66 | 0.047 | 1.37 | 0.58 | -0.82 |
| 1430470_at | 5730411F24Rik | RIKEN cDNA 5730411F24 gene | -1.63 | 0.236 | -1.64 | 0.062 | -1.4 | 0.008 | 1.08 | 0.617 | -0.9 |
| 1433347_at | A930036A04Rik | RIKEN cDNA A930036A04 gene | -1.5 | 0.553 | -1.08 | 0.893 | -2.89 | 0.04 | 1.33 | 0.659 | -1.04 |
| 1428089_at | Slitrk1 | SLIT and NTRK-like family, member 1 | -2.4 | 0.155 | -1.93 | 0.293 | 1 | 0.996 | -1.39 | 0.245 | -1.18 |
| 1457546_at | Cnot1 | CCR4-NOT transcription complex, subunit 1 | -1.56 | 0.403 | -1.26 | 0.634 | -1.99 | 0.11 | 1.43 | 0.271 | -0.84 |
| 1457894_at | --- | --- | -2.29 | 0.026 | -1.08 | 0.852 | -1.72 | 0.209 | 2.31 | 0.141 | -0.69 |
| 1444707_at | --- | Transcribed locus | 1.01 | 0.987 | -3.16 | 0.056 | -1.62 | 0.351 | -1.26 | 0.412 | -1.26 |
| 1432838_at | 6430598H11Rik | RIKEN cDNA 6430598H11 gene | 1 | 0.99 | -3.08 | 0.091 | -1.63 | 0.412 | -2.5 | 0.299 | -1.55 |
| 1431683_at | 4930473H19Rik | RIKEN cDNA 4930473H19 gene | 1.08 | 0.893 | -2.01 | 0.349 | -2.72 | 0.07 | -1.22 | 0.622 | -1.22 |
| 1453660_at | 5430420F09Rik | RIKEN cDNA 5430420F09 gene | -1.02 | 0.919 | -2.53 | 0.005 | -1.76 | 0.067 | 1.26 | 0.604 | -1.01 |
| 1439385_x_at | Slc13a3 | Solute carrier family 13 (sodium-dependent dicarboxylate transporter), member 3 | -1.87 | 0.377 | -1.44 | 0.355 | -1.4 | 0.325 | 3.19 | 0.004 | -0.38 |
| 1424855_at | Olah | oleoyl-ACP hydrolase | -1.87 | 0.074 | -1.84 | 0.525 | -1.15 | 0.752 | 1.06 | 0.731 | -0.95 |
| 1422312_a_at | Neurog3 | neurogenin 3 | -1.44 | 0.34 | -1.73 | 0.436 | -1.48 | 0.431 | 1.52 | 0.465 | -0.78 |
| 1437219_at | Igf1r | Insulin-like growth factor I receptor | -1.5 | 0.028 | -1.41 | 0.29 | -1.75 | 0.02 | 1.09 | 0.706 | -0.89 |
| 1449378_at | Krt27 | keratin 27 | -3.08 | 0.351 | 1.1 | 0.107 | -1.91 | 0.2 | -1.22 | 0.74 | -1.28 |
| 1444756_at | --- | Transcribed locus | -1.18 | 0.532 | -1.54 | 0.44 | -2.2 | 0.353 | 1.02 | 0.946 | -0.98 |
| 1421711_at | Zfp109 | zinc finger protein 109 | -2.59 | 0.218 | -1.77 | 0.067 | -1 | 0.988 | 1.15 | 0.768 | -1.05 |
| 1432294_at | 9330177L23Rik | RIKEN cDNA 9330177L23 gene | -1.23 | 0.58 | -2.6 | 0.292 | -1.34 | 0.277 | 1.19 | 0.829 | -0.99 |
| 1438387_x_at | Top3b | topoisomerase (DNA) III beta | -1.75 | 0.078 | -1.5 | 0.066 | -1.41 | 0.444 | 1.73 | 0.27 | -0.73 |
| 1444414_at | Apod | Apolipoprotein D | -2.28 | 0.356 | -1.02 | 0.961 | -1.88 | 0.121 | 1.26 | 0.738 | -0.98 |
| 1424848_at | Kcnma1 | potassium large conductance calcium-activated channel, subfamily M, alpha men | -1.05 | 0.938 | -1.69 | 0.381 | -2.49 | 0.03 | 2.55 | 0.062 | -0.67 |
| 1449513_at | Adam24 | a disintegrin and metallopeptidase domain 24 (testase 1) | -1.7 | 0.248 | -1.17 | 0.487 | -1.98 | 0.31 | 1.01 | 0.95 | -0.96 |
| 1450553_at | Hoxd12 | homeo box D12 | -1.17 | 0.65 | -2.99 | 0.259 | -1.32 | 0.421 | 1.01 | 0.977 | -1.12 |
| 1444565_at | BB166591 | expressed sequence BB166591 | -1.29 | 0.093 | -1.3 | 0.227 | -2.45 | 0.013 | 2.6 | 0.238 | -0.61 |
| 1438333_at | Prtg | protogenin homolog (Gallus gallus) | -2.36 | 0.431 | -2.86 | 0.041 | 1.18 | 0.783 | -1.51 | 0.341 | -1.39 |
| 1434501_at | --- | --- | -1.51 | 0.316 | -1.11 | 0.872 | -2.61 | 0.101 | 5.44 | 0.191 | 0.05 |
| 1447487_at | --- | --- | -3.59 | 0.322 | -2.24 | 0.129 | 1.22 | 0.648 | -1.03 | 0.962 | -1.41 |
| 1425976_x_at | Zfp353 | zinc finger protein 353 | -3.27 | 0.081 | 1.04 | 0.953 | -1.67 | 0.465 | -1.06 | 0.849 | -1.24 |
| 1432945_at | 5230400M06Rik | RIKEN cDNA 5230400M06 gene | -3.04 | 0.12 | -2.42 | 0.141 | 1.21 | 0.639 | -1.29 | 0.354 | -1.39 |
| 1444297_at | Serpina4-ps1 | serine (or cysteine) peptidase inhibitor, clade A, member 4, pseudogene 1 | 1.39 | 0.597 | -24.88 | 0.181 | -1.91 | 0.278 | -1.08 | 0.538 | -6.62 |
| 1453094_at | Ches1 | checkpoint suppressor 1 | -1.47 | 0.06 | -2.41 | 0 | -1.17 | 0.323 | 1.24 | 0.634 | -0.95 |
| 1456305_x_at | LOC666466 | similar to oocyte specific homeobox 2 | -1.4 | 0.355 | -1.53 | 0.208 | -1.71 | 0.559 | 1.43 | 0.536 | -0.8 |
| 1433578_at | Slc10a4 | solute carrier family 10 (sodium/bile acid cotransporter family), member 4 | -1.44 | 0.722 | -2.79 | 0.017 | -1.11 | 0.276 | 1.02 | 0.912 | -1.08 |
| 1459222_x_at | --- | --- | -2.45 | 0.029 | -1.02 | 0.957 | -1.78 | 0.229 | 1.5 | 0.428 | -0.94 |
| 1428804_at | Mfap3l | microfibrillar-associated protein 3-like | -1.13 | 0.513 | -1.61 | 0.256 | -2.23 | 0.001 | 1.65 | 0.414 | -0.83 |

| | | | | | | | | | | | |
|---|---|---|---|---|---|---|---|---|---|---|---|
| 1440199_at | AU042651 | expressed sequence AU042651 | -1.46 | 0.245 | -1.11 | 0.844 | -2.73 | 0.085 | 1.1 | 0.902 | -1.05 |
| 1421695_at | Strc | stereocilin | -1.26 | 0.636 | -1.93 | 0.256 | -1.56 | 0.226 | 1.69 | 0.257 | -0.76 |
| 1446382_at | --- | 0 day neonate cerebellum cDNA, RIKEN full-length enriched library, clone:C2300 | -1.86 | 0.32 | -1.48 | 0.209 | -1.35 | 0.06 | 1 | 0.996 | -0.92 |
| 1458102_at | Plxna2 | Plexin A2 | -1.1 | 0.62 | -1.68 | 0.231 | -2.24 | 0.002 | 1.93 | 0.098 | -0.77 |
| 1421202_at | Chrna4 | cholinergic receptor, nicotinic, alpha polypeptide 4 | -1.16 | 0.765 | -1.71 | 0.333 | -1.97 | 0.276 | 2.09 | 0.055 | -0.69 |
| 1439627_at | Zic1 | zinc finger protein of the cerebellum 1 | -1.2 | 0.718 | -1.68 | 0.56 | -1.9 | 0.305 | 1.22 | 0.729 | -0.89 |
| 1423012_at | Syt7 | synaptotagmin VII | -3.29 | 0.029 | -1.28 | 0.507 | -1.15 | 0.804 | 1.08 | 0.414 | -1.16 |
| 1441527_at | 4930558J18Rik | RIKEN cDNA 4930558J18 gene | -1.25 | 0.603 | -1.73 | 0.379 | -1.73 | 0.355 | 1.71 | 0.065 | -0.75 |
| 1456239_at | Fgf17 | fibroblast growth factor 17 | -2.45 | 0.507 | 1.05 | 0.776 | -1.99 | 0.081 | -2.12 | 0.449 | -1.38 |
| 1418297_at | Dpysl4 | dihydropyrimidinase-like 4 | -1.75 | 0.417 | -1.44 | 0.348 | -1.44 | 0.499 | 1.16 | 0.405 | -0.87 |
| 1458732_at | --- | --- | -2.73 | 0.331 | 1.02 | 0.975 | -1.75 | 0.127 | -1.55 | 0.358 | -1.25 |
| 1425564_at | Rest | RE1-silencing transcription factor | -1.33 | 0.389 | -2.31 | 0.256 | -1.3 | 0.498 | 1.18 | 0.718 | -0.94 |
| 1432362_at | Cenpp | centromere protein P | -1.38 | 0.404 | -1.96 | 0.119 | -1.39 | 0.444 | 2.48 | 0.299 | -0.56 |
| 1432340_at | 1700121N20Rik | RIKEN cDNA 1700121N20 gene | -1.86 | 0.54 | -1.67 | 0.55 | -1.21 | 0.417 | 1.16 | 0.191 | -0.9 |
| 1458046_at | --- | 0 day neonate kidney cDNA, RIKEN full-length enriched library, clone:D630027M | -1.05 | 0.819 | -2.1 | 0.033 | -1.88 | 0.005 | 1.53 | 0.073 | -0.87 |
| 1421806_at | Defb3 | defensin beta 3 | -1.16 | 0.6 | -3.55 | 0.132 | -1.22 | 0.707 | 1.24 | 0.696 | -1.18 |
| 1439728_at | D330027H18Rik | RIKEN cDNA D330027H18 gene | -2.24 | 0.116 | -1.88 | 0.207 | -1.02 | 0.892 | 1.1 | 0.746 | -1.01 |
| 1435963_at | --- | --- | -1.74 | 0.358 | -2.19 | 0.065 | -1.08 | 0.733 | 1.27 | 0.266 | -0.93 |
| 1425541_at | Ddx27 | DEAD (Asp-Glu-Ala-Asp) box polypeptide 27 | -2.26 | 0.339 | -1.17 | 0.5 | -1.5 | 0.125 | 2.48 | 0.105 | -0.61 |
| 1440960_at | --- | 3 days neonate thymus cDNA, RIKEN full-length enriched library, clone:A630060 | -2.74 | 0.464 | -2.96 | 0.256 | 1.26 | 0.634 | -2.22 | 0.405 | -1.66 |
| 1441125_at | Setd5 | SET domain containing 5 | -1.65 | 0.525 | -2.93 | 0.045 | 1.02 | 0.981 | -2.03 | 0.559 | -1.4 |
| 1444265_at | --- | --- | -1.43 | 0.51 | -7.62 | 0 | 1.13 | 0.276 | -1.65 | 0.486 | -2.39 |
| 1431585_at | 1700034P13Rik | RIKEN cDNA 1700034P13 gene | -1.53 | 0.267 | -2.13 | 0.005 | -1.19 | 0.714 | 1.31 | 0.514 | -0.88 |
| 1420268_x_at | Npm1 | Nucleophosmin 1 | -1.1 | 0.86 | -1.36 | 0.599 | -3.18 | 0.006 | 1.18 | 0.658 | -1.11 |
| 1455272_at | Grm5 | glutamate receptor, metabotropic 5 | -2.07 | 0.426 | -2.56 | 0.318 | 1.09 | 0.807 | -1.06 | 0.819 | -1.15 |
| 1421382_at | Prlr | prolactin receptor | -1.62 | 0.017 | -1.1 | 0.481 | -2.29 | 0.233 | 1.13 | 0.701 | -0.97 |
| 1460459_at | Paqr5 | progestin and adipoQ receptor family member V | 1.02 | 0.954 | -2.59 | 0.172 | -1.77 | 0.208 | -2.51 | 0.011 | -1.47 |
| 1432277_at | 4930565D16Rik | RIKEN cDNA 4930565D16 gene | -1.87 | 0.021 | -1.24 | 0.634 | -1.6 | 0.383 | 1.22 | 0.439 | -0.87 |
| 1459562_at | --- | --- | -1.12 | 0.779 | -1.83 | 0.095 | -1.9 | 0.047 | 1.29 | 0.396 | -0.89 |
| 1423477_at | Zic1 | zinc finger protein of the cerebellum 1 | -4.35 | 0.029 | 1.03 | 0.972 | -1.41 | 0.552 | -2.83 | 0.259 | -1.89 |
| 1440100_at | 4930527D15Rik | RIKEN cDNA 4930527D15 gene | -1.42 | 0.644 | -1.8 | 0.509 | -1.41 | 0.571 | 1.17 | 0.755 | -0.87 |
| 1438189_s_at | Epb4.9 | erythrocyte protein band 4.9 | -1.01 | 0.994 | -1.75 | 0.119 | -2.51 | 0.228 | 8.82 | 0.263 | 0.89 |
| 1449249_at | Pcdh7 | protocadherin 7 | -1.8 | 0.056 | -1.54 | 0.466 | -1.32 | 0.422 | 2.1 | 0.074 | -0.64 |
| 1447702_x_at | Igsf1 | immunoglobulin superfamily, member 1 | -1.5 | 0.535 | -1.62 | 0.436 | -1.47 | 0.178 | 1.54 | 0.408 | -0.76 |
| 1429833_at | Ly6g6e | lymphocyte antigen 6 complex, locus G6E | -1.88 | 0.218 | -1.3 | 0.473 | -1.49 | 0.145 | 1.08 | 0.708 | -0.9 |
| 1424941_at | BC016579 | cDNA sequence, BC016579 | -1.14 | 0.733 | -2.4 | 0.212 | -1.48 | 0.476 | 3.35 | 0.027 | -0.42 |
| 1459181_at | --- | PREDICTED: Mus musculus similar to Tubulin alpha-1 chain (LOC629601), mRN | -1.46 | 0.625 | -1.29 | 0.635 | -1.96 | 0.08 | 2.04 | 0.284 | -0.67 |
| 1433418_at | --- | --- | -1.54 | 0.461 | -1.63 | 0.304 | -1.41 | 0.308 | 1 | 1 | -0.9 |
| 1426189_at | Dusp15 | dual specificity phosphatase-like 15 | -1.06 | 0.512 | -2.74 | 0.125 | -1.52 | 0.187 | 1.85 | 0.378 | -0.87 |
| 1454150_at | 4930453O03Rik | RIKEN cDNA 4930453O03 gene | -1.7 | 0.22 | -1.6 | 0.423 | -1.31 | 0.154 | 1.09 | 0.82 | -0.88 |
| 1444854_at | --- | PREDICTED: Mus musculus similar to gonadotropin inducible ovarian transcriptic | -1.44 | 0.265 | -2.07 | 0.146 | -1.26 | 0.19 | 1.56 | 0.31 | -0.8 |
| 1444968_at | --- | --- | -1.45 | 0.623 | -2.34 | 0.324 | -1.17 | 0.581 | 1.12 | 0.673 | -0.96 |
| 1430712_at | Arhgap24 | Rho GTPase activating protein 24 | -1.33 | 0.464 | -2.34 | 0.092 | -1.26 | 0.316 | 1.07 | 0.775 | -0.97 |
| 1432063_at | 4833427F10Rik | RIKEN cDNA 4833427F10 gene | -1.99 | 0.08 | -1.16 | 0.747 | -1.65 | 0.378 | 2.01 | 0.194 | -0.7 |
| 1439172_at | Atp13a5 | ATPase type 13A5 | -2.62 | 0.255 | -1.41 | 0.604 | -1.14 | 0.705 | 1.41 | 0.462 | -0.94 |
| 1418065_at | Rag2 | recombination activating gene 2 | -1.19 | 0.199 | -1.63 | 0.359 | -1.92 | 0.247 | 1.1 | 0.685 | -0.91 |
| 1432961_at | 2610024D14Rik | RIKEN cDNA 2610024D14 gene | -1.84 | 0.256 | -1.51 | 0.4 | -1.3 | 0.443 | 1.32 | 0.45 | -0.83 |
| 1438357_at | Pfdn5 /// LOC43486 | prefoldin 5 /// prefoldin 5 pseudogene | -2.32 | 0.004 | 1.1 | 0.857 | -2.26 | 0.141 | -1.48 | 0.33 | -1.24 |
| 1433274_at | 2610200G18Rik | RIKEN cDNA 2610200G18 gene | -1.37 | 0.597 | -2.15 | 0.038 | -1.28 | 0.741 | 2.62 | 0.225 | -0.55 |
| 1451900_at | Mfn2 | mitofusin 2 | -1.37 | 0.243 | -1.74 | 0.283 | -1.49 | 0.12 | 1.14 | 0.727 | -0.86 |
| 1440108_at | Foxp2 | forkhead box P2 | -1.38 | 0.495 | -1.6 | 0.027 | -1.59 | 0.008 | 1.35 | 0.361 | -0.81 |
| 1438307_at | Hmgb2 | High mobility group box 2 | -1.91 | 0.177 | -1.39 | 0.595 | -1.36 | 0.201 | 2.48 | 0.249 | -0.55 |
| 1459169_at | Coro1c | Coronin, actin binding protein 1C | -1.77 | 0.439 | -1.39 | 0.668 | -1.44 | 0.496 | 1.45 | 0.391 | -0.79 |

| | | | | | | | | | | | |
|---|---|---|---|---|---|---|---|---|---|---|---|
| 1442443_at | Rbbp4 | Retinoblastoma binding protein 4 | -1.04 | 0.285 | -1.77 | 0.121 | -2.19 | 0.032 | 1.09 | 0.539 | -0.98 |
| 1432449_at | 4930564B18Rik | RIKEN cDNA 4930564B18 gene | -2.49 | 0.216 | -1.99 | 0.019 | 1.07 | 0.796 | -2.05 | 0.317 | -1.36 |
| 1445179_at | D10Ertd494e | DNA segment, Chr 10, ERATO Doi 494, expressed | -1.19 | 0.791 | -1.29 | 0.682 | -2.75 | 0.034 | 1.16 | 0.79 | -1.02 |
| 1446482_at | Cep63 | centrosomal protein 63 | -1.83 | 0.354 | -1.65 | 0.601 | -1.21 | 0.636 | 1.25 | 0.698 | -0.86 |
| 1444490_at | --- | --- | -1.63 | 0.198 | -2.68 | 0.078 | -1.01 | 0.977 | 1.73 | 0.376 | -0.9 |
| 1453542_at | 2610007B07Rik | RIKEN cDNA 2610007B07 gene | -1.36 | 0.479 | -1.56 | 0.225 | -1.65 | 0.074 | 1.78 | 0.008 | -0.7 |
| 1431508_at | 4930423M02Rik | RIKEN cDNA 4930423M02 gene | -1.57 | 0.206 | -2.8 | 0.037 | -1.01 | 0.964 | 1.04 | 0.954 | -1.09 |
| 1447123_at | --- | --- | -1.08 | 0.937 | -1.7 | 0.23 | -2.15 | 0.199 | 1.37 | 0.589 | -0.89 |
| 1432018_at | Ascl2 | achaete-scute complex homolog-like 2 (Drosophila) | -1.5 | 0.226 | -1.63 | 0.4 | -1.43 | 0.306 | 1.15 | 0.698 | -0.85 |
| 1449610_at | Ep400 | E1A binding protein p400 | -2.62 | 0.055 | -1.4 | 0.357 | -1.13 | 0.798 | 1.18 | 0.455 | -0.99 |
| 1445643_at | --- | PREDICTED: Mus musculus similar to spindle assembly associated Sfi1 homolog | -1.87 | 0.337 | -1.86 | 0.081 | -1.1 | 0.479 | 1.17 | 0.543 | -0.92 |
| 1458893_at | --- | Transcribed locus | -2.2 | 0.022 | -1.94 | 0.122 | 1.01 | 0.977 | -1.76 | 0.545 | -1.22 |
| 1439437_x_at | Cpe /// LOC677374 | carboxypeptidase E /// similar to carboxypeptidase E | -1.99 | 0.237 | 1.01 | 0.982 | -2.14 | 0 | -1.86 | 0.4 | -1.24 |
| 1447921_at | --- | Transcribed locus | -1.24 | 0.782 | -3.34 | 0.173 | -1.14 | 0.73 | 1.62 | 0.273 | -1.03 |
| 1435743_at | Klhl23 | kelch-like 23 (Drosophila) | -1.48 | 0.199 | -1.23 | 0.661 | -2.03 | 0.005 | 3.11 | 0.185 | -0.41 |
| 1432389_at | 4933438A12Rik | RIKEN cDNA 4933438A12 gene | -1.67 | 0.097 | -2.39 | 0.09 | -1.03 | 0.936 | 1.04 | 0.947 | -1.02 |
| 1438537_at | --- | --- | 1.2 | 0.365 | -2.66 | 0.053 | -2.48 | 0.32 | -1.33 | 0.615 | -1.32 |
| 1458561_at | Neurl | neuralized-like homolog (Drosophila) | -1.62 | 0.311 | -1.32 | 0.102 | -1.65 | 0.23 | 1.35 | 0.185 | -0.81 |
| 1447429_at | --- | Transcribed locus | 1.08 | 0.912 | -2.32 | 0.205 | -2.14 | 0.287 | -1.05 | 0.921 | -1.1 |
| 1418507_s_at | Socs2 | suppressor of cytokine signaling 2 | -1.19 | 0.763 | -1.93 | 0.269 | -1.6 | 0.003 | 1.03 | 0.904 | -0.92 |
| 1432936_at | 5330433J24Rik | RIKEN cDNA 5330433J24 gene | -1.16 | 0.387 | -1.44 | 0.133 | -2.33 | 0.231 | 1.1 | 0.721 | -0.96 |
| 1450207_at | Lifr | leukemia inhibitory factor receptor | -1.05 | 0.71 | -5.04 | 0.006 | -1.2 | 0.661 | 1.05 | 0.925 | -1.56 |
| 1459124_at | Pcsk5 | Proprotein convertase subtilisin/kexin type 5 | -2.12 | 0.225 | -1.32 | 0.557 | -1.32 | 0.158 | 1.32 | 0.325 | -0.86 |
| 1456690_at | --- | --- | -3.2 | 0.446 | -2.75 | 0.157 | 1.31 | 0.245 | -1.38 | 0.68 | -1.51 |
| 1458058_at | 7030407E18Rik | RIKEN cDNA 7030407E18 gene | -1.27 | 0.619 | -1.86 | 0.011 | -1.51 | 0.016 | 1.09 | 0.394 | -0.89 |
| 1439227_at | Uhrf1 | ubiquitin-like, containing PHD and RING finger domains, 1 | -1.82 | 0.084 | -1.79 | 0.078 | -1.14 | 0.196 | 2.01 | 0.308 | -0.68 |
| 1457160_at | Fjx1 | four jointed box 1 (Drosophila) | -1.8 | 0.559 | -2.03 | 0.239 | -1.06 | 0.907 | 1.4 | 0.456 | -0.87 |
| 1420228_at | --- | --- | -1.26 | 0.033 | -3.61 | 0.035 | -1.09 | 0.777 | 1.27 | 0.486 | -1.17 |
| 1454744_at | F630043A04Rik | RIKEN cDNA F630043A04 gene | -1.95 | 0.194 | -1.89 | 0.371 | -1.06 | 0.866 | 2.38 | 0.198 | -0.63 |
| 1439822_at | --- | --- | -1.12 | 0.314 | -1.25 | 0.406 | -3.34 | 0.075 | 1.28 | 0.453 | -1.11 |
| 1438589_at | Igsf1 | immunoglobulin superfamily, member 1 | 1.02 | 0.95 | -1.99 | 0.153 | -2.15 | 0.257 | -1.13 | 0.797 | -1.06 |
| 1438158_at | --- | --- | -1.94 | 0.301 | -1.58 | 0.49 | -1.19 | 0.728 | 1.09 | 0.659 | -0.91 |
| 1440535_at | Uros | uroporphyrinogen III synthase | -1.76 | 0.231 | -1.6 | 0.409 | -1.26 | 0.542 | 3.58 | 0.244 | -0.26 |
| 1418425_at | Sp7 | trans-acting transcription factor 7 | -1.68 | 0.27 | -1.74 | 0.009 | -1.22 | 0.644 | 1.1 | 0.694 | -0.88 |
| 1433146_at | Cald1 | caldesmon 1 | -1.28 | 0.455 | -1.79 | 0.059 | -1.53 | 0.08 | 1.03 | 0.942 | -0.89 |
| 1422448_at | Tff2 | trefoil factor 2 (spasmolytic protein 1) | -1.3 | 0.756 | -2 | 0.205 | -1.39 | 0.337 | 1.13 | 0.575 | -0.89 |
| 1437401_at | --- | --- | -1.32 | 0.38 | -1.18 | 0.026 | -2.56 | 0.041 | 1.32 | 0.368 | -0.94 |
| 1431856_a_at | C1qtnf6 | C1q and tumor necrosis factor related protein 6 | -2.1 | 0.47 | -1.67 | 0.551 | -1.09 | 0.831 | 1.11 | 0.328 | -0.94 |
| 1423412_at | BC013481 | cDNA sequence BC013481 | -1.92 | 0.313 | -1.51 | 0.294 | -1.24 | 0.257 | 1.01 | 0.989 | -0.91 |
| 1421458_at | Zfp112 | zinc finger protein 112 | -1.99 | 0.231 | -2.11 | 0.191 | 1.01 | 0.956 | -1.02 | 0.916 | -1.02 |
| 1438651_a_at | Agtrl1 | angiotensin receptor-like 1 | -1.37 | 0.443 | 1.05 | 0.942 | -4.79 | 0.035 | -1.98 | 0.098 | -1.77 |
| 1440843_at | A230051N06Rik | RIKEN cDNA A230051N06 gene | -1.9 | 0.024 | -1.79 | 0.01 | -1.1 | 0.601 | 1.11 | 0.696 | -0.92 |
| 1442963_at | Zhx3 | zinc fingers and homeoboxes 3 | -1.13 | 0.456 | -1.59 | 0.006 | -2.09 | 0.038 | 1.47 | 0.174 | -0.83 |
| 1427676_a_at | Grik1 | glutamate receptor, ionotropic, kainate 1 | -1.32 | 0.714 | -1.94 | 0.148 | -1.38 | 0.595 | 3.05 | 0.002 | -0.4 |
| 1430362_at | 5730409N24Rik | RIKEN cDNA 5730409N24 gene | -1.64 | 0.083 | -1.23 | 0.64 | -1.75 | 0.146 | 1.58 | 0.32 | -0.76 |
| 1444296_a_at | Serpina4-ps1 | serine (or cysteine) peptidase inhibitor, clade A, member 4, pseudogene 1 | 1.39 | 0.56 | -22.47 | 0.142 | -1.77 | 0.019 | -1.07 | 0.9 | -5.98 |
| 1445019_at | --- | --- | -1.2 | 0.822 | -1.41 | 0.511 | -2.22 | 0.244 | 1.87 | 0.502 | -0.74 |
| 1446471_at | --- | --- | 1.02 | 0.901 | -1.84 | 0.533 | -2.3 | 0.026 | -1.81 | 0.316 | -1.23 |
| 1416623_at | Thbs3 | thrombospondin 3 | 1.06 | 0.872 | -1.85 | 0.041 | -2.53 | 0.029 | -1.32 | 0.095 | -1.16 |
| 1458211_at | Nek1 | NIMA (never in mitosis gene a)-related expressed kinase 1 | -1.13 | 0.765 | -1.45 | 0.631 | -2.36 | 0.01 | 1.18 | 0.83 | -0.94 |
| 1428208_at | Bcl7a | B-cell CLL/lymphoma 7A | -1.62 | 0.29 | -1.67 | 0.127 | -1.28 | 0.067 | 1.02 | 0.922 | -0.89 |
| 1444654_at | Trerf1 | Transcriptional regulating factor 1 | -3.31 | 0.024 | -1.18 | 0.728 | -1.18 | 0.66 | 1.09 | 0.806 | -1.14 |
| 1429790_at | Brunol6 /// LOC669 | bruno-like 6, RNA binding protein (Drosophila) /// similar to bruno-like 6, RNA bind | -2.52 | 0.137 | -1.03 | 0.948 | -1.58 | 0.1 | 1.21 | 0.822 | -0.98 |

| | | | | | | | | | | | |
|---|---|---|---|---|---|---|---|---|---|---|---|
| 1459912_at | Map4k4 | mitogen-activated protein kinase kinase kinase kinase 4 | -1.75 | 0.323 | -1.2 | 0.686 | -1.68 | 0.325 | 1.69 | 0.175 | -0.74 |
| 1440415_at | --- | 0 day neonate thymus cDNA, RIKEN full-length enriched library, clone:A430095E | -1.4 | 0.533 | -1.54 | 0.037 | -1.58 | 0.213 | 1.29 | 0.508 | -0.81 |
| 1446588_at | --- | --- | -4.2 | 0.006 | -1.1 | 0.807 | -1.17 | 0.739 | 1.15 | 0.721 | -1.33 |
| 1422984_at | Cyln2 | cytoplasmic linker 2 | -1.25 | 0.811 | -2.21 | 0.304 | -1.34 | 0.672 | 1.59 | 0.099 | -0.8 |
| 1417898_a_at | Gzma | granzyme A | -1.81 | 0.521 | 1.09 | 0.749 | -2.78 | 0.054 | -5.15 | 0.032 | -2.16 |
| 1431946_a_at | Apba2bp | amyloid beta (A4) precursor protein-binding, family A, member 1 binding protein | -1.47 | 0.382 | -2.01 | 0.009 | -1.22 | 0.497 | 1.7 | 0.144 | -0.75 |
| 1446320_at | 2310010I16Rik | RIKEN cDNA 2310010I16 gene | -1.95 | 0.249 | -1.69 | 0.163 | -1.12 | 0.455 | 1.05 | 0.893 | -0.93 |
| 1448992_at | Ina | internexin neuronal intermediate filament protein, alpha | -1.01 | 0.994 | -1.6 | 0.246 | -2.64 | 0.065 | 4.75 | 0.079 | -0.12 |
| 1444807_at | --- | --- | -2.11 | 0.035 | -1.24 | 0.594 | -1.39 | 0.499 | 1.57 | 0.403 | -0.79 |
| 1419707_at | Krtap14 | keratin associated protein 14 | -1.49 | 0.508 | -1.42 | 0.339 | -1.59 | 0.311 | 1.08 | 0.883 | -0.86 |
| 1440468_at | D630023F18Rik | RIKEN cDNA D630023F18 gene | -1.45 | 0.081 | -1.11 | 0.862 | -2.43 | 0.149 | 1.03 | 0.959 | -0.99 |
| 1441103_at | D12Ertd551e | DNA segment, Chr 12, ERATO Doi 551, expressed | -3.29 | 0.066 | -1.03 | 0.964 | -1.39 | 0.262 | 1.7 | 0.204 | -1 |
| 1441269_at | Hydin | Hydrocephalus inducing | -1.4 | 0.698 | -1.29 | 0.649 | -1.96 | 0.147 | 1.4 | 0.43 | -0.81 |
| 1439889_at | Scn8a | sodium channel, voltage-gated, type VIII, alpha | -1.83 | 0.042 | -1.42 | 0.308 | -1.33 | 0.016 | 1.28 | 0.08 | -0.82 |
| 1429886_at | Zfp294 | zinc finger protein 294 | -2.54 | 0.156 | -1.88 | 0.036 | 1.08 | 0.879 | -1.34 | 0.538 | -1.17 |
| 1450797_a_at | Cbx1 | chromobox homolog 1 (Drosophila HP1 beta) | -1.58 | 0.388 | -1.79 | 0.334 | -1.23 | 0.347 | 1.38 | 0.654 | -0.81 |
| 1444055_at | Hs3st3b1 | Heparan sulfate (glucosamine) 3-O-sulfotransferase 3B1 | -1.54 | 0.511 | -1.51 | 0.129 | -1.45 | 0.454 | 1.68 | 0.052 | -0.7 |
| 1441655_at | Gm884 | gene model 884, (NCBI) | -2.03 | 0.446 | -1.68 | 0.524 | -1.09 | 0.885 | 1.68 | 0.435 | -0.78 |
| 1456075_at | Prkd2 | protein kinase D2 | -2 | 0.225 | -1.27 | 0.656 | -1.39 | 0.513 | 1.2 | 0.769 | -0.87 |
| 1445102_at | B930096M23Rik | RIKEN cDNA B930096M23 gene | -3.17 | 0.028 | -1.35 | 0.517 | -1.06 | 0.908 | 1.9 | 0.229 | -0.92 |
| 1438854_x_at | Pitpnm1 | phosphatidylinositol membrane-associated 1 | -3.36 | 0.43 | -1.64 | 0.268 | 1.1 | 0.646 | -1 | 0.944 | -1.23 |
| 1459535_at | Cdh13 | Cadherin 13 | -1.74 | 0.377 | -1.37 | 0.551 | -1.43 | 0.459 | 1.26 | 0.62 | -0.82 |
| 1432193_at | 4930412L05Rik | RIKEN cDNA 4930412L05 gene | -1.55 | 0.027 | -1.83 | 0.27 | -1.23 | 0.462 | 1.13 | 0.795 | -0.87 |
| 1458700_at | Lrrc8c | Leucine rich repeat containing 8 family, member C | -1.23 | 0.355 | -1.64 | 0.15 | -1.71 | 0.166 | 1.03 | 0.803 | -0.89 |
| 1415969_s_at | Kap | kidney androgen regulated protein | -1.27 | 0.008 | -27.5 | 0.13 | 1.19 | 0.864 | -3.01 | 0.211 | -7.65 |
| 1432219_at | Nol9 | nucleolar protein 9 | -3.39 | 0.06 | -1.76 | 0.28 | 1.14 | 0.788 | -1.25 | 0.569 | -1.31 |
| 1440473_at | Rap2a | RAS related protein 2a | -1.67 | 0.49 | -1.33 | 0.424 | -1.52 | 0.335 | 1.27 | 0.72 | -0.81 |
| 1458570_at | Aspscr1 | Alveolar soft part sarcoma chromosome region, candidate 1 (human) | -1.32 | 0.387 | -1.28 | 0.777 | -2.11 | 0.152 | 1.58 | 0.065 | -0.78 |
| 1441735_at | --- | --- | -2.57 | 0.008 | -1.06 | 0.893 | -1.48 | 0.355 | 1.52 | 0.348 | -0.9 |
| 1424939_at | Asz1 | ankyrin repeat, SAM and basic leucine zipper domain containing 1 | -1.62 | 0.405 | -1.87 | 0.069 | -1.17 | 0.648 | 1.06 | 0.848 | -0.9 |
| 1448068_at | Soat1 | sterol O-acyltransferase 1 | -1.41 | 0.271 | -2.07 | 0.166 | -1.23 | 0.416 | 1.75 | 0.017 | -0.74 |
| 1438707_at | Atp13a4 | ATPase type 13A4 | -1.11 | 0.748 | -1.69 | 0.584 | -1.95 | 0.04 | 1.14 | 0.848 | -0.9 |
| 1431816_at | Tex9 | testis expressed gene 9 | -3.16 | 0.097 | -1.71 | 0.289 | 1.11 | 0.59 | -1.35 | 0.421 | -1.28 |
| 1441353_at | 5330421F07Rik | RIKEN cDNA 5330421F07 gene | 1.11 | 0.205 | -1.9 | 0.26 | -2.67 | 0.094 | -1.16 | 0.475 | -1.15 |
| 1432346_a_at | Cdh23 | cadherin 23 (otocadherin) | -5.22 | 0.021 | -1.48 | 0.486 | 1.14 | 0.329 | -1.39 | 0.264 | -1.73 |
| 1437145_s_at | 2310002J15Rik | RIKEN cDNA 2310002J15 gene | -3.77 | 0.131 | -1.5 | 0.167 | 1.08 | 0.612 | -1.14 | 0.376 | -1.33 |
| 1444922_at | --- | PREDICTED: Mus musculus similar to microtubule-associated protein, RP/EB far | 1.08 | 0.832 | -2.38 | 0.043 | -1.97 | 0.293 | -2.06 | 0.449 | -1.33 |
| 1432923_at | 2810404I24Rik | RIKEN cDNA 2810404I24 gene | -1.14 | 0.617 | -1.97 | 0.274 | -1.59 | 0.316 | 1.11 | 0.788 | -0.9 |
| 1419379_x_at | Fxyd2 | FXYD domain-containing ion transport regulator 2 | -1.26 | 0.01 | -4.69 | 0.201 | 1.01 | 0.986 | -1.61 | 0.392 | -1.64 |
| 1419395_at | Acot12 | acyl-CoA thioesterase 12 | -1.14 | 0.423 | -1.44 | 0.033 | -2.25 | 0.185 | 1.76 | 0.112 | -0.77 |
| 1416689_at | Tuft1 | tuftelin 1 | -1.5 | 0.136 | -1.9 | 0.102 | -1.22 | 0.149 | 1.42 | 0.31 | -0.8 |
| 1418475_at | Scnn1b | sodium channel, nonvoltage-gated 1 beta | -1.52 | 0.115 | -2.52 | 0.002 | -1.04 | 0.795 | 1.23 | 0.309 | -0.96 |
| 1446084_at | Nexn | Nexilin | -2.26 | 0.243 | -1.89 | 0.289 | 1.04 | 0.916 | -1.46 | 0.33 | -1.14 |
| 1452851_at | Tnrc4 | trinucleotide repeat containing 4 | -1.46 | 0.063 | -1.74 | 0.291 | -1.33 | 0.349 | 1.3 | 0.427 | -0.81 |
| 1452460_at | Ankrd26 /// LOC66 | ankyrin repeat domain 26 /// similar to ankyrin repeat domain 26 | -1.56 | 0.305 | -1.51 | 0.055 | -1.41 | 0.238 | 1.43 | 0.025 | -0.76 |
| 1457801_at | 9930024M15Rik | RIKEN cDNA 9930024M15 gene | -1.51 | 0.028 | -1.5 | 0.229 | -1.46 | 0.146 | 1.04 | 0.888 | -0.86 |
| 1444813_at | Mast2 | Microtubule associated serine/threonine kinase 2 | -2.38 | 0.017 | -1.4 | 0.63 | -1.13 | 0.677 | 1.29 | 0.619 | -0.91 |
| 1447030_at | --- | --- | 1.16 | 0.427 | -2.52 | 0.08 | -2.18 | 0.224 | -1.01 | 0.982 | -1.14 |
| 1453930_at | 1110015M06Rik | RIKEN cDNA 1110015M06 gene | -2.98 | 0.01 | -1.5 | 0.139 | 1.01 | 0.939 | -2.11 | 0.206 | -1.39 |
| 1446246_at | Gm438 | gene model 438, (NCBI) | -1.31 | 0.66 | -1.86 | 0.018 | -1.39 | 0.099 | 1 | 0.992 | -0.89 |
| 1445936_at | Skiv2l2 | Superkiller viralicidic activity 2-like 2 (S. cerevisiae) | -1.69 | 0.063 | -1.74 | 0.027 | -1.17 | 0.518 | 1.03 | 0.89 | -0.89 |
| 1433673_at | E130309D14Rik | RIKEN cDNA E130309D14 gene | -1.84 | 0.291 | -1.84 | 0.005 | -1.07 | 0.744 | 4.53 | 0.112 | -0.06 |
| 1442556_at | --- | --- | 1 | 0.976 | -1.67 | 0.185 | -2.42 | 0.024 | -1.11 | 0.342 | -1.05 |

| | | | | | | | | | | | |
|---|---|---|---|---|---|---|---|---|---|---|---|
| 1419928_at | Lrig1 | leucine-rich repeats and immunoglobulin-like domains 1 | -1.36 | 0.288 | -1.9 | 0.075 | -1.33 | 0.423 | 1.02 | 0.933 | -0.89 |
| 1430269_at | Mybphl | myosin binding protein H-like | -5.5 | 0.027 | -1.79 | 0.144 | 1.28 | 0.379 | -1.79 | 0.388 | -1.95 |
| 1441279_at | C430002E04Rik | RIKEN cDNA C430002E04 gene | -1.5 | 0.458 | -2.11 | 0.001 | -1.14 | 0.735 | 1.02 | 0.969 | -0.93 |
| 1453706_at | 2900042A17Rik | RIKEN cDNA 2900042A17 gene | -1.45 | 0.443 | -1.7 | 0.237 | -1.35 | 0.576 | 1.04 | 0.895 | -0.87 |
| 1454429_at | 5830462O15Rik | RIKEN cDNA 5830462O15 gene | -1.45 | 0.346 | -1.68 | 0.161 | -1.36 | 0.289 | 1.16 | 0.706 | -0.83 |
| 1430224_at | Wfdc3 | WAP four-disulfide core domain 3 | -1.7 | 0.145 | -1.95 | 0.045 | -1.09 | 0.784 | 2.26 | 0.183 | -0.62 |
| 1442602_at | --- | --- | -1.5 | 0.335 | -1.02 | 0.977 | -2.67 | 0 | 1.57 | 0.609 | -0.91 |
| 1440789_at | Neo1 | neogenin | -1.45 | 0.287 | -1.55 | 0.379 | -1.45 | 0.055 | 1.26 | 0.721 | -0.8 |
| 1443053_at | Ptprd | Protein tyrosine phosphatase, receptor type, D | -1.09 | 0.769 | -2.79 | 0.026 | -1.34 | 0.354 | 1.76 | 0.216 | -0.87 |
| 1420111_at | Zfp334 | Zinc finger protein 334 | -1.6 | 0.355 | -1.82 | 0.145 | -1.18 | 0.431 | 1.62 | 0.2 | -0.74 |
| 1445377_at | Als2cr19 | Amyotrophic lateral sclerosis 2 (juvenile) chromosome region, candidate 19 (hum | -1.49 | 0.086 | -1.24 | 0.344 | -1.82 | 0.001 | 1.27 | 0.016 | -0.82 |
| 1456965_at | --- | --- | -2.58 | 0.162 | 1.05 | 0.911 | -1.72 | 0.117 | -1.24 | 0.552 | -1.12 |
| 1431762_at | Htra3 | HtrA serine peptidase 3 | -2.04 | 0.253 | -1.46 | 0.161 | -1.18 | 0.467 | 1.63 | 0.212 | -0.76 |
| 1434428_at | D330028D13Rik | RIKEN cDNA D330028D13 gene | -1.41 | 0.382 | -1.57 | 0.026 | -1.47 | 0.021 | 1.01 | 0.975 | -0.86 |
| 1453410_at | Angptl4 | angiopoietin-like 4 | -1.14 | 0.804 | -1.95 | 0.038 | -1.57 | 0.218 | 1.75 | 0.111 | -0.73 |
| 1455344_at | Efna3 | ephrin A3 | -2.09 | 0.219 | -1.99 | 0.082 | 1.04 | 0.834 | -1.94 | 0.291 | -1.25 |
| 1437966_at | Prrt3 | proline-rich transmembrane protein 3 | -2.53 | 0.014 | -1.22 | 0.554 | -1.24 | 0.615 | 1.21 | 0.81 | -0.95 |
| 1443336_at | Fbxl11 | F-box and leucine-rich repeat protein 11 | -2.1 | 0.472 | 1.15 | 0.783 | -2.55 | 0.178 | -1.26 | 0.797 | -1.19 |
| 1428371_at | 2610507N02Rik | RIKEN cDNA 2610507N02 gene | -1.41 | 0.224 | -1.45 | 0.014 | -1.59 | 0.146 | 1.2 | 0.261 | -0.81 |
| 1446254_at | Depdc6 | DEP domain containing 6 | -1.23 | 0.252 | -1.51 | 0.019 | -1.83 | 0.074 | 1.43 | 0.548 | -0.78 |
| 1440652_at | Ankrd44 | ankyrin repeat domain 44 | 1.06 | 0.942 | -2.79 | 0.256 | -1.66 | 0.151 | -2.05 | 0.228 | -1.36 |
| 1456072_at | Ppp1r9a | protein phosphatase 1, regulatory (inhibitor) subunit 9A | -1.34 | 0.06 | -1.42 | 0.058 | -1.75 | 0.004 | 1.31 | 0.597 | -0.8 |
| 1441158_at | Chn2 | Chimerin (chimaerin) 2 | -1.27 | 0.489 | -2.03 | 0.005 | -1.34 | 0.49 | 1.39 | 0.228 | -0.81 |
| 1441078_at | 1700023F02Rik | RIKEN cDNA 1700023F02 gene | -3.22 | 0.298 | -1.96 | 0.486 | 1.21 | 0.622 | -1.04 | 0.948 | -1.26 |
| 1454443_at | 4833411I10Rik | RIKEN cDNA 4833411I10 gene | -1.98 | 0.062 | -1.17 | 0.763 | -1.51 | 0.01 | 1.41 | 0.345 | -0.81 |
| 1447087_at | Cgrrf1 | Cell growth regulator with ring finger domain 1 | -2.22 | 0.006 | -1.39 | 0.17 | -1.17 | 0.526 | 1.13 | 0.683 | -0.91 |
| 1459581_at | Trp63 | transformation related protein 63 | 1.25 | 0.679 | -2.2 | 0.036 | -3.07 | 0.034 | -1.03 | 0.963 | -1.26 |
| 1421473_at | Il1a | interleukin 1 alpha | -1.84 | 0.023 | -1.48 | 0.465 | -1.24 | 0.464 | 1.14 | 0.639 | -0.85 |
| 1444794_at | --- | --- | -1.53 | 0.158 | -1.2 | 0.781 | -1.85 | 0.002 | 1.19 | 0.517 | -0.85 |
| 1431534_at | Pacsin3 | protein kinase C and casein kinase substrate in neurons 3 | -2.45 | 0.11 | -1.03 | 0.952 | -1.53 | 0.442 | 1.31 | 0.582 | -0.93 |
| 1447630_x_at | Gnb5 | guanine nucleotide binding protein, beta 5 | -1.18 | 0.402 | -2.36 | 0.121 | -1.32 | 0.224 | 1.88 | 0.05 | -0.74 |
| 1417701_at | Ppp1r14c | protein phosphatase 1, regulatory (inhibitor) subunit 14c | -1.36 | 0.31 | -1.52 | 0.21 | -1.57 | 0.044 | 1.1 | 0.585 | -0.84 |
| 1433371_at | 5430427N15Rik | RIKEN cDNA 5430427N15 gene | -1.18 | 0.495 | -1.38 | 0.66 | -2.18 | 0.279 | 1.05 | 0.945 | -0.92 |
| 1432611_at | 4932430A15Rik | RIKEN cDNA 4932430A15 gene | -1.13 | 0.77 | -2.2 | 0.128 | -1.44 | 0.352 | 1.15 | 0.799 | -0.91 |
| 1445830_at | Ctnnd1 | catenin (cadherin associated protein), delta 1 | 1 | 0.991 | -1.95 | 0.074 | -1.94 | 0.09 | -1.07 | 0.901 | -0.99 |
| 1447793_x_at | Rbx1 | Ring-box 1 | -1.45 | 0.426 | -1.48 | 0.006 | -1.5 | 0.115 | 1.1 | 0.77 | -0.83 |
| 1431172_at | Orc4l | origin recognition complex, subunit 4-like (S. cerevisiae) | 1.03 | 0.743 | -1.84 | 0.131 | -2.17 | 0.012 | -1.15 | 0.504 | -1.03 |
| 1447642_x_at | Dmwd | dystrophia myotonica-containing WD repeat motif | -1.38 | 0.64 | -1.41 | 0.595 | -1.67 | 0.234 | 1.5 | 0.206 | -0.74 |
| 1450469_at | Ddc8 | testis specific protein, Ddc8 | -1.1 | 0.922 | -2.42 | 0.102 | -1.42 | 0.307 | 1.39 | 0.317 | -0.89 |
| 1433326_at | 4930488N24Rik | RIKEN cDNA 4930488N24 gene | -3.98 | 0.425 | 1.19 | 0.663 | -1.7 | 0.532 | -2.23 | 0.033 | -1.68 |
| 1442550_at | 1110033M05Rik | RIKEN cDNA 1110033M05 gene | -3.07 | 0.255 | -1.33 | 0.649 | -1.04 | 0.895 | 1 | 0.998 | -1.11 |
| 1453801_at | Them5 | thioesterase superfamily member 5 | -1.98 | 0.268 | -1.47 | 0.432 | -1.18 | 0.543 | 1.9 | 0.428 | -0.68 |
| 1429343_at | 1700012H05Rik | RIKEN cDNA 1700012H05 gene | -1.36 | 0.457 | -1.49 | 0.121 | -1.59 | 0.126 | 1.1 | 0.712 | -0.84 |
| 1440809_at | Swap70 | SWA-70 protein | -3.03 | 0.229 | -1.48 | 0.383 | 1.03 | 0.959 | -1.44 | 0.392 | -1.23 |
| 1438891_at | Ctnnd2 | Catenin (cadherin associated protein), delta 2 | -1.12 | 0.756 | -2.32 | 0.148 | -1.4 | 0.382 | 1.57 | 0.108 | -0.82 |
| 1420746_at | Prlpi | prolactin-like protein I | -2.22 | 0.017 | -1.76 | 0.222 | 1.02 | 0.879 | -1.08 | 0.81 | -1.01 |
| 1432535_at | 5530400C23Rik | RIKEN cDNA 5530400C23 gene | -1.14 | 0.533 | -2.19 | 0.28 | -1.44 | 0.104 | 1.6 | 0.453 | -0.79 |
| 1456327_at | --- | Transcribed locus | -2.03 | 0.006 | -1.06 | 0.882 | -1.66 | 0.048 | 1.35 | 0.654 | -0.85 |
| 1458215_at | Setdb1 | SET domain, bifurcated 1 | -1.23 | 0.752 | -1.65 | 0.194 | -1.61 | 0.025 | 1.32 | 0.038 | -0.79 |
| 1457318_at | A330008L17Rik | RIKEN cDNA A330008L17 gene | -2.84 | 0.017 | -1.27 | 0.313 | -1.11 | 0.777 | 1.99 | 0.098 | -0.81 |
| 1457767_at | Nisch | Nischarin | -2.38 | 0.204 | -1.51 | 0.348 | -1.05 | 0.92 | 1.34 | 0.366 | -0.9 |
| 1443744_at | Dcc | Deleted in colorectal carcinoma | -1.7 | 0.358 | -1.19 | 0.693 | -1.64 | 0.501 | 1.28 | 0.52 | -0.81 |
| 1443315_at | --- | --- | -2.8 | 0.216 | -1.68 | 0.162 | 1.08 | 0.443 | -1.05 | 0.691 | -1.11 |

| | | | | | | | | | | | |
|---|---|---|---|---|---|---|---|---|---|---|---|
| 1442842_at | Hspbap1 | Hspb associated protein 1 | -1.24 | 0.815 | -1.93 | 0.073 | -1.4 | 0.575 | 1.96 | 0.294 | -0.65 |
| 1417554_at | Hsd3b4 | hydroxy-delta-5-steroid dehydrogenase, 3 beta- and steroid delta-isomerase 4 | -1.02 | 0.725 | -12.17 | 0.124 | -1.02 | 0.832 | 1.15 | 0.656 | -3.26 |
| 1437967_at | --- | Transcribed locus | -1.4 | 0.033 | -1.36 | 0.097 | -1.7 | 0.063 | 1.3 | 0.396 | -0.79 |
| 1458540_at | --- | Transcribed locus | -1.75 | 0.252 | -1.39 | 0.583 | -1.34 | 0.242 | 1.15 | 0.801 | -0.83 |
| 1457004_at | D15Wsu169e | DNA segment, Chr 15, Wayne State University 169, expressed | -2.95 | 0.002 | -1.42 | 0.315 | -1 | 0.994 | 2.3 | 0.012 | -0.77 |
| 1435770_at | Txndc13 | thioredoxin domain containing 13 | -1.26 | 0.529 | -1.96 | 0.151 | -1.36 | 0.301 | 1.34 | 0.589 | -0.81 |
| 1426758_s_at | Gtl2 | GTL2, imprinted maternally expressed untranslated mRNA | -1.18 | 0.34 | -2.38 | 0.192 | -1.29 | 0.427 | 1.29 | 0.398 | -0.89 |
| 1446080_at | Cutl2 | Cut-like 2 (Drosophila) | -1.54 | 0.299 | -1.55 | 0.388 | -1.34 | 0.329 | 1.02 | 0.889 | -0.85 |
| 1425181_at | Sgip1 | SH3-domain GRB2-like (endophilin) interacting protein 1 | -1.22 | 0.725 | -2.38 | 0.104 | -1.25 | 0.395 | 2.04 | 0.045 | -0.7 |
| 1446859_at | --- | --- | -1.51 | 0.407 | -1.5 | 0.439 | -1.4 | 0.039 | 1.74 | 0.109 | -0.67 |
| 1440285_at | Ppp1r9a | protein phosphatase 1, regulatory (inhibitor) subunit 9A | -1.41 | 0.012 | -1.39 | 0.098 | -1.63 | 0.029 | 1.55 | 0.228 | -0.72 |
| 1458495_at | Ppp3ca | Protein phosphatase 3, catalytic subunit, alpha isoform | -1.21 | 0.346 | -1.53 | 0.216 | -1.77 | 0.201 | 1 | 0.909 | -0.88 |
| 1430920_at | 2410075D05Rik | RIKEN cDNA 2410075D05 gene | -1.34 | 0.469 | -1.89 | 0.035 | -1.31 | 0.397 | 1.06 | 0.53 | -0.87 |
| 1421549_at | Pcdhb15 | protocadherin beta 15 | -1.38 | 0.298 | -1.74 | 0.127 | -1.35 | 0.561 | 2.01 | 0.399 | -0.61 |
| 1423671_at | Dner | delta/notch-like EGF-related receptor | -1.96 | 0.237 | -1.17 | 0.707 | -1.47 | 0.086 | 1.36 | 0.411 | -0.81 |
| 1442028_at | AI593864 | expressed sequence AI593864 | -1.96 | 0.177 | -1.37 | 0.101 | -1.24 | 0.679 | 1.03 | 0.943 | -0.89 |
| 1441563_at | 2410017P07Rik | RIKEN cDNA 2410017P07 gene | -1.26 | 0.333 | -1.35 | 0.333 | -1.95 | 0.116 | 1.2 | 0.737 | -0.84 |
| 1443363_at | A430107O13Rik | RIKEN cDNA A430107O13 gene | -1.46 | 0.447 | -1.13 | 0.784 | -2.12 | 0.072 | 1.03 | 0.95 | -0.92 |
| 1436550_at | Fbxo30 | F-box protein 30 | -1.14 | 0.511 | -1.59 | 0.07 | -1.87 | 0.014 | 2.65 | 0.264 | -0.49 |
| 1442952_at | Shrm | Shroom | 1.13 | 0.377 | -2.43 | 0.058 | -1.98 | 0.005 | -1.95 | 0.069 | -1.31 |
| 1446316_at | Lpin2 | Lipin 2 | -1 | 0.995 | -2.12 | 0.095 | -1.74 | 0.227 | 2.59 | 0.147 | -0.57 |
| 1431867_a_at | 1700007B13Rik | RIKEN cDNA 1700007B13 gene | -1.64 | 0.089 | -1.85 | 0.193 | -1.12 | 0.6 | 1.38 | 0.611 | -0.81 |
| 1435012_x_at | Ela3 /// LOC63841ɛ | elastase 3, pancreatic /// similar to elastase 3B, pancreatic | -3.75 | 0.043 | -1.36 | 0.583 | 1.04 | 0.909 | -1.9 | 0.644 | -1.49 |
| 1459485_at | Neo1 | Neogenin | -1.23 | 0.619 | -1.51 | 0.239 | -1.76 | 0.004 | 1.14 | 0.8 | -0.84 |
| 1444384_at | AI591476 | Expressed sequence AI591476 | -2.42 | 0.353 | 1.02 | 0.977 | -1.63 | 0.005 | -1.26 | 0.322 | -1.07 |
| 1460274_at | 4921530G04Rik | RIKEN cDNA 4921530G04 gene | -1.18 | 0.575 | -1.83 | 0.234 | -1.54 | 0.45 | 1.16 | 0.618 | -0.85 |
| 1437233_x_at | 1110049F12Rik | RIKEN cDNA 1110049F12 gene | -1.87 | 0.318 | -1.28 | 0.705 | -1.38 | 0.588 | 1.34 | 0.348 | -0.8 |
| 1438726_at | Mical2 | microtubule associated monoxygenase, calponin and LIM domain containing 2 | -1.53 | 0.073 | -1.3 | 0.324 | -1.6 | 0.152 | 1.41 | 0.242 | -0.76 |
| 1456249_x_at | Syp | Synaptophysin | -1.41 | 0.605 | -1.45 | 0.261 | -1.55 | 0.315 | 1.07 | 0.778 | -0.83 |
| 1454618_at | --- | --- | -1.5 | 0.701 | -3.48 | 0.137 | 1.09 | 0.855 | -1.21 | 0.004 | -1.28 |
| 1458520_at | Hipk2 | Homeodomain interacting protein kinase 2 | -1.4 | 0.144 | -1.27 | 0.446 | -1.84 | 0.055 | 1.79 | 0.397 | -0.68 |
| 1453245_at | 9130024F11Rik | RIKEN cDNA 9130024F11 gene | -1.17 | 0.713 | -1.78 | 0.312 | -1.6 | 0.397 | 1.4 | 0.495 | -0.79 |
| 1431231_at | Hist2h3c1 /// Hist1h | histone 2, H3c1 /// histone 1, H3f /// histone1, H3d /// histone 1, H3b /// histone 1, | -1.32 | 0.567 | -2.2 | 0.035 | -1.2 | 0.381 | 1.76 | 0.037 | -0.74 |
| 1441585_at | Spg7 | Spastic paraplegia 7 homolog (human) | -1.19 | 0.779 | -1.5 | 0.064 | -1.84 | 0.337 | 1.52 | 0.487 | -0.75 |
| 1443938_at | --- | Transcribed locus | -1.97 | 0.48 | -1.16 | 0.272 | -1.48 | 0.233 | 1.5 | 0.217 | -0.78 |
| 1430916_at | Fancd2 | Fanconi anemia, complementation group D2 | -3.34 | 0.062 | -1.12 | 0.699 | -1.17 | 0.509 | 1.23 | 0.085 | -1.1 |
| 1447574_s_at | Slc32a1 | Solute carrier family 32 (GABA vesicular transporter), member 1 | -1.74 | 0.133 | -1.08 | 0.779 | -1.83 | 0.155 | 1.39 | 0.435 | -0.82 |
| 1453957_a_at | Igf2bp3 | insulin-like growth factor 2 mRNA binding protein 3 | 1.01 | 0.974 | -1.73 | 0.353 | -2.19 | 0.282 | -1.18 | 0.746 | -1.02 |
| 1436201_x_at | Mbp | myelin basic protein | -1.28 | 0.28 | -2.7 | 0.147 | -1.11 | 0.841 | 1.81 | 0.522 | -0.82 |
| 1432247_at | 4633402D09Rik | RIKEN cDNA 4633402D09 gene | 1.16 | 0.689 | -2.15 | 0.163 | -2.37 | 0.294 | -1.07 | 0.804 | -1.11 |
| 1429368_at | Lrig3 | leucine-rich repeats and immunoglobulin-like domains 3 | -1.17 | 0.227 | -1.4 | 0.265 | -2.07 | 0.1 | 1.18 | 0.731 | -0.86 |
| 1440648_at | Coq10a | Coenzyme Q10 homolog A (yeast) | -1.51 | 0.393 | -1.56 | 0.279 | -1.34 | 0.438 | 1.04 | 0.78 | -0.84 |
| 1439280_at | B230396O12Rik | RIKEN cDNA B230396O12 gene | -1.83 | 0.463 | -2.03 | 0.22 | 1.01 | 0.961 | -1.19 | 0.418 | -1.01 |
| 1419378_a_at | Fxyd2 | FXYD domain-containing ion transport regulator 2 | -1.24 | 0.015 | -4.83 | 0.168 | 1.04 | 0.888 | -1.64 | 0.292 | -1.67 |
| 1438485_at | E130112L23Rik | RIKEN cDNA E130112L23 gene | -1.43 | 0.421 | -1.97 | 0.105 | -1.19 | 0.653 | 1.5 | 0.444 | -0.77 |
| 1424543_at | Nck1 | non-catalytic region of tyrosine kinase adaptor protein 1 | -1.81 | 0.052 | -1.17 | 0.556 | -1.55 | 0.148 | 1.17 | 0.519 | -0.84 |
| 1439929_at | Mfhas1 | Malignant fibrous histiocytoma amplified sequence 1 | -1.25 | 0.082 | -1.32 | 0.037 | -2.01 | 0.042 | 1.2 | 0.478 | -0.85 |
| 1454465_at | 5830435N06Rik | RIKEN cDNA 5830435N06 gene | -1.91 | 0.044 | -2.09 | 0.058 | 1.05 | 0.877 | -1.04 | 0.945 | -1 |
| 1416492_at | Ccne1 | cyclin E1 | -2.22 | 0.4 | -1.2 | 0.772 | -1.31 | 0.579 | 4.06 | 0.152 | -0.16 |
| 1438911_at | 7-Sep | Septin 7 | -1.46 | 0.108 | -1.41 | 0.044 | -1.52 | 0.121 | 1.09 | 0.772 | -0.82 |
| 1419986_at | Ccdc69 | Coiled-coil domain containing 69 | -2.56 | 0.069 | -1.79 | 0.369 | 1.1 | 0.874 | -3.12 | 0.022 | -1.59 |
| 1419510_at | Es22 | esterase 22 | -1.96 | 0.106 | -1.17 | 0.719 | -1.46 | 0.125 | 2.59 | 0.017 | -0.5 |
| 1458707_at | E430016F16Rik | RIKEN cDNA E430016F16 gene | -1.85 | 0.45 | -1.34 | 0.212 | -1.3 | 0.4 | 3.29 | 0.012 | -0.3 |

| | | | | | | | | | | | |
|---|---|---|---|---|---|---|---|---|---|---|---|
| 1422239_at | Hoxd13 | homeo box D13 | -1.1 | 0.171 | -1.93 | 0.098 | -1.59 | 0.422 | 1.29 | 0.741 | -0.83 |
| 1458841_at | Nudcd1 | NudC domain containing 1 | -1.36 | 0.415 | -1.51 | 0.195 | -1.53 | 0.187 | 1 | 0.992 | -0.85 |
| 1425818_at | --- | --- | -1.26 | 0.113 | -1.53 | 0.009 | -1.65 | 0.203 | 1.34 | 0.495 | -0.77 |
| 1431550_at | 4930488B22Rik | RIKEN cDNA 4930488B22 gene | 1.23 | 0.697 | -1.98 | 0.232 | -3.18 | 0.231 | -1.34 | 0.744 | -1.32 |
| 1427223_a_at | Acsm2 | acyl-CoA synthetase medium-chain family member 2 | -1.31 | 0.085 | -1.3 | 0.584 | -1.9 | 0.004 | 1.48 | 0.293 | -0.76 |
| 1425167_a_at | Gngt1 | guanine nucleotide binding protein (G protein), gamma transducing activity polype | -1.63 | 0.515 | -1.56 | 0.309 | -1.25 | 0.634 | 1.02 | 0.973 | -0.86 |
| 1459401_at | LOC545191 | Similar to zinc finger protein 616 | -1.8 | 0.378 | -1.24 | 0.759 | -1.44 | 0.231 | 1.06 | 0.85 | -0.86 |
| 1456655_at | Ext1 | Exostoses (multiple) 1 | -1.3 | 0.315 | -1.28 | 0.047 | -1.98 | 0.037 | 1.47 | 0.143 | -0.77 |
| 1433380_at | 4921509A06Rik | RIKEN cDNA 4921509A06 gene | -2.18 | 0.201 | -1.6 | 0.266 | -1.03 | 0.939 | 1.23 | 0.687 | -0.89 |
| 1426273_at | Lmbr1 | limb region 1 | -1.64 | 0.067 | -1.64 | 0.009 | -1.2 | 0.284 | 1.64 | 0.053 | -0.71 |
| 1439291_at | --- | Adult male corpus striatum cDNA, RIKEN full-length enriched library, clone:C030( | -1.64 | 0.514 | -1.63 | 0.093 | -1.21 | 0.643 | 1.36 | 0.52 | -0.78 |
| 1438374_x_at | App | amyloid beta (A4) precursor protein | -1.45 | 0.286 | -1.99 | 0.363 | -1.16 | 0.516 | 1.7 | 0.298 | -0.73 |
| 1459578_at | AA407175 | expressed sequence AA407175 | -1.4 | 0.577 | -1.88 | 0.132 | -1.24 | 0.549 | 3.12 | 0.246 | -0.35 |
| 1427023_at | Phyhipl | phytanoyl-CoA hydroxylase interacting protein-like | -1.43 | 0.117 | -4.29 | 0.161 | 1.13 | 0.616 | -4.32 | 0.184 | -2.23 |
| 1428665_at | --- | --- | -3.23 | 0.019 | -1.12 | 0.852 | -1.17 | 0.686 | 1.16 | 0.793 | -1.09 |
| 1423072_at | 6720475J19Rik /// L | RIKEN cDNA 6720475J19 gene /// similar to putative retrovirus-related gag protei | -1.5 | 0.346 | -2.57 | 0.005 | 1 | 0.996 | -1.18 | 0.593 | -1.06 |
| 1457114_at | Tor1b | torsin family 1, member B | -1.25 | 0.838 | -1.41 | 0.628 | -1.82 | 0.106 | 1.17 | 0.72 | -0.83 |
| 1437562_at | 4932422M17Rik | RIKEN cDNA 4932422M17 gene | -2.32 | 0.332 | -1.34 | 0.749 | -1.13 | 0.829 | 2.09 | 0.118 | -0.68 |
| 1430572_at | 1810042K04Rik | RIKEN cDNA 1810042K04 gene | -1.28 | 0.237 | -1.45 | 0.337 | -1.71 | 0.204 | 1.28 | 0.422 | -0.79 |
| 1459511_at | 3110004L20Rik | RIKEN cDNA 3110004L20 gene | -1.31 | 0.155 | -1.42 | 0.23 | -1.68 | 0.009 | 3.97 | 0.135 | -0.11 |
| 1433085_at | 5930427J20Rik | RIKEN cDNA 5930427J20 gene | -5.21 | 0.01 | 1.19 | 0.588 | -1.48 | 0.276 | -1.97 | 0.37 | -1.86 |
| 1425752_at | BC014805 | cDNA sequence BC014805 | -4.16 | 0.035 | 1.04 | 0.827 | -1.28 | 0.516 | -1.3 | 0.19 | -1.43 |
| 1447746_at | Lrch1 | Leucine-rich repeats and calponin homology (CH) domain containing 1 | -1.37 | 0.115 | -2.56 | 0.363 | -1.07 | 0.793 | 1.63 | 0.294 | -0.84 |
| 1429406_at | Slco6c1 | solute carrier organic anion transporter family, member 6c1 | -1.5 | 0.668 | -1.06 | 0.906 | -2.21 | 0.137 | 1.09 | 0.879 | -0.92 |
| 1437897_at | Klhl21 | kelch-like 21 (Drosophila) | -2.02 | 0.467 | -1.79 | 0.242 | 1.01 | 0.987 | -1.22 | 0.663 | -1.01 |
| 1446216_at | --- | Adult male cecum cDNA, RIKEN full-length enriched library, clone:9130202G13 p | -1.78 | 0.2 | -1.24 | 0.616 | -1.44 | 0.465 | 1.24 | 0.688 | -0.81 |
| 1440452_at | Drp2 | dystrophin related protein 2 | -1.33 | 0.491 | -1.46 | 0.557 | -1.59 | 0.338 | 1.1 | 0.62 | -0.82 |
| 1460263_at | Pdcd6ip | Programmed cell death 6 interacting protein | -1.08 | 0.44 | -2.17 | 0.166 | -1.49 | 0.215 | 1.28 | 0.144 | -0.86 |
| 1436754_at | AI839735 /// LOC63 | expressed sequence AI839735 /// hypothetical protein LOC638311 | -1.89 | 0.001 | -1.48 | 0.149 | -1.17 | 0.606 | 1.91 | 0.172 | -0.66 |
| 1419579_at | Slc7a12 | solute carrier family 7 (cationic amino acid transporter, y+ system), member 12 | -1.07 | 0.822 | -7.37 | 0.145 | -1.01 | 0.977 | 1.29 | 0.501 | -2.04 |
| 1458180_at | --- | --- | -1.7 | 0.091 | -1.44 | 0.032 | -1.28 | 0.038 | 1.07 | 0.781 | -0.84 |
| 1419496_at | Slco1a6 | solute carrier organic anion transporter family, member 1a6 | 1.08 | 0.728 | -5.64 | 0.156 | -1.24 | 0.707 | -1.3 | 0.559 | -1.78 |
| 1430708_a_at | Usp45 | ubiquitin specific petidase 45 | -1.32 | 0.324 | -2.27 | 0.144 | -1.16 | 0.721 | 1.08 | 0.761 | -0.92 |
| 1445099_at | --- | Transcribed locus | -2.09 | 0.47 | -1.4 | 0.215 | -1.15 | 0.14 | 1.32 | 0.62 | -0.83 |
| 1453477_at | Zc3h6 | zinc finger CCCH type containing 6 | -1.8 | 0.099 | -1.45 | 0.484 | -1.22 | 0.638 | 1.12 | 0.79 | -0.84 |
| 1432342_at | Nmnat3 | nicotinamide nucleotide adenylyltransferase 3 | -4.04 | 0.008 | 1.45 | 0.336 | -2.75 | 0.135 | -1.05 | 0.921 | -1.59 |
| 1455709_at | 4930422I22Rik | RIKEN cDNA 4930422I22 gene | -1.19 | 0.689 | -1.45 | 0.528 | -1.88 | 0.202 | 2.71 | 0.104 | -0.45 |
| 1449759_at | D11Ertd326e | DNA segment, Chr 11, ERATO Doi 326, expressed | -2.17 | 0.207 | -1.36 | 0.662 | -1.16 | 0.846 | 1.42 | 0.457 | -0.82 |
| 1453549_at | 4930500G05Rik | RIKEN cDNA 4930500G05 gene | -1.55 | 0.064 | -1.45 | 0.199 | -1.37 | 0.124 | 1.48 | 0.557 | -0.72 |
| 1439632_at | --- | 12 days embryo spinal ganglion cDNA, RIKEN full-length enriched library, clone:D | 1.06 | 0.729 | -1.82 | 0.12 | -2.18 | 0.017 | -1.5 | 0.346 | -1.11 |
| 1453478_at | Pou3f2 | POU domain, class 3, transcription factor 2 | -1.38 | 0.369 | -2.75 | 0.084 | -1.03 | 0.935 | 1.06 | 0.91 | -1.02 |
| 1455501_at | Slc2a12 | solute carrier family 2 (facilitated glucose transporter), member 12 | -1.35 | 0.133 | -2.37 | 0.245 | -1.11 | 0.726 | 1.03 | 0.975 | -0.95 |
| 1435506_at | --- | --- | -1.81 | 0.47 | -1.21 | 0.32 | -1.46 | 0.487 | 1.12 | 0.858 | -0.84 |
| 1458765_at | Rbm10 | RNA binding motif protein 10 | 1.02 | 0.897 | -1.85 | 0.377 | -1.98 | 0.159 | -1.25 | 0.157 | -1.02 |
| 1451438_s_at | Clec2h | C-type lectin domain family 2, member h | -1.13 | 0.767 | -3.04 | 0.032 | -1.17 | 0.647 | 1.23 | 0.301 | -1.03 |
| 1445516_at | --- | --- | -1.16 | 0.549 | -1.81 | 0.077 | -1.53 | 0.306 | 1.25 | 0.663 | -0.81 |
| 1458902_at | --- | --- | -2.33 | 0.136 | -1.17 | 0.54 | -1.28 | 0.382 | 2.2 | 0.237 | -0.64 |
| 1444768_at | --- | Transcribed locus | -1.09 | 0.914 | -3.44 | 0.078 | -1.16 | 0.693 | 1.5 | 0.092 | -1.05 |
| 1447399_at | A330019N05Rik | RIKEN cDNA A330019N05 gene | -1.58 | 0.149 | -1.21 | 0.395 | -1.65 | 0.038 | 1.07 | 0.705 | -0.84 |
| 1450278_at | Tacr3 | tachykinin receptor 3 | -1.28 | 0.803 | -1.01 | 0.986 | -3.38 | 0.126 | 1.56 | 0.433 | -1.03 |
| 1449166_at | S100a14 | S100 calcium binding protein A14 | -3.56 | 0.055 | -1.22 | 0.735 | -1.03 | 0.81 | 1.15 | 0.68 | -1.17 |
| 1449908_at | Gip | gastric inhibitory polypeptide | -2.14 | 0.042 | 1.16 | 0.685 | -2.27 | 0.309 | -3.35 | 0.182 | -1.65 |
| 1418055_at | Neurod4 | neurogenic differentiation 4 | -1.28 | 0.721 | -1.36 | 0.521 | -1.81 | 0.228 | 1.31 | 0.396 | -0.79 |

| | | | | | | | | | | | |
|---|---|---|---|---|---|---|---|---|---|---|---|
| 1433219_at | 6720482G16Rik | RIKEN cDNA 6720482G16 gene | -1.42 | 0.576 | -1.42 | 0.205 | -1.52 | 0.348 | 1.9 | 0.291 | -0.61 |
| 1456140_at | LOC624168 | similar to zinc finger protein of the cerebellum 5 | -1.61 | 0.159 | -2.62 | 0.273 | 1.07 | 0.91 | -2.51 | 0.323 | -1.42 |
| 1458296_at | Ext1 | Exostoses (multiple) 1 | -1.34 | 0.162 | -1.17 | 0.062 | -2.14 | 0.063 | 1.15 | 0.089 | -0.87 |
| 1431572_at | 2310015K22Rik | RIKEN cDNA 2310015K22 gene | -4.87 | 0.123 | 1.03 | 0.874 | -1.2 | 0.657 | -1.83 | 0.339 | -1.72 |
| 1458630_at | Vav3 | Vav 3 oncogene | 1.15 | 0.861 | -2.11 | 0.242 | -2.27 | 0.127 | -1.72 | 0.22 | -1.24 |
| 1444875_at | Ppp2ca | Protein phosphatase 2 (formerly 2A), catalytic subunit, alpha isoform | -1.95 | 0.028 | 1.06 | 0.879 | -2.03 | 0.045 | -1.14 | 0.638 | -1.01 |
| 1421085_at | Rs1h | retinoschisis 1 homolog (human) | -7.02 | 0.001 | -1.16 | 0.557 | 1.07 | 0.624 | -1.07 | 0.83 | -2.05 |
| 1441481_at | Mfap3l | microfibrillar-associated protein 3-like | -1.13 | 0.65 | -3.17 | 0.147 | -1.15 | 0.442 | 1.18 | 0.579 | -1.07 |
| 1427594_at | Zfp697 | zinc finger protein 697 | -1.56 | 0.387 | -1.12 | 0.69 | -1.87 | 0.113 | 1.27 | 0.622 | -0.82 |
| 1440422_at | Htr1f | 5-hydroxytryptamine (serotonin) receptor 1F | -1.45 | 0.527 | -1.12 | 0.695 | -2.05 | 0.356 | 1.3 | 0.402 | -0.83 |
| 1450995_at | Folr1 | folate receptor 1 (adult) | -1.07 | 0.572 | -2.35 | 0.303 | -1.4 | 0.296 | 1.59 | 0.251 | -0.81 |
| 1431235_at | --- | --- | -1.08 | 0.86 | -2.6 | 0.208 | -1.32 | 0.338 | 1.44 | 0.48 | -0.89 |
| 1446333_at | --- | --- | -2.77 | 0.307 | -1.17 | 0.363 | -1.16 | 0.781 | 1.54 | 0.472 | -0.89 |
| 1442451_at | Ttc15 | Tetratricopeptide repeat domain 15 | 1.02 | 0.925 | -1.71 | 0.166 | -2.16 | 0.011 | -2.22 | 0.048 | -1.27 |
| 1446167_at | Hipk2 | Homeodomain interacting protein kinase 2 | -1.28 | 0.044 | -1.22 | 0.308 | -2.12 | 0.014 | 2.44 | 0.247 | -0.55 |
| 1425841_at | Slc26a7 | solute carrier family 26, member 7 | -1.8 | 0.591 | -2.52 | 0.192 | 1.12 | 0.583 | -1.1 | 0.872 | -1.08 |
| 1457561_at | Itga5 | integrin alpha 5 (fibronectin receptor alpha) | -1.41 | 0.399 | -1.71 | 0.21 | -1.29 | 0.25 | 1.18 | 0.691 | -0.81 |
| 1431078_at | Fbxo3 | F-box only protein 3 | -1.56 | 0.309 | -1.28 | 0.405 | -1.53 | 0.212 | 1.39 | 0.577 | -0.75 |
| 1442439_at | W91776 | expressed sequence W91776 | 1.01 | 0.97 | -2.02 | 0.333 | -1.77 | 0.136 | -1.74 | 0.296 | -1.13 |
| 1424592_a_at | Dnase1 | deoxyribonuclease I | 1.17 | 0.545 | -2.22 | 0.293 | -2.22 | 0.227 | -1.86 | 0.141 | -1.28 |
| 1432990_at | 5830495A06Rik | RIKEN cDNA 5830495A06 gene | 1.08 | 0.876 | -3.28 | 0.009 | -1.45 | 0.047 | -1.13 | 0.798 | -1.2 |
| 1453069_at | Pik3cb | phosphatidylinositol 3-kinase, catalytic, beta polypeptide | -1.58 | 0.02 | -1.35 | 0.177 | -1.42 | 0.064 | 1.52 | 0.283 | -0.71 |
| 1446867_at | --- | --- | 1.01 | 0.99 | -3 | 0.087 | -1.36 | 0.519 | -1.82 | 0.023 | -1.29 |
| 1442815_at | Pex19 | Peroxisome biogenesis factor 19 | -1.94 | 0.092 | -1.26 | 0.303 | -1.3 | 0.381 | 1.01 | 0.974 | -0.87 |
| 1447524_at | Ap3m2 | adaptor-related protein complex 3, mu 2 subunit | -1.35 | 0.642 | -1.32 | 0.586 | -1.74 | 0.078 | 1.78 | 0.183 | -0.66 |
| 1450292_a_at | Hormad1 | HORMA domain containing 1 | -1.6 | 0.377 | -2.02 | 0.154 | -1.05 | 0.948 | 1.09 | 0.858 | -0.89 |
| 1443810_at | Tssk4 | testis-specific serine kinase 4 | -1.33 | 0.743 | -1.15 | 0.51 | -2.21 | 0.006 | 1.05 | 0.945 | -0.91 |
| 1429736_at | 1110003F05Rik | RIKEN cDNA 1110003F05 gene | -1.44 | 0.367 | -1.47 | 0.082 | -1.43 | 0.125 | 1.53 | 0.18 | -0.7 |
| 1424659_at | Slit2 | slit homolog 2 (Drosophila) | -1.26 | 0.222 | -1.88 | 0.288 | -1.33 | 0.285 | 1.59 | 0.129 | -0.72 |
| 1442450_at | D2Ertd501e | DNA segment, Chr 2, ERATO Doi 501, expressed | -1.78 | 0.515 | -2.23 | 0.314 | 1.07 | 0.876 | -1.9 | 0.062 | -1.21 |
| 1436978_at | Wnt9a | wingless-type MMTV integration site 9A | -1.56 | 0.217 | -3.14 | 0.056 | 1.12 | 0.753 | -1.32 | 0.701 | -1.23 |
| 1438137_at | 2810055G22Rik | RIKEN cDNA 2810055G22 gene | -1.64 | 0.376 | -1.67 | 0.155 | -1.15 | 0.723 | 1.1 | 0.843 | -0.84 |
| 1459425_at | --- | --- | -2.11 | 0.047 | -1.16 | 0.616 | -1.35 | 0.137 | 1.08 | 0.66 | -0.89 |
| 1442875_at | --- | --- | -1.92 | 0.455 | -2.18 | 0.06 | 1.1 | 0.83 | -1.76 | 0.11 | -1.19 |
| 1432633_at | 9430063H18Rik | RIKEN cDNA 9430063H18 gene | -1.01 | 0.979 | -1.82 | 0.088 | -1.86 | 0.324 | 2.92 | 0.006 | -0.44 |
| 1435983_at | 1700019P01Rik | RIKEN cDNA 1700019P01 gene | -2.82 | 0.303 | -2.7 | 0.116 | 1.35 | 0.408 | -1.31 | 0.613 | -1.37 |
| 1451440_at | Chodl | chondrolectin | -1.23 | 0.433 | -1.87 | 0.295 | -1.37 | 0.476 | 2.17 | 0.007 | -0.57 |
| 1453501_at | Hoxb1 | homeo box B1 | -2.04 | 0.148 | 1.03 | 0.929 | -1.8 | 0.093 | -1.22 | 0.693 | -1.01 |
| 1440000_at | E330013P04Rik | RIKEN cDNA E330013P04 gene | -2.76 | 0.065 | -1.34 | 0.615 | -1.03 | 0.954 | 1.01 | 0.982 | -1.03 |
| 1458859_at | --- | --- | -1.06 | 0.906 | -1.38 | 0.525 | -2.41 | 0.068 | 1.94 | 0.457 | -0.73 |
| 1427616_at | --- | Mus musculus, clone IMAGE:5362978 | -2.32 | 0.138 | -1.32 | 0.681 | -1.12 | 0.805 | 1.22 | 0.337 | -0.89 |
| 1444992_at | AI120166 | expressed sequence AI120166 | -1.24 | 0.058 | -1.49 | 0.434 | -1.67 | 0.019 | 1.65 | 0.075 | -0.69 |
| 1457822_at | --- | --- | -1.04 | 0.684 | -2.24 | 0.034 | -1.49 | 0.074 | 1.76 | 0.1 | -0.75 |
| 1445793_at | 1700011I03Rik | RIKEN cDNA 1700011I03 gene | -1.58 | 0.652 | -1.6 | 0.404 | -1.22 | 0.748 | 1.44 | 0.618 | -0.74 |
| 1437191_at | BC050789 | cDNA sequence BC050789 | -1.89 | 0.548 | -3.45 | 0.109 | 1.26 | 0.316 | -2 | 0.101 | -1.52 |
| 1432120_at | 4921513D11Rik | RIKEN cDNA 4921513D11 gene | -1.4 | 0.187 | -1.8 | 0.226 | -1.23 | 0.152 | 1.6 | 0.221 | -0.71 |
| 1432014_at | 6530409C15Rik | RIKEN cDNA 6530409C15 gene | -1.49 | 0.476 | -1.41 | 0.566 | -1.42 | 0.181 | 1.23 | 0.338 | -0.77 |
| 1445257_at | AU022531 | expressed sequence AU022531 | -2.45 | 0.062 | -2.69 | 0.139 | 1.3 | 0.73 | -2.73 | 0.21 | -1.64 |
| 1426339_at | Ak5 | adenylate kinase 5 | 1.14 | 0.601 | -2.44 | 0.025 | -1.87 | 0.123 | -1.66 | 0.309 | -1.21 |
| 1446157_at | A330019N05Rik | RIKEN cDNA A330019N05 gene | -2.51 | 0.11 | -1.53 | 0.482 | 1.03 | 0.949 | -1.39 | 0.485 | -1.1 |
| 1433283_s_at | --- | --- | -1.29 | 0.56 | -1.22 | 0.653 | -2.05 | 0.116 | 1.76 | 0.249 | -0.7 |
| 1429410_at | Eny2 | enhancer of yellow 2 homolog (Drosophila) | -1.25 | 0.088 | -1.32 | 0.261 | -1.89 | 0.009 | 1.33 | 0.234 | -0.78 |
| 1437739_a_at | 5430432N15Rik | RIKEN cDNA 5430432N15 gene | -1.9 | 0.336 | -1.45 | 0.34 | -1.15 | 0.748 | 1.05 | 0.949 | -0.87 |

| | | | | | | | | | | | |
|---|---|---|---|---|---|---|---|---|---|---|---|
| 1459477_at | Gabarapl2 | Gamma-aminobutyric acid (GABA-A) receptor-associated protein-like 2 | -4.63 | 0.075 | -1.02 | 0.916 | -1.12 | 0.457 | 1.16 | 0.763 | -1.4 |
| 1447407_at | Centd3 | centaurin, delta 3 | -1.21 | 0.743 | -1.57 | 0.437 | -1.6 | 0.119 | 1.16 | 0.692 | -0.81 |
| 1443583_at | C030010B13Rik | RIKEN cDNA C030010B13 gene | -1.29 | 0.051 | -1.53 | 0.045 | -1.53 | 0.15 | 1.49 | 0.057 | -0.71 |
| 1442895_at | --- | PREDICTED: Mus musculus similar to zinc finger protein 547 (LOC628043), mRN | -1.26 | 0.72 | -1.36 | 0.371 | -1.79 | 0.17 | 1.83 | 0.117 | -0.65 |
| 1427638_at | Zbtb16 | zinc finger and BTB domain containing 16 | -1.59 | 0.532 | -1.45 | 0.513 | -1.3 | 0.696 | 1.16 | 0.883 | -0.8 |
| 1441161_at | B230216G23Rik | RIKEN cDNA B230216G23 gene | -1.14 | 0.847 | -2.31 | 0.19 | -1.28 | 0.016 | 1.21 | 0.575 | -0.88 |
| 1444600_at | Nrbp | nuclear receptor binding protein | -1.95 | 0.07 | -1.16 | 0.833 | -1.4 | 0.542 | 1.09 | 0.738 | -0.86 |
| 1441340_at | Cep68 | centrosomal protein 68 | -1.51 | 0.107 | -4.16 | 0.075 | 1.18 | 0.75 | -1.45 | 0.067 | -1.48 |
| 1433876_at | Lrrc24 | leucine rich repeat containing 24 | -1.29 | 0.271 | -1.63 | 0.407 | -1.43 | 0.233 | 1.65 | 0.377 | -0.68 |
| 1431654_at | 1700112M01Rik | RIKEN cDNA 1700112M01 gene | -2.02 | 0.036 | -1.12 | 0.79 | -1.43 | 0.39 | 1.01 | 0.95 | -0.89 |
| 1422060_at | Parvb /// LOC67602 | parvin, beta /// similar to parvin, beta | -2.39 | 0.018 | -1.2 | 0.468 | -1.19 | 0.753 | 2.99 | 0.339 | -0.45 |
| 1442504_at | D18Ertd653e | DNA segment, Chr 18, ERATO Doi 653, expressed | 1.13 | 0.693 | -2.3 | 0.013 | -1.91 | 0.028 | -1.18 | 0.026 | -1.06 |
| 1422320_x_at | Phxr5 | per-hexamer repeat gene 5 | -2.11 | 0.101 | -1.22 | 0.227 | -1.26 | 0.274 | 1.05 | 0.802 | -0.88 |
| 1438345_at | --- | --- | -1.28 | 0.13 | -1.52 | 0.151 | -1.54 | 0.052 | 1.51 | 0.216 | -0.71 |
| 1442954_at | --- | Transcribed locus | -1.23 | 0.383 | -1.75 | 0.226 | -1.42 | 0.145 | 1.51 | 0.168 | -0.72 |
| 1431735_at | 4930469G21Rik | RIKEN cDNA 4930469G21 gene | -2.4 | 0.117 | -1.05 | 0.949 | -1.39 | 0.386 | 1.18 | 0.645 | -0.92 |
| 1453765_at | A330106J01Rik | RIKEN cDNA A330106J01 gene | -1.01 | 0.994 | -1.59 | 0.412 | -2.15 | 0.077 | 3.39 | 0.038 | -0.34 |
| 1440433_at | Nmt2 | N-myristoyltransferase 2 | -1.15 | 0.872 | -1.69 | 0.318 | -1.59 | 0.075 | 1.11 | 0.633 | -0.83 |
| 1440553_at | Mecr | mitochondrial trans-2-enoyl-CoA reductase | -1.39 | 0.525 | -2.73 | 0.067 | 1 | 0.984 | -1.99 | 0.238 | -1.28 |
| 1447225_at | Slc16a5 | solute carrier family 16 (monocarboxylic acid transporters), member 5 | -1.89 | 0.122 | -2.45 | 0.056 | 1.15 | 0.409 | -1.15 | 0.719 | -1.09 |
| 1429946_at | 2610301F02Rik | RIKEN cDNA 2610301F02 gene | -1.19 | 0.577 | -1.28 | 0.435 | -2.13 | 0.089 | 1.36 | 0.389 | -0.81 |
| 1443491_at | Ptprk | Protein tyrosine phosphatase, receptor type, K | -1.45 | 0.258 | -1.15 | 0.645 | -1.89 | 0.007 | 1 | 0.988 | -0.87 |
| 1457921_at | 1810037C20Rik | RIKEN cDNA 1810037C20 gene | -1.36 | 0.107 | -1.73 | 0.301 | -1.29 | 0.526 | 1.13 | 0.366 | -0.81 |
| 1458803_at | Slfn9 /// LOC67761 | schlafen 9 /// similar to schlafen 9 | -1.55 | 0.457 | -1.47 | 0.271 | -1.31 | 0.542 | 1.23 | 0.209 | -0.77 |
| 1458553_at | Plat | plasminogen activator, tissue | -1.45 | 0.286 | -1.78 | 0.076 | -1.19 | 0.246 | 1.36 | 0.471 | -0.77 |
| 1457251_x_at | Adcy1 | Adenylate cyclase 1 | 1.05 | 0.939 | -4.78 | 0 | -1.21 | 0.712 | -1.14 | 0.843 | -1.52 |
| 1440713_at | 4933407H18Rik | RIKEN cDNA 4933407H18 gene | -1.06 | 0.928 | -1.74 | 0.337 | -1.76 | 0.432 | 1.25 | 0.728 | -0.82 |
| 1446327_at | --- | --- | -1.04 | 0.884 | -1.25 | 0.429 | -3.08 | 0.003 | 1.48 | 0.088 | -0.97 |
| 1441134_at | 6820424L24Rik | RIKEN cDNA 6820424L24 gene | -2.27 | 0.101 | -1.4 | 0.612 | -1.07 | 0.85 | 1.46 | 0.301 | -0.82 |
| 1444578_at | Spop | Speckle-type POZ protein | -3.91 | 0.049 | 1.2 | 0.498 | -1.57 | 0.371 | -1.36 | 0.024 | -1.41 |
| 1443611_at | Dnajb4 | DnaJ (Hsp40) homolog, subfamily B, member 4 | -1.5 | 0.196 | -2.02 | 0.077 | -1.08 | 0.805 | 1.74 | 0.087 | -0.72 |
| 1442777_at | --- | Transcribed locus | -1.22 | 0.612 | -2.18 | 0.139 | -1.23 | 0.559 | 1.39 | 0.083 | -0.81 |
| 1424260_at | Slc12a1 | solute carrier family 12, member 1 | -1.06 | 0.583 | -5.43 | 0.185 | -1.03 | 0.945 | 1.3 | 0.43 | -1.56 |
| 1454586_at | 1500002K03Rik | RIKEN cDNA 1500002K03 gene | -2.21 | 0.253 | -1.94 | 0.126 | 1.12 | 0.777 | -2.14 | 0.461 | -1.29 |
| 1456974_at | Onecut1 | one cut domain, family member 1 | -1.19 | 0.734 | -2.83 | 0.005 | -1.11 | 0.888 | 1.62 | 0.452 | -0.88 |
| 1441778_at | Adcyap1 | adenylate cyclase activating polypeptide 1 | -1.26 | 0.646 | -1.68 | 0.159 | -1.43 | 0.399 | 1.55 | 0.067 | -0.7 |
| 1454397_at | 4632418H02Rik | RIKEN cDNA 4632418H02 gene | 1.01 | 0.901 | -1.78 | 0.067 | -1.94 | 0.015 | -1.41 | 0.022 | -1.03 |
| 1421505_at | Mixl1 | Mix1 homeobox-like 1 (Xenopus laevis) | -2.95 | 0.001 | -2.38 | 0.292 | 1.33 | 0.551 | -3.54 | 0.19 | -1.88 |
| 1416617_at | Acss1 | acyl-CoA synthetase short-chain family member 1 | -1.58 | 0.028 | -2.02 | 0.324 | -1.04 | 0.372 | 1.15 | 0.489 | -0.87 |
| 1444771_at | Abtb2 | ankyrin repeat and BTB (POZ) domain containing 2 | 1.06 | 0.819 | -2.57 | 0.02 | -1.56 | 0.289 | -1.01 | 0.99 | -1.02 |
| 1460461_at | 4921521K07Rik | RIKEN cDNA 4921521K07 gene | -2.29 | 0.152 | -2.6 | 0.016 | 1.27 | 0.642 | -1.86 | 0.088 | -1.37 |
| 1432046_at | 4930404K06Rik | RIKEN cDNA 4930404K06 gene | 1.24 | 0.404 | -4.32 | 0.005 | -1.61 | 0.353 | -1.52 | 0.291 | -1.55 |
| 1440635_at | Palld | palladin, cytoskeletal associated protein | -2.7 | 0.117 | -1.32 | 0.54 | -1.04 | 0.934 | 1.91 | 0.304 | -0.79 |
| 1444697_at | 4732490B19Rik | RIKEN cDNA 4732490B19 gene | -1.56 | 0.483 | -1.29 | 0.636 | -1.48 | 0.385 | 1.53 | 0.508 | -0.7 |
| 1425959_x_at | Klra1 /// Klra12 /// K | killer cell lectin-like receptor, subfamily A, member 1 /// killer cell lectin-like recepto | -1.36 | 0.193 | -2.2 | 0.271 | -1.11 | 0.847 | 1.23 | 0.776 | -0.86 |
| 1442409_at | D9Wsu90e | DNA segment, Chr 9, Wayne State University 90, expressed | 1 | 1 | -1.78 | 0.531 | -1.88 | 0.03 | -2.53 | 0.014 | -1.3 |
| 1458115_at | 4930512H18Rik | RIKEN cDNA 4930512H18 gene | -1.42 | 0.542 | -1.87 | 0.476 | -1.17 | 0.564 | 1.16 | 0.789 | -0.83 |
| 1449475_at | Atp12a | ATPase, H+/K+ transporting, nongastric, alpha polypeptide | 1.08 | 0.758 | -1.88 | 0.295 | -2.07 | 0.169 | -2.21 | 0.282 | -1.27 |
| 1442892_at | Mcc | Mutated in colorectal cancers | -1.91 | 0.351 | -1.33 | 0.385 | -1.22 | 0.45 | 1.2 | 0.766 | -0.82 |
| 1446267_at | D17Ertd657e | DNA segment, Chr 17, ERATO Doi 657, expressed | 1.05 | 0.961 | -2.3 | 0.418 | -1.63 | 0.083 | -2.55 | 0.408 | -1.36 |
| 1447461_at | --- | --- | -1.49 | 0.675 | -1.43 | 0.541 | -1.38 | 0.402 | 1.23 | 0.765 | -0.77 |
| 1456923_at | Trpm3 | transient receptor potential cation channel, subfamily M, member 3 | -1.45 | 0.05 | -1.66 | 0.161 | -1.24 | 0.671 | 1.8 | 0.457 | -0.64 |
| 1421596_s_at | H28 | histocompatibility 28 | -1.41 | 0.47 | -1.22 | 0.427 | -1.78 | 0.192 | 2.8 | 0.304 | -0.4 |

| | | | | | | | | | | | |
|---|---|---|---|---|---|---|---|---|---|---|---|
| 1437823_at | 2310040A07Rik | RIKEN cDNA 2310040A07 gene | -1.04 | 0.94 | -2.63 | 0.198 | -1.33 | 0.367 | 1.31 | 0.207 | -0.92 |
| 1431435_at | --- | --- | -2.94 | 0.026 | -1.65 | 0.228 | 1.15 | 0.649 | -1.86 | 0.151 | -1.32 |
| 1452905_at | Gtl2 /// Lphn1 | GTL2, imprinted maternally expressed untranslated mRNA /// latrophilin 1 | -1.15 | 0.521 | -3.42 | 0.028 | -1.07 | 0.657 | 1.39 | 0.239 | -1.06 |
| 1439380_x_at | Gtl2 | GTL2, imprinted maternally expressed untranslated mRNA | -1.23 | 0.406 | -2.19 | 0.15 | -1.22 | 0.133 | 1.79 | 0.197 | -0.71 |
| 1444469_at | 1110059H15Rik | RIKEN cDNA 1110059H15 gene | -1.39 | 0.472 | -2.04 | 0.073 | -1.13 | 0.654 | 1.35 | 0.445 | -0.8 |
| 1442735_at | Oaz2 | Ornithine decarboxylase antizyme 2 | -1.07 | 0.225 | -1.42 | 0.699 | -2.18 | 0.062 | 1.03 | 0.961 | -0.91 |
| 1459068_at | Tmem109 | Transmembrane protein 109 | 1.08 | 0.814 | -2.21 | 0.168 | -1.76 | 0.077 | -1.44 | 0.459 | -1.08 |
| 1449237_at | Aloxe3 | arachidonate lipoxygenase 3 | -1.55 | 0.281 | -1.37 | 0.141 | -1.38 | 0.219 | 1.02 | 0.893 | -0.82 |
| 1439991_a_at | Pabpnl1 | poly(A)binding protein nuclear-like 1 | -1.73 | 0.188 | 1.05 | 0.828 | -2.13 | 0.177 | -2.45 | 0.076 | -1.31 |
| 1442498_at | C78662 | EST C78662 | -1.64 | 0.141 | -1.12 | 0.752 | -1.68 | 0.194 | 1.18 | 0.366 | -0.81 |
| 1419376_at | 1110018M03Rik | RIKEN cDNA 1110018M03 gene | -1.25 | 0.113 | -1.17 | 0.45 | -2.23 | 0.016 | 1.04 | 0.942 | -0.9 |
| 1433005_at | 4833446E11Rik | RIKEN cDNA 4833446E11 gene | -1.85 | 0.116 | -1.4 | 0.479 | -1.18 | 0.534 | 1.89 | 0.144 | -0.64 |
| 1443428_at | --- | --- | -1.21 | 0.667 | -1.86 | 0.252 | -1.37 | 0.621 | 1.04 | 0.951 | -0.85 |
| 1439042_at | Adcyap1r1 | adenylate cyclase activating polypeptide 1 receptor 1 | -1.62 | 0.58 | -1.59 | 0.278 | -1.18 | 0.772 | 1.07 | 0.408 | -0.83 |
| 1442824_at | 8030497I03Rik | RIKEN cDNA 8030497I03 gene | -1.24 | 0.222 | -1.24 | 0.335 | -2.05 | 0.033 | 1.14 | 0.651 | -0.85 |
| 1440350_at | --- | --- | -1.77 | 0.219 | -1.4 | 0.475 | -1.22 | 0.575 | 1.05 | 0.885 | -0.83 |
| 1438775_at | Ppargc1b | Peroxisome proliferative activated receptor, gamma, coactivator 1 beta | -1.2 | 0.436 | -2.01 | 0.152 | -1.29 | 0.584 | 2.1 | 0.251 | -0.6 |
| 1441377_at | Kif6 | kinesin family member 6 | -2.48 | 0.146 | -1.21 | 0.533 | -1.15 | 0.563 | 1.98 | 0.373 | -0.71 |
| 1443803_x_at | Hoxa5 | homeo box A5 | -1.06 | 0.667 | -2.1 | 0.145 | -1.46 | 0.227 | 1.12 | 0.451 | -0.88 |
| 1422310_at | Snn | stannin | -1.63 | 0.339 | -1.62 | 0.242 | -1.15 | 0.399 | 1.98 | 0.15 | -0.61 |
| 1445780_at | --- | --- | 1.23 | 0.29 | -2.1 | 0.24 | -2.52 | 0.178 | -2.07 | 0.311 | -1.36 |
| 1442689_at | D9Ertd278e | DNA segment, Chr 9, ERATO Doi 278, expressed | -1.58 | 0.239 | -1.94 | 0.36 | -1.05 | 0.839 | 1.4 | 0.125 | -0.79 |
| 1441578_at | --- | --- | -1.78 | 0.081 | -1.91 | 0.023 | 1.02 | 0.941 | -1.21 | 0.539 | -0.97 |
| 1438438_at | 2900002H16Rik | RIKEN cDNA 2900002H16 gene | -1.65 | 0.123 | -1.23 | 0.303 | -1.46 | 0.047 | 1.09 | 0.684 | -0.81 |
| 1424471_at | Rapgef3 | Rap guanine nucleotide exchange factor (GEF) 3 | -1.12 | 0.594 | -2.9 | 0.183 | -1.16 | 0.521 | 1.01 | 0.974 | -1.04 |
| 1446599_at | --- | --- | -1.17 | 0.484 | -1.27 | 0.606 | -2.15 | 0.029 | 2.99 | 0.012 | -0.4 |
| 1457933_at | Gm1964 | Gene model 1964, (NCBI) | -1.1 | 0.829 | -2.16 | 0.126 | -1.37 | 0.074 | 5.35 | 0.198 | 0.18 |
| 1421482_at | Bsnd | Bartter syndrome, infantile, with sensorineural deafness (Barttin) | -1.08 | 0.823 | -1.58 | 0.406 | -1.83 | 0.381 | 1.38 | 0.551 | -0.78 |
| 1443035_at | --- | --- | -1.72 | 0.082 | -1.15 | 0.731 | -1.54 | 0.208 | 1.18 | 0.724 | -0.81 |
| 1432468_at | Lcn9 | lipocalin 9 | -1.88 | 0.286 | -1.81 | 0.39 | 1.02 | 0.975 | -1.03 | 0.945 | -0.93 |
| 1436432_at | B230343J05Rik | RIKEN cDNA B230343J05 gene | -1.5 | 0.574 | -1.38 | 0.494 | -1.41 | 0.144 | 1.03 | 0.974 | -0.81 |
| 1445699_at | --- | --- | -1.38 | 0.093 | -1.65 | 0.437 | -1.29 | 0.481 | 1.44 | 0.353 | -0.72 |
| 1428601_at | 1700003E16Rik | RIKEN cDNA 1700003E16 gene | -2.27 | 0.232 | -1.6 | 0.042 | 1.04 | 0.903 | -1.95 | 0.333 | -1.19 |
| 1458378_at | Grin3a | glutamate receptor ionotropic, NMDA3A | -2.22 | 0.124 | -1.97 | 0.171 | 1.15 | 0.814 | -2.12 | 0.343 | -1.29 |
| 1444533_at | Cugbp2 | CUG triplet repeat, RNA binding protein 2 | 1.13 | 0.607 | -3.96 | 0.182 | -1.38 | 0.391 | -1.15 | 0.867 | -1.34 |
| 1451526_at | Arhgap12 | Rho GTPase activating protein 12 | -1.39 | 0.147 | -1.37 | 0.204 | -1.52 | 0.037 | 1.11 | 0.751 | -0.79 |
| 1421548_at | Pcdhb2 | protocadherin beta 2 | -1.61 | 0.5 | -2.74 | 0.056 | 1.12 | 0.806 | -1.13 | 0.849 | -1.09 |
| 1457637_at | Prickle1 | Prickle like 1 (Drosophila) | -1.01 | 0.884 | -1.63 | 0.239 | -1.99 | 0.059 | 1.04 | 0.89 | -0.89 |
| 1421470_at | Grpr | gastrin releasing peptide receptor | -2.53 | 0.401 | -1.73 | 0.132 | 1.14 | 0.86 | -1.16 | 0.782 | -1.07 |
| 1442816_at | Hs3st3b1 | heparan sulfate (glucosamine) 3-O-sulfotransferase 3B1 | -1.51 | 0.239 | -1.16 | 0.665 | -1.72 | 0.397 | 1.42 | 0.481 | -0.74 |
| 1442193_at | A430028G04Rik | RIKEN cDNA A430028G04 gene | -2.46 | 0.095 | -1.42 | 0.538 | -1 | 0.998 | 1.87 | 0.392 | -0.75 |
| 1441236_at | Slc9a3 | solute carrier family 9 (sodium/hydrogen exchanger), member 3 | -1.26 | 0.265 | -2.1 | 0.082 | -1.19 | 0.559 | 1.08 | 0.558 | -0.87 |
| 1456418_at | --- | PREDICTED: Mus musculus similar to Inward rectifier potassium channel 13 (Pot | -1.48 | 0.02 | -1.34 | 0.527 | -1.46 | 0.084 | 1.05 | 0.72 | -0.81 |
| 1431679_at | 2510042H12Rik | RIKEN cDNA 2510042H12 gene | -1.07 | 0.91 | -3.17 | 0.13 | -1.16 | 0.767 | 1.44 | 0.357 | -0.99 |
| 1427528_a_at | Epha7 | Eph receptor A7 | -2.33 | 0.228 | -1.48 | 0.438 | 1.01 | 0.979 | -3.17 | 0.221 | -1.5 |
| 1429938_at | A930009E05Rik | RIKEN cDNA A930009E05 gene | -1.49 | 0.033 | -2.53 | 0.29 | 1.04 | 0.934 | -1.3 | 0.551 | -1.07 |
| 1457758_at | Eny2 | enhancer of yellow 2 homolog (Drosophila) | -1.14 | 0.876 | -1.41 | 0.362 | -1.9 | 0.084 | 1.06 | 0.758 | -0.85 |
| 1424063_at | Abpa | androgen binding protein alpha | -2.33 | 0.028 | -1.15 | 0.695 | -1.24 | 0.333 | 1.2 | 0.464 | -0.88 |
| 1427817_at | LOC641050 | hypothetical protein LOC641050 | -1.05 | 0.957 | -1.88 | 0.295 | -1.6 | 0.203 | 1.42 | 0.565 | -0.78 |
| 1454737_at | Dusp9 | dual specificity phosphatase 9 | -1.27 | 0.503 | -1.55 | 0.06 | -1.47 | 0.016 | 1.05 | 0.703 | -0.81 |
| 1450343_at | V1rd1 | vomeronasal 1 receptor, D1 | -2 | 0.301 | -1.47 | 0.547 | -1.07 | 0.846 | 1.19 | 0.812 | -0.84 |
| 1447115_at | C78409 | Expressed sequence C78409 | -1.13 | 0.883 | -2.41 | 0.071 | -1.23 | 0.574 | 1.53 | 0.546 | -0.81 |
| 1421573_at | 4930533N22Rik | RIKEN cDNA 4930533N22 gene | -2 | 0.066 | -1.33 | 0.722 | -1.16 | 0.518 | 1.16 | 0.843 | -0.83 |

| | | | | | | | | | | | |
|---|---|---|---|---|---|---|---|---|---|---|---|
| 1443726_at | Smyd1 | SET and MYND domain containing 1 | -1.86 | 0.14 | -1.59 | 0.123 | -1.06 | 0.918 | 1.43 | 0.061 | -0.77 |
| 1421091_at | Serpina12 | serine (or cysteine) peptidase inhibitor, clade A (alpha-1 antiproteinase, antitrypsi | -1.12 | 0.766 | -2.08 | 0.422 | -1.36 | 0.134 | 3 | 0.121 | -0.39 |
| 1427775_at | Defb10 | defensin beta 10 | -1.42 | 0.193 | -2.3 | 0.026 | -1.03 | 0.907 | 1.36 | 0.079 | -0.85 |
| 1449701_at | D6Ertd109e | DNA segment, Chr 6, ERATO Doi 109, expressed | -1.42 | 0.557 | -1.17 | 0.708 | -1.79 | 0.354 | 1.27 | 0.544 | -0.78 |
| 1444005_at | --- | --- | -1.38 | 0.334 | -1.44 | 0.468 | -1.44 | 0.095 | 1.5 | 0.439 | -0.69 |
| 1446323_at | --- | --- | -1.37 | 0.227 | -1.6 | 0.191 | -1.32 | 0.16 | 1.31 | 0.253 | -0.74 |
| 1439379_x_at | Prm1 | protamine 1 | -2.62 | 0.459 | -1.3 | 0.351 | -1.04 | 0.694 | 1.95 | 0.076 | -0.75 |
| 1417641_at | Galntl5 | UDP-N-acetyl-alpha-D-galactosamine:polypeptide N-acetylgalactosaminyltransfe | -1.34 | 0.562 | -3.4 | 0.235 | 1.07 | 0.817 | -1.59 | 0.337 | -1.31 |
| 1457294_at | Tmtc3 | Transmembrane and tetratricopeptide repeat containing 3 | -1.27 | 0.144 | -1.37 | 0.128 | -1.68 | 0.063 | 1.37 | 0.064 | -0.74 |
| 1459443_at | Kcnq1 | Potassium voltage-gated channel, subfamily Q, member 1 | -1.3 | 0.21 | -1.16 | 0.633 | -2.07 | 0.06 | 3.37 | 0.006 | -0.29 |
| 1430285_at | Ppp1r14c | protein phosphatase 1, regulatory (inhibitor) subunit 14c | -1.27 | 0.604 | -1.35 | 0.316 | -1.71 | 0.099 | 2.28 | 0.224 | -0.51 |
| 1441380_at | 2810439F02Rik | RIKEN cDNA 2810439F02 gene | -1.17 | 0.567 | -1.8 | 0.027 | -1.42 | 0.184 | 2.14 | 0.395 | -0.56 |
| 1448393_at | Cldn7 | claudin 7 | -1.2 | 0.099 | -2.77 | 0.178 | -1.08 | 0.481 | 1.84 | 0.088 | -0.81 |
| 1447242_at | Parl | Presenilin associated, rhomboid-like | 1 | 0.977 | -1.62 | 0.167 | -2.01 | 0.16 | -1.83 | 0.334 | -1.11 |
| 1439620_at | Car13 | carbonic anhydrase 13 | -2.08 | 0.295 | -1.17 | 0.708 | -1.28 | 0.528 | 1.74 | 0.118 | -0.7 |
| 1424596_s_at | Lmcd1 | LIM and cysteine-rich domains 1 | 1.1 | 0.726 | -3.15 | 0.19 | -1.42 | 0.02 | -1.08 | 0.433 | -1.14 |
| 1427436_at | Six2 | sine oculis-related homeobox 2 homolog (Drosophila) | -1.87 | 0.277 | -1.45 | 0.371 | -1.12 | 0.636 | 1.04 | 0.913 | -0.85 |
| 1435136_at | Whsc1 | Wolf-Hirschhorn syndrome candidate 1 (human) | -1.24 | 0.468 | -1.48 | 0.13 | -1.58 | 0.011 | 1.93 | 0.227 | -0.59 |
| 1438240_at | Ccin | calicin | -5 | 0.317 | 1.26 | 0.694 | -1.52 | 0.259 | -1.21 | 0.558 | -1.62 |
| 1459287_at | Wdfy3 | WD repeat and FYVE domain containing 3 | -1.89 | 0.057 | -1.91 | 0.188 | 1.07 | 0.91 | -1.21 | 0.619 | -0.99 |
| 1449892_at | Lyzl1 | lysozyme-like 1 | -1.17 | 0.048 | -1.6 | 0.582 | -1.57 | 0.406 | 3.66 | 0.242 | -0.17 |
| 1452900_at | LOC671160 | similar to Proline oxidase, mitochondrial precursor (Proline dehydrogenase) | -1.83 | 0.149 | -1 | 0.996 | -1.74 | 0.058 | 1.15 | 0.449 | -0.85 |
| 1442259_at | --- | --- | -1.42 | 0.478 | -1.27 | 0.701 | -1.59 | 0.038 | 1.14 | 0.787 | -0.79 |
| 1442780_at | 2610002J02Rik | RIKEN cDNA 2610002J02 gene | 1.13 | 0.854 | -2.87 | 0.137 | -1.56 | 0.417 | -1.69 | 0.358 | -1.25 |
| 1433630_at | Map6d1 | MAP6 domain containing 1 | 1.2 | 0.844 | -3.72 | 0.196 | -1.53 | 0.164 | -1.1 | 0.867 | -1.29 |
| 1446832_at | --- | --- | 1.04 | 0.864 | -1.48 | 0.182 | -2.46 | 0.052 | -1.11 | 0.707 | -1 |
| 1433289_at | 9530078B04Rik | RIKEN cDNA 9530078B04 gene | -1.59 | 0.37 | -1.06 | 0.906 | -1.83 | 0.042 | 1.02 | 0.944 | -0.86 |
| 1427356_at | 2310031A18Rik | RIKEN cDNA 2310031A18 gene | -1.29 | 0.544 | -1.62 | 0.156 | -1.38 | 0.343 | 1.26 | 0.778 | -0.76 |
| 1417569_at | Ncald | neurocalcin delta | -1.31 | 0.372 | -1.36 | 0.093 | -1.61 | 0.15 | 1.05 | 0.794 | -0.81 |
| 1441626_at | --- | --- | -1.14 | 0.727 | -2.24 | 0.261 | -1.26 | 0.652 | 1.16 | 0.615 | -0.87 |
| 1442244_at | Inadl | InaD-like (Drosophila) | 1.34 | 0.241 | -3.12 | 0.286 | -2.17 | 0.007 | -2.02 | 0.037 | -1.49 |
| 1438877_at | Trpm6 | transient receptor potential cation channel, subfamily M, member 6 | -1.06 | 0.859 | -2.12 | 0.006 | -1.41 | 0.007 | 1.11 | 0.747 | -0.87 |
| 1446643_at | 5330409N07Rik | RIKEN cDNA 5330409N07 gene | -1.75 | 0.585 | -2.56 | 0.01 | 1.16 | 0.827 | -1.26 | 0.66 | -1.1 |
| 1419997_at | --- | --- | -1.2 | 0.692 | -2.72 | 0.069 | -1.09 | 0.765 | 1.25 | 0.7 | -0.94 |
| 1435700_at | Tln2 | talin 2 | -1.12 | 0.728 | -1.74 | 0.075 | -1.53 | 0.023 | 1.29 | 0.074 | -0.78 |
| 1429906_at | A930035E12Rik | RIKEN cDNA A930035E12 gene | -1.18 | 0.716 | -1.73 | 0.374 | -1.43 | 0.19 | 1.22 | 0.513 | -0.78 |
| 1456568_at | Gsn | Gelsolin | -2.07 | 0.384 | -1.1 | 0.56 | -1.38 | 0.581 | 1.54 | 0.535 | -0.75 |
| 1451991_at | Epha7 | Eph receptor A7 | -1.85 | 0.357 | -1.08 | 0.852 | -1.52 | 0.455 | 1.28 | 0.73 | -0.79 |
| 1425852_at | A230107C01Rik | RIKEN cDNA A230107C01 gene | -1.3 | 0.445 | -2.14 | 0.517 | -1.13 | 0.783 | 1.18 | 0.795 | -0.85 |
| 1445470_at | LOC235580 /// LOC | similar to alpha 3 type VI collagen isoform 4 precursor /// similar to alpha 3 type V | -1.79 | 0.155 | -1.31 | 0.083 | -1.25 | 0.679 | 1.71 | 0.399 | -0.66 |
| 1446644_at | Brd3 | Bromodomain containing 3 | -1.34 | 0.206 | -1.3 | 0.44 | -1.64 | 0.175 | 1.06 | 0.715 | -0.81 |
| 1438906_at | --- | --- | -1.37 | 0.218 | -1.29 | 0.074 | -1.61 | 0.013 | 1.14 | 0.108 | -0.78 |
| 1436369_at | 2900076A07Rik | RIKEN cDNA 2900076A07 gene | -1.38 | 0.421 | -2.61 | 0.23 | 1.02 | 0.973 | -1.55 | 0.102 | -1.13 |
| 1419230_at | Krt12 | keratin 12 | 1.07 | 0.903 | -2.36 | 0.193 | -1.58 | 0.518 | -1.88 | 0.452 | -1.19 |
| 1441365_at | Foxp2 | forkhead box P2 | -2.15 | 0.055 | -1.17 | 0.12 | -1.24 | 0.649 | 1.19 | 0.618 | -0.84 |
| 1442897_at | 2610024E20Rik | RIKEN cDNA 2610024E20 gene | -1.3 | 0.694 | -1.5 | 0.371 | -1.45 | 0.324 | 1.4 | 0.412 | -0.71 |
| 1446395_at | Robo2 | Roundabout homolog 2 (Drosophila) | -1.21 | 0.467 | -1.74 | 0.068 | -1.39 | 0.354 | 1.28 | 0.701 | -0.76 |
| 1427703_at | Pafah1b1 | platelet-activating factor acetylhydrolase, isoform 1b, beta1 subunit | -1.03 | 0.915 | -1.42 | 0.24 | -2.22 | 0.026 | 1.24 | 0.425 | -0.86 |
| 1431478_at | 3110035G12Rik | RIKEN cDNA 3110035G12 gene | -1.07 | 0.918 | -1.3 | 0.616 | -2.37 | 0.057 | 1.52 | 0.402 | -0.81 |
| 1459883_at | --- | Adult male epididymis cDNA, RIKEN full-length enriched library, clone:9230001K | 1.01 | 0.948 | -2.58 | 0.164 | -1.37 | 0.379 | -1.02 | 0.913 | -0.99 |
| 1421856_at | S100a3 | S100 calcium binding protein A3 | 1.36 | 0.6 | -2.76 | 0.205 | -2.48 | 0.036 | -1.32 | 0.387 | -1.3 |
| 1444873_at | Hip1 | Huntingtin interacting protein 1 | -2.9 | 0.065 | -1.02 | 0.97 | -1.25 | 0.637 | 1.16 | 0.449 | -1 |
| 1428036_at | --- | MRNA similar to phosphatidylinositol glycan, class S (cDNA clone MGC:41618 IM | -1.21 | 0.493 | -1.44 | 0.512 | -1.64 | 0.237 | 1.09 | 0.836 | -0.8 |

| | | | | | | | | | | | |
|---|---|---|---|---|---|---|---|---|---|---|---|
| 1459293_at | C030038J10Rik | RIKEN cDNA C030038J10 gene | 1.02 | 0.955 | -1.35 | 0.027 | -2.69 | 0.003 | -1.27 | 0.66 | -1.07 |
| 1446848_at | --- | --- | 1.28 | 0.662 | -2.6 | 0.023 | -2.16 | 0.033 | -2.28 | 0.095 | -1.44 |
| 1443966_at | --- | 3 days neonate thymus cDNA, RIKEN full-length enriched library, clone:A630040 | -1.44 | 0.151 | -1.5 | 0.011 | -1.3 | 0.154 | 1.01 | 0.941 | -0.81 |
| 1430606_at | 2310020F24Rik | RIKEN cDNA 2310020F24 gene | -1.62 | 0.374 | -2.12 | 0.303 | 1.04 | 0.823 | -1.03 | 0.914 | -0.93 |
| 1450567_a_at | Col2a1 | procollagen, type II, alpha 1 | -1.16 | 0.219 | -2.02 | 0.425 | -1.29 | 0.146 | 1.48 | 0.453 | -0.75 |
| 1431979_at | 4930444M15Rik | RIKEN cDNA 4930444M15 gene | -1.61 | 0.679 | -1.19 | 0.833 | -1.51 | 0.36 | 2.17 | 0.21 | -0.53 |
| 1449722_at | --- | Transcribed locus | -1.22 | 0.19 | -1.69 | 0.085 | -1.4 | 0.145 | 1.79 | 0.011 | -0.63 |
| 1459533_at | D19Ertd79e | DNA segment, Chr 19, ERATO Doi 79, expressed | -1.49 | 0.322 | -1 | 0.999 | -2.19 | 0.316 | 1.06 | 0.873 | -0.9 |
| 1443018_at | Ncam1 | Neural cell adhesion molecule 1 | -1.42 | 0.601 | -1.22 | 0.701 | -1.66 | 0.05 | 1.01 | 0.971 | -0.82 |
| 1443546_at | Hdlbp | High density lipoprotein (HDL) binding protein | -1.41 | 0.172 | -1.3 | 0.3 | -1.54 | 0.003 | 1.25 | 0.19 | -0.75 |
| 1458603_at | --- | --- | -1.44 | 0.01 | -1.29 | 0.191 | -1.51 | 0.083 | 1.08 | 0.651 | -0.79 |
| 1442560_at | --- | Adult male testis cDNA, RIKEN full-length enriched library, clone:4933433O05 pr | -1.22 | 0.124 | -1.9 | 0.104 | -1.27 | 0.439 | 2.02 | 0.113 | -0.59 |
| 1449601_x_at | --- | Transcribed locus | -1.18 | 0.605 | -1.62 | 0.434 | -1.5 | 0.219 | 1.04 | 0.897 | -0.82 |
| 1439816_at | LOC433270 | Hypothetical gene supported by AK087915 | -1.79 | 0.043 | -1.74 | 0.101 | -1 | 0.984 | 1.43 | 0.334 | -0.78 |
| 1436099_at | AI836003 | expressed sequence AI836003 | -1.94 | 0.016 | -1.5 | 0.018 | -1.06 | 0.658 | 1.17 | 0.615 | -0.83 |
| 1449167_at | Epb4.1l4a | erythrocyte protein band 4.1-like 4a | -1.38 | 0.199 | -1.78 | 0.472 | -1.19 | 0.251 | 2.09 | 0.08 | -0.56 |
| 1455624_at | --- | --- | 1.06 | 0.651 | -2.18 | 0.09 | -1.64 | 0.542 | -1.71 | 0.331 | -1.12 |
| 1419981_at | Riok3 | RIO kinase 3 (yeast) | -1.38 | 0.605 | -1.6 | 0.226 | -1.28 | 0.21 | 1.11 | 0.569 | -0.79 |
| 1444675_at | AL023051 | expressed sequence AL023051 | -1.09 | 0.626 | -1.24 | 0.613 | -2.46 | 0.047 | 1.52 | 0.273 | -0.82 |
| 1420452_at | Krtap5-2 | keratin associated protein 5-2 | -2.37 | 0.067 | -1.24 | 0.241 | -1.11 | 0.609 | 2.22 | 0.039 | -0.63 |
| 1458279_at | Foxo3a | Forkhead box O3a | -1.19 | 0.592 | -1.44 | 0.191 | -1.69 | 0.044 | 1.62 | 0.249 | -0.67 |
| 1459816_x_at | --- | --- | -2.44 | 0.064 | -1.14 | 0.71 | -1.19 | 0.557 | 1.06 | 0.922 | -0.93 |
| 1457843_at | Lypd6 | LY6/PLAUR domain containing 6 | -1.32 | 0.395 | -1.3 | 0.592 | -1.65 | 0.403 | 1.07 | 0.874 | -0.8 |
| 1457881_at | Osbpl6 | oxysterol binding protein-like 6 | -1.1 | 0.313 | -2.65 | 0.197 | -1.18 | 0.252 | 1.01 | 0.956 | -0.98 |
| 1422840_at | 1700013N18Rik | RIKEN cDNA 1700013N18 gene | -2.66 | 0.415 | -1.19 | 0.754 | -1.09 | 0.845 | 1.49 | 0.539 | -0.87 |
| 1422339_at | Gja3 | gap junction membrane channel protein alpha 3 | -1.28 | 0.634 | -1.65 | 0.228 | -1.34 | 0.592 | 1.84 | 0.122 | -0.61 |
| 1442658_at | Ndufb2 | NADH dehydrogenase (ubiquinone) 1 beta subcomplex, 2 | 1.01 | 0.969 | -1.78 | 0.056 | -1.78 | 0.064 | -1.23 | 0.435 | -0.95 |
| 1457441_at | Ebf1 | early B-cell factor 1 | -2.53 | 0.095 | 1.11 | 0.738 | -1.59 | 0.342 | -2.3 | 0.012 | -1.33 |
| 1450574_at | Cyp11b2 | cytochrome P450, family 11, subfamily b, polypeptide 2 | -1.42 | 0.583 | -2.19 | 0.052 | -1.03 | 0.953 | 1.09 | 0.824 | -0.89 |
| 1457520_at | Grhl2 | Grainyhead-like 2 (Drosophila) | -1.64 | 0.162 | -1.32 | 0.385 | -1.31 | 0.353 | 1.07 | 0.764 | -0.8 |
| 1456601_x_at | Fxyd2 | FXYD domain-containing ion transport regulator 2 | -1.09 | 0.251 | -7.07 | 0.163 | 1.08 | 0.857 | -1.62 | 0.42 | -2.18 |
| 1420236_at | --- | Transcribed locus | -2.56 | 0.47 | 1.03 | 0.887 | -1.4 | 0.609 | -1 | 0.996 | -0.98 |
| 1447937_a_at | 4933409K07Rik /// | RIKEN cDNA 4933409K07 gene /// similar to 4933409K07Rik protein /// similar to | -2.04 | 0.271 | -1.53 | 0.131 | -1.01 | 0.967 | 2.25 | 0.075 | -0.58 |
| 1425449_at | Ppap2a | phosphatidic acid phosphatase 2a | -2.78 | 0.178 | -1.02 | 0.957 | -1.26 | 0.54 | 1.2 | 0.586 | -0.96 |
| 1445441_at | LOC380907 | similar to RIKEN cDNA 1700129C05 | -2.23 | 0.026 | -1.29 | 0.583 | -1.1 | 0.767 | 1.3 | 0.296 | -0.83 |
| 1422358_at | Fpr-rs4 | formyl peptide receptor, related sequence 4 | -1.42 | 0.095 | -1.03 | 0.88 | -2.19 | 0.006 | 1.11 | 0.422 | -0.88 |
| 1458251_at | AU022166 | expressed sequence AU022166 | -2.02 | 0.221 | -1.57 | 0.073 | 1 | 0.999 | -1.45 | 0.058 | -1.01 |
| 1434013_at | Ablim3 | actin binding LIM protein family, member 3 | -1.65 | 0.344 | -2.04 | 0.054 | 1.04 | 0.899 | -1.47 | 0.409 | -1.03 |
| 1428358_at | 1810010M01Rik | RIKEN cDNA 1810010M01 gene | -1.13 | 0.184 | -2.14 | 0.024 | -1.27 | 0.453 | 1.91 | 0.074 | -0.66 |
| 1457867_at | Sgpp2 | sphingosine-1-phosphate phosphotase 2 | -1.05 | 0.281 | -2.18 | 0.111 | -1.38 | 0.144 | 2.43 | 0.167 | -0.54 |
| 1456344_at | Tnc | Tenascin C | -1.18 | 0.337 | -1.06 | 0.867 | -2.9 | 0.299 | 1.06 | 0.88 | -1.02 |
| 1457412_at | Scn8a | sodium channel, voltage-gated, type VIII, alpha | 1 | 0.996 | -1.45 | 0.45 | -2.25 | 0.094 | -1.01 | 0.988 | -0.93 |
| 1456491_at | AI606861 | Expressed sequence AI606861 | -1.98 | 0.101 | -1.47 | 0.334 | -1.05 | 0.304 | 1.1 | 0.757 | -0.85 |
| 1458800_at | Tnrc6c | Trinucleotide repeat containing 6C | 1.1 | 0.729 | -1.89 | 0.15 | -1.97 | 0.056 | -1.06 | 0.896 | -0.95 |
| 1453376_at | 4921524J06Rik | RIKEN cDNA 4921524J06 gene | -1.45 | 0.572 | -3.37 | 0 | 1.15 | 0.775 | -1.86 | 0.473 | -1.38 |
| 1441649_at | Sesn1 | Sestrin 1 | 1.08 | 0.724 | -1.75 | 0.035 | -2.06 | 0.007 | -1.21 | 0.386 | -0.99 |
| 1442536_at | LOC619719 | hypothetical protein LOC619719 | -1.13 | 0.803 | -2.04 | 0.044 | -1.32 | 0.445 | 1.03 | 0.817 | -0.86 |
| 1438248_at | Pcsk5 | proprotein convertase subtilisin/kexin type 5 | -1.17 | 0.595 | -1.45 | 0.233 | -1.69 | 0.004 | 1.15 | 0.739 | -0.79 |
| 1450618_a_at | Sprr2a | small proline-rich protein 2A | -1.86 | 0.321 | -1.08 | 0.878 | -1.48 | 0.081 | 1.09 | 0.74 | -0.83 |
| 1435121_at | Dio3as | deiodinase, iodothyronine type III, antisense | -1.43 | 0.499 | -1.18 | 0.699 | -1.69 | 0.004 | 1.2 | 0.636 | -0.77 |
| 1439757_s_at | Epha4 | Eph receptor A4 | -1.58 | 0.635 | -2.02 | 0.379 | 1.01 | 0.981 | -1.17 | 0.736 | -0.94 |
| 1456130_at | LOC553091 | hypothetical LOC553091 | -1.58 | 0.3 | -1.01 | 0.989 | -1.95 | 0.031 | 1.01 | 0.974 | -0.88 |
| 1449766_at | Syt6 | synaptotagmin VI | -1.2 | 0.282 | -1.85 | 0.252 | -1.31 | 0.129 | 1.59 | 0.157 | -0.69 |

| | | | | | | | | | | | |
|---|---|---|---|---|---|---|---|---|---|---|---|
| 1439870_at | A330008L17Rik | RIKEN cDNA A330008L17 gene | -3.23 | 0.021 | 1.33 | 0.676 | -1.99 | 0.173 | -1.04 | 0.948 | -1.23 |
| 1457879_at | A230107O07Rik | RIKEN cDNA A230107O07 gene | -1.06 | 0.875 | -2.06 | 0.002 | -1.42 | 0.449 | 1.89 | 0.191 | -0.66 |
| 1418979_at | Akr1c14 | aldo-keto reductase family 1, member C14 | -3.56 | 0.021 | -1.45 | 0.149 | 1.17 | 0.602 | -1.22 | 0.826 | -1.27 |
| 1446225_at | Ssu72 | Ssu72 RNA polymerase II CTD phosphatase homolog (yeast) | -1.62 | 0.013 | -2.45 | 0.091 | 1.11 | 0.256 | -1.31 | 0.172 | -1.07 |
| 1457395_at | 5330421F07Rik | RIKEN cDNA 5330421F07 gene | -1.49 | 0.101 | -2.17 | 0.067 | 1 | 0.988 | -1.01 | 0.989 | -0.91 |
| 1429809_at | Tmtc2 | transmembrane and tetratricopeptide repeat containing 2 | -1.27 | 0.522 | -1.54 | 0.157 | -1.42 | 0.186 | 1.23 | 0.616 | -0.75 |
| 1427513_at | BC024137 | cDNA sequence BC024137 | -1.35 | 0.52 | -1.93 | 0.025 | -1.13 | 0.622 | 1 | 0.999 | -0.85 |
| 1431729_at | Spata17 | spermatogenesis associated 17 | -1.69 | 0.015 | -1.54 | 0.42 | -1.12 | 0.678 | 1.07 | 0.897 | -0.82 |
| 1442672_at | 0610040F04Rik | RIKEN cDNA 0610040F04 gene | -1.7 | 0.329 | -1.87 | 0.22 | 1.01 | 0.972 | -1.42 | 0.49 | -0.99 |
| 1438057_at | --- | Transcribed locus | -2.68 | 0.096 | -1.01 | 0.985 | -1.29 | 0.291 | 1.08 | 0.848 | -0.97 |
| 1447526_at | D5Ertd255e | DNA segment, Chr 5, ERATO Doi 255, expressed | -1.54 | 0.017 | -1.2 | 0.377 | -1.52 | 0.036 | 1.31 | 0.426 | -0.74 |
| 1431241_at | Chchd3 | coiled-coil-helix-coiled-coil-helix domain containing 3 | -1.63 | 0.088 | -1.39 | 0.128 | -1.24 | 0.154 | 1.16 | 0.676 | -0.77 |
| 1449547_at | Asb14 | ankyrin repeat and SOCS box-containing protein 14 | -2.19 | 0.398 | -1.4 | 0.527 | -1.03 | 0.869 | 1.29 | 0.627 | -0.83 |
| 1432966_at | 4921507H08Rik | RIKEN cDNA 4921507H08 gene | -1.05 | 0.922 | -1.11 | 0.879 | -3.45 | 0.014 | 1.1 | 0.681 | -1.13 |
| 1458034_at | 1200016B10Rik | RIKEN cDNA 1200016B10 gene | -1.08 | 0.839 | -1.69 | 0.379 | -1.6 | 0.386 | 1.03 | 0.919 | -0.84 |
| 1460098_at | --- | --- | -1.53 | 0.067 | -1.26 | 0.315 | -1.45 | 0.244 | 1.16 | 0.42 | -0.77 |
| 1438284_at | Ocrl | oculocerebrorenal syndrome of Lowe | -1.38 | 0.158 | -1.27 | 0.459 | -1.59 | 0.021 | 1.34 | 0.001 | -0.73 |
| 1421693_a_at | Gpr98 | G protein-coupled receptor 98 | -1.43 | 0.254 | 1.02 | 0.955 | -2.38 | 0.164 | -1.26 | 0.768 | -1.01 |
| 1454140_at | Ncam1 | Neural cell adhesion molecule 1 | -1.59 | 0.088 | -1.35 | 0.608 | -1.3 | 0.636 | 1.06 | 0.893 | -0.79 |
| 1432457_at | 4930448F12Rik | RIKEN cDNA 4930448F12 gene | -1.79 | 0.549 | -1.58 | 0.433 | -1.05 | 0.233 | 1.08 | 0.863 | -0.84 |
| 1447144_at | 9130011E15Rik | RIKEN cDNA 9130011E15 gene | -1.11 | 0.389 | -1.08 | 0.884 | -3.2 | 0.022 | 1.45 | 0.143 | -0.98 |
| 1454530_at | --- | --- | 1.09 | 0.84 | -2.77 | 0.156 | -1.45 | 0.375 | -1.15 | 0.664 | -1.07 |
| 1437157_at | Atp1b1 | ATPase, Na+/K+ transporting, beta 1 polypeptide | -2.69 | 0.049 | -2.02 | 0.141 | 1.28 | 0.665 | -1.45 | 0.694 | -1.22 |
| 1440747_at | Cpox | coproporphyrinogen oxidase | -1.52 | 0.386 | -1.79 | 0.119 | -1.08 | 0.709 | 5.48 | 0.153 | 0.27 |
| 1458729_at | Fkbp4 | FK506 binding protein 4 | 1.06 | 0.191 | -2.12 | 0.078 | -1.64 | 0.127 | -1.33 | 0.64 | -1.01 |
| 1447911_at | Tead1 | TEA domain family member 1 | -1.37 | 0.482 | -1.85 | 0.164 | -1.15 | 0.669 | 1.79 | 0.046 | -0.64 |
| 1419955_at | Zfand3 | Zinc finger, AN1-type domain 3 | -1.23 | 0.818 | -2.25 | 0.428 | -1.13 | 0.815 | 1.54 | 0.201 | -0.77 |
| 1425583_at | --- | Mammary gland RCB-0526 Jyg-MC(A) cDNA, RIKEN full-length enriched library, | -1.63 | 0.163 | -1.84 | 0.069 | -1.01 | 0.977 | 1.21 | 0.756 | -0.82 |
| 1455934_at | Ndufb9 | NADH dehydrogenase (ubiquinone) 1 beta subcomplex, 9 | -1 | 0.997 | -2.55 | 0.025 | -1.32 | 0.434 | 1.16 | 0.77 | -0.93 |
| 1447094_at | --- | --- | -1.2 | 0.497 | -1.25 | 0.203 | -1.95 | 0.038 | 1.79 | 0.152 | -0.65 |
| 1440094_at | BC030870 | cDNA sequence BC030870 | -2.53 | 0.304 | 1.07 | 0.795 | -1.46 | 0.261 | -1.62 | 0.227 | -1.14 |
| 1447906_at | Ptprg | Protein tyrosine phosphatase, receptor type, G | 1.12 | 0.819 | -1.56 | 0.111 | -2.57 | 0.11 | -1.44 | 0.317 | -1.11 |
| 1459436_at | Zranb3 | Zinc finger, RAN-binding domain containing 3 | -1.51 | 0.589 | -1.05 | 0.946 | -1.86 | 0.354 | 1.19 | 0.798 | -0.81 |
| 1440129_at | Aurka | Aurora kinase A | -1.16 | 0.756 | -2.07 | 0.16 | -1.25 | 0.572 | 1.86 | 0.314 | -0.65 |
| 1454564_at | 4933401J01Rik | RIKEN cDNA 4933401J01 gene | -1.48 | 0.623 | -1.32 | 0.637 | -1.4 | 0.392 | 1.25 | 0.272 | -0.74 |
| 1457727_at | LOC544864 | hypothetical protein LOC544864 | -1.33 | 0.133 | -2.09 | 0.3 | -1.09 | 0.757 | 1.39 | 0.27 | -0.78 |
| 1431850_at | 3200001D21Rik | RIKEN cDNA 3200001D21 gene | -1.67 | 0.359 | 1.07 | 0.857 | -2.1 | 0.099 | -1.99 | 0.064 | -1.17 |
| 1454999_at | Phf21b | PHD finger protein 21B | -2.78 | 0 | -2.55 | 0.393 | 1.4 | 0.505 | -2.02 | 0.44 | -1.49 |
| 1450467_at | Bsn | bassoon | -1.32 | 0.395 | -1.37 | 0.262 | -1.52 | 0.462 | 1.01 | 0.961 | -0.8 |
| 1443334_at | D430042O09Rik | RIKEN cDNA D430042O09 gene | -1.55 | 0.366 | -1.41 | 0.629 | -1.27 | 0.598 | 1.06 | 0.921 | -0.79 |
| 1444641_at | Adcy3 | Adenylate cyclase 3 | -1.32 | 0.045 | -1.43 | 0.117 | -1.45 | 0.183 | 1.15 | 0.614 | -0.76 |
| 1432733_at | 4933402E15Rik | RIKEN cDNA 4933402E15 gene | -3.44 | 0.071 | -1.2 | 0.746 | 1.03 | 0.954 | -1.08 | 0.785 | -1.18 |
| 1446932_at | --- | --- | -1.17 | 0.739 | -1.2 | 0.687 | -2.17 | 0.036 | 1.9 | 0.146 | -0.66 |
| 1440542_at | 7420416P09Rik | RIKEN cDNA 7420416P09 gene | -2.19 | 0.012 | -1.03 | 0.908 | -1.39 | 0.478 | 1.49 | 0.201 | -0.78 |
| 1419387_s_at | Muc13 | mucin 13, epithelial transmembrane | -1.37 | 0.327 | -1.51 | 0.128 | -1.32 | 0.195 | 1.87 | 0.304 | -0.58 |
| 1443511_at | Rora | RAR-related orphan receptor alpha | 1.01 | 0.934 | -1.41 | 0.571 | -2.33 | 0.09 | -1.02 | 0.946 | -0.94 |
| 1446478_at | D130067I03Rik | RIKEN cDNA D130067I03 gene | 1.03 | 0.952 | -1.97 | 0.357 | -1.63 | 0.494 | -1.11 | 0.703 | -0.92 |
| 1454404_at | 2610011E03Rik | RIKEN cDNA 2610011E03 gene | 1.05 | 0.818 | -3.07 | 0.08 | -1.29 | 0.397 | -2.28 | 0.072 | -1.4 |
| 1459697_at | Thada | Thyroid adenoma associated | -2.97 | 0.264 | -1.22 | 0.757 | -1.01 | 0.98 | 1.4 | 0.269 | -0.95 |
| 1443142_at | Btrc | Beta-transducin repeat containing protein | -1.51 | 0.053 | -1.54 | 0.148 | -1.2 | 0.101 | 3.99 | 0.196 | -0.06 |
| 1443241_at | Pus7 | Pseudouridylate synthase 7 homolog (S. cerevisiae) | -1.22 | 0.79 | -2.05 | 0.245 | -1.19 | 0.367 | 1.14 | 0.405 | -0.83 |
| 1441390_at | Spdya | speedy homolog A (Drosophila) | -2.28 | 0.156 | -1.54 | 0.479 | 1.06 | 0.929 | -1.1 | 0.709 | -0.96 |
| 1430111_a_at | Bcat1 | branched chain aminotransferase 1, cytosolic | -1.59 | 0.55 | -1.88 | 0.08 | -1.01 | 0.889 | 1.2 | 0.564 | -0.82 |

| | | | | | | | | | | | |
|---|---|---|---|---|---|---|---|---|---|---|---|
| 1435094_at | Kcnj16 | potassium inwardly-rectifying channel, subfamily J, member 16 | 1.15 | 0.073 | -1.97 | 0.357 | -2.03 | 0.255 | -1.52 | 0.289 | -1.09 |
| 1446473_at | --- | --- | -1.21 | 0.063 | -1.42 | 0.441 | -1.6 | 0.27 | 1.03 | 0.821 | -0.8 |
| 1444422_at | Pcdh19 | protocadherin 19 | -1.77 | 0.169 | -1.42 | 0.237 | -1.13 | 0.671 | 1.55 | 0.37 | -0.69 |
| 1458074_at | Rslcan24 | Regulator of sex-limitation candidate 24 | -1.42 | 0.251 | -1.32 | 0.363 | -1.45 | 0.418 | 1.15 | 0.809 | -0.76 |
| 1441252_at | 2010001A14Rik | RIKEN cDNA 2010001A14 gene | -1.17 | 0.72 | -1.47 | 0.309 | -1.61 | 0.071 | 1.15 | 0.574 | -0.78 |
| 1436988_at | 5430433G21Rik | RIKEN cDNA 5430433G21 gene | -1.07 | 0.782 | -2.73 | 0.128 | -1.18 | 0.616 | 1.09 | 0.81 | -0.97 |
| 1420055_at | Slc35c2 | solute carrier family 35, member C2 | -1.11 | 0.526 | -2.08 | 0.086 | -1.29 | 0.159 | 1.6 | 0.229 | -0.72 |
| 1455356_at | Camsap1 | calmodulin regulated spectrin-associated protein 1 | -1.41 | 0.042 | -1.24 | 0.157 | -1.58 | 0.07 | 1.02 | 0.931 | -0.8 |
| 1450549_s_at | Elk4 | ELK4, member of ETS oncogene family | -1.13 | 0.655 | -2.7 | 0.078 | -1.12 | 0.816 | 2 | 0.154 | -0.74 |
| 1420009_at | Wwc1 | WW, C2 and coiled-coil domain containing 1 | -1.29 | 0.202 | -1.95 | 0.292 | -1.16 | 0.729 | 3.3 | 0.094 | -0.27 |
| 1433387_at | 2900022M07Rik | RIKEN cDNA 2900022M07 gene | -2.04 | 0.424 | 1.11 | 0.627 | -1.81 | 0.06 | -1.01 | 0.984 | -0.94 |
| 1443006_at | Syn3 | Synapsin III | -1.46 | 0.243 | -1.53 | 0.034 | -1.23 | 0.438 | 1.46 | 0.072 | -0.69 |
| 1443983_at | Sorbs1 | Sorbin and SH3 domain containing 1 | -1.22 | 0.569 | -1.18 | 0.601 | -2.07 | 0.12 | 1.18 | 0.554 | -0.82 |
| 1419965_at | C79445 | expressed sequence C79445 | -3.12 | 0.29 | 1.06 | 0.842 | -1.3 | 0.662 | -1.13 | 0.65 | -1.12 |
| 1435962_at | Rps6 /// LOC21473 | ribosomal protein S6 /// similar to 40S ribosomal protein S6 /// similar to 40S ribos | -1.28 | 0.696 | -1.51 | 0.126 | -1.41 | 0.238 | 1.12 | 0.745 | -0.77 |
| 1448724_at | Cish | cytokine inducible SH2-containing protein | -1.39 | 0.028 | -1.72 | 0.321 | -1.18 | 0.213 | 2.31 | 0.254 | -0.49 |
| 1457902_at | --- | Transcribed locus, moderately similar to XP_576460.1 PREDICTED: similar to hy | 1.17 | 0.641 | -1.73 | 0.439 | -2.48 | 0.26 | -1.19 | 0.461 | -1.06 |
| 1430169_at | 1700007K09Rik | RIKEN cDNA 1700007K09 gene | -1.45 | 0.495 | -1.21 | 0.463 | -1.57 | 0.176 | 2.28 | 0.16 | -0.49 |
| 1446356_at | Ppp2r5e | Protein phosphatase 2, regulatory subunit B (B56), epsilon isoform | -1.15 | 0.103 | -1.47 | 0.151 | -1.65 | 0.019 | 1.26 | 0.463 | -0.75 |
| 1432677_at | Gbf1 | Golgi-specific brefeldin A-resistance factor 1 | -1.06 | 0.701 | -1.17 | 0.664 | -2.85 | 0.086 | 1.08 | 0.48 | -1 |
| 1438782_at | Cntn4 | contactin 4 | 1.01 | 0.986 | -1.55 | 0.304 | -2 | 0.095 | -2.63 | 0.177 | -1.29 |
| 1445676_at | Kcnn2 | Potassium intermediate/small conductance calcium-activated channel, subfamily | -1.98 | 0.031 | -1.11 | 0.699 | -1.33 | 0.297 | 2.16 | 0.156 | -0.57 |
| 1422906_at | Abcg2 | ATP-binding cassette, sub-family G (WHITE), member 2 | -1.22 | 0.03 | -1.38 | 0.008 | -1.63 | 0.204 | 3.99 | 0.179 | -0.06 |
| 1432750_at | 2810409C01Rik | RIKEN cDNA 2810409C01 gene | 1.01 | 0.967 | -1.52 | 0.424 | -2.07 | 0.022 | -1.63 | 0.299 | -1.05 |
| 1443656_at | Fut8 | fucosyltransferase 8 | -1.2 | 0.798 | -2.08 | 0.273 | -1.19 | 0.589 | 1.67 | 0.367 | -0.7 |
| 1447126_at | Cdyl2 | Chromodomain protein, Y chromosome-like 2 | -1.01 | 0.911 | -1.89 | 0.114 | -1.56 | 0.018 | 1.09 | 0.505 | -0.84 |
| 1437797_at | Atp2a2 | ATPase, Ca++ transporting, cardiac muscle, slow twitch 2 | -1.54 | 0.056 | -1.23 | 0.359 | -1.44 | 0.186 | 1.03 | 0.798 | -0.8 |
| 1444706_at | E430014L09Rik | RIKEN cDNA E430014L09 gene | -1.19 | 0.238 | -1.3 | 0.404 | -1.84 | 0.035 | 1.07 | 0.81 | -0.81 |
| 1456969_at | 9530092O11Rik | RIKEN cDNA 9530092O11 gene | -1.64 | 0.617 | -1.01 | 0.975 | -1.78 | 0.311 | 1.34 | 0.717 | -0.77 |
| 1447659_x_at | Atp6v1h | ATPase, H+ transporting, lysosomal V1 subunit H | -3.54 | 0.033 | 1.28 | 0.535 | -1.69 | 0.319 | -1.78 | 0.512 | -1.43 |
| 1431242_at | 6330575P09Rik | RIKEN cDNA 6330575P09 gene | -1.55 | 0.084 | -2.17 | 0.261 | 1.05 | 0.896 | -2.07 | 0.187 | -1.18 |
| 1458317_at | Eml1 | Echinoderm microtubule associated protein like 1 | -1.55 | 0.611 | -1.26 | 0.559 | -1.4 | 0.106 | 1.61 | 0.089 | -0.65 |
| 1459392_at | Phf8 | PHD finger protein 8 | -1.02 | 0.937 | -2.18 | 0.045 | -1.39 | 0.193 | 1.05 | 0.803 | -0.88 |
| 1449539_at | Ms4a11 | membrane-spanning 4-domains, subfamily A, member 11 | -1.25 | 0.606 | -1.2 | 0.812 | -1.91 | 0.222 | 1.88 | 0.358 | -0.62 |
| 1458760_at | --- | --- | -1.1 | 0.812 | -1.36 | 0.577 | -1.95 | 0.271 | 1.62 | 0.204 | -0.7 |
| 1432797_at | 2900060N12Rik | RIKEN cDNA 2900060N12 gene | -1.94 | 0.185 | -1.9 | 0.068 | 1.12 | 0.786 | -1.15 | 0.087 | -0.97 |
| 1449596_at | C78142 | expressed sequence C78142 | -1.02 | 0.974 | -1.61 | 0.153 | -1.79 | 0.091 | 2 | 0.226 | -0.61 |
| 1458913_at | 4831440E17Rik | RIKEN cDNA 4831440E17 gene | 1.15 | 0.659 | -2.41 | 0.116 | -1.67 | 0.145 | -1.82 | 0.065 | -1.19 |
| 1458993_at | AU022882 | expressed sequence AU022882 | 1.25 | 0.57 | -4.2 | 0.009 | -1.49 | 0.419 | -4.76 | 0.19 | -2.3 |
| 1457732_at | Pcmtd2 | Protein-L-isoaspartate (D-aspartate) O-methyltransferase domain containing 2 | -1.09 | 0.238 | -1.98 | 0.017 | -1.36 | 0.017 | 1 | 0.997 | -0.86 |
| 1449754_at | AA511261 | expressed sequence AA511261 | -1.59 | 0.239 | -1.27 | 0.583 | -1.34 | 0.427 | 1.39 | 0.612 | -0.7 |
| 1422426_at | Cmar | cell matrix adhesion regulator | -1.46 | 0.261 | -1.29 | 0.309 | -1.43 | 0.214 | 1.22 | 0.413 | -0.74 |
| 1444124_a_at | E330017L17Rik | RIKEN cDNA E330017L17 gene | -1.76 | 0.277 | -1.04 | 0.947 | -1.59 | 0.292 | 1.78 | 0.129 | -0.65 |
| 1420561_at | Trpc7 | transient receptor potential cation channel, subfamily C, member 7 | -1.35 | 0.562 | -1.17 | 0.593 | -1.77 | 0.21 | 2.34 | 0.36 | -0.49 |
| 1437548_at | Bicd1 | bicaudal D homolog 1 (Drosophila) | -1.51 | 0.003 | -1.48 | 0.524 | -1.21 | 0.248 | 1.8 | 0.118 | -0.6 |
| 1447220_at | 1700125H20Rik | RIKEN cDNA 1700125H20 gene | 1.27 | 0.79 | -3.36 | 0.002 | -1.7 | 0.264 | -1.83 | 0.325 | -1.4 |
| 1459363_at | Atxn2 | Ataxin 2 | -1.28 | 0.237 | -1.04 | 0.871 | -2.39 | 0.007 | 1.26 | 0.426 | -0.86 |
| 1431603_at | 4931407E12Rik | RIKEN cDNA 4931407E12 gene | -2.31 | 0.035 | 1.03 | 0.923 | -1.43 | 0.338 | -1.36 | 0.505 | -1.02 |
| 1425923_at | Mycn | v-myc myelocytomatosis viral related oncogene, neuroblastoma derived (avian) | -1.86 | 0.35 | -1.25 | 0.375 | -1.22 | 0.452 | 1.39 | 0.534 | -0.73 |
| 1453901_at | Aldoa-ps2 | aldolase 1, A isoform, pseudogene 2 | -1.34 | 0.609 | -1.11 | 0.809 | -1.95 | 0.057 | 1.86 | 0.135 | -0.63 |
| 1452516_at | LOC545814 /// 493 | sperm motility kinase 2-like /// RIKEN cDNA 4931427P12 gene | -1.48 | 0.493 | -2.39 | 0.034 | 1.07 | 0.899 | -1.78 | 0.078 | -1.15 |
| 1443720_s_at | Bmpr1b | bone morphogenetic protein receptor, type 1B | -2.47 | 0.039 | 1.03 | 0.966 | -1.38 | 0.328 | -1.16 | 0.818 | -0.99 |
| 1459790_x_at | Alx3 | aristaless 3 | -1.17 | 0.438 | -1.68 | 0.358 | -1.41 | 0.522 | 1.58 | 0.599 | -0.67 |

| | | | | | | | | | | | |
|---|---|---|---|---|---|---|---|---|---|---|---|
| 1418995_at | --- | --- | -1.52 | 0.126 | -1.68 | 0.273 | -1.1 | 0.556 | 1.04 | 0.385 | -0.82 |
| 1432910_at | Btbd7 | BTB (POZ) domain containing 7 | -1.29 | 0.445 | -1.3 | 0.229 | -1.62 | 0.002 | 1.13 | 0.241 | -0.77 |
| 1454720_at | Apba3 /// Hs3st5 | amyloid beta (A4) precursor protein-binding, family A, member 3 /// heparan sulfa | -3.38 | 0.268 | 1.11 | 0.745 | -1.32 | 0.55 | -1.32 | 0.346 | -1.23 |
| 1447927_at | Mpa2l /// LOC6265 | macrophage activation 2 like /// similar to macrophage activation 2 like /// similar t | 1.1 | 0.584 | -2.05 | 0.01 | -1.73 | 0.033 | -1.3 | 0.325 | -0.99 |
| 1427551_at | Usp29 | ubiquitin specific peptidase 29 | -1.67 | 0.344 | -1.05 | 0.937 | -1.64 | 0.308 | 2.59 | 0.058 | -0.44 |
| 1457137_at | --- | Transcribed locus | -2.28 | 0.167 | -1.17 | 0.695 | -1.15 | 0.722 | 1.45 | 0.595 | -0.79 |
| 1455919_at | Gm879 | Gene model 879, (NCBI) | -1.97 | 0.55 | -1.41 | 0.343 | -1.06 | 0.922 | 1.26 | 0.395 | -0.79 |
| 1432669_at | 9030420N05Rik | RIKEN cDNA 9030420N05 gene | -1.4 | 0.054 | -1.06 | 0.832 | -1.95 | 0.038 | 2.3 | 0.113 | -0.53 |
| 1439284_at | E230025E14Rik | RIKEN cDNA E230025E14 gene | -1.22 | 0.48 | -1.59 | 0.438 | -1.4 | 0.151 | 1.08 | 0.813 | -0.78 |
| 1439545_at | Nrf1 | Nuclear respiratory factor 1 | -1.28 | 0.02 | -1.47 | 0.029 | -1.42 | 0.05 | 1.34 | 0.139 | -0.71 |
| 1435206_at | Slc24a4 | solute carrier family 24 (sodium/potassium/calcium exchanger), member 4 | -1.92 | 0.212 | -2.24 | 0.005 | 1.2 | 0.227 | -2.07 | 0.283 | -1.26 |
| 1433322_at | 4930529F21Rik | RIKEN cDNA 4930529F21 gene | -1.82 | 0.263 | -1.35 | 0.66 | -1.15 | 0.707 | 1.44 | 0.199 | -0.72 |
| 1437472_at | Unc13a | unc-13 homolog A (C. elegans) | -1.09 | 0.778 | -1.45 | 0.628 | -1.8 | 0.356 | 2.24 | 0.077 | -0.53 |
| 1433168_x_at | 5830456J23Rik | RIKEN cDNA 5830456J23 gene | 1.08 | 0.803 | -2.46 | 0.039 | -1.48 | 0.614 | -1.62 | 0.217 | -1.12 |
| 1432090_at | Ccdc83 | coiled-coil domain containing 83 | -2.76 | 0.02 | -1.32 | 0.66 | 1.05 | 0.945 | -2.09 | 0.447 | -1.28 |
| 1428041_at | 1600002H07Rik | RIKEN cDNA 1600002H07 gene | -3.2 | 0.097 | 1.04 | 0.925 | -1.23 | 0.439 | -1.13 | 0.899 | -1.13 |
| 1459564_at | C87580 | expressed sequence C87580 | 1 | 0.997 | -2.55 | 0.057 | -1.29 | 0.336 | -1.68 | 0.176 | -1.13 |
| 1419831_at | AA416453 | expressed sequence AA416453 | 1.26 | 0.687 | -2.18 | 0.108 | -2.22 | 0.028 | -1.28 | 0.482 | -1.11 |
| 1440716_at | 6430604M11Rik | RIKEN cDNA 6430604M11 gene | -2.26 | 0.341 | -1.23 | 0.581 | -1.1 | 0.638 | 1.39 | 0.362 | -0.8 |
| 1422290_at | Htr1d | 5-hydroxytryptamine (serotonin) receptor 1D | -1.34 | 0.467 | -2.37 | 0.256 | -1 | 0.999 | 1.41 | 0.231 | -0.82 |
| 1445950_at | C87926 | cDNA sequence, C87926 | -1.82 | 0.397 | -1.9 | 0.271 | 1.09 | 0.711 | -1.53 | 0.108 | -1.04 |
| 1448016_at | Sass6 | spindle assembly 6 homolog (C. elegans) | -1.22 | 0.452 | -1.88 | 0.133 | -1.22 | 0.191 | 2.54 | 0.146 | -0.45 |
| 1422606_at | C1qtnf3 | C1q and tumor necrosis factor related protein 3 | -1 | 0.986 | -1.16 | 0.458 | -3.25 | 0.004 | 1.89 | 0.262 | -0.88 |
| 1431647_a_at | Ceacam13 | CEA-related cell adhesion molecule 13 | -3.3 | 0.105 | 1.1 | 0.828 | -1.3 | 0.678 | -2.13 | 0.464 | -1.41 |
| 1456122_at | --- | --- | -1.15 | 0.565 | -1.52 | 0.192 | -1.56 | 0.182 | 1.01 | 0.971 | -0.81 |
| 1439749_at | Zap70 | zeta-chain (TCR) associated protein kinase | -1.18 | 0.156 | -1.44 | 0.034 | -1.59 | 0.22 | 1.74 | 0.511 | -0.62 |
| 1421517_at | St6galnac1 | ST6 (alpha-N-acetyl-neuraminyl-2,3-beta-galactosyl-1,3)-N-acetylgalactosaminide | 1.01 | 0.987 | -1.21 | 0.366 | -2.99 | 0.062 | -1.43 | 0.379 | -1.16 |
| 1449071_at | Myl7 | myosin, light polypeptide 7, regulatory | -3.12 | 0.393 | 1.11 | 0.811 | -1.36 | 0.267 | -1.27 | 0.737 | -1.16 |
| 1457175_at | Numb | Numb gene homolog (Drosophila) | -1.31 | 0.148 | -1.52 | 0.1 | -1.35 | 0.119 | 1.1 | 0.683 | -0.77 |
| 1422298_at | 5530401N06Rik | RIKEN cDNA 5530401N06 gene | -1.43 | 0.358 | -1.14 | 0.795 | -1.69 | 0.021 | 1.63 | 0.257 | -0.66 |
| 1430352_at | 8430417A20Rik | RIKEN cDNA 8430417A20 gene | 1 | 0.997 | -1.27 | 0.272 | -2.64 | 0.194 | -1.46 | 0.295 | -1.09 |
| 1446303_at | Igf1r | insulin-like growth factor I receptor | -1.22 | 0.45 | -1.45 | 0.107 | -1.52 | 0.191 | 1.16 | 0.533 | -0.76 |
| 1430073_at | 2900016B01Rik | RIKEN cDNA 2900016B01 gene | -2.12 | 0.447 | -1.34 | 0.621 | -1.05 | 0.644 | 1.11 | 0.697 | -0.85 |
| 1431351_at | Pvt1 | plasmacytoma variant translocation 1 | -1.62 | 0.124 | -1.64 | 0.297 | -1.06 | 0.83 | 1.54 | 0.217 | -0.7 |
| 1442962_at | AU022052 | expressed sequence AU022052 | -1.59 | 0.173 | -1.36 | 0.232 | -1.24 | 0.633 | 2.21 | 0.135 | -0.5 |
| 1421769_at | Lmx1b | LIM homeobox transcription factor 1 beta | -1.74 | 0.186 | -1.95 | 0.206 | 1.08 | 0.77 | -1.32 | 0.497 | -0.98 |
| 1416626_at | Pla2g1b | phospholipase A2, group IB, pancreas | -1.25 | 0.607 | -1.52 | 0.442 | -1.4 | 0.24 | 1.11 | 0.883 | -0.77 |
| 1447049_at | Iqwd1 | IQ motif and WD repeats 1 | -1.19 | 0.661 | -1.74 | 0.047 | -1.33 | 0.116 | 1.73 | 0.136 | -0.63 |
| 1444711_at | Ankrd15 | ankyrin repeat domain 15 | -1.05 | 0.925 | -1.77 | 0.317 | -1.52 | 0.633 | 1.23 | 0.47 | -0.78 |
| 1454131_at | 4931413K12Rik | RIKEN cDNA 4931413K12 gene | -1.37 | 0.024 | -1.77 | 0.273 | -1.14 | 0.796 | 1.97 | 0.36 | -0.58 |
| 1433157_at | 6330562C20Rik | RIKEN cDNA 6330562C20 gene | -2.49 | 0.177 | -1.34 | 0.494 | 1.02 | 0.958 | -1.32 | 0.521 | -1.03 |
| 1455446_x_at | Acadsb | acyl-Coenzyme A dehydrogenase, short/branched chain | -1.2 | 0.007 | -1.35 | 0.014 | -1.68 | 0.013 | 1.13 | 0.478 | -0.78 |
| 1448991_a_at | Ina | internexin neuronal intermediate filament protein, alpha | 1.02 | 0.966 | -2.04 | 0.043 | -1.5 | 0.166 | -2.43 | 0.147 | -1.24 |
| 1447091_at | Jmjd2c | Jumonji domain containing 2C | -1.07 | 0.915 | -1.45 | 0.257 | -1.82 | 0.04 | 1.39 | 0.416 | -0.74 |
| 1427426_at | Kcnq5 | potassium voltage-gated channel, subfamily Q, member 5 | 1.12 | 0.514 | -3.42 | 0.05 | -1.33 | 0.548 | -1.22 | 0.557 | -1.21 |
| 1451823_at | Clca4 | chloride channel calcium activated 4 | -4.13 | 0.095 | 1.55 | 0.324 | -2.61 | 0.066 | -1.39 | 0.469 | -1.65 |
| 1447656_at | Zdhhc17 | zinc finger, DHHC domain containing 17 | -1.71 | 0.061 | -1.44 | 0.349 | -1.12 | 0.816 | 1.11 | 0.869 | -0.79 |
| 1446935_at | 9130011E15Rik | RIKEN cDNA 9130011E15 gene | -4.73 | 0.224 | -1.63 | 0.37 | 1.35 | 0.013 | -1.14 | 0.793 | -1.54 |
| 1447299_at | --- | --- | 1.14 | 0.884 | -1.72 | 0.341 | -2.2 | 0.025 | -1.02 | 0.896 | -0.95 |
| 1425208_at | Lbh | limb-bud and heart | -1.7 | 0.627 | -2.03 | 0.342 | 1.09 | 0.889 | -1.44 | 0.632 | -1.02 |
| 1454528_at | A930011E06Rik | RIKEN cDNA A930011E06 gene | -1.84 | 0.222 | -1.01 | 0.978 | -1.56 | 0.316 | 1.04 | 0.961 | -0.84 |
| 1432303_at | 9330198I05Rik | RIKEN cDNA 9330198I05 gene | -1.46 | 0.047 | -1.26 | 0.374 | -1.44 | 0.323 | 1.52 | 0.403 | -0.66 |
| 1444914_at | --- | --- | -1.61 | 0.301 | -2.83 | 0.243 | 1.2 | 0.641 | -1.64 | 0.275 | -1.22 |

| | | | | | | | | | | | |
|---|---|---|---|---|---|---|---|---|---|---|---|
| 1445837_at | Ga17 | Dendritic cell protein GA17 | -1.28 | 0.579 | 1.05 | 0.705 | -2.92 | 0.003 | -1.91 | 0.067 | -1.27 |
| 1447273_x_at | Atp10a | ATPase, class V, type 10A | -2.16 | 0.204 | -1.25 | 0.721 | -1.1 | 0.758 | 1.06 | 0.75 | -0.86 |
| 1437574_at | Adamts18 | a disintegrin-like and metallopeptidase (reprolysin type) with thrombospondin type | -1.53 | 0.194 | -3.02 | 0.404 | 1.19 | 0.672 | -1.47 | 0.618 | -1.21 |
| 1449593_at | --- | --- | 1.12 | 0.753 | -1.53 | 0.482 | -2.47 | 0.163 | -1.52 | 0.306 | -1.1 |
| 1443153_at | Trip11 | Thyroid hormone receptor interactor 11 | -1.52 | 0.323 | 1.01 | 0.957 | -1.99 | 0.005 | -1.79 | 0.04 | -1.07 |
| 1457539_at | D10Ertd709e | DNA segment, Chr 10, ERATO Doi 709, expressed | -1.2 | 0.329 | -1.58 | 0.169 | -1.41 | 0.09 | 1.15 | 0.287 | -0.76 |
| 1415846_a_at | Ldhc | lactate dehydrogenase C | -2.33 | 0.357 | -1.89 | 0.182 | 1.22 | 0.719 | -1.52 | 0.518 | -1.13 |
| 1449381_a_at | Pacsin1 | protein kinase C and casein kinase substrate in neurons 1 | -2.29 | 0.47 | 1.02 | 0.868 | -1.4 | 0.302 | -2.92 | 0.059 | -1.4 |
| 1430265_at | Appbp2 | amyloid beta precursor protein (cytoplasmic tail) binding protein 2 | -1.88 | 0.192 | 1.08 | 0.867 | -1.78 | 0.067 | -1.3 | 0.123 | -0.97 |
| 1446965_at | Arhgef12 | Rho guanine nucleotide exchange factor (GEF) 12 | -1.21 | 0.445 | -1.3 | 0.167 | -1.73 | 0.029 | 2.03 | 0.142 | -0.55 |
| 1432789_at | 8030476L19Rik | RIKEN cDNA 8030476L19 gene | -3.96 | 0.339 | -1.16 | 0.775 | 1.06 | 0.87 | -1.12 | 0.878 | -1.29 |
| 1445518_at | Zfhx1b | Zinc finger homeobox 1b | -1.15 | 0.498 | -1.32 | 0.264 | -1.82 | 0.057 | 1.12 | 0.717 | -0.79 |
| 1439669_at | 6430571L13Rik | RIKEN cDNA 6430571L13 gene | -1.16 | 0.568 | -1.25 | 0.73 | -1.95 | 0.263 | 2.22 | 0.181 | -0.53 |
| 1459133_at | Edem3 | ER degradation enhancer, mannosidase alpha-like 3 | 1.07 | 0.825 | -1.56 | 0.114 | -2.16 | 0.02 | -1.33 | 0.216 | -0.99 |
| 1459365_at | 4930429A22Rik | RIKEN cDNA 4930429A22 gene | -1.16 | 0.64 | -1.19 | 0.449 | -2.09 | 0.104 | 1.44 | 0.488 | -0.75 |
| 1439915_at | Mrg1 | Myeloid ecotropic viral integration site-related gene 1 | -1.23 | 0.228 | -1.6 | 0.289 | -1.35 | 0.465 | 1.43 | 0.153 | -0.69 |
| 1443062_at | Auts2 | autism susceptibility candidate 2 | 1.04 | 0.967 | -1.15 | 0.655 | -3.69 | 0.065 | -2.01 | 0.307 | -1.45 |
| 1426561_a_at | Npnt | nephronectin | 1.14 | 0.376 | -1.63 | 0.54 | -2.37 | 0.004 | -1.73 | 0.133 | -1.15 |
| 1445939_at | Mtus1 | Mitochondrial tumor suppressor 1 | -1.29 | 0.665 | -1.12 | 0.883 | -1.97 | 0.028 | 2.34 | 0.098 | -0.51 |
| 1441330_at | Crb1 | crumbs homolog 1 (Drosophila) | -1.52 | 0.294 | -1.02 | 0.963 | -1.84 | 0.009 | 2.48 | 0.28 | -0.48 |
| 1444509_at | --- | Transcribed locus | -1.14 | 0.741 | -1.94 | 0.294 | -1.28 | 0.434 | 1.79 | 0.135 | -0.64 |
| 1422296_at | 5730470L24Rik | RIKEN cDNA 5730470L24 gene | -1.67 | 0.063 | -1.26 | 0.664 | -1.28 | 0.422 | 1.34 | 0.691 | -0.71 |
| 1421396_at | Pcsk1 | proprotein convertase subtilisin/kexin type 1 | -1.36 | 0.767 | -1.67 | 0.429 | -1.18 | 0.217 | 1.17 | 0.501 | -0.76 |
| 1435537_at | Ptprd | protein tyrosine phosphatase, receptor type, D | -1.16 | 0.205 | -1.34 | 0.051 | -1.75 | 0.016 | 1.12 | 0.774 | -0.79 |
| 1441376_at | Gabarapl2 | gamma-aminobutyric acid (GABA-A) receptor-associated protein-like 2 | -1.14 | 0.083 | -1.56 | 0.012 | -1.52 | 0.074 | 1.45 | 0.201 | -0.69 |
| 1445381_at | --- | --- | -2.54 | 0.097 | -1.27 | 0.475 | -1.01 | 0.988 | 1.48 | 0.304 | -0.83 |
| 1431541_at | Stt3b | STT3, subunit of the oligosaccharyltransferase complex, homolog B (S. cerevisia | -1.55 | 0.479 | -1.92 | 0.394 | 1.01 | 0.967 | -1.19 | 0.76 | -0.91 |
| 1420260_at | Phf7 | PHD finger protein 7 | -1.36 | 0.256 | -1.37 | 0.532 | -1.4 | 0.495 | 1.07 | 0.803 | -0.77 |
| 1446576_at | 4931419K03Rik | RIKEN cDNA 4931419K03 gene | -1.68 | 0.512 | -1.57 | 0.606 | -1.06 | 0.93 | 3.3 | 0.337 | -0.25 |
| 1454940_at | Psma5 /// LOC5456 | proteasome (prosome, macropain) subunit, alpha type 5 /// similar to Proteasome | -1.11 | 0.645 | -1.87 | 0.158 | -1.34 | 0.258 | 1 | 0.994 | -0.83 |
| 1425126_at | Ncam1 | neural cell adhesion molecule 1 | -1.7 | 0.58 | 1.2 | 0.632 | -2.57 | 0.088 | -2.73 | 0.029 | -1.45 |
| 1425800_at | Rad9b | RAD9 homolog B (S. cerevisiae) | -1.17 | 0.541 | -2.24 | 0.084 | -1.14 | 0.522 | 1.66 | 0.269 | -0.72 |
| 1445515_at | --- | --- | -1.93 | 0.363 | -1.16 | 0.741 | -1.25 | 0.163 | 1.3 | 0.474 | -0.76 |
| 1442718_at | --- | 7 days neonate cerebellum cDNA, RIKEN full-length enriched library, clone:A730 | -1.82 | 0.222 | 1.09 | 0.799 | -1.86 | 0.307 | -1.42 | 0.237 | -1 |
| 1441754_at | --- | --- | 1.07 | 0.848 | -2.72 | 0.064 | -1.34 | 0.403 | -1 | 0.986 | -1 |
| 1442633_at | --- | Transcribed locus, weakly similar to XP_576460.1 PREDICTED: similar to hypoth | -1.03 | 0.82 | -2.1 | 0.049 | -1.36 | 0.083 | 1.44 | 0.589 | -0.76 |
| 1451032_at | Fhl4 | four and a half LIM domains 4 | -3.26 | 0.05 | -1.17 | 0.79 | 1.02 | 0.969 | -2.81 | 0.165 | -1.56 |
| 1432994_at | 2900057E15Rik | RIKEN cDNA 2900057E15 gene | 1.28 | 0.736 | -2.95 | 0.312 | -1.78 | 0.295 | -1.01 | 0.971 | -1.12 |
| 1445217_at | A730073F16Rik | RIKEN cDNA A730073F16 gene | 1.04 | 0.948 | -1.73 | 0.535 | -1.78 | 0.469 | -1.48 | 0.135 | -0.99 |
| 1444472_at | Snf1lk2 | SNF1-like kinase 2 | -1.3 | 0.399 | -1.32 | 0.324 | -1.53 | 0.022 | 1.15 | 0.551 | -0.75 |
| 1445327_at | Cyfip1 | Cytoplasmic FMR1 interacting protein 1 | -1.13 | 0.764 | -1.83 | 0.128 | -1.34 | 0.257 | 1.35 | 0.116 | -0.74 |
| 1447531_x_at | --- | --- | -1.21 | 0.607 | -1.4 | 0.379 | -1.56 | 0.073 | 1.18 | 0.687 | -0.75 |
| 1437827_s_at | Pfpl | Pore forming protein-like | -1.94 | 0.427 | -1.07 | 0.866 | -1.37 | 0.393 | 1.35 | 0.638 | -0.76 |
| 1442921_at | Mmp24 | Matrix metallopeptidase 24 | -1.72 | 0.254 | -2.22 | 0.066 | 1.15 | 0.689 | -1.37 | 0.653 | -1.04 |
| 1431851_at | 4930534P07Rik | RIKEN cDNA 4930534P07 gene | -4.01 | 0.08 | -2.17 | 0.152 | 1.47 | 0.17 | -1.32 | 0.554 | -1.51 |
| 1431260_at | 4833417C18Rik | RIKEN cDNA 4833417C18 gene | -1.3 | 0.534 | -1.78 | 0.059 | -1.18 | 0.603 | 1.15 | 0.292 | -0.78 |
| 1433160_at | 1700008N17Rik | RIKEN cDNA 1700008N17 gene | -1.11 | 0.892 | -1.78 | 0.022 | -1.39 | 0.482 | 1.21 | 0.716 | -0.77 |
| 1446114_at | Inpp5a | Inositol polyphosphate-5-phosphatase A | -2.3 | 0.269 | 1.14 | 0.835 | -1.64 | 0.1 | -2.8 | 0.225 | -1.4 |
| 1425759_at | Nobox | NOBOX oogenesis homeobox | -1.07 | 0.927 | -1.84 | 0.237 | -1.43 | 0.478 | 1.82 | 0.449 | -0.63 |
| 1436239_at | Slc5a5 | solute carrier family 5 (sodium iodide symporter), member 5 | -2.38 | 0.005 | -1.82 | 0.023 | 1.21 | 0.501 | -1.05 | 0.656 | -1.01 |
| 1458367_at | Gm94 | gene model 94, (NCBI) | -1.73 | 0.434 | -3.4 | 0.025 | 1.31 | 0.422 | -1.08 | 0.914 | -1.22 |
| 1418213_at | Krt23 | keratin 23 | -5 | 0.004 | 1.23 | 0.852 | -1.33 | 0.113 | -1.81 | 0.284 | -1.73 |
| 1439586_at | LOC548102 | hypothetical LOC548102 | -1.22 | 0.538 | -1.46 | 0.003 | -1.48 | 0.077 | 1.14 | 0.75 | -0.75 |

| | | | | | | | | | | | |
|---|---|---|---|---|---|---|---|---|---|---|---|
| 1427865_at | --- | --- | -1.61 | 0.495 | 1.26 | 0.297 | -3.35 | 0.025 | -2.56 | 0.364 | -1.56 |
| 1440345_at | AI450353 | expressed sequence AI450353 | -1.63 | 0.355 | -1.18 | 0.162 | -1.39 | 0.517 | 1.29 | 0.388 | -0.73 |
| 1450277_at | Avpr2 | arginine vasopressin receptor 2 | 1 | 0.961 | -2.07 | 0.02 | -1.44 | 0.602 | -1.09 | 0.726 | -0.9 |
| 1446526_at | 9330199F22Rik | RIKEN cDNA 9330199F22 gene | -1.25 | 0.103 | -1.97 | 0.283 | -1.14 | 0.553 | 1.02 | 0.965 | -0.84 |
| 1443613_x_at | Ascc2 | Activating signal cointegrator 1 complex subunit 2 | -1.46 | 0.717 | -1.39 | 0.09 | -1.28 | 0.263 | 1.03 | 0.966 | -0.78 |
| 1440506_at | Slc7a2 | Solute carrier family 7 (cationic amino acid transporter, y+ system), member 2 | -1.66 | 0.387 | -1.87 | 0.144 | 1.05 | 0.828 | -1.78 | 0.35 | -1.07 |
| 1438747_at | A930006K02Rik | RIKEN cDNA A930006K02 gene | -1.33 | 0.472 | -1.58 | 0.377 | -1.25 | 0.63 | 2.09 | 0.261 | -0.52 |
| 1440662_at | Rgl1 | Ral guanine nucleotide dissociation stimulator,-like 1 | -1.48 | 0.233 | -1.09 | 0.851 | -1.7 | 0.065 | 1.08 | 0.872 | -0.8 |
| 1440436_at | A730030A06 | hypothetical protein A730030A06 | -1.41 | 0.174 | -1.2 | 0.553 | -1.56 | 0.084 | 1.81 | 0.291 | -0.59 |
| 1458473_at | Pou6f2 | POU domain, class 6, transcription factor 2 | -1.48 | 0.433 | -1.28 | 0.517 | -1.37 | 0.114 | 1.02 | 0.886 | -0.78 |
| 1421048_a_at | Ypel1 | yippee-like 1 (Drosophila) | -1.29 | 0.402 | -1.89 | 0.15 | -1.14 | 0.333 | 1.03 | 0.692 | -0.82 |
| 1454457_at | 4930439G18Rik | RIKEN cDNA 4930439G18 gene | -1.56 | 0.324 | -1.41 | 0.139 | -1.2 | 0.519 | 1.11 | 0.78 | -0.76 |
| 1449463_at | Klk1b8 | kallikrein 1-related peptidase b8 | -1.54 | 0.463 | -1.28 | 0.731 | -1.32 | 0.521 | 1.46 | 0.302 | -0.67 |
| 1449881_a_at | Casr | calcium-sensing receptor | -1.13 | 0.314 | -1.46 | 0.387 | -1.62 | 0.094 | 1.57 | 0.577 | -0.66 |
| 1446909_at | Amotl1 | Angiomotin-like 1 | -2.08 | 0.248 | -1.01 | 0.987 | -1.4 | 0.569 | 1.52 | 0.155 | -0.74 |
| 1420585_a_at | Nxf2 | nuclear RNA export factor 2 | -1.39 | 0.227 | -1.21 | 0.567 | -1.55 | 0.024 | 1.24 | 0.477 | -0.73 |
| 1455782_at | Samd7 | sterile alpha motif domain containing 7 | -2.61 | 0.048 | 1.13 | 0.695 | -1.49 | 0.471 | -1.23 | 0.687 | -1.05 |
| 1448949_at | Car4 | carbonic anhydrase 4 | -1.13 | 0.394 | -1.53 | 0.481 | -1.53 | 0.006 | 1.87 | 0.113 | -0.58 |
| 1437049_at | --- | Transcribed locus | -1.26 | 0.07 | -1.42 | 0.315 | -1.46 | 0.109 | 1.57 | 0.102 | -0.64 |
| 1458472_at | 4932417H02Rik | RIKEN cDNA 4932417H02 gene | -1.2 | 0.538 | -2.99 | 0.153 | 1.02 | 0.928 | -1.3 | 0.307 | -1.12 |
| 1458817_at | --- | --- | -1.23 | 0.829 | -1.46 | 0.577 | -1.45 | 0.459 | 1.61 | 0.292 | -0.63 |
| 1444642_at | --- | Transcribed locus | -1.15 | 0.533 | -1.3 | 0.469 | -1.82 | 0.118 | 1.21 | 0.355 | -0.76 |
| 1450595_at | V1rc1 | vomeronasal 1 receptor, C1 | -1.54 | 0.183 | -1.62 | 0.368 | -1.08 | 0.729 | 1.62 | 0.205 | -0.66 |
| 1457565_at | C130052G03Rik | RIKEN cDNA C130052G03 gene | -2.24 | 0.056 | -1.19 | 0.715 | -1.11 | 0.795 | 2.3 | 0.269 | -0.56 |
| 1418171_at | Tceal8 | transcription elongation factor A (SII)-like 8 | -1.25 | 0.055 | -1.29 | 0.138 | -1.63 | 0.007 | 2.69 | 0.321 | -0.37 |
| 1445757_at | Tbx3 | T-box 3 | 1.22 | 0.648 | -2.16 | 0.07 | -1.98 | 0.007 | -1.16 | 0.414 | -1.02 |
| 1438743_at | Cyp7a1 | cytochrome P450, family 7, subfamily a, polypeptide 1 | -1.54 | 0.563 | -1.39 | 0.284 | -1.22 | 0.335 | 1.25 | 0.322 | -0.73 |
| 1459325_at | --- | 7 days neonate cerebellum cDNA, RIKEN full-length enriched library, clone:A730 | -1.49 | 0.172 | -1.36 | 0.177 | -1.28 | 0.082 | 1.21 | 0.683 | -0.73 |
| 1459248_at | Ptpn9 | Protein tyrosine phosphatase, non-receptor type 9 | -1.51 | 0.529 | 1.06 | 0.877 | -2.13 | 0.145 | -1.61 | 0.178 | -1.05 |
| 1432957_at | 5730407O05Rik | RIKEN cDNA 5730407O05 gene | -1.24 | 0.809 | -1.1 | 0.9 | -2.13 | 0.248 | 1.74 | 0.477 | -0.68 |
| 1433394_at | Mast4 | microtubule associated serine/threonine kinase family member 4 | -1.24 | 0.422 | -1.62 | 0.481 | -1.31 | 0.093 | 1.01 | 0.966 | -0.79 |
| 1440697_at | AI746446 | expressed sequence AI746446 | -1.24 | 0.415 | -2.7 | 0.065 | 1.01 | 0.98 | -1.07 | 0.883 | -1 |
| 1427812_at | Ids | iduronate 2-sulfatase | -1.77 | 0.531 | -1.38 | 0.548 | -1.11 | 0.72 | 1.16 | 0.85 | -0.77 |
| 1442862_at | --- | --- | 1.04 | 0.63 | -2.01 | 0.282 | -1.53 | 0.003 | -1.31 | 0.241 | -0.95 |
| 1430096_at | 2900017F05Rik | RIKEN cDNA 2900017F05 gene | -1.98 | 0.188 | -1.38 | 0.359 | -1.04 | 0.851 | 1.59 | 0.551 | -0.7 |
| 1422634_a_at | Vsig2 | V-set and immunoglobulin domain containing 2 | -1 | 0.995 | -1.78 | 0.144 | -1.59 | 0.003 | 1.17 | 0.738 | -0.8 |
| 1424296_at | Gclc | glutamate-cysteine ligase, catalytic subunit | -1.32 | 0.081 | -1.52 | 0.065 | -1.3 | 0.007 | 2.39 | 0.199 | -0.44 |
| 1431412_at | 2810455B08Rik | RIKEN cDNA 2810455B08 gene | 1.04 | 0.866 | -1.71 | 0.044 | -1.76 | 0.027 | -1.34 | 0.307 | -0.94 |
| 1456104_at | Psmd11 | proteasome (prosome, macropain) 26S subunit, non-ATPase, 11 | -1.76 | 0.07 | -1.54 | 0.207 | -1.03 | 0.787 | 1.07 | 0.645 | -0.81 |
| 1443557_at | --- | --- | -1.79 | 0.562 | -1.93 | 0.225 | 1.12 | 0.596 | -1.4 | 0.252 | -1 |
| 1440969_at | BC030308 | cDNA sequence BC030308 | -1.21 | 0.212 | -1.2 | 0.587 | -1.89 | 0.006 | 1.08 | 0.581 | -0.8 |
| 1435104_at | D130004H04Rik | RIKEN cDNA D130004H04 gene | -3.09 | 0.298 | -1.27 | 0.642 | 1.08 | 0.874 | -2.55 | 0.23 | -1.46 |
| 1444530_at | --- | Transcribed locus | -1.15 | 0.544 | -1.64 | 0.013 | -1.41 | 0.252 | 1.03 | 0.867 | -0.79 |
| 1459822_at | Rae1 | RAE1 RNA export 1 homolog (S. pombe) | -1.04 | 0.954 | -1.33 | 0.719 | -2.1 | 0.267 | 1.23 | 0.786 | -0.81 |
| 1446651_at | --- | --- | -2.14 | 0.399 | -1.26 | 0.688 | -1.07 | 0.897 | 2.16 | 0.34 | -0.58 |
| 1431770_at | Cherp | calcium homeostasis endoplasmic reticulum protein | -1.2 | 0.775 | -1.48 | 0.321 | -1.45 | 0.103 | 1.11 | 0.491 | -0.76 |
| 1443981_at | --- | --- | -1.04 | 0.927 | -2.17 | 0.003 | -1.29 | 0.014 | 1.18 | 0.66 | -0.83 |
| 1454235_a_at | Ing5 | inhibitor of growth family, member 5 | -1.48 | 0.487 | -1.92 | 0.287 | -1 | 0.995 | 1.02 | 0.957 | -0.85 |
| 1450046_at | Tmem59 | transmembrane protein 59 | -1.48 | 0.107 | -1.8 | 0.004 | -1.04 | 0.745 | 1.39 | 0.38 | -0.73 |
| 1459210_at | Tmem108 | transmembrane protein 108 | -2.25 | 0.125 | 1.19 | 0.576 | -1.8 | 0.002 | -1.69 | 0.195 | -1.14 |
| 1453625_at | 2210407P21Rik | RIKEN cDNA 2210407P21 gene | -1.52 | 0.372 | -1.42 | 0.222 | -1.2 | 0.642 | 1.65 | 0.438 | -0.62 |
| 1428994_s_at | 1110017I16Rik | RIKEN cDNA 1110017I16 gene | -1.02 | 0.976 | -3.59 | 0.351 | -1.07 | 0.892 | 1.11 | 0.848 | -1.14 |
| 1453966_at | 2610008G14Rik | RIKEN cDNA 2610008G14 gene | -1.25 | 0.529 | -1.14 | 0.76 | -1.95 | 0.329 | 1.22 | 0.783 | -0.78 |

| | | | | | | | | | | | |
|---|---|---|---|---|---|---|---|---|---|---|---|
| 1460122_at | Tmem41b | transmembrane protein 41B | -1.28 | 0.018 | -1.42 | 0.185 | -1.42 | 0.035 | 1.36 | 0.184 | -0.69 |
| 1453523_at | A030006P16Rik | RIKEN cDNA A030006P16 gene | 1.01 | 0.92 | -1.21 | 0.635 | -2.84 | 0.022 | -1.09 | 0.838 | -1.03 |
| 1456459_x_at | Ccdc53 | coiled-coil domain containing 53 | 1.13 | 0.922 | -2.54 | 0.108 | -1.49 | 0.466 | -1.58 | 0.533 | -1.12 |
| 1447532_at | 1700025G04Rik | RIKEN cDNA 1700025G04 gene | -1.62 | 0.703 | -1.62 | 0.424 | -1.04 | 0.832 | 1.19 | 0.231 | -0.77 |
| 1448044_a_at | --- | Adult male kidney cDNA, RIKEN full-length enriched library, clone:F530012N16 p | -1.31 | 0.154 | -1.72 | 0.055 | -1.18 | 0.716 | 2 | 0.196 | -0.55 |
| 1429052_at | Ptprd | protein tyrosine phosphatase, receptor type, D | -1.05 | 0.408 | -1.29 | 0.294 | -2.13 | 0.013 | 1.06 | 0.88 | -0.85 |
| 1455619_at | BC062258 | cDNA sequence BC062258 | 1 | 0.998 | -1.93 | 0.207 | -1.48 | 0.216 | -1.54 | 0.012 | -0.99 |
| 1445509_at | Atf7 | Activating transcription factor 7 | -1.57 | 0.431 | -1.72 | 0.112 | -1.02 | 0.93 | 1.26 | 0.072 | -0.76 |
| 1437579_at | Nek2 | NIMA (never in mitosis gene a)-related expressed kinase 2 | -1.24 | 0.186 | -1.78 | 0.126 | -1.2 | 0.283 | 1.47 | 0.37 | -0.69 |
| 1432788_at | 6720420G18Rik | RIKEN cDNA 6720420G18 gene | -1.95 | 0.475 | -1.33 | 0.554 | -1.07 | 0.801 | 1.53 | 0.229 | -0.71 |
| 1447835_at | 2610528K11Rik | RIKEN cDNA 2610528K11 gene | -1.58 | 0.408 | -1.28 | 0.686 | -1.28 | 0.545 | 1.92 | 0.131 | -0.55 |
| 1420071_at | Serpinb6e | serine (or cysteine) peptidase inhibitor, clade B, member 6e | -1.4 | 0.722 | -1.18 | 0.757 | -1.57 | 0.246 | 1.73 | 0.233 | -0.61 |
| 1433581_at | 1190002N15Rik | RIKEN cDNA 1190002N15 gene | -1.28 | 0.288 | -1.09 | 0.232 | -2 | 0.084 | 1.31 | 0.37 | -0.76 |
| 1441050_at | Strn3 | Striatin, calmodulin binding protein 3 | -1.04 | 0.87 | -1.31 | 0.182 | -2.11 | 0.047 | 1.03 | 0.858 | -0.86 |
| 1441366_at | --- | --- | -1.5 | 0.008 | -2.28 | 0.381 | 1.09 | 0.868 | -1.08 | 0.587 | -0.94 |
| 1445527_at | Kpna3 | Karyopherin (importin) alpha 3 | -1.57 | 0.515 | -1.11 | 0.903 | -1.51 | 0.128 | 1.08 | 0.479 | -0.78 |
| 1434319_at | Mdh1 | malate dehydrogenase 1, NAD (soluble) | -1.53 | 0.604 | -1.03 | 0.88 | -1.73 | 0.193 | 1.18 | 0.472 | -0.78 |
| 1457766_at | Wfdc6a | WAP four-disulfide core domain 6A | -1.26 | 0.61 | -1.01 | 0.984 | -2.38 | 0.039 | 1.45 | 0.271 | -0.8 |
| 1421390_at | Slc12a1 | solute carrier family 12, member 1 | -1.54 | 0.327 | -1.52 | 0.498 | -1.13 | 0.694 | 1.63 | 0.574 | -0.64 |
| 1458550_at | Myo1d | myosin ID | -1.15 | 0.597 | -1.3 | 0.404 | -1.78 | 0.025 | 2.38 | 0.239 | -0.46 |
| 1419763_at | 2610020O08Rik | RIKEN cDNA 2610020O08 gene | -1.56 | 0.042 | -2.13 | 0.054 | 1.09 | 0.691 | -1.08 | 0.844 | -0.92 |
| 1430633_s_at | C430045I18Rik | RIKEN cDNA C430045I18 gene | -1.44 | 0.213 | -1.44 | 0.294 | -1.23 | 0.288 | 1.44 | 0.166 | -0.67 |
| 1444046_at | D430041B17Rik | RIKEN cDNA D430041B17 gene | -1.76 | 0.115 | -1.7 | 0.128 | 1.04 | 0.917 | -1.01 | 0.969 | -0.86 |
| 1430899_at | Muc5ac | mucin 5, subtypes A and C, tracheobronchial/gastric | -1.74 | 0.365 | -1.09 | 0.855 | -1.41 | 0.629 | 1.24 | 0.651 | -0.75 |
| 1455145_at | --- | 14, 17 days embryo head cDNA, RIKEN full-length enriched library, clone:322240 | -1.14 | 0.47 | -1.37 | 0.256 | -1.68 | 0.034 | 2.09 | 0.076 | -0.53 |
| 1443892_at | LOC435970 /// LOC | hypothetical LOC435970 /// similar to gonadotropin inducible ovarian transcription | -2.82 | 0.071 | -1.26 | 0.592 | 1.05 | 0.933 | -1.13 | 0.909 | -1.04 |
| 1456834_at | Ibrdc2 | IBR domain containing 2 | -1.82 | 0.503 | 1.24 | 0.569 | -2.45 | 0.081 | -1.1 | 0.795 | -1.03 |
| 1456102_a_at | Cul5 | cullin 5 | -1.11 | 0.486 | -1.17 | 0.494 | -2.25 | 0.018 | 1.05 | 0.884 | -0.87 |
| 1452859_at | 1200016B10Rik | RIKEN cDNA 1200016B10 gene | -1.28 | 0.03 | -1.27 | 0.051 | -1.57 | 0.037 | 1.21 | 0.475 | -0.73 |
| 1442412_at | Dnahc9 | dynein, axonemal, heavy chain 9 | -1.28 | 0.466 | -1.57 | 0.385 | -1.28 | 0.317 | 1.49 | 0.569 | -0.66 |
| 1447077_at | Add3 | Adducin 3 (gamma) | 1.2 | 0.838 | -2.88 | 0.021 | -1.53 | 0.137 | -1.57 | 0.114 | -1.2 |
| 1459129_at | --- | Adult male testis cDNA, RIKEN full-length enriched library, clone:4930548A09 pro | -1.46 | 0.14 | -1.24 | 0.385 | -1.42 | 0.167 | 1.07 | 0.681 | -0.76 |
| 1445767_at | Ptprd | protein tyrosine phosphatase, receptor type, D | 1.04 | 0.965 | -1.41 | 0.553 | -2.18 | 0.215 | -2.59 | 0.296 | -1.29 |
| 1453213_at | Spata9 | spermatogenesis associated 9 | -2.26 | 0.167 | -1.13 | 0.77 | -1.14 | 0.392 | 1.1 | 0.879 | -0.86 |
| 1449716_s_at | Nrd1 | nardilysin, N-arginine dibasic convertase, NRD convertase 1 | -1.85 | 0.037 | -1.33 | 0.225 | -1.1 | 0.423 | 1.13 | 0.62 | -0.79 |
| 1437577_at | --- | --- | -1.27 | 0.146 | -1.19 | 0.08 | -1.75 | 0.002 | 1.1 | 0.393 | -0.78 |
| 1443715_at | LOC380843 /// LOC | similar to RNA binding motif protein 24 /// similar to RNA binding motif protein 24 | -1.2 | 0.661 | -1.16 | 0.79 | -1.96 | 0.171 | 1.39 | 0.524 | -0.73 |
| 1419196_at | Hamp1 | hepcidin antimicrobial peptide 1 | -1.18 | 0.051 | -1.78 | 0.273 | -1.25 | 0.325 | 2.14 | 0.096 | -0.52 |
| 1458314_at | Slc1a2 | Solute carrier family 1 (glial high affinity glutamate transporter), member 2 | -2.76 | 0.275 | -1.27 | 0.255 | 1.06 | 0.754 | -4.8 | 0.009 | -1.94 |
| 1459349_at | A930011G23Rik | RIKEN cDNA A930011G23 gene | -1.12 | 0.731 | -1.62 | 0.213 | -1.45 | 0.382 | 1.29 | 0.526 | -0.72 |
| 1439016_x_at | Sprr2a | small proline-rich protein 2A | -1.46 | 0.146 | -1.88 | 0.02 | -1.01 | 0.94 | 1.21 | 0.223 | -0.79 |
| 1447241_at | H2afy2 | H2A histone family, member Y2 | -1.42 | 0.238 | -1.43 | 0.341 | -1.25 | 0.177 | 1.37 | 0.121 | -0.68 |
| 1458428_at | Kcnt1 | potassium channel, subfamily T, member 1 | -1.58 | 0.013 | -1 | 0.992 | -1.74 | 0.142 | 1.15 | 0.502 | -0.79 |
| 1432739_at | 2900060K15Rik | RIKEN cDNA 2900060K15 gene | -2.46 | 0.112 | 1.05 | 0.947 | -1.33 | 0.4 | -2.57 | 0.252 | -1.33 |
| 1443589_at | DXErtd242e | DNA segment, Chr X, ERATO Doi 242, expressed | 1.23 | 0.633 | -1.63 | 0.388 | -2.82 | 0.083 | -1.26 | 0.199 | -1.12 |
| 1444067_at | Pde1c | phosphodiesterase 1C | -2.01 | 0.114 | -1.22 | 0.647 | -1.13 | 0.756 | 1.09 | 0.726 | -0.82 |
| 1420435_at | 1700011A15Rik | RIKEN cDNA 1700011A15 gene | -2.71 | 0.013 | -1.07 | 0.862 | -1.11 | 0.842 | 1.17 | 0.618 | -0.93 |
| 1445788_at | Igf2bp1 | Insulin-like growth factor 2, binding protein 1 | -1.33 | 0.724 | -1.32 | 0.659 | -1.43 | 0.159 | 1.09 | 0.905 | -0.75 |
| 1438912_at | Hdgfrp2 | hepatoma-derived growth factor, related protein 2 | -1.1 | 0.476 | -2.17 | 0.028 | -1.2 | 0.412 | 1.21 | 0.3 | -0.82 |
| 1430489_at | 5430416G10Rik | RIKEN cDNA 5430416G10 gene | -1.34 | 0.183 | -1.1 | 0.748 | -1.82 | 0.09 | 1.63 | 0.057 | -0.66 |
| 1423606_at | Postn | periostin, osteoblast specific factor | -1.37 | 0.371 | 1.06 | 0.714 | -2.41 | 0.014 | -1.7 | 0.135 | -1.11 |
| 1434297_at | E130304F04Rik | RIKEN cDNA E130304F04 gene | -1.95 | 0.207 | -1.76 | 0.14 | 1.12 | 0.773 | -2.72 | 0.261 | -1.33 |
| 1446789_at | Zcrb1 | Zinc finger CCHC-type and RNA binding motif 1 | -1.85 | 0.237 | -1.22 | 0.803 | -1.18 | 0.115 | 1.32 | 0.492 | -0.73 |

| | | | | | | | | | | | |
|---|---|---|---|---|---|---|---|---|---|---|---|
| 1429346_a_at | 4933440J22Rik | RIKEN cDNA 4933440J22 gene | -1.27 | 0.215 | -1.62 | 0.221 | -1.25 | 0.481 | 1.52 | 0.375 | -0.65 |
| 1436713_s_at | Gtl2 | GTL2, imprinted maternally expressed untranslated mRNA | -1.06 | 0.476 | -1.48 | 0.345 | -1.7 | 0.187 | 1.18 | 0.363 | -0.76 |
| 1432658_at | 2810403D21Rik | RIKEN cDNA 2810403D21 gene | -2.19 | 0.54 | -2.39 | 0.297 | 1.33 | 0.515 | -2.62 | 0.358 | -1.47 |
| 1432040_at | Kcnq1ot1 | KCNQ1 overlapping transcript 1 | -1.61 | 0.023 | -1.39 | 0.134 | -1.16 | 0.393 | 1 | 0.995 | -0.79 |
| 1419197_x_at | Hamp1 | hepcidin antimicrobial peptide 1 | -1.29 | 0.432 | -1.77 | 0.284 | -1.16 | 0.393 | 2.27 | 0.007 | -0.49 |
| 1443587_at | AK129128 | cDNA sequence AK129128 | -1.48 | 0.711 | -1.98 | 0.35 | 1.03 | 0.964 | -2.2 | 0.222 | -1.16 |
| 1447673_x_at | 1700015C15Rik | RIKEN cDNA 1700015C15 gene | -1.2 | 0.301 | -1.36 | 0.631 | -1.56 | 0.301 | 1.59 | 0.015 | -0.63 |
| 1429669_at | 4930488L21Rik | RIKEN cDNA 4930488L21 gene | -1.5 | 0.139 | -1.16 | 0.572 | -1.48 | 0.341 | 1.03 | 0.948 | -0.78 |
| 1453906_at | Thrap2 | thyroid hormone receptor associated protein 2 | -1.27 | 0.476 | -1.38 | 0.514 | -1.44 | 0.2 | 1.06 | 0.851 | -0.76 |
| 1442730_at | --- | --- | 1.15 | 0.508 | -2.42 | 0.243 | -1.56 | 0.461 | -1.67 | 0.199 | -1.13 |
| 1418683_at | Lin7b | lin-7 homolog B (C. elegans) | -1.44 | 0.746 | 1.04 | 0.89 | -2.11 | 0.058 | -2.95 | 0.107 | -1.37 |
| 1447065_at | 9630041C05 | hypothetical protein 9630041C05 | -2.37 | 0.414 | -1.62 | 0.153 | 1.17 | 0.658 | -2.82 | 0.015 | -1.41 |
| 1441163_at | Med12l | mediator of RNA polymerase II transcription, subunit 12 homolog (yeast)-like | -1.22 | 0.498 | -3.43 | 0.159 | 1.1 | 0.829 | -1.19 | 0.454 | -1.19 |
| 1429725_at | Atbf1 | AT motif binding factor 1 | -1.18 | 0.677 | -2.22 | 0.074 | -1.1 | 0.44 | 1.16 | 0.626 | -0.83 |
| 1459373_at | Epb4.1 | Erythrocyte protein band 4.1 | -1.12 | 0.712 | -1.34 | 0.286 | -1.75 | 0.184 | 3.04 | 0.239 | -0.29 |
| 1431868_at | Akap4 | A kinase (PRKA) anchor protein 4 | -3.22 | 0.103 | -1.4 | 0.394 | 1.19 | 0.559 | -1.74 | 0.222 | -1.29 |
| 1427587_at | Zfp28 | zinc finger protein 28 | -1.24 | 0.087 | -2.67 | 0.075 | 1.03 | 0.913 | -1.1 | 0.745 | -0.99 |
| 1447340_at | --- | Adult male kidney cDNA, RIKEN full-length enriched library, clone:F530214C21 p | -1.55 | 0.29 | -1.18 | 0.319 | -1.39 | 0.052 | 1.09 | 0.843 | -0.76 |
| 1451647_at | Slc24a1 | solute carrier family 24 (sodium/potassium/calcium exchanger), member 1 | -1.5 | 0.182 | -1.13 | 0.816 | -1.52 | 0.405 | 1.1 | 0.892 | -0.76 |
| 1459144_at | Fndc3a | Fibronectin type III domain containing 3a | -1.21 | 0.357 | -1.51 | 0.099 | -1.38 | 0.028 | 1.15 | 0.712 | -0.74 |
| 1455228_at | Whsc1 | Wolf-Hirschhorn syndrome candidate 1 (human) | -1.44 | 0.096 | -1.24 | 0.043 | -1.41 | 0.005 | 1.34 | 0.385 | -0.69 |
| 1446035_at | --- | PREDICTED: Mus musculus similar to Protein CXorf22 (LOC629131), mRNA | -3.35 | 0.067 | 1.3 | 0.635 | -1.63 | 0.214 | -3.07 | 0.129 | -1.69 |
| 1445427_at | Nlk | Nemo like kinase | 1.18 | 0.709 | -2.3 | 0.308 | -1.67 | 0.271 | -1.38 | 0.398 | -1.04 |
| 1459230_at | D530025C14Rik | RIKEN cDNA B430319G15 gene | -1.17 | 0.233 | -1.67 | 0.057 | -1.32 | 0.14 | 1.85 | 0.257 | -0.58 |
| 1421702_at | Rdh1 | retinol dehydrogenase 1 (all trans) | -1.49 | 0.507 | -1.72 | 0.248 | -1.04 | 0.939 | 1.26 | 0.633 | -0.75 |
| 1419802_at | Ccdc12 | coiled-coil domain containing 12 | -1.75 | 0.134 | -1.03 | 0.924 | -1.49 | 0.231 | 1.21 | 0.399 | -0.76 |
| 1455764_at | B930007L02Rik | RIKEN cDNA B930007L02 gene | -1.69 | 0.209 | -1.38 | 0.643 | -1.12 | 0.358 | 1.15 | 0.65 | -0.76 |
| 1442293_at | --- | Transcribed locus | -1.41 | 0.108 | -1.69 | 0.121 | -1.1 | 0.585 | 1.33 | 0.414 | -0.72 |
| 1433338_at | 6720460K10Rik | RIKEN cDNA 6720460K10 gene | -1.16 | 0.541 | -1.21 | 0.602 | -1.89 | 0.085 | 1.2 | 0.491 | -0.77 |
| 1418352_at | Hsd17b2 | hydroxysteroid (17-beta) dehydrogenase 2 | 1.04 | 0.775 | -1.65 | 0.033 | -1.78 | 0.286 | -2.23 | 0.222 | -1.15 |
| 1451886_at | Speg | SPEG complex locus | -2.76 | 0.031 | -1.11 | 0.862 | -1.05 | 0.93 | 1.53 | 0.148 | -0.85 |
| 1456345_at | A630057N01Rik | RIKEN cDNA A630057N01 gene | -1.43 | 0.455 | -1.11 | 0.841 | -1.64 | 0.305 | 1.47 | 0.25 | -0.68 |
| 1437940_at | Apba1 | amyloid beta (A4) precursor protein binding, family A, member 1 | -1.46 | 0.306 | -1.4 | 0.279 | -1.23 | 0.15 | 1.31 | 0.35 | -0.69 |
| 1427808_at | Nfya | nuclear transcription factor-Y alpha | -2.19 | 0.277 | -1.08 | 0.881 | -1.21 | 0.558 | 1.3 | 0.69 | -0.79 |
| 1458957_at | LOC623548 | Similar to pyridoxal (pyridoxine, vitamin B6) kinase | 1.11 | 0.894 | -2.17 | 0.157 | -1.56 | 0.441 | -1.13 | 0.778 | -0.94 |
| 1449661_at | Suz12 | Suppressor of zeste 12 homolog (Drosophila) | -1.21 | 0.425 | -1.36 | 0.492 | -1.53 | 0.237 | 1.73 | 0.157 | -0.59 |
| 1420269_at | Npm1 | Nucleophosmin 1 | -1.86 | 0.207 | -1.05 | 0.87 | -1.37 | 0.51 | 2.17 | 0.143 | -0.53 |
| 1431991_at | 2410004P03Rik | RIKEN cDNA 2410004P03 gene | -1.92 | 0.123 | -1.39 | 0.357 | -1.02 | 0.94 | 1.1 | 0.845 | -0.81 |
| 1436468_at | Zdhhc8 | zinc finger, DHHC domain containing 8 | -2.33 | 0.066 | -1.61 | 0.258 | 1.17 | 0.226 | -1.53 | 0.033 | -1.08 |
| 1448381_at | Gfm1 | G elongation factor, mitochondrial 1 | -1.29 | 0.404 | -1.35 | 0.072 | -1.44 | 0.002 | 1.24 | 0.448 | -0.71 |
| 1456960_at | Adk | Adenosine kinase | -1.23 | 0.351 | -1.19 | 0.327 | -1.77 | 0.009 | 1.38 | 0.322 | -0.7 |
| 1453642_at | 6530411M01Rik | RIKEN cDNA 6530411M01 gene | -1.96 | 0.512 | -1.52 | 0.489 | 1.05 | 0.705 | -1.68 | 0.276 | -1.03 |
| 1439132_at | Phf8 | PHD finger protein 8 | -1.08 | 0.445 | -1.51 | 0.345 | -1.6 | 0.22 | 1.22 | 0.694 | -0.74 |
| 1433357_at | 5730422E09Rik | RIKEN cDNA 5730422E09 gene | 1.16 | 0.028 | -1.72 | 0.397 | -2.11 | 0.066 | -1.3 | 0.274 | -0.99 |
| 1439691_at | D5Ertd579e | DNA segment, Chr 5, ERATO Doi 579, expressed | -1.31 | 0.06 | -1.36 | 0.217 | -1.39 | 0.065 | 1.36 | 0.193 | -0.67 |
| 1432210_at | 4933401H06Rik | RIKEN cDNA 4933401H06 gene | -2.77 | 0.087 | -1 | 0.994 | -1.16 | 0.611 | 1.08 | 0.796 | -0.96 |
| 1436089_at | Ints6 | integrator complex subunit 6 | -1.78 | 0.003 | -1.17 | 0.141 | -1.25 | 0.135 | 1.28 | 0.49 | -0.73 |
| 1456921_at | A2bp1 | Ataxin 2 binding protein 1 | -1.09 | 0.904 | -1.3 | 0.182 | -1.88 | 0.269 | 1.12 | 0.855 | -0.79 |
| 1452858_at | Elavl1 | ELAV (embryonic lethal, abnormal vision, Drosophila)-like 1 (Hu antigen R) | -1.38 | 0.101 | -1.52 | 0.179 | -1.2 | 0.188 | 1.5 | 0.374 | -0.65 |
| 1447421_at | --- | Transcribed locus | -1.53 | 0.002 | 1.03 | 0.908 | -1.87 | 0.032 | -1.23 | 0.364 | -0.9 |
| 1434910_at | --- | --- | -1.37 | 0.035 | -1.6 | 0.106 | -1.16 | 0.004 | 1.03 | 0.689 | -0.77 |
| 1455698_at | Tloc1 | translocation protein 1 | -1.13 | 0.606 | -1.42 | 0.18 | -1.6 | 0.141 | 1.15 | 0.625 | -0.75 |
| 1439449_at | Satb1 | special AT-rich sequence binding protein 1 | -2.38 | 0.081 | 1.18 | 0.731 | -1.61 | 0.262 | -4.48 | 0.01 | -1.82 |

| | | | | | | | | | | | |
|---|---|---|---|---|---|---|---|---|---|---|---|
| 1447732_x_at | Peg3 | paternally expressed 3 | -1.09 | 0.844 | -2.77 | 0.047 | -1.06 | 0.648 | 2.13 | 0.307 | -0.7 |
| 1458880_at | --- | --- | 1.14 | 0.779 | -1.45 | 0.434 | -2.55 | 0.025 | -1.72 | 0.342 | -1.15 |
| 1433090_at | --- | --- | -3.23 | 0.215 | -1.27 | 0.637 | 1.12 | 0.769 | -1.18 | 0.165 | -1.14 |
| 1438796_at | --- | --- | -2.05 | 0.419 | 1.49 | 0.113 | -4.11 | 0.059 | -2.29 | 0.034 | -1.74 |
| 1443637_at | 2900084M01Rik | RIKEN cDNA 2900084M01 gene | -1.29 | 0.597 | -2.21 | 0.177 | -1.01 | 0.985 | 1.24 | 0.721 | -0.82 |
| 1457925_at | C530049I24Rik | RIKEN cDNA C530049I24 gene | -1.04 | 0.762 | -1.45 | 0.134 | -1.75 | 0.408 | 1.03 | 0.969 | -0.8 |
| 1444502_at | Pet112l | PET112-like (yeast) | -1.6 | 0.072 | -1.03 | 0.926 | -1.6 | 0.142 | 1.71 | 0.194 | -0.63 |
| 1427363_at | Myo10 | Myosin X | -1.11 | 0.129 | -1.5 | 0.048 | -1.54 | 0.065 | 1.23 | 0.002 | -0.73 |
| 1436631_at | 2010010M04Rik | RIKEN cDNA 2010010M04 gene | -1.06 | 0.457 | -1.35 | 0.105 | -1.89 | 0.009 | 1.12 | 0.258 | -0.79 |
| 1446537_at | Ga17 | Dendritic cell protein GA17 | 1.24 | 0.365 | -1.71 | 0.123 | -2.55 | 0.001 | -1.53 | 0.232 | -1.14 |
| 1426035_at | LOC574418 | hypothetical LOC574418 | -1.06 | 0.947 | -1.37 | 0.492 | -1.84 | 0.076 | 1.55 | 0.457 | -0.68 |
| 1418547_at | Tfpi2 | tissue factor pathway inhibitor 2 | -1.38 | 0.203 | 1.02 | 0.846 | -2.09 | 0.038 | -1.35 | 0.284 | -0.95 |
| 1450500_at | Uhmk1 /// LOC6772 | U2AF homology motif (UHM) kinase 1 /// similar to U2AF homology motif (UHM) k | -1.27 | 0.657 | -1.84 | 0.159 | -1.12 | 0.783 | 1.79 | 0.167 | -0.61 |
| 1459878_a_at | A430107O13Rik | RIKEN cDNA A430107O13 gene | 1.25 | 0.349 | -2.14 | 0.025 | -1.99 | 0.047 | -1.1 | 0.628 | -0.99 |
| 1425369_a_at | Sox10 | SRY-box containing gene 10 | 1.13 | 0.489 | -1.53 | 0.075 | -2.29 | 0.291 | -1.43 | 0.221 | -1.03 |
| 1425839_at | Fkbp11 | FK506 binding protein 11 | -1.84 | 0.307 | -1.32 | 0.65 | -1.09 | 0.836 | 1.37 | 0.166 | -0.72 |
| 1431925_at | 4933433H22Rik | RIKEN cDNA 4933433H22 gene | -1.68 | 0.396 | -1.27 | 0.642 | -1.2 | 0.554 | 2.11 | 0.215 | -0.51 |
| 1443029_at | Ppfibp1 | PTPRF interacting protein, binding protein 1 (liprin beta 1) | -1.27 | 0.256 | -1.16 | 0.487 | -1.76 | 0.034 | 1.24 | 0.336 | -0.74 |
| 1428032_at | Acvr1c | activin A receptor, type IC | -1.2 | 0.438 | -1.52 | 0.118 | -1.37 | 0.161 | 1.11 | 0.718 | -0.74 |
| 1436531_at | Metap2 | methionine aminopeptidase 2 | -1.47 | 0.124 | -1.24 | 0.322 | -1.37 | 0.035 | 2.68 | 0.249 | -0.35 |
| 1439572_at | --- | --- | 1.01 | 0.91 | -1.8 | 0.002 | -1.53 | 0.069 | -1.05 | 0.829 | -0.84 |
| 1437512_x_at | Ebna1bp2 | EBNA1 binding protein 2 | -1.97 | 0.2 | -1.41 | 0.208 | 1 | 0.992 | -1.19 | 0.438 | -0.89 |
| 1449067_at | Slc2a2 | solute carrier family 2 (facilitated glucose transporter), member 2 | -1.12 | 0.438 | -1.43 | 0.337 | -1.6 | 0.246 | 1.16 | 0.867 | -0.75 |
| 1453184_at | 2310040C09Rik | RIKEN cDNA 2310040C09 gene | 1.13 | 0.643 | -3.31 | 0.22 | -1.26 | 0.502 | -2.2 | 0.427 | -1.41 |
| 1431803_at | Cyp2d13 | cytochrome P450, family 2, subfamily d, polypeptide 13 | -1.25 | 0.442 | -1.84 | 0 | -1.14 | 0.724 | 1.02 | 0.964 | -0.8 |
| 1453102_at | Flrt3 | fibronectin leucine rich transmembrane protein 3 | 1.18 | 0.54 | -2.2 | 0.363 | -1.71 | 0.098 | -2.15 | 0.127 | -1.22 |
| 1433026_at | --- | --- | -1.29 | 0.379 | -1.43 | 0.362 | -1.33 | 0.361 | 1.28 | 0.558 | -0.7 |
| 1433167_at | 4930444E06Rik | RIKEN cDNA 4930444E06 gene | 1 | 0.987 | -2.47 | 0.114 | -1.23 | 0.418 | -1.26 | 0.294 | -0.99 |
| 1460316_at | Acsl1 | acyl-CoA synthetase long-chain family member 1 | -1.31 | 0.057 | -1.9 | 0.017 | -1.07 | 0.729 | 1.55 | 0.108 | -0.68 |
| 1447785_x_at | D11Wsu99e | DNA segment, Chr 11, Wayne State University 99, expressed | -1.63 | 0.118 | -1.36 | 0.39 | -1.14 | 0.543 | 1.19 | 0.645 | -0.74 |
| 1440311_at | Sorbs1 | sorbin and SH3 domain containing 1 | -1.23 | 0.65 | -1.2 | 0.454 | -1.74 | 0.208 | 1.63 | 0.232 | -0.64 |
| 1457389_at | 9830166K06Rik | RIKEN cDNA 9830166K06 gene | -1.39 | 0.665 | -1.61 | 0.166 | -1.13 | 0.623 | 1.15 | 0.622 | -0.75 |
| 1436294_at | Ankrd29 | ankyrin repeat domain 29 | 1 | 0.999 | -1.69 | 0.278 | -1.59 | 0.082 | -1.51 | 0.081 | -0.95 |
| 1460588_at | --- | 10 days lactation, adult female mammary gland cDNA, RIKEN full-length enriched | -1.33 | 0.483 | -1.75 | 0.076 | -1.11 | 0.631 | 1.01 | 0.972 | -0.8 |
| 1427290_at | Krt81 | keratin 81 | -1.52 | 0.099 | -1.28 | 0.177 | -1.27 | 0.284 | 1.38 | 0.07 | -0.67 |
| 1452677_at | Pnpt1 /// LOC6355 | polyribonucleotide nucleotidyltransferase 1 /// similar to polyribonucleotide nucleo | -2.57 | 0.189 | -1.4 | 0.39 | 1.12 | 0.773 | -1.04 | 0.933 | -0.97 |
| 1441055_at | Palm2 | paralemmin 2 | 1.1 | 0.901 | -1.92 | 0.175 | -1.65 | 0.031 | -1.18 | 0.718 | -0.91 |
| 1438583_at | Ern1 | Endoplasmic reticulum (ER) to nucleus signalling 1 | -1.2 | 0.416 | -1.31 | 0.072 | -1.58 | 0.086 | 1.29 | 0.017 | -0.7 |
| 1446454_at | --- | --- | -1.13 | 0.899 | -2.33 | 0.275 | -1.09 | 0.803 | 1.49 | 0.289 | -0.77 |
| 1440157_at | Scml4 | Sex comb on midleg-like 4 (Drosophila) | 1.1 | 0.576 | -2.03 | 0.105 | -1.59 | 0.348 | -3.35 | 0.061 | -1.47 |
| 1436637_at | Wbscr1 | Williams-Beuren syndrome chromosome region 1 homolog (human) | -1.94 | 0.135 | -1.01 | 0.95 | -1.38 | 0.334 | 1.15 | 0.429 | -0.8 |
| 1416460_at | Miox | myo-inositol oxygenase | -1.15 | 0.22 | -12.46 | 0.211 | 1.27 | 0.74 | -3.06 | 0.351 | -3.85 |
| 1458598_at | Peg13 | paternally expressed 13 | -2.39 | 0.223 | -1.36 | 0.647 | 1.07 | 0.82 | -1.02 | 0.82 | -0.93 |
| 1431841_at | 4930434E21Rik | RIKEN cDNA 4930434E21 gene | -1.53 | 0.246 | -1.88 | 0.232 | 1.04 | 0.923 | -1.36 | 0.451 | -0.93 |
| 1446360_at | Rora | RAR-related orphan receptor alpha | 1.05 | 0.93 | -1.12 | 0.829 | -3.47 | 0.041 | -2.17 | 0.208 | -1.43 |
| 1442034_at | Fastkd1 | FAST kinase domains 1 | -2.05 | 0.258 | -1.74 | 0.289 | 1.17 | 0.698 | -1.46 | 0.636 | -1.02 |
| 1454001_at | --- | --- | -1.71 | 0.549 | -1.62 | 0.558 | 1.02 | 0.958 | -1.06 | 0.911 | -0.84 |
| 1444594_at | Tbx3 | T-box 3 | -1.15 | 0.668 | -1.3 | 0.331 | -1.7 | 0.055 | 1.19 | 0.413 | -0.74 |
| 1421788_x_at | Egfbp2 | epidermal growth factor binding protein type B | -1.25 | 0.699 | -2.2 | 0.02 | -1.03 | 0.927 | 2.06 | 0.097 | -0.61 |
| 1420385_at | Gna14 | guanine nucleotide binding protein, alpha 14 | -1.2 | 0.138 | -1.35 | 0.091 | -1.54 | 0.024 | 2.05 | 0.027 | -0.51 |
| 1429928_at | 1700030B21Rik | RIKEN cDNA 1700030B21 gene | -1.19 | 0.631 | -3.6 | 0.105 | 1.11 | 0.578 | -1.73 | 0.315 | -1.35 |
| 1432437_at | 4933413N12Rik | RIKEN cDNA 4933413N12 gene | -1.63 | 0.187 | -1.45 | 0.338 | -1.08 | 0.755 | 1.09 | 0.896 | -0.77 |
| 1447046_at | Dnajb13 | DnaJ (Hsp40) related, subfamily B, member 13 | -1.32 | 0.758 | -1.24 | 0.321 | -1.51 | 0.112 | 2.77 | 0.274 | -0.33 |

| | | | | | | | | | | | |
|---|---|---|---|---|---|---|---|---|---|---|---|
| 1447604_at | BC053393 | cDNA sequence BC053393 | -1 | 0.986 | -2.41 | 0.163 | -1.23 | 0.423 | 1.06 | 0.872 | -0.9 |
| 1430204_at | 5033425G24Rik | RIKEN cDNA 5033425G24 gene | 1.04 | 0.616 | -1.36 | 0.311 | -2.22 | 0.039 | -1.17 | 0.589 | -0.93 |
| 1446933_at | --- | --- | -1.3 | 0.443 | -1.27 | 0.407 | -1.48 | 0.076 | 1.02 | 0.921 | -0.76 |
| 1458897_at | Ust | uronyl-2-sulfotransferase | -1.76 | 0.1 | -1.15 | 0.377 | -1.26 | 0.122 | 1.05 | 0.892 | -0.78 |
| 1458323_at | 4930444A19Rik | RIKEN cDNA 4930444A19 gene | -1.13 | 0.79 | -1.19 | 0.604 | -1.98 | 0.272 | 1.67 | 0.423 | -0.66 |
| 1422173_at | Ipf1 | insulin promoter factor 1, homeodomain transcription factor | -3.61 | 0.016 | -1.04 | 0.915 | -1.01 | 0.957 | 2.11 | 0.167 | -0.89 |
| 1453965_at | 4930432O09Rik | RIKEN cDNA 4930432O09 gene | -1.7 | 0.219 | 1 | 0.999 | -1.56 | 0.232 | -1.32 | 0.61 | -0.9 |
| 1436304_at | C030003D03Rik | RIKEN cDNA C030003D03 gene | -1.17 | 0.825 | -1.65 | 0.48 | -1.31 | 0.45 | 2.7 | 0.109 | -0.36 |
| 1444495_at | --- | --- | -1.37 | 0.205 | -1.02 | 0.964 | -1.92 | 0.066 | 1.3 | 0.753 | -0.75 |
| 1431216_s_at | Dnajc6 | DnaJ (Hsp40) homolog, subfamily C, member 6 | 1.15 | 0.468 | -1.29 | 0.691 | -3.25 | 0.221 | -1.76 | 0.129 | -1.29 |
| 1444856_at | Crebbp | CREB binding protein | 1.03 | 0.885 | -1.72 | 0.055 | -1.6 | 0.03 | -1.46 | 0.148 | -0.94 |
| 1429812_at | 2610002D18Rik | RIKEN cDNA 2610002D18 gene | -1.03 | 0.9 | -1.67 | 0.369 | -1.51 | 0.201 | 2.25 | 0.247 | -0.49 |
| 1420676_at | Sprrl3 | small proline rich-like 3 | -1.49 | 0.425 | -1.5 | 0.515 | -1.12 | 0.799 | 2.47 | 0.366 | -0.41 |
| 1458340_at | --- | Transcribed locus | -1.54 | 0.492 | -2.62 | 0.112 | 1.2 | 0.641 | -1.77 | 0.218 | -1.18 |
| 1446982_at | Pard3 | Par-3 (partitioning defective 3) homolog (C. elegans) | -1.22 | 0.375 | -1.09 | 0.729 | -2.04 | 0.004 | 1.02 | 0.933 | -0.83 |
| 1440008_at | 2310043L19Rik | RIKEN cDNA 2310043L19 gene | -1.37 | 0.354 | 1 | 0.998 | -2 | 0.129 | -2.65 | 0.22 | -1.26 |
| 1438344_at | 4833424O15Rik | RIKEN cDNA 4833424O15 gene | -1.63 | 0.625 | 1.06 | 0.847 | -1.79 | 0.215 | -2.27 | 0.017 | -1.16 |
| 1437655_at | D430020J02Rik | RIKEN cDNA D430020J02 gene | -1.12 | 0.815 | -1.56 | 0.266 | -1.43 | 0.544 | 1.01 | 0.987 | -0.78 |
| 1443175_at | A830010M09Rik | RIKEN cDNA A830010M09 gene | -1.98 | 0.305 | -1.63 | 0.435 | 1.11 | 0.807 | -2.04 | 0.306 | -1.13 |
| 1439684_at | 4930570G19Rik | RIKEN cDNA 4930570G19 gene | -1.13 | 0.89 | -2.31 | 0.027 | -1.1 | 0.816 | 1.53 | 0.498 | -0.75 |
| 1459812_x_at | Tspan7 | tetraspanin 7 | -1.6 | 0.309 | -1.38 | 0.482 | -1.13 | 0.766 | 1.37 | 0.544 | -0.69 |
| 1455745_at | Cln8 | ceroid-lipofuscinosis, neuronal 8 | -1.43 | 0.444 | -1.47 | 0.086 | -1.18 | 0.311 | 2.78 | 0.109 | -0.32 |
| 1459100_at | AU015253 | expressed sequence AU015253 | -1.68 | 0.28 | -1.09 | 0.829 | -1.39 | 0.113 | 1.05 | 0.913 | -0.78 |
| 1430529_at | Csnk1a1 | casein kinase 1, alpha 1 | -1.17 | 0.34 | -1.61 | 0.067 | -1.32 | 0.178 | 1.1 | 0.501 | -0.75 |
| 1435435_at | Cttnbp2 | cortactin binding protein 2 | -1.14 | 0.489 | -1.06 | 0.92 | -2.42 | 0.185 | 1.07 | 0.208 | -0.89 |
| 1421674_at | --- | --- | -1.16 | 0.809 | -1.25 | 0.443 | -1.74 | 0.27 | 1.51 | 0.54 | -0.66 |
| 1456082_x_at | Cct4 | chaperonin subunit 4 (delta) | -1.3 | 0.303 | -1.36 | 0.234 | -1.37 | 0.21 | 1.12 | 0.524 | -0.73 |
| 1457099_at | D030011O10Rik | RIKEN cDNA D030011O10 gene | -1.56 | 0.134 | -2.08 | 0.072 | 1.11 | 0.502 | -1.43 | 0.326 | -0.99 |
| 1459493_at | Setbp1 | SET binding protein 1 | 1.01 | 0.878 | -1.56 | 0.55 | -1.72 | 0.017 | -1.22 | 0.062 | -0.87 |
| 1446888_at | --- | --- | -1.42 | 0.765 | -1.07 | 0.899 | -1.68 | 0.25 | 1.83 | 0.277 | -0.59 |
| 1442267_at | Stxbp4 | syntaxin binding protein 4 | -1.05 | 0.891 | -1.15 | 0.664 | -2.44 | 0.08 | 2.67 | 0.19 | -0.49 |
| 1443056_at | Ptprd | Protein tyrosine phosphatase, receptor type, D | 1 | 0.991 | -1.41 | 0.128 | -1.91 | 0.099 | -1.5 | 0.298 | -0.96 |
| 1436837_at | Mael | maelstrom homolog (Drosophila) | -1.07 | 0.909 | -1.74 | 0.341 | -1.38 | 0.372 | 1.14 | 0.558 | -0.76 |
| 1426712_at | Slc6a15 | solute carrier family 6 (neurotransmitter transporter), member 15 | -1.27 | 0.141 | -1.85 | 0.314 | -1.11 | 0.66 | 1.17 | 0.692 | -0.76 |
| 1436692_at | --- | 12 days embryo head cDNA, RIKEN full-length enriched library, clone:3000003N1 | -1.32 | 0.205 | -1.12 | 0.602 | -1.73 | 0.008 | 1.07 | 0.666 | -0.77 |
| 1450045_at | Srrm1 | serine/arginine repetitive matrix 1 | -1.22 | 0.11 | -1.4 | 0.01 | -1.43 | 0.038 | 1.1 | 0.649 | -0.74 |
| 1443663_at | Prkg1 | Protein kinase, cGMP-dependent, type I | -1.34 | 0.469 | 1.04 | 0.823 | -2.26 | 0.006 | -1.69 | 0.456 | -1.06 |
| 1424881_at | Trib1 | tribbles homolog 1 (Drosophila) | -1.29 | 0.313 | -1.62 | 0.107 | -1.18 | 0.453 | 1.62 | 0.308 | -0.62 |
| 1457947_at | Ddx49 | DEAD (Asp-Glu-Ala-Asp) box polypeptide 49 | -1.72 | 0.14 | -1.48 | 0.65 | -1.02 | 0.93 | 1.34 | 0.479 | -0.72 |
| 1430438_at | 8430439J12Rik | RIKEN cDNA 8430439J12 gene | -1.41 | 0.35 | -1.3 | 0.152 | -1.32 | 0.129 | 1.07 | 0.69 | -0.74 |
| 1445452_at | Traf1 | Tnf receptor-associated factor 1 | 1.14 | 0.566 | -2.01 | 0.242 | -1.66 | 0.043 | -1.97 | 0.143 | -1.13 |
| 1438690_at | Tyms | Thymidylate synthase | -1.31 | 0.119 | -1.19 | 0.661 | -1.59 | 0.262 | 4.17 | 0.186 | 0.02 |
| 1446503_at | --- | --- | -1.8 | 0.05 | -1.76 | 0.183 | 1.11 | 0.724 | -1.33 | 0.224 | -0.94 |
| 1438495_at | Top1 | topoisomerase (DNA) I | -1.32 | 0.036 | -1.31 | 0.221 | -1.39 | 0.174 | 1.79 | 0.139 | -0.56 |
| 1421453_at | Jph2 | junctophilin 2 | -1.33 | 0.202 | -1.51 | 0.606 | -1.21 | 0.508 | 1.18 | 0.252 | -0.72 |
| 1446361_at | Gprk5 | G protein-coupled receptor kinase 5 | -1.35 | 0.024 | -1.22 | 0.719 | -1.48 | 0.218 | 1.14 | 0.415 | -0.73 |
| 1429844_at | 2310043J07Rik | RIKEN cDNA 2310043J07 gene | -1.82 | 0.152 | -2.41 | 0.056 | 1.27 | 0.631 | -1.15 | 0.816 | -1.03 |
| 1448397_at | Gjb6 | gap junction membrane channel protein beta 6 | -1.69 | 0.335 | -1.32 | 0.46 | -1.13 | 0.707 | 1.05 | 0.822 | -0.77 |
| 1418689_at | Cts6 | cathepsin 6 | -1.95 | 0.433 | -1.2 | 0.674 | -1.12 | 0.696 | 1.45 | 0.292 | -0.71 |
| 1459167_at | Cdc42bpb | Cdc42 binding protein kinase beta | -1.13 | 0.431 | -1.55 | 0.19 | -1.41 | 0.342 | 1.05 | 0.923 | -0.76 |
| 1441871_at | 1810044D09Rik | RIKEN cDNA 1810044D09 gene | -2.41 | 0.002 | -1.18 | 0.414 | -1.02 | 0.892 | 1.23 | 0.457 | -0.85 |
| 1450192_at | Lhcgr | luteinizing hormone/choriogonadotropin receptor | 1.02 | 0.966 | -1.36 | 0.591 | -2.09 | 0.278 | -1.82 | 0.57 | -1.06 |
| 1459192_at | --- | Transcribed locus | -1.45 | 0.318 | -1.72 | 0.365 | -1.03 | 0.943 | 1.19 | 0.674 | -0.75 |

| | | | | | | | | | | | |
|---|---|---|---|---|---|---|---|---|---|---|---|
| 1453918_at | 4933426F18Rik | RIKEN cDNA 4933426F18 gene | 1.03 | 0.95 | -2.62 | 0.178 | -1.2 | 0.735 | -3.07 | 0.152 | -1.47 |
| 1416682_at | Ube3a | ubiquitin protein ligase E3A | -1.48 | 0.147 | -1.18 | 0.652 | -1.41 | 0.141 | 1.06 | 0.629 | -0.75 |
| 1442651_at | Wwox | WW domain-containing oxidoreductase | 1.06 | 0.846 | -1.88 | 0.074 | -1.54 | 0.115 | -1.29 | 0.555 | -0.91 |
| 1421850_at | Mtap1b | microtubule-associated protein 1 B | -1.78 | 0.173 | -1.33 | 0.423 | -1.08 | 0.752 | 1.03 | 0.966 | -0.79 |
| 1442625_at | Plekhc1 | Pleckstrin homology domain containing, family C (with FERM domain) member 1 | -1.01 | 0.986 | -1.28 | 0.037 | -2.16 | 0.037 | 1.01 | 0.986 | -0.86 |
| 1440307_at | Gtf3c2 | general transcription factor IIIC, polypeptide 2, beta | 1.06 | 0.63 | -1.77 | 0.02 | -1.62 | 0.201 | -1.51 | 0.05 | -0.96 |
| 1440220_at | AI503564 | expressed sequence AI503564 | -1.13 | 0.378 | -1.4 | 0.009 | -1.57 | 0.009 | 1.52 | 0.015 | -0.64 |
| 1450805_at | Sgcd | sarcoglycan, delta (dystrophin-associated glycoprotein) | -1.88 | 0.172 | -1.13 | 0.83 | -1.21 | 0.08 | 1.93 | 0.06 | -0.57 |
| 1426595_at | Slc18a1 | solute carrier family 18 (vesicular monoamine), member 1 | -1.43 | 0.21 | -1.14 | 0.434 | -1.51 | 0.305 | 1.78 | 0.157 | -0.57 |
| 1446956_at | BC004022 | cDNA sequence BC004022 | -1.25 | 0.344 | 1.02 | 0.875 | -2.42 | 0.226 | -1.11 | 0.771 | -0.94 |
| 1453528_at | Lta4h | leukotriene A4 hydrolase | 1.1 | 0.858 | -2.59 | 0.196 | -1.32 | 0.426 | -1.61 | 0.114 | -1.11 |
| 1446456_at | --- | 0 day neonate eyeball cDNA, RIKEN full-length enriched library, clone:E130003J | -1.84 | 0.25 | -2.92 | 0.117 | 1.35 | 0.304 | -1.73 | 0.173 | -1.28 |
| 1457682_at | 9030420J04Rik | RIKEN cDNA 9030420J04 gene | 1.02 | 0.91 | -1.18 | 0.147 | -2.64 | 0.009 | -1.22 | 0.53 | -1.01 |
| 1451662_x_at | Akap4 | A kinase (PRKA) anchor protein 4 | -1.68 | 0.332 | -1.51 | 0.462 | -1.02 | 0.947 | 1.97 | 0.254 | -0.56 |
| 1442691_at | --- | 0 day neonate lung cDNA, RIKEN full-length enriched library, clone:E030049N05 | -1.32 | 0.266 | -1.26 | 0.146 | -1.45 | 0.148 | 1.03 | 0.904 | -0.75 |
| 1435345_at | Ceecam1 | cerebral endothelial cell adhesion molecule 1 | -1.81 | 0.043 | -1.28 | 0.371 | -1.11 | 0.211 | 3.35 | 0.153 | -0.21 |
| 1430107_at | 9230116B18Rik | RIKEN cDNA 9230116B18 gene | -1.18 | 0.738 | -1.41 | 0.244 | -1.47 | 0.167 | 1.23 | 0.732 | -0.71 |
| 1431884_at | 1110019B22Rik | RIKEN cDNA 1110019B22 gene | 1.03 | 0.969 | -2.19 | 0.279 | -1.34 | 0.59 | -1.57 | 0.496 | -1.01 |
| 1430894_at | 5530401N12Rik | RIKEN cDNA 5530401N12 gene | -2.21 | 0.198 | -1.58 | 0.284 | 1.15 | 0.659 | -2.18 | 0.29 | -1.2 |
| 1454279_at | Oaz3 | ornithine decarboxylase antizyme 3 | -1.66 | 0.631 | -1.29 | 0.687 | -1.16 | 0.768 | 1.73 | 0.4 | -0.6 |
| 1451966_at | Mrap | melanocortin 2 receptor accessory protein | 1.03 | 0.97 | -1.87 | 0.044 | -1.47 | 0.34 | -2.5 | 0.299 | -1.2 |
| 1427607_at | Cacna1h | calcium channel, voltage-dependent, T type, alpha 1H subunit | -1.39 | 0.045 | -1.85 | 0.308 | -1.02 | 0.97 | 1.48 | 0.583 | -0.7 |
| 1443839_at | 1700010H22Rik | RIKEN cDNA 1700010H22 gene | -1.19 | 0.622 | -1.81 | 0.372 | -1.17 | 0.171 | 1.15 | 0.527 | -0.76 |
| 1440993_at | AW111846 | Expressed sequence AW111846 | -1.2 | 0.068 | -1.32 | 0.213 | -1.54 | 0.31 | 1.9 | 0.277 | -0.54 |
| 1432700_at | 2810047J09Rik | RIKEN cDNA 2810047J09 gene | -2.65 | 0 | -1.05 | 0.919 | -1.09 | 0.864 | 1.11 | 0.872 | -0.92 |
| 1437062_s_at | Phyhipl | phytanoyl-CoA hydroxylase interacting protein-like | 1.07 | 0.423 | -2.23 | 0.22 | -1.38 | 0.355 | -2.43 | 0.043 | -1.24 |
| 1438588_at | Plagl1 | Pleiomorphic adenoma gene-like 1 | -1.43 | 0.44 | -1.41 | 0.332 | -1.2 | 0.444 | 1.63 | 0.167 | -0.6 |
| 1445938_at | 5930427L02Rik | RIKEN cDNA 5930427L02 gene | 1.15 | 0.7 | -1.3 | 0.753 | -3.12 | 0.011 | -2.09 | 0.372 | -1.34 |
| 1435851_at | Lgi1 | leucine-rich repeat LGI family, member 1 | -1.16 | 0.675 | -1.94 | 0.341 | -1.15 | 0.705 | 1.3 | 0.647 | -0.74 |
| 1438676_at | Mpa2l /// LOC6265 | macrophage activation 2 like /// similar to macrophage activation 2 like /// similar t | -1.31 | 0.309 | -2.15 | 0.035 | 1.01 | 0.957 | -1.39 | 0.003 | -0.96 |
| 1456989_at | Oxgr1 | oxoglutarate (alpha-ketoglutarate) receptor 1 | -1.38 | 0.277 | -1.48 | 0.307 | -1.19 | 0.657 | 1.98 | 0.08 | -0.52 |
| 1454470_at | 4930404F17Rik | RIKEN cDNA 4930404F17 gene | -1.25 | 0.219 | -1.38 | 0.645 | -1.39 | 0.481 | 1.64 | 0.271 | -0.6 |
| 1458980_at | --- | --- | 1.05 | 0.897 | -1.47 | 0.552 | -1.95 | 0.083 | -1.06 | 0.811 | -0.86 |
| 1442743_at | --- | Adult male olfactory brain cDNA, RIKEN full-length enriched library, clone:643053 | -1.42 | 0.025 | -1.41 | 0.016 | -1.2 | 0.145 | 1.22 | 0.495 | -0.7 |
| 1458552_at | D430050E20Rik | RIKEN cDNA D430050E20 gene | -1.52 | 0.007 | -1.79 | 0.059 | 1.03 | 0.903 | -1.5 | 0.174 | -0.95 |
| 1443145_at | Apbb1ip | amyloid beta (A4) precursor protein-binding, family B, member 1 interacting prote | -1.64 | 0.33 | -1.43 | 0.075 | -1.07 | 0.748 | 1.09 | 0.69 | -0.76 |
| 1454025_at | Ddo | D-aspartate oxidase | -2.63 | 0.291 | -1.63 | 0.369 | 1.25 | 0.065 | -1 | 0.996 | -1 |
| 1437393_at | AI875142 | expressed sequence AI875142 | -1.44 | 0.011 | -1.22 | 0.156 | -1.36 | 0.134 | 1.07 | 0.415 | -0.74 |
| 1444554_at | Tnrc6b | Trinucleotide repeat containing 6b | -1.54 | 0.141 | -1.42 | 0.558 | -1.12 | 0.674 | 1.35 | 0.067 | -0.68 |
| 1427018_at | Tsnaxip1 | translin-associated factor X (Tsnax) interacting protein 1 | -1.18 | 0.742 | -1.59 | 0.628 | -1.3 | 0.316 | 1.32 | 0.465 | -0.69 |
| 1427286_at | D11Bwg0517e | DNA segment, Chr 11, Brigham & Women's Genetics 0517 expressed | -1.41 | 0.213 | 1.13 | 0.619 | -2.45 | 0.143 | -1.06 | 0.931 | -0.95 |
| 1445495_at | --- | --- | -1.77 | 0.118 | -1.51 | 0.351 | 1.02 | 0.938 | -2.26 | 0.013 | -1.13 |
| 1447809_x_at | --- | --- | -1.14 | 0.621 | -1.67 | 0.154 | -1.29 | 0.496 | 1.9 | 0.355 | -0.55 |
| 1453730_at | Samd8 | sterile alpha motif domain containing 8 | -1.47 | 0.069 | -1.35 | 0.544 | -1.22 | 0.588 | 1.4 | 0.265 | -0.66 |
| 1434200_at | BC010981 | cDNA sequence BC010981 | -1.29 | 0.453 | -1.32 | 0.316 | -1.4 | 0.038 | 1.17 | 0.545 | -0.71 |
| 1453215_at | Rnase10 | ribonuclease, RNase A family, 10 (non-active) | 1.12 | 0.779 | -1.47 | 0.107 | -2.25 | 0.028 | -1 | 0.997 | -0.9 |
| 1440762_at | Syn2 | synapsin II | -1.35 | 0.675 | -1.33 | 0.669 | -1.33 | 0.403 | 1.54 | 0.483 | -0.62 |
| 1456972_at | C80256 | expressed sequence C80256 | -1.42 | 0.599 | -1.12 | 0.822 | -1.54 | 0.084 | 1.39 | 0.5 | -0.67 |
| 1453563_at | Nmral1 | NmrA-like family domain containing 1 | 1.05 | 0.946 | -1.86 | 0.12 | -1.51 | 0.148 | -1.08 | 0.788 | -0.85 |
| 1444667_at | Brdt | bromodomain, testis-specific | 1.16 | 0.635 | -2.88 | 0.009 | -1.35 | 0.346 | -1.13 | 0.847 | -1.05 |
| 1450375_at | Pspn | persephin | -1.31 | 0.346 | -1.26 | 0.538 | -1.45 | 0.195 | 1.13 | 0.833 | -0.72 |
| 1441852_x_at | Atg16l1 | autophagy-related 16-like 1 (yeast) | -1.29 | 0.797 | -2.11 | 0.012 | -1 | 0.995 | 1.18 | 0.723 | -0.8 |
| 1425658_at | Cd109 | CD109 antigen | 1.27 | 0.735 | -1.76 | 0.108 | -2.43 | 0.043 | -1.48 | 0.577 | -1.1 |

| | | | | | | | | | | | |
|---|---|---|---|---|---|---|---|---|---|---|---|
| 1446593_at | Trio | Triple functional domain (PTPRF interacting) | 1.1 | 0.738 | -1.44 | 0.043 | -2.22 | 0.009 | -1.17 | 0.667 | -0.93 |
| 1427425_at | 9130208E07Rik | RIKEN cDNA 9130208E07 gene | -1.13 | 0.457 | -1.33 | 0.098 | -1.63 | 0.012 | 3.02 | 0.234 | -0.27 |
| 1428983_at | Scx | scleraxis | -1.4 | 0.612 | -1.01 | 0.983 | -1.84 | 0.038 | 1.01 | 0.974 | -0.81 |
| 1459368_at | --- | --- | -1.25 | 0.586 | -1.19 | 0.842 | -1.65 | 0.321 | 1.2 | 0.602 | -0.72 |
| 1448819_at | Eif2s2 | eukaryotic translation initiation factor 2, subunit 2 (beta) | -1.12 | 0.129 | -1.33 | 0.008 | -1.65 | 0.077 | 1.3 | 0.259 | -0.7 |
| 1459076_at | BC052040 | CDNA sequence BC052040 | 1.12 | 0.609 | -2.39 | 0.014 | -1.4 | 0.206 | -1.03 | 0.956 | -0.93 |
| 1441001_at | AI225934 | expressed sequence AI225934 | 1.37 | 0.158 | -1.78 | 0.008 | -3.1 | 0.028 | -1.02 | 0.934 | -1.13 |
| 1457800_at | 2900019G14Rik | RIKEN cDNA 2900019G14 gene | -1.81 | 0.033 | -1.07 | 0.611 | -1.3 | 0.369 | 1.57 | 0.2 | -0.65 |
| 1442265_at | Fancc | Fanconi anemia, complementation group C | -1.5 | 0.092 | -1.53 | 0.383 | -1.07 | 0.654 | 1.37 | 0.437 | -0.69 |
| 1442603_at | Pb1 | Polybromo 1 | -1.09 | 0.743 | -1.22 | 0.529 | -1.94 | 0.087 | 1.3 | 0.321 | -0.74 |
| 1459179_at | D8Ertd268e | DNA segment, Chr 8, ERATO Doi 268, expressed | -1.46 | 0.616 | -1.21 | 0.722 | -1.36 | 0.625 | 3.39 | 0.343 | -0.16 |
| 1421539_at | Zic4 | zinc finger protein of the cerebellum 4 | -1.67 | 0.404 | -1.02 | 0.898 | -1.48 | 0.338 | 1.7 | 0.394 | -0.62 |
| 1445277_at | --- | 16 days neonate thymus cDNA, RIKEN full-length enriched library, clone:A13000 | 1.03 | 0.854 | -1.34 | 0.318 | -2.11 | 0.024 | -2.46 | 0.025 | -1.22 |
| 1440079_at | 3632454L22Rik | RIKEN cDNA 3632454L22 gene | -1.59 | 0.63 | -1.11 | 0.817 | -1.38 | 0.056 | 1.31 | 0.327 | -0.69 |
| 1440474_at | --- | --- | -2.08 | 0.092 | -1.54 | 0.266 | 1.12 | 0.826 | -1.66 | 0.346 | -1.04 |
| 1442641_at | Ankhd1 | Ankyrin repeat and KH domain containing 1 | -1.12 | 0.616 | -1.5 | 0.111 | -1.44 | 0.16 | 1.09 | 0.714 | -0.74 |
| 1427328_a_at | Clasp2 | CLIP associating protein 2 | -1.77 | 0.16 | -1.44 | 0.368 | -1 | 0.992 | 1.79 | 0.533 | -0.61 |
| 1459851_x_at | Riok1 | RIO kinase 1 (yeast) | -1.22 | 0.151 | -2.34 | 0.04 | 1.01 | 0.981 | -1.07 | 0.858 | -0.91 |
| 1420416_at | Sema3a | sema domain, immunoglobulin domain (Ig), short basic domain, secreted, (semap | 1.04 | 0.811 | -1.86 | 0.332 | -1.48 | 0.025 | -1.09 | 0.71 | -0.85 |
| 1454414_at | Btbd7 | BTB (POZ) domain containing 7 | -1.11 | 0.734 | -1.17 | 0.672 | -2 | 0.154 | 1.22 | 0.178 | -0.77 |
| 1436985_at | Zfp644 | zinc finger protein 644 | -1.02 | 0.964 | -1.5 | 0.324 | -1.67 | 0.018 | 1.28 | 0.454 | -0.73 |
| 1424691_at | 5930434B04Rik | RIKEN cDNA 5930434B04 gene | -2.02 | 0.208 | 1.18 | 0.408 | -1.73 | 0.16 | -1.52 | 0.523 | -1.02 |
| 1446418_at | Atg7 | Autophagy-related 7 (yeast) | -1.28 | 0.286 | -1.34 | 0.169 | -1.37 | 0.047 | 1.1 | 0.307 | -0.72 |
| 1444372_at | Arcn1 | Archain 1 | 1.03 | 0.936 | -1.61 | 0.088 | -1.65 | 0.09 | -1.37 | 0.372 | -0.9 |
| 1443195_at | --- | --- | -1.11 | 0.71 | -1.22 | 0.655 | -1.88 | 0.063 | 1.56 | 0.123 | -0.66 |
| 1422238_at | Usmg1 | upregulated during skeletal muscle growth 1 | -1.44 | 0.446 | -3.55 | 0.007 | 1.27 | 0.096 | -1.07 | 0.641 | -1.19 |
| 1427517_at | Boc | biregional cell adhesion molecule-related/down-regulated by oncogenes (Cdon) b | -1.07 | 0.889 | -1.69 | 0.263 | -1.38 | 0.249 | 2.57 | 0.067 | -0.39 |
| 1439183_at | Asah3 | N-acylsphingosine amidohydrolase (alkaline ceramidase) 3 | -1.29 | 0.699 | -1.46 | 0.637 | -1.26 | 0.55 | 1.37 | #DIV/0! | -0.66 |
| 1454311_at | --- | --- | 1.02 | 0.904 | -1.89 | 0.021 | -1.42 | 0.037 | -1.48 | 0.084 | -0.94 |
| 1417577_at | Trpc3 | transient receptor potential cation channel, subfamily C, member 3 | -1.2 | 0.422 | -1.51 | 0.354 | -1.31 | 0.514 | 1.51 | 0.055 | -0.63 |
| 1438066_at | Gtf2b | General transcription factor IIB | 1.34 | 0.744 | -4.49 | 0 | -1.45 | 0.312 | -1.89 | 0.228 | -1.62 |
| 1444319_at | E2f8 | E2F transcription factor 8 | -1.17 | 0.585 | -1.85 | 0.198 | -1.16 | 0.6 | 2.55 | 0.388 | -0.41 |
| 1430942_at | 8430437O03Rik | RIKEN cDNA 8430437O03 gene | -1.21 | 0.195 | -1.28 | 0.193 | -1.55 | 0.095 | 1.35 | 0.282 | -0.67 |
| 1430113_at | 4933424C13Rik | RIKEN cDNA 4933424C13 gene | -1.14 | 0.864 | -1.46 | 0.392 | -1.45 | 0.564 | 1.06 | 0.928 | -0.75 |
| 1433255_at | 8430437B07Rik | RIKEN cDNA 8430437B07 gene | -1.28 | 0.438 | -1.32 | 0.665 | -1.4 | 0.063 | 1.27 | 0.118 | -0.68 |
| 1446059_at | St6galnac5 | ST6 (alpha-N-acetyl-neuraminyl-2,3-beta-galactosyl-1,3)-N-acetylgalactosaminide | -1.23 | 0.282 | -1.5 | 0.105 | -1.29 | 0.485 | 1.14 | 0.543 | -0.72 |
| 1420186_at | --- | --- | 1.01 | 0.974 | -2.01 | 0.266 | -1.33 | 0.191 | -1.22 | 0.393 | -0.89 |
| 1452967_at | 1700006H03Rik | RIKEN cDNA 1700006H03 gene | -1.2 | 0.517 | -2.08 | 0.033 | -1.06 | 0.82 | 1.43 | 0.483 | -0.73 |
| 1458792_at | D12Ertd208e | DNA segment, Chr 12, ERATO Doi 208, expressed | -1.39 | 0.658 | -1.27 | 0.605 | -1.34 | 0.517 | 1.99 | 0.352 | -0.5 |
| 1447267_at | C85319 | expressed sequence C85319 | -1.05 | 0.874 | -1.65 | 0.115 | -1.43 | 0.255 | 1.06 | 0.864 | -0.77 |
| 1446873_at | --- | --- | -2.3 | 0.372 | 1.11 | 0.855 | -1.41 | 0.439 | -2.12 | 0.355 | -1.18 |
| 1447171_at | Nedd4l | Neural precursor cell expressed, developmentally down-regulated gene 4-like | -1.6 | 0.099 | 1 | 0.999 | -1.59 | 0.271 | -1.87 | 0.215 | -1.01 |
| 1459990_at | Ddr1 | discoidin domain receptor family, member 1 | -1.33 | 0.293 | -1.54 | 0.068 | -1.17 | 0.648 | 1.17 | 0.606 | -0.72 |
| 1447782_x_at | Tbl3 | transducin (beta)-like 3 | -1.19 | 0.415 | -2 | 0.062 | -1.09 | 0.813 | 1.8 | 0.283 | -0.62 |
| 1423977_at | 4930453N24Rik /// | RIKEN cDNA 4930453N24 gene /// similar to CG13876-PA /// similar to CG13876 | -1.01 | 0.993 | -2.34 | 0.147 | -1.2 | 0.662 | 1.22 | 0.08 | -0.83 |
| 1437660_at | Nktr | natural killer tumor recognition sequence | -1.15 | 0.528 | -1.27 | 0.157 | -1.66 | 0.016 | 1.06 | 0.501 | -0.76 |
| 1420215_x_at | 1810012K16Rik | RIKEN cDNA 1810012K16 gene | -1.98 | 0.223 | 1.14 | 0.729 | -1.63 | 0.173 | -1.62 | 0.08 | -1.02 |
| 1457212_at | --- | Transcribed locus | -2.19 | 0.395 | -2.31 | 0.374 | 1.36 | 0.5 | -1.21 | 0.818 | -1.08 |
| 1430504_at | 1700108E19Rik | RIKEN cDNA 1700108E19 gene | -1.7 | 0.414 | -1.19 | 0.579 | -1.21 | 0.501 | 1.26 | 0.58 | -0.71 |
| 1418756_at | Trh | thyrotropin releasing hormone | -1.19 | 0.324 | -1.09 | 0.891 | -2.01 | 0.337 | 1.14 | 0.346 | -0.79 |
| 1443846_x_at | Pank2 | Pantothenate kinase 2 (Hallervorden-Spatz syndrome) | -1.26 | 0.559 | -1.45 | 0.469 | -1.29 | 0.104 | 1.32 | 0.303 | -0.67 |
| 1445046_at | D8Ertd317e | DNA segment, Chr 8, ERATO Doi 317, expressed | -2.82 | 0.226 | 1 | 0.999 | -1.11 | 0.793 | -1.06 | 0.914 | -1 |
| 1458797_at | --- | --- | -3.36 | 0.041 | 1.09 | 0.799 | -1.15 | 0.782 | -1.1 | 0.732 | -1.13 |

| | | | | | | | | | | | |
|---|---|---|---|---|---|---|---|---|---|---|---|
| 1442966_at | 5330421F07Rik | RIKEN cDNA 5330421F07 gene | -1.21 | 0.766 | -2.82 | 0.184 | 1.08 | 0.831 | -1.64 | 0.341 | -1.15 |
| 1430919_at | 4930525F21Rik | RIKEN cDNA 4930525F21 gene | -1.74 | 0.451 | -1.25 | 0.686 | -1.14 | 0.618 | 2.36 | 0.16 | -0.44 |
| 1421531_at | Akap3 | A kinase (PRKA) anchor protein 3 | -3.37 | 0.022 | -1.4 | 0.184 | 1.25 | 0.571 | -2.03 | 0.306 | -1.39 |
| 1447317_at | --- | Transcribed locus | -1.8 | 0.085 | -1.55 | 0.528 | 1.06 | 0.875 | -1.16 | 0.799 | -0.86 |
| 1421503_at | Pcdh15 | protocadherin 15 | -1.7 | 0.258 | 1.02 | 0.971 | -1.54 | 0.406 | -1.39 | 0.461 | -0.9 |
| 1432712_at | 4933425M03Rik | RIKEN cDNA 4933425M03 gene | -1.62 | 0.182 | -1.4 | 0.5 | -1.08 | 0.667 | 1.32 | 0.07 | -0.69 |
| 1427509_at | Baiap3 | BAI1-associated protein 3 | -1.3 | 0.112 | -1.59 | 0.175 | -1.17 | 0.442 | 1.51 | 0.477 | -0.64 |
| 1432972_at | 4921518B13Rik | RIKEN cDNA 4921518B13 gene | -1.22 | 0.228 | -1.99 | 0.02 | -1.07 | 0.904 | 1.29 | 0.543 | -0.75 |
| 1430457_at | Daf2 | decay accelerating factor 2 | -2.79 | 0.118 | -1.08 | 0.904 | -1.03 | 0.959 | 2.62 | 0.324 | -0.57 |
| 1449457_at | Acot12 | acyl-CoA thioesterase 12 | -1.19 | 0.088 | -1.67 | 0.004 | -1.22 | 0.605 | 2.71 | 0.004 | -0.34 |
| 1446094_at | Atbf1 | AT motif binding factor 1 | -1.05 | 0.748 | -1.32 | 0.14 | -1.84 | 0.006 | 1.08 | 0.733 | -0.78 |
| 1429602_at | Cd164l2 | D164 sialomucin-like 2 | -1.26 | 0.49 | -1.54 | 0.571 | -1.23 | 0.347 | 1.31 | 0.16 | -0.68 |
| 1428238_at | 2700059D21Rik | RIKEN cDNA 2700059D21 gene | -1.59 | 0.591 | -1.34 | 0.519 | -1.14 | 0.699 | 1.5 | 0.178 | -0.64 |
| 1430644_at | Wbscr25 | Williams Beuren syndrome chromosome region 25 (human) | -2.83 | 0.323 | 1.05 | 0.87 | -1.17 | 0.69 | -1.78 | 0.414 | -1.18 |
| 1452873_at | 5830415F09Rik | RIKEN cDNA 5830415F09 gene | -1.42 | 0.351 | -1.32 | 0.108 | -1.26 | 0.396 | 1.85 | 0.316 | -0.54 |
| 1420027_at | C80171 | expressed sequence C80171 | -1.83 | 0.507 | -1.17 | 0.694 | -1.16 | 0.766 | 1.85 | 0.28 | -0.58 |
| 1450323_at | 5730493B19Rik | RIKEN cDNA 5730493B19 gene | -1.2 | 0.346 | -1.5 | 0.221 | -1.32 | 0.049 | 1.38 | 0.122 | -0.66 |
| 1421194_at | Itga4 | integrin alpha 4 | -2.85 | 0.153 | 1.09 | 0.83 | -1.22 | 0.447 | -1.04 | 0.803 | -1.01 |
| 1446340_at | Ar | Androgen receptor | -1.01 | 0.984 | -1.15 | 0.694 | -2.47 | 0.193 | 1.56 | 0.504 | -0.77 |
| 1444777_at | Rai14 | Retinoic acid induced 14 | 1.02 | 0.881 | -1.25 | 0.09 | -2.31 | 0.003 | -1.09 | 0.557 | -0.9 |
| 1455053_a_at | Dcun1d1 | DCUN1D1 DCN1, defective in cullin neddylation 1, domain containing 1 (S. cerev | -1.26 | 0.235 | -1.23 | 0.22 | -1.52 | 0.041 | 1.53 | 0.451 | -0.62 |
| 1458768_at | Epb4.1l5 | Erythrocyte protein band 4.1-like 5 | -1.05 | 0.742 | -1.55 | 0.065 | -1.51 | 0.089 | 1.15 | 0.815 | -0.74 |
| 1416872_at | Tspan6 | tetraspanin 6 | -1.19 | 0.159 | -1.37 | 0.288 | -1.45 | 0.034 | 1.26 | 0.186 | -0.69 |
| 1443625_at | Nat11 | N-acetyltransferase 11 | -1.43 | 0.289 | -1.57 | 0.071 | -1.08 | 0.802 | 1.22 | 0.123 | -0.72 |
| 1441629_at | Grip1 | Glutamate receptor interacting protein 1 | -1.19 | 0.16 | -1.19 | 0.573 | -1.73 | 0.007 | 1.61 | 0.42 | -0.62 |
| 1447096_at | --- | Transcribed locus | -1.5 | 0.011 | 1.14 | 0.602 | -2.21 | 0.009 | -1.36 | 0.153 | -0.98 |
| 1432773_at | 2610105M22Rik | RIKEN cDNA 2610105M22 gene | -1.26 | 0.413 | -2.02 | 0.191 | -1.03 | 0.951 | 3.26 | 0.002 | -0.26 |
| 1431795_a_at | Sema3b | sema domain, immunoglobulin domain (Ig), short basic domain, secreted, (semap | -1.55 | 0.316 | -1.25 | 0.698 | -1.23 | 0.376 | 1.6 | 0.286 | -0.61 |
| 1442488_at | Gpc5 | Glypican 5 | 1.18 | 0.779 | -3.59 | 0.033 | -1.24 | 0.643 | -1.4 | 0.084 | -1.26 |
| 1458509_at | Mre11a | Meiotic recombination 11 homolog A (S. cerevisiae) | -1.11 | 0.715 | -2.14 | 0.032 | -1.12 | 0.298 | 1.07 | 0.867 | -0.82 |
| 1429783_at | Pdlim5 | PDZ and LIM domain 5 | -1.53 | 0.631 | -1.84 | 0.082 | 1.06 | 0.822 | -1.39 | 0.391 | -0.92 |
| 1440114_x_at | Ankrd47 | ankyrin repeat domain 47 | 1.07 | 0.232 | -1.92 | 0.035 | -1.49 | 0.019 | -2 | 0.227 | -1.08 |
| 1420768_a_at | D11Lgp2e | DNA segment, Chr 11, Lothar Hennighausen 2, expressed | -1.99 | 0.235 | -1.39 | 0.512 | 1.04 | 0.96 | -1.39 | 0.683 | -0.93 |
| 1430701_a_at | 5730528L13Rik | RIKEN cDNA 5730528L13 gene | -1.04 | 0.78 | -1.41 | 0.434 | -1.69 | 0.2 | 1.11 | 0.719 | -0.76 |
| 1420284_at | Bat4 | HLA-B associated transcript 4 | -2.47 | 0.108 | -1.74 | 0.004 | 1.28 | 0.46 | -1.28 | 0.235 | -1.05 |
| 1447955_at | LOC670127 | similar to heterochromatin protein 1, binding protein 3 | -1.28 | 0.322 | -1.81 | 0.531 | -1.08 | 0.427 | 1.39 | 0.269 | -0.69 |
| 1450315_at | V1rd14 | vomeronasal 1 receptor, D14 | -1.55 | 0.475 | 1.04 | 0.878 | -1.73 | 0.042 | -1.05 | 0.916 | -0.82 |
| 1450301_at | H28 | histocompatibility 28 | -1.84 | 0.513 | 1.08 | 0.923 | -1.57 | 0.476 | -1.99 | 0.375 | -1.08 |
| 1442522_at | --- | --- | -1.16 | 0.534 | -1.66 | 0.256 | -1.25 | 0.501 | 1.38 | 0.334 | -0.67 |
| 1420300_at | --- | --- | -1.99 | 0.567 | -1.22 | 0.785 | -1.06 | 0.889 | 1.2 | 0.725 | -0.77 |
| 1443342_at | --- | Transcribed locus | 1.01 | 0.981 | -2.11 | 0.257 | -1.29 | 0.499 | -1.08 | 0.76 | -0.87 |
| 1418461_at | Sh3d19 | SH3 domain protein D19 | -1.38 | 0.098 | -1.51 | 0.051 | -1.14 | 0.312 | 1.26 | 0.533 | -0.69 |
| 1443307_at | Psmc2 | Proteasome (prosome, macropain) 26S subunit, ATPase 2 | 1 | 0.993 | -1.46 | 0.237 | -1.73 | 0.021 | -1.03 | 0.877 | -0.8 |
| 1457671_at | 9330120H11Rik | RIKEN cDNA 9330120H11 gene | -1.3 | 0.639 | -1.39 | 0.125 | -1.29 | 0.367 | 1.56 | 0.16 | -0.61 |
| 1454088_at | 5330411O13Rik | RIKEN cDNA 5330411O13 gene | -2.37 | 0.279 | -1.24 | 0.651 | 1.04 | 0.947 | -1.09 | 0.679 | -0.92 |
| 1435707_at | Nars2 | asparaginyl-tRNA synthetase 2 (mitochondrial)(putative) | -1.23 | 0.395 | -1.68 | 0.119 | -1.17 | 0.436 | 1.01 | 0.959 | -0.77 |
| 1444555_at | Ches1 | Checkpoint suppressor 1 | -2.02 | 0.301 | 1.24 | 0.685 | -1.89 | 0.133 | -1.57 | 0.197 | -1.06 |
| 1440416_at | Usp46 | Ubiquitin specific peptidase 46 | -1.13 | 0.643 | -1.17 | 0.546 | -1.91 | 0.059 | 1.84 | 0.248 | -0.59 |
| 1453700_s_at | 4933403O03Rik /// | RIKEN cDNA 4933403O03 gene /// similar to double homeobox, 4 | -1.19 | 0.856 | -1.9 | 0.162 | -1.11 | 0.85 | 1.07 | 0.884 | -0.78 |
| 1458368_at | Myh4 | myosin, heavy polypeptide 4, skeletal muscle | -2.1 | 0.24 | -1.4 | 0.662 | 1.08 | 0.624 | -1.51 | 0.29 | -0.98 |
| 1421535_a_at | Pde4a | phosphodiesterase 4A, cAMP specific | 1.01 | 0.973 | -2.73 | 0.011 | -1.12 | 0.385 | -1 | 0.985 | -0.96 |
| 1430711_at | 4933416E03Rik | RIKEN cDNA 4933416E03 gene | -1.49 | 0.603 | -1.38 | 0.65 | -1.15 | 0.792 | 1.56 | 0.312 | -0.61 |
| 1457485_at | Fbxw11 | F-box and WD-40 domain protein 11 | -1.15 | 0.225 | -1.27 | 0.37 | -1.63 | 0.093 | 1.1 | 0.375 | -0.74 |

| | | | | | | | | | | | |
|---|---|---|---|---|---|---|---|---|---|---|---|
| 1459152_at | C78515 | expressed sequence C78515 | -1.19 | 0.74 | -1.42 | 0.272 | -1.38 | 0.65 | 1.63 | 0.56 | -0.59 |
| 1429150_at | Ccdc77 | coiled-coil domain containing 77 | -1.57 | 0.229 | -1.28 | 0.025 | -1.18 | 0.34 | 1.54 | 0.292 | -0.62 |
| 1438703_at | Ankrd26 | ankyrin repeat domain 26 | -1.2 | 0.311 | -1.5 | 0.118 | -1.3 | 0.033 | 1.11 | 0.689 | -0.72 |
| 1460022_at | Abcb8 | ATP-binding cassette, sub-family B (MDR/TAP), member 8 | -1.13 | 0.795 | -1.53 | 0.62 | -1.37 | 0.477 | 1.63 | 0.148 | -0.6 |
| 1458885_at | --- | --- | -2.14 | 0.173 | -1.22 | 0.288 | -1.02 | 0.899 | 1.16 | 0.739 | -0.81 |
| 1424454_at | Tmem87a | transmembrane protein 87A | -1.35 | 0.736 | -2.9 | 0.176 | 1.18 | 0.698 | -2.02 | 0.362 | -1.27 |
| 1450090_at | Zfp101 | zinc finger protein 101 | -1.22 | 0.27 | -1.57 | 0.016 | -1.24 | 0.267 | 1.51 | 0.255 | -0.63 |
| 1446963_at | --- | --- | -1.06 | 0.671 | -1.42 | 0.403 | -1.62 | 0.166 | 2.61 | 0.085 | -0.37 |
| 1443678_at | --- | 10 days neonate cerebellum cDNA, RIKEN full-length enriched library, clone:B93 | -1.37 | 0.763 | -1.13 | 0.856 | -1.54 | 0.219 | 1.29 | 0.457 | -0.69 |
| 1445431_at | Stk39 | Serine/threonine kinase 39, STE20/SPS1 homolog (yeast) | -1.47 | 0.03 | -1.79 | 0.176 | 1.03 | 0.953 | -1.38 | 0.221 | -0.9 |
| 1431182_at | Hspa8 /// LOC6212 | heat shock protein 8 /// similar to heat shock protein 8 /// similar to heat shock pro | -2.1 | 0.404 | -1.35 | 0.463 | 1.05 | 0.911 | -1.24 | 0.704 | -0.91 |
| 1460540_at | Nrxn1 | neurexin I | -2.29 | 0.032 | -1.15 | 0.752 | -1.04 | 0.903 | 1.12 | 0.63 | -0.84 |
| 1438028_at | 4930535B03Rik | RIKEN cDNA 4930535B03 gene | -1.27 | 0.042 | -1.27 | 0.007 | -1.46 | 0.02 | 1.04 | 0.568 | -0.74 |
| 1458794_at | --- | --- | -1.22 | 0.464 | -1.51 | 0.307 | -1.28 | 0.653 | 2.44 | 0.043 | -0.39 |
| 1437228_at | BC048651 | cDNA sequence BC048651 | -1.45 | 0.073 | -1.22 | 0.473 | -1.31 | 0.311 | 1.12 | 0.331 | -0.72 |
| 1449081_at | Ces3 | carboxylesterase 3 | -1.45 | 0.228 | -1.36 | 0.343 | -1.18 | 0.067 | 2.62 | 0.039 | -0.34 |
| 1459631_at | Rps12 /// LOC4328 | ribosomal protein S12 /// similar to ribosomal protein S12 /// similar to ribosomal p | -3.03 | 0.193 | -1.01 | 0.982 | -1.05 | 0.824 | 1.16 | 0.775 | -0.99 |
| 1427664_at | 4930453N24Rik | RIKEN cDNA 4930453N24 gene | -2.21 | 0.296 | 1.17 | 0.723 | -1.54 | 0.399 | -1.03 | 0.968 | -0.9 |
| 1442463_at | Rab10 | RAB10, member RAS oncogene family | -1.52 | 0.303 | -1.13 | 0.746 | -1.38 | 0.297 | 1.17 | 0.777 | -0.72 |
| 1457997_at | Leng4 | leukocyte receptor cluster (LRC) member 4 | -1.84 | 0.104 | 1 | 0.996 | -1.38 | 0.099 | -1.54 | 0.079 | -0.94 |
| 1455095_at | Hist2h2be | histone 2, H2be | -1.4 | 0.236 | -1.22 | 0.241 | -1.36 | 0.082 | 1.52 | 0.375 | -0.62 |
| 1424310_at | --- | --- | -1.04 | 0.843 | -1.49 | 0.034 | -1.58 | 0.042 | 1.01 | 0.466 | -0.77 |
| 1458078_at | Chd9 | chromodomain helicase DNA binding protein 9 | 1.07 | 0.882 | -1.77 | 0.083 | -1.58 | 0.072 | -1.46 | 0.212 | -0.93 |
| 1433329_at | 4930573C08Rik | RIKEN cDNA 4930573C08 gene | -2.03 | 0.183 | -1.76 | 0.277 | 1.21 | 0.362 | -1.02 | 0.905 | -0.9 |
| 1454302_at | 5730410E19Rik | RIKEN cDNA 5730410E19 gene | -1.81 | 0.185 | -1.22 | 0.7 | -1.11 | 0.823 | 1.05 | 0.848 | -0.77 |
| 1446855_at | Vps13d | vacuolar protein sorting 13 D (yeast) | -1.57 | 0.081 | -1.34 | 0.297 | -1.13 | 0.654 | 1.01 | 0.981 | -0.76 |
| 1450692_at | Kif4 | kinesin family member 4 | -1.94 | 0.407 | -1.16 | 0.855 | -1.12 | 0.675 | 3.69 | 0.157 | -0.13 |
| 1422269_at | Insm2 | insulinoma-associated 2 | 1.13 | 0.171 | -1.79 | 0.218 | -1.72 | 0.317 | -1.57 | 0.504 | -0.99 |
| 1449604_at | Acp1 /// LOC61999 | acid phosphatase 1, soluble /// similar to acid phosphatase 1, soluble /// similar to | -2.7 | 0.071 | 1.22 | 0.769 | -1.48 | 0.329 | -1.2 | 0.612 | -1.04 |
| 1445418_at | Smg6 | Smg-6 homolog, nonsense mediated mRNA decay factor (C. elegans) | -1.17 | 0.652 | -1.66 | 0.292 | -1.23 | 0.65 | 1.01 | 0.97 | -0.76 |
| 1422728_at | Inha | inhibin alpha | 1.14 | 0.103 | -2.11 | 0.058 | -1.51 | 0.105 | -1.31 | 0.597 | -0.95 |
| 1431002_x_at | Fahd2a | fumarylacetoacetate hydrolase domain containing 2A | -1.24 | 0.279 | -1.62 | 0.087 | -1.18 | 0.307 | 1.17 | 0.249 | -0.72 |
| 1431443_at | 1810008B01Rik | RIKEN cDNA 1810008B01 gene | -1.8 | 0.113 | -1.12 | 0.776 | -1.21 | 0.308 | 1.18 | 0.221 | -0.74 |
| 1430092_at | Serac1 | serine active site containing 1 | -1.05 | 0.736 | -1.32 | 0.287 | -1.78 | 0.128 | 1.08 | 0.843 | -0.77 |
| 1453568_at | 2310032F03Rik | RIKEN cDNA 2310032F03 gene | 1.02 | 0.946 | -1.47 | 0.439 | -1.74 | 0.311 | -3.18 | 0.098 | -1.35 |
| 1447325_at | Ches1 | Checkpoint suppressor 1 | 1.11 | 0.659 | -1.38 | 0.582 | -2.28 | 0.091 | -2.03 | 0.078 | -1.15 |
| 1421171_at | Adam12 | a disintegrin and metallopeptidase domain 12 (meltrin alpha) | -1.2 | 0.198 | -1.19 | 0.792 | -1.68 | 0.264 | 1.46 | 0.58 | -0.65 |
| 1425955_at | Cav2 | caveolin 2 | -1.12 | 0.7 | -1.17 | 0.582 | -1.92 | 0.026 | 1.15 | 0.532 | -0.76 |
| 1422169_a_at | Bdnf | brain derived neurotrophic factor | -1.24 | 0.489 | -1.27 | 0.733 | -1.48 | 0.13 | 1.49 | 0.505 | -0.62 |
| 1430432_at | Trim42 | tripartite motif-containing 42 | -1.01 | 0.989 | -1.2 | 0.757 | -2.24 | 0.068 | 1.51 | 0.479 | -0.73 |
| 1429051_s_at | Sox11 | SRY-box containing gene 11 | -1.03 | 0.905 | -1.25 | 0.663 | -2 | 0.081 | 1.46 | 0.561 | -0.7 |
| 1438305_at | Rims1 | regulating synaptic membrane exocytosis 1 | -2.21 | 0.164 | -1.15 | 0.824 | -1.05 | 0.855 | 2.07 | 0.098 | -0.59 |
| 1420270_at | --- | --- | -1.81 | 0.045 | -1.27 | 0.589 | -1.07 | 0.733 | 1.25 | 0.6 | -0.72 |
| 1439895_at | AU021025 | expressed sequence AU021025 | -1.33 | 0.137 | -1.09 | 0.627 | -1.68 | 0.023 | 1.09 | 0.569 | -0.75 |
| 1425075_at | Gatad2b | GATA zinc finger domain containing 2B | -1.39 | 0.22 | -1.28 | 0.638 | -1.3 | 0.305 | 1.36 | 0.251 | -0.65 |
| 1442527_at | C87583 | expressed sequence C87583 | -2.26 | 0.367 | -1.72 | 0.027 | 1.25 | 0.569 | -2.45 | 0.202 | -1.3 |
| 1457084_at | Nalp9b | NACHT, LRR and PYD containing protein 9b | -1.79 | 0.559 | -1.47 | 0.348 | 1.03 | 0.945 | -2.38 | 0.152 | -1.15 |
| 1440797_at | Dlx6os2 | Dlx6 opposite strand transcript 2 | -1.45 | 0.504 | -2.05 | 0.145 | 1.09 | 0.624 | -1.02 | 0.954 | -0.86 |
| 1431967_at | 1700006A11Rik | RIKEN cDNA 1700006A11 gene | -1 | 0.993 | -1.32 | 0.735 | -1.94 | 0.38 | 1.26 | 0.655 | -0.75 |
| 1455967_at | Sorbs1 | sorbin and SH3 domain containing 1 | -1.16 | 0.432 | -1.39 | 0.236 | -1.45 | 0.202 | 1.65 | 0.22 | -0.59 |
| 1430674_at | 1700016C15Rik | RIKEN cDNA 1700016C15 gene | 1.11 | 0.78 | -1.71 | 0.486 | -1.73 | 0.345 | -1.51 | 0.337 | -0.96 |
| 1419734_at | Actb | actin, beta, cytoplasmic | -1.04 | 0.953 | -1.07 | 0.854 | -2.69 | 0.375 | 1.89 | 0.108 | -0.73 |
| 1447822_x_at | 2700038N03Rik | RIKEN cDNA 2700038N03 gene | -1.56 | 0.031 | -1.37 | 0.223 | -1.11 | 0.496 | 2.06 | 0.16 | -0.5 |

| | | | | | | | | | | | |
|---|---|---|---|---|---|---|---|---|---|---|---|
| 1442852_at | --- | --- | -1.31 | 0.623 | -1.33 | 0.312 | -1.32 | 0.464 | 1.08 | 0.813 | -0.72 |
| 1455730_at | Dlg7 | discs, large homolog 7 (Drosophila) | -1.06 | 0.929 | -1.9 | 0.208 | -1.25 | 0.621 | 4.72 | 0.191 | 0.13 |
| 1443472_at | C77691 | expressed sequence C77691 | 1.23 | 0.844 | -2.18 | 0.033 | -1.71 | 0.247 | -1.41 | 0.299 | -1.02 |
| 1451685_at | Mllt6 /// LOC63949( | myeloid/lymphoid or mixed lineage-leukemia translocation to 6 homolog (Drosoph | -1.71 | 0.008 | -1.18 | 0.763 | -1.19 | 0.125 | 1.46 | 0.382 | -0.65 |
| 1432392_at | Kif2c | kinesin family member 2C | -1.2 | 0.645 | -1.54 | 0.089 | -1.27 | 0.524 | 1.44 | 0.276 | -0.64 |
| 1417682_a_at | Prss2 | protease, serine, 2 | -1.8 | 0.187 | -1.78 | 0.2 | 1.16 | 0.693 | -1.22 | 0.523 | -0.91 |
| 1441651_at | 4930414N06Rik | RIKEN cDNA 4930414N06 gene | -1.18 | 0.205 | -1.15 | 0.598 | -1.79 | 0.121 | 1.13 | 0.671 | -0.75 |
| 1427339_at | Slc30a2 | solute carrier family 30 (zinc transporter), member 2 | -1.24 | 0.232 | -1.62 | 0.371 | -1.17 | 0.69 | 1.16 | 0.19 | -0.72 |
| 1444981_at | Punc | Putative neuronal cell adhesion molecule | -1.49 | 0.144 | -1.25 | 0.308 | -1.25 | 0.29 | 1.02 | 0.716 | -0.74 |
| 1426887_at | Nudt11 | nudix (nucleoside diphosphate linked moiety X)-type motif 11 | -1.44 | 0.261 | -1.15 | 0.748 | -1.41 | 0.111 | 1.19 | 0.246 | -0.7 |
| 1424030_at | Grhl1 | grainyhead-like 1 (Drosophila) | -1.61 | 0.25 | -1.83 | 0.426 | 1.11 | 0.344 | -1.18 | 0.759 | -0.88 |
| 1445673_at | 2900052N01Rik | RIKEN cDNA 2900052N01 gene | -2.28 | 0.33 | -1.23 | 0.715 | 1.02 | 0.95 | -1.73 | 0.136 | -1.05 |
| 1456746_a_at | Cd99l2 | Cd99 antigen-like 2 | -1.13 | 0.571 | -1.27 | 0.11 | -1.66 | 0.114 | 1.35 | 0.145 | -0.68 |
| 1427634_at | Kcnh7 | potassium voltage-gated channel, subfamily H (eag-related), member 7 | -1.67 | 0.002 | -1.01 | 0.993 | -1.47 | 0.305 | 1.94 | 0.013 | -0.55 |
| 1433948_at | Pla2g1b | Phospholipase A2, group IB, pancreas | -2.85 | 0.211 | -1.04 | 0.952 | -1.04 | 0.945 | 1.16 | 0.677 | -0.94 |
| 1449639_at | 0610040J01Rik | RIKEN cDNA 0610040J01 gene | -1.29 | 0.436 | -1.6 | 0.088 | -1.15 | 0.397 | 1.1 | 0.835 | -0.73 |
| 1426136_x_at | Klra21 | killer cell lectin-like receptor subfamily A, member 21 | -2.69 | 0.154 | 1.11 | 0.351 | -1.27 | 0.305 | -1.07 | 0.89 | -0.98 |
| 1449854_at | Nr0b2 | nuclear receptor subfamily 0, group B, member 2 | -1.24 | 0.231 | -1.61 | 0.039 | -1.18 | 0.725 | 2.11 | 0.29 | -0.48 |
| 1432133_at | 2210022J03Rik | RIKEN cDNA 2210022J03 gene | -1.47 | 0.528 | -1.79 | 0.183 | 1.04 | 0.924 | -1.49 | 0.35 | -0.93 |
| 1453084_s_at | Col22a1 | collagen, type XXII, alpha 1 | -1.66 | 0.007 | -1.73 | 0.285 | 1.1 | 0.584 | -1.18 | 0.841 | -0.87 |
| 1436287_at | --- | 13 days embryo head cDNA, RIKEN full-length enriched library, clone:3110033I2 | 1.06 | 0.738 | -1.6 | 0.384 | -1.68 | 0.228 | -1.71 | 0.315 | -0.98 |
| 1416444_at | Elovl2 | elongation of very long chain fatty acids (FEN1/Elo2, SUR4/Elo3, yeast)-like 2 | -1.22 | 0.098 | -1.56 | 0.014 | -1.23 | 0.669 | 1.45 | 0.429 | -0.64 |
| 1419325_at | Nmu | neuromedin U | -1.01 | 0.662 | -1.62 | 0.049 | -1.48 | 0.171 | 1.35 | 0.172 | -0.69 |
| 1459757_x_at | Cnot10 | CCR4-NOT transcription complex, subunit 10 | -1.53 | 0.285 | -1.27 | 0.511 | -1.2 | 0.325 | 1.02 | 0.92 | -0.74 |
| 1450749_a_at | Nr4a2 | nuclear receptor subfamily 4, group A, member 2 | -1.03 | 0.662 | -2.13 | 0.031 | -1.19 | 0.409 | 1.32 | 0.545 | -0.76 |
| 1452345_at | Lmod2 | leiomodin 2 (cardiac) | -3.5 | 0.342 | -1.02 | 0.964 | 1.01 | 0.921 | -1.42 | 0.378 | -1.23 |
| 1432563_at | 1700116B05Rik | RIKEN cDNA 1700116B05 gene | -1.16 | 0.588 | -2.58 | 0.212 | 1.02 | 0.87 | -1.26 | 0.59 | -0.99 |
| 1458852_at | --- | --- | -1.98 | 0.019 | -1.68 | 0.055 | 1.18 | 0.445 | -1.61 | 0.121 | -1.03 |
| 1439734_at | Mmp15 | Matrix metallopeptidase 15 | -1.32 | 0.516 | -1.92 | 0.15 | -1 | 0.991 | 1.09 | 0.782 | -0.79 |
| 1459308_at | Kcnn3 | Potassium intermediate/small conductance calcium-activated channel, subfamily | -1.3 | 0.441 | -1.48 | 0.283 | -1.21 | 0.46 | 2 | 0.047 | -0.49 |
| 1441450_s_at | Jarid1c | jumonji, AT rich interactive domain 1C (Rbp2 like) | -1.18 | 0.371 | -1.4 | 0.22 | -1.4 | 0.207 | 1.18 | 0.452 | -0.7 |
| 1447295_at | Cutl1 | Cut-like 1 (Drosophila) | -1.39 | 0.042 | -1.17 | 0.293 | -1.42 | 0.247 | 1.05 | 0.778 | -0.73 |
| 1446610_at | Elmo1 | engulfment and cell motility 1, ced-12 homolog (C. elegans) | -3 | 0.179 | 1.35 | 0.432 | -1.68 | 0.344 | -1.84 | 0.282 | -1.29 |
| 1430953_at | 3200001G23Rik | RIKEN cDNA 3200001G23 gene | 1 | 0.973 | -1.05 | 0.792 | -3.08 | 0.134 | -1.45 | 0.02 | -1.15 |
| 1424897_at | Gpr85 | G protein-coupled receptor 85 | -1.2 | 0.497 | -2.28 | 0.025 | 1 | 0.988 | -1.11 | 0.836 | -0.9 |
| 1432626_at | 5730507A11Rik | RIKEN cDNA 5730507A11 gene | -1.44 | 0.145 | 1.06 | 0.832 | -1.92 | 0.024 | -1.4 | 0.449 | -0.92 |
| 1428965_at | Ccdc54 | coiled-coil domain containing 54 | -1.24 | 0.117 | -1.31 | 0.683 | -1.42 | 0.003 | 1.2 | 0.472 | -0.69 |
| 1458035_at | Sgpp2 | Sphingosine-1-phosphate phosphotase 2 | -1.55 | 0.2 | -1.15 | 0.853 | -1.32 | 0.381 | 1.36 | 0.588 | -0.66 |
| 1438269_at | Zbtb38 | zinc finger and BTB domain containing 38 | -1.83 | 0.22 | -1.04 | 0.892 | -1.31 | 0.452 | 1.26 | 0.345 | -0.73 |
| 1440472_at | Spag17 | sperm associated antigen 17 | -1.24 | 0.67 | -1.44 | 0.598 | -1.28 | 0.431 | 1.08 | 0.867 | -0.72 |
| 1447036_at | Chl1 | cell adhesion molecule with homology to L1CAM | -1.31 | 0.334 | -1.27 | 0.464 | -1.38 | 0.47 | 1.33 | 0.442 | -0.66 |
| 1440375_at | 5730419I09Rik | RIKEN cDNA 5730419I09 gene | -1.04 | 0.891 | -1.41 | 0.504 | -1.63 | 0.098 | 1.22 | 0.37 | -0.72 |
| 1422652_at | Cryga | crystallin, gamma A | -1.39 | 0.023 | -1.09 | 0.843 | -1.56 | 0.209 | 2.19 | 0.085 | -0.46 |
| 1424699_at | 4921511K06Rik | RIKEN cDNA 4921511K06 gene | 1.14 | 0.721 | -2.56 | 0.411 | -1.33 | 0.316 | -1.19 | 0.15 | -0.99 |
| 1423360_at | Yme1l1 | YME1-like 1 (S. cerevisiae) | -1.28 | 0.292 | -1.21 | 0.164 | -1.49 | 0.059 | 1.05 | 0.839 | -0.73 |
| 1423849_a_at | Clk3 | CDC-like kinase 3 | -1.31 | 0.105 | -1.28 | 0.033 | -1.36 | 0.202 | 1.3 | 0.442 | -0.66 |
| 1426622_a_at | Qpct /// LOC639942 | glutaminyl-peptide cyclotransferase (glutaminyl cyclase) /// similar to Glutaminyl-p | 1.1 | 0.848 | -1.69 | 0.006 | -1.69 | 0.052 | -1.13 | 0.437 | -0.85 |
| 1457714_at | 2310005N03Rik | RIKEN cDNA 2310005N03 gene | -3.19 | 0.25 | -1.09 | 0.907 | 1.05 | 0.876 | -2.4 | 0.287 | -1.41 |
| 1437424_at | Syde2 /// LOC6396 | synapse defective 1, Rho GTPase, homolog 2 (C. elegans) /// similar to synapse | -1.28 | 0.219 | -1.52 | 0.244 | -1.2 | 0.339 | 1.27 | 0.447 | -0.68 |
| 1435043_at | Plcb1 | phospholipase C, beta 1 | -1.11 | 0.343 | -1.17 | 0.315 | -1.89 | 0.067 | 1.24 | 0.415 | -0.73 |
| 1420595_at | Dlx4 | distal-less homeobox 4 | -1.53 | 0.017 | -1.46 | 0.047 | -1.06 | 0.789 | 1.01 | 0.974 | -0.76 |
| 1454223_at | 4933423P22Rik | RIKEN cDNA 4933423P22 gene | -1.48 | 0.225 | -1.37 | 0.086 | -1.14 | 0.379 | 1.11 | 0.676 | -0.72 |
| 1420848_at | Sufu | suppressor of fused homolog (Drosophila) | -1.83 | 0.131 | -1.39 | 0.472 | 1.01 | 0.964 | -1.11 | 0.293 | -0.83 |

| | | | | | | | | | | | |
|---|---|---|---|---|---|---|---|---|---|---|---|
| 1453122_at | 4921533L14Rik | RIKEN cDNA 4921533L14 gene | -1.17 | 0.094 | -1.31 | 0.082 | -1.52 | 0.031 | 1.16 | 0.639 | -0.71 |
| 1417678_at | Mmp24 | matrix metallopeptidase 24 | -1.43 | 0.618 | -1.32 | 0.472 | -1.22 | 0.421 | 1.06 | 0.79 | -0.73 |
| 1430494_at | 2010005J08Rik | RIKEN cDNA 2010005J08 gene | -1.24 | 0.505 | -1.52 | 0.002 | -1.22 | 0.55 | 1 | 0.984 | -0.75 |
| 1447854_s_at | Hist2h2be | histone 2, H2be | -1.33 | 0.177 | -1.2 | 0.486 | -1.43 | 0.008 | 1.33 | 0.426 | -0.66 |
| 1420151_at | --- | --- | 1.26 | 0.679 | -1.56 | 0.433 | -2.66 | 0.045 | -1.18 | 0.608 | -1.03 |
| 1425389_a_at | Runx2 | runt related transcription factor 2 | -1.54 | 0.443 | -1.29 | 0.449 | -1.17 | 0.318 | 1.43 | 0.435 | -0.64 |
| 1419663_at | Ogn | osteoglycin | 1.04 | 0.829 | -2.12 | 0.015 | -1.29 | 0.076 | -1.6 | 0.383 | -0.99 |
| 1426081_a_at | Dio2 | deiodinase, iodothyronine, type II | -1.46 | 0.078 | -1.06 | 0.878 | -1.54 | 0.372 | 1.41 | 0.512 | -0.66 |
| 1458884_at | --- | --- | 1.08 | 0.85 | -1.32 | 0.61 | -2.26 | 0.001 | -1.18 | 0.667 | -0.92 |
| 1431536_at | 4933432K03Rik | RIKEN cDNA 4933432K03 gene | -1.17 | 0.855 | -1.41 | 0.524 | -1.4 | 0.357 | 1.35 | 0.716 | -0.66 |
| 1443234_at | BC088983 | CDNA sequence BC088983 | -1.07 | 0.749 | -1.35 | 0.502 | -1.65 | 0.011 | 1.1 | 0.75 | -0.74 |
| 1446173_at | --- | --- | -2.01 | 0.039 | -1.85 | 0.25 | 1.24 | 0.548 | -1.85 | 0.372 | -1.12 |
| 1445634_at | Mapt | Microtubule-associated protein tau | -1.38 | 0.14 | -1.22 | 0.389 | -1.36 | 0.427 | 1.09 | 0.728 | -0.72 |
| 1435301_at | 1110004E09Rik | RIKEN cDNA 1110004E09 gene | -1.12 | 0.39 | -1.7 | 0.443 | -1.24 | 0.248 | 1.28 | 0.371 | -0.7 |
| 1420061_s_at | AA675344 | expressed sequence AA675344 | -1.05 | 0.9 | -1.46 | 0.468 | -1.55 | 0.302 | 1.33 | 0.406 | -0.68 |
| 1430721_at | 4931431C16Rik | RIKEN cDNA 4931431C16 gene | 1.45 | 0.424 | -2.43 | 0.185 | -2.39 | 0.073 | -1.04 | 0.951 | -1.1 |
| 1439245_at | Tnrc6a | trinucleotide repeat containing 6a | -1.34 | 0.419 | -1.31 | 0.275 | -1.29 | 0.438 | 1.11 | 0.678 | -0.71 |
| 1445804_at | 9630020C08Rik | RIKEN cDNA 9630020C08 gene | -1.14 | 0.486 | -1.67 | 0.532 | -1.24 | 0.59 | 1.02 | 0.977 | -0.76 |
| 1459568_at | Tmtc4 | Transmembrane and tetratricopeptide repeat containing 4 | 1.23 | 0.303 | -1.72 | 0.294 | -2.09 | 0.021 | -1.61 | 0.182 | -1.05 |
| 1438166_x_at | Ndufs4 | NADH dehydrogenase (ubiquinone) Fe-S protein 4 | -1.65 | 0.182 | -1.26 | 0.033 | -1.13 | 0.445 | 1.02 | 0.938 | -0.76 |
| 1423496_a_at | Punc | putative neuronal cell adhesion molecule | -1.42 | 0.524 | -1.09 | 0.807 | -1.52 | 0.174 | 1.07 | 0.752 | -0.74 |
| 1440788_at | Gas8 | Growth arrest specific 8 | -2.36 | 0.119 | -2.67 | 0.192 | 1.49 | 0.272 | -1.05 | 0.921 | -1.15 |
| 1431111_at | Eno1 /// LOC23894 | enolase 1, alpha non-neuron /// similar to enolase 1, alpha non-neuron /// similar t | 1.01 | 0.96 | -1.8 | 0.029 | -1.38 | 0.494 | -1.2 | 0.448 | -0.84 |
| 1459328_at | 4930429A22Rik | RIKEN cDNA 4930429A22 gene | -1.43 | 0.678 | -1.53 | 0.462 | -1.07 | 0.894 | 1.23 | 0.647 | -0.7 |
| 1439535_at | --- | --- | -1.28 | 0.197 | -1.06 | 0.872 | -1.79 | 0.042 | 1.27 | 0.492 | -0.72 |
| 1458672_at | Tcte2 | T-complex-associated testis expressed 2 | -1.99 | 0.327 | -1.1 | 0.741 | -1.15 | 0.456 | 1.09 | 0.778 | -0.79 |
| 1438251_x_at | Htra1 | HtrA serine peptidase 1 | -1.17 | 0.093 | -2.87 | 0.027 | 1.08 | 0.764 | -1.71 | 0.218 | -1.17 |
| 1456049_at | Rala | v-ral simian leukemia viral oncogene homolog A (ras related) | 1.08 | 0.912 | -1.6 | 0.481 | -1.71 | 0.276 | -1.9 | 0.315 | -1.03 |
| 1426153_a_at | Dsg2 | desmoglein 2 | -1.42 | 0.503 | -2.7 | 0.4 | 1.21 | 0.775 | -1.36 | 0.033 | -1.07 |
| 1455961_at | --- | --- | 1.01 | 0.949 | -2.14 | 0.099 | -1.23 | 0.067 | -1.43 | 0.158 | -0.95 |
| 1437781_at | --- | Transcribed locus | -1.3 | 0.183 | 1 | 0.996 | -1.94 | 0.051 | -2.16 | 0.533 | -1.1 |
| 1442389_at | Oas1f | 2'-5' oligoadenylate synthetase 1F | -1.62 | 0.267 | -1.81 | 0.252 | 1.11 | 0.889 | -1.56 | 0.323 | -0.97 |
| 1444593_at | Phlppl | PH domain and leucine rich repeat protein phosphatase-like | -1.35 | 0.589 | -1.08 | 0.867 | -1.6 | 0.082 | 1.61 | 0.196 | -0.61 |
| 1453727_at | 2610101J03Rik | RIKEN cDNA 2610101J03 gene | -1.49 | 0.288 | -1.36 | 0.193 | -1.14 | 0.202 | 1.09 | 0.77 | -0.72 |
| 1447670_at | Psmd9 | proteasome (prosome, macropain) 26S subunit, non-ATPase, 9 | -1.34 | 0.531 | -1.18 | 0.486 | -1.44 | 0.105 | 1.04 | 0.907 | -0.73 |
| 1446918_at | Lphn3 | Latrophilin 3 | -2.98 | 0.022 | 1.28 | 0.008 | -1.49 | 0.269 | -1.42 | 0.245 | -1.15 |
| 1456315_a_at | Ptpla | protein tyrosine phosphatase-like (proline instead of catalytic arginine), member a | -1.4 | 0.003 | -1.16 | 0.126 | -1.41 | 0.079 | 2.69 | 0.254 | -0.32 |
| 1429274_at | 2310010M24Rik | RIKEN cDNA 2310010M24 gene | 1.13 | 0.625 | -1.91 | 0.323 | -1.59 | 0.24 | -2.44 | 0.02 | -1.2 |
| 1441030_at | Rai14 | retinoic acid induced 14 | -1.16 | 0.686 | 1.01 | 0.968 | -2.43 | 0.006 | -1.78 | 0.099 | -1.09 |
| 1439652_at | --- | --- | 1.01 | 0.962 | -1.25 | 0.022 | -2.08 | 0.03 | -1.73 | 0.113 | -1.01 |
| 1457534_at | Ubap2l | Ubiquitin associated protein 2-like | 1.02 | 0.934 | -1.44 | 0.214 | -1.74 | 0.014 | -1.02 | 0.946 | -0.8 |
| 1449080_at | Hdac2 | histone deacetylase 2 | -1.24 | 0.287 | -1.24 | 0.256 | -1.48 | 0.043 | 1.01 | 0.957 | -0.74 |
| 1442843_at | 4933411D12Rik | RIKEN cDNA 4933411D12 gene | -1.34 | 0.029 | -1.1 | 0.527 | -1.6 | 0.02 | 2.42 | 0.24 | -0.4 |
| 1441042_at | Fgf1 | fibroblast growth factor 1 | -1.1 | 0.498 | -1.31 | 0.155 | -1.63 | 0.022 | 1.03 | 0.963 | -0.75 |
| 1436328_at | Rnmt | RNA (guanine-7-) methyltransferase | -1.12 | 0.41 | -1.39 | 0.146 | -1.47 | 0.05 | 1.02 | 0.848 | -0.74 |
| 1440677_at | LOC245305 | similar to hypothetical protein FLJ38281 | -1.8 | 0.511 | -1.6 | 0.305 | 1.11 | 0.784 | -1.11 | 0.684 | -0.85 |
| 1434915_s_at | Lrrc19 | leucine rich repeat containing 19 | -1.41 | 0.189 | -1.38 | 0.622 | -1.17 | 0.761 | 1.14 | 0.854 | -0.71 |
| 1434772_at | Adora2b | adenosine A2b receptor | -1.67 | 0.35 | -1.26 | 0.727 | -1.12 | 0.589 | 1.32 | 0.703 | -0.68 |
| 1449919_at | Krtap6-2 | keratin associated protein 6-2 | -1 | 0.998 | -1.25 | 0.65 | -2.04 | 0.245 | 1.5 | 0.442 | -0.7 |
| 1448186_at | Pnliprp2 | pancreatic lipase-related protein 2 | -1.42 | 0.206 | -1.39 | 0.393 | -1.15 | 0.712 | 1.22 | 0.676 | -0.69 |
| 1434197_at | Atrn | attractin | -1.46 | 0.038 | -1.37 | 0.004 | -1.14 | 0.438 | 1.22 | 0.531 | -0.69 |
| 1421782_a_at | Smr2 | submaxillary gland androgen regulated protein 2 | -1.56 | 0.25 | -1.21 | 0.628 | -1.22 | 0.641 | 1.01 | 0.989 | -0.74 |
| 1433270_at | 9530004M16Rik | RIKEN cDNA 9530004M16 gene | -1.2 | 0.026 | -1.43 | 0.054 | -1.32 | 0.598 | 1.09 | 0.902 | -0.71 |

| | | | | | | | | | | | |
|---|---|---|---|---|---|---|---|---|---|---|---|
| 1456831_at | Arid5b | AT rich interactive domain 5B (Mrf1 like) | 1 | 0.985 | -1.83 | 0.12 | -1.36 | 0.281 | -1.87 | 0.015 | -1.01 |
| 1457131_at | Fry | furry homolog (Drosophila) | -2.44 | 0.084 | -1.99 | 0.332 | 1.38 | 0.253 | -1.06 | 0.848 | -1.03 |
| 1450239_at | Glra3 | glycine receptor, alpha 3 subunit | -3.08 | 0.451 | 1.13 | 0.682 | -1.2 | 0.254 | -1.37 | 0.638 | -1.13 |
| 1441736_at | Itfg1 | Integrin alpha FG-GAP repeat containing 1 | -1.27 | 0.239 | -1.24 | 0.506 | -1.44 | 0.105 | 1.11 | 0.551 | -0.71 |
| 1431427_at | 5430427M07Rik | RIKEN cDNA 5430427M07 gene | -1.53 | 0.483 | -1.22 | 0.679 | -1.23 | 0.274 | 1.2 | 0.768 | -0.69 |
| 1446878_at | Lonrf2 | LON peptidase N-terminal domain and ring finger 2 | 1 | 0.997 | -2.07 | 0.297 | -1.25 | 0.496 | -1.31 | 0.569 | -0.91 |
| 1441883_at | 0610010O12Rik | RIKEN cDNA 0610010O12 gene | -1.05 | 0.669 | -1.92 | 0.014 | -1.22 | 0.075 | 2.58 | 0.227 | -0.4 |
| 1438139_at | Arhgap28 | Rho GTPase activating protein 28 | -1.68 | 0.476 | -1.85 | 0.093 | 1.15 | 0.595 | -1.67 | 0.491 | -1.01 |
| 1453227_at | Rhobtb3 | Rho-related BTB domain containing 3 | -1.25 | 0.464 | -1.43 | 0.263 | -1.26 | 0.627 | 2.89 | 0.001 | -0.26 |
| 1458423_at | Luc7l2 | LUC7-like 2 (S. cerevisiae) | 1.14 | 0.859 | -2.22 | 0.31 | -1.42 | 0.105 | -2.2 | 0.237 | -1.18 |
| 1424228_at | Polr3h | polymerase (RNA) III (DNA directed) polypeptide H | -1.25 | 0.623 | -1.52 | 0.273 | -1.21 | 0.129 | 1.07 | 0.871 | -0.73 |
| 1419429_at | Cntfr | ciliary neurotrophic factor receptor | -2.18 | 0.001 | -1.76 | 0.113 | 1.26 | 0.627 | -1.09 | 0.701 | -0.94 |
| 1444434_at | Wdr33 | WD repeat domain 33 | -1.15 | 0.688 | -2.08 | 0.262 | -1.06 | 0.92 | 1.53 | 0.318 | -0.69 |
| 1450263_at | Pcdhb5 | protocadherin beta 5 | -1.78 | 0.28 | -1.27 | 0.498 | -1.06 | 0.911 | 2.19 | 0.19 | -0.48 |
| 1434030_at | BC034507 | cDNA sequence BC034507 | -1.16 | 0.136 | -1.46 | 0.193 | -1.35 | 0.024 | 1.03 | 0.862 | -0.73 |
| 1459880_at | Hnrpab | heterogeneous nuclear ribonucleoprotein A/B | -1.08 | 0.864 | -1.96 | 0.017 | -1.17 | 0.619 | 1.59 | 0.124 | -0.65 |
| 1454129_at | 4933416M06Rik | RIKEN cDNA 4933416M06 gene | -1.08 | 0.172 | -1.59 | 0.244 | -1.36 | 0.593 | 1.09 | 0.887 | -0.74 |
| 1442102_at | Aco2 | Aconitase 2, mitochondrial | -1.16 | 0.544 | -1.43 | 0.316 | -1.36 | 0.062 | 1.09 | 0.692 | -0.72 |
| 1441534_at | C86753 | expressed sequence C86753 | -2.41 | 0.051 | 1.06 | 0.754 | -1.23 | 0.749 | -1.08 | 0.857 | -0.91 |
| 1449611_at | Cd82 | CD82 antigen | -2.8 | 0.143 | 1.06 | 0.852 | -1.14 | 0.726 | -1.22 | 0.577 | -1.03 |
| 1458953_at | D5Ertd215e | DNA segment, Chr 5, ERATO Doi 215, expressed | -1.36 | 0.424 | -2.08 | 0.496 | 1.07 | 0.902 | -1.11 | 0.665 | -0.87 |
| 1435489_at | LOC552912 | hypothetical LOC552912 | -1.14 | 0.679 | -1.96 | 0.139 | -1.11 | 0.711 | 1.05 | 0.778 | -0.79 |
| 1444896_at | --- | --- | -2.01 | 0.206 | -1.27 | 0.601 | 1.01 | 0.99 | -1.05 | 0.914 | -0.83 |
| 1456286_at | Cog3 | Component of oligomeric golgi complex 3 | -1.37 | 0.015 | -1.19 | 0.195 | -1.39 | 0.126 | 1.03 | 0.898 | -0.73 |
| 1429096_at | 2810455D13Rik | RIKEN cDNA 2810455D13 gene | -1.32 | 0.315 | -1.18 | 0.42 | -1.45 | 0.202 | 1.21 | 0.519 | -0.69 |
| 1420421_s_at | Klrb1b | killer cell lectin-like receptor subfamily B member 1B | -1.07 | 0.933 | -1.02 | 0.864 | -2.62 | 0.012 | 1.22 | 0.405 | -0.87 |
| 1435282_at | Gm967 | gene model 967, (NCBI) | -1.2 | 0.255 | -1.6 | 0.016 | -1.2 | 0.268 | 1.2 | 0.435 | -0.7 |
| 1439315_at | LOC628919 | Similar to shroom | -1.69 | 0.397 | 1.11 | 0.78 | -1.69 | 0.374 | -2.42 | 0.209 | -1.17 |
| 1432785_at | A930028O11Rik | RIKEN cDNA A930028O11 gene | 1.28 | 0.372 | -2.73 | 0.277 | -1.55 | 0.387 | -1.36 | 0.201 | -1.09 |
| 1460137_at | Fgf12 | Fibroblast growth factor 12 | -2.36 | 0.411 | -1.09 | 0.851 | -1.05 | 0.891 | 1.78 | 0.4 | -0.68 |
| 1445970_at | AU022526 | expressed sequence AU022526 | -1.5 | 0.561 | -1.62 | 0.329 | 1.01 | 0.988 | -1.13 | 0.775 | -0.81 |
| 1438752_at | A230058F20Rik | RIKEN cDNA A230058F20 gene | -1.4 | 0.004 | 1.18 | 0.744 | -2.5 | 0.243 | -1.23 | 0.511 | -0.99 |
| 1438471_at | Wtap | Wilms' tumour 1-associating protein | 1.01 | 0.977 | -1.52 | 0.5 | -1.6 | 0.039 | -1.24 | 0.578 | -0.84 |
| 1417838_at | Ssty2 /// LOC38213 | spermiogenesis specific transcript on the Y 2 /// similar to RIKEN cDNA 1700029I | -1.37 | 0.714 | -2.26 | 0.169 | 1.12 | 0.616 | -1.25 | 0.64 | -0.94 |
| 1455931_at | Chrna3 | cholinergic receptor, nicotinic, alpha polypeptide 3 | -2.13 | 0.498 | -1.53 | 0.37 | 1.17 | 0.457 | -1.09 | 0.666 | -0.89 |
| 1446443_at | B230213L16Rik | RIKEN cDNA B230213L16 gene | -1.39 | 0.124 | -1.35 | 0.102 | -1.2 | 0.471 | 1.04 | 0.781 | -0.73 |
| 1445185_at | Ttll10 | tubulin tyrosine ligase-like family, member 10 | -2.44 | 0.525 | -1.92 | 0.301 | 1.36 | 0.313 | -1.05 | 0.897 | -1.01 |
| 1429109_at | Msl2l1 | male-specific lethal 2-like 1 (Drosophila) | -1.73 | 0.536 | -1.56 | 0.385 | 1.07 | 0.858 | -1.22 | 0.747 | -0.86 |
| 1436835_at | Crk | v-crk sarcoma virus CT10 oncogene homolog (avian) | -1.15 | 0.844 | -1.38 | 0.133 | -1.44 | 0.286 | 1.25 | 0.091 | -0.68 |
| 1450484_a_at | Tyki | thymidylate kinase family LPS-inducible member | -1.36 | 0.19 | -1.48 | 0.137 | -1.13 | 0.428 | 1.64 | 0.292 | -0.58 |
| 1436653_at | 4833401D15Rik | RIKEN cDNA 4833401D15 gene | -1.73 | 0.559 | -1.59 | 0.204 | 1.09 | 0.661 | -1.21 | 0.432 | -0.86 |
| 1459180_at | --- | Transcribed locus | -1.39 | 0.719 | -2.39 | 0.071 | 1.16 | 0.704 | -1.21 | 0.607 | -0.96 |
| 1441379_at | AU045094 | expressed sequence AU045094 | 1.44 | 0.373 | -2.51 | 0.219 | -2.18 | 0.323 | -2.68 | 0.272 | -1.48 |
| 1453046_at | LOC623215 | similar to diacylglycerol kinase, eta isoform 2 | -2.63 | 0.292 | -1.03 | 0.918 | -1.06 | 0.913 | 1.91 | 0.355 | -0.7 |
| 1451677_at | Narf | nuclear prelamin A recognition factor | -1.11 | 0.704 | -1.18 | 0.594 | -1.83 | 0.374 | 3.93 | 0.287 | -0.05 |
| 1418366_at | Hist2h3c1 /// Hist2h | histone 2, H3c1 /// histone 2, H2aa1 /// histone 1, H2ad /// histone 1, H2an /// histo | -1.37 | 0.295 | -1.39 | 0.152 | -1.19 | 0.319 | 1.34 | 0.518 | -0.65 |
| 1440112_at | Lrrtm3 | leucine rich repeat transmembrane neuronal 3 | -1.32 | 0.765 | -1.54 | 0.128 | -1.13 | 0.813 | 2.09 | 0.029 | -0.47 |
| 1424499_s_at | 5730596K20Rik | RIKEN cDNA 5730596K20 gene | -1.12 | 0.271 | -1.9 | 0.147 | -1.14 | 0.185 | 1.37 | 0.381 | -0.7 |
| 1456416_at | Cbfa2t2h | core-binding factor, runt domain, alpha subunit 2, translocated to, 2 homolog (hur | -1.58 | 0.315 | 1.01 | 0.991 | -1.53 | 0.1 | -1.13 | 0.435 | -0.81 |
| 1431658_at | Hist1h4j | histone 1, H4j | -1.46 | 0.576 | -1.17 | 0.549 | -1.33 | 0.499 | 2.26 | 0.232 | -0.42 |
| 1451634_at | 2810051F02Rik | RIKEN cDNA 2810051F02 gene | 1.02 | 0.939 | -1.6 | 0.12 | -1.55 | 0.079 | -1.07 | 0.668 | -0.8 |
| 1449631_at | Eno3 | Enolase 3, beta muscle | -1.56 | 0.216 | -1.44 | 0.443 | -1.04 | 0.84 | 1.32 | 0.228 | -0.68 |
| 1451996_at | Tm2d1 | TM2 domain containing 1 | -1.38 | 0.092 | -1.14 | 0.211 | -1.44 | 0.043 | 1.01 | 0.902 | -0.74 |

| | | | | | | | | | | | |
|---|---|---|---|---|---|---|---|---|---|---|---|
| 1432714_at | 6430709C05Rik | RIKEN cDNA 6430709C05 gene | -2.22 | 0.292 | 1.29 | 0.703 | -1.81 | 0.302 | -3.05 | 0.111 | -1.44 |
| 1430400_at | 4930521A18Rik | RIKEN cDNA 4930521A18 gene | 1.04 | 0.915 | -1.28 | 0.616 | -2.12 | 0.048 | -1.59 | 0.497 | -0.99 |
| 1416136_at | Mmp2 | matrix metallopeptidase 2 | -1.01 | 0.872 | -1.22 | 0.137 | -2.04 | 0.06 | 1.68 | 0.012 | -0.65 |
| 1445263_at | Cst7 | Cystatin F (leukocystatin) | -1.18 | 0.268 | -1.34 | 0.009 | -1.43 | 0.167 | 1.23 | 0.355 | -0.68 |
| 1449844_at | Slco1a1 | solute carrier organic anion transporter family, member 1a1 | 1.53 | 0.058 | -58.47 | 0.077 | -1.33 | 0.393 | -1.04 | 0.946 | -14.83 |
| 1443069_at | Prkca | Protein kinase C, alpha | -1.19 | 0.467 | -1.41 | 0.108 | -1.34 | 0.119 | 1.14 | 0.577 | -0.7 |
| 1439064_at | BC030046 | cDNA sequence BC030046 | -1.82 | 0.524 | -1.38 | 0.536 | 1.02 | 0.958 | -1.52 | 0.524 | -0.93 |
| 1443614_at | --- | Transcribed locus | 1.04 | 0.927 | -1.54 | 0.507 | -1.64 | 0.141 | -1.21 | 0.808 | -0.84 |
| 1440554_at | --- | --- | -1.31 | 0.331 | -1.11 | 0.386 | -1.59 | 0.019 | 1.28 | 0.377 | -0.68 |
| 1433618_at | C330006A16Rik | RIKEN cDNA C330006A16 gene | -1.72 | 0.472 | -1.51 | 0.384 | 1.05 | 0.874 | -1.02 | 0.957 | -0.8 |
| 1421100_a_at | Dab1 | disabled homolog 1 (Drosophila) | -1.58 | 0.495 | 1.07 | 0.911 | -1.69 | 0.228 | -2.17 | 0.31 | -1.09 |
| 1442427_at | 9630026M06Rik | RIKEN cDNA 9630026M06 gene | -1.3 | 0.308 | -1.14 | 0.386 | -1.55 | 0.021 | 1.13 | 0.627 | -0.71 |
| 1432609_at | --- | --- | -1.55 | 0.515 | -1.99 | 0.237 | 1.15 | 0.346 | -1.16 | 0.082 | -0.89 |
| 1427538_at | Zfp369 | zinc finger protein 369 | -2.42 | 0.356 | 1.17 | 0.633 | -1.39 | 0.469 | -1.25 | 0.651 | -0.98 |
| 1449270_at | Plxdc2 | plexin domain containing 2 | -1.14 | 0.715 | -1.16 | 0.547 | -1.8 | 0.035 | 1.58 | 0.116 | -0.63 |
| 1439698_at | Zfp276 | Zinc finger protein (C2H2 type) 276 | -1.35 | 0.216 | -2.46 | 0.161 | 1.15 | 0.79 | -1.42 | 0.275 | -1.02 |
| 1454251_at | 1700034J04Rik | RIKEN cDNA 1700034J04 gene | -1.87 | 0.208 | -1.18 | 0.274 | -1.09 | 0.85 | 1.46 | 0.58 | -0.67 |
| 1424969_s_at | --- | --- | -1.32 | 0.726 | -1.21 | 0.667 | -1.4 | 0.62 | 1.03 | 0.938 | -0.73 |
| 1444119_at | B930006L02Rik | RIKEN cDNA B930006L02 gene | -1.14 | 0.548 | -1.1 | 0.635 | -1.93 | 0.012 | 1.31 | 0.289 | -0.72 |
| 1433054_at | Itgb2l | integrin beta 2-like | -1.61 | 0.212 | -1.22 | 0.813 | -1.17 | 0.735 | 1.19 | 0.706 | -0.7 |
| 1429702_at | 2900072G11Rik | RIKEN cDNA 2900072G11 gene | -1.18 | 0.305 | 1.02 | 0.902 | -2.34 | 0.025 | -1.28 | 0.103 | -0.94 |
| 1419425_at | Cnr1 | cannabinoid receptor 1 (brain) | -1.32 | 0.643 | -1.1 | 0.538 | -1.58 | 0.001 | 1.48 | 0.122 | -0.63 |
| 1453538_at | A330102K23Rik | RIKEN cDNA A330102K23 gene | -1.59 | 0.458 | -1.01 | 0.988 | -1.47 | 0.555 | 1.02 | 0.962 | -0.76 |
| 1420570_x_at | Tcl1b3 | T-cell leukemia/lymphoma 1B, 3 | -1.69 | 0.265 | -1.29 | 0.488 | -1.07 | 0.829 | 1.37 | 0.598 | -0.67 |
| 1439925_at | Tm4sf1 | transmembrane 4 superfamily member 1 | -1.05 | 0.813 | -1.48 | 0.328 | -1.5 | 0.03 | 1.04 | 0.89 | -0.74 |
| 1416745_x_at | Uap1 | UDP-N-acetylglucosamine pyrophosphorylase 1 | -1.05 | 0.766 | -1.55 | 0.37 | -1.42 | 0.139 | 2 | 0.185 | -0.51 |
| 1425362_at | Hrbl | HIV-1 Rev binding protein-like | -1.44 | 0.329 | -1.05 | 0.743 | -1.54 | 0.009 | 1.91 | 0.357 | -0.53 |
| 1447695_at | 2610035D17Rik | RIKEN cDNA 2610035D17 gene | -1.2 | 0.356 | -1.1 | 0.47 | -1.79 | 0.113 | 1.36 | 0.649 | -0.68 |
| 1440718_at | BC052328 | cDNA sequence BC052328 | 1.31 | 0.471 | -2.44 | 0.203 | -1.72 | 0.298 | -1.34 | 0.714 | -1.05 |
| 1459948_at | Ghr | Growth hormone receptor | 1.19 | 0.222 | -1.87 | 0.09 | -1.75 | 0.08 | -1.09 | 0.687 | -0.88 |
| 1447751_x_at | Dus2l | dihydrouridine synthase 2-like (SMM1, S. cerevisiae) | -3.64 | 0.098 | -1.04 | 0.928 | 1.06 | 0.892 | -3.76 | 0.019 | -1.84 |
| 1453011_at | Bdh2 | 3-hydroxybutyrate dehydrogenase, type 2 | -1.14 | 0.438 | -1.04 | 0.91 | -2.17 | 0.133 | 1.05 | 0.955 | -0.82 |
| 1440259_at | Man1a2 | Mannosidase, alpha, class 1A, member 2 | 1 | 0.99 | -1.41 | 0.206 | -1.7 | 0.019 | -1.24 | 0.137 | -0.84 |
| 1445254_at | --- | --- | -1.02 | 0.944 | -1.26 | 0.378 | -1.9 | 0.159 | 3.01 | 0.24 | -0.29 |
| 1429139_at | Otud7b | OTU domain containing 7B | -1.09 | 0.755 | -1.57 | 0.074 | -1.34 | 0.434 | 1.02 | 0.937 | -0.75 |
| 1459738_x_at | --- | --- | -1.31 | 0.633 | -1.29 | 0.542 | -1.32 | 0.133 | 1.06 | 0.935 | -0.71 |
| 1452017_at | Sox15 | SRY-box containing gene 15 | 1.08 | 0.446 | -2.24 | 0.267 | -1.3 | 0.526 | -1.78 | 0.273 | -1.06 |
| 1432431_s_at | 2900006F19Rik | RIKEN cDNA 2900006F19 gene | -1.22 | 0.064 | -1.12 | 0.477 | -1.71 | 0.019 | 1.45 | 0.476 | -0.65 |
| 1449456_a_at | Cma1 | chymase 1, mast cell | -1.47 | 0.239 | -1.68 | 0.264 | 1.03 | 0.94 | -1.04 | 0.891 | -0.79 |
| 1442996_x_at | --- | 0 day neonate kidney cDNA, RIKEN full-length enriched library, clone:D630027M | 1 | 0.992 | -1.26 | 0.195 | -2 | 0.005 | -1.13 | 0.673 | -0.84 |
| 1458939_at | Synj2bp | Synaptojanin 2 binding protein | -1.17 | 0.612 | -1.55 | 0.33 | -1.25 | 0.63 | 1.26 | 0.479 | -0.68 |
| 1439461_x_at | 2410003A14Rik | RIKEN cDNA 2410003A14 gene | -1.38 | 0.199 | -1.21 | 0.244 | -1.33 | 0.213 | 1.14 | 0.593 | -0.7 |
| 1417708_at | Syt3 | synaptotagmin III | -1.49 | 0.257 | -1.14 | 0.767 | -1.33 | 0.226 | 1.35 | 0.372 | -0.65 |
| 1441822_at | 1700008F19Rik | RIKEN cDNA 1700008F19 gene | -1.22 | 0.688 | -2.29 | 0.048 | 1.04 | 0.801 | -2.66 | 0.058 | -1.28 |
| 1439533_at | 1700125D06Rik | RIKEN cDNA 1700125D06 gene | -1.49 | 0.263 | -1.1 | 0.724 | -1.39 | 0.127 | 1.3 | 0.065 | -0.67 |
| 1439201_at | Usp14 | ubiquitin specific peptidase 14 | -1.39 | 0.106 | -1.49 | 0.046 | -1.1 | 0.711 | 1.44 | 0.283 | -0.64 |
| 1422162_at | Dcc | deleted in colorectal carcinoma | -1.16 | 0.617 | 1.04 | 0.905 | -2.51 | 0.006 | -1.57 | 0.262 | -1.05 |
| 1458070_at | Vtcn1 | V-set domain containing T cell activation inhibitor 1 | -1.03 | 0.861 | -2.77 | 0.126 | -1.03 | 0.935 | 1.11 | 0.708 | -0.93 |
| 1436513_at | Tanc2 | tetratricopeptide repeat, ankyrin repeat and coiled-coil containing 2 | -1.18 | 0.607 | -1.39 | 0.023 | -1.36 | 0.216 | 1.09 | 0.672 | -0.71 |
| 1444938_at | --- | Transcribed locus | -1.51 | 0.405 | -2.59 | 0.248 | 1.25 | 0.692 | -1.37 | 0.586 | -1.05 |
| 1443245_at | --- | --- | 1.32 | 0.731 | -2.15 | 0.19 | -1.95 | 0.199 | -1.02 | 0.928 | -0.95 |
| 1439249_at | Wac | WW domain containing adaptor with coiled-coil | -1.49 | 0.377 | -1.14 | 0.675 | -1.32 | 0.23 | 1.29 | 0.387 | -0.67 |
| 1439053_at | D10Bwg1070e | DNA segment, Chr 10, Brigham & Women's Genetics 1070 expressed | 1.09 | 0.913 | -2.13 | 0.033 | -1.34 | 0.214 | -1.41 | 0.116 | -0.95 |

| | | | | | | | | | | | |
|---|---|---|---|---|---|---|---|---|---|---|---|
| 1435594_at | Arl6ip2 | ADP-ribosylation factor-like 6 interacting protein 2 | -1.16 | 0.522 | -1.25 | 0.305 | -1.56 | 0.227 | 1.39 | 0.511 | -0.65 |
| 1450854_at | Pa2g4 | proliferation-associated 2G4 | -1.56 | 0.188 | -1.3 | 0.147 | -1.12 | 0.304 | 1.78 | 0.183 | -0.55 |
| 1447254_at | --- | --- | -1.28 | 0.589 | -1.51 | 0.555 | -1.16 | 0.405 | 1.5 | 0.113 | -0.61 |
| 1458970_at | Rad50 | RAD50 homolog (S. cerevisiae) | -3.62 | 0.002 | -1.23 | 0.366 | 1.21 | 0.335 | -1.03 | 0.903 | -1.17 |
| 1444973_at | Kcnma1 | Potassium large conductance calcium-activated channel, subfamily M, alpha mer | 1.03 | 0.858 | -1.81 | 0.233 | -1.39 | 0.066 | -1.26 | 0.03 | -0.86 |
| 1457017_at | --- | --- | -1.52 | 0.415 | 1 | 0.988 | -1.56 | 0.087 | -1.54 | 0.273 | -0.9 |
| 1421449_at | Csmd1 | CUB and Sushi multiple domains 1 | -1.12 | 0.874 | -1.28 | 0.691 | -1.59 | 0.452 | 1.19 | 0.696 | -0.7 |
| 1457584_at | AI848100 | expressed sequence AI848100 | -1.37 | 0.263 | -1.12 | 0.767 | -1.46 | 0.293 | 1.06 | 0.808 | -0.72 |
| 1432082_at | 4933436I20Rik | RIKEN cDNA 4933436I20 gene | -3.09 | 0.301 | 1.08 | 0.838 | -1.12 | 0.729 | -2.01 | 0.255 | -1.28 |
| 1440985_at | D130023J23Rik | RIKEN cDNA D130023J23 gene | -1.38 | 0.295 | -1.11 | 0.835 | -1.48 | 0.313 | 1.01 | 0.956 | -0.74 |
| 1442908_at | Tm9sf1 | Transmembrane 9 superfamily member 1 | -1.38 | 0.099 | -2.06 | 0.328 | 1.1 | 0.689 | -1.08 | 0.756 | -0.86 |
| 1456706_at | 4833441D16Rik | RIKEN cDNA 4833441D16 gene | -1.2 | 0.297 | -1.04 | 0.76 | -1.96 | 0.008 | 1.01 | 0.977 | -0.8 |
| 1429747_at | 1700009N14Rik | RIKEN cDNA 1700009N14 gene | -1.5 | 0.065 | -1.27 | 0.445 | -1.17 | 0.72 | 2.51 | 0.006 | -0.36 |
| 1426325_at | Kif1c | kinesin family member 1C | -1.45 | 0.508 | -2.88 | 0.014 | 1.27 | 0.617 | -1.22 | 0.694 | -1.07 |
| 1436561_at | Suv39h2 | suppressor of variegation 3-9 homolog 2 (Drosophila) | -1.85 | 0.349 | -1.19 | 0.76 | -1.08 | 0.85 | 6.55 | 0.097 | 0.61 |
| 1427690_a_at | Tpte | transmembrane phosphatase with tensin homology | 1.1 | 0.767 | -2.31 | 0.097 | -1.3 | 0.435 | -1.93 | 0.185 | -1.11 |
| 1436933_at | Akap13 | A kinase (PRKA) anchor protein 13 | -1.23 | 0.167 | -1.2 | 0.487 | -1.52 | 0.011 | 1.16 | 0.492 | -0.7 |
| 1446604_at | --- | --- | -1.5 | 0.316 | -1.32 | 0.165 | -1.13 | 0.535 | 1.07 | 0.875 | -0.72 |
| 1431923_at | 2810401C16Rik | RIKEN cDNA 2810401C16 gene | -1.22 | 0.504 | -1.61 | 0.469 | -1.16 | 0.766 | 1.13 | 0.114 | -0.71 |
| 1454199_at | 1700014N06Rik | RIKEN cDNA 1700014N06 gene | 1.06 | 0.619 | -2.26 | 0.104 | -1.24 | 0.421 | -1.49 | 0.611 | -0.98 |
| 1438720_at | 9330159F19Rik | RIKEN cDNA 9330159F19 gene | 1.07 | 0.857 | -1.46 | 0.301 | -1.81 | 0.232 | -2.45 | 0.11 | -1.16 |
| 1429685_at | C030002O17Rik | RIKEN cDNA C030002O17 gene | -1.09 | 0.677 | -1.43 | 0.572 | -1.45 | 0.256 | 1.23 | 0.8 | -0.69 |
| 1438592_at | Nek1 | NIMA (never in mitosis gene a)-related expressed kinase 1 | -1.19 | 0.161 | -1.31 | 0.204 | -1.42 | 0.01 | 1.1 | 0.769 | -0.71 |
| 1435227_at | --- | --- | -1.49 | 0.03 | -2.33 | 0.334 | 1.2 | 0.456 | -2.3 | 0.153 | -1.23 |
| 1432964_at | 2410049M19Rik | RIKEN cDNA 2410049M19 gene | -1.49 | 0.003 | 1.06 | 0.941 | -1.73 | 0.198 | -1.91 | 0.375 | -1.02 |
| 1455057_at | Gmps | guanine monphosphate synthetase | -1.12 | 0.565 | -1.44 | 0.06 | -1.39 | 0.048 | 1.01 | 0.959 | -0.74 |
| 1452696_a_at | 4933439C10Rik | RIKEN cDNA 4933439C10 gene | 1.01 | 0.863 | -1.97 | 0.154 | -1.26 | 0.23 | -1.73 | 0.16 | -0.99 |
| 1435120_at | --- | Transcribed locus | 1.12 | 0.389 | -1.51 | 0.039 | -1.9 | 0.039 | -1.13 | 0.58 | -0.86 |
| 1441517_at | Spred1 | Sprouty protein with EVH-1 domain 1, related sequence | -1.19 | 0.442 | -1.42 | 0.391 | -1.32 | 0.396 | 1.42 | 0.593 | -0.63 |
| 1432292_at | 4930504C09Rik | RIKEN cDNA 4930504C09 gene | -2.1 | 0.341 | -1.51 | 0.439 | 1.17 | 0.724 | -1.85 | 0.072 | -1.07 |
| 1446913_at | --- | --- | -1.31 | 0.695 | -1.04 | 0.954 | -1.73 | 0.235 | 1.51 | 0.503 | -0.64 |
| 1424558_a_at | Cabyr | calcium-binding tyrosine-(Y)-phosphorylation regulated (fibrousheathin 2) | -1.27 | 0.291 | -1.17 | 0.589 | -1.52 | 0.09 | 1.47 | 0.45 | -0.62 |
| 1443470_at | A530058N18Rik | RIKEN cDNA A530058N18 gene | -1.62 | 0.607 | -1.2 | 0.788 | -1.17 | 0.587 | 2.77 | 0.357 | -0.3 |
| 1458811_at | 9430047L24Rik | RIKEN cDNA 9430047L24 gene | -1.05 | 0.943 | -1.31 | 0.67 | -1.68 | 0.241 | 2.04 | 0.207 | -0.5 |
| 1429913_at | Kcnk16 | potassium channel, subfamily K, member 16 | -4.45 | 0.002 | -2.85 | 0.056 | 1.73 | 0.364 | -1.12 | 0.867 | -1.67 |
| 1431432_at | Cfl2 | cofilin 2, muscle | -1.09 | 0.836 | -1.66 | 0.293 | -1.27 | 0.057 | 1 | 0.998 | -0.76 |
| 1451441_at | 2210415F13Rik | RIKEN cDNA 2210415F13 gene | -1.67 | 0.186 | -1.03 | 0.95 | -1.36 | 0.322 | 1.82 | 0.474 | -0.56 |
| 1449584_at | Dgkg | diacylglycerol kinase, gamma | -1.05 | 0.561 | -2.48 | 0.008 | -1.05 | 0.639 | 1.21 | 0.303 | -0.84 |
| 1436274_at | --- | Transcribed locus | -1.26 | 0.602 | -2.32 | 0.232 | 1.09 | 0.867 | -1.24 | 0.741 | -0.94 |
| 1458309_at | --- | Transcribed locus | -1.2 | 0.387 | -1.1 | 0.524 | -1.75 | 0.01 | 1.11 | 0.694 | -0.74 |
| 1423485_at | Rad54l2 | Rad54 like 2 (S. cerevisiae) | -2 | 0.58 | -1.32 | 0.561 | 1.05 | 0.864 | -1.52 | 0.373 | -0.95 |
| 1454364_at | 9530025L08Rik | RIKEN cDNA 9530025L08 gene | -1.47 | 0.238 | -1.12 | 0.555 | -1.36 | 0.592 | 1.54 | 0.161 | -0.6 |
| 1447416_at | Gm239 | gene model 239, (NCBI) | -1.45 | 0.211 | -1.62 | 0.381 | 1 | 1 | -1.17 | 0.708 | -0.81 |
| 1453408_s_at | 4930519G04Rik | RIKEN cDNA 4930519G04 gene | -1.03 | 0.897 | -1.56 | 0.322 | -1.43 | 0.24 | 1.05 | 0.882 | -0.74 |
| 1420557_at | Epha5 | Eph receptor A5 | -1.58 | 0.156 | -1.02 | 0.954 | -1.45 | 0.064 | 1.43 | 0.113 | -0.65 |
| 1431341_at | Ppp2cb | Protein phosphatase 2 (formerly 2A), catalytic subunit, beta isoform | -1.29 | 0.336 | -1.99 | 0.005 | 1.03 | 0.925 | -1.14 | 0.041 | -0.85 |
| 1429426_at | Rnf139 | ring finger protein 139 | -1 | 0.994 | -1.35 | 0.298 | -1.75 | 0.015 | 1.62 | 0.375 | -0.62 |
| 1426959_at | Bdh1 | 3-hydroxybutyrate dehydrogenase, type 1 | -1.17 | 0.215 | -1.99 | 0.047 | -1.05 | 0.928 | 1.58 | 0.32 | -0.66 |
| 1432475_at | 1700014B07Rik | RIKEN cDNA 1700014B07 gene | 1.06 | 0.942 | -2.77 | 0.004 | -1.12 | 0.727 | -1.45 | 0.052 | -1.07 |
| 1446836_at | --- | --- | -1.87 | 0.279 | -1.44 | 0.582 | 1.08 | 0.9 | -2.99 | 0.063 | -1.31 |
| 1440958_at | --- | --- | 1.12 | 0.603 | -2.75 | 0.086 | -1.2 | 0.659 | -1.1 | 0.555 | -0.98 |
| 1458413_at | Fbxw8 | F-box and WD-40 domain protein 8 | 1.04 | 0.924 | -1.5 | 0.406 | -1.66 | 0.351 | -1.15 | 0.789 | -0.82 |
| 1435612_at | Opcml | opioid binding protein/cell adhesion molecule-like | 1.17 | 0.448 | -2.25 | 0.017 | -1.44 | 0.386 | -1.23 | 0.701 | -0.94 |

| | | | | | | | | | | | |
|---|---|---|---|---|---|---|---|---|---|---|---|
| 1418589_a_at | Mlf1 | myeloid leukemia factor 1 | -1.91 | 0.23 | -1.34 | 0.392 | 1.04 | 0.901 | -1.12 | 0.622 | -0.83 |
| 1439848_at | Bves | blood vessel epicardial substance | 1.16 | 0.438 | -2.09 | 0.354 | -1.49 | 0.571 | -2.81 | 0.085 | -1.31 |
| 1446760_at | Trip13 | thyroid hormone receptor interactor 13 | -1.27 | 0.665 | -1.29 | 0.452 | -1.34 | 0.483 | 1.25 | 0.483 | -0.66 |
| 1442062_at | 7120426M23Rik | RIKEN cDNA 7120426M23 gene | -1.3 | 0.203 | -1.17 | 0.193 | -1.46 | 0.25 | 1.25 | 0.312 | -0.67 |
| 1432337_at | Fancl | Fanconi anemia, complementation group L | -1.02 | 0.974 | -1.48 | 0.435 | -1.52 | 0.41 | 1.24 | 0.563 | -0.7 |
| 1441565_at | --- | --- | -1.41 | 0.446 | -1.91 | 0.01 | 1.08 | 0.187 | -1.17 | 0.309 | -0.85 |
| 1453187_at | Ociad2 | OCIA domain containing 2 | -1.24 | 0.056 | -1.49 | 0.144 | -1.2 | 0.318 | 1.84 | 0.132 | -0.52 |
| 1435719_at | AI448984 | expressed sequence AI448984 | -1.28 | 0.011 | -1.24 | 0.233 | -1.39 | 0.099 | 1.06 | 0.834 | -0.71 |
| 1457041_at | 1700026B20Rik | RIKEN cDNA 1700026B20 gene | -3.34 | 0.137 | -1.09 | 0.889 | 1.1 | 0.418 | -1.99 | 0.154 | -1.33 |
| 1440035_at | --- | --- | -1.07 | 0.705 | -1.29 | 0.701 | -1.65 | 0.005 | 1.06 | 0.828 | -0.74 |
| 1439404_x_at | Zfa /// Zfx | zinc finger protein, autosomal /// zinc finger protein X-linked | -1.51 | 0.076 | -1.06 | 0.833 | -1.41 | 0.2 | 1.66 | 0.081 | -0.58 |
| 1426720_at | Apbb2 | amyloid beta (A4) precursor protein-binding, family B, member 2 | 1.02 | 0.94 | -1.43 | 0.053 | -1.67 | 0.018 | -1.21 | 0.624 | -0.83 |
| 1432745_at | 9430092D12Rik | RIKEN cDNA 9430092D12 gene | -2.02 | 0.121 | 1.04 | 0.922 | -1.28 | 0.548 | -1.33 | 0.415 | -0.9 |
| 1426732_at | Des | desmin | -1.72 | 0.12 | -1.07 | 0.607 | -1.25 | 0.387 | 1.28 | 0.636 | -0.69 |
| 1418336_at | Afg3l1 | AFG3(ATPase family gene 3)-like 1 (yeast) | -1.24 | 0.504 | -2.17 | 0.056 | 1.05 | 0.813 | -1.08 | 0.572 | -0.86 |
| 1445379_at | Atp11c | Atpase, class VI, type 11C | -1.08 | 0.802 | -1.23 | 0.156 | -1.73 | 0.089 | 1.09 | 0.586 | -0.74 |
| 1459802_at | --- | --- | -1.73 | 0.447 | -2.39 | 0.03 | 1.32 | 0.449 | -1.2 | 0.753 | -1 |
| 1434413_at | --- | --- | -1.05 | 0.899 | -1.27 | 0.022 | -1.76 | 0.063 | 1.18 | 0.486 | -0.72 |
| 1454309_at | 2810002N01Rik | RIKEN cDNA 2810002N01 gene | -1.63 | 0.073 | -1.01 | 0.993 | -1.41 | 0.513 | 1.11 | 0.232 | -0.73 |
| 1431180_at | 1700123A16Rik /// | RIKEN cDNA 1700123A16 gene /// similar to zinc finger protein 709 | -1.3 | 0.653 | -1.6 | 0.025 | -1.09 | 0.584 | 1.11 | 0.742 | -0.72 |
| 1449691_at | Zfp644 | Zinc finger protein 644 | -1.12 | 0.801 | -1.44 | 0.182 | -1.39 | 0.287 | 1.19 | 0.417 | -0.69 |
| 1445493_at | AU022255 | expressed sequence AU022255 | -1.48 | 0.522 | 1.04 | 0.945 | -1.67 | 0.016 | -1.08 | 0.864 | -0.8 |
| 1459200_at | --- | --- | -1.29 | 0.735 | -1.09 | 0.871 | -1.61 | 0.227 | 1.37 | 0.55 | -0.66 |
| 1442365_at | Rtn3 | Reticulon 3 | -1.16 | 0.641 | -1.19 | 0.307 | -1.63 | 0.001 | 1.1 | 0.7 | -0.72 |
| 1427445_a_at | Ttn | titin | -2.44 | 0.141 | -1.61 | 0.147 | 1.28 | 0.645 | -1.04 | 0.936 | -0.95 |
| 1446972_at | D15Wsu126e | DNA segment, Chr 15, Wayne State University 126, expressed | -1.02 | 0.898 | -1.06 | 0.758 | -2.55 | 0.007 | 1.41 | 0.208 | -0.8 |
| 1445203_at | Pdzd2 | PDZ domain containing 2 | -1.17 | 0.69 | -1.25 | 0.458 | -1.51 | 0.014 | 1.35 | 0.082 | -0.65 |
| 1445704_x_at | 2900093B09Rik | RIKEN cDNA 2900093B09 gene | -1.18 | 0.694 | -1.16 | 0.788 | -1.65 | 0.234 | 1.19 | 0.75 | -0.7 |
| 1451939_a_at | Srpx | sushi-repeat-containing protein | 1.05 | 0.758 | -1.32 | 0.318 | -1.97 | 0.009 | -1.65 | 0.347 | -0.97 |
| 1445555_at | Trpm3 | Transient receptor potential cation channel, subfamily M, member 3 | -1.1 | 0.259 | -1.34 | 0.433 | -1.5 | 0.294 | 1.81 | 0.498 | -0.53 |
| 1445624_at | D8Ertd587e | DNA segment, Chr 8, ERATO Doi 587, expressed | -1.4 | 0.094 | -1.37 | 0.07 | -1.15 | 0.134 | 2.52 | 0.096 | -0.35 |
| 1447039_at | Ptch1 | Patched homolog 1 | 1.21 | 0.491 | -1.83 | 0.212 | -1.79 | 0.098 | -1.94 | 0.155 | -1.09 |
| 1456559_at | Emx2os | empty spiracles homolog 2 (Drosophila) opposite strand | -1.09 | 0.491 | -1.46 | 0.358 | -1.39 | 0.452 | 1.5 | 0.303 | -0.61 |
| 1430241_at | Fbxl17 | F-box and leucine-rich repeat protein 17 | -1.48 | 0.119 | -1.28 | 0.406 | -1.16 | 0.388 | 1.15 | 0.523 | -0.69 |
| 1438790_x_at | Tmem41b | transmembrane protein 41B | -1.22 | 0.173 | -1.33 | 0.032 | -1.34 | 0.137 | 1.28 | 0.436 | -0.65 |
| 1430564_at | Mobkl1a | MOB1, Mps One Binder kinase activator-like 1A (yeast) | 1.08 | 0.806 | -1.68 | 0.26 | -1.57 | 0.012 | -1.05 | 0.874 | -0.8 |
| 1443295_at | --- | Transcribed locus | -2.18 | 0.222 | 1.26 | 0.654 | -1.65 | 0.263 | -1.02 | 0.979 | -0.9 |
| 1441397_at | Pard3 | Par-3 (partitioning defective 3) homolog (C. elegans) | -1.06 | 0.762 | -1.07 | 0.839 | -2.28 | 0.02 | 1.22 | 0.365 | -0.8 |
| 1453685_at | Nudt7 | nudix (nucleoside diphosphate linked moiety X)-type motif 7 | -1.04 | 0.821 | -1.7 | 0.1 | -1.3 | 0.459 | 2.07 | 0.314 | -0.49 |
| 1446755_at | Scg5 | Secretogranin V | -2.71 | 0.053 | 1.05 | 0.747 | -1.12 | 0.639 | -1.18 | 0.536 | -0.99 |
| 1435844_at | A330009N23Rik | RIKEN cDNA A330009N23 gene | -1.55 | 0.032 | 1.02 | 0.935 | -1.54 | 0.031 | -1.22 | 0.483 | -0.82 |
| 1430851_at | Zfp444 | zinc finger protein 444 | -1.88 | 0.051 | -1.21 | 0.661 | -1.04 | 0.821 | 1 | 0.989 | -0.78 |
| 1417436_at | Large | like-glycosyltransferase | -1.44 | 0.695 | -2.75 | 0.203 | 1.26 | 0.637 | -2.45 | 0.221 | -1.34 |
| 1433425_at | 1700101I19Rik | RIKEN cDNA 1700101I19 gene | -1.38 | 0.007 | -1.13 | 0.603 | -1.42 | 0.008 | 1.03 | 0.855 | -0.72 |
| 1424673_at | Clec2h | C-type lectin domain family 2, member h | -1.15 | 0.56 | -5.85 | 0.017 | 1.28 | 0.268 | -1.03 | 0.967 | -1.69 |
| 1445761_at | D4Ertd628e | DNA segment, Chr 4, ERATO Doi 628, expressed | -1.44 | 0.117 | -1.27 | 0.5 | -1.2 | 0.495 | 1.26 | 0.547 | -0.66 |
| 1419426_s_at | Ccl21b /// Ccl21a /// | chemokine (C-C motif) ligand 21b /// chemokine (C-C motif) ligand 21a /// chemok | 1.26 | 0.049 | -2.81 | 0.109 | -1.43 | 0.024 | -2.83 | 0.06 | -1.45 |
| 1457328_at | Adra2c | adrenergic receptor, alpha 2c | -1.48 | 0.294 | -1.1 | 0.837 | -1.36 | 0.572 | 1.11 | 0.895 | -0.71 |
| 1436655_at | 1300002A08Rik | RIKEN cDNA 1300002A08 gene | -1.17 | 0.712 | -1.45 | 0.201 | -1.29 | 0.552 | 1.01 | 0.931 | -0.72 |
| 1454368_at | Psmd7 | proteasome (prosome, macropain) 26S subunit, non-ATPase, 7 | -2.52 | 0.144 | 1.18 | 0.714 | -1.34 | 0.643 | -1.19 | 0.598 | -0.97 |
| 1430918_at | 3-Mar | membrane-associated ring finger (C3HC4) 3 | -1.28 | 0.37 | -1.21 | 0.707 | -1.4 | 0.294 | 3.49 | 0.256 | -0.1 |
| 1425342_a_at | Kcnk3 | potassium channel, subfamily K, member 3 | -1.36 | 0.4 | -1.22 | 0.331 | -1.3 | 0.39 | 1.17 | 0.536 | -0.68 |
| 1443097_at | Nf1 | Neurofibromatosis 1 | 1.06 | 0.869 | -1.78 | 0.06 | -1.43 | 0.099 | -1.71 | 0.304 | -0.96 |

| | | | | | | | | | | | |
|---|---|---|---|---|---|---|---|---|---|---|---|
| 1430119_at | Fndc1 | fibronectin type III domain containing 1 | -1.23 | 0.394 | -1.91 | 0.218 | -1.02 | 0.949 | 1.33 | 0.214 | -0.71 |
| 1460157_at | --- | --- | -3.44 | 0.091 | 1.32 | 0.333 | -1.41 | 0.535 | -1.07 | 0.752 | -1.15 |
| 1442878_at | Prdx6 | peroxiredoxin 6 | -1.02 | 0.868 | -1.32 | 0.467 | -1.71 | 0.077 | 1.2 | 0.585 | -0.71 |
| 1432904_at | 3110037C07Rik | RIKEN cDNA 3110037C07 gene | 1.18 | 0.458 | -1.33 | 0.357 | -2.57 | 0.086 | -1.71 | 0.416 | -1.11 |
| 1441891_x_at | Elovl7 | ELOVL family member 7, elongation of long chain fatty acids (yeast) | -1.14 | 0.226 | -2.09 | 0.093 | -1.04 | 0.859 | 1.91 | 0.201 | -0.59 |
| 1450492_at | Cngb3 | cyclic nucleotide gated channel beta 3 | -1.09 | 0.755 | -1.65 | 0.218 | -1.25 | 0.569 | 1.09 | 0.832 | -0.73 |
| 1431723_at | 1600022D10Rik | RIKEN cDNA 1600022D10 gene | -1.84 | 0.418 | -1.08 | 0.898 | -1.18 | 0.709 | 1.03 | 0.949 | -0.77 |
| 1445588_at | Ankrd44 | ankyrin repeat domain 44 | -1.3 | 0.405 | 1.03 | 0.958 | -1.9 | 0.019 | -2.23 | 0.16 | -1.1 |
| 1445927_at | D13Ertd212e | DNA segment, Chr 13, ERATO Doi 212, expressed | -1.48 | 0.433 | -1.04 | 0.966 | -1.47 | 0.257 | 1.23 | 0.728 | -0.69 |
| 1429378_x_at | AY761184 | cDNA sequence AY761184 | -1.6 | 0.153 | -1.15 | 0.558 | -1.21 | 0.429 | 1.21 | 0.673 | -0.69 |
| 1421335_a_at | Egfl7 | EGF-like domain 7 | -1.34 | 0.154 | -1.13 | 0.499 | -1.45 | 0.119 | 1.05 | 0.773 | -0.72 |
| 1433311_at | D530031A16Rik | RIKEN cDNA D530031A16 gene | -1.65 | 0.438 | -1.27 | 0.448 | -1.08 | 0.853 | 1.21 | 0.743 | -0.7 |
| 1436491_at | 5830431A10Rik | RIKEN cDNA 5830431A10 gene | -1.39 | 0.664 | -1.8 | 0.422 | 1.04 | 0.6 | -4.39 | 0.032 | -1.63 |
| 1420578_at | Optc | opticin | 1 | 1 | -1.7 | 0.411 | -1.37 | 0.47 | -2.11 | 0.095 | -1.04 |
| 1434527_at | Nalp4b | NACHT, leucine rich repeat and PYD containing 4B | 1.02 | 0.955 | -1.06 | 0.935 | -2.81 | 0.077 | -1.54 | 0.379 | -1.1 |
| 1435607_at | Grm2 | G protein-coupled receptor, family C, group 1, member B | -1.39 | 0.2 | -1.83 | 0.407 | 1.06 | 0.934 | -1.01 | 0.958 | -0.8 |
| 1458376_at | B930025B16Rik | RIKEN cDNA B930025B16 gene | -1.42 | 0.081 | 1.18 | 0.138 | -2.29 | 0.003 | -1.51 | 0.297 | -1.01 |
| 1443406_at | Plscr4 | phospholipid scramblase 4 | -1.22 | 0.084 | -1.26 | 0.449 | -1.42 | 0.237 | 1.1 | 0.855 | -0.7 |
| 1442879_at | --- | --- | -1.35 | 0.39 | -1.65 | 0.487 | -1.02 | 0.926 | 1.11 | 0.757 | -0.73 |
| 1427772_at | Defb15 | defensin beta 15 | -2.38 | 0.166 | -1.5 | 0.382 | 1.23 | 0.742 | -2.38 | 0.547 | -1.26 |
| 1447263_at | Metap2 | Methionine aminopeptidase 2 | -1.02 | 0.917 | -1.56 | 0.039 | -1.42 | 0.167 | 2.56 | 0.201 | -0.36 |
| 1450296_at | Klrb1a | killer cell lectin-like receptor subfamily B member 1A | -2.53 | 0.306 | 1.39 | 0.674 | -1.85 | 0.244 | -1.64 | 0.162 | -1.16 |
| 1419953_at | C80678 | expressed sequence C80678 | 1.15 | 0.058 | -1.23 | 0.575 | -2.74 | 0.1 | -1.75 | 0.579 | -1.14 |
| 1425642_at | Cep290 | centrosomal protein 290 | -1.55 | 0.442 | -1.19 | 0.197 | -1.19 | 0.499 | 2.26 | 0.082 | -0.42 |
| 1456793_at | Cyt1 | cytokine like 1 | 1.1 | 0.784 | -1.57 | 0.184 | -1.72 | 0.251 | -1.25 | 0.571 | -0.86 |
| 1460604_at | Cybrd1 | cytochrome b reductase 1 | 1.02 | 0.951 | -1.25 | 0.769 | -2 | 0.054 | -2.07 | 0.151 | -1.07 |
| 1458862_at | C78704 | expressed sequence C78704 | -1.27 | 0.445 | -1.32 | 0.555 | -1.29 | 0.555 | 1.29 | 0.197 | -0.65 |
| 1422585_at | Odf1 | outer dense fiber of sperm tails 1 | -1.76 | 0.466 | 1.14 | 0.729 | -1.62 | 0.383 | -1.07 | 0.704 | -0.83 |
| 1445446_at | --- | Transcribed locus | -1.98 | 0.08 | -1.07 | 0.863 | -1.14 | 0.54 | 1.05 | 0.777 | -0.78 |
| 1459027_at | --- | Transcribed locus | -2.08 | 0.071 | -1.79 | 0.321 | 1.28 | 0.492 | -1.2 | 0.786 | -0.95 |
| 1431389_at | Tbkbp1 | TBK1 binding protein 1 | -1.23 | 0.768 | -1.36 | 0.509 | -1.3 | 0.126 | 1.02 | 0.939 | -0.71 |
| 1458663_at | Large | Like-glycosyltransferase | -1.16 | 0.27 | 1.03 | 0.939 | -2.31 | 0.009 | -1.18 | 0.541 | -0.91 |
| 1430763_at | 4930563E22Rik | RIKEN cDNA 4930563E22 gene | -1.78 | 0.035 | -1.3 | 0.066 | -1.01 | 0.972 | 1.1 | 0.388 | -0.75 |
| 1428778_at | Sfi1 | Sfi1 homolog, spindle assembly associated (yeast) | -1.39 | 0.234 | -1.49 | 0.16 | -1.07 | 0.769 | 1.22 | 0.502 | -0.68 |
| 1425301_at | Ncam2 | neural cell adhesion molecule 2 | -1.28 | 0.664 | -1.39 | 0.478 | -1.21 | 0.627 | 1.92 | 0.187 | -0.49 |
| 1449903_at | Crtam | cytotoxic and regulatory T cell molecule | 1.14 | 0.652 | -1.77 | 0.244 | -1.62 | 0.274 | -1.27 | 0.62 | -0.88 |
| 1450909_at | Eif4e | eukaryotic translation initiation factor 4E | -1.55 | 0.014 | -1.18 | 0.309 | -1.21 | 0.422 | 1.39 | 0.154 | -0.64 |
| 1445282_at | Mkln1 | Muskelin 1, intracellular mediator containing kelch motifs | -1.3 | 0.062 | -1.02 | 0.8 | -1.74 | 0.034 | 1.07 | 0.757 | -0.75 |
| 1448365_at | Exosc7 | exosome component 7 | -1.4 | 0.143 | -1.28 | 0.024 | -1.21 | 0.04 | 1.21 | 0.541 | -0.67 |
| 1440465_at | Cutl1 | Cut-like 1 (Drosophila) | -1.42 | 0.018 | -1.17 | 0.442 | -1.31 | 0.241 | 1.05 | 0.845 | -0.71 |
| 1444749_at | Apod | Apolipoprotein D | -1.68 | 0.466 | -1.84 | 0.486 | 1.19 | 0.639 | -1.31 | 0.518 | -0.91 |
| 1439286_at | Grik2 | Glutamate receptor, ionotropic, kainate 2 (beta 2) | -1.51 | 0.531 | -1.08 | 0.911 | -1.36 | 0.414 | 1.16 | 0.734 | -0.7 |
| 1430304_at | Wdr76 | WD repeat domain 76 | -1.36 | 0.126 | -1.86 | 0.12 | 1.05 | 0.889 | -1.04 | 0.829 | -0.8 |
| 1418212_at | Omg | oligodendrocyte myelin glycoprotein | -1.55 | 0.011 | 1.08 | 0.684 | -1.67 | 0.389 | -2.16 | 0.179 | -1.07 |
| 1442986_at | Elk3 | ELK3, member of ETS oncogene family | 1.25 | 0.687 | -2.27 | 0.212 | -1.59 | 0.086 | -1.67 | 0.185 | -1.07 |
| 1442551_at | --- | --- | -1.77 | 0.171 | -1.37 | 0.548 | 1.03 | 0.936 | -1.08 | 0.856 | -0.8 |
| 1417565_at | Abhd5 | abhydrolase domain containing 5 | -1.24 | 0.377 | -1.28 | 0.012 | -1.35 | 0.054 | 1.96 | 0.325 | -0.48 |
| 1453834_at | 1500002C15Rik | RIKEN cDNA 1500002C15 gene | 1.19 | 0.838 | -1.4 | 0.416 | -2.36 | 0.051 | -1.44 | 0.581 | -1 |
| 1439366_at | Grsf1 | G-rich RNA sequence binding factor 1 | -3.33 | 0.386 | 1.19 | 0.413 | -1.2 | 0.641 | -1.38 | 0.416 | -1.18 |
| 1447256_at | --- | 17 days embryo kidney cDNA, RIKEN full-length enriched library, clone:I920178F | -1.15 | 0.755 | -1.32 | 0.569 | -1.43 | 0.452 | 1.19 | 0.788 | -0.68 |
| 1433316_at | 4930477O15Rik | RIKEN cDNA 4930477O15 gene | -2.47 | 0.483 | -1.28 | 0.604 | 1.14 | 0.779 | -2.18 | 0.368 | -1.2 |
| 1454141_at | 5730416O20Rik | RIKEN cDNA 5730416O20 gene | -1.6 | 0.173 | 1.23 | 0.244 | -2.13 | 0.05 | -1.27 | 0.68 | -0.94 |
| 1444657_at | B3bp | Bcl3 binding protein | -1.42 | 0.531 | -1.47 | 0.347 | -1.07 | 0.86 | 1.24 | 0.475 | -0.68 |

| | | | | | | | | | | | |
|---|---|---|---|---|---|---|---|---|---|---|---|
| 1432056_at | Cpvl | carboxypeptidase, vitellogenic-like | -1.59 | 0.491 | -1.09 | 0.727 | -1.29 | 0.277 | 1.01 | 0.99 | -0.74 |
| 1450619_x_at | Cmar | cell matrix adhesion regulator | -1.21 | 0.619 | -1.71 | 0.039 | -1.09 | 0.488 | 1.24 | 0.486 | -0.69 |
| 1439897_at | Nebl | Nebulette | -4.53 | 0.065 | -1.1 | 0.876 | 1.2 | 0.31 | -1.81 | 0.092 | -1.56 |
| 1457262_at | 2610207I05Rik | RIKEN cDNA 2610207I05 gene | 1.21 | 0.122 | -1.92 | 0.353 | -1.68 | 0.117 | -2.28 | 0.108 | -1.17 |
| 1454511_at | 9430018C23Rik | RIKEN cDNA 9430018C23 gene | -1.15 | 0.855 | 1.05 | 0.919 | -2.48 | 0.2 | -1.18 | 0.747 | -0.94 |
| 1431219_at | B3gat3 | beta-1,3-glucuronyltransferase 3 (glucuronosyltransferase I) | 1.12 | 0.746 | -1.7 | 0.435 | -1.61 | 0.123 | -1.46 | 0.246 | -0.92 |
| 1445797_at | Pam | Peptidylglycine alpha-amidating monooxygenase | -2.15 | 0.058 | -1.18 | 0.744 | 1.01 | 0.977 | -1.06 | 0.871 | -0.84 |
| 1431662_at | 4930555F03Rik | RIKEN cDNA 4930555F03 gene | 1.07 | 0.921 | -2.06 | 0.267 | -1.3 | 0.637 | -1.19 | 0.756 | -0.87 |
| 1456317_at | Aldh3b2 | aldehyde dehydrogenase 3 family, member B2 | -1.27 | 0.088 | -1.21 | 0.648 | -1.4 | 0.096 | 1.9 | 0.045 | -0.5 |
| 1448855_at | Rassf1 | Ras association (RalGDS/AF-6) domain family 1 | -1.43 | 0.018 | -1.43 | 0.304 | -1.08 | 0.751 | 1.17 | 0.501 | -0.69 |
| 1440056_at | --- | Adult male hippocampus cDNA, RIKEN full-length enriched library, clone:C63000 | -1.04 | 0.755 | -2.86 | 0.023 | 1.01 | 0.968 | -1.58 | 0.396 | -1.12 |
| 1449279_at | Gpx2 | glutathione peroxidase 2 | -1.2 | 0.739 | -1.57 | 0.204 | -1.17 | 0.547 | 1.08 | 0.902 | -0.71 |
| 1420718_at | Odz2 /// LOC66949 | odd Oz/ten-m homolog 2 (Drosophila) /// similar to odd Oz/ten-m homolog 2 | -1.67 | 0.111 | -1.17 | 0.823 | -1.14 | 0.738 | 1.22 | 0.607 | -0.69 |
| 1439281_at | --- | PREDICTED: Mus musculus similar to solute carrier family 26, member 8 isoform | -1.18 | 0.582 | -1.11 | 0.858 | -1.72 | 0.125 | 1.12 | 0.854 | -0.72 |
| 1435685_x_at | Abcc5 | ATP-binding cassette, sub-family C (CFTR/MRP), member 5 | -1.37 | 0.083 | -1.3 | 0.237 | -1.21 | 0.614 | 1.27 | 0.341 | -0.65 |
| 1456213_x_at | Qars | glutaminyl-tRNA synthetase | -1.46 | 0.278 | -1.12 | 0.202 | -1.34 | 0.325 | 1.02 | 0.787 | -0.73 |
| 1430207_at | 4933437N03Rik | RIKEN cDNA 4933437N03 gene | -1.51 | 0.354 | -1.02 | 0.955 | -1.46 | 0.583 | 1.26 | 0.584 | -0.68 |
| 1450389_s_at | Pip5k1a | phosphatidylinositol-4-phosphate 5-kinase, type 1 alpha | -1.56 | 0.314 | -1.33 | 0.171 | -1.07 | 0.558 | 6.86 | 0.225 | 0.72 |
| 1424217_at | Papola | poly (A) polymerase alpha | -1.21 | 0.101 | -1.24 | 0.325 | -1.45 | 0.164 | 1.23 | 0.111 | -0.67 |
| 1443297_at | Tbc1d12 | TBC1D12: TBC1 domain family, member 12 | -1.07 | 0.898 | -1.36 | 0.509 | -1.52 | 0.342 | 2.84 | 0.09 | -0.28 |
| 1443121_at | --- | --- | 1.03 | 0.9 | -1.96 | 0.003 | -1.28 | 0.406 | -1.14 | 0.25 | -0.84 |
| 1459390_at | --- | Transcribed locus | -2.01 | 0.195 | -1.36 | 0.197 | 1.09 | 0.798 | -1.67 | 0.123 | -0.99 |
| 1448738_at | Calb1 | calbindin-28K | -1.38 | 0.024 | 1.1 | 0.715 | -1.99 | 0.032 | -1.34 | 0.269 | -0.9 |
| 1429822_at | 4633401B06Rik | RIKEN cDNA 4633401B06 gene | 1.1 | 0.653 | -1.42 | 0.251 | -1.92 | 0.004 | -1.16 | 0.609 | -0.85 |
| 1446431_at | Dnm3 | Dynamin 3 | -1.33 | 0.098 | -1.45 | 0.335 | -1.13 | 0.519 | 1.27 | 0.264 | -0.66 |
| 1428160_at | Ndufab1 | NADH dehydrogenase (ubiquinone) 1, alpha/beta subcomplex, 1 | -1.19 | 0.272 | -1.48 | 0.166 | -1.23 | 0.155 | 1.08 | 0.732 | -0.7 |
| 1444461_at | Zmynd11 | Zinc finger, MYND domain containing 11 | 1.16 | 0.786 | -1.55 | 0.148 | -1.9 | 0.037 | -3.08 | 0.034 | -1.34 |
| 1417888_at | Trim13 | tripartite motif protein 13 | -1.11 | 0.548 | -1.44 | 0.431 | -1.36 | 0.189 | 1.29 | 0.252 | -0.66 |
| 1443636_at | Abcb9 | ATP-binding cassette, sub-family B (MDR/TAP), member 9 | -1.45 | 0.348 | -1.34 | 0.106 | -1.12 | 0.64 | 1.13 | 0.457 | -0.7 |
| 1431995_at | Antxr1 | anthrax toxin receptor 1 | -1.11 | 0.697 | -1.17 | 0.797 | -1.74 | 0.019 | 1.25 | 0.168 | -0.69 |
| 1431677_at | 4631424J17Rik | RIKEN cDNA 4631424J17 gene | 1.04 | 0.828 | -1.43 | 0.084 | -1.69 | 0.213 | -1 | 0.986 | -0.77 |
| 1452794_x_at | Speer1-ps1 /// LOC | spermatogenesis associated glutamate (E)-rich protein 1, pseudogene 1 /// simila | -2.08 | 0.329 | -1.34 | 0.256 | 1.1 | 0.544 | -1.35 | 0.638 | -0.92 |
| 1460080_at | AI645535 | expressed sequence AI645535 | -1.53 | 0.186 | -1.28 | 0.171 | -1.12 | 0.417 | 1.16 | 0.663 | -0.69 |
| 1451909_a_at | Prpf4b | PRP4 pre-mRNA processing factor 4 homolog B (yeast) | -1.33 | 0.026 | -1.24 | 0.087 | -1.29 | 0.286 | 1.13 | 0.157 | -0.68 |
| 1441404_at | Pafah1b1 | Platelet-activating factor acetylhydrolase, isoform 1b, beta1 subunit | -1.22 | 0.167 | -1.36 | 0.263 | -1.29 | 0.514 | 1.1 | 0.567 | -0.69 |
| 1435986_x_at | Sdhc | succinate dehydrogenase complex, subunit C, integral membrane protein | -1.28 | 0.362 | -1.26 | 0.223 | -1.33 | 0.086 | 1.11 | 0.642 | -0.69 |
| 1426026_at | Prpf6 | PRP6 pre-mRNA splicing factor 6 homolog (yeast) | -1.37 | 0.06 | -2.15 | 0.267 | 1.13 | 0.761 | -1.03 | 0.949 | -0.85 |
| 1421279_at | Lamc2 | laminin, gamma 2 | -1.18 | 0.246 | 1.09 | 0.798 | -2.58 | 0.135 | -3.45 | 0.047 | -1.53 |
| 1441791_at | Oprm1 | Opioid receptor, mu 1 | -1.31 | 0.298 | -1.42 | 0.06 | -1.15 | 0.487 | 1.53 | 0.369 | -0.59 |
| 1442279_at | Epc1 | Enhancer of polycomb homolog 1 (Drosophila) | -1.13 | 0.725 | 1.02 | 0.745 | -2.35 | 0.033 | -1 | 0.983 | -0.87 |
| 1456446_at | 4930523C07Rik | RIKEN cDNA 4930523C07 gene | 1.08 | 0.774 | -1.37 | 0.398 | -1.95 | 0.011 | -1.95 | 0.026 | -1.04 |
| 1441406_at | --- | --- | -1.29 | 0.805 | -2.22 | 0.253 | 1.11 | 0.81 | -1.48 | 0.55 | -0.97 |
| 1434422_at | --- | --- | -1.12 | 0.515 | -1.36 | 0.026 | -1.42 | 0.133 | 1 | 0.996 | -0.73 |
| 1458390_at | Anapc10 | anaphase promoting complex subunit 10 | -1.41 | 0.147 | -1.52 | 0.519 | -1.04 | 0.842 | 1.32 | 0.077 | -0.66 |
| 1419877_x_at | --- | Transcribed locus | -1.93 | 0.476 | 1.12 | 0.731 | -1.46 | 0.279 | -1.44 | 0.298 | -0.92 |
| 1432932_at | --- | --- | -2.66 | 0.238 | 1.03 | 0.952 | -1.08 | 0.861 | -3 | 0.044 | -1.43 |
| 1455477_s_at | Pdzk1ip1 | PDZK1 interacting protein 1 | -1.13 | 0.065 | -1.46 | 0.085 | -1.31 | 0.238 | 4.23 | 0.168 | 0.08 |
| 1423465_at | Frrs1 /// LOC63599 | ferric-chelate reductase 1 /// similar to stromal cell derived factor receptor 2 | -1.21 | 0.146 | -1.22 | 0.183 | -1.47 | 0.057 | 1.59 | 0.2 | -0.58 |
| 1430369_at | Epb4.1 | erythrocyte protein band 4.1 | -1.15 | 0.779 | -1.76 | 0.115 | -1.12 | 0.735 | 2.98 | 0.245 | -0.26 |
| 1433760_a_at | Rhbdd3 | rhomboid domain containing 3 | -1.03 | 0.798 | -1.02 | 0.952 | -2.68 | 0.009 | 1.7 | 0.242 | -0.76 |
| 1441802_at | Spred2 | sprouty-related, EVH1 domain containing 2 | 1.21 | 0.357 | -1.29 | 0.273 | -2.92 | 0.038 | -3.43 | 0.037 | -1.61 |
| 1454440_at | 4930421C12Rik | RIKEN cDNA 4930421C12 gene | 1.3 | 0.525 | -2 | 0.247 | -1.87 | 0.141 | -2.36 | 0.045 | -1.23 |
| 1448261_at | Cdh1 | cadherin 1 | -1.04 | 0.738 | -3.8 | 0.02 | 1.11 | 0.249 | -1.38 | 0.041 | -1.28 |

| | | | | | | | | | | | |
|---|---|---|---|---|---|---|---|---|---|---|---|
| 1441437_at | Rbms1 | RNA binding motif, single stranded interacting protein 1 | 1.08 | 0.613 | -1.12 | 0.662 | -2.77 | 0.013 | -1.75 | 0.012 | -1.14 |
| 1416325_at | Crisp1 | cysteine-rich secretory protein 1 | -1.23 | 0.58 | -1.35 | 0.577 | -1.29 | 0.663 | 1.12 | 0.805 | -0.69 |
| 1444180_at | --- | 12 days embryo spinal ganglion cDNA, RIKEN full-length enriched library, clone:D | 1.02 | 0.954 | -1.32 | 0.095 | -1.79 | 0.009 | -1.7 | 0.103 | -0.95 |
| 1439923_at | Tubgcp5 | tubulin, gamma complex associated protein 5 | 1.49 | 0.571 | -2.61 | 0.198 | -2.18 | 0.101 | -1.07 | 0.904 | -1.09 |
| 1430795_at | 5830407F19Rik | RIKEN cDNA 5830407F19 gene | -1.3 | 0.06 | -1.52 | 0.287 | -1.11 | 0.637 | 1.22 | 0.26 | -0.68 |
| 1444604_at | Ezh1 | Enhancer of zeste homolog 1 (Drosophila) | -1.31 | 0.516 | -1.45 | 0.046 | -1.14 | 0.441 | 1.99 | 0.055 | -0.48 |
| 1444301_at | Pcdh10 | protocadherin 10 | -1.96 | 0.135 | -1.9 | 0.502 | 1.29 | 0.563 | -1.62 | 0.435 | -1.05 |
| 1444311_at | --- | --- | -1.96 | 0.134 | -1.5 | 0.504 | 1.16 | 0.468 | -1.08 | 0.809 | -0.85 |
| 1443561_at | Mrg1 | Myeloid ecotropic viral integration site-related gene 1 | -1.6 | 0.344 | 1.06 | 0.922 | -1.56 | 0.169 | -3.48 | 0.042 | -1.39 |
| 1438763_at | Dnahc2 /// Dnhd3 | dynein, axonemal, heavy chain 2 /// dynein heavy chain domain 3 | -1.55 | 0.354 | -1.72 | 0.112 | 1.11 | 0.547 | -1.04 | 0.93 | -0.8 |
| 1447020_at | Zfpm2 | Zinc finger protein, multitype 2 | -1.7 | 0.135 | 1.17 | 0.629 | -1.75 | 0.025 | -1.13 | 0.826 | -0.85 |
| 1440444_at | Fads1 | fatty acid desaturase 1 | -1.36 | 0.348 | -1.44 | 0.408 | -1.11 | 0.555 | 2.41 | 0.234 | -0.37 |
| 1423576_a_at | Fhl4 | four and a half LIM domains 4 | -1.11 | 0.85 | -1.04 | 0.963 | -2.14 | 0.387 | 1.1 | 0.794 | -0.8 |
| 1452567_at | Gcap8 | granule cell antiserum positive 8 | -3.11 | 0.054 | -1.01 | 0.975 | 1.02 | 0.964 | -1.13 | 0.55 | -1.06 |
| 1442059_at | Fxr1h | fragile X mental retardation gene 1, autosomal homolog | -1.45 | 0.485 | -1.05 | 0.818 | -1.44 | 0.08 | 1.13 | 0.561 | -0.7 |
| 1458213_at | Nfat5 | Nuclear factor of activated T-cells 5 | 1.09 | 0.789 | -1.73 | 0.087 | -1.5 | 0.124 | -1.56 | 0.117 | -0.93 |
| 1443642_at | Taok2 | TAO kinase 2 | -2.04 | 0.253 | -1.05 | 0.899 | -1.12 | 0.719 | 3.7 | 0.078 | -0.13 |
| 1444077_at | --- | Transcribed locus | -1.32 | 0.286 | 1.14 | 0.811 | -2.28 | 0.31 | -1.02 | 0.947 | -0.87 |
| 1458926_at | 5830415L20Rik | RIKEN cDNA 5830415L20 gene | -1.03 | 0.939 | -1.83 | 0.184 | -1.22 | 0.165 | 1.77 | 0.052 | -0.58 |
| 1445494_at | Aaas | Achalasia, adrenocortical insufficiency, alacrimia | -2.01 | 0.46 | 1.13 | 0.591 | -1.41 | 0.353 | -1.75 | 0.439 | -1.01 |
| 1445536_at | AW061096 | expressed sequence AW061096 | -1.6 | 0.231 | -1.08 | 0.763 | -1.28 | 0.459 | 1.36 | 0.499 | -0.65 |
| 1453028_at | 4631424J17Rik | RIKEN cDNA 4631424J17 gene | -1.2 | 0.07 | -1.24 | 0.339 | -1.44 | 0.139 | 1.06 | 0.827 | -0.71 |
| 1459543_at | AA407787 | expressed sequence AA407787 | -1.32 | 0.652 | -1.31 | 0.702 | -1.23 | 0.624 | 1.78 | 0.251 | -0.52 |
| 1458409_at | C86595 | expressed sequence C86595 | -1.8 | 0.212 | 1.4 | 0.538 | -2.65 | 0.073 | -1.01 | 0.973 | -1.01 |
| 1446185_at | Frap1 | FK506 binding protein 12-rapamycin associated protein 1 | -1.28 | 0.497 | -1.33 | 0.599 | -1.24 | 0.454 | 1.22 | 0.724 | -0.66 |
| 1446109_at | Wdfy3 | WD repeat and FYVE domain containing 3 | 1.48 | 0.003 | -2.81 | 0.247 | -1.99 | 0.267 | -1.27 | 0.69 | -1.15 |
| 1447631_at | Myst2 | MYST histone acetyltransferase 2 | -1.13 | 0.287 | -1.33 | 0.295 | -1.44 | 0.102 | 1.26 | 0.2 | -0.66 |
| 1421362_a_at | Frk | fyn-related kinase | -1.12 | 0.406 | -1.85 | 0.276 | -1.11 | 0.758 | 1.83 | 0.353 | -0.56 |
| 1434724_at | Usp31 | ubiquitin specific peptidase 31 | -1.12 | 0.417 | -1.46 | 0.001 | -1.32 | 0.404 | 1.29 | 0.454 | -0.65 |
| 1460389_at | Cdk8 | cyclin-dependent kinase 8 | -1.11 | 0.714 | -1.3 | 0.051 | -1.51 | 0.081 | 1.04 | 0.811 | -0.72 |
| 1432114_at | 1110035E04Rik | RIKEN cDNA 1110035E04 gene | -1.19 | 0.607 | -1.52 | 0.312 | -1.19 | 0.433 | 1.08 | 0.752 | -0.71 |
| 1458822_at | --- | --- | 1.23 | 0.83 | -1.31 | 0.409 | -2.91 | 0.058 | -1.58 | 0.481 | -1.14 |
| 1453809_at | Lypd5 | Ly6/Plaur domain containing 5 | -3.22 | 0.005 | -1.44 | 0.494 | 1.33 | 0.382 | -1.53 | 0.014 | -1.22 |
| 1445092_at | --- | --- | -1.25 | 0.433 | -1.51 | 0.405 | -1.14 | 0.591 | 1.11 | 0.792 | -0.7 |
| 1439926_at | 4632417D23Rik | RIKEN cDNA 4632417D23 gene | -1.54 | 0.312 | 1.13 | 0.759 | -1.81 | 0.153 | -1.67 | 0.225 | -0.97 |
| 1445606_a_at | 2900009J06Rik | RIKEN cDNA 2900009J06 gene | -1.15 | 0.781 | -1.47 | 0.308 | -1.27 | 0.333 | 1.53 | 0.323 | -0.59 |
| 1432512_at | 4931433A01Rik | RIKEN cDNA 4931433A01 gene | -1.83 | 0.412 | -1.08 | 0.851 | -1.16 | 0.628 | 1.34 | 0.293 | -0.68 |
| 1448209_a_at | Slc22a17 | solute carrier family 22 (organic cation transporter), member 17 | -1.25 | 0.479 | -1.62 | 0.014 | -1.09 | 0.169 | 1.02 | 0.957 | -0.74 |
| 1449073_at | Flnc | filamin C, gamma (actin binding protein 280) | 1.03 | 0.952 | -2.02 | 0.073 | -1.23 | 0.549 | -1.61 | 0.527 | -0.96 |
| 1424214_at | 9130213B05Rik | RIKEN cDNA 9130213B05 gene | -1.74 | 0.103 | -1.31 | 0.268 | 1 | 0.985 | -1.95 | 0.244 | -1 |
| 1418669_at | Hspg2 | perlecan (heparan sulfate proteoglycan 2) | -1.38 | 0.193 | -1.45 | 0.222 | -1.09 | 0.653 | 1.19 | 0.48 | -0.68 |
| 1447308_at | Lass5 | longevity assurance homolog 5 (S. cerevisiae) | -1.02 | 0.957 | -1.78 | 0.276 | -1.26 | 0.013 | 1.08 | 0.724 | -0.75 |
| 1438039_at | Hectd1 | HECT domain containing 1 | -1.16 | 0.624 | -1.45 | 0.023 | -1.27 | 0.115 | 1.2 | 0.438 | -0.67 |
| 1444651_at | LOC553089 | hypothetical LOC553089 | 1.04 | 0.882 | -1.2 | 0.434 | -2.14 | 0.015 | -1.11 | 0.736 | -0.85 |
| 1452597_at | 2310061C15Rik | RIKEN cDNA 2310061C15 gene | -1.17 | 0.086 | -1.22 | 0.242 | -1.52 | 0.002 | 2.03 | 0.177 | -0.47 |
| 1450346_at | Gpr50 | G-protein-coupled receptor 50 | -1.29 | 0.174 | -1.23 | 0.456 | -1.35 | 0.364 | 1.11 | 0.657 | -0.69 |
| 1446689_at | D230007K08Rik | RIKEN cDNA D230007K08 gene | -2.06 | 0.082 | 1.02 | 0.935 | -1.2 | 0.453 | -1.37 | 0.563 | -0.9 |
| 1453332_at | 2410002O22Rik | RIKEN cDNA 2410002O22 gene | -1.26 | 0.256 | -1.72 | 0.016 | -1.04 | 0.868 | 1.22 | 0.548 | -0.7 |
| 1430081_at | Phf15 | PHD finger protein 15 | 1.01 | 0.956 | -1.28 | 0.55 | -1.83 | 0.029 | -1.84 | 0.012 | -0.99 |
| 1419887_at | Nudcd1 | NudC domain containing 1 | -1.93 | 0.228 | -1.39 | 0.478 | 1.1 | 0.827 | -1.09 | 0.841 | -0.83 |
| 1452723_at | Klf9 | Kruppel-like factor 9 | -1.07 | 0.673 | -1.65 | 0.134 | -1.25 | 0.547 | 1.14 | 0.782 | -0.71 |
| 1421678_at | Itpr2 | inositol 1,4,5-triphosphate receptor 2 | -1.37 | 0.592 | -1.8 | 0.005 | 1.05 | 0.866 | -1.55 | 0.107 | -0.92 |
| 1459220_at | C78651 | expressed sequence C78651 | -1.08 | 0.853 | -1.71 | 0.106 | -1.21 | 0.541 | 1.43 | 0.443 | -0.64 |

| | | | | | | | | | | | |
|---|---|---|---|---|---|---|---|---|---|---|---|
| 1456756_at | E430016P22Rik | RIKEN cDNA E430016P22 gene | -1.16 | 0.513 | -1.83 | 0.256 | -1.08 | 0.803 | 1.03 | 0.94 | -0.76 |
| 1441153_at | Utrn | Utrophin | 1.12 | 0.353 | -1.41 | 0.014 | -1.98 | 0.013 | -1.66 | 0.304 | -0.98 |
| 1446829_at | D12Ertd216e | DNA segment, Chr 12, ERATO Doi 216, expressed | -1.14 | 0.74 | -2.77 | 0.314 | 1.1 | 0.886 | -1.87 | 0.338 | -1.17 |
| 1454537_at | 4930556A12Rik | RIKEN cDNA 4930556A12 gene | -2.5 | 0.433 | -3.18 | 0.014 | 1.62 | 0.303 | -2.07 | 0.478 | -1.53 |
| 1429986_at | Gpr173 | G-protein coupled receptor 173 | -3.39 | 0.094 | -1.49 | 0.567 | 1.37 | 0.534 | -1.5 | 0.494 | -1.25 |
| 1445525_at | Kif14 | kinesin family member 14 | -1.28 | 0.244 | -1.27 | 0.599 | -1.3 | 0.569 | 1.51 | 0.47 | -0.58 |
| 1460666_a_at | Ebf3 | early B-cell factor 3 | -1.05 | 0.883 | -1.2 | 0.738 | -1.82 | 0.078 | 1.7 | 0.342 | -0.59 |
| 1455179_at | Mpp7 | membrane protein, palmitoylated 7 (MAGUK p55 subfamily member 7) | -1.16 | 0.12 | -1.23 | 0.37 | -1.5 | 0.146 | 1.61 | 0.288 | -0.57 |
| 1460002_at | Tlk1 | tousled-like kinase 1 | -1.13 | 0.51 | -1.97 | 0.095 | -1.06 | 0.671 | 1.17 | 0.581 | -0.75 |
| 1449431_at | Trpc6 | transient receptor potential cation channel, subfamily C, member 6 | -1.11 | 0.894 | -1.12 | 0.861 | -1.86 | 0.039 | 1.4 | 0.582 | -0.67 |
| 1455716_at | --- | Adult male corpora quadrigemina cDNA, RIKEN full-length enriched library, clone | -1.1 | 0.748 | -1.42 | 0.629 | -1.38 | 0.428 | 1.05 | 0.912 | -0.71 |
| 1442242_at | C79127 | expressed sequence C79127 | -1.05 | 0.941 | -1.57 | 0.422 | -1.32 | 0.522 | 1.1 | 0.902 | -0.71 |
| 1430946_at | 2600014E21Rik | RIKEN cDNA 2600014E21 gene | 1.35 | 0.555 | -2.12 | 0.003 | -1.92 | 0.067 | -1.66 | 0.516 | -1.09 |
| 1436624_at | Dnm3 | dynamin 3 | -1.31 | 0.018 | -1.09 | 0.748 | -1.51 | 0.04 | 1.65 | 0.035 | -0.57 |
| 1440514_at | D3Ertd508e | DNA segment, Chr 3, ERATO Doi 508, expressed | 1.01 | 0.99 | -1.93 | 0.284 | -1.23 | 0.596 | -1.48 | 0.292 | -0.91 |
| 1447373_at | 1810015C04Rik | RIKEN cDNA 1810015C04 gene | -1.3 | 0.62 | -1.33 | 0.575 | -1.22 | 0.124 | 1.98 | 0.14 | -0.47 |
| 1423085_at | Efnb3 | ephrin B3 | -1.22 | 0.819 | -1.52 | 0.322 | -1.16 | 0.578 | 1.41 | 0.131 | -0.62 |
| 1446230_at | Gm944 | Gene model 944, (NCBI) | -1.09 | 0.747 | -1.16 | 0.433 | -1.78 | 0.09 | 5.7 | 0.179 | 0.42 |
| 1436404_at | Tlcd1 | TLC domain containing 1 | -1.2 | 0.023 | -1.19 | 0.262 | -1.49 | 0.031 | 3.81 | 0.228 | -0.02 |
| 1431596_at | --- | --- | 1.31 | 0.668 | -1.47 | 0.231 | -2.84 | 0.015 | -1.21 | 0.655 | -1.05 |
| 1445312_at | C530043G21Rik | RIKEN cDNA C530043G21 gene | -1.42 | 0.536 | -1.42 | 0.358 | -1.08 | 0.767 | 1.03 | 0.876 | -0.72 |
| 1426638_at | Six3 | sine oculis-related homeobox 3 homolog (Drosophila) | -2.19 | 0.088 | 1.21 | 0.379 | -1.5 | 0.077 | -1.91 | 0.426 | -1.09 |
| 1458865_at | --- | --- | -1.54 | 0.518 | -1.09 | 0.875 | -1.29 | 0.573 | 1.61 | 0.31 | -0.58 |
| 1451942_x_at | Klra20 | killer cell lectin-like receptor subfamily A, member 20 | 1.11 | 0.644 | -1.36 | 0.436 | -2.03 | 0.109 | -1.32 | 0.688 | -0.9 |
| 1440603_at | --- | Adult male corpus striatum cDNA, RIKEN full-length enriched library, clone:C030 | -2.75 | 0.39 | -1.01 | 0.981 | -1.01 | 0.954 | 1.01 | 0.989 | -0.94 |
| 1459160_at | Gm879 | Gene model 879, (NCBI) | -2.92 | 0.438 | 1.18 | 0.825 | -1.22 | 0.606 | -1.8 | 0.492 | -1.19 |
| 1432087_at | 4933406G16Rik | RIKEN cDNA 4933406G16 gene | -1.02 | 0.984 | -1.05 | 0.895 | -2.44 | 0.068 | 1.44 | 0.061 | -0.77 |
| 1454031_at | 2310029O18Rik | RIKEN cDNA 2310029O18 gene | -1.12 | 0.576 | -1.1 | 0.823 | -1.85 | 0.002 | 1.43 | 0.109 | -0.66 |
| 1459094_at | Snap91 | Synaptosomal-associated protein 91 | -1.89 | 0.392 | 1.06 | 0.924 | -1.33 | 0.355 | -1.19 | 0.617 | -0.84 |
| 1425868_at | Hist2h2bb | Histone 2, H2bb | 1.15 | 0.478 | -2.63 | 0.233 | -1.24 | 0.44 | -1.42 | 0.201 | -1.03 |
| 1440195_at | Serbp1 | Serpine1 mRNA binding protein 1 | -1.18 | 0.281 | -1.17 | 0.181 | -1.56 | 0.283 | 1.12 | 0.61 | -0.7 |
| 1439930_at | Smarca2 | SWI/SNF related, matrix associated, actin dependent regulator of chromatin, subf | 1.03 | 0.851 | -1.39 | 0.168 | -1.7 | 0.054 | -1.52 | 0.152 | -0.89 |
| 1458237_at | --- | --- | -2.95 | 0.411 | -1.08 | 0.905 | 1.08 | 0.921 | -1.15 | 0.879 | -1.03 |
| 1419991_at | Rab3il1 | RAB3A interacting protein (rabin3)-like 1 | 1.04 | 0.964 | -1.32 | 0.351 | -1.83 | 0.156 | -2 | 0.459 | -1.03 |
| 1418558_at | Rax | retina and anterior neural fold homeobox | -1.11 | 0.336 | -1.17 | 0.715 | -1.69 | 0.198 | 1.19 | 0.448 | -0.7 |
| 1437482_at | Srd5a2l2 | steroid 5 alpha-reductase 2-like 2 | -1.38 | 0.623 | -1.32 | 0.639 | -1.17 | 0.679 | 1.97 | 0.184 | -0.47 |
| 1445616_at | Eif4g3 | Eukaryotic translation initiation factor 4 gamma, 3 | -1.63 | 0.079 | -1.07 | 0.844 | -1.25 | 0.613 | 1.33 | 0.66 | -0.66 |
| 1443651_at | Phc3 | Polyhomeotic-like 3 (Drosophila) | 1.09 | 0.926 | -1.15 | 0.684 | -2.6 | 0.051 | -1.63 | 0.339 | -1.07 |
| 1429516_at | Ubr2 | ubiquitin protein ligase E3 component n-recognin 2 | -1.55 | 0.44 | -1.14 | 0.576 | -1.22 | 0.512 | 1.24 | 0.303 | -0.67 |
| 1423376_a_at | Dok4 | docking protein 4 | -1.02 | 0.929 | -2.16 | 0.398 | -1.11 | 0.639 | 1.28 | 0.454 | -0.75 |
| 1437315_at | Gpr172b | G protein-coupled receptor 172B | -1.14 | 0.114 | -1.08 | 0.777 | -1.84 | 0.175 | 1.78 | 0.043 | -0.57 |
| 1435286_at | AW125296 | expressed sequence AW125296 | -1.24 | 0.786 | -3.09 | 0.182 | 1.21 | 0.728 | -1.76 | 0.447 | -1.22 |
| 1440529_at | --- | Transcribed locus | -2.47 | 0.098 | -1.46 | 0.335 | 1.25 | 0.667 | -5.19 | 0.013 | -1.97 |
| 1430346_at | 5730507A09Rik | RIKEN cDNA 5730507A09 gene | -1.36 | 0.576 | -1.15 | 0.726 | -1.35 | 0.461 | 1.35 | 0.436 | -0.63 |
| 1446177_at | --- | --- | -1.87 | 0.041 | -2.04 | 0.051 | 1.32 | 0.538 | -1.08 | 0.699 | -0.92 |
| 1428896_at | Pdgfrl | platelet-derived growth factor receptor-like | -1.63 | 0.05 | 1.22 | 0.506 | -1.96 | 0.004 | -1.07 | 0.585 | -0.86 |
| 1450542_s_at | Magea1 /// Magea2 | melanoma antigen, family A, 1 /// melanoma antigen, family A, 2 /// melanoma ant | -1.46 | 0.03 | -1.4 | 0.547 | -1.06 | 0.899 | 1.38 | 0.38 | -0.63 |
| 1424152_at | --- | CDNA clone IMAGE:30031514 | -1.06 | 0.899 | 1.08 | 0.87 | -3.17 | 0.002 | -1.34 | 0.509 | -1.12 |
| 1458287_at | --- | Transcribed locus | -1.86 | 0.1 | -1.13 | 0.635 | -1.09 | 0.875 | 1.49 | 0.582 | -0.64 |
| 1446494_at | --- | Non-coding RNA Kis2-T1 mRNA, complete sequence, alternatively spliced | 1.07 | 0.827 | -1.42 | 0.336 | -1.77 | 0.176 | -1.17 | 0.771 | -0.82 |
| 1416771_at | Trappc3 | trafficking protein particle complex 3 | -1.83 | 0.352 | -1.31 | 0.526 | 1.03 | 0.943 | -2.3 | 0.211 | -1.1 |
| 1442202_at | 6430706D22Rik | RIKEN cDNA 6430706D22 gene | -1.41 | 0.43 | -1.3 | 0.485 | -1.15 | 0.664 | 1.43 | 0.346 | -0.61 |
| 1424794_at | Rnf186 | ring finger protein 186 | -1.27 | 0.42 | -1.4 | 0.303 | -1.19 | 0.375 | 1.05 | 0.888 | -0.7 |

| | | | | | | | | | | | |
|---|---|---|---|---|---|---|---|---|---|---|---|
| 1430428_at | 2310066F23Rik | RIKEN cDNA 2310066F23 gene | -1.43 | 0.03 | 1.03 | 0.854 | -1.63 | 0.062 | -1.32 | 0.04 | -0.84 |
| 1429976_at | Clasp2 | CLIP associating protein 2 | -1.3 | 0.799 | -1.75 | 0.272 | 1.01 | 0.974 | -1.17 | 0.661 | -0.8 |
| 1421246_at | Pax9 | paired box gene 9 | -1.66 | 0.394 | -1.61 | 0.297 | 1.12 | 0.85 | -1.73 | 0.468 | -0.97 |
| 1416697_at | Dpp4 | dipeptidylpeptidase 4 | 1.01 | 0.945 | -1.19 | 0.056 | -2.03 | 0.088 | -3.01 | 0.05 | -1.3 |
| 1451376_at | 5730596K20Rik | RIKEN cDNA 5730596K20 gene | -1.19 | 0.382 | -1.31 | 0.071 | -1.35 | 0.087 | 1.34 | 0.479 | -0.63 |
| 1444094_at | --- | --- | 1.02 | 0.904 | -1.72 | 0.028 | -1.35 | 0.36 | -1.89 | 0.173 | -0.98 |
| 1440908_at | D030063E12 | hypothetical protein D030063E12 | -1.21 | 0.145 | -1.46 | 0.308 | -1.2 | 0.46 | 1.19 | 0.372 | -0.67 |
| 1423892_at | Apbb1 | amyloid beta (A4) precursor protein-binding, family B, member 1 | -1.04 | 0.859 | -2.39 | 0.005 | -1.04 | 0.708 | 1.01 | 0.95 | -0.86 |
| 1444808_at | Pawr | PRKC, apoptosis, WT1, regulator | -1.19 | 0.283 | -1.14 | 0.526 | -1.59 | 0.149 | 1.07 | 0.893 | -0.71 |
| 1449790_at | --- | --- | 1.2 | 0.571 | -1.77 | 0.276 | -1.71 | 0.037 | -1.42 | 0.013 | -0.93 |
| 1446391_at | Snca | Synuclein, alpha | -1.27 | 0.052 | -1.21 | 0.654 | -1.36 | 0.359 | 6.6 | 0.178 | 0.69 |
| 1446807_at | Usp8 | ubiquitin specific peptidase 8 | 1.03 | 0.947 | -1.18 | 0.298 | -2.11 | 0.007 | -1.11 | 0.54 | -0.84 |
| 1439635_at | Rgs9 | regulator of G-protein signaling 9 | -2.91 | 0.002 | 1.11 | 0.821 | -1.13 | 0.767 | -2.12 | 0.307 | -1.26 |
| 1456360_at | Dcun1d1 | DCUN1D1 DCN1, defective in cullin neddylation 1, domain containing 1 (S. cerev | -1.47 | 0.369 | -1.47 | 0.104 | -1.01 | 0.972 | 1.97 | 0.265 | -0.5 |
| 1432572_at | 4931406H21Rik | RIKEN cDNA 4931406H21 gene | -1.75 | 0.259 | 1.02 | 0.775 | -1.33 | 0.134 | -1.02 | 0.913 | -0.77 |
| 1444804_at | D17Ertd96e | DNA segment, Chr 17, ERATO Doi 96, expressed | 1.3 | 0.712 | -1.45 | 0.43 | -2.81 | 0.016 | -1.37 | 0.33 | -1.08 |
| 1440149_at | Pan3 | PAN3 polyA specific ribonuclease subunit homolog (S. cerevisiae) | -2.25 | 0.456 | 1.02 | 0.982 | -1.13 | 0.795 | -2.62 | 0.204 | -1.25 |
| 1450863_a_at | Dcamkl1 | doublecortin and calcium/calmodulin-dependent protein kinase-like 1 | -1 | 0.967 | -4.04 | 0.004 | 1.1 | 0.64 | -1.58 | 0.221 | -1.38 |
| 1421281_at | Gabra1 | gamma-aminobutyric acid (GABA-A) receptor, subunit alpha 1 | -1.04 | 0.899 | -1.34 | 0.596 | -1.57 | 0.355 | 1.13 | 0.806 | -0.7 |
| 1431887_at | Rbm31y | RNA binding motif 31, Y-linked | -2.03 | 0.529 | -1.26 | 0.641 | 1.06 | 0.877 | -2.47 | 0.415 | -1.17 |
| 1443847_x_at | --- | Transcribed locus | 1.61 | 0.168 | -2.22 | 0.105 | -3.43 | 0.004 | -1.12 | 0.814 | -1.29 |
| 1457213_a_at | Dgkh | diacylglycerol kinase, eta | -1.35 | 0.168 | -1.54 | 0.148 | -1.04 | 0.759 | 1 | 0.991 | -0.73 |
| 1444183_at | C030013C21Rik | RIKEN cDNA C030013C21 gene | -1.37 | 0.169 | -1 | 0.984 | -1.6 | 0.175 | 2.97 | 0.06 | -0.25 |
| 1438414_at | Fkrp | fukutin related protein | -1.44 | 0.6 | -1.87 | 0.279 | 1.12 | 0.751 | -1.62 | 0.043 | -0.95 |
| 1441214_at | Exph5 | exophilin 5 | 1.12 | 0.517 | -1.58 | 0.019 | -1.7 | 0.028 | -1.49 | 0.243 | -0.91 |
| 1419043_a_at | Iigp1 | interferon inducible GTPase 1 | -1.07 | 0.804 | -1.31 | 0.064 | -1.54 | 0.126 | 1.1 | 0.66 | -0.7 |
| 1430819_at | Asahl | N-acylsphingosine amidohydrolase (acid ceramidase)-like | -1.71 | 0.175 | -1.13 | 0.839 | -1.13 | 0.662 | 1.35 | 0.358 | -0.66 |
| 1430983_at | Pdia6 | protein disulfide isomerase associated 6 | -1.06 | 0.83 | -1.31 | 0.524 | -1.57 | 0.065 | 1.24 | 0.63 | -0.67 |
| 1453385_at | 1110015C02Rik | RIKEN cDNA 1110015C02 gene | -1.63 | 0.149 | -1.3 | 0.221 | -1.03 | 0.91 | 1.07 | 0.777 | -0.72 |
| 1420402_at | Atp2b2 | ATPase, Ca++ transporting, plasma membrane 2 | -1.2 | 0.361 | 1.01 | 0.952 | -1.98 | 0.296 | -1.21 | 0.381 | -0.84 |
| 1432864_at | --- | --- | 1.03 | 0.931 | -1.42 | 0.157 | -1.62 | 0.19 | -1.36 | 0.393 | -0.84 |
| 1456203_at | 1110020A10Rik | RIKEN cDNA 1110020A10 gene | 1.15 | 0.614 | -1.59 | 0.276 | -1.76 | 0.113 | -1.39 | 0.493 | -0.9 |
| 1437654_at | 3110001K24Rik | RIKEN cDNA 3110001K24 gene | -1.31 | 0.231 | -1.82 | 0.032 | 1.04 | 0.786 | -1.42 | 0.274 | -0.88 |
| 1429693_at | Dab2 | disabled homolog 2 (Drosophila) | -1.38 | 0.09 | 1.05 | 0.894 | -1.72 | 0.086 | -1.2 | 0.643 | -0.81 |
| 1420245_x_at | Tagln2 /// LOC6724 | transgelin 2 /// similar to transgelin 2 | -1 | 0.995 | -1.79 | 0.222 | -1.26 | 0.584 | 1.07 | 0.861 | -0.75 |
| 1441505_at | --- | --- | -1.13 | 0.516 | -1.03 | 0.926 | -2.04 | 0.005 | 1.18 | 0.527 | -0.76 |
| 1452552_at | Npn2 | neoplastic progression 2 | -1.83 | 0.432 | 1.47 | 0.33 | -2.97 | 0.092 | -1.31 | 0.607 | -1.16 |
| 1418780_at | Cyp39a1 | cytochrome P450, family 39, subfamily a, polypeptide 1 | -2.05 | 0.3 | -1.55 | 0.106 | 1.22 | 0.592 | -3.18 | 0.09 | -1.39 |
| 1427326_at | 4732471D19Rik | RIKEN cDNA 4732471D19 gene | -1.43 | 0.015 | -1.34 | 0.457 | -1.1 | 0.636 | 1.07 | 0.782 | -0.7 |
| 1431092_at | Ppp1r12c | protein phosphatase 1, regulatory (inhibitor) subunit 12C | -1.43 | 0.362 | -2.66 | 0.207 | 1.28 | 0.326 | -1.11 | 0.812 | -0.98 |
| 1425236_at | Col20a1 | collagen, type XX, alpha 1 | -1.2 | 0.772 | -1.32 | 0.56 | -1.31 | 0.667 | 1.28 | 0.35 | -0.64 |
| 1456335_at | Gm106 | gene model 106, (NCBI) | -1.07 | 0.767 | -1.74 | 0.473 | -1.19 | 0.515 | 2.28 | 0.024 | -0.43 |
| 1431838_at | 1700128E19Rik | RIKEN cDNA 1700128E19 gene | -1.81 | 0.086 | -1.37 | 0.259 | 1.07 | 0.761 | -1.38 | 0.07 | -0.87 |
| 1425636_at | Hhat | hedgehog acyltransferase | 1.06 | 0.673 | -1.25 | 0.46 | -2.04 | 0.091 | -1.16 | 0.637 | -0.85 |
| 1441435_at | Tbl1x | Transducin (beta)-like 1 X-linked | 1.03 | 0.939 | -1.31 | 0.168 | -1.77 | 0.009 | -1.43 | 0.048 | -0.87 |
| 1420926_at | Arx | aristaless related homeobox gene (Drosophila) | -1.79 | 0.311 | 1.35 | 0.489 | -2.28 | 0.053 | -1.46 | 0.516 | -1.04 |
| 1429505_at | 2310076G13Rik | RIKEN cDNA 2310076G13 gene | 1.02 | 0.934 | -1.3 | 0.003 | -1.78 | 0.018 | -1.06 | 0.734 | -0.78 |
| 1445126_at | Ldb2 | LIM domain binding 2 | -1.28 | 0.572 | -1.09 | 0.867 | -1.54 | 0.059 | 1.13 | 0.821 | -0.69 |
| 1424308_at | Slc24a3 | solute carrier family 24 (sodium/potassium/calcium exchanger), member 3 | -1.14 | 0.611 | -1.9 | 0.034 | -1.06 | 0.801 | 1.09 | 0.76 | -0.75 |
| 1446198_at | Mier3 | Mesoderm induction early response 1, family member 3 | -1.73 | 0.251 | 1.08 | 0.732 | -1.43 | 0.164 | -1.31 | 0.111 | -0.85 |
| 1416790_a_at | Tdg /// LOC545124 | thymine DNA glycosylase /// similar to thymine DNA glycosylase isoform 2 /// simi | -1.22 | 0.074 | -1.6 | 0.001 | -1.1 | 0.268 | 1.32 | 0.264 | -0.65 |
| 1442586_at | Socs2 | suppressor of cytokine signaling 2 | -1.13 | 0.019 | -1.11 | 0.573 | -1.78 | 0.068 | 1.11 | 0.459 | -0.73 |
| 1418951_at | Txlnb | taxilin beta | -3.42 | 0.247 | -2.44 | 0.213 | 1.65 | 0.27 | -1.16 | 0.659 | -1.34 |

| | | | | | | | | | | | |
|---|---|---|---|---|---|---|---|---|---|---|---|
| 1435931_at | E130308A19Rik | RIKEN cDNA E130308A19 gene | -1.29 | 0.129 | -1.06 | 0.79 | -1.58 | 0.051 | 1.28 | 0.354 | -0.66 |
| 1422825_at | Cart | cocaine and amphetamine regulated transcript | -1.04 | 0.932 | -3.53 | 0.099 | 1.11 | 0.81 | -3.75 | 0.042 | -1.8 |
| 1440371_at | --- | 2 days neonate sympathetic ganglion cDNA, RIKEN full-length enriched library, c | -1.26 | 0.574 | -1.2 | 0.723 | -1.38 | 0.087 | 1.58 | 0.304 | -0.57 |
| 1446632_at | Cacnb2 | Calcium channel, voltage-dependent, beta 2 subunit | -1.13 | 0.657 | 1.01 | 0.952 | -2.18 | 0.04 | -1.26 | 0.543 | -0.89 |
| 1428622_at | Depdc6 | DEP domain containing 6 | -1.06 | 0.615 | -1.33 | 0.018 | -1.53 | 0.043 | 1.02 | 0.879 | -0.72 |
| 1452455_at | Fer1l3 | fer-1-like 3, myoferlin (C. elegans) | -1.51 | 0.643 | -1.01 | 0.986 | -1.43 | 0.514 | 1.85 | 0.155 | -0.53 |
| 1443276_at | Htr5a | 5-hydroxytryptamine (serotonin) receptor 5A | -1.35 | 0.576 | -1.21 | 0.708 | -1.28 | 0.326 | 1.53 | 0.385 | -0.58 |
| 1420803_at | Tex18 | testis expressed gene 18 | -2.27 | 0.176 | -1.2 | 0.626 | 1.08 | 0.855 | -1.28 | 0.722 | -0.92 |
| 1422076_at | Acot4 | acyl-CoA thioesterase 4 | 1.03 | 0.932 | -1.47 | 0.269 | -1.55 | 0.442 | -1.65 | 0.564 | -0.91 |
| 1438811_at | Dlg7 | discs, large homolog 7 (Drosophila) | -1.91 | 0.233 | -1.02 | 0.98 | -1.18 | 0.498 | 3.36 | 0.258 | -0.19 |
| 1423638_at | Galnt4 | UDP-N-acetyl-alpha-D-galactosamine:polypeptide N-acetylgalactosaminyltransfe | 1.05 | 0.89 | -1.58 | 0.031 | -1.5 | 0.038 | -1.05 | 0.909 | -0.77 |
| 1459218_at | D330001F17Rik | RIKEN cDNA D330001F17 gene | -1.02 | 0.98 | -1.25 | 0.529 | -1.75 | 0.083 | 1.03 | 0.959 | -0.75 |
| 1426544_a_at | Ttc14 | tetratricopeptide repeat domain 14 | 1.08 | 0.623 | -1.51 | 0.229 | -1.63 | 0.092 | -1.42 | 0.389 | -0.87 |
| 1430611_at | Zdhhc25 | zinc finger, DHHC domain containing 25 | -5.04 | 0.017 | 1.48 | 0.545 | -1.49 | 0.307 | -1.01 | 0.987 | -1.51 |
| 1428289_at | Klf9 | Kruppel-like factor 9 | -1.23 | 0.538 | -1.31 | 0.301 | -1.29 | 0.369 | 1.03 | 0.962 | -0.7 |
| 1459010_at | 5430437P03Rik | RIKEN cDNA 5430437P03 gene | 1.09 | 0.847 | -1.56 | 0.105 | -1.6 | 0.113 | -1.01 | 0.94 | -0.77 |
| 1434160_at | Zfp592 | zinc finger protein 592 | -1.36 | 0.403 | -1.42 | 0.055 | -1.1 | 0.608 | 1.07 | 0.691 | -0.7 |
| 1455591_at | --- | --- | -1.33 | 0.154 | 1.04 | 0.93 | -1.78 | 0.054 | -1.13 | 0.628 | -0.8 |
| 1420462_at | Il1rapl2 | interleukin 1 receptor accessory protein-like 2 | 1.25 | 0.417 | -2.15 | 0.19 | -1.56 | 0.286 | -1.89 | 0.513 | -1.09 |
| 1453117_at | Surf2 | surfeit gene 2 | -1.35 | 0.396 | -1.22 | 0.568 | -1.26 | 0.629 | 1.52 | 0.492 | -0.58 |
| 1422550_a_at | Mtap6 | microtubule-associated protein 6 | -1.38 | 0.41 | -1.61 | 0.45 | 1.01 | 0.966 | -1.08 | 0.832 | -0.77 |
| 1457166_at | AA536749 | Expressed sequence AA536749 | -1.25 | 0.278 | 1.1 | 0.828 | -2.17 | 0.016 | -1.24 | 0.474 | -0.89 |
| 1429377_at | 2410004A20Rik | RIKEN cDNA 2410004A20 gene | -1.06 | 0.818 | -2.44 | 0.045 | -1 | 0.995 | 1.15 | 0.205 | -0.84 |
| 1440420_at | Stk24 | Serine/threonine kinase 24 (STE20 homolog, yeast) | -2.04 | 0.039 | -1 | 0.999 | -1.15 | 0.791 | 1.73 | 0.244 | -0.62 |
| 1431908_at | 4933408J17Rik | RIKEN cDNA 4933408J17 gene | -1.6 | 0.562 | -1.12 | 0.836 | -1.19 | 0.729 | 1.52 | 0.611 | -0.6 |
| 1456862_at | Six4 | sine oculis-related homeobox 4 homolog (Drosophila) | -1.46 | 0.178 | -2.24 | 0.3 | 1.23 | 0.26 | -2.86 | 0.412 | -1.33 |
| 1444430_at | Armc8 | Armadillo repeat containing 8 | -1.15 | 0.563 | -1.09 | 0.734 | -1.77 | 0.07 | 1.13 | 0.178 | -0.72 |
| 1433814_at | Wnt5b | Wingless-related MMTV integration site 5B | -1 | 0.991 | -1.31 | 0.509 | -1.68 | 0.257 | 1.33 | 0.194 | -0.67 |
| 1444425_at | Zfp53 | Zinc finger protein 53 | 1.04 | 0.895 | -1.74 | 0.185 | -1.36 | 0.148 | -1.1 | 0.776 | -0.79 |
| 1439610_at | Rab27b | RAB27b, member RAS oncogene family | -1.05 | 0.633 | -1.19 | 0.647 | -1.78 | 0.006 | 2.56 | 0.332 | -0.37 |
| 1433312_at | 2700057C20Rik | RIKEN cDNA 2700057C20 gene | -1.95 | 0.295 | -1.25 | 0.75 | 1.04 | 0.944 | -2.53 | 0.15 | -1.17 |
| 1457988_at | Sec63 | SEC63-like (S. cerevisiae) | 1.06 | 0.746 | -1.48 | 0.147 | -1.62 | 0.008 | -1.2 | 0.549 | -0.81 |
| 1446260_at | Six6os1 | Six6 opposite strand transcript 1 | -1.88 | 0.365 | -1.26 | 0.646 | 1.03 | 0.948 | -1.31 | 0.215 | -0.85 |
| 1426250_s_at | 9130404D08Rik | RIKEN cDNA 9130404D08 gene | 1.02 | 0.935 | -2.58 | 0.036 | -1.05 | 0.815 | -1.02 | 0.899 | -0.91 |
| 1450993_at | Pask | PAS domain containing serine/threonine kinase | -1.03 | 0.967 | -1.27 | 0.61 | -1.67 | 0.094 | 2.09 | 0.279 | -0.47 |
| 1457477_at | Mbnl2 | Muscleblind-like 2 | -1.34 | 0.395 | 1.18 | 0.287 | -2.32 | 0.006 | -1.23 | 0.469 | -0.93 |
| 1460086_at | --- | --- | 1.02 | 0.939 | -1.49 | 0.374 | -1.51 | 0.262 | -1.05 | 0.813 | -0.76 |
| 1445074_at | --- | 16 days neonate heart cDNA, RIKEN full-length enriched library, clone:D830036I | -1.25 | 0.719 | -2.06 | 0.258 | 1.07 | 0.902 | -1.43 | 0.486 | -0.92 |
| 1454910_at | --- | 13 days embryo heart cDNA, RIKEN full-length enriched library, clone:D330008M | -1.83 | 0.326 | -1.6 | 0.149 | 1.18 | 0.543 | -1.39 | 0.074 | -0.91 |
| 1457760_at | A930004J17Rik | RIKEN cDNA A930004J17 gene | -1.02 | 0.871 | -1.24 | 0.225 | -1.74 | 0.137 | 1.41 | 0.143 | -0.65 |
| 1426122_a_at | Coro6 | coronin, actin binding protein 6 | -1.37 | 0.624 | -2.24 | 0.093 | 1.18 | 0.264 | -1.17 | 0.432 | -0.9 |
| 1441486_at | C430014M02Rik | RIKEN cDNA C430014M02 gene | -1.3 | 0.656 | -1.32 | 0.416 | -1.21 | 0.304 | 1 | 0.993 | -0.71 |
| 1458273_at | --- | Transcribed locus | -1.61 | 0.071 | -1.2 | 0.751 | -1.11 | 0.738 | 1.33 | 0.199 | -0.65 |
| 1442234_at | LOC667085 | hypothetical protein LOC667085 | -1.68 | 0.36 | -1.27 | 0.563 | -1.03 | 0.916 | 1.73 | 0.049 | -0.56 |
| 1420185_at | Ssx2ip | synovial sarcoma, X breakpoint 2 interacting protein | -1.64 | 0.593 | -1.16 | 0.575 | -1.13 | 0.342 | 1.77 | 0.151 | -0.54 |
| 1450783_at | --- | --- | 1.13 | 0.757 | -1.46 | 0.239 | -1.85 | 0.092 | -1.17 | 0.457 | -0.84 |
| 1429814_at | Pank1 | pantothenate kinase 1 | -1.08 | 0.866 | -1.5 | 0.197 | -1.31 | 0.671 | 1.22 | 0.763 | -0.67 |
| 1446444_at | Mnab | membrane associated DNA binding protein | 1.09 | 0.809 | -1.4 | 0.467 | -1.82 | 0.104 | -1.5 | 0.114 | -0.91 |
| 1421000_at | Cnot4 | CCR4-NOT transcription complex, subunit 4 | 1.05 | 0.79 | -1.3 | 0.049 | -1.87 | 0.016 | -1.39 | 0.089 | -0.88 |
| 1445913_at | A730090H04Rik | RIKEN cDNA A730090H04 gene | -1.39 | 0.19 | 1.25 | 0.726 | -2.6 | 0.344 | -1.24 | 0.761 | -0.99 |
| 1418903_at | Aqp2 | aquaporin 2 | 1.29 | 0.385 | -1.63 | 0.54 | -2.18 | 0.033 | -1.1 | 0.807 | -0.9 |
| 1416733_at | Mkln1 | muskelin 1, intracellular mediator containing kelch motifs | -1.47 | 0.068 | -1.06 | 0.645 | -1.35 | 0.034 | 1.24 | 0.186 | -0.66 |
| 1433161_at | 1700008N17Rik | RIKEN cDNA 1700008N17 gene | -1.04 | 0.963 | -1.62 | 0.065 | -1.29 | 0.387 | 1.08 | 0.797 | -0.72 |

| | | | | | | | | | | | |
|---|---|---|---|---|---|---|---|---|---|---|---|
| 1459576_at | --- | --- | -1.52 | 0.205 | 1.15 | 0.758 | -1.81 | 0.362 | -1.21 | 0.591 | -0.85 |
| 1421555_at | Adrb3 | adrenergic receptor, beta 3 | -2.27 | 0.136 | -1.58 | 0.408 | 1.28 | 0.473 | -1.19 | 0.807 | -0.94 |
| 1436428_at | Chrnb2 | cholinergic receptor, nicotinic, beta polypeptide 2 (neuronal) | -1.37 | 0.251 | -1.5 | 0.219 | -1.04 | 0.816 | 1.11 | 0.746 | -0.7 |
| 1437920_at | Epha5 | Eph receptor A5 | -2.18 | 0.3 | 1.25 | 0.747 | -1.54 | 0.522 | -1.8 | 0.478 | -1.07 |
| 1445400_at | Hbxip | Hepatitis B virus x interacting protein | -1.05 | 0.777 | -1.38 | 0.545 | -1.46 | 0.077 | 1.43 | 0.324 | -0.62 |
| 1458262_at | Exdl1 | Vexonuclease 3'-5' domain-like 1 | -1.19 | 0.758 | -1.55 | 0.069 | -1.15 | 0.8 | 1.05 | 0.935 | -0.71 |
| 1455811_at | Eif3s6ip | eukaryotic translation initiation factor 3, subunit 6 interacting protein | -2.25 | 0.099 | -1.53 | 0.444 | 1.26 | 0.329 | -1.25 | 0.673 | -0.94 |
| 1450221_at | 4933417A18Rik | RIKEN cDNA 4933417A18 gene | 1.23 | 0.707 | -1.33 | 0.56 | -2.62 | 0.225 | -1.82 | 0.199 | -1.14 |
| 1423032_at | Rpl39 | ribosomal protein L39 | -1.71 | 0.036 | -1.3 | 0.427 | 1.01 | 0.973 | -1.07 | 0.898 | -0.77 |
| 1459467_at | AA986715 | expressed sequence AA986715 | -1.32 | 0.22 | -1.03 | 0.937 | -1.58 | 0.122 | 1.54 | 0.476 | -0.6 |
| 1433135_at | Golgb1 | golgi autoantigen, golgin subfamily b, macrogolgin 1 | 1.15 | 0.75 | -2.62 | 0.35 | -1.21 | 0.513 | -1.38 | 0.496 | -1.01 |
| 1435846_x_at | --- | --- | -1.67 | 0.2 | -1.19 | 0.47 | -1.09 | 0.531 | 1.25 | 0.169 | -0.67 |
| 1417067_s_at | Cabc1 | chaperone, ABC1 activity of bc1 complex like (S. pombe) | 1.02 | 0.947 | -1.47 | 0.164 | -1.51 | 0.155 | -1.79 | 0.226 | -0.94 |
| 1420723_at | Vnn3 | vanin 3 | -1.38 | 0.619 | -1.56 | 0.097 | -1.01 | 0.959 | 1.51 | 0.172 | -0.61 |
| 1419131_at | F13b | coagulation factor XIII, beta subunit | -1.21 | 0.381 | -1.09 | 0.179 | -1.62 | 0.284 | 1.33 | 0.594 | -0.65 |
| 1433170_at | 2900034C19Rik | RIKEN cDNA 2900034C19 gene | -1.1 | 0.866 | -1.12 | 0.646 | -1.8 | 0.327 | 1.36 | 0.526 | -0.66 |
| 1422826_at | Igfals | insulin-like growth factor binding protein, acid labile subunit | 1.02 | 0.93 | -1.26 | 0.252 | -1.84 | 0.182 | -1.6 | 0.329 | -0.92 |
| 1457958_at | Ankrd26 | Ankyrin repeat domain 26 | -1.23 | 0.592 | -1.76 | 0.065 | -1.02 | 0.958 | 1.07 | 0.78 | -0.73 |
| 1438830_at | --- | In vitro fertilized eggs cDNA, RIKEN full-length enriched library, clone:7420422E1 | 1.32 | 0.669 | -1.91 | 0.127 | -1.94 | 0.288 | -1.58 | 0.339 | -1.03 |
| 1444250_at | --- | --- | -1.48 | 0.19 | -1.29 | 0.242 | -1.1 | 0.544 | 1.97 | 0.224 | -0.47 |
| 1443100_at | Thrb | Thyroid hormone receptor beta | 1.08 | 0.473 | -1.37 | 0.132 | -1.8 | 0.017 | -1.14 | 0.448 | -0.81 |
| 1424451_at | Acaa1b | acetyl-Coenzyme A acyltransferase 1B | -1.15 | 0.406 | -1.68 | 0.339 | -1.12 | 0.722 | 2.08 | 0.328 | -0.47 |
| 1435530_at | Camsap1 | calmodulin regulated spectrin-associated protein 1 | -1.03 | 0.831 | -1.27 | 0.384 | -1.67 | 0.062 | 1.27 | 0.432 | -0.67 |
| 1444671_at | Rasal2 | RAS protein activator like 2 | 1.21 | 0.621 | -1.55 | 0.352 | -1.99 | 0.04 | -1.35 | 0.327 | -0.92 |
| 1444790_at | 1810005K13Rik | RIKEN cDNA 1810005K13 gene | 1.21 | 0.495 | -2.1 | 0.048 | -1.48 | 0.023 | -1.7 | 0.504 | -1.02 |
| 1444002_at | Foxl2 | forkhead box L2 | 1.59 | 0.396 | -1.88 | 0.276 | -4.23 | 0.009 | -1.24 | 0.542 | -1.44 |
| 1415927_at | Actc1 | actin, alpha, cardiac | -1.55 | 0.527 | -1.57 | 0.353 | 1.08 | 0.809 | -1.13 | 0.711 | -0.79 |
| 1432161_a_at | Ptar1 | protein prenyltransferase alpha subunit repeat containing 1 | -1.18 | 0.813 | -1.16 | 0.672 | -1.55 | 0.199 | 1.26 | 0.518 | -0.65 |
| 1420564_at | Insrr | insulin receptor-related receptor | -1.93 | 0.061 | 1.12 | 0.314 | -1.38 | 0.456 | -1.48 | 0.474 | -0.92 |
| 1437890_at | 1500005I02Rik | RIKEN cDNA 1500005I02 gene | -1.59 | 0.285 | -1.41 | 0.288 | 1.02 | 0.913 | -1.16 | 0.546 | -0.78 |
| 1425353_at | Enpp3 | ectonucleotide pyrophosphatase/phosphodiesterase 3 | -1.33 | 0.512 | -1.03 | 0.948 | -1.57 | 0.517 | 1.48 | 0.419 | -0.61 |
| 1445921_at | D2Ertd397e | DNA segment, Chr 2, ERATO Doi 397, expressed | 1.22 | 0.371 | -1.64 | 0.358 | -1.89 | 0.366 | -2.25 | 0.409 | -1.14 |
| 1442874_at | Ascc1 | Activating signal cointegrator 1 complex subunit 1 | 1.09 | 0.659 | -1.47 | 0.244 | -1.7 | 0.057 | -2.05 | 0.033 | -1.03 |
| 1442320_at | LOC553096 | hypothetical LOC553096 | 1.09 | 0.733 | -1.2 | 0.18 | -2.26 | 0.002 | -1.49 | 0.229 | -0.97 |
| 1459370_at | Cacng5 | Calcium channel, voltage-dependent, gamma subunit 5 | -1.01 | 0.777 | -1.04 | 0.908 | -2.44 | 0.071 | 1.92 | 0.226 | -0.64 |
| 1432619_at | --- | --- | -1.72 | 0.484 | -1.28 | 0.547 | -1 | 0.997 | 1.01 | 0.972 | -0.75 |
| 1428740_a_at | Pigt | phosphatidylinositol glycan anchor biosynthesis, class T | -2.32 | 0.344 | -1.64 | 0.512 | 1.32 | 0.411 | -3.63 | 0.152 | -1.57 |
| 1443996_at | Fgfr2 | Fibroblast growth factor receptor 2 | -1.14 | 0.479 | -1.05 | 0.933 | -1.87 | 0.077 | 1.39 | 0.413 | -0.67 |
| 1441031_at | Padi1 | peptidyl arginine deiminase, type I | -1.36 | 0.528 | -1.65 | 0.289 | 1.02 | 0.956 | -3.6 | 0.003 | -1.4 |
| 1449178_at | Pdlim3 | PDZ and LIM domain 3 | -1.45 | 0.179 | -1.69 | 0.197 | 1.08 | 0.611 | -1.7 | 0.133 | -0.94 |
| 1439314_at | Clock | circadian locomoter output cycles kaput | -1.15 | 0.488 | 1.01 | 0.97 | -2.09 | 0.041 | -1.55 | 0.265 | -0.94 |
| 1442885_at | Scap | SREBP cleavage activating protein | 1.03 | 0.925 | -1.65 | 0.212 | -1.38 | 0.173 | -1.74 | 0.047 | -0.93 |
| 1447074_at | Zbtb24 | zinc finger and BTB domain containing 24 | 1.05 | 0.92 | -1.74 | 0.037 | -1.36 | 0.283 | -1.01 | 0.985 | -0.76 |
| 1417588_at | Galnt3 | UDP-N-acetyl-alpha-D-galactosamine:polypeptide N-acetylgalactosaminyltransfer | -1.22 | 0.282 | -1.27 | 0.605 | -1.32 | 0.115 | 1.11 | 0.705 | -0.68 |
| 1454050_at | 4921521D15Rik | RIKEN cDNA 4921521D15 gene | -1.14 | 0.593 | -1.82 | 0.315 | -1.07 | 0.661 | 1.14 | 0.46 | -0.72 |
| 1441488_at | --- | Transcribed locus | 1.06 | 0.918 | -1.77 | 0.378 | -1.34 | 0.423 | -1.09 | 0.909 | -0.79 |
| 1439743_at | AW048948 | expressed sequence AW048948 | -1.31 | 0.61 | -1.25 | 0.434 | -1.24 | 0.414 | 1.32 | 0.327 | -0.62 |
| 1432243_a_at | 4933433G15Rik | RIKEN cDNA 4933433G15 gene | -1.16 | 0.772 | -1.54 | 0.282 | -1.17 | 0.709 | 1.9 | 0.049 | -0.49 |
| 1434291_a_at | Serf1 | small EDRK-rich factor 1 | -1.47 | 0.396 | -1.01 | 0.967 | -1.44 | 0.004 | 1.03 | 0.909 | -0.72 |
| 1454188_at | 4921508D12Rik | RIKEN cDNA 4921508D12 gene | -1.11 | 0.706 | -1.67 | 0.22 | -1.15 | 0.688 | 1.1 | 0.723 | -0.71 |
| 1432709_at | 4930505O19Rik | RIKEN cDNA 4930505O19 gene | -1.52 | 0.34 | 1.17 | 0.856 | -1.88 | 0.278 | -2.37 | 0.266 | -1.15 |
| 1454867_at | Mn1 | meningioma 1 | 1.04 | 0.896 | -1.95 | 0.086 | -1.24 | 0.458 | -1.5 | 0.199 | -0.91 |
| 1425983_x_at | Hipk2 | homeodomain interacting protein kinase 2 | -1.17 | 0.17 | -1.39 | 0.145 | -1.26 | 0.288 | 1.8 | 0.226 | -0.51 |

| | | | | | | | | | | | |
|---|---|---|---|---|---|---|---|---|---|---|---|
| 1457730_at | Fancm | Fanconi anemia, complementation group M | -1.85 | 0.087 | -1.13 | 0.549 | -1.06 | 0.596 | 1.26 | 0.364 | -0.7 |
| 1444314_at | 4921506I22Rik | RIKEN cDNA 4921506I22 gene | -1.28 | 0.091 | -1.36 | 0.234 | -1.18 | 0.473 | 1.03 | 0.924 | -0.7 |
| 1458089_at | Fkbp5 | FK506 binding protein 5 | 1.08 | 0.912 | -1.4 | 0.552 | -1.77 | 0.285 | -1.42 | 0.036 | -0.87 |
| 1430066_at | 4930589M24Rik | RIKEN cDNA 4930589M24 gene | -1.2 | 0.183 | -1.21 | 0.666 | -1.42 | 0.375 | 2.19 | 0.075 | -0.41 |
| 1457607_at | AW046457 | expressed sequence AW046457 | -4.01 | 0.012 | 1.67 | 0.09 | -2.22 | 0.016 | -1.45 | 0.508 | -1.5 |
| 1432624_at | 5830487K18Rik | RIKEN cDNA 5830487K18 gene | -1.49 | 0.547 | 1.17 | 0.409 | -1.9 | 0.136 | -1.81 | 0.226 | -1.01 |
| 1446822_at | --- | --- | -1.73 | 0.123 | -1.3 | 0.16 | 1.02 | 0.909 | -1.3 | 0.649 | -0.83 |
| 1451266_at | Mrpl50 | mitochondrial ribosomal protein L50 | -1.32 | 0.147 | -1.11 | 0.192 | -1.41 | 0.205 | 1.04 | 0.864 | -0.7 |
| 1417057_a_at | Ppid /// Lamp3 /// L( | peptidylprolyl isomerase D (cyclophilin D) /// lysosomal-associated membrane prc | -1.14 | 0.424 | -1.23 | 0.019 | -1.48 | 0.058 | 1.13 | 0.627 | -0.68 |
| 1427873_at | Defcr15 | defensin related cryptdin 15 | -1.35 | 0.481 | 1.11 | 0.721 | -1.95 | 0.033 | -2.41 | 0.14 | -1.15 |
| 1449657_at | MGC107702 | similar to proteasome (prosome, macropain) subunit, beta type 7 | -1.17 | 0.293 | -1.38 | 0.328 | -1.27 | 0.123 | 1.03 | 0.874 | -0.7 |
| 1460312_at | Olfr157 | olfactory receptor 157 | -2.18 | 0.387 | -1.15 | 0.499 | 1.04 | 0.873 | -1.13 | 0.802 | -0.86 |
| 1439307_at | Cdh2 | Cadherin 2 | -1.46 | 0.27 | -1.62 | 0.014 | 1.06 | 0.495 | -1.06 | 0.597 | -0.77 |
| 1445613_at | Nr2c2 | Nuclear receptor subfamily 2, group C, member 2 | 1.03 | 0.785 | -1.3 | 0.23 | -1.76 | 0.101 | -1.49 | 0.118 | -0.88 |
| 1438764_at | Anxa7 | annexin A7 | -1.17 | 0.042 | -1.17 | 0.586 | -1.52 | 0.122 | 1.11 | 0.476 | -0.69 |
| 1439400_x_at | 5430433E21Rik | RIKEN cDNA 5430433E21 gene | -2.39 | 0.027 | -1.25 | 0.028 | 1.15 | 0.705 | -1.31 | 0.494 | -0.95 |
| 1440059_at | Map4k5 | Mitogen-activated protein kinase kinase kinase kinase 5 | -1.74 | 0.118 | -1.17 | 0.396 | -1.07 | 0.837 | 1.14 | 0.239 | -0.71 |
| 1441734_at | Camk2a | Calcium/calmodulin-dependent protein kinase II alpha | -2.46 | 0.198 | 1.05 | 0.921 | -1.1 | 0.791 | -2.19 | 0.384 | -1.17 |
| 1445082_at | Kif16b | Kinesin family member 16B | -1.29 | 0.251 | -1.24 | 0.49 | -1.27 | 0.078 | 1.05 | 0.918 | -0.69 |
| 1422420_at | Mb | myoglobin | -1.37 | 0.598 | -2.71 | 0.341 | 1.27 | 0.204 | -1.41 | 0.03 | -1.05 |
| 1447445_at | --- | Transcribed locus | -1.6 | 0.549 | 1.1 | 0.847 | -1.55 | 0.099 | -2.42 | 0.058 | -1.12 |
| 1430305_at | 2500004C02Rik | RIKEN cDNA 2500004C02 gene | -1.56 | 0.178 | -1.26 | 0.594 | -1.07 | 0.777 | 1.1 | 0.564 | -0.7 |
| 1451531_at | Cbwd1 | COBW domain containing 1 | -1.19 | 0.183 | -1.49 | 0.037 | -1.17 | 0.46 | 1.05 | 0.892 | -0.7 |
| 1454710_at | Spink2 | serine peptidase inhibitor, Kazal type 2 | -1.88 | 0.186 | -1.49 | 0.506 | 1.17 | 0.58 | -1.02 | 0.98 | -0.81 |
| 1425244_a_at | Theg | testicular haploid expressed gene | 1.02 | 0.98 | -1.15 | 0.557 | -2.08 | 0.345 | -1.04 | 0.947 | -0.81 |
| 1449602_at | Klhl5 | Kelch-like 5 (Drosophila) | -1.66 | 0.333 | 1.16 | 0.74 | -1.64 | 0.193 | -1.47 | 0.414 | -0.9 |
| 1427099_at | Maz | MYC-associated zinc finger protein (purine-binding transcription factor) | -1.21 | 0.364 | -1.42 | 0.085 | -1.2 | 0.459 | 1.37 | 0.234 | -0.61 |
| 1444970_at | C79870 | expressed sequence C79870 | -2.01 | 0.213 | -1.07 | 0.863 | -1.07 | 0.85 | 1.09 | 0.636 | -0.76 |
| 1443629_at | Nav1 | neuron navigator 1 | -1.11 | 0.837 | -1.88 | 0.064 | -1.07 | 0.866 | 1.09 | 0.799 | -0.74 |
| 1439796_at | AI747448 | expressed sequence AI747448 | 1.1 | 0.919 | -1.59 | 0.532 | -1.56 | 0.138 | -1.24 | 0.535 | -0.82 |
| 1441034_at | Itsn1 | Intersectin 1 (SH3 domain protein 1A) | -1.34 | 0.773 | -1.1 | 0.817 | -1.41 | 0.462 | 4.39 | 0.197 | 0.14 |
| 1438337_x_at | 9930032O22Rik | RIKEN cDNA 9930032O22 gene | -1.15 | 0.707 | -1.18 | 0.725 | -1.54 | 0.19 | 1.12 | 0.816 | -0.69 |
| 1444445_at | C77648 | expressed sequence C77648 | 1.02 | 0.924 | -1.17 | 0.189 | -2.02 | 0.009 | -1.33 | 0.24 | -0.88 |
| 1431336_at | Scara5 | scavenger receptor class A, member 5 (putative) | 1.26 | 0.732 | -2.36 | 0.171 | -1.47 | 0.245 | -1.18 | 0.789 | -0.94 |
| 1442146_at | Mta3 | Metastasis associated 3 | -1.28 | 0.754 | -2.28 | 0.054 | 1.15 | 0.298 | -1.01 | 0.914 | -0.86 |
| 1418876_at | Foxd1 | forkhead box D1 | -1.23 | 0.333 | -1.42 | 0.585 | -1.17 | 0.802 | 1.68 | 0.274 | -0.54 |
| 1441877_x_at | Tssc1 | tumor suppressing subtransferable candidate 1 | -1.1 | 0.882 | -2.07 | 0.066 | -1.02 | 0.96 | 1.33 | 0.137 | -0.72 |
| 1433194_at | 4930433M22Rik | RIKEN cDNA 4930433M22 gene | 1.03 | 0.816 | -1.33 | 0.378 | -1.69 | 0.401 | -1.28 | 0.724 | -0.82 |
| 1459960_at | --- | Transcribed locus | -1 | 0.97 | -3.78 | 0.016 | 1.11 | 0.857 | -1.06 | 0.916 | -1.18 |
| 1443265_at | Cd44 | CD44 antigen | -1.22 | 0.154 | -1.67 | 0.5 | -1.05 | 0.866 | 1.16 | 0.676 | -0.69 |
| 1427111_s_at | Raver1 | ribonucleoprotein, PTB-binding 1 | -1.18 | 0.724 | -1.84 | 0.135 | -1.02 | 0.86 | 1.15 | 0.692 | -0.72 |
| 1446102_at | D9Ertd292e | DNA segment, Chr 9, ERATO Doi 292, expressed | -1.56 | 0.316 | -1.13 | 0.789 | -1.18 | 0.199 | 1.12 | 0.759 | -0.69 |
| 1453339_at | 1700008I05Rik | RIKEN cDNA 1700008I05 gene | -1.62 | 0.572 | 1.12 | 0.823 | -1.59 | 0.545 | -1.75 | 0.142 | -0.96 |
| 1454453_at | 2610011I18Rik | RIKEN cDNA 2610011I18 gene | -1.83 | 0.444 | 1.02 | 0.963 | -1.24 | 0.195 | -1.5 | 0.064 | -0.89 |
| 1429694_at | 4930402H24Rik | RIKEN cDNA 4930402H24 gene | -1.15 | 0.54 | -1.46 | 0.183 | -1.23 | 0.215 | 1.13 | 0.552 | -0.68 |
| 1447366_at | --- | --- | -1.89 | 0.384 | -1.29 | 0.313 | 1.07 | 0.863 | -2.65 | 0.029 | -1.19 |
| 1460695_a_at | 2010111I01Rik | RIKEN cDNA 2010111I01 gene | -1.2 | 0.096 | -1.25 | 0.375 | -1.36 | 0.002 | 1.02 | 0.859 | -0.7 |
| 1441545_at | 9230115F04Rik | RIKEN cDNA 9230115F04 gene | 1.01 | 0.803 | -1.23 | 0.363 | -1.82 | 0.025 | -2.26 | 0.086 | -1.08 |
| 1435655_at | Snora65 | small nucleolar RNA, H/ACA box 65 | -1.4 | 0.07 | -1.76 | 0.018 | 1.09 | 0.673 | -1.77 | 0.025 | -0.96 |
| 1460226_at | Trap1a | tumor rejection antigen P1A | -1.21 | 0.635 | -1.41 | 0.486 | -1.2 | 0.107 | 1.46 | 0.462 | -0.59 |
| 1447518_at | Tpx2 | TPX2, microtubule-associated protein homolog (Xenopus laevis) | -1.4 | 0.162 | -1.4 | 0.354 | -1.06 | 0.62 | 1.11 | 0.495 | -0.69 |
| 1442085_at | Whsc1l1 | Wolf-Hirschhorn syndrome candidate 1-like 1 (human) | -1.71 | 0.293 | -1.53 | 0.136 | 1.13 | 0.685 | -1.16 | 0.513 | -0.82 |
| 1457949_at | --- | Transcribed locus | -1.27 | 0.056 | -1.22 | 0.142 | -1.31 | 0.11 | 1.1 | 0.734 | -0.67 |

| | | | | | | | | | | | |
|---|---|---|---|---|---|---|---|---|---|---|---|
| 1459188_at | Ryk | Receptor-like tyrosine kinase | -1.22 | 0.798 | 1.21 | 0.801 | -2.9 | 0.309 | -1.6 | 0.67 | -1.13 |
| 1429256_at | Gtl2 | GTL2, imprinted maternally expressed untranslated mRNA | -1.61 | 0.205 | -1.15 | 0.664 | -1.14 | 0.757 | 1.09 | 0.862 | -0.7 |
| 1446178_at | Bicc1 | Bicaudal C homolog 1 (Drosophila) | 1.04 | 0.796 | -1.07 | 0.919 | -2.51 | 0.044 | -2.82 | 0.013 | -1.34 |
| 1444247_at | C81557 | expressed sequence C81557 | -1.04 | 0.898 | -1.29 | 0.571 | -1.58 | 0.13 | 1.49 | 0.174 | -0.6 |
| 1446090_at | --- | 15 days embryo head cDNA, RIKEN full-length enriched library, clone:D930002E | -1.03 | 0.946 | -1.34 | 0.078 | -1.53 | 0.022 | 1.75 | 0.054 | -0.54 |
| 1455959_s_at | --- | --- | -1.37 | 0.028 | -1.38 | 0.036 | -1.09 | 0.289 | 2.58 | 0.206 | -0.31 |
| 1439813_at | Sppl3 | Signal peptide peptidase 3 | -1.07 | 0.72 | -1.1 | 0.04 | -1.9 | 0.016 | 1.21 | 0.38 | -0.71 |
| 1443696_s_at | Habp2 | hyaluronic acid binding protein 2 | 1.02 | 0.973 | -1.02 | 0.96 | -2.69 | 0.037 | -1.62 | 0.237 | -1.08 |
| 1431763_a_at | Ctrl | chymotrypsin-like | 1.18 | 0.558 | -2.97 | 0.13 | -1.16 | 0.429 | -1.49 | 0.209 | -1.11 |
| 1440297_at | 4931406I20Rik | RIKEN cDNA 4931406I20 gene | 1.12 | 0.814 | -1.43 | 0.599 | -1.79 | 0.214 | -1.57 | 0.017 | -0.92 |
| 1422816_a_at | Mutyh | mutY homolog (E. coli) | -1.08 | 0.724 | -3.03 | 0.043 | 1.12 | 0.366 | -1.53 | 0.07 | -1.13 |
| 1454243_at | Ick | intestinal cell kinase | -1.02 | 0.928 | -1.22 | 0.319 | -1.77 | 0.037 | 1.18 | 0.149 | -0.7 |
| 1424846_at | Cep68 | centrosomal protein 68 | -1.38 | 0.353 | -1.24 | 0.082 | -1.18 | 0.342 | 1.08 | 0.611 | -0.68 |
| 1448841_at | Pfpl | pore forming protein-like | -1.05 | 0.821 | 1.02 | 0.919 | -2.5 | 0.043 | -1.16 | 0.791 | -0.92 |
| 1443278_at | Dcc | Deleted in colorectal carcinoma | -1.56 | 0.05 | -1.14 | 0.801 | -1.17 | 0.825 | 1.2 | 0.354 | -0.67 |
| 1433182_at | 4930431N21Rik | RIKEN cDNA 4930431N21 gene | 1.13 | 0.235 | -1.63 | 0.464 | -1.58 | 0.269 | -1.1 | 0.154 | -0.79 |
| 1458551_at | Mef2c | Myocyte enhancer factor 2C | 1.23 | 0.566 | -1.77 | 0.371 | -1.71 | 0.191 | -1.84 | 0.176 | -1.02 |
| 1458142_at | Zdhhc9 | zinc finger, DHHC domain containing 9 | -1.56 | 0.256 | 1.02 | 0.95 | -1.4 | 0.215 | -1.06 | 0.811 | -0.75 |
| 1439638_at | Erbb2ip | Erbb2 interacting protein | -1.23 | 0.442 | 1.04 | 0.878 | -1.9 | 0.073 | -1.65 | 0.092 | -0.94 |
| 1441237_at | D5Ertd163e | DNA segment, Chr 5, ERATO Doi 163, expressed | -1.05 | 0.941 | -1.25 | 0.619 | -1.6 | 0.376 | 2.48 | 0.107 | -0.36 |
| 1438923_at | Fibp | Fibroblast growth factor (acidic) intracellular binding protein | -1.13 | 0.865 | -1.74 | 0.041 | -1.1 | 0.9 | 3.84 | 0.324 | -0.03 |
| 1421619_at | Kcnh3 | potassium voltage-gated channel, subfamily H (eag-related), member 3 | -1.39 | 0.19 | -1.54 | 0.289 | 1 | 0.995 | -1.03 | 0.87 | -0.74 |
| 1440391_at | LOC638038 | hypothetical protein LOC638038 | -1.34 | 0.027 | -1.38 | 0.226 | -1.1 | 0.499 | 1.02 | 0.914 | -0.7 |
| 1429793_at | 1600014K23Rik | RIKEN cDNA 1600014K23 gene | -1.06 | 0.821 | -1.12 | 0.753 | -1.84 | 0.16 | 2.68 | 0.046 | -0.34 |
| 1421726_at | Ap4b1 | adaptor-related protein complex AP-4, beta 1 | -1.39 | 0.085 | -1.42 | 0.183 | -1.05 | 0.726 | 1.16 | 0.425 | -0.67 |
| 1434597_at | Larp5 | La ribonucleoprotein domain family, member 5 | -1.12 | 0.223 | -1.33 | 0.046 | -1.36 | 0.007 | 1.27 | 0.195 | -0.64 |
| 1433273_at | 2610200G18Rik | RIKEN cDNA 2610200G18 gene | -1.64 | 0.174 | -1.2 | 0.74 | -1.07 | 0.592 | 1.38 | 0.49 | -0.63 |
| 1456252_x_at | Ap1s2 | adaptor-related protein complex 1, sigma 2 subunit | -1.42 | 0.247 | -1.05 | 0.92 | -1.39 | 0.388 | 1.61 | 0.559 | -0.56 |
| 1441143_at | E130309D14Rik | RIKEN cDNA E130309D14 gene | -1.87 | 0.231 | -1.99 | 0.393 | 1.34 | 0.131 | -1.12 | 0.531 | -0.91 |
| 1451515_s_at | Glyat | glycine-N-acyltransferase | -1.36 | 0.052 | -1.18 | 0.095 | -1.26 | 0.251 | 1.49 | 0.238 | -0.58 |
| 1430651_s_at | Zfp191 | zinc finger protein 191 | 1.06 | 0.734 | -1.34 | 0.514 | -1.76 | 0.103 | -1.2 | 0.337 | -0.81 |
| 1444109_at | C130009A20Rik | RIKEN cDNA C130009A20 gene | 1.06 | 0.892 | -1.5 | 0.277 | -1.54 | 0.106 | -1.1 | 0.498 | -0.77 |
| 1424992_at | Tprkb | Tp53rk binding protein | -1.11 | 0.729 | -1.19 | 0.344 | -1.57 | 0.328 | 1.27 | 0.189 | -0.65 |
| 1438823_at | --- | Transcribed locus | -1.68 | 0.062 | 1.1 | 0.836 | -1.47 | 0.457 | -2.18 | 0.177 | -1.06 |
| 1433379_at | 9430019H13Rik | RIKEN cDNA 9430019H13 gene | -1.13 | 0.28 | -1.47 | 0.304 | -1.23 | 0.054 | 1.3 | 0.516 | -0.63 |
| 1430178_at | 2810408A11Rik | RIKEN cDNA 2810408A11 gene | -1.45 | 0.245 | -1.55 | 0.382 | 1.04 | 0.714 | -1.26 | 0.627 | -0.8 |
| 1435523_s_at | 2700089E24Rik | RIKEN cDNA 2700089E24 gene | -1.62 | 0.106 | -1.32 | 0.467 | 1 | 0.983 | -1.16 | 0.388 | -0.78 |
| 1436228_at | --- | --- | -1.14 | 0.633 | -1.22 | 0.401 | -1.47 | 0.054 | 1.06 | 0.706 | -0.69 |
| 1435445_at | Ccnt2 | cyclin T2 | -1.08 | 0.813 | -1.2 | 0.36 | -1.62 | 0.091 | 1.01 | 0.972 | -0.72 |
| 1443722_at | 6030419C18Rik | RIKEN cDNA 6030419C18 gene | -1.3 | 0.1 | -1.04 | 0.857 | -1.55 | 0.07 | 1.08 | 0.827 | -0.7 |
| 1434840_at | Hrb | HIV-1 Rev binding protein | -1.11 | 0.477 | -1.3 | 0.014 | -1.42 | 0.045 | 1.13 | 0.611 | -0.67 |
| 1417952_at | Cyp2j6 | cytochrome P450, family 2, subfamily j, polypeptide 6 | -2.01 | 0.038 | 1.06 | 0.699 | -1.23 | 0.377 | -1.06 | 0.835 | -0.81 |
| 1416305_at | Sh3bp4 | SH3-domain binding protein 4 | -1.11 | 0.432 | -2.45 | 0.142 | 1.06 | 0.111 | -1.47 | 0.127 | -0.99 |
| 1458857_at | --- | Transcribed locus | -1.31 | 0.595 | 1.03 | 0.915 | -1.71 | 0.056 | -1.1 | 0.813 | -0.77 |
| 1459376_at | Hivep2 | Human immunodeficiency virus type I enhancer binding protein 2 | 1.03 | 0.912 | -1.19 | 0.325 | -1.97 | 0.012 | -1.71 | 0.167 | -0.96 |
| 1420136_a_at | --- | --- | -1.1 | 0.579 | -1.22 | 0.064 | -1.54 | 0.176 | 2.37 | 0.244 | -0.37 |
| 1446902_at | AU022538 | expressed sequence AU022538 | -1.16 | 0.524 | -1.55 | 0.317 | -1.15 | 0.647 | 2.43 | 0.071 | -0.36 |
| 1458500_at | AU021034 | expressed sequence AU021034 | -2.26 | 0.308 | -1.13 | 0.829 | 1.05 | 0.831 | -1.13 | 0.771 | -0.87 |
| 1444330_at | D2Ertd173e | DNA segment, Chr 2, ERATO Doi 173, expressed | -1.8 | 0.537 | -1.86 | 0.384 | 1.28 | 0.736 | -1.11 | 0.801 | -0.87 |
| 1450683_at | Tagln3 | transgelin 3 | -1.19 | 0.69 | 1.16 | 0.671 | -2.68 | 0.023 | -1.25 | 0.731 | -0.99 |
| 1445415_at | B230303A05 | Hypothetical protein B230303A05 | -2.57 | 0.396 | -1.34 | 0.319 | 1.24 | 0.359 | -1.51 | 0.233 | -1.05 |
| 1443402_at | Phka2 | Phosphorylase kinase alpha 2 | -1.53 | 0.365 | 1.07 | 0.811 | -1.53 | 0.163 | -1.25 | 0.252 | -0.81 |
| 1435765_at | E130114P18Rik | RIKEN cDNA E130114P18 gene | -1.57 | 0.58 | -1.06 | 0.837 | -1.25 | 0.196 | 2.31 | 0.022 | -0.39 |

| | | | | | | | | | | | |
|---|---|---|---|---|---|---|---|---|---|---|---|
| 1454833_at | Rpl35 | ribosomal protein L35 | -1.5 | 0.031 | -1.13 | 0.662 | -1.21 | 0.602 | 1.09 | 0.778 | -0.69 |
| 1436440_at | B230107K20Rik | RIKEN cDNA B230107K20 gene | -1.24 | 0.404 | -1.48 | 0.342 | -1.12 | 0.618 | 1.04 | 0.725 | -0.7 |
| 1447691_x_at | Bicc1 | Bicaudal C homolog 1 (Drosophila) | -1.17 | 0.502 | -1.22 | 0.621 | -1.42 | 0.059 | 1.1 | 0.598 | -0.68 |
| 1439420_x_at | Ggtla1 | gamma-glutamyltransferase-like activity 1 | -1.22 | 0.636 | -1.11 | 0.786 | -1.52 | 0.028 | 1.2 | 0.757 | -0.66 |
| 1445215_at | 2600003E23Rik | RIKEN cDNA 2600003E23 gene | -1.19 | 0.632 | 1.03 | 0.926 | -1.96 | 0.155 | -1 | 0.995 | -0.78 |
| 1423173_at | Napb | N-ethylmaleimide sensitive fusion protein attachment protein beta | -1.43 | 0.005 | 1.2 | 0.395 | -2.09 | 0.066 | -1.03 | 0.848 | -0.84 |
| 1459411_at | Sdccag10 | Serologically defined colon cancer antigen 10 | -1.31 | 0.078 | 1.05 | 0.834 | -1.78 | 0.114 | -1.21 | 0.506 | -0.81 |
| 1432723_at | 4933408K01Rik | RIKEN cDNA 4933408K01 gene | -1.22 | 0.591 | -1.05 | 0.915 | -1.64 | 0.399 | 1.4 | 0.314 | -0.63 |
| 1443778_at | LOC672274 | similar to Transcription factor SOX-4 | -1.54 | 0.519 | 1.19 | 0.167 | -1.85 | 0.236 | -1.23 | 0.75 | -0.86 |
| 1421689_at | Krtap8-2 | keratin associated protein 8-2 | -3.33 | 0.167 | -1.07 | 0.81 | 1.14 | 0.808 | -1.14 | 0.722 | -1.1 |
| 1447286_at | Emb | Embigin | -1.71 | 0.601 | 1.09 | 0.882 | -1.43 | 0.239 | -4.2 | 0.035 | -1.56 |
| 1439298_at | --- | --- | -1.09 | 0.535 | -1.28 | 0.009 | -1.47 | 0.23 | 1.01 | 0.95 | -0.71 |
| 1458757_at | Ptbp2 | Polypyrimidine tract binding protein 2 | -2.38 | 0.273 | -1.12 | 0.872 | 1.07 | 0.832 | -1.32 | 0.664 | -0.94 |
| 1457080_at | --- | Transcribed locus | 1.06 | 0.854 | -2.3 | 0.157 | -1.13 | 0.72 | -1.45 | 0.314 | -0.96 |
| 1425010_at | Zfp119 | zinc finger protein 119 | -1.88 | 0.028 | 1.11 | 0.621 | -1.36 | 0.495 | -1.04 | 0.908 | -0.79 |
| 1440565_at | Zbtb20 | Zinc finger and BTB domain containing 20 | -1.54 | 0.083 | -1.13 | 0.488 | -1.18 | 0.407 | 1.39 | 0.233 | -0.62 |
| 1434595_at | Trim9 | tripartite motif protein 9 | -1.25 | 0.707 | -1.21 | 0.532 | -1.32 | 0.136 | 1.32 | 0.323 | -0.62 |
| 1457970_at | --- | --- | 1.19 | 0.261 | -1.69 | 0.222 | -1.67 | 0.125 | -1.18 | 0.119 | -0.84 |
| 1451404_at | ORF19 | open reading frame 19 | -1.09 | 0.703 | -1.63 | 0.095 | -1.18 | 0.399 | 1.03 | 0.912 | -0.72 |
| 1435064_a_at | Tmem27 | transmembrane protein 27 | -1.11 | 0.367 | -14.72 | 0.127 | 1.41 | 0.502 | -1.27 | 0.235 | -3.92 |
| 1456701_at | B230208H17Rik | RIKEN cDNA B230208H17 gene | 1.05 | 0.924 | -1.43 | 0.413 | -1.57 | 0.105 | -1.97 | 0.178 | -0.98 |
| 1455536_at | A630023A22Rik | RIKEN cDNA A630023A22 gene | -1.12 | 0.823 | 1.05 | 0.901 | -2.32 | 0.022 | -2.11 | 0.353 | -1.12 |
| 1439150_x_at | Grtp1 | GH regulated TBC protein 1 | -1.11 | 0.571 | -1.15 | 0.518 | -1.64 | 0.415 | 3.06 | 0.073 | -0.21 |
| 1456496_at | Dapk3 | Death-associated kinase 3 | -2.63 | 0.086 | -1.17 | 0.655 | 1.15 | 0.748 | -1.03 | 0.929 | -0.92 |
| 1435260_at | --- | --- | 1.16 | 0.254 | -1.59 | 0.068 | -1.69 | 0.011 | -1.8 | 0.017 | -0.98 |
| 1435726_at | Lima1 | LIM domain and actin binding 1 | -1.42 | 0.497 | -1.4 | 0.14 | -1.04 | 0.938 | 2.95 | 0.049 | -0.23 |
| 1458477_at | --- | 3 days neonate thymus cDNA, RIKEN full-length enriched library, clone:A630098 | -1.38 | 0.442 | -1.58 | 0.466 | 1.02 | 0.963 | -1.2 | 0.495 | -0.78 |
| 1442126_at | 5830417C01Rik | RIKEN cDNA 5830417C01 gene | 1.12 | 0.715 | -1.25 | 0.234 | -2.17 | 0.051 | -2.35 | 0.061 | -1.16 |
| 1459845_at | Cbx5 | Chromobox homolog 5 (Drosophila HP1a) | -1.41 | 0.093 | -1.13 | 0.805 | -1.27 | 0.546 | 1.55 | 0.255 | -0.57 |
| 1435384_at | Ube2n | ubiquitin-conjugating enzyme E2N | -1.18 | 0.499 | -1.21 | 0.263 | -1.41 | 0.039 | 1.2 | 0.226 | -0.65 |
| 1456299_at | E330012B07Rik | RIKEN cDNA E330012B07 gene | 1.46 | 0.685 | -3.67 | 0.029 | -1.53 | 0.201 | -1.34 | 0.235 | -1.27 |
| 1432679_at | Itgav | integrin alpha V | -1.52 | 0.127 | 1.08 | 0.869 | -1.55 | 0.058 | -1.52 | 0.423 | -0.88 |
| 1449480_at | Sap18 | Sin3-associated polypeptide 18 | 1.01 | 0.966 | -1.46 | 0.053 | -1.44 | 0.015 | -1.35 | 0.032 | -0.81 |
| 1435000_at | Gspt1 | G1 to S phase transition 1 | -1.21 | 0.063 | -1.28 | 0.021 | -1.29 | 0.001 | 1.03 | 0.887 | -0.69 |
| 1424097_at | Elovl7 | ELOVL family member 7, elongation of long chain fatty acids (yeast) | -1.03 | 0.758 | -1.32 | 0.583 | -1.52 | 0.11 | 1.74 | 0.114 | -0.53 |
| 1445088_at | Magi1 | Membrane associated guanylate kinase, WW and PDZ domain containing 1 | -2.15 | 0.506 | 1.2 | 0.148 | -1.39 | 0.489 | -1.24 | 0.498 | -0.89 |
| 1432567_at | A430105D02Rik | RIKEN cDNA A430105D02 gene | -1.28 | 0.552 | -2.01 | 0.243 | 1.1 | 0.863 | -1.16 | 0.684 | -0.84 |
| 1415712_at | Zranb1 | zinc finger, RAN-binding domain containing 1 | 1.06 | 0.222 | -1.27 | 0.196 | -1.87 | 0.003 | -1 | 0.983 | -0.77 |
| 1430416_at | 4931431B13Rik | RIKEN cDNA 4931431B13 gene | -3.01 | 0.005 | 1.25 | 0.509 | -1.26 | 0.633 | -1.06 | 0.884 | -1.02 |
| 1440404_at | --- | --- | -1.13 | 0.552 | 1.09 | 0.816 | -2.44 | 0.03 | -1.32 | 0.412 | -0.95 |
| 1422332_at | Ifna11 | interferon alpha 11 | -1.86 | 0.186 | -1.88 | 0.142 | 1.32 | 0.47 | -1.13 | 0.421 | -0.89 |
| 1442958_at | --- | Lung RCB-0558 LLC cDNA, RIKEN full-length enriched library, clone:G730002L0 | -2.57 | 0.056 | 1.55 | 0.451 | -2.25 | 0.121 | -1.33 | 0.227 | -1.15 |
| 1424132_at | Hras1 | Harvey rat sarcoma virus oncogene 1 | -1.13 | 0.701 | -1.34 | 0.399 | -1.33 | 0.111 | 1.38 | 0.344 | -0.6 |
| 1444286_at | --- | Transcribed locus, weakly similar to XP_530798.1 PREDICTED: hypothetical pro | -1.44 | 0.498 | -1.08 | 0.841 | -1.31 | 0.529 | 1.04 | 0.908 | -0.7 |
| 1433147_at | Cald1 | caldesmon 1 | -1.05 | 0.924 | 1.05 | 0.82 | -2.63 | 0.06 | -1.7 | 0.125 | -1.08 |
| 1459551_at | --- | --- | 1.27 | 0.694 | -2.39 | 0.103 | -1.43 | 0.368 | -2.52 | 0.296 | -1.27 |
| 1446618_at | LOC432971 | hypothetical gene supported by AK038224 | -1.24 | 0.108 | -1.08 | 0.546 | -1.53 | 0.123 | 1.13 | 0.369 | -0.68 |
| 1459663_at | --- | --- | -1.56 | 0.381 | 1.22 | 0.648 | -1.91 | 0.076 | -1.62 | 0.041 | -0.97 |
| 1453388_at | 4933416M07Rik | RIKEN cDNA 4933416M07 gene | 1.06 | 0.869 | -1.53 | 0.44 | -1.49 | 0.147 | -1.83 | 0.433 | -0.95 |
| 1459461_at | --- | --- | -1.21 | 0.359 | -1.19 | 0.629 | -1.39 | 0.028 | 1.11 | 0.678 | -0.67 |
| 1432484_at | 2310061C15Rik | RIKEN cDNA 2310061C15 gene | -1.09 | 0.789 | -2.08 | 0.148 | -1.02 | 0.926 | 1.53 | 0.177 | -0.66 |
| 1438249_at | Usp7 | Ubiquitin specific peptidase 7 | -1.1 | 0.155 | -1.34 | 0.001 | -1.37 | 0.297 | 1.28 | 0.456 | -0.63 |
| 1425175_at | C1ql3 | C1q-like 3 | -1.54 | 0.324 | -1.52 | 0.122 | 1.08 | 0.866 | -1.02 | 0.941 | -0.75 |

| | | | | | | | | | | | |
|---|---|---|---|---|---|---|---|---|---|---|---|
| 1458984_at | E130308A19Rik | RIKEN cDNA E130308A19 gene | -1.05 | 0.685 | -1.4 | 0.22 | -1.39 | 0.009 | 1.37 | 0.28 | -0.62 |
| 1457177_at | Rora | RAR-related orphan receptor alpha | -1.11 | 0.678 | -1.02 | 0.903 | -1.99 | 0.044 | 1.14 | 0.694 | -0.74 |
| 1447336_at | --- | Adult male kidney cDNA, RIKEN full-length enriched library, clone:F530012N16 p | -1.18 | 0.817 | -1.64 | 0.117 | -1.08 | 0.894 | 1.79 | 0.361 | -0.53 |
| 1459622_at | Gm22 | gene model 22, (NCBI) | -1.58 | 0.191 | -1.09 | 0.762 | -1.21 | 0.362 | 1.07 | 0.815 | -0.7 |
| 1456535_at | Vps13c | vacuolar protein sorting 13C (yeast) | -1.53 | 0.049 | -1.28 | 0.56 | -1.05 | 0.904 | 1.21 | 0.71 | -0.66 |
| 1451150_at | Zfp410 | zinc finger protein 410 | -1.27 | 0.264 | -1.28 | 0.063 | -1.23 | 0.403 | 1.38 | 0.373 | -0.6 |
| 1450265_at | Smad9 | MAD homolog 9 (Drosophila) | -2.23 | 0.105 | 1.08 | 0.902 | -1.17 | 0.694 | -1.07 | 0.908 | -0.85 |
| 1460510_a_at | Coq10b | coenzyme Q10 homolog B (S. cerevisiae) | -1.27 | 0.506 | -1.29 | 0.462 | -1.22 | 0.273 | 1.44 | 0.381 | -0.58 |
| 1433098_at | --- | --- | -1.93 | 0.057 | 1.18 | 0.768 | -1.45 | 0.305 | -2.06 | 0.069 | -1.07 |
| 1454939_at | Phf20l1 | PHD finger protein 20-like 1 | -1.33 | 0.422 | -1.31 | 0.368 | -1.15 | 0.408 | 1.23 | 0.235 | -0.64 |
| 1454239_at | 4930578G10Rik | RIKEN cDNA 4930578G10 gene | -1.42 | 0.409 | 1.02 | 0.983 | -1.5 | 0.312 | -1.17 | 0.717 | -0.77 |
| 1456276_at | --- | --- | -1.3 | 0.557 | -1.2 | 0.69 | -1.28 | 0.677 | 1.25 | 0.571 | -0.63 |
| 1445067_at | Arfgap1 | ADP-ribosylation factor GTPase activating protein 1 | -1.12 | 0.11 | -1.23 | 0.281 | -1.47 | 0.058 | 1.17 | 0.218 | -0.66 |
| 1429169_at | Rbm3 | RNA binding motif protein 3 | -1.17 | 0.583 | -1.33 | 0.234 | -1.29 | 0.41 | 1.26 | 0.353 | -0.63 |
| 1439983_a_at | Accn3 | amiloride-sensitive cation channel 3 | -1.09 | 0.664 | -2.31 | 0.229 | 1.03 | 0.842 | -2.22 | 0.418 | -1.15 |
| 1440660_at | Nfia | Nuclear factor I/A | -1.27 | 0.072 | -1.06 | 0.737 | -1.52 | 0.087 | 1.49 | 0.052 | -0.59 |
| 1442990_at | --- | --- | -1.66 | 0.618 | -1.34 | 0.621 | 1.03 | 0.944 | -1.11 | 0.774 | -0.77 |
| 1420162_at | AA409749 | expressed sequence AA409749 | -2.13 | 0.195 | -3.27 | 0.059 | 1.61 | 0.214 | -1.63 | 0.473 | -1.35 |
| 1451539_at | Baiap2l1 | BAI1-associated protein 2-like 1 | -1.01 | 0.958 | -1.43 | 0.344 | -1.44 | 0.005 | 1.44 | 0.556 | -0.61 |
| 1459882_at | --- | --- | -1.3 | 0.198 | -1.43 | 0.052 | -1.09 | 0.706 | 1.49 | 0.07 | -0.58 |
| 1459360_at | Sp3 | Trans-acting transcription factor 3 | -1.32 | 0.21 | 1.08 | 0.629 | -1.82 | 0.04 | -1.3 | 0.211 | -0.84 |
| 1431199_at | 0610031G08Rik | RIKEN cDNA 0610031G08 gene | -1.51 | 0.096 | -1.42 | 0.046 | 1.02 | 0.965 | -1.41 | 0.482 | -0.83 |
| 1453137_at | Fbxo30 | F-box protein 30 | -1.14 | 0.455 | -1.31 | 0.202 | -1.34 | 0.002 | 5.13 | 0.204 | 0.34 |
| 1456804_at | Av381130 | hypothetical protein LOC627821 | -3.8 | 0.007 | -1.25 | 0.463 | 1.32 | 0.586 | -1.68 | 0.532 | -1.35 |
| 1453337_at | 1700013E18Rik | RIKEN cDNA 1700013E18 gene | -1.39 | 0.309 | 1.02 | 0.942 | -1.55 | 0.427 | -1.3 | 0.725 | -0.8 |
| 1437853_x_at | Ndn | necdin | 1.13 | 0.682 | -1.68 | 0.161 | -1.51 | 0.222 | -1.54 | 0.274 | -0.9 |
| 1429064_at | Dip2c | DIP2 disco-interacting protein 2 homolog C (Drosophila) | -1.24 | 0.031 | -1.09 | 0.434 | -1.51 | 0.088 | 1.21 | 0.403 | -0.66 |
| 1456242_at | Esgp | embryonic stem cell- and germ cell-specific protein | 1.02 | 0.949 | -1.94 | 0.413 | -1.17 | 0.822 | -1.27 | 0.726 | -0.84 |
| 1442943_at | Usp20 | Ubiquitin specific peptidase 20 | 1.01 | 0.994 | -1.43 | 0.586 | -1.46 | 0.414 | -1.13 | 0.751 | -0.75 |
| 1416489_at | Pi4k2b | phosphatidylinositol 4-kinase type 2 beta | -1.26 | 0.25 | -1.15 | 0.4 | -1.38 | 0.044 | 3.81 | 0.179 | 0 |
| 1428486_at | 1700123L14Rik | RIKEN cDNA 1700123L14 gene | -1.05 | 0.927 | 1.02 | 0.978 | -2.38 | 0.024 | -2.16 | 0.173 | -1.14 |
| 1451447_at | Cuedc1 | CUE domain containing 1 | -1.66 | 0.077 | -1.49 | 0.36 | 1.11 | 0.617 | -1.68 | 0.195 | -0.93 |
| 1441104_at | Myo5b | myosin Vb | 1.12 | 0.783 | -1.31 | 0.652 | -2 | 0.171 | -1.04 | 0.914 | -0.81 |
| 1427573_at | Chic1 | cysteine-rich hydrophobic domain 1 | -1.24 | 0.212 | -1.41 | 0.272 | -1.15 | 0.341 | 1.17 | 0.257 | -0.66 |
| 1427620_at | Parc | p53-associated parkin-like cytoplasmic protein | -1.56 | 0.058 | -1.77 | 0.052 | 1.18 | 0.087 | -1.47 | 0.326 | -0.9 |
| 1443066_at | --- | --- | -1.79 | 0.059 | -1.17 | 0.394 | -1.03 | 0.955 | 1.38 | 0.5 | -0.65 |
| 1459339_at | 1700049G17Rik | RIKEN cDNA 1700049G17 gene | -1.24 | 0.376 | -1.41 | 0.092 | -1.14 | 0.529 | 1.05 | 0.671 | -0.69 |
| 1451502_at | Pla2g10 | phospholipase A2, group X | -2.69 | 0.208 | -1.07 | 0.69 | 1.08 | 0.839 | -1.03 | 0.923 | -0.93 |
| 1443811_at | Prkg1 | Protein kinase, cGMP-dependent, type I | -1.27 | 0.713 | -1.03 | 0.938 | -1.58 | 0.122 | 1.21 | 0.764 | -0.67 |
| 1452530_a_at | Runx1 | runt related transcription factor 1 | -1.58 | 0.215 | -1.07 | 0.919 | -1.21 | 0.575 | 1.25 | 0.738 | -0.65 |
| 1433319_at | 5430437A18Rik | RIKEN cDNA 5430437A18 gene | -1.9 | 0.473 | -1.04 | 0.962 | -1.11 | 0.849 | 1.48 | 0.395 | -0.64 |
| 1447675_x_at | Myod1 | myogenic differentiation 1 | 1.09 | 0.898 | -1.3 | 0.664 | -1.87 | 0.08 | -2.22 | 0.288 | -1.08 |
| 1427151_at | Qser1 | glutamine and serine rich 1 | -1.28 | 0.301 | -1.18 | 0.505 | -1.31 | 0.11 | 1.05 | 0.582 | -0.68 |
| 1423343_at | Slco1c1 | solute carrier organic anion transporter family, member 1c1 | -1.86 | 0.23 | -2.27 | 0.285 | 1.41 | 0.338 | -1.22 | 0.617 | -0.98 |
| 1425685_at | 2310005E10Rik | RIKEN cDNA 2310005E10 gene | 1.07 | 0.452 | -1.8 | 0.383 | -1.31 | 0.482 | -1.05 | 0.91 | -0.77 |
| 1453926_at | Rad54l | RAD54 like (S. cerevisiae) | -1.05 | 0.846 | -1.35 | 0.631 | -1.43 | 0.61 | 2.3 | 0.321 | -0.38 |
| 1415917_at | Mthfd1 | methylenetetrahydrofolate dehydrogenase (NADP+ dependent), methenyltetrahy | -1.2 | 0.077 | -1.12 | 0.476 | -1.5 | 0.043 | 2.19 | 0.174 | -0.41 |
| 1440824_at | Fbxo45 | F-box protein 45 | 1.27 | 0.68 | -2.37 | 0.108 | -1.44 | 0.263 | -1.11 | 0.289 | -0.91 |
| 1457461_at | A630039O03Rik | RIKEN cDNA A630039O03 gene | 1.38 | 0.17 | -2.06 | 0.15 | -1.91 | 0.328 | -1.08 | 0.509 | -0.92 |
| 1427550_at | Peg10 | paternally expressed 10 | -1.15 | 0.848 | -1.16 | 0.713 | -1.51 | 0.057 | 1.19 | 0.767 | -0.66 |
| 1459727_at | AW125646 | expressed sequence AW125646 | -1.93 | 0.472 | 1.19 | 0.747 | -1.46 | 0.511 | -1.32 | 0.228 | -0.88 |
| 1416670_at | Setdb1 | SET domain, bifurcated 1 | -1.11 | 0.618 | -1.65 | 0.445 | -1.13 | 0.833 | 1.18 | 0.816 | -0.68 |
| 1426155_a_at | Osr2 | odd-skipped related 2 (Drosophila) | -1.32 | 0.498 | -1.11 | 0.136 | -1.36 | 0.629 | 1.24 | 0.77 | -0.64 |

| | | | | | | | | | | | |
|---|---|---|---|---|---|---|---|---|---|---|---|
| 1446684_at | Garnl1 | GTPase activating RANGAP domain-like 1 | 1.18 | 0.462 | -1.42 | 0.506 | -1.95 | 0.037 | -1.5 | 0.241 | -0.92 |
| 1458697_at | --- | Transcribed locus | -1.03 | 0.942 | -2.06 | 0.393 | -1.07 | 0.477 | 1.28 | 0.667 | -0.72 |
| 1440785_at | Rxfp1 | relaxin/insulin-like family peptide receptor 1 | -1.46 | 0.018 | 1.05 | 0.884 | -1.51 | 0.151 | -1.41 | 0.606 | -0.84 |
| 1442805_at | A130086G11Rik | RIKEN cDNA A130086G11 gene | -1.24 | 0.106 | -1.55 | 0.304 | -1.07 | 0.706 | 1.28 | 0.421 | -0.64 |
| 1431964_at | 4921518J05Rik | RIKEN cDNA 4921518J05 gene | -1.05 | 0.95 | -1.4 | 0.339 | -1.37 | 0.469 | 1.21 | 0.606 | -0.66 |
| 1442041_at | LOC552876 | hypothetical LOC552876 | 1.1 | 0.735 | -1.25 | 0.547 | -2.04 | 0.037 | -1.79 | 0.197 | -0.99 |
| 1444781_at | Xab1 | XPA binding protein 1 | 1.35 | 0.41 | -1.95 | 0.055 | -1.91 | 0.174 | -1.51 | 0.469 | -1 |
| 1459997_s_at | Tmem17 | transmembrane protein 17 | 1.18 | 0.376 | -2.09 | 0.442 | -1.37 | 0.149 | -1.49 | 0.475 | -0.94 |
| 1434097_at | --- | Transcribed locus | -1.18 | 0.333 | -1.44 | 0.036 | -1.18 | 0.038 | 1.04 | 0.806 | -0.69 |
| 1436057_at | Gtl2 | GTL2, imprinted maternally expressed untranslated mRNA | -1.05 | 0.92 | -1.62 | 0.454 | -1.22 | 0.546 | 1.07 | 0.829 | -0.71 |
| 1460050_x_at | 5630401D24Rik | RIKEN cDNA 5630401D24 gene | -1.23 | 0.71 | -1.38 | 0.152 | -1.17 | 0.389 | 1.12 | 0.283 | -0.66 |
| 1455754_at | Lmo3 | LIM domain only 3 | -1.17 | 0.631 | -1.33 | 0.383 | -1.28 | 0.609 | 1.06 | 0.833 | -0.68 |
| 1459556_at | BC013481 | CDNA sequence BC013481 | 1.22 | 0.356 | -1.55 | 0.25 | -1.89 | 0.002 | -1.22 | 0.714 | -0.86 |
| 1454304_at | Epn2 | epsin 2 | -1.26 | 0.445 | -1.05 | 0.922 | -1.56 | 0.092 | 1.43 | 0.523 | -0.61 |
| 1444926_at | --- | --- | -1.72 | 0.133 | 1.1 | 0.902 | -1.41 | 0.628 | -1.64 | 0.171 | -0.92 |
| 1435731_x_at | Stag1 | Stromal antigen 1 | -1.64 | 0.082 | -1.14 | 0.046 | -1.1 | 0.29 | 1.42 | 0.096 | -0.62 |
| 1456753_at | --- | --- | -1.19 | 0.223 | -1.16 | 0.547 | -1.44 | 0.03 | 1.99 | 0.166 | -0.45 |
| 1432503_a_at | Pdcl2 | phosducin-like 2 | -2.38 | 0.1 | -1.22 | 0.781 | 1.15 | 0.78 | -1.23 | 0.746 | -0.92 |
| 1445036_at | D11Ertd518e | DNA segment, Chr 11, ERATO Doi 518, expressed | -1.01 | 0.971 | -1.67 | 0.295 | -1.24 | 0.758 | 1.74 | 0.448 | -0.55 |
| 1441372_at | 5930405F01Rik | RIKEN cDNA 5930405F01 gene | 1 | 0.989 | -1.21 | 0.642 | -1.78 | 0.166 | -1.68 | 0.26 | -0.92 |
| 1429942_at | Gmeb1 | glucocorticoid modulatory element binding protein 1 | -1.35 | 0.2 | -1.26 | 0.256 | -1.17 | 0.411 | 1.15 | 0.424 | -0.66 |
| 1419126_at | Hoxd9 | homeo box D9 | -1.1 | 0.07 | -1.53 | 0.358 | -1.21 | 0.802 | 2.25 | 0.205 | -0.4 |
| 1417933_at | Igfbp6 | insulin-like growth factor binding protein 6 | -1.4 | 0.335 | -1.26 | 0.451 | -1.13 | 0.636 | 2.05 | 0.086 | -0.43 |
| 1446937_at | Falz | fetal Alzheimer antigen | -1.59 | 0.117 | -1.05 | 0.9 | -1.23 | 0.054 | 1.01 | 0.96 | -0.72 |
| 1420007_at | --- | --- | -1.14 | 0.741 | -1.49 | 0.02 | -1.18 | 0.559 | 1.45 | 0.189 | -0.59 |
| 1451270_at | Dusp18 | dual specificity phosphatase 18 | -1.24 | 0.634 | -1.83 | 0.173 | 1.04 | 0.826 | -2.98 | 0.062 | -1.25 |
| 1445982_at | Gpc6 | Glypican 6 | 1.25 | 0.765 | -1.41 | 0.113 | -2.29 | 0.262 | -2.82 | 0.257 | -1.32 |
| 1449597_at | LOC236874 | similar to odorant binding protein Ia | -1.07 | 0.86 | -1.23 | 0.788 | -1.55 | 0.588 | 2.99 | 0.194 | -0.21 |
| 1443401_at | Astn2 | Astrotactin 2 | -2.27 | 0.272 | -1.09 | 0.855 | 1.04 | 0.883 | -1.04 | 0.949 | -0.84 |
| 1417713_at | Eif2s2 | eukaryotic translation initiation factor 2, subunit 2 (beta) | -1.11 | 0.32 | -1.23 | 0.022 | -1.47 | 0.073 | 1.18 | 0.416 | -0.66 |
| 1444478_at | 2900057D21Rik | RIKEN cDNA 2900057D21 gene | -1.55 | 0.161 | -1.48 | 0.129 | 1.07 | 0.743 | -1.55 | 0.072 | -0.88 |
| 1426335_at | Kcnq2 | potassium voltage-gated channel, subfamily Q, member 2 | -1.27 | 0.443 | -2.07 | 0.32 | 1.13 | 0.497 | -1.74 | 0.376 | -0.99 |
| 1422161_at | Siglec1 | sialic acid binding Ig-like lectin 1, sialoadhesin | -1.66 | 0.517 | 1.06 | 0.859 | -1.37 | 0.353 | -1.73 | 0.332 | -0.92 |
| 1437430_at | Slc45a2 | solute carrier family 45, member 2 | -1.2 | 0.673 | 1.12 | 0.754 | -2.28 | 0.012 | -1.82 | 0.607 | -1.04 |
| 1427726_at | LOC633417 | similar to histocompatibility 2, T region locus 3 | -1.28 | 0.645 | -1.52 | 0.34 | -1.05 | 0.903 | 1.01 | 0.973 | -0.71 |
| 1435336_at | Celsr2 | cadherin EGF LAG seven-pass G-type receptor 2 | -1.17 | 0.051 | -2.29 | 0.127 | 1.1 | 0.602 | -1.1 | 0.596 | -0.86 |
| 1455328_at | Accn2 | amiloride-sensitive cation channel 2, neuronal | -2.68 | 0.208 | -1.97 | 0.071 | 1.51 | 0.025 | -1.31 | 0.437 | -1.11 |
| 1422195_s_at | Tbx15 | T-box 15 | 1.31 | 0.422 | -2.33 | 0.37 | -1.52 | 0.482 | -1.11 | 0.826 | -0.91 |
| 1453098_at | Gria2 | glutamate receptor, ionotropic, AMPA2 (alpha 2) | -1.84 | 0.078 | -1.08 | 0.817 | -1.08 | 0.804 | 1.16 | 0.303 | -0.71 |
| 1443026_at | Grip1 | Glutamate receptor interacting protein 1 | -1.16 | 0.559 | -1.24 | 0.754 | -1.38 | 0.011 | 1.07 | 0.903 | -0.68 |
| 1439549_at | Prrg3 | proline rich Gla (G-carboxyglutamic acid) 3 (transmembrane) | -1.07 | 0.681 | -1.24 | 0.394 | -1.53 | 0.058 | 1.18 | 0.12 | -0.67 |
| 1439762_x_at | Adra2c | adrenergic receptor, alpha 2c | -1.89 | 0.294 | -1.33 | 0.549 | 1.12 | 0.875 | -2.7 | 0.321 | -1.2 |
| 1449786_at | Zdhhc7 | zinc finger, DHHC domain containing 7 | 1.19 | 0.813 | -1.94 | 0.277 | -1.44 | 0.253 | -1.5 | 0.254 | -0.92 |
| 1450274_at | 1700108E19Rik | RIKEN cDNA 1700108E19 gene | -2.49 | 0.057 | -1.25 | 0.77 | 1.19 | 0.694 | -1.69 | 0.422 | -1.06 |
| 1429930_at | Ccdc19 | coiled-coil domain containing 19 | -1.45 | 0.203 | -1.26 | 0.371 | -1.1 | 0.57 | 1.18 | 0.642 | -0.66 |
| 1418657_at | Znhit4 | zinc finger, HIT type 4 | -1.6 | 0.201 | -1.35 | 0.384 | 1.03 | 0.883 | -1.77 | 0.103 | -0.92 |
| 1441953_at | Stox2 | Storkhead box 2 | -1.41 | 0.137 | -1.1 | 0.769 | -1.28 | 0.355 | 1.42 | 0.329 | -0.59 |
| 1420795_at | Fgf9 | fibroblast growth factor 9 | -1.37 | 0.264 | -1.1 | 0.679 | -1.32 | 0.238 | 1.22 | 0.379 | -0.64 |
| 1420931_at | Mapk8 | mitogen activated protein kinase 8 | -2.5 | 0.064 | -1.41 | 0.427 | 1.29 | 0.498 | -1.82 | 0.011 | -1.11 |
| 1456685_at | Nsg2 | neuron specific gene family member 2 | -1.56 | 0.474 | -1.59 | 0.305 | 1.13 | 0.853 | -1.95 | 0.194 | -0.99 |
| 1441702_at | Rfx3 | Regulatory factor X, 3 (influences HLA class II expression) | -2.11 | 0.25 | -1.61 | 0.397 | 1.3 | 0.153 | -1.34 | 0.084 | -0.94 |
| 1457233_at | Dnaja2 | DnaJ (Hsp40) homolog, subfamily A, member 2 | 1.1 | 0.485 | -1.46 | 0.229 | -1.64 | 0.028 | -1.66 | 0.174 | -0.91 |
| 1457630_at | Whsc1l1 | Wolf-Hirschhorn syndrome candidate 1-like 1 (human) | -2.31 | 0.339 | -1.45 | 0.54 | 1.27 | 0.728 | -2.73 | 0.207 | -1.3 |

| | | | | | | | | | | | |
|---|---|---|---|---|---|---|---|---|---|---|---|
| 1435667_at | Rims1 | regulating synaptic membrane exocytosis 1 | -1.51 | 0.187 | -1.49 | 0.373 | 1.06 | 0.881 | -3.41 | 0.018 | -1.34 |
| 1457469_at | --- | --- | -1.19 | 0.591 | -1.18 | 0.775 | -1.4 | 0.24 | 1.54 | 0.252 | -0.56 |
| 1430211_at | 4930415O20Rik | RIKEN cDNA 4930415O20 gene | -1.16 | 0.662 | -1.24 | 0.471 | -1.37 | 0.481 | 1.55 | 0.211 | -0.56 |
| 1454273_at | 1700025H01Rik | RIKEN cDNA 1700025H01 gene | 1.16 | 0.544 | -1.16 | 0.789 | -2.63 | 0.204 | -1.17 | 0.4 | -0.95 |
| 1422338_at | Gpr63 | G protein-coupled receptor 63 | 1.05 | 0.862 | -1.06 | 0.826 | -2.48 | 0.06 | -1.73 | 0.537 | -1.05 |
| 1458858_at | --- | --- | -1.24 | 0.317 | -1.64 | 0.576 | -1.02 | 0.966 | 1.8 | 0.184 | -0.52 |
| 1423406_at | Sv2a | synaptic vesicle glycoprotein 2 a | -1.5 | 0.109 | 1.09 | 0.859 | -1.56 | 0.315 | -1.29 | 0.62 | -0.81 |
| 1427502_at | Adam4 | a disintegrin and metallopeptidase domain 4 | -1.24 | 0.467 | -1.02 | 0.94 | -1.64 | 0.137 | 1.64 | 0.175 | -0.56 |
| 1459930_at | Ube2h | Ubiquitin-conjugating enzyme E2H | -1.32 | 0.575 | -1.23 | 0.79 | -1.21 | 0.587 | 1.3 | 0.176 | -0.62 |
| 1429044_at | Camsap1l1 | calmodulin regulated spectrin-associated protein 1-like 1 | -1.06 | 0.483 | -1.18 | 0.269 | -1.64 | 0.006 | 2.01 | 0.135 | -0.47 |
| 1435390_at | Exod1 | exonuclease domain containing 1 | -1.39 | 0.271 | -1.21 | 0.248 | -1.18 | 0.451 | 1.64 | 0.194 | -0.53 |
| 1446206_at | B230209C24Rik | RIKEN cDNA B230209C24 gene | -2.35 | 0.525 | 1.44 | 0.396 | -1.88 | 0.296 | -1.42 | 0.361 | -1.05 |
| 1433944_at | Hectd2 | HECT domain containing 2 | 1.02 | 0.956 | -2.03 | 0.078 | -1.14 | 0.681 | -1.4 | 0.396 | -0.89 |
| 1456689_at | Rnf10 | Ring finger protein 10 | -1.03 | 0.941 | -1.64 | 0.009 | -1.22 | 0.414 | 2.12 | 0.17 | -0.44 |
| 1447511_at | --- | Transcribed locus | 1.36 | 0.052 | -1.71 | 0.362 | -2.22 | 0.135 | -4.4 | 0.024 | -1.74 |
| 1418968_at | Rb1cc1 | RB1-inducible coiled-coil 1 | -1.11 | 0.507 | -1.18 | 0.138 | -1.54 | 0.074 | 1.11 | 0.629 | -0.68 |
| 1429401_at | Stox2 | storkhead box 2 | -1.32 | 0.245 | -1.13 | 0.532 | -1.32 | 0.013 | 1.25 | 0.442 | -0.63 |
| 1445940_at | D4Ertd298e | DNA segment, Chr 4, ERATO Doi 298, expressed | -1.3 | 0.328 | -1.24 | 0.421 | -1.22 | 0.614 | 1.22 | 0.336 | -0.63 |
| 1438255_at | Ches1 | checkpoint suppressor 1 | 1.03 | 0.206 | -1.43 | 0.026 | -1.5 | 0.061 | -1.34 | 0.536 | -0.81 |
| 1450685_at | Arpp19 | cAMP-regulated phosphoprotein 19 | -1.21 | 0.072 | -1.15 | 0.038 | -1.43 | 0.026 | 1.08 | 0.595 | -0.68 |
| 1416744_at | Uap1 | UDP-N-acetylglucosamine pyrophosphorylase 1 | -1.53 | 0.341 | -1.29 | 0.351 | -1.03 | 0.934 | 1.3 | 0.386 | -0.64 |
| 1449760_at | --- | Transcribed locus | -1.47 | 0.175 | -1.01 | 0.912 | -1.37 | 0.184 | 1.4 | 0.253 | -0.61 |
| 1458256_at | 5830446M03Rik | RIKEN cDNA 5830446M03 gene | -1.82 | 0.102 | 1.02 | 0.961 | -1.2 | 0.352 | -1.2 | 0.498 | -0.8 |
| 1427249_x_at | Mup3 | major urinary protein 3 | 1.68 | 0.235 | -4.66 | 0.007 | -1.97 | 0.129 | -1.05 | 0.743 | -1.5 |
| 1445306_at | Cugbp2 | CUG triplet repeat, RNA binding protein 2 | 1.1 | 0.887 | -2.11 | 0.056 | -1.21 | 0.326 | -2.13 | 0.081 | -1.09 |
| 1430033_at | 5330431K02Rik | RIKEN cDNA 5330431K02 gene | 1.2 | 0.287 | -2.05 | 0.07 | -1.41 | 0.089 | -1.54 | 0.162 | -0.95 |
| 1427883_a_at | Col3a1 | procollagen, type III, alpha 1 | -1.13 | 0.218 | 1.13 | 0.29 | -2.57 | 0.055 | -1.07 | 0.792 | -0.91 |
| 1453437_at | 2810407D09Rik | RIKEN cDNA 2810407D09 gene | -1.31 | 0.34 | -1.13 | 0.467 | -1.33 | 0.078 | 1.15 | 0.439 | -0.65 |
| 1420063_at | --- | Transcribed locus | -1.96 | 0.535 | -2.06 | 0.122 | 1.41 | 0.566 | -1.41 | 0.512 | -1.01 |
| 1422696_at | Ttyh1 | tweety homolog 1 (Drosophila) | -1.32 | 0.695 | -1.1 | 0.885 | -1.37 | 0.558 | 1.13 | 0.783 | -0.66 |
| 1446440_at | --- | CDNA clone IMAGE:40051559 | 1.08 | 0.362 | -1.15 | 0.8 | -2.23 | 0.007 | -1.29 | 0.454 | -0.9 |
| 1452529_a_at | Creb1 | cAMP responsive element binding protein 1 | -1.42 | 0.452 | -1.45 | 0.235 | 1.01 | 0.982 | -1.08 | 0.627 | -0.74 |
| 1427963_s_at | Rdh9 | retinol dehydrogenase 9 | -1 | 0.83 | -2.12 | 0.001 | -1.07 | 0.621 | 1.1 | 0.769 | -0.77 |
| 1451867_x_at | Arhgap6 | Rho GTPase activating protein 6 | -1.42 | 0.015 | -1.2 | 0.387 | -1.15 | 0.383 | 1.02 | 0.909 | -0.69 |
| 1442402_at | Sh3rf1 | SH3 domain containing ring finger 1 | 1.15 | 0.7 | -1.36 | 0.094 | -1.94 | 0.021 | -1.13 | 0.407 | -0.82 |
| 1458873_at | Aspm | asp (abnormal spindle)-like, microcephaly associated (Drosophila) | -1.47 | 0.145 | -1.25 | 0.462 | -1.09 | 0.749 | 1.17 | 0.36 | -0.66 |
| 1443109_at | 5830417C01Rik | RIKEN cDNA 5830417C01 gene | -1.4 | 0.255 | 1.01 | 0.977 | -1.46 | 0.017 | -1.93 | 0.144 | -0.95 |
| 1452808_at | Snupn | snurportin 1 | -1.33 | 0.225 | -1.36 | 0.477 | -1.09 | 0.438 | 1.01 | 0.964 | -0.69 |
| 1457281_at | 4930461P20Rik | RIKEN cDNA 4930461P20 gene | -1.07 | 0.821 | -1.38 | 0.117 | -1.35 | 0.47 | 1.47 | 0.215 | -0.58 |
| 1437382_at | Acvr2a | Activin receptor IIA | -1.03 | 0.455 | -1.04 | 0.694 | -2.11 | 0.014 | 1.08 | 0.778 | -0.78 |
| 1444446_at | --- | --- | 1 | 0.99 | -1.16 | 0.62 | -1.86 | 0.033 | -1.1 | 0.727 | -0.78 |
| 1436161_at | Aprin | androgen-induced proliferation inhibitor | -1.09 | 0.372 | -1.22 | 0.136 | -1.5 | 0.087 | 1.06 | 0.562 | -0.69 |
| 1438081_at | Mcc | mutated in colorectal cancers | -1.32 | 0.37 | -1.19 | 0.359 | -1.25 | 0.46 | 1.93 | 0.057 | -0.45 |
| 1420735_at | Gabrr2 | gamma-aminobutyric acid (GABA-C) receptor, subunit rho 2 | -1.12 | 0.816 | -1.04 | 0.928 | -1.83 | 0.309 | 1.08 | 0.866 | -0.73 |
| 1427371_at | Abca8a | ATP-binding cassette, sub-family A (ABC1), member 8a | 1.24 | 0.42 | -2.17 | 0.016 | -1.43 | 0.225 | -1.14 | 0.709 | -0.87 |
| 1439880_at | D630023F18Rik | RIKEN cDNA D630023F18 gene | -1.61 | 0.098 | -1.05 | 0.896 | -1.21 | 0.617 | 1.28 | 0.329 | -0.65 |
| 1439844_at | 8430426J06Rik | RIKEN cDNA 8430426J06 gene | -1.37 | 0.387 | -1.05 | 0.845 | -1.39 | 0.156 | 1.06 | 0.896 | -0.69 |
| 1446390_at | Elk1 | ELK1, member of ETS oncogene family | -1.04 | 0.864 | -1.85 | 0.402 | -1.11 | 0.478 | 1.31 | 0.679 | -0.67 |
| 1451913_a_at | Hyou1 | hypoxia up-regulated 1 | -1.63 | 0.386 | -1.63 | 0.351 | 1.18 | 0.632 | -1.39 | 0.224 | -0.87 |
| 1434627_at | Nrf1 | Nuclear respiratory factor 1 | -1.26 | 0.248 | -1.04 | 0.478 | -1.55 | 0.037 | 1.02 | 0.801 | -0.71 |
| 1431424_at | 2810055G20Rik | RIKEN cDNA 2810055G20 gene | 1.17 | 0.384 | -1.46 | 0.023 | -1.84 | 0.005 | -1.37 | 0.227 | -0.87 |
| 1446758_at | Bre | brain and reproductive organ-expressed protein | -1.22 | 0.524 | -1.09 | 0.617 | -1.5 | 0.221 | 1.02 | 0.926 | -0.7 |
| 1440448_at | --- | Transcribed locus | -1.05 | 0.732 | -1.12 | 0.739 | -1.79 | 0.062 | 1.11 | 0.491 | -0.71 |

| | | | | | | | | | | | |
|---|---|---|---|---|---|---|---|---|---|---|---|
| 1432250_at | Utp14a | UTP14, U3 small nucleolar ribonucleoprotein, homolog A (yeast) | -1.16 | 0.7 | -1.51 | 0.223 | -1.14 | 0.398 | 1.04 | 0.563 | -0.69 |
| 1454581_at | 5330425B07Rik | RIKEN cDNA 5330425B07 gene | -1.11 | 0.72 | -1.05 | 0.917 | -1.81 | 0.079 | 1.35 | 0.078 | -0.66 |
| 1422610_s_at | Igf2bp3 | insulin-like growth factor 2 mRNA binding protein 3 | -1.09 | 0.901 | -2.05 | 0.244 | 1 | 0.999 | -3.89 | 0.046 | -1.51 |
| 1440930_a_at | Scrt2 | scratch homolog 2, zinc finger protein (Drosophila) | 1.3 | 0.71 | -1.64 | 0.194 | -2.03 | 0.209 | -2.42 | 0.288 | -1.2 |
| 1432396_at | 3110018I06Rik | RIKEN cDNA 3110018I06 gene | 1.32 | 0.63 | -2.18 | 0.011 | -1.61 | 0.096 | -1.09 | 0.653 | -0.89 |
| 1429517_at | Zfyve20 | zinc finger, FYVE domain containing 20 | -1.76 | 0.17 | -2.17 | 0.101 | 1.38 | 0.494 | -1.45 | 0.386 | -1 |
| 1437015_x_at | Pla2g1b | phospholipase A2, group IB, pancreas | -1.15 | 0.835 | 1.12 | 0.873 | -2.39 | 0.011 | -1.93 | 0.395 | -1.09 |
| 1456842_at | --- | PREDICTED: Mus musculus hypothetical protein LOC628013 (LOC628013), mR | 1.1 | 0.846 | -2.2 | 0.207 | -1.17 | 0.788 | -2.15 | 0.453 | -1.11 |
| 1432556_a_at | 3100002J23Rik | RIKEN cDNA 3100002J23 gene | -1.06 | 0.905 | -2.26 | 0.355 | 1.02 | 0.942 | -1.64 | 0.612 | -0.99 |
| 1431173_at | A930008G19Rik | RIKEN cDNA A930008G19 gene | -1.28 | 0.31 | -1.05 | 0.808 | -1.49 | 0.08 | 1.42 | 0.342 | -0.6 |
| 1454854_at | Ostb | organic solute transporter beta | 1.01 | 0.9 | -1.45 | 0.418 | -1.42 | 0.373 | -1.6 | 0.208 | -0.86 |
| 1420929_at | Ctnnal1 | catenin (cadherin associated protein), alpha-like 1 | 1.04 | 0.875 | -1.83 | 0.016 | -1.22 | 0.371 | -1.34 | 0.409 | -0.84 |
| 1446352_at | 2410016O06Rik | RIKEN cDNA 2410016O06 gene | -1.72 | 0.511 | -1.08 | 0.895 | -1.11 | 0.76 | 2.59 | 0.113 | -0.33 |
| 1447515_at | --- | Transcribed locus | 1.23 | 0.688 | -1.6 | 0.092 | -1.82 | 0.16 | -1.11 | 0.711 | -0.82 |
| 1437605_at | Nphs2 | nephrosis 2 homolog, podocin (human) | 1.31 | 0.291 | -1.81 | 0.145 | -1.85 | 0.022 | -2.37 | 0.096 | -1.18 |
| 1442471_at | Ephb2 | Eph receptor B2 | -1.33 | 0.048 | -1.27 | 0.414 | -1.16 | 0.521 | 1.08 | 0.415 | -0.67 |
| 1425421_at | Rbbp6 | Retinoblastoma binding protein 6 | 1.05 | 0.659 | -1.23 | 0.207 | -1.86 | 0.02 | -1.36 | 0.214 | -0.85 |
| 1442914_at | --- | Transcribed locus, weakly similar to XP_858458.1 PREDICTED: hypothetical pro | -1.24 | 0.151 | 1.06 | 0.838 | -1.87 | 0.277 | -2.47 | 0.036 | -1.13 |
| 1453136_at | Fbxo30 | F-box protein 30 | -1.37 | 0.18 | -1.14 | 0.592 | -1.25 | 0.184 | 4.86 | 0.189 | 0.27 |
| 1447490_at | --- | Transcribed locus | -1.27 | 0.775 | -1.08 | 0.749 | -1.44 | 0.512 | 1.3 | 0.616 | -0.62 |
| 1431882_at | Als2cr12 | amyotrophic lateral sclerosis 2 (juvenile) chromosome region, candidate 12 (huma | -3.29 | 0.053 | -1.49 | 0.016 | 1.43 | 0.29 | -1.45 | 0.188 | -1.2 |
| 1421635_at | Cnnm4 | cyclin M4 | -1.85 | 0.443 | 1.1 | 0.72 | -1.3 | 0.591 | -1.34 | 0.707 | -0.85 |
| 1435370_a_at | Ces3 | carboxylesterase 3 | -1.35 | 0.391 | -1.26 | 0.393 | -1.15 | 0.008 | 4.84 | 0.057 | 0.27 |
| 1440454_at | A530088I07Rik | RIKEN cDNA A530088I07 gene | -1.43 | 0.238 | -1.13 | 0.433 | -1.21 | 0.542 | 1.11 | 0.672 | -0.67 |
| 1441270_at | Akap2 | A kinase (PRKA) anchor protein 2 | 1.24 | 0.746 | -4.55 | 0.088 | -1.06 | 0.837 | -1.27 | 0.105 | -1.41 |
| 1421472_at | 2900083I11Rik | RIKEN cDNA 2900083I11 gene | -1.47 | 0.079 | -1.43 | 0.588 | 1.03 | 0.763 | -1.02 | 0.901 | -0.72 |
| 1453280_at | Mageb5 /// LOC629 | melanoma antigen, family B, 5 /// similar to Melanoma-associated antigen B3 (MA | -1.15 | 0.872 | -1.01 | 0.989 | -1.85 | 0.385 | 1.03 | 0.855 | -0.74 |
| 1452836_at | Lpin2 | lipin 2 | -1.19 | 0.279 | -1.3 | 0.439 | -1.25 | 0.392 | 1.84 | 0.147 | -0.48 |
| 1440904_at | Senp5 | SUMO/sentrin specific peptidase 5 | -1.29 | 0.282 | -2.22 | 0.029 | 1.18 | 0.397 | -1.28 | 0.149 | -0.9 |
| 1433205_at | 2810436B12Rik | RIKEN cDNA 2810436B12 gene | 1.3 | 0.432 | -1.79 | 0.309 | -1.81 | 0.091 | -1.63 | 0.287 | -0.99 |
| 1436577_at | Arhgef9 | Cdc42 guanine nucleotide exchange factor (GEF) 9 | 1.13 | 0.839 | -2.06 | 0.091 | -1.27 | 0.479 | -1.33 | 0.593 | -0.88 |
| 1442532_at | --- | --- | -1.29 | 0.385 | 1.04 | 0.896 | -1.68 | 0.068 | -1.54 | 0.004 | -0.87 |
| 1427641_at | Dlx4 | Distal-less homeobox 4 | -1.32 | 0.741 | -2.48 | 0.078 | 1.25 | 0.498 | -2.28 | 0.012 | -1.21 |
| 1432640_at | 4633401L03Rik | RIKEN cDNA 4633401L03 gene | 1.22 | 0.065 | -1.31 | 0.686 | -2.37 | 0.115 | -1.34 | 0.551 | -0.95 |
| 1439472_at | Gcn1l1 | GCN1 general control of amino-acid synthesis 1-like 1 (yeast) | -2.21 | 0.186 | -1.06 | 0.912 | 1.01 | 0.979 | -3.08 | 0 | -1.33 |
| 1440979_at | Igf2r | Insulin-like growth factor 2 receptor | 1.18 | 0.245 | -1.74 | 0.247 | -1.53 | 0.056 | -1.89 | 0.183 | -0.99 |
| 1435891_x_at | 2610021A01Rik /// | RIKEN cDNA 2610021A01 gene /// similar to zinc finger protein 347 | -1.24 | 0.426 | -1.14 | 0.541 | -1.38 | 0.121 | 1.11 | 0.583 | -0.66 |
| 1441680_at | Rbms3 | RNA binding motif, single stranded interacting protein | -1.5 | 0.167 | 1.26 | 0.496 | -2.08 | 0.015 | -1.64 | 0.204 | -0.99 |
| 1456705_at | Zfp532 | Zinc finger protein 532 | -2.07 | 0.015 | 1.23 | 0.579 | -1.44 | 0.137 | -1.15 | 0.651 | -0.86 |
| 1440037_at | Pbx1 | pre B-cell leukemia transcription factor 1 | -1.12 | 0.744 | 1.03 | 0.841 | -2.07 | 0.005 | -1.12 | 0.671 | -0.82 |
| 1420458_at | Tac4 | tachykinin 4 | -1.53 | 0.322 | -1.04 | 0.84 | -1.26 | 0.591 | 1.08 | 0.823 | -0.69 |
| 1424949_at | Huwe1 | HECT, UBA and WWE domain containing 1 | -1.12 | 0.209 | -1.25 | 0.02 | -1.4 | 0.054 | 1 | 1 | -0.69 |
| 1458711_at | --- | --- | -1.2 | 0.793 | -1.54 | 0.405 | -1.08 | 0.907 | 1.57 | 0.042 | -0.56 |
| 1451144_at | Bxdc2 | brix domain containing 2 | -1.22 | 0.107 | -1.33 | 0.038 | -1.2 | 0.013 | 1.05 | 0.714 | -0.67 |
| 1419929_at | D15Ertd55e | DNA segment, Chr 15, ERATO Doi 55, expressed | -1.66 | 0.454 | 1.21 | 0.393 | -1.68 | 0.045 | -1.03 | 0.96 | -0.79 |
| 1445928_at | 6-Mar | membrane-associated ring finger (C3HC4) 6 | 1.11 | 0.794 | -1.36 | 0.367 | -1.76 | 0.072 | -1 | 0.996 | -0.75 |
| 1418445_at | --- | --- | -1.21 | 0.357 | -1.31 | 0.095 | -1.23 | 0.385 | 1.32 | 0.4 | -0.6 |
| 1427118_at | krt86 | keratin 86 | -2.06 | 0.264 | 1.01 | 0.984 | -1.09 | 0.874 | -3.31 | 0.101 | -1.36 |
| 1441021_at | Letmd1 | LETM1 domain containing 1 | -1.67 | 0.574 | -3.49 | 0.004 | 1.52 | 0.185 | -1.82 | 0.091 | -1.36 |
| 1442071_at | Abce1 | ATP-binding cassette, sub-family E (OABP), member 1 | -1.34 | 0.161 | -1.56 | 0.172 | 1.02 | 0.954 | -1.33 | 0.2 | -0.8 |
| 1437024_at | Smpd4 | sphingomyelin phosphodiesterase 4 | -1.03 | 0.886 | -1.62 | 0.163 | -1.23 | 0.439 | 2.24 | 0.28 | -0.41 |
| 1441902_x_at | --- | --- | -1.74 | 0.013 | -1.32 | 0.355 | 1.08 | 0.738 | -1.24 | 0.13 | -0.81 |
| 1453409_at | Cgrrf1 | cell growth regulator with ring finger domain 1 | -1.53 | 0.286 | -1.06 | 0.776 | -1.24 | 0.482 | 1.48 | 0.301 | -0.59 |

| | | | | | | | | | | | |
|---|---|---|---|---|---|---|---|---|---|---|---|
| 1450586_at | Bdkrb1 | bradykinin receptor, beta 1 | 1.03 | 0.93 | -1.32 | 0.3 | -1.61 | 0.064 | -1.23 | 0.515 | -0.78 |
| 1442396_at | A730020M07Rik | RIKEN cDNA A730020M07 gene | -1.59 | 0.017 | -1.33 | 0.515 | 1.03 | 0.907 | -1.61 | 0.315 | -0.88 |
| 1459938_at | C330024D21Rik | RIKEN cDNA C330024D21 gene | -3.03 | 0.007 | -1.42 | 0.479 | 1.38 | 0.155 | -1.55 | 0.458 | -1.16 |
| 1452096_s_at | D230025D16Rik | RIKEN cDNA D230025D16 gene | -1.08 | 0.372 | -1.49 | 0.033 | -1.23 | 0.187 | 1 | 0.993 | -0.7 |
| 1420024_s_at | Etf1 | eukaryotic translation termination factor 1 | -1.27 | 0.413 | -1.09 | 0.653 | -1.42 | 0.045 | 1.26 | 0.431 | -0.63 |
| 1439445_x_at | Acly | ATP citrate lyase | -1.73 | 0.384 | 1.18 | 0.506 | -1.52 | 0.167 | -1.06 | 0.532 | -0.79 |
| 1454759_at | Git1 | G protein-coupled receptor kinase-interactor 1 | -1.22 | 0.139 | -1.87 | 0.094 | 1.05 | 0.671 | -1.18 | 0.69 | -0.8 |
| 1460654_at | Slc30a3 | solute carrier family 30 (zinc transporter), member 3 | -2.14 | 0.253 | 1.03 | 0.942 | -1.1 | 0.824 | -1.29 | 0.203 | -0.87 |
| 1427732_s_at | Abcg4 | ATP-binding cassette, sub-family G (WHITE), member 4 | -1.49 | 0.35 | -1.08 | 0.899 | -1.23 | 0.469 | 3.84 | 0.225 | 0.01 |
| 1447218_at | 1110003F02Rik | RIKEN cDNA 1110003F02 gene | -1.71 | 0.478 | -1.11 | 0.82 | -1.08 | 0.594 | 2.31 | 0.187 | -0.4 |
| 1434264_at | Ank2 | ankyrin 2, brain | 1.04 | 0.906 | -1.56 | 0.197 | -1.38 | 0.445 | -1.53 | 0.419 | -0.86 |
| 1443313_at | A530058N18Rik | RIKEN cDNA A530058N18 gene | -1.28 | 0.364 | -1.3 | 0.471 | -1.16 | 0.552 | 1.47 | 0.308 | -0.57 |
| 1428698_at | 2310004I03Rik | RIKEN cDNA 2310004I03 gene | 1.04 | 0.76 | -1.19 | 0.379 | -1.88 | 0.001 | -1.08 | 0.75 | -0.78 |
| 1437141_x_at | Dym | dymeclin | -1.35 | 0.241 | -1.41 | 0.101 | -1.04 | 0.718 | 1.42 | 0.159 | -0.59 |
| 1443436_at | Map2k2 | Mitogen activated protein kinase kinase 2 | -1.31 | 0.246 | -1.15 | 0.505 | -1.29 | 0.205 | 1.06 | 0.647 | -0.67 |
| 1453815_at | Spbc25 | spindle pole body component 25 homolog (S. cerevisiae) | -1.51 | 0.153 | -1.15 | 0.583 | -1.14 | 0.648 | 3.23 | 0.149 | -0.14 |
| 1444597_at | --- | Transcribed locus | -1.52 | 0.293 | -1.49 | 0.341 | 1.08 | 0.865 | -1.47 | 0.594 | -0.85 |
| 1429838_at | Spag16 | sperm associated antigen 16 | 1.02 | 0.962 | -1.17 | 0.558 | -1.86 | 0.228 | -1.15 | 0.738 | -0.79 |
| 1434488_at | Arfrp1 | ADP-ribosylation factor related protein 1 | -1.52 | 0.12 | -1.45 | 0.054 | 1.06 | 0.67 | -1.34 | 0.057 | -0.81 |
| 1427766_at | Tcra-V8 | T-cell receptor alpha, variable 8 | -1.83 | 0.534 | 1.04 | 0.918 | -1.21 | 0.642 | -1.08 | 0.878 | -0.77 |
| 1425127_at | Hsd3b2 | hydroxy-delta-5-steroid dehydrogenase, 3 beta- and steroid delta-isomerase 2 | -1.67 | 0.136 | -3.15 | 0.022 | 1.5 | 0.458 | -1.95 | 0.18 | -1.32 |
| 1425595_at | Gabbr1 | gamma-aminobutyric acid (GABA-B) receptor, 1 | -1.33 | 0.631 | 1.03 | 0.944 | -1.6 | 0.274 | -1.16 | 0.725 | -0.76 |
| 1440740_at | Ctdspl2 | CTD (carboxy-terminal domain, RNA polymerase II, polypeptide A) small phospha | 1.08 | 0.696 | -1.46 | 0.022 | -1.55 | 0.024 | -1.08 | 0.396 | -0.75 |
| 1431163_at | 2700046G09Rik | RIKEN cDNA 2700046G09 gene | -1.22 | 0.109 | -1.06 | 0.58 | -1.54 | 0.171 | 1.02 | 0.922 | -0.7 |
| 1439963_x_at | 1700129I04Rik | RIKEN cDNA 1700129I04 gene | 1.17 | 0.635 | -2.76 | 0.14 | -1.14 | 0.85 | -1.55 | 0.585 | -1.07 |
| 1426060_at | --- | --- | -1.71 | 0.404 | 1.02 | 0.901 | -1.24 | 0.276 | -1.62 | 0.155 | -0.89 |
| 1434916_at | Vkorc1l1 | vitamin K epoxide reductase complex, subunit 1-like 1 | -1.39 | 0.153 | -1.15 | 0.254 | -1.22 | 0.126 | 1.8 | 0.226 | -0.49 |
| 1441017_at | Zcchc14 | zinc finger, CCHC domain containing 14 | 1 | 0.996 | -1.42 | 0.204 | -1.41 | 0.093 | -1.35 | 0.662 | -0.8 |
| 1453781_at | --- | --- | -1.29 | 0.025 | -1.64 | 0.054 | 1.03 | 0.895 | -1.05 | 0.851 | -0.74 |
| 1418846_at | Ap4m1 | adaptor-related protein complex AP-4, mu 1 | 1.01 | 0.975 | -1.81 | 0.111 | -1.18 | 0.433 | -1.16 | 0.243 | -0.78 |
| 1451869_at | Abca3 | ATP-binding cassette, sub-family A (ABC1), member 3 | -1.35 | 0.203 | -1.02 | 0.955 | -1.46 | 0.2 | 1.64 | 0.004 | -0.54 |
| 1420946_at | Atrx | alpha thalassemia/mental retardation syndrome X-linked homolog (human) | -1.02 | 0.952 | -1.22 | 0.481 | -1.63 | 0.237 | 1.05 | 0.838 | -0.7 |
| 1429572_at | Nsun7 | NOL1/NOP2/Sun domain family, member 7 | -2.05 | 0.411 | -1.18 | 0.571 | 1.08 | 0.884 | -1.59 | 0.498 | -0.94 |
| 1446975_at | Casz1 | Castor homolog 1, zinc finger (Drosophila) | -3.14 | 0.036 | -1.03 | 0.927 | 1.13 | 0.804 | -1.17 | 0.266 | -1.05 |
| 1427961_s_at | Ugt2b34 | UDP glucuronosyltransferase 2 family, polypeptide B34 | -1.12 | 0.106 | -1.24 | 0.038 | -1.4 | 0.329 | 1.88 | 0.099 | -0.47 |
| 1458338_x_at | Nr2c1 | nuclear receptor subfamily 2, group C, member 1 | -1.17 | 0.643 | -1.03 | 0.946 | -1.72 | 0.085 | 1.58 | 0.349 | -0.58 |
| 1433352_at | C430049A07Rik | RIKEN cDNA C430049A07 gene | -2.52 | 0.007 | -1.34 | 0.599 | 1.27 | 0.374 | -1.4 | 0.286 | -1 |
| 1445932_at | Celsr1 | Cadherin EGF LAG seven-pass G-type receptor 1 | 1.29 | 0.802 | -1.46 | 0.352 | -2.31 | 0.227 | -1.55 | 0.583 | -1.01 |
| 1432214_at | 1700017D01Rik | RIKEN cDNA 1700017D01 gene | 1.12 | 0.903 | -2.56 | 0.202 | -1.1 | 0.879 | -1.09 | 0.796 | -0.91 |
| 1444616_x_at | --- | Transcribed locus | -1.18 | 0.716 | -1.04 | 0.822 | -1.64 | 0.063 | 1.46 | 0.562 | -0.6 |
| 1433663_s_at | AU014645 | expressed sequence AU014645 | -1.25 | 0.128 | -1.12 | 0.417 | -1.39 | 0.146 | 1.02 | 0.919 | -0.69 |
| 1423277_at | Ptprk | protein tyrosine phosphatase, receptor type, K | -1.15 | 0.027 | -1.37 | 0.081 | -1.23 | 0.213 | 1.18 | 0.421 | -0.64 |
| 1436460_at | BC030440 | cDNA sequence BC030440 | -1.54 | 0.227 | -1.15 | 0.665 | -1.12 | 0.057 | 2.18 | 0.169 | -0.41 |
| 1421494_at | 1110033F04Rik | RIKEN cDNA 1110033F04 gene | -1.1 | 0.878 | -1.7 | 0.443 | -1.09 | 0.803 | 1.72 | 0.349 | -0.54 |
| 1427196_at | Wnk4 | WNK lysine deficient protein kinase 4 | -1.02 | 0.938 | -1.91 | 0.018 | -1.1 | 0.086 | 2.09 | 0.101 | -0.48 |
| 1435912_at | Ubxd7 | UBX domain containing 7 | -1.05 | 0.177 | -1.36 | 0.137 | -1.37 | 0 | 1.2 | 0.16 | -0.65 |
| 1445176_at | --- | --- | -1.35 | 0.674 | -1.22 | 0.676 | -1.18 | 0.803 | 1 | 0.975 | -0.68 |
| 1429486_at | Pfkfb2 | 6-phosphofructo-2-kinase/fructose-2,6-biphosphatase 2 | -1.31 | 0.302 | -1.03 | 0.898 | -1.47 | 0.072 | 1.49 | 0.293 | -0.58 |
| 1426395_s_at | Eif3s1 | eukaryotic translation initiation factor 3, subunit 1 alpha | -1.28 | 0.202 | -1.4 | 0.008 | -1.09 | 0.496 | 1.4 | 0.411 | -0.59 |
| 1417199_at | 1300007B12Rik | RIKEN cDNA 1300007B12 gene | -1.09 | 0.578 | -1.25 | 0.036 | -1.43 | 0.036 | 1.14 | 0.588 | -0.66 |
| 1437759_at | Pfkp | Phosphofructokinase, platelet | -1.04 | 0.856 | -2.65 | 0.036 | 1.08 | 0.81 | -1.13 | 0.654 | -0.93 |
| 1428203_at | C030002O17Rik | RIKEN cDNA C030002O17 gene | -1.79 | 0.293 | -3.02 | 0.042 | 1.52 | 0.366 | -1.02 | 0.897 | -1.07 |
| 1441668_at | E130107B13Rik | RIKEN cDNA E130107B13 gene | -1.54 | 0.209 | -1.32 | 0.196 | 1.01 | 0.939 | -1.4 | 0.076 | -0.81 |

| | | | | | | | | | | | |
|---|---|---|---|---|---|---|---|---|---|---|---|
| 1457434_s_at | Ptpla | protein tyrosine phosphatase-like (proline instead of catalytic arginine), member a | -1.19 | 0.381 | -1.04 | 0.747 | -1.63 | 0.011 | 2.83 | 0.262 | -0.26 |
| 1457216_at | Rc3h1 | RING CCCH (C3H) domains 1 | 1.05 | 0.852 | -1.43 | 0.211 | -1.51 | 0.039 | -1.01 | 0.949 | -0.72 |
| 1431800_at | 1300010F03Rik | RIKEN cDNA 1300010F03 gene | 1.01 | 0.846 | -1.4 | 0.242 | -1.44 | 0.136 | -1.65 | 0.345 | -0.87 |
| 1459313_at | --- | Transcribed locus | 1.34 | 0.719 | -1.84 | 0.405 | -1.87 | 0.014 | -2.5 | 0.291 | -1.22 |
| 1427237_at | 2410015M20Rik | RIKEN cDNA 2410015M20 gene | 1.03 | 0.968 | -2.39 | 0.26 | -1.03 | 0.932 | -2.27 | 0.372 | -1.17 |
| 1422921_at | Vpreb3 | pre-B lymphocyte gene 3 | -1.31 | 0.177 | -1.91 | 0.338 | 1.13 | 0.751 | -2.9 | 0.044 | -1.25 |
| 1459440_at | Nav3 | Neuron navigator 3 | -1.47 | 0.39 | 1.01 | 0.985 | -1.38 | 0.519 | -1.42 | 0.098 | -0.82 |
| 1416167_at | Prdx4 | peroxiredoxin 4 | -1.11 | 0.351 | -1.34 | 0.006 | -1.3 | 0.012 | 1.7 | 0.009 | -0.51 |
| 1428324_at | --- | --- | 1.11 | 0.647 | -1.67 | 0.087 | -1.41 | 0.035 | -1.39 | 0.111 | -0.84 |
| 1456922_at | 4933437K13Rik | RIKEN cDNA 4933437K13 gene | -1.71 | 0.103 | 1.13 | 0.829 | -1.42 | 0.175 | -2.13 | 0.017 | -1.03 |
| 1439540_at | 2-Mar | membrane-associated ring finger (C3HC4) 2 | -1.06 | 0.894 | -2 | 0.128 | -1.03 | 0.884 | 3.15 | 0.242 | -0.24 |
| 1420944_at | Zfp185 | zinc finger protein 185 | -1.25 | 0.568 | -1.38 | 0.293 | -1.12 | 0.755 | 1.22 | 0.727 | -0.63 |
| 1436701_at | Trim61 | tripartite motif-containing 61 | 1.08 | 0.926 | -1.65 | 0.506 | -1.38 | 0.295 | -1.34 | 0.591 | -0.82 |
| 1457580_at | Chd8 | chromodomain helicase DNA binding protein 8 | 1 | 0.992 | -1.17 | 0.613 | -1.79 | 0.162 | -1.43 | 0.241 | -0.85 |
| 1454016_at | --- | --- | -1.06 | 0.9 | -3.43 | 0.02 | 1.18 | 0.715 | -1.54 | 0.466 | -1.21 |
| 1456577_x_at | Pitrm1 | pitrilysin metallepetidase 1 | -1.18 | 0.58 | -1.26 | 0.434 | -1.28 | 0.154 | 1.91 | 0.303 | -0.45 |
| 1439344_at | --- | Transcribed locus | -1.14 | 0.588 | -1.14 | 0.505 | -1.51 | 0.051 | 1.08 | 0.631 | -0.68 |
| 1449828_at | Ptgfr | prostaglandin F receptor | -1.29 | 0.682 | -1.61 | 0.492 | 1.02 | 0.957 | -1.39 | 0.053 | -0.82 |
| 1423307_s_at | Tgoln1 /// Tgoln2 | trans-golgi network protein /// trans-golgi network protein 2 | -1.1 | 0.25 | -1.43 | 0.001 | -1.24 | 0.245 | 1.04 | 0.868 | -0.68 |
| 1416042_s_at | Nasp | nuclear autoantigenic sperm protein (histone-binding) | -1.32 | 0.13 | -1.32 | 0.026 | -1.11 | 0.458 | 1.69 | 0.202 | -0.51 |
| 1451186_at | Isg20l1 | interferon stimulated exonuclease gene 20-like 1 | -1.02 | 0.969 | -1.35 | 0.656 | -1.43 | 0.1 | 1.42 | 0.261 | -0.6 |
| 1437564_at | Polg | polymerase (DNA directed), gamma | 1.02 | 0.965 | -1.25 | 0.784 | -1.68 | 0.249 | -1.28 | 0.116 | -0.8 |
| 1442277_at | Chka | choline kinase alpha | -1.08 | 0.752 | 1.05 | 0.894 | -2.26 | 0.006 | -1.38 | 0.185 | -0.92 |
| 1443867_at | Ankrd12 | ankyrin repeat domain 12 | -1.34 | 0.272 | 1.02 | 0.923 | -1.53 | 0.205 | -1.22 | 0.604 | -0.77 |
| 1434766_at | --- | Mus musculus, clone IMAGE:1512359, mRNA | -1.09 | 0.339 | -1.32 | 0.052 | -1.35 | 0.008 | 1.4 | 0.425 | -0.59 |
| 1455094_s_at | Ube2g1 | ubiquitin-conjugating enzyme E2G 1 (UBC7 homolog, C. elegans) | -1.17 | 0.313 | -1.17 | 0.183 | -1.42 | 0.203 | 1.2 | 0.092 | -0.64 |
| 1420741_x_at | 2310069N01Rik | RIKEN cDNA 2310069N01 gene | -1.6 | 0.132 | -1.96 | 0.125 | 1.28 | 0.534 | -1.2 | 0.734 | -0.87 |
| 1455748_at | Gpr178 | G protein-coupled receptor 178 | -1.96 | 0.198 | 1.15 | 0.531 | -1.32 | 0.289 | -1.78 | 0.386 | -0.98 |
| 1445705_x_at | Dpp8 | dipeptidylpeptidase 8 | 1.37 | 0.327 | -1.6 | 0.495 | -2.36 | 0.353 | -1.46 | 0.594 | -1.01 |
| 1450610_at | Ucn | urocortin | -1.37 | 0.388 | -1.74 | 0.332 | 1.11 | 0.758 | -2.95 | 0.178 | -1.24 |
| 1427861_at | Arl6ip3 | ADP-ribosylation factor-like 6 interacting protein 3 | -1.18 | 0.587 | -1.49 | 0.466 | -1.11 | 0.519 | 1.08 | 0.714 | -0.67 |
| 1454866_s_at | Clic6 | chloride intracellular channel 6 | -3.79 | 0.005 | -1.45 | 0.381 | 1.47 | 0.148 | -1.26 | 0.784 | -1.26 |
| 1452138_a_at | Ace2 | angiotensin I converting enzyme (peptidyl-dipeptidase A) 2 | -1.15 | 0.471 | -2.54 | 0.049 | 1.15 | 0.621 | -1.47 | 0.562 | -1 |
| 1439294_at | D530008I22 | hypothetical protein D530008I22 | -1.11 | 0.628 | -1.61 | 0.418 | -1.12 | 0.55 | 1.1 | 0.671 | -0.68 |
| 1439203_at | Satb1 | Special AT-rich sequence binding protein 1 | 1.01 | 0.958 | -1.29 | 0.417 | -1.59 | 0.013 | -2.29 | 0.344 | -1.04 |
| 1435065_x_at | Vav2 | Vav2 oncogene | -1.29 | 0.367 | -2.86 | 0.05 | 1.29 | 0.287 | -1.26 | 0.515 | -1.03 |
| 1457586_at | Rasa2 | RAS p21 protein activator 2 | -1.19 | 0.403 | 1.12 | 0.757 | -2.21 | 0.001 | -1.65 | 0.142 | -0.98 |
| 1446120_at | Uhmk1 | U2AF homology motif (UHM) kinase 1 | -1.24 | 0.225 | 1.13 | 0.663 | -2.05 | 0.186 | -1.04 | 0.95 | -0.8 |
| 1449443_at | Decr1 | 2,4-dienoyl CoA reductase 1, mitochondrial | -1.02 | 0.894 | -1.84 | 0.127 | -1.11 | 0.603 | 1.24 | 0.085 | -0.68 |
| 1438516_at | Rif1 | Rap1 interacting factor 1 homolog (yeast) | -1.04 | 0.693 | -1.4 | 0.215 | -1.35 | 0.064 | 1.03 | 0.87 | -0.69 |
| 1456390_at | --- | --- | -1.08 | 0.608 | -1.19 | 0.061 | -1.53 | 0.011 | 1.09 | 0.483 | -0.68 |
| 1456384_at | Nlgn3 | neuroligin 3 | -1.17 | 0.826 | 1.18 | 0.78 | -2.62 | 0.09 | -1.54 | 0.643 | -1.04 |
| 1418802_at | R74862 | expressed sequence R74862 | -1.07 | 0.828 | -1.76 | 0.353 | -1.09 | 0.755 | 1.18 | 0.031 | -0.69 |
| 1456231_at | Pla2g3 | phospholipase A2, group III | -1.12 | 0.101 | -1.92 | 0.284 | 1.01 | 0.959 | -1.13 | 0.429 | -0.79 |
| 1446051_at | Gm1140 | gene model 1140, (NCBI) | -1.55 | 0.573 | -1.55 | 0.405 | 1.13 | 0.817 | -1.21 | 0.742 | -0.8 |
| 1418694_at | Kcmf1 | potassium channel modulatory factor 1 | -1.02 | 0.848 | -1.38 | 0.057 | -1.39 | 0.026 | 1.11 | 0.518 | -0.67 |
| 1418496_at | Foxa1 | forkhead box A1 | -1.21 | 0.254 | -2.44 | 0.014 | 1.18 | 0.235 | -1.2 | 0.754 | -0.92 |
| 1457515_at | Hipk1 | Homeodomain interacting protein kinase 1 | -1.16 | 0.5 | -1.43 | 0.325 | -1.16 | 0.435 | 1.81 | 0.232 | -0.49 |
| 1418537_at | 0610042E07Rik | RIKEN cDNA 0610042E07 gene | -1.56 | 0.116 | -1.3 | 0.041 | 1.01 | 0.937 | -1.39 | 0.165 | -0.81 |
| 1453004_at | 3110004L20Rik | RIKEN cDNA 3110004L20 gene | -1.23 | 0.297 | -1.19 | 0.053 | -1.31 | 0.166 | 3.98 | 0.162 | 0.06 |
| 1429431_at | Zfpn1a5 | zinc finger protein, subfamily 1A, 5 | -1.06 | 0.011 | -1.24 | 0.165 | -1.5 | 0.029 | 1.58 | 0.214 | -0.55 |
| 1454410_at | 9130414P19Rik | RIKEN cDNA 9130414P19 gene | -2.19 | 0.257 | -1.07 | 0.938 | 1.03 | 0.868 | -2.02 | 0.283 | -1.06 |
| 1419918_at | Tmed7 | transmembrane emp24 protein transport domain containing 7 | -1.07 | 0.783 | -1.09 | 0.492 | -1.75 | 0.043 | 1.19 | 0.158 | -0.68 |

| | | | | | | | | | | | |
|---|---|---|---|---|---|---|---|---|---|---|---|
| 1437589_x_at | Lime1 | Lck interacting transmembrane adaptor 1 | -1.77 | 0.003 | -1.22 | 0.167 | 1.03 | 0.755 | -1.42 | 0.334 | -0.84 |
| 1440901_at | Dgkb | diacylglycerol kinase, beta | -1.21 | 0.064 | -1.29 | 0.556 | -1.22 | 0.483 | 1.11 | 0.827 | -0.65 |
| 1453976_at | 4432414F05Rik | RIKEN cDNA 4432414F05 gene | -1.2 | 0.447 | -1.14 | 0.756 | -1.41 | 0.332 | 1.22 | 0.462 | -0.63 |
| 1451485_at | 3300001P08Rik | RIKEN cDNA 3300001P08 gene | -1.03 | 0.834 | -1.32 | 0.26 | -1.44 | 0.215 | 1.02 | 0.93 | -0.69 |
| 1446253_at | --- | Adult male medulla oblongata cDNA, RIKEN full-length enriched library, clone:63 | -1.56 | 0.038 | -1.38 | 0.198 | 1.05 | 0.217 | -1.19 | 0.674 | -0.77 |
| 1439330_at | D230040J21Rik | RIKEN cDNA D230040J21 gene | -1.44 | 0.01 | 1.12 | 0.712 | -1.66 | 0.085 | -1.27 | 0.076 | -0.81 |
| 1430538_at | 2210013O21Rik | RIKEN cDNA 2210013O21 gene | -1.05 | 0.607 | -1.23 | 0.047 | -1.52 | 0 | 1.05 | 0.744 | -0.69 |
| 1424405_at | Mbip | MAP3K12 binding inhibitory protein 1 | -1.35 | 0.18 | 1.05 | 0.6 | -1.59 | 0.002 | -1.2 | 0.158 | -0.77 |
| 1422099_a_at | Oprl1 | opioid receptor-like 1 | 1.21 | 0.836 | -1.6 | 0.289 | -1.71 | 0.317 | -1.21 | 0.743 | -0.83 |
| 1455808_at | 4922502D21Rik | RIKEN cDNA 4922502D21 gene | -1.16 | 0.473 | -1 | 1 | -1.78 | 0.374 | 1.25 | 0.384 | -0.67 |
| 1427061_at | Rbbp8 | retinoblastoma binding protein 8 | -1.39 | 0.17 | -1.03 | 0.819 | -1.37 | 0.048 | 1.7 | 0.14 | -0.52 |
| 1459304_at | A730054J21Rik | RIKEN cDNA A730054J21 gene | -1.55 | 0.475 | -1.24 | 0.187 | -1.03 | 0.909 | 2.08 | 0.273 | -0.43 |
| 1460126_at | Usp34 | Ubiquitin specific peptidase 34 | 1.21 | 0.184 | -1.63 | 0.019 | -1.66 | 0.027 | -1.14 | 0.651 | -0.81 |
| 1443681_at | AI595406 | Expressed sequence AI595406 | -1.29 | 0.483 | -1.2 | 0.565 | -1.24 | 0.383 | 1.24 | 0.431 | -0.62 |
| 1421617_at | Trpm8 | transient receptor potential cation channel, subfamily M, member 8 | -2.36 | 0.431 | 1.43 | 0.232 | -1.76 | 0.266 | -2.33 | 0.079 | -1.25 |
| 1428401_at | Zcchc3 | zinc finger, CCHC domain containing 3 | -1.16 | 0.242 | -1.4 | 0.158 | -1.18 | 0.031 | 1.26 | 0.329 | -0.62 |
| 1430790_at | 4930485G23Rik | RIKEN cDNA 4930485G23 gene | -2.1 | 0.403 | 1.14 | 0.623 | -1.24 | 0.67 | -1.94 | 0.351 | -1.03 |
| 1418517_at | Irx3 | Iroquois related homeobox 3 (Drosophila) | -1.28 | 0.573 | -3.28 | 0.019 | 1.34 | 0.215 | -1.53 | 0.254 | -1.19 |
| 1445896_at | 2810433D01Rik | RIKEN cDNA 2810433D01 gene | 1.07 | 0.505 | -1.25 | 0.73 | -1.8 | 0.047 | -4.05 | 0.008 | -1.51 |
| 1457447_at | Rb1 | Retinoblastoma 1 | -1.11 | 0.409 | -1.16 | 0.236 | -1.52 | 0.109 | 1.29 | 0.269 | -0.62 |
| 1456809_at | --- | Adult male thymus cDNA, RIKEN full-length enriched library, clone:5830435O18 | -1.41 | 0.015 | -1.52 | 0.145 | 1.06 | 0.873 | -1.03 | 0.878 | -0.73 |
| 1459887_at | --- | Transcribed locus | 1.13 | 0.738 | -1.68 | 0.003 | -1.44 | 0.139 | -1.19 | 0.456 | -0.79 |
| 1425492_at | Bmpr1a | bone morphogenetic protein receptor, type 1A | -1.07 | 0.663 | -1.24 | 0.011 | -1.46 | 0.016 | 1.11 | 0.557 | -0.67 |
| 1445512_at | --- | --- | 1.07 | 0.753 | -1.36 | 0.335 | -1.63 | 0.123 | -1.09 | 0.712 | -0.75 |
| 1434053_x_at | Atp5k | ATP synthase, H+ transporting, mitochondrial F1F0 complex, subunit e | -1.19 | 0.003 | -1.3 | 0.069 | -1.23 | 0.082 | 1.06 | 0.257 | -0.67 |
| 1432473_a_at | Abca14 | ATP-binding cassette, sub-family A (ABC1), member 14 | -2.35 | 0.063 | 1.15 | 0.546 | -1.18 | 0.653 | -1.3 | 0.658 | -0.92 |
| 1444759_at | Prickle1 | prickle like 1 (Drosophila) | -1.84 | 0.154 | -1.51 | 0.481 | 1.22 | 0.826 | -1.43 | 0.543 | -0.89 |
| 1447174_at | Dach1 | Dachshund 1 (Drosophila) | -1.26 | 0.319 | -1.02 | 0.982 | -1.55 | 0.13 | 1.48 | 0.482 | -0.59 |
| 1456408_x_at | 4933439C10Rik | RIKEN cDNA 4933439C10 gene | -1.66 | 0.022 | 1.05 | 0.922 | -1.29 | 0.224 | -1.7 | 0.232 | -0.9 |
| 1453667_at | 1700065D16Rik | RIKEN cDNA 1700065D16 gene | 1.37 | 0.589 | -2.19 | 0.269 | -1.67 | 0.242 | -1.17 | 0.726 | -0.91 |
| 1425314_at | Gpr98 | G protein-coupled receptor 98 | -1.58 | 0.418 | 1.07 | 0.786 | -1.38 | 0.088 | -1.17 | 0.806 | -0.77 |
| 1448042_s_at | Rnf2 | ring finger protein 2 | -1.59 | 0.05 | 1.06 | 0.904 | -1.35 | 0.537 | -1.87 | 0.547 | -0.94 |
| 1455807_at | Tspyl5 | testis-specific protein, Y-encoded-like 5 | -1.17 | 0.374 | -1.56 | 0.228 | -1.08 | 0.554 | 1.92 | 0.137 | -0.47 |
| 1447156_at | Chm | Choroidermia | 1.05 | 0.768 | -1.43 | 0.006 | -1.47 | 0.028 | -1.5 | 0.264 | -0.84 |
| 1440392_at | Akap13 | A kinase (PRKA) anchor protein 13 | -1.2 | 0.27 | -1.13 | 0.526 | -1.43 | 0.185 | 1.74 | 0.168 | -0.5 |
| 1459245_s_at | Grid2 | glutamate receptor, ionotropic, delta 2 | -2.24 | 0.295 | -1.01 | 0.965 | -1.01 | 0.746 | 1.73 | 0.345 | -0.63 |
| 1456717_at | Tead1 | TEA domain family member 1 | -1.04 | 0.961 | 1.12 | 0.84 | -2.98 | 0.209 | -1.32 | 0.129 | -1.05 |
| 1440264_at | --- | --- | 1.05 | 0.879 | -1.83 | 0.129 | -1.22 | 0.295 | -2.29 | 0.069 | -1.07 |
| 1443972_at | AI465300 | expressed sequence AI465300 | -1.55 | 0.426 | 1.03 | 0.969 | -1.33 | 0.489 | -2.47 | 0.017 | -1.08 |
| 1447606_x_at | Aqp11 | aquaporin 11 | -1.75 | 0.206 | -1.01 | 0.926 | -1.16 | 0.57 | 1.3 | 0.216 | -0.65 |
| 1454966_at | Itga8 | integrin alpha 8 | -1.13 | 0.505 | -1.16 | 0.26 | -1.48 | 0.045 | 1.11 | 0.78 | -0.66 |
| 1433109_at | Mif | macrophage migration inhibitory factor | -1.92 | 0.113 | -1.01 | 0.986 | -1.1 | 0.791 | 1.07 | 0.847 | -0.74 |
| 1454063_at | 4933412E24Rik | RIKEN cDNA 4933412E24 gene | -1.48 | 0.609 | -1.75 | 0.322 | 1.18 | 0.796 | -1.44 | 0.263 | -0.87 |
| 1420118_s_at | --- | --- | -1.54 | 0.648 | 1.14 | 0.842 | -1.56 | 0.142 | -1.01 | 0.964 | -0.74 |
| 1421363_at | Cyp2c39 | cytochrome P450, family 2, subfamily c, polypeptide 39 | -1.39 | 0.677 | 1.09 | 0.564 | -1.62 | 0.44 | -1.11 | 0.861 | -0.76 |
| 1438553_x_at | 4930453N24Rik | RIKEN cDNA 4930453N24 gene | -1.07 | 0.75 | -1.21 | 0.266 | -1.49 | 0.032 | 1.16 | 0.455 | -0.66 |
| 1424944_at | Pcp2 | Purkinje cell protein 2 (L7) | -1.14 | 0.832 | 1.08 | 0.903 | -2.15 | 0.251 | -1.23 | 0.525 | -0.86 |
| 1445815_at | Fzd8 | frizzled homolog 8 (Drosophila) | 1.39 | 0.211 | -1.97 | 0.128 | -1.9 | 0.055 | -1.58 | 0.351 | -1.02 |
| 1442352_at | 9430091N11Rik | RIKEN cDNA 9430091N11 gene | -1.49 | 0.568 | 1.42 | 0.515 | -3.01 | 0.074 | -1.34 | 0.492 | -1.1 |
| 1450750_a_at | Nr4a2 | nuclear receptor subfamily 4, group A, member 2 | -1.69 | 0.144 | -1.02 | 0.966 | -1.18 | 0.439 | 1.07 | 0.699 | -0.7 |
| 1453771_at | Gulp1 | GULP, engulfment adaptor PTB domain containing 1 | 1.12 | 0.701 | -1.82 | 0.105 | -1.32 | 0.133 | -1.3 | 0.204 | -0.83 |
| 1453656_a_at | 4933431G14Rik | RIKEN cDNA 4933431G14 gene | -1.01 | 0.961 | -1.98 | 0.06 | -1.07 | 0.886 | 1.16 | 0.661 | -0.73 |
| 1441707_at | --- | --- | -1.13 | 0.543 | 1.01 | 0.957 | -1.87 | 0.03 | -1.33 | 0.456 | -0.83 |

| | | | | | | | | | | | |
|---|---|---|---|---|---|---|---|---|---|---|---|
| 1447122_at | --- | --- | -1.06 | 0.44 | -1.24 | 0.064 | -1.47 | 0.06 | 1.14 | 0.393 | -0.66 |
| 1453716_at | 4930480K23Rik | RIKEN cDNA 4930480K23 gene | -1.24 | 0.522 | -1.94 | 0.087 | 1.1 | 0.67 | -1.19 | 0.003 | -0.82 |
| 1432593_at | 4933428P19Rik | RIKEN cDNA 4933428P19 gene | -1.34 | 0.116 | -1.22 | 0.497 | -1.16 | 0.422 | 2.17 | 0.243 | -0.39 |
| 1424717_at | Mis12 | MIS12 homolog (yeast) | -1.46 | 0.282 | -1.14 | 0.373 | -1.15 | 0.302 | 1.7 | 0.248 | -0.51 |
| 1460401_at | Edem3 | ER degradation enhancer, mannosidase alpha-like 3 | -1.13 | 0.229 | -1.21 | 0.235 | -1.39 | 0.059 | 1.05 | 0.272 | -0.67 |
| 1449030_at | Syn2 | synapsin II | -1.42 | 0.333 | -1.16 | 0.664 | -1.17 | 0.718 | 1.85 | 0.26 | -0.47 |
| 1431346_at | 6330405D24Rik | RIKEN cDNA 6330405D24 gene | -1.17 | 0.809 | -1.54 | 0.346 | -1.09 | 0.833 | 1.34 | 0.216 | -0.61 |
| 1440272_at | Eif2s1 | Eukaryotic translation initiation factor 2, subunit 1 alpha | -1.49 | 0.132 | 1.11 | 0.541 | -1.56 | 0.097 | -1.14 | 0.723 | -0.77 |
| 1439763_at | Dnajc11 | DnaJ (Hsp40) homolog, subfamily C, member 11 | -1 | 0.999 | -1.17 | 0.738 | -1.74 | 0.239 | 1.41 | 0.353 | -0.63 |
| 1441473_at | LOC666775 /// LOC | hypothetical protein LOC666775 /// hypothetical protein LOC671556 | -1.07 | 0.892 | -1.93 | 0.265 | -1.03 | 0.81 | 1.02 | 0.945 | -0.75 |
| 1420930_s_at | Ctnnal1 | catenin (cadherin associated protein), alpha-like 1 | -1.14 | 0.196 | -1.23 | 0.393 | -1.35 | 0.052 | 1.03 | 0.881 | -0.67 |
| 1445079_at | D15Ertd320e | DNA segment, Chr 15, ERATO Doi 320, expressed | -1.29 | 0.337 | -1.62 | 0.401 | 1.04 | 0.913 | -1.48 | 0.387 | -0.84 |
| 1443716_at | --- | --- | -1.05 | 0.86 | -1.39 | 0.651 | -1.33 | 0.207 | 1.15 | 0.48 | -0.65 |
| 1439177_at | --- | Transcribed locus | -1.22 | 0.435 | -1.18 | 0.681 | -1.31 | 0.24 | 1.13 | 0.864 | -0.65 |
| 1422349_at | Ccr1l1 | chemokine (C-C motif) receptor 1-like 1 | -1.52 | 0.022 | -1.41 | 0.452 | 1.06 | 0.896 | -1.08 | 0.886 | -0.74 |
| 1424087_at | 1810042K04Rik | RIKEN cDNA 1810042K04 gene | -1.41 | 0.277 | 1.03 | 0.878 | -1.46 | 0.297 | -1.08 | 0.575 | -0.73 |
| 1444308_at | --- | --- | -1.03 | 0.885 | -1.6 | 0.194 | -1.2 | 0.455 | 1.64 | 0.282 | -0.55 |
| 1447121_at | 0610038K03Rik | RIKEN cDNA 0610038K03 gene | 1 | 0.993 | -1.6 | 0.032 | -1.25 | 0.255 | -1.33 | 0.374 | -0.79 |
| 1425117_at | 0610012D14Rik | RIKEN cDNA 0610012D14 gene | -1.21 | 0.295 | -1.21 | 0.057 | -1.3 | 0.529 | 1.44 | 0.501 | -0.57 |
| 1423498_at | Aldoa-ps1 | aldolase 1, A isoform, pseudogene 1 | 1.14 | 0.508 | -2.06 | 0.29 | -1.25 | 0.616 | -1.08 | 0.709 | -0.81 |
| 1438300_at | 4921511I16Rik | RIKEN cDNA 4921511I16 gene | -1.33 | 0.431 | -1.05 | 0.879 | -1.39 | 0.08 | 1.14 | 0.059 | -0.66 |
| 1445974_at | --- | Transcribed locus | -1.13 | 0.749 | -1.19 | 0.796 | -1.43 | 0.601 | 1.07 | 0.834 | -0.67 |
| 1455454_at | Akr1c19 | aldo-keto reductase family 1, member C19 | -1.15 | 0.273 | -1.45 | 0.325 | -1.15 | 0.478 | 1 | 0.984 | -0.69 |
| 1457913_at | 5730601F06Rik | RIKEN cDNA 5730601F06 gene | 1.12 | 0.176 | -1.13 | 0.264 | -2.37 | 0.01 | -1.62 | 0.215 | -1 |
| 1441760_at | LOC243302 /// LOC | similar to 40S ribosomal protein S25 /// similar to 40S ribosomal protein S25 /// hy | 1.33 | 0.554 | -3.01 | 0.312 | -1.3 | 0.093 | -1.16 | 0.506 | -1.04 |
| 1448002_x_at | 2610001J05Rik | RIKEN cDNA 2610001J05 gene | -1.19 | 0.694 | -1.9 | 0.358 | 1.06 | 0.872 | -1.54 | 0.381 | -0.89 |
| 1453548_at | --- | --- | -1.43 | 0.007 | -1.62 | 0.359 | 1.11 | 0.757 | -1.33 | 0.602 | -0.82 |
| 1419620_at | Pttg1 | pituitary tumor-transforming 1 | -1.3 | 0.27 | -1.57 | 0.021 | 1.02 | 0.879 | -1.2 | 0.435 | -0.76 |
| 1440885_at | Evl | Ena-vasodilator stimulated phosphoprotein | -1.54 | 0.025 | 1 | 0.99 | -1.29 | 0.219 | -1.95 | 0.113 | -0.94 |
| 1455508_at | A530082C11Rik | RIKEN cDNA A530082C11 gene | -1.15 | 0.311 | -1.38 | 0.093 | -1.2 | 0.179 | 1.2 | 0.194 | -0.63 |
| 1440800_at | LOC665193 | hypothetical protein LOC665193 | -1.06 | 0.864 | -1.29 | 0.491 | -1.42 | 0.302 | 1.21 | 0.526 | -0.64 |
| 1438581_at | 4932439K10Rik | RIKEN cDNA 4932439K10 gene | -1.64 | 0.391 | 1.33 | 0.645 | -2.06 | 0.038 | -1.27 | 0.682 | -0.91 |
| 1457374_at | Nedd4l | neural precursor cell expressed, developmentally down-regulated gene 4-like | -1.15 | 0.303 | 1.09 | 0.649 | -2.15 | 0.016 | -1.43 | 0.198 | -0.91 |
| 1453987_at | Btbd5 | BTB (POZ) domain containing 5 | 1.17 | 0.852 | -2.13 | 0.067 | -1.28 | 0.173 | -1.66 | 0.011 | -0.97 |
| 1459020_at | Amigo1 | adhesion molecule with Ig like domain 1 | -1.5 | 0.145 | -1.04 | 0.923 | -1.25 | 0.221 | 1.37 | 0.511 | -0.61 |
| 1453779_at | 1700020L24Rik | RIKEN cDNA 1700020L24 gene | -1.26 | 0.624 | -4.99 | 0.012 | 1.43 | 0.361 | -1.45 | 0.366 | -1.56 |
| 1425768_at | Diablo | diablo homolog (Drosophila) | -2.14 | 0.149 | -1.12 | 0.73 | 1.07 | 0.672 | -1.05 | 0.868 | -0.81 |
| 1446413_at | 4930555G07Rik | RIKEN cDNA 4930555G07 gene | -1.08 | 0.836 | -1.28 | 0.448 | -1.39 | 0.52 | 1.84 | 0.284 | -0.48 |
| 1428561_at | 2610002J23Rik | RIKEN cDNA 2610002J23 gene | -1.21 | 0.017 | -1.23 | 0.157 | -1.27 | 0.055 | 1.02 | 0.842 | -0.67 |
| 1419694_at | St8sia1 | ST8 alpha-N-acetyl-neuraminide alpha-2,8-sialyltransferase 1 | 1.11 | 0.725 | -2.71 | 0.029 | -1.05 | 0.929 | -1.36 | 0.051 | -1 |
| 1457067_at | Vps4b | Vacuolar protein sorting 4b (yeast) | -1.1 | 0.495 | -1.39 | 0.395 | -1.25 | 0.14 | 1.1 | 0.57 | -0.66 |
| 1422605_at | Ppp1r1a | protein phosphatase 1, regulatory (inhibitor) subunit 1A | 1 | 0.987 | -2.1 | 0.127 | -1.05 | 0.881 | -1.89 | 0.282 | -1.01 |
| 1419453_at | Uchl5 | ubiquitin carboxyl-terminal esterase L5 | -1.22 | 0.542 | -1.2 | 0.558 | -1.29 | 0.075 | 1.06 | 0.809 | -0.66 |
| 1459168_at | Zfp521 | Zinc finger protein 521 | -1.35 | 0.392 | -1.1 | 0.778 | -1.29 | 0.17 | 1.34 | 0.437 | -0.6 |
| 1440586_at | B430203I24Rik | RIKEN cDNA B430203I24 gene | -1.16 | 0.015 | 1.11 | 0.744 | -2.18 | 0.022 | -1.12 | 0.553 | -0.84 |
| 1432817_x_at | 4930413G21Rik | RIKEN cDNA 4930413G21 gene | 1.2 | 0.644 | -1.57 | 0.086 | -1.7 | 0.277 | -1.19 | 0.78 | -0.81 |
| 1432110_at | 4930402F06Rik | RIKEN cDNA 4930402F06 gene | 1.14 | 0.895 | -1.34 | 0.394 | -1.84 | 0.143 | -1.15 | 0.432 | -0.8 |
| 1434905_at | BC064011 | cDNA sequence BC064011 | -1.01 | 0.976 | -1.13 | 0.769 | -1.8 | 0.073 | 2.2 | 0.027 | -0.43 |
| 1436253_at | Pex16 | peroxisome biogenesis factor 16 | 1.37 | 0.473 | -1.39 | 0.104 | -2.91 | 0.132 | -1.13 | 0.688 | -1.02 |
| 1422288_at | Htr1b | 5-hydroxytryptamine (serotonin) receptor 1B | -1.01 | 0.952 | -1.51 | 0.076 | -1.29 | 0.355 | 1.11 | 0.636 | -0.67 |
| 1446218_at | Ppfia2 | protein tyrosine phosphatase, receptor type, f polypeptide (PTPRF), interacting pı | -1.88 | 0.24 | -1.23 | 0.397 | 1.09 | 0.816 | -1.26 | 0.524 | -0.82 |
| 1445662_x_at | --- | Transcribed locus | -1.74 | 0.142 | 1.04 | 0.852 | -1.23 | 0.333 | -1.47 | 0.104 | -0.85 |
| 1458682_at | D130059P03Rik | RIKEN cDNA D130059P03 gene | 1.68 | 0.382 | -4.53 | 0.051 | -1.89 | 0.184 | -1.01 | 0.989 | -1.44 |

| | | | | | | | | | | | |
|---|---|---|---|---|---|---|---|---|---|---|---|
| 1444767_at | Gnas | GNAS (guanine nucleotide binding protein, alpha stimulating) complex locus | 1.24 | 0.726 | -1.59 | 0.289 | -1.78 | 0.097 | -1.43 | 0.334 | -0.89 |
| 1421423_at | Prlpo | prolactin-like protein O | -2.09 | 0.343 | -1.65 | 0.147 | 1.35 | 0.396 | -1.32 | 0.577 | -0.93 |
| 1442722_at | --- | Transcribed locus | -2.32 | 0.315 | -1.56 | 0.164 | 1.36 | 0.634 | -1.3 | 0.73 | -0.95 |
| 1439263_at | Fin15 | fibroblast growth factor inducible 15 | -1.13 | 0.481 | -1.27 | 0.059 | -1.32 | 0.376 | 1.16 | 0.504 | -0.64 |
| 1454010_a_at | 1700025E21Rik | RIKEN cDNA 1700025E21 gene | -1.31 | 0.22 | 1.08 | 0.884 | -1.69 | 0.214 | -2.2 | 0.059 | -1.03 |
| 1420554_a_at | Rac3 | RAS-related C3 botulinum substrate 3 | -1.27 | 0.267 | -2.01 | 0.058 | 1.14 | 0.709 | -1.14 | 0.539 | -0.82 |
| 1438927_x_at | LOC227054 /// Rpl2 | similar to 60S ribosomal protein L23a /// ribosomal protein L23a /// hypothetical ge | -1.88 | 0.44 | -1.65 | 0.48 | 1.29 | 0.461 | -2.21 | 0.1 | -1.11 |
| 1441556_at | Baz2b | Bromodomain adjacent to zinc finger domain, 2B | 1.07 | 0.37 | -1.36 | 0.083 | -1.59 | 0.021 | -1.72 | 0.015 | -0.9 |
| 1439840_at | A430088C08Rik | RIKEN cDNA A430088C08 gene | 1.01 | 0.955 | -1.28 | 0.286 | -1.56 | 0.088 | -1.77 | 0.304 | -0.9 |
| 1437347_at | Ednrb | endothelin receptor type B | 1.06 | 0.632 | -1.06 | 0.797 | -2.36 | 0.005 | -2.63 | 0.008 | -1.24 |
| 1433115_at | 5730552O08Rik | RIKEN cDNA 5730552O08 gene | -1.13 | 0.823 | -1.11 | 0.523 | -1.55 | 0.361 | 1.64 | 0.468 | -0.54 |
| 1456810_at | Vps54 | vacuolar protein sorting 54 (yeast) | -1.4 | 0.105 | 1.14 | 0.595 | -1.74 | 0.075 | -1.11 | 0.701 | -0.78 |
| 1431519_at | 4933406L23Rik | RIKEN cDNA 4933406L23 gene | 1.14 | 0.763 | -1.75 | 0.282 | -1.4 | 0.199 | -1.49 | 0.424 | -0.87 |
| 1447718_at | Pde4b | phosphodiesterase 4B, cAMP specific | -1.18 | 0.829 | -1.85 | 0.287 | 1.04 | 0.933 | -3.45 | 0.387 | -1.36 |
| 1437821_at | Diap1 | Diaphanous homolog 1 (Drosophila) | 1.08 | 0.493 | -1.31 | 0.313 | -1.71 | 0.007 | -1.33 | 0.316 | -0.82 |
| 1430622_at | 4833423F13Rik | RIKEN cDNA 4833423F13 gene | -1.11 | 0.729 | 1.07 | 0.836 | -2.15 | 0.033 | -2.67 | 0.053 | -1.22 |
| 1416324_s_at | Kctd20 | potassium channel tetramerisation domain containing 20 | -1.25 | 0.372 | -1.14 | 0.236 | -1.33 | 0.023 | 1.03 | 0.842 | -0.67 |
| 1445164_at | 2210418O10Rik | RIKEN cDNA 2210418O10 gene | -1.23 | 0.684 | -1.02 | 0.978 | -1.57 | 0.303 | 1.52 | 0.54 | -0.57 |
| 1446626_at | D16H22S680E | DNA segment, Chr 16, human D22S680E, expressed | -1.12 | 0.746 | -1.15 | 0.613 | -1.51 | 0.11 | 1.93 | 0.111 | -0.46 |
| 1425606_at | Slc5a8 | solute carrier family 5 (iodide transporter), member 8 | -1.31 | 0.473 | -1.31 | 0.558 | -1.1 | 0.775 | 2.14 | 0.415 | -0.4 |
| 1436396_at | D430033N04Rik | RIKEN cDNA D430033N04 gene | -1.23 | 0.436 | -1.51 | 0.392 | -1.05 | 0.823 | 2.86 | 0.092 | -0.23 |
| 1445505_at | Ndst1 | N-deacetylase/N-sulfotransferase (heparan glucosaminyl) 1 | -1.37 | 0.587 | 1.03 | 0.962 | -1.5 | 0.469 | -1.63 | 0.324 | -0.87 |
| 1443604_at | --- | --- | -2.17 | 0.467 | -1.64 | 0.282 | 1.36 | 0.405 | -1.79 | 0.156 | -1.06 |
| 1431674_at | 2610303G11Rik | RIKEN cDNA 2610303G11 gene | -1.27 | 0.558 | -1.64 | 0.022 | 1.04 | 0.866 | -1.74 | 0.443 | -0.9 |
| 1455002_at | Ptp4a1 | protein tyrosine phosphatase 4a1 | -1.15 | 0.326 | -1.21 | 0.304 | -1.36 | 0.099 | 1.31 | 0.278 | -0.6 |
| 1459518_at | Gm489 | Gene model 489, (NCBI) | -1.96 | 0.237 | -1.1 | 0.725 | 1.01 | 0.948 | -1.37 | 0.48 | -0.86 |
| 1446305_at | --- | Adult male corpora quadrigemina cDNA, RIKEN full-length enriched library, clone | -1.63 | 0.516 | 1.05 | 0.943 | -1.3 | 0.596 | -1.59 | 0.217 | -0.87 |
| 1441178_at | Dtwd2 | DTW domain containing 2 | -1.35 | 0.477 | -1.16 | 0.423 | -1.21 | 0.55 | 1.09 | 0.701 | -0.66 |
| 1441617_at | Pcqap | positive cofactor 2, multiprotein complex, glutamine/Q-rich-associated protein | -1.28 | 0.044 | 1.01 | 0.924 | -1.55 | 0.092 | -1.26 | 0.013 | -0.77 |
| 1418176_at | Vdr | vitamin D receptor | -1.19 | 0.031 | -1.53 | 0.397 | -1.07 | 0.521 | 1.3 | 0.283 | -0.62 |
| 1453104_at | Mapk1 | mitogen activated protein kinase 1 | -1.24 | 0.166 | -1.21 | 0.048 | -1.25 | 0.029 | 1.03 | 0.767 | -0.67 |
| 1438213_at | A830018L16Rik | RIKEN cDNA A830018L16 gene | -1.44 | 0.582 | -2.26 | 0.197 | 1.3 | 0.281 | -1.35 | 0.622 | -0.94 |
| 1429483_at | Ndp52 | nuclear domain 10 protein 52 | -1.45 | 0.564 | -1.49 | 0.509 | 1.07 | 0.85 | -1.32 | 0.479 | -0.8 |
| 1454653_at | Cpne9 | copine family member IX | -1.8 | 0.109 | -1.23 | 0.534 | 1.07 | 0.48 | -1.3 | 0.186 | -0.82 |
| 1458360_at | Spock2 | Sparc/osteonectin, cwcv and kazal-like domains proteoglycan 2 | -1.7 | 0.244 | -1.4 | 0.387 | 1.13 | 0.767 | -1.27 | 0.663 | -0.81 |
| 1455037_at | Plxna2 | plexin A2 | -1.23 | 0.342 | -1.19 | 0.31 | -1.28 | 0.351 | 1.24 | 0.567 | -0.62 |
| 1456986_at | Zbtb16 | Zinc finger and BTB domain containing 16 | 1.02 | 0.98 | -1.19 | 0.751 | -1.74 | 0.286 | -2.07 | 0.147 | -0.99 |
| 1446346_at | --- | --- | -1.23 | 0.608 | 1.01 | 0.983 | -1.61 | 0.361 | -1.42 | 0.423 | -0.81 |
| 1418668_at | Acsm1 | acyl-CoA synthetase medium-chain family member 1 | -1.19 | 0.527 | -1.1 | 0.19 | -1.46 | 0.218 | 1.75 | 0.201 | -0.5 |
| 1446161_at | Taf11 | TAF11 RNA polymerase II, TATA box binding protein (TBP)-associated factor | -1.3 | 0.575 | -1.52 | 0.335 | 1.01 | 0.982 | -1.04 | 0.953 | -0.71 |
| 1428519_at | 2610528E23Rik | RIKEN cDNA 2610528E23 gene | -1.08 | 0.755 | -1.46 | 0.144 | -1.22 | 0.167 | 1.26 | 0.373 | -0.62 |
| 1440213_a_at | 2010001M06Rik | RIKEN cDNA 2010001M06 gene | -1.1 | 0.693 | -1.26 | 0.465 | -1.38 | 0.151 | 1 | 0.99 | -0.68 |
| 1440703_at | 2810429I04Rik | RIKEN cDNA 2810429I04 gene | -1.83 | 0.219 | 1.21 | 0.546 | -1.47 | 0.306 | -1.79 | 0.215 | -0.97 |
| 1442426_at | Wapal | wings apart-like homolog (Drosophila) | -1.25 | 0.039 | -1.28 | 0.184 | -1.17 | 0.212 | 1.62 | 0.277 | -0.52 |
| 1433271_at | 9530004M16Rik | RIKEN cDNA 9530004M16 gene | 1.04 | 0.934 | -1.36 | 0.028 | -1.51 | 0.371 | -1.12 | 0.697 | -0.74 |
| 1421325_at | Pla2g2f | phospholipase A2, group IIF | -1.11 | 0.855 | -1.51 | 0.313 | -1.15 | 0.818 | 1.34 | 0.587 | -0.61 |
| 1431410_at | D16Ertd472e | DNA segment, Chr 16, ERATO Doi 472, expressed | 1.03 | 0.964 | -1.72 | 0.404 | -1.21 | 0.404 | -1.84 | 0.398 | -0.93 |
| 1442464_at | Fbxl20 | F-box and leucine-rich repeat protein 20 | -1.51 | 0.232 | -1.22 | 0.075 | -1.05 | 0.761 | 1.01 | 0.957 | -0.69 |
| 1444825_at | --- | --- | -3.35 | 0.094 | -1.35 | 0.315 | 1.4 | 0.29 | -1.02 | 0.963 | -1.08 |
| 1435510_at | Ppm1h | protein phosphatase 1H (PP2C domain containing) | -1.27 | 0.292 | -1.55 | 0.046 | 1 | 0.989 | -1.62 | 0.159 | -0.86 |
| 1441966_at | Trpm3 | transient receptor potential cation channel, subfamily M, member 3 | -1.45 | 0.411 | -1.13 | 0.759 | -1.16 | 0.213 | 1.33 | 0.101 | -0.6 |
| 1431788_at | 1700008G05Rik | RIKEN cDNA 1700008G05 gene | -1.58 | 0.111 | -1.07 | 0.78 | -1.15 | 0.208 | 1.08 | 0.853 | -0.68 |
| 1459982_a_at | --- | --- | -1.31 | 0.122 | -1.41 | 0.641 | -1.04 | 0.883 | 1.12 | 0.775 | -0.66 |

| | | | | | | | | | | | |
|---|---|---|---|---|---|---|---|---|---|---|---|
| 1449161_at | Edn2 | endothelin 2 | -1.38 | 0.588 | -1.03 | 0.941 | -1.35 | 0.323 | 1.45 | 0.2 | -0.58 |
| 1432735_at | 1700017H01Rik | RIKEN cDNA 1700017H01 gene | -1.12 | 0.721 | -2.07 | 0.319 | 1.06 | 0.725 | -1.25 | 0.203 | -0.84 |
| 1441863_x_at | --- | --- | -1.5 | 0.332 | -1.27 | 0.589 | -1.02 | 0.972 | 1.89 | 0.32 | -0.47 |
| 1427738_at | D0Kist2 | DNA segment, KIST 2 | -1.52 | 0.495 | 1.14 | 0.802 | -1.56 | 0.296 | -1.78 | 0.351 | -0.93 |
| 1457811_at | --- | --- | 1.17 | 0.036 | -1.51 | 0.008 | -1.67 | 0.102 | -1.24 | 0.326 | -0.81 |
| 1416490_at | Tmed6 | transmembrane emp24 protein transport domain containing 6 | -1.29 | 0.345 | 1.04 | 0.949 | -1.6 | 0.201 | -1.38 | 0.186 | -0.81 |
| 1444186_at | 2900006B13Rik | RIKEN cDNA 2900006B13 gene | -1.52 | 0.555 | -1.41 | 0.287 | 1.07 | 0.89 | -1.25 | 0.56 | -0.78 |
| 1443789_x_at | Cox8c | cytochrome c oxidase, subunit VIIIc | -1.55 | 0.477 | -2.08 | 0.302 | 1.31 | 0.382 | -1.32 | 0.647 | -0.91 |
| 1429904_at | 4930507A01Rik | RIKEN cDNA 4930507A01 gene | 1.08 | 0.898 | -1.99 | 0.333 | -1.17 | 0.382 | -1.25 | 0.285 | -0.83 |
| 1425172_at | Rho | rhodopsin | -1.14 | 0.703 | -1.47 | 0.55 | -1.13 | 0.788 | 1.35 | 0.515 | -0.6 |
| 1452315_at | Kif11 | kinesin family member 11 | -1.14 | 0.755 | -1.39 | 0.466 | -1.18 | 0.079 | 2.79 | 0.284 | -0.23 |
| 1420734_at | Ppp1r3f | protein phosphatase 1, regulatory (inhibitor) subunit 3F | -1.19 | 0.131 | -1.94 | 0.29 | 1.08 | 0.672 | -1.09 | 0.763 | -0.78 |
| 1453371_at | 4930535B03Rik | RIKEN cDNA 4930535B03 gene | 1.11 | 0.568 | -1.06 | 0.689 | -2.63 | 0.006 | -1.85 | 0.05 | -1.11 |
| 1427584_at | Amot | angiomotin | -1.61 | 0.132 | -1.39 | 0.489 | 1.1 | 0.751 | -1.27 | 0.452 | -0.79 |
| 1425246_at | 0610008F07Rik | RIKEN cDNA 0610008F07 gene | -1.22 | 0.433 | -1.36 | 0.134 | -1.13 | 0.652 | 1.14 | 0.491 | -0.64 |
| 1442080_at | Creb3l2 | CAMP responsive element binding protein 3-like 2 | -1.19 | 0.307 | -1.12 | 0.472 | -1.42 | 0.146 | 1.41 | 0.004 | -0.58 |
| 1420881_at | Nsd1 | nuclear receptor-binding SET-domain protein 1 | -1.18 | 0.85 | -2.08 | 0.287 | 1.11 | 0.793 | -1.87 | 0.191 | -1 |
| 1460714_at | Tcfcp2l1 | Transcription factor CP2-like 1 | -1.38 | 0.065 | -1.81 | 0.094 | 1.16 | 0.718 | -1.44 | 0.562 | -0.87 |
| 1426362_at | Tmem144 | transmembrane protein 144 | -1.28 | 0.073 | -1.48 | 0.018 | -1.02 | 0.927 | 1.47 | 0.322 | -0.58 |
| 1436155_at | Nmnat2 | nicotinamide nucleotide adenylyltransferase 2 | -1.08 | 0.891 | -1.27 | 0.243 | -1.38 | 0.081 | 1.33 | 0.31 | -0.6 |
| 1456682_at | Lonrf2 | LON peptidase N-terminal domain and ring finger 2 | 1.04 | 0.961 | -1.33 | 0.547 | -1.54 | 0.106 | -2.68 | 0.197 | -1.13 |
| 1430162_at | 3830417A13Rik | RIKEN cDNA 3830417A13 gene | -1.47 | 0.624 | -1.14 | 0.807 | -1.13 | 0.688 | 1.16 | 0.842 | -0.65 |
| 1450485_at | Sox3 | SRY-box containing gene 3 | -1.19 | 0.767 | -4.35 | 0.097 | 1.37 | 0.541 | -1.13 | 0.705 | -1.32 |
| 1434670_at | Kif5a | kinesin family member 5A | -1.06 | 0.923 | 1.05 | 0.906 | -2.24 | 0.175 | -1.43 | 0.185 | -0.92 |
| 1445184_at | 9130019O22Rik | RIKEN cDNA 9130019O22 gene | -1.09 | 0.708 | -1.91 | 0.345 | -1 | 0.997 | 1.2 | 0.771 | -0.7 |
| 1434020_at | Pdap1 | PDGFA associated protein 1 | -1.04 | 0.793 | -1.44 | 0.017 | -1.28 | 0.226 | 1.46 | 0.252 | -0.57 |
| 1460484_at | Ttl | tubulin tyrosine ligase | -1.16 | 0.214 | -1.59 | 0.505 | -1.05 | 0.806 | 1.07 | 0.867 | -0.69 |
| 1451739_at | Klf5 | Kruppel-like factor 5 | -1.09 | 0.524 | -1.44 | 0.085 | -1.21 | 0.185 | 1.52 | 0.489 | -0.56 |
| 1436082_at | Myef2 | Myelin basic protein expression factor 2, repressor | -1.34 | 0.477 | -1.03 | 0.934 | -1.38 | 0.256 | 1.71 | 0.369 | -0.51 |
| 1442509_at | Evi5 | Ecotropic viral integration site 5 | -1.19 | 0.013 | -1.07 | 0.71 | -1.5 | 0.014 | 3.03 | 0.232 | -0.18 |
| 1438548_x_at | Rhbdd3 | rhomboid domain containing 3 | -1.14 | 0.504 | -1.15 | 0.246 | -1.44 | 0.088 | 1.18 | 0.628 | -0.64 |
| 1421893_a_at | Tpp2 | tripeptidyl peptidase II | -1.34 | 0.304 | -1.86 | 0 | 1.15 | 0.52 | -1.11 | 0.612 | -0.79 |
| 1442192_at | Tyms | thymidylate synthase | -1.07 | 0.811 | -1.08 | 0.818 | -1.72 | 0.028 | 1.9 | 0.315 | -0.49 |
| 1453447_at | 1700109H08Rik | RIKEN cDNA 1700109H08 gene | -1.95 | 0.168 | 1.03 | 0.947 | -1.12 | 0.816 | -2.31 | 0.052 | -1.09 |
| 1424306_at | Elovl4 | elongation of very long chain fatty acids (FEN1/Elo2, SUR4/Elo3, yeast)-like 4 | -1.12 | 0.845 | 1.1 | 0.804 | -2.2 | 0.135 | -1.03 | 0.876 | -0.81 |
| 1431409_at | C030015A19Rik | RIKEN cDNA C030015A19 gene | -1.23 | 0.546 | -1.24 | 0.428 | -1.21 | 0.258 | 1.03 | 0.896 | -0.66 |
| 1452310_at | Tada2l | transcriptional adaptor 2 (ADA2 homolog, yeast)-like | -1.17 | 0.043 | -1.24 | 0.141 | -1.28 | 0.205 | 1.39 | 0.102 | -0.58 |
| 1434804_at | Sec15l2 | SEC15-like 2 (S. cerevisiae) | -1.11 | 0.51 | -1.05 | 0.509 | -1.71 | 0.001 | 1.03 | 0.81 | -0.71 |
| 1417347_at | Pycard | PYD and CARD domain containing | -1.44 | 0.173 | 1.05 | 0.884 | -1.44 | 0.054 | -2.4 | 0.245 | -1.06 |
| 1447481_at | Setd5 | SET domain containing 5 | 1.09 | 0.757 | -1.68 | 0.185 | -1.32 | 0.256 | -1.05 | 0.778 | -0.74 |
| 1422252_a_at | Cdc25c | cell division cycle 25 homolog C (S. cerevisiae) | -1.48 | 0.174 | -1 | 0.989 | -1.3 | 0.654 | 2.85 | 0.127 | -0.23 |
| 1446409_at | Gtdc1 | glycosyltransferase-like domain containing 1 | -2.18 | 0.21 | -1.37 | 0.379 | 1.25 | 0.554 | -1.01 | 0.978 | -0.83 |
| 1417508_at | Rnf19 | ring finger protein (C3HC4 type) 19 | -1.22 | 0.027 | -1.19 | 0.082 | -1.28 | 0.016 | 1.66 | 0.284 | -0.51 |
| 1424219_at | Mrm1 | mitochondrial rRNA methyltransferase 1 homolog (S. cerevisiae) | -1.21 | 0.286 | -1.6 | 0.269 | -1.01 | 0.956 | 2.14 | 0.133 | -0.42 |
| 1435564_at | C230078M08Rik | RIKEN cDNA C230078M08 gene | -2.15 | 0.277 | 1.14 | 0.812 | -1.19 | 0.621 | -2.66 | 0.197 | -1.21 |
| 1442452_at | Als2cr2 | amyotrophic lateral sclerosis 2 (juvenile) chromosome region, candidate 2 (huma | -1.19 | 0.525 | -1.5 | 0.321 | -1.07 | 0.636 | 2.91 | 0.186 | -0.21 |
| 1442481_at | LOC675578 | hypothetical protein LOC675578 | 1.25 | 0.515 | -3.05 | 0.03 | -1.16 | 0.251 | -1.12 | 0.4 | -1.02 |
| 1459106_at | --- | PREDICTED: Mus musculus hypothetical protein LOC622817 (LOC622817), mR | -1.6 | 0.559 | 1.09 | 0.911 | -1.38 | 0.461 | -1.14 | 0.418 | -0.76 |
| 1429955_at | 5031434O11Rik | RIKEN cDNA 5031434O11 gene | -1.23 | 0.705 | -1.63 | 0.407 | 1.02 | 0.931 | -1.08 | 0.742 | -0.73 |
| 1459385_at | Camsap1l1 | calmodulin regulated spectrin-associated protein 1-like 1 | -1.17 | 0.772 | -2.49 | 0.187 | 1.18 | 0.684 | -1.11 | 0.803 | -0.9 |
| 1457093_at | Gapvd1 | GTPase activating protein and VPS9 domains 1 | -1.3 | 0.152 | -1.28 | 0.191 | -1.12 | 0.373 | 1.1 | 0.394 | -0.65 |
| 1427042_at | Mal2 | mal, T-cell differentiation protein 2 | -1.19 | 0.305 | -1.21 | 0.282 | -1.3 | 0.037 | 1.58 | 0.622 | -0.53 |
| 1454272_at | 9630015K15Rik | RIKEN cDNA 9630015K15 gene | -3.62 | 0.279 | 1.29 | 0.633 | -1.14 | 0.808 | -1.88 | 0.024 | -1.34 |

| | | | | | | | | | | | |
|---|---|---|---|---|---|---|---|---|---|---|---|
| 1444541_at | Myo9a | Myosin IXa | -1.47 | 0.009 | -1.01 | 0.945 | -1.29 | 0.028 | 1.17 | 0.15 | -0.65 |
| 1441704_at | Plekha5 | Pleckstrin homology domain containing, family A member 5 | -1.2 | 0.664 | 1.11 | 0.818 | -2 | 0.019 | -1.58 | 0.323 | -0.92 |
| 1447468_at | --- | Transcribed locus | -1.35 | 0.638 | 1.3 | 0.545 | -2.51 | 0.19 | -1.43 | 0.657 | -1 |
| 1454262_at | 1700124P09Rik | RIKEN cDNA 1700124P09 gene | -1.62 | 0.186 | -1.13 | 0.752 | -1.06 | 0.739 | 1.1 | 0.637 | -0.68 |
| 1431184_a_at | 4930503B20Rik | RIKEN cDNA 4930503B20 gene | -1.24 | 0.681 | -1.62 | 0.133 | 1.02 | 0.976 | -1.54 | 0.415 | -0.85 |
| 1429647_at | 1700027L20Rik | RIKEN cDNA 1700027L20 gene | 1.46 | 0.611 | -1.79 | 0.272 | -2.37 | 0.044 | -1.71 | 0.373 | -1.1 |
| 1438788_at | D5Wsu152e | DNA segment, Chr 5, Wayne State University 152, expressed | 1.02 | 0.747 | -1.36 | 0.087 | -1.47 | 0.043 | -1.32 | 0.197 | -0.78 |
| 1432221_at | 5330417H12Rik | RIKEN cDNA 5330417H12 gene | -1.5 | 0.421 | -1.09 | 0.799 | -1.16 | 0.665 | 1.75 | 0.241 | -0.5 |
| 1441149_at | --- | --- | 1.3 | 0.052 | -1.38 | 0.42 | -2.41 | 0.023 | -1.41 | 0.682 | -0.97 |
| 1441035_at | Kcne1 | potassium voltage-gated channel, Isk-related subfamily, member 1 | -2.12 | 0.04 | 1.26 | 0.673 | -1.4 | 0.134 | -1.22 | 0.138 | -0.87 |
| 1458239_at | --- | --- | -1.24 | 0.543 | -1.29 | 0.592 | -1.16 | 0.379 | 1.35 | 0.281 | -0.59 |
| 1453539_at | Alkbh8 | alkB, alkylation repair homolog 8 (E. coli) | -1.09 | 0.562 | -1.72 | 0.31 | -1.06 | 0.723 | 1.35 | 0.59 | -0.63 |
| 1416970_a_at | Cox7a2 | cytochrome c oxidase, subunit VIIa 2 | -1.14 | 0.004 | -1.26 | 0.081 | -1.29 | 0 | 1.03 | 0.728 | -0.66 |
| 1447760_x_at | Ehf | ets homologous factor | 1.02 | 0.968 | -1.22 | 0.149 | -1.64 | 0.376 | -1.82 | 0.164 | -0.92 |
| 1458470_at | --- | --- | 1.04 | 0.945 | -1.59 | 0.398 | -1.29 | 0.114 | -1.28 | 0.357 | -0.78 |
| 1442681_at | Tsen2 | TRNA splicing endonuclease 2 homolog (SEN2, S. cerevisiae) | -1.26 | 0.351 | -1.22 | 0.616 | -1.2 | 0.384 | 1.35 | 0.245 | -0.58 |
| 1431809_at | 4932442L08Rik | RIKEN cDNA 4932442L08 gene | -1.01 | 0.979 | -1.27 | 0.667 | -1.5 | 0.409 | 1.6 | 0.242 | -0.55 |
| 1420968_at | Btbd14b | BTB (POZ) domain containing 14B | -1.12 | 0.884 | -1.73 | 0.005 | -1.03 | 0.943 | 1.45 | 0.451 | -0.61 |
| 1459656_at | --- | PREDICTED: Mus musculus similar to potassium voltage-gated channel, subfam | 1.39 | 0.689 | -2.14 | 0.191 | -1.71 | 0.264 | -2.27 | 0.135 | -1.18 |
| 1445530_at | 5330401P04Rik | RIKEN cDNA 5330401P04 gene | -1.32 | 0.541 | 1.2 | 0.242 | -2.03 | 0.292 | -1.4 | 0.413 | -0.89 |
| 1445910_at | C79999 | expressed sequence C79999 | -1.17 | 0.591 | -1.33 | 0.507 | -1.2 | 0.547 | 1.08 | 0.734 | -0.65 |
| 1440101_at | --- | --- | -1.3 | 0.02 | -1.19 | 0.723 | -1.2 | 0.698 | 2.01 | 0.164 | -0.42 |
| 1427276_at | Smc4 | structural maintenance of chromosomes 4 | -1.23 | 0.167 | -1.14 | 0.506 | -1.32 | 0.126 | 1.72 | 0.353 | -0.49 |
| 1456138_at | Lypd6 | LY6/PLAUR domain containing 6 | -1.02 | 0.821 | -1.74 | 0.312 | -1.13 | 0.552 | 1.14 | 0.537 | -0.69 |
| 1426408_at | Cugbp1 | CUG triplet repeat, RNA binding protein 1 | -1.1 | 0.123 | -1.3 | 0.104 | -1.3 | 0.134 | 1.2 | 0.461 | -0.63 |
| 1443185_at | Lhfpl2 | lipoma HMGIC fusion partner-like 2 | -1.33 | 0.071 | 1.07 | 0.863 | -1.61 | 0.102 | -2.19 | 0.145 | -1.02 |
| 1459683_at | LOC218695 | similar to spermatogenic cell-specific gene 2 | -2.26 | 0.174 | 1.26 | 0.587 | -1.35 | 0.364 | -1.1 | 0.814 | -0.86 |
| 1437801_at | Morf4l1 /// LOC433! | mortality factor 4 like 1 /// similar to mortality factor 4 like 1 isoform b /// similar to r | -1.04 | 0.806 | -1.27 | 0.224 | -1.43 | 0.319 | 1.02 | 0.856 | -0.68 |
| 1442949_at | Nfkb1 | Nuclear factor of kappa light chain gene enhancer in B-cells 1, p105 | -1.41 | 0.366 | -1.39 | 0.419 | 1.02 | 0.948 | -1.96 | 0.466 | -0.94 |
| 1450365_at | Myoz3 | myozenin 3 | -1.19 | 0.724 | -1.63 | 0.438 | -1.01 | 0.989 | 1.6 | 0.123 | -0.56 |
| 1444110_at | Tcn2 | transcobalamin 2 | -1.58 | 0.183 | 1.16 | 0.339 | -1.54 | 0.188 | -1.36 | 0.092 | -0.83 |
| 1420405_at | Slco1a4 | solute carrier organic anion transporter family, member 1a4 | -1.04 | 0.9 | -1.85 | 0.126 | -1.06 | 0.796 | 1.54 | 0.143 | -0.6 |
| 1434962_x_at | Ccl27 | chemokine (C-C motif) ligand 27 | -1.18 | 0.035 | -1.2 | 0.497 | -1.31 | 0.031 | 1.09 | 0.529 | -0.65 |
| 1426270_at | Smc5 | structural maintenance of chromosomes 5 | -1.55 | 0.111 | -1.06 | 0.661 | -1.17 | 0.397 | 1.23 | 0.283 | -0.64 |
| 1443953_at | Tex2 | testis expressed gene 2 | -1.25 | 0.473 | -1.19 | 0.16 | -1.24 | 0.014 | 1.13 | 0.329 | -0.64 |
| 1449637_at | Cdh4 | cadherin 4 | -1.7 | 0.557 | 1.32 | 0.692 | -1.87 | 0.345 | -1.39 | 0.287 | -0.91 |
| 1446735_at | Itsn2 | intersectin 2 | 1.08 | 0.223 | -1.25 | 0.301 | -1.76 | 0.01 | -1.81 | 0.074 | -0.94 |
| 1438492_at | Socs7 | Suppressor of cytokine signaling 7 | -1.72 | 0.432 | -1.12 | 0.8 | -1.03 | 0.945 | 2.32 | 0.054 | -0.39 |
| 1430109_at | 4833412E19Rik | RIKEN cDNA 4833412E19 gene | -1.18 | 0.369 | -1.06 | 0.764 | -1.51 | 0.11 | 1.82 | 0.154 | -0.48 |
| 1439093_at | --- | --- | -1.46 | 0.049 | -1.05 | 0.751 | -1.23 | 0.224 | 2.1 | 0.301 | -0.41 |
| 1454835_at | Epm2aip1 | EPM2A (laforin) interacting protein 1 | -1.23 | 0.077 | -1.17 | 0.287 | -1.28 | 0.224 | 1.01 | 0.973 | -0.67 |
| 1425540_at | Otc | ornithine transcarbamylase | -1.18 | 0.706 | -1.33 | 0.144 | -1.18 | 0.702 | 1.9 | 0.434 | -0.45 |
| 1439917_at | Pdzd8 | PDZ domain containing 8 | 1.08 | 0.597 | -1.39 | 0.091 | -1.54 | 0.048 | -1.29 | 0.113 | -0.78 |
| 1432726_at | Psmd11 | proteasome (prosome, macropain) 26S subunit, non-ATPase, 11 | -1.02 | 0.975 | -3.69 | 0.015 | 1.19 | 0.682 | -1.54 | 0.446 | -1.26 |
| 1453411_at | 2010110K16Rik | RIKEN cDNA 2010110K16 gene | -1.29 | 0.078 | 1.26 | 0.531 | -2.42 | 0.13 | -1.26 | 0.472 | -0.93 |
| 1452109_at | Il17re | interleukin 17 receptor E | -1.13 | 0.635 | -2.1 | 0.069 | 1.08 | 0.616 | -1.03 | 0.885 | -0.79 |
| 1447593_x_at | Gnaq | guanine nucleotide binding protein, alpha q polypeptide | -1.43 | 0.637 | 1.03 | 0.938 | -1.39 | 0.224 | -1.66 | 0.349 | -0.86 |
| 1445157_at | --- | --- | -1.29 | 0.745 | -1.2 | 0.273 | -1.2 | 0.59 | 1.02 | 0.948 | -0.67 |
| 1443157_at | Glis3 | GLIS family zinc finger 3 | -1.55 | 0.271 | 1.25 | 0.486 | -1.81 | 0.008 | -1.08 | 0.538 | -0.8 |
| 1458613_at | Arrdc1 | arrestin domain containing 1 | 1.06 | 0.525 | -1.26 | 0.489 | -1.69 | 0.176 | -1.28 | 0.552 | -0.79 |
| 1450496_a_at | 2810433K01Rik | RIKEN cDNA 2810433K01 gene | -1.07 | 0.931 | -1.95 | 0.452 | -1 | 0.999 | 4.53 | 0.209 | 0.13 |
| 1424364_a_at | 1110020P15Rik | RIKEN cDNA 1110020P15 gene | -1.18 | 0.011 | -1.29 | 0.074 | -1.21 | 0.234 | 1.33 | 0.119 | -0.59 |
| 1416265_at | Capn10 | calpain 10 | -1.22 | 0.271 | -1.38 | 0.295 | -1.11 | 0.414 | 1.01 | 0.919 | -0.67 |

| | | | | | | | | | | | |
|---|---|---|---|---|---|---|---|---|---|---|---|
| 1451934_at | H2-D1 | histocompatibility 2, D region locus 1 | -1.21 | 0.637 | -1.16 | 0.772 | -1.31 | 0.13 | 1.31 | 0.698 | -0.59 |
| 1443249_at | 3-Mar | Membrane-associated ring finger (C3HC4) 3 | -1.08 | 0.747 | -1.08 | 0.85 | -1.67 | 0.018 | 3.85 | 0.224 | 0 |
| 1439696_at | Nr2c2 | nuclear receptor subfamily 2, group C, member 2 | -1.43 | 0.419 | 1.15 | 0.724 | -1.65 | 0.041 | -1.44 | 0.475 | -0.85 |
| 1456258_at | Emx2 | empty spiracles homolog 2 (Drosophila) | -1.12 | 0.428 | 1.09 | 0.872 | -2.13 | 0.072 | -1.56 | 0.408 | -0.93 |
| 1450109_s_at | Abcc2 | ATP-binding cassette, sub-family C (CFTR/MRP), member 2 | -1.28 | 0.09 | -1.15 | 0.388 | -1.25 | 0.691 | 2.16 | 0.056 | -0.38 |
| 1460215_at | Rpo1-4 | RNA polymerase 1-4 | -1.06 | 0.24 | -1.31 | 0.753 | -1.35 | 0.355 | 1.05 | 0.836 | -0.67 |
| 1440460_at | 4930504O13Rik | RIKEN cDNA 4930504O13 gene | -1.97 | 0.022 | -1.1 | 0.599 | 1.03 | 0.856 | -1.15 | 0.296 | -0.8 |
| 1455982_at | Jmjd4 | jumonji domain containing 4 | -1.37 | 0.351 | -1.4 | 0.296 | 1.01 | 0.954 | -1.09 | 0.736 | -0.72 |
| 1443457_at | A230055J12Rik | RIKEN cDNA A230055J12 gene | 1.04 | 0.931 | -1.23 | 0.704 | -1.68 | 0.144 | -1.11 | 0.722 | -0.75 |
| 1422401_at | Sprr3 | small proline-rich protein 3 | -2.47 | 0.068 | -2.01 | 0.201 | 1.55 | 0.384 | -1.09 | 0.818 | -1.01 |
| 1458951_at | Vrk1 | vaccinia related kinase 1 | -1.63 | 0.155 | 1.04 | 0.869 | -1.27 | 0.016 | -1.02 | 0.845 | -0.72 |
| 1430034_at | Cct4 | chaperonin subunit 4 (delta) | -1.04 | 0.791 | -1.44 | 0.126 | -1.26 | 0.117 | 1.45 | 0.212 | -0.57 |
| 1426113_x_at | Tcra /// A430107P0 | T-cell receptor alpha chain /// RIKEN cDNA A430107P09 gene | -1.26 | 0.145 | -1.65 | 0.118 | 1.05 | 0.778 | -2.23 | 0.102 | -1.02 |
| 1459090_at | Ubxd4 | UBX domain containing 4 | -1.2 | 0.617 | -1.3 | 0.536 | -1.18 | 0.37 | 1.03 | 0.609 | -0.66 |
| 1425773_s_at | Nmnat1 | nicotinamide nucleotide adenylyltransferase 1 | -1.19 | 0.296 | -1.79 | 0.021 | 1.05 | 0.694 | -1.41 | 0.306 | -0.84 |
| 1444659_at | Trip11 | Thyroid hormone receptor interactor 11 | -1.49 | 0.413 | 1.15 | 0.678 | -1.59 | 0.244 | -1.55 | 0.137 | -0.87 |
| 1453424_at | Fyco1 | FYVE and coiled-coil domain containing 1 | 1.03 | 0.791 | -1.34 | 0.017 | -1.47 | 0.109 | -1.49 | 0.077 | -0.82 |
| 1437491_at | Bicd2 | bicaudal D homolog 2 (Drosophila) | -1.07 | 0.887 | -1.05 | 0.885 | -1.78 | 0.337 | 1.67 | 0.236 | -0.56 |
| 1438254_at | 1110007A13Rik | RIKEN cDNA 1110007A13 gene | -1.45 | 0.059 | 1.09 | 0.383 | -1.48 | 0.013 | -1.31 | 0.087 | -0.79 |
| 1454270_at | Grifin | galectin-related inter-fiber protein | -2.26 | 0.018 | -1.03 | 0.951 | 1.04 | 0.921 | -1.55 | 0.067 | -0.95 |
| 1431527_at | Cd164 | CD164 antigen | -2.65 | 0.163 | 1.1 | 0.912 | -1.02 | 0.939 | -1.46 | 0.51 | -1.01 |
| 1440943_at | B230208H17Rik | RIKEN cDNA B230208H17 gene | -1.04 | 0.907 | -1.13 | 0.644 | -1.66 | 0.09 | 1.05 | 0.831 | -0.7 |
| 1454393_at | 2310047C04Rik | RIKEN cDNA 2310047C04 gene | -1.21 | 0.466 | 1.2 | 0.696 | -2.35 | 0.064 | -1.26 | 0.141 | -0.9 |
| 1449726_at | Txnl2 /// LOC62001 | thioredoxin-like 2 /// similar to thioredoxin-like 2 | -1.1 | 0.913 | -1.07 | 0.851 | -1.64 | 0.081 | 1.13 | 0.605 | -0.67 |
| 1446240_at | Kns2 | Kinesin 2 | 1.09 | 0.648 | -1.61 | 0.384 | -1.35 | 0.204 | -1.37 | 0.484 | -0.81 |
| 1432369_at | 3010027C24Rik | RIKEN cDNA 3010027C24 gene | -1.31 | 0.483 | -1.39 | 0.361 | -1.04 | 0.947 | 1.02 | 0.94 | -0.68 |
| 1439238_at | Unc84b | unc-84 homolog B (C. elegans) | 1.11 | 0.437 | -1.68 | 0.17 | -1.33 | 0.148 | -1.1 | 0.491 | -0.75 |
| 1437594_x_at | Pigt | phosphatidylinositol glycan anchor biosynthesis, class T | -1.27 | 0.649 | -1.58 | 0.174 | 1.03 | 0.732 | -1.23 | 0.532 | -0.76 |
| 1423512_at | AW209491 | expressed sequence AW209491 | -1.14 | 0.241 | -1.2 | 0.045 | -1.35 | 0.096 | 1.12 | 0.6 | -0.64 |
| 1441301_at | Jakmip1 | Janus kinase and microtubule interacting protein 1 | -2.46 | 0.427 | -1.21 | 0.637 | 1.22 | 0.615 | -1.68 | 0.072 | -1.03 |
| 1430016_at | 4930584F24Rik | RIKEN cDNA 4930584F24 gene | -2.61 | 0.332 | 1.23 | 0.555 | -1.2 | 0.655 | -1.61 | 0.458 | -1.05 |
| 1458661_at | Morf4l1 | Mortality factor 4 like 1 | -1.08 | 0.584 | 1.06 | 0.697 | -2.13 | 0.017 | -1.1 | 0.631 | -0.81 |
| 1437604_x_at | Apcdd1 | adenomatosis polyposis coli down-regulated 1 | -1.51 | 0.209 | -1.07 | 0.88 | -1.17 | 0.164 | 1.01 | 0.968 | -0.69 |
| 1430502_at | 9130206N08Rik | RIKEN cDNA 9130206N08 gene | 1.18 | 0.753 | -1.64 | 0.404 | -1.52 | 0.406 | -1.54 | 0.443 | -0.88 |
| 1437474_at | Gatad2b | GATA zinc finger domain containing 2B | -1.17 | 0.139 | -1.19 | 0.207 | -1.32 | 0.187 | 1.05 | 0.758 | -0.66 |
| 1444448_at | 1300007F04Rik | RIKEN cDNA 1300007F04 gene | -4.47 | 0.017 | 1.53 | 0.509 | -1.43 | 0.478 | -2.17 | 0.109 | -1.64 |
| 1417233_at | Chchd4 | coiled-coil-helix-coiled-coil-helix domain containing 4 | -1.31 | 0.14 | -1.19 | 0.209 | -1.18 | 0.337 | 1.21 | 0.543 | -0.62 |
| 1431246_at | Iqce | IQ motif containing E | -1.31 | 0.04 | -1.27 | 0.568 | -1.11 | 0.73 | 1.12 | 0.194 | -0.64 |
| 1440294_at | Setd2 | SET domain containing 2 | 1.13 | 0.613 | -1.33 | 0.576 | -1.75 | 0.075 | -1.14 | 0.603 | -0.77 |
| 1433865_at | E330016L19Rik /// | RIKEN cDNA E330016L19 gene /// RIKEN cDNA E330010L02 gene | 1.2 | 0.83 | -2.09 | 0.098 | -1.3 | 0.531 | -1.4 | 0.489 | -0.9 |
| 1437373_at | Isg20l1 | interferon stimulated exonuclease gene 20-like 1 | -1.12 | 0.734 | -1.74 | 0.19 | -1.02 | 0.874 | 1.26 | 0.183 | -0.66 |
| 1435798_a_at | Sfrs14 | splicing factor, arginine/serine-rich 14 | -1.44 | 0.4 | -1.42 | 0.071 | 1.05 | 0.186 | -1.13 | 0.047 | -0.73 |
| 1442802_x_at | Bat2 | HLA-B associated transcript 2 | -1.31 | 0.501 | -1.03 | 0.926 | -1.4 | 0.044 | 1.03 | 0.729 | -0.68 |
| 1445688_at | Hcfc1 | host cell factor C1 | 1.24 | 0.425 | -1.65 | 0.433 | -1.64 | 0.415 | -2.04 | 0.254 | -1.02 |
| 1432748_at | 4933403J19Rik | RIKEN cDNA 4933403J19 gene | -2.3 | 0.122 | 1.2 | 0.583 | -1.22 | 0.651 | -1.43 | 0.232 | -0.94 |
| 1432903_at | --- | --- | 1.08 | 0.677 | -1.15 | 0.626 | -1.96 | 0.372 | -1.91 | 0.452 | -0.99 |
| 1458849_at | Asah2 | N-acylsphingosine amidohydrolase 2 | -1.45 | 0.132 | -1.1 | 0.623 | -1.17 | 0.377 | 1.28 | 0.292 | -0.61 |
| 1425692_a_at | Dnahc8 | dynein, axonemal, heavy chain 8 | -1.05 | 0.945 | -1.32 | 0.693 | -1.34 | 0.334 | 1.23 | 0.445 | -0.62 |
| 1434341_x_at | 1110020P15Rik /// | RIKEN cDNA 1110020P15 gene /// similar to ubiquinol-cytochrome c reductase c | -1.15 | 0.071 | -1.38 | 0.024 | -1.16 | 0.416 | 1.06 | 0.712 | -0.66 |
| 1439020_at | AW146020 | expressed sequence AW146020 | -1.34 | 0.476 | -1.21 | 0.465 | -1.14 | 0.573 | 1.22 | 0.407 | -0.62 |
| 1429325_at | Wdr51b | WD repeat domain 51B | -1.39 | 0.141 | -1.42 | 0.483 | 1.03 | 0.853 | -1.22 | 0.413 | -0.75 |
| 1455191_x_at | Pip5k1b | Phosphatidylinositol-4-phosphate 5-kinase, type 1 beta | -1.91 | 0.341 | -1.65 | 0.11 | 1.32 | 0.571 | -1.55 | 0.446 | -0.95 |
| 1460476_s_at | 1200015N20Rik | RIKEN cDNA 1200015N20 gene | -1.19 | 0.841 | -1.91 | 0.222 | 1.09 | 0.796 | -1.44 | 0.524 | -0.86 |

| | | | | | | | | | | | |
|---|---|---|---|---|---|---|---|---|---|---|---|
| 1452731_x_at | LOC544988 /// B93 | hypothetical protein LOC544988 /// RIKEN cDNA B930046C15 gene /// hypotheti | -1.03 | 0.943 | -2.31 | 0.252 | 1.05 | 0.713 | -1.08 | 0.856 | -0.84 |
| 1426063_a_at | Gem | GTP binding protein (gene overexpressed in skeletal muscle) | -1.88 | 0.503 | 1.24 | 0.539 | -1.46 | 0.41 | -1.34 | 0.225 | -0.86 |
| 1438834_at | Mospd2 | motile sperm domain containing 2 | 1.09 | 0.561 | -1.35 | 0.4 | -1.61 | 0.005 | -1.18 | 0.564 | -0.76 |
| 1448080_at | E2f8 | E2F transcription factor 8 | -1.14 | 0.882 | -1.6 | 0.062 | -1.05 | 0.892 | 1.3 | 0.736 | -0.62 |
| 1424347_at | Ppp6c | protein phosphatase 6, catalytic subunit | -1.12 | 0.254 | -1.1 | 0.127 | -1.53 | 0.009 | 1.14 | 0.183 | -0.65 |
| 1444764_at | A130022J21Rik | RIKEN cDNA A130022J21 gene | -1.14 | 0.612 | 1.02 | 0.945 | -1.79 | 0.15 | -1.05 | 0.887 | -0.74 |
| 1444353_at | C030040A22Rik | RIKEN cDNA C030040A22 gene | -1.25 | 0.671 | -1.27 | 0.67 | -1.15 | 0.664 | 1.04 | 0.953 | -0.66 |
| 1443014_at | 4833403I15Rik | RIKEN cDNA 4833403I15 gene | 1 | 0.99 | -1.13 | 0.737 | -1.76 | 0.282 | -1.44 | 0.44 | -0.83 |
| 1421564_at | Serpina3c | serine (or cysteine) peptidase inhibitor, clade A, member 3C | 1.14 | 0.831 | -1.97 | 0.222 | -1.24 | 0.411 | -1.47 | 0.317 | -0.89 |
| 1431989_at | 4930433E13Rik | RIKEN cDNA 4930433E13 gene | -1.04 | 0.69 | -1.34 | 0.464 | -1.34 | 0.123 | 1.07 | 0.842 | -0.66 |
| 1441724_at | --- | --- | -1.14 | 0.525 | -1.01 | 0.973 | -1.71 | 0.073 | 1.1 | 0.16 | -0.69 |
| 1433207_at | 5033430J17Rik | RIKEN cDNA 5033430J17 gene | -1.85 | 0.116 | 1.46 | 0.581 | -2.19 | 0.09 | -1.31 | 0.237 | -0.97 |
| 1431863_at | 4931430N09Rik | RIKEN cDNA 4931430N09 gene | -1.7 | 0.503 | -1.01 | 0.982 | -1.14 | 0.724 | 1.84 | 0.33 | -0.5 |
| 1446983_at | Cdc73 | Vcell division cycle 73, Paf1/RNA polymerase II complex component, homolog (S | 1.02 | 0.938 | -1.35 | 0.574 | -1.45 | 0.081 | -1.26 | 0.716 | -0.76 |
| 1436101_at | Rnf24 | ring finger protein 24 | -1.69 | 0.004 | 1.01 | 0.979 | -1.17 | 0.345 | -1.07 | 0.755 | -0.73 |
| 1449756_at | Dnajc17 | DnaJ (Hsp40) homolog, subfamily C, member 17 | -2.18 | 0.185 | 1.01 | 0.994 | -1.01 | 0.985 | -1.15 | 0.656 | -0.83 |
| 1447384_at | Abcc5 | ATP-binding cassette, sub-family C (CFTR/MRP), member 5 | 1.16 | 0.591 | -1.21 | 0.72 | -2.13 | 0.183 | -1.03 | 0.96 | -0.8 |
| 1439715_at | Osgepl1 | O-sialoglycoprotein endopeptidase-like 1 | 1.18 | 0.483 | -1.48 | 0.384 | -1.67 | 0.407 | -2.47 | 0.103 | -1.11 |
| 1445478_at | --- | --- | -1.22 | 0.525 | -1.22 | 0.519 | -1.23 | 0.276 | 1.06 | 0.792 | -0.65 |
| 1422373_at | Olfr71 | olfactory receptor 71 | -1.02 | 0.96 | -1.34 | 0.35 | -1.38 | 0.142 | 2.2 | 0.084 | -0.38 |
| 1455136_at | Atp1a2 | ATPase, Na+/K+ transporting, alpha 2 polypeptide | -2.75 | 0.089 | -1.15 | 0.795 | 1.22 | 0.619 | -1.07 | 0.93 | -0.94 |
| 1442929_at | St8sia5 | ST8 alpha-N-acetyl-neuraminide alpha-2,8-sialyltransferase 5 | -1.57 | 0.369 | 1.06 | 0.827 | -1.31 | 0.618 | -1.04 | 0.941 | -0.72 |
| 1457153_at | LOC552882 | hypothetical LOC552882 | 1.03 | 0.932 | -1.9 | 0.125 | -1.11 | 0.729 | -1.03 | 0.906 | -0.75 |
| 1432083_a_at | Lrrc23 | leucine rich repeat containing 23 | -1.84 | 0.436 | -1.2 | 0.821 | 1.08 | 0.81 | -1.64 | 0.11 | -0.9 |
| 1434817_s_at | 4930535B03Rik | RIKEN cDNA 4930535B03 gene | -1.28 | 0.101 | -1.46 | 0.029 | -1.01 | 0.943 | 1.33 | 0.259 | -0.6 |
| 1435588_at | Wdfy1 | WD repeat and FYVE domain containing 1 | -1.4 | 0.39 | -1.27 | 0.36 | -1.05 | 0.823 | 1.04 | 0.892 | -0.67 |
| 1442809_at | Scn9a /// LOC6718 | sodium channel, voltage-gated, type IX, alpha /// similar to sodium channel 25 | 1.05 | 0.936 | -1.05 | 0.945 | -2.24 | 0.233 | -1.16 | 0.794 | -0.85 |
| 1438818_at | Nmd3 | NMD3 homolog (S. cerevisiae) | -1.47 | 0.558 | 1.08 | 0.79 | -1.44 | 0.033 | -1.48 | 0.404 | -0.83 |
| 1416225_at | Adh1 | alcohol dehydrogenase 1 (class I) | -1.03 | 0.676 | -1.22 | 0.376 | -1.5 | 0.126 | 1.36 | 0.632 | -0.6 |
| 1438292_x_at | Adk | adenosine kinase | -1.04 | 0.235 | -1.1 | 0.548 | -1.71 | 0.008 | 1.89 | 0.083 | -0.49 |
| 1443005_at | Zfhx1a | zinc finger homeobox 1a | -1.37 | 0.193 | -1.16 | 0.49 | -1.15 | 0.371 | 1.06 | 0.788 | -0.66 |
| 1457120_at | Itk | IL2-inducible T-cell kinase | -2.05 | 0.528 | 1.12 | 0.85 | -1.17 | 0.568 | -2.42 | 0.116 | -1.13 |
| 1442045_at | --- | --- | -1.73 | 0.589 | 1.11 | 0.877 | -1.29 | 0.448 | -1.3 | 0.159 | -0.8 |
| 1418303_at | 1700113O17Rik | RIKEN cDNA 1700113O17 gene | -1.84 | 0.386 | -1.05 | 0.913 | -1.04 | 0.818 | 2.72 | 0.034 | -0.3 |
| 1457954_at | --- | --- | 1.07 | 0.801 | -2.08 | 0.184 | -1.1 | 0.748 | -1.22 | 0.416 | -0.83 |
| 1460354_a_at | Mrpl13 | mitochondrial ribosomal protein L13 | -1.15 | 0.264 | -1.12 | 0.348 | -1.45 | 0.072 | 1.02 | 0.921 | -0.67 |
| 1451699_at | LOC668468 /// LOC | similar to putative retrovirus-related gag protein /// hypothetical protein LOC66863 | -1.67 | 0.29 | -1.1 | 0.771 | -1.05 | 0.835 | 1.9 | 0.098 | -0.48 |
| 1441431_at | 1700041C02Rik | RIKEN cDNA 1700041C02 gene | -1.44 | 0.458 | -1.17 | 0.682 | -1.1 | 0.711 | 1.02 | 0.94 | -0.67 |
| 1442394_at | --- | 0 day neonate thymus cDNA, RIKEN full-length enriched library, clone:A430024H | 1.37 | 0.496 | -2.08 | 0.049 | -1.65 | 0.147 | -1.87 | 0.253 | -1.06 |
| 1441043_at | --- | Adult male testis cDNA, RIKEN full-length enriched library, clone:4930579G14 pr | 1.16 | 0.551 | -2.51 | 0.051 | -1.12 | 0.802 | -1.24 | 0.624 | -0.93 |
| 1415916_a_at | Mthfd1 | methylenetetrahydrofolate dehydrogenase (NADP+ dependent), methenyltetrahy | -1.23 | 0.037 | -1.13 | 0.366 | -1.32 | 0.043 | 2.36 | 0.15 | -0.33 |
| 1417996_at | Ngb | neuroglobin | 1.03 | 0.971 | -1.48 | 0.35 | -1.32 | 0.296 | -1.95 | 0.041 | -0.93 |
| 1421132_at | Pvrl3 | poliovirus receptor-related 3 | 1.04 | 0.848 | -1.37 | 0.352 | -1.45 | 0.1 | -2.46 | 0.222 | -1.06 |
| 1431849_at | 4933432B09Rik | RIKEN cDNA 4933432B09 gene | 1.1 | 0.893 | -2.45 | 0.204 | -1.05 | 0.619 | -2.16 | 0.357 | -1.14 |
| 1424968_at | 2210023G05Rik | RIKEN cDNA 2210023G05 gene | -1.14 | 0.734 | -1.15 | 0.319 | -1.41 | 0.061 | 4.3 | 0.18 | 0.15 |
| 1433365_at | 4930563N14Rik | RIKEN cDNA 4930563N14 gene | 1.19 | 0.228 | -1.46 | 0.672 | -1.71 | 0.352 | -1.02 | 0.98 | -0.75 |
| 1420154_at | --- | --- | -1.13 | 0.579 | -1.09 | 0.869 | -1.54 | 0.477 | 1.09 | 0.722 | -0.67 |
| 1418739_at | Sgk2 | serum/glucocorticoid regulated kinase 2 | -1.15 | 0.315 | -1.52 | 0.189 | -1.07 | 0.827 | 1.12 | 0.225 | -0.66 |
| 1435091_at | Zfp568 | zinc finger protein 568 | -1.35 | 0.132 | -1.17 | 0.106 | -1.16 | 0.203 | 1.25 | 0.34 | -0.61 |
| 1429581_at | Acad9 | acyl-Coenzyme A dehydrogenase family, member 9 | -1.38 | 0.233 | -1.54 | 0.071 | 1.08 | 0.686 | -1.3 | 0.244 | -0.78 |
| 1443081_at | Gata6 | GATA binding protein 6 | 1.07 | 0.916 | -1.14 | 0.832 | -1.95 | 0.026 | -1.39 | 0.156 | -0.85 |
| 1441273_at | Lrch2 | Leucine-rich repeats and calponin homology (CH) domain containing 2 | -2.75 | 0.365 | -2.61 | 0.191 | 1.71 | 0.121 | -1.97 | 0.078 | -1.4 |
| 1420348_at | Lhx5 | LIM homeobox protein 5 | -1.12 | 0.783 | -2.45 | 0.099 | 1.16 | 0.717 | -1.44 | 0.313 | -0.96 |

| | | | | | | | | | | | |
|---|---|---|---|---|---|---|---|---|---|---|---|
| 1446801_at | --- | --- | -1.7 | 0.241 | 1.34 | 0.363 | -1.89 | 0.185 | -1.52 | 0.61 | -0.94 |
| 1444598_at | Etv6 | Ets variant gene 6 (TEL oncogene) | 1.02 | 0.939 | -1.23 | 0.357 | -1.6 | 0.062 | -1.65 | 0.257 | -0.86 |
| 1441873_at | Prlpe | prolactin-like protein E | 1.31 | 0.585 | -1.39 | 0.637 | -2.35 | 0.358 | -2.08 | 0.477 | -1.13 |
| 1431094_at | 1110006E14Rik | RIKEN cDNA 1110006E14 gene | -1.1 | 0.877 | -1.01 | 0.986 | -1.79 | 0.005 | 1.74 | 0.217 | -0.54 |
| 1417848_at | Zfp704 | zinc finger protein 704 | -1.18 | 0.262 | -1.06 | 0.761 | -1.5 | 0.021 | 1.13 | 0.418 | -0.65 |
| 1439088_at | Pdzd8 | PDZ domain containing 8 | -1.15 | 0.607 | -1.19 | 0.497 | -1.34 | 0.055 | 1.09 | 0.744 | -0.65 |
| 1436731_at | Zfp533 | zinc finger protein 533 | -1.09 | 0.721 | -1.24 | 0.327 | -1.36 | 0.407 | 1.07 | 0.881 | -0.66 |
| 1442421_at | --- | --- | -1.51 | 0.195 | -1.07 | 0.844 | -1.15 | 0.687 | 1.19 | 0.604 | -0.64 |
| 1420044_at | --- | --- | -1.35 | 0.317 | -1.46 | 0.015 | 1.03 | 0.854 | -1.41 | 0.157 | -0.8 |
| 1439659_at | Trfp /// Usp49 | Trf (TATA binding protein-related factor)-proximal protein homolog (Drosophila) // | -1.06 | 0.808 | -1.54 | 0.028 | -1.15 | 0.389 | 1.69 | 0.142 | -0.52 |
| 1455864_at | Trim75 | tripartite motif-containing 75 | -1.23 | 0.703 | 1.26 | 0.753 | -2.59 | 0.129 | -1.23 | 0.664 | -0.95 |
| 1429540_at | Cnfn | cornifelin | -1.11 | 0.661 | -1.23 | 0.615 | -1.35 | 0.123 | 1.18 | 0.584 | -0.63 |
| 1432186_at | 1700028J19Rik | RIKEN cDNA 1700028J19 gene | 1.4 | 0.253 | -1.98 | 0.214 | -1.81 | 0.251 | -1.16 | 0.853 | -0.89 |
| 1445957_at | Evl | Ena-vasodilator stimulated phosphoprotein | -1.03 | 0.906 | -1.65 | 0.236 | -1.14 | 0.557 | 1.25 | 0.631 | -0.64 |
| 1457053_at | BC016423 | CDNA sequence BC016423 | 1.01 | 0.91 | -1.32 | 0.31 | -1.45 | 0.318 | -1.25 | 0.308 | -0.75 |
| 1455866_x_at | Txnl1 | thioredoxin-like 1 | -1.61 | 0.039 | -1.71 | 0.061 | 1.25 | 0.646 | -2.18 | 0.185 | -1.06 |
| 1431211_s_at | Them5 | thioesterase superfamily member 5 | -2.51 | 0.054 | 1.08 | 0.936 | -1.02 | 0.972 | -1.74 | 0.453 | -1.05 |
| 1454502_at | 2900022B07Rik | RIKEN cDNA 2900022B07 gene | -1.86 | 0.048 | -1.01 | 0.98 | -1.07 | 0.765 | 1.62 | 0.291 | -0.58 |
| 1450139_at | Ern2 | endoplasmic reticulum (ER) to nucleus signalling 2 | -1.78 | 0.358 | 1.54 | 0.329 | -2.79 | 0.015 | -1.63 | 0.433 | -1.17 |
| 1439333_at | Kcnv1 | potassium channel, subfamily V, member 1 | -2.58 | 0.281 | 1.67 | 0.46 | -2.49 | 0.022 | -1.9 | 0.117 | -1.33 |
| 1421050_at | Vps25 | vacuolar protein sorting 25 (yeast) | -1.1 | 0.55 | -1.38 | 0.338 | -1.21 | 0.359 | 1.13 | 0.546 | -0.64 |
| 1423753_at | Bambi | BMP and activin membrane-bound inhibitor, homolog (Xenopus laevis) | -1.2 | 0.207 | 1.07 | 0.653 | -1.8 | 0.002 | -1.46 | 0.031 | -0.85 |
| 1435961_at | BC047219 | cDNA sequence BC047219 | -1.07 | 0.858 | -1.93 | 0.162 | 1 | 0.974 | -1.82 | 0.044 | -0.95 |
| 1453620_at | 3010022N24Rik | RIKEN cDNA 3010022N24 gene | -2.22 | 0.111 | -1.46 | 0.419 | 1.32 | 0.497 | -1.01 | 0.989 | -0.84 |
| 1456202_at | BC094219 | cDNA sequence BC094219 | -2.73 | 0.495 | 1.29 | 0.58 | -1.25 | 0.721 | -5.27 | 0.05 | -1.99 |
| 1442155_at | 4632427E13Rik | RIKEN cDNA 4632427E13 gene | 1.12 | 0.622 | -1.11 | 0.581 | -2.26 | 0.002 | -1.25 | 0.164 | -0.87 |
| 1442887_at | AI853363 | expressed sequence AI853363 | -1.17 | 0.746 | -1.29 | 0.39 | -1.21 | 0.74 | 1.67 | 0.48 | -0.5 |
| 1440019_at | Gpr22 | G protein-coupled receptor 22 | -1.44 | 0.695 | 1.26 | 0.438 | -1.97 | 0.072 | -1.41 | 0.586 | -0.89 |
| 1453652_at | 4933400F21Rik | RIKEN cDNA 4933400F21 gene | -1.33 | 0.66 | -2.07 | 0.29 | 1.22 | 0.582 | -1.74 | 0.128 | -0.98 |
| 1416014_at | Abce1 | ATP-binding cassette, sub-family E (OABP), member 1 | -1.16 | 0.516 | -1.25 | 0.178 | -1.25 | 0.118 | 1.17 | 0.406 | -0.62 |
| 1435006_s_at | Abcb7 | ATP-binding cassette, sub-family B (MDR/TAP), member 7 | -1.32 | 0.013 | -1.18 | 0.106 | -1.17 | 0.226 | 1.47 | 0.095 | -0.55 |
| 1428411_at | 1700020I14Rik | RIKEN cDNA 1700020I14 gene | -1.14 | 0.009 | -1.11 | 0.388 | -1.46 | 0.054 | 1.08 | 0.553 | -0.66 |
| 1454466_at | 4933407I18Rik | RIKEN cDNA 4933407I18 gene | 1.09 | 0.924 | -2.34 | 0.424 | -1.06 | 0.886 | -1.13 | 0.832 | -0.86 |
| 1446922_at | --- | --- | -2.01 | 0.124 | -1.13 | 0.439 | 1.08 | 0.847 | -1.33 | 0.434 | -0.85 |
| 1419894_at | MGC107702 | Similar to proteasome (prosome, macropain) subunit, beta type 7 | 1.19 | 0.793 | -1.66 | 0.175 | -1.5 | 0.086 | -1.06 | 0.717 | -0.76 |
| 1450800_at | Syt8 | synaptotagmin VIII | -1.86 | 0.582 | 1.04 | 0.928 | -1.13 | 0.86 | -1.89 | 0.335 | -0.96 |
| 1450415_at | Pde6a | phosphodiesterase 6A, cGMP-specific, rod, alpha | 1.01 | 0.981 | -1.62 | 0.311 | -1.21 | 0.7 | -1.16 | 0.845 | -0.74 |
| 1452751_at | Ebf3 | early B-cell factor 3 | -1.04 | 0.466 | -1.39 | 0.635 | -1.29 | 0.609 | 1.33 | 0.378 | -0.6 |
| 1420103_at | --- | --- | -1.78 | 0.114 | -1.11 | 0.761 | -1 | 0.983 | 1.5 | 0.13 | -0.6 |
| 1447022_at | --- | Adult male testis cDNA, RIKEN full-length enriched library, clone:4930402K03 pro | 1.04 | 0.888 | -1.76 | 0.501 | -1.17 | 0.355 | -1.42 | 0.105 | -0.83 |
| 1444493_at | Tbc1d2b | TBC1 domain family, member 2B | -1.09 | 0.753 | -1.73 | 0.056 | -1.03 | 0.857 | 1.01 | 0.958 | -0.71 |
| 1459136_at | Bach2 | BTB and CNC homology 2 | 1.05 | 0.879 | -1.38 | 0.528 | -1.47 | 0.271 | -2.93 | 0.217 | -1.18 |
| 1420031_at | Chek1 | checkpoint kinase 1 homolog (S. pombe) | 1.26 | 0.723 | -1.25 | 0.738 | -2.46 | 0.067 | -1.9 | 0.359 | -1.09 |
| 1419937_at | AA536749 | expressed sequence AA536749 | 1.25 | 0.66 | -1.76 | 0.156 | -1.56 | 0.302 | -1.66 | 0.051 | -0.93 |
| 1445526_at | AY512915 | cDNA sequence AY512915 | -2.14 | 0.253 | 1.13 | 0.797 | -1.16 | 0.123 | -1.26 | 0.411 | -0.86 |
| 1454126_at | 0710001A04Rik | RIKEN cDNA 0710001A04 gene | -1.2 | 0.637 | -1.38 | 0.171 | -1.1 | 0.866 | 1.9 | 0.155 | -0.45 |
| 1428266_at | Myl3 | myosin, light polypeptide 3 | -1.67 | 0.318 | -1.76 | 0.131 | 1.3 | 0.527 | -1.12 | 0.619 | -0.81 |
| 1453551_at | Polq | polymerase (DNA directed), theta | -1.65 | 0.032 | -1.04 | 0.918 | -1.12 | 0.763 | 1.24 | 0.601 | -0.64 |
| 1439805_at | Nfat5 | Nuclear factor of activated T-cells 5 | 1.11 | 0.122 | -1.36 | 0.313 | -1.61 | 0.007 | -1.45 | 0.105 | -0.83 |
| 1447124_at | --- | Transcribed locus | -1.26 | 0.145 | -1.27 | 0.105 | -1.13 | 0.528 | 1.08 | 0.867 | -0.65 |
| 1419924_at | A730024A03Rik | RIKEN cDNA A730024A03 gene | -1.25 | 0.747 | -1.95 | 0.071 | 1.15 | 0.59 | -1.24 | 0.26 | -0.82 |
| 1423511_at | Asf1a | ASF1 anti-silencing function 1 homolog A (S. cerevisiae) | -1.26 | 0.064 | -1.13 | 0.343 | -1.28 | 0.022 | 1.59 | 0.269 | -0.52 |
| 1433300_at | 2900005I04Rik | RIKEN cDNA 2900005I04 gene | -1.52 | 0.21 | -3.34 | 0.127 | 1.5 | 0.632 | -1.71 | 0.255 | -1.27 |

| | | | | | | | | | | | |
|---|---|---|---|---|---|---|---|---|---|---|---|
| 1436871_at | Sfrs7 | splicing factor, arginine/serine-rich 7 | -1.63 | 0.261 | -1.22 | 0.301 | 1.03 | 0.78 | -1.23 | 0.597 | -0.76 |
| 1418953_at | Fbxo16 | F-box protein 16 | -1.4 | 0.142 | -1.62 | 0.074 | 1.13 | 0.473 | -1.05 | 0.872 | -0.74 |
| 1419478_at | Sectm1 | secreted and transmembrane 1 | 1.08 | 0.664 | -1.79 | 0.191 | -1.21 | 0.558 | -2.58 | 0.236 | -1.13 |
| 1419067_a_at | Rabgef1 | RAB guanine nucleotide exchange factor (GEF) 1 | -1.14 | 0.305 | -1.35 | 0.159 | -1.19 | 0.124 | 1.6 | 0.164 | -0.52 |
| 1444947_at | Lasp1 | LIM and SH3 protein 1 | -1.25 | 0.383 | -1.57 | 0.149 | 1.03 | 0.902 | -1.26 | 0.381 | -0.76 |
| 1424417_at | --- | --- | -1.41 | 0.146 | -1.19 | 0.542 | -1.09 | 0.748 | 1.02 | 0.856 | -0.67 |
| 1443392_at | Trpv1 | Transient receptor potential cation channel, subfamily V, member 1 | -1.46 | 0.354 | -1.19 | 0.637 | -1.07 | 0.763 | 1.35 | 0.621 | -0.59 |
| 1458222_at | --- | Transcribed locus | -1.44 | 0.105 | -1.15 | 0.485 | -1.11 | 0.373 | 1.03 | 0.903 | -0.67 |
| 1419628_at | Chx10 | C. elegans ceh-10 homeo domain containing homolog | -1.35 | 0.186 | -1.67 | 0.223 | 1.13 | 0.705 | -1.01 | 0.963 | -0.73 |
| 1455992_at | Vgll4 | vestigial like 4 (Drosophila) | -1.45 | 0.082 | -1.65 | 0.07 | 1.17 | 0.573 | -1.32 | 0.088 | -0.81 |
| 1440672_at | LOC638394 /// LOC | similar to zinc finger protein 541 /// similar to zinc finger protein 541 /// similar to z | -1.34 | 0.564 | -1.47 | 0.669 | 1.04 | 0.811 | -1.05 | 0.954 | -0.71 |
| 1457029_at | C030010B13Rik | RIKEN cDNA C030010B13 gene | -1.13 | 0.405 | -1.9 | 0.142 | 1.06 | 0.865 | -1.08 | 0.864 | -0.77 |
| 1422986_at | Esrrb | estrogen related receptor, beta | -1.11 | 0.811 | -1.59 | 0.205 | -1.07 | 0.738 | 1.4 | 0.125 | -0.59 |
| 1436755_at | Itih5 | inter-alpha (globulin) inhibitor H5 | 1.17 | 0.659 | -1.81 | 0.078 | -1.34 | 0.449 | -1.73 | 0.324 | -0.93 |
| 1456917_at | Arfgef1 | ADP-ribosylation factor guanine nucleotide-exchange factor 1(brefeldin A-inhibite | 1.07 | 0.446 | -1.28 | 0.317 | -1.63 | 0.006 | -1.85 | 0.081 | -0.92 |
| 1439173_at | Hook1 | hook homolog 1 (Drosophila) | 1.02 | 0.958 | -1.19 | 0.434 | -1.65 | 0.06 | -1.97 | 0.066 | -0.95 |
| 1436347_a_at | 5530601H04Rik | RIKEN cDNA 5530601H04 gene | -1.06 | 0.781 | -1.48 | 0.234 | -1.19 | 0.292 | 1.03 | 0.767 | -0.67 |
| 1455627_at | Col8a1 | procollagen, type VIII, alpha 1 | -1.43 | 0.35 | -1.12 | 0.642 | -1.14 | 0.612 | 1.28 | 0.108 | -0.6 |
| 1415719_s_at | Armc1 | armadillo repeat containing 1 | -1.17 | 0.306 | -1.1 | 0.117 | -1.42 | 0.103 | 1.33 | 0.226 | -0.59 |
| 1446048_at | --- | --- | 1.01 | 0.976 | -1.4 | 0.08 | -1.35 | 0.139 | -1.91 | 0.048 | -0.91 |
| 1428779_at | Zbtb41 | zinc finger and BTB domain containing 41 homolog | -1.16 | 0.311 | -1.26 | 0.007 | -1.24 | 0.085 | 1.06 | 0.792 | -0.65 |
| 1446619_at | A130038J17Rik | RIKEN cDNA A130038J17 gene | -1.33 | 0.232 | -1.3 | 0.578 | -1.07 | 0.847 | 1.25 | 0.559 | -0.61 |
| 1442880_at | --- | --- | 1.47 | 0.454 | -1.76 | 0.07 | -2.31 | 0.118 | -1.41 | 0.235 | -1 |
| 1427116_at | Setd1a | SET domain containing 1A | -1.54 | 0.184 | -1.2 | 0.125 | -1.02 | 0.925 | 1.13 | 0.535 | -0.66 |
| 1443042_at | Robo1 | Roundabout homolog 1 (Drosophila) | -1.05 | 0.928 | -1.03 | 0.925 | -1.83 | 0.133 | 1.73 | 0.028 | -0.55 |
| 1454090_at | Pdss1 | prenyl (solanesyl) diphosphate synthase, subunit 1 | -1.65 | 0.199 | 1.32 | 0.288 | -1.84 | 0.296 | -1.08 | 0.747 | -0.81 |
| 1458294_at | --- | Transcribed locus | -1.21 | 0.632 | -1.04 | 0.93 | -1.48 | 0.221 | 1.81 | 0.168 | -0.48 |
| 1457465_at | Shroom4 | shroom family member 4 | -1.01 | 0.942 | -1.39 | 0.381 | -1.34 | 0.277 | 1.03 | 0.844 | -0.67 |
| 1429832_at | Ppih /// LOC43306 | peptidyl prolyl isomerase H /// similar to peptidyl prolyl isomerase H /// hypothetica | 1.12 | 0.491 | -2.08 | 0.059 | -1.15 | 0.248 | -1.01 | 0.987 | -0.78 |
| 1449091_at | Cldn8 | claudin 8 | -1.23 | 0.216 | -1.35 | 0.135 | -1.1 | 0.793 | 1.88 | 0.35 | -0.45 |
| 1456059_at | Psmd11 | proteasome (prosome, macropain) 26S subunit, non-ATPase, 11 | -1.45 | 0.146 | -1.21 | 0.064 | -1.05 | 0.733 | 1.32 | 0.286 | -0.6 |
| 1450996_at | Fshb | follicle stimulating hormone beta | -1.51 | 0.338 | 1.14 | 0.131 | -1.51 | 0.633 | -1.44 | 0.484 | -0.83 |
| 1439765_x_at | Krt42 | keratin 42 | -1.4 | 0.163 | -1.12 | 0.557 | -1.17 | 0.405 | 1.09 | 0.789 | -0.65 |
| 1443489_at | C330002D13Rik | RIKEN cDNA C330002D13 gene | -1.14 | 0.536 | -1.15 | 0.338 | -1.39 | 0.156 | 1.18 | 0.015 | -0.62 |
| 1440457_at | Cltc | Clathrin, heavy polypeptide (Hc) | -1.22 | 0.445 | -1.19 | 0.247 | -1.24 | 0.254 | 1.4 | 0.22 | -0.56 |
| 1419553_a_at | Rabggtb | RAB geranylgeranyl transferase, b subunit | -1.4 | 0.053 | -1.26 | 0.058 | -1.05 | 0.717 | 1.38 | 0.408 | -0.58 |
| 1435443_at | Eya3 | eyes absent 3 homolog (Drosophila) | -1.05 | 0.724 | -1.21 | 0.27 | -1.45 | 0.144 | 1.11 | 0.552 | -0.65 |
| 1453418_at | Col24a1 | procollagen, type XXIV, alpha 1 | 1.36 | 0.251 | -1.19 | 0.54 | -3.8 | 0.002 | -2.34 | 0.304 | -1.49 |
| 1459379_at | --- | --- | 1.03 | 0.792 | -1.59 | 0.191 | -1.24 | 0.311 | -1.2 | 0.183 | -0.75 |
| 1460499_at | 9230110I02Rik | RIKEN cDNA 9230110I02 gene | -1.44 | 0.076 | 1.06 | 0.835 | -1.4 | 0.005 | -1.82 | 0.213 | -0.9 |
| 1451047_at | Itm2a | integral membrane protein 2A | -1.41 | 0.083 | 1.2 | 0.17 | -1.81 | 0.021 | -2.61 | 0.051 | -1.16 |
| 1433849_at | Cdc27 | cell division cycle 27 homolog (S. cerevisiae) | -1 | 0.993 | -1.42 | 0.14 | -1.31 | 0.265 | 1.84 | 0.301 | -0.47 |
| 1428638_at | Efhc2 | EF-hand domain (C-terminal) containing 2 | 1.16 | 0.109 | -3.05 | 0.29 | -1.02 | 0.921 | -1.16 | 0.81 | -1.02 |
| 1453859_at | 2410003P15Rik | RIKEN cDNA 2410003P15 gene | -2.14 | 0.164 | -1.53 | 0.397 | 1.35 | 0.634 | -1.06 | 0.95 | -0.85 |
| 1431238_at | Pqlc1 | PQ loop repeat containing 1 | -1.5 | 0.276 | -1.99 | 0.262 | 1.3 | 0.2 | -1.48 | 0.501 | -0.92 |
| 1460583_at | Golt1b | golgi transport 1 homolog B (S. cerevisiae) | -1.16 | 0.364 | -1.39 | 0.53 | -1.12 | 0.497 | 1.18 | 0.588 | -0.63 |
| 1437443_at | --- | PREDICTED: Mus musculus hypothetical protein LOC620382 (LOC620382), mR | -1.17 | 0.489 | -1.24 | 0.049 | -1.24 | 0.326 | 1.03 | 0.853 | -0.66 |
| 1443485_at | Epha7 | Eph receptor A7 | -1.04 | 0.948 | 1.03 | 0.905 | -2.11 | 0.022 | -1.55 | 0.331 | -0.92 |
| 1442033_at | C1qtnf9 | C1q and tumor necrosis factor related protein 9 | -1.35 | 0.141 | -1.31 | 0.493 | -1.03 | 0.919 | 1.71 | 0.136 | -0.5 |
| 1457641_at | Crebbp | CREB binding protein | -1.38 | 0.294 | 1.15 | 0.471 | -1.68 | 0.06 | -1.5 | 0.007 | -0.85 |
| 1457535_at | 4932438A13Rik | RIKEN cDNA 4932438A13 gene | 1.04 | 0.589 | -1.44 | 0.134 | -1.35 | 0.097 | -1.19 | 0.261 | -0.74 |
| 1440025_at | Kptn | kaptin | -1.8 | 0.211 | -1.14 | 0.685 | 1.03 | 0.86 | -1.29 | 0.287 | -0.8 |
| 1440972_at | Nsd1 | nuclear receptor-binding SET-domain protein 1 | 1.03 | 0.54 | -1.33 | 0.004 | -1.45 | 0.111 | -1.24 | 0.414 | -0.75 |

| | | | | | | | | | | | |
|---|---|---|---|---|---|---|---|---|---|---|---|
| 1430040_at | Hspa12a | heat shock protein 12A | -4.17 | 0.014 | -1.22 | 0.57 | 1.41 | 0.607 | -1.02 | 0.96 | -1.25 |
| 1438824_at | Slc20a1 | solute carrier family 20, member 1 | -1.17 | 0.545 | -1.01 | 0.978 | -1.6 | 0.089 | 1.81 | 0.254 | -0.49 |
| 1457462_at | --- | --- | 1.11 | 0.672 | -1.44 | 0.023 | -1.51 | 0.136 | -1.05 | 0.808 | -0.72 |
| 1460116_s_at | Spred1 | sprouty protein with EVH-1 domain 1, related sequence | 1.09 | 0.592 | -1.22 | 0.269 | -1.79 | 0.018 | -1.08 | 0.738 | -0.75 |
| 1454930_at | Lrrc35 | leucine rich repeat containing 35 | -1.14 | 0.376 | -1.36 | 0.263 | -1.16 | 0.053 | 2.24 | 0.207 | -0.36 |
| 1419938_s_at | Arhgef17 | Rho guanine nucleotide exchange factor (GEF) 17 | -1.31 | 0.137 | -1.65 | 0.075 | 1.1 | 0.805 | -1.55 | 0.093 | -0.85 |
| 1440516_at | --- | 13 days embryo heart cDNA, RIKEN full-length enriched library, clone:D330017K | -2.1 | 0.108 | -1.01 | 0.985 | 1.01 | 0.987 | -1.08 | 0.731 | -0.8 |
| 1418910_at | Bmp7 | bone morphogenetic protein 7 | 1.12 | 0.158 | -1.94 | 0.021 | -1.19 | 0.266 | -1.21 | 0.608 | -0.81 |
| 1424221_at | Susd4 | sushi domain containing 4 | 1.22 | 0.435 | -2.24 | 0.051 | -1.25 | 0.073 | -1.09 | 0.803 | -0.84 |
| 1450801_at | Adam21 | a disintegrin and metallopeptidase domain 21 | 1.11 | 0.715 | -1.64 | 0.233 | -1.33 | 0.517 | -1.44 | 0.555 | -0.83 |
| 1425000_s_at | 5430407P10Rik | RIKEN cDNA 5430407P10 gene | -1.8 | 0.137 | -1.03 | 0.921 | -1.06 | 0.82 | 1.02 | 0.963 | -0.72 |
| 1421361_at | Grk1 | G protein-coupled receptor kinase 1 | -1.03 | 0.888 | 1.1 | 0.74 | -2.53 | 0.315 | -1.06 | 0.795 | -0.88 |
| 1425004_s_at | Mocs1 | molybdenum cofactor synthesis 1 | -1.24 | 0.428 | -1.19 | 0.339 | -1.21 | 0.193 | 2.12 | 0.079 | -0.38 |
| 1426713_s_at | Eprs /// LOC63367 | glutamyl-prolyl-tRNA synthetase /// similar to Bifunctional aminoacyl-tRNA synthe | -1.19 | 0.104 | -1.16 | 0.021 | -1.31 | 0.118 | 1.2 | 0.468 | -0.61 |
| 1422727_at | Nme5 | expressed in non-metastatic cells 5 | -1.46 | 0.157 | -1.12 | 0.599 | -1.12 | 0.663 | 1.28 | 0.522 | -0.6 |
| 1446258_at | Sntb1 | Syntrophin, basic 1 | 1.16 | 0.127 | -1.72 | 0.107 | -1.37 | 0.125 | -1.45 | 0.279 | -0.85 |
| 1419150_at | Myf6 | myogenic factor 6 | -1.74 | 0.028 | -1.3 | 0.735 | 1.13 | 0.654 | -1.64 | 0.02 | -0.89 |
| 1424589_s_at | Rnpc3 | RNA-binding region (RNP1, RRM) containing 3 | -1.22 | 0.53 | -1.1 | 0.393 | -1.35 | 0.117 | 1.26 | 0.509 | -0.6 |
| 1443380_at | Adh5 | alcohol dehydrogenase 5 (class III), chi polypeptide | -1.44 | 0.758 | 1.08 | 0.848 | -1.43 | 0.523 | -1.3 | 0.618 | -0.77 |
| 1454208_at | 4930483O08Rik | RIKEN cDNA 4930483O08 gene | 1.03 | 0.834 | -2.05 | 0.174 | -1.05 | 0.903 | -1.1 | 0.597 | -0.79 |
| 1442920_at | Klf3 | Kruppel-like factor 3 (basic) | -1.16 | 0.349 | -1.31 | 0.06 | -1.18 | 0.284 | 1.33 | 0.374 | -0.58 |
| 1448925_at | Twist2 | twist homolog 2 (Drosophila) | -1.44 | 0.218 | -1.17 | 0.659 | -1.08 | 0.821 | 1.13 | 0.744 | -0.64 |
| 1435073_a_at | Armc9 | armadillo repeat containing 9 | -1.02 | 0.898 | -1.71 | 0.219 | -1.1 | 0.587 | 1.21 | 0.528 | -0.66 |
| 1451689_a_at | Sox10 | SRY-box containing gene 10 | -2.14 | 0.38 | 1.06 | 0.845 | -1.06 | 0.916 | -2.34 | 0.309 | -1.12 |
| 1443180_at | Ergic2 | ERGIC and golgi 2 | -1.33 | 0.179 | -1.18 | 0.446 | -1.15 | 0.416 | 1.19 | 0.381 | -0.62 |
| 1450837_at | Prh1 | proline rich protein HaeIII subfamily 1 | -1.15 | 0.473 | -1.1 | 0.658 | -1.43 | 0.574 | 1.36 | 0.089 | -0.58 |
| 1433044_at | Cspg2 | chondroitin sulfate proteoglycan 2 | 1.13 | 0.623 | -1.82 | 0.045 | -1.27 | 0.321 | -2 | 0.03 | -0.99 |
| 1418119_at | Rbm8a | RNA binding motif protein 8a | -1.06 | 0.653 | -1.29 | 0.064 | -1.33 | 0.023 | 1.08 | 0.589 | -0.65 |
| 1442577_at | KIAA0415 | KIAA0415 protein | -1.78 | 0.344 | -1.06 | 0.801 | -1.03 | 0.9 | 2.86 | 0.135 | -0.25 |
| 1458690_at | 7-Mar | membrane-associated ring finger (C3HC4) 7 | -1.07 | 0.738 | 1.01 | 0.97 | -1.9 | 0.003 | -1.78 | 0.085 | -0.94 |
| 1442973_at | C80865 | expressed sequence C80865 | 1.18 | 0.779 | -1.62 | 0.453 | -1.48 | 0.596 | -1.14 | 0.863 | -0.77 |
| 1449487_at | Ccdc70 | coiled-coil domain containing 70 | -1.08 | 0.229 | -2.51 | 0.121 | 1.15 | 0.35 | -1.28 | 0.7 | -0.93 |
| 1431156_at | 9430040K09Rik | RIKEN cDNA 9430040K09 gene | -1 | 0.98 | -1.1 | 0.698 | -1.79 | 0.04 | 1.12 | 0.815 | -0.69 |
| 1448694_at | Jun | Jun oncogene | -1.63 | 0.117 | -1.07 | 0.753 | -1.09 | 0.415 | 1.51 | 0.076 | -0.57 |
| 1419087_s_at | Sf3a1 | splicing factor 3a, subunit 1 | -1.18 | 0.382 | -1.36 | 0.349 | -1.13 | 0.287 | 1.66 | 0.237 | -0.5 |
| 1450680_at | Rag1 | recombination activating gene 1 | -1.11 | 0.262 | -1.04 | 0.937 | -1.64 | 0.593 | 1.44 | 0.346 | -0.59 |
| 1421542_at | Dzip1 | DAZ interacting protein 1 | -1.22 | 0.515 | -1.64 | 0.337 | 1.04 | 0.935 | -1.56 | 0.515 | -0.84 |
| 1441918_x_at | RP23-262J21.5 | similar to NK13 | -1.36 | 0.325 | 1.35 | 0.521 | -2.58 | 0.047 | -5.13 | 0.086 | -1.93 |
| 1443178_at | Rnf13 | ring finger protein 13 | -1.83 | 0.068 | -1.69 | 0.371 | 1.33 | 0.604 | -1.92 | 0.137 | -1.03 |
| 1421563_at | Atp7b | ATPase, Cu++ transporting, beta polypeptide | -1.34 | 0.335 | -1.3 | 0.328 | -1.05 | 0.762 | 1.43 | 0.12 | -0.56 |
| 1444073_at | Maf | avian musculoaponeurotic fibrosarcoma (v-maf) AS42 oncogene homolog | -1.28 | 0.423 | -1.08 | 0.66 | -1.3 | 0.463 | 1.21 | 0.699 | -0.62 |
| 1428639_at | Lin9 | lin-9 homolog (C. elegans) | -1.26 | 0.424 | -1.11 | 0.642 | -1.29 | 0.38 | 1.4 | 0.54 | -0.56 |
| 1433362_at | 4930527F18Rik | RIKEN cDNA 4930527F18 gene | -1.26 | 0.228 | -1.2 | 0.416 | -1.18 | 0.391 | 1.33 | 0.654 | -0.58 |
| 1444687_at | C1ql2 | complement component 1, q subcomponent-like 2 | -1.04 | 0.877 | -1.2 | 0.325 | -1.48 | 0.182 | 1.36 | 0.527 | -0.59 |
| 1453923_at | Ccdc83 | coiled-coil domain containing 83 | -1.38 | 0.439 | -1.12 | 0.813 | -1.18 | 0.52 | 1.31 | 0.436 | -0.59 |
| 1435398_at | Stxbp5 | syntaxin binding protein 5 (tomosyn) | -1.15 | 0.284 | -1.36 | 0.121 | -1.15 | 0.597 | 1.42 | 0.254 | -0.56 |
| 1435320_at | Ctdspl2 | CTD (carboxy-terminal domain, RNA polymerase II, polypeptide A) small phospha | -1.05 | 0.63 | -1.3 | 0.096 | -1.33 | 0.295 | 1.36 | 0.321 | -0.58 |
| 1442248_at | Trio | Triple functional domain (PTPRF interacting) | -1.37 | 0.267 | 1.08 | 0.714 | -1.51 | 0.095 | -1.6 | 0.156 | -0.85 |
| 1427169_at | Als2cr19 | amyotrophic lateral sclerosis 2 (juvenile) chromosome region, candidate 19 (huma | -1.02 | 0.839 | -1.22 | 0.451 | -1.5 | 0.032 | 1.34 | 0.291 | -0.6 |
| 1440417_at | D19Ertd409e | DNA segment, Chr 19, ERATO Doi 409, expressed | 1.02 | 0.771 | -1.14 | 0.237 | -1.74 | 0.046 | -1.41 | 0.222 | -0.82 |
| 1433898_at | AV025504 | Expressed sequence AV025504 | -1.03 | 0.543 | -2.48 | 0.008 | 1.1 | 0.463 | -1.21 | 0.264 | -0.91 |
| 1454792_s_at | Sephs1 | selenophosphate synthetase 1 | -1.04 | 0.094 | -1.27 | 0.049 | -1.38 | 0.007 | 1.32 | 0.234 | -0.59 |
| 1449640_at | Tlr7 | toll-like receptor 7 | -2.37 | 0.426 | 1.29 | 0.648 | -1.31 | 0.471 | -1.42 | 0.305 | -0.95 |

| | | | | | | | | | | | |
|---|---|---|---|---|---|---|---|---|---|---|---|
| 1450209_at | Hoxd4 | homeo box D4 | -2.2 | 0.498 | -1.07 | 0.9 | 1.08 | 0.8 | -1.32 | 0.497 | -0.87 |
| 1440418_at | --- | --- | -1.79 | 0.445 | -1.37 | 0.711 | 1.18 | 0.754 | -1.49 | 0.63 | -0.87 |
| 1439007_at | Alg6 | asparagine-linked glycosylation 6 homolog (yeast, alpha-1,3,-glucosyltransferase | -1.14 | 0.17 | -1.28 | 0.099 | -1.23 | 0.196 | 1.38 | 0.142 | -0.57 |
| 1457812_at | Trp53bp1 | transformation related protein 53 binding protein 1 | -1.06 | 0.859 | -1.15 | 0.42 | -1.51 | 0.083 | 1.09 | 0.675 | -0.66 |
| 1420562_at | Slurp1 | secreted Ly6/Plaur domain containing 1 | -1.03 | 0.961 | -1.62 | 0.454 | -1.13 | 0.816 | 1.02 | 0.968 | -0.69 |
| 1440524_at | Utx | ubiquitously transcribed tetratricopeptide repeat gene, X chromosome | -1.42 | 0.654 | 1.08 | 0.768 | -1.44 | 0.089 | -1.64 | 0.428 | -0.86 |
| 1439401_x_at | Ppp2r5e | Protein phosphatase 2, regulatory subunit B (B56), epsilon isoform | -1.4 | 0.345 | -1.23 | 0.385 | -1.06 | 0.713 | 1.16 | 0.014 | -0.63 |
| 1460455_at | 1110059H15Rik | RIKEN cDNA 1110059H15 gene | -1.23 | 0.153 | -1.11 | 0.201 | -1.32 | 0.17 | 1.27 | 0.141 | -0.6 |
| 1456912_at | Zfp691 | zinc finger protein 691 | -1.16 | 0.535 | -1.19 | 0.623 | -1.3 | 0.597 | 1.05 | 0.872 | -0.65 |
| 1419042_at | Iigp1 | interferon inducible GTPase 1 | -1.05 | 0.647 | -1.18 | 0.121 | -1.5 | 0.184 | 1.11 | 0.594 | -0.65 |
| 1455621_at | BC066107 | cDNA sequence BC066107 | -1.44 | 0.372 | 1.16 | 0.554 | -1.63 | 0.036 | -1.64 | 0.269 | -0.89 |
| 1451669_at | Ppm1b | protein phosphatase 1B, magnesium dependent, beta isoform | -1.16 | 0.531 | -1.21 | 0.042 | -1.26 | 0.14 | 1.24 | 0.16 | -0.6 |
| 1431114_at | Dock4 | dedicator of cytokinesis 4 | 1.02 | 0.965 | -1.01 | 0.965 | -2.15 | 0.037 | -1.37 | 0.169 | -0.88 |
| 1442561_at | Mamdc1 | MAM domain containing 1 | -1.6 | 0.567 | 1.16 | 0.857 | -1.46 | 0.345 | -1.7 | 0.488 | -0.9 |
| 1422149_at | Mllt7 | myeloid/lymphoid or mixed lineage-leukemia translocation to 7 homolog (Drosoph | -2.01 | 0.573 | -1.1 | 0.545 | 1.06 | 0.67 | -1.86 | 0.444 | -0.97 |
| 1423623_at | 2810021B07Rik | RIKEN cDNA 2810021B07 gene | -1.31 | 0.06 | -1.5 | 0.053 | 1.05 | 0.769 | -1.12 | 0.588 | -0.72 |
| 1453857_at | Rnf180 | ring finger protein 180 | -1.3 | 0.435 | -1.18 | 0.722 | -1.16 | 0.482 | 1.13 | 0.741 | -0.63 |
| 1458961_at | Eya3 | eyes absent 3 homolog (Drosophila) | 1.08 | 0.829 | -1.32 | 0.293 | -1.58 | 0.296 | -1.44 | 0.162 | -0.81 |
| 1423397_at | Ugt2b38 | UDP glucuronosyltransferase 2 family, polypeptide B38 | -2.36 | 0.003 | -5.41 | 0.031 | 1.87 | 0.106 | -1.58 | 0.459 | -1.87 |
| 1420293_at | Aytl2 | acyltransferase like 2 | -1.26 | 0.578 | -1.94 | 0.296 | 1.17 | 0.594 | -1.3 | 0.587 | -0.83 |
| 1433530_at | 2210411K19Rik | RIKEN cDNA 2210411K19 gene | -1.22 | 0.047 | -1.15 | 0.526 | -1.28 | 0.249 | 1.02 | 0.951 | -0.66 |
| 1444285_at | Morf4l1 | Mortality factor 4 like 1 | -1.69 | 0.016 | 1.36 | 0.526 | -1.93 | 0.008 | -1.48 | 0.04 | -0.93 |
| 1444744_at | Dido1 | Death inducer-obliterator 1 | -1.47 | 0.321 | -1.33 | 0.127 | 1.04 | 0.591 | -1.02 | 0.885 | -0.69 |
| 1423337_at | Orc4l | origin recognition complex, subunit 4-like (S. cerevisiae) | -1.03 | 0.807 | -1.32 | 0.06 | -1.34 | 0.139 | 1.02 | 0.926 | -0.67 |
| 1458169_at | Gm700 /// LOC668 | gene model 700, (NCBI) /// similar to Motilin receptor (G-protein coupled receptor | -1.39 | 0.318 | 1.11 | 0.715 | -1.54 | 0.179 | -1.29 | 0.459 | -0.78 |
| 1423193_at | Pspc1 | paraspeckle protein 1 | -1.42 | 0.212 | -1.31 | 0.252 | 1.01 | 0.962 | -1.08 | 0.511 | -0.7 |
| 1433375_at | D530037P16Rik | RIKEN cDNA D530037P16 gene | 1.02 | 0.883 | -1.03 | 0.916 | -2.08 | 0.354 | -1.08 | 0.887 | -0.79 |
| 1458829_at | --- | --- | 1.03 | 0.932 | -1.15 | 0.693 | -1.73 | 0.175 | -1.51 | 0.298 | -0.84 |
| 1460144_at | BC052040 | CDNA sequence BC052040 | -1.02 | 0.977 | -1.1 | 0.762 | -1.72 | 0.26 | 1.08 | 0.796 | -0.69 |
| 1420201_at | --- | --- | -1.25 | 0.811 | -2.57 | 0.16 | 1.28 | 0.19 | -1.11 | 0.811 | -0.91 |
| 1419828_at | Usp31 | ubiquitin specific peptidase 31 | -1.28 | 0.203 | -1.19 | 0.541 | -1.17 | 0.405 | 3.39 | 0.236 | -0.06 |
| 1440096_at | Ecm2 | extracellular matrix protein 2, female organ and adipocyte specific | -1 | 0.993 | 1.06 | 0.829 | -2.37 | 0.008 | -1.82 | 0.553 | -1.03 |
| 1449234_at | Car15 | carbonic anhydrase 15 | 1.14 | 0.414 | -2.27 | 0.104 | -1.12 | 0.631 | -1.26 | 0.234 | -0.88 |
| 1428458_at | Pop1 | processing of precursor 1, ribonuclease P/MRP family, (S. cerevisiae) | -1.32 | 0.264 | -1.32 | 0.409 | -1.04 | 0.893 | 1.28 | 0.438 | -0.6 |
| 1447186_at | Slc6a16 /// LOC672 | solute carrier family 6, member 16 /// similar to Orphan sodium- and chloride-depe | -1.33 | 0.313 | -1.32 | 0.566 | -1.04 | 0.922 | 1.25 | 0.557 | -0.61 |
| 1444574_at | Elf2 | E74-like factor 2 | 1.09 | 0.863 | -1.35 | 0.095 | -1.56 | 0.011 | -1.07 | 0.83 | -0.72 |
| 1459850_x_at | Glrb | glycine receptor, beta subunit | -2.05 | 0.514 | 1.22 | 0.31 | -1.3 | 0.24 | -1.38 | 0.064 | -0.88 |
| 1458554_at | 9330169B04Rik | RIKEN cDNA 9330169B04 gene | -1.86 | 0.2 | 1.23 | 0.598 | -1.41 | 0.366 | -1.48 | 0.345 | -0.88 |
| 1432317_at | 9430078K24Rik | RIKEN cDNA 9430078K24 gene | 1.07 | 0.741 | -2.28 | 0.018 | -1.03 | 0.896 | -2.36 | 0.273 | -1.15 |
| 1455216_at | Paqr6 | progestin and adipoQ receptor family member VI | -2.23 | 0.057 | 1.21 | 0.767 | -1.22 | 0.401 | -1.25 | 0.512 | -0.87 |
| 1439371_x_at | Timm44 | translocase of inner mitochondrial membrane 44 | -1.21 | 0.196 | -1.48 | 0.102 | -1.02 | 0.943 | 1.13 | 0.372 | -0.65 |
| 1423936_at | Kctd5 | potassium channel tetramerisation domain containing 5 | -1.12 | 0.389 | -1.56 | 0.049 | -1.06 | 0.643 | 1.09 | 0.175 | -0.66 |
| 1425424_at | MGC7817 | hypothetical protein LOC620031 | -1.36 | 0.058 | 1.13 | 0.556 | -1.65 | 0.093 | -1.46 | 0.171 | -0.83 |
| 1416272_at | Map2k1ip1 | mitogen-activated protein kinase kinase 1 interacting protein 1 | -1.24 | 0.182 | -1.08 | 0.391 | -1.34 | 0.175 | 1.05 | 0.561 | -0.65 |
| 1459268_at | 5330439C02Rik | RIKEN cDNA 5330439C02 gene | -1.16 | 0.707 | -1.21 | 0.374 | -1.27 | 0.629 | 1.85 | 0.322 | -0.45 |
| 1430138_at | Cd3eap | CD3E antigen, epsilon polypeptide associated protein | -1.54 | 0.31 | 1.17 | 0.64 | -1.52 | 0.134 | -1.32 | 0.126 | -0.8 |
| 1437175_at | Pdik1l | PDLIM1 interacting kinase 1 like | -1.02 | 0.784 | -1.26 | 0.118 | -1.42 | 0.005 | 1.28 | 0.37 | -0.61 |
| 1457189_at | Itpr1 | Inositol 1,4,5-triphosphate receptor 1 | -1.11 | 0.716 | 1.05 | 0.887 | -1.89 | 0.025 | -1.66 | 0.18 | -0.9 |
| 1442103_at | Nipbl | Nipped-B homolog (Drosophila) | -1.13 | 0.473 | 1.03 | 0.93 | -1.78 | 0.021 | -1.13 | 0.421 | -0.75 |
| 1425254_at | Foxn4 | forkhead box N4 | -2.23 | 0.103 | 1.25 | 0.708 | -1.28 | 0.675 | -1.14 | 0.835 | -0.85 |
| 1458342_at | Tmem90a | transmembrane protein 90a | -1.22 | 0.621 | 1.21 | 0.622 | -2.21 | 0.012 | -1.49 | 0.401 | -0.93 |
| 1458799_at | A330033J07Rik | RIKEN cDNA A330033J07 gene | -1.53 | 0.111 | 1.19 | 0.691 | -1.58 | 0.432 | -1.69 | 0.371 | -0.9 |
| 1447795_at | Bspry | B-box and SPRY domain containing | 1.04 | 0.731 | -2.02 | 0.491 | -1.07 | 0.427 | -2.11 | 0.106 | -1.04 |

| | | | | | | | | | | | |
|---|---|---|---|---|---|---|---|---|---|---|---|
| 1451202_at | C330007P06Rik | RIKEN cDNA C330007P06 gene | -1.13 | 0.208 | -1.19 | 0.056 | -1.33 | 0.002 | 1.2 | 0.06 | -0.61 |
| 1433521_at | Ankrd13c | ankyrin repeat domain 13c | -1.2 | 0.038 | -1.24 | 0.003 | -1.2 | 0.141 | 1.05 | 0.47 | -0.65 |
| 1456396_at | --- | --- | -1.62 | 0.251 | -1.3 | 0.095 | 1.09 | 0.661 | -1.15 | 0.653 | -0.74 |
| 1450222_x_at | Klk1b4 | kallikrein 1-related pepidase b4 | -1.3 | 0.365 | -1.07 | 0.85 | -1.29 | 0.544 | 1.41 | 0.552 | -0.56 |
| 1458111_at | C530043G21Rik | RIKEN cDNA C530043G21 gene | 1.14 | 0.711 | -1.31 | 0.572 | -1.73 | 0.032 | -1.43 | 0.149 | -0.83 |
| 1419785_at | AA516738 | expressed sequence AA516738 | -1.29 | 0.398 | -1.7 | 0.427 | 1.12 | 0.848 | -1.31 | 0.667 | -0.8 |
| 1457491_at | --- | --- | 1.09 | 0.456 | -1.22 | 0.427 | -1.75 | 0.004 | -1.54 | 0.05 | -0.86 |
| 1437720_at | Lgtn | Ligatin | -1.29 | 0.403 | -1.32 | 0.359 | -1.06 | 0.771 | 1.22 | 0.576 | -0.61 |
| 1457470_at | D3Ertd452e | DNA segment, Chr 3, ERATO Doi 452, expressed | -1.82 | 0.007 | 1.27 | 0.597 | -1.52 | 0.195 | -1.21 | 0.638 | -0.82 |
| 1430061_at | 1700105P06Rik | RIKEN cDNA 1700105P06 gene | -1.01 | 0.958 | 1.01 | 0.959 | -2.09 | 0.354 | -1.35 | 0.375 | -0.86 |
| 1450875_at | Gpr37 | G protein-coupled receptor 37 | 1.13 | 0.862 | -1.39 | 0.554 | -1.6 | 0.138 | -1.5 | 0.385 | -0.84 |
| 1460443_at | Brms1l | breast cancer metastasis-suppressor 1-like | -1.32 | 0.203 | -1.16 | 0.287 | -1.17 | 0.199 | 1.21 | 0.342 | -0.61 |
| 1445078_at | D7Ertd526e | DNA segment, Chr 7, ERATO Doi 526, expressed | -2.29 | 0.053 | -1.49 | 0.472 | 1.37 | 0.576 | -1.66 | 0.394 | -1.02 |
| 1447240_at | --- | --- | 1.1 | 0.695 | -1.09 | 0.596 | -2.16 | 0.017 | -1.36 | 0.345 | -0.88 |
| 1439488_at | --- | 7 days embryo whole body cDNA, RIKEN full-length enriched library, clone:C430 | -1.28 | 0.066 | -1.17 | 0.498 | -1.19 | 0.339 | 1.53 | 0.03 | -0.53 |
| 1434977_at | 4933403F05Rik | RIKEN cDNA 4933403F05 gene | -1.17 | 0.441 | -1.12 | 0.38 | -1.37 | 0.086 | 1.21 | 0.494 | -0.61 |
| 1435399_at | 2310068J10Rik | RIKEN cDNA 2310068J10 gene | 1.02 | 0.94 | -1.25 | 0.617 | -1.52 | 0.076 | -1.93 | 0.066 | -0.92 |
| 1419844_a_at | LOC545086 | hypothetical protein LOC545086 | 1.05 | 0.912 | -1.1 | 0.887 | -1.94 | 0.056 | -1.57 | 0.282 | -0.89 |
| 1458321_at | Pik3cd | phosphatidylinositol 3-kinase catalytic delta polypeptide | -1.03 | 0.781 | -1.51 | 0.394 | -1.18 | 0.48 | 1.74 | 0.304 | -0.5 |
| 1430662_at | 9430091E24Rik | RIKEN cDNA 9430091E24 gene | -1.5 | 0.19 | -1.24 | 0.639 | 1.01 | 0.979 | -1.41 | 0.006 | -0.79 |
| 1460016_at | Tmem164 | transmembrane protein 164 | -1.24 | 0.05 | 1.11 | 0.776 | -1.78 | 0.067 | -1.19 | 0.211 | -0.77 |
| 1441013_at | C81521 | expressed sequence C81521 | -1.12 | 0.606 | 1.22 | 0.457 | -2.76 | 0.008 | -1.29 | 0.462 | -0.99 |
| 1457851_at | D10Ertd276e | DNA segment, Chr 10, ERATO Doi 276, expressed | -1.06 | 0.893 | -1.28 | 0.132 | -1.33 | 0.02 | 5.57 | 0.14 | 0.48 |
| 1428756_at | Aasdhppt | aminoadipate-semialdehyde dehydrogenase-phosphopantetheinyl transferase | -1.18 | 0.02 | -1.2 | 0.096 | -1.26 | 0.036 | 1.12 | 0.587 | -0.63 |
| 1430989_a_at | 1700020I14Rik | RIKEN cDNA 1700020I14 gene | -1.09 | 0.665 | -1.04 | 0.631 | -1.67 | 0.018 | 1.24 | 0.35 | -0.64 |
| 1447106_at | Lrrk2 | Leucine-rich repeat kinase 2 | -1.9 | 0.468 | -1.08 | 0.614 | 1.03 | 0.956 | -1.5 | 0.699 | -0.86 |
| 1445464_at | --- | --- | -1.84 | 0.562 | 1.41 | 0.547 | -1.89 | 0.207 | -1.49 | 0.319 | -0.95 |
| 1434233_at | 2610030H06Rik | RIKEN cDNA 2610030H06 gene | -1.46 | 0.155 | -1.4 | 0.026 | 1.08 | 0.719 | -1.13 | 0.584 | -0.73 |
| 1440373_at | Elavl1 | ELAV (embryonic lethal, abnormal vision, Drosophila)-like 1 (Hu antigen R) | 1.01 | 0.966 | -1.09 | 0.636 | -1.83 | 0.014 | -1.05 | 0.744 | -0.74 |
| 1440691_at | Cyp2j6 | cytochrome P450, family 2, subfamily j, polypeptide 6 | -1.33 | 0.17 | -1.08 | 0.64 | -1.24 | 0.329 | 1.08 | 0.616 | -0.64 |
| 1438569_at | Ccdc38 | coiled-coil domain containing 38 | -1.21 | 0.788 | -1.14 | 0.708 | -1.29 | 0.62 | 1.71 | 0.373 | -0.48 |
| 1432402_at | 4930402F11Rik | RIKEN cDNA 4930402F11 gene | -1.78 | 0.188 | 1.32 | 0.533 | -1.67 | 0.176 | -1.03 | 0.931 | -0.79 |
| 1440929_at | --- | --- | -1.44 | 0.396 | -1.15 | 0.697 | -1.09 | 0.637 | 1.24 | 0.174 | -0.61 |
| 1457180_at | Dip2c | DIP2 disco-interacting protein 2 homolog C (Drosophila) | -1.01 | 0.969 | -1.11 | 0.702 | -1.7 | 0.119 | 1.73 | 0.312 | -0.52 |
| 1451319_at | Senp1 | SUMO1/sentrin specific peptidase 1 | -1.23 | 0.532 | -1.36 | 0.522 | -1.08 | 0.735 | 1.23 | 0.282 | -0.61 |
| 1443184_at | Cdc14a | CDC14 cell division cycle 14 homolog A (S. cerevisiae) | 1.09 | 0.746 | -1.7 | 0.188 | -1.24 | 0.321 | -1.27 | 0.178 | -0.78 |
| 1444207_at | Cml3 | Camello-like 3 | -1.07 | 0.78 | -1.61 | 0.038 | -1.08 | 0.753 | 1.61 | 0.203 | -0.54 |
| 1427147_at | F730047E07Rik | RIKEN cDNA F730047E07 gene | -1.23 | 0.529 | -1.37 | 0.195 | -1.07 | 0.688 | 2.21 | 0.186 | -0.37 |
| 1453415_at | Zc3h6 | zinc finger CCCH type containing 6 | -1.08 | 0.84 | -1.3 | 0.328 | -1.27 | 0.547 | 1.02 | 0.98 | -0.66 |
| 1452879_at | Synpo2 | synaptopodin 2 | 1.29 | 0.109 | -3.74 | 0.003 | -1.09 | 0.751 | -1.14 | 0.585 | -1.17 |
| 1435562_at | Pdzd8 | PDZ domain containing 8 | -1.09 | 0.405 | -1.48 | 0.115 | -1.13 | 0.415 | 1.23 | 0.475 | -0.62 |
| 1444356_at | Rab6ip2 | Rab6 interacting protein 2 | 1.03 | 0.913 | -1.48 | 0.39 | -1.29 | 0.654 | -1.38 | 0.614 | -0.78 |
| 1423979_a_at | Slc25a29 | solute carrier family 25 (mitochondrial carrier, palmitoylcarnitine transporter), men | -1.25 | 0.352 | -1.75 | 0.127 | 1.11 | 0.586 | -1.16 | 0.627 | -0.76 |
| 1432115_a_at | Pign | phosphatidylinositol glycan anchor biosynthesis, class N | -2.74 | 0.342 | 1.23 | 0.671 | -1.13 | 0.858 | -1.34 | 0.609 | -0.99 |
| 1443628_at | --- | --- | -1.12 | 0.693 | -1.07 | 0.763 | -1.53 | 0.21 | 1.16 | 0.469 | -0.64 |
| 1420298_at | --- | --- | -2.54 | 0.373 | -2.46 | 0.432 | 1.68 | 0.245 | -1.08 | 0.901 | -1.1 |
| 1425475_at | Col4a5 | procollagen, type IV, alpha 5 | -1.53 | 0.005 | -1.47 | 0.041 | 1.15 | 0.608 | -1.21 | 0.41 | -0.77 |
| 1424199_at | Seh1l | SEH1-like (S. cerevisiae | -1.26 | 0.025 | -1.25 | 0.03 | -1.13 | 0.321 | 1.45 | 0.196 | -0.55 |
| 1429106_at | 4921509J17Rik | RIKEN cDNA 4921509J17 gene | -1.37 | 0.053 | -1.17 | 0.492 | -1.12 | 0.518 | 2.01 | 0.255 | -0.41 |
| 1442417_at | Med8 | mediator of RNA polymerase II transcription, subunit 8 homolog (yeast) | -1.22 | 0.185 | -1.18 | 0.144 | -1.23 | 0.249 | 1.12 | 0.249 | -0.63 |
| 1420174_s_at | --- | --- | -1.28 | 0.49 | -1.13 | 0.449 | -1.22 | 0.178 | 1.02 | 0.898 | -0.65 |
| 1443838_x_at | Fads2 | fatty acid desaturase 2 | -1.21 | 0.214 | -1.36 | 0.451 | -1.09 | 0.841 | 1.12 | 0.847 | -0.63 |
| 1435167_at | Ranbp6 | RAN binding protein 6 | -1.15 | 0.549 | -1.17 | 0.291 | -1.32 | 0.051 | 1.17 | 0.448 | -0.62 |

| | | | | | | | | | | | |
|---|---|---|---|---|---|---|---|---|---|---|---|
| 1435162_at | Prkg2 | protein kinase, cGMP-dependent, type II | -1.05 | 0.828 | -1.41 | 0.12 | -1.22 | 0.066 | 1.14 | 0.831 | -0.64 |
| 1440221_at | AA408650 | expressed sequence AA408650 | -1.2 | 0.497 | -1.31 | 0.144 | -1.12 | 0.181 | 1.18 | 0.591 | -0.61 |
| 1447048_at | 6720458F09Rik | RIKEN cDNA 6720458F09 gene | -1.85 | 0.417 | -1.52 | 0.228 | 1.28 | 0.621 | -1.11 | 0.328 | -0.8 |
| 1432391_at | Ccdc21 | coiled-coil domain containing 21 | -1.01 | 0.949 | -1.09 | 0.673 | -1.73 | 0.207 | 1.06 | 0.504 | -0.69 |
| 1430500_s_at | Mtx2 | metaxin 2 | -1.3 | 0.005 | -1.31 | 0.008 | -1.06 | 0.327 | 1.04 | 0.825 | -0.66 |
| 1442844_at | A830052D11Rik | RIKEN cDNA A830052D11 gene | 1.1 | 0.801 | -1.38 | 0.405 | -1.53 | 0.461 | -1.12 | 0.616 | -0.73 |
| 1445428_at | --- | --- | 1.1 | 0.703 | -1.31 | 0.443 | -1.62 | 0.068 | -1.4 | 0.256 | -0.81 |
| 1444775_at | 9930033D15Rik | RIKEN cDNA 9930033D15 gene | 1.41 | 0.172 | -2.73 | 0.199 | -1.41 | 0.185 | -1.12 | 0.735 | -0.96 |
| 1451856_at | Tnrc15 | trinucleotide repeat containing 15 | -1.58 | 0.231 | -1.17 | 0.787 | -1.01 | 0.981 | 1.1 | 0.641 | -0.66 |
| 1450600_at | Olfr1508 | olfactory receptor 1508 | -1.09 | 0.608 | -1.3 | 0.391 | -1.26 | 0.172 | 1.4 | 0.013 | -0.56 |
| 1440567_at | D030051N19Rik | RIKEN cDNA D030051N19 gene | 1.12 | 0.428 | -1.37 | 0.299 | -1.56 | 0.192 | -1.24 | 0.267 | -0.77 |
| 1443008_at | Msi2 | Musashi homolog 2 (Drosophila) | -1.43 | 0.254 | -1.04 | 0.86 | -1.21 | 0.678 | 1.17 | 0.582 | -0.63 |
| 1457975_at | Gtpbp3 | GTP binding protein 3 | -1.87 | 0.213 | -1.07 | 0.828 | 1.01 | 0.983 | -1.2 | 0.477 | -0.78 |
| 1458007_at | Myo1b | myosin IB | -1 | 0.997 | -1.02 | 0.931 | -1.97 | 0.002 | 1.32 | 0.512 | -0.67 |
| 1446580_at | 9430083G14Rik | RIKEN cDNA 9430083G14 gene | 1.07 | 0.766 | -1.21 | 0.767 | -1.69 | 0.128 | -1.72 | 0.282 | -0.89 |
| 1418938_at | Dio2 | deiodinase, iodothyronine, type II | -1.18 | 0.36 | -1.56 | 0.355 | -1 | 0.998 | 1.14 | 0.758 | -0.65 |
| 1427848_at | Tcrb-V13 | T-cell receptor beta, variable 13 | -4.95 | 0.08 | -1.97 | 0 | 1.77 | 0.13 | -1.65 | 0.423 | -1.7 |
| 1439487_at | Lig4 | ligase IV, DNA, ATP-dependent | 1.19 | 0.48 | -1.68 | 0.051 | -1.44 | 0.008 | -1.29 | 0.301 | -0.8 |
| 1441466_at | Sfrs10 | splicing factor, arginine/serine-rich 10 (transformer 2 homolog, Drosophila) | -1.27 | 0.141 | -1.17 | 0.58 | -1.19 | 0.615 | 1.39 | 0.236 | -0.56 |
| 1444145_at | Mettl6 | Methyltransferase like 6 | -1.31 | 0.432 | -1.02 | 0.946 | -1.35 | 0.1 | 1.04 | 0.903 | -0.66 |
| 1449140_at | Nudcd2 | NudC domain containing 2 | -1.45 | 0.17 | -1.03 | 0.811 | -1.22 | 0.403 | 1.69 | 0.234 | -0.5 |
| 1421055_at | Lats2 /// Xpo4 | large tumor suppressor 2 /// exportin 4 | -1.15 | 0.457 | -1.12 | 0.547 | -1.37 | 0.029 | 1.33 | 0.273 | -0.58 |
| 1430183_at | Pbx1 | Pre B-cell leukemia transcription factor 1 | -1.01 | 0.982 | 1.08 | 0.798 | -2.41 | 0.018 | -1.39 | 0.124 | -0.93 |
| 1447874_x_at | Smpd1 | sphingomyelin phosphodiesterase 1, acid lysosomal | -1.41 | 0.339 | -1.01 | 0.942 | -1.28 | 0.248 | 1.29 | 0.088 | -0.6 |
| 1458925_at | Zfp82 | zinc finger protein 82 | -1.41 | 0.659 | -1.05 | 0.888 | -1.22 | 0.512 | 1.08 | 0.87 | -0.65 |
| 1454354_at | 8030476L19Rik | RIKEN cDNA 8030476L19 gene | -1.02 | 0.813 | -1.4 | 0.073 | -1.26 | 0.298 | 1.37 | 0.073 | -0.58 |
| 1457802_at | B930012P20Rik | RIKEN cDNA B930012P20 gene | -1.19 | 0.307 | 1.16 | 0.459 | -2.06 | 0.032 | -1.26 | 0.588 | -0.84 |
| 1444438_at | Cib3 | calcium and integrin binding family member 3 | 1.55 | 0.425 | -2.39 | 0.051 | -1.96 | 0.15 | -1.56 | 0.395 | -1.09 |
| 1458280_at | Ascc2 | Activating signal cointegrator 1 complex subunit 2 | -1.34 | 0.626 | -1.4 | 0.472 | 1.02 | 0.891 | -1.44 | 0.099 | -0.79 |
| 1460614_at | Mier3 | mesoderm induction early response 1, family member 3 | -1.19 | 0.053 | -1.05 | 0.367 | -1.46 | 0.005 | 2.05 | 0.243 | -0.41 |
| 1444746_at | Ptbp2 | Polypyrimidine tract binding protein 2 | 1.07 | 0.602 | -1.17 | 0.167 | -1.79 | 0.002 | -1.3 | 0.142 | -0.8 |
| 1433750_at | Slc31a1 | solute carrier family 31, member 1 | -1.14 | 0.161 | -1.03 | 0.612 | -1.57 | 0.045 | 1.33 | 0.444 | -0.6 |
| 1443505_at | Prok1 | Prokineticin 1 | 1.33 | 0.16 | -3.26 | 0.013 | -1.19 | 0.657 | -1.11 | 0.372 | -1.06 |
| 1449090_a_at | Yes1 | Yamaguchi sarcoma viral (v-yes) oncogene homolog 1 | -1.27 | 0.398 | -1.04 | 0.795 | -1.35 | 0.042 | 1.02 | 0.804 | -0.66 |
| 1430738_at | 4833419K08Rik | RIKEN cDNA 4833419K08 gene | -2.27 | 0.44 | -1.23 | 0.522 | 1.23 | 0.534 | -3.01 | 0.179 | -1.32 |
| 1438313_at | --- | Transcribed locus | 1.02 | 0.9 | -1.94 | 0.183 | -1.06 | 0.884 | -1.8 | 0.072 | -0.94 |
| 1441531_at | Plcb4 | phospholipase C, beta 4 | -1.05 | 0.712 | 1.11 | 0.682 | -2.36 | 0.001 | -1.58 | 0.049 | -0.97 |
| 1436728_s_at | Rtel1 | regulator of telomere elongation helicase 1 | -1.47 | 0.017 | -1.18 | 0.199 | -1.05 | 0.892 | 1.21 | 0.492 | -0.62 |
| 1435950_at | Hr | hairless | -1.68 | 0.05 | 1.11 | 0.757 | -1.29 | 0.353 | -1.75 | 0.08 | -0.9 |
| 1453377_at | Sh2d4a | SH2 domain containing 4A | -1.23 | 0.036 | -1.07 | 0.599 | -1.36 | 0.102 | 1.51 | 0.298 | -0.54 |
| 1424029_at | Tspyl4 | TSPY-like 4 | -1.11 | 0.423 | -1.95 | 0.107 | 1.07 | 0.386 | -1.69 | 0.089 | -0.92 |
| 1420208_at | --- | --- | -1.27 | 0.723 | -1.48 | 0.639 | 1.02 | 0.923 | -1.66 | 0.531 | -0.85 |
| 1443741_x_at | Whsc1 /// LOC6223 | Wolf-Hirschhorn syndrome candidate 1 (human) /// similar to Wolf-Hirschhorn syn | -1.13 | 0.686 | -1.16 | 0.484 | -1.35 | 0.299 | 1.06 | 0.796 | -0.65 |
| 1434733_at | Stk36 | serine/threonine kinase 36 (fused homolog, Drosophila) | -1.89 | 0.025 | -1.19 | 0.557 | 1.12 | 0.528 | -1.14 | 0.753 | -0.78 |
| 1429305_at | Ankrd10 | ankyrin repeat domain 10 | -1.12 | 0.222 | -1.43 | 0.096 | -1.13 | 0.27 | 1.87 | 0.345 | -0.45 |
| 1433088_at | 5330430C04Rik | RIKEN cDNA 5330430C04 gene | -1.79 | 0.077 | -1.11 | 0.564 | 1.02 | 0.938 | -1.23 | 0.418 | -0.78 |
| 1417957_a_at | Tspan1 | tetraspanin 1 | -1.04 | 0.708 | -1.64 | 0.121 | -1.1 | 0.705 | 1.34 | 0.162 | -0.61 |
| 1457385_at | --- | --- | -1.35 | 0.257 | -1.02 | 0.956 | -1.3 | 0.284 | 1.19 | 0.484 | -0.62 |
| 1449733_s_at | Siah1a | seven in absentia 1A | -1.18 | 0.334 | -1.1 | 0.101 | -1.38 | 0.042 | 1.1 | 0.611 | -0.64 |
| 1436744_x_at | Fbxw14 /// LOC668 | F-box and WD-40 domain protein 14 /// similar to F-box and WD-40 domain prote | -1.68 | 0.213 | -1.62 | 0.427 | 1.27 | 0.448 | -1.02 | 0.969 | -0.76 |
| 1436700_a_at | Ddx48 | DEAD (Asp-Glu-Ala-Asp) box polypeptide 48 | 1.29 | 0.644 | -1.98 | 0.056 | -1.45 | 0.303 | -1.39 | 0.665 | -0.88 |
| 1458361_at | Dclre1c | DNA cross-link repair 1C, PSO2 homolog (S. cerevisiae) | -1.49 | 0.281 | -1.23 | 0.629 | 1 | 0.992 | -1.21 | 0.431 | -0.73 |
| 1425286_at | Crygn | crystallin, gamma N | -1.38 | 0.077 | -1.24 | 0.705 | -1.05 | 0.928 | 1.42 | 0.585 | -0.56 |

| | | | | | | | | | | | |
|---|---|---|---|---|---|---|---|---|---|---|---|
| 1441211_at | Kcns2 | K+ voltage-gated channel, subfamily S, 2 | -1.41 | 0.496 | -1.3 | 0.664 | 1 | 0.989 | -1.54 | 0.363 | -0.81 |
| 1455100_at | Akr1d1 | aldo-keto reductase family 1, member D1 | -1.7 | 0.256 | -1.07 | 0.362 | -1.04 | 0.916 | 1.72 | 0.366 | -0.52 |
| 1442876_at | Mapk1 | Mitogen activated protein kinase 1 | -1.99 | 0.419 | 1.61 | 0.191 | -2.66 | 0.035 | -1.21 | 0.682 | -1.06 |
| 1417645_at | Sspn | sarcospan | -1.07 | 0.925 | -2.8 | 0.01 | 1.19 | 0.245 | -1.25 | 0.582 | -0.98 |
| 1429545_at | Ube2i /// F830028C | ubiquitin-conjugating enzyme E2I /// RIKEN cDNA F830028O17 gene /// similar tc | -1.33 | 0.028 | -1.15 | 0.729 | -1.15 | 0.094 | 1.02 | 0.898 | -0.65 |
| 1431570_at | 4930588G17Rik | RIKEN cDNA 4930588G17 gene | -1.57 | 0.435 | 1.1 | 0.834 | -1.33 | 0.538 | -1.01 | 0.977 | -0.7 |
| 1428221_at | Klhdc8b | kelch domain containing 8B | 1.08 | 0.852 | -1.37 | 0.437 | -1.49 | 0.033 | -1.2 | 0.76 | -0.74 |
| 1439882_at | Sec23ip | Sec23 interacting protein | -1.01 | 0.974 | -1.28 | 0.506 | -1.4 | 0.218 | 1.24 | 0.145 | -0.61 |
| 1424037_at | Itpka | inositol 1,4,5-trisphosphate 3-kinase A | -2.31 | 0.068 | -1.42 | 0.556 | 1.35 | 0.202 | -1.69 | 0.199 | -1.02 |
| 1421242_at | Rnf144 | ring finger protein 144 | -3.37 | 0.041 | -1.34 | 0.218 | 1.45 | 0.473 | -1.14 | 0.789 | -1.1 |
| 1422589_at | Rab3a | RAB3A, member RAS oncogene family | -1.12 | 0.7 | -2.38 | 0.361 | 1.18 | 0.372 | -1.12 | 0.787 | -0.86 |
| 1427754_a_at | Dnm1 | dynamin 1 | 1.1 | 0.654 | -1.69 | 0.341 | -1.26 | 0.231 | -1.23 | 0.352 | -0.77 |
| 1458908_at | Dtna | Dystrobrevin alpha | -1.03 | 0.954 | 1.08 | 0.811 | -2.31 | 0.118 | -2.27 | 0.165 | -1.13 |
| 1460006_at | Atbf1 | AT motif binding factor 1 | -1.01 | 0.959 | -1.08 | 0.684 | -1.74 | 0.035 | 1.12 | 0.666 | -0.68 |
| 1443952_at | Thra | thyroid hormone receptor alpha | -1.13 | 0.575 | -1.01 | 0.917 | -1.62 | 0.086 | 1.37 | 0.111 | -0.6 |
| 1436730_at | Clcc1 | chloride channel CLIC-like 1 | -1.23 | 0.04 | 1.09 | 0.789 | -1.71 | 0.093 | -1.08 | 0.706 | -0.73 |
| 1423220_at | Eif4e /// LOC63052 | eukaryotic translation initiation factor 4E /// hypothetical LOC630527 | -1.12 | 0.343 | -1.08 | 0.633 | -1.51 | 0.001 | 1.62 | 0.31 | -0.52 |
| 1429161_at | Arv1 | ARV1 homolog (yeast) | -1.36 | 0.138 | -1.4 | 0.16 | 1.04 | 0.851 | -1.45 | 0.086 | -0.79 |
| 1455675_a_at | Tial1 | Tial1 cytotoxic granule-associated RNA binding protein-like 1 | -1.24 | 0.247 | -1.16 | 0.288 | -1.22 | 0.202 | 1.26 | 0.256 | -0.59 |
| 1460533_at | 4933440K10Rik | RIKEN cDNA 4933440K10 gene | -1.77 | 0.532 | -1.77 | 0.419 | 1.36 | 0.486 | -2.33 | 0.303 | -1.13 |
| 1427320_at | Copg2as2 | coatomer protein complex, subunit gamma 2, antisense 2 | -1.16 | 0.63 | 1.15 | 0.315 | -2.1 | 0.001 | -1.37 | 0.341 | -0.87 |
| 1440847_at | Mtss1 | metastasis suppressor 1 | -1.8 | 0.005 | -1.18 | 0.043 | 1.09 | 0.818 | -1.9 | 0.124 | -0.95 |
| 1431387_at | Anxa11 | Annexin A11 | -2.7 | 0.464 | -1.65 | 0.431 | 1.51 | 0.357 | -1.25 | 0.412 | -1.02 |
| 1439597_at | 4932417H02Rik | RIKEN cDNA 4932417H02 gene | -1.41 | 0.149 | -1.06 | 0.838 | -1.2 | 0.436 | 1 | 0.984 | -0.67 |
| 1453845_at | 4733401D01Rik | RIKEN cDNA 4733401D01 gene | -1.8 | 0.37 | 1.1 | 0.874 | -1.2 | 0.716 | -1.39 | 0.629 | -0.82 |
| 1448426_at | Sardh | sarcosine dehydrogenase | -1.22 | 0.228 | -1.24 | 0.285 | -1.16 | 0.709 | 1.59 | 0.445 | -0.51 |
| 1455475_at | 3110057O12Rik | RIKEN cDNA 3110057O12 gene | -1.09 | 0.563 | -1.27 | 0.294 | -1.28 | 0.31 | 1.03 | 0.884 | -0.65 |
| 1446475_at | Mbnl2 | Muscleblind-like 2 | -1.34 | 0.382 | 1.25 | 0.34 | -2.03 | 0.033 | -1.54 | 0.146 | -0.91 |
| 1422241_a_at | Ndufa1 | NADH dehydrogenase (ubiquinone) 1 alpha subcomplex, 1 | -1.24 | 0.009 | -1.17 | 0.081 | -1.21 | 0.003 | 1.07 | 0.531 | -0.64 |
| 1437321_at | --- | --- | -2.77 | 0.108 | -1.26 | 0.446 | 1.34 | 0.579 | -1.2 | 0.845 | -0.97 |
| 1449771_at | --- | --- | -1.51 | 0.494 | 1.11 | 0.794 | -1.4 | 0.415 | -1.09 | 0.842 | -0.72 |
| 1444974_at | AU023617 | expressed sequence AU023617 | -1.34 | 0.691 | 1.06 | 0.715 | -1.46 | 0.103 | -1.34 | 0.581 | -0.77 |
| 1443777_at | --- | --- | -1.64 | 0.472 | -1.24 | 0.526 | 1.07 | 0.808 | -1.72 | 0.349 | -0.88 |
| 1460005_at | AF013969 /// LOC6 | expressed sequence AF013969 /// similar to CG5514-PB, isoform B /// region cor | -1.1 | 0.726 | -1.53 | 0.134 | -1.08 | 0.693 | 1.18 | 0.473 | -0.63 |
| 1446126_at | --- | Transcribed locus | -1.87 | 0.041 | 1.08 | 0.52 | -1.15 | 0.568 | -1.15 | 0.473 | -0.77 |
| 1428588_a_at | Mrpl41 | mitochondrial ribosomal protein L41 | -1.01 | 0.903 | -1.13 | 0.416 | -1.61 | 0.025 | 1.19 | 0.262 | -0.64 |
| 1422979_at | Suv39h2 | suppressor of variegation 3-9 homolog 2 (Drosophila) | -1.14 | 0.805 | -1.18 | 0.763 | -1.31 | 0.523 | 3.91 | 0.238 | 0.07 |
| 1431844_at | Kcnmb2 | potassium large conductance calcium-activated channel, subfamily M, beta meml | -1.61 | 0.021 | -1.6 | 0.574 | 1.25 | 0.221 | -1.75 | 0.218 | -0.93 |
| 1433437_at | Dock8 | dedicator of cytokinesis 8 | -1.97 | 0.087 | -1.54 | 0.108 | 1.33 | 0.666 | -2.25 | 0.385 | -1.11 |
| 1443819_x_at | Fis1 | fission 1 (mitochondrial outer membrane) homolog (yeast) | 1.03 | 0.85 | -1.13 | 0.403 | -1.73 | 0.053 | -1.24 | 0.436 | -0.77 |
| 1457178_at | Rabgap1l | RAB GTPase activating protein 1-like | -1.33 | 0.345 | -1.12 | 0.522 | -1.18 | 0.485 | 1.2 | 0.159 | -0.61 |
| 1427540_at | Zwint | ZW10 interactor | -1.14 | 0.176 | -1.26 | 0.418 | -1.21 | 0.252 | 1.33 | 0.47 | -0.57 |
| 1449676_at | Rab2 | RAB2, member RAS oncogene family | 1.11 | 0.682 | -1.15 | 0.552 | -1.97 | 0.045 | -1.9 | 0.172 | -0.98 |
| 1460668_at | Gal | galanin | -1.26 | 0.514 | 1.11 | 0.809 | -1.71 | 0.13 | -1.14 | 0.771 | -0.75 |
| 1426718_at | Skiv2l2 | superkiller viralicidic activity 2-like 2 (S. cerevisiae) | -1.12 | 0.597 | -1.25 | 0.013 | -1.25 | 0.06 | 1 | 0.98 | -0.65 |
| 1453396_at | 1700123D08Rik | RIKEN cDNA 1700123D08 gene | -1.68 | 0.278 | -1.3 | 0.447 | 1.13 | 0.861 | -1.43 | 0.534 | -0.82 |
| 1458460_at | Stim1 | Stromal interaction molecule 1 | -1.15 | 0.723 | 1 | 0.983 | -1.62 | 0.032 | -1.35 | 0.156 | -0.78 |
| 1420053_at | Psmb1 | Proteasome (prosome, macropain) subunit, beta type 1 | -1.15 | 0.663 | -1.13 | 0.548 | -1.35 | 0.342 | 1.04 | 0.883 | -0.65 |
| 1460577_at | AA591059 | Expressed sequence AA591059 | -1.19 | 0.048 | -1.13 | 0.367 | -1.31 | 0.097 | 1.4 | 0.263 | -0.56 |
| 1456866_x_at | 1700027D21Rik | RIKEN cDNA 1700027D21 gene | 1.19 | 0.585 | -1.76 | 0.133 | -1.37 | 0.591 | -1.19 | 0.646 | -0.78 |
| 1448532_at | Prlpc2 | prolactin-like protein C 2 | -2.25 | 0.271 | 1.55 | 0.249 | -2.01 | 0.252 | -1.31 | 0.618 | -1 |
| 1435160_at | 1110064P04Rik | RIKEN cDNA 1110064P04 gene | -1.05 | 0.847 | -1.07 | 0.697 | -1.64 | 0.023 | 1.08 | 0.52 | -0.67 |
| 1427053_at | Abi3bp | ABI gene family, member 3 (NESH) binding protein | 1.28 | 0.501 | -1.7 | 0.021 | -1.61 | 0.243 | -1.62 | 0.22 | -0.91 |

| | | | | | | | | | | | |
|---|---|---|---|---|---|---|---|---|---|---|---|
| 1457890_at | D6Ertd234e | DNA segment, Chr 6, ERATO Doi 234, expressed | -1.15 | 0.807 | -1.08 | 0.638 | -1.43 | 0.44 | 2.11 | 0.261 | -0.39 |
| 1444338_at | --- | --- | -3.87 | 0.009 | 1.26 | 0.763 | -1.02 | 0.951 | -1.23 | 0.696 | -1.22 |
| 1428761_a_at | Snapc3 | small nuclear RNA activating complex, polypeptide 3 | 1.22 | 0.446 | -1.6 | 0.07 | -1.54 | 0.011 | -1.41 | 0.431 | -0.83 |
| 1447748_x_at | Slc29a2 | solute carrier family 29 (nucleoside transporters), member 2 | 1.06 | 0.756 | -1.57 | 0.282 | -1.25 | 0.607 | -1.12 | 0.824 | -0.72 |
| 1417005_at | Kns2 | kinesin 2 | -1.05 | 0.856 | -1.46 | 0.034 | -1.17 | 0.526 | 1.08 | 0.661 | -0.65 |
| 1437362_at | A930028L21Rik | RIKEN cDNA A930028L21 gene | -1.16 | 0.504 | -1.6 | 0.22 | 1.01 | 0.981 | -1.4 | 0.163 | -0.79 |
| 1445646_at | Btbd1 | BTB (POZ) domain containing 1 | 1.12 | 0.426 | -1.22 | 0.259 | -1.81 | 0.022 | -1.69 | 0.228 | -0.9 |
| 1455510_at | Spop | Speckle-type POZ protein | -1.1 | 0.305 | -1.11 | 0.104 | -1.46 | 0.093 | 1.08 | 0.737 | -0.65 |
| 1424853_s_at | Cyp4a10 /// BC013 | cytochrome P450, family 4, subfamily a, polypeptide 10 /// cDNA sequence BC01 | -1 | 0.836 | -1.97 | 0.151 | -1.01 | 0.92 | 1.55 | 0.533 | -0.61 |
| 1445134_at | Mkl2 | MKL/myocardin-like 2 | 1.37 | 0.627 | -1.94 | 0.31 | -1.65 | 0.203 | -1.76 | 0.473 | -0.99 |
| 1434800_at | Sv2b | synaptic vesicle glycoprotein 2 b | -2.09 | 0.033 | 1.18 | 0.717 | -1.2 | 0.528 | -1.36 | 0.194 | -0.87 |
| 1432814_at | 2900064F13Rik | RIKEN cDNA 2900064F13 gene | 1 | 0.996 | -1.92 | 0.409 | -1.03 | 0.869 | -2.5 | 0.252 | -1.11 |
| 1460730_at | Eif2b1 | eukaryotic translation initiation factor 2B, subunit 1 (alpha) | -1.32 | 0.2 | -1.23 | 0.035 | -1.09 | 0.505 | 1.34 | 0.341 | -0.57 |
| 1453284_at | 4930405M20Rik | RIKEN cDNA 4930405M20 gene | -1.2 | 0.221 | -1.42 | 0.011 | -1.05 | 0.51 | 1.06 | 0.734 | -0.65 |
| 1431019_at | Lrrc28 | leucine rich repeat containing 28 | -1.25 | 0.384 | -1.22 | 0.442 | -1.15 | 0.688 | 1.29 | 0.687 | -0.58 |
| 1420183_at | Lor | Loricrin | -1.3 | 0.333 | -1.16 | 0.722 | -1.16 | 0.706 | 1.01 | 0.982 | -0.65 |
| 1443012_at | Tcf12 | Transcription factor 12 | -1.33 | 0.215 | 1.21 | 0.367 | -1.89 | 0.016 | -1.65 | 0.211 | -0.91 |
| 1443247_at | Gfod1 | Glucose-fructose oxidoreductase domain containing 1 | -1.79 | 0.281 | 1.28 | 0.411 | -1.53 | 0.369 | -1.44 | 0.19 | -0.87 |
| 1458494_at | --- | Transcribed locus | 1.25 | 0.679 | -2.23 | 0.259 | -1.25 | 0.629 | -1.37 | 0.597 | -0.9 |
| 1422668_at | Serpinb9b | serine (or cysteine) peptidase inhibitor, clade B, member 9b | -1.47 | 0.299 | -1.18 | 0.581 | -1.03 | 0.938 | 1.29 | 0.142 | -0.6 |
| 1434135_at | B3galnt2 | UDP-GalNAc:betaGlcNAc beta 1,3-galactosaminyltransferase, polypeptide 2 | -1.34 | 0.124 | -1.06 | 0.593 | -1.25 | 0.192 | 1.01 | 0.95 | -0.66 |
| 1452837_at | Lpin2 | lipin 2 | -1.16 | 0.305 | -1.28 | 0.472 | -1.18 | 0.208 | 1.95 | 0.104 | -0.42 |
| 1422307_at | --- | --- | -1.03 | 0.838 | -1.22 | 0.235 | -1.41 | 0.154 | 1.52 | 0.154 | -0.54 |
| 1442264_at | Rasgrp2 | RAS, guanyl releasing protein 2 | 1.03 | 0.558 | -1.14 | 0.46 | -1.7 | 0.006 | -1.74 | 0.094 | -0.89 |
| 1433912_at | Tiprl | TIP41, TOR signalling pathway regulator-like (S. cerevisiae) | -1.19 | 0.591 | -1.21 | 0.255 | -1.21 | 0.115 | 1.04 | 0.86 | -0.64 |
| 1447596_at | --- | Transcribed locus | -1.18 | 0.662 | -1.45 | 0.473 | -1.04 | 0.299 | 1.2 | 0.281 | -0.62 |
| 1447224_at | --- | Transcribed locus | -1.82 | 0.563 | 1.14 | 0.778 | -1.25 | 0.664 | -1.33 | 0.615 | -0.81 |
| 1454957_at | Nob1 | NIN1/RPN12 binding protein 1 homolog (S. cerevisiae) | -1.03 | 0.887 | -1.47 | 0.034 | -1.2 | 0.402 | 1.12 | 0.658 | -0.64 |
| 1430414_at | 5730406G12Rik | RIKEN cDNA 5730406G12 gene | -1.21 | 0.317 | -1.38 | 0.252 | -1.06 | 0.819 | 1.28 | 0.339 | -0.59 |
| 1441574_at | Pitpnc1 | Phosphatidylinositol transfer protein, cytoplasmic 1 | -1.35 | 0.074 | 1.05 | 0.862 | -1.44 | 0.237 | -1.34 | 0.238 | -0.77 |
| 1431731_at | Bnip2 | BCL2/adenovirus E1B interacting protein 1, NIP2 | 1.12 | #DIV/0! | -1.09 | 0.365 | -2.19 | 0.301 | -1.05 | 0.907 | -0.8 |
| 1437329_at | Ptplb | protein tyrosine phosphatase-like (proline instead of catalytic arginine), member b | -1.16 | 0.324 | -1.05 | 0.132 | -1.47 | 0.084 | 1.15 | 0.656 | -0.63 |
| 1424801_at | Enah | enabled homolog (Drosophila) | 1.07 | 0.377 | -1.61 | 0.343 | -1.25 | 0.207 | -1.4 | 0.107 | -0.8 |
| 1451545_at | Tdrd3 | tudor domain containing 3 | -1.11 | 0.396 | -1.14 | 0.493 | -1.4 | 0.024 | 1.27 | 0.385 | -0.59 |
| 1439647_at | LOC545611 | hypothetical LOC545611 | -1.39 | 0.394 | -1.26 | 0.116 | -1.02 | 0.899 | 1.06 | 0.534 | -0.65 |
| 1452157_at | Eprs /// LOC63367 | glutamyl-prolyl-tRNA synthetase /// similar to Bifunctional aminoacyl-tRNA synthe | -1.25 | 0.215 | -1.11 | 0.479 | -1.26 | 0.117 | 1.33 | 0.255 | -0.57 |
| 1444391_at | --- | Transcribed locus | 1.13 | 0.473 | -2.04 | 0.25 | -1.14 | 0.535 | -1.39 | 0.336 | -0.86 |
| 1444912_at | --- | --- | 1.25 | 0.56 | -1.54 | 0.242 | -1.69 | 0.107 | -1.45 | 0.22 | -0.86 |
| 1453975_a_at | 1700029M20Rik | RIKEN cDNA 1700029M20 gene | 1.14 | 0.77 | -1.18 | 0.818 | -1.97 | 0.272 | -1.21 | 0.17 | -0.81 |
| 1450475_at | Dlx3 | distal-less homeobox 3 | -2.99 | 0.215 | -1.23 | 0.631 | 1.35 | 0.52 | -1.76 | 0.132 | -1.16 |
| 1417182_at | Dnaja2 | DnaJ (Hsp40) homolog, subfamily A, member 2 | -1.24 | 0.076 | -1.18 | 0.244 | -1.19 | 0.368 | 1.04 | 0.891 | -0.64 |
| 1450193_at | Hcn1 | hyperpolarization-activated, cyclic nucleotide-gated K+ 1 | -2.72 | 0.026 | 1.55 | 0.477 | -1.73 | 0.082 | -1.2 | 0.626 | -1.03 |
| 1453869_at | LOC328277 | hypothetical protein LOC328277 | -1.26 | 0.578 | -1.18 | 0.525 | -1.16 | 0.187 | 1.13 | 0.723 | -0.62 |
| 1444956_at | Gabarapl2 | Gamma-aminobutyric acid (GABA-A) receptor-associated protein-like 2 | -1.22 | 0.482 | -1.32 | 0.612 | -1.09 | 0.458 | 2.07 | 0.179 | -0.39 |
| 1430758_x_at | Bcas1 | breast carcinoma amplified sequence 1 | -1.65 | 0.445 | -1.38 | 0.516 | 1.16 | 0.777 | -1.81 | 0.657 | -0.92 |
| 1457971_at | Cspp1 | Centrosome and spindle pole associated protein 1 | 1.27 | 0.555 | -1.74 | 0.113 | -1.53 | 0.074 | -2.02 | 0.032 | -1.01 |
| 1432645_at | Atrn | Attractin | 1 | 0.996 | -1.19 | 0.62 | -1.54 | 0.115 | -1.14 | 0.812 | -0.71 |
| 1432316_at | 4933439N14Rik | RIKEN cDNA 4933439N14 gene | 1.03 | 0.719 | -1.83 | 0.063 | -1.08 | 0.903 | -1.04 | 0.963 | -0.73 |
| 1449999_a_at | Cacna2d1 | calcium channel, voltage-dependent, alpha2/delta subunit 1 | -2.3 | 0.065 | 1.43 | 0.587 | -1.58 | 0.014 | -1.13 | 0.832 | -0.89 |
| 1428008_at | Dnm1l | dynamin 1-like | 1.35 | 0.598 | -2.01 | 0.357 | -1.54 | 0.135 | -1.09 | 0.893 | -0.82 |
| 1424098_at | Elovl7 | ELOVL family member 7, elongation of long chain fatty acids (yeast) | -1.01 | 0.853 | -1.47 | 0.324 | -1.21 | 0.332 | 2.3 | 0.015 | -0.35 |
| 1420181_at | 1700011M02Rik /// | RIKEN cDNA 1700011M02 gene /// hypothetical protein LOC668339 | -1.34 | 0.427 | 1.03 | 0.937 | -1.39 | 0.587 | -1.91 | 0.479 | -0.9 |
| 1451690_a_at | Pvrl4 | poliovirus receptor-related 4 | -1.06 | 0.923 | -1.41 | 0.456 | -1.19 | 0.54 | 2 | 0.403 | -0.41 |

| | | | | | | | | | | | |
|---|---|---|---|---|---|---|---|---|---|---|---|
| 1436977_at | Eps8 | Epidermal growth factor receptor pathway substrate 8 | -1.19 | 0.215 | 1.17 | 0.665 | -2.07 | 0.035 | -1.12 | 0.316 | -0.8 |
| 1458605_at | Arl15 | ADP-ribosylation factor-like 15 | -1.2 | 0.65 | 1.15 | 0.453 | -1.96 | 0.034 | -1.14 | 0.719 | -0.79 |
| 1446248_at | Slc18a1 | solute carrier family 18 (vesicular monoamine), member 1 | -1.07 | 0.845 | -1.46 | 0.196 | -1.14 | 0.261 | 1.42 | 0.012 | -0.56 |
| 1416681_at | Ube3a | ubiquitin protein ligase E3A | -1.04 | 0.782 | -1.24 | 0.111 | -1.37 | 0.183 | 1.15 | 0.679 | -0.63 |
| 1455055_at | 4930562D19Rik | RIKEN cDNA 4930562D19 gene | -1.26 | 0.221 | -1.72 | 0.004 | 1.12 | 0.511 | -1.11 | 0.022 | -0.74 |
| 1434598_at | Larp5 | La ribonucleoprotein domain family, member 5 | -1.27 | 0.159 | -1.18 | 0.03 | -1.16 | 0.142 | 1.14 | 0.5 | -0.62 |
| 1422159_at | Ppef2 | protein phosphatase, EF hand calcium-binding domain 2 | -1.23 | 0.376 | -1.53 | 0.447 | 1.03 | 0.881 | -1.21 | 0.202 | -0.74 |
| 1453161_at | Thrap1 | thyroid hormone receptor associated protein 1 | -1 | 1 | -2.29 | 0.062 | 1.06 | 0.863 | -2.14 | 0.103 | -1.09 |
| 1436895_at | Centd1 | centaurin, delta 1 | 1.17 | 0.694 | -1.28 | 0.236 | -1.82 | 0.044 | -1.56 | 0.362 | -0.87 |
| 1460292_a_at | Smarca1 | SWI/SNF related, matrix associated, actin dependent regulator of chromatin, subf | 1.14 | 0.787 | -1.2 | 0.738 | -1.88 | 0.074 | -1.17 | 0.724 | -0.78 |
| 1431104_at | 4930522H14Rik | RIKEN cDNA 4930522H14 gene | -2.2 | 0.446 | 1.06 | 0.815 | -1.02 | 0.904 | -2.59 | 0.006 | -1.19 |
| 1447331_at | Fbxo42 | F-box protein 42 | -1.41 | 0.5 | -1.2 | 0.773 | -1.05 | 0.925 | 1.72 | 0.411 | -0.49 |
| 1419409_at | Sprrl5 | small proline rich-like 5 | 1.02 | 0.969 | -1.31 | 0.658 | -1.41 | 0.269 | -1.05 | 0.946 | -0.69 |
| 1454599_at | 4930425F17Rik | RIKEN cDNA 4930425F17 gene | -1.08 | 0.801 | -1.47 | 0.109 | -1.12 | 0.502 | 1.3 | 0.283 | -0.59 |
| 1432409_at | Prkar2a | protein kinase, cAMP dependent regulatory, type II alpha | 1.14 | 0.411 | -1.84 | 0.322 | -1.23 | 0.673 | -1.04 | 0.932 | -0.74 |
| 1441467_at | Tspan5 | Tetraspanin 5 | -1.09 | 0.744 | 1.21 | 0.629 | -2.68 | 0.117 | -1.61 | 0.126 | -1.04 |
| 1427049_s_at | Smo | smoothened homolog (Drosophila) | -1.07 | 0.538 | -1.1 | 0.66 | -1.53 | 0.223 | 1.05 | 0.79 | -0.66 |
| 1454014_a_at | Mkks | McKusick-Kaufman syndrome protein | -1.11 | 0.502 | -1.18 | 0.135 | -1.34 | 0.027 | 1.01 | 0.949 | -0.65 |
| 1420522_at | Ccdc50 | coiled-coil domain containing 50 | -1.47 | 0.143 | -1.57 | 0.004 | 1.18 | 0.3 | -1.21 | 0.501 | -0.77 |
| 1438403_s_at | --- | --- | 1.19 | 0.811 | -1.27 | 0.17 | -1.91 | 0.212 | -1.16 | 0.695 | -0.79 |
| 1460149_at | Atp13a5 | ATPase type 13A5 | -1.16 | 0.586 | -1.01 | 0.977 | -1.56 | 0.449 | 2.39 | 0.308 | -0.33 |
| 1422297_at | Pfdn5 | prefoldin 5 | -1.71 | 0.208 | -1.28 | 0.652 | 1.13 | 0.85 | -1.58 | 0.283 | -0.86 |
| 1458044_at | Cml1 | camello-like 1 | -1.68 | 0.437 | -1.09 | 0.857 | -1.02 | 0.956 | 1.15 | 0.742 | -0.66 |
| 1421096_at | Trpc1 | transient receptor potential cation channel, subfamily C, member 1 | -1.17 | 0.624 | -1.06 | 0.878 | -1.44 | 0.199 | 1.02 | 0.855 | -0.66 |
| 1424371_at | Psmf1 | proteasome (prosome, macropain) inhibitor subunit 1 | -1.17 | 0.631 | -1.3 | 0.335 | -1.14 | 0.392 | 1.98 | 0.159 | -0.41 |
| 1444526_at | Ssr1 | signal sequence receptor, alpha | 1.05 | 0.94 | -1.58 | 0.342 | -1.23 | 0.525 | -1.16 | 0.698 | -0.73 |
| 1454489_at | 4930513L20Rik | RIKEN cDNA 4930513L20 gene | -1.31 | 0.63 | -1.1 | 0.848 | -1.22 | 0.622 | 1.57 | 0.47 | -0.51 |
| 1444993_at | LOC664949 /// LOC | similar to mortality factor 4 like 1 (predicted) /// similar to mortality factor 4 like 1 (p | -1.08 | 0.855 | -2.22 | 0.376 | 1.13 | 0.54 | -3.18 | 0.12 | -1.34 |
| 1436277_at | D330010C22Rik | RIKEN cDNA 330010C22 gene | -1.53 | 0.152 | -1.76 | 0.09 | 1.28 | 0.68 | -2.08 | 0.006 | -1.02 |
| 1434037_s_at | Pcaf | p300/CBP-associated factor | -1.08 | 0.579 | -1.3 | 0.011 | -1.25 | 0.102 | 1.18 | 0.132 | -0.61 |
| 1420259_at | Pkp2 | Plakophilin 2 | -1.84 | 0.236 | 1.35 | 0.138 | -1.67 | 0.156 | -1.15 | 0.67 | -0.83 |
| 1420037_at | Atp5a1 | ATP synthase, H+ transporting, mitochondrial F1 complex, alpha subunit, isoform | 1.05 | 0.856 | -1.09 | 0.358 | -1.87 | 0.054 | -1.24 | 0.586 | -0.79 |
| 1455590_at | Nqo2 | NAD(P)H dehydrogenase, quinone 2 | -1.15 | 0.159 | -1.16 | 0.565 | -1.31 | 0.04 | 1.02 | 0.617 | -0.65 |
| 1433089_at | --- | --- | -1.76 | 0.535 | 1.39 | 0.296 | -1.86 | 0.336 | -2.27 | 0.366 | -1.12 |
| 1444376_at | Sesn1 | Sestrin 1 | -1.08 | 0.457 | 1.1 | 0.756 | -2.09 | 0.049 | -1.71 | 0.081 | -0.94 |
| 1439415_x_at | LOC622373 | similar to 40S ribosomal protein S21 | -1.47 | 0.116 | 1.04 | 0.767 | -1.29 | 0.102 | -1.69 | 0.147 | -0.85 |
| 1419517_at | Cnih3 | cornichon homolog 3 (Drosophila) | 1.02 | 0.973 | -1.72 | 0.107 | -1.11 | 0.823 | -1.98 | 0.37 | -0.95 |
| 1419944_at | Ccnb1 | cyclin B1 | -1.15 | 0.836 | -1.34 | 0.318 | -1.13 | 0.823 | 2.59 | 0.107 | -0.26 |
| 1424672_at | Dmxl1 | Dmx-like 1 | -1.39 | 0.18 | 1.02 | 0.906 | -1.33 | 0.168 | -1.27 | 0.098 | -0.74 |
| 1419034_at | Csnk2a1 | casein kinase 2, alpha 1 polypeptide | -1.08 | 0.836 | -1.39 | 0.193 | -1.18 | 0.524 | 1.36 | 0.187 | -0.57 |
| 1451030_at | Akr1c21 | aldo-keto reductase family 1, member C21 | -1.22 | 0.206 | -2.55 | 0.198 | 1.28 | 0.457 | -1.45 | 0.077 | -0.98 |
| 1452617_at | Ssbp1 | single-stranded DNA binding protein 1 | -1.45 | 0.408 | -1.24 | 0.149 | 1 | 0.994 | -1.56 | 0.291 | -0.81 |
| 1452582_at | Galm | galactose mutarotase | -1.14 | 0.102 | -1.27 | 0.277 | -1.19 | 0.537 | 1.36 | 0.367 | -0.56 |
| 1459619_at | Epb4.1l2 | erythrocyte protein band 4.1-like 2 | 1.19 | 0.37 | -1.23 | 0.56 | -2.02 | 0.007 | -1.63 | 0.116 | -0.92 |
| 1449084_s_at | Sh3d19 | SH3 domain protein D19 | -1.42 | 0.311 | -1.23 | 0.253 | -1.02 | 0.931 | 1.34 | 0.234 | -0.58 |
| 1452344_at | Synj2 | synaptojanin 2 | -1.66 | 0.4 | -1.16 | 0.794 | 1.03 | 0.854 | -1.75 | 0.157 | -0.88 |
| 1451827_a_at | Nox4 | NADPH oxidase 4 | -1.14 | 0.299 | -1.4 | 0.189 | -1.11 | 0.706 | 1.72 | 0.267 | -0.48 |
| 1441275_at | Kbtbd8 | kelch repeat and BTB (POZ) domain containing 8 | -1.03 | 0.963 | -1.38 | 0.546 | -1.25 | 0.641 | 1.21 | 0.512 | -0.61 |
| 1427054_s_at | Abi3bp | ABI gene family, member 3 (NESH) binding protein | 1.22 | 0.18 | -1.51 | 0.096 | -1.63 | 0.111 | -1.52 | 0.286 | -0.86 |
| 1443711_at | Chd8 | Chromodomain helicase DNA binding protein 8 | 1.04 | 0.945 | -1.53 | 0.184 | -1.25 | 0.44 | -1.19 | 0.708 | -0.73 |
| 1453594_at | 4930451G09Rik | RIKEN cDNA 4930451G09 gene | -1.02 | 0.858 | -1.68 | 0.172 | -1.08 | 0.837 | 1.02 | 0.895 | -0.69 |
| 1457363_at | LOC654469 | hypothetical protein LOC654469 | -1.55 | 0.021 | -1.36 | 0.161 | 1.12 | 0.508 | -1.32 | 0.75 | -0.78 |
| 1454322_at | 4933404M09Rik | RIKEN cDNA 4933404M09 gene | 1.14 | 0.854 | -1.25 | 0.18 | -1.79 | 0.364 | -1.68 | 0.247 | -0.89 |

| | | | | | | | | | | | |
|---|---|---|---|---|---|---|---|---|---|---|---|
| 1422040_at | Sema7a | sema domain, immunoglobulin domain (Ig), and GPI membrane anchor, (semaph | -2.54 | 0.292 | -1.68 | 0.367 | 1.51 | 0.464 | -2.29 | 0.256 | -1.25 |
| 1446805_at | Gabra2 | Gamma-aminobutyric acid (GABA-A) receptor, subunit alpha 2 | -2.2 | 0.022 | -1 | 0.997 | 1.05 | 0.88 | -2.66 | 0.206 | -1.2 |
| 1447109_at | Elavl1 | ELAV (embryonic lethal, abnormal vision, Drosophila)-like 1 (Hu antigen R) | 1.05 | 0.612 | -1.16 | 0.594 | -1.71 | 0.061 | -1.18 | 0.311 | -0.75 |
| 1424390_at | Nupl1 | Nucleoporin like 1 | -1.27 | 0.11 | -1.22 | 0.261 | -1.12 | 0.551 | 1.61 | 0.242 | -0.5 |
| 1427955_a_at | Deb1 | differentially expressed in B16F10 1 | -1.19 | 0.018 | -1.07 | 0.292 | -1.37 | 0.077 | 1.19 | 0.324 | -0.61 |
| 1428523_at | Lphn3 | latrophilin 3 | -2.05 | 0.454 | 1.12 | 0.855 | -1.12 | 0.508 | -1.69 | 0.083 | -0.93 |
| 1438719_at | AI585793 | expressed sequence AI585793 | 1.04 | 0.947 | -1.31 | 0.216 | -1.44 | 0.251 | -1.09 | 0.793 | -0.7 |
| 1452975_at | Agxt2l1 | alanine-glyoxylate aminotransferase 2-like 1 | -1.09 | 0.758 | -2.08 | 0.036 | 1.1 | 0.571 | -1.45 | 0.178 | -0.88 |
| 1451262_a_at | Jtv1 | JTV1 gene | -1.38 | 0.143 | -1.09 | 0.55 | -1.17 | 0.045 | 1.04 | 0.849 | -0.65 |
| 1433400_at | 5033405D04Rik | RIKEN cDNA 5033405D04 gene | -1.4 | 0.165 | -1.08 | 0.712 | -1.16 | 0.394 | 1.06 | 0.894 | -0.65 |
| 1425372_at | Dnajc19 | DnaJ (Hsp40) homolog, subfamily C, member 19 | -1.36 | 0.489 | -1.42 | 0.155 | 1.06 | 0.761 | -1.16 | 0.5 | -0.72 |
| 1458030_at | --- | --- | -1.95 | 0.088 | 1.11 | 0.604 | -1.14 | 0.425 | -1.51 | 0.043 | -0.87 |
| 1440320_at | --- | --- | -1.25 | 0.225 | 1.1 | 0.692 | -1.68 | 0.282 | -1.22 | 0.31 | -0.76 |
| 1453087_at | 6330403L08Rik | RIKEN cDNA 6330403L08 gene | -1.12 | 0.223 | -2.03 | 0.123 | 1.11 | 0.715 | -1.27 | 0.417 | -0.82 |
| 1447565_at | 1700082C02Rik | RIKEN cDNA 1700082C02 gene | -1.06 | 0.852 | -1.39 | 0.511 | -1.2 | 0.781 | 1.03 | 0.974 | -0.65 |
| 1458950_at | --- | Transcribed locus | 1.19 | 0.438 | -1.57 | 0.544 | -1.49 | 0.334 | -1.2 | 0.724 | -0.77 |
| 1425243_at | Cd207 | CD 207 antigen | -2.59 | 0.337 | 1.76 | 0 | -2.82 | 0.249 | -1.61 | 0.168 | -1.32 |
| 1460711_at | 4930461P20Rik | RIKEN cDNA 4930461P20 gene | -1.15 | 0.173 | -1.31 | 0.011 | -1.15 | 0.206 | 1.17 | 0.267 | -0.61 |
| 1437289_at | Impad1 | inositol monophosphatase domain containing 1 | -1.05 | 0.496 | -1.01 | 0.89 | -1.77 | 0.059 | 1.02 | 0.745 | -0.7 |
| 1454113_at | 4930472D12Rik | RIKEN cDNA 4930472D12 gene | -2.63 | 0.17 | -1.09 | 0.624 | 1.2 | 0.756 | -1.36 | 0.29 | -0.97 |
| 1453715_at | Sv2c | synaptic vesicle glycoprotein 2c | -1.36 | 0.046 | -1.04 | 0.911 | -1.25 | 0.553 | 1.22 | 0.516 | -0.61 |
| 1458493_a_at | --- | --- | -1.04 | 0.853 | -1.19 | 0.688 | -1.43 | 0.335 | 1.56 | 0.34 | -0.52 |
| 1437273_at | Htra4 | HtrA serine peptidase 4 | -1.27 | 0.012 | -1.4 | 0.23 | 1 | 0.996 | -1.04 | 0.898 | -0.68 |
| 1429282_at | Thap1 | THAP domain containing, apoptosis associated protein 1 | -1.4 | 0.232 | -1.2 | 0.337 | -1.05 | 0.863 | 1.24 | 0.206 | -0.6 |
| 1454135_at | 4930458K08Rik | RIKEN cDNA 4930458K08 gene | -1.55 | 0.34 | -1.16 | 0.523 | -1 | 0.992 | 1.02 | 0.952 | -0.67 |
| 1444216_at | Emx1 | empty spiracles homolog 1 (Drosophila) | 1.03 | 0.957 | -2.08 | 0.069 | -1.01 | 0.968 | -1.14 | 0.874 | -0.8 |
| 1451472_at | 3230401I01Rik | RIKEN cDNA 3230401I01 gene | 1.1 | 0.79 | -1.51 | 0.628 | -1.36 | 0.691 | -1.64 | 0.369 | -0.85 |
| 1435360_at | Zfp651 | zinc finger protein 651 | -1.18 | 0.256 | -1.41 | 0.051 | -1.06 | 0.789 | 1.42 | 0.286 | -0.56 |
| 1431540_at | 4930511E03Rik | RIKEN cDNA 4930511E03 gene | -1.33 | 0.624 | -1.01 | 0.979 | -1.31 | 0.401 | 1.12 | 0.749 | -0.63 |
| 1433421_at | 4930527J03Rik | RIKEN cDNA 4930527J03 gene | -1.82 | 0.199 | -1.01 | 0.976 | -1.04 | 0.931 | 1.31 | 0.102 | -0.64 |
| 1416838_at | Mut | methylmalonyl-Coenzyme A mutase | -1.21 | 0.091 | -1.18 | 0.183 | -1.21 | 0.238 | 1.03 | 0.878 | -0.64 |
| 1457110_at | Pank1 | pantothenate kinase 1 | 1.21 | 0.647 | -1.52 | 0.059 | -1.57 | 0.06 | -1.21 | 0.394 | -0.77 |
| 1458135_at | G630014P10Rik | RIKEN cDNA G630014P10 gene | 1.35 | 0.566 | -1.74 | 0.526 | -1.73 | 0.115 | -1.26 | 0.371 | -0.85 |
| 1424849_at | Wdr62 | WD repeat domain 62 | -1.19 | 0.259 | -1.26 | 0.786 | -1.15 | 0.862 | 2.18 | 0.195 | -0.35 |
| 1424353_at | Lrpprc | leucine-rich PPR-motif containing | -1.35 | 0.117 | -1.26 | 0.074 | -1.04 | 0.782 | 1.25 | 0.276 | -0.6 |
| 1433370_at | 4930474A20Rik | RIKEN cDNA 4930474A20 gene | -1.29 | 0.659 | -1.65 | 0.22 | 1.12 | 0.428 | -1.46 | 0.285 | -0.82 |
| 1442123_at | 2010012P19Rik | RIKEN cDNA 2010012P19 gene | -1.45 | 0.405 | -1.09 | 0.829 | -1.11 | 0.652 | 1.88 | 0.134 | -0.44 |
| 1453734_at | Atrx | alpha thalassemia/mental retardation syndrome X-linked homolog (human) | -1.59 | 0.012 | 1.06 | 0.739 | -1.22 | 0.484 | -1.13 | 0.491 | -0.72 |
| 1440003_at | --- | Transcribed locus | -1.21 | 0.215 | -1.72 | 0.024 | 1.1 | 0.012 | -1.16 | 0.571 | -0.75 |
| 1430322_at | Arl13b | ADP-ribosylation factor-like 13B | -1.59 | 0.503 | -1.15 | 0.745 | 1 | 0.994 | -1.17 | 0.812 | -0.73 |
| 1440900_at | Gpr174 | G protein-coupled receptor 174 | -1.34 | 0.551 | -1.43 | 0.433 | 1.06 | 0.798 | -2.05 | 0.147 | -0.94 |
| 1448691_at | Ubqln4 | ubiquilin 4 | -1.23 | 0.196 | -1.14 | 0.535 | -1.23 | 0.449 | 1.12 | 0.282 | -0.62 |
| 1454899_at | Lpp | LIM domain containing preferred translocation partner in lipoma | -1.07 | 0.533 | 1.01 | 0.88 | -1.77 | 0.011 | -1.36 | 0.298 | -0.8 |
| 1460024_at | Tnrc6b | trinucleotide repeat containing 6b | -1.23 | 0.437 | -1.09 | 0.038 | -1.29 | 0.307 | 1.27 | 0.485 | -0.59 |
| 1443017_at | Cpeb2 | cytoplasmic polyadenylation element binding protein 2 | -1.07 | 0.795 | -1.59 | 0.107 | -1.07 | 0.846 | 1.03 | 0.883 | -0.67 |
| 1445449_at | Pde4b | Phosphodiesterase 4B, cAMP specific | 1.41 | 0.279 | -2.51 | 0.141 | -1.43 | 0.329 | -1.26 | 0.654 | -0.95 |
| 1452419_at | Heatr1 | HEAT repeat containing 1 | -1.79 | 0.279 | -1.7 | 0.298 | 1.36 | 0.724 | -1.35 | 0.468 | -0.87 |
| 1420140_at | Bcdin3 | bin3, bicoid-interacting 3, homolog (Drosophila) | -1.15 | 0.487 | -1.15 | 0.644 | -1.31 | 0.047 | 1.01 | 0.954 | -0.65 |
| 1435633_at | 4631424J17Rik | RIKEN cDNA 4631424J17 gene | -1.18 | 0.577 | -1.15 | 0.637 | -1.27 | 0.374 | 1.16 | 0.547 | -0.61 |
| 1435299_at | C230069N13 | hypothetical protein C230069N13 | -1.13 | 0.359 | -1.22 | 0.235 | -1.25 | 0.213 | 1.01 | 0.982 | -0.65 |
| 1444812_at | Prdm10 | PR domain containing 10 | 1.07 | 0.576 | -1.37 | 0.587 | -1.42 | 0.139 | -1.1 | 0.745 | -0.71 |
| 1452466_a_at | Rbm6 | RNA binding motif protein 6 | -1.23 | 0.416 | -1.1 | 0.236 | -1.28 | 0.267 | 1.26 | 0.196 | -0.59 |
| 1454654_at | Dirc2 | disrupted in renal carcinoma 2 (human) | -1.04 | 0.595 | -1.35 | 0.206 | -1.25 | 0.087 | 1.01 | 0.974 | -0.66 |

| | | | | | | | | | | | |
|---|---|---|---|---|---|---|---|---|---|---|---|
| 1458491_at | 4930422I07Rik | RIKEN cDNA 4930422I07 gene | -1.23 | 0.071 | -1.18 | 0.514 | -1.19 | 0.415 | 1.32 | 0.373 | -0.57 |
| 1426550_at | Sidt1 | SID1 transmembrane family, member 1 | 1.05 | 0.901 | -1.62 | 0.155 | -1.19 | 0.523 | -1.9 | 0.264 | -0.92 |
| 1436387_at | C330006P03Rik | RIKEN cDNA C330006P03 gene | -1.19 | 0.555 | -1.03 | 0.873 | -1.43 | 0.074 | 1.14 | 0.736 | -0.63 |
| 1429594_at | Slc38a2 | solute carrier family 38, member 2 | -1.25 | 0.028 | -1.45 | 0.047 | 1.01 | 0.95 | -1.77 | 0.023 | -0.86 |
| 1450058_at | Asph | aspartate-beta-hydroxylase | -1.09 | 0.637 | -1.17 | 0.198 | -1.38 | 0.179 | 1.61 | 0.392 | -0.5 |
| 1430804_at | Slc13a1 | solute carrier family 13 (sodium/sulphate symporters), member 1 | -1.11 | 0.806 | -1.13 | 0.809 | -1.4 | 0.511 | 1.36 | 0.554 | -0.57 |
| 1425909_at | --- | CDNA clone MGC:11898 IMAGE:3598523 | 1.01 | 0.985 | -1.28 | 0.548 | -1.4 | 0.089 | -1.3 | 0.278 | -0.74 |
| 1443581_at | St6gal1 | Beta galactoside alpha 2,6 sialyltransferase 1 | -1.5 | 0.396 | 1.05 | 0.912 | -1.26 | 0.322 | -1.63 | 0.156 | -0.84 |
| 1458290_at | R3hdm1 | R3H domain 1 (binds single-stranded nucleic acids) | 1.12 | 0.399 | -1.71 | 0.021 | -1.24 | 0.006 | -1.1 | 0.616 | -0.73 |
| 1420770_at | Klk1b24 | kallikrein 1-related peptidase b24 | -1.96 | 0.008 | 1.35 | 0.32 | -1.54 | 0.17 | -1.65 | 0.131 | -0.95 |
| 1447527_at | Irf2 | Interferon regulatory factor 2 | 1.24 | 0.135 | -1.26 | 0.042 | -2.15 | 0.008 | -1.73 | 0.201 | -0.97 |
| 1447975_a_at | LOC545261 | hypothetical protein LOC545261 | -1.5 | 0.477 | 1.22 | 0.339 | -1.61 | 0.039 | -1.1 | 0.766 | -0.75 |
| 1459932_at | D8Ertd28e | DNA segment, Chr 8, ERATO Doi 28, expressed | -1.38 | 0.359 | 1.15 | 0.862 | -1.59 | 0.152 | -2.26 | 0.117 | -1.02 |
| 1429086_at | Grhl2 | grainyhead-like 2 (Drosophila) | -1.06 | 0.619 | -1.61 | 0.215 | -1.07 | 0.777 | 1.01 | 0.973 | -0.68 |
| 1428429_at | Rgmb | RGM domain family, member B | -1.36 | 0.116 | -1.52 | 0.069 | 1.11 | 0.678 | -1.2 | 0.65 | -0.74 |
| 1444393_at | Rims1 | Regulating synaptic membrane exocytosis 1 | 1.03 | 0.633 | -1.99 | 0.039 | -1.03 | 0.942 | -1.11 | 0.91 | -0.77 |
| 1433799_at | Rdh13 | retinol dehydrogenase 13 (all-trans and 9-cis) | -1.39 | 0.173 | -1.02 | 0.891 | -1.23 | 0.075 | 1.02 | 0.86 | -0.66 |
| 1444483_at | 4931428D14Rik | RIKEN cDNA 4931428D14 gene | -1.26 | 0.013 | 1.05 | 0.949 | -1.51 | 0.015 | -1 | 0.986 | -0.68 |
| 1434267_at | Nek1 | NIMA (never in mitosis gene a)-related expressed kinase 1 | -1.24 | 0.166 | -1.25 | 0.137 | -1.11 | 0.335 | 1.72 | 0.21 | -0.47 |
| 1442369_at | 4832406H04Rik | RIKEN cDNA 4832406H04 gene | -1.67 | 0.451 | -1.99 | 0.238 | 1.41 | 0.066 | -1.91 | 0.348 | -1.04 |
| 1436114_at | Gm96 | gene model 96, (NCBI) | -1.59 | 0.602 | -1.36 | 0.633 | 1.14 | 0.752 | -1.15 | 0.731 | -0.74 |
| 1418173_at | Krt25 | keratin 25 | -1.46 | 0.461 | 1.07 | 0.834 | -1.33 | 0.477 | -1.6 | 0.313 | -0.83 |
| 1456071_a_at | Cycs /// LOC67071 | cytochrome c, somatic /// similar to Cytochrome c, somatic /// similar to Cytochron | -1.28 | 0.015 | -1.28 | 0.01 | -1.06 | 0.408 | 1.23 | 0.345 | -0.6 |
| 1427011_a_at | Lancl1 | LanC (bacterial lantibiotic synthetase component C)-like 1 | -1.37 | 0.09 | -1.34 | 0.009 | 1.03 | 0.859 | -1.44 | 0.039 | -0.78 |
| 1428359_s_at | 1810010M01Rik | RIKEN cDNA 1810010M01 gene | -1.28 | 0.693 | -1.35 | 0.554 | -1.02 | 0.968 | 3.21 | 0.013 | -0.11 |
| 1446119_at | --- | Adult male hypothalamus cDNA, RIKEN full-length enriched library, clone:A23005 | 1.12 | 0.64 | -1.68 | 0.288 | -1.26 | 0.559 | -1.34 | 0.24 | -0.79 |
| 1455810_a_at | E130112L23Rik /// | RIKEN cDNA E130112L23 gene /// similar to quattro | -1.08 | 0.751 | -1.31 | 0.68 | -1.23 | 0.351 | 1.73 | 0.28 | -0.47 |
| 1420736_at | Sim1 | single-minded homolog 1 (Drosophila) | -1.54 | 0.09 | -1.16 | 0.823 | -1 | 0.991 | 1.07 | 0.864 | -0.66 |
| 1453029_s_at | 1110059H15Rik | RIKEN cDNA 1110059H15 gene | -1.12 | 0.426 | -1.3 | 0.035 | -1.19 | 0.107 | 1.09 | 0.602 | -0.63 |
| 1448461_a_at | Thoc7 | THO complex 7 homolog (Drosophila) | -1.16 | 0.215 | -1.15 | 0.238 | -1.29 | 0.011 | 1.23 | 0.303 | -0.59 |
| 1435976_at | --- | --- | -1.66 | 0.2 | -1.18 | 0.305 | 1.06 | 0.76 | -1.48 | 0.195 | -0.81 |
| 1430972_x_at | Aqr | aquarius | -1.72 | 0.538 | 1.05 | 0.939 | -1.14 | 0.673 | -1.59 | 0.086 | -0.85 |
| 1445923_at | 1700055N04Rik | RIKEN cDNA 1700055N04 gene | -2.45 | 0.304 | 1.26 | 0.325 | -1.18 | 0.14 | -1.34 | 0.173 | -0.93 |
| 1457856_at | --- | --- | -1.48 | 0.333 | 1.13 | 0.811 | -1.42 | 0.146 | -1.25 | 0.292 | -0.76 |
| 1441465_at | Cnot3 | CCR4-NOT transcription complex, subunit 3 | -1.29 | 0.045 | -1.22 | 0.158 | -1.1 | 0.66 | 1.1 | 0.575 | -0.63 |
| 1455384_x_at | D030056L22Rik | RIKEN cDNA D030056L22 gene | -1.06 | 0.492 | -1.5 | 0.023 | -1.11 | 0.275 | 1.64 | 0.233 | -0.51 |
| 1457422_at | 9030205A07Rik | RIKEN cDNA 9030205A07 Gene | -2.03 | 0.465 | 1.3 | 0.561 | -1.39 | 0.639 | -1.31 | 0.032 | -0.86 |
| 1459922_at | AI646383 | expressed sequence AI646383 | 1.1 | 0.713 | -2.24 | 0.01 | -1.04 | 0.907 | -1.3 | 0.15 | -0.87 |
| 1440573_at | Erbb2ip | Erbb2 interacting protein | 1.04 | 0.849 | -1.36 | 0.018 | -1.36 | 0.056 | -1.41 | 0.281 | -0.77 |
| 1452254_at | Mtmr9 | myotubularin related protein 9 | -1.31 | 0.083 | -1.22 | 0.125 | -1.08 | 0.503 | 1.42 | 0.285 | -0.55 |
| 1423766_at | Pak1ip1 | PAK1 interacting protein 1 | -1.28 | 0.027 | -1.23 | 0.106 | -1.1 | 0.056 | 1.13 | 0.445 | -0.62 |
| 1432228_at | 4933406B15Rik | RIKEN cDNA 4933406B15 gene | -1.88 | 0.048 | 1.13 | 0.803 | -1.18 | 0.738 | -2.21 | 0.184 | -1.03 |
| 1442617_at | Cbfb | Core binding factor beta | 1.02 | 0.951 | -1.05 | 0.909 | -1.9 | 0.011 | -1.52 | 0.195 | -0.86 |
| 1429056_at | Narg1l | NMDA receptor regulated 1-like | -1.06 | 0.685 | -1.15 | 0.152 | -1.43 | 0.019 | 1.28 | 0.438 | -0.59 |
| 1440850_at | Rnf12 | ring finger protein 12 | 1.2 | 0.237 | -1.49 | 0.277 | -1.57 | 0.036 | -1.01 | 0.938 | -0.72 |
| 1452219_at | Tmem63b | transmembrane protein 63b | -1.07 | 0.456 | -1.33 | 0.044 | -1.22 | 0.383 | 1.01 | 0.938 | -0.65 |
| 1427595_at | Acaca /// LOC6722 | acetyl-Coenzyme A carboxylase alpha /// similar to acetyl-coenzyme A carboxylas | -3.12 | 0.076 | -1 | 0.998 | 1.19 | 0.634 | -1.38 | 0.366 | -1.08 |
| 1457716_at | Otud7b | OTU domain containing 7B | 1.02 | 0.878 | -1.25 | 0.31 | -1.46 | 0.161 | -1.24 | 0.067 | -0.73 |
| 1459430_at | Gpr158 | G protein-coupled receptor 158 | -1.09 | 0.867 | -1.2 | 0.16 | -1.31 | 0.646 | 1.12 | 0.78 | -0.62 |
| 1446914_at | C80425 | expressed sequence C80425 | -1.49 | 0.304 | -1.21 | 0.683 | 1.01 | 0.968 | -1.08 | 0.869 | -0.69 |
| 1430411_at | 6330525I24Rik | RIKEN cDNA 6330525I24 gene | -1.65 | 0.392 | -1.39 | 0.273 | 1.18 | 0.701 | -1.32 | 0.569 | -0.79 |
| 1459272_at | Peli1 | Pellino 1 | -1.41 | 0.246 | -1.22 | 0.517 | -1.02 | 0.906 | 1.04 | 0.9 | -0.65 |
| 1460318_at | Csrp3 | cysteine and glycine-rich protein 3 | -1.07 | 0.691 | -1.12 | 0.255 | -1.46 | 0.185 | 1.02 | 0.856 | -0.66 |

| | | | | | | | | | | | |
|---|---|---|---|---|---|---|---|---|---|---|---|
| 1459219_at | --- | --- | -1.08 | 0.408 | 1.12 | 0.759 | -2.18 | 0.011 | -1.97 | 0.006 | -1.02 |
| 1453483_at | 1700023E05Rik | RIKEN cDNA 1700023E05 gene | -1.49 | 0.115 | -1.18 | 0.061 | -1.01 | 0.953 | 1.19 | 0.189 | -0.62 |
| 1416337_at | Uqcrb | ubiquinol-cytochrome c reductase binding protein | -1.3 | 0.03 | -1.19 | 0.12 | -1.11 | 0.139 | 1.03 | 0.799 | -0.64 |
| 1441016_at | --- | Adult male corpora quadrigemina cDNA, RIKEN full-length enriched library, clone | -1.85 | 0.159 | -1.47 | 0.129 | 1.29 | 0.451 | -1.5 | 0.223 | -0.88 |
| 1455585_at | Rnf168 | ring fnger protein 168 | -1.02 | 0.612 | -1.24 | 0.156 | -1.38 | 0.033 | 1.59 | 0.169 | -0.51 |
| 1448944_at | Nrp1 | neuropilin 1 | -1.02 | 0.865 | -1.07 | 0.662 | -1.68 | 0.023 | 1.02 | 0.93 | -0.69 |
| 1427804_at | --- | --- | 1.26 | 0.585 | -1.11 | 0.878 | -2.87 | 0.038 | -1.16 | 0.605 | -0.97 |
| 1416578_at | Rbx1 | ring-box 1 | -1.13 | 0.462 | -1.18 | 0.575 | -1.29 | 0.011 | 1.1 | 0.546 | -0.62 |
| 1417202_s_at | Ube1c | ubiquitin-activating enzyme E1C | -1.18 | 0.042 | -1.12 | 0.297 | -1.3 | 0.047 | 1.01 | 0.92 | -0.65 |
| 1439798_at | Hoxc10 | homeo box C10 | -1.2 | 0.262 | -1.5 | 0.345 | 1.01 | 0.975 | -1.67 | 0.453 | -0.84 |
| 1432191_at | --- | --- | -2.88 | 0.124 | 1.23 | 0.747 | -1.07 | 0.888 | -1.9 | 0.419 | -1.16 |
| 1434133_s_at | Wdr42a | WD repeat domain 42A | -1.16 | 0.52 | -1.2 | 0.408 | -1.22 | 0.253 | 1.15 | 0.533 | -0.61 |
| 1432833_at | 4933435G04Rik | RIKEN cDNA 4933435G04 gene | -1.08 | 0.148 | -1.45 | 0.586 | -1.11 | 0.577 | 1.22 | 0.064 | -0.61 |
| 1423590_at | Napsa | napsin A aspartic peptidase | 1.04 | 0.433 | -2.02 | 0.209 | -1.03 | 0.853 | -1.85 | 0.201 | -0.96 |
| 1458868_at | Atp1a1 | ATPase, Na+/K+ transporting, alpha 1 polypeptide | 1.38 | 0.623 | -2.16 | 0.024 | -1.5 | 0.196 | -1.75 | 0.165 | -1.01 |
| 1442547_at | --- | Transcribed locus | 1.15 | 0.343 | -1.55 | 0.555 | -1.39 | 0.021 | -1.26 | 0.417 | -0.76 |
| 1441240_at | Rpa1 | Replication protein A1 | -1.66 | 0.61 | 1.14 | 0.735 | -1.29 | 0.441 | -1.17 | 0.472 | -0.75 |
| 1445915_at | H2-K1 | Histocompatibility 2, K1, K region | -1.26 | 0.167 | -1.11 | 0.23 | -1.23 | 0.128 | 1.1 | 0.46 | -0.62 |
| 1454692_x_at | Hnrpk | Heterogeneous nuclear ribonucleoprotein K | -1.39 | 0.334 | 1.14 | 0.154 | -1.54 | 0.105 | -1.1 | 0.099 | -0.72 |
| 1446991_at | Gcn1l1 | GCN1 general control of amino-acid synthesis 1-like 1 (yeast) | 1.18 | 0.447 | -2.47 | 0.216 | -1.08 | 0.878 | -1.38 | 0.148 | -0.94 |
| 1443877_a_at | Rapgef6 | Rap guanine nucleotide exchange factor (GEF) 6 | -1.7 | 0.059 | 1.17 | 0.682 | -1.32 | 0.263 | -1.21 | 0.652 | -0.77 |
| 1448646_at | Wdr12 | WD repeat domain 12 | -1.18 | 0.267 | -1.29 | 0.052 | -1.13 | 0.47 | 1.29 | 0.43 | -0.58 |
| 1445644_at | A830091I15Rik | RIKEN cDNA A830091I15 gene | -2.07 | 0.349 | 1.03 | 0.953 | -1 | 0.996 | -2.06 | 0.359 | -1.03 |
| 1443818_at | --- | --- | 1.21 | 0.487 | -1.72 | 0.459 | -1.39 | 0.589 | -1.01 | 0.979 | -0.73 |
| 1427353_at | Clasp1 | CLIP associating protein 1 | 1.01 | 0.947 | -1.68 | 0.029 | -1.1 | 0.521 | -1.54 | 0.074 | -0.83 |
| 1435179_at | C130071C03Rik | RIKEN cDNA C130071C03 gene | -1.06 | 0.829 | 1.28 | 0.601 | -3.58 | 0.021 | -3.09 | 0.165 | -1.61 |
| 1424909_at | Lrrc46 | leucine rich repeat containing 46 | -2.86 | 0.019 | -1.04 | 0.831 | 1.2 | 0.489 | -1.93 | 0.171 | -1.16 |
| 1444060_at | 2610027K06Rik | RIKEN cDNA 2610027K06 gene | -1.34 | 0.445 | -2.33 | 0.207 | 1.33 | 0.447 | -1.41 | 0.722 | -0.93 |
| 1444166_at | Thrsp | thyroid hormone responsive SPOT14 homolog (Rattus) | 1.02 | 0.908 | -1.37 | 0.656 | -1.33 | 0.553 | -1.15 | 0.844 | -0.7 |
| 1420264_at | --- | --- | -2.51 | 0.18 | -1.23 | 0.654 | 1.3 | 0.524 | -1.26 | 0.534 | -0.92 |
| 1438079_at | BC050078 | cDNA sequence BC050078 | -1.03 | 0.925 | -1.59 | 0.102 | -1.1 | 0.516 | 1.05 | 0.678 | -0.67 |
| 1442818_at | LOC671796 /// LOC | hypothetical protein LOC671796 /// hypothetical protein LOC675117 | -1.47 | 0.476 | -1.81 | 0.522 | 1.28 | 0.523 | -1.24 | 0.083 | -0.81 |
| 1441014_at | Atrx | Alpha thalassemia/mental retardation syndrome X-linked homolog (human) | 1.1 | 0.882 | -1.01 | 0.983 | -2.37 | 0.018 | -2.98 | 0.087 | -1.31 |
| 1457440_at | Sstr4 | somatostatin receptor 4 | -1.12 | 0.771 | -1.98 | 0.02 | 1.11 | 0.61 | -2.09 | 0.049 | -1.02 |
| 1450708_at | Scg2 | secretogranin II | -3.58 | 0.01 | 2.01 | 0.134 | -4.57 | 0.023 | -1.3 | 0.722 | -1.86 |
| 1458594_at | Shprh | SNF2 histone linker PHD RING helicase | -1.19 | 0.734 | -2.1 | 0.252 | 1.19 | 0.674 | -1.44 | 0.63 | -0.88 |
| 1452193_a_at | Wasl | Wiskott-Aldrich syndrome-like (human) | -1.1 | 0.584 | -1.21 | 0.059 | -1.29 | 0.13 | 1.02 | 0.953 | -0.65 |
| 1444454_at | Rb1 | Retinoblastoma 1 | -1.2 | 0.537 | -1.05 | 0.893 | -1.37 | 0.178 | 1.17 | 0.583 | -0.61 |
| 1455541_a_at | 4430402I18Rik | RIKEN cDNA 4430402I18 gene | -1.34 | 0.069 | -1.39 | 0.452 | 1.05 | 0.833 | -2.36 | 0.222 | -1.01 |
| 1431646_a_at | Stx6 | syntaxin 6 | -1.01 | 0.984 | -1.77 | 0.154 | -1.05 | 0.85 | 1.06 | 0.369 | -0.69 |
| 1421612_a_at | H2afy3 /// H2afy2 | H2A histone family, member Y3 /// H2A histone family, member Y2 | -1.19 | 0.149 | -1.11 | 0.826 | -1.29 | 0.147 | 1.38 | 0.318 | -0.56 |
| 1424442_a_at | Pja2 | praja 2, RING-H2 motif containing | -1.03 | 0.88 | -1.23 | 0.076 | -1.38 | 0.018 | 1.16 | 0.109 | -0.62 |
| 1460629_at | Trim16 | tripartite motif protein 16 | -1.03 | 0.699 | -1.41 | 0.386 | -1.21 | 0.426 | 1.74 | 0.115 | -0.48 |
| 1443226_at | 5730470L24Rik | RIKEN cDNA 5730470L24 gene | 1.03 | 0.881 | -1.28 | 0.474 | -1.43 | 0.067 | -1.63 | 0.247 | -0.83 |
| 1440849_at | 6330417G04Rik | RIKEN cDNA 6330417G04 gene | -1.16 | 0.225 | -1.14 | 0.658 | -1.29 | 0.162 | 1 | 0.994 | -0.65 |
| 1448989_a_at | Myo1b | myosin IB | -1.02 | 0.965 | -1.21 | 0.142 | -1.41 | 0.173 | 1.05 | 0.91 | -0.65 |
| 1453860_s_at | Nr3c1 | nuclear receptor subfamily 3, group C, member 1 | -1.53 | 0.484 | -1.24 | 0.401 | 1.05 | 0.768 | -1.01 | 0.985 | -0.68 |
| 1427152_at | Qser1 | glutamine and serine rich 1 | -1.29 | 0.159 | -1.05 | 0.828 | -1.27 | 0.112 | 1.01 | 0.959 | -0.65 |
| 1451638_s_at | Armc1 /// LOC6343 | armadillo repeat containing 1 /// similar to armadillo repeat-containing protein | -1.13 | 0.316 | -1.16 | 0.015 | -1.3 | 0.107 | 1.35 | 0.256 | -0.56 |
| 1424530_at | Sec14l2 | SEC14-like 2 (S. cerevisiae) | -1.01 | 0.953 | -1.43 | 0.051 | -1.22 | 0.303 | 4.28 | 0.173 | 0.16 |
| 1436061_at | --- | --- | 1.03 | 0.789 | -1.57 | 0.174 | -1.17 | 0.434 | -1.96 | 0.286 | -0.92 |
| 1443196_at | --- | --- | 1.07 | 0.76 | -1.6 | 0.127 | -1.23 | 0.469 | -1.42 | 0.389 | -0.79 |
| 1453287_at | 5730557B15Rik | RIKEN cDNA 5730557B15 gene | 1.18 | 0.291 | -1.61 | 0.075 | -1.41 | 0.076 | -1.4 | 0.018 | -0.81 |

| | | | | | | | | | | | |
|---|---|---|---|---|---|---|---|---|---|---|---|
| 1422611_s_at | Igf2bp3 | insulin-like growth factor 2 mRNA binding protein 3 | -1.31 | 0.606 | -1.85 | 0.174 | 1.21 | 0.726 | -1.18 | 0.788 | -0.78 |
| 1454263_at | Gscl | goosecoid-like | -1.8 | 0.411 | 1.07 | 0.837 | -1.13 | 0.626 | -1.44 | 0.508 | -0.82 |
| 1437774_at | 1700020I14Rik | RIKEN cDNA 1700020I14 gene | -1.04 | 0.559 | -1.1 | 0.639 | -1.54 | 0.09 | 1.71 | 0.106 | -0.5 |
| 1448059_at | Mipol1 | mirror-image polydactyly gene 1 homolog (human) | -2.21 | 0.428 | 1.46 | 0.475 | -1.65 | 0.159 | -1.04 | 0.961 | -0.86 |
| 1438369_x_at | Tmem85 | transmembrane protein 85 | 1 | 0.996 | -1.24 | 0.081 | -1.42 | 0.163 | -1.54 | 0.411 | -0.8 |
| 1445592_at | --- | --- | -1.09 | 0.905 | -1.25 | 0.297 | -1.25 | 0.672 | 1.27 | 0.765 | -0.58 |
| 1457999_at | 2410005O16Rik | RIKEN cDNA 2410005O16 gene | 1.04 | 0.862 | -1.22 | 0.204 | -1.54 | 0.079 | -1.15 | 0.666 | -0.71 |
| 1423137_at | Rala | v-ral simian leukemia viral oncogene homolog A (ras related) | 1.02 | 0.797 | -1.28 | 0.051 | -1.41 | 0.036 | -1.86 | 0.101 | -0.88 |
| 1424584_a_at | Ranbp10 | RAN binding protein 10 | -1.24 | 0.139 | -1.22 | 0.018 | -1.12 | 0.265 | 3.17 | 0.117 | -0.1 |
| 1447530_at | F8a | Factor 8-associated gene A | 1.09 | 0.909 | -1.21 | 0.755 | -1.68 | 0.297 | -1.54 | 0.58 | -0.83 |
| 1452885_at | Sfrs2ip | splicing factor, arginine/serine-rich 2, interacting protein | -1.17 | 0.452 | -1.3 | 0.076 | -1.12 | 0.745 | 1.29 | 0.224 | -0.58 |
| 1437156_at | Efcbp1 | EF hand calcium binding protein 1 | -1.02 | 0.97 | -1.21 | 0.22 | -1.41 | 0.234 | 2.24 | 0.021 | -0.35 |
| 1433420_at | 4930527J03Rik | RIKEN cDNA 4930527J03 gene | -1.11 | 0.679 | -1.01 | 0.961 | -1.59 | 0.013 | 1.33 | 0.263 | -0.59 |
| 1439337_at | Tom1l1 | target of myb1-like 1 (chicken) | -1.67 | 0.114 | -1.11 | 0.704 | 1.02 | 0.694 | -1.47 | 0.207 | -0.81 |
| 1460056_at | 1700109F18Rik | RIKEN cDNA 1700109F18 gene | 1.13 | 0.16 | -2.25 | 0.461 | -1.06 | 0.861 | -1.48 | 0.295 | -0.92 |
| 1455551_at | Uevld | UEV and lactate/malate dehyrogenase domains | -1.35 | 0.059 | -1.11 | 0.084 | -1.15 | 0.133 | 1.15 | 0.503 | -0.61 |
| 1429355_at | 3300001K11Rik | RIKEN cDNA 3300001K11 gene | -1.1 | 0.853 | 1.15 | 0.436 | -2.22 | 0.105 | -1.17 | 0.885 | -0.83 |
| 1415714_a_at | 2610209M04Rik | RIKEN cDNA 2610209M04 gene | -1.13 | 0.176 | -1.31 | 0.014 | -1.15 | 0.058 | 1.17 | 0.304 | -0.61 |
| 1457466_at | AA409368 | expressed sequence AA409368 | 1.14 | 0.429 | -1.03 | 0.944 | -2.49 | 0.025 | -1.26 | 0.616 | -0.91 |
| 1429555_at | Cldnd1 | claudin domain containing 1 | -1.17 | 0.244 | -1.08 | 0.229 | -1.36 | 0.174 | 1.12 | 0.367 | -0.62 |
| 1439450_x_at | A230046K03Rik | RIKEN cDNA A230046K03 gene | 1.02 | 0.927 | -1.24 | 0.133 | -1.45 | 0.011 | -2.17 | 0.131 | -0.96 |
| 1454403_at | 4930466I24Rik | RIKEN cDNA 4930466I24 gene | -1.74 | 0.225 | 1.38 | 0.393 | -1.79 | 0.167 | -1.17 | 0.559 | -0.83 |
| 1457932_at | Hivep2 | Human immunodeficiency virus type I enhancer binding protein 2 | 1.07 | 0.654 | -2.19 | 0.306 | -1.01 | 0.982 | -2.93 | 0.253 | -1.27 |
| 1422234_at | Tacr2 | tachykinin receptor 2 | -1.39 | 0.296 | -1.23 | 0.49 | -1.02 | 0.969 | 1.04 | 0.931 | -0.65 |
| 1441635_at | Olfml2a | Olfactomedin-like 2A | -1.4 | 0.094 | 1.01 | 0.949 | -1.26 | 0.203 | -1.27 | 0.075 | -0.73 |
| 1440015_at | --- | Transcribed locus | -1.21 | 0.164 | 1.12 | 0.744 | -1.77 | 0.043 | -2 | 0.004 | -0.96 |
| 1441497_at | AA388235 | expressed sequence AA388235 | -1.76 | 0.015 | -1.06 | 0.863 | -1 | 0.991 | 1.19 | 0.547 | -0.65 |
| 1432089_at | 4930550C17Rik | RIKEN cDNA 4930550C17 gene | 1.06 | 0.941 | -1.17 | 0.712 | -1.67 | 0.349 | -1.61 | 0.368 | -0.85 |
| 1447280_at | Dtna | Dystrobrevin alpha | -1.46 | 0.04 | 1.09 | 0.88 | -1.34 | 0.455 | -1.07 | 0.905 | -0.7 |
| 1441995_at | Ncam1 | Neural cell adhesion molecule 1 | -1.03 | 0.967 | -1.58 | 0.329 | -1.1 | 0.775 | 1.03 | 0.863 | -0.67 |
| 1444012_at | Yipf3 | Yip1 domain family, member 3 | -1.35 | 0.405 | -1.36 | 0.214 | 1.04 | 0.833 | -2.05 | 0.051 | -0.93 |
| 1429031_at | 4833408D11Rik | RIKEN cDNA 4833408D11 gene | 1.1 | 0.684 | -1.57 | 0.117 | -1.29 | 0.121 | -1.7 | 0.019 | -0.86 |
| 1446572_at | Usp53 | Ubiquitin specific peptidase 53 | 1.12 | 0.445 | -1.31 | 0.453 | -1.58 | 0.217 | -2.03 | 0.495 | -0.95 |
| 1449987_at | Alk | anaplastic lymphoma kinase | 1.07 | 0.505 | -1.44 | 0.492 | -1.33 | 0.115 | -1.34 | 0.34 | -0.76 |
| 1425806_a_at | Surb7 | SRB7 (suppressor of RNA polymerase B) homolog (S. cerevisiae) | -1.26 | 0.076 | -1.09 | 0.531 | -1.24 | 0.126 | 1.08 | 0.708 | -0.63 |
| 1445623_at | --- | --- | 1.6 | 0.342 | -1.97 | 0.277 | -2.47 | 0.004 | -1.03 | 0.849 | -0.97 |
| 1457990_at | Anks1b /// LOC544 | ankyrin repeat and sterile alpha motif domain containing 1B /// similar to AIDA-1b | 1.07 | 0.832 | -1.88 | 0.193 | -1.09 | 0.779 | -2.25 | 0.233 | -1.04 |
| 1429966_at | 3110057O12Rik | RIKEN cDNA 3110057O12 gene | -1.27 | 0.454 | -1.23 | 0.426 | -1.09 | 0.794 | 1.08 | 0.618 | -0.63 |
| 1433986_at | BC024659 | cDNA sequence BC024659 | -1.03 | 0.82 | -1.08 | 0.193 | -1.61 | 0.003 | 1.27 | 0.086 | -0.61 |
| 1439081_at | Mgea5 | meningioma expressed antigen 5 (hyaluronidase) | 1.13 | 0.531 | -1.36 | 0.336 | -1.54 | 0.106 | -1.52 | 0.05 | -0.82 |
| 1426773_at | Mfn1 | mitofusin 1 | -1.07 | 0.488 | -1.42 | 0.033 | -1.13 | 0.572 | 1.39 | 0.024 | -0.56 |
| 1435434_at | Braf | Braf transforming gene | -1.18 | 0.335 | -1.01 | 0.909 | -1.46 | 0.035 | 1.01 | 0.963 | -0.66 |
| 1435589_at | Ccdc85b | coiled-coil domain containing 85B | 1.08 | 0.741 | -1.57 | 0.074 | -1.25 | 0.351 | -1.55 | 0.156 | -0.82 |
| 1457220_at | --- | --- | 1 | 0.999 | -1.28 | 0.395 | -1.36 | 0.075 | -1.16 | 0.6 | -0.7 |
| 1444105_at | Acta2 | actin, alpha 2, smooth muscle, aorta | -1.46 | 0.148 | 1.05 | 0.822 | -1.29 | 0.543 | -4.08 | 0.22 | -1.44 |
| 1419613_at | Col7a1 | procollagen, type VII, alpha 1 | -1.18 | 0.298 | -1.1 | 0.615 | -1.31 | 0.502 | 1.12 | 0.522 | -0.62 |
| 1448362_at | Dnajc7 | DnaJ (Hsp40) homolog, subfamily C, member 7 | -1.98 | 0.467 | -1.74 | 0.201 | 1.44 | 0.187 | -2.49 | 0.201 | -1.19 |
| 1446472_at | Ahctf1 | AT hook containing transcription factor 1 | -1.07 | 0.621 | -1.26 | 0.235 | -1.26 | 0.223 | 1.54 | 0.018 | -0.51 |
| 1416544_at | Ezh2 | enhancer of zeste homolog 2 (Drosophila) | -1.23 | 0.03 | -1.18 | 0.16 | -1.17 | 0.472 | 2.01 | 0.235 | -0.39 |
| 1460612_at | Ranbp3 | RAN binding protein 3 | 1.08 | 0.824 | -1.54 | 0.187 | -1.27 | 0.393 | -1.12 | 0.145 | -0.71 |
| 1423362_at | Sort1 | sortilin 1 | -1.14 | 0.411 | -1.51 | 0.112 | -1.02 | 0.929 | 1.35 | 0.236 | -0.58 |
| 1458043_at | Btbd9 | BTB (POZ) domain containing 9 | -1.31 | 0.397 | 1.05 | 0.713 | -1.42 | 0.054 | -1.04 | 0.838 | -0.68 |
| 1435987_x_at | 1110059G02Rik | RIKEN cDNA 1110059G02 gene | -1.64 | 0.076 | 1.08 | 0.718 | -1.21 | 0.219 | -1.1 | 0.477 | -0.72 |

| | | | | | | | | | | | |
|---|---|---|---|---|---|---|---|---|---|---|---|
| 1438260_at | Kcnq2 | potassium voltage-gated channel, subfamily Q, member 2 | -1.08 | 0.477 | -1.19 | 0.628 | -1.34 | 0.225 | 2.31 | 0.246 | -0.32 |
| 1442761_at | Tpd52l2 | Tumor protein D52-like 2 | -2.18 | 0.061 | 1.14 | 0.679 | -1.09 | 0.608 | -1.24 | 0.523 | -0.84 |
| 1434211_at | Sh3bgrl2 | SH3 domain binding glutamic acid-rich protein like 2 | -1.28 | 0.112 | -1.2 | 0.374 | -1.1 | 0.635 | 1.04 | 0.661 | -0.64 |
| 1422579_at | Hspe1 /// LOC6284 | heat shock protein 1 (chaperonin 10) /// similar to 10 kDa heat shock protein, mitc | -1.36 | 0.156 | -1.34 | 0.039 | 1.04 | 0.865 | -1.39 | 0.117 | -0.76 |
| 1420810_at | 1500003O03Rik | RIKEN cDNA 1500003O03 gene | -1.07 | 0.513 | -1.48 | 0.076 | -1.1 | 0.619 | 1.09 | 0.649 | -0.64 |
| 1447411_at | Ugdh | UDP-glucose dehydrogenase | -1.33 | 0.422 | 1.08 | 0.647 | -1.46 | 0.177 | -1.51 | 0.01 | -0.8 |
| 1429691_at | 5430405N12Rik | RIKEN cDNA 5430405N12 gene | 1.07 | 0.672 | -1.29 | 0.265 | -1.49 | 0.071 | -1.08 | 0.87 | -0.7 |
| 1457483_at | Arid5b | AT rich interactive domain 5B (Mrf1 like) | 1.44 | 0.504 | -1.36 | 0.184 | -2.92 | 0.018 | -1.68 | 0.036 | -1.13 |
| 1459735_at | C430014M02Rik | RIKEN cDNA C430014M02 gene | -1.18 | 0.439 | 1.04 | 0.899 | -1.56 | 0.027 | -1.67 | 0.031 | -0.85 |
| 1424257_at | Cdk7 | cyclin-dependent kinase 7 (homolog of Xenopus MO15 cdk-activating kinase) | -1.19 | 0.094 | -1.28 | 0.379 | -1.11 | 0.524 | 1.11 | 0.547 | -0.62 |
| 1435892_at | Asxl3 | additional sex combs like 3 (Drosophila) | -1.41 | 0.329 | -1.69 | 0.432 | 1.22 | 0.625 | -2.68 | 0.315 | -1.14 |
| 1454145_at | Pla2r1 | phospholipase A2 receptor 1 | 1.67 | 0.362 | -2.67 | 0.163 | -2.09 | 0.177 | -1.12 | 0.64 | -1.05 |
| 1454639_x_at | Rpl41 /// 2210411K | ribosomal protein L41 /// RIKEN cDNA 2210411K19 gene | -1.36 | 0.069 | 1.11 | 0.589 | -1.48 | 0.143 | -1.13 | 0.671 | -0.72 |
| 1454286_at | 1110004M10Rik | RIKEN cDNA 1110004M10 gene | -1.22 | 0.295 | 1.01 | 0.949 | -1.45 | 0.127 | -2.1 | 0.044 | -0.94 |
| 1454519_at | 4930429L21Rik | RIKEN cDNA 4930429L21 gene | -2.37 | 0.085 | 1.2 | 0.834 | -1.11 | 0.796 | -1.05 | 0.8 | -0.83 |
| 1454201_a_at | 5730405I09Rik | RIKEN cDNA 5730405I09 gene | 1.14 | 0.765 | -1.45 | 0.169 | -1.45 | 0.251 | -1.52 | 0.615 | -0.82 |
| 1427759_a_at | Art5 | ADP-ribosyltransferase 5 | 1.26 | 0.658 | -1.26 | 0.706 | -2.12 | 0.28 | -1.09 | 0.81 | -0.8 |
| 1458807_at | Epb4.1 | erythrocyte protein band 4.1 | -1.01 | 0.983 | -1.05 | 0.746 | -1.74 | 0.135 | 2.9 | 0.199 | -0.22 |
| 1445607_at | 4833418A01Rik | RIKEN cDNA 4833418A01 gene | -1.38 | 0.185 | 1.13 | 0.566 | -1.49 | 0.055 | -1.29 | 0.383 | -0.76 |
| 1441083_at | Clptm1 | cleft lip and palate associated transmembrane protein 1 | -2.72 | 0.059 | 1.2 | 0.432 | -1.04 | 0.858 | -1.05 | 0.801 | -0.91 |
| 1418367_x_at | Hist2h3c1 /// Hist2h | histone 2, H3c1 /// histone 2, H2aa1 /// histone 1, H2ad /// histone 1, H2an /// histo | -1.15 | 0.228 | -1.37 | 0.027 | -1.08 | 0.517 | 1 | 0.985 | -0.65 |
| 1419600_at | Defb4 | defensin beta 4 | -1.42 | 0.464 | -1.26 | 0.582 | 1.02 | 0.964 | -1.14 | 0.82 | -0.7 |
| 1452052_s_at | Eif3s1 | eukaryotic translation initiation factor 3, subunit 1 alpha | -1.15 | 0.279 | -1.23 | 0.009 | -1.19 | 0.019 | 1.31 | 0.327 | -0.57 |
| 1416705_at | Rpe | ribulose-5-phosphate-3-epimerase | -1.08 | 0.329 | -1.34 | 0.045 | -1.18 | 0.164 | 1.04 | 0.844 | -0.64 |
| 1446530_at | --- | --- | -1.38 | 0.53 | -1.46 | 0.434 | 1.11 | 0.791 | -1.52 | 0.062 | -0.81 |
| 1445885_at | Ube2d2 | ubiquitin-conjugating enzyme E2D 2 | 1.11 | 0.79 | -1.17 | 0.611 | -1.8 | 0.025 | -1.01 | 0.972 | -0.72 |
| 1455447_at | D430019H16Rik | RIKEN cDNA D430019H16 gene | 1.08 | 0.76 | -1.2 | 0.58 | -1.63 | 0.259 | -1.71 | 0.048 | -0.87 |
| 1458067_at | A430041B07Rik | RIKEN cDNA A430041B07 gene | -1.4 | 0.06 | -1.36 | 0.483 | 1.08 | 0.639 | -2.18 | 0.008 | -0.97 |
| 1424346_at | Ppp6c | protein phosphatase 6, catalytic subunit | -1.15 | 0.286 | -1.13 | 0.07 | -1.3 | 0.063 | 1.15 | 0.409 | -0.61 |
| 1455045_at | Srr | serine racemase | -1.14 | 0.204 | -1.15 | 0.202 | -1.29 | 0.133 | 1.19 | 0.04 | -0.6 |
| 1451785_at | Rpgrip1 | retinitis pigmentosa GTPase regulator interacting protein 1 | -1.61 | 0.253 | -1.32 | 0.44 | 1.14 | 0.351 | -1.58 | 0.278 | -0.84 |
| 1443507_at | Slc4a1ap | Solute carrier family 4 (anion exchanger), member 1, adaptor protein | 1.37 | 0.589 | -1.45 | 0.223 | -2.15 | 0.044 | -1.34 | 0.438 | -0.89 |
| 1420312_s_at | --- | --- | 1.14 | 0.555 | -1.17 | 0.466 | -1.91 | 0.02 | -1.38 | 0.351 | -0.83 |
| 1445922_at | Xrcc4 | X-ray repair complementing defective repair in Chinese hamster cells 4 | -1.23 | 0.031 | 1 | 0.999 | -1.42 | 0 | -1.69 | 0.061 | -0.83 |
| 1447184_at | AU021760 | expressed sequence AU021760 | 1.04 | 0.393 | -1.22 | 0.446 | -1.52 | 0.13 | -1.29 | 0.32 | -0.75 |
| 1432363_at | 2410018E23Rik | RIKEN cDNA 2410018E23 gene | -1.39 | 0.468 | 1.05 | 0.944 | -1.33 | 0.531 | -1.04 | 0.925 | -0.68 |
| 1449576_at | Eif1ay | eukaryotic translation initiation factor 1A, Y-linked | -1.04 | 0.886 | -1.22 | 0.208 | -1.36 | 0.089 | 1.52 | 0.2 | -0.52 |
| 1434524_at | Eif2b3 | eukaryotic translation initiation factor 2B, subunit 3 | -1.26 | 0.034 | -1.19 | 0.171 | -1.12 | 0.32 | 1.65 | 0.319 | -0.48 |
| 1447556_x_at | 1700094D03Rik | RIKEN cDNA 1700094D03 gene | -1.53 | 0.274 | -1.21 | 0.69 | 1.04 | 0.911 | -1.46 | 0.315 | -0.79 |
| 1454160_at | 4930483C13Rik | RIKEN cDNA 4930483C13 gene | -1.41 | 0.533 | -1.16 | 0.613 | -1.05 | 0.92 | 1.05 | 0.882 | -0.64 |
| 1443267_at | Tanc2 | Tetratricopeptide repeat, ankyrin repeat and coiled-coil containing 2 | -1.14 | 0.656 | -1.17 | 0.685 | -1.27 | 0.186 | 1.24 | 0.505 | -0.58 |
| 1424183_at | Acat1 | acetyl-Coenzyme A acetyltransferase 1 | -1.22 | 0.073 | -1.47 | 0.044 | 1.03 | 0.796 | -1.03 | 0.685 | -0.68 |
| 1439075_at | Polr3f | polymerase (RNA) III (DNA directed) polypeptide F | -1.05 | 0.721 | -1.22 | 0.314 | -1.33 | 0.284 | 1.09 | 0.77 | -0.63 |
| 1455011_at | Stard4 | StAR-related lipid transfer (START) domain containing 4 | -1.06 | 0.632 | -1.07 | 0.643 | -1.55 | 0.007 | 1.01 | 0.985 | -0.67 |
| 1455287_at | Cdk6 | cyclin-dependent kinase 6 | -1.15 | 0.208 | -1.02 | 0.744 | -1.49 | 0.117 | 1.26 | 0.348 | -0.6 |
| 1457516_at | 1110033M05Rik | RIKEN cDNA 1110033M05 gene | -3.51 | 0.169 | -1.15 | 0.753 | 1.37 | 0.117 | -1.69 | 0.364 | -1.25 |
| 1458219_at | Bcas2 | breast carcinoma amplified sequence 2 | 1.07 | 0.864 | -1.59 | 0.218 | -1.22 | 0.297 | -1.1 | 0.729 | -0.71 |
| 1437039_at | Cops2 | COP9 (constitutive photomorphogenic) homolog, subunit 2 (Arabidopsis thaliana) | -1.14 | 0.235 | -1.12 | 0.212 | -1.33 | 0.028 | 1.42 | 0.308 | -0.54 |
| 1441133_at | Stx6 | Syntaxin 6 | 1.08 | 0.859 | -1.79 | 0.105 | -1.13 | 0.721 | -1.46 | 0.075 | -0.82 |
| 1455521_at | Klf12 | Kruppel-like factor 12 | 1.03 | 0.703 | -1.15 | 0.126 | -1.61 | 0.023 | -1.54 | 0.191 | -0.82 |
| 1429897_a_at | D16Ertd472e | DNA segment, Chr 16, ERATO Doi 472, expressed | -1.19 | 0.773 | -1.56 | 0.122 | 1.05 | 0.8 | -1.08 | 0.591 | -0.7 |
| 1458631_at | 4921533L14Rik | RIKEN cDNA 4921533L14 gene | -3.43 | 0.027 | 1.73 | 0.32 | -1.99 | 0.13 | -2.35 | 0.257 | -1.51 |
| 1419341_at | Epha8 | Eph receptor A8 | -2.52 | 0.207 | -1.46 | 0.46 | 1.44 | 0.358 | -1.05 | 0.921 | -0.9 |

| | | | | | | | | | | | |
|---|---|---|---|---|---|---|---|---|---|---|---|
| 1439868_at | --- | Adult male spinal cord cDNA, RIKEN full-length enriched library, clone:A330108B | -1.13 | 0.381 | -1.31 | 0.167 | -1.14 | 0.451 | 1.33 | 0.456 | -0.56 |
| 1457560_at | Ssbp3 | Single-stranded DNA binding protein 3 | -1.05 | 0.85 | -1.15 | 0.721 | -1.43 | 0.041 | 1.38 | 0.465 | -0.56 |
| 1417511_at | Lyar | Ly1 antibody reactive clone | -1.15 | 0.168 | -1.16 | 0.011 | -1.26 | 0.081 | 1.15 | 0.55 | -0.61 |
| 1454346_at | 4930540E01Rik | RIKEN cDNA 4930540E01 gene | -1.17 | 0.724 | -1.2 | 0.424 | -1.2 | 0.171 | 1.25 | 0.515 | -0.58 |
| 1422927_at | Yipf7 | Yip1 domain family, member 7 | -1.54 | 0.02 | -1.62 | 0.497 | 1.26 | 0.144 | -1.01 | 0.975 | -0.73 |
| 1417770_s_at | --- | --- | -1.2 | 0.207 | -1.11 | 0.363 | -1.26 | 0.187 | 1.39 | 0.374 | -0.55 |
| 1440152_x_at | Edf1 /// LOC66518 | endothelial differentiation-related factor 1 /// similar to Endothelial differentiation-r | -1.52 | 0.354 | -1.4 | 0.095 | 1.15 | 0.288 | -1.37 | 0.302 | -0.79 |
| 1442100_at | Inpp5f | inositol polyphosphate-5-phosphatase F | -1.25 | 0.177 | 1.09 | 0.666 | -1.58 | 0.136 | -1.81 | 0.109 | -0.89 |
| 1419028_at | Arpp21 | cyclic AMP-regulated phosphoprotein, 21 | -1.32 | 0.486 | -1.26 | 0.735 | -1.03 | 0.9 | 1.01 | 0.96 | -0.65 |
| 1441806_at | Chd1l | Chromodomain helicase DNA binding protein 1-like | -1.37 | 0.482 | -1.24 | 0.486 | -1.01 | 0.988 | 1.22 | 0.14 | -0.6 |
| 1423749_s_at | Rangap1 | RAN GTPase activating protein 1 | -1.3 | 0.369 | -1.31 | 0.192 | -1.01 | 0.94 | 1.5 | 0.15 | -0.53 |
| 1446145_at | Eif4g3 | eukaryotic translation initiation factor 4 gamma, 3 | -1.86 | 0.258 | 1.2 | 0.51 | -1.27 | 0.246 | -1.04 | 0.899 | -0.74 |
| 1417486_at | Shd | src homology 2 domain-containing transforming protein D | 1.19 | 0.258 | -2.35 | 0.286 | -1.1 | 0.786 | -1.44 | 0.616 | -0.93 |
| 1421114_a_at | Dspg3 | dermatan sulphate proteoglycan 3 | -2.67 | 0.042 | -1.45 | 0.359 | 1.46 | 0.063 | -1.25 | 0.617 | -0.98 |
| 1459635_at | Dlgh1 | Discs, large homolog 1 (Drosophila) | 1.04 | 0.871 | -1.05 | 0.832 | -1.91 | 0.021 | -1.26 | 0.252 | -0.79 |
| 1438496_a_at | 6330505F04Rik | RIKEN cDNA 6330505F04 gene | -1.2 | 0.498 | -1.02 | 0.954 | -1.41 | 0.034 | 1.4 | 0.284 | -0.56 |
| 1418800_at | Bhlhb8 | basic helix-loop-helix domain containing, class B, 8 | -1.09 | 0.869 | -1.12 | 0.808 | -1.4 | 0.235 | 1.55 | 0.195 | -0.51 |
| 1443554_at | Ssbp3 | Single-stranded DNA binding protein 3 | -1.17 | 0.278 | -1.17 | 0.706 | -1.22 | 0.47 | 1.7 | 0.267 | -0.47 |
| 1443820_x_at | Elovl1 | elongation of very long chain fatty acids (FEN1/Elo2, SUR4/Elo3, yeast)-like 1 | 1.12 | 0.586 | -1.3 | 0.298 | -1.56 | 0.014 | -1.58 | 0.286 | -0.83 |
| 1420094_at | Hnrpdl | Heterogeneous nuclear ribonucleoprotein D-like | -2.94 | 0.122 | -1.26 | 0.69 | 1.39 | 0.261 | -1.43 | 0.122 | -1.06 |
| 1417774_at | Nans | N-acetylneuraminic acid synthase (sialic acid synthase) | -1.02 | 0.941 | -1.27 | 0.357 | -1.32 | 0.171 | 1.25 | 0.29 | -0.59 |
| 1420360_at | Dkk1 | dickkopf homolog 1 (Xenopus laevis) | -1.55 | 0.159 | 1.28 | 0.566 | -1.67 | 0.224 | -1.47 | 0.345 | -0.85 |
| 1452475_at | Pcsk5 | proprotein convertase subtilisin/kexin type 5 | -1.36 | 0.153 | 1.02 | 0.897 | -1.29 | 0.205 | -1.37 | 0.125 | -0.75 |
| 1436431_at | 1700025G04Rik | RIKEN cDNA 1700025G04 gene | -1.01 | 0.876 | -1.36 | 0.502 | -1.25 | 0.443 | 1.16 | 0.711 | -0.61 |
| 1441652_at | Bmpr2 | bone morphogenic protein receptor, type II (serine/threonine kinase) | 1.04 | 0.927 | -1.29 | 0.701 | -1.4 | 0.087 | -1.1 | 0.879 | -0.69 |
| 1447456_x_at | LOC671351 | similar to common salivary protein 1 | -1.19 | 0.703 | -1.66 | 0.218 | 1.08 | 0.599 | -1.56 | 0.151 | -0.83 |
| 1455707_at | C130037N17Rik | RIKEN cDNA C130037N17 gene | 1.08 | 0.373 | -2.02 | 0.16 | -1.06 | 0.747 | -1.56 | 0.062 | -0.89 |
| 1438973_x_at | Gja1 | gap junction membrane channel protein alpha 1 | -1.55 | 0.202 | 1.01 | 0.923 | -1.15 | 0.741 | -1.2 | 0.235 | -0.72 |
| 1444900_at | D18Ertd289e | DNA segment, Chr 18, ERATO Doi 289, expressed | 1.12 | 0.74 | -1.32 | 0.423 | -1.55 | 0.275 | -1.06 | 0.855 | -0.7 |
| 1424182_at | Acat1 | acetyl-Coenzyme A acetyltransferase 1 | -1.25 | 0.125 | -1.51 | 0.204 | 1.07 | 0.663 | -1.13 | 0.657 | -0.71 |
| 1430214_a_at | 2610020H08Rik | RIKEN cDNA 2610020H08 gene | -1.09 | 0.697 | -1.22 | 0.421 | -1.27 | 0.4 | 1.09 | 0.674 | -0.62 |
| 1446964_at | --- | --- | 1.31 | 0.729 | -1.25 | 0.685 | -2.37 | 0.187 | -1.29 | 0.715 | -0.9 |
| 1429318_a_at | Qk | quaking | -1.12 | 0.514 | -1.11 | 0.396 | -1.37 | 0.05 | 1.27 | 0.096 | -0.58 |
| 1439338_at | LOC620417 | hypothetical protein LOC620416 | 1.14 | 0.868 | -1.45 | 0.586 | -1.43 | 0.405 | -1.2 | 0.787 | -0.73 |
| 1453141_at | 0610009L18Rik | RIKEN cDNA 0610009L18 gene | -1.13 | 0.414 | -1.64 | 0.026 | 1.03 | 0.573 | -1.61 | 0.23 | -0.84 |
| 1416678_at | Cops3 | COP9 (constitutive photomorphogenic) homolog, subunit 3 (Arabidopsis thaliana) | -1.14 | 0.094 | -1.17 | 0.028 | -1.26 | 0.183 | 1.06 | 0.7 | -0.63 |
| 1432003_a_at | Rnf41 | ring finger protein 41 | -1.57 | 0.016 | -1.23 | 0.065 | 1.08 | 0.577 | -1.46 | 0.012 | -0.8 |
| 1444087_at | Prpf38a | PRP38 pre-mRNA processing factor 38 (yeast) domain containing A | 1.12 | 0.815 | -1.48 | 0.33 | -1.37 | 0.073 | -1.06 | 0.771 | -0.7 |
| 1416539_at | Siae | sialic acid acetylesterase | -1.22 | 0.282 | -1.21 | 0.095 | -1.13 | 0.243 | 1.02 | 0.866 | -0.64 |
| 1439365_at | Myt1 | Myelin transcription factor 1 | -1.47 | 0.465 | -2.69 | 0.043 | 1.47 | 0.542 | -1.14 | 0.846 | -0.96 |
| 1444916_at | --- | PREDICTED: Mus musculus similar to Kinesin-Like Protein family member (klp-6 | -1.29 | 0.726 | -1.89 | 0.225 | 1.23 | 0.718 | -1.34 | 0.33 | -0.83 |
| 1447984_at | D1Ertd75e | DNA segment, Chr 1, ERATO Doi 75, expressed | 1.07 | 0.739 | -1.23 | 0.225 | -1.55 | 0.13 | -1.29 | 0.423 | -0.75 |
| 1454745_at | Arhgap29 | Rho GTPase activating protein 29 | 1.02 | 0.803 | -1.15 | 0.441 | -1.56 | 0.022 | -1.34 | 0.021 | -0.76 |
| 1459788_at | AI790205 | expressed sequence AI790205 | -1.65 | 0.221 | -1.06 | 0.752 | -1.03 | 0.846 | 1.12 | 0.543 | -0.65 |
| 1418974_at | Blzf1 | basic leucine zipper nuclear factor 1 | -1.53 | 0.111 | -1.28 | 0.464 | 1.09 | 0.816 | -1 | 0.994 | -0.68 |
| 1437789_at | Birc6 | baculoviral IAP repeat-containing 6 | 1.15 | 0.411 | -1.35 | 0.073 | -1.57 | 0.039 | -1.48 | 0.229 | -0.81 |
| 1459495_at | Tmem16a | Transmembrane protein 16A | -1.16 | 0.773 | -1.12 | 0.625 | -1.3 | 0.023 | 1.01 | 0.971 | -0.64 |
| 1448910_at | Pecr | peroxisomal trans-2-enoyl-CoA reductase | -1.42 | 0.094 | -1.35 | 0.027 | 1.08 | 0.699 | -1.08 | 0.451 | -0.69 |
| 1422299_a_at | Styxl1 | serine/threonine/tyrosine interacting-like 1 | -1.32 | 0.005 | -1.27 | 0.189 | -1.02 | 0.946 | 1.23 | 0.029 | -0.59 |
| 1458293_at | 5730403B10Rik | RIKEN cDNA 5730403B10 gene | -1.49 | 0.251 | -1.2 | 0.589 | 1.02 | 0.911 | -1.07 | 0.752 | -0.68 |
| 1446628_at | Zbtb24 | zinc finger and BTB domain containing 24 | 1.11 | 0.788 | -1.1 | 0.85 | -1.99 | 0.024 | -1.2 | 0.619 | -0.79 |
| 1433611_s_at | Bud31 | BUD31 homolog (yeast) | -1.09 | 0.186 | -1.12 | 0.204 | -1.41 | 0.054 | 1.12 | 0.57 | -0.62 |
| 1453823_a_at | A930028L21Rik | RIKEN cDNA A930028L21 gene | -1.02 | 0.975 | -2.18 | 0.112 | 1.08 | 0.678 | -1.25 | 0.656 | -0.84 |

| | | | | | | | | | | | |
|---|---|---|---|---|---|---|---|---|---|---|---|
| 1419725_at | Slc26a4 | solute carrier family 26, member 4 | -1.79 | 0.003 | -1.54 | 0.503 | 1.32 | 0.269 | -1.2 | 0.447 | -0.8 |
| 1451542_at | Ssbp2 | single-stranded DNA binding protein 2 | -1.27 | 0.596 | -1.02 | 0.936 | -1.32 | 0.023 | 1.19 | 0.486 | -0.6 |
| 1417778_at | Zfp35 | zinc finger protein 35 | -1.14 | 0.654 | -1.05 | 0.64 | -1.43 | 0.012 | 1.05 | 0.788 | -0.64 |
| 1451177_at | Dnajb4 | DnaJ (Hsp40) homolog, subfamily B, member 4 | -1.06 | 0.834 | -1.19 | 0.176 | -1.35 | 0.13 | 2.39 | 0.23 | -0.3 |
| 1446272_at | Pctk2 | PCTAIRE-motif protein kinase 2 | -1.49 | 0.077 | 1.18 | 0.585 | -1.48 | 0.04 | -1.63 | 0.169 | -0.86 |
| 1455584_at | Sdf4 | stromal cell derived factor 4 | 1.05 | 0.77 | -1.14 | 0.452 | -1.66 | 0.012 | -1.07 | 0.69 | -0.71 |
| 1418534_at | --- | --- | 1.17 | 0.652 | -1.75 | 0.338 | -1.27 | 0.348 | -1.08 | 0.829 | -0.73 |
| 1426727_s_at | Ppp1r10 | protein phosphatase 1, regulatory subunit 10 | -1.08 | 0.812 | -1.45 | 0.34 | -1.09 | 0.426 | 1.21 | 0.326 | -0.6 |
| 1442275_at | 4933402J07Rik | RIKEN cDNA 4933402J07 gene | -1.06 | 0.241 | -1.18 | 0.204 | -1.36 | 0.386 | 1.26 | 0.46 | -0.58 |
| 1422264_s_at | Klf9 | Kruppel-like factor 9 | -1.09 | 0.747 | -1.1 | 0.673 | -1.42 | 0.011 | 1.25 | 0.329 | -0.59 |
| 1437077_at | AW495713 | expressed sequence AW495713 | -1.18 | 0.107 | -1.4 | 0.021 | -1.03 | 0.857 | 1 | 0.98 | -0.65 |
| 1421234_at | Tcf1 | transcription factor 1 | -2.12 | 0.391 | 1.47 | 0.002 | -1.71 | 0.138 | -2.77 | 0.003 | -1.28 |
| 1432734_at | 4933402E15Rik | RIKEN cDNA 4933402E15 gene | -1.34 | 0.403 | 1.12 | 0.675 | -1.51 | 0.355 | -1.41 | 0.262 | -0.78 |
| 1426610_a_at | Ttf1 | transcription termination factor 1 | -1.24 | 0.535 | -1.12 | 0.371 | -1.2 | 0.259 | 1.14 | 0.354 | -0.61 |
| 1442513_at | BC016423 /// LOC6 | cDNA sequence BC016423 /// similar to cDNA sequence BC016423 | 1.06 | 0.934 | -1.27 | 0.219 | -1.46 | 0.233 | -1.19 | 0.726 | -0.72 |
| 1452888_at | 1110034G24Rik | RIKEN cDNA 1110034G24 gene | -1.08 | 0.539 | -1.04 | 0.787 | -1.54 | 0.085 | 1 | 0.956 | -0.67 |
| 1443293_at | --- | --- | 1.17 | 0.658 | -1.37 | 0.426 | -1.6 | 0.071 | -1.61 | 0.187 | -0.85 |
| 1419203_at | Gtlf3a | gene trap locus F3a | -1.06 | 0.86 | -1.14 | 0.531 | -1.41 | 0.095 | 2.05 | 0.239 | -0.39 |
| 1418111_at | 1810008K16Rik | RIKEN cDNA 1810008K16 gene | -1.28 | 0.693 | -1.57 | 0.226 | 1.11 | 0.844 | -1.25 | 0.765 | -0.75 |
| 1442284_at | --- | --- | -1.29 | 0.136 | -1.61 | 0.059 | 1.13 | 0.518 | -1.44 | 0.12 | -0.8 |
| 1454497_at | 5230401M06Rik | RIKEN cDNA 5230401M06 gene | -1.42 | 0.262 | -1.34 | 0.244 | 1.08 | 0.846 | -2.56 | 0.101 | -1.06 |
| 1454751_at | G430022H21Rik | RIKEN cDNA G430022H21 gene | -1.19 | 0.028 | -1.16 | 0.464 | -1.21 | 0.282 | 1.03 | 0.747 | -0.63 |
| 1448681_at | Il15ra | interleukin 15 receptor, alpha chain | -1.38 | 0.187 | -1.33 | 0.32 | 1.05 | 0.819 | -1.24 | 0.489 | -0.72 |
| 1429093_at | Ddi2 | DNA-damage inducible protein 2 | -1.27 | 0.113 | -1.05 | 0.447 | -1.26 | 0.138 | 1.53 | 0.123 | -0.51 |
| 1439124_at | 9530020G05Rik | RIKEN cDNA 9530020G05 gene | -1.26 | 0.316 | -1.15 | 0.509 | -1.15 | 0.572 | 1.31 | 0.241 | -0.56 |
| 1429219_at | 1200009F10Rik | RIKEN cDNA 1200009F10 gene | -1.1 | 0.054 | -1.04 | 0.85 | -1.51 | 0.003 | 1.29 | 0.167 | -0.59 |
| 1445357_at | AI851613 | expressed sequence AI851613 | -1.53 | 0.502 | 1.23 | 0.288 | -1.54 | 0.317 | -1.14 | 0.782 | -0.75 |
| 1434426_at | B130055D15Rik | RIKEN cDNA B130055D15 gene | -1.15 | 0.424 | -1.19 | 0.593 | -1.22 | 0.362 | 1.97 | 0.174 | -0.4 |
| 1429065_at | 1200009F10Rik | RIKEN cDNA 1200009F10 gene | -1.11 | 0.357 | 1.09 | 0.618 | -1.85 | 0.022 | -1.3 | 0.156 | -0.79 |
| 1416269_at | Atp5j2 | ATP synthase, H+ transporting, mitochondrial F0 complex, subunit f, isoform 2 | -1.14 | 0.193 | -1.28 | 0.063 | -1.15 | 0.33 | 1.12 | 0.417 | -0.61 |
| 1440632_at | Pcdhb4 | protocadherin beta 4 | 1.16 | 0.693 | -1.51 | 0.525 | -1.4 | 0.338 | -1.37 | 0.652 | -0.78 |
| 1440160_x_at | 2010321M09Rik | RIKEN cDNA 2010321M09 gene | -1.28 | 0.001 | -1.3 | 0.448 | -1.02 | 0.936 | 1.07 | 0.41 | -0.63 |
| 1416680_at | Ube3a | ubiquitin protein ligase E3A | 1.02 | 0.878 | -1.18 | 0.237 | -1.51 | 0.021 | -1.63 | 0.045 | -0.82 |
| 1423679_at | 2810432L12Rik | RIKEN cDNA 2810432L12 gene | -1.08 | 0.582 | -1.42 | 0.092 | -1.11 | 0.106 | 1.5 | 0.286 | -0.53 |
| 1431455_at | 4933401B01Rik | RIKEN cDNA 4933401B01 gene | -1.01 | 0.978 | -1.51 | 0.34 | -1.14 | 0.773 | 1.21 | 0.542 | -0.61 |
| 1430736_at | 9030411M15Rik /// | RIKEN cDNA 9030411M15 gene /// similar to CG8486-PA, isoform A /// similar to | -2.1 | 0.252 | 1.47 | 0.632 | -1.72 | 0.289 | -1.02 | 0.956 | -0.84 |
| 1417175_at | Csnk1e | casein kinase 1, epsilon | -1.17 | 0.652 | -1.18 | 0.553 | -1.21 | 0.215 | 1.15 | 0.701 | -0.6 |
| 1434923_at | 2810437L13Rik | RIKEN cDNA 2810437L13 gene | -1.2 | 0.283 | -1.13 | 0.161 | -1.24 | 0.136 | 1.24 | 0.348 | -0.58 |
| 1436914_at | Ctdspl2 | CTD (carboxy-terminal domain, RNA polymerase II, polypeptide A) small phospha | -1 | 0.92 | -1.13 | 0.264 | -1.53 | 0.002 | 1.23 | 0.353 | -0.61 |
| 1458469_at | Cblb | Casitas B-lineage lymphoma b | 1.04 | 0.88 | -1.1 | 0.432 | -1.72 | 0.034 | -1.94 | 0.086 | -0.93 |
| 1435664_at | Zfp397 | zinc finger protein 397 | 1.01 | 0.952 | -1.12 | 0.525 | -1.59 | 0.049 | -1.28 | 0.092 | -0.75 |
| 1429798_s_at | Cmtm5 | CKLF-like MARVEL transmembrane domain containing 5 | -1.29 | 0.032 | -1.05 | 0.804 | -1.24 | 0.511 | 1.22 | 0.578 | -0.59 |
| 1421903_at | Ixl | intersex-like (Drosophila) | -1.03 | 0.792 | -1.12 | 0.181 | -1.49 | 0.109 | 1.02 | 0.942 | -0.66 |
| 1442944_at | C76555 | expressed sequence C76555 | 1.26 | 0.64 | -1.42 | 0.247 | -1.76 | 0.178 | -1.15 | 0.362 | -0.77 |
| 1445781_at | Sh3d19 | SH3 domain protein D19 | 1.07 | 0.52 | -1.06 | 0.746 | -1.94 | 0.022 | -3.56 | 0.181 | -1.37 |
| 1448279_at | Arpc3 | actin related protein 2/3 complex, subunit 3 | -1.57 | 0.291 | -1.26 | 0.457 | 1.1 | 0.811 | -2.19 | 0.18 | -0.98 |
| 1442499_at | Cdv3 | carnitine deficiency-associated gene expressed in ventricle 3 | 1.06 | 0.418 | -1.3 | 0.278 | -1.42 | 0.065 | -1.25 | 0.516 | -0.73 |
| 1418349_at | Hbegf | heparin-binding EGF-like growth factor | -1.56 | 0.133 | 1.14 | 0.678 | -1.33 | 0.421 | -1.02 | 0.956 | -0.69 |
| 1457547_at | --- | Adult male spinal cord cDNA, RIKEN full-length enriched library, clone:A330081M | -1.24 | 0.536 | 1.04 | 0.931 | -1.46 | 0.282 | -1.1 | 0.815 | -0.69 |
| 1435277_x_at | Nme1 | expressed in non-metastatic cells 1, protein | -1.12 | 0.769 | 1.15 | 0.15 | -2.05 | 0.058 | -1.87 | 0.155 | -0.97 |
| 1429091_at | 1600002K03Rik | RIKEN cDNA 1600002K03 gene | 1.01 | 0.973 | -1.46 | 0.025 | -1.19 | 0.292 | -1.19 | 0.403 | -0.71 |
| 1443379_at | A430033K04Rik | RIKEN cDNA A430033K04 gene | 1.15 | 0.694 | -1.75 | 0.076 | -1.23 | 0.145 | -1.2 | 0.057 | -0.76 |
| 1421558_at | T2 | brachyury 2 | -1.7 | 0.128 | -1.37 | 0.677 | 1.21 | 0.147 | -2.74 | 0.243 | -1.15 |

| | | | | | | | | | | | |
|---|---|---|---|---|---|---|---|---|---|---|---|
| 1435809_at | Ankrd34 | ankyrin repeat domain 34 | -2.12 | 0.239 | -1.04 | 0.932 | 1.09 | 0.835 | -1.12 | 0.848 | -0.79 |
| 1460589_at | Zfp597 | zinc finger protein 597 | -1.4 | 0.004 | 1 | 0.989 | -1.23 | 0.448 | -1.68 | 0.029 | -0.83 |
| 1442056_at | Zfp608 | Zinc finger protein 608 | 1.12 | 0.097 | -1.06 | 0.848 | -2.17 | 0.076 | -2.28 | 0.068 | -1.1 |
| 1436331_at | Vps13d | vacuolar protein sorting 13 D (yeast) | -1.49 | 0.018 | -1.05 | 0.797 | -1.1 | 0.541 | 1.15 | 0.452 | -0.62 |
| 1426579_at | Gnl2 | guanine nucleotide binding protein-like 2 (nucleolar) | -1.22 | 0.128 | -1.08 | 0.631 | -1.27 | 0.028 | 1.14 | 0.563 | -0.61 |
| 1444663_at | Vezf1 | Vascular endothelial zinc finger 1 | 1.23 | 0.539 | -1.91 | 0.104 | -1.28 | 0.359 | -1.29 | 0.372 | -0.81 |
| 1417107_at | Tpd52l2 | tumor protein D52-like 2 | -1.11 | 0.136 | -1.13 | 0.284 | -1.34 | 0.025 | 1.22 | 0.338 | -0.59 |
| 1446737_a_at | Hook3 | hook homolog 3 (Drosophila) | -1.2 | 0.288 | -1.28 | 0.002 | -1.09 | 0.576 | 1.69 | 0.131 | -0.47 |
| 1438964_x_at | Tfpt | TCF3 (E2A) fusion partner | -1.36 | 0.421 | 1.06 | 0.878 | -1.36 | 0.142 | -1.12 | 0.44 | -0.69 |
| 1431313_at | 5730442P18Rik | RIKEN cDNA 5730442P18 gene | -1.26 | 0.581 | -1.38 | 0.583 | 1.02 | 0.959 | -1.13 | 0.825 | -0.69 |
| 1437330_at | Lrrk1 | leucine-rich repeat kinase 1 | -2.31 | 0.081 | -1.08 | 0.852 | 1.17 | 0.482 | -1.25 | 0.692 | -0.87 |
| 1433851_at | Ppp4r2 | protein phosphatase 4, regulatory subunit 2 | -1.13 | 0.491 | -1.17 | 0.335 | -1.26 | 0.147 | 1.04 | 0.761 | -0.63 |
| 1416227_at | Arpc1b /// LOC4347 | actin related protein 2/3 complex, subunit 1B /// similar to Actin-related protein 2/3 | -1.31 | 0.111 | 1.13 | 0.619 | -1.55 | 0.388 | -1.18 | 0.553 | -0.73 |
| 1441712_at | Trfp | Trf (TATA binding protein-related factor)-proximal protein homolog (Drosophila) | -1.11 | 0.737 | -1.04 | 0.916 | -1.49 | 0.053 | 1.04 | 0.94 | -0.65 |
| 1438229_at | Pggt1b | protein geranylgeranyltransferase type I, beta subunit | -1.19 | 0.086 | -1.08 | 0.139 | -1.3 | 0.092 | 1.12 | 0.305 | -0.61 |
| 1454071_at | Ly6k | lymphocyte antigen 6 complex, locus K | -2.07 | 0.347 | -1.95 | 0.209 | 1.54 | 0.192 | -1.35 | 0.638 | -0.96 |
| 1446329_at | --- | --- | -1.59 | 0.202 | 1.41 | 0.444 | -2 | 0.077 | -1.65 | 0.452 | -0.96 |
| 1456808_at | 4933426M11Rik | RIKEN cDNA 4933426M11 gene | -1.04 | 0.894 | 1.07 | 0.768 | -1.96 | 0.007 | -1.4 | 0.016 | -0.84 |
| 1432199_at | 4930568D16Rik | RIKEN cDNA 4930568D16 gene | 1.15 | 0.407 | -1.25 | 0.386 | -1.73 | 0.178 | -1.71 | 0.439 | -0.88 |
| 1448269_a_at | Klhl13 | kelch-like 13 (Drosophila) | -1.19 | 0.419 | -1.08 | 0.624 | -1.3 | 0.231 | 1.11 | 0.616 | -0.61 |
| 1440440_at | --- | --- | 1.36 | 0.374 | -1.35 | 0.386 | -2.29 | 0.044 | -2.81 | 0.237 | -1.27 |
| 1449719_at | 4930453N24Rik | RIKEN cDNA 4930453N24 gene | -1.12 | 0.83 | -1.42 | 0.342 | -1.07 | 0.822 | 1.49 | 0.334 | -0.53 |
| 1446953_at | Tcf4 | Transcription factor 4 | -1.16 | 0.115 | 1.05 | 0.447 | -1.61 | 0.085 | -1.33 | 0.275 | -0.76 |
| 1416098_at | Syngr3 | synaptogyrin 3 | -1.68 | 0.299 | 1.16 | 0.698 | -1.28 | 0.187 | -1 | 0.999 | -0.7 |
| 1441411_at | Lims1 | LIM and senescent cell antigen-like domains 1 | -1.03 | 0.875 | 1.03 | 0.919 | -1.86 | 0.004 | -1.18 | 0.222 | -0.76 |
| 1432736_at | 4933430H06Rik | RIKEN cDNA 4933430H06 gene | -1.06 | 0.682 | -1.43 | 0.288 | -1.12 | 0.539 | 1.52 | 0.311 | -0.52 |
| 1427119_at | Spink4 | serine peptidase inhibitor, Kazal type 4 | 1.31 | 0.57 | -1.81 | 0.091 | -1.49 | 0.146 | -1.74 | 0.315 | -0.93 |
| 1458108_at | Ptcd2 | Pentatricopeptide repeat domain 2 | 1.12 | 0.558 | -1.57 | 0.245 | -1.29 | 0.017 | -1.81 | 0.034 | -0.89 |
| 1422372_at | Olfr15 | olfactory receptor 15 | -1.72 | 0.063 | -1.6 | 0.484 | 1.33 | 0.327 | -1.32 | 0.368 | -0.83 |
| 1434565_at | Cgrrf1 | cell growth regulator with ring finger domain 1 | -1.16 | 0.354 | -1.07 | 0.362 | -1.35 | 0.084 | 1.34 | 0.244 | -0.56 |
| 1442905_at | Arf4 | ADP-ribosylation factor 4 | 1.02 | 0.927 | -1.33 | 0.301 | -1.32 | 0.237 | -1.21 | 0.525 | -0.71 |
| 1421422_at | AF397014 | cDNA sequence AF397014 | -1.12 | 0.601 | -1.73 | 0.052 | 1.06 | 0.847 | -2.08 | 0.215 | -0.97 |
| 1432410_a_at | Bmp7 | bone morphogenetic protein 7 | 1.24 | 0.427 | -1.42 | 0.497 | -1.69 | 0.092 | -1.78 | 0.408 | -0.91 |
| 1431243_at | 1700120J03Rik | RIKEN cDNA 1700120J03 gene | 1.04 | 0.536 | -1.57 | 0.42 | -1.17 | 0.616 | -2.32 | 0.15 | -1 |
| 1438122_at | 2900006K08Rik | RIKEN cDNA 2900006K08 gene | -1.38 | 0.334 | -1.37 | 0.492 | 1.09 | 0.746 | -1.06 | 0.566 | -0.68 |
| 1455566_s_at | 2810022L02Rik | RIKEN cDNA 2810022L02 gene | 1.13 | 0.525 | -1.11 | 0.598 | -1.98 | 0.031 | -1.15 | 0.38 | -0.78 |
| 1438882_at | Arhgap18 | Rho GTPase activating protein 18 | -1.26 | 0.019 | 1.07 | 0.788 | -1.48 | 0.06 | -1.36 | 0.226 | -0.76 |
| 1437497_a_at | Hsp90aa1 | heat shock protein 90kDa alpha (cytosolic), class A member 1 | -1.2 | 0.29 | -1.2 | 0.464 | -1.15 | 0.517 | 1.29 | 0.389 | -0.57 |
| 1422790_at | Nppc | natriuretic peptide precursor type C | -1.08 | 0.865 | -1 | 0.997 | -1.64 | 0.4 | 1.2 | 0.689 | -0.63 |
| 1442972_at | Coro1c | Coronin, actin binding protein 1C | -1.33 | 0.36 | 1.23 | 0.651 | -1.8 | 0.138 | -1 | 0.996 | -0.73 |
| 1427272_at | Btbd15 | BTB (POZ) domain containing 15 | -1.29 | 0.3 | -2.48 | 0.005 | 1.36 | 0.573 | -1.16 | 0.741 | -0.89 |
| 1430156_at | 4930520O04Rik | RIKEN cDNA 4930520O04 gene | -1.23 | 0.799 | -1.16 | 0.728 | -1.16 | 0.79 | 2.67 | 0.202 | -0.22 |
| 1445712_at | --- | Transcribed locus | 1.32 | 0.037 | -1.09 | 0.869 | -3.38 | 0.18 | -1.03 | 0.949 | -1.04 |
| 1424264_at | Med6 | mediator of RNA polymerase II transcription, subunit 6 homolog (yeast) | -1.15 | 0.564 | -1.04 | 0.769 | -1.42 | 0.042 | 1.34 | 0.039 | -0.57 |
| 1429009_at | Snrp70 | U1 small nuclear ribonucleoprotein polypeptide A | -1.17 | 0.249 | -1.07 | 0.692 | -1.34 | 0.139 | 1.01 | 0.97 | -0.64 |
| 1453514_at | Gpm6b | glycoprotein m6b | -1.27 | 0.731 | 1.28 | 0.562 | -2.15 | 0.112 | -1.4 | 0.371 | -0.88 |
| 1430710_at | --- | --- | -1.14 | 0.455 | -1.65 | 0.212 | 1.05 | 0.662 | -1.09 | 0.828 | -0.71 |
| 1446676_at | A130033P14 | hypothetical protein A130033P14 | -2.19 | 0.142 | 1.2 | 0.731 | -1.14 | 0.321 | -1.14 | 0.694 | -0.82 |
| 1441986_at | Zcchc6 | zinc finger, CCHC domain containing 6 | -1.22 | 0.27 | -1.25 | 0.374 | -1.09 | 0.526 | 2.26 | 0.172 | -0.33 |
| 1441321_at | LOC552911 | hypothetical LOC552911 | -1.12 | 0.143 | 1.25 | 0.413 | -2.54 | 0 | -1.13 | 0.649 | -0.88 |
| 1433272_at | A430110A21Rik | RIKEN cDNA A430110A21 gene | -1.78 | 0.312 | 1.06 | 0.915 | -1.09 | 0.859 | -1.26 | 0.443 | -0.77 |
| 1446317_at | Arid4b | AT rich interactive domain 4B (Rbp1 like) | -2.48 | 0.077 | -1.13 | 0.808 | 1.25 | 0.644 | -1.11 | 0.795 | -0.87 |
| 1416293_at | Nfib | nuclear factor I/B | -1.08 | 0.844 | -2.14 | 0.101 | 1.15 | 0.257 | -2.37 | 0.399 | -1.11 |

| | | | | | | | | | | | |
|---|---|---|---|---|---|---|---|---|---|---|---|
| 1440372_at | Arl15 | ADP-ribosylation factor-like 15 | -1.01 | 0.902 | 1.08 | 0.517 | -2.1 | 0.05 | -1.09 | 0.702 | -0.78 |
| 1442254_at | Dgkd | Diacylglycerol kinase, delta | 1.14 | 0.021 | -1.25 | 0.431 | -1.68 | 0.123 | -2.09 | 0.164 | -0.97 |
| 1434102_at | Nfib | nuclear factor I/B | 1.05 | 0.861 | -1.16 | 0.463 | -1.58 | 0.162 | -1.03 | 0.944 | -0.68 |
| 1419104_at | Abhd6 | abhydrolase domain containing 6 | 1 | 0.984 | -1.59 | 0.289 | -1.11 | 0.402 | -1.67 | 0.071 | -0.84 |
| 1448455_at | Cln8 | ceroid-lipofuscinosis, neuronal 8 | -1.4 | 0.205 | -1.09 | 0.641 | -1.1 | 0.601 | 2.68 | 0.17 | -0.23 |
| 1429240_at | Stard4 | StAR-related lipid transfer (START) domain containing 4 | -1.12 | 0.719 | -1.04 | 0.742 | -1.47 | 0.002 | 1.23 | 0.627 | -0.6 |
| 1443544_at | D18Ertd653e | DNA segment, Chr 18, ERATO Doi 653, expressed | -1.2 | 0.096 | 1.11 | 0.698 | -1.68 | 0.03 | -1.58 | 0.213 | -0.84 |
| 1431929_a_at | Stx17 | syntaxin 17 | -1.18 | 0.611 | -1.2 | 0.175 | -1.16 | 0.072 | 1.13 | 0.636 | -0.6 |
| 1438607_at | Zdhhc19 | zinc finger, DHHC domain containing 19 | 1.46 | 0.584 | -2.04 | 0.049 | -1.71 | 0.022 | -1.28 | 0.35 | -0.89 |
| 1445322_x_at | E430025E21Rik | RIKEN cDNA E430025E21 gene | -1.08 | 0.824 | -1.05 | 0.586 | -1.51 | 0.07 | 1.1 | 0.804 | -0.64 |
| 1458442_at | AI132709 | expressed sequence AI132709 | -1.17 | 0.677 | -1.6 | 0.175 | 1.06 | 0.901 | -1.17 | 0.443 | -0.72 |
| 1427928_s_at | Cdc91l1 | CDC91 cell division cycle 91-like 1 (S. cerevisiae) | -1.36 | 0.196 | -1.31 | 0.019 | 1.04 | 0.769 | -1.04 | 0.832 | -0.67 |
| 1459321_at | --- | --- | 1.22 | 0.664 | -2.25 | 0.035 | -1.14 | 0.033 | -1.75 | 0.032 | -0.98 |
| 1432882_at | 4932431P20Rik | RIKEN cDNA 4932431P20 gene | -1.28 | 0.435 | -1.06 | 0.584 | -1.23 | 0.38 | 1.01 | 0.964 | -0.64 |
| 1442104_at | Usp25 | Ubiquitin specific peptidase 25 | -1.03 | 0.948 | -1.56 | 0.195 | -1.08 | 0.804 | 2.41 | 0.115 | -0.32 |
| 1438713_at | Rassf8 | Ras association (RalGDS/AF-6) domain family 8 | -1.18 | 0.623 | -1.33 | 0.67 | -1.06 | 0.817 | 1.53 | 0.135 | -0.51 |
| 1418670_s_at | Hspg2 | perlecan (heparan sulfate proteoglycan 2) | -1.07 | 0.856 | 1.05 | 0.91 | -1.81 | 0.144 | -1.86 | 0.203 | -0.92 |
| 1436110_at | --- | 10 days neonate medulla oblongata cDNA, RIKEN full-length enriched library, clo | 1.01 | 0.982 | -1.35 | 0.222 | -1.27 | 0.704 | -3.94 | 0.4 | -1.39 |
| 1450959_at | D930014E17Rik | RIKEN cDNA D930014E17 gene | -1.15 | 0.263 | -1.33 | 0.157 | -1.1 | 0.598 | 1.55 | 0.278 | -0.5 |
| 1421074_at | Cyp7b1 | cytochrome P450, family 7, subfamily b, polypeptide 1 | -1.12 | 0.626 | -1.57 | 0.098 | 1 | 0.968 | -1.45 | 0.186 | -0.78 |
| 1453645_at | 2700046A07Rik | RIKEN cDNA 2700046A07 gene | -1.76 | 0.494 | -2.65 | 0.205 | 1.59 | 0.175 | -1.17 | 0.766 | -1 |
| 1444027_at | Slc30a8 | solute carrier family 30 (zinc transporter), member 8 | -2.33 | 0.508 | 1.59 | 0.165 | -1.94 | 0.139 | -1.14 | 0.876 | -0.95 |
| 1459571_at | Sh3bgrl | SH3-binding domain glutamic acid-rich protein like | 1.18 | 0.197 | -1.33 | 0.046 | -1.64 | 0.013 | -1.04 | 0.808 | -0.71 |
| 1458629_at | --- | --- | 1.07 | 0.918 | -1.45 | 0.614 | -1.29 | 0.349 | -2.46 | 0.169 | -1.03 |
| 1438865_at | H13 | histocompatibility 13 | -1.34 | 0.366 | -2.51 | 0.018 | 1.39 | 0.206 | -1.94 | 0.064 | -1.1 |
| 1443521_at | Ggnbp2 | Gametogenetin binding protein 2 | -1.11 | 0.466 | -1.09 | 0.521 | -1.38 | 0.249 | 1.12 | 0.448 | -0.62 |
| 1416755_at | Dnajb1 | DnaJ (Hsp40) homolog, subfamily B, member 1 | -1.06 | 0.484 | -1.23 | 0.144 | -1.28 | 0.399 | 1.28 | 0.647 | -0.57 |
| 1419345_at | Pth | parathyroid hormone | -1.28 | 0.796 | -1.08 | 0.858 | -1.2 | 0.796 | 1.34 | 0.278 | -0.56 |
| 1447898_s_at | Sfrs6 | splicing factor, arginine/serine-rich 6 | -1.52 | 0.014 | 1.08 | 0.67 | -1.25 | 0.028 | -1.06 | 0.671 | -0.69 |
| 1446064_at | --- | --- | -1.65 | 0.31 | -1.18 | 0.702 | 1.08 | 0.834 | -1.79 | 0.171 | -0.88 |
| 1435369_at | Fastkd5 | FAST kinase domains 5 | -1.02 | 0.843 | -1.35 | 0.006 | -1.22 | 0.038 | 1.02 | 0.941 | -0.64 |
| 1451290_at | Map1lc3a | microtubule-associated protein 1 light chain 3 alpha | -1.16 | 0.621 | -1.53 | 0.063 | 1.02 | 0.951 | -1.29 | 0.321 | -0.74 |
| 1442304_at | --- | 16 days neonate heart cDNA, RIKEN full-length enriched library, clone:D830044D | 1.09 | 0.84 | -2.03 | 0.404 | -1.04 | 0.889 | -2.42 | 0.435 | -1.1 |
| 1444015_at | Papola | Poly (A) polymerase alpha | -1.15 | 0.402 | -1.08 | 0.697 | -1.35 | 0.204 | 1.36 | 0.126 | -0.55 |
| 1429969_at | 4833403J16Rik | RIKEN cDNA 4833403J16 gene | -1.62 | 0.225 | 1.26 | 0.727 | -1.52 | 0.06 | -1.85 | 0.148 | -0.93 |
| 1439159_at | --- | --- | -1.22 | 0.567 | 1.1 | 0.513 | -1.61 | 0.078 | -1.46 | 0.164 | -0.8 |
| 1438259_at | --- | --- | -1.31 | 0.44 | 1.07 | 0.745 | -1.41 | 0.048 | -1.48 | 0.043 | -0.78 |
| 1446236_at | C230072F16Rik | RIKEN cDNA C230072F16 gene | -1.06 | 0.838 | -1.32 | 0.383 | -1.18 | 0.49 | 1.14 | 0.781 | -0.61 |
| 1418333_at | Mtf1 | metal response element binding transcription factor 1 | -1.34 | 0.006 | -1.03 | 0.873 | -1.22 | 0.023 | 1.17 | 0.505 | -0.6 |
| 1416725_at | Tcf4 | transcription factor 4 | -2.46 | 0.364 | -1.45 | 0.33 | 1.44 | 0.055 | -1.49 | 0.1 | -0.99 |
| 1428617_at | Hcfc2 | host cell factor C2 | -1.07 | 0.638 | -1.32 | 0.104 | -1.18 | 0.26 | 1.08 | 0.726 | -0.62 |
| 1434300_at | 2610101N10Rik | RIKEN cDNA 2610101N10 gene | -1.1 | 0.588 | -1.19 | 0.144 | -1.26 | 0.192 | 1.16 | 0.562 | -0.6 |
| 1420526_at | Dcbld2 | discoidin, CUB and LCCL domain containing 2 | 1.24 | 0.544 | -2.91 | 0.013 | -1.05 | 0.858 | -1.19 | 0.611 | -0.98 |
| 1438006_at | 4933439F18Rik | RIKEN cDNA 4933439F18 gene | -1.14 | 0.1 | -1.19 | 0.251 | -1.21 | 0.202 | 1.15 | 0.191 | -0.6 |
| 1447817_at | Hif1an | hypoxia-inducible factor 1, alpha subunit inhibitor | 1.11 | 0.895 | -2.08 | 0.225 | -1.06 | 0.862 | -1.25 | 0.639 | -0.82 |
| 1436891_at | Usp15 | ubiquitin specific peptidase 15 | -1.3 | 0.133 | -1.12 | 0.538 | -1.15 | 0.57 | 1.52 | 0.261 | -0.51 |
| 1420409_at | Krt35 | keratin 35 | 1.12 | 0.636 | -2.15 | 0.12 | -1.05 | 0.901 | -2.41 | 0.222 | -1.12 |
| 1429810_at | 4921505C17Rik | RIKEN cDNA 4921505C17 gene | 1.01 | 0.97 | -1.24 | 0.097 | -1.38 | 0.279 | -1.03 | 0.903 | -0.66 |
| 1444996_at | Depdc5 | DEP domain containing 5 | 1.19 | 0.814 | -1.28 | 0.378 | -1.76 | 0.241 | -1.36 | 0.613 | -0.8 |
| 1444092_at | 9430025M13Rik | RIKEN cDNA 9430025M13 gene | -1.51 | 0.023 | -1.09 | 0.647 | -1.04 | 0.831 | 1 | 0.981 | -0.66 |
| 1422336_at | Hoxa13 | homeo box A13 | -1.17 | 0.495 | -1.25 | 0.665 | -1.13 | 0.599 | 1.62 | 0.241 | -0.48 |
| 1419847_at | Pskh1 | Protein serine kinase H1 | -1.37 | 0.267 | 1.02 | 0.972 | -1.27 | 0.344 | -1.09 | 0.759 | -0.68 |
| 1420206_at | --- | --- | 1.12 | 0.908 | -1.81 | 0.435 | -1.15 | 0.805 | -1.42 | 0.46 | -0.81 |

| | | | | | | | | | | | |
|---|---|---|---|---|---|---|---|---|---|---|---|
| 1452795_at | 1110008B24Rik | RIKEN cDNA 1110008B24 gene | -1.14 | 0.157 | -1.3 | 0.046 | -1.12 | 0.203 | 1.02 | 0.885 | -0.63 |
| 1418250_at | Arfl4 | ADP-ribosylation factor 4-like | -1.27 | 0.167 | -1.16 | 0.595 | -1.13 | 0.533 | 1.28 | 0.425 | -0.57 |
| 1458049_at | --- | --- | -1.28 | 0.107 | -1.2 | 0.365 | -1.08 | 0.83 | 2.31 | 0.353 | -0.31 |
| 1441444_at | Nbeal1 | Neurobeachin like 1 | 1.01 | 0.943 | -1.05 | 0.791 | -1.73 | 0.014 | -1.43 | 0.086 | -0.8 |
| 1451038_at | Apln | apelin | -1.03 | 0.719 | -1.12 | 0.609 | -1.47 | 0.148 | 1.18 | 0.603 | -0.61 |
| 1425090_s_at | Kcnc4 | potassium voltage gated channel, Shaw-related subfamily, member 4 | -1.07 | 0.336 | -1.11 | 0.594 | -1.41 | 0.433 | 1.08 | 0.219 | -0.63 |
| 1455910_at | Klhdc3 | kelch domain containing 3 | -1.5 | 0.509 | -1.07 | 0.903 | -1.07 | 0.841 | 1.1 | 0.574 | -0.63 |
| 1417552_at | Fap | fibroblast activation protein | -1.26 | 0.549 | -1.18 | 0.674 | -1.11 | 0.528 | 1.23 | 0.44 | -0.58 |
| 1452809_at | 9030607L17Rik | RIKEN cDNA 9030607L17 gene | -1.52 | 0.052 | -1.14 | 0.643 | 1.01 | 0.952 | -1.27 | 0.455 | -0.73 |
| 1439136_at | Ssbp3 | Single-stranded DNA binding protein 3 | -1.21 | 0.169 | -1.15 | 0.487 | -1.19 | 0.664 | 1.63 | 0.307 | -0.48 |
| 1427069_at | Fbxo28 | F-box protein 28 | -1.13 | 0.46 | -1.13 | 0.431 | -1.29 | 0.092 | 1.14 | 0.336 | -0.6 |
| 1458385_at | Hspa4l | heat shock protein 4 like | -1.19 | 0.61 | -1.27 | 0.337 | -1.09 | 0.84 | 2.36 | 0.259 | -0.3 |
| 1428672_at | Snrpf | small nuclear ribonucleoprotein polypeptide F | -1.33 | 0.402 | 1.11 | 0.224 | -1.48 | 0.031 | -1.62 | 0.372 | -0.83 |
| 1420732_at | 2510027J23Rik | RIKEN cDNA 2510027J23 gene | -1.27 | 0.805 | 1.36 | 0.599 | -2.53 | 0.157 | -1.42 | 0.284 | -0.97 |
| 1424712_at | Ahctf1 | AT hook containing transcription factor 1 | -1.05 | 0.77 | -1.56 | 0.01 | -1.05 | 0.391 | 1.2 | 0.168 | -0.61 |
| 1437650_at | C730026J16 | hypothetical protein C730026J16 | -1.16 | 0.007 | -1.23 | 0.101 | -1.16 | 0.347 | 1.01 | 0.926 | -0.63 |
| 1429714_at | Sumf2 | sulfatase modifying factor 2 | -1.28 | 0.354 | -1.14 | 0.669 | -1.13 | 0.35 | 1.46 | 0.038 | -0.52 |
| 1442332_at | Tgfbr3 | Transforming growth factor, beta receptor III | 1.09 | 0.782 | -1.28 | 0.502 | -1.5 | 0.031 | -1.41 | 0.181 | -0.77 |
| 1439367_x_at | Arf4 | ADP-ribosylation factor 4 | -1.49 | 0.158 | -1.1 | 0.421 | -1.04 | 0.841 | 1.27 | 0.286 | -0.59 |
| 1447608_x_at | Btbd14a | BTB (POZ) domain containing 14A | -1.33 | 0.521 | -1.58 | 0.314 | 1.16 | 0.107 | -1.19 | 0.485 | -0.73 |
| 1447132_at | Cdkal1 | CDK5 regulatory subunit associated protein 1-like 1 | -1.44 | 0.458 | -1.26 | 0.561 | 1.05 | 0.407 | -1.12 | 0.851 | -0.69 |
| 1457513_at | 6330569M22Rik | RIKEN cDNA 6330569M22 gene | 1.07 | 0.141 | -1.19 | 0.705 | -1.58 | 0.013 | -1.45 | 0.052 | -0.79 |
| 1443883_at | 2610042O14Rik | RIKEN cDNA 2610042O14 gene | -1.3 | 0.449 | -1.35 | 0.484 | 1.03 | 0.895 | -1.63 | 0.11 | -0.81 |
| 1426993_at | Xpr1 | xenotropic and polytropic retrovirus receptor 1 | -1.17 | 0.742 | -1.35 | 0.364 | -1.06 | 0.921 | 1.36 | 0.568 | -0.55 |
| 1435391_at | 3200001K10Rik | RIKEN cDNA 3200001K10 gene | -1.24 | 0.315 | 1.01 | 0.95 | -1.37 | 0.286 | -1.31 | 0.486 | -0.73 |
| 1455579_at | Csn1s2a | casein alpha s2-like A | -1.17 | 0.574 | -1.63 | 0.148 | 1.08 | 0.867 | -2.07 | 0.255 | -0.95 |
| 1427964_at | Cmtm8 | CKLF-like MARVEL transmembrane domain containing 8 | -1.12 | 0.116 | -1.12 | 0.592 | -1.33 | 0.233 | 1.19 | 0.013 | -0.59 |
| 1420888_at | Bcl2l1 | Bcl2-like 1 | -1.2 | 0.256 | -1.2 | 0.298 | -1.14 | 0.555 | 2.4 | 0.206 | -0.29 |
| 1454698_at | Ptplad1 | protein tyrosine phosphatase-like A domain containing 1 | -1.15 | 0.309 | -1.07 | 0.607 | -1.35 | 0.025 | 1.23 | 0.099 | -0.59 |
| 1428146_s_at | Acaa2 | acetyl-Coenzyme A acyltransferase 2 (mitochondrial 3-oxoacyl-Coenzyme A thiol | -1.11 | 0.052 | -1.34 | 0.133 | -1.11 | 0.44 | 1.01 | 0.91 | -0.64 |
| 1424956_at | Ahdc1 | AT hook, DNA binding motif, containing 1 | 1.05 | 0.838 | -1.33 | 0.341 | -1.35 | 0.174 | -1.19 | 0.572 | -0.7 |
| 1437241_at | Tieg3 | TGFB inducible early growth response 3 | -1.2 | 0.531 | -1.34 | 0.305 | -1.03 | 0.911 | 1.87 | 0.102 | -0.43 |
| 1455903_at | Tbc1d4 | TBC1 domain family, member 4 | 1.12 | 0.774 | -1.66 | 0.465 | -1.22 | 0.397 | -1.21 | 0.415 | -0.74 |
| 1439601_at | 2700008B19Rik | RIKEN cDNA 2700008B19 gene | -1.38 | 0.321 | -1.43 | 0.059 | 1.12 | 0.418 | -1.52 | 0.119 | -0.8 |
| 1437126_at | Immt | Inner membrane protein, mitochondrial | 1.19 | 0.01 | -1.13 | 0.284 | -2.15 | 0.007 | -1.6 | 0.19 | -0.92 |
| 1417513_at | Evi5 | ecotropic viral integration site 5 | -1.05 | 0.721 | -1.28 | 0 | -1.24 | 0.051 | 2.44 | 0.146 | -0.28 |
| 1447858_x_at | Il4ra | interleukin 4 receptor, alpha | -1.29 | 0.41 | 1.04 | 0.905 | -1.37 | 0.459 | -1.34 | 0.45 | -0.74 |
| 1447028_at | 4930563E22Rik | RIKEN cDNA 4930563E22 gene | -1.45 | 0.107 | -1.24 | 0.579 | 1.05 | 0.765 | -1.64 | 0.147 | -0.82 |
| 1450540_x_at | Krtap5-1 | keratin associated protein 5-1 | -1.26 | 0.193 | -1.12 | 0.595 | -1.16 | 0.71 | 2.24 | 0.326 | -0.33 |
| 1443407_at | Rnf13 | ring finger protein 13 | 1.05 | 0.884 | -1.3 | 0.464 | -1.38 | 0.242 | -1.19 | 0.665 | -0.7 |
| 1453522_at | 6530401N04Rik | RIKEN cDNA 6530401N04 gene | -1.19 | 0.526 | -1.12 | 0.385 | -1.23 | 0.586 | 1.07 | 0.736 | -0.62 |
| 1429615_at | Zfp91 | zinc finger protein 91 | -1.15 | 0.127 | -1.14 | 0.118 | -1.25 | 0.061 | 1.16 | 0.518 | -0.6 |
| 1454117_at | 5330439B14Rik | RIKEN cDNA 5330439B14 gene | 1.03 | 0.965 | -1.35 | 0.422 | -1.3 | 0.61 | -1.29 | 0.519 | -0.73 |
| 1446428_at | D530005L17Rik | RIKEN cDNA D530005L17 gene | -1.21 | 0.673 | -1.05 | 0.923 | -1.3 | 0.209 | 1.31 | 0.107 | -0.56 |
| 1422244_at | Pkdrej | polycystic kidney disease (polycystin) and REJ (sperm receptor for egg jelly, sea | -1.65 | 0.321 | 1.17 | 0.73 | -1.3 | 0.195 | -1.42 | 0.376 | -0.8 |
| 1424237_at | Zfp639 | zinc finger protein 639 | -1.23 | 0.352 | -1.13 | 0.149 | -1.18 | 0.288 | 1.11 | 0.313 | -0.61 |
| 1426279_at | 5830415L20Rik | RIKEN cDNA 5830415L20 gene | -1.12 | 0.55 | -1.1 | 0.437 | -1.35 | 0.01 | 1.13 | 0.283 | -0.61 |
| 1442009_at | Eif2c3 | eukaryotic translation initiation factor 2C, 3 | 1.61 | 0.505 | -3.1 | 0.332 | -1.63 | 0.188 | -3.16 | 0.287 | -1.57 |
| 1420034_at | Ppp2r2d | Protein phosphatase 2, regulatory subunit B, delta isoform | -1.06 | 0.853 | -1.18 | 0.58 | -1.33 | 0.066 | 1.1 | 0.85 | -0.62 |
| 1439453_x_at | 1500026D16Rik | RIKEN cDNA 1500026D16 gene | -1.06 | 0.626 | -1.21 | 0.164 | -1.29 | 0.06 | 1.31 | 0.372 | -0.56 |
| 1439707_at | C230034O21Rik | RIKEN cDNA C230034O21 gene | -1.09 | 0.728 | -1.08 | 0.719 | -1.42 | 0.164 | 1.65 | 0.258 | -0.49 |
| 1438919_x_at | Fdft1 /// LOC62771 | farnesyl diphosphate farnesyl transferase 1 /// similar to farnesyl diphosphate farr | -1.45 | 0.266 | -1.49 | 0.221 | 1.18 | 0.687 | -1.27 | 0.463 | -0.76 |
| 1424476_at | Camkk2 | calcium/calmodulin-dependent protein kinase kinase 2, beta | -1.08 | 0.94 | -1.18 | 0.548 | -1.3 | 0.561 | 1.53 | 0.097 | -0.51 |

| | | | | | | | | | | | | |
|---|---|---|---|---|---|---|---|---|---|---|---|---|
| 1421662_a_at | Tusc3 | tumor suppressor candidate 3 | -1 | 0.978 | -1.39 | 0.035 | -1.21 | 0.131 | 1.12 | 0.677 | -0.62 |
| 1421343_at | Bach2 | BTB and CNC homology 2 | -1.47 | 0.4 | -1.43 | 0.496 | 1.16 | 0.768 | -1.07 | 0.907 | -0.7 |
| 1435134_at | Aadacl1 | arylacetamide deacetylase-like 1 | -1.05 | 0.262 | -1.7 | 0.05 | 1 | 0.989 | -1.43 | 0.078 | -0.79 |
| 1441024_at | Stim2 | stromal interaction molecule 2 | -1.26 | 0.35 | 1.02 | 0.859 | -1.38 | 0.056 | -1.05 | 0.885 | -0.67 |
| 1444815_at | C85328 | expressed sequence C85328 | 1.04 | 0.865 | -1.23 | 0.625 | -1.45 | 0.458 | -1.74 | 0.174 | -0.84 |
| 1443558_s_at | C630002B14Rik | RIKEN cDNA C630002B14 gene | -1.57 | 0.074 | -1.34 | 0.057 | 1.16 | 0.116 | -1.16 | 0.332 | -0.73 |
| 1436463_at | Jakmip2 | Janus kinase and microtubule interacting protein 2 | -2.5 | 0.45 | -2.75 | 0.219 | 1.78 | 0.208 | -1.32 | 0.319 | -1.2 |
| 1426668_at | Slc30a9 | solute carrier family 30 (zinc transporter), member 9 | -1.11 | 0.548 | -1.2 | 0.056 | -1.23 | 0.118 | 1.99 | 0.22 | -0.39 |
| 1439061_at | Ccdc84 | coiled-coil domain containing 84 | 1.01 | 0.976 | -1.46 | 0.041 | -1.18 | 0.121 | -1.24 | 0.231 | -0.72 |
| 1417737_at | Mrps31 | mitochondrial ribosomal protein S31 | -1.08 | 0.423 | -1.28 | 0.004 | -1.19 | 0.152 | 1.09 | 0.675 | -0.62 |
| 1419146_a_at | Gck | glucokinase | -1.42 | 0.01 | -1.5 | 0.478 | 1.17 | 0.769 | -1.67 | 0.465 | -0.85 |
| 1451459_at | Ahctf1 | AT hook containing transcription factor 1 | -1.01 | 0.907 | -1.35 | 0.016 | -1.22 | 0.117 | 1.04 | 0.757 | -0.64 |
| 1453078_at | 8430408J07Rik | RIKEN cDNA 8430408J07 gene | -1.19 | 0.133 | -1.03 | 0.771 | -1.37 | 0.01 | 1.1 | 0.493 | -0.62 |
| 1452247_at | Fxr1h | fragile X mental retardation gene 1, autosomal homolog | -1.11 | 0.528 | -1.07 | 0.371 | -1.41 | 0.034 | 1.03 | 0.834 | -0.64 |
| 1449797_x_at | Prph1 | Peripherin 1 | -1.54 | 0.396 | 1.19 | 0.784 | -1.42 | 0.407 | -1.01 | 0.982 | -0.69 |
| 1435130_at | LOC383103 | similar to RIKEN cDNA 1810036I24 | -1.43 | 0.357 | -1.31 | 0.585 | 1.08 | 0.841 | -1.08 | 0.769 | -0.68 |
| 1434785_at | Cacng5 | calcium channel, voltage-dependent, gamma subunit 5 | 1.12 | 0.771 | -2.08 | 0.001 | -1.06 | 0.793 | -1.67 | 0.113 | -0.92 |
| 1446653_at | --- | --- | 1.17 | 0.594 | -1.35 | 0.186 | -1.58 | 0.091 | -1.49 | 0.014 | -0.81 |
| 1416267_at | Scoc | short coiled-coil protein | -1.29 | 0.184 | -1 | 0.963 | -1.3 | 0.077 | 2.98 | 0.217 | -0.15 |
| 1421934_at | Cbx5 | chromobox homolog 5 (Drosophila HP1a) | -1.1 | 0.195 | -1.47 | 0.101 | -1.05 | 0.813 | 2.06 | 0.136 | -0.39 |
| 1420488_at | Mrps14 | mitochondrial ribosomal protein S14 | -1.45 | 0.091 | -1.02 | 0.647 | -1.14 | 0.658 | 1.05 | 0.622 | -0.64 |
| 1431012_a_at | Peci | peroxisomal delta3, delta2-enoyl-Coenzyme A isomerase | -1.06 | 0.764 | -1.52 | 0.069 | -1.06 | 0.577 | 1.03 | 0.747 | -0.65 |
| 1416823_a_at | Osbpl1a | oxysterol binding protein-like 1A | -1.17 | 0.43 | -1.23 | 0.208 | -1.14 | 0.414 | 1.68 | 0.053 | -0.46 |
| 1440580_at | C030014M07Rik /// | RIKEN cDNA C030014M07 gene /// similar to RIKEN cDNA C920006C10 | 1.29 | 0.59 | -2.59 | 0.017 | -1.14 | 0.768 | -3.37 | 0.107 | -1.45 |
| 1454712_at | Mcart1 | mitochondrial carrier triple repeat 1 | -1.19 | 0.125 | -1.12 | 0.306 | -1.23 | 0.02 | 2.76 | 0.223 | -0.2 |
| 1443886_at | Prkcz2 | protein kinase C, zeta 2 | -1.09 | 0.702 | 1.1 | 0.833 | -1.9 | 0.275 | -1.21 | 0.406 | -0.77 |
| 1416672_s_at | Mcoln1 | mucolipin 1 | -1.44 | 0.335 | -1.39 | 0.154 | 1.13 | 0.615 | -1.59 | 0.018 | -0.82 |
| 1454444_at | 5730406E14Rik | RIKEN cDNA 5730406E14 gene | -2.4 | 0.446 | -1.49 | 0.219 | 1.46 | 0.436 | -1.02 | 0.972 | -0.86 |
| 1426917_s_at | Scrn3 | secernin 3 | -1.14 | 0.547 | -1.18 | 0.287 | -1.22 | 0.283 | 3.52 | 0.22 | 0 |
| 1456110_at | Ankrd11 | ankyrin repeat domain 11 | 1.01 | 0.958 | -1.52 | 0.21 | -1.14 | 0.571 | -1.22 | 0.256 | -0.72 |
| 1453068_at | Prdm2 | PR domain containing 2, with ZNF domain | 1.08 | 0.784 | -1.44 | 0.094 | -1.3 | 0.355 | -1.08 | 0.639 | -0.69 |
| 1417769_at | --- | --- | -1.28 | 0.292 | -1.08 | 0.749 | -1.19 | 0.374 | 1.35 | 0.409 | -0.55 |
| 1422306_at | Lcn3 | lipocalin 3 | -1.34 | 0.478 | 1.02 | 0.963 | -1.28 | 0.205 | -3.5 | 0.012 | -1.28 |
| 1421565_at | Robo3 | roundabout homolog 3 (Drosophila) | 1.04 | 0.91 | -1.29 | 0.543 | -1.37 | 0.039 | -1.87 | 0.141 | -0.87 |
| 1440441_at | Fbxl7 | F-box and leucine-rich repeat protein 7 | -1.1 | 0.561 | 1.16 | 0.75 | -2.08 | 0 | -1.4 | 0.38 | -0.85 |
| 1455797_x_at | Lgtn | ligatin | -1.27 | 0.222 | -1.16 | 0.245 | -1.11 | 0.544 | 1.27 | 0.653 | -0.57 |
| 1457327_at | Shprh | SNF2 histone linker PHD RING helicase | 1.06 | 0.844 | -1.43 | 0.35 | -1.26 | 0.306 | -1.57 | 0.423 | -0.8 |
| 1417001_a_at | D4Wsu53e | DNA segment, Chr 4, Wayne State University 53, expressed | -1.21 | 0.133 | 1.07 | 0.702 | -1.52 | 0.048 | -1.75 | 0.049 | -0.85 |
| 1443527_at | Terf1 | telomeric repeat binding factor 1 | -1.23 | 0.483 | 1.05 | 0.894 | -1.46 | 0.087 | -1.01 | 0.971 | -0.66 |
| 1429306_at | Lzic | leucine zipper and CTNNBIP1 domain containing | -1.23 | 0.183 | 1.03 | 0.917 | -1.42 | 0.143 | -1.39 | 0.184 | -0.75 |
| 1431861_a_at | Odf2l | outer dense fiber of sperm tails 2-like | -1.19 | 0.609 | -1.41 | 0.12 | -1 | 0.996 | 1.09 | 0.737 | -0.63 |
| 1429746_at | 1700007I06Rik | RIKEN cDNA 1700007I06 gene | -1.2 | 0.726 | 1.1 | 0.908 | -1.62 | 0.445 | -1.75 | 0.444 | -0.87 |
| 1457912_at | E130317F20Rik | RIKEN cDNA E130317F20 gene | -1.36 | 0.692 | 1.06 | 0.906 | -1.32 | 0.515 | -3.3 | 0.216 | -1.23 |
| 1460706_s_at | Rer1 | RER1 retention in endoplasmic reticulum 1 homolog (S. cerevisiae) | -1.24 | 0.325 | -1.05 | 0.737 | -1.27 | 0.181 | 1.05 | 0.76 | -0.63 |
| 1439643_at | Acvr2a | Activin receptor IIA | 1.21 | 0.138 | -1.22 | 0.168 | -1.93 | 0.001 | -1.03 | 0.939 | -0.74 |
| 1447624_s_at | Stox2 | storkhead box 2 | -1.34 | 0.173 | -1.41 | 0.214 | 1.09 | 0.474 | -1.1 | 0.641 | -0.69 |
| 1444264_at | Tnfsf5ip1 | Tumor necrosis factor superfamily, member 5-induced protein 1 | -1.15 | 0.755 | -1.06 | 0.829 | -1.36 | 0.48 | 1.65 | 0.272 | -0.48 |
| 1425765_at | LOC622757 /// LOC | LOC622757 gene /// similar to transcription elongation factor B (SIII), polypeptide | -1.07 | 0.879 | -1.26 | 0.582 | -1.21 | 0.586 | 1.17 | 0.365 | -0.59 |
| 1420302_at | AA414903 | expressed sequence AA414903 | -2.64 | 0.302 | 1.57 | 0.517 | -1.66 | 0.206 | -2.95 | 0.036 | -1.42 |
| 1452299_at | Wwp1 | WW domain containing E3 ubiquitin protein ligase 1 | -1.07 | 0.623 | -1.12 | 0.09 | -1.39 | 0.035 | 1.34 | 0.401 | -0.56 |
| 1447878_s_at | Fgfrl1 | fibroblast growth factor receptor-like 1 | -1.25 | 0.263 | -1.07 | 0.788 | -1.22 | 0.196 | 1.88 | 0.189 | -0.42 |
| 1424750_at | Zbtb1 | zinc finger and BTB domain containing 1 | -1.1 | 0.226 | -1.22 | 0.202 | -1.23 | 0.282 | 1.08 | 0.509 | -0.61 |
| 1432835_at | Cpb2 | carboxypeptidase B2 (plasma) | -1.04 | 0.768 | -1.67 | 0.157 | -1.01 | 0.883 | 1.02 | 0.795 | -0.67 |

| | | | | | | | | | | | |
|---|---|---|---|---|---|---|---|---|---|---|---|
| 1437267_x_at | Hnrph1 | Heterogeneous nuclear ribonucleoprotein H1 | -1.5 | 0.172 | 1.03 | 0.827 | -1.18 | 0.133 | -1.13 | 0.511 | -0.69 |
| 1456733_x_at | Serpinh1 | serine (or cysteine) peptidase inhibitor, clade H, member 1 | -1.24 | 0.377 | 1.24 | 0.067 | -1.97 | 0.19 | -1.98 | 0.182 | -0.99 |
| 1435042_at | 9130004C02Rik | RIKEN cDNA 9130004C02 gene | 1.06 | 0.867 | -1.47 | 0.242 | -1.24 | 0.046 | -1.15 | 0.087 | -0.7 |
| 1455869_at | Camk2b | Calcium/calmodulin-dependent protein kinase II, beta | 1.2 | 0.499 | -1.75 | 0.375 | -1.28 | 0.372 | -1.94 | 0.098 | -0.94 |
| 1442216_at | Atp6v0d1 | ATPase, H+ transporting, lysosomal V0 subunit D1 | -1.17 | 0.543 | -1.22 | 0.195 | -1.14 | 0.569 | 1.04 | 0.714 | -0.62 |
| 1421997_s_at | Itga3 | integrin alpha 3 | 1.43 | 0.336 | -2.23 | 0.233 | -1.5 | 0.226 | -1.28 | 0.048 | -0.89 |
| 1459195_at | --- | --- | -1.64 | 0.225 | -1.42 | 0.538 | 1.24 | 0.662 | -1.73 | 0.365 | -0.89 |
| 1455432_at | Taok1 | TAO kinase 1 | -1.06 | 0.367 | -1.21 | 0.134 | -1.27 | 0.012 | 1.1 | 0.35 | -0.61 |
| 1458056_at | Sfrs12 | splicing factor, arginine/serine-rich 12 | -1.18 | 0.41 | -1.23 | 0.233 | -1.13 | 0.516 | 1.1 | 0.659 | -0.61 |
| 1421730_at | Nr5a1 | nuclear receptor subfamily 5, group A, member 1 | 1.65 | 0.433 | -2.31 | 0.229 | -2.16 | 0.053 | -1 | 0.99 | -0.95 |
| 1438026_at | Zfp560 | zinc finger protein 560 | -1.24 | 0.024 | 1.03 | 0.811 | -1.4 | 0.008 | -1.01 | 0.917 | -0.66 |
| 1435823_x_at | Egfl7 | EGF-like domain 7 | -1.19 | 0.216 | -1.14 | 0.125 | -1.2 | 0.279 | 1.15 | 0.314 | -0.6 |
| 1447089_at | Arl1 | ADP-ribosylation factor-like 1 | -1.59 | 0.413 | -1.15 | 0.81 | 1.05 | 0.807 | -2.89 | 0.216 | -1.14 |
| 1454274_at | 4930571C24Rik | RIKEN cDNA 4930571C24 gene | -1.28 | 0.277 | -1.45 | 0.013 | 1.08 | 0.864 | -1.15 | 0.363 | -0.7 |
| 1426417_at | Yipf4 | Yip1 domain family, member 4 | -1.19 | 0.441 | -1.09 | 0.587 | -1.27 | 0.328 | 1.42 | 0.289 | -0.53 |
| 1433567_at | Gmps | guanine monphosphate synthetase | -1.18 | 0.235 | -1.31 | 0.028 | -1.06 | 0.576 | 1.31 | 0.255 | -0.56 |
| 1430338_at | 4122401K19Rik | RIKEN cDNA 4122401K19 gene | -2.06 | 0.05 | -1.26 | 0.642 | 1.27 | 0.574 | -1.82 | 0.309 | -0.97 |
| 1450940_at | Gdap1 | ganglioside-induced differentiation-associated-protein 1 | -1.12 | 0.878 | -1.82 | 0.437 | 1.11 | 0.548 | -2.04 | 0.065 | -0.97 |
| 1455394_at | Pias4 | protein inhibitor of activated STAT 4 | -1.13 | 0.027 | -1.51 | 0.359 | 1.01 | 0.981 | -1.04 | 0.226 | -0.67 |
| 1420788_at | Klrg1 | killer cell lectin-like receptor subfamily G, member 1 | 1.14 | 0.584 | -1.51 | 0.312 | -1.35 | 0.249 | -1.26 | 0.214 | -0.74 |
| 1418152_at | Nsbp1 | nucleosome binding protein 1 | -1.08 | 0.585 | -1.19 | 0.001 | -1.28 | 0.014 | 1.29 | 0.112 | -0.56 |
| 1456420_at | Arid4a | AT rich interactive domain 4A (Rbp1 like) | -1.51 | 0.183 | -1.17 | 0.604 | 1.03 | 0.871 | -1.25 | 0.245 | -0.72 |
| 1448293_at | --- | --- | 1.07 | 0.153 | -1.54 | 0.342 | -1.21 | 0.509 | -2.8 | 0.066 | -1.12 |
| 1457626_at | D3Wsu106e | DNA segment, Chr 3, Wayne State University 106, expressed | -1.24 | 0.278 | 1.12 | 0.575 | -1.61 | 0.096 | -1.38 | 0.086 | -0.78 |
| 1419797_at | AA672641 | expressed sequence AA672641 | -1.14 | 0.892 | -1.93 | 0.094 | 1.15 | 0.809 | -1.31 | 0.716 | -0.81 |
| 1429728_at | 4930429M06Rik | RIKEN cDNA 4930429M06Rik | -1.14 | 0.624 | -1.39 | 0.313 | -1.05 | 0.854 | 1.08 | 0.706 | -0.62 |
| 1451786_at | Muc20 | mucin 20 | -1.16 | 0.404 | -1.04 | 0.952 | -1.37 | 0.141 | 1.76 | 0.149 | -0.45 |
| 1447463_at | --- | --- | -2.74 | 0.451 | -1.82 | 0.248 | 1.64 | 0.447 | -3.46 | 0.227 | -1.6 |
| 1419858_at | Ifi203 | Interferon activated gene 203 | -1.29 | 0.422 | -1.3 | 0.49 | 1.01 | 0.961 | -1.91 | 0.269 | -0.87 |
| 1440387_at | 2610005L07Rik | RIKEN cDNA 2610005L07 gene | -1.69 | 0.067 | -1.18 | 0.258 | 1.11 | 0.581 | -1.08 | 0.511 | -0.71 |
| 1429308_at | Prdm16 | PR domain containing 16 | -1.27 | 0.247 | -1.51 | 0.641 | 1.1 | 0.393 | -1.38 | 0.617 | -0.77 |
| 1418414_at | Kcnh1 | potassium voltage-gated channel, subfamily H (eag-related), member 1 | -1.7 | 0.385 | -1.09 | 0.87 | 1.04 | 0.87 | -1.18 | 0.514 | -0.73 |
| 1426647_at | 9130011J15Rik | RIKEN cDNA 9130011J15 gene | -1.05 | 0.837 | -1.27 | 0.129 | -1.23 | 0.074 | 1.19 | 0.509 | -0.59 |
| 1455766_at | Gabra1 | gamma-aminobutyric acid (GABA-A) receptor, subunit alpha 1 | 1.16 | 0.8 | -1.41 | 0.4 | -1.47 | 0.194 | -1.44 | 0.641 | -0.79 |
| 1437636_at | LOC623121 | Similar to Interferon-activatable protein 203 (Ifi-203) (Interferon-inducible protein p | -1.18 | 0.608 | 1.05 | 0.929 | -1.53 | 0.081 | -2.37 | 0.083 | -1.01 |
| 1425468_at | Plp1 | proteolipid protein (myelin) 1 | -1.37 | 0.586 | -1.38 | 0.343 | 1.1 | 0.726 | -1.91 | 0.296 | -0.89 |
| 1460496_at | LOC433492 | similar to RIKEN cDNA 5430413K10 | -1.23 | 0.737 | -1.85 | 0.013 | 1.19 | 0.637 | -1.79 | 0.165 | -0.92 |
| 1443765_at | 1810058N05Rik | RIKEN cDNA 1810058N05 gene | -3.07 | 0.03 | 1.67 | 0.138 | -1.81 | 0.105 | -1.73 | 0.339 | -1.23 |
| 1445236_at | LOC626410 | Hypothetical protein LOC626410 | -2.45 | 0.054 | 1.34 | 0.697 | -1.25 | 0.188 | -1.63 | 0.341 | -1 |
| 1431429_a_at | Arl4a | ADP-ribosylation factor-like 4A | -1.21 | 0.312 | -1.19 | 0.285 | -1.13 | 0.499 | 2.67 | 0.209 | -0.21 |
| 1441019_at | Fbxo3 | F-box only protein 3 | -1.37 | 0.265 | -1.09 | 0.582 | -1.1 | 0.352 | 1.69 | 0.083 | -0.47 |
| 1434460_at | Bbs4 | Bardet-Biedl syndrome 4 homolog (human) | -1.4 | 0.199 | -1.39 | 0.172 | 1.12 | 0.545 | -1.78 | 0.381 | -0.86 |
| 1426823_s_at | Psme4 | proteasome (prosome, macropain) activator subunit 4 | -1.12 | 0.341 | -1.2 | 0.037 | -1.21 | 0.139 | 1.32 | 0.125 | -0.55 |
| 1457181_at | D15Wsu75e | DNA segment, Chr 15, Wayne State University 75, expressed | -1.34 | 0.283 | -1.02 | 0.868 | -1.21 | 0.491 | 1.08 | 0.352 | -0.62 |
| 1435242_at | Aprin | androgen-induced proliferation inhibitor | -1.23 | 0.159 | -1.22 | 0.185 | -1.09 | 0.469 | 1.11 | 0.574 | -0.61 |
| 1417499_at | Timm10 | translocase of inner mitochondrial membrane 10 homolog (yeast) | -1.26 | 0.01 | -1.26 | 0.019 | -1.04 | 0.821 | 1.41 | 0.188 | -0.54 |
| 1446372_at | Cd8b1 | CD8 antigen, beta chain 1 | -1.42 | 0.073 | -1.27 | 0.234 | 1.06 | 0.43 | -1.52 | 0.102 | -0.79 |
| 1447635_at | Prkar1a | protein kinase, cAMP dependent regulatory, type I, alpha | -1.15 | 0.77 | -1.4 | 0.298 | -1.03 | 0.905 | 1.15 | 0.807 | -0.61 |
| 1439266_a_at | Polr3k | polymerase (RNA) III (DNA directed) polypeptide K | -1.38 | 0.291 | 1.03 | 0.85 | -1.25 | 0.351 | -1.62 | 0.498 | -0.8 |
| 1456191_x_at | Pacsin3 | protein kinase C and casein kinase substrate in neurons 3 | -1.24 | 0.27 | -1.23 | 0.113 | -1.08 | 0.708 | 1.6 | 0.055 | -0.49 |
| 1431305_at | Mcoln1 | mucolipin 1 | -1.52 | 0.357 | -1.24 | 0.545 | 1.08 | 0.667 | -1.76 | 0.314 | -0.86 |
| 1443888_at | AU023762 | Expressed sequence AU023762 | -1.05 | 0.554 | -1.07 | 0.667 | -1.5 | 0.041 | 1.13 | 0.245 | -0.62 |
| 1445434_at | Snupn | snurportin 1 | -1.09 | 0.864 | -1.38 | 0.249 | -1.1 | 0.566 | 1.09 | 0.849 | -0.62 |

| | | | | | | | | | | | |
|---|---|---|---|---|---|---|---|---|---|---|---|
| 1432426_a_at | Ube2f | ubiquitin-conjugating enzyme E2F (putative) | -1.46 | 0.217 | -1.07 | 0.551 | -1.07 | 0.672 | 1.3 | 0.461 | -0.57 |
| 1448134_at | X99384 | cDNA sequence X99384 | 1.02 | 0.912 | -1.19 | 0.058 | -1.45 | 0.024 | -1.01 | 0.969 | -0.66 |
| 1456919_at | A930013B10Rik | RIKEN cDNA A930013B10 gene | -1.44 | 0.161 | -1.31 | 0.46 | 1.09 | 0.75 | -1.79 | 0.094 | -0.86 |
| 1459701_x_at | --- | --- | 1.28 | 0.192 | -1.48 | 0.275 | -1.66 | 0.029 | -1.11 | 0.682 | -0.75 |
| 1455351_at | AU023006 | expressed sequence AU023006 | -1.11 | 0.583 | -1.03 | 0.736 | -1.46 | 0.019 | 1.13 | 0.6 | -0.62 |
| 1434434_s_at | Tcerg1 | transcription elongation regulator 1 (CA150) | -1.2 | 0.119 | -1.27 | 0.109 | -1.07 | 0.208 | 1.2 | 0.454 | -0.59 |
| 1443166_at | BC021395 | CDNA sequence BC021395 | 1.01 | 0.974 | -1.1 | 0.753 | -1.57 | 0.196 | -1.89 | 0.119 | -0.89 |
| 1420116_s_at | Golph3 | golgi phosphoprotein 3 | -1.14 | 0.489 | -1.13 | 0.39 | -1.27 | 0.083 | 1.22 | 0.164 | -0.58 |
| 1432230_at | Hip1 | huntingtin interacting protein 1 | -1.53 | 0.157 | 1.09 | 0.516 | -1.24 | 0.125 | -1.3 | 0.142 | -0.74 |
| 1427561_a_at | Afm | afamin | -1.4 | 0.033 | -1.25 | 0.244 | 1.04 | 0.755 | -2.16 | 0.388 | -0.94 |
| 1458971_at | --- | --- | -1.36 | 0.369 | -1.31 | 0.428 | 1.06 | 0.781 | -1.54 | 0.589 | -0.79 |
| 1457392_at | --- | Adult retina cDNA, RIKEN full-length enriched library, clone:A930031I16 product: | -1.08 | 0.608 | -1.27 | 0.163 | -1.18 | 0.484 | 1.08 | 0.664 | -0.62 |
| 1428982_at | D530031C13Rik | RIKEN cDNA D530031C13 gene | -1.2 | 0.258 | -1.15 | 0.129 | -1.18 | 0.265 | 1.21 | 0.397 | -0.58 |
| 1441480_at | Srp54 | Signal recognition particle 54 | -1.01 | 0.762 | 1.01 | 0.957 | -1.82 | 0.005 | -1.47 | 0.061 | -0.82 |
| 1447823_x_at | Mapk12 | mitogen-activated protein kinase 12 | 1.17 | 0.073 | -2.16 | 0.171 | -1.09 | 0.531 | -1.15 | 0.51 | -0.81 |
| 1453140_at | 9030612M13Rik | RIKEN cDNA 9030612M13 gene | 1.08 | 0.581 | -1.23 | 0.097 | -1.53 | 0.007 | -1.05 | 0.826 | -0.68 |
| 1444715_at | --- | Transcribed locus | -1.21 | 0.839 | 1.39 | 0.6 | -3 | 0.014 | -1.71 | 0.426 | -1.13 |
| 1426351_at | Hspd1 | heat shock protein 1 (chaperonin) | -1.21 | 0.215 | -1.28 | 0.071 | -1.05 | 0.677 | 1.18 | 0.395 | -0.59 |
| 1458161_at | Kcnq1ot1 | KCNQ1 overlapping transcript 1 | -1.06 | 0.554 | 1.07 | 0.708 | -1.84 | 0.028 | -1.04 | 0.826 | -0.72 |
| 1459151_x_at | Ifi35 | interferon-induced protein 35 | -1.15 | 0.193 | -1.14 | 0.404 | -1.24 | 0.257 | 1.4 | 0.334 | -0.53 |
| 1436844_at | 6330403M23Rik | RIKEN cDNA 6330403M23 gene | -1.12 | 0.402 | 1.05 | 0.717 | -1.62 | 0.005 | -1.44 | 0.176 | -0.78 |
| 1436770_x_at | Psma1 | proteasome (prosome, macropain) subunit, alpha type 1 | -1.26 | 0.651 | -1.24 | 0.119 | -1.05 | 0.74 | 1.18 | 0.352 | -0.59 |
| 1453042_at | Anks4b | ankyrin repeat and sterile alpha motif domain containing 4B | -1.38 | 0.063 | -1.19 | 0.467 | -1.02 | 0.959 | 1.57 | 0.347 | -0.5 |
| 1444421_at | Etohd2 | ethanol decreased 2 | -1.21 | 0.559 | -1.21 | 0.377 | -1.1 | 0.252 | 1.64 | 0.223 | -0.47 |
| 1417318_at | Dbc1 | deleted in bladder cancer 1 (human) | 1.19 | 0.809 | -2.12 | 0.422 | -1.12 | 0.738 | -1.08 | 0.891 | -0.78 |
| 1457514_at | Ylpm1 | YLP motif containing 1 | -1.23 | 0.502 | -1.22 | 0.247 | -1.08 | 0.878 | 1.4 | 0.305 | -0.53 |
| 1458710_at | Polr2a | Polymerase (RNA) II (DNA directed) polypeptide A | -1.04 | 0.858 | -1.24 | 0.555 | -1.28 | 0.354 | 1 | 0.997 | -0.64 |
| 1449789_x_at | Ly6g6c | lymphocyte antigen 6 complex, locus G6C | -1.55 | 0.295 | -1.2 | 0.299 | 1.08 | 0.796 | -1.24 | 0.405 | -0.73 |
| 1445999_at | D19Ertd132e | DNA segment, Chr 19, ERATO Doi 132, expressed | -1.1 | 0.757 | 1.01 | 0.974 | -1.57 | 0.008 | -1.29 | 0.169 | -0.74 |
| 1433484_at | D6Ertd527e | DNA segment, Chr 6, ERATO Doi 527, expressed | -1.99 | 0.396 | -1.06 | 0.931 | 1.11 | 0.823 | -1.08 | 0.844 | -0.76 |
| 1424649_a_at | Tspan8 | tetraspanin 8 | -1.04 | 0.789 | -1.21 | 0.458 | -1.31 | 0.128 | 5.4 | 0.229 | 0.46 |
| 1429143_at | Tekt3 | tektin 3 | -1.14 | 0.859 | -2.14 | 0.416 | 1.21 | 0.06 | -1.7 | 0.324 | -0.94 |
| 1440999_at | Zfp697 | zinc finger protein 697 | -1.13 | 0.466 | -1.69 | 0.006 | 1.07 | 0.722 | -1.02 | 0.922 | -0.69 |
| 1442751_at | Trio | Triple functional domain (PTPRF interacting) | -1.36 | 0.007 | -1.15 | 0.714 | -1.05 | 0.824 | 1.04 | 0.834 | -0.63 |
| 1424250_a_at | Arhgef3 | Rho guanine nucleotide exchange factor (GEF) 3 | -1.31 | 0.02 | 1.14 | 0.565 | -1.54 | 0.079 | -1.89 | 0.109 | -0.9 |
| 1421019_at | 1700021F05Rik | RIKEN cDNA 1700021F05 gene | -1.14 | 0.146 | -1.27 | 0.037 | -1.12 | 0.091 | 1.25 | 0.279 | -0.57 |
| 1445760_at | Rya3 | antimicrobial peptide RYA3 | -1.22 | 0.792 | 1.21 | 0.535 | -1.92 | 0.205 | -1.2 | 0.855 | -0.78 |
| 1422510_at | Ctdspl | CTD (carboxy-terminal domain, RNA polymerase II, polypeptide A) small phospha | 1.15 | 0.308 | -1.33 | 0.147 | -1.52 | 0.145 | -1.32 | 0.513 | -0.76 |
| 1421568_at | Kcna6 | potassium voltage-gated channel, shaker-related, subfamily, member 6 | 1.04 | 0.904 | -1.64 | 0.097 | -1.11 | 0.722 | -1.18 | 0.584 | -0.72 |
| 1444240_at | Shank1 | SH3/ankyrin domain gene 1 | 1.1 | 0.782 | -1.77 | 0.168 | -1.13 | 0.556 | -1.15 | 0.53 | -0.73 |
| 1456593_at | Sytl2 | Synaptotagmin-like 2 | 1.22 | 0.479 | -1.31 | 0.455 | -1.76 | 0.017 | -1.39 | 0.398 | -0.81 |
| 1452785_at | 1700034H14Rik | RIKEN cDNA 1700034H14 gene | -1.28 | 0.042 | -1.16 | 0.026 | -1.09 | 0.378 | 1.05 | 0.627 | -0.62 |
| 1438989_s_at | B130021B11Rik | RIKEN cDNA B130021B11 gene | 1.3 | 0.236 | -1.27 | 0.172 | -2.13 | 0.03 | -3.93 | 0.076 | -1.51 |
| 1448133_at | Nmd3 | NMD3 homolog (S. cerevisiae) | -1.18 | 0.666 | -1.47 | 0.027 | 1.03 | 0.825 | -1.21 | 0.204 | -0.71 |
| 1426016_a_at | Tro | trophinin | 1.13 | 0.857 | -1.42 | 0.44 | -1.38 | 0.24 | -1.28 | 0.619 | -0.74 |
| 1425614_x_at | H2-D1 | histocompatibility 2, D region locus 1 | -1.33 | 0.711 | -1.44 | 0.198 | 1.11 | 0.536 | -1.28 | 0.597 | -0.73 |
| 1445147_at | Psme4 | proteasome (prosome, macropain) activator subunit 4 | -1.19 | 0.677 | 1.15 | 0.284 | -1.78 | 0.02 | -1.11 | 0.552 | -0.73 |
| 1440733_at | 1200009O22Rik | RIKEN cDNA 1200009O22 gene | -2.7 | 0.131 | 1.32 | 0.306 | -1.15 | 0.354 | -1.12 | 0.706 | -0.91 |
| 1458439_a_at | 2310047C04Rik | RIKEN cDNA 2310047C04 gene | 1.07 | 0.564 | -1.1 | 0.717 | -1.75 | 0.01 | -1.08 | 0.667 | -0.71 |
| 1415764_at | Zc3h11a | zinc finger CCCH type containing 11A | 1.02 | 0.854 | -1.13 | 0.079 | -1.53 | 0.025 | -1.31 | 0.106 | -0.74 |
| 1449734_s_at | Bbs4 | Bardet-Biedl syndrome 4 homolog (human) | 1.15 | 0.518 | -1.47 | 0.051 | -1.38 | 0.062 | -1.74 | 0.055 | -0.86 |
| 1442609_at | Prkce | Protein kinase C, epsilon | 1.02 | 0.927 | -1.1 | 0.525 | -1.6 | 0.169 | -2.84 | 0.112 | -1.13 |
| 1458537_at | --- | --- | -1.32 | 0.086 | -1.07 | 0.798 | -1.16 | 0.259 | 1.12 | 0.746 | -0.61 |

| | | | | | | | | | | | |
|---|---|---|---|---|---|---|---|---|---|---|---|
| 1417200_at | 1300007B12Rik | RIKEN cDNA 1300007B12 gene | -1.09 | 0.144 | -1.4 | 0.024 | -1.08 | 0.702 | 1.35 | 0.344 | -0.55 |
| 1439619_at | Tcf12 | transcription factor 12 | -1.17 | 0.623 | 1.17 | 0.517 | -1.87 | 0.002 | -1.24 | 0.423 | -0.78 |
| 1432853_at | 2900057C01Rik | RIKEN cDNA 2900057C01 gene | -1.19 | 0.763 | -1.51 | 0.145 | 1.05 | 0.695 | -1.11 | 0.669 | -0.69 |
| 1420339_at | 0610016J10Rik | RIKEN cDNA 0610016J10 gene | -1.06 | 0.475 | -1.54 | 0.011 | -1.03 | 0.844 | 3.06 | 0.2 | -0.15 |
| 1460699_at | Rps27 | ribosomal protein S27 | -1.05 | 0.921 | -1.17 | 0.792 | -1.33 | 0.645 | 1.22 | 0.516 | -0.58 |
| 1442859_at | Spred1 | Sprouty protein with EVH-1 domain 1, related sequence | 1.49 | 0.581 | -3.02 | 0.239 | -1.37 | 0.66 | -1.56 | 0.34 | -1.11 |
| 1450219_at | Htr1a | 5-hydroxytryptamine (serotonin) receptor 1A | -1.42 | 0.059 | -1.49 | 0.417 | 1.18 | 0.78 | -1.48 | 0.607 | -0.8 |
| 1447452_at | --- | --- | 1.09 | 0.883 | -1.21 | 0.128 | -1.57 | 0.088 | -1.08 | 0.821 | -0.69 |
| 1446592_at | --- | --- | 1.13 | 0.613 | -1.34 | 0.036 | -1.48 | 0.068 | -1.34 | 0.232 | -0.76 |
| 1440791_x_at | Tcea2 | transcription elongation factor A (SII), 2 | -1.82 | 0.09 | -1.02 | 0.942 | 1.02 | 0.891 | -1.17 | 0.515 | -0.75 |
| 1422484_at | Cycs | cytochrome c, somatic | -1.24 | 0.175 | -1.19 | 0.03 | -1.1 | 0.511 | 1.09 | 0.603 | -0.61 |
| 1456386_at | Rnpc2 | RNA-binding region (RNP1, RRM) containing 2 | -1.53 | 0.439 | -1.19 | 0.594 | 1.06 | 0.793 | -1.91 | 0.18 | -0.89 |
| 1447219_at | --- | Transcribed locus | 1.24 | 0.422 | -1.62 | 0.189 | -1.44 | 0.132 | -1.19 | 0.638 | -0.75 |
| 1426031_a_at | Nfatc2 | nuclear factor of activated T-cells, cytoplasmic, calcineurin-dependent 2 | -1.21 | 0.203 | -1.35 | 0.482 | -1.01 | 0.964 | 1.53 | 0.405 | -0.51 |
| 1437518_at | --- | 10 days neonate cerebellum cDNA, RIKEN full-length enriched library, clone:653( | -1.02 | 0.822 | -2.25 | 0.341 | 1.13 | 0.767 | -2.39 | 0.15 | -1.13 |
| 1421892_at | --- | --- | -1.07 | 0.681 | -1.2 | 0.765 | -1.26 | 0.333 | 3.27 | 0.16 | -0.07 |
| 1432782_at | 4933435C09Rik | RIKEN cDNA 4933435C09 gene | -1.05 | 0.951 | -1.28 | 0.674 | -1.22 | 0.73 | 1.64 | 0.361 | -0.48 |
| 1438733_at | Zfp689 | zinc finger protein 689 | 1.31 | 0.434 | -1.76 | 0.381 | -1.48 | 0.364 | -1.16 | 0.776 | -0.77 |
| 1416361_a_at | Dync1i1 | dynein cytoplasmic 1 intermediate chain 1 | -1.07 | 0.779 | 1.05 | 0.879 | -1.74 | 0.121 | -1.34 | 0.476 | -0.78 |
| 1451734_a_at | Dbn1 | drebrin 1 | 1.02 | 0.897 | -1.49 | 0.612 | -1.16 | 0.763 | -2.51 | 0.093 | -1.03 |
| 1424527_at | Ppp2r2d | protein phosphatase 2, regulatory subunit B, delta isoform | -1.05 | 0.684 | -1.17 | 0.107 | -1.33 | 0.119 | 1.34 | 0.049 | -0.55 |
| 1436070_at | Glo1 | glyoxalase 1 | -1.03 | 0.79 | -1.23 | 0.018 | -1.29 | 0.014 | 1.64 | 0.2 | -0.48 |
| 1450998_at | Zfp110 | zinc finger protein 110 | 1.02 | 0.891 | -1.31 | 0.238 | -1.29 | 0.11 | -1.57 | 0.078 | -0.79 |
| 1454170_at | E130101E03Rik | RIKEN cDNA E130101E03 gene | -2.62 | 0.297 | -1.59 | 0.252 | 1.55 | 0.123 | -2.11 | 0.151 | -1.19 |
| 1454990_at | Arid2 | AT rich interactive domain 2 (Arid-rfx like) | -1.09 | 0.384 | -1.1 | 0.25 | -1.37 | 0.015 | 1.1 | 0.714 | -0.61 |
| 1437530_at | Pdcd10 | Programmed cell death 10 | -1.14 | 0.904 | -1.1 | 0.799 | -1.29 | 0.3 | 1.15 | 0.836 | -0.6 |
| 1431118_at | 6720427H10Rik | RIKEN cDNA 6720427H10 gene | 1.07 | 0.7 | -1.01 | 0.969 | -1.99 | 0.014 | -1.12 | 0.336 | -0.76 |
| 1427374_at | Muc3 /// LOC66633 | mucin 3, intestinal /// similar to mucin 17 /// similar to mucin 17 | 1.75 | 0.524 | -2.79 | 0.072 | -2.23 | 0.007 | -1.13 | 0.536 | -1.1 |
| 1444861_at | D13Ertd297e | DNA segment, Chr 13, ERATO Doi 297, expressed | -1.34 | 0.662 | -1.09 | 0.871 | -1.12 | 0.818 | 1.49 | 0.408 | -0.52 |
| 1438526_at | Gpr158 | G protein-coupled receptor 158 | -1.97 | 0.101 | -1.73 | 0.356 | 1.47 | 0.48 | -1.71 | 0.637 | -0.98 |
| 1439869_at | 2900054J07Rik | RIKEN cDNA 2900054J07 gene | -1.73 | 0.16 | -1.31 | 0.217 | 1.22 | 0.462 | -1.39 | 0.129 | -0.8 |
| 1431942_at | Wwox | WW domain-containing oxidoreductase | 1.17 | 0.767 | -1.3 | 0.52 | -1.61 | 0.134 | -1.47 | 0.235 | -0.8 |
| 1454038_at | 4833403I15Rik | RIKEN cDNA 4833403I15 gene | -1.68 | 0.289 | 1 | 0.999 | -1.04 | 0.804 | -1.34 | 0.127 | -0.77 |
| 1435092_at | Arl4a | ADP-ribosylation factor-like 4A | -1.08 | 0.808 | -1.27 | 0.4 | -1.18 | 0.35 | 2.29 | 0.252 | -0.31 |
| 1440354_at | Elovl7 | ELOVL family member 7, elongation of long chain fatty acids (yeast) | -1.13 | 0.285 | -1.45 | 0.516 | -1.01 | 0.947 | 2.82 | 0.103 | -0.2 |
| 1437718_x_at | Fmod | fibromodulin | -1.37 | 0.422 | -2.29 | 0.226 | 1.39 | 0.473 | -1.27 | 0.37 | -0.89 |
| 1456780_at | Btrc | Beta-transducin repeat containing protein | -1.14 | 0.691 | -1.1 | 0.753 | -1.29 | 0.405 | 1.8 | 0.084 | -0.43 |
| 1428646_at | LOC676870 | region containing RIKEN cDNA 2310056B04 gene; pre B-cell leukemia transcripti | -1.08 | 0.487 | -1.24 | 0.214 | -1.21 | 0.294 | 1.02 | 0.931 | -0.63 |
| 1434531_at | Mgat5b | mannoside acetylglucosaminyltransferase 5, isoenzyme B | 1.21 | 0.778 | -2.1 | 0.139 | -1.14 | 0.75 | -1.78 | 0.118 | -0.95 |
| 1428006_at | Scfd1 | sec1 family domain containing 1 | -1.67 | 0.24 | -1.34 | 0.131 | 1.21 | 0.7 | -1.01 | 0.964 | -0.7 |
| 1434173_s_at | D19Bwg1357e /// S | DNA segment, Chr 19, Brigham & Women's Genetics 1357 expressed /// SWI/SN | 1.09 | 0.188 | -1.48 | 0.046 | -1.26 | 0.075 | -1.14 | 0.135 | -0.7 |
| 1444349_at | --- | --- | 1.2 | 0.157 | -1.16 | 0.105 | -2.02 | 0.003 | -1.17 | 0.595 | -0.79 |
| 1430820_a_at | Bbx | bobby sox homolog (Drosophila) | 1.04 | 0.889 | -1.36 | 0.268 | -1.28 | 0.489 | -1.09 | 0.612 | -0.67 |
| 1450799_at | Adcyap1r1 | adenylate cyclase activating polypeptide 1 receptor 1 | -1.71 | 0.13 | -1.12 | 0.559 | 1.08 | 0.815 | -1.35 | 0.46 | -0.77 |
| 1445260_at | Dcun1d1 | DCUN1D1 DCN1, defective in cullin neddylation 1, domain containing 1 (S. cerev | -1.18 | 0.573 | -1.11 | 0.393 | -1.23 | 0.349 | 1.03 | 0.781 | -0.62 |
| 1446057_at | Rlf | rearranged L-myc fusion sequence | -1.14 | 0.773 | -1.02 | 0.909 | -1.42 | 0.031 | 1.02 | 0.701 | -0.64 |
| 1440368_at | Jmjd2b | Jumonji domain containing 2B | -1.22 | 0.428 | -1.37 | 0.543 | 1.01 | 0.972 | -1.39 | 0.37 | -0.74 |
| 1432019_at | Trub1 | TruB pseudouridine (psi) synthase homolog 1 (E. coli) | 1.08 | 0.899 | -2.06 | 0.151 | -1 | 0.994 | -2.6 | 0.291 | -1.15 |
| 1447064_at | --- | --- | 1.09 | 0.563 | -1.08 | 0.523 | -1.85 | 0.04 | -1.13 | 0.694 | -0.74 |
| 1442881_at | --- | --- | -1.09 | 0.409 | -1.57 | 0.118 | 1 | 0.967 | -1.76 | 0.312 | -0.85 |
| 1441247_at | Pla2g3 | Phospholipase A2, group III | -1.45 | 0.118 | 1.12 | 0.851 | -1.33 | 0.278 | -1.71 | 0.239 | -0.84 |
| 1444762_at | Ppp1r12a | Protein phosphatase 1, regulatory (inhibitor) subunit 12A | 1.02 | 0.882 | -1.14 | 0.642 | -1.51 | 0.011 | -1.5 | 0.006 | -0.78 |
| 1443471_at | Zbtb20 | zinc finger and BTB domain containing 20 | -1.18 | 0.238 | -1 | 0.993 | -1.4 | 0.1 | 1.23 | 0.444 | -0.59 |

| | | | | | | | | | | | |
|---|---|---|---|---|---|---|---|---|---|---|---|
| 1431678_at | 4930556N08Rik | RIKEN cDNA 4930556N08 gene | -1.5 | 0.087 | -1.16 | 0.714 | 1.03 | 0.804 | -1.19 | 0.689 | -0.71 |
| 1438570_at | --- | Transcribed locus | -1.74 | 0.294 | 1.17 | 0.575 | -1.23 | 0.604 | -3.13 | 0.081 | -1.23 |
| 1431862_x_at | Odf2l | outer dense fiber of sperm tails 2-like | -1.38 | 0.147 | -1.07 | 0.695 | -1.11 | 0.76 | 1.16 | 0.757 | -0.6 |
| 1441058_at | Itpkb | inositol 1,4,5-trisphosphate 3-kinase B | -1.26 | 0.149 | 1.1 | 0.8 | -1.5 | 0.222 | -2.05 | 0.122 | -0.93 |
| 1444819_at | C86090 | expressed sequence C86090 | -1.79 | 0.021 | -1.01 | 0.981 | 1.01 | 0.96 | -1.08 | 0.748 | -0.72 |
| 1440174_at | E130307A14Rik | RIKEN cDNA E130307A14 gene | 1.19 | 0.628 | -1.12 | 0.849 | -2.1 | 0.104 | -1.08 | 0.866 | -0.78 |
| 1439511_at | Cdk7 | Cyclin-dependent kinase 7 (homolog of Xenopus MO15 cdk-activating kinase) | 1 | 0.99 | -1.02 | 0.868 | -1.72 | 0.101 | -1.21 | 0.503 | -0.74 |
| 1457549_at | Ccnd3 | Cyclin D3 | 1.2 | 0.412 | -1.3 | 0.455 | -1.69 | 0.198 | -2.1 | 0.183 | -0.97 |
| 1448462_at | Tdg /// LOC545124 | thymine DNA glycosylase /// similar to thymine DNA glycosylase isoform 2 /// simi | -1.11 | 0.581 | -1.21 | 0.021 | -1.19 | 0.186 | 1.35 | 0.225 | -0.54 |
| 1436221_at | D1Ertd471e | DNA segment, Chr 1, ERATO Doi 471, expressed | -1.2 | 0.55 | -1.04 | 0.91 | -1.31 | 0.624 | 1.41 | 0.39 | -0.53 |
| 1451236_at | Rerg | RAS-like, estrogen-regulated, growth-inhibitor | -1.03 | 0.929 | 1.07 | 0.758 | -1.92 | 0.015 | -1.26 | 0.753 | -0.79 |
| 1442003_at | --- | Mus musculus, clone IMAGE:4219318, mRNA | -1.03 | 0.063 | -1.29 | 0.198 | -1.23 | 0.133 | 1.05 | 0.859 | -0.62 |
| 1433565_at | Prpf38a | PRP38 pre-mRNA processing factor 38 (yeast) domain containing A | -1.1 | 0.46 | -1.11 | 0.59 | -1.33 | 0.054 | 1.06 | 0.417 | -0.62 |
| 1441196_at | C230076A16Rik | RIKEN cDNA C230076A16 gene | 1.08 | 0.642 | -1.49 | 0.132 | -1.23 | 0.163 | -1.19 | 0.355 | -0.71 |
| 1432350_at | Sync | syncoilin | -1.48 | 0.482 | -1.71 | 0.169 | 1.3 | 0.408 | -1.24 | 0.474 | -0.78 |
| 1419290_at | Mettl6 | methyltransferase like 6 | -1.07 | 0.637 | -1.23 | 0.02 | -1.23 | 0.031 | 1.02 | 0.926 | -0.63 |
| 1447270_at | --- | --- | 1.06 | 0.678 | -1.06 | 0.553 | -1.81 | 0.103 | -1.06 | 0.707 | -0.72 |
| 1458724_at | E230008O15Rik | RIKEN cDNA E230008O15 gene | -1.11 | 0.525 | -1.14 | 0.599 | -1.28 | 0.302 | 1.56 | 0.244 | -0.49 |
| 1459977_x_at | Cox10 | COX10 homolog, cytochrome c oxidase assembly protein, heme A: farnesyltrans | -1.26 | 0.582 | -1.08 | 0.879 | -1.19 | 0.673 | 1.13 | 0.732 | -0.6 |
| 1429212_a_at | Lrrc51 | leucine rich repeat containing 51 | 1.1 | 0.776 | -1.89 | 0.006 | -1.07 | 0.815 | -2.33 | 0.045 | -1.05 |
| 1418044_at | A630042L21Rik | RIKEN cDNA A630042L21 gene | -1.23 | 0.079 | -1.03 | 0.779 | -1.29 | 0.221 | 1.18 | 0.47 | -0.59 |
| 1441045_at | --- | --- | -1.5 | 0.327 | 1.15 | 0.65 | -1.35 | 0.075 | -1.18 | 0.712 | -0.72 |
| 1458393_at | Srr | serine racemase | -1.26 | 0.044 | -1.22 | 0.25 | -1.06 | 0.648 | 1.05 | 0.755 | -0.62 |
| 1427105_at | 2610510J17Rik | RIKEN cDNA 2610510J17 gene | -1.26 | 0.327 | -1.27 | 0.426 | -1.02 | 0.901 | 3.06 | 0.207 | -0.12 |
| 1444928_at | Slc35f1 | Solute carrier family 35, member F1 | -1.07 | 0.914 | -1.28 | 0.238 | -1.18 | 0.469 | 1.34 | 0.239 | -0.55 |
| 1449948_at | Ero1lb | ERO1-like beta (S. cerevisiae) | -1.23 | 0.404 | -1.88 | 0.071 | 1.22 | 0.73 | -1.94 | 0.251 | -0.96 |
| 1449944_a_at | Sec61a2 | Sec61, alpha subunit 2 (S. cerevisiae) | -1.21 | 0.087 | -1.18 | 0.086 | -1.13 | 0.692 | 1.29 | 0.387 | -0.56 |
| 1445459_at | Sstr5 | somatostatin receptor 5 | -1.14 | 0.788 | -1.8 | 0.077 | 1.13 | 0.688 | -1.77 | 0.238 | -0.9 |
| 1451789_a_at | Ryk | receptor-like tyrosine kinase | -1.23 | 0.326 | -1.22 | 0.434 | -1.07 | 0.593 | 1.68 | 0.086 | -0.46 |
| 1426625_at | Zfp623 | zinc finger protein 623 | 1 | 0.959 | -1.21 | 0.026 | -1.37 | 0.042 | -1.12 | 0.295 | -0.67 |
| 1457923_at | C130045F17Rik | RIKEN cDNA C130045F17 gene | -1.48 | 0.025 | -1.58 | 0.292 | 1.25 | 0.339 | -1.11 | 0.736 | -0.73 |
| 1420033_s_at | Ppp2r2d | protein phosphatase 2, regulatory subunit B, delta isoform | -1.05 | 0.704 | -1.16 | 0.162 | -1.35 | 0.104 | 1.15 | 0.306 | -0.6 |
| 1427809_at | Lphn3 | Latrophilin 3 | -1.31 | 0.591 | 1.32 | 0.632 | -2.06 | 0.104 | -1.71 | 0.356 | -0.94 |
| 1421299_a_at | Lef1 | lymphoid enhancer binding factor 1 | -1.47 | 0.16 | 1.01 | 0.983 | -1.14 | 0.23 | -1.98 | 0.108 | -0.9 |
| 1423242_at | Mrps36 | mitochondrial ribosomal protein S36 | -1.27 | 0.413 | -1.27 | 0.008 | -1.02 | 0.903 | 1.42 | 0.331 | -0.53 |
| 1431628_at | 4930435H24Rik | RIKEN cDNA 4930435H24 gene | 1.04 | 0.906 | -1.47 | 0.356 | -1.19 | 0.378 | -1.22 | 0.435 | -0.71 |
| 1430429_at | Pgs1 | phosphatidylglycerophosphate synthase 1 | -1.39 | 0.102 | 1.09 | 0.778 | -1.31 | 0.058 | -1.67 | 0.042 | -0.82 |
| 1457336_at | R3hdm1 | R3H domain 1 (binds single-stranded nucleic acids) | 1.02 | 0.933 | -1.12 | 0.819 | -1.54 | 0.33 | -1.02 | 0.976 | -0.66 |
| 1441526_at | Mbtd1 | Mbt domain containing 1 | 1.04 | 0.251 | -1.23 | 0.55 | -1.4 | 0.27 | -1.48 | 0.201 | -0.77 |
| 1437164_x_at | Atp5o /// LOC43267 | ATP synthase, H+ transporting, mitochondrial F1 complex, O subunit /// similar to | -1.04 | 0.573 | -1.11 | 0.368 | -1.42 | 0.027 | 1.13 | 0.361 | -0.61 |
| 1440103_at | --- | Transcribed locus | 1.1 | 0.813 | -1.11 | 0.782 | -1.78 | 0.016 | -1.47 | 0.506 | -0.81 |
| 1434174_at | Lysmd3 | LysM, putative peptidoglycan-binding, domain containing 3 | -1.14 | 0.36 | -1.01 | 0.901 | -1.43 | 0.017 | 1.11 | 0.518 | -0.62 |
| 1435796_at | C530024P05Rik | RIKEN cDNA C530024P05 gene | -1.44 | 0.325 | -1.48 | 0.286 | 1.19 | 0.579 | -1.43 | 0.602 | -0.79 |
| 1459539_at | --- | --- | -1.21 | 0.606 | 1.26 | 0.683 | -2.09 | 0.056 | -2.02 | 0.168 | -1.02 |
| 1434479_at | Col5a1 | Procollagen, type V, alpha 1 | -1.03 | 0.836 | -1.03 | 0.816 | -1.62 | 0.012 | 2.35 | 0.291 | -0.33 |
| 1450607_s_at | Ucn2 | urocortin 2 | -2.11 | 0.311 | -2.6 | 0.146 | 1.7 | 0.253 | -2 | 0.042 | -1.25 |
| 1440707_at | Dmrt3 | doublesex and mab-3 related transcription factor 3 | -1.88 | 0.243 | 1.15 | 0.721 | -1.13 | 0.794 | -1.49 | 0.233 | -0.84 |
| 1441152_at | --- | --- | 1.13 | 0.141 | -1.27 | 0.235 | -1.54 | 0.017 | -1.37 | 0.086 | -0.76 |
| 1432982_at | 4930456J16Rik | RIKEN cDNA 4930456J16 gene | -1.64 | 0.149 | 1.25 | 0.539 | -1.42 | 0.358 | -1.05 | 0.822 | -0.71 |
| 1425901_at | Nfatc2 | nuclear factor of activated T-cells, cytoplasmic, calcineurin-dependent 2 | -1.35 | 0.21 | -1.25 | 0.269 | 1.02 | 0.858 | -1.79 | 0.056 | -0.84 |
| 1436951_x_at | Txndc9 | thioredoxin domain containing 9 | -1.27 | 0.133 | -1.09 | 0.195 | -1.16 | 0.232 | 1.06 | 0.535 | -0.62 |
| 1425921_a_at | 1810055G02Rik | RIKEN cDNA 1810055G02 gene | -1.02 | 0.932 | -1.36 | 0.492 | -1.18 | 0.427 | 2.09 | 0.213 | -0.37 |
| 1459254_at | Cdca2 | cell division cycle associated 2 | -1.12 | 0.84 | -1.16 | 0.783 | -1.23 | 0.496 | 1.81 | 0.302 | -0.43 |

| | | | | | | | | | | | |
|---|---|---|---|---|---|---|---|---|---|---|---|
| 1431067_at | 6330404A07Rik | RIKEN cDNA 6330404A07 gene | 1.22 | 0.577 | -1.54 | 0.227 | -1.45 | 0.124 | -1.57 | 0.218 | -0.83 |
| 1416934_at | Mtm1 | X-linked myotubular myopathy gene 1 | -1.36 | 0.265 | -1.2 | 0.316 | -1 | 0.997 | 1.16 | 0.644 | -0.6 |
| 1431405_a_at | 4930413O22Rik | RIKEN cDNA 4930413O22 gene | -1.02 | 0.941 | -1.08 | 0.685 | -1.53 | 0.176 | 1.03 | 0.906 | -0.65 |
| 1430650_at | Zfp191 | zinc finger protein 191 | 1.02 | 0.903 | -1.35 | 0.215 | -1.25 | 0.461 | -1.11 | 0.161 | -0.67 |
| 1440278_at | --- | --- | -1.29 | 0.196 | -1.06 | 0.941 | -1.19 | 0.295 | 1.03 | 0.888 | -0.62 |
| 1434073_at | Gprasp2 | G protein-coupled receptor associated sorting protein 2 | -1.12 | 0.759 | -2 | 0.096 | 1.17 | 0.517 | -1.05 | 0.889 | -0.75 |
| 1447865_x_at | Pdzd11 | PDZ domain containing 11 | -1.24 | 0.127 | -1.21 | 0.221 | -1.08 | 0.648 | 1.59 | 0.412 | -0.48 |
| 1460198_a_at | Psmb3 /// LOC2402 | proteasome (prosome, macropain) subunit, beta type 3 /// similar to proteasome b | 1.13 | 0.262 | -1.11 | 0.322 | -1.89 | 0.007 | -1.43 | 0.327 | -0.82 |
| 1433919_at | Asb4 | ankyrin repeat and SOCS box-containing protein 4 | 1.14 | 0.866 | -1.11 | 0.694 | -1.92 | 0.163 | -1.08 | 0.905 | -0.74 |
| 1448594_at | Wisp1 | WNT1 inducible signaling pathway protein 1 | -1.15 | 0.541 | 1.3 | 0.453 | -2.51 | 0.229 | -1.03 | 0.95 | -0.85 |
| 1458366_at | Asxl2 | Additional sex combs like 2 (Drosophila) | -1.76 | 0.233 | -1.18 | 0.521 | 1.15 | 0.529 | -1.28 | 0.22 | -0.77 |
| 1423039_a_at | --- | --- | -1.18 | 0.188 | 1.02 | 0.855 | -1.43 | 0.014 | -1.57 | 0.015 | -0.79 |
| 1426992_at | Xpr1 | xenotropic and polytropic retrovirus receptor 1 | -1.08 | 0.393 | -1.08 | 0.519 | -1.4 | 0.013 | 1.01 | 0.97 | -0.64 |
| 1428811_at | Wfikkn2 | WAP, follistatin/kazal, immunoglobulin, kunitz and netrin domain containing 2 | 1.14 | 0.854 | -1.97 | 0.29 | -1.09 | 0.797 | -2.68 | 0.161 | -1.15 |
| 1448807_at | Hrh3 | histamine receptor H 3 | 1.08 | 0.331 | -1.8 | 0.035 | -1.08 | 0.826 | -1.67 | 0.225 | -0.87 |
| 1434123_at | Fut11 | fucosyltransferase 11 | 1.1 | 0.537 | -1.5 | 0.125 | -1.25 | 0.366 | -1.31 | 0.189 | -0.74 |
| 1441525_at | Nek11 | NIMA (never in mitosis gene a)-related expressed kinase 11 | -1.23 | 0.729 | 1.09 | 0.629 | -1.51 | 0.395 | -1.65 | 0.572 | -0.83 |
| 1445540_at | Dnm3 | Dynamin 3 | -1.63 | 0.126 | -1.04 | 0.961 | -1.01 | 0.934 | 1.35 | 0.252 | -0.58 |
| 1440187_at | Taf3 | TAF3 RNA polymerase II, TATA box binding protein (TBP)-associated factor | 1.16 | 0.595 | -1.54 | 0.238 | -1.32 | 0.148 | -1.13 | 0.481 | -0.71 |
| 1421311_at | Pcdh10 | protocadherin 10 | -1.13 | 0.692 | -2.13 | 0.215 | 1.21 | 0.367 | -1.97 | 0.331 | -1.01 |
| 1425891_a_at | Grtp1 | GH regulated TBC protein 1 | -1.16 | 0.525 | -1.21 | 0.055 | -1.14 | 0.351 | 2.02 | 0.336 | -0.37 |
| 1437069_at | Osbpl8 | oxysterol binding protein-like 8 | -1 | 0.964 | -1.19 | 0.095 | -1.38 | 0.018 | 1.3 | 0.222 | -0.57 |
| 1441816_at | 2900056M20Rik | RIKEN cDNA 2900056M20 gene | -1.2 | 0.629 | 1.22 | 0.706 | -1.95 | 0.057 | -1.9 | 0.099 | -0.96 |
| 1454935_at | D930001I22Rik | RIKEN cDNA D930001I22 gene | -1.15 | 0.273 | -1.51 | 0.129 | 1.03 | 0.688 | -1.06 | 0.534 | -0.67 |
| 1445183_s_at | D7Ertd523e | DNA segment, Chr 7, ERATO Doi 523, expressed | -1.29 | 0.552 | -2.11 | 0.061 | 1.31 | 0.28 | -1.06 | 0.82 | -0.79 |
| 1456149_at | D11Bwg0517e | DNA segment, Chr 11, Brigham & Women's Genetics 0517 expressed | -1.35 | 0.623 | -1.07 | 0.91 | -1.13 | 0.557 | 1.05 | 0.832 | -0.62 |
| 1453190_at | Usp19 | ubiquitin specific peptidase 19 | -1.05 | 0.876 | 1.15 | 0.755 | -2.18 | 0.037 | -1.16 | 0.621 | -0.81 |
| 1454055_at | 1600029O15Rik | RIKEN cDNA 1600029O15 gene | 1.15 | 0.825 | -1.28 | 0.54 | -1.58 | 0.219 | -1.51 | 0.393 | -0.81 |
| 1423459_at | Cops2 | COP9 (constitutive photomorphogenic) homolog, subunit 2 (Arabidopsis thaliana) | -1.07 | 0.605 | -1.31 | 0.002 | -1.16 | 0.378 | 1.12 | 0.581 | -0.6 |
| 1456839_at | Gramd1b | GRAM domain containing 1B | 1.56 | 0.439 | -1.58 | 0.313 | -2.66 | 0.009 | -1.18 | 0.585 | -0.96 |
| 1432872_at | 4932702M13Rik | RIKEN cDNA 4932702M13 gene | -1.13 | 0.867 | -1.57 | 0.513 | 1.04 | 0.903 | -1.38 | 0.52 | -0.76 |
| 1432021_at | Cpne3 | copine III | -1.13 | 0.495 | -1.31 | 0.495 | -1.09 | 0.701 | 1.26 | 0.316 | -0.57 |
| 1458351_s_at | Klhl2 | kelch-like 2, Mayven (Drosophila) | 1.01 | 0.961 | -1.2 | 0.286 | -1.38 | 0.169 | -1.25 | 0.422 | -0.71 |
| 1419348_at | Psp | parotid secretory protein | -1.25 | 0.784 | -1.22 | 0.634 | -1.05 | 0.86 | 1.27 | 0.461 | -0.56 |
| 1440781_at | B830007D08Rik | RIKEN cDNA B830007D08 gene | -1.15 | 0.356 | 1.18 | 0.625 | -1.94 | 0.067 | -1.63 | 0.256 | -0.88 |
| 1437767_s_at | Fts | fused toes | -1.18 | 0.195 | 1.05 | 0.886 | -1.49 | 0.041 | -1.19 | 0.642 | -0.7 |
| 1456688_at | Top2b | Topoisomerase (DNA) II beta | -1.31 | 0.178 | 1.13 | 0.757 | -1.48 | 0 | -1.85 | 0.063 | -0.88 |
| 1438539_at | Ascc1 | Activating signal cointegrator 1 complex subunit 1 | -1.36 | 0.253 | 1.06 | 0.828 | -1.31 | 0.193 | -1.25 | 0.141 | -0.71 |
| 1441355_at | E030047P09Rik | RIKEN cDNA E030047P09 gene | 1.21 | 0.771 | -1.53 | 0.545 | -1.42 | 0.014 | -1.16 | 0.7 | -0.73 |
| 1442998_at | Ctps2 | Cytidine 5'-triphosphate synthase 2 | 1.06 | 0.823 | -1.35 | 0.186 | -1.31 | 0.25 | -2.31 | 0.249 | -0.98 |
| 1443002_at | Zfr | zinc finger RNA binding protein | -1.07 | 0.841 | 1.05 | 0.433 | -1.74 | 0.084 | -1.19 | 0.638 | -0.73 |
| 1459818_x_at | Zmym3 | zinc finger, MYM-type 3 | -1.32 | 0.139 | 1.03 | 0.943 | -1.28 | 0.339 | -1.02 | 0.959 | -0.65 |
| 1443918_at | 2700050L05Rik | RIKEN cDNA 2700050L05 gene | -1.16 | 0.607 | -1.07 | 0.429 | -1.3 | 0.078 | 1.15 | 0.625 | -0.6 |
| 1460712_s_at | Ap1g1 | adaptor protein complex AP-1, gamma 1 subunit | -1.41 | 0.188 | -1.17 | 0.245 | 1 | 0.984 | -1.21 | 0.055 | -0.7 |
| 1435514_at | Lztfl1 | leucine zipper transcription factor-like 1 | -1.3 | 0.226 | -1.29 | 0.386 | 1.03 | 0.924 | -1.18 | 0.327 | -0.69 |
| 1444170_at | 6030446N20Rik | RIKEN cDNA 6030446N20 gene | -1.79 | 0.321 | -1.56 | 0.327 | 1.37 | 0.311 | -2.03 | 0.286 | -1 |
| 1443624_at | BC013481 | CDNA sequence BC013481 | -1.1 | 0.158 | -1.09 | 0.369 | -1.35 | 0.128 | 1.29 | 0.205 | -0.56 |
| 1452196_a_at | Nckap1 | NCK-associated protein 1 | -1.05 | 0.685 | -1.09 | 0.132 | -1.44 | 0.088 | 1.35 | 0.153 | -0.56 |
| 1440060_at | LOC331480 | hypothetical LOC331480 | -1.36 | 0.034 | 1.13 | 0.45 | -1.42 | 0.138 | -1.26 | 0.241 | -0.73 |
| 1458850_at | Myh6 | Myosin, heavy polypeptide 6, cardiac muscle, alpha | -1.34 | 0.201 | -1.66 | 0.172 | 1.21 | 0.287 | -1.74 | 0.195 | -0.88 |
| 1425410_at | Tprkb | Tp53rk binding protein | -1.09 | 0.102 | -1.39 | 0.023 | -1.08 | 0.745 | 1.02 | 0.926 | -0.63 |
| 1443168_at | --- | Adult male colon cDNA, RIKEN full-length enriched library, clone:9030003C19 pr | -1.05 | 0.947 | -1.75 | 0.102 | 1.04 | 0.912 | -1.4 | 0.001 | -0.79 |
| 1432810_at | Ttc32 | tetratricopeptide repeat domain 32 | 1.43 | 0.574 | -4.1 | 0.214 | -1.12 | 0.683 | -1.02 | 0.867 | -1.2 |

| | | | | | | | | | | | |
|---|---|---|---|---|---|---|---|---|---|---|---|
| 1423823_at | 2610012O22Rik | RIKEN cDNA 2610012O22 gene | -1.32 | 0.059 | -1.21 | 0.027 | -1.01 | 0.949 | 1.31 | 0.344 | -0.56 |
| 1432836_at | 4833410I11Rik | RIKEN cDNA 4833410I11 gene | -1.75 | 0.309 | 1.53 | 0.44 | -2.16 | 0.169 | -2.09 | 0.182 | -1.12 |
| 1453921_at | 4930429F11Rik | RIKEN cDNA 4930429F11 gene | 1.47 | 0.487 | -2.67 | 0.065 | -1.39 | 0.47 | -1.45 | 0.628 | -1.01 |
| 1459884_at | Cox7c | cytochrome c oxidase, subunit VIIc | -1.24 | 0.494 | -1.53 | 0.193 | 1.11 | 0.542 | -1.2 | 0.302 | -0.72 |
| 1448445_at | Acp6 | acid phosphatase 6, lysophosphatidic | -1.15 | 0.393 | -1.46 | 0.051 | 1.02 | 0.93 | -3.48 | 0.028 | -1.27 |
| 1443559_at | 1500011J06Rik | RIKEN cDNA 1500011J06 gene | 1.37 | 0.445 | -2.02 | 0.144 | -1.43 | 0.547 | -2.02 | 0.303 | -1.02 |
| 1440626_at | Hoxd13 | Homeo box D13 | -1.4 | 0.642 | -1.73 | 0.052 | 1.28 | 0.492 | -1.27 | 0.486 | -0.78 |
| 1456686_at | Zfhx1a | Zinc finger homeobox 1a | 1.14 | 0.612 | -1.43 | 0.081 | -1.37 | 0.081 | -1.39 | 0.1 | -0.76 |
| 1428881_at | Kns2 | kinesin 2 | -1.25 | 0.15 | -1.31 | 0.185 | 1.01 | 0.977 | -1.14 | 0.262 | -0.67 |
| 1440995_at | 6430531B16Rik | RIKEN cDNA 6430531B16 gene | 1.62 | 0.599 | -1.9 | 0.242 | -2.35 | 0.056 | -1.27 | 0.618 | -0.98 |
| 1433997_at | 2410127E18Rik | RIKEN cDNA 2410127E18 gene | -1.13 | 0.356 | -1.25 | 0.057 | -1.13 | 0.341 | 1.37 | 0.321 | -0.54 |
| 1429458_at | 2410127L17Rik /// I | RIKEN cDNA 2410127L17 gene /// similar to RIKEN cDNA 2410127L17 /// similar | -1.25 | 0.063 | -1.28 | 0.099 | -1.01 | 0.923 | 1.45 | 0.283 | -0.52 |
| 1456521_at | --- | Transcribed locus | -2.25 | 0.072 | -1.06 | 0.354 | 1.18 | 0.643 | -1.17 | 0.796 | -0.82 |
| 1418774_a_at | Atp7a | ATPase, Cu++ transporting, alpha polypeptide | -1.17 | 0.326 | 1.16 | 0.412 | -1.8 | 0 | -1.22 | 0.631 | -0.76 |
| 1419096_at | Apom | apolipoprotein M | -1.24 | 0.405 | -1.01 | 0.904 | -1.3 | 0.508 | 1.17 | 0.676 | -0.59 |
| 1436305_at | Ibrdc1 | IBR domain containing 1 | -1.22 | 0.286 | -1.17 | 0.113 | -1.12 | 0.504 | 1.24 | 0.14 | -0.57 |
| 1450028_a_at | Lancl2 | LanC (bacterial lantibiotic synthetase component C)-like 2 | -1.22 | 0.063 | -1.14 | 0.053 | -1.15 | 0.179 | 2.06 | 0.208 | -0.36 |
| 1458229_at | Robo2 | roundabout homolog 2 (Drosophila) | -1.14 | 0.47 | 1.15 | 0.635 | -1.85 | 0.003 | -1.63 | 0.392 | -0.87 |
| 1457602_at | --- | 16 days neonate thymus cDNA, RIKEN full-length enriched library, clone:A13004 | -3.33 | 0.089 | 1.52 | 0.169 | -1.34 | 0.292 | -2.14 | 0.023 | -1.32 |
| 1444976_at | --- | --- | 1.15 | 0.834 | -1.49 | 0.483 | -1.35 | 0.281 | -1.51 | 0.571 | -0.8 |
| 1452411_at | Lrrc1 | leucine rich repeat containing 1 | 1.18 | 0.193 | -1.8 | 0.06 | -1.2 | 0.093 | -1.09 | 0.564 | -0.73 |
| 1457133_at | --- | Transcribed locus | -1.66 | 0.355 | 1.26 | 0.699 | -1.43 | 0.451 | -1 | 0.98 | -0.71 |
| 1448122_at | Tcp1 | t-complex protein 1 | -1.07 | 0.331 | -1.24 | 0.089 | -1.21 | 0.139 | 1.11 | 0.153 | -0.6 |
| 1446036_at | --- | --- | -1.52 | 0.654 | 1.2 | 0.543 | -1.41 | 0.14 | -3.26 | 0.21 | -1.25 |
| 1454638_a_at | Pah | phenylalanine hydroxylase | -1.24 | 0.009 | -1.31 | 0.312 | -1 | 0.992 | 2.93 | 0.337 | -0.15 |
| 1433913_at | C80913 | expressed sequence C80913 | -1.05 | 0.592 | -1.17 | 0.188 | -1.31 | 0.027 | 1.03 | 0.885 | -0.63 |
| 1441092_at | 9330159M07Rik | RIKEN cDNA 9330159M07 gene | -1.25 | 0.326 | -1.13 | 0.477 | -1.13 | 0.659 | 1.15 | 0.701 | -0.59 |
| 1423919_at | BC023882 | cDNA sequence BC023882 | -1.12 | 0.631 | -1.11 | 0.202 | -1.29 | 0.219 | 1.07 | 0.657 | -0.61 |
| 1417962_s_at | Ghr | growth hormone receptor | 1.1 | 0.032 | -1.47 | 0.019 | -1.27 | 0.4 | -1.15 | 0.605 | -0.7 |
| 1456817_at | --- | Adult retina cDNA, RIKEN full-length enriched library, clone:A930002A02 product | -1.6 | 0.432 | -1.49 | 0.508 | 1.27 | 0.187 | -1.81 | 0.299 | -0.91 |
| 1441018_at | Usp24 | ubiquitin specific peptidase 24 | 1.39 | 0.495 | -1.35 | 0.343 | -2.3 | 0.013 | -1.33 | 0.456 | -0.9 |
| 1438378_at | 1700013G23Rik | RIKEN cDNA 1700013G23 gene | -1.23 | 0.589 | -1.3 | 0.661 | -1.01 | 0.978 | 3.01 | 0.107 | -0.13 |
| 1442406_at | 9230104K21Rik | RIKEN cDNA 9230104K21 gene | -1.02 | 0.842 | -1.02 | 0.912 | -1.62 | 0.039 | 2.98 | 0.207 | -0.17 |
| 1459326_at | D9Ertd256e | DNA segment, Chr 9, ERATO Doi 256, expressed | -1.42 | 0.245 | 1.35 | 0.581 | -1.94 | 0.057 | -1.72 | 0.275 | -0.93 |
| 1456017_x_at | LOC666466 | similar to oocyte specific homeobox 2 | 1.32 | 0.252 | -1.87 | 0.244 | -1.4 | 0.664 | -1.89 | 0.321 | -0.96 |
| 1446193_at | --- | --- | -1.07 | 0.87 | -1.09 | 0.847 | -1.4 | 0.336 | 2.01 | 0.126 | -0.39 |
| 1430896_s_at | Nudt7 | nudix (nucleoside diphosphate linked moiety X)-type motif 7 | -1.05 | 0.699 | -2.26 | 0.115 | 1.18 | 0.407 | -1.49 | 0.061 | -0.91 |
| 1430847_a_at | Crem | cAMP responsive element modulator | 1 | 0.994 | -1.42 | 0.401 | -1.16 | 0.596 | -2.6 | 0.086 | -1.04 |
| 1425590_s_at | Aipl1 | aryl hydrocarbon receptor-interacting protein-like 1 | 1.09 | 0.687 | -1.54 | 0.453 | -1.2 | 0.482 | -1.51 | 0.587 | -0.79 |
| 1420949_at | Znrf1 | zinc and ring finger 1 | -1.43 | 0.416 | -1.24 | 0.417 | 1.07 | 0.604 | -1.11 | 0.142 | -0.68 |
| 1459439_at | Mrps5 | mitochondrial ribosomal protein S5 | 1.21 | 0.574 | -2.16 | 0.023 | -1.11 | 0.726 | -1.22 | 0.611 | -0.82 |
| 1451027_at | Baiap2 | brain-specific angiogenesis inhibitor 1-associated protein 2 | -1.16 | 0.522 | 1.02 | 0.947 | -1.45 | 0.053 | -1.01 | 0.964 | -0.65 |
| 1429753_at | Nxph4 | neurexophilin 4 | 1.07 | 0.926 | -1.35 | 0.276 | -1.32 | 0.532 | -1.06 | 0.916 | -0.67 |
| 1448003_at | --- | --- | -1.16 | 0.553 | 1.05 | 0.628 | -1.52 | 0.089 | -1.63 | 0.124 | -0.82 |
| 1453149_at | Slc25a32 | solute carrier family 25, member 32 | -1.2 | 0.486 | -1.25 | 0.277 | -1.07 | 0.444 | 1.11 | 0.474 | -0.6 |
| 1447040_at | Alcam | Activated leukocyte cell adhesion molecule | 1.08 | 0.859 | -1.08 | 0.762 | -1.77 | 0.003 | -1.3 | 0.396 | -0.77 |
| 1456669_at | --- | Transcribed locus | -1.28 | 0.445 | -1.51 | 0.475 | 1.13 | 0.808 | -1.3 | 0.71 | -0.74 |
| 1434513_at | Atp13a3 | ATPase type 13A3 | -1.07 | 0.551 | -1.2 | 0.233 | -1.24 | 0.123 | 1.2 | 0.342 | -0.58 |
| 1416918_at | Dlgh3 | discs, large homolog 3 (Drosophila) | -1.05 | 0.349 | -1.17 | 0.148 | -1.31 | 0.13 | 1.12 | 0.35 | -0.6 |
| 1446005_at | D2Ertd337e | DNA segment, Chr 2, ERATO Doi 337, expressed | -1.3 | 0.171 | -1.78 | 0.195 | 1.24 | 0.57 | -2.44 | 0.029 | -1.07 |
| 1418076_at | St14 | suppression of tumorigenicity 14 (colon carcinoma) | -1.01 | 0.978 | -1.83 | 0.38 | 1.03 | 0.882 | -1.72 | 0.327 | -0.88 |
| 1421496_at | Asah3l | N-acylsphingosine amidohydrolase 3-like | -1.07 | 0.919 | -1.88 | 0.198 | 1.1 | 0.836 | -1.97 | 0.04 | -0.95 |
| 1423266_at | 2810405K02Rik | RIKEN cDNA 2810405K02 gene | 1.01 | 0.673 | -1.32 | 0.003 | -1.25 | 0.048 | -1.21 | 0.44 | -0.69 |

| | | | | | | | | | | | |
|---|---|---|---|---|---|---|---|---|---|---|---|
| 1433982_at | Usp28 | ubiquitin specific peptidase 28 | -1.51 | 0.304 | 1.01 | 0.952 | -1.11 | 0.606 | -2.31 | 0.051 | -0.98 |
| 1446307_at | Papolg | Poly(A) polymerase gamma | -1.54 | 0.302 | 1.04 | 0.931 | -1.13 | 0.532 | -1.32 | 0.246 | -0.74 |
| 1456716_s_at | 3110002H16Rik | RIKEN cDNA 3110002H16 gene | -1.13 | 0.456 | -1.35 | 0.033 | -1.06 | 0.179 | 1.59 | 0.28 | -0.49 |
| 1440479_at | Cbx4 | Chromobox homolog 4 (Drosophila Pc class) /// 10 days lactation, adult female m | -1.53 | 0.586 | -1.68 | 0.435 | 1.32 | 0.02 | -1.35 | 0.452 | -0.81 |
| 1434356_a_at | Psma5 | proteasome (prosome, macropain) subunit, alpha type 5 | -1.11 | 0.095 | -1.15 | 0.029 | -1.25 | 0.051 | 1.15 | 0.365 | -0.59 |
| 1446883_at | 4933427D06Rik | RIKEN cDNA 4933427D06 gene | -1.11 | 0.87 | -1.98 | 0.209 | 1.17 | 0.756 | -1.56 | 0.291 | -0.87 |
| 1443135_at | Cog4 | component of oligomeric golgi complex 4 | 1 | 0.987 | -1.25 | 0.282 | -1.3 | 0.169 | -1.17 | 0.564 | -0.68 |
| 1439244_a_at | Tnrc6a | trinucleotide repeat containing 6a | 1.03 | 0.896 | -1.24 | 0.041 | -1.36 | 0.062 | -1.52 | 0.196 | -0.77 |
| 1430010_at | 2810465G24Rik | RIKEN cDNA 2810465G24 gene | -1.16 | 0.81 | -1.4 | 0.178 | -1 | 0.994 | 3.29 | 0.163 | -0.07 |
| 1420780_at | Ascl3 | achaete-scute complex homolog-like 3 (Drosophila) | -1.55 | 0.018 | -1.17 | 0.327 | 1.07 | 0.595 | -1.69 | 0.281 | -0.83 |
| 1444194_at | --- | --- | 1.3 | 0.088 | -1.19 | 0.558 | -2.29 | 0.01 | -1.2 | 0.417 | -0.85 |
| 1427383_at | Irx6 | Iroquois related homeobox 6 (Drosophila) | -2.52 | 0.141 | -1.37 | 0.32 | 1.44 | 0.288 | -1.13 | 0.838 | -0.89 |
| 1439232_at | 1500016L03Rik | RIKEN cDNA 1500016L03 gene | -1.06 | 0.776 | -1.49 | 0.596 | -1.04 | 0.901 | 2.14 | 0.341 | -0.36 |
| 1455817_x_at | LOC668166 | similar to Zinc finger X-linked protein ZXDB | -1.55 | 0.296 | 1.01 | 0.978 | -1.09 | 0.635 | -1.4 | 0.484 | -0.76 |
| 1459044_at | AU015558 | expressed sequence AU015558 | -1.23 | 0.624 | -1.43 | 0.584 | 1.06 | 0.863 | -1.19 | 0.607 | -0.7 |
| 1439790_at | Serpinb9 | serine (or cysteine) peptidase inhibitor, clade B, member 9 | 1.01 | 0.952 | -1.03 | 0.721 | -1.69 | 0.003 | -1.19 | 0.127 | -0.72 |
| 1418441_at | Col8a1 | procollagen, type VIII, alpha 1 | -2.07 | 0.492 | -1.19 | 0.681 | 1.25 | 0.492 | -1.01 | 0.971 | -0.76 |
| 1454352_at | A930028O11Rik | RIKEN cDNA A930028O11 gene | -1.61 | 0.534 | 1.33 | 0.516 | -1.6 | 0.223 | -1.36 | 0.462 | -0.81 |
| 1449021_at | Rpp21 | ribonuclease P 21 subunit (human) | 1 | 0.977 | -1.4 | 0.084 | -1.17 | 0.335 | -1.13 | 0.056 | -0.67 |
| 1425777_at | Cacnb1 | calcium channel, voltage-dependent, beta 1 subunit | -1.39 | 0.565 | -1.8 | 0.048 | 1.3 | 0.301 | -1.2 | 0.724 | -0.77 |
| 1456419_at | 5730455P16Rik | RIKEN cDNA 5730455P16 gene | 1.14 | 0.594 | -1.35 | 0.195 | -1.45 | 0.072 | -1.08 | 0.636 | -0.68 |
| 1430664_at | Zfp169 | zinc finger protein 169 | 1.26 | 0.702 | -1.73 | 0.229 | -1.36 | 0.025 | -2.84 | 0.211 | -1.17 |
| 1427863_at | Hist1h3d | histone1, H3d | 1 | 0.999 | -1.34 | 0.518 | -1.21 | 0.479 | -1.19 | 0.304 | -0.68 |
| 1450507_at | Pou2f3 | POU domain, class 2, transcription factor 3 | -2.33 | 0.137 | -1.21 | 0.659 | 1.32 | 0.176 | -1.06 | 0.849 | -0.82 |
| 1451708_at | Gpr33 | G protein-coupled receptor 33 | -1.25 | 0.385 | -1.28 | 0.216 | -1.01 | 0.988 | 1.09 | 0.661 | -0.61 |
| 1436320_at | --- | CDNA clone IMAGE:1328649 | 1.01 | 0.935 | -1.74 | 0.031 | -1.02 | 0.935 | -1.05 | 0.855 | -0.7 |
| 1447359_at | Zfp575 | zinc finger protein 575 | -1.32 | 0.229 | 1.28 | 0.481 | -1.87 | 0.215 | -1.15 | 0.457 | -0.76 |
| 1459811_at | Mtf2 | metal response element binding transcription factor 2 | -1.96 | 0.304 | 1.22 | 0.697 | -1.18 | 0.785 | -1.31 | 0.27 | -0.81 |
| 1423585_at | Igfbp7 /// LOC6215 | insulin-like growth factor binding protein 7 /// similar to O-acyltransferase (membra | 1.01 | 0.913 | -1.1 | 0.668 | -1.53 | 0.145 | -1.6 | 0.013 | -0.8 |
| 1428153_at | Mrps10 | mitochondrial ribosomal protein S10 | -1.11 | 0.268 | -1.25 | 0.236 | -1.15 | 0.112 | 1.01 | 0.925 | -0.62 |
| 1444488_at | Cadps | Ca<2+>dependent activator protein for secretion | 1.51 | 0.253 | -3.42 | 0.108 | -1.3 | 0.091 | -3.11 | 0.251 | -1.58 |
| 1439920_at | --- | Transcribed locus | 1.53 | 0.075 | -2.22 | 0.333 | -1.69 | 0.229 | -1.14 | 0.718 | -0.88 |
| 1448963_at | Nfyc | nuclear transcription factor-Y gamma | -1.03 | 0.798 | -1.18 | 0.278 | -1.32 | 0.053 | 1.09 | 0.602 | -0.61 |
| 1418928_a_at | 2310038H17Rik | RIKEN cDNA 2310038H17 gene | -1.49 | 0.036 | -1.21 | 0.072 | 1.08 | 0.615 | -1.19 | 0.495 | -0.7 |
| 1435708_at | Gls | Glutaminase | -1.22 | 0.315 | 1.04 | 0.943 | -1.41 | 0.163 | -1.45 | 0.338 | -0.76 |
| 1436129_at | 4930523C11Rik /// | RIKEN cDNA 4930523C11 gene /// a disintegrin and metallopeptidase domain 6 | -1.16 | 0.797 | 1.1 | 0.702 | -1.61 | 0.413 | -1.51 | 0.139 | -0.8 |
| 1448983_at | Cdrt4 | CMT1A duplicated region transcript 4 | -1.1 | 0.261 | -1.2 | 0.172 | -1.2 | 0.297 | 1.02 | 0.957 | -0.62 |
| 1452739_at | Fbxo7 | F-box only protein 7 | -1.26 | 0.003 | -1.24 | 0.026 | -1.03 | 0.844 | 2.17 | 0.211 | -0.34 |
| 1449629_s_at | Snrpd3 | Small nuclear ribonucleoprotein D3 | -1.09 | 0.475 | -1.31 | 0.07 | -1.13 | 0.31 | 1.11 | 0.664 | -0.6 |
| 1425211_at | 2010003O18Rik | RIKEN cDNA 2010003O18 gene | -1.11 | 0.338 | -1.08 | 0.399 | -1.34 | 0.005 | 1.32 | 0.178 | -0.55 |
| 1424735_at | Slc25a25 | solute carrier family 25 (mitochondrial carrier, phosphate carrier), member 25 | -1.29 | 0.468 | -1.17 | 0.681 | -1.06 | 0.879 | 1.18 | 0.479 | -0.58 |
| 1441319_at | Rbm5 | RNA binding motif protein 5 | -1.29 | 0.165 | 1.3 | 0.308 | -2 | 0.006 | -1.26 | 0.029 | -0.81 |
| 1440971_x_at | G630024C07Rik | RIKEN cDNA G630024C07 gene | -1.4 | 0.444 | -1.18 | 0.365 | 1.01 | 0.881 | -1.1 | 0.709 | -0.67 |
| 1454943_a_at | Paxip1 | PAX interacting (with transcription-activation domain) protein 1 | -1.3 | 0.08 | -1.16 | 0.571 | -1.06 | 0.746 | 1.64 | 0.304 | -0.47 |
| 1458981_at | --- | Transcribed locus | 1.12 | 0.831 | -1.52 | 0.196 | -1.26 | 0.326 | -1.32 | 0.419 | -0.74 |
| 1444437_at | Usp34 | ubiquitin specific peptidase 34 | -1.22 | 0.612 | 1.15 | 0.715 | -1.65 | 0.069 | -1.16 | 0.548 | -0.72 |
| 1449142_a_at | Yipf5 | Yip1 domain family, member 5 | -1.14 | 0.631 | -1.24 | 0.232 | -1.12 | 0.476 | 1.02 | 0.867 | -0.62 |
| 1442699_at | --- | --- | -1.62 | 0.378 | 1.18 | 0.361 | -1.29 | 0.05 | -1.17 | 0.169 | -0.72 |
| 1443750_s_at | Rpp40 | ribonuclease P 40 subunit (human) | -1.47 | 0.128 | -1.26 | 0.003 | 1.1 | 0.621 | -1.2 | 0.477 | -0.71 |
| 1434234_at | Zfp341 | zinc finger protein 341 | 1.08 | 0.785 | -1.3 | 0.53 | -1.39 | 0.358 | -1.03 | 0.85 | -0.66 |
| 1419736_a_at | Eif1ay | eukaryotic translation initiation factor 1A, Y-linked | -1.12 | 0.026 | -1.07 | 0.474 | -1.34 | 0.008 | 1.21 | 0.245 | -0.58 |
| 1441737_s_at | Rassf1 | Ras association (RalGDS/AF-6) domain family 1 | -1.25 | 0.065 | 1.01 | 0.858 | -1.32 | 0.087 | -1.12 | 0.259 | -0.67 |
| 1451059_at | Zfp474 | zinc finger protein 474 | -1.5 | 0.051 | -1.08 | 0.928 | -1.02 | 0.932 | 1.39 | 0.209 | -0.55 |

| | | | | | | | | | | | |
|---|---|---|---|---|---|---|---|---|---|---|---|
| 1419024_at | Ptp4a1 | protein tyrosine phosphatase 4a1 | -1.23 | 0.234 | -1.1 | 0.616 | -1.18 | 0.366 | 1.84 | 0.173 | -0.41 |
| 1420227_at | --- | Transcribed locus | 1.32 | 0.173 | -1.35 | 0.328 | -1.95 | 0.074 | -1.1 | 0.865 | -0.77 |
| 1444824_at | C76793 | expressed sequence C76793 | -1.29 | 0.538 | -1.26 | 0.559 | 1.01 | 0.956 | -1.97 | 0.369 | -0.88 |
| 1416282_at | Psmc3 | proteasome (prosome, macropain) 26S subunit, ATPase 3 | -1.03 | 0.388 | -1.16 | 0.009 | -1.35 | 0.07 | 1.1 | 0.602 | -0.61 |
| 1447724_x_at | Opa3 | Optic atrophy 3 (human) | -1.02 | 0.918 | -1.17 | 0.556 | -1.36 | 0.163 | 1.26 | 0.434 | -0.57 |
| 1423106_at | Ube2b | ubiquitin-conjugating enzyme E2B, RAD6 homology (S. cerevisiae) | -1.22 | 0.102 | -1.08 | 0.154 | -1.2 | 0.16 | 1.03 | 0.772 | -0.62 |
| 1420912_at | Aff4 | AF4/FMR2 family, member 4 | 1.11 | 0.6 | -1.27 | 0.216 | -1.46 | 0.024 | -1.85 | 0.044 | -0.87 |
| 1437462_x_at | Mmp15 | matrix metallopeptidase 15 | 1.06 | 0.504 | -1.58 | 0.192 | -1.13 | 0.374 | -1.25 | 0.653 | -0.73 |
| 1438222_at | D2Ertd612e | DNA segment, Chr 2, ERATO Doi 612, expressed | 1.02 | 0.894 | -1.18 | 0.247 | -1.42 | 0.02 | -1.07 | 0.723 | -0.66 |
| 1448514_at | Cox5b | cytochrome c oxidase, subunit Vb | -1.16 | 0.231 | -1.18 | 0.773 | -1.15 | 0.743 | 1.11 | 0.834 | -0.6 |
| 1432480_a_at | Cntd1 | cyclin N-terminal domain containing 1 | -1.35 | 0.477 | -1.15 | 0.829 | -1.03 | 0.883 | 1.95 | 0.088 | -0.4 |
| 1434190_at | Sms | spermine synthase | -1.1 | 0.52 | -1.08 | 0.7 | -1.34 | 0.175 | 1.39 | 0.03 | -0.54 |
| 1440706_at | Bmp8b | bone morphogenetic protein 8b | -1.77 | 0.282 | 1.25 | 0.661 | -1.32 | 0.39 | -1.28 | 0.112 | -0.78 |
| 1425660_at | Btbd3 | BTB (POZ) domain containing 3 | -1.21 | 0.566 | -1.25 | 0.105 | -1.05 | 0.794 | 1.09 | 0.814 | -0.61 |
| 1442117_at | --- | Adult retina cDNA, RIKEN full-length enriched library, clone:A930003C13 product | 1.12 | 0.889 | -1.05 | 0.869 | -1.97 | 0.176 | -1.37 | 0.262 | -0.82 |
| 1420659_at | Slamf6 | SLAM family member 6 | -1.55 | 0.345 | -1.18 | 0.344 | 1.08 | 0.706 | -2.02 | 0.136 | -0.92 |
| 1419864_x_at | --- | Transcribed locus | 1.43 | 0.144 | -1.47 | 0.002 | -2.15 | 0.084 | -1.64 | 0.362 | -0.96 |
| 1440957_at | Defb29 | defensin beta 29 | 1.15 | 0.034 | -1.34 | 0.487 | -1.47 | 0.283 | -1.28 | 0.427 | -0.74 |
| 1437965_at | Heatr1 | HEAT repeat containing 1 | 1.05 | 0.917 | -1.06 | 0.875 | -1.72 | 0.05 | -1.52 | 0.39 | -0.81 |
| 1440463_at | Ngly1 | N-glycanase 1 | 1.14 | 0.328 | -1.11 | 0.806 | -1.88 | 0.054 | -1.08 | 0.837 | -0.73 |
| 1455620_at | --- | PREDICTED: Mus musculus similar to heparan sulfate D-glucosaminyl 3-O-sulfot | -1.25 | 0.727 | -1.85 | 0.106 | 1.24 | 0.59 | -1.13 | 0.717 | -0.75 |
| 1445618_at | --- | --- | -1.21 | 0.205 | -1.45 | 0.554 | 1.06 | 0.854 | -1.28 | 0.505 | -0.72 |
| 1429978_at | 5830467E07Rik | RIKEN cDNA 5830467E07 gene | 1.12 | 0.778 | -1.13 | 0.541 | -1.76 | 0.171 | -1.4 | 0.542 | -0.79 |
| 1433075_at | 4931412I15Rik | RIKEN cDNA 4931412I15 gene | -1.26 | 0.632 | -2.98 | 0.039 | 1.45 | 0.455 | -1.42 | 0.59 | -1.05 |
| 1425240_at | BC011426 | cDNA sequence BC011426 | 1.06 | 0.5 | -1.84 | 0.066 | -1.02 | 0.903 | -1.27 | 0.334 | -0.77 |
| 1440427_at | BB124205 | expressed sequence BB124205 | -1.56 | 0.101 | -1.11 | 0.514 | 1.03 | 0.832 | -1.93 | 0.088 | -0.89 |
| 1426884_at | Rmnd5a | required for meiotic nuclear division 5 homolog A (S. cerevisiae) | -1.19 | 0.059 | -1.19 | 0.229 | -1.12 | 0.394 | 1.17 | 0.378 | -0.58 |
| 1417132_at | Cdc25a | cell division cycle 25 homolog A (S. cerevisiae) | -1.07 | 0.668 | -1.45 | 0.197 | -1.05 | 0.757 | 1.92 | 0.283 | -0.41 |
| 1429304_at | Ankrd10 | ankyrin repeat domain 10 | -1.26 | 0.167 | 1.04 | 0.878 | -1.35 | 0.139 | -1.68 | 0.071 | -0.81 |
| 1447650_at | Nmd3 | NMD3 homolog (S. cerevisiae) | -1.07 | 0.051 | -1.48 | 0.253 | -1.03 | 0.782 | 1.09 | 0.338 | -0.62 |
| 1440745_at | Prdm16 | PR domain containing 16 | -1.02 | 0.9 | -1.14 | 0.562 | -1.39 | 0.44 | 1.35 | 0.207 | -0.55 |
| 1419268_at | Agr2 | anterior gradient 2 (Xenopus laevis) | -1.22 | 0.741 | -1.25 | 0.53 | -1.04 | 0.912 | 1.75 | 0.4 | -0.44 |
| 1415875_at | 3010003L21Rik | RIKEN cDNA 3010003L21 gene | -1.52 | 0.499 | -1.28 | 0.411 | 1.14 | 0.734 | -1.19 | 0.812 | -0.71 |
| 1459695_at | --- | --- | 1.27 | 0.394 | -1.14 | 0.528 | -2.3 | 0 | -1.42 | 0.346 | -0.9 |
| 1429759_at | Rps6ka6 | ribosomal protein S6 kinase polypeptide 6 | 1.03 | 0.912 | -1.44 | 0.416 | -1.17 | 0.322 | -1.2 | 0.817 | -0.7 |
| 1427741_x_at | Nkx2-3 | NK2 transcription factor related, locus 3 (Drosophila) | 1.29 | 0.671 | -2.23 | 0.269 | -1.18 | 0.736 | -1.18 | 0.728 | -0.83 |
| 1428164_at | Nudt9 | nudix (nucleoside diphosphate linked moiety X)-type motif 9 | -1.27 | 0.294 | -1.11 | 0.524 | -1.13 | 0.182 | 1.46 | 0.223 | -0.51 |
| 1418526_at | Fusip1 | FUS interacting protein (serine-arginine rich) 1 | -1.06 | 0.524 | -1.26 | 0.006 | -1.19 | 0.216 | 1.51 | 0.295 | -0.5 |
| 1445550_at | --- | --- | -1.16 | 0.362 | -1.17 | 0.654 | -1.16 | 0.278 | 1.12 | 0.727 | -0.59 |
| 1451123_at | C330016O10Rik | RIKEN cDNA C330016O10 gene | 1.01 | 0.958 | -1.59 | 0.392 | -1.06 | 0.68 | -1.4 | 0.086 | -0.76 |
| 1456892_at | D030036P13Rik | RIKEN cDNA D030036P13 gene | 1.1 | 0.418 | -1.82 | 0.045 | -1.07 | 0.522 | -1.07 | 0.813 | -0.72 |
| 1415716_a_at | Rps27 | ribosomal protein S27 | 1.03 | 0.523 | -1.24 | 0.3 | -1.34 | 0.034 | -1.42 | 0.056 | -0.74 |
| 1432978_at | 9030607L02Rik | RIKEN cDNA 9030607L02 gene | -1.27 | 0.725 | -1.26 | 0.684 | 1 | 0.998 | -2.44 | 0.068 | -0.99 |
| 1424771_at | H2afj /// LOC63240 | H2A histone family, member J /// similar to H2A histone family, member J isoform | -1.3 | 0.404 | -1.04 | 0.827 | -1.18 | 0.124 | 1.24 | 0.003 | -0.57 |
| 1453338_at | Stam2 | signal transducing adaptor molecule (SH3 domain and ITAM motif) 2 | 1.14 | 0.731 | -1.7 | 0.177 | -1.18 | 0.403 | -1.75 | 0.118 | -0.87 |
| 1418519_at | Aadat | aminoadipate aminotransferase | 1.07 | 0.256 | -1.37 | 0.133 | -1.29 | 0.398 | -1.64 | 0.18 | -0.81 |
| 1426029_a_at | Nfat5 | nuclear factor of activated T-cells 5 | -1.57 | 0.354 | -1.21 | 0.601 | 1.11 | 0.87 | -1.4 | 0.212 | -0.76 |
| 1438725_at | Thrap1 | thyroid hormone receptor associated protein 1 | -1.45 | 0.292 | -1.45 | 0.06 | 1.2 | 0.106 | -1.21 | 0.227 | -0.73 |
| 1422446_x_at | Ins2 | insulin II | -2.24 | 0.422 | -1.05 | 0.635 | 1.18 | 0.728 | -1.28 | 0.45 | -0.85 |
| 1455102_at | D330037H05Rik /// | RIKEN cDNA D330037H05 gene /// similar to La-related protein 4 (La ribonucleop | -1.13 | 0.377 | -1.18 | 0.288 | -1.18 | 0.344 | 1.04 | 0.834 | -0.61 |
| 1416570_s_at | Gfm1 | G elongation factor, mitochondrial 1 | -1.27 | 0.066 | -1.27 | 0.049 | -1 | 0.99 | 1.13 | 0.489 | -0.6 |
| 1432325_at | 4930502M04Rik | RIKEN cDNA 4930502M04 gene | -1.02 | 0.969 | 1 | 0.995 | -1.66 | 0.219 | -1.03 | 0.959 | -0.68 |
| 1455289_at | Ankrd13b | ankyrin repeat domain 13b | -1.07 | 0.715 | -1.37 | 0.46 | -1.09 | 0.366 | 1.43 | 0.232 | -0.53 |

| | | | | | | | | | | | |
|---|---|---|---|---|---|---|---|---|---|---|---|
| 1434182_at | Ddx31 | DEAD/H (Asp-Glu-Ala-Asp/His) box polypeptide 31 | -1.19 | 0.576 | -1.46 | 0.026 | 1.06 | 0.782 | -1.21 | 0.452 | -0.7 |
| 1433613_at | --- | --- | 1.04 | 0.493 | -1.12 | 0.471 | -1.54 | 0.042 | -1.32 | 0.064 | -0.74 |
| 1451333_a_at | Acrbp | proacrosin binding protein | -1.42 | 0.238 | -1.22 | 0.56 | 1.06 | 0.831 | -1.54 | 0.33 | -0.78 |
| 1427901_at | Mrps18c | mitochondrial ribosomal protein S18C | -1.09 | 0.433 | -1.09 | 0.387 | -1.35 | 0.041 | 1.1 | 0.407 | -0.61 |
| 1453156_s_at | Zadh1 | zinc binding alcohol dehydrogenase, domain containing 1 | -1.15 | 0.381 | -1.13 | 0.57 | -1.2 | 0.127 | 1.14 | 0.622 | -0.59 |
| 1442070_at | Mllt10 | myeloid/lymphoid or mixed lineage-leukemia translocation to 10 homolog (Drosop | 1.01 | 0.967 | -1.41 | 0.313 | -1.16 | 0.445 | -1.12 | 0.74 | -0.67 |
| 1452634_at | Obox3 | oocyte specific homeobox 3 | -1.86 | 0.033 | 1.62 | 0.391 | -2.36 | 0.013 | -1.34 | 0.343 | -0.99 |
| 1421159_at | Cgnl1 /// LOC6774 | cingulin-like 1 /// similar to cingulin-like 1 isoform 1 | 1.29 | 0.796 | -2.19 | 0.092 | -1.2 | 0.8 | -1.73 | 0.272 | -0.96 |
| 1416366_at | LOC675851 | similar to NADH dehydrogenase (ubiquinone) 1, subcomplex unknown, 2 | -1.26 | 0.003 | -1.15 | 0.057 | -1.09 | 0.298 | 1.02 | 0.82 | -0.62 |
| 1417920_at | Amn | amnionless | -1.02 | 0.8 | -2.08 | 0.225 | 1.12 | 0.754 | -1.48 | 0.202 | -0.86 |
| 1432061_at | 4933406B17Rik | RIKEN cDNA 4933406B17 gene | -1.53 | 0.518 | 1.01 | 0.967 | -1.09 | 0.847 | -1.41 | 0.14 | -0.75 |
| 1445410_at | Mak10 | MAK10 homolog, amino-acid N-acetyltransferase subunit, (S. cerevisiae) | -1.51 | 0.417 | 1.18 | 0.644 | -1.36 | 0.074 | -1.13 | 0.56 | -0.71 |
| 1446022_at | Msi2 | Musashi homolog 2 (Drosophila) | -1.24 | 0.181 | 1 | 0.993 | -1.29 | 0.197 | -1.07 | 0.821 | -0.65 |
| 1454696_at | Gnb1 | guanine nucleotide binding protein, beta 1 | -1.26 | 0.509 | 1.04 | 0.812 | -1.35 | 0.34 | -1.05 | 0.854 | -0.65 |
| 1436301_at | Ripk5 | receptor interacting protein kinase 5 | -1.06 | 0.536 | -1.14 | 0.284 | -1.31 | 0.088 | 1.14 | 0.292 | -0.59 |
| 1416501_at | Pdpk1 | 3-phosphoinositide dependent protein kinase-1 | -1.02 | 0.906 | -1.19 | 0.032 | -1.32 | 0.312 | 1.36 | 0.208 | -0.54 |
| 1459153_at | Ston2 | stonin 2 | -1.09 | 0.667 | -2.28 | 0.091 | 1.22 | 0.488 | -2.45 | 0.265 | -1.15 |
| 1415860_at | Kpna2 /// LOC6705 | karyopherin (importin) alpha 2 /// similar to Importin alpha-2 subunit (Karyopherin | -1.09 | 0.689 | -1.18 | 0.15 | -1.23 | 0.214 | 1.78 | 0.193 | -0.43 |
| 1433372_at | 5330425B07Rik | RIKEN cDNA 5330425B07 gene | -1.23 | 0.802 | -1.25 | 0.733 | -1.03 | 0.935 | 1.83 | 0.427 | -0.42 |
| 1458739_at | --- | --- | -1.54 | 0.44 | 1.59 | 0.125 | -2.93 | 0.148 | -1.41 | 0.009 | -1.07 |
| 1416133_at | C920006C10Rik | RIKEN cDNA C920006C10 gene | -1.12 | 0.036 | -1.13 | 0.123 | -1.24 | 0.16 | 1.07 | 0.774 | -0.61 |
| 1426902_at | Coq6 | coenzyme Q6 homolog (yeast) | -1.11 | 0.6 | -1.2 | 0.49 | -1.19 | 0.093 | 1.04 | 0.811 | -0.61 |
| 1456799_at | E130110O22Rik | RIKEN cDNA E130110O22 gene | 1.2 | 0.091 | -1.35 | 0.171 | -1.56 | 0.211 | -3.12 | 0.039 | -1.21 |
| 1444615_x_at | Runx1t1 | runt-related transcription factor 1; translocated to, 1 (cyclin D-related) | 1.12 | 0.549 | -1.29 | 0.371 | -1.46 | 0.051 | -1.27 | 0.319 | -0.72 |
| 1430957_at | Arhgap26 | Rho GTPase activating protein 26 | -2.15 | 0.452 | -1.25 | 0.538 | 1.31 | 0.381 | -1.47 | 0.134 | -0.89 |
| 1416432_at | Pfkfb3 | 6-phosphofructo-2-kinase/fructose-2,6-biphosphatase 3 | -1.19 | 0.024 | -1.67 | 0.408 | 1.14 | 0.73 | -1.72 | 0.15 | -0.86 |
| 1455806_x_at | Ndufa12 /// LOC66 | NADH dehydrogenase (ubiquinone) 1 alpha subcomplex, 12 /// similar to 13kDa d | -1.14 | 0.365 | -1.26 | 0.038 | -1.1 | 0.133 | 1.23 | 0.066 | -0.57 |
| 1425199_a_at | Epb4.1l5 | erythrocyte protein band 4.1-like 5 | -1.3 | 0.068 | -1.12 | 0.297 | -1.09 | 0.625 | 1.06 | 0.865 | -0.61 |
| 1458825_at | Mier3 | Mesoderm induction early response 1, family member 3 | -1.27 | 0.428 | 1.02 | 0.946 | -1.28 | 0.437 | -1.19 | 0.542 | -0.68 |
| 1446688_at | Gnaq | Guanine nucleotide binding protein, alpha q polypeptide | 1.22 | 0.513 | -1.28 | 0.365 | -1.73 | 0.032 | -1.33 | 0.33 | -0.78 |
| 1424465_at | Ccdc58 | coiled-coil domain containing 58 | -1.31 | 0.009 | -1.22 | 0.004 | -1 | 0.994 | 1.13 | 0.708 | -0.6 |
| 1433001_at | 1700019L13Rik | RIKEN cDNA 1700019L13 gene | 1.3 | 0.733 | -2.3 | 0.167 | -1.18 | 0.717 | -1.41 | 0.576 | -0.9 |
| 1429126_at | 2600001M11Rik | RIKEN cDNA 2600001M11 gene | -1.21 | 0.068 | -1.19 | 0.214 | -1.09 | 0.652 | 1.54 | 0.16 | -0.49 |
| 1426953_at | Hmgb2l1 | high mobility group box 2-like 1 | -1.07 | 0.237 | -1.11 | 0.496 | -1.34 | 0.101 | 1.2 | 0.48 | -0.58 |
| 1448494_at | Gas1 | growth arrest specific 1 | -1.29 | 0.108 | -1.39 | 0.415 | 1.08 | 0.829 | -1.57 | 0.378 | -0.79 |
| 1416848_at | Ubl5 | ubiquitin-like 5 | -1.37 | 0.014 | -1.08 | 0.523 | -1.08 | 0.514 | 1.04 | 0.563 | -0.62 |
| 1429879_at | 0610037L13Rik | RIKEN cDNA 0610037L13 gene | -2.2 | 0.008 | 1.52 | 0.134 | -1.65 | 0.028 | -1.37 | 0.323 | -0.92 |
| 1437312_at | Bmpr1b | bone morphogenetic protein receptor, type 1B | -1.34 | 0.323 | 1.23 | 0.745 | -1.66 | 0.087 | -1.04 | 0.953 | -0.7 |
| 1425141_at | Lactb2 | lactamase, beta 2 | -1.1 | 0.042 | -1.26 | 0.014 | -1.14 | 0.017 | 1.15 | 0.631 | -0.59 |
| 1442946_at | Atxn7 | Ataxin 7 | -1 | 0.993 | 1.02 | 0.921 | -1.77 | 0.004 | -1.31 | 0.043 | -0.76 |
| 1429453_a_at | Mrpl55 | mitochondrial ribosomal protein L55 | -1.07 | 0.596 | -1.28 | 0.001 | -1.15 | 0.165 | 1.47 | 0.081 | -0.51 |
| 1417675_a_at | Mdn1 | midasin homolog (yeast) | -1.49 | 0.036 | 1.06 | 0.756 | -1.18 | 0.295 | -1.65 | 0.097 | -0.81 |
| 1445166_at | Pcgf3 | Polycomb group ring finger 3 | 1.06 | 0.939 | -1.4 | 0.12 | -1.24 | 0.404 | -1.9 | 0.242 | -0.87 |
| 1426685_a_at | Cnot6 | CCR4-NOT transcription complex, subunit 6 | -1.02 | 0.935 | -1.07 | 0.338 | -1.5 | 0.004 | 1.03 | 0.874 | -0.64 |
| 1445168_at | --- | --- | -1.2 | 0.766 | -1.31 | 0.477 | -1.01 | 0.95 | 1.15 | 0.75 | -0.59 |
| 1460410_at | 2310079F23Rik | RIKEN cDNA 2310079F23 gene | -1.11 | 0.829 | -1.29 | 0.473 | -1.1 | 0.72 | 1.13 | 0.682 | -0.59 |
| 1435539_at | Hdh | Huntington disease gene homolog | 1.01 | 0.975 | -1.39 | 0.076 | -1.18 | 0.355 | -1.17 | 0.309 | -0.68 |
| 1422910_s_at | Smc6 | structural maintenance of chromosomes 6 | -1.05 | 0.749 | -1.16 | 0.53 | -1.3 | 0.369 | 1.01 | 0.972 | -0.63 |
| 1433237_at | 3830422I06Rik | RIKEN cDNA 3830422I06 gene | -1.78 | 0.407 | 1.03 | 0.955 | -1.01 | 0.979 | -1.02 | 0.782 | -0.7 |
| 1444405_at | E430029J22Rik | RIKEN cDNA E430029J22 gene | -3.04 | 0.276 | 1.46 | 0.083 | -1.27 | 0.377 | -3.24 | 0.034 | -1.52 |
| 1451165_at | Lmbr1l | limb region 1 like | -1.79 | 0.363 | -1 | 0.994 | 1.03 | 0.939 | -1.87 | 0.373 | -0.91 |
| 1424170_at | Phf5a | PHD finger protein 5A | -1.22 | 0.231 | -1.18 | 0.052 | -1.1 | 0.468 | 1.47 | 0.319 | -0.51 |
| 1448793_a_at | Sdc4 | syndecan 4 | 1.13 | 0.525 | -1.16 | 0.476 | -1.68 | 0.078 | -1.61 | 0.072 | -0.83 |

| | | | | | | | | | | | |
|---|---|---|---|---|---|---|---|---|---|---|---|
| 1424959_at | Anxa13 | annexin A13 | -1.19 | 0.553 | -1.66 | 0.008 | 1.14 | 0.487 | -1.72 | 0.31 | -0.86 |
| 1449238_at | Psg16 | pregnancy specific glycoprotein 16 | -1.09 | 0.741 | -1.31 | 0.593 | -1.11 | 0.417 | 1.14 | 0.723 | -0.59 |
| 1458257_at | --- | --- | -1.48 | 0.073 | 1.13 | 0.446 | -1.28 | 0.089 | -1.13 | 0.524 | -0.69 |
| 1426105_a_at | Ubxd5 | UBX domain containing 5 | -1.59 | 0.198 | -1.65 | 0.523 | 1.35 | 0.064 | -1.4 | 0.009 | -0.82 |
| 1444524_at | --- | Activated spleen cDNA, RIKEN full-length enriched library, clone:F830206P12 pro | -1.73 | 0.263 | 1.33 | 0.627 | -1.48 | 0.093 | -1.01 | 0.909 | -0.72 |
| 1448578_at | Pafah1b1 | platelet-activating factor acetylhydrolase, isoform 1b, beta1 subunit | -1.54 | 0.141 | -1.16 | 0.435 | 1.07 | 0.762 | -1.4 | 0.327 | -0.76 |
| 1455218_at | 6330503K22Rik | RIKEN cDNA 6330503K22 gene | -1.05 | 0.522 | -1.24 | 0.521 | -1.21 | 0.221 | 2.98 | 0.165 | -0.13 |
| 1436290_at | --- | --- | 1.03 | 0.89 | -1.33 | 0.521 | -1.24 | 0.322 | -1.01 | 0.978 | -0.64 |
| 1440105_at | Jmjd2d | jumonji domain containing 2D | 1.01 | 0.982 | -1.04 | 0.913 | -1.63 | 0.452 | -1.16 | 0.622 | -0.71 |
| 1429441_at | Fbxo30 | F-box protein 30 | -1.23 | 0.288 | -1.21 | 0.235 | -1.06 | 0.163 | 3.26 | 0.218 | -0.06 |
| 1434669_at | Ralgps1 | Ral GEF with PH domain and SH3 binding motif 1 | -1.2 | 0.142 | -1.46 | 0.358 | 1.07 | 0.72 | -1.39 | 0.432 | -0.75 |
| 1449201_at | Star | steroidogenic acute regulatory protein | -1.24 | 0.298 | -3.16 | 0.288 | 1.46 | 0.484 | -1.32 | 0.388 | -1.07 |
| 1418179_at | Atg3 | autophagy-related 3 (yeast) | -1.17 | 0.565 | -1.15 | 0.656 | -1.16 | 0.523 | 1.31 | 0.01 | -0.54 |
| 1432438_at | 4930597L12Rik | RIKEN cDNA 4930597L12 gene | -1.11 | 0.438 | -1.31 | 0.322 | -1.09 | 0.849 | 1.25 | 0.194 | -0.56 |
| 1417624_at | Nab1 | Ngfi-A binding protein 1 | 1.03 | 0.876 | -1.07 | 0.707 | -1.62 | 0 | -1.32 | 0.264 | -0.74 |
| 1440226_at | Zfp760 | zinc finger protein 760 | -1.22 | 0.182 | 1.07 | 0.708 | -1.44 | 0.18 | -1.57 | 0.011 | -0.79 |
| 1440776_at | 3732412D22Rik | RIKEN cDNA 3732412D22 gene | 1.28 | 0.67 | -1.41 | 0.621 | -1.68 | 0.091 | -1.81 | 0.463 | -0.91 |
| 1437634_at | Thoc2 | THO complex 2 | -2.13 | 0.211 | -1.51 | 0.106 | 1.45 | 0.258 | -1.05 | 0.876 | -0.81 |
| 1425327_at | BC008163 | cDNA sequence BC008163 | -1.08 | 0.771 | -1.22 | 0.182 | -1.2 | 0.407 | 1.62 | 0.226 | -0.47 |
| 1425104_at | Kctd1 | potassium channel tetramerisation domain containing 1 | -1.36 | 0.098 | -1.2 | 0.299 | 1.02 | 0.932 | -1.04 | 0.655 | -0.65 |
| 1415941_s_at | Zfand2a | zinc finger, AN1-type domain 2A | -1.41 | 0.161 | 1.1 | 0.661 | -1.28 | 0.154 | -1.32 | 0.225 | -0.73 |
| 1429331_at | 4632427E13Rik | RIKEN cDNA 4632427E13 gene | 1.09 | 0.603 | -1.22 | 0.177 | -1.47 | 0.002 | -1.41 | 0.306 | -0.75 |
| 1438976_x_at | Mat2a | methionine adenosyltransferase II, alpha | -1.25 | 0.52 | 1.01 | 0.946 | -1.3 | 0.108 | -1.14 | 0.466 | -0.67 |
| 1429195_at | --- | --- | -1.63 | 0.647 | -1.04 | 0.947 | 1.01 | 0.99 | -1.44 | 0.206 | -0.77 |
| 1443256_at | Clca6 | Chloride channel calcium activated 6 | -1.31 | 0.501 | -1.06 | 0.937 | -1.14 | 0.742 | 1.14 | 0.812 | -0.59 |
| 1451596_a_at | Sphk1 | sphingosine kinase 1 | -1.01 | 0.969 | -1.55 | 0.226 | -1.05 | 0.829 | 3.68 | 0.123 | 0.02 |
| 1429466_s_at | Aph1c /// Aph1b | anterior pharynx defective 1c homolog (C. elegans) /// anterior pharynx defective | -1.33 | 0.057 | -1.33 | 0.599 | 1.08 | 0.759 | -1.36 | 0.344 | -0.73 |
| 1436156_at | Ccar1 | cell division cycle and apoptosis regulator 1 | -1.13 | 0.534 | -1.17 | 0.23 | -1.18 | 0.286 | 1.19 | 0.213 | -0.57 |
| 1452729_at | Dpm3 | dolichyl-phosphate mannosyltransferase polypeptide 3 | -1.51 | 0.04 | 1.06 | 0.888 | -1.17 | 0.691 | -1.11 | 0.818 | -0.68 |
| 1451084_at | Etfdh | electron transferring flavoprotein, dehydrogenase | -1.18 | 0.194 | -1.31 | 0.099 | -1.02 | 0.888 | 1.22 | 0.203 | -0.58 |
| 1447056_at | Slit2 | Slit homolog 2 (Drosophila) | -1.24 | 0.459 | -1.03 | 0.913 | -1.24 | 0.334 | 1.7 | 0.092 | -0.45 |
| 1423953_at | Cdkal1 | CDK5 regulatory subunit associated protein 1-like 1 | 1.07 | 0.617 | -1.31 | 0.069 | -1.33 | 0.118 | -1.48 | 0.041 | -0.76 |
| 1434339_at | Fnbp1l | formin binding protein 1-like | -1.28 | 0.224 | 1.02 | 0.855 | -1.28 | 0.137 | -1.43 | 0.045 | -0.74 |
| 1438451_at | Grit | Rho GTPase-activating protein | -1.19 | 0.353 | -1.39 | 0.054 | 1.02 | 0.912 | -1.62 | 0.247 | -0.79 |
| 1460055_at | Evi2a | Ecotropic viral integration site 2a | -1.66 | 0.076 | 1.08 | 0.768 | -1.11 | 0.712 | -1.4 | 0.382 | -0.77 |
| 1456617_a_at | Eif2s2 | eukaryotic translation initiation factor 2, subunit 2 (beta) | -1.22 | 0.002 | -1.01 | 0.922 | -1.3 | 0.113 | 1.05 | 0.79 | -0.62 |
| 1440287_at | 1110014D18Rik | RIKEN cDNA 1110014D18 gene | 1.01 | 0.933 | -1.18 | 0.359 | -1.38 | 0.184 | -1.45 | 0.035 | -0.75 |
| 1421208_at | Ikbkg | inhibitor of kappaB kinase gamma | -1.31 | 0.301 | -1.49 | 0.15 | 1.15 | 0.671 | -1.14 | 0.65 | -0.7 |
| 1418859_at | Rfxap | regulatory factor X-associated protein | -1.09 | 0.456 | -1.45 | 0.064 | -1.02 | 0.759 | 1.15 | 0.524 | -0.6 |
| 1455246_at | Smarcc1 | SWI/SNF related, matrix associated, actin dependent regulator of chromatin, subf | -1.12 | 0.756 | -1.12 | 0.716 | -1.25 | 0.503 | 1.16 | 0.552 | -0.58 |
| 1430108_at | 9030622M22Rik | RIKEN cDNA 9030622M22 gene | -1.17 | 0.61 | -1.04 | 0.848 | -1.3 | 0.502 | 1.63 | 0.062 | -0.47 |
| 1426314_at | Ednrb | endothelin receptor type B | 1.08 | 0.76 | -1.11 | 0.731 | -1.65 | 0.039 | -1.53 | 0.054 | -0.8 |
| 1424206_at | Smarca5 /// 923011 | SWI/SNF related, matrix associated, actin dependent regulator of chromatin, subf | -1.25 | 0.261 | -1.13 | 0.392 | -1.1 | 0.058 | 1.23 | 0.396 | -0.56 |
| 1456898_at | Pura | purine rich element binding protein A | 1.09 | 0.758 | -1.27 | 0.047 | -1.41 | 0.007 | -1.2 | 0.276 | -0.7 |
| 1440891_at | Gria4 | glutamate receptor, ionotropic, AMPA4 (alpha 4) | 1.79 | 0.041 | -2.15 | 0.055 | -3.06 | 0.013 | -1.54 | 0.578 | -1.24 |
| 1446095_at | Air | antisense Igf2r RNA | -1.2 | 0.792 | 1.38 | 0.616 | -2.64 | 0.015 | -1.05 | 0.869 | -0.88 |
| 1431031_at | Arid4b | AT rich interactive domain 4B (Rbp1 like) | -1.5 | 0.185 | -1.11 | 0.712 | 1.02 | 0.925 | -1.25 | 0.061 | -0.71 |
| 1455267_at | Esrrg | estrogen-related receptor gamma | -1.19 | 0.158 | -1.64 | 0.374 | 1.14 | 0.092 | -2.9 | 0.107 | -1.15 |
| 1453678_at | Mbd1 | methyl-CpG binding domain protein 1 | 1.19 | 0.384 | -1.64 | 0.31 | -1.27 | 0.15 | -1.27 | 0.276 | -0.75 |
| 1434506_at | Arid2 | AT rich interactive domain 2 (Arid-rfx like) | -1.15 | 0.576 | -1.09 | 0.623 | -1.26 | 0.213 | 1.47 | 0.186 | -0.51 |
| 1437282_at | Dock2 | Dedicator of cyto-kinesis 2 | -1.8 | 0.309 | -1.2 | 0.468 | 1.19 | 0.588 | -2.01 | 0.086 | -0.95 |
| 1420340_at | 4930413O22Rik | RIKEN cDNA 4930413O22 gene | -1.42 | 0.255 | -1.06 | 0.919 | -1.06 | 0.794 | 1.03 | 0.893 | -0.63 |
| 1418355_at | Nucb2 | nucleobindin 2 | 1.02 | 0.865 | -1.25 | 0.217 | -1.3 | 0.043 | -1.55 | 0.078 | -0.77 |

| | | | | | | | | | | | |
|---|---|---|---|---|---|---|---|---|---|---|---|
| 1423146_at | Hes5 | hairy and enhancer of split 5 (Drosophila) | -1.14 | 0.653 | 1.13 | 0.732 | -1.71 | 0.22 | -1.86 | 0.034 | -0.9 |
| 1453576_at | Nipbl | Nipped-B homolog (Drosophila) | 1.01 | 0.966 | -1.1 | 0.045 | -1.5 | 0.129 | -1.34 | 0.301 | -0.73 |
| 1426892_at | Utrn | utrophin | -1.15 | 0.501 | 1.13 | 0.685 | -1.7 | 0.165 | -1.81 | 0.23 | -0.88 |
| 1444463_at | Arhgap12 | Rho GTPase activating protein 12 | 1.49 | 0.264 | -1.4 | 0.556 | -2.59 | 0.033 | -3.21 | 0.013 | -1.43 |
| 1435246_at | Paqr8 | progestin and adipoQ receptor family member VIII | -1.67 | 0.586 | 1.08 | 0.807 | -1.1 | 0.524 | -2 | 0.27 | -0.92 |
| 1447217_at | Uhrf2 | ubiquitin-like, containing PHD and RING finger domains 2 | -1.08 | 0.596 | 1 | 0.982 | -1.53 | 0.114 | -1.15 | 0.566 | -0.69 |
| 1425143_a_at | Ndufs1 | NADH dehydrogenase (ubiquinone) Fe-S protein 1 | -1.17 | 0.084 | -1.29 | 0.051 | -1.04 | 0.772 | 1.05 | 0.781 | -0.61 |
| 1446380_at | 9430076C15Rik | RIKEN cDNA 9430076C15 gene | -1.17 | 0.87 | 1.11 | 0.887 | -1.62 | 0.253 | -1.47 | 0.651 | -0.79 |
| 1458960_at | Ryk | Receptor-like tyrosine kinase | -1 | 0.995 | 1.09 | 0.894 | -1.99 | 0.209 | -4.96 | 0.021 | -1.72 |
| 1423364_a_at | Fts | fused toes | 1.04 | 0.584 | -1.2 | 0.017 | -1.4 | 0.013 | -1.4 | 0.058 | -0.74 |
| 1426664_x_at | Slc45a3 | solute carrier family 45, member 3 | -1.29 | 0.22 | -1.02 | 0.927 | -1.2 | 0.17 | 1.22 | 0.34 | -0.57 |
| 1430576_at | Tpp2 | tripeptidyl peptidase II | 1.36 | 0.45 | -1.55 | 0.067 | -1.71 | 0.06 | -2.15 | 0.064 | -1.01 |
| 1451390_s_at | Zfand2b | zinc finger, AN1 type domain 2B | -1.24 | 0.035 | -1.03 | 0.791 | -1.23 | 0.314 | 1.19 | 0.495 | -0.58 |
| 1423250_a_at | Tgfb2 | transforming growth factor, beta 2 | -1.55 | 0.013 | 1.25 | 0.625 | -1.45 | 0.323 | -1.41 | 0.669 | -0.79 |
| 1446827_at | Ncoa2 | nuclear receptor coactivator 2 | -1.26 | 0.422 | 1 | 0.995 | -1.26 | 0.039 | -1.16 | 0.24 | -0.67 |
| 1441783_at | 2410022M11Rik | RIKEN cDNA 2410022M11 gene | 1.15 | 0.575 | -2.13 | 0.048 | -1.03 | 0.869 | -1.11 | 0.856 | -0.78 |
| 1445611_at | 1810044A24Rik | RIKEN cDNA 1810044A24 gene | 1.16 | 0.482 | -1.85 | 0.079 | -1.13 | 0.716 | -1.69 | 0.164 | -0.88 |
| 1441468_at | --- | PREDICTED: Mus musculus similar to dachsous 2 isoform 2 (LOC627094), mRN | -1.06 | 0.718 | -1.22 | 0.425 | -1.21 | 0.513 | 1.55 | 0.323 | -0.49 |
| 1420516_at | a | nonagouti | 1.03 | 0.935 | -1.08 | 0.678 | -1.59 | 0.132 | -1.01 | 0.965 | -0.66 |
| 1454978_at | Ttyh3 | tweety homolog 3 (Drosophila) | -1.09 | 0.563 | -1.5 | 0.412 | 1.01 | 0.978 | -1.12 | 0.6 | -0.68 |
| 1432798_at | 9530023I19Rik | RIKEN cDNA 9530023I19 gene | -1.3 | 0.565 | 1.05 | 0.81 | -1.3 | 0.311 | -1.2 | 0.173 | -0.69 |
| 1449158_at | Kcnk2 | potassium channel, subfamily K, member 2 | -1.06 | 0.706 | -1.25 | 0.67 | -1.19 | 0.477 | 1.61 | 0.256 | -0.47 |
| 1428423_at | Pcgf3 | polycomb group ring finger 3 | -1.12 | 0.116 | -1.06 | 0.615 | -1.33 | 0.067 | 1.55 | 0.2 | -0.49 |
| 1446948_at | Denr | Density-regulated protein | 1.19 | 0.481 | -1.37 | 0.328 | -1.51 | 0.328 | -1.35 | 0.614 | -0.76 |
| 1428933_at | Hdac8 | histone deacetylase 8 | -1.39 | 0.198 | -1.37 | 0.203 | 1.14 | 0.562 | -1.31 | 0.12 | -0.73 |
| 1450417_a_at | Rps20 /// LOC2456 | ribosomal protein S20 /// similar to 40S ribosomal protein S20 /// similar to 40S rit | -1.12 | 0.754 | -1.15 | 0.507 | -1.21 | 0.46 | 1.27 | 0.468 | -0.55 |
| 1421686_at | Rfrp | RFamide-related peptide | -1.55 | 0.242 | 1.21 | 0.545 | -1.37 | 0.127 | -1.27 | 0.28 | -0.74 |
| 1450401_at | Ncoa6ip | nuclear receptor coactivator 6 interacting protein | 1.04 | 0.899 | -1.16 | 0.273 | -1.45 | 0.073 | -1.06 | 0.854 | -0.66 |
| 1417286_at | Ndufa5 | NADH dehydrogenase (ubiquinone) 1 alpha subcomplex, 5 | -1.13 | 0.11 | -1.17 | 0.263 | -1.18 | 0.064 | 1.12 | 0.631 | -0.59 |
| 1446529_at | 1200003I07Rik | RIKEN cDNA 1200003I07 gene | -1.02 | 0.797 | 1.26 | 0.744 | -2.81 | 0.019 | -1.67 | 0.155 | -1.06 |
| 1438335_at | 6030413G23Rik | RIKEN cDNA 6030413G23 gene | -1.23 | 0.339 | -1.44 | 0.33 | 1.08 | 0.832 | -1.3 | 0.74 | -0.72 |
| 1437506_at | --- | --- | -1.49 | 0.575 | -1.47 | 0.557 | 1.24 | 0.483 | -2.61 | 0.132 | -1.08 |
| 1448102_a_at | Wdr61 | WD repeat domain 61 | -1.15 | 0.43 | -1.12 | 0.004 | -1.2 | 0.046 | 1.09 | 0.549 | -0.6 |
| 1426172_a_at | Cd209a | CD209a antigen | 1.16 | 0.8 | -1.13 | 0.835 | -1.82 | 0.141 | -2.73 | 0.027 | -1.13 |
| 1456789_at | Zfp462 | Zinc finger protein 462 | -1.18 | 0.253 | 1.14 | 0.714 | -1.66 | 0.177 | -1.21 | 0.378 | -0.73 |
| 1419036_at | Csnk2a1 | casein kinase 2, alpha 1 polypeptide | -1.07 | 0.629 | -1.11 | 0.135 | -1.32 | 0.047 | 1.15 | 0.262 | -0.59 |
| 1419155_a_at | Sox4 /// LOC67227 | SRY-box containing gene 4 /// similar to Transcription factor SOX-4 | 1.16 | 0.449 | -1.33 | 0.326 | -1.47 | 0.273 | -1.34 | 0.166 | -0.75 |
| 1457487_at | --- | Adult male liver tumor cDNA, RIKEN full-length enriched library, clone:C730014I0 | -1.86 | 0.204 | 1.09 | 0.731 | -1.04 | 0.905 | -2.61 | 0.03 | -1.1 |
| 1444838_at | C630010N09 | hypothetical protein C630010N09 | -1.61 | 0.012 | -1 | 0.996 | -1.03 | 0.925 | 1.34 | 0.057 | -0.57 |
| 1436864_at | A430090L17Rik | RIKEN cDNA A430090L17 gene | -1.13 | 0.576 | 1.01 | 0.98 | -1.43 | 0.205 | -1.06 | 0.606 | -0.65 |
| 1456611_at | D430015B01Rik | RIKEN cDNA D430015B01 gene | -1.62 | 0.143 | -1.84 | 0.037 | 1.43 | 0.253 | -1.62 | 0.145 | -0.91 |
| 1431079_at | C1qtnf2 | C1q and tumor necrosis factor related protein 2 | 1.03 | 0.934 | -1.1 | 0.785 | -1.53 | 0.125 | -1.12 | 0.693 | -0.68 |
| 1416793_at | Arl6ip2 | ADP-ribosylation factor-like 6 interacting protein 2 | -1.17 | 0.078 | -1.16 | 0.2 | -1.15 | 0.019 | 1.47 | 0.268 | -0.5 |
| 1452608_at | Mycbp | c-myc binding protein | -1.15 | 0.487 | -1.32 | 0.162 | -1.04 | 0.774 | 1.09 | 0.645 | -0.61 |
| 1432307_at | 1700086D15Rik | RIKEN cDNA 1700086D15 gene | -1.84 | 0.445 | -1.79 | 0.352 | 1.49 | 0.371 | -2.4 | 0.369 | -1.14 |
| 1436108_at | Txndc9 | thioredoxin domain containing 9 | -1.01 | 0.951 | -1.43 | 0.135 | -1.11 | 0.448 | 1.44 | 0.168 | -0.53 |
| 1440172_s_at | 1700113H08Rik | RIKEN cDNA 1700113H08 gene | 1.05 | 0.964 | -1.16 | 0.815 | -1.46 | 0.542 | -2.22 | 0.336 | -0.95 |
| 1419952_at | Stx5a | Syntaxin 5A | 1.05 | 0.949 | -1.44 | 0.211 | -1.18 | 0.489 | -1.1 | 0.823 | -0.67 |
| 1436446_at | 2310007O11Rik | RIKEN cDNA 2310007O11 gene | -1.18 | 0.423 | -1.01 | 0.972 | -1.34 | 0.006 | 1.8 | 0.076 | -0.43 |
| 1450728_at | Fjx1 | four jointed box 1 (Drosophila) | 1.34 | 0.7 | -2.12 | 0.134 | -1.28 | 0.277 | -1.07 | 0.828 | -0.78 |
| 1431479_at | 4921524L21Rik | RIKEN cDNA 4921524L21 gene | -1.41 | 0.532 | -1.02 | 0.971 | -1.11 | 0.87 | 1.19 | 0.79 | -0.59 |
| 1444528_at | --- | --- | -1.41 | 0.164 | -1.61 | 0.195 | 1.26 | 0.574 | -2.31 | 0.085 | -1.02 |
| 1459987_s_at | Cct3 | chaperonin subunit 3 (gamma) | -1.3 | 0.023 | -1.15 | 0.287 | -1.05 | 0.793 | 1.14 | 0.591 | -0.59 |

| | | | | | | | | | | | |
|---|---|---|---|---|---|---|---|---|---|---|---|
| 1443626_at | Chn2 | Chimerin (chimaerin) 2 | 1.31 | 0.161 | -1.3 | 0.505 | -1.94 | 0.032 | -1.33 | 0.144 | -0.82 |
| 1417362_at | Rhcg | Rhesus blood group-associated C glycoprotein | -1.35 | 0.242 | -1.47 | 0.37 | 1.17 | 0.456 | -1.43 | 0.065 | -0.77 |
| 1427243_at | AA536743 | expressed sequence AA536743 | -1.02 | 0.6 | -1.05 | 0.688 | -1.52 | 0.014 | 1.58 | 0.138 | -0.5 |
| 1454275_at | --- | --- | -1.1 | 0.862 | -1.29 | 0.619 | -1.1 | 0.751 | 1.06 | 0.879 | -0.61 |
| 1418034_at | Mrps9 | mitochondrial ribosomal protein S9 | 1.02 | 0.906 | -1.33 | 0.066 | -1.22 | 0.326 | -1.34 | 0.252 | -0.72 |
| 1418399_at | Kctd9 | potassium channel tetramerisation domain containing 9 | -1.19 | 0.24 | -1.15 | 0.373 | -1.13 | 0.209 | 2.1 | 0.263 | -0.34 |
| 1426798_a_at | --- | --- | -1.32 | 0.402 | -1.24 | 0.222 | 1.02 | 0.938 | -1.08 | 0.682 | -0.65 |
| 1431201_at | Slc25a27 | solute carrier family 25, member 27 | -1.4 | 0.577 | -1.16 | 0.781 | 1.01 | 0.968 | -1.32 | 0.442 | -0.71 |
| 1431531_at | 1700025D03Rik | RIKEN cDNA 1700025D03 gene | -1 | 0.994 | -1.15 | 0.756 | -1.39 | 0.027 | 1.06 | 0.725 | -0.62 |
| 1434000_at | Kras | v-Ki-ras2 Kirsten rat sarcoma viral oncogene homolog | -1.07 | 0.073 | -1.13 | 0.29 | -1.29 | 0.074 | 1.39 | 0.072 | -0.53 |
| 1441300_at | Kcnf1 | potassium voltage-gated channel, subfamily F, member 1 | -1.14 | 0.543 | -1.12 | 0.811 | -1.21 | 0.51 | 1.91 | 0.414 | -0.39 |
| 1429061_at | 1810063B05Rik | RIKEN cDNA 1810063B05 gene | -1.34 | 0.149 | -1.16 | 0.444 | -1.01 | 0.922 | 1.28 | 0.386 | -0.56 |
| 1440998_at | 2310021P13Rik | RIKEN cDNA 2310021P13 gene | 1.04 | 0.763 | -1.32 | 0.096 | -1.26 | 0.547 | -1.46 | 0.056 | -0.75 |
| 1425347_a_at | Zfp318 | zinc finger protein 318 | -1.06 | 0.723 | -1.72 | 0.121 | 1.06 | 0.74 | -2.62 | 0.035 | -1.08 |
| 1429899_at | 5730414N17Rik | RIKEN cDNA 5730414N17 gene | 1.03 | 0.877 | -1.44 | 0.091 | -1.15 | 0.385 | -1.53 | 0.242 | -0.77 |
| 1449098_a_at | Poli | polymerase (DNA directed), iota | -1.28 | 0.238 | -1.48 | 0.002 | 1.13 | 0.553 | -1.24 | 0.512 | -0.72 |
| 1448563_at | Phb | prohibitin | -1.21 | 0.176 | -1.35 | 0.039 | 1.03 | 0.697 | -1.22 | 0.329 | -0.69 |
| 1446421_at | Schip1 | Schwannomin interacting protein 1 | -1.05 | 0.913 | 1.17 | 0.767 | -2.15 | 0.026 | -1.29 | 0.421 | -0.83 |
| 1447815_x_at | 6430527G18Rik | RIKEN cDNA 6430527G18 gene | -1.26 | 0.708 | -1.51 | 0.437 | 1.13 | 0.566 | -3.53 | 0.033 | -1.29 |
| 1437177_at | D330037H05Rik /// | RIKEN cDNA D330037H05 gene /// similar to La-related protein 4 (La ribonucleop | -1.04 | 0.923 | -1.21 | 0.519 | -1.24 | 0.429 | 1.15 | 0.7 | -0.59 |
| 1439981_at | A430001F24Rik | RIKEN cDNA A430001F24 gene | -2.25 | 0.089 | -1.19 | 0.684 | 1.31 | 0.626 | -1.87 | 0.178 | -1 |
| 1417187_at | Hip2 | huntingtin interacting protein 2 | -1.45 | 0.271 | -1.05 | 0.891 | -1.05 | 0.943 | 3.2 | 0.017 | -0.09 |
| 1423184_at | Itsn2 | intersectin 2 | 1.2 | 0.681 | -1.55 | 0.301 | -1.33 | 0.401 | -1.33 | 0.533 | -0.75 |
| 1437818_at | 9430016H08Rik | RIKEN cDNA 9430016H08 gene | -1.23 | 0.27 | -1.21 | 0.074 | -1.05 | 0.724 | 1.31 | 0.465 | -0.55 |
| 1421778_at | V1rb2 | vomeronasal 1, receptor B2 | -1.43 | 0.121 | -1.35 | 0.319 | 1.15 | 0.392 | -2.29 | 0.321 | -0.98 |
| 1447141_at | AW107722 | expressed sequence AW107722 | 1.12 | 0.375 | -1 | 0.99 | -2.12 | 0.005 | -1.87 | 0.031 | -0.97 |
| 1420193_at | Krt17 | keratin 17 | -1.72 | 0.35 | 1.04 | 0.942 | -1.03 | 0.961 | -1.21 | 0.203 | -0.73 |
| 1430237_at | Cldn22 | claudin 22 | -1.55 | 0.116 | -1.27 | 0.355 | 1.16 | 0.51 | -1.14 | 0.028 | -0.7 |
| 1444395_at | Dixdc1 | DIX domain containing 1 | -1.55 | 0.358 | -1.15 | 0.52 | 1.08 | 0.827 | -1.27 | 0.665 | -0.72 |
| 1446912_at | Tmem28 | Transmembrane protein 28 | -2.32 | 0.457 | 1.4 | 0.443 | -1.31 | 0.498 | -1.2 | 0.203 | -0.86 |
| 1450361_at | Prop1 | paired like homeodomain factor 1 | -2.74 | 0.111 | 1.63 | 0.06 | -1.67 | 0.11 | -1.3 | 0.011 | -1.02 |
| 1446971_at | D10Wsu159e | DNA segment, Chr 10, Wayne State University 159, expressed | -1.31 | 0.306 | -1.01 | 0.984 | -1.19 | 0.438 | 1.04 | 0.666 | -0.62 |
| 1417400_at | Rai14 | retinoic acid induced 14 | -1.09 | 0.626 | 1.02 | 0.883 | -1.54 | 0.032 | -1.6 | 0.111 | -0.8 |
| 1422369_at | V1ra6 | vomeronasal 1 receptor, A6 | -1.32 | 0.537 | -2.33 | 0.009 | 1.4 | 0.44 | -2.64 | 0.07 | -1.22 |
| 1440082_at | Ptk2 | PTK2 protein tyrosine kinase 2 | 1.22 | 0.55 | -1.3 | 0.668 | -1.66 | 0.073 | -1.04 | 0.905 | -0.69 |
| 1418997_at | --- | --- | -1.23 | 0.326 | -1 | 1 | -1.28 | 0.304 | 1.1 | 0.454 | -0.6 |
| 1431378_at | --- | --- | -2.48 | 0.246 | 1.26 | 0.368 | -1.08 | 0.898 | -1.68 | 0.045 | -0.99 |
| 1448543_at | 2310042G06Rik | RIKEN cDNA 2310042G06 gene | -1.12 | 0.332 | -1.07 | 0.502 | -1.31 | 0.008 | 1.63 | 0.152 | -0.47 |
| 1447569_at | 1700081L11Rik | RIKEN cDNA 1700081L11 gene | -1.8 | 0.012 | 1.21 | 0.392 | -1.22 | 0.709 | -4.72 | 0.227 | -1.63 |
| 1441576_at | 2410002O22Rik | RIKEN cDNA 2410002O22 gene | -1.12 | 0.536 | -1.08 | 0.66 | -1.28 | 0.311 | 1.09 | 0.841 | -0.6 |
| 1424884_at | Fbxw2 | F-box and WD-40 domain protein 2 | -1.21 | 0.128 | -1.21 | 0.303 | -1.06 | 0.534 | 1.08 | 0.806 | -0.6 |
| 1424516_at | B230354K17Rik | RIKEN cDNA B230354K17 gene | -1.01 | 0.925 | -1.18 | 0.054 | -1.32 | 0.06 | 1.21 | 0.275 | -0.58 |
| 1426786_s_at | Dhx38 | DEAH (Asp-Glu-Ala-His) box polypeptide 38 | -1.36 | 0.085 | -1.19 | 0.276 | 1.02 | 0.962 | -1.12 | 0.613 | -0.66 |
| 1453009_at | Cpm | carboxypeptidase M | -1.29 | 0.309 | 1.27 | 0.638 | -1.81 | 0.03 | -4.64 | 0.042 | -1.62 |
| 1426958_at | Rps9 /// LOC27269 | ribosomal protein S9 /// similar to ribosomal protein S9 | -1.25 | 0.323 | -1.64 | 0.183 | 1.18 | 0.334 | -1.22 | 0.369 | -0.73 |
| 1418574_a_at | Shfm1 | split hand/foot malformation (ectrodactyly) type 1 | -1.12 | 0.539 | 1.02 | 0.868 | -1.47 | 0.007 | -1.32 | 0.18 | -0.72 |
| 1445085_at | Clstn1 | Calsyntenin 1 | -1.17 | 0.823 | 1.04 | 0.864 | -1.44 | 0.4 | -1.37 | 0.603 | -0.73 |
| 1454918_at | Agps | alkylglycerone phosphate synthase | -1.37 | 0.266 | 1.12 | 0.629 | -1.34 | 0.112 | -1.25 | 0.335 | -0.71 |
| 1455512_at | Gm879 | gene model 879, (NCBI) | -2.32 | 0.052 | 1.29 | 0.496 | -1.15 | 0.712 | -1.66 | 0.136 | -0.96 |
| 1418286_a_at | Efnb1 | ephrin B1 | -1.11 | 0.219 | -1.15 | 0.32 | -1.21 | 0.237 | 1.01 | 0.955 | -0.62 |
| 1434612_s_at | Sbno1 | sno, strawberry notch homolog 1 (Drosophila) | -1.15 | 0.179 | -1.1 | 0.359 | -1.23 | 0.188 | 1 | 0.999 | -0.62 |
| 1443605_at | C81272 | expressed sequence C81272 | 1.24 | 0.532 | -1.25 | 0.747 | -1.8 | 0.157 | -1.4 | 0.312 | -0.8 |
| 1439671_at | 4930466K18Rik | RIKEN cDNA 4930466K18 gene | -1.14 | 0.318 | -1.06 | 0.785 | -1.29 | 0.4 | 1.18 | 0.412 | -0.58 |

| | | | | | | | | | | | |
|---|---|---|---|---|---|---|---|---|---|---|---|
| 1456987_at | 4932418E24Rik | RIKEN cDNA 4932418E24 gene | -1.13 | 0.787 | -1.8 | 0.261 | 1.16 | 0.793 | -1.2 | 0.686 | -0.74 |
| 1439729_at | A930038B10Rik | RIKEN cDNA A930038B10 gene | -1.01 | 0.98 | -1.86 | 0.356 | 1.07 | 0.872 | -3.22 | 0.082 | -1.25 |
| 1448609_at | Tst | thiosulfate sulfurtransferase, mitochondrial | -1.19 | 0.353 | -1.14 | 0.249 | -1.15 | 0.545 | 1.63 | 0.205 | -0.46 |
| 1431416_a_at | Jam2 | junction adhesion molecule 2 | -1.47 | 0.369 | -1.29 | 0.75 | 1.14 | 0.579 | -1.38 | 0.692 | -0.75 |
| 1441733_s_at | Nup153 | nucleoporin 153 | 1.05 | 0.867 | -1.14 | 0.578 | -1.49 | 0.037 | -1.22 | 0.135 | -0.7 |
| 1452304_a_at | Arhgef5 | Rho guanine nucleotide exchange factor (GEF) 5 | -1.22 | 0.137 | -1.06 | 0.551 | -1.2 | 0.122 | 1.44 | 0.38 | -0.51 |
| 1449274_at | 2310038H17Rik | RIKEN cDNA 2310038H17 gene | -1.38 | 0.084 | -1.16 | 0.253 | 1.01 | 0.956 | -1.41 | 0.353 | -0.74 |
| 1419905_s_at | Hpgd | hydroxyprostaglandin dehydrogenase 15 (NAD) | 1.14 | 0.152 | -1.6 | 0.072 | -1.21 | 0.144 | -1.29 | 0.447 | -0.74 |
| 1431945_at | Arl13a | ADP-ribosylation factor-like 13A | -1.12 | 0.859 | -2.32 | 0.358 | 1.27 | 0.53 | -1.86 | 0.365 | -1.01 |
| 1436585_at | BB182297 | expressed sequence BB182297 | -1.01 | 0.95 | -1.11 | 0.511 | -1.42 | 0.003 | 1.08 | 0.7 | -0.61 |
| 1438944_at | 1700007J24Rik | RIKEN cDNA 1700007J24 gene | 1.26 | 0.063 | -2.39 | 0.012 | -1.09 | 0.767 | -1.37 | 0.573 | -0.9 |
| 1443922_at | Rcor3 | REST corepressor 3 | -1.16 | 0.681 | -1.1 | 0.7 | -1.22 | 0.413 | 2.39 | 0.031 | -0.27 |
| 1460536_at | 2310079F09Rik | RIKEN cDNA 2310079F09 gene | -1.32 | 0.129 | 1.03 | 0.784 | -1.24 | 0.445 | -1.57 | 0.109 | -0.78 |
| 1452821_at | Tial1 | Tial1 cytotoxic granule-associated RNA binding protein-like 1 | -1.06 | 0.706 | -1.04 | 0.729 | -1.45 | 0.177 | 1 | 0.987 | -0.64 |
| 1417771_a_at | Psmc6 | proteasome (prosome, macropain) 26S subunit, ATPase, 6 | -1.1 | 0.409 | -1.17 | 0.013 | -1.21 | 0.122 | 1.17 | 0.521 | -0.58 |
| 1420711_a_at | Pxmp3 | peroxisomal membrane protein 3 | -1.27 | 0.091 | -1.09 | 0.139 | -1.13 | 0.368 | 1.03 | 0.832 | -0.61 |
| 1440065_at | Polr2j | Polymerase (RNA) II (DNA directed) polypeptide J | 1.13 | 0.831 | -1.77 | 0.128 | -1.11 | 0.811 | -1.38 | 0.454 | -0.78 |
| 1454652_at | Zranb2 | zinc finger, RAN-binding domain containing 2 | -1.1 | 0.283 | -1.09 | 0.383 | -1.3 | 0.041 | 1.06 | 0.719 | -0.61 |
| 1439755_at | --- | --- | -1.19 | 0.188 | -1.55 | 0.207 | 1.11 | 0.767 | -1.53 | 0.457 | -0.79 |
| 1430309_at | Nipbl | Nipped-B homolog (Drosophila) | 1.02 | 0.433 | -1.15 | 0.04 | -1.41 | 0.073 | -1.15 | 0.476 | -0.67 |
| 1441740_at | --- | --- | 1.04 | 0.868 | -1.04 | 0.818 | -1.69 | 0.006 | -1.26 | 0.091 | -0.74 |
| 1439483_at | AI506816 | expressed sequence AI506816 | -1.25 | 0.61 | -1.02 | 0.973 | -1.23 | 0.585 | 1.88 | 0.244 | -0.4 |
| 1436341_at | Hmbox1 | homeobox containing 1 | 1.01 | 0.96 | -1.19 | 0.192 | -1.34 | 0.254 | -1.06 | 0.751 | -0.64 |
| 1443905_at | Sfrs8 | Splicing factor, arginine/serine-rich 8 | 1.17 | 0.799 | -1.32 | 0.654 | -1.49 | 0.239 | -1.29 | 0.359 | -0.73 |
| 1427839_at | Igh-VJ558 | Immunoglobulin heavy chain (J558 family) | 1.32 | 0.551 | -2.42 | 0.214 | -1.17 | 0.765 | -1.26 | 0.655 | -0.88 |
| 1455020_at | Snx25 | sorting nexin 25 | -1.18 | 0.418 | -1.09 | 0.27 | -1.21 | 0.282 | 1.08 | 0.806 | -0.6 |
| 1452275_at | Zfand3 | zinc finger, AN1-type domain 3 | 1.08 | 0.524 | -1.92 | 0.352 | -1.01 | 0.945 | -1.13 | 0.35 | -0.74 |
| 1448033_at | Tatdn1 | TatD DNase domain containing 1 | -1.14 | 0.217 | -1.16 | 0.042 | -1.17 | 0.546 | 1.5 | 0.39 | -0.49 |
| 1452712_at | Hnrpa3 | heterogeneous nuclear ribonucleoprotein A3 | -1.09 | 0.179 | -1.11 | 0.229 | -1.28 | 0.075 | 1.03 | 0.579 | -0.61 |
| 1432165_at | 8430422H06Rik | RIKEN cDNA 8430422H06 gene | -1.4 | 0.305 | -1.59 | 0.375 | 1.26 | 0.454 | -1.72 | 0.003 | -0.86 |
| 1423800_at | Dars | aspartyl-tRNA synthetase | -1.17 | 0.105 | -1.23 | 0.041 | -1.08 | 0.295 | 1.04 | 0.759 | -0.61 |
| 1418538_at | Kdelr3 | KDEL (Lys-Asp-Glu-Leu) endoplasmic reticulum protein retention receptor 3 | 1.24 | 0.083 | -1.18 | 0.751 | -1.96 | 0.002 | -1.23 | 0.562 | -0.78 |
| 1449313_at | Klk1b5 | kallikrein 1-related peptidase b5 | -1.68 | 0.164 | 1.07 | 0.906 | -1.07 | 0.748 | -2.08 | 0.326 | -0.94 |
| 1445881_at | 2310035P21Rik | RIKEN cDNA 2310035P21 gene | 1.01 | 0.982 | -1.15 | 0.673 | -1.4 | 0.467 | -1.54 | 0.133 | -0.77 |
| 1441696_at | Sec22a | SEC22 vesicle trafficking protein-like A (S. cerevisiae) | 1.09 | 0.375 | -1.2 | 0.208 | -1.48 | 0.017 | -1.44 | 0.2 | -0.76 |
| 1421661_at | Fprl1 | formyl peptide receptor-like 1 | -3.12 | 0.421 | 1.43 | 0.491 | -1.18 | 0.177 | -1.29 | 0.518 | -1.04 |
| 1441056_at | Usp3 | ubiquitin specific peptidase 3 | 1.23 | 0.501 | -1.49 | 0.008 | -1.43 | 0.006 | -1.43 | 0.015 | -0.78 |
| 1428812_at | 1700040L02Rik | RIKEN cDNA 1700040L02 gene | -1.15 | 0.138 | -1.66 | 0.285 | 1.13 | 0.615 | -1.68 | 0.522 | -0.84 |
| 1441784_at | --- | Adult male hypothalamus cDNA, RIKEN full-length enriched library, clone:A23007 | -1.45 | 0.483 | 1.07 | 0.774 | -1.2 | 0.41 | -1.33 | 0.384 | -0.73 |
| 1445037_at | 6430510B20Rik | RIKEN cDNA 6430510B20 gene | 1.08 | 0.387 | -1.27 | 0.342 | -1.38 | 0.277 | -1.44 | 0.079 | -0.75 |
| 1437875_at | Bicd2 | bicaudal D homolog 2 (Drosophila) | -1.19 | 0.18 | -1.12 | 0.525 | -1.15 | 0.184 | 1.16 | 0.321 | -0.58 |
| 1457873_at | --- | 11 days embryo gonad cDNA, RIKEN full-length enriched library, clone:7030407I | 1.2 | 0.828 | -2.2 | 0.17 | -1.06 | 0.844 | -1.33 | 0.637 | -0.85 |
| 1444127_at | Asb1 | ankyrin repeat and SOCS box-containing protein 1 | -1.13 | 0.881 | -1.46 | 0.273 | 1.03 | 0.951 | -1.03 | 0.941 | -0.65 |
| 1416397_at | Mesdc1 | mesoderm development candidate 1 | -1.45 | 0.235 | -1.1 | 0.619 | -1 | 0.952 | 1.8 | 0.123 | -0.44 |
| 1456421_at | 4930578M01Rik | RIKEN cDNA 4930578M01 gene | 1.21 | 0.463 | -1.14 | 0.569 | -1.97 | 0.352 | -1.13 | 0.632 | -0.76 |
| 1458167_at | --- | --- | -1.19 | 0.834 | 1.11 | 0.824 | -1.55 | 0.21 | -2.66 | 0.282 | -1.07 |
| 1447027_s_at | Lias | lipoic acid synthetase | -1.13 | 0.037 | -1.14 | 0.013 | -1.2 | 0.064 | 1.1 | 0.647 | -0.59 |
| 1436213_a_at | 1110028C15Rik | RIKEN cDNA 1110028C15 gene | -1.43 | 0.358 | 1.13 | 0.667 | -1.3 | 0.336 | -1.03 | 0.865 | -0.66 |
| 1434115_at | --- | --- | -1.23 | 0.361 | 1.08 | 0.714 | -1.42 | 0.162 | -1.3 | 0.492 | -0.72 |
| 1460199_a_at | Pafah1b1 | platelet-activating factor acetylhydrolase, isoform 1b, beta1 subunit | -1.12 | 0.528 | -1.1 | 0.27 | -1.26 | 0.033 | 1.28 | 0.408 | -0.55 |
| 1438808_at | Trp53 | transformation related protein 53 | 1.63 | 0.295 | -2.04 | 0.128 | -2.1 | 0.017 | -1.41 | 0.065 | -0.98 |
| 1418604_at | Avpr1a | arginine vasopressin receptor 1A | -1.59 | 0.229 | -1.14 | 0.655 | 1.09 | 0.759 | -1.5 | 0.014 | -0.78 |
| 1423375_at | 1700023B02Rik | RIKEN cDNA 1700023B02 gene | -1.45 | 0.116 | -1.04 | 0.713 | -1.06 | 0.723 | 1.59 | 0.283 | -0.49 |

| | | | | | | | | | | | |
|---|---|---|---|---|---|---|---|---|---|---|---|
| 1443556_at | 1110007F12Rik | RIKEN cDNA 1110007F12 gene | -1.26 | 0.562 | -2.03 | 0.153 | 1.31 | 0.41 | -1.21 | 0.816 | -0.8 |
| 1434242_at | Usp37 | ubiquitin specific peptidase 37 | -1.05 | 0.77 | -1.15 | 0.489 | -1.29 | 0.491 | 1.42 | 0.208 | -0.52 |
| 1434261_at | Sipa1l2 | signal-induced proliferation-associated 1 like 2 | 1.03 | 0.602 | -1.51 | 0.269 | -1.11 | 0.593 | -1.06 | 0.767 | -0.66 |
| 1451264_at | Frmd6 | FERM domain containing 6 | 1.09 | 0.252 | -1.3 | 0.165 | -1.36 | 0.125 | -1.67 | 0.13 | -0.81 |
| 1459979_x_at | Zfp68 | zinc finger protein 68 | -1.63 | 0.286 | 1.2 | 0.683 | -1.28 | 0.151 | -1.34 | 0.38 | -0.76 |
| 1446813_s_at | D5Ertd560e | DNA segment, Chr 5, ERATO Doi 560, expressed | -2.17 | 0.322 | -1.03 | 0.93 | 1.17 | 0.726 | -1.21 | 0.349 | -0.81 |
| 1432483_at | 1700124K17Rik | RIKEN cDNA 1700124K17 gene | -4.75 | 0.346 | -1.02 | 0.949 | 1.41 | 0.502 | -1.95 | 0.331 | -1.58 |
| 1435176_a_at | Id2 | inhibitor of DNA binding 2 | -1.43 | 0.197 | 1.13 | 0.549 | -1.31 | 0.048 | -1.55 | 0.171 | -0.79 |
| 1446566_at | Ap2b1 | Adaptor-related protein complex 2, beta 1 subunit | 1.16 | 0.382 | -1.03 | 0.868 | -2.13 | 0.045 | -1.28 | 0.384 | -0.82 |
| 1444085_at | Pdss2 | prenyl (solanesyl) diphosphate synthase, subunit 2 | -1.19 | 0.439 | -1.1 | 0.516 | -1.17 | 0.535 | 1.3 | 0.237 | -0.54 |
| 1424802_a_at | 3300001P08Rik | RIKEN cDNA 3300001P08 gene | -1.11 | 0.276 | 1.01 | 0.921 | -1.45 | 0.045 | -1.35 | 0.012 | -0.73 |
| 1457977_at | 2310005N01Rik | RIKEN cDNA 2310005N01 gene | -1.47 | 0.185 | -1.14 | 0.6 | 1.04 | 0.87 | -1.2 | 0.005 | -0.69 |
| 1435103_x_at | Farslb | phenylalanine-tRNA synthetase-like, beta subunit | -1.11 | 0.172 | -1.2 | 0.006 | -1.16 | 0.395 | 1.08 | 0.663 | -0.6 |
| 1447312_at | Ankib1 | Ankyrin repeat and IBR domain containing 1 | 1.01 | 0.961 | -1.29 | 0.285 | -1.23 | 0.367 | -1.02 | 0.894 | -0.63 |
| 1431330_at | 3110002H16Rik | RIKEN cDNA 3110002H16 gene | -1.02 | 0.94 | -1.42 | 0.373 | -1.09 | 0.67 | 1.42 | 0.394 | -0.53 |
| 1428353_at | Foxk2 | forkhead box K2 | -1.02 | 0.912 | -1.16 | 0.198 | -1.32 | 0.089 | 1.35 | 0.21 | -0.54 |
| 1436495_s_at | Zfp260 | zinc finger protein 260 | 1.03 | 0.598 | -1.12 | 0.12 | -1.48 | 0.021 | -1.52 | 0.235 | -0.77 |
| 1423309_at | Tgoln1 | trans-golgi network protein | 1.02 | 0.746 | -1.16 | 0.161 | -1.39 | 0.024 | -1.26 | 0.487 | -0.7 |
| 1419786_at | Ltbp1 | latent transforming growth factor beta binding protein 1 | 1.24 | 0.107 | -1.12 | 0.677 | -2.15 | 0.011 | -1.13 | 0.8 | -0.79 |
| 1446043_at | C81086 | expressed sequence C81086 | -1.18 | 0.663 | -1.03 | 0.947 | -1.28 | 0.508 | 1.61 | 0.382 | -0.47 |
| 1457978_at | Centg2 | Centaurin, gamma 2 | -2.4 | 0.11 | -1.45 | 0.456 | 1.5 | 0.431 | -2.59 | 0.154 | -1.24 |
| 1453312_at | Iqwd1 | IQ motif and WD repeats 1 | -1.02 | 0.838 | -1.21 | 0.407 | -1.26 | 0.236 | 1.84 | 0.164 | -0.41 |
| 1432239_at | 1700102J08Rik | RIKEN cDNA 1700102J08 gene | 1.4 | 0.525 | -1.93 | 0.3 | -1.47 | 0.548 | -1.9 | 0.149 | -0.97 |
| 1442553_at | Mapre2 | microtubule-associated protein, RP/EB family, member 2 | -1.29 | 0.586 | -1.07 | 0.794 | -1.12 | 0.454 | 1.47 | 0.231 | -0.5 |
| 1452955_at | Gtf2h5 | general transcription factor IIH, polypeptide 5 | -1.21 | 0.403 | -1.18 | 0.575 | -1.08 | 0.271 | 1.01 | 0.945 | -0.61 |
| 1421589_at | Krt31 | keratin 31 | -1.01 | 0.978 | 1.1 | 0.848 | -1.99 | 0.15 | -1.29 | 0.529 | -0.8 |
| 1430661_at | Myl6b | myosin, light polypeptide 6B | 1.32 | 0.313 | -1.59 | 0.39 | -1.55 | 0.257 | -1.59 | 0.025 | -0.85 |
| 1433730_at | Elmod2 | ELMO domain containing 2 | -1.21 | 0.194 | -1.19 | 0.064 | -1.06 | 0.62 | 1.13 | 0.514 | -0.59 |
| 1418088_a_at | Stx8 | syntaxin 8 | -1.06 | 0.454 | -1.25 | 0.046 | -1.17 | 0.103 | 1.03 | 0.811 | -0.61 |
| 1446374_at | Arhgef10 | Rho guanine nucleotide exchange factor (GEF) 10 | -1.05 | 0.884 | 1.07 | 0.901 | -1.74 | 0.065 | -1.65 | 0.267 | -0.84 |
| 1416743_at | Uap1 /// LOC64050 | UDP-N-acetylglucosamine pyrophosphorylase 1 /// similar to UDP-N-acetylhexosa | -1.21 | 0.556 | 1.06 | 0.693 | -1.4 | 0.165 | -1.03 | 0.807 | -0.64 |
| 1445245_at | B130065D12Rik | RIKEN cDNA B130065D12 gene | 1.43 | 0.551 | -1.44 | 0.418 | -2.1 | 0.047 | -1.21 | 0.505 | -0.83 |
| 1437211_x_at | Elovl5 | ELOVL family member 5, elongation of long chain fatty acids (yeast) | 1.14 | 0.154 | -1.34 | 0.148 | -1.4 | 0.061 | -1.37 | 0.293 | -0.74 |
| 1443738_at | Ptdss2 | Phosphatidylserine synthase 2 | -1.67 | 0.318 | -2.08 | 0.036 | 1.52 | 0.261 | -1.25 | 0.7 | -0.87 |
| 1440777_x_at | --- | --- | 1.14 | 0.765 | -1.21 | 0.636 | -1.58 | 0.194 | -1.48 | 0.302 | -0.78 |
| 1447391_at | Rufy1 | RUN and FYVE domain containing 1 | -1.06 | 0.88 | -1.74 | 0.114 | 1.08 | 0.794 | -1.01 | 0.922 | -0.68 |
| 1423275_at | Ints6 | integrator complex subunit 6 | 1.02 | 0.824 | -1.15 | 0.378 | -1.41 | 0.115 | -1.09 | 0.678 | -0.66 |
| 1428866_at | 2810037O22Rik | RIKEN cDNA 2810037O22 gene | -2.19 | 0.41 | -1.02 | 0.93 | 1.17 | 0.543 | -3.99 | 0.018 | -1.51 |
| 1456296_at | 5830418K08Rik | RIKEN cDNA 5830418K08 gene | 1.09 | 0.626 | -1.26 | 0.46 | -1.39 | 0.271 | -1.07 | 0.476 | -0.66 |
| 1452939_a_at | Pitpnc1 | phosphatidylinositol transfer protein, cytoplasmic 1 | -1.05 | 0.848 | 1.03 | 0.919 | -1.62 | 0.048 | -1.5 | 0.091 | -0.78 |
| 1460541_at | Slc7a6 | solute carrier family 7 (cationic amino acid transporter, y+ system), member 6 | -1.12 | 0.711 | -1.48 | 0.507 | 1.03 | 0.75 | -1.78 | 0.002 | -0.84 |
| 1433968_a_at | --- | --- | -1.17 | 0.558 | -1.02 | 0.927 | -1.31 | 0.17 | 1.74 | 0.421 | -0.44 |
| 1434184_s_at | 9430080K19Rik | RIKEN cDNA 9430080K19 gene | 1.02 | 0.906 | -1.21 | 0.544 | -1.32 | 0.232 | -2.49 | 0.039 | -1 |
| 1441561_at | Fbxl3 | F-box and leucine-rich repeat protein 3 | 1.42 | 0.354 | -2.02 | 0.151 | -1.45 | 0.001 | -1.03 | 0.852 | -0.77 |
| 1450560_a_at | Ppp2r5d | protein phosphatase 2, regulatory subunit B (B56), delta isoform | -1.07 | 0.532 | -1.28 | 0.119 | -1.14 | 0.324 | 1 | 0.979 | -0.62 |
| 1416119_at | Txn1 | thioredoxin 1 | -1.15 | 0.022 | -1.13 | 0.104 | -1.18 | 0.015 | 1.02 | 0.903 | -0.61 |
| 1420021_s_at | Suz12 | suppressor of zeste 12 homolog (Drosophila) | -1.06 | 0.55 | -1.08 | 0.148 | -1.37 | 0.062 | 1.49 | 0.146 | -0.5 |
| 1432506_at | Tmem86b | transmembrane protein 86B | -1.64 | 0.365 | 1.15 | 0.733 | -1.19 | 0.526 | -2.55 | 0.061 | -1.06 |
| 1419961_s_at | C330003B14Rik | RIKEN cDNA C330003B14 gene | -2.22 | 0.016 | 1.25 | 0.385 | -1.11 | 0.447 | -1.29 | 0.265 | -0.84 |
| 1429341_at | Oaz3 | ornithine decarboxylase antizyme 3 | -1.42 | 0.596 | -1.34 | 0.537 | 1.15 | 0.812 | -1.23 | 0.557 | -0.71 |
| 1427191_at | Npr2 | natriuretic peptide receptor 2 | 1.01 | 0.943 | -1.6 | 0.028 | -1.03 | 0.908 | -1.38 | 0.213 | -0.75 |
| 1437415_at | 4933427D06Rik | RIKEN cDNA 4933427D06 gene | -1.25 | 0.388 | -1.16 | 0.496 | -1.07 | 0.71 | 1.43 | 0.123 | -0.51 |
| 1445871_at | AI315376 | expressed sequence AI315376 | -2.1 | 0.242 | -1.32 | 0.659 | 1.37 | 0.462 | -1.89 | 0.111 | -0.99 |

| | | | | | | | | | | | |
|---|---|---|---|---|---|---|---|---|---|---|---|
| 1439481_at | Ipo9 | importin 9 | -1.12 | 0.444 | -1.02 | 0.837 | -1.36 | 0.063 | 1.1 | 0.393 | -0.6 |
| 1460611_at | C87499 | expressed sequence C87499 | -1.3 | 0.355 | 1.09 | 0.477 | -1.35 | 0.615 | -1.28 | 0.506 | -0.71 |
| 1453817_at | Abca6 | ATP-binding cassette, sub-family A (ABC1), member 6 | -1.45 | 0.71 | -1.01 | 0.966 | -1.08 | 0.72 | 1.24 | 0.617 | -0.58 |
| 1456606_a_at | Chst11 /// Phactr1 | carbohydrate sulfotransferase 11 /// phosphatase and actin regulator 1 | -1.18 | 0.493 | -1.38 | 0.056 | 1.03 | 0.885 | -1.42 | 0.074 | -0.74 |
| 1456863_at | Epha4 | Eph receptor A4 | 1.06 | 0.903 | -1.65 | 0.494 | -1.07 | 0.611 | -1.25 | 0.657 | -0.73 |
| 1432365_a_at | 4930556L07Rik | RIKEN cDNA 4930556L07 gene | -1.38 | 0.507 | 1.04 | 0.911 | -1.19 | 0.481 | -1.17 | 0.54 | -0.68 |
| 1436948_a_at | 6430550H21Rik | RIKEN cDNA 6430550H21 gene | -1.17 | 0.657 | -1.72 | 0.513 | 1.16 | 0.688 | -1.35 | 0.322 | -0.77 |
| 1421122_at | Cbll1 | Casitas B-lineage lymphoma-like 1 | -1.21 | 0.098 | -1.23 | 0.044 | -1.04 | 0.853 | 1.02 | 0.842 | -0.61 |
| 1436220_at | Zfp287 | zinc finger protein 287 | 1.16 | 0.044 | -1.19 | 0.688 | -1.64 | 0.115 | -1.28 | 0.538 | -0.74 |
| 1434088_at | Zfp496 | zinc finger protein 496 | -1.15 | 0.105 | -1.21 | 0.293 | -1.11 | 0.443 | 1.04 | 0.902 | -0.61 |
| 1417830_at | Smc1a | structural maintenance of chromosomes 1A | -1.12 | 0.321 | -1.12 | 0.078 | -1.22 | 0.115 | 1.24 | 0.186 | -0.56 |
| 1457882_at | 5730466H23Rik | RIKEN cDNA 5730466H23 gene | -1.28 | 0.607 | -1.14 | 0.48 | -1.06 | 0.762 | 1.21 | 0.21 | -0.57 |
| 1426448_at | Pja1 | praja1, RING-H2 motif containing | -1.13 | 0.368 | 1.01 | 0.953 | -1.4 | 0.081 | -1.51 | 0.285 | -0.76 |
| 1441184_at | Trpc4ap | Transient receptor potential cation channel, subfamily C, member 4 associated pr | -1.1 | 0.725 | -1.44 | 0.543 | -1 | 0.994 | 1.83 | 0.367 | -0.43 |
| 1429487_at | Ppp1r12a | protein phosphatase 1, regulatory (inhibitor) subunit 12A | -1.02 | 0.845 | -1.04 | 0.759 | -1.52 | 0.009 | 1.19 | 0.136 | -0.6 |
| 1416834_x_at | Ndufb2 | NADH dehydrogenase (ubiquinone) 1 beta subcomplex, 2 | -1.18 | 0.149 | -1.11 | 0.24 | -1.17 | 0.318 | 1.02 | 0.941 | -0.61 |
| 1432802_at | 9230106L01Rik | RIKEN cDNA 9230106L01 gene | -1.81 | 0.396 | 1.46 | 0.483 | -1.71 | 0.207 | -1.12 | 0.798 | -0.79 |
| 1450943_at | 2010012C16Rik | RIKEN cDNA 2010012C16 gene | -1.2 | 0.503 | -1.17 | 0.403 | -1.09 | 0.447 | 1.29 | 0.308 | -0.54 |
| 1460077_at | Ttc3 | tetratricopeptide repeat domain 3 | -1.24 | 0.482 | 1.29 | 0.65 | -1.97 | 0.022 | -1.92 | 0.167 | -0.96 |
| 1436560_at | AW124694 | expressed sequence AW124694 | -1.05 | 0.687 | -1.09 | 0.814 | -1.37 | 0.059 | 2.33 | 0.111 | -0.29 |
| 1435459_at | Fmo2 | flavin containing monooxygenase 2 | -1.22 | 0.231 | -1.22 | 0.357 | -1.03 | 0.754 | 2.47 | 0.012 | -0.25 |
| 1435921_at | D8Ertd587e | DNA segment, Chr 8, ERATO Doi 587, expressed | 1.06 | 0.745 | -1.31 | 0.028 | -1.29 | 0.365 | -1.58 | 0.078 | -0.78 |
| 1426009_a_at | Pip5k1b | phosphatidylinositol-4-phosphate 5-kinase, type 1 beta | -2.39 | 0.584 | -2.28 | 0.323 | 1.75 | 0.551 | -4.7 | 0.395 | -1.9 |
| 1444014_at | LOC670853 | hypothetical protein LOC670853 | 1.36 | 0.31 | -1.21 | 0.625 | -2.38 | 0.014 | -1.2 | 0.29 | -0.86 |
| 1455129_at | Mtdh | Metadherin | -1.11 | 0.199 | -1.23 | 0.025 | -1.12 | 0.452 | 1 | 0.993 | -0.62 |
| 1453688_at | Cwf19l2 | CWF19-like 2, cell cycle control (S. pombe) | -1.12 | 0.073 | 1.06 | 0.778 | -1.53 | 0.092 | -1.45 | 0.134 | -0.76 |
| 1427999_at | Snapc5 | small nuclear RNA activating complex, polypeptide 5 | 1.27 | 0.053 | -1.45 | 0.292 | -1.55 | 0.345 | -1.46 | 0.057 | -0.8 |
| 1418880_at | Gfra3 | glial cell line derived neurotrophic factor family receptor alpha 3 | -1.49 | 0.006 | -1.28 | 0.676 | 1.15 | 0.634 | -1.08 | 0.858 | -0.67 |
| 1451439_at | BC027231 | cDNA sequence BC027231 | -1.19 | 0.558 | -1.45 | 0.187 | 1.07 | 0.266 | -1.16 | 0.265 | -0.68 |
| 1456537_at | A930033H14Rik | RIKEN cDNA A930033H14 gene | -1.07 | 0.604 | -1.37 | 0.541 | -1.06 | 0.859 | 1.4 | 0.192 | -0.53 |
| 1449684_at | Oxsm | 3-oxoacyl-ACP synthase, mitochondrial | 1.58 | 0.555 | -2.08 | 0.348 | -1.85 | 0.303 | -1.03 | 0.943 | -0.85 |
| 1425639_at | Centa2 | centaurin, alpha 2 | -1.02 | 0.691 | -1.04 | 0.466 | -1.5 | 0.169 | 1.23 | 0.606 | -0.58 |
| 1435521_at | Msi2 | Musashi homolog 2 (Drosophila) | 1.2 | 0.517 | -1.03 | 0.94 | -2.34 | 0.047 | -1.27 | 0.56 | -0.86 |
| 1441814_s_at | Rpain | RPA interacting protein | -1.33 | 0.333 | -1.03 | 0.792 | -1.14 | 0.372 | 1.35 | 0.2 | -0.54 |
| 1459984_at | Mia3 | melanoma inhibitory activity 3 | 1.08 | 0.832 | -1.5 | 0.194 | -1.17 | 0.679 | -1.16 | 0.219 | -0.69 |
| 1418024_at | Narg1 | NMDA receptor-regulated gene 1 | 1.01 | 0.944 | -1.23 | 0.16 | -1.29 | 0.065 | -1.06 | 0.779 | -0.64 |
| 1460435_at | 1500002O20Rik | RIKEN cDNA 1500002O20 gene | -1.63 | 0.167 | -1.14 | 0.356 | 1.11 | 0.225 | -1.09 | 0.527 | -0.69 |
| 1441666_at | --- | Visual cortex cDNA, RIKEN full-length enriched library, clone:K530010N24 produ | -1.25 | 0.589 | -1.06 | 0.922 | -1.16 | 0.727 | 1.51 | 0.528 | -0.49 |
| 1442169_at | Vldlr | very low density lipoprotein receptor | -1.26 | 0.332 | 1.19 | 0.83 | -1.6 | 0.103 | -1.14 | 0.812 | -0.7 |
| 1420002_at | D7Ertd1e | DNA segment, Chr 7, ERATO Doi 1, expressed | 1.21 | 0.511 | -2.51 | 0.166 | -1.01 | 0.972 | -1.09 | 0.906 | -0.85 |
| 1420128_s_at | Pigb | phosphatidylinositol glycan anchor biosynthesis, class B | 1.36 | 0.723 | -1.89 | 0.232 | -1.4 | 0.525 | -1.69 | 0.027 | -0.91 |
| 1418543_s_at | Ccdc43 | coiled-coil domain containing 43 | -1.04 | 0.121 | 1.03 | 0.829 | -1.65 | 0.067 | -1.31 | 0.039 | -0.74 |
| 1446774_at | Syvn1 | synovial apoptosis inhibitor 1, synoviolin | -1.85 | 0.162 | -1.33 | 0.444 | 1.31 | 0.292 | -1.09 | 0.802 | -0.74 |
| 1436028_at | Tmem33 | transmembrane protein 33 | -1.12 | 0.412 | -1.14 | 0.142 | -1.19 | 0.157 | 1.38 | 0.137 | -0.52 |
| 1443171_at | --- | --- | -1.4 | 0.517 | -1.1 | 0.704 | -1.02 | 0.935 | 1.65 | 0.367 | -0.47 |
| 1425382_a_at | Aqp4 | aquaporin 4 | -1.03 | 0.933 | -1.32 | 0.62 | -1.15 | 0.672 | 1.33 | 0.321 | -0.54 |
| 1429301_at | Csnk2a2 | casein kinase 2, alpha prime polypeptide | -1.16 | 0.385 | -1.12 | 0.303 | -1.18 | 0.028 | 1.08 | 0.526 | -0.59 |
| 1445340_at | Phr1 | pam, highwire, rpm 1 | 1.01 | 0.985 | -1.08 | 0.67 | -1.5 | 0.06 | -1.98 | 0.043 | -0.89 |
| 1441323_at | --- | Transcribed locus | -1.11 | 0.327 | -1.26 | 0.43 | -1.1 | 0.155 | 1.23 | 0.51 | -0.56 |
| 1450568_at | Galr1 | galanin receptor 1 | -1.29 | 0.661 | -1.03 | 0.85 | -1.16 | 0.83 | 1.17 | 0.855 | -0.58 |
| 1438477_a_at | Mcee | methylmalonyl CoA epimerase | -1.31 | 0.011 | -1.24 | 0.185 | 1.03 | 0.852 | -1.12 | 0.191 | -0.66 |
| 1435319_at | Ihpk2 | inositol hexaphosphate kinase 2 | -1.24 | 0.386 | 1.09 | 0.736 | -1.41 | 0.246 | -1.22 | 0.237 | -0.7 |
| 1447917_x_at | Ntan1 | N-terminal Asn amidase | 1.05 | 0.396 | -1.15 | 0.399 | -1.45 | 0.018 | -1.26 | 0.187 | -0.7 |

| | | | | | | | | | | | |
|---|---|---|---|---|---|---|---|---|---|---|---|
| 1453595_at | 2900064B18Rik | RIKEN cDNA 2900064B18 gene | 1.14 | 0.765 | -1.14 | 0.7 | -1.68 | 0.118 | -2.16 | 0.043 | -0.96 |
| 1430012_at | 1110050K14Rik | RIKEN cDNA 1110050K14 gene | -1.15 | 0.691 | -1.04 | 0.876 | -1.3 | 0.514 | 1.5 | 0.369 | -0.5 |
| 1417693_a_at | Gab1 | growth factor receptor bound protein 2-associated protein 1 | -1.07 | 0.646 | -1.1 | 0.408 | -1.32 | 0.223 | 2 | 0.14 | -0.37 |
| 1440390_at | BC025575 | cDNA sequence BC025575 | 1.02 | 0.912 | -1.24 | 0.526 | -1.28 | 0.441 | -1.17 | 0.688 | -0.67 |
| 1445828_at | --- | --- | -1.54 | 0.562 | -2.89 | 0.091 | 1.61 | 0.409 | -2.06 | 0.27 | -1.22 |
| 1458424_at | Fa2h | Fatty acid 2-hydroxylase | -2.97 | 0.091 | 1.34 | 0.59 | -1.08 | 0.823 | -1.37 | 0.249 | -1.02 |
| 1431293_a_at | Cldnd1 | claudin domain containing 1 | 1.01 | 0.96 | -1.11 | 0.394 | -1.43 | 0.139 | -1.18 | 0.552 | -0.68 |
| 1427499_at | Zfp81 | zinc finger protein 81 | -1.06 | 0.884 | -1.19 | 0.742 | -1.22 | 0.34 | 1.1 | 0.751 | -0.59 |
| 1443077_at | 1700081L11Rik | RIKEN cDNA 1700081L11 gene | 1.13 | 0.7 | -1.15 | 0.738 | -1.66 | 0.025 | -1.19 | 0.304 | -0.72 |
| 1441335_at | 4930474N05Rik | RIKEN cDNA 4930474N05 gene | -1.51 | 0.498 | -1.12 | 0.596 | 1.05 | 0.654 | -1.01 | 0.981 | -0.65 |
| 1429083_at | Agl | amylo-1,6-glucosidase, 4-alpha-glucanotransferase | -1.19 | 0.387 | -1.16 | 0.527 | -1.11 | 0.728 | 1.42 | 0.042 | -0.51 |
| 1444017_at | Peli1 | Pellino 1 | -1.25 | 0.391 | 1.16 | 0.412 | -1.57 | 0.076 | -1.78 | 0.19 | -0.86 |
| 1434265_s_at | Ank2 | ankyrin 2, brain | 1.2 | 0.383 | -1.25 | 0.645 | -1.64 | 0.304 | -1.55 | 0.019 | -0.81 |
| 1435808_at | A230051G13Rik | RIKEN cDNA A230051G13 gene | -1.24 | 0.151 | -1.15 | 0.565 | -1.08 | 0.503 | 2.04 | 0.283 | -0.36 |
| 1437286_x_at | --- | --- | -1.3 | 0.31 | -1.17 | 0.331 | -1.02 | 0.922 | 2.44 | 0.242 | -0.26 |
| 1426256_at | Timm17a | translocase of inner mitochondrial membrane 17a | -1.07 | 0.293 | -1.19 | 0.203 | -1.21 | 0.035 | 1.12 | 0.529 | -0.59 |
| 1422891_at | H2-Ea | histocompatibility 2, class II antigen E alpha | -1.49 | 0.382 | -1.1 | 0.812 | 1.02 | 0.938 | -1.12 | 0.546 | -0.67 |
| 1434257_s_at | Pitpnb | phosphatidylinositol transfer protein, beta | -1.12 | 0.414 | -1.32 | 0.008 | -1.04 | 0.756 | 1.05 | 0.723 | -0.61 |
| 1442971_at | Baz2b | bromodomain adjacent to zinc finger domain, 2B | -1.1 | 0.817 | 1.22 | 0.56 | -2.12 | 0.019 | -1.78 | 0.085 | -0.94 |
| 1450371_at | Tshb | thyroid stimulating hormone, beta subunit | -1.24 | 0.618 | -1.61 | 0.442 | 1.17 | 0.598 | -1.2 | 0.666 | -0.72 |
| 1434033_at | Tle1 | transducin-like enhancer of split 1, homolog of Drosophila E(spl) | -1.08 | 0.653 | -1.49 | 0.011 | 1.01 | 0.89 | -1.25 | 0.103 | -0.7 |
| 1440536_at | Slc22a5 | Solute carrier family 22 (organic cation transporter), member 5 | 1.03 | 0.277 | -1.03 | 0.877 | -1.64 | 0.313 | -1.08 | 0.856 | -0.68 |
| 1433989_at | Slc6a11 | solute carrier family 6 (neurotransmitter transporter, GABA), member 11 | 1.03 | 0.974 | -1.17 | 0.752 | -1.38 | 0.524 | -2.96 | 0.218 | -1.12 |
| 1424786_s_at | Wdr45 | WD repeat domain 45 | -1 | 0.979 | -1.18 | 0.171 | -1.31 | 0.003 | 1.01 | 0.926 | -0.62 |
| 1456624_at | Wipi1 | WD repeat domain, phosphoinositide interacting 1 | -1.11 | 0.222 | -1.07 | 0.843 | -1.29 | 0.183 | 1.99 | 0.13 | -0.37 |
| 1458083_at | 4932438A13Rik | RIKEN cDNA 4932438A13 gene | 1.12 | 0.681 | -1.17 | 0.414 | -1.58 | 0.109 | -1.28 | 0.227 | -0.73 |
| 1419399_at | Mttp | microsomal triglyceride transfer protein | -1.15 | 0.417 | -1.2 | 0.071 | -1.1 | 0.732 | 1.28 | 0.679 | -0.55 |
| 1445394_at | 4933409K07Rik | RIKEN cDNA 4933409K07 gene | -1.5 | 0.325 | -1.09 | 0.824 | 1.02 | 0.95 | -1.4 | 0.126 | -0.74 |
| 1437442_at | --- | --- | -1.35 | 0.39 | 1.06 | 0.841 | -1.24 | 0.063 | -1.17 | 0.42 | -0.67 |
| 1429537_at | 5730406M06Rik | RIKEN cDNA 5730406M06 gene | 1.09 | 0.645 | -1.1 | 0.462 | -1.64 | 0.084 | -1.01 | 0.978 | -0.66 |
| 1447663_at | Akap13 | A kinase (PRKA) anchor protein 13 | -1.28 | 0.012 | 1.3 | 0.339 | -1.89 | 0.026 | -1.09 | 0.874 | -0.74 |
| 1436639_at | Bola2 | bolA-like 2 (E. coli) | -1.49 | 0.261 | -1.08 | 0.835 | 1.01 | 0.961 | -1.06 | 0.536 | -0.66 |
| 1429188_at | Cox11 | COX11 homolog, cytochrome c oxidase assembly protein (yeast) | -1.16 | 0.217 | -1.32 | 0.055 | -1.01 | 0.951 | 1.75 | 0.284 | -0.44 |
| 1430424_at | 4933433C11Rik | RIKEN cDNA 4933433C11 gene | -1.14 | 0.284 | 1.04 | 0.954 | -1.46 | 0.405 | -1.96 | 0.019 | -0.88 |
| 1444609_at | Exoc4 | Exocyst complex component 4 | -1.1 | 0.594 | -1.05 | 0.856 | -1.33 | 0.312 | 1.08 | 0.407 | -0.6 |
| 1430599_at | Myt1l | myelin transcription factor 1-like | -1.35 | 0.76 | -1.32 | 0.675 | 1.11 | 0.803 | -1.14 | 0.42 | -0.68 |
| 1430372_at | D730045B01Rik | RIKEN cDNA D730045B01 gene | -1.54 | 0.499 | 1.08 | 0.865 | -1.13 | 0.659 | -1.19 | 0.605 | -0.7 |
| 1441131_at | --- | --- | -1.03 | 0.674 | 1 | 0.999 | -1.57 | 0.056 | -1.31 | 0.01 | -0.73 |
| 1449010_at | Hspa4l | heat shock protein 4 like | -1.2 | 0.098 | -1.18 | 0.504 | -1.08 | 0.764 | 2.28 | 0.271 | -0.29 |
| 1433269_at | 4930553M12Rik | RIKEN cDNA 4930553M12 gene | -1.11 | 0.8 | -1.29 | 0.038 | -1.07 | 0.647 | 1.06 | 0.448 | -0.6 |
| 1454256_s_at | 1700113I22Rik | RIKEN cDNA 1700113I22 gene | -1.07 | 0.641 | -1.86 | 0.066 | 1.14 | 0.622 | -1.46 | 0.45 | -0.81 |
| 1427066_a_at | 4933439F18Rik | RIKEN cDNA 4933439F18 gene | -1.18 | 0.585 | -1.24 | 0.036 | -1.05 | 0.787 | 1.11 | 0.633 | -0.59 |
| 1442657_at | D1Ertd273e | DNA segment, Chr 1, ERATO Doi 273, expressed | -1.48 | 0.611 | -1.48 | 0.427 | 1.26 | 0.631 | -1.03 | 0.951 | -0.68 |
| 1420015_s_at | Ppt1 | palmitoyl-protein thioesterase 1 | -1.13 | 0.261 | -1.52 | 0.025 | 1.07 | 0.77 | -1.27 | 0.569 | -0.71 |
| 1436382_at | Zbtb12 | zinc finger and BTB domain containing 12 | -1.25 | 0.058 | -2.49 | 0.042 | 1.41 | 0.154 | -1.92 | 0.169 | -1.06 |
| 1442490_at | C130090I23Rik | RIKEN cDNA C130090I23 gene | 1.32 | 0.492 | -1.21 | 0.614 | -2.17 | 0.369 | -1.37 | 0.706 | -0.86 |
| 1446037_at | C230081A13Rik | RIKEN cDNA C230081A13 gene | -1.24 | 0.059 | -1.26 | 0.684 | 1.01 | 0.96 | -2.02 | 0.013 | -0.88 |
| 1430273_at | 2410087M07Rik | RIKEN cDNA 2410087M07 gene | -1.03 | 0.936 | -1.48 | 0.233 | -1.04 | 0.825 | 1.89 | 0.064 | -0.42 |
| 1428057_a_at | Ahnak | AHNAK nucleoprotein (desmoyokin) | 1.12 | 0.575 | -1.24 | 0.242 | -1.46 | 0.244 | -1.04 | 0.906 | -0.66 |
| 1440845_at | E030031F02Rik | RIKEN cDNA E030031F02 gene | 1.1 | 0.823 | -1.38 | 0.573 | -1.27 | 0.241 | -2.69 | 0.043 | -1.06 |
| 1419867_a_at | Ankhd1 | ankyrin repeat and KH domain containing 1 | 1.07 | 0.508 | -1.1 | 0.302 | -1.59 | 0 | -1.36 | 0.359 | -0.75 |
| 1427606_at | Usp27x | ubiquitin specific peptidase 27, X chromosome | -1.09 | 0.902 | -1.87 | 0.284 | 1.16 | 0.717 | -1.21 | 0.798 | -0.75 |
| 1429740_at | Ttc7 | tetratricopeptide repeat domain 7 | -1.04 | 0.857 | -1.33 | 0.417 | -1.12 | 0.407 | 1.37 | 0.32 | -0.53 |

| | | | | | | | | | | | |
|---|---|---|---|---|---|---|---|---|---|---|---|
| 1439532_s_at | Kif13a | kinesin family member 13A | -1.01 | 0.987 | -1.45 | 0.578 | -1.08 | 0.352 | 1.02 | 0.953 | -0.63 |
| 1419273_at | C80913 | expressed sequence C80913 | 1.02 | 0.447 | -1.22 | 0.08 | -1.3 | 0.009 | -1.27 | 0.147 | -0.69 |
| 1447210_at | --- | Transcribed locus | -1.29 | 0.482 | -2.29 | 0.119 | 1.4 | 0.272 | -1.16 | 0.477 | -0.84 |
| 1454982_at | Arfgef2 /// LOC6707 | ADP-ribosylation factor guanine nucleotide-exchange factor 2 (brefeldin A-inhibite | -1.17 | 0.435 | -1.51 | 0.033 | 1.1 | 0.722 | -1.06 | 0.415 | -0.66 |
| 1421905_at | Ncoa6ip | nuclear receptor coactivator 6 interacting protein | -1.04 | 0.949 | -1.09 | 0.82 | -1.37 | 0.305 | 1.26 | 0.287 | -0.56 |
| 1420787_at | 4933436I01Rik | RIKEN cDNA 4933436I01 gene | -1.02 | 0.958 | -1.64 | 0.364 | 1.02 | 0.952 | -1.3 | 0.029 | -0.74 |
| 1454119_at | Mknk1 | MAP kinase-interacting serine/threonine kinase 1 | -2.72 | 0.368 | 1.28 | 0.672 | -1.04 | 0.939 | -1.68 | 0.246 | -1.04 |
| 1447538_at | Crtc1 | CREB regulated transcription coactivator 1 | -1.21 | 0.686 | -1.2 | 0.485 | -1.05 | 0.88 | 1.04 | 0.524 | -0.61 |
| 1439623_at | Otud1 | OTU domain containing 1 | 1.15 | 0.545 | -1.76 | 0.101 | -1.12 | 0.509 | -1.72 | 0.163 | -0.86 |
| 1434447_at | AI838057 | expressed sequence AI838057 | -1.08 | 0.534 | 1.12 | 0.269 | -1.78 | 0.003 | -1.42 | 0.271 | -0.79 |
| 1445097_at | Pik3r3 | Phosphatidylinositol 3 kinase, regulatory subunit, polypeptide 3 (p55) | -1.41 | 0.456 | 1.21 | 0.414 | -1.44 | 0.483 | -1.39 | 0.165 | -0.76 |
| 1458947_at | 2010111I01Rik | RIKEN cDNA 2010111I01 gene | -1.12 | 0.443 | 1.18 | 0.177 | -1.86 | 0.028 | -1.06 | 0.746 | -0.72 |
| 1428624_at | 2810482I07Rik | RIKEN cDNA 2810482I07 gene | -1.14 | 0.339 | -1.14 | 0.019 | -1.17 | 0.197 | 1.18 | 0.186 | -0.57 |
| 1455254_at | 4833420G11Rik | RIKEN cDNA 4833420G11 gene | -1.14 | 0.044 | -1.13 | 0.328 | -1.18 | 0.09 | 1.99 | 0.204 | -0.37 |
| 1418585_at | Ccnh | cyclin H | -1.04 | 0.583 | -1.15 | 0.259 | -1.29 | 0.039 | 1.07 | 0.816 | -0.6 |
| 1440996_at | Nedd8 | Neural precursor cell expressed, developmentally down-regulated gene 8 | -1.07 | 0.632 | -1.66 | 0.041 | 1.07 | 0.618 | -1.34 | 0.25 | -0.75 |
| 1422116_at | Aff2 | AF4/FMR2 family, member 2 | -1.63 | 0.607 | 1.05 | 0.937 | -1.05 | 0.873 | -1.67 | 0.071 | -0.83 |
| 1432204_at | 4930570B17Rik | RIKEN cDNA 4930570B17 gene | 1.29 | 0.492 | -1.8 | 0.328 | -1.32 | 0.383 | -2.01 | 0.175 | -0.96 |
| 1426796_at | Kpna6 | karyopherin (importin) alpha 6 | -1.31 | 0.216 | -1.12 | 0.579 | -1.05 | 0.844 | 1.07 | 0.784 | -0.6 |
| 1428570_at | Ccnc | cyclin C | -1.28 | 0.224 | -1.13 | 0.558 | -1.05 | 0.774 | 1.16 | 0.325 | -0.58 |
| 1454320_at | 5830420C07Rik | RIKEN cDNA 5830420C07 gene | -1.35 | 0.582 | -1.28 | 0.587 | 1.09 | 0.839 | -1.01 | 0.975 | -0.64 |
| 1424091_at | AI449175 | expressed sequence AI449175 | -1.11 | 0.199 | -1.44 | 0.03 | 1.01 | 0.923 | -1.13 | 0.036 | -0.67 |
| 1441445_at | Per3 | period homolog 3 (Drosophila) | 1.08 | 0.866 | -1.1 | 0.903 | -1.62 | 0.345 | -1.09 | 0.766 | -0.68 |
| 1429558_a_at | Larp7 | La ribonucleoprotein domain family, member 7 | -1.19 | 0.292 | -1.15 | 0.166 | -1.11 | 0.079 | 1.01 | 0.956 | -0.61 |
| 1455182_at | Kif1b | kinesin family member 1B | 1.04 | 0.866 | -1.13 | 0.608 | -1.45 | 0.266 | -1.5 | 0.18 | -0.76 |
| 1423206_s_at | 2310003F16Rik | RIKEN cDNA 2310003F16 gene | -1.2 | 0.068 | -1.09 | 0.232 | -1.16 | 0.306 | 1.02 | 0.888 | -0.61 |
| 1426977_at | Usp47 | ubiquitin specific peptidase 47 | -1.15 | 0.074 | -1.13 | 0.073 | -1.16 | 0.06 | 1.02 | 0.844 | -0.61 |
| 1458119_at | Slc25a27 | solute carrier family 25, member 27 | 1.07 | 0.891 | -1.18 | 0.806 | -1.45 | 0.289 | -1.18 | 0.754 | -0.68 |
| 1443673_x_at | --- | --- | -1.02 | 0.149 | -1.05 | 0.779 | -1.47 | 0.093 | 5.08 | 0.197 | 0.38 |
| 1442977_at | Col3a1 | Procollagen, type III, alpha 1 | -1.24 | 0.213 | 1.23 | 0.568 | -1.74 | 0.085 | -1.35 | 0.083 | -0.77 |
| 1451124_at | Sod1 | superoxide dismutase 1, soluble | -1.09 | 0.495 | -1.11 | 0.564 | -1.26 | 0.096 | 1.02 | 0.819 | -0.61 |
| 1442337_at | Adcyap1r1 | Adenylate cyclase activating polypeptide 1 receptor 1 | -1.94 | 0.42 | -2.32 | 0.421 | 1.66 | 0.251 | -1.34 | 0.669 | -0.98 |
| 1416031_s_at | Mcm7 | minichromosome maintenance deficient 7 (S. cerevisiae) | -1.21 | 0.354 | -1.21 | 0.011 | -1.05 | 0.714 | 2.19 | 0.204 | -0.32 |
| 1416965_at | Pcsk1n | proprotein convertase subtilisin/kexin type 1 inhibitor | 1.48 | 0.616 | -2.13 | 0.046 | -1.5 | 0.322 | -1.02 | 0.977 | -0.79 |
| 1435613_x_at | Cox5b | cytochrome c oxidase, subunit Vb | -1.11 | 0.078 | -1.34 | 0.106 | -1.04 | 0.588 | 1.27 | 0.132 | -0.55 |
| 1457628_at | Ccdc53 | coiled-coil domain containing 53 | -1.11 | 0.62 | 1.07 | 0.745 | -1.55 | 0.086 | -1.61 | 0.07 | -0.8 |
| 1442298_at | Bicc1 | Bicaudal C homolog 1 (Drosophila) | -1.38 | 0.101 | 1.28 | 0.139 | -1.64 | 0.063 | -1.04 | 0.647 | -0.7 |
| 1445983_at | Nkrf | NF-kappaB repressing factor | -1.67 | 0.45 | -1.24 | 0.752 | 1.21 | 0.604 | -2.05 | 0.455 | -0.94 |
| 1425687_at | Cflar | CASP8 and FADD-like apoptosis regulator | 1.23 | 0.509 | -1.32 | 0.169 | -1.61 | 0.063 | -1.42 | 0.209 | -0.78 |
| 1446994_at | Exosc2 | Exosome component 2 | -2.55 | 0.061 | 1.6 | 0.34 | -1.61 | 0.538 | -1.63 | 0.508 | -1.05 |
| 1426181_a_at | Il24 | interleukin 24 | -4.25 | 0.109 | 1.83 | 0.215 | -1.82 | 0.338 | -1.28 | 0.346 | -1.38 |
| 1454533_at | 9430085L16Rik | RIKEN cDNA 9430085L16 gene | 1.66 | 0.008 | -2.28 | 0.106 | -1.95 | 0.014 | -2.85 | 0.406 | -1.36 |
| 1438442_at | AI450236 | expressed sequence AI450236 | -1.36 | 0.252 | 1.22 | 0.377 | -1.52 | 0.056 | -1.86 | 0.273 | -0.88 |
| 1443560_at | --- | Adult male testis cDNA, RIKEN full-length enriched library, clone:4930506M03 pr | -1.08 | 0.547 | -1.26 | 0.274 | -1.12 | 0.54 | 1.21 | 0.397 | -0.56 |
| 1445856_at | 6030443O07Rik | RIKEN cDNA 6030443O07 gene | -1.37 | 0.498 | 1.02 | 0.964 | -1.16 | 0.506 | -1.29 | 0.476 | -0.7 |
| 1444531_at | Sod2 | Superoxide dismutase 2, mitochondrial | -1.08 | 0.756 | -1.05 | 0.762 | -1.35 | 0.116 | 1.16 | 0.555 | -0.58 |
| 1428157_at | Gng2 | guanine nucleotide binding protein (G protein), gamma 2 subunit | 1.08 | 0.658 | -1.24 | 0.526 | -1.39 | 0.125 | -1.23 | 0.6 | -0.69 |
| 1422590_at | Cdk5 | cyclin-dependent kinase 5 | -1.33 | 0.119 | -1.38 | 0.168 | 1.14 | 0.563 | -1.35 | 0.507 | -0.73 |
| 1432314_at | 4930554C24Rik | RIKEN cDNA 4930554C24 gene | -1.07 | 0.8 | 1.14 | 0.237 | -1.86 | 0.205 | -1.15 | 0.532 | -0.73 |
| 1445724_at | Iqgap1 | IQ motif containing GTPase activating protein 1 | 1.15 | 0.442 | -1.13 | 0.78 | -1.72 | 0.122 | -2.26 | 0.083 | -0.99 |
| 1418144_a_at | Pip5k1b | phosphatidylinositol-4-phosphate 5-kinase, type 1 beta | -1.09 | 0.484 | -1.15 | 0.308 | -1.21 | 0.385 | 1.17 | 0.323 | -0.57 |
| 1415891_at | Suclg1 | succinate-CoA ligase, GDP-forming, alpha subunit | -1.2 | 0.044 | -1.33 | 0.08 | 1.03 | 0.77 | -1.34 | 0.221 | -0.71 |
| 1446655_at | Scyl3 | SCY1-like 3 (S. cerevisiae) | -3.79 | 0.313 | 1.39 | 0.545 | -1.04 | 0.938 | -2.99 | 0.021 | -1.61 |

| | | | | | | | | | | | |
|---|---|---|---|---|---|---|---|---|---|---|---|
| 1430468_at | 2810449G22Rik | RIKEN cDNA 2810449G22 gene | -1.02 | 0.979 | -1.02 | 0.984 | -1.55 | 0.346 | 1.12 | 0.801 | -0.62 |
| 1458637_x_at | Ube3c | Ubiquitin protein ligase E3C | 1.03 | 0.908 | -1.04 | 0.897 | -1.62 | 0.082 | -1.1 | 0.554 | -0.68 |
| 1429908_at | 6530403A03Rik | RIKEN cDNA 6530403A03 gene | -1.29 | 0.188 | -1.42 | 0.089 | 1.13 | 0.229 | -1.1 | 0.706 | -0.67 |
| 1424647_at | Gabrp | gamma-aminobutyric acid (GABA-A) receptor, pi | -1.63 | 0.233 | -1.05 | 0.896 | 1.04 | 0.909 | -1.42 | 0.108 | -0.76 |
| 1429926_at | 6720473G16Rik | RIKEN cDNA 6720473G16 gene | 1.03 | 0.919 | -1.5 | 0.056 | -1.09 | 0.342 | -1.06 | 0.665 | -0.66 |
| 1430163_at | 1810048P08Rik | RIKEN cDNA 1810048P08 gene | -1.33 | 0.538 | -1.04 | 0.887 | -1.11 | 0.672 | 1.03 | 0.89 | -0.61 |
| 1445045_at | Maml1 | Mastermind like 1 (Drosophila) | -2.56 | 0.346 | 1.29 | 0.67 | -1.07 | 0.898 | -1.25 | 0.747 | -0.9 |
| 1418681_at | Glt28d1 | glycosyltransferase 28 domain containing 1 | -1.18 | 0.027 | -1.2 | 0.322 | -1.07 | 0.402 | 1.18 | 0.429 | -0.57 |
| 1437988_x_at | 1700003E24Rik | RIKEN cDNA 1700003E24 gene | 1.1 | 0.822 | -1.85 | 0.209 | -1.03 | 0.947 | -1.54 | 0.461 | -0.83 |
| 1438463_x_at | Zdhhc6 | zinc finger, DHHC domain containing 6 | -1.12 | 0.162 | -1.1 | 0.158 | -1.23 | 0.093 | 1.43 | 0.198 | -0.51 |
| 1419237_at | Usp29 | ubiquitin specific peptidase 29 | 1.4 | 0.592 | -2.3 | 0.139 | -1.28 | 0.639 | -2 | 0.235 | -1.04 |
| 1446670_at | Cugbp2 | CUG triplet repeat, RNA binding protein 2 | -1.02 | 0.942 | 1.12 | 0.708 | -1.95 | 0.108 | -1.9 | 0.242 | -0.94 |
| 1415986_at | Clcn4-2 | chloride channel 4-2 | -1.23 | 0.151 | 1.03 | 0.836 | -1.3 | 0.188 | -1.11 | 0.739 | -0.65 |
| 1425235_s_at | Col20a1 | collagen, type XX, alpha 1 | -1.59 | 0.541 | 1.05 | 0.949 | -1.07 | 0.92 | -4.32 | 0.144 | -1.48 |
| 1418616_at | Mafk | v-maf musculoaponeurotic fibrosarcoma oncogene family, protein K (avian) | -1.27 | 0.247 | -1.03 | 0.861 | -1.17 | 0.448 | 1.86 | 0.215 | -0.4 |
| 1437085_at | D630039A03Rik | RIKEN cDNA D630039A03 gene | -1.29 | 0.452 | -1.57 | 0.063 | 1.2 | 0.27 | -1.46 | 0.475 | -0.78 |
| 1423024_at | Sh2d1b1 | SH2 domain protein 1B1 | -1.03 | 0.888 | -1.37 | 0.597 | -1.1 | 0.725 | 1.44 | 0.306 | -0.51 |
| 1454424_at | 2610040L17Rik | RIKEN cDNA 2610040L17 gene | -2.05 | 0.319 | -1.07 | 0.855 | 1.19 | 0.766 | -1.23 | 0.329 | -0.79 |
| 1426110_a_at | Edg2 | endothelial differentiation, lysophosphatidic acid G-protein-coupled receptor, 2 | -1.36 | 0.047 | -1.34 | 0.532 | 1.14 | 0.559 | -1.58 | 0.205 | -0.79 |
| 1427246_at | Magi1 | membrane associated guanylate kinase, WW and PDZ domain containing 1 | -2.06 | 0.262 | 1.24 | 0.419 | -1.12 | 0.715 | -2.06 | 0.234 | -1 |
| 1446544_at | Arntl | Aryl hydrocarbon receptor nuclear translocator-like | -1.41 | 0.146 | -1.66 | 0.359 | 1.3 | 0.394 | -4 | 0.048 | -1.44 |
| 1429253_at | Zmym4 | zinc finger, MYM-type 4 | 1.06 | 0.653 | -1.12 | 0.581 | -1.53 | 0.009 | -1.17 | 0.313 | -0.69 |
| 1450337_a_at | Nek8 | NIMA (never in mitosis gene a)-related expressed kinase 8 | -1.16 | 0.46 | -1.19 | 0.419 | -1.1 | 0.531 | 1.43 | 0.393 | -0.5 |
| 1428989_at | 0710001D07Rik | RIKEN cDNA 0710001D07 gene | -1.32 | 0.087 | -1.21 | 0.34 | 1.03 | 0.799 | -1.15 | 0.355 | -0.66 |
| 1454727_at | AI173486 | expressed sequence AI173486 | -1.14 | 0.389 | 1.03 | 0.889 | -1.41 | 0.09 | -1.51 | 0.072 | -0.76 |
| 1424868_at | Glyat | glycine-N-acyltransferase | -1.27 | 0.272 | -1.48 | 0.042 | 1.15 | 0.577 | -1.24 | 0.579 | -0.71 |
| 1428229_at | Prkcn | protein kinase C, nu | 1.1 | 0.167 | -1.15 | 0.199 | -1.55 | 0.002 | -1.61 | 0.003 | -0.8 |
| 1425388_a_at | Tpk1 | thiamine pyrophosphokinase | -1.18 | 0.189 | -1.18 | 0.002 | -1.09 | 0.265 | 1.12 | 0.126 | -0.58 |
| 1419906_at | Hpgd | Hydroxyprostaglandin dehydrogenase 15 (NAD) | 1.37 | 0.302 | -1.81 | 0.148 | -1.44 | 0.237 | -1.52 | 0.223 | -0.85 |
| 1436438_s_at | --- | --- | -1.38 | 0.256 | -1.24 | 0.236 | 1.08 | 0.739 | -1.34 | 0.206 | -0.72 |
| 1447338_at | --- | Transcribed locus | -1.64 | 0.342 | -1.01 | 0.964 | 1.01 | 0.97 | -1.12 | 0.658 | -0.69 |
| 1435797_at | D5Wsu178e | DNA segment, Chr 5, Wayne State University 178, expressed | -1.01 | 0.93 | -1.37 | 0.089 | -1.12 | 0.418 | 1.13 | 0.58 | -0.59 |
| 1459712_at | Zfp182 | zinc finger protein 182 | -1.15 | 0.015 | -1.04 | 0.867 | -1.28 | 0.247 | 1.03 | 0.961 | -0.61 |
| 1454862_at | Phldb2 | pleckstrin homology-like domain, family B, member 2 | -1.04 | 0.705 | 1.01 | 0.955 | -1.54 | 0.011 | -1.31 | 0.361 | -0.72 |
| 1426135_a_at | Park2 | parkin | -1.57 | 0.262 | -1.45 | 0.33 | 1.29 | 0.593 | -1.85 | 0.126 | -0.9 |
| 1459968_at | Rbms1 | RNA binding motif, single stranded interacting protein 1 | 1.2 | 0.636 | -1.46 | 0.509 | -1.37 | 0.161 | -1.61 | 0.319 | -0.81 |
| 1424103_at | Atg4b | autophagy-related 4B (yeast) | -1.25 | 0.16 | -1.12 | 0.343 | -1.08 | 0.76 | 1.14 | 0.686 | -0.58 |
| 1445438_at | Ddhd1 | DDHD domain containing 1 | 1.07 | 0.615 | -1.07 | 0.843 | -1.65 | 0.089 | -1.15 | 0.472 | -0.7 |
| 1433503_at | Zadh1 | zinc binding alcohol dehydrogenase, domain containing 1 | -1.17 | 0.099 | -1.12 | 0.32 | -1.16 | 0.219 | 1.16 | 0.24 | -0.57 |
| 1435105_at | 1110061N23Rik | RIKEN cDNA 1110061N23 gene | -1.43 | 0.377 | -1.23 | 0.708 | 1.1 | 0.641 | -1.18 | 0.507 | -0.69 |
| 1437576_at | 2810427A07Rik | RIKEN cDNA 2810427A07 gene | 1.17 | 0.561 | -1.14 | 0.354 | -1.77 | 0.084 | -1.23 | 0.242 | -0.74 |
| 1436241_s_at | Hira | histone cell cycle regulation defective homolog A (S. cerevisiae) | -2.05 | 0.012 | -1.33 | 0.451 | 1.38 | 0.009 | -1.02 | 0.946 | -0.76 |
| 1456218_at | Snx22 | sorting nexin 22 | -1.15 | 0.421 | -1.16 | 0.564 | -1.14 | 0.141 | 1.99 | 0.243 | -0.36 |
| 1451178_at | Mrpl53 | mitochondrial ribosomal protein L53 | -1.18 | 0.136 | -1.13 | 0.141 | -1.13 | 0.143 | 1.36 | 0.374 | -0.52 |
| 1453169_a_at | Gtf2h1 | general transcription factor II H, polypeptide 1 | -1 | 0.996 | -1.19 | 0.021 | -1.29 | 0.011 | 1.02 | 0.919 | -0.62 |
| 1440464_at | Elavl1 | ELAV (embryonic lethal, abnormal vision, Drosophila)-like 1 (Hu antigen R) | 1.03 | 0.878 | -1.24 | 0.482 | -1.28 | 0.447 | -1.68 | 0.14 | -0.79 |
| 1451412_a_at | Ift20 | intraflagellar transport 20 homolog (Chlamydomonas) | -1.18 | 0.052 | -1.1 | 0.114 | -1.17 | 0.277 | 1.03 | 0.796 | -0.6 |
| 1437735_at | Ppp1r12a | protein phosphatase 1, regulatory (inhibitor) subunit 12A | -1.21 | 0.314 | 1.05 | 0.65 | -1.35 | 0.013 | -1.42 | 0.133 | -0.73 |
| 1429430_at | --- | --- | -1.35 | 0.083 | -1.18 | 0.358 | 1.02 | 0.908 | -1.63 | 0.166 | -0.78 |
| 1427101_at | Metrn | meteorin, glial cell differentiation regulator | 1.32 | 0.37 | -2.16 | 0.413 | -1.2 | 0.667 | -1.17 | 0.702 | -0.8 |
| 1431947_at | Ldlr | low density lipoprotein receptor | 1.06 | 0.946 | -1.12 | 0.684 | -1.52 | 0.202 | -1.85 | 0.058 | -0.86 |
| 1443751_at | Cacnb4 | Calcium channel, voltage-dependent, beta 4 subunit | -1.09 | 0.665 | -1.12 | 0.413 | -1.24 | 0.675 | 2.22 | 0.281 | -0.31 |
| 1423969_at | Nup37 | nucleoporin 37 | -1.24 | 0.379 | -1.11 | 0.44 | -1.11 | 0.357 | 1.28 | 0.408 | -0.54 |

| | | | | | | | | | | | |
|---|---|---|---|---|---|---|---|---|---|---|---|
| 1451393_at | Pex26 | peroxisome biogenesis factor 26 | -1.16 | 0.033 | -1.34 | 0.065 | 1.01 | 0.934 | -1.08 | 0.604 | -0.64 |
| 1435420_at | C330016K18Rik | RIKEN cDNA C330016K18 gene | -1.26 | 0.63 | -1.69 | 0.23 | 1.23 | 0.378 | -1.2 | 0.552 | -0.73 |
| 1419876_at | 2810449G22Rik | RIKEN cDNA 2810449G22 gene | -1.47 | 0.441 | 1.06 | 0.668 | -1.15 | 0.363 | -1.54 | 0.556 | -0.77 |
| 1427384_at | Chd6 | chromodomain helicase DNA binding protein 6 | 1.02 | 0.886 | -1.38 | 0.192 | -1.14 | 0.361 | -1.73 | 0.074 | -0.81 |
| 1458809_at | --- | --- | 1.18 | 0.771 | -1.19 | 0.798 | -1.67 | 0.039 | -1.05 | 0.825 | -0.68 |
| 1436073_at | A630007B06Rik /// | RIKEN cDNA A630007B06 gene /// similar to oocyte-testis gene 1 | 1.08 | 0.64 | -1.25 | 0.148 | -1.36 | 0.248 | -1.12 | 0.409 | -0.66 |
| 1440302_at | Slc25a3 | solute carrier family 25 (mitochondrial carrier, phosphate carrier), member 3 | -1.12 | 0.628 | -1.41 | 0.065 | 1.02 | 0.941 | -1.67 | 0.254 | -0.8 |
| 1455119_at | Ppapdc1a | phosphatidic acid phosphatase type 2 domain containing 1A | -1.3 | 0.697 | -2.55 | 0.167 | 1.45 | 0.216 | -2.65 | 0.324 | -1.26 |
| 1441607_at | AU015603 | expressed sequence AU015603 | -1.27 | 0.581 | -1.23 | 0.059 | 1.02 | 0.905 | -1.26 | 0.322 | -0.69 |
| 1456200_at | Ipmk | inositol polyphosphate multikinase | -1.04 | 0.767 | -1.08 | 0.505 | -1.36 | 0.062 | 1.39 | 0.298 | -0.52 |
| 1451536_at | Mtfr1 | mitochondrial fission regulator 1 | -1.11 | 0.536 | -1.16 | 0.003 | -1.17 | 0.148 | 3.1 | 0.177 | -0.09 |
| 1415723_at | Eif5 | eukaryotic translation initiation factor 5 | -1.07 | 0.777 | -1.19 | 0.209 | -1.2 | 0.254 | 1.34 | 0.219 | -0.53 |
| 1448150_at | Nup62 | nucleoporin 62 | 1.05 | 0.943 | -1.17 | 0.821 | -1.41 | 0.471 | -1.22 | 0.06 | -0.69 |
| 1416090_at | Pdhb | pyruvate dehydrogenase (lipoamide) beta | -1.31 | 0.043 | -1.23 | 0.044 | 1.04 | 0.739 | -1.06 | 0.696 | -0.64 |
| 1453640_at | 2210019G11Rik | RIKEN cDNA 2210019G11 gene | 1.05 | 0.907 | -1.16 | 0.805 | -1.42 | 0.409 | -1.91 | 0.168 | -0.86 |
| 1432183_at | Tmem116 | transmembrane protein 116 | -1.49 | 0.218 | -1.02 | 0.911 | -1.04 | 0.792 | 1.29 | 0.446 | -0.56 |
| 1427765_a_at | Tcrb-V13 /// LOC38 | T-cell receptor beta, variable 13 /// similar to T-cell receptor beta chain V region C | 1.05 | 0.907 | -1.42 | 0.532 | -1.15 | 0.783 | -2.83 | 0.321 | -1.09 |
| 1426932_at | Smarca2 | SWI/SNF related, matrix associated, actin dependent regulator of chromatin, subf | -1.04 | 0.565 | -1.09 | 0.435 | -1.36 | 0.091 | 1.11 | 0.534 | -0.59 |
| 1451272_a_at | Ube2f | ubiquitin-conjugating enzyme E2F (putative) | -1.13 | 0.167 | -1.11 | 0.414 | -1.2 | 0.123 | 1.21 | 0.27 | -0.56 |
| 1451865_at | Cxadr | coxsackievirus and adenovirus receptor | -1.08 | 0.913 | -1.42 | 0.047 | -1.02 | 0.961 | 1.58 | 0.264 | -0.48 |
| 1429921_at | 9530068E07Rik | RIKEN cDNA 9530068E07 gene | 1.08 | 0.82 | -1.29 | 0.663 | -1.32 | 0.129 | -1.6 | 0.271 | -0.78 |
| 1438915_at | 6720401G13Rik | RIKEN cDNA 6720401G13 gene | 1.09 | 0.664 | -1.08 | 0.723 | -1.65 | 0.032 | -1.49 | 0.343 | -0.78 |
| 1418294_at | Epb4.1l4b | erythrocyte protein band 4.1-like 4b | 1.05 | 0.188 | -1.56 | 0.023 | -1.08 | 0.528 | -1.13 | 0.815 | -0.68 |
| 1428738_a_at | D14Ertd449e | DNA segment, Chr 14, ERATO Doi 449, expressed | -1.33 | 0.062 | -1.16 | 0.07 | 1.01 | 0.979 | -1.11 | 0.531 | -0.65 |
| 1428179_at | Ndufv2 | NADH dehydrogenase (ubiquinone) flavoprotein 2 | -1.14 | 0.081 | -1.19 | 0.005 | -1.11 | 0.15 | 1.05 | 0.513 | -0.6 |
| 1458655_at | Wwtr1 | WW domain containing transcription regulator 1 | -1.19 | 0.724 | 1.26 | 0.628 | -1.93 | 0.026 | -1.05 | 0.717 | -0.73 |
| 1427945_at | Dpyd | dihydropyrimidine dehydrogenase | -1.21 | 0.516 | 1.04 | 0.592 | -1.34 | 0.185 | -1.91 | 0.002 | -0.85 |
| 1456934_at | --- | --- | -1.47 | 0.059 | 1.41 | 0.615 | -1.89 | 0.069 | -1.11 | 0.876 | -0.76 |
| 1423819_s_at | Arl6ip1 | ADP-ribosylation factor-like 6 interacting protein 1 | -1.07 | 0.51 | -1.26 | 0.184 | -1.12 | 0.548 | 1.32 | 0.301 | -0.53 |
| 1458638_at | Mtdh | Metadherin | -1.83 | 0.481 | 1.65 | 0.235 | -2.35 | 0.163 | -1.14 | 0.808 | -0.92 |
| 1430303_at | 4921537D05Rik | RIKEN cDNA 4921537D05 gene | -1.21 | 0.324 | -1.21 | 0.206 | -1.03 | 0.913 | 1.4 | 0.332 | -0.51 |
| 1459335_at | --- | --- | -1.16 | 0.501 | 1.17 | 0.737 | -1.71 | 0.108 | -1.2 | 0.169 | -0.73 |
| 1437082_at | Akap9 | A kinase (PRKA) anchor protein (yotiao) 9 | 1.1 | 0.348 | -1.22 | 0.323 | -1.43 | 0.12 | -1.27 | 0.564 | -0.71 |
| 1438354_x_at | Cnn3 | Calponin 3, acidic | 1.05 | 0.819 | -1.06 | 0.796 | -1.61 | 0.063 | -2.29 | 0.105 | -0.98 |
| 1441477_at | Calu | calumenin | 1.06 | 0.825 | -1.06 | 0.88 | -1.62 | 0.046 | -1.27 | 0.452 | -0.72 |
| 1420225_at | --- | --- | -1.03 | #DIV/0! | 1 | 0.995 | -1.53 | 0.347 | -3.79 | 0.189 | -1.34 |
| 1434528_at | Aard | alanine and arginine rich domain containing protein | 1.25 | 0.555 | -1.13 | 0.466 | -2.04 | 0 | -2.2 | 0.267 | -1.03 |
| 1424042_at | Tmem5 | transmembrane protein 5 | -1.04 | 0.569 | -1.24 | 0.008 | -1.18 | 0.231 | 1.05 | 0.714 | -0.6 |
| 1458801_at | Fus | fusion, derived from t(12;16) malignant liposarcoma (human) | -1.01 | 0.976 | -1 | 0.995 | -1.58 | 0.094 | 1.63 | 0.481 | -0.49 |
| 1435112_a_at | Atp5h | ATP synthase, H+ transporting, mitochondrial F0 complex, subunit d | -1.03 | 0.328 | -1.21 | 0.135 | -1.21 | 0.015 | 1.14 | 0.045 | -0.58 |
| 1451041_at | Rock2 | Rho-associated coiled-coil containing protein kinase 2 | -1.17 | 0.157 | 1.03 | 0.847 | -1.37 | 0.004 | -1.03 | 0.858 | -0.63 |
| 1444860_at | Lama3 | Laminin, alpha 3 | 1.16 | 0.697 | -1.05 | 0.931 | -1.96 | 0.338 | -1.71 | 0.458 | -0.89 |
| 1449349_at | Nudt1 | nudix (nucleoside diphosphate linked moiety X)-type motif 1 | -1.08 | 0.509 | -1.24 | 0.02 | -1.12 | 0.348 | 1.46 | 0.108 | -0.5 |
| 1420495_a_at | Vps26a | vacuolar protein sorting 26 homolog A (yeast) | -1.15 | 0.299 | -1.15 | 0.262 | -1.14 | 0.255 | 1.07 | 0.27 | -0.59 |
| 1426183_a_at | Cd209d | CD209d antigen | -1.64 | 0.369 | -1.61 | 0.222 | 1.39 | 0.456 | -2.19 | 0.042 | -1.01 |
| 1455197_at | Rnd1 | Rho family GTPase 1 | -1.13 | 0.734 | -1.48 | 0.123 | 1.06 | 0.817 | -1.71 | 0.139 | -0.82 |
| 1459975_at | --- | Transcribed locus | -1.43 | 0.042 | -1.03 | 0.813 | -1.05 | 0.807 | 1.01 | 0.935 | -0.63 |
| 1442733_at | --- | --- | -1.26 | 0.319 | -1.21 | 0.337 | -1.01 | 0.957 | 1.08 | 0.763 | -0.6 |
| 1433743_at | Dach1 | dachshund 1 (Drosophila) | -1.49 | 0.315 | -1.05 | 0.891 | -1.01 | 0.957 | 1.75 | 0.41 | -0.45 |
| 1434660_at | Alkbh1 | alkB, alkylation repair homolog 1 (E. coli) | -1.19 | 0.275 | -1.28 | 0.164 | 1 | 0.994 | -1.19 | 0.063 | -0.67 |
| 1428906_at | Gtf2h5 | general transcription factor IIH, polypeptide 5 | -1.16 | 0.22 | -1.08 | 0.062 | -1.21 | 0.168 | 1.13 | 0.406 | -0.58 |
| 1458824_at | Unc45a | unc-45 homolog A (C. elegans) | -1.19 | 0.325 | 1.04 | 0.963 | -1.35 | 0.625 | -1.91 | 0.114 | -0.85 |
| 1440200_at | 9630031F12Rik | RIKEN cDNA 9630031F12 gene | 1.06 | 0.811 | -1.69 | 0.382 | -1.03 | 0.848 | -1.35 | 0.195 | -0.75 |

| | | | | | | | | | | | |
|---|---|---|---|---|---|---|---|---|---|---|---|
| 1446683_at | Eps15l1 | epidermal growth factor receptor pathway substrate 15-like 1 | -1.08 | 0.63 | -1.11 | 0.774 | -1.25 | 0.314 | 1.01 | 0.929 | -0.61 |
| 1438992_x_at | Atf4 | activating transcription factor 4 | -1.23 | 0.477 | -1.02 | 0.861 | -1.21 | 0.139 | 1.06 | 0.714 | -0.6 |
| 1452197_at | Smc4 | structural maintenance of chromosomes 4 | -1.1 | 0.64 | -1.06 | 0.276 | -1.3 | 0.052 | 1.29 | 0.388 | -0.54 |
| 1452895_at | Fbxo45 | F-box protein 45 | -1.05 | 0.657 | -1.27 | 0.146 | -1.13 | 0.45 | 1.37 | 0.276 | -0.52 |
| 1428791_at | Ube2h | ubiquitin-conjugating enzyme E2H | -1.2 | 0.058 | -1.26 | 0.199 | -1 | 0.987 | 1.27 | 0.147 | -0.55 |
| 1423845_at | Csdc2 | cold shock domain containing C2, RNA binding | 1.37 | 0.422 | -2.25 | 0.047 | -1.24 | 0.618 | -2.14 | 0.118 | -1.06 |
| 1452005_at | Dlat | dihydrolipoamide S-acetyltransferase (E2 component of pyruvate dehydrogenase | -1.21 | 0.286 | -1.33 | 0.231 | 1.04 | 0.87 | -1.01 | 0.947 | -0.63 |
| 1434778_at | --- | Transcribed locus | -1.1 | 0.484 | -1.05 | 0.574 | -1.33 | 0.014 | 1.33 | 0.342 | -0.53 |
| 1441630_at | Ep400 | E1A binding protein p400 | -1.06 | 0.856 | -1.28 | 0.471 | -1.11 | 0.72 | 1.07 | 0.383 | -0.6 |
| 1447342_at | --- | --- | -2.06 | 0.492 | 1.6 | 0.403 | -1.89 | 0.335 | -2.45 | 0.224 | -1.2 |
| 1420985_at | Ash1l | ash1 (absent, small, or homeotic)-like (Drosophila) | 1.04 | 0.823 | -1.09 | 0.463 | -1.5 | 0.032 | -1.15 | 0.484 | -0.68 |
| 1426640_s_at | Trib2 | tribbles homolog 2 (Drosophila) | -1.47 | 0.045 | 1.16 | 0.481 | -1.27 | 0.013 | -2.01 | 0.06 | -0.9 |
| 1446894_at | Arnt2 | Aryl hydrocarbon receptor nuclear translocator 2 | -1.17 | 0.816 | -1.24 | 0.71 | -1.04 | 0.923 | 1.22 | 0.756 | -0.56 |
| 1458333_x_at | Utf1 | undifferentiated embryonic cell transcription factor 1 | 1.07 | 0.792 | -1.24 | 0.743 | -1.35 | 0.627 | -2.35 | 0.256 | -0.97 |
| 1422094_a_at | Zfp329 | zinc finger protein 329 | -1.06 | 0.75 | 1.1 | 0.432 | -1.73 | 0.012 | -1.83 | 0.015 | -0.88 |
| 1446144_at | Pex2 | peroxin 2 | -1.64 | 0.065 | 1.07 | 0.821 | -1.06 | 0.829 | -1.25 | 0.581 | -0.72 |
| 1453208_at | 2700089E24Rik | RIKEN cDNA 2700089E24 gene | 1.04 | 0.626 | -1.47 | 0.043 | -1.11 | 0.422 | -1.04 | 0.862 | -0.65 |
| 1441392_at | --- | Transcribed locus | -1.85 | 0.306 | 1.36 | 0.262 | -1.4 | 0.241 | -1.2 | 0.69 | -0.77 |
| 1460670_at | Riok3 | RIO kinase 3 (yeast) | -1.14 | 0.058 | -1.1 | 0.25 | -1.19 | 0.21 | 1.06 | 0.53 | -0.59 |
| 1419458_at | Rgnef | Rho-guanine nucleotide exchange factor | -1.17 | 0.511 | -1.01 | 0.911 | -1.28 | 0.103 | 1.92 | 0.218 | -0.39 |
| 1458208_s_at | --- | --- | 1.03 | 0.805 | -1.15 | 0.385 | -1.39 | 0.404 | -1.7 | 0.235 | -0.8 |
| 1450422_a_at | Kdelc1 | KDEL (Lys-Asp-Glu-Leu) containing 1 | -1.01 | 0.914 | -1.15 | 0.307 | -1.31 | 0.247 | 1.16 | 0.393 | -0.58 |
| 1437668_at | Ccrl1 | Chemokine (C-C motif) receptor-like 1 | 1.24 | 0.463 | -1.41 | 0.218 | -1.49 | 0.039 | -1.57 | 0.227 | -0.81 |
| 1429251_at | Prdm2 | PR domain containing 2, with ZNF domain | -1.2 | 0.562 | -1.5 | 0.021 | 1.12 | 0.386 | -1.24 | 0.248 | -0.7 |
| 1435768_at | Arid4b | AT rich interactive domain 4B (Rbp1 like) | -1.12 | 0.358 | 1.01 | 0.965 | -1.38 | 0.112 | -1.32 | 0.359 | -0.7 |
| 1424079_x_at | 2900073H19Rik | RIKEN cDNA 2900073H19 gene | 1.05 | 0.612 | -1.27 | 0.313 | -1.27 | 0.076 | -1.36 | 0.088 | -0.71 |
| 1444676_at | --- | --- | -2.13 | 0.16 | 1.27 | 0.422 | -1.14 | 0.67 | -1.26 | 0.579 | -0.81 |
| 1428872_at | 4121402D02Rik | RIKEN cDNA 4121402D02 gene | -1.84 | 0.306 | -1.5 | 0.239 | 1.42 | 0.483 | -1.96 | 0.104 | -0.97 |
| 1458200_at | Btbd14b | BTB (POZ) domain containing 14B | -1.19 | 0.543 | -1.19 | 0.713 | -1.06 | 0.654 | 1.25 | 0.376 | -0.55 |
| 1417857_at | Mmaa | methylmalonic aciduria (cobalamin deficiency) type A | -1.33 | 0.306 | -1.18 | 0.021 | 1.02 | 0.915 | -1.26 | 0.251 | -0.69 |
| 1433545_s_at | Acad11 | acyl-Coenzyme A dehydrogenase family, member 11 | -1.05 | 0.695 | -1.49 | 0.061 | 1 | 1 | -1.32 | 0.281 | -0.72 |
| 1444685_at | --- | --- | -1.23 | 0.375 | -1.42 | 0.083 | 1.1 | 0.686 | -1.15 | 0.583 | -0.67 |
| 1428454_at | Bcas3 | breast carcinoma amplified sequence 3 | -1.38 | 0.41 | -1.04 | 0.843 | -1.08 | 0.831 | 1.07 | 0.824 | -0.6 |
| 1438836_at | Eftud2 | Elongation factor Tu GTP binding domain containing 2 | -1.81 | 0.27 | -1.58 | 0.505 | 1.44 | 0.418 | -2.25 | 0.136 | -1.05 |
| 1443885_at | --- | 7 days neonate cerebellum cDNA, RIKEN full-length enriched library, clone:A730 | -3.44 | 0.042 | -1.19 | 0.803 | 1.49 | 0.417 | -1.57 | 0.428 | -1.18 |
| 1449094_at | Gja7 | gap junction membrane channel protein alpha 7 | 1.1 | 0.561 | -1.27 | 0.484 | -1.37 | 0.025 | -1.79 | 0.242 | -0.83 |
| 1440956_at | Ccdc94 | coiled-coil domain containing 94 | -1.15 | 0.391 | -1.31 | 0.122 | -1.01 | 0.945 | 1.03 | 0.872 | -0.61 |
| 1416850_s_at | Zcd1 | zinc finger, CDGSH-type domain 1 | -1.13 | 0.004 | -1.25 | 0.217 | -1.06 | 0.607 | 1.19 | 0.41 | -0.56 |
| 1429488_at | Zdhhc21 | zinc finger, DHHC domain containing 21 | -1.09 | 0.68 | 1.01 | 0.875 | -1.45 | 0.141 | -1.23 | 0.487 | -0.69 |
| 1458830_at | Fgf14 | Fibroblast growth factor 14 | -1.17 | 0.444 | 1.08 | 0.901 | -1.45 | 0.555 | -1.92 | 0.238 | -0.86 |
| 1437120_at | Snx30 | sorting nexin family member 30 | 1.02 | 0.648 | -1.21 | 0.371 | -1.29 | 0.262 | -1.51 | 0.247 | -0.75 |
| 1438769_a_at | Thyn1 | thymocyte nuclear protein 1 | -1.29 | 0.302 | -1.09 | 0.46 | -1.07 | 0.676 | 1.01 | 0.936 | -0.61 |
| 1431746_a_at | Ube1c | ubiquitin-activating enzyme E1C | -1.18 | 0.34 | -1.29 | 0.229 | -1 | 1 | 1.12 | 0.709 | -0.59 |
| 1427491_at | Nsun6 | NOL1/NOP2/Sun domain family 6 | 1.02 | 0.937 | -1.17 | 0.361 | -1.33 | 0.33 | -1.29 | 0.233 | -0.69 |
| 1457301_at | AI931714 | expressed sequence AI931714 | 1.26 | 0.644 | -1.37 | 0.531 | -1.56 | 0.117 | -1.59 | 0.374 | -0.82 |
| 1429800_at | 9130221H12Rik | RIKEN cDNA 9130221H12 gene | -1.24 | 0.199 | -1.23 | 0.262 | 1 | 0.981 | -1.06 | 0.376 | -0.63 |
| 1459973_x_at | Dpp4 | dipeptidylpeptidase 4 | 1.14 | 0.455 | -1.1 | 0.703 | -1.74 | 0.013 | -3.18 | 0.026 | -1.22 |
| 1432678_at | Itgav | integrin alpha V | -1.19 | 0.831 | -1.07 | 0.903 | -1.18 | 0.74 | 1.14 | 0.612 | -0.57 |
| 1433853_at | Mib1 | mindbomb homolog 1 (Drosophila) | -1.33 | 0.633 | -1.39 | 0.344 | 1.15 | 0.543 | -1.51 | 0.133 | -0.77 |
| 1418820_s_at | Zcchc10 | zinc finger, CCHC domain containing 10 | -1.16 | 0.56 | -1.15 | 0.362 | -1.11 | 0.511 | 1 | 0.994 | -0.61 |
| 1420152_at | --- | --- | -1.15 | 0.74 | -1.31 | 0.644 | -1.01 | 0.978 | 1.24 | 0.158 | -0.56 |
| 1435947_at | 2810455D13Rik | RIKEN cDNA 2810455D13 gene | -1.04 | 0.709 | -1.01 | 0.98 | -1.49 | 0.015 | 1.26 | 0.524 | -0.57 |
| 1450744_at | Ell2 | elongation factor RNA polymerase II 2 | -1.27 | 0.402 | -1.11 | 0.564 | -1.06 | 0.603 | 1.82 | 0.033 | -0.41 |

| | | | | | | | | | | | |
|---|---|---|---|---|---|---|---|---|---|---|---|
| 1452159_at | 2310001A20Rik | RIKEN cDNA 2310001A20 gene | -1.16 | 0.068 | -1.07 | 0.444 | -1.22 | 0.098 | 1.13 | 0.518 | -0.58 |
| 1444691_at | Prkcn | protein kinase C, nu | -1.05 | 0.903 | -1.54 | 0.047 | 1.02 | 0.961 | -1.35 | 0.599 | -0.73 |
| 1448236_at | Rdx | radixin | -1.06 | 0.639 | -1.1 | 0.491 | -1.3 | 0.211 | 1.15 | 0.239 | -0.58 |
| 1435643_x_at | Ubb /// Gm1821 | ubiquitin B /// gene model 1821, (NCBI) | 1.01 | 0.878 | -1.19 | 0.347 | -1.29 | 0.025 | -1.14 | 0.034 | -0.65 |
| 1424253_at | 1810073G14Rik | RIKEN cDNA 1810073G14 gene | -1.19 | 0.469 | 1.06 | 0.583 | -1.38 | 0.121 | -1.09 | 0.725 | -0.65 |
| 1424074_at | Btf3l4 | basic transcription factor 3-like 4 | -1.17 | 0.11 | -1.03 | 0.863 | -1.25 | 0.173 | 1.23 | 0.367 | -0.56 |
| 1426738_at | Dgkz | diacylglycerol kinase zeta | -1.25 | 0.383 | -1.33 | 0.291 | 1.08 | 0.797 | -2.09 | 0.06 | -0.9 |
| 1421953_at | Crkl | v-crk sarcoma virus CT10 oncogene homolog (avian)-like | -1.2 | 0.661 | -1.92 | 0.445 | 1.27 | 0.724 | -1.51 | 0.402 | -0.84 |
| 1441220_at | --- | --- | -1.29 | 0.177 | 1.05 | 0.938 | -1.25 | 0.296 | -1.27 | 0.613 | -0.69 |
| 1458555_at | --- | --- | 1.06 | 0.917 | -1.52 | 0.532 | -1.1 | 0.814 | -2.22 | 0.398 | -0.95 |
| 1429454_at | Gapvd1 | GTPase activating protein and VPS9 domains 1 | -1.1 | 0.31 | -1.23 | 0.005 | -1.11 | 0.257 | 1.36 | 0.227 | -0.52 |
| 1449917_at | Pitx3 | paired-like homeodomain transcription factor 3 | -1.69 | 0.39 | -1.39 | 0.5 | 1.31 | 0.574 | -1.68 | 0.42 | -0.86 |
| 1455616_at | AI586002 | expressed sequence AI586002 | -1.11 | 0.496 | 1.1 | 0.497 | -1.62 | 0.094 | -1.16 | 0.492 | -0.7 |
| 1457634_at | 4930488E11Rik /// | RIKEN cDNA 4930488E11 gene /// similar to thymosin beta-like protein /// similar | -1.3 | 0.584 | 1.08 | 0.864 | -1.29 | 0.574 | -1.25 | 0.613 | -0.69 |
| 1419960_at | C330003B14Rik | RIKEN cDNA C330003B14 gene | -1.51 | 0.648 | 1.28 | 0.558 | -1.48 | 0.421 | -1.03 | 0.96 | -0.68 |
| 1427539_a_at | Zwint | ZW10 interactor | -1.05 | 0.808 | -1.05 | 0.664 | -1.4 | 0.048 | 1.44 | 0.349 | -0.51 |
| 1460359_at | Armcx3 | armadillo repeat containing, X-linked 3 | -1.05 | 0.69 | 1.01 | 0.953 | -1.53 | 0.054 | -1.31 | 0.197 | -0.72 |
| 1433265_at | 2810416A17Rik | RIKEN cDNA 2810416A17 gene | 1.41 | 0.541 | -1.3 | 0.723 | -2.25 | 0.288 | -1.95 | 0.254 | -1.02 |
| 1449403_at | Pde9a | phosphodiesterase 9A | -1.1 | 0.109 | -1.26 | 0.053 | -1.08 | 0.66 | 1.1 | 0.652 | -0.58 |
| 1429885_at | 4930431P19Rik | RIKEN cDNA 4930431P19 gene | -2.04 | 0.325 | 1.25 | 0.162 | -1.12 | 0.762 | -1.26 | 0.045 | -0.79 |
| 1447676_x_at | S100a16 | S100 calcium binding protein A16 | -1.05 | 0.136 | 1.06 | 0.57 | -1.65 | 0.01 | -1.21 | 0.503 | -0.71 |
| 1419975_at | --- | --- | -1.14 | 0.542 | 1.08 | 0.7 | -1.51 | 0.195 | -1.29 | 0.349 | -0.71 |
| 1424549_at | Degs2 | degenerative spermatocyte homolog 2 (Drosophila), lipid desaturase | -1.12 | 0.474 | -2.11 | 0.328 | 1.26 | 0.045 | -2.08 | 0.227 | -1.01 |
| 1429491_s_at | Rif1 /// LOC671598 | Rap1 interacting factor 1 homolog (yeast) /// similar to Telomere-associated prote | -1.08 | 0.246 | -1.07 | 0.7 | -1.31 | 0.079 | 1.39 | 0.153 | -0.52 |
| 1450938_at | --- | --- | -1.21 | 0.169 | 1.02 | 0.914 | -1.28 | 0.086 | -1.11 | 0.633 | -0.64 |
| 1450714_at | Azin1 | antizyme inhibitor 1 | -1.13 | 0.595 | -1.13 | 0.508 | -1.17 | 0.002 | 2.57 | 0.127 | -0.22 |
| 1435262_at | --- | Transcribed locus | -1.27 | 0.458 | -1.12 | 0.22 | -1.05 | 0.696 | 1.12 | 0.465 | -0.58 |
| 1420158_s_at | Abcf1 | ATP-binding cassette, sub-family F (GCN20), member 1 | -1.39 | 0.238 | -1.28 | 0.602 | 1.12 | 0.628 | -1.64 | 0.098 | -0.8 |
| 1432042_a_at | Smu1 | smu-1 suppressor of mec-8 and unc-52 homolog (C. elegans) | -1.11 | 0.43 | -1.41 | 0.09 | 1.01 | 0.952 | -1.25 | 0.335 | -0.69 |
| 1418792_at | Sh3gl2 | SH3-domain GRB2-like 2 | 1.07 | 0.641 | -1.37 | 0.713 | -1.21 | 0.467 | -1.08 | 0.909 | -0.65 |
| 1448288_at | Nfib | nuclear factor I/B | 1.15 | 0.43 | -1.13 | 0.481 | -1.69 | 0.022 | -1.1 | 0.828 | -0.69 |
| 1441266_at | Strn3 | Striatin, calmodulin binding protein 3 | 1.08 | 0.131 | -1.09 | 0.372 | -1.59 | 0.001 | -1.06 | 0.535 | -0.67 |
| 1455652_at | AW124694 | expressed sequence AW124694 | -1.1 | 0.571 | -1.26 | 0.707 | -1.08 | 0.332 | 1.08 | 0.702 | -0.59 |
| 1459678_at | LOC497255 | hypothetical LOC497255 | 1.07 | 0.626 | -1.29 | 0.162 | -1.29 | 0.012 | -1.23 | 0.377 | -0.68 |
| 1425998_at | Sytl4 | synaptotagmin-like 4 | 1.73 | 0.33 | -2.33 | 0.323 | -2.13 | 0.239 | -1.57 | 0.588 | -1.08 |
| 1426969_at | Trim23 | tripartite motif protein 23 | -1.14 | 0.592 | -1.1 | 0.199 | -1.19 | 0.33 | 1.27 | 0.292 | -0.54 |
| 1460299_at | Hlxb9 | homeobox gene HB9 | -2.04 | 0.046 | -1.07 | 0.893 | 1.2 | 0.739 | -1.99 | 0.196 | -0.97 |
| 1448322_a_at | Cox4i1 | cytochrome c oxidase subunit IV isoform 1 | -1.16 | 0.098 | -1.33 | 0.047 | 1.01 | 0.797 | -1.07 | 0.394 | -0.64 |
| 1435471_at | Zfp708 | zinc finger protein 708 | -1.41 | 0.124 | 1.04 | 0.832 | -1.14 | 0.454 | -2 | 0.066 | -0.88 |
| 1424166_at | Msh3 | mutS homolog 3 (E. coli) | -1.21 | 0.473 | -1.11 | 0.66 | -1.11 | 0.633 | 1.08 | 0.579 | -0.59 |
| 1445234_at | C130030K03Rik | RIKEN cDNA C130030K03 gene | -1.34 | 0.096 | -1.99 | 0.348 | 1.38 | 0.389 | -2.06 | 0.263 | -1 |
| 1455744_at | Gpr178 | G protein-coupled receptor 178 | -1.16 | 0.025 | -1.11 | 0.708 | -1.17 | 0.582 | 1.11 | 0.63 | -0.58 |
| 1420950_at | Znrf1 | zinc and ring finger 1 | 1.1 | 0.539 | -1.44 | 0.116 | -1.21 | 0.137 | -1.36 | 0.344 | -0.72 |
| 1420471_at | Hcrt | hypocretin | -1.34 | 0.319 | -1.24 | 0.711 | 1.07 | 0.69 | -1.26 | 0.673 | -0.69 |
| 1444903_at | 6-Mar | Membrane-associated ring finger (C3HC4) 6 | 1.16 | 0.629 | -1.4 | 0.249 | -1.32 | 0.329 | -1.34 | 0.444 | -0.73 |
| 1444793_at | --- | --- | -2.22 | 0.532 | 1.35 | 0.497 | -1.21 | 0.637 | -1.25 | 0.696 | -0.83 |
| 1455300_at | E130014J05Rik | RIKEN cDNA E130014J05 gene | -1.09 | 0.737 | 1 | 0.934 | -1.41 | 0.021 | -1.3 | 0.473 | -0.7 |
| 1447790_at | --- | --- | -1.57 | 0.449 | 1 | 0.999 | -1.01 | 0.985 | -1.62 | 0.17 | -0.8 |
| 1455181_at | Rasa2 | RAS p21 protein activator 2 | 1.02 | 0.887 | -1.06 | 0.581 | -1.51 | 0 | -1.81 | 0.101 | -0.84 |
| 1437184_at | Guf1 | GUF1 GTPase homolog (S. cerevisiae) | -1.35 | 0.088 | 1.07 | 0.82 | -1.23 | 0.474 | -1.08 | 0.848 | -0.65 |
| 1438908_at | Map3k12 | mitogen activated protein kinase kinase kinase 12 | -1.27 | 0.607 | 1.14 | 0.633 | -1.43 | 0.32 | -1.1 | 0.788 | -0.66 |
| 1442539_at | Wwc2 | WW, C2 and coiled-coil domain containing 2 | 1.14 | 0.255 | -1.35 | 0.494 | -1.34 | 0.373 | -1.34 | 0.684 | -0.72 |
| 1428348_at | Zfp336 | zinc finger protein 336 | -1.31 | 0.111 | -1.02 | 0.636 | -1.13 | 0.234 | 1.13 | 0.358 | -0.58 |

| | | | | | | | | | | | |
|---|---|---|---|---|---|---|---|---|---|---|---|
| 1453212_at | Zfp383 | zinc finger protein 383 | -1.16 | 0.197 | 1.03 | 0.864 | -1.36 | 0.095 | -1.81 | 0.105 | -0.82 |
| 1453131_at | Cd300lg | CD300 antigen like family member G | 1.03 | 0.914 | -1.25 | 0.142 | -1.25 | 0.497 | -1.67 | 0.33 | -0.78 |
| 1425512_at | Map2k7 | mitogen activated protein kinase kinase 7 | -1.01 | 0.927 | -1.19 | 0.118 | -1.26 | 0.364 | 1.1 | 0.703 | -0.59 |
| 1422447_at | Ins1 | insulin I | -1.33 | 0.44 | -1.28 | 0.23 | 1.09 | 0.785 | -1.06 | 0.88 | -0.64 |
| 1446396_at | Angptl1 | Angiopoietin-like 1 | -1.7 | 0.523 | -1.23 | 0.566 | 1.23 | 0.672 | -2 | 0.218 | -0.93 |
| 1442898_at | LOC671579 | hypothetical protein LOC671579 | -1.02 | 0.953 | -1.46 | 0.127 | -1.04 | 0.913 | 1.12 | 0.865 | -0.6 |
| 1444722_at | --- | --- | -1.03 | 0.701 | -1.01 | 0.962 | -1.48 | 0.041 | 1.15 | 0.211 | -0.6 |
| 1421659_at | Adra1a | adrenergic receptor, alpha 1a | -1.25 | 0.028 | -1.4 | 0.452 | 1.11 | 0.79 | -1.76 | 0.434 | -0.82 |
| 1438995_at | 6330500D04Rik | RIKEN cDNA 6330500D04 gene | -1.27 | 0.82 | -1.19 | 0.688 | 1.01 | 0.984 | -2.3 | 0.074 | -0.94 |
| 1443619_at | Tmem30a | transmembrane protein 30A | 1.16 | 0.387 | -1.15 | 0.472 | -1.66 | 0.026 | -1.36 | 0.221 | -0.75 |
| 1440138_at | Ahdc1 | AT hook, DNA binding motif, containing 1 | -1.8 | 0.501 | -1.91 | 0.103 | 1.55 | 0.384 | -2 | 0.137 | -1.04 |
| 1441075_at | Nostrin | nitric oxide synthase trafficker | -1.07 | 0.677 | 1.1 | 0.63 | -1.69 | 0.005 | -1.69 | 0.177 | -0.84 |
| 1417535_at | Fbxo25 | F-box only protein 25 | 1 | 0.977 | -1.09 | 0.488 | -1.41 | 0.011 | -1.1 | 0.151 | -0.65 |
| 1441410_at | D2Ertd112e | DNA segment, Chr 2, ERATO Doi 112, expressed | 1.01 | 0.985 | -1.4 | 0.162 | -1.11 | 0.47 | -1.51 | 0.067 | -0.75 |
| 1452449_at | Hmbox1 | homeobox containing 1 | -1.45 | 0.333 | -1.28 | 0.074 | 1.16 | 0.337 | -1.09 | 0.426 | -0.67 |
| 1448147_at | Tnfrsf19 | tumor necrosis factor receptor superfamily, member 19 | -1.17 | 0.278 | -1.02 | 0.97 | -1.25 | 0.318 | 1.33 | 0.416 | -0.53 |
| 1444291_at | Lysmd4 | LysM, putative peptidoglycan-binding, domain containing 4 | 1.14 | 0.027 | -1.57 | 0.33 | -1.17 | 0.799 | -1.14 | 0.79 | -0.68 |
| 1423885_at | Lamc1 | laminin, gamma 1 | 1.02 | 0.957 | -1.08 | 0.76 | -1.46 | 0.091 | -1.07 | 0.636 | -0.65 |
| 1437469_at | Zfp750 | zinc finger protein 750 | -1.06 | 0.888 | -1.55 | 0.288 | 1.04 | 0.824 | -2.78 | 0.301 | -1.09 |
| 1441219_at | --- | 11 days pregnant adult female ovary and uterus cDNA, RIKEN full-length enriche | -2.09 | 0.156 | -1.97 | 0.257 | 1.64 | 0.354 | -1.02 | 0.937 | -0.86 |
| 1431535_at | Plb1 | phospholipase B1 | 1.07 | 0.893 | -1.16 | 0.677 | -1.43 | 0.113 | -1.5 | 0.168 | -0.76 |
| 1421982_a_at | Unc50 | unc-50 homolog (C. elegans) | -1.13 | 0.149 | -1.15 | 0.058 | -1.15 | 0.213 | 1.03 | 0.693 | -0.6 |
| 1419462_s_at | Gtl3 | gene trap locus 3 | -1.19 | 0.222 | -1.27 | 0.109 | -1 | 0.981 | 1.39 | 0.083 | -0.52 |
| 1446925_at | Btrc | beta-transducin repeat containing protein | -1.73 | 0.414 | 1.06 | 0.756 | -1.01 | 0.984 | -1.44 | 0.198 | -0.78 |
| 1448931_at | F2rl1 | coagulation factor II (thrombin) receptor-like 1 | 1.02 | 0.887 | -1.29 | 0.694 | -1.19 | 0.289 | -1.59 | 0.278 | -0.76 |
| 1432762_at | 9530025H10Rik | RIKEN cDNA 9530025H10 gene | -1.04 | 0.96 | -1.16 | 0.524 | -1.25 | 0.464 | 1.35 | 0.422 | -0.52 |
| 1417479_at | 4930511A21Rik | RIKEN cDNA 4930511A21 gene | -1.21 | 0.33 | -1.19 | 0.515 | -1.04 | 0.807 | 1.13 | 0.541 | -0.58 |
| 1452211_at | Psme4 | proteasome (prosome, macropain) activator subunit 4 | -1.2 | 0.163 | -1.16 | 0.002 | -1.08 | 0.208 | 1.32 | 0.444 | -0.53 |
| 1437854_at | Lrrc18 | Leucine rich repeat containing 18 | -1.24 | 0.54 | 1.12 | 0.685 | -1.43 | 0.619 | -1.97 | 0.216 | -0.88 |
| 1453805_at | Fuca1 | Fucosidase, alpha-L- 1, tissue | -1.08 | 0.908 | 1.19 | 0.497 | -1.96 | 0.281 | -1.4 | 0.349 | -0.81 |
| 1437995_x_at | 7-Sep | septin 7 | -1.47 | 0.117 | 1.02 | 0.894 | -1.08 | 0.686 | -1.18 | 0.583 | -0.68 |
| 1456599_at | Nxt2 | nuclear transport factor 2-like export factor 2 | -1.18 | 0.582 | -1.23 | 0.04 | -1.03 | 0.871 | 1.52 | 0.412 | -0.48 |
| 1439751_at | Zfp708 | zinc finger protein 708 | 1.09 | 0.632 | -1.31 | 0.418 | -1.29 | 0.64 | -1.28 | 0.468 | -0.7 |
| 1452360_a_at | Jarid1a | jumonji, AT rich interactive domain 1A (Rbp2 like) | -1.04 | 0.872 | -1.05 | 0.827 | -1.4 | 0.312 | 1.11 | 0.468 | -0.59 |
| 1433489_s_at | Fgfr2 | fibroblast growth factor receptor 2 | -1.06 | 0.805 | 1.01 | 0.936 | -1.48 | 0.037 | -1.38 | 0.213 | -0.73 |
| 1450638_at | Pdcd5 | programmed cell death 5 | 1.01 | 0.934 | -1.27 | 0.003 | -1.2 | 0.087 | -1.23 | 0.137 | -0.67 |
| 1460018_at | D11Ertd759e | DNA segment, Chr 11, ERATO Doi 759, expressed | -1.11 | 0.683 | -1.14 | 0.699 | -1.18 | 0.517 | 1.14 | 0.322 | -0.57 |
| 1433758_at | Nisch | nischarin | -1.07 | 0.586 | 1.05 | 0.506 | -1.55 | 0.084 | -1.25 | 0.282 | -0.7 |
| 1437207_at | Rnf170 | ring finger protein 170 | 1.02 | 0.905 | -1.2 | 0.699 | -1.29 | 0.196 | -1.02 | 0.907 | -0.62 |
| 1451920_a_at | Recc1 | replication factor C 1 | -1.08 | 0.024 | -1.09 | 0.352 | -1.26 | 0.21 | 1.14 | 0.539 | -0.57 |
| 1443947_at | Lims1 | LIM and senescent cell antigen-like domains 1 | 1.1 | 0.748 | -1.1 | 0.858 | -1.62 | 0.108 | -1.19 | 0.245 | -0.7 |
| 1456362_at | Cyp11b1 | cytochrome P450, family 11, subfamily b, polypeptide 1 | -1.32 | 0.308 | 1.12 | 0.874 | -1.33 | 0.21 | -1.28 | 0.585 | -0.7 |
| 1433017_at | 8430420C20Rik | RIKEN cDNA 8430420C20 gene | -1.87 | 0.333 | -1.19 | 0.672 | 1.26 | 0.704 | -2.28 | 0.337 | -1.02 |
| 1436024_at | A930025D01Rik | RIKEN cDNA A930025D01 gene | -1.17 | 0.56 | -1.7 | 0.208 | 1.19 | 0.743 | -1.01 | 0.974 | -0.67 |
| 1415897_a_at | Mgst1 | microsomal glutathione S-transferase 1 | -1.01 | 0.939 | -1.16 | 0.524 | -1.29 | 0.003 | 1.06 | 0.636 | -0.6 |
| 1450448_at | Stc1 | stanniocalcin 1 | -1.21 | 0.362 | -1.17 | 0.807 | -1.05 | 0.653 | 1.33 | 0.447 | -0.53 |
| 1434134_at | Wdr42a | WD repeat domain 42A | -1.08 | 0.312 | -1.29 | 0.164 | -1.08 | 0.375 | 1 | 0.991 | -0.61 |
| 1456543_at | Prokr1 | prokineticin receptor 1 | -1.02 | 0.982 | -1.39 | 0.708 | -1.08 | 0.838 | 4.63 | 0.226 | 0.29 |
| 1440941_at | LOC552912 | hypothetical LOC552912 | -1.31 | 0.347 | -1.13 | 0.55 | -1.02 | 0.934 | 1.13 | 0.714 | -0.58 |
| 1429423_at | 4930518I15Rik | RIKEN cDNA 4930518I15 gene | 1.01 | 0.958 | -1.23 | 0.326 | -1.24 | 0.229 | -1.32 | 0.237 | -0.69 |
| 1449069_at | Zfp148 | zinc finger protein 148 | 1.11 | 0.702 | -1.21 | 0.247 | -1.43 | 0.063 | -1.4 | 0.129 | -0.73 |
| 1424204_at | Mrpl13 | mitochondrial ribosomal protein L13 | -1.17 | 0.003 | -1.16 | 0.072 | -1.09 | 0.121 | 1.01 | 0.906 | -0.6 |
| 1451892_at | Kl | klotho | -1.43 | 0.18 | 1.22 | 0.138 | -1.4 | 0.517 | -1.61 | 0.196 | -0.81 |

| | | | | | | | | | | | |
|---|---|---|---|---|---|---|---|---|---|---|---|
| 1432617_at | 4933424C09Rik | RIKEN cDNA 4933424C09 gene | -1.25 | 0.674 | -1.07 | 0.918 | -1.12 | 0.75 | 1.43 | 0.461 | -0.5 |
| 1432201_a_at | Zswim2 | zinc finger, SWIM domain containing 2 | -1.19 | 0.369 | -1.07 | 0.758 | -1.17 | 0.574 | 1.08 | 0.844 | -0.59 |
| 1426388_s_at | Ryk | receptor-like tyrosine kinase | -1.08 | 0.632 | -1.24 | 0.037 | -1.11 | 0.479 | 1.97 | 0.274 | -0.36 |
| 1442215_at | Smo | smoothened homolog (Drosophila) | -1.36 | 0.573 | 1.43 | 0.22 | -2.15 | 0.336 | -1.17 | 0.657 | -0.81 |
| 1453639_s_at | 0610037D15Rik | RIKEN cDNA 0610037D15 gene | -1.15 | 0.801 | 1.05 | 0.677 | -1.39 | 0.338 | -1.54 | 0.31 | -0.76 |
| 1459877_x_at | Rnf185 | Ring finger protein 185 | -1.3 | 0.173 | 1.02 | 0.813 | -1.19 | 0.074 | -1.17 | 0.497 | -0.66 |
| 1454156_at | A230104H11Rik | RIKEN cDNA A230104H11 gene | -1.35 | 0.525 | 1.16 | 0.601 | -1.37 | 0.356 | -1.13 | 0.374 | -0.67 |
| 1442640_at | Slfn5 | Schlafen 5 | 1.21 | 0.723 | -1.32 | 0.499 | -1.52 | 0.168 | -1.64 | 0.266 | -0.81 |
| 1434764_at | Akap11 | A kinase (PRKA) anchor protein 11 | -1.17 | 0.276 | -1.01 | 0.801 | -1.27 | 0.012 | 1.01 | 0.922 | -0.61 |
| 1437479_x_at | Tbx3 | T-box 3 | -1.3 | 0.423 | 1 | 0.997 | -1.16 | 0.658 | -1.35 | 0.56 | -0.7 |
| 1447025_at | Ube2e2 | Ubiquitin-conjugating enzyme E2E 2 (UBC4/5 homolog, yeast) | 1.34 | 0.5 | -1.81 | 0.068 | -1.35 | 0.282 | -1.61 | 0.072 | -0.86 |
| 1422397_a_at | Il15ra | interleukin 15 receptor, alpha chain | -1.47 | 0.015 | -1.39 | 0.099 | 1.23 | 0.31 | -1.14 | 0.329 | -0.69 |
| 1447101_at | Rnf122 | ring finger protein 122 | -1.45 | 0.094 | 1.18 | 0.818 | -1.3 | 0.477 | -1.99 | 0.234 | -0.89 |
| 1440289_at | Gabpb2 | GA repeat binding protein, beta 2 | -1.22 | 0.838 | -1.33 | 0.299 | 1.06 | 0.844 | -1.16 | 0.368 | -0.66 |
| 1445853_at | --- | --- | -1.49 | 0.497 | -2.39 | 0.326 | 1.54 | 0.202 | -1.92 | 0.238 | -1.06 |
| 1436705_at | Tmem32 | transmembrane protein 32 | -1.07 | 0.068 | -1.06 | 0.611 | -1.33 | 0.027 | 1.14 | 0.164 | -0.58 |
| 1438454_at | B430203M17Rik | RIKEN cDNA B430203M17 gene | 1.02 | 0.934 | -1.04 | 0.821 | -1.53 | 0.004 | -1.77 | 0.1 | -0.83 |
| 1459019_at | Sprn | shadow of prion protein | -1.18 | 0.707 | -1.26 | 0.507 | -1.01 | 0.945 | 1 | 0.998 | -0.61 |
| 1440841_at | Ywhae | Tyrosine 3-monooxygenase/tryptophan 5-monooxygenase activation protein, eps | 1.01 | 0.971 | -1.14 | 0.087 | -1.35 | 0.104 | -1.1 | 0.811 | -0.65 |
| 1435497_at | 5730590G19Rik | RIKEN cDNA 5730590G19 gene | -1.26 | 0.037 | -1.03 | 0.885 | -1.15 | 0.695 | 3.82 | 0.187 | 0.1 |
| 1418191_at | Usp18 | ubiquitin specific peptidase 18 | -1.23 | 0.546 | -1.45 | 0.093 | 1.13 | 0.612 | -1.42 | 0.103 | -0.74 |
| 1454061_at | Thumpd3 | THUMP domain containing 3 | 1.17 | 0.437 | -1.5 | 0.283 | -1.25 | 0.146 | -1.22 | 0.022 | -0.7 |
| 1460392_a_at | Eny2 | enhancer of yellow 2 homolog (Drosophila) | -1.16 | 0.044 | -1.03 | 0.583 | -1.25 | 0.004 | 1.35 | 0.262 | -0.52 |
| 1423339_s_at | Ccdc16 | coiled-coil domain containing 16 | 1.05 | 0.653 | -1.14 | 0.79 | -1.43 | 0.091 | -1 | 0.993 | -0.63 |
| 1445397_at | --- | --- | -1.08 | 0.577 | -1.29 | 0.652 | -1.07 | 0.836 | 1.08 | 0.825 | -0.59 |
| 1457467_at | Pknox1 | Pbx/knotted 1 homeobox | -1.48 | 0.039 | -1.09 | 0.64 | 1.04 | 0.843 | -1.04 | 0.828 | -0.64 |
| 1448809_at | Cse1l | chromosome segregation 1-like (S. cerevisiae) | -1.13 | 0.054 | -1.07 | 0.363 | -1.24 | 0.042 | 1.01 | 0.944 | -0.61 |
| 1453277_at | 3021401N23Rik | RIKEN cDNA 3021401N23 gene | 1.1 | 0.707 | -1.45 | 0.294 | -1.18 | 0.753 | -1.34 | 0.411 | -0.72 |
| 1443465_at | --- | --- | -1.01 | 0.92 | 1.08 | 0.843 | -1.77 | 0.149 | -1.14 | 0.507 | -0.71 |
| 1453728_a_at | Mrps17 | mitochondrial ribosomal protein S17 | -1.16 | 0.165 | -1.12 | 0.075 | -1.13 | 0.312 | 1 | 0.971 | -0.6 |
| 1430837_a_at | Mbd1 | methyl-CpG binding domain protein 1 | -1.49 | 0.104 | -1.1 | 0.852 | 1.06 | 0.851 | -1.56 | 0.104 | -0.78 |
| 1416987_at | Elp4 | elongation protein 4 homolog (S. cerevisiae) | -1.01 | 0.953 | -1.5 | 0.063 | -1.03 | 0.935 | 1.24 | 0.627 | -0.57 |
| 1451748_a_at | Setd3 | SET domain containing 3 | -1.29 | 0.179 | -1.15 | 0.071 | -1.01 | 0.928 | 1.31 | 0.294 | -0.53 |
| 1457268_at | Dot1l | DOT1-like, histone H3 methyltransferase (S. cerevisiae) | -1.13 | 0.637 | -1.22 | 0.108 | -1.08 | 0.705 | 1.1 | 0.722 | -0.58 |
| 1424402_at | Rufy3 | RUN and FYVE domain containing 3 | -1.11 | 0.62 | 1.04 | 0.84 | -1.45 | 0.087 | -1.11 | 0.454 | -0.66 |
| 1421127_at | Tmem42 | transmembrane protein 42 | 1.04 | 0.884 | -1.17 | 0.042 | -1.36 | 0.01 | -1.87 | 0.063 | -0.84 |
| 1451791_at | Tfpi | tissue factor pathway inhibitor | -1.29 | 0.018 | 1.2 | 0.323 | -1.52 | 0.006 | -1.31 | 0.504 | -0.73 |
| 1428822_a_at | Snx24 | sorting nexing 24 | -1.26 | 0.26 | 1.11 | 0.235 | -1.36 | 0.003 | -1.06 | 0.753 | -0.65 |
| 1459530_at | Hsd17b12 | Hydroxysteroid (17-beta) dehydrogenase 12 | 1.36 | 0.693 | -1.5 | 0.091 | -1.64 | 0.265 | -1.72 | 0.102 | -0.88 |
| 1422154_at | Gpr27 | G protein-coupled receptor 27 | -1.09 | 0.874 | -1.92 | 0.372 | 1.19 | 0.409 | -1.13 | 0.59 | -0.74 |
| 1428607_at | Araf | v-raf murine sarcoma 3611 viral oncogene homolog | -1.1 | 0.492 | -1.4 | 0.174 | 1.01 | 0.925 | -1.29 | 0.013 | -0.69 |
| 1458263_at | Cugbp2 | CUG triplet repeat, RNA binding protein 2 | 1.04 | 0.946 | -1.16 | 0.694 | -1.37 | 0.164 | -2.34 | 0.041 | -0.96 |
| 1452474_a_at | Art3 | ADP-ribosyltransferase 3 | -1.02 | 0.844 | -1.19 | 0.52 | -1.23 | 0.513 | 2.18 | 0.004 | -0.31 |
| 1444855_at | Bcl9l | B-cell CLL/lymphoma 9-like | -1.21 | 0.31 | 1.08 | 0.752 | -1.37 | 0.339 | -1.3 | 0.362 | -0.7 |
| 1445800_at | Auh | AU RNA binding protein/enoyl-coenzyme A hydratase | -1.28 | 0.514 | -1.31 | 0.675 | 1.09 | 0.802 | -2.37 | 0.078 | -0.97 |
| 1437358_at | Wdfy1 | WD repeat and FYVE domain containing 1 | -1.05 | 0.2 | -1.13 | 0.515 | -1.26 | 0.071 | 1.14 | 0.513 | -0.57 |
| 1442072_at | C230081A13Rik | RIKEN cDNA C230081A13 gene | -1.03 | 0.95 | -1.4 | 0.464 | -1.05 | 0.875 | 1.27 | 0.594 | -0.55 |
| 1422250_at | Map3k2 | mitogen activated protein kinase kinase kinase 2 | -1.65 | 0.464 | -1.22 | 0.619 | 1.21 | 0.467 | -1.16 | 0.836 | -0.71 |
| 1437695_at | Prokr2 | prokineticin receptor 2 | -1.79 | 0.245 | 1.15 | 0.753 | -1.08 | 0.837 | -1.62 | 0.3 | -0.84 |
| 1457764_at | A530058O07Rik | RIKEN cDNA A530058O07 gene | -1.12 | 0.705 | 1.09 | 0.81 | -1.53 | 0.223 | -1.46 | 0.597 | -0.75 |
| 1428073_a_at | Nup88 | nucleoporin 88 | -1.12 | 0.073 | -1.22 | 0.001 | -1.08 | 0.124 | 1.1 | 0.553 | -0.58 |
| 1448212_at | Tnfsf5ip1 | tumor necrosis factor superfamily, member 5-induced protein 1 | -1.07 | 0.556 | -1.15 | 0.167 | -1.2 | 0.162 | 1.25 | 0.25 | -0.54 |
| 1426293_at | 6330581L23Rik | RIKEN cDNA 6330581L23 gene | 1.15 | 0.412 | -1.15 | 0.413 | -1.63 | 0.011 | -1.77 | 0.077 | -0.85 |

| | | | | | | | | | | | |
|---|---|---|---|---|---|---|---|---|---|---|---|
| 1420661_a_at | 4933439F18Rik | RIKEN cDNA 4933439F18 gene | -1.32 | 0.398 | -1.24 | 0.054 | 1.07 | 0.139 | -1.03 | 0.875 | -0.63 |
| 1459271_at | --- | Transcribed locus | -1.28 | 0.22 | -1.6 | 0.296 | 1.23 | 0.409 | -1.74 | 0.251 | -0.85 |
| 1444940_at | C76411 | expressed sequence C76411 | -1.75 | 0.135 | -2.98 | 0.137 | 1.73 | 0.212 | -1.72 | 0.297 | -1.18 |
| 1447279_at | --- | Transcribed locus | -1.24 | 0.402 | -1.05 | 0.919 | -1.14 | 0.794 | 1.07 | 0.716 | -0.59 |
| 1433616_a_at | 2310028O11Rik | RIKEN cDNA 2310028O11 gene | -1.1 | 0.133 | -1.24 | 0.032 | -1.08 | 0.406 | 1.19 | 0.279 | -0.56 |
| 1446549_at | Kbtbd11 | Kelch repeat and BTB (POZ) domain containing 11 | -1.31 | 0.311 | -2.47 | 0.053 | 1.47 | 0.262 | -1.26 | 0.578 | -0.89 |
| 1418748_at | Casp14 | caspase 14 | 1 | 0.986 | -1.21 | 0.098 | -1.24 | 0.489 | -1.41 | 0.418 | -0.71 |
| 1418351_a_at | Dnmt3b | DNA methyltransferase 3B | -1.03 | 0.888 | -1.12 | 0.733 | -1.29 | 0.245 | 1.42 | 0.224 | -0.51 |
| 1428308_at | Pdrg1 | p53 and DNA damage regulated 1 | -1.29 | 0.074 | -1.18 | 0.376 | 1.01 | 0.951 | -1.42 | 0.282 | -0.72 |
| 1449776_at | --- | --- | -1.46 | 0.276 | 1.01 | 0.985 | -1.07 | 0.742 | -1.79 | 0.267 | -0.83 |
| 1442158_at | Mast4 | microtubule associated serine/threonine kinase family member 4 | -2.49 | 0.004 | 1.78 | 0.384 | -2.18 | 0.257 | -1.4 | 0.566 | -1.07 |
| 1425300_at | Dak | dihydroxyacetone kinase 2 homolog (yeast) | -1.12 | 0.756 | -1.24 | 0.316 | -1.06 | 0.8 | 1.64 | 0.379 | -0.45 |
| 1440489_at | Btbd4 | BTB (POZ) domain containing 4 | -1.18 | 0.307 | -1.06 | 0.842 | -1.19 | 0.048 | 1.09 | 0.674 | -0.58 |
| 1434293_at | Hectd3 | HECT domain containing 3 | 1.08 | 0.686 | -1.28 | 0.118 | -1.28 | 0.197 | -1.3 | 0.052 | -0.7 |
| 1427636_at | Tspy-ps | testis specific protein-Y encoded, pseudogene | -1.24 | 0.438 | 1.06 | 0.823 | -1.31 | 0.559 | -1 | 0.991 | -0.62 |
| 1440090_at | Slc25a27 | Solute carrier family 25, member 27 | -1.05 | 0.354 | 1.07 | 0.526 | -1.62 | 0.031 | -1.51 | 0.136 | -0.78 |
| 1453147_at | Polr3e | polymerase (RNA) III (DNA directed) polypeptide E | 1.1 | 0.687 | -1.37 | 0.254 | -1.24 | 0.127 | -1.11 | 0.57 | -0.65 |
| 1416155_at | Hmgb3 | high mobility group box 3 | -1.06 | 0.614 | -1.35 | 0.121 | -1.05 | 0.818 | 4.09 | 0.158 | 0.16 |
| 1454504_at | Kcnip1 | Kv channel-interacting protein 1 | -1.1 | 0.771 | -2.56 | 0.023 | 1.34 | 0.463 | -1.3 | 0.449 | -0.91 |
| 1446533_at | Crtc1 | CREB regulated transcription coactivator 1 | -1.61 | 0.203 | -1.1 | 0.836 | 1.11 | 0.58 | -1.1 | 0.722 | -0.68 |
| 1435303_at | Taf4b | TAF4B RNA polymerase II, TATA box binding protein (TBP)-associated factor | 1.06 | 0.418 | -1.19 | 0.545 | -1.35 | 0.047 | -1.36 | 0.041 | -0.71 |
| 1424210_at | Spfh1 | SPFH domain family, member 1 | -1.2 | 0.309 | 1.05 | 0.394 | -1.33 | 0.09 | -1.09 | 0.351 | -0.64 |
| 1457163_at | D730035F11Rik | RIKEN cDNA D730035F11 gene | -1.23 | 0.456 | 1.14 | 0.689 | -1.46 | 0.12 | -1.85 | 0.259 | -0.85 |
| 1417657_s_at | Dnajc2 | DnaJ (Hsp40) homolog, subfamily C, member 2 | 1.03 | 0.538 | -1.28 | 0.182 | -1.21 | 0.228 | -1.15 | 0.075 | -0.65 |
| 1428323_at | Gpd2 | glycerol phosphate dehydrogenase 2, mitochondrial | -1.3 | 0.437 | -1.37 | 0.3 | 1.14 | 0.622 | -1.11 | 0.237 | -0.66 |
| 1435080_x_at | 5730406M06Rik | RIKEN cDNA 5730406M06 gene | -1.17 | 0.14 | 1.01 | 0.919 | -1.29 | 0.101 | -1.33 | 0.362 | -0.69 |
| 1444450_at | 9530096D07Rik | RIKEN cDNA 9530096D07 gene | -1.28 | 0.356 | -1.35 | 0.281 | 1.11 | 0.215 | -1.31 | 0.416 | -0.71 |
| 1438299_at | 3100002L24Rik /// ! | RIKEN cDNA 3100002L24 gene /// RIKEN cDNA 9230108I15 gene /// similar to g | 1.04 | 0.85 | -1.09 | 0.401 | -1.47 | 0.218 | -1.12 | 0.037 | -0.66 |
| 1420390_s_at | Zfp354a | zinc finger protein 354A | -1.14 | 0.665 | 1.06 | 0.72 | -1.44 | 0.026 | -1.19 | 0.669 | -0.68 |
| 1420851_at | Pard6g | par-6 partitioning defective 6 homolog gamma (C. elegans) | 1.03 | 0.636 | -1.29 | 0.512 | -1.2 | 0.189 | -1.12 | 0.701 | -0.65 |
| 1433691_at | Ppp1r3c | protein phosphatase 1, regulatory (inhibitor) subunit 3C | 1.13 | 0.85 | -1.62 | 0.005 | -1.12 | 0.593 | -1.05 | 0.904 | -0.67 |
| 1429782_at | Grcc10 | gene rich cluster, C10 gene | 1.04 | 0.912 | -1.18 | 0.661 | -1.33 | 0.146 | -1.99 | 0.148 | -0.86 |
| 1460678_at | Klhdc2 | kelch domain containing 2 | -1.24 | 0.214 | -1.1 | 0.343 | -1.08 | 0.586 | 1.72 | 0.234 | -0.43 |
| 1442623_at | Lysmd4 | LysM, putative peptidoglycan-binding, domain containing 4 | 1.07 | 0.858 | -1.14 | 0.539 | -1.45 | 0.219 | -1.4 | 0.299 | -0.73 |
| 1455261_at | --- | PREDICTED: Mus musculus similar to Hemoglobin alpha subunit (Hemoglobin al | 1.09 | 0.453 | -1.26 | 0.24 | -1.32 | 0.149 | -1.26 | 0.195 | -0.69 |
| 1456976_at | Wnt5a | wingless-related MMTV integration site 5A | 1.24 | 0.431 | -1.68 | 0.227 | -1.25 | 0.318 | -1.15 | 0.698 | -0.71 |
| 1427899_at | Rnf6 | ring finger protein (C3H2C3 type) 6 | -1.26 | 0.433 | 1.01 | 0.932 | -1.19 | 0.172 | -1.29 | 0.45 | -0.68 |
| 1418610_at | Slc17a6 | solute carrier family 17 (sodium-dependent inorganic phosphate cotransporter), m | 1.2 | 0.498 | -1.15 | 0.534 | -1.78 | 0.509 | -1.36 | 0.572 | -0.77 |
| 1439964_at | D8Bwg1414e | DNA segment, Chr 8, Brigham & Women's Genetics 1414 expressed | -1.12 | 0.504 | -1.07 | 0.59 | -1.23 | 0.34 | 1.15 | 0.247 | -0.57 |
| 1429168_at | Btbd4 | BTB (POZ) domain containing 4 | -1.16 | 0.564 | 1.03 | 0.863 | -1.35 | 0.16 | -1.08 | 0.652 | -0.64 |
| 1434996_at | Slc25a16 | solute carrier family 25 (mitochondrial carrier, Graves disease autoantigen), mem | -1.06 | 0.663 | -1.29 | 0.224 | -1.09 | 0.603 | 1.01 | 0.964 | -0.61 |
| 1417596_at | Eppb9 | endothelial precursor protein B9 | 1.05 | 0.678 | -1.58 | 0.351 | -1.04 | 0.865 | -1.27 | 0.188 | -0.71 |
| 1428810_at | 2700097O09Rik | RIKEN cDNA 2700097O09 gene | -1.2 | 0.504 | -1.07 | 0.351 | -1.15 | 0.18 | 1.56 | 0.194 | -0.46 |
| 1425938_a_at | Fkbp11 | FK506 binding protein 11 | -2.2 | 0.111 | -1.13 | 0.842 | 1.3 | 0.654 | -2.18 | 0.068 | -1.05 |
| 1438397_a_at | Rbm39 | RNA binding motif protein 39 | -1.3 | 0.6 | -1.2 | 0.327 | 1.04 | 0.904 | -2.18 | 0.1 | -0.91 |
| 1452551_at | --- | --- | 1.29 | 0.451 | -1.39 | 0.47 | -1.6 | 0.272 | -2.26 | 0.255 | -0.99 |
| 1419211_s_at | 4933424B01Rik | RIKEN cDNA 4933424B01 gene | -1.33 | 0.137 | -1.03 | 0.732 | -1.09 | 0.323 | 1.22 | 0.616 | -0.56 |
| 1438809_at | Atp5c1 | ATP synthase, H+ transporting, mitochondrial F1 complex, gamma polypeptide 1 | -1.38 | 0.221 | 1.08 | 0.798 | -1.2 | 0.36 | -1.63 | 0.279 | -0.78 |
| 1429122_a_at | 1700040I03Rik | RIKEN cDNA 1700040I03 gene | -1.11 | 0.468 | -1.09 | 0.402 | -1.22 | 0.019 | 1.3 | 0.389 | -0.53 |
| 1420922_at | Usp9x | ubiquitin specific peptidase 9, X chromosome | -1.15 | 0.062 | -1.15 | 0.155 | -1.12 | 0.347 | 1.26 | 0.174 | -0.54 |
| 1454958_at | Gsk3b | glycogen synthase kinase 3 beta | -1.11 | 0.015 | -1.08 | 0.628 | -1.22 | 0.374 | 1.14 | 0.514 | -0.57 |
| 1418378_at | Bpil1 | bactericidal/permeability-increasing protein-like 1 | -1.12 | 0.751 | -1.25 | 0.279 | -1.06 | 0.709 | 1.07 | 0.848 | -0.59 |
| 1459929_at | Zfp568 | zinc finger protein 568 | -1.05 | 0.954 | -1.09 | 0.791 | -1.3 | 0.464 | 1.26 | 0.321 | -0.54 |

| | | | | | | | | | | | |
|---|---|---|---|---|---|---|---|---|---|---|---|
| 1418359_at | Wbscr27 | Williams Beuren syndrome chromosome region 27 (human) | -1.21 | 0.317 | -1.11 | 0.336 | -1.09 | 0.517 | 2.98 | 0.231 | -0.11 |
| 1455486_at | Pias1 | protein inhibitor of activated STAT 1 | -1.17 | 0.66 | 1.07 | 0.576 | -1.41 | 0.046 | -1.21 | 0.51 | -0.68 |
| 1456933_at | Smg7 | Smg-7 homolog, nonsense mediated mRNA decay factor (C. elegans) | -1.08 | 0.831 | 1.21 | 0.508 | -2.02 | 0.005 | -1.41 | 0.444 | -0.82 |
| 1424326_at | Lemd2 | LEM domain containing 2 | -1.16 | 0.439 | -1.29 | 0.086 | -1 | 0.997 | 1.05 | 0.18 | -0.6 |
| 1438089_a_at | Bclaf1 | BCL2-associated transcription factor 1 | 1.09 | 0.452 | -1.28 | 0.247 | -1.31 | 0.063 | -1.18 | 0.175 | -0.67 |
| 1453252_at | Dus4l | dihydrouridine synthase 4-like (S. cerevisiae) | -1.49 | 0.327 | -1.36 | 0.356 | 1.23 | 0.669 | -1.92 | 0.286 | -0.88 |
| 1441298_at | --- | --- | -1.14 | 0.742 | -1.18 | 0.65 | -1.09 | 0.657 | 1.52 | 0.469 | -0.47 |
| 1444908_at | Habp4 /// LOC6717 | hyaluronic acid binding protein 4 /// similar to hyaluronic acid binding protein 4 | -1.23 | 0.11 | -1.21 | 0.257 | 1 | 0.996 | -1.25 | 0.067 | -0.67 |
| 1458031_at | B230315F11Rik | RIKEN cDNA B230315F11 gene | 1.15 | 0.026 | -1.18 | 0.678 | -1.57 | 0.068 | -1.03 | 0.789 | -0.66 |
| 1425457_a_at | Grb10 | growth factor receptor bound protein 10 | -1.1 | 0.67 | -2.07 | 0.236 | 1.24 | 0.706 | -1.02 | 0.958 | -0.73 |
| 1423513_at | Neo1 /// E030041M | neogenin /// RIKEN cDNA E030041M21 gene | -1.16 | 0.288 | -1.04 | 0.787 | -1.22 | 0.195 | 1.07 | 0.635 | -0.59 |
| 1450264_a_at | Chka | choline kinase alpha | -1.19 | 0.015 | 1.09 | 0.809 | -1.4 | 0.159 | -1.52 | 0.159 | -0.76 |
| 1446490_at | Ptbp2 | Polypyrimidine tract binding protein 2 | 1.12 | 0.491 | -1.35 | 0.272 | -1.28 | 0.219 | -1.23 | 0.401 | -0.69 |
| 1444611_at | U2af1-rs2 | U2 small nuclear ribonucleoprotein auxiliary factor (U2AF) 1, related sequence 2 | -1.04 | 0.781 | 1.01 | 0.954 | -1.5 | 0.003 | -1.15 | 0.206 | -0.67 |
| 1451397_at | --- | --- | -1.81 | 0.406 | -1.22 | 0.466 | 1.27 | 0.498 | -1.33 | 0.475 | -0.77 |
| 1457793_a_at | Whsc1l1 | Wolf-Hirschhorn syndrome candidate 1-like 1 (human) | 1.06 | 0.611 | -1.24 | 0.435 | -1.29 | 0.076 | -1 | 0.988 | -0.62 |
| 1416035_at | --- | --- | -1.34 | 0.221 | 1.02 | 0.847 | -1.15 | 0.091 | -1.31 | 0.226 | -0.69 |
| 1454950_at | B930006L02Rik | RIKEN cDNA B930006L02 gene | -1.03 | 0.915 | -1.17 | 0.716 | -1.23 | 0.632 | 1.12 | 0.368 | -0.58 |
| 1443400_at | Mier1 | Mesoderm induction early response 1 homolog (Xenopus laevis | 1.15 | 0.066 | -1.21 | 0.262 | -1.52 | 0.031 | -1.05 | 0.591 | -0.66 |
| 1458204_at | Sparc | secreted acidic cysteine rich glycoprotein | 1.37 | 0.145 | -1.44 | 0.631 | -1.72 | 0.155 | -1 | 0.988 | -0.7 |
| 1434771_at | 0610011F06Rik | RIKEN cDNA 0610011F06 gene | 1.02 | 0.857 | -1.57 | 0.085 | -1.02 | 0.926 | -1.22 | 0.291 | -0.7 |
| 1449799_s_at | Pkp2 | plakophilin 2 | -1.08 | 0.591 | -1.12 | 0.296 | -1.21 | 0.219 | 1.65 | 0.279 | -0.44 |
| 1436826_at | Tmtc3 | transmembrane and tetratricopeptide repeat containing 3 | -1.34 | 0.175 | -1.08 | 0.68 | -1.03 | 0.865 | 1.32 | 0.148 | -0.53 |
| 1444839_at | --- | --- | -1.48 | 0.426 | 1.39 | 0.495 | -1.72 | 0.411 | -1.63 | 0.529 | -0.86 |
| 1418454_at | Mfap5 | microfibrillar associated protein 5 | 1.14 | 0.723 | -1.03 | 0.923 | -1.89 | 0.048 | -1.48 | 0.283 | -0.81 |
| 1459283_at | 6430510B20Rik | RIKEN cDNA 6430510B20 gene | -1.07 | 0.694 | 1.1 | 0.758 | -1.65 | 0.058 | -1.15 | 0.582 | -0.69 |
| 1457367_at | Amy2 | Amylase 2, pancreatic | 1.01 | 0.918 | -1.21 | 0.275 | -1.25 | 0.361 | -1.21 | 0.287 | -0.66 |
| 1425990_a_at | Nfatc2 | nuclear factor of activated T-cells, cytoplasmic, calcineurin-dependent 2 | -2.4 | 0.031 | -1.01 | 0.974 | 1.23 | 0.384 | -1.34 | 0.528 | -0.88 |
| 1440091_at | Mrg1 | myeloid ecotropic viral integration site-related gene 1 | -1.19 | 0.052 | 1.16 | 0.099 | -1.56 | 0.247 | -1.07 | 0.781 | -0.67 |
| 1458183_at | 2810039B14Rik | RIKEN cDNA 2810039B14 gene | -1.15 | 0.102 | -1.14 | 0.61 | -1.11 | 0.331 | 1.41 | 0.453 | -0.5 |
| 1434607_at | Ddx52 | DEAD (Asp-Glu-Ala-Asp) box polypeptide 52 | 1.08 | 0.71 | -1.2 | 0.114 | -1.37 | 0.166 | -1.78 | 0.127 | -0.82 |
| 1443698_at | Fbxo39 | F-box protein 39 | -1.1 | 0.594 | 1.05 | 0.623 | -1.46 | 0.013 | -1.26 | 0.302 | -0.69 |
| 1435919_at | Atp1a1 | ATPase, Na+/K+ transporting, alpha 1 polypeptide | -1.08 | 0.449 | -1.55 | 0.467 | 1.07 | 0.477 | -1.06 | 0.581 | -0.66 |
| 1427139_at | Adamts10 | a disintegrin-like and metallopeptidase (reprolysin type) with thrombospondin type | -1.03 | 0.627 | 1.06 | 0.359 | -1.62 | 0.021 | -1.71 | 0.172 | -0.83 |
| 1433364_at | A930036I15Rik | RIKEN cDNA A930036I15 gene | -2.16 | 0.072 | -1.61 | 0.466 | 1.56 | 0.495 | -1.01 | 0.967 | -0.81 |
| 1417116_at | Slc6a8 | solute carrier family 6 (neurotransmitter transporter, creatine), member 8 | -1.31 | 0.011 | 1.04 | 0.72 | -1.19 | 0.333 | -1.13 | 0.538 | -0.65 |
| 1460233_at | Guca2b | guanylate cyclase activator 2b (retina) | 1.24 | 0.122 | -1.39 | 0.468 | -1.46 | 0.31 | -1.48 | 0.144 | -0.77 |
| 1452297_at | Ccnt2 | cyclin T2 | -1.07 | 0.68 | -1.32 | 0.204 | -1.05 | 0.818 | 1.29 | 0.383 | -0.54 |
| 1416319_at | Adk | adenosine kinase | -1.13 | 0.202 | -1.01 | 0.898 | -1.31 | 0.155 | 1.9 | 0.163 | -0.39 |
| 1429708_at | Ndufa11 | NADH dehydrogenase (ubiquinone) 1 alpha subcomplex 11 | -1.22 | 0.312 | -1.06 | 0.281 | -1.14 | 0.072 | 1.08 | 0.509 | -0.58 |
| 1443959_at | C230095G01Rik | RIKEN cDNA C230095G01 gene | -1.17 | 0.647 | 1.29 | 0.599 | -2 | 0.113 | -1.44 | 0.191 | -0.83 |
| 1456995_at | 2700045P11Rik | RIKEN cDNA 2700045P11 gene | -1.15 | 0.894 | -1.13 | 0.554 | -1.12 | 0.499 | 1.23 | 0.562 | -0.54 |
| 1436877_at | Lrch2 | leucine-rich repeats and calponin homology (CH) domain containing 2 | -1.5 | 0.334 | 1.15 | 0.799 | -1.21 | 0.606 | -2.17 | 0.036 | -0.93 |
| 1419010_x_at | Klk1b5 | kallikrein 1-related peptidase b5 | -1.35 | 0.411 | -1.14 | 0.661 | 1.02 | 0.962 | -1.51 | 0.517 | -0.74 |
| 1416367_at | 1110001J03Rik | RIKEN cDNA 1110001J03 gene | -1.13 | 0.31 | -1.24 | 0.101 | -1.05 | 0.768 | 1.1 | 0.443 | -0.58 |
| 1416369_at | Hiatl1 | hippocampus abundant transcript-like 1 | -1.18 | 0.096 | -1.17 | 0.118 | -1.07 | 0.297 | 1.18 | 0.402 | -0.56 |
| 1431736_at | 4933405D12Rik | RIKEN cDNA 4933405D12 gene | 1.03 | 0.957 | -1.18 | 0.723 | -1.3 | 0.605 | -1.03 | 0.828 | -0.62 |
| 1445031_at | Tbc1d8 | TBC1 domain family, member 8 | 1.07 | 0.889 | -1.02 | 0.951 | -1.69 | 0.285 | -1.19 | 0.386 | -0.71 |
| 1421148_a_at | Tial1 | Tial1 cytotoxic granule-associated RNA binding protein-like 1 | -1.19 | 0.235 | -1.25 | 0.206 | 1 | 1 | -1.08 | 0.738 | -0.63 |
| 1418885_a_at | Idh3b | isocitrate dehydrogenase 3 (NAD+) beta | -1.18 | 0.056 | -1.28 | 0.08 | 1.01 | 0.889 | -1.43 | 0.048 | -0.72 |
| 1428925_at | Senp1 | SUMO1/sentrin specific peptidase 1 | -1.19 | 0.57 | -1.07 | 0.688 | -1.16 | 0.474 | 1.34 | 0.206 | -0.52 |
| 1420035_at | Aqp6 | Aquaporin 6 | -1.03 | 0.823 | -1.21 | 0.636 | -1.18 | 0.715 | 1.03 | 0.959 | -0.6 |
| 1441071_at | Kcnq5 | potassium voltage-gated channel, subfamily Q, member 5 | 1.65 | 0.566 | -1.48 | 0.461 | -3.1 | 0.014 | -1.35 | 0.512 | -1.07 |

| | | | | | | | | | | | |
|---|---|---|---|---|---|---|---|---|---|---|---|
| 1443929_at | --- | In vitro fertilized eggs cDNA, RIKEN full-length enriched library, clone:7420432F0 | -1.39 | 0.199 | 1 | 0.989 | -1.09 | 0.808 | -1.15 | 0.426 | -0.66 |
| 1443057_at | --- | --- | -1.05 | 0.435 | -1.01 | 0.97 | -1.43 | 0.034 | 1.07 | 0.716 | -0.61 |
| 1421621_at | Rasgrf2 | RAS protein-specific guanine nucleotide-releasing factor 2 | -1.45 | 0.565 | -1.15 | 0.653 | 1.08 | 0.788 | -1.49 | 0.133 | -0.75 |
| 1439513_at | Pnrc1 | proline-rich nuclear receptor coactivator 1 | -1.12 | 0.842 | -2.25 | 0.045 | 1.31 | 0.552 | -1.26 | 0.449 | -0.83 |
| 1441487_at | Trim2 | tripartite motif protein 2 | -1.04 | 0.944 | -1.12 | 0.808 | -1.26 | 0.324 | 1.42 | 0.202 | -0.5 |
| 1425474_a_at | Vps39 | vacuolar protein sorting 39 (yeast) | 1.16 | 0.72 | -1.33 | 0.374 | -1.36 | 0.247 | -1.41 | 0.192 | -0.74 |
| 1429539_at | Bcl2l13 | BCL2-like 13 (apoptosis facilitator) | -1.09 | 0.313 | -1.17 | 0.028 | -1.14 | 0.239 | 2.13 | 0.103 | -0.32 |
| 1457243_at | 1110032O16Rik | RIKEN cDNA 1110032O16 gene | -1.32 | 0.36 | 1.04 | 0.8 | -1.18 | 0.543 | -1.15 | 0.371 | -0.65 |
| 1454995_at | Ddah1 | dimethylarginine dimethylaminohydrolase 1 | -1.18 | 0.301 | -1.38 | 0.321 | 1.07 | 0.723 | -1.38 | 0.053 | -0.72 |
| 1434005_at | Rbms1 | RNA binding motif, single stranded interacting protein 1 | 1.02 | 0.872 | -1.14 | 0.274 | -1.34 | 0.039 | -1.63 | 0.073 | -0.77 |
| 1427850_x_at | Igh-VJ558 | immunoglobulin heavy chain (J558 family) | -1.19 | 0.369 | -1.08 | 0.846 | -1.15 | 0.773 | 1.48 | 0.493 | -0.48 |
| 1458567_at | D130017N08Rik | RIKEN cDNA D130017N08 gene | -1.42 | 0.18 | 1.18 | 0.496 | -1.31 | 0.058 | -1.3 | 0.417 | -0.71 |
| 1424932_at | Egfr | epidermal growth factor receptor | -1.07 | 0.806 | 1.13 | 0.789 | -1.73 | 0.025 | -1.61 | 0.096 | -0.82 |
| 1424434_at | BC024814 | cDNA sequence BC024814 | -1.11 | 0.592 | -1.36 | 0.192 | 1.01 | 0.895 | -1.03 | 0.754 | -0.62 |
| 1442903_at | 4732423E21Rik | RIKEN cDNA 4732423E21 gene | 1.07 | 0.512 | -1.09 | 0.765 | -1.53 | 0.068 | -1.01 | 0.941 | -0.64 |
| 1458432_at | Nck2 | Non-catalytic region of tyrosine kinase adaptor protein 2 | 1.21 | 0.465 | -1.09 | 0.429 | -1.93 | 0.055 | -1.58 | 0.177 | -0.85 |
| 1424085_at | Ndufa4 | NADH dehydrogenase (ubiquinone) 1 alpha subcomplex, 4 | -1.16 | 0.219 | -1.17 | 0.157 | -1.08 | 0.748 | 1.32 | 0.104 | -0.52 |
| 1423136_at | Fgf1 | fibroblast growth factor 1 | 1.23 | 0.038 | -1.45 | 0.053 | -1.38 | 0.078 | -1.71 | 0.014 | -0.83 |
| 1434375_at | B930006L02Rik | RIKEN cDNA B930006L02 gene | 1.08 | 0.501 | -1.14 | 0.309 | -1.45 | 0.011 | -1.11 | 0.66 | -0.66 |
| 1453830_at | --- | --- | -1.72 | 0.245 | 1.25 | 0.534 | -1.23 | 0.622 | -1.52 | 0.449 | -0.8 |
| 1445106_at | Mbnl1 | Muscleblind-like 1 (Drosophila) | -1.16 | 0.148 | 1.07 | 0.831 | -1.41 | 0.12 | -1.31 | 0.316 | -0.7 |
| 1452694_at | Ihpk1 | inositol hexaphosphate kinase 1 | -1.32 | 0.262 | -1.05 | 0.52 | -1.07 | 0.758 | 1.05 | 0.752 | -0.6 |
| 1457761_at | C79607 | expressed sequence C79607 | -1.07 | 0.809 | -1.03 | 0.885 | -1.34 | 0.176 | 1.07 | 0.648 | -0.6 |
| 1451367_at | Cops6 | COP9 (constitutive photomorphogenic) homolog, subunit 6 (Arabidopsis thaliana) | -1.34 | 0.249 | -1.2 | 0.423 | 1.06 | 0.614 | -1.26 | 0.141 | -0.68 |
| 1423725_at | Pls3 | plastin 3 (T-isoform) | -1.12 | 0.002 | 1 | 0.953 | -1.34 | 0.004 | -1.47 | 0.285 | -0.73 |
| 1428490_at | C1galt1 | core 1 UDP-galactose:N-acetylgalactosamine-alpha-R beta 1,3-galactosyltransfe | -1.26 | 0.12 | -1.04 | 0.745 | -1.13 | 0.352 | 1.08 | 0.741 | -0.59 |
| 1421227_at | Gzme | granzyme E | 1.08 | 0.768 | -1.01 | 0.976 | -1.74 | 0.119 | -1.71 | 0.354 | -0.85 |
| 1445453_at | --- | --- | -1.08 | 0.774 | 1.07 | 0.716 | -1.55 | 0.235 | -1.27 | 0.657 | -0.71 |
| 1457656_s_at | C230085N15Rik | RIKEN cDNA C230085N15 gene | 1.36 | 0.39 | -1.23 | 0.24 | -2.13 | 0.043 | -4.38 | 0.059 | -1.6 |
| 1424601_at | Xrcc4 | X-ray repair complementing defective repair in Chinese hamster cells 4 | -1.27 | 0.309 | 1.03 | 0.678 | -1.21 | 0.099 | -1.03 | 0.802 | -0.62 |
| 1455778_at | Zfp192 | zinc finger protein 192 | -1.05 | 0.65 | -1.17 | 0.256 | -1.2 | 0.203 | 1.02 | 0.948 | -0.6 |
| 1435546_a_at | 1810013L24Rik | RIKEN cDNA 1810013L24 gene | -1.18 | 0.192 | -1.07 | 0.584 | -1.16 | 0.206 | 1.19 | 0.43 | -0.55 |
| 1451572_a_at | 5230400G24Rik /// | RIKEN cDNA 5230400G24 gene /// hypothetical protein LOC637796 | -1.12 | 0.461 | -1.04 | 0.614 | -1.27 | 0.003 | 1.11 | 0.684 | -0.58 |
| 1419869_s_at | Hdlbp | high density lipoprotein (HDL) binding protein | -1.17 | 0.073 | -1.04 | 0.456 | -1.2 | 0.131 | 1.04 | 0.817 | -0.59 |
| 1416662_at | Sardh | sarcosine dehydrogenase | 1.04 | 0.899 | -1.16 | 0.481 | -1.34 | 0.505 | -1.43 | 0.402 | -0.72 |
| 1457983_s_at | Rwdd4a | RWD domain containing 4A | 1.03 | 0.87 | -1.1 | 0.411 | -1.42 | 0.048 | -1.01 | 0.94 | -0.62 |
| 1436214_at | 1110028C15Rik | RIKEN cDNA 1110028C15 gene | -1.2 | 0.335 | 1.13 | 0.43 | -1.46 | 0.061 | -1.08 | 0.279 | -0.65 |
| 1453796_a_at | Ergic2 | ERGIC and golgi 2 | -1.04 | 0.61 | -1.26 | 0.258 | -1.12 | 0.409 | 1.4 | 0.254 | -0.51 |
| 1458831_at | Nsdhl | NAD(P) dependent steroid dehydrogenase-like | 1.1 | 0.562 | -1.27 | 0.492 | -1.32 | 0.204 | -1.44 | 0.147 | -0.73 |
| 1436865_at | Slc26a11 | solute carrier family 26, member 11 | -1.73 | 0.084 | -1.21 | 0.393 | 1.24 | 0.278 | -1.23 | 0.568 | -0.73 |
| 1419959_s_at | C330003B14Rik | RIKEN cDNA C330003B14 gene | -1.27 | 0.782 | -1.16 | 0.586 | -1 | 0.997 | 1.13 | 0.66 | -0.58 |
| 1450891_at | Srp19 | signal recognition particle 19 | -1.04 | 0.448 | -1.08 | 0.238 | -1.32 | 0.029 | 1.2 | 0.035 | -0.56 |
| 1422110_at | Hr | hairless | -1.35 | 0.274 | 1.05 | 0.803 | -1.16 | 0.367 | -1.2 | 0.444 | -0.67 |
| 1438890_at | --- | --- | -1.1 | 0.711 | -1.03 | 0.798 | -1.3 | 0.174 | 1.04 | 0.78 | -0.6 |
| 1458359_at | --- | Transcribed locus | 1.09 | 0.847 | -1.19 | 0.697 | -1.41 | 0.546 | -2.68 | 0.049 | -1.05 |
| 1431577_at | --- | --- | -1.4 | 0.622 | 1.07 | 0.902 | -1.16 | 0.52 | -1.84 | 0.213 | -0.83 |
| 1452848_at | Gpr178 | G protein-coupled receptor 178 | -1.31 | 0.066 | 1.09 | 0.669 | -1.26 | 0.447 | -1.31 | 0.27 | -0.7 |
| 1418931_at | Reg4 | regenerating islet-derived family, member 4 | -1.65 | 0.16 | -1.36 | 0.248 | 1.31 | 0.522 | -1.14 | 0.707 | -0.71 |
| 1442038_at | Rbm26 | RNA binding motif protein 26 | -1.17 | 0.661 | -1.13 | 0.352 | -1.1 | 0.706 | 1.12 | 0.557 | -0.57 |
| 1428228_at | Pgm3 | phosphoglucomutase 3 | 1.08 | 0.669 | -1.14 | 0.473 | -1.45 | 0.156 | -1.3 | 0.4 | -0.7 |
| 1457827_at | Arsj | arylsulfatase J | 1.48 | 0.363 | -1.57 | 0.478 | -1.87 | 0.213 | -1.68 | 0.003 | -0.91 |
| 1442087_at | H3f3a | H3 histone, family 3A | -1.02 | 0.924 | -1.36 | 0.317 | -1.08 | 0.62 | 1.35 | 0.336 | -0.53 |
| 1441632_at | C130079B09Rik | RIKEN cDNA C130079B09 gene | 1.44 | 0.24 | -1.52 | 0.23 | -1.81 | 0.032 | -1.16 | 0.679 | -0.76 |

| | | | | | | | | | | | |
|---|---|---|---|---|---|---|---|---|---|---|---|
| 1443236_at | B230118H07Rik | RIKEN cDNA B230118H07 gene | -1.59 | 0.177 | -1.3 | 0.562 | 1.25 | 0.627 | -1.79 | 0.512 | -0.86 |
| 1424469_a_at | Cpsf4 | cleavage and polyadenylation specific factor 4 | -1.03 | 0.683 | -1.06 | 0.71 | -1.37 | 0.204 | 1.05 | 0.827 | -0.6 |
| 1449961_at | Rph3a | rabphilin 3A | -1.3 | 0.357 | 1.01 | 0.931 | -1.16 | 0.495 | -1.56 | 0.461 | -0.75 |
| 1444549_at | --- | Adult male adrenal gland cDNA, RIKEN full-length enriched library, clone:733040 | 1.3 | 0.535 | -2.26 | 0.21 | -1.11 | 0.866 | -1.18 | 0.828 | -0.81 |
| 1441455_at | Arpp19 | CAMP-regulated phosphoprotein 19 | -1.22 | 0.364 | 1.05 | 0.875 | -1.28 | 0.124 | -1.17 | 0.412 | -0.66 |
| 1446383_at | Efna5 | Ephrin A5 | -1.17 | 0.616 | 1.08 | 0.791 | -1.4 | 0.164 | -1.83 | 0.305 | -0.83 |
| 1428204_at | C030002O17Rik | RIKEN cDNA C030002O17 gene | -1.31 | 0.678 | -1.05 | 0.921 | -1.07 | 0.896 | 1.47 | 0.575 | -0.49 |
| 1429664_at | Cdkl1 | cyclin-dependent kinase-like 1 (CDC2-related kinase) | -1.11 | 0.829 | -1.14 | 0.678 | -1.16 | 0.616 | 1.35 | 0.311 | -0.51 |
| 1437408_at | Gpr126 | G protein-coupled receptor 126 | 1.5 | 0.299 | -1.52 | 0.447 | -2.03 | 0.053 | -1.82 | 0.262 | -0.97 |
| 1426126_a_at | MGC30332 | hypothetical protein MGC30332 | -1.39 | 0.188 | -1.17 | 0.374 | 1.07 | 0.735 | -1.47 | 0.174 | -0.74 |
| 1457443_at | --- | --- | 1.04 | 0.906 | -1.44 | 0.291 | -1.1 | 0.46 | -1.06 | 0.814 | -0.64 |
| 1430908_at | 4933429O19Rik | RIKEN cDNA 4933429O19 gene | -1.96 | 0.12 | 1.28 | 0.167 | -1.17 | 0.392 | -1.3 | 0.202 | -0.79 |
| 1431949_at | 4930544F09Rik | RIKEN cDNA 4930544F09 gene | -1.62 | 0.61 | 1.35 | 0.529 | -1.47 | 0.398 | -2.01 | 0.304 | -0.94 |
| 1438848_at | Rbpsuh | Recombining binding protein suppressor of hairless (Drosophila) | -1.26 | 0.407 | 1.19 | 0.631 | -1.49 | 0.108 | -1.22 | 0.158 | -0.7 |
| 1434380_at | Gbp7 | guanylate binding protein 7 | 1.22 | 0.475 | -1.32 | 0.07 | -1.49 | 0.08 | -1.19 | 0.288 | -0.7 |
| 1435209_at | BC057079 | cDNA sequence BC057079 | -1.3 | 0.281 | -1.02 | 0.74 | -1.11 | 0.171 | 1.16 | 0.598 | -0.57 |
| 1441898_at | E530011F12Rik | RIKEN cDNA E530011F12 gene | 1.31 | 0.492 | -1.79 | 0.231 | -1.29 | 0.538 | -1.02 | 0.957 | -0.7 |
| 1443331_at | Plekhg1 | Pleckstrin homology domain containing, family G (with RhoGef domain) member | -1.28 | 0.059 | 1.26 | 0.07 | -1.65 | 0.03 | -1.09 | 0.683 | -0.69 |
| 1416782_s_at | Praf2 | PRA1 domain family 2 | -1.06 | 0.468 | -1.42 | 0.009 | 1 | 0.986 | -1.05 | 0.818 | -0.63 |
| 1436410_at | AI449023 /// Gm941 | expressed sequence AI449023 /// gene model 941, (NCBI) | -1.52 | 0.331 | -1.22 | 0.665 | 1.17 | 0.766 | -2.34 | 0.092 | -0.98 |
| 1442419_at | --- | Transcribed locus | -1.17 | 0.684 | -2.04 | 0.024 | 1.3 | 0.503 | -1.17 | 0.769 | -0.77 |
| 1449874_at | Ly96 | lymphocyte antigen 96 | -1.37 | 0.101 | 1.08 | 0.61 | -1.19 | 0.559 | -1.15 | 0.589 | -0.66 |
| 1434313_at | 6330407D12Rik | RIKEN cDNA 6330407D12 gene | -1.49 | 0.296 | 1.1 | 0.774 | -1.13 | 0.516 | -1.21 | 0.516 | -0.69 |
| 1428186_at | Kctd6 | potassium channel tetramerisation domain containing 6 | -1.19 | 0.118 | -1.02 | 0.895 | -1.22 | 0.027 | 1.01 | 0.949 | -0.6 |
| 1456027_at | Rbm41 | RNA binding motif protein 41 | -1.36 | 0.097 | 1.03 | 0.802 | -1.14 | 0.215 | -1.27 | 0.343 | -0.68 |
| 1440982_at | LOC552909 | hypothetical LOC552909 | 1.13 | 0.78 | -1.11 | 0.709 | -1.61 | 0.052 | -1.3 | 0.285 | -0.72 |
| 1433582_at | 1190002N15Rik | RIKEN cDNA 1190002N15 gene | -1.16 | 0.498 | -1.05 | 0.669 | -1.2 | 0.482 | 1.66 | 0.175 | -0.44 |
| 1453867_at | Fbxw2 | F-box and WD-40 domain protein 2 | 1.18 | 0.462 | -1.07 | 0.902 | -1.87 | 0.379 | -1.02 | 0.958 | -0.7 |
| 1445658_at | --- | Transcribed locus | -1.31 | 0.171 | -1.08 | 0.766 | -1.04 | 0.925 | 1.37 | 0.211 | -0.52 |
| 1432650_at | 2510019K15Rik | RIKEN cDNA 2510019K15 gene | -1.43 | 0.452 | -1.35 | 0.207 | 1.21 | 0.534 | -1.23 | 0.164 | -0.7 |
| 1451105_at | Vash2 | vasohibin 2 | 1.34 | 0.62 | -2.17 | 0.074 | -1.18 | 0.53 | -1.79 | 0.168 | -0.95 |
| 1449370_at | Sox4 /// LOC67227 | SRY-box containing gene 4 /// similar to Transcription factor SOX-4 | 1.3 | 0.521 | -1.42 | 0.481 | -1.55 | 0.256 | -1.24 | 0.293 | -0.73 |
| 1432899_at | 4833408G04Rik | RIKEN cDNA 4833408G04 gene | -1.58 | 0.34 | 1.1 | 0.772 | -1.09 | 0.816 | -1.51 | 0.123 | -0.77 |
| 1429777_at | Dnajb6 | DnaJ (Hsp40) homolog, subfamily B, member 6 | -1.18 | 0.76 | -1.37 | 0.244 | 1.07 | 0.746 | -1.64 | 0.192 | -0.78 |
| 1434329_s_at | Adipor2 | adiponectin receptor 2 | -1.11 | 0.129 | -1.24 | 0.171 | -1.06 | 0.716 | 1.14 | 0.183 | -0.57 |
| 1416824_at | B230118H07Rik | RIKEN cDNA B230118H07 gene | -1.1 | 0.13 | -1.03 | 0.294 | -1.29 | 0.096 | 1.09 | 0.615 | -0.58 |
| 1458910_at | --- | --- | 1.21 | 0.847 | -1.25 | 0.452 | -1.56 | 0.254 | -1.41 | 0.514 | -0.75 |
| 1421320_a_at | Qtrtd1 | queuine tRNA-ribosyltransferase domain containing 1 | 1.2 | 0.833 | -2.15 | 0.281 | -1.01 | 0.98 | -1.52 | 0.005 | -0.87 |
| 1438117_x_at | Tmem41b | transmembrane protein 41B | -1.21 | 0.014 | -1.16 | 0.191 | -1.05 | 0.797 | 1.17 | 0.433 | -0.56 |
| 1442682_at | --- | --- | -1.03 | 0.888 | -1.28 | 0.544 | -1.12 | 0.671 | 1.57 | 0.296 | -0.46 |
| 1460736_at | LOC665775 /// LOC | similar to CG5514-PB, isoform B /// region containing expressed sequence AF01 | -1.17 | 0.16 | 1.03 | 0.899 | -1.31 | 0.159 | -1.1 | 0.561 | -0.64 |
| 1437128_a_at | A630033E08Rik | RIKEN cDNA A630033E08 gene | -1.03 | 0.824 | 1.02 | 0.856 | -1.5 | 0.061 | -1.3 | 0.484 | -0.71 |
| 1459939_at | --- | Transcribed locus | -1.73 | 0.182 | 1.15 | 0.702 | -1.09 | 0.812 | -1.55 | 0.328 | -0.8 |
| 1450903_at | Rad23b | RAD23b homolog (S. cerevisiae) | 1.06 | 0.732 | -1.26 | 0.008 | -1.24 | 0.063 | -1.17 | 0.143 | -0.65 |
| 1446398_at | LOC433197 | hypothetical gene supported by AK028463 | 1.53 | 0.111 | -1.97 | 0.355 | -1.62 | 0.453 | -1.02 | 0.951 | -0.77 |
| 1446960_at | LOC626082 | Hypothetical protein LOC626082 | 1.12 | 0.829 | -1.14 | 0.781 | -1.54 | 0.488 | -1.29 | 0.543 | -0.71 |
| 1432578_at | 4933440H19Rik | RIKEN cDNA 4933440H19 gene | -1.1 | 0.757 | -1.04 | 0.86 | -1.29 | 0.053 | 2.36 | 0.001 | -0.27 |
| 1424761_at | BC011487 | cDNA sequence BC011487 | 1.09 | 0.676 | -1.41 | 0.482 | -1.17 | 0.341 | -1.34 | 0.014 | -0.71 |
| 1437023_at | Zfp12 | zinc finger protein 12 | 1.05 | 0.654 | -1.16 | 0.064 | -1.34 | 0.088 | -1.09 | 0.715 | -0.64 |
| 1458217_s_at | 2210018M11Rik | RIKEN cDNA 2210018M11 gene | -1.29 | 0.391 | 1.04 | 0.867 | -1.19 | 0.166 | -1.1 | 0.157 | -0.64 |
| 1449819_at | Dmc1h | disrupted meiotic cDNA 1 homolog | 1.1 | 0.921 | -1.52 | 0.534 | -1.12 | 0.818 | -1.88 | 0.602 | -0.85 |
| 1436266_x_at | Cbx1 | chromobox homolog 1 (Drosophila HP1 beta) | -1.08 | 0.414 | -1.23 | 0.133 | -1.09 | 0.469 | 1.07 | 0.7 | -0.58 |
| 1428037_at | Otoa | otoancorin | -1.24 | 0.238 | 1.05 | 0.915 | -1.26 | 0.657 | -1.15 | 0.752 | -0.65 |

| | | | | | | | | | | | |
|---|---|---|---|---|---|---|---|---|---|---|---|
| 1423534_at | Pdcd2 | programmed cell death 2 | 1.02 | 0.944 | -1.36 | 0.016 | -1.11 | 0.333 | -1.68 | 0.008 | -0.78 |
| 1436521_at | Slc36a2 | solute carrier family 36 (proton/amino acid symporter), member 2 | -1.6 | 0.241 | 1.06 | 0.875 | -1.04 | 0.882 | -1.97 | 0.119 | -0.89 |
| 1435812_at | Unc50 | unc-50 homolog (C. elegans) | -1.46 | 0.293 | -1.37 | 0.067 | 1.23 | 0.147 | -1.07 | 0.847 | -0.66 |
| 1451077_at | Rpl5 /// LOC38274( | ribosomal protein L5 /// similar to 60S ribosomal protein L5 /// similar to 60S ribos( | -1.07 | 0.001 | 1.02 | 0.916 | -1.42 | 0.102 | -1.14 | 0.475 | -0.65 |
| 1424726_at | Tmem150 | transmembrane protein 150 | -1.1 | 0.118 | 1.04 | 0.788 | -1.42 | 0.037 | -1.29 | 0.19 | -0.69 |
| 1455075_at | Pigv | phosphatidylinositol glycan anchor biosynthesis, class V | -1.33 | 0.339 | -1.19 | 0.662 | 1.06 | 0.542 | -1.37 | 0.089 | -0.71 |
| 1449203_at | Slco1a5 | solute carrier organic anion transporter family, member 1a5 | 1.29 | 0.527 | -1.14 | 0.65 | -2.07 | 0.016 | -1.26 | 0.674 | -0.79 |
| 1437658_a_at | Snord22 | small nucleolar RNA, C/D box 22 | -1.53 | 0.37 | -1.09 | 0.696 | 1.08 | 0.464 | -1.06 | 0.876 | -0.65 |
| 1439024_at | Bag4 | BCL2-associated athanogene 4 | 1.02 | 0.927 | -1.34 | 0.13 | -1.12 | 0.096 | -1.08 | 0.519 | -0.63 |
| 1428279_a_at | Atxn7l4 | ataxin 7-like 4 | -1.05 | 0.771 | -1.23 | 0.243 | -1.13 | 0.369 | 1.13 | 0.483 | -0.57 |
| 1442188_at | --- | 0 day neonate cerebellum cDNA, RIKEN full-length enriched library, clone:C2300 | -1.41 | 0.163 | 1.26 | 0.347 | -1.47 | 0.121 | -1.8 | 0.144 | -0.85 |
| 1420296_at | Clcn5 | chloride channel 5 | -1.19 | 0.205 | -1.27 | 0.238 | 1.02 | 0.935 | -1.68 | 0.315 | -0.78 |
| 1457538_at | 5930422O12Rik | RIKEN cDNA 5930422O12 gene | -1.08 | 0.513 | -1.06 | 0.826 | -1.28 | 0.113 | 2.19 | 0.09 | -0.31 |
| 1457629_at | BC060631 | cDNA sequence BC060631 | -1.13 | 0.343 | -1.97 | 0.068 | 1.26 | 0.523 | -1.12 | 0.819 | -0.74 |
| 1420507_a_at | 3110031B13Rik | RIKEN cDNA 3110031B13 gene | -1.07 | 0.427 | -1.09 | 0.403 | -1.25 | 0.153 | 1.09 | 0.538 | -0.58 |
| 1448544_at | Crat | carnitine acetyltransferase | -1.18 | 0.634 | -1.07 | 0.856 | -1.14 | 0.666 | 1.8 | 0.257 | -0.4 |
| 1438061_at | 4930523C07Rik | RIKEN cDNA 4930523C07 gene | 1.1 | 0.216 | -1.43 | 0.381 | -1.18 | 0.191 | -1.85 | 0.011 | -0.84 |
| 1438113_at | Zmat4 | zinc finger, matrin type 4 | -1.2 | 0.183 | 1.1 | 0.874 | -1.4 | 0.524 | -1.1 | 0.869 | -0.65 |
| 1434792_at | 2010320M18Rik | RIKEN cDNA 2010320M18 gene | 1 | 0.932 | -1.13 | 0.476 | -1.31 | 0.183 | -1.18 | 0.476 | -0.65 |
| 1457100_at | AW552889 | expressed sequence AW552889 | 1.47 | 0.583 | -1.5 | 0.34 | -1.92 | 0.23 | -1.02 | 0.971 | -0.74 |
| 1456962_at | Cntn2 | contactin 2 | -1.96 | 0.17 | -1.12 | 0.886 | 1.25 | 0.506 | -1.82 | 0.406 | -0.91 |
| 1430966_at | Cml3 | camello-like 3 | -1.19 | 0.601 | -1 | 0.999 | -1.24 | 0.536 | 1.23 | 0.293 | -0.55 |
| 1458027_at | Mrpl17 | mitochondrial ribosomal protein L17 | -1.97 | 0.106 | 1.2 | 0.84 | -1.06 | 0.791 | -1.63 | 0.326 | -0.86 |
| 1415791_at | Rnf34 | ring finger protein 34 | -1.55 | 0.011 | -1.09 | 0.313 | 1.09 | 0.566 | -1.2 | 0.088 | -0.69 |
| 1452945_at | 2610020C11Rik | RIKEN cDNA 2610020C11 gene | -1.1 | 0.337 | 1.12 | 0.313 | -1.62 | 0.134 | -1.85 | 0.09 | -0.86 |
| 1428776_at | Slc10a6 | solute carrier family 10 (sodium/bile acid cotransporter family), member 6 | -1.05 | 0.872 | 1.04 | 0.784 | -1.52 | 0.008 | -1.2 | 0.177 | -0.68 |
| 1452263_at | Slc35f4 | solute carrier family 35, member F4 | 1.14 | 0.351 | -1.33 | 0.398 | -1.31 | 0.498 | -1.4 | 0.701 | -0.73 |
| 1430921_at | 9130015G15Rik | RIKEN cDNA 9130015G15 gene | -1.24 | 0.701 | -1.13 | 0.775 | -1.04 | 0.924 | 1.65 | 0.319 | -0.44 |
| 1429720_at | Mak10 | MAK10 homolog, amino-acid N-acetyltransferase subunit, (S. cerevisiae) | -1.08 | 0.476 | -1.48 | 0.027 | 1.05 | 0.815 | -1.02 | 0.89 | -0.63 |
| 1457418_at | Speer1-ps1 | Spermatogenesis associated glutamate (E)-rich protein 1, pseudogene 1 | -1.75 | 0.321 | 1.6 | 0.286 | -2.08 | 0.115 | -1.17 | 0.698 | -0.85 |
| 1415689_s_at | Zfp306 | zinc finger protein 306 | -1.18 | 0.143 | 1.01 | 0.937 | -1.25 | 0.158 | -1.18 | 0.336 | -0.65 |
| 1417671_at | Scly | selenocysteine lyase | -1.09 | 0.828 | -1.21 | 0.331 | -1.1 | 0.129 | 1.61 | 0.076 | -0.45 |
| 1442209_at | --- | 7 days neonate cerebellum cDNA, RIKEN full-length enriched library, clone:A730 | -1.57 | 0.435 | -1.92 | 0.253 | 1.5 | 0.265 | -2 | 0.134 | -1 |
| 1457345_at | Polr3b | Polymerase (RNA) III (DNA directed) polypeptide B | -1.11 | 0.711 | -1.08 | 0.901 | -1.21 | 0.702 | 1.05 | 0.889 | -0.59 |
| 1421844_at | --- | --- | -1.55 | 0.033 | -1.01 | 0.886 | 1.02 | 0.895 | -1.1 | 0.614 | -0.66 |
| 1435374_at | --- | Transcribed locus | -1.07 | 0.78 | 1.18 | 0.558 | -1.86 | 0.032 | -1.7 | 0.262 | -0.86 |
| 1428694_at | 5033413D16Rik | RIKEN cDNA 5033413D16 gene | -1.1 | 0.709 | -1.32 | 0.309 | -1.02 | 0.942 | 1.12 | 0.662 | -0.58 |
| 1428233_at | Cpsf6 | cleavage and polyadenylation specific factor 6 | -1.17 | 0.553 | -1.21 | 0.143 | -1.03 | 0.774 | 1.17 | 0.454 | -0.56 |
| 1431270_a_at | Aste1 | asteroid homolog 1 (Drosophila) | -1.13 | 0.748 | -1.34 | 0.412 | 1.02 | 0.964 | -1.03 | 0.923 | -0.62 |
| 1438646_x_at | 2510039O18Rik | RIKEN cDNA 2510039O18 gene | -1.42 | 0.258 | -1.11 | 0.452 | 1.05 | 0.705 | -1.36 | 0.101 | -0.71 |
| 1438916_x_at | 6720401G13Rik | RIKEN cDNA 6720401G13 gene | 1.05 | 0.766 | -1.12 | 0.585 | -1.41 | 0.066 | -1.14 | 0.626 | -0.65 |
| 1427946_s_at | Dpyd | dihydropyrimidine dehydrogenase | 1.01 | 0.953 | -1.04 | 0.661 | -1.47 | 0.21 | -1.47 | 0.378 | -0.74 |
| 1446772_at | Sema5a | Sema domain, seven thrombospondin repeats (type 1 and type 1-like), transmem | -1.37 | 0.297 | 1.4 | 0.448 | -1.93 | 0.306 | -1.3 | 0.372 | -0.8 |
| 1423036_at | Txnl5 | thioredoxin-like 5 | -1.05 | 0.782 | -1.21 | 0.529 | -1.15 | 0.369 | 1.04 | 0.897 | -0.59 |
| 1443050_at | BC032265 | CDNA sequence BC032265 | -1.19 | 0.243 | 1.22 | 0.655 | -1.67 | 0.187 | -2.01 | 0.099 | -0.91 |
| 1419165_at | Zfp260 /// LOC635( | zinc finger protein 260 /// similar to Zinc finger protein OZF (Zinc finger protein 26 | 1.03 | 0.14 | -1.09 | 0.25 | -1.41 | 0.062 | -1.55 | 0.043 | -0.76 |
| 1417966_at | Mrpl39 | mitochondrial ribosomal protein L39 | -1.17 | 0.294 | -1.38 | 0.034 | 1.07 | 0.615 | -1.19 | 0.144 | -0.67 |
| 1459728_at | 5830446M03Rik | RIKEN cDNA 5830446M03 gene | -1.2 | 0.504 | 1.04 | 0.74 | -1.29 | 0.29 | -1.01 | 0.943 | -0.61 |
| 1435170_at | Tsr2 | TSR2, 20S rRNA accumulation, homolog (S. cerevisiae) | -1.19 | 0.501 | -1.24 | 0.434 | 1.01 | 0.977 | -1.07 | 0.84 | -0.62 |
| 1425095_at | BC002059 | cDNA sequence BC002059 | -1.28 | 0.245 | -1.22 | 0.264 | 1.05 | 0.463 | -1.21 | 0.046 | -0.66 |
| 1448547_at | Rassf3 | Ras association (RalGDS/AF-6) domain family 3 | -1.16 | 0.217 | -1.26 | 0.148 | 1 | 0.999 | -1.34 | 0.196 | -0.69 |
| 1454790_at | Wdr35 | WD repeat domain 35 | -1.08 | 0.201 | -1.32 | 0.479 | -1.03 | 0.768 | 1.12 | 0.616 | -0.58 |
| 1431063_at | --- | --- | -1.13 | 0.621 | -1.14 | 0.716 | -1.12 | 0.622 | 1.14 | 0.258 | -0.56 |

| | | | | | | | | | | | |
|---|---|---|---|---|---|---|---|---|---|---|---|
| 1456579_x_at | 1200003M09Rik | RIKEN cDNA 1200003M09 gene | -1.47 | 0.115 | 1.14 | 0.755 | -1.21 | 0.417 | -1.06 | 0.898 | -0.65 |
| 1420222_at | --- | --- | -1.86 | 0.564 | 1.4 | 0.577 | -1.4 | 0.456 | -1.97 | 0.348 | -0.96 |
| 1432771_at | Slc9a3 | solute carrier family 9 (sodium/hydrogen exchanger), member 3 | -1.99 | 0.093 | -1.74 | 0.17 | 1.58 | 0.09 | -1.11 | 0.847 | -0.82 |
| 1452255_at | Fbxo38 | F-box protein 38 | -1.25 | 0.137 | -1.18 | 0.4 | 1.01 | 0.963 | -1.11 | 0.51 | -0.63 |
| 1428196_a_at | 1200015F23Rik | RIKEN cDNA 1200015F23 gene | -1.12 | 0.158 | -1.11 | 0.021 | -1.16 | 0.218 | 1.34 | 0.454 | -0.51 |
| 1445867_at | AL023008 | expressed sequence AL023008 | -1.01 | 0.779 | 1.02 | 0.805 | -1.56 | 0.016 | -1.57 | 0.038 | -0.78 |
| 1454976_at | Sod2 | superoxide dismutase 2, mitochondrial | -1.19 | 0.448 | -1.06 | 0.706 | -1.14 | 0.332 | 1.4 | 0.219 | -0.5 |
| 1435576_at | AW413774 | expressed sequence AW413774 | -1.04 | 0.786 | -1.19 | 0.189 | -1.17 | 0.413 | 1.03 | 0.901 | -0.59 |
| 1435135_at | Aadacl1 | arylacetamide deacetylase-like 1 | 1.06 | 0.211 | -1.41 | 0.049 | -1.13 | 0.204 | -1.6 | 0.123 | -0.77 |
| 1449565_at | Cyp2g1 | cytochrome P450, family 2, subfamily g, polypeptide 1 | -1.92 | 0.241 | -1.4 | 0.331 | 1.42 | 0.323 | -1.1 | 0.867 | -0.75 |
| 1455608_at | Sclt1 | sodium channel and clathrin linker 1 | -1.29 | 0.275 | -1.19 | 0.104 | 1.04 | 0.735 | -1.2 | 0.206 | -0.66 |
| 1443954_at | Rad18 | RAD18 homolog (S. cerevisiae) | -1.04 | 0.872 | -1.1 | 0.768 | -1.28 | 0.383 | 1.18 | 0.478 | -0.56 |
| 1425197_at | Ptpn2 | protein tyrosine phosphatase, non-receptor type 2 | -1.21 | 0.524 | -1.08 | 0.868 | -1.11 | 0.448 | 1.31 | 0.306 | -0.52 |
| 1436326_at | Rora | RAR-related orphan receptor alpha | 1.17 | 0.174 | -1.26 | 0.051 | -1.44 | 0.037 | -1.3 | 0.331 | -0.71 |
| 1442442_at | Thoc7 | THO complex 7 homolog (Drosophila) | -1.29 | 0.403 | -1.16 | 0.543 | 1.02 | 0.847 | -1.01 | 0.877 | -0.61 |
| 1424623_at | Serpinb5 | serine (or cysteine) peptidase inhibitor, clade B, member 5 | 1.07 | 0.816 | -1.13 | 0.804 | -1.44 | 0.199 | -1.82 | 0.236 | -0.83 |
| 1439758_at | Als2cr12 | amyotrophic lateral sclerosis 2 (juvenile) chromosome region, candidate 12 (huma | -1.26 | 0.232 | 1.22 | 0.597 | -1.56 | 0.156 | -1.37 | 0.271 | -0.74 |
| 1450953_at | Wdr39 | WD repeat domain 39 | -1.27 | 0.092 | -1.24 | 0.094 | 1.06 | 0.485 | -1.06 | 0.514 | -0.63 |
| 1443163_at | Slc39a2 | solute carrier family 39 (zinc transporter), member 2 | -1.13 | 0.826 | -1.38 | 0.079 | 1.04 | 0.85 | -1.13 | 0.885 | -0.65 |
| 1417573_at | 2010311D03Rik | RIKEN cDNA 2010311D03 gene | -1.09 | 0.283 | -1.18 | 0.015 | -1.12 | 0.182 | 1.29 | 0.362 | -0.53 |
| 1454988_s_at | Rab22a | RAB22A, member RAS oncogene family | -1.08 | 0.733 | -1.06 | 0.821 | -1.27 | 0.46 | 1.02 | 0.919 | -0.6 |
| 1445677_x_at | Slc35f2 | solute carrier family 35, member F2 | -1.23 | 0.778 | 1.02 | 0.943 | -1.22 | 0.499 | -1.15 | 0.757 | -0.64 |
| 1430723_at | Pdss2 | prenyl (solanesyl) diphosphate synthase, subunit 2 | 1.25 | 0.237 | -1.4 | 0.525 | -1.44 | 0.416 | -2.08 | 0.453 | -0.92 |
| 1456932_at | B430201F14 | Hypothetical protein B430201F14 | -1.4 | 0.507 | -1.14 | 0.837 | 1.06 | 0.723 | -2.69 | 0.04 | -1.04 |
| 1422443_at | Xpnpep1 | X-prolyl aminopeptidase (aminopeptidase P) 1, soluble | -1.11 | 0.352 | -1.21 | 0.026 | -1.07 | 0.47 | 1.7 | 0.297 | -0.43 |
| 1431959_x_at | Psg21 | pregnancy-specific glycoprotein 21 | -1.45 | 0.105 | 1.04 | 0.918 | -1.08 | 0.83 | -1.34 | 0.259 | -0.71 |
| 1430627_at | 5330420P17Rik | RIKEN cDNA 5330420P17 gene | 1 | 0.989 | -1.23 | 0.437 | -1.19 | 0.461 | -1.47 | 0.197 | -0.72 |
| 1434435_s_at | Cox17 | cytochrome c oxidase, subunit XVII assembly protein homolog (yeast) | -1.11 | 0.037 | -1.02 | 0.895 | -1.3 | 0.056 | 1.22 | 0.319 | -0.55 |
| 1439968_x_at | --- | Adult male corpora quadrigemina cDNA, RIKEN full-length enriched library, clone | 1.18 | 0.816 | -1.41 | 0.657 | -1.3 | 0.206 | -1.26 | 0.292 | -0.7 |
| 1442786_s_at | Rufy3 | RUN and FYVE domain containing 3 | -1.31 | 0.032 | -1.08 | 0.631 | -1.03 | 0.934 | 1.09 | 0.852 | -0.58 |
| 1447085_s_at | Nfatc1 | nuclear factor of activated T-cells, cytoplasmic, calcineurin-dependent 1 | -1.25 | 0.469 | -1.36 | 0.316 | 1.12 | 0.636 | -1.34 | 0.142 | -0.71 |
| 1428314_at | Pcnp | PEST proteolytic signal containing nuclear protein | -1 | 0.929 | -1.24 | 0.157 | -1.17 | 0.149 | 1.09 | 0.471 | -0.58 |
| 1457202_at | --- | --- | -1.54 | 0.191 | -2.14 | 0.18 | 1.54 | 0.429 | -1.02 | 0.953 | -0.79 |
| 1442622_at | Strbp | Spermatid perinuclear RNA binding protein | 1.07 | 0.649 | -1.07 | 0.603 | -1.52 | 0.064 | -2.3 | 0.063 | -0.96 |
| 1451995_at | Taf11 | TAF11 RNA polymerase II, TATA box binding protein (TBP)-associated factor | -1.05 | 0.828 | -1.3 | 0.137 | -1.08 | 0.732 | 1.09 | 0.716 | -0.58 |
| 1433442_at | Klhl9 | Kelch-like 9 (Drosophila) | -1.03 | 0.827 | 1.03 | 0.738 | -1.53 | 0 | -1.13 | 0.554 | -0.66 |
| 1455612_at | AI848218 | expressed sequence AI848218 | -1.17 | 0.448 | -1.18 | 0.542 | -1.05 | 0.845 | 1.33 | 0.461 | -0.52 |
| 1456749_at | --- | Transcribed locus | 1.32 | 0.177 | -1.33 | 0.57 | -1.7 | 0.248 | -1.93 | 0.306 | -0.91 |
| 1434978_at | 4933403F05Rik | RIKEN cDNA 4933403F05 gene | -1.06 | 0.717 | -1.1 | 0.502 | -1.25 | 0.191 | 1.32 | 0.482 | -0.52 |
| 1435079_at | 5730406M06Rik | RIKEN cDNA 5730406M06 gene | -1.15 | 0.41 | 1.05 | 0.719 | -1.36 | 0.06 | -1.22 | 0.258 | -0.67 |
| 1429218_at | Det1 | de-etiolated homolog 1 (Arabidopsis) | -1.43 | 0.155 | -1.21 | 0.287 | 1.13 | 0.477 | -1.13 | 0.355 | -0.66 |
| 1446075_at | --- | --- | -1.03 | 0.882 | -1.04 | 0.892 | -1.38 | 0.024 | 1.5 | 0.134 | -0.49 |
| 1440522_at | B930075F07 | hypothetical protein B930075F07 | -1.21 | 0.045 | -1.02 | 0.891 | -1.17 | 0.411 | 1.09 | 0.568 | -0.58 |
| 1433910_at | Zcchc6 | zinc finger, CCHC domain containing 6 | -1.05 | 0.292 | -1.12 | 0.139 | -1.23 | 0.035 | 1.31 | 0.229 | -0.52 |
| 1447060_at | Smyd3 | SET and MYND domain containing 3 | -1.28 | 0.225 | 1.05 | 0.68 | -1.21 | 0.417 | -1.73 | 0.043 | -0.79 |
| 1437510_x_at | Rps17 | ribosomal protein S17 | -1.11 | 0.086 | 1.03 | 0.61 | -1.38 | 0.018 | -1.39 | 0.02 | -0.71 |
| 1430425_at | Sdk2 | sidekick homolog 2 (chicken) | 1.07 | 0.751 | -1.22 | 0.456 | -1.31 | 0.334 | -1.05 | 0.22 | -0.63 |
| 1451558_at | Fbxw7 | F-box and WD-40 domain protein 7, archipelago homolog (Drosophila) | -1.14 | 0.28 | -1.04 | 0.806 | -1.22 | 0.295 | 1.09 | 0.143 | -0.58 |
| 1426488_at | Bfar | bifunctional apoptosis regulator | 1 | 0.997 | -1.03 | 0.707 | -1.45 | 0.012 | -1.23 | 0.305 | -0.68 |
| 1429191_at | Dhx33 | DEAH (Asp-Glu-Ala-His) box polypeptide 33 | -1.18 | 0.088 | -1.41 | 0.041 | 1.1 | 0.678 | -1.1 | 0.298 | -0.65 |
| 1446816_at | --- | Transcribed locus | 1.4 | 0.706 | -1.48 | 0.536 | -1.71 | 0.223 | -1.18 | 0.623 | -0.74 |
| 1455632_at | Gnb5 | Guanine nucleotide binding protein, beta 5 | -1.03 | 0.914 | 1.16 | 0.586 | -1.9 | 0.09 | -1.87 | 0.385 | -0.91 |
| 1458696_at | Actr1a | ARP1 actin-related protein 1 homolog A (yeast) | -1.3 | 0.505 | -2.68 | 0.225 | 1.52 | 0.2 | -1.59 | 0.445 | -1.02 |

| | | | | | | | | | | | |
|---|---|---|---|---|---|---|---|---|---|---|---|
| 1449723_at | Serpinb6e | serine (or cysteine) peptidase inhibitor, clade B, member 6e | 1.22 | 0.667 | -1.31 | 0.405 | -1.47 | 0.09 | -2.21 | 0.364 | -0.94 |
| 1439068_at | Arts1 | type 1 tumor necrosis factor receptor shedding aminopeptidase regulator | 1.03 | 0.908 | -1.09 | 0.21 | -1.4 | 0.011 | -1.31 | 0.08 | -0.69 |
| 1449880_s_at | Bglap-rs1 /// Bglap1 | bone gamma-carboxyglutamate protein, related sequence 1 /// bone gamma carb | -1.07 | 0.784 | -1.17 | 0.442 | -1.16 | 0.205 | 1.58 | 0.29 | -0.45 |
| 1422665_a_at | Pcmt1 | protein-L-isoaspartate (D-aspartate) O-methyltransferase 1 | -1.02 | 0.906 | -1.01 | 0.956 | -1.47 | 0.066 | 1.15 | 0.509 | -0.59 |
| 1456382_at | Atad1 | ATPase family, AAA domain containing 1 | -1.03 | 0.864 | -1.1 | 0.829 | -1.28 | 0.478 | 1.07 | 0.873 | -0.59 |
| 1452994_at | Pip5k3 | phosphatidylinositol-3-phosphate/phosphatidylinositol 5-kinase, type III | -1.39 | 0.133 | 1.07 | 0.123 | -1.15 | 0.378 | -1.29 | 0.417 | -0.69 |
| 1451621_at | 5830417C01Rik | RIKEN cDNA 5830417C01 gene | -1.06 | 0.769 | 1.04 | 0.893 | -1.47 | 0.064 | -1.24 | 0.403 | -0.68 |
| 1428256_at | 2310047H23Rik | RIKEN cDNA 2310047H23 gene | 1.02 | 0.634 | -1.19 | 0.017 | -1.25 | 0.106 | -1.37 | 0.217 | -0.7 |
| 1425033_at | Slc17a2 | solute carrier family 17 (sodium phosphate), member 2 | 1.18 | 0.398 | -1.39 | 0.004 | -1.32 | 0.364 | -1.15 | 0.119 | -0.67 |
| 1459946_at | --- | Adult male diencephalon cDNA, RIKEN full-length enriched library, clone:933015 | -1.13 | 0.838 | 1.08 | 0.876 | -1.44 | 0.212 | -1.51 | 0.589 | -0.75 |
| 1452505_at | Lamc2 | laminin, gamma 2 | 1.44 | 0.689 | -1.6 | 0.406 | -1.7 | 0.431 | -1.04 | 0.947 | -0.72 |
| 1431886_at | Ern1 | endoplasmic reticulum (ER) to nucleus signalling 1 | 1.12 | 0.146 | -1.27 | 0.665 | -1.34 | 0.151 | -1.26 | 0.658 | -0.68 |
| 1432905_at | 3110037C07Rik | RIKEN cDNA 3110037C07 gene | 1.04 | 0.945 | -1.42 | 0.559 | -1.09 | 0.778 | -1.55 | 0.246 | -0.76 |
| 1456336_at | A330102K23Rik | RIKEN cDNA A330102K23 gene | -1.12 | 0.49 | -1.31 | 0.752 | 1 | 0.991 | -2.29 | 0.252 | -0.93 |
| 1432153_at | 4933426B08Rik | RIKEN cDNA 4933426B08 gene | 1.04 | 0.523 | -1.24 | 0.399 | -1.23 | 0.499 | -1.38 | 0.52 | -0.7 |
| 1419110_at | Riok1 | RIO kinase 1 (yeast) | -1.17 | 0.546 | -1.2 | 0.401 | -1.03 | 0.698 | 1.11 | 0.226 | -0.57 |
| 1458327_x_at | Slc26a1 | solute carrier family 26 (sulfate transporter), member 1 | -1.16 | 0.66 | -1.15 | 0.512 | -1.08 | 0.91 | 3.72 | 0.041 | 0.08 |
| 1436780_at | Ogt | O-linked N-acetylglucosamine (GlcNAc) transferase (UDP-N-acetylglucosamine:p | 1.13 | 0.785 | -1.03 | 0.9 | -1.81 | 0.081 | -1.02 | 0.966 | -0.68 |
| 1437885_at | D030029J20Rik | RIKEN cDNA D030029J20 gene | -1.09 | 0.528 | 1.03 | 0.721 | -1.4 | 0.142 | -1.06 | 0.827 | -0.63 |
| 1426965_at | Rap2a | RAS related protein 2a | 1.03 | 0.728 | -1.34 | 0.193 | -1.14 | 0.517 | -1.1 | 0.557 | -0.64 |
| 1455080_at | Ppp1r16b | protein phosphatase 1, regulatory (inhibitor) subunit 16B | 1.1 | 0.795 | -1.35 | 0.274 | -1.23 | 0.308 | -1.26 | 0.351 | -0.68 |
| 1438407_at | 9330132E09Rik | RIKEN cDNA 9330132E09 gene | -1.17 | 0.605 | 1.2 | 0.339 | -1.66 | 0.011 | -1.22 | 0.396 | -0.71 |
| 1418158_at | Trp63 | transformation related protein 63 | -1.18 | 0.617 | 1.13 | 0.594 | -1.46 | 0.289 | -1.53 | 0.318 | -0.76 |
| 1434924_at | Phf2 | PHD finger protein 2 | -1.06 | 0.658 | -1.45 | 0.053 | 1.03 | 0.917 | -1.1 | 0.648 | -0.65 |
| 1455677_s_at | Clcnka /// LOC6390 | chloride channel Ka /// similar to Chloride channel protein ClC-Ka (Chloride chanr | 1.29 | 0.336 | -1.26 | 0.759 | -1.72 | 0.097 | -1.49 | 0.486 | -0.8 |
| 1437486_at | Gprc5a | G protein-coupled receptor, family C, group 5, member A | -1.07 | 0.571 | -1.16 | 0.672 | -1.15 | 0.288 | 1.43 | 0.538 | -0.49 |
| 1416997_a_at | Hap1 | huntingtin-associated protein 1 | -1.26 | 0.626 | -1.43 | 0.203 | 1.17 | 0.345 | -1.17 | 0.722 | -0.67 |
| 1442473_at | --- | --- | 1.21 | 0.416 | -1.25 | 0.276 | -1.53 | 0.018 | -1.57 | 0.393 | -0.79 |
| 1446010_at | LOC668019 /// LOC | similar to F-box only protein 34 /// similar to F-box only protein 34 /// similar to F-b | 1.12 | 0.909 | -1.7 | 0.189 | -1.05 | 0.898 | -1.44 | 0.714 | -0.77 |
| 1438159_x_at | Ndufv2 | NADH dehydrogenase (ubiquinone) flavoprotein 2 | 1.02 | 0.806 | -1.33 | 0.115 | -1.13 | 0.631 | -1.6 | 0.269 | -0.76 |
| 1445349_at | --- | 0 day neonate lung cDNA, RIKEN full-length enriched library, clone:E030049A20 | 1.26 | 0.669 | -2.27 | 0.285 | -1.04 | 0.922 | -1.1 | 0.89 | -0.79 |
| 1422636_at | Dmtf1 | cyclin D binding myb-like transcription factor 1 | -1.33 | 0.118 | 1.13 | 0.361 | -1.28 | 0.011 | -1.55 | 0.074 | -0.76 |
| 1445541_at | Jarid1a | jumonji, AT rich interactive domain 1A (Rbp2 like) | -1.71 | 0.64 | 1.33 | 0.657 | -1.34 | 0.298 | -1.3 | 0.23 | -0.75 |
| 1434518_at | Phka2 | phosphorylase kinase alpha 2 | -1.22 | 0.002 | -1.1 | 0.161 | -1.07 | 0.661 | 1.17 | 0.196 | -0.56 |
| 1422032_a_at | Zfand6 | zinc finger, AN1-type domain 6 | -1.07 | 0.437 | -1.3 | 0.006 | -1.04 | 0.529 | 1.32 | 0.205 | -0.52 |
| 1426976_at | Usp47 | ubiquitin specific peptidase 47 | -1.12 | 0.356 | -1.05 | 0.389 | -1.23 | 0.034 | 1.24 | 0.234 | -0.54 |
| 1453373_at | 6330532G10Rik | RIKEN cDNA 6330532G10 gene | 1.29 | 0.732 | -2.16 | 0.224 | -1.11 | 0.82 | -1.29 | 0.703 | -0.82 |
| 1419693_at | Colec12 | collectin sub-family member 12 | 1.05 | 0.82 | -1.03 | 0.771 | -1.56 | 0.054 | -1.06 | 0.87 | -0.65 |
| 1433585_at | Tnpo1 | transportin 1 | -1.17 | 0.292 | 1.03 | 0.742 | -1.29 | 0.115 | -1.19 | 0.315 | -0.65 |
| 1437896_at | Zcrb1 | Zinc finger CCHC-type and RNA binding motif 1 | -1.19 | 0.549 | -1.03 | 0.779 | -1.18 | 0.466 | 1.15 | 0.434 | -0.56 |
| 1440948_at | Frk | Fyn-related kinase | -1.4 | 0.088 | -1.19 | 0.54 | 1.11 | 0.716 | -1.37 | 0.426 | -0.71 |
| 1449883_at | Fxyd2 | FXYD domain-containing ion transport regulator 2 | 1.01 | 0.975 | -1.15 | 0.252 | -1.28 | 0.349 | -1.21 | 0.752 | -0.66 |
| 1425893_a_at | Fhit | fragile histidine triad gene | -1.23 | 0.204 | -1.28 | 0.326 | 1.06 | 0.847 | -1.21 | 0.35 | -0.66 |
| 1433268_at | 4930553M12Rik | RIKEN cDNA 4930553M12 gene | -3.01 | 0.012 | -1.29 | 0.3 | 1.55 | 0.422 | -1.2 | 0.812 | -0.99 |
| 1457631_at | 2310047N11Rik | RIKEN cDNA 2310047N11 gene | 1.12 | 0.857 | -1.11 | 0.831 | -1.56 | 0.012 | -1.83 | 0.288 | -0.84 |
| 1418128_at | Adcy6 | adenylate cyclase 6 | -1.13 | 0.338 | -1.26 | 0.388 | -1.02 | 0.936 | 2.71 | 0.256 | -0.17 |
| 1432246_at | 4922502N22Rik | RIKEN cDNA 4922502N22 gene | 1.35 | 0.761 | -1.88 | 0.211 | -1.28 | 0.624 | -1.57 | 0.512 | -0.85 |
| 1444417_at | 9330101J02Rik /// L | RIKEN cDNA 9330101J02 gene /// similar to Protein C10orf93 /// similar to Protei | -1.26 | 0.689 | -1.5 | 0.406 | 1.2 | 0.563 | -1.15 | 0.772 | -0.68 |
| 1429335_at | Snapc1 | small nuclear RNA activating complex, polypeptide 1 | -1.4 | 0.312 | 1.09 | 0.535 | -1.16 | 0.1 | -1.55 | 0.126 | -0.76 |
| 1437047_at | Zfp664 | zinc finger protein 664 | 1.21 | 0.694 | -1.11 | 0.67 | -1.84 | 0.238 | -1.46 | 0.461 | -0.8 |
| 1457269_at | Gm1123 /// LOC67 | gene model 1123, (NCBI) /// similar to coxsackie-adenovirus receptor-like | -1.26 | 0.305 | 1.02 | 0.954 | -1.18 | 0.712 | -1.02 | 0.955 | -0.61 |
| 1455765_a_at | Abcc8 | ATP-binding cassette, sub-family C (CFTR/MRP), member 8 | -1.34 | 0.489 | -1.51 | 0.385 | 1.25 | 0.337 | -1.62 | 0.298 | -0.81 |
| 1416958_at | Nr1d2 | nuclear receptor subfamily 1, group D, member 2 | 1 | 0.995 | -1.16 | 0.618 | -1.26 | 0.521 | -1.12 | 0.837 | -0.63 |

| | | | | | | | | | | | |
|---|---|---|---|---|---|---|---|---|---|---|---|
| 1435711_at | --- | --- | -1.24 | 0.066 | -1.09 | 0.684 | -1.07 | 0.733 | 1.14 | 0.652 | -0.56 |
| 1451455_at | BC051244 | cDNA sequence BC051244 | -1.3 | 0.201 | -1.1 | 0.641 | -1.02 | 0.89 | 1.47 | 0.589 | -0.49 |
| 1452619_a_at | Agbl3 | ATP/GTP binding protein-like 3 | -1.01 | 0.943 | -1.26 | 0.541 | -1.14 | 0.68 | 1.31 | 0.441 | -0.52 |
| 1454680_at | D5Ertd579e | DNA segment, Chr 5, ERATO Doi 579, expressed | -1.05 | 0.79 | -1.2 | 0.237 | -1.14 | 0.267 | 1.64 | 0.151 | -0.44 |
| 1416285_at | Ndufc1 | NADH dehydrogenase (ubiquinone) 1, subcomplex unknown, 1 | -1.11 | 0.143 | -1.16 | 0.014 | -1.11 | 0.168 | 1.17 | 0.473 | -0.55 |
| 1458575_at | Setbp1 | SET binding protein 1 | -1.04 | 0.889 | 1.14 | 0.436 | -1.8 | 0.02 | -1.22 | 0.68 | -0.73 |
| 1452615_s_at | Trpt1 | tRNA phosphotransferase 1 | -1.07 | 0.15 | -1.07 | 0.701 | -1.26 | 0.007 | 1.18 | 0.06 | -0.56 |
| 1425341_at | Kcnk3 | potassium channel, subfamily K, member 3 | -2.1 | 0.205 | -1.06 | 0.894 | 1.24 | 0.317 | -1.65 | 0.031 | -0.89 |
| 1416770_at | Stk25 | serine/threonine kinase 25 (yeast) | -1.04 | 0.818 | -1.07 | 0.64 | -1.31 | 0.251 | 1.19 | 0.402 | -0.56 |
| 1452639_at | Enpp4 | ectonucleotide pyrophosphatase/phosphodiesterase 4 | -1.46 | 0.545 | 1.02 | 0.949 | -1.04 | 0.895 | -1.22 | 0.577 | -0.68 |
| 1442314_at | Nup188 | nucleoporin 188 | -1.2 | 0.486 | -1.09 | 0.684 | -1.1 | 0.778 | 1.46 | 0.118 | -0.48 |
| 1421868_a_at | Pnlip | pancreatic lipase | -1.22 | 0.113 | -1.5 | 0.356 | 1.18 | 0.786 | -1.34 | 0.258 | -0.72 |
| 1444765_at | Rbpms | RNA binding protein gene with multiple splicing | -1.08 | 0.664 | 1.13 | 0.656 | -1.64 | 0.164 | -2.32 | 0.156 | -0.98 |
| 1441866_s_at | Ptdss1 | phosphatidylserine synthase 1 | -1.11 | 0.197 | -1.1 | 0.394 | -1.17 | 0.127 | 1.06 | 0.391 | -0.58 |
| 1433513_x_at | Ndufa12 | NADH dehydrogenase (ubiquinone) 1 alpha subcomplex, 12 | -1.13 | 0.217 | -1.25 | 0.01 | -1.03 | 0.474 | 1.2 | 0.123 | -0.55 |
| 1434876_at | Glt8d3 | glycosyltransferase 8 domain containing 3 | 1.01 | 0.916 | -1.06 | 0.476 | -1.42 | 0.031 | -1.3 | 0.106 | -0.69 |
| 1433902_at | Kbtbd8 | kelch repeat and BTB (POZ) domain containing 8 | -1.15 | 0.054 | -1.17 | 0.369 | -1.07 | 0.551 | 1.08 | 0.775 | -0.58 |
| 1458576_at | Nat11 | N-acetyltransferase 11 | 1.11 | 0.665 | -1.41 | 0.573 | -1.19 | 0.684 | -1.2 | 0.681 | -0.67 |
| 1424970_at | Purg | purine-rich element binding protein G | -1.05 | 0.532 | -1.09 | 0.581 | -1.25 | 0.066 | 1.16 | 0.455 | -0.56 |
| 1417147_at | B230317C12Rik | RIKEN cDNA B230317C12 gene | -1.09 | 0.738 | -1.14 | 0.788 | -1.15 | 0.318 | 1.08 | 0.845 | -0.58 |
| 1460163_at | C1qtnf7 | C1q and tumor necrosis factor related protein 7 | -1.04 | 0.736 | 1.14 | 0.171 | -1.79 | 0.019 | -1.84 | 0.215 | -0.88 |
| 1425691_at | B3gat1 | beta-1,3-glucuronyltransferase 1 (glucuronosyltransferase P) | -1.07 | 0.93 | -1.41 | 0.65 | 1.02 | 0.975 | -1.27 | 0.775 | -0.68 |
| 1437342_x_at | Pttg1ip | pituitary tumor-transforming 1 interacting protein | 1.01 | 0.933 | -1.27 | 0.072 | -1.15 | 0.395 | -1.13 | 0.248 | -0.64 |
| 1424807_at | Lama4 | laminin, alpha 4 | -1.02 | 0.961 | 1.07 | 0.884 | -1.63 | 0.009 | -2.03 | 0.007 | -0.9 |
| 1441484_at | Tcf2 | Transcription factor 2 | -1.19 | 0.234 | 1.05 | 0.91 | -1.29 | 0.334 | -1.88 | 0.102 | -0.83 |
| 1457288_at | Ank3 | ankyrin 3, epithelial | -1.15 | 0.54 | 1.31 | 0.446 | -2.09 | 0.142 | -1.5 | 0.335 | -0.86 |
| 1418973_at | Blzf1 | basic leucine zipper nuclear factor 1 | -1.08 | 0.484 | -1.04 | 0.821 | -1.29 | 0.259 | 1.12 | 0.542 | -0.57 |
| 1453027_at | Dlgap1 | discs, large (Drosophila) homolog-associated protein 1 | -1.18 | 0.25 | 1.3 | 0.509 | -1.95 | 0.247 | -1.14 | 0.542 | -0.74 |
| 1425375_at | Tdpoz1 | TD and POZ domain containing 1 | -1.45 | 0.135 | -1.05 | 0.926 | 1.02 | 0.953 | -1.18 | 0.789 | -0.67 |
| 1417975_at | Kpna4 | karyopherin (importin) alpha 4 | -1.14 | 0.64 | 1.02 | 0.971 | -1.3 | 0.563 | -1.59 | 0.279 | -0.75 |
| 1433735_a_at | Tmem64 | transmembrane protein 64 | -1.61 | 0.181 | -1.05 | 0.799 | 1.08 | 0.612 | -1.05 | 0.778 | -0.65 |
| 1433759_at | Dpy19l1 | dpy-19-like 1 (C. elegans) | -1.12 | 0.137 | -1.25 | 0.155 | -1.03 | 0.833 | 1.05 | 0.734 | -0.59 |
| 1439950_at | Dync1h1 | dynein cytoplasmic 1 heavy chain 1 | 1.14 | 0.375 | -1.32 | 0.203 | -1.31 | 0.535 | -1.07 | 0.742 | -0.64 |
| 1424154_a_at | Hbld1 | HESB like domain containing 1 | -1.09 | 0.288 | -1.16 | 0.122 | -1.13 | 0.327 | 1.44 | 0.384 | -0.49 |
| 1448860_at | Rem2 | rad and gem related GTP binding protein 2 | -1.73 | 0.272 | 1.23 | 0.453 | -1.16 | 0.595 | -1.04 | 0.884 | -0.68 |
| 1439273_at | Ripk1 | receptor (TNFRSF)-interacting serine-threonine kinase 1 | 1 | 0.989 | -1.1 | 0.568 | -1.32 | 0.008 | -1.03 | 0.851 | -0.62 |
| 1447393_at | --- | PREDICTED: Mus musculus hypothetical LOC434436 (LOC434436), mRNA | -1.06 | 0.252 | -1.23 | 0.636 | -1.1 | 0.851 | 2.59 | 0.108 | -0.2 |
| 1436130_s_at | 4930523C11Rik /// | RIKEN cDNA 4930523C11 gene /// a disintegrin and metallopeptidase domain 6 | -1.11 | 0.876 | -1.98 | 0.224 | 1.26 | 0.665 | -1.22 | 0.417 | -0.76 |
| 1433889_at | Sox9 | SRY-box containing gene 9 | 1.07 | 0.893 | -1.37 | 0.626 | -1.16 | 0.758 | -2.41 | 0.433 | -0.97 |
| 1444059_at | 6330415G19Rik | RIKEN cDNA 6330415G19 gene | -1.06 | 0.87 | -2.04 | 0.058 | 1.23 | 0.373 | -1.35 | 0.305 | -0.8 |
| 1455625_at | --- | --- | -1.24 | 0.258 | -1.08 | 0.326 | -1.08 | 0.379 | 1.04 | 0.823 | -0.59 |
| 1453352_at | 9030605H24Rik | RIKEN cDNA 9030605H24 gene | 1.06 | 0.903 | -1.01 | 0.964 | -1.64 | 0.333 | -1.62 | 0.365 | -0.8 |
| 1441409_at | Gnn | Grp94 neighboring nucleotidase variant 4 | -1.24 | 0.652 | -1.07 | 0.826 | -1.08 | 0.48 | 1.42 | 0.356 | -0.49 |
| 1417434_at | Gpd2 | glycerol phosphate dehydrogenase 2, mitochondrial | -1.14 | 0.641 | -1.55 | 0.101 | 1.14 | 0.54 | -1.3 | 0.316 | -0.71 |
| 1438866_at | Grin3a | glutamate receptor ionotropic, NMDA3A | -1.47 | 0.586 | 1.17 | 0.849 | -1.23 | 0.583 | -1.77 | 0.141 | -0.83 |
| 1416070_a_at | Ddx18 | DEAD (Asp-Glu-Ala-Asp) box polypeptide 18 | 1.07 | 0.707 | -1.27 | 0.004 | -1.23 | 0.09 | -1.47 | 0.033 | -0.73 |
| 1449934_at | Pura | purine rich element binding protein A | 1.04 | 0.794 | -1.2 | 0.022 | -1.27 | 0.101 | -1.05 | 0.839 | -0.62 |
| 1459962_at | 4930523C07Rik | RIKEN cDNA 4930523C07 gene | 1.16 | 0.154 | -1.1 | 0.745 | -1.67 | 0.027 | -2.45 | 0.021 | -1.02 |
| 1448586_at | Hspa14 | heat shock protein 14 | -1.11 | 0.254 | -1.16 | 0.246 | -1.11 | 0.15 | 1.1 | 0.704 | -0.57 |
| 1449474_a_at | Nelf | nasal embryonic LHRH factor | -1.15 | 0.291 | -1.07 | 0.588 | -1.16 | 0.325 | 1.02 | 0.708 | -0.59 |
| 1440917_at | 2900093K20Rik | RIKEN cDNA 2900093K20 gene | 1.09 | 0.178 | -1.27 | 0.341 | -1.28 | 0.264 | -2.27 | 0.033 | -0.93 |
| 1440459_at | Setx | senataxin | -1.08 | 0.702 | 1.04 | 0.882 | -1.44 | 0.078 | -1.12 | 0.499 | -0.65 |
| 1435890_at | 5730596K20Rik | RIKEN cDNA 5730596K20 gene | -1.17 | 0.017 | -1.08 | 0.135 | -1.13 | 0.132 | 1.03 | 0.832 | -0.59 |

| | | | | | | | | | | | |
|---|---|---|---|---|---|---|---|---|---|---|---|
| 1429742_at | Rcbtb2 | regulator of chromosome condensation (RCC1) and BTB (POZ) domain containir | -1.09 | 0.854 | -1.5 | 0.522 | 1.08 | 0.886 | -1.03 | 0.937 | -0.63 |
| 1445430_at | Arhgap28 | Rho GTPase activating protein 28 | -1.11 | 0.065 | 1.02 | 0.66 | -1.33 | 0.461 | -1.34 | 0.244 | -0.69 |
| 1437083_at | A830020B06Rik | RIKEN cDNA A830020B06 gene | -2.03 | 0.053 | 1.4 | 0.55 | -1.3 | 0.671 | -1.22 | 0.771 | -0.79 |
| 1436149_at | Cox5b | cytochrome c oxidase, subunit Vb | -1.03 | 0.87 | -1.49 | 0.119 | 1.02 | 0.906 | -1.06 | 0.734 | -0.64 |
| 1434836_at | Nfatc2ip | nuclear factor of activated T-cells, cytoplasmic, calcineurin-dependent 2 interactir | -1.08 | 0.541 | -1.25 | 0.129 | -1.06 | 0.3 | 1.09 | 0.623 | -0.58 |
| 1454108_at | 4930524O08Rik | RIKEN cDNA 4930524O08 gene | -1.51 | 0.413 | 1.29 | 0.712 | -1.4 | 0.445 | -1.1 | 0.863 | -0.68 |
| 1419547_at | Fahd1 | fumarylacetoacetate hydrolase domain containing 1 | -1.07 | 0.173 | -1.35 | 0.051 | -1.01 | 0.931 | 3.72 | 0.202 | 0.07 |
| 1427543_s_at | Ube1y1 | ubiquitin-activating enzyme E1, Chr Y 1 | -1.52 | 0.107 | -1.17 | 0.501 | 1.15 | 0.651 | -1.09 | 0.766 | -0.65 |
| 1458484_at | A730020M07Rik | RIKEN cDNA A730020M07 gene | -1.3 | 0.164 | -1.53 | 0.222 | 1.24 | 0.369 | -1.46 | 0.034 | -0.76 |
| 1456161_at | 0610040B10Rik | RIKEN cDNA 0610040B10 gene | -1.06 | 0.82 | -1.45 | 0.181 | 1.03 | 0.688 | -1.09 | 0.857 | -0.64 |
| 1457479_at | --- | --- | -1.55 | 0.053 | 1.3 | 0.028 | -1.39 | 0.195 | -1.77 | 0.1 | -0.85 |
| 1443230_at | 5330421F07Rik | RIKEN cDNA 5330421F07 gene | 1.17 | 0.247 | -1.12 | 0.844 | -1.66 | 0.102 | -1.18 | 0.479 | -0.7 |
| 1450770_at | 3632451O06Rik | RIKEN cDNA 3632451O06 gene | -1.31 | 0.546 | -1.07 | 0.865 | -1.04 | 0.861 | 1.63 | 0.484 | -0.45 |
| 1458179_at | --- | --- | 1.74 | 0.314 | -1.72 | 0.108 | -2.92 | 0.034 | -1.11 | 0.785 | -1 |
| 1445684_s_at | Hdac2 | histone deacetylase 2 | -1.08 | 0.177 | -1.03 | 0.701 | -1.3 | 0.014 | 1.26 | 0.148 | -0.54 |
| 1429771_at | 3110073H01Rik | RIKEN cDNA 3110073H01 gene | 1.05 | 0.392 | -1.07 | 0.192 | -1.47 | 0 | -1.01 | 0.929 | -0.63 |
| 1444140_at | --- | --- | 1.61 | 0.153 | -1.71 | 0.264 | -2.11 | 0.044 | -1.02 | 0.943 | -0.81 |
| 1430753_at | 8030491N06Rik | RIKEN cDNA 8030491N06 gene | -1.48 | 0.367 | 1.16 | 0.52 | -1.21 | 0.419 | -1.28 | 0.575 | -0.7 |
| 1421222_at | Fip1l1 | FIP1 like 1 (S. cerevisiae) | -1.39 | 0.603 | -1.24 | 0.498 | 1.14 | 0.663 | -1.17 | 0.327 | -0.66 |
| 1439631_at | --- | --- | -1.09 | 0.679 | 1.04 | 0.622 | -1.41 | 0.042 | -2.13 | 0.065 | -0.9 |
| 1436498_at | Arih1 | ariadne ubiquitin-conjugating enzyme E2 binding protein homolog 1 (Drosophila) | -1.37 | 0.34 | -1.28 | 0.141 | 1.16 | 0.566 | -1.34 | 0.291 | -0.71 |
| 1431154_at | Znrf2 | zinc and ring finger 2 | -1.28 | 0.522 | 1 | 0.99 | -1.13 | 0.432 | -1.21 | 0.557 | -0.66 |
| 1451370_at | LOC622404 | hypothetical protein LOC622403 | 1.02 | 0.849 | -1.32 | 0.092 | -1.12 | 0.235 | -1.3 | 0.042 | -0.68 |
| 1460171_at | Cops5 | COP9 (constitutive photomorphogenic) homolog, subunit 5 (Arabidopsis thaliana) | -1.07 | 0.789 | -1.13 | 0.282 | -1.18 | 0.411 | 1.19 | 0.566 | -0.55 |
| 1433789_at | Snhg3 /// LOC6704 | small nucleolar RNA host gene (non-protein coding) 3 /// similar to RNA, U17d sn | -1.04 | 0.649 | -1.2 | 0.678 | -1.15 | 0.624 | 1.18 | 0.576 | -0.55 |
| 1428100_at | Sfrs1 | splicing factor, arginine/serine-rich 1 (ASF/SF2) | -1.35 | 0.139 | -1.03 | 0.781 | -1.05 | 0.696 | 1.04 | 0.891 | -0.6 |
| 1433243_at | 2810421E14Rik | RIKEN cDNA 2810421E14 gene | 1.01 | 0.929 | -1.27 | 0.651 | -1.15 | 0.72 | -1.05 | 0.929 | -0.62 |
| 1447026_at | Mas1 | MAS1 oncogene | 1.35 | 0.192 | -1.48 | 0.073 | -1.54 | 0.011 | -1.16 | 0.591 | -0.71 |
| 1456999_at | Wipi2 | WD repeat domain, phosphoinositide interacting 2 | -1.27 | 0.221 | -1.03 | 0.887 | -1.1 | 0.606 | 1.49 | 0.345 | -0.48 |
| 1459583_at | Grip1 | Glutamate receptor interacting protein 1 | -1.14 | 0.408 | 1.15 | 0.511 | -1.58 | 0.363 | -1.13 | 0.438 | -0.67 |
| 1434244_x_at | Htf9c | HpaII tiny fragments locus 9c | -1.48 | 0.043 | 1.04 | 0.512 | -1.05 | 0.64 | -1.3 | 0.007 | -0.7 |
| 1436514_at | Gpc4 | glypican 4 | -1.1 | 0.61 | -1.22 | 0.083 | -1.07 | 0.79 | 1.26 | 0.643 | -0.53 |
| 1458418_at | --- | Mus musculus, clone IMAGE:5368497, mRNA | -1.2 | 0.415 | -1.32 | 0.117 | 1.08 | 0.871 | -1.91 | 0.23 | -0.84 |
| 1457085_at | LOC665934 /// LOC | hypothetical protein LOC665934 /// hypothetical protein LOC669392 /// hypothetic | 1.05 | 0.841 | -1.34 | 0.546 | -1.15 | 0.7 | -1.23 | 0.159 | -0.67 |
| 1418954_at | Camkk1 | calcium/calmodulin-dependent protein kinase kinase 1, alpha | -1.36 | 0.635 | -1.03 | 0.908 | -1.04 | 0.77 | 1.08 | 0.749 | -0.59 |
| 1416652_at | Aspn | asporin | 1.06 | 0.842 | -1.49 | 0.145 | -1.06 | 0.876 | -2.12 | 0.212 | -0.91 |
| 1426919_at | --- | --- | -1.56 | 0.401 | 1.23 | 0.485 | -1.26 | 0.313 | -1.05 | 0.91 | -0.66 |
| 1447786_at | Pscd1 | pleckstrin homology, Sec7 and coiled-coil domains 1 | -1.25 | 0.731 | 1.1 | 0.774 | -1.31 | 0.56 | -1.03 | 0.867 | -0.62 |
| 1418388_s_at | 4930548G07Rik | RIKEN cDNA 4930548G07 gene | 1.02 | 0.881 | -1.08 | 0.584 | -1.4 | 0.096 | -1.25 | 0.079 | -0.68 |
| 1435388_at | Ndor1 | NADPH dependent diflavin oxidoreductase 1 | 1.27 | 0.398 | -1.57 | 0.115 | -1.31 | 0.309 | -1.06 | 0.639 | -0.67 |
| 1416946_a_at | Acaa1a /// Acaa1b | acetyl-Coenzyme A acyltransferase 1A /// acetyl-Coenzyme A acyltransferase 1B | -1 | 0.99 | -1.74 | 0.232 | 1.1 | 0.271 | -1.5 | 0.044 | -0.79 |
| 1454864_at | Zfp592 | zinc finger protein 592 | 1.01 | 0.98 | -1.29 | 0.017 | -1.13 | 0.437 | -1.15 | 0.517 | -0.64 |
| 1459445_at | --- | --- | 1.04 | 0.917 | -1.3 | 0.674 | -1.16 | 0.662 | -3.96 | 0.324 | -1.35 |
| 1430895_at | 2010109A12Rik | RIKEN cDNA 2010109A12 gene | -1.02 | 0.953 | -1.51 | 0.196 | 1.03 | 0.86 | -1.38 | 0.042 | -0.72 |
| 1427553_at | LOC628926 | hypothetical protein LOC628926 | -1.34 | 0.61 | -2.65 | 0.217 | 1.54 | 0.114 | -1.5 | 0.399 | -0.99 |
| 1442044_at | --- | Transcribed locus | 1.06 | 0.799 | -1.19 | 0.565 | -1.3 | 0.263 | -1.75 | 0.085 | -0.79 |
| 1453407_at | 1700018L24Rik | RIKEN cDNA 1700018L24 gene | -1.27 | 0.295 | -1.36 | 0.433 | 1.14 | 0.341 | -1.02 | 0.965 | -0.63 |
| 1442910_at | AL033314 | Expressed sequence AL033314 | 1.51 | 0.554 | -2.31 | 0.064 | -1.37 | 0.364 | -2.53 | 0.17 | -1.17 |
| 1442967_at | AU020147 | expressed sequence AU020147 | -1.03 | 0.909 | 1.02 | 0.967 | -1.49 | 0.402 | -1.08 | 0.776 | -0.64 |
| 1442348_at | --- | --- | -1.09 | 0.618 | -1.23 | 0.237 | -1.07 | 0.616 | 1.87 | 0.259 | -0.38 |
| 1418308_at | Hus1 | Hus1 homolog (S. pombe) | -1.09 | 0.598 | -1.05 | 0.719 | -1.25 | 0.149 | 1.18 | 0.297 | -0.55 |
| 1434549_at | --- | --- | -1.05 | 0.63 | -1.05 | 0.735 | -1.32 | 0.007 | 1.04 | 0.868 | -0.59 |
| 1429665_at | 6230416J20Rik | RIKEN cDNA 6230416J20 gene | -1 | 0.965 | -1.04 | 0.89 | -1.4 | 0.103 | 1.13 | 0.68 | -0.58 |

| | | | | | | | | | | | |
|---|---|---|---|---|---|---|---|---|---|---|---|
| 1431477_at | 4921515L22Rik | RIKEN cDNA 4921515L22 gene | -2.39 | 0.517 | -1.21 | 0.718 | 1.42 | 0.217 | -1.02 | 0.94 | -0.8 |
| 1423709_s_at | Farslb | phenylalanine-tRNA synthetase-like, beta subunit | -1.03 | 0.741 | -1.13 | 0.068 | -1.24 | 0.132 | 1.01 | 0.979 | -0.6 |
| 1453684_s_at | 2610312B22Rik | RIKEN cDNA 2610312B22 gene | 1 | 0.988 | -1.18 | 0.187 | -1.22 | 0.115 | -1.37 | 0.448 | -0.69 |
| 1449063_at | Sec22b | SEC22 vesicle trafficking protein homolog B (S. cerevisiae) | -1.19 | 0.062 | 1 | 0.995 | -1.21 | 0.112 | -1.15 | 0.369 | -0.64 |
| 1457532_at | Garnl1 | GTPase activating RANGAP domain-like 1 | -1.29 | 0.585 | 1.15 | 0.555 | -1.34 | 0.405 | -1.59 | 0.04 | -0.77 |
| 1458463_at | --- | --- | -2.11 | 0.166 | 1.82 | 0.15 | -2.67 | 0.176 | -1.97 | 0.56 | -1.23 |
| 1446280_at | Foxp1 | Forkhead box P1 | 1.14 | 0.261 | -1.07 | 0.636 | -1.7 | 0.085 | -2.02 | 0.121 | -0.91 |
| 1438291_x_at | Rpl37 /// LOC3834 | ribosomal protein L37 /// similar to ribosomal protein L37 | -1.13 | 0.665 | 1.05 | 0.656 | -1.36 | 0.005 | -2.93 | 0.26 | -1.09 |
| 1450702_at | Hfe | hemochromatosis | -1.18 | 0.642 | 1.14 | 0.197 | -1.47 | 0.03 | -1.05 | 0.77 | -0.64 |
| 1424521_at | Zfand2b | zinc finger, AN1 type domain 2B | -1.18 | 0.073 | -1.09 | 0.16 | -1.11 | 0.456 | 1.29 | 0.385 | -0.52 |
| 1425557_x_at | Tsc22d3 | TSC22 domain family 3 | 1 | 0.997 | -1.17 | 0.419 | -1.23 | 0.479 | -1.2 | 0.215 | -0.65 |
| 1457574_at | LOC635668 /// LOC | similar to F-box and WD-40 domain protein FBXW14 /// similar to F-box and WD- | 1.03 | 0.937 | -1.04 | 0.807 | -1.48 | 0.364 | -1.42 | 0.499 | -0.73 |
| 1443250_at | --- | --- | -1.34 | 0.496 | 1.25 | 0.595 | -1.48 | 0.336 | -1.59 | 0.346 | -0.79 |
| 1441885_s_at | --- | Adult male urinary bladder cDNA, RIKEN full-length enriched library, clone:95300 | 1.37 | 0.661 | -1.7 | 0.424 | -1.41 | 0.428 | -1.56 | 0.515 | -0.82 |
| 1449523_at | Bcl7c | B-cell CLL/lymphoma 7C | -1.25 | 0.113 | -1.03 | 0.702 | -1.11 | 0.502 | 1.03 | 0.905 | -0.59 |
| 1432445_at | 2310016G11Rik | RIKEN cDNA 2310016G11 gene | 1.05 | 0.869 | -1.26 | 0.648 | -1.21 | 0.456 | -1.07 | 0.762 | -0.62 |
| 1427123_s_at | Copg2as2 | coatomer protein complex, subunit gamma 2, antisense 2 | -1.04 | 0.907 | 1.22 | 0.129 | -2.05 | 0.006 | -1.89 | 0.206 | -0.94 |
| 1448085_at | D5Ertd102e | DNA segment, Chr 5, ERATO Doi 102, expressed | 1.27 | 0.535 | -1.11 | 0.789 | -1.97 | 0.245 | -1.13 | 0.673 | -0.74 |
| 1435718_at | Ap3s2 | adaptor-related protein complex 3, sigma 2 subunit | -1.26 | 0.25 | -1.18 | 0.382 | 1.03 | 0.928 | -1.02 | 0.948 | -0.61 |
| 1434503_s_at | Lamp2 | lysosomal membrane glycoprotein 2 | -1.01 | 0.968 | -1.01 | 0.936 | -1.46 | 0.109 | 1.1 | 0.59 | -0.59 |
| 1449213_at | Lage3 | L antigen family, member 3 | 1.03 | 0.883 | -1.19 | 0.194 | -1.25 | 0.052 | -1.31 | 0.311 | -0.68 |
| 1455047_at | Fbxo3 | F-box only protein 3 | -1.18 | 0.173 | -1.04 | 0.454 | -1.15 | 0.126 | 1.94 | 0.245 | -0.36 |
| 1433111_at | Nhsl1 | NHS-like 1 | -1.72 | 0.021 | 1.16 | 0.704 | -1.07 | 0.808 | -1.02 | 0.964 | -0.66 |
| 1435058_x_at | Stxbp3a | syntaxin binding protein 3A | -1.94 | 0.156 | 1.22 | 0.479 | -1.07 | 0.743 | -1.28 | 0.331 | -0.77 |
| 1458039_at | Ncoa3 | Nuclear receptor coactivator 3 | -1.09 | 0.236 | 1.24 | 0.606 | -1.95 | 0.013 | -3.01 | 0.028 | -1.2 |
| 1423042_at | Ddx3x | DEAD/H (Asp-Glu-Ala-Asp/His) box polypeptide 3, X-linked | -1.11 | 0.377 | 1.04 | 0.396 | -1.38 | 0.011 | -1.11 | 0.542 | -0.64 |
| 1454226_at | 4931403M11Rik | RIKEN cDNA 4931403M11 gene | -1.34 | 0.557 | 1.32 | 0.65 | -1.65 | 0.467 | -1.03 | 0.934 | -0.68 |
| 1423236_at | Galnt1 | UDP-N-acetyl-alpha-D-galactosamine:polypeptide N-acetylgalactosaminyltransfe | -1.04 | 0.319 | -1.08 | 0.126 | -1.29 | 0.021 | 1.25 | 0.186 | -0.54 |
| 1441992_at | Rab14 | RAB14, member RAS oncogene family | 1.09 | 0.794 | -1.06 | 0.866 | -1.58 | 0.051 | -1.23 | 0.563 | -0.69 |
| 1426461_at | Ugp2 | UDP-glucose pyrophosphorylase 2 | -1.15 | 0.143 | -1.32 | 0.262 | 1.04 | 0.734 | -1.06 | 0.58 | -0.62 |
| 1444879_at | --- | PREDICTED: Mus musculus similar to long interspersed repetitive DNA sequenc | 1.56 | 0.51 | -2.38 | 0.354 | -1.44 | 0.508 | -2.26 | 0.212 | -1.13 |
| 1429670_a_at | Lrriq2 | leucine-rich repeats and IQ motif containing 2 | -1.29 | 0.009 | 1.16 | 0.83 | -1.35 | 0.239 | -1.18 | 0.556 | -0.67 |
| 1435478_at | Wdr26 | WD repeat domain 26 | -1.04 | 0.745 | -1.19 | 0.226 | -1.15 | 0.182 | 1.61 | 0.188 | -0.44 |
| 1432955_at | 4930444K16Rik | RIKEN cDNA 4930444K16 gene | -1.53 | 0.686 | 1.08 | 0.761 | -1.07 | 0.866 | -1.05 | 0.935 | -0.64 |
| 1448597_at | Cstf1 | cleavage stimulation factor, 3' pre-RNA, subunit 1 | -1.21 | 0.416 | -1.26 | 0.021 | 1.05 | 0.65 | -1.02 | 0.919 | -0.61 |
| 1442834_at | Ppp4r2 | Protein phosphatase 4, regulatory subunit 2 | 1 | 0.982 | -1.01 | 0.941 | -1.48 | 0.014 | -1.12 | 0.687 | -0.65 |
| 1442993_at | Itgb3bp | integrin beta 3 binding protein (beta3-endonexin) | -1.49 | 0.372 | 1.06 | 0.887 | -1.06 | 0.866 | -2.08 | 0.451 | -0.89 |
| 1427143_at | Jarid1b | jumonji, AT rich interactive domain 1B (Rbp2 like) | 1.02 | 0.903 | -1.26 | 0.152 | -1.16 | 0.126 | -1.4 | 0.22 | -0.7 |
| 1459634_at | A230048G03Rik | RIKEN cDNA A230048G03 gene | 1.07 | 0.831 | -1.56 | 0.127 | -1.04 | 0.708 | -1.5 | 0.411 | -0.76 |
| 1420483_at | Cnnm3 | cyclin M3 | -1.03 | 0.964 | -1.54 | 0.171 | 1.05 | 0.798 | -1.17 | 0.366 | -0.67 |
| 1418404_at | Rad9 | RAD9 homolog (S. pombe) | -1.56 | 0.239 | 1.14 | 0.439 | -1.12 | 0.386 | -1.04 | 0.788 | -0.65 |
| 1438785_at | Enpp6 | ectonucleotide pyrophosphatase/phosphodiesterase 6 | -1.39 | 0.12 | 1.15 | 0.624 | -1.25 | 0.619 | -1 | 0.996 | -0.62 |
| 1460335_at | Lysmd3 | LysM, putative peptidoglycan-binding, domain containing 3 | -1.05 | 0.664 | -1.03 | 0.733 | -1.34 | 0.103 | 1.07 | 0.808 | -0.59 |
| 1437507_at | Gatad2b | GATA zinc finger domain containing 2B | -1.48 | 0.254 | -1.08 | 0.734 | 1.07 | 0.863 | -1.6 | 0.142 | -0.77 |
| 1416861_at | Stam | signal transducing adaptor molecule (SH3 domain and ITAM motif) 1 | -1.05 | 0.375 | -1.03 | 0.511 | -1.34 | 0.053 | 1.67 | 0.204 | -0.44 |
| 1420571_at | Prlpb | prolactin-like protein B | -1.07 | 0.851 | 1.07 | 0.885 | -1.5 | 0.235 | -1.07 | 0.826 | -0.64 |
| 1426460_a_at | Ugp2 | UDP-glucose pyrophosphorylase 2 | -1.03 | 0.771 | -1.33 | 0.358 | -1.05 | 0.764 | 1.03 | 0.914 | -0.6 |
| 1443152_at | Ptcd3 | Pentatricopeptide repeat domain 3 | 1.2 | 0.648 | -1.79 | 0.189 | -1.09 | 0.806 | -1.02 | 0.859 | -0.68 |
| 1428194_at | Usp9x | ubiquitin specific peptidase 9, X chromosome | -1.01 | 0.88 | -1.1 | 0.028 | -1.3 | 0.006 | 1.39 | 0.019 | -0.5 |
| 1444714_at | 2510006D16Rik | RIKEN cDNA 2510006D16 gene | -1.12 | 0.554 | 1.06 | 0.878 | -1.39 | 0 | -1.2 | 0.065 | -0.66 |
| 1451740_at | Paip1 | polyadenylate binding protein-interacting protein 1 | -1.13 | 0.406 | -1.12 | 0.443 | -1.12 | 0.336 | 1.1 | 0.542 | -0.57 |
| 1418658_at | 2410005O16Rik | RIKEN cDNA 2410005O16 gene | -1.1 | 0.076 | -1.33 | 0.043 | 1.02 | 0.911 | -1.24 | 0.252 | -0.67 |
| 1458515_at | Zfp128 | zinc finger protein 128 | -1.34 | 0.155 | 1.07 | 0.744 | -1.17 | 0.354 | -1.69 | 0.032 | -0.78 |

| | | | | | | | | | | | |
|---|---|---|---|---|---|---|---|---|---|---|---|
| 1437323_a_at | Iapp | islet amyloid polypeptide | -1.53 | 0.304 | 1.3 | 0.697 | -1.38 | 0.077 | -1.19 | 0.142 | -0.7 |
| 1452937_s_at | Ccdc28b | coiled coil domain containing 28B | -1.19 | 0.065 | -1.33 | 0.25 | 1.08 | 0.722 | -1.14 | 0.321 | -0.65 |
| 1426053_a_at | Xpr1 | xenotropic and polytropic retrovirus receptor 1 | -1.13 | 0.724 | -1.08 | 0.882 | -1.16 | 0.478 | 1.16 | 0.317 | -0.55 |
| 1453613_at | A130006I12Rik | RIKEN cDNA A130006I12 gene | -1.5 | 0.348 | 1.27 | 0.524 | -1.36 | 0.347 | -1.27 | 0.09 | -0.71 |
| 1423191_at | Fnbp4 | formin binding protein 4 | -1.23 | 0.084 | -1.21 | 0.01 | 1.03 | 0.867 | -1.23 | 0.42 | -0.66 |
| 1426925_at | Mnab | membrane associated DNA binding protein | 1.21 | 0.39 | -1.2 | 0.619 | -1.58 | 0.073 | -1.65 | 0.252 | -0.81 |
| 1441263_a_at | A930005H10Rik | RIKEN cDNA A930005H10 gene | -1.26 | 0.04 | -1.17 | 0.315 | 1.03 | 0.852 | -1.99 | 0.046 | -0.85 |
| 1452124_at | Ank3 /// 2900054D( | ankyrin 3, epithelial /// RIKEN cDNA 2900054D09 gene | -1.11 | 0.536 | 1.29 | 0.231 | -2.08 | 0.002 | -1.56 | 0.106 | -0.86 |
| 1424901_at | Gcnt3 | glucosaminyl (N-acetyl) transferase 3, mucin type | -1.23 | 0.64 | -1.76 | 0.487 | 1.29 | 0.497 | -1.12 | 0.806 | -0.7 |
| 1436616_at | R74740 | expressed sequence R74740 | -1.35 | 0.017 | 1.07 | 0.753 | -1.16 | 0.455 | -1.15 | 0.716 | -0.65 |
| 1428096_at | Ipo11 | importin 11 | -1.22 | 0.142 | -1.08 | 0.61 | -1.08 | 0.643 | 1.22 | 0.537 | -0.54 |
| 1415733_a_at | 1110019J04Rik | RIKEN cDNA 1110019J04 gene | -1.3 | 0.081 | 1.02 | 0.794 | -1.13 | 0.344 | -1.24 | 0.303 | -0.66 |
| 1451434_s_at | 5430405G24Rik | RIKEN cDNA 5430405G24 gene | -1.14 | 0.062 | -1 | 0.996 | -1.26 | 0.111 | 1.12 | 0.474 | -0.57 |
| 1447019_at | Cmah | cytidine monophospho-N-acetylneuraminic acid hydroxylase | -1.5 | 0.332 | -1.28 | 0.168 | 1.23 | 0.516 | -1.25 | 0.323 | -0.7 |
| 1434913_at | Hmgcll1 | 3-hydroxymethyl-3-methylglutaryl-Coenzyme A lyase-like 1 | -1.1 | 0.65 | 1 | 1 | -1.3 | 0.118 | -1.42 | 0.394 | -0.71 |
| 1426747_at | Abcf3 | ATP-binding cassette, sub-family F (GCN20), member 3 | -1.14 | 0.351 | -1.3 | 0.029 | 1.02 | 0.831 | -1.4 | 0.079 | -0.7 |
| 1444104_at | --- | --- | 1.13 | 0.91 | -1.55 | 0.281 | -1.11 | 0.841 | -1.09 | 0.798 | -0.65 |
| 1443804_at | Ppp2r2c | Protein phosphatase 2 (formerly 2A), regulatory subunit B (PR 52), gamma isofor | -1.68 | 0.586 | 1.23 | 0.681 | -1.17 | 0.391 | -1.65 | 0.5 | -0.82 |
| 1434266_at | AI847670 | expressed sequence AI847670 | -1.17 | 0.066 | -1.15 | 0.491 | -1.05 | 0.752 | 1 | 0.967 | -0.59 |
| 1455708_at | Tmod3 | Tropomodulin 3 | -1.68 | 0.303 | -1.04 | 0.947 | 1.12 | 0.768 | -1.16 | 0.77 | -0.69 |
| 1428592_s_at | Usp38 | ubiquitin specific peptidase 38 | -1.2 | 0.68 | -1.26 | 0.041 | 1.05 | 0.728 | -1.33 | 0.181 | -0.69 |
| 1448304_a_at | Rab6 | RAB6, member RAS oncogene family | -1.21 | 0.092 | -1.08 | 0.159 | -1.08 | 0.265 | 1.4 | 0.288 | -0.49 |
| 1443114_at | A730059M13Rik | RIKEN cDNA A730059M13 gene | -1.18 | 0.535 | 1.19 | 0.115 | -1.57 | 0.049 | -1.1 | 0.609 | -0.67 |
| 1419866_s_at | Atxn2 | ataxin 2 | -1.08 | 0.2 | -1.15 | 0.364 | -1.13 | 0.477 | 1.07 | 0.783 | -0.58 |
| 1425250_a_at | Slc14a2 | solute carrier family 14 (urea transporter), member 2 | -1.07 | 0.644 | -2.1 | 0.012 | 1.26 | 0.386 | -1.1 | 0.415 | -0.75 |
| 1443219_at | --- | --- | -1.26 | 0.611 | -1.05 | 0.858 | -1.07 | 0.728 | 1.22 | 0.344 | -0.54 |
| 1424609_a_at | BC003993 /// LOC5 | cDNA sequence BC003993 /// hypothetical LOC544818 | 1.01 | 0.977 | -1.17 | 0.032 | -1.23 | 0.207 | -1.06 | 0.312 | -0.61 |
| 1457459_at | AU014973 | expressed sequence AU014973 | 1.05 | 0.77 | -1.38 | 0.299 | -1.12 | 0.414 | -1.08 | 0.696 | -0.63 |
| 1455033_at | B430201A12Rik | RIKEN cDNA B430201A12 gene | 1.01 | 0.969 | -1.05 | 0.887 | -1.41 | 0.173 | -1.06 | 0.735 | -0.63 |
| 1436688_x_at | Rpl14 | ribosomal protein L14 | -1.39 | 0.443 | 1.07 | 0.363 | -1.12 | 0.184 | -1.53 | 0.102 | -0.75 |
| 1431257_at | 6230409E13Rik | RIKEN cDNA 6230409E13 gene | -1.94 | 0.205 | -1.27 | 0.63 | 1.37 | 0.424 | -1.32 | 0.549 | -0.79 |
| 1429727_at | Slc16a9 | solute carrier family 16 (monocarboxylic acid transporters), member 9 | -1.01 | 0.981 | 1.13 | 0.576 | -1.82 | 0.062 | -1.02 | 0.95 | -0.68 |
| 1452288_at | BB128963 | expressed sequence BB128963 | 1.1 | 0.504 | -1.22 | 0.044 | -1.33 | 0.174 | -1.09 | 0.424 | -0.63 |
| 1426871_at | Fbxo33 | F-box only protein 33 | -1.09 | 0.188 | 1.04 | 0.847 | -1.38 | 0.085 | -1.04 | 0.765 | -0.62 |
| 1440083_at | A430061O12Rik | RIKEN cDNA A430061O12 gene | 1.19 | 0.296 | -1.22 | 0.31 | -1.5 | 0.025 | -1.62 | 0.084 | -0.79 |
| 1416047_at | Fuca2 | fucosidase, alpha-L- 2, plasma | 1.1 | 0.869 | -1.1 | 0.82 | -1.49 | 0.323 | -1.22 | 0.406 | -0.68 |
| 1438019_at | Ippk | inositol 1,3,4,5,6-pentakisphosphate 2-kinase | -1.32 | 0.366 | -1.09 | 0.284 | -1 | 0.96 | 1.3 | 0.227 | -0.53 |
| 1436720_s_at | Oog3 | oogenesin 3 | -1.09 | 0.534 | -2.04 | 0.089 | 1.26 | 0.601 | -2.66 | 0.008 | -1.13 |
| 1455206_at | C130006E23 | Hypothetical protein C130006E23 | -1.36 | 0.141 | 1.09 | 0.596 | -1.18 | 0.301 | -1.3 | 0.397 | -0.69 |
| 1451747_a_at | Atg12 | autophagy-related 12 (yeast) | -1.12 | 0.38 | -1.15 | 0.307 | -1.1 | 0.376 | 1.01 | 0.913 | -0.59 |
| 1421432_at | Arl10 | ADP-ribosylation factor-like 10 | 1.05 | 0.745 | -1.34 | 0.47 | -1.14 | 0.545 | -1.81 | 0.296 | -0.81 |
| 1438615_x_at | 2010317E24Rik | RIKEN cDNA 2010317E24 gene | -1.02 | 0.933 | -1.19 | 0.526 | -1.17 | 0.483 | 1.82 | 0.041 | -0.39 |
| 1458397_at | LOC622208 | hypothetical LOC622208 | 1.14 | 0.385 | -1.44 | 0.286 | -1.19 | 0.601 | -1.54 | 0.19 | -0.76 |
| 1426089_a_at | BC003331 | cDNA sequence BC003331 | -1.06 | 0.587 | -1.19 | 0.009 | -1.13 | 0.335 | 1.01 | 0.949 | -0.59 |
| 1451320_at | 3110043J09Rik | RIKEN cDNA 3110043J09 gene | 1.03 | 0.932 | -1.41 | 0.623 | -1.07 | 0.665 | -1.51 | 0.61 | -0.74 |
| 1438755_at | C80068 | expressed sequence C80068 | -1.35 | 0.237 | 1.01 | 0.959 | -1.09 | 0.777 | -1.06 | 0.816 | -0.62 |
| 1440403_at | Arhgef1 | Rho guanine nucleotide exchange factor (GEF) 1 | -1.11 | 0.833 | -1.39 | 0.055 | 1.06 | 0.789 | -2.28 | 0.05 | -0.93 |
| 1455208_at | Pex19 | peroxisome biogenesis factor 19 | 1.04 | 0.167 | -1.11 | 0.544 | -1.37 | 0.039 | -1.14 | 0.414 | -0.64 |
| 1448652_at | Ift88 | intraflagellar transport 88 homolog (Chlamydomonas) | -1.09 | 0.395 | -1.46 | 0.081 | 1.07 | 0.75 | -1.21 | 0.225 | -0.67 |
| 1443032_at | Mrps22 | Mitochondrial ribosomal protein S22 | -1.01 | 0.976 | -1.11 | 0.776 | -1.27 | 0.264 | 1.15 | 0.594 | -0.56 |
| 1448312_at | Pcsk2 | proprotein convertase subtilisin/kexin type 2 | -1.07 | 0.917 | -1.05 | 0.882 | -1.28 | 0.555 | 1.44 | 0.6 | -0.49 |
| 1447672_x_at | Pskh1 | protein serine kinase H1 | -1.1 | 0.662 | -1 | 0.977 | -1.3 | 0.336 | 1.06 | 0.491 | -0.59 |
| 1423881_at | Saps3 | SAPS domain family, member 3 | -1.27 | 0.075 | -1.05 | 0.459 | -1.07 | 0.619 | 1.16 | 0.346 | -0.56 |

| | | | | | | | | | | | |
|---|---|---|---|---|---|---|---|---|---|---|---|
| 1439033_at | Zcchc7 | zinc finger, CCHC domain containing 7 | 1.08 | 0.046 | -1.34 | 0.019 | -1.18 | 0.537 | -1.39 | 0.342 | -0.71 |
| 1459967_at | --- | Transcribed locus | -1.19 | 0.698 | -1.11 | 0.713 | -1.07 | 0.862 | 1.05 | 0.893 | -0.58 |
| 1431273_at | 2810417H13Rik | RIKEN cDNA 2810417H13 gene | 1.24 | 0.737 | -2.17 | 0.302 | -1.03 | 0.889 | -1.6 | 0.482 | -0.89 |
| 1443737_at | Xpo6 | Exportin 6 | -2.73 | 0.296 | -1.58 | 0.449 | 1.68 | 0.435 | -1.08 | 0.893 | -0.93 |
| 1436203_a_at | 1110059G02Rik | RIKEN cDNA 1110059G02 gene | -1.21 | 0.108 | -1.02 | 0.895 | -1.15 | 0.175 | 1.12 | 0.366 | -0.56 |
| 1429369_at | Tnpo3 | transportin 3 | -1.07 | 0.322 | -1.43 | 0.051 | 1.04 | 0.586 | -1.13 | 0.21 | -0.65 |
| 1424955_at | Ccdc5 | coiled-coil domain containing 5 | -1.04 | 0.714 | -1.23 | 0.236 | -1.11 | 0.397 | 1.84 | 0.235 | -0.39 |
| 1452282_at | Crsp7 | cofactor required for Sp1 transcriptional activation, subunit 7 | -1.24 | 0.236 | -1.35 | 0.251 | 1.13 | 0.571 | -1.03 | 0.718 | -0.62 |
| 1429732_at | Ppp1r1c | protein phosphatase 1, regulatory (inhibitor) subunit 1C | -1.2 | 0.769 | 1.21 | 0.672 | -1.59 | 0.253 | -1.53 | 0.116 | -0.78 |
| 1415781_a_at | Sumo2 /// LOC672 | SMT3 suppressor of mif two 3 homolog 2 (yeast) /// similar to SMT3 suppressor o | 1.05 | 0.192 | -1.19 | 0.096 | -1.27 | 0.002 | -1.05 | 0.345 | -0.61 |
| 1432761_at | 4930551I15Rik | RIKEN cDNA 4930551I15 gene | 1.44 | 0.594 | -2.49 | 0.216 | -1.2 | 0.267 | -1.42 | 0.021 | -0.92 |
| 1423684_at | Hnrpk | heterogeneous nuclear ribonucleoprotein K | -1.1 | 0.267 | -1.01 | 0.959 | -1.3 | 0.099 | 1.06 | 0.62 | -0.59 |
| 1442668_at | --- | --- | -2.34 | 0.159 | -1.15 | 0.791 | 1.38 | 0.567 | -1.2 | 0.551 | -0.83 |
| 1457321_at | LOC667963 | hypothetical protein LOC667963 | -1.01 | 0.977 | -1.23 | 0.435 | -1.14 | 0.305 | 1.18 | 0.591 | -0.55 |
| 1427805_at | --- | --- | -1.06 | 0.379 | -1.12 | 0.568 | -1.2 | 0.499 | 1.29 | 0.362 | -0.52 |
| 1432926_at | 2900069M18Rik | RIKEN cDNA 2900069M18 gene | -1.71 | 0.466 | -1.45 | 0.366 | 1.4 | 0.193 | -2.69 | 0.05 | -1.11 |
| 1424820_a_at | Ndfip1 | Nedd4 family interacting protein 1 | -1.16 | 0.206 | -1.24 | 0.026 | 1.01 | 0.932 | -1.34 | 0.335 | -0.68 |
| 1439553_s_at | Nutf2 /// LOC6218 | nuclear transport factor 2 /// similar to Nuclear transport factor 2 (NTF-2) /// simila | -1.08 | 0.623 | -1.04 | 0.712 | -1.27 | 0.059 | 1.26 | 0.396 | -0.53 |
| 1443861_at | Cgnl1 /// LOC6774 | cingulin-like 1 /// similar to cingulin-like 1 isoform 1 | -1.05 | 0.83 | -1.42 | 0.576 | 1.02 | 0.979 | -3.58 | 0.075 | -1.26 |
| 1419568_at | Mapk1 | mitogen activated protein kinase 1 | -1.72 | 0.408 | -1.19 | 0.57 | 1.26 | 0.515 | -1.19 | 0.784 | -0.71 |
| 1458355_x_at | AI553587 | expressed sequence AI553587 | -1.24 | 0.325 | -1.21 | 0.334 | 1.05 | 0.762 | -1.11 | 0.435 | -0.63 |
| 1438045_at | Eea1 | early endosome antigen 1 | -1.07 | 0.594 | -1.16 | 0.137 | -1.15 | 0.459 | 1.2 | 0.021 | -0.54 |
| 1450922_a_at | Tgfb2 | transforming growth factor, beta 2 | 1.1 | 0.66 | -1.57 | 0.375 | -1.06 | 0.835 | -1.58 | 0.308 | -0.78 |
| 1435407_at | Zfp483 | Zinc finger protein 483 | 1.1 | 0.546 | -1.26 | 0.63 | -1.27 | 0.49 | -1.59 | 0.156 | -0.76 |
| 1423251_at | Luc7l2 | LUC7-like 2 (S. cerevisiae) | -1.06 | 0.378 | -1.12 | 0.484 | -1.19 | 0.235 | 1.25 | 0.262 | -0.53 |
| 1452880_at | Znhit3 | zinc finger, HIT type 3 | -1.16 | 0.354 | -1.19 | 0.208 | -1.02 | 0.83 | 1.36 | 0.408 | -0.5 |
| 1425618_at | Dhx9 | DEAH (Asp-Glu-Ala-His) box polypeptide 9 | -3.14 | 0.06 | -1.85 | 0.214 | 1.82 | 0.145 | -1.21 | 0.553 | -1.09 |
| 1433679_at | Fubp3 | far upstream element (FUSE) binding protein 3 | -1.07 | 0.718 | -1.08 | 0.416 | -1.21 | 0.139 | 1 | 0.986 | -0.59 |
| 1426413_at | Neurod1 | neurogenic differentiation 1 | 1.19 | 0.755 | -1.59 | 0.307 | -1.16 | 0.746 | -1.48 | 0.469 | -0.76 |
| 1420108_at | --- | --- | -1.5 | 0.51 | 1.16 | 0.71 | -1.19 | 0.717 | -2.79 | 0.004 | -1.08 |
| 1434075_at | BC030336 | cDNA sequence BC030336 | 1.04 | 0.764 | -1.13 | 0.357 | -1.33 | 0.093 | -1 | 0.985 | -0.61 |
| 1452573_a_at | Sr278 | stretch responsive protein 278 | -1.16 | 0.066 | -1.1 | 0.516 | -1.11 | 0.514 | 1.27 | 0.113 | -0.52 |
| 1451754_a_at | Wdr45 | WD repeat domain 45 | -1.09 | 0.695 | -1.18 | 0.011 | -1.1 | 0.421 | 1.08 | 0.14 | -0.57 |
| 1438353_at | --- | --- | 1.04 | 0.825 | -1.09 | 0.526 | -1.39 | 0.083 | -1.2 | 0.598 | -0.66 |
| 1420935_a_at | Srrm1 | serine/arginine repetitive matrix 1 | -1.13 | 0.693 | -1.2 | 0.506 | -1.04 | 0.881 | 1.79 | 0.003 | -0.4 |
| 1440127_a_at | --- | Transcribed locus | -1.35 | 0.542 | -1.27 | 0.14 | 1.15 | 0.729 | -2.07 | 0.136 | -0.88 |
| 1452385_at | Usp53 | ubiquitin specific peptidase 53 | -1.16 | 0.626 | 1 | 0.992 | -1.23 | 0.305 | -1.86 | 0.15 | -0.81 |
| 1438227_at | She | src homology 2 domain-containing transforming protein E | -1.1 | 0.855 | 1.06 | 0.855 | -1.4 | 0.317 | -2.23 | 0.069 | -0.92 |
| 1428494_a_at | Polr2i | polymerase (RNA) II (DNA directed) polypeptide I | -1.02 | 0.868 | -1.17 | 0.088 | -1.2 | 0.406 | 1.09 | 0.071 | -0.57 |
| 1427830_at | Zfp260 | zinc finger protein 260 | -1.26 | 0.752 | 1.13 | 0.802 | -1.34 | 0.319 | -1.5 | 0.095 | -0.74 |
| 1428554_a_at | 1810035L17Rik | RIKEN cDNA 1810035L17 gene | -1.06 | 0.545 | 1 | 0.95 | -1.37 | 0.035 | -1.08 | 0.749 | -0.63 |
| 1422237_at | Mc3r | melanocortin 3 receptor | 1.04 | 0.673 | -1.46 | 0.004 | -1.05 | 0.813 | -1.16 | 0.008 | -0.66 |
| 1445645_at | Adam22 | A disintegrin and metallopeptidase domain 22 | 1.38 | 0.663 | -1.85 | 0.117 | -1.32 | 0.381 | -1.35 | 0.295 | -0.79 |
| 1452974_at | Nol8 | nucleolar protein 8 | -1.16 | 0.501 | 1.09 | 0.738 | -1.38 | 0.141 | -1.41 | 0.018 | -0.71 |
| 1435854_at | Tmem10 | transmembrane protein 10 | 1.06 | 0.912 | -1.15 | 0.609 | -1.34 | 0.292 | -1.41 | 0.553 | -0.71 |
| 1450622_at | Bcar1 | breast cancer anti-estrogen resistance 1 | -1.14 | 0.078 | -1.22 | 0.223 | -1.02 | 0.938 | 1.07 | 0.497 | -0.58 |
| 1458114_at | Samd12 | sterile alpha motif domain containing 12 | -1.23 | 0.659 | 1.02 | 0.957 | -1.19 | 0.427 | -1.1 | 0.671 | -0.62 |
| 1429494_at | Trim35 | tripartite motif-containing 35 | 1.02 | 0.918 | -1.04 | 0.94 | -1.43 | 0.135 | -1.97 | 0.073 | -0.86 |
| 1422433_s_at | Idh1 | isocitrate dehydrogenase 1 (NADP+), soluble | -1.38 | 0.259 | -1.13 | 0.078 | 1.07 | 0.659 | -1.15 | 0.388 | -0.65 |
| 1429383_at | Csnk1g3 | casein kinase 1, gamma 3 | 1.03 | 0.915 | -1.02 | 0.848 | -1.48 | 0.008 | -1.64 | 0.095 | -0.78 |
| 1428384_at | D4Bwg0951e | DNA segment, Chr 4, Brigham & Women's Genetics 0951 expressed | 1.04 | 0.86 | -1.09 | 0.679 | -1.38 | 0.028 | -1.86 | 0.273 | -0.82 |
| 1458744_at | Taf1 | TAF1 RNA polymerase II, TATA box binding protein (TBP)-associated factor | -2.53 | 0.438 | -1.22 | 0.017 | 1.46 | 0.625 | -1.06 | 0.931 | -0.84 |
| 1447223_at | --- | Transcribed locus | -1.15 | 0.179 | -1.19 | 0.09 | -1.03 | 0.773 | 1.09 | 0.527 | -0.57 |

| | | | | | | | | | | | |
|---|---|---|---|---|---|---|---|---|---|---|---|
| 1443490_at | D3Ertd34e | DNA segment, Chr 3, ERATO Doi 34, expressed | 1.02 | 0.768 | -1.17 | 0.254 | -1.24 | 0.272 | -1.41 | 0.273 | -0.7 |
| 1446748_at | 2010007H06Rik | RIKEN cDNA 2010007H06 gene | 1.28 | 0.03 | -2.29 | 0.055 | -1.04 | 0.759 | -1.79 | 0.243 | -0.96 |
| 1460691_at | Zfp598 | zinc finger protein 598 | 1.05 | 0.737 | -1.28 | 0.016 | -1.18 | 0.189 | -1.02 | 0.875 | -0.61 |
| 1459307_at | Sec24b | SEC24 related gene family, member B (S. cerevisiae) | -1.08 | 0.805 | 1.18 | 0.655 | -1.73 | 0.053 | -1.17 | 0.699 | -0.7 |
| 1460032_at | 4933407C03Rik | RIKEN cDNA 4933407C03 gene | -1.35 | 0.297 | 1.16 | 0.471 | -1.28 | 0.152 | -1.02 | 0.923 | -0.62 |
| 1438647_x_at | Cetn2 | centrin 2 | -1.13 | 0.329 | 1.02 | 0.827 | -1.29 | 0.187 | -1.27 | 0.491 | -0.67 |
| 1443467_at | Dock11 | dedicator of cytokinesis 11 | 1.21 | 0.474 | -1.58 | 0.167 | -1.19 | 0.194 | -1.8 | 0.235 | -0.84 |
| 1433103_at | Hyls1 | hydrolethalus syndrome 1 | -1.73 | 0.166 | 1.27 | 0.57 | -1.2 | 0.733 | -1.92 | 0.338 | -0.9 |
| 1455597_at | AI585793 | Expressed sequence AI585793 | 1.08 | 0.468 | -1.03 | 0.735 | -1.59 | 0.013 | -1.35 | 0.201 | -0.72 |
| 1447477_at | 3110006E14Rik | RIKEN cDNA 3110006E14 gene | -1.54 | 0.135 | -1.05 | 0.795 | 1.08 | 0.865 | -2.62 | 0.14 | -1.03 |
| 1443898_at | Bcl7c | B-cell CLL/lymphoma 7C | -1.29 | 0.401 | 1.01 | 0.957 | -1.12 | 0.528 | -1.07 | 0.764 | -0.62 |
| 1437368_at | Gtf2h4 /// LOC6403 | general transcription factor II H, polypeptide 4 /// similar to general transcription fa | -1.33 | 0.369 | -1.52 | 0.312 | 1.27 | 0.592 | -2.24 | 0.313 | -0.95 |
| 1430592_at | Tmem106b | transmembrane protein 106B | 1.26 | 0.495 | -1.37 | 0.09 | -1.46 | 0.055 | -1.16 | 0.563 | -0.68 |
| 1416727_a_at | Cyb5 | cytochrome b-5 | -1.09 | 0.384 | -1.14 | 0.46 | -1.13 | 0.26 | 1.3 | 0.187 | -0.52 |
| 1452579_at | Nifun | NifU-like N-terminal domain containing | 1.01 | 0.942 | -1.17 | 0.09 | -1.23 | 0.129 | -1.09 | 0.655 | -0.62 |
| 1423854_a_at | Rasl11b | RAS-like, family 11, member B | 1.02 | 0.808 | -1.02 | 0.867 | -1.48 | 0.072 | -1.18 | 0.71 | -0.66 |
| 1441830_x_at | --- | --- | 1.14 | 0.508 | -1.31 | 0.291 | -1.29 | 0.201 | -2.4 | 0.014 | -0.96 |
| 1437550_at | Dhx36 | DEAH (Asp-Glu-Ala-His) box polypeptide 36 | 1.07 | 0.586 | -1.27 | 0.099 | -1.22 | 0.34 | -1.23 | 0.574 | -0.66 |
| 1451983_at | Irx1 | Iroquois related homeobox 1 (Drosophila) | -1.3 | 0.019 | 1.27 | 0.502 | -1.57 | 0.158 | -1.41 | 0.189 | -0.75 |
| 1417965_at | Plekha1 | pleckstrin homology domain containing, family A (phosphoinositide binding specif | -1.19 | 0.059 | 1.03 | 0.703 | -1.24 | 0.098 | -1.56 | 0.164 | -0.74 |
| 1419156_at | Sox4 /// LOC67227 | SRY-box containing gene 4 /// similar to Transcription factor SOX-4 | 1.16 | 0.378 | -1.12 | 0.762 | -1.6 | 0.078 | -1.45 | 0.314 | -0.75 |
| 1453225_at | A930038C07Rik | RIKEN cDNA A930038C07 gene | -2.71 | 0.131 | 1.6 | 0.35 | -1.41 | 0.249 | -1.27 | 0.732 | -0.95 |
| 1455303_at | --- | --- | -1.33 | 0.21 | -1.11 | 0.169 | 1.03 | 0.848 | -1.08 | 0.551 | -0.62 |
| 1450112_a_at | Gas2 | growth arrest specific 2 | -1.23 | 0.026 | -1.01 | 0.967 | -1.15 | 0.332 | 1.86 | 0.023 | -0.38 |
| 1431983_at | Myo3a | myosin IIIA | -1.32 | 0.716 | -1.29 | 0.47 | 1.15 | 0.716 | -1.2 | 0.459 | -0.67 |
| 1429593_at | Slc38a2 | solute carrier family 38, member 2 | -1.25 | 0.322 | 1.08 | 0.727 | -1.25 | 0.18 | -1.23 | 0.263 | -0.66 |
| 1437295_at | Pkn2 | protein kinase N2 | -1.05 | 0.438 | -1.03 | 0.726 | -1.33 | 0.026 | 1.6 | 0.31 | -0.45 |
| 1448896_at | Pigf | phosphatidylinositol glycan anchor biosynthesis, class F | -1.13 | 0.101 | 1.09 | 0.414 | -1.41 | 0.123 | -1.27 | 0.115 | -0.68 |
| 1449746_s_at | Glipr1 | GLI pathogenesis-related 1 (glioma) | -1.64 | 0.106 | 1.35 | 0.363 | -1.39 | 0.222 | -1.23 | 0.267 | -0.73 |
| 1459114_at | Anks1 | Ankyrin repeat and SAM domain containing 1 | -1.39 | 0.753 | -1.09 | 0.904 | 1.04 | 0.926 | -1.44 | 0.16 | -0.72 |
| 1427925_at | Stx17 | syntaxin 17 | -1.1 | 0.57 | -1.07 | 0.744 | -1.19 | 0.289 | 1.15 | 0.579 | -0.55 |
| 1456145_at | Dleu2 | deleted in lymphocytic leukemia, 2 | -1.03 | 0.83 | -1.34 | 0.097 | -1.04 | 0.714 | 2.22 | 0.208 | -0.3 |
| 1456698_s_at | Hnrpdl | heterogeneous nuclear ribonucleoprotein D-like | -1.25 | 0.283 | 1.05 | 0.78 | -1.21 | 0.176 | -1.48 | 0.074 | -0.72 |
| 1446275_at | Tnrc15 | Trinucleotide repeat containing 15 | -1.17 | 0.726 | -1.42 | 0.561 | 1.12 | 0.832 | -2.1 | 0.446 | -0.89 |
| 1437876_at | Fndc6 | fibronectin type III domain containing 6 | -1.28 | 0.479 | -1.29 | 0.63 | 1.13 | 0.427 | -1.2 | 0.705 | -0.66 |
| 1455295_at | BC031853 | cDNA sequence BC031853 | -1.34 | 0.192 | -1.07 | 0.635 | 1 | 0.956 | -1.2 | 0.53 | -0.65 |
| 1457504_at | --- | --- | -1.62 | 0.446 | 1.16 | 0.56 | -1.11 | 0.763 | -1.06 | 0.88 | -0.66 |
| 1443920_at | Ccdc66 | coiled-coil domain containing 66 | -1.04 | 0.776 | -1.09 | 0.89 | -1.26 | 0.291 | 1.06 | 0.622 | -0.58 |
| 1458894_at | --- | --- | -1.23 | 0.546 | -1.91 | 0.028 | 1.34 | 0.436 | -1.96 | 0.081 | -0.94 |
| 1456217_at | D7Wsu128e | DNA segment, Chr 7, Wayne State University 128, expressed | -1.06 | 0.85 | -1.38 | 0.262 | 1.01 | 0.952 | -1.14 | 0.522 | -0.64 |
| 1454000_s_at | 4930578N16Rik | RIKEN cDNA 4930578N16 gene | -1.01 | 0.989 | -1.21 | 0.475 | -1.16 | 0.459 | 1.01 | 0.987 | -0.59 |
| 1419074_at | 2510006C20Rik | RIKEN cDNA 2510006C20 gene | -1.11 | 0.617 | -1.19 | 0.127 | -1.06 | 0.539 | 4.37 | 0.205 | 0.25 |
| 1418886_s_at | Idh3b | isocitrate dehydrogenase 3 (NAD+) beta | -1.18 | 0.022 | -1.3 | 0.076 | 1.07 | 0.524 | -1.42 | 0.032 | -0.71 |
| 1419108_at | Ophn1 | oligophrenin 1 | -1.03 | 0.904 | -1.17 | 0.523 | -1.16 | 0.544 | 1.11 | 0.506 | -0.56 |
| 1424709_at | Sc5d | sterol-C5-desaturase (fungal ERG3, delta-5-desaturase) homolog (S. cerevisae) | -1.16 | 0.503 | -1.41 | 0.66 | 1.1 | 0.667 | -1.45 | 0.33 | -0.73 |
| 1424636_at | 2610204L23Rik | RIKEN cDNA 2610204L23 gene | -1.42 | 0.214 | -1.01 | 0.917 | -1.01 | 0.962 | 1.13 | 0.65 | -0.58 |
| 1437206_at | Setd5 | SET domain containing 5 | -1.73 | 0.374 | -1.66 | 0.202 | 1.5 | 0.327 | -1.98 | 0.184 | -0.97 |
| 1430461_at | 1700120E14Rik | RIKEN cDNA 1700120E14 gene | 1.14 | 0.871 | -1.03 | 0.958 | -1.76 | 0.414 | -1.61 | 0.08 | -0.81 |
| 1438038_at | 4930402H24Rik | RIKEN cDNA 4930402H24 gene | -1.08 | 0.016 | -1.25 | 0.071 | -1.05 | 0.848 | 1.17 | 0.235 | -0.55 |
| 1440426_at | Nfatc2 | nuclear factor of activated T-cells, cytoplasmic, calcineurin-dependent 2 | -1.37 | 0.017 | -1.42 | 0.378 | 1.25 | 0.515 | -1.36 | 0.036 | -0.73 |
| 1416362_a_at | Fkbp4 | FK506 binding protein 4 | 1.01 | 0.898 | -1.15 | 0.14 | -1.24 | 0.209 | -1.02 | 0.907 | -0.6 |
| 1439907_at | Rdx | Radixin | 1.07 | 0.8 | -1.14 | 0.69 | -1.37 | 0.193 | -1.4 | 0.436 | -0.71 |
| 1455999_at | C330003B14Rik | RIKEN cDNA C330003B14 gene | -1.12 | 0.421 | -1.04 | 0.908 | -1.22 | 0.629 | 1.17 | 0.227 | -0.55 |

| | | | | | | | | | | | |
|---|---|---|---|---|---|---|---|---|---|---|---|
| 1416452_at | Oat | ornithine aminotransferase | 1.03 | 0.825 | -1.48 | 0.196 | -1.02 | 0.915 | -1.16 | 0.497 | -0.66 |
| 1442068_at | --- | Mus musculus, clone IMAGE:5363897, mRNA | 1.04 | 0.913 | -1.05 | 0.917 | -1.46 | 0.294 | -1.12 | 0.691 | -0.65 |
| 1446341_at | 6030403N03Rik | RIKEN cDNA 6030403N03 gene | -1.23 | 0.433 | 1.08 | 0.61 | -1.27 | 0.127 | -1.12 | 0.687 | -0.63 |
| 1450144_at | Pla2r1 | phospholipase A2 receptor 1 | -1.05 | 0.754 | -1.04 | 0.885 | -1.31 | 0.067 | 1.5 | 0.069 | -0.47 |
| 1439626_at | Tmod3 | tropomodulin 3 | 1.19 | 0.683 | -1.34 | 0.248 | -1.34 | 0.189 | -2.49 | 0.021 | -1 |
| 1451512_s_at | Hibch | 3-hydroxyisobutyryl-Coenzyme A hydrolase | -1.05 | 0.615 | -1.17 | 0.21 | -1.14 | 0.409 | 1.12 | 0.593 | -0.56 |
| 1436435_at | 9530026P05Rik | RIKEN cDNA 9530026P05 gene | 1.12 | 0.615 | -1.08 | 0.892 | -1.55 | 0.165 | -1.43 | 0.225 | -0.74 |
| 1426367_at | Cab39l | calcium binding protein 39-like | 1.03 | 0.647 | -1.27 | 0.003 | -1.15 | 0.098 | -1.11 | 0.377 | -0.63 |
| 1443926_at | Mrg1 | Myeloid ecotropic viral integration site-related gene 1 | 1.01 | 0.963 | -1.21 | 0.452 | -1.19 | 0.565 | -2.07 | 0.064 | -0.86 |
| 1419438_at | Sim2 | single-minded homolog 2 (Drosophila) | 1.44 | 0.229 | -1.53 | 0.139 | -1.72 | 0.118 | -1.49 | 0.479 | -0.82 |
| 1455558_at | Gm114 | gene model 114, (NCBI) | -1.04 | 0.805 | -1.22 | 0.293 | -1.1 | 0.238 | 1.14 | 0.475 | -0.56 |
| 1436308_at | Zfp292 | zinc finger protein 292 | -1.09 | 0.628 | 1.04 | 0.809 | -1.38 | 0.053 | -1.38 | 0.162 | -0.7 |
| 1429461_at | Ints2 | integrator complex subunit 2 | -1.18 | 0.269 | -1.12 | 0.644 | -1.06 | 0.774 | 1.18 | 0.289 | -0.54 |
| 1443551_at | Atp2a2 | ATPase, Ca++ transporting, cardiac muscle, slow twitch 2 | -1.43 | 0.241 | -1.09 | 0.86 | 1.07 | 0.775 | -1.37 | 0.28 | -0.71 |
| 1439678_at | Gls | Glutaminase | -1.63 | 0.091 | 1.39 | 0.25 | -1.47 | 0.078 | -1.4 | 0.16 | -0.78 |
| 1421282_at | Bmp5 | bone morphogenetic protein 5 | -1.27 | 0.349 | 1.14 | 0.575 | -1.33 | 0.291 | -2 | 0.575 | -0.86 |
| 1420266_at | --- | --- | -1.63 | 0.192 | -1.08 | 0.686 | 1.14 | 0.456 | -1.27 | 0.247 | -0.71 |
| 1434832_at | Foxo3a | forkhead box O3a | -1.14 | 0.739 | -1.01 | 0.97 | -1.23 | 0.264 | 2.01 | 0.171 | -0.34 |
| 1429749_at | 9330180L21Rik | RIKEN cDNA 9330180L21 gene | -1.11 | 0.457 | -1.19 | 0.139 | -1.06 | 0.611 | 1.24 | 0.468 | -0.53 |
| 1447594_at | 1810012K16Rik | RIKEN cDNA 1810012K16 gene | 1.1 | 0.891 | -1.29 | 0.721 | -1.24 | 0.614 | -1.08 | 0.859 | -0.63 |
| 1430519_a_at | Cnot7 | CCR4-NOT transcription complex, subunit 7 | -1.05 | 0.684 | -1.28 | 0.081 | -1.05 | 0.855 | 1.1 | 0.726 | -0.57 |
| 1424566_s_at | Polr3d | polymerase (RNA) III (DNA directed) polypeptide D | -1.42 | 0.358 | -1.07 | 0.861 | 1.05 | 0.895 | -1.08 | 0.397 | -0.63 |
| 1449983_a_at | Nqo2 | NAD(P)H dehydrogenase, quinone 2 | -1.17 | 0.308 | -1.15 | 0.525 | -1.04 | 0.817 | 1.03 | 0.836 | -0.58 |
| 1423517_at | Cct6a | chaperonin subunit 6a (zeta) | -1.07 | 0.51 | -1.15 | 0.019 | -1.14 | 0.107 | 1.06 | 0.653 | -0.57 |
| 1437891_at | Frs2 | fibroblast growth factor receptor substrate 2 | -1.14 | 0.281 | -1.01 | 0.917 | -1.22 | 0.072 | 1.03 | 0.83 | -0.59 |
| 1425014_at | Nr2c2 | nuclear receptor subfamily 2, group C, member 2 | -1.23 | 0.733 | -1.15 | 0.721 | -1 | 0.991 | 1.04 | 0.914 | -0.58 |
| 1429146_at | 6620401M08Rik | RIKEN cDNA 6620401M08 gene | -1.02 | 0.883 | -1.1 | 0.283 | -1.26 | 0.195 | 3.6 | 0.22 | 0.06 |
| 1418005_at | Sdhb | succinate dehydrogenase complex, subunit B, iron sulfur (Ip) | -1.19 | 0.302 | -1.3 | 0.083 | 1.07 | 0.394 | -1.14 | 0.1 | -0.64 |
| 1434621_at | BC054438 | cDNA sequence BC054438 | -1.02 | 0.912 | 1.06 | 0.67 | -1.56 | 0.047 | -1.8 | 0.113 | -0.83 |
| 1432034_at | Neurog3 | neurogenin 3 | -1.05 | 0.723 | -1.47 | 0.325 | 1.05 | 0.919 | -1.08 | 0.589 | -0.64 |
| 1460246_at | Mecp2 | methyl CpG binding protein 2 | -1.02 | 0.898 | -1.15 | 0.504 | -1.2 | 0.533 | 1.17 | 0.582 | -0.55 |
| 1459499_at | --- | Transcribed locus | 1.34 | 0.081 | -2.32 | 0.377 | -1.1 | 0.646 | -1.79 | 0.53 | -0.97 |
| 1443375_at | Gnas | GNAS (guanine nucleotide binding protein, alpha stimulating) complex locus | -1.04 | 0.917 | -1.16 | 0.681 | -1.16 | 0.165 | 1.05 | 0.886 | -0.58 |
| 1457836_at | 2600014M03Rik | RIKEN cDNA 2600014M03 gene | 1.02 | 0.978 | -1.3 | 0.304 | -1.12 | 0.781 | -1.1 | 0.531 | -0.62 |
| 1446751_s_at | Impact | imprinted and ancient | 1.16 | 0.802 | -1.36 | 0.596 | -1.26 | 0.367 | -1.48 | 0.321 | -0.74 |
| 1420396_at | Cd160 | CD160 antigen | -1.43 | 0.244 | 1 | 0.988 | -1.02 | 0.897 | -1.43 | 0.152 | -0.72 |
| 1447848_at | Eps15l1 | epidermal growth factor receptor pathway substrate 15-like 1 | 1.1 | 0.903 | -1.24 | 0.742 | -1.28 | 0.48 | -1.85 | 0.231 | -0.82 |
| 1457369_at | Phactr4 | Phosphatase and actin regulator 4 | -1.04 | 0.92 | -1.11 | 0.422 | -1.21 | 0.504 | 1.26 | 0.45 | -0.53 |
| 1453807_at | 6330563C09Rik | RIKEN cDNA 6330563C09 gene | -1.01 | 0.992 | -1.25 | 0.665 | -1.12 | 0.266 | 2.1 | 0.23 | -0.32 |
| 1435609_at | --- | --- | -1.23 | 0.081 | -1.05 | 0.938 | -1.09 | 0.617 | 1.12 | 0.56 | -0.56 |
| 1427282_a_at | Fxn | frataxin | -1.15 | 0.431 | -1.2 | 0.189 | -1.02 | 0.842 | 2.54 | 0.231 | -0.21 |
| 1423474_at | Top1 | topoisomerase (DNA) I | -1.09 | 0.716 | -1.06 | 0.488 | -1.22 | 0.005 | 1.78 | 0.121 | -0.4 |
| 1419159_at | Golga3 | golgi autoantigen, golgin subfamily a, 3 | 1 | 0.984 | -1.21 | 0.265 | -1.17 | 0.553 | -1.29 | 0.086 | -0.67 |
| 1460044_at | --- | 0 day neonate cerebellum cDNA, RIKEN full-length enriched library, clone:C2300 | 1.03 | 0.975 | -1.18 | 0.204 | -1.23 | 0.582 | -1.36 | 0.538 | -0.69 |
| 1424520_at | 2010305A19Rik | RIKEN cDNA 2010305A19 gene | -1.09 | 0.503 | 1.01 | 0.957 | -1.32 | 0.316 | -1.03 | 0.865 | -0.61 |
| 1455336_at | Thap2 | THAP domain containing, apoptosis associated protein 2 | -1.13 | 0.052 | 1.08 | 0.608 | -1.39 | 0.009 | -1.05 | 0.431 | -0.62 |
| 1458057_at | Cald1 | Caldesmon 1 | -1.01 | 0.979 | 1.19 | 0.312 | -1.99 | 0.136 | -1.2 | 0.044 | -0.75 |
| 1441920_x_at | Htf9c | HpaII tiny fragments locus 9c | 1.06 | 0.902 | -1.22 | 0.698 | -1.25 | 0.654 | -1.62 | 0.224 | -0.76 |
| 1425870_a_at | Kcnip2 | Kv channel-interacting protein 2 | 1.32 | 0.144 | -2.38 | 0.031 | -1.06 | 0.924 | -1.25 | 0.548 | -0.84 |
| 1442299_at | --- | --- | -1.24 | 0.586 | -1.13 | 0.637 | -1.01 | 0.974 | 1.36 | 0.143 | -0.51 |
| 1417937_at | Dact1 | dapper homolog 1, antagonist of beta-catenin (xenopus) | 1.17 | 0.26 | -1.36 | 0.364 | -1.28 | 0.175 | -1.15 | 0.559 | -0.65 |
| 1427670_a_at | Tcf12 | transcription factor 12 | -1.19 | 0.312 | 1.07 | 0.614 | -1.29 | 0.002 | -1.24 | 0.154 | -0.66 |
| 1425052_at | --- | CDNA, clone:Y2G0147N17, strand:unspecified | -1.05 | 0.69 | -1.12 | 0.183 | -1.19 | 0.165 | 1 | 0.937 | -0.59 |

| | | | | | | | | | | | |
|---|---|---|---|---|---|---|---|---|---|---|---|
| 1432334_at | 4930488P18Rik | RIKEN cDNA 4930488P18 gene | -3.36 | 0.017 | 1.72 | 0.426 | -1.5 | 0.334 | -2.04 | 0.301 | -1.3 |
| 1458535_at | --- | Adult male spinal cord cDNA, RIKEN full-length enriched library, clone:A330040D | -1.33 | 0.073 | 1.45 | 0.488 | -2.07 | 0.054 | -1.17 | 0.587 | -0.78 |
| 1443909_at | Cstf3 | Cleavage stimulation factor, 3' pre-RNA, subunit 3 | 1.09 | 0.847 | -1.01 | 0.968 | -1.65 | 0.003 | -1.42 | 0.261 | -0.75 |
| 1431175_at | 1810019D21Rik | RIKEN cDNA 1810019D21 gene | -1.1 | 0.61 | 1.06 | 0.834 | -1.39 | 0.025 | -1.35 | 0.204 | -0.7 |
| 1444563_at | B4galt5 | UDP-Gal:betaGlcNAc beta 1,4-galactosyltransferase, polypeptide 5 | -1.31 | 0.577 | -1.32 | 0.613 | 1.16 | 0.729 | -1.61 | 0.24 | -0.77 |
| 1416373_at | Nfs1 | nitrogen fixation gene 1 (S. cerevisiae) | -1.13 | 0.324 | -1.15 | 0.121 | -1.07 | 0.362 | 1.21 | 0.303 | -0.54 |
| 1457564_at | Dffa | DNA fragmentation factor, alpha subunit | 1.02 | 0.941 | -1.1 | 0.538 | -1.32 | 0.407 | -1.09 | 0.66 | -0.62 |
| 1447232_at | --- | --- | -1.09 | 0.869 | -1.15 | 0.493 | -1.12 | 0.765 | 1.62 | 0.2 | -0.43 |
| 1456902_at | Satb1 | Special AT-rich sequence binding protein 1 | -1.03 | 0.488 | -1.19 | 0.403 | -1.14 | 0.302 | 1.03 | 0.932 | -0.58 |
| 1442125_at | Krit1 | KRIT1, ankyrin repeat containing | -1.07 | 0.84 | 1.01 | 0.962 | -1.34 | 0.229 | -1.4 | 0.162 | -0.7 |
| 1428121_at | 2610528K11Rik | RIKEN cDNA 2610528K11 gene | -1.11 | 0.48 | -1.13 | 0.231 | -1.11 | 0.493 | 1.33 | 0.391 | -0.51 |
| 1455705_at | Tmem161a | transmembrane protein 161A | 1.17 | 0.323 | -1.51 | 0.044 | -1.17 | 0.58 | -1.19 | 0.406 | -0.68 |
| 1452132_at | Tlcd1 | TLC domain containing 1 | -1.16 | 0.198 | -1.11 | 0.647 | -1.08 | 0.489 | 3.68 | 0.183 | 0.08 |
| 1427950_at | Zfp294 | zinc finger protein 294 | -1.14 | 0.642 | -1.04 | 0.923 | -1.17 | 0.741 | 1.12 | 0.778 | -0.56 |
| 1450101_a_at | Magi3 | membrane associated guanylate kinase, WW and PDZ domain containing 3 | 1.12 | 0.533 | -1.74 | 0.282 | -1.01 | 0.985 | -1 | 0.99 | -0.66 |
| 1450386_at | Kpna3 | karyopherin (importin) alpha 3 | -1.12 | 0.131 | -1.09 | 0.056 | -1.15 | 0.096 | 1.29 | 0.236 | -0.52 |
| 1416972_at | Nhp2l1 /// LOC434 | NHP2 non-histone chromosome protein 2-like 1 (S. cerevisiae) /// similar to Nhp2 | -1.08 | 0.472 | 1.01 | 0.89 | -1.34 | 0.011 | -1.08 | 0.385 | -0.62 |
| 1453045_at | 4921537D05Rik | RIKEN cDNA 4921537D05 gene | -1.03 | 0.814 | -1.21 | 0.054 | -1.13 | 0.32 | 1.18 | 0.511 | -0.55 |
| 1423071_x_at | LOC270335 /// LOC | hypothetical gene supported by BC019681; BC027236 /// hypothetical LOC43432 | -1.22 | 0.582 | 1.02 | 0.919 | -1.19 | 0.481 | -1.89 | 0.149 | -0.82 |
| 1425020_at | Ubxd4 /// LOC6216 | UBX domain containing 4 /// similar to UBX domain containing 4 /// similar to UBX | -1.09 | 0.366 | -1.12 | 0.236 | -1.15 | 0.254 | 1.29 | 0.241 | -0.52 |
| 1451114_at | Cmtm6 | CKLF-like MARVEL transmembrane domain containing 6 | -1.23 | 0.17 | -1.22 | 0.019 | 1.05 | 0.606 | -1.66 | 0.082 | -0.76 |
| 1445103_at | Tmem23 | Transmembrane protein 23 | -1.19 | 0.393 | -1.13 | 0.659 | -1.04 | 0.826 | 1.14 | 0.592 | -0.56 |
| 1457316_at | Mtap6 | microtubule-associated protein 6 | -1.2 | 0.632 | 1.08 | 0.84 | -1.29 | 0.06 | -1.81 | 0.397 | -0.81 |
| 1433834_at | 6-Mar | membrane-associated ring finger (C3HC4) 6 | -1.08 | 0.358 | -1.07 | 0.173 | -1.21 | 0.145 | 1.24 | 0.094 | -0.53 |
| 1434035_at | Dnajb6 | DnaJ (Hsp40) homolog, subfamily B, member 6 | -1.27 | 0.438 | -1.09 | 0.649 | -1.02 | 0.943 | 1.29 | 0.365 | -0.52 |
| 1417158_at | Zxdc | ZXD family zinc finger C | -1.35 | 0.01 | 1.07 | 0.579 | -1.14 | 0.568 | -1.62 | 0.183 | -0.76 |
| 1444845_at | --- | --- | 1.11 | 0.655 | -1.35 | 0.11 | -1.2 | 0.286 | -1.43 | 0.052 | -0.72 |
| 1449773_s_at | Gadd45b | growth arrest and DNA-damage-inducible 45 beta | 1.1 | 0.221 | -1.12 | 0.53 | -1.44 | 0.153 | -1.45 | 0.389 | -0.73 |
| 1415726_at | Ankrd17 | ankyrin repeat domain 17 | -1.09 | 0.376 | -1.18 | 0.12 | -1.08 | 0.502 | 1.15 | 0.053 | -0.55 |
| 1420253_at | D2Ertd63e | DNA segment, Chr 2, ERATO Doi 63, expressed | 1.22 | 0.248 | -1.48 | 0.234 | -1.27 | 0.289 | -1.03 | 0.886 | -0.64 |
| 1441893_at | Tex24 | testis expressed gene 24 | 1.11 | 0.825 | -1.18 | 0.721 | -1.36 | 0.648 | -1.01 | 0.985 | -0.61 |
| 1420105_at | D13Ertd205e | DNA segment, Chr 13, ERATO Doi 205, expressed | -2.01 | 0.539 | -1.42 | 0.302 | 1.49 | 0.106 | -1.47 | 0.395 | -0.85 |
| 1416963_at | Ubadc1 | ubiquitin associated domain containing 1 | -1.19 | 0.31 | -1.07 | 0.416 | -1.09 | 0.402 | 3.58 | 0.156 | 0.06 |
| 1449641_at | --- | --- | -1.05 | 0.911 | -1.2 | 0.267 | -1.11 | 0.73 | 1.64 | 0.169 | -0.43 |
| 1439251_at | --- | Transcribed locus | -1.21 | 0.266 | -1.05 | 0.756 | -1.11 | 0.61 | 1.85 | 0.204 | -0.38 |
| 1455661_at | --- | 0 day neonate thymus cDNA, RIKEN full-length enriched library, clone:A430106B | -1.06 | 0.861 | -1.49 | 0.035 | 1.07 | 0.768 | -1.13 | 0.29 | -0.65 |
| 1437586_at | Cnot4 | CCR4-NOT transcription complex, subunit 4 | -1.15 | 0.304 | -1.19 | 0.263 | -1.02 | 0.93 | 1.32 | 0.23 | -0.51 |
| 1425196_a_at | Hint2 | histidine triad nucleotide binding protein 2 | 1.01 | 0.978 | -1.15 | 0.507 | -1.24 | 0.43 | -1.14 | 0.593 | -0.63 |
| 1424641_a_at | Thoc1 | THO complex 1 | -1.21 | 0.304 | 1.11 | 0.211 | -1.32 | 0.077 | -1.33 | 0.038 | -0.69 |
| 1432080_s_at | Myst1 | MYST histone acetyltransferase 1 | -1.21 | 0.003 | -1.21 | 0.035 | 1.03 | 0.829 | -1.28 | 0.211 | -0.67 |
| 1454624_at | Dennd2a | DENN/MADD domain containing 2A | -1.15 | 0.844 | -1.21 | 0.683 | -1.01 | 0.982 | 1.1 | 0.534 | -0.57 |
| 1440466_at | BC003965 | cDNA sequence BC003965 | -1.26 | 0.35 | 1.07 | 0.775 | -1.21 | 0.673 | -1.34 | 0.638 | -0.68 |
| 1451504_at | Chchd3 | coiled-coil-helix-coiled-coil-helix domain containing 3 | -1.09 | 0.555 | -1.27 | 0.057 | -1.01 | 0.888 | 1.3 | 0.313 | -0.52 |
| 1427259_at | Trim24 | tripartite motif protein 24 | -1.24 | 0.366 | -1.08 | 0.814 | -1.05 | 0.796 | 1.06 | 0.868 | -0.58 |
| 1448292_at | Uqcr | ubiquinol-cytochrome c reductase (6.4kD) subunit | -1.13 | 0.254 | -1.11 | 0.238 | -1.11 | 0.151 | 1.13 | 0.509 | -0.55 |
| 1422679_s_at | Ctr9 | Ctr9, Paf1/RNA polymerase II complex component, homolog (S. cerevisiae) | -1.14 | 0.246 | 1.01 | 0.867 | -1.26 | 0.023 | -1.13 | 0.311 | -0.63 |
| 1427675_at | V1ra2 | vomeronasal 1 receptor, A2 | -1.66 | 0.033 | 1.35 | 0.655 | -1.37 | 0.592 | -1.54 | 0.57 | -0.8 |
| 1455204_at | Pitpnc1 | phosphatidylinositol transfer protein, cytoplasmic 1 | -1.18 | 0.081 | 1.11 | 0.454 | -1.36 | 0.196 | -1.11 | 0.286 | -0.64 |
| 1437172_x_at | Hadhb | hydroxyacyl-Coenzyme A dehydrogenase/3-ketoacyl-Coenzyme A thiolase/enoyl | -1.1 | 0.264 | -1.26 | 0.274 | -1.02 | 0.866 | 1.02 | 0.852 | -0.59 |
| 1438567_at | Vwa2 | von Willebrand factor A domain containing 2 | 1.44 | 0.201 | -1.15 | 0.707 | -2.6 | 0.034 | -1.08 | 0.926 | -0.85 |
| 1426411_a_at | Strbp /// C230082I2 | spermatid perinuclear RNA binding protein /// RIKEN cDNA C230082I21 gene | -1.15 | 0.444 | -1.29 | 0.059 | 1.04 | 0.64 | -1.44 | 0.026 | -0.71 |
| 1458562_at | --- | --- | -1.23 | 0.369 | -1.03 | 0.93 | -1.11 | 0.07 | 1.04 | 0.921 | -0.58 |
| 1457914_at | --- | Adult retina cDNA, RIKEN full-length enriched library, clone:A930016I24 product: | -1.13 | 0.151 | -1.02 | 0.936 | -1.22 | 0.332 | 2.78 | 0.101 | -0.15 |

| | | | | | | | | | | | |
|---|---|---|---|---|---|---|---|---|---|---|---|
| 1427685_a_at | Synj2 | synaptojanin 2 | -1.44 | 0.261 | -1.23 | 0.439 | 1.18 | 0.069 | -1.37 | 0.016 | -0.72 |
| 1446795_at | Vav3 | Vav 3 oncogene | -1.49 | 0.396 | 1.23 | 0.569 | -1.27 | 0.63 | -1.6 | 0.484 | -0.78 |
| 1423238_at | Itgb1bp2 | integrin beta 1 binding protein 2 | -1.07 | 0.811 | -2.68 | 0.122 | 1.38 | 0.563 | -1.15 | 0.77 | -0.88 |
| 1438099_at | Trio | triple functional domain (PTPRF interacting) | 1.53 | 0.493 | -2.22 | 0.332 | -1.41 | 0.448 | -1.69 | 0.36 | -0.95 |
| 1431395_a_at | Iqgap1 | IQ motif containing GTPase activating protein 1 | -1.34 | 0.551 | -1.76 | 0.13 | 1.37 | 0.407 | -2.92 | 0.187 | -1.16 |
| 1447884_x_at | 1300010M03Rik | RIKEN cDNA 1300010M03 gene | -1.12 | 0.814 | -1.4 | 0.295 | 1.08 | 0.867 | -1.25 | 0.399 | -0.67 |
| 1447827_x_at | Spop | speckle-type POZ protein | -1.2 | 0.301 | -1.17 | 0.352 | 1 | 0.964 | -1.36 | 0.22 | -0.68 |
| 1459053_at | Centg2 | centaurin, gamma 2 | 1.05 | 0.626 | -1.16 | 0.466 | -1.29 | 0.589 | -1.01 | 0.982 | -0.6 |
| 1419889_at | 1110033M05Rik | RIKEN cDNA 1110033M05 gene | 1.24 | 0.107 | -1.55 | 0.459 | -1.24 | 0.554 | -1.48 | 0.403 | -0.76 |
| 1416816_at | Nek7 | NIMA (never in mitosis gene a)-related expressed kinase 7 | 1.02 | 0.839 | -1.13 | 0.409 | -1.27 | 0.182 | -1.03 | 0.922 | -0.6 |
| 1432435_s_at | C030004A17Rik | RIKEN cDNA C030004A17 gene | -1.26 | 0.407 | 1.03 | 0.925 | -1.16 | 0.019 | -1.06 | 0.353 | -0.61 |
| 1421880_at | Mtmr1 | myotubularin related protein 1 | -1.19 | 0.186 | -1.28 | 0.042 | 1.07 | 0.505 | -1.29 | 0.251 | -0.67 |
| 1443474_at | D7Ertd495e | DNA segment, Chr 7, ERATO Doi 495, expressed | -1.05 | 0.897 | -1.1 | 0.883 | -1.21 | 0.581 | 1.24 | 0.242 | -0.53 |
| 1438460_at | Zmym4 | zinc finger, MYM-type 4 | -1.15 | 0.393 | 1.06 | 0.809 | -1.32 | 0.044 | -1.4 | 0.333 | -0.7 |
| 1438578_a_at | Btbd10 | BTB (POZ) domain containing 10 | -1.06 | 0.707 | -1.14 | 0.522 | -1.15 | 0.095 | 1.08 | 0.498 | -0.57 |
| 1416542_at | Phf1 | PHD finger protein 1 | -1.17 | 0.298 | -1.4 | 0.155 | 1.12 | 0.501 | -1.25 | 0.296 | -0.67 |
| 1435786_at | Klhl12 | kelch-like 12 (Drosophila) | -1.22 | 0.294 | -1.13 | 0.392 | -1.01 | 0.907 | 3.01 | 0.153 | -0.09 |
| 1456177_x_at | Zfp706 | zinc finger protein 706 | -1.22 | 0.129 | -1.22 | 0.09 | 1.05 | 0.641 | -1.06 | 0.827 | -0.61 |
| 1444516_at | Ppfibp2 | Protein tyrosine phosphatase, receptor-type, F interacting protein, binding protein | -1.13 | 0.532 | 1.12 | 0.544 | -1.45 | 0.148 | -1.56 | 0.133 | -0.76 |
| 1431671_at | 4930447A16Rik | RIKEN cDNA 4930447A16 gene | -2 | 0.067 | -1.06 | 0.822 | 1.25 | 0.446 | -1.53 | 0.031 | -0.84 |
| 1456574_at | AA407452 | EST AA407452 | -1.16 | 0.681 | -1.05 | 0.83 | -1.14 | 0.212 | 1.05 | 0.823 | -0.58 |
| 1454412_at | 2810410P21Rik | RIKEN cDNA 2810410P21 gene | 1.12 | 0.387 | -1.35 | 0.248 | -1.21 | 0.406 | -1.22 | 0.614 | -0.66 |
| 1426285_at | Lama2 | laminin, alpha 2 | -1.29 | 0.057 | 1.25 | 0.418 | -1.5 | 0.147 | -1.73 | 0.161 | -0.82 |
| 1416176_at | Hmgb1 | high mobility group box 1 | -1.04 | 0.498 | -1.07 | 0.335 | -1.25 | 0.001 | 1.35 | 0.11 | -0.51 |
| 1419551_s_at | Stk39 | serine/threonine kinase 39, STE20/SPS1 homolog (yeast) | 1.09 | 0.751 | -1.46 | 0.254 | -1.09 | 0.764 | -1.1 | 0.782 | -0.64 |
| 1443251_at | Prdm16 | PR domain containing 16 | -1.96 | 0.027 | 1.45 | 0.455 | -1.38 | 0.157 | -1.1 | 0.852 | -0.75 |
| 1450362_at | --- | --- | -1.15 | 0.855 | 1.33 | 0.645 | -2.04 | 0.164 | -1.01 | 0.973 | -0.72 |
| 1442174_at | Tspan18 | tetraspanin 18 | -1.17 | 0.083 | 1.06 | 0.849 | -1.3 | 0.179 | -1.23 | 0.42 | -0.66 |
| 1425076_at | Dnajc18 | DnaJ (Hsp40) homolog, subfamily C, member 18 | -1.29 | 0.054 | 1.05 | 0.737 | -1.15 | 0.356 | -1.19 | 0.506 | -0.65 |
| 1415910_s_at | Ciapin1 | cytokine induced apoptosis inhibitor 1 | -1.11 | 0.405 | -1.09 | 0.552 | -1.15 | 0.151 | 1.74 | 0.149 | -0.4 |
| 1447355_at | Acsl1 | acyl-CoA synthetase long-chain family member 1 | -2.06 | 0.283 | -2.25 | 0.042 | 1.76 | 0.232 | -1.72 | 0.504 | -1.07 |
| 1416720_at | Sfrs6 | splicing factor, arginine/serine-rich 6 | -1.11 | 0.562 | -1.23 | 0.015 | -1.02 | 0.675 | 1.18 | 0.598 | -0.55 |
| 1441512_at | 6030445D17Rik | RIKEN cDNA 6030445D17 gene | -1.35 | 0.697 | 1.1 | 0.86 | -1.18 | 0.629 | -2.21 | 0.236 | -0.91 |
| 1456094_at | Usp36 | ubiquitin specific peptidase 36 | -1.04 | 0.9 | -1.32 | 0.634 | -1.03 | 0.917 | 1.32 | 0.24 | -0.52 |
| 1428931_a_at | Parp6 | poly (ADP-ribose) polymerase family, member 6 | 1.03 | 0.76 | -1.36 | 0.219 | -1.08 | 0.461 | -1.95 | 0.022 | -0.84 |
| 1425402_at | Thop1 | thimet oligopeptidase 1 | 1.62 | 0.518 | -1.75 | 0.442 | -2 | 0.259 | -1.23 | 0.788 | -0.84 |
| 1460367_at | Hbp1 | high mobility group box transcription factor 1 | -1.05 | 0.22 | -1.07 | 0.355 | -1.24 | 0.353 | 1.08 | 0.719 | -0.57 |
| 1432827_x_at | Ubc | ubiquitin C | -1.17 | 0.227 | -1.24 | 0.263 | 1.03 | 0.748 | -1.04 | 0.508 | -0.61 |
| 1452283_at | Rassf8 | Ras association (RalGDS/AF-6) domain family 8 | 1.07 | 0.727 | -1.21 | 0.211 | -1.26 | 0.288 | -1.47 | 0.045 | -0.72 |
| 1451384_at | Jmjd5 | jumonji domain containing 5 | -1.04 | 0.553 | -1.57 | 0.065 | 1.1 | 0.539 | -1.05 | 0.77 | -0.64 |
| 1455096_at | --- | --- | -1.34 | 0.387 | -1.4 | 0.37 | 1.23 | 0.626 | -1.1 | 0.551 | -0.65 |
| 1436426_at | 5730509K17Rik | RIKEN cDNA 5730509K17 gene | -1.13 | 0.313 | -1.32 | 0.239 | 1.05 | 0.67 | -1.63 | 0.138 | -0.76 |
| 1428939_s_at | Gnaq | guanine nucleotide binding protein, alpha q polypeptide | -1.11 | 0.626 | 1.06 | 0.6 | -1.36 | 0.125 | -1.56 | 0.059 | -0.74 |
| 1417875_at | Ddx50 | DEAD (Asp-Glu-Ala-Asp) box polypeptide 50 | -1.11 | 0.453 | 1 | 0.971 | -1.27 | 0.218 | -1.35 | 0.091 | -0.68 |
| 1458791_at | AU015849 | expressed sequence AU015849 | -1.66 | 0.054 | 1.27 | 0.485 | -1.22 | 0.621 | -2.07 | 0.547 | -0.92 |
| 1437638_at | Srrm2 | serine/arginine repetitive matrix 2 | 1.25 | 0.35 | -1.26 | 0.415 | -1.54 | 0.094 | -1.19 | 0.639 | -0.69 |
| 1449899_at | Grin3b | glutamate receptor, ionotropic, NMDA3B | -1.18 | 0.392 | 1.29 | 0.415 | -1.82 | 0.219 | -1.02 | 0.966 | -0.68 |
| 1444760_at | Ncor1 | nuclear receptor co-repressor 1 | 1.09 | 0.827 | -1.1 | 0.608 | -1.44 | 0.272 | -1.25 | 0.502 | -0.68 |
| 1438863_at | A530079E22Rik | RIKEN cDNA A530079E22 gene | -1.15 | 0.694 | 1.14 | 0.794 | -1.46 | 0.003 | -1.92 | 0.135 | -0.85 |
| 1459601_at | Snf1lk | SNF1-like kinase | -1.3 | 0.395 | 1.13 | 0.794 | -1.26 | 0.277 | -1.96 | 0.059 | -0.85 |
| 1449782_at | AA517650 | expressed sequence AA517650 | -1.21 | 0.709 | -1.56 | 0.217 | 1.23 | 0.628 | -1.58 | 0.31 | -0.78 |
| 1447381_at | Cpsf6 | Cleavage and polyadenylation specific factor 6 | 1.04 | 0.665 | -1.11 | 0.588 | -1.33 | 0.382 | -1.37 | 0.34 | -0.69 |
| 1417251_at | Palmd | palmdelphin | -1.01 | 0.934 | -1.44 | 0.12 | 1.01 | 0.968 | -1.29 | 0.044 | -0.68 |

| | | | | | | | | | | | |
|---|---|---|---|---|---|---|---|---|---|---|---|
| 1417159_at | --- | --- | -1.04 | 0.967 | -1.06 | 0.888 | -1.28 | 0.615 | 1.3 | 0.51 | -0.52 |
| 1454709_at | Tmem64 | transmembrane protein 64 | -1.4 | 0.139 | -1.07 | 0.633 | 1.04 | 0.653 | -1.17 | 0.404 | -0.65 |
| 1440071_at | Magi1 | membrane associated guanylate kinase, WW and PDZ domain containing 1 | -1.45 | 0.451 | 1.06 | 0.864 | -1.07 | 0.858 | -1.06 | 0.848 | -0.63 |
| 1460033_at | C030002C11Rik | RIKEN cDNA C030002C11 gene | -1.05 | 0.875 | -1.46 | 0.289 | 1.05 | 0.879 | -1.22 | 0.672 | -0.67 |
| 1426151_a_at | Stx3 | syntaxin 3 | -1.14 | 0.64 | -1.05 | 0.86 | -1.15 | 0.455 | 1.19 | 0.556 | -0.54 |
| 1444321_at | Psmd9 | Proteasome (prosome, macropain) 26S subunit, non-ATPase, 9 | -1.11 | 0.744 | 1.08 | 0.849 | -1.4 | 0.18 | -1.1 | 0.802 | -0.63 |
| 1423472_at | 2-Sep | septin 2 | -1.21 | 0.367 | 1.04 | 0.742 | -1.21 | 0.392 | -1.2 | 0.311 | -0.65 |
| 1433967_at | LOC433492 | similar to RIKEN cDNA 5430413K10 | -1.3 | 0.495 | 1.28 | 0.611 | -1.55 | 0.173 | -2.27 | 0.213 | -0.96 |
| 1448542_at | Bccip | BRCA2 and CDKN1A interacting protein | -1.11 | 0.031 | -1.17 | 0.031 | -1.07 | 0.337 | 1.01 | 0.968 | -0.59 |
| 1425875_a_at | Lepr | leptin receptor | 1.21 | 0.682 | -1.67 | 0.007 | -1.14 | 0.393 | -1.13 | 0.248 | -0.68 |
| 1442110_at | Smyd3 | SET and MYND domain containing 3 | -1.69 | 0.348 | 1.21 | 0.64 | -1.12 | 0.692 | -1.42 | 0.333 | -0.75 |
| 1434262_at | --- | --- | 1.06 | 0.599 | -1.46 | 0.364 | -1.05 | 0.786 | -1.45 | 0.243 | -0.73 |
| 1428434_at | Zcchc12 | zinc finger, CCHC domain containing 12 | -1.2 | 0.418 | 1.02 | 0.971 | -1.19 | 0.1 | -1.33 | 0.455 | -0.67 |
| 1417421_at | S100a1 | S100 calcium binding protein A1 | -1.13 | 0.183 | 1 | 0.982 | -1.24 | 0.095 | -1.2 | 0.357 | -0.64 |
| 1416484_at | Ttc3 | tetratricopeptide repeat domain 3 | 1.05 | 0.834 | -1.38 | 0.128 | -1.09 | 0.661 | -1.63 | 0.367 | -0.76 |
| 1452931_at | 2810408M09Rik | RIKEN cDNA 2810408M09 gene | -1.41 | 0.184 | 1.13 | 0.565 | -1.18 | 0.525 | -1.7 | 0.096 | -0.79 |
| 1453991_at | Cenpo | centromere protein O | 1.08 | 0.8 | -1.19 | 0.543 | -1.29 | 0.301 | -1.7 | 0.42 | -0.78 |
| 1448331_at | Ndufb7 | NADH dehydrogenase (ubiquinone) 1 beta subcomplex, 7 | -1.04 | 0.264 | -1.23 | 0.019 | -1.1 | 0.413 | 1.15 | 0.442 | -0.55 |
| 1457704_at | Zfp533 | zinc finger protein 533 | -1.03 | 0.864 | -1.14 | 0.654 | -1.19 | 0.466 | 1.01 | 0.962 | -0.59 |
| 1447405_at | AI449023 | Expressed sequence AI449023 | 1.5 | 0.087 | -4.61 | 0 | -1.02 | 0.972 | -1.12 | 0.814 | -1.31 |
| 1416909_at | 1810008A14Rik | RIKEN cDNA 1810008A14 gene | -1.03 | 0.818 | -1.01 | 0.899 | -1.37 | 0.169 | 1.02 | 0.743 | -0.6 |
| 1416313_at | Mllt11 | myeloid/lymphoid or mixed-lineage leukemia (trithorax homolog, Drosophila); tran | 1.01 | 0.968 | -1.03 | 0.939 | -1.41 | 0.004 | -1.6 | 0.053 | -0.76 |
| 1452054_at | Ube2w | ubiquitin-conjugating enzyme E2W (putative) | -1.24 | 0.386 | 1.15 | 0.325 | -1.35 | 0.077 | -1.36 | 0.174 | -0.7 |
| 1429513_at | 1700019M22Rik | RIKEN cDNA 1700019M22 gene | -1.15 | 0.204 | -1.44 | 0.305 | 1.13 | 0.587 | -1.13 | 0.458 | -0.65 |
| 1428052_a_at | Zmym1 | zinc finger, MYM domain containing 1 | 1.03 | 0.8 | -1.16 | 0.414 | -1.25 | 0.073 | -1.1 | 0.573 | -0.62 |
| 1446585_at | Dlgh2 | Discs, large homolog 2 (Drosophila) | -2.67 | 0 | -1.4 | 0.262 | 1.6 | 0.158 | -1.49 | 0.343 | -0.99 |
| 1424403_a_at | Rufy3 | RUN and FYVE domain containing 3 | -1.11 | 0.003 | -1.52 | 0.146 | 1.13 | 0.558 | -1.04 | 0.862 | -0.63 |
| 1441238_at | 9030416H16Rik | RIKEN cDNA 9030416H16 gene | -1.04 | 0.874 | -1.41 | 0.051 | 1.02 | 0.964 | -1.13 | 0.774 | -0.64 |
| 1442232_at | C530025M09Rik | RIKEN cDNA C530025M09 gene | -1.17 | 0.596 | -1.38 | 0.243 | 1.11 | 0.617 | -1.94 | 0.159 | -0.84 |
| 1458133_at | --- | --- | 1.2 | 0.242 | -1.71 | 0.008 | -1.11 | 0.583 | -1.54 | 0.081 | -0.79 |
| 1435894_at | C030014L02 | hypothetical protein C030014L02 | -1.05 | 0.957 | -1.35 | 0.725 | 1 | 0.987 | -1.08 | 0.929 | -0.62 |
| 1435029_at | --- | --- | -1.06 | 0.607 | -1.04 | 0.662 | -1.28 | 0.237 | 1.59 | 0.096 | -0.45 |
| 1426671_a_at | Rbm39 | RNA binding motif protein 39 | -1.11 | 0.559 | 1.01 | 0.912 | -1.28 | 0.1 | -1.31 | 0.086 | -0.67 |
| 1442669_at | --- | --- | -1.65 | 0.49 | 1.11 | 0.803 | -1.03 | 0.962 | -1.29 | 0.712 | -0.71 |
| 1431518_at | Ptchd3 | patched domain containing 3 | -1.83 | 0.161 | -1.31 | 0.532 | 1.38 | 0.547 | -1.05 | 0.832 | -0.7 |
| 1435395_s_at | Atp5j2 | ATP synthase, H+ transporting, mitochondrial F0 complex, subunit f, isoform 2 | -1.13 | 0.017 | -1.03 | 0.724 | -1.2 | 0.057 | 1.09 | 0.094 | -0.57 |
| 1459004_at | --- | --- | -1.16 | 0.617 | -1.72 | 0.051 | 1.25 | 0.221 | -1.46 | 0.277 | -0.77 |
| 1418115_s_at | Ifrg15 | interferon alpha responsive gene | -1.1 | 0.085 | -1.11 | 0.47 | -1.14 | 0.331 | 1.07 | 0.713 | -0.57 |
| 1443193_at | Ttll1 | tubulin tyrosine ligase-like 1 | 1.21 | 0.561 | -1.73 | 0.441 | -1.1 | 0.801 | -2.19 | 0.075 | -0.95 |
| 1420367_at | Denr | density-regulated protein | -1.17 | 0.008 | 1.05 | 0.643 | -1.26 | 0.235 | -1.03 | 0.885 | -0.6 |
| 1429731_at | 6530403G13Rik | RIKEN cDNA 6530403G13 gene | -1.05 | 0.919 | -1.09 | 0.793 | -1.21 | 0.574 | 1.41 | 0.264 | -0.49 |
| 1454541_at | 4921522E08Rik | RIKEN cDNA 4921522E08 gene | 1.31 | 0.511 | -1.5 | 0.361 | -1.4 | 0.269 | -1.52 | 0.49 | -0.78 |
| 1447143_at | Phf14 | PHD finger protein 14 | 1.18 | 0.752 | -1.31 | 0.458 | -1.33 | 0.323 | -1.27 | 0.372 | -0.68 |
| 1441209_at | Rin2 | Ras and Rab interactor 2 | 1.05 | 0.888 | -1.11 | 0.777 | -1.35 | 0.438 | -1.56 | 0.066 | -0.74 |
| 1436029_at | Bicc1 | bicaudal C homolog 1 (Drosophila) | -1.06 | 0.751 | 1.11 | 0.49 | -1.56 | 0.115 | -1.4 | 0.166 | -0.73 |
| 1424478_at | Bbs2 | Bardet-Biedl syndrome 2 homolog (human) | 1.11 | 0.333 | -1.64 | 0.401 | -1.02 | 0.726 | -1.32 | 0.071 | -0.72 |
| 1435754_at | 2810482G21Rik | RIKEN cDNA 2810482G21 gene | -1.17 | 0.068 | 1.06 | 0.431 | -1.27 | 0.159 | -1.7 | 0.152 | -0.77 |
| 1448839_at | Ankrd47 | ankyrin repeat domain 47 | 1.07 | 0.785 | -1.24 | 0.012 | -1.22 | 0.376 | -1.3 | 0.391 | -0.67 |
| 1441935_at | Ankra2 | ankyrin repeat, family A (RFXANK-like), 2 | -1.49 | 0.218 | -1.52 | 0.327 | 1.36 | 0.265 | -1.11 | 0.38 | -0.69 |
| 1448936_at | Stx12 | syntaxin 12 | -1.06 | 0.532 | -1.08 | 0.351 | -1.22 | 0.127 | 1.09 | 0.527 | -0.56 |
| 1442745_x_at | C79248 | expressed sequence C79248 | -1.06 | 0.787 | 1.07 | 0.666 | -1.46 | 0.009 | -1.84 | 0.078 | -0.82 |
| 1428385_at | 8-Mar | membrane-associated ring finger (C3HC4) 8 | -1.18 | 0.34 | -1.11 | 0.508 | -1.06 | 0.647 | 3.9 | 0.153 | 0.14 |
| 1439435_x_at | Pgk1 /// LOC43359 | phosphoglycerate kinase 1 /// phosphoglycerate kinase 1-like /// phosphoglycerat | -1.2 | 0.416 | -1.5 | 0.02 | 1.19 | 0.455 | -1.21 | 0.439 | -0.68 |

| | | | | | | | | | | | |
|---|---|---|---|---|---|---|---|---|---|---|---|
| 1426752_at | Phf17 | PHD finger protein 17 | -1.03 | 0.735 | -1.13 | 0.414 | -1.2 | 0.163 | 1.1 | 0.533 | -0.56 |
| 1426878_at | Pb1 | polybromo 1 | -1.01 | 0.953 | -1.08 | 0.575 | -1.29 | 0.072 | 1.36 | 0.413 | -0.5 |
| 1439716_at | Piwil4 | piwi-like homolog 4 (Drosophila) | -1.48 | 0.44 | 1.34 | 0.613 | -1.48 | 0.095 | -2.14 | 0.335 | -0.94 |
| 1455979_at | Arid1b | AT rich interactive domain 1B (Swi1 like) | -1.13 | 0.495 | 1.05 | 0.683 | -1.32 | 0.054 | -1.44 | 0.166 | -0.71 |
| 1459871_x_at | 2-Mar | membrane-associated ring finger (C3HC4) 2 | -1.09 | 0.576 | -1.14 | 0.359 | -1.11 | 0.771 | 2.89 | 0.286 | -0.11 |
| 1433798_a_at | E330034G19Rik | RIKEN cDNA E330034G19 gene | -1.02 | 0.941 | -1.03 | 0.922 | -1.34 | 0.368 | 1.01 | 0.979 | -0.6 |
| 1425383_a_at | Pbx1 /// LOC67687 | pre B-cell leukemia transcription factor 1 /// region containing RIKEN cDNA 2310 | -1.1 | 0.758 | -1.12 | 0.585 | -1.12 | 0.647 | 1.22 | 0.502 | -0.53 |
| 1428961_a_at | Sfrs16 | splicing factor, arginine/serine-rich 16 (suppressor-of-white-apricot homolog, Dros | -1.47 | 0.102 | -1.03 | 0.789 | 1.05 | 0.649 | -1.22 | 0.406 | -0.67 |
| 1450581_at | Galr3 | galanin receptor 3 | -1.91 | 0.104 | -1.11 | 0.544 | 1.27 | 0.224 | -1.42 | 0.309 | -0.79 |
| 1425108_a_at | BC004728 | cDNA sequence BC004728 | 1.11 | 0.526 | -1.26 | 0.034 | -1.27 | 0.052 | -1.04 | 0.863 | -0.61 |
| 1450347_at | Syt10 | synaptotagmin X | -2.42 | 0.089 | 1.34 | 0.526 | -1.07 | 0.828 | -1.44 | 0.209 | -0.89 |
| 1432521_at | Mxra8 | matrix-remodelling associated 8 | -1.24 | 0.028 | -1.25 | 0.49 | 1.09 | 0.523 | -1.14 | 0.606 | -0.64 |
| 1447244_at | Cybasc3 | cytochrome b, ascorbate dependent 3 | 1.5 | 0.363 | -1.37 | 0.362 | -2.13 | 0.106 | -2.2 | 0.271 | -1.05 |
| 1436012_s_at | Scrn2 | secernin 2 | 1.15 | 0.338 | -1.6 | 0.136 | -1.09 | 0.73 | -1.22 | 0.186 | -0.69 |
| 1415816_at | Cct7 | chaperonin subunit 7 (eta) | -1.12 | 0.398 | -1.19 | 0.006 | -1.04 | 0.589 | 1.05 | 0.712 | -0.58 |
| 1417247_at | AI597479 | expressed sequence AI597479 | -1.14 | 0.02 | 1.03 | 0.752 | -1.27 | 0.129 | -1.19 | 0.478 | -0.64 |
| 1442942_at | Wbscr1 | Williams-Beuren syndrome chromosome region 1 homolog (human) | -1.06 | 0.875 | -1.61 | 0.435 | 1.13 | 0.68 | -2.26 | 0.095 | -0.95 |
| 1423649_at | Tmem68 | transmembrane protein 68 | 1.06 | 0.601 | -1.1 | 0.225 | -1.38 | 0.082 | -1.09 | 0.196 | -0.63 |
| 1427389_at | BC021785 | cDNA sequence BC021785 | -1.14 | 0.428 | 1.1 | 0.88 | -1.4 | 0.164 | -1.31 | 0.015 | -0.69 |
| 1437582_at | Acss1 | acyl-CoA synthetase short-chain family member 1 | 1.12 | 0.827 | -1.36 | 0.58 | -1.19 | 0.77 | -2.93 | 0.16 | -1.09 |
| 1436610_at | Ankrd12 | ankyrin repeat domain 12 | 1.11 | 0.349 | -1.28 | 0.38 | -1.25 | 0.556 | -1.01 | 0.964 | -0.61 |
| 1416610_a_at | Clcn3 | chloride channel 3 | -1.12 | 0.059 | -1.09 | 0.088 | -1.13 | 0.32 | 2.46 | 0.224 | -0.22 |
| 1454161_s_at | 0610007P14Rik | RIKEN cDNA 0610007P14 gene | -1.21 | 0.531 | -1.05 | 0.592 | -1.09 | 0.748 | 1.09 | 0.642 | -0.57 |
| 1442928_at | Kpna4 | Karyopherin (importin) alpha 4 | 1.5 | 0.075 | -1.67 | 0.012 | -1.67 | 0 | -1.46 | 0.061 | -0.83 |
| 1458776_at | --- | --- | -1.13 | 0.741 | -1.1 | 0.747 | -1.12 | 0.387 | 1.32 | 0.169 | -0.51 |
| 1455773_at | Picalm | Phosphatidylinositol binding clathrin assembly protein | -1.03 | 0.91 | -1.2 | 0.09 | -1.13 | 0.54 | 1.9 | 0.12 | -0.36 |
| 1441389_at | --- | Transcribed locus | 1.03 | 0.89 | -1.14 | 0.764 | -1.26 | 0.288 | -1.18 | 0.371 | -0.64 |
| 1425813_at | Pign | phosphatidylinositol glycan anchor biosynthesis, class N | -1.2 | 0.743 | -1.39 | 0.653 | 1.14 | 0.743 | -1.24 | 0.696 | -0.67 |
| 1423159_at | Dld | dihydrolipoamide dehydrogenase | -1.18 | 0.224 | -1.14 | 0.034 | -1.03 | 0.877 | 1.09 | 0.569 | -0.57 |
| 1428352_at | Arrdc2 | arrestin domain containing 2 | -1.09 | 0.775 | -1.25 | 0.457 | -1.02 | 0.967 | 1.13 | 0.613 | -0.56 |
| 1443202_at | Cyb5b | Cytochrome b5 type B | -1.01 | 0.987 | -1.06 | 0.846 | -1.33 | 0.204 | 1.07 | 0.815 | -0.58 |
| 1452669_at | 2810012G03Rik | RIKEN cDNA 2810012G03 gene | -1.25 | 0.338 | 1.03 | 0.728 | -1.16 | 0.451 | -1.04 | 0.538 | -0.6 |
| 1418560_at | Pdha1 | pyruvate dehydrogenase E1 alpha 1 | -1.18 | 0.028 | -1.25 | 0.157 | 1.05 | 0.837 | -1.03 | 0.855 | -0.6 |
| 1433249_at | 4931409D07Rik | RIKEN cDNA 4931409D07 gene | 1.43 | 0.145 | -1.89 | 0.343 | -1.36 | 0.468 | -1.28 | 0.773 | -0.77 |
| 1459289_at | Pde4d | Phosphodiesterase 4D, cAMP specific | -1.56 | 0.318 | 1.58 | 0.495 | -2.1 | 0.072 | -1.44 | 0.381 | -0.88 |
| 1443809_at | Ptn | Pleiotrophin | -1.34 | 0.739 | -1.28 | 0.594 | 1.17 | 0.771 | -2.01 | 0.247 | -0.87 |
| 1424525_at | Grp | gastrin releasing peptide | -1.2 | 0.735 | 1.38 | 0.438 | -2.1 | 0.06 | -1.43 | 0.437 | -0.83 |
| 1434587_x_at | Ptdss2 | phosphatidylserine synthase 2 | -1.07 | 0.338 | -1.25 | 0.214 | -1.04 | 0.82 | 4.07 | 0.192 | 0.18 |
| 1429291_at | Psmd1 | proteasome (prosome, macropain) 26S subunit, non-ATPase, 1 | -1.1 | 0.19 | -1.17 | 0.026 | -1.07 | 0.186 | 1.47 | 0.149 | -0.47 |
| 1417086_at | Pafah1b1 | platelet-activating factor acetylhydrolase, isoform 1b, beta1 subunit | -1.27 | 0.331 | -1.24 | 0.064 | 1.1 | 0.663 | -1.07 | 0.74 | -0.62 |
| 1436724_a_at | Lgtn | ligatin | -1.02 | 0.611 | -1.18 | 0.117 | -1.16 | 0.134 | 1.1 | 0.385 | -0.56 |
| 1446623_at | --- | --- | -1.04 | 0.867 | -3.85 | 0.133 | 1.47 | 0.514 | -2.12 | 0.346 | -1.38 |
| 1435143_at | Pctk2 | PCTAIRE-motif protein kinase 2 | 1.16 | 0.217 | -1.28 | 0.397 | -1.33 | 0.075 | -1.57 | 0.097 | -0.75 |
| 1447437_at | Acsm1 | Acyl-CoA synthetase medium-chain family member 1 | -1.13 | 0.747 | -1.25 | 0.293 | 1.01 | 0.978 | -1.12 | 0.794 | -0.62 |
| 1421271_at | Sh3rf1 | SH3 domain containing ring finger 1 | -1.21 | 0.432 | -1.52 | 0.428 | 1.21 | 0.545 | -1.04 | 0.916 | -0.64 |
| 1443729_at | Mtss1 | metastasis suppressor 1 | -1.17 | 0.305 | 1.07 | 0.112 | -1.3 | 0.231 | -1.31 | 0.163 | -0.68 |
| 1443055_at | B230369F24Rik | RIKEN cDNA B230369F24 gene | -3.25 | 0.005 | 1.46 | 0.701 | -1.08 | 0.901 | -2.42 | 0.296 | -1.32 |
| 1458018_at | Ppm1h | Protein phosphatase 1H (PP2C domain containing) | -1.07 | 0.838 | 1.18 | 0.619 | -1.73 | 0.03 | -1.28 | 0.477 | -0.72 |
| 1454760_at | Htatsf1 | HIV TAT specific factor 1 | -1.17 | 0.457 | 1.04 | 0.734 | -1.25 | 0.126 | -1.07 | 0.607 | -0.61 |
| 1442477_at | --- | --- | -1.48 | 0.224 | 1.29 | 0.376 | -1.36 | 0.042 | -1.29 | 0.036 | -0.71 |
| 1433486_at | Clcn3 | chloride channel 3 | -1.03 | 0.819 | -1.05 | 0.648 | -1.3 | 0.013 | 2.4 | 0.188 | -0.24 |
| 1431641_at | 9330161L09Rik | RIKEN cDNA 9330161L09 gene | -1.12 | 0.865 | -1.84 | 0.247 | 1.26 | 0.468 | -1.13 | 0.515 | -0.71 |
| 1455943_at | Zfp451 | zinc finger protein 451 | -1.08 | 0.778 | 1.03 | 0.882 | -1.36 | 0.317 | -1.1 | 0.549 | -0.63 |

| | | | | | | | | | | | |
|---|---|---|---|---|---|---|---|---|---|---|---|
| 1451145_s_at | Tmem111 | transmembrane protein 111 | -1.08 | 0.102 | -1.01 | 0.854 | -1.28 | 0.001 | 1.06 | 0.474 | -0.58 |
| 1416812_at | Tia1 | cytotoxic granule-associated RNA binding protein 1 | -1.07 | 0.47 | 1.02 | 0.88 | -1.34 | 0.049 | -1.54 | 0.15 | -0.73 |
| 1426618_a_at | Pomgnt1 | protein O-linked mannose beta1,2-N-acetylglucosaminyltransferase | 1.01 | 0.912 | -1.23 | 0.018 | -1.14 | 0.109 | -1.4 | 0.079 | -0.69 |
| 1452343_at | D18Ertd653e | DNA segment, Chr 18, ERATO Doi 653, expressed | -1.06 | 0.711 | -1.44 | 0.149 | 1.06 | 0.782 | -1.5 | 0.122 | -0.73 |
| 1438644_x_at | Commd9 | COMM domain containing 9 | -1.14 | 0.382 | 1.08 | 0.36 | -1.35 | 0.055 | -1.27 | 0.111 | -0.67 |
| 1458407_s_at | AI429294 | Expressed sequence AI429294 | -1.07 | 0.766 | -1.14 | 0.812 | -1.13 | 0.407 | 1.05 | 0.79 | -0.57 |
| 1458568_at | Tusc3 | Tumor suppressor candidate 3 | 1.15 | 0.791 | -1.32 | 0.61 | -1.27 | 0.511 | -1.03 | 0.959 | -0.62 |
| 1444086_at | E030049G20Rik | RIKEN cDNA E030049G20 gene | -1.13 | 0.594 | 1.02 | 0.96 | -1.27 | 0.254 | -1.48 | 0.307 | -0.71 |
| 1456146_at | Osbpl10 | oxysterol binding protein-like 10 | -1.31 | 0.156 | -1.36 | 0.478 | 1.2 | 0.167 | -1.21 | 0.197 | -0.67 |
| 1458524_at | Fndc3a | fibronectin type III domain containing 3a | -1.18 | 0.508 | 1.04 | 0.849 | -1.24 | 0.134 | -1.47 | 0.277 | -0.71 |
| 1445165_at | 1200003I07Rik | RIKEN cDNA 1200003I07 gene | 1.33 | 0.725 | -1.17 | 0.815 | -1.94 | 0.05 | -1.55 | 0.048 | -0.83 |
| 1422742_at | Hivep1 | human immunodeficiency virus type I enhancer binding protein 1 | -1.13 | 0.544 | -1.28 | 0.011 | 1.03 | 0.851 | -1.31 | 0.062 | -0.67 |
| 1419459_a_at | 2610529C04Rik | RIKEN cDNA 2610529C04 gene | -1.03 | 0.896 | -1.12 | 0.543 | -1.2 | 0.438 | 1.63 | 0.031 | -0.43 |
| 1420422_at | Pcdhb21 | protocadherin beta 21 | 1.21 | 0.834 | -1.39 | 0.605 | -1.31 | 0.062 | -1.36 | 0.182 | -0.71 |
| 1424026_s_at | BC013529 | cDNA sequence BC013529 | -1.13 | 0.16 | -1.1 | 0.582 | -1.11 | 0.047 | 1.03 | 0.525 | -0.58 |
| 1460072_at | --- | Transcribed locus | -1.6 | 0.263 | 1.41 | 0.273 | -1.52 | 0.205 | -1.55 | 0.346 | -0.81 |
| 1448882_at | Tmem93 | transmembrane protein 93 | 1.01 | 0.658 | -1.08 | 0.138 | -1.32 | 0.075 | -1.21 | 0.188 | -0.65 |
| 1424121_at | Commd1 | COMM domain containing 1 | -1.06 | 0.75 | -1.03 | 0.798 | -1.28 | 0.277 | 1.13 | 0.581 | -0.56 |
| 1439236_at | D15Ertd509e | DNA segment, Chr 15, ERATO Doi 509, expressed | 1.27 | 0.637 | -1.48 | 0.188 | -1.32 | 0.096 | -1.91 | 0.041 | -0.86 |
| 1430091_at | 1700001P01Rik | RIKEN cDNA 1700001P01 gene | -1.38 | 0.244 | 1.1 | 0.416 | -1.15 | 0.551 | -1.17 | 0.594 | -0.65 |
| 1426017_a_at | 0610011L14Rik | RIKEN cDNA 0610011L14 gene | -1.17 | 0.326 | -1.19 | 0.225 | 1 | 0.971 | -1.02 | 0.817 | -0.6 |
| 1422285_at | Otp | orthopedia homolog (Drosophila) | -2.25 | 0.096 | -1.68 | 0.059 | 1.66 | 0.181 | -1.94 | 0.375 | -1.05 |
| 1451251_at | Appbp2 | amyloid beta precursor protein (cytoplasmic tail) binding protein 2 | -1.06 | 0.357 | -1.08 | 0.524 | -1.21 | 0.225 | 1.04 | 0.814 | -0.58 |
| 1424586_at | Ehbp1 | EH domain binding protein 1 | -1.13 | 0.487 | -1.1 | 0.363 | -1.1 | 0.685 | 1.07 | 0.816 | -0.57 |
| 1428274_s_at | Abhd13 | abhydrolase domain containing 13 | 1.04 | 0.859 | -1.24 | 0.451 | -1.18 | 0.177 | -1.13 | 0.566 | -0.63 |
| 1449007_at | Btg3 /// LOC64041 | B-cell translocation gene 3 /// similar to BTG3 protein (Tob5 protein) (Abundant in | -1.14 | 0.306 | -1.06 | 0.707 | -1.14 | 0.218 | 1.6 | 0.204 | -0.43 |
| 1443529_at | Rutbc2 | RUN and TBC1 domain containing 2 | -1.06 | 0.059 | 1.01 | 0.994 | -1.33 | 0.568 | -1.21 | 0.776 | -0.65 |
| 1427100_at | Metrn | meteorin, glial cell differentiation regulator | -1.82 | 0.346 | -1.55 | 0.348 | 1.5 | 0.255 | -1.69 | 0.329 | -0.89 |
| 1444172_at | --- | Transcribed locus | 1.29 | 0.761 | -1.34 | 0.536 | -1.5 | 0.058 | -1.28 | 0.651 | -0.71 |
| 1454739_at | Cdc27 | cell division cycle 27 homolog (S. cerevisiae) | -1.09 | 0.518 | -1.01 | 0.899 | -1.27 | 0.014 | 1.33 | 0.216 | -0.51 |
| 1442025_a_at | --- | --- | 1.02 | 0.987 | -1.14 | 0.809 | -1.24 | 0.718 | -2.06 | 0.287 | -0.86 |
| 1456752_at | --- | 13 days embryo heart cDNA, RIKEN full-length enriched library, clone:D330038P | 1.08 | 0.893 | -1.54 | 0.477 | -1.03 | 0.916 | -1.03 | 0.899 | -0.63 |
| 1446153_at | Zfp608 | Zinc finger protein 608 | -1.5 | 0.674 | 1.21 | 0.552 | -1.22 | 0.53 | -1.21 | 0.763 | -0.68 |
| 1443712_x_at | Ccdc82 | Coiled-coil domain containing 82 | 1.05 | 0.94 | -1.12 | 0.817 | -1.32 | 0.393 | -2.11 | 0.161 | -0.87 |
| 1451870_a_at | Brd4 | bromodomain containing 4 | 1.17 | 0.705 | -1.06 | 0.815 | -1.69 | 0.308 | -1.31 | 0.122 | -0.72 |
| 1439830_at | LOC675366 | similar to mitogen activated protein kinase kinase kinase 5 | -1.26 | 0.609 | -1.5 | 0.246 | 1.24 | 0.34 | -1.34 | 0.194 | -0.71 |
| 1428187_at | Cd47 | CD47 antigen (Rh-related antigen, integrin-associated signal transducer) | -1.01 | 0.963 | -1.11 | 0.574 | -1.25 | 0.315 | 1.09 | 0.687 | -0.57 |
| 1439213_at | 4921524J06Rik | RIKEN cDNA 4921524J06 gene | 1.37 | 0.006 | -1.25 | 0.558 | -1.89 | 0.006 | -1.41 | 0.15 | -0.8 |
| 1437962_at | Gm443 | gene model 443, (NCBI) | -2.97 | 0.334 | 1.43 | 0.391 | -1.07 | 0.876 | -1.2 | 0.707 | -0.95 |
| 1435498_at | Arfip2 | ADP-ribosylation factor interacting protein 2 | 1.07 | 0.453 | -1.06 | 0.648 | -1.46 | 0.045 | -1.06 | 0.656 | -0.63 |
| 1435567_at | Phka1 | phosphorylase kinase alpha 1 | -1.28 | 0.065 | -1.04 | 0.599 | -1.05 | 0.791 | 1.13 | 0.593 | -0.56 |
| 1431501_at | 1700037C18Rik | RIKEN cDNA 1700037C18 gene | -1.07 | 0.626 | -1.74 | 0.294 | 1.19 | 0.486 | -1.49 | 0.134 | -0.78 |
| 1429751_at | Zswim2 | zinc finger, SWIM domain containing 2 | -1.24 | 0.576 | -1.02 | 0.961 | -1.1 | 0.84 | 1.52 | 0.014 | -0.46 |
| 1423605_a_at | Mdm2 | transformed mouse 3T3 cell double minute 2 | -1.12 | 0.547 | -1.01 | 0.849 | -1.23 | 0.065 | 1.12 | 0.226 | -0.56 |
| 1441573_at | Scmh1 | Sex comb on midleg homolog 1 | 1.13 | 0.217 | -1.11 | 0.468 | -1.48 | 0.045 | -1.64 | 0.163 | -0.78 |
| 1423175_s_at | Pard6b | par-6 (partitioning defective 6) homolog beta (C. elegans) | -1.14 | 0.077 | -1.42 | 0.335 | 1.11 | 0.701 | -1.16 | 0.599 | -0.65 |
| 1428410_at | Nat13 | N-acetyltransferase 13 | -1.11 | 0.441 | -1.07 | 0.466 | -1.16 | 0.29 | 1.26 | 0.334 | -0.52 |
| 1416003_at | Cldn11 | claudin 11 | -1.29 | 0.581 | -1.75 | 0.494 | 1.35 | 0.416 | -1.42 | 0.598 | -0.78 |
| 1432474_a_at | Krtcap3 | keratinocyte associated protein 3 | 1 | 0.999 | -1.07 | 0.824 | -1.3 | 0.252 | -1.35 | 0.132 | -0.68 |
| 1456992_at | Ube3c | ubiquitin protein ligase E3C | 1.05 | 0.818 | -1 | 0.996 | -1.53 | 0.031 | -1.11 | 0.62 | -0.65 |
| 1440627_at | Atp8a2 | ATPase, aminophospholipid transporter-like, class I, type 8A, member 2 | -2.03 | 0.127 | -1.37 | 0.16 | 1.48 | 0.216 | -1.21 | 0.417 | -0.78 |
| 1444829_at | C78532 | expressed sequence C78532 | 1.61 | 0.485 | -3.09 | 0.016 | -1.3 | 0.41 | -1.04 | 0.864 | -0.96 |
| 1444022_at | --- | --- | -1.37 | 0.5 | -1.13 | 0.712 | 1.08 | 0.734 | -1.5 | 0.105 | -0.73 |

| | | | | | | | | | | | |
|---|---|---|---|---|---|---|---|---|---|---|---|
| 1415934_at | Cops8 | COP9 (constitutive photomorphogenic) homolog, subunit 8 (Arabidopsis thaliana) | 1 | 0.981 | -1.13 | 0.342 | -1.23 | 0.076 | -1.19 | 0.128 | -0.64 |
| 1449354_at | U2af1-rs1 | U2 small nuclear ribonucleoprotein auxiliary factor (U2AF) 1, related sequence 1 | -1.13 | 0.251 | -1.22 | 0.047 | -1.01 | 0.942 | 1.1 | 0.709 | -0.56 |
| 1438183_x_at | Sord | sorbitol dehydrogenase | -1.12 | 0.604 | -1.2 | 0.421 | -1.03 | 0.763 | 2.1 | 0.259 | -0.31 |
| 1438520_at | Slc25a36 | solute carrier family 25, member 36 | -1.15 | 0.4 | 1.09 | 0.779 | -1.35 | 0.096 | -1.24 | 0.404 | -0.66 |
| 1437265_at | 5330438D12Rik | RIKEN cDNA 5330438D12 gene | -1.13 | 0.114 | -1.35 | 0.276 | 1.07 | 0.729 | -1.68 | 0.178 | -0.77 |
| 1436852_at | E130308A19Rik | RIKEN cDNA E130308A19 gene | -1.05 | 0.71 | -1.1 | 0.639 | -1.2 | 0.225 | 1.26 | 0.523 | -0.52 |
| 1455244_at | Daam1 | dishevelled associated activator of morphogenesis 1 | -1.01 | 0.85 | -1.07 | 0.665 | -1.29 | 0.111 | 2.77 | 0.229 | -0.15 |
| 1430583_at | C130076O07Rik | RIKEN cDNA C130076O07 gene | 1.3 | 0.494 | -1.11 | 0.719 | -1.98 | 0.087 | -1.12 | 0.867 | -0.73 |
| 1458838_at | 4732468M13Rik | RIKEN cDNA 4732468M13 gene | -1.74 | 0.344 | 1.38 | 0.381 | -1.35 | 0.316 | -1.6 | 0.333 | -0.82 |
| 1441108_at | --- | 12 days embryo eyeball cDNA, RIKEN full-length enriched library, clone:D230035 | -1.02 | 0.923 | 1.12 | 0.125 | -1.69 | 0.021 | -1.18 | 0.47 | -0.69 |
| 1418083_at | 0610009B22Rik | RIKEN cDNA 0610009B22 gene | -1.15 | 0.191 | -1.03 | 0.779 | -1.16 | 0.262 | 1.26 | 0.295 | -0.52 |
| 1430951_at | 2810011L19Rik | RIKEN cDNA 2810011L19 gene | -1.02 | 0.97 | 1 | 0.995 | -1.4 | 0.215 | -1.27 | 0.625 | -0.67 |
| 1423481_at | Riok2 | RIO kinase 2 (yeast) | -1.23 | 0.362 | -1.17 | 0.097 | 1.03 | 0.825 | -1.42 | 0.025 | -0.7 |
| 1427443_at | LOC625794 | Hypothetical protein LOC625794 | -1.95 | 0.038 | 1.34 | 0.571 | -1.18 | 0.694 | -1.01 | 0.989 | -0.7 |
| 1434025_at | --- | --- | -1.12 | 0.698 | -1.16 | 0.761 | -1.06 | 0.626 | 5.7 | 0.26 | 0.59 |
| 1441571_at | 2410089E03Rik | RIKEN cDNA 2410089E03 gene | -1.1 | 0.819 | 1.15 | 0.601 | -1.57 | 0.011 | -1.2 | 0.491 | -0.68 |
| 1437861_s_at | --- | --- | -1.19 | 0.187 | 1.04 | 0.747 | -1.22 | 0.267 | -1.52 | 0.075 | -0.72 |
| 1418996_a_at | 4930469P12Rik | RIKEN cDNA 4930469P12 gene | -1.07 | 0.693 | -1.31 | 0.014 | 1 | 0.992 | -1.12 | 0.482 | -0.62 |
| 1427444_at | Abcf1 | ATP-binding cassette, sub-family F (GCN20), member 1 | -1.13 | 0.715 | -1.14 | 0.772 | -1.07 | 0.829 | 1.39 | 0.287 | -0.49 |
| 1433755_at | Mier1 | mesoderm induction early response 1 homolog (Xenopus laevis | -1.01 | 0.873 | -1.02 | 0.836 | -1.36 | 0.04 | 1.23 | 0.319 | -0.54 |
| 1444758_at | Traf3 | Tnf receptor-associated factor 3 | 1.04 | 0.895 | -1.1 | 0.813 | -1.33 | 0.368 | -1.35 | 0.399 | -0.69 |
| 1423010_at | Sacs | sacsin | -1.39 | 0.511 | -1.12 | 0.226 | 1.09 | 0.459 | -1.04 | 0.906 | -0.62 |
| 1451696_at | Zfp64 | zinc finger protein 64 | -2.06 | 0.257 | -1 | 0.998 | 1.22 | 0.559 | -2.31 | 0.485 | -1.04 |
| 1452845_at | Hif1an | hypoxia-inducible factor 1, alpha subunit inhibitor | 1.43 | 0.075 | -1.24 | 0.543 | -2.16 | 0.084 | -2.26 | 0.348 | -1.06 |
| 1458563_at | Zfp668 | zinc finger protein 668 | -1.14 | 0.738 | 1.21 | 0.61 | -1.63 | 0.432 | -1.42 | 0.619 | -0.74 |
| 1430393_at | 2510017J16Rik | RIKEN cDNA 2510017J16 gene | -1.22 | 0.329 | 1.24 | 0.462 | -1.56 | 0.165 | -1.18 | 0.29 | -0.68 |
| 1440081_at | 4631422C13Rik | RIKEN cDNA 4631422C13 gene | 1.09 | 0.818 | -1.56 | 0.228 | -1.03 | 0.778 | -1.33 | 0.104 | -0.71 |
| 1454734_at | --- | --- | 1.11 | 0.581 | -1.29 | 0.482 | -1.22 | 0.332 | -5.73 | 0.043 | -1.78 |
| 1431355_s_at | Trpm7 | transient receptor potential cation channel, subfamily M, member 7 | 1.13 | 0.346 | -1.04 | 0.851 | -1.63 | 0.082 | -1.11 | 0.655 | -0.66 |
| 1435159_at | 9430038I01Rik | RIKEN cDNA 9430038I01 gene | -1.47 | 0.035 | 1.13 | 0.674 | -1.12 | 0.578 | -1.31 | 0.172 | -0.69 |
| 1446506_at | B930041G04 | hypothetical protein B930041G04 | -1.26 | 0.597 | -1.22 | 0.589 | 1.09 | 0.72 | -1.85 | 0.12 | -0.81 |
| 1434970_a_at | --- | --- | -1.12 | 0.289 | -1.03 | 0.74 | -1.2 | 0.176 | 1.07 | 0.731 | -0.57 |
| 1442654_at | A530054K11Rik | RIKEN cDNA A530054K11 gene | 1.04 | 0.836 | -1.09 | 0.417 | -1.34 | 0.266 | -1.49 | 0.038 | -0.72 |
| 1443825_x_at | Spaca3 | sperm acrosome associated 3 | -1.31 | 0.67 | 1.32 | 0.601 | -1.61 | 0.306 | -1.07 | 0.838 | -0.67 |
| 1441370_at | H2afz | H2A histone family, member Z | -1.2 | 0.577 | -1.02 | 0.906 | -1.13 | 0.654 | 1 | 0.994 | -0.59 |
| 1458154_at | --- | Transcribed locus | 1.39 | 0.665 | -1.65 | 0.56 | -1.41 | 0.556 | -1.84 | 0.377 | -0.88 |
| 1450519_a_at | Prkaca | protein kinase, cAMP dependent, catalytic, alpha | -1.15 | 0.751 | -1.07 | 0.6 | -1.11 | 0.621 | 1.47 | 0.009 | -0.47 |
| 1423190_at | 1810020G14Rik | RIKEN cDNA 1810020G14 gene | 1.25 | 0.343 | -1.36 | 0.239 | -1.41 | 0.009 | -1.87 | 0.069 | -0.84 |
| 1418575_at | Shfm1 | split hand/foot malformation (ectrodactyly) type 1 | -1.06 | 0.511 | 1.02 | 0.847 | -1.37 | 0.009 | -1.22 | 0.127 | -0.65 |
| 1439425_x_at | BC024814 | cDNA sequence BC024814 | 1.12 | 0.723 | -1.32 | 0.451 | -1.2 | 0.617 | -1.39 | 0.304 | -0.7 |
| 1456668_at | Zcchc11 | Zinc finger, CCHC domain containing 11 | 1.27 | 0.116 | -1.47 | 0.096 | -1.32 | 0.199 | -2.13 | 0.124 | -0.91 |
| 1420375_at | Kif3a | kinesin family member 3A | -1.04 | 0.727 | -1.17 | 0.395 | -1.13 | 0.376 | 1.45 | 0.142 | -0.47 |
| 1424951_at | Baiap2l1 | BAI1-associated protein 2-like 1 | -1.01 | 0.956 | -1.28 | 0.21 | -1.07 | 0.533 | 1.04 | 0.81 | -0.58 |
| 1452399_at | Rgs6 | regulator of G-protein signaling 6 | 1.57 | 0.05 | -1.5 | 0.18 | -2.15 | 0.047 | -1.03 | 0.91 | -0.78 |
| 1453554_a_at | Wdr33 | WD repeat domain 33 | -1.23 | 0.259 | -1.13 | 0.571 | 1 | 0.981 | -1.18 | 0.585 | -0.63 |
| 1444673_at | E030031F02Rik | RIKEN cDNA E030031F02 gene | 1.03 | 0.959 | -1.35 | 0.569 | -1.07 | 0.714 | -1.49 | 0.332 | -0.72 |
| 1451718_at | Plp1 | proteolipid protein (myelin) 1 | 1.01 | 0.974 | -1.19 | 0.528 | -1.17 | 0.491 | -1.1 | 0.755 | -0.61 |
| 1427278_at | Rsnl2 | restin-like 2 | -1.37 | 0.003 | -1.02 | 0.9 | -1.01 | 0.966 | 1.1 | 0.733 | -0.57 |
| 1448629_at | Hps4 | Hermansky-Pudlak syndrome 4 homolog (human) | -1.1 | 0.422 | -1.13 | 0.15 | -1.09 | 0.366 | 1.08 | 0.57 | -0.56 |
| 1428587_at | Tmem41b | transmembrane protein 41B | -1.1 | 0.201 | -1.06 | 0.713 | -1.18 | 0.273 | 1.44 | 0.325 | -0.47 |
| 1421587_at | Klk1b27 | kallikrein 1-related peptidase b27 | -1.1 | 0.842 | 1.1 | 0.574 | -1.44 | 0.506 | -1.28 | 0.386 | -0.68 |
| 1450616_at | Ear5 | eosinophil-associated, ribonuclease A family, member 5 | -2.61 | 0.039 | 1.52 | 0.444 | -1.25 | 0.471 | -1.28 | 0.581 | -0.91 |
| 1418320_at | Prss8 | protease, serine, 8 (prostasin) | 1.02 | 0.882 | -1.16 | 0.711 | -1.21 | 0.179 | -2.34 | 0.164 | -0.92 |

| | | | | | | | | | | | |
|---|---|---|---|---|---|---|---|---|---|---|---|
| 1431375_s_at | Parva | parvin, alpha | 1.02 | 0.893 | -1.1 | 0.348 | -1.3 | 0.107 | -1.61 | 0.228 | -0.75 |
| 1449841_at | Kif3a | kinesin family member 3A | -1.18 | 0.07 | -1 | 0.983 | -1.17 | 0.261 | 1.28 | 0.291 | -0.52 |
| 1425237_at | Krtap16-8 | keratin associated protein 16-8 | 1.44 | 0.364 | -1.64 | 0.364 | -1.52 | 0.196 | -1.55 | 0.514 | -0.82 |
| 1416499_a_at | Dctn6 | dynactin 6 | -1.21 | 0.336 | 1.18 | 0.085 | -1.44 | 0.192 | -2.15 | 0.19 | -0.9 |
| 1443344_at | 4932409I22Rik | RIKEN cDNA 4932409I22 gene | 1.18 | 0.728 | -1.52 | 0.071 | -1.15 | 0.574 | -1.05 | 0.822 | -0.64 |
| 1460286_at | 6-Sep | septin 6 | -1.05 | 0.753 | -1.83 | 0.13 | 1.2 | 0.554 | -2.09 | 0.038 | -0.94 |
| 1445086_at | Cyp2j5 | Cytochrome P450, family 2, subfamily j, polypeptide 5 | 1.17 | 0.794 | -1.26 | 0.235 | -1.35 | 0.554 | -1.07 | 0.767 | -0.63 |
| 1437997_x_at | Mrpl48 | mitochondrial ribosomal protein L48 | -1.13 | 0.537 | 1.08 | 0.267 | -1.35 | 0.13 | -1.11 | 0.619 | -0.63 |
| 1448834_at | Foxm1 | forkhead box M1 | -1.08 | 0.786 | -1.21 | 0.487 | -1.05 | 0.658 | 4.25 | 0.133 | 0.23 |
| 1453890_at | --- | --- | -1.25 | 0.245 | 1.02 | 0.774 | -1.14 | 0.453 | -1.07 | 0.751 | -0.61 |
| 1421029_a_at | Hbs1l | Hbs1-like (S. cerevisiae) | -1.23 | 0.03 | -1.08 | 0.328 | -1.03 | 0.887 | 1.21 | 0.489 | -0.54 |
| 1442933_at | Foxk2 | forkhead box K2 | 1.1 | 0.744 | -1.15 | 0.683 | -1.37 | 0.25 | -1.06 | 0.717 | -0.62 |
| 1435524_at | 2010109N14Rik | RIKEN cDNA 2010109N14 gene | -1.05 | 0.506 | 1.02 | 0.843 | -1.36 | 0.108 | -1.19 | 0.154 | -0.65 |
| 1425361_at | Zfp397 | zinc finger protein 397 | -1.56 | 0.055 | -2.21 | 0 | 1.61 | 0 | -1.21 | 0.142 | -0.84 |
| 1435066_at | Pitpnc1 | phosphatidylinositol transfer protein, cytoplasmic 1 | -1.16 | 0.332 | 1.2 | 0.416 | -1.56 | 0.061 | -1.11 | 0.687 | -0.66 |
| 1420134_at | RP23-289L21.1 | Hypothetical protein LOC433719 | -1.41 | 0.262 | 1.13 | 0.887 | -1.16 | 0.737 | -1.28 | 0.244 | -0.68 |
| 1446496_at | Sfrs10 | splicing factor, arginine/serine-rich 10 (transformer 2 homolog, Drosophila) | 1.27 | 0.446 | -1.49 | 0.165 | -1.32 | 0.656 | -1.24 | 0.556 | -0.69 |
| 1449085_at | Phf10 | PHD finger protein 10 | -1.03 | 0.892 | -1.24 | 0.065 | -1.08 | 0.58 | 2.33 | 0.213 | -0.25 |
| 1422329_a_at | Ntrk3 | neurotrophic tyrosine kinase, receptor, type 3 | -1.09 | 0.712 | -1.5 | 0.074 | 1.12 | 0.684 | -1.45 | 0.394 | -0.73 |
| 1428674_at | Prpf38b | PRP38 pre-mRNA processing factor 38 (yeast) domain containing B | -1.18 | 0.268 | -1.23 | 0.068 | 1.04 | 0.743 | -1.4 | 0.197 | -0.69 |
| 1423563_at | Prrt1 | proline-rich transmembrane protein 1 | -1.21 | 0.552 | -1.52 | 0.098 | 1.22 | 0.151 | -1.35 | 0.333 | -0.72 |
| 1418466_at | Rnf141 | ring finger protein 141 | -1.6 | 0.363 | -1.43 | 0.133 | 1.38 | 0.284 | -1.19 | 0.541 | -0.71 |
| 1452645_x_at | Klra22 | killer cell lectin-like receptor subfamily A, member 22 | -1.33 | 0.737 | -1.2 | 0.308 | 1.12 | 0.575 | -1.53 | 0.631 | -0.74 |
| 1444458_at | Mgea6 | Meningioma expressed antigen 6 (coiled-coil proline-rich) | 1.13 | 0.359 | -1.02 | 0.92 | -1.68 | 0.002 | -1.29 | 0.184 | -0.72 |
| 1434187_at | --- | --- | 1.14 | 0.11 | -1.18 | 0.2 | -1.39 | 0.006 | -1.23 | 0.152 | -0.67 |
| 1458086_at | 4732471D19Rik | RIKEN cDNA 4732471D19 gene | 1.42 | 0.078 | -1.5 | 0.208 | -1.62 | 0.076 | -1.28 | 0.446 | -0.75 |
| 1447576_at | 2010001K21Rik | RIKEN cDNA 2010001K21 gene | -1.02 | 0.983 | -1.51 | 0.464 | 1.06 | 0.65 | -1.28 | 0.746 | -0.69 |
| 1457550_at | 9530059O14Rik | RIKEN cDNA 9530059O14 gene | 1.59 | 0.538 | -1.58 | 0.45 | -2.07 | 0.358 | -1.8 | 0.23 | -0.96 |
| 1436803_a_at | Ndufb9 | NADH dehydrogenase (ubiquinone) 1 beta subcomplex, 9 | -1.04 | 0.442 | -1.2 | 0.184 | -1.1 | 0.135 | 1.33 | 0.163 | -0.5 |
| 1455465_at | Snx12 | Sorting nexin 12 | -1.13 | 0.046 | 1.03 | 0.914 | -1.26 | 0.152 | -1.3 | 0.472 | -0.67 |
| 1429468_at | 1110018F16Rik | RIKEN cDNA 1110018F16 gene | -1.15 | 0.142 | 1.22 | 0.353 | -1.63 | 0.037 | -1.23 | 0.442 | -0.7 |
| 1433656_a_at | Gnl3 | guanine nucleotide binding protein-like 3 (nucleolar) | -1.05 | 0.451 | -1.2 | 0.218 | -1.08 | 0.501 | 1.11 | 0.588 | -0.56 |
| 1432868_at | 2310047C04Rik | RIKEN cDNA 2310047C04 gene | 1.15 | 0.616 | -1.19 | 0.62 | -1.4 | 0.401 | -1.05 | 0.82 | -0.62 |
| 1448822_at | Psmb6 | proteasome (prosome, macropain) subunit, beta type 6 | 1.03 | 0.476 | -1.24 | 0.028 | -1.15 | 0.155 | -1.1 | 0.368 | -0.61 |
| 1426272_at | Lmbr1 | limb region 1 | -1 | 0.984 | -1.07 | 0.515 | -1.3 | 0.048 | 1.13 | 0.468 | -0.56 |
| 1426360_at | Zc3h11a | zinc finger CCCH type containing 11A | 1.03 | 0.87 | -1.22 | 0.161 | -1.18 | 0.431 | -1.18 | 0.656 | -0.63 |
| 1445405_at | --- | --- | 1.12 | 0.69 | -1.34 | 0.546 | -1.19 | 0.297 | -1.32 | 0.477 | -0.68 |
| 1455769_at | Dcun1d5 | DCN1, defective in cullin neddylation 1, domain containing 5 (S. cerevisiae) | 1.01 | 0.941 | -1.17 | 0.672 | -1.19 | 0.392 | -1.81 | 0.284 | -0.79 |
| 1430006_x_at | 1700024B05Rik | RIKEN cDNA 1700024B05 gene | -1.87 | 0.187 | -1.76 | 0.389 | 1.6 | 0.252 | -1.29 | 0.456 | -0.83 |
| 1437402_x_at | Tmem41b | transmembrane protein 41B | -1.22 | 0.306 | -1.09 | 0.322 | -1.03 | 0.873 | 1.21 | 0.453 | -0.53 |
| 1436033_at | BC031353 | cDNA sequence BC031353 | -1.32 | 0.344 | -1.57 | 0.123 | 1.31 | 0.447 | -1.34 | 0.539 | -0.73 |
| 1436279_at | --- | Transcribed locus | -1.16 | 0.691 | 1.06 | 0.873 | -1.28 | 0.366 | -1.41 | 0.719 | -0.7 |
| 1449783_at | --- | --- | -1.21 | 0.203 | 1.03 | 0.867 | -1.18 | 0.502 | -1.5 | 0.411 | -0.71 |
| 1420058_s_at | 2410166I05Rik | RIKEN cDNA 2410166I05 gene | -1.25 | 0.198 | 1.06 | 0.416 | -1.18 | 0.163 | -1.27 | 0.065 | -0.66 |
| 1432636_at | --- | --- | -1.57 | 0.018 | 1.25 | 0.567 | -1.22 | 0.668 | -1.31 | 0.211 | -0.71 |
| 1451719_at | Crsp6 | cofactor required for Sp1 transcriptional activation, subunit 6 | 1.4 | 0.521 | -2.34 | 0.308 | -1.14 | 0.477 | -2.79 | 0.013 | -1.22 |
| 1441341_at | --- | Transcribed locus | -1.22 | 0.426 | -1.19 | 0.695 | 1.05 | 0.697 | -1.4 | 0.523 | -0.69 |
| 1452144_a_at | Mrpl44 | mitochondrial ribosomal protein L44 | -1.19 | 0.088 | -1.18 | 0.046 | 1.02 | 0.882 | -1.14 | 0.143 | -0.62 |
| 1451420_at | 2610204L23Rik | RIKEN cDNA 2610204L23 gene | -1.14 | 0.318 | -1.23 | 0.219 | 1.01 | 0.881 | -1.31 | 0.048 | -0.67 |
| 1429307_s_at | Lzic | leucine zipper and CTNNBIP1 domain containing | -1.43 | 0.005 | 1.05 | 0.811 | -1.05 | 0.39 | -1.08 | 0.229 | -0.63 |
| 1419070_at | Cys1 | cystin 1 | -1.1 | 0.643 | -1.49 | 0.271 | 1.13 | 0.733 | -1.38 | 0.598 | -0.71 |
| 1437038_x_at | 1700029I08Rik | RIKEN cDNA 1700029I08 gene | -1.07 | 0.934 | 1.19 | 0.707 | -1.72 | 0.165 | -1.14 | 0.74 | -0.69 |
| 1424325_at | Esco1 | establishment of cohesion 1 homolog 1 (S. cerevisiae) | -1.15 | 0.405 | -2.26 | 0.12 | 1.4 | 0.387 | -1.71 | 0.252 | -0.93 |

| | | | | | | | | | | | |
|---|---|---|---|---|---|---|---|---|---|---|---|
| 1457673_at | 2610005L07Rik | RIKEN cDNA 2610005L07 gene | -1.3 | 0.269 | 1.04 | 0.89 | -1.12 | 0.571 | -1.36 | 0.32 | -0.68 |
| 1457734_at | Pou3f2 | POU domain, class 3, transcription factor 2 | 1.39 | 0.718 | -3 | 0.276 | -1.01 | 0.978 | -1.06 | 0.75 | -0.92 |
| 1447472_at | --- | Transcribed locus | 1.21 | 0.647 | -1.49 | 0.531 | -1.22 | 0.216 | -1.9 | 0.189 | -0.85 |
| 1428937_at | Atp2b1 | ATPase, Ca++ transporting, plasma membrane 1 | -1.15 | 0.167 | 1.06 | 0.71 | -1.28 | 0.211 | -1.36 | 0.437 | -0.68 |
| 1427933_at | Vps33b | vacuolar protein sorting 33B (yeast) | -1.05 | 0.82 | 1.08 | 0.806 | -1.49 | 0.064 | -1.2 | 0.392 | -0.66 |
| 1433675_at | Snord22 | small nucleolar RNA, C/D box 22 | -1.05 | 0.872 | -1.47 | 0.097 | 1.08 | 0.778 | -1.03 | 0.918 | -0.62 |
| 1435794_at | BC050254 | cDNA sequence BC050254 | -1.3 | 0.059 | -1.12 | 0.505 | 1.04 | 0.607 | -1.15 | 0.433 | -0.63 |
| 1455524_at | 2810046L04Rik | RIKEN cDNA 2810046L04 gene | -1.28 | 0.29 | -1.17 | 0.384 | 1.07 | 0.642 | -1.04 | 0.547 | -0.61 |
| 1435225_s_at | Brpf3 | bromodomain and PHD finger containing, 3 | -1.16 | 0.422 | -1.16 | 0.405 | -1.02 | 0.952 | 3.94 | 0.105 | 0.15 |
| 1425149_a_at | Pdcl | phosducin-like | -1.13 | 0.472 | 1.03 | 0.796 | -1.26 | 0.055 | -1.12 | 0.378 | -0.62 |
| 1418700_at | Lias | lipoic acid synthetase | -1.12 | 0.304 | -1.16 | 0.05 | -1.05 | 0.269 | 1.12 | 0.413 | -0.55 |
| 1446315_at | Acly | ATP citrate lyase | -1.3 | 0.664 | -1.62 | 0.089 | 1.32 | 0.529 | -1.34 | 0.523 | -0.74 |
| 1437192_x_at | Vdac1 | voltage-dependent anion channel 1 | -1.1 | 0.15 | -1.18 | 0.228 | -1.05 | 0.749 | 1.21 | 0.309 | -0.53 |
| 1459833_x_at | Cib2 | calcium and integrin binding family member 2 | -1.08 | 0.874 | -1.14 | 0.849 | -1.11 | 0.838 | 3.66 | 0.093 | 0.08 |
| 1460404_at | Sr278 | stretch responsive protein 278 | -1.03 | 0.427 | -1.12 | 0.293 | -1.18 | 0.263 | 1.32 | 0.021 | -0.5 |
| 1418763_at | Nit2 | nitrilase family, member 2 | -1.16 | 0.167 | -1.27 | 0.03 | 1.06 | 0.688 | -1.21 | 0.217 | -0.65 |
| 1424780_a_at | D10Ucla1 | DNA segment, Chr 10, University of California at Los Angeles 1 | 1 | 0.977 | -1.07 | 0.111 | -1.3 | 0.065 | -1.72 | 0.183 | -0.77 |
| 1426613_a_at | Snrpb2 | U2 small nuclear ribonucleoprotein B | 1.12 | 0.664 | -1.28 | 0.213 | -1.24 | 0.269 | -1.48 | 0.121 | -0.72 |
| 1457060_at | Anks1b /// LOC544 | ankyrin repeat and sterile alpha motif domain containing 1B /// similar to AIDA-1b | -1.1 | 0.142 | -1.21 | 0.063 | -1.03 | 0.235 | 1.04 | 0.614 | -0.57 |
| 1420916_at | Prpf40a | PRP40 pre-mRNA processing factor 40 homolog A (yeast) | -1.15 | 0.248 | -1.41 | 0.014 | 1.13 | 0.53 | -1.12 | 0.544 | -0.64 |
| 1457945_at | Eif2s3y | Eukaryotic translation initiation factor 2, subunit 3, structural gene Y-linked | -1.39 | 0.575 | -1.44 | 0.262 | 1.3 | 0.304 | -1.51 | 0.219 | -0.76 |
| 1431734_a_at | Dnajb4 | DnaJ (Hsp40) homolog, subfamily B, member 4 | -1.17 | 0.413 | -1.13 | 0.696 | -1.03 | 0.924 | 3.26 | 0.236 | -0.02 |
| 1454345_at | 1700081H04Rik | RIKEN cDNA 1700081H04 gene | -1.09 | 0.92 | 1.08 | 0.852 | -1.41 | 0.095 | -1.2 | 0.465 | -0.66 |
| 1436573_at | Scrn3 | secernin 3 | -1.07 | 0.652 | -1.19 | 0.093 | -1.07 | 0.554 | 2.52 | 0.265 | -0.2 |
| 1426406_at | Setd8 | SET domain containing (lysine methyltransferase) 8 | -1.15 | 0.598 | -1.06 | 0.363 | -1.12 | 0.154 | 2.17 | 0.085 | -0.29 |
| 1452761_a_at | 8430436O14Rik | RIKEN cDNA 8430436O14 gene | -1 | 0.981 | 1.15 | 0.539 | -1.78 | 0.024 | -1.86 | 0.124 | -0.87 |
| 1432284_at | Mipol1 | mirror-image polydactyly gene 1 homolog (human) | 1.22 | 0.636 | -1.41 | 0.622 | -1.28 | 0.411 | -1.25 | 0.486 | -0.68 |
| 1429763_at | Cnih4 | cornichon homolog 4 (Drosophila) | -1.11 | 0.207 | -1.03 | 0.745 | -1.19 | 0.209 | 1.14 | 0.544 | -0.55 |
| 1432427_at | Ndufb4 | NADH dehydrogenase (ubiquinone) 1 beta subcomplex 4 | -1.36 | 0.249 | -1.11 | 0.725 | 1.08 | 0.758 | -1.15 | 0.769 | -0.64 |
| 1423976_at | 4930453N24Rik | RIKEN cDNA 4930453N24 gene | -1.05 | 0.806 | -1.04 | 0.842 | -1.27 | 0.145 | 1.01 | 0.872 | -0.59 |
| 1428883_at | Tmem57 | transmembrane protein 57 | -1.06 | 0.816 | -1.2 | 0.255 | -1.08 | 0.555 | 1.04 | 0.857 | -0.57 |
| 1444100_at | Pdpn | podoplanin | -1.11 | 0.505 | 1.02 | 0.956 | -1.27 | 0.097 | -1.87 | 0.145 | -0.81 |
| 1459707_at | Pacs1 | Phosphofurin acidic cluster sorting protein 1 | 1.15 | 0.309 | -1 | 0.998 | -1.8 | 0.053 | -1.39 | 0.208 | -0.76 |
| 1434988_x_at | Aldh2 | aldehyde dehydrogenase 2, mitochondrial | -1.51 | 0.475 | -1.53 | 0.168 | 1.4 | 0.35 | -1.01 | 0.96 | -0.67 |
| 1417444_at | E2f5 | E2F transcription factor 5 | -1.31 | 0.052 | 1.07 | 0.517 | -1.14 | 0.368 | -1.41 | 0.014 | -0.7 |
| 1447636_x_at | Tmco4 | transmembrane and coiled-coil domains 4 | -1.24 | 0.506 | 1.1 | 0.47 | -1.25 | 0.389 | -1.31 | 0.073 | -0.68 |
| 1459681_at | --- | --- | -1.75 | 0.331 | 1.16 | 0.796 | -1.02 | 0.937 | -1.57 | 0.295 | -0.8 |
| 1447188_at | --- | Transcribed locus | -1.33 | 0.498 | 1.12 | 0.568 | -1.19 | 0.752 | -1.15 | 0.452 | -0.64 |
| 1456355_s_at | 2810002G02Rik /// | RIKEN cDNA 2810002G02 gene /// region containing RIKEN cDNA 2810002G02 | -1.11 | 0.634 | -1.11 | 0.477 | -1.11 | 0.688 | 1.3 | 0.195 | -0.51 |
| 1444299_at | A430093F15Rik | RIKEN cDNA A430093F15 gene | -1.01 | 0.872 | -1.9 | 0.215 | 1.19 | 0.686 | -1.36 | 0.472 | -0.77 |
| 1452812_at | Lphn1 | latrophilin 1 | -1.73 | 0.016 | -1.07 | 0.893 | 1.19 | 0.456 | -1.16 | 0.634 | -0.69 |
| 1440790_x_at | 5033414K04Rik | RIKEN cDNA 5033414K04 gene | -1.01 | 0.976 | 1.35 | 0.443 | -2.71 | 0.027 | -1.94 | 0.105 | -1.08 |
| 1430271_x_at | Josd3 | Josephin domain containing 3 | -1.04 | 0.852 | -1.75 | 0.114 | 1.17 | 0.255 | -1.44 | 0.274 | -0.76 |
| 1435715_x_at | Tdg /// LOC545124 | thymine DNA glycosylase /// similar to thymine DNA glycosylase isoform 2 /// simi | -1.08 | 0.566 | -1.15 | 0.028 | -1.1 | 0.501 | 1.33 | 0.211 | -0.5 |
| 1440699_at | Mtap2 | microtubule-associated protein 2 | -2 | 0.309 | 1.32 | 0.622 | -1.12 | 0.829 | -3.61 | 0.059 | -1.35 |
| 1426495_at | 2410042D21Rik | RIKEN cDNA 2410042D21 gene | -1.04 | 0.853 | -1.14 | 0.533 | -1.16 | 0.491 | 1.22 | 0.588 | -0.53 |
| 1444251_x_at | D4Ertd111e | DNA segment, Chr 4, ERATO Doi 111, expressed | -1.23 | 0.58 | 1.25 | 0.395 | -1.56 | 0.091 | -1.1 | 0.774 | -0.66 |
| 1441415_at | Spred2 | Sprouty-related, EVH1 domain containing 2 | -1.02 | 0.882 | -1.11 | 0.725 | -1.21 | 0.651 | 1.1 | 0.695 | -0.56 |
| 1433531_at | Acsl4 | acyl-CoA synthetase long-chain family member 4 | -1.1 | 0.358 | -1.03 | 0.749 | -1.2 | 0.1 | 1.07 | 0.419 | -0.57 |
| 1429477_at | D15Ertd785e | DNA segment, Chr 15, ERATO Doi 785, expressed | -1.1 | 0.715 | -1.07 | 0.776 | -1.16 | 0.597 | 1.23 | 0.471 | -0.53 |
| 1455427_at | Angpt4 | angiopoietin 4 | -1.04 | 0.876 | 1.03 | 0.889 | -1.41 | 0.064 | -1.31 | 0.465 | -0.68 |
| 1459203_at | Ipo8 | importin 8 | 1.06 | 0.851 | -1.15 | 0.68 | -1.28 | 0.294 | -1.31 | 0.213 | -0.67 |
| 1419643_s_at | 2310057J18Rik /// 2 | RIKEN cDNA 2310057J18 gene /// RIKEN cDNA 2310065H11 gene | -2.68 | 0.069 | -1.14 | 0.635 | 1.46 | 0.413 | -1.23 | 0.65 | -0.9 |

| | | | | | | | | | | | |
|---|---|---|---|---|---|---|---|---|---|---|---|
| 1416170_at | Trap1 | TNF receptor-associated protein 1 | -1.1 | 0.639 | -1.22 | 0.125 | -1.02 | 0.836 | 1.12 | 0.508 | -0.56 |
| 1454593_at | Fgd3 | FYVE, RhoGEF and PH domain containing 3 | -1.18 | 0.746 | -1.2 | 0.263 | 1.03 | 0.934 | -1.49 | 0.256 | -0.71 |
| 1436911_at | Ss18l1 | synovial sarcoma translocation gene on chromosome 18-like 1 | -1.16 | 0.436 | -1.27 | 0.076 | 1.06 | 0.825 | -1.6 | 0.259 | -0.74 |
| 1425363_at | B4galnt1 | beta-1,4-N-acetyl-galactosaminyl transferase 1 | -1.11 | 0.731 | -1.28 | 0.727 | 1.03 | 0.941 | -1.76 | 0.005 | -0.78 |
| 1422350_at | Fpr-rs3 | formyl peptide receptor, related sequence 3 | -1.24 | 0.601 | -1.05 | 0.931 | -1.06 | 0.882 | 1.69 | 0.069 | -0.41 |
| 1437613_s_at | AW456874 | expressed sequence AW456874 | -1.18 | 0.205 | -1.11 | 0.851 | -1.04 | 0.864 | 1.03 | 0.924 | -0.58 |
| 1459976_s_at | Sod1 | superoxide dismutase 1, soluble | -1.02 | 0.884 | -1.02 | 0.885 | -1.33 | 0.01 | 1.01 | 0.957 | -0.59 |
| 1433196_at | 9530006O14Rik | RIKEN cDNA 9530006O14 gene | -1.16 | 0.61 | -1.03 | 0.93 | -1.15 | 0.44 | 1.23 | 0.601 | -0.53 |
| 1450169_at | Slc4a4 | solute carrier family 4 (anion exchanger), member 4 | -2.06 | 0.27 | 1.25 | 0.713 | -1.03 | 0.959 | -1.27 | 0.797 | -0.78 |
| 1415704_a_at | Cdv3 | carnitine deficiency-associated gene expressed in ventricle 3 | -1.07 | 0.586 | -1.11 | 0.459 | -1.15 | 0.493 | 1.04 | 0.721 | -0.57 |
| 1453982_at | Mdm4 | transformed mouse 3T3 cell double minute 4 | 1.05 | 0.444 | -1.45 | 0.449 | -1.03 | 0.896 | -1.37 | 0.527 | -0.7 |
| 1440557_at | Ipw | imprinted gene in the Prader-Willi syndrome region | 1.2 | 0.778 | -1.88 | 0.016 | -1.02 | 0.967 | -1.17 | 0.788 | -0.72 |
| 1426729_at | 2900046G09Rik | RIKEN cDNA 2900046G09 gene | 1.57 | 0.402 | -2.89 | 0.004 | -1.26 | 0.156 | -1.17 | 0.657 | -0.94 |
| 1436762_x_at | Elp3 | Elongation protein 3 homolog (S. cerevisiae) | 1.01 | 0.874 | -1.1 | 0.326 | -1.27 | 0.111 | -1.35 | 0.121 | -0.68 |
| 1442128_at | Reep5 | Receptor accessory protein 5 | -1.24 | 0.438 | 1.12 | 0.705 | -1.28 | 0.178 | -1.35 | 0.335 | -0.69 |
| 1457057_at | 1700042G07Rik | RIKEN cDNA 1700042G07 gene | 1.05 | 0.901 | -1.12 | 0.86 | -1.31 | 0.298 | -1.03 | 0.952 | -0.6 |
| 1440153_at | --- | Adult male brain cDNA, RIKEN full-length enriched library, clone:3526403L10 prc | -1.07 | 0.465 | 1.18 | 0.519 | -1.68 | 0.02 | -1.33 | 0.304 | -0.72 |
| 1456297_at | Tscot | thymic stromal cotransporter | -2.04 | 0.409 | 1.42 | 0.206 | -1.25 | 0.438 | -1.65 | 0.404 | -0.88 |
| 1435362_at | Foxj3 | forkhead box J3 | 1.01 | 0.917 | -1.18 | 0.217 | -1.17 | 0.279 | -1.07 | 0.747 | -0.6 |
| 1457840_at | Plxna4 | plexin A4 | -1.02 | 0.973 | -2.32 | 0.094 | 1.3 | 0.471 | -2.84 | 0.458 | -1.22 |
| 1450185_a_at | Kcnj15 | potassium inwardly-rectifying channel, subfamily J, member 15 | 1.15 | 0.329 | -1.23 | 0.553 | -1.35 | 0.333 | -1.3 | 0.755 | -0.68 |
| 1435483_x_at | Slc25a32 | solute carrier family 25, member 32 | -1.55 | 0.024 | -1.18 | 0.394 | 1.22 | 0.391 | -1.15 | 0.622 | -0.66 |
| 1446462_at | Hars2 | Histidyl tRNA synthetase 2 | -1.5 | 0.375 | -1.19 | 0.744 | 1.2 | 0.461 | -3.17 | 0.26 | -1.16 |
| 1441388_at | --- | Adult male hippocampus cDNA, RIKEN full-length enriched library, clone:C63002 | 1.03 | 0.629 | -1.19 | 0.099 | -1.2 | 0.342 | -1.06 | 0.728 | -0.6 |
| 1423591_at | Fgfr1op2 | FGFR1 oncogene partner 2 | -1.02 | 0.689 | -1.13 | 0.086 | -1.18 | 0.099 | 1.04 | 0.672 | -0.57 |
| 1442414_at | Rnf103 | Ring finger protein 103 | 1.32 | 0.166 | -1.66 | 0.105 | -1.27 | 0.517 | -1.04 | 0.856 | -0.66 |
| 1433995_s_at | D16Bwg1543e | DNA segment, Chr 16, Brigham & Women's Genetics 1543 expressed | -1.26 | 0.03 | -1.13 | 0.126 | 1.04 | 0.865 | -1.56 | 0.198 | -0.73 |
| 1417841_at | Pxmp2 | peroxisomal membrane protein 2 | -1.05 | 0.705 | -1.22 | 0.063 | -1.07 | 0.659 | 1.92 | 0.155 | -0.35 |
| 1426382_at | Ppm1b | protein phosphatase 1B, magnesium dependent, beta isoform | -1.03 | 0.8 | -1.25 | 0.051 | -1.06 | 0.266 | 1.31 | 0.263 | -0.51 |
| 1445946_at | Pde4a | Phosphodiesterase 4A, cAMP specific | 1.2 | 0.628 | -1.48 | 0.1 | -1.2 | 0.617 | -1.14 | 0.607 | -0.66 |
| 1458395_at | B930054O08 | hypothetical protein B930054O08 | -1.27 | 0.349 | -1.07 | 0.554 | -1.01 | 0.966 | 1.38 | 0.421 | -0.49 |
| 1454803_a_at | Hdac11 | histone deacetylase 11 | -1.17 | 0.599 | -1.29 | 0.093 | 1.09 | 0.52 | -1.05 | 0.86 | -0.61 |
| 1437561_at | Trat1 | T cell receptor associated transmembrane adaptor 1 | -2.03 | 0.233 | -1.28 | 0.501 | 1.43 | 0.43 | -4 | 0.028 | -1.47 |
| 1423856_at | Popdc3 | popeye domain containing 3 | -1.45 | 0.572 | -1.03 | 0.955 | 1.05 | 0.699 | -1.26 | 0.425 | -0.67 |
| 1455594_at | Exoc3 | exocyst complex component 3 | -1.29 | 0.093 | 1.09 | 0.46 | -1.18 | 0.152 | -1.45 | 0.157 | -0.71 |
| 1452676_a_at | Pnpt1 | polyribonucleotide nucleotidyltransferase 1 | -1.11 | 0.026 | -1.06 | 0.387 | -1.15 | 0.138 | 1.05 | 0.751 | -0.57 |
| 1448922_at | Dusp19 | dual specificity phosphatase 19 | -1.24 | 0.207 | -1.11 | 0.172 | 1 | 0.997 | -1.18 | 0.536 | -0.63 |
| 1418370_at | Tnnc1 | troponin C, cardiac/slow skeletal | -1.03 | 0.954 | -1.26 | 0.087 | -1.06 | 0.625 | 1.14 | 0.728 | -0.55 |
| 1418654_at | Hao3 | hydroxyacid oxidase (glycolate oxidase) 3 | -1.08 | 0.417 | 1.09 | 0.932 | -1.43 | 0.488 | -1.57 | 0.177 | -0.75 |
| 1421910_at | Tcf20 | transcription factor 20 | 1.01 | 0.912 | -1.22 | 0.391 | -1.14 | 0.378 | -1.11 | 0.636 | -0.61 |
| 1442311_at | Zfr | Zinc finger RNA binding protein | 1.07 | 0.706 | -1.02 | 0.78 | -1.5 | 0.134 | -1.03 | 0.879 | -0.62 |
| 1421724_at | V1rd7 | vomeronasal 1 receptor, D7 | 1.16 | 0.697 | -1.74 | 0.217 | -1.02 | 0.943 | -1.13 | 0.759 | -0.68 |
| 1451868_at | Kcnj6 | potassium inwardly-rectifying channel, subfamily J, member 6 | -1.63 | 0.49 | 1.45 | 0.532 | -1.55 | 0.344 | -1.15 | 0.823 | -0.72 |
| 1445305_at | --- | --- | -1.18 | 0.616 | 1.21 | 0.699 | -1.53 | 0.257 | -1.27 | 0.324 | -0.69 |
| 1437275_at | Ctnna1 | Catenin (cadherin associated protein), alpha 1 | 1.51 | 0.605 | -2.39 | 0.409 | -1.27 | 0.307 | -1.51 | 0.52 | -0.92 |
| 1446041_at | --- | Transcribed locus | 1.16 | 0.756 | -1.18 | 0.784 | -1.42 | 0.492 | -1.09 | 0.817 | -0.63 |
| 1430589_at | Pigh | phosphatidylinositol glycan anchor biosynthesis, class H | -1.04 | 0.932 | -1.65 | 0.385 | 1.15 | 0.761 | -1.25 | 0.296 | -0.7 |
| 1426688_at | Sdha | succinate dehydrogenase complex, subunit A, flavoprotein (Fp) | -1.23 | 0.265 | -1.28 | 0.105 | 1.12 | 0.16 | -1.15 | 0.025 | -0.64 |
| 1418242_at | Faf1 | Fas-associated factor 1 | -1.11 | 0.337 | -1.23 | 0.134 | -1.01 | 0.95 | 1.37 | 0.241 | -0.49 |
| 1434721_at | D5Ertd585e | DNA segment, Chr 5, ERATO Doi 585, expressed | 1 | 0.993 | -1.17 | 0.138 | -1.17 | 0.039 | -1.27 | 0.311 | -0.65 |
| 1416565_at | Cox6b1 | cytochrome c oxidase, subunit VIb polypeptide 1 | -1.1 | 0.388 | -1.12 | 0.143 | -1.1 | 0.351 | 1.03 | 0.814 | -0.57 |
| 1418387_at | 4930548G07Rik | RIKEN cDNA 4930548G07 gene | -1.05 | 0.539 | -1.04 | 0.875 | -1.25 | 0.425 | 1.13 | 0.253 | -0.55 |
| 1446001_at | Reps1 | RalBP1 associated Eps domain containing protein | 1.23 | 0.081 | -1.23 | 0.298 | -1.49 | 0.046 | -1.43 | 0.425 | -0.73 |

| | | | | | | | | | | | |
|---|---|---|---|---|---|---|---|---|---|---|---|
| 1452223_s_at | Gcap14 | granule cell antiserum positive 14 | -1.27 | 0.064 | 1.18 | 0.248 | -1.35 | 0.265 | -1.55 | 0.37 | -0.75 |
| 1418485_at | Slc4a3 | solute carrier family 4 (anion exchanger), member 3 | 1.22 | 0.279 | -1.49 | 0.023 | -1.23 | 0.108 | -1.51 | 0.403 | -0.75 |
| 1453693_at | Wdr20 | WD repeat domain 20 | -1.65 | 0.325 | -1.42 | 0.414 | 1.4 | 0.206 | -1.35 | 0.025 | -0.75 |
| 1423168_at | Prei3 | preimplantation protein 3 | 1.03 | 0.82 | -1.06 | 0.504 | -1.36 | 0.024 | -1.17 | 0.538 | -0.64 |
| 1424837_at | Rnf113a1 | ring finger protein 113A1 | 1.04 | 0.888 | -1.22 | 0.508 | -1.17 | 0.381 | -1.01 | 0.983 | -0.59 |
| 1426563_at | Zfp553 | zinc finger protein 553 | 1.2 | 0.707 | -1.45 | 0.274 | -1.21 | 0.63 | -1.3 | 0.325 | -0.69 |
| 1448574_at | Nme6 | expressed in non-metastatic cells 6, protein | -1.05 | 0.64 | -1.22 | 0.166 | -1.07 | 0.543 | 1.48 | 0.258 | -0.46 |
| 1424952_at | Ociad1 | OCIA domain containing 1 | -1.05 | 0.896 | -1.03 | 0.928 | -1.28 | 0.282 | 1.13 | 0.837 | -0.56 |
| 1423169_at | Taf7 | TAF7 RNA polymerase II, TATA box binding protein (TBP)-associated factor | 1.08 | 0.752 | -1.36 | 0.429 | -1.12 | 0.294 | -1.01 | 0.963 | -0.6 |
| 1431271_at | 2810428J06Rik | RIKEN cDNA 2810428J06 gene | 1.32 | 0.703 | -1.63 | 0.452 | -1.28 | 0.367 | -1.4 | 0.209 | -0.75 |
| 1448938_at | Rpa3 | replication protein A3 | -1.16 | 0.329 | -1.12 | 0.277 | -1.04 | 0.711 | 1.4 | 0.299 | -0.48 |
| 1432968_at | 4930456A14Rik | RIKEN cDNA 4930456A14 gene | -1.3 | 0.1 | 1.01 | 0.919 | -1.08 | 0.849 | -1.44 | 0.137 | -0.7 |
| 1439369_x_at | Slc9a3r2 | solute carrier family 9 (sodium/hydrogen exchanger), isoform 3 regulator 2 | 1.08 | 0.812 | -1.25 | 0.29 | -1.2 | 0.555 | -1.45 | 0.296 | -0.71 |
| 1456244_x_at | Txnl2 | thioredoxin-like 2 | -1.17 | 0.456 | 1 | 0.988 | -1.17 | 0.217 | -1.51 | 0.046 | -0.71 |
| 1452055_at | Ctdsp1 | CTD (carboxy-terminal domain, RNA polymerase II, polypeptide A) small phospha | 1.06 | 0.724 | -1.2 | 0.46 | -1.22 | 0.237 | -1.01 | 0.972 | -0.59 |
| 1451330_a_at | Inpp5b | inositol polyphosphate-5-phosphatase B | -1.02 | 0.946 | -1.08 | 0.047 | -1.24 | 0.029 | 1.18 | 0.412 | -0.54 |
| 1432109_at | 4930578I07Rik | RIKEN cDNA 4930578I07 gene | -1.12 | 0.681 | -1.25 | 0.683 | 1.02 | 0.963 | -1.3 | 0.647 | -0.66 |
| 1425624_at | Epm2aip1 | EPM2A (laforin) interacting protein 1 | -1.36 | 0.225 | -1.1 | 0.832 | 1.07 | 0.857 | -1.16 | 0.603 | -0.64 |
| 1442495_at | Birc6 | Baculoviral IAP repeat-containing 6 | 1.24 | 0.405 | -1.2 | 0.562 | -1.58 | 0.058 | -1.08 | 0.855 | -0.65 |
| 1419452_at | Uchl5 | ubiquitin carboxyl-terminal esterase L5 | -1.07 | 0.379 | -1.16 | 0.008 | -1.08 | 0.524 | 1.13 | 0.512 | -0.55 |
| 1449666_at | Atrnl1 | Attractin like 1 | 1.53 | 0.522 | -2.16 | 0.232 | -1.39 | 0.047 | -1.08 | 0.637 | -0.78 |
| 1438535_at | Phip | pleckstrin homology domain interacting protein | 1.13 | 0.494 | -1.18 | 0.411 | -1.35 | 0.058 | -1.63 | 0.006 | -0.76 |
| 1435152_at | Leng8 | leukocyte receptor cluster (LRC) member 8 | -1.01 | 0.934 | -1.13 | 0.528 | -1.19 | 0.456 | 1.15 | 0.699 | -0.55 |
| 1459589_at | Cryl1 | crystallin, lamda 1 | -1.09 | 0.764 | -1.42 | 0.279 | 1.09 | 0.848 | -1.27 | 0.726 | -0.67 |
| 1451348_at | Depdc6 | DEP domain containing 6 | 1.02 | 0.666 | -1.29 | 0.041 | -1.09 | 0.622 | -1.47 | 0.038 | -0.71 |
| 1420326_s_at | Cramp1l | Crm, cramped-like (Drosophila) | -1.16 | 0.23 | -1.13 | 0.266 | -1.04 | 0.773 | 1.15 | 0.474 | -0.54 |
| 1449525_at | Fmo3 | flavin containing monooxygenase 3 | -1.2 | 0.765 | -1.23 | 0.353 | 1.07 | 0.815 | -1.62 | 0.144 | -0.74 |
| 1421849_at | Stag2 | stromal antigen 2 | -1.08 | 0.458 | -1.04 | 0.561 | -1.21 | 0.173 | 1.11 | 0.262 | -0.56 |
| 1438143_s_at | Atxn2 | ataxin 2 | -1.18 | 0.264 | -1.02 | 0.91 | -1.13 | 0.352 | 1.01 | 0.956 | -0.58 |
| 1439824_at | Chm | choroidermia | -1 | 0.939 | 1.05 | 0.431 | -1.49 | 0.045 | -1.28 | 0.466 | -0.68 |
| 1442997_at | Odz3 | Odd Oz/ten-m homolog 3 (Drosophila) | 1.32 | 0.704 | -2.32 | 0.221 | -1.04 | 0.866 | -1.49 | 0.473 | -0.88 |
| 1450659_at | Rgs7 | regulator of G protein signaling 7 | -1.28 | 0.045 | -2.3 | 0.32 | 1.5 | 0.652 | -2.02 | 0.272 | -1.03 |
| 1416555_at | Ei24 | etoposide induced 2.4 mRNA | 1.03 | 0.572 | -1.08 | 0.557 | -1.31 | 0.012 | -1.02 | 0.795 | -0.6 |
| 1417096_at | 2810430M08Rik | RIKEN cDNA 2810430M08 gene | -1.38 | 0.009 | -1.16 | 0.296 | 1.13 | 0.48 | -1.08 | 0.882 | -0.62 |
| 1442642_at | 4930413O22Rik | RIKEN cDNA 4930413O22 gene | 1.04 | 0.766 | -1.19 | 0.811 | -1.2 | 0.682 | -1.34 | 0.645 | -0.67 |
| 1442294_at | A530053G22Rik | RIKEN cDNA A530053G22 gene | -2.48 | 0.132 | -1.05 | 0.941 | 1.36 | 0.722 | -1.57 | 0.616 | -0.94 |
| 1452930_at | Dock3 | dedicator of cyto-kinesis 3 | 1.02 | 0.974 | -1.38 | 0.104 | -1.04 | 0.953 | -1.04 | 0.956 | -0.61 |
| 1423438_at | Kptn | kaptin | -1.16 | 0.249 | -1.19 | 0.274 | 1.01 | 0.962 | -1.4 | 0.111 | -0.69 |
| 1448428_at | Nbl1 | neuroblastoma, suppression of tumorigenicity 1 | 1.43 | 0.279 | -1.9 | 0.364 | -1.32 | 0.173 | -1.08 | 0.762 | -0.72 |
| 1438275_at | C130046K22Rik | RIKEN cDNA C130046K22 gene | 1.07 | 0.305 | -1.5 | 0.009 | -1.02 | 0.916 | -1.2 | 0.219 | -0.66 |
| 1430448_at | 6720418B01Rik | RIKEN cDNA 6720418B01 gene | -1.7 | 0.37 | 1.59 | 0.165 | -1.85 | 0.13 | -3.54 | 0.02 | -1.38 |
| 1438136_at | Astl | astacin-like metalloendopeptidase (M12 family) | -2.26 | 0.105 | 1.53 | 0.512 | -1.35 | 0.452 | -1.16 | 0.387 | -0.81 |
| 1453097_a_at | Ubtf | upstream binding transcription factor, RNA polymerase I | -1.27 | 0.102 | -1.01 | 0.913 | -1.06 | 0.798 | 1.25 | 0.169 | -0.52 |
| 1419350_at | Hook2 | hook homolog 2 (Drosophila) | -1.08 | 0.705 | -1.53 | 0.181 | 1.14 | 0.556 | -1.03 | 0.914 | -0.63 |
| 1456502_at | Dyrk2 | dual-specificity tyrosine-(Y)-phosphorylation regulated kinase 2 | -1.2 | 0.132 | -1.14 | 0.369 | 1 | 0.985 | -1.9 | 0.24 | -0.81 |
| 1433774_x_at | Cog1 | component of oligomeric golgi complex 1 | 1.05 | 0.738 | -1.28 | 0.155 | -1.13 | 0.49 | -1.09 | 0.514 | -0.61 |
| 1446565_at | Man1a2 | Mannosidase, alpha, class 1A, member 2 | -1.07 | 0.153 | -1.11 | 0.747 | -1.13 | 0.62 | 1.35 | 0.078 | -0.49 |
| 1445273_at | D10Ertd638e | DNA segment, Chr 10, ERATO Doi 638, expressed | 1.14 | 0.511 | -1.08 | 0.729 | -1.53 | 0.138 | -1.09 | 0.619 | -0.64 |
| 1432585_at | 4931428A05Rik | RIKEN cDNA 4931428A05 gene | 1.55 | 0.33 | -1.79 | 0.194 | -1.65 | 0.102 | -1.27 | 0.389 | -0.79 |
| 1437566_at | LOC633966 | similar to guanine nucleotide binding protein-like 2 (nucleolar) | 1.05 | 0.873 | -1.36 | 0.181 | -1.08 | 0.499 | -1.27 | 0.262 | -0.66 |
| 1435815_at | Ldoc1 | leucine zipper, down-regulated in cancer 1 | -1.31 | 0.702 | 1.12 | 0.484 | -1.21 | 0.694 | -1.72 | 0.106 | -0.78 |
| 1433891_at | Lgr4 /// LOC669588 | leucine-rich repeat-containing G protein-coupled receptor 4 /// similar to Leucine-r | -1.19 | 0.384 | 1.06 | 0.574 | -1.23 | 0.436 | -1.22 | 0.254 | -0.65 |
| 1452607_at | 2610030H06Rik | RIKEN cDNA 2610030H06 gene | -1.07 | 0.488 | -1.13 | 0.026 | -1.11 | 0.285 | 1.38 | 0.003 | -0.48 |

| | | | | | | | | | | | |
|---|---|---|---|---|---|---|---|---|---|---|---|
| 1429245_at | 2510022D24Rik | RIKEN cDNA 2510022D24 gene | -1.04 | 0.764 | -1.11 | 0.703 | -1.17 | 0.484 | 1.12 | 0.158 | -0.55 |
| 1440708_at | Myh9 | myosin, heavy polypeptide 9, non-muscle | 1.12 | 0.226 | -1.04 | 0.921 | -1.57 | 0.219 | -1.13 | 0.49 | -0.66 |
| 1460019_at | LOC627607 | similar to leucine rich repeat containing 45 | 1.46 | 0.655 | -1.78 | 0.561 | -1.44 | 0.488 | -1.4 | 0.565 | -0.79 |
| 1450465_at | Cdk5r2 | cyclin-dependent kinase 5, regulatory subunit 2 (p39) | 1.01 | 0.965 | -1.08 | 0.737 | -1.28 | 0.379 | -1.41 | 0.326 | -0.69 |
| 1454834_at | Nfib | nuclear factor I/B | 1.16 | 0.023 | -1.03 | 0.79 | -1.71 | 0.013 | -1.21 | 0.458 | -0.7 |
| 1444317_at | Pcdh15 | protocadherin 15 | -1.46 | 0.257 | -1.07 | 0.927 | 1.09 | 0.895 | -1.49 | 0.465 | -0.73 |
| 1447709_at | Epb4.1l3 | Erythrocyte protein band 4.1-like 3 | 1.1 | 0.89 | -1.11 | 0.787 | -1.39 | 0.588 | -2.59 | 0.29 | -1 |
| 1434865_a_at | Exoc7 | exocyst complex component 7 | -1.26 | 0.001 | -1.13 | 0.313 | 1.04 | 0.713 | -1.31 | 0.154 | -0.66 |
| 1435418_at | Slc22a8 | solute carrier family 22 (organic anion transporter), member 8 | -1.06 | 0.748 | 1.1 | 0.801 | -1.49 | 0.268 | -1.28 | 0.572 | -0.68 |
| 1428552_at | 2610001J05Rik | RIKEN cDNA 2610001J05 gene | -1.21 | 0.038 | 1.03 | 0.655 | -1.15 | 0.308 | -1.26 | 0.279 | -0.65 |
| 1416849_at | Zcd1 | zinc finger, CDGSH-type domain 1 | -1.16 | 0.123 | -1.13 | 0.347 | -1.03 | 0.816 | 1.28 | 0.359 | -0.51 |
| 1420142_s_at | Pa2g4 | proliferation-associated 2G4 | -1.01 | 0.969 | -1.32 | 0.081 | -1.03 | 0.834 | 1.26 | 0.4 | -0.53 |
| 1453512_at | 5830407P18Rik | RIKEN cDNA 5830407P18 gene | -1.02 | 0.889 | 1.13 | 0.684 | -1.66 | 0.017 | -1.44 | 0.203 | -0.75 |
| 1435881_at | Pcbp2 | poly(rC) binding protein 2 | -1.07 | 0.451 | -1.01 | 0.914 | -1.25 | 0.22 | 1.33 | 0.111 | -0.5 |
| 1431370_at | 4930512B01Rik | RIKEN cDNA 4930512B01 gene | -1.23 | 0.463 | -1.46 | 0.182 | 1.22 | 0.659 | -1.12 | 0.615 | -0.65 |
| 1442316_x_at | Trp53bp1 | transformation related protein 53 binding protein 1 | -1.16 | 0.64 | 1.16 | 0.527 | -1.45 | 0.13 | -1.04 | 0.92 | -0.62 |
| 1433894_at | AI591476 | expressed sequence AI591476 | -1.12 | 0.64 | -1.03 | 0.955 | -1.17 | 0.537 | 1.07 | 0.832 | -0.56 |
| 1444731_at | Pde4b | phosphodiesterase 4B, cAMP specific | 1.04 | 0.888 | -1.2 | 0.476 | -1.19 | 0.281 | -1.66 | 0.013 | -0.75 |
| 1437433_at | B3galt2 | UDP-Gal:betaGlcNAc beta 1,3-galactosyltransferase, polypeptide 2 | -2.08 | 0.374 | -1.44 | 0.254 | 1.54 | 0.515 | -1.27 | 0.63 | -0.81 |
| 1431413_at | 9130218E19Rik | RIKEN cDNA 9130218E19 gene | -1.45 | 0.471 | -1.27 | 0.497 | 1.24 | 0.577 | -1.76 | 0.154 | -0.81 |
| 1442027_at | Nbeal1 | neurobeachin like 1 | 1.21 | 0.443 | -1.23 | 0.38 | -1.45 | 0.271 | -1.45 | 0.489 | -0.73 |
| 1440574_at | Rag1 | Recombination activating gene 1 | -1.34 | 0.107 | -1.16 | 0.725 | 1.11 | 0.709 | -1.2 | 0.627 | -0.65 |
| 1441023_at | Eif2s2 | eukaryotic translation initiation factor 2, subunit 2 (beta) | 1.16 | 0.572 | -1.3 | 0.011 | -1.27 | 0.282 | -1.02 | 0.94 | -0.61 |
| 1415937_s_at | Pdcd6ip | programmed cell death 6 interacting protein | -1.09 | 0.126 | -1.4 | 0.079 | 1.09 | 0.721 | -1.35 | 0.282 | -0.69 |
| 1419920_s_at | Usp7 | ubiquitin specific peptidase 7 | -1.05 | 0.039 | -1.09 | 0.471 | -1.18 | 0.205 | 1.6 | 0.308 | -0.43 |
| 1436950_at | Crkl | v-crk sarcoma virus CT10 oncogene homolog (avian)-like | -1.07 | 0.591 | -1.09 | 0.482 | -1.15 | 0.218 | 1.08 | 0.713 | -0.56 |
| 1428383_a_at | 2310021P13Rik | RIKEN cDNA 2310021P13 gene | 1.01 | 0.955 | -1.06 | 0.653 | -1.3 | 0.254 | -1.24 | 0.576 | -0.65 |
| 1446013_at | Slc38a3 | solute carrier family 38, member 3 | -1.39 | 0.758 | 1.19 | 0.146 | -1.24 | 0.711 | -1.75 | 0.356 | -0.8 |
| 1438657_x_at | Ptp4a1 /// LOC4334 | protein tyrosine phosphatase 4a1 /// protein tyrosine phosphatase 4a1-like /// prot | 1.03 | 0.815 | -1.16 | 0.392 | -1.21 | 0.033 | -1.05 | 0.64 | -0.6 |
| 1433769_at | Als2cl | ALS2 C-terminal like | -1.11 | 0.411 | 1.02 | 0.625 | -1.27 | 0.054 | -3.22 | 0.024 | -1.14 |
| 1451444_s_at | 2700008B19Rik | RIKEN cDNA 2700008B19 gene | -1.22 | 0.049 | -1.73 | 0.001 | 1.32 | 0.087 | -1.3 | 0.044 | -0.73 |
| 1428159_s_at | Ndufab1 | NADH dehydrogenase (ubiquinone) 1, alpha/beta subcomplex, 1 | -1.07 | 0.201 | -1.19 | 0.021 | -1.06 | 0.199 | 1.2 | 0.376 | -0.53 |
| 1435089_at | 2010111I01Rik | RIKEN cDNA 2010111I01 gene | 1.06 | 0.744 | -1.07 | 0.807 | -1.37 | 0.212 | -1.17 | 0.453 | -0.64 |
| 1446429_at | P2rx4 | purinergic receptor P2X, ligand-gated ion channel 4 | 1.33 | 0.454 | -2.3 | 0.054 | -1.05 | 0.861 | -1.04 | 0.94 | -0.76 |
| 1418725_at | Dnajc5b | DnaJ (Hsp40) homolog, subfamily C, member 5 beta | -1.02 | 0.929 | -1.74 | 0.221 | 1.16 | 0.626 | -1.6 | 0.316 | -0.8 |
| 1451493_at | Ndfip1 | Nedd4 family interacting protein 1 | -1.05 | 0.452 | -1.42 | 0.054 | 1.06 | 0.745 | -1.29 | 0.415 | -0.68 |
| 1454552_at | 4930456K20Rik | RIKEN cDNA 4930456K20 gene | 1.2 | 0.765 | -1.33 | 0.235 | -1.31 | 0.503 | -1.52 | 0.003 | -0.74 |
| 1428117_x_at | Gpr178 | G protein-coupled receptor 178 | 1.02 | 0.937 | -1.31 | 0.607 | -1.07 | 0.708 | -1.54 | 0.216 | -0.73 |
| 1451994_s_at | 3930401K13Rik | RIKEN cDNA 3930401K13 gene | -1.88 | 0.291 | -1.32 | 0.262 | 1.43 | 0.273 | -2.03 | 0.187 | -0.95 |
| 1434753_at | Nfrkb | nuclear factor related to kappa B binding protein | -1.34 | 0.502 | -1.34 | 0.188 | 1.23 | 0.426 | -2.09 | 0.251 | -0.89 |
| 1423062_at | Igfbp3 | insulin-like growth factor binding protein 3 | -1.13 | 0.516 | 1.36 | 0.45 | -2.1 | 0.035 | -2.12 | 0.208 | -1 |
| 1429652_at | 1190002C06Rik | RIKEN cDNA 1190002C06 gene | -1.14 | 0.126 | 1.04 | 0.813 | -1.25 | 0.003 | -1.12 | 0.347 | -0.62 |
| 1451242_a_at | Ppp5c | protein phosphatase 5, catalytic subunit | -1.24 | 0.188 | -1.46 | 0.045 | 1.23 | 0.182 | -1.13 | 0.376 | -0.65 |
| 1425035_s_at | Dnmt3l | DNA (cytosine-5-)-methyltransferase 3-like | 1.12 | 0.804 | -1.33 | 0.495 | -1.18 | 0.548 | -1.46 | 0.656 | -0.71 |
| 1423210_a_at | Nola3 | nucleolar protein family A, member 3 | -1.11 | 0.444 | 1.04 | 0.591 | -1.28 | 0.08 | -1.12 | 0.083 | -0.62 |
| 1441790_at | --- | Transcribed locus | -1.08 | 0.909 | -1.75 | 0.524 | 1.23 | 0.314 | -1.18 | 0.765 | -0.7 |
| 1434889_at | Plekha7 | pleckstrin homology domain containing, family A member 7 | -1.33 | 0.195 | -1.65 | 0.162 | 1.37 | 0.317 | -1.31 | 0.551 | -0.73 |
| 1423889_at | MGC5739 | similar to hypothetical protein FLJ20010 | -1.21 | 0.173 | -1.31 | 0.167 | 1.13 | 0.667 | -1.09 | 0.749 | -0.62 |
| 1416663_at | Ndufa9 | NADH dehydrogenase (ubiquinone) 1 alpha subcomplex, 9 | -1.14 | 0.017 | -1.16 | 0.105 | -1.03 | 0.613 | 1.07 | 0.554 | -0.56 |
| 1444562_at | Ube3c | Ubiquitin protein ligase E3C | -1.05 | 0.838 | 1.15 | 0.667 | -1.62 | 0.059 | -1.3 | 0.021 | -0.7 |
| 1442648_at | D130054H01 | hypothetical protein D130054H01 | -1.33 | 0.268 | -1.09 | 0.784 | 1.05 | 0.848 | -1.18 | 0.661 | -0.64 |
| 1429752_x_at | Rsnl2 | restin-like 2 | -1.54 | 0.256 | 1.25 | 0.701 | -1.22 | 0.115 | -2.03 | 0.346 | -0.88 |
| 1447383_at | BC005471 | CDNA sequence BC005471 | -1.17 | 0.755 | 1.12 | 0.691 | -1.35 | 0.455 | -1.29 | 0.292 | -0.67 |

| | | | | | | | | | | | |
|---|---|---|---|---|---|---|---|---|---|---|---|
| 1430088_at | 3000002G13Rik | RIKEN cDNA 3000002G13 gene | -1.19 | 0.496 | 1.14 | 0.636 | -1.36 | 0.3 | -1.57 | 0.194 | -0.74 |
| 1439077_at | Zxda /// LOC66817 | zinc finger, X-linked, duplicated A /// similar to Zinc finger X-linked protein ZXDA | -1.09 | 0.139 | -1.56 | 0.189 | 1.16 | 0.552 | -1.56 | 0.355 | -0.76 |
| 1449143_at | Rtp4 | receptor transporter protein 4 | -1.53 | 0.358 | 1.32 | 0.52 | -1.34 | 0.233 | -1.88 | 0.286 | -0.86 |
| 1439153_at | Ibrdc2 | IBR domain containing 2 | -1.11 | 0.637 | -1.11 | 0.548 | -1.09 | 0.633 | 1.3 | 0.397 | -0.5 |
| 1432347_at | 4931428L18Rik | RIKEN cDNA 4931428L18 gene | -1.19 | 0.722 | 1.1 | 0.741 | -1.29 | 0.573 | -2.47 | 0.148 | -0.96 |
| 1447294_at | Fbxw11 | F-box and WD-40 domain protein 11 | -1.08 | 0.295 | 1.04 | 0.88 | -1.32 | 0.203 | -1.04 | 0.777 | -0.6 |
| 1418814_s_at | Ndufa12 | NADH dehydrogenase (ubiquinone) 1 alpha subcomplex, 12 | -1.07 | 0.608 | -1.16 | 0.173 | -1.09 | 0.589 | 1.26 | 0.178 | -0.51 |
| 1457156_at | Trhde | TRH-degrading enzyme | -3.93 | 0.005 | -1.03 | 0.936 | 1.5 | 0.467 | -1.85 | 0.282 | -1.33 |
| 1438658_a_at | Edg3 | endothelial differentiation, sphingolipid G-protein-coupled receptor, 3 | 1.19 | 0.27 | -1.28 | 0.059 | -1.33 | 0.119 | -2.39 | 0.097 | -0.95 |
| 1442203_at | LOC552880 | hypothetical LOC552880 | -2.34 | 0.095 | 1.41 | 0.553 | -1.13 | 0.818 | -2.44 | 0.053 | -1.13 |
| 1460450_at | E230022H04Rik | RIKEN cDNA E230022H04 gene | -1.07 | 0.535 | -1.18 | 0.107 | -1.07 | 0.645 | 1.1 | 0.061 | -0.55 |
| 1430515_s_at | Aasdhppt | aminoadipate-semialdehyde dehydrogenase-phosphopantetheinyl transferase | -1 | 0.989 | -1.31 | 0.011 | -1.04 | 0.657 | 1.03 | 0.73 | -0.58 |
| 1448692_at | Ubqln4 | ubiquilin 4 | -1.29 | 0.344 | -1.03 | 0.875 | -1.03 | 0.799 | 1.53 | 0.169 | -0.45 |
| 1459095_at | --- | --- | 1.28 | 0.763 | -1.6 | 0.541 | -1.23 | 0.485 | -1.96 | 0.303 | -0.88 |
| 1455972_x_at | Hadhsc | L-3-hydroxyacyl-Coenzyme A dehydrogenase, short chain | -1.2 | 0.056 | -1.31 | 0.343 | 1.12 | 0.302 | -1.42 | 0.032 | -0.7 |
| 1423818_a_at | Arl6ip1 | ADP-ribosylation factor-like 6 interacting protein 1 | -1.18 | 0.085 | -1.06 | 0.379 | -1.08 | 0.6 | 1.26 | 0.291 | -0.51 |
| 1434861_at | --- | Transcribed locus | 1.05 | 0.551 | -1.07 | 0.644 | -1.35 | 0.078 | -1.19 | 0.379 | -0.64 |
| 1456958_at | C230072F16Rik | RIKEN cDNA C230072F16 gene | -1.21 | 0.477 | 1.08 | 0.412 | -1.23 | 0.3 | -1.21 | 0.606 | -0.64 |
| 1440456_at | LOC381255 | similar to KIAA0944 protein | -1.01 | 0.976 | -1.33 | 0.579 | -1.02 | 0.938 | 1.15 | 0.791 | -0.55 |
| 1434615_x_at | --- | --- | -1.11 | 0.864 | 1.03 | 0.96 | -1.28 | 0.654 | -1.01 | 0.96 | -0.59 |
| 1452371_at | Sfrs11 | splicing factor, arginine/serine-rich 11 | -1.16 | 0.233 | -1.18 | 0.013 | 1.01 | 0.964 | -1.1 | 0.502 | -0.61 |
| 1416731_at | Top2b | topoisomerase (DNA) II beta | -1.15 | 0.669 | 1.12 | 0.266 | -1.37 | 0.03 | -1.36 | 0.054 | -0.69 |
| 1429847_a_at | 4833418A01Rik | RIKEN cDNA 4833418A01 gene | -1.05 | 0.675 | -1.42 | 0.041 | 1.06 | 0.755 | -1.38 | 0.01 | -0.7 |
| 1457292_at | Josd3 | Josephin domain containing 3 | -1.16 | 0.452 | -1.13 | 0.335 | -1.02 | 0.939 | 1.03 | 0.857 | -0.57 |
| 1432965_at | 4921507H08Rik | RIKEN cDNA 4921507H08 gene | -2 | 0.391 | 1.22 | 0.707 | -1 | 0.996 | -1.62 | 0.611 | -0.85 |
| 1416211_a_at | Ptn | pleiotrophin | 1.2 | 0.409 | -1.26 | 0.492 | -1.37 | 0.081 | -1.11 | 0.116 | -0.64 |
| 1428884_at | Tmem57 | transmembrane protein 57 | -1.1 | 0.582 | -1.17 | 0.209 | -1.05 | 0.453 | 1.31 | 0.287 | -0.5 |
| 1417241_at | X83328 | EST X83328 | -1.15 | 0.086 | -1.08 | 0.339 | -1.08 | 0.38 | 1.2 | 0.111 | -0.53 |
| 1444848_at | R3hdm1 | R3H domain 1 (binds single-stranded nucleic acids) | 1.27 | 0.047 | -1.26 | 0.449 | -1.51 | 0.028 | -1.31 | 0.154 | -0.7 |
| 1435556_at | Zfp597 | zinc finger protein 597 | -1.08 | 0.535 | -1.33 | 0.114 | 1.04 | 0.583 | -1.48 | 0.02 | -0.71 |
| 1427010_s_at | Lama5 /// LOC6719 | laminin, alpha 5 /// similar to Laminin alpha-5 chain precursor | -1.2 | 0.057 | -1.15 | 0.612 | 1.02 | 0.918 | -1.72 | 0.464 | -0.76 |
| 1448474_at | Nek7 | NIMA (never in mitosis gene a)-related expressed kinase 7 | 1.12 | 0.04 | -1.11 | 0.173 | -1.43 | 0.162 | -1.04 | 0.879 | -0.62 |
| 1460702_at | Triap1 | TP53 regulated inhibitor of apoptosis 1 | 1.03 | 0.864 | -1.07 | 0.489 | -1.32 | 0.138 | -1.16 | 0.653 | -0.63 |
| 1448905_at | Nme3 | expressed in non-metastatic cells 3 | -1.04 | 0.69 | -1.17 | 0.07 | -1.1 | 0.569 | 1.5 | 0.379 | -0.45 |
| 1451893_s_at | Magi1 | membrane associated guanylate kinase, WW and PDZ domain containing 1 | -1.44 | 0.263 | 1.08 | 0.595 | -1.06 | 0.813 | -1.67 | 0.113 | -0.77 |
| 1423445_at | Rock1 | Rho-associated coiled-coil containing protein kinase 1 | -1.09 | 0.36 | -1.16 | 0.129 | -1.06 | 0.542 | 1.11 | 0.349 | -0.55 |
| 1417512_at | Evi5 | ecotropic viral integration site 5 | -1.09 | 0.537 | -1.06 | 0.563 | -1.16 | 0.26 | 2.23 | 0.171 | -0.27 |
| 1434305_at | U2af1l4 | U2 small nuclear RNA auxiliary factor 1-like 4 | -1.08 | 0.634 | -1.22 | 0.041 | -1.02 | 0.846 | 1.01 | 0.975 | -0.58 |
| 1430463_a_at | Sncaip | synuclein, alpha interacting protein (synphilin) | -1.38 | 0.538 | -1.34 | 0.575 | 1.25 | 0.616 | -1.44 | 0.334 | -0.73 |
| 1450497_at | Apc2 | adenomatosis polyposis coli 2 | -1.2 | 0.179 | -1.08 | 0.781 | -1.04 | 0.863 | 1.38 | 0.347 | -0.49 |
| 1428582_at | 2010208K18Rik | RIKEN cDNA 2010208K18 gene | 1.02 | 0.606 | -1.02 | 0.751 | -1.39 | 0.038 | -1.36 | 0.068 | -0.69 |
| 1451125_at | BC017133 | cDNA sequence BC017133 | -1.14 | 0.504 | 1 | 0.92 | -1.19 | 0.141 | -1.5 | 0.026 | -0.71 |
| 1423200_at | Ncor1 | nuclear receptor co-repressor 1 | 1.01 | 0.922 | -1.13 | 0.017 | -1.21 | 0.065 | -1.14 | 0.025 | -0.62 |
| 1422215_at | 4930542C12Rik | RIKEN cDNA 4930542C12 gene | -1.03 | 0.639 | -1.81 | 0.136 | 1.2 | 0.547 | -1.12 | 0.392 | -0.69 |
| 1428382_at | Smarcc2 | SWI/SNF related, matrix associated, actin dependent regulator of chromatin, subf | -1.26 | 0.214 | -1.06 | 0.834 | -1.02 | 0.948 | 1.21 | 0.368 | -0.53 |
| 1454109_a_at | Ptdsr | phosphatidylserine receptor | -1.11 | 0.05 | -1.07 | 0.28 | -1.13 | 0.666 | 1.03 | 0.944 | -0.57 |
| 1436910_at | Rasal2 | RAS protein activator like 2 | -1.02 | 0.893 | 1.13 | 0.69 | -1.65 | 0.018 | -1.37 | 0.51 | -0.73 |
| 1427264_at | Crygb | crystallin, gamma B | -2.47 | 0.218 | 1.47 | 0.474 | -1.18 | 0.472 | -3.22 | 0.248 | -1.35 |
| 1436401_at | --- | --- | -1.12 | 0.637 | -1.28 | 0.373 | 1.05 | 0.773 | -1.28 | 0.06 | -0.66 |
| 1448492_a_at | Psmd12 | proteasome (prosome, macropain) 26S subunit, non-ATPase, 12 | -1.04 | 0.517 | -1.16 | 0.04 | -1.12 | 0.415 | 1.19 | 0.485 | -0.53 |
| 1426425_at | Sugt1 | SGT1, suppressor of G2 allele of SKP1 (S. cerevisiae) | -1.21 | 0.166 | -1.16 | 0.056 | 1.03 | 0.885 | -1.62 | 0.087 | -0.74 |
| 1458912_at | Rbm6 | RNA binding motif protein 6 | -1.05 | 0.849 | 1.08 | 0.237 | -1.46 | 0.025 | -1.09 | 0.627 | -0.63 |
| 1444112_at | --- | 6 days neonate head cDNA, RIKEN full-length enriched library, clone:5430409O0 | -1.14 | 0.399 | -1.14 | 0.355 | -1.03 | 0.814 | 1.02 | 0.942 | -0.57 |

| | | | | | | | | | | | |
|---|---|---|---|---|---|---|---|---|---|---|---|
| 1452700_s_at | Kbtbd7 | kelch repeat and BTB (POZ) domain containing 7 | -1.13 | 0.322 | -1.16 | 0.079 | -1.02 | 0.783 | 1.11 | 0.594 | -0.55 |
| 1438179_s_at | Statip1 | Signal transducer and activator of transcription interacting protein 1 | -1.02 | 0.807 | -1.13 | 0.182 | -1.17 | 0.14 | 1.02 | 0.899 | -0.57 |
| 1446525_at | Gpc3 | Glypican 3 | 1.18 | 0.832 | -1.04 | 0.898 | -1.73 | 0.004 | -1.96 | 0.166 | -0.89 |
| 1423105_a_at | Yeats4 | YEATS domain containing 4 | -1.14 | 0.05 | 1 | 0.965 | -1.19 | 0.21 | -1.32 | 0.015 | -0.66 |
| 1444863_at | Nhlrc2 | NHL repeat containing 2 | -1.27 | 0.275 | -1.83 | 0.355 | 1.39 | 0.17 | -1.05 | 0.803 | -0.69 |
| 1448774_at | Stoml2 | stomatin (Epb7.2)-like 2 | -1.1 | 0.02 | -1.08 | 0.151 | -1.13 | 0.151 | 1.11 | 0.662 | -0.55 |
| 1441591_at | --- | --- | -1.46 | 0.523 | 1.25 | 0.63 | -1.26 | 0.596 | -1.08 | 0.87 | -0.64 |
| 1460507_at | 4930552P12Rik | RIKEN cDNA 4930552P12 gene | -2.02 | 0.272 | -1.1 | 0.826 | 1.32 | 0.486 | -1.06 | 0.934 | -0.72 |
| 1433552_a_at | Polr2b | polymerase (RNA) II (DNA directed) polypeptide B | 1.04 | 0.803 | -1.21 | 0.031 | -1.16 | 0.131 | -1.16 | 0.3 | -0.62 |
| 1453120_at | Txndc13 | thioredoxin domain containing 13 | -1.05 | 0.822 | -1.19 | 0.204 | -1.08 | 0.504 | 1.99 | 0.213 | -0.33 |
| 1428499_at | 2810454L23Rik | RIKEN cDNA 2810454L23 gene | -1.14 | 0.299 | 1.13 | 0.441 | -1.41 | 0.068 | -1.03 | 0.94 | -0.61 |
| 1441959_s_at | 1200003C05Rik | RIKEN cDNA 1200003C05 gene | -1.05 | 0.087 | 1.05 | 0.279 | -1.38 | 0.005 | -1.56 | 0.058 | -0.74 |
| 1418260_at | Hunk /// LOC63056 | hormonally upregulated Neu-associated kinase /// similar to hormonally upregulat | -1.44 | 0.074 | 1.25 | 0.376 | -1.28 | 0.102 | -1.14 | 0.061 | -0.65 |
| 1430221_at | 9130008F23Rik | RIKEN cDNA 9130008F23 gene | -1.17 | 0.411 | -1.04 | 0.915 | -1.11 | 0.554 | 1.01 | 0.909 | -0.57 |
| 1447536_at | Lysmd2 | LysM, putative peptidoglycan-binding, domain containing 2 | 1.39 | 0.644 | -1.35 | 0.329 | -1.68 | 0.038 | -1.01 | 0.972 | -0.66 |
| 1435818_at | --- | Adult male corpus striatum cDNA, RIKEN full-length enriched library, clone:C030( | -1.17 | 0.204 | 1.05 | 0.69 | -1.22 | 0.069 | -1.11 | 0.527 | -0.61 |
| 1416859_at | Fkbp3 | FK506 binding protein 3 | -1.02 | 0.866 | 1 | 0.975 | -1.34 | 0.058 | -1.87 | 0.058 | -0.81 |
| 1441049_at | Kcna6 | potassium voltage-gated channel, shaker-related, subfamily, member 6 | -1.28 | 0.716 | -1.01 | 0.96 | -1.05 | 0.901 | 1.5 | 0.304 | -0.46 |
| 1434188_at | Slc16a12 | solute carrier family 16 (monocarboxylic acid transporters), member 12 | -1.36 | 0.231 | 1 | 0.982 | -1.02 | 0.931 | -1.02 | 0.958 | -0.6 |
| 1433793_s_at | Nrip2 | nuclear receptor interacting protein 2 | -1.6 | 0.664 | 1.29 | 0.477 | -1.23 | 0.761 | -1.6 | 0.422 | -0.79 |
| 1427065_at | 4933439F18Rik | RIKEN cDNA 4933439F18 gene | -1.36 | 0.165 | -1.04 | 0.772 | 1.03 | 0.833 | -1.17 | 0.559 | -0.64 |
| 1426717_at | Nipa2 | non imprinted in Prader-Willi/Angelman syndrome 2 homolog (human) | -1.28 | 0.014 | 1.07 | 0.454 | -1.14 | 0.189 | -1.02 | 0.682 | -0.59 |
| 1452177_at | Abcf3 | ATP-binding cassette, sub-family F (GCN20), member 3 | -1.2 | 0.394 | -1.49 | 0.198 | 1.22 | 0.143 | -1.5 | 0.006 | -0.74 |
| 1446647_at | --- | --- | -1.13 | 0.136 | -1.31 | 0.486 | 1.07 | 0.838 | -1.06 | 0.909 | -0.61 |
| 1445937_at | A630001G21Rik | RIKEN cDNA A630001G21 gene | 1.31 | 0.503 | -1.49 | 0.473 | -1.35 | 0.344 | -1.1 | 0.501 | -0.66 |
| 1451336_at | Lgals4 | lectin, galactose binding, soluble 4 | -1.01 | 0.965 | -1.39 | 0.541 | 1.02 | 0.949 | -1.09 | 0.747 | -0.62 |
| 1429257_at | Gtl2 | GTL2, imprinted maternally expressed untranslated mRNA | 1.35 | 0.603 | -1.6 | 0.441 | -1.34 | 0.088 | -1.27 | 0.573 | -0.71 |
| 1433674_a_at | Snord22 | small nucleolar RNA, C/D box 22 | -1.1 | 0.567 | -1.24 | 0.181 | 1.01 | 0.973 | -1.11 | 0.644 | -0.61 |
| 1431728_at | 2310010I16Rik | RIKEN cDNA 2310010I16 gene | -1.82 | 0.097 | 1.65 | 0.261 | -1.92 | 0.048 | -1.03 | 0.951 | -0.78 |
| 1422693_a_at | Sub1 | SUB1 homolog (S. cerevisia | -1.14 | 0.316 | -1.19 | 0.026 | 1.01 | 0.969 | -1.27 | 0.175 | -0.65 |
| 1440282_at | Tulp4 /// 2210038L1 | tubby like protein 4 /// RIKEN cDNA 2210038L17 gene | 1.26 | 0.665 | -1.24 | 0.642 | -1.52 | 0.511 | -1.47 | 0.569 | -0.74 |
| 1418439_at | D10Ertd322e | DNA segment, Chr 10, ERATO Doi 322, expressed | -1.1 | 0.342 | -1.11 | 0.212 | -1.1 | 0.297 | 1.26 | 0.22 | -0.51 |
| 1450588_at | Olfr67 | olfactory receptor 67 | -1.15 | 0.754 | -1.62 | 0.042 | 1.24 | 0.667 | -1.03 | 0.892 | -0.64 |
| 1436711_at | Actr5 | ARP5 actin-related protein 5 homolog (yeast) | -1.33 | 0.099 | -1.07 | 0.771 | 1.04 | 0.841 | -1.23 | 0.006 | -0.65 |
| 1455784_at | Sec1 | secretory blood group 1 | -1.25 | 0.717 | 1.03 | 0.883 | -1.11 | 0.8 | -1.12 | 0.835 | -0.62 |
| 1428094_at | Lamp2 | lysosomal membrane glycoprotein 2 | -1.03 | 0.745 | -1.04 | 0.725 | -1.27 | 0.063 | 1.27 | 0.303 | -0.52 |
| 1442917_at | --- | Adult male vesicular gland cDNA, RIKEN full-length enriched library, clone:G630( | 1.15 | 0.86 | -1.29 | 0.706 | -1.24 | 0.502 | -4.38 | 0.237 | -1.44 |
| 1433671_at | A130022J15Rik | RIKEN cDNA A130022J15 gene | 1.04 | 0.55 | -1.09 | 0.697 | -1.3 | 0.08 | -1.02 | 0.902 | -0.59 |
| 1426180_a_at | Smr2 | submaxillary gland androgen regulated protein 2 | -1.26 | 0.696 | -1.23 | 0.387 | 1.12 | 0.367 | -1.04 | 0.759 | -0.6 |
| 1431466_at | 4930553D19Rik | RIKEN cDNA 4930553D19 gene | -1.36 | 0.714 | -1.02 | 0.981 | 1.01 | 0.989 | -1.17 | 0.864 | -0.64 |
| 1421877_at | Mapk9 | mitogen activated protein kinase 9 | -1.1 | 0.133 | -1.15 | 0.264 | -1.06 | 0.589 | 1.07 | 0.345 | -0.56 |
| 1425526_a_at | Prrx1 | paired related homeobox 1 | 1.3 | 0.633 | -1.75 | 0.223 | -1.18 | 0.651 | -1.08 | 0.837 | -0.68 |
| 1427943_at | Acyp2 | acylphosphatase 2, muscle type | 1 | 0.977 | -1.09 | 0.642 | -1.24 | 0.229 | -1.03 | 0.85 | -0.59 |
| 1457926_at | Rnpepl1 | arginyl aminopeptidase (aminopeptidase B)-like 1 | -1.6 | 0.056 | -1.19 | 0.735 | 1.26 | 0.423 | -1.14 | 0.688 | -0.67 |
| 1457951_at | 4930434E21Rik | RIKEN cDNA 4930434E21 gene | -2.08 | 0.33 | 1.65 | 0.542 | -1.68 | 0.293 | -2.91 | 0.074 | -1.25 |
| 1428502_at | Actr6 | ARP6 actin-related protein 6 homolog (yeast) | -1.34 | 0.389 | -1.05 | 0.647 | 1.03 | 0.772 | -1.2 | 0.108 | -0.64 |
| 1416494_at | Ndufs5 | NADH dehydrogenase (ubiquinone) Fe-S protein 5 | -1.08 | 0.276 | -1.29 | 0.024 | 1.02 | 0.648 | -1.05 | 0.717 | -0.6 |
| 1424910_at | Kif12 | kinesin family member 12 | -1.55 | 0.244 | 1.22 | 0.238 | -1.17 | 0.547 | -1.41 | 0.498 | -0.73 |
| 1423833_a_at | Brp44 | brain protein 44 | -1.14 | 0.259 | -1.16 | 0.045 | -1.02 | 0.799 | 1.49 | 0.306 | -0.46 |
| 1438806_at | Bcl9l | B-cell CLL/lymphoma 9-like | -1.4 | 0.475 | 1.27 | 0.674 | -1.35 | 0.66 | -2.77 | 0.115 | -1.06 |
| 1419988_at | Map3k7 | mitogen activated protein kinase kinase kinase 7 | -1.2 | 0.256 | -1.03 | 0.855 | -1.09 | 0.392 | 1.1 | 0.305 | -0.55 |
| 1447043_at | Erbb4 | V-erb-a erythroblastic leukemia viral oncogene homolog 4 (avian) | 1.34 | 0.315 | -1.57 | 0.511 | -1.33 | 0.539 | -1.11 | 0.848 | -0.67 |
| 1428768_at | Pan3 | PAN3 polyA specific ribonuclease subunit homolog (S. cerevisiae) | 1.01 | 0.933 | -1.18 | 0.557 | -1.16 | 0.569 | -1.01 | 0.978 | -0.58 |

| | | | | | | | | | | | |
|---|---|---|---|---|---|---|---|---|---|---|---|
| 1443314_at | 2410042D21Rik | RIKEN cDNA 2410042D21 gene | -1.05 | 0.743 | 1.09 | 0.656 | -1.47 | 0.043 | -1.37 | 0.111 | -0.7 |
| 1446406_at | Paqr8 | Progestin and adipoQ receptor family member VIII | -2.85 | 0.164 | 1.82 | 0.37 | -1.8 | 0.067 | -1.99 | 0.142 | -1.2 |
| 1433659_at | Trp53bp1 | Transformation related protein 53 binding protein 1 | 1.01 | 0.896 | -1.22 | 0.016 | -1.12 | 0.393 | -1.25 | 0.169 | -0.65 |
| 1436898_at | Sfpq | splicing factor proline/glutamine rich (polypyrimidine tract binding protein associat | 1.23 | 0.257 | -1.07 | 0.739 | -1.81 | 0.027 | -1.03 | 0.931 | -0.67 |
| 1435893_at | Vldlr | very low density lipoprotein receptor | -1.11 | 0.402 | 1.2 | 0.822 | -1.59 | 0.065 | -1.22 | 0.691 | -0.68 |
| 1451916_s_at | Trim15 | tripartite motif protein 15 | 1.63 | 0.416 | -3.42 | 0.059 | -1.25 | 0.508 | -1 | 0.993 | -1.01 |
| 1450791_at | Nppb | natriuretic peptide precursor type B | -3 | 0.352 | -1.66 | 0.1 | 1.79 | 0.118 | -1.12 | 0.751 | -1 |
| 1458787_at | Map3k7 | Mitogen activated protein kinase kinase kinase 7 | -4.14 | 0.006 | -3.35 | 0.133 | 2.19 | 0 | -1.29 | 0.73 | -1.65 |
| 1448399_at | Tax1bp1 | Tax1 (human T-cell leukemia virus type I) binding protein 1 | -1.14 | 0.436 | -1.15 | 0.087 | -1.02 | 0.729 | 1.02 | 0.914 | -0.57 |
| 1456415_at | Zfp451 | zinc finger protein 451 | -1.22 | 0.053 | 1.14 | 0.398 | -1.32 | 0.082 | -1.28 | 0.211 | -0.67 |
| 1434898_at | Tnrc6a | trinucleotide repeat containing 6a | 1.04 | 0.779 | -1.05 | 0.715 | -1.36 | 0.016 | -1.23 | 0.415 | -0.65 |
| 1443790_x_at | 4930414L22Rik | RIKEN cDNA 4930414L22 gene | -1.27 | 0.2 | -1.18 | 0.223 | 1.09 | 0.513 | -1.22 | 0.115 | -0.65 |
| 1429081_at | Gcc2 | GRIP and coiled-coil domain containing 2 | -1.05 | 0.369 | 1.02 | 0.527 | -1.33 | 0.036 | -1.42 | 0.121 | -0.69 |
| 1430118_at | 2700046A07Rik | RIKEN cDNA 2700046A07 gene | -1.03 | 0.971 | -2.51 | 0.186 | 1.36 | 0.452 | -1.01 | 0.983 | -0.8 |
| 1433293_at | 1500032O14Rik | RIKEN cDNA 1500032O14 gene | 1.26 | 0.307 | -1.23 | 0.52 | -1.54 | 0.149 | -1.09 | 0.653 | -0.65 |
| 1432729_at | 5830415G21Rik | RIKEN cDNA 5830415G21 gene | -1.47 | 0.391 | -1.19 | 0.655 | 1.21 | 0.364 | -1.72 | 0.519 | -0.79 |
| 1456762_at | --- | --- | 1.25 | 0.35 | -1.67 | 0.143 | -1.15 | 0.805 | -1.44 | 0.434 | -0.75 |
| 1441349_at | LOC552904 | hypothetical LOC552904 | 1.5 | 0.097 | -1.53 | 0.174 | -1.75 | 0.058 | -1.38 | 0.396 | -0.79 |
| 1444144_at | 5530400K22Rik | RIKEN cDNA 5530400K22 gene | 1.2 | 0.673 | -1.17 | 0.549 | -1.49 | 0.032 | -1.14 | 0.69 | -0.65 |
| 1437520_a_at | Nup85 | nucleoporin 85 | -1.03 | 0.922 | -1.14 | 0.577 | -1.14 | 0.424 | 1.42 | 0.415 | -0.47 |
| 1439497_at | 4933415E08Rik | RIKEN cDNA 4933415E08 gene | -1.03 | 0.782 | -1.88 | 0 | 1.22 | 0.256 | -1.18 | 0.025 | -0.72 |
| 1420384_at | Col4a3bp | procollagen, type IV, alpha 3 (Goodpasture antigen) binding protein | -1.16 | 0.462 | -1.3 | 0.052 | 1.1 | 0.698 | -1.13 | 0.556 | -0.62 |
| 1434892_x_at | Rbbp4 | retinoblastoma binding protein 4 | -1.08 | 0.063 | -1.16 | 0.023 | -1.06 | 0.404 | 1.29 | 0.166 | -0.5 |
| 1448570_at | Gmfb | glia maturation factor, beta | -1.13 | 0.16 | 1.02 | 0.671 | -1.21 | 0.098 | -1.4 | 0.183 | -0.68 |
| 1415740_at | Psmc5 | protease (prosome, macropain) 26S subunit, ATPase 5 | -1.05 | 0.197 | -1.23 | 0.012 | -1.04 | 0.726 | 1.12 | 0.288 | -0.55 |
| 1433355_at | 2610021J01Rik | RIKEN cDNA 2610021J01 gene | -2.31 | 0.103 | -1.57 | 0.457 | 1.66 | 0.247 | -3.12 | 0.219 | -1.33 |
| 1426722_at | Slc38a2 | solute carrier family 38, member 2 | -1.08 | 0.776 | -1.14 | 0.271 | -1.08 | 0.705 | 1.05 | 0.39 | -0.56 |
| 1418630_at | Khdrbs1 | KH domain containing, RNA binding, signal transduction associated 1 | 1.13 | 0.597 | -1.17 | 0.776 | -1.34 | 0.196 | -1.72 | 0.031 | -0.78 |
| 1426372_a_at | Bet1l | blocked early in transport 1 homolog (S. cerevisiae)-like | 1.11 | 0.581 | -1.04 | 0.658 | -1.52 | 0.012 | -1.35 | 0.004 | -0.7 |
| 1434833_at | Map4k2 | Mitogen activated protein kinase kinase kinase kinase 2 | -1.15 | 0.26 | -1.27 | 0.35 | 1.07 | 0.81 | -2.58 | 0.075 | -0.98 |
| 1422819_at | Mrpl36 | mitochondrial ribosomal protein L36 | -1.27 | 0.312 | -1.01 | 0.764 | -1.05 | 0.742 | 1.02 | 0.898 | -0.58 |
| 1423458_at | Slc35a5 | solute carrier family 35, member A5 | -1.16 | 0.422 | 1.16 | 0.532 | -1.41 | 0.057 | -1.07 | 0.848 | -0.62 |
| 1460355_at | Bud13 | BUD13 homolog (yeast) | -1.12 | 0.743 | -1.36 | 0.251 | 1.1 | 0.053 | -1.05 | 0.75 | -0.61 |
| 1425631_at | Ppp1r3c | protein phosphatase 1, regulatory (inhibitor) subunit 3C | -1.46 | 0.459 | -1.34 | 0.421 | 1.3 | 0.567 | -1.75 | 0.089 | -0.81 |
| 1419316_s_at | Gnb1l | guanine nucleotide binding protein (G protein), beta polypeptide 1-like | -1.24 | 0.309 | -1.2 | 0.169 | 1.08 | 0.801 | -1 | 0.995 | -0.59 |
| 1424642_at | Thoc1 | THO complex 1 | -1.15 | 0.254 | 1.08 | 0.585 | -1.28 | 0.042 | -1.32 | 0.021 | -0.67 |
| 1447364_x_at | Myo1b | myosin IB | 1.14 | 0.773 | -1.11 | 0.35 | -1.45 | 0.101 | -1.36 | 0.443 | -0.7 |
| 1453931_at | Col14a1 | procollagen, type XIV, alpha 1 | -1.01 | 0.827 | -1.34 | 0.059 | -1.01 | 0.902 | 1.06 | 0.693 | -0.57 |
| 1440171_x_at | 1700049G17Rik | RIKEN cDNA 1700049G17 gene | -1.24 | 0.166 | 1.14 | 0.592 | -1.27 | 0.258 | -1.16 | 0.214 | -0.63 |
| 1453999_at | LOC670473 | similar to Nucleolar preribosomal-associated protein 1 | 1.63 | 0.509 | -1.58 | 0.105 | -2.16 | 0.316 | -1.92 | 0.336 | -1.01 |
| 1432534_at | 3300002I08Rik | RIKEN cDNA 3300002I08 gene | -1.45 | 0.311 | -1.12 | 0.832 | 1.14 | 0.695 | -1.03 | 0.854 | -0.61 |
| 1432190_at | Sox5 | SRY-box containing gene 5 | -2.19 | 0.087 | 1.54 | 0.132 | -1.36 | 0.437 | -2.3 | 0.145 | -1.08 |
| 1422711_a_at | Pnck | pregnancy upregulated non-ubiquitously expressed CaM kinase | -1.55 | 0.62 | -1.2 | 0.234 | 1.25 | 0.427 | -1.4 | 0.56 | -0.72 |
| 1450765_a_at | Pde6h | phosphodiesterase 6H, cGMP-specific, cone, gamma | -1.16 | 0.807 | -1.1 | 0.683 | -1.04 | 0.873 | 1.04 | 0.937 | -0.57 |
| 1451090_a_at | Eif2s3x | eukaryotic translation initiation factor 2, subunit 3, structural gene X-linked | -1.16 | 0.167 | -1.06 | 0.481 | -1.08 | 0.662 | 1.21 | 0.147 | -0.52 |
| 1439784_at | 2310046O06Rik | RIKEN cDNA 2310046O06 gene | -1.14 | 0.029 | 1.02 | 0.934 | -1.2 | 0.291 | -1.42 | 0.221 | -0.69 |
| 1423724_at | Zwint | ZW10 interactor | -1.11 | 0.24 | -1.03 | 0.792 | -1.17 | 0.189 | 1.64 | 0.113 | -0.42 |
| 1426447_at | Nup35 | nucleoporin 35 | -1.16 | 0.058 | -1.09 | 0.401 | -1.05 | 0.614 | 1.51 | 0.188 | -0.45 |
| 1423502_at | Brd2 | bromodomain containing 2 | -1.34 | 0.012 | -1.12 | 0.159 | 1.09 | 0.552 | -1.29 | 0.011 | -0.67 |
| 1425503_at | Gcnt2 | glucosaminyl (N-acetyl) transferase 2, I-branching enzyme | -1.01 | 0.975 | -1.13 | 0.477 | -1.17 | 0.102 | 1.11 | 0.733 | -0.55 |
| 1459306_at | Bfsp2 | Beaded filament structural protein 2, phakinin | -1.32 | 0.403 | -1.39 | 0.593 | 1.25 | 0.688 | -1.18 | 0.768 | -0.66 |
| 1436766_at | Luc7l2 | LUC7-like 2 (S. cerevisiae) | 1.15 | 0.678 | -1.09 | 0.609 | -1.51 | 0.11 | -1.25 | 0.457 | -0.67 |
| 1424838_at | A330049M08Rik | RIKEN cDNA A330049M08 gene | -1.67 | 0.408 | 1.31 | 0.084 | -1.23 | 0.704 | -1.53 | 0.007 | -0.78 |

| | | | | | | | | | | | |
|---|---|---|---|---|---|---|---|---|---|---|---|
| 1445542_at | 4930579C12Rik | RIKEN cDNA 4930579C12 gene | -1.02 | 0.978 | 1.22 | 0.771 | -1.91 | 0.376 | -2.06 | 0.377 | -0.94 |
| 1427174_at | 3100002L24Rik /// 9 | RIKEN cDNA 3100002L24 gene /// RIKEN cDNA 9230108I15 gene /// hypothetica | -1.12 | 0.091 | 1.01 | 0.919 | -1.21 | 0.393 | -1.88 | 0.103 | -0.8 |
| 1429077_x_at | Rpl21 /// LOC43312 | ribosomal protein L21 /// similar to 60S ribosomal protein L21 /// similar to ribosom | -1.13 | 0.135 | 1.08 | 0.344 | -1.31 | 0.013 | -1.35 | 0.022 | -0.68 |
| 1434049_at | Entpd3 | ectonucleoside triphosphate diphosphohydrolase 3 | -1.97 | 0.191 | -1.27 | 0.647 | 1.43 | 0.122 | -2.49 | 0.078 | -1.07 |
| 1420410_at | Nr5a2 | nuclear receptor subfamily 5, group A, member 2 | 1.18 | 0.818 | -1.1 | 0.341 | -1.56 | 0.372 | -1.17 | 0.767 | -0.66 |
| 1450528_at | B3galt5 | UDP-Gal:betaGlcNAc beta 1,3-galactosyltransferase, polypeptide 5 | -1.02 | 0.979 | 1.42 | 0.647 | -3.06 | 0.107 | -1.03 | 0.968 | -0.92 |
| 1427270_a_at | Bsdc1 | BSD domain containing 1 | -1.07 | 0.35 | -1.18 | 0.213 | -1.06 | 0.763 | 2.35 | 0.236 | -0.24 |
| 1427680_a_at | Nfib | nuclear factor I/B | 1.11 | 0.542 | -1.1 | 0.575 | -1.41 | 0.25 | -1.2 | 0.666 | -0.65 |
| 1437674_at | Rac1 | RAS-related C3 botulinum substrate 1 | -1.27 | 0.459 | -1.08 | 0.792 | 1.02 | 0.834 | -1.41 | 0.286 | -0.69 |
| 1424459_at | Aytl2 | acyltransferase like 2 | -1.1 | 0.371 | -1.05 | 0.784 | -1.14 | 0.566 | 1.34 | 0.287 | -0.49 |
| 1442806_at | 9430030N17Rik | RIKEN cDNA 9430030N17 gene | -1.1 | 0.897 | -1.84 | 0.309 | 1.27 | 0.223 | -1.47 | 0.15 | -0.78 |
| 1451343_at | Vps36 | vacuolar protein sorting 36 (yeast) | -1.19 | 0.249 | 1.05 | 0.682 | -1.2 | 0.369 | -1.7 | 0.007 | -0.76 |
| 1427171_at | Rlf | rearranged L-myc fusion sequence | -1.13 | 0.766 | -1.15 | 0.142 | -1.03 | 0.916 | 1.05 | 0.799 | -0.56 |
| 1456845_at | Oxa1l | Oxidase assembly 1-like | 1.34 | 0.735 | -1.84 | 0.397 | -1.19 | 0.75 | -1.87 | 0.081 | -0.89 |
| 1452805_at | D11Wsu47e | DNA segment, Chr 11, Wayne State University 47, expressed | -1.3 | 0.081 | -1.3 | 0.067 | 1.19 | 0.395 | -1.06 | 0.817 | -0.62 |
| 1442324_at | --- | PREDICTED: Mus musculus hypothetical LOC547070 (LOC547070), mRNA | 1 | 0.937 | -1.16 | 0.488 | -1.16 | 0.229 | -1.61 | 0.171 | -0.73 |
| 1452911_at | Spred1 | sprouty protein with EVH-1 domain 1, related sequence | 1.21 | 0.169 | -1.07 | 0.823 | -1.7 | 0.014 | -1.03 | 0.9 | -0.65 |
| 1422404_x_at | Ifna1 /// Ifna2 /// Ifna | interferon alpha 1 /// interferon alpha 2 /// interferon alpha 4 /// interferon alpha 5 / | 1.07 | 0.22 | -1.06 | 0.923 | -1.39 | 0.552 | -1.1 | 0.843 | -0.62 |
| 1435934_at | Ndufab1 | NADH dehydrogenase (ubiquinone) 1, alpha/beta subcomplex, 1 | -1.36 | 0.041 | -1.03 | 0.773 | 1.03 | 0.909 | -1.52 | 0.087 | -0.72 |
| 1448515_at | Tsn | translin | -1.12 | 0.05 | -1.11 | 0.017 | -1.06 | 0.069 | 1.23 | 0.193 | -0.52 |
| 1419913_at | Strap | Serine/threonine kinase receptor associated protein | -1.03 | 0.941 | -1.09 | 0.605 | -1.2 | 0.467 | 1.34 | 0.309 | -0.49 |
| 1454959_s_at | Gnai1 | guanine nucleotide binding protein, alpha inhibiting 1 | 1.02 | 0.849 | -1.01 | 0.962 | -1.39 | 0.191 | -1.08 | 0.694 | -0.62 |
| 1452209_at | Pkp4 | plakophilin 4 | 1.02 | 0.872 | -1.08 | 0.546 | -1.27 | 0.058 | -1.13 | 0.689 | -0.62 |
| 1416976_at | Stam2 | signal transducing adaptor molecule (SH3 domain and ITAM motif) 2 | -1.24 | 0.163 | -1.15 | 0.108 | 1.05 | 0.547 | -1.16 | 0.157 | -0.62 |
| 1438903_at | Maea | Macrophage erythroblast attacher | -1.42 | 0.427 | -1.14 | 0.763 | 1.15 | 0.773 | -2.53 | 0.305 | -0.99 |
| 1446134_at | C78115 | expressed sequence C78115 | -1.1 | 0.73 | 1.26 | 0.278 | -1.76 | 0 | -1.01 | 0.987 | -0.65 |
| 1448628_at | Scg3 | secretogranin III | -1.05 | 0.758 | -1.24 | 0.277 | -1.03 | 0.961 | 1.08 | 0.902 | -0.56 |
| 1440273_at | --- | 10 days neonate cerebellum cDNA, RIKEN full-length enriched library, clone:B93 | -1.41 | 0.227 | -1.17 | 0.552 | 1.17 | 0.34 | -1.06 | 0.652 | -0.62 |
| 1444937_at | Aff1 | AF4/FMR2 family, member 1 | -1.28 | 0.304 | 1.3 | 0.388 | -1.54 | 0.104 | -1.52 | 0.144 | -0.76 |
| 1425152_s_at | Serpini2 | serine (or cysteine) peptidase inhibitor, clade I, member 2 | -1.01 | 0.904 | 1.02 | 0.939 | -1.39 | 0.3 | -2.12 | 0.345 | -0.87 |
| 1430105_at | 1700042O10Rik | RIKEN cDNA 1700042O10 gene | 1.08 | 0.819 | -1 | 0.987 | -1.54 | 0.371 | -1.21 | 0.707 | -0.67 |
| 1452898_at | --- | --- | -1.17 | 0.164 | 1.12 | 0.176 | -1.32 | 0.021 | -1.34 | 0.003 | -0.68 |
| 1425991_a_at | Ankrd25 | ankyrin repeat domain 25 | 1.19 | 0.656 | -1.35 | 0.582 | -1.25 | 0.234 | -1.02 | 0.944 | -0.61 |
| 1426645_at | Hsp90aa1 | heat shock protein 90kDa alpha (cytosolic), class A member 1 | -1.07 | 0.359 | -1.08 | 0.709 | -1.16 | 0.304 | 1.3 | 0.211 | -0.5 |
| 1452683_at | Dnajc8 /// LOC6692 | DnaJ (Hsp40) homolog, subfamily C, member 8 /// similar to DnaJ (Hsp40) homol | -1.23 | 0.023 | 1.07 | 0.083 | -1.18 | 0.164 | -1.05 | 0.603 | -0.6 |
| 1426287_at | Atxn7 | ataxin 7 | 1.08 | 0.863 | -1.17 | 0.559 | -1.26 | 0.543 | -1.24 | 0.405 | -0.65 |
| 1435737_a_at | Nde1 | nuclear distribution gene E homolog 1 (A nidulans) | -1.15 | 0.493 | -1.08 | 0.597 | -1.07 | 0.662 | 1.39 | 0.294 | -0.48 |
| 1430597_at | Jakmip1 | janus kinase and microtubule interacting protein 1 | -1.18 | 0.609 | -1.29 | 0.667 | 1.11 | 0.876 | -2.37 | 0.127 | -0.93 |
| 1455578_x_at | Rpl41 | ribosomal protein L41 | -1.1 | 0.112 | 1.05 | 0.263 | -1.29 | 0.001 | -1.27 | 0.002 | -0.65 |
| 1449471_at | Kcnmb4 | potassium large conductance calcium-activated channel, subfamily M, beta meml | -1.34 | 0.592 | -1.12 | 0.756 | 1.09 | 0.707 | -1.06 | 0.854 | -0.61 |
| 1454675_at | Thra | thyroid hormone receptor alpha | -1.11 | 0.582 | -1.03 | 0.835 | -1.16 | 0.073 | 1.43 | 0.044 | -0.47 |
| 1429507_at | LOC671838 | similar to naked cuticle 1 homolog | -1.3 | 0.259 | 1.1 | 0.639 | -1.16 | 0.479 | -1.01 | 0.981 | -0.59 |
| 1441293_at | --- | Adult male accessory axillary lymph node cDNA, RIKEN full-length enriched librar | -1.07 | 0.793 | 1.06 | 0.718 | -1.36 | 0.061 | -1.46 | 0.225 | -0.71 |
| 1435311_s_at | Fbxo7 | F-box only protein 7 | -1.11 | 0.795 | -1.13 | 0.63 | -1.06 | 0.793 | 1.33 | 0.17 | -0.49 |
| 1431411_a_at | Rai12 | retinoic acid induced 12 | -1.09 | 0.178 | -1.06 | 0.38 | -1.16 | 0.19 | 1.13 | 0.429 | -0.54 |
| 1438486_at | Tssk4 | testis-specific serine kinase 4 | -2.33 | 0.384 | 1.43 | 0.686 | -1.15 | 0.712 | -2.34 | 0.241 | -1.1 |
| 1430133_at | Tbc1d8b | TBC1 domain family, member 8B | -1.01 | 0.922 | 1.04 | 0.666 | -1.42 | 0.015 | -1.07 | 0.698 | -0.62 |
| 1439106_at | Zfp462 | zinc finger protein 462 | -1.07 | 0.352 | 1.13 | 0.789 | -1.5 | 0.153 | -1.14 | 0.673 | -0.64 |
| 1429981_a_at | 4933426K21Rik | RIKEN cDNA 4933426K21 gene | -1.31 | 0.232 | -2.76 | 0.033 | 1.6 | 0.26 | -1.06 | 0.908 | -0.88 |
| 1451957_at | Il1f10 | interleukin 1 family, member 10 | -1.28 | 0.638 | -1.33 | 0.419 | 1.2 | 0.601 | -1.03 | 0.86 | -0.61 |
| 1452497_a_at | Nfatc3 | nuclear factor of activated T-cells, cytoplasmic, calcineurin-dependent 3 | 1.21 | 0.326 | -1.42 | 0.106 | -1.23 | 0.444 | -1.69 | 0.35 | -0.78 |
| 1457957_at | --- | Transcribed locus | -1.06 | 0.888 | -1.02 | 0.933 | -1.24 | 0.496 | 1.12 | 0.706 | -0.55 |
| 1455767_x_at | Rpl21 | ribosomal protein L21 | -1.08 | 0.418 | 1.08 | 0.292 | -1.39 | 0.001 | -1.5 | 0.029 | -0.72 |

| | | | | | | | | | | | |
|---|---|---|---|---|---|---|---|---|---|---|---|
| 1442346_at | --- | Transcribed locus | -1.22 | 0.844 | 1.47 | 0.46 | -2.29 | 0.011 | -1.95 | 0.322 | -1 |
| 1448181_at | Klf15 | Kruppel-like factor 15 | 1.05 | 0.876 | -1.17 | 0.35 | -1.21 | 0.604 | -1.96 | 0.505 | -0.82 |
| 1428189_at | 5730494M16Rik | RIKEN cDNA 5730494M16 gene | -1.13 | 0.145 | 1.01 | 0.903 | -1.2 | 0.218 | -1.33 | 0.289 | -0.66 |
| 1419262_at | Acad8 | acyl-Coenzyme A dehydrogenase family, member 8 | -1.08 | 0.703 | -1.23 | 0.018 | -1.01 | 0.952 | 1.07 | 0.793 | -0.56 |
| 1428915_at | Sirt5 | sirtuin 5 (silent mating type information regulation 2 homolog) 5 (S. cerevisiae) | -1.15 | 0.411 | -1.26 | 0.095 | 1.07 | 0.741 | -1.39 | 0.463 | -0.68 |
| 1449044_at | Eef1e1 | eukaryotic translation elongation factor 1 epsilon 1 | -1.14 | 0.511 | -1.16 | 0.334 | -1.01 | 0.974 | 1.47 | 0.274 | -0.46 |
| 1427285_s_at | Malat1 | metastasis associated lung adenocarcinoma transcript 1 (non-coding RNA) | -1.39 | 0.165 | -1.58 | 0.227 | 1.38 | 0.117 | -1.23 | 0.388 | -0.71 |
| 1452671_s_at | Lman1 | lectin, mannose-binding, 1 | -1.11 | 0.119 | -1.01 | 0.662 | -1.19 | 0.132 | 1.34 | 0.056 | -0.49 |
| 1428408_a_at | D12Ertd551e | DNA segment, Chr 12, ERATO Doi 551, expressed | 1.01 | 0.765 | -1.02 | 0.864 | -1.35 | 0.04 | -1.05 | 0.739 | -0.6 |
| 1424804_at | BC020002 | cDNA sequence BC020002 | -1.28 | 0.105 | 1.02 | 0.851 | -1.07 | 0.625 | -1.02 | 0.892 | -0.59 |
| 1452923_at | Ndufa12l | Ndufa12-like | -1.01 | 0.854 | -1.05 | 0.621 | -1.27 | 0.133 | 1.05 | 0.872 | -0.57 |
| 1417305_at | Speg | SPEG complex locus | -1.46 | 0.453 | -1.23 | 0.665 | 1.24 | 0.321 | -1.04 | 0.939 | -0.62 |
| 1417211_a_at | 1110032A03Rik | RIKEN cDNA 1110032A03 gene | -1.04 | 0.774 | -1.08 | 0.465 | -1.18 | 0.284 | 1.14 | 0.5 | -0.54 |
| 1449940_a_at | Eif2b4 | eukaryotic translation initiation factor 2B, subunit 4 delta | -1.16 | 0.417 | -1.2 | 0.249 | 1.03 | 0.747 | -1.12 | 0.572 | -0.61 |
| 1424678_at | Actl7b | actin-like 7b | -1.36 | 0.127 | -1.23 | 0.421 | 1.18 | 0.47 | -1.13 | 0.782 | -0.63 |
| 1452398_at | Plce1 | phospholipase C, epsilon 1 | 1.01 | 0.884 | -1.02 | 0.907 | -1.36 | 0.153 | -1.05 | 0.781 | -0.6 |
| 1436943_at | Cyb5d2 | cytochrome b5 domain containing 2 | -1.05 | 0.839 | 1.03 | 0.683 | -1.33 | 0.366 | -1.09 | 0.738 | -0.61 |
| 1441856_x_at | Tysnd1 | trypsin domain containing 1 | -1.12 | 0.665 | -1.13 | 0.453 | -1.05 | 0.8 | 1.19 | 0.494 | -0.53 |
| 1434888_a_at | Matr3 | matrin 3 | -1.02 | 0.744 | 1.04 | 0.408 | -1.41 | 0.047 | -1.05 | 0.672 | -0.61 |
| AFFX-r2-P1-cre-5_a | --- | --- | 1.03 | 0.835 | -1.03 | 0.888 | -1.36 | 0.05 | -1.07 | 0.53 | -0.61 |
| 1416425_at | Pex19 | peroxisome biogenesis factor 19 | -1.07 | 0.641 | -1.19 | 0.248 | -1.05 | 0.638 | 1 | 0.975 | -0.58 |
| 1439446_at | BC048507 | cDNA sequence BC048507 | -1.35 | 0.54 | 1.33 | 0.705 | -1.51 | 0.439 | -1.07 | 0.934 | -0.65 |
| 1457846_at | Cox11 | COX11 homolog, cytochrome c oxidase assembly protein (yeast) | 1.2 | 0.737 | -1.01 | 0.984 | -1.85 | 0.302 | -1.76 | 0.2 | -0.85 |
| 1448358_s_at | Snrpg /// LOC6666( | small nuclear ribonucleoprotein polypeptide G /// similar to small nuclear ribonucle | -1.12 | 0.044 | 1.05 | 0.38 | -1.27 | 0.01 | -1.22 | 0.092 | -0.64 |
| 1425089_at | Kcnc4 | potassium voltage gated channel, Shaw-related subfamily, member 4 | 1.59 | 0.147 | -1.74 | 0.391 | -1.76 | 0.118 | -1.17 | 0.508 | -0.77 |
| 1429544_at | 1700029J11Rik | RIKEN cDNA 1700029J11 gene | -2.21 | 0.28 | -1.31 | 0.658 | 1.52 | 0.327 | -1.25 | 0.394 | -0.81 |
| 1451954_at | --- | --- | -2.42 | 0.035 | -1 | 0.993 | 1.32 | 0.495 | -2.01 | 0.139 | -1.03 |
| 1417782_at | Lass4 | longevity assurance homolog 4 (S. cerevisiae) | -1.15 | 0.276 | 1.02 | 0.941 | -1.19 | 0.309 | -1.68 | 0.1 | -0.75 |
| 1438182_x_at | Asb6 | ankyrin repeat and SOCS box-containing protein 6 | -1.38 | 0.592 | -1.2 | 0.557 | 1.17 | 0.698 | -1.74 | 0.109 | -0.78 |
| 1437500_at | Slc39a12 | Solute carrier family 39 (zinc transporter), member 12 | -1.03 | 0.816 | -1.11 | 0.601 | -1.17 | 0.415 | 1.01 | 0.856 | -0.57 |
| 1459016_at | Igf2bp3 | Insulin-like growth factor 2, binding protein 3 | 1.72 | 0.527 | -1.73 | 0.115 | -2.28 | 0.071 | -1.16 | 0.762 | -0.86 |
| 1436204_at | 1110059G02Rik | RIKEN cDNA 1110059G02 gene | -1.02 | 0.936 | -1.11 | 0.533 | -1.18 | 0.435 | 1.13 | 0.771 | -0.54 |
| 1430288_x_at | Rps21 | ribosomal protein S21 | -1.1 | 0.268 | 1.07 | 0.244 | -1.34 | 0.007 | -1.44 | 0.027 | -0.7 |
| 1446531_at | --- | --- | 1.09 | 0.93 | -1.05 | 0.954 | -1.44 | 0.395 | -1.34 | 0.554 | -0.69 |
| 1427113_s_at | 2700049H19Rik | RIKEN cDNA 2700049H19 gene | -1.22 | 0.318 | 1.05 | 0.806 | -1.16 | 0.091 | -1.13 | 0.382 | -0.62 |
| 1437944_at | Shc2 | Src homology 2 domain-containing transforming protein C2 | -1.26 | 0.066 | 1.18 | 0.678 | -1.31 | 0.638 | -1.01 | 0.988 | -0.6 |
| 1427131_s_at | Lrrc58 | leucine rich repeat containing 58 | -1.08 | 0.657 | 1.06 | 0.579 | -1.35 | 0.043 | -1.13 | 0.573 | -0.62 |
| 1441141_at | C730024G19Rik | RIKEN cDNA C730024G19 gene | -1.07 | 0.35 | 1.04 | 0.857 | -1.33 | 0.149 | -1.32 | 0.26 | -0.67 |
| 1440760_at | Lef1 | Lymphoid enhancer binding factor 1 | -1.15 | 0.409 | 1.36 | 0.573 | -1.96 | 0.09 | -3.32 | 0.166 | -1.27 |
| 1419320_at | Chst5 | carbohydrate (N-acetylglucosamine 6-O) sulfotransferase 5 | 1.4 | 0.644 | -2.29 | 0.279 | -1.11 | 0.584 | -1.55 | 0.197 | -0.89 |
| 1437679_a_at | Glrx2 | glutaredoxin 2 (thioltransferase) | -1.03 | 0.345 | -1.13 | 0.041 | -1.13 | 0.286 | 1.01 | 0.962 | -0.57 |
| 1439079_a_at | Erbb2ip | Erbb2 interacting protein | -1.02 | 0.849 | 1.06 | 0.757 | -1.45 | 0.006 | -1.41 | 0.128 | -0.7 |
| 1460251_at | Fas | Fas (TNF receptor superfamily member) | -1.17 | 0.661 | 1.1 | 0.742 | -1.29 | 0.374 | -1.25 | 0.346 | -0.65 |
| 1433866_x_at | Prdx1 | peroxiredoxin 1 | -1.27 | 0.206 | 1.1 | 0.087 | -1.18 | 0.062 | -1.47 | 0.148 | -0.71 |
| 1447904_s_at | Fnta | farnesyltransferase, CAAX box, alpha | 1.01 | 0.898 | -1.05 | 0.504 | -1.3 | 0.021 | -1.22 | 0.147 | -0.64 |
| 1453240_a_at | Gcap14 | granule cell antiserum positive 14 | -1.05 | 0.77 | 1.02 | 0.628 | -1.32 | 0.033 | -1.19 | 0.715 | -0.63 |
| 1447212_at | Cnot2 | CCR4-NOT transcription complex, subunit 2 | -1.66 | 0.102 | -1.09 | 0.867 | 1.21 | 0.583 | -1.86 | 0.143 | -0.85 |
| 1451192_a_at | Ttc4 | tetratricopeptide repeat domain 4 | 1 | 0.979 | -1.04 | 0.547 | -1.31 | 0.165 | -1.11 | 0.611 | -0.61 |
| 1440122_at | LOC666504 /// LOC | hypothetical protein LOC666504 /// hypothetical protein LOC672638 /// hypothetic | 1.11 | 0.89 | -1.04 | 0.918 | -1.5 | 0.583 | -1.27 | 0.408 | -0.67 |
| 1435580_at | C230081A13Rik | RIKEN cDNA C230081A13 gene | -1.04 | 0.15 | 1.08 | 0.479 | -1.44 | 0.203 | -1.24 | 0.48 | -0.66 |
| 1415840_at | Elovl5 | ELOVL family member 5, elongation of long chain fatty acids (yeast) | 1.15 | 0.126 | -1.25 | 0.149 | -1.28 | 0.093 | -1.47 | 0.292 | -0.71 |
| 1454971_x_at | Tsc22d1 | TSC22 domain family, member 1 | 1.14 | 0.664 | -1.22 | 0.155 | -1.29 | 0.009 | -1.36 | 0.137 | -0.68 |
| 1427895_at | 2310004N24Rik | RIKEN cDNA 2310004N24 gene | -1.01 | 0.913 | -1.28 | 0.582 | -1.04 | 0.811 | 1.07 | 0.668 | -0.56 |

| | | | | | | | | | | | |
|---|---|---|---|---|---|---|---|---|---|---|---|
| 1452026_a_at | Pla2g12a | phospholipase A2, group XIIA | -1.02 | 0.911 | -1.32 | 0.168 | -1 | 0.969 | 2.34 | 0.306 | -0.25 |
| 1434907_at | Tbpl1 | TATA box binding protein-like 1 | -1.25 | 0.366 | 1.05 | 0.642 | -1.13 | 0.101 | -1.02 | 0.805 | -0.59 |
| 1424057_at | Gdap2 | ganglioside-induced differentiation-associated-protein 2 | -1.16 | 0.397 | -1.06 | 0.681 | -1.07 | 0.48 | 1.09 | 0.448 | -0.55 |
| 1460635_at | Fastk | Fas-activated serine/threonine kinase | -1.26 | 0.314 | -1.02 | 0.911 | -1.05 | 0.748 | 1.02 | 0.896 | -0.58 |
| 1458823_at | Lrig1 | Leucine-rich repeats and immunoglobulin-like domains 1 | 1.4 | 0.622 | -1.21 | 0.768 | -1.95 | 0.186 | -1.01 | 0.986 | -0.69 |
| 1454967_at | --- | Transcribed locus | 1.01 | 0.847 | -1.06 | 0.578 | -1.29 | 0.288 | -1.29 | 0.577 | -0.65 |
| 1455376_at | 1300010F03Rik | RIKEN cDNA 1300010F03 gene | 1.04 | 0.774 | -1.4 | 0.246 | -1.02 | 0.857 | -1.19 | 0.236 | -0.64 |
| 1438502_x_at | Rps17 /// LOC3830 | ribosomal protein S17 /// similar to 40S ribosomal protein S17 | -1.43 | 0.258 | -1.01 | 0.962 | 1.05 | 0.674 | -1.63 | 0.083 | -0.76 |
| 1443513_at | --- | Transcribed locus | -1.03 | 0.88 | -1.03 | 0.898 | -1.25 | 0.543 | 1.39 | 0.172 | -0.48 |
| 1452787_a_at | Prmt1 | protein arginine N-methyltransferase 1 | -1.28 | 0.365 | -1.15 | 0.37 | 1.08 | 0.678 | -1.55 | 0.227 | -0.73 |
| 1423118_at | 1200014J11Rik | RIKEN cDNA 1200014J11 gene | -1.17 | 0.262 | 1.03 | 0.89 | -1.18 | 0.163 | -1.39 | 0.104 | -0.68 |
| 1417239_at | Cetn3 | centrin 3 | -1.21 | 0.058 | -1.02 | 0.789 | -1.08 | 0.273 | 1.08 | 0.72 | -0.56 |
| 1435675_at | Tbc1d12 | TBC1D12: TBC1 domain family, member 12 | -1.02 | 0.817 | -1.1 | 0.197 | -1.17 | 0.236 | 1.28 | 0.353 | -0.5 |
| 1415708_at | Tug1 | taurine upregulated gene 1 | 1.01 | 0.92 | -1.02 | 0.889 | -1.34 | 0.022 | -1.2 | 0.295 | -0.64 |
| 1424615_at | Frag1 | FGF receptor activating protein 1 | 1.04 | 0.441 | -1.11 | 0.287 | -1.26 | 0.137 | -1.29 | 0.43 | -0.66 |
| 1453969_at | Prlpc4 | prolactin-like protein C 4 | -2.13 | 0.386 | -1.02 | 0.752 | 1.29 | 0.319 | -1.12 | 0.083 | -0.75 |
| 1444217_at | Mrpl38 | mitochondrial ribosomal protein L38 | -1.06 | 0.88 | -1.21 | 0.218 | -1.04 | 0.844 | 1.09 | 0.518 | -0.55 |
| 1452374_at | Zfp322a | zinc finger protein 322a | -1.08 | 0.411 | 1.04 | 0.58 | -1.3 | 0.001 | -1.25 | 0.173 | -0.65 |
| 1439368_a_at | Slc9a3r2 | solute carrier family 9 (sodium/hydrogen exchanger), isoform 3 regulator 2 | 1.01 | 0.958 | -1.12 | 0.508 | -1.2 | 0.195 | -1.06 | 0.884 | -0.59 |
| 1428076_s_at | Ndufb4 | NADH dehydrogenase (ubiquinone) 1 beta subcomplex 4 | -1.1 | 0.175 | -1.05 | 0.399 | -1.15 | 0.242 | 1.04 | 0.85 | -0.56 |
| 1426003_at | Ntrk3 | neurotrophic tyrosine kinase, receptor, type 3 | 1.02 | 0.931 | -1.11 | 0.708 | -1.23 | 0.622 | -1.04 | 0.952 | -0.59 |
| 1458847_at | --- | --- | -1.14 | 0.723 | 1.17 | 0.585 | -1.45 | 0.097 | -1.17 | 0.573 | -0.65 |
| 1456247_x_at | Plp2 /// LOC620648 | proteolipid protein 2 /// similar to Proteolipid protein 2 /// similar to Proteolipid prot | -1.32 | 0.561 | 1.32 | 0.31 | -1.51 | 0.411 | -1.24 | 0.708 | -0.69 |
| 1423148_at | Skp1a | S-phase kinase-associated protein 1A | -1.03 | 0.779 | -1.14 | 0.105 | -1.12 | 0.249 | 1.07 | 0.575 | -0.56 |
| 1437878_s_at | Ccdc39 /// Ttc14 | coiled-coil domain containing 39 /// tetratricopeptide repeat domain 14 | 1.08 | 0.623 | -1.2 | 0.206 | -1.22 | 0.073 | -1.15 | 0.467 | -0.62 |
| 1448082_at | Clcn1 | Chloride channel 1 | -2.57 | 0.036 | 1.72 | 0.365 | -1.61 | 0.505 | -1.9 | 0.278 | -1.09 |
| 1455213_at | 4930488E11Rik | RIKEN cDNA 4930488E11 gene | -1.22 | 0.283 | -1.1 | 0.7 | 1.01 | 0.976 | -2 | 0.08 | -0.83 |
| 1445523_at | Gje1 | Gap junction membrane channel protein epsilon 1 | -1.89 | 0.281 | 1.52 | 0.028 | -1.46 | 0.42 | -1.76 | 0.082 | -0.9 |
| 1438271_at | Lpp | LIM domain containing preferred translocation partner in lipoma | 1.05 | 0.785 | -1.01 | 0.966 | -1.44 | 0.103 | -1.21 | 0.396 | -0.65 |
| 1429237_at | Stx16 | syntaxin 16 | -1.28 | 0.11 | -1.1 | 0.139 | 1.04 | 0.727 | -1.67 | 0.164 | -0.75 |
| 1444013_at | 5930412G12Rik | RIKEN cDNA 5930412G12 gene | -4.22 | 0.076 | 1.72 | 0.244 | -1.28 | 0.759 | -1.13 | 0.845 | -1.23 |
| 1430780_a_at | Pmm1 | phosphomannomutase 1 | -1.18 | 0.2 | -1.1 | 0.879 | -1.02 | 0.935 | 1.49 | 0.326 | -0.45 |
| 1415691_at | Dlgh1 | discs, large homolog 1 (Drosophila) | 1.01 | 0.747 | -1.04 | 0.514 | -1.31 | 0.021 | -1.02 | 0.885 | -0.59 |
| 1437357_at | Ythdc2 | YTH domain containing 2 | -1.18 | 0.202 | -1.03 | 0.905 | -1.09 | 0.28 | 1.07 | 0.649 | -0.56 |
| 1444963_at | --- | --- | -1.54 | 0.375 | -1.21 | 0.445 | 1.26 | 0.27 | -1.87 | 0.059 | -0.84 |
| 1430290_at | Rps15a /// LOC434 | ribosomal protein S15a /// similar to ribosomal protein S15a /// similar to ribosoma | -1.25 | 0.304 | 1.35 | 0.569 | -1.7 | 0.269 | -1.69 | 0.209 | -0.82 |
| 1443455_at | Cuedc1 | CUE domain containing 1 | -1.07 | 0.754 | 1.22 | 0.688 | -1.73 | 0.153 | -2.18 | 0.225 | -0.94 |
| 1419302_at | Heyl | hairy/enhancer-of-split related with YRPW motif-like | -1.34 | 0.31 | -1.08 | 0.77 | 1.07 | 0.632 | -1.15 | 0.099 | -0.63 |
| 1452111_at | Mrps35 | mitochondrial ribosomal protein S35 | -1.29 | 0.106 | -1.35 | 0.02 | 1.22 | 0.217 | -1.06 | 0.588 | -0.62 |
| 1453435_a_at | Fmo2 | flavin containing monooxygenase 2 | -1.25 | 0.143 | -1.13 | 0.398 | 1.05 | 0.77 | -1.16 | 0.455 | -0.62 |
| 1443775_x_at | Zfp406 | zinc finger protein 406 | -1.48 | 0.568 | -1.06 | 0.876 | 1.12 | 0.665 | -1.38 | 0.652 | -0.7 |
| 1426133_a_at | Mitd1 | MIT, microtubule interacting and transport, domain containing 1 | -1.23 | 0.415 | 1.06 | 0.712 | -1.17 | 0.264 | -1.35 | 0.16 | -0.67 |
| 1416106_at | Kti12 | KTI12 homolog, chromatin associated (S. cerevisiae) | -1.2 | 0.128 | -1.23 | 0.053 | 1.09 | 0.481 | -1.09 | 0.587 | -0.61 |
| 1451277_at | Zadh2 | zinc binding alcohol dehydrogenase, domain containing 2 | 1.02 | 0.72 | -1.33 | 0.087 | -1.04 | 0.745 | -1.24 | 0.136 | -0.65 |
| 1460184_at | Hadhsc | L-3-hydroxyacyl-Coenzyme A dehydrogenase, short chain | -1.17 | 0.033 | -1.37 | 0.033 | 1.15 | 0.303 | -1.28 | 0.145 | -0.67 |
| 1430334_at | Zfp192 | zinc finger protein 192 | 1.2 | 0.67 | -1.7 | 0.177 | -1.06 | 0.922 | -1.51 | 0.469 | -0.77 |
| 1456431_at | Zfp64 | zinc finger protein 64 | -1.29 | 0.573 | 1.19 | 0.711 | -1.3 | 0.319 | -2.15 | 0.018 | -0.89 |
| 1449113_at | Gpbp1l1 | GC-rich promoter binding protein 1-like 1 | -1.07 | 0.709 | -1.12 | 0.348 | -1.1 | 0.632 | 1.05 | 0.57 | -0.56 |
| 1428315_at | Ebna1bp2 | EBNA1 binding protein 2 | -1.13 | 0.351 | -1.22 | 0.025 | 1.03 | 0.74 | -1.25 | 0.131 | -0.64 |
| 1458302_at | --- | Transcribed locus | -1.11 | 0.544 | -1.22 | 0.342 | 1.02 | 0.883 | -1.35 | 0.335 | -0.67 |
| 1440115_at | Olfm1 | Olfactomedin 1 | -1.09 | 0.854 | -1.38 | 0.615 | 1.1 | 0.833 | -1.41 | 0.136 | -0.7 |
| 1417694_at | Gab1 | growth factor receptor bound protein 2-associated protein 1 | -1.12 | 0.302 | -1.04 | 0.78 | -1.13 | 0.677 | 1.99 | 0.207 | -0.33 |
| 1428277_at | Otud6b | OTU domain containing 6B | -1.12 | 0.09 | -1.02 | 0.826 | -1.16 | 0.275 | 1.12 | 0.512 | -0.54 |

| | | | | | | | | | | | |
|---|---|---|---|---|---|---|---|---|---|---|---|
| 1423599_a_at | Pdcl | phosducin-like | -1.12 | 0.379 | -1.08 | 0.573 | -1.09 | 0.438 | 1.1 | 0.496 | -0.55 |
| 1443046_at | Abcd3 | ATP-binding cassette, sub-family D (ALD), member 3 | 1.29 | 0.537 | -1.46 | 0.542 | -1.31 | 0.422 | -3.52 | 0.01 | -1.25 |
| 1457030_at | Mirg | miRNA containing gene | -1.23 | 0.718 | 1.38 | 0.635 | -1.82 | 0.333 | -1.36 | 0.7 | -0.76 |
| 1428061_at | Hat1 | histone aminotransferase 1 | -1.12 | 0.39 | -1.19 | 0.229 | -1 | 0.994 | 1.36 | 0.329 | -0.49 |
| 1431785_at | Rnaset2 | ribonuclease T2 | -1.15 | 0.661 | -1.58 | 0.096 | 1.24 | 0.582 | -1.07 | 0.838 | -0.64 |
| 1427120_at | Zfp26 | zinc finger protein 26 | 1.16 | 0.109 | -1.17 | 0.109 | -1.38 | 0.055 | -1.2 | 0.196 | -0.65 |
| 1446674_at | Kns2 | Kinesin 2 | -1.01 | 0.835 | 1.05 | 0.91 | -1.43 | 0.145 | -1.7 | 0.233 | -0.77 |
| 1452703_at | 4631427C17Rik | RIKEN cDNA 4631427C17 gene | -1.11 | 0.028 | -1.13 | 0.093 | -1.05 | 0.516 | 1.07 | 0.449 | -0.55 |
| 1447831_s_at | Mtmr7 | myotubularin related protein 7 | -1.18 | 0.261 | -1.05 | 0.855 | -1.06 | 0.465 | 1.23 | 0.303 | -0.52 |
| 1418048_at | 1110059G10Rik | RIKEN cDNA 1110059G10 gene | 1 | 0.823 | -1.08 | 0.194 | -1.24 | 0.057 | -1.17 | 0.176 | -0.62 |
| 1420377_at | St8sia2 | ST8 alpha-N-acetyl-neuraminide alpha-2,8-sialyltransferase 2 | -1.5 | 0.017 | -1.23 | 0.484 | 1.26 | 0.419 | -1.15 | 0.673 | -0.66 |
| 1435581_at | Bace2 | beta-site APP-cleaving enzyme 2 | -1.21 | 0.816 | 1.07 | 0.766 | -1.19 | 0.489 | -1.46 | 0.121 | -0.7 |
| 1451379_at | Rab22a | RAB22A, member RAS oncogene family | -1.04 | 0.714 | -1.13 | 0.185 | -1.12 | 0.309 | 1.18 | 0.232 | -0.53 |
| 1441221_at | 1700008J07Rik | RIKEN cDNA 1700008J07 gene | 1.45 | 0.359 | -1.43 | 0.29 | -1.71 | 0.332 | -1.09 | 0.521 | -0.69 |
| 1452179_at | Phf17 | PHD finger protein 17 | -1.07 | 0.755 | -1.08 | 0.703 | -1.14 | 0.116 | 1.01 | 0.954 | -0.57 |
| 1457183_at | Slc6a1 | solute carrier family 6 (neurotransmitter transporter, GABA), member 1 | -1.3 | 0.744 | -1.18 | 0.793 | 1.12 | 0.773 | -1.81 | 0.149 | -0.79 |
| 1441829_s_at | Akap10 | A kinase (PRKA) anchor protein 10 | 1.15 | 0.449 | -1.2 | 0.188 | -1.33 | 0.128 | -1.33 | 0.091 | -0.68 |
| 1430419_at | 2310031A07Rik | RIKEN cDNA 2310031A07 gene | -1.47 | 0.111 | -1.04 | 0.878 | 1.1 | 0.727 | -1.59 | 0.186 | -0.75 |
| 1432695_at | 4921527H02Rik | RIKEN cDNA 4921527H02 gene | 1.04 | 0.953 | -1.15 | 0.713 | -1.21 | 0.281 | -3.76 | 0.016 | -1.27 |
| 1449752_at | Spsb1 | SplA/ryanodine receptor domain and SOCS box containing 1 | -1.15 | 0.29 | 1.47 | 0.396 | -2.54 | 0.089 | -2.12 | 0.221 | -1.08 |
| 1457232_at | Fbxl21 | F-box and leucine-rich repeat protein 21 | -1.34 | 0.002 | 1 | 0.999 | -1.01 | 0.987 | -1.2 | 0.805 | -0.64 |
| 1438265_at | --- | --- | -1.31 | 0.6 | -1.2 | 0.538 | 1.14 | 0.791 | -1.7 | 0.455 | -0.77 |
| 1436053_at | Tbc1d22b | TBC1 domain family, member 22B | -1.02 | 0.935 | 1.05 | 0.858 | -1.4 | 0.059 | -1.08 | 0.087 | -0.61 |
| 1418165_at | Itlna | intelectin a | 1.07 | 0.874 | -1.12 | 0.558 | -1.29 | 0.183 | -1.41 | 0.13 | -0.69 |
| 1423584_at | Igfbp7 | insulin-like growth factor binding protein 7 | -1.15 | 0.07 | 1.19 | 0.054 | -1.48 | 0.038 | -1.43 | 0.184 | -0.72 |
| 1443903_at | Ppm1a | Protein phosphatase 1A, magnesium dependent, alpha isoform | -1.62 | 0.18 | 1.24 | 0.626 | -1.13 | 0.72 | -1.63 | 0.389 | -0.79 |
| 1439782_s_at | D930048N14Rik | RIKEN cDNA D930048N14 gene | -1.09 | 0.648 | -1.22 | 0.764 | 1.01 | 0.991 | -2.35 | 0.267 | -0.91 |
| 1443172_at | Orc1l | origin recognition complex, subunit 1-like (S.cereviaiae) | -1.1 | 0.652 | -1.05 | 0.876 | -1.14 | 0.628 | 3.44 | 0.276 | 0.04 |
| 1457069_at | Ascc3 | activating signal cointegrator 1 complex subunit 3 | -1.1 | 0.828 | -1.06 | 0.718 | -1.13 | 0.42 | 1.04 | 0.737 | -0.56 |
| 1432353_at | 4933421B21Rik | RIKEN cDNA 4933421B21 gene | -1.25 | 0.294 | 1.07 | 0.879 | -1.15 | 0.658 | -1.3 | 0.597 | -0.66 |
| 1459669_at | --- | Transcribed locus | -1.04 | 0.864 | -1.4 | 0.514 | 1.06 | 0.86 | -1.15 | 0.38 | -0.63 |
| 1424996_at | Cflar | CASP8 and FADD-like apoptosis regulator | 1.06 | 0.646 | -1.03 | 0.828 | -1.4 | 0.176 | -1.51 | 0.129 | -0.72 |
| 1449229_a_at | Cdkl2 | cyclin-dependent kinase-like 2 (CDC2-related kinase) | -1.11 | 0.506 | 1.07 | 0.594 | -1.29 | 0.016 | -1.11 | 0.76 | -0.61 |
| 1458786_at | C79250 | expressed sequence C79250 | -1.2 | 0.829 | -2.03 | 0.518 | 1.42 | 0.316 | -1.59 | 0.032 | -0.85 |
| 1426468_at | 0610037L13Rik | RIKEN cDNA 0610037L13 gene | -1.1 | 0.005 | 1.05 | 0.126 | -1.28 | 0.024 | -1.15 | 0.066 | -0.62 |
| 1433835_at | Ppp3cb | protein phosphatase 3, catalytic subunit, beta isoform | -1.19 | 0.288 | -1.11 | 0.027 | -1 | 0.994 | 1.67 | 0.219 | -0.41 |
| 1452743_at | --- | --- | -1.07 | 0.777 | -1.05 | 0.679 | -1.17 | 0.215 | 2.52 | 0.16 | -0.19 |
| 1419809_s_at | Cog4 | component of oligomeric golgi complex 4 | 1.07 | 0.562 | -1.25 | 0.26 | -1.15 | 0.236 | -1.02 | 0.799 | -0.59 |
| 1441085_at | Rora | RAR-related orphan receptor alpha | -1.66 | 0.331 | 1.29 | 0.72 | -1.18 | 0.74 | -1.09 | 0.667 | -0.66 |
| 1447298_at | C130071C03Rik | RIKEN cDNA C130071C03 gene | -1.04 | 0.967 | 1.08 | 0.924 | -1.43 | 0.434 | -3.15 | 0.117 | -1.14 |
| 1448372_a_at | Tmem4 | transmembrane protein 4 | -1.11 | 0.205 | -1.11 | 0.017 | -1.07 | 0.526 | 1.2 | 0.086 | -0.52 |
| 1440820_x_at | Tmco2 | transmembrane and coiled-coil domains 2 | 1.37 | 0.354 | -1.11 | 0.814 | -2.16 | 0.059 | -1.06 | 0.785 | -0.74 |
| 1423994_at | Kif1b | kinesin family member 1B | -1.06 | 0.484 | -1.3 | 0.029 | 1.03 | 0.809 | -1.24 | 0.032 | -0.64 |
| 1427642_at | Trpm6 | transient receptor potential cation channel, subfamily M, member 6 | 1.23 | 0.357 | -1.69 | 0.204 | -1.09 | 0.721 | -1.33 | 0.574 | -0.72 |
| 1438850_at | Cldn1 | claudin 1 | -1.02 | 0.935 | 1.07 | 0.683 | -1.44 | 0.561 | -1.94 | 0.38 | -0.83 |
| 1437983_at | --- | --- | -1.11 | 0.401 | 1.19 | 0.41 | -1.54 | 0.352 | -1.46 | 0.435 | -0.73 |
| 1434131_at | Rufy1 | RUN and FYVE domain containing 1 | -1.17 | 0.337 | -1.14 | 0.222 | 1.01 | 0.806 | -1.54 | 0.098 | -0.71 |
| 1427335_at | 6720456H20Rik | RIKEN cDNA 6720456H20 gene | -1.16 | 0.582 | -1.27 | 0.375 | 1.09 | 0.742 | -1.16 | 0.566 | -0.62 |
| 1441544_at | Cnnm3 | Cyclin M3 | -1.1 | 0.831 | -1.58 | 0.011 | 1.19 | 0.489 | -1.21 | 0.382 | -0.67 |
| 1428628_at | 1190002A17Rik | RIKEN cDNA 1190002A17 gene | -1.4 | 0.482 | -1.5 | 0.62 | 1.36 | 0.386 | -1.43 | 0.426 | -0.74 |
| 1434383_at | Pja2 | praja 2, RING-H2 motif containing | -1.05 | 0.602 | -1.01 | 0.76 | -1.25 | 0.003 | 1.06 | 0.543 | -0.56 |
| 1438740_at | Nmt2 | N-myristoyltransferase 2 | -1.26 | 0.254 | -1.19 | 0.572 | 1.11 | 0.462 | -1.13 | 0.607 | -0.62 |
| 1452828_at | Fbxo21 | F-box only protein 21 | -1.31 | 0.08 | -1.26 | 0.207 | 1.18 | 0.496 | -1.54 | 0.155 | -0.73 |

| | | | | | | | | | | | |
|---|---|---|---|---|---|---|---|---|---|---|---|
| 1434688_x_at | Drg1 | developmentally regulated GTP binding protein 1 | -1.03 | 0.871 | -1.08 | 0.297 | -1.18 | 0.246 | 1.08 | 0.659 | -0.55 |
| 1448007_at | Baz2b | bromodomain adjacent to zinc finger domain, 2B | 1.12 | 0.624 | -1.27 | 0.189 | -1.2 | 0.413 | -1.3 | 0.136 | -0.66 |
| 1450626_at | Manba | mannosidase, beta A, lysosomal | -1.2 | 0.093 | 1.01 | 0.957 | -1.11 | 0.447 | -1.38 | 0.261 | -0.67 |
| 1448970_at | 1200007B05Rik | RIKEN cDNA 1200007B05 gene | -1.07 | 0.319 | -1.11 | 0.327 | -1.11 | 0.36 | 1.03 | 0.719 | -0.56 |
| 1431754_at | 4930578N16Rik | RIKEN cDNA 4930578N16 gene | -1.06 | 0.465 | -1.47 | 0.202 | 1.12 | 0.728 | -1.43 | 0.17 | -0.71 |
| 1448486_at | Mut | methylmalonyl-Coenzyme A mutase | -1.1 | 0.18 | -1.11 | 0.214 | -1.08 | 0.432 | 1.25 | 0.241 | -0.51 |
| 1439025_at | Zfp446 | zinc finger protein 446 | -1.23 | 0.41 | -1.14 | 0.65 | 1.05 | 0.703 | -1.06 | 0.611 | -0.6 |
| 1420907_at | Cd2ap | CD2-associated protein | -1.12 | 0.482 | 1.07 | 0.527 | -1.29 | 0.072 | -1.38 | 0.173 | -0.68 |
| 1428366_at | 1600027N09Rik | RIKEN cDNA 1600027N09 gene | -1.15 | 0.314 | 1.02 | 0.909 | -1.17 | 0.354 | -1.29 | 0.276 | -0.65 |
| 1417865_at | Tnfaip1 | tumor necrosis factor, alpha-induced protein 1 (endothelial) | -1.04 | 0.801 | 1.06 | 0.599 | -1.4 | 0.133 | -1.44 | 0.36 | -0.71 |
| 1441661_at | --- | --- | -1.26 | 0.3 | 1.18 | 0.627 | -1.3 | 0.571 | -1.19 | 0.652 | -0.64 |
| 1449889_a_at | Ociad1 | OCIA domain containing 1 | -1.31 | 0.214 | -1.34 | 0.062 | 1.24 | 0.39 | -1.38 | 0.248 | -0.7 |
| 1446354_at | C130098B18Rik | RIKEN cDNA C130098B18 gene | -1.34 | 0.147 | 1.19 | 0.608 | -1.24 | 0.568 | -1.13 | 0.785 | -0.63 |
| 1432705_at | 5330428N10Rik | RIKEN cDNA 5330428N10 gene | -1.07 | 0.637 | -1.11 | 0.65 | -1.1 | 0.85 | 1.23 | 0.732 | -0.51 |
| 1457826_a_at | 1810043H04Rik | RIKEN cDNA 1810043H04 gene | -1.09 | 0.383 | 1.07 | 0.092 | -1.34 | 0.071 | -1.48 | 0.209 | -0.71 |
| 1439224_at | Ankib1 | Ankyrin repeat and IBR domain containing 1 | 1.44 | 0.57 | -1.36 | 0.595 | -1.77 | 0.182 | -1.47 | 0.533 | -0.79 |
| 1425819_at | Zbtb7c | zinc finger and BTB domain containing 7C | -1.37 | 0.222 | -1.3 | 0.642 | 1.24 | 0.528 | -1.12 | 0.861 | -0.64 |
| 1452507_at | Dlx6 | distal-less homeobox 6 | 1.56 | 0.488 | -1.89 | 0.141 | -1.53 | 0.356 | -1.07 | 0.834 | -0.73 |
| 1423764_s_at | Mrpl37 | mitochondrial ribosomal protein L37 | -1.13 | 0.494 | -1.07 | 0.53 | -1.08 | 0.468 | 1.01 | 0.939 | -0.57 |
| 1456996_at | 1190005F20Rik | RIKEN cDNA 1190005F20 gene | -1.01 | 0.932 | -1.42 | 0.261 | 1.04 | 0.6 | -1.2 | 0.071 | -0.64 |
| 1420095_s_at | Zipro1 | zinc finger proliferation 1 | -1.03 | 0.527 | 1.06 | 0.576 | -1.41 | 0.015 | -1.02 | 0.842 | -0.6 |
| 1420576_at | Edg4 | endothelial differentiation, lysophosphatidic acid G-protein-coupled receptor 4 | 1.1 | 0.629 | -1.44 | 0.666 | -1.06 | 0.876 | -1.39 | 0.289 | -0.7 |
| 1425665_a_at | Srp54 /// LOC6651 | signal recognition particle 54 /// similar to signal recognition particle 54 | -1.38 | 0.137 | -1 | 0.992 | 1.02 | 0.806 | -1.21 | 0.11 | -0.64 |
| 1444221_at | Tbc1d12 | TBC1D12: TBC1 domain family, member 12 | 1.06 | 0.766 | -1.31 | 0.266 | -1.09 | 0.603 | -1.04 | 0.785 | -0.59 |
| 1435413_x_at | 2700060E02Rik | RIKEN cDNA 2700060E02 gene | 1 | 0.966 | -1.03 | 0.629 | -1.31 | 0.025 | -1.55 | 0.044 | -0.72 |
| 1423208_at | Tmem167 | transmembrane protein 167 | -1.11 | 0.176 | -1.05 | 0.82 | -1.13 | 0.459 | 1.01 | 0.949 | -0.57 |
| 1447618_at | B630019K06Rik | RIKEN cDNA B630019K06 gene | 1.04 | 0.058 | -1.26 | 0.057 | -1.1 | 0.308 | -1.24 | 0.548 | -0.64 |
| 1446775_at | --- | --- | -1.45 | 0.224 | -1.1 | 0.352 | 1.14 | 0.365 | -1.38 | 0.501 | -0.7 |
| 1417690_at | Prkag1 | protein kinase, AMP-activated, gamma 1 non-catalytic subunit | -1.21 | 0.315 | -1.06 | 0.736 | -1.03 | 0.813 | 1.8 | 0.174 | -0.37 |
| 1437124_at | A630052C17Rik | RIKEN cDNA A630052C17 gene | -1.06 | 0.57 | -1.27 | 0.153 | 1.01 | 0.972 | -1.22 | 0.109 | -0.63 |
| 1448509_at | 3110001A13Rik | RIKEN cDNA 3110001A13 gene | -1.14 | 0.501 | -1.5 | 0.137 | 1.2 | 0.007 | -1.83 | 0.055 | -0.82 |
| 1424433_at | Msrb2 | methionine sulfoxide reductase B2 | -1.02 | 0.867 | -1.32 | 0.01 | 1.01 | 0.978 | -1.5 | 0.085 | -0.71 |
| 1436551_at | Fgfr1 | fibroblast growth factor receptor 1 | -1.24 | 0.274 | -1.19 | 0.781 | 1.1 | 0.48 | -1.37 | 0.547 | -0.68 |
| 1455602_x_at | 1110028C15Rik | RIKEN cDNA 1110028C15 gene | -1.36 | 0.142 | 1.21 | 0.197 | -1.25 | 0.04 | -1.2 | 0.168 | -0.65 |
| 1420454_at | Rai1 | retinoic acid induced 1 | 1.34 | 0.502 | -1.31 | 0.742 | -1.56 | 0.083 | -1.01 | 0.967 | -0.64 |
| 1416585_at | Ruvbl1 | RuvB-like protein 1 | -1.14 | 0.359 | -1.17 | 0.02 | 1.01 | 0.876 | -1.06 | 0.728 | -0.59 |
| 1447626_x_at | Arid3b | AT rich interactive domain 3B (Bright like) | 1.07 | 0.208 | -1.33 | 0.652 | -1.09 | 0.633 | -2.38 | 0.481 | -0.93 |
| 1445641_at | Elovl6 | ELOVL family member 6, elongation of long chain fatty acids (yeast) | 1.08 | 0.67 | -1.12 | 0.777 | -1.29 | 0.263 | -1.19 | 0.018 | -0.63 |
| 1437912_at | Iqsec3 | IQ motif and Sec7 domain 3 | -1.3 | 0.521 | 1.11 | 0.196 | -1.15 | 0.207 | -1.01 | 0.913 | -0.59 |
| 1446176_at | Mrg1 | Myeloid ecotropic viral integration site-related gene 1 | 1.18 | 0.402 | -1.21 | 0.818 | -1.36 | 0.488 | -1.14 | 0.831 | -0.63 |
| 1445857_at | BC026585 | cDNA sequence BC026585 | -1.24 | 0.17 | -1.39 | 0.475 | 1.22 | 0.618 | -1.28 | 0.518 | -0.67 |
| 1437113_s_at | Pld1 | phospholipase D1 | -1.03 | 0.802 | 1.15 | 0.419 | -1.62 | 0.136 | -1.29 | 0.626 | -0.7 |
| 1422559_at | Ube2n | ubiquitin-conjugating enzyme E2N | -1.2 | 0.13 | 1.02 | 0.789 | -1.12 | 0.317 | -1.03 | 0.834 | -0.58 |
| 1450948_a_at | Mrpl1 | mitochondrial ribosomal protein L1 | -1.15 | 0.213 | -1.18 | 0.124 | 1.03 | 0.837 | -1.09 | 0.552 | -0.6 |
| 1440936_at | Serac1 | serine active site containing 1 | -1.12 | 0.24 | 1.06 | 0.807 | -1.26 | 0.248 | -1.11 | 0.565 | -0.61 |
| 1416059_at | Sec23b | SEC23B (S. cerevisiae) | -1.09 | 0.073 | -1.14 | 0.426 | -1.05 | 0.588 | 1.03 | 0.213 | -0.56 |
| 1433718_a_at | Cbx1 | chromobox homolog 1 (Drosophila HP1 beta) | -1.07 | 0.146 | 1.01 | 0.926 | -1.26 | 0.099 | -1.11 | 0.171 | -0.61 |
| 1441136_at | --- | 7 days neonate cerebellum cDNA, RIKEN full-length enriched library, clone:A730 | -1.01 | 0.965 | -1.52 | 0.228 | 1.09 | 0.707 | -1.24 | 0.201 | -0.67 |
| 1449536_at | Kcnn1 | potassium intermediate/small conductance calcium-activated channel, subfamily | -1.34 | 0.292 | 1.09 | 0.853 | -1.1 | 0.672 | -1.11 | 0.875 | -0.62 |
| 1452081_a_at | 9130017N09Rik | RIKEN cDNA 9130017N09 gene | 1.18 | 0.615 | -1.69 | 0.193 | -1.02 | 0.903 | -1.56 | 0.245 | -0.77 |
| 1437868_at | BC023892 | cDNA sequence BC023892 | 1.02 | 0.957 | -1.21 | 0.506 | -1.12 | 0.39 | -1.64 | 0.13 | -0.74 |
| 1441305_at | --- | Adult male corpus striatum cDNA, RIKEN full-length enriched library, clone:C030 | 1.03 | 0.815 | -1.04 | 0.891 | -1.32 | 0.25 | -1.38 | 0.543 | -0.68 |
| 1453288_at | Atf6 | activating transcription factor 6 | -1.1 | 0.635 | -1.42 | 0.194 | 1.13 | 0.8 | -1.07 | 0.792 | -0.62 |

| | | | | | | | | | | | |
|---|---|---|---|---|---|---|---|---|---|---|---|
| 1429479_at | 6430590I03Rik | RIKEN cDNA 6430590I03 gene | -1.13 | 0.672 | -1 | 0.992 | -1.17 | 0.513 | 1.19 | 0.345 | -0.52 |
| 1426682_at | Cnot6 | CCR4-NOT transcription complex, subunit 6 | -1.03 | 0.702 | -1.01 | 0.894 | -1.28 | 0.049 | 1.14 | 0.205 | -0.54 |
| 1444921_at | D15Ertd466e | DNA segment, Chr 15, ERATO Doi 466, expressed | 1.09 | 0.618 | -1.3 | 0.611 | -1.13 | 0.458 | -1.45 | 0.008 | -0.7 |
| 1436075_at | Sfrp5 | secreted frizzled-related sequence protein 5 | -1.06 | 0.804 | -1.44 | 0.371 | 1.11 | 0.672 | -1.45 | 0.335 | -0.71 |
| 1420902_at | St6galnac3 | ST6 (alpha-N-acetyl-neuraminyl-2,3-beta-galactosyl-1,3)-N-acetylgalactosaminide | 1.22 | 0.688 | -1.27 | 0.44 | -1.36 | 0.309 | -1.08 | 0.908 | -0.62 |
| 1456089_at | Trim23 | tripartite motif protein 23 | -1.16 | 0.156 | 1.03 | 0.907 | -1.17 | 0.56 | -1 | 0.998 | -0.58 |
| 1426266_s_at | Zbtb8os | zinc finger and BTB domain containing 8 opposite strand | -1.13 | 0.047 | -1.17 | 0.058 | 1 | 0.999 | -1.56 | 0.055 | -0.71 |
| 1422686_s_at | Exoc4 | exocyst complex component 4 | -1.06 | 0.54 | -1.1 | 0.253 | -1.13 | 0.504 | 1.22 | 0.005 | -0.52 |
| 1451602_at | Snx6 | sorting nexin 6 | 1.01 | 0.971 | -1.05 | 0.412 | -1.29 | 0.022 | -1.26 | 0.104 | -0.64 |
| 1460628_at | Mapk8ip3 | Mitogen-activated protein kinase 8 interacting protein 3 | -1.16 | 0.149 | -1.34 | 0.344 | 1.14 | 0.408 | -1.5 | 0.052 | -0.72 |
| 1439186_at | E130319B15Rik | RIKEN cDNA E130319B15 gene | 1.05 | 0.872 | -1.3 | 0.171 | -1.08 | 0.712 | -1.03 | 0.959 | -0.59 |
| 1457040_at | Lgi2 | leucine-rich repeat LGI family, member 2 | 1.14 | 0.79 | -1.23 | 0.547 | -1.27 | 0.229 | -1.12 | 0.266 | -0.62 |
| 1457865_at | Phr1 | Pam, highwire, rpm 1 | -1.93 | 0.368 | 1.63 | 0.01 | -1.67 | 0.478 | -1.56 | 0.558 | -0.88 |
| 1458177_at | Ebpl | emopamil binding protein-like | -1 | 0.998 | 1.3 | 0.449 | -2.26 | 0.079 | -1.78 | 0.098 | -0.94 |
| 1434446_at | D630014A15Rik | RIKEN cDNA D630014A15 gene | -1.12 | 0.648 | 1 | 0.991 | -1.18 | 0.116 | -1.04 | 0.897 | -0.58 |
| 1427905_at | 1810063B07Rik | RIKEN cDNA 1810063B07 gene | -1.1 | 0.051 | 1.05 | 0.618 | -1.27 | 0.073 | -1.37 | 0.267 | -0.67 |
| 1454490_at | 4930423C22Rik | RIKEN cDNA 4930423C22 gene | -1.21 | 0.45 | -1.18 | 0.581 | 1.07 | 0.659 | -1.3 | 0.509 | -0.65 |
| 1428574_a_at | Chn2 | chimerin (chimaerin) 2 | -1.43 | 0.116 | -1.23 | 0.097 | 1.23 | 0.565 | -1.2 | 0.425 | -0.66 |
| 1455317_at | Epc2 | enhancer of polycomb homolog 2 (Drosophila) | 1.05 | 0.715 | -1.03 | 0.715 | -1.4 | 0.009 | -1.29 | 0.026 | -0.66 |
| 1454725_at | Tra2a | transformer 2 alpha homolog (Drosophila) | -1.12 | 0.631 | 1.04 | 0.789 | -1.24 | 0.034 | -1.15 | 0.227 | -0.62 |
| 1430342_at | 4930518J20Rik | RIKEN cDNA 4930518J20 gene | 1.63 | 0.419 | -1.79 | 0.411 | -1.79 | 0.203 | -1.25 | 0.48 | -0.8 |
| 1416924_at | Bri3 | brain protein I3 | 1.02 | 0.89 | -1.14 | 0.558 | -1.17 | 0.035 | -1.24 | 0.51 | -0.63 |
| 1423744_x_at | Eif2s3x | eukaryotic translation initiation factor 2, subunit 3, structural gene X-linked | -1.03 | 0.536 | -1.12 | 0.313 | -1.13 | 0.421 | 1.16 | 0.302 | -0.53 |
| 1429448_s_at | Cxxc6 | CXXC finger 6 | -1.34 | 0.552 | 1.36 | 0.61 | -1.57 | 0.294 | -2.39 | 0.219 | -0.98 |
| 1443232_at | --- | Transcribed locus | 1.02 | 0.929 | -1.07 | 0.66 | -1.27 | 0.28 | -1.29 | 0.528 | -0.65 |
| 1433676_at | Wnk1 | WNK lysine deficient protein kinase 1 | -1.14 | 0.007 | -1.04 | 0.087 | -1.11 | 0.387 | 1.21 | 0.218 | -0.52 |
| 1424002_at | Pdcl3 | phosducin-like 3 | -1.11 | 0.005 | 1.03 | 0.696 | -1.23 | 0.042 | -1.36 | 0.004 | -0.67 |
| 1436313_at | Scyl2 | SCY1-like 2 (S. cerevisiae) | -1.1 | 0.36 | -1.21 | 0.499 | 1.01 | 0.896 | -1.05 | 0.841 | -0.59 |
| 1429723_at | 6330409N04Rik | RIKEN cDNA 6330409N04 gene | -1.04 | 0.671 | -1.07 | 0.508 | -1.19 | 0.332 | 1.31 | 0.454 | -0.5 |
| 1457854_at | AU018877 | expressed sequence AU018877 | 1.22 | 0.648 | -1.23 | 0.505 | -1.41 | 0.378 | -1.57 | 0.088 | -0.75 |
| 1459156_at | --- | --- | -1.16 | 0.403 | 1.3 | 0.564 | -1.72 | 0.173 | -1.92 | 0.105 | -0.88 |
| 1435790_at | Olfm2 | olfactomedin 2 | 1.37 | 0.505 | -1.46 | 0.497 | -1.44 | 0.117 | -1.33 | 0.195 | -0.72 |
| 1431318_at | 4833420G17Rik | RIKEN cDNA 4833420G17 gene | -1.83 | 0.534 | -1.27 | 0.651 | 1.42 | 0.161 | -1.28 | 0.489 | -0.74 |
| 1431337_a_at | 1810055E12Rik | RIKEN cDNA 1810055E12 gene | -1.13 | 0.138 | 1.09 | 0.418 | -1.29 | 0.097 | -1.01 | 0.895 | -0.59 |
| 1459002_at | Rpl21 /// LOC26867 | ribosomal protein L21 /// similar to 60S ribosomal protein L21 /// similar to riboson | 1.56 | 0.415 | -1.73 | 0.074 | -1.65 | 0.381 | -2.07 | 0.094 | -0.97 |
| 1456872_a_at | D230010M03Rik | RIKEN cDNA D230010M03 gene | -1.11 | 0.584 | -1.03 | 0.79 | -1.15 | 0.652 | 1.56 | 0.13 | -0.43 |
| 1442978_at | Mpdz | Multiple PDZ domain protein | 1.05 | 0.821 | -1.24 | 0.63 | -1.13 | 0.441 | -1.1 | 0.816 | -0.6 |
| 1434663_at | 2410129H14Rik | RIKEN cDNA 2410129H14 gene | -1.11 | 0.421 | -1.08 | 0.574 | -1.09 | 0.459 | 2.34 | 0.158 | -0.23 |
| 1431666_at | 2610021K21Rik | RIKEN cDNA 2610021K21 gene | -1.63 | 0.296 | 1.3 | 0.584 | -1.2 | 0.717 | -1.65 | 0.614 | -0.8 |
| 1422730_at | Limd1 | LIM domains containing 1 | 1.06 | 0.729 | -1.13 | 0.51 | -1.25 | 0.376 | -1.03 | 0.845 | -0.59 |
| 1433077_at | 2700078F05Rik | RIKEN cDNA 2700078F05 gene | -1.2 | 0.717 | -1.1 | 0.602 | 1 | 0.953 | -1.04 | 0.807 | -0.58 |
| 1455222_a_at | Ubp1 | upstream binding protein 1 | -1.22 | 0.054 | -1.02 | 0.836 | -1.06 | 0.698 | 1.45 | 0.132 | -0.46 |
| 1429085_at | Vezf1 | vascular endothelial zinc finger 1 | 1.01 | 0.946 | -1.19 | 0.002 | -1.11 | 0.026 | -1.31 | 0.14 | -0.65 |
| 1423861_at | Plekhf2 | pleckstrin homology domain containing, family F (with FYVE domain) member 2 | -1.14 | 0.481 | -1.03 | 0.941 | -1.12 | 0.555 | 1.46 | 0.24 | -0.46 |
| 1445174_at | --- | --- | -1.27 | 0.724 | -1.53 | 0.478 | 1.3 | 0.654 | -3.18 | 0.311 | -1.17 |
| 1456894_at | C130039O16Rik | RIKEN cDNA C130039O16 gene | -1.12 | 0.834 | -1.08 | 0.817 | -1.08 | 0.848 | 1.41 | 0.451 | -0.47 |
| 1440641_at | Vps29 | vacuolar protein sorting 29 (S. pombe) | -1.3 | 0.288 | 1.17 | 0.432 | -1.24 | 0.418 | -1.43 | 0.177 | -0.7 |
| 1446950_at | Tox | Thymocyte selection-associated HMG box gene | -1.02 | 0.981 | -1.43 | 0.223 | 1.07 | 0.861 | -1.49 | 0.357 | -0.72 |
| 1458298_at | Cadps | Ca<2+>dependent activator protein for secretion | -1.25 | 0.57 | -1.1 | 0.85 | 1.04 | 0.94 | -1.28 | 0.709 | -0.65 |
| 1442295_at | Arpc2 | Actin related protein 2/3 complex, subunit 2 | 1.13 | 0.598 | -1.34 | 0.546 | -1.14 | 0.617 | -1.55 | 0.199 | -0.73 |
| 1418400_at | Larp6 | La ribonucleoprotein domain family, member 6 | 1.2 | 0.536 | -1.62 | 0.409 | -1.08 | 0.289 | -1.2 | 0.487 | -0.68 |
| 1456849_at | --- | --- | 1.2 | 0.546 | -1.33 | 0.144 | -1.25 | 0.276 | -1.24 | 0.334 | -0.66 |
| 1415744_at | H2-Ke2 | H2-K region expressed gene 2 | -1.06 | 0.385 | -1.18 | 0.061 | -1.04 | 0.694 | 1.1 | 0.543 | -0.55 |

| | | | | | | | | | | | |
|---|---|---|---|---|---|---|---|---|---|---|---|
| 1448864_at | Snrk | SNF related kinase | 1.06 | 0.792 | -1.3 | 0.386 | -1.1 | 0.749 | -1.57 | 0.111 | -0.72 |
| 1432336_at | 5033404E19Rik | RIKEN cDNA 5033404E19 gene | -1.7 | 0.105 | 1.38 | 0.438 | -1.28 | 0.529 | -1.34 | 0.299 | -0.74 |
| 1454388_at | 2900046F13Rik | RIKEN cDNA 2900046F13 gene | -1.09 | 0.833 | 1.24 | 0.59 | -1.7 | 0.114 | -1.28 | 0.273 | -0.71 |
| 1455377_at | 4921517B04Rik | RIKEN cDNA 4921517B04 gene | 1.07 | 0.765 | -1.04 | 0.923 | -1.41 | 0.16 | -1 | 0.985 | -0.59 |
| 1434973_at | Car7 | carbonic anhydrase 7 | -1.16 | 0.748 | -1.23 | 0.525 | 1.07 | 0.923 | -1.12 | 0.665 | -0.61 |
| 1424082_at | Tbc1d13 | TBC1 domain family, member 13 | 1.01 | 0.947 | -1.14 | 0.477 | -1.17 | 0.389 | -1.17 | 0.017 | -0.62 |
| 1439896_at | Limk2 | LIM motif-containing protein kinase 2 | -1.34 | 0.674 | 1.24 | 0.462 | -1.32 | 0.25 | -2.7 | 0.103 | -1.03 |
| 1423299_at | Txnl1 | thioredoxin-like 1 | -1.1 | 0.253 | -1.19 | 0.009 | -1 | 0.974 | 1.55 | 0.212 | -0.44 |
| 1445005_at | C79491 | expressed sequence C79491 | -1.36 | 0.047 | -1.08 | 0.452 | 1.08 | 0.615 | -1.14 | 0.477 | -0.62 |
| 1429119_at | 4833421E05Rik | RIKEN cDNA 4833421E05 gene | 1.06 | 0.481 | -1.05 | 0.502 | -1.35 | 0.045 | -1.35 | 0.202 | -0.67 |
| 1449689_at | C81600 | expressed sequence C81600 | -1.25 | 0.712 | -1.54 | 0.473 | 1.3 | 0.391 | -1.46 | 0.391 | -0.74 |
| 1431822_a_at | Azi2 | 5-azacytidine induced gene 2 | -1.14 | 0.229 | -1.06 | 0.151 | -1.07 | 0.18 | 1.04 | 0.807 | -0.56 |
| 1419460_at | Rpp14 | ribonuclease P 14 subunit (human) | -1.06 | 0.599 | -1.05 | 0.436 | -1.18 | 0.19 | 1.02 | 0.873 | -0.57 |
| 1443874_at | 2810002I04Rik | RIKEN cDNA 2810002I04 gene | -1.16 | 0.612 | -1.28 | 0.001 | 1.1 | 0.755 | -1.06 | 0.745 | -0.6 |
| 1424828_a_at | Fh1 | fumarate hydratase 1 | -1.12 | 0.39 | 1.02 | 0.83 | -1.2 | 0.315 | -1.59 | 0.105 | -0.72 |
| 1438880_at | 1700012D14Rik | RIKEN cDNA 1700012D14 gene | 1.06 | 0.806 | -1.27 | 0.321 | -1.12 | 0.513 | -1.45 | 0.139 | -0.69 |
| 1416747_at | D15Mgi27 | DNA Segment, Chr 15, Mouse Genome Informatics 27 | -1.07 | 0.528 | -1.09 | 0.115 | -1.11 | 0.427 | 1.03 | 0.686 | -0.56 |
| 1458517_at | Akr1e1 | Aldo-keto reductase family 1, member E1 | -1.17 | 0.04 | 1.04 | 0.877 | -1.17 | 0.659 | -1.16 | 0.52 | -0.62 |
| 1453174_at | 2310076G13Rik | RIKEN cDNA 2310076G13 gene | -1.21 | 0.284 | 1.09 | 0.51 | -1.21 | 0.146 | -1.33 | 0.445 | -0.66 |
| 1416005_at | Psmc1 | protease (prosome, macropain) 26S subunit, ATPase 1 | -1.05 | 0.597 | -1.1 | 0.009 | -1.13 | 0.243 | 1.06 | 0.698 | -0.55 |
| 1419798_at | 2610019E17Rik | RIKEN cDNA 2610019E17 gene | 1.11 | 0.704 | -1.56 | 0.164 | -1 | 0.997 | -1.14 | 0.715 | -0.65 |
| 1452364_at | Suz12 | suppressor of zeste 12 homolog (Drosophila) | -1.11 | 0.593 | -1.02 | 0.888 | -1.16 | 0.15 | 1.72 | 0.12 | -0.39 |
| 1439430_x_at | 4930403C10Rik | RIKEN cDNA 4930403C10 gene | 1.02 | 0.959 | -1.33 | 0.297 | -1.02 | 0.913 | -1.43 | 0.121 | -0.69 |
| 1423538_at | Ntan1 | N-terminal Asn amidase | 1.07 | 0.165 | -1.11 | 0.409 | -1.29 | 0.054 | -1.27 | 0.177 | -0.65 |
| 1456626_a_at | 1110005A23Rik /// | RIKEN cDNA 1110005A23 gene /// similar to cytokine induced protein 29 kDa /// s | -1.25 | 0.236 | 1.07 | 0.498 | -1.14 | 0.239 | -1.18 | 0.702 | -0.62 |
| 1418567_a_at | Srp14 | signal recognition particle 14 | 1.02 | 0.862 | -1.08 | 0.288 | -1.23 | 0.061 | -1.42 | 0.038 | -0.68 |
| 1434625_at | 4930432O21Rik | RIKEN cDNA 4930432O21 gene | 1.08 | 0.749 | -1.13 | 0.224 | -1.28 | 0.086 | -1.13 | 0.404 | -0.61 |
| 1426352_s_at | Tial1 | Tial1 cytotoxic granule-associated RNA binding protein-like 1 | 1.28 | 0.427 | -1.44 | 0.396 | -1.29 | 0.09 | -1.6 | 0.01 | -0.76 |
| 1430797_at | Xpo4 | exportin 4 | 1.41 | 0.375 | -2.07 | 0.031 | -1.16 | 0.575 | -1.03 | 0.962 | -0.71 |
| 1446414_at | 0910001A06Rik | RIKEN cDNA 0910001A06 gene | 1.35 | 0.341 | -1.37 | 0.483 | -1.5 | 0.538 | -1.89 | 0.314 | -0.85 |
| 1450561_a_at | Surf1 | surfeit gene 1 | 1.03 | 0.84 | -1.22 | 0.013 | -1.12 | 0.037 | -1.26 | 0.271 | -0.64 |
| 1455375_at | LOC669787 | similar to Protein enabled homolog (NPC-derived proline-rich protein 1) (NDPP-1) | -1.33 | 0.488 | 1.06 | 0.783 | -1.07 | 0.714 | -1.38 | 0.084 | -0.68 |
| 1429497_s_at | Snx6 | sorting nexin 6 | -1.21 | 0.181 | 1.1 | 0.39 | -1.22 | 0.012 | -1.45 | 0.09 | -0.69 |
| 1429166_s_at | Clmn | calmin | -1.15 | 0.487 | -1.29 | 0.258 | 1.11 | 0.744 | -3.26 | 0.337 | -1.15 |
| 1424200_s_at | Seh1l | SEH1-like (S. cerevisiae | -1.04 | 0.545 | -1 | 0.976 | -1.26 | 0.003 | 1.09 | 0.452 | -0.55 |
| 1418584_at | Ccnh /// LOC67265 | cyclin H /// similar to cyclin H | -1.07 | 0.713 | -1.15 | 0.266 | -1.06 | 0.575 | 1.07 | 0.638 | -0.55 |
| 1439144_at | Cwf19l1 | CWF19-like 1, cell cycle control (S. pombe) | -1.73 | 0.012 | 1.22 | 0.527 | -1.05 | 0.813 | -1.95 | 0.039 | -0.88 |
| 1441927_at | Syt7 | Synaptotagmin VII | -1.86 | 0.425 | 1.24 | 0.476 | -1.03 | 0.919 | -1.54 | 0.473 | -0.8 |
| 1430518_at | 5430402E10Rik | RIKEN cDNA 5430402E10 gene | -1.13 | 0.867 | 1.31 | 0.46 | -1.8 | 0.301 | -1.47 | 0.621 | -0.77 |
| 1456981_at | Tmc7 | transmembrane channel-like gene family 7 | -1.6 | 0.38 | -1.5 | 0.441 | 1.46 | 0.211 | -1.03 | 0.883 | -0.67 |
| 1456740_x_at | Cog1 | component of oligomeric golgi complex 1 | -1.25 | 0.569 | 1.03 | 0.844 | -1.09 | 0.721 | -1.3 | 0.486 | -0.65 |
| 1444154_at | Ndufa11 | NADH dehydrogenase (ubiquinone) 1 alpha subcomplex 11 | -1.22 | 0.243 | -1.06 | 0.851 | -1.02 | 0.743 | 2.02 | 0.161 | -0.32 |
| 1438776_x_at | Rps17 /// LOC2686 | ribosomal protein S17 /// similar to ribosomal protein S17 /// similar to 40S ribosor | -1.09 | 0.581 | 1.04 | 0.724 | -1.26 | 0.031 | -1.52 | 0.09 | -0.71 |
| 1416952_at | Atp6v1d | ATPase, H+ transporting, lysosomal V1 subunit D | -1.09 | 0.397 | -1.18 | 0.033 | -1.02 | 0.79 | 1.02 | 0.867 | -0.57 |
| 1454793_x_at | Ddx5 | DEAD (Asp-Glu-Ala-Asp) box polypeptide 5 | -1.23 | 0.321 | 1.03 | 0.653 | -1.1 | 0.267 | -1.68 | 0.102 | -0.75 |
| 1440240_at | Npb | neuropeptide B | -1 | 0.998 | -1.43 | 0.439 | 1.05 | 0.732 | -1.52 | 0.431 | -0.72 |
| 1420890_at | Hccs | holocytochrome c synthetase | -1.09 | 0.018 | -1.13 | 0.265 | -1.05 | 0.656 | 1.09 | 0.395 | -0.55 |
| 1438839_a_at | Ywhae | tyrosine 3-monooxygenase/tryptophan 5-monooxygenase activation protein, epsil | -1.08 | 0.493 | -1.05 | 0.622 | -1.15 | 0.058 | 1.04 | 0.799 | -0.56 |
| 1457054_a_at | D130020L05Rik | RIKEN cDNA D130020L05 gene | -1.41 | 0.055 | 1.06 | 0.785 | -1.02 | 0.65 | -1.55 | 0.127 | -0.73 |
| 1431557_at | 4930488N15Rik | RIKEN cDNA 4930488N15 gene | -1.35 | 0.45 | 1.22 | 0.68 | -1.27 | 0.471 | -1.76 | 0.433 | -0.79 |
| 1438420_at | Rbm39 | RNA binding motif protein 39 | 1.14 | 0.295 | -1.04 | 0.728 | -1.52 | 0.046 | -1.28 | 0.227 | -0.68 |
| 1435267_at | A430108E01Rik | RIKEN cDNA A430108E01 gene | 1.09 | 0.606 | -1.19 | 0.135 | -1.22 | 0.315 | -1.31 | 0.319 | -0.66 |
| 1448670_at | Ube2e3 | ubiquitin-conjugating enzyme E2E 3, UBC4/5 homolog (yeast) | -1.08 | 0.02 | -1.09 | 0.11 | -1.1 | 0.313 | 1.55 | 0.246 | -0.43 |

| | | | | | | | | | | | |
|---|---|---|---|---|---|---|---|---|---|---|---|
| 1435004_at | Pank4 | pantothenate kinase 4 | -1.08 | 0.385 | -1.41 | 0.067 | 1.12 | 0.003 | -1.18 | 0.281 | -0.64 |
| 1431830_at | Zfp329 | zinc finger protein 329 | -1.03 | 0.796 | 1.14 | 0.594 | -1.56 | 0.134 | -1.51 | 0.222 | -0.74 |
| 1458121_at | A430107O13Rik | RIKEN cDNA A430107O13 gene | 1.62 | 0.062 | -1.97 | 0.149 | -1.61 | 0.193 | -1.1 | 0.705 | -0.77 |
| 1421388_at | Mef2d | myocyte enhancer factor 2D | -1.07 | 0.627 | -1.15 | 0.185 | -1.06 | 0.868 | 1.21 | 0.07 | -0.52 |
| 1460689_at | D15Wsu75e | DNA segment, Chr 15, Wayne State University 75, expressed | -1.08 | 0.105 | -1.06 | 0.697 | -1.13 | 0.545 | 1.15 | 0.587 | -0.53 |
| 1434312_at | AI788669 | expressed sequence AI788669 | -1.14 | 0.444 | -1 | 0.988 | -1.14 | 0.595 | 1 | 0.965 | -0.57 |
| 1440838_at | AI852064 | Expressed sequence AI852064 | -1.1 | 0.731 | -1.04 | 0.951 | -1.14 | 0.625 | 1.33 | 0.059 | -0.49 |
| 1432456_at | 4930533B18Rik | RIKEN cDNA 4930533B18 gene | 1.59 | 0.59 | -3.48 | 0.002 | -1.14 | 0.36 | -1.68 | 0.513 | -1.18 |
| 1415936_at | Bcar3 | breast cancer anti-estrogen resistance 3 | -1.29 | 0.218 | -1.21 | 0.355 | 1.15 | 0.402 | -1.96 | 0.074 | -0.83 |
| 1439922_at | 1190002C06Rik | RIKEN cDNA 1190002C06 gene | -1.33 | 0.493 | 1.11 | 0.774 | -1.13 | 0.691 | -1.07 | 0.718 | -0.6 |
| 1434317_s_at | Tex10 | testis expressed gene 10 | -1.09 | 0.198 | 1.01 | 0.875 | -1.22 | 0.144 | -1.2 | 0.061 | -0.62 |
| 1450341_at | Pcdhb8 | protocadherin beta 8 | 1.24 | 0.35 | -1.37 | 0.558 | -1.28 | 0.546 | -1.18 | 0.817 | -0.65 |
| 1457078_at | Zfp473 | zinc finger protein 473 | -1.15 | 0.854 | 1.05 | 0.885 | -1.2 | 0.566 | -1.08 | 0.821 | -0.6 |
| 1424588_at | Srgap3 | SLIT-ROBO Rho GTPase activating protein 3 | -1.04 | 0.867 | -1.45 | 0.228 | 1.1 | 0.686 | -1.06 | 0.726 | -0.61 |
| 1460580_at | Pcnx | pecanex homolog (Drosophila) | -1.05 | 0.741 | 1.02 | 0.915 | -1.28 | 0.253 | -1.43 | 0.15 | -0.68 |
| 1437911_at | 6330416L07Rik | RIKEN cDNA 6330416L07 gene | -1.34 | 0.176 | -1.05 | 0.834 | 1.06 | 0.643 | -1.09 | 0.575 | -0.61 |
| 1436896_at | D14Ertd231e | DNA segment, Chr 14, ERATO Doi 231, expressed | -1.25 | 0.157 | -1.08 | 0.26 | 1.02 | 0.773 | -1.12 | 0.149 | -0.61 |
| 1453515_at | 4930511A21Rik | RIKEN cDNA 4930511A21 gene | 1.75 | 0.386 | -1.69 | 0.255 | -2.42 | 0.28 | -1.02 | 0.971 | -0.85 |
| 1441265_at | Plekhm2 | Pleckstrin homology domain containing, family M (with RUN domain) member 2 | -1.06 | 0.813 | -1.13 | 0.496 | -1.08 | 0.749 | 1.29 | 0.13 | -0.5 |
| 1435380_at | Cox10 | COX10 homolog, cytochrome c oxidase assembly protein, heme A: farnesyltrans | -1.19 | 0.385 | -1.1 | 0.678 | -1 | 0.999 | 1.08 | 0.348 | -0.55 |
| 1454860_x_at | Dad1 | defender against cell death 1 | 1.15 | 0.43 | -1.03 | 0.739 | -1.58 | 0.006 | -1.22 | 0.44 | -0.67 |
| 1433377_at | 5830445D09Rik | RIKEN cDNA 5830445D09 gene | -2.12 | 0.089 | 1.41 | 0.312 | -1.15 | 0.631 | -1.24 | 0.258 | -0.78 |
| 1442757_at | Lrch1 | leucine-rich repeats and calponin homology (CH) domain containing 1 | -1.04 | 0.863 | 1.08 | 0.528 | -1.42 | 0.088 | -1.35 | 0.379 | -0.68 |
| 1449624_at | Gm118 | gene model 118, (NCBI) | 1.05 | 0.568 | -1.17 | 0.737 | -1.18 | 0.553 | -1.2 | 0.414 | -0.63 |
| 1457264_at | Phf20l1 | PHD finger protein 20-like 1 | 1.28 | 0.11 | -1.19 | 0.465 | -1.59 | 0.022 | -1.74 | 0.052 | -0.81 |
| 1434731_x_at | Prdx1 | peroxiredoxin 1 | -1.13 | 0.214 | 1.01 | 0.899 | -1.18 | 0.104 | -1.29 | 0.151 | -0.65 |
| 1443620_at | Gpc4 | Glypican 4 | -1.04 | 0.806 | -1.05 | 0.878 | -1.2 | 0.339 | 1.5 | 0.046 | -0.45 |
| 1450757_at | Cdh11 | cadherin 11 | 1.16 | 0.404 | -1.32 | 0.18 | -1.2 | 0.472 | -1.95 | 0.075 | -0.83 |
| 1457073_at | Ogt | O-linked N-acetylglucosamine (GlcNAc) transferase (UDP-N-acetylglucosamine:p | 1.29 | 0.15 | -1.02 | 0.959 | -2.07 | 0.008 | -1.02 | 0.916 | -0.71 |
| 1460574_at | Fat4 | FAT tumor suppressor homolog 4 (Drosophila) | 1.17 | 0.506 | -1.62 | 0.098 | -1.04 | 0.902 | -1.39 | 0.268 | -0.72 |
| 1422647_at | Ring1 | ring finger protein 1 | -1.07 | 0.827 | 1 | 0.968 | -1.23 | 0.285 | -1.33 | 0.359 | -0.66 |
| 1455495_at | AA536808 | Expressed sequence AA536808 | 1.02 | 0.881 | -1.07 | 0.306 | -1.25 | 0.167 | -1.52 | 0.123 | -0.71 |
| 1447866_x_at | 2300009A05Rik | RIKEN cDNA 2300009A05 gene | 1.12 | 0.621 | -1.41 | 0.332 | -1.09 | 0.862 | -1.39 | 0.205 | -0.69 |
| 1419899_at | Zc3h7a | Zinc finger CCCH type containing 7 A | 1.63 | 0.094 | -1.35 | 0.519 | -2.66 | 0.191 | -1.45 | 0.472 | -0.96 |
| 1434093_at | Lysmd4 | LysM, putative peptidoglycan-binding, domain containing 4 | -1.04 | 0.872 | 1.01 | 0.95 | -1.28 | 0.328 | -1.12 | 0.565 | -0.61 |
| 1458820_at | Zfp560 | zinc finger protein 560 | 1.05 | 0.786 | -1.03 | 0.934 | -1.36 | 0.116 | -1.22 | 0.334 | -0.64 |
| 1438373_at | App | Amyloid beta (A4) precursor protein | -1.84 | 0.049 | -1.2 | 0.73 | 1.38 | 0.311 | -1.1 | 0.843 | -0.69 |
| 1445416_at | Tsga14 | Testis specific gene A14 | -1.59 | 0.09 | 1.33 | 0.67 | -1.26 | 0.406 | -2.87 | 0.048 | -1.1 |
| 1424993_at | 8430410K20Rik | RIKEN cDNA 8430410K20 gene | -1.19 | 0.073 | 1.08 | 0.51 | -1.21 | 0.099 | -1.09 | 0.629 | -0.6 |
| 1436622_at | Iqsec2 | IQ motif and Sec7 domain 2 | -1.07 | 0.664 | -1.04 | 0.762 | -1.17 | 0.41 | 1 | 0.995 | -0.57 |
| 1450721_at | Acp1 | acid phosphatase 1, soluble | -1.14 | 0.258 | -1.05 | 0.185 | -1.09 | 0.368 | 1.56 | 0.29 | -0.43 |
| 1443059_at | Dhrs8 | dehydrogenase/reductase (SDR family) member 8 | -1.4 | 0.634 | -1.12 | 0.257 | 1.15 | 0.639 | -1.31 | 0.227 | -0.67 |
| 1459234_at | Alox5ap | Arachidonate 5-lipoxygenase activating protein | -1.35 | 0.038 | -1 | 0.995 | 1.01 | 0.911 | -1.14 | 0.633 | -0.62 |
| 1420936_s_at | Cpsf2 | cleavage and polyadenylation specific factor 2 | -1.19 | 0.171 | -1.02 | 0.822 | -1.07 | 0.69 | 1.36 | 0.334 | -0.48 |
| 1425742_a_at | Tsc22d1 | TSC22 domain family, member 1 | 1.11 | 0.75 | -1.16 | 0.319 | -1.28 | 0.025 | -1.29 | 0.316 | -0.66 |
| 1419354_at | Klf7 | Kruppel-like factor 7 (ubiquitous) | 1.08 | 0.616 | -1.05 | 0.557 | -1.39 | 0.069 | -1.12 | 0.454 | -0.62 |
| 1447850_x_at | Zfand3 | zinc finger, AN1-type domain 3 | -1.05 | 0.668 | 1.08 | 0.749 | -1.4 | 0.36 | -2.41 | 0.093 | -0.94 |
| 1417709_at | Cyp46a1 | cytochrome P450, family 46, subfamily a, polypeptide 1 | 1.26 | 0.108 | -1.37 | 0.306 | -1.31 | 0.231 | -1.49 | 0.432 | -0.73 |
| 1443150_at | Dnm2 | Dynamin 2 | 1.07 | 0.831 | -1.32 | 0.25 | -1.08 | 0.747 | -1.03 | 0.925 | -0.59 |
| 1458585_at | --- | Transcribed locus | -1.15 | 0.691 | 1.15 | 0.779 | -1.37 | 0.079 | -1.13 | 0.764 | -0.62 |
| 1449694_s_at | Commd5 | COMM domain containing 5 | -1.24 | 0.167 | -1.08 | 0.577 | 1.02 | 0.904 | -1.19 | 0.248 | -0.62 |
| 1442987_at | --- | --- | -1.49 | 0.524 | 1.26 | 0.618 | -1.21 | 0.736 | -1.02 | 0.968 | -0.62 |
| 1424135_at | Rspry1 | ring finger and SPRY domain containing 1 | -1.11 | 0.423 | -1.1 | 0.19 | -1.06 | 0.705 | 1.05 | 0.405 | -0.56 |

| | | | | | | | | | | | |
|---|---|---|---|---|---|---|---|---|---|---|---|
| 1427248_at | Whsc2 | Wolf-Hirschhorn syndrome candidate 2 (human) | -1.09 | 0.702 | -1.09 | 0.374 | -1.09 | 0.645 | 1.02 | 0.925 | -0.56 |
| 1455703_at | Akt2 | thymoma viral proto-oncogene 2 | -1.11 | 0.025 | -1.23 | 0.018 | 1.04 | 0.687 | -1.16 | 0.11 | -0.61 |
| 1433627_at | Sec23ip | Sec23 interacting protein | -1.19 | 0.319 | -1.08 | 0.367 | -1.02 | 0.398 | 1.08 | 0.143 | -0.55 |
| 1424904_at | 1300010F03Rik | RIKEN cDNA 1300010F03 gene | -1.04 | 0.844 | -1.83 | 0.057 | 1.24 | 0.236 | -1.15 | 0.683 | -0.69 |
| 1455039_a_at | Sin3b | transcriptional regulator, SIN3B (yeast) | 1.04 | 0.814 | -1.16 | 0.179 | -1.17 | 0.308 | -1.51 | 0.252 | -0.7 |
| 1434605_at | Eif5b | eukaryotic translation initiation factor 5B | -1.04 | 0.746 | -1.16 | 0.045 | -1.08 | 0.646 | 1.13 | 0.375 | -0.54 |
| 1433848_at | Cdc27 | cell division cycle 27 homolog (S. cerevisiae) | -1 | 0.992 | -1.1 | 0.42 | -1.18 | 0.208 | 2.11 | 0.125 | -0.29 |
| 1435625_at | Entpd7 | ectonucleoside triphosphate diphosphohydrolase 7 | -1.23 | 0.619 | -1.44 | 0.16 | 1.24 | 0.315 | -1.51 | 0.1 | -0.73 |
| 1420115_at | Exosc8 | exosome component 8 | -1.24 | 0.555 | -1.3 | 0.367 | 1.17 | 0.581 | -1.11 | 0.565 | -0.62 |
| 1458538_at | Sirt1 | Sirtuin 1 ((silent mating type information regulation 2, homolog) 1 (S. cerevisiae) | 1.04 | 0.639 | -1.16 | 0.269 | -1.17 | 0.29 | -1.15 | 0.489 | -0.61 |
| 1425460_at | Mtmr2 | myotubularin related protein 2 | -1.1 | 0.511 | 1.08 | 0.582 | -1.31 | 0.001 | -1.18 | 0.102 | -0.63 |
| 1415995_at | Casp6 | caspase 6 | -1.09 | 0.293 | -1.27 | 0.214 | 1.05 | 0.69 | -1.29 | 0.169 | -0.65 |
| 1449744_at | Slc19a1 | Solute carrier family 19 (sodium/hydrogen exchanger), member 1 | -1.23 | 0.177 | 1.02 | 0.95 | -1.08 | 0.746 | -1.88 | 0.346 | -0.79 |
| 1433909_at | Syt17 | synaptotagmin XVII | -1.02 | 0.725 | -1.03 | 0.953 | -1.26 | 0.405 | 1.31 | 0.351 | -0.5 |
| 1454769_at | Tatdn2 | TatD DNase domain containing 2 | -2.11 | 0.299 | -1.31 | 0.527 | 1.51 | 0.219 | -2.06 | 0.278 | -0.99 |
| 1445346_at | Ank2 | Ankyrin 2, brain | -1.37 | 0.538 | 1.31 | 0.631 | -1.41 | 0.292 | -1.14 | 0.817 | -0.65 |
| 1417424_at | Ier3ip1 | immediate early response 3 interacting protein 1 | -1.02 | 0.455 | 1.01 | 0.924 | -1.31 | 0.021 | -1.37 | 0.181 | -0.67 |
| 1455988_a_at | Cct6a | chaperonin subunit 6a (zeta) | -1.02 | 0.427 | -1.06 | 0.334 | -1.21 | 0.105 | 1.03 | 0.819 | -0.56 |
| 1429623_at | Zfp644 | zinc finger protein 644 | -1.04 | 0.785 | -1.1 | 0.441 | -1.14 | 0.333 | 1.02 | 0.945 | -0.56 |
| 1460176_at | Crk | v-crk sarcoma virus CT10 oncogene homolog (avian) | 1.13 | 0.583 | -1.23 | 0.102 | -1.24 | 0.199 | -1.06 | 0.65 | -0.6 |
| 1454644_at | 6330569M22Rik | RIKEN cDNA 6330569M22 gene | -1.16 | 0.284 | 1.04 | 0.785 | -1.18 | 0.222 | -1.59 | 0.104 | -0.72 |
| 1446343_at | --- | Transcribed locus | -2.04 | 0.085 | -1.58 | 0.033 | 1.63 | 0.082 | -1.28 | 0.489 | -0.82 |
| 1424472_at | Nol6 | nucleolar protein family 6 (RNA-associated) | -1.45 | 0.188 | -1.24 | 0.019 | 1.26 | 0.289 | -1.04 | 0.66 | -0.62 |
| 1427921_s_at | 2310061C15Rik | RIKEN cDNA 2310061C15 gene | -1.1 | 0.275 | -1.1 | 0.417 | -1.07 | 0.169 | 2.01 | 0.229 | -0.32 |
| 1416732_at | Top2b | topoisomerase (DNA) II beta | -1.13 | 0.425 | -1.28 | 0.326 | 1.09 | 0.698 | -1.28 | 0.449 | -0.65 |
| 1459414_at | Wdfy3 | WD repeat and FYVE domain containing 3 | -1.1 | 0.589 | -1.09 | 0.48 | -1.08 | 0.834 | 1.6 | 0.168 | -0.42 |
| 1450425_a_at | 2700062C07Rik | RIKEN cDNA 2700062C07 gene | -1.21 | 0.316 | -1.17 | 0.113 | 1.07 | 0.716 | -1.11 | 0.294 | -0.6 |
| 1423769_at | Ptcd2 | pentatricopeptide repeat domain 2 | -1.18 | 0.089 | -1.14 | 0.031 | 1.03 | 0.822 | -1.32 | 0.036 | -0.65 |
| 1417672_at | Slc4a10 | solute carrier family 4, sodium bicarbonate cotransporter-like, member 10 | -1.02 | 0.856 | 1.22 | 0.497 | -1.82 | 0.208 | -3.04 | 0.223 | -1.16 |
| 1430558_at | Zfp318 | zinc finger protein 318 | -1.18 | 0.248 | 1.09 | 0.556 | -1.23 | 0.306 | -1.81 | 0.138 | -0.78 |
| 1424240_at | Arfip2 | ADP-ribosylation factor interacting protein 2 | -1.03 | 0.823 | -1.03 | 0.762 | -1.22 | 0.429 | 1.19 | 0.226 | -0.53 |
| 1428871_at | 4121402D02Rik | RIKEN cDNA 4121402D02 gene | -1.13 | 0.255 | 1.06 | 0.605 | -1.23 | 0.331 | -1.21 | 0.375 | -0.63 |
| 1436152_a_at | Hbxip | hepatitis B virus x interacting protein | -1.15 | 0.024 | 1.04 | 0.587 | -1.18 | 0.258 | -1.39 | 0.137 | -0.67 |
| 1415813_at | Api5 | apoptosis inhibitor 5 | -1.1 | 0.236 | -1.09 | 0.256 | -1.08 | 0.253 | 1.1 | 0.513 | -0.54 |
| 1423490_at | Fbxo3 | F-box only protein 3 | -1.11 | 0.356 | -1.1 | 0.247 | -1.06 | 0.565 | 1.76 | 0.196 | -0.38 |
| 1450583_s_at | Fprl1 | formyl peptide receptor-like 1 | 1.08 | 0.775 | -1.12 | 0.679 | -1.28 | 0.66 | -1.28 | 0.382 | -0.65 |
| 1427990_at | Usp45 | ubiquitin specific petidase 45 | -1.11 | 0.531 | -1.03 | 0.855 | -1.13 | 0.396 | 1.87 | 0.242 | -0.35 |
| 1436684_a_at | Riok2 | RIO kinase 2 (yeast) | -1.16 | 0.638 | 1.09 | 0.425 | -1.25 | 0.193 | -1.22 | 0.39 | -0.63 |
| 1426581_at | Ptpmt1 | protein tyrosine phosphatase, mitochondrial 1 | -1.08 | 0.545 | -1.19 | 0.175 | -1.01 | 0.94 | 1.06 | 0.733 | -0.56 |
| 1435624_at | 9030409G11Rik | RIKEN cDNA 9030409G11 gene | 1.1 | 0.898 | -1.36 | 0.399 | -1.08 | 0.816 | -1.2 | 0.78 | -0.64 |
| 1448771_a_at | Fth1 | ferritin heavy chain 1 | -1.08 | 0.11 | -1.09 | 0.641 | -1.09 | 0.03 | 1.03 | 0.726 | -0.56 |
| 1418693_at | Hnrpc /// LOC6208 | heterogeneous nuclear ribonucleoprotein C /// similar to heterogeneous nuclear ri | -1.08 | 0.4 | -1.1 | 0.108 | -1.09 | 0.323 | 1.17 | 0.229 | -0.52 |
| 1434517_at | Wdfy2 | WD repeat and FYVE domain containing 2 | -1.24 | 0.351 | 1.14 | 0.409 | -1.24 | 0.164 | -1.46 | 0.132 | -0.7 |
| 1444280_at | A930001M12Rik | RIKEN cDNA A930001M12 gene | -1.5 | 0.605 | 1.44 | 0.493 | -1.53 | 0.531 | -1.1 | 0.859 | -0.68 |
| 1417227_at | Mccc1 | methylcrotonoyl-Coenzyme A carboxylase 1 (alpha) | -1.16 | 0.132 | -1.31 | 0.077 | 1.13 | 0.118 | -1.3 | 0.049 | -0.66 |
| 1459900_at | C79468 | expressed sequence C79468 | 1.06 | 0.756 | -1 | 0.991 | -1.44 | 0.036 | -1.69 | 0.175 | -0.77 |
| 1440084_at | AI663975 | Expressed sequence AI663975 | -1.17 | 0.42 | 1.11 | 0.879 | -1.27 | 0.455 | -1.72 | 0.37 | -0.76 |
| 1424740_at | Creb3 | cAMP responsive element binding protein 3 | 1.01 | 0.897 | -1.04 | 0.423 | -1.27 | 0.098 | -1.29 | 0.216 | -0.65 |
| 1426696_at | Lrpap1 | low density lipoprotein receptor-related protein associated protein 1 | 1.02 | 0.822 | -1.09 | 0.305 | -1.23 | 0.07 | -1.01 | 0.943 | -0.58 |
| 1438157_s_at | Nfkbia | nuclear factor of kappa light chain gene enhancer in B-cells inhibitor, alpha | -1.28 | 0.571 | 1.01 | 0.953 | -1.03 | 0.81 | -2.22 | 0.067 | -0.88 |
| 1428307_at | Zdhhc13 | zinc finger, DHHC domain containing 13 | -1.19 | 0.216 | 1 | 0.986 | -1.1 | 0.379 | -1.39 | 0.171 | -0.67 |
| 1455505_at | Gatad2a | GATA zinc finger domain containing 2A | -1.23 | 0.331 | 1.1 | 0.623 | -1.19 | 0.178 | -1.03 | 0.877 | -0.59 |
| 1448253_at | Glud1 | glutamate dehydrogenase 1 | -1.21 | 0.042 | -1.12 | 0.052 | 1.04 | 0.736 | -1.54 | 0.198 | -0.71 |

| | | | | | | | | | | | |
|---|---|---|---|---|---|---|---|---|---|---|---|
| 1442953_at | --- | Transcribed locus | -1.06 | 0.878 | 1.08 | 0.61 | -1.38 | 0.049 | -1.35 | 0.442 | -0.68 |
| 1457370_at | Rabgap1 | RAB GTPase activating protein 1 | -1.03 | 0.915 | 1.21 | 0.33 | -1.73 | 0.062 | -1.44 | 0.202 | -0.75 |
| 1431538_at | 4931415C17Rik | RIKEN cDNA 4931415C17 gene | 1.09 | 0.817 | -1.19 | 0.302 | -1.21 | 0.534 | -1.2 | 0.819 | -0.63 |
| 1435536_at | Rsc1a1 | regulatory solute carrier protein, family 1, member 1 | -1.02 | 0.756 | -1.09 | 0.152 | -1.17 | 0.07 | 1.21 | 0.24 | -0.52 |
| 1433427_at | 1700111A04Rik | RIKEN cDNA 1700111A04 gene | -1.26 | 0.529 | -1.26 | 0.257 | 1.17 | 0.403 | -1.88 | 0.007 | -0.81 |
| 1445367_at | --- | Transcribed locus | 1.4 | 0.221 | -1.52 | 0.217 | -1.43 | 0.024 | -1.23 | 0.27 | -0.7 |
| 1460168_at | Slbp | stem-loop binding protein | -1.14 | 0.005 | -1.07 | 0.108 | -1.06 | 0.576 | 2.31 | 0.167 | -0.24 |
| 1450701_a_at | Gtf2h2 | general transcription factor II H, polypeptide 2 | -1.16 | 0.545 | -1.04 | 0.678 | -1.08 | 0.278 | 1.02 | 0.895 | -0.56 |
| 1454817_at | Utp18 | UTP18, small subunit (SSU) processome component, homolog (yeast) | -1.3 | 0.177 | -1.26 | 0.032 | 1.19 | 0.533 | -1.09 | 0.514 | -0.61 |
| 1447753_at | Cdc37l1 | Cell division cycle 37 homolog (S. cerevisiae)-like 1 | -1.41 | 0.345 | 1.25 | 0.254 | -1.25 | 0.315 | -1.17 | 0.71 | -0.65 |
| 1417652_a_at | Tbca | tubulin cofactor a | -1.01 | 0.83 | 1.01 | 0.751 | -1.33 | 0.02 | -1.28 | 0.096 | -0.65 |
| 1434579_x_at | Ndufs8 | NADH dehydrogenase (ubiquinone) Fe-S protein 8 | -1.02 | 0.817 | -1.08 | 0.194 | -1.18 | 0.118 | 1.03 | 0.787 | -0.56 |
| 1454975_at | Psrc2 | proline/serine-rich coiled-coil 2 | 1.01 | 0.96 | -1.04 | 0.751 | -1.28 | 0.192 | -1.16 | 0.534 | -0.62 |
| 1426957_at | Trp53bp1 | transformation related protein 53 binding protein 1 | -1.23 | 0.134 | -1.09 | 0.453 | 1.03 | 0.82 | -1.06 | 0.697 | -0.59 |
| 1423143_at | Gtpbp4 | GTP binding protein 4 | 1.07 | 0.814 | -1.32 | 0.104 | -1.08 | 0.628 | -1.39 | 0.261 | -0.68 |
| 1433991_x_at | Dbi | diazepam binding inhibitor | -1.07 | 0.501 | -1.04 | 0.803 | -1.16 | 0.639 | 1.05 | 0.86 | -0.56 |
| 1428261_at | 2310042L06Rik | RIKEN cDNA 2310042L06 gene | -1.33 | 0.02 | -1.22 | 0.054 | 1.18 | 0.137 | -1.13 | 0.518 | -0.62 |
| 1428876_at | Srp72 | signal recognition particle 72 | -1.07 | 0.512 | -1.06 | 0.389 | -1.13 | 0.146 | 1.15 | 0.165 | -0.53 |
| 1452126_at | Zfp160 | zinc finger protein 160 | -1.22 | 0.143 | -1.07 | 0.285 | 1 | 0.991 | -1.25 | 0.095 | -0.63 |
| 1425007_at | Zfp566 | zinc finger protein 566 | -1.35 | 0.251 | -1.02 | 0.95 | 1.03 | 0.808 | -1.41 | 0.324 | -0.69 |
| 1449583_at | Pcdhb20 | protocadherin beta 20 | 1.64 | 0.275 | -1.74 | 0.169 | -1.86 | 0.025 | -1.5 | 0.445 | -0.86 |
| 1434412_x_at | Stub1 | STIP1 homology and U-Box containing protein 1 | 1.08 | 0.769 | -1.05 | 0.804 | -1.38 | 0.15 | -1.34 | 0.068 | -0.67 |
| 1417992_a_at | Svs3 /// 9530004A2 | seminal vesicle secretion 3 /// RIKEN cDNA 9530004A22 | -2.11 | 0.242 | -1.03 | 0.97 | 1.31 | 0.626 | -1.22 | 0.745 | -0.76 |
| 1457371_at | 5230400G24Rik | RIKEN cDNA 5230400G24 gene | -1.28 | 0.433 | 1.14 | 0.504 | -1.2 | 0.118 | -1.14 | 0.194 | -0.62 |
| 1460350_at | Osbp | oxysterol binding protein | -1.14 | 0.262 | -1.12 | 0.341 | -1.01 | 0.808 | 1.03 | 0.56 | -0.56 |
| 1454613_at | Dpysl3 | dihydropyrimidinase-like 3 | 1.33 | 0.01 | -1.25 | 0.541 | -1.6 | 0.06 | -1.18 | 0.246 | -0.67 |
| 1427450_x_at | Myo1b | myosin IB | 1.07 | 0.848 | -1.16 | 0.327 | -1.22 | 0.28 | -1.04 | 0.883 | -0.59 |
| 1448234_at | Dnajb6 | DnaJ (Hsp40) homolog, subfamily B, member 6 | -1.04 | 0.373 | -1.07 | 0.081 | -1.17 | 0.162 | 1.12 | 0.362 | -0.54 |
| 1446442_at | Rnmt | RNA (guanine-7-) methyltransferase | 1.65 | 0.089 | -1.73 | 0.361 | -1.87 | 0.143 | -1.46 | 0.562 | -0.86 |
| 1434337_at | --- | Transcribed locus | -1.08 | 0.416 | -1.21 | 0.309 | 1.01 | 0.902 | -1.11 | 0.636 | -0.6 |
| 1427448_at | Rabep1 | rabaptin, RAB GTPase binding effector protein 1 | -1.11 | 0.621 | 1.03 | 0.859 | -1.21 | 0.142 | -1.29 | 0.077 | -0.65 |
| 1426394_at | Eif3s1 | eukaryotic translation initiation factor 3, subunit 1 alpha | -1.02 | 0.857 | -1.16 | 0 | -1.1 | 0.061 | 1.37 | 0.428 | -0.48 |
| 1454214_a_at | 2410019A14Rik | RIKEN cDNA 2410019A14 gene | -1.17 | 0.139 | -1.15 | 0.015 | 1.03 | 0.801 | -1.4 | 0.02 | -0.67 |
| 1444601_at | --- | --- | -1.21 | 0.021 | -1.09 | 0.773 | 1.01 | 0.934 | -1.43 | 0.363 | -0.68 |
| 1451532_s_at | Steap1 | six transmembrane epithelial antigen of the prostate 1 | -1.19 | 0.02 | -1.21 | 0.685 | 1.09 | 0.688 | -1.17 | 0.691 | -0.62 |
| 1445111_at | Exoc4 | Exocyst complex component 4 | 1.46 | 0.148 | -1.14 | 0.558 | -2.39 | 0.002 | -1.14 | 0.63 | -0.8 |
| 1441936_x_at | 4930447M23Rik | RIKEN cDNA 4930447M23 gene | 1.13 | 0.69 | -1.06 | 0.905 | -1.45 | 0.514 | -1.67 | 0.085 | -0.76 |
| 1448111_at | Ctps2 | cytidine 5'-triphosphate synthase 2 | 1.01 | 0.948 | -1.16 | 0.388 | -1.13 | 0.074 | -1.72 | 0.223 | -0.75 |
| 1437982_x_at | Cox15 | COX15 homolog, cytochrome c oxidase assembly protein (yeast) | -1.39 | 0.153 | -1.12 | 0.532 | 1.14 | 0.379 | -1.23 | 0.211 | -0.65 |
| 1452222_at | Utrn | utrophin | 1.09 | 0.132 | -1.15 | 0.178 | -1.26 | 0.392 | -1.64 | 0.107 | -0.74 |
| 1437991_x_at | Rusc1 | RUN and SH3 domain containing 1 | -1.17 | 0.481 | -1.22 | 0.314 | 1.09 | 0.58 | -1.52 | 0.102 | -0.71 |
| 1424069_at | Napg | N-ethylmaleimide sensitive fusion protein attachment protein gamma | -1.17 | 0.037 | 1 | 0.997 | -1.11 | 0.305 | -1.19 | 0.398 | -0.62 |
| 1426933_at | Oxsr1 | oxidative-stress responsive 1 | -1.15 | 0.505 | -1.13 | 0.492 | -1 | 0.992 | 1.56 | 0.133 | -0.43 |
| 1416860_s_at | Ing1 | inhibitor of growth family, member 1 | 1.05 | 0.852 | -1.09 | 0.26 | -1.27 | 0.258 | -1.39 | 0.311 | -0.67 |
| 1419755_at | Mfi2 | antigen p97 (melanoma associated) identified by monoclonal antibodies 133.2 an | -1.19 | 0.8 | -2.89 | 0.049 | 1.57 | 0.102 | -2.25 | 0.018 | -1.19 |
| 1446342_at | 2310001H17Rik | RIKEN cDNA 2310001H17 gene | 1.01 | 0.971 | -1.29 | 0.181 | -1.03 | 0.944 | -1.64 | 0.295 | -0.74 |
| 1435810_at | 5730455O13Rik | RIKEN cDNA 5730455O13 gene | 1.06 | 0.444 | -1.14 | 0.161 | -1.22 | 0.037 | -1.5 | 0.217 | -0.7 |
| 1448366_at | Stx1a | syntaxin 1A (brain) | 1.35 | 0.034 | -1.65 | 0.126 | -1.25 | 0.119 | -1.55 | 0.259 | -0.77 |
| 1439550_at | --- | --- | 1.15 | 0.216 | -1.27 | 0.282 | -1.22 | 0.263 | -2.51 | 0.069 | -0.96 |
| 1453682_at | Gabpb2 | GA repeat binding protein, beta 2 | 1.03 | 0.436 | -1.09 | 0.156 | -1.24 | 0.034 | -1.19 | 0.368 | -0.62 |
| 1434052_at | AI593442 | expressed sequence AI593442 | 1.38 | 0.18 | -1.65 | 0.512 | -1.3 | 0.604 | -1.19 | 0.247 | -0.69 |
| 1449492_a_at | Lect2 | leukocyte cell-derived chemotaxin 2 | 1.11 | 0.896 | -1.53 | 0.093 | -1 | 0.997 | -1.94 | 0.272 | -0.84 |
| 1422263_at | Bdkrb2 | bradykinin receptor, beta 2 | -1.62 | 0.527 | -1.7 | 0.42 | 1.55 | 0.384 | -1.1 | 0.875 | -0.72 |

| | | | | | | | | | | | |
|---|---|---|---|---|---|---|---|---|---|---|---|
| 1451524_at | Fbxw2 | F-box and WD-40 domain protein 2 | -1.22 | 0.155 | -1.03 | 0.855 | -1.04 | 0.823 | 2.42 | 0.245 | -0.22 |
| 1454459_at | 4933423L19Rik | RIKEN cDNA 4933423L19 gene | 1.33 | 0.032 | -1.25 | 0.635 | -1.59 | 0.342 | -1.08 | 0.881 | -0.65 |
| 1448654_at | Mtch2 | mitochondrial carrier homolog 2 (C. elegans) | -1.09 | 0.237 | -1.06 | 0.328 | -1.1 | 0.436 | 1.27 | 0.485 | -0.5 |
| 1420368_at | Denr | density-regulated protein | 1.04 | 0.735 | -1.12 | 0.306 | -1.22 | 0.191 | -1.22 | 0.247 | -0.63 |
| 1451152_a_at | Atp1b1 | ATPase, Na+/K+ transporting, beta 1 polypeptide | 1.01 | 0.958 | -1.23 | 0.303 | -1.06 | 0.738 | -2.82 | 0.011 | -1.03 |
| 1441592_at | Jmjd1c | Jumonji domain containing 1C | 1.27 | 0.421 | -1.07 | 0.782 | -1.82 | 0 | -1.78 | 0.121 | -0.85 |
| 1415966_a_at | Ndufv1 | NADH dehydrogenase (ubiquinone) flavoprotein 1 | -1.02 | 0.779 | -1.2 | 0.118 | -1.06 | 0.646 | 1.12 | 0.55 | -0.54 |
| 1453313_at | Sesn3 | sestrin 3 | 1.13 | 0.846 | -1.41 | 0.269 | -1.09 | 0.892 | -3.27 | 0.152 | -1.16 |
| 1424787_a_at | Nrf1 | nuclear respiratory factor 1 | -1.04 | 0.869 | -1.1 | 0.677 | -1.12 | 0.292 | 1.14 | 0.713 | -0.53 |
| 1419723_at | Opn1mw | opsin 1 (cone pigments), medium-wave-sensitive (color blindness, deutan) | -1.15 | 0.358 | 1.34 | 0.683 | -1.81 | 0.286 | -1.98 | 0.545 | -0.9 |
| 1443630_at | --- | --- | 1.25 | 0.48 | -1.09 | 0.813 | -1.71 | 0.077 | -2 | 0.104 | -0.89 |
| 1422756_at | Slc32a1 | solute carrier family 32 (GABA vesicular transporter), member 1 | -1.99 | 0.412 | 1.34 | 0.258 | -1.09 | 0.758 | -2.08 | 0.541 | -0.96 |
| 1422968_at | Ihpk1 | inositol hexaphosphate kinase 1 | -1.28 | 0.196 | -1.04 | 0.88 | 1.02 | 0.921 | -1.45 | 0.119 | -0.69 |
| 1439748_at | Dpp6 | dipeptidylpeptidase 6 | -1.19 | 0.402 | -1.2 | 0.838 | 1.08 | 0.587 | -1.15 | 0.725 | -0.61 |
| 1436990_s_at | Ndg2 | Nur77 downstream gene 2 | -1.13 | 0.01 | -1.33 | 0.242 | 1.12 | 0.39 | -1.46 | 0.139 | -0.7 |
| 1448262_at | Psmb2 | proteasome (prosome, macropain) subunit, beta type 2 | -1.02 | 0.93 | -1.17 | 0.298 | -1.08 | 0.705 | 1.39 | 0.112 | -0.47 |
| 1440793_at | 1700029M03Rik | RIKEN cDNA 1700029M03 gene | 1.26 | 0.592 | -1.56 | 0.107 | -1.16 | 0.727 | -1.1 | 0.872 | -0.64 |
| 1454047_a_at | 2410017P07Rik | RIKEN cDNA 2410017P07 gene | -1.06 | 0.744 | 1.02 | 0.787 | -1.25 | 0.282 | -1.07 | 0.61 | -0.59 |
| 1447974_s_at | Aqp6 | aquaporin 6 | -1.28 | 0.429 | 1 | 0.992 | -1.02 | 0.915 | -1.11 | 0.854 | -0.6 |
| 1423285_at | Coch | coagulation factor C homolog (Limulus polyphemus) | -1.44 | 0.371 | -1.42 | 0.385 | 1.36 | 0.284 | -2.06 | 0.201 | -0.89 |
| 1453285_at | Tmem88 | transmembrane protein 88 | -1.23 | 0.469 | -1.01 | 0.984 | -1.05 | 0.805 | 1.22 | 0.204 | -0.52 |
| 1438447_at | Ddx11 | DEAD/H (Asp-Glu-Ala-Asp/His) box polypeptide 11 (CHL1-like helicase homolog, | -1.17 | 0.255 | -1.03 | 0.733 | -1.07 | 0.805 | 1.33 | 0.118 | -0.49 |
| 1423697_at | Psmd6 | proteasome (prosome, macropain) 26S subunit, non-ATPase, 6 | -1.04 | 0.092 | -1.11 | 0.191 | -1.12 | 0.178 | 1.07 | 0.562 | -0.55 |
| 1420393_at | Nos2 | nitric oxide synthase 2, inducible, macrophage | -1.16 | 0.266 | -1.45 | 0.027 | 1.2 | 0.233 | -1.04 | 0.936 | -0.61 |
| 1427379_at | Nte | Neuropathy target esterase | -1.1 | 0.6 | -1.06 | 0.685 | -1.1 | 0.74 | 1.19 | 0.382 | -0.52 |
| 1416566_at | Strap | serine/threonine kinase receptor associated protein | -1.09 | 0.099 | -1.01 | 0.85 | -1.17 | 0.075 | 1.13 | 0.451 | -0.54 |
| 1421370_a_at | Il1f5 | interleukin 1 family, member 5 (delta) | 1.19 | 0.199 | -1.23 | 0.661 | -1.32 | 0.376 | -1.26 | 0.68 | -0.66 |
| 1441429_at | Irs4 | insulin receptor substrate 4 | -1.44 | 0.181 | 1.5 | 0.406 | -1.75 | 0.188 | -1.77 | 0.292 | -0.87 |
| 1431563_at | 2010003K10Rik | RIKEN cDNA 2010003K10 gene | -1.45 | 0.68 | 1.15 | 0.67 | -1.08 | 0.824 | -1.13 | 0.875 | -0.63 |
| 1427194_a_at | Ercc8 | excision repaiross-complementing rodent repair deficiency, complementation grou | -1.22 | 0.421 | -1 | 0.961 | -1.06 | 0.828 | 1.18 | 0.586 | -0.52 |
| 1420786_a_at | Rbmy1a1 | RNA binding motif protein, Y chromosome, family 1, member A1 | 1.26 | 0.633 | -1.15 | 0.823 | -1.58 | 0.593 | -1.07 | 0.94 | -0.64 |
| 1438505_s_at | Rnasen | ribonuclease III, nuclear | -1.28 | 0.09 | 1.06 | 0.694 | -1.08 | 0.521 | -1.06 | 0.723 | -0.59 |
| 1423863_at | Abcf2 | ATP-binding cassette, sub-family F (GCN20), member 2 | -1.07 | 0.634 | -1.05 | 0.624 | -1.14 | 0.655 | 1.16 | 0.603 | -0.53 |
| 1427150_at | Mll3 | myeloid/lymphoid or mixed-lineage leukemia 3 | 1.04 | 0.658 | -1.25 | 0.163 | -1.09 | 0.668 | -1.34 | 0.315 | -0.66 |
| 1434870_at | 2810004N23Rik | RIKEN cDNA 2810004N23 gene | 1.05 | 0.642 | -1.25 | 0.004 | -1.09 | 0.404 | -1.17 | 0.375 | -0.62 |
| 1448392_at | Sparc | secreted acidic cysteine rich glycoprotein | -1.1 | 0.542 | 1.14 | 0.314 | -1.41 | 0.363 | -1.38 | 0.266 | -0.69 |
| 1451129_at | Calb2 | calbindin 2 | -1.21 | 0.284 | -1.33 | 0.308 | 1.18 | 0.713 | -1.92 | 0.552 | -0.82 |
| 1448783_at | Slc7a9 | solute carrier family 7 (cationic amino acid transporter, y+ system), member 9 | -1.07 | 0.568 | -2.21 | 0.311 | 1.38 | 0.454 | -1.87 | 0.12 | -0.94 |
| 1436578_at | A330104H05Rik | RIKEN cDNA A330104H05 gene | -1.16 | 0.816 | -1.08 | 0.82 | -1.03 | 0.954 | 2.49 | 0.031 | -0.19 |
| 1454778_x_at | Tia1 | Cytotoxic granule-associated RNA binding protein 1 | -1.08 | 0.429 | 1.05 | 0.63 | -1.27 | 0.029 | -1.2 | 0.03 | -0.63 |
| 1457884_at | 4732496C06Rik | RIKEN cDNA 4732496C06 gene | -1.12 | 0.63 | -1.37 | 0.233 | 1.14 | 0.575 | -1.33 | 0.289 | -0.67 |
| 1421938_at | Serhl | serine hydrolase-like | -1.08 | 0.69 | -1.03 | 0.794 | -1.16 | 0.112 | 1.01 | 0.946 | -0.56 |
| 1428090_at | Ptcd3 | Pentatricopeptide repeat domain 3 | -1.12 | 0.155 | -1.16 | 0.066 | 1.01 | 0.94 | -1.06 | 0.11 | -0.58 |
| 1417725_a_at | Sssca1 | Sjogren's syndrome/scleroderma autoantigen 1 homolog (human) | 1.03 | 0.921 | -1.15 | 0.42 | -1.16 | 0.286 | -1.36 | 0.326 | -0.66 |
| 1460102_at | Clasp1 | CLIP associating protein 1 | -1.11 | 0.11 | 1.21 | 0.547 | -1.53 | 0.02 | -1.41 | 0.127 | -0.71 |
| 1439726_at | 4432406C05Rik | RIKEN cDNA 4432406C05 gene | -1.26 | 0.513 | -1.53 | 0.118 | 1.31 | 0.184 | -1.11 | 0.542 | -0.65 |
| 1425600_a_at | Plcb1 | phospholipase C, beta 1 | -1.09 | 0.653 | -1.17 | 0.377 | -1.01 | 0.973 | 1.74 | 0.389 | -0.38 |
| 1452271_at | Xpr1 | xenotropic and polytropic retrovirus receptor 1 | -1.17 | 0.259 | -1.11 | 0.122 | 1 | 0.98 | -1.01 | 0.876 | -0.57 |
| 1447052_at | --- | --- | 1.01 | 0.949 | -1.02 | 0.935 | -1.29 | 0.306 | -1.81 | 0.169 | -0.78 |
| 1446819_at | Ran | RAN, member RAS oncogene family | -1.2 | 0.803 | -1.14 | 0.68 | 1.05 | 0.89 | -1.87 | 0.455 | -0.79 |
| 1456215_at | Tfam | transcription factor A, mitochondrial | -1.07 | 0.194 | -1.2 | 0.157 | -1.01 | 0.962 | 1.11 | 0.68 | -0.54 |
| 1435348_at | D930009K15Rik | RIKEN cDNA D930009K15 gene | 1.07 | 0.74 | -1.22 | 0.168 | -1.15 | 0.262 | -1.15 | 0.616 | -0.61 |
| 1452833_at | Rapgef2 | Rap guanine nucleotide exchange factor (GEF) 2 | -1.16 | 0.582 | 1.03 | 0.795 | -1.15 | 0.502 | -1.4 | 0.363 | -0.67 |

| | | | | | | | | | | | |
|---|---|---|---|---|---|---|---|---|---|---|---|
| 1446778_at | Hunk | Hormonally upregulated Neu-associated kinase | 1.55 | 0.374 | -1.32 | 0.472 | -2.22 | 0.103 | -2.14 | 0.159 | -1.03 |
| 1436836_x_at | Cnn3 | calponin 3, acidic | 1.02 | 0.58 | -1.03 | 0.649 | -1.3 | 0.019 | -2.45 | 0.038 | -0.94 |
| 1416413_at | Ctsj | cathepsin J | -3.56 | 0.223 | 1.52 | 0.552 | -1.04 | 0.896 | -1.04 | 0.681 | -1.03 |
| 1455957_x_at | Ceacam11 | CEA-related cell adhesion molecule 11 | -1.54 | 0.447 | -1.53 | 0.479 | 1.46 | 0.567 | -1.06 | 0.914 | -0.67 |
| 1422033_a_at | Cntf /// Zfp91 | ciliary neurotrophic factor /// zinc finger protein 91 | -1.02 | 0.894 | -1.25 | 0.375 | -1.01 | 0.947 | 1.47 | 0.427 | -0.46 |
| 1433296_at | 2310045N14Rik | RIKEN cDNA 2310045N14 gene | -1.16 | 0.658 | 1.03 | 0.946 | -1.15 | 0.781 | -1.13 | 0.584 | -0.6 |
| 1437261_at | 2900024O10Rik | RIKEN cDNA 2900024O10 gene | -1.34 | 0.095 | -1.1 | 0.645 | 1.1 | 0.562 | -1.24 | 0.488 | -0.64 |
| 1449390_at | Gpatc4 | G patch domain containing 4 | 1.1 | 0.748 | -1.11 | 0.777 | -1.3 | 0.166 | -1.47 | 0.001 | -0.7 |
| 1415789_a_at | Ublcp1 /// LOC6656 | ubiquitin-like domain containing CTD phosphatase 1 /// similar to ubiquitin-like dor | 1.14 | 0.66 | -1.26 | 0.449 | -1.21 | 0.552 | -1.32 | 0.175 | -0.66 |
| 1423901_at | Trip12 | thyroid hormone receptor interactor 12 | -1.11 | 0.413 | -1.1 | 0.41 | -1.05 | 0.68 | 1.11 | 0.649 | -0.54 |
| 1455298_at | --- | Transcribed locus | -1.11 | 0.77 | 1.04 | 0.862 | -1.21 | 0.118 | -1.47 | 0.203 | -0.69 |
| 1457675_at | 2510002D24Rik | RIKEN cDNA 2510002D24 gene | -1.02 | 0.898 | -1.29 | 0.191 | 1.01 | 0.951 | -1.24 | 0.383 | -0.64 |
| 1429630_at | Ezh1 | enhancer of zeste homolog 1 (Drosophila) | 1.33 | 0.076 | -1.77 | 0.363 | -1.16 | 0.767 | -1.24 | 0.721 | -0.71 |
| 1439942_at | Prep | prolyl endopeptidase | -1.33 | 0.32 | -1.04 | 0.87 | 1.04 | 0.647 | -1.04 | 0.855 | -0.59 |
| 1448537_at | Ttc1 | tetratricopeptide repeat domain 1 | -1.1 | 0.419 | -1.02 | 0.86 | -1.15 | 0.367 | 1.2 | 0.461 | -0.52 |
| 1455000_at | Gpr68 | G protein-coupled receptor 68 | -1.05 | 0.881 | -1.27 | 0.25 | 1.02 | 0.893 | -1.88 | 0.089 | -0.8 |
| 1432064_at | Ubox5 | U box domain containing 5 | -1.1 | 0.558 | 1.05 | 0.894 | -1.24 | 0.511 | -1.08 | 0.514 | -0.59 |
| 1423385_at | Actr8 | ARP8 actin-related protein 8 homolog (S. cerevisiae) | -1.16 | 0.257 | -1.22 | 0.004 | 1.08 | 0.372 | -1.19 | 0.191 | -0.62 |
| 1420178_at | --- | Transcribed locus | -1.65 | 0.327 | -1.32 | 0.567 | 1.4 | 0.509 | -1.13 | 0.62 | -0.67 |
| 1446389_at | Nrip1 | Nuclear receptor interacting protein 1 | 1.07 | 0.908 | -1.04 | 0.902 | -1.38 | 0.121 | -1.73 | 0.18 | -0.77 |
| 1457647_x_at | 1600023N17Rik | RIKEN cDNA 1600023N17 gene | -1.54 | 0.437 | 1.2 | 0.762 | -1.09 | 0.815 | -1.09 | 0.627 | -0.63 |
| 1438235_at | --- | --- | 1.3 | 0.265 | -1.14 | 0.639 | -1.71 | 0.041 | -1.66 | 0.071 | -0.8 |
| 1448465_at | Nipsnap1 | 4-nitrophenylphosphatase domain and non-neuronal SNAP25-like protein homolc | -1.14 | 0.355 | -1.17 | 0.136 | 1.03 | 0.784 | -1.84 | 0.08 | -0.78 |
| 1429018_at | 3830408D24Rik | RIKEN cDNA 3830408D24 gene | -1.23 | 0.226 | 1.19 | 0.547 | -1.32 | 0.121 | -2.16 | 0.33 | -0.88 |
| 1448463_at | 4933434E20Rik | RIKEN cDNA 4933434E20 gene | 1.01 | 0.85 | -1.08 | 0.075 | -1.2 | 0.041 | -1.3 | 0.096 | -0.65 |
| 1432372_a_at | Spr | sepiapterin reductase | -1.7 | 0.258 | -1.26 | 0.255 | 1.38 | 0.381 | -1.98 | 0.069 | -0.89 |
| 1439977_at | 4930515I15 | hypothetical protein 4930515I15 | -2.15 | 0.21 | 1.45 | 0.609 | -1.18 | 0.562 | -1.66 | 0.179 | -0.89 |
| 1434423_at | --- | --- | -1.12 | 0.227 | 1.17 | 0.692 | -1.43 | 0.068 | -2.04 | 0.046 | -0.86 |
| 1448193_at | 5730403B10Rik | RIKEN cDNA 5730403B10 gene | -1.26 | 0.344 | -1.11 | 0.692 | 1.07 | 0.738 | -1.06 | 0.738 | -0.59 |
| 1426048_s_at | Hrc /// Tcfap2a | histidine rich calcium binding protein /// transcription factor AP-2, alpha | 1.36 | 0.125 | -2.1 | 0.202 | -1.08 | 0.723 | -2.07 | 0.066 | -0.97 |
| 1435492_at | Socs6 | suppressor of cytokine signaling 6 | -1.05 | 0.612 | 1.06 | 0.604 | -1.34 | 0.099 | -1.61 | 0.147 | -0.73 |
| 1428576_at | Hif1an | hypoxia-inducible factor 1, alpha subunit inhibitor | -1.06 | 0.627 | 1.02 | 0.795 | -1.26 | 0.186 | -1.23 | 0.092 | -0.63 |
| 1449710_s_at | Atp5a1 | ATP synthase, H+ transporting, mitochondrial F1 complex, alpha subunit, isoform | -1.14 | 0.052 | -1.16 | 0.021 | 1.02 | 0.743 | -1.1 | 0.149 | -0.59 |
| 1452823_at | Gstk1 | glutathione S-transferase kappa 1 | 1.03 | 0.772 | -1.13 | 0.349 | -1.17 | 0.396 | -1.21 | 0.54 | -0.62 |
| 1437162_at | Gpiap1 | GPI-anchored membrane protein 1 | 1.01 | 0.788 | -1.09 | 0.475 | -1.2 | 0.305 | -1.05 | 0.582 | -0.58 |
| 1431051_at | 4930577N17Rik | RIKEN cDNA 4930577N17 gene | -1.12 | 0.546 | -1.62 | 0.145 | 1.26 | 0.165 | -1.44 | 0.252 | -0.73 |
| 1452941_at | 0610038F07Rik | RIKEN cDNA 0610038F07 gene | -1.25 | 0.44 | -1.1 | 0.458 | 1.06 | 0.507 | -1.27 | 0.023 | -0.64 |
| 1417768_at | Pnpla8 | patatin-like phospholipase domain containing 8 | -1.1 | 0.725 | -1.04 | 0.611 | -1.12 | 0.48 | 1.02 | 0.917 | -0.56 |
| 1446148_x_at | C79248 | expressed sequence C79248 | -1.01 | 0.973 | 1.11 | 0.369 | -1.5 | 0.038 | -1.17 | 0.393 | -0.64 |
| 1453005_at | Prpf31 | PRP31 pre-mRNA processing factor 31 homolog (yeast) | -1.11 | 0.622 | 1.23 | 0.339 | -1.58 | 0.034 | -1.07 | 0.761 | -0.63 |
| 1456759_at | --- | 16 days neonate cerebellum cDNA, RIKEN full-length enriched library, clone:963( | -1.38 | 0.633 | 1.85 | 0.33 | -5.27 | 0.014 | -2.31 | 0.106 | -1.78 |
| 1426187_a_at | Hax1 | HCLS1 associated X-1 | -1.09 | 0.414 | -1.07 | 0.305 | -1.09 | 0.398 | 1.04 | 0.828 | -0.55 |
| 1435030_at | Upf2 | UPF2 regulator of nonsense transcripts homolog (yeast) | -1.01 | 0.971 | -1.13 | 0.323 | -1.13 | 0.235 | 1.03 | 0.771 | -0.56 |
| 1458577_at | --- | --- | -1.31 | 0.521 | -1.64 | 0.488 | 1.39 | 0.079 | -1.47 | 0.287 | -0.76 |
| 1430199_at | 1700007E05Rik | RIKEN cDNA 1700007E05 gene | -1.05 | 0.633 | 1 | 0.997 | -1.24 | 0.734 | -1.94 | 0.466 | -0.81 |
| 1416519_at | LOC622534 | similar to ribosomal protein L36 | -1.15 | 0.117 | 1.03 | 0.625 | -1.16 | 0.181 | -1.39 | 0.003 | -0.67 |
| 1436325_at | Rora | RAR-related orphan receptor alpha | -1.01 | 0.959 | 1.08 | 0.527 | -1.43 | 0.148 | -1.27 | 0.206 | -0.66 |
| 1427085_at | 2810432D09Rik | RIKEN cDNA 2810432D09 gene | 1.02 | 0.899 | -1.03 | 0.739 | -1.3 | 0.073 | -1.06 | 0.524 | -0.59 |
| 1440193_at | Ankrd12 | ankyrin repeat domain 12 | 1.06 | 0.847 | -1.07 | 0.612 | -1.3 | 0.326 | -1.33 | 0.387 | -0.66 |
| 1433002_at | 9030613N10Rik | RIKEN cDNA 9030613N10 gene | 1.05 | 0.938 | -1.07 | 0.685 | -1.28 | 0.58 | -1.89 | 0.566 | -0.8 |
| 1420096_at | Zipro1 | Zinc finger proliferation 1 | 1.23 | 0.498 | -1.39 | 0.28 | -1.24 | 0.362 | -1.19 | 0.611 | -0.64 |
| 1418102_at | Hes1 | hairy and enhancer of split 1 (Drosophila) | -1.21 | 0.436 | -1.07 | 0.746 | 1 | 0.987 | -1.11 | 0.51 | -0.6 |
| 1435474_at | Taf5 | TAF5 RNA polymerase II, TATA box binding protein (TBP)-associated factor | -1.13 | 0.536 | -1.01 | 0.967 | -1.13 | 0.584 | 1.52 | 0.322 | -0.44 |

| | | | | | | | | | | | |
|---|---|---|---|---|---|---|---|---|---|---|---|
| 1434706_at | Vcpip1 | valosin containing protein (p97)/p47 complex interacting protein 1 | -1.02 | 0.921 | -1.15 | 0.218 | -1.1 | 0.268 | 1.15 | 0.341 | -0.53 |
| 1428836_at | 2300009A05Rik | RIKEN cDNA 2300009A05 gene | -1.05 | 0.83 | -1.02 | 0.875 | -1.2 | 0.263 | 1.06 | 0.224 | -0.55 |
| 1455876_at | Slc4a7 | solute carrier family 4, sodium bicarbonate cotransporter, member 7 | 1.36 | 0.216 | -1.52 | 0.026 | -1.35 | 0.245 | -1.16 | 0.613 | -0.66 |
| 1415747_s_at | Riok3 | RIO kinase 3 (yeast) | -1.13 | 0.092 | -1.09 | 0.256 | -1.03 | 0.726 | 1.4 | 0.15 | -0.46 |
| 1441059_at | 1700049G17Rik | RIKEN cDNA 1700049G17 gene | -1.22 | 0.728 | 1.08 | 0.739 | -1.16 | 0.339 | -1.25 | 0.642 | -0.64 |
| 1434170_at | Wdr40b | WD repeat domain 40B | -1.47 | 0.149 | -1.07 | 0.707 | 1.15 | 0.577 | -1.06 | 0.922 | -0.61 |
| 1416892_s_at | 3110001A13Rik | RIKEN cDNA 3110001A13 gene | -1.11 | 0.412 | -1.52 | 0.086 | 1.2 | 0.388 | -1.37 | 0.207 | -0.7 |
| 1432589_at | --- | --- | 1.33 | 0.391 | -1.34 | 0.538 | -1.45 | 0.081 | -1.51 | 0.203 | -0.74 |
| 1431266_at | 5830433G22Rik | RIKEN cDNA 5830433G22 gene | -1.15 | 0.508 | 1.11 | 0.766 | -1.28 | 0.295 | -1.33 | 0.397 | -0.66 |
| 1419661_at | 1600012F09Rik /// l | RIKEN cDNA 1600012F09 gene /// hypothetical LOC624446 | 1.02 | 0.905 | -1.3 | 0.475 | -1.03 | 0.825 | -1.02 | 0.945 | -0.58 |
| 1426407_at | Cugbp1 | CUG triplet repeat, RNA binding protein 1 | -1 | 0.979 | -1.1 | 0.327 | -1.17 | 0.197 | 1.19 | 0.178 | -0.52 |
| 1451842_a_at | Chrnb3 | cholinergic receptor, nicotinic, beta polypeptide 3 | -1.81 | 0.585 | 1.45 | 0.164 | -1.31 | 0.609 | -1.31 | 0.745 | -0.75 |
| 1448343_a_at | Nbr1 | neighbor of Brca1 gene 1 | -1.07 | 0.475 | -1.06 | 0.341 | -1.12 | 0.49 | 1.18 | 0.326 | -0.52 |
| 1424759_at | Arrdc4 | arrestin domain containing 4 | -1.15 | 0.331 | 1.08 | 0.37 | -1.23 | 0.447 | -1.38 | 0.047 | -0.67 |
| 1454044_a_at | Pex3 | peroxisomal biogenesis factor 3 | 1.04 | 0.44 | -1.14 | 0.179 | -1.18 | 0.129 | -1.09 | 0.598 | -0.59 |
| 1431889_x_at | Psg21 | pregnancy-specific glycoprotein 21 | -1.14 | 0.554 | 1.1 | 0.731 | -1.27 | 0.166 | -1.06 | 0.861 | -0.59 |
| 1436651_at | --- | Transcribed locus | -1.29 | 0.299 | 1.01 | 0.98 | -1.02 | 0.923 | -1.58 | 0.043 | -0.72 |
| 1458962_at | Tmed5 | transmembrane emp24 protein transport domain containing 5 | -1.18 | 0.644 | -1.17 | 0.279 | 1.06 | 0.897 | -1.21 | 0.122 | -0.63 |
| 1416451_s_at | Tbn | taube nuss | -1.05 | 0.872 | -1.38 | 0.266 | 1.09 | 0.625 | -1.08 | 0.645 | -0.61 |
| 1436424_at | 1600020E01Rik | RIKEN cDNA 1600020E01 gene | -1.01 | 0.784 | 1.01 | 0.913 | -1.3 | 0.109 | -1.26 | 0.222 | -0.64 |
| 1437690_x_at | Csnk1d | casein kinase 1, delta | -1.09 | 0.575 | -1.33 | 0.046 | 1.1 | 0.54 | -1.09 | 0.509 | -0.6 |
| 1453593_at | 1700110N18Rik | RIKEN cDNA 1700110N18 gene | 1.27 | 0.494 | -1.4 | 0.564 | -1.28 | 0.342 | -1.72 | 0.091 | -0.78 |
| 1425506_at | Mylk | myosin, light polypeptide kinase | 1.06 | 0.708 | -1.2 | 0.167 | -1.15 | 0.486 | -1.04 | 0.799 | -0.58 |
| 1457847_at | Rfxdc2 | Regulatory factor X domain containing 2 homolog (human) | 1.14 | 0.635 | -1.08 | 0.71 | -1.41 | 0.076 | -1.85 | 0.171 | -0.8 |
| 1453271_at | Phf14 | PHD finger protein 14 | -1.11 | 0.565 | 1.03 | 0.864 | -1.2 | 0.267 | -1.37 | 0.171 | -0.66 |
| 1437900_at | 4930523C07Rik | RIKEN cDNA 4930523C07 gene | 1.09 | 0.103 | -1.19 | 0.507 | -1.19 | 0.183 | -1.85 | 0.156 | -0.79 |
| 1448172_at | Mdh1 | malate dehydrogenase 1, NAD (soluble) | -1.22 | 0.002 | -1.29 | 0.017 | 1.17 | 0.234 | -1.32 | 0.201 | -0.67 |
| 1425044_at | Kcnj6 | potassium inwardly-rectifying channel, subfamily J, member 6 | -1.34 | 0.274 | -1.76 | 0.15 | 1.45 | 0.272 | -2.87 | 0.236 | -1.13 |
| 1416716_at | Efs | embryonal Fyn-associated substrate | -1.15 | 0.495 | -1.13 | 0.797 | 1.01 | 0.969 | -1.88 | 0.171 | -0.79 |
| 1434220_at | Nup98 | nucleoporin 98 | -1.07 | 0.509 | -1.14 | 0.363 | -1.05 | 0.754 | 1.08 | 0.537 | -0.54 |
| 1420241_at | --- | --- | -2.26 | 0.372 | -2.11 | 0.23 | 1.85 | 0.22 | -1.02 | 0.95 | -0.88 |
| 1438328_at | Hcfc2 | host cell factor C2 | 1.13 | 0.631 | -1.16 | 0.394 | -1.3 | 0.122 | -1.3 | 0.232 | -0.66 |
| 1456334_s_at | A230106D06Rik | RIKEN cDNA A230106D06 gene | -1.54 | 0.083 | 1.39 | 0.607 | -1.37 | 0.429 | -2.15 | 0.239 | -0.92 |
| 1452740_at | Myh10 | myosin, heavy polypeptide 10, non-muscle | -1.1 | 0.304 | -1.12 | 0.446 | -1.04 | 0.894 | 5.72 | 0.129 | 0.62 |
| 1459787_at | --- | --- | 1.16 | 0.184 | -1.34 | 0.494 | -1.16 | 0.692 | -1.56 | 0.111 | -0.73 |
| 1433655_at | Rnf141 | ring finger protein 141 | -1.01 | 0.876 | -1.01 | 0.876 | -1.27 | 0.095 | 1.1 | 0.679 | -0.55 |
| 1436252_at | Slc45a1 | solute carrier family 45, member 1 | -1.38 | 0.424 | 1.05 | 0.836 | -1 | 0.985 | -1.62 | 0.13 | -0.74 |
| 1417034_at | Trappc6a | trafficking protein particle complex 6A | 1.02 | 0.911 | -1.12 | 0.423 | -1.17 | 0.126 | -1.51 | 0.313 | -0.69 |
| 1442700_at | Pde4b | phosphodiesterase 4B, cAMP specific | 1.15 | 0.629 | -1.37 | 0.241 | -1.13 | 0.571 | -2.58 | 0.106 | -0.98 |
| 1439464_s_at | Tex10 | testis expressed gene 10 | -1.13 | 0.584 | 1.04 | 0.726 | -1.18 | 0.25 | -1.46 | 0.162 | -0.68 |
| 1418614_at | Kcnj1 | potassium inwardly-rectifying channel, subfamily J, member 1 | 1.19 | 0.738 | -1.32 | 0.603 | -1.22 | 0.723 | -2.01 | 0.38 | -0.84 |
| 1446949_at | 4930431P19Rik | RIKEN cDNA 4930431P19 gene | 1.33 | 0.131 | -1.7 | 0.006 | -1.18 | 0.221 | -1.67 | 0.006 | -0.8 |
| 1438545_at | Slc25a5 /// LOC433 | solute carrier family 25 (mitochondrial carrier, adenine nucleotide translocator), m | -1.3 | 0.488 | -1.13 | 0.477 | 1.11 | 0.694 | -1.09 | 0.75 | -0.6 |
| 1435173_at | Ate1 | arginine-tRNA-protein transferase 1 | 1.07 | 0.744 | -1.09 | 0.707 | -1.29 | 0.133 | -1.33 | 0.088 | -0.66 |
| 1448674_at | Rnf25 | ring finger protein 25 | -1.51 | 0.535 | -1.26 | 0.633 | 1.31 | 0.589 | -2.08 | 0.214 | -0.88 |
| 1452659_at | Dek | DEK oncogene (DNA binding) | -1.08 | 0.48 | -1.04 | 0.354 | -1.14 | 0.141 | 1.66 | 0.15 | -0.4 |
| 1426480_at | Sbds | Shwachman-Bodian-Diamond syndrome homolog (human) | -1.07 | 0.378 | -1.24 | 0.016 | 1.03 | 0.416 | -1.05 | 0.702 | -0.58 |
| 1430242_at | Rwdd1 | RWD domain containing 1 | 1.23 | 0.677 | -1.78 | 0.425 | -1.03 | 0.944 | -1.42 | 0.281 | -0.75 |
| 1427603_at | Atf7ip2 | activating transcription factor 7 interacting protein 2 | -1.61 | 0.128 | -1.44 | 0.282 | 1.45 | 0.381 | -1.02 | 0.962 | -0.65 |
| 1417011_at | Sdc2 | syndecan 2 | 1.15 | 0.31 | -1.03 | 0.551 | -1.55 | 0.136 | -1.29 | 0.517 | -0.68 |
| 1421918_at | Anp32a | acidic (leucine-rich) nuclear phosphoprotein 32 family, member A | 1 | 0.997 | -1.12 | 0.591 | -1.15 | 0.432 | -1.22 | 0.087 | -0.62 |
| 1437978_at | Slit2 | Slit homolog 2 (Drosophila) | -1.09 | 0.886 | -1.1 | 0.776 | -1.06 | 0.885 | 1.21 | 0.811 | -0.51 |
| 1427310_at | Falz | fetal Alzheimer antigen | 1.01 | 0.946 | -1.2 | 0.081 | -1.08 | 0.681 | -1.63 | 0.084 | -0.73 |

| | | | | | | | | | | | |
|---|---|---|---|---|---|---|---|---|---|---|---|
| 1435126_at | Dusp15 | Dual specificity phosphatase-like 15 | -1.44 | 0.312 | 1.14 | 0.809 | -1.07 | 0.896 | -1.44 | 0.37 | -0.7 |
| 1452085_at | Gatad1 | GATA zinc finger domain containing 1 | -1.09 | 0.609 | -1.09 | 0.502 | -1.07 | 0.211 | 1.36 | 0.228 | -0.47 |
| 1425971_at | --- | (ORF 1) and reverse transcriptase (ORF 2) genes | -1.31 | 0.511 | -1.04 | 0.94 | 1.04 | 0.903 | -1.08 | 0.604 | -0.6 |
| 1451101_a_at | Rps28 | ribosomal protein S28 | -1.07 | 0.24 | 1.02 | 0.789 | -1.24 | 0.062 | -1.22 | 0.016 | -0.63 |
| 1442229_at | Pax9 | Paired box gene 9 | 1.47 | 0.416 | -1.33 | 0.266 | -1.85 | 0.114 | -1.93 | 0.105 | -0.91 |
| 1435755_at | 1110001A16Rik | RIKEN cDNA 1110001A16 gene | 1.01 | 0.93 | -1.07 | 0.47 | -1.22 | 0.077 | -1.17 | 0.622 | -0.61 |
| 1415863_at | Eif4g2 | eukaryotic translation initiation factor 4, gamma 2 | -1.17 | 0.117 | 1.05 | 0.491 | -1.16 | 0.016 | -1.04 | 0.478 | -0.58 |
| 1433711_s_at | Sesn1 | sestrin 1 | -1.03 | 0.923 | 1.16 | 0.29 | -1.58 | 0.158 | -2 | 0.158 | -0.86 |
| 1418187_at | Ramp2 | receptor (calcitonin) activity modifying protein 2 | -1.14 | 0.494 | 1.03 | 0.834 | -1.16 | 0.24 | -1.05 | 0.794 | -0.58 |
| 1444735_at | --- | --- | 1.09 | 0.373 | -1.04 | 0.626 | -1.39 | 0.405 | -2.03 | 0.028 | -0.84 |
| 1459783_s_at | Cno | cappuccino | 1.01 | 0.892 | -1.11 | 0.352 | -1.16 | 0.358 | -1.27 | 0.345 | -0.63 |
| 1436958_x_at | Tpm3 | Tropomyosin 3, gamma | 1.11 | 0.602 | -1.12 | 0.347 | -1.3 | 0.355 | -1.51 | 0.168 | -0.71 |
| 1458082_at | Cd200 | Cd200 antigen | -1.31 | 0.161 | 1.35 | 0.341 | -1.53 | 0.405 | -2.27 | 0.127 | -0.94 |
| 1452668_x_at | Rab2b | RAB2B, member RAS oncogene family | 1.08 | 0.467 | -1.33 | 0.118 | -1.06 | 0.694 | -1.36 | 0.102 | -0.67 |
| 1435836_at | Pdk1 | pyruvate dehydrogenase kinase, isoenzyme 1 | -1.3 | 0.403 | -1.18 | 0.491 | 1.15 | 0.479 | -1.71 | 0.151 | -0.76 |
| 1442342_at | Sp6 | trans-acting transcription factor 6 | -1.32 | 0.565 | 1.09 | 0.704 | -1.09 | 0.779 | -1.16 | 0.642 | -0.62 |
| 1452012_a_at | Exosc1 | exosome component 1 | 1.19 | 0.045 | -1.21 | 0.259 | -1.33 | 0.013 | -1.48 | 0.102 | -0.71 |
| 1431802_a_at | D5Wsu178e | DNA segment, Chr 5, Wayne State University 178, expressed | -1.06 | 0.569 | -1.08 | 0.539 | -1.11 | 0.454 | 1.38 | 0.243 | -0.47 |
| 1439888_at | --- | Mammary gland RCB-0527 Jyg-MC(B) cDNA, RIKEN full-length enriched library, | 1.26 | 0.732 | -1.62 | 0.465 | -1.12 | 0.785 | -1.28 | 0.766 | -0.69 |
| 1442762_at | Ttll10 | tubulin tyrosine ligase-like family, member 10 | -1.06 | 0.884 | -1.61 | 0.31 | 1.21 | 0.332 | -1 | 0.997 | -0.62 |
| 1418382_at | Apcdd1 | adenomatosis polyposis coli down-regulated 1 | -1.24 | 0.23 | 1.16 | 0.856 | -1.25 | 0.417 | -1.12 | 0.365 | -0.61 |
| 1435116_at | 4933403G14Rik | RIKEN cDNA 4933403G14 gene | 1.07 | 0.841 | -1.28 | 0.114 | -1.09 | 0.663 | -1.46 | 0.064 | -0.69 |
| 1430516_at | 4930428B01Rik | RIKEN cDNA 4930428B01 gene | -1.26 | 0.344 | 1.26 | 0.399 | -1.4 | 0.053 | -1.31 | 0.185 | -0.68 |
| 1451381_at | 1810020D17Rik | RIKEN cDNA 1810020D17 gene | -1.15 | 0.295 | 1.06 | 0.462 | -1.19 | 0.195 | -1.28 | 0.323 | -0.64 |
| 1439936_at | --- | --- | -1.51 | 0.537 | 1.48 | 0.447 | -1.6 | 0.333 | -1.15 | 0.825 | -0.69 |
| 1426020_at | Tmpo | thymopoietin | -1.11 | 0.496 | -1.12 | 0.813 | -1.03 | 0.918 | 2.01 | 0.198 | -0.31 |
| 1416481_s_at | Higd1a | HIG1 domain family, member 1A | -1.01 | 0.941 | 1.01 | 0.949 | -1.3 | 0.048 | -1.18 | 0.539 | -0.62 |
| 1436893_a_at | 7-Mar | membrane-associated ring finger (C3HC4) 7 | 1.01 | 0.942 | -1.02 | 0.898 | -1.28 | 0.198 | -1.56 | 0.018 | -0.71 |
| 1427040_at | Mdfic | MyoD family inhibitor domain containing | 1.07 | 0.643 | -1.07 | 0.026 | -1.3 | 0.086 | -1.27 | 0.457 | -0.64 |
| 1431151_at | LOC628236 /// LOC | similar to Gastric triacylglycerol lipase precursor (Gastric lipase) (GL) /// similar to | -1.69 | 0.325 | -1.23 | 0.802 | 1.36 | 0.345 | -1.26 | 0.564 | -0.7 |
| 1426182_a_at | Klrc1 | killer cell lectin-like receptor subfamily C, member 1 | -1.24 | 0.028 | -1.33 | 0.557 | 1.21 | 0.733 | -1.04 | 0.948 | -0.6 |
| 1436177_at | --- | Transcribed locus | 1.12 | 0.206 | -1.12 | 0.539 | -1.33 | 0.063 | -1.78 | 0.117 | -0.78 |
| 1420414_at | Hoxa11 | homeo box A11 | 1.06 | 0.597 | -1.05 | 0.886 | -1.33 | 0.584 | -1.24 | 0.532 | -0.64 |
| 1446465_at | Atp6v1b2 | ATPase, H+ transporting, lysosomal V1 subunit B2 | -1.84 | 0.397 | 1.55 | 0.205 | -1.47 | 0.345 | -1.4 | 0.178 | -0.79 |
| 1438413_at | Senp7 | SUMO1/sentrin specific peptidase 7 | -1.32 | 0.096 | 1.14 | 0.411 | -1.15 | 0.254 | -1.33 | 0.031 | -0.67 |
| 1452495_at | Adam28 | a disintegrin and metallopeptidase domain 28 | -1.94 | 0.027 | -1.11 | 0.789 | 1.35 | 0.337 | -2.25 | 0.228 | -0.99 |
| 1434686_at | --- | --- | 1.02 | 0.949 | -1.27 | 0.138 | -1.04 | 0.717 | -1.44 | 0.061 | -0.68 |
| 1415979_x_at | Rpl7 | ribosomal protein L7 | -1.06 | 0.152 | 1.02 | 0.457 | -1.25 | 0.004 | -1.33 | 0.013 | -0.65 |
| 1456141_x_at | Brcc3 | BRCA1/BRCA2-containing complex, subunit 3 | -1.21 | 0.243 | 1.14 | 0.344 | -1.24 | 0.385 | -1.13 | 0.508 | -0.61 |
| 1444375_at | --- | Transcribed locus | -1.94 | 0.188 | -1.2 | 0.522 | 1.42 | 0.445 | -1.4 | 0.02 | -0.78 |
| 1430472_at | Armc1 | armadillo repeat containing 1 | -1.06 | 0.774 | -1.71 | 0.327 | 1.24 | 0.7 | -1.58 | 0.049 | -0.78 |
| 1423864_at | Nrbp | nuclear receptor binding protein | 1.09 | 0.79 | -1 | 0.983 | -1.46 | 0.053 | -1.1 | 0.629 | -0.62 |
| 1433812_at | Lix1l | Lix1-like | 1.26 | 0.239 | -1.1 | 0.684 | -1.67 | 0.109 | -1.24 | 0.155 | -0.69 |
| 1428510_at | Lphn1 | latrophilin 1 | -1.13 | 0.124 | -1.19 | 0.016 | 1.04 | 0.895 | -1.17 | 0.276 | -0.61 |
| 1440380_at | Capza1 | Capping protein (actin filament) muscle Z-line, alpha 1 | -1.14 | 0.464 | 1.22 | 0.277 | -1.48 | 0.155 | -1.35 | 0.122 | -0.69 |
| 1451724_at | Ankmy2 | ankyrin repeat and MYND domain containing 2 | -1.02 | 0.716 | -1.4 | 0.12 | 1.07 | 0.567 | -1.05 | 0.747 | -0.6 |
| 1460519_a_at | Mettl5 | methyltransferase like 5 | -1.03 | 0.373 | -1.22 | 0.021 | -1.02 | 0.876 | 1.24 | 0.501 | -0.51 |
| 1432571_at | --- | --- | -1.34 | 0.649 | 1.3 | 0.238 | -1.38 | 0.545 | -1.01 | 0.988 | -0.61 |
| 1452340_at | 6820424L24Rik | RIKEN cDNA 6820424L24 gene | 1.01 | 0.93 | -1.04 | 0.855 | -1.26 | 0.103 | -1.17 | 0.153 | -0.61 |
| 1441909_s_at | 9530066K23Rik | RIKEN cDNA 9530066K23 gene | -1.34 | 0.677 | -1.44 | 0.287 | 1.33 | 0.504 | -3.31 | 0.024 | -1.19 |
| 1434691_at | Sfrs2ip | splicing factor, arginine/serine-rich 2, interacting protein | -1.11 | 0.586 | -1.11 | 0.235 | -1.03 | 0.585 | 1.15 | 0.244 | -0.53 |
| 1438185_at | --- | --- | -1.01 | 0.992 | -1.4 | 0.251 | 1.06 | 0.792 | -4.13 | 0.054 | -1.37 |
| 1455485_x_at | Rpl13a | ribosomal protein L13a | -1.03 | 0.827 | 1.01 | 0.913 | -1.27 | 0.045 | -1.45 | 0.027 | -0.68 |

| | | | | | | | | | | | |
|---|---|---|---|---|---|---|---|---|---|---|---|
| 1440818_s_at | Sf3b1 | splicing factor 3b, subunit 1 | -1.31 | 0.283 | 1.04 | 0.76 | -1.03 | 0.805 | -1.52 | 0.033 | -0.71 |
| 1452576_at | Igh | Immunoglobulin heavy chain complex | -1.35 | 0.685 | -1.8 | 0.102 | 1.47 | 0.301 | -2.15 | 0.135 | -0.96 |
| 1419492_s_at | Defb1 | defensin beta 1 | -1.08 | 0.294 | -2.4 | 0.008 | 1.43 | 0.439 | -2.26 | 0.156 | -1.08 |
| 1429469_at | A930013F10Rik | RIKEN cDNA A930013F10 gene | -1.04 | 0.828 | 1.2 | 0.071 | -1.64 | 0.031 | -1.39 | 0.23 | -0.72 |
| 1428847_a_at | Macf1 | microtubule-actin crosslinking factor 1 | -1.12 | 0.125 | 1.08 | 0.545 | -1.24 | 0.263 | -1.69 | 0.27 | -0.74 |
| 1437450_x_at | 2700060E02Rik | RIKEN cDNA 2700060E02 gene | -1.02 | 0.844 | 1.02 | 0.812 | -1.29 | 0.009 | -1.48 | 0.082 | -0.69 |
| 1438226_at | AU022252 | expressed sequence AU022252 | -1.23 | 0.254 | -1.14 | 0.315 | 1.09 | 0.738 | -1.01 | 0.977 | -0.57 |
| 1425537_at | Ppm1a | protein phosphatase 1A, magnesium dependent, alpha isoform | -1.02 | 0.837 | -1.1 | 0.421 | -1.14 | 0.435 | 1.03 | 0.839 | -0.56 |
| 1416671_a_at | Mcoln1 | mucolipin 1 | -1.84 | 0.115 | -1.34 | 0.293 | 1.48 | 0.282 | -1.31 | 0.344 | -0.75 |
| 1451654_x_at | BC012278 | cDNA sequence BC012278 | -1.31 | 0.4 | -1.03 | 0.306 | 1.04 | 0.88 | -1.07 | 0.413 | -0.59 |
| 1456737_x_at | Acaa1a | acetyl-Coenzyme A acyltransferase 1A | -1.51 | 0.009 | -1.2 | 0.321 | 1.27 | 0.467 | -1.17 | 0.488 | -0.65 |
| 1416158_at | Nr2f2 | nuclear receptor subfamily 2, group F, member 2 | 1.16 | 0.327 | -1.05 | 0.803 | -1.51 | 0.079 | -1.52 | 0.337 | -0.73 |
| 1434084_at | 5730601F06Rik | RIKEN cDNA 5730601F06 gene | -1 | 0.966 | 1.13 | 0.378 | -1.55 | 0.004 | -1.06 | 0.24 | -0.62 |
| 1439526_at | --- | Adult male cecum cDNA, RIKEN full-length enriched library, clone:9130022D06 p | -1.63 | 0.113 | -1.29 | 0.593 | 1.38 | 0.252 | -1.76 | 0.333 | -0.82 |
| 1453783_at | Pura | purine rich element binding protein A | 1.16 | 0.372 | -1.4 | 0.403 | -1.12 | 0.637 | -1.3 | 0.252 | -0.66 |
| 1431938_a_at | Pmm2 | phosphomannomutase 2 | -1.02 | 0.905 | -1.07 | 0.435 | -1.16 | 0.337 | 1.18 | 0.318 | -0.52 |
| 1433432_x_at | Rps12 /// LOC6707 | ribosomal protein S12 /// similar to ribosomal protein S12 /// similar to ribosomal p | -1.09 | 0.493 | 1.09 | 0.061 | -1.31 | 0.013 | -1.32 | 0.02 | -0.66 |
| 1432461_at | Eya4 | eyes absent 4 homolog (Drosophila) | 1.24 | 0.764 | -1.14 | 0.284 | -1.52 | 0.183 | -1.07 | 0.859 | -0.62 |
| 1425568_a_at | Tmem33 | transmembrane protein 33 | -1.08 | 0.487 | -1.11 | 0.255 | -1.06 | 0.762 | 1.36 | 0.25 | -0.47 |
| 1434581_at | --- | --- | 1.18 | 0.68 | -1.29 | 0.473 | -1.22 | 0.544 | -1.04 | 0.822 | -0.59 |
| 1448536_at | Lsm3 | LSM3 homolog, U6 small nuclear RNA associated (S. cerevisiae) | -1.19 | 0.13 | -1.04 | 0.465 | -1.03 | 0.868 | 1.03 | 0.778 | -0.56 |
| 1415715_at | Tmem129 | transmembrane protein 129 | -1.11 | 0.413 | -1.01 | 0.903 | -1.13 | 0.439 | 1.26 | 0.172 | -0.5 |
| 1450191_a_at | Sox13 | SRY-box containing gene 13 | -1.13 | 0.403 | -1.14 | 0.726 | 1.01 | 0.966 | -1.22 | 0.465 | -0.62 |
| 1421675_at | 1700123K08Rik | RIKEN cDNA 1700123K08 gene | -1.24 | 0.546 | 1.09 | 0.763 | -1.13 | 0.522 | -1.11 | 0.182 | -0.6 |
| 1435257_at | Klhl17 | kelch-like 17 (Drosophila) | -1.33 | 0.137 | -1.12 | 0.646 | 1.12 | 0.53 | -1.03 | 0.797 | -0.59 |
| 1426771_at | Tug1 | taurine upregulated gene 1 | -1.41 | 0.038 | -1.1 | 0.532 | 1.16 | 0.303 | -1.01 | 0.94 | -0.59 |
| 1420642_a_at | 2010100O12Rik | RIKEN cDNA 2010100O12 gene | -1.06 | 0.392 | 1.02 | 0.826 | -1.24 | 0.137 | -1.19 | 0.311 | -0.62 |
| 1437511_x_at | Clcc1 | chloride channel CLIC-like 1 | -1.15 | 0.234 | 1.02 | 0.805 | -1.13 | 0.293 | -1.13 | 0.49 | -0.6 |
| 1417105_at | Trappc2l | trafficking protein particle complex 2-like | -1.03 | 0.801 | 1 | 0.958 | -1.26 | 0.162 | -1.08 | 0.575 | -0.59 |
| 1457249_at | Ubap2l | Ubiquitin associated protein 2-like | -1.06 | 0.841 | 1.19 | 0.538 | -1.55 | 0.104 | -1.84 | 0.035 | -0.82 |
| 1416476_a_at | Ube2d2 | ubiquitin-conjugating enzyme E2D 2 | -1.38 | 0.238 | 1.1 | 0.458 | -1.06 | 0.761 | -1.13 | 0.318 | -0.62 |
| 1454923_at | AW214353 | expressed sequence AW214353 | 1.03 | 0.454 | -1.1 | 0.39 | -1.2 | 0.1 | -1.08 | 0.783 | -0.59 |
| 1420043_s_at | Thoc1 | THO complex 1 | 1.05 | 0.941 | -1.11 | 0.801 | -1.21 | 0.74 | -1.03 | 0.846 | -0.58 |
| 1448813_at | Aadac | arylacetamide deacetylase (esterase) | -1.13 | 0.38 | -1.57 | 0.007 | 1.25 | 0.548 | -1.01 | 0.971 | -0.62 |
| 1451431_a_at | Dbndd2 | dysbindin (dystrobrevin binding protein 1) domain containing 2 | -1.07 | 0.315 | 1.16 | 0.257 | -1.48 | 0.056 | -1.37 | 0.044 | -0.69 |
| 1418210_at | Pfn2 | profilin 2 | -1.14 | 0.42 | 1.05 | 0.847 | -1.18 | 0.246 | -1.29 | 0.456 | -0.64 |
| 1423201_at | Ncor1 | nuclear receptor co-repressor 1 | -1.13 | 0.499 | -1.18 | 0.054 | 1.04 | 0.703 | -1.17 | 0.491 | -0.61 |
| 1452563_a_at | Jarid1d | jumonji, AT rich interactive domain 1D (Rbp2 like) | -1.01 | 0.994 | -1.38 | 0.501 | 1.05 | 0.887 | -1.12 | 0.763 | -0.61 |
| 1421965_s_at | Notch3 | Notch gene homolog 3 (Drosophila) | 1.01 | 0.929 | -1.25 | 0.456 | -1.04 | 0.803 | -1.36 | 0.345 | -0.66 |
| 1426504_a_at | Rnf121 | ring finger protein 121 | 1.04 | 0.034 | -1.09 | 0.276 | -1.23 | 0.196 | -1.18 | 0.154 | -0.61 |
| 1433561_at | Centb2 | centaurin, beta 2 | -1.2 | 0.272 | 1.04 | 0.605 | -1.12 | 0.502 | -1.49 | 0.107 | -0.69 |
| 1447414_at | --- | --- | -2.61 | 0.21 | 1.56 | 0.523 | -1.2 | 0.215 | -1.24 | 0.469 | -0.87 |
| 1442739_at | BC031441 | cDNA sequence BC031441 | -1.24 | 0.626 | 1.16 | 0.566 | -1.24 | 0.453 | -1.76 | 0.137 | -0.77 |
| 1439399_a_at | Snord22 | small nucleolar RNA, C/D box 22 | -1.11 | 0.601 | -1.08 | 0.626 | -1.05 | 0.833 | 1.17 | 0.613 | -0.52 |
| 1458402_at | Pts | 6-pyruvoyl-tetrahydropterin synthase | -1.27 | 0.439 | 1.04 | 0.931 | -1.06 | 0.712 | -1.92 | 0.364 | -0.8 |
| 1452125_at | Thrap3 | thyroid hormone receptor associated protein 3 | 1.17 | 0.38 | -1.06 | 0.664 | -1.52 | 0.06 | -1.26 | 0.019 | -0.67 |
| 1417248_at | Ralbp1 | ralA binding protein 1 | 1.05 | 0.665 | -1.06 | 0.24 | -1.28 | 0.129 | -1.12 | 0.648 | -0.6 |
| 1431498_at | 9530097N15Rik | RIKEN cDNA 9530097N15 gene | -1.88 | 0.233 | -1.26 | 0.051 | 1.45 | 0.374 | -1.22 | 0.802 | -0.73 |
| 1435400_at | 2900075B16Rik | RIKEN cDNA 2900075B16 gene | -1.06 | 0.796 | 1.09 | 0.7 | -1.35 | 0.038 | -1.5 | 0.28 | -0.7 |
| 1422976_x_at | Ndufa7 | NADH dehydrogenase (ubiquinone) 1 alpha subcomplex, 7 (B14.5a) | 1.04 | 0.602 | -1.26 | 0.22 | -1.06 | 0.635 | -1.1 | 0.786 | -0.6 |
| 1448740_at | 2400006H24Rik | RIKEN cDNA 2400006H24 gene | -1.16 | 0.42 | -1.09 | 0.143 | -1 | 0.994 | 1.88 | 0.274 | -0.34 |
| 1455816_a_at | Kctd3 | potassium channel tetramerisation domain containing 3 | -1.18 | 0.452 | 1 | 0.979 | -1.08 | 0.454 | -1.13 | 0.681 | -0.6 |
| 1423572_at | Bcl2l2 | Bcl2-like 2 | -1.03 | 0.11 | -1.08 | 0.483 | -1.14 | 0.551 | 1.02 | 0.915 | -0.56 |

| | | | | | | | | | | | |
|---|---|---|---|---|---|---|---|---|---|---|---|
| 1447868_x_at | Txnl2 /// LOC62001 | thioredoxin-like 2 /// similar to thioredoxin-like 2 | -1.18 | 0.765 | -1.29 | 0.229 | 1.15 | 0.142 | -1.29 | 0.31 | -0.65 |
| 1424293_s_at | Tmem55a | transmembrane protein 55A | -1.08 | 0.427 | -1.03 | 0.756 | -1.14 | 0.204 | 1.17 | 0.072 | -0.52 |
| 1460737_at | Igbp1 | immunoglobulin (CD79A) binding protein 1 | -1.17 | 0.239 | -1.1 | 0.381 | 1.01 | 0.911 | -1.5 | 0.171 | -0.69 |
| 1457271_at | Gm131 /// LOC634 | gene model 131, (NCBI) /// similar to prochymosin | 1.11 | 0.386 | -1.12 | 0.658 | -1.3 | 0.146 | -1.22 | 0.422 | -0.63 |
| 1419231_s_at | Krt12 | keratin 12 | -1.26 | 0.698 | -1.44 | 0.445 | 1.29 | 0.61 | -1.76 | 0.462 | -0.79 |
| 1455607_at | Rspo3 | R-spondin 3 homolog (Xenopus laevis) | -1.22 | 0.553 | 1.1 | 0.385 | -1.17 | 0.607 | -1.8 | 0.169 | -0.77 |
| 1454823_at | Wdr37 | WD repeat domain 37 | -1.24 | 0.503 | -1.39 | 0.174 | 1.25 | 0.259 | -1 | 0.989 | -0.6 |
| 1419016_at | 1700034I23Rik | RIKEN cDNA 1700034I23 gene | 1.01 | 0.952 | -1.25 | 0.664 | -1.04 | 0.913 | -1.34 | 0.7 | -0.65 |
| 1441615_at | Cbfa2t2h | core-binding factor, runt domain, alpha subunit 2, translocated to, 2 homolog (hur | 1.12 | 0.52 | -1.09 | 0.671 | -1.36 | 0.061 | -1.3 | 0.143 | -0.66 |
| 1434323_at | Eif3s3 /// LOC6250 | eukaryotic translation initiation factor 3, subunit 3 (gamma) /// similar to eukaryotic | -1.45 | 0.45 | -1.83 | 0.429 | 1.54 | 0.18 | -1.1 | 0.834 | -0.71 |
| 1449827_at | Agc1 | aggrecan 1 | -2.12 | 0.079 | 1.44 | 0.092 | -1.15 | 0.645 | -1.16 | 0.765 | -0.75 |
| 1416115_at | Orc3l | origin recognition complex, subunit 3-like (S. cerevisiae) | -1.15 | 0.24 | -1.24 | 0.103 | 1.1 | 0.114 | -1.14 | 0.073 | -0.61 |
| 1437925_at | 2210018M03Rik | RIKEN cDNA 2210018M03 gene | -1.01 | 0.995 | -1.93 | 0.243 | 1.26 | 0.563 | -1.13 | 0.416 | -0.7 |
| 1416125_at | Fkbp5 | FK506 binding protein 5 | -1.31 | 0.782 | -1.23 | 0.556 | 1.19 | 0.679 | -1.2 | 0.714 | -0.64 |
| 1434776_at | --- | --- | 1.19 | 0.039 | -1.35 | 0.062 | -1.18 | 0.243 | -1.35 | 0.485 | -0.67 |
| 1426557_at | Mesp1 | mesoderm posterior 1 | -1.27 | 0.414 | 1.33 | 0.282 | -1.52 | 0.026 | -1.2 | 0.323 | -0.67 |
| 1416825_at | Snta1 | syntrophin, acidic 1 | 1.05 | 0.773 | -1.04 | 0.601 | -1.3 | 0.303 | -1.56 | 0.147 | -0.71 |
| 1427904_s_at | 2410091C18Rik | RIKEN cDNA 2410091C18 gene | -1.09 | 0.339 | -1.11 | 0.31 | -1.05 | 0.77 | 1.16 | 0.157 | -0.52 |
| 1422654_at | Sgca | sarcoglycan, alpha (dystrophin-associated glycoprotein) | -1.96 | 0.466 | -1.18 | 0.123 | 1.42 | 0.269 | -1.62 | 0.429 | -0.83 |
| 1434222_at | Sipa1l1 | signal-induced proliferation-associated 1 like 1 | -1.12 | 0.264 | -1.35 | 0.01 | 1.14 | 0.538 | -1.26 | 0.422 | -0.65 |
| 1457612_at | LOC14433 /// LOC2 | similar to glyceraldehyde-3-phosphate dehydrogenase /// similar to glyceraldehyd | -1.46 | 0.272 | -1.02 | 0.878 | 1.11 | 0.635 | -1.3 | 0.009 | -0.67 |
| 1438257_at | Zfp313 | Zinc finger protein 313 | -1.13 | 0.178 | 1.02 | 0.889 | -1.15 | 0.402 | -1.56 | 0.067 | -0.7 |
| 1415925_a_at | Nup62 | nucleoporin 62 | -1.05 | 0.786 | -1.21 | 0.088 | -1 | 0.983 | 1.19 | 0.589 | -0.52 |
| 1415772_at | Ncl | nucleolin | 1.03 | 0.876 | -1.05 | 0.823 | -1.25 | 0.047 | -1.07 | 0.636 | -0.59 |
| 1425873_a_at | Lepr | leptin receptor | 1.28 | 0.541 | -1.63 | 0.526 | -1.14 | 0.545 | -1.07 | 0.821 | -0.64 |
| 1446215_at | Zfhx2as | zinc finger homeobox 2, antisense | -1.36 | 0.668 | -1.08 | 0.718 | 1.11 | 0.569 | -1.49 | 0.091 | -0.7 |
| 1427257_at | Cspg2 | chondroitin sulfate proteoglycan 2 | -1.05 | 0.871 | -1.11 | 0.407 | -1.09 | 0.581 | 3.32 | 0.155 | 0.02 |
| 1415790_at | Ublcp1 | ubiquitin-like domain containing CTD phosphatase 1 | 1.02 | 0.913 | -1.1 | 0.687 | -1.18 | 0.423 | -1.29 | 0.207 | -0.64 |
| 1447563_at | Rps3 | ribosomal protein S3 | -2.29 | 0.363 | 1.59 | 0.378 | -1.34 | 0.503 | -1.05 | 0.787 | -0.77 |
| 1434016_at | Znrf2 | zinc and ring finger 2 | -1.13 | 0.07 | -1.14 | 0.068 | 1.01 | 0.898 | -1.23 | 0.398 | -0.62 |
| 1418327_at | 1110058L19Rik | RIKEN cDNA 1110058L19 gene | -1.07 | 0.507 | 1.02 | 0.779 | -1.21 | 0.055 | -1.1 | 0.288 | -0.59 |
| 1429519_at | Fpgt | fucose-1-phosphate guanylyltransferase | -1.1 | 0.693 | 1.04 | 0.708 | -1.21 | 0.173 | -1.37 | 0.093 | -0.66 |
| 1446906_at | C81615 | expressed sequence C81615 | -1.12 | 0.303 | 1.36 | 0.179 | -1.9 | 0.042 | -1.21 | 0.101 | -0.72 |
| 1444485_at | Shq1 | SHQ1 homolog (S. cerevisiae) | -1.12 | 0.644 | -1.66 | 0.072 | 1.28 | 0.045 | -1.16 | 0.449 | -0.67 |
| 1456462_x_at | Ppp1cb | protein phosphatase 1, catalytic subunit, beta isoform | -1.1 | 0.779 | -1.04 | 0.853 | -1.1 | 0.451 | 1.33 | 0.375 | -0.48 |
| 1420846_at | Mrps2 | mitochondrial ribosomal protein S2 | -1.27 | 0.003 | -1.11 | 0.225 | 1.09 | 0.235 | -1.3 | 0.18 | -0.65 |
| 1441824_at | Mboat1 | membrane bound O-acyltransferase domain containing 1 | 1.41 | 0.651 | -1.72 | 0.388 | -1.27 | 0.606 | -1.25 | 0.618 | -0.71 |
| 1457173_at | Ywhae | Tyrosine 3-monooxygenase/tryptophan 5-monooxygenase activation protein, eps | -1.41 | 0.338 | 1.42 | 0.193 | -1.55 | 0.241 | -1.25 | 0.455 | -0.7 |
| 1416627_at | Spint1 | serine protease inhibitor, Kunitz type 1 | 1 | 0.959 | -1.23 | 0.386 | -1.04 | 0.447 | -1.01 | 0.915 | -0.57 |
| 1460631_at | Ogt | O-linked N-acetylglucosamine (GlcNAc) transferase (UDP-N-acetylglucosamine:p | -1.07 | 0.522 | 1.01 | 0.965 | -1.19 | 0.168 | -1.25 | 0.378 | -0.63 |
| 1455236_x_at | Serf2 | small EDRK-rich factor 2 | -1.13 | 0.437 | 1.05 | 0.578 | -1.19 | 0.047 | -1.36 | 0.054 | -0.66 |
| 1444670_at | A130004G11Rik | RIKEN cDNA A130004G11 gene | -1.08 | 0.692 | 1.08 | 0.818 | -1.29 | 0.034 | -1.71 | 0.18 | -0.75 |
| 1434289_at | Nsun5 | NOL1/NOP2/Sun domain family, member 5 | -1.02 | 0.939 | -1.42 | 0.164 | 1.1 | 0.74 | -1.18 | 0.365 | -0.63 |
| 1444842_at | Jarid1a | jumonji, AT rich interactive domain 1A (Rbp2 like) | 1.25 | 0.544 | -1.22 | 0.625 | -1.41 | 0.042 | -1.03 | 0.93 | -0.6 |
| 1446088_at | 9430081I23Rik | RIKEN cDNA 9430081I23 gene | 1.35 | 0.179 | -1.34 | 0.437 | -1.46 | 0.058 | -1.95 | 0.186 | -0.85 |
| 1438368_a_at | Matr3 | matrin 3 | -1.05 | 0.435 | 1.04 | 0.254 | -1.29 | 0.035 | -1.2 | 0.108 | -0.62 |
| 1427897_s_at | 2400003N08Rik | RIKEN cDNA 2400003N08 gene | -1.08 | 0.504 | -1.17 | 0.08 | 1 | 0.993 | -1.02 | 0.92 | -0.57 |
| 1418618_at | En1 | engrailed 1 | 1.54 | 0.107 | -2.3 | 0.048 | -1.25 | 0.664 | -1.23 | 0.781 | -0.81 |
| 1443666_at | Mid1 | Midline 1 | 1.64 | 0.484 | -1.42 | 0.511 | -2.29 | 0.159 | -1.8 | 0.393 | -0.97 |
| 1425209_at | Zfp84 | zinc finger protein 84 | -1 | 0.996 | -1.69 | 0.072 | 1.19 | 0.579 | -1.21 | 0.277 | -0.68 |
| 1453896_at | Pdzd2 | PDZ domain containing 2 | -1.17 | 0.538 | 1.01 | 0.984 | -1.09 | 0.773 | -1.01 | 0.981 | -0.57 |
| 1448473_at | Bub3 | budding uninhibited by benzimidazoles 3 homolog (S. cerevisiae) | -1.14 | 0.444 | -1.04 | 0.798 | -1.06 | 0.799 | 1.49 | 0.298 | -0.44 |
| 1457936_at | Mapk8 | mitogen activated protein kinase 8 | -1.04 | 0.924 | 1.04 | 0.894 | -1.28 | 0.25 | -2.11 | 0.017 | -0.85 |

| | | | | | | | | | | | |
|---|---|---|---|---|---|---|---|---|---|---|---|
| 1450427_at | Chrna6 | cholinergic receptor, nicotinic, alpha polypeptide 6 | 1.46 | 0.634 | -1.28 | 0.293 | -1.85 | 0.112 | -1.69 | 0.441 | -0.84 |
| 1454730_at | 4932414K18Rik | RIKEN cDNA 4932414K18 gene | -1.1 | 0.25 | -1.01 | 0.864 | -1.14 | 0.187 | 1.7 | 0.176 | -0.39 |
| 1433737_at | LOC677213 | similar to U2AF homology motif (UHM) kinase 1 | 1.03 | 0.807 | -1.05 | 0.682 | -1.26 | 0.074 | -1.07 | 0.535 | -0.59 |
| 1417591_at | Ptges2 | prostaglandin E synthase 2 | -1.18 | 0.359 | -1 | 0.982 | -1.07 | 0.591 | 1.32 | 0.262 | -0.48 |
| 1448305_at | Rab6 | RAB6, member RAS oncogene family | -1.09 | 0.281 | -1.08 | 0.249 | -1.08 | 0.553 | 1.31 | 0.069 | -0.48 |
| 1437799_at | 4933407H18Rik | RIKEN cDNA 4933407H18 gene | -1.29 | 0.459 | 1.14 | 0.7 | -1.16 | 0.588 | -1.39 | 0.312 | -0.68 |
| 1444548_at | 1600025M17Rik | RIKEN cDNA 1600025M17 gene | -2.46 | 0.314 | 1.52 | 0.594 | -1.17 | 0.824 | -1.53 | 0.519 | -0.91 |
| 1426836_s_at | Metap1 | methionyl aminopeptidase 1 | -1.07 | 0.296 | -1.05 | 0.727 | -1.12 | 0.118 | 1.03 | 0.901 | -0.55 |
| 1427001_s_at | Hnf4a | hepatic nuclear factor 4, alpha | -1.08 | 0.501 | -1.11 | 0.543 | -1.05 | 0.875 | 2.13 | 0.218 | -0.28 |
| 1433926_at | Dync1li2 | dynein, cytoplasmic 1 light intermediate chain 2 | -1.05 | 0.544 | -1.07 | 0.608 | -1.13 | 0.332 | 1.05 | 0.705 | -0.55 |
| 1460492_at | 8030498B09Rik | RIKEN cDNA 8030498B09 gene | -1.4 | 0.439 | 1.12 | 0.735 | -1.06 | 0.932 | -2.05 | 0.545 | -0.85 |
| 1435801_at | Fcmd | Fukuyama type congenital muscular dystrophy homolog (human) | 1.01 | 0.689 | -1.12 | 0.515 | -1.14 | 0.546 | -1.03 | 0.861 | -0.57 |
| 1429446_at | Sdccag1 | serologically defined colon cancer antigen 1 | -1.12 | 0.579 | 1 | 0.956 | -1.13 | 0.236 | -1.34 | 0.123 | -0.65 |
| 1440881_at | Brwd3 | bromodomain and WD repeat domain containing 3 | 1.11 | 0.509 | -1.27 | 0.498 | -1.14 | 0.516 | -1.2 | 0.476 | -0.62 |
| 1442357_at | Arhgef17 | Rho guanine nucleotide exchange factor (GEF) 17 | -3.06 | 0.057 | -1.36 | 0.602 | 1.72 | 0.079 | -1.53 | 0.382 | -1.06 |
| 1453230_at | Zfp74 | zinc finger protein 74 | -1.08 | 0.766 | 1.16 | 0.168 | -1.45 | 0.07 | -1.57 | 0.157 | -0.73 |
| 1438192_s_at | Baz2a | bromodomain adjacent to zinc finger domain, 2A | -1.48 | 0.065 | -1.03 | 0.815 | 1.13 | 0.576 | -1.13 | 0.608 | -0.63 |
| 1448259_at | Fstl1 | follistatin-like 1 | -1.01 | 0.916 | 1.29 | 0.198 | -2 | 0.02 | -1.39 | 0.319 | -0.78 |
| 1422609_at | Arpp19 | cAMP-regulated phosphoprotein 19 | -1.15 | 0.031 | -1.01 | 0.959 | -1.09 | 0.755 | 1 | 0.99 | -0.56 |
| 1428286_at | --- | --- | -1.06 | 0.409 | -1.04 | 0.717 | -1.14 | 0.431 | 1.09 | 0.629 | -0.54 |
| 1436385_at | Ganc | glucosidase, alpha; neutral C | -1.18 | 0.044 | -1.14 | 0.459 | 1.06 | 0.835 | -1.61 | 0.008 | -0.72 |
| 1456402_at | A330076H08Rik | RIKEN cDNA A330076H08 gene | 1.18 | 0.255 | -1.06 | 0.939 | -1.52 | 0.054 | -1.56 | 0.601 | -0.74 |
| 1427731_at | Gnrhr | gonadotropin releasing hormone receptor | -1.57 | 0.372 | -1.02 | 0.949 | 1.17 | 0.601 | -1.16 | 0.67 | -0.65 |
| 1423383_a_at | Osbpl9 | oxysterol binding protein-like 9 | -1.26 | 0.621 | 1.14 | 0.141 | -1.18 | 0.224 | -1.62 | 0.143 | -0.73 |
| 1418711_at | Pdgfa | platelet derived growth factor, alpha | -1.24 | 0.26 | 1.19 | 0.626 | -1.28 | 0.372 | -1.06 | 0.741 | -0.6 |
| 1446430_at | Hectd2 | HECT domain containing 2 | -1.64 | 0.232 | -1.01 | 0.961 | 1.18 | 0.641 | -1.35 | 0.584 | -0.71 |
| 1424977_at | 4930418G15Rik | RIKEN cDNA 4930418G15 gene | -1.41 | 0.607 | 1.23 | 0.754 | -1.19 | 0.585 | -1.03 | 0.952 | -0.6 |
| 1426827_at | A730098D12Rik | RIKEN cDNA A730098D12 gene | -1.13 | 0.127 | -1.12 | 0.49 | 1.01 | 0.952 | -1.17 | 0.284 | -0.6 |
| 1443669_at | Zfyve28 | Zinc finger, FYVE domain containing 28 | -1.08 | 0.838 | -1.73 | 0.259 | 1.27 | 0.521 | -1.3 | 0.553 | -0.71 |
| 1421272_at | Vav2 | Vav2 oncogene | -1.08 | 0.887 | -1.31 | 0.618 | 1.09 | 0.705 | -1.96 | 0.399 | -0.81 |
| 1426621_a_at | Ppp2r2b | protein phosphatase 2 (formerly 2A), regulatory subunit B (PR 52), beta isoform | -1.3 | 0.083 | 1.32 | 0.436 | -1.45 | 0.09 | -1.88 | 0.271 | -0.83 |
| 1424147_at | Ahsa1 | AHA1, activator of heat shock protein ATPase homolog 1 (yeast) | -1.19 | 0.182 | -1.14 | 0.132 | 1.06 | 0.677 | -1.03 | 0.856 | -0.57 |
| 1435491_at | 9830167H18Rik | RIKEN cDNA 9830167H18 gene | -1.01 | 0.96 | 1.13 | 0.226 | -1.52 | 0.008 | -1.09 | 0.679 | -0.62 |
| 1416417_a_at | Ndufb7 | NADH dehydrogenase (ubiquinone) 1 beta subcomplex, 7 | -1.01 | 0.894 | -1.19 | 0.266 | -1.06 | 0.291 | 1.08 | 0.404 | -0.54 |
| 1439217_at | Nalp9c | NACHT, LRR and PYD containing protein 9c | -1.02 | 0.976 | -1.15 | 0.795 | -1.08 | 0.503 | 1.9 | 0.165 | -0.34 |
| 1433983_at | Cnksr3 | Cnksr family member 3 | 1 | 0.986 | -1.18 | 0.29 | -1.07 | 0.352 | -1.39 | 0.071 | -0.66 |
| 1437907_a_at | Tbca | tubulin cofactor a | -1.06 | 0.074 | 1.04 | 0.661 | -1.26 | 0.003 | -1.39 | 0.111 | -0.67 |
| 1428281_at | Trub1 | TruB pseudouridine (psi) synthase homolog 1 (E. coli) | -1.18 | 0.525 | -1.32 | 0.088 | 1.17 | 0.229 | -1.08 | 0.725 | -0.6 |
| 1423144_at | Pik3ca | phosphatidylinositol 3-kinase, catalytic, alpha polypeptide | -1.05 | 0.651 | 1.02 | 0.872 | -1.23 | 0.37 | -1.33 | 0.291 | -0.65 |
| 1430849_a_at | Cgrrf1 | cell growth regulator with ring finger domain 1 | -1.03 | 0.852 | -1.14 | 0.382 | -1.08 | 0.68 | 1.41 | 0.352 | -0.46 |
| 1435140_at | Ide | insulin degrading enzyme | -1.03 | 0.686 | -1.11 | 0.119 | -1.11 | 0.376 | 1.13 | 0.294 | -0.53 |
| 1434523_x_at | Eif3s6 | eukaryotic translation initiation factor 3, subunit 6 | 1.02 | 0.873 | -1.06 | 0.371 | -1.22 | 0.219 | -1.32 | 0.068 | -0.65 |
| 1420840_at | Plekha3 | pleckstrin homology domain-containing, family A (phosphoinositide binding specif | -1.24 | 0.007 | -1.03 | 0.749 | 1.01 | 0.94 | -1.47 | 0.086 | -0.68 |
| 1458972_at | 9330112F22Rik | RIKEN cDNA 9330112F22 gene | -1.25 | 0.397 | 1.25 | 0.515 | -1.37 | 0.221 | -1.1 | 0.654 | -0.62 |
| 1440236_at | A830039H05Rik | RIKEN cDNA A830039H05 gene | 1.26 | 0.224 | -1.12 | 0.581 | -1.59 | 0.11 | -1.31 | 0.432 | -0.69 |
| 1430148_at | Rab19 | RAB19, member RAS oncogene family | 1.04 | 0.878 | -1.32 | 0.704 | -1.02 | 0.888 | -1.62 | 0.049 | -0.73 |
| 1415760_s_at | Atox1 | ATX1 (antioxidant protein 1) homolog 1 (yeast) | -1.07 | 0.536 | -1.03 | 0.338 | -1.14 | 0.298 | 1.05 | 0.894 | -0.55 |
| 1451307_at | Mrpl14 | mitochondrial ribosomal protein L14 | -1 | 0.982 | -1.17 | 0.286 | -1.08 | 0.536 | 1.37 | 0.205 | -0.47 |
| 1447323_at | LOC621954 | Hypothetical protein LOC621954 | -1.63 | 0.405 | 1.57 | 0.476 | -1.67 | 0.237 | -1.38 | 0.563 | -0.78 |
| 1435698_at | 4921505C17Rik | RIKEN cDNA 4921505C17 gene | -1.01 | 0.904 | 1.06 | 0.635 | -1.36 | 0.287 | -1.16 | 0.554 | -0.62 |
| 1439994_at | --- | Adult male corpora quadrigemina cDNA, RIKEN full-length enriched library, clone | 1.01 | 0.981 | -1.28 | 0.683 | -1.01 | 0.983 | -1.01 | 0.968 | -0.57 |
| 1460567_at | Rfxdc2 | regulatory factor X domain containing 2 homolog (human) | 1.12 | 0.489 | -1.18 | 0.437 | -1.24 | 0.295 | -1.98 | 0.134 | -0.82 |
| 1448317_at | Tmem128 | transmembrane protein 128 | -1.22 | 0.092 | 1.03 | 0.586 | -1.07 | 0.428 | -1.13 | 0.146 | -0.6 |

| | | | | | | | | | | | |
|---|---|---|---|---|---|---|---|---|---|---|---|
| 1416653_at | Stxbp3a | syntaxin binding protein 3A | -1.05 | 0.39 | 1.07 | 0.535 | -1.32 | 0.039 | -1.46 | 0.082 | -0.69 |
| 1446847_at | 0610010D24Rik | RIKEN cDNA 0610010D24 gene | 1.12 | 0.837 | -1.13 | 0.712 | -1.29 | 0.183 | -1.33 | 0.054 | -0.66 |
| 1454814_s_at | AU021838 | expressed sequence AU021838 | -1.03 | 0.856 | -1.1 | 0.193 | -1.11 | 0.296 | 1.17 | 0.494 | -0.52 |
| 1434737_at | Obfc1 | oligonucleotide/oligosaccharide-binding fold containing 1 | -1.31 | 0.091 | 1.07 | 0.798 | -1.06 | 0.51 | -1.27 | 0.158 | -0.64 |
| 1456318_at | Clec1a | C-type lectin domain family 1, member a | -1.23 | 0.415 | 1.36 | 0.401 | -1.64 | 0.055 | -1.06 | 0.853 | -0.64 |
| 1419246_s_at | Rab14 | RAB14, member RAS oncogene family | -1.06 | 0.475 | -1.25 | 0.021 | 1.04 | 0.575 | -1.01 | 0.914 | -0.57 |
| 1428797_at | 0610039J04Rik | RIKEN cDNA 0610039J04 gene | -1.13 | 0.592 | -1.21 | 0.114 | 1.07 | 0.443 | -1.12 | 0.499 | -0.6 |
| 1423152_at | Vapb | vesicle-associated membrane protein, associated protein B and C | -2 | 0.47 | -1.45 | 0.367 | 1.59 | 0.46 | -2.34 | 0.335 | -1.05 |
| 1436390_a_at | Clcc1 | chloride channel CLIC-like 1 | -1.23 | 0.321 | 1.11 | 0.176 | -1.17 | 0.245 | -1.21 | 0.148 | -0.62 |
| 1416631_at | Ap4b1 | adaptor-related protein complex AP-4, beta 1 | -1.16 | 0.584 | -1.05 | 0.685 | -1.04 | 0.803 | 1.07 | 0.752 | -0.54 |
| 1459859_x_at | Chrac1 | chromatin accessibility complex 1 | 1.04 | 0.698 | -1.02 | 0.846 | -1.33 | 0.085 | -1.42 | 0.183 | -0.68 |
| 1446362_at | --- | --- | 1.17 | 0.13 | -1.57 | 0.342 | -1.03 | 0.933 | -2.99 | 0.351 | -1.1 |
| 1426402_at | Syncrip | synaptotagmin binding, cytoplasmic RNA interacting protein | -1.03 | 0.892 | 1.01 | 0.947 | -1.25 | 0.126 | -1.16 | 0.272 | -0.61 |
| 1430573_s_at | Etohd2 | ethanol decreased 2 | -1.16 | 0.639 | 1.2 | 0.418 | -1.39 | 0.133 | -1.12 | 0.575 | -0.62 |
| 1428051_a_at | Cacna1d | calcium channel, voltage-dependent, L type, alpha 1D subunit | -1.21 | 0.619 | -1.44 | 0.579 | 1.26 | 0.057 | -1.24 | 0.538 | -0.66 |
| 1454952_s_at | B130055D15Rik | RIKEN cDNA B130055D15 gene | -1.16 | 0.343 | -1.02 | 0.831 | -1.06 | 0.574 | 1.99 | 0.158 | -0.31 |
| 1456374_x_at | Eif3s8 | eukaryotic translation initiation factor 3, subunit 8 | 1.03 | 0.826 | -1.11 | 0.072 | -1.17 | 0.097 | -1.21 | 0.163 | -0.62 |
| 1425867_at | Plekha4 | pleckstrin homology domain containing, family A (phosphoinositide binding specif | -1.14 | 0.809 | 1.29 | 0.618 | -1.63 | 0.137 | -1.6 | 0.491 | -0.77 |
| 1422998_a_at | Glrx2 | glutaredoxin 2 (thioltransferase) | -1.02 | 0.757 | -1.04 | 0.598 | -1.2 | 0.27 | 1.24 | 0.349 | -0.5 |
| 1446158_at | Sec15l2 | SEC15-like 2 (S. cerevisiae) | -1.21 | 0.363 | 1.39 | 0.205 | -1.76 | 0.063 | -1.51 | 0.392 | -0.77 |
| 1422407_s_at | Hras1 | Harvey rat sarcoma virus oncogene 1 | -1.01 | 0.943 | -1.09 | 0.263 | -1.15 | 0.268 | 2.17 | 0.179 | -0.27 |
| 1416426_at | Rab5a | RAB5A, member RAS oncogene family | -1.15 | 0.183 | -1.05 | 0.559 | -1.04 | 0.49 | 1.07 | 0.317 | -0.54 |
| 1429278_at | Nubpl | nucleotide binding protein-like | -1.17 | 0.542 | 1.08 | 0.643 | -1.18 | 0.379 | -1.25 | 0.306 | -0.63 |
| 1417593_at | Tusc2 | tumor suppressor candidate 2 | -1.15 | 0.32 | -1.13 | 0.048 | 1.03 | 0.547 | -1.04 | 0.829 | -0.57 |
| 1453997_a_at | Nes | nestin | -1.37 | 0.628 | -1.67 | 0.349 | 1.45 | 0.506 | -2.28 | 0.465 | -0.97 |
| 1428968_at | Cep57 | centrosomal protein 57 | -1.06 | 0.762 | -1.03 | 0.85 | -1.15 | 0.421 | 1.46 | 0.246 | -0.45 |
| 1428715_at | 2810423A18Rik | RIKEN cDNA 2810423A18 gene | 1.03 | 0.58 | -1.07 | 0.671 | -1.22 | 0.02 | -1.15 | 0.458 | -0.6 |
| 1430125_s_at | Pqlc1 | PQ loop repeat containing 1 | -1.05 | 0.651 | -1.12 | 0.557 | -1.07 | 0.446 | 2.56 | 0.159 | -0.17 |
| 1442309_at | Dnmt3a | DNA methyltransferase 3A | -1.06 | 0.791 | 1.19 | 0.578 | -1.55 | 0.127 | -1.42 | 0.354 | -0.71 |
| 1437951_at | 4930429A22Rik | RIKEN cDNA 4930429A22 gene | 1.15 | 0.602 | -1.04 | 0.829 | -1.48 | 0.03 | -1.27 | 0.338 | -0.66 |
| 1427432_a_at | Sfrs10 | splicing factor, arginine/serine-rich 10 (transformer 2 homolog, Drosophila) | -1.05 | 0.656 | -1.02 | 0.856 | -1.18 | 0.139 | 1.04 | 0.696 | -0.55 |
| 1443796_at | Il16 | interleukin 16 | -1.6 | 0.346 | -1.01 | 0.989 | 1.16 | 0.237 | -1.72 | 0.124 | -0.79 |
| 1435265_at | --- | Adult male cecum cDNA, RIKEN full-length enriched library, clone:9130204C03 p | -1.27 | 0.344 | 1.08 | 0.681 | -1.09 | 0.797 | -1.39 | 0.247 | -0.67 |
| 1422911_at | Cbln3 | cerebellin 3 precursor protein | -1.11 | 0.862 | -1.3 | 0.243 | 1.11 | 0.78 | -1 | 0.987 | -0.58 |
| 1426467_s_at | 0610037L13Rik | RIKEN cDNA 0610037L13 gene | 1.11 | 0.381 | -1.1 | 0.413 | -1.3 | 0.144 | -1.08 | 0.761 | -0.59 |
| 1424220_a_at | Porcn | porcupine homolog (Drosophila) | -1.07 | 0.785 | -1.08 | 0.704 | -1.09 | 0.411 | 1.05 | 0.796 | -0.55 |
| 1427397_at | 2810046L04Rik | RIKEN cDNA 2810046L04 gene | -1.18 | 0.569 | -1.13 | 0.281 | 1.05 | 0.545 | -1.3 | 0.202 | -0.64 |
| 1453590_at | Arl5b | ADP-ribosylation factor-like 5B | -1.35 | 0.392 | -1.52 | 0.262 | 1.38 | 0.104 | -1.52 | 0.086 | -0.75 |
| 1428707_at | Ptms | parathymosin | 1.03 | 0.918 | -1.11 | 0.64 | -1.16 | 0.184 | -1.06 | 0.818 | -0.58 |
| 1457293_at | Zbtb4 | Zinc finger and BTB domain containing 4 | -1.01 | 0.983 | 1.13 | 0.764 | -1.51 | 0.023 | -1.73 | 0.23 | -0.78 |
| 1448055_at | Fndc7 | fibronectin type III domain containing 7 | -1.09 | 0.875 | 1.07 | 0.901 | -1.25 | 0.67 | -1.22 | 0.684 | -0.62 |
| 1456473_x_at | Arf2 | ADP-ribosylation factor 2 | -1.15 | 0.751 | 1 | 0.993 | -1.1 | 0.841 | -1.52 | 0.433 | -0.69 |
| 1437208_at | 10-Sep | septin 10 | -1.08 | 0.306 | 1.11 | 0.521 | -1.34 | 0.286 | -1.08 | 0.318 | -0.6 |
| 1427593_at | Trim8 | tripartite motif protein 8 | -1.29 | 0.692 | -2.18 | 0.063 | 1.55 | 0.392 | -1.63 | 0.348 | -0.89 |
| 1435811_a_at | Unc50 | unc-50 homolog (C. elegans) | -1.1 | 0.186 | 1.09 | 0.519 | -1.28 | 0.109 | -1.1 | 0.63 | -0.6 |
| 1459980_x_at | Rab3a | RAB3A, member RAS oncogene family | -1.14 | 0.03 | 1.05 | 0.287 | -1.17 | 0.041 | -1.11 | 0.411 | -0.59 |
| 1432577_at | --- | --- | -1.77 | 0.015 | -1.44 | 0.579 | 1.52 | 0.508 | -1.43 | 0.524 | -0.78 |
| 1430167_a_at | Rwdd3 | RWD domain containing 3 | -1.49 | 0.121 | -1.12 | 0.277 | 1.22 | 0.212 | -1.09 | 0.735 | -0.62 |
| 1426398_at | Ube2w | ubiquitin-conjugating enzyme E2W (putative) | -1.04 | 0.785 | -1.22 | 0.034 | 1 | 0.973 | -1.39 | 0.022 | -0.66 |
| 1445267_at | LOC626854 | Similar to zinc finger protein 709 | 1.22 | 0.515 | -1.39 | 0.187 | -1.18 | 0.166 | -1.61 | 0.067 | -0.74 |
| 1445775_at | --- | --- | -1.27 | 0.487 | 1.13 | 0.798 | -1.15 | 0.51 | -1.05 | 0.488 | -0.59 |
| 1418576_at | Yipf5 | Yip1 domain family, member 5 | -1.08 | 0.237 | 1.04 | 0.815 | -1.21 | 0.129 | -1.11 | 0.571 | -0.59 |
| 1425022_at | Usp3 | ubiquitin specific peptidase 3 | -1.11 | 0.184 | -1.21 | 0.069 | 1.05 | 0.791 | -1.33 | 0.081 | -0.65 |

| | | | | | | | | | | | |
|---|---|---|---|---|---|---|---|---|---|---|---|
| 1437850_a_at | Cnbp | cellular nucleic acid binding protein | -1.23 | 0.289 | 1.02 | 0.855 | -1.05 | 0.728 | -1.15 | 0.262 | -0.6 |
| 1434443_at | --- | --- | -1.25 | 0.254 | 1.04 | 0.848 | -1.06 | 0.793 | -1.16 | 0.453 | -0.61 |
| 1447897_x_at | Anapc11 | Anaphase promoting complex subunit 11 homolog (yeast) | 1.18 | 0.756 | -1.01 | 0.96 | -1.63 | 0.003 | -1.54 | 0.244 | -0.75 |
| 1435937_at | Sptlc2 | serine palmitoyltransferase, long chain base subunit 2 | -1.08 | 0.147 | 1.04 | 0.856 | -1.22 | 0.182 | -1.01 | 0.948 | -0.57 |
| 1438003_at | Pols | polymerase (DNA directed) sigma | -1.17 | 0.218 | -1.07 | 0.526 | -1.01 | 0.941 | 1.72 | 0.182 | -0.38 |
| 1444336_at | Kcnj3 | Potassium inwardly-rectifying channel, subfamily J, member 3 | -2.45 | 0.116 | -1.07 | 0.894 | 1.44 | 0.437 | -1.1 | 0.302 | -0.8 |
| 1447652_at | Slc35a4 /// LOC545 | solute carrier family 35, member A4 /// similar to solute carrier family 35, member | -2.66 | 0.047 | 1.76 | 0.404 | -1.56 | 0.462 | -2.91 | 0.109 | -1.34 |
| 1422156_a_at | Rps2 /// LOC54487 | ribosomal protein S2 /// similar to 40S ribosomal protein S2 /// similar to 40S ribos | -1.2 | 0.066 | -1.07 | 0.621 | 1.02 | 0.792 | -1.13 | 0.014 | -0.6 |
| 1438786_a_at | 2610021A01Rik /// | RIKEN cDNA 2610021A01 gene /// similar to zinc finger protein 347 | -1.15 | 0.419 | 1.14 | 0.64 | -1.28 | 0.081 | -1.3 | 0.289 | -0.65 |
| 1455914_at | AI987944 | expressed sequence AI987944 | -1.1 | 0.693 | 1 | 0.979 | -1.15 | 0.125 | -1.82 | 0.056 | -0.77 |
| 1439508_at | A730055L17Rik | RIKEN cDNA A730055L17 gene | -1.23 | 0.695 | 1.03 | 0.881 | -1.07 | 0.639 | -1.15 | 0.629 | -0.6 |
| 1431372_at | Srpk2 | serine/arginine-rich protein specific kinase 2 | -1.16 | 0.674 | 1.24 | 0.47 | -1.47 | 0.054 | -1.26 | 0.54 | -0.66 |
| 1456205_x_at | Tbca | tubulin cofactor a | -1.02 | 0.039 | 1.03 | 0.642 | -1.29 | 0.006 | -1.23 | 0.038 | -0.63 |
| 1439978_at | D030022P06Rik | RIKEN cDNA D030022P06 gene | -1.34 | 0.325 | 1.27 | 0.54 | -1.3 | 0.373 | -1.45 | 0.259 | -0.71 |
| 1454699_at | Sesn1 | sestrin 1 | 1.01 | 0.975 | -1.01 | 0.966 | -1.28 | 0.334 | -1.9 | 0.144 | -0.79 |
| 1440263_at | Nrp | neural regeneration protein | 1.05 | 0.836 | -1.22 | 0.535 | -1.1 | 0.602 | -1.15 | 0.363 | -0.6 |
| 1423028_at | Ifna2 | interferon alpha 2 | -1.16 | 0.872 | -1.33 | 0.435 | 1.17 | 0.543 | -2.25 | 0.191 | -0.89 |
| 1429754_a_at | 4933406F09Rik | RIKEN cDNA 4933406F09 gene | 1.16 | 0.84 | -1.43 | 0.553 | -1.09 | 0.907 | -2.33 | 0.091 | -0.92 |
| 1446279_at | Negr1 | Neuronal growth regulator 1 | 1.46 | 0.449 | -1.77 | 0.26 | -1.31 | 0.561 | -1.33 | 0.728 | -0.74 |
| 1435994_at | Kcnh1 | potassium voltage-gated channel, subfamily H (eag-related), member 1 | -1.27 | 0.427 | -1.04 | 0.886 | 1.03 | 0.685 | -1.4 | 0.265 | -0.67 |
| 1423594_a_at | Ednrb | endothelin receptor type B | 1.3 | 0.32 | -1.36 | 0.023 | -1.34 | 0.003 | -1.13 | 0.602 | -0.63 |
| 1451817_at | Suv420h1 | suppressor of variegation 4-20 homolog 1 (Drosophila) | -1.72 | 0.042 | -1.78 | 0.338 | 1.64 | 0.253 | -1.14 | 0.642 | -0.75 |
| 1448861_at | Traf5 /// LOC62260 | Tnf receptor-associated factor 5 /// similar to TNF receptor-associated factor 5 | -1.32 | 0.256 | -1.15 | 0.594 | 1.16 | 0.367 | -1.81 | 0.025 | -0.78 |
| 1417307_at | Dmd | dystrophin, muscular dystrophy | -1.07 | 0.654 | 1.03 | 0.74 | -1.23 | 0.132 | -1.26 | 0.397 | -0.63 |
| 1417431_a_at | Sphk2 | sphingosine kinase 2 | -1.36 | 0.165 | -1.25 | 0.238 | 1.25 | 0.077 | -1.39 | 0.104 | -0.69 |
| 1437683_x_at | Serf2 | small EDRK-rich factor 2 | -1.3 | 0.282 | 1.15 | 0.505 | -1.16 | 0.486 | -1.36 | 0.129 | -0.67 |
| 1417062_at | 2810037C14Rik | RIKEN cDNA 2810037C14 gene | -1.14 | 0.115 | 1.05 | 0.676 | -1.16 | 0.207 | -1.65 | 0.091 | -0.73 |
| 1438650_x_at | Gja1 | gap junction membrane channel protein alpha 1 | -1.39 | 0.176 | 1.39 | 0.159 | -1.48 | 0.136 | -1.5 | 0.026 | -0.74 |
| 1450095_a_at | Acyp1 | acylphosphatase 1, erythrocyte (common) type | -1.36 | 0.095 | -1.01 | 0.933 | 1.06 | 0.694 | -1.36 | 0.23 | -0.67 |
| 1417532_at | Cyp2j5 | cytochrome P450, family 2, subfamily j, polypeptide 5 | -1.03 | 0.892 | -1.32 | 0.006 | 1.06 | 0.918 | -1.02 | 0.967 | -0.58 |
| 1453292_at | 4921501E09Rik | RIKEN cDNA 4921501E09 gene | -2.93 | 0.187 | 1.85 | 0.308 | -1.68 | 0.269 | -1.02 | 0.939 | -0.94 |
| 1435255_at | Plxnb1 | plexin B1 | -1.24 | 0.298 | -1.16 | 0.147 | 1.12 | 0.377 | -1.24 | 0.249 | -0.63 |
| 1418781_at | Nat9 | NAT9 | -1.02 | 0.884 | -1.1 | 0.66 | -1.12 | 0.561 | 1.12 | 0.56 | -0.53 |
| 1419129_at | Slc22a13 | solute carrier family 22 (organic cation transporter), member 13 | 1.49 | 0.172 | -1.37 | 0.309 | -1.77 | 0.238 | -1.36 | 0.522 | -0.75 |
| 1416864_at | Surf6 | surfeit gene 6 | -1.11 | 0.815 | 1.01 | 0.921 | -1.15 | 0.473 | -1.15 | 0.58 | -0.6 |
| 1439309_at | 9630044O09Rik | RIKEN cDNA 9630044O09 gene | -1.1 | 0.872 | 1.01 | 0.977 | -1.15 | 0.484 | -1.9 | 0.078 | -0.79 |
| 1457805_at | --- | --- | 1.27 | 0.558 | -1.06 | 0.647 | -1.73 | 0.073 | -1.37 | 0.375 | -0.72 |
| 1453725_a_at | Mrps7 | mitchondrial ribosomal protein S7 | -1.06 | 0.617 | -1.2 | 0.02 | 1.01 | 0.954 | -1.03 | 0.858 | -0.57 |
| 1431737_at | LOC629820 | hypothetical LOC629820 | 1.3 | 0.04 | -1.34 | 0.574 | -1.34 | 0.184 | -1.16 | 0.589 | -0.64 |
| 1440990_at | BC056349 | cDNA sequence BC056349 | -1.09 | 0.766 | 1.02 | 0.932 | -1.18 | 0.392 | -2.35 | 0.314 | -0.9 |
| 1430655_at | 4631405K08Rik | RIKEN cDNA 4631405K08 gene | 1.46 | 0.618 | -1.64 | 0.111 | -1.4 | 0.507 | -1.08 | 0.823 | -0.66 |
| 1424394_at | Selm | selenoprotein M | 1.04 | 0.622 | -1 | 0.995 | -1.34 | 0.071 | -1.02 | 0.889 | -0.58 |
| 1420231_at | --- | --- | -1.56 | 0.621 | 1.29 | 0.592 | -1.17 | 0.734 | -1.66 | 0.431 | -0.78 |
| 1445360_at | Tgfb1 | Transforming growth factor, beta 1 | -1.09 | 0.594 | -1.23 | 0.528 | 1.06 | 0.656 | -1.57 | 0.017 | -0.71 |
| 1434768_at | Tpp1 | tripeptidyl peptidase I | -1.13 | 0.063 | 1.04 | 0.437 | -1.16 | 0.33 | -1.02 | 0.897 | -0.57 |
| 1450671_at | Cryba1 | crystallin, beta A1 | 1.41 | 0.389 | -1.4 | 0.483 | -1.51 | 0.588 | -1.96 | 0.525 | -0.87 |
| 1457877_at | Esr1 | Estrogen receptor 1 (alpha) | -2.13 | 0.21 | 1.72 | 0.221 | -1.67 | 0.287 | -1.58 | 0.002 | -0.92 |
| 1421374_a_at | Fxyd1 | FXYD domain-containing ion transport regulator 1 | 1.23 | 0.538 | -1.16 | 0.037 | -1.45 | 0.029 | -1.11 | 0.711 | -0.62 |
| 1416814_at | Tia1 | cytotoxic granule-associated RNA binding protein 1 | -1.19 | 0.238 | 1.22 | 0.243 | -1.38 | 0.147 | -1.33 | 0.407 | -0.67 |
| 1427021_s_at | Fth1 | ferritin heavy chain 1 | -1.11 | 0.256 | -1.09 | 0.596 | -1.04 | 0.334 | 1.15 | 0.248 | -0.52 |
| 1449534_at | Sycp3 | synaptonemal complex protein 3 | 1.18 | 0.553 | -1.2 | 0.671 | -1.29 | 0.566 | -1.02 | 0.895 | -0.58 |
| 1429799_at | Zfp606 | zinc finger protein 606 | 1.14 | 0.621 | -1.49 | 0.09 | -1.03 | 0.948 | -1 | 0.996 | -0.59 |
| 1460545_at | Thrap3 | thyroid hormone receptor associated protein 3 | -1.18 | 0.095 | -1.01 | 0.789 | -1.05 | 0.508 | 1.1 | 0.167 | -0.54 |

| | | | | | | | | | | | |
|---|---|---|---|---|---|---|---|---|---|---|---|
| 1454527_at | 8430437B07Rik | RIKEN cDNA 8430437B07 gene | 1.45 | 0.437 | -1.65 | 0.361 | -1.37 | 0.58 | -1.46 | 0.594 | -0.76 |
| 1455237_at | Usp36 | ubiquitin specific peptidase 36 | -1.39 | 0.023 | -1.17 | 0.313 | 1.21 | 0.105 | -1.16 | 0.416 | -0.63 |
| 1458091_at | Dpp10 | Dipeptidylpeptidase 10 | 1.68 | 0.269 | -1.6 | 0.268 | -2.07 | 0.127 | -2.12 | 0.043 | -1.03 |
| 1438917_x_at | Nup62 | nucleoporin 62 | -1.05 | 0.112 | -1.1 | 0.453 | -1.08 | 0.587 | 1.15 | 0.627 | -0.52 |
| 1440634_at | Gpc6 | Glypican 6 | -1.05 | 0.81 | 1.1 | 0.66 | -1.36 | 0.311 | -1.49 | 0.5 | -0.7 |
| 1440424_at | Tnnt2 | Troponin T2, cardiac | -1.1 | 0.748 | -1.13 | 0.67 | -1 | 0.998 | 1.17 | 0.384 | -0.52 |
| 1425009_at | Tcte2 | t-complex-associated testis expressed 2 | -1.51 | 0.165 | -1.4 | 0.633 | 1.41 | 0.599 | -1.29 | 0.712 | -0.7 |
| 1423937_at | Kctd5 | potassium channel tetramerisation domain containing 5 | -1.18 | 0.443 | -1.07 | 0.508 | 1.01 | 0.937 | -1 | 0.978 | -0.56 |
| 1420486_at | Nol7 | nucleolar protein 7 | -1.21 | 0.015 | -1.08 | 0.428 | 1.03 | 0.818 | -1.51 | 0.061 | -0.69 |
| 1442484_at | D9Ertd306e | DNA segment, Chr 9, ERATO Doi 306, expressed | 1.25 | 0.263 | -1.07 | 0.578 | -1.64 | 0.1 | -1.13 | 0.486 | -0.65 |
| 1449567_at | Tktl1 | transketolase-like 1 | -1.11 | 0.77 | -1.13 | 0.623 | 1 | 0.997 | -1.17 | 0.751 | -0.6 |
| 1428063_at | Ankrd46 | ankyrin repeat domain 46 | -1.03 | 0.164 | -1.18 | 0.15 | -1.03 | 0.828 | 1.17 | 0.371 | -0.52 |
| 1435093_at | Zfyve20 | zinc finger, FYVE domain containing 20 | 1.02 | 0.912 | -1.03 | 0.86 | -1.25 | 0.052 | -1.15 | 0.527 | -0.6 |
| 1416276_a_at | Rps4x | ribosomal protein S4, X-linked | -1.04 | 0.424 | 1.04 | 0.598 | -1.27 | 0.013 | -1.35 | 0.051 | -0.65 |
| 1442869_at | Dok7 | docking protein 7 | -1.48 | 0.456 | -1.01 | 0.986 | 1.12 | 0.221 | -1.33 | 0.358 | -0.67 |
| 1456210_at | 5430407P10Rik | RIKEN cDNA 5430407P10 gene | -1.02 | 0.926 | -1.23 | 0.491 | -1.01 | 0.955 | 1.13 | 0.631 | -0.53 |
| 1428963_at | Rwdd2 | RWD domain containing 2 | -1.11 | 0.709 | -1.21 | 0.174 | 1.06 | 0.655 | -1.3 | 0.404 | -0.64 |
| 1426770_at | Pex5 | peroxisome biogenesis factor 5 | -1.12 | 0.525 | -1.15 | 0.213 | 1.02 | 0.926 | -1 | 0.99 | -0.56 |
| 1453795_at | Fahd2a | fumarylacetoacetate hydrolase domain containing 2A | -1.16 | 0.313 | -1.43 | 0.049 | 1.23 | 0.187 | -1.23 | 0.418 | -0.65 |
| 1450694_at | Fkbp2 | FK506 binding protein 2 | -1.01 | 0.935 | -1.15 | 0.004 | -1.08 | 0.269 | 1.16 | 0.191 | -0.52 |
| 1439022_at | Phactr1 | phosphatase and actin regulator 1 | -1.18 | 0.47 | 1.07 | 0.85 | -1.15 | 0.729 | -1.51 | 0.028 | -0.69 |
| 1418310_a_at | Rlbp1 | retinaldehyde binding protein 1 | 1.14 | 0.442 | -1.47 | 0.331 | -1.03 | 0.9 | -1.07 | 0.879 | -0.61 |
| 1454627_a_at | Rpl29 /// LOC4333 | ribosomal protein L29 /// similar to 60S ribosomal protein L29 /// similar to 60S rib | -1.06 | 0.728 | 1.05 | 0.506 | -1.26 | 0.03 | -1.21 | 0.016 | -0.62 |
| 1423035_s_at | Txnl5 | thioredoxin-like 5 | -1.13 | 0.124 | 1.02 | 0.755 | -1.13 | 0.082 | -1.13 | 0.32 | -0.59 |
| 1455205_a_at | Usp19 | ubiquitin specific peptidase 19 | -1.17 | 0.031 | -1.19 | 0.037 | 1.09 | 0.497 | -1.17 | 0.388 | -0.61 |
| 1439117_at | Clmn | calmin | -1.02 | 0.781 | -1.27 | 0.373 | 1.02 | 0.925 | -1.42 | 0.117 | -0.67 |
| 1417580_s_at | Selenbp1 | selenium binding protein 1 | -1.08 | 0.483 | -1.09 | 0.583 | -1.06 | 0.68 | 2.08 | 0.108 | -0.29 |
| 1417126_a_at | Rpl22l1 | ribosomal protein L22 like 1 | -1.15 | 0.127 | 1.02 | 0.856 | -1.12 | 0.208 | -1.47 | 0.007 | -0.68 |
| 1440851_at | 4933407N01Rik | RIKEN cDNA 4933407N01 gene | 1.16 | 0.445 | -1 | 0.995 | -1.58 | 0.088 | -1.15 | 0.266 | -0.64 |
| 1434331_at | Eif2c1 | eukaryotic translation initiation factor 2C, 1 | 1.01 | 0.895 | -1.02 | 0.889 | -1.25 | 0.262 | -1.4 | 0.299 | -0.67 |
| 1433886_at | Eif2b5 | eukaryotic translation initiation factor 2B, subunit 5 epsilon | -1.07 | 0.72 | -1.13 | 0.028 | -1.04 | 0.442 | 1.02 | 0.922 | -0.55 |
| 1416027_at | Pdcd6 | programmed cell death 6 | -1.09 | 0.214 | 1.01 | 0.855 | -1.16 | 0.182 | -1.03 | 0.772 | -0.57 |
| 1452777_a_at | Nub1 | negative regulator of ubiquitin-like proteins 1 | 1.06 | 0.356 | -1.36 | 0.248 | -1.01 | 0.975 | -1.56 | 0.015 | -0.72 |
| 1419935_s_at | Csnk2a2 | casein kinase 2, alpha prime polypeptide | -1.01 | 0.951 | -1.02 | 0.934 | -1.22 | 0.302 | 1.17 | 0.434 | -0.52 |
| 1417530_a_at | Srp9 | signal recognition particle 9 | -1.01 | 0.81 | 1.03 | 0.368 | -1.3 | 0.137 | -1.25 | 0.291 | -0.63 |
| 1438966_x_at | Fmod | fibromodulin | -1.33 | 0.7 | -1.13 | 0.757 | 1.15 | 0.668 | -1.88 | 0.126 | -0.8 |
| 1428239_at | Ankrd16 | ankyrin repeat domain 16 | -1.32 | 0.06 | 1.07 | 0.376 | -1.04 | 0.81 | -1.5 | 0.323 | -0.7 |
| 1430151_at | Nisch | nischarin | 1.14 | 0.421 | -1.05 | 0.812 | -1.44 | 0.234 | -1.55 | 0.005 | -0.72 |
| 1448258_a_at | Spcs1 | signal peptidase complex subunit 1 homolog (S. cerevisiae) | -1.03 | 0.661 | -1.06 | 0.427 | -1.15 | 0.049 | 1.12 | 0.001 | -0.53 |
| 1418874_a_at | Psmd4 | proteasome (prosome, macropain) 26S subunit, non-ATPase, 4 | -1.05 | 0.382 | -1.16 | 0.022 | -1.02 | 0.829 | 1.1 | 0.585 | -0.53 |
| 1435235_at | Txnl1 | thioredoxin-like 1 | -1.02 | 0.692 | -1.04 | 0.648 | -1.18 | 0.266 | 1.48 | 0.142 | -0.44 |
| 1451137_a_at | Brd8 | bromodomain containing 8 | -1.12 | 0.56 | -1.21 | 0.197 | 1.07 | 0.579 | -1.03 | 0.818 | -0.57 |
| 1444923_at | --- | Adult male diencephalon cDNA, RIKEN full-length enriched library, clone:933015 | 1.2 | 0.676 | -1.08 | 0.167 | -1.5 | 0.399 | -1.71 | 0.199 | -0.77 |
| 1417099_at | Ftsj1 | Ftsj homolog 1 (E. coli) | -1.07 | 0.184 | -1.16 | 0.192 | -1.01 | 0.941 | 1.87 | 0.23 | -0.34 |
| 1416581_at | Wdr5 | WD repeat domain 5 | -1.08 | 0.492 | -1.09 | 0.408 | -1.06 | 0.703 | 1.05 | 0.642 | -0.55 |
| 1420622_a_at | Hspa8 /// LOC6212 | heat shock protein 8 /// similar to heat shock protein 8 /// heat shock protein 8 pse | -1.43 | 0.228 | -1.08 | 0.666 | 1.16 | 0.253 | -1.3 | 0.238 | -0.66 |
| 1453153_at | Lins2 | lines homolog 2 (Drosophila) | -1.05 | 0.845 | -1.45 | 0.305 | 1.15 | 0.163 | -1.53 | 0.16 | -0.72 |
| 1424413_at | Ogfrl1 | opioid growth factor receptor-like 1 | -1.14 | 0.67 | 1.09 | 0.46 | -1.23 | 0.103 | -1.07 | 0.73 | -0.59 |
| 1446493_at | Cdc14a | CDC14 cell division cycle 14 homolog A (S. cerevisiae) | -1.45 | 0.414 | -1.45 | 0.287 | 1.41 | 0.542 | -1.03 | 0.923 | -0.63 |
| 1452635_x_at | Josd3 | Josephin domain containing 3 | -1.05 | 0.835 | -1.31 | 0.242 | 1.07 | 0.703 | -1.08 | 0.782 | -0.59 |
| 1434637_x_at | Sin3b | transcriptional regulator, SIN3B (yeast) | 1.04 | 0.796 | -1.18 | 0.077 | -1.11 | 0.369 | -1.3 | 0.225 | -0.64 |
| 1430959_at | 4930546H06Rik | RIKEN cDNA 4930546H06 gene | -1.16 | 0.754 | 1.52 | 0.374 | -2.45 | 0.378 | -1.27 | 0.354 | -0.84 |
| 1417145_at | Nfxl1 | nuclear transcription factor, X-box binding-like 1 | -1.07 | 0.708 | -1.02 | 0.823 | -1.14 | 0.555 | 1.4 | 0.171 | -0.46 |

| | | | | | | | | | | | |
|---|---|---|---|---|---|---|---|---|---|---|---|
| 1441072_at | Cugbp2 | CUG triplet repeat, RNA binding protein 2 | 1.61 | 0.076 | -1.43 | 0.219 | -2.1 | 0.021 | -1.12 | 0.543 | -0.76 |
| 1419101_at | Sin3a | transcriptional regulator, SIN3A (yeast) | 1.14 | 0.484 | -1.14 | 0.389 | -1.3 | 0.1 | -1.21 | 0.193 | -0.63 |
| 1448517_at | Timm22 | translocase of inner mitochondrial membrane 22 homolog (yeast) | -1.05 | 0.619 | -1.14 | 0.331 | -1.04 | 0.667 | 1.04 | 0.766 | -0.55 |
| 1424653_at | LOC669875 | similar to Tetraspanin-15 (Tspan-15) (Transmembrane 4 superfamily member 15) | -1.18 | 0.333 | 1.13 | 0.662 | -1.22 | 0.396 | -1.16 | 0.275 | -0.61 |
| 1427875_a_at | 1100001I22Rik | RIKEN cDNA 1100001I22 gene | -1.08 | 0.347 | 1.02 | 0.664 | -1.19 | 0.024 | -1.31 | 0.003 | -0.64 |
| 1417306_at | Tyk2 | tyrosine kinase 2 | -1.33 | 0.052 | -1.06 | 0.549 | 1.1 | 0.555 | -1.32 | 0.316 | -0.65 |
| 1439009_at | Gnl3l | guanine nucleotide binding protein-like 3 (nucleolar)-like | -1.11 | 0.353 | -1.03 | 0.796 | -1.09 | 0.367 | 1.17 | 0.054 | -0.51 |
| 1424235_at | Ormdl2 | ORM1-like 2 (S. cerevisiae) | -1.02 | 0.477 | -1.07 | 0.496 | -1.14 | 0.401 | 1.2 | 0.433 | -0.51 |
| 1435243_at | Zfp746 | zinc finger protein 746 | -1.25 | 0.323 | 1.1 | 0.673 | -1.13 | 0.341 | -1.71 | 0.098 | -0.75 |
| 1428322_a_at | Ndufb10 | NADH dehydrogenase (ubiquinone) 1 beta subcomplex, 10 | -1.02 | 0.883 | -1.2 | 0.181 | -1.03 | 0.477 | 1.01 | 0.968 | -0.56 |
| 1434510_at | Papss2 | 3'-phosphoadenosine 5'-phosphosulfate synthase 2 | -1.12 | 0.437 | -1.21 | 0.408 | 1.07 | 0.833 | -1.59 | 0.252 | -0.71 |
| 1430382_at | 4833413G10Rik | RIKEN cDNA 4833413G10 gene | 1.04 | 0.847 | -1.14 | 0.525 | -1.15 | 0.433 | -1.15 | 0.596 | -0.6 |
| 1447625_at | E2f5 | E2F transcription factor 5 | -1.11 | 0.717 | -1.03 | 0.927 | -1.09 | 0.884 | 1.39 | 0.441 | -0.46 |
| 1445596_at | Adar | Adenosine deaminase, RNA-specific | -1.1 | 0.802 | 1.03 | 0.958 | -1.18 | 0.74 | -1.06 | 0.889 | -0.58 |
| 1438812_x_at | Usp19 | ubiquitin specific peptidase 19 | -1.43 | 0.218 | -1.22 | 0.692 | 1.27 | 0.512 | -1.01 | 0.968 | -0.6 |
| 1429645_at | 1700017G19Rik | RIKEN cDNA 1700017G19 gene | -2 | 0.411 | -1.31 | 0.67 | 1.53 | 0.503 | -1.26 | 0.558 | -0.76 |
| 1430187_at | Zfp60 | zinc finger protein 60 | -1.24 | 0.452 | 1.2 | 0.585 | -1.29 | 0.301 | -1.63 | 0.292 | -0.74 |
| 1417103_at | Ddt | D-dopachrome tautomerase | -1.27 | 0.047 | -1.18 | 0.267 | 1.16 | 0.209 | -1.16 | 0.628 | -0.62 |
| 1455722_at | --- | --- | 1.61 | 0.099 | -3.99 | 0.115 | -1.08 | 0.813 | -1.51 | 0.255 | -1.24 |
| 1429588_at | 2810474O19Rik | RIKEN cDNA 2810474O19 gene | -1.07 | 0.471 | -1.03 | 0.874 | -1.13 | 0.511 | 1.21 | 0.221 | -0.51 |
| 1451514_at | Usp43 | ubiquitin specific peptidase 43 | -1.64 | 0.014 | -1.65 | 0.416 | 1.57 | 0.219 | -1.06 | 0.332 | -0.69 |
| 1456925_at | --- | --- | -2.59 | 0.082 | 1.46 | 0.143 | -1.06 | 0.849 | -1.52 | 0.398 | -0.93 |
| 1447704_s_at | D530033C11Rik | RIKEN cDNA D530033C11 gene | -1.16 | 0.56 | 1.13 | 0.378 | -1.26 | 0.112 | -1.07 | 0.754 | -0.59 |
| 1442139_at | --- | Adult male diencephalon cDNA, RIKEN full-length enriched library, clone:933014 | -1.07 | 0.382 | 1.13 | 0.51 | -1.37 | 0.185 | -1.18 | 0.518 | -0.62 |
| 1454060_a_at | Nras | neuroblastoma ras oncogene | -1.12 | 0.445 | 1.02 | 0.936 | -1.13 | 0.19 | -1.23 | 0.266 | -0.62 |
| 1429612_at | Eml4 | echinoderm microtubule associated protein like 4 | -1.08 | 0.332 | 1.03 | 0.85 | -1.2 | 0.12 | -1.54 | 0.212 | -0.7 |
| 1452104_at | 2600005N12Rik | RIKEN cDNA 2600005N12 gene | 1.47 | 0.525 | -1.39 | 0.577 | -1.66 | 0.194 | -1.39 | 0.505 | -0.74 |
| 1452993_at | 5430416O09Rik | RIKEN cDNA 5430416O09 gene | 1.12 | 0.769 | -1.15 | 0.776 | -1.24 | 0.242 | -1.15 | 0.592 | -0.61 |
| 1433962_at | 6720458F09Rik | RIKEN cDNA 6720458F09 gene | -1.24 | 0.432 | -1.02 | 0.957 | 1 | 0.982 | -1.2 | 0.479 | -0.62 |
| 1422642_at | Cdc42ep3 | CDC42 effector protein (Rho GTPase binding) 3 | -1.02 | 0.933 | -1.08 | 0.612 | -1.12 | 0.342 | 1.62 | 0.083 | -0.4 |
| 1428772_at | Xpot | exportin, tRNA (nuclear export receptor for tRNAs) | -1.09 | 0.009 | -1.1 | 0.115 | -1.04 | 0.551 | 1.21 | 0.259 | -0.5 |
| 1418636_at | Etv3 | ets variant gene 3 | -1.31 | 0.273 | -1.05 | 0.753 | 1.08 | 0.777 | -1.57 | 0.168 | -0.71 |
| 1454716_x_at | Cox5b | cytochrome c oxidase, subunit Vb | -1.05 | 0.565 | -1.13 | 0.374 | -1.05 | 0.724 | 1.16 | 0.293 | -0.52 |
| 1428128_at | 4921506J03Rik | RIKEN cDNA 4921506J03 gene | -1.16 | 0.001 | 1.09 | 0.463 | -1.2 | 0.042 | -1.07 | 0.313 | -0.58 |
| 1435943_at | Dpep1 | dipeptidase 1 (renal) | -1.02 | 0.896 | -1.11 | 0.811 | -1.1 | 0.601 | 1.45 | 0.598 | -0.44 |
| 1458617_at | Prkcbp1 | protein kinase C binding protein 1 | -1.2 | 0.048 | 1.06 | 0.483 | -1.11 | 0.605 | -1.13 | 0.68 | -0.6 |
| 1433960_at | Isg20l2 | interferon stimulated exonuclease gene 20-like 2 | -1.11 | 0.39 | 1 | 0.996 | -1.12 | 0.494 | -1.11 | 0.2 | -0.59 |
| 1440894_at | LOC670793 | similar to CG4050-PA, isoform A | -1.29 | 0.312 | 1.04 | 0.933 | -1.02 | 0.969 | -1.23 | 0.624 | -0.63 |
| 1425505_at | Mylk | myosin, light polypeptide kinase | -1.05 | 0.869 | -1.57 | 0.022 | 1.2 | 0.635 | -1.48 | 0.22 | -0.72 |
| 1430998_at | Sqrdl | sulfide quinone reductase-like (yeast) | -5.4 | 0.25 | 1.99 | 0.274 | -1.61 | 0.429 | -1.2 | 0.756 | -1.56 |
| 1456068_at | Nfasc | neurofascin | 1.45 | 0.582 | -1.81 | 0.31 | -1.27 | 0.253 | -1.43 | 0.251 | -0.76 |
| 1423424_at | Zic3 | zinc finger protein of the cerebellum 3 | -1.02 | 0.767 | 1.12 | 0.751 | -1.45 | 0.152 | -1.19 | 0.689 | -0.63 |
| 1433786_x_at | Serf2 | small EDRK-rich factor 2 | -1.1 | 0.412 | 1.04 | 0.459 | -1.19 | 0.095 | -1.2 | 0.059 | -0.61 |
| 1439140_at | 1700019P01Rik | RIKEN cDNA 1700019P01 gene | 1.05 | 0.932 | -1.17 | 0.752 | -1.13 | 0.659 | -1.11 | 0.749 | -0.59 |
| 1426678_at | Zfp706 | zinc finger protein 706 | 1.02 | 0.869 | -1.18 | 0.207 | -1.08 | 0.154 | -1.2 | 0.546 | -0.61 |
| 1423792_a_at | Cmtm6 | CKLF-like MARVEL transmembrane domain containing 6 | -1.37 | 0.294 | -1 | 0.991 | 1.06 | 0.617 | -1.79 | 0.11 | -0.77 |
| 1423902_s_at | Arhgef12 | Rho guanine nucleotide exchange factor (GEF) 12 | -1.14 | 0.167 | -1 | 0.987 | -1.1 | 0.656 | 1.97 | 0.169 | -0.32 |
| 1440933_at | 4732460I02Rik | RIKEN cDNA 4732460I02 gene | -1.27 | 0.428 | 1.05 | 0.77 | -1.05 | 0.615 | -1.28 | 0.166 | -0.64 |
| 1427695_a_at | Pou2f1 | POU domain, class 2, transcription factor 1 | -1.2 | 0.772 | -1.06 | 0.92 | 1.01 | 0.969 | -1.44 | 0.296 | -0.67 |
| 1444204_at | Hoxb2 | Homeo box B2 | -1.08 | 0.609 | -1.13 | 0.778 | -1.02 | 0.944 | 1.35 | 0.541 | -0.47 |
| 1429252_at | 0610010K14Rik | RIKEN cDNA 0610010K14 gene | -1.12 | 0.546 | 1.14 | 0.36 | -1.32 | 0.157 | -1.93 | 0.244 | -0.81 |
| 1455286_at | Btbd1 | BTB (POZ) domain containing 1 | 1.01 | 0.958 | -1.05 | 0.224 | -1.19 | 0.144 | -1.51 | 0.073 | -0.69 |
| 1431071_at | Mtx3 | metaxin 3 | 1.07 | 0.675 | -1.31 | 0.194 | -1.04 | 0.715 | -1.1 | 0.414 | -0.6 |

| | | | | | | | | | | | |
|---|---|---|---|---|---|---|---|---|---|---|---|
| 1427833_at | Spi16 | serine protease inhibitor 16 | -1.24 | 0.549 | -3.83 | 0.044 | 1.73 | 0.093 | -1.14 | 0.659 | -1.12 |
| 1435101_at | Derl2 | Der1-like domain family, member 2 | 1.17 | 0.025 | -1.22 | 0.191 | -1.24 | 0.368 | -1.1 | 0.463 | -0.6 |
| 1442266_at | AI662175 | expressed sequence AI662175 | -2.08 | 0.106 | -1.22 | 0.763 | 1.49 | 0.407 | -1.55 | 0.13 | -0.84 |
| 1430944_at | A930030B08Rik | RIKEN cDNA A930030B08 gene | -1.75 | 0.141 | -1.12 | 0.52 | 1.33 | 0.173 | -1.19 | 0.692 | -0.68 |
| 1435845_at | --- | B16 F10Y cells cDNA, RIKEN full-length enriched library, clone:G370036C05 pro | -1.82 | 0.398 | -1.34 | 0.197 | 1.5 | 0.414 | -1.26 | 0.639 | -0.73 |
| 1453318_at | Tmem102 | transmembrane protein 102 | -1 | 0.99 | -1.19 | 0.375 | -1.05 | 0.928 | 3.05 | 0.011 | -0.05 |
| 1444881_at | Taok3 | TAO kinase 3 | -1.15 | 0.679 | 1.34 | 0.268 | -1.71 | 0.006 | -1.3 | 0.256 | -0.71 |
| 1422457_s_at | Sumo3 | SMT3 suppressor of mif two 3 homolog 3 (yeast) | -1.1 | 0.385 | -1.03 | 0.605 | -1.09 | 0.2 | 1.1 | 0.162 | -0.53 |
| 1432429_at | 8430437N05Rik | RIKEN cDNA 8430437N05 gene | -1.25 | 0.784 | -1.1 | 0.821 | 1.08 | 0.876 | -2.2 | 0.314 | -0.87 |
| 1460156_at | Aoah | Acyloxyacyl hydrolase | 1.29 | 0.479 | -1.41 | 0.653 | -1.27 | 0.631 | -1.25 | 0.575 | -0.66 |
| 1455763_at | Rnf41 | ring finger protein 41 | -1.24 | 0.063 | -1.3 | 0.185 | 1.22 | 0.3 | -1.05 | 0.771 | -0.59 |
| 1454540_at | 4930504E06Rik | RIKEN cDNA 4930504E06 gene | -1.03 | 0.897 | 1.02 | 0.941 | -1.25 | 0.102 | -1.38 | 0.276 | -0.66 |
| 1453263_at | Mak10 | MAK10 homolog, amino-acid N-acetyltransferase subunit, (S. cerevisiae) | -1.04 | 0.872 | -1.52 | 0.157 | 1.17 | 0.588 | -1.3 | 0.447 | -0.67 |
| 1434118_at | 0610009K11Rik | RIKEN cDNA 0610009K11 gene | 1.03 | 0.919 | -1.06 | 0.806 | -1.22 | 0.257 | -1.16 | 0.265 | -0.6 |
| 1456009_x_at | Top3b | topoisomerase (DNA) III beta | 1.01 | 0.979 | -1.21 | 0.045 | -1.05 | 0.776 | -1.01 | 0.946 | -0.56 |
| 1417615_a_at | Rpl11 | ribosomal protein L11 | -1.06 | 0.659 | 1.03 | 0.713 | -1.22 | 0.03 | -1.56 | 0.041 | -0.7 |
| 1460705_at | Rps6kb1 | ribosomal protein S6 kinase, polypeptide 1 | -1.11 | 0.51 | -1 | 0.981 | -1.12 | 0.668 | 1.06 | 0.801 | -0.54 |
| 1452781_a_at | Gtf3c2 | general transcription factor IIIC, polypeptide 2, beta | -1.33 | 0.1 | 1.15 | 0.232 | -1.11 | 0.268 | -1.04 | 0.542 | -0.59 |
| 1430238_at | Got1l1 | glutamic-oxaloacetic transaminase 1-like 1 | -1.3 | 0.444 | -1.89 | 0.158 | 1.5 | 0.241 | -1.31 | 0.672 | -0.75 |
| 1437250_at | Mreg | melanoregulin | -1.37 | 0.097 | -1.21 | 0.156 | 1.23 | 0.455 | -1.3 | 0.452 | -0.66 |
| 1434310_at | Bmpr2 | bone morphogenic protein receptor, type II (serine/threonine kinase) | -1.06 | 0.543 | 1.1 | 0.205 | -1.33 | 0.047 | -1.15 | 0.622 | -0.61 |
| 1447337_at | Dapp1 | Dual adaptor for phosphotyrosine and 3-phosphoinositides 1 | 1.33 | 0.331 | -2.07 | 0.235 | -1.02 | 0.949 | -1.28 | 0.191 | -0.76 |
| 1437007_x_at | Usp39 | ubiquitin specific peptidase 39 | -1.05 | 0.707 | -1 | 0.983 | -1.19 | 0.002 | 1.21 | 0.417 | -0.51 |
| 1435163_at | 9030612M13Rik | RIKEN cDNA 9030612M13 gene | 1.42 | 0.53 | -1.34 | 0.333 | -1.6 | 0.253 | -1.4 | 0.531 | -0.73 |
| 1424781_at | D10Ucla1 | DNA segment, Chr 10, University of California at Los Angeles 1 | 1.07 | 0.669 | -1.11 | 0.155 | -1.21 | 0.051 | -1.16 | 0.651 | -0.6 |
| 1424660_s_at | Crtc2 | CREB regulated transcription coactivator 2 | -1.15 | 0.676 | -1.32 | 0.083 | 1.17 | 0.513 | -2.1 | 0.054 | -0.85 |
| 1428935_at | Canx | calnexin | 1.15 | 0.131 | -1.22 | 0.105 | -1.21 | 0.219 | -1.03 | 0.812 | -0.58 |
| 1428697_at | Dpp8 | dipeptidylpeptidase 8 | -1.5 | 0.148 | -1.28 | 0.375 | 1.34 | 0.464 | -1.26 | 0.4 | -0.67 |
| 1417685_at | Ankfy1 | ankyrin repeat and FYVE domain containing 1 | -1.13 | 0.186 | 1.06 | 0.606 | -1.17 | 0.21 | -1.17 | 0.112 | -0.6 |
| 1421865_at | Dbil5 | diazepam binding inhibitor-like 5 | 1.42 | 0.552 | -2.53 | 0.027 | -1.03 | 0.954 | -1.09 | 0.811 | -0.8 |
| 1437533_at | Birc4 | baculoviral IAP repeat-containing 4 | 1.01 | 0.834 | -1.06 | 0.421 | -1.2 | 0.041 | -1.18 | 0.364 | -0.6 |
| 1418816_at | Chmp1b | chromatin modifying protein 1B | -1.14 | 0.313 | 1.01 | 0.948 | -1.1 | 0.567 | -1.03 | 0.865 | -0.56 |
| 1448009_at | Ugcgl1 | UDP-glucose ceramide glucosyltransferase-like 1 | -1.17 | 0.329 | -1.22 | 0.298 | 1.12 | 0.39 | -1.16 | 0.547 | -0.61 |
| 1454742_at | Rasgef1b | RasGEF domain family, member 1B | -1.29 | 0.467 | 1.24 | 0.273 | -1.28 | 0.171 | -1.83 | 0.149 | -0.79 |
| 1435036_at | A530050D06Rik | RIKEN cDNA A530050D06 gene | -1.17 | 0.614 | -1.06 | 0.503 | -1 | 0.988 | 1.19 | 0.775 | -0.51 |
| 1448633_at | Prpf31 | PRP31 pre-mRNA processing factor 31 homolog (yeast) | -1.17 | 0.287 | -1.06 | 0.733 | -1.01 | 0.935 | 1.09 | 0.742 | -0.54 |
| 1416131_s_at | C920006C10Rik | RIKEN cDNA C920006C10 gene | -1.19 | 0.149 | 1.03 | 0.744 | -1.08 | 0.375 | -1.34 | 0.161 | -0.65 |
| 1447546_s_at | Maml2 | Mastermind like 2 (Drosophila) | 1.3 | 0.173 | -1.09 | 0.68 | -1.72 | 0.099 | -3.27 | 0.009 | -1.2 |
| 1446596_at | C130009A20Rik | RIKEN cDNA C130009A20 gene | -1.43 | 0.073 | 1.62 | 0.191 | -2.1 | 0.009 | -1.21 | 0.614 | -0.78 |
| 1430764_at | 1700023F06Rik | RIKEN cDNA 1700023F06 gene | -1.55 | 0.38 | -1.19 | 0.741 | 1.31 | 0.385 | -1.03 | 0.958 | -0.62 |
| 1428852_at | Dock3 | dedicator of cyto-kinesis 3 | -1.18 | 0.805 | -1.5 | 0.175 | 1.28 | 0.69 | -1.29 | 0.493 | -0.67 |
| 1447214_at | LOC666827 /// LOC | hypothetical protein LOC666827 /// hypothetical protein LOC670370 /// hypothetic | -1.75 | 0.613 | -1.73 | 0.139 | 1.64 | 0.349 | -1.29 | 0.715 | -0.78 |
| 1458769_at | D7Ertd558e | DNA segment, Chr 7, ERATO Doi 558, expressed | -1.59 | 0.552 | -1.02 | 0.983 | 1.19 | 0.71 | -1.53 | 0.281 | -0.74 |
| 1444740_at | 4930550L11Rik | RIKEN cDNA 4930550L11 gene | 1.2 | 0.792 | -1.35 | 0.638 | -1.17 | 0.608 | -2.18 | 0.06 | -0.88 |
| 1436462_at | LOC626832 /// LOC | similar to gonadotropin inducible ovarian transcription factor 1 /// similar to zinc fir | -1.07 | 0.704 | 1.02 | 0.861 | -1.19 | 0.248 | -1.08 | 0.69 | -0.58 |
| 1426326_at | Zfp91 | zinc finger protein 91 | -1.25 | 0.554 | 1.12 | 0.244 | -1.14 | 0.632 | -1.1 | 0.76 | -0.59 |
| 1451730_at | Zfp62 | zinc finger protein 62 | -1.15 | 0.63 | -1.18 | 0.361 | 1.08 | 0.736 | -1.55 | 0.2 | -0.7 |
| 1436447_at | A630026N12Rik | RIKEN cDNA A630026N12 gene | -1.02 | 0.823 | -1.47 | 0.071 | 1.13 | 0.629 | -1.57 | 0.142 | -0.73 |
| 1430294_at | Ssbp1 | single-stranded DNA binding protein 1 | -1.11 | 0.588 | -1.19 | 0.254 | 1.06 | 0.818 | -1.26 | 0.158 | -0.63 |
| 1433614_at | Snx27 | sorting nexin family member 27 | -1.23 | 0.332 | -1.26 | 0.276 | 1.19 | 0.26 | -1.13 | 0.396 | -0.61 |
| 1435759_at | Ctps2 | cytidine 5'-triphosphate synthase 2 | 1.18 | 0.761 | -1.13 | 0.729 | -1.38 | 0.207 | -1.59 | 0.114 | -0.73 |
| 1445398_at | 3732412D22Rik | RIKEN cDNA 3732412D22 gene | -1.07 | 0.705 | 1.49 | 0.336 | -2.69 | 0.009 | -1.87 | 0.261 | -1.04 |
| 1428597_at | Tbc1d9b | TBC1 domain family, member 9B | -1.01 | 0.957 | -1.02 | 0.9 | -1.21 | 0.237 | 1.39 | 0.053 | -0.46 |

| | | | | | | | | | | | |
|---|---|---|---|---|---|---|---|---|---|---|---|
| 1415767_at | Ythdf1 | YTH domain family 1 | -1.04 | 0.693 | -1.16 | 0.261 | -1.03 | 0.815 | 1.1 | 0.459 | -0.53 |
| 1419397_at | Pola1 | polymerase (DNA directed), alpha 1 | -1.04 | 0.414 | -1.15 | 0.377 | -1.03 | 0.83 | 2.71 | 0.184 | -0.13 |
| 1442109_at | --- | --- | 1.43 | 0.081 | -1.35 | 0.466 | -1.61 | 0.088 | -1.31 | 0.434 | -0.71 |
| 1444365_at | Tsc22d3 | TSC22 domain family 3 | -1.3 | 0.267 | 1.36 | 0.504 | -1.49 | 0.129 | -1.27 | 0.476 | -0.68 |
| 1441041_at | Zfp746 | Zinc finger protein 746 | 1.11 | 0.697 | -1.14 | 0.263 | -1.23 | 0.439 | -1.15 | 0.462 | -0.6 |
| 1452797_at | Fastkd3 | FAST kinase domains 3 | -1.11 | 0.39 | -1.19 | 0.433 | 1.06 | 0.683 | -1.07 | 0.783 | -0.58 |
| 1440480_at | AB182283 | cDNA sequence AB182283 | -1.39 | 0.127 | -1.34 | 0.492 | 1.33 | 0.166 | -1.44 | 0.052 | -0.71 |
| 1447447_s_at | Srrm1 | serine/arginine repetitive matrix 1 | -1.09 | 0.495 | -1.03 | 0.781 | -1.1 | 0.515 | 1.07 | 0.47 | -0.54 |
| 1420681_at | 1700120K04Rik | RIKEN cDNA 1700120K04 gene | -1.82 | 0.2 | 1.38 | 0.535 | -1.16 | 0.339 | -1.17 | 0.208 | -0.69 |
| 1452914_at | 2410024N18Rik | RIKEN cDNA 2410024N18 gene | -1.47 | 0.607 | -1.52 | 0.043 | 1.46 | 0.07 | -1.09 | 0.631 | -0.66 |
| 1442257_at | B130021B11Rik | RIKEN cDNA B130021B11 gene | 1.37 | 0.403 | -1.29 | 0.342 | -1.53 | 0.054 | -1.56 | 0.004 | -0.75 |
| 1435038_s_at | Aak1 | AP2 associated kinase 1 | -1.6 | 0.136 | 1.41 | 0.103 | -1.31 | 0.396 | -1.47 | 0.019 | -0.75 |
| 1460477_at | 1700010C24Rik | RIKEN cDNA 1700010C24 gene | -1.06 | 0.7 | 1.15 | 0.842 | -1.42 | 0.32 | -1.17 | 0.818 | -0.63 |
| 1450231_a_at | Birc4 | baculoviral IAP repeat-containing 4 | -1.39 | 0.206 | -1.44 | 0.593 | 1.38 | 0.498 | -1.08 | 0.851 | -0.63 |
| 1416567_s_at | Atp5e | ATP synthase, H+ transporting, mitochondrial F1 complex, epsilon subunit | 1.01 | 0.807 | -1.14 | 0.013 | -1.09 | 0.261 | -1.34 | 0.102 | -0.64 |
| 1417801_a_at | Ppfibp2 | protein tyrosine phosphatase, receptor-type, F interacting protein, binding protein | -1.28 | 0.024 | 1.05 | 0.824 | -1.04 | 0.694 | -1.29 | 0.277 | -0.64 |
| 1433441_at | Fbxl5 | F-box and leucine-rich repeat protein 5 | -1.22 | 0.195 | -1.04 | 0.572 | 1.01 | 0.918 | -1.35 | 0.176 | -0.65 |
| 1431368_at | 4930404N11Rik | RIKEN cDNA 4930404N11 gene | -1.8 | 0.245 | 1.26 | 0.162 | -1.02 | 0.967 | -1.75 | 0.036 | -0.83 |
| 1450464_at | E4f1 | E4F transcription factor 1 | -1.18 | 0.338 | -1.14 | 0.538 | 1.07 | 0.511 | -1.44 | 0.111 | -0.67 |
| 1439557_s_at | Ldb2 | LIM domain binding 2 | -1.06 | 0.849 | 1.14 | 0.761 | -1.39 | 0.262 | -1.27 | 0.469 | -0.65 |
| 1428551_at | 2410075D05Rik | RIKEN cDNA 2410075D05 gene | -1.13 | 0.678 | -1.06 | 0.389 | -1.03 | 0.556 | 1.21 | 0.286 | -0.5 |
| 1419353_at | Dpm1 | dolichol-phosphate (beta-D) mannosyltransferase 1 | -1.15 | 0.236 | -1.23 | 0.347 | 1.11 | 0.264 | -1.13 | 0.614 | -0.6 |
| 1425334_at | Gorasp1 | golgi reassembly stacking protein 1 | -1.29 | 0.246 | -1.41 | 0.029 | 1.31 | 0.114 | -1.71 | 0.349 | -0.77 |
| 1420428_at | Ager | advanced glycosylation end product-specific receptor | -1.29 | 0.679 | 1.17 | 0.543 | -1.18 | 0.166 | -2.14 | 0.016 | -0.86 |
| 1454372_at | Cd80 | CD80 antigen | -1.01 | 0.965 | -1.38 | 0.421 | 1.08 | 0.853 | -1.22 | 0.655 | -0.63 |
| 1428075_at | Ndufb4 | NADH dehydrogenase (ubiquinone) 1 beta subcomplex 4 | -1.04 | 0.609 | -1.04 | 0.268 | -1.15 | 0.155 | 1.01 | 0.932 | -0.55 |
| 1438223_at | Vps54 | Vacuolar protein sorting 54 (yeast) | -1.43 | 0.269 | 1.19 | 0.403 | -1.1 | 0.636 | -1.5 | 0.075 | -0.71 |
| 1439438_a_at | 1110005A23Rik /// | RIKEN cDNA 1110005A23 gene /// similar to cytokine induced protein 29 kDa /// s | -1.04 | 0.436 | 1.06 | 0.287 | -1.29 | 0.087 | -1.11 | 0.276 | -0.6 |
| 1437749_s_at | Mrpl9 | mitochondrial ribosomal protein L9 | -1.03 | 0.838 | -1.13 | 0.302 | -1.06 | 0.626 | 1.04 | 0.838 | -0.54 |
| 1453251_at | Dhx30 | DEAH (Asp-Glu-Ala-His) box polypeptide 30 | 1.26 | 0.283 | -1.5 | 0.114 | -1.15 | 0.304 | -1.24 | 0.087 | -0.66 |
| 1444700_at | Nrxn3 | Neurexin III | -1.25 | 0.755 | -1.35 | 0.486 | 1.26 | 0.584 | -1.7 | 0.192 | -0.76 |
| 1439528_at | 4833423E24Rik | RIKEN cDNA 4833423E24 gene | -1.14 | 0.609 | 1.11 | 0.72 | -1.24 | 0.498 | -1.37 | 0.526 | -0.66 |
| 1441687_at | --- | --- | -1.33 | 0.586 | -1.57 | 0.196 | 1.4 | 0.16 | -1.48 | 0.308 | -0.74 |
| 1455334_at | 9830167H18Rik | RIKEN cDNA 9830167H18 gene | -1.09 | 0.538 | 1.02 | 0.896 | -1.16 | 0.191 | -1.06 | 0.584 | -0.57 |
| 1436479_a_at | Dpp7 | dipeptidylpeptidase 7 | -1.25 | 0.005 | -1.07 | 0.745 | 1.06 | 0.368 | -1.48 | 0.169 | -0.68 |
| 1433687_at | AI662250 | expressed sequence AI662250 | 1.08 | 0.621 | -1.18 | 0.044 | -1.14 | 0.573 | -1.18 | 0.406 | -0.61 |
| 1459944_at | Utp11l | UTP11-like, U3 small nucleolar ribonucleoprotein, (yeast) | -1.2 | 0.791 | 1.14 | 0.721 | -1.22 | 0.375 | -1.21 | 0.102 | -0.62 |
| 1455804_x_at | Oxct1 | 3-oxoacid CoA transferase 1 | -1.51 | 0.172 | 1.2 | 0.523 | -1.06 | 0.61 | -1.14 | 0.26 | -0.63 |
| 1453282_at | Cxadr | coxsackievirus and adenovirus receptor | 1.08 | 0.528 | -1.01 | 0.974 | -1.38 | 0.093 | -1.3 | 0.416 | -0.65 |
| 1417185_at | Ly6a | lymphocyte antigen 6 complex, locus A | -1.21 | 0.301 | 1.12 | 0.568 | -1.17 | 0.112 | -1.22 | 0.389 | -0.62 |
| 1450418_a_at | Yipf4 | Yip1 domain family, member 4 | -1.14 | 0.643 | -1.05 | 0.792 | -1.04 | 0.729 | 1.77 | 0.192 | -0.36 |
| 1447828_x_at | 2310005P05Rik | RIKEN cDNA 2310005P05 gene | -1.65 | 0.508 | 1.45 | 0.441 | -1.35 | 0.509 | -1.17 | 0.677 | -0.68 |
| 1451193_x_at | Ttc4 | tetratricopeptide repeat domain 4 | 1.03 | 0.648 | -1.02 | 0.849 | -1.26 | 0.273 | -1.11 | 0.499 | -0.59 |
| 1433204_at | 2810436B12Rik | RIKEN cDNA 2810436B12 gene | -1.1 | 0.851 | 1.14 | 0.481 | -1.34 | 0.462 | -1.14 | 0.772 | -0.61 |
| 1447349_s_at | Ep400 | E1A binding protein p400 | -1.01 | 0.943 | -1.15 | 0.515 | -1.07 | 0.815 | 1.06 | 0.654 | -0.54 |
| 1452820_at | Brwd2 | bromodomain and WD repeat domain containing 2 | -1.16 | 0.333 | -1.12 | 0.346 | 1.04 | 0.8 | -1.35 | 0.358 | -0.65 |
| 1428011_a_at | Erbb2ip | Erbb2 interacting protein | -1.13 | 0.305 | 1.08 | 0.499 | -1.19 | 0.08 | -1.36 | 0.037 | -0.65 |
| 1438793_x_at | 1200007D18Rik | RIKEN cDNA 1200007D18 gene | 1.05 | 0.874 | -1.14 | 0.424 | -1.15 | 0.567 | -1.03 | 0.869 | -0.57 |
| 1433762_at | C630043F03Rik | RIKEN cDNA C630043F03 gene | 1.25 | 0.32 | -1.68 | 0.033 | -1.05 | 0.815 | -1.05 | 0.777 | -0.63 |
| 1428212_x_at | Rpl31 /// LOC54486 | ribosomal protein L31 /// similar to ribosomal protein L31 /// similar to ribosomal pr | -1.08 | 0.011 | 1.04 | 0.204 | -1.2 | 0.035 | -1.23 | 0 | -0.62 |
| 1426860_at | Ep400 | E1A binding protein p400 | -1.21 | 0.13 | -1.26 | 0.104 | 1.18 | 0.338 | -1.14 | 0.461 | -0.61 |
| 1439107_a_at | Mll5 | myeloid/lymphoid or mixed-lineage leukemia 5 | -1.15 | 0.682 | -1.35 | 0.117 | 1.18 | 0.557 | -1.25 | 0.205 | -0.64 |
| 1448546_at | Rassf3 | Ras association (RalGDS/AF-6) domain family 3 | 1.07 | 0.61 | -1.36 | 0.264 | -1 | 0.986 | -1.37 | 0.286 | -0.67 |

| | | | | | | | | | | | |
|---|---|---|---|---|---|---|---|---|---|---|---|
| 1441260_a_at | --- | --- | -2.08 | 0.071 | -1.21 | 0.694 | 1.49 | 0.203 | -2.24 | 0.073 | -1.01 |
| 1426759_at | Map4k3 /// LOC675 | mitogen-activated protein kinase kinase kinase 3 /// similar to mitogen-acti | -1.03 | 0.834 | 1.04 | 0.693 | -1.27 | 0.231 | -1.33 | 0.493 | -0.65 |
| 1431287_at | Pcm1 | pericentriolar material 1 | -1.41 | 0.158 | 1.15 | 0.847 | -1.06 | 0.791 | -1.45 | 0.255 | -0.69 |
| 1451259_at | Rexo2 | REX2, RNA exonuclease 2 homolog (S. cerevisiae) | -1.05 | 0.617 | -1.09 | 0.109 | -1.07 | 0.559 | 1.6 | 0.086 | -0.4 |
| 1453317_a_at | Khdrbs3 | KH domain containing, RNA binding, signal transduction associated 3 | -1.02 | 0.881 | -1.35 | 0.172 | 1.08 | 0.754 | -1.43 | 0.167 | -0.68 |
| 1433598_at | 9430010O03Rik | RIKEN cDNA 9430010O03 gene | -1.16 | 0.586 | -1.25 | 0.183 | 1.13 | 0.537 | -1.04 | 0.731 | -0.58 |
| 1431423_a_at | Med8 | mediator of RNA polymerase II transcription, subunit 8 homolog (yeast) | -1.15 | 0.181 | -1.01 | 0.957 | -1.07 | 0.623 | 1.21 | 0.307 | -0.5 |
| 1421882_a_at | Elavl2 | ELAV (embryonic lethal, abnormal vision, Drosophila)-like 2 (Hu antigen B) | 1.99 | 0.509 | -1.8 | 0.295 | -3.97 | 0 | -2.31 | 0.021 | -1.52 |
| 1455826_a_at | Bace1 | beta-site APP cleaving enzyme 1 | 1.48 | 0.025 | -1.48 | 0.052 | -1.54 | 0.141 | -1.28 | 0.126 | -0.71 |
| 1431453_at | 4933406K04Rik | RIKEN cDNA 4933406K04 gene | -1.08 | 0.92 | 1.01 | 0.986 | -1.16 | 0.704 | -1.2 | 0.431 | -0.61 |
| 1425338_at | Plcb4 | phospholipase C, beta 4 | -1.09 | 0.821 | -1.28 | 0.695 | 1.1 | 0.852 | -1.53 | 0.195 | -0.7 |
| 1449879_at | 1700008F21Rik | RIKEN cDNA 1700008F21 gene | 1.78 | 0.396 | -2.71 | 0.199 | -1.55 | 0.203 | -1.59 | 0.409 | -1.02 |
| 1448711_at | Mcm3ap | minichromosome maintenance deficient 3 (S. cerevisiae) associated protein | -1.1 | 0.706 | -1.15 | 0.16 | 1.02 | 0.908 | -1.27 | 0.245 | -0.63 |
| 1452612_at | Zfp294 | zinc finger protein 294 | 1.06 | 0.515 | -1.28 | 0.06 | -1.04 | 0.443 | -1.08 | 0.584 | -0.59 |
| 1424531_a_at | Tcea3 | transcription elongation factor A (SII), 3 | -1.11 | 0.514 | -1.4 | 0.162 | 1.18 | 0.24 | -1.85 | 0.303 | -0.79 |
| 1444695_at | --- | Adult male colon cDNA, RIKEN full-length enriched library, clone:9030003C19 pr | 1.32 | 0.225 | -1.42 | 0.026 | -1.29 | 0.09 | -1.29 | 0.304 | -0.67 |
| 1446993_at | Plin | Perilipin | -1.56 | 0.062 | 1.4 | 0.552 | -1.33 | 0.521 | -1.39 | 0.463 | -0.72 |
| 1450275_x_at | Ube2f | ubiquitin-conjugating enzyme E2F (putative) | -1.05 | 0.481 | -1.05 | 0.642 | -1.11 | 0.446 | 1.14 | 0.596 | -0.52 |
| 1451622_at | Lmbrd1 | LMBR1 domain containing 1 | -1.15 | 0.31 | -1.14 | 0.097 | 1.05 | 0.564 | -1.67 | 0.098 | -0.73 |
| 1434532_at | BC035295 | cDNA sequence BC035295 | -1.03 | 0.832 | -1.07 | 0.388 | -1.12 | 0.223 | 1.03 | 0.859 | -0.55 |
| 1450433_at | Rbpsuhl | recombining binding protein suppressor of hairless-like (Drosophila) | -1.87 | 0.587 | -1.08 | 0.718 | 1.34 | 0.344 | -1.12 | 0.307 | -0.68 |
| 1416300_a_at | Slc25a3 | solute carrier family 25 (mitochondrial carrier, phosphate carrier), member 3 | -1.2 | 0.014 | -1.26 | 0.029 | 1.17 | 0.021 | -1.02 | 0.844 | -0.58 |
| 1415680_at | Anapc1 | anaphase promoting complex subunit 1 | -1.09 | 0.444 | -1.06 | 0.326 | -1.06 | 0.644 | 1.2 | 0.402 | -0.5 |
| 1450732_a_at | Bicd2 | bicaudal D homolog 2 (Drosophila) | 1.07 | 0.794 | -1.05 | 0.355 | -1.28 | 0.407 | -1.1 | 0.761 | -0.59 |
| 1448848_at | Tor1b | torsin family 1, member B | -1.01 | 0.921 | 1.02 | 0.893 | -1.27 | 0.045 | -1.17 | 0.121 | -0.61 |
| 1425370_a_at | Erg | avian erythroblastosis virus E-26 (v-ets) oncogene related | 1.07 | 0.814 | -1.23 | 0.239 | -1.08 | 0.875 | -1.12 | 0.35 | -0.59 |
| 1458988_at | --- | --- | -1.7 | 0.378 | 1.47 | 0.323 | -1.34 | 0.354 | -1.4 | 0.574 | -0.74 |
| 1435898_x_at | Gdi2 | guanosine diphosphate (GDP) dissociation inhibitor 2 | 1.02 | 0.527 | -1.01 | 0.943 | -1.28 | 0.073 | -1.31 | 0.255 | -0.64 |
| 1435364_at | Cyhr1 | cysteine and histidine rich 1 | -1.01 | 0.864 | -1.1 | 0.303 | -1.11 | 0.246 | 1.21 | 0.35 | -0.5 |
| 1416402_at | Abcb10 | ATP-binding cassette, sub-family B (MDR/TAP), member 10 | -1.04 | 0.711 | -1.15 | 0.328 | -1.03 | 0.837 | 5.08 | 0.17 | 0.47 |
| 1426368_at | Rin2 | Ras and Rab interactor 2 | -1.19 | 0.469 | 1.11 | 0.48 | -1.18 | 0.481 | -1.16 | 0.635 | -0.6 |
| 1453942_at | 1700123I01Rik | RIKEN cDNA 1700123I01 gene | -1.56 | 0.605 | -1.97 | 0.312 | 1.65 | 0.229 | -1.34 | 0.453 | -0.8 |
| 1432693_at | 4930517G19Rik | RIKEN cDNA 4930517G19 gene | 1.18 | 0.577 | -1.1 | 0.86 | -1.39 | 0.343 | -1.55 | 0.056 | -0.72 |
| 1445698_at | Capn11 | calpain 11 | -1.36 | 0.383 | 1.25 | 0.296 | -1.24 | 0.559 | -1.31 | 0.303 | -0.66 |
| 1442487_at | D14Ertd24e | DNA segment, Chr 14, ERATO Doi 24, expressed | -1.11 | 0.454 | 1.2 | 0.571 | -1.43 | 0.172 | -1.15 | 0.809 | -0.62 |
| 1434646_s_at | Sap18 | Sin3-associated polypeptide 18 | -1.05 | 0.41 | -1.11 | 0.019 | -1.05 | 0.646 | 1.08 | 0.666 | -0.53 |
| 1419611_at | --- | --- | -1.23 | 0.704 | 1.01 | 0.982 | -1.02 | 0.937 | -1.21 | 0.352 | -0.61 |
| 1421204_a_at | Nudt16 | nudix (nucleoside diphosphate linked moiety X)-type motif 16 | -1.16 | 0.314 | -1.23 | 0.243 | 1.12 | 0.564 | -1.3 | 0.529 | -0.64 |
| 1443448_x_at | 2010110K16Rik | RIKEN cDNA 2010110K16 gene | -1.01 | 0.975 | 1.25 | 0.378 | -1.79 | 0.22 | -1.22 | 0.73 | -0.69 |
| 1436038_a_at | Pigp | phosphatidylinositol glycan anchor biosynthesis, class P | -1.03 | 0.778 | 1.04 | 0.724 | -1.25 | 0.238 | -1.56 | 0.03 | -0.7 |
| 1457842_at | Zfp292 | Zinc finger protein 292 | 1.08 | 0.406 | -1.02 | 0.931 | -1.35 | 0.111 | -1.67 | 0.003 | -0.74 |
| 1437680_x_at | Glrx2 | glutaredoxin 2 (thioltransferase) | -1.29 | 0.104 | -1.03 | 0.854 | 1.05 | 0.776 | -1.05 | 0.752 | -0.58 |
| 1418491_a_at | Pus3 | pseudouridine synthase 3 | -1.23 | 0.02 | -1.04 | 0.746 | 1.03 | 0.838 | -1.46 | 0.23 | -0.68 |
| 1438819_at | --- | Transcribed locus | 1.4 | 0.508 | -1.33 | 0.237 | -1.56 | 0.07 | -2.16 | 0.054 | -0.91 |
| 1449438_at | Dpm1 | dolichol-phosphate (beta-D) mannosyltransferase 1 | -1.1 | 0.362 | -1.15 | 0.006 | 1.02 | 0.825 | -1.09 | 0.376 | -0.58 |
| 1420791_x_at | Speer4f | spermatogenesis associated glutamate (E)-rich protein 4f | 1.36 | 0.609 | -2.03 | 0.425 | -1.05 | 0.763 | -1.67 | 0.467 | -0.85 |
| 1425776_a_at | C87436 | expressed sequence C87436 | 1.02 | 0.921 | -1.01 | 0.9 | -1.26 | 0.096 | -1.02 | 0.723 | -0.57 |
| 1424574_at | Tmed5 | transmembrane emp24 protein transport domain containing 5 | 1.01 | 0.962 | -1.23 | 0.102 | -1.03 | 0.707 | -1.17 | 0.371 | -0.6 |
| 1439403_x_at | Rnf12 | ring finger protein 12 | -1.13 | 0.665 | 1.15 | 0.581 | -1.3 | 0.295 | -1.32 | 0.173 | -0.65 |
| 1422751_at | Tle1 | transducin-like enhancer of split 1, homolog of Drosophila E(spl) | 1.44 | 0.247 | -1.53 | 0.169 | -1.41 | 0.179 | -1.06 | 0.778 | -0.64 |
| 1428802_at | Mgat3 | mannoside acetylglucosaminyltransferase 3 | -1.35 | 0.477 | 1.21 | 0.84 | -1.18 | 0.627 | -1.22 | 0.482 | -0.63 |
| 1451613_at | Hrnr | hornerin | -1.03 | 0.821 | 1.27 | 0.161 | -1.77 | 0.322 | -1.87 | 0.362 | -0.85 |
| 1417660_s_at | Vps29 | vacuolar protein sorting 29 (S. pombe) | -1.1 | 0.22 | 1.05 | 0.572 | -1.18 | 0.144 | -1.42 | 0.043 | -0.66 |

| | | | | | | | | | | | |
|---|---|---|---|---|---|---|---|---|---|---|---|
| 1438754_at | --- | --- | -1.05 | 0.562 | 1.1 | 0.843 | -1.34 | 0.176 | -1.44 | 0.246 | -0.68 |
| 1458527_at | 2610005L07Rik | RIKEN cDNA 2610005L07 gene | -1.35 | 0.021 | -1.07 | 0.84 | 1.12 | 0.426 | -1.45 | 0.348 | -0.69 |
| 1460175_at | Rps23 | ribosomal protein S23 | -1.09 | 0.318 | 1.05 | 0.242 | -1.21 | 0.011 | -1.31 | 0.021 | -0.64 |
| 1459244_at | AU014756 | expressed sequence AU014756 | -1.02 | 0.844 | -1.08 | 0.496 | -1.12 | 0.804 | 2.09 | 0.157 | -0.28 |
| 1453146_at | Gapvd1 | GTPase activating protein and VPS9 domains 1 | -1.14 | 0.075 | -1.07 | 0.355 | -1 | 0.984 | 1.86 | 0.202 | -0.34 |
| 1429327_at | Sdccag1 | serologically defined colon cancer antigen 1 | 1.01 | 0.972 | -1.07 | 0.825 | -1.17 | 0.618 | -1.16 | 0.716 | -0.6 |
| 1424274_at | Vdp | vesicle docking protein | -1.1 | 0.451 | 1.02 | 0.878 | -1.15 | 0.216 | -1.15 | 0.175 | -0.59 |
| 1442037_at | LOC668580 | hypothetical protein LOC668580 | 1.19 | 0.628 | -1.06 | 0.774 | -1.49 | 0.482 | -1.65 | 0.535 | -0.75 |
| 1458197_x_at | Mapkapk5 /// Adam | MAP kinase-activated protein kinase 5 /// a disintegrin and metallopeptidase dom | -1.67 | 0.355 | -1.24 | 0.767 | 1.4 | 0.522 | -1.43 | 0.36 | -0.74 |
| 1439147_at | 1110049B09Rik | RIKEN cDNA 1110049B09 gene | -1.29 | 0.786 | -1.67 | 0.404 | 1.43 | 0.025 | -2.09 | 0.078 | -0.91 |
| 1426994_at | Phlpp | PH domain and leucine rich repeat protein phosphatase | -1.01 | 0.879 | -1.29 | 0.156 | 1.03 | 0.849 | -1.35 | 0.25 | -0.65 |
| 1423225_at | Selk | selenoprotein K | -1.14 | 0.017 | 1.15 | 0.247 | -1.29 | 0.041 | -1.1 | 0.486 | -0.59 |
| 1438599_at | --- | --- | -1.36 | 0.64 | 1.1 | 0.5 | -1.04 | 0.907 | -2.05 | 0.431 | -0.83 |
| 1422174_at | Ipf1 | insulin promoter factor 1, homeodomain transcription factor | -2.54 | 0.431 | -1.36 | 0.454 | 1.68 | 0.34 | -2.94 | 0.201 | -1.29 |
| 1430510_at | B130050I23Rik | RIKEN cDNA B130050I23 gene | -1.55 | 0.595 | 1.53 | 0.203 | -1.59 | 0.281 | -1.3 | 0.225 | -0.73 |
| 1451735_at | Arfrp1 | ADP-ribosylation factor related protein 1 | -1.25 | 0.191 | -1.42 | 0.14 | 1.3 | 0.232 | -1.43 | 0.124 | -0.7 |
| 1449672_s_at | Tufm /// LOC66983 | Tu translation elongation factor, mitochondrial /// similar to Tu translation elongati | -1.97 | 0.068 | -1.01 | 0.983 | 1.3 | 0.593 | -2.68 | 0.038 | -1.09 |
| 1419839_x_at | Prpf19 | PRP19/PSO4 pre-mRNA processing factor 19 homolog (S. cerevisiae) | -1.13 | 0.28 | -1.12 | 0.037 | 1.02 | 0.862 | -1.49 | 0.056 | -0.68 |
| 1419278_at | Usp48 | ubiquitin specific peptidase 48 | -1.14 | 0.54 | -1.11 | 0.06 | 1.02 | 0.769 | -1.03 | 0.879 | -0.56 |
| 1438242_at | Usp40 | ubiquitin specific peptidase 40 | 1 | 0.99 | -1.17 | 0.093 | -1.05 | 0.526 | -1.18 | 0.439 | -0.6 |
| 1455887_at | Alg8 | asparagine-linked glycosylation 8 homolog (yeast, alpha-1,3-glucosyltransferase) | -1.69 | 0.318 | -1.27 | 0.439 | 1.42 | 0.349 | -1.21 | 0.551 | -0.69 |
| 1423759_a_at | Tmco1 | transmembrane and coiled-coil domains 1 | -1.05 | 0.605 | -1.01 | 0.777 | -1.16 | 0.081 | 1.1 | 0.074 | -0.53 |
| 1454908_x_at | Serf1 | small EDRK-rich factor 1 | -1.09 | 0.474 | -1.09 | 0.675 | -1.03 | 0.931 | 1.1 | 0.809 | -0.53 |
| 1425774_at | 1500001A10Rik | RIKEN cDNA 1500001A10 gene | -1.12 | 0.803 | 1.27 | 0.575 | -1.54 | 0.181 | -1.01 | 0.985 | -0.6 |
| 1453904_at | 4930528G09Rik | RIKEN cDNA 4930528G09 gene | -5.97 | 0.03 | -1.21 | 0.423 | 1.81 | 0.32 | -1 | 0.999 | -1.59 |
| 1425231_a_at | Zfp46 | zinc finger protein 46 | -1.2 | 0.25 | 1.07 | 0.639 | -1.12 | 0.297 | -1.17 | 0.454 | -0.6 |
| 1459350_at | Socs6 | Suppressor of cytokine signaling 6 | -1.09 | 0.821 | -1.23 | 0.486 | 1.07 | 0.79 | -1.25 | 0.292 | -0.62 |
| 1456441_at | Ccdc87 | coiled-coil domain containing 87 | -1.53 | 0.252 | 1.2 | 0.396 | -1.05 | 0.823 | -1.14 | 0.569 | -0.63 |
| 1425174_at | --- | CDNA clone MGC:18942 IMAGE:3979577 | 1.01 | 0.979 | -1.04 | 0.915 | -1.2 | 0.665 | -1.3 | 0.044 | -0.63 |
| 1455922_at | Rab3gap1 | RAB3 GTPase activating protein subunit 1 | -1.17 | 0.086 | -1.06 | 0.454 | 1 | 0.978 | -1.32 | 0.137 | -0.64 |
| 1422548_at | Dhdds | dehydrodolichyl diphosphate synthase | -1.11 | 0.716 | -1.06 | 0.279 | -1.04 | 0.837 | 1.35 | 0.006 | -0.47 |
| 1455083_at | Atp11c | Atpase, class VI, type 11C | 1.02 | 0.944 | -1.05 | 0.643 | -1.2 | 0.309 | -1.11 | 0.249 | -0.58 |
| 1422624_at | Rev1l | REV1-like (S. cerevisiae) | -1.14 | 0.714 | -1.02 | 0.946 | -1.06 | 0.762 | 1.1 | 0.696 | -0.53 |
| 1429010_at | Snrp70 | U1 small nuclear ribonucleoprotein polypeptide A | 1.49 | 0.68 | -1.74 | 0.32 | -1.34 | 0.642 | -2.46 | 0.43 | -1.02 |
| 1416944_a_at | Tlk2 | tousled-like kinase 2 (Arabidopsis) | 1.13 | 0.323 | -1.15 | 0.046 | -1.26 | 0.153 | -1.23 | 0.189 | -0.62 |
| 1428732_at | 1700008J07Rik | RIKEN cDNA 1700008J07 gene | -1.04 | 0.445 | -1.26 | 0.007 | 1.05 | 0.584 | -1.23 | 0.046 | -0.62 |
| 1437559_at | D13Bwg1146e | DNA segment, Chr 13, Brigham & Women's Genetics 1146 expressed | -1.06 | 0.913 | 1.16 | 0.758 | -1.42 | 0.244 | -1.07 | 0.778 | -0.6 |
| 1434507_at | Npepl1 | aminopeptidase-like 1 | -1.18 | 0.154 | -1.08 | 0.403 | 1.03 | 0.876 | -1.3 | 0.304 | -0.63 |
| 1422594_at | 5730470L24Rik | RIKEN cDNA 5730470L24 gene | -1.13 | 0.44 | 1.09 | 0.238 | -1.21 | 0.104 | -1.74 | 0.164 | -0.75 |
| 1448357_at | Snrpg /// LOC6666( | small nuclear ribonucleoprotein polypeptide G /// similar to small nuclear ribonucle | -1.01 | 0.913 | 1.01 | 0.889 | -1.25 | 0.168 | -1.2 | 0.104 | -0.61 |
| 1424920_at | Slc37a3 | solute carrier family 37 (glycerol-3-phosphate transporter), member 3 | -1.17 | 0.434 | -1.16 | 0.26 | 1.09 | 0.666 | -1.98 | 0.038 | -0.81 |
| 1436968_x_at | Klhl24 | kelch-like 24 (Drosophila) | -1.1 | 0.373 | -1.14 | 0.562 | 1.02 | 0.936 | -1.82 | 0.154 | -0.76 |
| 1416884_at | Cbx3 /// LOC62957 | chromobox homolog 3 (Drosophila HP1 gamma) /// similar to chromobox homolog | -1.03 | 0.77 | -1 | 0.995 | -1.2 | 0.356 | 1.22 | 0.145 | -0.5 |
| 1457055_at | Nubp1 | Nucleotide binding protein 1 | 1.07 | 0.488 | -1.06 | 0.886 | -1.26 | 0.087 | -1.3 | 0.05 | -0.64 |
| 1459926_at | C77068 | expressed sequence C77068 | 1.62 | 0.227 | -1.31 | 0.654 | -2.36 | 0.109 | -1.42 | 0.721 | -0.87 |
| 1444932_at | --- | --- | -1.02 | 0.967 | 1.16 | 0.197 | -1.51 | 0.353 | -1.26 | 0.51 | -0.66 |
| 1451338_at | Nisch | nischarin | -1.23 | 0.057 | -1.06 | 0.577 | 1.05 | 0.5 | -1.26 | 0.454 | -0.63 |
| 1445035_at | Igk-V | immunoglobulin kappa chain complex variable region | 1.23 | 0.682 | -1.51 | 0.279 | -1.09 | 0.778 | -1.88 | 0.305 | -0.81 |
| 1439228_at | Zfp407 | zinc finger protein 407 | 1.3 | 0.788 | -1.53 | 0.401 | -1.18 | 0.779 | -5.07 | 0 | -1.62 |
| 1417245_at | Gpr180 | G protein-coupled receptor 180 | -1.12 | 0.085 | 1.09 | 0.343 | -1.21 | 0.29 | -1.43 | 0.202 | -0.67 |
| 1455372_at | Cpeb3 | cytoplasmic polyadenylation element binding protein 3 | -1.21 | 0.573 | -1.01 | 0.976 | -1.02 | 0.943 | 3.96 | 0.085 | 0.18 |
| 1449148_a_at | Phtf1 | putative homeodomain transcription factor 1 | -1.22 | 0.107 | 1.06 | 0.597 | -1.08 | 0.515 | -1.11 | 0.591 | -0.59 |
| 1437709_x_at | Ttc13 | tetratricopeptide repeat domain 13 | -1.07 | 0.492 | 1.02 | 0.802 | -1.18 | 0.075 | -1.38 | 0.18 | -0.65 |

| | | | | | | | | | | | |
|---|---|---|---|---|---|---|---|---|---|---|---|
| 1433875_at | 4732418C07Rik | RIKEN cDNA 4732418C07 gene | -1.07 | 0.288 | 1.08 | 0.248 | -1.26 | 0.029 | -1.04 | 0.722 | -0.57 |
| 1427159_at | Pcf11 | cleavage and polyadenylation factor subunit homolog (S. cerevisiae) | -1.24 | 0.205 | -1 | 0.984 | 1 | 0.976 | -1.05 | 0.602 | -0.57 |
| 1453470_a_at | Gna13 | guanine nucleotide binding protein, alpha 13 | -1.35 | 0.237 | -1.11 | 0.599 | 1.16 | 0.53 | -1.29 | 0.569 | -0.65 |
| 1418135_at | Aff1 | AF4/FMR2 family, member 1 | -1.01 | 0.942 | -1.13 | 0.189 | -1.07 | 0.616 | 1.36 | 0.156 | -0.46 |
| 1423491_at | Fbxo3 | F-box only protein 3 | -1.01 | 0.969 | -1.12 | 0.469 | -1.08 | 0.746 | 1.43 | 0.342 | -0.45 |
| 1450178_at | Brdt | bromodomain, testis-specific | 1.06 | 0.921 | -1.01 | 0.97 | -1.31 | 0.189 | -2.24 | 0.038 | -0.88 |
| 1419375_at | Wbp4 | WW domain binding protein 4 | -1.01 | 0.937 | -1.05 | 0.36 | -1.16 | 0.142 | 1.11 | 0.269 | -0.53 |
| 1455185_s_at | --- | --- | -1.04 | 0.879 | -1.08 | 0.674 | -1.09 | 0.625 | 1.88 | 0.107 | -0.33 |
| 1439184_s_at | Txnl5 | thioredoxin-like 5 | -1.08 | 0.581 | 1.05 | 0.641 | -1.2 | 0.137 | -1.2 | 0.365 | -0.61 |
| 1458932_at | Pex2 | peroxin 2 | -1.84 | 0.156 | 1.27 | 0.554 | -1 | 0.992 | -1.09 | 0.874 | -0.67 |
| 1440261_at | Ap4e1 | adaptor-related protein complex AP-4, epsilon 1 | 1.02 | 0.899 | -1.02 | 0.747 | -1.24 | 0.032 | -1.03 | 0.889 | -0.57 |
| 1422487_at | Smad4 | MAD homolog 4 (Drosophila) | 1.05 | 0.501 | -1.04 | 0.321 | -1.26 | 0.023 | -1.69 | 0.108 | -0.74 |
| 1426588_at | Tspan10 | tetraspanin 10 | -1.25 | 0.326 | -1.56 | 0.311 | 1.37 | 0.583 | -2.02 | 0.271 | -0.87 |
| 1437154_at | Cep170 | centrosomal protein 170 | -1.6 | 0.231 | 1.22 | 0.408 | -1.04 | 0.722 | -1.18 | 0.401 | -0.65 |
| 1419134_at | Rhbg | Rhesus blood group-associated B glycoprotein | -1.44 | 0.292 | -1 | 0.99 | 1.11 | 0.601 | -1.19 | 0.037 | -0.63 |
| 1436537_at | Zfp629 | zinc finger protein 629 | 1.02 | 0.922 | -1.07 | 0.705 | -1.17 | 0.238 | -1.25 | 0.061 | -0.62 |
| 1446717_at | --- | --- | -1 | 0.977 | -1.16 | 0.662 | -1.06 | 0.891 | 1.03 | 0.917 | -0.55 |
| 1441212_at | 6720467C03Rik | RIKEN cDNA 6720467C03 gene | 1.32 | 0.11 | -1.37 | 0.3 | -1.32 | 0.373 | -1.21 | 0.468 | -0.64 |
| 1447494_at | D7Bwg0826e | DNA segment, Chr 7, Brigham & Women's Genetics 0826 expressed | -1.24 | 0.233 | 1.11 | 0.755 | -1.12 | 0.725 | -1.47 | 0.043 | -0.68 |
| 1441780_at | --- | Transcribed locus | -2.32 | 0.405 | -1.26 | 0.726 | 1.58 | 0.415 | -1.6 | 0.509 | -0.9 |
| 1417072_at | Slc22a6 | solute carrier family 22 (organic anion transporter), member 6 | -1.05 | 0.558 | -1.44 | 0.328 | 1.15 | 0.873 | -1.2 | 0.732 | -0.63 |
| 1451848_a_at | Cryz | crystallin, zeta | -1.14 | 0.255 | -1.22 | 0.205 | 1.11 | 0.528 | -1.13 | 0.519 | -0.6 |
| 1437881_at | 6330416L07Rik | RIKEN cDNA 6330416L07 gene | -1.18 | 0.043 | 1.02 | 0.957 | -1.07 | 0.872 | -1.12 | 0.509 | -0.58 |
| 1438612_a_at | Clps | colipase, pancreatic | 1.46 | 0.575 | -1.48 | 0.472 | -1.48 | 0.41 | -1.81 | 0.642 | -0.83 |
| 1455906_at | 6030446N20Rik | RIKEN cDNA 6030446N20 gene | -1.09 | 0.75 | 1.09 | 0.73 | -1.24 | 0.148 | -1.04 | 0.702 | -0.57 |
| 1444218_at | D19Ertd737e | DNA segment, Chr 19, ERATO Doi 737, expressed | 1.09 | 0.435 | -1.02 | 0.746 | -1.34 | 0.147 | -1.52 | 0.23 | -0.7 |
| 1437276_at | --- | Transcribed locus | -1.27 | 0.635 | 1.05 | 0.814 | -1.04 | 0.876 | -1.08 | 0.686 | -0.58 |
| 1433705_at | Zfp213 | zinc finger protein 213 | 1.06 | 0.716 | -1.26 | 0.209 | -1.05 | 0.702 | -1.2 | 0.443 | -0.61 |
| 1436523_s_at | 1810022K09Rik | RIKEN cDNA 1810022K09 gene | -1.11 | 0.27 | 1.03 | 0.755 | -1.15 | 0.146 | -1.3 | 0.059 | -0.63 |
| 1446337_at | Hdh | Huntington disease gene homolog | 1.14 | 0.637 | -1.34 | 0.341 | -1.09 | 0.853 | -1.05 | 0.854 | -0.58 |
| 1417006_at | Commd4 | COMM domain containing 4 | -1.2 | 0.374 | -1.11 | 0.066 | 1.07 | 0.241 | -1.5 | 0.1 | -0.68 |
| 1460260_s_at | Kpna1 | karyopherin (importin) alpha 1 | -1.17 | 0.095 | 1.01 | 0.926 | -1.06 | 0.637 | -1.02 | 0.938 | -0.56 |
| 1415887_at | Tfg | Trk-fused gene | -1.11 | 0.447 | -1.04 | 0.699 | -1.06 | 0.141 | 1.33 | 0.15 | -0.47 |
| 1436855_at | Ptprs | protein tyrosine phosphatase, receptor type, S | -1.01 | 0.963 | -1.11 | 0.69 | -1.09 | 0.646 | 1.56 | 0.222 | -0.41 |
| 1440828_x_at | Phf7 | PHD finger protein 7 | 1.04 | 0.913 | -1.16 | 0.744 | -1.1 | 0.81 | -1.14 | 0.853 | -0.59 |
| 1458620_at | LOC332993 | similar to ubiquitin A-52 residue ribosomal protein fusion product 1 | 1.56 | 0.469 | -1.71 | 0.102 | -1.52 | 0.439 | -2.16 | 0.29 | -0.96 |
| 1428806_at | Csnk1g1 | casein kinase 1, gamma 1 | 1.01 | 0.954 | -1.06 | 0.752 | -1.16 | 0.302 | -1.17 | 0.296 | -0.6 |
| 1457893_at | Ptplad2 | Protein tyrosine phosphatase-like A domain containing 2 | -1.3 | 0.71 | 1.06 | 0.823 | -1.02 | 0.935 | -2.1 | 0.066 | -0.84 |
| 1420167_at | --- | --- | -1.4 | 0.265 | -1.18 | 0.728 | 1.24 | 0.605 | -1.2 | 0.299 | -0.63 |
| 1435525_at | Kctd17 | potassium channel tetramerisation domain containing 17 | -1.22 | 0.216 | -1.01 | 0.924 | -1.01 | 0.961 | 1.06 | 0.465 | -0.54 |
| 1450055_at | Vsnl1 | visinin-like 1 | 1.31 | 0.102 | -1.09 | 0.808 | -1.72 | 0.105 | -1.52 | 0.404 | -0.75 |
| 1435237_at | 2310009A05Rik | RIKEN cDNA 2310009A05 gene | 1.06 | 0.498 | -1.06 | 0.305 | -1.25 | 0.193 | -1.29 | 0.069 | -0.63 |
| 1420811_a_at | Ctnnb1 | catenin (cadherin associated protein), beta 1 | 1 | 0.986 | -1.01 | 0.835 | -1.22 | 0.043 | -1.17 | 0.121 | -0.6 |
| 1451479_a_at | Tmem53 | transmembrane protein 53 | -1.09 | 0.682 | 1.14 | 0.333 | -1.34 | 0.102 | -1.17 | 0.691 | -0.61 |
| 1456199_x_at | Ranbp9 | RAN binding protein 9 | -1.2 | 0.113 | -1.12 | 0.044 | 1.08 | 0.619 | -1.09 | 0.629 | -0.58 |
| 1419038_a_at | Csnk2a1 | casein kinase 2, alpha 1 polypeptide | 1.19 | 0.671 | -1.14 | 0.577 | -1.36 | 0.39 | -1.03 | 0.953 | -0.58 |
| 1450898_at | Hiat1 | hippocampus abundant gene transcript 1 | -1.2 | 0.067 | -1.13 | 0.508 | 1.09 | 0.62 | -1.11 | 0.529 | -0.59 |
| 1431059_x_at | Htatsf1 | HIV TAT specific factor 1 | -1.41 | 0.333 | -1.23 | 0.406 | 1.29 | 0.379 | -1.22 | 0.612 | -0.64 |
| 1442922_at | Chfr | checkpoint with forkhead and ring finger domains | -1.04 | 0.856 | -2.09 | 0.085 | 1.37 | 0.523 | -2.71 | 0.079 | -1.12 |
| 1433534_a_at | Cct2 | chaperonin subunit 2 (beta) | -1.19 | 0.393 | -1.05 | 0.776 | 1.02 | 0.898 | -1.23 | 0.171 | -0.61 |
| 1416582_a_at | Bad | Bcl-associated death promoter | -1.2 | 0.426 | 1.05 | 0.833 | -1.08 | 0.567 | -1.05 | 0.771 | -0.57 |
| 1460025_at | Lrig2 | leucine-rich repeats and immunoglobulin-like domains 2 | -1.33 | 0.134 | 1.14 | 0.471 | -1.09 | 0.218 | -1.3 | 0.047 | -0.64 |
| 1431310_s_at | Ttll10 | tubulin tyrosine ligase-like family, member 10 | 1.28 | 0.196 | -1.2 | 0.732 | -1.45 | 0.052 | -1.63 | 0.493 | -0.75 |

| | | | | | | | | | | | |
|---|---|---|---|---|---|---|---|---|---|---|---|
| 1456492_at | 9130404D08Rik | RIKEN cDNA 9130404D08 gene | 1.17 | 0.56 | -1.18 | 0.516 | -1.26 | 0.031 | -1.02 | 0.139 | -0.57 |
| 1424557_at | Zcchc9 | zinc finger, CCHC domain containing 9 | 1.03 | 0.814 | -1.2 | 0.112 | -1.06 | 0.356 | -1.09 | 0.37 | -0.58 |
| 1435266_at | A930041I02Rik | RIKEN cDNA A930041I02 gene | -1.1 | 0.371 | -1.15 | 0.606 | 1.03 | 0.859 | -1.29 | 0.38 | -0.63 |
| 1425746_at | D730039F16Rik | RIKEN cDNA D730039F16 gene | -1.03 | 0.659 | 1.08 | 0.544 | -1.32 | 0.482 | -1.01 | 0.984 | -0.57 |
| 1428085_at | 1110057K04Rik | RIKEN cDNA 1110057K04 gene | -1.14 | 0.335 | -1.05 | 0.496 | -1.02 | 0.815 | 1.1 | 0.219 | -0.53 |
| 1439500_at | --- | --- | 1.23 | 0.307 | -1.27 | 0.631 | -1.28 | 0.124 | -1.13 | 0.707 | -0.61 |
| 1424173_at | Tmem48 | transmembrane protein 48 | -1.04 | 0.446 | -1.06 | 0.906 | -1.11 | 0.76 | 1.94 | 0.317 | -0.32 |
| 1456160_at | Nxn | Nucleoredoxin | 1.43 | 0.003 | -1.34 | 0.694 | -1.57 | 0.185 | -1.25 | 0.035 | -0.68 |
| 1450869_at | Fgf1 | fibroblast growth factor 1 | 1.13 | 0.429 | -1.28 | 0.002 | -1.12 | 0.478 | -1.09 | 0.456 | -0.59 |
| 1417900_a_at | Vldlr | very low density lipoprotein receptor | -1.11 | 0.254 | 1.18 | 0.805 | -1.38 | 0.258 | -1.33 | 0.393 | -0.66 |
| 1459869_x_at | 4930402E16Rik | RIKEN cDNA 4930402E16 gene | 1.04 | 0.735 | -1.24 | 0.306 | -1.04 | 0.877 | -1.16 | 0.459 | -0.6 |
| 1455207_at | 2410017P09Rik | RIKEN cDNA 2410017P09 gene | -1.15 | 0.224 | 1.07 | 0.399 | -1.15 | 0.346 | -1.12 | 0.536 | -0.59 |
| 1420054_s_at | Slc35c2 | solute carrier family 35, member C2 | -1.19 | 0.604 | 1.06 | 0.873 | -1.1 | 0.414 | -1.36 | 0.335 | -0.65 |
| 1426715_s_at | D11Ertd18e | DNA segment, Chr 11, ERATO Doi 18, expressed | -1.05 | 0.741 | -1.13 | 0.162 | -1.03 | 0.928 | 1.33 | 0.511 | -0.47 |
| 1417016_at | Mapkapk5 | MAP kinase-activated protein kinase 5 | -1.11 | 0.465 | -1.11 | 0.689 | 1 | 0.973 | -1.07 | 0.759 | -0.57 |
| 1416456_a_at | Chia | chitinase, acidic | -1.01 | 0.966 | -1.39 | 0.327 | 1.11 | 0.329 | -1.39 | 0.673 | -0.67 |
| 1429993_s_at | Speer4b | spermatogenesis associated glutamate (E)-rich protein 4b | -1 | 0.999 | -1.1 | 0.476 | -1.11 | 0.736 | 2.73 | 0.288 | -0.12 |
| 1436846_x_at | Ywhaq /// LOC5461 | tyrosine 3-monooxygenase/tryptophan 5-monooxygenase activation protein, theta | -1.06 | 0.496 | -1.16 | 0.07 | 1.01 | 0.946 | -1.07 | 0.778 | -0.57 |
| 1459879_at | 4921513D23Rik | RIKEN cDNA 4921513D23 gene | 1.29 | 0.395 | -1.06 | 0.808 | -1.74 | 0.201 | -1.03 | 0.636 | -0.63 |
| 1454937_at | B630005N14Rik | RIKEN cDNA B630005N14 gene | -1.23 | 0.227 | -1.13 | 0.197 | 1.11 | 0.52 | -1.39 | 0.254 | -0.66 |
| 1460632_at | --- | --- | -1.03 | 0.857 | 1.03 | 0.796 | -1.23 | 0.11 | -1.15 | 0.659 | -0.6 |
| 1451141_at | Mettl8 | methyltransferase like 8 | -1.06 | 0.732 | -1.01 | 0.943 | -1.14 | 0.238 | 1.16 | 0.409 | -0.51 |
| 1424222_s_at | Rad23b | RAD23b homolog (S. cerevisiae) | -1.1 | 0.159 | -1.19 | 0.042 | 1.06 | 0.441 | -1.18 | 0.204 | -0.6 |
| 1422933_at | Xlr5d /// LOC62708 | X-linked lymphocyte-regulated 5D /// similar to X-linked lymphocyte-regulated 5C | -1.2 | 0.613 | 1.15 | 0.531 | -1.21 | 0.723 | -1.21 | 0.452 | -0.62 |
| 1437110_at | 2810474O19Rik | RIKEN cDNA 2810474O19 gene | -1 | 0.986 | -1.09 | 0.52 | -1.11 | 0.26 | 1.43 | 0.106 | -0.45 |
| 1427907_at | 1110037F02Rik | RIKEN cDNA 1110037F02 gene | -1.26 | 0.438 | 1.05 | 0.663 | -1.04 | 0.78 | -1.2 | 0.485 | -0.61 |
| 1446034_at | --- | Adult male cortex cDNA, RIKEN full-length enriched library, clone:B530045B12 p | -1.2 | 0.297 | -1.12 | 0.704 | 1.09 | 0.272 | -1.16 | 0.035 | -0.6 |
| 1429606_at | 4930527B16Rik | RIKEN cDNA 4930527B16 gene | 1.32 | 0.591 | -1.48 | 0.412 | -1.23 | 0.13 | -1.09 | 0.761 | -0.62 |
| 1455013_at | Arih2 | ariadne homolog 2 (Drosophila) | -1.05 | 0.724 | -1.08 | 0.071 | -1.07 | 0.366 | 1.14 | 0.43 | -0.52 |
| 1446679_at | Grm5 | Glutamate receptor, metabotropic 5 | 1.39 | 0.582 | -1.15 | 0.699 | -1.8 | 0.146 | -1.34 | 0.582 | -0.73 |
| 1426107_at | Prdm9 | VPR domain containing 9 | -1.21 | 0.595 | 1.41 | 0.617 | -1.73 | 0.363 | -1.4 | 0.233 | -0.73 |
| 1456432_at | Grap2 | GRB2-related adaptor protein 2 | -1.23 | 0.299 | 1.2 | 0.756 | -1.26 | 0.33 | -2.47 | 0.111 | -0.94 |
| 1423456_at | Bzw2 | basic leucine zipper and W2 domains 2 | -1.26 | 0.22 | 1.13 | 0.483 | -1.13 | 0.455 | -1.07 | 0.808 | -0.58 |
| 1446379_at | C79296 | expressed sequence C79296 | -1.73 | 0.288 | 1.32 | 0.232 | -1.09 | 0.819 | -1.38 | 0.517 | -0.72 |
| 1427330_at | Cars | cysteinyl-tRNA synthetase | -1.24 | 0.007 | -1.29 | 0.585 | 1.23 | 0.536 | -1.74 | 0.376 | -0.76 |
| 1446020_at | --- | --- | -1.12 | 0.4 | 1.13 | 0.499 | -1.27 | 0.322 | -1.08 | 0.596 | -0.58 |
| 1428917_at | Stx17 | syntaxin 17 | 1.12 | 0.37 | -1.19 | 0.224 | -1.18 | 0.259 | -1.03 | 0.873 | -0.57 |
| 1451880_at | LOC638575 | hypothetical LOC638575 | 1.54 | 0.59 | -2.28 | 0.293 | -1.21 | 0.711 | -1.13 | 0.793 | -0.77 |
| 1453924_a_at | Ptgfr | prostaglandin F receptor | -1.01 | 0.941 | -1.19 | 0.516 | -1.02 | 0.924 | 1.36 | 0.537 | -0.47 |
| 1426292_at | 6330581L23Rik | RIKEN cDNA 6330581L23 gene | 1.11 | 0.038 | -1.16 | 0.064 | -1.19 | 0.173 | -1.37 | 0.296 | -0.65 |
| 1442647_at | Yipf1 | Yip1 domain family, member 1 | -1.13 | 0.778 | 1.16 | 0.668 | -1.31 | 0.132 | -1.45 | 0.165 | -0.68 |
| 1420727_a_at | Tmlhe | trimethyllysine hydroxylase, epsilon | -1.07 | 0.758 | 1.01 | 0.95 | -1.16 | 0.332 | -1.01 | 0.976 | -0.56 |
| 1439599_at | Gal3st2 | galactose-3-O-sulfotransferase 2 | 1.16 | 0.812 | -1.21 | 0.473 | -1.22 | 0.172 | -1.91 | 0.091 | -0.79 |
| 1434316_at | Chsy1 | carbohydrate (chondroitin) synthase 1 | -1.14 | 0.708 | -1.03 | 0.876 | -1.04 | 0.274 | 1.14 | 0.486 | -0.52 |
| 1428476_a_at | Elac2 | elaC homolog 2 (E. coli) | -1.24 | 0.398 | -1.25 | 0.009 | 1.2 | 0.17 | -1.04 | 0.676 | -0.58 |
| 1436536_at | C730015A04Rik | RIKEN cDNA C730015A04 gene | 1.1 | 0.535 | -1.13 | 0.445 | -1.21 | 0.407 | -1 | 0.984 | -0.56 |
| 1439670_at | B020006M18Rik | RIKEN cDNA B020006M18 gene | 1.48 | 0.615 | -1.62 | 0.417 | -1.41 | 0.442 | -2.38 | 0.322 | -0.98 |
| 1456006_at | Bcl2l11 | BCL2-like 11 (apoptosis facilitator) | -1.09 | 0.754 | -1.06 | 0.767 | -1.06 | 0.506 | 1.2 | 0.524 | -0.5 |
| 1433430_s_at | Cdc23 | CDC23 (cell division cycle 23, yeast, homolog) | -1.04 | 0.832 | -1.06 | 0.592 | -1.1 | 0.569 | 1.12 | 0.504 | -0.52 |
| 1418529_at | Osgep | O-sialoglycoprotein endopeptidase | -1.12 | 0.133 | 1.01 | 0.873 | -1.11 | 0.4 | -1.28 | 0.162 | -0.62 |
| 1458557_at | Bcas3 | breast carcinoma amplified sequence 3 | -1.68 | 0.119 | -1.04 | 0.937 | 1.26 | 0.484 | -1.59 | 0.505 | -0.76 |
| 1448235_s_at | Hmgb1 /// 4932431 | high mobility group box 1 /// RIKEN cDNA 4932431P20 gene /// similar to High mo | -1.04 | 0.786 | -1.08 | 0.665 | -1.08 | 0.667 | 1.15 | 0.516 | -0.51 |
| 1430051_at | 4930486L24Rik | RIKEN cDNA 4930486L24 gene | 1.22 | 0.772 | -1.25 | 0.666 | -1.26 | 0.439 | -1.28 | 0.509 | -0.64 |

| | | | | | | | | | | | |
|---|---|---|---|---|---|---|---|---|---|---|---|
| 1434408_at | Mjd | Machado-Joseph disease (spinocerebellar ataxia 3, olivopontocerebellar ataxia 3 | 1.18 | 0.635 | -1.07 | 0.631 | -1.43 | 0.028 | -1.09 | 0.158 | -0.6 |
| 1434585_at | 2210038L17Rik | RIKEN cDNA 2210038L17 gene | 1.06 | 0.656 | -1.13 | 0.231 | -1.16 | 0.157 | -1.25 | 0.248 | -0.62 |
| 1446861_at | Gns | glucosamine (N-acetyl)-6-sulfatase | -1.15 | 0.093 | -1.15 | 0.437 | 1.07 | 0.731 | -1.05 | 0.822 | -0.57 |
| 1450473_at | Pcdh12 | protocadherin 12 | -1.34 | 0.575 | 1.3 | 0.532 | -1.3 | 0.451 | -1.06 | 0.863 | -0.6 |
| 1458665_at | Clic1 | Chloride intracellular channel 1 | 1.75 | 0.094 | -4.02 | 0.197 | -1.23 | 0.622 | -1.02 | 0.936 | -1.13 |
| 1434452_x_at | Eif2a | eukaryotic translation initiation factor 2a | -1.2 | 0.565 | 1.01 | 0.956 | -1.03 | 0.834 | -1.1 | 0.582 | -0.58 |
| 1421480_a_at | Adarb1 | adenosine deaminase, RNA-specific, B1 | -1.5 | 0.362 | -1.26 | 0.453 | 1.35 | 0.067 | -1.12 | 0.754 | -0.63 |
| 1455159_at | 2900057D21Rik | RIKEN cDNA 2900057D21 gene | -1.13 | 0.337 | 1.17 | 0.288 | -1.32 | 0.146 | -1.78 | 0.13 | -0.76 |
| 1460336_at | Ppargc1a | peroxisome proliferative activated receptor, gamma, coactivator 1 alpha | -1.27 | 0.227 | -1.28 | 0.161 | 1.24 | 0.089 | -1.88 | 0.253 | -0.8 |
| 1439886_at | 4632415L05Rik | RIKEN cDNA 4632415L05 gene | -1.23 | 0.144 | -1.21 | 0.219 | 1.17 | 0.363 | -1.28 | 0.055 | -0.64 |
| 1440899_at | Fmo5 | flavin containing monooxygenase 5 | -1.17 | 0.687 | -1.14 | 0.486 | 1.08 | 0.644 | -1.31 | 0.352 | -0.63 |
| 1443216_at | Tbc1d5 | TBC1 domain family, member 5 | -1.16 | 0.538 | 1.16 | 0.252 | -1.27 | 0.292 | -1.8 | 0.232 | -0.77 |
| 1426620_at | Chst10 | carbohydrate sulfotransferase 10 | -1.12 | 0.482 | -1.3 | 0.02 | 1.15 | 0.283 | -1.56 | 0.07 | -0.71 |
| 1430788_at | --- | --- | -1.25 | 0.796 | 1.09 | 0.488 | -1.08 | 0.888 | -2.12 | 0.27 | -0.84 |
| 1447090_s_at | Arl1 | ADP-ribosylation factor-like 1 | -1.05 | 0.681 | 1.06 | 0.402 | -1.26 | 0.006 | -1.36 | 0.166 | -0.65 |
| 1437280_s_at | Serbp1 | Serpine1 mRNA binding protein 1 | 1.02 | 0.855 | -1.07 | 0.395 | -1.17 | 0.255 | -1.08 | 0.109 | -0.57 |
| 1416951_a_at | Atp6v1d | ATPase, H+ transporting, lysosomal V1 subunit D | -1.07 | 0.529 | -1.03 | 0.787 | -1.1 | 0.504 | 1.05 | 0.404 | -0.54 |
| 1421115_a_at | Zdhhc16 | zinc finger, DHHC domain containing 16 | -1.03 | 0.29 | -1.12 | 0.142 | -1.05 | 0.715 | 1.04 | 0.83 | -0.54 |
| 1448802_at | Nufip1 | nuclear fragile X mental retardation protein interacting protein 1 | -1.35 | 0.129 | 1.14 | 0.513 | -1.08 | 0.612 | -1.19 | 0.172 | -0.62 |
| 1458349_s_at | --- | 10 days neonate skin cDNA, RIKEN full-length enriched library, clone:4732488M1 | 1.24 | 0.12 | -1.72 | 0.206 | -1.01 | 0.977 | -1.22 | 0.286 | -0.68 |
| 1441401_at | C79329 | expressed sequence C79329 | 1.2 | 0.781 | -1.06 | 0.91 | -1.48 | 0.407 | -3.19 | 0.067 | -1.13 |
| 1434104_at | 6030458H05 | hypothetical protein 6030458H05 | -1.02 | 0.895 | -1.17 | 0.431 | -1.02 | 0.915 | 1.59 | 0.201 | -0.41 |
| 1426894_s_at | C230093N12Rik | RIKEN cDNA C230093N12 gene | 1.02 | 0.916 | -1.24 | 0.387 | -1.01 | 0.879 | -1.76 | 0.155 | -0.75 |
| 1438856_x_at | Serpinb5 | serine (or cysteine) peptidase inhibitor, clade B, member 5 | 1.16 | 0.713 | -1.11 | 0.265 | -1.33 | 0.381 | -1.05 | 0.841 | -0.58 |
| 1433555_at | Eaf1 | ELL associated factor 1 | -1.06 | 0.352 | -1.12 | 0.148 | -1.03 | 0.877 | 1.16 | 0.466 | -0.51 |
| 1428263_a_at | Tceb2 | transcription elongation factor B (SIII), polypeptide 2 | -1.03 | 0.367 | -1.05 | 0.064 | -1.13 | 0.031 | 1.08 | 0.676 | -0.53 |
| 1448352_at | Luzp1 | leucine zipper protein 1 | -1.14 | 0.598 | 1.1 | 0.773 | -1.19 | 0.585 | -1.06 | 0.691 | -0.57 |
| 1426354_at | Bap1 | Brca1 associated protein 1 | -1.23 | 0.159 | -1.03 | 0.741 | 1.03 | 0.817 | -1.05 | 0.745 | -0.57 |
| 1424280_at | Mospd1 | motile sperm domain containing 1 | -1.05 | 0.526 | -1.02 | 0.847 | -1.13 | 0.361 | 1.69 | 0.108 | -0.38 |
| 1444178_at | LOC433237 | hypothetical gene supported by AK028012 | -1.18 | 0.365 | -1.03 | 0.936 | -1 | 0.996 | 3.98 | 0.204 | 0.19 |
| 1421733_a_at | Tpst1 | protein-tyrosine sulfotransferase 1 | 1.05 | 0.863 | -1.1 | 0.566 | -1.17 | 0.519 | -1.25 | 0.174 | -0.62 |
| 1437499_at | Ankrd39 | ankyrin repeat domain 39 | -1.21 | 0.698 | 1.02 | 0.93 | -1.04 | 0.906 | -1.62 | 0.063 | -0.71 |
| 1433897_at | AI597468 | expressed sequence AI597468 | -1.05 | 0.856 | -1.01 | 0.954 | -1.14 | 0.153 | 1.11 | 0.068 | -0.53 |
| 1421489_a_at | 2010106E10Rik | RIKEN cDNA 2010106E10 gene | 1.64 | 0.302 | -2.22 | 0.388 | -1.38 | 0.225 | -2.29 | 0.239 | -1.06 |
| 1416891_at | Numb | numb gene homolog (Drosophila) | -1.07 | 0.083 | -1.25 | 0.071 | 1.08 | 0.54 | -1.26 | 0.216 | -0.62 |
| 1434482_at | D4Ertd22e | DNA segment, Chr 4, ERATO Doi 22, expressed | -1.24 | 0.41 | -1.06 | 0.598 | 1.06 | 0.759 | -1.49 | 0.039 | -0.68 |
| 1417642_at | Aldh1a3 | aldehyde dehydrogenase family 1, subfamily A3 | -2.13 | 0.199 | -1.01 | 0.979 | 1.36 | 0.35 | -2.26 | 0.18 | -1.01 |
| 1448527_at | Pdcd10 | programmed cell death 10 | -1.16 | 0.127 | 1.07 | 0.425 | -1.12 | 0.163 | -1.33 | 0.043 | -0.64 |
| 1456143_at | Prkcbp1 | protein kinase C binding protein 1 | -1.09 | 0.551 | -1.1 | 0.313 | -1.01 | 0.97 | 1.02 | 0.907 | -0.55 |
| 1426271_at | Smc5 | structural maintenance of chromosomes 5 | -1.08 | 0.646 | -1.02 | 0.841 | -1.1 | 0.283 | 1.12 | 0.427 | -0.52 |
| 1418894_s_at | Pbx2 | pre B-cell leukemia transcription factor 2 | -1.07 | 0.715 | -1.23 | 0.498 | 1.07 | 0.778 | -1.78 | 0.134 | -0.75 |
| 1427233_at | Tshz1 | teashirt zinc finger family member 1 | 1.08 | 0.368 | -1.15 | 0.217 | -1.15 | 0.193 | -1.07 | 0.7 | -0.57 |
| 1448484_at | Amd1 | S-adenosylmethionine decarboxylase 1 | -1.08 | 0.227 | -1.09 | 0.189 | -1.04 | 0.639 | 1.97 | 0.216 | -0.31 |
| 1452628_at | Bag5 | BCL2-associated athanogene 5 | -1 | 0.976 | -1.07 | 0.435 | -1.13 | 0.361 | 1.06 | 0.773 | -0.54 |
| 1460674_at | Paqr7 | progestin and adipoQ receptor family member VII | 1.07 | 0.807 | -1.24 | 0.604 | -1.07 | 0.386 | -1.19 | 0.204 | -0.61 |
| 1426212_s_at | Tmem161a | transmembrane protein 161A | -1.06 | 0.526 | -1.02 | 0.842 | -1.12 | 0.074 | 1.01 | 0.928 | -0.55 |
| 1420124_s_at | Tcta | T-cell leukemia translocation altered gene | -1.08 | 0.54 | 1.06 | 0.684 | -1.21 | 0.004 | -1.13 | 0.608 | -0.59 |
| 1459705_at | Olfm3 | Olfactomedin 3 | 1.14 | 0.836 | -1.16 | 0.821 | -1.24 | 0.559 | -1.31 | 0.607 | -0.64 |
| 1428946_at | Ube1l2 | ubiquitin-activating enzyme E1-like 2 | 1.02 | 0.905 | -1.1 | 0.606 | -1.13 | 0.654 | -1.05 | 0.839 | -0.57 |
| 1436814_at | Tpcn2 | Two pore segment channel 2 | 1.04 | 0.954 | -1.05 | 0.918 | -1.21 | 0.248 | -2.66 | 0.123 | -0.97 |
| 1432869_at | --- | --- | -1 | 0.999 | -2.06 | 0.149 | 1.33 | 0.412 | -2.6 | 0.311 | -1.08 |
| 1454246_at | St3gal6 | ST3 beta-galactoside alpha-2,3-sialyltransferase 6 | -1.22 | 0.821 | -1.55 | 0.069 | 1.35 | 0.678 | -1.6 | 0.115 | -0.76 |
| 1441454_at | Zhx3 | zinc fingers and homeoboxes 3 | -1.1 | 0.673 | 1.06 | 0.732 | -1.18 | 0.51 | -1.14 | 0.711 | -0.59 |

| | | | | | | | | | | | |
|---|---|---|---|---|---|---|---|---|---|---|---|
| 1416074_a_at | Rpl28 | ribosomal protein L28 | -1.05 | 0.683 | 1.03 | 0.779 | -1.2 | 0.139 | -1.17 | 0.056 | -0.6 |
| 1430838_x_at | Mbd1 | methyl-CpG binding domain protein 1 | -1.13 | 0.022 | -1.15 | 0.431 | 1.07 | 0.716 | -1.26 | 0.442 | -0.62 |
| 1419371_s_at | Gosr2 | golgi SNAP receptor complex member 2 | -1.13 | 0.433 | 1.03 | 0.63 | -1.11 | 0.225 | -1.29 | 0.155 | -0.63 |
| 1417635_at | Spa17 | sperm autoantigenic protein 17 | -1.01 | 0.927 | -1.01 | 0.983 | -1.19 | 0.478 | 1.23 | 0.617 | -0.5 |
| 1433554_at | AU022870 | expressed sequence AU022870 | -1.03 | 0.241 | 1.03 | 0.364 | -1.23 | 0.051 | -1 | 0.983 | -0.56 |
| 1455350_at | Tmem62 | transmembrane protein 62 | -1.16 | 0.021 | 1.09 | 0.665 | -1.15 | 0.226 | -1.29 | 0.187 | -0.63 |
| 1453086_at | 6330408A02Rik | RIKEN cDNA 6330408A02 gene | -1.03 | 0.932 | -1.48 | 0.213 | 1.17 | 0.439 | -1.28 | 0.133 | -0.66 |
| 1429968_at | 2310047L11Rik | RIKEN cDNA 2310047L11 gene | -1 | 0.981 | 1.12 | 0.65 | -1.43 | 0.11 | -1.43 | 0.398 | -0.69 |
| 1440974_at | 9030203C11Rik | RIKEN cDNA 9030203C11 gene | 1.21 | 0.662 | -1.2 | 0.329 | -1.3 | 0.078 | -1.42 | 0.258 | -0.68 |
| 1442182_at | Dnajc19 | DnaJ (Hsp40) homolog, subfamily C, member 19 | 1.08 | 0.62 | -1.2 | 0.39 | -1.1 | 0.635 | -1.26 | 0.229 | -0.62 |
| 1417476_at | Fbxw5 | F-box and WD-40 domain protein 5 | -1.1 | 0.555 | -1.11 | 0.478 | 1.01 | 0.958 | -1.16 | 0.336 | -0.59 |
| 1448770_a_at | Atpif1 | ATPase inhibitory factor 1 | -1.06 | 0.27 | -1.01 | 0.884 | -1.14 | 0.255 | 2.38 | 0.159 | -0.21 |
| 1424582_at | Mulk /// LOC66726( | multiple substrate lipid kinase /// similar to multi-substrate lipid kinase /// similar to | -1.17 | 0.439 | -1.01 | 0.926 | -1.03 | 0.855 | 1.01 | 0.959 | -0.55 |
| 1454605_a_at | Pi4k2a | phosphatidylinositol 4-kinase type 2 alpha | -1.25 | 0.217 | -1.01 | 0.921 | 1.02 | 0.47 | -1.07 | 0.596 | -0.58 |
| 1453944_at | 5430435K18Rik | RIKEN cDNA 5430435K18 gene | -1.36 | 0.152 | 1.09 | 0.821 | -1.01 | 0.989 | -1.24 | 0.563 | -0.63 |
| 1435561_at | Erf | Ets2 repressor factor | -1.43 | 0.555 | -1.07 | 0.817 | 1.18 | 0.562 | -1.73 | 0.26 | -0.76 |
| 1435690_at | 2310008H09Rik | RIKEN cDNA 2310008H09 gene | -1.05 | 0.762 | -1.13 | 0.646 | -1.02 | 0.92 | 1.12 | 0.629 | -0.52 |
| 1415984_at | Acadm | acyl-Coenzyme A dehydrogenase, medium chain | -1.09 | 0.411 | -1.45 | 0.14 | 1.21 | 0.288 | -1.39 | 0.228 | -0.68 |
| 1425698_a_at | Zf | HCF-binding transcription factor Zhangfei | -1.26 | 0.561 | -1.5 | 0.283 | 1.36 | 0.004 | -1.45 | 0.185 | -0.71 |
| 1452810_at | Ints10 /// LOC6748( | integrator complex subunit 10 /// similar to integrator complex subunit 10 | -1.02 | 0.928 | -1.18 | 0.201 | -1.01 | 0.894 | 1.05 | 0.325 | -0.54 |
| 1421466_at | Asb10 | ankyrin repeat and SOCS box-containing protein 10 | -1.01 | 0.985 | -1.02 | 0.958 | -1.19 | 0.335 | 1.09 | 0.361 | -0.53 |
| 1417703_at | Pvrl2 | poliovirus receptor-related 2 | 1.1 | 0.473 | -1.19 | 0.286 | -1.15 | 0.203 | -1.1 | 0.441 | -0.58 |
| 1419242_at | 9530002B09Rik | RIKEN cDNA 9530002B09 gene | -1.4 | 0.295 | -1.09 | 0.82 | 1.18 | 0.654 | -1.51 | 0.495 | -0.7 |
| 1432863_a_at | Nkx2-4 | NK2 transcription factor related, locus 4 (Drosophila) | -1.17 | 0.368 | 1.15 | 0.515 | -1.24 | 0.279 | -2.13 | 0.502 | -0.85 |
| 1418106_at | Hey2 | hairy/enhancer-of-split related with YRPW motif 2 | -1.18 | 0.408 | -1.05 | 0.901 | 1.01 | 0.962 | -1.69 | 0.036 | -0.73 |
| 1450531_at | H2-Bl | histocompatibility 2, blastocyst | -1.66 | 0.646 | 1.39 | 0.004 | -1.21 | 0.452 | -1.02 | 0.976 | -0.62 |
| 1455087_at | D7Ertd715e | DNA segment, Chr 7, ERATO Doi 715, expressed | -1.24 | 0.125 | 1.13 | 0.723 | -1.14 | 0.594 | -1.04 | 0.744 | -0.57 |
| 1422969_s_at | Ihpk1 | inositol hexaphosphate kinase 1 | -1.29 | 0.303 | -1.79 | 0.268 | 1.49 | 0.506 | -1.18 | 0.603 | -0.7 |
| 1438969_x_at | Dhx30 | DEAH (Asp-Glu-Ala-His) box polypeptide 30 | -1.1 | 0.684 | -1.14 | 0.341 | 1.03 | 0.705 | -1.85 | 0.071 | -0.76 |
| 1437067_at | Phtf2 | putative homeodomain transcription factor 2 | -1.15 | 0.567 | -1.07 | 0.722 | 1.01 | 0.922 | -1.65 | 0.052 | -0.71 |
| 1454766_at | C730024G19Rik | RIKEN cDNA C730024G19 gene | 1.04 | 0.543 | -1.07 | 0.744 | -1.19 | 0.263 | -1.1 | 0.633 | -0.58 |
| 1435740_at | Rai17 | Retinoic acid induced 17 | -1.07 | 0.226 | -1.2 | 0.303 | 1.05 | 0.787 | -1.4 | 0.347 | -0.66 |
| 1446911_at | Itsn2 | Intersectin 2 | 1.08 | 0.723 | -1.2 | 0.403 | -1.11 | 0.764 | -1.43 | 0.369 | -0.67 |
| 1421836_at | --- | --- | -1.23 | 0.07 | -1.3 | 0.032 | 1.23 | 0.027 | -1.27 | 0.188 | -0.64 |
| 1423940_at | Yif1a | Yip1 interacting factor homolog A (S. cerevisiae) | -1.14 | 0.64 | -1.1 | 0.527 | 1.04 | 0.671 | -1.15 | 0.074 | -0.59 |
| 1449441_a_at | Wbp1 | WW domain binding protein 1 | -1.03 | 0.886 | -1.1 | 0.388 | -1.07 | 0.655 | 1.24 | 0.239 | -0.49 |
| 1419285_s_at | Cyhr1 | cysteine and histidine rich 1 | 1.08 | 0.733 | -1.26 | 0.169 | -1.06 | 0.347 | -1.14 | 0.283 | -0.59 |
| 1417932_at | Il18 | interleukin 18 | -1.13 | 0.588 | -1.13 | 0.535 | 1.05 | 0.408 | -1.08 | 0.776 | -0.57 |
| 1459686_at | --- | --- | 1.11 | 0.728 | -1.26 | 0.518 | -1.09 | 0.82 | -1.29 | 0.423 | -0.63 |
| 1458844_at | 4933415L06Rik | RIKEN cDNA 4933415L06 gene | 1.18 | 0.172 | -1.21 | 0.671 | -1.23 | 0.2 | -2.36 | 0.318 | -0.91 |
| 1420271_at | --- | 3 days neonate thymus cDNA, RIKEN full-length enriched library, clone:A630030 | -1.95 | 0.444 | 1.76 | 0.446 | -1.82 | 0.336 | -1.04 | 0.961 | -0.77 |
| 1454954_at | Wdr61 | WD repeat domain 61 | -1.04 | 0.854 | -1.08 | 0.737 | -1.08 | 0.735 | 1.22 | 0.326 | -0.49 |
| 1460743_at | Tigd5 | tigger transposable element derived 5 | -1.11 | 0.768 | -1.16 | 0.774 | 1.05 | 0.758 | -1.07 | 0.896 | -0.57 |
| 1446336_at | --- | --- | -1.37 | 0.214 | -1.05 | 0.865 | 1.14 | 0.69 | -2.05 | 0.1 | -0.83 |
| 1430731_at | Krtap2-4 | keratin associated protein 2-4 | 1.81 | 0.394 | -1.63 | 0.393 | -2.52 | 0.165 | -1.36 | 0.487 | -0.92 |
| 1449350_at | Osr1 | odd-skipped related 1 (Drosophila) | -1.03 | 0.918 | 1.18 | 0.774 | -1.52 | 0.141 | -1.52 | 0.503 | -0.72 |
| 1423821_at | Tmem168 | transmembrane protein 168 | -1.18 | 0.404 | 1.06 | 0.271 | -1.1 | 0.45 | -1.38 | 0.179 | -0.65 |
| 1422255_at | Kcna4 | potassium voltage-gated channel, shaker-related subfamily, member 4 | 1.15 | 0.738 | -1.38 | 0.382 | -1.06 | 0.745 | -1.5 | 0.477 | -0.7 |
| 1425036_a_at | Tnrc6a | trinucleotide repeat containing 6a | 1.03 | 0.801 | -1.03 | 0.771 | -1.22 | 0.229 | -1.58 | 0.045 | -0.7 |
| 1449953_at | Itgb3bp | integrin beta 3 binding protein (beta3-endonexin) | -1.43 | 0.024 | 1.13 | 0.702 | -1.01 | 0.974 | -1.32 | 0.544 | -0.66 |
| 1436593_at | 1700016K19Rik | RIKEN cDNA 1700016K19 gene | -1.2 | 0.462 | 1.02 | 0.929 | -1.04 | 0.866 | -2.03 | 0.369 | -0.81 |
| 1446711_at | A130040G06Rik | RIKEN cDNA A130040G06 gene | 1.08 | 0.685 | -1.25 | 0.764 | -1.07 | 0.826 | -2.3 | 0.05 | -0.88 |
| 1442001_at | Prkab2 | protein kinase, AMP-activated, beta 2 non-catalytic subunit | -1.26 | 0.327 | -1.72 | 0.332 | 1.44 | 0.146 | -1.24 | 0.325 | -0.69 |

| | | | | | | | | | | | |
|---|---|---|---|---|---|---|---|---|---|---|---|
| AFFX-r2-Bs-dap-3_a | --- | --- | 1.08 | 0.853 | -1.17 | 0.148 | -1.13 | 0.417 | -1.23 | 0.348 | -0.61 |
| 1428817_at | Mon2 | MON2 homolog (yeast) | -1.15 | 0.254 | -1.14 | 0.119 | 1.07 | 0.495 | -1.36 | 0.17 | -0.64 |
| 1446091_at | Anp32b | Acidic nuclear phosphoprotein 32 family, member B | -1.31 | 0.616 | 1.06 | 0.834 | -1.01 | 0.964 | -1.22 | 0.125 | -0.62 |
| 1426257_a_at | Sars | seryl-aminoacyl-tRNA synthetase | -1.07 | 0.693 | -1.08 | 0.321 | -1.05 | 0.735 | 1.26 | 0.014 | -0.48 |
| 1449732_at | Zipro1 | zinc finger proliferation 1 | 1.26 | 0.528 | -1.04 | 0.807 | -1.69 | 0.013 | -1.6 | 0.255 | -0.77 |
| 1435729_at | Mapbpip | mitogen activated protein binding protein interacting protein | 1.03 | 0.336 | -1.26 | 0.454 | -1 | 0.984 | -1.51 | 0.561 | -0.69 |
| 1435956_at | A930004K21Rik | RIKEN cDNA A930004K21 gene | -2 | 0.149 | -1.63 | 0.153 | 1.7 | 0.14 | -1.92 | 0.36 | -0.96 |
| 1418998_at | Kmo | kynurenine 3-monooxygenase (kynurenine 3-hydroxylase) | 1.08 | 0.598 | -1.04 | 0.679 | -1.28 | 0.042 | -3.1 | 0.044 | -1.09 |
| 1432164_a_at | Gcsh /// LOC67432 | glycine cleavage system protein H (aminomethyl carrier) /// similar to Glycine clea | -1.01 | 0.883 | 1.01 | 0.849 | -1.22 | 0.07 | -1.09 | 0.564 | -0.58 |
| 1427413_a_at | Cugbp1 | CUG triplet repeat, RNA binding protein 1 | -1.03 | 0.881 | -1.13 | 0.314 | -1.04 | 0.789 | 1.01 | 0.952 | -0.55 |
| 1434230_at | Polb /// A430088C0 | polymerase (DNA directed), beta /// RIKEN cDNA A430088C08 gene | 1.08 | 0.498 | -1.04 | 0.644 | -1.3 | 0.073 | -1.26 | 0.194 | -0.63 |
| 1428668_at | Acbd3 | acyl-Coenzyme A binding domain containing 3 | -1.06 | 0.334 | 1 | 0.993 | -1.14 | 0.209 | -1.33 | 0.029 | -0.63 |
| 1458580_at | --- | Transcribed locus | -1.39 | 0.524 | 1.21 | 0.617 | -1.13 | 0.787 | -1.35 | 0.274 | -0.66 |
| 1418228_at | Hirip5 | histone cell cycle regulation defective interacting protein 5 | -1.11 | 0.66 | -1.11 | 0.362 | 1.01 | 0.934 | -1.25 | 0.241 | -0.61 |
| 1436519_a_at | 1110057K04Rik | RIKEN cDNA 1110057K04 gene | -1.15 | 0.678 | -1.17 | 0.248 | 1.1 | 0.602 | -1.01 | 0.982 | -0.56 |
| 1424833_at | Itpr2 | inositol 1,4,5-triphosphate receptor 2 | -1.43 | 0.215 | -1.19 | 0.646 | 1.28 | 0.271 | -1.36 | 0.42 | -0.67 |
| 1450654_a_at | Dhdds | dehydrodolichyl diphosphate synthase | -1.08 | 0.581 | -1.14 | 0.35 | 1.01 | 0.908 | -1.02 | 0.84 | -0.55 |
| 1419003_at | Bves | blood vessel epicardial substance | -1.19 | 0.595 | -1.27 | 0.755 | 1.19 | 0.462 | -1.5 | 0.597 | -0.69 |
| 1416453_x_at | Rps12 /// LOC4328 | ribosomal protein S12 /// similar to ribosomal protein S12 /// similar to ribosomal p | -1.07 | 0.484 | 1.02 | 0.842 | -1.16 | 0.102 | -1.32 | 0.001 | -0.63 |
| 1440888_at | Oxtr | oxytocin receptor | -1.47 | 0.521 | -1.24 | 0.53 | 1.34 | 0.519 | -1.76 | 0.17 | -0.78 |
| 1425713_a_at | Rnf146 | ring finger protein 146 | -1.52 | 0.294 | -1.26 | 0.187 | 1.36 | 0.279 | -1.24 | 0.461 | -0.66 |
| 1416904_at | Mbnl1 | muscleblind-like 1 (Drosophila) | 1.03 | 0.754 | -1.06 | 0.087 | -1.18 | 0.158 | -1.27 | 0.056 | -0.62 |
| 1448149_at | Ctnna1 | catenin (cadherin associated protein), alpha 1 | -1.02 | 0.613 | -1.01 | 0.822 | -1.19 | 0.059 | 1.26 | 0.208 | -0.49 |
| 1423931_s_at | Anapc4 | anaphase promoting complex subunit 4 | -1.13 | 0.457 | 1.14 | 0.109 | -1.26 | 0.167 | -1.27 | 0.359 | -0.63 |
| 1442137_at | 2010000I03Rik | RIKEN cDNA 2010000I03 gene | -1.1 | 0.521 | -1.08 | 0.685 | -1.01 | 0.853 | 1.17 | 0.675 | -0.51 |
| 1440414_at | Mtf1 | metal response element binding transcription factor 1 | -1.39 | 0.393 | -1.31 | 0.575 | 1.33 | 0.229 | -1.36 | 0.206 | -0.68 |
| 1456530_x_at | Elovl1 | elongation of very long chain fatty acids (FEN1/Elo2, SUR4/Elo3, yeast)-like 1 | -1.2 | 0.021 | 1.15 | 0.237 | -1.21 | 0.245 | -1.03 | 0.768 | -0.57 |
| 1456347_at | Lnpep | Leucyl/cystinyl aminopeptidase | -1.22 | 0.443 | -1.01 | 0.989 | 1.01 | 0.959 | -1.31 | 0.417 | -0.63 |
| 1432012_a_at | Nsun6 | NOL1/NOP2/Sun domain family 6 | 1.04 | 0.957 | -1.24 | 0.747 | -1.03 | 0.89 | -1.14 | 0.759 | -0.59 |
| 1436007_a_at | Thumpd1 | THUMP domain containing 1 | -1.16 | 0.466 | -1.11 | 0.62 | 1.05 | 0.736 | -1.33 | 0.036 | -0.63 |
| 1418392_a_at | Gbp4 | guanylate nucleotide binding protein 4 | -1.27 | 0.075 | -1.37 | 0.271 | 1.3 | 0.474 | -1.19 | 0.421 | -0.63 |
| 1434894_at | Zc3h13 | zinc finger CCCH type containing 13 | 1.02 | 0.909 | -1.09 | 0.582 | -1.13 | 0.572 | -1.1 | 0.601 | -0.58 |
| 1451510_s_at | Olah | oleoyl-ACP hydrolase | -1.24 | 0.364 | -1 | 0.998 | 1.01 | 0.953 | -1.01 | 0.977 | -0.56 |
| 1460257_a_at | Mthfs | 5, 10-methenyltetrahydrofolate synthetase | -1.17 | 0.306 | -1.11 | 0.077 | 1.06 | 0.725 | -1.56 | 0.093 | -0.69 |
| 1444979_at | Proc | protein C | -1.08 | 0.917 | -1.24 | 0.663 | 1.09 | 0.804 | -1.15 | 0.595 | -0.6 |
| 1440605_at | Fscn2 | fascin homolog 2, actin-bundling protein, retinal (Strongylocentrotus purpuratus) | 1.03 | 0.264 | -1.13 | 0.774 | -1.11 | 0.414 | -1.06 | 0.68 | -0.57 |
| 1438653_x_at | Atxn10 | ataxin 10 | -1.12 | 0.46 | 1.04 | 0.178 | -1.13 | 0.175 | -1.23 | 0.157 | -0.61 |
| 1430124_x_at | Akr1a4 | aldo-keto reductase family 1, member A4 (aldehyde reductase) | -1.22 | 0.155 | -1.06 | 0.095 | 1.06 | 0.375 | -1.1 | 0.512 | -0.58 |
| 1430129_a_at | Commd8 | COMM domain containing 8 | -1.23 | 0.199 | 1.04 | 0.706 | -1.03 | 0.759 | -1.39 | 0.144 | -0.65 |
| 1453457_at | 2900070H08Rik | RIKEN cDNA 2900070H08 gene | -1.11 | 0.609 | 1.12 | 0.509 | -1.26 | 0.125 | -1.44 | 0.005 | -0.67 |
| 1452013_at | Atp10a | ATPase, class V, type 10A | -1.07 | 0.409 | -1.75 | 0.333 | 1.32 | 0.379 | -1.93 | 0.107 | -0.86 |
| 1448596_at | Slc6a8 | solute carrier family 6 (neurotransmitter transporter, creatine), member 8 | -1.42 | 0.151 | -1.18 | 0.646 | 1.27 | 0.384 | -1 | 0.998 | -0.58 |
| 1434500_at | Ttyh2 | tweety homolog 2 (Drosophila) | -1.12 | 0.387 | 1.05 | 0.82 | -1.14 | 0.515 | -1.03 | 0.788 | -0.56 |
| 1422072_a_at | Gstm6 | glutathione S-transferase, mu 6 | 1.19 | 0.573 | -1.47 | 0.403 | -1.05 | 0.892 | -1.79 | 0.065 | -0.78 |
| 1449263_at | Ufm1 | ubiquitin-fold modifier 1 | 1.02 | 0.867 | -1.06 | 0.38 | -1.17 | 0.11 | -1.06 | 0.657 | -0.57 |
| 1453536_at | 1700034K16Rik | RIKEN cDNA 1700034K16 gene | -1.03 | 0.91 | 1.18 | 0.717 | -1.48 | 0.396 | -2.4 | 0.035 | -0.94 |
| 1416908_s_at | Tsn | translin | -1.03 | 0.599 | -1.08 | 0.444 | -1.09 | 0.394 | 1.14 | 0.506 | -0.51 |
| 1454365_at | 4930554P06Rik | RIKEN cDNA 4930554P06 gene | -1.25 | 0.405 | 1.05 | 0.877 | -1.03 | 0.863 | -1.31 | 0.154 | -0.63 |
| 1425179_at | Shmt1 | serine hydroxymethyl transferase 1 (soluble) | -1.16 | 0.134 | -1.19 | 0.259 | 1.12 | 0.547 | -1.07 | 0.232 | -0.58 |
| 1438047_at | Zfp384 | zinc finger protein 384 | -1.01 | 0.871 | 1.02 | 0.741 | -1.24 | 0.167 | -1.19 | 0.089 | -0.6 |
| 1454647_at | Acad11 | acyl-Coenzyme A dehydrogenase family, member 11 | -1.14 | 0.595 | -1.45 | 0.064 | 1.25 | 0.26 | -1.05 | 0.496 | -0.6 |
| 1441988_at | Ppm1k | protein phosphatase 1K (PP2C domain containing) | 1.22 | 0.185 | -1.24 | 0.099 | -1.26 | 0.425 | -1.08 | 0.785 | -0.59 |
| 1458718_at | --- | PREDICTED: Mus musculus hypothetical LOC243025 (LOC243025), mRNA | -2.07 | 0.363 | 1.47 | 0.037 | -1.16 | 0.584 | -1.9 | 0.116 | -0.91 |

| | | | | | | | | | | | |
|---|---|---|---|---|---|---|---|---|---|---|---|
| 1457731_at | Snapc3 | small nuclear RNA activating complex, polypeptide 3 | 1.13 | 0.513 | -1.26 | 0.365 | -1.12 | 0.549 | -1.27 | 0.127 | -0.63 |
| 1419743_s_at | Carm1 | coactivator-associated arginine methyltransferase 1 | -1.09 | 0.463 | -1.06 | 0.225 | -1.04 | 0.843 | 1.86 | 0.092 | -0.33 |
| 1437204_a_at | Tcf25 | transcription factor 25 (basic helix-loop-helix) | -1.09 | 0.668 | -1.19 | 0.44 | 1.06 | 0.74 | -1.63 | 0.158 | -0.71 |
| 1446640_at | --- | Transcribed locus | -1.1 | 0.658 | -1.29 | 0.35 | 1.14 | 0.797 | -1.17 | 0.513 | -0.61 |
| 1453105_at | Zfp263 | zinc finger protein 263 | -1.31 | 0.012 | -1.02 | 0.878 | 1.08 | 0.652 | -1.78 | 0.151 | -0.76 |
| 1424646_at | Uckl1 | uridine-cytidine kinase 1-like 1 | -1.11 | 0.282 | -1.12 | 0.151 | 1.03 | 0.11 | -1.41 | 0.087 | -0.65 |
| 1430047_at | Brctd1 | BRCT domain containing 1 | -1.16 | 0.576 | 1.14 | 0.432 | -1.22 | 0.311 | -1.21 | 0.548 | -0.61 |
| 1438557_x_at | Dnpep | aspartyl aminopeptidase | -1.23 | 0.058 | -1.05 | 0.473 | 1.06 | 0.686 | -1.12 | 0.298 | -0.59 |
| 1424045_at | 5730437N04Rik | RIKEN cDNA 5730437N04 gene | -1.37 | 0.348 | -1.42 | 0.103 | 1.39 | 0.36 | -2.16 | 0.19 | -0.89 |
| 1448947_at | 2810004N23Rik | RIKEN cDNA 2810004N23 gene | -1.13 | 0.272 | -1.16 | 0.196 | 1.07 | 0.526 | -1.12 | 0.667 | -0.58 |
| 1416485_at | Timm23 | translocase of inner mitochondrial membrane 23 homolog (yeast) | -1.06 | 0.431 | -1.07 | 0.301 | -1.06 | 0.391 | 1.1 | 0.484 | -0.52 |
| 1454795_at | Cobll1 | Cobl-like 1 | -1.05 | 0.733 | -1.18 | 0.28 | 1.02 | 0.819 | -1.52 | 0.439 | -0.68 |
| 1451310_a_at | Ctsl | cathepsin L | -1.16 | 0.209 | 1.08 | 0.522 | -1.14 | 0.279 | -1.6 | 0.165 | -0.7 |
| 1416175_a_at | Vdac3 | voltage-dependent anion channel 3 | -1.05 | 0.048 | -1.07 | 0.295 | -1.08 | 0.276 | 1.13 | 0.383 | -0.51 |
| 1435816_at | Rps6 /// LOC21473 | ribosomal protein S6 /// similar to 40S ribosomal protein S6 /// similar to 40S ribos | 1.01 | 0.936 | -1.03 | 0.87 | -1.19 | 0.453 | -1.49 | 0.024 | -0.67 |
| 1441033_at | Tmtc2 | transmembrane and tetratricopeptide repeat containing 2 | 1.25 | 0.014 | -1.16 | 0.458 | -1.41 | 0.319 | -1.2 | 0.524 | -0.63 |
| 1435534_a_at | Tomm20 | translocase of outer mitochondrial membrane 20 homolog (yeast) | -1.19 | 0.208 | -1.01 | 0.856 | -1 | 0.979 | 1 | 0.984 | -0.55 |
| 1450612_a_at | Pemt | phosphatidylethanolamine N-methyltransferase | 1.05 | 0.793 | -1.03 | 0.853 | -1.25 | 0.394 | -1.17 | 0.245 | -0.6 |
| 1421066_at | --- | --- | -1.05 | 0.627 | 1.02 | 0.871 | -1.17 | 0.101 | -1.27 | 0.26 | -0.62 |
| 1456279_a_at | Bcap31 | B-cell receptor-associated protein 31 | 1.02 | 0.291 | -1.03 | 0.466 | -1.21 | 0.103 | -1.25 | 0.385 | -0.62 |
| 1419963_at | Depdc6 | DEP domain containing 6 | 1.15 | 0.777 | -1.21 | 0.615 | -1.18 | 0.594 | -1.08 | 0.908 | -0.58 |
| 1436480_at | Dpp7 | dipeptidylpeptidase 7 | -1.02 | 0.961 | -1.23 | 0.069 | 1.03 | 0.875 | -1.52 | 0.093 | -0.69 |
| 1419922_s_at | Atrnl1 | attractin like 1 | 1.06 | 0.765 | -1.04 | 0.67 | -1.24 | 0.367 | -1.45 | 0.295 | -0.67 |
| 1447773_x_at | 6330409D20Rik | RIKEN cDNA 6330409D20 gene | 1.54 | 0.617 | -2.48 | 0.021 | -1.13 | 0.748 | -1.25 | 0.737 | -0.83 |
| 1458042_at | --- | --- | -1.08 | 0.933 | -1.51 | 0.34 | 1.23 | 0.728 | -1.17 | 0.803 | -0.63 |
| 1443911_at | --- | --- | -1.58 | 0.067 | 1.65 | 0.431 | -1.84 | 0.067 | -1.88 | 0.218 | -0.91 |
| AFFX-PheX-3_at | --- | --- | 1.08 | 0.829 | -1.13 | 0.211 | -1.16 | 0.574 | -1.67 | 0.366 | -0.72 |
| 1449256_a_at | Rab11a | RAB11a, member RAS oncogene family | 1.03 | 0.636 | -1.07 | 0.321 | -1.16 | 0.14 | -1.16 | 0.034 | -0.59 |
| 1450972_at | 3110040N11Rik | RIKEN cDNA 3110040N11 gene | -1.03 | 0.765 | -1.02 | 0.929 | -1.15 | 0.22 | 1.04 | 0.738 | -0.54 |
| 1422209_s_at | Krtap9-1 /// LOC43 | keratin associated protein 9-1 /// similar to keratin associated protein 9-1 /// simila | 1.1 | 0.769 | -1.34 | 0.714 | -1.03 | 0.913 | -1.02 | 0.947 | -0.57 |
| 1434461_at | Zfp715 | zinc finger protein 715 | -1.01 | 0.76 | 1 | 0.973 | -1.21 | 0.135 | -1.46 | 0.042 | -0.67 |
| AFFX-DapX-3_at | --- | --- | 1.09 | 0.862 | -1.34 | 0.045 | -1.02 | 0.932 | -1.41 | 0.206 | -0.67 |
| 1437779_at | Foxh1 | forkhead box H1 | 1.54 | 0.531 | -1.66 | 0.381 | -1.48 | 0.057 | -1.53 | 0.509 | -0.78 |
| 1435967_s_at | Hibadh | 3-hydroxyisobutyrate dehydrogenase | -1.14 | 0.38 | -1.11 | 0.485 | 1.04 | 0.393 | -1.25 | 0.194 | -0.61 |
| 1424689_at | Prss32 | protease, serine, 32 | 1.81 | 0.359 | -1.53 | 0.337 | -2.75 | 0.06 | -1.19 | 0.637 | -0.92 |
| 1437198_at | Lig3 | ligase III, DNA, ATP-dependent | -1.02 | 0.939 | -1.06 | 0.754 | -1.12 | 0.663 | 1.05 | 0.842 | -0.54 |
| 1456357_at | A930041I02Rik | RIKEN cDNA A930041I02 gene | 1.21 | 0.556 | -1.41 | 0.366 | -1.1 | 0.694 | -1.06 | 0.088 | -0.59 |
| 1459837_at | Cyb561d1 | Cytochrome b-561 domain containing 1 | -1.14 | 0.696 | -1.26 | 0.374 | 1.15 | 0.238 | -2.41 | 0.011 | -0.91 |
| 1446141_at | Tgfb2 | Transforming growth factor, beta 2 | 1.78 | 0.361 | -1.47 | 0.434 | -2.78 | 0.09 | -1.74 | 0.544 | -1.05 |
| 1445152_at | Cask | Calcium/calmodulin-dependent serine protein kinase (MAGUK family) | -1.02 | 0.955 | 1.16 | 0.531 | -1.47 | 0.072 | -1.21 | 0.519 | -0.64 |
| 1460551_at | Ran | RAN, member RAS oncogene family | -1.01 | 0.828 | -1.15 | 0.076 | -1.03 | 0.788 | 1.52 | 0.197 | -0.42 |
| 1426375_s_at | Oxnad1 | oxidoreductase NAD-binding domain containing 1 | -1.08 | 0.723 | -1.09 | 0.571 | -1.02 | 0.79 | 1.44 | 0.307 | -0.44 |
| 1453417_at | 4933407K13Rik | RIKEN cDNA 4933407K13 gene | -1.13 | 0.743 | -1.13 | 0.699 | 1.05 | 0.752 | -1.26 | 0.009 | -0.62 |
| 1452086_at | Trmt5 | TRM5 tRNA methyltransferase 5 homolog (S. cerevisiae) | -1.1 | 0.568 | 1.01 | 0.981 | -1.1 | 0.402 | -1.09 | 0.449 | -0.57 |
| 1428167_a_at | Mpzl1 | myelin protein zero-like 1 | -1.13 | 0.539 | 1.17 | 0.48 | -1.31 | 0.435 | -1.9 | 0.189 | -0.79 |
| 1416788_a_at | Idh3g | isocitrate dehydrogenase 3 (NAD+), gamma | -1.07 | 0.265 | -1.13 | 0.238 | 1.01 | 0.699 | -1 | 0.992 | -0.55 |
| 1427253_s_at | Suz12 | suppressor of zeste 12 homolog (Drosophila) | -1.11 | 0.217 | -1.05 | 0.176 | -1.03 | 0.668 | 1.72 | 0.094 | -0.37 |
| 1416470_a_at | Rpn1 | ribophorin I | -1.04 | 0.558 | 1.03 | 0.82 | -1.2 | 0.064 | -1.14 | 0.53 | -0.59 |
| 1423717_at | Ak3 | adenylate kinase 3 | -1.09 | 0.151 | -1.06 | 0.328 | -1.03 | 0.606 | 1.08 | 0.494 | -0.53 |
| 1439300_at | --- | --- | 1.18 | 0.666 | -1.33 | 0.327 | -1.13 | 0.599 | -1.27 | 0.578 | -0.63 |
| 1417505_s_at | Il11ra1 /// Il11ra2 /// | interleukin 11 receptor, alpha chain 1 /// interleukin 11 receptor, alpha chain 2 /// s | 1.05 | 0.575 | -1 | 0.946 | -1.28 | 0.044 | -1.51 | 0.059 | -0.69 |
| 1447943_x_at | Yeats2 | YEATS domain containing 2 | 1.02 | 0.821 | -1.03 | 0.863 | -1.19 | 0.196 | -1.25 | 0.149 | -0.61 |
| 1427451_a_at | BC018473 | cDNA sequence BC018473 | 1.19 | 0.798 | -1.15 | 0.792 | -1.3 | 0.669 | -2.1 | 0.232 | -0.84 |

| | | | | | | | | | | | |
|---|---|---|---|---|---|---|---|---|---|---|---|
| 1421195_at | Cckar | cholecystokinin A receptor | -1.14 | 0.007 | 1.02 | 0.98 | -1.08 | 0.694 | -2.13 | 0.499 | -0.83 |
| 1426795_at | Ptprs | protein tyrosine phosphatase, receptor type, S | -2.14 | 0.163 | -1.34 | 0.646 | 1.61 | 0.152 | -2.61 | 0.234 | -1.12 |
| 1450725_s_at | Car14 | carbonic anhydrase 14 | -1.4 | 0.115 | -1.52 | 0.053 | 1.45 | 0.121 | -1.37 | 0.484 | -0.71 |
| 1456552_at | --- | 2 days pregnant adult female ovary cDNA, RIKEN full-length enriched library, clo | 1.4 | 0.55 | -1.27 | 0.609 | -1.56 | 0.123 | -1.11 | 0.614 | -0.64 |
| 1418817_at | Chmp1b | chromatin modifying protein 1B | -1.09 | 0.352 | 1.06 | 0.541 | -1.19 | 0.113 | -1.12 | 0.437 | -0.58 |
| 1451233_at | Traf2 | Tnf receptor-associated factor 2 | -1.12 | 0.555 | -1.21 | 0.085 | 1.1 | 0.346 | -1.71 | 0.089 | -0.73 |
| 1447014_at | --- | --- | 1.51 | 0.611 | -1.68 | 0.316 | -1.39 | 0.099 | -1.15 | 0.271 | -0.68 |
| 1438643_at | Camk1d | Calcium/calmodulin-dependent protein kinase ID | -1.2 | 0.622 | 1.35 | 0.341 | -1.56 | 0.077 | -2.47 | 0.152 | -0.97 |
| 1416146_at | Hspa4 | heat shock protein 4 | 1.1 | 0.476 | -1.21 | 0.013 | -1.11 | 0.567 | -1.11 | 0.303 | -0.58 |
| 1452325_at | Trp73 | transformation related protein 73 | -1.31 | 0.366 | -1.54 | 0.419 | 1.42 | 0.077 | -1.42 | 0.344 | -0.71 |
| 1425377_at | Wnt1 | wingless-related MMTV integration site 1 | -1.89 | 0.205 | 1.87 | 0.308 | -2.35 | 0.152 | -1.13 | 0.629 | -0.87 |
| 1423692_at | Ndufa8 | NADH dehydrogenase (ubiquinone) 1 alpha subcomplex, 8 | -1.12 | 0.099 | -1.1 | 0.232 | 1.02 | 0.843 | -1.13 | 0.431 | -0.58 |
| 1436116_x_at | 2900057D21Rik | RIKEN cDNA 2900057D21 gene | -1.6 | 0.524 | 1.29 | 0.557 | -1.1 | 0.406 | -2.31 | 0.053 | -0.93 |
| 1442476_at | Ift81 | Intraflagellar transport 81 homolog (Chlamydomonas) | 1.09 | 0.373 | -1.2 | 0.675 | -1.11 | 0.758 | -1.18 | 0.358 | -0.6 |
| 1426449_a_at | Pja1 | praja1, RING-H2 motif containing | 1.03 | 0.704 | -1.06 | 0.633 | -1.17 | 0.289 | -1.15 | 0.511 | -0.59 |
| 1424662_at | Pmpca | peptidase (mitochondrial processing) alpha | -1.01 | 0.857 | -1.17 | 0.201 | -1.01 | 0.902 | 1.15 | 0.462 | -0.51 |
| 1425838_at | Atp9a | ATPase, class II, type 9A | -1.21 | 0.733 | 1.26 | 0.61 | -1.36 | 0.519 | -2.78 | 0.128 | -1.02 |
| 1417320_at | Grpel1 | GrpE-like 1, mitochondrial | -1.02 | 0.279 | -1.05 | 0.2 | -1.12 | 0.298 | 1.12 | 0.683 | -0.52 |
| 1435081_at | Sypl | synaptophysin-like protein | -1.09 | 0.331 | -1.11 | 0.094 | 1.01 | 0.854 | -1.04 | 0.784 | -0.56 |
| 1449635_at | Prpf19 | PRP19/PSO4 pre-mRNA processing factor 19 homolog (S. cerevisiae) | -1.14 | 0.333 | -1.09 | 0.101 | 1.03 | 0.768 | -1.43 | 0.292 | -0.66 |
| 1455897_x_at | Hmgn1 | high mobility group nucleosomal binding domain 1 | -1.13 | 0.338 | 1.1 | 0.313 | -1.19 | 0.025 | -1.42 | 0.01 | -0.66 |
| 1419169_at | Mapk6 | mitogen-activated protein kinase 6 | -1.18 | 0.111 | -1.01 | 0.83 | -1.01 | 0.928 | 1.11 | 0.677 | -0.52 |
| 1426882_at | Ube3c | ubiquitin protein ligase E3C | -1.07 | 0.328 | -1.07 | 0.329 | -1.05 | 0.789 | 1.26 | 0.349 | -0.48 |
| 1458168_at | --- | Adult male adrenal gland cDNA, RIKEN full-length enriched library, clone:733044 | 1.1 | 0.177 | -1.04 | 0.888 | -1.31 | 0.341 | -1.32 | 0.39 | -0.64 |
| 1426826_at | Rbm16 | RNA binding motif protein 16 | -1.1 | 0.165 | -1.11 | 0.291 | 1.01 | 0.919 | -1.43 | 0.097 | -0.65 |
| 1458099_at | --- | --- | -1.25 | 0.051 | -1.36 | 0.042 | 1.29 | 0.631 | -2 | 0.105 | -0.83 |
| 1447611_at | 1700073E17Rik | RIKEN cDNA 1700073E17 gene | 1.09 | 0.183 | -1.3 | 0.115 | -1.04 | 0.704 | -1.07 | 0.663 | -0.58 |
| 1428225_s_at | Hnrpdl | heterogeneous nuclear ribonucleoprotein D-like | 1.1 | 0.467 | -1.09 | 0.573 | -1.23 | 0.309 | -1.58 | 0.046 | -0.7 |
| 1434260_at | Fchsd2 | FCH and double SH3 domains 2 | 1.01 | 0.954 | -1.18 | 0.321 | -1.03 | 0.817 | -2.15 | 0.042 | -0.84 |
| 1430326_s_at | Uqcrq | ubiquinol-cytochrome c reductase, complex III subunit VII | -1.02 | 0.815 | -1.05 | 0.56 | -1.13 | 0.323 | 1.17 | 0.487 | -0.5 |
| 1437014_x_at | LOC545161 /// LOC | similar to Peroxiredoxin 1 (Thioredoxin peroxidase 2) (Thioredoxin-dependent pe | -1.4 | 0.214 | 1.2 | 0.107 | -1.1 | 0.397 | -1.08 | 0.403 | -0.59 |
| 1455109_at | Tbl1xr1 | transducin (beta)-like 1X-linked receptor 1 | 1.24 | 0.585 | -1.17 | 0.439 | -1.36 | 0.443 | -1.48 | 0.113 | -0.69 |
| 1460607_at | Igsf11 | immunoglobulin superfamily, member 11 | -1.22 | 0.248 | -1.01 | 0.965 | 1.01 | 0.979 | -1.69 | 0.161 | -0.73 |
| 1429074_at | 1700026D08Rik | RIKEN cDNA 1700026D08 gene | -1.47 | 0.665 | -1.13 | 0.835 | 1.26 | 0.53 | -1.41 | 0.226 | -0.69 |
| 1426019_at | Plaa | phospholipase A2, activating protein | 1.47 | 0.557 | -1.24 | 0.805 | -1.82 | 0.427 | -1.48 | 0.524 | -0.77 |
| 1438507_x_at | Rpl14 | ribosomal protein L14 | -1.1 | 0.535 | 1.03 | 0.743 | -1.12 | 0.206 | -1.47 | 0.098 | -0.67 |
| 1417083_at | Sec61b | Sec61 beta subunit | -1.06 | 0.432 | 1.08 | 0.291 | -1.24 | 0.048 | -1.33 | 0.182 | -0.64 |
| 1425397_at | --- | CDNA clone MGC:6909 IMAGE:2655941 | -1.48 | 0.535 | 1.32 | 0.507 | -1.21 | 0.576 | -1.64 | 0.054 | -0.75 |
| 1458371_at | E130006N16Rik | RIKEN cDNA E130006N16 gene | 1.13 | 0.797 | -1.21 | 0.485 | -1.14 | 0.47 | -1.36 | 0.218 | -0.65 |
| 1423951_at | Tm2d3 | TM2 domain containing 3 | 1.09 | 0.346 | -1.12 | 0.27 | -1.19 | 0.258 | -1.41 | 0.059 | -0.66 |
| 1448199_at | Ankrd10 | ankyrin repeat domain 10 | -1.22 | 0.028 | 1.16 | 0.535 | -1.19 | 0.314 | -1.53 | 0.056 | -0.69 |
| 1439082_at | Ddx50 | DEAD (Asp-Glu-Ala-Asp) box polypeptide 50 | 1.08 | 0.746 | -1.16 | 0.683 | -1.14 | 0.63 | -1.15 | 0.646 | -0.59 |
| 1427983_at | Suhw3 | suppressor of hairy wing homolog 3 (Drosophila) | -1.15 | 0.293 | 1.05 | 0.557 | -1.1 | 0.49 | -1.09 | 0.789 | -0.57 |
| 1447619_at | A830039H05Rik | RIKEN cDNA A830039H05 gene | -1.02 | 0.984 | 1.12 | 0.848 | -1.38 | 0.71 | -1.73 | 0.175 | -0.75 |
| 1426478_at | Rasa1 | RAS p21 protein activator 1 | 1.11 | 0.365 | -1.3 | 0.449 | -1.06 | 0.721 | -1.16 | 0.453 | -0.6 |
| 1455570_x_at | Cnn3 | calponin 3, acidic | -1.08 | 0.531 | 1.14 | 0.456 | -1.3 | 0.01 | -2.56 | 0.071 | -0.95 |
| 1445390_at | Pawr | PRKC, apoptosis, WT1, regulator | -1.26 | 0.568 | 1.44 | 0.285 | -1.69 | 0.007 | -1.05 | 0.897 | -0.64 |
| 1420291_at | --- | --- | -1.72 | 0.394 | 1.67 | 0.219 | -1.73 | 0.346 | -1.2 | 0.785 | -0.74 |
| 1437807_x_at | Ctnna1 | catenin (cadherin associated protein), alpha 1 | -1.1 | 0.592 | 1.09 | 0.454 | -1.2 | 0.06 | -1.05 | 0.688 | -0.57 |
| 1417197_at | Wwc2 | WW, C2 and coiled-coil domain containing 2 | 1.03 | 0.871 | -1.2 | 0.71 | -1.04 | 0.87 | -1.39 | 0.521 | -0.65 |
| 1449638_at | --- | --- | -1.52 | 0.211 | 1.35 | 0.445 | -1.21 | 0.592 | -1.07 | 0.889 | -0.62 |
| 1451351_at | Ttc13 | tetratricopeptide repeat domain 13 | -1.12 | 0.146 | 1.03 | 0.852 | -1.11 | 0.511 | -1.19 | 0.16 | -0.6 |
| 1439843_at | Camk4 | calcium/calmodulin-dependent protein kinase IV | -1.03 | 0.953 | 1.18 | 0.406 | -1.48 | 0.215 | -3.67 | 0.048 | -1.25 |

| | | | | | | | | | | | |
|---|---|---|---|---|---|---|---|---|---|---|---|
| 1448979_at | Muted | muted | -1.03 | 0.276 | -1.04 | 0.658 | -1.12 | 0.203 | 1.29 | 0.344 | -0.48 |
| 1452045_at | Zfp281 | zinc finger protein 281 | 1.01 | 0.869 | -1.03 | 0.772 | -1.19 | 0.23 | -1.18 | 0.245 | -0.6 |
| 1439092_at | Nxf1 | nuclear RNA export factor 1 homolog (S. cerevisiae) | -1.03 | 0.944 | 1.03 | 0.937 | -1.21 | 0.494 | -1.35 | 0.137 | -0.64 |
| 1454789_x_at | Prpf6 | PRP6 pre-mRNA splicing factor 6 homolog (yeast) | -1.15 | 0.629 | -1.15 | 0.322 | 1.09 | 0.222 | -1.38 | 0.125 | -0.65 |
| 1444160_at | Pkd1l3 | Polycystic kidney disease 1 like 3 | -1.28 | 0.56 | -1.45 | 0.329 | 1.36 | 0.382 | -1.06 | 0.852 | -0.61 |
| 1425740_at | --- | --- | -1.78 | 0.518 | 1.5 | 0.223 | -1.3 | 0.33 | -1.47 | 0.22 | -0.76 |
| 1440967_at | Bri3 /// Baiap2l1 | Brain protein I3 /// BAI1-associated protein 2-like 1 | 1.11 | 0.864 | -1.11 | 0.735 | -1.22 | 0.252 | -1.61 | 0.167 | -0.71 |
| 1445716_at | --- | --- | -1.28 | 0.159 | -1.77 | 0.24 | 1.48 | 0.41 | -2.03 | 0.118 | -0.9 |
| 1455923_at | Kctd8 | potassium channel tetramerisation domain containing 8 | -1.17 | 0.752 | 1.03 | 0.948 | -1.06 | 0.795 | -1.46 | 0.334 | -0.66 |
| 1452053_a_at | Tmem33 | transmembrane protein 33 | -1.02 | 0.843 | -1.12 | 0.282 | -1.05 | 0.642 | 1.44 | 0.276 | -0.44 |
| 1420842_at | Ptprf | protein tyrosine phosphatase, receptor type, F | 1 | 0.987 | -1.01 | 0.75 | -1.19 | 0.413 | -1.04 | 0.861 | -0.56 |
| 1441360_at | Rps6ka3 | Ribosomal protein S6 kinase polypeptide 3 | -1.03 | 0.771 | 1.17 | 0.707 | -1.45 | 0.085 | -1.26 | 0.594 | -0.64 |
| 1446989_at | --- | Transcribed locus | 1.38 | 0.17 | -1.1 | 0.845 | -1.86 | 0.207 | -2.01 | 0.439 | -0.9 |
| 1439014_at | 1600021P15Rik | RIKEN cDNA 1600021P15 gene | -1.06 | 0.874 | 1.21 | 0.329 | -1.49 | 0.21 | -2.97 | 0.092 | -1.08 |
| 1448214_at | Pdhb /// LOC62000 | pyruvate dehydrogenase (lipoamide) beta /// similar to pyruvate dehydrogenase (l | -1.18 | 0.169 | -1.22 | 0.264 | 1.16 | 0.362 | -1.05 | 0.835 | -0.57 |
| 1429781_s_at | Ccdc39 | coiled-coil domain containing 39 | 1 | 0.995 | -1.15 | 0.72 | -1.04 | 0.881 | -1.28 | 0.278 | -0.62 |
| 1440234_at | 1810012P15Rik | RIKEN cDNA 1810012P15 gene | -1.19 | 0.385 | -1.01 | 0.981 | -1 | 0.985 | 1.13 | 0.677 | -0.52 |
| 1434647_at | AU040377 | expressed sequence AU040377 | 1.38 | 0.018 | -1.29 | 0.355 | -1.49 | 0.204 | -1.36 | 0.344 | -0.69 |
| 1450932_s_at | Dock9 /// LOC6703 | dedicator of cytokinesis 9 /// similar to Dedicator of cytokinesis protein 9 (Cdc42 g | -1.16 | 0.445 | -1.06 | 0.633 | 1.02 | 0.935 | -1.27 | 0.354 | -0.62 |
| 1436402_at | Dohh | deoxyhypusine hydroxylase/monooxygenase | -1.4 | 0.112 | -1.09 | 0.702 | 1.2 | 0.356 | -1.51 | 0.053 | -0.7 |
| 1439959_at | Fgf11 | fibroblast growth factor 11 | -1.08 | 0.749 | -1.05 | 0.949 | -1.05 | 0.788 | 1.13 | 0.436 | -0.51 |
| 1449886_a_at | Timm9 | translocase of inner mitochondrial membrane 9 homolog (yeast) | -1.14 | 0.742 | -1.07 | 0.598 | 1.02 | 0.855 | -1.21 | 0.54 | -0.6 |
| 1441682_s_at | Xpot | exportin, tRNA (nuclear export receptor for tRNAs) | -1.05 | 0.666 | -1.09 | 0.275 | -1.04 | 0.603 | 1.07 | 0.615 | -0.53 |
| 1449236_at | Dll3 | delta-like 3 (Drosophila) | 1.45 | 0.564 | -2.16 | 0.263 | -1.09 | 0.846 | -1.3 | 0.432 | -0.78 |
| 1425115_at | Rbbp6 | retinoblastoma binding protein 6 | -1.18 | 0.313 | -1.14 | 0.504 | 1.1 | 0.518 | -1.65 | 0.164 | -0.72 |
| 1457562_at | Rps6kb1 | ribosomal protein S6 kinase, polypeptide 1 | 1.53 | 0.137 | -1.52 | 0.291 | -1.57 | 0.001 | -1.61 | 0.203 | -0.79 |
| 1416151_at | Sfrs3 | splicing factor, arginine/serine-rich 3 (SRp20) | -1.02 | 0.804 | 1.07 | 0.519 | -1.28 | 0.003 | -1.02 | 0.871 | -0.56 |
| 1431600_at | 4930564C03Rik | RIKEN cDNA 4930564C03 gene | -1.19 | 0.748 | -1.26 | 0.748 | 1.19 | 0.442 | -1.31 | 0.248 | -0.64 |
| 1440964_s_at | AI465270 | expressed sequence AI465270 | -1.13 | 0.547 | -1.07 | 0.698 | 1.01 | 0.925 | -1.23 | 0.144 | -0.61 |
| 1429610_a_at | Zfp511 | zinc finger protein 511 | 1.03 | 0.763 | -1.12 | 0.57 | -1.1 | 0.33 | -1.16 | 0.156 | -0.59 |
| 1438930_s_at | Mecp2 | methyl CpG binding protein 2 | 1.12 | 0.398 | -1.11 | 0.584 | -1.23 | 0.166 | -1.06 | 0.837 | -0.57 |
| 1454133_s_at | 4930523O13Rik | RIKEN cDNA 4930523O13 gene | -1.24 | 0.537 | -1.2 | 0.537 | 1.19 | 0.327 | -1.52 | 0.181 | -0.69 |
| 1453665_at | 4930529M08Rik | RIKEN cDNA 4930529M08 gene | -1.67 | 0.086 | -1.93 | 0.036 | 1.71 | 0.147 | -1.1 | 0.854 | -0.75 |
| 1446752_at | C78200 | expressed sequence C78200 | -1.04 | 0.875 | -1.27 | 0.714 | 1.08 | 0.885 | -1.43 | 0.645 | -0.66 |
| 1440742_at | Epc1 | Enhancer of polycomb homolog 1 (Drosophila) | -1.03 | 0.864 | -1.23 | 0.379 | 1.05 | 0.677 | -1.13 | 0.38 | -0.59 |
| 1442557_at | Syt1 | Synaptotagmin I | -2.79 | 0.027 | -1.69 | 0.339 | 1.88 | 0.317 | -1.14 | 0.809 | -0.93 |
| 1444136_at | Rai12 | Retinoic acid induced 12 | -1.25 | 0.46 | 1.2 | 0.227 | -1.21 | 0.407 | -1.22 | 0.231 | -0.62 |
| 1454682_at | A430005L14Rik | RIKEN cDNA A430005L14 gene | -1.04 | 0.408 | -1.06 | 0.522 | -1.07 | 0.394 | 1.75 | 0.232 | -0.36 |
| 1424050_s_at | Fgfr1 | fibroblast growth factor receptor 1 | 1.01 | 0.866 | -1.07 | 0.759 | -1.13 | 0.424 | -1.04 | 0.84 | -0.56 |
| 1437871_at | Pgm5 | phosphoglucomutase 5 | -1 | 0.995 | 1.01 | 0.991 | -1.21 | 0.443 | -2.51 | 0.347 | -0.93 |
| 1448368_at | Dctn6 | dynactin 6 | -1.13 | 0.023 | 1.02 | 0.762 | -1.08 | 0.326 | -1.05 | 0.587 | -0.56 |
| 1446397_at | Nup98 | Nucleoporin 98 | 1.26 | 0.137 | -1.2 | 0.543 | -1.36 | 0.061 | -1.2 | 0.453 | -0.63 |
| 1455741_a_at | Ece1 | endothelin converting enzyme 1 | 1.02 | 0.827 | -1.04 | 0.601 | -1.19 | 0.478 | -1.08 | 0.702 | -0.57 |
| 1454615_x_at | Srp14 | signal recognition particle 14 | -1.04 | 0.512 | 1.03 | 0.571 | -1.19 | 0.152 | -1.2 | 0.203 | -0.6 |
| 1423733_a_at | Fiz1 | Flt3 interacting zinc finger protein 1 | -1.08 | 0.362 | -1.16 | 0.04 | 1.04 | 0.37 | -1.08 | 0.242 | -0.57 |
| 1445330_at | Pdia3 | protein disulfide isomerase associated 3 | -1.47 | 0.155 | 1.24 | 0.303 | -1.1 | 0.627 | -1.09 | 0.686 | -0.61 |
| 1432296_a_at | Itgav | integrin alpha V | -1.4 | 0.218 | 1.24 | 0.345 | -1.13 | 0.755 | -1.19 | 0.395 | -0.62 |
| 1426655_a_at | 4930504E06Rik | RIKEN cDNA 4930504E06 gene | -1.12 | 0.466 | -1.09 | 0.56 | 1.02 | 0.908 | -1.07 | 0.787 | -0.56 |
| 1457689_at | Sbf2 | SET binding factor 2 | -1.18 | 0.537 | 1.29 | 0.425 | -1.45 | 0.143 | -1.16 | 0.614 | -0.62 |
| 1460324_at | Dnmt3a | DNA methyltransferase 3A | -1.25 | 0.277 | -1.04 | 0.892 | 1.07 | 0.77 | -1.28 | 0.387 | -0.62 |
| 1424887_at | Klhdc4 | kelch domain containing 4 | -1.14 | 0.294 | -1.08 | 0.598 | 1.03 | 0.835 | -1.08 | 0.613 | -0.57 |
| 1439352_at | Trim7 | tripartite motif protein 7 | -1.11 | 0.769 | -1.76 | 0.073 | 1.36 | 0.512 | -2.5 | 0.033 | -1 |
| 1430755_at | 4930452G13Rik | RIKEN cDNA 4930452G13 gene | 1.46 | 0.564 | -1.59 | 0.002 | -1.35 | 0.013 | -2.34 | 0.015 | -0.96 |

| | | | | | | | | | | | |
|---|---|---|---|---|---|---|---|---|---|---|---|
| 1429821_at | 2810046L04Rik | RIKEN cDNA 2810046L04 gene | -1.07 | 0.799 | 1.22 | 0.35 | -1.49 | 0.228 | -1.39 | 0.197 | -0.68 |
| 1458505_at | LOC552901 | hypothetical LOC552901 | -1.26 | 0.705 | 1.52 | 0.141 | -1.95 | 0.003 | -1.22 | 0.345 | -0.73 |
| 1442489_at | D1Ertd564e | DNA segment, Chr 1, ERATO Doi 564, expressed | 1.35 | 0.413 | -1.21 | 0.433 | -1.53 | 0.109 | -1.05 | 0.818 | -0.61 |
| 1436596_at | H2afv | H2A histone family, member V | -1.03 | 0.713 | -1.11 | 0.342 | -1.04 | 0.833 | 1.37 | 0.324 | -0.45 |
| 1428483_a_at | 2610039C10Rik | RIKEN cDNA 2610039C10 gene | -1.08 | 0.848 | 1.04 | 0.768 | -1.15 | 0.137 | -1.03 | 0.812 | -0.56 |
| 1444941_at | --- | --- | 1.48 | 0.387 | -1.2 | 0.332 | -1.95 | 0.023 | -1.08 | 0.839 | -0.69 |
| 1418342_at | Recc1 | replication factor C 1 | 1.13 | 0.419 | -1.05 | 0.64 | -1.34 | 0.07 | -1.08 | 0.38 | -0.58 |
| 1427896_at | 2400003N08Rik | RIKEN cDNA 2400003N08 gene | -1.02 | 0.899 | -1.1 | 0.464 | -1.06 | 0.319 | 1.02 | 0.688 | -0.54 |
| 1453877_at | 4930467D21Rik | RIKEN cDNA 4930467D21 gene | 1.11 | 0.643 | -1.03 | 0.969 | -1.33 | 0.269 | -1.18 | 0.753 | -0.61 |
| 1443346_at | --- | --- | -1.16 | 0.528 | 1.24 | 0.251 | -1.38 | 0.221 | -1.31 | 0.656 | -0.65 |
| 1457121_at | AW822216 | expressed sequence AW822216 | -1.58 | 0.442 | -1.01 | 0.976 | 1.21 | 0.523 | -3.93 | 0.096 | -1.33 |
| 1420717_at | A930018P22Rik | RIKEN cDNA A930018P22 gene | 1.33 | 0.302 | -1.16 | 0.729 | -1.55 | 0.536 | -1.61 | 0.381 | -0.75 |
| 1452674_a_at | Eif3s12 | eukaryotic translation initiation factor 3, subunit 12 | 1.04 | 0.778 | -1.11 | 0.096 | -1.13 | 0.448 | -1.24 | 0.106 | -0.61 |
| 1443945_at | C130073F10Rik | RIKEN cDNA C130073F10 gene | -1.01 | 0.99 | 1.1 | 0.75 | -1.34 | 0.522 | -1.95 | 0.275 | -0.8 |
| 1457098_at | D2Ertd624e | DNA segment, Chr 2, ERATO Doi 624, expressed | 1.24 | 0.486 | -1.07 | 0.884 | -1.53 | 0.019 | -1.52 | 0.517 | -0.72 |
| 1455155_at | Lsm14b | LSM14 homolog B (SCD6, S. cerevisiae) | -1.09 | 0.05 | -1.21 | 0.214 | 1.09 | 0.749 | -1.38 | 0.158 | -0.65 |
| 1423741_at | Rbm10 | RNA binding motif protein 10 | -1.13 | 0.698 | -1.09 | 0.407 | 1.03 | 0.836 | -1.11 | 0.304 | -0.58 |
| 1418964_at | Pigm | phosphatidylinositol glycan anchor biosynthesis, class M | 1.05 | 0.766 | -1.09 | 0.75 | -1.15 | 0.268 | -1.19 | 0.188 | -0.6 |
| 1419716_a_at | Pou2f1 | POU domain, class 2, transcription factor 1 | 1.06 | 0.657 | -1.1 | 0.461 | -1.16 | 0.506 | -1.26 | 0.197 | -0.62 |
| 1450140_a_at | Cdkn2a | cyclin-dependent kinase inhibitor 2A | -1.94 | 0.078 | -1.21 | 0.687 | 1.49 | 0.039 | -1.2 | 0.661 | -0.72 |
| 1418687_at | Arc | activity regulated cytoskeletal-associated protein | 1.05 | 0.941 | -1.08 | 0.775 | -1.17 | 0.624 | -1.13 | 0.667 | -0.58 |
| 1425702_a_at | Enpp5 | ectonucleotide pyrophosphatase/phosphodiesterase 5 | -1.25 | 0.238 | -1.12 | 0.374 | 1.14 | 0.368 | -1.31 | 0.253 | -0.63 |
| 1419659_s_at | Chic2 | cysteine-rich hydrophobic domain 2 | -1.07 | 0.2 | -1.11 | 0.194 | 1 | 0.998 | -1.07 | 0.354 | -0.56 |
| 1418672_at | Akr1c13 | aldo-keto reductase family 1, member C13 | 1.02 | 0.937 | -1.17 | 0.226 | -1.04 | 0.853 | -1.1 | 0.689 | -0.57 |
| 1433277_at | D730048A03Rik | RIKEN cDNA D730048A03 gene | 1.41 | 0.659 | -1.41 | 0.551 | -1.4 | 0.351 | -1.37 | 0.316 | -0.69 |
| 1454559_at | 5430437A18Rik | RIKEN cDNA 5430437A18 gene | -3.4 | 0.103 | 1.59 | 0.344 | -1.05 | 0.909 | -2.04 | 0.07 | -1.23 |
| 1451256_at | Gpr172b | G protein-coupled receptor 172B | -1.12 | 0.612 | -1 | 0.997 | -1.06 | 0.775 | 1.23 | 0.181 | -0.49 |
| 1432494_a_at | 1700019E19Rik | RIKEN cDNA 1700019E19 gene | -1.15 | 0.277 | -1.05 | 0.697 | 1.01 | 0.964 | -1.56 | 0.143 | -0.69 |
| 1435807_at | Cdc42 | cell division cycle 42 homolog (S. cerevisiae) | -1.09 | 0.623 | -1.16 | 0.742 | 1.05 | 0.887 | -1.37 | 0.317 | -0.64 |
| 1417371_at | Peli1 | pellino 1 | 1.04 | 0.406 | -1.06 | 0.503 | -1.18 | 0.046 | -1.94 | 0.022 | -0.79 |
| 1454123_at | 4933406K04Rik | RIKEN cDNA 4933406K04 gene | -1.36 | 0.459 | 1.55 | 0.224 | -1.81 | 0.053 | -1.79 | 0.059 | -0.85 |
| 1427410_at | Dleu2 | deleted in lymphocytic leukemia, 2 | 1.13 | 0.485 | -1.01 | 0.934 | -1.4 | 0.116 | -1.27 | 0.117 | -0.64 |
| 1418646_at | Gna-rs1 | guanine nucleotide binding protein, related sequence 1 | -1.15 | 0.536 | -1.1 | 0.62 | 1.05 | 0.736 | -1.55 | 0.063 | -0.69 |
| 1456019_at | Cwf19l2 | CWF19-like 2, cell cycle control (S. pombe) | -1.04 | 0.665 | 1.06 | 0.419 | -1.23 | 0.122 | -1.34 | 0.244 | -0.64 |
| 1457875_at | Hcfc1 | host cell factor C1 | -1.88 | 0.449 | -2.13 | 0.367 | 1.83 | 0.205 | -1.07 | 0.655 | -0.81 |
| 1420613_at | Ptp4a2 | protein tyrosine phosphatase 4a2 | 1.1 | 0.585 | -1.11 | 0.021 | -1.19 | 0.067 | -1.3 | 0.167 | -0.63 |
| 1425011_x_at | Stx18 | syntaxin 18 | 1.04 | 0.745 | -1.14 | 0.442 | -1.09 | 0.338 | -1.37 | 0.167 | -0.64 |
| 1423993_at | Atp6v1f | ATPase, H+ transporting, lysosomal V1 subunit F | -1.16 | 0.291 | 1.02 | 0.709 | -1.05 | 0.751 | -1.25 | 0.361 | -0.61 |
| 1417186_at | Hip2 | huntingtin interacting protein 2 | -1.03 | 0.692 | -1.03 | 0.721 | -1.12 | 0.187 | 1.07 | 0.564 | -0.53 |
| 1424366_at | Tmem15 | transmembrane protein 15 | -1.01 | 0.947 | -1.2 | 0.093 | 1.01 | 0.941 | -1.01 | 0.935 | -0.55 |
[truncated: 3,942,721 more chars]
